# Paired metabolomics identify associations between fecal and serum metabolites in cardiovascular-kidney-metabolic syndrome

**DOI:** 10.3389/fendo.2026.1982623

**Published:** 2026-10-27

**Authors:** Zihan Zhao, Jinjie Dai, Yuqing Zou, Xueying An, Yunxi Zhang, Congcong Zhu, Lixiao Hao, Linpei Jia

**Affiliations:** 1 Department of Nephrology, Xuanwu Hospital, Capital Medical University, Beijing, China; 2 Department of Nephrology, The First Hospital of Fangshan District, Beijing, China; 3 Department of General Practice, Xuanwu Hospital, Capital Medical University, Beijing, China; 4 State Key Laboratory of Kidney Diseases, National Clinical Research Center for Kidney Diseases, Beijing, China

**Keywords:** cardiovascular-kidney-metabolic syndrome, fecal microbiome profiling, metabolomics, proteomic analysis, uridine

## Abstract

**Background:**

Whether fecal metabolic alterations are associated with circulating metabolic changes in cardiovascular-kidney-metabolic (CKM) syndrome remains unclear. We compared serum and fecal metabolites between different CKM stages and investigated fecal-serum associations.

**Methods:**

Forty-four participants participated in the cross-sectional study, including 19 subjects in CKM stages 1–2 and 25 subjects in CKM stages 3-4. All participants underwent untargeted serum and fecal metabolomics and fecal microbiome profiling. Exploratory serum proteomics was available for 35 participants. Candidate metabolites were selected according to group differences, annotation confidence and redundancy. CKM-adjusted partial Spearman correlations were calculated for paired metabolites. Bootstrap and leave-one-participant-out analyses assessed stability. Serum-fecal KEGG pathway scores and clinical associations were also examined.

**Results:**

Serum analysis identified 470 annotated, non-exogenous features. Different features were involved in lipid-related, organic acid and steroid-related metabolites. Fecal analysis identified six features after Benjamini-Hochberg (BH) correction. Paired analysis of 17 fecal and 48 serum metabolites identified exploratory eight associations. Serum uridine was associated with fecal 1-palmitoyl lysophosphatidic acid, Asp Ala Phe and PE (16:0/0:0). Serum and fecal pyrimidine metabolism scores were positively correlated after adjustment for CKM group (partial rho = 0.429, *P* = 0.0041, BH-FDR = 0.0246). Selected metabolites were mainly associated with renal function indicators. The fecal microbiome showed greater genus-level dispersion in stages 3-4. Proteomic analysis identified 221 differential proteins enriched in complement and coagulation, inflammatory and lipid-related pathways.

**Conclusions:**

Paired serum and fecal metabolomics identified a positive association between serum and fecal pyrimidine metabolism scores in CKM syndrome, accompanied by the uridine as an exploratory candidate associated with multiple fecal metabolites.

## 1 Introduction

Cardiovascular-kidney-metabolic (CKM) syndrome is defined as a systemic disorder attributable to the pathophysiological interactions among metabolic, renal and the cardiovascular systems (1, 2). Metabolic dysfunction is defined as the initiator and core pathogenesis of CKM syndrome, which contributes to functional abnormalities across the cardiovascular and renal systems. The American Heart Association (AHA) classifies CKM into stages 0–4 according to the presence and severity of adiposity, metabolic risk factors, chronic kidney disease (CKD) and cardiovascular disease. The staging is used to predict the prognosis and perform stage-based management of CKM patients (2). Among the five stages, CKM stages 0–2 represent states without identified cardiovascular disease, whereas stages 3–4 are defined by the presence of subclinical or clinical cardiovascular disease, respectively (3). Therefore, the critical distinction between stages 0–2 and 3–4 depends on whether metabolic dysfunction has caused cardiovascular damage. Thus, characterizing the metabolic differences between stages 0–2 and 3–4 may clarify the pathology as well as the potential biomarkers associated with cardiovascular involvement in CKM syndrome.

Metabolic alterations in CKM syndrome involve several biochemical processes rather than a single metabolite or pathway. Current evidence indicates that CKM-related dysfunctions are driven by complicated inflammatory and metabolomic dysregulations, especially in lipid, amino acid and bile acid pathways (4, 5). Recent CKM metabolomics studies further showed that higher-risk CKM stages are associated with lipid-related metabolic patterns, including triglycerides, fatty acids, phosphatidylcholines, sphingolipids and acylcarnitines (5). Individuals within the same advanced CKM stage may also have metabolic heterogeneity. Other population-based analyses have suggested that selected amino acids, fatty acids and lipid-related signatures may partly account for the associations between CKM stages and organ outcomes (6, 7). However, most CKM metabolomics studies have only analyzed circulating metabolites or proteins. Given the systemic complexity of metabolic perturbations in CKM, single-system omics are insufficient to capture the global metabolic landscape. Thus, integrative multi-omics analysis of CKM is necessary.

The intestine is another important system involved in metabolic regulation. Intestinal microbial metabolism contributes to the production or transformation of short-chain fatty acids, bile acids, indole derivatives and trimethylamine-related metabolites, which have been implicated in metabolic, renal and cardiovascular dysfunction (8). However, studies of intestinal metabolism in CKM syndrome remain limited. Previous studies have mainly reported whether dietary index for gut microbiota is associated with CKM classification, systemic inflammation and mortality (9, 10). Studies on CKD and cardiovascular diseases have integrated fecal and circulating metabolomic data, revealing associations between gut and systemic metabolic features (11, 12). However, whether fecal metabolic alterations are related to circulating metabolic changes in CKM syndrome remains unclear. A paired analysis of fecal and serum metabolomics is necessary to determine the association between intestinal and systemic metabolism.

This study aimed to investigate whether fecal metabolic alterations are related to circulating metabolic changes in CKM syndrome. We performed untargeted serum and fecal metabolomics in participants classified as CKM stages 1–2 or stages 3-4. Serum and fecal metabolic differences between the two groups were first analyzed separately. Paired fecal and serum metabolomic data were then integrated to identify fecal metabolites associated with circulating metabolic features. We further examined whether the selected metabolites were related to CKM classification and clinical indicators. Fecal microbiome and exploratory serum proteomic data were also analyzed to assess the biological context of these metabolic associations. Our study may help indicate fecal-serum metabolic associations in CKM syndrome and provide candidate metabolic features for future targeted validation.

## 2 Materials and methods

### 2.1 Study design and participants

We performed the cross-sectional study, which enrolled subjects at Xuanwu Hospital, Capital Medical University from January 2021 to December 2022. Subjects were aged 18–85 years. Patients were excluded if they were receiving hemodialysis, adhering to a special diet, or had severe infection, malignancy, acute heart failure, recent acute cardiovascular events, a life expectancy of less than six months, or recent antibiotic exposure. The study was approved by the Ethics Board of Xuanwu Hospital, Capital Medical University (KS2021048). All participants provided written informed consent.

Sixty-four subjects agreed to participate in the study. Fasting blood and fecal samples from 44 subjects were collected according to standardized protocols for the serum metabolomics, fecal metabolomics and fecal 16S rRNA sequencing analysis. Serum proteomics were available for 35 subjects.

### 2.2 CKM classification and clinical variables

CKM stages were classified according to the AHA Presidential Advisory (2). Stage 0 indicates the absence of CKM risk factors. Stage 1 was defined by excess or dysfunctional adiposity without additional metabolic risk factors or CKD. Stage 2 was defined by the presence of metabolic risk factors, including hypertension, diabetes, hypertriglyceridemia or metabolic syndrome, or moderate- to high-risk CKD. Stage 3 is defined as very-high-risk CKD or a predicted 10-year total cardiovascular disease risk of ≥ 20% calculated by the PREVENT equation (13, 14). Stage 4 represents clinical cardiovascular disease. Since the primary aim was to characterize metabolic differences according to cardiovascular involvement and cardiovascular risk burden, participants were grouped as CKM stages 1–2 and CKM stages 3-4.

Demographic data, body mass index, blood pressure, serum creatinine, albumin-to-creatinine ratio, 24-h urinary protein and histories of diseases were obtained from clinical records. Estimated glomerular filtration rate (eGFR) was calculated by the CKD-EPI creatinine equation (15).

### 2.3 Untargeted serum and fecal metabolomics

Fasting venous blood was collected into serum separation tubes, centrifuged at 3,000 rpm for 15 min at 4 °C. Then serum was separated. Metabolites were extracted from 100 μL of serum using 400 μL of acetonitrile:methanol (1:1, v/v) containing 0.02 mg/mL L-2-chlorophenylalanine as an internal standard. After protein precipitation and centrifugation, the supernatant was dried under nitrogen and reconstituted in acetonitrile:water (1:1, v/v). Fecal samples were stored at -80 °C. Metabolites were extracted from 50 mg feces using methanol:water (4:1, v/v) containing the same internal standard, followed by homogenization, ultrasonication, protein precipitation, and centrifugation.

Untargeted metabolomic profiling was performed using a UHPLC-Q Exactive HF-X mass spectrometry system (Thermo Fisher Scientific, Waltham, MA, USA) equipped with an HSS T3 column (100 mm × 2.1 mm, 1.8 μm; Waters Corporation, Milford, MA, USA). Data were acquired in positive and negative electrospray ionization modes over an m/z range of 70–1050 using data-dependent acquisition. Pooled quality-control samples, prepared by combining equal aliquots of all sample extracts, were analyzed after every 5–15 study samples.

Raw data were processed using Progenesis QI (Waters Corporation) for peak detection, integration and retention-time alignment. Feature intensities were normalized by total signal intensity and log10-transformed. Features with a relative standard deviation greater than 30% in pooled quality-control samples were excluded. Metabolic features were putatively annotated using accurate mass, retention time, adduct information and MS/MS fragmentation evidence by searches in HMDB, METLIN (11) and the Majorbio in-house database. After quality control, serum and fecal metabolic features were obtained. Fecal features with more than 30% missing values were further excluded. The remaining missing values were replaced with one-half of the minimum non-missing value for the corresponding feature.

### 2.4 Fecal microbiome profiling

Total microbial DNA was extracted from fecal samples using the FastPure Stool DNA Isolation Kit (MJYH, Shanghai, China). DNA concentration and purity were assessed using a NanoDrop 2000 spectrophotometer. DNA integrity was evaluated by 1% agarose gel electrophoresis. The V3-V4 region of the bacterial 16S rRNA gene was amplified using primers 338F (5′-ACTCCTACGGGAGGCAGCAG-3′) and 806R (5′-GGACTACHVGGGTWTCTAAT-3′). Sequencing libraries were prepared using the NEXTFLEX Rapid DNA-Seq Kit and sequenced on an Illumina NextSeq 2000 platform at Majorbio Bio-Pharm Technology Co. Ltd. (Shanghai, China). Raw paired-end reads were quality-filtered using fastp (version 0.19.6) and merged using FLASH (version 1.2.11). Merged reads were processed using DADA2 in R for amplicon sequence variant inference and chimera removal. Taxonomy was assigned against the SILVA database (version 138.1) using the RDP classifier with a minimum bootstrap value of 50. Mitochondrial, chloroplast and unclassified-kingdom sequences were removed. Amplicon sequence variants were aggregated at the genus level. Genera present in at least 20% of samples and with a mean relative abundance of at least 0.05% were retained, yielding 91 genera for subsequent analysis. Microbial functional potential was predicted from the ASV abundance table using PICRUSt2 (version 2.6.3). Predicted enzyme commission numbers, KEGG orthologs and MetaCyc pathway abundances were generated using PICRUSt2 (version 2.6.3). The MetaCyc pathway database was version 24. Partial Spearman correlations between predicted pathway abundances and the corresponding measured metabolites were calculated after adjustment for CKM group. *P* values were corrected using the BH method.

### 2.5 Exploratory serum proteomic profiling

Serum proteomic profiling was performed as a complementary analysis to provide additional biological context for the metabolomic findings. Sufficient residual serum was selected for proteomic analysis. Serum proteins were extracted in lysis buffer containing 8 M urea, 1% sodium dodecyl sulfate and protease inhibitors. Protein concentrations were determined using a BCA Protein Assay Kit (Thermo Fisher Scientific, Waltham, MA, USA). Proteins were reduced with tris (2-carboxyethyl) phosphine, alkylated with iodoacetamide and precipitated with pre-cooled acetone. The protein pellets were resuspended in triethylammonium bicarbonate buffer. Proteins were digested overnight with trypsin at an enzyme-to-protein ratio of 1:50. The resulting peptides were desalted using HLB cartridges. Peptides were separated using a Vanquish Neo liquid chromatography system equipped with a uPAC High Throughput column (Thermo Fisher Scientific). Mass spectrometry was performed on an Orbitrap Astral mass spectrometer in data-independent acquisition mode. Data acquisition was controlled using Xcalibur software (version 4.7). Raw data were processed using Spectronaut software (version 19; Biognosys, Schlieren, Switzerland) in the UniProt human reference proteome (version 2022.09). Carbamidomethylation of cysteine was specified as a fixed modification. Oxidation of methionine and acetylation of the protein N-terminus were specified as variable modifications. Peptide- and protein-level false discovery rates were controlled at 1%. Protein abundance was quantified using the MaxLFQ algorithm.

### 2.6 Statistical analyses

Continuous variables are presented as medians with interquartile ranges. Differences between CKM stages 1–2 and 3–4 were assessed using the Mann-Whitney U test. Categorical variables are presented as numbers with percentages. Fisher’s exact test was used for categorical comparisons.

Grouped comparisons were performed for each omics dataset. Metabolomic features, microbial genera and proteins were compared using the Mann-Whitney U test. Cliff’s delta was calculated for metabolomic and microbiome data. Rank-biserial correlation was analyzed for proteomic data. For the single-omics metabolomic analyses, annotated features with a *P* < 0.05 and an absolute Cliff’s delta ≥ 0.33 were retained. Benjamini-Hochberg (BH) correction was performed within each omics dataset. Microbial alpha diversity was compared using the Mann-Whitney U test. Beta diversity was assessed from Bray-Curtis dissimilarities using principal coordinates analysis. Group differences were evaluated with PERMANOVA. PERMDISP was used to assess differences in within-group dispersion. Proteins with at least five available measurements in each CKM group were included in the differential analysis.

For the paired fecal-serum metabolomics analysis, candidate features were selected before correlation analysis. Eligible features were required with an available metabolite annotation, exclusion of clearly exogenous compounds, a group-comparison *P* < 0.05, an absolute Cliff’s delta ≥ 0.50 and high or moderate annotation confidence. Duplicate and highly redundant metabolites within the same data layer were reduced to one representative feature when the absolute Spearman correlation was ≥ 0.95. A pair was selected when the absolute partial Spearman correlation coefficient was ≥ 0.40, the *P* value < 0.005, the 1000-resample bootstrap 95% confidence interval excluding zero and the association with no direction reversal in leave-one-participant-out analyses. BH-FDR values were calculated across all 816 feature-level fecal-serum metabolite comparisons to control for multiple testing. Pairs meeting the combined effect-size, nominal *P*-value and resampling-stability criteria were retained as exploratory candidate associations.

For the paired KEGG pathway analysis, only high- or moderate-confidence annotations with a fragmentation score ≥ 40 and valid KEGG compound identifiers were selected. Pathways containing at least five unique compounds in both serum and feces were defined as the primary pathway. Pathways with at least three compounds and fewer than five compounds were defined as the secondary pathway. For each pathway, z score was calculated. The primary model adjusted for CKM group. The expanded model additionally adjusted for age, sex and eGFR. For the pathway-level analysis, BH correction was applied.

Associations between selected serum-fecal paired metabolites and 12 clinical indicators, including eGFR, serum creatinine, blood urea nitrogen, albumin, hemoglobin, systolic blood pressure, diastolic blood pressure, total cholesterol, triglycerides, LDL cholesterol, BMI and diabetes history, were evaluated by Spearman correlation. CKM-adjusted partial Spearman correlations were calculated using rank residualization. All statistical tests were two-sided. Analyses were performed using Python 3.12 and R 4.6.0.

## 3 Results

### 3.1 Study population characteristics

Sixty-four participants provided written informed consent. Twenty participants were excluded according to exclusion criteria, including 4 who followed a vegetarian diet, 14 who had received antibiotics and 2 who were expected to start maintenance hemodialysis within six months (**Supplementary Figure S1**). Finally, 44 participants were selected, including 3 participants in CKM stage 1, 16 participants in CKM stage 2, 13 participants in CKM stage 3 and 12 participants in CKM stage 4 (**Figure 1**). Among the 44 participants in the primary cohort, 35 had sufficient residual serum available for proteomic analysis and constituted a nested proteomic sub-cohort, including 17 participants with CKM stages 1–2 and 18 participants with CKM stages 3-4. Median age was 59.0 (50.5-65.0) years in CKM stages 1–2 and 61.0 (47.0-67.0) years in CKM stages 3-4 (*P* = 0.915). Male accounted for 47.4% and 64.0% of the two groups, respectively (*P* = 0.361). CKM stages 3–4 showed higher blood pressure. Median systolic blood pressure was 146.0 (140.0-159.0) mmHg in CKM stages 3–4 and 126.0 (117.0-143.5) mmHg in CKM stages 1-2 (*P* = 0.002). Median diastolic blood pressure was 84.0 (78.0-93.0) mmHg and 72.0 (68.0-82.5) mmHg, respectively (*P* = 0.010). Participants with CKM stages 3–4 had higher serum creatinine [183.0 (105.0-336.0) μmol/L in CKM stage 3–4 vs 67.0 (55.0-85.5) μmol/L in CKM stage 1-2, *P* < 0.001] and lower eGFR [27.3 (16.5-69.2) mL/min/1.73 m² in CKM stage 3–4 vs 97.7 (86.8-104.7) mL/min/1.73 m² in CKM stage 1-2, *P* < 0.001]. CKD was present in 96.0% of participants with CKM stages 3–4 and 21.1% of those with CKM stages 1-2 (*P* < 0.001). Diabetes mellitus was present in 60.0% in CKM stage 3-4, which was higher than that of participants in CKM stage 1-2 (*P* = 0.035). Hypertension was more common in CKM stages 3–4 than in stages 1-2 (96.0% vs 42.1%, *P* < 0.001). Clinical cardiovascular disease was present in 48.0% of participants with CKM stages 3-4.

**Figure 1:**
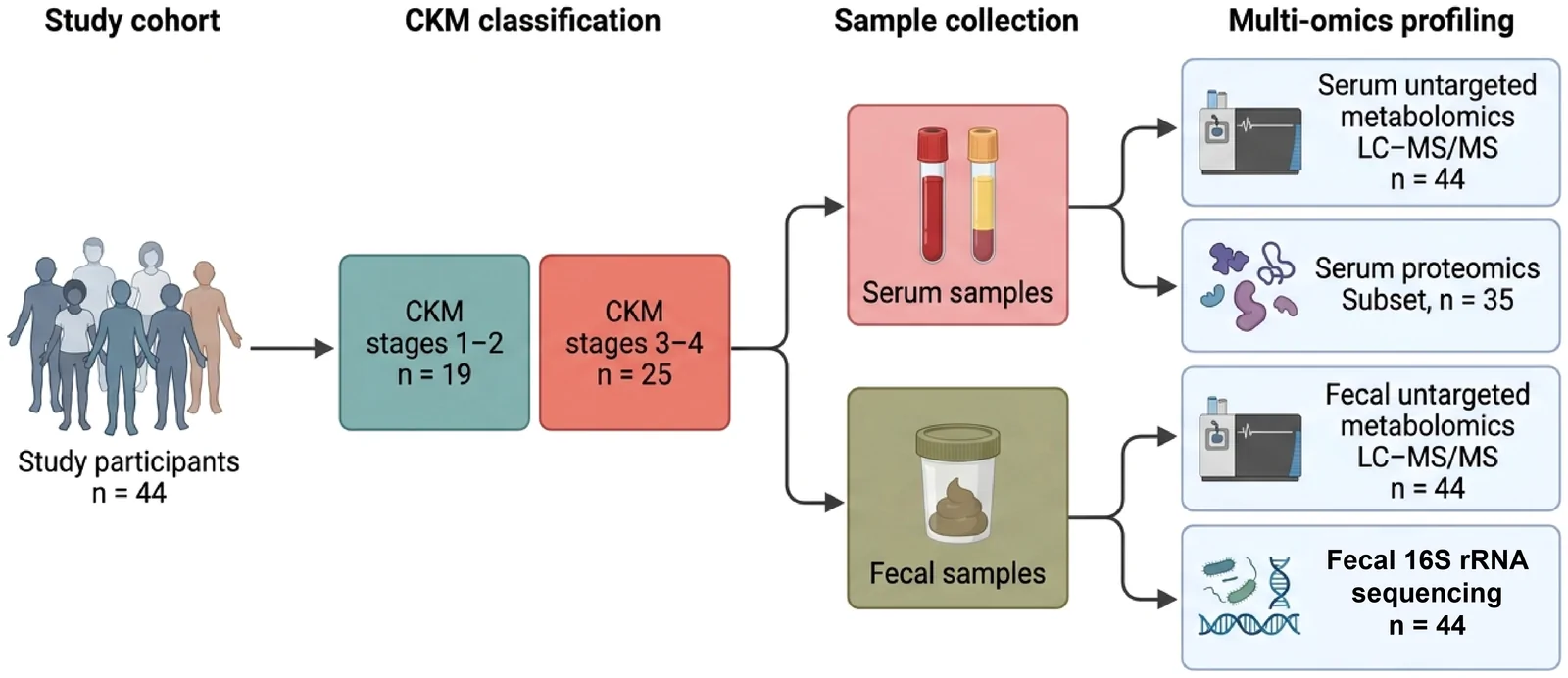
Study design and multi-omics profiling. Forty-four participants participated in the study, including 19 with CKM stages 1–2 and 25 with stages 3–4. All participants underwent serum and fecal untargeted metabolomics and fecal microbiome profiling. Serum proteomics was performed in a subset of 35 participants. CKM, cardiovascular-kidney-metabolic; LC-MS/MS, liquid chromatography-tandem mass spectrometry.

Participants with CKM stages 3–4 had lower hemoglobin [114.0 (86.0-129.0) g/L vs 132.0 (117.5-141.5) g/L, *P* = 0.005], albumin [35.22 (30.85-38.63) vs 41.81 (38.36-42.73) g/L, *P* < 0.001] and serum calcium [2.17 (1.97-2.28) versus 2.29 (2.25-2.38) mmol/L, *P* = 0.002]. Alanine aminotransferase and aspartate aminotransferase were also lower in CKM stages 3-4 (*P* < 0.05). No significance of participants between CKM stage 1–2 and CKM stage 3–4 was observed in BMI, blood glucose, total cholesterol, triglycerides, LDL cholesterol, white blood cell count, platelet count or the remaining measured electrolytes (**Table 1**). Medication use was also summarized in **Table 1**. Antihypertensive therapy was more common in CKM stages 3–4 than in stages 1-2 (88.0% vs 36.8%, P < 0.001), whereas statin therapy and oral hypoglycemic agents did not differ significantly between two groups.

**Table 1:** Clinical characteristics of the study population.

Characteristics	Overall (n = 44)	CKM stages 1–2 (n = 19)	CKM stages 3–4 (n = 25)	*P* value
Demographic and anthropometric characteristics				
Age (years)	60.0 (49.5-66.2)	59.0 (50.5-65.0)	61.0 (47.0-67.0)	0.915
Male, n (%)	25 (56.8%)	9 (47.4%)	16 (64.0%)	0.361
BMI (kg/m²)	24.5 (23.5-27.3)	25.4 (23.6-28.4)	24.4 (23.6-26.4)	0.236
Comorbidities, n (%)				
Chronic kidney disease	28 (63.6%)	4 (21.1%)	24 (96.0%)	<0.001
Diabetes mellitus	20 (45.5%)	5 (26.3%)	15 (60.0%)	0.035
Hypertension	32 (72.7%)	8 (42.1%)	24 (96.0%)	<0.001
Clinical cardiovascular disease	12 (27.3%)	0 (0.0%)	12 (48.0%)	<0.001
Clinical measurements, median (IQR)				
Systolic blood pressure (mmHg)	140.0 (125.5-150.8)	126.0 (117.0-143.5)	146.0 (140.0-159.0)	0.002
Diastolic blood pressure (mmHg)	81.5 (72.0-88.2)	72.0 (68.0-82.5)	84.0 (78.0-93.0)	0.010
Pulse (beats/min)	77.0 (72.0-80.5)	76.0 (70.0-84.5)	78.0 (72.0-80.0)	0.886
Kidney function and urinary measures, median (IQR)				
Serum creatinine (μmol/L)	98.5 (64.0-227.5)	67.0 (55.0-85.5)	183.0 (105.0-336.0)	<0.001
eGFR (mL/min/1.73 m²)	76.6 (25.9-101.4)	97.7 (86.8-104.7)	27.3 (16.5-69.2)	<0.001
Blood urea nitrogen (mmol/L)	7.57 (4.57-14.25)	4.64 (4.38-6.52)	14.09 (7.55-18.11)	<0.001
Uric acid (μmol/L)	377.5 (310.8-432.2)	333.0 (297.0-405.0)	391.0 (346.0-433.0)	0.124
Metabolic measures, median (IQR)				
Blood glucose (mmol/L)	5.05 (4.31-5.61)	5.17 (4.78-5.53)	4.73 (4.19-5.83)	0.292
Total cholesterol (mmol/L)	4.71 (3.80-5.20)	4.53 (3.87-5.03)	4.99 (3.79-6.04)	0.286
Triglycerides (mmol/L)	1.81 (1.29-2.30)	1.87 (1.37-2.28)	1.75 (1.27-2.28)	0.840
LDL cholesterol (mmol/L)	2.66 (2.17-3.25)	2.83 (2.20-3.07)	2.58 (2.18-3.39)	0.934
Hematologic measures, median (IQR)				
White blood cell count (×10^9^/L)	5.91 (4.79-6.91)	5.34 (4.73-6.61)	5.94 (5.22-7.23)	0.265
Hemoglobin (g/L)	120.0 (108.0-134.8)	132.0 (117.5-141.5)	114.0 (86.0-129.0)	0.005
Platelet count (×10^9^/L)	225.0 (181.2-259.8)	236.0 (209.0-255.5)	219.0 (163.0-299.0)	0.653
Liver function and nutritional measures, median (IQR)				
Albumin (g/L)	38.19 (32.49-41.26)	41.81 (38.36-42.73)	35.22 (30.85-38.63)	<0.001
Alanine aminotransferase (U/L)	18.0 (13.0-22.0)	21.0 (16.5-23.5)	14.0 (11.0-22.0)	0.016
Aspartate aminotransferase (U/L)	19.0 (16.0-21.5)	20.0 (18.5-25.0)	18.0 (16.0-20.0)	0.018
Electrolytes, median (IQR)				
Potassium (mmol/L)	4.04 (3.73-4.27)	3.85 (3.72-4.11)	4.12 (3.74-4.56)	0.115
Sodium (mmol/L)	142.0 (140.0-143.0)	142.0 (141.5-143.0)	141.0 (140.0-143.0)	0.106
Calcium (mmol/L)	2.25 (2.15-2.33)	2.29 (2.25-2.38)	2.17 (1.97-2.28)	0.002
Phosphorus (mmol/L)	1.32 (1.20-1.45)	1.25 (1.20-1.40)	1.33 (1.20-1.54)	0.275
Medication use, n (%)				
Antiplatelet therapy	7 (15.9%)	2 (10.5%)	5 (20.0%)	0.680
Insulin	12 (27.3%)	1 (5.3%)	11 (44.0%)	0.006
Traditional Chinese medicines	13 (29.5%)	2 (10.5%)	11 (44.0%)	0.021
Proton pump inhibitors	1 (2.3%)	1 (5.3%)	0 (0.0%)	0.432
Antihypertensive medication	29 (65.9%)	7 (36.8%)	22 (88.0%)	<0.001
Statin therapy	19 (43.2%)	8 (42.1%)	11 (44.0%)	1.000
oral antidiabetic drugs	14 (31.8%)	4 (21.1%)	10 (40.0%)	0.211

BMI, body mass index; CKD, chronic kidney disease; CKM, cardiovascular–kidney–metabolic; eGFR, estimated glomerular filtration rate; IQR, interquartile range; LDL, low-density lipoprotein.

### 3.2 Serum metabolomics identifies metabolic differences between CKM stages 1–2 and stages 3-4

Quality-control samples showed a total of 93.6% of features had a coefficient of variation below 30% (**Figure 2A**). Among the 8862 detected features, the median coefficient of variation was 5.63% (**Figure 2B**). After quality-control filtering, 8294 features were retained (**Supplementary Table S1**). Principal component analysis (PCA) showed partial separation of the two CKM groups in the first principal component (**Figure 2C**). Differential analysis identified 589 annotated, non-exogenous serum metabolomic features with a *P* < 0.05 and an absolute Cliff’s delta ≥ 0.33. Among these features, 324 were higher and 265 were lower in CKM stages 3-4 (**Figure 2D**). The heatmap showed 24 selected serum metabolite features. Pseudouridine, deoxyribose, glutamine and hydroxyproline were more abundant in CKM stages 3-4. Tryptophan, carnitine, uridine and several steroid- and bile-acid-related were generally lower in CKM stages 3-4 (**Figure 2E**).

**Figure 2:**
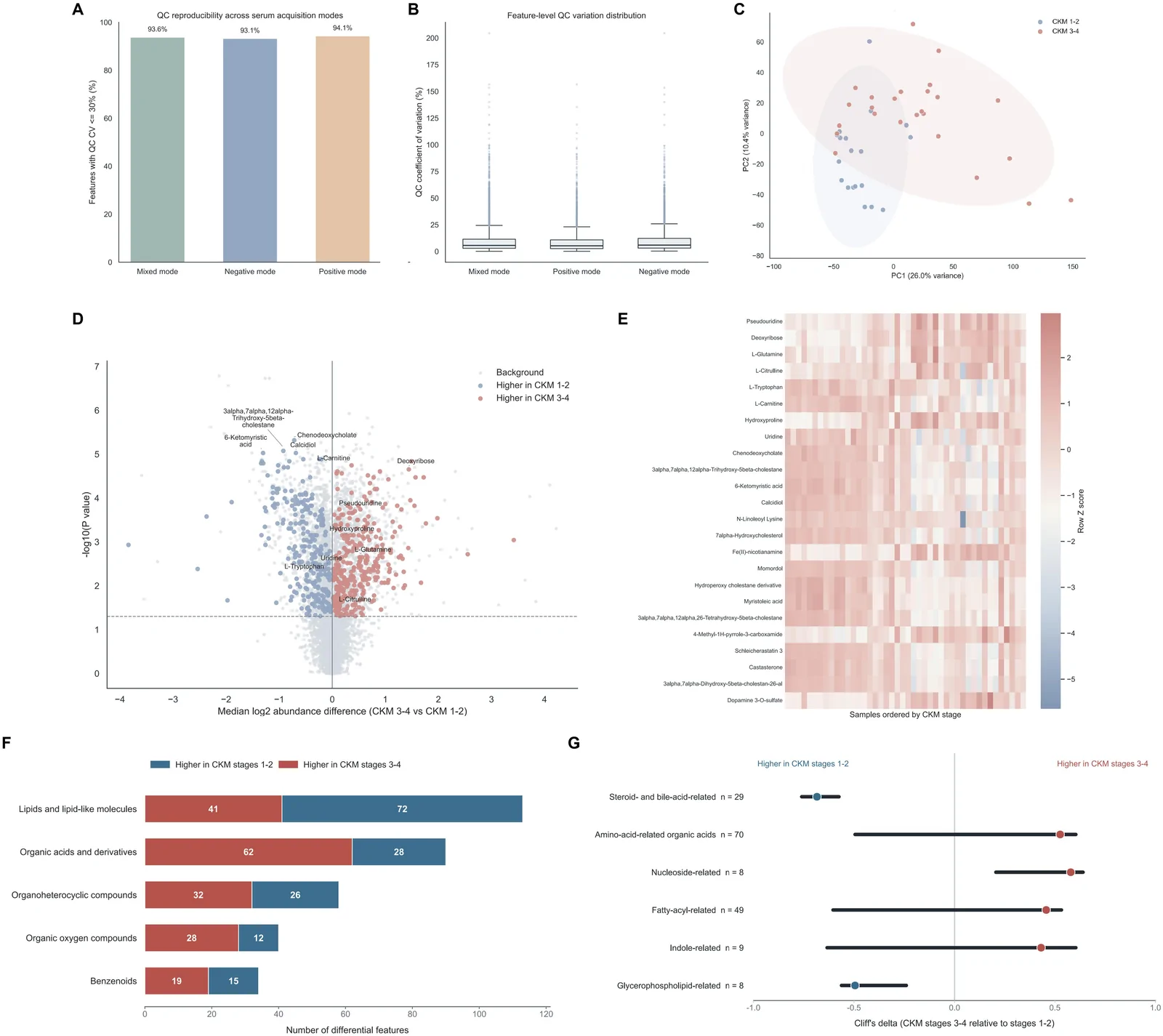
Serum metabolomic differences between CKM stages 1–2 and 3-4. **(A)** Proportion of features with a CV below 30% in quality-control samples. **(B)** Distribution of feature-level CVs. **(C)** Principal component analysis of serum metabolomic profiles. Each point represents one participant. **(D)** Volcano plot of differential features. Colored points indicate 589 annotated, non-exogenous features with *P* < 0.05 and an absolute Cliff’s delta ≥0.33. **(E)** Heatmap of 24 selected features. Abundances were standardized as row Z scores. **(F)** HMDB superclass distribution of the 470 features that remained significant after BH correction. **(G)** Effect-size distributions across six metabolite classes. Points and horizontal lines indicate the median and interquartile range of Cliff’s delta, respectively. Negative values indicate higher abundance in CKM stages 1-2. Positive values indicate higher abundance in stages 3-4. BH, Benjamini–Hochberg; CKM, cardiovascular-kidney-metabolic; CV, coefficient of variation; HMDB, Human Metabolome Database.

Among the 589 serum metabolomic features, 470 differed between CKM stages 1–2 and stages 3–4 after BH correction. Lipids and lipid-like molecules represented the five most represented HMDB superclass, including 113 features, followed by organic acids and derivatives (n = 90), organoheterocyclic compounds (n = 58), organic oxygen compounds (n = 40) and benzenoids (n = 34). Most lipid and lipid-like features were lower in CKM stages 3-4, while most organic acids and derivatives, organoheterocyclic compounds and organic oxygen compounds were higher (**Figure 2F**). Several steroid-related features were lower in CKM stages 3-4, including bile acids, bile-acid derivatives and cholestane steroids (**Figure 2G**). The chenodeoxycholic-acid isomer feature was among the most prominent annotated differential features with a difference in median normalized abundance of -0.716, a Cliff’s delta of -0.815 and a BH-FDR of 0.002.

### 3.3 Fecal metabolomics identifies metabolic differences between CKM stages 1–2 and stages 3-4

A total of 12869 features were identified in the fecal metabolomics (**Supplementary Table S2**). Among these metabolites, 91.98% had a coefficient of variation below 30% in quality-control samples. The median coefficient of variation was 4.99% (**Figures 3A, B**). After quality-control filtering, 11837 features were selected. The first two principal components explained 16.0% and 11.4% of the total variance, respectively. The two CKM groups overlapped in PC1, while their distributions differed mainly in PC2 (**Figure 3C**).

**Figure 3:**
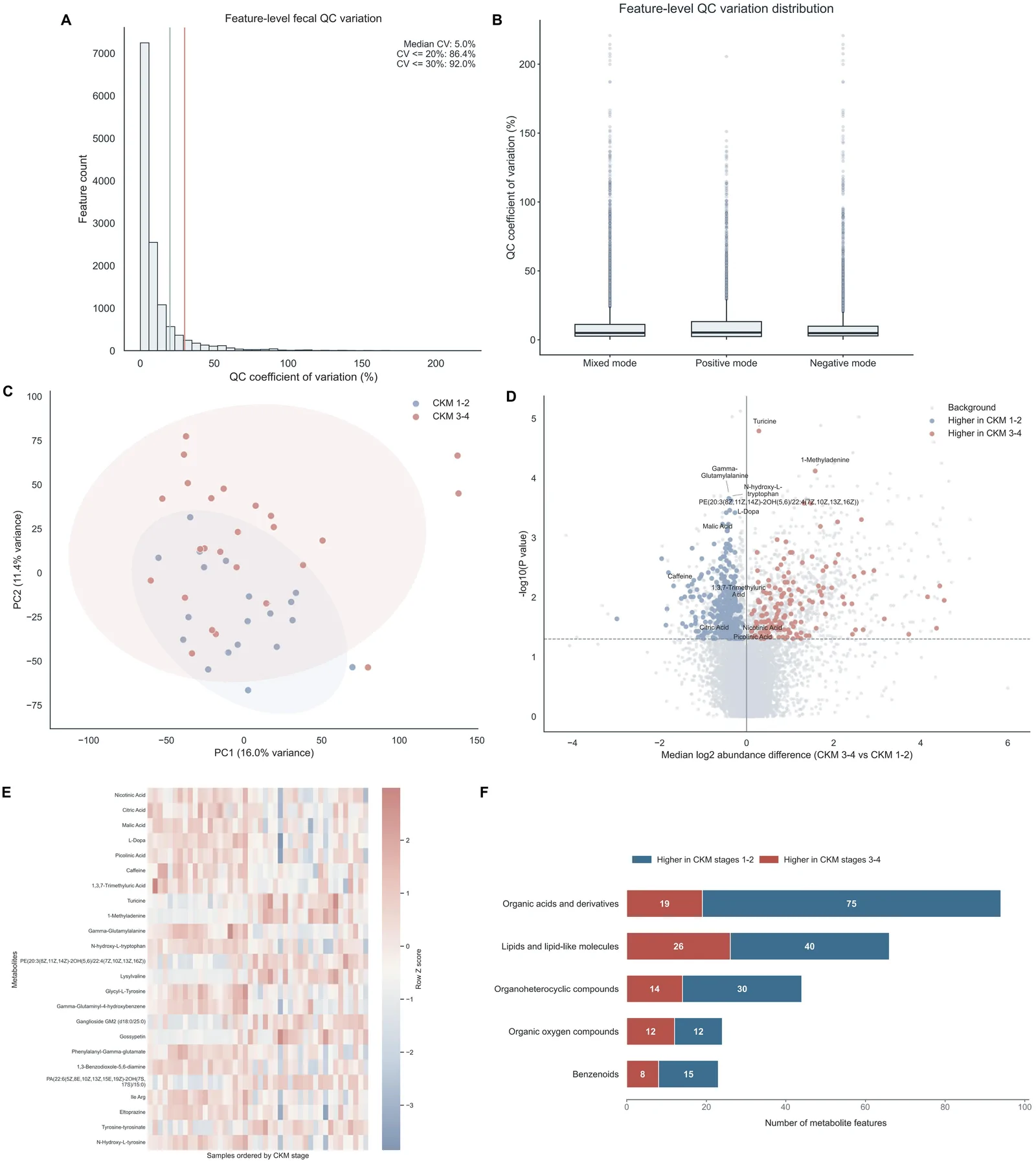
Fecal metabolomic differences between CKM stages 1–2 and stages 3-4. **(A)** Distribution of CVs in quality-control samples. **(B)** Feature-level CV distributions across the combined, positive-ion and negative-ion modes. **(C)** Principal component analysis of fecal metabolomic profiles. Each point represents one participant. **(D)** Volcano plot of differential features. Colored points indicate 498 annotated, non-exogenous features with *P* < 0.05 and an absolute Cliff’s delta ≥ 0.33. **(E)** Heatmap of 24 selected features. Abundances were standardized as row Z scores. **(F)** HMDB superclass distribution of the 498 differential features. Colors indicate higher abundance in CKM stages 1–2 or stages 3-4. CKM, cardiovascular-kidney-metabolic; CV, coefficient of variation; HMDB, Human Metabolome Database.

Differential analysis identified 498 annotated, non-exogenous fecal metabolomic features according to the exploratory criteria of *P* < 0.05 and an absolute Cliff’s delta ≥ 0.33. Among these features, 339 were lower and 159 were higher in CKM stages 3-4. Malic acid, L-Dopa, gamma-glutamylalanine and N-hydroxy-L-tryptophan had lower abundance in CKM stages 3-4 (**Figure 3D**). The heatmap included 24 important metabolites. Malic acid, L-Dopa, gamma-glutamylalanine, N-hydroxy-L-tryptophan, nicotinic acid and citric acid were lower in CKM stages 3-4. In contrast, Turicine, 1-methyladenine, lysylvaline, gossypetin and ganglioside GM2 were generally higher (**Figure 3E**). Organic acids and derivatives constituted the largest superclass among the metabolite features, with 75 lower and 19 higher in CKM stages 3-4. Lipids and lipid-like molecules included 40 lower and 26 higher features. Organoheterocyclic compounds included 30 lower and 14 higher features (**Figure 3F**). After BH correction, six features remained significant. Four were higher and two were lower in CKM stages 3-4. Five of these features lacked a metabolite annotation. The remaining feature was annotated as Turicine, which was higher in CKM stages 3-4. The median abundance difference was 0.283, with a Cliff’s delta of 0.768 and a BH-FDR of 0.038 of Turicine.

### 3.4 Paired fecal and serum metabolomics identifies correlated metabolites

Based on the differential analysis, features with an available metabolite annotation were retained after excluding exogenous compounds. The selection further required a *P* < 0.05, an absolute Cliff’s delta ≥ 0.50 and high or moderate annotation confidence. One representative feature was selected among duplicate metabolites and highly correlated features. Finally, 17 fecal and 48 serum metabolites were selected (**Figure 4A**).

**Figure 4:**
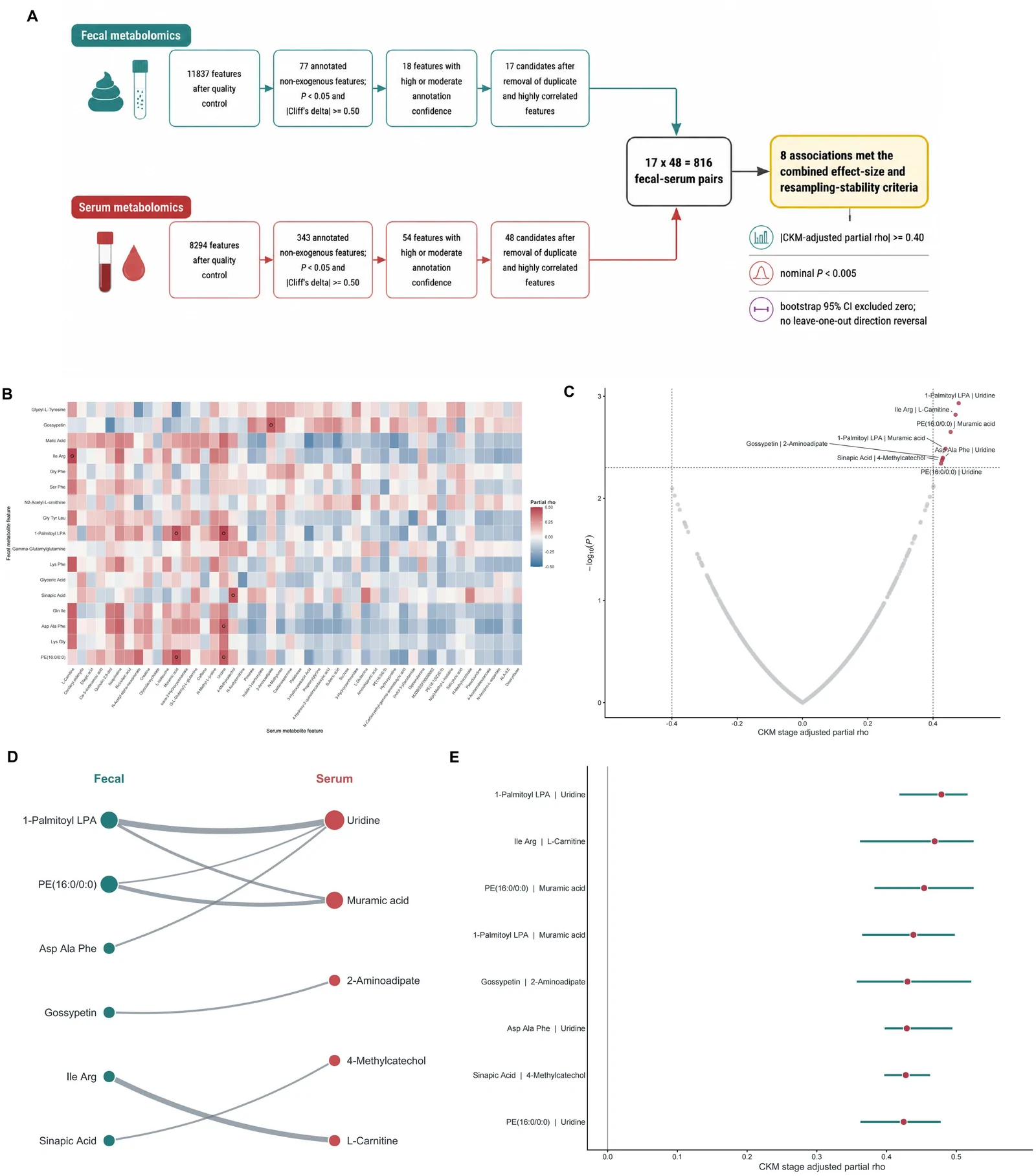
Exploratory paired analysis of fecal and serum metabolites. **(A)** Selection of 17 fecal and 48 serum metabolite candidates for 816 paired tests. None of the individual correlations remained significant after BH correction across all 816 tests. Eight exploratory candidate pairs met the combined criteria of an absolute CKM-adjusted partial rho ≥ 0.40, a nominal *P* < 0.005, a bootstrap 95% confidence interval excluding zero and no direction reversal in leave-one-participant-out analysis. **(B)** CKM-adjusted partial Spearman correlations for all 816 fecal–serum pairs. Open circles indicate the eight exploratory selected associations. **(C)** Distribution of partial correlation coefficients and *P* values. Dashed lines indicate the selection thresholds. **(D)** Bipartite network of the eight selected associations. Node size represents the number of associations. Edge width represents the absolute partial correlation coefficient. **(E)** Leave-one-out stability analysis. Points indicate full-cohort estimates. Horizontal lines indicate the range of estimates after omitting each participant. CKM, cardiovascular-kidney-metabolic.

Partial Spearman correlation analysis was performed after adjustment for the CKM group. Among the 816 pairs, the partial correlation coefficients ranged from -0.399 to 0.479. The median absolute partial correlation coefficient was 0.129. A total of 383 correlations were positive and 433 were negative (**Figure 4B**). None of the 816 correlations remained statistically significant after BH correction (minimum BH-FDR = 0.465, **Supplementary Table S3**). Eight fecal-serum pairs met the combined exploratory candidate-selection criteria of an absolute partial rho ≥ 0.40 and a *P* < 0.005. The 1000-resample bootstrap 95% confidence intervals excluded zero, with no direction reversal in leave-one-out analyses. The primary model was adjusted by CKM group (**Figure 4C**; **Table 2**). After further adjustment for age, sex and eGFR, the associations remained positive, with partial correlation coefficients ranging from 0.360 to 0.516. Among candidate pairs, the largest partial correlation was observed between fecal 1-palmitoyl lysophosphatidic acid and serum uridine (partial rho = 0.479, nominal *P* = 0.0012). Fecal Ile Arg was correlated with serum L-carnitine (partial rho = 0.469, *P* = 0.0015). Fecal PE (16:0/0:0) was correlated with serum muramic acid (partial rho = 0.454, *P* = 0.0022). Several metabolites occurred in more than one pair. Serum uridine was associated with fecal 1-palmitoyl lysophosphatidic acid, Asp Ala Phe and PE (16:0/0:0). Serum muramic acid was associated with fecal PE (16:0/0:0) and 1-palmitoyl lysophosphatidic acid. Thus, serum uridine, serum muramic acid, fecal PE (16:0/0:0) and fecal 1-palmitoyl lysophosphatidic acid emerged as central nodes within the bipartite correlation network (**Figure 4D**). The bootstrap 95% confidence intervals of the eight CKM stage adjusted partial correlations ranged from 0.094-0.712. Leave-one-out estimates remained positive for all eight pairs. The overall leave-one-out coefficients ranged from 0.357 to 0.525 (**Figure 4E**). After further adjustment for age, sex and eGFR, the associations were positive, with partial correlation coefficients ranging from 0.360 to 0.516 (**Table 2**).

**Table 2:** Exploratory candidate associations between fecal and serum metabolites.

Fecal metabolite feature	Fecal annotation confidence	Serum metabolite feature	Serum annotation confidence	Crude rho	CKM-adjusted partial rho	*P*	Bootstrap 95% CI	Leave-one-out rho range	Fully adjusted rho	Adjusted *P* value
1-Palmitoyl Lysophosphatidic Acid	Moderate	Uridine	High	0.585	0.479	0.001	0.197 to 0.653	0.418 to 0.516	0.501	0.001
Ile Arg	Moderate	L-Carnitine	High	0.643	0.469	0.002	0.094 to 0.712	0.362 to 0.525	0.516	0.001
PE(16:0/0:0)	High	Muramic acid	Moderate	0.567	0.454	0.002	0.140 to 0.700	0.383 to 0.525	0.401	0.010
1-Palmitoyl Lysophosphatidic Acid	Moderate	Muramic acid	Moderate	0.563	0.438	0.003	0.139 to 0.644	0.365 to 0.498	0.397	0.011
Gossypetin	High	L-2-Aminoadipate	Moderate	0.560	0.430	0.004	0.114 to 0.656	0.357 to 0.521	0.513	0.001
Asp Ala Phe	Moderate	Uridine	High	0.538	0.429	0.004	0.178 to 0.638	0.397 to 0.494	0.390	0.013
Sinapic Acid	High	4-Methylcatechol	Moderate	0.537	0.427	0.004	0.181 to 0.618	0.397 to 0.462	0.360	0.022
PE(16:0/0:0)	High	Uridine	High	0.534	0.424	0.005	0.160 to 0.649	0.363 to 0.478	0.421	0.007

To determine whether the paired associations of metabolites assigned to the same pathway, we calculated serum and fecal abundance scores for KEGG pathways with sufficient compound coverage. Twelve pathways met the primary criterion and 12 pathways met the secondary criterion (**Supplementary Figure S2**). Among the 24 pathways, serum and fecal pyrimidine metabolism scores were positively correlated after adjustment for CKM group (partial rho = 0.429, *P* = 0.0041, BH-FDR = 0.0246). The bootstrap 95% confidence interval ranged from 0.092 to 0.688. Leave-one-out coefficients ranged from 0.388 to 0.500. The association remained positive after further adjustment for age, sex and eGFR (partial rho = 0.406, *P* = 0.0093). Caffeine metabolism also showed a positive serum-fecal association after BH correction (partial rho = 0.608, *P* < 0.001, BH-FDR < 0.001). The contributing metabolites included caffeine and methylxanthine-related compounds, which was considered to be related to dietary or medication.

### 3.5 Clinical associations of the paired fecal-serum metabolite features

The 11 metabolite features involved in the eight exploratory fecal-serum associations were examined in relation to 12 clinical indicators. We first performed unadjusted Spearman correlation analysis, and then calculated partial Spearman correlations after adjustment for CKM group. The main clinical associations involved serum L-carnitine and renal function. In the unadjusted analysis, serum L-carnitine was positively correlated with eGFR (rho = 0.752, BH-FDR < 0.001). Serum L-carnitine was negatively correlated with serum creatinine (rho = -0.746, BH-FDR < 0.001) and blood urea nitrogen (rho = -0.724, BH-FDR < 0.001). Serum 4-methylcatechol was positively correlated with blood urea nitrogen (rho = 0.609, BH-FDR < 0.001, **Figure 5A**; **Supplementary Table S4**). After adjustment for CKM group, four associations remained significant after BH correction **(Figures 5B, C**). Serum L-carnitine and serum 4-methylcatechol remained the same association with indicators of renal function (**Figure 5D**). No association with total cholesterol, triglycerides, LDL cholesterol, BMI or diabetes history was significant after BH correction. In the separate sensitivity analysis, the eight fecal-serum metabolite associations were positive after the adjustment of age, sex, eGFR, diabetes history, BMI, triglycerides and LDL cholesterol (**Figure 5E**). Thus, the clinical associations were concentrated in renal function indicators.

**Figure 5:**
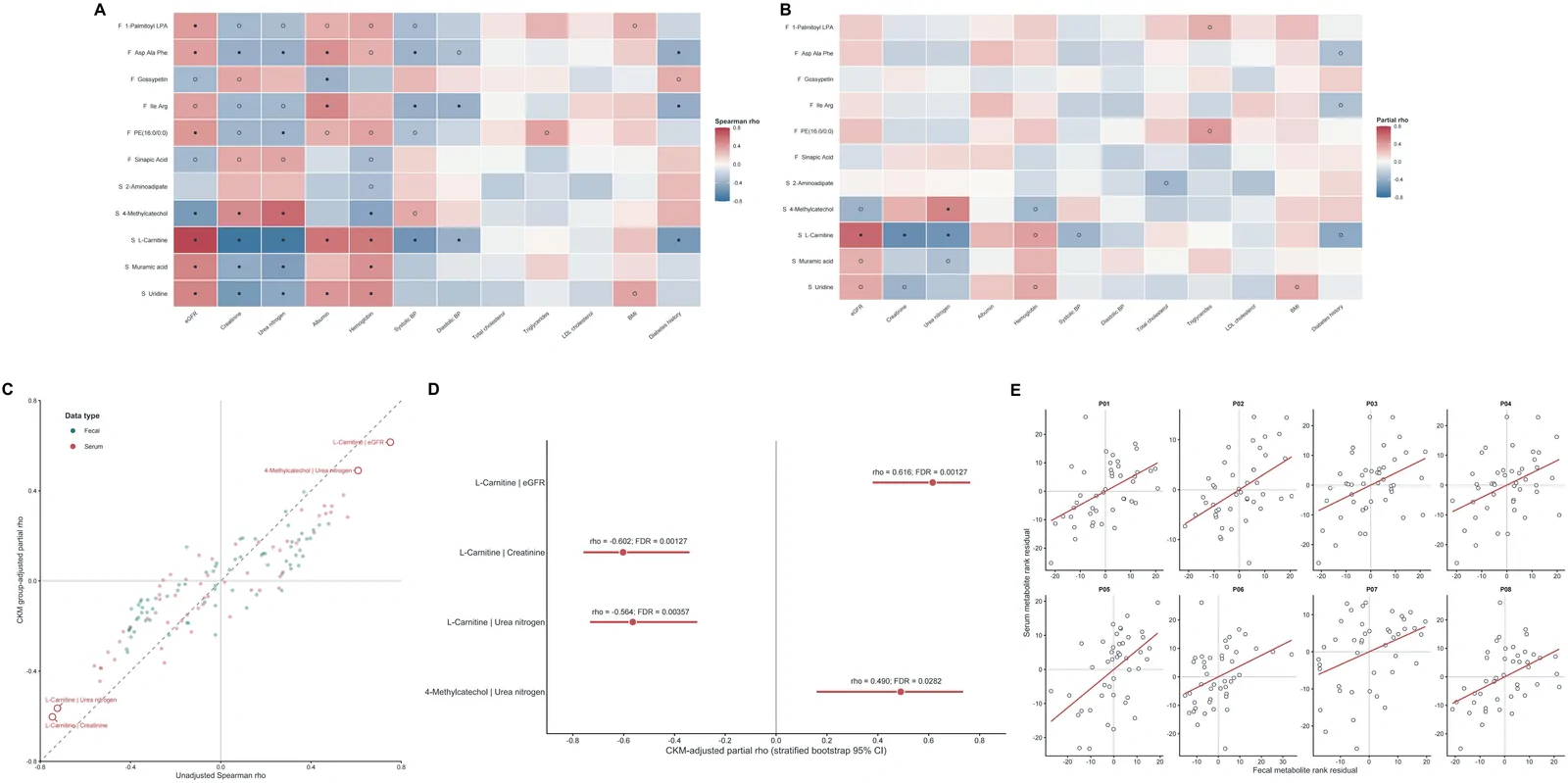
Clinical associations of metabolites selected from the paired fecal-serum analysis. **(A)** Unadjusted Spearman correlations between 11 metabolite features and 12 clinical indicators. **(B)** Partial Spearman correlations adjusted for CKM group. Open and solid circles indicate *P* < 0.05 and BH-FDR < 0.05, respectively. **(C)** Comparison of unadjusted and CKM-adjusted correlation coefficients. Solid points indicate associations with an adjusted BH-FDR < 0.05. **(D)** CKM-adjusted associations that remained significant after BH correction. Points indicate partial correlation coefficients. Horizontal lines indicate bootstrap 95% confidence intervals. **(E)** Rank-residual plots of the eight fecal-serum associations after adjustment for CKM group, age, sex, eGFR, diabetes history, BMI, triglycerides and LDL cholesterol. BH-FDR, Benjamini–Hochberg false discovery rate; CKM, cardiovascular-kidney-metabolic; eGFR, estimated glomerular filtration rate; LDL, low-density lipoprotein.

### 3.6 Greater interindividual variation of fecal microbiome in CKM stages 3-4

The Shannon and Simpson indices were compared between CKM stages 1–2 and stages 3-4 (*P* = 0.776 vs *P* = 0.286, **Figures 6A, B**). Bray-Curtis principal coordinate analysis showed substantial overlap between the two groups. PERMANOVA showed no significant difference in overall microbial composition (R² = 0.022, *P* = 0.506, **Figure 6C**). PERMDISP showed greater multivariate dispersion of genus-level microbial composition in CKM stages 3–4 than in stages 1-2 (mean Bray-Curtis distance to the group centroid, 0.513 vs 0.436; *P* = 0.011, **Figure 6D**). Results indicated greater compositional heterogeneity in CKM stages 3–4 without a detectable difference in overall microbial composition between the two groups. Among the 91 genera, 12 showed significant differences between groups (*P* < 0.05), but none remained significant after BH correction (**Figure 6E**). In CKM group adjusted analyses of 546 genus-metabolite pairs, 77 associations had *P* < 0.05, but none survived BH correction (**Figure 6F**). PICRUSt2-based analyses of pyrimidine-, phospholipid-, peptidoglycan- and lysine-related functional sets did not identify a significant association with the measured metabolites after the correction for multiple testing.

**Figure 6:**
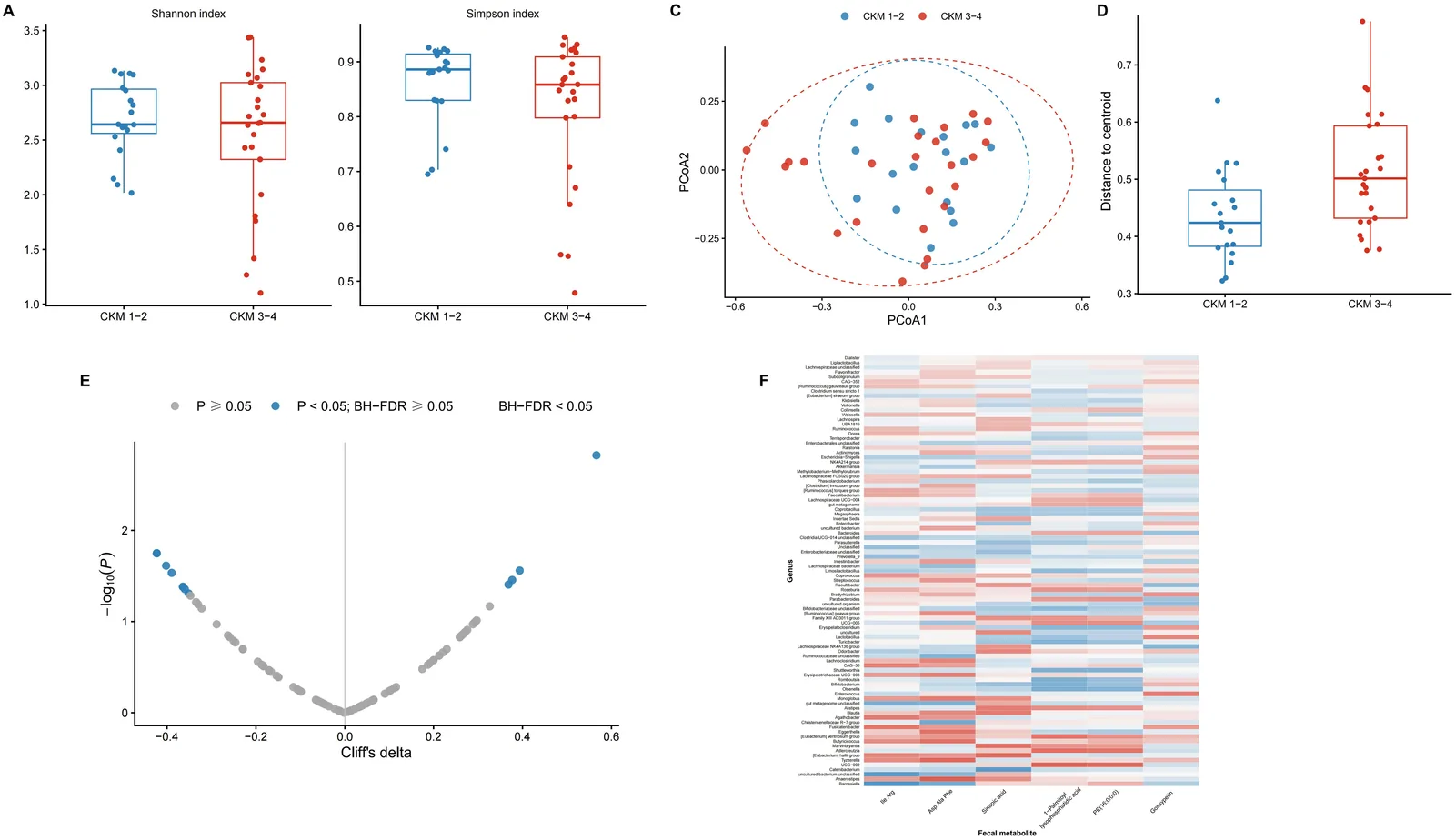
Fecal microbiome profiles in CKM stages 1–2 and stages 3-4. **(A, B)** Shannon and Simpson diversity indices. **(C)** Principal coordinate analysis based on Bray-Curtis distances. **(D)** Multivariate dispersion measured as the Bray-Curtis distance to the group centroid. **(E)** Genus-level differences between the two CKM groups. Colors indicate statistical evidence before and after BH correction. **(F)** CKM-adjusted partial Spearman correlations between 91 genera and the six fecal metabolites included in the paired metabolomics analysis. BH-FDR, Benjamini–Hochberg false discovery rate; CKM, cardiovascular-kidney-metabolic; PCoA, principal coordinate analysis.

### 3.7 Serum proteomics provides protein-level context for paired metabolite associations

As a complementary analysis, serum proteomic profiling was performed in the nested sub-cohort of 35 participants for exploration. A total of 6030 proteins were selected for differential analysis. Principal component analysis showed partial separation between the two CKM groups (**Figure 7A**). Differential analysis identified 221 proteins with a BH-FDR < 0.05. Among these proteins, 81 were higher and 140 were lower in CKM stages 3–4 than in stages 1-2 (**Figure 7B**; **Supplementary Table S5**).

**Figure 7:**
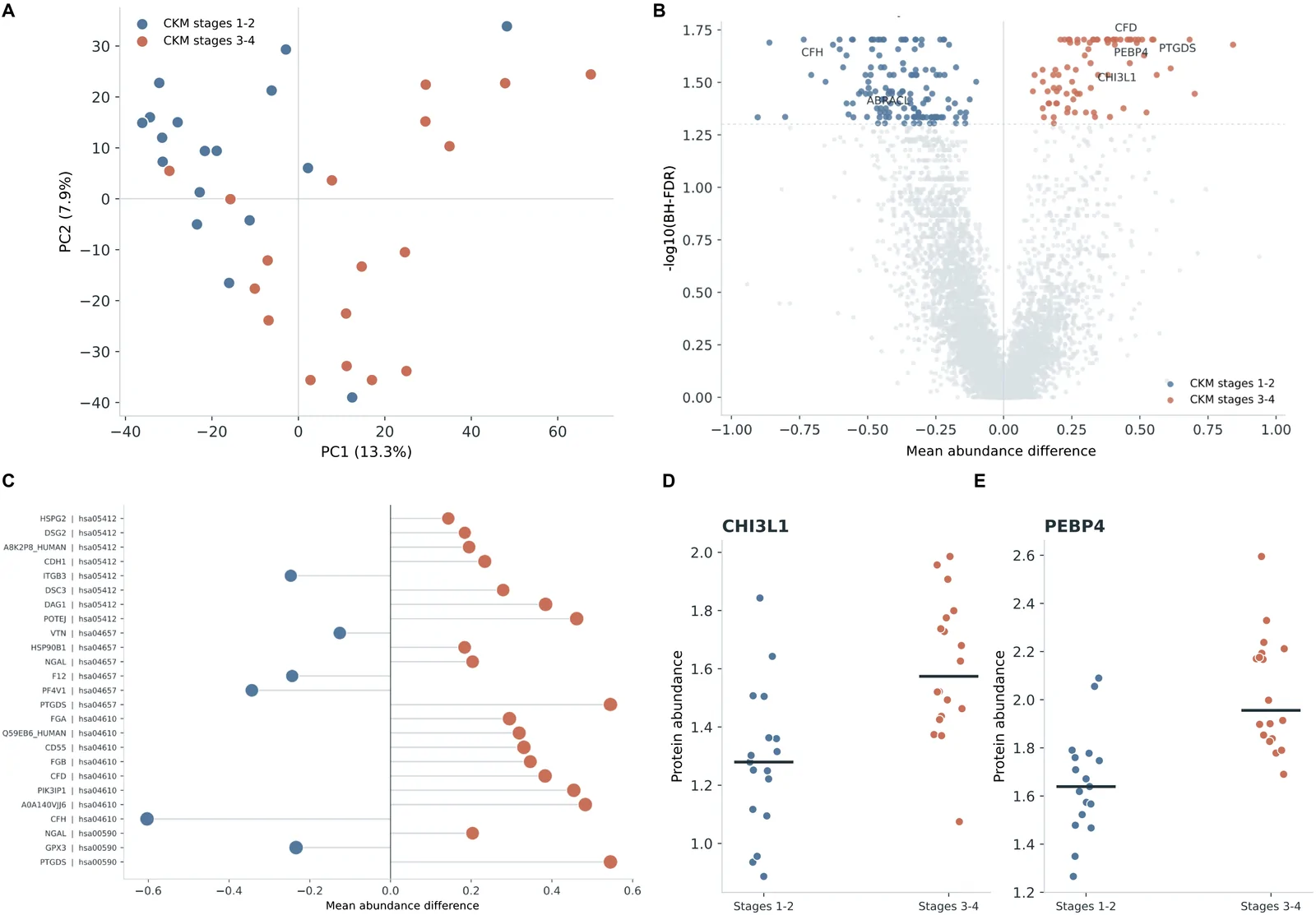
Serum proteomic differences between CKM stages 1–2 and stages 3-4. **(A)** Principal component analysis of 6030 serum proteins in 35 participants. **(B)** Differential protein analysis. Colors indicate the direction of differences among proteins with a BH-FDR < 0.05. **(C)** Representative differential proteins contributing to the four enriched KEGG pathways. **(D, E)** Abundance distributions of CHI3L1 and PEBP4, respectively. Points represent individual participants. Horizontal lines indicate medians. BH-FDR, Benjamini–Hochberg false discovery rate; CKM, cardiovascular-kidney-metabolic; KEGG, Kyoto Encyclopedia of Genes and Genomes.

KEGG over-representation analysis identified four enriched pathways among the differential proteins, including complement and coagulation cascades (n = 19), arrhythmogenic right ventricular cardiomyopathy (n = 8), IL-17 signaling (n = 6) and arachidonic acid metabolism (n = 3). The pathway-associated proteins showed both higher and lower abundance in CKM stages 3-4 (**Figure 7C**).

CHI3L1 and PEBP4 were abundant in CKM stages 3-4. The individual abundance distributions and between-group differences of two proteins are shown in **Figures 7D, E**. We next examined the associations between the 221 differential proteins and the five serum metabolites. No association remained significant after BH correction. CHI3L1 was annotated to amino sugar and nucleotide sugar metabolism, which is related to the biochemical context of serum muramic acid. The CKM-adjusted correlation between CHI3L1 and serum muramic acid was not statistically significant (partial rho = -0.289, *P* = 0.098). PEBP4, a member of the phosphatidylethanolamine-binding protein family, was not associated with fecal PE (16:0/0:0) after adjustment for CKM group (partial rho = 0.038, *P* = 0.831).

Thus, the complementary proteomic analysis identified stage-related changes in circulating protein processes, whereas the exploratory protein-metabolite analyses did not identify FDR-significant cross-omics associations.

## 4 Discussion

In the cross-sectional study, CKM stages 3–4 were associated with more significant differences in serum metabolites than in fecal metabolites. In the paired analysis of serum and fecal metabolomics, exploratory candidate selection based on effect size and resampling stability identified eight pairs, with serum uridine recurring in three pairs. At the KEGG pathway level, serum and fecal pyrimidine metabolism scores were positively correlated after BH correction. Our results identified pyrimidine metabolism as the most prominent feature in the paired fecal-serum metabolite associations.

In our research, serum differences between CKM stages 1–2 and stages 3–4 involved multiple chemical classes, particularly lipid-related molecules, organic acids and steroid-related metabolites. The predominance of lipid-related features is consistent with the clinical composition of CKM syndrome, which integrates adiposity, insulin resistance, diabetes, dyslipidemia, kidney dysfunction and cardiovascular disease (2). These conditions converge on circulating lipid transport, fatty-acid utilization, phospholipid remodeling and sphingolipid metabolism, making lipid-related metabolites sensitive indicators of the combined metabolic and organ burden of CKM (16). A recent CKM metabolomics study similarly reported that higher-risk CKM profiles were enriched in glycerophospholipids, fatty acyls, glycolipids, triglycerides, fatty acids, phosphatidylcholines, sphingolipids and acylcarnitines, with substantial metabolic heterogeneity among individuals with advanced CKM stages (5). The lipid-related pattern observed in our cohort is consistent with previous CKM metabolomics findings rather than representing a difference restricted to a single lipid subclass. The changes in steroid- and bile-acid-related features further support the involvement of lipid-derived metabolites in the metabolic differences between CKM stages.

Evidence from paired fecal and circulating metabolomics remains limited in CKM. Studies in related cardiometabolic conditions have provided evidence that intestinal microbial features and fecal metabolites are associated with circulating metabolic changes. In patients with stable coronary artery disease and type 2 diabetes, integrated analyses of fecal microbiota, fecal metabolites and serum metabolites identified differences in aromatic amino acid and carbohydrate metabolism (17). A study of metabolic syndrome also revealed extensive changes in the fecal lipidome. One fecal phospholipid feature was correlated with serum triglycerides, HDL cholesterol and circulating TNF-α, suggesting that intestinal metabolic alterations may accompany systemic cardiometabolic abnormalities (18). For CKD patients, Wang et al. identified coordinated changes in fecal and serum metabolites involving arginine and proline metabolism, arachidonic acid metabolism, glutathione metabolism and ubiquinone-related pathways (11). Our study examined fecal-serum metabolic associations in CKM patients and suggest that the magnitude and distribution of metabolic differences may vary between fecal and circulating compartments.

Pyrimidine metabolism was the most recurrent circulating pathway in the fecal-serum paired analysis. Uridine is a major pyrimidine nucleoside involved in nucleotide synthesis and salvage. The metabolism uridine is associated with glucose, lipid and amino acid homeostasis (19). Previous studies have linked circulating uridine and other pyrimidine metabolites, suggesting that pyrimidine metabolism is responsive to both systemic metabolic dysfunctions and kidney injury (20, 21). The concordant metabolite-level and pathway-level findings highlight pyrimidine metabolism as a candidate feature of the observed fecal–serum associations in CKM.

The three uridine-associated fecal metabolites represented different components of intestinal metabolism. 1-Palmitoyl lysophosphatidic acid and PE (16:0/0:0) are related to glycerophospholipid metabolism and membrane lipid remodeling. Asp Ala Phe reflects intestinal peptide turnover. Pyrimidine metabolism is biochemically associated with phospholipid synthesis through CTP-dependent pathways. Nucleotide and peptide metabolism are both influenced by nutrient utilization and cellular turnover (22). The associations of fecal metabolites with serum uridine showed that the paired metabolic pattern may not only be restricted to nucleotide metabolites. Instead, uridine was related to concurrent variations in intestinal nucleotide metabolism, membrane lipid metabolism and peptide turnover. Thus pyrimidine metabolism with circulating uridine could be a pathway-level association between fecal and circulating metabolomes in CKM.

Among the clinical variables, metabolites selected from the fecal–serum paired analysis were predominantly associated with indicators of renal function. Serum L-carnitine and 4-methylcatechol were correlated with eGFR, serum creatinine, and blood urea nitrogen. These findings are consistent with previous metabolomics studies showing that carnitine- and nucleotide-related metabolites are associated with glomerular filtration and kidney disease progression (20, 21). Reduced kidney function may affect these metabolites through changes in filtration, tubular handling, and systemic metabolic regulation. The corresponding eGFR-adjusted analyses retained consistent directions of association, suggesting that the observed associations were not solely attributable to differences in eGFR. However, kidney dysfunction is itself an important component of the CKM staging framework, particularly for CKM stage 3. Therefore, these findings should be interpreted as metabolic features of the integrated metabolic and cardiorenal phenotype of advanced CKM rather than as CKM-related changes independent of kidney function. In particular, the associations of serum L-carnitine with renal indicators suggest that renal dysfunction contributes to the metabolic pattern observed in advanced CKM.

The fecal microbiome did not show a significant difference in alpha diversity or overall microbial composition between CKM stages 1–2 and stages 3-4. However, microbial composition showed an increasing interindividual heterogeneity at more advanced CKM stages. Previous large-scale studies have shown that gut microbial and metabolic profiles varied across the cardiometabolic disease spectrum. Most changes are presented in individuals with obesity or diabetes before the development of clinically overt cardiovascular disease (23). Gut microbiome-metabolome relationships are also influenced by diet and host metabolic status (24). In patients with coronary artery disease, microbiome-related metabolic deviations showed person-specific patterns (25). Previous studies provide a relevant clue for the significant microbial heterogeneity observed in our cohort, because participants within the same CKM stage may have different combinations of metabolic, renal and cardiovascular abnormalities.

Previous paired multi-omics studies have identified specific microbial features in cardiometabolic and kidney diseases. Wang et al. reported progressive changes in microbial species and metabolic pathways across CKD groups (11). A recent longitudinal CKD study further linked disease progression to the enrichment of microbial species involved in uremic-toxin production (26). In the Framingham Heart Study, matched fecal metagenomic and metabolomic analyses identified microbial pathways related to cholesterol metabolism and connected with fecal and circulating lipid (27). In our cohort, the microbial contribution was expressed primarily as significant community heterogeneity rather than a shared genus-level signature. Our results suggest that similar metabolic outputs may occur across different microbial community configurations in CKM.

Serum proteomics identified multiple protein differences between CKM stages 1–2 and stages 3-4. The differential proteins were enriched in complement and coagulation cascades, which were IL-17 signaling and arachidonic acid metabolism. Proteins assigned to a cardiac structural pathway were also enriched. The metabolic changes of proteins were major in immune regulation, coagulation, lipid-mediator metabolism and cardiovascular structural proteins. Recent large-scale studies have shown that circulating protein profiles are associated with CKM progression, cardiovascular outcomes and mortality risk (28). Proteomic heterogeneity has also been observed among clinically defined CKM subtypes, indicating that patients within the same broad CKM framework may have distinct circulating molecular profiles (29). In our cohort, CHI3L1 and PEBP4 were representative proteins that were more abundant in CKM stages 3-4. The proteomic findings therefore provide a complementary protein-level context for the stage-related metabolic differences and support the molecular heterogeneity of CKM.

Several limitations should be considered. First, we only performed a single-center cross-sectional study with a relatively small sample size. Future multicenter, prospective studies with larger cohorts across individual CKM stages 0 to 4 are needed to validate these metabolic patterns and changes with disease progression or intervention. Second, 16S rRNA amplicon sequencing provided limited species-level resolution, and PICRUSt2 estimated microbial functional potential rather than directly measuring microbial genes or pathway activity. Shotgun metagenomic and metatranscriptomic analyses are needed to identify species-level and functional contributors to the paired metabolic associations. Third, serum proteomics captured changes in circulating proteins but did not determine the origin of tissue. And the identities of the key metabolites were not confirmed using authentic standards or targeted assays. Further validation should be carried out in targeted metabolomic and proteomics studies. Mechanism studies should be performed to illustrate the metabolic alterations in CKM syndrome. Finally, candidate metabolites were selected and tested in the same small cohort. This data-dependent procedure may introduce selection-related bias and lead to optimistic effect estimates. Future studies should prespecify candidate metabolites in a larger discovery cohort and validation should be done in an independent validation cohort.

In this cross-sectional CKM cohort, stages 3–4 showed broader serum metabolic differences than stages 1-2, particularly involving lipid-related, organic acid and steroid-related metabolites. The positive association between serum and fecal pyrimidine metabolism scores was the main cross-system finding. Serum uridine was the most recurrent feature among the selected fecal-serum associations and warrants targeted validation.

## Data Availability

The datasets presented in this study can be found in online repositories. The names of the repository/repositories and accession number(s) can be found below:https://ngdc.cncb.ac.cn/gsa-human, HRA020686https://ngdc.cncb.ac.cn/omix/, OMIX020007https://ngdc.cncb.ac.cn/omix/, OMIX020006https://ngdc.cncb.ac.cn/omix/, OMIX020011.
