# Supplementary material for: Paired metabolomics identify associations between fecal and serum metabolites in cardiovascular-kidney-metabolic syndrome
Source: Front Endocrinol (Lausanne). 2026 Oct 27;17:1982623. doi: 10.3389/fendo.2026.1982623 (PMC13747780; doi:10.3389/fendo.2026.1982623)
Supplement: Supplementary file 1 [file Table1.docx]

**Supplementary Figure S1. Participant selection flowchart.** Sixty-four participants signed the informed consents. Twenty participants were excluded according to the prespecified eligibility criteria, including a vegetarian diet (n = 4), recent antibiotic exposure (n = 14) and expected initiation of maintenance hemodialysis within six months (n = 2). The remaining 44 participants were selected.

**Supplementary Figure S2. KEGG pathway coverage in serum and fecal metabolomics.** The number of KEGG compounds contributing to each pathway score is shown for serum and fecal metabolomics. Primary pathways included at least five compounds in both datasets, whereas secondary pathways included at least three compounds in both datasets. KEGG, Kyoto Encyclopedia of Genes and Genomes.
