# Supplementary material for: Paired metabolomics identify associations between fecal and serum metabolites in cardiovascular-kidney-metabolic syndrome
Source: Front Endocrinol (Lausanne). 2026 Oct 27;17:1982623. doi: 10.3389/fendo.2026.1982623 (PMC13747780; doi:10.3389/fendo.2026.1982623)
Supplement: Supplementary file 2 [file Table2.docx]

**Supplementary File**

**Table S1. Differential analysis of serum metabolomic features**

| **Feature ID** | **Putative annotation** | **Ion** | **m/z** | **RT** | **Median 1-2** | **Median 3-4** | **Median difference** | **Cliff's delta** | **P value** | **BH-FDR** | **Confidence** | **Superclass** | **Class** |
| --- | --- | --- | --- | --- | --- | --- | --- | --- | --- | --- | --- | --- | --- |
| metab_8129 | Unannotated feature | neg | 266.8891 | 6.157 | 5.651 | 3.519 | -2.131 | -0.933 | 1.638E-07 | 5.146E-04 | Unannotated | NA | NA |
| metab_7219 | 3,5-Dichloro-2,6-dihydroxybenzoic acid | neg | 220.9414 | 5.953 | 5.914 | 4.729 | -1.185 | -0.928 | 1.861E-07 | 5.146E-04 | Tentative | Benzenoids | Benzene and substituted derivatives |
| metab_8210 | Unannotated feature | neg | 222.9385 | 5.953 | 5.663 | 4.399 | -1.264 | -0.928 | 1.861E-07 | 5.146E-04 | Unannotated | NA | NA |
| metab_7279 | 3-Bromo-5-chloro-2,6-dihydroxybenzoic acid | neg | 264.8913 | 6.157 | 5.501 | 3.541 | -1.960 | -0.916 | 2.724E-07 | 5.649E-04 | Tentative | Benzenoids | Benzene and substituted derivatives |
| metab_5477 | Unannotated feature | neg | 292.8185 | 0.692 | 5.093 | 5.413 | 0.320 | 0.865 | 1.190E-06 | 0.002 | Unannotated | NA | NA |
| metab_4887 | Unannotated feature | pos | 200.1282 | 1.949 | 3.959 | 4.623 | 0.664 | 0.857 | 1.510E-06 | 0.002 | Unannotated | NA | NA |
| metab_7322 | Unannotated feature | neg | 310.8389 | 6.305 | 5.520 | 3.229 | -2.291 | -0.840 | 2.416E-06 | 0.002 | Unannotated | NA | NA |
| metab_10415 | Unannotated feature | neg | 352.7741 | 0.684 | 4.185 | 4.939 | 0.754 | 0.840 | 2.416E-06 | 0.002 | Unannotated | NA | NA |
| metab_10143 | Unannotated feature | neg | 164.9471 | 0.936 | 4.533 | 4.984 | 0.452 | 0.827 | 3.417E-06 | 0.002 | Unannotated | NA | NA |
| metab_7323 | Unannotated feature | neg | 308.8410 | 6.312 | 5.166 | 3.133 | -2.033 | -0.827 | 3.417E-06 | 0.002 | Unannotated | NA | NA |
| metab_6550 | Unannotated feature | neg | 348.0474 | 0.786 | 3.449 | 4.151 | 0.702 | 0.827 | 3.417E-06 | 0.002 | Unannotated | NA | NA |
| metab_11221 | Unannotated feature | neg | 221.1547 | 5.961 | 4.668 | 4.832 | 0.164 | 0.823 | 3.831E-06 | 0.002 | Unannotated | NA | NA |
| metab_8065 | Unannotated feature | neg | 312.8369 | 6.312 | 5.087 | 3.319 | -1.768 | -0.823 | 3.831E-06 | 0.002 | Unannotated | NA | NA |
| metab_847 | Unannotated feature | pos | 267.0426 | 3.313 | 4.664 | 4.041 | -0.623 | -0.819 | 4.294E-06 | 0.002 | Unannotated | NA | NA |
| metab_7251 | Chenodeoxycholic-acid isomer feature | neg | 391.2862 | 6.040 | 6.006 | 5.290 | -0.716 | -0.815 | 4.809E-06 | 0.002 | Class-level only | Lipids and lipid-like molecules | Steroids and steroid derivatives |
| metab_2525 | Unannotated feature | pos | 770.5353 | 5.879 | 5.932 | 5.719 | -0.214 | -0.811 | 5.384E-06 | 0.002 | Unannotated | NA | NA |
| metab_5503 | Unannotated feature | neg | 156.9152 | 0.747 | 4.114 | 4.797 | 0.683 | 0.806 | 6.024E-06 | 0.002 | Unannotated | NA | NA |
| metab_811 | Unannotated feature | pos | 258.1700 | 3.074 | 4.381 | 4.831 | 0.450 | 0.806 | 6.024E-06 | 0.002 | Unannotated | NA | NA |
| metab_10316 | Unannotated feature | neg | 176.9003 | 0.794 | 4.613 | 4.821 | 0.207 | 0.802 | 6.736E-06 | 0.002 | Unannotated | NA | NA |
| metab_623 | Unannotated feature | pos | 154.0499 | 2.187 | 2.767 | 4.015 | 1.247 | 0.802 | 6.736E-06 | 0.002 | Unannotated | NA | NA |
| metab_3446 | Unannotated feature | pos | 180.1019 | 2.319 | 3.726 | 4.183 | 0.457 | 0.798 | 7.529E-06 | 0.002 | Unannotated | NA | NA |
| metab_10325 | Unannotated feature | neg | 164.9471 | 0.786 | 4.381 | 4.915 | 0.534 | 0.798 | 7.529E-06 | 0.002 | Unannotated | NA | NA |
| metab_806 | Unannotated feature | pos | 267.0427 | 3.051 | 4.496 | 4.037 | -0.459 | -0.798 | 7.529E-06 | 0.002 | Unannotated | NA | NA |
| metab_4500 | Unannotated feature | pos | 187.0578 | 0.551 | 6.053 | 5.940 | -0.113 | -0.794 | 8.410E-06 | 0.002 | Unannotated | NA | NA |
| metab_7270 | 3alpha,7alpha,12alpha-Trihydroxy-5beta-cholestane | neg | 465.3594 | 6.110 | 5.937 | 5.016 | -0.921 | -0.794 | 8.410E-06 | 0.002 | Tentative | Lipids and lipid-like molecules | Steroids and steroid derivatives |
| metab_6343 | Unannotated feature | neg | 236.8565 | 0.699 | 5.004 | 5.408 | 0.404 | 0.794 | 8.410E-06 | 0.002 | Unannotated | NA | NA |
| metab_7243 | 6-Ketomyristic acid | neg | 483.3701 | 6.016 | 5.548 | 4.244 | -1.303 | -0.789 | 9.390E-06 | 0.002 | Tentative | Lipids and lipid-like molecules | Fatty Acyls |
| metab_8160 | Unannotated feature | neg | 399.3277 | 6.047 | 4.200 | 3.747 | -0.453 | -0.789 | 9.390E-06 | 0.002 | Unannotated | NA | NA |
| metab_6958 | Unannotated feature | neg | 198.1134 | 5.699 | 3.554 | 4.127 | 0.574 | 0.789 | 9.390E-06 | 0.002 | Unannotated | NA | NA |
| metab_8154 | Calcidiol | neg | 445.3331 | 6.071 | 6.408 | 5.722 | -0.685 | -0.789 | 9.390E-06 | 0.002 | Tentative | Lipids and lipid-like molecules | Steroids and steroid derivatives |
| metab_11226 | Unannotated feature | neg | 479.3387 | 5.977 | 5.590 | 4.075 | -1.515 | -0.789 | 9.390E-06 | 0.002 | Unannotated | NA | NA |
| metab_3000 | Unannotated feature | pos | 265.9811 | 4.378 | 4.367 | 3.996 | -0.370 | -0.785 | 1.048E-05 | 0.002 | Unannotated | NA | NA |
| metab_11265 | Unannotated feature | neg | 449.3646 | 6.180 | 6.081 | 5.199 | -0.882 | -0.785 | 1.048E-05 | 0.002 | Unannotated | NA | NA |
| metab_45 | Unannotated feature | pos | 196.0969 | 1.956 | 4.197 | 4.528 | 0.331 | 0.785 | 1.048E-05 | 0.002 | Unannotated | NA | NA |
| metab_431 | Unannotated feature | pos | 443.2337 | 5.717 | 4.571 | 5.325 | 0.753 | 0.781 | 1.169E-05 | 0.002 | Unannotated | NA | NA |
| metab_9272 | Unannotated feature | neg | 187.0140 | 3.827 | 4.298 | 3.692 | -0.606 | -0.781 | 1.169E-05 | 0.002 | Unannotated | NA | NA |
| metab_4952 | L-Carnitine | pos | 162.1125 | 0.644 | 7.200 | 6.997 | -0.204 | -0.777 | 1.302E-05 | 0.002 | Strong putative | Organic nitrogen compounds | Organonitrogen compounds |
| metab_6431 | Unannotated feature | neg | 89.4877 | 4.199 | 2.788 | 1.918 | -0.870 | -0.777 | 1.302E-05 | 0.002 | Unannotated | NA | NA |
| metab_8030 | Unannotated feature | neg | 168.9888 | 6.576 | 3.917 | 2.588 | -1.329 | -0.777 | 1.302E-05 | 0.002 | Unannotated | NA | NA |
| metab_2674 | N-Linoleoyl Lysine | pos | 472.3476 | 5.778 | 5.020 | 4.776 | -0.244 | -0.777 | 1.302E-05 | 0.002 | Tentative | NA | NA |
| metab_7275 | 7alpha-Hydroxycholesterol | neg | 447.3490 | 6.118 | 6.203 | 5.657 | -0.546 | -0.777 | 1.302E-05 | 0.002 | Tentative | Lipids and lipid-like molecules | Steroids and steroid derivatives |
| metab_10445 | Unannotated feature | neg | 257.0549 | 0.660 | 3.500 | 4.634 | 1.134 | 0.777 | 1.302E-05 | 0.002 | Unannotated | NA | NA |
| metab_3379 | Unannotated feature | pos | 231.0839 | 2.521 | 4.238 | 3.822 | -0.417 | -0.777 | 1.302E-05 | 0.002 | Unannotated | NA | NA |
| metab_1307 | Unannotated feature | pos | 158.1540 | 5.879 | 4.939 | 5.013 | 0.074 | 0.777 | 1.302E-05 | 0.002 | Unannotated | NA | NA |
| metab_236 | Unannotated feature | pos | 163.1158 | 0.644 | 6.104 | 5.880 | -0.223 | -0.777 | 1.302E-05 | 0.002 | Unannotated | NA | NA |
| metab_11242 | Momordol | neg | 439.3438 | 6.110 | 5.709 | 4.900 | -0.809 | -0.773 | 1.451E-05 | 0.002 | Tentative | Lipids and lipid-like molecules | Fatty Acyls |
| metab_11260 | Unannotated feature | neg | 493.3908 | 6.188 | 6.086 | 5.426 | -0.661 | -0.773 | 1.451E-05 | 0.002 | Unannotated | NA | NA |
| metab_3856 | Unannotated feature | pos | 301.1410 | 1.025 | 5.710 | 5.586 | -0.124 | -0.773 | 1.451E-05 | 0.002 | Unannotated | NA | NA |
| metab_3855 | Unannotated feature | pos | 83.4486 | 1.033 | 3.420 | 3.684 | 0.264 | 0.773 | 1.451E-05 | 0.002 | Unannotated | NA | NA |
| metab_9508 | Fe(II)-nicotianamine | neg | 302.1362 | 3.240 | 3.114 | 4.605 | 1.491 | 0.773 | 1.451E-05 | 0.002 | Tentative | Organic acids and derivatives | Carboxylic acids and derivatives |
| metab_8161 | (8S,9S,10R,13R,14S,17R)-5,6-Dihydroperoxy-10,13-dimethyl-17-[(2R)-6-methylheptan-2-yl]-1,2,3,4,6,7,8,9,11,12,14,15,16,17-tetradecahydrocyclopenta[a]phenanthren-3-ol | neg | 451.3438 | 6.040 | 5.002 | 3.676 | -1.326 | -0.773 | 1.451E-05 | 0.002 | Tentative | Lipids and lipid-like molecules | Steroids and steroid derivatives |
| metab_10477 | Unannotated feature | neg | 253.0099 | 0.644 | 3.436 | 4.490 | 1.054 | 0.773 | 1.451E-05 | 0.002 | Unannotated | NA | NA |
| metab_2721 | Unannotated feature | pos | 545.3767 | 5.733 | 5.320 | 5.132 | -0.188 | -0.773 | 1.451E-05 | 0.002 | Unannotated | NA | NA |
| metab_3860 | Unannotated feature | pos | 142.9387 | 0.963 | 4.550 | 4.799 | 0.249 | 0.773 | 1.451E-05 | 0.002 | Unannotated | NA | NA |
| metab_7271 | Myristoleic acid | neg | 511.4011 | 6.110 | 5.346 | 4.047 | -1.300 | -0.768 | 1.616E-05 | 0.002 | Tentative | Lipids and lipid-like molecules | Fatty Acyls |
| metab_11254 | Unannotated feature | neg | 425.3645 | 6.180 | 5.840 | 5.112 | -0.728 | -0.768 | 1.616E-05 | 0.002 | Unannotated | NA | NA |
| metab_8135 | N-(3-(Dimethylamino)propyl)acrylamide | neg | 467.3751 | 6.118 | 6.110 | 5.126 | -0.984 | -0.768 | 1.616E-05 | 0.002 | Tentative | Organic acids and derivatives | Carboxylic acids and derivatives |
| metab_4726 | Unannotated feature | pos | 784.5489 | 5.933 | 5.872 | 5.589 | -0.283 | -0.768 | 1.616E-05 | 0.002 | Unannotated | NA | NA |
| metab_8185 | 3alpha,7alpha,12alpha,26-Tetrahydroxy-5beta-cholestane | neg | 481.3545 | 5.993 | 5.796 | 4.456 | -1.340 | -0.768 | 1.616E-05 | 0.002 | Tentative | Lipids and lipid-like molecules | Steroids and steroid derivatives |
| metab_370 | Unannotated feature | pos | 308.8786 | 0.885 | 4.961 | 4.751 | -0.210 | -0.768 | 1.616E-05 | 0.002 | Unannotated | NA | NA |
| metab_3624 | 4-Methyl-1H-pyrrole-3-carboxamide | pos | 125.0712 | 1.964 | 3.958 | 4.321 | 0.364 | 0.764 | 1.798E-05 | 0.002 | Tentative | Organoheterocyclic compounds | Pyrroles |
| metab_9278 | Unannotated feature | neg | 274.9871 | 3.819 | 4.332 | 3.910 | -0.423 | -0.764 | 1.798E-05 | 0.002 | Unannotated | NA | NA |
| metab_7926 | Unannotated feature | neg | 244.9085 | 7.109 | 5.143 | 4.629 | -0.514 | -0.764 | 1.798E-05 | 0.002 | Unannotated | NA | NA |
| metab_7287 | Schleicherastatin 3 | neg | 491.3751 | 6.180 | 5.958 | 4.940 | -1.018 | -0.764 | 1.798E-05 | 0.002 | Tentative | Lipids and lipid-like molecules | Steroids and steroid derivatives |
| metab_8031 | Unannotated feature | neg | 368.9772 | 6.572 | 6.626 | 5.709 | -0.917 | -0.764 | 1.798E-05 | 0.002 | Unannotated | NA | NA |
| metab_393 | Unannotated feature | pos | 413.2660 | 1.025 | 5.362 | 5.194 | -0.168 | -0.764 | 1.798E-05 | 0.002 | Unannotated | NA | NA |
| metab_665 | Nevirapine | pos | 284.1492 | 2.405 | 3.399 | 3.946 | 0.547 | 0.764 | 1.798E-05 | 0.002 | Tentative | Organic nitrogen compounds | Organonitrogen compounds |
| metab_3108 | Castasterone | pos | 447.3470 | 6.049 | 5.941 | 5.105 | -0.836 | -0.760 | 2.000E-05 | 0.002 | Tentative | Lipids and lipid-like molecules | Steroids and steroid derivatives |
| metab_9280 | Unannotated feature | neg | 214.0332 | 3.819 | 5.159 | 4.838 | -0.321 | -0.760 | 2.000E-05 | 0.002 | Unannotated | NA | NA |
| metab_1859 | Unannotated feature | pos | 119.0817 | 7.602 | 4.632 | 4.751 | 0.119 | 0.760 | 2.000E-05 | 0.002 | Unannotated | NA | NA |
| metab_7356 | Perfluorooctanoic acid | neg | 412.9673 | 6.576 | 6.505 | 5.571 | -0.934 | -0.760 | 2.000E-05 | 0.002 | Strong putative | Organohalogen compounds | Acyl halides |
| metab_11139 | Unannotated feature | neg | 433.2088 | 5.723 | 3.292 | 4.843 | 1.551 | 0.760 | 2.000E-05 | 0.002 | Unannotated | NA | NA |
| metab_34 | Unannotated feature | pos | 216.1231 | 1.956 | 4.408 | 4.599 | 0.191 | 0.760 | 2.000E-05 | 0.002 | Unannotated | NA | NA |
| metab_5003 | Unannotated feature | pos | 176.1282 | 0.792 | 5.272 | 4.988 | -0.283 | -0.760 | 2.000E-05 | 0.002 | Unannotated | NA | NA |
| metab_8187 | 3alpha,7alpha-Dihydroxy-5beta-cholestan-26-al | neg | 463.3439 | 5.993 | 6.185 | 5.275 | -0.911 | -0.760 | 2.000E-05 | 0.002 | Tentative | Lipids and lipid-like molecules | Steroids and steroid derivatives |
| metab_8110 | Geosmin | neg | 423.3488 | 6.180 | 5.245 | 4.261 | -0.984 | -0.756 | 2.223E-05 | 0.002 | Tentative | Organic oxygen compounds | Organooxygen compounds |
| metab_10202 | Deoxyribose-isomer feature | neg | 133.0500 | 0.873 | 3.141 | 4.584 | 1.443 | 0.756 | 2.223E-05 | 0.002 | Moderate putative | Organic oxygen compounds | Organooxygen compounds |
| metab_5617 | Unannotated feature | neg | 156.9153 | 0.912 | 4.351 | 4.817 | 0.466 | 0.756 | 2.223E-05 | 0.002 | Unannotated | NA | NA |
| metab_3177 | Unannotated feature | pos | 83.6587 | 3.399 | 3.728 | 3.961 | 0.233 | 0.756 | 2.223E-05 | 0.002 | Unannotated | NA | NA |
| metab_2248 | 2-Hydroxydodecyl methacrylate | pos | 563.4284 | 6.203 | 5.442 | 3.956 | -1.486 | -0.756 | 2.223E-05 | 0.002 | Tentative | Lipids and lipid-like molecules | Fatty Acyls |
| metab_2313 | Unannotated feature | pos | 249.1849 | 6.049 | 5.289 | 4.364 | -0.925 | -0.756 | 2.223E-05 | 0.002 | Moderate putative | NA | NA |
| metab_71 | Unannotated feature | pos | 280.9954 | 0.403 | 4.947 | 4.798 | -0.149 | -0.756 | 2.223E-05 | 0.002 | Unannotated | NA | NA |
| metab_1346 | Unannotated feature | pos | 799.5547 | 5.894 | 5.725 | 5.474 | -0.252 | -0.756 | 2.223E-05 | 0.002 | Unannotated | NA | NA |
| metab_3401 | Unannotated feature | pos | 201.9910 | 2.467 | 3.885 | 4.442 | 0.557 | 0.756 | 2.223E-05 | 0.002 | Unannotated | NA | NA |
| metab_8036 | Unannotated feature | neg | 133.0499 | 6.527 | 4.153 | 4.350 | 0.196 | 0.752 | 2.470E-05 | 0.002 | Unannotated | NA | NA |
| metab_9955 | Dopamine 3-O-sulfate | neg | 232.0287 | 2.111 | 2.859 | 3.758 | 0.899 | 0.752 | 2.470E-05 | 0.002 | Tentative | Organic acids and derivatives | Organic sulfuric acids and derivatives |
| metab_1471 | Unannotated feature | pos | 632.6621 | 0.575 | 5.407 | 5.331 | -0.077 | -0.752 | 2.470E-05 | 0.002 | Unannotated | NA | NA |
| metab_9273 | Unannotated feature | neg | 404.0420 | 3.819 | 4.697 | 3.440 | -1.256 | -0.752 | 2.470E-05 | 0.002 | Unannotated | NA | NA |
| metab_6281 | Unannotated feature | neg | 214.0333 | 3.570 | 4.811 | 4.566 | -0.246 | -0.752 | 2.470E-05 | 0.002 | Unannotated | NA | NA |
| metab_10157 | Unannotated feature | neg | 380.8153 | 0.912 | 4.841 | 4.566 | -0.275 | -0.752 | 2.470E-05 | 0.002 | Unannotated | NA | NA |
| metab_8229 | Unannotated feature | neg | 349.9041 | 5.922 | 5.123 | 5.274 | 0.151 | 0.752 | 2.470E-05 | 0.002 | Unannotated | NA | NA |
| metab_8153 | 8-Methyl-6-nonenoic acid | neg | 509.3855 | 6.079 | 5.433 | 4.410 | -1.023 | -0.752 | 2.470E-05 | 0.002 | Tentative | Lipids and lipid-like molecules | Fatty Acyls |
| metab_1364 | Dhelwangin | pos | 189.0911 | 5.910 | 4.894 | 4.992 | 0.098 | 0.752 | 2.470E-05 | 0.002 | Tentative | NA | NA |
| metab_8085 | Unannotated feature | neg | 451.3800 | 6.196 | 5.123 | 3.813 | -1.310 | -0.752 | 2.470E-05 | 0.002 | Unannotated | NA | NA |
| metab_5732 | Unannotated feature | neg | 222.9919 | 1.649 | 2.463 | 4.783 | 2.320 | 0.752 | 2.470E-05 | 0.002 | Unannotated | NA | NA |
| metab_9284 | Unannotated feature | neg | 128.6527 | 3.819 | 4.824 | 4.412 | -0.412 | -0.752 | 2.470E-05 | 0.002 | Unannotated | NA | NA |
| metab_75 | Unannotated feature | pos | 144.1020 | 0.434 | 4.771 | 4.822 | 0.051 | 0.752 | 2.470E-05 | 0.002 | Unannotated | NA | NA |
| metab_2660 | Unannotated feature | pos | 146.0812 | 1.925 | 5.161 | 5.605 | 0.445 | 0.747 | 2.743E-05 | 0.002 | Unannotated | NA | NA |
| metab_3635 | Isohumulinone A | pos | 212.0918 | 1.956 | 4.530 | 4.709 | 0.179 | 0.747 | 2.743E-05 | 0.002 | Tentative | Lipids and lipid-like molecules | Prenol lipids |
| metab_9860 | Unannotated feature | neg | 144.0660 | 2.445 | 3.925 | 4.248 | 0.323 | 0.747 | 2.743E-05 | 0.002 | Unannotated | NA | NA |
| metab_2321 | Chenodeoxycholylarginine | pos | 590.4265 | 6.026 | 6.134 | 6.216 | 0.083 | 0.747 | 2.743E-05 | 0.002 | Tentative | NA | NA |
| metab_10419 | Unannotated feature | neg | 212.8773 | 0.684 | 3.897 | 4.807 | 0.909 | 0.747 | 2.743E-05 | 0.002 | Unannotated | NA | NA |
| metab_1485 | Unannotated feature | pos | 463.3631 | 6.049 | 4.016 | 5.328 | 1.312 | 0.747 | 2.743E-05 | 0.002 | Unannotated | NA | NA |
| metab_9663 | Unannotated feature | neg | 197.0431 | 3.819 | 5.306 | 5.059 | -0.247 | -0.747 | 2.743E-05 | 0.002 | Unannotated | NA | NA |
| metab_3842 | Unannotated feature | pos | 215.0526 | 1.150 | 4.705 | 4.427 | -0.278 | -0.747 | 2.743E-05 | 0.002 | Unannotated | NA | NA |
| metab_6442 | Unannotated feature | neg | 201.1130 | 4.248 | 2.625 | 3.650 | 1.025 | 0.743 | 3.044E-05 | 0.002 | Moderate putative | NA | NA |
| metab_9275 | Unannotated feature | neg | 293.8920 | 3.819 | 5.074 | 4.648 | -0.426 | -0.743 | 3.044E-05 | 0.002 | Unannotated | NA | NA |
| metab_10217 | Unannotated feature | neg | 281.0550 | 0.865 | 3.724 | 4.517 | 0.793 | 0.743 | 3.044E-05 | 0.002 | Unannotated | NA | NA |
| metab_6348 | Unannotated feature | neg | 281.1012 | 3.827 | 4.650 | 3.756 | -0.894 | -0.743 | 3.044E-05 | 0.002 | Unannotated | NA | NA |
| metab_7228 | Unannotated feature | neg | 319.2648 | 5.993 | 4.676 | 3.531 | -1.145 | -0.743 | 3.044E-05 | 0.002 | Unannotated | NA | NA |
| metab_3328 | Unannotated feature | pos | 156.5765 | 2.637 | 5.365 | 5.457 | 0.092 | 0.743 | 3.044E-05 | 0.002 | Unannotated | NA | NA |
| metab_7555 | Unannotated feature | neg | 172.0612 | 2.119 | 2.643 | 3.802 | 1.159 | 0.743 | 3.044E-05 | 0.002 | Unannotated | NA | NA |
| metab_3270 | Unannotated feature | pos | 128.1072 | 2.934 | 3.607 | 3.897 | 0.290 | 0.743 | 3.044E-05 | 0.002 | Unannotated | NA | NA |
| metab_1243 | Unannotated feature | pos | 177.0884 | 5.832 | 5.389 | 5.457 | 0.068 | 0.743 | 3.044E-05 | 0.002 | Unannotated | NA | NA |
| metab_498 | Coprine | pos | 185.0921 | 1.894 | 3.858 | 4.530 | 0.671 | 0.743 | 3.044E-05 | 0.002 | Tentative | Organic acids and derivatives | Carboxylic acids and derivatives |
| metab_7267 | 3alpha,7alpha-Dihydroxycoprostanic acid | neg | 433.3329 | 6.102 | 4.860 | 3.643 | -1.217 | -0.743 | 3.044E-05 | 0.002 | Tentative | Lipids and lipid-like molecules | Steroids and steroid derivatives |
| metab_4737 | Unannotated feature | pos | 390.2850 | 6.049 | 4.982 | 1.390 | -3.592 | -0.743 | 3.044E-05 | 0.002 | Unannotated | NA | NA |
| metab_1521 | Unannotated feature | pos | 484.3994 | 6.172 | 5.260 | 4.964 | -0.296 | -0.739 | 3.377E-05 | 0.002 | Unannotated | NA | NA |
| metab_3205 | 6-Benzylaminopurine | pos | 243.1340 | 3.221 | 3.908 | 4.688 | 0.780 | 0.739 | 3.377E-05 | 0.002 | Tentative | Organoheterocyclic compounds | Imidazopyrimidines |
| metab_8887 | Prenyl arabinosyl-(1->6)-glucoside | neg | 379.1618 | 5.199 | 3.191 | 4.914 | 1.723 | 0.739 | 3.377E-05 | 0.002 | Tentative | Lipids and lipid-like molecules | Fatty Acyls |
| metab_7974 | Unannotated feature | neg | 479.2844 | 7.093 | 4.495 | 3.706 | -0.789 | -0.739 | 3.377E-05 | 0.002 | Unannotated | NA | NA |
| metab_520 | Unannotated feature | pos | 160.1333 | 1.949 | 3.113 | 3.656 | 0.544 | 0.739 | 3.377E-05 | 0.002 | Unannotated | NA | NA |
| metab_11137 | Unannotated feature | neg | 319.9842 | 3.819 | 5.642 | 5.309 | -0.333 | -0.739 | 3.377E-05 | 0.002 | Unannotated | NA | NA |
| metab_3616 | Unannotated feature | pos | 187.1264 | 1.980 | 4.718 | 4.795 | 0.077 | 0.739 | 3.377E-05 | 0.002 | Unannotated | NA | NA |
| metab_4727 | Unannotated feature | pos | 722.5049 | 6.018 | 5.834 | 5.929 | 0.095 | 0.739 | 3.377E-05 | 0.002 | Unannotated | NA | NA |
| metab_9612 | Dihydrocaffeic acid 3-O-glucuronide | neg | 357.0835 | 3.136 | 3.104 | 4.673 | 1.570 | 0.739 | 3.377E-05 | 0.002 | Tentative | Organic oxygen compounds | Organooxygen compounds |
| metab_583 | Lotaustralin | pos | 294.1546 | 2.065 | 5.833 | 5.416 | -0.416 | -0.739 | 3.377E-05 | 0.002 | Tentative | Organic oxygen compounds | Organooxygen compounds |
| metab_11236 | Unannotated feature | neg | 437.3281 | 6.063 | 5.861 | 5.153 | -0.708 | -0.735 | 3.744E-05 | 0.002 | Unannotated | NA | NA |
| metab_11233 | Unannotated feature | neg | 489.3595 | 6.024 | 6.050 | 5.184 | -0.866 | -0.735 | 3.744E-05 | 0.002 | Unannotated | NA | NA |
| metab_10178 | Unannotated feature | neg | 264.8978 | 0.904 | 5.167 | 4.958 | -0.209 | -0.735 | 3.744E-05 | 0.002 | Unannotated | NA | NA |
| metab_9048 | 2-acetyl-1,4,5,6-tetrahydropyridine | neg | 170.0819 | 4.627 | 2.639 | 3.697 | 1.057 | 0.735 | 3.744E-05 | 0.002 | Tentative | Organoheterocyclic compounds | Pyridines and derivatives |
| metab_5513 | Unannotated feature | neg | 347.0757 | 0.754 | 3.592 | 4.539 | 0.947 | 0.735 | 3.744E-05 | 0.002 | Unannotated | NA | NA |
| metab_9283 | Unannotated feature | neg | 129.0820 | 3.819 | 4.059 | 3.579 | -0.481 | -0.735 | 3.744E-05 | 0.002 | Unannotated | NA | NA |
| metab_10768 | Unannotated feature | neg | 312.8758 | 0.570 | 4.404 | 4.672 | 0.268 | 0.735 | 3.744E-05 | 0.002 | Unannotated | NA | NA |
| metab_1873 | Unannotated feature | pos | 84.5070 | 7.409 | 4.340 | 4.546 | 0.206 | 0.735 | 3.744E-05 | 0.002 | Unannotated | NA | NA |
| metab_766 | Unannotated feature | pos | 128.1072 | 2.849 | 3.414 | 3.938 | 0.524 | 0.735 | 3.744E-05 | 0.002 | Unannotated | NA | NA |
| metab_11228 | Unannotated feature | neg | 461.3283 | 6.000 | 5.475 | 4.428 | -1.047 | -0.735 | 3.744E-05 | 0.002 | Unannotated | NA | NA |
| metab_311 | Ethyl methanesulfonate | pos | 107.0165 | 0.784 | 2.419 | 3.288 | 0.869 | 0.735 | 3.744E-05 | 0.002 | Tentative | Organic acids and derivatives | Organic sulfonic acids and derivatives |
| metab_9654 | Unannotated feature | neg | 289.8978 | 3.819 | 5.814 | 5.365 | -0.449 | -0.735 | 3.744E-05 | 0.002 | Unannotated | NA | NA |
| metab_6520 | Unannotated feature | neg | 210.9626 | 0.794 | 3.280 | 4.041 | 0.761 | 0.735 | 3.744E-05 | 0.002 | Unannotated | NA | NA |
| metab_8094 | Unannotated feature | neg | 495.4066 | 6.188 | 5.642 | 4.273 | -1.369 | -0.731 | 4.148E-05 | 0.002 | Unannotated | NA | NA |
| metab_6346 | Unannotated feature | neg | 291.9893 | 3.819 | 5.080 | 4.676 | -0.404 | -0.731 | 4.148E-05 | 0.002 | Unannotated | NA | NA |
| metab_7346 | Unannotated feature | neg | 353.8271 | 6.493 | 4.877 | 3.319 | -1.559 | -0.731 | 4.148E-05 | 0.002 | Unannotated | NA | NA |
| metab_4445 | Unannotated feature | pos | 232.9079 | 0.551 | 5.146 | 5.534 | 0.388 | 0.731 | 4.148E-05 | 0.002 | Unannotated | NA | NA |
| metab_1896 | Unannotated feature | pos | 238.0403 | 7.331 | 4.023 | 4.471 | 0.448 | 0.731 | 4.148E-05 | 0.002 | Unannotated | NA | NA |
| metab_1082 | Myosmine | pos | 310.2012 | 5.267 | 5.987 | 6.329 | 0.342 | 0.731 | 4.148E-05 | 0.002 | Tentative | Organoheterocyclic compounds | Pyridines and derivatives |
| metab_8682 | Unannotated feature | neg | 186.9802 | 5.619 | 3.760 | 2.829 | -0.931 | -0.731 | 4.148E-05 | 0.002 | Unannotated | NA | NA |
| metab_809 | Unannotated feature | pos | 138.9954 | 3.066 | 4.654 | 4.612 | -0.042 | -0.731 | 4.148E-05 | 0.002 | Unannotated | NA | NA |
| metab_2302 | Unannotated feature | pos | 477.3790 | 6.072 | 5.097 | 5.644 | 0.547 | 0.731 | 4.148E-05 | 0.002 | Unannotated | NA | NA |
| metab_8093 | Diacylglycerol | neg | 519.4063 | 6.188 | 5.995 | 5.440 | -0.555 | -0.731 | 4.148E-05 | 0.002 | Tentative | Lipids and lipid-like molecules | Glycerolipids |
| metab_219 | Unannotated feature | pos | 164.9206 | 0.551 | 5.295 | 5.674 | 0.379 | 0.726 | 4.594E-05 | 0.002 | Unannotated | NA | NA |
| metab_6220 | Unannotated feature | neg | 108.0209 | 3.388 | 1.813 | 3.243 | 1.430 | 0.726 | 4.594E-05 | 0.002 | Unannotated | NA | NA |
| metab_7348 | Unannotated feature | neg | 137.0237 | 6.522 | 5.626 | 5.713 | 0.087 | 0.726 | 4.594E-05 | 0.002 | Unannotated | NA | NA |
| metab_7242 | Unannotated feature | neg | 345.2806 | 6.016 | 4.348 | 3.555 | -0.793 | -0.726 | 4.594E-05 | 0.002 | Unannotated | NA | NA |
| metab_2037 | Unannotated feature | pos | 391.2621 | 7.261 | 4.723 | 4.951 | 0.228 | 0.726 | 4.594E-05 | 0.002 | Unannotated | NA | NA |
| metab_6784 | Unannotated feature | neg | 226.0908 | 5.543 | 3.075 | 3.645 | 0.569 | 0.726 | 4.594E-05 | 0.002 | Unannotated | NA | NA |
| metab_6530 | Unannotated feature | neg | 208.9656 | 0.794 | 4.047 | 4.645 | 0.598 | 0.726 | 4.594E-05 | 0.002 | Unannotated | NA | NA |
| metab_1445 | Unannotated feature | pos | 202.0444 | 5.972 | 4.820 | 4.920 | 0.100 | 0.726 | 4.594E-05 | 0.002 | Unannotated | NA | NA |
| metab_6576 | Unannotated feature | neg | 177.0666 | 4.758 | 3.987 | 3.374 | -0.612 | -0.726 | 4.594E-05 | 0.002 | Unannotated | NA | NA |
| metab_9291 | Gamma-Glutamylisoleucine | neg | 241.1196 | 3.789 | 3.739 | 4.450 | 0.711 | 0.726 | 4.594E-05 | 0.002 | Tentative | Organic acids and derivatives | Carboxylic acids and derivatives |
| metab_4706 | Unannotated feature | pos | 256.2272 | 5.925 | 4.712 | 4.812 | 0.100 | 0.726 | 4.594E-05 | 0.002 | Unannotated | NA | NA |
| metab_4847 | Unannotated feature | pos | 517.3870 | 6.288 | 5.162 | 4.628 | -0.534 | -0.726 | 4.594E-05 | 0.002 | Unannotated | NA | NA |
| metab_296 | Unannotated feature | pos | 142.9387 | 0.753 | 3.994 | 4.328 | 0.335 | 0.726 | 4.594E-05 | 0.002 | Unannotated | NA | NA |
| metab_11214 | Unannotated feature | neg | 261.7756 | 5.922 | 6.098 | 5.959 | -0.139 | -0.722 | 5.085E-05 | 0.002 | Unannotated | NA | NA |
| metab_4563 | Unannotated feature | pos | 333.1444 | 5.512 | 6.362 | 5.967 | -0.395 | -0.722 | 5.085E-05 | 0.002 | Unannotated | NA | NA |
| metab_6341 | Unannotated feature | neg | 126.8804 | 3.819 | 4.147 | 4.041 | -0.106 | -0.722 | 5.085E-05 | 0.002 | Unannotated | NA | NA |
| metab_11023 | Unannotated feature | neg | 569.7147 | 0.013 | 4.667 | 4.503 | -0.165 | -0.722 | 5.085E-05 | 0.002 | Unannotated | NA | NA |
| metab_1535 | Germacrone | pos | 251.2006 | 6.249 | 4.962 | 3.645 | -1.317 | -0.722 | 5.085E-05 | 0.002 | Tentative | Lipids and lipid-like molecules | Prenol lipids |
| metab_5429 | Unannotated feature | neg | 195.0510 | 0.644 | 5.000 | 5.598 | 0.599 | 0.722 | 5.085E-05 | 0.002 | Unannotated | NA | NA |
| metab_7221 | Unannotated feature | neg | 244.9084 | 5.969 | 6.285 | 5.659 | -0.626 | -0.722 | 5.085E-05 | 0.002 | Unannotated | NA | NA |
| metab_2240 | Unannotated feature | pos | 495.4045 | 6.242 | 4.796 | 3.666 | -1.130 | -0.722 | 5.085E-05 | 0.002 | Unannotated | NA | NA |
| metab_10150 | Unannotated feature | neg | 212.8773 | 0.920 | 3.705 | 4.481 | 0.776 | 0.722 | 5.085E-05 | 0.002 | Unannotated | NA | NA |
| metab_7255 | Bisabolol oxide B | neg | 535.4013 | 6.047 | 5.733 | 4.956 | -0.777 | -0.722 | 5.085E-05 | 0.002 | Tentative | Organoheterocyclic compounds | Tetrahydrofurans |
| metab_4846 | Unannotated feature | pos | 523.4357 | 6.288 | 5.494 | 4.399 | -1.095 | -0.722 | 5.085E-05 | 0.002 | Unannotated | NA | NA |
| metab_10864 | Unannotated feature | neg | 495.8522 | 0.554 | 0.482 | 4.580 | 4.098 | 0.722 | 5.085E-05 | 0.002 | Unannotated | NA | NA |
| metab_3329 | Unannotated feature | pos | 155.5040 | 2.637 | 5.355 | 5.448 | 0.093 | 0.722 | 5.085E-05 | 0.002 | Unannotated | NA | NA |
| metab_4819 | Unannotated feature | pos | 114.9844 | 7.967 | 4.500 | 4.678 | 0.177 | 0.722 | 5.085E-05 | 0.002 | Unannotated | NA | NA |
| metab_3654 | Tranexamic acid | pos | 122.0967 | 1.956 | 3.783 | 3.966 | 0.183 | 0.722 | 5.085E-05 | 0.002 | Tentative | Organic acids and derivatives | Carboxylic acids and derivatives |
| metab_9646 | Unannotated feature | neg | 291.8948 | 3.819 | 5.741 | 5.283 | -0.458 | -0.722 | 5.085E-05 | 0.002 | Unannotated | NA | NA |
| metab_1446 | Unannotated feature | pos | 239.1615 | 5.972 | 4.560 | 4.628 | 0.068 | 0.722 | 5.085E-05 | 0.002 | Unannotated | NA | NA |
| metab_4721 | Unannotated feature | pos | 138.9954 | 5.979 | 5.006 | 4.766 | -0.240 | -0.718 | 5.626E-05 | 0.002 | Unannotated | NA | NA |
| metab_10243 | Unannotated feature | neg | 88.7930 | 0.865 | 6.012 | 5.814 | -0.199 | -0.718 | 5.626E-05 | 0.002 | Unannotated | NA | NA |
| metab_584 | Prolyl-Arginine | pos | 316.1366 | 2.065 | 4.418 | 3.299 | -1.119 | -0.718 | 5.626E-05 | 0.002 | Tentative | Organic acids and derivatives | Carboxylic acids and derivatives |
| metab_9918 | Unannotated feature | neg | 257.9842 | 2.119 | 3.873 | 3.327 | -0.546 | -0.718 | 5.626E-05 | 0.002 | Unannotated | NA | NA |
| metab_237 | Unannotated feature | pos | 175.0577 | 0.644 | 4.913 | 5.345 | 0.432 | 0.718 | 5.626E-05 | 0.002 | Unannotated | NA | NA |
| metab_2816 | Citral propylene glycol acetal | pos | 228.1959 | 5.832 | 6.502 | 6.544 | 0.042 | 0.718 | 5.626E-05 | 0.002 | Tentative | NA | NA |
| metab_4879 | Unannotated feature | pos | 750.5758 | 0.591 | 5.695 | 5.597 | -0.098 | -0.718 | 5.626E-05 | 0.002 | Unannotated | NA | NA |
| metab_2528 | Unannotated feature | pos | 665.4596 | 5.879 | 5.335 | 5.084 | -0.252 | -0.718 | 5.626E-05 | 0.002 | Unannotated | NA | NA |
| metab_8092 | Unannotated feature | neg | 521.4220 | 6.188 | 5.888 | 4.964 | -0.924 | -0.718 | 5.626E-05 | 0.002 | Unannotated | NA | NA |
| metab_6875 | Unannotated feature | neg | 322.8566 | 0.904 | 4.955 | 4.679 | -0.276 | -0.718 | 5.626E-05 | 0.002 | Unannotated | NA | NA |
| metab_8218 | Unannotated feature | neg | 198.9415 | 5.937 | 3.948 | 4.196 | 0.248 | 0.718 | 5.626E-05 | 0.002 | Unannotated | NA | NA |
| metab_4256 | Unannotated feature | pos | 245.0996 | 0.598 | 4.312 | 4.052 | -0.259 | -0.718 | 5.626E-05 | 0.002 | Unannotated | NA | NA |
| metab_1080 | Hydroxypioglitazone | pos | 355.1111 | 5.244 | 3.888 | 2.870 | -1.018 | -0.718 | 5.626E-05 | 0.002 | Tentative | Benzenoids | Phenol ethers |
| metab_4691 | Unannotated feature | pos | 607.0933 | 5.894 | 5.123 | 4.813 | -0.310 | -0.718 | 5.626E-05 | 0.002 | Unannotated | NA | NA |
| metab_10414 | Unannotated feature | neg | 112.0509 | 0.692 | 2.737 | 3.960 | 1.222 | 0.718 | 5.626E-05 | 0.002 | Unannotated | NA | NA |
| metab_6639 | Unannotated feature | neg | 177.0666 | 5.017 | 3.709 | 2.719 | -0.990 | -0.718 | 5.626E-05 | 0.002 | Unannotated | NA | NA |
| metab_2533 | Unannotated feature | pos | 407.3017 | 5.879 | 4.566 | 4.894 | 0.327 | 0.714 | 6.221E-05 | 0.002 | Unannotated | NA | NA |
| metab_6144 | 3-Hydroxyindolin-2-one-sulfate | neg | 227.9973 | 3.224 | 3.668 | 4.508 | 0.840 | 0.714 | 6.221E-05 | 0.002 | Tentative | Organoheterocyclic compounds | Indoles and derivatives |
| metab_5867 | Unannotated feature | neg | 142.0504 | 2.431 | 3.785 | 4.053 | 0.267 | 0.714 | 6.221E-05 | 0.002 | Unannotated | NA | NA |
| metab_7272 | 9-Oxohexadecanoic acid | neg | 539.4327 | 6.110 | 6.192 | 5.181 | -1.011 | -0.714 | 6.221E-05 | 0.002 | Tentative | Lipids and lipid-like molecules | Fatty Acyls |
| metab_7195 | Unannotated feature | neg | 317.8226 | 5.922 | 5.575 | 5.416 | -0.160 | -0.714 | 6.221E-05 | 0.002 | Unannotated | NA | NA |
| metab_10182 | Unannotated feature | neg | 204.9415 | 0.896 | 5.585 | 5.443 | -0.143 | -0.714 | 6.221E-05 | 0.002 | Unannotated | NA | NA |
| metab_9279 | Unannotated feature | neg | 228.9811 | 3.819 | 4.629 | 4.023 | -0.606 | -0.714 | 6.221E-05 | 0.002 | Unannotated | NA | NA |
| metab_4646 | Unannotated feature | pos | 481.8152 | 5.740 | 4.798 | 4.490 | -0.308 | -0.714 | 6.221E-05 | 0.002 | Unannotated | NA | NA |
| metab_7252 | (10betaH,11xi)-11-Hydroxy-13-nor-6-eremophilen-8-one | neg | 443.3175 | 6.040 | 5.228 | 4.179 | -1.049 | -0.714 | 6.221E-05 | 0.002 | Tentative | Lipids and lipid-like molecules | Prenol lipids |
| metab_2812 | Indolophenanthridine | pos | 292.1808 | 5.573 | 4.540 | 5.342 | 0.802 | 0.714 | 6.221E-05 | 0.002 | Tentative | Organoheterocyclic compounds | Quinolines and derivatives |
| metab_4145 | Unannotated feature | pos | 196.0032 | 0.667 | 3.753 | 4.271 | 0.518 | 0.714 | 6.221E-05 | 0.002 | Unannotated | NA | NA |
| metab_10865 | Unannotated feature | neg | 474.8540 | 0.554 | 4.572 | 4.857 | 0.286 | 0.714 | 6.221E-05 | 0.002 | Unannotated | NA | NA |
| metab_9194 | Unannotated feature | neg | 89.4876 | 4.167 | 3.118 | 2.221 | -0.897 | -0.714 | 6.221E-05 | 0.002 | Unannotated | NA | NA |
| metab_1479 | (3S,3'S,5R,5'R,6R)-3,6-Epoxy-5,6-dihydro-3',5,8'-trihydroxy-beta,kappa-caroten-6'-one | pos | 634.4526 | 6.026 | 6.063 | 6.163 | 0.100 | 0.714 | 6.221E-05 | 0.002 | Tentative | NA | NA |
| metab_5582 | Unannotated feature | neg | 89.2550 | 0.865 | 6.043 | 5.829 | -0.213 | -0.714 | 6.221E-05 | 0.002 | Unannotated | NA | NA |
| metab_10849 | Unannotated feature | neg | 610.8290 | 0.554 | 4.844 | 5.070 | 0.226 | 0.714 | 6.221E-05 | 0.002 | Unannotated | NA | NA |
| metab_1499 | Unannotated feature | pos | 467.3735 | 6.095 | 5.274 | 4.230 | -1.043 | -0.714 | 6.221E-05 | 0.002 | Unannotated | NA | NA |
| metab_5615 | Unannotated feature | neg | 382.8125 | 0.904 | 4.501 | 4.034 | -0.467 | -0.714 | 6.221E-05 | 0.002 | Unannotated | NA | NA |
| metab_1138 | Unannotated feature | pos | 509.3480 | 5.649 | 4.746 | 4.500 | -0.246 | -0.714 | 6.221E-05 | 0.002 | Unannotated | NA | NA |
| metab_6342 | Unannotated feature | neg | 129.0550 | 3.819 | 7.076 | 6.738 | -0.338 | -0.714 | 6.221E-05 | 0.002 | Unannotated | NA | NA |
| metab_7222 | Unannotated feature | neg | 248.9025 | 5.969 | 5.724 | 5.011 | -0.713 | -0.714 | 6.221E-05 | 0.002 | Unannotated | NA | NA |
| metab_6927 | Unannotated feature | neg | 292.8185 | 0.912 | 4.339 | 4.693 | 0.354 | 0.714 | 6.221E-05 | 0.002 | Unannotated | NA | NA |
| metab_2972 | Unannotated feature | pos | 321.1080 | 4.539 | 4.438 | 3.289 | -1.150 | -0.714 | 6.221E-05 | 0.002 | Unannotated | NA | NA |
| metab_2703 | Unannotated feature | pos | 457.3238 | 5.748 | 5.306 | 5.087 | -0.219 | -0.714 | 6.221E-05 | 0.002 | Unannotated | NA | NA |
| metab_5310 | Unannotated feature | neg | 720.8009 | 0.562 | 4.753 | 5.048 | 0.295 | 0.709 | 6.875E-05 | 0.002 | Unannotated | NA | NA |
| metab_317 | Unannotated feature | pos | 254.9441 | 0.792 | 3.351 | 4.103 | 0.751 | 0.709 | 6.875E-05 | 0.002 | Unannotated | NA | NA |
| metab_373 | Unannotated feature | pos | 192.9616 | 0.892 | 4.972 | 4.824 | -0.148 | -0.709 | 6.875E-05 | 0.002 | Unannotated | NA | NA |
| metab_6098 | Unannotated feature | neg | 165.0400 | 0.644 | 4.978 | 5.415 | 0.437 | 0.709 | 6.875E-05 | 0.002 | Unannotated | NA | NA |
| metab_8182 | 4-Methylpentanal | neg | 389.2705 | 6.000 | 4.932 | 4.163 | -0.769 | -0.709 | 6.875E-05 | 0.002 | Tentative | Organic oxygen compounds | Organooxygen compounds |
| metab_10840 | Unannotated feature | neg | 678.8167 | 0.554 | 4.904 | 5.092 | 0.188 | 0.709 | 6.875E-05 | 0.002 | Unannotated | NA | NA |
| metab_3319 | Unannotated feature | pos | 201.6131 | 2.644 | 4.771 | 5.069 | 0.298 | 0.709 | 6.875E-05 | 0.002 | Unannotated | NA | NA |
| metab_4684 | Unannotated feature | pos | 436.3171 | 5.871 | 5.508 | 5.666 | 0.158 | 0.709 | 6.875E-05 | 0.002 | Unannotated | NA | NA |
| metab_5585 | Unannotated feature | neg | 98.9551 | 0.549 | 4.184 | 4.566 | 0.382 | 0.709 | 6.875E-05 | 0.002 | Unannotated | NA | NA |
| metab_21 | Unannotated feature | pos | 634.6587 | 0.583 | 5.829 | 5.728 | -0.101 | -0.709 | 6.875E-05 | 0.002 | Unannotated | NA | NA |
| metab_2239 | Unannotated feature | pos | 391.3570 | 6.249 | 5.127 | 4.062 | -1.066 | -0.709 | 6.875E-05 | 0.002 | Unannotated | NA | NA |
| metab_8213 | 27-Norcholestanehexol | neg | 453.3231 | 5.945 | 5.254 | 4.368 | -0.886 | -0.709 | 6.875E-05 | 0.002 | Tentative | Lipids and lipid-like molecules | Steroids and steroid derivatives |
| metab_2737 | Unannotated feature | pos | 430.7811 | 5.710 | 4.542 | 4.168 | -0.374 | -0.709 | 6.875E-05 | 0.002 | Unannotated | NA | NA |
| metab_3202 | Unannotated feature | pos | 83.6586 | 3.259 | 3.463 | 3.688 | 0.225 | 0.709 | 6.875E-05 | 0.002 | Unannotated | NA | NA |
| metab_9586 | Unannotated feature | neg | 257.9842 | 3.827 | 4.376 | 3.682 | -0.695 | -0.709 | 6.875E-05 | 0.002 | Unannotated | NA | NA |
| metab_1336 | Unannotated feature | pos | 460.2697 | 5.894 | 4.858 | 5.695 | 0.836 | 0.709 | 6.875E-05 | 0.002 | Unannotated | NA | NA |
| metab_3940 | ALA-ILE | pos | 203.1391 | 0.854 | 4.898 | 5.261 | 0.363 | 0.705 | 7.593E-05 | 0.002 | Moderate putative | Organic acids and derivatives | Carboxylic acids and derivatives |
| metab_1436 | Unannotated feature | pos | 165.0883 | 5.964 | 5.511 | 5.609 | 0.099 | 0.705 | 7.593E-05 | 0.002 | Unannotated | NA | NA |
| metab_1405 | Unannotated feature | pos | 772.5317 | 5.925 | 5.031 | 4.570 | -0.461 | -0.705 | 7.593E-05 | 0.002 | Unannotated | NA | NA |
| metab_2569 | Unannotated feature | pos | 504.8577 | 0.551 | 5.771 | 6.044 | 0.272 | 0.705 | 7.593E-05 | 0.002 | Unannotated | NA | NA |
| metab_3917 | Unannotated feature | pos | 215.1391 | 0.861 | 4.156 | 4.680 | 0.524 | 0.705 | 7.593E-05 | 0.002 | Unannotated | NA | NA |
| metab_6391 | Unannotated feature | neg | 79.9565 | 4.059 | 2.371 | 3.427 | 1.056 | 0.705 | 7.593E-05 | 0.002 | Unannotated | NA | NA |
| metab_310 | Unannotated feature | pos | 322.8110 | 0.776 | 4.679 | 4.532 | -0.147 | -0.705 | 7.593E-05 | 0.002 | Unannotated | NA | NA |
| metab_8037 | Unannotated feature | neg | 448.9345 | 6.522 | 5.310 | 4.136 | -1.174 | -0.705 | 7.593E-05 | 0.002 | Unannotated | NA | NA |
| metab_8220 | Unannotated feature | neg | 319.8177 | 5.930 | 6.633 | 6.511 | -0.122 | -0.705 | 7.593E-05 | 0.002 | Unannotated | NA | NA |
| metab_8199 | Unannotated feature | neg | 246.9055 | 5.969 | 6.425 | 5.834 | -0.591 | -0.705 | 7.593E-05 | 0.002 | Unannotated | NA | NA |
| metab_8169 | Alpha-Tocopheryloxyacetic acid | neg | 533.3856 | 6.024 | 4.993 | 3.876 | -1.117 | -0.705 | 7.593E-05 | 0.002 | Tentative | Lipids and lipid-like molecules | Prenol lipids |
| metab_8053 | Unannotated feature | neg | 398.9373 | 6.392 | 6.239 | 4.793 | -1.446 | -0.705 | 7.593E-05 | 0.002 | Unannotated | NA | NA |
| metab_972 | Embelin | pos | 333.1444 | 4.301 | 4.789 | 3.749 | -1.040 | -0.705 | 7.593E-05 | 0.002 | Tentative | Organic oxygen compounds | Organooxygen compounds |
| metab_7333 | Unannotated feature | neg | 89.0236 | 0.865 | 7.532 | 7.373 | -0.159 | -0.705 | 7.593E-05 | 0.002 | Unannotated | NA | NA |
| metab_2262 | 13(S)-HOT | pos | 277.2163 | 6.188 | 5.371 | 4.869 | -0.502 | -0.705 | 7.593E-05 | 0.002 | Tentative | Lipids and lipid-like molecules | Fatty Acyls |
| metab_1766 | Unannotated feature | pos | 216.0682 | 7.339 | 4.891 | 5.115 | 0.224 | 0.705 | 7.593E-05 | 0.002 | Unannotated | NA | NA |
| metab_3667 | Unannotated feature | pos | 186.0762 | 1.949 | 3.727 | 3.989 | 0.261 | 0.705 | 7.593E-05 | 0.002 | Unannotated | NA | NA |
| metab_3648 | 1-Phenylbiguanide | pos | 160.0969 | 1.956 | 4.571 | 5.278 | 0.707 | 0.705 | 7.593E-05 | 0.002 | Tentative | Organic nitrogen compounds | Organonitrogen compounds |
| metab_10176 | Unannotated feature | neg | 320.8596 | 0.904 | 4.418 | 4.126 | -0.292 | -0.701 | 8.382E-05 | 0.003 | Unannotated | NA | NA |
| metab_11068 | Unannotated feature | neg | 317.1148 | 5.563 | 5.274 | 4.686 | -0.589 | -0.701 | 8.382E-05 | 0.003 | Unannotated | NA | NA |
| metab_9285 | 3-Methylbutanoic acid | neg | 101.0600 | 3.819 | 4.652 | 4.278 | -0.374 | -0.701 | 8.382E-05 | 0.003 | Tentative | NA | NA |
| metab_10162 | Unannotated feature | neg | 276.8332 | 0.912 | 5.082 | 4.997 | -0.084 | -0.701 | 8.382E-05 | 0.003 | Unannotated | NA | NA |
| metab_3330 | Unannotated feature | pos | 153.5172 | 2.637 | 5.958 | 6.044 | 0.086 | 0.701 | 8.382E-05 | 0.003 | Unannotated | NA | NA |
| metab_8142 | Soyasapogenol D | neg | 517.3907 | 6.110 | 5.998 | 5.439 | -0.559 | -0.701 | 8.382E-05 | 0.003 | Tentative | Lipids and lipid-like molecules | Prenol lipids |
| metab_4172 | Unannotated feature | pos | 162.6815 | 0.644 | 5.168 | 4.957 | -0.210 | -0.701 | 8.382E-05 | 0.003 | Unannotated | NA | NA |
| metab_3670 | Unannotated feature | pos | 133.5444 | 1.949 | 3.918 | 3.614 | -0.304 | -0.701 | 8.382E-05 | 0.003 | Unannotated | NA | NA |
| metab_1679 | Unannotated feature | pos | 377.2466 | 7.261 | 4.933 | 5.098 | 0.165 | 0.701 | 8.382E-05 | 0.003 | Unannotated | NA | NA |
| metab_5276 | Unannotated feature | neg | 806.8051 | 0.554 | 4.515 | 4.903 | 0.387 | 0.701 | 8.382E-05 | 0.003 | Unannotated | NA | NA |
| metab_5592 | Unannotated feature | neg | 89.0082 | 0.873 | 4.933 | 4.699 | -0.234 | -0.701 | 8.382E-05 | 0.003 | Unannotated | NA | NA |
| metab_4487 | Unannotated feature | pos | 572.8448 | 0.551 | 5.519 | 5.807 | 0.288 | 0.701 | 8.382E-05 | 0.003 | Unannotated | NA | NA |
| metab_3902 | Unannotated feature | pos | 314.0845 | 0.861 | 3.714 | 4.277 | 0.563 | 0.701 | 8.382E-05 | 0.003 | Unannotated | NA | NA |
| metab_2885 | Sobetirome | pos | 351.1550 | 5.107 | 5.945 | 5.453 | -0.493 | -0.701 | 8.382E-05 | 0.003 | Tentative | Benzenoids | Benzene and substituted derivatives |
| metab_5597 | Unannotated feature | neg | 160.0611 | 0.873 | 3.904 | 4.644 | 0.741 | 0.701 | 8.382E-05 | 0.003 | Unannotated | NA | NA |
| metab_1231 | Unannotated feature | pos | 603.4181 | 5.809 | 5.532 | 5.320 | -0.212 | -0.701 | 8.382E-05 | 0.003 | Unannotated | NA | NA |
| metab_2316 | Unannotated feature | pos | 546.3999 | 6.034 | 6.022 | 6.126 | 0.103 | 0.701 | 8.382E-05 | 0.003 | Unannotated | NA | NA |
| metab_9939 | Unannotated feature | neg | 157.0767 | 2.119 | 3.559 | 3.908 | 0.349 | 0.701 | 8.382E-05 | 0.003 | Unannotated | NA | NA |
| metab_6631 | Unannotated feature | neg | 204.8696 | 4.977 | 3.337 | 3.571 | 0.234 | 0.701 | 8.382E-05 | 0.003 | Unannotated | NA | NA |
| metab_8697 | Unannotated feature | neg | 243.0778 | 5.611 | 2.641 | 3.498 | 0.856 | 0.701 | 8.382E-05 | 0.003 | Unannotated | NA | NA |
| metab_6893 | Unannotated feature | neg | 277.7706 | 5.651 | 4.218 | 4.055 | -0.163 | -0.701 | 8.382E-05 | 0.003 | Unannotated | NA | NA |
| metab_1342 | Unannotated feature | pos | 685.1423 | 5.894 | 5.233 | 4.836 | -0.396 | -0.701 | 8.382E-05 | 0.003 | Unannotated | NA | NA |
| metab_7298 | alpha-Cedrol | neg | 503.4114 | 6.188 | 5.438 | 4.827 | -0.611 | -0.701 | 8.382E-05 | 0.003 | Tentative | Lipids and lipid-like molecules | Prenol lipids |
| metab_9721 | Unannotated feature | neg | 233.9469 | 2.804 | 5.497 | 5.389 | -0.108 | -0.701 | 8.382E-05 | 0.003 | Unannotated | NA | NA |
| metab_5001 | Salicylamide glucuronide | pos | 314.0845 | 0.776 | 4.360 | 4.966 | 0.606 | 0.697 | 9.249E-05 | 0.003 | Tentative | Organic oxygen compounds | Organooxygen compounds |
| metab_1892 | Unannotated feature | pos | 330.9156 | 7.331 | 4.873 | 5.096 | 0.223 | 0.697 | 9.249E-05 | 0.003 | Unannotated | NA | NA |
| metab_4771 | Unannotated feature | pos | 300.8953 | 0.551 | 5.376 | 5.761 | 0.385 | 0.697 | 9.249E-05 | 0.003 | Unannotated | NA | NA |
| metab_4079 | Ecgonine | pos | 186.1125 | 0.737 | 4.679 | 3.984 | -0.695 | -0.697 | 9.249E-05 | 0.003 | Tentative | Alkaloids and derivatives | Tropane alkaloids |
| metab_686 | Unannotated feature | pos | 180.9899 | 2.475 | 5.187 | 5.143 | -0.044 | -0.697 | 9.249E-05 | 0.003 | Unannotated | NA | NA |
| metab_999 | Unannotated feature | pos | 474.3272 | 4.508 | 5.187 | 5.035 | -0.152 | -0.697 | 9.249E-05 | 0.003 | Unannotated | NA | NA |
| metab_2234 | Unannotated feature | pos | 102.1249 | 6.265 | 5.419 | 5.472 | 0.053 | 0.697 | 9.249E-05 | 0.003 | Unannotated | NA | NA |
| metab_2925 | Unannotated feature | pos | 444.3166 | 4.869 | 5.498 | 5.309 | -0.189 | -0.697 | 9.249E-05 | 0.003 | Unannotated | NA | NA |
| metab_4984 | Unannotated feature | pos | 173.0421 | 0.721 | 4.800 | 5.512 | 0.712 | 0.697 | 9.249E-05 | 0.003 | Unannotated | NA | NA |
| metab_1815 | Unannotated feature | pos | 543.8599 | 0.551 | 4.674 | 5.015 | 0.341 | 0.697 | 9.249E-05 | 0.003 | Unannotated | NA | NA |
| metab_5889 | Unannotated feature | neg | 154.9981 | 2.504 | 2.374 | 3.707 | 1.333 | 0.697 | 9.249E-05 | 0.003 | Unannotated | NA | NA |
| metab_835 | Unannotated feature | pos | 182.9852 | 7.331 | 7.044 | 7.136 | 0.091 | 0.697 | 9.249E-05 | 0.003 | Unannotated | NA | NA |
| metab_6344 | Unannotated feature | neg | 171.9465 | 3.819 | 4.195 | 4.050 | -0.145 | -0.697 | 9.249E-05 | 0.003 | Unannotated | NA | NA |
| metab_6347 | Unannotated feature | neg | 259.0141 | 3.827 | 4.918 | 4.634 | -0.284 | -0.697 | 9.249E-05 | 0.003 | Unannotated | NA | NA |
| metab_3888 | Chlorocitric acid | pos | 270.9581 | 0.869 | 5.498 | 5.418 | -0.080 | -0.697 | 9.249E-05 | 0.003 | Tentative | NA | NA |
| metab_6997 | Unannotated feature | neg | 284.9358 | 0.881 | 5.055 | 4.949 | -0.106 | -0.697 | 9.249E-05 | 0.003 | Unannotated | NA | NA |
| metab_11157 | Unannotated feature | neg | 151.0065 | 0.786 | 4.413 | 4.944 | 0.531 | 0.697 | 9.249E-05 | 0.003 | Unannotated | NA | NA |
| metab_5240 | Unannotated feature | neg | 492.8740 | 0.554 | 4.522 | 5.079 | 0.557 | 0.697 | 9.249E-05 | 0.003 | Unannotated | NA | NA |
| metab_5565 | Unannotated feature | neg | 196.0283 | 0.849 | 0.023 | 3.746 | 3.723 | 0.697 | 9.249E-05 | 0.003 | Unannotated | NA | NA |
| metab_8976 | Unannotated feature | neg | 255.1354 | 4.845 | 3.171 | 4.249 | 1.078 | 0.697 | 9.249E-05 | 0.003 | Unannotated | NA | NA |
| metab_9576 | Unannotated feature | neg | 271.9317 | 3.819 | 4.362 | 3.598 | -0.764 | -0.693 | 1.020E-04 | 0.003 | Unannotated | NA | NA |
| metab_3937 | Unannotated feature | pos | 247.1290 | 0.854 | 4.894 | 4.742 | -0.152 | -0.693 | 1.020E-04 | 0.003 | Unannotated | NA | NA |
| metab_2246 | Unannotated feature | pos | 415.3572 | 6.211 | 4.734 | 3.571 | -1.163 | -0.693 | 1.020E-04 | 0.003 | Unannotated | NA | NA |
| metab_4806 | Unannotated feature | pos | 165.9829 | 7.347 | 4.877 | 5.039 | 0.162 | 0.693 | 1.020E-04 | 0.003 | Unannotated | NA | NA |
| metab_2270 | Dehydroeburicoic acid | pos | 501.3947 | 6.157 | 5.321 | 4.425 | -0.897 | -0.693 | 1.020E-04 | 0.003 | Tentative | Lipids and lipid-like molecules | Steroids and steroid derivatives |
| metab_4645 | Unannotated feature | pos | 683.2556 | 5.717 | 5.995 | 5.393 | -0.602 | -0.693 | 1.020E-04 | 0.003 | Unannotated | NA | NA |
| metab_731 | Unannotated feature | pos | 117.7133 | 2.629 | 4.373 | 4.544 | 0.171 | 0.693 | 1.020E-04 | 0.003 | Unannotated | NA | NA |
| metab_729 | Unannotated feature | pos | 469.3888 | 6.203 | 5.905 | 5.049 | -0.855 | -0.693 | 1.020E-04 | 0.003 | Unannotated | NA | NA |
| metab_5494 | Unannotated feature | neg | 149.0450 | 0.731 | 4.447 | 5.403 | 0.955 | 0.693 | 1.020E-04 | 0.003 | Unannotated | NA | NA |
| metab_11218 | Unannotated feature | neg | 323.8117 | 5.937 | 6.135 | 5.990 | -0.145 | -0.693 | 1.020E-04 | 0.003 | Unannotated | NA | NA |
| metab_1908 | Unannotated feature | pos | 334.9113 | 7.324 | 4.679 | 4.778 | 0.099 | 0.693 | 1.020E-04 | 0.003 | Unannotated | NA | NA |
| metab_1872 | Unannotated feature | pos | 196.0168 | 7.409 | 5.296 | 5.426 | 0.130 | 0.693 | 1.020E-04 | 0.003 | Unannotated | NA | NA |
| metab_7297 | Unannotated feature | neg | 437.3644 | 6.188 | 4.541 | 3.842 | -0.699 | -0.693 | 1.020E-04 | 0.003 | Unannotated | NA | NA |
| metab_85 | Unannotated feature | pos | 219.0063 | 0.473 | 4.646 | 4.571 | -0.075 | -0.693 | 1.020E-04 | 0.003 | Unannotated | NA | NA |
| metab_4493 | Unannotated feature | pos | 436.8704 | 0.551 | 5.470 | 5.736 | 0.266 | 0.693 | 1.020E-04 | 0.003 | Unannotated | NA | NA |
| metab_7229 | Varanic acid | neg | 435.3124 | 5.993 | 5.420 | 4.290 | -1.130 | -0.693 | 1.020E-04 | 0.003 | Tentative | Lipids and lipid-like molecules | Steroids and steroid derivatives |
| metab_6945 | Unannotated feature | neg | 614.6848 | 0.578 | 4.616 | 4.759 | 0.143 | 0.693 | 1.020E-04 | 0.003 | Unannotated | NA | NA |
| metab_7022 | Unannotated feature | neg | 647.2374 | 5.739 | 4.172 | 3.283 | -0.889 | -0.693 | 1.020E-04 | 0.003 | Unannotated | NA | NA |
| metab_10628 | Unannotated feature | neg | 482.6852 | 0.594 | 5.435 | 5.346 | -0.089 | -0.693 | 1.020E-04 | 0.003 | Unannotated | NA | NA |
| metab_4094 | Unannotated feature | pos | 400.0234 | 0.721 | 4.479 | 4.204 | -0.275 | -0.693 | 1.020E-04 | 0.003 | Unannotated | NA | NA |
| metab_787 | Estriol | pos | 333.1444 | 2.950 | 5.731 | 5.338 | -0.393 | -0.693 | 1.020E-04 | 0.003 | Tentative | Lipids and lipid-like molecules | Steroids and steroid derivatives |
| metab_1242 | 2-Hydroxyestrone-4-S-glutathione | pos | 633.2555 | 5.824 | 6.659 | 6.280 | -0.380 | -0.693 | 1.020E-04 | 0.003 | Tentative | Organic acids and derivatives | Carboxylic acids and derivatives |
| metab_5714 | Unannotated feature | neg | 736.7873 | 0.554 | 5.247 | 5.719 | 0.472 | 0.693 | 1.020E-04 | 0.003 | Unannotated | NA | NA |
| metab_5725 | Unannotated feature | neg | 186.9291 | 0.554 | 5.479 | 5.834 | 0.356 | 0.693 | 1.020E-04 | 0.003 | Unannotated | NA | NA |
| metab_2280 | Unannotated feature | pos | 493.3887 | 6.126 | 5.025 | 3.776 | -1.248 | -0.693 | 1.020E-04 | 0.003 | Unannotated | NA | NA |
| metab_5238 | Unannotated feature | neg | 464.8365 | 0.554 | 4.791 | 5.445 | 0.654 | 0.693 | 1.020E-04 | 0.003 | Unannotated | NA | NA |
| metab_8150 | Unannotated feature | neg | 207.0040 | 0.731 | 4.006 | 5.162 | 1.156 | 0.693 | 1.020E-04 | 0.003 | Unannotated | NA | NA |
| metab_138 | Unannotated feature | pos | 640.8319 | 0.551 | 5.524 | 5.787 | 0.263 | 0.688 | 1.124E-04 | 0.003 | Unannotated | NA | NA |
| metab_1864 | Unannotated feature | pos | 119.0256 | 7.525 | 3.683 | 3.887 | 0.204 | 0.688 | 1.124E-04 | 0.003 | Unannotated | NA | NA |
| metab_5682 | Unannotated feature | neg | 96.9593 | 0.554 | 5.575 | 5.934 | 0.360 | 0.688 | 1.124E-04 | 0.003 | Unannotated | NA | NA |
| metab_2651 | Pseudomonine | pos | 683.2555 | 5.801 | 5.844 | 5.179 | -0.666 | -0.688 | 1.124E-04 | 0.003 | Tentative | Organic acids and derivatives | Carboxylic acids and derivatives |
| metab_3968 | Dihydrolipoate | pos | 231.0476 | 0.839 | 4.406 | 3.948 | -0.458 | -0.688 | 1.124E-04 | 0.003 | Tentative | Lipids and lipid-like molecules | Fatty Acyls |
| metab_5039 | Unannotated feature | pos | 234.9815 | 0.861 | 5.322 | 5.225 | -0.097 | -0.688 | 1.124E-04 | 0.003 | Unannotated | NA | NA |
| metab_8165 | 1-(cis-13-Docosenoyl)-rac-glycerol | neg | 457.3543 | 6.032 | 4.811 | 4.066 | -0.745 | -0.688 | 1.124E-04 | 0.003 | Tentative | Lipids and lipid-like molecules | Glycerolipids |
| metab_1893 | Unannotated feature | pos | 238.0636 | 7.331 | 4.742 | 4.970 | 0.229 | 0.688 | 1.124E-04 | 0.003 | Unannotated | NA | NA |
| metab_1897 | Unannotated feature | pos | 197.0372 | 7.331 | 6.391 | 6.488 | 0.097 | 0.688 | 1.124E-04 | 0.003 | Unannotated | NA | NA |
| metab_4853 | Unannotated feature | pos | 171.1175 | 6.041 | 5.027 | 4.774 | -0.253 | -0.688 | 1.124E-04 | 0.003 | Unannotated | NA | NA |
| metab_4805 | Unannotated feature | pos | 200.9957 | 7.339 | 6.089 | 6.181 | 0.093 | 0.688 | 1.124E-04 | 0.003 | Unannotated | NA | NA |
| metab_191 | Unannotated feature | pos | 615.8186 | 0.567 | 4.577 | 4.474 | -0.103 | -0.688 | 1.124E-04 | 0.003 | Unannotated | NA | NA |
| metab_10872 | Unannotated feature | neg | 271.9075 | 0.554 | 3.410 | 3.929 | 0.519 | 0.688 | 1.124E-04 | 0.003 | Unannotated | NA | NA |
| metab_10792 | Unannotated feature | neg | 340.0655 | 5.255 | 4.393 | 3.026 | -1.367 | -0.688 | 1.124E-04 | 0.003 | Unannotated | NA | NA |
| metab_5289 | Unannotated feature | neg | 971.7646 | 0.554 | 2.173 | 4.291 | 2.118 | 0.688 | 1.124E-04 | 0.003 | Unannotated | NA | NA |
| metab_3798 | V-PYRRO/NO | pos | 158.0927 | 1.515 | 3.950 | 4.594 | 0.644 | 0.688 | 1.124E-04 | 0.003 | Tentative | Organoheterocyclic compounds | Pyrrolidines |
| metab_4425 | Unannotated feature | pos | 632.8366 | 0.559 | 4.275 | 4.536 | 0.261 | 0.688 | 1.124E-04 | 0.003 | Unannotated | NA | NA |
| metab_1534 | Unannotated feature | pos | 405.3726 | 6.242 | 5.248 | 4.483 | -0.765 | -0.688 | 1.124E-04 | 0.003 | Unannotated | NA | NA |
| metab_2554 | Unannotated feature | pos | 763.5276 | 5.863 | 5.856 | 5.717 | -0.140 | -0.688 | 1.124E-04 | 0.003 | Unannotated | NA | NA |
| metab_473 | L-Hydroxylysine | pos | 204.1343 | 1.801 | 3.639 | 5.111 | 1.472 | 0.688 | 1.124E-04 | 0.003 | Tentative | Organic acids and derivatives | Carboxylic acids and derivatives |
| metab_11213 | Unannotated feature | neg | 263.7727 | 5.922 | 5.990 | 5.844 | -0.146 | -0.688 | 1.124E-04 | 0.003 | Unannotated | NA | NA |
| metab_11225 | Unannotated feature | neg | 363.2549 | 5.985 | 4.779 | 3.961 | -0.818 | -0.688 | 1.124E-04 | 0.003 | Unannotated | NA | NA |
| metab_3007 | Unannotated feature | pos | 430.3466 | 5.949 | 6.981 | 7.124 | 0.143 | 0.688 | 1.124E-04 | 0.003 | Unannotated | NA | NA |
| metab_6678 | Camonagrel | neg | 317.1148 | 5.184 | 5.377 | 4.858 | -0.519 | -0.688 | 1.124E-04 | 0.003 | Tentative | Benzenoids | Indanes |
| metab_10875 | Unannotated feature | neg | 164.9471 | 0.554 | 5.229 | 5.545 | 0.317 | 0.684 | 1.238E-04 | 0.003 | Unannotated | NA | NA |
| metab_131 | Unannotated feature | pos | 577.8533 | 0.551 | 4.775 | 5.022 | 0.247 | 0.684 | 1.238E-04 | 0.003 | Unannotated | NA | NA |
| metab_8173 | 3alpha,7alpha,12alpha-Trihydroxy-5beta-cholestanoate | neg | 449.3275 | 6.016 | 4.829 | 2.933 | -1.895 | -0.684 | 1.238E-04 | 0.003 | Tentative | Lipids and lipid-like molecules | Steroids and steroid derivatives |
| metab_9856 | Unannotated feature | neg | 292.1408 | 2.445 | 4.528 | 4.016 | -0.512 | -0.684 | 1.238E-04 | 0.003 | Unannotated | NA | NA |
| metab_3416 | Unannotated feature | pos | 202.0162 | 2.420 | 5.573 | 5.545 | -0.029 | -0.684 | 1.238E-04 | 0.003 | Unannotated | NA | NA |
| metab_7176 | Bile salt | neg | 407.2811 | 5.898 | 5.285 | 4.432 | -0.853 | -0.684 | 1.238E-04 | 0.003 | Tentative | Lipids and lipid-like molecules | Steroids and steroid derivatives |
| metab_9363 | Unannotated feature | neg | 299.9266 | 3.562 | 5.236 | 5.071 | -0.165 | -0.684 | 1.238E-04 | 0.003 | Unannotated | NA | NA |
| metab_2803 | 2-(5'-Methylthio)pentylmalic acid | pos | 514.1739 | 5.588 | 4.799 | 4.208 | -0.591 | -0.684 | 1.238E-04 | 0.003 | Tentative | Organic acids and derivatives | Hydroxy acids and derivatives |
| metab_8866 | Unannotated feature | neg | 343.0864 | 5.255 | 3.004 | 3.832 | 0.828 | 0.684 | 1.238E-04 | 0.003 | Unannotated | NA | NA |
| metab_9931 | Unannotated feature | neg | 192.9359 | 2.119 | 3.624 | 2.530 | -1.095 | -0.684 | 1.238E-04 | 0.003 | Unannotated | NA | NA |
| metab_6726 | Unannotated feature | neg | 136.0761 | 5.377 | 3.567 | 2.849 | -0.717 | -0.684 | 1.238E-04 | 0.003 | Unannotated | NA | NA |
| metab_1806 | Unannotated feature | pos | 113.9640 | 7.952 | 5.144 | 5.201 | 0.057 | 0.684 | 1.238E-04 | 0.003 | Unannotated | NA | NA |
| metab_4308 | 1-Hydroxy-2,2,5,5-tetramethylpyrrolidine-3-carboxylic acid | pos | 205.1548 | 0.583 | 3.726 | 4.469 | 0.743 | 0.684 | 1.238E-04 | 0.003 | Tentative | Organoheterocyclic compounds | Pyrrolidines |
| metab_2871 | Indole-3-acetyl-beta-1-D-glucoside | pos | 302.1022 | 5.160 | 5.447 | 4.873 | -0.574 | -0.684 | 1.238E-04 | 0.003 | Tentative | Organoheterocyclic compounds | Indoles and derivatives |
| metab_4883 | Unannotated feature | pos | 290.1597 | 2.058 | 4.914 | 5.718 | 0.804 | 0.684 | 1.238E-04 | 0.003 | Unannotated | NA | NA |
| metab_24 | Unannotated feature | pos | 175.0036 | 0.784 | 4.836 | 5.287 | 0.452 | 0.684 | 1.238E-04 | 0.003 | Unannotated | NA | NA |
| metab_11240 | Unannotated feature | neg | 163.0607 | 0.865 | 3.630 | 4.672 | 1.042 | 0.684 | 1.238E-04 | 0.003 | Unannotated | NA | NA |
| metab_6864 | Unannotated feature | neg | 206.9386 | 0.896 | 5.586 | 5.413 | -0.173 | -0.684 | 1.238E-04 | 0.003 | Unannotated | NA | NA |
| metab_63 | Unannotated feature | pos | 102.3761 | 0.108 | 4.333 | 4.411 | 0.077 | 0.684 | 1.238E-04 | 0.003 | Unannotated | NA | NA |
| metab_7521 | Unannotated feature | neg | 378.2423 | 7.164 | 4.616 | 4.437 | -0.179 | -0.680 | 1.363E-04 | 0.003 | Unannotated | NA | NA |
| metab_9076 | Unannotated feature | neg | 99.0444 | 3.282 | 3.961 | 4.564 | 0.603 | 0.680 | 1.363E-04 | 0.003 | Unannotated | NA | NA |
| metab_755 | Unannotated feature | pos | 204.9540 | 7.261 | 5.238 | 5.386 | 0.148 | 0.680 | 1.363E-04 | 0.003 | Unannotated | NA | NA |
| metab_2903 | D-4'-Phosphopantothenate | pos | 341.1108 | 4.969 | 5.060 | 4.423 | -0.636 | -0.680 | 1.363E-04 | 0.003 | Tentative | Organic acids and derivatives | Carboxylic acids and derivatives |
| metab_1194 | Endomorphin-1 | pos | 649.2498 | 5.748 | 5.257 | 4.176 | -1.081 | -0.680 | 1.363E-04 | 0.003 | Tentative | Organic acids and derivatives | Carboxylic acids and derivatives |
| metab_7091 | Ichangin 4-glucoside | neg | 631.2425 | 5.803 | 5.915 | 5.375 | -0.541 | -0.680 | 1.363E-04 | 0.003 | Tentative | Lipids and lipid-like molecules | Prenol lipids |
| metab_1758 | Unannotated feature | pos | 98.5124 | 7.339 | 4.897 | 5.071 | 0.174 | 0.680 | 1.363E-04 | 0.003 | Unannotated | NA | NA |
| metab_1719 | Unannotated feature | pos | 144.9822 | 7.300 | 5.269 | 5.337 | 0.067 | 0.680 | 1.363E-04 | 0.003 | Unannotated | NA | NA |
| metab_9610 | 3-(3,5-dihydroxyphenyl)-1-propanoic acid sulphate | neg | 261.0079 | 3.144 | 4.265 | 5.265 | 1.000 | 0.680 | 1.363E-04 | 0.003 | Tentative | Benzenoids | Phenols |
| metab_6349 | Unannotated feature | neg | 336.9745 | 3.827 | 5.110 | 4.779 | -0.331 | -0.680 | 1.363E-04 | 0.003 | Unannotated | NA | NA |
| metab_8460 | Unannotated feature | neg | 663.2325 | 5.747 | 4.339 | 3.396 | -0.943 | -0.680 | 1.363E-04 | 0.003 | Unannotated | NA | NA |
| metab_9667 | Unannotated feature | neg | 246.9921 | 2.961 | 3.629 | 5.010 | 1.381 | 0.680 | 1.363E-04 | 0.003 | Unannotated | NA | NA |
| metab_7209 | Unannotated feature | neg | 321.8146 | 5.930 | 6.634 | 6.511 | -0.123 | -0.680 | 1.363E-04 | 0.003 | Unannotated | NA | NA |
| metab_2831 | Unannotated feature | pos | 546.3845 | 5.474 | 5.449 | 5.264 | -0.186 | -0.680 | 1.363E-04 | 0.003 | Unannotated | NA | NA |
| metab_1175 | Unannotated feature | pos | 567.3898 | 5.725 | 5.143 | 4.845 | -0.299 | -0.680 | 1.363E-04 | 0.003 | Unannotated | NA | NA |
| metab_1853 | Unannotated feature | pos | 84.5070 | 7.726 | 3.189 | 3.507 | 0.318 | 0.680 | 1.363E-04 | 0.003 | Unannotated | NA | NA |
| metab_7057 | Ancymidol | neg | 255.1143 | 5.779 | 5.474 | 4.781 | -0.693 | -0.680 | 1.363E-04 | 0.003 | Tentative | Benzenoids | Phenol ethers |
| metab_3012 | Hpmpa | pos | 321.1080 | 4.286 | 4.484 | 3.453 | -1.030 | -0.680 | 1.363E-04 | 0.003 | Tentative | Organoheterocyclic compounds | Imidazopyrimidines |
| metab_1887 | Unannotated feature | pos | 137.0001 | 7.339 | 4.154 | 4.422 | 0.268 | 0.680 | 1.363E-04 | 0.003 | Unannotated | NA | NA |
| metab_11103 | Unannotated feature | neg | 455.9960 | 5.659 | 4.779 | 4.042 | -0.737 | -0.676 | 1.500E-04 | 0.003 | Unannotated | NA | NA |
| metab_5249 | Unannotated feature | neg | 600.8119 | 0.554 | 4.860 | 5.485 | 0.625 | 0.676 | 1.500E-04 | 0.003 | Unannotated | NA | NA |
| metab_10909 | Unannotated feature | neg | 273.1250 | 5.406 | 5.537 | 5.018 | -0.519 | -0.676 | 1.500E-04 | 0.003 | Unannotated | NA | NA |
| metab_2509 | Unannotated feature | pos | 821.5676 | 5.886 | 5.556 | 5.333 | -0.223 | -0.676 | 1.500E-04 | 0.003 | Unannotated | NA | NA |
| metab_7206 | Unannotated feature | neg | 265.7698 | 5.930 | 5.328 | 5.187 | -0.141 | -0.676 | 1.500E-04 | 0.003 | Unannotated | NA | NA |
| metab_7217 | Unannotated feature | neg | 463.3075 | 5.945 | 4.333 | 3.836 | -0.497 | -0.676 | 1.500E-04 | 0.003 | Unannotated | NA | NA |
| metab_3232 | Beta-D-Glucopyranosylurea | pos | 486.2023 | 3.136 | 4.692 | 3.578 | -1.114 | -0.676 | 1.500E-04 | 0.003 | Tentative | Organic oxygen compounds | Organooxygen compounds |
| metab_11234 | Unannotated feature | neg | 417.3019 | 6.024 | 5.300 | 4.167 | -1.133 | -0.676 | 1.500E-04 | 0.003 | Unannotated | NA | NA |
| metab_8828 | Dihydrophaseic acid | neg | 317.1149 | 5.377 | 5.657 | 5.160 | -0.497 | -0.676 | 1.500E-04 | 0.003 | Tentative | Lipids and lipid-like molecules | Prenol lipids |
| metab_1895 | Unannotated feature | pos | 243.0426 | 7.331 | 4.851 | 5.033 | 0.182 | 0.676 | 1.500E-04 | 0.003 | Unannotated | NA | NA |
| metab_9331 | Unannotated feature | neg | 197.0432 | 3.554 | 4.996 | 4.823 | -0.173 | -0.676 | 1.500E-04 | 0.003 | Unannotated | NA | NA |
| metab_4495 | Unannotated feature | pos | 316.8694 | 0.551 | 3.929 | 4.398 | 0.469 | 0.676 | 1.500E-04 | 0.003 | Unannotated | NA | NA |
| metab_11076 | 2-(p-Acetamidophenyl)-2-ethylglutarimide | neg | 255.1143 | 5.563 | 5.425 | 5.013 | -0.412 | -0.676 | 1.500E-04 | 0.003 | Tentative | Organoheterocyclic compounds | Piperidines |
| metab_8188 | 5alpha-Pregnane-3alpha,20alpha-diol | neg | 365.2704 | 5.993 | 5.499 | 5.114 | -0.385 | -0.676 | 1.500E-04 | 0.003 | Tentative | Lipids and lipid-like molecules | Steroids and steroid derivatives |
| metab_10842 | Unannotated feature | neg | 670.8276 | 0.554 | 4.846 | 5.143 | 0.297 | 0.676 | 1.500E-04 | 0.003 | Unannotated | NA | NA |
| metab_490 | Lysyl-Glycine | pos | 245.1608 | 1.871 | 3.410 | 4.635 | 1.224 | 0.676 | 1.500E-04 | 0.003 | Tentative | Organic acids and derivatives | Carboxylic acids and derivatives |
| metab_749 | Fagomine | pos | 333.1443 | 2.726 | 4.985 | 4.229 | -0.756 | -0.676 | 1.500E-04 | 0.003 | Tentative | Organoheterocyclic compounds | Piperidines |
| metab_379 | Ribothymidine | pos | 259.0925 | 0.815 | 4.634 | 5.128 | 0.494 | 0.676 | 1.500E-04 | 0.003 | Tentative | Nucleosides, nucleotides, and analogues | Pyrimidine nucleosides |
| metab_4964 | N-Amidino-L-aspartate | pos | 176.0667 | 0.667 | 3.170 | 4.719 | 1.549 | 0.676 | 1.500E-04 | 0.003 | Moderate putative | Organic acids and derivatives | Carboxylic acids and derivatives |
| metab_2478 | Harmaline | pos | 446.2537 | 5.902 | 5.895 | 6.187 | 0.291 | 0.676 | 1.500E-04 | 0.003 | Tentative | Alkaloids and derivatives | Harmala alkaloids |
| metab_1614 | Unannotated feature | pos | 102.6984 | 6.991 | 3.678 | 3.858 | 0.180 | 0.676 | 1.500E-04 | 0.003 | Unannotated | NA | NA |
| metab_716 | Unannotated feature | pos | 144.0445 | 2.583 | 4.412 | 4.180 | -0.232 | -0.676 | 1.500E-04 | 0.003 | Unannotated | NA | NA |
| metab_10353 | Unannotated feature | neg | 296.8123 | 0.747 | 4.233 | 4.467 | 0.234 | 0.672 | 1.650E-04 | 0.003 | Unannotated | NA | NA |
| metab_2829 | Pinolidoxin | pos | 377.1343 | 5.489 | 4.917 | 4.435 | -0.482 | -0.672 | 1.650E-04 | 0.003 | Tentative | Organoheterocyclic compounds | Oxocins |
| metab_1884 | Unannotated feature | pos | 195.0419 | 7.339 | 5.065 | 5.194 | 0.129 | 0.672 | 1.650E-04 | 0.003 | Unannotated | NA | NA |
| metab_1788 | Unannotated feature | pos | 111.5412 | 7.556 | 3.894 | 3.717 | -0.177 | -0.672 | 1.650E-04 | 0.003 | Unannotated | NA | NA |
| metab_10829 | Unannotated feature | neg | 773.3164 | 0.554 | 3.819 | 4.343 | 0.524 | 0.672 | 1.650E-04 | 0.003 | Unannotated | NA | NA |
| metab_9712 | Alpha-Muramic acid | neg | 232.0828 | 2.852 | 2.942 | 3.894 | 0.952 | 0.672 | 1.650E-04 | 0.003 | Tentative | Organic oxygen compounds | Organooxygen compounds |
| metab_10151 | Unannotated feature | neg | 176.9003 | 0.920 | 3.671 | 4.320 | 0.649 | 0.672 | 1.650E-04 | 0.003 | Unannotated | NA | NA |
| metab_4358 | Fluvoxamino acid | pos | 301.1183 | 4.961 | 5.957 | 5.467 | -0.490 | -0.672 | 1.650E-04 | 0.003 | Tentative | Benzenoids | Benzene and substituted derivatives |
| metab_2536 | Unannotated feature | pos | 741.5154 | 5.871 | 5.935 | 5.824 | -0.111 | -0.672 | 1.650E-04 | 0.003 | Unannotated | NA | NA |
| metab_2257 | Pachymic acid | pos | 561.4133 | 6.188 | 5.094 | 3.825 | -1.268 | -0.672 | 1.650E-04 | 0.003 | Tentative | Lipids and lipid-like molecules | Prenol lipids |
| metab_8170 | 3-Methyl-5-pentyl-2-furantridecanoic acid | neg | 409.2966 | 6.024 | 4.774 | 3.689 | -1.085 | -0.672 | 1.650E-04 | 0.003 | Tentative | Lipids and lipid-like molecules | Fatty Acyls |
| metab_1402 | Unannotated feature | pos | 692.4788 | 5.925 | 4.951 | 4.647 | -0.304 | -0.672 | 1.650E-04 | 0.003 | Unannotated | NA | NA |
| metab_9635 | Unannotated feature | neg | 326.9457 | 3.819 | 5.129 | 4.842 | -0.288 | -0.672 | 1.650E-04 | 0.003 | Unannotated | NA | NA |
| metab_5243 | Unannotated feature | neg | 560.8616 | 0.554 | 4.956 | 5.437 | 0.481 | 0.672 | 1.650E-04 | 0.003 | Unannotated | NA | NA |
| metab_4802 | Unannotated feature | pos | 312.9428 | 7.331 | 5.155 | 5.315 | 0.160 | 0.672 | 1.650E-04 | 0.003 | Unannotated | NA | NA |
| metab_518 | Cinnamamide | pos | 130.0653 | 1.941 | 3.909 | 4.249 | 0.339 | 0.672 | 1.650E-04 | 0.003 | Tentative | Phenylpropanoids and polyketides | Cinnamic acids and derivatives |
| metab_1442 | Unannotated feature | pos | 151.0728 | 5.972 | 4.464 | 4.573 | 0.109 | 0.672 | 1.650E-04 | 0.003 | Unannotated | NA | NA |
| metab_129 | Unannotated feature | pos | 569.8663 | 0.551 | 5.433 | 5.630 | 0.197 | 0.672 | 1.650E-04 | 0.003 | Unannotated | NA | NA |
| metab_5693 | Unannotated feature | neg | 188.9250 | 0.554 | 4.180 | 4.545 | 0.365 | 0.672 | 1.650E-04 | 0.003 | Unannotated | NA | NA |
| metab_11105 | Unannotated feature | neg | 493.9697 | 5.659 | 4.652 | 3.623 | -1.029 | -0.672 | 1.650E-04 | 0.003 | Unannotated | NA | NA |
| metab_6951 | Unannotated feature | neg | 405.1924 | 5.691 | 4.413 | 3.957 | -0.455 | -0.672 | 1.650E-04 | 0.003 | Unannotated | NA | NA |
| metab_9615 | Unannotated feature | neg | 383.9845 | 3.819 | 4.106 | 3.185 | -0.921 | -0.672 | 1.650E-04 | 0.003 | Unannotated | NA | NA |
| metab_4801 | Unannotated feature | pos | 138.9954 | 7.331 | 5.960 | 6.073 | 0.113 | 0.667 | 1.814E-04 | 0.003 | Unannotated | NA | NA |
| metab_9446 | Unannotated feature | neg | 319.1040 | 3.348 | 4.176 | 4.559 | 0.383 | 0.667 | 1.814E-04 | 0.003 | Unannotated | NA | NA |
| metab_11284 | Unannotated feature | neg | 244.8883 | 0.570 | 4.433 | 4.735 | 0.302 | 0.667 | 1.814E-04 | 0.003 | Unannotated | NA | NA |
| metab_1137 | Unannotated feature | pos | 465.3218 | 5.649 | 4.931 | 4.750 | -0.181 | -0.667 | 1.814E-04 | 0.003 | Unannotated | NA | NA |
| metab_9940 | 4-Acetamidobutanoate | neg | 144.0661 | 2.119 | 4.240 | 4.768 | 0.529 | 0.667 | 1.814E-04 | 0.003 | Moderate putative | Organic acids and derivatives | Carboxylic acids and derivatives |
| metab_5907 | Xylobiose | neg | 327.0951 | 2.523 | 2.935 | 4.238 | 1.303 | 0.667 | 1.814E-04 | 0.003 | Tentative | Organic oxygen compounds | Organooxygen compounds |
| metab_9884 | Unannotated feature | neg | 277.9735 | 2.302 | 4.686 | 4.486 | -0.200 | -0.667 | 1.814E-04 | 0.003 | Unannotated | NA | NA |
| metab_2118 | Unannotated feature | pos | 88.5693 | 6.983 | 3.375 | 3.762 | 0.386 | 0.667 | 1.814E-04 | 0.003 | Unannotated | NA | NA |
| metab_5787 | Unannotated feature | neg | 359.9494 | 2.061 | 3.108 | 4.095 | 0.987 | 0.667 | 1.814E-04 | 0.003 | Unannotated | NA | NA |
| metab_4491 | Unannotated feature | pos | 501.8793 | 0.551 | 5.375 | 5.602 | 0.227 | 0.667 | 1.814E-04 | 0.003 | Unannotated | NA | NA |
| metab_5271 | Unannotated feature | neg | 767.8025 | 0.554 | 3.887 | 4.937 | 1.049 | 0.667 | 1.814E-04 | 0.003 | Unannotated | NA | NA |
| metab_8627 | Cromakalim | neg | 331.1307 | 5.651 | 5.815 | 5.360 | -0.455 | -0.667 | 1.814E-04 | 0.003 | Tentative | Organoheterocyclic compounds | Benzopyrans |
| metab_978 | Unannotated feature | pos | 283.0077 | 4.370 | 4.770 | 4.499 | -0.271 | -0.667 | 1.814E-04 | 0.003 | Unannotated | NA | NA |
| metab_2187 | Unannotated feature | pos | 301.1183 | 4.739 | 5.758 | 5.171 | -0.587 | -0.667 | 1.814E-04 | 0.003 | Unannotated | NA | NA |
| metab_6519 | Unannotated feature | neg | 150.0555 | 4.497 | 3.795 | 4.020 | 0.226 | 0.667 | 1.814E-04 | 0.003 | Unannotated | NA | NA |
| metab_9274 | Unannotated feature | neg | 316.9167 | 3.819 | 4.830 | 4.453 | -0.377 | -0.667 | 1.814E-04 | 0.003 | Unannotated | NA | NA |
| metab_9360 | Unannotated feature | neg | 128.6528 | 3.570 | 4.477 | 4.198 | -0.279 | -0.667 | 1.814E-04 | 0.003 | Unannotated | NA | NA |
| metab_1350 | Atropaldehyde | pos | 133.0650 | 5.902 | 4.460 | 4.595 | 0.135 | 0.667 | 1.814E-04 | 0.003 | Tentative | Benzenoids | Benzene and substituted derivatives |
| metab_5622 | Unannotated feature | neg | 236.8565 | 0.912 | 5.038 | 5.196 | 0.158 | 0.667 | 1.814E-04 | 0.003 | Unannotated | NA | NA |
| metab_10048 | Unannotated feature | neg | 160.0611 | 1.438 | 4.009 | 4.549 | 0.540 | 0.667 | 1.814E-04 | 0.003 | Unannotated | NA | NA |
| metab_1027 | Melannin | pos | 302.1023 | 4.739 | 4.864 | 4.159 | -0.705 | -0.667 | 1.814E-04 | 0.003 | Tentative | Phenylpropanoids and polyketides | Neoflavonoids |
| metab_3051 | Pentisomide | pos | 342.2486 | 4.086 | 4.933 | 4.745 | -0.188 | -0.667 | 1.814E-04 | 0.003 | Tentative | Organic nitrogen compounds | Organonitrogen compounds |
| metab_1390 | Unannotated feature | pos | 736.5065 | 5.917 | 5.067 | 4.723 | -0.345 | -0.667 | 1.814E-04 | 0.003 | Unannotated | NA | NA |
| metab_7065 | Unannotated feature | neg | 286.9710 | 0.865 | 4.715 | 5.168 | 0.454 | 0.667 | 1.814E-04 | 0.003 | Unannotated | NA | NA |
| metab_2936 | Coniferyl aldehyde | pos | 179.0703 | 4.800 | 4.658 | 4.142 | -0.516 | -0.667 | 1.814E-04 | 0.003 | Moderate putative | Benzenoids | Phenols |
| metab_4745 | DG(15:0/18:3(9,11,15)-OH(13)/0:0) | pos | 557.4571 | 6.288 | 5.899 | 5.424 | -0.475 | -0.667 | 1.814E-04 | 0.003 | Tentative | NA | NA |
| metab_669 | Unannotated feature | pos | 521.4191 | 6.180 | 6.231 | 5.417 | -0.813 | -0.667 | 1.814E-04 | 0.003 | Unannotated | NA | NA |
| metab_1915 | Unannotated feature | pos | 227.0703 | 7.324 | 5.679 | 5.650 | -0.029 | -0.667 | 1.814E-04 | 0.003 | Unannotated | NA | NA |
| metab_3886 | Unannotated feature | pos | 416.0549 | 0.869 | 4.513 | 4.285 | -0.228 | -0.663 | 1.993E-04 | 0.003 | Unannotated | NA | NA |
| metab_4482 | Unannotated feature | pos | 611.8471 | 0.551 | 4.695 | 5.046 | 0.351 | 0.663 | 1.993E-04 | 0.003 | Unannotated | NA | NA |
| metab_4914 | Unannotated feature | pos | 870.4866 | 0.591 | 5.276 | 5.113 | -0.163 | -0.663 | 1.993E-04 | 0.003 | Unannotated | NA | NA |
| metab_1053 | Genkwanin | pos | 302.1023 | 4.969 | 5.429 | 4.862 | -0.568 | -0.663 | 1.993E-04 | 0.003 | Tentative | Phenylpropanoids and polyketides | Flavonoids |
| metab_10330 | Unannotated feature | neg | 340.7830 | 0.778 | 4.445 | 4.007 | -0.438 | -0.663 | 1.993E-04 | 0.003 | Unannotated | NA | NA |
| metab_8975 | Unannotated feature | neg | 280.9936 | 4.845 | 4.445 | 4.133 | -0.312 | -0.663 | 1.993E-04 | 0.003 | Unannotated | NA | NA |
| metab_4430 | Unannotated feature | pos | 564.8504 | 0.559 | 4.098 | 4.359 | 0.261 | 0.663 | 1.993E-04 | 0.003 | Unannotated | NA | NA |
| metab_1501 | Unannotated feature | pos | 281.2476 | 6.103 | 4.229 | 3.344 | -0.885 | -0.663 | 1.993E-04 | 0.003 | Unannotated | NA | NA |
| metab_11100 | Unannotated feature | neg | 445.2078 | 5.651 | 4.416 | 5.120 | 0.704 | 0.663 | 1.993E-04 | 0.003 | Unannotated | NA | NA |
| metab_4701 | Unannotated feature | pos | 758.5235 | 5.910 | 5.479 | 4.951 | -0.527 | -0.663 | 1.993E-04 | 0.003 | Unannotated | NA | NA |
| metab_10451 | Unannotated feature | neg | 157.0268 | 0.660 | 3.134 | 4.147 | 1.014 | 0.663 | 1.993E-04 | 0.003 | Unannotated | NA | NA |
| metab_4855 | Unannotated feature | pos | 479.3374 | 5.740 | 5.408 | 5.264 | -0.144 | -0.663 | 1.993E-04 | 0.003 | Unannotated | NA | NA |
| metab_1129 | N2-Succinyl-L-ornithine | pos | 233.1132 | 1.949 | 4.183 | 4.610 | 0.427 | 0.663 | 1.993E-04 | 0.003 | Tentative | Organic acids and derivatives | Carboxylic acids and derivatives |
| metab_4012 | Unannotated feature | pos | 301.1410 | 0.807 | 4.790 | 4.674 | -0.115 | -0.663 | 1.993E-04 | 0.003 | Unannotated | NA | NA |
| metab_5161 | Unannotated feature | neg | 204.8788 | 0.505 | 4.455 | 4.566 | 0.111 | 0.663 | 1.993E-04 | 0.003 | Unannotated | NA | NA |
| metab_9362 | Unannotated feature | neg | 319.9842 | 3.562 | 5.431 | 5.203 | -0.227 | -0.663 | 1.993E-04 | 0.003 | Unannotated | NA | NA |
| metab_9885 | Unannotated feature | neg | 115.0393 | 2.298 | 6.033 | 5.783 | -0.250 | -0.663 | 1.993E-04 | 0.003 | Unannotated | NA | NA |
| metab_2588 | Unannotated feature | pos | 470.8637 | 0.551 | 3.027 | 4.349 | 1.323 | 0.663 | 1.993E-04 | 0.003 | Unannotated | NA | NA |
| metab_4890 | Unannotated feature | pos | 281.1132 | 2.179 | 4.223 | 5.494 | 1.271 | 0.663 | 1.993E-04 | 0.003 | Unannotated | NA | NA |
| metab_4817 | Unannotated feature | pos | 141.9587 | 7.944 | 6.149 | 6.206 | 0.058 | 0.663 | 1.993E-04 | 0.003 | Unannotated | NA | NA |
| metab_1150 | Unannotated feature | pos | 183.1129 | 1.964 | 4.965 | 5.319 | 0.354 | 0.663 | 1.993E-04 | 0.003 | Unannotated | NA | NA |
| metab_9093 | Unannotated feature | neg | 379.0349 | 3.301 | 3.445 | 5.047 | 1.602 | 0.663 | 1.993E-04 | 0.003 | Unannotated | NA | NA |
| metab_1811 | Unannotated feature | pos | 114.0736 | 7.975 | 4.929 | 4.904 | -0.026 | -0.663 | 1.993E-04 | 0.003 | Unannotated | NA | NA |
| metab_4298 | Unannotated feature | pos | 760.6038 | 0.583 | 5.297 | 5.214 | -0.084 | -0.663 | 1.993E-04 | 0.003 | Unannotated | NA | NA |
| metab_3352 | Bethanechol | pos | 206.1005 | 2.583 | 6.498 | 6.278 | -0.219 | -0.663 | 1.993E-04 | 0.003 | Tentative | Organic nitrogen compounds | Organonitrogen compounds |
| metab_4071 | Unannotated feature | pos | 189.0734 | 0.745 | 4.527 | 5.093 | 0.566 | 0.663 | 1.993E-04 | 0.003 | Unannotated | NA | NA |
| metab_2202 | Unannotated feature | pos | 181.1587 | 6.303 | 4.121 | 3.079 | -1.042 | -0.663 | 1.993E-04 | 0.003 | Unannotated | NA | NA |
| metab_1861 | Unannotated feature | pos | 199.9753 | 7.540 | 5.672 | 5.752 | 0.080 | 0.663 | 1.993E-04 | 0.003 | Unannotated | NA | NA |
| metab_3327 | Unannotated feature | pos | 199.2712 | 2.637 | 6.463 | 6.536 | 0.073 | 0.663 | 1.993E-04 | 0.003 | Unannotated | NA | NA |
| metab_9597 | Unannotated feature | neg | 111.0192 | 3.843 | 4.382 | 4.237 | -0.146 | -0.663 | 1.993E-04 | 0.003 | Unannotated | NA | NA |
| metab_11264 | Diacylglycerol | neg | 545.4221 | 6.188 | 5.400 | 4.151 | -1.249 | -0.663 | 1.993E-04 | 0.003 | Tentative | Lipids and lipid-like molecules | Glycerolipids |
| metab_5662 | Unannotated feature | neg | 258.9971 | 1.086 | 4.288 | 3.831 | -0.457 | -0.659 | 2.188E-04 | 0.003 | Unannotated | NA | NA |
| metab_5250 | Unannotated feature | neg | 602.8407 | 0.554 | 4.686 | 4.971 | 0.284 | 0.659 | 2.188E-04 | 0.003 | Unannotated | NA | NA |
| metab_1035 | Unannotated feature | pos | 400.2904 | 4.808 | 5.388 | 5.220 | -0.168 | -0.659 | 2.188E-04 | 0.003 | Unannotated | NA | NA |
| metab_8639 | Unannotated feature | neg | 375.1206 | 5.643 | 5.143 | 4.724 | -0.419 | -0.659 | 2.188E-04 | 0.003 | Unannotated | NA | NA |
| metab_8191 | Taurolithocholate sulfate | neg | 600.2047 | 5.985 | 4.667 | 5.037 | 0.370 | 0.659 | 2.188E-04 | 0.003 | Tentative | Lipids and lipid-like molecules | Steroids and steroid derivatives |
| metab_4761 | Unannotated feature | pos | 912.7809 | 0.551 | 5.553 | 5.778 | 0.225 | 0.659 | 2.188E-04 | 0.003 | Unannotated | NA | NA |
| metab_1857 | Unannotated feature | pos | 708.8190 | 0.551 | 5.876 | 6.084 | 0.208 | 0.659 | 2.188E-04 | 0.003 | Unannotated | NA | NA |
| metab_1054 | Unannotated feature | pos | 144.9930 | 0.784 | 4.126 | 4.514 | 0.388 | 0.659 | 2.188E-04 | 0.003 | Unannotated | NA | NA |
| metab_1865 | Unannotated feature | pos | 111.5411 | 7.471 | 3.790 | 3.554 | -0.236 | -0.659 | 2.188E-04 | 0.003 | Unannotated | NA | NA |
| metab_8891 | Unannotated feature | neg | 136.0762 | 5.184 | 3.793 | 3.143 | -0.650 | -0.659 | 2.188E-04 | 0.003 | Unannotated | NA | NA |
| metab_8870 | Unannotated feature | neg | 257.0929 | 5.247 | 4.001 | 2.789 | -1.212 | -0.659 | 2.188E-04 | 0.003 | Unannotated | NA | NA |
| metab_4978 | Unannotated feature | pos | 367.1210 | 0.721 | 5.405 | 4.989 | -0.415 | -0.659 | 2.188E-04 | 0.003 | Unannotated | NA | NA |
| metab_10193 | Unannotated feature | neg | 146.9825 | 0.881 | 6.401 | 6.243 | -0.158 | -0.659 | 2.188E-04 | 0.003 | Unannotated | NA | NA |
| metab_4501 | Unannotated feature | pos | 166.9164 | 0.551 | 3.777 | 4.189 | 0.412 | 0.659 | 2.188E-04 | 0.003 | Unannotated | NA | NA |
| metab_8672 | Unannotated feature | neg | 165.0917 | 5.627 | 4.037 | 4.324 | 0.287 | 0.659 | 2.188E-04 | 0.003 | Unannotated | NA | NA |
| metab_316 | Unannotated feature | pos | 301.1183 | 5.168 | 5.694 | 5.058 | -0.637 | -0.659 | 2.188E-04 | 0.003 | Unannotated | NA | NA |
| metab_2851 | 4-Hydroxyestradiol-17beta | pos | 333.1444 | 5.275 | 6.211 | 5.798 | -0.413 | -0.659 | 2.188E-04 | 0.003 | Tentative | Lipids and lipid-like molecules | Steroids and steroid derivatives |
| metab_4615 | Unannotated feature | pos | 201.9911 | 0.002 | 4.494 | 4.755 | 0.261 | 0.659 | 2.188E-04 | 0.003 | Unannotated | NA | NA |
| metab_4987 | Unannotated feature | pos | 318.1295 | 0.745 | 5.132 | 4.753 | -0.379 | -0.659 | 2.188E-04 | 0.003 | Unannotated | NA | NA |
| metab_7218 | Aspartyl-Glutamate | neg | 243.0626 | 0.865 | 5.288 | 5.607 | 0.318 | 0.659 | 2.188E-04 | 0.003 | Tentative | Organic acids and derivatives | Carboxylic acids and derivatives |
| metab_2487 | Unannotated feature | pos | 786.5413 | 5.894 | 5.511 | 5.046 | -0.465 | -0.659 | 2.188E-04 | 0.003 | Unannotated | NA | NA |
| metab_9138 | Unannotated feature | neg | 246.9921 | 3.217 | 3.594 | 5.005 | 1.411 | 0.659 | 2.188E-04 | 0.003 | Unannotated | NA | NA |
| metab_10861 | Unannotated feature | neg | 532.8243 | 0.554 | 4.837 | 5.448 | 0.610 | 0.659 | 2.188E-04 | 0.003 | Unannotated | NA | NA |
| metab_7345 | Unannotated feature | neg | 75.0079 | 6.493 | 5.096 | 5.156 | 0.060 | 0.659 | 2.188E-04 | 0.003 | Unannotated | NA | NA |
| metab_5663 | Unannotated feature | neg | 254.9172 | 0.554 | 5.888 | 6.147 | 0.258 | 0.659 | 2.188E-04 | 0.003 | Unannotated | NA | NA |
| metab_4572 | Unannotated feature | pos | 91.0578 | 0.457 | 5.040 | 5.014 | -0.026 | -0.659 | 2.188E-04 | 0.003 | Unannotated | NA | NA |
| metab_10104 | Pseudouridine | neg | 243.0627 | 1.119 | 4.922 | 5.309 | 0.387 | 0.659 | 2.188E-04 | 0.003 | Moderate putative | Nucleosides, nucleotides, and analogues | Nucleoside and nucleotide analogues |
| metab_8023 | Unannotated feature | neg | 498.9313 | 6.628 | 6.977 | 6.411 | -0.566 | -0.659 | 2.188E-04 | 0.003 | Unannotated | NA | NA |
| metab_6375 | Unannotated feature | neg | 215.0826 | 3.980 | 3.858 | 2.785 | -1.073 | -0.659 | 2.188E-04 | 0.003 | Unannotated | NA | NA |
| metab_1415 | Unannotated feature | pos | 813.5666 | 5.933 | 5.170 | 4.868 | -0.303 | -0.659 | 2.188E-04 | 0.003 | Unannotated | NA | NA |
| metab_5625 | Unannotated feature | neg | 382.7220 | 0.912 | 5.016 | 4.950 | -0.066 | -0.655 | 2.401E-04 | 0.004 | Unannotated | NA | NA |
| metab_4642 | Unannotated feature | pos | 662.4689 | 5.725 | 5.350 | 5.140 | -0.210 | -0.655 | 2.401E-04 | 0.004 | Unannotated | NA | NA |
| metab_3210 | 3-O-Methylniveusin A | pos | 409.1870 | 2.583 | 6.142 | 5.819 | -0.323 | -0.655 | 2.401E-04 | 0.004 | Tentative | Lipids and lipid-like molecules | Prenol lipids |
| metab_1513 | 1-Acyl-sn-glycero-3-phosphocholine | pos | 468.3085 | 6.149 | 6.656 | 6.459 | -0.196 | -0.655 | 2.401E-04 | 0.004 | Tentative | Lipids and lipid-like molecules | Glycerophospholipids |
| metab_10237 | Unannotated feature | neg | 126.9975 | 0.865 | 5.071 | 4.902 | -0.170 | -0.655 | 2.401E-04 | 0.004 | Unannotated | NA | NA |
| metab_5452 | Unannotated feature | neg | 245.0436 | 0.660 | 3.406 | 3.924 | 0.518 | 0.655 | 2.401E-04 | 0.004 | Unannotated | NA | NA |
| metab_4820 | Pyramid | pos | 126.0551 | 7.944 | 5.165 | 5.205 | 0.041 | 0.655 | 2.401E-04 | 0.004 | Tentative | NA | NA |
| metab_2339 | Unannotated feature | pos | 535.4002 | 5.995 | 5.686 | 5.495 | -0.191 | -0.655 | 2.401E-04 | 0.004 | Unannotated | NA | NA |
| metab_6900 | Aminoglutethimide | neg | 231.1141 | 5.659 | 4.134 | 3.438 | -0.696 | -0.655 | 2.401E-04 | 0.004 | Tentative | Benzenoids | Benzene and substituted derivatives |
| metab_4484 | Unannotated feature | pos | 603.8599 | 0.551 | 5.417 | 5.581 | 0.164 | 0.655 | 2.401E-04 | 0.004 | Unannotated | NA | NA |
| metab_2358 | Unannotated feature | pos | 355.2456 | 5.972 | 4.857 | 4.966 | 0.110 | 0.655 | 2.401E-04 | 0.004 | Unannotated | NA | NA |
| metab_3783 | Unannotated feature | pos | 136.0619 | 1.585 | 4.735 | 4.675 | -0.060 | -0.655 | 2.401E-04 | 0.004 | Unannotated | NA | NA |
| metab_10418 | Unannotated feature | neg | 350.7770 | 0.684 | 3.801 | 4.894 | 1.093 | 0.655 | 2.401E-04 | 0.004 | Unannotated | NA | NA |
| metab_7236 | Unannotated feature | neg | 563.2691 | 6.000 | 4.423 | 3.789 | -0.634 | -0.655 | 2.401E-04 | 0.004 | Unannotated | NA | NA |
| metab_4552 | Unannotated feature | pos | 368.8827 | 0.551 | 5.423 | 5.779 | 0.357 | 0.655 | 2.401E-04 | 0.004 | Unannotated | NA | NA |
| metab_6353 | Unannotated feature | neg | 468.6917 | 0.715 | 4.157 | 5.146 | 0.989 | 0.655 | 2.401E-04 | 0.004 | Unannotated | NA | NA |
| metab_4260 | Unannotated feature | pos | 810.5310 | 0.591 | 5.644 | 5.550 | -0.094 | -0.655 | 2.401E-04 | 0.004 | Unannotated | NA | NA |
| metab_4677 | Unannotated feature | pos | 578.4031 | 5.863 | 5.615 | 5.423 | -0.193 | -0.655 | 2.401E-04 | 0.004 | Unannotated | NA | NA |
| metab_4655 | Unannotated feature | pos | 301.1183 | 5.793 | 5.694 | 4.905 | -0.789 | -0.655 | 2.401E-04 | 0.004 | Unannotated | NA | NA |
| metab_261 | Trigonelline (N-methylnicotinate) | pos | 138.0549 | 0.691 | 5.286 | 5.458 | 0.172 | 0.655 | 2.401E-04 | 0.004 | Strong putative | NA | NA |
| metab_8151 | Unannotated feature | neg | 545.2586 | 6.087 | 4.390 | 3.947 | -0.443 | -0.655 | 2.401E-04 | 0.004 | Unannotated | NA | NA |
| metab_235 | Unannotated feature | pos | 161.5464 | 0.644 | 5.086 | 4.899 | -0.187 | -0.655 | 2.401E-04 | 0.004 | Unannotated | NA | NA |
| metab_6242 | Unannotated feature | neg | 233.9470 | 3.467 | 4.851 | 4.720 | -0.131 | -0.655 | 2.401E-04 | 0.004 | Unannotated | NA | NA |
| metab_4809 | Unannotated feature | pos | 97.9692 | 7.331 | 4.763 | 4.878 | 0.115 | 0.655 | 2.401E-04 | 0.004 | Unannotated | NA | NA |
| metab_946 | Unannotated feature | pos | 272.1856 | 4.093 | 4.274 | 4.847 | 0.573 | 0.655 | 2.401E-04 | 0.004 | Unannotated | NA | NA |
| metab_9880 | Unannotated feature | neg | 111.0080 | 2.350 | 2.741 | 3.454 | 0.713 | 0.655 | 2.401E-04 | 0.004 | Unannotated | NA | NA |
| metab_6338 | Unannotated feature | neg | 130.2727 | 3.811 | 3.262 | 2.496 | -0.766 | -0.655 | 2.401E-04 | 0.004 | Unannotated | NA | NA |
| metab_10910 | Unannotated feature | neg | 554.9056 | 0.543 | 4.951 | 4.736 | -0.216 | -0.655 | 2.401E-04 | 0.004 | Unannotated | NA | NA |
| metab_1226 | Unannotated feature | pos | 384.2964 | 5.793 | 5.054 | 4.899 | -0.155 | -0.655 | 2.401E-04 | 0.004 | Unannotated | NA | NA |
| metab_6676 | Unannotated feature | neg | 136.0398 | 5.184 | 3.703 | 3.194 | -0.509 | -0.655 | 2.401E-04 | 0.004 | Unannotated | NA | NA |
| metab_776 | Jasmolone glucoside | pos | 360.2015 | 2.904 | 4.098 | 4.305 | 0.206 | 0.655 | 2.401E-04 | 0.004 | Tentative | Organic oxygen compounds | Organooxygen compounds |
| metab_8250 | Unannotated feature | neg | 259.7806 | 5.906 | 4.238 | 3.840 | -0.398 | -0.655 | 2.401E-04 | 0.004 | Unannotated | NA | NA |
| metab_1257 | CDP-DG(20:2(11Z,14Z)/LTE4) | pos | 617.2603 | 5.840 | 5.076 | 4.518 | -0.558 | -0.651 | 2.634E-04 | 0.004 | Tentative | NA | NA |
| metab_9342 | Unannotated feature | neg | 129.0550 | 3.562 | 6.708 | 6.543 | -0.165 | -0.651 | 2.634E-04 | 0.004 | Unannotated | NA | NA |
| metab_268 | Unannotated feature | pos | 606.5693 | 0.698 | 5.298 | 5.172 | -0.126 | -0.651 | 2.634E-04 | 0.004 | Unannotated | NA | NA |
| metab_10868 | Unannotated feature | neg | 396.8488 | 0.554 | 4.724 | 5.361 | 0.637 | 0.651 | 2.634E-04 | 0.004 | Unannotated | NA | NA |
| metab_1421 | Unannotated feature | pos | 621.4186 | 5.941 | 5.044 | 4.864 | -0.180 | -0.651 | 2.634E-04 | 0.004 | Unannotated | NA | NA |
| metab_10502 | 2-Hydroxyethanesulfonate | neg | 124.9907 | 0.636 | 3.672 | 4.355 | 0.682 | 0.651 | 2.634E-04 | 0.004 | Tentative | Organic acids and derivatives | Organic sulfonic acids and derivatives |
| metab_5028 | 5-Aminopentanoate | pos | 162.0502 | 0.846 | 5.259 | 5.022 | -0.237 | -0.651 | 2.634E-04 | 0.004 | Tentative | Organic acids and derivatives | Carboxylic acids and derivatives |
| metab_4473 | Unannotated feature | pos | 679.8343 | 0.551 | 4.942 | 5.122 | 0.180 | 0.651 | 2.634E-04 | 0.004 | Unannotated | NA | NA |
| metab_8147 | Unannotated feature | neg | 453.3593 | 6.102 | 4.496 | 3.836 | -0.660 | -0.651 | 2.634E-04 | 0.004 | Unannotated | NA | NA |
| metab_8783 | Unannotated feature | neg | 689.1922 | 5.518 | 4.181 | 4.721 | 0.540 | 0.651 | 2.634E-04 | 0.004 | Unannotated | NA | NA |
| metab_1906 | Unannotated feature | pos | 110.0204 | 7.331 | 6.274 | 6.381 | 0.107 | 0.651 | 2.634E-04 | 0.004 | Unannotated | NA | NA |
| metab_1274 | Unannotated feature | pos | 405.3674 | 5.855 | 5.922 | 5.999 | 0.077 | 0.651 | 2.634E-04 | 0.004 | Unannotated | NA | NA |
| metab_7199 | Unannotated feature | neg | 363.9564 | 5.922 | 5.144 | 5.302 | 0.159 | 0.651 | 2.634E-04 | 0.004 | Unannotated | NA | NA |
| metab_1795 | Unannotated feature | pos | 172.0056 | 7.788 | 4.690 | 4.756 | 0.066 | 0.651 | 2.634E-04 | 0.004 | Unannotated | NA | NA |
| metab_1379 | Unannotated feature | pos | 828.5730 | 5.910 | 5.573 | 5.287 | -0.286 | -0.651 | 2.634E-04 | 0.004 | Unannotated | NA | NA |
| metab_5817 | Unannotated feature | neg | 263.8824 | 2.119 | 3.994 | 3.677 | -0.317 | -0.651 | 2.634E-04 | 0.004 | Unannotated | NA | NA |
| metab_400 | Unannotated feature | pos | 269.0743 | 0.854 | 4.844 | 5.286 | 0.442 | 0.651 | 2.634E-04 | 0.004 | Unannotated | NA | NA |
| metab_312 | Unannotated feature | pos | 153.0217 | 0.784 | 3.985 | 4.458 | 0.473 | 0.651 | 2.634E-04 | 0.004 | Unannotated | NA | NA |
| metab_7225 | Deoxycorticosterone acetate | neg | 353.2129 | 5.969 | 6.429 | 4.061 | -2.368 | -0.651 | 2.634E-04 | 0.004 | Tentative | Lipids and lipid-like molecules | Steroids and steroid derivatives |
| metab_10607 | Unannotated feature | neg | 477.6699 | 0.598 | 5.498 | 5.425 | -0.073 | -0.651 | 2.634E-04 | 0.004 | Unannotated | NA | NA |
| metab_1164 | Unannotated feature | pos | 494.3426 | 5.695 | 5.194 | 4.951 | -0.243 | -0.651 | 2.634E-04 | 0.004 | Unannotated | NA | NA |
| metab_741 | Unannotated feature | pos | 200.8280 | 2.644 | 6.435 | 6.500 | 0.065 | 0.651 | 2.634E-04 | 0.004 | Unannotated | NA | NA |
| metab_5569 | Unannotated feature | neg | 260.8737 | 0.554 | 4.572 | 5.307 | 0.734 | 0.651 | 2.634E-04 | 0.004 | Unannotated | NA | NA |
| metab_11191 | Unannotated feature | neg | 517.3025 | 5.859 | 4.535 | 3.548 | -0.987 | -0.651 | 2.634E-04 | 0.004 | Unannotated | NA | NA |
| metab_1362 | Unannotated feature | pos | 184.0605 | 2.273 | 3.519 | 4.596 | 1.078 | 0.651 | 2.634E-04 | 0.004 | Unannotated | NA | NA |
| metab_353 | Unannotated feature | pos | 330.0783 | 0.854 | 4.713 | 4.385 | -0.328 | -0.651 | 2.634E-04 | 0.004 | Unannotated | NA | NA |
| metab_5605 | Unannotated feature | neg | 148.9796 | 0.881 | 5.891 | 5.759 | -0.131 | -0.651 | 2.634E-04 | 0.004 | Unannotated | NA | NA |
| metab_3072 | Unannotated feature | pos | 84.5071 | 3.970 | 3.436 | 3.619 | 0.184 | 0.651 | 2.634E-04 | 0.004 | Unannotated | NA | NA |
| metab_86 | Unannotated feature | pos | 149.0447 | 0.488 | 4.146 | 4.075 | -0.071 | -0.646 | 2.888E-04 | 0.004 | Unannotated | NA | NA |
| metab_9474 | Dihydrocaffeic acid 3-sulfate | neg | 261.0079 | 3.282 | 2.899 | 4.880 | 1.981 | 0.646 | 2.888E-04 | 0.004 | Tentative | Organic acids and derivatives | Organic sulfuric acids and derivatives |
| metab_4927 | Unannotated feature | pos | 800.5024 | 0.591 | 5.673 | 5.564 | -0.109 | -0.646 | 2.888E-04 | 0.004 | Unannotated | NA | NA |
| metab_4705 | Unannotated feature | pos | 728.5042 | 5.917 | 5.191 | 4.747 | -0.445 | -0.646 | 2.888E-04 | 0.004 | Unannotated | NA | NA |
| metab_11120 | Unannotated feature | neg | 431.2292 | 5.675 | 3.955 | 4.728 | 0.773 | 0.646 | 2.888E-04 | 0.004 | Unannotated | NA | NA |
| metab_2323 | Phosphatidylethanolamine | pos | 678.4787 | 6.018 | 5.974 | 6.078 | 0.104 | 0.646 | 2.888E-04 | 0.004 | Tentative | NA | NA |
| metab_3218 | L-Allothreonine | pos | 84.0450 | 3.167 | 4.918 | 5.245 | 0.327 | 0.646 | 2.888E-04 | 0.004 | Tentative | Organic acids and derivatives | Carboxylic acids and derivatives |
| metab_6372 | Unannotated feature | neg | 277.0835 | 3.966 | 4.082 | 3.492 | -0.591 | -0.646 | 2.888E-04 | 0.004 | Unannotated | NA | NA |
| metab_717 | 1,5-Naphthalenediamine | pos | 159.0918 | 2.583 | 5.957 | 5.779 | -0.179 | -0.646 | 2.888E-04 | 0.004 | Moderate putative | Benzenoids | Naphthalenes |
| metab_901 | Unannotated feature | pos | 131.5336 | 7.324 | 5.758 | 5.843 | 0.085 | 0.646 | 2.888E-04 | 0.004 | Unannotated | NA | NA |
| metab_2838 | Unannotated feature | pos | 502.3584 | 5.390 | 5.482 | 5.310 | -0.173 | -0.646 | 2.888E-04 | 0.004 | Unannotated | NA | NA |
| metab_5435 | Unannotated feature | neg | 132.8672 | 0.652 | 3.984 | 4.219 | 0.235 | 0.646 | 2.888E-04 | 0.004 | Unannotated | NA | NA |
| metab_4068 | Unannotated feature | pos | 305.9662 | 0.745 | 4.384 | 4.071 | -0.313 | -0.646 | 2.888E-04 | 0.004 | Unannotated | NA | NA |
| metab_3441 | Unannotated feature | pos | 83.6586 | 2.342 | 3.432 | 3.710 | 0.278 | 0.646 | 2.888E-04 | 0.004 | Unannotated | NA | NA |
| metab_1334 | Chavicol | pos | 117.0702 | 5.894 | 4.675 | 4.805 | 0.130 | 0.646 | 2.888E-04 | 0.004 | Tentative | Benzenoids | Phenols |
| metab_4496 | Unannotated feature | pos | 234.9039 | 0.551 | 3.475 | 3.878 | 0.403 | 0.646 | 2.888E-04 | 0.004 | Unannotated | NA | NA |
| metab_1742 | Unannotated feature | pos | 223.9884 | 7.324 | 4.503 | 4.690 | 0.187 | 0.646 | 2.888E-04 | 0.004 | Unannotated | NA | NA |
| metab_11274 | Unannotated feature | neg | 173.0087 | 0.865 | 3.715 | 4.225 | 0.511 | 0.646 | 2.888E-04 | 0.004 | Unannotated | NA | NA |
| metab_10459 | Unannotated feature | neg | 431.9040 | 0.652 | 4.531 | 4.045 | -0.486 | -0.646 | 2.888E-04 | 0.004 | Unannotated | NA | NA |
| metab_4575 | Unannotated feature | pos | 202.0161 | 0.449 | 6.242 | 6.219 | -0.023 | -0.646 | 2.888E-04 | 0.004 | Unannotated | NA | NA |
| metab_4015 | Unannotated feature | pos | 201.1598 | 0.807 | 2.846 | 3.666 | 0.819 | 0.646 | 2.888E-04 | 0.004 | Unannotated | NA | NA |
| metab_11082 | Unannotated feature | neg | 370.0786 | 3.136 | 2.711 | 5.086 | 2.375 | 0.646 | 2.888E-04 | 0.004 | Unannotated | NA | NA |
| metab_2466 | Unannotated feature | pos | 453.1675 | 5.910 | 4.409 | 4.757 | 0.347 | 0.646 | 2.888E-04 | 0.004 | Unannotated | NA | NA |
| metab_8415 | Unannotated feature | neg | 111.0192 | 0.723 | 2.813 | 4.023 | 1.210 | 0.646 | 2.888E-04 | 0.004 | Unannotated | NA | NA |
| metab_8581 | Unannotated feature | neg | 124.0067 | 5.683 | 3.360 | 2.450 | -0.910 | -0.646 | 2.888E-04 | 0.004 | Unannotated | NA | NA |
| metab_525 | Unannotated feature | pos | 376.2594 | 5.871 | 6.703 | 6.843 | 0.141 | 0.646 | 2.888E-04 | 0.004 | Unannotated | NA | NA |
| metab_4337 | Unannotated feature | pos | 402.8243 | 0.575 | 5.698 | 5.646 | -0.051 | -0.646 | 2.888E-04 | 0.004 | Unannotated | NA | NA |
| metab_8146 | Unannotated feature | neg | 537.4170 | 6.102 | 6.301 | 5.697 | -0.604 | -0.646 | 2.888E-04 | 0.004 | Unannotated | NA | NA |
| metab_142 | Unannotated feature | pos | 674.8255 | 0.551 | 4.424 | 5.176 | 0.752 | 0.646 | 2.888E-04 | 0.004 | Unannotated | NA | NA |
| metab_6741 | Unannotated feature | neg | 173.0717 | 5.470 | 3.892 | 2.926 | -0.966 | -0.646 | 2.888E-04 | 0.004 | Unannotated | NA | NA |
| metab_2712 | Unannotated feature | pos | 618.4426 | 5.740 | 5.215 | 4.957 | -0.258 | -0.646 | 2.888E-04 | 0.004 | Unannotated | NA | NA |
| metab_2748 | Unannotated feature | pos | 538.3689 | 5.695 | 4.957 | 4.635 | -0.322 | -0.646 | 2.888E-04 | 0.004 | Unannotated | NA | NA |
| metab_133 | Unannotated feature | pos | 606.8384 | 0.551 | 4.477 | 5.058 | 0.581 | 0.642 | 3.164E-04 | 0.004 | Unannotated | NA | NA |
| metab_10727 | Unannotated feature | neg | 273.1251 | 5.184 | 4.987 | 4.290 | -0.697 | -0.642 | 3.164E-04 | 0.004 | Unannotated | NA | NA |
| metab_149 | Unannotated feature | pos | 747.8217 | 0.551 | 4.925 | 5.145 | 0.221 | 0.642 | 3.164E-04 | 0.004 | Unannotated | NA | NA |
| metab_7374 | Unannotated feature | neg | 185.0284 | 6.925 | 5.356 | 5.437 | 0.081 | 0.642 | 3.164E-04 | 0.004 | Unannotated | NA | NA |
| metab_2896 | Unannotated feature | pos | 532.3689 | 5.015 | 5.364 | 5.196 | -0.168 | -0.642 | 3.164E-04 | 0.004 | Unannotated | NA | NA |
| metab_9086 | Salicyluric acid | neg | 194.0457 | 4.497 | 3.928 | 4.401 | 0.472 | 0.642 | 3.164E-04 | 0.004 | Strong putative | Benzenoids | Benzene and substituted derivatives |
| metab_3378 | (6Z)-Oct-6-enedioylcarnitine | pos | 316.1754 | 2.521 | 4.458 | 5.073 | 0.615 | 0.642 | 3.164E-04 | 0.004 | Tentative | Lipids and lipid-like molecules | Fatty Acyls |
| metab_1420 | Unannotated feature | pos | 577.3927 | 5.941 | 5.030 | 4.852 | -0.178 | -0.642 | 3.164E-04 | 0.004 | Unannotated | NA | NA |
| metab_3361 | 2-Naphthylamine | pos | 144.0809 | 2.576 | 6.225 | 6.049 | -0.176 | -0.642 | 3.164E-04 | 0.004 | Moderate putative | Benzenoids | Naphthalenes |
| metab_1801 | Unannotated feature | pos | 130.9792 | 7.889 | 4.494 | 4.551 | 0.057 | 0.642 | 3.164E-04 | 0.004 | Unannotated | NA | NA |
| metab_5024 | Unannotated feature | pos | 452.7512 | 0.591 | 5.680 | 5.613 | -0.067 | -0.642 | 3.164E-04 | 0.004 | Unannotated | NA | NA |
| metab_8119 | Unannotated feature | neg | 414.0016 | 0.731 | 4.459 | 3.859 | -0.600 | -0.642 | 3.164E-04 | 0.004 | Unannotated | NA | NA |
| metab_734 | Unannotated feature | pos | 154.5691 | 2.637 | 5.962 | 6.028 | 0.066 | 0.642 | 3.164E-04 | 0.004 | Unannotated | NA | NA |
| metab_6734 | Unannotated feature | neg | 380.1108 | 5.406 | 4.509 | 3.674 | -0.835 | -0.642 | 3.164E-04 | 0.004 | Unannotated | NA | NA |
| metab_5048 | Unannotated feature | pos | 135.0030 | 0.869 | 6.161 | 6.048 | -0.114 | -0.642 | 3.164E-04 | 0.004 | Unannotated | NA | NA |
| metab_2699 | Mytilin A | pos | 665.2450 | 5.748 | 5.758 | 4.838 | -0.921 | -0.642 | 3.164E-04 | 0.004 | Tentative | Organic acids and derivatives | Carboxylic acids and derivatives |
| metab_2039 | Unannotated feature | pos | 644.6874 | 0.575 | 5.704 | 5.614 | -0.090 | -0.642 | 3.164E-04 | 0.004 | Unannotated | NA | NA |
| metab_1752 | Unannotated feature | pos | 153.0110 | 7.331 | 5.416 | 5.523 | 0.106 | 0.642 | 3.164E-04 | 0.004 | Unannotated | NA | NA |
| metab_3707 | Unannotated feature | pos | 300.1078 | 1.894 | 3.043 | 3.785 | 0.743 | 0.642 | 3.164E-04 | 0.004 | Unannotated | NA | NA |
| metab_2489 | Unannotated feature | pos | 726.5098 | 5.894 | 6.187 | 6.096 | -0.091 | -0.642 | 3.164E-04 | 0.004 | Unannotated | NA | NA |
| metab_10824 | Unannotated feature | neg | 814.7921 | 0.554 | 4.923 | 5.106 | 0.183 | 0.642 | 3.164E-04 | 0.004 | Unannotated | NA | NA |
| metab_8013 | Unannotated feature | neg | 462.9643 | 6.714 | 5.542 | 4.846 | -0.696 | -0.642 | 3.164E-04 | 0.004 | Strong putative | NA | NA |
| metab_5861 | Unannotated feature | neg | 137.0350 | 2.423 | 2.430 | 3.310 | 0.880 | 0.642 | 3.164E-04 | 0.004 | Unannotated | NA | NA |
| metab_2568 | Unannotated feature | pos | 405.5992 | 5.863 | 5.117 | 5.216 | 0.099 | 0.642 | 3.164E-04 | 0.004 | Unannotated | NA | NA |
| metab_6268 | Unannotated feature | neg | 144.0449 | 3.546 | 4.108 | 4.484 | 0.375 | 0.642 | 3.164E-04 | 0.004 | Unannotated | NA | NA |
| metab_1505 | Unannotated feature | pos | 389.3414 | 6.118 | 4.498 | 3.522 | -0.977 | -0.642 | 3.164E-04 | 0.004 | Unannotated | NA | NA |
| metab_10881 | Unannotated feature | neg | 96.6973 | 0.554 | 3.437 | 3.950 | 0.513 | 0.642 | 3.164E-04 | 0.004 | Unannotated | NA | NA |
| metab_6620 | Ellagic acid | neg | 300.9996 | 4.953 | 4.977 | 4.721 | -0.256 | -0.642 | 3.164E-04 | 0.004 | Strong putative | Phenylpropanoids and polyketides | Tannins |
| metab_3793 | Unannotated feature | pos | 75.0446 | 1.546 | 4.523 | 4.579 | 0.056 | 0.642 | 3.164E-04 | 0.004 | Unannotated | NA | NA |
| metab_3414 | Unannotated feature | pos | 244.1292 | 2.428 | 4.424 | 3.901 | -0.523 | -0.642 | 3.164E-04 | 0.004 | Unannotated | NA | NA |
| metab_4044 | Unannotated feature | pos | 264.8525 | 0.776 | 4.686 | 4.607 | -0.079 | -0.642 | 3.164E-04 | 0.004 | Unannotated | NA | NA |
| metab_1125 | Unannotated feature | pos | 200.1282 | 5.626 | 3.090 | 3.768 | 0.678 | 0.642 | 3.164E-04 | 0.004 | Unannotated | NA | NA |
| metab_10026 | Unannotated feature | neg | 279.7677 | 1.793 | 4.419 | 4.312 | -0.107 | -0.642 | 3.164E-04 | 0.004 | Unannotated | NA | NA |
| metab_2180 | Unannotated feature | pos | 597.4581 | 6.373 | 5.482 | 5.204 | -0.278 | -0.642 | 3.164E-04 | 0.004 | Unannotated | NA | NA |
| metab_4229 | N(pi)-Methyl-L-histidine | pos | 170.0925 | 0.614 | 4.938 | 5.419 | 0.481 | 0.642 | 3.164E-04 | 0.004 | Strong putative | Organic acids and derivatives | Carboxylic acids and derivatives |
| metab_4479 | Unannotated feature | pos | 641.8344 | 0.551 | 4.472 | 4.898 | 0.426 | 0.642 | 3.164E-04 | 0.004 | Unannotated | NA | NA |
| metab_4910 | Unannotated feature | pos | 570.6655 | 0.591 | 5.626 | 5.514 | -0.111 | -0.642 | 3.164E-04 | 0.004 | Unannotated | NA | NA |
| metab_4232 | Unannotated feature | pos | 124.0872 | 0.614 | 3.906 | 4.366 | 0.461 | 0.642 | 3.164E-04 | 0.004 | Unannotated | NA | NA |
| metab_3550 | Unannotated feature | pos | 125.4981 | 2.073 | 5.178 | 5.259 | 0.081 | 0.642 | 3.164E-04 | 0.004 | Unannotated | NA | NA |
| metab_2058 | Unannotated feature | pos | 146.9804 | 7.223 | 4.627 | 4.762 | 0.135 | 0.638 | 3.465E-04 | 0.004 | Unannotated | NA | NA |
| metab_8471 | Unannotated feature | neg | 629.2267 | 5.739 | 4.114 | 3.393 | -0.721 | -0.638 | 3.465E-04 | 0.004 | Unannotated | NA | NA |
| metab_10590 | Unannotated feature | neg | 375.1304 | 5.001 | 3.693 | 4.161 | 0.468 | 0.638 | 3.465E-04 | 0.004 | Unannotated | NA | NA |
| metab_6026 | Unannotated feature | neg | 278.0708 | 2.953 | 2.932 | 4.150 | 1.218 | 0.638 | 3.465E-04 | 0.004 | Unannotated | NA | NA |
| metab_2907 | 5-Methoxycanthin-6-one | pos | 283.1077 | 4.938 | 4.800 | 4.224 | -0.576 | -0.638 | 3.465E-04 | 0.004 | Tentative | Alkaloids and derivatives | Indolonaphthyridine alkaloids |
| metab_1512 | Unannotated feature | pos | 128.1072 | 6.149 | 4.593 | 4.631 | 0.038 | 0.638 | 3.465E-04 | 0.004 | Unannotated | NA | NA |
| metab_6946 | Unannotated feature | neg | 352.7738 | 0.904 | 4.150 | 4.434 | 0.284 | 0.638 | 3.465E-04 | 0.004 | Unannotated | NA | NA |
| metab_5227 | Unannotated feature | neg | 315.4019 | 0.546 | 4.757 | 4.647 | -0.110 | -0.638 | 3.465E-04 | 0.004 | Unannotated | NA | NA |
| metab_2968 | Unannotated feature | pos | 162.1125 | 2.265 | 5.067 | 4.953 | -0.114 | -0.638 | 3.465E-04 | 0.004 | Unannotated | NA | NA |
| metab_1088 | beta-D-Glucuronoside | pos | 377.1342 | 5.328 | 4.966 | 4.412 | -0.555 | -0.638 | 3.465E-04 | 0.004 | Tentative | Organic oxygen compounds | Organooxygen compounds |
| metab_841 | Unannotated feature | pos | 267.1802 | 3.290 | 4.680 | 4.553 | -0.127 | -0.638 | 3.465E-04 | 0.004 | Unannotated | NA | NA |
| metab_2470 | Unannotated feature | pos | 703.4877 | 5.902 | 4.827 | 4.432 | -0.395 | -0.638 | 3.465E-04 | 0.004 | Unannotated | NA | NA |
| metab_1760 | Unannotated feature | pos | 243.1340 | 3.453 | 4.340 | 5.049 | 0.709 | 0.638 | 3.465E-04 | 0.004 | Unannotated | NA | NA |
| metab_10826 | Unannotated feature | neg | 804.7748 | 0.554 | 4.822 | 5.447 | 0.625 | 0.638 | 3.465E-04 | 0.004 | Unannotated | NA | NA |
| metab_9883 | Unannotated feature | neg | 305.9686 | 2.302 | 5.214 | 5.009 | -0.204 | -0.638 | 3.465E-04 | 0.004 | Unannotated | NA | NA |
| metab_8485 | Unannotated feature | neg | 220.0980 | 5.739 | 3.081 | 3.628 | 0.547 | 0.638 | 3.465E-04 | 0.004 | Unannotated | NA | NA |
| metab_1526 | Indoleacrylic acid | pos | 188.0707 | 2.576 | 7.765 | 7.584 | -0.181 | -0.638 | 3.465E-04 | 0.004 | Tentative | Organoheterocyclic compounds | Indoles and derivatives |
| metab_1899 | Unannotated feature | pos | 180.9898 | 7.331 | 5.781 | 5.871 | 0.090 | 0.638 | 3.465E-04 | 0.004 | Unannotated | NA | NA |
| metab_1784 | Unannotated feature | pos | 171.0057 | 7.509 | 4.360 | 4.495 | 0.135 | 0.638 | 3.465E-04 | 0.004 | Unannotated | NA | NA |
| metab_1314 | Unannotated feature | pos | 636.4429 | 5.879 | 5.327 | 4.876 | -0.452 | -0.638 | 3.465E-04 | 0.004 | Unannotated | NA | NA |
| metab_3138 | 6-[3-(2-carboxyethyl)-5-hydroxyphenoxy]-3,4,5-trihydroxyoxane-2-carboxylic acid | pos | 376.1237 | 2.506 | 3.310 | 4.451 | 1.141 | 0.638 | 3.465E-04 | 0.004 | Tentative | NA | NA |
| metab_1233 | Unannotated feature | pos | 199.1077 | 2.065 | 4.310 | 5.160 | 0.850 | 0.638 | 3.465E-04 | 0.004 | Unannotated | NA | NA |
| metab_1881 | Unannotated feature | pos | 158.0264 | 7.347 | 3.885 | 4.295 | 0.410 | 0.638 | 3.465E-04 | 0.004 | Unannotated | NA | NA |
| metab_3356 | Unannotated feature | pos | 143.0731 | 2.583 | 5.486 | 5.275 | -0.211 | -0.638 | 3.465E-04 | 0.004 | Unannotated | NA | NA |
| metab_1877 | Unannotated feature | pos | 201.9757 | 7.355 | 4.846 | 4.901 | 0.055 | 0.638 | 3.465E-04 | 0.004 | Unannotated | NA | NA |
| metab_2974 | Panaxytriol | pos | 317.1495 | 4.532 | 4.877 | 4.331 | -0.546 | -0.638 | 3.465E-04 | 0.004 | Tentative | Lipids and lipid-like molecules | Fatty Acyls |
| metab_512 | Unannotated feature | pos | 152.5460 | 1.925 | 3.659 | 4.092 | 0.434 | 0.638 | 3.465E-04 | 0.004 | Unannotated | NA | NA |
| metab_4470 | Unannotated feature | pos | 713.8281 | 0.551 | 5.014 | 5.201 | 0.187 | 0.638 | 3.465E-04 | 0.004 | Unannotated | NA | NA |
| metab_8338 | Unannotated feature | neg | 441.2138 | 5.827 | 3.490 | 4.488 | 0.998 | 0.638 | 3.465E-04 | 0.004 | Unannotated | NA | NA |
| metab_5256 | Unannotated feature | neg | 662.8430 | 0.554 | 5.917 | 6.135 | 0.218 | 0.638 | 3.465E-04 | 0.004 | Unannotated | NA | NA |
| metab_2585 | Unannotated feature | pos | 529.7057 | 5.855 | 5.126 | 4.954 | -0.171 | -0.634 | 3.793E-04 | 0.004 | Unannotated | NA | NA |
| metab_2689 | N-(3-Methyl-2-pyridyl)-3-phenylsuccinimide | pos | 299.1390 | 5.763 | 6.105 | 5.615 | -0.489 | -0.634 | 3.793E-04 | 0.004 | Tentative | Organoheterocyclic compounds | Pyrrolidines |
| metab_1558 | Cis-5-dodecenoic acid | pos | 199.1694 | 6.311 | 4.112 | 3.081 | -1.031 | -0.634 | 3.793E-04 | 0.004 | Moderate putative | Lipids and lipid-like molecules | Fatty Acyls |
| metab_1769 | Unannotated feature | pos | 213.9829 | 7.347 | 4.629 | 4.753 | 0.124 | 0.634 | 3.793E-04 | 0.004 | Unannotated | NA | NA |
| metab_6930 | Unannotated feature | neg | 155.1072 | 5.683 | 2.985 | 3.666 | 0.681 | 0.634 | 3.793E-04 | 0.004 | Unannotated | NA | NA |
| metab_1272 | trans-Zeatin riboside | pos | 396.1232 | 5.855 | 3.417 | 4.204 | 0.788 | 0.634 | 3.793E-04 | 0.004 | Tentative | Nucleosides, nucleotides, and analogues | Purine nucleosides |
| metab_9372 | Unannotated feature | neg | 289.8978 | 3.554 | 5.423 | 5.270 | -0.153 | -0.634 | 3.793E-04 | 0.004 | Unannotated | NA | NA |
| metab_3385 | Unannotated feature | pos | 127.1119 | 2.514 | 3.398 | 3.734 | 0.336 | 0.634 | 3.793E-04 | 0.004 | Unannotated | NA | NA |
| metab_9623 | Unannotated feature | neg | 219.8918 | 3.128 | 4.769 | 4.707 | -0.061 | -0.634 | 3.793E-04 | 0.004 | Unannotated | NA | NA |
| metab_2744 | Unannotated feature | pos | 472.3305 | 5.703 | 5.309 | 5.092 | -0.217 | -0.634 | 3.793E-04 | 0.004 | Unannotated | NA | NA |
| metab_315 | Unannotated feature | pos | 222.8791 | 0.792 | 4.668 | 4.823 | 0.155 | 0.634 | 3.793E-04 | 0.004 | Unannotated | NA | NA |
| metab_2935 | Helminthosporol | pos | 305.1132 | 4.800 | 4.724 | 4.036 | -0.687 | -0.634 | 3.793E-04 | 0.004 | Tentative | Organoheterocyclic compounds | Dioxepanes |
| metab_6340 | Unannotated feature | neg | 85.0651 | 3.819 | 3.436 | 3.009 | -0.427 | -0.634 | 3.793E-04 | 0.004 | Unannotated | NA | NA |
| metab_813 | 3-Succinoylpyridine | pos | 221.0921 | 3.097 | 4.289 | 3.675 | -0.614 | -0.634 | 3.793E-04 | 0.004 | Tentative | Organic acids and derivatives | Keto acids and derivatives |
| metab_1777 | Unannotated feature | pos | 144.1020 | 7.409 | 4.766 | 4.848 | 0.081 | 0.634 | 3.793E-04 | 0.004 | Unannotated | NA | NA |
| metab_4061 | Unannotated feature | pos | 257.1495 | 4.370 | 3.347 | 4.476 | 1.129 | 0.634 | 3.793E-04 | 0.004 | Unannotated | NA | NA |
| metab_4810 | Unannotated feature | pos | 169.9773 | 7.509 | 5.722 | 5.790 | 0.068 | 0.634 | 3.793E-04 | 0.004 | Unannotated | NA | NA |
| metab_930 | Unannotated feature | pos | 83.6585 | 3.970 | 3.764 | 3.935 | 0.170 | 0.634 | 3.793E-04 | 0.004 | Unannotated | NA | NA |
| metab_3953 | Unannotated feature | pos | 112.0510 | 0.854 | 3.277 | 3.710 | 0.433 | 0.634 | 3.793E-04 | 0.004 | Unannotated | NA | NA |
| metab_4158 | 2-Amino-2-methyl-1,3-propandiol | pos | 106.0866 | 0.660 | 3.948 | 4.613 | 0.665 | 0.634 | 3.793E-04 | 0.004 | Tentative | Organic nitrogen compounds | Organonitrogen compounds |
| metab_9935 | Unannotated feature | neg | 170.9024 | 2.119 | 3.882 | 3.687 | -0.194 | -0.634 | 3.793E-04 | 0.004 | Unannotated | NA | NA |
| metab_4031 | Unannotated feature | pos | 105.0427 | 0.792 | 4.182 | 4.109 | -0.073 | -0.634 | 3.793E-04 | 0.004 | Unannotated | NA | NA |
| metab_4725 | Unannotated feature | pos | 330.2639 | 6.010 | 5.173 | 5.269 | 0.096 | 0.634 | 3.793E-04 | 0.004 | Unannotated | NA | NA |
| metab_9824 | Epijasminoside A | neg | 365.1361 | 2.515 | 4.709 | 5.204 | 0.495 | 0.634 | 3.793E-04 | 0.004 | Tentative | Organic oxygen compounds | Organooxygen compounds |
| metab_11140 | Unannotated feature | neg | 681.2431 | 5.723 | 5.208 | 4.488 | -0.720 | -0.634 | 3.793E-04 | 0.004 | Unannotated | NA | NA |
| metab_2388 | Unannotated feature | pos | 849.5050 | 5.941 | 4.336 | 5.098 | 0.762 | 0.634 | 3.793E-04 | 0.004 | Unannotated | NA | NA |
| metab_1095 | Metyrosine | pos | 237.1234 | 5.382 | 4.594 | 3.811 | -0.783 | -0.634 | 3.793E-04 | 0.004 | Tentative | Phenylpropanoids and polyketides | Phenylpropanoic acids |
| metab_6897 | Ibuprofen glucuronide | neg | 427.1613 | 5.651 | 2.868 | 4.633 | 1.765 | 0.634 | 3.793E-04 | 0.004 | Tentative | Organic oxygen compounds | Organooxygen compounds |
| metab_5623 | Unannotated feature | neg | 390.8925 | 0.554 | 5.807 | 6.033 | 0.226 | 0.634 | 3.793E-04 | 0.004 | Unannotated | NA | NA |
| metab_513 | Unannotated feature | pos | 153.5883 | 1.925 | 3.900 | 4.229 | 0.328 | 0.634 | 3.793E-04 | 0.004 | Unannotated | NA | NA |
| metab_3257 | Indole-3-acetaldehyde | pos | 142.0653 | 2.583 | 5.475 | 5.272 | -0.203 | -0.634 | 3.793E-04 | 0.004 | Tentative | Organoheterocyclic compounds | Indoles and derivatives |
| metab_2268 | PE(18:1(9Z)/0:0) | pos | 480.3089 | 6.165 | 5.715 | 5.880 | 0.165 | 0.634 | 3.793E-04 | 0.004 | Strong putative | NA | NA |
| metab_4219 | Unannotated feature | pos | 490.6523 | 0.614 | 6.058 | 5.983 | -0.076 | -0.634 | 3.793E-04 | 0.004 | Unannotated | NA | NA |
| metab_3041 | Quinolin-2,8-diol | pos | 179.0815 | 4.140 | 4.097 | 3.163 | -0.933 | -0.634 | 3.793E-04 | 0.004 | Moderate putative | Organoheterocyclic compounds | Quinolines and derivatives |
| metab_10751 | Unannotated feature | neg | 584.8269 | 0.570 | 4.766 | 5.004 | 0.238 | 0.634 | 3.793E-04 | 0.004 | Unannotated | NA | NA |
| metab_28 | Unannotated feature | pos | 518.7417 | 0.583 | 5.814 | 5.740 | -0.073 | -0.634 | 3.793E-04 | 0.004 | Unannotated | NA | NA |
| metab_4238 | Unannotated feature | pos | 239.0914 | 4.792 | 6.023 | 5.322 | -0.701 | -0.629 | 4.149E-04 | 0.005 | Unannotated | NA | NA |
| metab_4907 | Unannotated feature | pos | 392.7955 | 0.583 | 5.974 | 5.908 | -0.065 | -0.629 | 4.149E-04 | 0.005 | Unannotated | NA | NA |
| metab_4908 | Unannotated feature | pos | 576.7003 | 0.583 | 5.842 | 5.765 | -0.078 | -0.629 | 4.149E-04 | 0.005 | Unannotated | NA | NA |
| metab_1682 | Unannotated feature | pos | 239.2248 | 7.269 | 4.699 | 4.587 | -0.112 | -0.629 | 4.149E-04 | 0.005 | Unannotated | NA | NA |
| metab_5247 | Unannotated feature | neg | 594.8555 | 0.554 | 5.937 | 6.156 | 0.219 | 0.629 | 4.149E-04 | 0.005 | Unannotated | NA | NA |
| metab_8916 | Unannotated feature | neg | 159.0117 | 7.117 | 5.171 | 5.253 | 0.081 | 0.629 | 4.149E-04 | 0.005 | Unannotated | NA | NA |
| metab_9999 | Unannotated feature | neg | 290.9295 | 1.935 | 4.151 | 3.790 | -0.361 | -0.629 | 4.149E-04 | 0.005 | Unannotated | NA | NA |
| metab_7144 | Unannotated feature | neg | 279.1606 | 5.867 | 3.423 | 3.935 | 0.512 | 0.629 | 4.149E-04 | 0.005 | Unannotated | NA | NA |
| metab_8701 | Unannotated feature | neg | 455.9959 | 5.603 | 4.063 | 3.246 | -0.817 | -0.629 | 4.149E-04 | 0.005 | Unannotated | NA | NA |
| metab_4036 | Unannotated feature | pos | 159.0628 | 0.784 | 4.692 | 5.017 | 0.325 | 0.629 | 4.149E-04 | 0.005 | Unannotated | NA | NA |
| metab_10869 | Unannotated feature | neg | 322.9047 | 0.554 | 5.818 | 6.065 | 0.247 | 0.629 | 4.149E-04 | 0.005 | Unannotated | NA | NA |
| metab_4571 | Unannotated feature | pos | 177.9565 | 0.449 | 4.257 | 4.094 | -0.163 | -0.629 | 4.149E-04 | 0.005 | Unannotated | NA | NA |
| metab_10833 | Unannotated feature | neg | 738.8150 | 0.554 | 4.764 | 5.102 | 0.338 | 0.629 | 4.149E-04 | 0.005 | Unannotated | NA | NA |
| metab_5761 | Unannotated feature | neg | 147.0657 | 1.935 | 3.321 | 4.028 | 0.707 | 0.629 | 4.149E-04 | 0.005 | Unannotated | NA | NA |
| metab_3992 | Unannotated feature | pos | 222.9721 | 0.823 | 3.867 | 4.259 | 0.392 | 0.629 | 4.149E-04 | 0.005 | Unannotated | NA | NA |
| metab_5135 | Indole-3-carboxaldehyde | pos | 146.0601 | 2.583 | 6.394 | 6.223 | -0.171 | -0.629 | 4.149E-04 | 0.005 | Strong putative | Nucleosides, nucleotides, and analogues | Purine nucleosides |
| metab_8014 | 1H-Perfluorooctane | neg | 418.9743 | 6.714 | 5.674 | 5.098 | -0.577 | -0.629 | 4.149E-04 | 0.005 | Tentative | Organohalogen compounds | Organofluorides |
| metab_377 | Unannotated feature | pos | 341.9623 | 0.900 | 4.890 | 4.699 | -0.192 | -0.629 | 4.149E-04 | 0.005 | Unannotated | NA | NA |
| metab_10819 | Unannotated feature | neg | 869.7834 | 0.554 | 2.863 | 4.754 | 1.891 | 0.629 | 4.149E-04 | 0.005 | Unannotated | NA | NA |
| metab_10170 | Unannotated feature | neg | 562.5893 | 0.904 | 5.437 | 5.307 | -0.130 | -0.629 | 4.149E-04 | 0.005 | Unannotated | NA | NA |
| metab_4620 | MJDBISSP00000002 | pos | 403.3611 | 5.855 | 7.498 | 7.568 | 0.070 | 0.629 | 4.149E-04 | 0.005 | Strong putative | NA | NA |
| metab_1489 | Unannotated feature | pos | 129.7517 | 6.072 | 4.129 | 4.237 | 0.109 | 0.629 | 4.149E-04 | 0.005 | Unannotated | NA | NA |
| metab_659 | Unannotated feature | pos | 244.1293 | 2.358 | 4.377 | 3.671 | -0.706 | -0.629 | 4.149E-04 | 0.005 | Unannotated | NA | NA |
| metab_4524 | Unannotated feature | pos | 684.9256 | 0.538 | 4.793 | 4.480 | -0.313 | -0.629 | 4.149E-04 | 0.005 | Unannotated | NA | NA |
| metab_4849 | 9,10-Epoxy-18-hydroxystearate | pos | 279.2318 | 6.242 | 6.195 | 5.988 | -0.207 | -0.629 | 4.149E-04 | 0.005 | Tentative | NA | NA |
| metab_4848 | Unannotated feature | pos | 332.8399 | 0.583 | 5.800 | 5.760 | -0.040 | -0.629 | 4.149E-04 | 0.005 | Unannotated | NA | NA |
| metab_1101 | Unannotated feature | pos | 590.4106 | 5.489 | 5.385 | 5.180 | -0.205 | -0.629 | 4.149E-04 | 0.005 | Unannotated | NA | NA |
| metab_249 | Unannotated feature | pos | 113.7322 | 0.667 | 4.633 | 5.108 | 0.474 | 0.629 | 4.149E-04 | 0.005 | Unannotated | NA | NA |
| metab_9662 | Unannotated feature | neg | 189.0766 | 2.993 | 2.692 | 3.732 | 1.040 | 0.629 | 4.149E-04 | 0.005 | Unannotated | NA | NA |
| metab_6290 | Unannotated feature | neg | 540.9473 | 0.684 | 4.919 | 4.453 | -0.465 | -0.629 | 4.149E-04 | 0.005 | Unannotated | NA | NA |
| metab_271 | 1,7-Dimethylxanthine | pos | 244.0791 | 0.714 | 5.399 | 5.556 | 0.157 | 0.629 | 4.149E-04 | 0.005 | Tentative | Organoheterocyclic compounds | Imidazopyrimidines |
| metab_3661 | Unannotated feature | pos | 271.0924 | 1.949 | 4.853 | 5.268 | 0.415 | 0.629 | 4.149E-04 | 0.005 | Unannotated | NA | NA |
| metab_3358 | Isoquinoline | pos | 130.0653 | 2.583 | 5.216 | 5.038 | -0.178 | -0.629 | 4.149E-04 | 0.005 | Strong putative | Organoheterocyclic compounds | Isoquinolines and derivatives |
| metab_6362 | Unannotated feature | neg | 127.0394 | 3.902 | 3.475 | 4.221 | 0.746 | 0.629 | 4.149E-04 | 0.005 | Unannotated | NA | NA |
| metab_5037 | D-Citrulline | pos | 217.1296 | 0.861 | 4.516 | 4.806 | 0.290 | 0.629 | 4.149E-04 | 0.005 | Tentative | Organic acids and derivatives | Carboxylic acids and derivatives |
| metab_1407 | Diphenylamine | pos | 170.0965 | 5.933 | 4.632 | 4.682 | 0.051 | 0.629 | 4.149E-04 | 0.005 | Moderate putative | NA | NA |
| metab_4970 | Unannotated feature | pos | 178.0088 | 0.698 | 4.467 | 4.894 | 0.427 | 0.629 | 4.149E-04 | 0.005 | Unannotated | NA | NA |
| metab_4733 | Unannotated feature | pos | 537.4141 | 6.103 | 5.230 | 4.703 | -0.527 | -0.629 | 4.149E-04 | 0.005 | Unannotated | NA | NA |
| metab_642 | Unannotated feature | pos | 83.6582 | 2.257 | 3.123 | 3.455 | 0.332 | 0.625 | 4.537E-04 | 0.005 | Unannotated | NA | NA |
| metab_4576 | Unannotated feature | pos | 163.0601 | 0.449 | 5.303 | 5.322 | 0.020 | 0.625 | 4.537E-04 | 0.005 | Unannotated | NA | NA |
| metab_5777 | Unannotated feature | neg | 872.7628 | 0.554 | 5.104 | 5.511 | 0.406 | 0.625 | 4.537E-04 | 0.005 | Unannotated | NA | NA |
| metab_3266 | Unannotated feature | pos | 132.0809 | 2.583 | 5.856 | 5.653 | -0.203 | -0.625 | 4.537E-04 | 0.005 | Unannotated | NA | NA |
| metab_2331 | Unannotated feature | pos | 146.0813 | 6.010 | 4.715 | 4.777 | 0.062 | 0.625 | 4.537E-04 | 0.005 | Unannotated | NA | NA |
| metab_7304 | Unannotated feature | neg | 549.4534 | 6.196 | 5.542 | 4.323 | -1.219 | -0.625 | 4.537E-04 | 0.005 | Unannotated | NA | NA |
| metab_4613 | Unannotated feature | pos | 285.1539 | 0.005 | 4.700 | 4.772 | 0.071 | 0.625 | 4.537E-04 | 0.005 | Unannotated | NA | NA |
| metab_7995 | Unannotated feature | neg | 100.9043 | 6.983 | 4.040 | 4.132 | 0.092 | 0.625 | 4.537E-04 | 0.005 | Unannotated | NA | NA |
| metab_361 | Unannotated feature | pos | 241.1546 | 0.861 | 4.794 | 5.281 | 0.486 | 0.625 | 4.537E-04 | 0.005 | Unannotated | NA | NA |
| metab_8989 | Buprofezin | neg | 350.1517 | 4.782 | 5.202 | 4.908 | -0.294 | -0.625 | 4.537E-04 | 0.005 | Tentative | Organoheterocyclic compounds | Thiadiazinanes |
| metab_9924 | Unannotated feature | neg | 218.0493 | 2.119 | 2.813 | 4.507 | 1.694 | 0.625 | 4.537E-04 | 0.005 | Unannotated | NA | NA |
| metab_8830 | Unannotated feature | neg | 136.0398 | 5.377 | 3.660 | 3.119 | -0.540 | -0.625 | 4.537E-04 | 0.005 | Unannotated | NA | NA |
| metab_6275 | Unannotated feature | neg | 291.9893 | 3.554 | 4.954 | 4.704 | -0.250 | -0.625 | 4.537E-04 | 0.005 | Unannotated | NA | NA |
| metab_7203 | Unannotated feature | neg | 201.9406 | 5.930 | 4.583 | 4.670 | 0.088 | 0.625 | 4.537E-04 | 0.005 | Unannotated | NA | NA |
| metab_298 | N-(L-Arginino)succinate | pos | 335.0947 | 0.753 | 4.746 | 5.378 | 0.632 | 0.625 | 4.537E-04 | 0.005 | Tentative | Organic acids and derivatives | Carboxylic acids and derivatives |
| metab_10844 | Unannotated feature | neg | 663.8456 | 0.554 | 4.975 | 5.220 | 0.245 | 0.625 | 4.537E-04 | 0.005 | Unannotated | NA | NA |
| metab_10848 | Unannotated feature | neg | 628.8491 | 0.554 | 5.148 | 5.618 | 0.470 | 0.625 | 4.537E-04 | 0.005 | Unannotated | NA | NA |
| metab_9364 | Unannotated feature | neg | 293.8919 | 3.562 | 4.814 | 4.650 | -0.164 | -0.625 | 4.537E-04 | 0.005 | Unannotated | NA | NA |
| metab_4460 | Unannotated feature | pos | 810.8002 | 0.551 | 4.350 | 5.014 | 0.664 | 0.625 | 4.537E-04 | 0.005 | Unannotated | NA | NA |
| metab_1743 | Unannotated feature | pos | 226.0121 | 7.324 | 5.755 | 5.809 | 0.054 | 0.625 | 4.537E-04 | 0.005 | Unannotated | NA | NA |
| metab_10554 | Unannotated feature | neg | 274.7927 | 0.621 | 5.537 | 5.481 | -0.056 | -0.625 | 4.537E-04 | 0.005 | Unannotated | NA | NA |
| metab_4024 | Unannotated feature | pos | 146.0925 | 0.800 | 3.356 | 4.043 | 0.687 | 0.625 | 4.537E-04 | 0.005 | Unannotated | NA | NA |
| metab_7898 | Unannotated feature | neg | 168.0298 | 2.700 | 2.637 | 4.339 | 1.702 | 0.625 | 4.537E-04 | 0.005 | Unannotated | NA | NA |
| metab_8091 | (9Z)-Hexadecenoic acid | neg | 567.4637 | 6.188 | 5.298 | 4.029 | -1.270 | -0.625 | 4.537E-04 | 0.005 | Tentative | Lipids and lipid-like molecules | Fatty Acyls |
| metab_74 | Unannotated feature | pos | 121.4900 | 0.434 | 5.402 | 5.439 | 0.037 | 0.625 | 4.537E-04 | 0.005 | Unannotated | NA | NA |
| metab_89 | Unannotated feature | pos | 147.0252 | 0.504 | 3.937 | 4.288 | 0.351 | 0.625 | 4.537E-04 | 0.005 | Unannotated | NA | NA |
| metab_2620 | Unannotated feature | pos | 171.0265 | 0.551 | 5.218 | 5.160 | -0.058 | -0.625 | 4.537E-04 | 0.005 | Unannotated | NA | NA |
| metab_272 | Unannotated feature | pos | 277.0894 | 0.714 | 3.969 | 5.090 | 1.120 | 0.625 | 4.537E-04 | 0.005 | Unannotated | NA | NA |
| metab_4395 | Unannotated feature | pos | 426.8408 | 0.567 | 4.430 | 4.616 | 0.186 | 0.625 | 4.537E-04 | 0.005 | Unannotated | NA | NA |
| metab_4989 | Unannotated feature | pos | 636.6558 | 0.591 | 5.641 | 5.559 | -0.082 | -0.625 | 4.537E-04 | 0.005 | Unannotated | NA | NA |
| metab_9590 | Unannotated feature | neg | 727.1715 | 3.160 | 5.488 | 4.903 | -0.585 | -0.625 | 4.537E-04 | 0.005 | Unannotated | NA | NA |
| metab_4087 | Unannotated feature | pos | 302.9750 | 0.729 | 4.533 | 3.550 | -0.983 | -0.625 | 4.537E-04 | 0.005 | Unannotated | NA | NA |
| metab_11286 | Unannotated feature | neg | 176.9003 | 0.570 | 3.704 | 4.159 | 0.454 | 0.625 | 4.537E-04 | 0.005 | Unannotated | NA | NA |
| metab_5018 | Unannotated feature | pos | 280.9307 | 0.823 | 3.714 | 4.289 | 0.574 | 0.625 | 4.537E-04 | 0.005 | Unannotated | NA | NA |
| metab_1869 | Unannotated feature | pos | 776.8061 | 0.551 | 5.828 | 6.023 | 0.195 | 0.625 | 4.537E-04 | 0.005 | Unannotated | NA | NA |
| metab_1284 | Unannotated feature | pos | 757.5217 | 5.855 | 5.231 | 4.873 | -0.359 | -0.625 | 4.537E-04 | 0.005 | Unannotated | NA | NA |
| metab_5977 | Unannotated feature | neg | 279.0991 | 2.780 | 3.853 | 4.863 | 1.010 | 0.625 | 4.537E-04 | 0.005 | Unannotated | NA | NA |
| metab_10774 | Unannotated feature | neg | 255.0878 | 5.247 | 6.068 | 5.309 | -0.759 | -0.625 | 4.537E-04 | 0.005 | Unannotated | NA | NA |
| metab_214 | Unannotated feature | pos | 171.0959 | 0.614 | 3.510 | 4.103 | 0.593 | 0.625 | 4.537E-04 | 0.005 | Unannotated | NA | NA |
| metab_8930 | Gibberellin A43 | neg | 439.1619 | 5.001 | 4.364 | 4.968 | 0.604 | 0.625 | 4.537E-04 | 0.005 | Tentative | Lipids and lipid-like molecules | Prenol lipids |
| metab_7369 | Unannotated feature | neg | 468.9711 | 6.816 | 5.039 | 3.856 | -1.183 | -0.625 | 4.537E-04 | 0.005 | Unannotated | NA | NA |
| metab_9631 | Quinolin-2-yl hydrogen sulfate | neg | 270.0083 | 3.104 | 2.795 | 4.072 | 1.277 | 0.625 | 4.537E-04 | 0.005 | Tentative | Organoheterocyclic compounds | Quinolines and derivatives |
| metab_11261 | Unannotated feature | neg | 547.4375 | 6.188 | 5.223 | 3.986 | -1.237 | -0.625 | 4.537E-04 | 0.005 | Unannotated | NA | NA |
| metab_629 | Unannotated feature | pos | 359.2881 | 5.941 | 7.033 | 7.130 | 0.097 | 0.621 | 4.958E-04 | 0.005 | Unannotated | NA | NA |
| metab_3318 | Unannotated feature | pos | 202.8367 | 2.644 | 5.896 | 5.960 | 0.063 | 0.621 | 4.958E-04 | 0.005 | Unannotated | NA | NA |
| metab_9358 | Unannotated feature | neg | 336.9745 | 3.570 | 5.033 | 4.812 | -0.221 | -0.621 | 4.958E-04 | 0.005 | Unannotated | NA | NA |
| metab_9271 | Unannotated feature | neg | 276.0042 | 3.827 | 4.646 | 4.313 | -0.333 | -0.621 | 4.958E-04 | 0.005 | Unannotated | NA | NA |
| metab_6287 | Unannotated feature | neg | 129.0507 | 3.602 | 2.775 | 2.140 | -0.635 | -0.621 | 4.958E-04 | 0.005 | Unannotated | NA | NA |
| metab_5036 | Unannotated feature | pos | 203.0350 | 0.854 | 4.601 | 5.180 | 0.579 | 0.621 | 4.958E-04 | 0.005 | Unannotated | NA | NA |
| metab_8048 | Unannotated feature | neg | 232.9319 | 6.441 | 5.024 | 5.149 | 0.125 | 0.621 | 4.958E-04 | 0.005 | Unannotated | NA | NA |
| metab_1423 | Unannotated feature | pos | 769.5378 | 5.941 | 5.269 | 5.022 | -0.248 | -0.621 | 4.958E-04 | 0.005 | Unannotated | NA | NA |
| metab_7223 | Unannotated feature | neg | 277.8751 | 5.969 | 5.778 | 5.723 | -0.055 | -0.621 | 4.958E-04 | 0.005 | Unannotated | NA | NA |
| metab_2905 | Unannotated feature | pos | 488.3428 | 4.961 | 5.487 | 5.299 | -0.188 | -0.621 | 4.958E-04 | 0.005 | Unannotated | NA | NA |
| metab_3652 | Unannotated feature | pos | 140.0707 | 1.956 | 4.089 | 4.305 | 0.216 | 0.621 | 4.958E-04 | 0.005 | Unannotated | NA | NA |
| metab_5023 | Unannotated feature | pos | 510.7101 | 0.591 | 5.740 | 5.674 | -0.066 | -0.621 | 4.958E-04 | 0.005 | Unannotated | NA | NA |
| metab_10794 | Unannotated feature | neg | 652.8136 | 0.562 | 4.677 | 4.915 | 0.238 | 0.621 | 4.958E-04 | 0.005 | Unannotated | NA | NA |
| metab_10205 | Unannotated feature | neg | 89.0389 | 0.873 | 4.687 | 4.514 | -0.173 | -0.621 | 4.958E-04 | 0.005 | Unannotated | NA | NA |
| metab_5480 | Unannotated feature | neg | 408.7359 | 0.699 | 1.645 | 4.277 | 2.633 | 0.621 | 4.958E-04 | 0.005 | Unannotated | NA | NA |
| metab_3702 | Unannotated feature | pos | 195.0765 | 1.902 | 3.354 | 4.072 | 0.718 | 0.621 | 4.958E-04 | 0.005 | Unannotated | NA | NA |
| metab_5104 | Unannotated feature | pos | 430.6966 | 0.591 | 6.436 | 6.375 | -0.060 | -0.621 | 4.958E-04 | 0.005 | Unannotated | NA | NA |
| metab_4605 | Unannotated feature | pos | 199.9753 | 0.025 | 5.442 | 5.421 | -0.022 | -0.621 | 4.958E-04 | 0.005 | Unannotated | NA | NA |
| metab_2535 | Unannotated feature | pos | 814.5600 | 5.871 | 5.411 | 5.130 | -0.281 | -0.621 | 4.958E-04 | 0.005 | Unannotated | NA | NA |
| metab_2108 | Unannotated feature | pos | 101.5626 | 7.061 | 4.643 | 4.817 | 0.174 | 0.621 | 4.958E-04 | 0.005 | Unannotated | NA | NA |
| metab_5869 | Unannotated feature | neg | 359.0561 | 2.431 | 0.501 | 4.720 | 4.220 | 0.621 | 4.958E-04 | 0.005 | Unannotated | NA | NA |
| metab_4268 | Unannotated feature | pos | 616.5983 | 0.591 | 5.512 | 5.411 | -0.100 | -0.621 | 4.958E-04 | 0.005 | Unannotated | NA | NA |
| metab_5686 | Unannotated feature | neg | 279.0351 | 1.286 | 4.944 | 4.734 | -0.210 | -0.621 | 4.958E-04 | 0.005 | Unannotated | NA | NA |
| metab_2376 | Unannotated feature | pos | 747.5305 | 5.956 | 5.387 | 5.246 | -0.141 | -0.621 | 4.958E-04 | 0.005 | Unannotated | NA | NA |
| metab_2402 | Unannotated feature | pos | 792.0547 | 5.933 | 5.023 | 4.647 | -0.376 | -0.621 | 4.958E-04 | 0.005 | Unannotated | NA | NA |
| metab_9233 | Glutamylisoleucine | neg | 241.1196 | 4.017 | 4.740 | 5.199 | 0.459 | 0.621 | 4.958E-04 | 0.005 | Tentative | Organic acids and derivatives | Carboxylic acids and derivatives |
| metab_11125 | Unannotated feature | neg | 394.1264 | 5.651 | 5.145 | 4.834 | -0.311 | -0.621 | 4.958E-04 | 0.005 | Unannotated | NA | NA |
| metab_6919 | Elymoclavine | neg | 253.1350 | 5.675 | 5.376 | 4.832 | -0.544 | -0.621 | 4.958E-04 | 0.005 | Tentative | Alkaloids and derivatives | Ergoline and derivatives |
| metab_5224 | Unannotated feature | neg | 996.8246 | 0.543 | 5.967 | 5.817 | -0.150 | -0.621 | 4.958E-04 | 0.005 | Unannotated | NA | NA |
| metab_4475 | Unannotated feature | pos | 671.8470 | 0.551 | 5.473 | 5.656 | 0.184 | 0.621 | 4.958E-04 | 0.005 | Unannotated | NA | NA |
| metab_4477 | Unannotated feature | pos | 645.8406 | 0.551 | 4.891 | 5.133 | 0.242 | 0.621 | 4.958E-04 | 0.005 | Unannotated | NA | NA |
| metab_1024 | Ricinoleic acid | pos | 619.4906 | 6.296 | 6.319 | 5.500 | -0.820 | -0.621 | 4.958E-04 | 0.005 | Moderate putative | Lipids and lipid-like molecules | Fatty Acyls |
| metab_8872 | 3-Hydroxy-p-mentha-1,8-dien-7-al | neg | 211.0975 | 5.247 | 5.232 | 4.453 | -0.779 | -0.621 | 4.958E-04 | 0.005 | Tentative | Lipids and lipid-like molecules | Prenol lipids |
| metab_2309 | N-Arachidonoyl Arginine | pos | 502.3739 | 6.049 | 5.833 | 5.910 | 0.077 | 0.621 | 4.958E-04 | 0.005 | Tentative | NA | NA |
| metab_4261 | Unannotated feature | pos | 752.5728 | 0.591 | 5.500 | 5.413 | -0.087 | -0.621 | 4.958E-04 | 0.005 | Unannotated | NA | NA |
| metab_4265 | Unannotated feature | pos | 684.5855 | 0.591 | 5.543 | 5.431 | -0.112 | -0.621 | 4.958E-04 | 0.005 | Unannotated | NA | NA |
| metab_4253 | Unannotated feature | pos | 434.6907 | 0.598 | 5.863 | 5.792 | -0.071 | -0.621 | 4.958E-04 | 0.005 | Unannotated | NA | NA |
| metab_9975 | Unannotated feature | neg | 179.8823 | 2.061 | 3.376 | 4.093 | 0.716 | 0.621 | 4.958E-04 | 0.005 | Unannotated | NA | NA |
| metab_9923 | Unannotated feature | neg | 219.0776 | 2.119 | 4.251 | 4.509 | 0.258 | 0.617 | 5.415E-04 | 0.005 | Unannotated | NA | NA |
| metab_3808 | Unannotated feature | pos | 184.0581 | 1.414 | 3.847 | 4.515 | 0.669 | 0.617 | 5.415E-04 | 0.005 | Unannotated | NA | NA |
| metab_5251 | Unannotated feature | neg | 606.7680 | 0.554 | 3.896 | 4.763 | 0.868 | 0.617 | 5.415E-04 | 0.005 | Unannotated | NA | NA |
| metab_6931 | Unannotated feature | neg | 194.0458 | 5.683 | 4.428 | 4.962 | 0.534 | 0.617 | 5.415E-04 | 0.005 | Moderate putative | NA | NA |
| metab_6179 | Unannotated feature | neg | 547.8212 | 0.652 | 4.767 | 4.146 | -0.620 | -0.617 | 5.415E-04 | 0.005 | Unannotated | NA | NA |
| metab_2550 | Unannotated feature | pos | 463.3638 | 5.871 | 4.988 | 5.323 | 0.335 | 0.617 | 5.415E-04 | 0.005 | Unannotated | NA | NA |
| metab_5892 | Unannotated feature | neg | 788.7885 | 0.570 | 4.666 | 5.032 | 0.366 | 0.617 | 5.415E-04 | 0.005 | Unannotated | NA | NA |
| metab_1143 | Unannotated feature | pos | 421.2955 | 5.657 | 4.970 | 4.785 | -0.185 | -0.617 | 5.415E-04 | 0.005 | Unannotated | NA | NA |
| metab_7488 | Unannotated feature | neg | 205.0717 | 2.119 | 2.414 | 3.721 | 1.307 | 0.617 | 5.415E-04 | 0.005 | Unannotated | NA | NA |
| metab_4996 | Gamma-Glutamylglycine | pos | 205.0819 | 0.768 | 3.898 | 4.530 | 0.632 | 0.617 | 5.415E-04 | 0.005 | Tentative | Organic acids and derivatives | Carboxylic acids and derivatives |
| metab_5046 | Unannotated feature | pos | 403.0097 | 0.861 | 5.909 | 5.797 | -0.113 | -0.617 | 5.415E-04 | 0.005 | Unannotated | NA | NA |
| metab_10785 | Unannotated feature | neg | 720.8592 | 0.562 | 4.564 | 4.048 | -0.516 | -0.617 | 5.415E-04 | 0.005 | Unannotated | NA | NA |
| metab_2220 | Unannotated feature | pos | 245.2265 | 6.296 | 4.409 | 3.425 | -0.984 | -0.617 | 5.415E-04 | 0.005 | Unannotated | NA | NA |
| metab_587 | Unannotated feature | pos | 124.7282 | 2.073 | 5.188 | 5.268 | 0.080 | 0.617 | 5.415E-04 | 0.005 | Unannotated | NA | NA |
| metab_4041 | Unannotated feature | pos | 120.0770 | 0.784 | 4.902 | 5.098 | 0.197 | 0.617 | 5.415E-04 | 0.005 | Unannotated | NA | NA |
| metab_2256 | Unannotated feature | pos | 610.5042 | 6.188 | 5.461 | 4.909 | -0.552 | -0.617 | 5.415E-04 | 0.005 | Unannotated | NA | NA |
| metab_4060 | 4-Amino-3-hydroxybutanoate | pos | 120.0658 | 0.760 | 5.226 | 5.383 | 0.157 | 0.617 | 5.415E-04 | 0.005 | Tentative | NA | NA |
| metab_397 | 4-hydroxy-5-[4-hydroxy-3-(sulfooxy)phenyl]pentanoic acid | pos | 289.0406 | 1.087 | 3.627 | 4.503 | 0.877 | 0.617 | 5.415E-04 | 0.005 | Tentative | Lipids and lipid-like molecules | Fatty Acyls |
| metab_2634 | Unannotated feature | pos | 109.1016 | 5.832 | 4.789 | 4.885 | 0.096 | 0.617 | 5.415E-04 | 0.005 | Unannotated | NA | NA |
| metab_61 | Unannotated feature | pos | 136.0619 | 0.038 | 4.856 | 4.809 | -0.047 | -0.617 | 5.415E-04 | 0.005 | Unannotated | NA | NA |
| metab_9467 | Unannotated feature | neg | 360.8721 | 3.308 | 4.858 | 4.734 | -0.124 | -0.617 | 5.415E-04 | 0.005 | Unannotated | NA | NA |
| metab_1449 | Unannotated feature | pos | 384.7638 | 0.591 | 5.947 | 5.876 | -0.070 | -0.617 | 5.415E-04 | 0.005 | Unannotated | NA | NA |
| metab_2828 | Unannotated feature | pos | 634.4364 | 5.489 | 5.049 | 4.787 | -0.262 | -0.617 | 5.415E-04 | 0.005 | Unannotated | NA | NA |
| metab_718 | Unannotated feature | pos | 190.0771 | 2.583 | 5.075 | 4.885 | -0.189 | -0.617 | 5.415E-04 | 0.005 | Unannotated | NA | NA |
| metab_3662 | Unannotated feature | pos | 241.1183 | 1.949 | 3.077 | 3.719 | 0.642 | 0.617 | 5.415E-04 | 0.005 | Unannotated | NA | NA |
| metab_4248 | Unannotated feature | pos | 428.6996 | 0.598 | 5.792 | 5.713 | -0.079 | -0.617 | 5.415E-04 | 0.005 | Unannotated | NA | NA |
| metab_6929 | Unannotated feature | neg | 106.0655 | 5.683 | 3.103 | 3.830 | 0.727 | 0.617 | 5.415E-04 | 0.005 | Unannotated | NA | NA |
| metab_6770 | Unannotated feature | neg | 666.7227 | 5.518 | 4.282 | 4.744 | 0.463 | 0.613 | 5.912E-04 | 0.005 | Unannotated | NA | NA |
| metab_10362 | Unannotated feature | neg | 471.9601 | 0.731 | 4.359 | 3.938 | -0.421 | -0.613 | 5.912E-04 | 0.005 | Unannotated | NA | NA |
| metab_1863 | Unannotated feature | pos | 154.9902 | 7.525 | 6.316 | 6.394 | 0.078 | 0.613 | 5.912E-04 | 0.005 | Unannotated | NA | NA |
| metab_2112 | Unannotated feature | pos | 603.2993 | 7.022 | 5.170 | 4.950 | -0.220 | -0.613 | 5.912E-04 | 0.005 | Unannotated | NA | NA |
| metab_8977 | Unannotated feature | neg | 282.9917 | 4.838 | 4.421 | 4.201 | -0.220 | -0.613 | 5.912E-04 | 0.005 | Unannotated | NA | NA |
| metab_5280 | Unannotated feature | neg | 835.7904 | 0.554 | 4.052 | 4.960 | 0.908 | 0.613 | 5.912E-04 | 0.005 | Unannotated | NA | NA |
| metab_3673 | (Indol-3-yl)acetamide | pos | 157.0762 | 1.941 | 3.030 | 3.614 | 0.585 | 0.613 | 5.912E-04 | 0.005 | Strong putative | Organoheterocyclic compounds | Indoles and derivatives |
| metab_1678 | Unannotated feature | pos | 363.2308 | 7.261 | 5.591 | 5.664 | 0.073 | 0.613 | 5.912E-04 | 0.005 | Unannotated | NA | NA |
| metab_467 | L-trans-5-Hydroxy-2-piperidinecarboxylic acid | pos | 168.0631 | 1.786 | 2.912 | 4.091 | 1.179 | 0.613 | 5.912E-04 | 0.005 | Tentative | Organic acids and derivatives | Carboxylic acids and derivatives |
| metab_2595 | Unannotated feature | pos | 559.0565 | 5.848 | 4.958 | 4.723 | -0.235 | -0.613 | 5.912E-04 | 0.005 | Unannotated | NA | NA |
| metab_4639 | Unannotated feature | pos | 474.8073 | 5.703 | 4.776 | 4.421 | -0.355 | -0.613 | 5.912E-04 | 0.005 | Unannotated | NA | NA |
| metab_6475 | Unannotated feature | neg | 217.0176 | 4.337 | 3.636 | 4.405 | 0.769 | 0.613 | 5.912E-04 | 0.005 | Unannotated | NA | NA |
| metab_4798 | Unannotated feature | pos | 197.0220 | 7.324 | 5.667 | 5.739 | 0.072 | 0.613 | 5.912E-04 | 0.005 | Unannotated | NA | NA |
| metab_8181 | Unannotated feature | neg | 455.3388 | 6.000 | 4.289 | 3.600 | -0.689 | -0.613 | 5.912E-04 | 0.005 | Unannotated | NA | NA |
| metab_4913 | Unannotated feature | pos | 804.4960 | 0.591 | 5.408 | 5.291 | -0.117 | -0.613 | 5.912E-04 | 0.005 | Unannotated | NA | NA |
| metab_5241 | Unannotated feature | neg | 529.8460 | 0.554 | 3.918 | 4.908 | 0.990 | 0.613 | 5.912E-04 | 0.005 | Unannotated | NA | NA |
| metab_6554 | Unannotated feature | neg | 411.0661 | 4.635 | 4.627 | 4.242 | -0.385 | -0.613 | 5.912E-04 | 0.005 | Unannotated | NA | NA |
| metab_7650 | Unannotated feature | neg | 137.0350 | 2.119 | 2.880 | 3.754 | 0.874 | 0.613 | 5.912E-04 | 0.005 | Unannotated | NA | NA |
| metab_6108 | Unannotated feature | neg | 271.9999 | 3.176 | 4.166 | 3.886 | -0.280 | -0.613 | 5.912E-04 | 0.005 | Unannotated | NA | NA |
| metab_2840 | Unannotated feature | pos | 458.3321 | 5.375 | 5.381 | 5.144 | -0.237 | -0.613 | 5.912E-04 | 0.005 | Unannotated | NA | NA |
| metab_1930 | Unannotated feature | pos | 143.9810 | 7.324 | 4.882 | 4.990 | 0.108 | 0.613 | 5.912E-04 | 0.005 | Unannotated | NA | NA |
| metab_4957 | Unannotated feature | pos | 122.0756 | 0.644 | 4.455 | 4.966 | 0.510 | 0.613 | 5.912E-04 | 0.005 | Unannotated | NA | NA |
| metab_5645 | Unannotated feature | neg | 526.8678 | 0.554 | 5.977 | 6.213 | 0.236 | 0.613 | 5.912E-04 | 0.005 | Unannotated | NA | NA |
| metab_4070 | Deoxyuridine | pos | 246.1084 | 0.745 | 3.834 | 4.563 | 0.728 | 0.613 | 5.912E-04 | 0.005 | Tentative | Nucleosides, nucleotides, and analogues | Pyrimidine nucleosides |
| metab_2593 | Obacunone 17-O-beta-D-glucoside | pos | 599.2499 | 5.848 | 4.720 | 4.001 | -0.719 | -0.613 | 5.912E-04 | 0.005 | Tentative | NA | NA |
| metab_2773 | Unannotated feature | pos | 472.3477 | 5.657 | 5.112 | 4.955 | -0.157 | -0.613 | 5.912E-04 | 0.005 | Unannotated | NA | NA |
| metab_9270 | Unannotated feature | neg | 318.9138 | 3.827 | 4.599 | 4.229 | -0.370 | -0.613 | 5.912E-04 | 0.005 | Unannotated | NA | NA |
| metab_7060 | Unannotated feature | neg | 498.1450 | 5.779 | 4.314 | 2.211 | -2.104 | -0.613 | 5.912E-04 | 0.005 | Unannotated | NA | NA |
| metab_2791 | Indoprofen | pos | 299.1390 | 5.626 | 6.137 | 5.675 | -0.462 | -0.613 | 5.912E-04 | 0.005 | Tentative | Phenylpropanoids and polyketides | Phenylpropanoic acids |
| metab_1248 | Isomasticadienonalic acid | pos | 486.3625 | 5.832 | 5.543 | 5.419 | -0.124 | -0.613 | 5.912E-04 | 0.005 | Tentative | Lipids and lipid-like molecules | Prenol lipids |
| metab_981 | Unannotated feature | pos | 285.0056 | 4.378 | 4.731 | 4.454 | -0.277 | -0.613 | 5.912E-04 | 0.005 | Unannotated | NA | NA |
| metab_7370 | Unannotated feature | neg | 512.9611 | 6.823 | 4.983 | 3.783 | -1.200 | -0.613 | 5.912E-04 | 0.005 | Unannotated | NA | NA |
| metab_3979 | Unannotated feature | pos | 249.1082 | 0.831 | 4.129 | 4.463 | 0.335 | 0.613 | 5.912E-04 | 0.005 | Moderate putative | NA | NA |
| metab_5811 | Unannotated feature | neg | 221.9555 | 2.119 | 4.123 | 3.828 | -0.295 | -0.613 | 5.912E-04 | 0.005 | Unannotated | NA | NA |
| metab_1438 | Unannotated feature | pos | 442.7225 | 0.591 | 5.931 | 5.846 | -0.085 | -0.613 | 5.912E-04 | 0.005 | Unannotated | NA | NA |
| metab_3535 | Unannotated feature | pos | 192.1053 | 3.120 | 3.899 | 4.620 | 0.721 | 0.613 | 5.912E-04 | 0.005 | Unannotated | NA | NA |
| metab_485 | Unannotated feature | pos | 311.1829 | 5.840 | 4.724 | 4.928 | 0.204 | 0.608 | 6.450E-04 | 0.006 | Unannotated | NA | NA |
| metab_19 | Unannotated feature | pos | 704.6433 | 0.583 | 5.508 | 5.372 | -0.136 | -0.608 | 6.450E-04 | 0.006 | Unannotated | NA | NA |
| metab_10831 | Unannotated feature | neg | 764.8240 | 0.554 | 5.170 | 5.454 | 0.284 | 0.608 | 6.450E-04 | 0.006 | Unannotated | NA | NA |
| metab_2480 | Unannotated feature | pos | 412.8936 | 5.902 | 5.039 | 5.277 | 0.238 | 0.608 | 6.450E-04 | 0.006 | Unannotated | NA | NA |
| metab_2549 | Unannotated feature | pos | 477.3801 | 5.871 | 5.170 | 5.472 | 0.301 | 0.608 | 6.450E-04 | 0.006 | Unannotated | NA | NA |
| metab_5129 | Estrone 3-sulfate | pos | 315.1338 | 2.950 | 4.721 | 4.176 | -0.545 | -0.608 | 6.450E-04 | 0.006 | Tentative | Lipids and lipid-like molecules | Steroids and steroid derivatives |
| metab_5744 | Unannotated feature | neg | 867.8082 | 0.554 | 4.965 | 5.231 | 0.267 | 0.608 | 6.450E-04 | 0.006 | Unannotated | NA | NA |
| metab_10922 | Unannotated feature | neg | 360.9748 | 0.539 | 4.440 | 4.178 | -0.261 | -0.608 | 6.450E-04 | 0.006 | Unannotated | NA | NA |
| metab_5621 | Unannotated feature | neg | 218.8741 | 0.912 | 5.139 | 5.059 | -0.081 | -0.608 | 6.450E-04 | 0.006 | Unannotated | NA | NA |
| metab_9353 | Unannotated feature | neg | 326.9457 | 3.562 | 4.984 | 4.823 | -0.162 | -0.608 | 6.450E-04 | 0.006 | Unannotated | NA | NA |
| metab_336 | Unannotated feature | pos | 282.9278 | 0.823 | 3.581 | 3.988 | 0.407 | 0.608 | 6.450E-04 | 0.006 | Unannotated | NA | NA |
| metab_4387 | Unannotated feature | pos | 562.8159 | 0.567 | 4.199 | 4.477 | 0.279 | 0.608 | 6.450E-04 | 0.006 | Unannotated | NA | NA |
| metab_3957 | Unannotated feature | pos | 81.0453 | 0.854 | 2.126 | 3.047 | 0.922 | 0.608 | 6.450E-04 | 0.006 | Unannotated | NA | NA |
| metab_10867 | Unannotated feature | neg | 458.8803 | 0.554 | 5.843 | 6.069 | 0.225 | 0.608 | 6.450E-04 | 0.006 | Unannotated | NA | NA |
| metab_4305 | Unannotated feature | pos | 450.7542 | 0.583 | 5.824 | 5.763 | -0.060 | -0.608 | 6.450E-04 | 0.006 | Unannotated | NA | NA |
| metab_1537 | Unannotated feature | pos | 88.1126 | 6.257 | 7.464 | 7.508 | 0.043 | 0.608 | 6.450E-04 | 0.006 | Unannotated | NA | NA |
| metab_3705 | Unannotated feature | pos | 175.1271 | 1.902 | 3.725 | 4.752 | 1.027 | 0.608 | 6.450E-04 | 0.006 | Unannotated | NA | NA |
| metab_983 | Unannotated feature | pos | 430.3010 | 4.394 | 5.327 | 5.180 | -0.147 | -0.608 | 6.450E-04 | 0.006 | Unannotated | NA | NA |
| metab_1768 | Unannotated feature | pos | 172.9564 | 7.347 | 5.536 | 5.586 | 0.050 | 0.608 | 6.450E-04 | 0.006 | Unannotated | NA | NA |
| metab_2738 | Unannotated feature | pos | 428.3032 | 5.710 | 5.135 | 4.986 | -0.149 | -0.608 | 6.450E-04 | 0.006 | Unannotated | NA | NA |
| metab_3863 | Unannotated feature | pos | 399.9208 | 0.908 | 4.888 | 4.614 | -0.274 | -0.608 | 6.450E-04 | 0.006 | Unannotated | NA | NA |
| metab_6764 | Unannotated feature | neg | 248.9221 | 5.518 | 3.761 | 4.001 | 0.240 | 0.608 | 6.450E-04 | 0.006 | Unannotated | NA | NA |
| metab_4492 | Unannotated feature | pos | 452.8442 | 0.551 | 4.175 | 4.554 | 0.378 | 0.608 | 6.450E-04 | 0.006 | Unannotated | NA | NA |
| metab_289 | Unannotated feature | pos | 219.1703 | 0.745 | 3.562 | 4.575 | 1.013 | 0.608 | 6.450E-04 | 0.006 | Unannotated | NA | NA |
| metab_2749 | Unannotated feature | pos | 516.3578 | 5.695 | 5.122 | 4.768 | -0.353 | -0.608 | 6.450E-04 | 0.006 | Unannotated | NA | NA |
| metab_11238 | Unannotated feature | neg | 478.2948 | 6.094 | 5.546 | 5.779 | 0.233 | 0.608 | 6.450E-04 | 0.006 | Unannotated | NA | NA |
| metab_1294 | Unannotated feature | pos | 631.7731 | 5.863 | 5.042 | 4.745 | -0.297 | -0.608 | 6.450E-04 | 0.006 | Unannotated | NA | NA |
| metab_1256 | Unannotated feature | pos | 574.0664 | 5.840 | 4.894 | 4.602 | -0.292 | -0.608 | 6.450E-04 | 0.006 | Unannotated | NA | NA |
| metab_1363 | Unannotated feature | pos | 751.5171 | 5.902 | 5.156 | 4.698 | -0.458 | -0.608 | 6.450E-04 | 0.006 | Unannotated | NA | NA |
| metab_155 | Unannotated feature | pos | 940.8188 | 0.551 | 5.864 | 5.717 | -0.147 | -0.608 | 6.450E-04 | 0.006 | Unannotated | NA | NA |
| metab_170 | Unannotated feature | pos | 666.8305 | 0.559 | 4.393 | 4.634 | 0.241 | 0.608 | 6.450E-04 | 0.006 | Unannotated | NA | NA |
| metab_5016 | Unannotated feature | pos | 143.0316 | 0.823 | 4.947 | 5.166 | 0.220 | 0.608 | 6.450E-04 | 0.006 | Unannotated | NA | NA |
| metab_5630 | Unannotated feature | neg | 165.0400 | 0.920 | 3.913 | 4.223 | 0.310 | 0.608 | 6.450E-04 | 0.006 | Unannotated | NA | NA |
| metab_1823 | Unannotated feature | pos | 90.9481 | 7.990 | 4.799 | 4.836 | 0.038 | 0.608 | 6.450E-04 | 0.006 | Unannotated | NA | NA |
| metab_1891 | Unannotated feature | pos | 89.5072 | 7.339 | 5.346 | 5.432 | 0.086 | 0.608 | 6.450E-04 | 0.006 | Unannotated | NA | NA |
| metab_3884 | Acetylhomoserine | pos | 206.0400 | 0.877 | 3.805 | 4.306 | 0.501 | 0.604 | 7.034E-04 | 0.006 | Tentative | Organic acids and derivatives | Carboxylic acids and derivatives |
| metab_3016 | 1-Hydroxy-2,2,5,5-tetramethyl-2,5-dihydro-1h-pyrrole-3-carboxamide | pos | 386.2747 | 4.248 | 5.270 | 5.135 | -0.135 | -0.604 | 7.034E-04 | 0.006 | Tentative | Organoheterocyclic compounds | Pyrrolines |
| metab_6051 | Unannotated feature | neg | 307.1040 | 3.057 | 2.938 | 3.868 | 0.930 | 0.604 | 7.034E-04 | 0.006 | Unannotated | NA | NA |
| metab_2878 | 3-(Aminomethyl)-2,5,9-trimethyl-7H-furo[3,2-g]chromen-7-one | pos | 299.1391 | 5.122 | 5.067 | 3.733 | -1.334 | -0.604 | 7.034E-04 | 0.006 | Tentative | Phenylpropanoids and polyketides | Coumarins and derivatives |
| metab_4991 | N-Acetyl-alpha-neuraminate | pos | 310.1132 | 0.753 | 5.420 | 5.033 | -0.387 | -0.604 | 7.034E-04 | 0.006 | Moderate putative | Organic oxygen compounds | Organooxygen compounds |
| metab_4895 | N-Hydroxy-L-tryptophan | pos | 221.0921 | 1.941 | 5.219 | 5.491 | 0.272 | 0.604 | 7.034E-04 | 0.006 | Tentative | Organoheterocyclic compounds | Indoles and derivatives |
| metab_4953 | Unannotated feature | pos | 241.0302 | 0.644 | 4.965 | 5.235 | 0.269 | 0.604 | 7.034E-04 | 0.006 | Unannotated | NA | NA |
| metab_3872 | 1-Cyano-N-[4-(trifluoromethoxy)phenyl]formohydrazide | pos | 284.0037 | 0.900 | 5.043 | 4.843 | -0.200 | -0.604 | 7.034E-04 | 0.006 | Tentative | Benzenoids | Benzene and substituted derivatives |
| metab_1375 | Unannotated feature | pos | 743.5135 | 5.910 | 5.181 | 4.828 | -0.352 | -0.604 | 7.034E-04 | 0.006 | Unannotated | NA | NA |
| metab_3964 | Unannotated feature | pos | 333.1444 | 4.201 | 5.059 | 4.446 | -0.613 | -0.604 | 7.034E-04 | 0.006 | Unannotated | NA | NA |
| metab_9374 | Unannotated feature | neg | 291.8949 | 3.554 | 5.489 | 5.368 | -0.121 | -0.604 | 7.034E-04 | 0.006 | Unannotated | NA | NA |
| metab_2418 | Unannotated feature | pos | 767.5185 | 5.925 | 5.355 | 5.053 | -0.302 | -0.604 | 7.034E-04 | 0.006 | Unannotated | NA | NA |
| metab_9794 | Unannotated feature | neg | 197.9625 | 2.549 | 2.890 | 3.826 | 0.936 | 0.604 | 7.034E-04 | 0.006 | Unannotated | NA | NA |
| metab_9818 | (S)-3-Hydroxybutanoate | neg | 149.0463 | 2.523 | 3.620 | 3.964 | 0.345 | 0.604 | 7.034E-04 | 0.006 | Tentative | Organic acids and derivatives | Hydroxy acids and derivatives |
| metab_4306 | Unannotated feature | pos | 334.8369 | 0.583 | 5.672 | 5.621 | -0.051 | -0.604 | 7.034E-04 | 0.006 | Unannotated | NA | NA |
| metab_11036 | Unannotated feature | neg | 322.8514 | 0.013 | 4.466 | 4.383 | -0.082 | -0.604 | 7.034E-04 | 0.006 | Unannotated | NA | NA |
| metab_550 | Unannotated feature | pos | 122.5474 | 2.019 | 3.523 | 3.357 | -0.166 | -0.604 | 7.034E-04 | 0.006 | Unannotated | NA | NA |
| metab_5257 | Unannotated feature | neg | 665.8207 | 0.554 | 4.720 | 5.114 | 0.394 | 0.604 | 7.034E-04 | 0.006 | Unannotated | NA | NA |
| metab_5258 | Unannotated feature | neg | 668.7994 | 0.554 | 4.970 | 5.636 | 0.666 | 0.604 | 7.034E-04 | 0.006 | Unannotated | NA | NA |
| metab_4468 | Unannotated feature | pos | 742.8127 | 0.551 | 4.529 | 5.100 | 0.571 | 0.604 | 7.034E-04 | 0.006 | Unannotated | NA | NA |
| metab_11083 | Azelaic acid | neg | 187.0973 | 5.619 | 5.125 | 5.311 | 0.186 | 0.604 | 7.034E-04 | 0.006 | Tentative | Lipids and lipid-like molecules | Fatty Acyls |
| metab_2218 | 9(S)-HODE | pos | 297.2425 | 6.296 | 5.164 | 4.754 | -0.410 | -0.604 | 7.034E-04 | 0.006 | Tentative | Lipids and lipid-like molecules | Fatty Acyls |
| metab_8558 | Unannotated feature | neg | 301.6097 | 5.691 | 4.118 | 3.507 | -0.611 | -0.604 | 7.034E-04 | 0.006 | Unannotated | NA | NA |
| metab_8873 | Unannotated feature | neg | 173.0717 | 5.239 | 3.721 | 2.613 | -1.107 | -0.604 | 7.034E-04 | 0.006 | Unannotated | NA | NA |
| metab_4304 | Unannotated feature | pos | 458.7859 | 0.583 | 5.536 | 5.483 | -0.053 | -0.604 | 7.034E-04 | 0.006 | Unannotated | NA | NA |
| metab_8159 | Unannotated feature | neg | 491.2117 | 6.047 | 3.958 | 3.762 | -0.195 | -0.604 | 7.034E-04 | 0.006 | Unannotated | NA | NA |
| metab_3678 | Pro-leu | pos | 229.1547 | 1.933 | 5.897 | 6.299 | 0.402 | 0.604 | 7.034E-04 | 0.006 | Tentative | Organic acids and derivatives | Carboxylic acids and derivatives |
| metab_384 | Unannotated feature | pos | 401.9179 | 0.908 | 4.690 | 4.434 | -0.256 | -0.604 | 7.034E-04 | 0.006 | Unannotated | NA | NA |
| metab_7958 | Unannotated feature | neg | 82.9533 | 7.109 | 4.038 | 3.964 | -0.073 | -0.604 | 7.034E-04 | 0.006 | Unannotated | NA | NA |
| metab_7690 | Unannotated feature | neg | 244.8849 | 7.221 | 4.401 | 4.336 | -0.065 | -0.604 | 7.034E-04 | 0.006 | Unannotated | NA | NA |
| metab_1207 | 3-(Hydroxymethyl)-5,5-diphenylimidazolidine-2,4-dione | pos | 315.1339 | 5.771 | 5.456 | 5.162 | -0.294 | -0.604 | 7.034E-04 | 0.006 | Tentative | Organoheterocyclic compounds | Azolidines |
| metab_8620 | Unannotated feature | neg | 609.2544 | 5.651 | 3.944 | 4.681 | 0.738 | 0.604 | 7.034E-04 | 0.006 | Unannotated | NA | NA |
| metab_5814 | Unannotated feature | neg | 245.9471 | 2.119 | 4.484 | 4.227 | -0.257 | -0.604 | 7.034E-04 | 0.006 | Unannotated | NA | NA |
| metab_10569 | Unannotated feature | neg | 790.4307 | 0.613 | 5.712 | 5.649 | -0.063 | -0.604 | 7.034E-04 | 0.006 | Unannotated | NA | NA |
| metab_6315 | Unannotated feature | neg | 264.2817 | 3.726 | 3.219 | 4.440 | 1.221 | 0.604 | 7.034E-04 | 0.006 | Unannotated | NA | NA |
| metab_9079 | Unannotated feature | neg | 275.1043 | 4.513 | 4.284 | 3.726 | -0.558 | -0.604 | 7.034E-04 | 0.006 | Unannotated | NA | NA |
| metab_4743 | Unannotated feature | pos | 130.1592 | 6.257 | 6.981 | 7.029 | 0.048 | 0.604 | 7.034E-04 | 0.006 | Unannotated | NA | NA |
| metab_5673 | Unannotated feature | neg | 634.8060 | 0.554 | 4.419 | 4.886 | 0.467 | 0.604 | 7.034E-04 | 0.006 | Unannotated | NA | NA |
| metab_10437 | Unannotated feature | neg | 179.0558 | 0.668 | 5.368 | 5.572 | 0.204 | 0.604 | 7.034E-04 | 0.006 | Unannotated | NA | NA |
| metab_10469 | Unannotated feature | neg | 187.0376 | 0.652 | 4.161 | 4.855 | 0.694 | 0.604 | 7.034E-04 | 0.006 | Unannotated | NA | NA |
| metab_3575 | Morphine | pos | 211.1077 | 2.058 | 4.449 | 4.682 | 0.233 | 0.604 | 7.034E-04 | 0.006 | Tentative | Organoheterocyclic compounds | Oxazinanes |
| metab_4284 | Unannotated feature | pos | 370.7409 | 0.591 | 5.906 | 5.836 | -0.071 | -0.604 | 7.034E-04 | 0.006 | Unannotated | NA | NA |
| metab_4113 | Unannotated feature | pos | 782.4416 | 0.698 | 5.406 | 5.241 | -0.165 | -0.604 | 7.034E-04 | 0.006 | Unannotated | NA | NA |
| metab_1927 | Unannotated feature | pos | 178.9907 | 7.324 | 5.381 | 5.485 | 0.104 | 0.604 | 7.034E-04 | 0.006 | Unannotated | NA | NA |
| metab_14 | Unannotated feature | pos | 256.8207 | 0.591 | 6.595 | 6.543 | -0.052 | -0.604 | 7.034E-04 | 0.006 | Unannotated | NA | NA |
| metab_6938 | Nafazatrom | neg | 313.1201 | 5.683 | 5.147 | 4.815 | -0.332 | -0.604 | 7.034E-04 | 0.006 | Tentative | Benzenoids | Naphthalenes |
| metab_1173 | Unannotated feature | pos | 706.4945 | 5.717 | 5.412 | 5.208 | -0.204 | -0.604 | 7.034E-04 | 0.006 | Unannotated | NA | NA |
| metab_1145 | Unannotated feature | pos | 604.4270 | 5.657 | 5.582 | 5.413 | -0.169 | -0.604 | 7.034E-04 | 0.006 | Unannotated | NA | NA |
| metab_2065 | Unannotated feature | pos | 144.9822 | 7.215 | 5.335 | 5.469 | 0.134 | 0.604 | 7.034E-04 | 0.006 | Unannotated | NA | NA |
| metab_7803 | Unannotated feature | neg | 538.3165 | 7.164 | 4.502 | 4.112 | -0.390 | -0.600 | 7.667E-04 | 0.006 | Unannotated | NA | NA |
| metab_4732 | Unannotated feature | pos | 458.3475 | 6.057 | 5.423 | 5.680 | 0.257 | 0.600 | 7.667E-04 | 0.006 | Unannotated | NA | NA |
| metab_10052 | Unannotated feature | neg | 240.0491 | 1.398 | 4.262 | 3.818 | -0.444 | -0.600 | 7.667E-04 | 0.006 | Unannotated | NA | NA |
| metab_10110 | Unannotated feature | neg | 214.9704 | 1.094 | 4.835 | 4.729 | -0.106 | -0.600 | 7.667E-04 | 0.006 | Unannotated | NA | NA |
| metab_448 | 5-Acetamidopentanoate | pos | 160.0969 | 1.694 | 4.093 | 4.916 | 0.824 | 0.600 | 7.667E-04 | 0.006 | Tentative | Lipids and lipid-like molecules | Fatty Acyls |
| metab_10775 | Unannotated feature | neg | 923.8238 | 0.562 | 4.608 | 4.131 | -0.477 | -0.600 | 7.667E-04 | 0.006 | Unannotated | NA | NA |
| metab_6668 | Unannotated feature | neg | 142.0656 | 5.139 | 2.426 | 3.655 | 1.229 | 0.600 | 7.667E-04 | 0.006 | Unannotated | NA | NA |
| metab_1283 | Unannotated feature | pos | 712.4962 | 5.855 | 5.962 | 5.844 | -0.119 | -0.600 | 7.667E-04 | 0.006 | Unannotated | NA | NA |
| metab_9961 | Unannotated feature | neg | 185.9623 | 2.111 | 4.742 | 4.552 | -0.191 | -0.600 | 7.667E-04 | 0.006 | Unannotated | NA | NA |
| metab_8195 | Unannotated feature | neg | 259.9012 | 5.977 | 5.469 | 5.582 | 0.112 | 0.600 | 7.667E-04 | 0.006 | Unannotated | NA | NA |
| metab_9366 | Unannotated feature | neg | 228.9811 | 3.562 | 4.271 | 4.019 | -0.252 | -0.600 | 7.667E-04 | 0.006 | Unannotated | NA | NA |
| metab_5222 | Unannotated feature | neg | 962.8310 | 0.543 | 5.853 | 5.685 | -0.168 | -0.600 | 7.667E-04 | 0.006 | Unannotated | NA | NA |
| metab_4429 | Unannotated feature | pos | 567.3901 | 0.559 | 4.934 | 4.832 | -0.102 | -0.600 | 7.667E-04 | 0.006 | Unannotated | NA | NA |
| metab_6800 | Unannotated feature | neg | 380.1109 | 5.563 | 4.274 | 3.789 | -0.485 | -0.600 | 7.667E-04 | 0.006 | Unannotated | NA | NA |
| metab_9137 | Unannotated feature | neg | 193.0868 | 4.306 | 3.867 | 3.169 | -0.698 | -0.600 | 7.667E-04 | 0.006 | Moderate putative | NA | NA |
| metab_10309 | Unannotated feature | neg | 450.7096 | 0.794 | 4.584 | 4.219 | -0.365 | -0.600 | 7.667E-04 | 0.006 | Unannotated | NA | NA |
| metab_4250 | Unannotated feature | pos | 554.6456 | 0.598 | 5.010 | 4.902 | -0.107 | -0.600 | 7.667E-04 | 0.006 | Unannotated | NA | NA |
| metab_8694 | Unannotated feature | neg | 269.1512 | 5.611 | 4.807 | 5.345 | 0.539 | 0.600 | 7.667E-04 | 0.006 | Unannotated | NA | NA |
| metab_3805 | Unannotated feature | pos | 133.0497 | 1.421 | 4.132 | 4.237 | 0.105 | 0.600 | 7.667E-04 | 0.006 | Unannotated | NA | NA |
| metab_3862 | Unannotated feature | pos | 90.9771 | 0.916 | 4.153 | 4.239 | 0.086 | 0.600 | 7.667E-04 | 0.006 | Unannotated | NA | NA |
| metab_10391 | Unannotated feature | neg | 256.0597 | 0.715 | 4.345 | 4.644 | 0.300 | 0.600 | 7.667E-04 | 0.006 | Unannotated | NA | NA |
| metab_15 | N-Carboxyethyl-gamma-aminobutyric acid | pos | 158.0813 | 1.801 | 3.959 | 4.533 | 0.574 | 0.600 | 7.667E-04 | 0.006 | Moderate putative | Organic acids and derivatives | Carboxylic acids and derivatives |
| metab_12 | Unannotated feature | pos | 258.8178 | 0.591 | 6.453 | 6.397 | -0.056 | -0.600 | 7.667E-04 | 0.006 | Unannotated | NA | NA |
| metab_1755 | Unannotated feature | pos | 202.0162 | 7.331 | 5.500 | 5.628 | 0.128 | 0.600 | 7.667E-04 | 0.006 | Unannotated | NA | NA |
| metab_10879 | Unannotated feature | neg | 97.2222 | 0.554 | 3.404 | 3.929 | 0.525 | 0.600 | 7.667E-04 | 0.006 | Unannotated | NA | NA |
| metab_1886 | Unannotated feature | pos | 156.0106 | 7.339 | 5.668 | 5.755 | 0.086 | 0.600 | 7.667E-04 | 0.006 | Unannotated | NA | NA |
| metab_10219 | (2S,3S,4S,5R)-3,4,5-Trihydroxy-6-sulfooxyoxane-2-carboxylic acid | neg | 254.9820 | 0.865 | 3.765 | 4.434 | 0.669 | 0.600 | 7.667E-04 | 0.006 | Tentative | Organic oxygen compounds | Organooxygen compounds |
| metab_2207 | Unannotated feature | pos | 592.4932 | 6.296 | 4.949 | 3.945 | -1.004 | -0.600 | 7.667E-04 | 0.006 | Unannotated | NA | NA |
| metab_5226 | Unannotated feature | neg | 1030.8183 | 0.543 | 5.520 | 5.260 | -0.260 | -0.600 | 7.667E-04 | 0.006 | Unannotated | NA | NA |
| metab_4936 | Unannotated feature | pos | 694.6143 | 0.591 | 5.776 | 5.677 | -0.099 | -0.600 | 7.667E-04 | 0.006 | Unannotated | NA | NA |
| metab_4918 | Unannotated feature | pos | 548.6109 | 0.598 | 6.165 | 6.073 | -0.092 | -0.600 | 7.667E-04 | 0.006 | Unannotated | NA | NA |
| metab_5038 | Unannotated feature | pos | 229.1547 | 0.861 | 5.756 | 6.218 | 0.462 | 0.600 | 7.667E-04 | 0.006 | Unannotated | NA | NA |
| metab_2732 | Unannotated feature | pos | 633.4283 | 5.717 | 4.530 | 4.001 | -0.529 | -0.600 | 7.667E-04 | 0.006 | Unannotated | NA | NA |
| metab_9643 | 4-Hydroxyphenylacetic acid sulfate | neg | 230.9970 | 3.072 | 4.080 | 5.219 | 1.139 | 0.600 | 7.667E-04 | 0.006 | Tentative | Benzenoids | Phenols |
| metab_263 | Unannotated feature | pos | 233.0632 | 0.691 | 4.143 | 4.539 | 0.396 | 0.600 | 7.667E-04 | 0.006 | Unannotated | NA | NA |
| metab_3194 | Unannotated feature | pos | 289.1620 | 3.283 | 4.517 | 4.352 | -0.165 | -0.600 | 7.667E-04 | 0.006 | Unannotated | NA | NA |
| metab_4868 | Unannotated feature | pos | 682.5886 | 0.591 | 5.641 | 5.507 | -0.135 | -0.600 | 7.667E-04 | 0.006 | Unannotated | NA | NA |
| metab_4640 | Unannotated feature | pos | 794.5461 | 5.710 | 5.004 | 4.576 | -0.428 | -0.600 | 7.667E-04 | 0.006 | Unannotated | NA | NA |
| metab_3544 | Unannotated feature | pos | 170.5036 | 2.073 | 5.653 | 5.719 | 0.066 | 0.600 | 7.667E-04 | 0.006 | Unannotated | NA | NA |
| metab_4712 | Unannotated feature | pos | 674.5067 | 5.949 | 6.478 | 6.395 | -0.083 | -0.600 | 7.667E-04 | 0.006 | Unannotated | NA | NA |
| metab_8198 | Unannotated feature | neg | 250.1452 | 5.969 | 5.324 | 5.437 | 0.113 | 0.600 | 7.667E-04 | 0.006 | Unannotated | NA | NA |
| metab_2726 | Unannotated feature | pos | 589.4025 | 5.725 | 5.089 | 4.829 | -0.261 | -0.600 | 7.667E-04 | 0.006 | Unannotated | NA | NA |
| metab_3698 | Unannotated feature | pos | 108.0447 | 1.910 | 3.841 | 4.013 | 0.171 | 0.600 | 7.667E-04 | 0.006 | Unannotated | NA | NA |
| metab_325 | Unannotated feature | pos | 129.1024 | 0.815 | 3.313 | 3.693 | 0.380 | 0.600 | 7.667E-04 | 0.006 | Unannotated | NA | NA |
| metab_2592 | Unannotated feature | pos | 607.7548 | 5.848 | 5.236 | 4.950 | -0.286 | -0.600 | 7.667E-04 | 0.006 | Unannotated | NA | NA |
| metab_6701 | Unannotated feature | neg | 359.1256 | 5.271 | 4.047 | 3.068 | -0.978 | -0.600 | 7.667E-04 | 0.006 | Unannotated | NA | NA |
| metab_1542 | Diacylglycerol | pos | 575.4669 | 6.288 | 5.960 | 5.587 | -0.374 | -0.596 | 8.352E-04 | 0.006 | Tentative | Lipids and lipid-like molecules | Fatty Acyls |
| metab_372 | Unannotated feature | pos | 115.0635 | 0.892 | 3.029 | 4.144 | 1.115 | 0.596 | 8.352E-04 | 0.006 | Unannotated | NA | NA |
| metab_8847 | Unannotated feature | neg | 321.8146 | 5.338 | 4.630 | 4.763 | 0.132 | 0.596 | 8.352E-04 | 0.006 | Unannotated | NA | NA |
| metab_417 | Unannotated feature | pos | 230.1580 | 1.290 | 5.015 | 5.475 | 0.460 | 0.596 | 8.352E-04 | 0.006 | Unannotated | NA | NA |
| metab_3980 | Unannotated feature | pos | 231.1704 | 0.831 | 2.896 | 3.649 | 0.753 | 0.596 | 8.352E-04 | 0.006 | Unannotated | NA | NA |
| metab_5274 | Unannotated feature | neg | 799.8207 | 0.554 | 4.980 | 5.140 | 0.160 | 0.596 | 8.352E-04 | 0.006 | Unannotated | NA | NA |
| metab_10639 | Unannotated feature | neg | 657.5600 | 0.590 | 5.402 | 5.253 | -0.150 | -0.596 | 8.352E-04 | 0.006 | Unannotated | NA | NA |
| metab_7881 | Unannotated feature | neg | 173.9258 | 7.140 | 5.167 | 5.072 | -0.095 | -0.596 | 8.352E-04 | 0.006 | Unannotated | NA | NA |
| metab_9859 | Unannotated feature | neg | 504.6308 | 0.621 | 6.583 | 6.519 | -0.064 | -0.596 | 8.352E-04 | 0.006 | Unannotated | NA | NA |
| metab_2318 | Unannotated feature | pos | 766.7759 | 0.559 | 4.289 | 4.550 | 0.260 | 0.596 | 8.352E-04 | 0.006 | Unannotated | NA | NA |
| metab_5221 | Unannotated feature | neg | 936.8257 | 0.543 | 5.576 | 5.463 | -0.113 | -0.596 | 8.352E-04 | 0.006 | Unannotated | NA | NA |
| metab_1298 | Unannotated feature | pos | 529.2080 | 5.871 | 4.614 | 3.829 | -0.786 | -0.596 | 8.352E-04 | 0.006 | Unannotated | NA | NA |
| metab_2778 | Unannotated feature | pos | 531.3608 | 5.649 | 4.623 | 4.384 | -0.240 | -0.596 | 8.352E-04 | 0.006 | Unannotated | NA | NA |
| metab_9505 | Unannotated feature | neg | 454.0325 | 3.734 | 3.799 | 4.813 | 1.014 | 0.596 | 8.352E-04 | 0.006 | Unannotated | NA | NA |
| metab_4919 | Unannotated feature | pos | 802.4999 | 0.598 | 5.651 | 5.539 | -0.112 | -0.596 | 8.352E-04 | 0.006 | Unannotated | NA | NA |
| metab_3926 | Hydroxyproline-isomer feature | pos | 114.0553 | 0.861 | 4.418 | 4.643 | 0.225 | 0.596 | 8.352E-04 | 0.006 | Feature-level only | Organic acids and derivatives | Carboxylic acids and derivatives |
| metab_2967 | Unannotated feature | pos | 94.0655 | 4.570 | 4.136 | 4.296 | 0.160 | 0.596 | 8.352E-04 | 0.006 | Unannotated | NA | NA |
| metab_5044 | Aspartylhydroxyproline | pos | 247.0924 | 0.721 | 3.796 | 4.709 | 0.913 | 0.596 | 8.352E-04 | 0.006 | Tentative | Organic acids and derivatives | Carboxylic acids and derivatives |
| metab_3928 | 3-Aminoisobutyric acid | pos | 86.0605 | 0.861 | 4.061 | 4.357 | 0.296 | 0.596 | 8.352E-04 | 0.006 | Tentative | Organic acids and derivatives | Carboxylic acids and derivatives |
| metab_4894 | Unannotated feature | pos | 228.2030 | 1.941 | 3.575 | 4.170 | 0.595 | 0.596 | 8.352E-04 | 0.006 | Unannotated | NA | NA |
| metab_4392 | Unannotated feature | pos | 517.8522 | 0.567 | 4.393 | 4.226 | -0.167 | -0.596 | 8.352E-04 | 0.006 | Unannotated | NA | NA |
| metab_247 | Unannotated feature | pos | 316.1114 | 0.660 | 4.983 | 5.424 | 0.442 | 0.596 | 8.352E-04 | 0.006 | Unannotated | NA | NA |
| metab_2746 | Unannotated feature | pos | 450.3164 | 5.703 | 5.178 | 4.997 | -0.181 | -0.596 | 8.352E-04 | 0.006 | Unannotated | NA | NA |
| metab_5060 | Unannotated feature | pos | 219.0717 | 0.908 | 4.174 | 4.636 | 0.462 | 0.596 | 8.352E-04 | 0.006 | Unannotated | NA | NA |
| metab_8510 | Unannotated feature | neg | 167.1073 | 5.723 | 2.577 | 3.576 | 1.000 | 0.596 | 8.352E-04 | 0.006 | Unannotated | NA | NA |
| metab_11235 | Unannotated feature | neg | 515.3748 | 6.047 | 4.506 | 3.495 | -1.011 | -0.596 | 8.352E-04 | 0.006 | Unannotated | NA | NA |
| metab_1837 | Unannotated feature | pos | 131.9744 | 7.959 | 5.150 | 5.195 | 0.045 | 0.596 | 8.352E-04 | 0.006 | Unannotated | NA | NA |
| metab_5660 | Unannotated feature | neg | 201.0380 | 1.086 | 5.427 | 5.092 | -0.335 | -0.596 | 8.352E-04 | 0.006 | Unannotated | NA | NA |
| metab_4285 | Unannotated feature | pos | 316.7764 | 0.591 | 5.936 | 5.886 | -0.050 | -0.596 | 8.352E-04 | 0.006 | Unannotated | NA | NA |
| metab_1773 | Unannotated feature | pos | 83.6587 | 7.362 | 4.441 | 4.579 | 0.138 | 0.596 | 8.352E-04 | 0.006 | Unannotated | NA | NA |
| metab_1804 | Unannotated feature | pos | 538.8510 | 0.551 | 4.316 | 5.021 | 0.706 | 0.596 | 8.352E-04 | 0.006 | Unannotated | NA | NA |
| metab_853 | Unannotated feature | pos | 188.9786 | 7.324 | 5.165 | 5.235 | 0.070 | 0.596 | 8.352E-04 | 0.006 | Unannotated | NA | NA |
| metab_2915 | 3-Hydroxy-5-methoxy-6-prenylstilbene-2-carboxylic acid | pos | 361.1393 | 4.900 | 4.731 | 3.533 | -1.197 | -0.596 | 8.352E-04 | 0.006 | Tentative | Phenylpropanoids and polyketides | Stilbenes |
| metab_10972 | Unannotated feature | neg | 269.9069 | 0.505 | 4.735 | 4.662 | -0.073 | -0.596 | 8.352E-04 | 0.006 | Unannotated | NA | NA |
| metab_9268 | Unannotated feature | neg | 366.0111 | 0.668 | 4.738 | 4.136 | -0.602 | -0.596 | 8.352E-04 | 0.006 | Unannotated | NA | NA |
| metab_10296 | Unannotated feature | neg | 374.1365 | 4.635 | 5.437 | 5.126 | -0.311 | -0.596 | 8.352E-04 | 0.006 | Unannotated | NA | NA |
| metab_11009 | Unannotated feature | neg | 516.7703 | 0.016 | 4.690 | 4.649 | -0.041 | -0.596 | 8.352E-04 | 0.006 | Unannotated | NA | NA |
| metab_4826 | Unannotated feature | pos | 876.7387 | 0.567 | 5.080 | 4.932 | -0.147 | -0.596 | 8.352E-04 | 0.006 | Unannotated | NA | NA |
| metab_10158 | Unannotated feature | neg | 294.8154 | 0.912 | 4.355 | 4.778 | 0.423 | 0.596 | 8.352E-04 | 0.006 | Unannotated | NA | NA |
| metab_3398 | Unannotated feature | pos | 577.4827 | 6.249 | 6.527 | 6.104 | -0.423 | -0.596 | 8.352E-04 | 0.006 | Unannotated | NA | NA |
| metab_5795 | Unannotated feature | neg | 147.0657 | 2.111 | 4.148 | 4.765 | 0.617 | 0.596 | 8.352E-04 | 0.006 | Unannotated | NA | NA |
| metab_3429 | Vomitoxin | pos | 314.1597 | 2.381 | 3.746 | 4.339 | 0.592 | 0.596 | 8.352E-04 | 0.006 | Tentative | Lipids and lipid-like molecules | Prenol lipids |
| metab_2733 | Unannotated feature | pos | 406.2903 | 5.717 | 4.797 | 4.646 | -0.151 | -0.592 | 9.094E-04 | 0.007 | Unannotated | NA | NA |
| metab_31 | Unannotated feature | pos | 241.1546 | 1.886 | 5.741 | 6.191 | 0.450 | 0.592 | 9.094E-04 | 0.007 | Unannotated | NA | NA |
| metab_8938 | Unannotated feature | neg | 348.9897 | 4.969 | 3.799 | 4.251 | 0.452 | 0.592 | 9.094E-04 | 0.007 | Unannotated | NA | NA |
| metab_6107 | Unannotated feature | neg | 526.0890 | 3.168 | 4.723 | 4.063 | -0.660 | -0.592 | 9.094E-04 | 0.007 | Unannotated | NA | NA |
| metab_407 | Unannotated feature | pos | 117.0549 | 1.196 | 4.944 | 4.992 | 0.048 | 0.592 | 9.094E-04 | 0.007 | Unannotated | NA | NA |
| metab_9439 | Unannotated feature | neg | 127.0394 | 3.372 | 3.091 | 3.586 | 0.495 | 0.592 | 9.094E-04 | 0.007 | Strong putative | NA | NA |
| metab_2449 | Unannotated feature | pos | 777.5421 | 5.910 | 5.924 | 5.871 | -0.053 | -0.592 | 9.094E-04 | 0.007 | Unannotated | NA | NA |
| metab_7299 | Diacylglycerol | neg | 575.4691 | 6.188 | 6.279 | 5.949 | -0.330 | -0.592 | 9.094E-04 | 0.007 | Tentative | Lipids and lipid-like molecules | Fatty Acyls |
| metab_10412 | Unannotated feature | neg | 174.8392 | 0.692 | 6.031 | 5.886 | -0.145 | -0.592 | 9.094E-04 | 0.007 | Unannotated | NA | NA |
| metab_10896 | Unannotated feature | neg | 894.8435 | 0.543 | 6.003 | 5.813 | -0.189 | -0.592 | 9.094E-04 | 0.007 | Unannotated | NA | NA |
| metab_5668 | Unannotated feature | neg | 174.0017 | 1.103 | 5.556 | 5.421 | -0.135 | -0.592 | 9.094E-04 | 0.007 | Unannotated | NA | NA |
| metab_1119 | Unannotated feature | pos | 157.0609 | 1.949 | 4.277 | 4.630 | 0.353 | 0.592 | 9.094E-04 | 0.007 | Unannotated | NA | NA |
| metab_3443 | 3,4-dimethylidenehexanedioylcarnitine | pos | 296.1492 | 2.327 | 4.017 | 4.716 | 0.700 | 0.592 | 9.094E-04 | 0.007 | Tentative | Lipids and lipid-like molecules | Fatty Acyls |
| metab_7994 | Unannotated feature | neg | 98.9061 | 6.987 | 5.126 | 5.170 | 0.044 | 0.592 | 9.094E-04 | 0.007 | Unannotated | NA | NA |
| metab_11173 | 5-Butyltetrahydro-2-oxo-3-furancarboxylic acid | neg | 431.1934 | 5.699 | 1.139 | 4.560 | 3.421 | 0.592 | 9.094E-04 | 0.007 | Tentative | Organoheterocyclic compounds | Lactones |
| metab_2359 | Unannotated feature | pos | 225.1459 | 5.972 | 4.568 | 4.637 | 0.068 | 0.592 | 9.094E-04 | 0.007 | Unannotated | NA | NA |
| metab_5146 | N-Acetylneuraminate | pos | 354.0771 | 0.760 | 4.685 | 4.200 | -0.484 | -0.592 | 9.094E-04 | 0.007 | Tentative | Organic oxygen compounds | Organooxygen compounds |
| metab_7184 | Unannotated feature | neg | 287.8537 | 5.906 | 4.735 | 4.464 | -0.272 | -0.592 | 9.094E-04 | 0.007 | Unannotated | NA | NA |
| metab_5260 | Unannotated feature | neg | 696.8364 | 0.554 | 5.176 | 5.518 | 0.342 | 0.592 | 9.094E-04 | 0.007 | Unannotated | NA | NA |
| metab_10654 | Unannotated feature | neg | 545.6577 | 0.587 | 5.452 | 5.354 | -0.097 | -0.592 | 9.094E-04 | 0.007 | Unannotated | NA | NA |
| metab_2227 | Diacylglycerol | pos | 617.4750 | 6.288 | 5.936 | 5.401 | -0.535 | -0.592 | 9.094E-04 | 0.007 | Tentative | Lipids and lipid-like molecules | Glycerolipids |
| metab_4695 | Unannotated feature | pos | 588.4272 | 5.863 | 5.392 | 5.223 | -0.169 | -0.592 | 9.094E-04 | 0.007 | Unannotated | NA | NA |
| metab_1227 | Unannotated feature | pos | 389.2509 | 5.793 | 5.054 | 4.988 | -0.066 | -0.592 | 9.094E-04 | 0.007 | Unannotated | NA | NA |
| metab_10258 | Unannotated feature | neg | 201.9887 | 0.849 | 3.855 | 4.236 | 0.380 | 0.592 | 9.094E-04 | 0.007 | Unannotated | NA | NA |
| metab_2018 | Unannotated feature | pos | 702.6460 | 0.575 | 5.391 | 5.331 | -0.060 | -0.592 | 9.094E-04 | 0.007 | Unannotated | NA | NA |
| metab_5552 | Unannotated feature | neg | 286.8622 | 0.825 | 4.307 | 4.003 | -0.303 | -0.592 | 9.094E-04 | 0.007 | Unannotated | NA | NA |
| metab_7678 | Unannotated feature | neg | 71.0130 | 7.253 | 4.839 | 4.793 | -0.046 | -0.592 | 9.094E-04 | 0.007 | Unannotated | NA | NA |
| metab_9238 | Unannotated feature | neg | 242.0131 | 3.997 | 3.870 | 4.348 | 0.478 | 0.592 | 9.094E-04 | 0.007 | Unannotated | NA | NA |
| metab_4441 | Unannotated feature | pos | 1048.7555 | 0.551 | 4.139 | 4.717 | 0.578 | 0.587 | 9.896E-04 | 0.007 | Unannotated | NA | NA |
| metab_5101 | Unannotated feature | pos | 448.7571 | 0.591 | 5.720 | 5.667 | -0.053 | -0.587 | 9.896E-04 | 0.007 | Unannotated | NA | NA |
| metab_2492 | Unannotated feature | pos | 655.7918 | 5.894 | 4.794 | 4.432 | -0.361 | -0.587 | 9.896E-04 | 0.007 | Unannotated | NA | NA |
| metab_4692 | Unannotated feature | pos | 710.4900 | 5.894 | 5.172 | 4.861 | -0.310 | -0.587 | 9.896E-04 | 0.007 | Unannotated | NA | NA |
| metab_1463 | Unannotated feature | pos | 576.4106 | 5.995 | 4.934 | 5.176 | 0.243 | 0.587 | 9.896E-04 | 0.007 | Unannotated | NA | NA |
| metab_9916 | Unannotated feature | neg | 265.8792 | 2.119 | 3.991 | 3.804 | -0.187 | -0.587 | 9.896E-04 | 0.007 | Unannotated | NA | NA |
| metab_3887 | Unannotated feature | pos | 272.9552 | 0.869 | 4.898 | 4.794 | -0.104 | -0.587 | 9.896E-04 | 0.007 | Unannotated | NA | NA |
| metab_3546 | Unannotated feature | pos | 171.7351 | 2.073 | 5.642 | 5.720 | 0.078 | 0.587 | 9.896E-04 | 0.007 | Unannotated | NA | NA |
| metab_6794 | Unannotated feature | neg | 411.0915 | 0.865 | 4.143 | 4.712 | 0.569 | 0.587 | 9.896E-04 | 0.007 | Unannotated | NA | NA |
| metab_2575 | Unannotated feature | pos | 729.1696 | 5.855 | 5.092 | 4.652 | -0.440 | -0.587 | 9.896E-04 | 0.007 | Unannotated | NA | NA |
| metab_9716 | Unannotated feature | neg | 388.7132 | 0.629 | 6.312 | 6.249 | -0.063 | -0.587 | 9.896E-04 | 0.007 | Unannotated | NA | NA |
| metab_4939 | Unannotated feature | pos | 332.0385 | 0.629 | 5.084 | 4.702 | -0.382 | -0.587 | 9.896E-04 | 0.007 | Unannotated | NA | NA |
| metab_4221 | sn-Glycero-3-phosphocholine | pos | 280.0921 | 0.614 | 5.930 | 5.497 | -0.433 | -0.587 | 9.896E-04 | 0.007 | Tentative | Lipids and lipid-like molecules | Glycerophospholipids |
| metab_4752 | Unannotated feature | pos | 579.4973 | 6.319 | 5.469 | 4.390 | -1.079 | -0.587 | 9.896E-04 | 0.007 | Unannotated | NA | NA |
| metab_4891 | Unannotated feature | pos | 624.6301 | 0.591 | 5.853 | 5.739 | -0.114 | -0.587 | 9.896E-04 | 0.007 | Unannotated | NA | NA |
| metab_5022 | Creatine | pos | 132.0769 | 0.691 | 6.502 | 6.094 | -0.409 | -0.587 | 9.896E-04 | 0.007 | Strong putative | Organic acids and derivatives | Carboxylic acids and derivatives |
| metab_125 | Unannotated feature | pos | 752.9129 | 0.545 | 4.648 | 4.305 | -0.342 | -0.587 | 9.896E-04 | 0.007 | Unannotated | NA | NA |
| metab_2400 | Unannotated feature | pos | 145.1013 | 5.941 | 4.381 | 4.494 | 0.113 | 0.587 | 9.896E-04 | 0.007 | Unannotated | NA | NA |
| metab_8785 | Unannotated feature | neg | 636.7130 | 5.518 | 4.362 | 4.775 | 0.413 | 0.587 | 9.896E-04 | 0.007 | Unannotated | NA | NA |
| metab_1270 | Unannotated feature | pos | 656.4521 | 5.848 | 5.309 | 4.822 | -0.486 | -0.587 | 9.896E-04 | 0.007 | Unannotated | NA | NA |
| metab_3069 | Octa-3,6-dienoylcarnitine | pos | 284.1856 | 3.985 | 4.558 | 4.936 | 0.378 | 0.587 | 9.896E-04 | 0.007 | Tentative | Lipids and lipid-like molecules | Fatty Acyls |
| metab_4982 | Unannotated feature | pos | 304.9720 | 0.729 | 4.574 | 3.314 | -1.260 | -0.587 | 9.896E-04 | 0.007 | Unannotated | NA | NA |
| metab_737 | 1-[(2R,3R,4S,5R)-3,4-Dihydroxy-5-(hydroxymethyl)oxolan-2-yl]oxypyrimidin-2-one | pos | 267.0587 | 0.854 | 5.795 | 6.077 | 0.282 | 0.587 | 9.896E-04 | 0.007 | Tentative | Organic oxygen compounds | Organooxygen compounds |
| metab_10626 | Unannotated feature | neg | 603.6170 | 0.594 | 4.982 | 4.793 | -0.188 | -0.587 | 9.896E-04 | 0.007 | Unannotated | NA | NA |
| metab_7617 | Unannotated feature | neg | 155.8736 | 7.309 | 4.705 | 4.776 | 0.071 | 0.587 | 9.896E-04 | 0.007 | Unannotated | NA | NA |
| metab_5468 | Unannotated feature | neg | 172.8421 | 0.692 | 5.985 | 5.849 | -0.136 | -0.587 | 9.896E-04 | 0.007 | Unannotated | NA | NA |
| metab_5521 | Unannotated feature | neg | 512.6620 | 0.778 | 5.024 | 4.806 | -0.219 | -0.587 | 9.896E-04 | 0.007 | Unannotated | NA | NA |
| metab_5798 | Unannotated feature | neg | 225.9097 | 2.111 | 3.492 | 2.943 | -0.549 | -0.587 | 9.896E-04 | 0.007 | Unannotated | NA | NA |
| metab_10487 | Unannotated feature | neg | 344.0195 | 0.636 | 4.821 | 4.514 | -0.307 | -0.587 | 9.896E-04 | 0.007 | Unannotated | NA | NA |
| metab_4560 | Unannotated feature | pos | 157.9739 | 0.481 | 4.468 | 4.452 | -0.016 | -0.587 | 9.896E-04 | 0.007 | Unannotated | NA | NA |
| metab_7745 | Unannotated feature | neg | 530.3030 | 7.181 | 4.756 | 4.550 | -0.206 | -0.587 | 9.896E-04 | 0.007 | Unannotated | NA | NA |
| metab_3332 | Unannotated feature | pos | 201.8371 | 2.629 | 5.172 | 5.273 | 0.101 | 0.587 | 9.896E-04 | 0.007 | Unannotated | NA | NA |
| metab_4808 | Unannotated feature | pos | 132.0353 | 7.331 | 4.332 | 4.432 | 0.100 | 0.587 | 9.896E-04 | 0.007 | Unannotated | NA | NA |
| metab_6488 | Unannotated feature | neg | 181.5046 | 4.371 | 3.847 | 3.659 | -0.189 | -0.587 | 9.896E-04 | 0.007 | Unannotated | NA | NA |
| metab_4490 | Unannotated feature | pos | 509.8663 | 0.551 | 4.611 | 4.915 | 0.304 | 0.587 | 9.896E-04 | 0.007 | Unannotated | NA | NA |
| metab_4075 | Unannotated feature | pos | 143.6273 | 0.745 | 4.043 | 4.561 | 0.518 | 0.587 | 9.896E-04 | 0.007 | Unannotated | NA | NA |
| metab_4814 | Unannotated feature | pos | 141.9834 | 7.324 | 5.952 | 6.033 | 0.081 | 0.587 | 9.896E-04 | 0.007 | Unannotated | NA | NA |
| metab_4821 | Unannotated feature | pos | 142.9912 | 7.324 | 5.546 | 5.606 | 0.060 | 0.587 | 9.896E-04 | 0.007 | Unannotated | NA | NA |
| metab_10838 | Unannotated feature | neg | 699.8146 | 0.554 | 4.486 | 5.173 | 0.688 | 0.587 | 9.896E-04 | 0.007 | Unannotated | NA | NA |
| metab_1187 | Unannotated feature | pos | 640.4371 | 5.740 | 4.846 | 4.562 | -0.284 | -0.587 | 9.896E-04 | 0.007 | Unannotated | NA | NA |
| metab_2251 | Unannotated feature | pos | 465.3563 | 6.203 | 5.200 | 4.951 | -0.249 | -0.583 | 0.001 | 0.008 | Unannotated | NA | NA |
| metab_3871 | Unannotated feature | pos | 114.4024 | 0.908 | 2.621 | 3.979 | 1.358 | 0.583 | 0.001 | 0.008 | Unannotated | NA | NA |
| metab_6365 | Unannotated feature | neg | 217.0718 | 3.935 | 4.651 | 3.876 | -0.776 | -0.583 | 0.001 | 0.008 | Unannotated | NA | NA |
| metab_11101 | Unannotated feature | neg | 626.2444 | 5.651 | 3.658 | 4.628 | 0.970 | 0.583 | 0.001 | 0.008 | Unannotated | NA | NA |
| metab_1677 | Unannotated feature | pos | 335.1996 | 7.261 | 4.847 | 4.995 | 0.149 | 0.583 | 0.001 | 0.008 | Unannotated | NA | NA |
| metab_9917 | Unannotated feature | neg | 446.6719 | 0.621 | 6.469 | 6.412 | -0.058 | -0.583 | 0.001 | 0.008 | Unannotated | NA | NA |
| metab_1083 | Unannotated feature | pos | 187.0578 | 0.721 | 6.564 | 6.283 | -0.281 | -0.583 | 0.001 | 0.008 | Unannotated | NA | NA |
| metab_10853 | Unannotated feature | neg | 595.8582 | 0.554 | 5.186 | 5.353 | 0.167 | 0.583 | 0.001 | 0.008 | Unannotated | NA | NA |
| metab_3677 | Unannotated feature | pos | 231.1600 | 1.933 | 3.170 | 3.984 | 0.814 | 0.583 | 0.001 | 0.008 | Unannotated | NA | NA |
| metab_1354 | Unannotated feature | pos | 417.5494 | 5.902 | 5.144 | 5.350 | 0.206 | 0.583 | 0.001 | 0.008 | Unannotated | NA | NA |
| metab_5209 | Unannotated feature | neg | 622.8931 | 0.543 | 5.891 | 5.812 | -0.079 | -0.583 | 0.001 | 0.008 | Unannotated | NA | NA |
| metab_2179 | Unannotated feature | pos | 102.6983 | 6.381 | 3.551 | 3.775 | 0.224 | 0.583 | 0.001 | 0.008 | Unannotated | NA | NA |
| metab_3683 | Prenyl-L-cysteine | pos | 154.0693 | 1.925 | 4.858 | 5.180 | 0.322 | 0.583 | 0.001 | 0.008 | Tentative | Organic acids and derivatives | Carboxylic acids and derivatives |
| metab_3778 | Tenuazonic acid | pos | 215.1391 | 1.647 | 4.487 | 5.006 | 0.519 | 0.583 | 0.001 | 0.008 | Tentative | Organoheterocyclic compounds | Pyrrolines |
| metab_4264 | Unannotated feature | pos | 709.5490 | 0.591 | 4.275 | 4.001 | -0.274 | -0.583 | 0.001 | 0.008 | Unannotated | NA | NA |
| metab_1976 | Unannotated feature | pos | 440.7254 | 0.591 | 5.964 | 5.875 | -0.090 | -0.583 | 0.001 | 0.008 | Unannotated | NA | NA |
| metab_6148 | Unannotated feature | neg | 143.0707 | 3.232 | 3.083 | 3.615 | 0.532 | 0.583 | 0.001 | 0.008 | Unannotated | NA | NA |
| metab_661 | Unannotated feature | pos | 127.1119 | 2.374 | 3.597 | 3.863 | 0.266 | 0.583 | 0.001 | 0.008 | Unannotated | NA | NA |
| metab_650 | Unannotated feature | pos | 474.3794 | 6.103 | 5.667 | 5.437 | -0.230 | -0.583 | 0.001 | 0.008 | Unannotated | NA | NA |
| metab_8612 | Unannotated feature | neg | 527.2873 | 5.659 | 3.599 | 4.755 | 1.156 | 0.583 | 0.001 | 0.008 | Unannotated | NA | NA |
| metab_4288 | Unannotated feature | pos | 266.8496 | 0.591 | 5.996 | 5.920 | -0.076 | -0.583 | 0.001 | 0.008 | Unannotated | NA | NA |
| metab_4143 | Unannotated feature | pos | 228.1284 | 0.667 | 4.613 | 5.000 | 0.387 | 0.583 | 0.001 | 0.008 | Unannotated | NA | NA |
| metab_9044 | Unannotated feature | neg | 313.1474 | 4.635 | 4.745 | 4.489 | -0.256 | -0.583 | 0.001 | 0.008 | Unannotated | NA | NA |
| metab_2527 | Unannotated feature | pos | 878.7874 | 0.551 | 4.780 | 5.110 | 0.330 | 0.583 | 0.001 | 0.008 | Unannotated | NA | NA |
| metab_2772 | Unannotated feature | pos | 560.4004 | 5.657 | 5.565 | 5.377 | -0.188 | -0.583 | 0.001 | 0.008 | Unannotated | NA | NA |
| metab_6099 | Unannotated feature | neg | 578.0211 | 3.160 | 4.823 | 4.432 | -0.391 | -0.583 | 0.001 | 0.008 | Unannotated | NA | NA |
| metab_10613 | Unannotated feature | neg | 336.7891 | 0.598 | 6.261 | 6.193 | -0.068 | -0.583 | 0.001 | 0.008 | Unannotated | NA | NA |
| metab_1852 | Unannotated feature | pos | 83.6586 | 7.749 | 3.262 | 3.516 | 0.254 | 0.583 | 0.001 | 0.008 | Unannotated | NA | NA |
| metab_250 | Unannotated feature | pos | 114.0665 | 0.667 | 6.649 | 7.000 | 0.350 | 0.583 | 0.001 | 0.008 | Unannotated | NA | NA |
| metab_4069 | Unannotated feature | pos | 257.1607 | 0.745 | 3.850 | 4.578 | 0.727 | 0.583 | 0.001 | 0.008 | Unannotated | NA | NA |
| metab_4442 | Unannotated feature | pos | 1042.8002 | 0.551 | 5.659 | 5.537 | -0.122 | -0.583 | 0.001 | 0.008 | Unannotated | NA | NA |
| metab_864 | Unannotated feature | pos | 328.2329 | 3.514 | 4.691 | 4.543 | -0.148 | -0.583 | 0.001 | 0.008 | Unannotated | NA | NA |
| metab_30 | Unannotated feature | pos | 460.7830 | 0.583 | 5.773 | 5.712 | -0.062 | -0.583 | 0.001 | 0.008 | Unannotated | NA | NA |
| metab_10 | Unannotated feature | pos | 324.8081 | 0.591 | 6.040 | 5.966 | -0.075 | -0.583 | 0.001 | 0.008 | Unannotated | NA | NA |
| metab_2040 | Unannotated feature | pos | 349.2151 | 7.261 | 5.510 | 5.594 | 0.084 | 0.583 | 0.001 | 0.008 | Strong putative | NA | NA |
| metab_8010 | Unannotated feature | neg | 143.0245 | 6.823 | 5.160 | 5.234 | 0.075 | 0.583 | 0.001 | 0.008 | Unannotated | NA | NA |
| metab_1880 | Unannotated feature | pos | 193.0428 | 7.347 | 4.363 | 4.552 | 0.189 | 0.579 | 0.001 | 0.008 | Unannotated | NA | NA |
| metab_5540 | Unannotated feature | neg | 570.6210 | 0.802 | 4.792 | 4.325 | -0.467 | -0.579 | 0.001 | 0.008 | Unannotated | NA | NA |
| metab_3353 | Sterigmatocystin | pos | 289.0494 | 2.583 | 4.681 | 4.556 | -0.125 | -0.579 | 0.001 | 0.008 | Tentative | Phenylpropanoids and polyketides | Sterigmatocystins |
| metab_8167 | 5alpha-Pregnan-20alpha-ol-3-one | neg | 339.2335 | 6.032 | 6.998 | 3.158 | -3.840 | -0.579 | 0.001 | 0.008 | Tentative | Lipids and lipid-like molecules | Steroids and steroid derivatives |
| metab_10423 | Unannotated feature | neg | 148.0277 | 0.676 | 3.323 | 4.155 | 0.832 | 0.579 | 0.001 | 0.008 | Unannotated | NA | NA |
| metab_8687 | Unannotated feature | neg | 125.0965 | 5.619 | 3.300 | 3.544 | 0.244 | 0.579 | 0.001 | 0.008 | Unannotated | NA | NA |
| metab_4651 | Isokobusone | pos | 223.1693 | 5.763 | 4.195 | 4.744 | 0.549 | 0.579 | 0.001 | 0.008 | Tentative | Organic oxygen compounds | Organooxygen compounds |
| metab_5015 | Unannotated feature | pos | 125.0712 | 0.784 | 3.875 | 4.173 | 0.298 | 0.579 | 0.001 | 0.008 | Unannotated | NA | NA |
| metab_9658 | Atractylenolide III | neg | 269.1148 | 3.009 | 3.750 | 4.180 | 0.430 | 0.579 | 0.001 | 0.008 | Tentative | Lipids and lipid-like molecules | Prenol lipids |
| metab_6419 | Unannotated feature | neg | 198.5751 | 4.167 | 5.193 | 5.097 | -0.096 | -0.579 | 0.001 | 0.008 | Unannotated | NA | NA |
| metab_10468 | Unannotated feature | neg | 196.0744 | 0.652 | 4.680 | 4.470 | -0.210 | -0.579 | 0.001 | 0.008 | Unannotated | NA | NA |
| metab_4880 | Voriconazole | pos | 367.1499 | 2.065 | 5.106 | 5.617 | 0.511 | 0.579 | 0.001 | 0.008 | Tentative | Benzenoids | Benzene and substituted derivatives |
| metab_11021 | Unannotated feature | neg | 94.9163 | 0.016 | 3.742 | 3.702 | -0.040 | -0.579 | 0.001 | 0.008 | Unannotated | NA | NA |
| metab_1008 | Unannotated feature | pos | 151.0755 | 4.555 | 2.943 | 3.746 | 0.803 | 0.579 | 0.001 | 0.008 | Moderate putative | NA | NA |
| metab_3173 | Unannotated feature | pos | 319.1651 | 3.421 | 4.918 | 4.320 | -0.599 | -0.579 | 0.001 | 0.008 | Unannotated | NA | NA |
| metab_7984 | Unannotated feature | neg | 213.8191 | 7.085 | 3.715 | 3.923 | 0.207 | 0.579 | 0.001 | 0.008 | Unannotated | NA | NA |
| metab_7527 | Unannotated feature | neg | 91.4823 | 7.170 | 3.368 | 3.250 | -0.118 | -0.579 | 0.001 | 0.008 | Unannotated | NA | NA |
| metab_10539 | Unannotated feature | neg | 384.7190 | 0.621 | 6.532 | 6.471 | -0.061 | -0.579 | 0.001 | 0.008 | Unannotated | NA | NA |
| metab_6696 | Salacinol | neg | 355.0154 | 5.255 | 4.316 | 3.059 | -1.258 | -0.579 | 0.001 | 0.008 | Tentative | Organic acids and derivatives | Organic sulfuric acids and derivatives |
| metab_3873 | Unannotated feature | pos | 206.8939 | 0.900 | 4.962 | 4.918 | -0.044 | -0.579 | 0.001 | 0.008 | Unannotated | NA | NA |
| metab_6190 | Unannotated feature | neg | 358.8751 | 3.308 | 4.625 | 4.506 | -0.119 | -0.579 | 0.001 | 0.008 | Unannotated | NA | NA |
| metab_4101 | Unannotated feature | pos | 257.0744 | 0.714 | 3.824 | 4.368 | 0.544 | 0.579 | 0.001 | 0.008 | Unannotated | NA | NA |
| metab_2998 | Unannotated feature | pos | 276.1805 | 2.327 | 4.205 | 4.888 | 0.684 | 0.579 | 0.001 | 0.008 | Unannotated | NA | NA |
| metab_4960 | Unannotated feature | pos | 115.0698 | 0.667 | 5.204 | 5.562 | 0.358 | 0.579 | 0.001 | 0.008 | Unannotated | NA | NA |
| metab_2978 | Unannotated feature | pos | 125.1076 | 2.288 | 5.045 | 5.283 | 0.238 | 0.579 | 0.001 | 0.008 | Unannotated | NA | NA |
| metab_4915 | Unannotated feature | pos | 189.1598 | 0.598 | 4.918 | 5.258 | 0.340 | 0.579 | 0.001 | 0.008 | Strong putative | NA | NA |
| metab_1144 | Unannotated feature | pos | 516.3739 | 5.657 | 5.415 | 5.267 | -0.148 | -0.579 | 0.001 | 0.008 | Unannotated | NA | NA |
| metab_9480 | L-beta-aspartyl-L-leucine | neg | 245.1146 | 3.274 | 2.900 | 3.843 | 0.943 | 0.579 | 0.001 | 0.008 | Tentative | Organic acids and derivatives | Peptidomimetics |
| metab_5943 | Unannotated feature | neg | 225.0882 | 2.652 | 4.125 | 4.993 | 0.868 | 0.579 | 0.001 | 0.008 | Unannotated | NA | NA |
| metab_6559 | Unannotated feature | neg | 321.8146 | 4.643 | 4.874 | 4.942 | 0.068 | 0.579 | 0.001 | 0.008 | Unannotated | NA | NA |
| metab_5824 | Unannotated feature | neg | 174.0768 | 2.127 | 2.914 | 3.365 | 0.450 | 0.579 | 0.001 | 0.008 | Unannotated | NA | NA |
| metab_6974 | Unannotated feature | neg | 179.1075 | 5.707 | 2.760 | 3.732 | 0.972 | 0.579 | 0.001 | 0.008 | Unannotated | NA | NA |
| metab_7566 | Unannotated feature | neg | 171.0270 | 2.119 | 4.615 | 4.405 | -0.209 | -0.579 | 0.001 | 0.008 | Unannotated | NA | NA |
| metab_1652 | Unannotated feature | pos | 844.7935 | 0.551 | 6.051 | 6.128 | 0.077 | 0.579 | 0.001 | 0.008 | Unannotated | NA | NA |
| metab_4480 | Unannotated feature | pos | 637.8535 | 0.551 | 5.463 | 5.621 | 0.159 | 0.579 | 0.001 | 0.008 | Unannotated | NA | NA |
| metab_4459 | Unannotated feature | pos | 815.8090 | 0.551 | 4.912 | 5.087 | 0.174 | 0.579 | 0.001 | 0.008 | Unannotated | NA | NA |
| metab_7407 | Unannotated feature | neg | 267.9723 | 7.101 | 4.767 | 4.705 | -0.062 | -0.579 | 0.001 | 0.008 | Unannotated | NA | NA |
| metab_6 | Unannotated feature | pos | 326.8052 | 0.591 | 5.609 | 5.561 | -0.048 | -0.579 | 0.001 | 0.008 | Unannotated | NA | NA |
| metab_4689 | Unannotated feature | pos | 612.4246 | 5.863 | 5.656 | 5.431 | -0.225 | -0.579 | 0.001 | 0.008 | Unannotated | NA | NA |
| metab_8869 | Unannotated feature | neg | 400.0106 | 5.247 | 4.139 | 3.069 | -1.070 | -0.579 | 0.001 | 0.008 | Unannotated | NA | NA |
| metab_1816 | Unannotated feature | pos | 101.0602 | 7.983 | 4.677 | 4.698 | 0.021 | 0.579 | 0.001 | 0.008 | Unannotated | NA | NA |
| metab_1249 | Unannotated feature | pos | 539.7091 | 5.832 | 4.801 | 4.629 | -0.172 | -0.579 | 0.001 | 0.008 | Unannotated | NA | NA |
| metab_7202 | Unannotated feature | neg | 194.9240 | 5.930 | 3.885 | 4.125 | 0.240 | 0.579 | 0.001 | 0.008 | Unannotated | NA | NA |
| metab_6885 | Unannotated feature | neg | 560.5923 | 0.904 | 5.707 | 5.629 | -0.078 | -0.579 | 0.001 | 0.008 | Unannotated | NA | NA |
| metab_1825 | Unannotated feature | pos | 510.8145 | 0.551 | 3.792 | 4.665 | 0.873 | 0.579 | 0.001 | 0.008 | Unannotated | NA | NA |
| metab_1328 | Unannotated feature | pos | 817.5650 | 5.886 | 5.863 | 6.024 | 0.161 | 0.579 | 0.001 | 0.008 | Unannotated | NA | NA |
| metab_8657 | Unannotated feature | neg | 243.1240 | 5.635 | 4.114 | 4.655 | 0.541 | 0.575 | 0.001 | 0.008 | Moderate putative | NA | NA |
| metab_6672 | Unannotated feature | neg | 855.3186 | 5.139 | 4.205 | 4.788 | 0.583 | 0.575 | 0.001 | 0.008 | Unannotated | NA | NA |
| metab_3337 | Unannotated feature | pos | 118.4191 | 2.622 | 4.413 | 4.587 | 0.174 | 0.575 | 0.001 | 0.008 | Unannotated | NA | NA |
| metab_2722 | Unannotated feature | pos | 523.8655 | 5.733 | 4.733 | 4.505 | -0.229 | -0.575 | 0.001 | 0.008 | Unannotated | NA | NA |
| metab_3736 | Unannotated feature | pos | 87.1003 | 1.809 | 5.328 | 5.269 | -0.059 | -0.575 | 0.001 | 0.008 | Unannotated | NA | NA |
| metab_1004 | Unannotated feature | pos | 451.6819 | 4.532 | 5.210 | 5.429 | 0.219 | 0.575 | 0.001 | 0.008 | Unannotated | NA | NA |
| metab_2238 | Decanoic acid | pos | 435.3468 | 6.249 | 5.061 | 4.546 | -0.515 | -0.575 | 0.001 | 0.008 | Tentative | Lipids and lipid-like molecules | Fatty Acyls |
| metab_9793 | Unannotated feature | neg | 324.9141 | 2.549 | 4.399 | 4.280 | -0.118 | -0.575 | 0.001 | 0.008 | Unannotated | NA | NA |
| metab_7446 | Unannotated feature | neg | 258.0040 | 2.119 | 4.128 | 3.609 | -0.519 | -0.575 | 0.001 | 0.008 | Unannotated | NA | NA |
| metab_3375 | Unannotated feature | pos | 101.8085 | 2.529 | 3.772 | 3.903 | 0.131 | 0.575 | 0.001 | 0.008 | Unannotated | NA | NA |
| metab_2472 | Unannotated feature | pos | 191.0767 | 0.559 | 5.962 | 5.864 | -0.098 | -0.575 | 0.001 | 0.008 | Unannotated | NA | NA |
| metab_4489 | Unannotated feature | pos | 532.8950 | 0.551 | 5.462 | 5.367 | -0.095 | -0.575 | 0.001 | 0.008 | Unannotated | NA | NA |
| metab_9959 | (R)C(R)S-S-Propylcysteine sulfoxide | neg | 216.0091 | 2.111 | 4.177 | 3.745 | -0.432 | -0.575 | 0.001 | 0.008 | Tentative | Organic acids and derivatives | Carboxylic acids and derivatives |
| metab_215 | Unannotated feature | pos | 221.0193 | 0.614 | 4.641 | 4.178 | -0.462 | -0.575 | 0.001 | 0.008 | Unannotated | NA | NA |
| metab_4279 | Unannotated feature | pos | 438.7284 | 0.591 | 5.541 | 5.446 | -0.095 | -0.575 | 0.001 | 0.008 | Unannotated | NA | NA |
| metab_2401 | Glycodeoxycholate | pos | 899.6349 | 5.933 | 4.805 | 4.270 | -0.535 | -0.575 | 0.001 | 0.008 | Strong putative | Lipids and lipid-like molecules | Steroids and steroid derivatives |
| metab_2821 | Unannotated feature | pos | 83.4480 | 5.543 | 3.004 | 3.241 | 0.237 | 0.575 | 0.001 | 0.008 | Unannotated | NA | NA |
| metab_9594 | Glycylphenylalanylleucylglycine | neg | 429.1551 | 3.160 | 4.863 | 4.295 | -0.569 | -0.575 | 0.001 | 0.008 | Tentative | Organic acids and derivatives | Carboxylic acids and derivatives |
| metab_5109 | 2-Amino-3-hydroxy-3-phenylpropanoic acid | pos | 204.0632 | 1.740 | 5.536 | 5.325 | -0.210 | -0.575 | 0.001 | 0.008 | Tentative | Organic acids and derivatives | Carboxylic acids and derivatives |
| metab_314 | Unannotated feature | pos | 312.9027 | 0.784 | 3.108 | 3.586 | 0.478 | 0.575 | 0.001 | 0.008 | Unannotated | NA | NA |
| metab_8429 | (+)-Lysergic acid | neg | 313.1201 | 5.771 | 5.158 | 4.823 | -0.334 | -0.575 | 0.001 | 0.008 | Tentative | Alkaloids and derivatives | Ergoline and derivatives |
| metab_1515 | Unannotated feature | pos | 264.8526 | 0.591 | 6.137 | 6.074 | -0.063 | -0.575 | 0.001 | 0.008 | Unannotated | NA | NA |
| metab_4977 | Unannotated feature | pos | 121.0723 | 0.644 | 6.386 | 6.805 | 0.419 | 0.575 | 0.001 | 0.008 | Unannotated | NA | NA |
| metab_1978 | Unannotated feature | pos | 744.8510 | 7.293 | 4.866 | 4.746 | -0.120 | -0.575 | 0.001 | 0.008 | Unannotated | NA | NA |
| metab_1564 | Unannotated feature | pos | 129.7516 | 6.327 | 4.150 | 4.323 | 0.173 | 0.575 | 0.001 | 0.008 | Unannotated | NA | NA |
| metab_785 | 4-[(2,4-Dihydroxy-3,3-dimethylbutanoyl)amino]butanoic acid | pos | 234.1336 | 2.942 | 3.840 | 4.685 | 0.845 | 0.575 | 0.001 | 0.008 | Tentative | Organic acids and derivatives | Carboxylic acids and derivatives |
| metab_1958 | Unannotated feature | pos | 220.9346 | 7.308 | 4.794 | 4.758 | -0.036 | -0.575 | 0.001 | 0.008 | Unannotated | NA | NA |
| metab_7645 | Unannotated feature | neg | 184.8895 | 7.740 | 4.658 | 4.726 | 0.068 | 0.575 | 0.001 | 0.008 | Unannotated | NA | NA |
| metab_11117 | Lumirubin | neg | 583.2576 | 5.675 | 5.408 | 4.704 | -0.703 | -0.575 | 0.001 | 0.008 | Tentative | NA | NA |
| metab_1146 | Unannotated feature | pos | 648.4528 | 5.657 | 5.526 | 5.390 | -0.136 | -0.575 | 0.001 | 0.008 | Unannotated | NA | NA |
| metab_4234 | Unannotated feature | pos | 103.8158 | 0.614 | 4.588 | 4.755 | 0.167 | 0.575 | 0.001 | 0.008 | Unannotated | NA | NA |
| metab_10328 | Unannotated feature | neg | 456.7008 | 0.778 | 4.690 | 4.363 | -0.326 | -0.575 | 0.001 | 0.008 | Unannotated | NA | NA |
| metab_7257 | Unannotated feature | neg | 393.3021 | 6.055 | 4.685 | 4.318 | -0.367 | -0.575 | 0.001 | 0.008 | Unannotated | NA | NA |
| metab_10338 | Unannotated feature | neg | 366.0580 | 0.762 | 4.443 | 3.984 | -0.459 | -0.575 | 0.001 | 0.008 | Unannotated | NA | NA |
| metab_605 | Unannotated feature | pos | 295.1287 | 2.103 | 4.537 | 3.357 | -1.180 | -0.575 | 0.001 | 0.008 | Moderate putative | NA | NA |
| metab_3629 | Unannotated feature | pos | 301.1396 | 1.956 | 3.788 | 4.051 | 0.263 | 0.575 | 0.001 | 0.008 | Unannotated | NA | NA |
| metab_151 | Unannotated feature | pos | 781.8153 | 0.551 | 4.913 | 5.089 | 0.176 | 0.571 | 0.001 | 0.009 | Unannotated | NA | NA |
| metab_2225 | Unannotated feature | pos | 109.1016 | 6.296 | 4.658 | 4.496 | -0.162 | -0.571 | 0.001 | 0.009 | Unannotated | NA | NA |
| metab_9691 | Unannotated feature | neg | 79.9565 | 2.914 | 2.058 | 2.916 | 0.859 | 0.571 | 0.001 | 0.009 | Unannotated | NA | NA |
| metab_3684 | N1-Methyl-4-pyridone-5-carboxamide | pos | 153.0659 | 1.925 | 5.974 | 6.200 | 0.227 | 0.571 | 0.001 | 0.009 | Tentative | Organoheterocyclic compounds | Pyridines and derivatives |
| metab_5496 | Unannotated feature | neg | 354.0458 | 0.731 | 4.882 | 4.679 | -0.202 | -0.571 | 0.001 | 0.009 | Unannotated | NA | NA |
| metab_10008 | Unannotated feature | neg | 130.0503 | 1.871 | 4.959 | 5.143 | 0.184 | 0.571 | 0.001 | 0.009 | Unannotated | NA | NA |
| metab_5049 | Unannotated feature | pos | 593.0199 | 0.861 | 5.458 | 5.234 | -0.225 | -0.571 | 0.001 | 0.009 | Unannotated | NA | NA |
| metab_2702 | Unannotated feature | pos | 574.4066 | 5.748 | 5.306 | 5.077 | -0.229 | -0.571 | 0.001 | 0.009 | Unannotated | NA | NA |
| metab_7591 | Unannotated feature | neg | 261.8752 | 7.221 | 4.098 | 4.000 | -0.098 | -0.571 | 0.001 | 0.009 | Unannotated | NA | NA |
| metab_10240 | Unannotated feature | neg | 89.4873 | 0.865 | 4.393 | 3.957 | -0.436 | -0.571 | 0.001 | 0.009 | Unannotated | NA | NA |
| metab_11178 | Unannotated feature | neg | 401.9318 | 5.827 | 5.009 | 3.879 | -1.130 | -0.571 | 0.001 | 0.009 | Unannotated | NA | NA |
| metab_1888 | Unannotated feature | pos | 111.9847 | 7.339 | 4.836 | 4.905 | 0.069 | 0.571 | 0.001 | 0.009 | Unannotated | NA | NA |
| metab_1373 | Unannotated feature | pos | 185.1286 | 2.288 | 6.374 | 6.595 | 0.220 | 0.571 | 0.001 | 0.009 | Unannotated | NA | NA |
| metab_9559 | Unannotated feature | neg | 203.7998 | 3.198 | 4.085 | 4.020 | -0.065 | -0.571 | 0.001 | 0.009 | Unannotated | NA | NA |
| metab_287 | Unannotated feature | pos | 144.5788 | 0.745 | 4.070 | 4.540 | 0.471 | 0.571 | 0.001 | 0.009 | Unannotated | NA | NA |
| metab_9933 | Unannotated feature | neg | 180.0524 | 2.119 | 2.789 | 3.910 | 1.122 | 0.571 | 0.001 | 0.009 | Unannotated | NA | NA |
| metab_4095 | Unannotated feature | pos | 252.9826 | 0.721 | 3.208 | 4.279 | 1.071 | 0.571 | 0.001 | 0.009 | Unannotated | NA | NA |
| metab_2561 | Unannotated feature | pos | 547.2188 | 5.863 | 4.809 | 4.360 | -0.449 | -0.571 | 0.001 | 0.009 | Unannotated | NA | NA |
| metab_8149 | PE(16:0/0:0) | neg | 452.2791 | 6.094 | 5.553 | 5.780 | 0.228 | 0.571 | 0.001 | 0.009 | Moderate putative | Lipids and lipid-like molecules | Glycerophospholipids |
| metab_2266 | Unannotated feature | pos | 379.2838 | 6.172 | 4.743 | 4.459 | -0.284 | -0.571 | 0.001 | 0.009 | Unannotated | NA | NA |
| metab_4912 | Unannotated feature | pos | 566.6715 | 0.591 | 5.775 | 5.711 | -0.064 | -0.571 | 0.001 | 0.009 | Unannotated | NA | NA |
| metab_2247 | Unannotated feature | pos | 253.2405 | 6.211 | 4.655 | 4.272 | -0.383 | -0.571 | 0.001 | 0.009 | Unannotated | NA | NA |
| metab_2237 | Unannotated feature | pos | 89.1159 | 6.257 | 6.157 | 6.194 | 0.037 | 0.571 | 0.001 | 0.009 | Unannotated | NA | NA |
| metab_8808 | Unannotated feature | neg | 203.7990 | 5.477 | 5.074 | 5.009 | -0.065 | -0.571 | 0.001 | 0.009 | Unannotated | NA | NA |
| metab_10884 | Unannotated feature | neg | 679.8295 | 0.549 | 3.946 | 4.345 | 0.399 | 0.571 | 0.001 | 0.009 | Unannotated | NA | NA |
| metab_2084 | Unannotated feature | pos | 586.7291 | 0.575 | 5.789 | 5.715 | -0.074 | -0.571 | 0.001 | 0.009 | Unannotated | NA | NA |
| metab_738 | Unannotated feature | pos | 201.2567 | 2.637 | 5.520 | 5.591 | 0.071 | 0.571 | 0.001 | 0.009 | Unannotated | NA | NA |
| metab_3876 | Unannotated feature | pos | 226.0451 | 0.892 | 5.983 | 5.838 | -0.145 | -0.571 | 0.001 | 0.009 | Unannotated | NA | NA |
| metab_253 | Unannotated feature | pos | 194.0069 | 0.667 | 4.139 | 4.654 | 0.515 | 0.571 | 0.001 | 0.009 | Unannotated | NA | NA |
| metab_255 | (-)-Betonicine | pos | 160.0969 | 0.675 | 3.478 | 4.911 | 1.433 | 0.571 | 0.001 | 0.009 | Tentative | Organic acids and derivatives | Carboxylic acids and derivatives |
| metab_5895 | 2-Furoate | neg | 111.0080 | 2.510 | 3.312 | 3.770 | 0.458 | 0.571 | 0.001 | 0.009 | Tentative | Organoheterocyclic compounds | Furans |
| metab_1330 | Unannotated feature | pos | 209.0809 | 2.234 | 4.765 | 4.617 | -0.147 | -0.571 | 0.001 | 0.009 | Unannotated | NA | NA |
| metab_2966 | 3-Deoxyestrone | pos | 299.1389 | 4.570 | 5.424 | 4.611 | -0.813 | -0.571 | 0.001 | 0.009 | Tentative | Lipids and lipid-like molecules | Steroids and steroid derivatives |
| metab_3776 | 8-Amino-7-oxononanoate | pos | 229.1547 | 1.663 | 6.145 | 6.547 | 0.402 | 0.571 | 0.001 | 0.009 | Tentative | Lipids and lipid-like molecules | Fatty Acyls |
| metab_7373 | Unannotated feature | neg | 138.0554 | 2.119 | 3.229 | 3.655 | 0.426 | 0.571 | 0.001 | 0.009 | Unannotated | NA | NA |
| metab_10103 | Unannotated feature | neg | 282.0059 | 1.127 | 3.563 | 4.540 | 0.977 | 0.571 | 0.001 | 0.009 | Unannotated | NA | NA |
| metab_5659 | Unannotated feature | neg | 269.0883 | 1.079 | 4.958 | 4.749 | -0.209 | -0.571 | 0.001 | 0.009 | Unannotated | NA | NA |
| metab_1417 | Unannotated feature | pos | 115.0635 | 0.667 | 4.651 | 5.043 | 0.392 | 0.571 | 0.001 | 0.009 | Unannotated | NA | NA |
| metab_4419 | Unannotated feature | pos | 724.7923 | 0.559 | 4.581 | 4.800 | 0.219 | 0.571 | 0.001 | 0.009 | Unannotated | NA | NA |
| metab_10489 | Unannotated feature | neg | 286.0609 | 0.636 | 4.929 | 4.607 | -0.322 | -0.571 | 0.001 | 0.009 | Unannotated | NA | NA |
| metab_1023 | Unannotated feature | pos | 857.3297 | 4.708 | 4.388 | 5.136 | 0.748 | 0.571 | 0.001 | 0.009 | Unannotated | NA | NA |
| metab_1737 | Unannotated feature | pos | 181.0269 | 7.324 | 4.553 | 4.623 | 0.070 | 0.571 | 0.001 | 0.009 | Unannotated | NA | NA |
| metab_8088 | Unannotated feature | neg | 577.4843 | 6.188 | 5.447 | 4.625 | -0.823 | -0.571 | 0.001 | 0.009 | Unannotated | NA | NA |
| metab_11247 | Unannotated feature | neg | 564.3319 | 6.157 | 6.836 | 6.947 | 0.111 | 0.571 | 0.001 | 0.009 | Unannotated | NA | NA |
| metab_6792 | Unannotated feature | neg | 380.0726 | 5.547 | 1.931 | 2.603 | 0.672 | 0.571 | 0.001 | 0.009 | Unannotated | NA | NA |
| metab_6400 | Unannotated feature | neg | 287.1253 | 4.079 | 2.896 | 3.866 | 0.971 | 0.566 | 0.001 | 0.009 | Unannotated | NA | NA |
| metab_3994 | Unannotated feature | pos | 127.0504 | 0.823 | 3.207 | 3.696 | 0.489 | 0.566 | 0.001 | 0.009 | Unannotated | NA | NA |
| metab_4432 | Unannotated feature | pos | 520.8313 | 0.559 | 4.014 | 4.645 | 0.631 | 0.566 | 0.001 | 0.009 | Unannotated | NA | NA |
| metab_8942 | Unannotated feature | neg | 158.0607 | 4.969 | 4.177 | 4.523 | 0.347 | 0.566 | 0.001 | 0.009 | Unannotated | NA | NA |
| metab_2199 | Unannotated feature | pos | 810.7487 | 0.567 | 5.070 | 4.955 | -0.115 | -0.566 | 0.001 | 0.009 | Unannotated | NA | NA |
| metab_4845 | Unannotated feature | pos | 341.3050 | 6.296 | 5.765 | 5.476 | -0.289 | -0.566 | 0.001 | 0.009 | Unannotated | NA | NA |
| metab_7137 | Rhodamine B | neg | 478.1994 | 5.859 | 4.979 | 4.246 | -0.733 | -0.566 | 0.001 | 0.009 | Tentative | Organoheterocyclic compounds | Benzopyrans |
| metab_7856 | Unannotated feature | neg | 129.9357 | 7.148 | 4.141 | 4.069 | -0.072 | -0.566 | 0.001 | 0.009 | Unannotated | NA | NA |
| metab_10318 | 9-O-Acetylneuraminic acid | neg | 290.0888 | 0.786 | 3.581 | 4.527 | 0.946 | 0.566 | 0.001 | 0.009 | Tentative | Organic oxygen compounds | Organooxygen compounds |
| metab_649 | Unannotated feature | pos | 186.1318 | 2.288 | 5.256 | 5.462 | 0.206 | 0.566 | 0.001 | 0.009 | Unannotated | NA | NA |
| metab_9688 | 4-hydroxybenzoic acid-4-O-sulphate | neg | 216.9812 | 2.914 | 4.312 | 5.414 | 1.102 | 0.566 | 0.001 | 0.009 | Tentative | Organic acids and derivatives | Organic sulfuric acids and derivatives |
| metab_610 | Unannotated feature | pos | 844.5496 | 5.941 | 5.822 | 6.039 | 0.216 | 0.566 | 0.001 | 0.009 | Unannotated | NA | NA |
| metab_712 | Cinnamyl alcohol | pos | 117.0701 | 2.576 | 4.473 | 4.267 | -0.206 | -0.566 | 0.001 | 0.009 | Tentative | Phenylpropanoids and polyketides | Cinnamyl alcohols |
| metab_4515 | Unannotated feature | pos | 531.0137 | 0.541 | 5.160 | 5.065 | -0.095 | -0.566 | 0.001 | 0.009 | Unannotated | NA | NA |
| metab_3724 | Dopamine 4-O-sulfate | pos | 216.0326 | 1.847 | 3.763 | 4.428 | 0.665 | 0.566 | 0.001 | 0.009 | Tentative | Organic acids and derivatives | Organic sulfuric acids and derivatives |
| metab_7342 | Unannotated feature | neg | 187.9780 | 2.111 | 4.129 | 3.546 | -0.583 | -0.566 | 0.001 | 0.009 | Unannotated | NA | NA |
| metab_5286 | Unannotated feature | neg | 937.7715 | 0.554 | 2.239 | 4.588 | 2.348 | 0.566 | 0.001 | 0.009 | Unannotated | NA | NA |
| metab_3004 | Epanolol | pos | 352.1655 | 4.324 | 5.889 | 5.610 | -0.278 | -0.566 | 0.001 | 0.009 | Tentative | Benzenoids | Phenol ethers |
| metab_9695 | Unannotated feature | neg | 448.6689 | 0.629 | 5.998 | 5.936 | -0.061 | -0.566 | 0.001 | 0.009 | Unannotated | NA | NA |
| metab_1104 | Metipranolol | pos | 310.2012 | 5.520 | 6.221 | 6.472 | 0.251 | 0.566 | 0.001 | 0.009 | Tentative | Benzenoids | Phenol esters |
| metab_1443 | Unannotated feature | pos | 165.9830 | 5.972 | 4.343 | 4.190 | -0.153 | -0.566 | 0.001 | 0.009 | Unannotated | NA | NA |
| metab_9965 | Unannotated feature | neg | 216.0336 | 2.103 | 1.215 | 3.759 | 2.544 | 0.566 | 0.001 | 0.009 | Unannotated | NA | NA |
| metab_8019 | Unannotated feature | neg | 532.8936 | 6.683 | 5.442 | 4.065 | -1.377 | -0.566 | 0.001 | 0.009 | Unannotated | NA | NA |
| metab_11128 | Unannotated feature | neg | 477.2350 | 5.699 | 3.489 | 4.416 | 0.927 | 0.566 | 0.001 | 0.009 | Unannotated | NA | NA |
| metab_9563 | Midodrine | neg | 299.1254 | 3.192 | 4.039 | 4.831 | 0.792 | 0.566 | 0.001 | 0.009 | Tentative | Benzenoids | Benzene and substituted derivatives |
| metab_1332 | Unannotated feature | pos | 865.5930 | 5.886 | 5.086 | 4.837 | -0.249 | -0.566 | 0.001 | 0.009 | Unannotated | NA | NA |
| metab_4177 | Unannotated feature | pos | 121.0974 | 0.644 | 4.962 | 5.184 | 0.222 | 0.566 | 0.001 | 0.009 | Moderate putative | NA | NA |
| metab_2641 | Unannotated feature | pos | 581.4054 | 5.817 | 5.508 | 5.339 | -0.169 | -0.566 | 0.001 | 0.009 | Unannotated | NA | NA |
| metab_1397 | Unannotated feature | pos | 447.2926 | 5.925 | 5.933 | 5.991 | 0.058 | 0.566 | 0.001 | 0.009 | Unannotated | NA | NA |
| metab_3459 | Unannotated feature | pos | 184.4374 | 2.288 | 3.989 | 4.344 | 0.355 | 0.566 | 0.001 | 0.009 | Unannotated | NA | NA |
| metab_1210 | Unannotated feature | pos | 647.9462 | 5.771 | 4.982 | 4.402 | -0.580 | -0.566 | 0.001 | 0.009 | Unannotated | NA | NA |
| metab_8196 | Unannotated feature | neg | 232.9763 | 3.104 | 1.894 | 3.935 | 2.041 | 0.566 | 0.001 | 0.009 | Unannotated | NA | NA |
| metab_1814 | Unannotated feature | pos | 202.0162 | 7.975 | 6.082 | 6.124 | 0.042 | 0.566 | 0.001 | 0.009 | Unannotated | NA | NA |
| metab_2083 | Unannotated feature | pos | 382.7667 | 0.591 | 6.118 | 6.038 | -0.079 | -0.566 | 0.001 | 0.009 | Unannotated | NA | NA |
| metab_251 | Unannotated feature | pos | 864.4094 | 4.532 | 6.325 | 6.520 | 0.196 | 0.566 | 0.001 | 0.009 | Unannotated | NA | NA |
| metab_1746 | Unannotated feature | pos | 287.6331 | 7.324 | 3.791 | 4.117 | 0.326 | 0.566 | 0.001 | 0.009 | Unannotated | NA | NA |
| metab_8557 | Unannotated feature | neg | 329.1767 | 5.691 | 4.427 | 4.083 | -0.344 | -0.566 | 0.001 | 0.009 | Unannotated | NA | NA |
| metab_9181 | 2-Octenedioic acid | neg | 171.0659 | 4.193 | 3.351 | 4.098 | 0.746 | 0.566 | 0.001 | 0.009 | Tentative | Lipids and lipid-like molecules | Fatty Acyls |
| metab_3176 | 5'-Deoxy-5-fluorouridine | pos | 279.0975 | 3.399 | 4.647 | 4.292 | -0.355 | -0.566 | 0.001 | 0.009 | Tentative | Nucleosides, nucleotides, and analogues | 5''-deoxyribonucleosides |
| metab_1384 | Unannotated feature | pos | 216.0867 | 2.303 | 5.567 | 5.275 | -0.292 | -0.566 | 0.001 | 0.009 | Unannotated | NA | NA |
| metab_1613 | Unannotated feature | pos | 87.6588 | 6.983 | 2.545 | 2.847 | 0.302 | 0.566 | 0.001 | 0.009 | Unannotated | NA | NA |
| metab_27 | Unannotated feature | pos | 262.0380 | 1.847 | 4.883 | 5.355 | 0.473 | 0.566 | 0.001 | 0.009 | Unannotated | NA | NA |
| metab_3068 | Unannotated feature | pos | 148.1121 | 2.397 | 2.765 | 3.931 | 1.166 | 0.562 | 0.002 | 0.010 | Moderate putative | NA | NA |
| metab_10905 | Unannotated feature | neg | 725.8777 | 0.543 | 5.863 | 5.763 | -0.100 | -0.562 | 0.002 | 0.010 | Unannotated | NA | NA |
| metab_10906 | Unannotated feature | neg | 690.8807 | 0.543 | 6.176 | 6.055 | -0.121 | -0.562 | 0.002 | 0.010 | Unannotated | NA | NA |
| metab_891 | Unannotated feature | pos | 139.9880 | 7.324 | 6.385 | 6.451 | 0.066 | 0.562 | 0.002 | 0.010 | Unannotated | NA | NA |
| metab_6324 | Unannotated feature | neg | 132.0296 | 0.707 | 4.311 | 4.686 | 0.375 | 0.562 | 0.002 | 0.010 | Unannotated | NA | NA |
| metab_3935 | Unannotated feature | pos | 269.1243 | 0.854 | 4.570 | 4.993 | 0.423 | 0.562 | 0.002 | 0.010 | Unannotated | NA | NA |
| metab_11263 | omega-Cyclohexylundecanoic acid | neg | 595.4954 | 6.188 | 6.532 | 5.999 | -0.533 | -0.562 | 0.002 | 0.010 | Tentative | Lipids and lipid-like molecules | Fatty Acyls |
| metab_2103 | Unannotated feature | pos | 116.4887 | 7.084 | 5.026 | 5.130 | 0.104 | 0.562 | 0.002 | 0.010 | Unannotated | NA | NA |
| metab_2043 | Unannotated feature | pos | 252.2281 | 7.261 | 4.785 | 4.714 | -0.071 | -0.562 | 0.002 | 0.010 | Unannotated | NA | NA |
| metab_5103 | Unannotated feature | pos | 432.6937 | 0.591 | 6.365 | 6.299 | -0.065 | -0.562 | 0.002 | 0.010 | Unannotated | NA | NA |
| metab_11282 | Unannotated feature | neg | 366.8753 | 0.570 | 5.382 | 5.422 | 0.040 | 0.562 | 0.002 | 0.010 | Unannotated | NA | NA |
| metab_862 | Unannotated feature | pos | 184.9856 | 7.324 | 6.191 | 6.257 | 0.066 | 0.562 | 0.002 | 0.010 | Unannotated | NA | NA |
| metab_957 | L-Isoleucine | pos | 132.1020 | 1.321 | 7.330 | 7.282 | -0.048 | -0.562 | 0.002 | 0.010 | Strong putative | NA | NA |
| metab_6345 | Unannotated feature | neg | 202.0719 | 3.819 | 3.202 | 3.790 | 0.588 | 0.562 | 0.002 | 0.010 | Unannotated | NA | NA |
| metab_10893 | Unannotated feature | neg | 970.8178 | 0.543 | 5.495 | 5.358 | -0.137 | -0.562 | 0.002 | 0.010 | Unannotated | NA | NA |
| metab_6623 | Unannotated feature | neg | 261.7757 | 4.961 | 3.790 | 4.030 | 0.240 | 0.562 | 0.002 | 0.010 | Unannotated | NA | NA |
| metab_8183 | Unannotated feature | neg | 150.0013 | 6.000 | 5.103 | 4.119 | -0.984 | -0.562 | 0.002 | 0.010 | Unannotated | NA | NA |
| metab_7259 | Unannotated feature | neg | 315.2547 | 6.071 | 3.989 | 3.468 | -0.520 | -0.562 | 0.002 | 0.010 | Unannotated | NA | NA |
| metab_11230 | Phenylalanylphenylalanine | neg | 311.1407 | 4.635 | 6.577 | 6.339 | -0.238 | -0.562 | 0.002 | 0.010 | Tentative | Organic acids and derivatives | Carboxylic acids and derivatives |
| metab_7666 | Unannotated feature | neg | 307.8565 | 7.269 | 5.728 | 5.802 | 0.074 | 0.562 | 0.002 | 0.010 | Unannotated | NA | NA |
| metab_1273 | Unannotated feature | pos | 401.1413 | 5.855 | 5.226 | 5.307 | 0.081 | 0.562 | 0.002 | 0.010 | Unannotated | NA | NA |
| metab_10772 | Unannotated feature | neg | 145.0977 | 0.570 | 3.730 | 3.470 | -0.260 | -0.562 | 0.002 | 0.010 | Unannotated | NA | NA |
| metab_1224 | Prolyl-Asparagine | pos | 230.1134 | 1.941 | 4.532 | 4.829 | 0.297 | 0.562 | 0.002 | 0.010 | Tentative | Organic acids and derivatives | Carboxylic acids and derivatives |
| metab_2198 | Unannotated feature | pos | 267.2561 | 6.303 | 4.626 | 4.028 | -0.598 | -0.562 | 0.002 | 0.010 | Unannotated | NA | NA |
| metab_2162 | Unannotated feature | pos | 424.9582 | 6.427 | 5.111 | 5.207 | 0.096 | 0.562 | 0.002 | 0.010 | Unannotated | NA | NA |
| metab_5214 | Unannotated feature | neg | 758.8683 | 0.543 | 6.185 | 6.077 | -0.108 | -0.562 | 0.002 | 0.010 | Unannotated | NA | NA |
| metab_7669 | Unannotated feature | neg | 557.3220 | 7.261 | 5.073 | 4.929 | -0.145 | -0.562 | 0.002 | 0.010 | Unannotated | NA | NA |
| metab_10047 | Unannotated feature | neg | 98.0239 | 1.462 | 2.718 | 3.683 | 0.965 | 0.562 | 0.002 | 0.010 | Unannotated | NA | NA |
| metab_4867 | Unannotated feature | pos | 170.0602 | 4.677 | 3.347 | 4.840 | 1.493 | 0.562 | 0.002 | 0.010 | Unannotated | NA | NA |
| metab_3199 | 6-Oxopiperidine-2-carboxylic acid | pos | 304.1502 | 2.552 | 4.007 | 4.316 | 0.309 | 0.562 | 0.002 | 0.010 | Tentative | Organic acids and derivatives | Carboxylic acids and derivatives |
| metab_9580 | Unannotated feature | neg | 462.1006 | 3.168 | 4.397 | 3.913 | -0.484 | -0.562 | 0.002 | 0.010 | Unannotated | NA | NA |
| metab_7614 | Unannotated feature | neg | 152.8945 | 7.301 | 4.838 | 4.887 | 0.050 | 0.562 | 0.002 | 0.010 | Unannotated | NA | NA |
| metab_10276 | Unannotated feature | neg | 176.9933 | 0.841 | 4.530 | 4.992 | 0.462 | 0.562 | 0.002 | 0.010 | Unannotated | NA | NA |
| metab_519 | 3-Hydroxy-L-kynurenine | pos | 257.1131 | 1.941 | 4.992 | 5.373 | 0.381 | 0.562 | 0.002 | 0.010 | Tentative | Organic oxygen compounds | Organooxygen compounds |
| metab_5089 | Unannotated feature | pos | 141.0716 | 0.846 | 4.050 | 3.806 | -0.244 | -0.562 | 0.002 | 0.010 | Unannotated | NA | NA |
| metab_9724 | Unannotated feature | neg | 386.7161 | 0.629 | 6.693 | 6.640 | -0.053 | -0.562 | 0.002 | 0.010 | Unannotated | NA | NA |
| metab_1949 | Unannotated feature | pos | 310.1621 | 7.316 | 3.961 | 4.340 | 0.378 | 0.562 | 0.002 | 0.010 | Unannotated | NA | NA |
| metab_9045 | Unannotated feature | neg | 309.6358 | 4.635 | 4.391 | 4.061 | -0.330 | -0.562 | 0.002 | 0.010 | Unannotated | NA | NA |
| metab_3847 | Unannotated feature | pos | 284.0199 | 1.095 | 3.829 | 4.707 | 0.878 | 0.562 | 0.002 | 0.010 | Unannotated | NA | NA |
| metab_9298 | Gamma-Glutamyltyrosine | neg | 331.0918 | 3.742 | 3.745 | 4.697 | 0.953 | 0.558 | 0.002 | 0.010 | Tentative | Organic acids and derivatives | Carboxylic acids and derivatives |
| metab_10273 | Unannotated feature | neg | 234.9524 | 0.841 | 3.702 | 4.310 | 0.608 | 0.558 | 0.002 | 0.010 | Unannotated | NA | NA |
| metab_10847 | Unannotated feature | neg | 633.8578 | 0.554 | 4.739 | 4.898 | 0.159 | 0.558 | 0.002 | 0.010 | Unannotated | NA | NA |
| metab_4543 | Unannotated feature | pos | 135.0442 | 0.512 | 3.537 | 3.644 | 0.107 | 0.558 | 0.002 | 0.010 | Unannotated | NA | NA |
| metab_8945 | Unannotated feature | neg | 552.2381 | 4.961 | 5.488 | 5.671 | 0.183 | 0.558 | 0.002 | 0.010 | Unannotated | NA | NA |
| metab_1734 | Unannotated feature | pos | 98.9617 | 7.324 | 4.355 | 4.457 | 0.102 | 0.558 | 0.002 | 0.010 | Unannotated | NA | NA |
| metab_640 | Muramic acid | pos | 274.0897 | 0.660 | 5.866 | 5.552 | -0.314 | -0.558 | 0.002 | 0.010 | Moderate putative | Organic oxygen compounds | Organooxygen compounds |
| metab_10470 | Unannotated feature | neg | 146.0817 | 0.652 | 4.340 | 4.121 | -0.219 | -0.558 | 0.002 | 0.010 | Unannotated | NA | NA |
| metab_7891 | Unannotated feature | neg | 271.9999 | 2.111 | 4.966 | 4.404 | -0.563 | -0.558 | 0.002 | 0.010 | Unannotated | NA | NA |
| metab_6778 | Unannotated feature | neg | 82.9533 | 5.538 | 3.980 | 4.048 | 0.068 | 0.558 | 0.002 | 0.010 | Unannotated | NA | NA |
| metab_2503 | 4-Propylphenol | pos | 119.0858 | 5.894 | 6.290 | 6.422 | 0.132 | 0.558 | 0.002 | 0.010 | Tentative | Benzenoids | Benzene and substituted derivatives |
| metab_4447 | Unannotated feature | pos | 974.8129 | 0.551 | 6.014 | 5.914 | -0.100 | -0.558 | 0.002 | 0.010 | Unannotated | NA | NA |
| metab_10107 | Unannotated feature | neg | 243.9683 | 1.103 | 4.127 | 3.446 | -0.682 | -0.558 | 0.002 | 0.010 | Unannotated | NA | NA |
| metab_4561 | Unannotated feature | pos | 221.0472 | 0.473 | 5.585 | 5.559 | -0.026 | -0.558 | 0.002 | 0.010 | Unannotated | NA | NA |
| metab_4283 | Unannotated feature | pos | 376.7320 | 0.591 | 5.703 | 5.632 | -0.071 | -0.558 | 0.002 | 0.010 | Unannotated | NA | NA |
| metab_10081 | Unannotated feature | neg | 620.5483 | 0.613 | 6.364 | 6.288 | -0.077 | -0.558 | 0.002 | 0.010 | Unannotated | NA | NA |
| metab_4443 | Unannotated feature | pos | 1008.8060 | 0.551 | 5.632 | 5.460 | -0.172 | -0.558 | 0.002 | 0.010 | Unannotated | NA | NA |
| metab_10901 | Unannotated feature | neg | 794.8670 | 0.543 | 5.011 | 4.870 | -0.141 | -0.558 | 0.002 | 0.010 | Unannotated | NA | NA |
| metab_6967 | Unannotated feature | neg | 400.8532 | 0.881 | 5.163 | 4.958 | -0.205 | -0.558 | 0.002 | 0.010 | Unannotated | NA | NA |
| metab_9365 | Unannotated feature | neg | 257.9841 | 3.562 | 3.974 | 3.715 | -0.259 | -0.558 | 0.002 | 0.010 | Unannotated | NA | NA |
| metab_2942 | Acetoxyacetylaminofluorene | pos | 299.1390 | 4.769 | 5.481 | 4.805 | -0.676 | -0.558 | 0.002 | 0.010 | Tentative | Benzenoids | Fluorenes |
| metab_5315 | Unannotated feature | neg | 821.8415 | 0.562 | 4.889 | 4.377 | -0.511 | -0.558 | 0.002 | 0.010 | Unannotated | NA | NA |
| metab_6686 | Unannotated feature | neg | 497.2053 | 5.215 | 3.662 | 4.463 | 0.801 | 0.558 | 0.002 | 0.010 | Unannotated | NA | NA |
| metab_2671 | Unannotated feature | pos | 164.0740 | 1.925 | 4.904 | 5.209 | 0.305 | 0.558 | 0.002 | 0.010 | Unannotated | NA | NA |
| metab_8381 | (2S,4R)-p-Mentha-1(7),5-dien-2-ol acetate | neg | 239.1291 | 5.803 | 4.083 | 4.362 | 0.279 | 0.558 | 0.002 | 0.010 | Tentative | Lipids and lipid-like molecules | Prenol lipids |
| metab_10823 | Unannotated feature | neg | 832.8117 | 0.554 | 5.050 | 5.323 | 0.274 | 0.558 | 0.002 | 0.010 | Unannotated | NA | NA |
| metab_9001 | 3,4-Dihydroxyphenylethanol-4-diglucoside | neg | 459.1519 | 4.742 | 3.174 | 4.277 | 1.102 | 0.558 | 0.002 | 0.010 | Tentative | Organic oxygen compounds | Organooxygen compounds |
| metab_10033 | Unannotated feature | neg | 233.9470 | 1.664 | 5.274 | 5.180 | -0.094 | -0.558 | 0.002 | 0.010 | Unannotated | NA | NA |
| metab_1627 | Unannotated feature | pos | 239.2248 | 7.084 | 5.364 | 5.323 | -0.041 | -0.558 | 0.002 | 0.010 | Unannotated | NA | NA |
| metab_5073 | Unannotated feature | pos | 203.1391 | 1.142 | 4.931 | 5.298 | 0.367 | 0.558 | 0.002 | 0.010 | Unannotated | NA | NA |
| metab_11133 | Unannotated feature | neg | 223.0977 | 5.715 | 3.887 | 4.418 | 0.531 | 0.558 | 0.002 | 0.010 | Unannotated | NA | NA |
| metab_9702 | 3-Acetylphenol sulfate | neg | 261.0079 | 2.875 | 3.796 | 4.720 | 0.924 | 0.558 | 0.002 | 0.010 | Tentative | Organic oxygen compounds | Organooxygen compounds |
| metab_8018 | Unannotated feature | neg | 530.8967 | 6.690 | 6.065 | 5.287 | -0.778 | -0.558 | 0.002 | 0.010 | Unannotated | NA | NA |
| metab_3644 | Unannotated feature | pos | 174.1490 | 1.956 | 2.704 | 3.818 | 1.114 | 0.558 | 0.002 | 0.010 | Unannotated | NA | NA |
| metab_505 | Unannotated feature | pos | 113.0600 | 1.910 | 4.484 | 4.569 | 0.084 | 0.558 | 0.002 | 0.010 | Unannotated | NA | NA |
| metab_4663 | Unannotated feature | pos | 442.3364 | 5.840 | 5.759 | 5.726 | -0.033 | -0.558 | 0.002 | 0.010 | Unannotated | NA | NA |
| metab_260 | Unannotated feature | pos | 133.0802 | 0.691 | 5.087 | 4.633 | -0.454 | -0.558 | 0.002 | 0.010 | Unannotated | NA | NA |
| metab_2405 | Unannotated feature | pos | 227.1251 | 0.667 | 6.094 | 6.363 | 0.269 | 0.558 | 0.002 | 0.010 | Unannotated | NA | NA |
| metab_2104 | Unannotated feature | pos | 102.6984 | 7.084 | 3.961 | 4.163 | 0.203 | 0.558 | 0.002 | 0.010 | Unannotated | NA | NA |
| metab_2446 | Unannotated feature | pos | 225.1962 | 5.917 | 4.106 | 4.294 | 0.188 | 0.558 | 0.002 | 0.010 | Unannotated | NA | NA |
| metab_4859 | Unannotated feature | pos | 508.7130 | 0.591 | 5.865 | 5.796 | -0.070 | -0.558 | 0.002 | 0.010 | Unannotated | NA | NA |
| metab_7908 | Unannotated feature | neg | 259.9994 | 2.111 | 5.623 | 5.194 | -0.429 | -0.558 | 0.002 | 0.010 | Unannotated | NA | NA |
| metab_8858 | Unannotated feature | neg | 217.1081 | 5.279 | 3.276 | 3.908 | 0.632 | 0.554 | 0.002 | 0.011 | Unannotated | NA | NA |
| metab_7158 | Unannotated feature | neg | 224.9798 | 1.286 | 5.979 | 5.895 | -0.084 | -0.554 | 0.002 | 0.011 | Unannotated | NA | NA |
| metab_8298 | Unannotated feature | neg | 506.0902 | 3.160 | 4.831 | 4.262 | -0.570 | -0.554 | 0.002 | 0.011 | Unannotated | NA | NA |
| metab_10558 | Unannotated feature | neg | 204.0665 | 4.969 | 5.731 | 6.020 | 0.289 | 0.554 | 0.002 | 0.011 | Unannotated | NA | NA |
| metab_4889 | Unannotated feature | pos | 198.0973 | 1.949 | 4.769 | 4.531 | -0.238 | -0.554 | 0.002 | 0.011 | Unannotated | NA | NA |
| metab_2694 | Unannotated feature | pos | 669.4572 | 5.756 | 5.026 | 4.749 | -0.277 | -0.554 | 0.002 | 0.011 | Unannotated | NA | NA |
| metab_1374 | Unannotated feature | pos | 733.5168 | 5.910 | 6.248 | 6.196 | -0.052 | -0.554 | 0.002 | 0.011 | Unannotated | NA | NA |
| metab_10495 | Unannotated feature | neg | 242.0520 | 0.636 | 5.178 | 5.376 | 0.199 | 0.554 | 0.002 | 0.011 | Unannotated | NA | NA |
| metab_8363 | Ansamitocin P-3 | neg | 615.2475 | 5.811 | 4.408 | 3.705 | -0.703 | -0.554 | 0.002 | 0.011 | Tentative | NA | NA |
| metab_5217 | Unannotated feature | neg | 826.8561 | 0.543 | 6.114 | 5.963 | -0.151 | -0.554 | 0.002 | 0.011 | Unannotated | NA | NA |
| metab_4141 | Unannotated feature | pos | 469.0105 | 0.667 | 4.523 | 3.921 | -0.602 | -0.554 | 0.002 | 0.011 | Unannotated | NA | NA |
| metab_4717 | Unannotated feature | pos | 732.5475 | 5.964 | 6.360 | 6.275 | -0.085 | -0.554 | 0.002 | 0.011 | Unannotated | NA | NA |
| metab_6404 | Unannotated feature | neg | 173.0452 | 4.099 | 3.818 | 3.706 | -0.111 | -0.554 | 0.002 | 0.011 | Unannotated | NA | NA |
| metab_597 | Unannotated feature | pos | 336.1964 | 2.081 | 5.322 | 5.204 | -0.118 | -0.554 | 0.002 | 0.011 | Unannotated | NA | NA |
| metab_5375 | Unannotated feature | neg | 599.6009 | 0.594 | 5.537 | 5.450 | -0.087 | -0.554 | 0.002 | 0.011 | Unannotated | NA | NA |
| metab_1481 | Unannotated feature | pos | 449.2876 | 6.034 | 4.254 | 4.592 | 0.338 | 0.554 | 0.002 | 0.011 | Unannotated | NA | NA |
| metab_7276 | 9(S)-HODE | neg | 591.4641 | 6.118 | 6.253 | 5.869 | -0.384 | -0.554 | 0.002 | 0.011 | Tentative | Lipids and lipid-like molecules | Fatty Acyls |
| metab_4013 | 4,5-seco-Dopa | pos | 247.0925 | 0.807 | 1.296 | 3.849 | 2.553 | 0.554 | 0.002 | 0.011 | Tentative | Organic acids and derivatives | Carboxylic acids and derivatives |
| metab_1546 | Unannotated feature | pos | 226.2126 | 6.296 | 4.875 | 4.492 | -0.383 | -0.554 | 0.002 | 0.011 | Unannotated | NA | NA |
| metab_3704 | 6-Methoxymellein | pos | 209.0809 | 3.383 | 4.862 | 4.783 | -0.080 | -0.554 | 0.002 | 0.011 | Tentative | NA | NA |
| metab_4578 | Unannotated feature | pos | 285.1539 | 0.442 | 5.162 | 5.233 | 0.071 | 0.554 | 0.002 | 0.011 | Unannotated | NA | NA |
| metab_2475 | Unannotated feature | pos | 602.4481 | 5.902 | 5.377 | 5.253 | -0.123 | -0.554 | 0.002 | 0.011 | Unannotated | NA | NA |
| metab_415 | Unannotated feature | pos | 172.0403 | 1.266 | 4.735 | 4.985 | 0.250 | 0.554 | 0.002 | 0.011 | Unannotated | NA | NA |
| metab_5628 | Unannotated feature | neg | 618.5510 | 0.912 | 4.764 | 4.555 | -0.209 | -0.554 | 0.002 | 0.011 | Unannotated | NA | NA |
| metab_10114 | Unannotated feature | neg | 228.9423 | 1.086 | 5.153 | 5.012 | -0.142 | -0.554 | 0.002 | 0.011 | Unannotated | NA | NA |
| metab_1741 | Unannotated feature | pos | 199.0174 | 7.324 | 5.170 | 5.261 | 0.091 | 0.554 | 0.002 | 0.011 | Unannotated | NA | NA |
| metab_3487 | Unannotated feature | pos | 158.0489 | 2.506 | 4.883 | 4.801 | -0.082 | -0.554 | 0.002 | 0.011 | Unannotated | NA | NA |
| metab_2508 | Unannotated feature | pos | 843.5799 | 5.886 | 5.181 | 4.956 | -0.225 | -0.554 | 0.002 | 0.011 | Unannotated | NA | NA |
| metab_5055 | (5-L-Glutamyl)-L-glutamine | pos | 276.1190 | 0.721 | 5.653 | 5.451 | -0.202 | -0.554 | 0.002 | 0.011 | Tentative | Organic acids and derivatives | Carboxylic acids and derivatives |
| metab_7178 | Unannotated feature | neg | 318.0461 | 0.865 | 5.395 | 5.287 | -0.109 | -0.554 | 0.002 | 0.011 | Unannotated | NA | NA |
| metab_7494 | LysoPI(20:4(5Z,8Z,11Z,14Z)/0:0) | neg | 619.2903 | 7.148 | 5.192 | 4.966 | -0.226 | -0.554 | 0.002 | 0.011 | Tentative | Lipids and lipid-like molecules | Glycerophospholipids |
| metab_1618 | Unannotated feature | pos | 129.7517 | 7.030 | 5.099 | 5.175 | 0.076 | 0.554 | 0.002 | 0.011 | Unannotated | NA | NA |
| metab_9992 | Apigenin 7,4'-dimethyl ether | neg | 333.0523 | 1.965 | 5.100 | 4.761 | -0.339 | -0.554 | 0.002 | 0.011 | Tentative | Phenylpropanoids and polyketides | Flavonoids |
| metab_6168 | Unannotated feature | neg | 572.6182 | 0.594 | 6.315 | 6.272 | -0.043 | -0.554 | 0.002 | 0.011 | Unannotated | NA | NA |
| metab_5253 | Unannotated feature | neg | 631.8272 | 0.554 | 4.689 | 5.087 | 0.399 | 0.554 | 0.002 | 0.011 | Unannotated | NA | NA |
| metab_10889 | Unannotated feature | neg | 318.5060 | 0.546 | 4.777 | 4.670 | -0.107 | -0.554 | 0.002 | 0.011 | Unannotated | NA | NA |
| metab_5150 | Phenol | pos | 95.0496 | 1.740 | 5.424 | 5.319 | -0.106 | -0.554 | 0.002 | 0.011 | Moderate putative | NA | NA |
| metab_4857 | Unannotated feature | pos | 358.2588 | 5.710 | 5.213 | 5.586 | 0.373 | 0.554 | 0.002 | 0.011 | Unannotated | NA | NA |
| metab_7628 | Unannotated feature | neg | 90.0270 | 7.680 | 5.407 | 5.328 | -0.079 | -0.554 | 0.002 | 0.011 | Unannotated | NA | NA |
| metab_3496 | 5'-N-Ethylcarboxamidoadenosine | pos | 309.1291 | 0.667 | 6.190 | 5.964 | -0.226 | -0.554 | 0.002 | 0.011 | Tentative | NA | NA |
| metab_4401 | Unannotated feature | pos | 519.2238 | 5.122 | 4.536 | 3.708 | -0.828 | -0.549 | 0.002 | 0.011 | Unannotated | NA | NA |
| metab_20 | Unannotated feature | pos | 762.6018 | 0.583 | 5.620 | 5.544 | -0.076 | -0.549 | 0.002 | 0.011 | Unannotated | NA | NA |
| metab_7544 | Unannotated feature | neg | 197.0567 | 2.119 | 4.338 | 4.725 | 0.387 | 0.549 | 0.002 | 0.011 | Unannotated | NA | NA |
| metab_3369 | Unannotated feature | pos | 245.0995 | 2.545 | 4.390 | 4.168 | -0.223 | -0.549 | 0.002 | 0.011 | Unannotated | NA | NA |
| metab_5992 | Aminohippuric acid | neg | 193.0617 | 2.852 | 3.197 | 3.842 | 0.645 | 0.549 | 0.002 | 0.011 | Moderate putative | Benzenoids | Benzene and substituted derivatives |
| metab_9430 | Buflomedil | neg | 328.1520 | 3.388 | 3.986 | 4.236 | 0.251 | 0.549 | 0.002 | 0.011 | Tentative | Organic oxygen compounds | Organooxygen compounds |
| metab_6769 | Unannotated feature | neg | 639.7206 | 5.518 | 4.762 | 5.128 | 0.367 | 0.549 | 0.002 | 0.011 | Unannotated | NA | NA |
| metab_1107 | Piroxantrone | pos | 475.2075 | 5.543 | 4.961 | 4.340 | -0.621 | -0.549 | 0.002 | 0.011 | Tentative | Benzenoids | Anthracenes |
| metab_2371 | 10-hydroxy-(2E,8E)-decadien-4-ynoic Acid | pos | 163.0756 | 5.964 | 4.253 | 4.326 | 0.073 | 0.549 | 0.002 | 0.011 | Tentative | Lipids and lipid-like molecules | Fatty Acyls |
| metab_394 | Unannotated feature | pos | 133.0497 | 1.048 | 3.579 | 3.700 | 0.120 | 0.549 | 0.002 | 0.011 | Unannotated | NA | NA |
| metab_10532 | Unannotated feature | neg | 616.5540 | 0.621 | 6.135 | 6.053 | -0.082 | -0.549 | 0.002 | 0.011 | Unannotated | NA | NA |
| metab_4983 | Unannotated feature | pos | 251.1115 | 0.729 | 2.228 | 4.058 | 1.830 | 0.549 | 0.002 | 0.011 | Unannotated | NA | NA |
| metab_1912 | Unannotated feature | pos | 288.1601 | 7.324 | 4.998 | 4.899 | -0.098 | -0.549 | 0.002 | 0.011 | Unannotated | NA | NA |
| metab_5215 | Unannotated feature | neg | 792.8622 | 0.543 | 6.594 | 6.507 | -0.087 | -0.549 | 0.002 | 0.011 | Unannotated | NA | NA |
| metab_10324 | Unannotated feature | neg | 379.1283 | 4.643 | 4.944 | 4.617 | -0.327 | -0.549 | 0.002 | 0.011 | Unannotated | NA | NA |
| metab_8359 | Unannotated feature | neg | 197.0009 | 5.819 | 3.611 | 2.978 | -0.634 | -0.549 | 0.002 | 0.011 | Unannotated | NA | NA |
| metab_2887 | Unannotated feature | pos | 576.3949 | 5.068 | 5.125 | 4.879 | -0.246 | -0.549 | 0.002 | 0.011 | Unannotated | NA | NA |
| metab_5218 | Unannotated feature | neg | 860.8497 | 0.543 | 6.389 | 6.304 | -0.085 | -0.549 | 0.002 | 0.011 | Unannotated | NA | NA |
| metab_5159 | Unannotated feature | neg | 203.0824 | 3.160 | 6.438 | 6.124 | -0.314 | -0.549 | 0.002 | 0.011 | Unannotated | NA | NA |
| metab_10111 | Unannotated feature | neg | 178.9200 | 1.094 | 5.147 | 5.066 | -0.081 | -0.549 | 0.002 | 0.011 | Unannotated | NA | NA |
| metab_4436 | Unannotated feature | pos | 327.0515 | 0.559 | 5.357 | 5.248 | -0.109 | -0.549 | 0.002 | 0.011 | Unannotated | NA | NA |
| metab_3537 | Ornaline | pos | 227.1026 | 2.081 | 3.934 | 5.226 | 1.292 | 0.549 | 0.002 | 0.011 | Tentative | Organic acids and derivatives | Carboxylic acids and derivatives |
| metab_428 | (R)-3-hydroxybutyrylcarnitine | pos | 248.1492 | 1.499 | 3.693 | 4.586 | 0.893 | 0.549 | 0.002 | 0.011 | Tentative | Lipids and lipid-like molecules | Fatty Acyls |
| metab_4465 | Unannotated feature | pos | 773.8276 | 0.551 | 5.486 | 5.617 | 0.130 | 0.549 | 0.002 | 0.011 | Unannotated | NA | NA |
| metab_7227 | Unannotated feature | neg | 264.1609 | 5.993 | 3.736 | 4.057 | 0.321 | 0.549 | 0.002 | 0.011 | Unannotated | NA | NA |
| metab_1324 | Unannotated feature | pos | 667.4605 | 5.886 | 4.923 | 4.462 | -0.461 | -0.549 | 0.002 | 0.011 | Unannotated | NA | NA |
| metab_10841 | Unannotated feature | neg | 673.8073 | 0.554 | 3.814 | 4.368 | 0.553 | 0.549 | 0.002 | 0.011 | Unannotated | NA | NA |
| metab_1192 | Unannotated feature | pos | 459.8019 | 5.748 | 4.601 | 4.253 | -0.348 | -0.549 | 0.002 | 0.011 | Unannotated | NA | NA |
| metab_4604 | Unannotated feature | pos | 99.5316 | 0.028 | 4.553 | 4.637 | 0.084 | 0.549 | 0.002 | 0.011 | Unannotated | NA | NA |
| metab_2616 | 3-Methylguanine | pos | 207.0988 | 5.840 | 4.792 | 4.867 | 0.075 | 0.545 | 0.002 | 0.012 | Tentative | NA | NA |
| metab_5077 | Unannotated feature | pos | 204.0439 | 0.745 | 5.693 | 5.497 | -0.196 | -0.545 | 0.002 | 0.012 | Unannotated | NA | NA |
| metab_3217 | Glutamine | pos | 129.0660 | 3.167 | 4.216 | 4.742 | 0.525 | 0.545 | 0.002 | 0.012 | Strong putative | Organic acids and derivatives | Carboxylic acids and derivatives |
| metab_3357 | Unannotated feature | pos | 261.1445 | 2.764 | 4.706 | 5.181 | 0.475 | 0.545 | 0.002 | 0.012 | Unannotated | NA | NA |
| metab_5284 | Unannotated feature | neg | 900.7994 | 0.554 | 4.941 | 5.163 | 0.222 | 0.545 | 0.002 | 0.012 | Unannotated | NA | NA |
| metab_5268 | Unannotated feature | neg | 733.8083 | 0.554 | 4.592 | 5.152 | 0.560 | 0.545 | 0.002 | 0.012 | Unannotated | NA | NA |
| metab_1917 | Unannotated feature | pos | 208.0393 | 7.324 | 5.466 | 5.432 | -0.035 | -0.545 | 0.002 | 0.012 | Unannotated | NA | NA |
| metab_4921 | Unannotated feature | pos | 192.0745 | 0.606 | 4.473 | 4.946 | 0.473 | 0.545 | 0.002 | 0.012 | Unannotated | NA | NA |
| metab_2241 | Avenoleic acid | pos | 593.4769 | 6.226 | 5.411 | 4.711 | -0.700 | -0.545 | 0.002 | 0.012 | Tentative | Lipids and lipid-like molecules | Fatty Acyls |
| metab_1939 | Unannotated feature | pos | 110.5418 | 7.324 | 4.708 | 4.623 | -0.085 | -0.545 | 0.002 | 0.012 | Unannotated | NA | NA |
| metab_9514 | Unannotated feature | neg | 145.0613 | 3.734 | 4.750 | 5.459 | 0.709 | 0.545 | 0.002 | 0.012 | Unannotated | NA | NA |
| metab_5242 | Unannotated feature | neg | 538.7805 | 0.554 | 2.280 | 4.506 | 2.226 | 0.545 | 0.002 | 0.012 | Unannotated | NA | NA |
| metab_10650 | Unannotated feature | neg | 636.6125 | 0.587 | 5.608 | 5.563 | -0.045 | -0.545 | 0.002 | 0.012 | Unannotated | NA | NA |
| metab_7210 | Unannotated feature | neg | 427.2167 | 5.930 | 4.954 | 4.116 | -0.838 | -0.545 | 0.002 | 0.012 | Unannotated | NA | NA |
| metab_788 | 4,8-Dihydroxyquinoline | pos | 162.0550 | 2.965 | 3.745 | 4.408 | 0.663 | 0.545 | 0.002 | 0.012 | Tentative | Organoheterocyclic compounds | Quinolines and derivatives |
| metab_4680 | Unannotated feature | pos | 564.8949 | 5.871 | 5.075 | 4.921 | -0.153 | -0.545 | 0.002 | 0.012 | Unannotated | NA | NA |
| metab_10194 | Unannotated feature | neg | 396.7447 | 0.598 | 5.953 | 5.857 | -0.096 | -0.545 | 0.002 | 0.012 | Unannotated | NA | NA |
| metab_9752 | Unannotated feature | neg | 136.8937 | 2.716 | 4.404 | 4.342 | -0.062 | -0.545 | 0.002 | 0.012 | Unannotated | NA | NA |
| metab_10327 | Unannotated feature | neg | 510.6649 | 0.778 | 4.933 | 4.455 | -0.479 | -0.545 | 0.002 | 0.012 | Unannotated | NA | NA |
| metab_6628 | Unannotated feature | neg | 142.0656 | 4.977 | 3.439 | 3.842 | 0.402 | 0.545 | 0.002 | 0.012 | Unannotated | NA | NA |
| metab_10340 | 3h-Sialic acid | neg | 308.0993 | 0.762 | 4.763 | 4.443 | -0.320 | -0.545 | 0.002 | 0.012 | Tentative | Organic oxygen compounds | Organooxygen compounds |
| metab_2486 | Unannotated feature | pos | 851.3967 | 5.894 | 5.214 | 5.525 | 0.312 | 0.545 | 0.002 | 0.012 | Unannotated | NA | NA |
| metab_10116 | Unannotated feature | neg | 88.5635 | 1.086 | 3.277 | 2.950 | -0.327 | -0.545 | 0.002 | 0.012 | Unannotated | NA | NA |
| metab_390 | Unannotated feature | pos | 146.0449 | 0.839 | 3.950 | 4.389 | 0.440 | 0.545 | 0.002 | 0.012 | Unannotated | NA | NA |
| metab_4979 | Unannotated feature | pos | 686.5826 | 0.591 | 5.352 | 5.228 | -0.125 | -0.545 | 0.002 | 0.012 | Unannotated | NA | NA |
| metab_4969 | Unannotated feature | pos | 226.1186 | 0.698 | 4.583 | 3.763 | -0.820 | -0.545 | 0.002 | 0.012 | Unannotated | NA | NA |
| metab_3279 | Unannotated feature | pos | 113.9640 | 2.896 | 5.955 | 5.939 | -0.016 | -0.545 | 0.002 | 0.012 | Unannotated | NA | NA |
| metab_4825 | Unannotated feature | pos | 111.5193 | 7.324 | 4.743 | 4.823 | 0.080 | 0.545 | 0.002 | 0.012 | Unannotated | NA | NA |
| metab_4866 | Indolylacryloylglycine | pos | 245.0921 | 4.677 | 3.461 | 4.715 | 1.254 | 0.545 | 0.002 | 0.012 | Tentative | Organic acids and derivatives | Carboxylic acids and derivatives |
| metab_4289 | Unannotated feature | pos | 260.8148 | 0.591 | 5.475 | 5.414 | -0.060 | -0.545 | 0.002 | 0.012 | Unannotated | NA | NA |
| metab_4943 | Unannotated feature | pos | 205.0682 | 0.636 | 4.647 | 5.444 | 0.797 | 0.545 | 0.002 | 0.012 | Unannotated | NA | NA |
| metab_6052 | Unannotated feature | neg | 107.0494 | 3.065 | 2.293 | 3.490 | 1.197 | 0.545 | 0.002 | 0.012 | Unannotated | NA | NA |
| metab_1412 | Unannotated feature | pos | 585.9182 | 5.933 | 4.702 | 4.504 | -0.198 | -0.545 | 0.002 | 0.012 | Unannotated | NA | NA |
| metab_6013 | Succinyladenosine | neg | 382.1012 | 2.914 | 4.220 | 4.787 | 0.566 | 0.545 | 0.002 | 0.012 | Tentative | Nucleosides, nucleotides, and analogues | Purine nucleosides |
| metab_6007 | Unannotated feature | neg | 203.0560 | 2.906 | 4.170 | 3.415 | -0.755 | -0.545 | 0.002 | 0.012 | Unannotated | NA | NA |
| metab_4339 | Unannotated feature | pos | 276.8784 | 0.575 | 5.597 | 5.562 | -0.035 | -0.545 | 0.002 | 0.012 | Unannotated | NA | NA |
| metab_6305 | Unannotated feature | neg | 482.9884 | 0.676 | 5.135 | 4.769 | -0.366 | -0.545 | 0.002 | 0.012 | Unannotated | NA | NA |
| metab_11209 | Unannotated feature | neg | 280.8273 | 0.590 | 6.290 | 6.230 | -0.060 | -0.545 | 0.002 | 0.012 | Unannotated | NA | NA |
| metab_8527 | Unannotated feature | neg | 159.9783 | 7.109 | 6.380 | 6.321 | -0.059 | -0.545 | 0.002 | 0.012 | Unannotated | NA | NA |
| metab_4990 | Unannotated feature | pos | 628.6241 | 0.591 | 5.452 | 5.330 | -0.122 | -0.545 | 0.002 | 0.012 | Unannotated | NA | NA |
| metab_910 | Unannotated feature | pos | 111.0205 | 7.324 | 6.576 | 6.650 | 0.074 | 0.545 | 0.002 | 0.012 | Unannotated | NA | NA |
| metab_10099 | Unannotated feature | neg | 260.0239 | 1.182 | 4.915 | 5.405 | 0.489 | 0.545 | 0.002 | 0.012 | Unannotated | NA | NA |
| metab_2874 | 5-Hydroxydec-6-enoylcarnitine | pos | 330.2274 | 5.153 | 4.202 | 5.148 | 0.947 | 0.545 | 0.002 | 0.012 | Tentative | Lipids and lipid-like molecules | Fatty Acyls |
| metab_3162 | Unannotated feature | pos | 128.1072 | 3.507 | 3.090 | 3.579 | 0.489 | 0.545 | 0.002 | 0.012 | Unannotated | NA | NA |
| metab_2116 | Unannotated feature | pos | 400.8274 | 0.575 | 5.885 | 5.845 | -0.041 | -0.545 | 0.002 | 0.012 | Unannotated | NA | NA |
| metab_6097 | Unannotated feature | neg | 558.0526 | 3.160 | 4.571 | 4.201 | -0.370 | -0.545 | 0.002 | 0.012 | Unannotated | NA | NA |
| metab_10471 | Unannotated feature | neg | 134.8645 | 0.652 | 3.944 | 4.165 | 0.220 | 0.545 | 0.002 | 0.012 | Unannotated | NA | NA |
| metab_6536 | Unannotated feature | neg | 131.0707 | 4.574 | 5.406 | 5.207 | -0.199 | -0.545 | 0.002 | 0.012 | Unannotated | NA | NA |
| metab_10088 | Unannotated feature | neg | 284.9359 | 1.286 | 4.421 | 4.280 | -0.141 | -0.545 | 0.002 | 0.012 | Unannotated | NA | NA |
| metab_4307 | Unannotated feature | pos | 278.8754 | 0.583 | 4.736 | 4.673 | -0.063 | -0.545 | 0.002 | 0.012 | Unannotated | NA | NA |
| metab_4514 | Unannotated feature | pos | 650.9320 | 0.541 | 4.760 | 4.470 | -0.290 | -0.545 | 0.002 | 0.012 | Unannotated | NA | NA |
| metab_571 | Paramethadione | pos | 140.0707 | 2.058 | 3.246 | 3.762 | 0.516 | 0.545 | 0.002 | 0.012 | Tentative | Organoheterocyclic compounds | Azolidines |
| metab_3696 | 3-Hydroxyanthranilate | pos | 154.0499 | 1.910 | 5.095 | 5.274 | 0.179 | 0.545 | 0.002 | 0.012 | Strong putative | Benzenoids | Benzene and substituted derivatives |
| metab_10895 | Unannotated feature | neg | 928.8375 | 0.543 | 6.217 | 6.077 | -0.141 | -0.545 | 0.002 | 0.012 | Unannotated | NA | NA |
| metab_186 | Unannotated feature | pos | 548.8280 | 0.567 | 5.188 | 5.137 | -0.050 | -0.545 | 0.002 | 0.012 | Unannotated | NA | NA |
| metab_2642 | Unannotated feature | pos | 565.2293 | 5.817 | 4.656 | 3.919 | -0.738 | -0.541 | 0.002 | 0.013 | Unannotated | NA | NA |
| metab_7288 | 4alpha-hydroxymethyl,4beta,14alpha-dimethyl-9beta,19-cyclo-5alpha-ergost-24(241)-en-3beta-ol | neg | 501.3959 | 6.180 | 5.696 | 5.313 | -0.383 | -0.541 | 0.002 | 0.013 | Tentative | Lipids and lipid-like molecules | Steroids and steroid derivatives |
| metab_2457 | Unannotated feature | pos | 694.8186 | 5.910 | 4.666 | 4.045 | -0.620 | -0.541 | 0.002 | 0.013 | Unannotated | NA | NA |
| metab_281 | Unannotated feature | pos | 362.9303 | 0.729 | 4.219 | 3.449 | -0.770 | -0.541 | 0.002 | 0.013 | Unannotated | NA | NA |
| metab_1664 | Unannotated feature | pos | 121.0651 | 0.279 | 5.266 | 5.238 | -0.028 | -0.541 | 0.002 | 0.013 | Unannotated | NA | NA |
| metab_2501 | Psilostachyin | pos | 281.1383 | 5.894 | 5.898 | 6.045 | 0.147 | 0.541 | 0.002 | 0.013 | Tentative | Lipids and lipid-like molecules | Prenol lipids |
| metab_10148 | Unannotated feature | neg | 295.9844 | 0.920 | 4.686 | 4.496 | -0.190 | -0.541 | 0.002 | 0.013 | Unannotated | NA | NA |
| metab_8882 | Solavetivone | neg | 253.1350 | 5.207 | 4.349 | 3.520 | -0.829 | -0.541 | 0.002 | 0.013 | Tentative | Lipids and lipid-like molecules | Prenol lipids |
| metab_1232 | Unannotated feature | pos | 165.0883 | 5.817 | 4.799 | 4.850 | 0.051 | 0.541 | 0.002 | 0.013 | Unannotated | NA | NA |
| metab_9599 | Unannotated feature | neg | 227.1038 | 3.160 | 3.841 | 4.395 | 0.554 | 0.541 | 0.002 | 0.013 | Unannotated | NA | NA |
| metab_5701 | Unannotated feature | neg | 116.9241 | 1.334 | 3.749 | 3.530 | -0.218 | -0.541 | 0.002 | 0.013 | Unannotated | NA | NA |
| metab_6306 | Ferulic acid 4-O-sulfate | neg | 273.0079 | 3.710 | 2.868 | 4.194 | 1.326 | 0.541 | 0.002 | 0.013 | Tentative | Phenylpropanoids and polyketides | Cinnamic acids and derivatives |
| metab_4851 | Unannotated feature | pos | 125.0963 | 6.196 | 4.362 | 4.269 | -0.093 | -0.541 | 0.002 | 0.013 | Unannotated | NA | NA |
| metab_2690 | Unannotated feature | pos | 286.1285 | 1.949 | 4.732 | 5.156 | 0.424 | 0.541 | 0.002 | 0.013 | Unannotated | NA | NA |
| metab_8777 | Unannotated feature | neg | 644.1945 | 5.523 | 5.165 | 5.601 | 0.436 | 0.541 | 0.002 | 0.013 | Unannotated | NA | NA |
| metab_2682 | Unannotated feature | pos | 316.7445 | 5.771 | 5.265 | 5.375 | 0.110 | 0.541 | 0.002 | 0.013 | Unannotated | NA | NA |
| metab_5482 | Unannotated feature | neg | 738.4624 | 0.699 | 5.155 | 4.911 | -0.244 | -0.541 | 0.002 | 0.013 | Unannotated | NA | NA |
| metab_5068 | Unannotated feature | pos | 158.1176 | 0.854 | 4.275 | 4.674 | 0.400 | 0.541 | 0.002 | 0.013 | Unannotated | NA | NA |
| metab_1444 | Unannotated feature | pos | 178.9908 | 5.972 | 4.979 | 4.930 | -0.049 | -0.541 | 0.002 | 0.013 | Unannotated | NA | NA |
| metab_335 | Unannotated feature | pos | 214.0322 | 0.823 | 2.807 | 3.636 | 0.830 | 0.541 | 0.002 | 0.013 | Unannotated | NA | NA |
| metab_1925 | Unannotated feature | pos | 182.0222 | 7.324 | 5.559 | 5.616 | 0.057 | 0.541 | 0.002 | 0.013 | Unannotated | NA | NA |
| metab_8307 | Unannotated feature | neg | 349.0133 | 3.160 | 4.362 | 4.132 | -0.230 | -0.541 | 0.002 | 0.013 | Unannotated | NA | NA |
| metab_11384 | Aflatoxin M1 | neg | 363.0298 | 3.734 | 4.261 | 5.115 | 0.854 | 0.541 | 0.002 | 0.013 | Tentative | Phenylpropanoids and polyketides | Coumarins and derivatives |
| metab_10782 | Unannotated feature | neg | 753.8534 | 0.562 | 4.929 | 4.721 | -0.208 | -0.541 | 0.002 | 0.013 | Unannotated | NA | NA |
| metab_4922 | Unannotated feature | pos | 676.5537 | 0.598 | 5.439 | 5.290 | -0.148 | -0.541 | 0.002 | 0.013 | Unannotated | NA | NA |
| metab_10097 | Unannotated feature | neg | 117.0186 | 1.206 | 5.058 | 4.944 | -0.114 | -0.541 | 0.002 | 0.013 | Strong putative | NA | NA |
| metab_3081 | Unannotated feature | pos | 101.8086 | 3.916 | 3.994 | 4.079 | 0.086 | 0.541 | 0.002 | 0.013 | Unannotated | NA | NA |
| metab_3848 | Unannotated feature | pos | 306.0017 | 1.087 | 3.090 | 4.351 | 1.261 | 0.541 | 0.002 | 0.013 | Unannotated | NA | NA |
| metab_1493 | Unannotated feature | pos | 372.7380 | 0.591 | 6.270 | 6.217 | -0.053 | -0.541 | 0.002 | 0.013 | Unannotated | NA | NA |
| metab_1371 | Unannotated feature | pos | 701.4852 | 5.910 | 4.715 | 4.349 | -0.366 | -0.541 | 0.002 | 0.013 | Unannotated | NA | NA |
| metab_3794 | Unannotated feature | pos | 280.9954 | 1.530 | 4.644 | 4.591 | -0.053 | -0.541 | 0.002 | 0.013 | Unannotated | NA | NA |
| metab_2636 | Unannotated feature | pos | 530.3880 | 5.824 | 5.404 | 5.297 | -0.107 | -0.541 | 0.002 | 0.013 | Unannotated | NA | NA |
| metab_10547 | Unannotated feature | neg | 862.3978 | 4.953 | 5.824 | 5.991 | 0.166 | 0.541 | 0.002 | 0.013 | Unannotated | NA | NA |
| metab_6109 | Unannotated feature | neg | 578.1482 | 3.176 | 4.659 | 3.918 | -0.740 | -0.541 | 0.002 | 0.013 | Unannotated | NA | NA |
| metab_3921 | Unannotated feature | pos | 166.0724 | 0.861 | 4.026 | 4.164 | 0.138 | 0.537 | 0.003 | 0.013 | Unannotated | NA | NA |
| metab_2695 | Unannotated feature | pos | 435.3111 | 5.756 | 5.162 | 4.994 | -0.167 | -0.537 | 0.003 | 0.013 | Unannotated | NA | NA |
| metab_5916 | Unannotated feature | neg | 169.0139 | 2.541 | 4.108 | 3.934 | -0.174 | -0.537 | 0.003 | 0.013 | Unannotated | NA | NA |
| metab_5318 | Unannotated feature | neg | 855.8356 | 0.562 | 4.842 | 4.408 | -0.434 | -0.537 | 0.003 | 0.013 | Unannotated | NA | NA |
| metab_10630 | Unannotated feature | neg | 338.7859 | 0.594 | 6.066 | 5.992 | -0.074 | -0.537 | 0.003 | 0.013 | Unannotated | NA | NA |
| metab_8311 | Unannotated feature | neg | 534.2288 | 5.851 | 5.335 | 5.418 | 0.082 | 0.537 | 0.003 | 0.013 | Unannotated | NA | NA |
| metab_4994 | Sucrose | pos | 365.1054 | 0.760 | 4.634 | 5.160 | 0.527 | 0.537 | 0.003 | 0.013 | Strong putative | Organic oxygen compounds | Organooxygen compounds |
| metab_6669 | Unannotated feature | neg | 203.9858 | 0.849 | 3.120 | 3.768 | 0.649 | 0.537 | 0.003 | 0.013 | Unannotated | NA | NA |
| metab_3832 | Unannotated feature | pos | 270.9582 | 1.227 | 5.269 | 5.151 | -0.118 | -0.537 | 0.003 | 0.013 | Unannotated | NA | NA |
| metab_4338 | Unannotated feature | pos | 274.8813 | 0.575 | 5.778 | 5.732 | -0.046 | -0.537 | 0.003 | 0.013 | Unannotated | NA | NA |
| metab_245 | Unannotated feature | pos | 82.8144 | 0.660 | 2.831 | 3.387 | 0.556 | 0.537 | 0.003 | 0.013 | Unannotated | NA | NA |
| metab_1787 | Unannotated feature | pos | 244.9981 | 7.548 | 5.688 | 5.734 | 0.047 | 0.537 | 0.003 | 0.013 | Unannotated | NA | NA |
| metab_16 | Unannotated feature | pos | 254.8237 | 0.591 | 6.425 | 6.383 | -0.042 | -0.537 | 0.003 | 0.013 | Unannotated | NA | NA |
| metab_6906 | Unannotated feature | neg | 444.6748 | 0.904 | 5.897 | 5.827 | -0.070 | -0.537 | 0.003 | 0.013 | Unannotated | NA | NA |
| metab_3061 | N-Heptanoylglycine | pos | 170.1176 | 4.024 | 4.605 | 4.699 | 0.094 | 0.537 | 0.003 | 0.013 | Tentative | NA | NA |
| metab_4911 | Unannotated feature | pos | 790.4737 | 0.591 | 5.421 | 5.354 | -0.067 | -0.537 | 0.003 | 0.013 | Unannotated | NA | NA |
| metab_6553 | Unannotated feature | neg | 312.6565 | 4.635 | 4.309 | 3.990 | -0.320 | -0.537 | 0.003 | 0.013 | Unannotated | NA | NA |
| metab_3817 | Unannotated feature | pos | 87.1003 | 1.321 | 5.151 | 5.111 | -0.040 | -0.537 | 0.003 | 0.013 | Unannotated | NA | NA |
| metab_1304 | Unannotated feature | pos | 699.8183 | 5.871 | 5.270 | 4.864 | -0.405 | -0.537 | 0.003 | 0.013 | Unannotated | NA | NA |
| metab_3479 | D-Glucarate | pos | 252.0727 | 2.241 | 3.997 | 4.659 | 0.662 | 0.537 | 0.003 | 0.013 | Tentative | Organic oxygen compounds | Organooxygen compounds |
| metab_4277 | Unannotated feature | pos | 444.7195 | 0.591 | 5.353 | 5.254 | -0.099 | -0.537 | 0.003 | 0.013 | Unannotated | NA | NA |
| metab_3523 | Unannotated feature | pos | 302.1597 | 2.110 | 3.176 | 4.814 | 1.639 | 0.537 | 0.003 | 0.013 | Unannotated | NA | NA |
| metab_10135 | Unannotated feature | neg | 177.8597 | 0.960 | 3.931 | 4.149 | 0.218 | 0.537 | 0.003 | 0.013 | Unannotated | NA | NA |
| metab_5885 | Unannotated feature | neg | 117.9786 | 2.492 | 2.566 | 2.104 | -0.462 | -0.537 | 0.003 | 0.013 | Unannotated | NA | NA |
| metab_7076 | Unannotated feature | neg | 226.9769 | 1.286 | 5.510 | 5.468 | -0.041 | -0.537 | 0.003 | 0.013 | Unannotated | NA | NA |
| metab_5534 | Unannotated feature | neg | 392.7509 | 0.794 | 4.741 | 4.393 | -0.348 | -0.537 | 0.003 | 0.013 | Unannotated | NA | NA |
| metab_9700 | Unannotated feature | neg | 131.0343 | 2.890 | 4.633 | 4.737 | 0.104 | 0.537 | 0.003 | 0.013 | Unannotated | NA | NA |
| metab_3869 | Unannotated feature | pos | 148.9354 | 0.908 | 4.141 | 4.073 | -0.068 | -0.537 | 0.003 | 0.013 | Unannotated | NA | NA |
| metab_10224 | Unannotated feature | neg | 201.0378 | 0.865 | 5.075 | 4.925 | -0.150 | -0.537 | 0.003 | 0.013 | Unannotated | NA | NA |
| metab_11000 | Unannotated feature | neg | 178.9199 | 0.036 | 4.364 | 4.244 | -0.120 | -0.537 | 0.003 | 0.013 | Unannotated | NA | NA |
| metab_3355 | Unannotated feature | pos | 187.3631 | 2.583 | 5.391 | 5.257 | -0.134 | -0.537 | 0.003 | 0.013 | Unannotated | NA | NA |
| metab_403 | Unannotated feature | pos | 130.0581 | 1.165 | 3.817 | 3.657 | -0.161 | -0.537 | 0.003 | 0.013 | Unannotated | NA | NA |
| metab_3981 | Unannotated feature | pos | 165.0135 | 0.831 | 4.672 | 5.114 | 0.442 | 0.537 | 0.003 | 0.013 | Unannotated | NA | NA |
| metab_1197 | Unannotated feature | pos | 413.2982 | 5.756 | 4.622 | 4.379 | -0.243 | -0.537 | 0.003 | 0.013 | Unannotated | NA | NA |
| metab_1193 | Unannotated feature | pos | 596.4108 | 5.748 | 5.216 | 4.984 | -0.232 | -0.537 | 0.003 | 0.013 | Unannotated | NA | NA |
| metab_152 | Unannotated feature | pos | 804.8441 | 0.551 | 6.037 | 5.918 | -0.120 | -0.537 | 0.003 | 0.013 | Unannotated | NA | NA |
| metab_3974 | N6-Methyl-L-lysine | pos | 143.1180 | 0.839 | 4.171 | 4.805 | 0.633 | 0.537 | 0.003 | 0.013 | Tentative | Organic acids and derivatives | Carboxylic acids and derivatives |
| metab_5233 | Unannotated feature | neg | 79.9565 | 0.554 | 2.823 | 3.269 | 0.446 | 0.537 | 0.003 | 0.013 | Unannotated | NA | NA |
| metab_4078 | Unannotated feature | pos | 221.0456 | 0.737 | 3.755 | 4.208 | 0.453 | 0.537 | 0.003 | 0.013 | Unannotated | NA | NA |
| metab_9951 | Unannotated feature | neg | 273.8879 | 2.111 | 5.181 | 4.995 | -0.186 | -0.537 | 0.003 | 0.013 | Unannotated | NA | NA |
| metab_9066 | Unannotated feature | neg | 201.1130 | 4.536 | 3.989 | 4.271 | 0.282 | 0.533 | 0.003 | 0.014 | Unannotated | NA | NA |
| metab_2038 | Unannotated feature | pos | 374.9090 | 7.261 | 4.780 | 4.903 | 0.123 | 0.533 | 0.003 | 0.014 | Unannotated | NA | NA |
| metab_2111 | Unannotated feature | pos | 87.8855 | 7.045 | 4.431 | 4.507 | 0.076 | 0.533 | 0.003 | 0.014 | Unannotated | NA | NA |
| metab_3243 | Unannotated feature | pos | 172.0969 | 3.043 | 3.876 | 3.457 | -0.419 | -0.533 | 0.003 | 0.014 | Unannotated | NA | NA |
| metab_7258 | MG(24:1(15Z)/0:0/0:0) | neg | 485.3855 | 6.063 | 4.251 | 3.678 | -0.573 | -0.533 | 0.003 | 0.014 | Tentative | Lipids and lipid-like molecules | Glycerolipids |
| metab_7007 | Unannotated feature | neg | 282.9388 | 0.873 | 5.360 | 5.247 | -0.113 | -0.533 | 0.003 | 0.014 | Unannotated | NA | NA |
| metab_2771 | Unannotated feature | pos | 692.4785 | 5.657 | 5.472 | 5.273 | -0.199 | -0.533 | 0.003 | 0.014 | Unannotated | NA | NA |
| metab_10344 | Unannotated feature | neg | 334.7920 | 0.594 | 6.102 | 6.028 | -0.074 | -0.533 | 0.003 | 0.014 | Unannotated | NA | NA |
| metab_851 | Unannotated feature | pos | 84.1315 | 3.360 | 3.452 | 3.606 | 0.155 | 0.533 | 0.003 | 0.014 | Unannotated | NA | NA |
| metab_430 | Unannotated feature | pos | 322.8111 | 0.591 | 5.926 | 5.863 | -0.063 | -0.533 | 0.003 | 0.014 | Unannotated | NA | NA |
| metab_1360 | Unannotated feature | pos | 675.1387 | 5.902 | 4.463 | 3.987 | -0.475 | -0.533 | 0.003 | 0.014 | Unannotated | NA | NA |
| metab_4266 | Unannotated feature | pos | 684.8029 | 0.567 | 5.178 | 5.095 | -0.083 | -0.533 | 0.003 | 0.014 | Unannotated | NA | NA |
| metab_9306 | Unannotated feature | neg | 265.1099 | 3.734 | 3.636 | 4.675 | 1.039 | 0.533 | 0.003 | 0.014 | Unannotated | NA | NA |
| metab_827 | Unannotated feature | pos | 131.0534 | 3.167 | 3.410 | 4.341 | 0.931 | 0.533 | 0.003 | 0.014 | Unannotated | NA | NA |
| metab_3264 | 4-Hydroxy-2-quinolinecarboxylic acid | pos | 190.0499 | 2.965 | 4.907 | 5.419 | 0.512 | 0.533 | 0.003 | 0.014 | Strong putative | Organoheterocyclic compounds | Quinolines and derivatives |
| metab_162 | N-carbamoylglycine | pos | 259.0641 | 0.559 | 4.772 | 4.697 | -0.075 | -0.533 | 0.003 | 0.014 | Tentative | NA | NA |
| metab_3638 | Unannotated feature | pos | 195.0652 | 1.956 | 3.832 | 3.963 | 0.131 | 0.533 | 0.003 | 0.014 | Unannotated | NA | NA |
| metab_4414 | Unannotated feature | pos | 769.8105 | 0.559 | 4.038 | 4.254 | 0.216 | 0.533 | 0.003 | 0.014 | Unannotated | NA | NA |
| metab_1933 | Unannotated feature | pos | 342.8688 | 0.575 | 5.711 | 5.677 | -0.035 | -0.533 | 0.003 | 0.014 | Unannotated | NA | NA |
| metab_536 | Unannotated feature | pos | 208.8910 | 0.583 | 6.151 | 6.103 | -0.049 | -0.533 | 0.003 | 0.014 | Unannotated | NA | NA |
| metab_1966 | Unannotated feature | pos | 306.8946 | 7.300 | 5.111 | 5.151 | 0.041 | 0.533 | 0.003 | 0.014 | Unannotated | NA | NA |
| metab_3354 | Unannotated feature | pos | 204.2916 | 2.583 | 5.341 | 5.187 | -0.154 | -0.533 | 0.003 | 0.014 | Unannotated | NA | NA |
| metab_4467 | Unannotated feature | pos | 271.1652 | 5.306 | 4.702 | 5.375 | 0.673 | 0.533 | 0.003 | 0.014 | Unannotated | NA | NA |
| metab_6671 | Unannotated feature | neg | 243.0778 | 5.139 | 3.902 | 4.757 | 0.855 | 0.533 | 0.003 | 0.014 | Unannotated | NA | NA |
| metab_6844 | Unannotated feature | neg | 153.0916 | 5.619 | 2.587 | 3.553 | 0.965 | 0.533 | 0.003 | 0.014 | Unannotated | NA | NA |
| metab_6609 | Suberic acid | neg | 173.0816 | 4.901 | 4.524 | 4.734 | 0.210 | 0.533 | 0.003 | 0.014 | Moderate putative | Lipids and lipid-like molecules | Fatty Acyls |
| metab_4523 | Unannotated feature | pos | 718.9191 | 0.538 | 4.730 | 4.529 | -0.201 | -0.533 | 0.003 | 0.014 | Unannotated | NA | NA |
| metab_396 | Unannotated feature | pos | 269.0742 | 1.064 | 2.985 | 3.993 | 1.008 | 0.533 | 0.003 | 0.014 | Unannotated | NA | NA |
| metab_4614 | 17alpha,21-Dihydroxypregnenolone | pos | 331.2268 | 5.581 | 4.018 | 4.826 | 0.808 | 0.533 | 0.003 | 0.014 | Tentative | Lipids and lipid-like molecules | Steroids and steroid derivatives |
| metab_3560 | Biotin | pos | 245.0953 | 2.065 | 3.719 | 4.350 | 0.631 | 0.533 | 0.003 | 0.014 | Tentative | Organoheterocyclic compounds | Biotin and derivatives |
| metab_8784 | Unannotated feature | neg | 662.1832 | 5.518 | 4.101 | 4.600 | 0.499 | 0.533 | 0.003 | 0.014 | Unannotated | NA | NA |
| metab_2823 | 3-(5'-Methylthio)pentylmalic acid | pos | 514.1743 | 5.520 | 4.360 | 3.736 | -0.624 | -0.533 | 0.003 | 0.014 | Tentative | Organic acids and derivatives | Hydroxy acids and derivatives |
| metab_2295 | Unannotated feature | pos | 518.3692 | 6.080 | 4.370 | 1.078 | -3.292 | -0.533 | 0.003 | 0.014 | Unannotated | NA | NA |
| metab_5199 | Unannotated feature | neg | 597.3937 | 0.539 | 4.810 | 4.695 | -0.115 | -0.533 | 0.003 | 0.014 | Unannotated | NA | NA |
| metab_4303 | Unannotated feature | pos | 516.7447 | 0.583 | 5.426 | 5.349 | -0.077 | -0.533 | 0.003 | 0.014 | Unannotated | NA | NA |
| metab_11141 | Unannotated feature | neg | 213.1132 | 5.731 | 4.918 | 5.220 | 0.302 | 0.533 | 0.003 | 0.014 | Moderate putative | NA | NA |
| metab_9602 | Unannotated feature | neg | 116.0499 | 3.160 | 5.009 | 4.809 | -0.200 | -0.533 | 0.003 | 0.014 | Unannotated | NA | NA |
| metab_5019 | Unannotated feature | pos | 568.6685 | 0.591 | 5.808 | 5.721 | -0.087 | -0.533 | 0.003 | 0.014 | Unannotated | NA | NA |
| metab_6269 | Alpha-Cyano-4-hydroxycinnamic acid | neg | 188.0351 | 3.546 | 4.148 | 4.663 | 0.515 | 0.533 | 0.003 | 0.014 | Tentative | Phenylpropanoids and polyketides | Cinnamic acids and derivatives |
| metab_2398 | Unannotated feature | pos | 341.2779 | 5.941 | 4.975 | 5.083 | 0.108 | 0.533 | 0.003 | 0.014 | Unannotated | NA | NA |
| metab_2817 | Unannotated feature | pos | 230.1387 | 2.058 | 3.807 | 4.342 | 0.535 | 0.533 | 0.003 | 0.014 | Unannotated | NA | NA |
| metab_1349 | Unannotated feature | pos | 115.0546 | 5.902 | 4.656 | 4.759 | 0.104 | 0.533 | 0.003 | 0.014 | Unannotated | NA | NA |
| metab_7835 | Unannotated feature | neg | 480.3106 | 7.153 | 5.896 | 5.805 | -0.092 | -0.533 | 0.003 | 0.014 | Unannotated | NA | NA |
| metab_4326 | Unannotated feature | pos | 649.7020 | 0.575 | 4.443 | 4.181 | -0.262 | -0.533 | 0.003 | 0.014 | Unannotated | NA | NA |
| metab_5794 | Unannotated feature | neg | 103.0201 | 2.111 | 3.786 | 3.259 | -0.527 | -0.533 | 0.003 | 0.014 | Unannotated | NA | NA |
| metab_6878 | Unannotated feature | neg | 543.2819 | 5.635 | 4.764 | 5.380 | 0.617 | 0.533 | 0.003 | 0.014 | Unannotated | NA | NA |
| metab_9538 | Unannotated feature | neg | 242.0522 | 3.217 | 4.803 | 4.533 | -0.270 | -0.528 | 0.003 | 0.015 | Unannotated | NA | NA |
| metab_79 | Unannotated feature | pos | 130.0500 | 3.167 | 5.261 | 5.777 | 0.516 | 0.528 | 0.003 | 0.015 | Unannotated | NA | NA |
| metab_2306 | Unannotated feature | pos | 109.0651 | 6.065 | 3.991 | 3.539 | -0.452 | -0.528 | 0.003 | 0.015 | Unannotated | NA | NA |
| metab_0 | Unannotated feature | pos | 265.1182 | 3.159 | 6.365 | 6.985 | 0.620 | 0.528 | 0.003 | 0.015 | Strong putative | NA | NA |
| metab_6907 | Unannotated feature | neg | 665.2482 | 5.659 | 4.508 | 3.974 | -0.534 | -0.528 | 0.003 | 0.015 | Unannotated | NA | NA |
| metab_5188 | Unannotated feature | neg | 462.8792 | 0.530 | 5.141 | 5.029 | -0.111 | -0.528 | 0.003 | 0.015 | Unannotated | NA | NA |
| metab_7360 | Unannotated feature | neg | 182.9884 | 6.633 | 5.391 | 5.444 | 0.053 | 0.528 | 0.003 | 0.015 | Unannotated | NA | NA |
| metab_2793 | CD 1790 | pos | 496.2078 | 5.619 | 4.848 | 4.129 | -0.719 | -0.528 | 0.003 | 0.015 | Tentative | Organoheterocyclic compounds | Imidazopyrimidines |
| metab_5328 | Unannotated feature | neg | 574.8018 | 0.570 | 4.185 | 4.407 | 0.222 | 0.528 | 0.003 | 0.015 | Unannotated | NA | NA |
| metab_11168 | Unannotated feature | neg | 263.1041 | 3.734 | 6.023 | 6.677 | 0.654 | 0.528 | 0.003 | 0.015 | Unannotated | NA | NA |
| metab_1414 | Unannotated feature | pos | 791.5550 | 5.933 | 5.225 | 4.972 | -0.253 | -0.528 | 0.003 | 0.015 | Unannotated | NA | NA |
| metab_8089 | Diacylglycerol | neg | 573.4535 | 6.188 | 6.297 | 5.950 | -0.348 | -0.528 | 0.003 | 0.015 | Tentative | Lipids and lipid-like molecules | Fatty Acyls |
| metab_5397 | Unannotated feature | neg | 154.0617 | 0.613 | 5.274 | 5.185 | -0.089 | -0.528 | 0.003 | 0.015 | Unannotated | NA | NA |
| metab_9835 | Unannotated feature | neg | 329.9454 | 2.504 | 4.397 | 4.252 | -0.145 | -0.528 | 0.003 | 0.015 | Unannotated | NA | NA |
| metab_1215 | Unannotated feature | pos | 508.8603 | 5.778 | 4.958 | 4.654 | -0.305 | -0.528 | 0.003 | 0.015 | Unannotated | NA | NA |
| metab_4504 | Unannotated feature | pos | 81.0316 | 0.551 | 4.085 | 4.050 | -0.034 | -0.528 | 0.003 | 0.015 | Unannotated | NA | NA |
| metab_3748 | N6-Acetyl-L-lysine | pos | 171.1128 | 1.778 | 5.220 | 4.855 | -0.365 | -0.528 | 0.003 | 0.015 | Tentative | Organic acids and derivatives | Carboxylic acids and derivatives |
| metab_262 | Unannotated feature | pos | 193.0860 | 4.792 | 4.472 | 3.837 | -0.635 | -0.528 | 0.003 | 0.015 | Unannotated | NA | NA |
| metab_2499 | Neocuproine | pos | 480.2131 | 5.894 | 5.519 | 4.902 | -0.618 | -0.528 | 0.003 | 0.015 | Tentative | Organoheterocyclic compounds | Phenanthrolines |
| metab_1036 | Unannotated feature | pos | 118.0865 | 0.854 | 7.100 | 7.032 | -0.068 | -0.528 | 0.003 | 0.015 | Unannotated | NA | NA |
| metab_3605 | Unannotated feature | pos | 159.9692 | 2.019 | 4.683 | 4.666 | -0.017 | -0.528 | 0.003 | 0.015 | Unannotated | NA | NA |
| metab_3752 | Propionylglycine | pos | 132.0658 | 1.755 | 4.790 | 5.074 | 0.284 | 0.528 | 0.003 | 0.015 | Moderate putative | Organic acids and derivatives | Carboxylic acids and derivatives |
| metab_4116 | Unannotated feature | pos | 131.6603 | 0.698 | 4.374 | 3.811 | -0.563 | -0.528 | 0.003 | 0.015 | Unannotated | NA | NA |
| metab_9587 | Unannotated feature | neg | 203.8803 | 3.168 | 4.155 | 3.810 | -0.345 | -0.528 | 0.003 | 0.015 | Unannotated | NA | NA |
| metab_4163 | Unannotated feature | pos | 84.0254 | 0.652 | 3.621 | 3.993 | 0.372 | 0.528 | 0.003 | 0.015 | Unannotated | NA | NA |
| metab_5680 | Unannotated feature | neg | 174.9776 | 1.214 | 4.116 | 3.750 | -0.366 | -0.528 | 0.003 | 0.015 | Unannotated | NA | NA |
| metab_4001 | Unannotated feature | pos | 283.8397 | 0.815 | 4.717 | 4.666 | -0.051 | -0.528 | 0.003 | 0.015 | Unannotated | NA | NA |
| metab_8698 | Unannotated feature | neg | 197.0818 | 5.611 | 3.770 | 4.567 | 0.797 | 0.528 | 0.003 | 0.015 | Strong putative | NA | NA |
| metab_449 | Unannotated feature | pos | 175.0479 | 1.694 | 3.820 | 4.536 | 0.716 | 0.528 | 0.003 | 0.015 | Unannotated | NA | NA |
| metab_8592 | beta-D-Glucuronoside | neg | 297.0985 | 5.675 | 3.106 | 3.744 | 0.638 | 0.528 | 0.003 | 0.015 | Tentative | Organic oxygen compounds | Organooxygen compounds |
| metab_11169 | alpha,alpha-Trehalose | neg | 377.0863 | 0.778 | 3.685 | 4.514 | 0.828 | 0.528 | 0.003 | 0.015 | Tentative | Organic oxygen compounds | Organooxygen compounds |
| metab_8340 | Unannotated feature | neg | 399.9350 | 5.827 | 4.778 | 3.846 | -0.933 | -0.528 | 0.003 | 0.015 | Unannotated | NA | NA |
| metab_9109 | Unannotated feature | neg | 81.9523 | 4.386 | 3.825 | 4.041 | 0.216 | 0.528 | 0.003 | 0.015 | Unannotated | NA | NA |
| metab_5107 | Unannotated feature | pos | 91.0547 | 1.740 | 5.525 | 5.420 | -0.105 | -0.528 | 0.003 | 0.015 | Unannotated | NA | NA |
| metab_7664 | Unannotated feature | neg | 205.9232 | 7.293 | 4.778 | 4.854 | 0.076 | 0.528 | 0.003 | 0.015 | Unannotated | NA | NA |
| metab_366 | Unannotated feature | pos | 394.0734 | 0.869 | 4.521 | 4.367 | -0.154 | -0.528 | 0.003 | 0.015 | Unannotated | NA | NA |
| metab_5300 | Unannotated feature | neg | 617.8787 | 0.562 | 4.741 | 4.438 | -0.303 | -0.528 | 0.003 | 0.015 | Unannotated | NA | NA |
| metab_10334 | Unannotated feature | neg | 524.6879 | 0.590 | 5.652 | 5.575 | -0.076 | -0.528 | 0.003 | 0.015 | Unannotated | NA | NA |
| metab_3556 | Unannotated feature | pos | 267.1239 | 3.167 | 3.680 | 4.864 | 1.184 | 0.528 | 0.003 | 0.015 | Unannotated | NA | NA |
| metab_2219 | Unannotated feature | pos | 253.2524 | 6.296 | 4.469 | 3.379 | -1.090 | -0.528 | 0.003 | 0.015 | Unannotated | NA | NA |
| metab_9090 | Unannotated feature | neg | 277.1562 | 4.465 | 5.396 | 5.059 | -0.337 | -0.528 | 0.003 | 0.015 | Unannotated | NA | NA |
| metab_11108 | 3-Hydroxysebacic Acid | neg | 199.0974 | 5.675 | 4.549 | 4.891 | 0.342 | 0.528 | 0.003 | 0.015 | Moderate putative | Organic acids and derivatives | Hydroxy acids and derivatives |
| metab_7644 | Unannotated feature | neg | 88.7930 | 7.888 | 4.925 | 4.840 | -0.085 | -0.528 | 0.003 | 0.015 | Unannotated | NA | NA |
| metab_4088 | Unannotated feature | pos | 245.0164 | 0.729 | 4.524 | 3.875 | -0.649 | -0.528 | 0.003 | 0.015 | Unannotated | NA | NA |
| metab_8746 | Indeloxazine | neg | 230.1188 | 5.563 | 4.030 | 3.726 | -0.303 | -0.528 | 0.003 | 0.015 | Tentative | Benzenoids | Indenes and isoindenes |
| metab_4635 | (4E,15Z)-Bilirubin | pos | 585.2707 | 5.672 | 6.302 | 5.845 | -0.457 | -0.528 | 0.003 | 0.015 | Tentative | NA | NA |
| metab_5237 | Unannotated feature | neg | 407.8826 | 0.554 | 3.607 | 4.031 | 0.423 | 0.528 | 0.003 | 0.015 | Unannotated | NA | NA |
| metab_343 | 3-Hydroxy-3-methylglutarate | pos | 163.0602 | 0.831 | 4.665 | 4.599 | -0.066 | -0.528 | 0.003 | 0.015 | Tentative | NA | NA |
| metab_5053 | Unannotated feature | pos | 342.0420 | 0.861 | 5.469 | 5.335 | -0.133 | -0.528 | 0.003 | 0.015 | Unannotated | NA | NA |
| metab_8642 | Unannotated feature | neg | 216.8806 | 5.643 | 4.477 | 4.302 | -0.174 | -0.528 | 0.003 | 0.015 | Unannotated | NA | NA |
| metab_1959 | Unannotated feature | pos | 209.9461 | 7.308 | 3.925 | 4.139 | 0.214 | 0.528 | 0.003 | 0.015 | Unannotated | NA | NA |
| metab_5112 | trans-2-Hydroxycinnamate | pos | 182.0813 | 1.740 | 7.101 | 6.979 | -0.122 | -0.524 | 0.003 | 0.015 | Strong putative | Phenylpropanoids and polyketides | Cinnamic acids and derivatives |
| metab_5610 | Unannotated feature | neg | 172.8421 | 0.904 | 4.994 | 4.882 | -0.111 | -0.524 | 0.003 | 0.015 | Unannotated | NA | NA |
| metab_9868 | Unannotated feature | neg | 502.6337 | 0.621 | 6.755 | 6.702 | -0.054 | -0.524 | 0.003 | 0.015 | Unannotated | NA | NA |
| metab_6232 | Unannotated feature | neg | 423.9700 | 0.668 | 4.706 | 3.846 | -0.860 | -0.524 | 0.003 | 0.015 | Unannotated | NA | NA |
| metab_4840 | Unannotated feature | pos | 838.8380 | 7.285 | 5.656 | 5.577 | -0.079 | -0.524 | 0.003 | 0.015 | Unannotated | NA | NA |
| metab_5403 | Unannotated feature | neg | 421.7978 | 0.016 | 4.752 | 4.706 | -0.045 | -0.524 | 0.003 | 0.015 | Unannotated | NA | NA |
| metab_7609 | Unannotated feature | neg | 452.7066 | 0.598 | 6.267 | 6.197 | -0.070 | -0.524 | 0.003 | 0.015 | Unannotated | NA | NA |
| metab_6101 | Unannotated feature | neg | 202.2885 | 3.168 | 4.205 | 3.790 | -0.415 | -0.524 | 0.003 | 0.015 | Unannotated | NA | NA |
| metab_6102 | Unannotated feature | neg | 205.0889 | 3.168 | 3.805 | 3.243 | -0.562 | -0.524 | 0.003 | 0.015 | Unannotated | NA | NA |
| metab_4716 | Unannotated feature | pos | 834.6152 | 5.964 | 6.140 | 6.068 | -0.072 | -0.524 | 0.003 | 0.015 | Unannotated | NA | NA |
| metab_1 | Unannotated feature | pos | 415.2116 | 5.894 | 7.660 | 7.787 | 0.127 | 0.524 | 0.003 | 0.015 | Unannotated | NA | NA |
| metab_4941 | Unannotated feature | pos | 554.7983 | 0.629 | 5.218 | 5.092 | -0.126 | -0.524 | 0.003 | 0.015 | Unannotated | NA | NA |
| metab_9593 | Unannotated feature | neg | 552.0962 | 3.160 | 4.779 | 4.442 | -0.337 | -0.524 | 0.003 | 0.015 | Unannotated | NA | NA |
| metab_1456 | Unannotated feature | pos | 790.5882 | 5.979 | 6.051 | 5.992 | -0.059 | -0.524 | 0.003 | 0.015 | Unannotated | NA | NA |
| metab_546 | Unannotated feature | pos | 112.0760 | 2.011 | 4.165 | 4.278 | 0.113 | 0.524 | 0.003 | 0.015 | Unannotated | NA | NA |
| metab_1439 | Tanacetol B | pos | 297.2034 | 5.964 | 5.586 | 5.656 | 0.071 | 0.524 | 0.003 | 0.015 | Tentative | NA | NA |
| metab_10566 | Unannotated feature | neg | 792.4276 | 0.613 | 6.210 | 6.133 | -0.076 | -0.524 | 0.003 | 0.015 | Unannotated | NA | NA |
| metab_9905 | Unannotated feature | neg | 187.0278 | 2.127 | 2.741 | 3.419 | 0.678 | 0.524 | 0.003 | 0.015 | Unannotated | NA | NA |
| metab_1931 | Unannotated feature | pos | 141.9991 | 7.324 | 4.164 | 4.252 | 0.087 | 0.524 | 0.003 | 0.015 | Unannotated | NA | NA |
| metab_7314 | Unannotated feature | neg | 260.0239 | 2.061 | 5.208 | 5.680 | 0.472 | 0.524 | 0.003 | 0.015 | Unannotated | NA | NA |
| metab_2491 | Unannotated feature | pos | 689.8139 | 5.894 | 4.719 | 4.380 | -0.339 | -0.524 | 0.003 | 0.015 | Unannotated | NA | NA |
| metab_814 | Unannotated feature | pos | 140.9958 | 7.324 | 5.862 | 5.908 | 0.046 | 0.524 | 0.003 | 0.015 | Unannotated | NA | NA |
| metab_10947 | Unannotated feature | neg | 157.8716 | 0.530 | 3.616 | 3.798 | 0.182 | 0.524 | 0.003 | 0.015 | Unannotated | NA | NA |
| metab_9312 | Unannotated feature | neg | 261.9343 | 3.726 | 3.661 | 4.459 | 0.798 | 0.524 | 0.003 | 0.015 | Unannotated | NA | NA |
| metab_4564 | Unannotated feature | pos | 103.0394 | 0.473 | 4.485 | 4.551 | 0.066 | 0.524 | 0.003 | 0.015 | Unannotated | NA | NA |
| metab_9224 | Unannotated feature | neg | 279.0991 | 3.459 | 3.262 | 3.993 | 0.732 | 0.524 | 0.003 | 0.015 | Unannotated | NA | NA |
| metab_10629 | Unannotated feature | neg | 541.6418 | 0.594 | 5.343 | 5.265 | -0.078 | -0.524 | 0.003 | 0.015 | Unannotated | NA | NA |
| metab_626 | Unannotated feature | pos | 187.0941 | 2.202 | 3.982 | 3.462 | -0.519 | -0.524 | 0.003 | 0.015 | Unannotated | NA | NA |
| metab_5212 | Unannotated feature | neg | 472.7613 | 0.013 | 5.567 | 5.518 | -0.049 | -0.524 | 0.003 | 0.015 | Unannotated | NA | NA |
| metab_4241 | Unannotated feature | pos | 348.0794 | 0.606 | 4.293 | 3.398 | -0.896 | -0.524 | 0.003 | 0.015 | Unannotated | NA | NA |
| metab_6976 | Unannotated feature | neg | 216.9658 | 5.707 | 3.444 | 2.805 | -0.639 | -0.524 | 0.003 | 0.015 | Unannotated | NA | NA |
| metab_10406 | Palatinose | neg | 377.0864 | 0.692 | 4.798 | 5.294 | 0.497 | 0.524 | 0.003 | 0.015 | Moderate putative | Organic oxygen compounds | Organooxygen compounds |
| metab_2731 | Unannotated feature | pos | 750.5199 | 5.717 | 5.335 | 5.076 | -0.259 | -0.524 | 0.003 | 0.015 | Unannotated | NA | NA |
| metab_3456 | 4-Amino-2-methylenebutanoic acid | pos | 98.0605 | 2.296 | 4.457 | 4.574 | 0.117 | 0.524 | 0.003 | 0.015 | Tentative | Organic acids and derivatives | Carboxylic acids and derivatives |
| metab_3180 | L-Nicotianine | pos | 266.1137 | 2.537 | 4.794 | 4.560 | -0.234 | -0.524 | 0.003 | 0.015 | Tentative | Organic acids and derivatives | Carboxylic acids and derivatives |
| metab_6898 | Unannotated feature | neg | 473.1938 | 5.651 | 4.768 | 4.160 | -0.608 | -0.524 | 0.003 | 0.015 | Unannotated | NA | NA |
| metab_1326 | Unannotated feature | pos | 715.4912 | 5.886 | 4.543 | 4.207 | -0.337 | -0.524 | 0.003 | 0.015 | Unannotated | NA | NA |
| metab_199 | Unannotated feature | pos | 313.1547 | 4.163 | 7.296 | 7.104 | -0.192 | -0.524 | 0.003 | 0.015 | Strong putative | NA | NA |
| metab_7127 | Unannotated feature | neg | 209.1546 | 5.851 | 4.146 | 4.249 | 0.103 | 0.524 | 0.003 | 0.015 | Unannotated | NA | NA |
| metab_2333 | Unannotated feature | pos | 262.2530 | 6.003 | 4.695 | 4.605 | -0.091 | -0.524 | 0.003 | 0.015 | Unannotated | NA | NA |
| metab_5320 | Unannotated feature | neg | 889.8294 | 0.562 | 4.504 | 4.108 | -0.396 | -0.524 | 0.003 | 0.015 | Unannotated | NA | NA |
| metab_9443 | Unannotated feature | neg | 91.4822 | 3.364 | 3.772 | 3.601 | -0.171 | -0.524 | 0.003 | 0.015 | Unannotated | NA | NA |
| metab_9132 | Unannotated feature | neg | 275.9054 | 4.329 | 3.937 | 4.507 | 0.570 | 0.524 | 0.003 | 0.015 | Unannotated | NA | NA |
| metab_3347 | Dethiobiotin | pos | 197.1286 | 2.591 | 3.402 | 4.461 | 1.059 | 0.524 | 0.003 | 0.015 | Tentative | Lipids and lipid-like molecules | Fatty Acyls |
| metab_4456 | Unannotated feature | pos | 849.8027 | 0.551 | 4.853 | 4.978 | 0.125 | 0.524 | 0.003 | 0.015 | Unannotated | NA | NA |
| metab_1517 | Unannotated feature | pos | 456.3685 | 6.157 | 4.610 | 4.189 | -0.421 | -0.524 | 0.003 | 0.015 | Unannotated | NA | NA |
| metab_9766 | Unannotated feature | neg | 293.1149 | 2.660 | 4.030 | 3.512 | -0.519 | -0.524 | 0.003 | 0.015 | Unannotated | NA | NA |
| metab_4185 | Unannotated feature | pos | 445.8408 | 0.636 | 4.731 | 4.198 | -0.533 | -0.524 | 0.003 | 0.015 | Unannotated | NA | NA |
| metab_6522 | Unannotated feature | neg | 93.0338 | 4.505 | 3.244 | 3.440 | 0.196 | 0.520 | 0.004 | 0.016 | Unannotated | NA | NA |
| metab_8959 | Unannotated feature | neg | 135.0292 | 4.923 | 3.963 | 3.842 | -0.121 | -0.520 | 0.004 | 0.016 | Unannotated | NA | NA |
| metab_4421 | Unannotated feature | pos | 666.9885 | 0.559 | 4.931 | 4.822 | -0.109 | -0.520 | 0.004 | 0.016 | Unannotated | NA | NA |
| metab_6333 | Unannotated feature | neg | 188.0205 | 0.707 | 4.095 | 3.245 | -0.850 | -0.520 | 0.004 | 0.016 | Unannotated | NA | NA |
| metab_2452 | Unannotated feature | pos | 755.5312 | 5.910 | 6.054 | 5.949 | -0.105 | -0.520 | 0.004 | 0.016 | Unannotated | NA | NA |
| metab_11355 | Unannotated feature | neg | 671.6490 | 0.016 | 4.264 | 4.068 | -0.197 | -0.520 | 0.004 | 0.016 | Unannotated | NA | NA |
| metab_4194 | Unannotated feature | pos | 494.8427 | 0.629 | 5.939 | 5.881 | -0.059 | -0.520 | 0.004 | 0.016 | Unannotated | NA | NA |
| metab_83 | Unannotated feature | pos | 284.2944 | 0.465 | 5.169 | 5.211 | 0.042 | 0.520 | 0.004 | 0.016 | Unannotated | NA | NA |
| metab_47 | Castanospermine | pos | 190.1074 | 2.019 | 4.299 | 4.425 | 0.127 | 0.520 | 0.004 | 0.016 | Strong putative | NA | NA |
| metab_6276 | Unannotated feature | neg | 318.9137 | 3.554 | 4.226 | 4.056 | -0.170 | -0.520 | 0.004 | 0.016 | Unannotated | NA | NA |
| metab_6647 | 2-Propyl-2,4-pentadienoic acid | neg | 185.0817 | 5.071 | 3.395 | 4.177 | 0.783 | 0.520 | 0.004 | 0.016 | Tentative | Lipids and lipid-like molecules | Fatty Acyls |
| metab_2364 | Unannotated feature | pos | 635.4344 | 5.964 | 5.068 | 4.883 | -0.184 | -0.520 | 0.004 | 0.016 | Unannotated | NA | NA |
| metab_8087 | 18-Hydroxyoleate | neg | 593.4798 | 6.188 | 6.656 | 6.360 | -0.295 | -0.520 | 0.004 | 0.016 | Tentative | Lipids and lipid-like molecules | Fatty Acyls |
| metab_2677 | Unannotated feature | pos | 530.8733 | 5.771 | 4.868 | 4.649 | -0.219 | -0.520 | 0.004 | 0.016 | Unannotated | NA | NA |
| metab_10733 | Unannotated feature | neg | 278.8302 | 0.587 | 6.467 | 6.409 | -0.057 | -0.520 | 0.004 | 0.016 | Unannotated | NA | NA |
| metab_9040 | Unannotated feature | neg | 456.0635 | 4.635 | 5.100 | 4.801 | -0.299 | -0.520 | 0.004 | 0.016 | Unannotated | NA | NA |
| metab_391 | Unannotated feature | pos | 134.0449 | 1.002 | 4.412 | 4.590 | 0.178 | 0.520 | 0.004 | 0.016 | Unannotated | NA | NA |
| metab_11176 | 3-Hydroxydodecanedioic acid | neg | 245.1397 | 5.827 | 3.562 | 4.356 | 0.794 | 0.520 | 0.004 | 0.016 | Tentative | Organic acids and derivatives | Hydroxy acids and derivatives |
| metab_9039 | Unannotated feature | neg | 473.0535 | 4.635 | 4.541 | 4.266 | -0.275 | -0.520 | 0.004 | 0.016 | Unannotated | NA | NA |
| metab_401 | (S)-Methylmalonate semialdehyde | pos | 147.0030 | 1.150 | 4.124 | 3.850 | -0.274 | -0.520 | 0.004 | 0.016 | Tentative | Organic oxygen compounds | Organooxygen compounds |
| metab_3666 | 8-Hydroxy-7-methylguanine | pos | 182.0674 | 1.949 | 4.213 | 4.752 | 0.539 | 0.520 | 0.004 | 0.016 | Tentative | Organoheterocyclic compounds | Imidazopyrimidines |
| metab_136 | Unannotated feature | pos | 616.9386 | 0.551 | 4.624 | 4.455 | -0.169 | -0.520 | 0.004 | 0.016 | Unannotated | NA | NA |
| metab_714 | Unannotated feature | pos | 213.1648 | 2.576 | 4.210 | 4.360 | 0.150 | 0.520 | 0.004 | 0.016 | Unannotated | NA | NA |
| metab_4958 | Unannotated feature | pos | 496.8398 | 0.629 | 5.815 | 5.736 | -0.078 | -0.520 | 0.004 | 0.016 | Unannotated | NA | NA |
| metab_7309 | Unannotated feature | neg | 175.0720 | 0.865 | 3.772 | 4.182 | 0.411 | 0.520 | 0.004 | 0.016 | Unannotated | NA | NA |
| metab_4783 | Unannotated feature | pos | 129.0548 | 0.457 | 6.087 | 6.113 | 0.027 | 0.520 | 0.004 | 0.016 | Unannotated | NA | NA |
| metab_9496 | Unannotated feature | neg | 326.1000 | 3.734 | 4.711 | 5.511 | 0.801 | 0.520 | 0.004 | 0.016 | Unannotated | NA | NA |
| metab_4098 | Dihydrourocanate | pos | 141.0659 | 0.721 | 4.343 | 5.208 | 0.865 | 0.520 | 0.004 | 0.016 | Tentative | Organoheterocyclic compounds | Azoles |
| metab_3866 | Unannotated feature | pos | 256.8207 | 0.908 | 5.669 | 5.625 | -0.044 | -0.520 | 0.004 | 0.016 | Unannotated | NA | NA |
| metab_10274 | Unannotated feature | neg | 178.9904 | 0.841 | 3.945 | 4.501 | 0.556 | 0.520 | 0.004 | 0.016 | Unannotated | NA | NA |
| metab_2299 | Unannotated feature | pos | 614.4837 | 6.072 | 4.746 | 4.915 | 0.170 | 0.516 | 0.004 | 0.017 | Unannotated | NA | NA |
| metab_7156 | Unannotated feature | neg | 241.7936 | 5.883 | 4.007 | 3.528 | -0.478 | -0.516 | 0.004 | 0.017 | Unannotated | NA | NA |
| metab_2953 | Unannotated feature | pos | 518.3531 | 4.616 | 5.003 | 4.751 | -0.252 | -0.516 | 0.004 | 0.017 | Unannotated | NA | NA |
| metab_4032 | N-Methylurea | pos | 75.0559 | 0.792 | 3.580 | 3.988 | 0.408 | 0.516 | 0.004 | 0.017 | Moderate putative | NA | NA |
| metab_10858 | Unannotated feature | neg | 563.8395 | 0.554 | 4.428 | 4.937 | 0.509 | 0.516 | 0.004 | 0.017 | Unannotated | NA | NA |
| metab_7263 | Unannotated feature | neg | 224.9797 | 0.865 | 6.071 | 6.025 | -0.047 | -0.516 | 0.004 | 0.017 | Unannotated | NA | NA |
| metab_3912 | Unannotated feature | pos | 226.0687 | 0.861 | 3.930 | 4.497 | 0.567 | 0.516 | 0.004 | 0.017 | Unannotated | NA | NA |
| metab_6063 | Aspartyl-Isoleucine | neg | 245.1146 | 3.104 | 3.513 | 4.374 | 0.861 | 0.516 | 0.004 | 0.017 | Tentative | Organic acids and derivatives | Carboxylic acids and derivatives |
| metab_1744 | Unannotated feature | pos | 230.0051 | 7.324 | 4.107 | 4.233 | 0.126 | 0.516 | 0.004 | 0.017 | Unannotated | NA | NA |
| metab_1796 | Unannotated feature | pos | 201.0082 | 7.804 | 5.566 | 5.609 | 0.043 | 0.516 | 0.004 | 0.017 | Unannotated | NA | NA |
| metab_7919 | Unannotated feature | neg | 205.0353 | 2.095 | 3.418 | 3.769 | 0.350 | 0.516 | 0.004 | 0.017 | Unannotated | NA | NA |
| metab_1010 | Methyldopa anhydrous | pos | 194.0813 | 4.570 | 4.433 | 4.932 | 0.498 | 0.516 | 0.004 | 0.017 | Tentative | Phenylpropanoids and polyketides | Phenylpropanoic acids |
| metab_4136 | Unannotated feature | pos | 233.0510 | 0.675 | 4.172 | 3.647 | -0.525 | -0.516 | 0.004 | 0.017 | Unannotated | NA | NA |
| metab_459 | Unannotated feature | pos | 230.1863 | 1.740 | 3.071 | 3.710 | 0.639 | 0.516 | 0.004 | 0.017 | Unannotated | NA | NA |
| metab_11148 | Unannotated feature | neg | 259.0140 | 3.562 | 4.695 | 4.561 | -0.134 | -0.516 | 0.004 | 0.017 | Unannotated | NA | NA |
| metab_3898 | Unannotated feature | pos | 418.9841 | 0.861 | 4.265 | 3.913 | -0.352 | -0.516 | 0.004 | 0.017 | Unannotated | NA | NA |
| metab_5158 | Unannotated feature | neg | 570.6213 | 0.594 | 6.693 | 6.613 | -0.080 | -0.516 | 0.004 | 0.017 | Unannotated | NA | NA |
| metab_2622 | Unannotated feature | pos | 559.3922 | 5.832 | 5.739 | 5.627 | -0.112 | -0.516 | 0.004 | 0.017 | Unannotated | NA | NA |
| metab_5563 | Unannotated feature | neg | 247.0939 | 0.841 | 3.413 | 3.921 | 0.508 | 0.516 | 0.004 | 0.017 | Unannotated | NA | NA |
| metab_1960 | Unannotated feature | pos | 568.3376 | 7.300 | 4.406 | 3.947 | -0.459 | -0.516 | 0.004 | 0.017 | Unannotated | NA | NA |
| metab_3732 | Unannotated feature | pos | 134.1088 | 1.809 | 4.349 | 4.250 | -0.099 | -0.516 | 0.004 | 0.017 | Unannotated | NA | NA |
| metab_10207 | Unannotated feature | neg | 292.8715 | 0.865 | 4.973 | 4.795 | -0.178 | -0.516 | 0.004 | 0.017 | Unannotated | NA | NA |
| metab_5466 | Unannotated feature | neg | 308.0992 | 0.684 | 4.103 | 4.653 | 0.550 | 0.516 | 0.004 | 0.017 | Unannotated | NA | NA |
| metab_1929 | Unannotated feature | pos | 144.9888 | 7.324 | 4.250 | 4.338 | 0.088 | 0.516 | 0.004 | 0.017 | Unannotated | NA | NA |
| metab_7240 | 5,9,11-trihydroxyprosta-6E,14Z-dien-1-oate | neg | 576.2045 | 6.008 | 4.738 | 5.149 | 0.411 | 0.516 | 0.004 | 0.017 | Tentative | Lipids and lipid-like molecules | Prenol lipids |
| metab_2045 | Unannotated feature | pos | 146.9121 | 7.261 | 3.992 | 4.118 | 0.126 | 0.516 | 0.004 | 0.017 | Unannotated | NA | NA |
| metab_9029 | Unannotated feature | neg | 363.0763 | 4.679 | 4.083 | 4.864 | 0.780 | 0.516 | 0.004 | 0.017 | Unannotated | NA | NA |
| metab_2653 | Unannotated feature | pos | 286.3105 | 5.801 | 4.504 | 4.592 | 0.088 | 0.516 | 0.004 | 0.017 | Unannotated | NA | NA |
| metab_688 | Unannotated feature | pos | 291.1339 | 2.475 | 4.389 | 4.006 | -0.384 | -0.516 | 0.004 | 0.017 | Unannotated | NA | NA |
| metab_7045 | Unannotated feature | neg | 223.9181 | 1.182 | 6.057 | 5.917 | -0.140 | -0.516 | 0.004 | 0.017 | Unannotated | NA | NA |
| metab_4269 | Unannotated feature | pos | 574.7032 | 0.591 | 5.292 | 5.225 | -0.067 | -0.516 | 0.004 | 0.017 | Unannotated | NA | NA |
| metab_5372 | Unannotated feature | neg | 298.8358 | 0.016 | 4.983 | 4.934 | -0.049 | -0.516 | 0.004 | 0.017 | Unannotated | NA | NA |
| metab_2629 | Unannotated feature | pos | 250.1778 | 5.832 | 5.530 | 5.636 | 0.105 | 0.516 | 0.004 | 0.017 | Unannotated | NA | NA |
| metab_713 | Unannotated feature | pos | 188.7822 | 2.576 | 5.357 | 5.213 | -0.143 | -0.516 | 0.004 | 0.017 | Unannotated | NA | NA |
| metab_5486 | Unannotated feature | neg | 515.7884 | 0.707 | 4.351 | 3.887 | -0.464 | -0.516 | 0.004 | 0.017 | Unannotated | NA | NA |
| metab_736 | Unannotated feature | pos | 206.8940 | 0.583 | 6.343 | 6.298 | -0.045 | -0.516 | 0.004 | 0.017 | Unannotated | NA | NA |
| metab_5122 | D-Apiofuranose | pos | 115.0392 | 2.303 | 4.162 | 4.269 | 0.106 | 0.516 | 0.004 | 0.017 | Tentative | NA | NA |
| metab_3773 | Indole-3-carboxylate | pos | 162.0550 | 3.769 | 3.452 | 4.537 | 1.085 | 0.512 | 0.004 | 0.018 | Strong putative | Organoheterocyclic compounds | Indoles and derivatives |
| metab_3764 | 4-Hydroxybenzaldehyde | pos | 123.0443 | 1.740 | 5.903 | 5.781 | -0.121 | -0.512 | 0.004 | 0.018 | Strong putative | Organic oxygen compounds | Organooxygen compounds |
| metab_1722 | Unannotated feature | pos | 146.9804 | 7.308 | 4.876 | 4.937 | 0.061 | 0.512 | 0.004 | 0.018 | Unannotated | NA | NA |
| metab_3488 | L-2-Aminoadipate | pos | 126.0551 | 2.234 | 4.379 | 4.420 | 0.041 | 0.512 | 0.004 | 0.018 | Moderate putative | NA | NA |
| metab_5106 | Unannotated feature | pos | 268.8466 | 0.591 | 5.515 | 5.460 | -0.055 | -0.512 | 0.004 | 0.018 | Unannotated | NA | NA |
| metab_6370 | Unannotated feature | neg | 275.9942 | 3.959 | 3.416 | 3.862 | 0.445 | 0.512 | 0.004 | 0.018 | Unannotated | NA | NA |
| metab_1965 | Unannotated feature | pos | 888.5477 | 0.575 | 4.920 | 4.826 | -0.095 | -0.512 | 0.004 | 0.018 | Unannotated | NA | NA |
| metab_2780 | Unannotated feature | pos | 487.3347 | 5.649 | 4.826 | 4.564 | -0.262 | -0.512 | 0.004 | 0.018 | Unannotated | NA | NA |
| metab_6278 | Unannotated feature | neg | 130.2729 | 3.562 | 2.894 | 2.593 | -0.301 | -0.512 | 0.004 | 0.018 | Unannotated | NA | NA |
| metab_1767 | Unannotated feature | pos | 108.0231 | 7.347 | 4.914 | 4.993 | 0.080 | 0.512 | 0.004 | 0.018 | Unannotated | NA | NA |
| metab_662 | Streptidine | pos | 304.1754 | 2.381 | 4.690 | 5.383 | 0.693 | 0.512 | 0.004 | 0.018 | Tentative | Organic oxygen compounds | Organooxygen compounds |
| metab_8141 | (5-L-Glutamyl)-L-glutamine | neg | 274.1050 | 0.731 | 5.386 | 5.135 | -0.252 | -0.512 | 0.004 | 0.018 | Moderate putative | Organic acids and derivatives | Carboxylic acids and derivatives |
| metab_3411 | Unannotated feature | pos | 199.9753 | 2.436 | 5.157 | 5.115 | -0.042 | -0.512 | 0.004 | 0.018 | Unannotated | NA | NA |
| metab_5298 | Unannotated feature | neg | 342.8447 | 0.013 | 5.356 | 5.323 | -0.033 | -0.512 | 0.004 | 0.018 | Unannotated | NA | NA |
| metab_1361 | Unannotated feature | pos | 709.1607 | 5.902 | 5.109 | 4.949 | -0.160 | -0.512 | 0.004 | 0.018 | Unannotated | NA | NA |
| metab_2311 | LysoPE(20:4(8Z,11Z,14Z,17Z)/0:0) | pos | 502.2931 | 6.049 | 6.501 | 6.601 | 0.100 | 0.512 | 0.004 | 0.018 | Tentative | NA | NA |
| metab_10457 | Unannotated feature | neg | 545.8241 | 0.652 | 4.279 | 3.935 | -0.344 | -0.512 | 0.004 | 0.018 | Unannotated | NA | NA |
| metab_3250 | Caffeine | pos | 195.0877 | 3.019 | 6.145 | 3.609 | -2.536 | -0.512 | 0.004 | 0.018 | Strong putative | Organoheterocyclic compounds | Imidazopyrimidines |
| metab_9454 | Unannotated feature | neg | 259.1303 | 3.332 | 4.366 | 4.645 | 0.279 | 0.512 | 0.004 | 0.018 | Unannotated | NA | NA |
| metab_6834 | Unannotated feature | neg | 134.0605 | 5.611 | 4.054 | 3.564 | -0.490 | -0.512 | 0.004 | 0.018 | Unannotated | NA | NA |
| metab_5786 | Unannotated feature | neg | 499.7805 | 0.013 | 5.854 | 5.820 | -0.034 | -0.512 | 0.004 | 0.018 | Unannotated | NA | NA |
| metab_2957 | Theobromine | pos | 198.0972 | 2.241 | 4.560 | 4.279 | -0.281 | -0.512 | 0.004 | 0.018 | Tentative | Organoheterocyclic compounds | Imidazopyrimidines |
| metab_4593 | Unannotated feature | pos | 120.7566 | 0.317 | 4.786 | 4.829 | 0.043 | 0.512 | 0.004 | 0.018 | Unannotated | NA | NA |
| metab_1454 | Unannotated feature | pos | 586.4536 | 5.979 | 6.104 | 6.059 | -0.045 | -0.512 | 0.004 | 0.018 | Unannotated | NA | NA |
| metab_4518 | N-Methyl-L-proline | pos | 147.1129 | 0.541 | 5.673 | 5.604 | -0.070 | -0.512 | 0.004 | 0.018 | Strong putative | NA | NA |
| metab_4512 | Unannotated feature | pos | 997.4749 | 0.541 | 4.635 | 4.384 | -0.251 | -0.512 | 0.004 | 0.018 | Unannotated | NA | NA |
| metab_3432 | Cerulenin | pos | 224.1281 | 2.374 | 3.411 | 4.415 | 1.005 | 0.512 | 0.004 | 0.018 | Tentative | Organoheterocyclic compounds | Epoxides |
| metab_10990 | Unannotated feature | neg | 221.9029 | 0.140 | 5.033 | 5.006 | -0.027 | -0.512 | 0.004 | 0.018 | Unannotated | NA | NA |
| metab_4117 | Unannotated feature | pos | 132.4955 | 0.698 | 4.320 | 3.613 | -0.707 | -0.512 | 0.004 | 0.018 | Unannotated | NA | NA |
| metab_3942 | Unannotated feature | pos | 178.1187 | 0.854 | 4.086 | 4.383 | 0.297 | 0.512 | 0.004 | 0.018 | Unannotated | NA | NA |
| metab_5175 | Unannotated feature | neg | 184.9156 | 0.522 | 3.975 | 4.157 | 0.182 | 0.512 | 0.004 | 0.018 | Unannotated | NA | NA |
| metab_3595 | L-Galactose | pos | 198.0973 | 2.034 | 4.134 | 3.956 | -0.178 | -0.512 | 0.004 | 0.018 | Tentative | NA | NA |
| metab_4295 | Unannotated feature | pos | 820.5596 | 0.583 | 5.427 | 5.318 | -0.108 | -0.512 | 0.004 | 0.018 | Unannotated | NA | NA |
| metab_305 | Unannotated feature | pos | 619.7515 | 5.107 | 6.534 | 6.865 | 0.331 | 0.512 | 0.004 | 0.018 | Unannotated | NA | NA |
| metab_2591 | Unannotated feature | pos | 808.0553 | 5.848 | 4.976 | 4.666 | -0.310 | -0.512 | 0.004 | 0.018 | Unannotated | NA | NA |
| metab_739 | Dimethyl-3-hydroxypyrid-4-one | pos | 104.0499 | 2.644 | 4.985 | 5.005 | 0.019 | 0.512 | 0.004 | 0.018 | Tentative | NA | NA |
| metab_3 | Unannotated feature | pos | 136.0758 | 1.740 | 6.369 | 6.250 | -0.119 | -0.512 | 0.004 | 0.018 | Unannotated | NA | NA |
| metab_9956 | Unannotated feature | neg | 229.9022 | 2.111 | 5.056 | 4.827 | -0.229 | -0.512 | 0.004 | 0.018 | Unannotated | NA | NA |
| metab_8245 | Unannotated feature | neg | 670.2064 | 5.906 | 4.289 | 4.661 | 0.372 | 0.512 | 0.004 | 0.018 | Unannotated | NA | NA |
| metab_4090 | Unannotated feature | pos | 186.3563 | 0.729 | 4.059 | 3.318 | -0.741 | -0.512 | 0.004 | 0.018 | Unannotated | NA | NA |
| metab_9203 | Unannotated feature | neg | 365.0739 | 0.676 | 5.388 | 5.166 | -0.222 | -0.512 | 0.004 | 0.018 | Unannotated | NA | NA |
| metab_3613 | Unannotated feature | pos | 138.9953 | 1.995 | 4.548 | 4.522 | -0.026 | -0.512 | 0.004 | 0.018 | Unannotated | NA | NA |
| metab_5108 | Uridine | pos | 267.0587 | 1.724 | 5.090 | 4.971 | -0.119 | -0.512 | 0.004 | 0.018 | Strong putative | Nucleosides, nucleotides, and analogues | Pyrimidine nucleosides |
| metab_6879 | o-Cresol | neg | 107.0494 | 5.643 | 3.207 | 3.964 | 0.756 | 0.512 | 0.004 | 0.018 | Tentative | Benzenoids | Phenols |
| metab_8247 | Unannotated feature | neg | 441.8293 | 5.906 | 5.487 | 5.383 | -0.104 | -0.507 | 0.004 | 0.019 | Unannotated | NA | NA |
| metab_7665 | Unannotated feature | neg | 216.0012 | 7.285 | 3.056 | 3.289 | 0.233 | 0.507 | 0.004 | 0.019 | Unannotated | NA | NA |
| metab_3547 | Unannotated feature | pos | 169.8925 | 2.073 | 3.905 | 4.248 | 0.344 | 0.507 | 0.004 | 0.019 | Unannotated | NA | NA |
| metab_8100 | Mdo-npa | neg | 288.1403 | 6.188 | 5.230 | 5.083 | -0.147 | -0.507 | 0.004 | 0.019 | Tentative | NA | NA |
| metab_10250 | Unannotated feature | neg | 698.5656 | 0.594 | 5.489 | 5.400 | -0.088 | -0.507 | 0.004 | 0.019 | Unannotated | NA | NA |
| metab_46 | N-Acetylornithine | pos | 197.0897 | 0.784 | 5.609 | 5.778 | 0.169 | 0.507 | 0.004 | 0.019 | Moderate putative | NA | NA |
| metab_9140 | 3,4-Dihydroxyphenylvaleric acid | neg | 255.0878 | 4.298 | 4.752 | 4.350 | -0.402 | -0.507 | 0.004 | 0.019 | Tentative | NA | NA |
| metab_3694 | Unannotated feature | pos | 209.0809 | 1.910 | 4.818 | 4.724 | -0.093 | -0.507 | 0.004 | 0.019 | Unannotated | NA | NA |
| metab_6367 | Pimelate | neg | 159.0658 | 3.946 | 4.296 | 4.698 | 0.402 | 0.507 | 0.004 | 0.019 | Moderate putative | Lipids and lipid-like molecules | Fatty Acyls |
| metab_4869 | Unannotated feature | pos | 234.0972 | 2.257 | 3.461 | 4.245 | 0.784 | 0.507 | 0.004 | 0.019 | Unannotated | NA | NA |
| metab_6396 | Unannotated feature | neg | 360.0863 | 4.071 | 4.999 | 4.775 | -0.224 | -0.507 | 0.004 | 0.019 | Unannotated | NA | NA |
| metab_2495 | Unannotated feature | pos | 622.9367 | 5.894 | 4.903 | 4.673 | -0.230 | -0.507 | 0.004 | 0.019 | Unannotated | NA | NA |
| metab_10936 | Unannotated feature | neg | 530.8666 | 0.530 | 5.131 | 5.042 | -0.088 | -0.507 | 0.004 | 0.019 | Unannotated | NA | NA |
| metab_2483 | Unannotated feature | pos | 1011.7840 | 0.551 | 5.272 | 5.428 | 0.157 | 0.507 | 0.004 | 0.019 | Unannotated | NA | NA |
| metab_8745 | Unannotated feature | neg | 259.0283 | 5.563 | 4.334 | 4.472 | 0.138 | 0.507 | 0.004 | 0.019 | Unannotated | NA | NA |
| metab_3997 | L-Histidinol | pos | 124.0872 | 0.823 | 2.835 | 3.620 | 0.785 | 0.507 | 0.004 | 0.019 | Tentative | Organic nitrogen compounds | Organonitrogen compounds |
| metab_5208 | Unannotated feature | neg | 589.9024 | 0.543 | 5.960 | 5.890 | -0.070 | -0.507 | 0.004 | 0.019 | Unannotated | NA | NA |
| metab_4900 | Unannotated feature | pos | 195.1016 | 0.481 | 5.120 | 5.147 | 0.026 | 0.507 | 0.004 | 0.019 | Unannotated | NA | NA |
| metab_2341 | Unannotated feature | pos | 484.3206 | 5.995 | 4.817 | 4.295 | -0.521 | -0.507 | 0.004 | 0.019 | Unannotated | NA | NA |
| metab_2370 | Unannotated feature | pos | 184.1123 | 5.964 | 4.356 | 4.395 | 0.039 | 0.507 | 0.004 | 0.019 | Unannotated | NA | NA |
| metab_5964 | Unannotated feature | neg | 173.1040 | 0.613 | 4.544 | 4.700 | 0.157 | 0.507 | 0.004 | 0.019 | Unannotated | NA | NA |
| metab_5100 | Unannotated feature | pos | 212.9804 | 0.839 | 1.983 | 3.559 | 1.576 | 0.507 | 0.004 | 0.019 | Unannotated | NA | NA |
| metab_950 | Unannotated feature | pos | 83.4492 | 4.124 | 3.030 | 3.265 | 0.235 | 0.507 | 0.004 | 0.019 | Unannotated | NA | NA |
| metab_5985 | Unannotated feature | neg | 155.9805 | 2.812 | 3.503 | 3.597 | 0.095 | 0.507 | 0.004 | 0.019 | Unannotated | NA | NA |
| metab_8158 | Unannotated feature | neg | 199.0375 | 0.731 | 5.884 | 5.480 | -0.404 | -0.507 | 0.004 | 0.019 | Unannotated | NA | NA |
| metab_1721 | Unannotated feature | pos | 308.8900 | 7.300 | 4.627 | 4.675 | 0.048 | 0.507 | 0.004 | 0.019 | Unannotated | NA | NA |
| metab_6670 | Unannotated feature | neg | 198.9981 | 5.139 | 3.940 | 3.243 | -0.697 | -0.507 | 0.004 | 0.019 | Unannotated | NA | NA |
| metab_10057 | Unannotated feature | neg | 290.8745 | 1.382 | 5.422 | 5.333 | -0.089 | -0.507 | 0.004 | 0.019 | Unannotated | NA | NA |
| metab_1305 | Unannotated feature | pos | 720.0035 | 5.871 | 5.774 | 5.711 | -0.063 | -0.507 | 0.004 | 0.019 | Unannotated | NA | NA |
| metab_9371 | Unannotated feature | neg | 290.9008 | 3.554 | 3.753 | 3.335 | -0.417 | -0.507 | 0.004 | 0.019 | Unannotated | NA | NA |
| metab_4397 | Unannotated feature | pos | 317.0229 | 0.567 | 3.812 | 3.609 | -0.202 | -0.507 | 0.004 | 0.019 | Unannotated | NA | NA |
| metab_5954 | Unannotated feature | neg | 512.6623 | 0.598 | 6.283 | 6.221 | -0.062 | -0.507 | 0.004 | 0.019 | Unannotated | NA | NA |
| metab_8885 | Unannotated feature | neg | 680.2285 | 5.199 | 3.819 | 4.339 | 0.520 | 0.507 | 0.004 | 0.019 | Unannotated | NA | NA |
| metab_4162 | Unannotated feature | pos | 140.9803 | 0.652 | 4.141 | 4.525 | 0.384 | 0.507 | 0.004 | 0.019 | Unannotated | NA | NA |
| metab_10568 | Unannotated feature | neg | 116.0498 | 4.969 | 3.820 | 4.056 | 0.235 | 0.507 | 0.004 | 0.019 | Unannotated | NA | NA |
| metab_2287 | Unannotated feature | pos | 526.4312 | 6.103 | 5.038 | 5.174 | 0.136 | 0.507 | 0.004 | 0.019 | Unannotated | NA | NA |
| metab_5860 | Temocaprilat | neg | 469.0911 | 2.417 | 4.835 | 5.061 | 0.226 | 0.507 | 0.004 | 0.019 | Tentative | Organic acids and derivatives | Carboxylic acids and derivatives |
| metab_1628 | Unannotated feature | pos | 225.2092 | 7.091 | 5.454 | 5.383 | -0.070 | -0.507 | 0.004 | 0.019 | Unannotated | NA | NA |
| metab_4967 | Lactulose | pos | 365.1054 | 0.691 | 5.006 | 5.551 | 0.545 | 0.507 | 0.004 | 0.019 | Moderate putative | Organic oxygen compounds | Organooxygen compounds |
| metab_1552 | (4-Hydroxybenzoyl)choline | pos | 490.2904 | 6.296 | 5.195 | 4.972 | -0.223 | -0.507 | 0.004 | 0.019 | Tentative | Benzenoids | Benzene and substituted derivatives |
| metab_132 | Unannotated feature | pos | 600.8822 | 0.551 | 5.997 | 5.919 | -0.078 | -0.507 | 0.004 | 0.019 | Unannotated | NA | NA |
| metab_5767 | Unannotated feature | neg | 130.0867 | 1.951 | 4.619 | 4.547 | -0.072 | -0.507 | 0.004 | 0.019 | Unannotated | NA | NA |
| metab_539 | Unannotated feature | pos | 122.5474 | 1.980 | 3.603 | 3.497 | -0.105 | -0.507 | 0.004 | 0.019 | Unannotated | NA | NA |
| metab_1178 | Unannotated feature | pos | 501.3505 | 5.733 | 5.386 | 5.214 | -0.173 | -0.507 | 0.004 | 0.019 | Unannotated | NA | NA |
| metab_5190 | Unannotated feature | neg | 292.9872 | 0.534 | 4.304 | 4.137 | -0.167 | -0.503 | 0.005 | 0.020 | Unannotated | NA | NA |
| metab_10435 | Unannotated feature | neg | 217.1081 | 4.782 | 3.104 | 4.155 | 1.051 | 0.503 | 0.005 | 0.020 | Unannotated | NA | NA |
| metab_1302 | Unannotated feature | pos | 651.1202 | 5.871 | 5.286 | 4.974 | -0.312 | -0.503 | 0.005 | 0.020 | Unannotated | NA | NA |
| metab_4271 | Unannotated feature | pos | 562.6338 | 0.591 | 4.876 | 4.759 | -0.117 | -0.503 | 0.005 | 0.020 | Unannotated | NA | NA |
| metab_10221 | Unannotated feature | neg | 340.7830 | 0.598 | 5.553 | 5.482 | -0.071 | -0.503 | 0.005 | 0.020 | Unannotated | NA | NA |
| metab_6687 | Unannotated feature | neg | 119.0342 | 0.857 | 5.156 | 5.573 | 0.418 | 0.503 | 0.005 | 0.020 | Unannotated | NA | NA |
| metab_5478 | Unannotated feature | neg | 88.0396 | 0.699 | 4.044 | 3.730 | -0.314 | -0.503 | 0.005 | 0.020 | Unannotated | NA | NA |
| metab_9041 | Unannotated feature | neg | 436.0059 | 4.635 | 4.191 | 3.234 | -0.957 | -0.503 | 0.005 | 0.020 | Unannotated | NA | NA |
| metab_7682 | Unannotated feature | neg | 163.9283 | 7.245 | 5.277 | 5.323 | 0.045 | 0.503 | 0.005 | 0.020 | Unannotated | NA | NA |
| metab_2628 | Bisabolol oxide A | pos | 271.2268 | 5.832 | 5.234 | 5.263 | 0.028 | 0.503 | 0.005 | 0.020 | Tentative | NA | NA |
| metab_4906 | Tiglic acid | pos | 83.0497 | 0.442 | 5.570 | 5.599 | 0.029 | 0.503 | 0.005 | 0.020 | Tentative | NA | NA |
| metab_11183 | Unannotated feature | neg | 509.2762 | 5.834 | 4.473 | 4.810 | 0.338 | 0.503 | 0.005 | 0.020 | Unannotated | NA | NA |
| metab_3579 | Unannotated feature | pos | 146.0601 | 2.058 | 4.735 | 4.944 | 0.208 | 0.503 | 0.005 | 0.020 | Strong putative | NA | NA |
| metab_2673 | Unannotated feature | pos | 625.4315 | 5.778 | 5.376 | 5.176 | -0.201 | -0.503 | 0.005 | 0.020 | Unannotated | NA | NA |
| metab_1548 | Unannotated feature | pos | 301.1394 | 2.568 | 3.802 | 4.985 | 1.183 | 0.503 | 0.005 | 0.020 | Unannotated | NA | NA |
| metab_3220 | Bis-noryangonin | pos | 248.0917 | 3.159 | 3.768 | 4.843 | 1.076 | 0.503 | 0.005 | 0.020 | Tentative | Benzenoids | Benzene and substituted derivatives |
| metab_1938 | Unannotated feature | pos | 111.5222 | 7.324 | 5.366 | 5.444 | 0.078 | 0.503 | 0.005 | 0.020 | Unannotated | NA | NA |
| metab_2511 | Unannotated feature | pos | 738.3415 | 5.886 | 4.883 | 4.447 | -0.436 | -0.503 | 0.005 | 0.020 | Unannotated | NA | NA |
| metab_2512 | Unannotated feature | pos | 714.4925 | 5.886 | 5.204 | 4.899 | -0.306 | -0.503 | 0.005 | 0.020 | Unannotated | NA | NA |
| metab_3993 | Asparaginyl-Proline | pos | 212.1030 | 0.823 | 4.011 | 4.727 | 0.717 | 0.503 | 0.005 | 0.020 | Tentative | Organic acids and derivatives | Carboxylic acids and derivatives |
| metab_5210 | Unannotated feature | neg | 657.8901 | 0.543 | 5.881 | 5.813 | -0.068 | -0.503 | 0.005 | 0.020 | Unannotated | NA | NA |
| metab_9370 | Unannotated feature | neg | 316.9168 | 3.554 | 4.674 | 4.536 | -0.138 | -0.503 | 0.005 | 0.020 | Unannotated | NA | NA |
| metab_6042 | Unannotated feature | neg | 136.8937 | 3.025 | 3.896 | 3.840 | -0.056 | -0.503 | 0.005 | 0.020 | Unannotated | NA | NA |
| metab_9222 | 4-Methylcatechol | neg | 123.0444 | 4.059 | 3.993 | 4.641 | 0.648 | 0.503 | 0.005 | 0.020 | Moderate putative | Benzenoids | Phenols |
| metab_3426 | Unannotated feature | pos | 159.9692 | 2.888 | 4.963 | 4.892 | -0.071 | -0.503 | 0.005 | 0.020 | Unannotated | NA | NA |
| metab_6626 | Sepiapterin | neg | 272.0545 | 4.969 | 3.927 | 4.283 | 0.357 | 0.503 | 0.005 | 0.020 | Tentative | Organoheterocyclic compounds | Pteridines and derivatives |
| metab_1061 | para-Cresidine | pos | 120.0811 | 5.122 | 4.166 | 4.332 | 0.166 | 0.503 | 0.005 | 0.020 | Tentative | NA | NA |
| metab_5340 | Unannotated feature | neg | 571.7104 | 0.013 | 5.384 | 5.334 | -0.050 | -0.503 | 0.005 | 0.020 | Unannotated | NA | NA |
| metab_6773 | Unannotated feature | neg | 617.7386 | 5.523 | 7.009 | 7.326 | 0.317 | 0.503 | 0.005 | 0.020 | Unannotated | NA | NA |
| metab_7965 | Unannotated feature | neg | 170.9843 | 7.101 | 5.210 | 5.172 | -0.038 | -0.503 | 0.005 | 0.020 | Unannotated | NA | NA |
| metab_3592 | Glutaconylcarnitine | pos | 256.1178 | 2.042 | 3.452 | 4.047 | 0.594 | 0.503 | 0.005 | 0.020 | Tentative | Lipids and lipid-like molecules | Fatty Acyls |
| metab_4746 | Unannotated feature | pos | 239.2248 | 6.296 | 5.962 | 5.670 | -0.292 | -0.503 | 0.005 | 0.020 | Unannotated | NA | NA |
| metab_5944 | Unannotated feature | neg | 241.7937 | 2.660 | 4.428 | 4.366 | -0.061 | -0.503 | 0.005 | 0.020 | Unannotated | NA | NA |
| metab_8948 | Unannotated feature | neg | 321.8146 | 4.953 | 5.064 | 5.125 | 0.061 | 0.503 | 0.005 | 0.020 | Unannotated | NA | NA |
| metab_9796 | Unannotated feature | neg | 370.8964 | 2.541 | 4.567 | 4.451 | -0.117 | -0.503 | 0.005 | 0.020 | Unannotated | NA | NA |
| metab_9920 | Unannotated feature | neg | 245.9674 | 2.119 | 4.117 | 3.913 | -0.204 | -0.503 | 0.005 | 0.020 | Unannotated | NA | NA |
| metab_5851 | Unannotated feature | neg | 117.6255 | 2.363 | 2.885 | 2.716 | -0.169 | -0.503 | 0.005 | 0.020 | Unannotated | NA | NA |
| metab_36 | 3,4-Methylenesebacic acid | pos | 290.1346 | 1.956 | 4.183 | 5.043 | 0.861 | 0.503 | 0.005 | 0.020 | Tentative | Lipids and lipid-like molecules | Fatty Acyls |
| metab_9451 | Unannotated feature | neg | 411.1279 | 3.332 | 3.960 | 4.595 | 0.635 | 0.503 | 0.005 | 0.020 | Unannotated | NA | NA |
| metab_8306 | Unannotated feature | neg | 323.1870 | 5.859 | 4.056 | 3.618 | -0.438 | -0.503 | 0.005 | 0.020 | Unannotated | NA | NA |
| metab_572 | Unannotated feature | pos | 151.0615 | 2.058 | 4.959 | 5.404 | 0.445 | 0.503 | 0.005 | 0.020 | Unannotated | NA | NA |
| metab_2984 | Unannotated feature | pos | 130.0653 | 4.501 | 4.319 | 4.483 | 0.164 | 0.503 | 0.005 | 0.020 | Unannotated | NA | NA |
| metab_9226 | Carmofur | neg | 302.1152 | 4.037 | 3.407 | 4.754 | 1.347 | 0.503 | 0.005 | 0.020 | Tentative | Organoheterocyclic compounds | Diazines |
| metab_1673 | Unannotated feature | pos | 238.2125 | 7.261 | 4.162 | 3.958 | -0.203 | -0.503 | 0.005 | 0.020 | Unannotated | NA | NA |
| metab_9985 | Unannotated feature | neg | 188.9861 | 2.002 | 2.885 | 4.059 | 1.174 | 0.503 | 0.005 | 0.020 | Unannotated | NA | NA |
| metab_4161 | 6-Amino-2-oxohexanoate | pos | 146.0813 | 0.652 | 4.557 | 4.731 | 0.174 | 0.499 | 0.005 | 0.021 | Tentative | Organic acids and derivatives | Keto acids and derivatives |
| metab_5813 | Unannotated feature | neg | 232.9982 | 2.119 | 4.727 | 4.556 | -0.171 | -0.499 | 0.005 | 0.021 | Unannotated | NA | NA |
| metab_3403 | 4,6-Diamino-5-formamidopyrimidine | pos | 136.0619 | 2.467 | 4.520 | 4.656 | 0.136 | 0.499 | 0.005 | 0.021 | Tentative | NA | NA |
| metab_4472 | Unannotated feature | pos | 705.8406 | 0.551 | 5.494 | 5.657 | 0.163 | 0.499 | 0.005 | 0.021 | Unannotated | NA | NA |
| metab_11084 | Unannotated feature | neg | 439.1618 | 5.611 | 4.593 | 5.138 | 0.545 | 0.499 | 0.005 | 0.021 | Unannotated | NA | NA |
| metab_9984 | Unannotated feature | neg | 229.0695 | 2.002 | 3.462 | 2.727 | -0.735 | -0.499 | 0.005 | 0.021 | Unannotated | NA | NA |
| metab_5667 | Unannotated feature | neg | 157.0114 | 1.103 | 5.690 | 5.604 | -0.086 | -0.499 | 0.005 | 0.021 | Unannotated | NA | NA |
| metab_8803 | Unannotated feature | neg | 157.0113 | 5.498 | 4.340 | 4.229 | -0.112 | -0.499 | 0.005 | 0.021 | Unannotated | NA | NA |
| metab_8143 | 3beta-Hydroxy-4beta-methyl-5alpha-cholest-7-ene-4alpha-carbaldehyde | neg | 473.3644 | 6.110 | 5.834 | 5.480 | -0.354 | -0.499 | 0.005 | 0.021 | Tentative | Lipids and lipid-like molecules | Steroids and steroid derivatives |
| metab_7264 | Plazomicin | neg | 637.3420 | 6.087 | 4.885 | 3.907 | -0.978 | -0.499 | 0.005 | 0.021 | Tentative | Organic acids and derivatives | Carboxylic acids and derivatives |
| metab_3897 | Unannotated feature | pos | 554.0342 | 0.861 | 4.586 | 4.264 | -0.322 | -0.499 | 0.005 | 0.021 | Unannotated | NA | NA |
| metab_1681 | Unannotated feature | pos | 225.2092 | 7.269 | 4.719 | 4.651 | -0.068 | -0.499 | 0.005 | 0.021 | Unannotated | NA | NA |
| metab_10734 | Unannotated feature | neg | 710.7758 | 0.570 | 4.154 | 4.417 | 0.263 | 0.499 | 0.005 | 0.021 | Unannotated | NA | NA |
| metab_1333 | 1-Cyclohexenecarboxylic acid | pos | 91.0547 | 5.894 | 5.595 | 5.675 | 0.080 | 0.499 | 0.005 | 0.021 | Tentative | NA | NA |
| metab_4267 | Unannotated feature | pos | 558.6397 | 0.591 | 5.567 | 5.473 | -0.094 | -0.499 | 0.005 | 0.021 | Unannotated | NA | NA |
| metab_9925 | Unannotated feature | neg | 214.0470 | 2.119 | 3.103 | 3.690 | 0.587 | 0.499 | 0.005 | 0.021 | Unannotated | NA | NA |
| metab_10836 | Unannotated feature | neg | 731.8329 | 0.554 | 5.101 | 5.224 | 0.123 | 0.499 | 0.005 | 0.021 | Unannotated | NA | NA |
| metab_2222 | Unannotated feature | pos | 227.2062 | 6.296 | 5.378 | 5.094 | -0.284 | -0.499 | 0.005 | 0.021 | Unannotated | NA | NA |
| metab_7352 | Unannotated feature | neg | 245.9835 | 2.111 | 4.652 | 4.447 | -0.205 | -0.499 | 0.005 | 0.021 | Unannotated | NA | NA |
| metab_7790 | Unannotated feature | neg | 187.9286 | 7.170 | 4.336 | 4.273 | -0.063 | -0.499 | 0.005 | 0.021 | Unannotated | NA | NA |
| metab_5654 | Unannotated feature | neg | 384.9361 | 0.554 | 6.798 | 6.742 | -0.056 | -0.499 | 0.005 | 0.021 | Unannotated | NA | NA |
| metab_5669 | Unannotated feature | neg | 253.8604 | 1.103 | 5.040 | 4.875 | -0.164 | -0.499 | 0.005 | 0.021 | Unannotated | NA | NA |
| metab_8262 | Unannotated feature | neg | 194.0820 | 5.898 | 6.633 | 6.685 | 0.052 | 0.499 | 0.005 | 0.021 | Unannotated | NA | NA |
| metab_1281 | Unannotated feature | pos | 675.7998 | 5.855 | 5.068 | 4.666 | -0.402 | -0.499 | 0.005 | 0.021 | Unannotated | NA | NA |
| metab_2001 | Unannotated feature | pos | 85.0766 | 7.293 | 3.925 | 3.980 | 0.055 | 0.499 | 0.005 | 0.021 | Unannotated | NA | NA |
| metab_10203 | Unannotated feature | neg | 394.7477 | 0.598 | 5.969 | 5.920 | -0.050 | -0.499 | 0.005 | 0.021 | Unannotated | NA | NA |
| metab_1315 | Unannotated feature | pos | 697.4894 | 5.879 | 6.177 | 6.120 | -0.057 | -0.499 | 0.005 | 0.021 | Unannotated | NA | NA |
| metab_2762 | Unannotated feature | pos | 356.2431 | 5.680 | 4.295 | 5.041 | 0.745 | 0.499 | 0.005 | 0.021 | Unannotated | NA | NA |
| metab_10914 | Unannotated feature | neg | 862.8538 | 0.539 | 4.737 | 4.536 | -0.201 | -0.499 | 0.005 | 0.021 | Unannotated | NA | NA |
| metab_8034 | Unannotated feature | neg | 195.0854 | 6.549 | 4.571 | 4.696 | 0.125 | 0.499 | 0.005 | 0.021 | Unannotated | NA | NA |
| metab_4115 | Unannotated feature | pos | 245.1359 | 0.698 | 3.876 | 3.193 | -0.684 | -0.499 | 0.005 | 0.021 | Unannotated | NA | NA |
| metab_1497 | 2-aminooctadec-4-ene-1,3-diol | pos | 264.2686 | 6.095 | 4.881 | 4.822 | -0.059 | -0.499 | 0.005 | 0.021 | Tentative | NA | NA |
| metab_2579 | Unannotated feature | pos | 671.4622 | 5.855 | 5.130 | 4.667 | -0.463 | -0.499 | 0.005 | 0.021 | Unannotated | NA | NA |
| metab_10982 | Unannotated feature | neg | 203.9367 | 0.469 | 4.776 | 4.739 | -0.037 | -0.499 | 0.005 | 0.021 | Unannotated | NA | NA |
| metab_9656 | Unannotated feature | neg | 149.0239 | 3.025 | 3.655 | 4.489 | 0.834 | 0.499 | 0.005 | 0.021 | Unannotated | NA | NA |
| metab_288 | Unannotated feature | pos | 145.1054 | 0.745 | 5.086 | 5.528 | 0.441 | 0.499 | 0.005 | 0.021 | Unannotated | NA | NA |
| metab_285 | Unannotated feature | pos | 141.0272 | 0.737 | 2.897 | 3.696 | 0.800 | 0.499 | 0.005 | 0.021 | Unannotated | NA | NA |
| metab_5604 | Unannotated feature | neg | 316.9482 | 0.554 | 6.832 | 6.782 | -0.049 | -0.499 | 0.005 | 0.021 | Unannotated | NA | NA |
| metab_8616 | Unannotated feature | neg | 281.1038 | 5.659 | 3.510 | 4.163 | 0.653 | 0.499 | 0.005 | 0.021 | Unannotated | NA | NA |
| metab_4280 | Unannotated feature | pos | 669.6501 | 0.575 | 4.631 | 4.443 | -0.188 | -0.499 | 0.005 | 0.021 | Unannotated | NA | NA |
| metab_2453 | Unannotated feature | pos | 745.5144 | 5.910 | 5.111 | 4.687 | -0.423 | -0.499 | 0.005 | 0.021 | Unannotated | NA | NA |
| metab_4275 | Unannotated feature | pos | 500.6811 | 0.591 | 5.654 | 5.560 | -0.094 | -0.499 | 0.005 | 0.021 | Unannotated | NA | NA |
| metab_3152 | Esculetin | pos | 220.0605 | 3.576 | 3.190 | 4.393 | 1.202 | 0.495 | 0.006 | 0.023 | Tentative | Phenylpropanoids and polyketides | Coumarins and derivatives |
| metab_3147 | Unannotated feature | pos | 209.0809 | 3.584 | 4.826 | 4.734 | -0.092 | -0.495 | 0.006 | 0.023 | Unannotated | NA | NA |
| metab_5632 | Unannotated feature | neg | 238.0254 | 0.920 | 4.742 | 4.543 | -0.199 | -0.495 | 0.006 | 0.023 | Unannotated | NA | NA |
| metab_178 | Unannotated feature | pos | 225.1097 | 4.024 | 3.770 | 4.529 | 0.758 | 0.495 | 0.006 | 0.023 | Unannotated | NA | NA |
| metab_3816 | Unannotated feature | pos | 86.0969 | 1.321 | 6.405 | 6.369 | -0.036 | -0.495 | 0.006 | 0.023 | Unannotated | NA | NA |
| metab_8801 | Unannotated feature | neg | 259.8166 | 5.498 | 5.586 | 5.506 | -0.080 | -0.495 | 0.006 | 0.023 | Unannotated | NA | NA |
| metab_3273 | L-Tryptophan | pos | 246.1237 | 2.911 | 5.554 | 5.300 | -0.255 | -0.495 | 0.006 | 0.023 | Strong putative | Organoheterocyclic compounds | Indoles and derivatives |
| metab_10093 | Unannotated feature | neg | 172.8676 | 1.222 | 4.422 | 4.532 | 0.111 | 0.495 | 0.006 | 0.023 | Unannotated | NA | NA |
| metab_9994 | Unannotated feature | neg | 170.9023 | 1.951 | 4.253 | 4.131 | -0.122 | -0.495 | 0.006 | 0.023 | Unannotated | NA | NA |
| metab_10004 | Unannotated feature | neg | 248.0544 | 1.886 | 4.571 | 4.395 | -0.176 | -0.495 | 0.006 | 0.023 | Unannotated | NA | NA |
| metab_1549 | Unannotated feature | pos | 253.2405 | 6.296 | 5.009 | 4.640 | -0.369 | -0.495 | 0.006 | 0.023 | Unannotated | NA | NA |
| metab_2224 | Unannotated feature | pos | 177.0884 | 6.296 | 6.095 | 6.142 | 0.047 | 0.495 | 0.006 | 0.023 | Unannotated | NA | NA |
| metab_627 | Benazeprilat | pos | 414.2022 | 2.202 | 5.491 | 5.012 | -0.479 | -0.495 | 0.006 | 0.023 | Tentative | Organic acids and derivatives | Carboxylic acids and derivatives |
| metab_9618 | Unannotated feature | neg | 153.0552 | 3.136 | 3.770 | 2.582 | -1.188 | -0.495 | 0.006 | 0.023 | Unannotated | NA | NA |
| metab_9598 | Benzenebutanoic acid, alpha-(acetylamino)-2-amino-gamma-oxo- | neg | 271.0706 | 3.160 | 5.062 | 4.855 | -0.207 | -0.495 | 0.006 | 0.023 | Tentative | Organic acids and derivatives | Carboxylic acids and derivatives |
| metab_10462 | Unannotated feature | neg | 292.0033 | 0.652 | 3.847 | 4.360 | 0.513 | 0.495 | 0.006 | 0.023 | Unannotated | NA | NA |
| metab_6380 | Unannotated feature | neg | 273.0920 | 4.004 | 3.670 | 4.194 | 0.524 | 0.495 | 0.006 | 0.023 | Unannotated | NA | NA |
| metab_3958 | O-Butylhydroxylamine | pos | 72.0814 | 0.854 | 6.051 | 5.983 | -0.068 | -0.495 | 0.006 | 0.023 | Tentative | NA | NA |
| metab_1527 | 1-Acyl-sn-glycero-3-phosphocholine | pos | 494.3241 | 6.196 | 7.078 | 6.951 | -0.127 | -0.495 | 0.006 | 0.023 | Tentative | Lipids and lipid-like molecules | Glycerophospholipids |
| metab_4862 | Unannotated feature | pos | 452.7941 | 5.703 | 4.809 | 4.564 | -0.245 | -0.495 | 0.006 | 0.023 | Unannotated | NA | NA |
| metab_267 | Unannotated feature | pos | 249.1557 | 0.698 | 4.322 | 3.400 | -0.921 | -0.495 | 0.006 | 0.023 | Unannotated | NA | NA |
| metab_1799 | Unannotated feature | pos | 174.0212 | 7.866 | 4.803 | 4.840 | 0.037 | 0.495 | 0.006 | 0.023 | Unannotated | NA | NA |
| metab_4711 | Unannotated feature | pos | 361.2948 | 5.941 | 5.343 | 5.437 | 0.095 | 0.495 | 0.006 | 0.023 | Unannotated | NA | NA |
| metab_6659 | Unannotated feature | neg | 144.0296 | 0.849 | 4.034 | 4.451 | 0.417 | 0.495 | 0.006 | 0.023 | Unannotated | NA | NA |
| metab_9099 | Unannotated feature | neg | 265.8515 | 4.425 | 4.784 | 4.732 | -0.052 | -0.495 | 0.006 | 0.023 | Unannotated | NA | NA |
| metab_2469 | Unannotated feature | pos | 716.4929 | 5.902 | 4.979 | 4.561 | -0.417 | -0.495 | 0.006 | 0.023 | Unannotated | NA | NA |
| metab_2473 | Unannotated feature | pos | 674.4695 | 5.902 | 4.981 | 4.839 | -0.142 | -0.495 | 0.006 | 0.023 | Unannotated | NA | NA |
| metab_10161 | Unannotated feature | neg | 562.5894 | 0.613 | 6.885 | 6.820 | -0.065 | -0.495 | 0.006 | 0.023 | Unannotated | NA | NA |
| metab_398 | Unannotated feature | pos | 174.9914 | 1.118 | 4.544 | 4.440 | -0.104 | -0.495 | 0.006 | 0.023 | Unannotated | NA | NA |
| metab_2534 | Unannotated feature | pos | 378.2658 | 5.879 | 4.661 | 4.820 | 0.160 | 0.495 | 0.006 | 0.023 | Unannotated | NA | NA |
| metab_5551 | Unannotated feature | neg | 452.9237 | 0.543 | 6.807 | 6.758 | -0.049 | -0.495 | 0.006 | 0.023 | Unannotated | NA | NA |
| metab_3733 | L-Norleucine | pos | 132.1020 | 1.809 | 7.680 | 7.631 | -0.048 | -0.495 | 0.006 | 0.023 | Strong putative | NA | NA |
| metab_3978 | Unannotated feature | pos | 271.0902 | 0.831 | 3.691 | 3.991 | 0.300 | 0.495 | 0.006 | 0.023 | Unannotated | NA | NA |
| metab_2632 | 2,5-Heptadien-1-ol | pos | 95.0860 | 5.832 | 4.863 | 4.902 | 0.039 | 0.495 | 0.006 | 0.023 | Tentative | NA | NA |
| metab_3545 | L-Furosine | pos | 296.1604 | 3.167 | 4.074 | 4.682 | 0.608 | 0.495 | 0.006 | 0.023 | Tentative | Organic acids and derivatives | Carboxylic acids and derivatives |
| metab_2530 | Unannotated feature | pos | 616.4283 | 5.879 | 4.974 | 4.790 | -0.184 | -0.495 | 0.006 | 0.023 | Unannotated | NA | NA |
| metab_9482 | Unannotated feature | neg | 157.0502 | 3.274 | 4.116 | 4.653 | 0.538 | 0.495 | 0.006 | 0.023 | Unannotated | NA | NA |
| metab_7 | Unannotated feature | pos | 231.1452 | 1.763 | 4.215 | 4.693 | 0.478 | 0.495 | 0.006 | 0.023 | Unannotated | NA | NA |
| metab_4797 | (2R)-2-{[(9Z)-Hexadec-9-enoyl]oxy}-3-hydroxypropyl 2-(trimethylazaniumyl)ethyl phosphate | pos | 494.3244 | 7.300 | 4.911 | 4.785 | -0.126 | -0.495 | 0.006 | 0.023 | Tentative | Lipids and lipid-like molecules | Glycerophospholipids |
| metab_6899 | Unannotated feature | neg | 541.2669 | 5.651 | 5.887 | 6.218 | 0.331 | 0.495 | 0.006 | 0.023 | Unannotated | NA | NA |
| metab_2300 | Unannotated feature | pos | 516.3322 | 6.072 | 4.911 | 5.272 | 0.361 | 0.491 | 0.006 | 0.024 | Unannotated | NA | NA |
| metab_69 | Unannotated feature | pos | 99.0557 | 0.364 | 3.812 | 3.887 | 0.075 | 0.491 | 0.006 | 0.024 | Unannotated | NA | NA |
| metab_2559 | Unannotated feature | pos | 583.0753 | 5.863 | 5.208 | 5.046 | -0.162 | -0.491 | 0.006 | 0.024 | Unannotated | NA | NA |
| metab_5485 | Unannotated feature | neg | 305.9358 | 0.707 | 3.838 | 3.534 | -0.303 | -0.491 | 0.006 | 0.024 | Unannotated | NA | NA |
| metab_126 | Unannotated feature | pos | 83.0473 | 0.551 | 5.440 | 5.417 | -0.024 | -0.491 | 0.006 | 0.024 | Unannotated | NA | NA |
| metab_3454 | 4-Aminosalicylate | pos | 195.0765 | 2.296 | 4.535 | 4.710 | 0.175 | 0.491 | 0.006 | 0.024 | Tentative | Benzenoids | Benzene and substituted derivatives |
| metab_9178 | Unannotated feature | neg | 423.0328 | 0.676 | 5.251 | 5.001 | -0.251 | -0.491 | 0.006 | 0.024 | Unannotated | NA | NA |
| metab_1199 | Unannotated feature | pos | 691.4702 | 5.756 | 4.957 | 4.688 | -0.269 | -0.491 | 0.006 | 0.024 | Unannotated | NA | NA |
| metab_11322 | Unannotated feature | neg | 196.8848 | 7.317 | 5.554 | 5.600 | 0.046 | 0.491 | 0.006 | 0.024 | Unannotated | NA | NA |
| metab_3914 | Stachydrine | pos | 144.1020 | 0.745 | 6.197 | 6.583 | 0.386 | 0.491 | 0.006 | 0.024 | Moderate putative | Organic acids and derivatives | Carboxylic acids and derivatives |
| metab_4668 | Unannotated feature | pos | 330.2462 | 5.824 | 4.888 | 4.612 | -0.275 | -0.491 | 0.006 | 0.024 | Unannotated | NA | NA |
| metab_2604 | Unannotated feature | pos | 705.4861 | 5.840 | 5.792 | 5.613 | -0.178 | -0.491 | 0.006 | 0.024 | Unannotated | NA | NA |
| metab_2951 | Unannotated feature | pos | 102.3761 | 4.647 | 4.261 | 4.324 | 0.062 | 0.491 | 0.006 | 0.024 | Unannotated | NA | NA |
| metab_644 | Unannotated feature | pos | 125.0235 | 2.280 | 3.301 | 3.626 | 0.325 | 0.491 | 0.006 | 0.024 | Unannotated | NA | NA |
| metab_5220 | Unannotated feature | neg | 902.8311 | 0.543 | 5.639 | 5.512 | -0.128 | -0.491 | 0.006 | 0.024 | Unannotated | NA | NA |
| metab_10693 | Unannotated feature | neg | 146.9825 | 0.582 | 4.397 | 4.280 | -0.117 | -0.491 | 0.006 | 0.024 | Unannotated | NA | NA |
| metab_6492 | Unannotated feature | neg | 259.8166 | 4.371 | 4.828 | 4.665 | -0.162 | -0.491 | 0.006 | 0.024 | Unannotated | NA | NA |
| metab_6258 | Unannotated feature | neg | 319.8177 | 3.507 | 4.267 | 4.301 | 0.034 | 0.491 | 0.006 | 0.024 | Unannotated | NA | NA |
| metab_9811 | Tropolone | neg | 167.0346 | 2.530 | 3.145 | 3.500 | 0.355 | 0.491 | 0.006 | 0.024 | Tentative | Hydrocarbon derivatives | Tropones |
| metab_5443 | Unannotated feature | neg | 290.0062 | 0.652 | 4.288 | 4.659 | 0.371 | 0.491 | 0.006 | 0.024 | Unannotated | NA | NA |
| metab_8008 | Unannotated feature | neg | 118.0291 | 6.884 | 4.166 | 4.253 | 0.088 | 0.491 | 0.006 | 0.024 | Unannotated | NA | NA |
| metab_11093 | Unannotated feature | neg | 804.4531 | 0.594 | 6.245 | 6.185 | -0.060 | -0.491 | 0.006 | 0.024 | Unannotated | NA | NA |
| metab_651 | Unannotated feature | pos | 120.0447 | 2.296 | 4.113 | 4.495 | 0.383 | 0.491 | 0.006 | 0.024 | Unannotated | NA | NA |
| metab_9373 | Unannotated feature | neg | 276.0042 | 3.554 | 4.156 | 4.037 | -0.119 | -0.491 | 0.006 | 0.024 | Unannotated | NA | NA |
| metab_5326 | Unannotated feature | neg | 516.8390 | 0.570 | 4.539 | 4.745 | 0.205 | 0.491 | 0.006 | 0.024 | Unannotated | NA | NA |
| metab_2170 | Unannotated feature | pos | 446.9959 | 6.420 | 4.874 | 4.997 | 0.124 | 0.491 | 0.006 | 0.024 | Unannotated | NA | NA |
| metab_2221 | Unannotated feature | pos | 800.7197 | 0.567 | 5.137 | 5.064 | -0.074 | -0.491 | 0.006 | 0.024 | Unannotated | NA | NA |
| metab_5312 | Unannotated feature | neg | 754.8532 | 0.562 | 4.420 | 4.132 | -0.288 | -0.491 | 0.006 | 0.024 | Unannotated | NA | NA |
| metab_11199 | Unannotated feature | neg | 454.7037 | 0.594 | 6.164 | 6.107 | -0.057 | -0.491 | 0.006 | 0.024 | Unannotated | NA | NA |
| metab_6392 | Sulfuric acid 4-methoxyphenyl ester | neg | 203.0018 | 4.059 | 5.463 | 6.023 | 0.560 | 0.491 | 0.006 | 0.024 | Tentative | Organic acids and derivatives | Organic sulfuric acids and derivatives |
| metab_4896 | Unannotated feature | pos | 222.0795 | 1.933 | 4.328 | 4.658 | 0.330 | 0.491 | 0.006 | 0.024 | Unannotated | NA | NA |
| metab_4424 | Unannotated feature | pos | 656.8045 | 0.559 | 4.564 | 4.749 | 0.185 | 0.491 | 0.006 | 0.024 | Unannotated | NA | NA |
| metab_11268 | Unannotated feature | neg | 687.7574 | 0.570 | 4.962 | 4.857 | -0.105 | -0.491 | 0.006 | 0.024 | Unannotated | NA | NA |
| metab_7351 | Unannotated feature | neg | 181.0715 | 6.541 | 4.202 | 4.420 | 0.218 | 0.491 | 0.006 | 0.024 | Unannotated | NA | NA |
| metab_1472 | Unannotated feature | pos | 488.3589 | 6.010 | 5.472 | 5.274 | -0.198 | -0.491 | 0.006 | 0.024 | Unannotated | NA | NA |
| metab_8812 | Nitrendipine | neg | 359.1257 | 5.462 | 4.836 | 4.360 | -0.477 | -0.491 | 0.006 | 0.024 | Tentative | Organoheterocyclic compounds | Pyridines and derivatives |
| metab_10369 | Unannotated feature | neg | 201.0347 | 0.731 | 5.336 | 4.910 | -0.426 | -0.491 | 0.006 | 0.024 | Unannotated | NA | NA |
| metab_93 | Unannotated feature | pos | 128.1072 | 0.512 | 3.462 | 3.580 | 0.118 | 0.491 | 0.006 | 0.024 | Unannotated | NA | NA |
| metab_1797 | Unannotated feature | pos | 136.0619 | 7.835 | 4.984 | 4.955 | -0.029 | -0.491 | 0.006 | 0.024 | Unannotated | NA | NA |
| metab_1309 | Unannotated feature | pos | 312.3625 | 5.879 | 4.486 | 4.599 | 0.113 | 0.491 | 0.006 | 0.024 | Unannotated | NA | NA |
| metab_1299 | Unannotated feature | pos | 568.3997 | 5.871 | 5.192 | 5.056 | -0.136 | -0.491 | 0.006 | 0.024 | Unannotated | NA | NA |
| metab_1576 | Unannotated feature | pos | 429.8194 | 6.420 | 4.937 | 5.035 | 0.098 | 0.491 | 0.006 | 0.024 | Unannotated | NA | NA |
| metab_10055 | Unannotated feature | neg | 288.8775 | 1.390 | 5.436 | 5.341 | -0.094 | -0.491 | 0.006 | 0.024 | Unannotated | NA | NA |
| metab_5157 | Triethylenemelamine | pos | 237.1458 | 6.041 | 5.387 | 5.439 | 0.052 | 0.491 | 0.006 | 0.024 | Tentative | NA | NA |
| metab_2888 | Unannotated feature | pos | 125.1124 | 5.060 | 4.107 | 4.158 | 0.051 | 0.486 | 0.006 | 0.025 | Unannotated | NA | NA |
| metab_808 | Tyrosylhydroxyproline | pos | 295.1288 | 3.058 | 5.293 | 5.518 | 0.225 | 0.486 | 0.006 | 0.025 | Tentative | NA | NA |
| metab_307 | Unannotated feature | pos | 218.9198 | 0.776 | 4.299 | 4.194 | -0.105 | -0.486 | 0.006 | 0.025 | Unannotated | NA | NA |
| metab_9790 | Aminocatechol N-acetate sulfate | neg | 227.9973 | 2.565 | 3.878 | 4.732 | 0.854 | 0.486 | 0.006 | 0.025 | Tentative | NA | NA |
| metab_3215 | Unannotated feature | pos | 193.0629 | 3.167 | 3.701 | 4.374 | 0.673 | 0.486 | 0.006 | 0.025 | Unannotated | NA | NA |
| metab_3786 | Unannotated feature | pos | 187.1264 | 1.577 | 4.962 | 4.892 | -0.070 | -0.486 | 0.006 | 0.025 | Unannotated | NA | NA |
| metab_3415 | Hypoglycin | pos | 106.0655 | 2.428 | 3.075 | 3.705 | 0.630 | 0.486 | 0.006 | 0.025 | Tentative | Organic acids and derivatives | Carboxylic acids and derivatives |
| metab_1223 | Unannotated feature | pos | 418.1932 | 1.956 | 6.637 | 6.795 | 0.158 | 0.486 | 0.006 | 0.025 | Unannotated | NA | NA |
| metab_2637 | Unannotated feature | pos | 464.3325 | 5.824 | 5.355 | 5.291 | -0.064 | -0.486 | 0.006 | 0.025 | Unannotated | NA | NA |
| metab_1029 | Unannotated feature | pos | 685.2478 | 4.761 | 4.287 | 4.905 | 0.618 | 0.486 | 0.006 | 0.025 | Unannotated | NA | NA |
| metab_11216 | Unannotated feature | neg | 233.0376 | 5.834 | 3.040 | 2.436 | -0.604 | -0.486 | 0.006 | 0.025 | Unannotated | NA | NA |
| metab_3859 | Unannotated feature | pos | 99.5316 | 0.986 | 3.969 | 4.088 | 0.120 | 0.486 | 0.006 | 0.025 | Unannotated | NA | NA |
| metab_8939 | Unannotated feature | neg | 328.9322 | 4.969 | 3.013 | 3.849 | 0.836 | 0.486 | 0.006 | 0.025 | Unannotated | NA | NA |
| metab_4272 | Unannotated feature | pos | 564.6744 | 0.591 | 5.286 | 5.202 | -0.085 | -0.486 | 0.006 | 0.025 | Unannotated | NA | NA |
| metab_5608 | Unannotated feature | neg | 174.8392 | 0.896 | 4.972 | 4.871 | -0.101 | -0.486 | 0.006 | 0.025 | Unannotated | NA | NA |
| metab_970 | Unannotated feature | pos | 143.0340 | 4.294 | 4.351 | 4.309 | -0.043 | -0.486 | 0.006 | 0.025 | Unannotated | NA | NA |
| metab_8133 | Unannotated feature | neg | 75.0079 | 6.149 | 2.950 | 3.291 | 0.341 | 0.486 | 0.006 | 0.025 | Unannotated | NA | NA |
| metab_2553 | Rishitin | pos | 240.1959 | 5.871 | 4.757 | 4.800 | 0.043 | 0.486 | 0.006 | 0.025 | Tentative | NA | NA |
| metab_4955 | Unannotated feature | pos | 124.0483 | 0.652 | 4.250 | 4.562 | 0.312 | 0.486 | 0.006 | 0.025 | Unannotated | NA | NA |
| metab_1975 | Unannotated feature | pos | 770.8505 | 7.293 | 5.642 | 5.594 | -0.048 | -0.486 | 0.006 | 0.025 | Unannotated | NA | NA |
| metab_10860 | Unannotated feature | neg | 534.8537 | 0.554 | 3.755 | 4.142 | 0.387 | 0.486 | 0.006 | 0.025 | Unannotated | NA | NA |
| metab_2098 | Unannotated feature | pos | 115.8001 | 7.107 | 4.765 | 4.804 | 0.040 | 0.486 | 0.006 | 0.025 | Unannotated | NA | NA |
| metab_9601 | Unannotated feature | neg | 142.0656 | 3.160 | 4.505 | 4.204 | -0.300 | -0.486 | 0.006 | 0.025 | Unannotated | NA | NA |
| metab_9368 | Unannotated feature | neg | 129.0820 | 3.562 | 4.125 | 3.999 | -0.125 | -0.486 | 0.006 | 0.025 | Unannotated | NA | NA |
| metab_1856 | Unannotated feature | pos | 182.1024 | 7.672 | 4.859 | 4.791 | -0.068 | -0.486 | 0.006 | 0.025 | Unannotated | NA | NA |
| metab_922 | L-Hexanoylcarnitine | pos | 260.1856 | 3.900 | 5.863 | 5.784 | -0.079 | -0.486 | 0.006 | 0.025 | Strong putative | NA | NA |
| metab_949 | Unannotated feature | pos | 177.0871 | 0.854 | 4.244 | 4.470 | 0.225 | 0.486 | 0.006 | 0.025 | Unannotated | NA | NA |
| metab_8343 | Unannotated feature | neg | 321.1715 | 5.827 | 4.240 | 3.787 | -0.454 | -0.486 | 0.006 | 0.025 | Unannotated | NA | NA |
| metab_8428 | Unannotated feature | neg | 349.1680 | 5.771 | 4.758 | 3.887 | -0.871 | -0.486 | 0.006 | 0.025 | Unannotated | NA | NA |
| metab_4963 | Unannotated feature | pos | 309.9239 | 0.675 | 3.352 | 4.029 | 0.678 | 0.486 | 0.006 | 0.025 | Unannotated | NA | NA |
| metab_1148 | Unannotated feature | pos | 574.3794 | 5.664 | 4.453 | 3.942 | -0.510 | -0.486 | 0.006 | 0.025 | Unannotated | NA | NA |
| metab_1798 | Unannotated feature | pos | 120.7567 | 7.843 | 4.459 | 4.490 | 0.031 | 0.486 | 0.006 | 0.025 | Unannotated | NA | NA |
| metab_5394 | Unannotated feature | neg | 325.8549 | 0.016 | 5.233 | 5.202 | -0.031 | -0.486 | 0.006 | 0.025 | Unannotated | NA | NA |
| metab_2293 | Unannotated feature | pos | 512.4159 | 6.088 | 5.088 | 5.182 | 0.094 | 0.486 | 0.006 | 0.025 | Unannotated | NA | NA |
| metab_1473 | Unannotated feature | pos | 702.5361 | 6.010 | 5.483 | 5.393 | -0.090 | -0.486 | 0.006 | 0.025 | Unannotated | NA | NA |
| metab_4505 | Unannotated feature | pos | 599.0011 | 0.545 | 4.928 | 4.843 | -0.086 | -0.486 | 0.006 | 0.025 | Unannotated | NA | NA |
| metab_4448 | Unannotated feature | pos | 948.8059 | 0.551 | 5.315 | 5.233 | -0.082 | -0.486 | 0.006 | 0.025 | Unannotated | NA | NA |
| metab_10065 | Unannotated feature | neg | 149.0450 | 1.366 | 3.552 | 3.903 | 0.351 | 0.486 | 0.006 | 0.025 | Unannotated | NA | NA |
| metab_1808 | Unannotated feature | pos | 139.0390 | 7.959 | 4.573 | 4.514 | -0.060 | -0.486 | 0.006 | 0.025 | Unannotated | NA | NA |
| metab_3597 | Unannotated feature | pos | 340.1755 | 3.275 | 3.373 | 4.378 | 1.005 | 0.486 | 0.006 | 0.025 | Unannotated | NA | NA |
| metab_6650 | Phloretate | neg | 211.0612 | 5.077 | 3.389 | 4.016 | 0.627 | 0.486 | 0.006 | 0.025 | Tentative | Phenylpropanoids and polyketides | Phenylpropanoic acids |
| metab_4379 | Unannotated feature | pos | 610.8029 | 0.567 | 4.642 | 4.494 | -0.148 | -0.486 | 0.006 | 0.025 | Unannotated | NA | NA |
| metab_388 | Unannotated feature | pos | 222.8792 | 0.978 | 4.734 | 4.833 | 0.099 | 0.486 | 0.006 | 0.025 | Unannotated | NA | NA |
| metab_2485 | Unannotated feature | pos | 103.0546 | 5.902 | 3.877 | 4.009 | 0.132 | 0.486 | 0.006 | 0.025 | Unannotated | NA | NA |
| metab_3844 | (2E)-3-[3-(sulfooxy)phenyl]prop-2-enoic acid | pos | 262.0380 | 1.134 | 3.769 | 4.580 | 0.811 | 0.486 | 0.006 | 0.025 | Tentative | Phenylpropanoids and polyketides | Cinnamic acids and derivatives |
| metab_2619 | Unannotated feature | pos | 749.5123 | 5.832 | 5.355 | 5.177 | -0.178 | -0.486 | 0.006 | 0.025 | Unannotated | NA | NA |
| metab_4671 | Unannotated feature | pos | 641.7772 | 5.855 | 5.515 | 5.303 | -0.211 | -0.486 | 0.006 | 0.025 | Unannotated | NA | NA |
| metab_6608 | Unannotated feature | neg | 236.9495 | 0.833 | 3.361 | 4.329 | 0.968 | 0.486 | 0.006 | 0.025 | Unannotated | NA | NA |
| metab_2994 | 5-Hydroxyconiferyl alcohol | pos | 197.0809 | 4.401 | 3.364 | 3.770 | 0.407 | 0.486 | 0.006 | 0.025 | Tentative | Benzenoids | Phenols |
| metab_165 | Unannotated feature | pos | 588.8177 | 0.559 | 4.607 | 4.835 | 0.229 | 0.486 | 0.006 | 0.025 | Unannotated | NA | NA |
| metab_3710 | D-Norvaline | pos | 159.1129 | 1.894 | 5.721 | 5.454 | -0.267 | -0.486 | 0.006 | 0.025 | Tentative | Organic acids and derivatives | Carboxylic acids and derivatives |
| metab_5492 | Unannotated feature | neg | 164.9746 | 0.723 | 4.246 | 4.018 | -0.228 | -0.486 | 0.006 | 0.025 | Unannotated | NA | NA |
| metab_2781 | Unannotated feature | pos | 443.3085 | 5.649 | 4.947 | 4.808 | -0.138 | -0.486 | 0.006 | 0.025 | Unannotated | NA | NA |
| metab_410 | Unannotated feature | pos | 175.0037 | 1.227 | 2.995 | 3.744 | 0.749 | 0.486 | 0.006 | 0.025 | Unannotated | NA | NA |
| metab_2041 | Unannotated feature | pos | 289.9314 | 7.261 | 5.009 | 5.114 | 0.106 | 0.482 | 0.007 | 0.026 | Unannotated | NA | NA |
| metab_5052 | Unannotated feature | pos | 231.9725 | 0.877 | 4.676 | 4.530 | -0.146 | -0.482 | 0.007 | 0.026 | Unannotated | NA | NA |
| metab_9808 | Dihydroxyphenylacetic acid sulfate | neg | 246.9922 | 2.530 | 3.986 | 4.214 | 0.228 | 0.482 | 0.007 | 0.026 | Tentative | NA | NA |
| metab_5308 | Unannotated feature | neg | 714.8455 | 0.562 | 5.378 | 5.291 | -0.087 | -0.482 | 0.007 | 0.026 | Unannotated | NA | NA |
| metab_5381 | Unannotated feature | neg | 595.5846 | 0.598 | 5.342 | 5.282 | -0.061 | -0.482 | 0.007 | 0.026 | Unannotated | NA | NA |
| metab_2678 | Unannotated feature | pos | 488.8227 | 5.771 | 4.283 | 3.883 | -0.399 | -0.482 | 0.007 | 0.026 | Unannotated | NA | NA |
| metab_4059 | 1-Pyrroline-4-hydroxy-2-carboxylate | pos | 171.0765 | 0.760 | 4.221 | 4.916 | 0.695 | 0.482 | 0.007 | 0.026 | Tentative | Organoheterocyclic compounds | Pyrrolines |
| metab_1040 | Unannotated feature | pos | 347.2174 | 4.869 | 4.754 | 4.707 | -0.047 | -0.482 | 0.007 | 0.026 | Unannotated | NA | NA |
| metab_9583 | Unannotated feature | neg | 288.0609 | 3.168 | 4.454 | 4.038 | -0.416 | -0.482 | 0.007 | 0.026 | Unannotated | NA | NA |
| metab_4892 | 3,4-Methyleneadipic acid | pos | 188.0918 | 1.949 | 4.561 | 4.639 | 0.079 | 0.482 | 0.007 | 0.026 | Tentative | NA | NA |
| metab_4043 | Unannotated feature | pos | 266.8494 | 0.776 | 4.630 | 4.565 | -0.065 | -0.482 | 0.007 | 0.026 | Unannotated | NA | NA |
| metab_5128 | Bis(2-ethylhexyl)phthalate | pos | 391.2841 | 7.835 | 5.708 | 5.679 | -0.030 | -0.482 | 0.007 | 0.026 | Strong putative | NA | NA |
| metab_3956 | Unannotated feature | pos | 315.1611 | 4.171 | 5.478 | 5.286 | -0.192 | -0.482 | 0.007 | 0.026 | Unannotated | NA | NA |
| metab_5434 | Unannotated feature | neg | 105.9351 | 0.016 | 3.983 | 3.916 | -0.067 | -0.482 | 0.007 | 0.026 | Unannotated | NA | NA |
| metab_6774 | Unannotated feature | neg | 628.7296 | 5.523 | 5.944 | 6.150 | 0.206 | 0.482 | 0.007 | 0.026 | Unannotated | NA | NA |
| metab_10620 | Unannotated feature | neg | 816.4779 | 0.594 | 5.445 | 5.329 | -0.116 | -0.482 | 0.007 | 0.026 | Unannotated | NA | NA |
| metab_163 | Unannotated feature | pos | 463.0263 | 0.559 | 4.928 | 4.799 | -0.129 | -0.482 | 0.007 | 0.026 | Unannotated | NA | NA |
| metab_1955 | Unannotated feature | pos | 772.6305 | 0.575 | 5.244 | 5.134 | -0.110 | -0.482 | 0.007 | 0.026 | Unannotated | NA | NA |
| metab_11109 | Unannotated feature | neg | 239.1291 | 5.675 | 3.896 | 4.275 | 0.379 | 0.482 | 0.007 | 0.026 | Unannotated | NA | NA |
| metab_4594 | Unannotated feature | pos | 111.1171 | 0.279 | 3.767 | 3.893 | 0.126 | 0.482 | 0.007 | 0.026 | Unannotated | NA | NA |
| metab_558 | Indan-1-ol | pos | 135.0806 | 5.894 | 6.018 | 6.102 | 0.084 | 0.482 | 0.007 | 0.026 | Tentative | NA | NA |
| metab_147 | Unannotated feature | pos | 736.8568 | 0.551 | 6.109 | 6.016 | -0.094 | -0.482 | 0.007 | 0.026 | Unannotated | NA | NA |
| metab_4028 | Unannotated feature | pos | 204.0867 | 0.792 | 3.622 | 3.882 | 0.260 | 0.482 | 0.007 | 0.026 | Unannotated | NA | NA |
| metab_7278 | Unannotated feature | neg | 678.3092 | 6.149 | 5.016 | 5.349 | 0.332 | 0.482 | 0.007 | 0.026 | Unannotated | NA | NA |
| metab_5541 | Unannotated feature | neg | 520.9111 | 0.543 | 6.839 | 6.787 | -0.052 | -0.482 | 0.007 | 0.026 | Unannotated | NA | NA |
| metab_1500 | Unannotated feature | pos | 570.4577 | 6.095 | 4.828 | 5.007 | 0.179 | 0.482 | 0.007 | 0.026 | Unannotated | NA | NA |
| metab_4262 | Unannotated feature | pos | 754.5707 | 0.591 | 5.226 | 5.147 | -0.079 | -0.482 | 0.007 | 0.026 | Unannotated | NA | NA |
| metab_4357 | Unannotated feature | pos | 742.7615 | 0.567 | 5.131 | 5.072 | -0.059 | -0.482 | 0.007 | 0.026 | Unannotated | NA | NA |
| metab_387 | Unannotated feature | pos | 83.4486 | 0.963 | 2.947 | 3.118 | 0.171 | 0.482 | 0.007 | 0.026 | Unannotated | NA | NA |
| metab_1988 | Unannotated feature | pos | 780.6619 | 0.575 | 5.483 | 5.399 | -0.084 | -0.482 | 0.007 | 0.026 | Unannotated | NA | NA |
| metab_2279 | Sphinganine (C20) | pos | 394.3529 | 6.134 | 3.927 | 4.506 | 0.579 | 0.482 | 0.007 | 0.026 | Tentative | Organic oxygen compounds | Organooxygen compounds |
| metab_1128 | 12(S)-HETrE | pos | 345.2382 | 5.626 | 6.155 | 6.187 | 0.033 | 0.482 | 0.007 | 0.026 | Tentative | NA | NA |
| metab_8561 | (S)-9-Hydroxy-10-undecenoic acid | neg | 245.1397 | 5.691 | 4.053 | 4.390 | 0.337 | 0.482 | 0.007 | 0.026 | Tentative | Organic acids and derivatives | Hydroxy acids and derivatives |
| metab_6890 | Unannotated feature | neg | 227.1289 | 5.651 | 3.675 | 3.892 | 0.218 | 0.482 | 0.007 | 0.026 | Unannotated | NA | NA |
| metab_3057 | Unannotated feature | pos | 187.1264 | 4.055 | 4.407 | 4.513 | 0.106 | 0.482 | 0.007 | 0.026 | Unannotated | NA | NA |
| metab_5846 | Unannotated feature | neg | 177.0224 | 2.302 | 4.300 | 4.781 | 0.481 | 0.482 | 0.007 | 0.026 | Unannotated | NA | NA |
| metab_1303 | Unannotated feature | pos | 670.4645 | 5.871 | 5.168 | 4.879 | -0.289 | -0.482 | 0.007 | 0.026 | Unannotated | NA | NA |
| metab_10897 | Unannotated feature | neg | 868.8389 | 0.543 | 5.670 | 5.540 | -0.130 | -0.482 | 0.007 | 0.026 | Unannotated | NA | NA |
| metab_7832 | Unannotated feature | neg | 541.3354 | 7.153 | 5.862 | 5.759 | -0.103 | -0.482 | 0.007 | 0.026 | Unannotated | NA | NA |
| metab_10400 | Unannotated feature | neg | 130.0615 | 0.707 | 4.106 | 3.573 | -0.532 | -0.482 | 0.007 | 0.026 | Unannotated | NA | NA |
| metab_4413 | Unannotated feature | pos | 799.8296 | 0.559 | 4.846 | 4.715 | -0.130 | -0.482 | 0.007 | 0.026 | Unannotated | NA | NA |
| metab_9653 | Unannotated feature | neg | 500.6366 | 0.621 | 6.683 | 6.645 | -0.038 | -0.482 | 0.007 | 0.026 | Unannotated | NA | NA |
| metab_1211 | 3H-Pyrazol-3-one, 1,2-dihydro-4-(hydroxymethyl)-1,5-dimethyl-2-phenyl- | pos | 251.1391 | 5.778 | 4.087 | 3.415 | -0.672 | -0.482 | 0.007 | 0.026 | Tentative | Organoheterocyclic compounds | Azoles |
| metab_5362 | Unannotated feature | neg | 487.6989 | 0.587 | 5.388 | 5.294 | -0.094 | -0.482 | 0.007 | 0.026 | Unannotated | NA | NA |
| metab_1509 | Unannotated feature | pos | 437.3262 | 6.134 | 5.069 | 4.956 | -0.113 | -0.478 | 0.007 | 0.027 | Unannotated | NA | NA |
| metab_805 | 2,3,4,5-Tetrahydrodipicolinate | pos | 202.0710 | 3.043 | 2.977 | 4.035 | 1.059 | 0.478 | 0.007 | 0.027 | Tentative | NA | NA |
| metab_7541 | Unannotated feature | neg | 227.8945 | 7.173 | 4.820 | 4.795 | -0.025 | -0.478 | 0.007 | 0.027 | Unannotated | NA | NA |
| metab_5121 | Bis(2-ethylhexyl)phthalate | pos | 391.2841 | 0.411 | 5.751 | 5.769 | 0.019 | 0.478 | 0.007 | 0.027 | Moderate putative | NA | NA |
| metab_4189 | 1,1,1,2,2-Pentafluoro-7-phenylheptan-3-one | pos | 263.0849 | 0.636 | 5.362 | 5.539 | 0.178 | 0.478 | 0.007 | 0.027 | Tentative | Benzenoids | Benzene and substituted derivatives |
| metab_10648 | Unannotated feature | neg | 663.5729 | 0.587 | 5.064 | 4.883 | -0.182 | -0.478 | 0.007 | 0.027 | Unannotated | NA | NA |
| metab_4782 | Unannotated feature | pos | 347.0874 | 4.224 | 3.255 | 6.889 | 3.634 | 0.478 | 0.007 | 0.027 | Unannotated | NA | NA |
| metab_1279 | Unannotated feature | pos | 593.0794 | 5.855 | 5.210 | 5.109 | -0.100 | -0.478 | 0.007 | 0.027 | Unannotated | NA | NA |
| metab_1422 | Unannotated feature | pos | 707.4888 | 5.941 | 5.395 | 5.150 | -0.245 | -0.478 | 0.007 | 0.027 | Unannotated | NA | NA |
| metab_10212 | P-Cresol glucuronide | neg | 283.0828 | 4.513 | 4.573 | 5.153 | 0.580 | 0.478 | 0.007 | 0.027 | Tentative | Organic oxygen compounds | Organooxygen compounds |
| metab_9970 | Unannotated feature | neg | 286.8972 | 2.087 | 4.601 | 4.668 | 0.067 | 0.478 | 0.007 | 0.027 | Unannotated | NA | NA |
| metab_1641 | Unannotated feature | pos | 165.0758 | 0.403 | 5.102 | 5.121 | 0.020 | 0.478 | 0.007 | 0.027 | Unannotated | NA | NA |
| metab_2573 | Androstanediol | pos | 257.2264 | 5.863 | 4.236 | 5.055 | 0.819 | 0.478 | 0.007 | 0.027 | Tentative | Lipids and lipid-like molecules | Steroids and steroid derivatives |
| metab_1529 | PC(14:0/0:0) | pos | 490.2902 | 6.203 | 4.788 | 4.547 | -0.242 | -0.478 | 0.007 | 0.027 | Moderate putative | Lipids and lipid-like molecules | Glycerophospholipids |
| metab_5264 | Unannotated feature | neg | 707.8019 | 0.554 | 3.874 | 4.245 | 0.371 | 0.478 | 0.007 | 0.027 | Unannotated | NA | NA |
| metab_2317 | Unannotated feature | pos | 531.2743 | 6.034 | 4.818 | 4.942 | 0.124 | 0.478 | 0.007 | 0.027 | Unannotated | NA | NA |
| metab_7319 | Unannotated feature | neg | 139.0063 | 6.289 | 3.366 | 3.578 | 0.212 | 0.478 | 0.007 | 0.027 | Unannotated | NA | NA |
| metab_720 | 1-Acyl-sn-glycero-3-phosphocholine | pos | 496.3399 | 6.389 | 8.436 | 8.386 | -0.050 | -0.478 | 0.007 | 0.027 | Strong putative | NA | NA |
| metab_5385 | Unannotated feature | neg | 92.6813 | 0.601 | 5.121 | 5.066 | -0.055 | -0.478 | 0.007 | 0.027 | Unannotated | NA | NA |
| metab_2701 | Unannotated feature | pos | 618.9257 | 5.748 | 4.417 | 4.122 | -0.295 | -0.478 | 0.007 | 0.027 | Unannotated | NA | NA |
| metab_7244 | Unannotated feature | neg | 226.9768 | 0.865 | 5.643 | 5.593 | -0.051 | -0.478 | 0.007 | 0.027 | Unannotated | NA | NA |
| metab_2085 | Unannotated feature | pos | 603.2993 | 7.192 | 5.394 | 5.242 | -0.151 | -0.478 | 0.007 | 0.027 | Unannotated | NA | NA |
| metab_3253 | Icofungipen | pos | 106.0655 | 3.004 | 3.282 | 3.736 | 0.455 | 0.478 | 0.007 | 0.027 | Tentative | Organic acids and derivatives | Carboxylic acids and derivatives |
| metab_2352 | Unannotated feature | pos | 243.0195 | 5.979 | 4.888 | 4.848 | -0.040 | -0.478 | 0.007 | 0.027 | Unannotated | NA | NA |
| metab_4838 | Diethanolamine | pos | 88.0762 | 7.293 | 5.135 | 5.161 | 0.026 | 0.478 | 0.007 | 0.027 | Strong putative | NA | NA |
| metab_620 | Unannotated feature | pos | 630.4804 | 5.964 | 6.410 | 6.358 | -0.052 | -0.478 | 0.007 | 0.027 | Unannotated | NA | NA |
| metab_8171 | Unannotated feature | neg | 377.2706 | 6.024 | 4.355 | 4.014 | -0.341 | -0.478 | 0.007 | 0.027 | Unannotated | NA | NA |
| metab_8971 | Gentiobiosyl 2-methyl-6-oxo-2E,4E-heptadienoate | neg | 459.1519 | 4.853 | 4.082 | 4.723 | 0.641 | 0.478 | 0.007 | 0.027 | Tentative | Organic oxygen compounds | Organooxygen compounds |
| metab_4065 | Unannotated feature | pos | 142.9007 | 0.753 | 4.626 | 4.569 | -0.056 | -0.478 | 0.007 | 0.027 | Unannotated | NA | NA |
| metab_10096 | Unannotated feature | neg | 144.9913 | 1.206 | 3.664 | 2.811 | -0.852 | -0.478 | 0.007 | 0.027 | Unannotated | NA | NA |
| metab_1577 | Unannotated feature | pos | 441.8389 | 6.420 | 4.840 | 4.940 | 0.100 | 0.478 | 0.007 | 0.027 | Unannotated | NA | NA |
| metab_3483 | 2-Ethyl-3-methylpyrazine, 9CI | pos | 123.0919 | 2.241 | 4.628 | 4.135 | -0.493 | -0.478 | 0.007 | 0.027 | Tentative | Organoheterocyclic compounds | Diazines |
| metab_11160 | Unannotated feature | neg | 222.1137 | 5.787 | 4.309 | 4.510 | 0.201 | 0.478 | 0.007 | 0.027 | Unannotated | NA | NA |
| metab_3604 | Unannotated feature | pos | 172.0969 | 2.019 | 4.323 | 4.489 | 0.166 | 0.478 | 0.007 | 0.027 | Unannotated | NA | NA |
| metab_279 | Unannotated feature | pos | 187.7636 | 0.729 | 4.060 | 3.164 | -0.897 | -0.478 | 0.007 | 0.027 | Unannotated | NA | NA |
| metab_6742 | Unannotated feature | neg | 219.8918 | 5.470 | 5.961 | 5.941 | -0.020 | -0.478 | 0.007 | 0.027 | Unannotated | NA | NA |
| metab_1348 | Unannotated feature | pos | 104.0625 | 5.902 | 4.350 | 4.484 | 0.133 | 0.478 | 0.007 | 0.027 | Unannotated | NA | NA |
| metab_10358 | Ascorbic acid 2-sulfate | neg | 254.9820 | 0.739 | 4.295 | 4.647 | 0.352 | 0.478 | 0.007 | 0.027 | Tentative | Organoheterocyclic compounds | Dihydrofurans |
| metab_7610 | Unannotated feature | neg | 147.0657 | 2.445 | 3.593 | 4.248 | 0.655 | 0.478 | 0.007 | 0.027 | Unannotated | NA | NA |
| metab_3878 | Unannotated feature | pos | 133.0801 | 0.892 | 4.032 | 3.642 | -0.390 | -0.478 | 0.007 | 0.027 | Unannotated | NA | NA |
| metab_6092 | Unannotated feature | neg | 130.0656 | 3.160 | 3.326 | 3.035 | -0.292 | -0.478 | 0.007 | 0.027 | Unannotated | NA | NA |
| metab_9322 | Unannotated feature | neg | 624.7385 | 0.636 | 5.493 | 5.356 | -0.137 | -0.478 | 0.007 | 0.027 | Unannotated | NA | NA |
| metab_81 | Unannotated feature | pos | 180.0656 | 0.465 | 4.978 | 5.010 | 0.032 | 0.478 | 0.007 | 0.027 | Unannotated | NA | NA |
| metab_3777 | Pentan-2-one | pos | 128.1072 | 1.663 | 4.138 | 4.420 | 0.282 | 0.478 | 0.007 | 0.027 | Tentative | Organic oxygen compounds | Organooxygen compounds |
| metab_1172 | Unannotated feature | pos | 611.4160 | 5.717 | 4.573 | 4.287 | -0.286 | -0.478 | 0.007 | 0.027 | Unannotated | NA | NA |
| metab_4310 | Unannotated feature | pos | 936.4763 | 0.575 | 4.842 | 4.587 | -0.255 | -0.478 | 0.007 | 0.027 | Unannotated | NA | NA |
| metab_4332 | Unannotated feature | pos | 528.7705 | 0.575 | 5.457 | 5.406 | -0.051 | -0.478 | 0.007 | 0.027 | Unannotated | NA | NA |
| metab_6064 | Unannotated feature | neg | 365.1361 | 3.104 | 4.397 | 4.177 | -0.220 | -0.474 | 0.008 | 0.029 | Unannotated | NA | NA |
| metab_26 | Unannotated feature | pos | 262.1285 | 1.847 | 3.911 | 4.320 | 0.409 | 0.474 | 0.008 | 0.029 | Unannotated | NA | NA |
| metab_6469 | Unannotated feature | neg | 336.0732 | 4.306 | 3.709 | 4.565 | 0.856 | 0.474 | 0.008 | 0.029 | Unannotated | NA | NA |
| metab_4302 | Unannotated feature | pos | 663.2673 | 4.769 | 6.033 | 6.319 | 0.286 | 0.474 | 0.008 | 0.029 | Unannotated | NA | NA |
| metab_6756 | Unannotated feature | neg | 223.0460 | 5.506 | 4.269 | 4.378 | 0.109 | 0.474 | 0.008 | 0.029 | Unannotated | NA | NA |
| metab_203 | Unannotated feature | pos | 287.9000 | 0.606 | 4.168 | 3.894 | -0.275 | -0.474 | 0.008 | 0.029 | Unannotated | NA | NA |
| metab_4391 | Unannotated feature | pos | 638.7250 | 5.114 | 6.111 | 6.429 | 0.318 | 0.474 | 0.008 | 0.029 | Unannotated | NA | NA |
| metab_4799 | Unannotated feature | pos | 200.0958 | 7.324 | 5.624 | 5.599 | -0.025 | -0.474 | 0.008 | 0.029 | Unannotated | NA | NA |
| metab_924 | Unannotated feature | pos | 102.3761 | 3.916 | 3.931 | 4.036 | 0.106 | 0.474 | 0.008 | 0.029 | Unannotated | NA | NA |
| metab_2438 | Unannotated feature | pos | 630.4452 | 5.917 | 4.585 | 4.311 | -0.274 | -0.474 | 0.008 | 0.029 | Unannotated | NA | NA |
| metab_2044 | Unannotated feature | pos | 206.9696 | 7.261 | 4.577 | 4.684 | 0.107 | 0.474 | 0.008 | 0.029 | Unannotated | NA | NA |
| metab_2097 | Unannotated feature | pos | 211.1936 | 7.114 | 4.576 | 4.510 | -0.066 | -0.474 | 0.008 | 0.029 | Unannotated | NA | NA |
| metab_4453 | Unannotated feature | pos | 872.8316 | 0.551 | 5.890 | 5.828 | -0.063 | -0.474 | 0.008 | 0.029 | Unannotated | NA | NA |
| metab_8130 | Unannotated feature | neg | 144.4441 | 6.157 | 3.672 | 3.363 | -0.309 | -0.474 | 0.008 | 0.029 | Unannotated | NA | NA |
| metab_7630 | Unannotated feature | neg | 91.7225 | 7.932 | 2.689 | 2.519 | -0.171 | -0.474 | 0.008 | 0.029 | Unannotated | NA | NA |
| metab_3672 | Unannotated feature | pos | 105.0427 | 1.949 | 3.985 | 3.896 | -0.090 | -0.474 | 0.008 | 0.029 | Unannotated | NA | NA |
| metab_322 | L-Valine, N-(2-hydroxy-3-butenyl)- | pos | 188.1282 | 0.807 | 5.430 | 5.118 | -0.312 | -0.474 | 0.008 | 0.029 | Tentative | Organic acids and derivatives | Carboxylic acids and derivatives |
| metab_324 | Unannotated feature | pos | 413.2660 | 0.807 | 4.509 | 4.464 | -0.046 | -0.474 | 0.008 | 0.029 | Unannotated | NA | NA |
| metab_8492 | Unannotated feature | neg | 269.1399 | 5.731 | 4.142 | 4.449 | 0.307 | 0.474 | 0.008 | 0.029 | Unannotated | NA | NA |
| metab_5940 | Unannotated feature | neg | 173.0816 | 2.636 | 2.990 | 3.771 | 0.781 | 0.474 | 0.008 | 0.029 | Unannotated | NA | NA |
| metab_1237 | Unannotated feature | pos | 676.4625 | 5.817 | 5.296 | 5.057 | -0.239 | -0.474 | 0.008 | 0.029 | Unannotated | NA | NA |
| metab_3927 | Dimethyl sulfone | pos | 95.0166 | 0.861 | 3.845 | 4.268 | 0.423 | 0.474 | 0.008 | 0.029 | Moderate putative | Organosulfur compounds | Sulfonyls |
| metab_4803 | Unannotated feature | pos | 260.1532 | 7.331 | 4.482 | 4.341 | -0.142 | -0.474 | 0.008 | 0.029 | Unannotated | NA | NA |
| metab_4259 | Unannotated feature | pos | 868.4894 | 0.591 | 5.090 | 4.959 | -0.131 | -0.474 | 0.008 | 0.029 | Unannotated | NA | NA |
| metab_5303 | Unannotated feature | neg | 652.8717 | 0.562 | 4.289 | 4.161 | -0.128 | -0.474 | 0.008 | 0.029 | Unannotated | NA | NA |
| metab_7556 | Unannotated feature | neg | 82.9711 | 7.189 | 4.886 | 4.860 | -0.026 | -0.474 | 0.008 | 0.029 | Unannotated | NA | NA |
| metab_2894 | beta-D-Glucuronoside | pos | 361.1392 | 5.038 | 4.513 | 4.046 | -0.468 | -0.474 | 0.008 | 0.029 | Tentative | Organic oxygen compounds | Organooxygen compounds |
| metab_5070 | (-)-Betonicine | pos | 142.0864 | 0.854 | 4.899 | 5.018 | 0.119 | 0.474 | 0.008 | 0.029 | Tentative | NA | NA |
| metab_2661 | 10-Hydroxycarbazepine | pos | 255.1128 | 5.793 | 3.984 | 3.552 | -0.432 | -0.474 | 0.008 | 0.029 | Tentative | Organoheterocyclic compounds | Benzazepines |
| metab_6151 | Unannotated feature | neg | 187.0608 | 3.232 | 3.777 | 4.115 | 0.338 | 0.474 | 0.008 | 0.029 | Unannotated | NA | NA |
| metab_5455 | Unannotated feature | neg | 373.9452 | 0.660 | 4.083 | 3.805 | -0.278 | -0.474 | 0.008 | 0.029 | Unannotated | NA | NA |
| metab_10190 | Unannotated feature | neg | 398.8563 | 0.881 | 4.947 | 4.803 | -0.144 | -0.474 | 0.008 | 0.029 | Unannotated | NA | NA |
| metab_5105 | Unannotated feature | pos | 210.8881 | 0.591 | 5.184 | 5.143 | -0.041 | -0.474 | 0.008 | 0.029 | Unannotated | NA | NA |
| metab_2301 | LysoPE(18:2(9Z,12Z)/0:0) | pos | 500.2754 | 6.072 | 4.802 | 5.032 | 0.230 | 0.474 | 0.008 | 0.029 | Tentative | Lipids and lipid-like molecules | Glycerophospholipids |
| metab_119 | Unannotated feature | pos | 1016.7924 | 0.541 | 5.101 | 4.877 | -0.224 | -0.474 | 0.008 | 0.029 | Unannotated | NA | NA |
| metab_4104 | Unannotated feature | pos | 150.9979 | 0.714 | 4.767 | 4.653 | -0.114 | -0.474 | 0.008 | 0.029 | Unannotated | NA | NA |
| metab_3722 | 3-Hydroxypropyl methacrylate | pos | 186.1125 | 1.855 | 3.922 | 4.288 | 0.365 | 0.474 | 0.008 | 0.029 | Tentative | Organic acids and derivatives | Carboxylic acids and derivatives |
| metab_1519 | Ibuprofen | pos | 454.2927 | 6.165 | 5.764 | 5.912 | 0.148 | 0.469 | 0.009 | 0.030 | Tentative | Phenylpropanoids and polyketides | Phenylpropanoic acids |
| metab_4992 | Unannotated feature | pos | 618.5952 | 0.591 | 5.594 | 5.526 | -0.068 | -0.469 | 0.009 | 0.030 | Unannotated | NA | NA |
| metab_3746 | Unannotated feature | pos | 132.9411 | 1.786 | 3.887 | 4.003 | 0.116 | 0.469 | 0.009 | 0.030 | Unannotated | NA | NA |
| metab_8915 | Unannotated feature | neg | 245.0934 | 5.085 | 3.212 | 3.742 | 0.530 | 0.469 | 0.009 | 0.030 | Unannotated | NA | NA |
| metab_11220 | Unannotated feature | neg | 229.0485 | 0.857 | 3.736 | 4.010 | 0.274 | 0.469 | 0.009 | 0.030 | Unannotated | NA | NA |
| metab_1034 | Unannotated feature | pos | 84.7211 | 4.800 | 3.148 | 3.364 | 0.216 | 0.469 | 0.009 | 0.030 | Unannotated | NA | NA |
| metab_338 | Unannotated feature | pos | 109.0321 | 0.831 | 2.726 | 3.322 | 0.596 | 0.469 | 0.009 | 0.030 | Unannotated | NA | NA |
| metab_1883 | Unannotated feature | pos | 215.9836 | 7.339 | 4.446 | 4.371 | -0.075 | -0.469 | 0.009 | 0.030 | Unannotated | NA | NA |
| metab_1482 | Unannotated feature | pos | 380.7697 | 0.591 | 5.825 | 5.762 | -0.063 | -0.469 | 0.009 | 0.030 | Unannotated | NA | NA |
| metab_6889 | Unannotated feature | neg | 615.2474 | 5.643 | 4.376 | 4.003 | -0.372 | -0.469 | 0.009 | 0.030 | Unannotated | NA | NA |
| metab_3881 | Unannotated feature | pos | 121.0286 | 3.985 | 3.160 | 3.578 | 0.418 | 0.469 | 0.009 | 0.030 | Unannotated | NA | NA |
| metab_7471 | Unannotated feature | neg | 157.0113 | 7.140 | 4.087 | 3.995 | -0.093 | -0.469 | 0.009 | 0.030 | Unannotated | NA | NA |
| metab_7174 | Unannotated feature | neg | 257.0782 | 5.898 | 4.680 | 4.587 | -0.093 | -0.469 | 0.009 | 0.030 | Unannotated | NA | NA |
| metab_3569 | (2S,3R)-3-hydroxy-2-methylpentanedioylcarnitine | pos | 288.1441 | 2.058 | 3.065 | 4.112 | 1.047 | 0.469 | 0.009 | 0.030 | Tentative | Lipids and lipid-like molecules | Fatty Acyls |
| metab_857 | 3,4-Dihydroxyphenylethyleneglycol | pos | 212.0918 | 3.421 | 4.105 | 3.770 | -0.335 | -0.469 | 0.009 | 0.030 | Tentative | Benzenoids | Phenols |
| metab_10873 | Unannotated feature | neg | 157.0113 | 0.554 | 4.763 | 4.637 | -0.127 | -0.469 | 0.009 | 0.030 | Unannotated | NA | NA |
| metab_3507 | Unannotated feature | pos | 125.0599 | 2.179 | 3.460 | 3.605 | 0.145 | 0.469 | 0.009 | 0.030 | Unannotated | NA | NA |
| metab_6274 | 3-[4-methoxy-3-(sulfooxy)phenyl]propanoic acid | neg | 275.0237 | 3.554 | 3.714 | 4.700 | 0.985 | 0.469 | 0.009 | 0.030 | Tentative | Organic acids and derivatives | Organic sulfuric acids and derivatives |
| metab_4291 | Unannotated feature | pos | 200.8592 | 0.591 | 5.585 | 5.542 | -0.043 | -0.469 | 0.009 | 0.030 | Unannotated | NA | NA |
| metab_9425 | Unannotated feature | neg | 216.0877 | 3.412 | 3.877 | 3.639 | -0.239 | -0.469 | 0.009 | 0.030 | Unannotated | NA | NA |
| metab_3889 | Unannotated feature | pos | 248.9762 | 0.869 | 5.192 | 5.113 | -0.079 | -0.469 | 0.009 | 0.030 | Unannotated | NA | NA |
| metab_1910 | Unannotated feature | pos | 296.1464 | 7.324 | 4.910 | 5.022 | 0.112 | 0.469 | 0.009 | 0.030 | Unannotated | NA | NA |
| metab_1352 | Unannotated feature | pos | 255.0975 | 2.241 | 3.864 | 4.451 | 0.588 | 0.469 | 0.009 | 0.030 | Unannotated | NA | NA |
| metab_2326 | Unannotated feature | pos | 804.6029 | 6.010 | 5.154 | 5.042 | -0.112 | -0.469 | 0.009 | 0.030 | Unannotated | NA | NA |
| metab_3785 | Methylmalonylcarnitine | pos | 262.1285 | 1.577 | 4.906 | 5.104 | 0.198 | 0.469 | 0.009 | 0.030 | Tentative | Lipids and lipid-like molecules | Fatty Acyls |
| metab_4784 | Unannotated feature | pos | 343.1313 | 7.269 | 5.243 | 5.279 | 0.036 | 0.469 | 0.009 | 0.030 | Unannotated | NA | NA |
| metab_4452 | Unannotated feature | pos | 883.7960 | 0.551 | 4.635 | 4.806 | 0.171 | 0.469 | 0.009 | 0.030 | Unannotated | NA | NA |
| metab_5906 | Unannotated feature | neg | 242.0520 | 2.523 | 4.495 | 4.367 | -0.127 | -0.469 | 0.009 | 0.030 | Unannotated | NA | NA |
| metab_8481 | Unannotated feature | neg | 281.9777 | 5.739 | 3.648 | 3.980 | 0.332 | 0.469 | 0.009 | 0.030 | Unannotated | NA | NA |
| metab_4450 | Unannotated feature | pos | 914.8123 | 0.551 | 5.306 | 5.131 | -0.175 | -0.469 | 0.009 | 0.030 | Unannotated | NA | NA |
| metab_4215 | Unannotated feature | pos | 105.1107 | 0.621 | 5.322 | 5.441 | 0.120 | 0.469 | 0.009 | 0.030 | Unannotated | NA | NA |
| metab_5764 | Unannotated feature | neg | 432.0301 | 1.935 | 4.557 | 4.327 | -0.230 | -0.469 | 0.009 | 0.030 | Unannotated | NA | NA |
| metab_9957 | Unannotated feature | neg | 227.9050 | 2.111 | 5.338 | 5.168 | -0.170 | -0.469 | 0.009 | 0.030 | Unannotated | NA | NA |
| metab_7113 | Blumenol C glucoside | neg | 417.2140 | 5.827 | 4.045 | 4.978 | 0.933 | 0.469 | 0.009 | 0.030 | Tentative | Lipids and lipid-like molecules | Fatty Acyls |
| metab_2548 | Unannotated feature | pos | 841.8150 | 0.551 | 5.425 | 5.537 | 0.112 | 0.469 | 0.009 | 0.030 | Unannotated | NA | NA |
| metab_5454 | Unannotated feature | neg | 308.0524 | 0.660 | 4.851 | 4.599 | -0.252 | -0.469 | 0.009 | 0.030 | Unannotated | NA | NA |
| metab_2598 | Unannotated feature | pos | 484.8401 | 5.848 | 4.596 | 4.436 | -0.160 | -0.469 | 0.009 | 0.030 | Unannotated | NA | NA |
| metab_50 | Unannotated feature | pos | 108.0447 | 0.008 | 4.621 | 4.649 | 0.028 | 0.469 | 0.009 | 0.030 | Unannotated | NA | NA |
| metab_8281 | Unannotated feature | neg | 550.0805 | 3.160 | 4.526 | 4.200 | -0.327 | -0.469 | 0.009 | 0.030 | Unannotated | NA | NA |
| metab_1126 | Tanacetol A | pos | 312.2168 | 5.626 | 5.622 | 5.911 | 0.289 | 0.469 | 0.009 | 0.030 | Tentative | Lipids and lipid-like molecules | Prenol lipids |
| metab_6832 | Perindopril | neg | 405.1775 | 5.603 | 3.339 | 5.011 | 1.672 | 0.469 | 0.009 | 0.030 | Tentative | Organic acids and derivatives | Carboxylic acids and derivatives |
| metab_6830 | Unannotated feature | neg | 301.9117 | 0.881 | 4.668 | 4.411 | -0.257 | -0.469 | 0.009 | 0.030 | Unannotated | NA | NA |
| metab_507 | Unannotated feature | pos | 206.1176 | 1.910 | 3.820 | 3.544 | -0.276 | -0.469 | 0.009 | 0.030 | Unannotated | NA | NA |
| metab_9745 | Unannotated feature | neg | 161.0815 | 2.732 | 3.773 | 4.237 | 0.464 | 0.469 | 0.009 | 0.030 | Unannotated | NA | NA |
| metab_2574 | Unannotated feature | pos | 147.1169 | 5.863 | 3.617 | 3.918 | 0.301 | 0.469 | 0.009 | 0.030 | Unannotated | NA | NA |
| metab_8027 | Unannotated feature | neg | 243.0625 | 1.886 | 5.678 | 5.580 | -0.097 | -0.469 | 0.009 | 0.030 | Unannotated | NA | NA |
| metab_264 | Unannotated feature | pos | 318.7734 | 0.691 | 4.639 | 4.582 | -0.056 | -0.469 | 0.009 | 0.030 | Unannotated | NA | NA |
| metab_5388 | Unannotated feature | neg | 516.6564 | 0.601 | 5.538 | 5.440 | -0.098 | -0.469 | 0.009 | 0.030 | Unannotated | NA | NA |
| metab_10786 | Unannotated feature | neg | 719.8597 | 0.562 | 4.927 | 4.749 | -0.177 | -0.469 | 0.009 | 0.030 | Unannotated | NA | NA |
| metab_11081 | Unannotated feature | neg | 137.0238 | 5.619 | 5.040 | 5.262 | 0.222 | 0.469 | 0.009 | 0.030 | Unannotated | NA | NA |
| metab_29 | Aciclovir | pos | 226.0934 | 1.879 | 3.268 | 2.898 | -0.370 | -0.469 | 0.009 | 0.030 | Strong putative | NA | NA |
| metab_3493 | Unannotated feature | pos | 156.0107 | 2.218 | 4.549 | 4.491 | -0.058 | -0.469 | 0.009 | 0.030 | Unannotated | NA | NA |
| metab_5211 | Unannotated feature | neg | 698.8680 | 0.543 | 5.755 | 5.671 | -0.084 | -0.469 | 0.009 | 0.030 | Unannotated | NA | NA |
| metab_1491 | Glycolithocholate | pos | 478.2929 | 6.072 | 6.386 | 6.546 | 0.159 | 0.465 | 0.009 | 0.032 | Tentative | NA | NA |
| metab_1335 | Unannotated feature | pos | 418.2208 | 5.894 | 4.756 | 4.912 | 0.156 | 0.465 | 0.009 | 0.032 | Unannotated | NA | NA |
| metab_3428 | Unannotated feature | pos | 84.5070 | 2.389 | 3.865 | 3.987 | 0.122 | 0.465 | 0.009 | 0.032 | Unannotated | NA | NA |
| metab_1946 | Unannotated feature | pos | 580.2916 | 7.316 | 4.643 | 4.358 | -0.286 | -0.465 | 0.009 | 0.032 | Unannotated | NA | NA |
| metab_2210 | Unannotated feature | pos | 808.7508 | 0.567 | 4.943 | 4.846 | -0.096 | -0.465 | 0.009 | 0.032 | Unannotated | NA | NA |
| metab_5306 | Unannotated feature | neg | 686.8655 | 0.562 | 4.497 | 4.150 | -0.347 | -0.465 | 0.009 | 0.032 | Unannotated | NA | NA |
| metab_10003 | Unannotated feature | neg | 367.9279 | 1.892 | 4.684 | 4.577 | -0.107 | -0.465 | 0.009 | 0.032 | Unannotated | NA | NA |
| metab_532 | Unannotated feature | pos | 234.6312 | 1.956 | 3.775 | 3.293 | -0.482 | -0.465 | 0.009 | 0.032 | Unannotated | NA | NA |
| metab_10985 | Unannotated feature | neg | 856.7763 | 0.570 | 4.616 | 4.779 | 0.163 | 0.465 | 0.009 | 0.032 | Unannotated | NA | NA |
| metab_371 | Unannotated feature | pos | 92.0581 | 0.892 | 3.542 | 3.364 | -0.177 | -0.465 | 0.009 | 0.032 | Unannotated | NA | NA |
| metab_3458 | 7,8-Diaminononanoate | pos | 230.1863 | 2.288 | 3.885 | 4.116 | 0.231 | 0.465 | 0.009 | 0.032 | Tentative | Lipids and lipid-like molecules | Fatty Acyls |
| metab_3646 | Unannotated feature | pos | 169.9774 | 1.956 | 5.004 | 4.971 | -0.032 | -0.465 | 0.009 | 0.032 | Unannotated | NA | NA |
| metab_6589 | Unannotated feature | neg | 189.0766 | 4.806 | 4.064 | 3.533 | -0.531 | -0.465 | 0.009 | 0.032 | Unannotated | NA | NA |
| metab_8665 | Unannotated feature | neg | 93.0338 | 5.635 | 3.930 | 4.299 | 0.369 | 0.465 | 0.009 | 0.032 | Unannotated | NA | NA |
| metab_364 | Unannotated feature | pos | 231.0839 | 0.869 | 6.282 | 6.186 | -0.096 | -0.465 | 0.009 | 0.032 | Unannotated | NA | NA |
| metab_5912 | (+)-(1R,2R)-1,2-Diphenylethane-1,2-diol | neg | 487.2168 | 2.530 | 4.401 | 4.699 | 0.298 | 0.465 | 0.009 | 0.032 | Tentative | Phenylpropanoids and polyketides | Stilbenes |
| metab_2680 | Unannotated feature | pos | 319.8682 | 5.771 | 5.255 | 5.335 | 0.080 | 0.465 | 0.009 | 0.032 | Unannotated | NA | NA |
| metab_3839 | Unannotated feature | pos | 133.0496 | 1.173 | 3.903 | 3.997 | 0.094 | 0.465 | 0.009 | 0.032 | Unannotated | NA | NA |
| metab_9903 | Unannotated feature | neg | 469.0911 | 2.159 | 4.387 | 4.587 | 0.200 | 0.465 | 0.009 | 0.032 | Unannotated | NA | NA |
| metab_5071 | Unannotated feature | pos | 85.0289 | 1.118 | 5.455 | 5.394 | -0.061 | -0.465 | 0.009 | 0.032 | Unannotated | NA | NA |
| metab_3976 | Unannotated feature | pos | 117.0550 | 0.839 | 4.134 | 4.178 | 0.044 | 0.465 | 0.009 | 0.032 | Unannotated | NA | NA |
| metab_9674 | Unannotated feature | neg | 515.1139 | 2.938 | 4.358 | 3.708 | -0.650 | -0.465 | 0.009 | 0.032 | Unannotated | NA | NA |
| metab_4134 | Unannotated feature | pos | 115.0369 | 0.683 | 5.217 | 4.977 | -0.240 | -0.465 | 0.009 | 0.032 | Unannotated | NA | NA |
| metab_7605 | Unannotated feature | neg | 228.9447 | 7.261 | 4.493 | 4.525 | 0.032 | 0.465 | 0.009 | 0.032 | Unannotated | NA | NA |
| metab_5364 | Unannotated feature | neg | 638.6092 | 0.587 | 5.773 | 5.717 | -0.056 | -0.465 | 0.009 | 0.032 | Unannotated | NA | NA |
| metab_4690 | Unannotated feature | pos | 571.9030 | 5.894 | 5.370 | 5.228 | -0.142 | -0.465 | 0.009 | 0.032 | Unannotated | NA | NA |
| metab_8 | S-Adenosyl-L-homocysteine | pos | 193.0680 | 1.778 | 3.871 | 4.544 | 0.673 | 0.465 | 0.009 | 0.032 | Tentative | Organoheterocyclic compounds | Lactones |
| metab_4309 | Unannotated feature | pos | 150.9324 | 0.583 | 5.066 | 5.014 | -0.052 | -0.465 | 0.009 | 0.032 | Unannotated | NA | NA |
| metab_8190 | Unannotated feature | neg | 256.8743 | 5.993 | 4.583 | 4.710 | 0.126 | 0.465 | 0.009 | 0.032 | Unannotated | NA | NA |
| metab_1730 | Unannotated feature | pos | 302.1759 | 7.316 | 4.394 | 4.248 | -0.146 | -0.465 | 0.009 | 0.032 | Unannotated | NA | NA |
| metab_3514 | H-D-Asp(OtBu)-OH | pos | 231.1339 | 2.148 | 4.541 | 3.894 | -0.647 | -0.465 | 0.009 | 0.032 | Tentative | Organic acids and derivatives | Carboxylic acids and derivatives |
| metab_9628 | Unannotated feature | neg | 145.0500 | 3.120 | 4.215 | 4.345 | 0.130 | 0.465 | 0.009 | 0.032 | Unannotated | NA | NA |
| metab_3482 | (S)-2,3,4,5-Tetrahydropyridine-2-carboxylate | pos | 169.0972 | 2.241 | 5.494 | 5.142 | -0.352 | -0.465 | 0.009 | 0.032 | Tentative | Organic acids and derivatives | Carboxylic acids and derivatives |
| metab_637 | Fenvalerate | pos | 384.1149 | 2.241 | 4.645 | 5.178 | 0.533 | 0.465 | 0.009 | 0.032 | Tentative | Lipids and lipid-like molecules | Fatty Acyls |
| metab_5637 | Unannotated feature | neg | 297.9814 | 0.928 | 4.368 | 4.211 | -0.158 | -0.465 | 0.009 | 0.032 | Unannotated | NA | NA |
| metab_977 | Unannotated feature | pos | 171.0765 | 0.854 | 4.921 | 5.073 | 0.152 | 0.465 | 0.009 | 0.032 | Unannotated | NA | NA |
| metab_7364 | Unannotated feature | neg | 100.9515 | 6.690 | 3.374 | 3.492 | 0.118 | 0.465 | 0.009 | 0.032 | Unannotated | NA | NA |
| metab_4884 | Unannotated feature | pos | 283.1036 | 2.058 | 4.489 | 5.024 | 0.536 | 0.465 | 0.009 | 0.032 | Unannotated | NA | NA |
| metab_2236 | Unannotated feature | pos | 132.1659 | 6.257 | 3.763 | 3.875 | 0.112 | 0.461 | 0.010 | 0.034 | Unannotated | NA | NA |
| metab_1459 | Unannotated feature | pos | 752.5186 | 5.987 | 4.804 | 4.639 | -0.165 | -0.461 | 0.010 | 0.034 | Unannotated | NA | NA |
| metab_1247 | Unannotated feature | pos | 442.3193 | 5.832 | 5.254 | 5.179 | -0.075 | -0.461 | 0.010 | 0.034 | Unannotated | NA | NA |
| metab_8194 | Unannotated feature | neg | 325.2390 | 5.977 | 4.156 | 3.871 | -0.285 | -0.461 | 0.010 | 0.034 | Unannotated | NA | NA |
| metab_8490 | Unannotated feature | neg | 382.7219 | 0.629 | 6.274 | 6.231 | -0.043 | -0.461 | 0.010 | 0.034 | Unannotated | NA | NA |
| metab_3909 | Unannotated feature | pos | 245.0633 | 0.861 | 4.610 | 4.472 | -0.138 | -0.461 | 0.010 | 0.034 | Unannotated | NA | NA |
| metab_9016 | Hydroxydehydro Nifedipine Carboxylate | neg | 345.0734 | 4.710 | 4.360 | 5.729 | 1.369 | 0.461 | 0.010 | 0.034 | Tentative | Organoheterocyclic compounds | Pyridines and derivatives |
| metab_7192 | Unannotated feature | neg | 235.9263 | 5.922 | 6.802 | 6.832 | 0.030 | 0.461 | 0.010 | 0.034 | Unannotated | NA | NA |
| metab_11295 | Unannotated feature | neg | 218.8741 | 0.590 | 6.289 | 6.236 | -0.053 | -0.461 | 0.010 | 0.034 | Unannotated | NA | NA |
| metab_10172 | Unannotated feature | neg | 510.6650 | 0.598 | 6.127 | 6.061 | -0.066 | -0.461 | 0.010 | 0.034 | Unannotated | NA | NA |
| metab_9025 | Unannotated feature | neg | 338.0889 | 3.255 | 4.322 | 4.637 | 0.315 | 0.461 | 0.010 | 0.034 | Unannotated | NA | NA |
| metab_3231 | Unannotated feature | pos | 227.0792 | 2.599 | 5.901 | 5.802 | -0.099 | -0.461 | 0.010 | 0.034 | Unannotated | NA | NA |
| metab_2504 | Unannotated feature | pos | 909.8027 | 0.551 | 5.255 | 5.436 | 0.181 | 0.461 | 0.010 | 0.034 | Unannotated | NA | NA |
| metab_790 | 3-methylheptanedioylcarnitine | pos | 318.1910 | 2.973 | 4.838 | 5.363 | 0.525 | 0.461 | 0.010 | 0.034 | Tentative | Lipids and lipid-like molecules | Fatty Acyls |
| metab_11092 | Unannotated feature | neg | 343.0863 | 5.635 | 4.212 | 4.698 | 0.486 | 0.461 | 0.010 | 0.034 | Unannotated | NA | NA |
| metab_10658 | Unannotated feature | neg | 466.7294 | 0.587 | 5.500 | 5.436 | -0.064 | -0.461 | 0.010 | 0.034 | Unannotated | NA | NA |
| metab_9248 | Unannotated feature | neg | 282.9557 | 3.959 | 3.862 | 4.033 | 0.171 | 0.461 | 0.010 | 0.034 | Unannotated | NA | NA |
| metab_4565 | Unannotated feature | pos | 89.0238 | 0.473 | 3.840 | 3.797 | -0.043 | -0.461 | 0.010 | 0.034 | Unannotated | NA | NA |
| metab_4713 | Unannotated feature | pos | 616.4635 | 5.949 | 6.362 | 6.311 | -0.051 | -0.461 | 0.010 | 0.034 | Unannotated | NA | NA |
| metab_8951 | Unannotated feature | neg | 226.0180 | 4.945 | 3.775 | 5.016 | 1.241 | 0.461 | 0.010 | 0.034 | Unannotated | NA | NA |
| metab_1253 | Unannotated feature | pos | 478.1972 | 5.840 | 4.570 | 3.896 | -0.674 | -0.461 | 0.010 | 0.034 | Unannotated | NA | NA |
| metab_6096 | Unannotated feature | neg | 394.0112 | 3.160 | 5.031 | 4.827 | -0.204 | -0.461 | 0.010 | 0.034 | Unannotated | NA | NA |
| metab_4227 | Unannotated feature | pos | 682.2405 | 4.761 | 5.395 | 5.728 | 0.333 | 0.461 | 0.010 | 0.034 | Unannotated | NA | NA |
| metab_6883 | Unannotated feature | neg | 269.1036 | 5.643 | 2.820 | 3.472 | 0.651 | 0.461 | 0.010 | 0.034 | Unannotated | NA | NA |
| metab_4844 | Unannotated feature | pos | 643.2849 | 7.269 | 5.450 | 5.199 | -0.250 | -0.461 | 0.010 | 0.034 | Unannotated | NA | NA |
| metab_241 | Unannotated feature | pos | 655.2804 | 4.578 | 6.083 | 6.369 | 0.286 | 0.461 | 0.010 | 0.034 | Unannotated | NA | NA |
| metab_10125 | Unannotated feature | neg | 189.0402 | 0.999 | 4.166 | 4.570 | 0.404 | 0.461 | 0.010 | 0.034 | Unannotated | NA | NA |
| metab_10146 | Unannotated feature | neg | 180.8593 | 0.928 | 4.167 | 4.321 | 0.154 | 0.461 | 0.010 | 0.034 | Unannotated | NA | NA |
| metab_8776 | Unannotated feature | neg | 608.7338 | 5.528 | 3.868 | 4.391 | 0.522 | 0.461 | 0.010 | 0.034 | Unannotated | NA | NA |
| metab_5447 | Unannotated feature | neg | 136.8910 | 0.660 | 6.047 | 6.161 | 0.114 | 0.461 | 0.010 | 0.034 | Unannotated | NA | NA |
| metab_616 | 7-Aminomethyl-7-carbaguanine | pos | 180.0880 | 2.164 | 4.774 | 5.060 | 0.286 | 0.461 | 0.010 | 0.034 | Tentative | Organoheterocyclic compounds | Pyrrolopyrimidines |
| metab_7384 | Unannotated feature | neg | 163.0608 | 2.119 | 3.856 | 3.974 | 0.118 | 0.461 | 0.010 | 0.034 | Unannotated | NA | NA |
| metab_7118 | Kukoamine C | neg | 511.2920 | 5.834 | 5.308 | 5.822 | 0.514 | 0.461 | 0.010 | 0.034 | Tentative | Benzenoids | Phenols |
| metab_10811 | Unannotated feature | neg | 940.7502 | 0.554 | 4.196 | 5.081 | 0.884 | 0.461 | 0.010 | 0.034 | Unannotated | NA | NA |
| metab_6842 | Unannotated feature | neg | 342.8945 | 0.888 | 5.136 | 5.026 | -0.110 | -0.461 | 0.010 | 0.034 | Unannotated | NA | NA |
| metab_4920 | Unannotated feature | pos | 134.0190 | 0.606 | 4.942 | 4.845 | -0.098 | -0.461 | 0.010 | 0.034 | Unannotated | NA | NA |
| metab_5065 | Unannotated feature | pos | 172.0969 | 0.854 | 3.672 | 4.040 | 0.367 | 0.461 | 0.010 | 0.034 | Unannotated | NA | NA |
| metab_5088 | L-Homocitrulline | pos | 190.1187 | 0.753 | 4.669 | 5.084 | 0.415 | 0.461 | 0.010 | 0.034 | Tentative | Organic acids and derivatives | Carboxylic acids and derivatives |
| metab_7213 | Unannotated feature | neg | 233.1548 | 5.937 | 4.518 | 4.600 | 0.083 | 0.461 | 0.010 | 0.034 | Unannotated | NA | NA |
| metab_3641 | Thiomorpholine 3-carboxylate | pos | 189.0693 | 1.956 | 2.801 | 3.073 | 0.272 | 0.461 | 0.010 | 0.034 | Tentative | NA | NA |
| metab_3943 | Unannotated feature | pos | 207.1419 | 0.846 | 3.417 | 3.773 | 0.356 | 0.457 | 0.010 | 0.036 | Unannotated | NA | NA |
| metab_3830 | N-Acetyl-L-Alanine | pos | 132.0658 | 1.243 | 5.275 | 5.400 | 0.125 | 0.457 | 0.010 | 0.036 | Moderate putative | NA | NA |
| metab_10827 | Unannotated feature | neg | 801.7959 | 0.554 | 4.446 | 4.917 | 0.470 | 0.457 | 0.010 | 0.036 | Unannotated | NA | NA |
| metab_9074 | Unannotated feature | neg | 686.5394 | 0.594 | 6.098 | 6.055 | -0.043 | -0.457 | 0.010 | 0.036 | Unannotated | NA | NA |
| metab_6266 | Ethyl 2-furanacrylate | neg | 211.0611 | 3.538 | 3.565 | 4.109 | 0.544 | 0.457 | 0.010 | 0.036 | Tentative | Lipids and lipid-like molecules | Fatty Acyls |
| metab_2775 | 4-(3-Hydroxybutyl)-3,3,5-trimethylcyclohexanone | pos | 251.1390 | 5.657 | 4.070 | 3.462 | -0.608 | -0.457 | 0.010 | 0.036 | Tentative | Lipids and lipid-like molecules | Prenol lipids |
| metab_10780 | Unannotated feature | neg | 782.8332 | 0.562 | 5.169 | 5.061 | -0.108 | -0.457 | 0.010 | 0.036 | Unannotated | NA | NA |
| metab_3814 | Unannotated feature | pos | 268.1404 | 1.344 | 4.793 | 4.716 | -0.078 | -0.457 | 0.010 | 0.036 | Unannotated | NA | NA |
| metab_5047 | Unannotated feature | pos | 136.0758 | 0.869 | 5.890 | 5.787 | -0.103 | -0.457 | 0.010 | 0.036 | Unannotated | NA | NA |
| metab_10721 | Unannotated feature | neg | 302.8469 | 0.578 | 3.801 | 4.148 | 0.347 | 0.457 | 0.010 | 0.036 | Unannotated | NA | NA |
| metab_256 | Unannotated feature | pos | 411.0516 | 0.675 | 4.637 | 4.421 | -0.217 | -0.457 | 0.010 | 0.036 | Unannotated | NA | NA |
| metab_2506 | m-Xylene | pos | 107.0859 | 5.894 | 5.308 | 5.394 | 0.086 | 0.457 | 0.010 | 0.036 | Strong putative | NA | NA |
| metab_2557 | Unannotated feature | pos | 617.0969 | 5.863 | 4.878 | 4.741 | -0.136 | -0.457 | 0.010 | 0.036 | Unannotated | NA | NA |
| metab_2633 | Unannotated feature | pos | 123.1171 | 5.832 | 4.179 | 4.258 | 0.078 | 0.457 | 0.010 | 0.036 | Unannotated | NA | NA |
| metab_10770 | Unannotated feature | neg | 246.8853 | 0.570 | 4.346 | 4.434 | 0.088 | 0.457 | 0.010 | 0.036 | Unannotated | NA | NA |
| metab_756 | N-Acetyl-DL-Valine | pos | 160.0970 | 2.772 | 4.634 | 4.715 | 0.080 | 0.457 | 0.010 | 0.036 | Moderate putative | NA | NA |
| metab_2484 | Unannotated feature | pos | 106.0655 | 5.902 | 4.439 | 4.486 | 0.046 | 0.457 | 0.010 | 0.036 | Unannotated | NA | NA |
| metab_8522 | Unannotated feature | neg | 171.9466 | 5.715 | 4.577 | 4.520 | -0.057 | -0.457 | 0.010 | 0.036 | Unannotated | NA | NA |
| metab_5519 | Unannotated feature | neg | 688.9996 | 0.539 | 5.187 | 5.043 | -0.145 | -0.457 | 0.010 | 0.036 | Unannotated | NA | NA |
| metab_2285 | Unannotated feature | pos | 482.4046 | 6.111 | 5.018 | 5.200 | 0.182 | 0.457 | 0.010 | 0.036 | Unannotated | NA | NA |
| metab_1581 | Unannotated feature | pos | 472.4364 | 6.420 | 6.033 | 6.141 | 0.107 | 0.457 | 0.010 | 0.036 | Unannotated | NA | NA |
| metab_1991 | Unannotated feature | pos | 444.8649 | 7.293 | 4.310 | 4.455 | 0.144 | 0.457 | 0.010 | 0.036 | Unannotated | NA | NA |
| metab_7703 | Unannotated feature | neg | 147.9264 | 7.213 | 3.618 | 3.697 | 0.079 | 0.457 | 0.010 | 0.036 | Unannotated | NA | NA |
| metab_9221 | Unannotated feature | neg | 204.9977 | 4.059 | 3.698 | 4.552 | 0.854 | 0.457 | 0.010 | 0.036 | Unannotated | NA | NA |
| metab_5338 | Unannotated feature | neg | 681.7422 | 0.570 | 4.504 | 4.269 | -0.236 | -0.457 | 0.010 | 0.036 | Unannotated | NA | NA |
| metab_5808 | 4-Pyridoxate | neg | 182.0456 | 2.119 | 3.906 | 4.184 | 0.278 | 0.457 | 0.010 | 0.036 | Strong putative | Organoheterocyclic compounds | Pyridines and derivatives |
| metab_10073 | Unannotated feature | neg | 278.9988 | 1.302 | 3.984 | 3.827 | -0.157 | -0.457 | 0.010 | 0.036 | Unannotated | NA | NA |
| metab_2417 | Unannotated feature | pos | 1003.7916 | 0.559 | 4.529 | 4.210 | -0.319 | -0.457 | 0.010 | 0.036 | Unannotated | NA | NA |
| metab_4842 | Unannotated feature | pos | 357.1469 | 7.269 | 5.694 | 5.715 | 0.021 | 0.457 | 0.010 | 0.036 | Unannotated | NA | NA |
| metab_11050 | Unannotated feature | neg | 346.8178 | 0.582 | 6.120 | 6.065 | -0.055 | -0.457 | 0.010 | 0.036 | Unannotated | NA | NA |
| metab_641 | Unannotated feature | pos | 182.0813 | 0.869 | 6.620 | 6.507 | -0.113 | -0.457 | 0.010 | 0.036 | Unannotated | NA | NA |
| metab_10464 | Unannotated feature | neg | 232.0471 | 0.652 | 4.588 | 4.890 | 0.302 | 0.457 | 0.010 | 0.036 | Unannotated | NA | NA |
| metab_11102 | (+/-)-Rollipyrrole | neg | 269.1305 | 5.659 | 4.514 | 4.192 | -0.322 | -0.457 | 0.010 | 0.036 | Tentative | Organoheterocyclic compounds | Pyrrolines |
| metab_496 | Dopamine glucuronide | pos | 312.1110 | 1.886 | 4.544 | 4.276 | -0.268 | -0.457 | 0.010 | 0.036 | Tentative | NA | NA |
| metab_1904 | Unannotated feature | pos | 133.9932 | 7.331 | 4.398 | 4.478 | 0.080 | 0.457 | 0.010 | 0.036 | Unannotated | NA | NA |
| metab_1785 | Unannotated feature | pos | 123.0405 | 7.525 | 4.300 | 4.250 | -0.049 | -0.457 | 0.010 | 0.036 | Unannotated | NA | NA |
| metab_3918 | Unannotated feature | pos | 194.0224 | 0.861 | 4.488 | 4.316 | -0.172 | -0.457 | 0.010 | 0.036 | Unannotated | NA | NA |
| metab_6921 | Unannotated feature | neg | 285.1346 | 5.675 | 3.011 | 3.725 | 0.713 | 0.457 | 0.010 | 0.036 | Unannotated | NA | NA |
| metab_11385 | Androsterone glucuronide | neg | 465.2503 | 5.819 | 5.188 | 5.758 | 0.570 | 0.457 | 0.010 | 0.036 | Tentative | Lipids and lipid-like molecules | Steroids and steroid derivatives |
| metab_72 | Unannotated feature | pos | 123.0807 | 0.418 | 4.774 | 4.799 | 0.025 | 0.457 | 0.010 | 0.036 | Unannotated | NA | NA |
| metab_4724 | Unannotated feature | pos | 848.6283 | 5.995 | 5.511 | 5.442 | -0.069 | -0.457 | 0.010 | 0.036 | Unannotated | NA | NA |
| metab_7138 | (3beta,17alpha,23R)-17,23-Epoxy-3,29-dihydroxy-27-norlanost-8-ene-15,24-dione | neg | 517.3170 | 5.859 | 4.875 | 4.082 | -0.793 | -0.457 | 0.010 | 0.036 | Tentative | NA | NA |
| metab_10652 | Unannotated feature | neg | 582.6471 | 0.587 | 5.876 | 5.792 | -0.084 | -0.453 | 0.011 | 0.038 | Unannotated | NA | NA |
| metab_5962 | Unannotated feature | neg | 167.0822 | 2.740 | 4.160 | 3.719 | -0.441 | -0.453 | 0.011 | 0.038 | Unannotated | NA | NA |
| metab_2605 | Unannotated feature | pos | 622.4290 | 5.840 | 5.147 | 4.872 | -0.275 | -0.453 | 0.011 | 0.038 | Unannotated | NA | NA |
| metab_5231 | Unannotated feature | neg | 815.8010 | 0.549 | 4.040 | 4.365 | 0.325 | 0.453 | 0.011 | 0.038 | Unannotated | NA | NA |
| metab_5383 | Unannotated feature | neg | 343.8208 | 0.016 | 5.844 | 5.816 | -0.029 | -0.453 | 0.011 | 0.038 | Unannotated | NA | NA |
| metab_5313 | Unannotated feature | neg | 787.8475 | 0.562 | 4.830 | 4.479 | -0.351 | -0.453 | 0.011 | 0.038 | Unannotated | NA | NA |
| metab_5147 | Indole-3-acetate | pos | 171.1129 | 0.869 | 4.770 | 4.502 | -0.268 | -0.453 | 0.011 | 0.038 | Moderate putative | NA | NA |
| metab_6235 | Unannotated feature | neg | 250.8494 | 3.443 | 4.125 | 4.299 | 0.173 | 0.453 | 0.011 | 0.038 | Unannotated | NA | NA |
| metab_6283 | cis-beta-D-Glucosyl-2-hydroxycinnamate | neg | 371.0990 | 3.570 | 3.311 | 4.654 | 1.344 | 0.453 | 0.011 | 0.038 | Tentative | Organic oxygen compounds | Organooxygen compounds |
| metab_5302 | Unannotated feature | neg | 651.8724 | 0.562 | 4.671 | 4.555 | -0.116 | -0.453 | 0.011 | 0.038 | Unannotated | NA | NA |
| metab_10608 | Unannotated feature | neg | 458.6978 | 0.598 | 5.256 | 5.194 | -0.062 | -0.453 | 0.011 | 0.038 | Unannotated | NA | NA |
| metab_6722 | Unannotated feature | neg | 723.2295 | 5.346 | 5.072 | 5.536 | 0.465 | 0.453 | 0.011 | 0.038 | Unannotated | NA | NA |
| metab_2404 | Unannotated feature | pos | 723.4904 | 5.933 | 5.231 | 5.100 | -0.131 | -0.453 | 0.011 | 0.038 | Unannotated | NA | NA |
| metab_5139 | Lawsone | pos | 192.0656 | 4.977 | 4.712 | 4.888 | 0.176 | 0.453 | 0.011 | 0.038 | Tentative | Benzenoids | Naphthalenes |
| metab_4521 | Unannotated feature | pos | 929.4876 | 0.538 | 4.673 | 4.506 | -0.167 | -0.453 | 0.011 | 0.038 | Unannotated | NA | NA |
| metab_7214 | 10-HDoHE | neg | 343.2285 | 5.937 | 4.503 | 4.002 | -0.500 | -0.453 | 0.011 | 0.038 | Tentative | Lipids and lipid-like molecules | Fatty Acyls |
| metab_433 | Unannotated feature | pos | 121.0399 | 1.562 | 3.261 | 3.813 | 0.552 | 0.453 | 0.011 | 0.038 | Unannotated | NA | NA |
| metab_4180 | Unannotated feature | pos | 113.0712 | 0.644 | 3.766 | 3.998 | 0.232 | 0.453 | 0.011 | 0.038 | Unannotated | NA | NA |
| metab_9168 | Unannotated feature | neg | 480.9915 | 0.676 | 5.181 | 4.932 | -0.249 | -0.453 | 0.011 | 0.038 | Unannotated | NA | NA |
| metab_3503 | Unannotated feature | pos | 127.0755 | 2.195 | 3.160 | 3.901 | 0.741 | 0.453 | 0.011 | 0.038 | Unannotated | NA | NA |
| metab_6317 | Unannotated feature | neg | 146.0646 | 3.734 | 3.840 | 4.164 | 0.324 | 0.453 | 0.011 | 0.038 | Unannotated | NA | NA |
| metab_10908 | Unannotated feature | neg | 621.0115 | 0.543 | 4.884 | 4.724 | -0.159 | -0.453 | 0.011 | 0.038 | Unannotated | NA | NA |
| metab_530 | Unannotated feature | pos | 218.1024 | 1.956 | 3.554 | 4.082 | 0.527 | 0.453 | 0.011 | 0.038 | Unannotated | NA | NA |
| metab_4297 | Unannotated feature | pos | 878.5175 | 0.583 | 5.240 | 5.106 | -0.134 | -0.453 | 0.011 | 0.038 | Unannotated | NA | NA |
| metab_5616 | Unannotated feature | neg | 620.5482 | 0.904 | 4.708 | 4.514 | -0.194 | -0.453 | 0.011 | 0.038 | Unannotated | NA | NA |
| metab_4925 | Unannotated feature | pos | 556.6427 | 0.598 | 5.619 | 5.522 | -0.098 | -0.453 | 0.011 | 0.038 | Unannotated | NA | NA |
| metab_3838 | Unannotated feature | pos | 184.9803 | 1.181 | 3.691 | 3.110 | -0.580 | -0.453 | 0.011 | 0.038 | Unannotated | NA | NA |
| metab_6874 | Glucosyl 6-hydroxy-2,6-dimethyl-2E,7-octadienoate | neg | 345.1562 | 5.635 | 3.042 | 4.198 | 1.156 | 0.453 | 0.011 | 0.038 | Tentative | Organic oxygen compounds | Organooxygen compounds |
| metab_6095 | Unannotated feature | neg | 351.0139 | 3.160 | 4.322 | 4.160 | -0.163 | -0.453 | 0.011 | 0.038 | Unannotated | NA | NA |
| metab_8024 | Unannotated feature | neg | 173.1179 | 6.606 | 4.371 | 4.450 | 0.079 | 0.453 | 0.011 | 0.038 | Unannotated | NA | NA |
| metab_8928 | Unannotated feature | neg | 679.7238 | 5.001 | 5.055 | 5.277 | 0.222 | 0.453 | 0.011 | 0.038 | Unannotated | NA | NA |
| metab_11281 | Unannotated feature | neg | 209.0947 | 6.470 | 5.861 | 5.925 | 0.064 | 0.453 | 0.011 | 0.038 | Unannotated | NA | NA |
| metab_213 | Unannotated feature | pos | 169.9859 | 0.614 | 5.314 | 5.203 | -0.111 | -0.453 | 0.011 | 0.038 | Unannotated | NA | NA |
| metab_1776 | Unannotated feature | pos | 102.3761 | 7.385 | 4.940 | 4.989 | 0.048 | 0.453 | 0.011 | 0.038 | Unannotated | NA | NA |
| metab_7191 | Unannotated feature | neg | 209.9267 | 5.922 | 4.789 | 4.889 | 0.100 | 0.448 | 0.012 | 0.039 | Unannotated | NA | NA |
| metab_5248 | Unannotated feature | neg | 597.8333 | 0.554 | 4.696 | 5.107 | 0.411 | 0.448 | 0.012 | 0.039 | Unannotated | NA | NA |
| metab_5972 | Unannotated feature | neg | 145.0501 | 2.764 | 3.850 | 4.065 | 0.215 | 0.448 | 0.012 | 0.039 | Unannotated | NA | NA |
| metab_9867 | Unannotated feature | neg | 516.1050 | 2.417 | 4.885 | 5.030 | 0.146 | 0.448 | 0.012 | 0.039 | Unannotated | NA | NA |
| metab_10192 | Unannotated feature | neg | 185.9936 | 0.881 | 5.470 | 5.404 | -0.066 | -0.448 | 0.012 | 0.039 | Unannotated | NA | NA |
| metab_7701 | Unannotated feature | neg | 162.9133 | 7.213 | 3.452 | 3.576 | 0.124 | 0.448 | 0.012 | 0.039 | Unannotated | NA | NA |
| metab_3658 | Unannotated feature | pos | 415.8514 | 1.949 | 4.028 | 4.318 | 0.289 | 0.448 | 0.012 | 0.039 | Unannotated | NA | NA |
| metab_10174 | Unannotated feature | neg | 384.7190 | 0.904 | 5.493 | 5.460 | -0.032 | -0.448 | 0.012 | 0.039 | Unannotated | NA | NA |
| metab_11011 | Unannotated feature | neg | 369.8433 | 0.016 | 4.895 | 4.988 | 0.094 | 0.448 | 0.012 | 0.039 | Unannotated | NA | NA |
| metab_4209 | Unannotated feature | pos | 328.9985 | 0.621 | 4.947 | 4.836 | -0.111 | -0.448 | 0.012 | 0.039 | Unannotated | NA | NA |
| metab_10520 | Unannotated feature | neg | 179.9024 | 0.629 | 3.184 | 3.422 | 0.238 | 0.448 | 0.012 | 0.039 | Unannotated | NA | NA |
| metab_6657 | Unannotated feature | neg | 79.9565 | 5.116 | 2.672 | 3.204 | 0.532 | 0.448 | 0.012 | 0.039 | Unannotated | NA | NA |
| metab_9882 | Ethylmalonic Acid | neg | 131.0343 | 2.310 | 4.820 | 4.703 | -0.117 | -0.448 | 0.012 | 0.039 | Tentative | NA | NA |
| metab_8796 | Unannotated feature | neg | 257.8196 | 5.506 | 6.162 | 6.104 | -0.058 | -0.448 | 0.012 | 0.039 | Unannotated | NA | NA |
| metab_2448 | Unannotated feature | pos | 816.5599 | 5.910 | 4.871 | 4.223 | -0.648 | -0.448 | 0.012 | 0.039 | Unannotated | NA | NA |
| metab_8952 | Unannotated feature | neg | 79.9565 | 4.945 | 3.051 | 3.413 | 0.361 | 0.448 | 0.012 | 0.039 | Unannotated | NA | NA |
| metab_2537 | Unannotated feature | pos | 850.7512 | 0.551 | 4.062 | 4.757 | 0.695 | 0.448 | 0.012 | 0.039 | Unannotated | NA | NA |
| metab_5975 | Unannotated feature | neg | 798.4182 | 0.613 | 6.073 | 5.988 | -0.085 | -0.448 | 0.012 | 0.039 | Unannotated | NA | NA |
| metab_2461 | Unannotated feature | pos | 527.8804 | 0.559 | 4.529 | 4.655 | 0.126 | 0.448 | 0.012 | 0.039 | Unannotated | NA | NA |
| metab_10682 | Unannotated feature | neg | 491.7148 | 0.582 | 4.564 | 4.522 | -0.042 | -0.448 | 0.012 | 0.039 | Unannotated | NA | NA |
| metab_10928 | Unannotated feature | neg | 647.8609 | 0.534 | 4.532 | 4.462 | -0.071 | -0.448 | 0.012 | 0.039 | Unannotated | NA | NA |
| metab_4775 | Unannotated feature | pos | 472.4363 | 7.200 | 6.225 | 6.280 | 0.054 | 0.448 | 0.012 | 0.039 | Unannotated | NA | NA |
| metab_2684 | Unannotated feature | pos | 273.0291 | 5.771 | 5.208 | 5.264 | 0.057 | 0.448 | 0.012 | 0.039 | Unannotated | NA | NA |
| metab_3038 | Tyramine | pos | 120.0811 | 4.163 | 6.118 | 5.962 | -0.156 | -0.448 | 0.012 | 0.039 | Moderate putative | NA | NA |
| metab_7017 | Unannotated feature | neg | 215.8863 | 1.094 | 6.104 | 6.002 | -0.102 | -0.448 | 0.012 | 0.039 | Unannotated | NA | NA |
| metab_2977 | 2,3-Dimethoxyphenol sulfate | pos | 217.0166 | 4.524 | 4.697 | 4.224 | -0.473 | -0.448 | 0.012 | 0.039 | Tentative | NA | NA |
| metab_2962 | Unannotated feature | pos | 663.7933 | 4.578 | 4.192 | 4.739 | 0.547 | 0.448 | 0.012 | 0.039 | Unannotated | NA | NA |
| metab_4966 | Unannotated feature | pos | 498.6841 | 0.591 | 5.790 | 5.707 | -0.083 | -0.448 | 0.012 | 0.039 | Unannotated | NA | NA |
| metab_1802 | Unannotated feature | pos | 107.0859 | 7.928 | 4.282 | 4.240 | -0.041 | -0.448 | 0.012 | 0.039 | Unannotated | NA | NA |
| metab_10428 | trans-3-Hydroxy-L-proline | neg | 307.1153 | 0.676 | 5.458 | 5.272 | -0.185 | -0.448 | 0.012 | 0.039 | Tentative | NA | NA |
| metab_9581 | Unannotated feature | neg | 333.0412 | 3.168 | 4.450 | 4.286 | -0.164 | -0.448 | 0.012 | 0.039 | Unannotated | NA | NA |
| metab_576 | Encelin | pos | 489.2303 | 2.058 | 4.792 | 5.005 | 0.214 | 0.448 | 0.012 | 0.039 | Tentative | Lipids and lipid-like molecules | Prenol lipids |
| metab_11154 | Unannotated feature | neg | 181.1231 | 5.771 | 3.912 | 4.330 | 0.419 | 0.448 | 0.012 | 0.039 | Unannotated | NA | NA |
| metab_9589 | Unannotated feature | neg | 157.0767 | 3.168 | 3.562 | 3.168 | -0.394 | -0.448 | 0.012 | 0.039 | Unannotated | NA | NA |
| metab_9582 | Unannotated feature | neg | 304.0162 | 3.168 | 4.249 | 4.001 | -0.248 | -0.448 | 0.012 | 0.039 | Unannotated | NA | NA |
| metab_1487 | Metholone | pos | 337.2736 | 6.065 | 5.387 | 5.485 | 0.098 | 0.448 | 0.012 | 0.039 | Tentative | NA | NA |
| metab_10606 | Unannotated feature | neg | 508.6662 | 0.598 | 5.659 | 5.601 | -0.058 | -0.448 | 0.012 | 0.039 | Unannotated | NA | NA |
| metab_204 | Unannotated feature | pos | 345.8586 | 0.606 | 4.410 | 4.257 | -0.153 | -0.448 | 0.012 | 0.039 | Unannotated | NA | NA |
| metab_1100 | Unannotated feature | pos | 84.7211 | 5.474 | 3.023 | 3.262 | 0.239 | 0.448 | 0.012 | 0.039 | Unannotated | NA | NA |
| metab_1268 | Unannotated feature | pos | 573.7319 | 5.848 | 4.788 | 4.672 | -0.117 | -0.448 | 0.012 | 0.039 | Unannotated | NA | NA |
| metab_8422 | (E)-3,7-Dimethylocta-1,6-diene-3,8-diol | neg | 215.1289 | 5.779 | 4.380 | 4.460 | 0.080 | 0.448 | 0.012 | 0.039 | Tentative | Lipids and lipid-like molecules | Prenol lipids |
| metab_8629 | Unannotated feature | neg | 279.7677 | 5.651 | 4.347 | 4.260 | -0.087 | -0.448 | 0.012 | 0.039 | Unannotated | NA | NA |
| metab_333 | Unannotated feature | pos | 125.0235 | 0.823 | 4.256 | 4.141 | -0.115 | -0.444 | 0.013 | 0.041 | Unannotated | NA | NA |
| metab_283 | Unannotated feature | pos | 698.7858 | 4.915 | 6.748 | 7.019 | 0.271 | 0.444 | 0.013 | 0.041 | Unannotated | NA | NA |
| metab_7414 | Unannotated feature | neg | 220.0650 | 2.119 | 2.946 | 3.517 | 0.571 | 0.444 | 0.013 | 0.041 | Unannotated | NA | NA |
| metab_1204 | Aminopentol | pos | 428.3332 | 5.763 | 4.925 | 5.082 | 0.158 | 0.444 | 0.013 | 0.041 | Tentative | NA | NA |
| metab_6219 | O-methoxycatechol-O-sulphate | neg | 203.0018 | 3.380 | 5.089 | 5.491 | 0.402 | 0.444 | 0.013 | 0.041 | Tentative | Organic acids and derivatives | Organic sulfuric acids and derivatives |
| metab_6928 | Unannotated feature | neg | 607.2390 | 5.675 | 3.481 | 3.920 | 0.439 | 0.444 | 0.013 | 0.041 | Unannotated | NA | NA |
| metab_1013 | Unannotated feature | pos | 674.2538 | 4.578 | 5.178 | 5.456 | 0.278 | 0.444 | 0.013 | 0.041 | Unannotated | NA | NA |
| metab_10790 | Unannotated feature | neg | 685.8661 | 0.562 | 5.027 | 4.854 | -0.173 | -0.444 | 0.013 | 0.041 | Unannotated | NA | NA |
| metab_1260 | Unannotated feature | pos | 281.2951 | 5.848 | 4.875 | 4.946 | 0.071 | 0.444 | 0.013 | 0.041 | Unannotated | NA | NA |
| metab_9671 | Unannotated feature | neg | 118.0583 | 2.946 | 3.445 | 3.878 | 0.432 | 0.444 | 0.013 | 0.041 | Unannotated | NA | NA |
| metab_7620 | Unannotated feature | neg | 143.0344 | 7.369 | 4.143 | 4.099 | -0.044 | -0.444 | 0.013 | 0.041 | Unannotated | NA | NA |
| metab_8703 | Unannotated feature | neg | 453.0870 | 5.595 | 4.396 | 3.786 | -0.611 | -0.444 | 0.013 | 0.041 | Unannotated | NA | NA |
| metab_5756 | Unannotated feature | neg | 306.0586 | 1.878 | 4.852 | 4.638 | -0.214 | -0.444 | 0.013 | 0.041 | Unannotated | NA | NA |
| metab_10511 | Unannotated feature | neg | 376.8970 | 0.629 | 4.869 | 4.750 | -0.119 | -0.444 | 0.013 | 0.041 | Unannotated | NA | NA |
| metab_4171 | Unannotated feature | pos | 165.1313 | 0.644 | 4.416 | 4.188 | -0.228 | -0.444 | 0.013 | 0.041 | Unannotated | NA | NA |
| metab_7568 | Unannotated feature | neg | 163.8464 | 7.197 | 3.979 | 3.920 | -0.059 | -0.444 | 0.013 | 0.041 | Unannotated | NA | NA |
| metab_9252 | Unannotated feature | neg | 259.1303 | 3.491 | 5.284 | 5.155 | -0.129 | -0.444 | 0.013 | 0.041 | Unannotated | NA | NA |
| metab_6397 | Unannotated feature | neg | 107.0495 | 4.079 | 3.067 | 3.893 | 0.826 | 0.444 | 0.013 | 0.041 | Unannotated | NA | NA |
| metab_375 | Unannotated feature | pos | 169.9859 | 0.900 | 4.421 | 4.303 | -0.118 | -0.444 | 0.013 | 0.041 | Unannotated | NA | NA |
| metab_4208 | Unannotated feature | pos | 338.0504 | 0.621 | 4.502 | 4.226 | -0.276 | -0.444 | 0.013 | 0.041 | Unannotated | NA | NA |
| metab_5317 | Unannotated feature | neg | 850.8208 | 0.562 | 4.910 | 4.816 | -0.094 | -0.444 | 0.013 | 0.041 | Unannotated | NA | NA |
| metab_381 | Unannotated feature | pos | 196.8651 | 0.908 | 5.359 | 5.331 | -0.028 | -0.444 | 0.013 | 0.041 | Unannotated | NA | NA |
| metab_9175 | Unannotated feature | neg | 165.0763 | 4.219 | 3.958 | 3.917 | -0.041 | -0.444 | 0.013 | 0.041 | Unannotated | NA | NA |
| metab_1539 | Unannotated feature | pos | 101.0602 | 6.265 | 4.371 | 4.426 | 0.054 | 0.444 | 0.013 | 0.041 | Unannotated | NA | NA |
| metab_5229 | Unannotated feature | neg | 247.8838 | 0.549 | 4.627 | 4.587 | -0.040 | -0.444 | 0.013 | 0.041 | Unannotated | NA | NA |
| metab_3484 | Unannotated feature | pos | 499.6425 | 2.234 | 5.099 | 4.582 | -0.517 | -0.444 | 0.013 | 0.041 | Unannotated | NA | NA |
| metab_9844 | Unannotated feature | neg | 255.0449 | 2.492 | 4.178 | 3.800 | -0.378 | -0.444 | 0.013 | 0.041 | Unannotated | NA | NA |
| metab_5945 | Unannotated feature | neg | 630.5775 | 0.598 | 5.697 | 5.649 | -0.048 | -0.444 | 0.013 | 0.041 | Unannotated | NA | NA |
| metab_4509 | Unannotated feature | pos | 247.1305 | 0.545 | 4.717 | 4.673 | -0.044 | -0.444 | 0.013 | 0.041 | Unannotated | NA | NA |
| metab_9348 | 4-Methoxybenzoate | neg | 151.0395 | 3.618 | 4.237 | 4.425 | 0.188 | 0.444 | 0.013 | 0.041 | Moderate putative | NA | NA |
| metab_4665 | Unannotated feature | pos | 672.7767 | 0.567 | 5.354 | 5.256 | -0.097 | -0.444 | 0.013 | 0.041 | Unannotated | NA | NA |
| metab_3158 | Coumafuryl | pos | 362.1000 | 3.514 | 5.685 | 5.521 | -0.164 | -0.444 | 0.013 | 0.041 | Tentative | NA | NA |
| metab_3867 | Unannotated feature | pos | 198.8622 | 0.908 | 5.336 | 5.281 | -0.055 | -0.444 | 0.013 | 0.041 | Unannotated | NA | NA |
| metab_3991 | Unannotated feature | pos | 235.0092 | 0.823 | 3.707 | 3.953 | 0.245 | 0.444 | 0.013 | 0.041 | Unannotated | NA | NA |
| metab_5989 | N6-Methyl-2'-deoxyadenosine | neg | 310.1163 | 2.836 | 3.830 | 4.287 | 0.457 | 0.444 | 0.013 | 0.041 | Tentative | Nucleosides, nucleotides, and analogues | Purine nucleosides |
| metab_8006 | Unannotated feature | neg | 82.9533 | 6.903 | 3.803 | 3.855 | 0.052 | 0.444 | 0.013 | 0.041 | Unannotated | NA | NA |
| metab_10688 | Unannotated feature | neg | 160.9150 | 0.582 | 5.703 | 5.663 | -0.040 | -0.444 | 0.013 | 0.041 | Unannotated | NA | NA |
| metab_1389 | Unannotated feature | pos | 723.8364 | 5.917 | 4.622 | 4.345 | -0.276 | -0.444 | 0.013 | 0.041 | Unannotated | NA | NA |
| metab_6066 | Unannotated feature | neg | 89.9249 | 3.112 | 3.340 | 3.278 | -0.063 | -0.444 | 0.013 | 0.041 | Unannotated | NA | NA |
| metab_5572 | N-Acetylserine | neg | 146.0453 | 0.857 | 4.994 | 5.186 | 0.192 | 0.444 | 0.013 | 0.041 | Tentative | NA | NA |
| metab_7142 | Unannotated feature | neg | 185.9937 | 1.390 | 5.368 | 5.270 | -0.098 | -0.444 | 0.013 | 0.041 | Unannotated | NA | NA |
| metab_581 | Unannotated feature | pos | 173.1244 | 2.065 | 4.849 | 4.894 | 0.045 | 0.444 | 0.013 | 0.041 | Unannotated | NA | NA |
| metab_9579 | Unannotated feature | neg | 664.1759 | 3.168 | 4.018 | 3.299 | -0.720 | -0.444 | 0.013 | 0.041 | Unannotated | NA | NA |
| metab_4997 | Unannotated feature | pos | 246.0951 | 0.760 | 4.562 | 4.763 | 0.202 | 0.444 | 0.013 | 0.041 | Unannotated | NA | NA |
| metab_1836 | Unannotated feature | pos | 646.7889 | 0.551 | 4.163 | 4.704 | 0.540 | 0.444 | 0.013 | 0.041 | Unannotated | NA | NA |
| metab_1757 | Unannotated feature | pos | 331.9737 | 7.331 | 4.378 | 4.571 | 0.193 | 0.444 | 0.013 | 0.041 | Unannotated | NA | NA |
| metab_8341 | Unannotated feature | neg | 363.9572 | 5.827 | 5.233 | 5.052 | -0.180 | -0.444 | 0.013 | 0.041 | Unannotated | NA | NA |
| metab_9549 | 3-Methylcrotonylglycine | neg | 156.0661 | 3.209 | 3.441 | 3.805 | 0.364 | 0.444 | 0.013 | 0.041 | Moderate putative | NA | NA |
| metab_6996 | 3-((4-(5-(Hydroxymethyl)-2-oxo-3-oxazolidinyl)phenoxy)methyl)benzonitrile | neg | 359.0813 | 5.723 | 4.025 | 4.617 | 0.592 | 0.444 | 0.013 | 0.041 | Tentative | Benzenoids | Phenol ethers |
| metab_6989 | Unannotated feature | neg | 486.0677 | 5.715 | 4.539 | 4.373 | -0.166 | -0.440 | 0.014 | 0.044 | Unannotated | NA | NA |
| metab_8083 | Sterol | neg | 555.4427 | 6.196 | 5.231 | 4.517 | -0.714 | -0.440 | 0.014 | 0.044 | Tentative | NA | NA |
| metab_3035 | Unannotated feature | pos | 311.6359 | 4.163 | 5.107 | 4.858 | -0.249 | -0.440 | 0.014 | 0.044 | Unannotated | NA | NA |
| metab_9511 | Unannotated feature | neg | 342.0949 | 3.232 | 4.608 | 4.180 | -0.428 | -0.440 | 0.014 | 0.044 | Unannotated | NA | NA |
| metab_9475 | Unannotated feature | neg | 143.0344 | 3.282 | 4.589 | 4.638 | 0.049 | 0.440 | 0.014 | 0.044 | Unannotated | NA | NA |
| metab_6917 | Unannotated feature | neg | 157.0113 | 5.675 | 4.629 | 4.507 | -0.122 | -0.440 | 0.014 | 0.044 | Unannotated | NA | NA |
| metab_4986 | Unannotated feature | pos | 203.1503 | 0.737 | 5.677 | 5.745 | 0.068 | 0.440 | 0.014 | 0.044 | Unannotated | NA | NA |
| metab_10393 | Unannotated feature | neg | 162.9775 | 0.715 | 4.803 | 4.724 | -0.079 | -0.440 | 0.014 | 0.044 | Unannotated | NA | NA |
| metab_457 | p-Tolualdehyde | pos | 121.0651 | 1.740 | 4.691 | 4.612 | -0.079 | -0.440 | 0.014 | 0.044 | Moderate putative | NA | NA |
| metab_11118 | Unannotated feature | neg | 494.1943 | 5.683 | 5.044 | 4.641 | -0.403 | -0.440 | 0.014 | 0.044 | Unannotated | NA | NA |
| metab_3852 | Unannotated feature | pos | 172.1333 | 1.048 | 4.494 | 4.238 | -0.256 | -0.440 | 0.014 | 0.044 | Unannotated | NA | NA |
| metab_1937 | Unannotated feature | pos | 120.0258 | 7.324 | 4.324 | 4.396 | 0.071 | 0.440 | 0.014 | 0.044 | Unannotated | NA | NA |
| metab_10113 | Unannotated feature | neg | 125.8725 | 1.094 | 6.303 | 6.251 | -0.051 | -0.440 | 0.014 | 0.044 | Unannotated | NA | NA |
| metab_5755 | L-Tyrosine | neg | 180.0663 | 1.878 | 5.644 | 5.531 | -0.113 | -0.440 | 0.014 | 0.044 | Strong putative | NA | NA |
| metab_6418 | Unannotated feature | neg | 412.0045 | 0.731 | 4.637 | 4.246 | -0.391 | -0.440 | 0.014 | 0.044 | Unannotated | NA | NA |
| metab_7649 | Unannotated feature | neg | 198.5751 | 7.517 | 5.192 | 5.157 | -0.035 | -0.440 | 0.014 | 0.044 | Unannotated | NA | NA |
| metab_11018 | Unannotated feature | neg | 196.9262 | 0.016 | 4.195 | 4.128 | -0.067 | -0.440 | 0.014 | 0.044 | Unannotated | NA | NA |
| metab_7193 | Unannotated feature | neg | 245.8562 | 5.922 | 4.066 | 4.281 | 0.216 | 0.440 | 0.014 | 0.044 | Unannotated | NA | NA |
| metab_6453 | Unannotated feature | neg | 462.7355 | 0.582 | 6.047 | 6.008 | -0.039 | -0.440 | 0.014 | 0.044 | Unannotated | NA | NA |
| metab_1464 | Unannotated feature | pos | 746.5622 | 5.995 | 5.870 | 5.801 | -0.068 | -0.440 | 0.014 | 0.044 | Unannotated | NA | NA |
| metab_6094 | Unannotated feature | neg | 241.0565 | 3.160 | 3.482 | 2.816 | -0.666 | -0.440 | 0.014 | 0.044 | Unannotated | NA | NA |
| metab_5058 | Unannotated feature | pos | 254.8236 | 0.900 | 5.753 | 5.708 | -0.045 | -0.440 | 0.014 | 0.044 | Unannotated | NA | NA |
| metab_8961 | PGP(6 keto-PGF1alpha/a-13:0) | neg | 919.4187 | 4.901 | 4.960 | 5.169 | 0.208 | 0.440 | 0.014 | 0.044 | Tentative | NA | NA |
| metab_10742 | Unannotated feature | neg | 276.8331 | 0.587 | 6.468 | 6.424 | -0.044 | -0.440 | 0.014 | 0.044 | Unannotated | NA | NA |
| metab_1468 | Unannotated feature | pos | 479.3357 | 6.003 | 4.599 | 4.281 | -0.318 | -0.440 | 0.014 | 0.044 | Unannotated | NA | NA |
| metab_5324 | Unannotated feature | neg | 213.0856 | 0.570 | 4.056 | 3.801 | -0.255 | -0.440 | 0.014 | 0.044 | Unannotated | NA | NA |
| metab_4837 | Unannotated feature | pos | 216.9228 | 0.575 | 6.523 | 6.494 | -0.029 | -0.440 | 0.014 | 0.044 | Unannotated | NA | NA |
| metab_2416 | Unannotated feature | pos | 782.5380 | 5.925 | 4.873 | 4.607 | -0.266 | -0.440 | 0.014 | 0.044 | Unannotated | NA | NA |
| metab_7095 | Unannotated feature | neg | 273.1463 | 5.811 | 3.420 | 2.983 | -0.437 | -0.440 | 0.014 | 0.044 | Unannotated | NA | NA |
| metab_5831 | Unannotated feature | neg | 516.1049 | 2.159 | 4.467 | 4.714 | 0.247 | 0.440 | 0.014 | 0.044 | Unannotated | NA | NA |
| metab_8836 | Unannotated feature | neg | 707.7644 | 5.346 | 5.661 | 5.953 | 0.291 | 0.440 | 0.014 | 0.044 | Unannotated | NA | NA |
| metab_10965 | Unannotated feature | neg | 114.9333 | 0.515 | 4.123 | 4.260 | 0.137 | 0.440 | 0.014 | 0.044 | Unannotated | NA | NA |
| metab_103 | Unannotated feature | pos | 131.0898 | 0.538 | 3.776 | 3.688 | -0.088 | -0.440 | 0.014 | 0.044 | Unannotated | NA | NA |
| metab_11367 | Unannotated feature | neg | 159.8782 | 0.182 | 5.546 | 5.519 | -0.027 | -0.440 | 0.014 | 0.044 | Unannotated | NA | NA |
| metab_1005 | Unannotated feature | pos | 573.2245 | 4.532 | 4.411 | 4.869 | 0.458 | 0.440 | 0.014 | 0.044 | Unannotated | NA | NA |
| metab_1282 | Pepsinogen (1-12) | pos | 690.4768 | 5.855 | 5.878 | 5.793 | -0.086 | -0.440 | 0.014 | 0.044 | Tentative | NA | NA |
| metab_2787 | Unannotated feature | pos | 173.1283 | 2.050 | 4.123 | 4.347 | 0.224 | 0.440 | 0.014 | 0.044 | Unannotated | NA | NA |
| metab_990 | Oxytocin 1-8 | pos | 480.1925 | 4.470 | 4.319 | 4.729 | 0.409 | 0.440 | 0.014 | 0.044 | Tentative | Organic acids and derivatives | Carboxylic acids and derivatives |
| metab_5753 | Unannotated feature | neg | 90.5274 | 1.878 | 3.063 | 2.950 | -0.113 | -0.436 | 0.015 | 0.046 | Unannotated | NA | NA |
| metab_10175 | Unannotated feature | neg | 330.7543 | 0.904 | 5.192 | 5.156 | -0.036 | -0.436 | 0.015 | 0.046 | Unannotated | NA | NA |
| metab_10084 | Unannotated feature | neg | 185.0565 | 1.294 | 4.516 | 4.050 | -0.467 | -0.436 | 0.015 | 0.046 | Unannotated | NA | NA |
| metab_10622 | Unannotated feature | neg | 717.5157 | 0.594 | 4.438 | 4.211 | -0.227 | -0.436 | 0.015 | 0.046 | Unannotated | NA | NA |
| metab_4002 | Carnosine | pos | 209.1034 | 0.815 | 3.893 | 4.229 | 0.336 | 0.436 | 0.015 | 0.046 | Tentative | Organic acids and derivatives | Peptidomimetics |
| metab_5509 | Unannotated feature | neg | 673.8771 | 0.534 | 5.685 | 5.627 | -0.058 | -0.436 | 0.015 | 0.046 | Unannotated | NA | NA |
| metab_11352 | Unannotated feature | neg | 311.8440 | 0.016 | 4.229 | 4.181 | -0.049 | -0.436 | 0.015 | 0.046 | Unannotated | NA | NA |
| metab_7577 | Casimiroedine | neg | 416.1796 | 2.417 | 5.993 | 6.108 | 0.115 | 0.436 | 0.015 | 0.046 | Tentative | NA | NA |
| metab_8524 | DN-isobutylamide | neg | 481.2454 | 5.707 | 5.133 | 5.721 | 0.588 | 0.436 | 0.015 | 0.046 | Tentative | Organic acids and derivatives | Carboxylic acids and derivatives |
| metab_1455 | Unannotated feature | pos | 664.4630 | 5.979 | 5.092 | 5.164 | 0.072 | 0.436 | 0.015 | 0.046 | Unannotated | NA | NA |
| metab_7014 | Unannotated feature | neg | 259.1556 | 5.739 | 3.076 | 3.943 | 0.867 | 0.436 | 0.015 | 0.046 | Unannotated | NA | NA |
| metab_8227 | Unannotated feature | neg | 443.8242 | 5.922 | 6.457 | 6.393 | -0.064 | -0.436 | 0.015 | 0.046 | Unannotated | NA | NA |
| metab_10935 | Unannotated feature | neg | 802.8176 | 0.530 | 4.773 | 4.637 | -0.136 | -0.436 | 0.015 | 0.046 | Unannotated | NA | NA |
| metab_2646 | Unannotated feature | pos | 104.0959 | 5.817 | 3.313 | 2.973 | -0.340 | -0.436 | 0.015 | 0.046 | Unannotated | NA | NA |
| metab_3929 | Unannotated feature | pos | 549.0560 | 0.854 | 4.905 | 4.775 | -0.130 | -0.436 | 0.015 | 0.046 | Unannotated | NA | NA |
| metab_4735 | Unannotated feature | pos | 519.4046 | 6.126 | 5.482 | 5.271 | -0.211 | -0.436 | 0.015 | 0.046 | Unannotated | NA | NA |
| metab_1117 | Lactodifucotetraose | pos | 667.2602 | 5.588 | 4.663 | 3.990 | -0.674 | -0.436 | 0.015 | 0.046 | Tentative | Lipids and lipid-like molecules | Fatty Acyls |
| metab_2788 | Unannotated feature | pos | 102.3761 | 5.634 | 5.176 | 5.219 | 0.043 | 0.436 | 0.015 | 0.046 | Unannotated | NA | NA |
| metab_2596 | Unannotated feature | pos | 515.3662 | 5.848 | 5.760 | 5.695 | -0.066 | -0.436 | 0.015 | 0.046 | Unannotated | NA | NA |
| metab_244 | Unannotated feature | pos | 335.9838 | 0.652 | 3.675 | 4.138 | 0.462 | 0.436 | 0.015 | 0.046 | Unannotated | NA | NA |
| metab_5148 | 4-Coumarate | pos | 165.0547 | 0.869 | 5.767 | 5.665 | -0.102 | -0.436 | 0.015 | 0.046 | Moderate putative | Phenylpropanoids and polyketides | Cinnamic acids and derivatives |
| metab_10032 | Succinate | neg | 117.0186 | 1.715 | 5.420 | 5.353 | -0.067 | -0.436 | 0.015 | 0.046 | Strong putative | Organic acids and derivatives | Carboxylic acids and derivatives |
| metab_9910 | Unannotated feature | neg | 364.8907 | 2.119 | 4.736 | 4.656 | -0.080 | -0.436 | 0.015 | 0.046 | Unannotated | NA | NA |
| metab_9922 | Unannotated feature | neg | 220.9762 | 2.119 | 2.882 | 3.920 | 1.038 | 0.436 | 0.015 | 0.046 | Unannotated | NA | NA |
| metab_631 | Fluoro-beta-alanine | pos | 125.0712 | 2.226 | 4.343 | 4.468 | 0.125 | 0.436 | 0.015 | 0.046 | Tentative | NA | NA |
| metab_6434 | Unannotated feature | neg | 236.0566 | 4.211 | 3.859 | 4.032 | 0.172 | 0.436 | 0.015 | 0.046 | Unannotated | NA | NA |
| metab_6366 | Unannotated feature | neg | 202.0720 | 3.942 | 4.139 | 4.351 | 0.212 | 0.436 | 0.015 | 0.046 | Unannotated | NA | NA |
| metab_859 | Unannotated feature | pos | 130.0653 | 3.468 | 3.468 | 3.926 | 0.458 | 0.436 | 0.015 | 0.046 | Unannotated | NA | NA |
| metab_182 | Unannotated feature | pos | 935.8049 | 0.559 | 4.581 | 4.408 | -0.174 | -0.436 | 0.015 | 0.046 | Unannotated | NA | NA |
| metab_1205 | Unannotated feature | pos | 552.3846 | 5.763 | 5.340 | 5.221 | -0.119 | -0.436 | 0.015 | 0.046 | Unannotated | NA | NA |
| metab_209 | Unannotated feature | pos | 103.0546 | 4.163 | 5.126 | 4.948 | -0.178 | -0.436 | 0.015 | 0.046 | Unannotated | NA | NA |
| metab_8806 | Unannotated feature | neg | 336.8954 | 5.477 | 3.015 | 3.638 | 0.624 | 0.436 | 0.015 | 0.046 | Unannotated | NA | NA |
| metab_11251 | Unannotated feature | neg | 414.9908 | 0.865 | 5.647 | 5.552 | -0.095 | -0.436 | 0.015 | 0.046 | Unannotated | NA | NA |
| metab_6768 | Unannotated feature | neg | 613.5368 | 5.518 | 4.787 | 5.174 | 0.386 | 0.436 | 0.015 | 0.046 | Unannotated | NA | NA |
| metab_3636 | Unannotated feature | pos | 190.1438 | 1.956 | 3.860 | 4.129 | 0.269 | 0.432 | 0.016 | 0.048 | Unannotated | NA | NA |
| metab_4435 | Unannotated feature | pos | 395.0390 | 0.559 | 4.181 | 4.049 | -0.132 | -0.432 | 0.016 | 0.048 | Unannotated | NA | NA |
| metab_8004 | Unannotated feature | neg | 114.9891 | 6.925 | 4.499 | 4.554 | 0.056 | 0.432 | 0.016 | 0.048 | Unannotated | NA | NA |
| metab_2516 | Unannotated feature | pos | 906.8254 | 0.551 | 6.234 | 6.171 | -0.063 | -0.432 | 0.016 | 0.048 | Unannotated | NA | NA |
| metab_5056 | Unannotated feature | pos | 231.1451 | 0.861 | 3.741 | 4.162 | 0.420 | 0.432 | 0.016 | 0.048 | Unannotated | NA | NA |
| metab_1351 | Unannotated feature | pos | 295.1179 | 5.902 | 4.819 | 4.925 | 0.106 | 0.432 | 0.016 | 0.048 | Unannotated | NA | NA |
| metab_9123 | Unannotated feature | neg | 242.0938 | 4.355 | 4.736 | 4.240 | -0.496 | -0.432 | 0.016 | 0.048 | Unannotated | NA | NA |
| metab_6024 | Unannotated feature | neg | 90.9869 | 2.946 | 5.083 | 5.029 | -0.055 | -0.432 | 0.016 | 0.048 | Unannotated | NA | NA |
| metab_9698 | Unannotated feature | neg | 87.0443 | 2.898 | 3.907 | 4.352 | 0.445 | 0.432 | 0.016 | 0.048 | Unannotated | NA | NA |
| metab_2538 | Unannotated feature | pos | 719.5020 | 5.871 | 5.830 | 5.764 | -0.066 | -0.432 | 0.016 | 0.048 | Unannotated | NA | NA |
| metab_5870 | Unannotated feature | neg | 127.0394 | 2.437 | 2.567 | 3.145 | 0.579 | 0.432 | 0.016 | 0.048 | Unannotated | NA | NA |
| metab_4195 | Unannotated feature | pos | 492.8456 | 0.629 | 5.640 | 5.586 | -0.055 | -0.432 | 0.016 | 0.048 | Unannotated | NA | NA |
| metab_2390 | Unannotated feature | pos | 718.5330 | 5.941 | 6.356 | 6.334 | -0.022 | -0.432 | 0.016 | 0.048 | Unannotated | NA | NA |
| metab_1950 | Unannotated feature | pos | 297.1466 | 7.316 | 4.932 | 5.017 | 0.085 | 0.432 | 0.016 | 0.048 | Unannotated | NA | NA |
| metab_1826 | Unannotated feature | pos | 149.0962 | 7.983 | 3.927 | 3.835 | -0.092 | -0.432 | 0.016 | 0.048 | Unannotated | NA | NA |
| metab_3367 | Unannotated feature | pos | 247.1288 | 2.545 | 4.518 | 5.114 | 0.597 | 0.432 | 0.016 | 0.048 | Strong putative | NA | NA |
| metab_344 | Unannotated feature | pos | 258.9333 | 0.831 | 4.612 | 4.475 | -0.138 | -0.432 | 0.016 | 0.048 | Unannotated | NA | NA |
| metab_8551 | Unannotated feature | neg | 422.1830 | 5.691 | 4.438 | 4.207 | -0.231 | -0.432 | 0.016 | 0.048 | Unannotated | NA | NA |
| metab_2127 | Unannotated feature | pos | 148.9354 | 0.575 | 5.620 | 5.584 | -0.037 | -0.432 | 0.016 | 0.048 | Unannotated | NA | NA |
| metab_8451 | D,L-Buthionine | neg | 427.1973 | 5.755 | 4.226 | 4.796 | 0.570 | 0.432 | 0.016 | 0.048 | Tentative | Organic acids and derivatives | Carboxylic acids and derivatives |
| metab_141 | Unannotated feature | pos | 668.8695 | 0.551 | 6.116 | 6.023 | -0.092 | -0.432 | 0.016 | 0.048 | Unannotated | NA | NA |
| metab_11136 | Unannotated feature | neg | 201.1131 | 5.723 | 4.985 | 5.103 | 0.118 | 0.432 | 0.016 | 0.048 | Unannotated | NA | NA |
| metab_232 | Unannotated feature | pos | 77.0796 | 0.644 | 3.844 | 4.325 | 0.481 | 0.432 | 0.016 | 0.048 | Unannotated | NA | NA |
| metab_211 | Unannotated feature | pos | 112.0372 | 0.614 | 4.573 | 4.434 | -0.138 | -0.432 | 0.016 | 0.048 | Unannotated | NA | NA |
| metab_1321 | Unannotated feature | pos | 539.0493 | 5.886 | 5.022 | 4.970 | -0.052 | -0.432 | 0.016 | 0.048 | Unannotated | NA | NA |
| metab_2863 | Unannotated feature | pos | 101.8086 | 5.198 | 4.396 | 4.428 | 0.031 | 0.432 | 0.016 | 0.048 | Unannotated | NA | NA |
| metab_2704 | Unannotated feature | pos | 448.8108 | 5.748 | 4.520 | 4.187 | -0.333 | -0.432 | 0.016 | 0.048 | Unannotated | NA | NA |
| metab_1109 | Unannotated feature | pos | 414.2879 | 5.565 | 4.398 | 4.003 | -0.395 | -0.432 | 0.016 | 0.048 | Unannotated | NA | NA |
| metab_3554 | Unannotated feature | pos | 110.1016 | 2.073 | 4.249 | 4.324 | 0.075 | 0.432 | 0.016 | 0.048 | Unannotated | NA | NA |
| metab_10899 | Unannotated feature | neg | 834.8436 | 0.543 | 5.743 | 5.622 | -0.121 | -0.432 | 0.016 | 0.048 | Unannotated | NA | NA |
| metab_10837 | Unannotated feature | neg | 701.8455 | 0.554 | 5.182 | 5.239 | 0.057 | 0.432 | 0.016 | 0.048 | Unannotated | NA | NA |
| metab_10885 | Unannotated feature | neg | 450.2845 | 0.549 | 4.614 | 4.500 | -0.114 | -0.432 | 0.016 | 0.048 | Unannotated | NA | NA |
| metab_44 | Unannotated feature | pos | 180.1020 | 1.956 | 4.402 | 4.559 | 0.157 | 0.432 | 0.016 | 0.048 | Unannotated | NA | NA |
| metab_2658 | Unannotated feature | pos | 345.2247 | 5.793 | 4.504 | 4.363 | -0.141 | -0.432 | 0.016 | 0.048 | Unannotated | NA | NA |
| metab_9952 | Unannotated feature | neg | 264.9532 | 2.111 | 4.985 | 4.850 | -0.135 | -0.432 | 0.016 | 0.048 | Unannotated | NA | NA |
| metab_5996 | Unannotated feature | neg | 322.9324 | 0.621 | 4.608 | 4.509 | -0.100 | -0.432 | 0.016 | 0.048 | Unannotated | NA | NA |
| metab_2482 | Unannotated feature | pos | 118.7292 | 5.902 | 3.367 | 3.550 | 0.183 | 0.432 | 0.016 | 0.048 | Unannotated | NA | NA |
| metab_11210 | Dehydroepiandrosterone sulfate | neg | 367.1590 | 5.890 | 7.103 | 6.716 | -0.386 | -0.432 | 0.016 | 0.048 | Moderate putative | NA | NA |
| metab_1474 | 19(S)-HETE | pos | 303.2319 | 6.018 | 4.707 | 4.444 | -0.264 | -0.432 | 0.016 | 0.048 | Tentative | Lipids and lipid-like molecules | Fatty Acyls |
| metab_10659 | Unannotated feature | neg | 408.7706 | 0.587 | 5.073 | 5.028 | -0.045 | -0.432 | 0.016 | 0.048 | Unannotated | NA | NA |
| metab_8124 | Unannotated feature | neg | 145.4041 | 6.164 | 3.615 | 3.195 | -0.420 | -0.432 | 0.016 | 0.048 | Unannotated | NA | NA |
| metab_3269 | 5-Hydroxyindoleacetate | pos | 192.0655 | 2.934 | 3.383 | 3.814 | 0.432 | 0.432 | 0.016 | 0.048 | Strong putative | Organoheterocyclic compounds | Indoles and derivatives |
| metab_5636 | Unannotated feature | neg | 181.9657 | 0.928 | 4.212 | 3.892 | -0.320 | -0.432 | 0.016 | 0.048 | Unannotated | NA | NA |
| metab_7573 | Unannotated feature | neg | 121.9433 | 7.205 | 3.718 | 3.666 | -0.053 | -0.432 | 0.016 | 0.048 | Unannotated | NA | NA |
| metab_3344 | Cinnamyl alcohol | pos | 291.1338 | 2.599 | 4.593 | 4.211 | -0.381 | -0.432 | 0.016 | 0.048 | Tentative | NA | NA |
| metab_8321 | Unannotated feature | neg | 309.1020 | 5.842 | 3.139 | 4.639 | 1.500 | 0.432 | 0.016 | 0.048 | Unannotated | NA | NA |
| metab_11028 | Unannotated feature | neg | 404.7766 | 0.582 | 6.016 | 5.973 | -0.044 | -0.432 | 0.016 | 0.048 | Unannotated | NA | NA |
| metab_10342 | Unannotated feature | neg | 161.8753 | 0.762 | 4.417 | 4.492 | 0.075 | 0.432 | 0.016 | 0.048 | Unannotated | NA | NA |
| metab_5830 | Gabaculine | neg | 416.1796 | 2.159 | 5.617 | 5.714 | 0.097 | 0.432 | 0.016 | 0.048 | Tentative | NA | NA |
| metab_702 | Unannotated feature | pos | 102.3761 | 2.529 | 3.755 | 3.945 | 0.190 | 0.432 | 0.016 | 0.048 | Unannotated | NA | NA |
| metab_563 | Unannotated feature | pos | 150.0552 | 2.050 | 3.021 | 3.414 | 0.393 | 0.432 | 0.016 | 0.048 | Unannotated | NA | NA |
| metab_6840 | Unannotated feature | neg | 332.1466 | 5.611 | 2.398 | 3.595 | 1.197 | 0.432 | 0.016 | 0.048 | Unannotated | NA | NA |
| metab_4961 | Unannotated feature | pos | 203.0154 | 0.675 | 4.955 | 5.078 | 0.123 | 0.432 | 0.016 | 0.048 | Unannotated | NA | NA |
| metab_3891 | Unannotated feature | pos | 174.0139 | 0.869 | 5.083 | 5.019 | -0.064 | -0.427 | 0.017 | 0.050 | Unannotated | NA | NA |
| metab_10312 | Unannotated feature | neg | 231.0519 | 0.794 | 3.637 | 3.832 | 0.195 | 0.427 | 0.017 | 0.050 | Unannotated | NA | NA |
| metab_8888 | Hypoglycin B | neg | 599.2526 | 5.184 | 4.756 | 4.208 | -0.548 | -0.427 | 0.017 | 0.050 | Tentative | NA | NA |
| metab_4486 | Unannotated feature | pos | 367.1400 | 5.367 | 5.976 | 5.544 | -0.433 | -0.427 | 0.017 | 0.050 | Unannotated | NA | NA |
| metab_4233 | Pyrazole | pos | 110.0716 | 0.614 | 4.672 | 4.593 | -0.079 | -0.427 | 0.017 | 0.050 | Tentative | NA | NA |
| metab_7043 | Unannotated feature | neg | 369.1748 | 5.763 | 5.857 | 5.650 | -0.208 | -0.427 | 0.017 | 0.050 | Unannotated | NA | NA |
| metab_4210 | Unannotated feature | pos | 281.8427 | 0.621 | 3.804 | 4.113 | 0.309 | 0.427 | 0.017 | 0.050 | Unannotated | NA | NA |
| metab_7404 | Unannotated feature | neg | 215.1038 | 2.119 | 3.233 | 3.822 | 0.588 | 0.427 | 0.017 | 0.050 | Unannotated | NA | NA |
| metab_1492 | Unannotated feature | pos | 556.4415 | 6.072 | 5.069 | 5.141 | 0.072 | 0.427 | 0.017 | 0.050 | Unannotated | NA | NA |
| metab_339 | Unannotated feature | pos | 367.1401 | 5.275 | 5.720 | 5.195 | -0.525 | -0.427 | 0.017 | 0.050 | Unannotated | NA | NA |
| metab_360 | Unannotated feature | pos | 367.1401 | 5.512 | 5.951 | 5.500 | -0.450 | -0.427 | 0.017 | 0.050 | Unannotated | NA | NA |
| metab_4582 | Unannotated feature | pos | 265.2525 | 0.434 | 4.785 | 4.816 | 0.030 | 0.427 | 0.017 | 0.050 | Unannotated | NA | NA |
| metab_6665 | Unannotated feature | neg | 125.0157 | 5.131 | 4.031 | 3.778 | -0.253 | -0.427 | 0.017 | 0.050 | Unannotated | NA | NA |
| metab_3708 | 4-Isopropyl-3-cyclohexene-1-carboxylic acid | pos | 186.1489 | 1.894 | 4.409 | 4.107 | -0.301 | -0.427 | 0.017 | 0.050 | Tentative | Lipids and lipid-like molecules | Prenol lipids |
| metab_4341 | Unannotated feature | pos | 218.9199 | 0.575 | 5.938 | 5.902 | -0.036 | -0.427 | 0.017 | 0.050 | Unannotated | NA | NA |
| metab_10119 | Unannotated feature | neg | 286.9329 | 4.402 | 4.699 | 5.169 | 0.470 | 0.427 | 0.017 | 0.050 | Unannotated | NA | NA |
| metab_5283 | Unannotated feature | neg | 874.7935 | 0.554 | 4.211 | 4.562 | 0.351 | 0.427 | 0.017 | 0.050 | Unannotated | NA | NA |
| metab_10609 | Unannotated feature | neg | 450.7095 | 0.598 | 5.718 | 5.656 | -0.062 | -0.427 | 0.017 | 0.050 | Unannotated | NA | NA |
| metab_6448 | Unannotated feature | neg | 362.0924 | 4.256 | 4.513 | 3.860 | -0.653 | -0.427 | 0.017 | 0.050 | Unannotated | NA | NA |
| metab_7183 | Unannotated feature | neg | 627.1511 | 5.898 | 4.758 | 4.955 | 0.198 | 0.427 | 0.017 | 0.050 | Unannotated | NA | NA |
| metab_6587 | Unannotated feature | neg | 226.1247 | 4.798 | 3.464 | 3.767 | 0.302 | 0.427 | 0.017 | 0.050 | Unannotated | NA | NA |
| metab_10215 | Unannotated feature | neg | 290.8744 | 0.865 | 5.463 | 5.380 | -0.083 | -0.427 | 0.017 | 0.050 | Unannotated | NA | NA |
| metab_5437 | Carbamoyl (2R)-2,5-diaminopentanoate | neg | 174.0881 | 0.652 | 4.987 | 5.225 | 0.238 | 0.427 | 0.017 | 0.050 | Tentative | NA | NA |
| metab_653 | 6-Aminohexanoate | pos | 173.1286 | 2.327 | 5.862 | 5.668 | -0.194 | -0.427 | 0.017 | 0.050 | Tentative | NA | NA |
| metab_11029 | Unannotated feature | neg | 425.7795 | 0.013 | 4.956 | 4.916 | -0.039 | -0.427 | 0.017 | 0.050 | Unannotated | NA | NA |
| metab_689 | (1r,2s)-2-Aminocyclopentanecarboxylic acid | pos | 112.0760 | 2.482 | 3.706 | 3.785 | 0.079 | 0.427 | 0.017 | 0.050 | Tentative | Organic acids and derivatives | Carboxylic acids and derivatives |
| metab_8346 | 2,4-Hexadien-1-ol | neg | 241.1447 | 5.827 | 4.649 | 4.991 | 0.342 | 0.427 | 0.017 | 0.050 | Tentative | NA | NA |
| metab_8778 | Unannotated feature | neg | 621.9845 | 5.523 | 4.711 | 5.176 | 0.465 | 0.427 | 0.017 | 0.050 | Unannotated | NA | NA |
| metab_5712 | Unannotated feature | neg | 292.8715 | 1.390 | 4.780 | 4.671 | -0.110 | -0.427 | 0.017 | 0.050 | Unannotated | NA | NA |
| metab_1666 | Unannotated feature | pos | 678.4782 | 7.223 | 5.019 | 5.088 | 0.069 | 0.427 | 0.017 | 0.050 | Unannotated | NA | NA |
| metab_1633 | Unannotated feature | pos | 349.2151 | 7.107 | 4.709 | 4.863 | 0.154 | 0.427 | 0.017 | 0.050 | Unannotated | NA | NA |
| metab_7768 | Unannotated feature | neg | 178.8647 | 7.173 | 4.412 | 4.362 | -0.050 | -0.427 | 0.017 | 0.050 | Unannotated | NA | NA |
| metab_7598 | Unannotated feature | neg | 190.0508 | 2.431 | 3.987 | 4.151 | 0.164 | 0.427 | 0.017 | 0.050 | Unannotated | NA | NA |
| metab_698 | Unannotated feature | pos | 185.0809 | 2.521 | 2.908 | 3.677 | 0.769 | 0.427 | 0.017 | 0.050 | Unannotated | NA | NA |
| metab_697 | Unannotated feature | pos | 181.7441 | 2.521 | 4.044 | 3.751 | -0.293 | -0.427 | 0.017 | 0.050 | Unannotated | NA | NA |
| metab_8334 | Glucocochlearin | neg | 420.0635 | 3.176 | 4.732 | 4.385 | -0.347 | -0.427 | 0.017 | 0.050 | Tentative | Organic oxygen compounds | Organooxygen compounds |
| metab_66 | Unannotated feature | pos | 79.0184 | 0.286 | 4.496 | 4.514 | 0.018 | 0.427 | 0.017 | 0.050 | Unannotated | NA | NA |
| metab_3600 | Unannotated feature | pos | 199.9754 | 2.026 | 4.882 | 4.829 | -0.053 | -0.427 | 0.017 | 0.050 | Unannotated | NA | NA |
| metab_3582 | Unannotated feature | pos | 595.1444 | 2.050 | 4.328 | 4.174 | -0.153 | -0.427 | 0.017 | 0.050 | Unannotated | NA | NA |
| metab_3196 | Unannotated feature | pos | 128.1072 | 3.275 | 3.797 | 3.982 | 0.186 | 0.427 | 0.017 | 0.050 | Unannotated | NA | NA |
| metab_2665 | Gamma-Dodecalactone | pos | 163.1483 | 5.793 | 4.194 | 4.306 | 0.112 | 0.427 | 0.017 | 0.050 | Tentative | NA | NA |
| metab_9640 | Unannotated feature | neg | 164.0713 | 3.080 | 3.734 | 3.495 | -0.239 | -0.427 | 0.017 | 0.050 | Unannotated | NA | NA |
| metab_2761 | Unannotated feature | pos | 133.0649 | 1.956 | 3.417 | 3.616 | 0.199 | 0.427 | 0.017 | 0.050 | Unannotated | NA | NA |
| metab_6371 | Unannotated feature | neg | 280.8928 | 0.723 | 4.317 | 3.760 | -0.557 | -0.427 | 0.017 | 0.050 | Unannotated | NA | NA |
| metab_4758 | PC(P-16:0/0:0) | pos | 480.3450 | 6.652 | 6.445 | 6.367 | -0.078 | -0.427 | 0.017 | 0.050 | Moderate putative | NA | NA |
| metab_6263 | Unannotated feature | neg | 426.8208 | 0.676 | 4.351 | 3.797 | -0.554 | -0.427 | 0.017 | 0.050 | Unannotated | NA | NA |
| metab_10904 | Unannotated feature | neg | 732.8604 | 0.543 | 5.817 | 5.744 | -0.072 | -0.427 | 0.017 | 0.050 | Unannotated | NA | NA |
| metab_227 | Unannotated feature | pos | 331.9203 | 0.636 | 4.317 | 3.972 | -0.345 | -0.427 | 0.017 | 0.050 | Unannotated | NA | NA |
| metab_210 | Unannotated feature | pos | 105.1136 | 0.614 | 3.458 | 3.650 | 0.192 | 0.427 | 0.017 | 0.050 | Unannotated | NA | NA |
| metab_8831 | 4-Vinylcyclohexene diepoxide | neg | 185.0817 | 5.361 | 3.197 | 3.985 | 0.788 | 0.427 | 0.017 | 0.050 | Tentative | Organoheterocyclic compounds | Oxepanes |
| metab_23 | Unannotated feature | pos | 365.1739 | 1.840 | 4.058 | 4.560 | 0.502 | 0.423 | 0.018 | 0.053 | Unannotated | NA | NA |
| metab_6988 | Unannotated feature | neg | 348.7638 | 0.967 | 5.390 | 5.362 | -0.028 | -0.423 | 0.018 | 0.053 | Unannotated | NA | NA |
| metab_9566 | Unannotated feature | neg | 187.9782 | 3.192 | 1.901 | 2.464 | 0.563 | 0.423 | 0.018 | 0.053 | Unannotated | NA | NA |
| metab_865 | Unannotated feature | pos | 364.0968 | 3.514 | 5.122 | 4.899 | -0.223 | -0.423 | 0.018 | 0.053 | Unannotated | NA | NA |
| metab_1311 | Unannotated feature | pos | 553.7246 | 5.879 | 5.109 | 5.020 | -0.089 | -0.423 | 0.018 | 0.053 | Unannotated | NA | NA |
| metab_773 | Unannotated feature | pos | 84.5069 | 2.904 | 3.712 | 3.786 | 0.074 | 0.423 | 0.018 | 0.053 | Unannotated | NA | NA |
| metab_1246 | Unannotated feature | pos | 319.0845 | 5.832 | 5.095 | 4.956 | -0.139 | -0.423 | 0.018 | 0.053 | Unannotated | NA | NA |
| metab_1218 | Unannotated feature | pos | 486.3455 | 5.786 | 5.487 | 5.376 | -0.111 | -0.423 | 0.018 | 0.053 | Unannotated | NA | NA |
| metab_7186 | Unannotated feature | neg | 327.7215 | 5.906 | 5.517 | 5.448 | -0.069 | -0.423 | 0.018 | 0.053 | Unannotated | NA | NA |
| metab_9085 | Eprodisate | neg | 238.9444 | 4.497 | 4.465 | 4.537 | 0.072 | 0.423 | 0.018 | 0.053 | Tentative | NA | NA |
| metab_1610 | Unannotated feature | pos | 102.1954 | 6.891 | 3.346 | 3.498 | 0.152 | 0.423 | 0.018 | 0.053 | Unannotated | NA | NA |
| metab_7453 | Unannotated feature | neg | 238.9319 | 7.132 | 4.662 | 4.610 | -0.052 | -0.423 | 0.018 | 0.053 | Unannotated | NA | NA |
| metab_7478 | Unannotated feature | neg | 218.9970 | 2.119 | 3.135 | 4.080 | 0.944 | 0.423 | 0.018 | 0.053 | Unannotated | NA | NA |
| metab_1406 | Pentaethylene glycol | pos | 239.1489 | 2.366 | 6.001 | 5.839 | -0.162 | -0.423 | 0.018 | 0.053 | Strong putative | NA | NA |
| metab_6053 | Unannotated feature | neg | 174.0591 | 3.065 | 3.480 | 2.581 | -0.898 | -0.423 | 0.018 | 0.053 | Unannotated | NA | NA |
| metab_10753 | Unannotated feature | neg | 558.5953 | 0.613 | 6.708 | 6.666 | -0.042 | -0.423 | 0.018 | 0.053 | Unannotated | NA | NA |
| metab_5687 | Unannotated feature | neg | 309.9579 | 1.286 | 4.618 | 4.537 | -0.081 | -0.423 | 0.018 | 0.053 | Unannotated | NA | NA |
| metab_3788 | Unannotated feature | pos | 101.0794 | 1.577 | 3.036 | 3.387 | 0.351 | 0.423 | 0.018 | 0.053 | Unannotated | NA | NA |
| metab_1692 | Diethylene glycol | pos | 148.0969 | 7.285 | 5.284 | 5.311 | 0.027 | 0.423 | 0.018 | 0.053 | Tentative | NA | NA |
| metab_618 | Tuliposide B | pos | 312.1300 | 2.164 | 4.885 | 5.283 | 0.398 | 0.423 | 0.018 | 0.053 | Tentative | Organic oxygen compounds | Organooxygen compounds |
| metab_10517 | Unannotated feature | neg | 242.0802 | 0.629 | 4.750 | 4.504 | -0.246 | -0.423 | 0.018 | 0.053 | Unannotated | NA | NA |
| metab_11172 | Unannotated feature | neg | 225.1133 | 5.755 | 4.805 | 5.025 | 0.220 | 0.423 | 0.018 | 0.053 | Unannotated | NA | NA |
| metab_11195 | Unannotated feature | neg | 207.1390 | 5.875 | 4.827 | 4.902 | 0.075 | 0.423 | 0.018 | 0.053 | Unannotated | NA | NA |
| metab_6683 | Unannotated feature | neg | 216.8803 | 5.207 | 4.207 | 4.158 | -0.049 | -0.423 | 0.018 | 0.053 | Unannotated | NA | NA |
| metab_6547 | Unannotated feature | neg | 359.1255 | 4.605 | 4.260 | 3.990 | -0.270 | -0.423 | 0.018 | 0.053 | Unannotated | NA | NA |
| metab_1900 | Unannotated feature | pos | 946.7745 | 0.551 | 4.462 | 4.871 | 0.410 | 0.423 | 0.018 | 0.053 | Unannotated | NA | NA |
| metab_8568 | Unannotated feature | neg | 411.2027 | 5.683 | 2.953 | 3.412 | 0.458 | 0.423 | 0.018 | 0.053 | Unannotated | NA | NA |
| metab_6636 | Unannotated feature | neg | 653.2680 | 5.001 | 6.411 | 6.628 | 0.217 | 0.423 | 0.018 | 0.053 | Unannotated | NA | NA |
| metab_1750 | Unannotated feature | pos | 132.0628 | 7.331 | 3.858 | 3.595 | -0.263 | -0.423 | 0.018 | 0.053 | Unannotated | NA | NA |
| metab_5661 | Unannotated feature | neg | 208.9736 | 1.086 | 4.862 | 5.006 | 0.143 | 0.423 | 0.018 | 0.053 | Unannotated | NA | NA |
| metab_8678 | Takinib | neg | 321.1363 | 5.619 | 5.629 | 5.189 | -0.441 | -0.423 | 0.018 | 0.053 | Tentative | Organoheterocyclic compounds | Benzimidazoles |
| metab_3400 | 5'-Methylthioadenosine | pos | 298.0969 | 2.467 | 4.953 | 5.189 | 0.236 | 0.423 | 0.018 | 0.053 | Strong putative | Nucleosides, nucleotides, and analogues | 5''-deoxyribonucleosides |
| metab_5330 | Unannotated feature | neg | 554.7201 | 0.013 | 5.679 | 5.651 | -0.028 | -0.423 | 0.018 | 0.053 | Unannotated | NA | NA |
| metab_7831 | Unannotated feature | neg | 540.3320 | 7.153 | 6.363 | 6.284 | -0.079 | -0.423 | 0.018 | 0.053 | Unannotated | NA | NA |
| metab_2157 | Unannotated feature | pos | 101.8448 | 6.474 | 6.099 | 6.164 | 0.065 | 0.423 | 0.018 | 0.053 | Unannotated | NA | NA |
| metab_2194 | Unannotated feature | pos | 213.1907 | 6.311 | 4.781 | 4.544 | -0.236 | -0.423 | 0.018 | 0.053 | Unannotated | NA | NA |
| metab_4029 | Unannotated feature | pos | 119.0732 | 4.163 | 3.979 | 3.733 | -0.246 | -0.423 | 0.018 | 0.053 | Unannotated | NA | NA |
| metab_6735 | 4-Methoxybenzenepropanol 1-(2-sulfoglucoside) | neg | 389.0921 | 5.421 | 4.924 | 5.181 | 0.257 | 0.423 | 0.018 | 0.053 | Tentative | Lipids and lipid-like molecules | Fatty Acyls |
| metab_6758 | Unannotated feature | neg | 255.8226 | 5.506 | 5.509 | 5.444 | -0.065 | -0.423 | 0.018 | 0.053 | Unannotated | NA | NA |
| metab_8931 | Unannotated feature | neg | 316.0598 | 5.001 | 3.670 | 2.957 | -0.714 | -0.423 | 0.018 | 0.053 | Unannotated | NA | NA |
| metab_3955 | Unannotated feature | pos | 117.7342 | 0.854 | 5.124 | 5.022 | -0.103 | -0.423 | 0.018 | 0.053 | Unannotated | NA | NA |
| metab_2664 | Unannotated feature | pos | 200.2374 | 5.793 | 4.136 | 4.248 | 0.112 | 0.423 | 0.018 | 0.053 | Unannotated | NA | NA |
| metab_9681 | Unannotated feature | neg | 171.1136 | 2.922 | 3.814 | 3.530 | -0.283 | -0.423 | 0.018 | 0.053 | Unannotated | NA | NA |
| metab_2607 | Unannotated feature | pos | 537.3795 | 5.840 | 5.628 | 5.567 | -0.061 | -0.423 | 0.018 | 0.053 | Unannotated | NA | NA |
| metab_5102 | Unannotated feature | pos | 196.8652 | 0.591 | 6.097 | 6.071 | -0.027 | -0.423 | 0.018 | 0.053 | Unannotated | NA | NA |
| metab_8838 | Unannotated feature | neg | 696.7734 | 5.346 | 6.879 | 7.127 | 0.248 | 0.423 | 0.018 | 0.053 | Unannotated | NA | NA |
| metab_5061 | Unannotated feature | pos | 258.8178 | 0.908 | 5.517 | 5.476 | -0.040 | -0.423 | 0.018 | 0.053 | Unannotated | NA | NA |
| metab_5115 | APC | pos | 601.2655 | 4.754 | 5.555 | 5.213 | -0.343 | -0.423 | 0.018 | 0.053 | Tentative | NA | NA |
| metab_7724 | Unannotated feature | neg | 183.8343 | 7.197 | 5.078 | 5.043 | -0.034 | -0.423 | 0.018 | 0.053 | Unannotated | NA | NA |
| metab_10305 | Unannotated feature | neg | 188.0674 | 0.802 | 3.047 | 3.659 | 0.612 | 0.423 | 0.018 | 0.053 | Unannotated | NA | NA |
| metab_4109 | Indolelactate | pos | 206.0813 | 4.493 | 5.223 | 5.434 | 0.211 | 0.423 | 0.018 | 0.053 | Strong putative | Organoheterocyclic compounds | Indoles and derivatives |
| metab_4818 | Unannotated feature | pos | 129.0548 | 7.959 | 5.451 | 5.469 | 0.018 | 0.423 | 0.018 | 0.053 | Unannotated | NA | NA |
| metab_2932 | D-Arabinono-1,4-lactone | pos | 181.0720 | 2.195 | 5.351 | 4.588 | -0.762 | -0.423 | 0.018 | 0.053 | Tentative | Organic oxygen compounds | Organooxygen compounds |
| metab_2890 | Unannotated feature | pos | 145.0649 | 5.053 | 3.598 | 3.775 | 0.177 | 0.423 | 0.018 | 0.053 | Unannotated | NA | NA |
| metab_239 | Unannotated feature | pos | 290.0846 | 0.644 | 4.380 | 3.845 | -0.535 | -0.423 | 0.018 | 0.053 | Unannotated | NA | NA |
| metab_6779 | Unannotated feature | neg | 291.0210 | 5.538 | 4.788 | 5.043 | 0.254 | 0.423 | 0.018 | 0.053 | Unannotated | NA | NA |
| metab_6894 | 3-Hydroxy-1,4-benzodiazepine | neg | 365.1262 | 5.651 | 5.741 | 5.283 | -0.458 | -0.423 | 0.018 | 0.053 | Tentative | Organoheterocyclic compounds | Benzodiazepines |
| metab_3829 | Unannotated feature | pos | 166.0863 | 3.885 | 4.630 | 4.856 | 0.226 | 0.419 | 0.019 | 0.055 | Unannotated | NA | NA |
| metab_991 | PI(5-iso PGF2VI/16:2(9Z,12Z)) | pos | 921.4303 | 4.470 | 5.578 | 5.716 | 0.138 | 0.419 | 0.019 | 0.055 | Tentative | NA | NA |
| metab_956 | Unannotated feature | pos | 163.1118 | 4.171 | 4.518 | 4.342 | -0.176 | -0.419 | 0.019 | 0.055 | Moderate putative | NA | NA |
| metab_1644 | Unannotated feature | pos | 590.4265 | 7.192 | 5.370 | 5.411 | 0.041 | 0.419 | 0.019 | 0.055 | Unannotated | NA | NA |
| metab_10745 | Unannotated feature | neg | 636.8306 | 0.570 | 5.290 | 5.379 | 0.089 | 0.419 | 0.019 | 0.055 | Unannotated | NA | NA |
| metab_1404 | Unannotated feature | pos | 763.0378 | 5.925 | 5.316 | 5.211 | -0.104 | -0.419 | 0.019 | 0.055 | Unannotated | NA | NA |
| metab_1367 | Unannotated feature | pos | 600.9238 | 5.910 | 4.665 | 4.524 | -0.142 | -0.419 | 0.019 | 0.055 | Unannotated | NA | NA |
| metab_4046 | Unannotated feature | pos | 222.8678 | 0.776 | 4.403 | 4.292 | -0.111 | -0.419 | 0.019 | 0.055 | Unannotated | NA | NA |
| metab_2785 | Unannotated feature | pos | 237.1460 | 5.634 | 4.832 | 4.870 | 0.038 | 0.419 | 0.019 | 0.055 | Unannotated | NA | NA |
| metab_304 | Unannotated feature | pos | 129.0548 | 0.776 | 4.960 | 4.933 | -0.027 | -0.419 | 0.019 | 0.055 | Unannotated | NA | NA |
| metab_4652 | Unannotated feature | pos | 362.3264 | 5.771 | 7.003 | 7.043 | 0.039 | 0.419 | 0.019 | 0.055 | Unannotated | NA | NA |
| metab_4603 | Unannotated feature | pos | 367.1401 | 5.107 | 6.188 | 5.784 | -0.404 | -0.419 | 0.019 | 0.055 | Unannotated | NA | NA |
| metab_7892 | Unannotated feature | neg | 444.8913 | 7.132 | 4.511 | 4.326 | -0.185 | -0.419 | 0.019 | 0.055 | Unannotated | NA | NA |
| metab_9369 | Unannotated feature | neg | 126.8803 | 3.562 | 4.122 | 4.091 | -0.031 | -0.419 | 0.019 | 0.055 | Unannotated | NA | NA |
| metab_8304 | Unannotated feature | neg | 495.2975 | 5.859 | 4.475 | 4.837 | 0.362 | 0.419 | 0.019 | 0.055 | Unannotated | NA | NA |
| metab_516 | Unannotated feature | pos | 99.5316 | 1.933 | 4.784 | 4.843 | 0.059 | 0.419 | 0.019 | 0.055 | Unannotated | NA | NA |
| metab_4559 | Unannotated feature | pos | 132.9949 | 0.496 | 4.681 | 4.664 | -0.017 | -0.419 | 0.019 | 0.055 | Unannotated | NA | NA |
| metab_10430 | Unannotated feature | neg | 187.0721 | 0.676 | 4.417 | 4.255 | -0.162 | -0.419 | 0.019 | 0.055 | Unannotated | NA | NA |
| metab_10394 | Unannotated feature | neg | 517.7852 | 0.707 | 4.297 | 3.990 | -0.307 | -0.419 | 0.019 | 0.055 | Unannotated | NA | NA |
| metab_4321 | Unannotated feature | pos | 683.6950 | 0.575 | 4.381 | 4.181 | -0.200 | -0.419 | 0.019 | 0.055 | Unannotated | NA | NA |
| metab_5416 | Unannotated feature | neg | 214.8364 | 0.629 | 5.580 | 5.562 | -0.018 | -0.419 | 0.019 | 0.055 | Unannotated | NA | NA |
| metab_2080 | 2-Aminopurine | pos | 177.0884 | 7.200 | 5.655 | 5.671 | 0.016 | 0.419 | 0.019 | 0.055 | Tentative | NA | NA |
| metab_5329 | Unannotated feature | neg | 578.8702 | 0.570 | 5.590 | 5.517 | -0.073 | -0.419 | 0.019 | 0.055 | Unannotated | NA | NA |
| metab_8268 | Unannotated feature | neg | 369.1021 | 5.890 | 4.723 | 4.173 | -0.550 | -0.419 | 0.019 | 0.055 | Unannotated | NA | NA |
| metab_8276 | Unannotated feature | neg | 459.2983 | 5.883 | 3.005 | 2.045 | -0.960 | -0.419 | 0.019 | 0.055 | Unannotated | NA | NA |
| metab_5988 | Unannotated feature | neg | 195.0521 | 2.828 | 3.866 | 3.513 | -0.354 | -0.419 | 0.019 | 0.055 | Unannotated | NA | NA |
| metab_5951 | Unannotated feature | neg | 199.8991 | 2.692 | 5.244 | 5.198 | -0.046 | -0.419 | 0.019 | 0.055 | Unannotated | NA | NA |
| metab_7424 | Unannotated feature | neg | 188.9858 | 7.109 | 4.869 | 4.929 | 0.060 | 0.419 | 0.019 | 0.055 | Unannotated | NA | NA |
| metab_8122 | Unannotated feature | neg | 229.0902 | 6.172 | 5.167 | 4.988 | -0.179 | -0.419 | 0.019 | 0.055 | Unannotated | NA | NA |
| metab_592 | Unannotated feature | pos | 341.2279 | 2.073 | 4.931 | 4.693 | -0.238 | -0.419 | 0.019 | 0.055 | Unannotated | NA | NA |
| metab_10493 | Unannotated feature | neg | 266.0183 | 4.893 | 6.828 | 6.559 | -0.269 | -0.419 | 0.019 | 0.055 | Unannotated | NA | NA |
| metab_5973 | Unannotated feature | neg | 90.5272 | 2.780 | 2.808 | 2.556 | -0.252 | -0.419 | 0.019 | 0.055 | Unannotated | NA | NA |
| metab_3528 | L-Phenylalanine | pos | 331.1650 | 2.095 | 4.879 | 4.571 | -0.308 | -0.419 | 0.019 | 0.055 | Strong putative | Organic acids and derivatives | Carboxylic acids and derivatives |
| metab_7518 | Unannotated feature | neg | 178.9575 | 7.164 | 4.933 | 4.913 | -0.020 | -0.419 | 0.019 | 0.055 | Unannotated | NA | NA |
| metab_1728 | Unannotated feature | pos | 295.6480 | 7.316 | 3.546 | 3.341 | -0.205 | -0.419 | 0.019 | 0.055 | Unannotated | NA | NA |
| metab_8084 | Unannotated feature | neg | 529.4271 | 6.196 | 5.095 | 4.590 | -0.505 | -0.419 | 0.019 | 0.055 | Unannotated | NA | NA |
| metab_1073 | Unannotated feature | pos | 102.3760 | 5.198 | 4.379 | 4.421 | 0.042 | 0.419 | 0.019 | 0.055 | Unannotated | NA | NA |
| metab_4944 | Unannotated feature | pos | 273.9618 | 0.636 | 5.083 | 4.896 | -0.186 | -0.419 | 0.019 | 0.055 | Unannotated | NA | NA |
| metab_4940 | Unannotated feature | pos | 380.9223 | 0.629 | 5.440 | 5.384 | -0.056 | -0.419 | 0.019 | 0.055 | Unannotated | NA | NA |
| metab_9464 | Unannotated feature | neg | 171.9465 | 3.316 | 4.641 | 4.506 | -0.135 | -0.419 | 0.019 | 0.055 | Unannotated | NA | NA |
| metab_17 | Unannotated feature | pos | 198.8622 | 0.591 | 6.095 | 6.063 | -0.032 | -0.419 | 0.019 | 0.055 | Unannotated | NA | NA |
| metab_4426 | Unannotated feature | pos | 598.8437 | 0.559 | 4.317 | 4.406 | 0.089 | 0.419 | 0.019 | 0.055 | Unannotated | NA | NA |
| metab_708 | Unannotated feature | pos | 100.1125 | 2.560 | 4.156 | 4.383 | 0.227 | 0.415 | 0.020 | 0.058 | Unannotated | NA | NA |
| metab_1461 | Unannotated feature | pos | 417.2822 | 5.995 | 4.836 | 4.893 | 0.057 | 0.415 | 0.020 | 0.058 | Unannotated | NA | NA |
| metab_1609 | Unannotated feature | pos | 740.7639 | 0.567 | 5.407 | 5.335 | -0.072 | -0.415 | 0.020 | 0.058 | Unannotated | NA | NA |
| metab_6975 | Elemicin | neg | 207.1026 | 5.707 | 3.468 | 4.047 | 0.580 | 0.415 | 0.020 | 0.058 | Tentative | NA | NA |
| metab_6147 | Unannotated feature | neg | 283.9456 | 0.644 | 5.230 | 5.034 | -0.196 | -0.415 | 0.020 | 0.058 | Unannotated | NA | NA |
| metab_3559 | G-Glu-Val | pos | 247.1288 | 2.065 | 5.285 | 5.395 | 0.110 | 0.415 | 0.020 | 0.058 | Moderate putative | NA | NA |
| metab_4201 | Unannotated feature | pos | 134.0449 | 0.629 | 3.793 | 3.986 | 0.193 | 0.415 | 0.020 | 0.058 | Unannotated | NA | NA |
| metab_3435 | Unannotated feature | pos | 243.1339 | 2.366 | 3.966 | 3.662 | -0.304 | -0.415 | 0.020 | 0.058 | Unannotated | NA | NA |
| metab_5774 | Unannotated feature | neg | 90.5271 | 2.002 | 3.390 | 3.215 | -0.175 | -0.415 | 0.020 | 0.058 | Unannotated | NA | NA |
| metab_799 | Unannotated feature | pos | 196.0910 | 3.019 | 4.964 | 1.418 | -3.545 | -0.415 | 0.020 | 0.058 | Unannotated | NA | NA |
| metab_6328 | Unannotated feature | neg | 195.0596 | 3.758 | 4.204 | 4.138 | -0.066 | -0.415 | 0.020 | 0.058 | Unannotated | NA | NA |
| metab_10907 | Unannotated feature | neg | 630.8805 | 0.543 | 5.130 | 5.038 | -0.093 | -0.415 | 0.020 | 0.058 | Unannotated | NA | NA |
| metab_1317 | Unannotated feature | pos | 836.5903 | 5.879 | 5.456 | 5.367 | -0.089 | -0.415 | 0.020 | 0.058 | Unannotated | NA | NA |
| metab_10810 | Unannotated feature | neg | 968.7865 | 0.554 | 4.648 | 4.849 | 0.201 | 0.415 | 0.020 | 0.058 | Unannotated | NA | NA |
| metab_8901 | Unannotated feature | neg | 197.0010 | 5.131 | 4.804 | 4.558 | -0.246 | -0.415 | 0.020 | 0.058 | Moderate putative | NA | NA |
| metab_5384 | Unannotated feature | neg | 711.5028 | 0.598 | 4.932 | 4.829 | -0.103 | -0.415 | 0.020 | 0.058 | Unannotated | NA | NA |
| metab_5439 | Unannotated feature | neg | 198.0714 | 0.652 | 3.918 | 3.584 | -0.335 | -0.415 | 0.020 | 0.058 | Unannotated | NA | NA |
| metab_5506 | Unannotated feature | neg | 317.9469 | 0.747 | 3.945 | 3.592 | -0.353 | -0.415 | 0.020 | 0.058 | Unannotated | NA | NA |
| metab_5677 | Unannotated feature | neg | 171.9465 | 1.190 | 4.768 | 4.635 | -0.133 | -0.415 | 0.020 | 0.058 | Unannotated | NA | NA |
| metab_5666 | Unannotated feature | neg | 159.8596 | 1.094 | 5.965 | 5.931 | -0.034 | -0.415 | 0.020 | 0.058 | Unannotated | NA | NA |
| metab_1858 | Unannotated feature | pos | 458.4200 | 7.633 | 5.758 | 5.715 | -0.043 | -0.415 | 0.020 | 0.058 | Unannotated | NA | NA |
| metab_5361 | Unannotated feature | neg | 468.7264 | 0.587 | 4.688 | 4.558 | -0.130 | -0.415 | 0.020 | 0.058 | Unannotated | NA | NA |
| metab_3572 | Tolmetin | pos | 240.1018 | 2.058 | 4.739 | 4.483 | -0.256 | -0.415 | 0.020 | 0.058 | Tentative | Organic oxygen compounds | Organooxygen compounds |
| metab_10887 | Unannotated feature | neg | 485.1675 | 5.354 | 5.170 | 5.438 | 0.267 | 0.415 | 0.020 | 0.058 | Unannotated | NA | NA |
| metab_1280 | Unannotated feature | pos | 661.4604 | 5.855 | 5.938 | 5.875 | -0.063 | -0.415 | 0.020 | 0.058 | Unannotated | NA | NA |
| metab_121 | Unannotated feature | pos | 270.9777 | 0.545 | 4.889 | 4.692 | -0.198 | -0.415 | 0.020 | 0.058 | Unannotated | NA | NA |
| metab_8423 | Unannotated feature | neg | 199.1338 | 5.779 | 2.716 | 3.152 | 0.436 | 0.415 | 0.020 | 0.058 | Unannotated | NA | NA |
| metab_4947 | Unannotated feature | pos | 271.9648 | 0.636 | 5.535 | 5.406 | -0.129 | -0.415 | 0.020 | 0.058 | Unannotated | NA | NA |
| metab_4926 | Unannotated feature | pos | 502.6781 | 0.598 | 5.419 | 5.349 | -0.070 | -0.415 | 0.020 | 0.058 | Unannotated | NA | NA |
| metab_3754 | Unannotated feature | pos | 202.1186 | 1.747 | 3.171 | 4.317 | 1.146 | 0.415 | 0.020 | 0.058 | Unannotated | NA | NA |
| metab_2676 | Unannotated feature | pos | 275.5278 | 5.778 | 5.130 | 5.223 | 0.092 | 0.415 | 0.020 | 0.058 | Unannotated | NA | NA |
| metab_4877 | Unannotated feature | pos | 286.1032 | 2.073 | 3.672 | 4.517 | 0.845 | 0.415 | 0.020 | 0.058 | Unannotated | NA | NA |
| metab_2494 | Unannotated feature | pos | 880.8190 | 0.551 | 5.495 | 5.401 | -0.094 | -0.415 | 0.020 | 0.058 | Unannotated | NA | NA |
| metab_9797 | Unannotated feature | neg | 310.0028 | 2.541 | 4.647 | 4.554 | -0.093 | -0.415 | 0.020 | 0.058 | Unannotated | NA | NA |
| metab_6720 | Unannotated feature | neg | 701.8584 | 5.346 | 3.842 | 4.678 | 0.836 | 0.415 | 0.020 | 0.058 | Unannotated | NA | NA |
| metab_6682 | Unannotated feature | neg | 687.7106 | 5.199 | 4.886 | 5.239 | 0.353 | 0.415 | 0.020 | 0.058 | Unannotated | NA | NA |
| metab_2804 | Unannotated feature | pos | 300.2169 | 5.588 | 5.055 | 5.256 | 0.201 | 0.415 | 0.020 | 0.058 | Unannotated | NA | NA |
| metab_222 | sn-Glycero-3-phosphocholine | pos | 258.1102 | 0.621 | 5.480 | 5.320 | -0.160 | -0.411 | 0.022 | 0.061 | Moderate putative | Lipids and lipid-like molecules | Glycerophospholipids |
| metab_3581 | Aniline | pos | 94.0656 | 2.058 | 4.676 | 4.783 | 0.107 | 0.411 | 0.022 | 0.061 | Moderate putative | NA | NA |
| metab_196 | Unannotated feature | pos | 926.4477 | 0.591 | 4.843 | 4.675 | -0.168 | -0.411 | 0.022 | 0.061 | Unannotated | NA | NA |
| metab_11249 | Unannotated feature | neg | 822.7313 | 0.570 | 4.936 | 4.687 | -0.248 | -0.411 | 0.022 | 0.061 | Unannotated | NA | NA |
| metab_8131 | Trans-3-Feruloylcorosolic acid | neg | 693.4045 | 6.149 | 4.968 | 4.187 | -0.781 | -0.411 | 0.022 | 0.061 | Tentative | Lipids and lipid-like molecules | Prenol lipids |
| metab_9260 | Unannotated feature | neg | 425.9670 | 0.668 | 4.342 | 3.910 | -0.432 | -0.411 | 0.022 | 0.061 | Unannotated | NA | NA |
| metab_3382 | Theophylline | pos | 181.0720 | 2.521 | 6.077 | 4.103 | -1.974 | -0.411 | 0.022 | 0.061 | Strong putative | Organoheterocyclic compounds | Imidazopyrimidines |
| metab_8618 | (1beta,2beta,5beta)-p-Menth-3-ene-1,2,5-triol | neg | 231.1239 | 5.659 | 4.299 | 4.485 | 0.186 | 0.411 | 0.022 | 0.061 | Tentative | Lipids and lipid-like molecules | Prenol lipids |
| metab_1432 | Unannotated feature | pos | 776.5738 | 5.956 | 6.348 | 6.306 | -0.043 | -0.411 | 0.022 | 0.061 | Unannotated | NA | NA |
| metab_1391 | Unannotated feature | pos | 783.5406 | 5.917 | 4.868 | 4.620 | -0.249 | -0.411 | 0.022 | 0.061 | Unannotated | NA | NA |
| metab_6698 | Unannotated feature | neg | 158.0455 | 0.857 | 3.823 | 4.117 | 0.293 | 0.411 | 0.022 | 0.061 | Unannotated | NA | NA |
| metab_1208 | Unannotated feature | pos | 364.3327 | 5.771 | 5.263 | 5.327 | 0.064 | 0.411 | 0.022 | 0.061 | Unannotated | NA | NA |
| metab_11179 | Unannotated feature | neg | 522.6912 | 0.590 | 6.022 | 5.997 | -0.025 | -0.411 | 0.022 | 0.061 | Unannotated | NA | NA |
| metab_3100 | Unannotated feature | pos | 102.5360 | 3.846 | 4.516 | 4.491 | -0.025 | -0.411 | 0.022 | 0.061 | Unannotated | NA | NA |
| metab_6243 | Allantoin | neg | 157.0362 | 0.676 | 4.901 | 5.001 | 0.100 | 0.411 | 0.022 | 0.061 | Moderate putative | NA | NA |
| metab_6229 | 3-hydroxy-3-(3-hydroxyphenyl)propanoic acid-O-sulphate | neg | 261.0079 | 3.420 | 4.024 | 4.808 | 0.784 | 0.411 | 0.022 | 0.061 | Tentative | Organic acids and derivatives | Hydroxy acids and derivatives |
| metab_5802 | Unannotated feature | neg | 246.9299 | 2.111 | 3.388 | 2.951 | -0.437 | -0.411 | 0.022 | 0.061 | Unannotated | NA | NA |
| metab_7552 | Unannotated feature | neg | 172.8298 | 7.181 | 4.972 | 4.944 | -0.027 | -0.411 | 0.022 | 0.061 | Unannotated | NA | NA |
| metab_7511 | Unannotated feature | neg | 204.9651 | 2.119 | 4.496 | 4.318 | -0.178 | -0.411 | 0.022 | 0.061 | Unannotated | NA | NA |
| metab_10567 | Unannotated feature | neg | 794.4248 | 0.613 | 6.271 | 6.214 | -0.057 | -0.411 | 0.022 | 0.061 | Unannotated | NA | NA |
| metab_2718 | Unannotated feature | pos | 85.3008 | 5.740 | 3.892 | 3.983 | 0.092 | 0.411 | 0.022 | 0.061 | Unannotated | NA | NA |
| metab_10504 | Unannotated feature | neg | 453.0871 | 4.917 | 6.035 | 5.732 | -0.303 | -0.411 | 0.022 | 0.061 | Unannotated | NA | NA |
| metab_4988 | L-Citrulline | pos | 198.0850 | 0.644 | 5.967 | 6.164 | 0.198 | 0.411 | 0.022 | 0.061 | Strong putative | NA | NA |
| metab_4999 | Unannotated feature | pos | 324.1377 | 5.832 | 3.062 | 2.486 | -0.576 | -0.411 | 0.022 | 0.061 | Unannotated | NA | NA |
| metab_4973 | Unannotated feature | pos | 275.9517 | 0.706 | 4.769 | 4.714 | -0.055 | -0.411 | 0.022 | 0.061 | Unannotated | NA | NA |
| metab_3915 | Isoleucylhydroxyproline | pos | 245.1496 | 3.993 | 4.184 | 4.700 | 0.516 | 0.411 | 0.022 | 0.061 | Tentative | Organic acids and derivatives | Carboxylic acids and derivatives |
| metab_7121 | Unannotated feature | neg | 251.9990 | 1.294 | 5.958 | 5.924 | -0.035 | -0.411 | 0.022 | 0.061 | Unannotated | NA | NA |
| metab_11145 | Unannotated feature | neg | 491.1207 | 5.715 | 5.056 | 5.021 | -0.035 | -0.411 | 0.022 | 0.061 | Unannotated | NA | NA |
| metab_3029 | Unannotated feature | pos | 84.1316 | 4.201 | 4.013 | 4.096 | 0.083 | 0.411 | 0.022 | 0.061 | Unannotated | NA | NA |
| metab_3235 | 4-hydroxylamino-2,6-dinitrotoluene-O-glucoside | pos | 340.0807 | 3.112 | 5.180 | 5.349 | 0.168 | 0.411 | 0.022 | 0.061 | Tentative | NA | NA |
| metab_4874 | Unannotated feature | pos | 178.0897 | 2.195 | 4.299 | 4.762 | 0.464 | 0.411 | 0.022 | 0.061 | Unannotated | NA | NA |
| metab_6509 | Unannotated feature | neg | 157.5366 | 4.433 | 3.564 | 2.586 | -0.978 | -0.411 | 0.022 | 0.061 | Unannotated | NA | NA |
| metab_9064 | Unannotated feature | neg | 249.8923 | 4.559 | 3.940 | 4.071 | 0.131 | 0.411 | 0.022 | 0.061 | Unannotated | NA | NA |
| metab_10218 | Unannotated feature | neg | 257.0531 | 0.865 | 4.501 | 4.372 | -0.129 | -0.411 | 0.022 | 0.061 | Unannotated | NA | NA |
| metab_3586 | Unannotated feature | pos | 194.1037 | 2.050 | 3.552 | 3.834 | 0.281 | 0.411 | 0.022 | 0.061 | Unannotated | NA | NA |
| metab_3685 | Unannotated feature | pos | 128.1072 | 1.925 | 4.149 | 4.209 | 0.060 | 0.411 | 0.022 | 0.061 | Unannotated | NA | NA |
| metab_8941 | Unannotated feature | neg | 203.2677 | 4.969 | 3.237 | 3.524 | 0.287 | 0.411 | 0.022 | 0.061 | Unannotated | NA | NA |
| metab_7812 | Unannotated feature | neg | 176.9609 | 7.164 | 3.877 | 3.811 | -0.066 | -0.411 | 0.022 | 0.061 | Unannotated | NA | NA |
| metab_7826 | Unannotated feature | neg | 560.5924 | 0.621 | 6.884 | 6.846 | -0.038 | -0.411 | 0.022 | 0.061 | Unannotated | NA | NA |
| metab_4463 | Unannotated feature | pos | 778.8377 | 0.551 | 5.607 | 5.546 | -0.061 | -0.411 | 0.022 | 0.061 | Unannotated | NA | NA |
| metab_4033 | Unannotated feature | pos | 217.1296 | 0.784 | 3.715 | 3.918 | 0.204 | 0.411 | 0.022 | 0.061 | Unannotated | NA | NA |
| metab_5277 | Unannotated feature | neg | 308.8647 | 0.013 | 5.810 | 5.789 | -0.021 | -0.411 | 0.022 | 0.061 | Unannotated | NA | NA |
| metab_10355 | Unannotated feature | neg | 440.6806 | 0.621 | 6.222 | 6.187 | -0.035 | -0.411 | 0.022 | 0.061 | Unannotated | NA | NA |
| metab_10345 | Unannotated feature | neg | 259.9882 | 0.754 | 4.067 | 3.936 | -0.131 | -0.411 | 0.022 | 0.061 | Unannotated | NA | NA |
| metab_7000 | Unannotated feature | neg | 169.8336 | 5.731 | 3.917 | 3.829 | -0.088 | -0.411 | 0.022 | 0.061 | Unannotated | NA | NA |
| metab_11386 | Cefpodoxime proxetil | neg | 578.1022 | 4.885 | 4.831 | 4.300 | -0.530 | -0.411 | 0.022 | 0.061 | Tentative | NA | NA |
| metab_3627 | Unannotated feature | pos | 394.1343 | 1.956 | 4.492 | 4.431 | -0.061 | -0.411 | 0.022 | 0.061 | Unannotated | NA | NA |
| metab_6977 | Unannotated feature | neg | 202.8399 | 0.967 | 5.478 | 5.560 | 0.082 | 0.411 | 0.022 | 0.061 | Unannotated | NA | NA |
| metab_10448 | Unannotated feature | neg | 250.0935 | 0.660 | 4.714 | 4.491 | -0.222 | -0.411 | 0.022 | 0.061 | Unannotated | NA | NA |
| metab_5216 | Unannotated feature | neg | 800.8496 | 0.543 | 5.781 | 5.713 | -0.068 | -0.411 | 0.022 | 0.061 | Unannotated | NA | NA |
| metab_537 | N-Undecanoylglycine | pos | 226.1802 | 5.793 | 6.032 | 6.059 | 0.027 | 0.411 | 0.022 | 0.061 | Tentative | NA | NA |
| metab_2909 | Fructosyl valine | pos | 280.1391 | 0.854 | 6.080 | 5.951 | -0.129 | -0.406 | 0.023 | 0.064 | Tentative | NA | NA |
| metab_10832 | Unannotated feature | neg | 742.7437 | 0.554 | 4.241 | 4.695 | 0.454 | 0.406 | 0.023 | 0.064 | Unannotated | NA | NA |
| metab_2973 | Unannotated feature | pos | 459.6679 | 4.532 | 4.318 | 4.532 | 0.214 | 0.406 | 0.023 | 0.064 | Unannotated | NA | NA |
| metab_9449 | Unannotated feature | neg | 243.9179 | 3.340 | 3.193 | 2.748 | -0.445 | -0.406 | 0.023 | 0.064 | Unannotated | NA | NA |
| metab_8850 | Unannotated feature | neg | 241.7936 | 5.330 | 4.175 | 4.252 | 0.078 | 0.406 | 0.023 | 0.064 | Unannotated | NA | NA |
| metab_7226 | Unannotated feature | neg | 219.8328 | 5.977 | 5.414 | 5.392 | -0.022 | -0.406 | 0.023 | 0.064 | Unannotated | NA | NA |
| metab_10535 | Unannotated feature | neg | 629.1198 | 4.945 | 6.264 | 5.907 | -0.357 | -0.406 | 0.023 | 0.064 | Unannotated | NA | NA |
| metab_926 | Unannotated feature | pos | 279.1702 | 3.955 | 6.138 | 5.862 | -0.276 | -0.406 | 0.023 | 0.064 | Strong putative | NA | NA |
| metab_880 | Unannotated feature | pos | 124.0759 | 3.638 | 3.695 | 3.440 | -0.255 | -0.406 | 0.023 | 0.064 | Unannotated | NA | NA |
| metab_508 | Unannotated feature | pos | 89.5072 | 1.917 | 5.181 | 5.152 | -0.029 | -0.406 | 0.023 | 0.064 | Unannotated | NA | NA |
| metab_2992 | Nalidixic acid | pos | 215.0816 | 4.409 | 4.147 | 3.898 | -0.249 | -0.406 | 0.023 | 0.064 | Tentative | Organoheterocyclic compounds | Diazanaphthalenes |
| metab_7538 | Unannotated feature | neg | 136.8716 | 7.173 | 4.300 | 4.268 | -0.032 | -0.406 | 0.023 | 0.064 | Unannotated | NA | NA |
| metab_9112 | Unannotated feature | neg | 107.0494 | 4.379 | 5.527 | 6.134 | 0.607 | 0.406 | 0.023 | 0.064 | Unannotated | NA | NA |
| metab_11030 | Unannotated feature | neg | 423.7846 | 0.013 | 3.939 | 3.827 | -0.112 | -0.406 | 0.023 | 0.064 | Unannotated | NA | NA |
| metab_2414 | Unannotated feature | pos | 311.1835 | 5.933 | 4.803 | 4.853 | 0.050 | 0.406 | 0.023 | 0.064 | Unannotated | NA | NA |
| metab_8596 | 2,3-Dihydroxypropyl 4-phenylbutanoate | neg | 283.1193 | 5.675 | 5.012 | 5.205 | 0.193 | 0.406 | 0.023 | 0.064 | Tentative | Lipids and lipid-like molecules | Glycerolipids |
| metab_7604 | Unannotated feature | neg | 170.8837 | 7.261 | 4.706 | 4.744 | 0.038 | 0.406 | 0.023 | 0.064 | Unannotated | NA | NA |
| metab_7268 | Unannotated feature | neg | 160.5329 | 6.110 | 5.118 | 5.031 | -0.087 | -0.406 | 0.023 | 0.064 | Unannotated | NA | NA |
| metab_3998 | Unannotated feature | pos | 85.0653 | 0.823 | 3.262 | 3.216 | -0.046 | -0.406 | 0.023 | 0.064 | Unannotated | NA | NA |
| metab_2337 | Unannotated feature | pos | 644.4945 | 5.995 | 5.933 | 5.900 | -0.032 | -0.406 | 0.023 | 0.064 | Unannotated | NA | NA |
| metab_10256 | Unannotated feature | neg | 690.5330 | 0.594 | 5.210 | 5.069 | -0.141 | -0.406 | 0.023 | 0.064 | Unannotated | NA | NA |
| metab_8754 | Unannotated feature | neg | 378.0755 | 5.547 | 0.122 | 3.958 | 3.836 | 0.406 | 0.023 | 0.064 | Unannotated | NA | NA |
| metab_815 | Unannotated feature | pos | 83.6587 | 3.105 | 3.588 | 3.487 | -0.101 | -0.406 | 0.023 | 0.064 | Unannotated | NA | NA |
| metab_4273 | Unannotated feature | pos | 560.6368 | 0.591 | 5.378 | 5.285 | -0.093 | -0.406 | 0.023 | 0.064 | Unannotated | NA | NA |
| metab_673 | Dopamine quinone | pos | 134.0602 | 2.428 | 3.454 | 4.201 | 0.747 | 0.406 | 0.023 | 0.064 | Tentative | Organic oxygen compounds | Organooxygen compounds |
| metab_1650 | Unannotated feature | pos | 157.1224 | 7.207 | 4.816 | 4.845 | 0.028 | 0.406 | 0.023 | 0.064 | Moderate putative | NA | NA |
| metab_1621 | Unannotated feature | pos | 363.2308 | 7.053 | 4.988 | 5.051 | 0.063 | 0.406 | 0.023 | 0.064 | Unannotated | NA | NA |
| metab_8613 | (3-{[3-(2-Amino-2-Oxoethyl)-1-Benzyl-2-Ethyl-1h-Indol-5-Yl]oxy}propyl)phosphonic Acid | neg | 451.1399 | 5.659 | 4.917 | 4.606 | -0.311 | -0.406 | 0.023 | 0.064 | Tentative | NA | NA |
| metab_11237 | Unannotated feature | neg | 588.3320 | 6.047 | 6.073 | 6.231 | 0.159 | 0.406 | 0.023 | 0.064 | Unannotated | NA | NA |
| metab_1320 | Unannotated feature | pos | 519.7023 | 5.886 | 4.891 | 4.811 | -0.080 | -0.406 | 0.023 | 0.064 | Unannotated | NA | NA |
| metab_10854 | Unannotated feature | neg | 591.8773 | 0.554 | 5.380 | 5.464 | 0.084 | 0.406 | 0.023 | 0.064 | Unannotated | NA | NA |
| metab_3865 | Unannotated feature | pos | 309.1291 | 0.908 | 4.731 | 4.542 | -0.189 | -0.406 | 0.023 | 0.064 | Unannotated | NA | NA |
| metab_4830 | Unannotated feature | pos | 465.3342 | 7.293 | 5.541 | 5.489 | -0.052 | -0.406 | 0.023 | 0.064 | Unannotated | NA | NA |
| metab_1180 | Unannotated feature | pos | 200.0918 | 1.956 | 4.396 | 4.581 | 0.185 | 0.406 | 0.023 | 0.064 | Unannotated | NA | NA |
| metab_6165 | Unannotated feature | neg | 600.3012 | 3.247 | 4.514 | 4.792 | 0.279 | 0.406 | 0.023 | 0.064 | Unannotated | NA | NA |
| metab_1575 | Unannotated feature | pos | 101.0602 | 6.420 | 3.757 | 3.885 | 0.128 | 0.406 | 0.023 | 0.064 | Unannotated | NA | NA |
| metab_8965 | Unannotated feature | neg | 267.2159 | 4.893 | 4.028 | 3.581 | -0.447 | -0.406 | 0.023 | 0.064 | Unannotated | NA | NA |
| metab_8292 | Unannotated feature | neg | 195.1389 | 5.875 | 4.482 | 4.573 | 0.090 | 0.406 | 0.023 | 0.064 | Unannotated | NA | NA |
| metab_3800 | Unannotated feature | pos | 198.0973 | 1.499 | 5.583 | 5.506 | -0.078 | -0.406 | 0.023 | 0.064 | Unannotated | NA | NA |
| metab_4738 | Unannotated feature | pos | 441.3576 | 6.188 | 5.696 | 5.581 | -0.115 | -0.406 | 0.023 | 0.064 | Unannotated | NA | NA |
| metab_7651 | Unannotated feature | neg | 193.8154 | 7.517 | 5.957 | 5.930 | -0.028 | -0.406 | 0.023 | 0.064 | Unannotated | NA | NA |
| metab_2007 | Unannotated feature | pos | 584.7319 | 0.575 | 5.282 | 5.235 | -0.047 | -0.406 | 0.023 | 0.064 | Unannotated | NA | NA |
| metab_5495 | Unannotated feature | neg | 155.0457 | 0.731 | 3.578 | 3.866 | 0.288 | 0.406 | 0.023 | 0.064 | Unannotated | NA | NA |
| metab_295 | Unannotated feature | pos | 127.0867 | 0.753 | 2.734 | 3.413 | 0.679 | 0.406 | 0.023 | 0.064 | Unannotated | NA | NA |
| metab_8300 | Unannotated feature | neg | 144.4442 | 5.867 | 3.779 | 3.828 | 0.049 | 0.406 | 0.023 | 0.064 | Unannotated | NA | NA |
| metab_8121 | Unannotated feature | neg | 99.6509 | 6.180 | 4.214 | 4.090 | -0.125 | -0.406 | 0.023 | 0.064 | Unannotated | NA | NA |
| metab_4836 | Dihydropyrimidine | pos | 100.0873 | 7.293 | 4.348 | 4.381 | 0.033 | 0.406 | 0.023 | 0.064 | Tentative | NA | NA |
| metab_5799 | Unannotated feature | neg | 229.0695 | 2.111 | 4.238 | 3.900 | -0.338 | -0.402 | 0.024 | 0.067 | Unannotated | NA | NA |
| metab_2611 | Unannotated feature | pos | 387.2352 | 5.840 | 4.928 | 5.019 | 0.091 | 0.402 | 0.024 | 0.067 | Unannotated | NA | NA |
| metab_5790 | Unannotated feature | neg | 278.8652 | 2.087 | 4.046 | 4.219 | 0.173 | 0.402 | 0.024 | 0.067 | Unannotated | NA | NA |
| metab_531 | Unannotated feature | pos | 228.5738 | 1.956 | 4.849 | 5.019 | 0.170 | 0.402 | 0.024 | 0.067 | Unannotated | NA | NA |
| metab_9390 | Unannotated feature | neg | 80.9643 | 3.522 | 3.234 | 3.634 | 0.401 | 0.402 | 0.024 | 0.067 | Unannotated | NA | NA |
| metab_5715 | Unannotated feature | neg | 213.0129 | 1.398 | 4.845 | 4.737 | -0.108 | -0.402 | 0.024 | 0.067 | Unannotated | NA | NA |
| metab_5087 | Unannotated feature | pos | 172.1332 | 0.846 | 4.500 | 4.348 | -0.151 | -0.402 | 0.024 | 0.067 | Unannotated | NA | NA |
| metab_3178 | Glycyl-Tryptophan | pos | 303.1451 | 3.391 | 3.815 | 3.606 | -0.209 | -0.402 | 0.024 | 0.067 | Tentative | Organic acids and derivatives | Carboxylic acids and derivatives |
| metab_4946 | Unannotated feature | pos | 378.9254 | 0.636 | 5.973 | 5.940 | -0.033 | -0.402 | 0.024 | 0.067 | Unannotated | NA | NA |
| metab_2908 | II3Neu5-Acggose4-2-d-erythro-1,3-dihydroxy-2-dichloroacetamide-4-trans-octadacene | pos | 717.7595 | 4.915 | 5.637 | 5.973 | 0.336 | 0.402 | 0.024 | 0.067 | Tentative | Lipids and lipid-like molecules | Sphingolipids |
| metab_1092 | 3-hydroxynonanoyl carnitine | pos | 300.2169 | 5.359 | 4.742 | 5.178 | 0.436 | 0.402 | 0.024 | 0.067 | Tentative | Lipids and lipid-like molecules | Fatty Acyls |
| metab_5966 | Unannotated feature | neg | 251.8225 | 2.740 | 5.221 | 5.169 | -0.052 | -0.402 | 0.024 | 0.067 | Unannotated | NA | NA |
| metab_5857 | Unannotated feature | neg | 111.0192 | 2.417 | 3.989 | 4.038 | 0.050 | 0.402 | 0.024 | 0.067 | Unannotated | NA | NA |
| metab_3187 | Unannotated feature | pos | 211.1441 | 3.321 | 4.369 | 4.227 | -0.143 | -0.402 | 0.024 | 0.067 | Unannotated | NA | NA |
| metab_7397 | Unannotated feature | neg | 618.7685 | 0.570 | 5.363 | 5.427 | 0.063 | 0.402 | 0.024 | 0.067 | Unannotated | NA | NA |
| metab_6137 | Unannotated feature | neg | 92.7875 | 3.217 | 2.697 | 3.213 | 0.515 | 0.402 | 0.024 | 0.067 | Unannotated | NA | NA |
| metab_7782 | Unannotated feature | neg | 445.0588 | 2.537 | 4.621 | 4.493 | -0.129 | -0.402 | 0.024 | 0.067 | Unannotated | NA | NA |
| metab_2106 | Unannotated feature | pos | 470.8119 | 0.575 | 5.670 | 5.631 | -0.039 | -0.402 | 0.024 | 0.067 | Unannotated | NA | NA |
| metab_8177 | Unannotated feature | neg | 476.2792 | 6.008 | 6.046 | 6.198 | 0.152 | 0.402 | 0.024 | 0.067 | Strong putative | NA | NA |
| metab_5387 | Unannotated feature | neg | 94.6704 | 0.601 | 4.927 | 4.899 | -0.028 | -0.402 | 0.024 | 0.067 | Unannotated | NA | NA |
| metab_1781 | Unannotated feature | pos | 226.9750 | 7.455 | 4.212 | 4.266 | 0.054 | 0.402 | 0.024 | 0.067 | Unannotated | NA | NA |
| metab_8807 | Unannotated feature | neg | 334.8984 | 5.477 | 3.051 | 3.780 | 0.729 | 0.402 | 0.024 | 0.067 | Unannotated | NA | NA |
| metab_4981 | Unannotated feature | pos | 102.0554 | 0.729 | 4.838 | 4.897 | 0.059 | 0.402 | 0.024 | 0.067 | Unannotated | NA | NA |
| metab_10642 | Unannotated feature | neg | 517.6766 | 0.590 | 5.035 | 4.984 | -0.050 | -0.402 | 0.024 | 0.067 | Unannotated | NA | NA |
| metab_9202 | Unannotated feature | neg | 206.8603 | 4.130 | 3.910 | 3.975 | 0.065 | 0.402 | 0.024 | 0.067 | Unannotated | NA | NA |
| metab_8416 | Unannotated feature | neg | 429.2137 | 5.779 | 3.808 | 4.513 | 0.705 | 0.402 | 0.024 | 0.067 | Unannotated | NA | NA |
| metab_9276 | Unannotated feature | neg | 226.0720 | 3.819 | 1.628 | 3.730 | 2.102 | 0.402 | 0.024 | 0.067 | Unannotated | NA | NA |
| metab_6134 | Unannotated feature | neg | 402.1235 | 3.209 | 4.470 | 4.038 | -0.432 | -0.402 | 0.024 | 0.067 | Unannotated | NA | NA |
| metab_4770 | LysoPC(20:3(8Z,11Z,14Z)/0:0) | pos | 546.3556 | 6.296 | 7.022 | 6.923 | -0.098 | -0.402 | 0.024 | 0.067 | Tentative | NA | NA |
| metab_3779 | Unannotated feature | pos | 125.1075 | 1.647 | 3.508 | 4.734 | 1.226 | 0.402 | 0.024 | 0.067 | Unannotated | NA | NA |
| metab_679 | Unannotated feature | pos | 121.4900 | 2.451 | 5.000 | 5.067 | 0.067 | 0.402 | 0.024 | 0.067 | Unannotated | NA | NA |
| metab_5367 | Unannotated feature | neg | 640.6065 | 0.590 | 5.552 | 5.477 | -0.074 | -0.402 | 0.024 | 0.067 | Unannotated | NA | NA |
| metab_1920 | Unannotated feature | pos | 192.9763 | 7.324 | 5.035 | 4.983 | -0.052 | -0.402 | 0.024 | 0.067 | Unannotated | NA | NA |
| metab_5557 | Prolylhydroxyproline | neg | 227.1038 | 0.833 | 3.736 | 3.880 | 0.144 | 0.402 | 0.024 | 0.067 | Strong putative | NA | NA |
| metab_1778 | Unannotated feature | pos | 94.0655 | 7.424 | 4.438 | 4.486 | 0.048 | 0.402 | 0.024 | 0.067 | Unannotated | NA | NA |
| metab_2223 | Unannotated feature | pos | 225.2092 | 6.296 | 5.892 | 5.681 | -0.211 | -0.402 | 0.024 | 0.067 | Unannotated | NA | NA |
| metab_6483 | 2-Methyl-5-hydroxytryptamine | neg | 171.0925 | 4.363 | 4.068 | 3.014 | -1.054 | -0.402 | 0.024 | 0.067 | Tentative | Organoheterocyclic compounds | Indoles and derivatives |
| metab_2518 | Unannotated feature | pos | 587.7474 | 5.886 | 4.961 | 4.788 | -0.172 | -0.402 | 0.024 | 0.067 | Unannotated | NA | NA |
| metab_6500 | Unannotated feature | neg | 186.9596 | 4.394 | 4.192 | 4.907 | 0.715 | 0.402 | 0.024 | 0.067 | Unannotated | NA | NA |
| metab_2328 | Unannotated feature | pos | 403.2327 | 6.010 | 4.789 | 4.857 | 0.068 | 0.402 | 0.024 | 0.067 | Unannotated | NA | NA |
| metab_5665 | Unannotated feature | neg | 127.8695 | 1.094 | 6.050 | 6.004 | -0.046 | -0.402 | 0.024 | 0.067 | Unannotated | NA | NA |
| metab_2481 | Unannotated feature | pos | 149.1326 | 5.902 | 4.526 | 4.615 | 0.089 | 0.402 | 0.024 | 0.067 | Unannotated | NA | NA |
| metab_7175 | Unannotated feature | neg | 365.2316 | 5.898 | 4.747 | 4.468 | -0.279 | -0.402 | 0.024 | 0.067 | Unannotated | NA | NA |
| metab_5386 | Unannotated feature | neg | 93.1741 | 0.601 | 4.940 | 4.908 | -0.032 | -0.402 | 0.024 | 0.067 | Unannotated | NA | NA |
| metab_8963 | Unannotated feature | neg | 144.9228 | 7.109 | 6.867 | 6.891 | 0.024 | 0.402 | 0.024 | 0.067 | Unannotated | NA | NA |
| metab_10370 | Unannotated feature | neg | 202.0382 | 0.731 | 3.566 | 3.141 | -0.425 | -0.398 | 0.026 | 0.070 | Unannotated | NA | NA |
| metab_157 | Unannotated feature | pos | 951.7839 | 0.551 | 4.495 | 4.661 | 0.166 | 0.398 | 0.026 | 0.070 | Unannotated | NA | NA |
| metab_8364 | Unannotated feature | neg | 278.8956 | 0.723 | 4.102 | 3.926 | -0.176 | -0.398 | 0.026 | 0.070 | Unannotated | NA | NA |
| metab_10442 | Unannotated feature | neg | 135.0292 | 0.668 | 5.793 | 5.951 | 0.158 | 0.398 | 0.026 | 0.070 | Unannotated | NA | NA |
| metab_5589 | Unannotated feature | neg | 173.9622 | 0.865 | 4.328 | 4.030 | -0.297 | -0.398 | 0.026 | 0.070 | Unannotated | NA | NA |
| metab_602 | Unannotated feature | pos | 188.0683 | 2.095 | 5.145 | 5.045 | -0.100 | -0.398 | 0.026 | 0.070 | Unannotated | NA | NA |
| metab_7606 | Unannotated feature | neg | 143.0245 | 7.269 | 4.486 | 4.465 | -0.022 | -0.398 | 0.026 | 0.070 | Unannotated | NA | NA |
| metab_6510 | Unannotated feature | neg | 303.0052 | 4.433 | 4.048 | 3.190 | -0.859 | -0.398 | 0.026 | 0.070 | Unannotated | NA | NA |
| metab_7780 | Unannotated feature | neg | 349.8676 | 7.170 | 4.078 | 4.123 | 0.045 | 0.398 | 0.026 | 0.070 | Unannotated | NA | NA |
| metab_8920 | Unannotated feature | neg | 919.4183 | 5.057 | 4.414 | 4.715 | 0.301 | 0.398 | 0.026 | 0.070 | Unannotated | NA | NA |
| metab_10216 | Unannotated feature | neg | 288.8774 | 0.865 | 5.471 | 5.360 | -0.111 | -0.398 | 0.026 | 0.070 | Unannotated | NA | NA |
| metab_10186 | Unannotated feature | neg | 433.9636 | 0.888 | 4.539 | 4.223 | -0.316 | -0.398 | 0.026 | 0.070 | Unannotated | NA | NA |
| metab_3622 | Unannotated feature | pos | 182.1388 | 1.964 | 4.227 | 3.978 | -0.248 | -0.398 | 0.026 | 0.070 | Unannotated | NA | NA |
| metab_1842 | Unannotated feature | pos | 123.0807 | 7.944 | 4.449 | 4.414 | -0.035 | -0.398 | 0.026 | 0.070 | Unannotated | NA | NA |
| metab_8044 | Unannotated feature | neg | 160.0814 | 6.470 | 3.279 | 3.046 | -0.233 | -0.398 | 0.026 | 0.070 | Unannotated | NA | NA |
| metab_2578 | Unannotated feature | pos | 686.4703 | 5.855 | 4.424 | 4.029 | -0.395 | -0.398 | 0.026 | 0.070 | Unannotated | NA | NA |
| metab_2545 | Unannotated feature | pos | 544.4061 | 5.871 | 5.467 | 5.353 | -0.114 | -0.398 | 0.026 | 0.070 | Unannotated | NA | NA |
| metab_7253 | Unannotated feature | neg | 324.9898 | 1.886 | 4.808 | 4.650 | -0.158 | -0.398 | 0.026 | 0.070 | Unannotated | NA | NA |
| metab_1120 | Unannotated feature | pos | 84.7210 | 5.603 | 3.229 | 3.397 | 0.169 | 0.398 | 0.026 | 0.070 | Unannotated | NA | NA |
| metab_5933 | Unannotated feature | neg | 124.0397 | 2.597 | 4.042 | 4.082 | 0.040 | 0.398 | 0.026 | 0.070 | Unannotated | NA | NA |
| metab_5914 | Unannotated feature | neg | 398.7417 | 0.598 | 5.471 | 5.433 | -0.039 | -0.398 | 0.026 | 0.070 | Unannotated | NA | NA |
| metab_4243 | Unannotated feature | pos | 202.0064 | 0.606 | 3.294 | 2.971 | -0.323 | -0.398 | 0.026 | 0.070 | Unannotated | NA | NA |
| metab_6953 | Unannotated feature | neg | 525.2717 | 5.691 | 5.036 | 5.320 | 0.284 | 0.398 | 0.026 | 0.070 | Unannotated | NA | NA |
| metab_8879 | Unannotated feature | neg | 171.0659 | 5.223 | 4.501 | 4.472 | -0.029 | -0.398 | 0.026 | 0.070 | Unannotated | NA | NA |
| metab_4791 | Unannotated feature | pos | 498.9011 | 7.293 | 5.936 | 5.902 | -0.034 | -0.398 | 0.026 | 0.070 | Unannotated | NA | NA |
| metab_11398 | Nordephrine | neg | 164.0713 | 4.205 | 3.938 | 4.060 | 0.121 | 0.398 | 0.026 | 0.070 | Tentative | NA | NA |
| metab_212 | Unannotated feature | pos | 148.0040 | 0.614 | 4.909 | 4.750 | -0.159 | -0.398 | 0.026 | 0.070 | Unannotated | NA | NA |
| metab_11319 | Unannotated feature | neg | 214.8799 | 7.261 | 6.938 | 6.963 | 0.025 | 0.398 | 0.026 | 0.070 | Unannotated | NA | NA |
| metab_11393 | Hydroquinone | neg | 109.0286 | 1.150 | 3.046 | 3.191 | 0.146 | 0.398 | 0.026 | 0.070 | Strong putative | NA | NA |
| metab_1323 | Unannotated feature | pos | 641.1162 | 5.886 | 4.585 | 4.369 | -0.217 | -0.398 | 0.026 | 0.070 | Unannotated | NA | NA |
| metab_6110 | Unannotated feature | neg | 171.9466 | 3.184 | 3.736 | 3.577 | -0.159 | -0.398 | 0.026 | 0.070 | Unannotated | NA | NA |
| metab_2277 | Florbenazine | pos | 366.2427 | 6.141 | 3.472 | 3.404 | -0.068 | -0.398 | 0.026 | 0.070 | Tentative | Organoheterocyclic compounds | Tetrahydroisoquinolines |
| metab_10991 | Unannotated feature | neg | 161.8753 | 0.128 | 5.247 | 5.222 | -0.025 | -0.398 | 0.026 | 0.070 | Unannotated | NA | NA |
| metab_8788 | Enoxacin | neg | 365.1262 | 5.518 | 4.890 | 4.515 | -0.375 | -0.398 | 0.026 | 0.070 | Tentative | NA | NA |
| metab_127 | Unannotated feature | pos | 91.2162 | 0.551 | 4.295 | 4.330 | 0.035 | 0.398 | 0.026 | 0.070 | Unannotated | NA | NA |
| metab_2081 | Xi-3-Methyl-3-cyclohexen-1-ol | pos | 95.0860 | 7.200 | 4.706 | 4.752 | 0.046 | 0.398 | 0.026 | 0.070 | Tentative | NA | NA |
| metab_3233 | (-)-trans-3,4-Dihydro-4,8-dihydroxy-3-methyl-1H-2-benzopyran-1-one | pos | 212.0918 | 3.136 | 3.372 | 2.997 | -0.375 | -0.398 | 0.026 | 0.070 | Tentative | Organoheterocyclic compounds | Benzopyrans |
| metab_3316 | Unannotated feature | pos | 200.2322 | 2.659 | 4.442 | 4.648 | 0.206 | 0.398 | 0.026 | 0.070 | Unannotated | NA | NA |
| metab_11131 | Cortisol | neg | 407.2083 | 5.707 | 5.566 | 5.377 | -0.189 | -0.398 | 0.026 | 0.070 | Tentative | NA | NA |
| metab_6613 | Unannotated feature | neg | 688.7864 | 4.923 | 5.189 | 5.467 | 0.278 | 0.398 | 0.026 | 0.070 | Unannotated | NA | NA |
| metab_8255 | Unannotated feature | neg | 479.2938 | 5.898 | 4.721 | 4.966 | 0.244 | 0.398 | 0.026 | 0.070 | Unannotated | NA | NA |
| metab_2692 | Unannotated feature | pos | 84.7211 | 5.763 | 3.551 | 3.658 | 0.107 | 0.398 | 0.026 | 0.070 | Unannotated | NA | NA |
| metab_2834 | Unannotated feature | pos | 84.1228 | 5.459 | 3.504 | 3.725 | 0.221 | 0.398 | 0.026 | 0.070 | Unannotated | NA | NA |
| metab_4181 | Unannotated feature | pos | 103.0394 | 0.644 | 4.366 | 4.286 | -0.080 | -0.398 | 0.026 | 0.070 | Unannotated | NA | NA |
| metab_6597 | Unannotated feature | neg | 247.8927 | 4.838 | 4.943 | 5.005 | 0.062 | 0.398 | 0.026 | 0.070 | Unannotated | NA | NA |
| metab_2686 | Unannotated feature | pos | 532.8495 | 5.763 | 4.439 | 4.125 | -0.314 | -0.398 | 0.026 | 0.070 | Unannotated | NA | NA |
| metab_10416 | 3-Methoxy-4-hydroxymandelate | neg | 165.0553 | 4.774 | 5.390 | 5.491 | 0.101 | 0.398 | 0.026 | 0.070 | Moderate putative | NA | NA |
| metab_681 | Unannotated feature | pos | 155.0703 | 2.451 | 2.830 | 3.447 | 0.618 | 0.398 | 0.026 | 0.070 | Unannotated | NA | NA |
| metab_8294 | Unannotated feature | neg | 433.2816 | 5.867 | 4.537 | 3.923 | -0.614 | -0.398 | 0.026 | 0.070 | Unannotated | NA | NA |
| metab_3599 | Unannotated feature | pos | 391.2841 | 2.026 | 5.232 | 5.214 | -0.019 | -0.398 | 0.026 | 0.070 | Unannotated | NA | NA |
| metab_7720 | Unannotated feature | neg | 267.8483 | 7.197 | 4.614 | 4.590 | -0.024 | -0.398 | 0.026 | 0.070 | Unannotated | NA | NA |
| metab_4160 | Unannotated feature | pos | 278.0254 | 0.652 | 4.020 | 4.319 | 0.299 | 0.394 | 0.028 | 0.073 | Unannotated | NA | NA |
| metab_10918 | Unannotated feature | neg | 665.3808 | 0.539 | 5.362 | 5.282 | -0.079 | -0.394 | 0.028 | 0.073 | Unannotated | NA | NA |
| metab_5086 | Unannotated feature | pos | 149.0186 | 1.321 | 0.508 | 3.640 | 3.131 | 0.394 | 0.028 | 0.073 | Unannotated | NA | NA |
| metab_4662 | Unannotated feature | pos | 181.0860 | 5.840 | 5.446 | 5.053 | -0.393 | -0.394 | 0.028 | 0.073 | Moderate putative | NA | NA |
| metab_1171 | Unannotated feature | pos | 253.1183 | 1.956 | 5.018 | 4.896 | -0.123 | -0.394 | 0.028 | 0.073 | Unannotated | NA | NA |
| metab_4856 | Unannotated feature | pos | 292.1906 | 5.740 | 4.805 | 4.394 | -0.411 | -0.394 | 0.028 | 0.073 | Unannotated | NA | NA |
| metab_3346 | Unannotated feature | pos | 305.0217 | 2.591 | 4.424 | 4.391 | -0.033 | -0.394 | 0.028 | 0.073 | Unannotated | NA | NA |
| metab_8480 | Unannotated feature | neg | 283.9756 | 5.739 | 3.490 | 3.875 | 0.385 | 0.394 | 0.028 | 0.073 | Unannotated | NA | NA |
| metab_6062 | Apocynin | neg | 211.0612 | 3.096 | 2.708 | 3.093 | 0.385 | 0.394 | 0.028 | 0.073 | Tentative | Organic oxygen compounds | Organooxygen compounds |
| metab_3299 | 3-Methoxypropylamine | pos | 72.0813 | 2.787 | 3.842 | 3.891 | 0.049 | 0.394 | 0.028 | 0.073 | Tentative | NA | NA |
| metab_7493 | Unannotated feature | neg | 187.9780 | 7.148 | 4.432 | 4.389 | -0.043 | -0.394 | 0.028 | 0.073 | Unannotated | NA | NA |
| metab_7428 | Unannotated feature | neg | 201.6439 | 7.109 | 4.237 | 4.389 | 0.151 | 0.394 | 0.028 | 0.073 | Unannotated | NA | NA |
| metab_10813 | Unannotated feature | neg | 931.8151 | 0.554 | 5.521 | 5.470 | -0.050 | -0.394 | 0.028 | 0.073 | Unannotated | NA | NA |
| metab_1203 | Unannotated feature | pos | 406.3527 | 5.763 | 6.107 | 6.156 | 0.049 | 0.394 | 0.028 | 0.073 | Unannotated | NA | NA |
| metab_3470 | Unannotated feature | pos | 128.1072 | 2.265 | 3.603 | 3.738 | 0.135 | 0.394 | 0.028 | 0.073 | Unannotated | NA | NA |
| metab_6119 | Unannotated feature | neg | 201.9938 | 3.192 | 3.875 | 3.613 | -0.262 | -0.394 | 0.028 | 0.073 | Unannotated | NA | NA |
| metab_1142 | Unannotated feature | pos | 412.2844 | 5.657 | 4.187 | 3.658 | -0.529 | -0.394 | 0.028 | 0.073 | Unannotated | NA | NA |
| metab_9627 | Unannotated feature | neg | 221.0932 | 3.120 | 4.777 | 4.627 | -0.150 | -0.394 | 0.028 | 0.073 | Unannotated | NA | NA |
| metab_4949 | Trimethylamine N-oxide | pos | 76.0763 | 0.644 | 5.504 | 5.940 | 0.436 | 0.394 | 0.028 | 0.073 | Strong putative | Organic nitrogen compounds | Organonitrogen compounds |
| metab_4570 | Unannotated feature | pos | 87.0810 | 0.465 | 4.453 | 4.464 | 0.012 | 0.394 | 0.028 | 0.073 | Unannotated | NA | NA |
| metab_705 | Unannotated feature | pos | 157.0861 | 2.545 | 3.717 | 3.951 | 0.234 | 0.394 | 0.028 | 0.073 | Unannotated | NA | NA |
| metab_7190 | Unannotated feature | neg | 187.9780 | 5.922 | 4.810 | 4.899 | 0.089 | 0.394 | 0.028 | 0.073 | Unannotated | NA | NA |
| metab_5219 | Unannotated feature | neg | 876.8237 | 0.543 | 5.364 | 5.305 | -0.059 | -0.394 | 0.028 | 0.073 | Unannotated | NA | NA |
| metab_6577 | Unannotated feature | neg | 119.0495 | 4.774 | 3.909 | 4.037 | 0.128 | 0.394 | 0.028 | 0.073 | Unannotated | NA | NA |
| metab_10446 | Unannotated feature | neg | 324.7631 | 0.598 | 6.536 | 6.513 | -0.024 | -0.394 | 0.028 | 0.073 | Unannotated | NA | NA |
| metab_3973 | Unannotated feature | pos | 148.0040 | 0.839 | 3.598 | 3.261 | -0.337 | -0.394 | 0.028 | 0.073 | Unannotated | NA | NA |
| metab_7622 | Unannotated feature | neg | 146.9607 | 7.494 | 4.735 | 4.785 | 0.050 | 0.394 | 0.028 | 0.073 | Unannotated | NA | NA |
| metab_5259 | Unannotated feature | neg | 674.7554 | 0.554 | 4.324 | 4.756 | 0.431 | 0.394 | 0.028 | 0.073 | Unannotated | NA | NA |
| metab_5322 | Unannotated feature | neg | 924.7666 | 0.562 | 4.151 | 4.324 | 0.174 | 0.394 | 0.028 | 0.073 | Unannotated | NA | NA |
| metab_5230 | Unannotated feature | neg | 455.5867 | 0.549 | 4.559 | 4.512 | -0.047 | -0.394 | 0.028 | 0.073 | Unannotated | NA | NA |
| metab_6486 | Unannotated feature | neg | 79.9565 | 4.371 | 4.595 | 5.228 | 0.634 | 0.394 | 0.028 | 0.073 | Unannotated | NA | NA |
| metab_3723 | Unannotated feature | pos | 156.1384 | 1.855 | 2.908 | 3.274 | 0.365 | 0.394 | 0.028 | 0.073 | Unannotated | NA | NA |
| metab_5475 | Unannotated feature | neg | 200.1238 | 0.692 | 4.313 | 4.070 | -0.243 | -0.394 | 0.028 | 0.073 | Unannotated | NA | NA |
| metab_290 | Unannotated feature | pos | 248.9989 | 0.745 | 4.093 | 4.005 | -0.088 | -0.394 | 0.028 | 0.073 | Unannotated | NA | NA |
| metab_168 | Unannotated feature | pos | 633.8356 | 0.559 | 4.053 | 4.207 | 0.154 | 0.394 | 0.028 | 0.073 | Unannotated | NA | NA |
| metab_3789 | Unannotated feature | pos | 163.0602 | 1.570 | 4.575 | 4.597 | 0.023 | 0.394 | 0.028 | 0.073 | Unannotated | NA | NA |
| metab_137 | Unannotated feature | pos | 635.8791 | 0.551 | 5.640 | 5.597 | -0.043 | -0.394 | 0.028 | 0.073 | Unannotated | NA | NA |
| metab_113 | Unannotated feature | pos | 952.1495 | 0.538 | 4.740 | 4.473 | -0.266 | -0.389 | 0.029 | 0.077 | Unannotated | NA | NA |
| metab_9070 | Unannotated feature | neg | 171.0659 | 4.529 | 3.534 | 3.934 | 0.400 | 0.389 | 0.029 | 0.077 | Strong putative | NA | NA |
| metab_3619 | Unannotated feature | pos | 196.0605 | 2.342 | 4.361 | 4.906 | 0.545 | 0.389 | 0.029 | 0.077 | Unannotated | NA | NA |
| metab_10201 | Unannotated feature | neg | 240.0491 | 0.873 | 4.340 | 4.136 | -0.204 | -0.389 | 0.029 | 0.077 | Unannotated | NA | NA |
| metab_8770 | Unannotated feature | neg | 289.0239 | 5.538 | 5.314 | 5.560 | 0.246 | 0.389 | 0.029 | 0.077 | Unannotated | NA | NA |
| metab_5535 | Unannotated feature | neg | 102.0189 | 0.802 | 4.310 | 4.506 | 0.196 | 0.389 | 0.029 | 0.077 | Unannotated | NA | NA |
| metab_7840 | Unannotated feature | neg | 332.1746 | 7.153 | 4.113 | 3.973 | -0.140 | -0.389 | 0.029 | 0.077 | Unannotated | NA | NA |
| metab_10058 | Unannotated feature | neg | 187.9908 | 1.382 | 4.532 | 4.446 | -0.085 | -0.389 | 0.029 | 0.077 | Unannotated | NA | NA |
| metab_4089 | Zalcitabine | pos | 229.1296 | 0.729 | 4.434 | 4.724 | 0.290 | 0.389 | 0.029 | 0.077 | Tentative | Nucleosides, nucleotides, and analogues | Pyrimidine nucleosides |
| metab_9934 | Gallate | neg | 169.0139 | 2.119 | 4.188 | 4.156 | -0.032 | -0.389 | 0.029 | 0.077 | Strong putative | NA | NA |
| metab_7148 | Unannotated feature | neg | 145.4040 | 5.875 | 3.664 | 3.789 | 0.125 | 0.389 | 0.029 | 0.077 | Unannotated | NA | NA |
| metab_4331 | Unannotated feature | pos | 596.7578 | 0.575 | 5.305 | 5.257 | -0.048 | -0.389 | 0.029 | 0.077 | Unannotated | NA | NA |
| metab_5144 | Methylprednisolone acetate | pos | 458.2537 | 5.902 | 6.164 | 6.288 | 0.124 | 0.389 | 0.029 | 0.077 | Tentative | NA | NA |
| metab_11061 | L-Aspartyl-L-Phenylalanine | neg | 279.0991 | 3.224 | 5.699 | 5.521 | -0.178 | -0.389 | 0.029 | 0.077 | Strong putative | Organic acids and derivatives | Carboxylic acids and derivatives |
| metab_8789 | 5-Hydroxyxanthotoxin | neg | 321.1363 | 5.518 | 5.735 | 5.405 | -0.329 | -0.389 | 0.029 | 0.077 | Tentative | NA | NA |
| metab_6259 | Unannotated feature | neg | 81.9523 | 3.522 | 3.701 | 3.745 | 0.044 | 0.389 | 0.029 | 0.077 | Unannotated | NA | NA |
| metab_5067 | Unannotated feature | pos | 73.0847 | 0.854 | 4.862 | 4.789 | -0.072 | -0.389 | 0.029 | 0.077 | Unannotated | NA | NA |
| metab_10478 | Unannotated feature | neg | 219.0454 | 0.644 | 4.117 | 4.581 | 0.464 | 0.389 | 0.029 | 0.077 | Unannotated | NA | NA |
| metab_7576 | Unannotated feature | neg | 266.0183 | 7.205 | 4.816 | 4.684 | -0.131 | -0.389 | 0.029 | 0.077 | Unannotated | NA | NA |
| metab_10933 | Unannotated feature | neg | 469.9141 | 0.534 | 5.597 | 5.561 | -0.036 | -0.389 | 0.029 | 0.077 | Unannotated | NA | NA |
| metab_3962 | Unannotated feature | pos | 217.0684 | 0.846 | 5.033 | 5.216 | 0.183 | 0.389 | 0.029 | 0.077 | Unannotated | NA | NA |
| metab_9673 | Unannotated feature | neg | 225.9865 | 0.636 | 5.609 | 5.467 | -0.142 | -0.389 | 0.029 | 0.077 | Unannotated | NA | NA |
| metab_5924 | Unannotated feature | neg | 536.6274 | 0.598 | 5.488 | 5.445 | -0.043 | -0.389 | 0.029 | 0.077 | Unannotated | NA | NA |
| metab_4850 | Unannotated feature | pos | 443.3732 | 6.196 | 5.235 | 5.133 | -0.102 | -0.389 | 0.029 | 0.077 | Unannotated | NA | NA |
| metab_511 | Unannotated feature | pos | 87.0638 | 1.925 | 3.314 | 3.603 | 0.289 | 0.389 | 0.029 | 0.077 | Unannotated | NA | NA |
| metab_2986 | Unannotated feature | pos | 101.8086 | 4.478 | 4.886 | 4.913 | 0.026 | 0.389 | 0.029 | 0.077 | Unannotated | NA | NA |
| metab_4951 | Unannotated feature | pos | 145.0474 | 0.644 | 4.614 | 4.757 | 0.144 | 0.389 | 0.029 | 0.077 | Unannotated | NA | NA |
| metab_8857 | Unannotated feature | neg | 679.7812 | 5.287 | 5.728 | 5.883 | 0.155 | 0.389 | 0.029 | 0.077 | Unannotated | NA | NA |
| metab_4829 | Unannotated feature | pos | 141.1135 | 7.300 | 5.896 | 5.932 | 0.036 | 0.389 | 0.029 | 0.077 | Unannotated | NA | NA |
| metab_2872 | Trans-4-Aminocyclohexanecarboxylic acid | pos | 108.0811 | 2.133 | 5.849 | 5.884 | 0.035 | 0.389 | 0.029 | 0.077 | Tentative | NA | NA |
| metab_4147 | D-Ornithine | pos | 115.0869 | 0.667 | 3.989 | 4.133 | 0.144 | 0.389 | 0.029 | 0.077 | Tentative | NA | NA |
| metab_5025 | Unannotated feature | pos | 177.0193 | 0.839 | 4.139 | 4.562 | 0.423 | 0.389 | 0.029 | 0.077 | Unannotated | NA | NA |
| metab_6721 | Unannotated feature | neg | 715.7475 | 5.346 | 4.285 | 4.872 | 0.587 | 0.389 | 0.029 | 0.077 | Unannotated | NA | NA |
| metab_4389 | Unannotated feature | pos | 552.8441 | 0.567 | 4.305 | 4.237 | -0.068 | -0.389 | 0.029 | 0.077 | Unannotated | NA | NA |
| metab_6681 | Unannotated feature | neg | 672.2458 | 5.199 | 5.211 | 5.451 | 0.240 | 0.389 | 0.029 | 0.077 | Unannotated | NA | NA |
| metab_8886 | Unannotated feature | neg | 661.2550 | 5.199 | 6.548 | 6.775 | 0.227 | 0.389 | 0.029 | 0.077 | Unannotated | NA | NA |
| metab_9229 | Unannotated feature | neg | 124.0397 | 4.037 | 4.611 | 4.541 | -0.070 | -0.389 | 0.029 | 0.077 | Unannotated | NA | NA |
| metab_1545 | Unannotated feature | pos | 211.1936 | 6.296 | 5.386 | 5.194 | -0.192 | -0.389 | 0.029 | 0.077 | Unannotated | NA | NA |
| metab_10641 | Unannotated feature | neg | 826.5074 | 0.587 | 4.914 | 4.798 | -0.116 | -0.389 | 0.029 | 0.077 | Unannotated | NA | NA |
| metab_1212 | 5-Hydroxyindoleacetate | pos | 209.0921 | 2.058 | 5.991 | 6.059 | 0.068 | 0.389 | 0.029 | 0.077 | Strong putative | NA | NA |
| metab_9142 | Unannotated feature | neg | 209.0455 | 4.290 | 4.621 | 4.580 | -0.041 | -0.389 | 0.029 | 0.077 | Unannotated | NA | NA |
| metab_1325 | Unannotated feature | pos | 704.4888 | 5.886 | 5.524 | 5.399 | -0.125 | -0.389 | 0.029 | 0.077 | Unannotated | NA | NA |
| metab_7059 | Unannotated feature | neg | 347.8378 | 5.779 | 3.830 | 3.666 | -0.164 | -0.389 | 0.029 | 0.077 | Unannotated | NA | NA |
| metab_9101 | Unannotated feature | neg | 158.6298 | 4.425 | 3.528 | 2.972 | -0.556 | -0.389 | 0.029 | 0.077 | Unannotated | NA | NA |
| metab_6116 | Unannotated feature | neg | 517.7775 | 0.644 | 5.165 | 4.952 | -0.213 | -0.389 | 0.029 | 0.077 | Unannotated | NA | NA |
| metab_4675 | Unannotated feature | pos | 460.2388 | 3.275 | 4.919 | 5.148 | 0.229 | 0.389 | 0.029 | 0.077 | Unannotated | NA | NA |
| metab_7893 | Unannotated feature | neg | 374.9072 | 7.132 | 4.876 | 4.836 | -0.040 | -0.385 | 0.031 | 0.081 | Unannotated | NA | NA |
| metab_10472 | Unannotated feature | neg | 131.0820 | 0.652 | 4.857 | 5.070 | 0.214 | 0.385 | 0.031 | 0.081 | Unannotated | NA | NA |
| metab_8055 | Unannotated feature | neg | 134.9868 | 6.378 | 4.719 | 4.772 | 0.052 | 0.385 | 0.031 | 0.081 | Unannotated | NA | NA |
| metab_3970 | L-4-Hydroxyglutamate semialdehyde | pos | 192.0245 | 0.839 | 4.691 | 4.821 | 0.130 | 0.385 | 0.031 | 0.081 | Tentative | NA | NA |
| metab_1668 | Unannotated feature | pos | 446.7511 | 7.231 | 6.245 | 6.286 | 0.040 | 0.385 | 0.031 | 0.081 | Unannotated | NA | NA |
| metab_9736 | Unannotated feature | neg | 342.8255 | 2.748 | 5.533 | 5.475 | -0.058 | -0.385 | 0.031 | 0.081 | Unannotated | NA | NA |
| metab_1830 | Unannotated feature | pos | 135.0806 | 7.975 | 4.106 | 4.034 | -0.072 | -0.385 | 0.031 | 0.081 | Unannotated | NA | NA |
| metab_2502 | Unannotated feature | pos | 210.1854 | 5.894 | 4.471 | 4.501 | 0.030 | 0.385 | 0.031 | 0.081 | Unannotated | NA | NA |
| metab_10253 | Unannotated feature | neg | 134.8940 | 0.857 | 6.341 | 6.306 | -0.035 | -0.385 | 0.031 | 0.081 | Unannotated | NA | NA |
| metab_10941 | Unannotated feature | neg | 520.6940 | 0.582 | 5.984 | 5.954 | -0.031 | -0.385 | 0.031 | 0.081 | Unannotated | NA | NA |
| metab_2315 | Psychosine | pos | 444.3322 | 6.041 | 5.659 | 5.583 | -0.076 | -0.385 | 0.031 | 0.081 | Tentative | NA | NA |
| metab_1986 | Unannotated feature | pos | 510.8574 | 7.293 | 4.641 | 4.720 | 0.080 | 0.385 | 0.031 | 0.081 | Unannotated | NA | NA |
| metab_5553 | Unannotated feature | neg | 338.7144 | 0.825 | 4.353 | 4.189 | -0.163 | -0.385 | 0.031 | 0.081 | Unannotated | NA | NA |
| metab_764 | Unannotated feature | pos | 130.0864 | 7.277 | 5.292 | 5.314 | 0.022 | 0.385 | 0.031 | 0.081 | Unannotated | NA | NA |
| metab_2121 | Unannotated feature | pos | 590.4263 | 6.945 | 5.795 | 5.737 | -0.058 | -0.385 | 0.031 | 0.081 | Unannotated | NA | NA |
| metab_7671 | Unannotated feature | neg | 214.0163 | 7.261 | 4.134 | 4.222 | 0.089 | 0.385 | 0.031 | 0.081 | Unannotated | NA | NA |
| metab_10197 | Unannotated feature | neg | 281.0364 | 0.873 | 4.689 | 4.534 | -0.155 | -0.385 | 0.031 | 0.081 | Unannotated | NA | NA |
| metab_276 | Unannotated feature | pos | 162.0762 | 0.721 | 4.441 | 4.357 | -0.083 | -0.385 | 0.031 | 0.081 | Unannotated | NA | NA |
| metab_4730 | Unannotated feature | pos | 385.2584 | 6.057 | 5.110 | 4.737 | -0.372 | -0.385 | 0.031 | 0.081 | Unannotated | NA | NA |
| metab_1392 | Unannotated feature | pos | 812.5580 | 5.917 | 4.323 | 3.888 | -0.435 | -0.385 | 0.031 | 0.081 | Unannotated | NA | NA |
| metab_9108 | Unannotated feature | neg | 179.8824 | 4.386 | 4.510 | 5.167 | 0.658 | 0.385 | 0.031 | 0.081 | Unannotated | NA | NA |
| metab_3500 | Unannotated feature | pos | 158.0271 | 2.195 | 2.891 | 3.889 | 0.998 | 0.385 | 0.031 | 0.081 | Unannotated | NA | NA |
| metab_10541 | Unannotated feature | neg | 472.6563 | 0.621 | 5.681 | 5.618 | -0.063 | -0.385 | 0.031 | 0.081 | Unannotated | NA | NA |
| metab_527 | Azvudine | pos | 144.0479 | 1.956 | 3.083 | 2.627 | -0.456 | -0.385 | 0.031 | 0.081 | Tentative | NA | NA |
| metab_10091 | Unannotated feature | neg | 397.0040 | 4.371 | 5.067 | 6.066 | 0.998 | 0.385 | 0.031 | 0.081 | Unannotated | NA | NA |
| metab_5131 | D-Glucosamine | pos | 162.0762 | 1.406 | 5.071 | 5.147 | 0.076 | 0.385 | 0.031 | 0.081 | Tentative | NA | NA |
| metab_8513 | Unannotated feature | neg | 524.0416 | 5.715 | 4.206 | 3.980 | -0.225 | -0.385 | 0.031 | 0.081 | Unannotated | NA | NA |
| metab_321 | Unannotated feature | pos | 148.9354 | 0.807 | 4.566 | 4.524 | -0.042 | -0.385 | 0.031 | 0.081 | Unannotated | NA | NA |
| metab_6023 | Unannotated feature | neg | 497.1256 | 2.938 | 4.988 | 4.488 | -0.500 | -0.385 | 0.031 | 0.081 | Unannotated | NA | NA |
| metab_5829 | Unannotated feature | neg | 324.1307 | 2.151 | 4.171 | 3.687 | -0.484 | -0.385 | 0.031 | 0.081 | Unannotated | NA | NA |
| metab_1569 | Penbutolol | pos | 583.4444 | 6.365 | 5.078 | 4.830 | -0.248 | -0.385 | 0.031 | 0.081 | Tentative | Benzenoids | Phenol ethers |
| metab_3292 | Xanthurenic acid | pos | 206.0448 | 2.841 | 3.487 | 3.994 | 0.507 | 0.385 | 0.031 | 0.081 | Strong putative | Organoheterocyclic compounds | Quinolines and derivatives |
| metab_4408 | Unannotated feature | pos | 833.8231 | 0.559 | 4.905 | 4.796 | -0.109 | -0.385 | 0.031 | 0.081 | Unannotated | NA | NA |
| metab_2594 | Unannotated feature | pos | 588.4127 | 5.848 | 5.700 | 5.622 | -0.077 | -0.381 | 0.033 | 0.084 | Unannotated | NA | NA |
| metab_760 | Kynurenine | pos | 209.0920 | 2.795 | 4.233 | 3.760 | -0.473 | -0.381 | 0.033 | 0.084 | Tentative | Organic oxygen compounds | Organooxygen compounds |
| metab_7811 | Unannotated feature | neg | 189.0079 | 7.164 | 4.916 | 4.873 | -0.044 | -0.381 | 0.033 | 0.084 | Unannotated | NA | NA |
| metab_166 | Unannotated feature | pos | 629.8613 | 0.559 | 4.887 | 4.775 | -0.113 | -0.381 | 0.033 | 0.084 | Unannotated | NA | NA |
| metab_3642 | Unannotated feature | pos | 182.1023 | 1.956 | 4.422 | 4.396 | -0.025 | -0.381 | 0.033 | 0.084 | Unannotated | NA | NA |
| metab_6761 | Unannotated feature | neg | 169.8337 | 5.514 | 3.400 | 3.549 | 0.149 | 0.381 | 0.033 | 0.084 | Unannotated | NA | NA |
| metab_4719 | Unannotated feature | pos | 172.1697 | 5.941 | 5.076 | 4.905 | -0.171 | -0.381 | 0.033 | 0.084 | Unannotated | NA | NA |
| metab_2493 | Unannotated feature | pos | 645.4499 | 5.894 | 4.559 | 4.320 | -0.239 | -0.381 | 0.033 | 0.084 | Unannotated | NA | NA |
| metab_9791 | Unannotated feature | neg | 676.5099 | 0.621 | 6.075 | 6.027 | -0.048 | -0.381 | 0.033 | 0.084 | Unannotated | NA | NA |
| metab_5201 | Unannotated feature | neg | 661.5390 | 0.539 | 4.472 | 4.335 | -0.137 | -0.381 | 0.033 | 0.084 | Unannotated | NA | NA |
| metab_4533 | Unannotated feature | pos | 515.8919 | 0.534 | 5.004 | 4.947 | -0.057 | -0.381 | 0.033 | 0.084 | Unannotated | NA | NA |
| metab_2357 | Hypusine | pos | 484.3837 | 5.972 | 5.815 | 5.783 | -0.032 | -0.381 | 0.033 | 0.084 | Tentative | NA | NA |
| metab_792 | Unannotated feature | pos | 223.0601 | 2.981 | 4.262 | 3.997 | -0.265 | -0.381 | 0.033 | 0.084 | Moderate putative | NA | NA |
| metab_10346 | Unannotated feature | neg | 202.0290 | 0.754 | 4.225 | 4.076 | -0.150 | -0.381 | 0.033 | 0.084 | Unannotated | NA | NA |
| metab_7911 | Unannotated feature | neg | 337.2061 | 7.117 | 4.685 | 4.611 | -0.074 | -0.381 | 0.033 | 0.084 | Unannotated | NA | NA |
| metab_9960 | Unannotated feature | neg | 194.0491 | 2.111 | 2.890 | 3.322 | 0.431 | 0.381 | 0.033 | 0.084 | Unannotated | NA | NA |
| metab_11066 | Unannotated feature | neg | 286.8620 | 0.582 | 6.069 | 6.033 | -0.036 | -0.381 | 0.033 | 0.084 | Unannotated | NA | NA |
| metab_9684 | Unannotated feature | neg | 564.7826 | 0.629 | 5.228 | 5.159 | -0.069 | -0.381 | 0.033 | 0.084 | Unannotated | NA | NA |
| metab_6264 | Unannotated feature | neg | 374.9359 | 3.522 | 4.043 | 4.568 | 0.526 | 0.381 | 0.033 | 0.084 | Unannotated | NA | NA |
| metab_1706 | Unannotated feature | pos | 540.8893 | 7.293 | 5.056 | 5.014 | -0.042 | -0.381 | 0.033 | 0.084 | Unannotated | NA | NA |
| metab_7557 | Unannotated feature | neg | 143.8646 | 7.189 | 3.945 | 3.968 | 0.023 | 0.381 | 0.033 | 0.084 | Unannotated | NA | NA |
| metab_4937 | Unannotated feature | pos | 188.1395 | 0.629 | 4.374 | 4.046 | -0.328 | -0.381 | 0.033 | 0.084 | Unannotated | NA | NA |
| metab_3944 | N-(3-Hydroxypropyl)valine | pos | 217.1547 | 0.846 | 3.316 | 3.892 | 0.576 | 0.381 | 0.033 | 0.084 | Tentative | Organic acids and derivatives | Carboxylic acids and derivatives |
| metab_2836 | Unannotated feature | pos | 207.0877 | 2.081 | 5.427 | 5.171 | -0.256 | -0.381 | 0.033 | 0.084 | Unannotated | NA | NA |
| metab_8039 | Unannotated feature | neg | 471.2432 | 6.518 | 4.217 | 3.285 | -0.932 | -0.381 | 0.033 | 0.084 | Unannotated | NA | NA |
| metab_10525 | Unannotated feature | neg | 523.7271 | 4.931 | 5.915 | 6.113 | 0.198 | 0.381 | 0.033 | 0.084 | Unannotated | NA | NA |
| metab_10563 | Taurine | neg | 124.0067 | 0.621 | 5.132 | 5.003 | -0.129 | -0.381 | 0.033 | 0.084 | Strong putative | NA | NA |
| metab_2923 | Unannotated feature | pos | 284.8750 | 4.884 | 4.049 | 4.288 | 0.238 | 0.381 | 0.033 | 0.084 | Unannotated | NA | NA |
| metab_3896 | Unannotated feature | pos | 457.0689 | 0.861 | 4.582 | 4.723 | 0.141 | 0.381 | 0.033 | 0.084 | Unannotated | NA | NA |
| metab_4444 | Unannotated feature | pos | 982.7985 | 0.551 | 5.040 | 4.863 | -0.177 | -0.381 | 0.033 | 0.084 | Unannotated | NA | NA |
| metab_453 | Unannotated feature | pos | 318.3003 | 5.771 | 7.425 | 7.460 | 0.034 | 0.381 | 0.033 | 0.084 | Unannotated | NA | NA |
| metab_455 | 3-Dehydroquinate | pos | 191.0550 | 1.724 | 3.699 | 3.971 | 0.272 | 0.381 | 0.033 | 0.084 | Tentative | Organic acids and derivatives | Hydroxy acids and derivatives |
| metab_6614 | Unannotated feature | neg | 534.7179 | 4.931 | 3.860 | 4.354 | 0.493 | 0.381 | 0.033 | 0.084 | Unannotated | NA | NA |
| metab_3341 | 3-Hydroxymethylantipyrine | pos | 243.0531 | 2.606 | 4.709 | 4.650 | -0.059 | -0.381 | 0.033 | 0.084 | Tentative | NA | NA |
| metab_9404 | Unannotated feature | neg | 269.0672 | 3.491 | 3.441 | 4.131 | 0.690 | 0.381 | 0.033 | 0.084 | Unannotated | NA | NA |
| metab_9520 | Unannotated feature | neg | 280.9558 | 3.224 | 4.519 | 4.307 | -0.212 | -0.381 | 0.033 | 0.084 | Unannotated | NA | NA |
| metab_3125 | Unannotated feature | pos | 102.5360 | 3.700 | 5.223 | 5.203 | -0.020 | -0.381 | 0.033 | 0.084 | Unannotated | NA | NA |
| metab_10736 | Unannotated feature | neg | 686.7563 | 0.570 | 5.128 | 5.060 | -0.068 | -0.381 | 0.033 | 0.084 | Unannotated | NA | NA |
| metab_11174 | Unannotated feature | neg | 511.2561 | 5.811 | 4.134 | 4.510 | 0.376 | 0.381 | 0.033 | 0.084 | Unannotated | NA | NA |
| metab_1470 | Unannotated feature | pos | 378.2399 | 6.010 | 4.996 | 4.918 | -0.078 | -0.381 | 0.033 | 0.084 | Unannotated | NA | NA |
| metab_365 | Unannotated feature | pos | 250.9732 | 0.869 | 4.634 | 4.573 | -0.060 | -0.381 | 0.033 | 0.084 | Unannotated | NA | NA |
| metab_4247 | Unannotated feature | pos | 120.0035 | 0.606 | 4.265 | 4.333 | 0.067 | 0.381 | 0.033 | 0.084 | Unannotated | NA | NA |
| metab_7350 | Unannotated feature | neg | 634.3354 | 6.527 | 5.060 | 5.261 | 0.201 | 0.381 | 0.033 | 0.084 | Unannotated | NA | NA |
| metab_10787 | Unannotated feature | neg | 715.8464 | 0.562 | 4.597 | 4.459 | -0.138 | -0.381 | 0.033 | 0.084 | Unannotated | NA | NA |
| metab_10799 | Unannotated feature | neg | 583.8845 | 0.562 | 4.467 | 4.240 | -0.227 | -0.381 | 0.033 | 0.084 | Unannotated | NA | NA |
| metab_3508 | Unannotated feature | pos | 362.2281 | 2.374 | 4.648 | 4.755 | 0.107 | 0.381 | 0.033 | 0.084 | Unannotated | NA | NA |
| metab_11257 | Dodecylbenzenesulfonic Acid | neg | 325.1848 | 6.188 | 4.466 | 3.890 | -0.576 | -0.381 | 0.033 | 0.084 | Strong putative | NA | NA |
| metab_229 | Unannotated feature | pos | 447.8375 | 0.636 | 4.801 | 4.459 | -0.342 | -0.381 | 0.033 | 0.084 | Unannotated | NA | NA |
| metab_2314 | Unannotated feature | pos | 507.3295 | 6.041 | 4.574 | 4.650 | 0.076 | 0.381 | 0.033 | 0.084 | Unannotated | NA | NA |
| metab_22 | Unannotated feature | pos | 606.6769 | 0.583 | 4.605 | 4.473 | -0.132 | -0.381 | 0.033 | 0.084 | Unannotated | NA | NA |
| metab_3686 | Unannotated feature | pos | 119.0164 | 1.925 | 2.405 | 2.705 | 0.301 | 0.377 | 0.035 | 0.088 | Unannotated | NA | NA |
| metab_3675 | O-Succinyl-L-homoserine | pos | 184.0605 | 1.941 | 4.472 | 4.692 | 0.220 | 0.377 | 0.035 | 0.088 | Tentative | Organic acids and derivatives | Carboxylic acids and derivatives |
| metab_10702 | Unannotated feature | neg | 637.7191 | 0.578 | 4.619 | 4.467 | -0.152 | -0.377 | 0.035 | 0.088 | Unannotated | NA | NA |
| metab_6014 | Unannotated feature | neg | 109.0287 | 2.922 | 4.540 | 4.701 | 0.161 | 0.377 | 0.035 | 0.088 | Unannotated | NA | NA |
| metab_18 | Unannotated feature | pos | 140.9036 | 0.591 | 5.836 | 5.810 | -0.026 | -0.377 | 0.035 | 0.088 | Unannotated | NA | NA |
| metab_7341 | Unannotated feature | neg | 131.0343 | 6.465 | 5.051 | 5.087 | 0.035 | 0.377 | 0.035 | 0.088 | Unannotated | NA | NA |
| metab_1636 | Unannotated feature | pos | 463.3029 | 7.168 | 5.167 | 5.129 | -0.038 | -0.377 | 0.035 | 0.088 | Unannotated | NA | NA |
| metab_1632 | Unannotated feature | pos | 102.1956 | 7.107 | 3.638 | 3.770 | 0.132 | 0.377 | 0.035 | 0.088 | Unannotated | NA | NA |
| metab_8394 | Unannotated feature | neg | 230.9970 | 3.209 | 4.882 | 5.368 | 0.486 | 0.377 | 0.035 | 0.088 | Unannotated | NA | NA |
| metab_5900 | Unannotated feature | neg | 245.1146 | 2.515 | 4.730 | 4.827 | 0.096 | 0.377 | 0.035 | 0.088 | Unannotated | NA | NA |
| metab_8398 | Traumatic acid | neg | 227.1290 | 5.795 | 5.106 | 5.207 | 0.101 | 0.377 | 0.035 | 0.088 | Moderate putative | Lipids and lipid-like molecules | Fatty Acyls |
| metab_5853 | Unannotated feature | neg | 303.8873 | 2.379 | 5.307 | 5.378 | 0.070 | 0.377 | 0.035 | 0.088 | Unannotated | NA | NA |
| metab_1200 | Unannotated feature | pos | 808.5620 | 5.756 | 4.676 | 4.321 | -0.355 | -0.377 | 0.035 | 0.088 | Unannotated | NA | NA |
| metab_4242 | Unannotated feature | pos | 285.9031 | 0.606 | 4.394 | 4.264 | -0.130 | -0.377 | 0.035 | 0.088 | Unannotated | NA | NA |
| metab_1441 | Unannotated feature | pos | 533.3663 | 5.964 | 4.861 | 4.728 | -0.133 | -0.377 | 0.035 | 0.088 | Unannotated | NA | NA |
| metab_10670 | Unannotated feature | neg | 613.6456 | 0.582 | 5.050 | 4.958 | -0.092 | -0.377 | 0.035 | 0.088 | Unannotated | NA | NA |
| metab_8736 | Unannotated feature | neg | 343.0457 | 5.571 | 3.684 | 3.946 | 0.262 | 0.377 | 0.035 | 0.088 | Unannotated | NA | NA |
| metab_8296 | Unannotated feature | neg | 211.0360 | 5.867 | 4.245 | 3.949 | -0.296 | -0.377 | 0.035 | 0.088 | Unannotated | NA | NA |
| metab_2255 | N-Stearoyl Arginine | pos | 479.3346 | 6.196 | 5.191 | 5.017 | -0.174 | -0.377 | 0.035 | 0.088 | Tentative | NA | NA |
| metab_1747 | Unannotated feature | pos | 332.9159 | 7.324 | 4.693 | 4.749 | 0.056 | 0.377 | 0.035 | 0.088 | Unannotated | NA | NA |
| metab_920 | Unannotated feature | pos | 226.0798 | 0.854 | 4.458 | 4.583 | 0.125 | 0.377 | 0.035 | 0.088 | Unannotated | NA | NA |
| metab_6292 | Gamma-Glutamylphenylalanine | neg | 293.1150 | 3.634 | 4.906 | 5.040 | 0.135 | 0.377 | 0.035 | 0.088 | Tentative | NA | NA |
| metab_1711 | Unannotated feature | pos | 696.8456 | 7.293 | 4.293 | 4.069 | -0.224 | -0.377 | 0.035 | 0.088 | Unannotated | NA | NA |
| metab_2288 | Unannotated feature | pos | 472.3634 | 6.103 | 5.976 | 5.910 | -0.066 | -0.377 | 0.035 | 0.088 | Unannotated | NA | NA |
| metab_1705 | PC(O-16:0/0:0) | pos | 504.3423 | 7.293 | 4.997 | 4.925 | -0.072 | -0.377 | 0.035 | 0.088 | Strong putative | NA | NA |
| metab_9592 | Unannotated feature | neg | 569.0862 | 3.160 | 4.401 | 3.920 | -0.481 | -0.377 | 0.035 | 0.088 | Unannotated | NA | NA |
| metab_9641 | Unannotated feature | neg | 346.0800 | 3.072 | 3.903 | 3.597 | -0.306 | -0.377 | 0.035 | 0.088 | Unannotated | NA | NA |
| metab_9675 | 4-Hydroxyhippuric Acid | neg | 194.0457 | 2.938 | 4.536 | 4.971 | 0.434 | 0.377 | 0.035 | 0.088 | Moderate putative | Benzenoids | Benzene and substituted derivatives |
| metab_7997 | Unannotated feature | neg | 160.6404 | 6.976 | 3.666 | 3.411 | -0.255 | -0.377 | 0.035 | 0.088 | Unannotated | NA | NA |
| metab_2621 | Unannotated feature | pos | 632.4370 | 5.832 | 5.548 | 5.471 | -0.077 | -0.377 | 0.035 | 0.088 | Unannotated | NA | NA |
| metab_7150 | Unannotated feature | neg | 128.0346 | 1.390 | 6.439 | 6.370 | -0.069 | -0.377 | 0.035 | 0.088 | Unannotated | NA | NA |
| metab_2515 | Unannotated feature | pos | 267.1226 | 5.596 | 3.482 | 4.215 | 0.733 | 0.377 | 0.035 | 0.088 | Unannotated | NA | NA |
| metab_9393 | Unannotated feature | neg | 622.7414 | 0.636 | 5.550 | 5.486 | -0.064 | -0.377 | 0.035 | 0.088 | Unannotated | NA | NA |
| metab_3283 | Unannotated feature | pos | 266.1386 | 2.880 | 4.216 | 4.904 | 0.687 | 0.377 | 0.035 | 0.088 | Unannotated | NA | NA |
| metab_382 | Unannotated feature | pos | 314.7792 | 0.908 | 5.152 | 5.123 | -0.030 | -0.377 | 0.035 | 0.088 | Unannotated | NA | NA |
| metab_405 | Unannotated feature | pos | 280.1391 | 1.181 | 4.802 | 4.627 | -0.176 | -0.377 | 0.035 | 0.088 | Unannotated | NA | NA |
| metab_3386 | Unannotated feature | pos | 150.0583 | 2.506 | 3.864 | 3.969 | 0.105 | 0.377 | 0.035 | 0.088 | Unannotated | NA | NA |
| metab_9388 | Unannotated feature | neg | 276.8683 | 3.522 | 5.861 | 5.919 | 0.058 | 0.377 | 0.035 | 0.088 | Unannotated | NA | NA |
| metab_6606 | Unannotated feature | neg | 187.0875 | 4.877 | 3.653 | 3.478 | -0.175 | -0.377 | 0.035 | 0.088 | Unannotated | NA | NA |
| metab_8116 | Unannotated feature | neg | 115.5774 | 6.180 | 5.371 | 5.326 | -0.045 | -0.377 | 0.035 | 0.088 | Unannotated | NA | NA |
| metab_2900 | 5-Hydroxydec-7-enoylcarnitine | pos | 330.2274 | 4.977 | 4.102 | 5.022 | 0.920 | 0.377 | 0.035 | 0.088 | Tentative | Lipids and lipid-like molecules | Fatty Acyls |
| metab_9253 | Unannotated feature | neg | 130.0867 | 3.942 | 4.197 | 4.321 | 0.123 | 0.377 | 0.035 | 0.088 | Unannotated | NA | NA |
| metab_11224 | Unannotated feature | neg | 195.1025 | 5.827 | 5.767 | 5.296 | -0.471 | -0.377 | 0.035 | 0.088 | Unannotated | NA | NA |
| metab_9111 | Unannotated feature | neg | 187.0067 | 4.379 | 7.191 | 7.779 | 0.589 | 0.377 | 0.035 | 0.088 | Strong putative | NA | NA |
| metab_4722 | Unannotated feature | pos | 688.5210 | 5.987 | 6.187 | 6.159 | -0.028 | -0.377 | 0.035 | 0.088 | Unannotated | NA | NA |
| metab_6612 | Unannotated feature | neg | 533.0440 | 4.917 | 4.518 | 4.047 | -0.470 | -0.373 | 0.037 | 0.092 | Unannotated | NA | NA |
| metab_3916 | Unannotated feature | pos | 204.9276 | 0.861 | 4.562 | 4.706 | 0.144 | 0.373 | 0.037 | 0.092 | Unannotated | NA | NA |
| metab_7179 | Unannotated feature | neg | 474.3018 | 5.898 | 4.585 | 4.728 | 0.143 | 0.373 | 0.037 | 0.092 | Unannotated | NA | NA |
| metab_2016 | Unannotated feature | pos | 122.0967 | 7.285 | 3.634 | 3.553 | -0.082 | -0.373 | 0.037 | 0.092 | Unannotated | NA | NA |
| metab_10062 | Unannotated feature | neg | 215.0826 | 4.363 | 4.529 | 3.864 | -0.665 | -0.373 | 0.037 | 0.092 | Unannotated | NA | NA |
| metab_10444 | 6-(1,2,3,4-Tetrahydroxybutyl)oxane-2,3,4,5-tetrol | neg | 315.0939 | 0.660 | 4.519 | 4.626 | 0.107 | 0.373 | 0.037 | 0.092 | Tentative | NA | NA |
| metab_5327 | Unannotated feature | neg | 525.8177 | 0.570 | 4.057 | 3.759 | -0.298 | -0.373 | 0.037 | 0.092 | Unannotated | NA | NA |
| metab_1928 | Unannotated feature | pos | 112.3451 | 7.324 | 3.139 | 3.292 | 0.154 | 0.373 | 0.037 | 0.092 | Unannotated | NA | NA |
| metab_8347 | Unannotated feature | neg | 241.0979 | 5.827 | 4.525 | 3.767 | -0.758 | -0.373 | 0.037 | 0.092 | Unannotated | NA | NA |
| metab_1069 | Unannotated feature | pos | 288.2170 | 5.160 | 4.005 | 4.505 | 0.500 | 0.373 | 0.037 | 0.092 | Unannotated | NA | NA |
| metab_1000 | Docosenoyl-CoA | pos | 544.7139 | 4.508 | 5.381 | 5.490 | 0.109 | 0.373 | 0.037 | 0.092 | Tentative | Lipids and lipid-like molecules | Fatty Acyls |
| metab_5603 | Unannotated feature | neg | 474.9464 | 0.873 | 4.818 | 4.649 | -0.169 | -0.373 | 0.037 | 0.092 | Unannotated | NA | NA |
| metab_6855 | Unannotated feature | neg | 225.0767 | 5.627 | 3.730 | 4.164 | 0.434 | 0.373 | 0.037 | 0.092 | Unannotated | NA | NA |
| metab_4216 | Cyclopentanol | pos | 104.1074 | 0.621 | 6.687 | 6.740 | 0.053 | 0.373 | 0.037 | 0.092 | Tentative | NA | NA |
| metab_11323 | Unannotated feature | neg | 207.9186 | 7.301 | 6.025 | 6.045 | 0.021 | 0.373 | 0.037 | 0.092 | Unannotated | NA | NA |
| metab_8966 | Unannotated feature | neg | 264.8291 | 4.893 | 4.182 | 3.782 | -0.401 | -0.373 | 0.037 | 0.092 | Unannotated | NA | NA |
| metab_6821 | Unannotated feature | neg | 472.9494 | 0.873 | 5.130 | 5.043 | -0.088 | -0.373 | 0.037 | 0.092 | Unannotated | NA | NA |
| metab_8929 | Unannotated feature | neg | 664.2588 | 5.001 | 5.177 | 5.379 | 0.201 | 0.373 | 0.037 | 0.092 | Unannotated | NA | NA |
| metab_10649 | Unannotated feature | neg | 662.5746 | 0.587 | 4.747 | 4.562 | -0.185 | -0.373 | 0.037 | 0.092 | Unannotated | NA | NA |
| metab_11395 | L-Kynurenine | neg | 207.0774 | 2.431 | 4.044 | 4.138 | 0.094 | 0.373 | 0.037 | 0.092 | Tentative | Organic oxygen compounds | Organooxygen compounds |
| metab_11339 | Unannotated feature | neg | 282.9924 | 5.547 | 4.696 | 5.236 | 0.540 | 0.373 | 0.037 | 0.092 | Unannotated | NA | NA |
| metab_3618 | N-Acetylmuramate | pos | 326.1444 | 1.964 | 4.633 | 4.206 | -0.428 | -0.373 | 0.037 | 0.092 | Tentative | NA | NA |
| metab_3531 | Unannotated feature | pos | 166.0797 | 2.088 | 4.382 | 3.995 | -0.386 | -0.373 | 0.037 | 0.092 | Unannotated | NA | NA |
| metab_3498 | Unannotated feature | pos | 85.0652 | 2.202 | 2.480 | 2.771 | 0.292 | 0.373 | 0.037 | 0.092 | Unannotated | NA | NA |
| metab_7058 | Unannotated feature | neg | 271.1556 | 5.779 | 3.696 | 4.264 | 0.568 | 0.373 | 0.037 | 0.092 | Unannotated | NA | NA |
| metab_11180 | (3beta,19alpha)-3,19,23,24-Tetrahydroxy-12-oleanen-28-oic acid | neg | 503.3388 | 5.827 | 4.067 | 3.632 | -0.435 | -0.373 | 0.037 | 0.092 | Tentative | NA | NA |
| metab_300 | Unannotated feature | pos | 155.0540 | 0.760 | 2.665 | 2.979 | 0.314 | 0.373 | 0.037 | 0.092 | Unannotated | NA | NA |
| metab_4676 | Unannotated feature | pos | 606.7866 | 0.567 | 5.546 | 5.530 | -0.016 | -0.373 | 0.037 | 0.092 | Unannotated | NA | NA |
| metab_8156 | Unannotated feature | neg | 153.8680 | 6.063 | 5.217 | 5.247 | 0.029 | 0.373 | 0.037 | 0.092 | Unannotated | NA | NA |
| metab_11187 | Unannotated feature | neg | 183.1387 | 5.851 | 4.950 | 5.166 | 0.217 | 0.373 | 0.037 | 0.092 | Unannotated | NA | NA |
| metab_6745 | Unannotated feature | neg | 344.8208 | 0.582 | 5.899 | 5.858 | -0.041 | -0.373 | 0.037 | 0.092 | Unannotated | NA | NA |
| metab_3431 | 6-Hydroxyhexanoic acid | pos | 174.1126 | 2.381 | 4.820 | 4.772 | -0.047 | -0.373 | 0.037 | 0.092 | Tentative | NA | NA |
| metab_2663 | Unannotated feature | pos | 214.2530 | 5.793 | 4.286 | 4.379 | 0.093 | 0.373 | 0.037 | 0.092 | Unannotated | NA | NA |
| metab_5138 | L-Glutamate | pos | 148.0605 | 0.629 | 5.859 | 5.744 | -0.115 | -0.373 | 0.037 | 0.092 | Moderate putative | Organic acids and derivatives | Carboxylic acids and derivatives |
| metab_9783 | Unannotated feature | neg | 119.9068 | 2.597 | 4.324 | 4.293 | -0.030 | -0.373 | 0.037 | 0.092 | Unannotated | NA | NA |
| metab_3170 | Unannotated feature | pos | 196.0605 | 2.529 | 4.534 | 5.086 | 0.552 | 0.373 | 0.037 | 0.092 | Unannotated | NA | NA |
| metab_8097 | Unannotated feature | neg | 355.1588 | 6.188 | 5.432 | 5.136 | -0.295 | -0.373 | 0.037 | 0.092 | Unannotated | NA | NA |
| metab_4959 | Unannotated feature | pos | 376.9284 | 0.629 | 6.210 | 6.184 | -0.026 | -0.373 | 0.037 | 0.092 | Unannotated | NA | NA |
| metab_6499 | Unannotated feature | neg | 254.9949 | 4.386 | 4.861 | 5.400 | 0.539 | 0.373 | 0.037 | 0.092 | Unannotated | NA | NA |
| metab_8834 | Unannotated feature | neg | 728.2714 | 5.346 | 4.315 | 4.613 | 0.298 | 0.373 | 0.037 | 0.092 | Unannotated | NA | NA |
| metab_8041 | Unannotated feature | neg | 113.9937 | 6.493 | 4.884 | 4.910 | 0.026 | 0.373 | 0.037 | 0.092 | Unannotated | NA | NA |
| metab_6979 | Boldenone | neg | 331.1922 | 5.707 | 4.986 | 4.848 | -0.138 | -0.368 | 0.039 | 0.097 | Tentative | NA | NA |
| metab_1127 | Cardanolmonoene | pos | 341.2224 | 5.626 | 5.164 | 5.136 | -0.029 | -0.368 | 0.039 | 0.097 | Tentative | NA | NA |
| metab_8233 | Unannotated feature | neg | 249.9785 | 5.922 | 6.822 | 6.844 | 0.022 | 0.368 | 0.039 | 0.097 | Unannotated | NA | NA |
| metab_11366 | Unannotated feature | neg | 248.9222 | 0.128 | 5.810 | 5.787 | -0.024 | -0.368 | 0.039 | 0.097 | Unannotated | NA | NA |
| metab_8463 | Unannotated feature | neg | 412.2111 | 5.747 | 4.396 | 3.852 | -0.545 | -0.368 | 0.039 | 0.097 | Unannotated | NA | NA |
| metab_3737 | Piperidine | pos | 86.0969 | 1.809 | 6.828 | 6.796 | -0.032 | -0.368 | 0.039 | 0.097 | Tentative | NA | NA |
| metab_7306 | Unannotated feature | neg | 258.9011 | 6.203 | 6.445 | 6.309 | -0.136 | -0.368 | 0.039 | 0.097 | Unannotated | NA | NA |
| metab_1918 | Unannotated feature | pos | 200.0355 | 7.324 | 3.821 | 3.981 | 0.160 | 0.368 | 0.039 | 0.097 | Unannotated | NA | NA |
| metab_10674 | Unannotated feature | neg | 592.6758 | 0.582 | 5.451 | 5.405 | -0.046 | -0.368 | 0.039 | 0.097 | Unannotated | NA | NA |
| metab_5941 | Unannotated feature | neg | 90.9328 | 2.644 | 3.699 | 3.758 | 0.059 | 0.368 | 0.039 | 0.097 | Unannotated | NA | NA |
| metab_2267 | Unannotated feature | pos | 102.1954 | 6.172 | 3.593 | 3.702 | 0.109 | 0.368 | 0.039 | 0.097 | Unannotated | NA | NA |
| metab_2230 | Unannotated feature | pos | 465.3554 | 6.288 | 4.978 | 4.825 | -0.153 | -0.368 | 0.039 | 0.097 | Unannotated | NA | NA |
| metab_10031 | Unannotated feature | neg | 261.9422 | 1.715 | 4.304 | 4.219 | -0.085 | -0.368 | 0.039 | 0.097 | Unannotated | NA | NA |
| metab_7858 | Unannotated feature | neg | 962.8311 | 7.140 | 4.838 | 4.714 | -0.124 | -0.368 | 0.039 | 0.097 | Unannotated | NA | NA |
| metab_8301 | Unannotated feature | neg | 625.3606 | 5.859 | 4.565 | 4.282 | -0.283 | -0.368 | 0.039 | 0.097 | Unannotated | NA | NA |
| metab_10166 | Unannotated feature | neg | 200.5835 | 0.912 | 4.887 | 4.831 | -0.056 | -0.368 | 0.039 | 0.097 | Unannotated | NA | NA |
| metab_6369 | Unannotated feature | neg | 131.0707 | 3.959 | 4.831 | 4.907 | 0.076 | 0.368 | 0.039 | 0.097 | Unannotated | NA | NA |
| metab_10940 | Unannotated feature | neg | 401.9262 | 0.530 | 5.554 | 5.515 | -0.038 | -0.368 | 0.039 | 0.097 | Unannotated | NA | NA |
| metab_4099 | Pyrrolidine | pos | 72.0814 | 0.721 | 2.817 | 3.205 | 0.388 | 0.368 | 0.039 | 0.097 | Tentative | Organoheterocyclic compounds | Pyrrolidines |
| metab_1041 | Unannotated feature | pos | 163.1329 | 4.884 | 4.422 | 4.392 | -0.031 | -0.368 | 0.039 | 0.097 | Unannotated | NA | NA |
| metab_4568 | Unannotated feature | pos | 163.0754 | 0.465 | 4.784 | 4.801 | 0.017 | 0.368 | 0.039 | 0.097 | Unannotated | NA | NA |
| metab_5692 | Unannotated feature | neg | 306.9394 | 1.294 | 5.942 | 5.837 | -0.105 | -0.368 | 0.039 | 0.097 | Unannotated | NA | NA |
| metab_10548 | Unannotated feature | neg | 417.7952 | 0.621 | 3.883 | 3.678 | -0.206 | -0.368 | 0.039 | 0.097 | Unannotated | NA | NA |
| metab_2432 | Unannotated feature | pos | 815.5608 | 5.917 | 4.443 | 4.174 | -0.270 | -0.368 | 0.039 | 0.097 | Unannotated | NA | NA |
| metab_5092 | Unannotated feature | pos | 207.1380 | 1.515 | 5.106 | 5.087 | -0.019 | -0.368 | 0.039 | 0.097 | Unannotated | NA | NA |
| metab_9680 | Unannotated feature | neg | 188.9861 | 2.922 | 5.682 | 5.954 | 0.271 | 0.368 | 0.039 | 0.097 | Unannotated | NA | NA |
| metab_8099 | Unannotated feature | neg | 942.3468 | 2.938 | 6.267 | 5.919 | -0.348 | -0.368 | 0.039 | 0.097 | Unannotated | NA | NA |
| metab_11106 | Unannotated feature | neg | 453.1774 | 5.659 | 4.094 | 4.288 | 0.194 | 0.368 | 0.039 | 0.097 | Unannotated | NA | NA |
| metab_2924 | Unannotated feature | pos | 102.5360 | 4.884 | 4.884 | 4.868 | -0.016 | -0.368 | 0.039 | 0.097 | Unannotated | NA | NA |
| metab_2902 | Unannotated feature | pos | 146.0601 | 4.977 | 4.128 | 4.222 | 0.094 | 0.368 | 0.039 | 0.097 | Unannotated | NA | NA |
| metab_3947 | Unannotated feature | pos | 148.0428 | 0.854 | 4.354 | 4.645 | 0.291 | 0.368 | 0.039 | 0.097 | Unannotated | NA | NA |
| metab_5143 | Methyl methacrylate | pos | 218.1388 | 1.917 | 6.452 | 6.320 | -0.131 | -0.368 | 0.039 | 0.097 | Tentative | NA | NA |
| metab_3256 | Indolin-2-one | pos | 134.0602 | 2.989 | 5.296 | 5.656 | 0.360 | 0.368 | 0.039 | 0.097 | Strong putative | Organoheterocyclic compounds | Indoles and derivatives |
| metab_8252 | Unannotated feature | neg | 571.2893 | 5.898 | 5.420 | 5.494 | 0.074 | 0.368 | 0.039 | 0.097 | Unannotated | NA | NA |
| metab_8877 | Unannotated feature | neg | 384.0158 | 5.819 | 5.277 | 4.889 | -0.389 | -0.368 | 0.039 | 0.097 | Unannotated | NA | NA |
| metab_4794 | Unannotated feature | pos | 85.0289 | 0.457 | 5.891 | 5.908 | 0.017 | 0.368 | 0.039 | 0.097 | Unannotated | NA | NA |
| metab_4787 | Unannotated feature | pos | 218.9198 | 7.285 | 4.672 | 4.650 | -0.022 | -0.368 | 0.039 | 0.097 | Unannotated | NA | NA |
| metab_3577 | Unannotated feature | pos | 172.3556 | 2.058 | 3.865 | 4.036 | 0.171 | 0.368 | 0.039 | 0.097 | Unannotated | NA | NA |
| metab_273 | Unannotated feature | pos | 95.0496 | 3.159 | 4.973 | 5.215 | 0.242 | 0.368 | 0.039 | 0.097 | Unannotated | NA | NA |
| metab_4481 | Unannotated feature | pos | 634.8760 | 0.551 | 6.563 | 6.509 | -0.054 | -0.364 | 0.042 | 0.101 | Unannotated | NA | NA |
| metab_10984 | Unannotated feature | neg | 184.8895 | 0.440 | 4.903 | 4.958 | 0.055 | 0.364 | 0.042 | 0.101 | Unannotated | NA | NA |
| metab_746 | Unannotated feature | pos | 254.1248 | 0.854 | 4.922 | 4.788 | -0.133 | -0.364 | 0.042 | 0.101 | Unannotated | NA | NA |
| metab_4520 | Unannotated feature | pos | 117.0742 | 0.541 | 3.894 | 3.806 | -0.088 | -0.364 | 0.042 | 0.101 | Unannotated | NA | NA |
| metab_3373 | Unannotated feature | pos | 138.9954 | 2.529 | 5.026 | 5.009 | -0.016 | -0.364 | 0.042 | 0.101 | Unannotated | NA | NA |
| metab_10007 | Unannotated feature | neg | 110.0240 | 1.878 | 3.683 | 3.572 | -0.111 | -0.364 | 0.042 | 0.101 | Unannotated | NA | NA |
| metab_1520 | Unannotated feature | pos | 496.4208 | 6.165 | 4.651 | 4.718 | 0.067 | 0.364 | 0.042 | 0.101 | Unannotated | NA | NA |
| metab_7440 | Unannotated feature | neg | 382.1314 | 7.109 | 4.372 | 4.281 | -0.091 | -0.364 | 0.042 | 0.101 | Unannotated | NA | NA |
| metab_8331 | Unannotated feature | neg | 253.1449 | 5.834 | 4.918 | 5.108 | 0.190 | 0.364 | 0.042 | 0.101 | Moderate putative | NA | NA |
| metab_6987 | 11-Oxo-androsterone glucuronide | neg | 479.2296 | 5.715 | 4.622 | 5.167 | 0.544 | 0.364 | 0.042 | 0.101 | Tentative | Lipids and lipid-like molecules | Glycerolipids |
| metab_11279 | Unannotated feature | neg | 566.3477 | 6.403 | 6.748 | 6.841 | 0.092 | 0.364 | 0.042 | 0.101 | Unannotated | NA | NA |
| metab_8709 | Unannotated feature | neg | 387.1856 | 5.587 | 4.522 | 4.990 | 0.468 | 0.364 | 0.042 | 0.101 | Unannotated | NA | NA |
| metab_2435 | Unannotated feature | pos | 732.5077 | 5.917 | 4.534 | 4.325 | -0.209 | -0.364 | 0.042 | 0.101 | Unannotated | NA | NA |
| metab_6489 | Unannotated feature | neg | 186.3053 | 4.371 | 5.224 | 5.826 | 0.602 | 0.364 | 0.042 | 0.101 | Unannotated | NA | NA |
| metab_9459 | Unannotated feature | neg | 116.0710 | 3.332 | 3.664 | 3.599 | -0.065 | -0.364 | 0.042 | 0.101 | Unannotated | NA | NA |
| metab_7895 | Unannotated feature | neg | 308.9167 | 7.132 | 4.396 | 4.324 | -0.072 | -0.364 | 0.042 | 0.101 | Unannotated | NA | NA |
| metab_10931 | Unannotated feature | neg | 605.8899 | 0.534 | 5.713 | 5.667 | -0.046 | -0.364 | 0.042 | 0.101 | Unannotated | NA | NA |
| metab_1818 | Unannotated feature | pos | 167.0704 | 7.983 | 4.268 | 4.219 | -0.049 | -0.364 | 0.042 | 0.101 | Unannotated | NA | NA |
| metab_1843 | Unannotated feature | pos | 79.0548 | 7.944 | 3.969 | 3.993 | 0.024 | 0.364 | 0.042 | 0.101 | Unannotated | NA | NA |
| metab_9834 | Unannotated feature | neg | 375.9279 | 2.504 | 4.560 | 4.489 | -0.070 | -0.364 | 0.042 | 0.101 | Unannotated | NA | NA |
| metab_5766 | Unannotated feature | neg | 934.7932 | 0.554 | 5.820 | 5.901 | 0.081 | 0.364 | 0.042 | 0.101 | Unannotated | NA | NA |
| metab_2467 | Unannotated feature | pos | 240.2321 | 5.910 | 3.796 | 4.050 | 0.255 | 0.364 | 0.042 | 0.101 | Unannotated | NA | NA |
| metab_7567 | Unannotated feature | neg | 161.8494 | 7.197 | 3.953 | 3.896 | -0.057 | -0.364 | 0.042 | 0.101 | Unannotated | NA | NA |
| metab_4006 | L-Canaline | pos | 176.1031 | 0.815 | 4.902 | 5.163 | 0.261 | 0.364 | 0.042 | 0.101 | Tentative | NA | NA |
| metab_5093 | Unannotated feature | pos | 125.0235 | 1.538 | 4.613 | 4.550 | -0.063 | -0.364 | 0.042 | 0.101 | Unannotated | NA | NA |
| metab_3601 | Unannotated feature | pos | 139.0179 | 2.026 | 4.649 | 4.619 | -0.030 | -0.364 | 0.042 | 0.101 | Unannotated | NA | NA |
| metab_3827 | Unannotated feature | pos | 248.9762 | 1.259 | 5.117 | 5.072 | -0.045 | -0.364 | 0.042 | 0.101 | Unannotated | NA | NA |
| metab_3085 | Unannotated feature | pos | 232.9855 | 3.885 | 4.783 | 5.215 | 0.432 | 0.364 | 0.042 | 0.101 | Unannotated | NA | NA |
| metab_5096 | Unannotated feature | pos | 282.1196 | 1.570 | 5.445 | 5.537 | 0.091 | 0.364 | 0.042 | 0.101 | Unannotated | NA | NA |
| metab_3879 | Unannotated feature | pos | 96.0529 | 0.892 | 3.174 | 2.947 | -0.227 | -0.364 | 0.042 | 0.101 | Unannotated | NA | NA |
| metab_6067 | Unannotated feature | neg | 434.8629 | 0.570 | 5.307 | 5.359 | 0.051 | 0.364 | 0.042 | 0.101 | Unannotated | NA | NA |
| metab_1251 | 3-Oxododecanoic acid | pos | 237.1473 | 5.840 | 4.733 | 4.822 | 0.089 | 0.364 | 0.042 | 0.101 | Tentative | NA | NA |
| metab_4731 | Unannotated feature | pos | 88.0763 | 6.065 | 4.963 | 4.984 | 0.021 | 0.364 | 0.042 | 0.101 | Unannotated | NA | NA |
| metab_7627 | Unannotated feature | neg | 239.0774 | 7.541 | 4.460 | 4.428 | -0.032 | -0.364 | 0.042 | 0.101 | Unannotated | NA | NA |
| metab_5657 | Unannotated feature | neg | 154.8980 | 1.031 | 4.336 | 4.532 | 0.196 | 0.364 | 0.042 | 0.101 | Unannotated | NA | NA |
| metab_6300 | Unannotated feature | neg | 102.9560 | 3.680 | 4.283 | 4.236 | -0.047 | -0.364 | 0.042 | 0.101 | Unannotated | NA | NA |
| metab_3792 | Unannotated feature | pos | 112.0760 | 1.546 | 4.266 | 4.309 | 0.043 | 0.364 | 0.042 | 0.101 | Unannotated | NA | NA |
| metab_548 | Unannotated feature | pos | 274.2741 | 5.771 | 7.347 | 7.385 | 0.038 | 0.364 | 0.042 | 0.101 | Moderate putative | NA | NA |
| metab_435 | Unannotated feature | pos | 123.0807 | 1.577 | 3.981 | 4.038 | 0.057 | 0.364 | 0.042 | 0.101 | Unannotated | NA | NA |
| metab_3009 | Unannotated feature | pos | 158.0489 | 4.309 | 6.137 | 6.122 | -0.015 | -0.364 | 0.042 | 0.101 | Unannotated | NA | NA |
| metab_4968 | Unannotated feature | pos | 203.0526 | 0.629 | 7.834 | 7.776 | -0.058 | -0.364 | 0.042 | 0.101 | Unannotated | NA | NA |
| metab_5877 | Unannotated feature | neg | 161.8753 | 2.461 | 3.989 | 3.969 | -0.021 | -0.364 | 0.042 | 0.101 | Unannotated | NA | NA |
| metab_1794 | Unannotated feature | pos | 180.1019 | 7.718 | 5.018 | 5.058 | 0.040 | 0.364 | 0.042 | 0.101 | Unannotated | NA | NA |
| metab_1765 | Unannotated feature | pos | 183.6683 | 7.339 | 4.014 | 4.167 | 0.154 | 0.364 | 0.042 | 0.101 | Unannotated | NA | NA |
| metab_10900 | Unannotated feature | neg | 808.8363 | 0.543 | 5.751 | 5.688 | -0.063 | -0.360 | 0.044 | 0.106 | Unannotated | NA | NA |
| metab_7197 | Unannotated feature | neg | 325.7244 | 5.922 | 5.627 | 5.559 | -0.069 | -0.360 | 0.044 | 0.106 | Unannotated | NA | NA |
| metab_8237 | Unannotated feature | neg | 199.8509 | 5.922 | 5.901 | 5.932 | 0.031 | 0.360 | 0.044 | 0.106 | Unannotated | NA | NA |
| metab_4003 | Unannotated feature | pos | 244.1040 | 0.815 | 3.649 | 3.268 | -0.380 | -0.360 | 0.044 | 0.106 | Unannotated | NA | NA |
| metab_7172 | Unannotated feature | neg | 208.0978 | 5.898 | 3.817 | 4.022 | 0.206 | 0.360 | 0.044 | 0.106 | Unannotated | NA | NA |
| metab_4047 | Unannotated feature | pos | 218.1248 | 0.776 | 3.914 | 4.216 | 0.303 | 0.360 | 0.044 | 0.106 | Unannotated | NA | NA |
| metab_3149 | Unannotated feature | pos | 121.0287 | 2.521 | 4.491 | 4.978 | 0.488 | 0.360 | 0.044 | 0.106 | Unannotated | NA | NA |
| metab_10988 | Unannotated feature | neg | 334.8804 | 0.320 | 4.517 | 4.413 | -0.104 | -0.360 | 0.044 | 0.106 | Unannotated | NA | NA |
| metab_2269 | Unannotated feature | pos | 339.2895 | 6.165 | 4.842 | 4.887 | 0.045 | 0.360 | 0.044 | 0.106 | Unannotated | NA | NA |
| metab_1355 | Unannotated feature | pos | 434.3216 | 5.902 | 5.522 | 5.599 | 0.077 | 0.360 | 0.044 | 0.106 | Unannotated | NA | NA |
| metab_10585 | Unannotated feature | neg | 212.0208 | 0.613 | 4.372 | 4.294 | -0.078 | -0.360 | 0.044 | 0.106 | Unannotated | NA | NA |
| metab_1318 | Unannotated feature | pos | 456.3526 | 5.886 | 5.668 | 5.638 | -0.030 | -0.360 | 0.044 | 0.106 | Unannotated | NA | NA |
| metab_171 | Unannotated feature | pos | 667.8291 | 0.559 | 4.078 | 4.220 | 0.142 | 0.360 | 0.044 | 0.106 | Unannotated | NA | NA |
| metab_8693 | Unannotated feature | neg | 284.0449 | 5.611 | 3.483 | 3.595 | 0.112 | 0.360 | 0.044 | 0.106 | Unannotated | NA | NA |
| metab_159 | Unannotated feature | pos | 1014.7617 | 0.551 | 3.530 | 4.550 | 1.020 | 0.360 | 0.044 | 0.106 | Unannotated | NA | NA |
| metab_150 | Unannotated feature | pos | 771.8538 | 0.551 | 5.734 | 5.661 | -0.074 | -0.360 | 0.044 | 0.106 | Unannotated | NA | NA |
| metab_10625 | Unannotated feature | neg | 653.5441 | 0.594 | 5.364 | 5.284 | -0.080 | -0.360 | 0.044 | 0.106 | Unannotated | NA | NA |
| metab_10404 | Unannotated feature | neg | 199.8565 | 0.699 | 4.332 | 4.431 | 0.099 | 0.360 | 0.044 | 0.106 | Unannotated | NA | NA |
| metab_4499 | Unannotated feature | pos | 226.0139 | 0.551 | 5.013 | 5.046 | 0.033 | 0.360 | 0.044 | 0.106 | Unannotated | NA | NA |
| metab_1551 | Unannotated feature | pos | 472.4363 | 6.296 | 5.635 | 5.824 | 0.189 | 0.360 | 0.044 | 0.106 | Unannotated | NA | NA |
| metab_9516 | Unannotated feature | neg | 134.8940 | 3.232 | 6.103 | 6.065 | -0.038 | -0.360 | 0.044 | 0.106 | Unannotated | NA | NA |
| metab_615 | Unannotated feature | pos | 160.0969 | 2.164 | 4.494 | 4.699 | 0.205 | 0.360 | 0.044 | 0.106 | Unannotated | NA | NA |
| metab_4753 | Unannotated feature | pos | 607.4445 | 6.303 | 5.024 | 4.874 | -0.150 | -0.360 | 0.044 | 0.106 | Unannotated | NA | NA |
| metab_4666 | CMPF | pos | 223.0966 | 5.840 | 5.146 | 4.665 | -0.481 | -0.360 | 0.044 | 0.106 | Strong putative | NA | NA |
| metab_998 | Indole-3-acetaldehyde | pos | 160.0758 | 4.508 | 3.780 | 3.948 | 0.168 | 0.360 | 0.044 | 0.106 | Strong putative | Organoheterocyclic compounds | Indoles and derivatives |
| metab_5066 | Unannotated feature | pos | 130.1089 | 0.737 | 2.781 | 4.276 | 1.495 | 0.360 | 0.044 | 0.106 | Unannotated | NA | NA |
| metab_7086 | Unannotated feature | neg | 286.9709 | 1.294 | 5.087 | 5.172 | 0.085 | 0.360 | 0.044 | 0.106 | Unannotated | NA | NA |
| metab_6146 | Unannotated feature | neg | 136.8910 | 3.232 | 5.617 | 5.573 | -0.044 | -0.360 | 0.044 | 0.106 | Unannotated | NA | NA |
| metab_6213 | Unannotated feature | neg | 333.9346 | 3.364 | 4.395 | 4.268 | -0.127 | -0.360 | 0.044 | 0.106 | Unannotated | NA | NA |
| metab_2779 | Unannotated feature | pos | 284.1491 | 2.042 | 3.606 | 3.689 | 0.083 | 0.360 | 0.044 | 0.106 | Unannotated | NA | NA |
| metab_8206 | Unannotated feature | neg | 429.3018 | 5.961 | 4.704 | 4.534 | -0.170 | -0.360 | 0.044 | 0.106 | Unannotated | NA | NA |
| metab_1181 | Unannotated feature | pos | 547.8543 | 5.733 | 4.320 | 4.096 | -0.224 | -0.360 | 0.044 | 0.106 | Unannotated | NA | NA |
| metab_7596 | Unannotated feature | neg | 188.9126 | 7.245 | 5.266 | 5.295 | 0.029 | 0.360 | 0.044 | 0.106 | Unannotated | NA | NA |
| metab_8521 | Unannotated feature | neg | 211.0976 | 5.715 | 3.579 | 3.999 | 0.420 | 0.360 | 0.044 | 0.106 | Moderate putative | NA | NA |
| metab_2597 | Unannotated feature | pos | 495.6831 | 5.848 | 4.508 | 4.400 | -0.109 | -0.360 | 0.044 | 0.106 | Unannotated | NA | NA |
| metab_8818 | Unannotated feature | neg | 190.0508 | 5.428 | 3.724 | 3.944 | 0.220 | 0.360 | 0.044 | 0.106 | Unannotated | NA | NA |
| metab_2107 | Unannotated feature | pos | 128.1072 | 7.061 | 4.419 | 4.439 | 0.021 | 0.360 | 0.044 | 0.106 | Unannotated | NA | NA |
| metab_9314 | Unannotated feature | neg | 82.9533 | 3.726 | 3.727 | 3.761 | 0.034 | 0.360 | 0.044 | 0.106 | Unannotated | NA | NA |
| metab_41 | Threonylproline | pos | 217.1183 | 1.964 | 4.522 | 4.888 | 0.366 | 0.360 | 0.044 | 0.106 | Tentative | Organic acids and derivatives | Carboxylic acids and derivatives |
| metab_11341 | Unannotated feature | neg | 161.9330 | 0.013 | 4.252 | 4.293 | 0.041 | 0.360 | 0.044 | 0.106 | Unannotated | NA | NA |
| metab_524 | Unannotated feature | pos | 113.0349 | 1.956 | 3.799 | 3.839 | 0.040 | 0.360 | 0.044 | 0.106 | Unannotated | NA | NA |
| metab_3880 | Unannotated feature | pos | 88.0762 | 0.892 | 3.472 | 3.546 | 0.075 | 0.356 | 0.047 | 0.111 | Unannotated | NA | NA |
| metab_2789 | Tetrahydrocortisone | pos | 347.2217 | 5.626 | 3.693 | 4.267 | 0.574 | 0.356 | 0.047 | 0.111 | Tentative | Lipids and lipid-like molecules | Steroids and steroid derivatives |
| metab_8936 | Acetaminophen cystein | neg | 289.0416 | 4.977 | 3.846 | 4.161 | 0.314 | 0.356 | 0.047 | 0.111 | Tentative | Organic acids and derivatives | Carboxylic acids and derivatives |
| metab_1943 | Unannotated feature | pos | 90.5263 | 7.324 | 4.928 | 4.956 | 0.028 | 0.356 | 0.047 | 0.111 | Unannotated | NA | NA |
| metab_5704 | Unannotated feature | neg | 248.9606 | 0.554 | 6.683 | 6.649 | -0.034 | -0.356 | 0.047 | 0.111 | Unannotated | NA | NA |
| metab_2544 | Unannotated feature | pos | 586.9080 | 5.871 | 4.475 | 4.290 | -0.185 | -0.356 | 0.047 | 0.111 | Unannotated | NA | NA |
| metab_3594 | (2S)-2-Methylspiro[1,3-oxathiolane-5,3'-1-azabicyclo[2.2.2]octane] | pos | 111.5496 | 2.042 | 3.688 | 3.922 | 0.234 | 0.356 | 0.047 | 0.111 | Tentative | Organoheterocyclic compounds | Azaspirodecane derivatives |
| metab_2763 | Anabaseine | pos | 338.2324 | 5.680 | 5.087 | 5.281 | 0.194 | 0.356 | 0.047 | 0.111 | Tentative | Organoheterocyclic compounds | Pyridines and derivatives |
| metab_2790 | Unannotated feature | pos | 344.2424 | 5.626 | 3.715 | 3.825 | 0.109 | 0.356 | 0.047 | 0.111 | Unannotated | NA | NA |
| metab_1926 | Unannotated feature | pos | 181.0648 | 7.324 | 5.873 | 5.856 | -0.017 | -0.356 | 0.047 | 0.111 | Unannotated | NA | NA |
| metab_5664 | Unannotated feature | neg | 604.8730 | 0.554 | 5.738 | 5.695 | -0.043 | -0.356 | 0.047 | 0.111 | Unannotated | NA | NA |
| metab_7061 | Unannotated feature | neg | 165.8941 | 5.787 | 4.397 | 4.337 | -0.059 | -0.356 | 0.047 | 0.111 | Unannotated | NA | NA |
| metab_3143 | Unannotated feature | pos | 84.1227 | 3.607 | 3.384 | 3.523 | 0.140 | 0.356 | 0.047 | 0.111 | Unannotated | NA | NA |
| metab_10692 | Unannotated feature | neg | 162.9120 | 0.582 | 5.635 | 5.613 | -0.023 | -0.356 | 0.047 | 0.111 | Unannotated | NA | NA |
| metab_6016 | Unannotated feature | neg | 190.9819 | 2.922 | 4.051 | 4.437 | 0.386 | 0.356 | 0.047 | 0.111 | Unannotated | NA | NA |
| metab_7607 | Unannotated feature | neg | 215.7490 | 7.269 | 4.388 | 4.453 | 0.065 | 0.356 | 0.047 | 0.111 | Unannotated | NA | NA |
| metab_10245 | Unannotated feature | neg | 312.0593 | 0.857 | 3.027 | 3.474 | 0.448 | 0.356 | 0.047 | 0.111 | Unannotated | NA | NA |
| metab_5803 | Unannotated feature | neg | 247.9628 | 2.111 | 5.897 | 5.807 | -0.090 | -0.356 | 0.047 | 0.111 | Unannotated | NA | NA |
| metab_6068 | Unannotated feature | neg | 270.0176 | 0.636 | 4.669 | 4.612 | -0.057 | -0.356 | 0.047 | 0.111 | Unannotated | NA | NA |
| metab_2542 | Unannotated feature | pos | 646.4516 | 5.871 | 5.814 | 5.752 | -0.063 | -0.356 | 0.047 | 0.111 | Unannotated | NA | NA |
| metab_2786 | Unannotated feature | pos | 177.0884 | 5.634 | 5.682 | 5.692 | 0.009 | 0.356 | 0.047 | 0.111 | Unannotated | NA | NA |
| metab_4945 | Unannotated feature | pos | 214.0063 | 0.629 | 5.890 | 5.767 | -0.123 | -0.356 | 0.047 | 0.111 | Unannotated | NA | NA |
| metab_429 | Unannotated feature | pos | 159.9692 | 1.538 | 5.630 | 5.615 | -0.015 | -0.356 | 0.047 | 0.111 | Unannotated | NA | NA |
| metab_9167 | 3-Carboxy-4-Methyl-5-Propyl-2-Furanpropanoic Acid | neg | 239.0926 | 5.819 | 6.618 | 6.184 | -0.435 | -0.356 | 0.047 | 0.111 | Strong putative | NA | NA |
| metab_903 | 5-Hydroxy-p-mentha-6,8-dien-2-one | pos | 208.1333 | 3.792 | 4.193 | 3.876 | -0.317 | -0.356 | 0.047 | 0.111 | Tentative | Lipids and lipid-like molecules | Prenol lipids |
| metab_313 | Unannotated feature | pos | 158.9642 | 0.784 | 4.403 | 4.357 | -0.046 | -0.356 | 0.047 | 0.111 | Unannotated | NA | NA |
| metab_10559 | Unannotated feature | neg | 189.9886 | 0.621 | 3.251 | 3.611 | 0.359 | 0.356 | 0.047 | 0.111 | Unannotated | NA | NA |
| metab_4638 | Metaxalone | pos | 443.2216 | 5.687 | 4.813 | 4.896 | 0.082 | 0.356 | 0.047 | 0.111 | Tentative | NA | NA |
| metab_4815 | Unannotated feature | pos | 678.7901 | 0.567 | 4.796 | 4.722 | -0.074 | -0.356 | 0.047 | 0.111 | Unannotated | NA | NA |
| metab_6478 | Unannotated feature | neg | 181.5058 | 4.347 | 3.670 | 3.591 | -0.078 | -0.356 | 0.047 | 0.111 | Unannotated | NA | NA |
| metab_8405 | Unannotated feature | neg | 368.9727 | 3.209 | 2.658 | 4.296 | 1.638 | 0.356 | 0.047 | 0.111 | Unannotated | NA | NA |
| metab_8675 | Unannotated feature | neg | 428.1222 | 5.619 | 4.404 | 3.792 | -0.612 | -0.356 | 0.047 | 0.111 | Unannotated | NA | NA |
| metab_4612 | Unannotated feature | pos | 172.9564 | 0.008 | 4.603 | 4.630 | 0.027 | 0.356 | 0.047 | 0.111 | Unannotated | NA | NA |
| metab_10543 | Unannotated feature | neg | 471.6571 | 0.621 | 4.780 | 4.718 | -0.061 | -0.356 | 0.047 | 0.111 | Unannotated | NA | NA |
| metab_2017 | Unannotated feature | pos | 105.3396 | 7.285 | 3.501 | 3.591 | 0.090 | 0.356 | 0.047 | 0.111 | Unannotated | NA | NA |
| metab_2053 | Unannotated feature | pos | 433.3865 | 7.231 | 4.801 | 4.759 | -0.042 | -0.356 | 0.047 | 0.111 | Unannotated | NA | NA |
| metab_10932 | Unannotated feature | neg | 579.8734 | 0.534 | 4.497 | 4.422 | -0.075 | -0.356 | 0.047 | 0.111 | Unannotated | NA | NA |
| metab_4286 | Unannotated feature | pos | 318.7735 | 0.591 | 5.081 | 5.014 | -0.067 | -0.352 | 0.049 | 0.116 | Unannotated | NA | NA |
| metab_1807 | Unannotated feature | pos | 85.0289 | 7.959 | 5.221 | 5.234 | 0.013 | 0.352 | 0.049 | 0.116 | Unannotated | NA | NA |
| metab_4092 | Unannotated feature | pos | 188.0707 | 4.401 | 5.126 | 5.061 | -0.066 | -0.352 | 0.049 | 0.116 | Unannotated | NA | NA |
| metab_886 | Unannotated feature | pos | 84.1316 | 3.646 | 3.767 | 3.855 | 0.088 | 0.352 | 0.049 | 0.116 | Unannotated | NA | NA |
| metab_6728 | Unannotated feature | neg | 128.0346 | 0.865 | 6.219 | 6.146 | -0.074 | -0.352 | 0.049 | 0.116 | Unannotated | NA | NA |
| metab_11353 | Unannotated feature | neg | 362.8760 | 0.016 | 4.738 | 4.718 | -0.020 | -0.352 | 0.049 | 0.116 | Unannotated | NA | NA |
| metab_2114 | Unannotated feature | pos | 203.1278 | 7.007 | 4.346 | 4.291 | -0.055 | -0.352 | 0.049 | 0.116 | Unannotated | NA | NA |
| metab_10581 | Unannotated feature | neg | 556.5982 | 0.613 | 5.828 | 5.787 | -0.041 | -0.352 | 0.049 | 0.116 | Unannotated | NA | NA |
| metab_1018 | PI(16:2(9Z,12Z)/5-iso PGF2VI) | pos | 921.4296 | 4.624 | 4.857 | 5.293 | 0.436 | 0.352 | 0.049 | 0.116 | Tentative | NA | NA |
| metab_4378 | Unannotated feature | pos | 619.8329 | 0.567 | 4.566 | 4.432 | -0.134 | -0.352 | 0.049 | 0.116 | Unannotated | NA | NA |
| metab_472 | (-)-Salsolinol | pos | 180.1019 | 1.801 | 3.748 | 3.896 | 0.148 | 0.352 | 0.049 | 0.116 | Tentative | Organoheterocyclic compounds | Tetrahydroisoquinolines |
| metab_8204 | Unannotated feature | neg | 103.0393 | 2.111 | 6.831 | 6.706 | -0.124 | -0.352 | 0.049 | 0.116 | Unannotated | NA | NA |
| metab_2895 | Unannotated feature | pos | 130.0653 | 5.038 | 4.209 | 4.506 | 0.297 | 0.352 | 0.049 | 0.116 | Unannotated | NA | NA |
| metab_2346 | Unannotated feature | pos | 223.9885 | 5.987 | 4.860 | 4.837 | -0.023 | -0.352 | 0.049 | 0.116 | Unannotated | NA | NA |
| metab_7968 | Unannotated feature | neg | 141.9231 | 7.101 | 4.117 | 4.031 | -0.086 | -0.352 | 0.049 | 0.116 | Unannotated | NA | NA |
| metab_3046 | Unannotated feature | pos | 675.0054 | 4.101 | 3.882 | 3.725 | -0.157 | -0.352 | 0.049 | 0.116 | Unannotated | NA | NA |
| metab_3797 | Unannotated feature | pos | 79.0184 | 1.523 | 4.575 | 4.594 | 0.019 | 0.352 | 0.049 | 0.116 | Unannotated | NA | NA |
| metab_413 | Unannotated feature | pos | 265.0230 | 1.243 | 4.522 | 4.398 | -0.123 | -0.352 | 0.049 | 0.116 | Unannotated | NA | NA |
| metab_11075 | Unannotated feature | neg | 318.0504 | 5.587 | 3.849 | 4.025 | 0.176 | 0.352 | 0.049 | 0.116 | Unannotated | NA | NA |
| metab_2148 | PC(16:1/0:0) | pos | 516.3061 | 6.575 | 5.432 | 5.205 | -0.227 | -0.352 | 0.049 | 0.116 | Strong putative | NA | NA |
| metab_4813 | Unannotated feature | pos | 158.9862 | 7.324 | 4.261 | 4.293 | 0.032 | 0.352 | 0.049 | 0.116 | Unannotated | NA | NA |
| metab_5626 | Unannotated feature | neg | 446.6719 | 0.912 | 5.332 | 5.306 | -0.027 | -0.352 | 0.049 | 0.116 | Unannotated | NA | NA |
| metab_7670 | Unannotated feature | neg | 216.8769 | 7.261 | 6.316 | 6.338 | 0.022 | 0.352 | 0.049 | 0.116 | Unannotated | NA | NA |
| metab_11270 | Unannotated feature | neg | 580.8674 | 0.570 | 5.135 | 5.067 | -0.067 | -0.352 | 0.049 | 0.116 | Unannotated | NA | NA |
| metab_2000 | Unannotated feature | pos | 641.5115 | 7.285 | 4.530 | 4.430 | -0.100 | -0.352 | 0.049 | 0.116 | Unannotated | NA | NA |
| metab_11012 | Unannotated feature | neg | 349.8677 | 0.016 | 5.114 | 5.095 | -0.019 | -0.352 | 0.049 | 0.116 | Unannotated | NA | NA |
| metab_3121 | Unannotated feature | pos | 84.1227 | 3.730 | 3.374 | 3.532 | 0.158 | 0.352 | 0.049 | 0.116 | Unannotated | NA | NA |
| metab_677 | Unannotated feature | pos | 84.5068 | 2.451 | 3.853 | 3.920 | 0.066 | 0.352 | 0.049 | 0.116 | Unannotated | NA | NA |
| metab_9851 | Unannotated feature | neg | 506.6279 | 0.621 | 6.017 | 5.984 | -0.034 | -0.352 | 0.049 | 0.116 | Unannotated | NA | NA |
| metab_6621 | Unannotated feature | neg | 98.9384 | 4.961 | 4.879 | 4.894 | 0.015 | 0.352 | 0.049 | 0.116 | Unannotated | NA | NA |
| metab_10890 | Unannotated feature | neg | 250.0445 | 0.546 | 4.654 | 4.585 | -0.069 | -0.352 | 0.049 | 0.116 | Unannotated | NA | NA |
| metab_4320 | Unannotated feature | pos | 708.6599 | 0.575 | 4.205 | 4.076 | -0.129 | -0.352 | 0.049 | 0.116 | Unannotated | NA | NA |
| metab_1775 | Unannotated feature | pos | 84.1227 | 7.385 | 3.187 | 3.427 | 0.240 | 0.347 | 0.052 | 0.121 | Unannotated | NA | NA |
| metab_8840 | Unannotated feature | neg | 304.0348 | 5.346 | 4.282 | 4.418 | 0.136 | 0.347 | 0.052 | 0.121 | Unannotated | NA | NA |
| metab_6633 | Unannotated feature | neg | 277.7706 | 4.985 | 3.384 | 3.602 | 0.218 | 0.347 | 0.052 | 0.121 | Unannotated | NA | NA |
| metab_4354 | Unannotated feature | pos | 761.7102 | 0.567 | 3.899 | 4.170 | 0.271 | 0.347 | 0.052 | 0.121 | Unannotated | NA | NA |
| metab_2042 | Unannotated feature | pos | 266.2437 | 7.261 | 4.733 | 4.695 | -0.037 | -0.347 | 0.052 | 0.121 | Unannotated | NA | NA |
| metab_70 | Unannotated feature | pos | 182.1024 | 0.372 | 4.784 | 4.768 | -0.016 | -0.347 | 0.052 | 0.121 | Unannotated | NA | NA |
| metab_9874 | Unannotated feature | neg | 286.8972 | 2.385 | 5.541 | 5.567 | 0.026 | 0.347 | 0.052 | 0.121 | Unannotated | NA | NA |
| metab_2015 | Triethanolamine | pos | 150.1126 | 7.285 | 5.759 | 5.777 | 0.018 | 0.347 | 0.052 | 0.121 | Strong putative | NA | NA |
| metab_11015 | Unannotated feature | neg | 299.8247 | 0.016 | 4.614 | 4.583 | -0.031 | -0.347 | 0.052 | 0.121 | Unannotated | NA | NA |
| metab_11317 | Unannotated feature | neg | 574.6153 | 0.587 | 5.833 | 5.791 | -0.042 | -0.347 | 0.052 | 0.121 | Unannotated | NA | NA |
| metab_2165 | 5-Valerolactone | pos | 83.0497 | 6.427 | 4.202 | 4.228 | 0.026 | 0.347 | 0.052 | 0.121 | Tentative | NA | NA |
| metab_9263 | Unannotated feature | neg | 261.1249 | 3.858 | 3.856 | 4.088 | 0.232 | 0.347 | 0.052 | 0.121 | Unannotated | NA | NA |
| metab_7198 | Unannotated feature | neg | 350.8590 | 5.922 | 5.056 | 5.133 | 0.077 | 0.347 | 0.052 | 0.121 | Unannotated | NA | NA |
| metab_2178 | Unannotated feature | pos | 496.3063 | 6.389 | 5.089 | 5.001 | -0.088 | -0.347 | 0.052 | 0.121 | Unannotated | NA | NA |
| metab_7769 | Unannotated feature | neg | 178.9488 | 7.173 | 3.835 | 3.801 | -0.034 | -0.347 | 0.052 | 0.121 | Unannotated | NA | NA |
| metab_1561 | Unannotated feature | pos | 573.4300 | 6.311 | 4.599 | 4.298 | -0.300 | -0.347 | 0.052 | 0.121 | Unannotated | NA | NA |
| metab_8407 | Unannotated feature | neg | 369.2290 | 5.787 | 4.894 | 4.440 | -0.455 | -0.347 | 0.052 | 0.121 | Unannotated | NA | NA |
| metab_8671 | Unannotated feature | neg | 224.9868 | 5.627 | 4.396 | 4.236 | -0.160 | -0.347 | 0.052 | 0.121 | Unannotated | NA | NA |
| metab_10050 | Unannotated feature | neg | 201.9060 | 1.430 | 5.753 | 5.719 | -0.034 | -0.347 | 0.052 | 0.121 | Unannotated | NA | NA |
| metab_557 | Unannotated feature | pos | 339.1550 | 2.034 | 3.769 | 3.342 | -0.427 | -0.347 | 0.052 | 0.121 | Unannotated | NA | NA |
| metab_9551 | Unannotated feature | neg | 717.0716 | 3.201 | 4.815 | 4.334 | -0.480 | -0.347 | 0.052 | 0.121 | Unannotated | NA | NA |
| metab_10802 | Unannotated feature | neg | 128.9589 | 0.562 | 4.823 | 4.852 | 0.029 | 0.347 | 0.052 | 0.121 | Unannotated | NA | NA |
| metab_5084 | 2-Amino-2-(5-methyl-3-oxo-1,2-oxazol-4-yl)propanoic Acid | pos | 187.0713 | 1.274 | 4.687 | 4.289 | -0.398 | -0.347 | 0.052 | 0.121 | Tentative | Organic acids and derivatives | Carboxylic acids and derivatives |
| metab_7377 | Unannotated feature | neg | 121.9433 | 6.962 | 4.741 | 4.718 | -0.022 | -0.347 | 0.052 | 0.121 | Unannotated | NA | NA |
| metab_10961 | Unannotated feature | neg | 243.9564 | 0.515 | 3.574 | 3.727 | 0.153 | 0.347 | 0.052 | 0.121 | Unannotated | NA | NA |
| metab_193 | Unannotated feature | pos | 630.8027 | 0.567 | 4.307 | 4.530 | 0.222 | 0.347 | 0.052 | 0.121 | Unannotated | NA | NA |
| metab_1516 | Unannotated feature | pos | 313.2737 | 6.157 | 4.491 | 4.573 | 0.082 | 0.347 | 0.052 | 0.121 | Unannotated | NA | NA |
| metab_10540 | Unannotated feature | neg | 450.6660 | 0.621 | 5.055 | 4.944 | -0.111 | -0.347 | 0.052 | 0.121 | Unannotated | NA | NA |
| metab_1476 | Unannotated feature | pos | 548.2754 | 6.018 | 4.868 | 4.995 | 0.127 | 0.347 | 0.052 | 0.121 | Unannotated | NA | NA |
| metab_3198 | Ritodrine | pos | 252.1390 | 3.267 | 4.547 | 4.519 | -0.028 | -0.347 | 0.052 | 0.121 | Tentative | NA | NA |
| metab_2463 | Unannotated feature | pos | 536.3896 | 5.910 | 6.283 | 6.381 | 0.098 | 0.347 | 0.052 | 0.121 | Unannotated | NA | NA |
| metab_7943 | Unannotated feature | neg | 155.8737 | 7.109 | 5.578 | 5.630 | 0.052 | 0.347 | 0.052 | 0.121 | Unannotated | NA | NA |
| metab_2079 | 3-Methylazelaic acid | pos | 185.1174 | 7.200 | 5.645 | 5.676 | 0.031 | 0.347 | 0.052 | 0.121 | Tentative | NA | NA |
| metab_4598 | 1,2,5-Trihydroxypyrrole-3-sulfonic acid | pos | 159.9692 | 0.100 | 6.043 | 6.031 | -0.011 | -0.347 | 0.052 | 0.121 | Tentative | NA | NA |
| metab_329 | Unannotated feature | pos | 163.0278 | 0.815 | 4.185 | 4.079 | -0.106 | -0.347 | 0.052 | 0.121 | Unannotated | NA | NA |
| metab_1344 | Unannotated feature | pos | 744.5111 | 5.894 | 4.779 | 4.460 | -0.319 | -0.347 | 0.052 | 0.121 | Unannotated | NA | NA |
| metab_10902 | Unannotated feature | neg | 766.8559 | 0.543 | 5.812 | 5.707 | -0.105 | -0.347 | 0.052 | 0.121 | Unannotated | NA | NA |
| metab_1622 | Unannotated feature | pos | 102.1954 | 7.061 | 3.456 | 3.559 | 0.103 | 0.347 | 0.052 | 0.121 | Unannotated | NA | NA |
| metab_6256 | Unannotated feature | neg | 247.8538 | 3.507 | 5.011 | 5.048 | 0.037 | 0.347 | 0.052 | 0.121 | Unannotated | NA | NA |
| metab_10762 | Unannotated feature | neg | 510.8823 | 0.570 | 5.635 | 5.581 | -0.055 | -0.347 | 0.052 | 0.121 | Unannotated | NA | NA |
| metab_2556 | Unannotated feature | pos | 630.9365 | 5.863 | 4.344 | 3.935 | -0.409 | -0.347 | 0.052 | 0.121 | Unannotated | NA | NA |
| metab_1971 | Unannotated feature | pos | 906.8250 | 7.293 | 5.374 | 5.321 | -0.053 | -0.347 | 0.052 | 0.121 | Unannotated | NA | NA |
| metab_9569 | 2-(1,2,3,4-Tetrahydroxybutyl)thiazolidine-4-carboxylic acid | neg | 288.0308 | 3.184 | 4.270 | 3.894 | -0.376 | -0.347 | 0.052 | 0.121 | Tentative | Organic acids and derivatives | Carboxylic acids and derivatives |
| metab_9573 | Unannotated feature | neg | 199.9781 | 3.184 | 4.583 | 4.451 | -0.132 | -0.347 | 0.052 | 0.121 | Unannotated | NA | NA |
| metab_2911 | Unannotated feature | pos | 707.2990 | 4.915 | 5.276 | 5.630 | 0.354 | 0.347 | 0.052 | 0.121 | Unannotated | NA | NA |
| metab_5576 | Unannotated feature | neg | 276.9137 | 0.857 | 4.226 | 3.928 | -0.298 | -0.347 | 0.052 | 0.121 | Unannotated | NA | NA |
| metab_9619 | Unannotated feature | neg | 144.5426 | 3.136 | 3.715 | 3.426 | -0.289 | -0.347 | 0.052 | 0.121 | Unannotated | NA | NA |
| metab_1874 | Unannotated feature | pos | 101.8086 | 7.385 | 4.877 | 4.913 | 0.036 | 0.347 | 0.052 | 0.121 | Unannotated | NA | NA |
| metab_5695 | Unannotated feature | neg | 414.9908 | 1.294 | 4.686 | 4.639 | -0.048 | -0.347 | 0.052 | 0.121 | Unannotated | NA | NA |
| metab_3982 | Unannotated feature | pos | 163.0755 | 0.831 | 3.644 | 3.598 | -0.046 | -0.343 | 0.055 | 0.126 | Unannotated | NA | NA |
| metab_1502 | Unannotated feature | pos | 424.3631 | 6.111 | 4.835 | 4.935 | 0.101 | 0.343 | 0.055 | 0.126 | Unannotated | NA | NA |
| metab_2336 | Unannotated feature | pos | 754.5372 | 5.995 | 5.042 | 4.868 | -0.175 | -0.343 | 0.055 | 0.126 | Unannotated | NA | NA |
| metab_10536 | Unannotated feature | neg | 564.5866 | 0.621 | 6.184 | 6.161 | -0.023 | -0.343 | 0.055 | 0.126 | Unannotated | NA | NA |
| metab_174 | Unannotated feature | pos | 735.8170 | 0.559 | 4.075 | 4.265 | 0.190 | 0.343 | 0.055 | 0.126 | Unannotated | NA | NA |
| metab_4420 | Unannotated feature | pos | 697.8485 | 0.559 | 4.963 | 4.844 | -0.119 | -0.343 | 0.055 | 0.126 | Unannotated | NA | NA |
| metab_2476 | Unannotated feature | pos | 558.4033 | 5.902 | 5.952 | 6.039 | 0.087 | 0.343 | 0.055 | 0.126 | Unannotated | NA | NA |
| metab_4789 | Unannotated feature | pos | 371.1626 | 7.277 | 5.606 | 5.637 | 0.031 | 0.343 | 0.055 | 0.126 | Unannotated | NA | NA |
| metab_10516 | Unannotated feature | neg | 267.5935 | 0.629 | 4.582 | 4.489 | -0.093 | -0.343 | 0.055 | 0.126 | Unannotated | NA | NA |
| metab_10750 | Unannotated feature | neg | 593.8055 | 0.570 | 4.530 | 4.406 | -0.124 | -0.343 | 0.055 | 0.126 | Unannotated | NA | NA |
| metab_8771 | Unannotated feature | neg | 568.3634 | 7.153 | 6.113 | 6.016 | -0.097 | -0.343 | 0.055 | 0.126 | Unannotated | NA | NA |
| metab_5408 | Unannotated feature | neg | 181.9657 | 0.621 | 5.133 | 4.953 | -0.180 | -0.343 | 0.055 | 0.126 | Unannotated | NA | NA |
| metab_9795 | Unannotated feature | neg | 119.9462 | 2.549 | 3.509 | 3.462 | -0.047 | -0.343 | 0.055 | 0.126 | Unannotated | NA | NA |
| metab_11396 | L-Glutamate | neg | 146.0453 | 0.636 | 5.603 | 5.482 | -0.121 | -0.343 | 0.055 | 0.126 | Strong putative | NA | NA |
| metab_2271 | Unannotated feature | pos | 443.3345 | 6.157 | 4.210 | 4.537 | 0.327 | 0.343 | 0.055 | 0.126 | Unannotated | NA | NA |
| metab_10012 | Unannotated feature | neg | 189.0402 | 1.855 | 4.539 | 4.660 | 0.122 | 0.343 | 0.055 | 0.126 | Unannotated | NA | NA |
| metab_10195 | Unannotated feature | neg | 376.0049 | 0.873 | 4.935 | 4.811 | -0.124 | -0.343 | 0.055 | 0.126 | Unannotated | NA | NA |
| metab_6106 | Unannotated feature | neg | 411.0016 | 3.168 | 4.465 | 4.315 | -0.151 | -0.343 | 0.055 | 0.126 | Unannotated | NA | NA |
| metab_5866 | Unannotated feature | neg | 637.8312 | 0.570 | 4.421 | 4.649 | 0.228 | 0.343 | 0.055 | 0.126 | Unannotated | NA | NA |
| metab_5078 | Unannotated feature | pos | 131.0898 | 0.854 | 4.828 | 4.600 | -0.228 | -0.343 | 0.055 | 0.126 | Unannotated | NA | NA |
| metab_8217 | Unannotated feature | neg | 204.9803 | 5.937 | 4.707 | 4.747 | 0.040 | 0.343 | 0.055 | 0.126 | Unannotated | NA | NA |
| metab_4965 | Unannotated feature | pos | 318.9698 | 0.629 | 6.223 | 6.201 | -0.022 | -0.343 | 0.055 | 0.126 | Unannotated | NA | NA |
| metab_6637 | Unannotated feature | neg | 672.2423 | 5.001 | 3.929 | 4.370 | 0.441 | 0.343 | 0.055 | 0.126 | Unannotated | NA | NA |
| metab_7182 | Thienorphine | neg | 556.2289 | 5.898 | 6.069 | 6.122 | 0.054 | 0.343 | 0.055 | 0.126 | Tentative | NA | NA |
| metab_2655 | 8-(5-Butylfuran-2-yl)octanoylcarnitine | pos | 392.2793 | 5.793 | 4.535 | 3.969 | -0.566 | -0.343 | 0.055 | 0.126 | Tentative | NA | NA |
| metab_6638 | Unannotated feature | neg | 703.2303 | 5.001 | 3.824 | 4.212 | 0.388 | 0.343 | 0.055 | 0.126 | Unannotated | NA | NA |
| metab_570 | Unannotated feature | pos | 120.0447 | 2.058 | 4.373 | 4.593 | 0.220 | 0.343 | 0.055 | 0.126 | Unannotated | NA | NA |
| metab_2072 | N-Oleoyl Arginine | pos | 477.3190 | 7.200 | 6.303 | 6.268 | -0.035 | -0.343 | 0.055 | 0.126 | Tentative | NA | NA |
| metab_1020 | Gibberellin A8-catabolite | pos | 742.3034 | 4.639 | 4.824 | 5.108 | 0.284 | 0.343 | 0.055 | 0.126 | Tentative | NA | NA |
| metab_7452 | Unannotated feature | neg | 170.9438 | 7.132 | 4.131 | 4.058 | -0.073 | -0.343 | 0.055 | 0.126 | Unannotated | NA | NA |
| metab_6260 | Unannotated feature | neg | 179.8823 | 3.522 | 4.361 | 4.658 | 0.297 | 0.343 | 0.055 | 0.126 | Unannotated | NA | NA |
| metab_3691 | Unannotated feature | pos | 109.0227 | 1.917 | 4.103 | 4.075 | -0.028 | -0.343 | 0.055 | 0.126 | Unannotated | NA | NA |
| metab_5700 | Unannotated feature | neg | 318.0461 | 1.326 | 4.608 | 4.446 | -0.162 | -0.343 | 0.055 | 0.126 | Unannotated | NA | NA |
| metab_692 | Unannotated feature | pos | 102.0343 | 2.490 | 5.977 | 5.951 | -0.026 | -0.343 | 0.055 | 0.126 | Unannotated | NA | NA |
| metab_10156 | Unannotated feature | neg | 388.7131 | 0.912 | 5.120 | 5.098 | -0.023 | -0.343 | 0.055 | 0.126 | Unannotated | NA | NA |
| metab_11329 | Unannotated feature | neg | 182.8593 | 7.173 | 4.449 | 4.414 | -0.035 | -0.343 | 0.055 | 0.126 | Unannotated | NA | NA |
| metab_1739 | Unannotated feature | pos | 181.9883 | 7.324 | 5.435 | 5.457 | 0.022 | 0.339 | 0.058 | 0.131 | Unannotated | NA | NA |
| metab_2919 | 1-Acetylproline | pos | 202.0444 | 4.892 | 4.833 | 4.926 | 0.093 | 0.339 | 0.058 | 0.131 | Tentative | NA | NA |
| metab_7762 | Unannotated feature | neg | 334.8241 | 7.173 | 4.135 | 4.088 | -0.047 | -0.339 | 0.058 | 0.131 | Unannotated | NA | NA |
| metab_5424 | Unannotated feature | neg | 276.8682 | 0.505 | 4.443 | 4.520 | 0.077 | 0.339 | 0.058 | 0.131 | Unannotated | NA | NA |
| metab_1049 | Unannotated feature | pos | 728.7502 | 4.915 | 4.276 | 4.917 | 0.641 | 0.339 | 0.058 | 0.131 | Unannotated | NA | NA |
| metab_10863 | Unannotated feature | neg | 523.8895 | 0.554 | 4.660 | 4.782 | 0.122 | 0.339 | 0.058 | 0.131 | Unannotated | NA | NA |
| metab_9565 | Unannotated feature | neg | 227.9836 | 0.636 | 4.930 | 4.724 | -0.205 | -0.339 | 0.058 | 0.131 | Unannotated | NA | NA |
| metab_9560 | Unannotated feature | neg | 573.0984 | 3.192 | 4.202 | 3.860 | -0.343 | -0.339 | 0.058 | 0.131 | Unannotated | NA | NA |
| metab_2700 | Unannotated feature | pos | 88.0762 | 1.956 | 4.264 | 4.355 | 0.091 | 0.339 | 0.058 | 0.131 | Unannotated | NA | NA |
| metab_3833 | Unannotated feature | pos | 170.1176 | 1.227 | 4.023 | 3.892 | -0.131 | -0.339 | 0.058 | 0.131 | Unannotated | NA | NA |
| metab_7302 | Unannotated feature | neg | 187.0723 | 0.865 | 3.767 | 4.112 | 0.345 | 0.339 | 0.058 | 0.131 | Unannotated | NA | NA |
| metab_7112 | S-(-)-ureidoglycolate | neg | 398.0678 | 5.827 | 4.693 | 4.310 | -0.384 | -0.339 | 0.058 | 0.131 | Tentative | NA | NA |
| metab_5051 | Unannotated feature | pos | 105.0427 | 0.877 | 5.324 | 5.315 | -0.008 | -0.339 | 0.058 | 0.131 | Unannotated | NA | NA |
| metab_8070 | Unannotated feature | neg | 135.3259 | 6.281 | 3.926 | 3.873 | -0.053 | -0.339 | 0.058 | 0.131 | Unannotated | NA | NA |
| metab_10475 | Unannotated feature | neg | 403.8571 | 0.644 | 4.629 | 4.465 | -0.164 | -0.339 | 0.058 | 0.131 | Unannotated | NA | NA |
| metab_5155 | Styrene | pos | 105.0703 | 5.894 | 5.108 | 5.142 | 0.033 | 0.339 | 0.058 | 0.131 | Strong putative | NA | NA |
| metab_7109 | Norzolmitripan | neg | 240.1138 | 5.827 | 4.117 | 3.825 | -0.292 | -0.339 | 0.058 | 0.131 | Tentative | NA | NA |
| metab_632 | Unannotated feature | pos | 491.6566 | 2.226 | 5.677 | 5.335 | -0.341 | -0.339 | 0.058 | 0.131 | Unannotated | NA | NA |
| metab_1603 | Unannotated feature | pos | 81.0316 | 6.737 | 3.768 | 3.841 | 0.073 | 0.339 | 0.058 | 0.131 | Unannotated | NA | NA |
| metab_678 | L-Prolinamide | pos | 115.0869 | 2.451 | 3.748 | 3.976 | 0.227 | 0.339 | 0.058 | 0.131 | Tentative | NA | NA |
| metab_5409 | Unannotated feature | neg | 239.9247 | 0.621 | 4.095 | 3.930 | -0.165 | -0.339 | 0.058 | 0.131 | Unannotated | NA | NA |
| metab_5410 | Unannotated feature | neg | 265.6093 | 0.621 | 3.982 | 3.878 | -0.105 | -0.339 | 0.058 | 0.131 | Unannotated | NA | NA |
| metab_7962 | Unannotated feature | neg | 210.9262 | 7.101 | 4.462 | 4.436 | -0.027 | -0.339 | 0.058 | 0.131 | Unannotated | NA | NA |
| metab_8236 | Unannotated feature | neg | 233.9309 | 5.922 | 5.540 | 5.566 | 0.026 | 0.339 | 0.058 | 0.131 | Unannotated | NA | NA |
| metab_7294 | Unannotated feature | neg | 339.2005 | 6.188 | 4.772 | 4.485 | -0.287 | -0.339 | 0.058 | 0.131 | Unannotated | NA | NA |
| metab_4632 | Unannotated feature | pos | 222.1465 | 5.657 | 5.529 | 5.567 | 0.038 | 0.339 | 0.058 | 0.131 | Unannotated | NA | NA |
| metab_1295 | Unannotated feature | pos | 680.8051 | 5.863 | 4.717 | 4.466 | -0.251 | -0.339 | 0.058 | 0.131 | Unannotated | NA | NA |
| metab_1250 | Cardiolipin | pos | 654.4524 | 5.832 | 5.515 | 5.446 | -0.068 | -0.339 | 0.058 | 0.131 | Tentative | NA | NA |
| metab_2488 | Unannotated feature | pos | 748.5370 | 5.894 | 6.084 | 6.055 | -0.029 | -0.339 | 0.058 | 0.131 | Unannotated | NA | NA |
| metab_8254 | Unannotated feature | neg | 149.0272 | 3.128 | 4.135 | 4.341 | 0.207 | 0.339 | 0.058 | 0.131 | Unannotated | NA | NA |
| metab_4762 | Unannotated feature | pos | 1019.7019 | 6.737 | 6.065 | 5.870 | -0.196 | -0.339 | 0.058 | 0.131 | Unannotated | NA | NA |
| metab_913 | Anthranilate | pos | 138.0551 | 3.846 | 3.704 | 3.896 | 0.192 | 0.339 | 0.058 | 0.131 | Moderate putative | NA | NA |
| metab_707 | Salicin | pos | 328.1389 | 2.552 | 4.682 | 4.579 | -0.103 | -0.339 | 0.058 | 0.131 | Tentative | NA | NA |
| metab_709 | L-Tyrosine methyl ester | pos | 178.0863 | 2.560 | 3.230 | 3.903 | 0.672 | 0.339 | 0.058 | 0.131 | Tentative | Organic acids and derivatives | Carboxylic acids and derivatives |
| metab_9288 | Unannotated feature | neg | 279.9902 | 3.522 | 4.325 | 4.555 | 0.231 | 0.339 | 0.058 | 0.131 | Unannotated | NA | NA |
| metab_4828 | Unannotated feature | pos | 442.8694 | 7.300 | 4.911 | 4.961 | 0.050 | 0.339 | 0.058 | 0.131 | Unannotated | NA | NA |
| metab_10992 | Unannotated feature | neg | 264.9153 | 0.114 | 4.943 | 4.932 | -0.011 | -0.339 | 0.058 | 0.131 | Unannotated | NA | NA |
| metab_7802 | Unannotated feature | neg | 544.8219 | 7.164 | 4.807 | 4.767 | -0.041 | -0.339 | 0.058 | 0.131 | Unannotated | NA | NA |
| metab_7410 | Unannotated feature | neg | 84.9379 | 7.109 | 5.346 | 5.324 | -0.022 | -0.339 | 0.058 | 0.131 | Unannotated | NA | NA |
| metab_5805 | Unannotated feature | neg | 646.8580 | 0.562 | 5.595 | 5.538 | -0.057 | -0.339 | 0.058 | 0.131 | Unannotated | NA | NA |
| metab_185 | Unannotated feature | pos | 488.8725 | 0.567 | 5.645 | 5.625 | -0.020 | -0.339 | 0.058 | 0.131 | Unannotated | NA | NA |
| metab_5796 | Unannotated feature | neg | 579.8730 | 0.562 | 4.610 | 4.651 | 0.041 | 0.339 | 0.058 | 0.131 | Unannotated | NA | NA |
| metab_10039 | Unannotated feature | neg | 216.8805 | 1.577 | 5.246 | 5.230 | -0.016 | -0.339 | 0.058 | 0.131 | Unannotated | NA | NA |
| metab_4483 | Unannotated feature | pos | 608.8694 | 0.551 | 5.496 | 5.422 | -0.074 | -0.339 | 0.058 | 0.131 | Unannotated | NA | NA |
| metab_6882 | Unannotated feature | neg | 254.0912 | 5.643 | 3.883 | 3.527 | -0.355 | -0.339 | 0.058 | 0.131 | Unannotated | NA | NA |
| metab_2057 | Unannotated feature | pos | 335.2192 | 7.223 | 4.884 | 4.834 | -0.050 | -0.339 | 0.058 | 0.131 | Unannotated | NA | NA |
| metab_82 | Isofraxidin | pos | 223.0600 | 0.465 | 4.485 | 4.502 | 0.017 | 0.339 | 0.058 | 0.131 | Moderate putative | NA | NA |
| metab_6912 | Unannotated feature | neg | 281.0107 | 5.667 | 3.854 | 3.298 | -0.556 | -0.339 | 0.058 | 0.131 | Unannotated | NA | NA |
| metab_231 | Unannotated feature | pos | 616.9471 | 0.636 | 5.072 | 4.997 | -0.075 | -0.339 | 0.058 | 0.131 | Unannotated | NA | NA |
| metab_3160 | Unannotated feature | pos | 244.1544 | 2.521 | 5.178 | 5.482 | 0.304 | 0.339 | 0.058 | 0.131 | Unannotated | NA | NA |
| metab_8295 | Unannotated feature | neg | 267.1607 | 5.867 | 4.037 | 4.143 | 0.105 | 0.339 | 0.058 | 0.131 | Unannotated | NA | NA |
| metab_10189 | Unannotated feature | neg | 243.9528 | 0.888 | 4.475 | 4.340 | -0.135 | -0.339 | 0.058 | 0.131 | Unannotated | NA | NA |
| metab_9408 | Unannotated feature | neg | 261.8751 | 3.467 | 5.563 | 5.607 | 0.044 | 0.339 | 0.058 | 0.131 | Unannotated | NA | NA |
| metab_3449 | Unannotated feature | pos | 231.1703 | 2.303 | 4.585 | 4.297 | -0.288 | -0.339 | 0.058 | 0.131 | Moderate putative | NA | NA |
| metab_3511 | Unannotated feature | pos | 169.0496 | 2.164 | 3.091 | 3.966 | 0.875 | 0.335 | 0.061 | 0.137 | Unannotated | NA | NA |
| metab_6502 | Unannotated feature | neg | 316.9654 | 4.402 | 4.457 | 4.996 | 0.539 | 0.335 | 0.061 | 0.137 | Unannotated | NA | NA |
| metab_8957 | Unannotated feature | neg | 550.1831 | 4.923 | 3.764 | 4.268 | 0.503 | 0.335 | 0.061 | 0.137 | Unannotated | NA | NA |
| metab_11337 | Unannotated feature | neg | 190.8908 | 7.170 | 4.317 | 4.283 | -0.034 | -0.335 | 0.061 | 0.137 | Unannotated | NA | NA |
| metab_3826 | Tectorigenin 7-sulfate | pos | 403.0095 | 1.259 | 5.310 | 5.208 | -0.101 | -0.335 | 0.061 | 0.137 | Tentative | NA | NA |
| metab_6149 | L-Normetanephrine | neg | 164.0712 | 3.232 | 4.530 | 4.309 | -0.221 | -0.335 | 0.061 | 0.137 | Tentative | Benzenoids | Phenols |
| metab_2679 | Unannotated feature | pos | 320.3067 | 5.771 | 5.644 | 5.673 | 0.029 | 0.335 | 0.061 | 0.137 | Unannotated | NA | NA |
| metab_7273 | Unannotated feature | neg | 275.0104 | 1.935 | 5.624 | 5.563 | -0.061 | -0.335 | 0.061 | 0.137 | Unannotated | NA | NA |
| metab_8224 | Unannotated feature | neg | 197.8564 | 5.930 | 4.386 | 4.463 | 0.077 | 0.335 | 0.061 | 0.137 | Unannotated | NA | NA |
| metab_1161 | Butenoylcarnitine | pos | 262.1649 | 1.956 | 5.526 | 5.618 | 0.092 | 0.335 | 0.061 | 0.137 | Tentative | NA | NA |
| metab_1845 | Unannotated feature | pos | 171.1492 | 7.897 | 4.354 | 4.380 | 0.025 | 0.335 | 0.061 | 0.137 | Unannotated | NA | NA |
| metab_5151 | Phenyl acetate | pos | 137.0598 | 4.516 | 4.919 | 4.697 | -0.222 | -0.335 | 0.061 | 0.137 | Tentative | NA | NA |
| metab_10740 | Unannotated feature | neg | 671.7132 | 0.570 | 4.494 | 4.379 | -0.115 | -0.335 | 0.061 | 0.137 | Unannotated | NA | NA |
| metab_7663 | Unannotated feature | neg | 241.8992 | 7.293 | 7.103 | 7.126 | 0.023 | 0.335 | 0.061 | 0.137 | Unannotated | NA | NA |
| metab_8787 | Unannotated feature | neg | 428.1220 | 5.518 | 4.759 | 4.390 | -0.369 | -0.335 | 0.061 | 0.137 | Unannotated | NA | NA |
| metab_10584 | Unannotated feature | neg | 475.7540 | 0.613 | 4.146 | 3.827 | -0.319 | -0.335 | 0.061 | 0.137 | Unannotated | NA | NA |
| metab_11065 | Unannotated feature | neg | 288.8591 | 0.582 | 6.086 | 6.047 | -0.039 | -0.335 | 0.061 | 0.137 | Unannotated | NA | NA |
| metab_303 | Unannotated feature | pos | 233.1245 | 0.768 | 3.643 | 4.081 | 0.438 | 0.335 | 0.061 | 0.137 | Unannotated | NA | NA |
| metab_4768 | Unannotated feature | pos | 485.4312 | 6.296 | 5.551 | 5.459 | -0.092 | -0.335 | 0.061 | 0.137 | Unannotated | NA | NA |
| metab_6157 | Unannotated feature | neg | 198.0239 | 3.240 | 3.406 | 3.677 | 0.270 | 0.335 | 0.061 | 0.137 | Unannotated | NA | NA |
| metab_8844 | Unannotated feature | neg | 141.0915 | 5.346 | 3.998 | 4.172 | 0.174 | 0.335 | 0.061 | 0.137 | Unannotated | NA | NA |
| metab_9082 | Hexanoylglutamine | neg | 243.1353 | 4.505 | 4.308 | 4.673 | 0.366 | 0.335 | 0.061 | 0.137 | Tentative | Organic acids and derivatives | Carboxylic acids and derivatives |
| metab_9748 | Unannotated feature | neg | 268.8129 | 2.724 | 5.289 | 5.254 | -0.035 | -0.335 | 0.061 | 0.137 | Unannotated | NA | NA |
| metab_374 | Unannotated feature | pos | 376.7319 | 0.892 | 4.535 | 4.472 | -0.063 | -0.335 | 0.061 | 0.137 | Unannotated | NA | NA |
| metab_5530 | Unannotated feature | neg | 877.8381 | 0.539 | 5.248 | 5.197 | -0.051 | -0.335 | 0.061 | 0.137 | Unannotated | NA | NA |
| metab_2082 | Unannotated feature | pos | 130.0864 | 0.854 | 6.080 | 5.996 | -0.085 | -0.335 | 0.061 | 0.137 | Unannotated | NA | NA |
| metab_1047 | Unannotated feature | pos | 110.0716 | 0.854 | 4.749 | 4.908 | 0.159 | 0.335 | 0.061 | 0.137 | Strong putative | NA | NA |
| metab_1038 | Unannotated feature | pos | 681.7932 | 4.854 | 4.749 | 5.084 | 0.336 | 0.335 | 0.061 | 0.137 | Unannotated | NA | NA |
| metab_6252 | Tryptophyl-Isoleucine | neg | 338.1464 | 3.499 | 4.285 | 4.003 | -0.282 | -0.335 | 0.061 | 0.137 | Tentative | NA | NA |
| metab_1854 | Unannotated feature | pos | 111.5496 | 7.718 | 5.005 | 4.990 | -0.014 | -0.335 | 0.061 | 0.137 | Unannotated | NA | NA |
| metab_4471 | Unannotated feature | pos | 710.8494 | 0.551 | 5.616 | 5.573 | -0.043 | -0.335 | 0.061 | 0.137 | Unannotated | NA | NA |
| metab_7454 | Unannotated feature | neg | 306.9196 | 7.132 | 4.923 | 4.901 | -0.022 | -0.335 | 0.061 | 0.137 | Unannotated | NA | NA |
| metab_9476 | Unannotated feature | neg | 113.9938 | 3.282 | 3.876 | 3.975 | 0.099 | 0.335 | 0.061 | 0.137 | Unannotated | NA | NA |
| metab_3475 | Glutaminylleucine | pos | 242.1499 | 2.249 | 3.984 | 4.240 | 0.256 | 0.335 | 0.061 | 0.137 | Tentative | Organic acids and derivatives | Carboxylic acids and derivatives |
| metab_4207 | Unannotated feature | pos | 424.8717 | 0.621 | 4.915 | 4.824 | -0.091 | -0.335 | 0.061 | 0.137 | Unannotated | NA | NA |
| metab_1504 | Unannotated feature | pos | 82.9454 | 0.591 | 6.174 | 6.152 | -0.022 | -0.335 | 0.061 | 0.137 | Unannotated | NA | NA |
| metab_2344 | Unannotated feature | pos | 620.4370 | 5.987 | 4.810 | 4.892 | 0.082 | 0.335 | 0.061 | 0.137 | Unannotated | NA | NA |
| metab_8337 | Unannotated feature | neg | 500.9436 | 5.827 | 4.124 | 3.610 | -0.515 | -0.335 | 0.061 | 0.137 | Unannotated | NA | NA |
| metab_2174 | Unannotated feature | pos | 119.0688 | 6.420 | 3.316 | 3.455 | 0.139 | 0.335 | 0.061 | 0.137 | Unannotated | NA | NA |
| metab_6463 | 1-(1,2,3,4,5-Pentahydroxypent-1-yl)-1,2,3,4-tetrahydro-beta-carboline-3-carboxylate | neg | 411.1425 | 4.282 | 4.074 | 3.575 | -0.498 | -0.335 | 0.061 | 0.137 | Tentative | Organoheterocyclic compounds | Benzopyrans |
| metab_4457 | Unannotated feature | pos | 846.8258 | 0.551 | 5.511 | 5.362 | -0.150 | -0.335 | 0.061 | 0.137 | Unannotated | NA | NA |
| metab_8464 | Unannotated feature | neg | 357.0624 | 5.747 | 5.083 | 5.052 | -0.031 | -0.331 | 0.065 | 0.143 | Unannotated | NA | NA |
| metab_8391 | CDP-DG(18:3(9,11,15)-OH(13)/20:2(11Z,14Z)) | neg | 346.8368 | 5.795 | 5.196 | 5.145 | -0.052 | -0.331 | 0.065 | 0.143 | Tentative | NA | NA |
| metab_5350 | Unannotated feature | neg | 619.6585 | 0.578 | 4.648 | 4.542 | -0.105 | -0.331 | 0.065 | 0.143 | Unannotated | NA | NA |
| metab_4225 | Unannotated feature | pos | 176.1223 | 0.614 | 4.753 | 4.827 | 0.075 | 0.331 | 0.065 | 0.143 | Unannotated | NA | NA |
| metab_409 | Nicotinamide | pos | 123.0555 | 1.227 | 4.778 | 4.607 | -0.171 | -0.331 | 0.065 | 0.143 | Strong putative | NA | NA |
| metab_115 | Unannotated feature | pos | 449.3601 | 0.541 | 5.718 | 5.708 | -0.009 | -0.331 | 0.065 | 0.143 | Unannotated | NA | NA |
| metab_2586 | Unannotated feature | pos | 462.8268 | 5.855 | 4.591 | 4.409 | -0.182 | -0.331 | 0.065 | 0.143 | Unannotated | NA | NA |
| metab_1293 | Unannotated feature | pos | 617.4345 | 5.863 | 6.036 | 6.010 | -0.025 | -0.331 | 0.065 | 0.143 | Unannotated | NA | NA |
| metab_2254 | Unannotated feature | pos | 551.3556 | 6.196 | 5.023 | 4.837 | -0.186 | -0.331 | 0.065 | 0.143 | Unannotated | NA | NA |
| metab_840 | Unannotated feature | pos | 104.0710 | 3.290 | 3.462 | 3.560 | 0.098 | 0.331 | 0.065 | 0.143 | Unannotated | NA | NA |
| metab_2261 | Unannotated feature | pos | 449.2875 | 6.188 | 4.031 | 3.489 | -0.542 | -0.331 | 0.065 | 0.143 | Unannotated | NA | NA |
| metab_2754 | Unannotated feature | pos | 315.7694 | 5.695 | 4.074 | 4.338 | 0.264 | 0.331 | 0.065 | 0.143 | Unannotated | NA | NA |
| metab_6172 | 6-Epi-7-isocucurbic acid glucoside | neg | 411.1418 | 3.260 | 4.644 | 4.156 | -0.488 | -0.331 | 0.065 | 0.143 | Tentative | NA | NA |
| metab_870 | Unannotated feature | pos | 84.1315 | 3.584 | 3.818 | 3.882 | 0.063 | 0.331 | 0.065 | 0.143 | Unannotated | NA | NA |
| metab_4962 | Unannotated feature | pos | 296.9255 | 0.675 | 4.084 | 4.369 | 0.285 | 0.331 | 0.065 | 0.143 | Unannotated | NA | NA |
| metab_9536 | Unannotated feature | neg | 334.9448 | 0.636 | 4.962 | 4.882 | -0.080 | -0.331 | 0.065 | 0.143 | Unannotated | NA | NA |
| metab_1400 | Unannotated feature | pos | 565.4108 | 5.925 | 6.299 | 6.380 | 0.081 | 0.331 | 0.065 | 0.143 | Unannotated | NA | NA |
| metab_10892 | Unannotated feature | neg | 1004.8108 | 0.543 | 5.361 | 5.264 | -0.098 | -0.331 | 0.065 | 0.143 | Unannotated | NA | NA |
| metab_934 | Unannotated feature | pos | 102.5360 | 4.009 | 4.368 | 4.349 | -0.018 | -0.331 | 0.065 | 0.143 | Unannotated | NA | NA |
| metab_2742 | Unannotated feature | pos | 139.1118 | 5.710 | 3.972 | 4.015 | 0.043 | 0.331 | 0.065 | 0.143 | Unannotated | NA | NA |
| metab_4595 | Unannotated feature | pos | 101.8086 | 0.240 | 4.066 | 4.102 | 0.037 | 0.331 | 0.065 | 0.143 | Unannotated | NA | NA |
| metab_1716 | Unannotated feature | pos | 880.8252 | 7.293 | 4.531 | 4.455 | -0.076 | -0.331 | 0.065 | 0.143 | Unannotated | NA | NA |
| metab_1667 | Unannotated feature | pos | 261.1309 | 7.231 | 4.663 | 4.649 | -0.014 | -0.331 | 0.065 | 0.143 | Unannotated | NA | NA |
| metab_10876 | Unannotated feature | neg | 181.6440 | 0.554 | 4.154 | 4.104 | -0.050 | -0.331 | 0.065 | 0.143 | Unannotated | NA | NA |
| metab_8677 | Unannotated feature | neg | 411.1319 | 5.619 | 4.399 | 4.088 | -0.311 | -0.331 | 0.065 | 0.143 | Unannotated | NA | NA |
| metab_2458 | Unannotated feature | pos | 660.4637 | 5.910 | 5.567 | 5.522 | -0.045 | -0.331 | 0.065 | 0.143 | Unannotated | NA | NA |
| metab_4781 | Unannotated feature | pos | 479.3344 | 7.192 | 5.681 | 5.670 | -0.011 | -0.331 | 0.065 | 0.143 | Unannotated | NA | NA |
| metab_3506 | Unannotated feature | pos | 223.1077 | 2.179 | 4.034 | 3.711 | -0.323 | -0.331 | 0.065 | 0.143 | Unannotated | NA | NA |
| metab_595 | Unannotated feature | pos | 120.4438 | 2.081 | 5.235 | 5.286 | 0.051 | 0.331 | 0.065 | 0.143 | Unannotated | NA | NA |
| metab_8228 | Unannotated feature | neg | 352.8543 | 5.922 | 6.231 | 6.249 | 0.018 | 0.331 | 0.065 | 0.143 | Unannotated | NA | NA |
| metab_7053 | Unannotated feature | neg | 327.2184 | 5.771 | 3.691 | 3.883 | 0.193 | 0.331 | 0.065 | 0.143 | Unannotated | NA | NA |
| metab_5527 | Unannotated feature | neg | 173.0928 | 0.794 | 4.713 | 4.837 | 0.124 | 0.331 | 0.065 | 0.143 | Unannotated | NA | NA |
| metab_7581 | Unannotated feature | neg | 116.5807 | 7.213 | 4.083 | 4.044 | -0.039 | -0.331 | 0.065 | 0.143 | Unannotated | NA | NA |
| metab_6896 | Unannotated feature | neg | 502.6336 | 0.904 | 5.666 | 5.642 | -0.024 | -0.331 | 0.065 | 0.143 | Unannotated | NA | NA |
| metab_5670 | Unannotated feature | neg | 123.8772 | 1.111 | 5.080 | 5.030 | -0.050 | -0.331 | 0.065 | 0.143 | Unannotated | NA | NA |
| metab_4386 | Unannotated feature | pos | 580.8286 | 0.567 | 4.423 | 4.585 | 0.162 | 0.331 | 0.065 | 0.143 | Unannotated | NA | NA |
| metab_5650 | Unannotated feature | neg | 166.8352 | 0.991 | 5.351 | 5.400 | 0.049 | 0.331 | 0.065 | 0.143 | Unannotated | NA | NA |
| metab_7739 | Unannotated feature | neg | 183.9592 | 7.189 | 3.908 | 3.953 | 0.045 | 0.331 | 0.065 | 0.143 | Unannotated | NA | NA |
| metab_7698 | Unannotated feature | neg | 179.8920 | 7.213 | 3.623 | 3.557 | -0.066 | -0.331 | 0.065 | 0.143 | Unannotated | NA | NA |
| metab_4385 | Unannotated feature | pos | 586.8375 | 0.567 | 4.555 | 4.444 | -0.110 | -0.331 | 0.065 | 0.143 | Unannotated | NA | NA |
| metab_5688 | Unannotated feature | neg | 111.0080 | 1.294 | 3.592 | 3.778 | 0.186 | 0.331 | 0.065 | 0.143 | Unannotated | NA | NA |
| metab_1780 | Unannotated feature | pos | 99.0808 | 7.455 | 4.269 | 4.251 | -0.018 | -0.331 | 0.065 | 0.143 | Unannotated | NA | NA |
| metab_10246 | Unannotated feature | neg | 278.9107 | 0.857 | 4.271 | 4.087 | -0.184 | -0.331 | 0.065 | 0.143 | Unannotated | NA | NA |
| metab_5154 | Salicylate | pos | 121.0287 | 2.342 | 4.794 | 4.996 | 0.202 | 0.331 | 0.065 | 0.143 | Moderate putative | Benzenoids | Benzene and substituted derivatives |
| metab_3761 | Unannotated feature | pos | 181.4072 | 1.740 | 4.772 | 4.647 | -0.124 | -0.326 | 0.068 | 0.149 | Unannotated | NA | NA |
| metab_8057 | Rumenic acid | neg | 559.4744 | 6.368 | 5.810 | 5.637 | -0.173 | -0.326 | 0.068 | 0.149 | Tentative | NA | NA |
| metab_9876 | Unannotated feature | neg | 278.8653 | 2.379 | 5.487 | 5.549 | 0.062 | 0.326 | 0.068 | 0.149 | Unannotated | NA | NA |
| metab_10694 | Unannotated feature | neg | 105.2496 | 0.582 | 3.481 | 3.386 | -0.095 | -0.326 | 0.068 | 0.149 | Unannotated | NA | NA |
| metab_6870 | Unannotated feature | neg | 258.0559 | 5.635 | 4.955 | 4.844 | -0.111 | -0.326 | 0.068 | 0.149 | Unannotated | NA | NA |
| metab_5287 | Unannotated feature | neg | 965.8083 | 0.554 | 5.421 | 5.350 | -0.071 | -0.326 | 0.068 | 0.149 | Unannotated | NA | NA |
| metab_11051 | Unannotated feature | neg | 100.9527 | 0.013 | 4.432 | 4.451 | 0.019 | 0.326 | 0.068 | 0.149 | Unannotated | NA | NA |
| metab_9391 | Unannotated feature | neg | 203.8559 | 3.514 | 4.322 | 4.402 | 0.079 | 0.326 | 0.068 | 0.149 | Unannotated | NA | NA |
| metab_3986 | Unannotated feature | pos | 158.0449 | 0.831 | 3.242 | 3.362 | 0.120 | 0.326 | 0.068 | 0.149 | Unannotated | NA | NA |
| metab_7884 | Unannotated feature | neg | 113.9882 | 7.140 | 5.455 | 5.489 | 0.034 | 0.326 | 0.068 | 0.149 | Unannotated | NA | NA |
| metab_11042 | Unannotated feature | neg | 196.8847 | 0.013 | 5.633 | 5.665 | 0.032 | 0.326 | 0.068 | 0.149 | Unannotated | NA | NA |
| metab_7872 | Unannotated feature | neg | 677.8805 | 7.140 | 4.314 | 4.194 | -0.120 | -0.326 | 0.068 | 0.149 | Unannotated | NA | NA |
| metab_5957 | Unannotated feature | neg | 158.0818 | 2.724 | 4.169 | 4.296 | 0.127 | 0.326 | 0.068 | 0.149 | Unannotated | NA | NA |
| metab_5007 | Unannotated feature | pos | 350.0879 | 0.800 | 4.619 | 4.411 | -0.208 | -0.326 | 0.068 | 0.149 | Unannotated | NA | NA |
| metab_4192 | Unannotated feature | pos | 150.0874 | 0.636 | 3.550 | 3.846 | 0.296 | 0.326 | 0.068 | 0.149 | Unannotated | NA | NA |
| metab_10500 | Unannotated feature | neg | 166.0175 | 0.636 | 3.862 | 3.443 | -0.419 | -0.326 | 0.068 | 0.149 | Unannotated | NA | NA |
| metab_4097 | Imidazole lactate | pos | 157.0608 | 0.721 | 4.441 | 4.533 | 0.091 | 0.326 | 0.068 | 0.149 | Strong putative | Organoheterocyclic compounds | Azoles |
| metab_10718 | Unannotated feature | neg | 230.9001 | 0.578 | 5.639 | 5.613 | -0.025 | -0.326 | 0.068 | 0.149 | Unannotated | NA | NA |
| metab_1222 | Unannotated feature | pos | 302.2148 | 5.793 | 5.291 | 5.092 | -0.199 | -0.326 | 0.068 | 0.149 | Unannotated | NA | NA |
| metab_8598 | Unannotated feature | neg | 214.1449 | 5.675 | 4.913 | 4.947 | 0.033 | 0.326 | 0.068 | 0.149 | Unannotated | NA | NA |
| metab_3687 | N-Methyl-2-pyrrolidinone | pos | 86.0605 | 1.925 | 5.027 | 5.143 | 0.116 | 0.326 | 0.068 | 0.149 | Moderate putative | NA | NA |
| metab_1046 | Unannotated feature | pos | 227.1277 | 4.900 | 4.454 | 4.610 | 0.156 | 0.326 | 0.068 | 0.149 | Unannotated | NA | NA |
| metab_556 | Unannotated feature | pos | 184.0030 | 2.034 | 4.501 | 4.439 | -0.062 | -0.326 | 0.068 | 0.149 | Unannotated | NA | NA |
| metab_145 | Unannotated feature | pos | 546.2172 | 3.854 | 4.929 | 5.447 | 0.519 | 0.326 | 0.068 | 0.149 | Unannotated | NA | NA |
| metab_4367 | Unannotated feature | pos | 659.8393 | 0.567 | 4.452 | 4.626 | 0.174 | 0.326 | 0.068 | 0.149 | Unannotated | NA | NA |
| metab_8344 | Unannotated feature | neg | 300.0312 | 3.184 | 4.668 | 4.495 | -0.173 | -0.326 | 0.068 | 0.149 | Unannotated | NA | NA |
| metab_9981 | Unannotated feature | neg | 73.0287 | 2.009 | 3.676 | 3.639 | -0.037 | -0.326 | 0.068 | 0.149 | Unannotated | NA | NA |
| metab_2244 | LysoPC(0:0/18:2(9Z,12Z)) | pos | 1039.6709 | 6.211 | 6.452 | 6.587 | 0.135 | 0.326 | 0.068 | 0.149 | Tentative | NA | NA |
| metab_2666 | Unannotated feature | pos | 561.8701 | 5.786 | 4.205 | 3.989 | -0.216 | -0.326 | 0.068 | 0.149 | Unannotated | NA | NA |
| metab_9239 | Unannotated feature | neg | 363.1205 | 3.992 | 4.281 | 4.176 | -0.104 | -0.326 | 0.068 | 0.149 | Unannotated | NA | NA |
| metab_3241 | Unannotated feature | pos | 139.0390 | 3.051 | 4.037 | 4.099 | 0.063 | 0.326 | 0.068 | 0.149 | Unannotated | NA | NA |
| metab_9693 | Unannotated feature | neg | 241.9206 | 2.906 | 3.981 | 4.148 | 0.166 | 0.326 | 0.068 | 0.149 | Unannotated | NA | NA |
| metab_1942 | Unannotated feature | pos | 99.5127 | 7.324 | 4.687 | 4.733 | 0.046 | 0.326 | 0.068 | 0.149 | Unannotated | NA | NA |
| metab_6941 | Unannotated feature | neg | 169.1230 | 5.691 | 4.182 | 4.403 | 0.221 | 0.326 | 0.068 | 0.149 | Unannotated | NA | NA |
| metab_8583 | Unannotated feature | neg | 455.1911 | 5.715 | 3.743 | 4.327 | 0.584 | 0.326 | 0.068 | 0.149 | Unannotated | NA | NA |
| metab_10576 | Unannotated feature | neg | 626.5383 | 0.613 | 5.207 | 5.275 | 0.068 | 0.326 | 0.068 | 0.149 | Unannotated | NA | NA |
| metab_6630 | Unannotated feature | neg | 172.0976 | 4.977 | 2.550 | 2.976 | 0.425 | 0.326 | 0.068 | 0.149 | Unannotated | NA | NA |
| metab_2410 | Unannotated feature | pos | 567.8885 | 5.933 | 4.527 | 4.747 | 0.220 | 0.326 | 0.068 | 0.149 | Unannotated | NA | NA |
| metab_3213 | Unannotated feature | pos | 171.0992 | 3.174 | 3.973 | 3.549 | -0.423 | -0.326 | 0.068 | 0.149 | Unannotated | NA | NA |
| metab_7616 | Unannotated feature | neg | 96.9418 | 7.309 | 3.237 | 3.297 | 0.060 | 0.326 | 0.068 | 0.149 | Unannotated | NA | NA |
| metab_6123 | Unannotated feature | neg | 572.9713 | 3.192 | 4.212 | 3.906 | -0.306 | -0.326 | 0.068 | 0.149 | Unannotated | NA | NA |
| metab_7295 | Melleolide | neg | 381.1746 | 6.188 | 6.022 | 5.657 | -0.365 | -0.326 | 0.068 | 0.149 | Tentative | NA | NA |
| metab_2122 | Venlafaxine | pos | 341.2221 | 6.930 | 5.825 | 5.812 | -0.013 | -0.326 | 0.068 | 0.149 | Tentative | NA | NA |
| metab_7810 | Unannotated feature | neg | 189.9313 | 7.164 | 5.124 | 5.157 | 0.034 | 0.326 | 0.068 | 0.149 | Unannotated | NA | NA |
| metab_1484 | (4Z,7Z,10Z,13Z,16Z,19Z)-Docosahexaenoic acid | pos | 361.2737 | 6.041 | 4.991 | 5.113 | 0.122 | 0.322 | 0.072 | 0.155 | Tentative | NA | NA |
| metab_8361 | Trans-p-Menth-2-ene-1,4-diol | neg | 151.1123 | 5.819 | 4.698 | 4.348 | -0.350 | -0.322 | 0.072 | 0.155 | Tentative | NA | NA |
| metab_9386 | Unannotated feature | neg | 311.9282 | 3.522 | 4.392 | 4.660 | 0.268 | 0.322 | 0.072 | 0.155 | Unannotated | NA | NA |
| metab_9384 | Unannotated feature | neg | 509.0405 | 3.610 | 4.498 | 5.236 | 0.739 | 0.322 | 0.072 | 0.155 | Unannotated | NA | NA |
| metab_4159 | Unannotated feature | pos | 337.1716 | 0.652 | 5.002 | 4.650 | -0.353 | -0.322 | 0.072 | 0.155 | Unannotated | NA | NA |
| metab_1341 | Unannotated feature | pos | 944.3583 | 2.234 | 6.312 | 5.945 | -0.367 | -0.322 | 0.072 | 0.155 | Unannotated | NA | NA |
| metab_8197 | Unannotated feature | neg | 279.8724 | 5.969 | 4.901 | 4.833 | -0.068 | -0.322 | 0.072 | 0.155 | Unannotated | NA | NA |
| metab_1403 | Unannotated feature | pos | 740.5239 | 5.925 | 5.770 | 5.719 | -0.051 | -0.322 | 0.072 | 0.155 | Unannotated | NA | NA |
| metab_8984 | Unannotated feature | neg | 128.9278 | 7.109 | 6.016 | 5.991 | -0.026 | -0.322 | 0.072 | 0.155 | Unannotated | NA | NA |
| metab_8309 | Unannotated feature | neg | 211.1339 | 5.859 | 3.690 | 3.792 | 0.101 | 0.322 | 0.072 | 0.155 | Unannotated | NA | NA |
| metab_6198 | Unannotated feature | neg | 158.0818 | 3.332 | 4.861 | 4.897 | 0.036 | 0.322 | 0.072 | 0.155 | Unannotated | NA | NA |
| metab_207 | Unannotated feature | pos | 606.5695 | 0.606 | 5.373 | 5.267 | -0.106 | -0.322 | 0.072 | 0.155 | Unannotated | NA | NA |
| metab_5948 | Unannotated feature | neg | 324.1306 | 2.676 | 4.894 | 4.586 | -0.309 | -0.322 | 0.072 | 0.155 | Unannotated | NA | NA |
| metab_1431 | Unannotated feature | pos | 725.5134 | 5.956 | 5.372 | 5.286 | -0.086 | -0.322 | 0.072 | 0.155 | Unannotated | NA | NA |
| metab_6804 | Unannotated feature | neg | 282.0470 | 5.571 | 3.468 | 3.816 | 0.348 | 0.322 | 0.072 | 0.155 | Unannotated | NA | NA |
| metab_3065 | Pro-Ile | pos | 211.1442 | 4.001 | 4.384 | 4.140 | -0.244 | -0.322 | 0.072 | 0.155 | Tentative | Organic acids and derivatives | Carboxylic acids and derivatives |
| metab_3541 | Unannotated feature | pos | 119.7198 | 2.081 | 5.225 | 5.287 | 0.063 | 0.322 | 0.072 | 0.155 | Unannotated | NA | NA |
| metab_1136 | Unannotated feature | pos | 399.2825 | 5.649 | 4.755 | 4.637 | -0.118 | -0.322 | 0.072 | 0.155 | Unannotated | NA | NA |
| metab_1987 | Unannotated feature | pos | 499.9047 | 7.293 | 4.849 | 4.815 | -0.034 | -0.322 | 0.072 | 0.155 | Unannotated | NA | NA |
| metab_3623 | 6-Acetamido-2-oxohexanoate | pos | 152.0707 | 1.964 | 4.066 | 4.120 | 0.054 | 0.322 | 0.072 | 0.155 | Tentative | NA | NA |
| metab_9210 | Unannotated feature | neg | 90.2910 | 4.093 | 2.470 | 2.179 | -0.291 | -0.322 | 0.072 | 0.155 | Unannotated | NA | NA |
| metab_3211 | Tyrosyl-Lysine | pos | 310.1760 | 3.174 | 3.455 | 4.245 | 0.791 | 0.322 | 0.072 | 0.155 | Tentative | Organic acids and derivatives | Carboxylic acids and derivatives |
| metab_6853 | Unannotated feature | neg | 402.8503 | 0.888 | 4.666 | 4.579 | -0.087 | -0.322 | 0.072 | 0.155 | Unannotated | NA | NA |
| metab_1860 | Unannotated feature | pos | 84.5070 | 7.556 | 3.848 | 3.916 | 0.068 | 0.322 | 0.072 | 0.155 | Unannotated | NA | NA |
| metab_4417 | Unannotated feature | pos | 734.8181 | 0.559 | 4.435 | 4.529 | 0.095 | 0.322 | 0.072 | 0.155 | Unannotated | NA | NA |
| metab_2117 | Unannotated feature | pos | 451.9915 | 6.983 | 5.944 | 5.997 | 0.053 | 0.322 | 0.072 | 0.155 | Unannotated | NA | NA |
| metab_3882 | Unannotated feature | pos | 289.9310 | 0.885 | 4.124 | 4.042 | -0.082 | -0.322 | 0.072 | 0.155 | Unannotated | NA | NA |
| metab_4654 | Unannotated feature | pos | 674.7741 | 0.567 | 5.301 | 5.283 | -0.018 | -0.322 | 0.072 | 0.155 | Unannotated | NA | NA |
| metab_4852 | Unannotated feature | pos | 458.3839 | 6.180 | 6.361 | 6.315 | -0.046 | -0.322 | 0.072 | 0.155 | Unannotated | NA | NA |
| metab_10123 | Unannotated feature | neg | 441.6821 | 1.007 | 6.172 | 6.109 | -0.063 | -0.322 | 0.072 | 0.155 | Unannotated | NA | NA |
| metab_5116 | (S)-2-Aminobutanoate | pos | 86.0605 | 3.066 | 3.614 | 3.655 | 0.040 | 0.322 | 0.072 | 0.155 | Tentative | NA | NA |
| metab_3239 | Unannotated feature | pos | 364.0968 | 2.583 | 5.581 | 5.756 | 0.175 | 0.322 | 0.072 | 0.155 | Moderate putative | NA | NA |
| metab_3293 | Arctiopicrin | pos | 333.1703 | 2.834 | 4.633 | 4.498 | -0.135 | -0.322 | 0.072 | 0.155 | Tentative | NA | NA |
| metab_1536 | Unannotated feature | pos | 362.0999 | 2.583 | 6.252 | 6.348 | 0.096 | 0.322 | 0.072 | 0.155 | Unannotated | NA | NA |
| metab_6718 | Unannotated feature | neg | 373.1412 | 5.346 | 3.873 | 3.492 | -0.381 | -0.322 | 0.072 | 0.155 | Unannotated | NA | NA |
| metab_9422 | Unannotated feature | neg | 401.8601 | 0.636 | 5.277 | 5.042 | -0.234 | -0.322 | 0.072 | 0.155 | Unannotated | NA | NA |
| metab_9387 | D-Fructose 1-phosphate | neg | 296.9804 | 3.522 | 4.920 | 5.133 | 0.213 | 0.322 | 0.072 | 0.155 | Tentative | NA | NA |
| metab_3812 | Homoserine lactone | pos | 84.0449 | 1.375 | 5.503 | 5.540 | 0.037 | 0.322 | 0.072 | 0.155 | Tentative | NA | NA |
| metab_6719 | Unannotated feature | neg | 691.7423 | 5.346 | 3.774 | 4.545 | 0.771 | 0.322 | 0.072 | 0.155 | Unannotated | NA | NA |
| metab_7433 | Unannotated feature | neg | 241.9588 | 7.109 | 4.761 | 4.703 | -0.058 | -0.322 | 0.072 | 0.155 | Unannotated | NA | NA |
| metab_5399 | Unannotated feature | neg | 531.6131 | 0.613 | 5.440 | 5.391 | -0.049 | -0.322 | 0.072 | 0.155 | Unannotated | NA | NA |
| metab_3364 | Unannotated feature | pos | 146.1176 | 2.560 | 4.866 | 5.002 | 0.136 | 0.322 | 0.072 | 0.155 | Unannotated | NA | NA |
| metab_775 | Unannotated feature | pos | 201.0083 | 2.904 | 5.002 | 4.993 | -0.009 | -0.322 | 0.072 | 0.155 | Unannotated | NA | NA |
| metab_5606 | Unannotated feature | neg | 187.9908 | 0.881 | 4.914 | 4.843 | -0.071 | -0.322 | 0.072 | 0.155 | Unannotated | NA | NA |
| metab_7535 | Unannotated feature | neg | 486.7902 | 7.170 | 4.530 | 4.568 | 0.038 | 0.322 | 0.072 | 0.155 | Unannotated | NA | NA |
| metab_2423 | Unannotated feature | pos | 556.8976 | 5.925 | 4.974 | 4.853 | -0.121 | -0.322 | 0.072 | 0.155 | Unannotated | NA | NA |
| metab_2995 | Isocarbostyril | pos | 187.0866 | 4.401 | 4.684 | 4.650 | -0.034 | -0.322 | 0.072 | 0.155 | Tentative | NA | NA |
| metab_1839 | Unannotated feature | pos | 84.9601 | 7.959 | 4.597 | 4.607 | 0.010 | 0.322 | 0.072 | 0.155 | Unannotated | NA | NA |
| metab_9297 | Unannotated feature | neg | 212.0022 | 3.530 | 6.719 | 6.965 | 0.246 | 0.322 | 0.072 | 0.155 | Unannotated | NA | NA |
| metab_11365 | Unannotated feature | neg | 191.8548 | 0.088 | 5.343 | 5.321 | -0.022 | -0.322 | 0.072 | 0.155 | Unannotated | NA | NA |
| metab_9821 | Unannotated feature | neg | 636.5222 | 0.621 | 5.213 | 5.278 | 0.066 | 0.322 | 0.072 | 0.155 | Unannotated | NA | NA |
| metab_7611 | Unannotated feature | neg | 195.9059 | 7.293 | 5.815 | 5.841 | 0.026 | 0.322 | 0.072 | 0.155 | Unannotated | NA | NA |
| metab_5974 | Unannotated feature | neg | 167.0345 | 2.780 | 3.056 | 3.242 | 0.186 | 0.322 | 0.072 | 0.155 | Unannotated | NA | NA |
| metab_1365 | Unannotated feature | pos | 485.3558 | 5.910 | 6.118 | 6.217 | 0.099 | 0.318 | 0.076 | 0.162 | Unannotated | NA | NA |
| metab_1907 | Unannotated feature | pos | 104.0307 | 7.331 | 4.036 | 3.962 | -0.074 | -0.318 | 0.076 | 0.162 | Unannotated | NA | NA |
| metab_224 | Unannotated feature | pos | 366.9131 | 0.621 | 5.044 | 4.984 | -0.060 | -0.318 | 0.076 | 0.162 | Unannotated | NA | NA |
| metab_1559 | Unannotated feature | pos | 80.9484 | 0.591 | 6.676 | 6.660 | -0.016 | -0.318 | 0.076 | 0.162 | Unannotated | NA | NA |
| metab_10090 | Unannotated feature | neg | 167.6158 | 1.278 | 5.187 | 5.156 | -0.031 | -0.318 | 0.076 | 0.162 | Unannotated | NA | NA |
| metab_7066 | Unannotated feature | neg | 348.8337 | 5.787 | 4.948 | 4.887 | -0.060 | -0.318 | 0.076 | 0.162 | Unannotated | NA | NA |
| metab_4318 | Unannotated feature | pos | 737.6375 | 0.575 | 4.204 | 4.031 | -0.172 | -0.318 | 0.076 | 0.162 | Unannotated | NA | NA |
| metab_4314 | Unannotated feature | pos | 828.5918 | 0.575 | 4.828 | 4.750 | -0.078 | -0.318 | 0.076 | 0.162 | Unannotated | NA | NA |
| metab_9104 | Alpha-(Chloromethyl)-2-hydroxymethyl-5-nitroimidazole-1-ethanol | neg | 271.9851 | 4.409 | 5.489 | 5.789 | 0.299 | 0.318 | 0.076 | 0.162 | Tentative | NA | NA |
| metab_1458 | Unannotated feature | pos | 608.4475 | 5.987 | 5.297 | 5.359 | 0.062 | 0.318 | 0.076 | 0.162 | Unannotated | NA | NA |
| metab_7992 | Unannotated feature | neg | 142.8960 | 7.000 | 4.259 | 4.315 | 0.056 | 0.318 | 0.076 | 0.162 | Unannotated | NA | NA |
| metab_7859 | Unannotated feature | neg | 203.8443 | 2.629 | 5.767 | 5.735 | -0.032 | -0.318 | 0.076 | 0.162 | Unannotated | NA | NA |
| metab_146 | Unannotated feature | pos | 716.8031 | 0.551 | 4.490 | 4.766 | 0.276 | 0.318 | 0.076 | 0.162 | Unannotated | NA | NA |
| metab_438 | Unannotated feature | pos | 105.0550 | 1.585 | 3.256 | 3.581 | 0.324 | 0.318 | 0.076 | 0.162 | Unannotated | NA | NA |
| metab_7998 | Unannotated feature | neg | 127.0370 | 6.976 | 4.827 | 4.866 | 0.039 | 0.318 | 0.076 | 0.162 | Unannotated | NA | NA |
| metab_6624 | Unannotated feature | neg | 156.0120 | 4.969 | 4.169 | 4.097 | -0.072 | -0.318 | 0.076 | 0.162 | Unannotated | NA | NA |
| metab_354 | Unannotated feature | pos | 74.0242 | 0.861 | 3.480 | 3.585 | 0.104 | 0.318 | 0.076 | 0.162 | Unannotated | NA | NA |
| metab_635 | Naphthazarin | pos | 223.0600 | 2.241 | 4.050 | 4.006 | -0.044 | -0.318 | 0.076 | 0.162 | Tentative | NA | NA |
| metab_10911 | Unannotated feature | neg | 1038.8044 | 0.539 | 4.943 | 4.778 | -0.165 | -0.318 | 0.076 | 0.162 | Unannotated | NA | NA |
| metab_8075 | Diacylglycerol | neg | 557.4586 | 6.250 | 6.357 | 6.267 | -0.090 | -0.318 | 0.076 | 0.162 | Tentative | NA | NA |
| metab_9540 | Unannotated feature | neg | 179.8822 | 3.217 | 4.610 | 4.902 | 0.292 | 0.318 | 0.076 | 0.162 | Unannotated | NA | NA |
| metab_1963 | Unannotated feature | pos | 424.8968 | 7.300 | 4.740 | 4.714 | -0.026 | -0.318 | 0.076 | 0.162 | Unannotated | NA | NA |
| metab_5192 | Unannotated feature | neg | 537.9021 | 0.534 | 5.670 | 5.621 | -0.049 | -0.318 | 0.076 | 0.162 | Unannotated | NA | NA |
| metab_2983 | Unannotated feature | pos | 690.7986 | 4.501 | 4.858 | 5.128 | 0.270 | 0.318 | 0.076 | 0.162 | Unannotated | NA | NA |
| metab_1944 | Unannotated feature | pos | 712.6747 | 0.575 | 5.637 | 5.612 | -0.025 | -0.318 | 0.076 | 0.162 | Unannotated | NA | NA |
| metab_4454 | Unannotated feature | pos | 875.8092 | 0.551 | 5.349 | 5.456 | 0.107 | 0.318 | 0.076 | 0.162 | Unannotated | NA | NA |
| metab_7753 | Unannotated feature | neg | 170.8329 | 7.181 | 5.202 | 5.177 | -0.024 | -0.314 | 0.080 | 0.168 | Unannotated | NA | NA |
| metab_6449 | Unannotated feature | neg | 630.2886 | 4.256 | 4.620 | 4.803 | 0.183 | 0.314 | 0.080 | 0.168 | Unannotated | NA | NA |
| metab_5186 | Unannotated feature | neg | 418.9167 | 0.530 | 4.260 | 4.331 | 0.071 | 0.314 | 0.080 | 0.168 | Unannotated | NA | NA |
| metab_11351 | Unannotated feature | neg | 159.8863 | 0.016 | 3.975 | 3.926 | -0.050 | -0.314 | 0.080 | 0.168 | Unannotated | NA | NA |
| metab_1847 | Unannotated feature | pos | 703.8664 | 0.551 | 5.768 | 5.734 | -0.034 | -0.314 | 0.080 | 0.168 | Unannotated | NA | NA |
| metab_1827 | 3-Hydroxyadipic acid 3,6-lactone | pos | 109.0287 | 7.983 | 4.548 | 4.522 | -0.027 | -0.314 | 0.080 | 0.168 | Tentative | NA | NA |
| metab_2075 | Unannotated feature | pos | 451.9911 | 7.200 | 5.843 | 5.873 | 0.030 | 0.314 | 0.080 | 0.168 | Unannotated | NA | NA |
| metab_5420 | Unannotated feature | neg | 672.6823 | 0.629 | 4.863 | 4.709 | -0.154 | -0.314 | 0.080 | 0.168 | Unannotated | NA | NA |
| metab_5020 | Unannotated feature | pos | 228.1456 | 0.831 | 3.616 | 3.322 | -0.294 | -0.314 | 0.080 | 0.168 | Unannotated | NA | NA |
| metab_9647 | 2(5H)-Furanone | neg | 167.0345 | 3.065 | 2.745 | 3.109 | 0.364 | 0.314 | 0.080 | 0.168 | Tentative | Organoheterocyclic compounds | Dihydrofurans |
| metab_9136 | Nicardipine | neg | 478.1993 | 4.314 | 4.544 | 4.202 | -0.343 | -0.314 | 0.080 | 0.168 | Tentative | NA | NA |
| metab_6261 | Unannotated feature | neg | 213.9981 | 3.522 | 5.279 | 5.543 | 0.264 | 0.314 | 0.080 | 0.168 | Unannotated | NA | NA |
| metab_6605 | 1H-Imidazole, 2-(2-benzofuranyl)-4,5-dihydro- | neg | 231.0777 | 4.869 | 5.579 | 5.538 | -0.041 | -0.314 | 0.080 | 0.168 | Tentative | NA | NA |
| metab_7953 | Unannotated feature | neg | 115.5774 | 7.109 | 5.157 | 5.207 | 0.050 | 0.314 | 0.080 | 0.168 | Unannotated | NA | NA |
| metab_2981 | Unannotated feature | pos | 536.7307 | 4.508 | 5.092 | 5.238 | 0.146 | 0.314 | 0.080 | 0.168 | Unannotated | NA | NA |
| metab_8342 | Unannotated feature | neg | 322.0159 | 5.827 | 4.236 | 3.829 | -0.407 | -0.314 | 0.080 | 0.168 | Unannotated | NA | NA |
| metab_11142 | Unannotated feature | neg | 167.0710 | 5.731 | 3.897 | 3.687 | -0.210 | -0.314 | 0.080 | 0.168 | Unannotated | NA | NA |
| metab_6648 | Unannotated feature | neg | 402.7795 | 0.582 | 5.691 | 5.648 | -0.044 | -0.314 | 0.080 | 0.168 | Unannotated | NA | NA |
| metab_8794 | Unannotated feature | neg | 167.8363 | 5.514 | 2.636 | 3.044 | 0.408 | 0.314 | 0.080 | 0.168 | Unannotated | NA | NA |
| metab_4617 | Unannotated feature | pos | 171.1177 | 3.846 | 4.645 | 4.777 | 0.132 | 0.314 | 0.080 | 0.168 | Unannotated | NA | NA |
| metab_7246 | Icosatrienoic acid | neg | 351.2546 | 6.024 | 4.736 | 4.552 | -0.184 | -0.314 | 0.080 | 0.168 | Tentative | Lipids and lipid-like molecules | Fatty Acyls |
| metab_111 | Unannotated feature | pos | 581.8239 | 0.559 | 4.989 | 4.978 | -0.011 | -0.314 | 0.080 | 0.168 | Unannotated | NA | NA |
| metab_4139 | Unannotated feature | pos | 197.9808 | 0.675 | 3.776 | 3.342 | -0.434 | -0.314 | 0.080 | 0.168 | Unannotated | NA | NA |
| metab_6999 | Unannotated feature | neg | 137.8913 | 5.731 | 4.049 | 4.019 | -0.031 | -0.314 | 0.080 | 0.168 | Unannotated | NA | NA |
| metab_2381 | Unannotated feature | pos | 966.6916 | 5.949 | 5.446 | 5.376 | -0.070 | -0.314 | 0.080 | 0.168 | Unannotated | NA | NA |
| metab_9890 | Unannotated feature | neg | 498.6395 | 0.621 | 6.055 | 6.046 | -0.009 | -0.314 | 0.080 | 0.168 | Unannotated | NA | NA |
| metab_4562 | Unannotated feature | pos | 179.0002 | 0.473 | 4.907 | 4.895 | -0.011 | -0.314 | 0.080 | 0.168 | Unannotated | NA | NA |
| metab_907 | Unannotated feature | pos | 111.1172 | 3.831 | 3.813 | 3.990 | 0.177 | 0.314 | 0.080 | 0.168 | Unannotated | NA | NA |
| metab_6088 | Unannotated feature | neg | 577.7333 | 0.636 | 5.175 | 5.062 | -0.113 | -0.314 | 0.080 | 0.168 | Unannotated | NA | NA |
| metab_5162 | Unannotated feature | neg | 225.8975 | 0.505 | 5.615 | 5.599 | -0.016 | -0.314 | 0.080 | 0.168 | Unannotated | NA | NA |
| metab_7990 | 3,4-Dihydroxybenzoate | neg | 153.0188 | 7.038 | 5.409 | 5.443 | 0.034 | 0.314 | 0.080 | 0.168 | Strong putative | NA | NA |
| metab_10732 | Unannotated feature | neg | 715.7358 | 0.570 | 4.281 | 4.007 | -0.274 | -0.314 | 0.080 | 0.168 | Unannotated | NA | NA |
| metab_9972 | Unannotated feature | neg | 295.8318 | 2.079 | 4.101 | 4.160 | 0.059 | 0.314 | 0.080 | 0.168 | Unannotated | NA | NA |
| metab_4517 | Unannotated feature | pos | 201.0162 | 0.541 | 3.542 | 3.711 | 0.169 | 0.314 | 0.080 | 0.168 | Unannotated | NA | NA |
| metab_4932 | Unannotated feature | pos | 221.9880 | 0.621 | 4.381 | 4.487 | 0.106 | 0.314 | 0.080 | 0.168 | Unannotated | NA | NA |
| metab_5359 | Unannotated feature | neg | 364.7889 | 0.587 | 4.403 | 4.456 | 0.053 | 0.314 | 0.080 | 0.168 | Unannotated | NA | NA |
| metab_2113 | Unannotated feature | pos | 634.4523 | 7.014 | 5.662 | 5.639 | -0.022 | -0.314 | 0.080 | 0.168 | Unannotated | NA | NA |
| metab_11399 | Glycocholate | neg | 500.2794 | 5.985 | 6.086 | 6.219 | 0.133 | 0.314 | 0.080 | 0.168 | Tentative | NA | NA |
| metab_2560 | Unannotated feature | pos | 559.7252 | 5.863 | 3.942 | 3.750 | -0.192 | -0.314 | 0.080 | 0.168 | Unannotated | NA | NA |
| metab_4832 | Unannotated feature | pos | 172.0945 | 7.293 | 6.021 | 6.008 | -0.014 | -0.314 | 0.080 | 0.168 | Unannotated | NA | NA |
| metab_2618 | Unannotated feature | pos | 93.0703 | 5.840 | 3.419 | 3.495 | 0.077 | 0.314 | 0.080 | 0.168 | Unannotated | NA | NA |
| metab_1560 | Unannotated feature | pos | 266.2437 | 6.311 | 4.934 | 4.667 | -0.266 | -0.314 | 0.080 | 0.168 | Unannotated | NA | NA |
| metab_7809 | Unannotated feature | neg | 235.0852 | 7.164 | 4.337 | 4.310 | -0.028 | -0.314 | 0.080 | 0.168 | Unannotated | NA | NA |
| metab_6963 | Unannotated feature | neg | 280.5668 | 5.699 | 3.612 | 3.273 | -0.338 | -0.314 | 0.080 | 0.168 | Unannotated | NA | NA |
| metab_6706 | Unannotated feature | neg | 233.0673 | 5.310 | 3.654 | 4.156 | 0.502 | 0.314 | 0.080 | 0.168 | Unannotated | NA | NA |
| metab_8914 | Unannotated feature | neg | 740.2916 | 5.085 | 4.693 | 4.917 | 0.224 | 0.314 | 0.080 | 0.168 | Unannotated | NA | NA |
| metab_8890 | Unannotated feature | neg | 312.1096 | 5.184 | 4.509 | 4.313 | -0.196 | -0.314 | 0.080 | 0.168 | Unannotated | NA | NA |
| metab_648 | S-Carboxymethyl-L-cysteine | pos | 162.0220 | 2.288 | 2.841 | 2.922 | 0.081 | 0.314 | 0.080 | 0.168 | Tentative | NA | NA |
| metab_6136 | Unannotated feature | neg | 635.6921 | 0.644 | 5.017 | 4.826 | -0.191 | -0.314 | 0.080 | 0.168 | Unannotated | NA | NA |
| metab_1278 | Unannotated feature | pos | 558.3860 | 5.855 | 4.837 | 4.675 | -0.163 | -0.314 | 0.080 | 0.168 | Unannotated | NA | NA |
| metab_1940 | Unannotated feature | pos | 109.5219 | 7.324 | 4.819 | 4.848 | 0.029 | 0.309 | 0.084 | 0.175 | Unannotated | NA | NA |
| metab_1450 | Unannotated feature | pos | 591.4080 | 5.972 | 4.766 | 4.648 | -0.118 | -0.309 | 0.084 | 0.175 | Unannotated | NA | NA |
| metab_514 | Unannotated feature | pos | 217.2550 | 1.925 | 4.167 | 4.028 | -0.139 | -0.309 | 0.084 | 0.175 | Unannotated | NA | NA |
| metab_3669 | (2E)-2-ethylidene-4-hydroxy-5-methyl-3(2H)-furanone | pos | 122.0365 | 1.949 | 4.440 | 4.355 | -0.085 | -0.309 | 0.084 | 0.175 | Tentative | NA | NA |
| metab_7560 | Unannotated feature | neg | 189.8578 | 7.189 | 5.371 | 5.395 | 0.024 | 0.309 | 0.084 | 0.175 | Unannotated | NA | NA |
| metab_4373 | Unannotated feature | pos | 648.8158 | 0.567 | 4.477 | 4.640 | 0.162 | 0.309 | 0.084 | 0.175 | Unannotated | NA | NA |
| metab_11010 | Unannotated feature | neg | 398.7607 | 0.016 | 4.497 | 4.470 | -0.027 | -0.309 | 0.084 | 0.175 | Unannotated | NA | NA |
| metab_7216 | Unannotated feature | neg | 376.2266 | 5.945 | 4.347 | 4.111 | -0.236 | -0.309 | 0.084 | 0.175 | Unannotated | NA | NA |
| metab_10995 | Unannotated feature | neg | 228.9030 | 0.578 | 5.852 | 5.819 | -0.033 | -0.309 | 0.084 | 0.175 | Unannotated | NA | NA |
| metab_6916 | Unannotated feature | neg | 324.7632 | 0.912 | 5.504 | 5.478 | -0.026 | -0.309 | 0.084 | 0.175 | Unannotated | NA | NA |
| metab_5601 | Unannotated feature | neg | 279.0394 | 0.873 | 5.254 | 5.167 | -0.087 | -0.309 | 0.084 | 0.175 | Unannotated | NA | NA |
| metab_6942 | Unannotated feature | neg | 183.0660 | 5.691 | 3.495 | 3.366 | -0.129 | -0.309 | 0.084 | 0.175 | Unannotated | NA | NA |
| metab_1754 | Unannotated feature | pos | 194.9751 | 7.331 | 4.684 | 4.656 | -0.028 | -0.309 | 0.084 | 0.175 | Unannotated | NA | NA |
| metab_5178 | Unannotated feature | neg | 303.9139 | 0.522 | 3.829 | 3.875 | 0.046 | 0.309 | 0.084 | 0.175 | Unannotated | NA | NA |
| metab_8660 | Unannotated feature | neg | 223.1425 | 5.635 | 4.622 | 4.523 | -0.099 | -0.309 | 0.084 | 0.175 | Unannotated | NA | NA |
| metab_4203 | Unannotated feature | pos | 201.4701 | 0.629 | 3.723 | 3.947 | 0.224 | 0.309 | 0.084 | 0.175 | Unannotated | NA | NA |
| metab_2229 | Unannotated feature | pos | 502.3296 | 6.288 | 4.528 | 4.355 | -0.173 | -0.309 | 0.084 | 0.175 | Unannotated | NA | NA |
| metab_1130 | Glucosyl-limonin | pos | 633.2551 | 5.626 | 4.729 | 4.071 | -0.658 | -0.309 | 0.084 | 0.175 | Tentative | NA | NA |
| metab_7526 | Unannotated feature | neg | 688.2633 | 7.164 | 4.107 | 4.022 | -0.085 | -0.309 | 0.084 | 0.175 | Unannotated | NA | NA |
| metab_1139 | Unannotated feature | pos | 203.1391 | 1.956 | 5.415 | 5.541 | 0.126 | 0.309 | 0.084 | 0.175 | Unannotated | NA | NA |
| metab_7393 | Unannotated feature | neg | 256.8743 | 7.093 | 5.325 | 5.390 | 0.066 | 0.309 | 0.084 | 0.175 | Unannotated | NA | NA |
| metab_7128 | (6R,8Z)-6-Hydroxy-3-oxotetradecenoic acid | neg | 255.1606 | 5.851 | 5.012 | 5.113 | 0.101 | 0.309 | 0.084 | 0.175 | Tentative | NA | NA |
| metab_7362 | Unannotated feature | neg | 338.1465 | 2.111 | 5.359 | 5.098 | -0.260 | -0.309 | 0.084 | 0.175 | Unannotated | NA | NA |
| metab_947 | Unannotated feature | pos | 84.1314 | 4.109 | 3.578 | 3.638 | 0.060 | 0.309 | 0.084 | 0.175 | Unannotated | NA | NA |
| metab_10661 | Unannotated feature | neg | 224.8653 | 0.587 | 4.428 | 4.395 | -0.033 | -0.309 | 0.084 | 0.175 | Unannotated | NA | NA |
| metab_9529 | Unannotated feature | neg | 173.0328 | 3.224 | 4.031 | 4.419 | 0.388 | 0.309 | 0.084 | 0.175 | Unannotated | NA | NA |
| metab_10878 | Unannotated feature | neg | 112.9059 | 0.554 | 2.807 | 3.094 | 0.288 | 0.309 | 0.084 | 0.175 | Unannotated | NA | NA |
| metab_6249 | Unannotated feature | neg | 211.1559 | 3.499 | 4.584 | 4.852 | 0.268 | 0.309 | 0.084 | 0.175 | Unannotated | NA | NA |
| metab_1051 | Unannotated feature | pos | 99.5316 | 4.946 | 4.445 | 4.469 | 0.023 | 0.309 | 0.084 | 0.175 | Unannotated | NA | NA |
| metab_8240 | Unannotated feature | neg | 299.7768 | 5.914 | 4.573 | 4.681 | 0.108 | 0.309 | 0.084 | 0.175 | Unannotated | NA | NA |
| metab_1700 | Unannotated feature | pos | 488.8724 | 7.285 | 4.928 | 4.894 | -0.034 | -0.309 | 0.084 | 0.175 | Unannotated | NA | NA |
| metab_10079 | Unannotated feature | neg | 292.8945 | 1.294 | 4.729 | 4.674 | -0.055 | -0.309 | 0.084 | 0.175 | Unannotated | NA | NA |
| metab_7827 | Unannotated feature | neg | 380.9252 | 2.557 | 4.952 | 4.937 | -0.014 | -0.309 | 0.084 | 0.175 | Unannotated | NA | NA |
| metab_7324 | Unannotated feature | neg | 116.0346 | 0.865 | 4.996 | 5.056 | 0.060 | 0.309 | 0.084 | 0.175 | Unannotated | NA | NA |
| metab_4888 | Unannotated feature | pos | 220.0639 | 1.949 | 4.712 | 5.201 | 0.489 | 0.309 | 0.084 | 0.175 | Unannotated | NA | NA |
| metab_6714 | Unannotated feature | neg | 181.0058 | 5.346 | 3.388 | 3.772 | 0.384 | 0.309 | 0.084 | 0.175 | Unannotated | NA | NA |
| metab_10776 | Unannotated feature | neg | 892.8045 | 0.562 | 4.406 | 4.230 | -0.176 | -0.309 | 0.084 | 0.175 | Unannotated | NA | NA |
| metab_11177 | Unannotated feature | neg | 339.0183 | 5.827 | 4.999 | 4.589 | -0.410 | -0.309 | 0.084 | 0.175 | Unannotated | NA | NA |
| metab_8676 | Unannotated feature | neg | 429.1966 | 5.619 | 3.859 | 4.149 | 0.290 | 0.309 | 0.084 | 0.175 | Unannotated | NA | NA |
| metab_8611 | Phaseolotoxin | neg | 561.2024 | 5.659 | 4.592 | 4.696 | 0.104 | 0.309 | 0.084 | 0.175 | Tentative | Organic acids and derivatives | Carboxylic acids and derivatives |
| metab_4299 | Unannotated feature | pos | 728.6091 | 0.583 | 4.311 | 4.199 | -0.112 | -0.309 | 0.084 | 0.175 | Unannotated | NA | NA |
| metab_10112 | Unannotated feature | neg | 129.8666 | 1.094 | 5.422 | 5.357 | -0.064 | -0.309 | 0.084 | 0.175 | Unannotated | NA | NA |
| metab_8466 | Unannotated feature | neg | 349.1985 | 5.747 | 4.436 | 4.404 | -0.033 | -0.309 | 0.084 | 0.175 | Unannotated | NA | NA |
| metab_78 | (E)-2-Hexenal | pos | 99.0809 | 0.465 | 4.699 | 4.672 | -0.027 | -0.305 | 0.088 | 0.182 | Tentative | NA | NA |
| metab_6943 | Unannotated feature | neg | 215.0384 | 5.691 | 2.759 | 2.573 | -0.186 | -0.305 | 0.088 | 0.182 | Unannotated | NA | NA |
| metab_11336 | Unannotated feature | neg | 148.9386 | 7.170 | 4.307 | 4.277 | -0.029 | -0.305 | 0.088 | 0.182 | Unannotated | NA | NA |
| metab_9735 | Unannotated feature | neg | 357.8396 | 0.629 | 4.701 | 4.483 | -0.218 | -0.305 | 0.088 | 0.182 | Unannotated | NA | NA |
| metab_2500 | Elocalcitol | pos | 456.3356 | 5.894 | 5.819 | 5.918 | 0.099 | 0.305 | 0.088 | 0.182 | Tentative | NA | NA |
| metab_4276 | Unannotated feature | pos | 466.7282 | 0.591 | 5.019 | 4.963 | -0.056 | -0.305 | 0.088 | 0.182 | Unannotated | NA | NA |
| metab_10109 | Unannotated feature | neg | 161.8567 | 1.103 | 5.344 | 5.309 | -0.036 | -0.305 | 0.088 | 0.182 | Unannotated | NA | NA |
| metab_6156 | Unannotated feature | neg | 132.8995 | 3.240 | 4.638 | 4.605 | -0.033 | -0.305 | 0.088 | 0.182 | Unannotated | NA | NA |
| metab_10977 | Unannotated feature | neg | 162.8930 | 0.505 | 5.267 | 5.291 | 0.023 | 0.305 | 0.088 | 0.182 | Unannotated | NA | NA |
| metab_3114 | Unannotated feature | pos | 84.1316 | 3.769 | 4.020 | 4.067 | 0.047 | 0.305 | 0.088 | 0.182 | Unannotated | NA | NA |
| metab_1292 | Unannotated feature | pos | 549.0529 | 5.863 | 5.189 | 5.134 | -0.055 | -0.305 | 0.088 | 0.182 | Unannotated | NA | NA |
| metab_5042 | Unannotated feature | pos | 326.1445 | 0.861 | 4.954 | 4.735 | -0.219 | -0.305 | 0.088 | 0.182 | Unannotated | NA | NA |
| metab_6178 | N-Acetyl-DL-Methionine | neg | 190.0541 | 3.274 | 4.497 | 4.623 | 0.126 | 0.305 | 0.088 | 0.182 | Moderate putative | NA | NA |
| metab_7789 | Unannotated feature | neg | 187.9416 | 7.170 | 4.847 | 4.825 | -0.022 | -0.305 | 0.088 | 0.182 | Unannotated | NA | NA |
| metab_825 | Unannotated feature | pos | 190.9792 | 7.324 | 5.244 | 5.260 | 0.016 | 0.305 | 0.088 | 0.182 | Unannotated | NA | NA |
| metab_8897 | Phenoxyacetate | neg | 151.0395 | 5.139 | 4.192 | 4.156 | -0.036 | -0.305 | 0.088 | 0.182 | Strong putative | NA | NA |
| metab_1684 | Unannotated feature | pos | 456.1401 | 7.269 | 4.756 | 4.818 | 0.063 | 0.305 | 0.088 | 0.182 | Unannotated | NA | NA |
| metab_2758 | Furfuryl alcohol | pos | 197.0809 | 5.695 | 4.586 | 4.462 | -0.123 | -0.305 | 0.088 | 0.182 | Tentative | NA | NA |
| metab_265 | Unannotated feature | pos | 116.4156 | 0.698 | 4.946 | 4.975 | 0.029 | 0.305 | 0.088 | 0.182 | Unannotated | NA | NA |
| metab_10053 | Unannotated feature | neg | 131.0343 | 1.398 | 4.581 | 4.553 | -0.028 | -0.305 | 0.088 | 0.182 | Unannotated | NA | NA |
| metab_7887 | Unannotated feature | neg | 112.6553 | 7.140 | 4.487 | 4.449 | -0.039 | -0.305 | 0.088 | 0.182 | Unannotated | NA | NA |
| metab_5017 | Unannotated feature | pos | 232.0669 | 0.823 | 3.729 | 3.927 | 0.199 | 0.305 | 0.088 | 0.182 | Unannotated | NA | NA |
| metab_7883 | Unannotated feature | neg | 121.0288 | 7.140 | 5.047 | 5.066 | 0.019 | 0.305 | 0.088 | 0.182 | Unannotated | NA | NA |
| metab_2201 | (S)-Oleuropeic acid | pos | 207.0989 | 6.303 | 5.044 | 4.996 | -0.048 | -0.305 | 0.088 | 0.182 | Tentative | NA | NA |
| metab_6658 | 4-Hydroxystyrene | neg | 119.0495 | 5.116 | 4.374 | 4.635 | 0.260 | 0.305 | 0.088 | 0.182 | Moderate putative | Benzenoids | Benzene and substituted derivatives |
| metab_10974 | Unannotated feature | neg | 209.9490 | 0.505 | 4.880 | 4.912 | 0.032 | 0.305 | 0.088 | 0.182 | Unannotated | NA | NA |
| metab_6506 | Unannotated feature | neg | 381.0769 | 4.417 | 4.423 | 4.071 | -0.352 | -0.305 | 0.088 | 0.182 | Unannotated | NA | NA |
| metab_318 | N-Acetyl-beta-alanine | pos | 132.0657 | 0.800 | 4.975 | 5.125 | 0.150 | 0.305 | 0.088 | 0.182 | Tentative | NA | NA |
| metab_2290 | Unannotated feature | pos | 531.3868 | 6.095 | 4.400 | 4.487 | 0.086 | 0.305 | 0.088 | 0.182 | Unannotated | NA | NA |
| metab_3047 | Unannotated feature | pos | 272.1353 | 2.389 | 4.895 | 5.042 | 0.147 | 0.305 | 0.088 | 0.182 | Unannotated | NA | NA |
| metab_5837 | Unannotated feature | neg | 378.7793 | 2.189 | 5.055 | 5.097 | 0.042 | 0.305 | 0.088 | 0.182 | Unannotated | NA | NA |
| metab_1467 | Graphinone | pos | 279.1591 | 6.003 | 4.968 | 4.925 | -0.043 | -0.305 | 0.088 | 0.182 | Tentative | NA | NA |
| metab_5598 | Unannotated feature | neg | 166.4288 | 0.873 | 5.208 | 5.143 | -0.064 | -0.305 | 0.088 | 0.182 | Unannotated | NA | NA |
| metab_7537 | Unannotated feature | neg | 134.8746 | 7.173 | 4.147 | 4.106 | -0.041 | -0.305 | 0.088 | 0.182 | Unannotated | NA | NA |
| metab_4767 | 2-Hydroxy-p-mentha-1,8-dien-6-one | pos | 149.0962 | 6.427 | 4.932 | 4.977 | 0.045 | 0.305 | 0.088 | 0.182 | Tentative | NA | NA |
| metab_4123 | Unannotated feature | pos | 115.7276 | 0.698 | 4.977 | 5.016 | 0.040 | 0.305 | 0.088 | 0.182 | Unannotated | NA | NA |
| metab_9899 | Unannotated feature | neg | 136.8937 | 2.189 | 4.583 | 4.546 | -0.037 | -0.305 | 0.088 | 0.182 | Unannotated | NA | NA |
| metab_7934 | Unannotated feature | neg | 199.8508 | 7.109 | 5.669 | 5.716 | 0.047 | 0.305 | 0.088 | 0.182 | Unannotated | NA | NA |
| metab_10191 | Unannotated feature | neg | 246.9619 | 0.881 | 4.730 | 4.679 | -0.051 | -0.305 | 0.088 | 0.182 | Unannotated | NA | NA |
| metab_7637 | Unannotated feature | neg | 162.8930 | 7.974 | 5.162 | 5.170 | 0.008 | 0.305 | 0.088 | 0.182 | Unannotated | NA | NA |
| metab_5234 | Unannotated feature | neg | 222.9137 | 0.013 | 5.708 | 5.689 | -0.019 | -0.305 | 0.088 | 0.182 | Unannotated | NA | NA |
| metab_427 | Unannotated feature | pos | 107.0859 | 1.491 | 4.533 | 4.513 | -0.020 | -0.305 | 0.088 | 0.182 | Unannotated | NA | NA |
| metab_10485 | Unannotated feature | neg | 401.9781 | 0.636 | 4.082 | 3.848 | -0.234 | -0.305 | 0.088 | 0.182 | Unannotated | NA | NA |
| metab_10598 | Unannotated feature | neg | 832.4513 | 0.598 | 4.278 | 4.397 | 0.119 | 0.305 | 0.088 | 0.182 | Unannotated | NA | NA |
| metab_3343 | Unannotated feature | pos | 130.1592 | 2.606 | 5.301 | 5.313 | 0.013 | 0.305 | 0.088 | 0.182 | Unannotated | NA | NA |
| metab_10491 | Unannotated feature | neg | 285.9427 | 0.636 | 4.868 | 4.782 | -0.086 | -0.305 | 0.088 | 0.182 | Unannotated | NA | NA |
| metab_1418 | Unannotated feature | pos | 516.8544 | 5.941 | 4.849 | 5.032 | 0.183 | 0.305 | 0.088 | 0.182 | Unannotated | NA | NA |
| metab_8073 | Unannotated feature | neg | 145.4038 | 6.258 | 3.666 | 3.488 | -0.178 | -0.305 | 0.088 | 0.182 | Unannotated | NA | NA |
| metab_6572 | Unannotated feature | neg | 211.1136 | 4.726 | 5.166 | 5.114 | -0.052 | -0.305 | 0.088 | 0.182 | Unannotated | NA | NA |
| metab_3522 | Unannotated feature | pos | 360.2014 | 2.118 | 4.777 | 4.596 | -0.181 | -0.305 | 0.088 | 0.182 | Unannotated | NA | NA |
| metab_1727 | Unannotated feature | pos | 288.6334 | 7.316 | 4.515 | 4.662 | 0.147 | 0.305 | 0.088 | 0.182 | Unannotated | NA | NA |
| metab_4995 | Unannotated feature | pos | 131.0817 | 0.768 | 5.054 | 4.956 | -0.098 | -0.305 | 0.088 | 0.182 | Unannotated | NA | NA |
| metab_8101 | Terephthalate | neg | 165.0189 | 6.188 | 6.401 | 6.443 | 0.042 | 0.301 | 0.093 | 0.188 | Strong putative | NA | NA |
| metab_3137 | Irilone | pos | 340.0807 | 3.638 | 5.125 | 5.214 | 0.089 | 0.301 | 0.093 | 0.188 | Tentative | NA | NA |
| metab_9402 | Unannotated feature | neg | 321.8147 | 3.491 | 4.069 | 4.120 | 0.051 | 0.301 | 0.093 | 0.188 | Unannotated | NA | NA |
| metab_9392 | Unannotated feature | neg | 303.8873 | 3.507 | 5.551 | 5.622 | 0.071 | 0.301 | 0.093 | 0.188 | Unannotated | NA | NA |
| metab_2606 | Unannotated feature | pos | 610.4265 | 5.840 | 5.661 | 5.613 | -0.048 | -0.301 | 0.093 | 0.188 | Unannotated | NA | NA |
| metab_8239 | Unannotated feature | neg | 366.9062 | 5.914 | 5.331 | 5.346 | 0.015 | 0.301 | 0.093 | 0.188 | Unannotated | NA | NA |
| metab_5930 | Unannotated feature | neg | 228.9048 | 2.581 | 4.210 | 4.279 | 0.070 | 0.301 | 0.093 | 0.188 | Unannotated | NA | NA |
| metab_10061 | Unannotated feature | neg | 119.9068 | 7.213 | 4.572 | 4.618 | 0.047 | 0.301 | 0.093 | 0.188 | Unannotated | NA | NA |
| metab_11315 | Unannotated feature | neg | 216.8538 | 7.213 | 4.659 | 4.690 | 0.031 | 0.301 | 0.093 | 0.188 | Unannotated | NA | NA |
| metab_11037 | Unannotated feature | neg | 297.8277 | 0.013 | 5.745 | 5.730 | -0.015 | -0.301 | 0.093 | 0.188 | Unannotated | NA | NA |
| metab_3389 | 2-n-Propylthiazolidine-4-carboxylic acid | pos | 176.0741 | 2.498 | 5.363 | 4.907 | -0.455 | -0.301 | 0.093 | 0.188 | Tentative | Organic acids and derivatives | Carboxylic acids and derivatives |
| metab_4630 | Unannotated feature | pos | 736.5045 | 5.649 | 5.185 | 5.093 | -0.091 | -0.301 | 0.093 | 0.188 | Unannotated | NA | NA |
| metab_1478 | Polygonal | pos | 223.1693 | 6.026 | 6.219 | 6.255 | 0.036 | 0.301 | 0.093 | 0.188 | Tentative | NA | NA |
| metab_951 | Unannotated feature | pos | 172.0969 | 4.124 | 3.412 | 3.772 | 0.360 | 0.301 | 0.093 | 0.188 | Unannotated | NA | NA |
| metab_1182 | Unannotated feature | pos | 101.8086 | 5.740 | 5.139 | 5.170 | 0.031 | 0.301 | 0.093 | 0.188 | Unannotated | NA | NA |
| metab_2769 | Cortisol | pos | 363.2165 | 5.664 | 5.645 | 5.485 | -0.160 | -0.301 | 0.093 | 0.188 | Strong putative | NA | NA |
| metab_2687 | Polyglycerol esters of fatty acids | pos | 450.3786 | 5.763 | 5.050 | 5.121 | 0.071 | 0.301 | 0.093 | 0.188 | Tentative | NA | NA |
| metab_2774 | (3S,5R,6S,7E,9x)-7-Megastigmene-3,6,9-triol 9-glucoside | pos | 413.2119 | 5.657 | 5.744 | 5.774 | 0.030 | 0.301 | 0.093 | 0.188 | Tentative | NA | NA |
| metab_7413 | Unannotated feature | neg | 115.9199 | 7.109 | 7.070 | 7.095 | 0.025 | 0.301 | 0.093 | 0.188 | Unannotated | NA | NA |
| metab_6862 | Unannotated feature | neg | 144.0661 | 5.635 | 3.888 | 3.966 | 0.077 | 0.301 | 0.093 | 0.188 | Unannotated | NA | NA |
| metab_3099 | Unannotated feature | pos | 125.1124 | 3.846 | 4.395 | 4.555 | 0.160 | 0.301 | 0.093 | 0.188 | Unannotated | NA | NA |
| metab_4929 | Unannotated feature | pos | 199.9907 | 0.614 | 4.122 | 4.313 | 0.191 | 0.301 | 0.093 | 0.188 | Unannotated | NA | NA |
| metab_10619 | Unannotated feature | neg | 96.6595 | 0.598 | 3.392 | 3.299 | -0.094 | -0.301 | 0.093 | 0.188 | Unannotated | NA | NA |
| metab_5299 | Unannotated feature | neg | 586.8621 | 0.562 | 3.980 | 4.184 | 0.205 | 0.301 | 0.093 | 0.188 | Unannotated | NA | NA |
| metab_3477 | 2-(beta-D-Mannopyranosyl)-L-tryptophan | pos | 367.1500 | 2.506 | 5.669 | 5.553 | -0.116 | -0.301 | 0.093 | 0.188 | Tentative | NA | NA |
| metab_3141 | Unannotated feature | pos | 130.0653 | 3.638 | 4.058 | 4.151 | 0.093 | 0.301 | 0.093 | 0.188 | Unannotated | NA | NA |
| metab_8797 | Unannotated feature | neg | 445.1913 | 5.498 | 3.716 | 4.139 | 0.422 | 0.301 | 0.093 | 0.188 | Unannotated | NA | NA |
| metab_5771 | Unannotated feature | neg | 117.6266 | 1.981 | 3.147 | 3.008 | -0.138 | -0.301 | 0.093 | 0.188 | Unannotated | NA | NA |
| metab_7097 | Unannotated feature | neg | 279.2335 | 5.811 | 4.554 | 4.456 | -0.098 | -0.301 | 0.093 | 0.188 | Unannotated | NA | NA |
| metab_4510 | Unannotated feature | pos | 156.0826 | 0.545 | 4.156 | 4.059 | -0.097 | -0.301 | 0.093 | 0.188 | Unannotated | NA | NA |
| metab_3090 | (3b,16a,20R)-25-Acetoxy-3,16,20,22-tetrahydroxy-5-cucurbiten-11-one 3-glucoside | pos | 749.3825 | 3.854 | 6.036 | 6.124 | 0.088 | 0.301 | 0.093 | 0.188 | Tentative | NA | NA |
| metab_10981 | Unannotated feature | neg | 362.8760 | 0.476 | 5.547 | 5.524 | -0.023 | -0.301 | 0.093 | 0.188 | Unannotated | NA | NA |
| metab_1206 | Unannotated feature | pos | 276.2807 | 5.771 | 5.404 | 5.438 | 0.034 | 0.301 | 0.093 | 0.188 | Unannotated | NA | NA |
| metab_11381 | 4-Hydroxyphenylacetate | neg | 151.0395 | 4.322 | 4.600 | 4.775 | 0.176 | 0.301 | 0.093 | 0.188 | Strong putative | Benzenoids | Phenol esters |
| metab_8579 | Indole-3-acetate | neg | 174.0557 | 5.683 | 2.962 | 3.841 | 0.879 | 0.301 | 0.093 | 0.188 | Moderate putative | Organoheterocyclic compounds | Indoles and derivatives |
| metab_1567 | 1-Acyl-sn-glycero-3-phosphocholine | pos | 482.3242 | 6.350 | 6.335 | 6.285 | -0.051 | -0.301 | 0.093 | 0.188 | Tentative | NA | NA |
| metab_2291 | Unannotated feature | pos | 385.2946 | 6.095 | 5.074 | 5.132 | 0.058 | 0.301 | 0.093 | 0.188 | Unannotated | NA | NA |
| metab_10528 | Unannotated feature | neg | 684.4972 | 0.621 | 4.781 | 5.004 | 0.222 | 0.301 | 0.093 | 0.188 | Unannotated | NA | NA |
| metab_5683 | Unannotated feature | neg | 126.8803 | 1.222 | 4.769 | 4.843 | 0.074 | 0.301 | 0.093 | 0.188 | Unannotated | NA | NA |
| metab_1056 | Indole-3-acetate | pos | 176.0707 | 5.038 | 4.471 | 4.695 | 0.224 | 0.301 | 0.093 | 0.188 | Strong putative | Organoheterocyclic compounds | Indoles and derivatives |
| metab_11072 | 2-Hydroxypentanoic Acid | neg | 117.0549 | 3.192 | 6.516 | 6.397 | -0.120 | -0.301 | 0.093 | 0.188 | Tentative | NA | NA |
| metab_11287 | Unannotated feature | neg | 191.9460 | 0.349 | 5.822 | 5.839 | 0.017 | 0.301 | 0.093 | 0.188 | Unannotated | NA | NA |
| metab_1639 | Unannotated feature | pos | 171.1129 | 7.184 | 4.088 | 4.058 | -0.030 | -0.301 | 0.093 | 0.188 | Unannotated | NA | NA |
| metab_3538 | Unannotated feature | pos | 165.4990 | 2.081 | 5.640 | 5.692 | 0.052 | 0.301 | 0.093 | 0.188 | Unannotated | NA | NA |
| metab_9092 | Unannotated feature | neg | 545.1986 | 4.449 | 4.391 | 3.852 | -0.539 | -0.301 | 0.093 | 0.188 | Unannotated | NA | NA |
| metab_7688 | Unannotated feature | neg | 224.0619 | 7.229 | 5.293 | 5.266 | -0.027 | -0.301 | 0.093 | 0.188 | Unannotated | NA | NA |
| metab_7633 | Unannotated feature | neg | 198.9362 | 7.946 | 4.791 | 4.815 | 0.024 | 0.301 | 0.093 | 0.188 | Unannotated | NA | NA |
| metab_8759 | Unannotated feature | neg | 327.9506 | 5.543 | 4.298 | 4.363 | 0.065 | 0.301 | 0.093 | 0.188 | Unannotated | NA | NA |
| metab_5840 | Unannotated feature | neg | 317.9217 | 2.237 | 4.320 | 4.144 | -0.177 | -0.301 | 0.093 | 0.188 | Unannotated | NA | NA |
| metab_1048 | Bisacurone epoxide | pos | 286.2013 | 4.900 | 6.732 | 6.869 | 0.137 | 0.301 | 0.093 | 0.188 | Tentative | NA | NA |
| metab_4034 | Glutamate | pos | 189.0870 | 0.784 | 4.335 | 4.174 | -0.161 | -0.301 | 0.093 | 0.188 | Tentative | Organic acids and derivatives | Carboxylic acids and derivatives |
| metab_216 | Unannotated feature | pos | 504.6291 | 0.614 | 4.595 | 4.729 | 0.134 | 0.301 | 0.093 | 0.188 | Unannotated | NA | NA |
| metab_11347 | Unannotated feature | neg | 482.7902 | 0.013 | 5.917 | 5.909 | -0.008 | -0.301 | 0.093 | 0.188 | Unannotated | NA | NA |
| metab_1952 | Unannotated feature | pos | 267.6469 | 7.316 | 4.749 | 4.699 | -0.050 | -0.301 | 0.093 | 0.188 | Unannotated | NA | NA |
| metab_8186 | Unannotated feature | neg | 436.7986 | 0.723 | 4.394 | 3.965 | -0.430 | -0.301 | 0.093 | 0.188 | Unannotated | NA | NA |
| metab_1453 | Unannotated feature | pos | 528.3917 | 5.979 | 5.567 | 5.628 | 0.061 | 0.301 | 0.093 | 0.188 | Unannotated | NA | NA |
| metab_711 | 4-Hydroxynonenal | pos | 139.1118 | 6.420 | 6.283 | 6.308 | 0.025 | 0.301 | 0.093 | 0.188 | Tentative | NA | NA |
| metab_10115 | Unannotated feature | neg | 208.9534 | 1.086 | 4.325 | 4.282 | -0.043 | -0.301 | 0.093 | 0.188 | Unannotated | NA | NA |
| metab_411 | Unannotated feature | pos | 152.0682 | 0.861 | 4.510 | 4.295 | -0.214 | -0.301 | 0.093 | 0.188 | Unannotated | NA | NA |
| metab_3315 | 5-Hydroxyindoleacetylglycine | pos | 281.1132 | 2.666 | 5.820 | 5.655 | -0.166 | -0.301 | 0.093 | 0.188 | Tentative | Organic acids and derivatives | Carboxylic acids and derivatives |
| metab_3222 | Unannotated feature | pos | 137.0962 | 3.159 | 3.937 | 3.682 | -0.255 | -0.297 | 0.097 | 0.195 | Unannotated | NA | NA |
| metab_10996 | Unannotated feature | neg | 187.8608 | 0.055 | 6.372 | 6.353 | -0.018 | -0.297 | 0.097 | 0.195 | Unannotated | NA | NA |
| metab_7462 | Unannotated feature | neg | 646.8579 | 7.132 | 4.937 | 4.903 | -0.034 | -0.297 | 0.097 | 0.195 | Unannotated | NA | NA |
| metab_4317 | Unannotated feature | pos | 746.6689 | 0.575 | 4.317 | 4.210 | -0.107 | -0.297 | 0.097 | 0.195 | Unannotated | NA | NA |
| metab_99 | Unannotated feature | pos | 583.8794 | 0.534 | 5.076 | 5.051 | -0.026 | -0.297 | 0.097 | 0.195 | Unannotated | NA | NA |
| metab_35 | Unannotated feature | pos | 147.9982 | 0.792 | 4.416 | 4.490 | 0.074 | 0.297 | 0.097 | 0.195 | Unannotated | NA | NA |
| metab_2577 | Unannotated feature | pos | 498.9012 | 0.551 | 6.508 | 6.471 | -0.037 | -0.297 | 0.097 | 0.195 | Unannotated | NA | NA |
| metab_7763 | Unannotated feature | neg | 326.8305 | 7.173 | 4.684 | 4.718 | 0.034 | 0.297 | 0.097 | 0.195 | Unannotated | NA | NA |
| metab_7162 | Unannotated feature | neg | 503.3241 | 5.883 | 4.354 | 4.019 | -0.335 | -0.297 | 0.097 | 0.195 | Unannotated | NA | NA |
| metab_4602 | Unannotated feature | pos | 142.0864 | 0.031 | 4.061 | 4.078 | 0.017 | 0.297 | 0.097 | 0.195 | Unannotated | NA | NA |
| metab_5716 | Unannotated feature | neg | 227.9606 | 1.406 | 4.513 | 4.475 | -0.038 | -0.297 | 0.097 | 0.195 | Unannotated | NA | NA |
| metab_6230 | Unannotated feature | neg | 204.0301 | 3.428 | 3.721 | 3.868 | 0.147 | 0.297 | 0.097 | 0.195 | Unannotated | NA | NA |
| metab_3517 | Unannotated feature | pos | 231.0839 | 2.141 | 4.202 | 4.098 | -0.103 | -0.297 | 0.097 | 0.195 | Unannotated | NA | NA |
| metab_939 | (2S)-3-Hydroxy-1-[(2S)-pyrrolidine-2-carbonyl]pyrrolidine-2-carboxylic acid | pos | 229.1183 | 0.815 | 5.182 | 5.257 | 0.075 | 0.297 | 0.097 | 0.195 | Tentative | NA | NA |
| metab_9532 | p-Benzosemiquinone | neg | 109.0287 | 3.224 | 4.179 | 4.241 | 0.061 | 0.297 | 0.097 | 0.195 | Tentative | NA | NA |
| metab_5442 | Unannotated feature | neg | 342.8151 | 0.019 | 4.595 | 4.547 | -0.048 | -0.297 | 0.097 | 0.195 | Unannotated | NA | NA |
| metab_3566 | Unannotated feature | pos | 98.0684 | 2.065 | 5.162 | 5.179 | 0.017 | 0.297 | 0.097 | 0.195 | Unannotated | NA | NA |
| metab_3583 | Unannotated feature | pos | 333.1444 | 2.050 | 3.658 | 3.260 | -0.397 | -0.297 | 0.097 | 0.195 | Unannotated | NA | NA |
| metab_7631 | Unannotated feature | neg | 143.0344 | 2.119 | 4.510 | 4.554 | 0.044 | 0.297 | 0.097 | 0.195 | Unannotated | NA | NA |
| metab_10287 | Unannotated feature | neg | 684.5428 | 0.590 | 5.116 | 4.998 | -0.118 | -0.297 | 0.097 | 0.195 | Unannotated | NA | NA |
| metab_10966 | Unannotated feature | neg | 192.9587 | 0.509 | 4.374 | 4.414 | 0.040 | 0.297 | 0.097 | 0.195 | Unannotated | NA | NA |
| metab_647 | Peramine | pos | 311.1596 | 2.280 | 3.978 | 4.052 | 0.074 | 0.297 | 0.097 | 0.195 | Tentative | NA | NA |
| metab_11208 | Unannotated feature | neg | 779.4731 | 5.906 | 4.855 | 4.647 | -0.209 | -0.297 | 0.097 | 0.195 | Unannotated | NA | NA |
| metab_7431 | Unannotated feature | neg | 228.8538 | 7.109 | 5.052 | 4.973 | -0.079 | -0.297 | 0.097 | 0.195 | Unannotated | NA | NA |
| metab_10020 | Unannotated feature | neg | 230.0169 | 1.825 | 4.883 | 4.784 | -0.099 | -0.297 | 0.097 | 0.195 | Unannotated | NA | NA |
| metab_5936 | Unannotated feature | neg | 165.0414 | 2.613 | 4.204 | 3.710 | -0.494 | -0.297 | 0.097 | 0.195 | Unannotated | NA | NA |
| metab_8661 | Unannotated feature | neg | 222.0021 | 5.635 | 3.792 | 3.877 | 0.085 | 0.297 | 0.097 | 0.195 | Unannotated | NA | NA |
| metab_8974 | Unannotated feature | neg | 132.9228 | 7.109 | 6.815 | 6.849 | 0.034 | 0.297 | 0.097 | 0.195 | Unannotated | NA | NA |
| metab_9962 | Unannotated feature | neg | 130.0867 | 2.111 | 4.677 | 4.626 | -0.051 | -0.297 | 0.097 | 0.195 | Unannotated | NA | NA |
| metab_5471 | Unannotated feature | neg | 394.8913 | 0.530 | 5.504 | 5.477 | -0.027 | -0.297 | 0.097 | 0.195 | Unannotated | NA | NA |
| metab_5825 | Unannotated feature | neg | 772.8066 | 0.562 | 5.385 | 5.437 | 0.052 | 0.297 | 0.097 | 0.195 | Unannotated | NA | NA |
| metab_9926 | Unannotated feature | neg | 436.8529 | 0.621 | 5.501 | 5.417 | -0.083 | -0.297 | 0.097 | 0.195 | Unannotated | NA | NA |
| metab_1594 | Unannotated feature | pos | 459.2482 | 6.590 | 6.394 | 6.354 | -0.040 | -0.297 | 0.097 | 0.195 | Unannotated | NA | NA |
| metab_1134 | Unannotated feature | pos | 419.2612 | 5.634 | 5.751 | 5.767 | 0.016 | 0.297 | 0.097 | 0.195 | Unannotated | NA | NA |
| metab_4030 | Unannotated feature | pos | 131.1180 | 0.792 | 3.620 | 3.739 | 0.118 | 0.297 | 0.097 | 0.195 | Unannotated | NA | NA |
| metab_2496 | Unannotated feature | pos | 592.4178 | 5.894 | 4.793 | 4.596 | -0.197 | -0.297 | 0.097 | 0.195 | Unannotated | NA | NA |
| metab_10092 | Unannotated feature | neg | 174.0405 | 1.238 | 3.915 | 4.053 | 0.138 | 0.297 | 0.097 | 0.195 | Unannotated | NA | NA |
| metab_1261 | Unannotated feature | pos | 403.2669 | 5.848 | 4.510 | 4.436 | -0.074 | -0.297 | 0.097 | 0.195 | Unannotated | NA | NA |
| metab_6075 | Unannotated feature | neg | 150.0555 | 3.136 | 3.908 | 4.066 | 0.158 | 0.297 | 0.097 | 0.195 | Unannotated | NA | NA |
| metab_5342 | Unannotated feature | neg | 742.7180 | 0.570 | 4.409 | 4.323 | -0.086 | -0.297 | 0.097 | 0.195 | Unannotated | NA | NA |
| metab_9780 | Unannotated feature | neg | 703.5760 | 0.621 | 4.649 | 4.530 | -0.120 | -0.297 | 0.097 | 0.195 | Unannotated | NA | NA |
| metab_2555 | Unannotated feature | pos | 637.9410 | 5.863 | 4.415 | 4.080 | -0.335 | -0.297 | 0.097 | 0.195 | Unannotated | NA | NA |
| metab_11380 | O-Phospho-L-serine | neg | 415.0136 | 3.198 | 4.548 | 4.328 | -0.221 | -0.297 | 0.097 | 0.195 | Tentative | Organic acids and derivatives | Carboxylic acids and derivatives |
| metab_3840 | Unannotated feature | pos | 733.3309 | 3.893 | 6.454 | 6.560 | 0.106 | 0.297 | 0.097 | 0.195 | Unannotated | NA | NA |
| metab_7777 | Unannotated feature | neg | 484.7904 | 7.170 | 4.411 | 4.451 | 0.040 | 0.297 | 0.097 | 0.195 | Unannotated | NA | NA |
| metab_7220 | Unannotated feature | neg | 323.2235 | 5.961 | 4.247 | 4.039 | -0.208 | -0.297 | 0.097 | 0.195 | Unannotated | NA | NA |
| metab_6363 | 4-Methyl-1,4-dihydropyridine-3,5-dicarbaldehyde | neg | 150.0555 | 3.910 | 3.349 | 3.433 | 0.083 | 0.297 | 0.097 | 0.195 | Tentative | Organoheterocyclic compounds | Pyridines and derivatives |
| metab_7786 | (S)-3-Sulfolactate | neg | 204.9569 | 7.170 | 4.511 | 4.500 | -0.011 | -0.297 | 0.097 | 0.195 | Tentative | NA | NA |
| metab_4415 | Unannotated feature | pos | 766.8353 | 0.559 | 4.417 | 4.226 | -0.191 | -0.297 | 0.097 | 0.195 | Unannotated | NA | NA |
| metab_5465 | Unannotated feature | neg | 195.7606 | 0.684 | 4.568 | 4.601 | 0.033 | 0.293 | 0.102 | 0.203 | Unannotated | NA | NA |
| metab_3504 | Prenalterol | pos | 208.1332 | 2.187 | 5.089 | 5.556 | 0.467 | 0.293 | 0.102 | 0.203 | Tentative | NA | NA |
| metab_589 | Unannotated feature | pos | 134.0681 | 2.073 | 4.788 | 4.820 | 0.032 | 0.293 | 0.102 | 0.203 | Unannotated | NA | NA |
| metab_989 | Unannotated feature | pos | 102.0343 | 4.440 | 6.107 | 6.085 | -0.021 | -0.293 | 0.102 | 0.203 | Unannotated | NA | NA |
| metab_7625 | Unannotated feature | neg | 198.9075 | 7.533 | 5.159 | 5.131 | -0.029 | -0.293 | 0.102 | 0.203 | Unannotated | NA | NA |
| metab_5720 | Unannotated feature | neg | 242.0520 | 1.462 | 4.821 | 4.763 | -0.058 | -0.293 | 0.102 | 0.203 | Unannotated | NA | NA |
| metab_7697 | Unannotated feature | neg | 177.9082 | 7.213 | 3.318 | 3.411 | 0.093 | 0.293 | 0.102 | 0.203 | Unannotated | NA | NA |
| metab_5498 | Unannotated feature | neg | 702.8802 | 0.534 | 4.506 | 4.527 | 0.021 | 0.293 | 0.102 | 0.203 | Unannotated | NA | NA |
| metab_5005 | Unannotated feature | pos | 82.0269 | 0.792 | 4.798 | 4.748 | -0.050 | -0.293 | 0.102 | 0.203 | Unannotated | NA | NA |
| metab_1357 | Unannotated feature | pos | 558.9047 | 5.902 | 5.710 | 5.809 | 0.099 | 0.293 | 0.102 | 0.203 | Unannotated | NA | NA |
| metab_5050 | Unannotated feature | pos | 324.1029 | 0.869 | 4.451 | 4.267 | -0.184 | -0.293 | 0.102 | 0.203 | Unannotated | NA | NA |
| metab_1475 | Unannotated feature | pos | 492.3086 | 6.018 | 5.135 | 5.031 | -0.103 | -0.293 | 0.102 | 0.203 | Unannotated | NA | NA |
| metab_2283 | [14]-Gingerol | pos | 439.3419 | 6.118 | 5.815 | 5.771 | -0.044 | -0.293 | 0.102 | 0.203 | Tentative | NA | NA |
| metab_7502 | Unannotated feature | neg | 264.9349 | 7.153 | 4.139 | 4.069 | -0.070 | -0.293 | 0.102 | 0.203 | Unannotated | NA | NA |
| metab_11132 | Unannotated feature | neg | 527.2148 | 5.707 | 5.225 | 5.136 | -0.089 | -0.293 | 0.102 | 0.203 | Unannotated | NA | NA |
| metab_9399 | Unannotated feature | neg | 213.8732 | 3.499 | 5.000 | 4.978 | -0.022 | -0.293 | 0.102 | 0.203 | Unannotated | NA | NA |
| metab_9412 | Unannotated feature | neg | 459.8189 | 0.636 | 5.126 | 4.941 | -0.184 | -0.293 | 0.102 | 0.203 | Unannotated | NA | NA |
| metab_11031 | Unannotated feature | neg | 406.7947 | 0.013 | 4.370 | 4.301 | -0.068 | -0.293 | 0.102 | 0.203 | Unannotated | NA | NA |
| metab_6649 | Unannotated feature | neg | 388.8287 | 0.841 | 4.111 | 3.955 | -0.156 | -0.293 | 0.102 | 0.203 | Unannotated | NA | NA |
| metab_1154 | Unannotated feature | pos | 255.1591 | 5.687 | 4.223 | 4.518 | 0.294 | 0.293 | 0.102 | 0.203 | Unannotated | NA | NA |
| metab_6171 | Unannotated feature | neg | 338.1464 | 3.255 | 5.066 | 4.752 | -0.314 | -0.293 | 0.102 | 0.203 | Unannotated | NA | NA |
| metab_8902 | Unannotated feature | neg | 127.0127 | 5.131 | 2.572 | 2.329 | -0.243 | -0.293 | 0.102 | 0.203 | Unannotated | NA | NA |
| metab_10791 | Unannotated feature | neg | 672.8606 | 0.562 | 5.696 | 5.652 | -0.044 | -0.293 | 0.102 | 0.203 | Unannotated | NA | NA |
| metab_10591 | Unannotated feature | neg | 88.0396 | 0.613 | 3.402 | 3.123 | -0.278 | -0.293 | 0.102 | 0.203 | Unannotated | NA | NA |
| metab_368 | (3S,4R,5R)-1,3,4,5,6-Pentahydroxy-1-morpholin-4-ylhexan-2-one | pos | 266.1234 | 0.815 | 4.989 | 4.819 | -0.170 | -0.293 | 0.102 | 0.203 | Tentative | NA | NA |
| metab_5034 | Unannotated feature | pos | 98.0968 | 0.861 | 4.351 | 4.413 | 0.063 | 0.293 | 0.102 | 0.203 | Unannotated | NA | NA |
| metab_2305 | Unannotated feature | pos | 600.4682 | 6.065 | 5.399 | 5.362 | -0.038 | -0.293 | 0.102 | 0.203 | Unannotated | NA | NA |
| metab_7981 | Unannotated feature | neg | 100.9142 | 7.093 | 4.661 | 4.635 | -0.026 | -0.293 | 0.102 | 0.203 | Unannotated | NA | NA |
| metab_9894 | Unannotated feature | neg | 117.6255 | 2.221 | 2.969 | 2.758 | -0.211 | -0.293 | 0.102 | 0.203 | Unannotated | NA | NA |
| metab_1597 | 17-AAG | pos | 586.3091 | 6.660 | 6.607 | 6.663 | 0.056 | 0.293 | 0.102 | 0.203 | Tentative | NA | NA |
| metab_8503 | Unannotated feature | neg | 360.2400 | 5.723 | 3.739 | 3.470 | -0.269 | -0.293 | 0.102 | 0.203 | Unannotated | NA | NA |
| metab_4893 | Unannotated feature | pos | 151.0966 | 1.949 | 5.525 | 5.390 | -0.135 | -0.293 | 0.102 | 0.203 | Unannotated | NA | NA |
| metab_1709 | Unannotated feature | pos | 634.8760 | 7.293 | 5.731 | 5.696 | -0.034 | -0.293 | 0.102 | 0.203 | Unannotated | NA | NA |
| metab_3197 | Unannotated feature | pos | 245.1860 | 3.275 | 4.541 | 4.419 | -0.122 | -0.293 | 0.102 | 0.203 | Unannotated | NA | NA |
| metab_3951 | Unannotated feature | pos | 136.0620 | 0.854 | 3.641 | 3.786 | 0.145 | 0.293 | 0.102 | 0.203 | Unannotated | NA | NA |
| metab_383 | Unannotated feature | pos | 372.7379 | 0.908 | 4.972 | 4.938 | -0.033 | -0.293 | 0.102 | 0.203 | Unannotated | NA | NA |
| metab_3864 | Unannotated feature | pos | 318.9699 | 0.908 | 5.148 | 5.107 | -0.042 | -0.293 | 0.102 | 0.203 | Unannotated | NA | NA |
| metab_9403 | Unannotated feature | neg | 575.7363 | 0.636 | 5.236 | 5.003 | -0.232 | -0.293 | 0.102 | 0.203 | Unannotated | NA | NA |
| metab_1964 | Unannotated feature | pos | 356.9092 | 7.300 | 4.611 | 4.556 | -0.055 | -0.293 | 0.102 | 0.203 | Unannotated | NA | NA |
| metab_4066 | Unannotated feature | pos | 97.0401 | 0.753 | 3.257 | 3.603 | 0.347 | 0.293 | 0.102 | 0.203 | Unannotated | NA | NA |
| metab_8354 | Unannotated feature | neg | 338.8514 | 0.723 | 4.118 | 3.906 | -0.212 | -0.293 | 0.102 | 0.203 | Unannotated | NA | NA |
| metab_4558 | Unannotated feature | pos | 160.9897 | 0.496 | 5.349 | 5.338 | -0.012 | -0.293 | 0.102 | 0.203 | Unannotated | NA | NA |
| metab_941 | Unannotated feature | pos | 84.1315 | 4.070 | 3.982 | 4.005 | 0.023 | 0.293 | 0.102 | 0.203 | Unannotated | NA | NA |
| metab_9701 | Unannotated feature | neg | 116.0710 | 2.882 | 4.057 | 4.024 | -0.033 | -0.293 | 0.102 | 0.203 | Unannotated | NA | NA |
| metab_10597 | Unannotated feature | neg | 92.9435 | 0.601 | 4.232 | 4.184 | -0.048 | -0.293 | 0.102 | 0.203 | Unannotated | NA | NA |
| metab_3371 | Unannotated feature | pos | 74.0970 | 2.537 | 4.012 | 4.049 | 0.037 | 0.293 | 0.102 | 0.203 | Unannotated | NA | NA |
| metab_4449 | Unannotated feature | pos | 943.7968 | 0.551 | 5.243 | 5.290 | 0.047 | 0.293 | 0.102 | 0.203 | Unannotated | NA | NA |
| metab_5125 | Glycodeoxycholate | pos | 414.3003 | 5.933 | 6.279 | 6.471 | 0.192 | 0.293 | 0.102 | 0.203 | Strong putative | NA | NA |
| metab_2049 | Unannotated feature | pos | 615.7080 | 0.575 | 4.791 | 4.734 | -0.057 | -0.293 | 0.102 | 0.203 | Unannotated | NA | NA |
| metab_10577 | Unannotated feature | neg | 597.5593 | 0.613 | 5.270 | 5.393 | 0.123 | 0.293 | 0.102 | 0.203 | Unannotated | NA | NA |
| metab_478 | Unannotated feature | pos | 132.0973 | 1.809 | 4.067 | 3.767 | -0.300 | -0.288 | 0.107 | 0.210 | Unannotated | NA | NA |
| metab_614 | 4-Hydroxyproline galactoside | pos | 326.1444 | 2.148 | 4.920 | 4.617 | -0.303 | -0.288 | 0.107 | 0.210 | Tentative | NA | NA |
| metab_9493 | Unannotated feature | neg | 171.9102 | 3.260 | 4.401 | 4.310 | -0.091 | -0.288 | 0.107 | 0.210 | Unannotated | NA | NA |
| metab_5561 | Unannotated feature | neg | 333.9387 | 0.530 | 5.569 | 5.536 | -0.033 | -0.288 | 0.107 | 0.210 | Unannotated | NA | NA |
| metab_929 | Unannotated feature | pos | 204.1231 | 0.854 | 6.673 | 6.795 | 0.122 | 0.288 | 0.107 | 0.210 | Unannotated | NA | NA |
| metab_966 | 6-Hydroxyoctanoylcarnitine | pos | 304.2118 | 4.263 | 5.241 | 5.745 | 0.504 | 0.288 | 0.107 | 0.210 | Tentative | Lipids and lipid-like molecules | Fatty Acyls |
| metab_4976 | Unannotated feature | pos | 231.1340 | 0.706 | 4.248 | 4.152 | -0.097 | -0.288 | 0.107 | 0.210 | Unannotated | NA | NA |
| metab_5197 | Unannotated feature | neg | 821.8413 | 0.534 | 4.437 | 4.345 | -0.092 | -0.288 | 0.107 | 0.210 | Unannotated | NA | NA |
| metab_10719 | Unannotated feature | neg | 754.5257 | 0.582 | 5.173 | 5.100 | -0.072 | -0.288 | 0.107 | 0.210 | Unannotated | NA | NA |
| metab_5689 | Unannotated feature | neg | 166.4286 | 1.294 | 5.240 | 5.201 | -0.039 | -0.288 | 0.107 | 0.210 | Unannotated | NA | NA |
| metab_5213 | Unannotated feature | neg | 740.8483 | 0.543 | 5.673 | 5.625 | -0.048 | -0.288 | 0.107 | 0.210 | Unannotated | NA | NA |
| metab_4540 | Unannotated feature | pos | 107.9672 | 0.527 | 4.509 | 4.527 | 0.019 | 0.288 | 0.107 | 0.210 | Unannotated | NA | NA |
| metab_7695 | Unannotated feature | neg | 203.7990 | 7.213 | 4.138 | 4.120 | -0.018 | -0.288 | 0.107 | 0.210 | Unannotated | NA | NA |
| metab_7714 | Unannotated feature | neg | 358.8420 | 7.205 | 4.253 | 4.308 | 0.055 | 0.288 | 0.107 | 0.210 | Unannotated | NA | NA |
| metab_426 | Unannotated feature | pos | 172.9564 | 1.421 | 4.672 | 4.649 | -0.023 | -0.288 | 0.107 | 0.210 | Unannotated | NA | NA |
| metab_5653 | Unannotated feature | neg | 439.6851 | 0.999 | 6.078 | 6.021 | -0.057 | -0.288 | 0.107 | 0.210 | Unannotated | NA | NA |
| metab_7783 | Unannotated feature | neg | 335.8625 | 7.170 | 4.428 | 4.476 | 0.048 | 0.288 | 0.107 | 0.210 | Unannotated | NA | NA |
| metab_10605 | Unannotated feature | neg | 486.6544 | 0.598 | 4.106 | 4.294 | 0.188 | 0.288 | 0.107 | 0.210 | Unannotated | NA | NA |
| metab_11019 | Unannotated feature | neg | 700.6512 | 0.013 | 4.716 | 4.698 | -0.018 | -0.288 | 0.107 | 0.210 | Unannotated | NA | NA |
| metab_6131 | Unannotated feature | neg | 381.0085 | 3.201 | 4.639 | 4.524 | -0.116 | -0.288 | 0.107 | 0.210 | Unannotated | NA | NA |
| metab_1924 | Unannotated feature | pos | 184.0281 | 7.324 | 4.426 | 4.379 | -0.048 | -0.288 | 0.107 | 0.210 | Unannotated | NA | NA |
| metab_6543 | Unannotated feature | neg | 369.8879 | 4.582 | 5.169 | 5.135 | -0.034 | -0.288 | 0.107 | 0.210 | Unannotated | NA | NA |
| metab_10572 | Unannotated feature | neg | 732.5554 | 0.613 | 4.489 | 4.252 | -0.237 | -0.288 | 0.107 | 0.210 | Unannotated | NA | NA |
| metab_4081 | Unannotated feature | pos | 233.0921 | 4.401 | 5.480 | 5.473 | -0.007 | -0.288 | 0.107 | 0.210 | Unannotated | NA | NA |
| metab_11089 | Unannotated feature | neg | 245.0492 | 5.635 | 4.609 | 5.172 | 0.563 | 0.288 | 0.107 | 0.210 | Unannotated | NA | NA |
| metab_4106 | Unannotated feature | pos | 446.6706 | 0.706 | 4.639 | 4.536 | -0.103 | -0.288 | 0.107 | 0.210 | Unannotated | NA | NA |
| metab_2387 | Allocholic acid | pos | 472.3035 | 5.949 | 4.728 | 5.130 | 0.402 | 0.288 | 0.107 | 0.210 | Tentative | NA | NA |
| metab_2407 | Unannotated feature | pos | 616.9467 | 5.933 | 5.803 | 5.878 | 0.075 | 0.288 | 0.107 | 0.210 | Unannotated | NA | NA |
| metab_6009 | Unannotated feature | neg | 90.5271 | 2.914 | 2.673 | 2.541 | -0.132 | -0.288 | 0.107 | 0.210 | Unannotated | NA | NA |
| metab_10038 | Unannotated feature | neg | 297.8837 | 0.621 | 4.396 | 4.053 | -0.342 | -0.288 | 0.107 | 0.210 | Unannotated | NA | NA |
| metab_7308 | Unannotated feature | neg | 628.7975 | 0.570 | 5.612 | 5.566 | -0.045 | -0.288 | 0.107 | 0.210 | Unannotated | NA | NA |
| metab_357 | Unannotated feature | pos | 130.4953 | 0.861 | 3.508 | 3.266 | -0.242 | -0.288 | 0.107 | 0.210 | Unannotated | NA | NA |
| metab_2638 | Unannotated feature | pos | 372.3111 | 5.824 | 5.386 | 5.327 | -0.058 | -0.288 | 0.107 | 0.210 | Unannotated | NA | NA |
| metab_8652 | Unannotated feature | neg | 266.5512 | 5.635 | 4.685 | 4.484 | -0.201 | -0.288 | 0.107 | 0.210 | Unannotated | NA | NA |
| metab_4606 | Unannotated feature | pos | 100.0417 | 0.025 | 4.115 | 4.148 | 0.033 | 0.288 | 0.107 | 0.210 | Unannotated | NA | NA |
| metab_3463 | Canin | pos | 311.1511 | 2.280 | 4.464 | 4.513 | 0.049 | 0.288 | 0.107 | 0.210 | Tentative | NA | NA |
| metab_4774 | Isophorone | pos | 139.1118 | 7.200 | 6.088 | 6.121 | 0.032 | 0.288 | 0.107 | 0.210 | Moderate putative | NA | NA |
| metab_10846 | Unannotated feature | neg | 167.0346 | 5.346 | 4.687 | 4.458 | -0.229 | -0.288 | 0.107 | 0.210 | Unannotated | NA | NA |
| metab_10713 | Unannotated feature | neg | 559.7024 | 0.578 | 4.444 | 4.340 | -0.105 | -0.288 | 0.107 | 0.210 | Unannotated | NA | NA |
| metab_9803 | Unannotated feature | neg | 232.0593 | 2.537 | 4.387 | 4.336 | -0.051 | -0.288 | 0.107 | 0.210 | Unannotated | NA | NA |
| metab_2258 | Unannotated feature | pos | 528.4628 | 6.188 | 5.477 | 5.426 | -0.051 | -0.288 | 0.107 | 0.210 | Unannotated | NA | NA |
| metab_2185 | Unannotated feature | pos | 89.0690 | 6.342 | 3.207 | 3.455 | 0.248 | 0.288 | 0.107 | 0.210 | Unannotated | NA | NA |
| metab_10027 | Unannotated feature | neg | 318.9383 | 0.621 | 5.365 | 5.302 | -0.062 | -0.288 | 0.107 | 0.210 | Unannotated | NA | NA |
| metab_10934 | Unannotated feature | neg | 314.9329 | 0.534 | 4.420 | 4.378 | -0.043 | -0.288 | 0.107 | 0.210 | Unannotated | NA | NA |
| metab_863 | Unannotated feature | pos | 169.9774 | 3.499 | 4.231 | 4.210 | -0.021 | -0.288 | 0.107 | 0.210 | Unannotated | NA | NA |
| metab_6611 | Unannotated feature | neg | 90.9869 | 4.909 | 5.105 | 5.064 | -0.041 | -0.288 | 0.107 | 0.210 | Unannotated | NA | NA |
| metab_10017 | Unannotated feature | neg | 320.9353 | 0.621 | 5.174 | 5.104 | -0.070 | -0.288 | 0.107 | 0.210 | Unannotated | NA | NA |
| metab_6814 | Unannotated feature | neg | 223.0072 | 5.587 | 3.234 | 3.610 | 0.376 | 0.288 | 0.107 | 0.210 | Unannotated | NA | NA |
| metab_10894 | Unannotated feature | neg | 944.8110 | 0.543 | 5.278 | 5.141 | -0.137 | -0.288 | 0.107 | 0.210 | Unannotated | NA | NA |
| metab_5123 | beta-D-Fructose | pos | 198.0973 | 7.990 | 4.738 | 4.719 | -0.019 | -0.288 | 0.107 | 0.210 | Moderate putative | NA | NA |
| metab_3908 | Unannotated feature | pos | 248.1493 | 0.861 | 3.717 | 4.338 | 0.621 | 0.288 | 0.107 | 0.210 | Unannotated | NA | NA |
| metab_6484 | Unannotated feature | neg | 266.9652 | 4.363 | 4.470 | 4.555 | 0.086 | 0.288 | 0.107 | 0.210 | Unannotated | NA | NA |
| metab_5097 | Unannotated feature | pos | 279.1590 | 1.530 | 5.056 | 5.041 | -0.015 | -0.288 | 0.107 | 0.210 | Unannotated | NA | NA |
| metab_10420 | Unannotated feature | neg | 196.5664 | 0.684 | 6.034 | 6.028 | -0.006 | -0.288 | 0.107 | 0.210 | Unannotated | NA | NA |
| metab_6117 | Unannotated feature | neg | 437.0537 | 3.184 | 4.309 | 4.151 | -0.158 | -0.288 | 0.107 | 0.210 | Unannotated | NA | NA |
| metab_8445 | Unannotated feature | neg | 200.1291 | 5.763 | 4.355 | 4.532 | 0.177 | 0.284 | 0.112 | 0.218 | Unannotated | NA | NA |
| metab_4406 | Unannotated feature | pos | 870.7925 | 0.559 | 4.242 | 4.457 | 0.215 | 0.284 | 0.112 | 0.218 | Unannotated | NA | NA |
| metab_1753 | Unannotated feature | pos | 171.0057 | 7.331 | 4.296 | 4.294 | -0.002 | -0.284 | 0.112 | 0.218 | Unannotated | NA | NA |
| metab_7451 | Unannotated feature | neg | 543.1636 | 7.124 | 4.228 | 4.034 | -0.194 | -0.284 | 0.112 | 0.218 | Unannotated | NA | NA |
| metab_5400 | Unannotated feature | neg | 589.5718 | 0.613 | 6.011 | 5.966 | -0.046 | -0.284 | 0.112 | 0.218 | Unannotated | NA | NA |
| metab_7458 | Unannotated feature | neg | 376.9039 | 7.132 | 4.351 | 4.304 | -0.047 | -0.284 | 0.112 | 0.218 | Unannotated | NA | NA |
| metab_7785 | Unannotated feature | neg | 230.9561 | 7.170 | 3.934 | 3.912 | -0.021 | -0.284 | 0.112 | 0.218 | Unannotated | NA | NA |
| metab_2109 | Unannotated feature | pos | 551.3554 | 7.053 | 5.496 | 5.475 | -0.021 | -0.284 | 0.112 | 0.218 | Unannotated | NA | NA |
| metab_10106 | Unannotated feature | neg | 179.9023 | 1.111 | 4.760 | 4.830 | 0.070 | 0.284 | 0.112 | 0.218 | Unannotated | NA | NA |
| metab_7532 | Unannotated feature | neg | 203.0601 | 7.170 | 4.130 | 4.096 | -0.034 | -0.284 | 0.112 | 0.218 | Unannotated | NA | NA |
| metab_9919 | Unannotated feature | neg | 256.1056 | 2.119 | 4.273 | 4.128 | -0.146 | -0.284 | 0.112 | 0.218 | Unannotated | NA | NA |
| metab_10188 | Unannotated feature | neg | 359.8705 | 0.888 | 4.014 | 3.900 | -0.114 | -0.284 | 0.112 | 0.218 | Unannotated | NA | NA |
| metab_7696 | Unannotated feature | neg | 199.8503 | 7.213 | 4.765 | 4.784 | 0.018 | 0.284 | 0.112 | 0.218 | Unannotated | NA | NA |
| metab_9846 | Unannotated feature | neg | 117.6260 | 2.492 | 2.844 | 2.701 | -0.142 | -0.284 | 0.112 | 0.218 | Unannotated | NA | NA |
| metab_2409 | Unannotated feature | pos | 616.4416 | 5.933 | 4.933 | 5.051 | 0.118 | 0.284 | 0.112 | 0.218 | Unannotated | NA | NA |
| metab_2411 | Unannotated feature | pos | 492.3636 | 5.933 | 6.151 | 6.210 | 0.059 | 0.284 | 0.112 | 0.218 | Unannotated | NA | NA |
| metab_7358 | Unannotated feature | neg | 71.0130 | 6.606 | 4.706 | 4.686 | -0.019 | -0.284 | 0.112 | 0.218 | Unannotated | NA | NA |
| metab_5488 | D-Glycerate | neg | 105.0186 | 0.715 | 4.988 | 4.928 | -0.060 | -0.284 | 0.112 | 0.218 | Strong putative | NA | NA |
| metab_10542 | Unannotated feature | neg | 473.7572 | 0.621 | 4.173 | 3.824 | -0.349 | -0.284 | 0.112 | 0.218 | Unannotated | NA | NA |
| metab_6118 | Unannotated feature | neg | 185.0429 | 3.192 | 4.282 | 4.169 | -0.113 | -0.284 | 0.112 | 0.218 | Unannotated | NA | NA |
| metab_10763 | Unannotated feature | neg | 490.8249 | 0.570 | 5.264 | 5.253 | -0.011 | -0.284 | 0.112 | 0.218 | Unannotated | NA | NA |
| metab_6169 | Unannotated feature | neg | 716.8434 | 0.562 | 5.000 | 4.887 | -0.112 | -0.284 | 0.112 | 0.218 | Unannotated | NA | NA |
| metab_1190 | Unannotated feature | pos | 437.7887 | 5.748 | 4.366 | 4.079 | -0.287 | -0.284 | 0.112 | 0.218 | Unannotated | NA | NA |
| metab_1044 | Unannotated feature | pos | 288.2072 | 4.892 | 4.606 | 4.836 | 0.229 | 0.284 | 0.112 | 0.218 | Unannotated | NA | NA |
| metab_5743 | Unannotated feature | neg | 313.9092 | 1.831 | 4.846 | 4.738 | -0.108 | -0.284 | 0.112 | 0.218 | Unannotated | NA | NA |
| metab_964 | Unannotated feature | pos | 213.1282 | 4.248 | 4.953 | 4.915 | -0.038 | -0.284 | 0.112 | 0.218 | Unannotated | NA | NA |
| metab_979 | 3h-Adrenaline | pos | 186.1125 | 4.378 | 4.037 | 3.961 | -0.076 | -0.284 | 0.112 | 0.218 | Tentative | NA | NA |
| metab_982 | Unannotated feature | pos | 226.1548 | 4.386 | 3.645 | 3.728 | 0.083 | 0.284 | 0.112 | 0.218 | Unannotated | NA | NA |
| metab_7503 | Unannotated feature | neg | 396.8919 | 7.153 | 5.500 | 5.467 | -0.033 | -0.284 | 0.112 | 0.218 | Unannotated | NA | NA |
| metab_8058 | Unannotated feature | neg | 295.9589 | 2.906 | 3.808 | 4.079 | 0.271 | 0.284 | 0.112 | 0.218 | Unannotated | NA | NA |
| metab_844 | Unannotated feature | pos | 111.0395 | 7.331 | 5.970 | 5.956 | -0.014 | -0.284 | 0.112 | 0.218 | Unannotated | NA | NA |
| metab_9396 | Methoxyphenylacetic acid | neg | 165.0553 | 3.507 | 3.991 | 4.035 | 0.044 | 0.284 | 0.112 | 0.218 | Moderate putative | NA | NA |
| metab_9156 | Phthalate | neg | 165.0189 | 4.262 | 5.640 | 5.617 | -0.023 | -0.284 | 0.112 | 0.218 | Moderate putative | NA | NA |
| metab_8937 | Formylanthranilate | neg | 164.0349 | 4.977 | 3.660 | 3.774 | 0.114 | 0.284 | 0.112 | 0.218 | Moderate putative | NA | NA |
| metab_6655 | Unannotated feature | neg | 199.0069 | 5.108 | 5.544 | 5.785 | 0.240 | 0.284 | 0.112 | 0.218 | Unannotated | NA | NA |
| metab_8253 | Unannotated feature | neg | 511.2314 | 5.898 | 4.588 | 4.670 | 0.083 | 0.284 | 0.112 | 0.218 | Unannotated | NA | NA |
| metab_8234 | Unannotated feature | neg | 247.9831 | 5.922 | 5.418 | 5.436 | 0.018 | 0.284 | 0.112 | 0.218 | Unannotated | NA | NA |
| metab_306 | Unannotated feature | pos | 217.1547 | 0.776 | 3.720 | 3.838 | 0.118 | 0.284 | 0.112 | 0.218 | Unannotated | NA | NA |
| metab_8322 | Unannotated feature | neg | 265.1450 | 5.842 | 4.075 | 4.176 | 0.101 | 0.284 | 0.112 | 0.218 | Unannotated | NA | NA |
| metab_4193 | Unannotated feature | pos | 261.0112 | 0.629 | 6.187 | 6.144 | -0.043 | -0.284 | 0.112 | 0.218 | Unannotated | NA | NA |
| metab_164 | Unannotated feature | pos | 562.8731 | 0.559 | 4.437 | 4.352 | -0.085 | -0.284 | 0.112 | 0.218 | Unannotated | NA | NA |
| metab_8663 | Unannotated feature | neg | 179.0711 | 5.635 | 4.304 | 3.952 | -0.352 | -0.284 | 0.112 | 0.218 | Moderate putative | NA | NA |
| metab_11309 | Unannotated feature | neg | 195.8551 | 7.197 | 4.960 | 4.940 | -0.020 | -0.284 | 0.112 | 0.218 | Unannotated | NA | NA |
| metab_10164 | Unannotated feature | neg | 222.8682 | 0.912 | 4.324 | 4.222 | -0.102 | -0.284 | 0.112 | 0.218 | Unannotated | NA | NA |
| metab_4458 | Unannotated feature | pos | 812.8313 | 0.551 | 5.532 | 5.478 | -0.054 | -0.284 | 0.112 | 0.218 | Unannotated | NA | NA |
| metab_95 | Unannotated feature | pos | 295.1500 | 0.519 | 3.949 | 3.849 | -0.100 | -0.284 | 0.112 | 0.218 | Unannotated | NA | NA |
| metab_6924 | Unannotated feature | neg | 343.2243 | 5.675 | 4.622 | 4.595 | -0.026 | -0.284 | 0.112 | 0.218 | Unannotated | NA | NA |
| metab_8507 | Unannotated feature | neg | 274.0395 | 5.723 | 3.751 | 4.066 | 0.314 | 0.280 | 0.118 | 0.225 | Unannotated | NA | NA |
| metab_6877 | 2-Acetamidofluorene | neg | 445.1922 | 5.635 | 4.591 | 4.421 | -0.170 | -0.280 | 0.118 | 0.225 | Tentative | NA | NA |
| metab_1584 | Contignasterol | pos | 1017.6867 | 6.443 | 6.895 | 6.840 | -0.055 | -0.280 | 0.118 | 0.225 | Tentative | NA | NA |
| metab_7750 | Unannotated feature | neg | 174.8286 | 7.181 | 4.670 | 4.643 | -0.027 | -0.280 | 0.118 | 0.225 | Unannotated | NA | NA |
| metab_2214 | Unannotated feature | pos | 359.2036 | 6.296 | 5.438 | 5.376 | -0.062 | -0.280 | 0.118 | 0.225 | Unannotated | NA | NA |
| metab_7420 | Unannotated feature | neg | 156.9806 | 7.109 | 6.668 | 6.692 | 0.023 | 0.280 | 0.118 | 0.225 | Unannotated | NA | NA |
| metab_9799 | Unannotated feature | neg | 612.7263 | 0.621 | 5.328 | 5.267 | -0.061 | -0.280 | 0.118 | 0.225 | Unannotated | NA | NA |
| metab_6090 | Unannotated feature | neg | 243.9921 | 3.152 | 4.358 | 4.202 | -0.156 | -0.280 | 0.118 | 0.225 | Unannotated | NA | NA |
| metab_2392 | Unannotated feature | pos | 594.4319 | 5.941 | 6.193 | 6.241 | 0.047 | 0.280 | 0.118 | 0.225 | Unannotated | NA | NA |
| metab_7924 | Unannotated feature | neg | 256.9066 | 7.109 | 5.503 | 5.543 | 0.040 | 0.280 | 0.118 | 0.225 | Unannotated | NA | NA |
| metab_2426 | Unannotated feature | pos | 465.8208 | 5.925 | 4.472 | 4.581 | 0.109 | 0.280 | 0.118 | 0.225 | Unannotated | NA | NA |
| metab_1448 | Unannotated feature | pos | 528.4096 | 5.972 | 6.186 | 6.172 | -0.014 | -0.280 | 0.118 | 0.225 | Unannotated | NA | NA |
| metab_5462 | Unannotated feature | neg | 326.9039 | 0.530 | 5.205 | 5.163 | -0.041 | -0.280 | 0.118 | 0.225 | Unannotated | NA | NA |
| metab_1993 | Unannotated feature | pos | 310.9129 | 7.293 | 4.082 | 4.018 | -0.064 | -0.280 | 0.118 | 0.225 | Unannotated | NA | NA |
| metab_1498 | Sphingosine 1-phosphate | pos | 380.2560 | 6.095 | 5.254 | 5.199 | -0.055 | -0.280 | 0.118 | 0.225 | Strong putative | NA | NA |
| metab_7399 | Unannotated feature | neg | 86.7798 | 7.101 | 4.452 | 4.417 | -0.036 | -0.280 | 0.118 | 0.225 | Unannotated | NA | NA |
| metab_2440 | Unannotated feature | pos | 587.4244 | 5.917 | 6.378 | 6.447 | 0.069 | 0.280 | 0.118 | 0.225 | Unannotated | NA | NA |
| metab_2631 | Unannotated feature | pos | 119.0556 | 0.504 | 3.985 | 4.028 | 0.043 | 0.280 | 0.118 | 0.225 | Unannotated | NA | NA |
| metab_2862 | Unannotated feature | pos | 793.4060 | 5.198 | 5.194 | 5.000 | -0.194 | -0.280 | 0.118 | 0.225 | Unannotated | NA | NA |
| metab_6212 | Unannotated feature | neg | 252.0211 | 3.364 | 3.778 | 3.676 | -0.102 | -0.280 | 0.118 | 0.225 | Unannotated | NA | NA |
| metab_9541 | Unannotated feature | neg | 93.0337 | 3.217 | 5.445 | 5.715 | 0.270 | 0.280 | 0.118 | 0.225 | Unannotated | NA | NA |
| metab_9555 | Unannotated feature | neg | 99.9250 | 7.109 | 7.001 | 7.018 | 0.017 | 0.280 | 0.118 | 0.225 | Unannotated | NA | NA |
| metab_4861 | Trifolirhizin | pos | 469.1106 | 5.703 | 5.596 | 5.622 | 0.026 | 0.280 | 0.118 | 0.225 | Tentative | NA | NA |
| metab_9584 | Unannotated feature | neg | 265.1200 | 3.168 | 3.735 | 3.442 | -0.293 | -0.280 | 0.118 | 0.225 | Unannotated | NA | NA |
| metab_5033 | Unannotated feature | pos | 215.0527 | 0.714 | 5.319 | 5.226 | -0.094 | -0.280 | 0.118 | 0.225 | Unannotated | NA | NA |
| metab_8011 | Unannotated feature | neg | 101.6505 | 6.798 | 2.871 | 2.748 | -0.124 | -0.280 | 0.118 | 0.225 | Unannotated | NA | NA |
| metab_8056 | Unannotated feature | neg | 250.9763 | 6.373 | 5.193 | 5.243 | 0.050 | 0.280 | 0.118 | 0.225 | Unannotated | NA | NA |
| metab_9660 | Unannotated feature | neg | 159.0771 | 3.001 | 4.300 | 4.511 | 0.211 | 0.280 | 0.118 | 0.225 | Unannotated | NA | NA |
| metab_5110 | (S)-1-Pyrroline-5-carboxylate | pos | 268.1292 | 2.065 | 4.559 | 4.629 | 0.070 | 0.280 | 0.118 | 0.225 | Tentative | NA | NA |
| metab_2464 | Unannotated feature | pos | 543.7213 | 5.910 | 4.689 | 4.488 | -0.201 | -0.280 | 0.118 | 0.225 | Unannotated | NA | NA |
| metab_1686 | Unannotated feature | pos | 501.4085 | 7.269 | 5.140 | 5.106 | -0.034 | -0.280 | 0.118 | 0.225 | Unannotated | NA | NA |
| metab_690 | 1,2-Dihydroisoquinoline | pos | 132.0809 | 2.482 | 3.612 | 3.206 | -0.406 | -0.280 | 0.118 | 0.225 | Tentative | NA | NA |
| metab_9055 | Unannotated feature | neg | 371.8848 | 4.582 | 5.228 | 5.203 | -0.026 | -0.280 | 0.118 | 0.225 | Unannotated | NA | NA |
| metab_3640 | Gentianine | pos | 193.0973 | 1.956 | 3.027 | 3.568 | 0.542 | 0.280 | 0.118 | 0.225 | Tentative | NA | NA |
| metab_4728 | Unannotated feature | pos | 660.4892 | 5.933 | 6.219 | 6.197 | -0.022 | -0.280 | 0.118 | 0.225 | Unannotated | NA | NA |
| metab_446 | Unannotated feature | pos | 133.0318 | 1.663 | 4.250 | 4.140 | -0.111 | -0.280 | 0.118 | 0.225 | Unannotated | NA | NA |
| metab_8283 | Unannotated feature | neg | 611.3813 | 5.875 | 4.867 | 4.480 | -0.386 | -0.280 | 0.118 | 0.225 | Unannotated | NA | NA |
| metab_10357 | Unannotated feature | neg | 197.8586 | 0.747 | 4.350 | 4.391 | 0.041 | 0.280 | 0.118 | 0.225 | Unannotated | NA | NA |
| metab_11248 | Unannotated feature | neg | 104.9531 | 0.578 | 6.002 | 5.982 | -0.020 | -0.280 | 0.118 | 0.225 | Unannotated | NA | NA |
| metab_172 | Unannotated feature | pos | 698.8476 | 0.559 | 4.406 | 4.359 | -0.047 | -0.280 | 0.118 | 0.225 | Unannotated | NA | NA |
| metab_5631 | Unannotated feature | neg | 180.0663 | 0.920 | 4.410 | 4.331 | -0.079 | -0.280 | 0.118 | 0.225 | Unannotated | NA | NA |
| metab_6926 | Unannotated feature | neg | 443.1762 | 5.675 | 3.674 | 2.900 | -0.774 | -0.280 | 0.118 | 0.225 | Unannotated | NA | NA |
| metab_8606 | 3-Allylphenol sulfate | neg | 213.0227 | 5.667 | 6.085 | 5.902 | -0.183 | -0.280 | 0.118 | 0.225 | Tentative | NA | NA |
| metab_11357 | Unannotated feature | neg | 258.9164 | 0.019 | 5.256 | 5.270 | 0.014 | 0.280 | 0.118 | 0.225 | Unannotated | NA | NA |
| metab_4164 | Unannotated feature | pos | 117.0662 | 0.652 | 4.592 | 4.465 | -0.127 | -0.280 | 0.118 | 0.225 | Unannotated | NA | NA |
| metab_8653 | Unannotated feature | neg | 736.8305 | 7.148 | 4.850 | 4.905 | 0.055 | 0.280 | 0.118 | 0.225 | Unannotated | NA | NA |
| metab_6859 | Unannotated feature | neg | 404.1372 | 5.627 | 3.903 | 3.843 | -0.060 | -0.280 | 0.118 | 0.225 | Unannotated | NA | NA |
| metab_8603 | Cytochlor | neg | 298.0009 | 5.667 | 4.557 | 4.234 | -0.323 | -0.280 | 0.118 | 0.225 | Tentative | NA | NA |
| metab_3945 | Unannotated feature | pos | 145.0973 | 0.854 | 3.795 | 3.908 | 0.113 | 0.280 | 0.118 | 0.225 | Unannotated | NA | NA |
| metab_3451 | Unannotated feature | pos | 142.0864 | 2.303 | 5.993 | 5.969 | -0.024 | -0.280 | 0.118 | 0.225 | Unannotated | NA | NA |
| metab_11045 | Unannotated feature | neg | 178.9041 | 0.013 | 5.130 | 5.121 | -0.010 | -0.280 | 0.118 | 0.225 | Unannotated | NA | NA |
| metab_4777 | Unannotated feature | pos | 357.2999 | 7.200 | 5.745 | 5.729 | -0.015 | -0.280 | 0.118 | 0.225 | Unannotated | NA | NA |
| metab_6603 | Cyperotundone | neg | 255.1143 | 4.861 | 3.902 | 3.498 | -0.404 | -0.280 | 0.118 | 0.225 | Tentative | NA | NA |
| metab_8235 | Unannotated feature | neg | 243.9139 | 5.922 | 4.884 | 4.922 | 0.038 | 0.280 | 0.118 | 0.225 | Unannotated | NA | NA |
| metab_434 | Unannotated feature | pos | 151.0388 | 1.562 | 4.125 | 4.089 | -0.036 | -0.280 | 0.118 | 0.225 | Unannotated | NA | NA |
| metab_3920 | Unannotated feature | pos | 171.0628 | 0.861 | 3.322 | 3.780 | 0.458 | 0.280 | 0.118 | 0.225 | Unannotated | NA | NA |
| metab_3405 | Unannotated feature | pos | 102.3761 | 2.459 | 4.532 | 4.566 | 0.034 | 0.280 | 0.118 | 0.225 | Unannotated | NA | NA |
| metab_5698 | Unannotated feature | neg | 311.9445 | 1.302 | 4.598 | 4.656 | 0.058 | 0.280 | 0.118 | 0.225 | Unannotated | NA | NA |
| metab_3296 | Unannotated feature | pos | 114.0917 | 2.803 | 5.798 | 5.810 | 0.012 | 0.280 | 0.118 | 0.225 | Unannotated | NA | NA |
| metab_2378 | Unannotated feature | pos | 572.4374 | 5.956 | 6.358 | 6.345 | -0.013 | -0.276 | 0.124 | 0.233 | Unannotated | NA | NA |
| metab_6056 | N-Formylmethionine | neg | 176.0384 | 3.072 | 5.061 | 5.141 | 0.080 | 0.276 | 0.124 | 0.233 | Moderate putative | NA | NA |
| metab_10735 | Unannotated feature | neg | 691.7711 | 0.570 | 4.174 | 4.028 | -0.146 | -0.276 | 0.124 | 0.233 | Unannotated | NA | NA |
| metab_2008 | Unannotated feature | pos | 420.8849 | 7.285 | 5.083 | 5.052 | -0.031 | -0.276 | 0.124 | 0.233 | Unannotated | NA | NA |
| metab_7403 | Unannotated feature | neg | 171.9806 | 7.101 | 5.885 | 5.863 | -0.022 | -0.276 | 0.124 | 0.233 | Unannotated | NA | NA |
| metab_6451 | Succinic anhydride | neg | 99.0080 | 4.267 | 4.752 | 4.507 | -0.246 | -0.276 | 0.124 | 0.233 | Strong putative | NA | NA |
| metab_4864 | 16,17-Dihydro-16alpha,17-dihydroxygibberellin A4 17-glucoside | pos | 546.2546 | 5.695 | 4.808 | 4.608 | -0.200 | -0.276 | 0.124 | 0.233 | Tentative | NA | NA |
| metab_10926 | Unannotated feature | neg | 741.8642 | 0.534 | 5.576 | 5.555 | -0.021 | -0.276 | 0.124 | 0.233 | Unannotated | NA | NA |
| metab_3097 | Unannotated feature | pos | 214.1235 | 3.846 | 4.601 | 4.741 | 0.140 | 0.276 | 0.124 | 0.233 | Unannotated | NA | NA |
| metab_8081 | Unannotated feature | neg | 278.9688 | 2.906 | 4.383 | 4.560 | 0.176 | 0.276 | 0.124 | 0.233 | Unannotated | NA | NA |
| metab_909 | Ac-Tyr-OEt | pos | 188.0707 | 3.838 | 5.208 | 5.057 | -0.151 | -0.276 | 0.124 | 0.233 | Tentative | NA | NA |
| metab_9547 | Unannotated feature | neg | 272.9172 | 3.209 | 4.106 | 4.440 | 0.334 | 0.276 | 0.124 | 0.233 | Unannotated | NA | NA |
| metab_2975 | Unannotated feature | pos | 175.0754 | 4.532 | 3.757 | 4.198 | 0.441 | 0.276 | 0.124 | 0.233 | Unannotated | NA | NA |
| metab_6226 | Unannotated feature | neg | 144.0449 | 3.412 | 3.513 | 3.572 | 0.059 | 0.276 | 0.124 | 0.233 | Unannotated | NA | NA |
| metab_6141 | 2H-1-Benzopyran-2-one, 7-(sulfooxy)- | neg | 240.9790 | 3.217 | 3.496 | 4.032 | 0.536 | 0.276 | 0.124 | 0.233 | Tentative | Phenylpropanoids and polyketides | Coumarins and derivatives |
| metab_10239 | 2-Furoate | neg | 111.0080 | 0.865 | 4.072 | 4.178 | 0.105 | 0.276 | 0.124 | 0.233 | Moderate putative | NA | NA |
| metab_2477 | Unannotated feature | pos | 507.3687 | 5.902 | 6.323 | 6.376 | 0.053 | 0.276 | 0.124 | 0.233 | Unannotated | NA | NA |
| metab_5082 | Unannotated feature | pos | 84.0449 | 0.854 | 5.129 | 5.151 | 0.022 | 0.276 | 0.124 | 0.233 | Unannotated | NA | NA |
| metab_8029 | Unannotated feature | neg | 91.2420 | 6.581 | 5.144 | 5.189 | 0.045 | 0.276 | 0.124 | 0.233 | Unannotated | NA | NA |
| metab_2683 | Unannotated feature | pos | 300.1992 | 5.771 | 4.727 | 4.595 | -0.132 | -0.276 | 0.124 | 0.233 | Unannotated | NA | NA |
| metab_10808 | Unannotated feature | neg | 1002.7803 | 0.554 | 5.428 | 5.523 | 0.095 | 0.276 | 0.124 | 0.233 | Unannotated | NA | NA |
| metab_5132 | 7-Ethoxycoumarin | pos | 209.0809 | 5.717 | 4.787 | 4.761 | -0.027 | -0.276 | 0.124 | 0.233 | Tentative | NA | NA |
| metab_9774 | Unannotated feature | neg | 189.8702 | 2.629 | 4.548 | 4.535 | -0.013 | -0.276 | 0.124 | 0.233 | Unannotated | NA | NA |
| metab_1528 | Unannotated feature | pos | 374.3267 | 6.203 | 4.285 | 4.232 | -0.054 | -0.276 | 0.124 | 0.233 | Unannotated | NA | NA |
| metab_5321 | Unannotated feature | neg | 918.8088 | 0.562 | 4.577 | 4.515 | -0.061 | -0.276 | 0.124 | 0.233 | Unannotated | NA | NA |
| metab_2087 | Unannotated feature | pos | 424.9594 | 7.192 | 4.747 | 4.800 | 0.053 | 0.276 | 0.124 | 0.233 | Unannotated | NA | NA |
| metab_7797 | Unannotated feature | neg | 102.9480 | 7.170 | 5.124 | 5.119 | -0.005 | -0.276 | 0.124 | 0.233 | Unannotated | NA | NA |
| metab_10594 | Unannotated feature | neg | 544.6140 | 0.601 | 3.765 | 3.917 | 0.152 | 0.276 | 0.124 | 0.233 | Unannotated | NA | NA |
| metab_4380 | Unannotated feature | pos | 646.7108 | 5.114 | 5.573 | 5.733 | 0.160 | 0.276 | 0.124 | 0.233 | Unannotated | NA | NA |
| metab_55 | Unannotated feature | pos | 90.5263 | 0.025 | 5.357 | 5.375 | 0.018 | 0.276 | 0.124 | 0.233 | Unannotated | NA | NA |
| metab_48 | Unannotated feature | pos | 179.0849 | 2.019 | 3.930 | 4.411 | 0.481 | 0.276 | 0.124 | 0.233 | Unannotated | NA | NA |
| metab_2167 | Unannotated feature | pos | 485.4311 | 6.420 | 5.487 | 5.404 | -0.083 | -0.276 | 0.124 | 0.233 | Unannotated | NA | NA |
| metab_7387 | Unannotated feature | neg | 176.8169 | 7.078 | 4.222 | 4.145 | -0.077 | -0.276 | 0.124 | 0.233 | Unannotated | NA | NA |
| metab_6574 | Alpha-Hydroxy-1-methyl-1H-indole-3-propanoic acid | neg | 264.0882 | 4.742 | 3.586 | 3.995 | 0.409 | 0.276 | 0.124 | 0.233 | Tentative | Organoheterocyclic compounds | Indoles and derivatives |
| metab_11041 | Unannotated feature | neg | 205.9233 | 0.013 | 5.735 | 5.756 | 0.021 | 0.276 | 0.124 | 0.233 | Unannotated | NA | NA |
| metab_4790 | Unannotated feature | pos | 362.9262 | 7.293 | 6.154 | 6.148 | -0.006 | -0.276 | 0.124 | 0.233 | Unannotated | NA | NA |
| metab_3480 | Unannotated feature | pos | 216.1231 | 2.241 | 3.901 | 3.980 | 0.079 | 0.276 | 0.124 | 0.233 | Unannotated | NA | NA |
| metab_3959 | Unannotated feature | pos | 71.9133 | 0.854 | 4.284 | 4.246 | -0.038 | -0.276 | 0.124 | 0.233 | Unannotated | NA | NA |
| metab_3948 | Acetylcysteine | pos | 146.0271 | 0.854 | 4.948 | 5.100 | 0.152 | 0.276 | 0.124 | 0.233 | Tentative | Organic acids and derivatives | Carboxylic acids and derivatives |
| metab_420 | Unannotated feature | pos | 174.0138 | 1.367 | 4.629 | 4.569 | -0.060 | -0.276 | 0.124 | 0.233 | Unannotated | NA | NA |
| metab_419 | Unannotated feature | pos | 160.0311 | 1.344 | 4.568 | 4.497 | -0.071 | -0.276 | 0.124 | 0.233 | Unannotated | NA | NA |
| metab_10292 | Unannotated feature | neg | 201.0990 | 0.825 | 3.786 | 3.895 | 0.109 | 0.276 | 0.124 | 0.233 | Unannotated | NA | NA |
| metab_9056 | Unannotated feature | neg | 282.0761 | 4.582 | 3.998 | 3.751 | -0.247 | -0.276 | 0.124 | 0.233 | Unannotated | NA | NA |
| metab_4729 | Unannotated feature | pos | 284.9101 | 0.567 | 5.795 | 5.777 | -0.018 | -0.276 | 0.124 | 0.233 | Unannotated | NA | NA |
| metab_693 | Unannotated feature | pos | 276.1805 | 2.490 | 4.196 | 4.500 | 0.303 | 0.276 | 0.124 | 0.233 | Unannotated | NA | NA |
| metab_685 | (+/-)-Ribaline | pos | 317.1495 | 2.467 | 4.589 | 4.507 | -0.082 | -0.276 | 0.124 | 0.233 | Tentative | NA | NA |
| metab_3392 | 4-(2-Aminoethyl)cyclohexa-3,5-diene-1,2-diol | pos | 156.1020 | 2.482 | 3.504 | 3.808 | 0.304 | 0.276 | 0.124 | 0.233 | Tentative | NA | NA |
| metab_3302 | Unannotated feature | pos | 143.0341 | 2.764 | 4.104 | 4.038 | -0.067 | -0.276 | 0.124 | 0.233 | Unannotated | NA | NA |
| metab_7211 | Unannotated feature | neg | 198.8642 | 5.937 | 5.461 | 5.371 | -0.089 | -0.276 | 0.124 | 0.233 | Unannotated | NA | NA |
| metab_6738 | Unannotated feature | neg | 196.5664 | 5.443 | 5.311 | 5.286 | -0.025 | -0.276 | 0.124 | 0.233 | Unannotated | NA | NA |
| metab_225 | Unannotated feature | pos | 482.8304 | 0.621 | 5.043 | 4.981 | -0.062 | -0.276 | 0.124 | 0.233 | Unannotated | NA | NA |
| metab_4592 | Unannotated feature | pos | 167.0704 | 0.364 | 4.875 | 4.888 | 0.013 | 0.276 | 0.124 | 0.233 | Unannotated | NA | NA |
| metab_302 | Unannotated feature | pos | 188.0918 | 0.768 | 3.724 | 3.852 | 0.129 | 0.276 | 0.124 | 0.233 | Unannotated | NA | NA |
| metab_11303 | Unannotated feature | neg | 166.9239 | 7.189 | 4.375 | 4.320 | -0.055 | -0.276 | 0.124 | 0.233 | Unannotated | NA | NA |
| metab_11266 | Unannotated feature | neg | 734.6859 | 0.570 | 5.285 | 5.226 | -0.059 | -0.276 | 0.124 | 0.233 | Unannotated | NA | NA |
| metab_4135 | Unannotated feature | pos | 104.0710 | 0.683 | 5.572 | 5.605 | 0.033 | 0.276 | 0.124 | 0.233 | Unannotated | NA | NA |
| metab_8723 | Unannotated feature | neg | 260.9167 | 7.156 | 5.517 | 5.499 | -0.018 | -0.276 | 0.124 | 0.233 | Unannotated | NA | NA |
| metab_8305 | Unannotated feature | neg | 353.2341 | 5.859 | 4.289 | 4.057 | -0.232 | -0.276 | 0.124 | 0.233 | Unannotated | NA | NA |
| metab_8367 | DG(2:0/0:0/20:4(6E,8Z,11Z,13E)-2OH(5S,15S)) | neg | 497.2768 | 5.811 | 5.327 | 5.093 | -0.235 | -0.276 | 0.124 | 0.233 | Tentative | NA | NA |
| metab_11320 | Unannotated feature | neg | 131.0343 | 7.293 | 5.522 | 5.510 | -0.012 | -0.276 | 0.124 | 0.233 | Unannotated | NA | NA |
| metab_11383 | 9,10-Dihydroxystearate | neg | 337.2392 | 5.977 | 4.892 | 4.731 | -0.162 | -0.272 | 0.129 | 0.241 | Tentative | Lipids and lipid-like molecules | Fatty Acyls |
| metab_5691 | Unannotated feature | neg | 294.8929 | 1.294 | 4.670 | 4.581 | -0.089 | -0.272 | 0.129 | 0.241 | Unannotated | NA | NA |
| metab_4102 | 3-Ureidoisobutyrate | pos | 188.1031 | 0.714 | 4.731 | 4.565 | -0.166 | -0.272 | 0.129 | 0.241 | Tentative | NA | NA |
| metab_4416 | Unannotated feature | pos | 765.8356 | 0.559 | 4.889 | 4.794 | -0.096 | -0.272 | 0.129 | 0.241 | Unannotated | NA | NA |
| metab_4422 | Unannotated feature | pos | 664.8546 | 0.559 | 4.390 | 4.256 | -0.134 | -0.272 | 0.129 | 0.241 | Unannotated | NA | NA |
| metab_348 | L-Proline | pos | 157.0973 | 0.839 | 5.445 | 5.356 | -0.089 | -0.272 | 0.129 | 0.241 | Moderate putative | NA | NA |
| metab_4619 | Unannotated feature | pos | 171.1175 | 6.041 | 4.624 | 4.654 | 0.029 | 0.272 | 0.129 | 0.241 | Unannotated | NA | NA |
| metab_278 | 1-Pyrrolidineethanol | pos | 116.1073 | 0.729 | 4.614 | 4.562 | -0.052 | -0.272 | 0.129 | 0.241 | Tentative | NA | NA |
| metab_7074 | Unannotated feature | neg | 329.8468 | 5.795 | 5.148 | 5.080 | -0.068 | -0.272 | 0.129 | 0.241 | Unannotated | NA | NA |
| metab_3990 | Unannotated feature | pos | 248.1129 | 0.823 | 3.634 | 3.791 | 0.156 | 0.272 | 0.129 | 0.241 | Unannotated | NA | NA |
| metab_3674 | L-Methionine | pos | 191.0850 | 1.941 | 4.356 | 4.140 | -0.216 | -0.272 | 0.129 | 0.241 | Moderate putative | NA | NA |
| metab_8979 | Unannotated feature | neg | 215.0019 | 4.838 | 3.312 | 3.821 | 0.509 | 0.272 | 0.129 | 0.241 | Unannotated | NA | NA |
| metab_3625 | Unannotated feature | pos | 100.1125 | 1.964 | 2.966 | 3.203 | 0.237 | 0.272 | 0.129 | 0.241 | Unannotated | NA | NA |
| metab_9036 | Unannotated feature | neg | 376.0812 | 4.643 | 3.609 | 4.034 | 0.424 | 0.272 | 0.129 | 0.241 | Unannotated | NA | NA |
| metab_3448 | UDP-N-acetyl-D-galactosamine | pos | 306.1195 | 2.303 | 4.433 | 4.575 | 0.142 | 0.272 | 0.129 | 0.241 | Tentative | NA | NA |
| metab_9193 | Unannotated feature | neg | 196.5664 | 4.167 | 5.110 | 5.079 | -0.030 | -0.272 | 0.129 | 0.241 | Unannotated | NA | NA |
| metab_4788 | Unannotated feature | pos | 430.9138 | 7.285 | 6.138 | 6.119 | -0.019 | -0.272 | 0.129 | 0.241 | Unannotated | NA | NA |
| metab_1916 | Unannotated feature | pos | 225.1443 | 7.324 | 4.482 | 4.457 | -0.025 | -0.272 | 0.129 | 0.241 | Unannotated | NA | NA |
| metab_3310 | Unannotated feature | pos | 139.0310 | 2.718 | 3.339 | 3.583 | 0.244 | 0.272 | 0.129 | 0.241 | Unannotated | NA | NA |
| metab_8145 | Unannotated feature | neg | 132.5022 | 6.110 | 4.776 | 4.673 | -0.103 | -0.272 | 0.129 | 0.241 | Unannotated | NA | NA |
| metab_9347 | Unannotated feature | neg | 203.8443 | 3.618 | 6.046 | 6.029 | -0.017 | -0.272 | 0.129 | 0.241 | Unannotated | NA | NA |
| metab_9466 | Unannotated feature | neg | 338.8930 | 3.718 | 4.425 | 5.124 | 0.698 | 0.272 | 0.129 | 0.241 | Unannotated | NA | NA |
| metab_3044 | Unannotated feature | pos | 506.7575 | 4.117 | 4.439 | 4.098 | -0.340 | -0.272 | 0.129 | 0.241 | Unannotated | NA | NA |
| metab_9490 | Unannotated feature | neg | 485.9623 | 3.260 | 4.591 | 4.433 | -0.158 | -0.272 | 0.129 | 0.241 | Unannotated | NA | NA |
| metab_9495 | Unannotated feature | neg | 307.9874 | 3.255 | 4.084 | 3.726 | -0.358 | -0.272 | 0.129 | 0.241 | Unannotated | NA | NA |
| metab_6846 | Unannotated feature | neg | 233.0674 | 5.619 | 4.873 | 5.120 | 0.247 | 0.272 | 0.129 | 0.241 | Unannotated | NA | NA |
| metab_6231 | Unannotated feature | neg | 138.0554 | 3.436 | 4.142 | 4.053 | -0.089 | -0.272 | 0.129 | 0.241 | Unannotated | NA | NA |
| metab_6234 | Unannotated feature | neg | 195.0594 | 3.436 | 4.051 | 4.108 | 0.056 | 0.272 | 0.129 | 0.241 | Unannotated | NA | NA |
| metab_8069 | Unannotated feature | neg | 383.1900 | 6.281 | 3.992 | 3.831 | -0.161 | -0.272 | 0.129 | 0.241 | Unannotated | NA | NA |
| metab_9552 | Unannotated feature | neg | 568.0485 | 3.201 | 4.766 | 4.524 | -0.241 | -0.272 | 0.129 | 0.241 | Unannotated | NA | NA |
| metab_6802 | Unannotated feature | neg | 381.0307 | 0.865 | 5.089 | 5.223 | 0.134 | 0.272 | 0.129 | 0.241 | Unannotated | NA | NA |
| metab_7245 | Unannotated feature | neg | 206.9431 | 6.024 | 3.505 | 3.735 | 0.231 | 0.272 | 0.129 | 0.241 | Unannotated | NA | NA |
| metab_2847 | beta-D-Glucuronoside | pos | 591.3176 | 5.328 | 5.208 | 3.926 | -1.282 | -0.272 | 0.129 | 0.241 | Tentative | NA | NA |
| metab_1941 | Unannotated feature | pos | 102.0342 | 7.324 | 6.320 | 6.305 | -0.015 | -0.272 | 0.129 | 0.241 | Unannotated | NA | NA |
| metab_1698 | Unannotated feature | pos | 379.9163 | 7.285 | 4.565 | 4.614 | 0.049 | 0.272 | 0.129 | 0.241 | Unannotated | NA | NA |
| metab_1707 | Unannotated feature | pos | 231.0350 | 0.504 | 4.643 | 4.614 | -0.030 | -0.272 | 0.129 | 0.241 | Unannotated | NA | NA |
| metab_2800 | (-)-Perillyl alcohol | pos | 135.1169 | 5.603 | 2.958 | 3.230 | 0.272 | 0.272 | 0.129 | 0.241 | Tentative | Lipids and lipid-like molecules | Prenol lipids |
| metab_1074 | 1-Methyladenosine | pos | 304.1002 | 0.737 | 5.485 | 5.436 | -0.049 | -0.272 | 0.129 | 0.241 | Tentative | NA | NA |
| metab_1201 | Unannotated feature | pos | 173.0785 | 1.956 | 5.840 | 5.696 | -0.144 | -0.272 | 0.129 | 0.241 | Unannotated | NA | NA |
| metab_1096 | 14,15-DHET | pos | 361.2328 | 5.390 | 4.627 | 4.572 | -0.055 | -0.272 | 0.129 | 0.241 | Tentative | NA | NA |
| metab_9744 | Unannotated feature | neg | 283.9578 | 2.732 | 3.545 | 3.842 | 0.297 | 0.272 | 0.129 | 0.241 | Moderate putative | NA | NA |
| metab_5142 | meso-2,6-Diaminoheptanedioate | pos | 191.1026 | 1.871 | 2.709 | 3.118 | 0.408 | 0.272 | 0.129 | 0.241 | Tentative | Organic acids and derivatives | Carboxylic acids and derivatives |
| metab_6078 | Unannotated feature | neg | 343.9015 | 0.636 | 5.223 | 5.040 | -0.183 | -0.272 | 0.129 | 0.241 | Unannotated | NA | NA |
| metab_5160 | Unannotated feature | neg | 224.0619 | 5.635 | 6.793 | 6.739 | -0.055 | -0.272 | 0.129 | 0.241 | Unannotated | NA | NA |
| metab_1372 | Unannotated feature | pos | 724.1707 | 5.910 | 4.318 | 4.020 | -0.297 | -0.272 | 0.129 | 0.241 | Unannotated | NA | NA |
| metab_10708 | Unannotated feature | neg | 604.6155 | 0.587 | 5.303 | 5.273 | -0.031 | -0.272 | 0.129 | 0.241 | Unannotated | NA | NA |
| metab_5177 | Unannotated feature | neg | 258.9163 | 0.522 | 4.856 | 4.816 | -0.039 | -0.272 | 0.129 | 0.241 | Unannotated | NA | NA |
| metab_9889 | Unannotated feature | neg | 255.0083 | 2.245 | 4.037 | 3.736 | -0.301 | -0.272 | 0.129 | 0.241 | Unannotated | NA | NA |
| metab_7416 | Unannotated feature | neg | 117.9356 | 7.109 | 5.884 | 5.916 | 0.032 | 0.272 | 0.129 | 0.241 | Unannotated | NA | NA |
| metab_1460 | Unannotated feature | pos | 161.0922 | 2.459 | 4.734 | 4.956 | 0.221 | 0.272 | 0.129 | 0.241 | Unannotated | NA | NA |
| metab_2200 | (1R,3S)-3-(6-Aminopurin-9-yl)cyclopentan-1-ol | pos | 237.1458 | 6.303 | 5.149 | 5.082 | -0.067 | -0.272 | 0.129 | 0.241 | Tentative | NA | NA |
| metab_10021 | Unannotated feature | neg | 384.9877 | 1.817 | 0.907 | 0.259 | -0.648 | -0.272 | 0.129 | 0.241 | Unannotated | NA | NA |
| metab_5292 | Unannotated feature | neg | 112.6553 | 0.562 | 4.795 | 4.776 | -0.019 | -0.272 | 0.129 | 0.241 | Unannotated | NA | NA |
| metab_124 | Unannotated feature | pos | 540.8828 | 0.545 | 4.819 | 4.775 | -0.044 | -0.272 | 0.129 | 0.241 | Unannotated | NA | NA |
| metab_128 | Unannotated feature | pos | 231.0477 | 0.551 | 4.809 | 4.802 | -0.007 | -0.272 | 0.129 | 0.241 | Unannotated | NA | NA |
| metab_5886 | 5-Acetylamino-6-amino-3-methyluracil | neg | 197.0678 | 2.492 | 4.011 | 3.716 | -0.294 | -0.272 | 0.129 | 0.241 | Tentative | NA | NA |
| metab_5760 | Unannotated feature | neg | 387.9856 | 1.900 | 4.592 | 4.536 | -0.056 | -0.272 | 0.129 | 0.241 | Unannotated | NA | NA |
| metab_7528 | Unannotated feature | neg | 136.9830 | 7.170 | 3.623 | 3.570 | -0.053 | -0.272 | 0.129 | 0.241 | Unannotated | NA | NA |
| metab_5464 | Unannotated feature | neg | 152.0018 | 0.684 | 4.810 | 4.714 | -0.097 | -0.272 | 0.129 | 0.241 | Unannotated | NA | NA |
| metab_5507 | Unannotated feature | neg | 338.0631 | 0.747 | 4.685 | 4.466 | -0.219 | -0.272 | 0.129 | 0.241 | Unannotated | NA | NA |
| metab_10231 | Unannotated feature | neg | 706.5969 | 0.582 | 5.272 | 5.246 | -0.026 | -0.272 | 0.129 | 0.241 | Unannotated | NA | NA |
| metab_1774 | Unannotated feature | pos | 97.1016 | 7.378 | 4.528 | 4.512 | -0.016 | -0.272 | 0.129 | 0.241 | Unannotated | NA | NA |
| metab_11371 | Unannotated feature | neg | 90.9869 | 0.440 | 4.642 | 4.671 | 0.029 | 0.267 | 0.136 | 0.249 | Unannotated | NA | NA |
| metab_4374 | Unannotated feature | pos | 631.7500 | 0.567 | 3.938 | 4.070 | 0.131 | 0.267 | 0.136 | 0.249 | Unannotated | NA | NA |
| metab_5737 | Unannotated feature | neg | 278.8416 | 1.739 | 5.362 | 5.323 | -0.039 | -0.267 | 0.136 | 0.249 | Unannotated | NA | NA |
| metab_8417 | Unannotated feature | neg | 411.2400 | 5.779 | 3.965 | 3.782 | -0.183 | -0.267 | 0.136 | 0.249 | Unannotated | NA | NA |
| metab_1620 | Unannotated feature | pos | 209.0809 | 0.380 | 5.603 | 5.615 | 0.012 | 0.267 | 0.136 | 0.249 | Unannotated | NA | NA |
| metab_6736 | Unannotated feature | neg | 185.0565 | 0.865 | 4.829 | 4.721 | -0.108 | -0.267 | 0.136 | 0.249 | Unannotated | NA | NA |
| metab_10545 | Unannotated feature | neg | 438.8499 | 0.621 | 5.072 | 4.997 | -0.075 | -0.267 | 0.136 | 0.249 | Unannotated | NA | NA |
| metab_9539 | Unannotated feature | neg | 192.9594 | 3.217 | 3.933 | 4.077 | 0.144 | 0.267 | 0.136 | 0.249 | Unannotated | NA | NA |
| metab_148 | Unannotated feature | pos | 744.8440 | 0.551 | 5.630 | 5.585 | -0.044 | -0.267 | 0.136 | 0.249 | Unannotated | NA | NA |
| metab_1043 | Unannotated feature | pos | 156.0389 | 4.892 | 4.590 | 4.709 | 0.119 | 0.267 | 0.136 | 0.249 | Unannotated | NA | NA |
| metab_7652 | Unannotated feature | neg | 88.9872 | 7.390 | 5.451 | 5.444 | -0.007 | -0.267 | 0.136 | 0.249 | Unannotated | NA | NA |
| metab_4350 | Unannotated feature | pos | 790.7994 | 0.567 | 4.373 | 4.286 | -0.087 | -0.267 | 0.136 | 0.249 | Unannotated | NA | NA |
| metab_1640 | Unannotated feature | pos | 500.4675 | 7.184 | 5.278 | 5.246 | -0.032 | -0.267 | 0.136 | 0.249 | Unannotated | NA | NA |
| metab_4588 | Unannotated feature | pos | 193.0860 | 0.403 | 4.856 | 4.840 | -0.017 | -0.267 | 0.136 | 0.249 | Unannotated | NA | NA |
| metab_7624 | Unannotated feature | neg | 75.0079 | 7.527 | 5.310 | 5.321 | 0.011 | 0.267 | 0.136 | 0.249 | Unannotated | NA | NA |
| metab_9228 | Unannotated feature | neg | 194.0457 | 4.037 | 3.605 | 3.700 | 0.094 | 0.267 | 0.136 | 0.249 | Unannotated | NA | NA |
| metab_9633 | 1,7-Dimethyluric acid | neg | 195.0523 | 3.104 | 4.801 | 4.275 | -0.527 | -0.267 | 0.136 | 0.249 | Moderate putative | NA | NA |
| metab_7513 | Unannotated feature | neg | 328.9044 | 7.156 | 5.372 | 5.358 | -0.014 | -0.267 | 0.136 | 0.249 | Unannotated | NA | NA |
| metab_10313 | Unannotated feature | neg | 228.8462 | 0.794 | 4.390 | 4.142 | -0.248 | -0.267 | 0.136 | 0.249 | Unannotated | NA | NA |
| metab_5041 | Unannotated feature | pos | 282.1196 | 0.861 | 4.193 | 4.289 | 0.096 | 0.267 | 0.136 | 0.249 | Unannotated | NA | NA |
| metab_8843 | Unannotated feature | neg | 169.8336 | 5.346 | 2.651 | 3.032 | 0.381 | 0.267 | 0.136 | 0.249 | Unannotated | NA | NA |
| metab_10816 | Unannotated feature | neg | 897.8214 | 0.554 | 5.536 | 5.517 | -0.019 | -0.267 | 0.136 | 0.249 | Unannotated | NA | NA |
| metab_1068 | 10,16-Dihydroxyhexadecanoic acid | pos | 333.2017 | 5.153 | 5.090 | 5.121 | 0.031 | 0.267 | 0.136 | 0.249 | Tentative | NA | NA |
| metab_6507 | Unannotated feature | neg | 158.0818 | 4.425 | 5.970 | 5.781 | -0.189 | -0.267 | 0.136 | 0.249 | Unannotated | NA | NA |
| metab_7773 | Unannotated feature | neg | 134.9139 | 7.173 | 3.373 | 3.457 | 0.084 | 0.267 | 0.136 | 0.249 | Unannotated | NA | NA |
| metab_5923 | Unannotated feature | neg | 397.9154 | 2.549 | 4.863 | 4.828 | -0.035 | -0.267 | 0.136 | 0.249 | Unannotated | NA | NA |
| metab_4157 | Homoarginine | pos | 189.1347 | 0.660 | 5.112 | 5.253 | 0.142 | 0.267 | 0.136 | 0.249 | Moderate putative | NA | NA |
| metab_9644 | Unannotated feature | neg | 170.8704 | 3.072 | 4.261 | 4.252 | -0.009 | -0.267 | 0.136 | 0.249 | Unannotated | NA | NA |
| metab_6140 | Unannotated feature | neg | 173.9943 | 3.217 | 5.815 | 6.081 | 0.265 | 0.267 | 0.136 | 0.249 | Unannotated | NA | NA |
| metab_2760 | (1(10)E,4a,5E)-1(10),5-Germacradiene-12-acetoxy-4,11-diol | pos | 314.2325 | 5.687 | 6.622 | 6.773 | 0.151 | 0.267 | 0.136 | 0.249 | Tentative | NA | NA |
| metab_2644 | Polyoxyethylene 40 monostearate | pos | 346.3316 | 5.817 | 5.094 | 5.137 | 0.043 | 0.267 | 0.136 | 0.249 | Tentative | NA | NA |
| metab_337 | Unannotated feature | pos | 306.8630 | 0.823 | 4.017 | 3.628 | -0.388 | -0.267 | 0.136 | 0.249 | Unannotated | NA | NA |
| metab_500 | Unannotated feature | pos | 316.1366 | 1.894 | 4.184 | 4.132 | -0.052 | -0.267 | 0.136 | 0.249 | Unannotated | NA | NA |
| metab_3117 | Lablaboside D | pos | 632.3014 | 3.746 | 5.485 | 5.575 | 0.090 | 0.267 | 0.136 | 0.249 | Tentative | NA | NA |
| metab_3499 | Unannotated feature | pos | 198.1238 | 2.195 | 4.456 | 3.986 | -0.470 | -0.267 | 0.136 | 0.249 | Unannotated | NA | NA |
| metab_7300 | Unannotated feature | neg | 101.2123 | 6.196 | 5.780 | 5.755 | -0.025 | -0.267 | 0.136 | 0.249 | Unannotated | NA | NA |
| metab_11196 | Unannotated feature | neg | 239.1291 | 5.875 | 4.867 | 4.907 | 0.041 | 0.267 | 0.136 | 0.249 | Unannotated | NA | NA |
| metab_2510 | Unannotated feature | pos | 792.5635 | 5.886 | 5.851 | 5.811 | -0.041 | -0.267 | 0.136 | 0.249 | Unannotated | NA | NA |
| metab_6111 | Unannotated feature | neg | 202.0332 | 3.184 | 4.013 | 3.869 | -0.144 | -0.267 | 0.136 | 0.249 | Unannotated | NA | NA |
| metab_4672 | Isopiperitenone | pos | 151.1119 | 5.832 | 3.708 | 3.758 | 0.050 | 0.267 | 0.136 | 0.249 | Tentative | NA | NA |
| metab_11112 | Unannotated feature | neg | 672.3077 | 5.675 | 4.880 | 4.496 | -0.384 | -0.267 | 0.136 | 0.249 | Unannotated | NA | NA |
| metab_4245 | Unannotated feature | pos | 175.1442 | 0.606 | 4.107 | 4.167 | 0.060 | 0.267 | 0.136 | 0.249 | Unannotated | NA | NA |
| metab_4488 | Unannotated feature | pos | 430.9139 | 0.551 | 6.679 | 6.661 | -0.018 | -0.267 | 0.136 | 0.249 | Unannotated | NA | NA |
| metab_3234 | Unannotated feature | pos | 179.0703 | 3.120 | 4.270 | 4.127 | -0.143 | -0.267 | 0.136 | 0.249 | Unannotated | NA | NA |
| metab_3201 | Unannotated feature | pos | 188.1283 | 3.259 | 3.582 | 2.946 | -0.636 | -0.267 | 0.136 | 0.249 | Unannotated | NA | NA |
| metab_8912 | 2(3H)-Furanone | neg | 167.0346 | 5.108 | 4.319 | 4.736 | 0.417 | 0.267 | 0.136 | 0.249 | Tentative | Organoheterocyclic compounds | Dihydrofurans |
| metab_611 | O-Butanoylcarnitine | pos | 232.1543 | 2.141 | 6.218 | 6.382 | 0.164 | 0.267 | 0.136 | 0.249 | Moderate putative | NA | NA |
| metab_9172 | Unannotated feature | neg | 277.8848 | 4.227 | 3.331 | 4.076 | 0.744 | 0.267 | 0.136 | 0.249 | Unannotated | NA | NA |
| metab_51 | Unannotated feature | pos | 94.0655 | 0.012 | 4.496 | 4.521 | 0.025 | 0.267 | 0.136 | 0.249 | Unannotated | NA | NA |
| metab_10498 | Unannotated feature | neg | 215.9026 | 0.636 | 5.379 | 5.346 | -0.033 | -0.267 | 0.136 | 0.249 | Unannotated | NA | NA |
| metab_1983 | Unannotated feature | pos | 608.8767 | 7.293 | 5.083 | 5.059 | -0.025 | -0.267 | 0.136 | 0.249 | Unannotated | NA | NA |
| metab_1957 | Unannotated feature | pos | 95.0443 | 7.316 | 3.710 | 3.669 | -0.041 | -0.267 | 0.136 | 0.249 | Unannotated | NA | NA |
| metab_3660 | Unannotated feature | pos | 153.1275 | 3.476 | 3.555 | 3.277 | -0.278 | -0.267 | 0.136 | 0.249 | Unannotated | NA | NA |
| metab_3657 | Unannotated feature | pos | 98.0605 | 1.956 | 4.687 | 4.739 | 0.052 | 0.267 | 0.136 | 0.249 | Moderate putative | NA | NA |
| metab_7558 | Unannotated feature | neg | 159.8782 | 7.189 | 4.478 | 4.498 | 0.020 | 0.267 | 0.136 | 0.249 | Unannotated | NA | NA |
| metab_7159 | Unannotated feature | neg | 467.0845 | 5.883 | 4.671 | 4.346 | -0.325 | -0.267 | 0.136 | 0.249 | Unannotated | NA | NA |
| metab_4018 | Unannotated feature | pos | 102.9707 | 0.449 | 6.074 | 6.095 | 0.021 | 0.263 | 0.142 | 0.258 | Unannotated | NA | NA |
| metab_11026 | Unannotated feature | neg | 497.7842 | 0.013 | 4.369 | 4.343 | -0.026 | -0.263 | 0.142 | 0.258 | Unannotated | NA | NA |
| metab_1715 | Unannotated feature | pos | 858.2577 | 7.293 | 4.582 | 4.568 | -0.014 | -0.263 | 0.142 | 0.258 | Unannotated | NA | NA |
| metab_7499 | Unannotated feature | neg | 814.4822 | 0.594 | 5.821 | 5.799 | -0.023 | -0.263 | 0.142 | 0.258 | Unannotated | NA | NA |
| metab_7010 | Unannotated feature | neg | 182.9884 | 5.739 | 4.612 | 4.589 | -0.022 | -0.263 | 0.142 | 0.258 | Unannotated | NA | NA |
| metab_7081 | N-[(3a,5b,7a,12a)-3,7-dihydroxy-24-oxo-12-(sulfooxy)cholan-24-yl]-Glycine | neg | 526.2494 | 5.795 | 4.671 | 4.474 | -0.197 | -0.263 | 0.142 | 0.258 | Tentative | NA | NA |
| metab_6188 | Unannotated feature | neg | 155.8650 | 3.308 | 4.177 | 4.123 | -0.054 | -0.263 | 0.142 | 0.258 | Unannotated | NA | NA |
| metab_1791 | Unannotated feature | pos | 171.1493 | 7.703 | 4.201 | 4.234 | 0.033 | 0.263 | 0.142 | 0.258 | Unannotated | NA | NA |
| metab_6050 | Unannotated feature | neg | 266.9688 | 3.057 | 4.653 | 4.607 | -0.046 | -0.263 | 0.142 | 0.258 | Unannotated | NA | NA |
| metab_9411 | 5-Aminopentanoate | neg | 116.0710 | 3.459 | 4.133 | 4.051 | -0.082 | -0.263 | 0.142 | 0.258 | Strong putative | NA | NA |
| metab_7760 | Unannotated feature | neg | 265.0048 | 2.530 | 4.883 | 4.810 | -0.073 | -0.263 | 0.142 | 0.258 | Unannotated | NA | NA |
| metab_2327 | 1-Acyl-sn-glycero-3-phosphocholine | pos | 542.3241 | 6.010 | 6.351 | 6.216 | -0.136 | -0.263 | 0.142 | 0.258 | Tentative | NA | NA |
| metab_10701 | Unannotated feature | neg | 661.6847 | 0.578 | 4.117 | 4.296 | 0.179 | 0.263 | 0.142 | 0.258 | Unannotated | NA | NA |
| metab_5141 | 1-Acyl-sn-glycero-3-phosphocholine | pos | 524.3712 | 6.745 | 7.987 | 7.937 | -0.050 | -0.263 | 0.142 | 0.258 | Tentative | NA | NA |
| metab_11276 | Unannotated feature | neg | 558.8124 | 0.570 | 5.560 | 5.537 | -0.023 | -0.263 | 0.142 | 0.258 | Unannotated | NA | NA |
| metab_7470 | Unannotated feature | neg | 127.0370 | 7.140 | 4.330 | 4.308 | -0.022 | -0.263 | 0.142 | 0.258 | Unannotated | NA | NA |
| metab_1704 | Unannotated feature | pos | 472.9021 | 7.293 | 4.969 | 4.935 | -0.034 | -0.263 | 0.142 | 0.258 | Unannotated | NA | NA |
| metab_7085 | DNOC | neg | 197.0203 | 5.803 | 4.584 | 4.490 | -0.094 | -0.263 | 0.142 | 0.258 | Tentative | NA | NA |
| metab_6084 | Unannotated feature | neg | 229.0444 | 3.144 | 3.758 | 3.478 | -0.280 | -0.263 | 0.142 | 0.258 | Unannotated | NA | NA |
| metab_2521 | Unannotated feature | pos | 478.3480 | 5.886 | 5.848 | 5.952 | 0.104 | 0.263 | 0.142 | 0.258 | Unannotated | NA | NA |
| metab_10796 | Unannotated feature | neg | 621.8547 | 0.562 | 3.985 | 4.149 | 0.164 | 0.263 | 0.142 | 0.258 | Unannotated | NA | NA |
| metab_2645 | Sphinganine | pos | 302.3053 | 5.817 | 6.118 | 6.165 | 0.047 | 0.263 | 0.142 | 0.258 | Moderate putative | NA | NA |
| metab_4437 | Unannotated feature | pos | 227.8948 | 0.559 | 4.978 | 4.998 | 0.020 | 0.263 | 0.142 | 0.258 | Unannotated | NA | NA |
| metab_10524 | Unannotated feature | neg | 806.4059 | 0.621 | 4.614 | 4.798 | 0.184 | 0.263 | 0.142 | 0.258 | Unannotated | NA | NA |
| metab_9063 | 3-(4-Hydroxyphenyl)lactate | neg | 181.0503 | 3.274 | 5.753 | 5.826 | 0.073 | 0.263 | 0.142 | 0.258 | Moderate putative | NA | NA |
| metab_9958 | Unannotated feature | neg | 290.8544 | 7.213 | 5.063 | 5.083 | 0.020 | 0.263 | 0.142 | 0.258 | Unannotated | NA | NA |
| metab_7947 | Unannotated feature | neg | 132.8995 | 7.109 | 4.383 | 4.360 | -0.024 | -0.263 | 0.142 | 0.258 | Unannotated | NA | NA |
| metab_5742 | Unannotated feature | neg | 161.8753 | 1.831 | 4.472 | 4.500 | 0.028 | 0.263 | 0.142 | 0.258 | Unannotated | NA | NA |
| metab_1828 | Unannotated feature | pos | 85.0652 | 7.983 | 4.369 | 4.364 | -0.005 | -0.263 | 0.142 | 0.258 | Unannotated | NA | NA |
| metab_8760 | Unannotated feature | neg | 608.3197 | 7.153 | 5.486 | 5.438 | -0.047 | -0.263 | 0.142 | 0.258 | Unannotated | NA | NA |
| metab_7667 | Unannotated feature | neg | 255.9639 | 7.269 | 4.348 | 4.378 | 0.030 | 0.263 | 0.142 | 0.258 | Unannotated | NA | NA |
| metab_7593 | Unannotated feature | neg | 129.0187 | 7.237 | 5.176 | 5.163 | -0.014 | -0.263 | 0.142 | 0.258 | Unannotated | NA | NA |
| metab_2921 | Salidroside | pos | 342.1560 | 2.179 | 5.332 | 5.199 | -0.132 | -0.263 | 0.142 | 0.258 | Tentative | NA | NA |
| metab_7011 | Unannotated feature | neg | 201.9255 | 5.739 | 3.980 | 3.897 | -0.084 | -0.263 | 0.142 | 0.258 | Unannotated | NA | NA |
| metab_3408 | Unannotated feature | pos | 119.0732 | 2.644 | 6.715 | 6.730 | 0.015 | 0.263 | 0.142 | 0.258 | Unannotated | NA | NA |
| metab_6785 | Unannotated feature | neg | 335.0500 | 0.865 | 6.904 | 6.930 | 0.026 | -0.263 | 0.142 | 0.258 | Unannotated | NA | NA |
| metab_3034 | Unannotated feature | pos | 93.0703 | 4.171 | 3.556 | 3.469 | -0.087 | -0.263 | 0.142 | 0.258 | Unannotated | NA | NA |
| metab_5809 | Unannotated feature | neg | 201.1244 | 2.119 | 4.332 | 4.523 | 0.191 | 0.263 | 0.142 | 0.258 | Unannotated | NA | NA |
| metab_1090 | Unannotated feature | pos | 102.5360 | 5.352 | 4.544 | 4.521 | -0.023 | -0.259 | 0.148 | 0.267 | Unannotated | NA | NA |
| metab_10814 | Unannotated feature | neg | 197.0818 | 5.255 | 4.666 | 4.859 | 0.193 | 0.259 | 0.148 | 0.267 | Unannotated | NA | NA |
| metab_294 | Enterolactone | pos | 299.1278 | 5.053 | 3.295 | 3.908 | 0.613 | 0.259 | 0.148 | 0.267 | Strong putative | NA | NA |
| metab_5719 | Unannotated feature | neg | 166.9239 | 1.438 | 3.856 | 3.996 | 0.140 | 0.259 | 0.148 | 0.267 | Unannotated | NA | NA |
| metab_5004 | Unannotated feature | pos | 212.0642 | 0.792 | 4.466 | 4.684 | 0.218 | 0.259 | 0.148 | 0.267 | Unannotated | NA | NA |
| metab_1078 | Unannotated feature | pos | 257.0686 | 5.229 | 4.477 | 4.633 | 0.156 | 0.259 | 0.148 | 0.267 | Unannotated | NA | NA |
| metab_4657 | Unannotated feature | pos | 198.1853 | 5.801 | 4.967 | 4.980 | 0.013 | 0.259 | 0.148 | 0.267 | Moderate putative | NA | NA |
| metab_4625 | Unannotated feature | pos | 566.8886 | 0.559 | 6.766 | 6.738 | -0.028 | -0.259 | 0.148 | 0.267 | Unannotated | NA | NA |
| metab_3987 | 5-Methyl-2-furaldehyde | pos | 111.0444 | 0.831 | 4.911 | 4.895 | -0.016 | -0.259 | 0.148 | 0.267 | Moderate putative | NA | NA |
| metab_2497 | Unannotated feature | pos | 580.4159 | 5.894 | 6.272 | 6.362 | 0.089 | 0.259 | 0.148 | 0.267 | Unannotated | NA | NA |
| metab_5931 | Unannotated feature | neg | 170.8704 | 2.589 | 4.120 | 4.150 | 0.030 | 0.259 | 0.148 | 0.267 | Unannotated | NA | NA |
| metab_10575 | Unannotated feature | neg | 647.5306 | 0.613 | 5.995 | 5.949 | -0.046 | -0.259 | 0.148 | 0.267 | Unannotated | NA | NA |
| metab_1230 | Arlacel | pos | 446.3475 | 5.809 | 5.423 | 5.378 | -0.045 | -0.259 | 0.148 | 0.267 | Tentative | NA | NA |
| metab_2599 | Unannotated feature | pos | 185.0421 | 0.545 | 6.412 | 6.395 | -0.017 | -0.259 | 0.148 | 0.267 | Unannotated | NA | NA |
| metab_1235 | Unannotated feature | pos | 569.0599 | 5.817 | 4.728 | 4.455 | -0.274 | -0.259 | 0.148 | 0.267 | Unannotated | NA | NA |
| metab_1296 | Unannotated feature | pos | 500.3618 | 5.871 | 5.726 | 5.792 | 0.066 | 0.259 | 0.148 | 0.267 | Unannotated | NA | NA |
| metab_2529 | Unannotated feature | pos | 626.9197 | 5.879 | 4.464 | 4.306 | -0.158 | -0.259 | 0.148 | 0.267 | Unannotated | NA | NA |
| metab_4748 | Glycyl-Serine | pos | 195.0990 | 6.303 | 5.051 | 5.009 | -0.042 | -0.259 | 0.148 | 0.267 | Tentative | NA | NA |
| metab_4439 | Unannotated feature | pos | 159.5169 | 0.559 | 4.239 | 4.291 | 0.052 | 0.259 | 0.148 | 0.267 | Unannotated | NA | NA |
| metab_3005 | Unannotated feature | pos | 99.5316 | 4.324 | 4.923 | 4.944 | 0.021 | 0.259 | 0.148 | 0.267 | Unannotated | NA | NA |
| metab_1626 | Unannotated feature | pos | 116.1437 | 7.084 | 7.286 | 7.307 | 0.021 | 0.259 | 0.148 | 0.267 | Strong putative | NA | NA |
| metab_11335 | Unannotated feature | neg | 206.9727 | 7.170 | 4.742 | 4.724 | -0.017 | -0.259 | 0.148 | 0.267 | Unannotated | NA | NA |
| metab_2965 | Unannotated feature | pos | 102.5360 | 4.578 | 4.324 | 4.310 | -0.014 | -0.259 | 0.148 | 0.267 | Unannotated | NA | NA |
| metab_6990 | Unannotated feature | neg | 171.1387 | 5.723 | 3.930 | 3.840 | -0.090 | -0.259 | 0.148 | 0.267 | Unannotated | NA | NA |
| metab_8547 | Unannotated feature | neg | 219.8919 | 5.699 | 4.146 | 4.136 | -0.010 | -0.259 | 0.148 | 0.267 | Unannotated | NA | NA |
| metab_2897 | Unannotated feature | pos | 98.9846 | 5.015 | 4.060 | 4.073 | 0.013 | 0.259 | 0.148 | 0.267 | Unannotated | NA | NA |
| metab_5010 | Unannotated feature | pos | 109.0288 | 0.815 | 5.083 | 5.071 | -0.012 | -0.259 | 0.148 | 0.267 | Unannotated | NA | NA |
| metab_5014 | Unannotated feature | pos | 102.9707 | 0.823 | 4.605 | 4.564 | -0.040 | -0.259 | 0.148 | 0.267 | Unannotated | NA | NA |
| metab_167 | Unannotated feature | pos | 217.1071 | 3.955 | 5.726 | 5.763 | 0.037 | 0.259 | 0.148 | 0.267 | Unannotated | NA | NA |
| metab_11262 | Unannotated feature | neg | 615.1040 | 4.845 | 6.251 | 6.018 | -0.233 | -0.259 | 0.148 | 0.267 | Unannotated | NA | NA |
| metab_6781 | Unannotated feature | neg | 387.8732 | 5.538 | 4.711 | 4.597 | -0.114 | -0.259 | 0.148 | 0.267 | Unannotated | NA | NA |
| metab_60 | Unannotated feature | pos | 180.9899 | 0.031 | 5.194 | 5.182 | -0.012 | -0.259 | 0.148 | 0.267 | Unannotated | NA | NA |
| metab_5674 | Unannotated feature | neg | 182.9165 | 1.166 | 4.117 | 4.033 | -0.084 | -0.259 | 0.148 | 0.267 | Unannotated | NA | NA |
| metab_4121 | Unannotated feature | pos | 145.0496 | 0.457 | 6.935 | 6.919 | -0.015 | -0.259 | 0.148 | 0.267 | Moderate putative | NA | NA |
| metab_2272 | Unannotated feature | pos | 282.2216 | 6.157 | 4.724 | 4.776 | 0.052 | 0.259 | 0.148 | 0.267 | Unannotated | NA | NA |
| metab_8623 | Unannotated feature | neg | 399.1491 | 5.651 | 3.482 | 4.160 | 0.678 | 0.259 | 0.148 | 0.267 | Unannotated | NA | NA |
| metab_1533 | Solutol HS 15 | pos | 362.3265 | 6.226 | 5.180 | 5.194 | 0.014 | 0.259 | 0.148 | 0.267 | Tentative | NA | NA |
| metab_585 | Levetiracetam | pos | 379.1755 | 2.065 | 4.593 | 4.407 | -0.186 | -0.259 | 0.148 | 0.267 | Tentative | NA | NA |
| metab_2460 | Unannotated feature | pos | 609.4369 | 5.910 | 6.321 | 6.381 | 0.060 | 0.259 | 0.148 | 0.267 | Unannotated | NA | NA |
| metab_477 | Unannotated feature | pos | 131.6854 | 1.809 | 5.412 | 5.380 | -0.032 | -0.259 | 0.148 | 0.267 | Unannotated | NA | NA |
| metab_804 | Unannotated feature | pos | 340.1601 | 3.035 | 4.774 | 4.567 | -0.207 | -0.259 | 0.148 | 0.267 | Moderate putative | NA | NA |
| metab_8090 | Unannotated feature | neg | 261.9786 | 2.906 | 4.804 | 4.993 | 0.189 | 0.259 | 0.148 | 0.267 | Unannotated | NA | NA |
| metab_1337 | Unannotated feature | pos | 529.3820 | 5.894 | 6.210 | 6.271 | 0.061 | 0.259 | 0.148 | 0.267 | Unannotated | NA | NA |
| metab_9477 | Unannotated feature | neg | 340.8900 | 3.718 | 4.504 | 5.019 | 0.515 | 0.259 | 0.148 | 0.267 | Unannotated | NA | NA |
| metab_11314 | Unannotated feature | neg | 256.9399 | 7.229 | 4.700 | 4.735 | 0.034 | 0.259 | 0.148 | 0.267 | Unannotated | NA | NA |
| metab_8685 | Unannotated feature | neg | 92.9282 | 7.170 | 4.131 | 4.087 | -0.044 | -0.259 | 0.148 | 0.267 | Unannotated | NA | NA |
| metab_8106 | Unannotated feature | neg | 100.9514 | 6.188 | 5.033 | 5.090 | 0.057 | 0.259 | 0.148 | 0.267 | Unannotated | NA | NA |
| metab_3171 | Unannotated feature | pos | 216.0601 | 3.453 | 4.664 | 4.691 | 0.027 | 0.259 | 0.148 | 0.267 | Unannotated | NA | NA |
| metab_3142 | Unannotated feature | pos | 146.0601 | 3.623 | 3.612 | 3.893 | 0.282 | 0.259 | 0.148 | 0.267 | Strong putative | NA | NA |
| metab_810 | Unannotated feature | pos | 113.0964 | 3.074 | 3.862 | 4.055 | 0.193 | 0.259 | 0.148 | 0.267 | Unannotated | NA | NA |
| metab_545 | Unannotated feature | pos | 102.0343 | 2.011 | 5.904 | 5.888 | -0.016 | -0.259 | 0.148 | 0.267 | Unannotated | NA | NA |
| metab_7961 | Unannotated feature | neg | 268.9686 | 7.101 | 5.608 | 5.584 | -0.024 | -0.259 | 0.148 | 0.267 | Unannotated | NA | NA |
| metab_6566 | Zerumbone | neg | 255.1143 | 4.679 | 3.690 | 3.492 | -0.198 | -0.259 | 0.148 | 0.267 | Tentative | NA | NA |
| metab_5200 | Unannotated feature | neg | 631.3872 | 0.539 | 5.186 | 5.140 | -0.045 | -0.259 | 0.148 | 0.267 | Unannotated | NA | NA |
| metab_7957 | Unannotated feature | neg | 87.2263 | 7.109 | 4.495 | 4.473 | -0.022 | -0.259 | 0.148 | 0.267 | Unannotated | NA | NA |
| metab_9103 | Unannotated feature | neg | 174.9868 | 3.224 | 5.589 | 5.848 | 0.259 | 0.259 | 0.148 | 0.267 | Unannotated | NA | NA |
| metab_10503 | Unannotated feature | neg | 102.0553 | 0.636 | 4.084 | 3.897 | -0.187 | -0.259 | 0.148 | 0.267 | Unannotated | NA | NA |
| metab_10677 | Unannotated feature | neg | 822.5139 | 0.587 | 5.579 | 5.538 | -0.041 | -0.259 | 0.148 | 0.267 | Unannotated | NA | NA |
| metab_6908 | Indan-1-ol | neg | 133.0652 | 5.667 | 4.766 | 4.516 | -0.250 | -0.259 | 0.148 | 0.267 | Moderate putative | NA | NA |
| metab_5789 | Unannotated feature | neg | 278.8417 | 2.079 | 5.063 | 5.100 | 0.037 | 0.259 | 0.148 | 0.267 | Unannotated | NA | NA |
| metab_4313 | Unannotated feature | pos | 725.7451 | 4.915 | 4.712 | 5.032 | 0.320 | 0.259 | 0.148 | 0.267 | Unannotated | NA | NA |
| metab_1820 | Unannotated feature | pos | 99.0808 | 7.990 | 4.562 | 4.544 | -0.018 | -0.259 | 0.148 | 0.267 | Unannotated | NA | NA |
| metab_1771 | Unannotated feature | pos | 184.0029 | 7.355 | 4.769 | 4.739 | -0.030 | -0.259 | 0.148 | 0.267 | Unannotated | NA | NA |
| metab_1630 | Unannotated feature | pos | 70.0132 | 0.084 | 4.873 | 4.893 | 0.020 | 0.255 | 0.155 | 0.276 | Unannotated | NA | NA |
| metab_10533 | Unannotated feature | neg | 610.7292 | 0.621 | 5.084 | 5.001 | -0.083 | -0.255 | 0.155 | 0.276 | Unannotated | NA | NA |
| metab_10506 | Unannotated feature | neg | 514.6134 | 0.629 | 4.502 | 4.610 | 0.109 | 0.255 | 0.155 | 0.276 | Unannotated | NA | NA |
| metab_1672 | Unannotated feature | pos | 116.4887 | 7.261 | 3.920 | 3.979 | 0.058 | 0.255 | 0.155 | 0.276 | Unannotated | NA | NA |
| metab_7531 | Unannotated feature | neg | 181.0715 | 7.170 | 3.854 | 3.917 | 0.063 | 0.255 | 0.155 | 0.276 | Unannotated | NA | NA |
| metab_5797 | Unannotated feature | neg | 170.0455 | 2.111 | 2.793 | 2.985 | 0.192 | 0.255 | 0.155 | 0.276 | Unannotated | NA | NA |
| metab_11403 | Aryl sulfate | neg | 172.9910 | 3.217 | 6.951 | 7.201 | 0.250 | 0.255 | 0.155 | 0.276 | Tentative | Organic acids and derivatives | Organic sulfuric acids and derivatives |
| metab_11363 | Unannotated feature | neg | 189.8578 | 0.055 | 5.373 | 5.357 | -0.016 | -0.255 | 0.155 | 0.276 | Unannotated | NA | NA |
| metab_9900 | Unannotated feature | neg | 496.8088 | 0.621 | 5.430 | 5.377 | -0.052 | -0.255 | 0.155 | 0.276 | Unannotated | NA | NA |
| metab_7406 | Unannotated feature | neg | 226.9630 | 7.101 | 6.490 | 6.519 | 0.029 | 0.255 | 0.155 | 0.276 | Unannotated | NA | NA |
| metab_11104 | Unannotated feature | neg | 648.6381 | 0.582 | 5.804 | 5.782 | -0.022 | -0.255 | 0.155 | 0.276 | Unannotated | NA | NA |
| metab_3836 | Unannotated feature | pos | 111.5497 | 1.196 | 4.372 | 4.345 | -0.028 | -0.255 | 0.155 | 0.276 | Unannotated | NA | NA |
| metab_7860 | Unannotated feature | neg | 894.8437 | 7.140 | 4.986 | 5.003 | 0.017 | 0.255 | 0.155 | 0.276 | Unannotated | NA | NA |
| metab_6010 | Unannotated feature | neg | 218.9770 | 2.914 | 3.343 | 3.630 | 0.288 | 0.255 | 0.155 | 0.276 | Unannotated | NA | NA |
| metab_11115 | Unannotated feature | neg | 460.7385 | 0.587 | 5.747 | 5.716 | -0.031 | -0.255 | 0.155 | 0.276 | Unannotated | NA | NA |
| metab_8947 | Unannotated feature | neg | 132.8672 | 4.961 | 3.652 | 3.672 | 0.020 | 0.255 | 0.155 | 0.276 | Unannotated | NA | NA |
| metab_7986 | 6-isobutyl-4-hydroxy-2-pyrone | neg | 333.1361 | 7.062 | 4.125 | 4.002 | -0.123 | -0.255 | 0.155 | 0.276 | Tentative | NA | NA |
| metab_4899 | Unannotated feature | pos | 136.9533 | 0.496 | 5.528 | 5.516 | -0.012 | -0.255 | 0.155 | 0.276 | Unannotated | NA | NA |
| metab_7334 | Unannotated feature | neg | 864.5082 | 6.378 | 4.596 | 3.906 | -0.690 | -0.255 | 0.155 | 0.276 | Unannotated | NA | NA |
| metab_122 | Unannotated feature | pos | 381.0233 | 0.545 | 4.419 | 4.362 | -0.057 | -0.255 | 0.155 | 0.276 | Unannotated | NA | NA |
| metab_4142 | Unannotated feature | pos | 238.9671 | 0.667 | 4.234 | 3.986 | -0.248 | -0.255 | 0.155 | 0.276 | Unannotated | NA | NA |
| metab_10834 | Unannotated feature | neg | 735.8393 | 0.554 | 5.212 | 5.252 | 0.041 | 0.255 | 0.155 | 0.276 | Unannotated | NA | NA |
| metab_5382 | Unannotated feature | neg | 644.5539 | 0.598 | 4.764 | 4.865 | 0.101 | 0.255 | 0.155 | 0.276 | Unannotated | NA | NA |
| metab_5883 | Unannotated feature | neg | 764.7736 | 0.570 | 5.007 | 4.854 | -0.153 | -0.255 | 0.155 | 0.276 | Unannotated | NA | NA |
| metab_9749 | Unannotated feature | neg | 115.0393 | 2.724 | 5.429 | 5.201 | -0.228 | -0.255 | 0.155 | 0.276 | Unannotated | NA | NA |
| metab_8134 | Unannotated feature | neg | 100.6545 | 6.125 | 5.859 | 5.837 | -0.022 | -0.255 | 0.155 | 0.276 | Unannotated | NA | NA |
| metab_807 | Unannotated feature | pos | 99.5316 | 3.058 | 5.339 | 5.361 | 0.022 | 0.255 | 0.155 | 0.276 | Unannotated | NA | NA |
| metab_6616 | Unannotated feature | neg | 595.7543 | 4.938 | 5.772 | 5.839 | 0.067 | 0.255 | 0.155 | 0.276 | Unannotated | NA | NA |
| metab_7444 | Unannotated feature | neg | 119.9463 | 7.124 | 5.415 | 5.386 | -0.029 | -0.255 | 0.155 | 0.276 | Unannotated | NA | NA |
| metab_5006 | Unannotated feature | pos | 202.0823 | 0.800 | 4.058 | 4.233 | 0.175 | 0.255 | 0.155 | 0.276 | Unannotated | NA | NA |
| metab_6677 | Unannotated feature | neg | 348.9303 | 0.841 | 4.406 | 4.035 | -0.371 | -0.255 | 0.155 | 0.276 | Unannotated | NA | NA |
| metab_4008 | Unannotated feature | pos | 159.9693 | 0.815 | 3.696 | 3.631 | -0.065 | -0.255 | 0.155 | 0.276 | Unannotated | NA | NA |
| metab_7522 | Unannotated feature | neg | 315.9247 | 2.245 | 4.331 | 4.113 | -0.217 | -0.255 | 0.155 | 0.276 | Unannotated | NA | NA |
| metab_5684 | Unannotated feature | neg | 339.8983 | 1.222 | 4.864 | 4.790 | -0.074 | -0.255 | 0.155 | 0.276 | Unannotated | NA | NA |
| metab_1782 | Unannotated feature | pos | 101.0713 | 7.471 | 5.295 | 5.274 | -0.021 | -0.255 | 0.155 | 0.276 | Unannotated | NA | NA |
| metab_6428 | Unannotated feature | neg | 239.0774 | 4.187 | 4.074 | 4.058 | -0.015 | -0.255 | 0.155 | 0.276 | Unannotated | NA | NA |
| metab_10983 | Unannotated feature | neg | 309.8572 | 0.455 | 4.820 | 4.850 | 0.030 | 0.255 | 0.155 | 0.276 | Unannotated | NA | NA |
| metab_4835 | Isoxazoline | pos | 113.0713 | 7.293 | 5.109 | 5.146 | 0.037 | 0.255 | 0.155 | 0.276 | Tentative | NA | NA |
| metab_1935 | Unannotated feature | pos | 139.5335 | 7.324 | 3.578 | 3.696 | 0.117 | 0.255 | 0.155 | 0.276 | Unannotated | NA | NA |
| metab_7572 | Unannotated feature | neg | 323.8846 | 7.197 | 4.090 | 3.927 | -0.164 | -0.255 | 0.155 | 0.276 | Unannotated | NA | NA |
| metab_10473 | Unannotated feature | neg | 395.0969 | 0.644 | 4.677 | 4.599 | -0.078 | -0.255 | 0.155 | 0.276 | Unannotated | NA | NA |
| metab_7580 | Unannotated feature | neg | 101.9406 | 7.213 | 3.668 | 3.665 | -0.003 | -0.255 | 0.155 | 0.276 | Unannotated | NA | NA |
| metab_1689 | Unannotated feature | pos | 435.3082 | 7.277 | 4.157 | 4.073 | -0.084 | -0.255 | 0.155 | 0.276 | Unannotated | NA | NA |
| metab_4800 | Unannotated feature | pos | 314.9432 | 7.324 | 4.906 | 4.884 | -0.022 | -0.255 | 0.155 | 0.276 | Unannotated | NA | NA |
| metab_1625 | Unannotated feature | pos | 131.1625 | 7.076 | 6.249 | 6.256 | 0.008 | 0.255 | 0.155 | 0.276 | Unannotated | NA | NA |
| metab_5390 | Unannotated feature | neg | 661.5306 | 0.601 | 4.391 | 4.620 | 0.230 | 0.251 | 0.162 | 0.285 | Unannotated | NA | NA |
| metab_9122 | Unannotated feature | neg | 212.1088 | 4.363 | 4.163 | 4.044 | -0.119 | -0.251 | 0.162 | 0.285 | Unannotated | NA | NA |
| metab_6143 | Unannotated feature | neg | 172.3671 | 3.224 | 5.005 | 5.259 | 0.253 | 0.251 | 0.162 | 0.285 | Unannotated | NA | NA |
| metab_8683 | Unannotated feature | neg | 159.0294 | 5.619 | 4.003 | 3.961 | -0.041 | -0.251 | 0.162 | 0.285 | Unannotated | NA | NA |
| metab_2110 | Unannotated feature | pos | 142.1591 | 7.053 | 5.714 | 5.737 | 0.023 | 0.251 | 0.162 | 0.285 | Unannotated | NA | NA |
| metab_8978 | Unannotated feature | neg | 231.9178 | 4.838 | 4.298 | 4.272 | -0.026 | -0.251 | 0.162 | 0.285 | Unannotated | NA | NA |
| metab_487 | Unannotated feature | pos | 185.0421 | 1.855 | 3.240 | 3.311 | 0.070 | 0.251 | 0.162 | 0.285 | Unannotated | NA | NA |
| metab_633 | Unannotated feature | pos | 97.0288 | 2.241 | 4.889 | 4.895 | 0.006 | 0.251 | 0.162 | 0.285 | Unannotated | NA | NA |
| metab_4876 | Gibberellin A4 | pos | 333.1703 | 2.073 | 5.629 | 5.719 | 0.090 | 0.251 | 0.162 | 0.285 | Moderate putative | NA | NA |
| metab_8144 | 6-(2-Hydroxyethoxy)-6-oxohexanoic acid | neg | 379.1589 | 6.110 | 5.529 | 5.233 | -0.296 | -0.251 | 0.162 | 0.285 | Tentative | NA | NA |
| metab_6361 | Unannotated feature | neg | 260.9254 | 0.723 | 4.274 | 4.084 | -0.190 | -0.251 | 0.162 | 0.285 | Unannotated | NA | NA |
| metab_1221 | Unannotated feature | pos | 212.2374 | 5.793 | 3.867 | 3.600 | -0.267 | -0.251 | 0.162 | 0.285 | Unannotated | NA | NA |
| metab_1122 | Indole-3-Carbinol | pos | 130.0653 | 5.619 | 4.062 | 4.387 | 0.326 | 0.251 | 0.162 | 0.285 | Moderate putative | NA | NA |
| metab_2740 | Unannotated feature | pos | 161.0791 | 1.956 | 4.488 | 4.399 | -0.090 | -0.251 | 0.162 | 0.285 | Unannotated | NA | NA |
| metab_5413 | Unannotated feature | neg | 175.9684 | 0.509 | 4.832 | 4.893 | 0.060 | 0.251 | 0.162 | 0.285 | Unannotated | NA | NA |
| metab_9969 | Unannotated feature | neg | 369.8445 | 2.087 | 5.095 | 5.134 | 0.039 | 0.251 | 0.162 | 0.285 | Unannotated | NA | NA |
| metab_2439 | Unannotated feature | pos | 830.8071 | 0.559 | 4.795 | 4.891 | 0.096 | 0.251 | 0.162 | 0.285 | Unannotated | NA | NA |
| metab_6019 | Unannotated feature | neg | 214.0721 | 2.930 | 4.635 | 4.566 | -0.069 | -0.251 | 0.162 | 0.285 | Unannotated | NA | NA |
| metab_3835 | Unannotated feature | pos | 176.0295 | 1.204 | 4.209 | 4.197 | -0.012 | 0.251 | 0.162 | 0.285 | Unannotated | NA | NA |
| metab_1191 | Unannotated feature | pos | 282.1196 | 2.050 | 5.054 | 5.115 | 0.061 | 0.251 | 0.162 | 0.285 | Unannotated | NA | NA |
| metab_7719 | Unannotated feature | neg | 355.8502 | 7.197 | 4.137 | 4.152 | 0.015 | 0.251 | 0.162 | 0.285 | Unannotated | NA | NA |
| metab_11020 | Unannotated feature | neg | 118.9430 | 0.016 | 4.324 | 4.338 | 0.014 | 0.251 | 0.162 | 0.285 | Unannotated | NA | NA |
| metab_4017 | Unannotated feature | pos | 144.5702 | 0.807 | 3.012 | 2.991 | -0.021 | 0.251 | 0.162 | 0.285 | Unannotated | NA | NA |
| metab_2639 | Unannotated feature | pos | 102.1250 | 5.824 | 3.704 | 3.736 | 0.031 | 0.251 | 0.162 | 0.285 | Unannotated | NA | NA |
| metab_7280 | Unannotated feature | neg | 346.9408 | 1.943 | 5.350 | 5.311 | -0.039 | -0.251 | 0.162 | 0.285 | Unannotated | NA | NA |
| metab_1898 | Unannotated feature | pos | 185.0234 | 7.331 | 5.532 | 5.523 | -0.009 | -0.251 | 0.162 | 0.285 | Unannotated | NA | NA |
| metab_293 | Vigabatrin | pos | 94.0656 | 0.753 | 3.313 | 3.440 | 0.127 | 0.251 | 0.162 | 0.285 | Tentative | Organic acids and derivatives | Carboxylic acids and derivatives |
| metab_11003 | Unannotated feature | neg | 128.8774 | 0.023 | 3.977 | 3.995 | 0.018 | 0.251 | 0.162 | 0.285 | Unannotated | NA | NA |
| metab_4833 | Unannotated feature | pos | 140.0683 | 7.293 | 4.373 | 4.397 | 0.024 | 0.251 | 0.162 | 0.285 | Unannotated | NA | NA |
| metab_4583 | Unannotated feature | pos | 143.0340 | 0.434 | 5.433 | 5.425 | -0.008 | -0.251 | 0.162 | 0.285 | Unannotated | NA | NA |
| metab_3280 | Unannotated feature | pos | 81.5211 | 2.896 | 6.114 | 6.096 | -0.017 | -0.251 | 0.162 | 0.285 | Unannotated | NA | NA |
| metab_6937 | Unannotated feature | neg | 280.8272 | 0.912 | 4.984 | 4.988 | 0.004 | -0.251 | 0.162 | 0.285 | Unannotated | NA | NA |
| metab_11299 | Unannotated feature | neg | 128.8774 | 7.181 | 4.793 | 4.786 | -0.008 | -0.251 | 0.162 | 0.285 | Unannotated | NA | NA |
| metab_6286 | Unannotated feature | neg | 338.1464 | 3.594 | 5.175 | 4.838 | -0.337 | -0.251 | 0.162 | 0.285 | Unannotated | NA | NA |
| metab_10797 | Unannotated feature | neg | 618.8780 | 0.562 | 4.122 | 4.100 | -0.022 | -0.251 | 0.162 | 0.285 | Unannotated | NA | NA |
| metab_2003 | Unannotated feature | pos | 760.8216 | 7.285 | 4.725 | 4.684 | -0.041 | -0.251 | 0.162 | 0.285 | Unannotated | NA | NA |
| metab_1424 | Phosphatidate | pos | 540.3662 | 5.949 | 5.287 | 5.376 | 0.089 | 0.251 | 0.162 | 0.285 | Tentative | NA | NA |
| metab_11346 | Unannotated feature | neg | 408.7894 | 0.013 | 5.380 | 5.364 | -0.016 | -0.251 | 0.162 | 0.285 | Unannotated | NA | NA |
| metab_1297 | Unannotated feature | pos | 195.1227 | 2.118 | 6.351 | 6.211 | -0.141 | -0.251 | 0.162 | 0.285 | Unannotated | NA | NA |
| metab_7855 | Unannotated feature | neg | 141.8943 | 7.148 | 3.328 | 3.428 | 0.101 | 0.251 | 0.162 | 0.285 | Unannotated | NA | NA |
| metab_6833 | Unannotated feature | neg | 90.2904 | 5.611 | 2.519 | 2.322 | -0.197 | -0.251 | 0.162 | 0.285 | Unannotated | NA | NA |
| metab_7472 | Unannotated feature | neg | 189.9209 | 7.140 | 4.033 | 3.974 | -0.059 | -0.251 | 0.162 | 0.285 | Unannotated | NA | NA |
| metab_9611 | Unannotated feature | neg | 221.8425 | 3.144 | 3.284 | 3.426 | 0.142 | 0.251 | 0.162 | 0.285 | Unannotated | NA | NA |
| metab_1645 | Unannotated feature | pos | 165.0884 | 7.200 | 5.131 | 5.153 | 0.022 | 0.251 | 0.162 | 0.285 | Unannotated | NA | NA |
| metab_4054 | Unannotated feature | pos | 130.0694 | 0.768 | 3.231 | 3.364 | 0.134 | 0.251 | 0.162 | 0.285 | Unannotated | NA | NA |
| metab_11364 | Unannotated feature | neg | 215.7488 | 0.063 | 5.088 | 5.073 | -0.015 | -0.251 | 0.162 | 0.285 | Unannotated | NA | NA |
| metab_5591 | Unannotated feature | neg | 737.0538 | 0.865 | 5.461 | 5.474 | 0.013 | -0.251 | 0.162 | 0.285 | Unannotated | NA | NA |
| metab_9534 | Unannotated feature | neg | 299.9555 | 3.217 | 4.982 | 4.914 | -0.068 | -0.251 | 0.162 | 0.285 | Unannotated | NA | NA |
| metab_2861 | Unannotated feature | pos | 328.1389 | 2.118 | 5.825 | 5.848 | 0.023 | 0.251 | 0.162 | 0.285 | Unannotated | NA | NA |
| metab_4993 | Unannotated feature | pos | 274.1873 | 0.760 | 4.825 | 4.940 | 0.114 | 0.251 | 0.162 | 0.285 | Moderate putative | NA | NA |
| metab_1055 | Triethyl Phosphate | pos | 183.0782 | 5.015 | 4.906 | 4.920 | 0.014 | 0.251 | 0.162 | 0.285 | Moderate putative | NA | NA |
| metab_2351 | Unannotated feature | pos | 559.8363 | 0.567 | 4.141 | 4.331 | 0.190 | 0.251 | 0.162 | 0.285 | Unannotated | NA | NA |
| metab_11331 | Unannotated feature | neg | 227.8945 | 7.986 | 5.877 | 5.870 | -0.007 | -0.251 | 0.162 | 0.285 | Unannotated | NA | NA |
| metab_3811 | Unannotated feature | pos | 146.0419 | 1.375 | 3.610 | 3.495 | -0.115 | -0.251 | 0.162 | 0.285 | Unannotated | NA | NA |
| metab_7618 | Unannotated feature | neg | 123.9457 | 7.339 | 5.589 | 5.607 | 0.018 | 0.251 | 0.162 | 0.285 | Unannotated | NA | NA |
| metab_7942 | Unannotated feature | neg | 157.8717 | 7.109 | 5.203 | 5.219 | 0.015 | 0.251 | 0.162 | 0.285 | Unannotated | NA | NA |
| metab_2183 | Rishitin | pos | 508.3399 | 6.358 | 5.612 | 5.524 | -0.088 | -0.251 | 0.162 | 0.285 | Tentative | NA | NA |
| metab_6036 | L-Valine | neg | 116.0710 | 3.001 | 4.596 | 4.723 | 0.127 | 0.251 | 0.162 | 0.285 | Strong putative | NA | NA |
| metab_4679 | Unannotated feature | pos | 491.8479 | 5.871 | 5.054 | 4.962 | -0.092 | -0.246 | 0.169 | 0.294 | Unannotated | NA | NA |
| metab_7817 | Unannotated feature | neg | 610.8106 | 7.156 | 4.289 | 4.218 | -0.071 | -0.246 | 0.169 | 0.294 | Unannotated | NA | NA |
| metab_10155 | Unannotated feature | neg | 448.6689 | 0.912 | 4.352 | 4.312 | -0.040 | -0.246 | 0.169 | 0.294 | Unannotated | NA | NA |
| metab_5406 | Unannotated feature | neg | 127.0506 | 0.621 | 4.427 | 4.483 | 0.055 | 0.246 | 0.169 | 0.294 | Unannotated | NA | NA |
| metab_7461 | Unannotated feature | neg | 510.8825 | 7.132 | 4.993 | 4.974 | -0.020 | -0.246 | 0.169 | 0.294 | Unannotated | NA | NA |
| metab_6082 | Unannotated feature | neg | 144.0660 | 3.144 | 5.900 | 5.766 | -0.134 | -0.246 | 0.169 | 0.294 | Unannotated | NA | NA |
| metab_4905 | Unannotated feature | pos | 113.0600 | 0.449 | 5.071 | 5.081 | 0.010 | 0.246 | 0.169 | 0.294 | Unannotated | NA | NA |
| metab_3690 | Unannotated feature | pos | 131.0705 | 1.917 | 3.537 | 3.598 | 0.062 | 0.246 | 0.169 | 0.294 | Unannotated | NA | NA |
| metab_2160 | Vinylacetylene | pos | 105.0703 | 6.435 | 4.191 | 4.235 | 0.045 | 0.246 | 0.169 | 0.294 | Tentative | NA | NA |
| metab_1962 | Unannotated feature | pos | 374.8820 | 7.300 | 4.800 | 4.828 | 0.029 | 0.246 | 0.169 | 0.294 | Unannotated | NA | NA |
| metab_1961 | Unannotated feature | pos | 502.2928 | 7.300 | 4.194 | 4.392 | 0.198 | 0.246 | 0.169 | 0.294 | Unannotated | NA | NA |
| metab_10277 | Unannotated feature | neg | 209.0455 | 4.582 | 6.205 | 6.188 | -0.017 | -0.246 | 0.169 | 0.294 | Unannotated | NA | NA |
| metab_11368 | Unannotated feature | neg | 75.0079 | 0.352 | 5.160 | 5.180 | 0.019 | 0.246 | 0.169 | 0.294 | Unannotated | NA | NA |
| metab_4062 | Unannotated feature | pos | 74.0970 | 0.760 | 3.716 | 3.697 | -0.019 | -0.246 | 0.169 | 0.294 | Unannotated | NA | NA |
| metab_7293 | 2-Oxoglutarate | neg | 191.0194 | 0.865 | 5.781 | 5.850 | 0.069 | 0.246 | 0.169 | 0.294 | Tentative | NA | NA |
| metab_9775 | Unannotated feature | neg | 226.9093 | 2.621 | 5.353 | 5.377 | 0.024 | 0.246 | 0.169 | 0.294 | Unannotated | NA | NA |
| metab_4607 | Unannotated feature | pos | 97.9918 | 0.025 | 5.022 | 5.039 | 0.017 | 0.246 | 0.169 | 0.294 | Unannotated | NA | NA |
| metab_3390 | L-Thyronine | pos | 315.1338 | 2.490 | 5.034 | 4.975 | -0.059 | -0.246 | 0.169 | 0.294 | Tentative | NA | NA |
| metab_499 | Unannotated feature | pos | 231.0840 | 1.894 | 4.502 | 4.374 | -0.128 | -0.246 | 0.169 | 0.294 | Unannotated | NA | NA |
| metab_2442 | Unannotated feature | pos | 505.8637 | 5.917 | 5.184 | 5.130 | -0.054 | -0.246 | 0.169 | 0.294 | Unannotated | NA | NA |
| metab_3306 | Unannotated feature | pos | 156.0389 | 2.644 | 7.539 | 7.552 | 0.013 | 0.246 | 0.169 | 0.294 | Unannotated | NA | NA |
| metab_5644 | Unannotated feature | neg | 159.8447 | 0.960 | 4.635 | 4.674 | 0.039 | 0.246 | 0.169 | 0.294 | Unannotated | NA | NA |
| metab_5296 | Unannotated feature | neg | 196.9468 | 0.562 | 4.107 | 4.156 | 0.050 | 0.246 | 0.169 | 0.294 | Unannotated | NA | NA |
| metab_8946 | Unannotated feature | neg | 245.0934 | 4.961 | 4.630 | 4.551 | -0.079 | -0.246 | 0.169 | 0.294 | Moderate putative | NA | NA |
| metab_5647 | Unannotated feature | neg | 338.7148 | 0.960 | 4.503 | 4.458 | -0.046 | -0.246 | 0.169 | 0.294 | Unannotated | NA | NA |
| metab_11390 | Dodecanedioic acid | neg | 229.1447 | 5.819 | 4.844 | 5.077 | 0.233 | 0.246 | 0.169 | 0.294 | Moderate putative | NA | NA |
| metab_5467 | Unannotated feature | neg | 131.0092 | 0.692 | 3.524 | 3.675 | 0.150 | 0.246 | 0.169 | 0.294 | Unannotated | NA | NA |
| metab_9572 | Unannotated feature | neg | 243.9179 | 3.184 | 4.054 | 4.198 | 0.144 | 0.246 | 0.169 | 0.294 | Unannotated | NA | NA |
| metab_5196 | Unannotated feature | neg | 783.8403 | 0.534 | 3.931 | 4.074 | 0.144 | 0.246 | 0.169 | 0.294 | Unannotated | NA | NA |
| metab_4254 | Unannotated feature | pos | 390.7089 | 0.598 | 5.279 | 5.240 | -0.039 | -0.246 | 0.169 | 0.294 | Unannotated | NA | NA |
| metab_10679 | Unannotated feature | neg | 534.7169 | 0.582 | 5.231 | 5.185 | -0.046 | -0.246 | 0.169 | 0.294 | Unannotated | NA | NA |
| metab_7972 | Unannotated feature | neg | 87.0026 | 7.101 | 6.718 | 6.731 | 0.013 | 0.246 | 0.169 | 0.294 | Unannotated | NA | NA |
| metab_9117 | Unannotated feature | neg | 193.8153 | 4.371 | 5.005 | 4.965 | -0.039 | -0.246 | 0.169 | 0.294 | Unannotated | NA | NA |
| metab_6939 | Unannotated feature | neg | 385.1701 | 5.683 | 5.336 | 5.436 | 0.101 | 0.246 | 0.169 | 0.294 | Unannotated | NA | NA |
| metab_7640 | Unannotated feature | neg | 261.8752 | 7.937 | 5.375 | 5.392 | 0.017 | 0.246 | 0.169 | 0.294 | Unannotated | NA | NA |
| metab_9892 | Unannotated feature | neg | 155.0821 | 2.237 | 4.916 | 4.788 | -0.128 | -0.246 | 0.169 | 0.294 | Unannotated | NA | NA |
| metab_5706 | Unannotated feature | neg | 303.9216 | 1.358 | 5.705 | 5.676 | -0.029 | -0.246 | 0.169 | 0.294 | Unannotated | NA | NA |
| metab_5451 | Unannotated feature | neg | 213.9166 | 0.033 | 6.553 | 6.567 | 0.014 | 0.246 | 0.169 | 0.294 | Unannotated | NA | NA |
| metab_2071 | Unannotated feature | pos | 597.7576 | 0.575 | 5.022 | 4.982 | -0.040 | -0.246 | 0.169 | 0.294 | Unannotated | NA | NA |
| metab_8474 | Unannotated feature | neg | 467.1933 | 5.739 | 3.547 | 4.165 | 0.618 | 0.246 | 0.169 | 0.294 | Unannotated | NA | NA |
| metab_1661 | Unannotated feature | pos | 458.3838 | 7.215 | 5.367 | 5.352 | -0.015 | -0.246 | 0.169 | 0.294 | Unannotated | NA | NA |
| metab_5000 | Unannotated feature | pos | 129.0660 | 0.675 | 5.874 | 5.988 | 0.114 | 0.246 | 0.169 | 0.294 | Unannotated | NA | NA |
| metab_1984 | 1-Acyl-sn-glycero-3-phosphocholine | pos | 546.3531 | 7.293 | 6.340 | 6.265 | -0.075 | -0.246 | 0.169 | 0.294 | Strong putative | NA | NA |
| metab_8279 | Unannotated feature | neg | 281.1764 | 5.883 | 4.320 | 4.462 | 0.142 | 0.246 | 0.169 | 0.294 | Unannotated | NA | NA |
| metab_10408 | Unannotated feature | neg | 201.8019 | 0.692 | 6.820 | 6.841 | 0.021 | 0.246 | 0.169 | 0.294 | Unannotated | NA | NA |
| metab_8345 | Unannotated feature | neg | 281.2491 | 5.827 | 4.460 | 4.379 | -0.082 | -0.246 | 0.169 | 0.294 | Unannotated | NA | NA |
| metab_130 | Unannotated feature | pos | 574.8751 | 0.551 | 5.276 | 5.230 | -0.045 | -0.246 | 0.169 | 0.294 | Unannotated | NA | NA |
| metab_636 | Imidazolone A | pos | 360.1902 | 2.241 | 5.588 | 5.575 | -0.013 | -0.246 | 0.169 | 0.294 | Tentative | NA | NA |
| metab_2028 | Unannotated feature | pos | 529.4399 | 7.269 | 4.967 | 4.939 | -0.028 | -0.246 | 0.169 | 0.294 | Unannotated | NA | NA |
| metab_4342 | Unannotated feature | pos | 216.0475 | 0.575 | 4.262 | 4.306 | 0.045 | 0.246 | 0.169 | 0.294 | Unannotated | NA | NA |
| metab_5414 | Unannotated feature | neg | 163.4116 | 0.629 | 4.568 | 4.522 | -0.046 | -0.246 | 0.169 | 0.294 | Unannotated | NA | NA |
| metab_10610 | Unannotated feature | neg | 400.7388 | 0.598 | 4.687 | 4.637 | -0.050 | -0.246 | 0.169 | 0.294 | Unannotated | NA | NA |
| metab_6162 | 5-Hydroxy-N-formylkynurenine | neg | 251.0678 | 3.247 | 3.849 | 3.317 | -0.532 | -0.246 | 0.169 | 0.294 | Tentative | Organic oxygen compounds | Organooxygen compounds |
| metab_5694 | 4-hydroxytriazolam | neg | 357.0321 | 1.294 | 5.960 | 5.864 | -0.096 | -0.246 | 0.169 | 0.294 | Tentative | NA | NA |
| metab_10565 | Unannotated feature | neg | 823.4040 | 0.613 | 5.068 | 4.984 | -0.083 | -0.246 | 0.169 | 0.294 | Unannotated | NA | NA |
| metab_7723 | Unannotated feature | neg | 171.9806 | 7.197 | 4.427 | 4.453 | 0.026 | 0.246 | 0.169 | 0.294 | Unannotated | NA | NA |
| metab_10152 | Unannotated feature | neg | 110.8983 | 0.920 | 4.149 | 4.228 | 0.078 | 0.242 | 0.177 | 0.303 | Unannotated | NA | NA |
| metab_7554 | Unannotated feature | neg | 332.8270 | 7.181 | 4.358 | 4.338 | -0.020 | -0.242 | 0.177 | 0.303 | Unannotated | NA | NA |
| metab_5724 | Unannotated feature | neg | 117.2763 | 1.510 | 5.610 | 5.592 | -0.018 | -0.242 | 0.177 | 0.303 | Unannotated | NA | NA |
| metab_5549 | Unannotated feature | neg | 177.8597 | 0.825 | 3.955 | 4.059 | 0.104 | 0.242 | 0.177 | 0.303 | Unannotated | NA | NA |
| metab_5872 | Unannotated feature | neg | 231.8829 | 2.445 | 4.963 | 4.981 | 0.019 | 0.242 | 0.177 | 0.303 | Unannotated | NA | NA |
| metab_9249 | Unannotated feature | neg | 255.9365 | 3.959 | 4.636 | 4.708 | 0.073 | 0.242 | 0.177 | 0.303 | Unannotated | NA | NA |
| metab_9750 | Unannotated feature | neg | 285.9096 | 2.716 | 5.433 | 5.413 | -0.020 | -0.242 | 0.177 | 0.303 | Unannotated | NA | NA |
| metab_2551 | Unannotated feature | pos | 427.3140 | 5.871 | 5.221 | 5.276 | 0.054 | 0.242 | 0.177 | 0.303 | Unannotated | NA | NA |
| metab_1538 | Unannotated feature | pos | 145.1781 | 6.257 | 3.634 | 3.678 | 0.044 | 0.242 | 0.177 | 0.303 | Unannotated | NA | NA |
| metab_9119 | Unannotated feature | neg | 185.6077 | 4.371 | 3.932 | 4.487 | 0.555 | 0.242 | 0.177 | 0.303 | Unannotated | NA | NA |
| metab_5187 | Unannotated feature | neg | 439.8892 | 0.530 | 4.392 | 4.438 | 0.046 | 0.242 | 0.177 | 0.303 | Unannotated | NA | NA |
| metab_1240 | L-Palmitoylcarnitine | pos | 422.3264 | 5.824 | 5.200 | 5.065 | -0.135 | -0.242 | 0.177 | 0.303 | Tentative | NA | NA |
| metab_6113 | Unannotated feature | neg | 261.9786 | 3.184 | 4.602 | 4.514 | -0.087 | -0.242 | 0.177 | 0.303 | Unannotated | NA | NA |
| metab_3368 | Unannotated feature | pos | 137.0153 | 2.644 | 6.011 | 6.031 | 0.020 | 0.242 | 0.177 | 0.303 | Unannotated | NA | NA |
| metab_3096 | Unannotated feature | pos | 139.0179 | 2.467 | 5.676 | 5.660 | -0.015 | -0.242 | 0.177 | 0.303 | Unannotated | NA | NA |
| metab_8095 | Stearoylglycerone phosphate | neg | 457.2371 | 6.188 | 6.543 | 6.344 | -0.199 | -0.242 | 0.177 | 0.303 | Tentative | NA | NA |
| metab_4222 | Unannotated feature | pos | 248.9990 | 0.614 | 5.163 | 5.091 | -0.072 | -0.242 | 0.177 | 0.303 | Unannotated | NA | NA |
| metab_11122 | Unannotated feature | neg | 425.1653 | 5.691 | 4.120 | 4.296 | 0.176 | 0.242 | 0.177 | 0.303 | Unannotated | NA | NA |
| metab_9345 | Unannotated feature | neg | 183.0296 | 3.642 | 3.983 | 4.045 | 0.062 | 0.242 | 0.177 | 0.303 | Unannotated | NA | NA |
| metab_10950 | Unannotated feature | neg | 379.9318 | 0.522 | 3.820 | 3.991 | 0.171 | 0.242 | 0.177 | 0.303 | Unannotated | NA | NA |
| metab_2395 | Unannotated feature | pos | 521.3847 | 5.941 | 6.144 | 6.182 | 0.038 | 0.242 | 0.177 | 0.303 | Unannotated | NA | NA |
| metab_53 | Unannotated feature | pos | 113.9640 | 0.018 | 5.286 | 5.293 | 0.007 | 0.242 | 0.177 | 0.303 | Unannotated | NA | NA |
| metab_8743 | Unannotated feature | neg | 326.9039 | 7.156 | 5.441 | 5.419 | -0.022 | -0.242 | 0.177 | 0.303 | Unannotated | NA | NA |
| metab_8446 | Unannotated feature | neg | 169.0138 | 3.224 | 4.228 | 4.118 | -0.110 | -0.242 | 0.177 | 0.303 | Unannotated | NA | NA |
| metab_1980 | Unannotated feature | pos | 682.3275 | 7.293 | 4.993 | 4.903 | -0.090 | -0.242 | 0.177 | 0.303 | Unannotated | NA | NA |
| metab_5279 | Unannotated feature | neg | 829.8339 | 0.554 | 5.703 | 5.676 | -0.028 | -0.242 | 0.177 | 0.303 | Unannotated | NA | NA |
| metab_3741 | D-2-Aminobutyrate | pos | 86.0605 | 1.801 | 5.743 | 5.304 | -0.439 | -0.242 | 0.177 | 0.303 | Tentative | NA | NA |
| metab_1974 | Unannotated feature | pos | 790.2706 | 7.293 | 4.818 | 4.779 | -0.039 | -0.242 | 0.177 | 0.303 | Unannotated | NA | NA |
| metab_7539 | Unannotated feature | neg | 138.8688 | 7.173 | 3.688 | 3.653 | -0.035 | -0.242 | 0.177 | 0.303 | Unannotated | NA | NA |
| metab_3767 | 3-Hydroxybutyrylcarnitine | pos | 248.1492 | 1.724 | 4.315 | 4.662 | 0.347 | 0.242 | 0.177 | 0.303 | Tentative | NA | NA |
| metab_1522 | (R)-3-Hydroxydodecanoic acid | pos | 455.3365 | 6.188 | 5.033 | 4.967 | -0.066 | -0.242 | 0.177 | 0.303 | Tentative | NA | NA |
| metab_4843 | Unannotated feature | pos | 80.9484 | 7.277 | 5.011 | 4.974 | -0.037 | -0.242 | 0.177 | 0.303 | Unannotated | NA | NA |
| metab_11044 | Unannotated feature | neg | 188.9329 | 0.013 | 6.093 | 6.108 | 0.015 | 0.242 | 0.177 | 0.303 | Unannotated | NA | NA |
| metab_10835 | Unannotated feature | neg | 646.6410 | 0.582 | 5.639 | 5.617 | -0.022 | -0.242 | 0.177 | 0.303 | Unannotated | NA | NA |
| metab_5365 | Unannotated feature | neg | 701.5824 | 0.587 | 4.474 | 4.323 | -0.151 | -0.242 | 0.177 | 0.303 | Unannotated | NA | NA |
| metab_1606 | Unannotated feature | pos | 105.0427 | 6.768 | 6.637 | 6.673 | 0.036 | 0.242 | 0.177 | 0.303 | Unannotated | NA | NA |
| metab_10571 | Unannotated feature | neg | 734.4688 | 0.613 | 5.460 | 5.398 | -0.062 | -0.242 | 0.177 | 0.303 | Unannotated | NA | NA |
| metab_3281 | Unannotated feature | pos | 212.1394 | 2.888 | 4.637 | 4.271 | -0.367 | -0.242 | 0.177 | 0.303 | Unannotated | NA | NA |
| metab_5627 | Unannotated feature | neg | 498.6394 | 0.912 | 4.526 | 4.386 | -0.141 | -0.242 | 0.177 | 0.303 | Unannotated | NA | NA |
| metab_3078 | (E)-2,6-Dimethyl-2,5-heptadienoic acid | pos | 137.0962 | 2.397 | 3.535 | 3.120 | -0.415 | -0.242 | 0.177 | 0.303 | Tentative | NA | NA |
| metab_1936 | Unannotated feature | pos | 122.5474 | 7.324 | 5.188 | 5.161 | -0.027 | -0.242 | 0.177 | 0.303 | Unannotated | NA | NA |
| metab_8769 | Unannotated feature | neg | 325.9535 | 5.538 | 4.914 | 5.001 | 0.087 | 0.242 | 0.177 | 0.303 | Unannotated | NA | NA |
| metab_4596 | Unannotated feature | pos | 98.0968 | 0.193 | 5.068 | 5.063 | -0.005 | -0.242 | 0.177 | 0.303 | Unannotated | NA | NA |
| metab_2716 | Unannotated feature | pos | 212.1646 | 5.740 | 4.607 | 4.618 | 0.011 | 0.242 | 0.177 | 0.303 | Unannotated | NA | NA |
| metab_2720 | Unannotated feature | pos | 170.0813 | 1.956 | 4.813 | 4.821 | 0.008 | 0.242 | 0.177 | 0.303 | Unannotated | NA | NA |
| metab_1712 | Unannotated feature | pos | 736.8572 | 7.293 | 4.684 | 4.604 | -0.080 | -0.242 | 0.177 | 0.303 | Unannotated | NA | NA |
| metab_638 | Unannotated feature | pos | 108.0447 | 2.249 | 3.425 | 3.563 | 0.138 | 0.242 | 0.177 | 0.303 | Unannotated | NA | NA |
| metab_1862 | Unannotated feature | pos | 111.0808 | 7.532 | 4.389 | 4.413 | 0.024 | 0.242 | 0.177 | 0.303 | Unannotated | NA | NA |
| metab_10455 | Unannotated feature | neg | 130.0503 | 0.660 | 4.109 | 3.986 | -0.123 | -0.242 | 0.177 | 0.303 | Unannotated | NA | NA |
| metab_2783 | Allyl isovalerate | pos | 302.2326 | 5.649 | 5.404 | 5.545 | 0.141 | 0.242 | 0.177 | 0.303 | Tentative | NA | NA |
| metab_3861 | Unannotated feature | pos | 261.0112 | 0.955 | 5.107 | 5.060 | -0.047 | -0.242 | 0.177 | 0.303 | Unannotated | NA | NA |
| metab_9706 | Unannotated feature | neg | 415.7982 | 0.629 | 4.423 | 4.325 | -0.099 | -0.242 | 0.177 | 0.303 | Unannotated | NA | NA |
| metab_2630 | Senkyunolide N | pos | 209.1173 | 5.832 | 4.050 | 4.165 | 0.114 | 0.242 | 0.177 | 0.303 | Tentative | Organoheterocyclic compounds | Isobenzofurans |
| metab_1800 | Unannotated feature | pos | 152.0707 | 7.882 | 4.507 | 4.490 | -0.017 | -0.242 | 0.177 | 0.303 | Unannotated | NA | NA |
| metab_2926 | Unannotated feature | pos | 266.1959 | 4.869 | 5.102 | 5.077 | -0.025 | -0.242 | 0.177 | 0.303 | Unannotated | NA | NA |
| metab_2857 | Unannotated feature | pos | 102.5360 | 5.229 | 4.348 | 4.331 | -0.017 | -0.242 | 0.177 | 0.303 | Unannotated | NA | NA |
| metab_7654 | Unannotated feature | neg | 180.9312 | 7.339 | 5.341 | 5.356 | 0.015 | 0.242 | 0.177 | 0.303 | Unannotated | NA | NA |
| metab_4184 | Unannotated feature | pos | 499.0331 | 0.636 | 5.347 | 5.249 | -0.098 | -0.242 | 0.177 | 0.303 | Unannotated | NA | NA |
| metab_9567 | Unannotated feature | neg | 148.9696 | 3.192 | 3.349 | 2.992 | -0.357 | -0.242 | 0.177 | 0.303 | Unannotated | NA | NA |
| metab_4972 | Unannotated feature | pos | 174.0197 | 0.629 | 3.632 | 3.941 | 0.309 | 0.242 | 0.177 | 0.303 | Unannotated | NA | NA |
| metab_7656 | Unannotated feature | neg | 96.9593 | 7.317 | 6.679 | 6.694 | 0.015 | 0.242 | 0.177 | 0.303 | Unannotated | NA | NA |
| metab_8664 | 1-Heneicosanoyl-glycero-3-phosphoserine | neg | 566.3479 | 7.153 | 5.701 | 5.686 | -0.015 | -0.242 | 0.177 | 0.303 | Tentative | NA | NA |
| metab_254 | Unannotated feature | pos | 130.0694 | 0.675 | 4.518 | 4.609 | 0.090 | 0.242 | 0.177 | 0.303 | Unannotated | NA | NA |
| metab_1659 | (R)-1-O-[b-D-Apiofuranosyl-(1->2)-b-D-glucopyranoside]-1,3-octanediol | pos | 419.2615 | 7.215 | 6.488 | 6.478 | -0.010 | -0.242 | 0.177 | 0.303 | Tentative | NA | NA |
| metab_38 | Unannotated feature | pos | 340.1602 | 1.956 | 5.647 | 5.491 | -0.156 | -0.242 | 0.177 | 0.303 | Unannotated | NA | NA |
| metab_7586 | Unannotated feature | neg | 197.9631 | 7.213 | 4.077 | 4.047 | -0.030 | -0.238 | 0.185 | 0.312 | Unannotated | NA | NA |
| metab_4466 | Unannotated feature | pos | 752.8305 | 0.551 | 4.296 | 4.025 | -0.270 | -0.238 | 0.185 | 0.312 | Unannotated | NA | NA |
| metab_11348 | Unannotated feature | neg | 489.7515 | 0.013 | 4.804 | 4.794 | -0.010 | -0.238 | 0.185 | 0.312 | Unannotated | NA | NA |
| metab_7003 | Unannotated feature | neg | 340.1408 | 5.731 | 4.136 | 4.242 | 0.106 | 0.238 | 0.185 | 0.312 | Unannotated | NA | NA |
| metab_5648 | Unannotated feature | neg | 232.8459 | 0.967 | 5.240 | 5.238 | -0.002 | -0.238 | 0.185 | 0.312 | Unannotated | NA | NA |
| metab_6972 | Unannotated feature | neg | 113.3159 | 5.707 | 3.876 | 3.789 | -0.086 | -0.238 | 0.185 | 0.312 | Unannotated | NA | NA |
| metab_2960 | 13(S)-HODE | pos | 319.2224 | 4.586 | 4.816 | 4.840 | 0.025 | 0.238 | 0.185 | 0.312 | Tentative | NA | NA |
| metab_4197 | Unannotated feature | pos | 337.9052 | 0.629 | 4.524 | 4.438 | -0.087 | -0.238 | 0.185 | 0.312 | Unannotated | NA | NA |
| metab_3036 | 2',3'-Dideoxy-3'-fluorouridine | pos | 248.1029 | 4.163 | 4.142 | 3.827 | -0.315 | -0.238 | 0.185 | 0.312 | Tentative | Nucleosides, nucleotides, and analogues | Pyrimidine nucleosides |
| metab_3421 | Pirbuterol | pos | 241.1545 | 2.397 | 3.621 | 3.326 | -0.296 | -0.238 | 0.185 | 0.312 | Tentative | NA | NA |
| metab_9502 | 6-Hydroxy-3,4-dihydro-2(1H)-quinolinone | neg | 208.0615 | 3.247 | 5.434 | 5.325 | -0.109 | -0.238 | 0.185 | 0.312 | Tentative | NA | NA |
| metab_4935 | Unannotated feature | pos | 364.9161 | 0.621 | 5.270 | 5.237 | -0.033 | -0.238 | 0.185 | 0.312 | Unannotated | NA | NA |
| metab_3093 | Hordatine B glucoside | pos | 394.1684 | 3.854 | 4.472 | 4.699 | 0.227 | 0.238 | 0.185 | 0.312 | Tentative | NA | NA |
| metab_5555 | Unannotated feature | neg | 170.8705 | 0.833 | 4.169 | 4.068 | -0.100 | -0.238 | 0.185 | 0.312 | Unannotated | NA | NA |
| metab_4329 | Unannotated feature | pos | 620.7226 | 0.575 | 4.514 | 4.466 | -0.047 | -0.238 | 0.185 | 0.312 | Unannotated | NA | NA |
| metab_8544 | Unannotated feature | neg | 158.9750 | 7.109 | 8.043 | 8.066 | 0.023 | 0.238 | 0.185 | 0.312 | Unannotated | NA | NA |
| metab_1866 | Unannotated feature | pos | 112.0760 | 7.463 | 3.788 | 3.851 | 0.063 | 0.238 | 0.185 | 0.312 | Unannotated | NA | NA |
| metab_1140 | Unannotated feature | pos | 107.9672 | 5.657 | 4.951 | 4.984 | 0.033 | 0.238 | 0.185 | 0.312 | Unannotated | NA | NA |
| metab_2624 | 2-Propenyl octanoate | pos | 386.3260 | 5.832 | 4.372 | 4.210 | -0.162 | -0.238 | 0.185 | 0.312 | Tentative | NA | NA |
| metab_8696 | Unannotated feature | neg | 146.9381 | 7.170 | 5.866 | 5.853 | -0.013 | -0.238 | 0.185 | 0.312 | Unannotated | NA | NA |
| metab_3453 | Thymol Sulfate | pos | 248.0951 | 2.296 | 4.240 | 4.553 | 0.313 | 0.238 | 0.185 | 0.312 | Tentative | Organic acids and derivatives | Organic sulfuric acids and derivatives |
| metab_9771 | Unannotated feature | neg | 231.8829 | 2.636 | 5.261 | 5.280 | 0.019 | 0.238 | 0.185 | 0.312 | Unannotated | NA | NA |
| metab_1262 | Unannotated feature | pos | 420.3063 | 5.848 | 5.030 | 4.987 | -0.044 | -0.238 | 0.185 | 0.312 | Unannotated | NA | NA |
| metab_4816 | Unannotated feature | pos | 153.9934 | 7.324 | 4.063 | 4.104 | 0.042 | 0.238 | 0.185 | 0.312 | Unannotated | NA | NA |
| metab_4822 | Unannotated feature | pos | 238.9072 | 7.308 | 4.774 | 4.796 | 0.022 | 0.238 | 0.185 | 0.312 | Unannotated | NA | NA |
| metab_3102 | 5-Methoxytryptophan | pos | 217.0972 | 3.838 | 4.609 | 4.492 | -0.117 | -0.238 | 0.185 | 0.312 | Tentative | NA | NA |
| metab_3497 | Unannotated feature | pos | 169.0496 | 3.027 | 4.399 | 2.993 | -1.406 | -0.238 | 0.185 | 0.312 | Unannotated | NA | NA |
| metab_5163 | Unannotated feature | neg | 289.9495 | 0.505 | 2.849 | 3.256 | 0.407 | 0.238 | 0.185 | 0.312 | Unannotated | NA | NA |
| metab_9815 | Unannotated feature | neg | 389.1099 | 2.523 | 4.694 | 4.603 | -0.091 | -0.238 | 0.185 | 0.312 | Unannotated | NA | NA |
| metab_1386 | Unannotated feature | pos | 578.9107 | 5.917 | 4.957 | 4.909 | -0.049 | -0.238 | 0.185 | 0.312 | Unannotated | NA | NA |
| metab_6640 | Unannotated feature | neg | 330.8698 | 0.841 | 4.305 | 4.127 | -0.178 | -0.238 | 0.185 | 0.312 | Unannotated | NA | NA |
| metab_1409 | Unannotated feature | pos | 461.8371 | 5.933 | 4.585 | 4.537 | -0.048 | -0.238 | 0.185 | 0.312 | Unannotated | NA | NA |
| metab_2425 | Unannotated feature | pos | 514.3783 | 5.925 | 6.378 | 6.441 | 0.062 | 0.238 | 0.185 | 0.312 | Unannotated | NA | NA |
| metab_2384 | Unannotated feature | pos | 478.8437 | 0.567 | 5.593 | 5.585 | -0.008 | -0.238 | 0.185 | 0.312 | Unannotated | NA | NA |
| metab_1437 | Unannotated feature | pos | 146.0634 | 1.956 | 4.385 | 4.726 | 0.341 | 0.238 | 0.185 | 0.312 | Unannotated | NA | NA |
| metab_2394 | Unannotated feature | pos | 412.8532 | 0.567 | 5.705 | 5.695 | -0.010 | -0.238 | 0.185 | 0.312 | Unannotated | NA | NA |
| metab_8201 | Unannotated feature | neg | 624.2046 | 5.961 | 4.532 | 4.680 | 0.148 | 0.238 | 0.185 | 0.312 | Unannotated | NA | NA |
| metab_3743 | Alloxanthine | pos | 153.0407 | 1.794 | 3.787 | 3.891 | 0.105 | 0.238 | 0.185 | 0.312 | Strong putative | NA | NA |
| metab_3750 | Butirosina | pos | 278.6455 | 1.763 | 4.610 | 4.867 | 0.257 | 0.238 | 0.185 | 0.312 | Tentative | NA | NA |
| metab_5752 | Unannotated feature | neg | 245.9675 | 1.863 | 3.856 | 4.186 | 0.330 | 0.238 | 0.185 | 0.312 | Unannotated | NA | NA |
| metab_2213 | 6-n-Octylaminouracil | pos | 479.3344 | 6.296 | 4.952 | 4.915 | -0.037 | -0.238 | 0.185 | 0.312 | Tentative | NA | NA |
| metab_8634 | Unannotated feature | neg | 183.0296 | 5.651 | 3.801 | 3.699 | -0.102 | -0.238 | 0.185 | 0.312 | Unannotated | NA | NA |
| metab_4667 | Unannotated feature | pos | 94.0655 | 5.840 | 3.677 | 3.641 | -0.036 | -0.238 | 0.185 | 0.312 | Unannotated | NA | NA |
| metab_3701 | Unannotated feature | pos | 284.0989 | 1.902 | 3.899 | 4.172 | 0.273 | 0.238 | 0.185 | 0.312 | Unannotated | NA | NA |
| metab_10082 | Citrate | neg | 191.0195 | 1.294 | 5.426 | 5.546 | 0.120 | 0.238 | 0.185 | 0.312 | Strong putative | NA | NA |
| metab_5331 | Unannotated feature | neg | 584.7751 | 0.570 | 4.187 | 4.076 | -0.111 | -0.238 | 0.185 | 0.312 | Unannotated | NA | NA |
| metab_4228 | Unannotated feature | pos | 174.1238 | 0.614 | 3.503 | 3.349 | -0.154 | -0.238 | 0.185 | 0.312 | Unannotated | NA | NA |
| metab_3952 | L-2-Amino-5-hydroxypentanoic acid | pos | 134.0813 | 0.854 | 3.384 | 3.383 | -0.001 | 0.238 | 0.185 | 0.312 | Tentative | NA | NA |
| metab_3781 | Unannotated feature | pos | 73.0290 | 1.624 | 3.720 | 3.702 | -0.018 | -0.238 | 0.185 | 0.312 | Unannotated | NA | NA |
| metab_10142 | Unannotated feature | neg | 290.8050 | 0.936 | 4.698 | 4.651 | -0.047 | -0.238 | 0.185 | 0.312 | Unannotated | NA | NA |
| metab_2102 | Unannotated feature | pos | 117.1470 | 7.091 | 5.930 | 5.949 | 0.019 | 0.238 | 0.185 | 0.312 | Unannotated | NA | NA |
| metab_7427 | Unannotated feature | neg | 200.0779 | 7.109 | 4.200 | 4.270 | 0.070 | 0.238 | 0.185 | 0.312 | Unannotated | NA | NA |
| metab_5378 | Unannotated feature | neg | 156.8773 | 0.598 | 4.979 | 4.966 | -0.014 | -0.238 | 0.185 | 0.312 | Unannotated | NA | NA |
| metab_320 | Unannotated feature | pos | 225.8811 | 0.800 | 4.280 | 4.265 | -0.015 | -0.238 | 0.185 | 0.312 | Unannotated | NA | NA |
| metab_10614 | Unannotated feature | neg | 228.8135 | 0.598 | 4.826 | 4.875 | 0.049 | 0.238 | 0.185 | 0.312 | Unannotated | NA | NA |
| metab_2035 | Unannotated feature | pos | 260.9318 | 7.269 | 4.441 | 4.421 | -0.020 | -0.238 | 0.185 | 0.312 | Unannotated | NA | NA |
| metab_2036 | Unannotated feature | pos | 115.8001 | 7.269 | 3.914 | 3.962 | 0.049 | 0.238 | 0.185 | 0.312 | Unannotated | NA | NA |
| metab_5839 | Unannotated feature | neg | 128.0710 | 2.237 | 4.564 | 4.455 | -0.109 | -0.238 | 0.185 | 0.312 | Unannotated | NA | NA |
| metab_8767 | Unannotated feature | neg | 388.8671 | 5.538 | 5.785 | 5.674 | -0.110 | -0.238 | 0.185 | 0.312 | Unannotated | NA | NA |
| metab_8768 | Unannotated feature | neg | 386.8701 | 5.538 | 5.660 | 5.568 | -0.092 | -0.238 | 0.185 | 0.312 | Unannotated | NA | NA |
| metab_3258 | Unannotated feature | pos | 197.1285 | 2.981 | 5.083 | 5.143 | 0.060 | 0.238 | 0.185 | 0.312 | Moderate putative | NA | NA |
| metab_7049 | Unannotated feature | neg | 171.1023 | 5.771 | 4.007 | 4.122 | 0.115 | 0.238 | 0.185 | 0.312 | Unannotated | NA | NA |
| metab_1763 | Unannotated feature | pos | 151.0053 | 7.339 | 4.509 | 4.489 | -0.020 | -0.238 | 0.185 | 0.312 | Unannotated | NA | NA |
| metab_11343 | Unannotated feature | neg | 182.8963 | 0.013 | 5.761 | 5.746 | -0.015 | -0.234 | 0.193 | 0.322 | Unannotated | NA | NA |
| metab_7601 | Unannotated feature | neg | 168.8864 | 7.253 | 5.260 | 5.272 | 0.013 | 0.234 | 0.193 | 0.322 | Unannotated | NA | NA |
| metab_7608 | Unannotated feature | neg | 264.8729 | 7.269 | 4.085 | 4.128 | 0.043 | 0.234 | 0.193 | 0.322 | Unannotated | NA | NA |
| metab_1764 | Unannotated feature | pos | 164.0663 | 7.339 | 4.437 | 4.437 | 0.000 | -0.234 | 0.193 | 0.322 | Unannotated | NA | NA |
| metab_3262 | 2-Hydroxyacetophenone | pos | 137.0598 | 2.973 | 3.749 | 3.843 | 0.094 | 0.234 | 0.193 | 0.322 | Tentative | NA | NA |
| metab_11017 | Unannotated feature | neg | 802.6736 | 0.578 | 4.763 | 4.663 | -0.101 | -0.234 | 0.193 | 0.322 | Unannotated | NA | NA |
| metab_4834 | Unannotated feature | pos | 124.0735 | 7.293 | 5.229 | 5.273 | 0.044 | 0.234 | 0.193 | 0.322 | Unannotated | NA | NA |
| metab_836 | Indole-3-ethanol | pos | 144.0808 | 3.244 | 3.685 | 3.875 | 0.190 | 0.234 | 0.193 | 0.322 | Moderate putative | NA | NA |
| metab_3107 | AMPA | pos | 209.0535 | 0.854 | 5.216 | 5.107 | -0.109 | -0.234 | 0.193 | 0.322 | Tentative | NA | NA |
| metab_3396 | Unannotated feature | pos | 184.0030 | 2.475 | 5.249 | 5.232 | -0.017 | -0.234 | 0.193 | 0.322 | Unannotated | NA | NA |
| metab_11001 | Unannotated feature | neg | 239.9039 | 0.033 | 6.433 | 6.453 | 0.020 | 0.234 | 0.193 | 0.322 | Unannotated | NA | NA |
| metab_9595 | Unannotated feature | neg | 419.9362 | 3.160 | 4.138 | 3.980 | -0.158 | -0.234 | 0.193 | 0.322 | Unannotated | NA | NA |
| metab_954 | (2S)-2-Amino-3-(4-hydroxyphenyl)propane-1,1-diol | pos | 166.0863 | 4.163 | 5.018 | 4.948 | -0.070 | -0.234 | 0.193 | 0.322 | Tentative | NA | NA |
| metab_8649 | Feruloylagmatine | neg | 351.1644 | 5.635 | 5.013 | 4.922 | -0.091 | -0.234 | 0.193 | 0.322 | Tentative | NA | NA |
| metab_4980 | Unannotated feature | pos | 165.0759 | 0.729 | 4.837 | 4.884 | 0.047 | 0.234 | 0.193 | 0.322 | Unannotated | NA | NA |
| metab_996 | Unannotated feature | pos | 363.4783 | 4.501 | 3.955 | 4.174 | 0.220 | 0.234 | 0.193 | 0.322 | Unannotated | NA | NA |
| metab_5021 | Unannotated feature | pos | 237.0958 | 0.831 | 4.229 | 4.316 | 0.087 | 0.234 | 0.193 | 0.322 | Unannotated | NA | NA |
| metab_2916 | 1-(2-Hydroxyethoxy)methyl-5-methyluracil | pos | 245.0502 | 4.900 | 4.610 | 4.733 | 0.123 | 0.234 | 0.193 | 0.322 | Tentative | NA | NA |
| metab_2777 | 2-Methyl-1,3-cyclohexadiene | pos | 95.0860 | 5.657 | 4.612 | 4.625 | 0.013 | 0.234 | 0.193 | 0.322 | Tentative | NA | NA |
| metab_2601 | Unannotated feature | pos | 406.6084 | 5.848 | 4.270 | 4.308 | 0.037 | 0.234 | 0.193 | 0.322 | Unannotated | NA | NA |
| metab_6216 | Unannotated feature | neg | 232.0940 | 3.372 | 4.001 | 3.746 | -0.255 | -0.234 | 0.193 | 0.322 | Unannotated | NA | NA |
| metab_5085 | Unannotated feature | pos | 130.0501 | 1.367 | 6.537 | 6.509 | -0.028 | -0.234 | 0.193 | 0.322 | Strong putative | NA | NA |
| metab_3650 | p-Cymene | pos | 135.1170 | 3.484 | 3.125 | 2.788 | -0.337 | -0.234 | 0.193 | 0.322 | Moderate putative | NA | NA |
| metab_9058 | N-Acetyl-L-leucine | neg | 172.0976 | 4.582 | 4.700 | 4.648 | -0.052 | -0.234 | 0.193 | 0.322 | Moderate putative | NA | NA |
| metab_7378 | Unannotated feature | neg | 132.4467 | 6.976 | 3.591 | 3.540 | -0.051 | -0.234 | 0.193 | 0.322 | Unannotated | NA | NA |
| metab_8934 | Unannotated feature | neg | 136.8910 | 7.117 | 5.400 | 5.381 | -0.019 | -0.234 | 0.193 | 0.322 | Unannotated | NA | NA |
| metab_4720 | Unannotated feature | pos | 506.3798 | 5.979 | 5.827 | 5.865 | 0.038 | 0.234 | 0.193 | 0.322 | Unannotated | NA | NA |
| metab_2444 | Unannotated feature | pos | 417.2823 | 5.917 | 4.838 | 4.909 | 0.071 | 0.234 | 0.193 | 0.322 | Unannotated | NA | NA |
| metab_11098 | Unannotated feature | neg | 183.0045 | 5.651 | 4.393 | 4.469 | 0.076 | 0.234 | 0.193 | 0.322 | Unannotated | NA | NA |
| metab_6080 | Unannotated feature | neg | 288.9896 | 3.136 | 4.422 | 4.305 | -0.117 | -0.234 | 0.193 | 0.322 | Unannotated | NA | NA |
| metab_5083 | Unannotated feature | pos | 236.9982 | 1.274 | 4.666 | 4.736 | 0.070 | 0.234 | 0.193 | 0.322 | Unannotated | NA | NA |
| metab_9149 | Unannotated feature | neg | 329.9405 | 3.364 | 4.986 | 4.933 | -0.053 | -0.234 | 0.193 | 0.322 | Unannotated | NA | NA |
| metab_8605 | Unannotated feature | neg | 292.0499 | 7.132 | 4.006 | 3.869 | -0.138 | -0.234 | 0.193 | 0.322 | Unannotated | NA | NA |
| metab_7766 | Unannotated feature | neg | 225.8974 | 7.173 | 5.030 | 5.020 | -0.010 | -0.234 | 0.193 | 0.322 | Unannotated | NA | NA |
| metab_2048 | Unannotated feature | pos | 528.4986 | 7.254 | 6.739 | 6.731 | -0.008 | -0.234 | 0.193 | 0.322 | Unannotated | NA | NA |
| metab_7692 | Unannotated feature | neg | 243.9533 | 7.213 | 4.137 | 4.172 | 0.035 | 0.234 | 0.193 | 0.322 | Unannotated | NA | NA |
| metab_10171 | Unannotated feature | neg | 506.6275 | 0.904 | 4.763 | 4.706 | -0.056 | -0.234 | 0.193 | 0.322 | Unannotated | NA | NA |
| metab_1624 | Hexylamine | pos | 102.1281 | 7.076 | 8.045 | 8.066 | 0.020 | 0.234 | 0.193 | 0.322 | Tentative | NA | NA |
| metab_2027 | Unannotated feature | pos | 543.4555 | 7.269 | 4.554 | 4.544 | -0.010 | -0.234 | 0.193 | 0.322 | Unannotated | NA | NA |
| metab_10627 | Unannotated feature | neg | 578.6087 | 0.594 | 4.651 | 4.721 | 0.070 | 0.234 | 0.193 | 0.322 | Unannotated | NA | NA |
| metab_3988 | Unannotated feature | pos | 75.0446 | 0.831 | 4.140 | 4.106 | -0.034 | -0.234 | 0.193 | 0.322 | Unannotated | NA | NA |
| metab_8409 | Unannotated feature | neg | 310.1644 | 5.787 | 3.467 | 3.588 | 0.121 | 0.234 | 0.193 | 0.322 | Unannotated | NA | NA |
| metab_8484 | Unannotated feature | neg | 239.0857 | 5.739 | 5.058 | 5.015 | -0.044 | -0.234 | 0.193 | 0.322 | Unannotated | NA | NA |
| metab_9 | Unannotated feature | pos | 287.6507 | 1.755 | 5.537 | 5.683 | 0.146 | 0.234 | 0.193 | 0.322 | Unannotated | NA | NA |
| metab_10501 | Unannotated feature | neg | 161.4041 | 0.636 | 4.638 | 4.596 | -0.042 | -0.234 | 0.193 | 0.322 | Unannotated | NA | NA |
| metab_1631 | Unannotated feature | pos | 252.2281 | 7.099 | 4.493 | 4.437 | -0.056 | -0.234 | 0.193 | 0.322 | Unannotated | NA | NA |
| metab_1623 | Octylamine | pos | 130.1592 | 7.061 | 7.291 | 7.302 | 0.011 | 0.234 | 0.193 | 0.322 | Tentative | NA | NA |
| metab_1634 | Unannotated feature | pos | 74.0970 | 7.122 | 5.666 | 5.682 | 0.016 | 0.234 | 0.193 | 0.322 | Unannotated | NA | NA |
| metab_6712 | Unannotated feature | neg | 330.1929 | 5.338 | 3.442 | 3.818 | 0.376 | 0.234 | 0.193 | 0.322 | Unannotated | NA | NA |
| metab_5905 | Unannotated feature | neg | 135.8941 | 2.523 | 4.729 | 4.711 | -0.018 | -0.234 | 0.193 | 0.322 | Unannotated | NA | NA |
| metab_4218 | Unannotated feature | pos | 780.4450 | 0.614 | 5.130 | 5.013 | -0.117 | -0.234 | 0.193 | 0.322 | Unannotated | NA | NA |
| metab_8374 | Propylparaben | neg | 179.0710 | 5.811 | 2.845 | 3.281 | 0.436 | 0.234 | 0.193 | 0.322 | Moderate putative | NA | NA |
| metab_4503 | Unannotated feature | pos | 90.7389 | 0.551 | 4.305 | 4.319 | 0.014 | 0.234 | 0.193 | 0.322 | Unannotated | NA | NA |
| metab_10086 | Unannotated feature | neg | 124.0145 | 1.294 | 4.914 | 4.881 | -0.033 | -0.234 | 0.193 | 0.322 | Unannotated | NA | NA |
| metab_3825 | Unannotated feature | pos | 215.0162 | 1.266 | 5.339 | 5.457 | 0.118 | 0.234 | 0.193 | 0.322 | Unannotated | NA | NA |
| metab_8907 | Unannotated feature | neg | 283.9852 | 5.116 | 3.264 | 3.999 | 0.736 | 0.234 | 0.193 | 0.322 | Unannotated | NA | NA |
| metab_7390 | Unannotated feature | neg | 301.9559 | 7.085 | 3.653 | 3.869 | 0.216 | 0.229 | 0.201 | 0.332 | Unannotated | NA | NA |
| metab_10490 | Unannotated feature | neg | 292.0729 | 0.636 | 4.331 | 4.016 | -0.315 | -0.229 | 0.201 | 0.332 | Unannotated | NA | NA |
| metab_9717 | Unannotated feature | neg | 218.1034 | 2.828 | 5.045 | 5.167 | 0.121 | 0.229 | 0.201 | 0.332 | Unannotated | NA | NA |
| metab_522 | Unannotated feature | pos | 89.0602 | 1.956 | 3.981 | 3.870 | -0.110 | -0.229 | 0.201 | 0.332 | Unannotated | NA | NA |
| metab_594 | 2-Methylidene-4-(oxiran-2-yl)butanoic Acid | pos | 107.0495 | 2.081 | 5.499 | 5.534 | 0.035 | 0.229 | 0.201 | 0.332 | Tentative | NA | NA |
| metab_5133 | 4-Methylpentanal | pos | 142.1227 | 7.061 | 6.147 | 6.163 | 0.016 | 0.229 | 0.201 | 0.332 | Tentative | NA | NA |
| metab_7157 | Unannotated feature | neg | 321.1478 | 5.883 | 4.423 | 4.572 | 0.149 | 0.229 | 0.201 | 0.332 | Unannotated | NA | NA |
| metab_7152 | Unannotated feature | neg | 481.2818 | 5.875 | 5.505 | 5.210 | -0.295 | -0.229 | 0.201 | 0.332 | Unannotated | NA | NA |
| metab_7547 | Unannotated feature | neg | 136.8939 | 7.181 | 4.609 | 4.584 | -0.025 | -0.229 | 0.201 | 0.332 | Unannotated | NA | NA |
| metab_7468 | Unannotated feature | neg | 113.0069 | 7.140 | 3.537 | 3.491 | -0.047 | -0.229 | 0.201 | 0.332 | Unannotated | NA | NA |
| metab_326 | Unannotated feature | pos | 145.0173 | 0.815 | 3.533 | 3.485 | -0.048 | -0.229 | 0.201 | 0.332 | Unannotated | NA | NA |
| metab_7083 | Unannotated feature | neg | 147.0922 | 5.803 | 4.052 | 4.229 | 0.176 | 0.229 | 0.201 | 0.332 | Unannotated | NA | NA |
| metab_470 | N(pi)-Methyl-L-histidine | pos | 339.1762 | 1.794 | 4.612 | 4.394 | -0.218 | -0.229 | 0.201 | 0.332 | Tentative | NA | NA |
| metab_5918 | Unannotated feature | neg | 312.0030 | 2.541 | 4.726 | 4.690 | -0.036 | -0.229 | 0.201 | 0.332 | Unannotated | NA | NA |
| metab_11152 | Unannotated feature | neg | 304.0753 | 5.755 | 4.249 | 3.882 | -0.367 | -0.229 | 0.201 | 0.332 | Unannotated | NA | NA |
| metab_10127 | Unannotated feature | neg | 174.8868 | 0.991 | 6.192 | 6.181 | -0.010 | -0.229 | 0.201 | 0.332 | Unannotated | NA | NA |
| metab_8302 | Unannotated feature | neg | 558.1206 | 5.859 | 5.232 | 5.308 | 0.076 | 0.229 | 0.201 | 0.332 | Unannotated | NA | NA |
| metab_2217 | Unannotated feature | pos | 311.1826 | 6.296 | 4.668 | 4.611 | -0.057 | -0.229 | 0.201 | 0.332 | Unannotated | NA | NA |
| metab_7899 | Unannotated feature | neg | 137.0238 | 7.132 | 4.421 | 4.401 | -0.020 | -0.229 | 0.201 | 0.332 | Unannotated | NA | NA |
| metab_2250 | Unannotated feature | pos | 477.3189 | 6.203 | 5.100 | 4.950 | -0.149 | -0.229 | 0.201 | 0.332 | Unannotated | NA | NA |
| metab_1413 | Unannotated feature | pos | 719.0130 | 5.933 | 5.320 | 5.268 | -0.052 | -0.229 | 0.201 | 0.332 | Unannotated | NA | NA |
| metab_3565 | Unannotated feature | pos | 99.0746 | 2.065 | 4.658 | 4.672 | 0.014 | 0.229 | 0.201 | 0.332 | Unannotated | NA | NA |
| metab_11130 | Unannotated feature | neg | 251.0929 | 5.707 | 3.416 | 3.932 | 0.516 | 0.229 | 0.201 | 0.332 | Unannotated | NA | NA |
| metab_9806 | Unannotated feature | neg | 448.0671 | 2.530 | 4.384 | 4.282 | -0.102 | -0.229 | 0.201 | 0.332 | Unannotated | NA | NA |
| metab_9779 | Unannotated feature | neg | 206.8606 | 2.605 | 4.897 | 4.921 | 0.024 | 0.229 | 0.201 | 0.332 | Unannotated | NA | NA |
| metab_4760 | 1-Acyl-sn-glycero-3-phosphocholine | pos | 510.3556 | 6.675 | 6.719 | 6.596 | -0.123 | -0.229 | 0.201 | 0.332 | Tentative | NA | NA |
| metab_4773 | Unannotated feature | pos | 432.2381 | 6.296 | 5.141 | 5.080 | -0.060 | -0.229 | 0.201 | 0.332 | Unannotated | NA | NA |
| metab_7140 | Unannotated feature | neg | 796.4218 | 0.613 | 6.402 | 6.394 | -0.008 | -0.229 | 0.201 | 0.332 | Unannotated | NA | NA |
| metab_2451 | Unannotated feature | pos | 775.5300 | 5.910 | 4.107 | 3.967 | -0.141 | -0.229 | 0.201 | 0.332 | Unannotated | NA | NA |
| metab_4756 | Unannotated feature | pos | 518.3219 | 6.567 | 8.074 | 8.055 | -0.019 | -0.229 | 0.201 | 0.332 | Unannotated | NA | NA |
| metab_10154 | Unannotated feature | neg | 564.5863 | 0.912 | 4.958 | 4.948 | -0.010 | -0.229 | 0.201 | 0.332 | Unannotated | NA | NA |
| metab_5652 | Unannotated feature | neg | 293.8235 | 0.991 | 5.142 | 5.130 | -0.012 | -0.229 | 0.201 | 0.332 | Unannotated | NA | NA |
| metab_56 | Unannotated feature | pos | 142.9618 | 0.025 | 4.289 | 4.304 | 0.015 | 0.229 | 0.201 | 0.332 | Unannotated | NA | NA |
| metab_8211 | 12-Hydroxydodecanoic acid | neg | 215.1652 | 5.953 | 4.370 | 4.276 | -0.094 | -0.229 | 0.201 | 0.332 | Tentative | NA | NA |
| metab_445 | Unannotated feature | pos | 113.9640 | 1.655 | 6.134 | 6.122 | -0.012 | -0.229 | 0.201 | 0.332 | Unannotated | NA | NA |
| metab_4055 | 1F-beta-D-Fructosylsucrose | pos | 527.1583 | 0.760 | 4.678 | 4.715 | 0.038 | 0.229 | 0.201 | 0.332 | Strong putative | NA | NA |
| metab_1979 | Unannotated feature | pos | 722.2836 | 7.293 | 5.098 | 5.045 | -0.052 | -0.229 | 0.201 | 0.332 | Unannotated | NA | NA |
| metab_6940 | Unannotated feature | neg | 517.0998 | 5.683 | 4.237 | 4.227 | -0.010 | -0.229 | 0.201 | 0.332 | Unannotated | NA | NA |
| metab_6910 | Unannotated feature | neg | 212.1699 | 5.667 | 3.819 | 3.543 | -0.277 | -0.229 | 0.201 | 0.332 | Unannotated | NA | NA |
| metab_2061 | Unannotated feature | pos | 457.2770 | 7.215 | 6.166 | 6.138 | -0.028 | -0.229 | 0.201 | 0.332 | Unannotated | NA | NA |
| metab_4971 | N(5)-Acetylornithine | pos | 175.1078 | 0.698 | 4.667 | 4.571 | -0.096 | -0.229 | 0.201 | 0.332 | Tentative | NA | NA |
| metab_3083 | 3'-Deoxythymidine | pos | 209.0921 | 3.893 | 4.431 | 4.707 | 0.276 | 0.229 | 0.201 | 0.332 | Tentative | NA | NA |
| metab_917 | Unannotated feature | pos | 174.1126 | 3.861 | 4.349 | 4.359 | 0.010 | 0.229 | 0.201 | 0.332 | Unannotated | NA | NA |
| metab_10923 | Unannotated feature | neg | 831.8337 | 0.534 | 4.414 | 4.330 | -0.084 | -0.229 | 0.201 | 0.332 | Unannotated | NA | NA |
| metab_3247 | Unannotated feature | pos | 300.2016 | 2.606 | 4.992 | 4.831 | -0.161 | -0.229 | 0.201 | 0.332 | Unannotated | NA | NA |
| metab_1834 | Sinapoyl aldehyde | pos | 209.0809 | 7.959 | 5.304 | 5.294 | -0.009 | -0.229 | 0.201 | 0.332 | Moderate putative | NA | NA |
| metab_10882 | Unannotated feature | neg | 1033.7954 | 0.549 | 4.965 | 4.919 | -0.046 | -0.229 | 0.201 | 0.332 | Unannotated | NA | NA |
| metab_9227 | Unannotated feature | neg | 241.0566 | 4.037 | 4.588 | 4.515 | -0.072 | -0.229 | 0.201 | 0.332 | Unannotated | NA | NA |
| metab_3394 | Unannotated feature | pos | 94.0456 | 2.482 | 4.176 | 4.138 | -0.038 | -0.229 | 0.201 | 0.332 | Unannotated | NA | NA |
| metab_2602 | all-trans-13,14-Dihydroretinol | pos | 289.2526 | 5.848 | 4.128 | 4.393 | 0.265 | 0.229 | 0.201 | 0.332 | Tentative | NA | NA |
| metab_9831 | Unannotated feature | neg | 622.5452 | 0.621 | 5.811 | 5.793 | -0.017 | -0.225 | 0.209 | 0.343 | Unannotated | NA | NA |
| metab_3774 | Unannotated feature | pos | 169.0972 | 1.694 | 4.704 | 4.769 | 0.065 | 0.225 | 0.209 | 0.343 | Unannotated | NA | NA |
| metab_2260 | Unannotated feature | pos | 457.3512 | 6.188 | 4.960 | 4.876 | -0.084 | -0.225 | 0.209 | 0.343 | Unannotated | NA | NA |
| metab_2209 | Isoetharine | pos | 542.3218 | 6.296 | 7.344 | 7.404 | 0.060 | 0.225 | 0.209 | 0.343 | Tentative | NA | NA |
| metab_7553 | Unannotated feature | neg | 229.9374 | 7.181 | 4.113 | 4.096 | -0.017 | -0.225 | 0.209 | 0.343 | Unannotated | NA | NA |
| metab_9838 | 4-Methylene-2-oxoglutarate | neg | 315.0342 | 2.498 | 4.713 | 4.695 | -0.018 | -0.225 | 0.209 | 0.343 | Tentative | NA | NA |
| metab_8062 | Unannotated feature | neg | 435.2525 | 6.332 | 4.722 | 4.745 | 0.024 | 0.225 | 0.209 | 0.343 | Unannotated | NA | NA |
| metab_187 | Unannotated feature | pos | 557.8611 | 0.567 | 4.635 | 4.609 | -0.026 | -0.225 | 0.209 | 0.343 | Unannotated | NA | NA |
| metab_4554 | Unannotated feature | pos | 132.9982 | 0.504 | 4.227 | 4.261 | 0.034 | 0.225 | 0.209 | 0.343 | Unannotated | NA | NA |
| metab_3076 | Unannotated feature | pos | 121.0844 | 3.955 | 3.784 | 3.557 | -0.227 | -0.225 | 0.209 | 0.343 | Unannotated | NA | NA |
| metab_1714 | Unannotated feature | pos | 804.8443 | 7.293 | 4.774 | 4.683 | -0.091 | -0.225 | 0.209 | 0.343 | Unannotated | NA | NA |
| metab_7201 | Unannotated feature | neg | 101.9406 | 5.930 | 4.920 | 4.939 | 0.019 | 0.225 | 0.209 | 0.343 | Unannotated | NA | NA |
| metab_9888 | Unannotated feature | neg | 300.0056 | 2.245 | 4.175 | 3.945 | -0.230 | -0.225 | 0.209 | 0.343 | Unannotated | NA | NA |
| metab_7415 | Unannotated feature | neg | 117.2762 | 7.109 | 4.727 | 4.752 | 0.025 | 0.225 | 0.209 | 0.343 | Unannotated | NA | NA |
| metab_7901 | Adb-chminaca, (+/-)- | neg | 407.1881 | 7.124 | 5.101 | 5.060 | -0.041 | -0.225 | 0.209 | 0.343 | Tentative | NA | NA |
| metab_2566 | 13-Hydroxyoctadecanoylcarnitine | pos | 426.3578 | 5.863 | 6.564 | 6.524 | -0.040 | -0.225 | 0.209 | 0.343 | Tentative | NA | NA |
| metab_6060 | Unannotated feature | neg | 270.1213 | 3.088 | 4.287 | 4.138 | -0.149 | -0.225 | 0.209 | 0.343 | Unannotated | NA | NA |
| metab_1388 | Unannotated feature | pos | 660.9726 | 5.917 | 5.782 | 5.822 | 0.039 | 0.225 | 0.209 | 0.343 | Unannotated | NA | NA |
| metab_501 | 2,6-Diamino-4-hydroxy-5-(N-methyl)formamidopyrimidine | pos | 152.0568 | 1.902 | 4.148 | 4.301 | 0.153 | 0.225 | 0.209 | 0.343 | Tentative | Organic nitrogen compounds | Organonitrogen compounds |
| metab_2422 | Unannotated feature | pos | 607.9316 | 5.925 | 4.489 | 4.457 | -0.033 | -0.225 | 0.209 | 0.343 | Unannotated | NA | NA |
| metab_5997 | Unannotated feature | neg | 211.0836 | 2.867 | 3.386 | 3.196 | -0.190 | -0.225 | 0.209 | 0.343 | Unannotated | NA | NA |
| metab_580 | D-Glucaro-1,4-lactone | pos | 215.0163 | 0.861 | 5.569 | 5.667 | 0.099 | 0.225 | 0.209 | 0.343 | Tentative | NA | NA |
| metab_10180 | Unannotated feature | neg | 195.7605 | 0.904 | 4.282 | 4.331 | 0.049 | 0.225 | 0.209 | 0.343 | Unannotated | NA | NA |
| metab_7517 | Unannotated feature | neg | 175.5939 | 7.164 | 3.405 | 3.312 | -0.093 | -0.225 | 0.209 | 0.343 | Unannotated | NA | NA |
| metab_4618 | Unannotated feature | pos | 171.1177 | 4.255 | 4.729 | 4.743 | 0.014 | 0.225 | 0.209 | 0.343 | Unannotated | NA | NA |
| metab_11192 | Unannotated feature | neg | 540.3320 | 5.859 | 6.381 | 6.330 | -0.051 | -0.225 | 0.209 | 0.343 | Unannotated | NA | NA |
| metab_5884 | Unannotated feature | neg | 261.9786 | 2.485 | 4.321 | 4.394 | 0.073 | 0.225 | 0.209 | 0.343 | Unannotated | NA | NA |
| metab_4534 | Unannotated feature | pos | 379.9163 | 0.534 | 4.872 | 4.846 | -0.025 | -0.225 | 0.209 | 0.343 | Unannotated | NA | NA |
| metab_9524 | Unannotated feature | neg | 199.2539 | 3.224 | 4.218 | 4.189 | -0.029 | -0.225 | 0.209 | 0.343 | Unannotated | NA | NA |
| metab_6485 | Unannotated feature | neg | 544.2042 | 4.363 | 5.908 | 5.838 | -0.070 | -0.225 | 0.209 | 0.343 | Unannotated | NA | NA |
| metab_7582 | Unannotated feature | neg | 117.2762 | 7.213 | 4.069 | 4.049 | -0.020 | -0.225 | 0.209 | 0.343 | Unannotated | NA | NA |
| metab_3103 | 1-Naphthylamine | pos | 144.0809 | 3.838 | 4.688 | 4.605 | -0.083 | -0.225 | 0.209 | 0.343 | Moderate putative | NA | NA |
| metab_2054 | Unannotated feature | pos | 131.0857 | 7.231 | 4.176 | 4.230 | 0.054 | 0.225 | 0.209 | 0.343 | Unannotated | NA | NA |
| metab_7497 | Unannotated feature | neg | 812.2826 | 7.148 | 4.781 | 4.702 | -0.079 | -0.225 | 0.209 | 0.343 | Unannotated | NA | NA |
| metab_1612 | Unannotated feature | pos | 88.0762 | 6.953 | 4.550 | 4.586 | 0.036 | 0.225 | 0.209 | 0.343 | Unannotated | NA | NA |
| metab_5910 | Unannotated feature | neg | 384.0788 | 2.530 | 4.287 | 4.155 | -0.132 | -0.225 | 0.209 | 0.343 | Unannotated | NA | NA |
| metab_1556 | Unannotated feature | pos | 252.2282 | 6.303 | 5.011 | 4.865 | -0.147 | -0.225 | 0.209 | 0.343 | Unannotated | NA | NA |
| metab_10184 | Unannotated feature | neg | 197.7568 | 0.896 | 4.495 | 4.436 | -0.059 | -0.225 | 0.209 | 0.343 | Unannotated | NA | NA |
| metab_7754 | Unannotated feature | neg | 168.8359 | 7.181 | 4.977 | 4.966 | -0.011 | -0.225 | 0.209 | 0.343 | Unannotated | NA | NA |
| metab_3689 | 3-Aminopicolinaldehyde | pos | 140.0819 | 1.917 | 2.680 | 3.150 | 0.469 | 0.225 | 0.209 | 0.343 | Tentative | Organoheterocyclic compounds | Pyridines and derivatives |
| metab_7402 | Unannotated feature | neg | 158.4251 | 7.101 | 6.030 | 6.048 | 0.018 | 0.225 | 0.209 | 0.343 | Unannotated | NA | NA |
| metab_8889 | Unannotated feature | neg | 396.1469 | 7.117 | 5.292 | 5.267 | -0.025 | -0.225 | 0.209 | 0.343 | Unannotated | NA | NA |
| metab_3695 | Unannotated feature | pos | 175.0479 | 1.910 | 4.660 | 4.746 | 0.085 | 0.225 | 0.209 | 0.343 | Unannotated | NA | NA |
| metab_10238 | Unannotated feature | neg | 111.0192 | 0.865 | 3.103 | 3.363 | 0.260 | 0.225 | 0.209 | 0.343 | Unannotated | NA | NA |
| metab_4114 | Unannotated feature | pos | 277.9488 | 0.698 | 4.408 | 4.323 | -0.085 | -0.225 | 0.209 | 0.343 | Unannotated | NA | NA |
| metab_2105 | Unannotated feature | pos | 103.1314 | 7.076 | 6.774 | 6.792 | 0.019 | 0.225 | 0.209 | 0.343 | Unannotated | NA | NA |
| metab_7889 | Unannotated feature | neg | 714.8452 | 7.132 | 4.905 | 4.932 | 0.026 | 0.225 | 0.209 | 0.343 | Unannotated | NA | NA |
| metab_3809 | Unannotated feature | pos | 133.0318 | 1.414 | 4.034 | 4.016 | -0.018 | -0.225 | 0.209 | 0.343 | Unannotated | NA | NA |
| metab_10634 | Unannotated feature | neg | 589.2338 | 5.065 | 4.688 | 4.559 | -0.129 | -0.225 | 0.209 | 0.343 | Unannotated | NA | NA |
| metab_9840 | Unannotated feature | neg | 101.9404 | 2.498 | 4.187 | 4.206 | 0.019 | 0.221 | 0.218 | 0.354 | Unannotated | NA | NA |
| metab_5183 | Unannotated feature | neg | 479.8694 | 0.522 | 4.265 | 4.250 | -0.015 | -0.221 | 0.218 | 0.354 | Unannotated | NA | NA |
| metab_5181 | Unannotated feature | neg | 384.8629 | 0.522 | 4.108 | 4.057 | -0.050 | -0.221 | 0.218 | 0.354 | Unannotated | NA | NA |
| metab_9826 | Unannotated feature | neg | 123.9012 | 2.515 | 3.901 | 3.869 | -0.031 | -0.221 | 0.218 | 0.354 | Unannotated | NA | NA |
| metab_11086 | Unannotated feature | neg | 433.1144 | 5.619 | 4.898 | 4.720 | -0.178 | -0.221 | 0.218 | 0.354 | Unannotated | NA | NA |
| metab_10959 | Unannotated feature | neg | 132.8672 | 0.522 | 5.597 | 5.609 | 0.012 | 0.221 | 0.218 | 0.354 | Unannotated | NA | NA |
| metab_6387 | Unannotated feature | neg | 130.0867 | 4.037 | 4.579 | 4.613 | 0.034 | 0.221 | 0.218 | 0.354 | Unannotated | NA | NA |
| metab_11342 | Unannotated feature | neg | 163.9283 | 0.013 | 5.758 | 5.771 | 0.013 | 0.221 | 0.218 | 0.354 | Unannotated | NA | NA |
| metab_687 | Indolebutyric acid | pos | 236.1281 | 2.475 | 4.592 | 4.175 | -0.418 | -0.221 | 0.218 | 0.354 | Tentative | NA | NA |
| metab_11349 | Unannotated feature | neg | 599.7181 | 0.013 | 4.612 | 4.571 | -0.041 | -0.221 | 0.218 | 0.354 | Unannotated | NA | NA |
| metab_7950 | Unannotated feature | neg | 272.9110 | 0.841 | 4.011 | 3.834 | -0.177 | -0.221 | 0.218 | 0.354 | Unannotated | NA | NA |
| metab_5986 | Unannotated feature | neg | 203.0204 | 0.621 | 5.444 | 5.412 | -0.032 | -0.221 | 0.218 | 0.354 | Unannotated | NA | NA |
| metab_628 | Unannotated feature | pos | 89.5072 | 2.218 | 4.275 | 4.257 | -0.017 | -0.221 | 0.218 | 0.354 | Unannotated | NA | NA |
| metab_1252 | 6-Ethylchenodeoxycholic acid | pos | 385.3105 | 5.840 | 4.428 | 4.546 | 0.118 | 0.221 | 0.218 | 0.354 | Tentative | Lipids and lipid-like molecules | Steroids and steroid derivatives |
| metab_10758 | Unannotated feature | neg | 550.7807 | 0.570 | 5.470 | 5.458 | -0.012 | -0.221 | 0.218 | 0.354 | Unannotated | NA | NA |
| metab_1216 | Unannotated feature | pos | 230.2479 | 5.786 | 5.884 | 5.918 | 0.033 | 0.221 | 0.218 | 0.354 | Strong putative | NA | NA |
| metab_7296 | Unannotated feature | neg | 397.2060 | 6.188 | 5.800 | 5.584 | -0.216 | -0.221 | 0.218 | 0.354 | Unannotated | NA | NA |
| metab_4178 | Betaine | pos | 118.0865 | 0.644 | 6.812 | 6.733 | -0.079 | -0.221 | 0.218 | 0.354 | Strong putative | NA | NA |
| metab_8045 | Unannotated feature | neg | 129.0187 | 6.460 | 5.063 | 5.082 | 0.019 | 0.221 | 0.218 | 0.354 | Unannotated | NA | NA |
| metab_11057 | Unannotated feature | neg | 169.1026 | 4.363 | 4.478 | 4.329 | -0.149 | -0.221 | 0.218 | 0.354 | Unannotated | NA | NA |
| metab_10717 | Unannotated feature | neg | 498.7670 | 0.578 | 4.108 | 4.223 | 0.115 | 0.221 | 0.218 | 0.354 | Unannotated | NA | NA |
| metab_3406 | Unannotated feature | pos | 241.1595 | 2.451 | 4.488 | 4.172 | -0.315 | -0.221 | 0.218 | 0.354 | Unannotated | NA | NA |
| metab_1919 | Unannotated feature | pos | 198.0802 | 7.324 | 4.692 | 4.673 | -0.019 | -0.221 | 0.218 | 0.354 | Unannotated | NA | NA |
| metab_9448 | Unannotated feature | neg | 408.0887 | 3.340 | 4.682 | 4.579 | -0.103 | -0.221 | 0.218 | 0.354 | Unannotated | NA | NA |
| metab_6307 | Unannotated feature | neg | 277.9765 | 3.710 | 3.656 | 4.244 | 0.588 | 0.221 | 0.218 | 0.354 | Unannotated | NA | NA |
| metab_3101 | Vasicinone | pos | 244.1081 | 3.838 | 5.345 | 5.241 | -0.104 | -0.221 | 0.218 | 0.354 | Tentative | NA | NA |
| metab_672 | Unannotated feature | pos | 99.5316 | 2.428 | 4.951 | 4.984 | 0.033 | 0.221 | 0.218 | 0.354 | Unannotated | NA | NA |
| metab_3387 | Unannotated feature | pos | 344.2278 | 2.826 | 4.920 | 4.871 | -0.049 | -0.221 | 0.218 | 0.354 | Unannotated | NA | NA |
| metab_10953 | Unannotated feature | neg | 311.9443 | 0.522 | 3.522 | 3.689 | 0.166 | 0.221 | 0.218 | 0.354 | Unannotated | NA | NA |
| metab_786 | Unannotated feature | pos | 174.0213 | 2.950 | 4.362 | 4.348 | -0.014 | -0.221 | 0.218 | 0.354 | Unannotated | NA | NA |
| metab_6984 | Unannotated feature | neg | 248.0804 | 5.715 | 5.018 | 5.076 | 0.059 | 0.221 | 0.218 | 0.354 | Unannotated | NA | NA |
| metab_6296 | alpha-Isopropylmalate | neg | 175.0609 | 3.674 | 3.912 | 3.778 | -0.133 | -0.221 | 0.218 | 0.354 | Tentative | NA | NA |
| metab_4526 | Unannotated feature | pos | 575.9366 | 0.538 | 4.412 | 4.241 | -0.171 | -0.221 | 0.218 | 0.354 | Unannotated | NA | NA |
| metab_1894 | Unannotated feature | pos | 255.9442 | 7.331 | 4.270 | 4.241 | -0.029 | -0.221 | 0.218 | 0.354 | Unannotated | NA | NA |
| metab_7256 | Unannotated feature | neg | 702.3094 | 6.047 | 5.139 | 5.265 | 0.126 | 0.221 | 0.218 | 0.354 | Unannotated | NA | NA |
| metab_6211 | Unannotated feature | neg | 405.9235 | 0.652 | 4.442 | 4.622 | 0.180 | 0.221 | 0.218 | 0.354 | Unannotated | NA | NA |
| metab_10820 | Unannotated feature | neg | 863.8272 | 0.554 | 5.641 | 5.627 | -0.013 | -0.221 | 0.218 | 0.354 | Unannotated | NA | NA |
| metab_4319 | Unannotated feature | pos | 717.6884 | 0.575 | 4.398 | 4.379 | -0.019 | -0.221 | 0.218 | 0.354 | Unannotated | NA | NA |
| metab_202 | Unannotated feature | pos | 202.0686 | 0.606 | 3.773 | 3.893 | 0.119 | 0.221 | 0.218 | 0.354 | Unannotated | NA | NA |
| metab_259 | Unannotated feature | pos | 128.6635 | 0.691 | 2.938 | 3.240 | 0.302 | 0.221 | 0.218 | 0.354 | Unannotated | NA | NA |
| metab_7509 | Unannotated feature | neg | 138.8595 | 7.156 | 4.276 | 4.249 | -0.027 | -0.221 | 0.218 | 0.354 | Unannotated | NA | NA |
| metab_1583 | Unannotated feature | pos | 446.7502 | 6.435 | 4.806 | 4.853 | 0.048 | 0.221 | 0.218 | 0.354 | Unannotated | NA | NA |
| metab_7481 | Unannotated feature | neg | 725.3761 | 7.140 | 4.602 | 4.513 | -0.089 | -0.221 | 0.218 | 0.354 | Unannotated | NA | NA |
| metab_4628 | Unannotated feature | pos | 504.2075 | 4.907 | 4.279 | 4.580 | 0.302 | 0.221 | 0.218 | 0.354 | Unannotated | NA | NA |
| metab_8827 | Unannotated feature | neg | 536.8854 | 7.148 | 4.908 | 4.868 | -0.041 | -0.221 | 0.218 | 0.354 | Unannotated | NA | NA |
| metab_8393 | Unannotated feature | neg | 554.7674 | 0.629 | 5.236 | 5.182 | -0.054 | -0.221 | 0.218 | 0.354 | Unannotated | NA | NA |
| metab_10094 | Unannotated feature | neg | 90.9869 | 1.222 | 3.600 | 3.552 | -0.048 | -0.221 | 0.218 | 0.354 | Unannotated | NA | NA |
| metab_1525 | Unannotated feature | pos | 542.4777 | 6.188 | 5.374 | 5.283 | -0.091 | -0.221 | 0.218 | 0.354 | Unannotated | NA | NA |
| metab_8489 | Unannotated feature | neg | 459.0944 | 5.731 | 4.184 | 4.130 | -0.055 | -0.221 | 0.218 | 0.354 | Unannotated | NA | NA |
| metab_42 | Unannotated feature | pos | 166.0475 | 1.964 | 4.390 | 4.462 | 0.072 | 0.221 | 0.218 | 0.354 | Unannotated | NA | NA |
| metab_37 | Unannotated feature | pos | 472.1123 | 1.956 | 4.781 | 4.796 | 0.015 | 0.217 | 0.227 | 0.364 | Unannotated | NA | NA |
| metab_4103 | Tropinone | pos | 184.0718 | 0.714 | 4.772 | 4.723 | -0.049 | -0.217 | 0.227 | 0.364 | Tentative | NA | NA |
| metab_4585 | Mellein | pos | 179.0703 | 0.426 | 4.682 | 4.694 | 0.012 | 0.217 | 0.227 | 0.364 | Tentative | NA | NA |
| metab_323 | Pegorgotein | pos | 217.1183 | 0.807 | 4.042 | 3.900 | -0.142 | -0.217 | 0.227 | 0.364 | Tentative | NA | NA |
| metab_8802 | Unannotated feature | neg | 201.0226 | 5.498 | 5.374 | 5.222 | -0.152 | -0.217 | 0.227 | 0.364 | Unannotated | NA | NA |
| metab_8192 | Unannotated feature | neg | 449.2554 | 5.985 | 5.344 | 5.512 | 0.168 | 0.217 | 0.227 | 0.364 | Unannotated | NA | NA |
| metab_11099 | Dihydronaringenin-O-sulphate | neg | 353.0344 | 5.651 | 5.348 | 4.849 | -0.499 | -0.217 | 0.227 | 0.364 | Tentative | NA | NA |
| metab_493 | Unannotated feature | pos | 172.9564 | 1.886 | 4.544 | 4.531 | -0.013 | -0.217 | 0.227 | 0.364 | Unannotated | NA | NA |
| metab_7122 | Unannotated feature | neg | 180.1027 | 5.842 | 3.769 | 3.706 | -0.063 | -0.217 | 0.227 | 0.364 | Unannotated | NA | NA |
| metab_9102 | Unannotated feature | neg | 207.0775 | 4.417 | 4.132 | 3.979 | -0.154 | -0.217 | 0.227 | 0.364 | Unannotated | NA | NA |
| metab_3534 | Cyclohexa-2,4-dienylmethanol | pos | 93.0703 | 2.088 | 5.171 | 5.191 | 0.020 | 0.217 | 0.227 | 0.364 | Tentative | NA | NA |
| metab_3509 | Unannotated feature | pos | 111.0808 | 2.171 | 3.257 | 3.498 | 0.241 | 0.217 | 0.227 | 0.364 | Unannotated | NA | NA |
| metab_4785 | Unannotated feature | pos | 385.1781 | 7.277 | 5.305 | 5.344 | 0.039 | 0.217 | 0.227 | 0.364 | Unannotated | NA | NA |
| metab_11055 | MJDBISSN00000003 | neg | 411.3062 | 5.890 | 6.869 | 6.903 | 0.034 | 0.217 | 0.227 | 0.364 | Strong putative | NA | NA |
| metab_4531 | Unannotated feature | pos | 719.8534 | 0.534 | 5.157 | 5.132 | -0.025 | -0.217 | 0.227 | 0.364 | Unannotated | NA | NA |
| metab_6775 | Unannotated feature | neg | 547.0428 | 0.865 | 5.830 | 5.901 | 0.071 | -0.217 | 0.227 | 0.364 | Unannotated | NA | NA |
| metab_3417 | Unannotated feature | pos | 100.1125 | 2.412 | 3.773 | 3.870 | 0.097 | 0.217 | 0.227 | 0.364 | Unannotated | NA | NA |
| metab_11038 | Unannotated feature | neg | 224.9089 | 0.013 | 6.591 | 6.611 | 0.020 | 0.217 | 0.227 | 0.364 | Unannotated | NA | NA |
| metab_9336 | Unannotated feature | neg | 220.8143 | 3.680 | 4.154 | 4.132 | -0.023 | -0.217 | 0.227 | 0.364 | Unannotated | NA | NA |
| metab_7194 | Unannotated feature | neg | 287.9041 | 5.922 | 4.625 | 4.659 | 0.034 | 0.217 | 0.227 | 0.364 | Unannotated | NA | NA |
| metab_4831 | Unannotated feature | pos | 226.1051 | 7.293 | 4.166 | 4.132 | -0.033 | -0.217 | 0.227 | 0.364 | Unannotated | NA | NA |
| metab_765 | Unannotated feature | pos | 253.0738 | 2.841 | 4.741 | 4.804 | 0.063 | 0.217 | 0.227 | 0.364 | Unannotated | NA | NA |
| metab_11005 | Unannotated feature | neg | 314.8179 | 0.019 | 5.743 | 5.738 | -0.005 | -0.217 | 0.227 | 0.364 | Unannotated | NA | NA |
| metab_759 | Unannotated feature | pos | 115.0950 | 2.795 | 4.312 | 4.358 | 0.046 | 0.217 | 0.227 | 0.364 | Unannotated | NA | NA |
| metab_9456 | Unannotated feature | neg | 134.0605 | 3.710 | 4.482 | 4.861 | 0.379 | 0.217 | 0.227 | 0.364 | Unannotated | NA | NA |
| metab_10957 | Unannotated feature | neg | 156.9750 | 0.522 | 3.696 | 3.722 | 0.026 | 0.217 | 0.227 | 0.364 | Unannotated | NA | NA |
| metab_9441 | Unannotated feature | neg | 331.9375 | 3.364 | 4.921 | 4.882 | -0.039 | -0.217 | 0.227 | 0.364 | Unannotated | NA | NA |
| metab_10913 | Unannotated feature | neg | 899.8211 | 0.539 | 4.271 | 4.138 | -0.133 | -0.217 | 0.227 | 0.364 | Unannotated | NA | NA |
| metab_4954 | Unannotated feature | pos | 159.0765 | 0.644 | 5.219 | 5.256 | 0.037 | 0.217 | 0.227 | 0.364 | Unannotated | NA | NA |
| metab_9568 | Unannotated feature | neg | 71.0494 | 3.192 | 3.316 | 3.171 | -0.145 | -0.217 | 0.227 | 0.364 | Unannotated | NA | NA |
| metab_4190 | Unannotated feature | pos | 221.1860 | 0.636 | 4.654 | 4.531 | -0.123 | -0.217 | 0.227 | 0.364 | Unannotated | NA | NA |
| metab_2941 | Unannotated feature | pos | 690.2260 | 4.769 | 4.302 | 4.412 | 0.110 | 0.217 | 0.227 | 0.364 | Unannotated | NA | NA |
| metab_2917 | Unannotated feature | pos | 119.0732 | 4.900 | 3.124 | 3.268 | 0.144 | 0.217 | 0.227 | 0.364 | Unannotated | NA | NA |
| metab_2824 | Therafectin | pos | 270.1699 | 5.520 | 3.763 | 3.362 | -0.401 | -0.217 | 0.227 | 0.364 | Tentative | Organic oxygen compounds | Organooxygen compounds |
| metab_5008 | Unannotated feature | pos | 126.0276 | 0.807 | 4.246 | 4.131 | -0.114 | -0.217 | 0.227 | 0.364 | Unannotated | NA | NA |
| metab_1070 | Unannotated feature | pos | 130.0653 | 5.191 | 4.659 | 4.722 | 0.063 | 0.217 | 0.227 | 0.364 | Unannotated | NA | NA |
| metab_2782 | (8R,9S,10S,13S,14S,17R)-16-Fluoro-17-hydroxy-10,13-dimethyl-1,2,4,5,6,7,8,9,11,12,14,15,16,17-tetradecahydrocyclopenta[a]phenanthren-3-one | pos | 331.2050 | 5.649 | 4.491 | 4.286 | -0.205 | -0.217 | 0.227 | 0.364 | Tentative | NA | NA |
| metab_2609 | Unannotated feature | pos | 217.0684 | 0.551 | 5.072 | 5.052 | -0.021 | -0.217 | 0.227 | 0.364 | Unannotated | NA | NA |
| metab_5126 | Dermatan | pos | 374.1444 | 2.187 | 4.878 | 4.521 | -0.357 | -0.217 | 0.227 | 0.364 | Tentative | NA | NA |
| metab_2474 | Unannotated feature | pos | 633.9276 | 5.902 | 4.892 | 4.777 | -0.115 | -0.217 | 0.227 | 0.364 | Unannotated | NA | NA |
| metab_5168 | Unannotated feature | neg | 130.9830 | 0.515 | 3.933 | 4.040 | 0.107 | 0.217 | 0.227 | 0.364 | Unannotated | NA | NA |
| metab_5172 | Unannotated feature | neg | 131.9794 | 0.522 | 2.857 | 2.909 | 0.052 | 0.217 | 0.227 | 0.364 | Unannotated | NA | NA |
| metab_10686 | Unannotated feature | neg | 118.9300 | 0.582 | 4.274 | 4.286 | 0.011 | 0.217 | 0.227 | 0.364 | Unannotated | NA | NA |
| metab_10675 | Unannotated feature | neg | 555.6865 | 0.582 | 5.075 | 5.047 | -0.028 | -0.217 | 0.227 | 0.364 | Unannotated | NA | NA |
| metab_2383 | Unannotated feature | pos | 696.4990 | 5.949 | 5.714 | 5.742 | 0.029 | 0.217 | 0.227 | 0.364 | Unannotated | NA | NA |
| metab_5935 | Unannotated feature | neg | 820.4284 | 0.598 | 5.511 | 5.543 | 0.032 | 0.217 | 0.227 | 0.364 | Unannotated | NA | NA |
| metab_10657 | Unannotated feature | neg | 486.7002 | 0.587 | 4.830 | 4.716 | -0.114 | -0.217 | 0.227 | 0.364 | Unannotated | NA | NA |
| metab_9997 | Unannotated feature | neg | 363.9311 | 1.935 | 4.637 | 4.565 | -0.072 | -0.217 | 0.227 | 0.364 | Unannotated | NA | NA |
| metab_2181 | Choline phosphate | pos | 184.0734 | 6.373 | 5.934 | 5.918 | -0.017 | -0.217 | 0.227 | 0.364 | Moderate putative | NA | NA |
| metab_5887 | Unannotated feature | neg | 199.9781 | 2.498 | 3.761 | 3.829 | 0.068 | 0.217 | 0.227 | 0.364 | Unannotated | NA | NA |
| metab_2101 | Unannotated feature | pos | 88.1125 | 7.099 | 7.665 | 7.680 | 0.015 | 0.217 | 0.227 | 0.364 | Unannotated | NA | NA |
| metab_2034 | Unannotated feature | pos | 339.1576 | 7.269 | 4.276 | 4.342 | 0.066 | 0.217 | 0.227 | 0.364 | Unannotated | NA | NA |
| metab_2024 | Unannotated feature | pos | 111.0920 | 7.277 | 3.796 | 3.854 | 0.058 | 0.217 | 0.227 | 0.364 | Unannotated | NA | NA |
| metab_1619 | Unannotated feature | pos | 144.1748 | 7.045 | 5.861 | 5.871 | 0.010 | 0.217 | 0.227 | 0.364 | Unannotated | NA | NA |
| metab_10179 | Unannotated feature | neg | 223.8859 | 0.904 | 4.362 | 4.307 | -0.054 | -0.217 | 0.227 | 0.364 | Unannotated | NA | NA |
| metab_2012 | Unannotated feature | pos | 284.9101 | 7.285 | 4.467 | 4.456 | -0.011 | -0.217 | 0.227 | 0.364 | Unannotated | NA | NA |
| metab_2011 | Unannotated feature | pos | 311.9289 | 7.285 | 4.620 | 4.589 | -0.031 | -0.217 | 0.227 | 0.364 | Unannotated | NA | NA |
| metab_1649 | Unannotated feature | pos | 546.4003 | 7.200 | 5.189 | 5.138 | -0.051 | -0.217 | 0.227 | 0.364 | Unannotated | NA | NA |
| metab_1968 | Unannotated feature | pos | 145.9856 | 7.300 | 3.152 | 3.200 | 0.047 | 0.217 | 0.227 | 0.364 | Unannotated | NA | NA |
| metab_7584 | Unannotated feature | neg | 153.8681 | 7.213 | 4.489 | 4.476 | -0.013 | -0.217 | 0.227 | 0.364 | Unannotated | NA | NA |
| metab_1690 | Unannotated feature | pos | 485.4312 | 7.277 | 5.313 | 5.297 | -0.017 | -0.217 | 0.227 | 0.364 | Unannotated | NA | NA |
| metab_5570 | Unannotated feature | neg | 265.9510 | 0.530 | 5.224 | 5.201 | -0.024 | -0.217 | 0.227 | 0.364 | Unannotated | NA | NA |
| metab_1922 | Unannotated feature | pos | 683.8062 | 0.567 | 4.681 | 4.708 | 0.028 | 0.217 | 0.227 | 0.364 | Unannotated | NA | NA |
| metab_8560 | Unannotated feature | neg | 264.0551 | 5.691 | 3.861 | 3.979 | 0.118 | 0.217 | 0.227 | 0.364 | Unannotated | NA | NA |
| metab_7587 | Unannotated feature | neg | 226.9629 | 7.213 | 4.108 | 4.124 | 0.017 | 0.217 | 0.227 | 0.364 | Unannotated | NA | NA |
| metab_6783 | Unannotated feature | neg | 455.0606 | 5.538 | 3.997 | 4.066 | 0.070 | 0.213 | 0.236 | 0.375 | Unannotated | NA | NA |
| metab_10482 | Unannotated feature | neg | 626.7355 | 0.636 | 5.169 | 5.069 | -0.100 | -0.213 | 0.236 | 0.375 | Unannotated | NA | NA |
| metab_2060 | Unannotated feature | pos | 611.6922 | 0.575 | 4.507 | 4.423 | -0.084 | -0.213 | 0.236 | 0.375 | Unannotated | NA | NA |
| metab_2135 | Unannotated feature | pos | 750.5582 | 6.729 | 5.859 | 5.820 | -0.040 | -0.213 | 0.236 | 0.375 | Unannotated | NA | NA |
| metab_5768 | Unannotated feature | neg | 220.8346 | 1.951 | 4.232 | 4.252 | 0.021 | 0.213 | 0.236 | 0.375 | Unannotated | NA | NA |
| metab_5835 | Unannotated feature | neg | 723.8362 | 0.562 | 4.323 | 4.438 | 0.115 | 0.213 | 0.236 | 0.375 | Unannotated | NA | NA |
| metab_2139 | Morpholine-4-carboxamide | pos | 113.0713 | 6.675 | 4.784 | 4.812 | 0.028 | 0.213 | 0.236 | 0.375 | Tentative | NA | NA |
| metab_242 | Unannotated feature | pos | 144.1020 | 0.652 | 3.804 | 3.933 | 0.130 | 0.213 | 0.236 | 0.375 | Unannotated | NA | NA |
| metab_10206 | Unannotated feature | neg | 416.9878 | 0.865 | 4.957 | 4.867 | -0.090 | -0.213 | 0.236 | 0.375 | Unannotated | NA | NA |
| metab_2216 | Unannotated feature | pos | 387.2350 | 6.296 | 4.997 | 4.946 | -0.051 | -0.213 | 0.236 | 0.375 | Unannotated | NA | NA |
| metab_7496 | Unannotated feature | neg | 744.2951 | 7.148 | 5.062 | 4.949 | -0.113 | -0.213 | 0.236 | 0.375 | Unannotated | NA | NA |
| metab_5206 | Unannotated feature | neg | 843.3373 | 0.539 | 4.697 | 4.790 | 0.093 | 0.213 | 0.236 | 0.375 | Unannotated | NA | NA |
| metab_5301 | Unannotated feature | neg | 620.8557 | 0.562 | 4.316 | 4.360 | 0.044 | 0.213 | 0.236 | 0.375 | Unannotated | NA | NA |
| metab_7098 | Unannotated feature | neg | 443.1570 | 5.811 | 4.568 | 4.722 | 0.154 | 0.213 | 0.236 | 0.375 | Unannotated | NA | NA |
| metab_1740 | Unannotated feature | pos | 192.0616 | 7.324 | 4.193 | 4.159 | -0.034 | -0.213 | 0.236 | 0.375 | Unannotated | NA | NA |
| metab_9096 | Unannotated feature | neg | 322.9184 | 4.433 | 4.060 | 3.922 | -0.138 | -0.213 | 0.236 | 0.375 | Unannotated | NA | NA |
| metab_447 | Unannotated feature | pos | 132.0480 | 1.671 | 3.498 | 3.526 | 0.028 | 0.213 | 0.236 | 0.375 | Unannotated | NA | NA |
| metab_439 | Unannotated feature | pos | 147.0653 | 1.593 | 4.808 | 4.835 | 0.027 | 0.213 | 0.236 | 0.375 | Unannotated | NA | NA |
| metab_3603 | L-Lysine | pos | 111.0920 | 2.026 | 5.150 | 4.999 | -0.151 | -0.213 | 0.236 | 0.375 | Tentative | NA | NA |
| metab_8157 | Unannotated feature | neg | 327.2334 | 6.055 | 4.011 | 3.796 | -0.215 | -0.213 | 0.236 | 0.375 | Unannotated | NA | NA |
| metab_4608 | Unannotated feature | pos | 141.9587 | 0.021 | 5.606 | 5.624 | 0.018 | 0.213 | 0.236 | 0.375 | Unannotated | NA | NA |
| metab_8303 | Unannotated feature | neg | 540.2743 | 5.859 | 4.065 | 3.634 | -0.431 | -0.213 | 0.236 | 0.375 | Unannotated | NA | NA |
| metab_2373 | Unannotated feature | pos | 536.8023 | 0.567 | 5.547 | 5.526 | -0.021 | -0.213 | 0.236 | 0.375 | Unannotated | NA | NA |
| metab_6030 | Unannotated feature | neg | 262.9872 | 2.961 | 4.142 | 4.893 | 0.751 | 0.213 | 0.236 | 0.375 | Unannotated | NA | NA |
| metab_4923 | 3-Oxopropanoate | pos | 130.0500 | 0.621 | 5.973 | 5.935 | -0.037 | -0.213 | 0.236 | 0.375 | Tentative | NA | NA |
| metab_10647 | Unannotated feature | neg | 708.5939 | 0.587 | 5.029 | 4.978 | -0.051 | -0.213 | 0.236 | 0.375 | Unannotated | NA | NA |
| metab_9113 | Unannotated feature | neg | 121.0288 | 3.332 | 4.492 | 4.535 | 0.043 | 0.213 | 0.236 | 0.375 | Unannotated | NA | NA |
| metab_8020 | Unannotated feature | neg | 498.2905 | 6.679 | 4.558 | 5.145 | 0.587 | 0.213 | 0.236 | 0.375 | Unannotated | NA | NA |
| metab_11025 | Unannotated feature | neg | 498.7842 | 0.013 | 4.416 | 4.415 | -0.001 | -0.213 | 0.236 | 0.375 | Unannotated | NA | NA |
| metab_3820 | Unannotated feature | pos | 131.6854 | 1.313 | 5.042 | 5.022 | -0.020 | -0.213 | 0.236 | 0.375 | Unannotated | NA | NA |
| metab_6203 | (4As,5aR,6R,12aR)-1,10,11,12a-tetrahydroxy-6-methyl-3,12-dioxo-4a,5,5a,6-tetrahydro-4H-tetracene-2-carboxamide | neg | 406.0919 | 3.332 | 5.328 | 5.210 | -0.118 | -0.213 | 0.236 | 0.375 | Tentative | NA | NA |
| metab_4674 | Unannotated feature | pos | 512.3349 | 5.863 | 6.178 | 6.295 | 0.117 | 0.213 | 0.236 | 0.375 | Unannotated | NA | NA |
| metab_8706 | Unannotated feature | neg | 265.0391 | 5.595 | 4.062 | 4.218 | 0.156 | 0.213 | 0.236 | 0.375 | Unannotated | NA | NA |
| metab_8587 | Tetrahydroaldosterone-3-glucuronide | neg | 539.2511 | 5.675 | 5.709 | 5.828 | 0.119 | 0.213 | 0.236 | 0.375 | Tentative | NA | NA |
| metab_4300 | Unannotated feature | pos | 906.6070 | 0.575 | 4.479 | 4.446 | -0.033 | -0.213 | 0.236 | 0.375 | Unannotated | NA | NA |
| metab_7200 | Unannotated feature | neg | 445.8212 | 5.922 | 6.245 | 6.201 | -0.045 | -0.213 | 0.236 | 0.375 | Unannotated | NA | NA |
| metab_8766 | Unannotated feature | neg | 392.8610 | 5.538 | 4.655 | 4.548 | -0.107 | -0.213 | 0.236 | 0.375 | Unannotated | NA | NA |
| metab_606 | 7-Hydroxyoctanoic acid | pos | 202.1438 | 2.110 | 4.424 | 4.224 | -0.200 | -0.213 | 0.236 | 0.375 | Tentative | Organic acids and derivatives | Hydroxy acids and derivatives |
| metab_108 | Unannotated feature | pos | 711.9113 | 0.538 | 4.412 | 4.326 | -0.086 | -0.213 | 0.236 | 0.375 | Unannotated | NA | NA |
| metab_1064 | Deferiprone | pos | 184.0339 | 5.122 | 4.496 | 4.554 | 0.058 | 0.213 | 0.236 | 0.375 | Tentative | NA | NA |
| metab_4021 | Unannotated feature | pos | 304.2610 | 0.800 | 5.320 | 5.314 | -0.006 | -0.213 | 0.236 | 0.375 | Unannotated | NA | NA |
| metab_4330 | Unannotated feature | pos | 591.7434 | 0.575 | 4.497 | 4.453 | -0.044 | -0.213 | 0.236 | 0.375 | Unannotated | NA | NA |
| metab_5713 | Unannotated feature | neg | 128.4340 | 1.398 | 3.811 | 3.751 | -0.060 | -0.213 | 0.236 | 0.375 | Unannotated | NA | NA |
| metab_3195 | Unannotated feature | pos | 122.0603 | 3.283 | 3.124 | 3.218 | 0.094 | 0.213 | 0.236 | 0.375 | Unannotated | NA | NA |
| metab_2088 | 2-Amino-6-methoxypurine | pos | 207.0989 | 7.192 | 5.062 | 5.072 | 0.010 | 0.213 | 0.236 | 0.375 | Tentative | NA | NA |
| metab_2086 | Unannotated feature | pos | 465.3549 | 7.192 | 5.179 | 5.150 | -0.028 | -0.213 | 0.236 | 0.375 | Unannotated | NA | NA |
| metab_7612 | Unannotated feature | neg | 239.9038 | 7.293 | 5.939 | 5.963 | 0.023 | 0.213 | 0.236 | 0.375 | Unannotated | NA | NA |
| metab_3225 | Unannotated feature | pos | 270.0891 | 3.151 | 5.376 | 5.176 | -0.200 | -0.213 | 0.236 | 0.375 | Unannotated | NA | NA |
| metab_6253 | Unannotated feature | neg | 180.8306 | 3.507 | 4.020 | 4.050 | 0.030 | 0.213 | 0.236 | 0.375 | Unannotated | NA | NA |
| metab_9738 | Unannotated feature | neg | 286.8972 | 2.740 | 5.888 | 5.914 | 0.026 | 0.213 | 0.236 | 0.375 | Unannotated | NA | NA |
| metab_7890 | Unannotated feature | neg | 578.8703 | 7.132 | 5.142 | 5.124 | -0.019 | -0.208 | 0.246 | 0.386 | Unannotated | NA | NA |
| metab_7823 | Unannotated feature | neg | 255.2333 | 7.156 | 4.031 | 4.006 | -0.026 | -0.208 | 0.246 | 0.386 | Unannotated | NA | NA |
| metab_7757 | Unannotated feature | neg | 140.8866 | 7.181 | 3.752 | 3.726 | -0.026 | -0.208 | 0.246 | 0.386 | Unannotated | NA | NA |
| metab_11245 | Unannotated feature | neg | 414.8054 | 0.578 | 5.622 | 5.606 | -0.016 | -0.208 | 0.246 | 0.386 | Unannotated | NA | NA |
| metab_9286 | Unannotated feature | neg | 161.8753 | 3.811 | 4.258 | 4.276 | 0.019 | 0.208 | 0.246 | 0.386 | Unannotated | NA | NA |
| metab_9282 | Unannotated feature | neg | 173.0816 | 3.819 | 2.946 | 3.237 | 0.291 | 0.208 | 0.246 | 0.386 | Unannotated | NA | NA |
| metab_9352 | Unannotated feature | neg | 211.0612 | 5.763 | 3.919 | 3.849 | -0.070 | -0.208 | 0.246 | 0.386 | Unannotated | NA | NA |
| metab_5479 | Unannotated feature | neg | 283.0084 | 0.699 | 3.950 | 4.078 | 0.128 | 0.208 | 0.246 | 0.386 | Unannotated | NA | NA |
| metab_5319 | Unannotated feature | neg | 340.8370 | 0.013 | 4.897 | 4.889 | -0.008 | -0.208 | 0.246 | 0.386 | Unannotated | NA | NA |
| metab_2164 | Toluene | pos | 93.0703 | 6.427 | 4.165 | 4.200 | 0.036 | 0.208 | 0.246 | 0.386 | Tentative | NA | NA |
| metab_2191 | Unannotated feature | pos | 363.2310 | 6.319 | 4.755 | 4.661 | -0.094 | -0.208 | 0.246 | 0.386 | Unannotated | NA | NA |
| metab_10883 | Unannotated feature | neg | 939.8022 | 0.549 | 5.089 | 5.067 | -0.022 | -0.208 | 0.246 | 0.386 | Unannotated | NA | NA |
| metab_1380 | Unannotated feature | pos | 952.6754 | 5.910 | 5.336 | 5.301 | -0.035 | -0.208 | 0.246 | 0.386 | Unannotated | NA | NA |
| metab_54 | Unannotated feature | pos | 139.9633 | 0.021 | 4.981 | 4.970 | -0.011 | -0.208 | 0.246 | 0.386 | Unannotated | NA | NA |
| metab_4795 | Unannotated feature | pos | 518.3218 | 7.293 | 6.585 | 6.546 | -0.040 | -0.208 | 0.246 | 0.386 | Unannotated | NA | NA |
| metab_4793 | Unannotated feature | pos | 566.8886 | 7.293 | 6.055 | 6.041 | -0.014 | -0.208 | 0.246 | 0.386 | Unannotated | NA | NA |
| metab_4715 | Unannotated feature | pos | 908.6510 | 5.933 | 6.032 | 6.104 | 0.072 | 0.208 | 0.246 | 0.386 | Unannotated | NA | NA |
| metab_8207 | 12(R)-HETE | neg | 319.2284 | 5.961 | 5.702 | 5.476 | -0.226 | -0.208 | 0.246 | 0.386 | Moderate putative | NA | NA |
| metab_8108 | Unannotated feature | neg | 243.9178 | 2.906 | 4.029 | 4.122 | 0.092 | 0.208 | 0.246 | 0.386 | Unannotated | NA | NA |
| metab_1017 | Unannotated feature | pos | 84.1227 | 4.624 | 3.614 | 3.718 | 0.103 | 0.208 | 0.246 | 0.386 | Unannotated | NA | NA |
| metab_3606 | Unannotated feature | pos | 112.0953 | 2.019 | 3.805 | 3.658 | -0.147 | -0.208 | 0.246 | 0.386 | Unannotated | NA | NA |
| metab_2350 | Unannotated feature | pos | 455.3454 | 5.979 | 5.046 | 5.107 | 0.061 | 0.208 | 0.246 | 0.386 | Unannotated | NA | NA |
| metab_7435 | Inosine | neg | 267.0740 | 2.119 | 5.360 | 5.641 | 0.281 | 0.208 | 0.246 | 0.386 | Strong putative | Nucleosides, nucleotides, and analogues | Purine nucleosides |
| metab_5911 | Unannotated feature | neg | 431.0767 | 2.530 | 4.491 | 4.424 | -0.067 | -0.208 | 0.246 | 0.386 | Unannotated | NA | NA |
| metab_3485 | Unannotated feature | pos | 483.6682 | 2.234 | 4.411 | 3.852 | -0.559 | -0.208 | 0.246 | 0.386 | Unannotated | NA | NA |
| metab_11164 | Bromide | neg | 528.2651 | 5.755 | 5.465 | 5.314 | -0.151 | -0.208 | 0.246 | 0.386 | Tentative | NA | NA |
| metab_843 | Unannotated feature | pos | 190.1074 | 3.306 | 3.997 | 3.773 | -0.224 | -0.208 | 0.246 | 0.386 | Unannotated | NA | NA |
| metab_839 | Unannotated feature | pos | 251.1551 | 3.259 | 4.683 | 4.413 | -0.269 | -0.208 | 0.246 | 0.386 | Unannotated | NA | NA |
| metab_8025 | Unannotated feature | neg | 160.0843 | 6.600 | 3.397 | 3.341 | -0.056 | -0.208 | 0.246 | 0.386 | Unannotated | NA | NA |
| metab_8022 | 3-Hydroxymethyl-picumast | neg | 437.1625 | 6.633 | 4.871 | 4.352 | -0.518 | -0.208 | 0.246 | 0.386 | Tentative | NA | NA |
| metab_9944 | Unannotated feature | neg | 114.0553 | 2.119 | 4.189 | 4.314 | 0.125 | 0.208 | 0.246 | 0.386 | Unannotated | NA | NA |
| metab_1657 | Unannotated feature | pos | 149.0963 | 7.215 | 3.973 | 3.985 | 0.013 | 0.208 | 0.246 | 0.386 | Unannotated | NA | NA |
| metab_3001 | Unannotated feature | pos | 199.0966 | 4.378 | 4.103 | 4.079 | -0.024 | -0.208 | 0.246 | 0.386 | Unannotated | NA | NA |
| metab_11256 | Unannotated feature | neg | 281.2490 | 6.188 | 4.744 | 4.369 | -0.375 | -0.208 | 0.246 | 0.386 | Unannotated | NA | NA |
| metab_6782 | Unannotated feature | neg | 390.8640 | 5.538 | 5.443 | 5.364 | -0.080 | -0.208 | 0.246 | 0.386 | Unannotated | NA | NA |
| metab_11359 | Unannotated feature | neg | 241.8990 | 0.036 | 7.586 | 7.604 | 0.018 | 0.208 | 0.246 | 0.386 | Unannotated | NA | NA |
| metab_10859 | Unannotated feature | neg | 557.8831 | 0.554 | 5.154 | 5.229 | 0.076 | 0.208 | 0.246 | 0.386 | Unannotated | NA | NA |
| metab_10424 | Unannotated feature | neg | 175.0721 | 0.676 | 4.227 | 4.313 | 0.086 | 0.208 | 0.246 | 0.386 | Unannotated | NA | NA |
| metab_8372 | Unannotated feature | neg | 223.0977 | 5.811 | 3.994 | 4.204 | 0.210 | 0.208 | 0.246 | 0.386 | Unannotated | NA | NA |
| metab_1824 | Unannotated feature | pos | 180.0867 | 7.983 | 5.114 | 5.106 | -0.008 | -0.208 | 0.246 | 0.386 | Unannotated | NA | NA |
| metab_552 | NIPECOTIC ACID | pos | 130.0864 | 2.026 | 5.433 | 5.314 | -0.119 | -0.208 | 0.246 | 0.386 | Moderate putative | NA | NA |
| metab_8535 | Unannotated feature | neg | 163.0396 | 5.707 | 3.848 | 3.890 | 0.043 | 0.208 | 0.246 | 0.386 | Unannotated | NA | NA |
| metab_11107 | N-Palmitoyl Asparagine | neg | 259.0287 | 5.667 | 3.986 | 4.034 | 0.048 | 0.208 | 0.246 | 0.386 | Tentative | NA | NA |
| metab_9810 | Unannotated feature | neg | 670.6853 | 0.621 | 5.260 | 5.207 | -0.053 | -0.208 | 0.246 | 0.386 | Unannotated | NA | NA |
| metab_1523 | 9,9-Dimethyl-1-(sulfinylamino)decane | pos | 463.3397 | 6.188 | 5.955 | 5.900 | -0.055 | -0.208 | 0.246 | 0.386 | Tentative | NA | NA |
| metab_7330 | Unannotated feature | neg | 369.1748 | 6.368 | 6.780 | 6.522 | -0.258 | -0.208 | 0.246 | 0.386 | Unannotated | NA | NA |
| metab_8739 | Unannotated feature | neg | 269.0340 | 5.571 | 4.086 | 4.234 | 0.148 | 0.208 | 0.246 | 0.386 | Unannotated | NA | NA |
| metab_102 | Unannotated feature | pos | 748.8553 | 0.534 | 4.705 | 4.654 | -0.051 | -0.208 | 0.246 | 0.386 | Unannotated | NA | NA |
| metab_2612 | (-)-Menthol | pos | 330.3367 | 5.840 | 5.440 | 5.548 | 0.108 | 0.208 | 0.246 | 0.386 | Tentative | NA | NA |
| metab_3824 | Unannotated feature | pos | 168.4327 | 1.274 | 4.518 | 4.584 | 0.066 | 0.208 | 0.246 | 0.386 | Unannotated | NA | NA |
| metab_2546 | Unannotated feature | pos | 534.3770 | 5.871 | 5.183 | 5.131 | -0.052 | -0.208 | 0.246 | 0.386 | Unannotated | NA | NA |
| metab_7170 | Unannotated feature | neg | 193.3404 | 5.898 | 4.158 | 4.247 | 0.089 | 0.208 | 0.246 | 0.386 | Unannotated | NA | NA |
| metab_7166 | Unannotated feature | neg | 412.0472 | 5.890 | 4.463 | 4.667 | 0.204 | 0.208 | 0.246 | 0.386 | Unannotated | NA | NA |
| metab_10538 | Unannotated feature | neg | 508.6246 | 0.621 | 5.562 | 5.543 | -0.018 | -0.208 | 0.246 | 0.386 | Unannotated | NA | NA |
| metab_5696 | Unannotated feature | neg | 547.0428 | 1.294 | 4.710 | 4.485 | -0.225 | -0.208 | 0.246 | 0.386 | Unannotated | NA | NA |
| metab_7570 | Unannotated feature | neg | 226.9093 | 7.197 | 5.137 | 5.158 | 0.021 | 0.208 | 0.246 | 0.386 | Unannotated | NA | NA |
| metab_9463 | Ac-Tyr-OEt | neg | 222.0773 | 3.316 | 4.265 | 4.214 | -0.051 | -0.208 | 0.246 | 0.386 | Moderate putative | NA | NA |
| metab_5681 | Unannotated feature | neg | 341.8953 | 1.214 | 4.814 | 4.695 | -0.119 | -0.208 | 0.246 | 0.386 | Unannotated | NA | NA |
| metab_2095 | Unannotated feature | pos | 567.7783 | 0.575 | 4.477 | 4.578 | 0.101 | 0.208 | 0.246 | 0.386 | Unannotated | NA | NA |
| metab_7613 | Unannotated feature | neg | 96.6972 | 7.301 | 4.585 | 4.593 | 0.008 | 0.208 | 0.246 | 0.386 | Unannotated | NA | NA |
| metab_11267 | Unannotated feature | neg | 749.7286 | 0.570 | 4.288 | 4.228 | -0.060 | -0.204 | 0.255 | 0.397 | Unannotated | NA | NA |
| metab_1547 | Arabinosylhypoxanthine | pos | 269.0880 | 1.910 | 5.149 | 5.407 | 0.258 | 0.204 | 0.255 | 0.397 | Tentative | NA | NA |
| metab_4244 | Unannotated feature | pos | 179.0622 | 0.606 | 3.621 | 3.607 | -0.014 | -0.204 | 0.255 | 0.397 | Unannotated | NA | NA |
| metab_7188 | 15-Keto-prostaglandin F2alpha | neg | 397.2238 | 5.914 | 4.634 | 4.391 | -0.243 | -0.204 | 0.255 | 0.397 | Tentative | NA | NA |
| metab_11253 | Unannotated feature | neg | 102.9370 | 6.180 | 5.040 | 5.020 | -0.020 | -0.204 | 0.255 | 0.397 | Unannotated | NA | NA |
| metab_169 | Unannotated feature | pos | 663.8550 | 0.559 | 4.905 | 4.892 | -0.013 | -0.204 | 0.255 | 0.397 | Unannotated | NA | NA |
| metab_8868 | Unannotated feature | neg | 224.0619 | 7.124 | 5.257 | 5.249 | -0.008 | -0.204 | 0.255 | 0.397 | Unannotated | NA | NA |
| metab_7674 | Unannotated feature | neg | 118.0291 | 7.261 | 5.391 | 5.400 | 0.009 | 0.204 | 0.255 | 0.397 | Unannotated | NA | NA |
| metab_9802 | Unannotated feature | neg | 143.9090 | 2.537 | 4.187 | 4.129 | -0.058 | -0.204 | 0.255 | 0.397 | Unannotated | NA | NA |
| metab_9718 | Patulin | neg | 153.0188 | 2.820 | 5.385 | 5.372 | -0.013 | -0.204 | 0.255 | 0.397 | Moderate putative | NA | NA |
| metab_10100 | Unannotated feature | neg | 678.5070 | 0.613 | 6.053 | 6.018 | -0.035 | -0.204 | 0.255 | 0.397 | Unannotated | NA | NA |
| metab_7792 | Unannotated feature | neg | 157.9308 | 7.170 | 3.360 | 3.464 | 0.104 | 0.204 | 0.255 | 0.397 | Unannotated | NA | NA |
| metab_10765 | Unannotated feature | neg | 374.9072 | 0.570 | 5.595 | 5.572 | -0.023 | -0.204 | 0.255 | 0.397 | Unannotated | NA | NA |
| metab_10602 | Unannotated feature | neg | 607.5879 | 0.598 | 4.567 | 4.657 | 0.090 | 0.204 | 0.255 | 0.397 | Unannotated | NA | NA |
| metab_3936 | Unannotated feature | pos | 160.0969 | 3.923 | 4.940 | 4.826 | -0.114 | -0.204 | 0.255 | 0.397 | Unannotated | NA | NA |
| metab_3900 | Unannotated feature | pos | 287.6508 | 0.861 | 4.355 | 4.604 | 0.249 | 0.204 | 0.255 | 0.397 | Unannotated | NA | NA |
| metab_6223 | 4-Hydroxy-3-methoxy-benzaldehyde | neg | 151.0395 | 3.396 | 4.075 | 4.029 | -0.046 | -0.204 | 0.255 | 0.397 | Strong putative | NA | NA |
| metab_2563 | Unannotated feature | pos | 513.8607 | 5.863 | 5.035 | 4.972 | -0.064 | -0.204 | 0.255 | 0.397 | Unannotated | NA | NA |
| metab_10809 | Unannotated feature | neg | 973.7959 | 0.554 | 4.805 | 4.737 | -0.068 | -0.204 | 0.255 | 0.397 | Unannotated | NA | NA |
| metab_2519 | Unannotated feature | pos | 551.3949 | 5.886 | 6.197 | 6.251 | 0.054 | 0.204 | 0.255 | 0.397 | Unannotated | NA | NA |
| metab_9760 | 3-Hydroxyhippuric acid sulfate | neg | 274.0032 | 2.692 | 4.487 | 4.828 | 0.341 | 0.204 | 0.255 | 0.397 | Tentative | Benzenoids | Benzene and substituted derivatives |
| metab_9769 | Unannotated feature | neg | 525.0355 | 4.065 | 4.409 | 3.803 | -0.606 | -0.204 | 0.255 | 0.397 | Unannotated | NA | NA |
| metab_553 | N5-Ethyl-L-glutamine | pos | 157.0972 | 2.026 | 5.758 | 5.618 | -0.140 | -0.204 | 0.255 | 0.397 | Tentative | NA | NA |
| metab_5176 | Unannotated feature | neg | 226.9661 | 0.522 | 4.527 | 4.552 | 0.026 | 0.204 | 0.255 | 0.397 | Unannotated | NA | NA |
| metab_4507 | Unannotated feature | pos | 304.2611 | 0.545 | 5.681 | 5.669 | -0.011 | -0.204 | 0.255 | 0.397 | Unannotated | NA | NA |
| metab_10183 | Unannotated feature | neg | 131.0707 | 4.465 | 5.202 | 5.152 | -0.050 | -0.204 | 0.255 | 0.397 | Unannotated | NA | NA |
| metab_6562 | Unannotated feature | neg | 246.0887 | 4.651 | 4.116 | 4.022 | -0.095 | -0.204 | 0.255 | 0.397 | Unannotated | NA | NA |
| metab_3768 | Unannotated feature | pos | 160.1081 | 1.724 | 4.624 | 4.667 | 0.043 | 0.204 | 0.255 | 0.397 | Unannotated | NA | NA |
| metab_3700 | Unannotated feature | pos | 307.0439 | 1.902 | 4.319 | 4.152 | -0.168 | -0.204 | 0.255 | 0.397 | Unannotated | NA | NA |
| metab_3804 | Unannotated feature | pos | 169.0583 | 1.429 | 4.735 | 4.790 | 0.055 | 0.204 | 0.255 | 0.397 | Unannotated | NA | NA |
| metab_3679 | Unannotated feature | pos | 110.5221 | 1.933 | 4.444 | 4.433 | -0.011 | -0.204 | 0.255 | 0.397 | Unannotated | NA | NA |
| metab_816 | 4-Amino-4-deoxychorismate | pos | 226.0710 | 3.112 | 4.715 | 4.679 | -0.036 | -0.204 | 0.255 | 0.397 | Tentative | NA | NA |
| metab_7834 | Unannotated feature | neg | 526.3526 | 7.153 | 4.527 | 4.515 | -0.012 | -0.204 | 0.255 | 0.397 | Unannotated | NA | NA |
| metab_7836 | Unannotated feature | neg | 466.8747 | 7.153 | 4.639 | 4.616 | -0.023 | -0.204 | 0.255 | 0.397 | Unannotated | NA | NA |
| metab_7515 | Unannotated feature | neg | 101.9511 | 7.164 | 3.302 | 3.269 | -0.033 | -0.204 | 0.255 | 0.397 | Unannotated | NA | NA |
| metab_914 | Anthranilate | pos | 120.0446 | 3.854 | 4.004 | 4.078 | 0.074 | 0.204 | 0.255 | 0.397 | Moderate putative | NA | NA |
| metab_7946 | Unannotated feature | neg | 134.8940 | 7.109 | 5.900 | 5.885 | -0.015 | -0.204 | 0.255 | 0.397 | Unannotated | NA | NA |
| metab_3033 | 6-Methoxymellein | pos | 188.0706 | 4.171 | 4.044 | 3.999 | -0.045 | -0.204 | 0.255 | 0.397 | Tentative | Organoheterocyclic compounds | Benzopyrans |
| metab_7930 | Unannotated feature | neg | 328.8732 | 0.841 | 4.151 | 4.049 | -0.102 | -0.204 | 0.255 | 0.397 | Unannotated | NA | NA |
| metab_466 | Unannotated feature | pos | 133.3641 | 1.786 | 3.707 | 3.781 | 0.074 | 0.204 | 0.255 | 0.397 | Unannotated | NA | NA |
| metab_11304 | Unannotated feature | neg | 197.8520 | 7.189 | 5.142 | 5.133 | -0.009 | -0.204 | 0.255 | 0.397 | Unannotated | NA | NA |
| metab_9886 | Unannotated feature | neg | 197.0453 | 2.261 | 4.381 | 4.419 | 0.039 | 0.204 | 0.255 | 0.397 | Unannotated | NA | NA |
| metab_5970 | Unannotated feature | neg | 156.0661 | 2.756 | 3.726 | 3.813 | 0.087 | 0.204 | 0.255 | 0.397 | Unannotated | NA | NA |
| metab_10011 | Unannotated feature | neg | 273.8879 | 1.855 | 4.931 | 5.198 | 0.267 | 0.204 | 0.255 | 0.397 | Unannotated | NA | NA |
| metab_4611 | Unannotated feature | pos | 114.9844 | 0.015 | 4.521 | 4.501 | -0.020 | -0.204 | 0.255 | 0.397 | Unannotated | NA | NA |
| metab_8280 | 10-Hydroxydecanoic acid | neg | 187.1337 | 5.883 | 5.007 | 4.974 | -0.033 | -0.204 | 0.255 | 0.397 | Tentative | NA | NA |
| metab_6027 | Unannotated feature | neg | 205.0174 | 0.629 | 4.758 | 4.712 | -0.046 | -0.204 | 0.255 | 0.397 | Unannotated | NA | NA |
| metab_4796 | Unannotated feature | pos | 288.9219 | 7.300 | 5.229 | 5.236 | 0.007 | 0.204 | 0.255 | 0.397 | Unannotated | NA | NA |
| metab_7467 | Unannotated feature | neg | 142.0504 | 2.127 | 4.810 | 4.830 | 0.020 | 0.204 | 0.255 | 0.397 | Unannotated | NA | NA |
| metab_10019 | Unannotated feature | neg | 315.9060 | 1.831 | 4.794 | 4.662 | -0.132 | -0.204 | 0.255 | 0.397 | Unannotated | NA | NA |
| metab_8779 | 5-(3',4',5'-Trihydroxyphenyl)-gamma-valerolactone-O-methyl-O-sulphate | neg | 315.0153 | 5.523 | 3.454 | 3.098 | -0.356 | -0.204 | 0.255 | 0.397 | Tentative | Benzenoids | Phenols |
| metab_9497 | Unannotated feature | neg | 186.0558 | 3.255 | 4.708 | 4.823 | 0.115 | 0.204 | 0.255 | 0.397 | Strong putative | NA | NA |
| metab_2927 | Disulfiram | pos | 169.0496 | 4.869 | 4.907 | 4.328 | -0.580 | -0.204 | 0.255 | 0.397 | Tentative | NA | NA |
| metab_10551 | Unannotated feature | neg | 359.8367 | 0.621 | 3.969 | 3.893 | -0.077 | -0.204 | 0.255 | 0.397 | Unannotated | NA | NA |
| metab_1970 | Unannotated feature | pos | 94.0655 | 7.300 | 3.952 | 3.978 | 0.026 | 0.204 | 0.255 | 0.397 | Unannotated | NA | NA |
| metab_11013 | Unannotated feature | neg | 320.8870 | 0.016 | 5.753 | 5.765 | 0.012 | 0.204 | 0.255 | 0.397 | Unannotated | NA | NA |
| metab_7718 | Unannotated feature | neg | 139.0064 | 7.205 | 4.240 | 4.232 | -0.009 | -0.200 | 0.265 | 0.409 | Unannotated | NA | NA |
| metab_8644 | Unannotated feature | neg | 143.1072 | 5.643 | 3.676 | 3.549 | -0.126 | -0.200 | 0.265 | 0.409 | Unannotated | NA | NA |
| metab_3972 | Cys-Gly | pos | 161.0380 | 0.839 | 5.127 | 5.272 | 0.145 | 0.200 | 0.265 | 0.409 | Tentative | NA | NA |
| metab_8501 | Unannotated feature | neg | 131.9149 | 7.101 | 4.101 | 4.142 | 0.041 | 0.200 | 0.265 | 0.409 | Unannotated | NA | NA |
| metab_11069 | Unannotated feature | neg | 302.0368 | 5.571 | 4.672 | 4.744 | 0.072 | 0.200 | 0.265 | 0.409 | Unannotated | NA | NA |
| metab_4366 | Unannotated feature | pos | 682.8064 | 0.567 | 5.321 | 5.343 | 0.022 | 0.200 | 0.265 | 0.409 | Unannotated | NA | NA |
| metab_8514 | Unannotated feature | neg | 488.1937 | 5.715 | 4.304 | 4.068 | -0.236 | -0.200 | 0.265 | 0.409 | Unannotated | NA | NA |
| metab_5450 | Unannotated feature | neg | 166.8328 | 0.660 | 5.256 | 5.296 | 0.040 | 0.200 | 0.265 | 0.409 | Unannotated | NA | NA |
| metab_11311 | Unannotated feature | neg | 145.9304 | 7.213 | 4.717 | 4.737 | 0.021 | 0.200 | 0.265 | 0.409 | Unannotated | NA | NA |
| metab_7441 | Unannotated feature | neg | 162.9818 | 7.117 | 4.931 | 4.906 | -0.025 | -0.200 | 0.265 | 0.409 | Unannotated | NA | NA |
| metab_7970 | Unannotated feature | neg | 143.0344 | 0.833 | 4.281 | 4.214 | -0.067 | -0.200 | 0.265 | 0.409 | Unannotated | NA | NA |
| metab_5917 | Unannotated feature | neg | 294.0302 | 2.541 | 4.672 | 4.680 | 0.008 | -0.200 | 0.265 | 0.409 | Unannotated | NA | NA |
| metab_4686 | Unannotated feature | pos | 593.9159 | 5.886 | 5.150 | 5.098 | -0.053 | -0.200 | 0.265 | 0.409 | Unannotated | NA | NA |
| metab_4807 | Unannotated feature | pos | 245.0430 | 7.331 | 4.872 | 4.891 | 0.020 | 0.200 | 0.265 | 0.409 | Unannotated | NA | NA |
| metab_4650 | Unannotated feature | pos | 227.1254 | 5.748 | 4.665 | 4.649 | -0.016 | -0.200 | 0.265 | 0.409 | Unannotated | NA | NA |
| metab_8411 | Thymyl acetate | neg | 237.1134 | 5.787 | 3.769 | 3.991 | 0.223 | 0.200 | 0.265 | 0.409 | Tentative | NA | NA |
| metab_7928 | Unannotated feature | neg | 229.0902 | 7.109 | 4.099 | 4.103 | 0.004 | -0.200 | 0.265 | 0.409 | Unannotated | NA | NA |
| metab_7084 | Unannotated feature | neg | 169.0866 | 5.803 | 3.946 | 3.863 | -0.084 | -0.200 | 0.265 | 0.409 | Unannotated | NA | NA |
| metab_3803 | Unannotated feature | pos | 109.0287 | 1.460 | 5.159 | 5.150 | -0.009 | -0.200 | 0.265 | 0.409 | Unannotated | NA | NA |
| metab_9813 | Unannotated feature | neg | 505.0305 | 2.523 | 5.269 | 5.244 | -0.025 | -0.200 | 0.265 | 0.409 | Unannotated | NA | NA |
| metab_91 | Unannotated feature | pos | 161.9697 | 0.504 | 4.577 | 4.563 | -0.013 | -0.200 | 0.265 | 0.409 | Unannotated | NA | NA |
| metab_10987 | Unannotated feature | neg | 198.9363 | 0.365 | 5.040 | 5.051 | 0.012 | 0.200 | 0.265 | 0.409 | Unannotated | NA | NA |
| metab_340 | Unannotated feature | pos | 118.9429 | 0.831 | 3.516 | 3.537 | 0.020 | 0.200 | 0.265 | 0.409 | Unannotated | NA | NA |
| metab_11194 | Isofenphos | neg | 257.1762 | 5.867 | 4.969 | 5.209 | 0.241 | 0.200 | 0.265 | 0.409 | Strong putative | NA | NA |
| metab_11273 | Unannotated feature | neg | 178.0506 | 3.710 | 5.873 | 6.230 | 0.357 | 0.200 | 0.265 | 0.409 | Moderate putative | NA | NA |
| metab_1266 | Unannotated feature | pos | 510.3565 | 5.848 | 5.012 | 4.990 | -0.023 | -0.200 | 0.265 | 0.409 | Unannotated | NA | NA |
| metab_4362 | Unannotated feature | pos | 707.7684 | 0.567 | 4.073 | 4.003 | -0.070 | -0.200 | 0.265 | 0.409 | Unannotated | NA | NA |
| metab_11275 | Unannotated feature | neg | 500.8538 | 0.570 | 5.568 | 5.546 | -0.021 | -0.200 | 0.265 | 0.409 | Unannotated | NA | NA |
| metab_10075 | Unannotated feature | neg | 381.0308 | 1.294 | 3.743 | 4.062 | 0.318 | 0.200 | 0.265 | 0.409 | Unannotated | NA | NA |
| metab_5470 | Unannotated feature | neg | 158.9781 | 0.509 | 5.220 | 5.277 | 0.057 | 0.200 | 0.265 | 0.409 | Unannotated | NA | NA |
| metab_7676 | Unannotated feature | neg | 143.8645 | 7.253 | 4.261 | 4.243 | -0.018 | -0.200 | 0.265 | 0.409 | Unannotated | NA | NA |
| metab_9801 | Unannotated feature | neg | 499.9991 | 2.537 | 4.986 | 4.907 | -0.079 | -0.200 | 0.265 | 0.409 | Unannotated | NA | NA |
| metab_7733 | Unannotated feature | neg | 203.9226 | 7.189 | 4.488 | 4.504 | 0.016 | 0.200 | 0.265 | 0.409 | Unannotated | NA | NA |
| metab_2022 | Unannotated feature | pos | 243.9417 | 7.277 | 5.196 | 5.216 | 0.020 | 0.200 | 0.265 | 0.409 | Unannotated | NA | NA |
| metab_5537 | Unannotated feature | neg | 161.9749 | 0.802 | 3.605 | 3.683 | 0.077 | 0.200 | 0.265 | 0.409 | Unannotated | NA | NA |
| metab_10122 | Unannotated feature | neg | 176.8839 | 1.023 | 5.569 | 5.554 | -0.014 | -0.200 | 0.265 | 0.409 | Unannotated | NA | NA |
| metab_6962 | Unannotated feature | neg | 272.0718 | 5.699 | 4.199 | 4.041 | -0.158 | -0.200 | 0.265 | 0.409 | Unannotated | NA | NA |
| metab_5690 | Unannotated feature | neg | 279.7676 | 1.294 | 5.619 | 5.637 | 0.018 | 0.200 | 0.265 | 0.409 | Unannotated | NA | NA |
| metab_3227 | Ent-Lamivudine | pos | 212.0473 | 3.151 | 4.441 | 4.192 | -0.250 | -0.200 | 0.265 | 0.409 | Tentative | NA | NA |
| metab_65 | Unannotated feature | pos | 137.9875 | 0.217 | 4.245 | 4.263 | 0.018 | 0.200 | 0.265 | 0.409 | Unannotated | NA | NA |
| metab_9438 | Unannotated feature | neg | 169.0979 | 3.372 | 5.173 | 5.058 | -0.114 | -0.200 | 0.265 | 0.409 | Unannotated | NA | NA |
| metab_997 | Unannotated feature | pos | 178.9928 | 0.831 | 5.028 | 4.895 | -0.133 | -0.200 | 0.265 | 0.409 | Unannotated | NA | NA |
| metab_9323 | Unannotated feature | neg | 246.0387 | 3.710 | 3.951 | 4.366 | 0.414 | 0.200 | 0.265 | 0.409 | Unannotated | NA | NA |
| metab_820 | Unannotated feature | pos | 166.0419 | 3.151 | 3.978 | 3.731 | -0.248 | -0.200 | 0.265 | 0.409 | Unannotated | NA | NA |
| metab_826 | Unannotated feature | pos | 119.0688 | 3.159 | 3.355 | 3.562 | 0.207 | 0.200 | 0.265 | 0.409 | Unannotated | NA | NA |
| metab_1012 | Loliolide | pos | 197.1173 | 4.578 | 4.293 | 4.350 | 0.057 | 0.200 | 0.265 | 0.409 | Moderate putative | NA | NA |
| metab_6918 | Allantoate | neg | 211.0248 | 5.675 | 4.327 | 4.175 | -0.152 | -0.200 | 0.265 | 0.409 | Tentative | NA | NA |
| metab_3172 | Unannotated feature | pos | 214.0629 | 3.445 | 5.229 | 5.265 | 0.036 | 0.200 | 0.265 | 0.409 | Strong putative | NA | NA |
| metab_2884 | 3'-Hydroxy-4',5,6,7,8-pentamethoxyflavone | pos | 406.1503 | 5.107 | 4.680 | 4.404 | -0.276 | -0.200 | 0.265 | 0.409 | Tentative | NA | NA |
| metab_1921 | Unannotated feature | pos | 183.0974 | 7.324 | 4.914 | 4.897 | -0.017 | -0.196 | 0.276 | 0.421 | Unannotated | NA | NA |
| metab_1599 | Unannotated feature | pos | 654.2965 | 6.660 | 5.512 | 5.603 | 0.091 | 0.196 | 0.276 | 0.421 | Unannotated | NA | NA |
| metab_2124 | 24-Acetyl-25-cinnamoylvulgaroside | pos | 631.3299 | 6.884 | 5.482 | 5.449 | -0.033 | -0.196 | 0.276 | 0.421 | Tentative | NA | NA |
| metab_7653 | Unannotated feature | neg | 97.2224 | 7.353 | 4.556 | 4.564 | 0.007 | 0.196 | 0.276 | 0.421 | Unannotated | NA | NA |
| metab_1720 | Unannotated feature | pos | 240.9027 | 7.300 | 4.285 | 4.312 | 0.028 | 0.196 | 0.276 | 0.421 | Unannotated | NA | NA |
| metab_9519 | Unannotated feature | neg | 379.0248 | 3.224 | 3.925 | 3.582 | -0.343 | -0.196 | 0.276 | 0.421 | Unannotated | NA | NA |
| metab_1772 | L-Ornithine | pos | 133.0973 | 0.541 | 5.511 | 5.490 | -0.022 | -0.196 | 0.276 | 0.421 | Strong putative | NA | NA |
| metab_5174 | Unannotated feature | neg | 146.9654 | 0.522 | 5.034 | 5.089 | 0.055 | 0.196 | 0.276 | 0.421 | Unannotated | NA | NA |
| metab_2175 | 1-Methyl-1,3-cyclohexadiene | pos | 95.0860 | 6.420 | 5.050 | 5.048 | -0.002 | 0.196 | 0.276 | 0.421 | Tentative | NA | NA |
| metab_10067 | Unannotated feature | neg | 160.9983 | 1.342 | 3.073 | 3.622 | 0.550 | 0.196 | 0.276 | 0.421 | Unannotated | NA | NA |
| metab_4292 | Unannotated feature | pos | 243.0531 | 4.892 | 5.276 | 5.416 | 0.139 | 0.196 | 0.276 | 0.421 | Unannotated | NA | NA |
| metab_7740 | Unannotated feature | neg | 181.9172 | 7.189 | 4.387 | 4.362 | -0.025 | -0.196 | 0.276 | 0.421 | Unannotated | NA | NA |
| metab_8573 | Unannotated feature | neg | 258.0560 | 7.124 | 4.268 | 4.222 | -0.046 | -0.196 | 0.276 | 0.421 | Unannotated | NA | NA |
| metab_1229 | Hexadecanedioic acid mono-L-carnitine ester | pos | 394.2952 | 5.809 | 5.175 | 5.083 | -0.093 | -0.196 | 0.276 | 0.421 | Tentative | NA | NA |
| metab_7575 | Unannotated feature | neg | 228.9048 | 7.205 | 4.631 | 4.652 | 0.021 | 0.196 | 0.276 | 0.421 | Unannotated | NA | NA |
| metab_2141 | Unannotated feature | pos | 612.3247 | 6.660 | 5.451 | 5.649 | 0.198 | 0.196 | 0.276 | 0.421 | Unannotated | NA | NA |
| metab_7215 | Unannotated feature | neg | 401.8815 | 5.937 | 5.009 | 5.047 | 0.038 | 0.196 | 0.276 | 0.421 | Unannotated | NA | NA |
| metab_7569 | Unannotated feature | neg | 211.9144 | 7.197 | 4.363 | 4.346 | -0.016 | -0.196 | 0.276 | 0.421 | Unannotated | NA | NA |
| metab_7600 | Unannotated feature | neg | 141.9120 | 7.253 | 5.145 | 5.150 | 0.005 | 0.196 | 0.276 | 0.421 | Unannotated | NA | NA |
| metab_5676 | Unannotated feature | neg | 277.7706 | 1.182 | 5.670 | 5.681 | 0.011 | 0.196 | 0.276 | 0.421 | Unannotated | NA | NA |
| metab_11277 | Unannotated feature | neg | 492.8219 | 0.570 | 5.584 | 5.567 | -0.017 | -0.196 | 0.276 | 0.421 | Unannotated | NA | NA |
| metab_7548 | Unannotated feature | neg | 160.8415 | 7.181 | 5.755 | 5.739 | -0.016 | -0.196 | 0.276 | 0.421 | Unannotated | NA | NA |
| metab_2615 | Unannotated feature | pos | 229.1800 | 5.840 | 4.376 | 4.395 | 0.019 | 0.196 | 0.276 | 0.421 | Unannotated | NA | NA |
| metab_9909 | Unannotated feature | neg | 392.1209 | 2.119 | 4.293 | 4.320 | 0.028 | 0.196 | 0.276 | 0.421 | Unannotated | NA | NA |
| metab_2368 | Unannotated feature | pos | 499.3715 | 5.964 | 6.002 | 6.037 | 0.034 | 0.196 | 0.276 | 0.421 | Unannotated | NA | NA |
| metab_8002 | Unannotated feature | neg | 197.9630 | 6.939 | 5.642 | 5.666 | 0.024 | 0.196 | 0.276 | 0.421 | Unannotated | NA | NA |
| metab_7434 | Unannotated feature | neg | 243.9533 | 7.109 | 6.546 | 6.560 | 0.014 | 0.196 | 0.276 | 0.421 | Unannotated | NA | NA |
| metab_2393 | Unannotated feature | pos | 545.8753 | 5.941 | 4.642 | 4.777 | 0.135 | 0.196 | 0.276 | 0.421 | Unannotated | NA | NA |
| metab_8392 | Unannotated feature | neg | 331.8436 | 5.795 | 4.790 | 4.751 | -0.039 | -0.196 | 0.276 | 0.421 | Unannotated | NA | NA |
| metab_10948 | Unannotated feature | neg | 112.9060 | 0.530 | 2.423 | 2.661 | 0.237 | 0.196 | 0.276 | 0.421 | Unannotated | NA | NA |
| metab_4786 | Unannotated feature | pos | 113.1076 | 7.285 | 3.833 | 3.885 | 0.052 | 0.196 | 0.276 | 0.421 | Unannotated | NA | NA |
| metab_3548 | Unannotated feature | pos | 167.1370 | 2.073 | 4.630 | 4.644 | 0.014 | 0.196 | 0.276 | 0.421 | Unannotated | NA | NA |
| metab_8348 | Unannotated feature | neg | 641.3560 | 5.819 | 4.222 | 3.917 | -0.305 | -0.196 | 0.276 | 0.421 | Unannotated | NA | NA |
| metab_1732 | Unannotated feature | pos | 73.5324 | 7.324 | 3.156 | 2.985 | -0.171 | -0.196 | 0.276 | 0.421 | Unannotated | NA | NA |
| metab_10976 | Unannotated feature | neg | 194.9562 | 0.505 | 3.210 | 3.283 | 0.073 | 0.196 | 0.276 | 0.421 | Unannotated | NA | NA |
| metab_399 | Unannotated feature | pos | 163.0278 | 1.134 | 4.682 | 4.656 | -0.026 | -0.196 | 0.276 | 0.421 | Unannotated | NA | NA |
| metab_7960 | Unannotated feature | neg | 190.9727 | 0.841 | 4.537 | 4.429 | -0.108 | -0.196 | 0.276 | 0.421 | Unannotated | NA | NA |
| metab_1985 | Unannotated feature | pos | 530.3583 | 7.293 | 4.770 | 4.649 | -0.121 | -0.196 | 0.276 | 0.421 | Unannotated | NA | NA |
| metab_444 | 6,8-Dihydroxypurine | pos | 153.0408 | 1.647 | 5.328 | 5.280 | -0.048 | -0.196 | 0.276 | 0.421 | Tentative | NA | NA |
| metab_77 | Unannotated feature | pos | 153.0547 | 0.442 | 4.412 | 4.421 | 0.009 | 0.196 | 0.276 | 0.421 | Unannotated | NA | NA |
| metab_10678 | Unannotated feature | neg | 550.6720 | 0.582 | 5.287 | 5.262 | -0.025 | -0.196 | 0.276 | 0.421 | Unannotated | NA | NA |
| metab_10921 | Unannotated feature | neg | 606.9961 | 0.539 | 4.725 | 4.620 | -0.105 | -0.196 | 0.276 | 0.421 | Unannotated | NA | NA |
| metab_11296 | Unannotated feature | neg | 320.8869 | 7.170 | 5.493 | 5.501 | 0.008 | 0.196 | 0.276 | 0.421 | Unannotated | NA | NA |
| metab_1457 | Unannotated feature | pos | 475.3240 | 5.987 | 6.031 | 6.063 | 0.033 | 0.196 | 0.276 | 0.421 | Unannotated | NA | NA |
| metab_57 | Queuine | pos | 278.1234 | 0.807 | 4.802 | 4.676 | -0.126 | -0.196 | 0.276 | 0.421 | Tentative | NA | NA |
| metab_9319 | Unannotated feature | neg | 178.7057 | 3.718 | 3.601 | 4.042 | 0.441 | 0.196 | 0.276 | 0.421 | Unannotated | NA | NA |
| metab_59 | Unannotated feature | pos | 114.0736 | 0.031 | 4.746 | 4.722 | -0.023 | -0.196 | 0.276 | 0.421 | Unannotated | NA | NA |
| metab_6703 | Unannotated feature | neg | 218.8173 | 5.279 | 4.359 | 4.327 | -0.032 | -0.196 | 0.276 | 0.421 | Unannotated | NA | NA |
| metab_6982 | Unannotated feature | neg | 209.0948 | 5.715 | 5.243 | 5.229 | -0.014 | -0.196 | 0.276 | 0.421 | Unannotated | NA | NA |
| metab_10967 | Unannotated feature | neg | 166.9403 | 0.509 | 3.331 | 3.422 | 0.091 | 0.192 | 0.286 | 0.433 | Unannotated | NA | NA |
| metab_9335 | Unannotated feature | neg | 338.0669 | 3.687 | 4.707 | 4.751 | 0.043 | 0.192 | 0.286 | 0.433 | Unannotated | NA | NA |
| metab_9484 | Unannotated feature | neg | 232.0941 | 3.266 | 4.442 | 4.354 | -0.088 | -0.192 | 0.286 | 0.433 | Unannotated | NA | NA |
| metab_4093 | Tulipalin A | pos | 99.0445 | 0.729 | 5.050 | 5.008 | -0.042 | -0.192 | 0.286 | 0.433 | Moderate putative | NA | NA |
| metab_4948 | Unannotated feature | pos | 501.0302 | 0.636 | 5.130 | 5.054 | -0.076 | -0.192 | 0.286 | 0.433 | Unannotated | NA | NA |
| metab_76 | Unannotated feature | pos | 138.9919 | 0.442 | 4.756 | 4.753 | -0.003 | -0.192 | 0.286 | 0.433 | Unannotated | NA | NA |
| metab_117 | Unannotated feature | pos | 652.9726 | 0.541 | 4.882 | 4.824 | -0.058 | -0.192 | 0.286 | 0.433 | Unannotated | NA | NA |
| metab_6038 | Unannotated feature | neg | 728.6442 | 0.629 | 4.983 | 4.889 | -0.094 | -0.192 | 0.286 | 0.433 | Unannotated | NA | NA |
| metab_2374 | Unannotated feature | pos | 922.6666 | 5.956 | 5.832 | 5.811 | -0.022 | -0.192 | 0.286 | 0.433 | Unannotated | NA | NA |
| metab_5026 | Unannotated feature | pos | 349.1105 | 0.839 | 4.318 | 4.371 | 0.053 | 0.192 | 0.286 | 0.433 | Unannotated | NA | NA |
| metab_2292 | Unannotated feature | pos | 288.1992 | 6.095 | 3.763 | 3.477 | -0.286 | -0.192 | 0.286 | 0.433 | Unannotated | NA | NA |
| metab_10286 | Unannotated feature | neg | 170.9438 | 0.833 | 4.100 | 4.075 | -0.025 | -0.192 | 0.286 | 0.433 | Unannotated | NA | NA |
| metab_6163 | Unannotated feature | neg | 350.9252 | 3.247 | 4.767 | 4.733 | -0.034 | -0.192 | 0.286 | 0.433 | Unannotated | NA | NA |
| metab_1792 | Unannotated feature | pos | 86.0605 | 7.718 | 3.626 | 3.617 | -0.009 | 0.192 | 0.286 | 0.433 | Unannotated | NA | NA |
| metab_2020 | Unannotated feature | pos | 439.1501 | 7.277 | 4.928 | 4.956 | 0.028 | 0.192 | 0.286 | 0.433 | Unannotated | NA | NA |
| metab_1713 | Unannotated feature | pos | 750.3149 | 7.293 | 4.835 | 4.699 | -0.136 | -0.192 | 0.286 | 0.433 | Unannotated | NA | NA |
| metab_7853 | Unannotated feature | neg | 468.8974 | 7.148 | 4.593 | 4.557 | -0.036 | -0.192 | 0.286 | 0.433 | Unannotated | NA | NA |
| metab_1356 | Unannotated feature | pos | 509.8468 | 5.902 | 4.701 | 4.760 | 0.059 | 0.192 | 0.286 | 0.433 | Unannotated | NA | NA |
| metab_1540 | Unannotated feature | pos | 171.1130 | 6.273 | 4.525 | 4.512 | -0.013 | -0.192 | 0.286 | 0.433 | Unannotated | NA | NA |
| metab_10748 | Unannotated feature | neg | 613.7544 | 0.570 | 4.440 | 4.400 | -0.040 | -0.192 | 0.286 | 0.433 | Unannotated | NA | NA |
| metab_634 | Pantothenol | pos | 206.1387 | 2.241 | 4.534 | 4.513 | -0.021 | -0.192 | 0.286 | 0.433 | Moderate putative | NA | NA |
| metab_2211 | Amastatin | pos | 516.3064 | 6.296 | 5.822 | 5.781 | -0.041 | -0.192 | 0.286 | 0.433 | Tentative | NA | NA |
| metab_1995 | Unannotated feature | pos | 216.1207 | 7.293 | 4.607 | 4.597 | -0.010 | -0.192 | 0.286 | 0.433 | Unannotated | NA | NA |
| metab_345 | Unannotated feature | pos | 276.9939 | 0.831 | 4.267 | 4.140 | -0.127 | -0.192 | 0.286 | 0.433 | Unannotated | NA | NA |
| metab_1503 | Unannotated feature | pos | 547.8489 | 6.111 | 4.887 | 5.033 | 0.145 | 0.192 | 0.286 | 0.433 | Unannotated | NA | NA |
| metab_4474 | Unannotated feature | pos | 676.8564 | 0.551 | 5.642 | 5.602 | -0.040 | -0.192 | 0.286 | 0.433 | Unannotated | NA | NA |
| metab_4537 | Unannotated feature | pos | 476.9068 | 0.527 | 4.875 | 4.739 | -0.135 | -0.192 | 0.286 | 0.433 | Unannotated | NA | NA |
| metab_7988 | Unannotated feature | neg | 173.9653 | 7.054 | 4.861 | 4.842 | -0.019 | -0.192 | 0.286 | 0.433 | Unannotated | NA | NA |
| metab_1889 | Unannotated feature | pos | 626.8445 | 0.559 | 5.200 | 5.163 | -0.037 | -0.192 | 0.286 | 0.433 | Unannotated | NA | NA |
| metab_8242 | Unannotated feature | neg | 283.1920 | 5.914 | 4.431 | 4.516 | 0.084 | 0.192 | 0.286 | 0.433 | Unannotated | NA | NA |
| metab_6661 | Unannotated feature | neg | 291.9005 | 5.116 | 2.929 | 3.425 | 0.496 | 0.192 | 0.286 | 0.433 | Unannotated | NA | NA |
| metab_4610 | Unannotated feature | pos | 81.5211 | 0.018 | 5.075 | 5.091 | 0.015 | 0.192 | 0.286 | 0.433 | Unannotated | NA | NA |
| metab_7234 | Unannotated feature | neg | 264.9531 | 1.847 | 5.331 | 5.533 | 0.202 | 0.192 | 0.286 | 0.433 | Unannotated | NA | NA |
| metab_173 | Unannotated feature | pos | 732.8419 | 0.559 | 4.396 | 4.327 | -0.069 | -0.192 | 0.286 | 0.433 | Unannotated | NA | NA |
| metab_9715 | Unannotated feature | neg | 224.9090 | 2.844 | 4.223 | 4.228 | 0.005 | -0.192 | 0.286 | 0.433 | Unannotated | NA | NA |
| metab_4144 | Unannotated feature | pos | 510.7591 | 3.669 | 6.302 | 6.336 | 0.033 | 0.192 | 0.286 | 0.433 | Unannotated | NA | NA |
| metab_8630 | Unannotated feature | neg | 255.0880 | 5.651 | 4.029 | 4.167 | 0.138 | 0.192 | 0.286 | 0.433 | Unannotated | NA | NA |
| metab_8695 | Unannotated feature | neg | 252.0437 | 5.611 | 4.414 | 4.332 | -0.082 | -0.192 | 0.286 | 0.433 | Unannotated | NA | NA |
| metab_10387 | Unannotated feature | neg | 92.9107 | 0.723 | 3.049 | 3.016 | -0.033 | -0.192 | 0.286 | 0.433 | Unannotated | NA | NA |
| metab_4236 | Unannotated feature | pos | 608.5665 | 0.606 | 5.203 | 5.139 | -0.064 | -0.192 | 0.286 | 0.433 | Unannotated | NA | NA |
| metab_2714 | (10E)-8-Hydroxydodec-10-enoylcarnitine | pos | 340.2483 | 5.740 | 5.272 | 5.545 | 0.273 | 0.192 | 0.286 | 0.433 | Tentative | NA | NA |
| metab_963 | Unannotated feature | pos | 125.1124 | 4.240 | 4.849 | 4.858 | 0.010 | 0.192 | 0.286 | 0.433 | Unannotated | NA | NA |
| metab_7561 | Unannotated feature | neg | 201.9255 | 7.189 | 5.004 | 4.986 | -0.018 | -0.192 | 0.286 | 0.433 | Unannotated | NA | NA |
| metab_5402 | Unannotated feature | neg | 854.3803 | 0.613 | 4.650 | 4.764 | 0.114 | 0.192 | 0.286 | 0.433 | Unannotated | NA | NA |
| metab_4293 | Unannotated feature | pos | 202.8563 | 0.591 | 4.457 | 4.527 | 0.069 | 0.192 | 0.286 | 0.433 | Unannotated | NA | NA |
| metab_2089 | Unannotated feature | pos | 508.3763 | 7.184 | 5.071 | 5.022 | -0.050 | -0.192 | 0.286 | 0.433 | Unannotated | NA | NA |
| metab_6385 | Unannotated feature | neg | 180.0663 | 4.025 | 4.095 | 4.140 | 0.045 | 0.192 | 0.286 | 0.433 | Unannotated | NA | NA |
| metab_6452 | 3,4-Dihydroxymandelate | neg | 183.0296 | 4.267 | 5.836 | 5.469 | -0.367 | -0.192 | 0.286 | 0.433 | Moderate putative | NA | NA |
| metab_7629 | Unannotated feature | neg | 248.9222 | 7.800 | 3.915 | 3.977 | 0.062 | 0.192 | 0.286 | 0.433 | Unannotated | NA | NA |
| metab_10704 | Unannotated feature | neg | 623.6741 | 0.578 | 4.645 | 4.630 | -0.015 | -0.192 | 0.286 | 0.433 | Unannotated | NA | NA |
| metab_8520 | Unannotated feature | neg | 219.1140 | 5.715 | 3.861 | 3.995 | 0.134 | 0.192 | 0.286 | 0.433 | Unannotated | NA | NA |
| metab_8472 | Unannotated feature | neg | 522.0327 | 5.739 | 4.195 | 3.977 | -0.219 | -0.192 | 0.286 | 0.433 | Unannotated | NA | NA |
| metab_4736 | Unannotated feature | pos | 503.3562 | 6.041 | 5.481 | 5.493 | 0.012 | 0.187 | 0.297 | 0.444 | Unannotated | NA | NA |
| metab_8284 | Unannotated feature | neg | 516.2747 | 5.875 | 3.943 | 3.720 | -0.223 | -0.187 | 0.297 | 0.444 | Unannotated | NA | NA |
| metab_8272 | Unannotated feature | neg | 304.9087 | 3.144 | 4.927 | 4.853 | -0.075 | -0.187 | 0.297 | 0.444 | Unannotated | NA | NA |
| metab_9822 | Unannotated feature | neg | 130.9284 | 2.520 | 3.501 | 3.572 | 0.071 | 0.187 | 0.297 | 0.444 | Unannotated | NA | NA |
| metab_2296 | Glycyl-D-Alanine | pos | 147.0765 | 0.614 | 6.758 | 6.721 | -0.037 | -0.187 | 0.297 | 0.444 | Tentative | NA | NA |
| metab_5193 | Unannotated feature | neg | 167.0206 | 1.294 | 7.324 | 7.277 | -0.048 | -0.187 | 0.297 | 0.444 | Unannotated | NA | NA |
| metab_7371 | Unannotated feature | neg | 174.0016 | 6.903 | 5.190 | 5.208 | 0.018 | 0.187 | 0.297 | 0.444 | Unannotated | NA | NA |
| metab_2274 | 7-Aminoheptanoic acid | pos | 184.0734 | 6.149 | 5.501 | 5.532 | 0.031 | 0.187 | 0.297 | 0.444 | Tentative | NA | NA |
| metab_6959 | Unannotated feature | neg | 221.8889 | 5.699 | 4.179 | 4.142 | -0.037 | -0.187 | 0.297 | 0.444 | Unannotated | NA | NA |
| metab_8200 | Unannotated feature | neg | 241.1811 | 5.969 | 4.293 | 4.071 | -0.222 | -0.187 | 0.297 | 0.444 | Unannotated | NA | NA |
| metab_116 | Unannotated feature | pos | 643.9240 | 0.541 | 4.552 | 4.464 | -0.087 | -0.187 | 0.297 | 0.444 | Unannotated | NA | NA |
| metab_7405 | Unannotated feature | neg | 197.8564 | 7.101 | 4.500 | 4.552 | 0.051 | 0.187 | 0.297 | 0.444 | Unannotated | NA | NA |
| metab_6194 | Unannotated feature | neg | 101.9405 | 3.324 | 3.642 | 3.566 | -0.075 | -0.187 | 0.297 | 0.444 | Unannotated | NA | NA |
| metab_8401 | Cholic acid glucuronide | neg | 583.3139 | 5.787 | 4.346 | 4.691 | 0.345 | 0.187 | 0.297 | 0.444 | Tentative | Lipids and lipid-like molecules | Steroids and steroid derivatives |
| metab_803 | Unannotated feature | pos | 107.9672 | 7.261 | 5.899 | 5.911 | 0.012 | 0.187 | 0.297 | 0.444 | Unannotated | NA | NA |
| metab_7249 | Unannotated feature | neg | 378.2422 | 6.032 | 4.576 | 4.550 | -0.026 | -0.187 | 0.297 | 0.444 | Unannotated | NA | NA |
| metab_1703 | Unannotated feature | pos | 413.2663 | 7.293 | 4.772 | 4.751 | -0.021 | -0.187 | 0.297 | 0.444 | Unannotated | NA | NA |
| metab_8319 | Unannotated feature | neg | 337.1430 | 5.842 | 4.497 | 4.451 | -0.047 | -0.187 | 0.297 | 0.444 | Unannotated | NA | NA |
| metab_1331 | Unannotated feature | pos | 185.1173 | 2.195 | 3.844 | 3.893 | 0.049 | 0.187 | 0.297 | 0.444 | Unannotated | NA | NA |
| metab_7936 | Unannotated feature | neg | 185.9109 | 7.109 | 5.263 | 5.241 | -0.022 | -0.187 | 0.297 | 0.444 | Unannotated | NA | NA |
| metab_11027 | Unannotated feature | neg | 486.7677 | 0.013 | 4.679 | 4.629 | -0.050 | -0.187 | 0.297 | 0.444 | Unannotated | NA | NA |
| metab_899 | Unannotated feature | pos | 101.8086 | 3.769 | 4.192 | 4.211 | 0.019 | 0.187 | 0.297 | 0.444 | Unannotated | NA | NA |
| metab_1518 | Unannotated feature | pos | 540.4467 | 6.157 | 4.479 | 4.506 | 0.027 | 0.187 | 0.297 | 0.444 | Unannotated | NA | NA |
| metab_3501 | Unannotated feature | pos | 173.1285 | 2.195 | 5.628 | 5.491 | -0.137 | -0.187 | 0.297 | 0.444 | Unannotated | NA | NA |
| metab_3492 | Unannotated feature | pos | 123.0807 | 2.226 | 3.961 | 3.927 | -0.034 | -0.187 | 0.297 | 0.444 | Unannotated | NA | NA |
| metab_9155 | Unannotated feature | neg | 83.0130 | 4.267 | 3.722 | 3.244 | -0.478 | -0.187 | 0.297 | 0.444 | Unannotated | NA | NA |
| metab_9979 | Unannotated feature | neg | 345.8690 | 4.274 | 4.965 | 4.622 | -0.343 | -0.187 | 0.297 | 0.444 | Unannotated | NA | NA |
| metab_740 | Chlorobenzene | pos | 154.0419 | 2.644 | 7.992 | 8.001 | 0.009 | 0.187 | 0.297 | 0.444 | Tentative | NA | NA |
| metab_883 | Unannotated feature | pos | 158.0489 | 7.324 | 5.599 | 5.593 | -0.006 | -0.187 | 0.297 | 0.444 | Unannotated | NA | NA |
| metab_2876 | Unannotated feature | pos | 250.0242 | 5.130 | 4.541 | 4.383 | -0.158 | -0.187 | 0.297 | 0.444 | Unannotated | NA | NA |
| metab_3780 | Unannotated feature | pos | 100.0248 | 1.647 | 4.257 | 4.277 | 0.020 | 0.187 | 0.297 | 0.444 | Unannotated | NA | NA |
| metab_887 | Cyclo(L-leucyl-L-phenylalanyl) | pos | 261.1593 | 3.684 | 3.928 | 3.940 | 0.011 | 0.187 | 0.297 | 0.444 | Tentative | NA | NA |
| metab_7388 | Unannotated feature | neg | 216.9341 | 7.085 | 5.435 | 5.423 | -0.013 | -0.187 | 0.297 | 0.444 | Unannotated | NA | NA |
| metab_4881 | Unannotated feature | pos | 108.0859 | 2.065 | 5.417 | 5.429 | 0.012 | 0.187 | 0.297 | 0.444 | Unannotated | NA | NA |
| metab_9879 | Unannotated feature | neg | 494.8117 | 0.621 | 5.363 | 5.323 | -0.040 | -0.187 | 0.297 | 0.444 | Unannotated | NA | NA |
| metab_2356 | Unannotated feature | pos | 579.4264 | 5.972 | 5.826 | 5.856 | 0.030 | 0.187 | 0.297 | 0.444 | Unannotated | NA | NA |
| metab_9908 | 2,3-Dihydroxybenzoate | neg | 153.0188 | 4.227 | 5.397 | 5.546 | 0.149 | 0.187 | 0.297 | 0.444 | Strong putative | NA | NA |
| metab_10937 | Unannotated feature | neg | 498.9166 | 0.530 | 4.280 | 4.444 | 0.164 | 0.187 | 0.297 | 0.444 | Unannotated | NA | NA |
| metab_5915 | Unannotated feature | neg | 610.1381 | 2.537 | 4.373 | 4.257 | -0.116 | -0.187 | 0.297 | 0.444 | Unannotated | NA | NA |
| metab_7120 | Unannotated feature | neg | 627.3764 | 5.834 | 5.384 | 5.332 | -0.052 | -0.187 | 0.297 | 0.444 | Unannotated | NA | NA |
| metab_3206 | Unannotated feature | pos | 84.1315 | 3.213 | 3.239 | 3.362 | 0.122 | 0.187 | 0.297 | 0.444 | Unannotated | NA | NA |
| metab_3064 | 3',4',5'-O-Trimethyltricetin | pos | 408.1055 | 4.001 | 4.897 | 4.895 | -0.001 | -0.187 | 0.297 | 0.444 | Tentative | NA | NA |
| metab_6829 | Unannotated feature | neg | 279.0547 | 5.603 | 3.513 | 3.617 | 0.104 | 0.187 | 0.297 | 0.444 | Unannotated | NA | NA |
| metab_7594 | Unannotated feature | neg | 285.9108 | 7.237 | 4.593 | 4.586 | -0.008 | -0.187 | 0.297 | 0.444 | Unannotated | NA | NA |
| metab_7795 | Unannotated feature | neg | 144.9035 | 7.170 | 3.370 | 3.322 | -0.048 | -0.187 | 0.297 | 0.444 | Unannotated | NA | NA |
| metab_7801 | Unannotated feature | neg | 624.7638 | 7.164 | 4.291 | 4.244 | -0.048 | -0.187 | 0.297 | 0.444 | Unannotated | NA | NA |
| metab_8553 | Unannotated feature | neg | 401.8815 | 7.109 | 5.503 | 5.561 | 0.059 | 0.187 | 0.297 | 0.444 | Unannotated | NA | NA |
| metab_10051 | Unannotated feature | neg | 272.9582 | 1.406 | 4.511 | 4.517 | 0.006 | 0.187 | 0.297 | 0.444 | Unannotated | NA | NA |
| metab_4133 | Unannotated feature | pos | 597.7668 | 4.516 | 5.220 | 5.388 | 0.167 | 0.187 | 0.297 | 0.444 | Unannotated | NA | NA |
| metab_197 | Unannotated feature | pos | 112.8960 | 0.598 | 4.388 | 4.378 | -0.010 | 0.187 | 0.297 | 0.444 | Unannotated | NA | NA |
| metab_2693 | Unannotated feature | pos | 777.6935 | 5.756 | 5.066 | 4.919 | -0.147 | -0.187 | 0.297 | 0.444 | Unannotated | NA | NA |
| metab_2759 | Dihydroroseoside | pos | 421.2405 | 5.687 | 5.157 | 5.187 | 0.030 | 0.187 | 0.297 | 0.444 | Tentative | NA | NA |
| metab_6454 | Unannotated feature | neg | 396.0218 | 0.747 | 4.210 | 3.986 | -0.224 | -0.187 | 0.297 | 0.444 | Unannotated | NA | NA |
| metab_2713 | Unannotated feature | pos | 426.8051 | 5.740 | 3.782 | 3.711 | -0.071 | -0.187 | 0.297 | 0.444 | Unannotated | NA | NA |
| metab_5583 | D-Norvaline | neg | 116.0710 | 0.865 | 4.613 | 4.560 | -0.053 | -0.187 | 0.297 | 0.444 | Moderate putative | NA | NA |
| metab_4240 | Unannotated feature | pos | 436.6878 | 0.606 | 4.703 | 4.659 | -0.044 | -0.187 | 0.297 | 0.444 | Unannotated | NA | NA |
| metab_5587 | Urate | neg | 167.0205 | 0.865 | 7.125 | 7.123 | -0.002 | -0.187 | 0.297 | 0.444 | Strong putative | NA | NA |
| metab_1600 | Unannotated feature | pos | 780.0023 | 6.683 | 5.289 | 5.107 | -0.182 | -0.187 | 0.297 | 0.444 | Unannotated | NA | NA |
| metab_10555 | Unannotated feature | neg | 268.0072 | 0.621 | 4.127 | 4.050 | -0.077 | -0.187 | 0.297 | 0.444 | Unannotated | NA | NA |
| metab_8734 | Unannotated feature | neg | 564.3320 | 7.156 | 5.871 | 5.815 | -0.056 | -0.187 | 0.297 | 0.444 | Unannotated | NA | NA |
| metab_2914 | ASARYLALDEHYDE | pos | 197.0809 | 4.915 | 4.198 | 4.386 | 0.188 | 0.187 | 0.297 | 0.444 | Moderate putative | NA | NA |
| metab_3520 | Unannotated feature | pos | 89.0602 | 2.133 | 3.467 | 3.221 | -0.246 | -0.187 | 0.297 | 0.444 | Unannotated | NA | NA |
| metab_9015 | Unannotated feature | neg | 200.0296 | 3.224 | 6.524 | 6.508 | -0.016 | -0.187 | 0.297 | 0.444 | Unannotated | NA | NA |
| metab_2355 | Unannotated feature | pos | 630.9620 | 5.972 | 5.073 | 5.117 | 0.043 | 0.187 | 0.297 | 0.444 | Unannotated | NA | NA |
| metab_7040 | Unannotated feature | neg | 90.5273 | 5.763 | 2.925 | 2.850 | -0.075 | -0.187 | 0.297 | 0.444 | Unannotated | NA | NA |
| metab_1629 | Unannotated feature | pos | 89.1159 | 7.099 | 6.424 | 6.433 | 0.009 | 0.183 | 0.308 | 0.456 | Unannotated | NA | NA |
| metab_8775 | Unannotated feature | neg | 183.0296 | 5.532 | 3.727 | 3.700 | -0.027 | -0.183 | 0.308 | 0.456 | Unannotated | NA | NA |
| metab_7004 | Apigenin 7-arabinoside | neg | 401.0887 | 5.731 | 5.722 | 5.713 | -0.009 | -0.183 | 0.308 | 0.456 | Tentative | NA | NA |
| metab_774 | Unannotated feature | pos | 90.9771 | 7.293 | 6.112 | 6.123 | 0.010 | 0.183 | 0.308 | 0.456 | Unannotated | NA | NA |
| metab_7114 | Unannotated feature | neg | 266.9468 | 1.294 | 6.556 | 6.490 | -0.067 | -0.183 | 0.308 | 0.456 | Unannotated | NA | NA |
| metab_7134 | Unannotated feature | neg | 209.1182 | 5.859 | 3.585 | 3.567 | -0.019 | 0.183 | 0.308 | 0.456 | Unannotated | NA | NA |
| metab_7132 | Unannotated feature | neg | 165.0049 | 1.294 | 5.094 | 5.039 | -0.055 | -0.183 | 0.308 | 0.456 | Unannotated | NA | NA |
| metab_9046 | Unannotated feature | neg | 130.0867 | 4.635 | 4.353 | 4.306 | -0.047 | -0.183 | 0.308 | 0.456 | Unannotated | NA | NA |
| metab_9570 | Unannotated feature | neg | 270.9369 | 3.184 | 3.363 | 3.503 | 0.141 | 0.183 | 0.308 | 0.456 | Unannotated | NA | NA |
| metab_7339 | Unannotated feature | neg | 100.9516 | 6.441 | 3.297 | 3.334 | 0.036 | 0.183 | 0.308 | 0.456 | Unannotated | NA | NA |
| metab_8715 | Unannotated feature | neg | 257.0517 | 5.587 | 3.807 | 3.825 | 0.018 | 0.183 | 0.308 | 0.456 | Unannotated | NA | NA |
| metab_6215 | Unannotated feature | neg | 142.0868 | 3.372 | 4.831 | 4.737 | -0.094 | -0.183 | 0.308 | 0.456 | Unannotated | NA | NA |
| metab_8457 | Unannotated feature | neg | 260.9158 | 5.755 | 3.514 | 3.461 | -0.053 | -0.183 | 0.308 | 0.456 | Unannotated | NA | NA |
| metab_6164 | Unannotated feature | neg | 517.9853 | 3.247 | 4.829 | 4.771 | -0.058 | -0.183 | 0.308 | 0.456 | Unannotated | NA | NA |
| metab_526 | Unannotated feature | pos | 139.0391 | 1.956 | 3.773 | 3.687 | -0.085 | -0.183 | 0.308 | 0.456 | Unannotated | NA | NA |
| metab_7281 | LysoPE(P-16:0/0:0) | neg | 436.2838 | 6.172 | 4.042 | 4.280 | 0.239 | 0.183 | 0.308 | 0.456 | Tentative | NA | NA |
| metab_1724 | Unannotated feature | pos | 96.9723 | 7.316 | 3.254 | 3.364 | 0.109 | 0.183 | 0.308 | 0.456 | Unannotated | NA | NA |
| metab_3326 | Unannotated feature | pos | 204.0502 | 2.637 | 5.484 | 5.491 | 0.007 | 0.183 | 0.308 | 0.456 | Unannotated | NA | NA |
| metab_6180 | Unannotated feature | neg | 233.0127 | 3.274 | 3.807 | 3.822 | 0.015 | 0.183 | 0.308 | 0.456 | Unannotated | NA | NA |
| metab_3822 | Unannotated feature | pos | 381.0277 | 1.274 | 4.547 | 4.450 | -0.097 | -0.183 | 0.308 | 0.456 | Unannotated | NA | NA |
| metab_10029 | Unannotated feature | neg | 116.9278 | 1.746 | 7.555 | 7.568 | 0.012 | 0.183 | 0.308 | 0.456 | Unannotated | NA | NA |
| metab_7734 | Unannotated feature | neg | 199.8865 | 7.189 | 5.042 | 5.054 | 0.012 | 0.183 | 0.308 | 0.456 | Unannotated | NA | NA |
| metab_8384 | Unannotated feature | neg | 166.0505 | 5.803 | 4.029 | 4.076 | 0.047 | 0.183 | 0.308 | 0.456 | Unannotated | NA | NA |
| metab_8351 | Unannotated feature | neg | 470.3495 | 5.819 | 4.086 | 3.962 | -0.123 | -0.183 | 0.308 | 0.456 | Unannotated | NA | NA |
| metab_10023 | Unannotated feature | neg | 117.6264 | 1.817 | 3.322 | 3.167 | -0.155 | -0.183 | 0.308 | 0.456 | Unannotated | NA | NA |
| metab_10746 | Unannotated feature | neg | 623.7833 | 0.570 | 4.175 | 4.019 | -0.156 | -0.183 | 0.308 | 0.456 | Unannotated | NA | NA |
| metab_9989 | Unannotated feature | neg | 343.8721 | 4.274 | 5.083 | 4.689 | -0.394 | -0.183 | 0.308 | 0.456 | Unannotated | NA | NA |
| metab_9845 | 3-Methylxanthine | neg | 165.0414 | 2.492 | 4.296 | 3.988 | -0.308 | -0.183 | 0.308 | 0.456 | Moderate putative | NA | NA |
| metab_11006 | Unannotated feature | neg | 411.1319 | 5.518 | 4.629 | 4.577 | -0.053 | -0.183 | 0.308 | 0.456 | Unannotated | NA | NA |
| metab_1419 | Unannotated feature | pos | 543.3978 | 5.941 | 6.331 | 6.386 | 0.056 | 0.183 | 0.308 | 0.456 | Unannotated | NA | NA |
| metab_9105 | Unannotated feature | neg | 164.0712 | 4.409 | 4.569 | 4.437 | -0.132 | -0.183 | 0.308 | 0.456 | Unannotated | NA | NA |
| metab_9814 | Unannotated feature | neg | 453.0985 | 2.523 | 4.690 | 4.665 | -0.024 | -0.183 | 0.308 | 0.456 | Unannotated | NA | NA |
| metab_3489 | Unannotated feature | pos | 120.0238 | 2.234 | 4.018 | 3.990 | -0.028 | -0.183 | 0.308 | 0.456 | Unannotated | NA | NA |
| metab_3003 | Unannotated feature | pos | 84.1227 | 4.363 | 3.473 | 3.563 | 0.089 | 0.183 | 0.308 | 0.456 | Unannotated | NA | NA |
| metab_3088 | Isoketocamphoric acid | pos | 199.0965 | 3.877 | 4.672 | 4.612 | -0.060 | -0.183 | 0.308 | 0.456 | Tentative | NA | NA |
| metab_5074 | Unannotated feature | pos | 150.0584 | 1.134 | 6.161 | 6.125 | -0.036 | -0.183 | 0.308 | 0.456 | Unannotated | NA | NA |
| metab_8105 | Unannotated feature | neg | 116.9277 | 6.188 | 7.370 | 7.387 | 0.016 | 0.183 | 0.308 | 0.456 | Unannotated | NA | NA |
| metab_992 | 5-Hydroxyoctanoylcarnitine | pos | 304.2118 | 4.478 | 4.952 | 5.182 | 0.230 | 0.183 | 0.308 | 0.456 | Tentative | NA | NA |
| metab_2278 | 1-Acyl-sn-glycero-3-phosphocholine | pos | 570.3557 | 6.134 | 6.309 | 6.275 | -0.034 | -0.183 | 0.308 | 0.456 | Tentative | NA | NA |
| metab_408 | Unannotated feature | pos | 203.8510 | 1.204 | 3.746 | 3.728 | -0.018 | -0.183 | 0.308 | 0.456 | Unannotated | NA | NA |
| metab_11167 | Unannotated feature | neg | 239.9496 | 5.795 | 5.071 | 5.033 | -0.038 | -0.183 | 0.308 | 0.456 | Unannotated | NA | NA |
| metab_5012 | Unannotated feature | pos | 269.0260 | 0.815 | 4.618 | 4.709 | 0.091 | 0.183 | 0.308 | 0.456 | Unannotated | NA | NA |
| metab_10673 | Unannotated feature | neg | 609.6296 | 0.582 | 5.225 | 5.176 | -0.049 | -0.183 | 0.308 | 0.456 | Unannotated | NA | NA |
| metab_11297 | Unannotated feature | neg | 188.9329 | 7.173 | 5.512 | 5.519 | 0.007 | 0.183 | 0.308 | 0.456 | Unannotated | NA | NA |
| metab_7677 | Unannotated feature | neg | 99.0080 | 7.253 | 5.028 | 5.021 | -0.007 | -0.183 | 0.308 | 0.456 | Unannotated | NA | NA |
| metab_258 | Unannotated feature | pos | 242.1247 | 0.683 | 3.349 | 3.664 | 0.315 | 0.183 | 0.308 | 0.456 | Unannotated | NA | NA |
| metab_7680 | Unannotated feature | neg | 189.8578 | 7.245 | 6.057 | 6.042 | -0.014 | -0.183 | 0.308 | 0.456 | Unannotated | NA | NA |
| metab_7728 | Unannotated feature | neg | 144.8710 | 7.197 | 3.806 | 3.823 | 0.017 | 0.183 | 0.308 | 0.456 | Unannotated | NA | NA |
| metab_10886 | Unannotated feature | neg | 112.9812 | 0.549 | 3.553 | 3.621 | 0.068 | 0.183 | 0.308 | 0.456 | Unannotated | NA | NA |
| metab_1562 | Unannotated feature | pos | 349.2153 | 6.319 | 4.473 | 4.414 | -0.059 | -0.183 | 0.308 | 0.456 | Unannotated | NA | NA |
| metab_3846 | Unannotated feature | pos | 118.4403 | 1.118 | 4.491 | 4.448 | -0.044 | -0.183 | 0.308 | 0.456 | Unannotated | NA | NA |
| metab_7775 | Unannotated feature | neg | 92.9192 | 7.173 | 4.297 | 4.285 | -0.012 | -0.183 | 0.308 | 0.456 | Unannotated | NA | NA |
| metab_2547 | Unannotated feature | pos | 500.8628 | 5.871 | 5.390 | 5.418 | 0.028 | 0.183 | 0.308 | 0.456 | Unannotated | NA | NA |
| metab_7742 | Unannotated feature | neg | 163.0608 | 7.189 | 4.763 | 4.752 | -0.011 | -0.183 | 0.308 | 0.456 | Unannotated | NA | NA |
| metab_7793 | Unannotated feature | neg | 428.0681 | 2.537 | 5.215 | 5.165 | -0.050 | -0.183 | 0.308 | 0.456 | Unannotated | NA | NA |
| metab_7181 | 2-Amino-N-[1-[[2-[[1-(2-hydroxyethylamino)-1-oxo-3-phenylpropan-2-yl]-methylamino]-2-oxoethyl]amino]-1-oxopropan-2-yl]-3-(4-hydroxyphenyl)propanamide | neg | 548.2331 | 5.898 | 5.401 | 5.608 | 0.207 | 0.183 | 0.308 | 0.456 | Tentative | NA | NA |
| metab_7655 | Unannotated feature | neg | 90.5272 | 7.333 | 3.020 | 2.947 | -0.074 | -0.183 | 0.308 | 0.456 | Unannotated | NA | NA |
| metab_9707 | Phenylacetylglycine | neg | 192.0664 | 2.867 | 4.034 | 3.932 | -0.102 | -0.183 | 0.308 | 0.456 | Tentative | NA | NA |
| metab_5545 | Unannotated feature | neg | 164.8392 | 0.818 | 4.162 | 4.131 | -0.031 | -0.183 | 0.308 | 0.456 | Unannotated | NA | NA |
| metab_11400 | Nandrolone phenpropionate | neg | 427.2259 | 5.747 | 4.216 | 4.099 | -0.117 | -0.179 | 0.320 | 0.468 | Tentative | NA | NA |
| metab_1913 | Unannotated feature | pos | 280.6547 | 7.324 | 3.979 | 3.906 | -0.074 | -0.179 | 0.320 | 0.468 | Unannotated | NA | NA |
| metab_10129 | Unannotated feature | neg | 164.8391 | 0.983 | 4.566 | 4.548 | -0.018 | -0.179 | 0.320 | 0.468 | Unannotated | NA | NA |
| metab_9307 | Unannotated feature | neg | 245.0128 | 3.734 | 4.478 | 4.604 | 0.126 | 0.179 | 0.320 | 0.468 | Unannotated | NA | NA |
| metab_1359 | Unannotated feature | pos | 646.4742 | 5.902 | 5.227 | 5.186 | -0.042 | -0.179 | 0.320 | 0.468 | Unannotated | NA | NA |
| metab_9506 | Unannotated feature | neg | 488.9679 | 3.240 | 4.324 | 4.248 | -0.076 | -0.179 | 0.320 | 0.468 | Unannotated | NA | NA |
| metab_9705 | Unannotated feature | neg | 219.0776 | 2.867 | 4.515 | 4.368 | -0.147 | -0.179 | 0.320 | 0.468 | Unannotated | NA | NA |
| metab_9254 | Diacetone Alcohol | neg | 115.0757 | 3.935 | 3.813 | 3.840 | 0.028 | 0.179 | 0.320 | 0.468 | Moderate putative | NA | NA |
| metab_2129 | 1-Acyl-sn-glycero-3-phosphocholine | pos | 550.3867 | 6.768 | 5.130 | 5.154 | 0.023 | 0.179 | 0.320 | 0.468 | Tentative | NA | NA |
| metab_10603 | Unannotated feature | neg | 602.5736 | 0.598 | 5.034 | 5.056 | 0.022 | 0.179 | 0.320 | 0.468 | Unannotated | NA | NA |
| metab_1982 | Unannotated feature | pos | 614.3403 | 7.293 | 5.455 | 5.415 | -0.041 | -0.179 | 0.320 | 0.468 | Unannotated | NA | NA |
| metab_10611 | Unannotated feature | neg | 168.8573 | 0.598 | 4.566 | 4.576 | 0.010 | 0.179 | 0.320 | 0.468 | Unannotated | NA | NA |
| metab_9942 | Unannotated feature | neg | 135.0115 | 2.119 | 3.740 | 3.808 | 0.068 | 0.179 | 0.320 | 0.468 | Unannotated | NA | NA |
| metab_617 | Unannotated feature | pos | 224.1442 | 2.164 | 3.593 | 3.309 | -0.284 | -0.179 | 0.320 | 0.468 | Unannotated | NA | NA |
| metab_10397 | Unannotated feature | neg | 629.1196 | 4.742 | 4.827 | 4.708 | -0.119 | -0.179 | 0.320 | 0.468 | Unannotated | NA | NA |
| metab_10737 | Unannotated feature | neg | 683.8282 | 0.570 | 4.071 | 4.127 | 0.056 | 0.179 | 0.320 | 0.468 | Unannotated | NA | NA |
| metab_10529 | Unannotated feature | neg | 510.6211 | 0.621 | 5.057 | 5.098 | 0.041 | 0.179 | 0.320 | 0.468 | Unannotated | NA | NA |
| metab_7445 | Unannotated feature | neg | 231.9318 | 7.124 | 5.890 | 5.889 | -0.001 | -0.179 | 0.320 | 0.468 | Unannotated | NA | NA |
| metab_10476 | Unannotated feature | neg | 345.8984 | 0.644 | 4.089 | 3.823 | -0.266 | -0.179 | 0.320 | 0.468 | Unannotated | NA | NA |
| metab_4551 | Unannotated feature | pos | 145.0348 | 0.504 | 3.807 | 3.869 | 0.062 | 0.179 | 0.320 | 0.468 | Unannotated | NA | NA |
| metab_201 | Unannotated feature | pos | 155.0316 | 0.606 | 3.857 | 3.957 | 0.100 | 0.179 | 0.320 | 0.468 | Unannotated | NA | NA |
| metab_2971 | 3-Methoxytyramine | pos | 132.0809 | 4.555 | 3.471 | 3.609 | 0.138 | 0.179 | 0.320 | 0.468 | Tentative | NA | NA |
| metab_4363 | Unannotated feature | pos | 693.8355 | 0.567 | 5.167 | 5.165 | -0.002 | -0.179 | 0.320 | 0.468 | Unannotated | NA | NA |
| metab_2063 | Latrunculin B | pos | 413.2118 | 7.215 | 6.644 | 6.623 | -0.022 | -0.179 | 0.320 | 0.468 | Tentative | NA | NA |
| metab_3845 | O-Acetylcarnitine | pos | 204.1231 | 1.118 | 6.833 | 6.897 | 0.064 | 0.179 | 0.320 | 0.468 | Strong putative | NA | NA |
| metab_10307 | Unannotated feature | neg | 159.9778 | 0.802 | 4.179 | 4.252 | 0.073 | 0.179 | 0.320 | 0.468 | Unannotated | NA | NA |
| metab_6744 | Unannotated feature | neg | 321.2034 | 5.477 | 4.766 | 4.752 | -0.014 | -0.179 | 0.320 | 0.468 | Unannotated | NA | NA |
| metab_4546 | Unannotated feature | pos | 172.0455 | 0.504 | 3.967 | 3.946 | -0.021 | -0.179 | 0.320 | 0.468 | Unannotated | NA | NA |
| metab_3491 | Unannotated feature | pos | 182.0763 | 2.226 | 4.680 | 4.540 | -0.140 | -0.179 | 0.320 | 0.468 | Unannotated | NA | NA |
| metab_7419 | Unannotated feature | neg | 153.9915 | 7.109 | 4.513 | 4.484 | -0.029 | -0.179 | 0.320 | 0.468 | Unannotated | NA | NA |
| metab_3796 | Unannotated feature | pos | 128.0195 | 1.530 | 4.889 | 4.870 | -0.019 | -0.179 | 0.320 | 0.468 | Unannotated | NA | NA |
| metab_8414 | Unannotated feature | neg | 173.9259 | 5.787 | 5.513 | 5.499 | -0.014 | -0.179 | 0.320 | 0.468 | Unannotated | NA | NA |
| metab_10954 | Unannotated feature | neg | 299.9197 | 0.522 | 3.971 | 4.036 | 0.065 | 0.179 | 0.320 | 0.468 | Unannotated | NA | NA |
| metab_2190 | 7,8-Dihydro-3b,6a-dihydroxy-alpha-ionol 9-glucoside | pos | 413.2119 | 6.319 | 5.128 | 5.218 | 0.090 | 0.179 | 0.320 | 0.468 | Tentative | NA | NA |
| metab_10643 | Unannotated feature | neg | 280.1153 | 0.590 | 3.946 | 3.828 | -0.118 | -0.179 | 0.320 | 0.468 | Unannotated | NA | NA |
| metab_11219 | 9,10,13-TriHOME | neg | 311.2234 | 5.945 | 5.542 | 5.481 | -0.061 | -0.179 | 0.320 | 0.468 | Tentative | NA | NA |
| metab_8259 | Unannotated feature | neg | 354.0658 | 5.898 | 5.230 | 5.193 | -0.037 | -0.179 | 0.320 | 0.468 | Unannotated | NA | NA |
| metab_1819 | Unannotated feature | pos | 182.1024 | 7.983 | 4.353 | 4.326 | -0.027 | -0.179 | 0.320 | 0.468 | Unannotated | NA | NA |
| metab_7505 | (11E,13E)-9-Hydroxyoctadeca-11,13-dienoylcarnitine | neg | 476.2793 | 7.153 | 4.682 | 4.703 | 0.021 | 0.179 | 0.320 | 0.468 | Tentative | NA | NA |
| metab_10949 | Unannotated feature | neg | 411.8817 | 0.522 | 4.639 | 4.611 | -0.027 | -0.179 | 0.320 | 0.468 | Unannotated | NA | NA |
| metab_3760 | Unannotated feature | pos | 189.0071 | 1.740 | 4.243 | 4.389 | 0.146 | 0.179 | 0.320 | 0.468 | Unannotated | NA | NA |
| metab_4626 | Phensuximide | pos | 190.0863 | 5.626 | 4.321 | 4.488 | 0.168 | 0.179 | 0.320 | 0.468 | Tentative | NA | NA |
| metab_1155 | Unannotated feature | pos | 316.2391 | 5.687 | 5.289 | 5.446 | 0.157 | 0.179 | 0.320 | 0.468 | Unannotated | NA | NA |
| metab_2031 | Unannotated feature | pos | 487.3926 | 7.269 | 4.770 | 4.742 | -0.029 | -0.179 | 0.320 | 0.468 | Unannotated | NA | NA |
| metab_120 | Unannotated feature | pos | 245.0997 | 0.545 | 4.418 | 4.373 | -0.045 | -0.179 | 0.320 | 0.468 | Unannotated | NA | NA |
| metab_11330 | Unannotated feature | neg | 180.8618 | 7.173 | 4.663 | 4.662 | -0.001 | -0.179 | 0.320 | 0.468 | Unannotated | NA | NA |
| metab_8155 | Unannotated feature | neg | 155.8651 | 6.063 | 4.695 | 4.662 | -0.034 | -0.179 | 0.320 | 0.468 | Unannotated | NA | NA |
| metab_3895 | Unannotated feature | pos | 154.0475 | 0.869 | 4.610 | 4.451 | -0.159 | -0.179 | 0.320 | 0.468 | Unannotated | NA | NA |
| metab_1637 | Unannotated feature | pos | 87.6592 | 7.176 | 2.724 | 2.814 | 0.090 | 0.179 | 0.320 | 0.468 | Unannotated | NA | NA |
| metab_6809 | Unannotated feature | neg | 338.9666 | 5.579 | 3.712 | 3.879 | 0.167 | 0.179 | 0.320 | 0.468 | Unannotated | NA | NA |
| metab_10136 | Unannotated feature | neg | 146.9461 | 0.960 | 4.224 | 4.235 | 0.011 | -0.179 | 0.320 | 0.468 | Unannotated | NA | NA |
| metab_2073 | Unannotated feature | pos | 470.4204 | 7.200 | 6.372 | 6.360 | -0.012 | -0.179 | 0.320 | 0.468 | Unannotated | NA | NA |
| metab_10396 | Unannotated feature | neg | 201.0157 | 0.707 | 4.310 | 4.282 | -0.028 | -0.179 | 0.320 | 0.468 | Unannotated | NA | NA |
| metab_4359 | Unannotated feature | pos | 726.8271 | 0.567 | 4.668 | 4.632 | -0.036 | -0.179 | 0.320 | 0.468 | Unannotated | NA | NA |
| metab_5711 | Unannotated feature | neg | 256.8467 | 1.390 | 5.265 | 5.259 | -0.006 | -0.179 | 0.320 | 0.468 | Unannotated | NA | NA |
| metab_1396 | PA(a-25:0/22:6(4Z,7Z,10Z,13E,15E,19Z)-OH(17)) | pos | 432.3111 | 5.925 | 5.475 | 5.616 | 0.141 | 0.179 | 0.320 | 0.468 | Tentative | NA | NA |
| metab_995 | Fusaric acid | pos | 180.1020 | 4.501 | 4.653 | 4.627 | -0.026 | -0.179 | 0.320 | 0.468 | Moderate putative | NA | NA |
| metab_2589 | Erucic acid | pos | 356.3523 | 5.855 | 4.880 | 4.992 | 0.112 | 0.179 | 0.320 | 0.468 | Tentative | NA | NA |
| metab_824 | Unannotated feature | pos | 272.0861 | 3.151 | 4.778 | 4.499 | -0.279 | -0.179 | 0.320 | 0.468 | Unannotated | NA | NA |
| metab_4528 | Unannotated feature | pos | 302.2454 | 0.538 | 4.925 | 4.922 | -0.003 | -0.175 | 0.331 | 0.480 | Unannotated | NA | NA |
| metab_3360 | Unannotated feature | pos | 188.0631 | 2.576 | 3.515 | 3.364 | -0.150 | -0.175 | 0.331 | 0.480 | Unannotated | NA | NA |
| metab_1783 | Unannotated feature | pos | 215.0526 | 0.541 | 5.222 | 5.203 | -0.019 | -0.175 | 0.331 | 0.480 | Unannotated | NA | NA |
| metab_3850 | Unannotated feature | pos | 501.8721 | 3.893 | 4.407 | 4.562 | 0.154 | 0.175 | 0.331 | 0.480 | Unannotated | NA | NA |
| metab_3960 | Unannotated feature | pos | 219.0839 | 0.846 | 3.194 | 3.309 | 0.115 | 0.175 | 0.331 | 0.480 | Unannotated | NA | NA |
| metab_7185 | 5-O-Methylembelin | neg | 307.1921 | 5.906 | 4.235 | 4.165 | -0.070 | -0.175 | 0.331 | 0.480 | Tentative | NA | NA |
| metab_7506 | Unannotated feature | neg | 598.8548 | 7.153 | 4.670 | 4.718 | 0.048 | 0.175 | 0.331 | 0.480 | Unannotated | NA | NA |
| metab_4649 | Unannotated feature | pos | 384.2745 | 5.748 | 4.680 | 4.960 | 0.280 | 0.175 | 0.331 | 0.480 | Unannotated | NA | NA |
| metab_8264 | Unannotated feature | neg | 306.9058 | 3.144 | 4.815 | 4.746 | -0.069 | -0.175 | 0.331 | 0.480 | Unannotated | NA | NA |
| metab_4009 | Unannotated feature | pos | 132.9874 | 0.815 | 4.652 | 4.594 | -0.057 | -0.175 | 0.331 | 0.480 | Unannotated | NA | NA |
| metab_7501 | Unannotated feature | neg | 241.0724 | 7.153 | 3.992 | 3.962 | -0.030 | -0.175 | 0.331 | 0.480 | Unannotated | NA | NA |
| metab_2032 | Unannotated feature | pos | 442.1246 | 7.269 | 4.803 | 4.811 | 0.008 | 0.175 | 0.331 | 0.480 | Unannotated | NA | NA |
| metab_10801 | Unannotated feature | neg | 180.9727 | 0.562 | 6.275 | 6.252 | -0.023 | -0.175 | 0.331 | 0.480 | Unannotated | NA | NA |
| metab_3697 | 9H-Purine-9-ol | pos | 137.0459 | 1.910 | 5.387 | 5.588 | 0.202 | 0.175 | 0.331 | 0.480 | Tentative | NA | NA |
| metab_630 | Unannotated feature | pos | 162.0584 | 2.218 | 4.253 | 4.201 | -0.052 | -0.175 | 0.331 | 0.480 | Unannotated | NA | NA |
| metab_3610 | Unannotated feature | pos | 111.0395 | 2.011 | 5.038 | 5.025 | -0.013 | -0.175 | 0.331 | 0.480 | Unannotated | NA | NA |
| metab_9225 | Formyllysine | neg | 173.0928 | 4.043 | 4.105 | 4.167 | 0.063 | 0.175 | 0.331 | 0.480 | Tentative | NA | NA |
| metab_4985 | Unannotated feature | pos | 214.1299 | 0.737 | 4.870 | 4.915 | 0.045 | 0.175 | 0.331 | 0.480 | Unannotated | NA | NA |
| metab_9817 | Unannotated feature | neg | 151.9408 | 2.523 | 4.081 | 4.135 | 0.054 | 0.175 | 0.331 | 0.480 | Unannotated | NA | NA |
| metab_3590 | tRNA containing 2'-O-methylguanosine | pos | 298.1145 | 2.042 | 4.507 | 4.543 | 0.036 | 0.175 | 0.331 | 0.480 | Strong putative | NA | NA |
| metab_2307 | Unannotated feature | pos | 590.3218 | 6.057 | 5.113 | 5.116 | 0.003 | 0.175 | 0.331 | 0.480 | Unannotated | NA | NA |
| metab_3263 | Unannotated feature | pos | 360.1906 | 2.965 | 4.365 | 4.338 | -0.027 | -0.175 | 0.331 | 0.480 | Unannotated | NA | NA |
| metab_5748 | Unannotated feature | neg | 247.9628 | 1.847 | 5.673 | 5.871 | 0.198 | 0.175 | 0.331 | 0.480 | Unannotated | NA | NA |
| metab_1923 | Unannotated feature | pos | 186.0438 | 7.324 | 6.262 | 6.255 | -0.008 | -0.175 | 0.331 | 0.480 | Unannotated | NA | NA |
| metab_9820 | Unannotated feature | neg | 436.1081 | 2.520 | 4.793 | 4.743 | -0.050 | -0.175 | 0.331 | 0.480 | Unannotated | NA | NA |
| metab_7536 | Unannotated feature | neg | 94.9163 | 7.173 | 3.689 | 3.698 | 0.009 | 0.175 | 0.331 | 0.480 | Unannotated | NA | NA |
| metab_2324 | LysoPA(22:5(7Z,10Z,13Z,16Z,19Z)/0:0) | pos | 526.2930 | 6.018 | 6.447 | 6.455 | 0.008 | 0.175 | 0.331 | 0.480 | Tentative | NA | NA |
| metab_9800 | Unannotated feature | neg | 113.9255 | 2.541 | 4.164 | 4.197 | 0.033 | 0.175 | 0.331 | 0.480 | Unannotated | NA | NA |
| metab_10271 | Unannotated feature | neg | 270.9139 | 0.841 | 4.273 | 4.117 | -0.156 | -0.175 | 0.331 | 0.480 | Unannotated | NA | NA |
| metab_9929 | Unannotated feature | neg | 202.1084 | 2.119 | 3.844 | 3.837 | -0.008 | -0.175 | 0.331 | 0.480 | Unannotated | NA | NA |
| metab_646 | 1-(beta-D-Ribofuranosyl)-1,4-dihydronicotinamide | pos | 221.0921 | 2.280 | 5.129 | 5.076 | -0.052 | -0.175 | 0.331 | 0.480 | Tentative | NA | NA |
| metab_10009 | Unannotated feature | neg | 232.9981 | 1.863 | 3.628 | 3.903 | 0.275 | 0.175 | 0.331 | 0.480 | Unannotated | NA | NA |
| metab_1108 | Unannotated feature | pos | 223.0965 | 5.565 | 3.916 | 3.808 | -0.108 | -0.175 | 0.331 | 0.480 | Unannotated | NA | NA |
| metab_11377 | Unannotated feature | neg | 90.9328 | 0.505 | 3.684 | 3.713 | 0.029 | 0.175 | 0.331 | 0.480 | Unannotated | NA | NA |
| metab_9746 | Unannotated feature | neg | 378.8942 | 0.629 | 5.350 | 5.291 | -0.059 | -0.175 | 0.331 | 0.480 | Unannotated | NA | NA |
| metab_6857 | Unannotated feature | neg | 244.9857 | 5.627 | 4.131 | 4.176 | 0.046 | 0.175 | 0.331 | 0.480 | Unannotated | NA | NA |
| metab_11401 | Oxalate | neg | 88.9872 | 6.281 | 5.583 | 5.603 | 0.020 | 0.175 | 0.331 | 0.480 | Moderate putative | NA | NA |
| metab_7520 | Unannotated feature | neg | 237.0807 | 7.164 | 3.811 | 3.797 | -0.014 | -0.175 | 0.331 | 0.480 | Unannotated | NA | NA |
| metab_10638 | Unannotated feature | neg | 691.5531 | 0.590 | 4.864 | 4.735 | -0.129 | -0.175 | 0.331 | 0.480 | Unannotated | NA | NA |
| metab_8325 | Unannotated feature | neg | 241.9208 | 3.176 | 4.461 | 4.568 | 0.106 | 0.175 | 0.331 | 0.480 | Unannotated | NA | NA |
| metab_9571 | Unannotated feature | neg | 259.9632 | 3.184 | 3.627 | 3.537 | -0.090 | -0.175 | 0.331 | 0.480 | Unannotated | NA | NA |
| metab_1152 | Unannotated feature | pos | 618.4064 | 5.672 | 4.306 | 4.099 | -0.207 | -0.175 | 0.331 | 0.480 | Unannotated | NA | NA |
| metab_5120 | Benzaldehyde | pos | 105.0338 | 3.151 | 5.110 | 5.558 | 0.448 | 0.175 | 0.331 | 0.480 | Strong putative | NA | NA |
| metab_2004 | Unannotated feature | pos | 521.1534 | 7.285 | 4.499 | 4.507 | 0.008 | 0.175 | 0.331 | 0.480 | Unannotated | NA | NA |
| metab_6498 | Unannotated feature | neg | 180.8335 | 4.386 | 3.696 | 3.673 | -0.023 | -0.175 | 0.331 | 0.480 | Unannotated | NA | NA |
| metab_9147 | Unannotated feature | neg | 347.8660 | 4.274 | 4.215 | 3.704 | -0.511 | -0.175 | 0.331 | 0.480 | Unannotated | NA | NA |
| metab_230 | Hippurate | pos | 180.0656 | 3.159 | 5.574 | 5.966 | 0.392 | 0.175 | 0.331 | 0.480 | Strong putative | NA | NA |
| metab_9325 | Unannotated feature | neg | 196.5664 | 3.710 | 4.647 | 4.697 | 0.050 | 0.175 | 0.331 | 0.480 | Unannotated | NA | NA |
| metab_4811 | Unannotated feature | pos | 478.2929 | 7.308 | 4.400 | 4.485 | 0.085 | 0.175 | 0.331 | 0.480 | Unannotated | NA | NA |
| metab_7033 | Unannotated feature | neg | 775.6815 | 5.747 | 4.734 | 4.647 | -0.087 | -0.175 | 0.331 | 0.480 | Unannotated | NA | NA |
| metab_4064 | Unannotated feature | pos | 145.0173 | 0.753 | 4.032 | 3.893 | -0.139 | -0.175 | 0.331 | 0.480 | Unannotated | NA | NA |
| metab_10505 | Unannotated feature | neg | 682.5007 | 0.629 | 5.182 | 5.119 | -0.064 | -0.175 | 0.331 | 0.480 | Unannotated | NA | NA |
| metab_11388 | D-Glucose | neg | 225.0617 | 0.644 | 5.930 | 5.893 | -0.037 | -0.175 | 0.331 | 0.480 | Tentative | NA | NA |
| metab_6227 | Alpha-Furyl methyl diketone | neg | 183.0296 | 3.412 | 4.543 | 4.447 | -0.095 | -0.175 | 0.331 | 0.480 | Tentative | NA | NA |
| metab_604 | Unannotated feature | pos | 101.0794 | 2.103 | 4.932 | 4.715 | -0.217 | -0.175 | 0.331 | 0.480 | Unannotated | NA | NA |
| metab_205 | Unannotated feature | pos | 386.7149 | 0.606 | 4.355 | 4.469 | 0.114 | 0.171 | 0.343 | 0.492 | Unannotated | NA | NA |
| metab_9757 | Unannotated feature | neg | 194.9241 | 7.164 | 5.532 | 5.520 | -0.011 | -0.171 | 0.343 | 0.492 | Unannotated | NA | NA |
| metab_2064 | Valylserine | pos | 237.1458 | 7.215 | 5.148 | 5.152 | 0.004 | 0.171 | 0.343 | 0.492 | Tentative | NA | NA |
| metab_9556 | Unannotated feature | neg | 330.9507 | 0.636 | 5.555 | 5.532 | -0.022 | -0.171 | 0.343 | 0.492 | Unannotated | NA | NA |
| metab_2938 | Unannotated feature | pos | 199.0965 | 4.792 | 4.308 | 4.328 | 0.020 | 0.171 | 0.343 | 0.492 | Unannotated | NA | NA |
| metab_5739 | Unannotated feature | neg | 90.5271 | 1.777 | 3.185 | 3.131 | -0.054 | -0.171 | 0.343 | 0.492 | Unannotated | NA | NA |
| metab_4557 | Unannotated feature | pos | 77.0089 | 0.504 | 3.671 | 3.676 | 0.004 | 0.171 | 0.343 | 0.492 | Unannotated | NA | NA |
| metab_32 | Unannotated feature | pos | 181.0584 | 1.956 | 4.396 | 4.373 | -0.023 | -0.171 | 0.343 | 0.492 | Unannotated | NA | NA |
| metab_10561 | Unannotated feature | neg | 151.3970 | 0.621 | 3.679 | 3.712 | 0.033 | 0.171 | 0.343 | 0.492 | Unannotated | NA | NA |
| metab_9436 | Unannotated feature | neg | 314.0214 | 3.372 | 3.883 | 3.666 | -0.218 | -0.171 | 0.343 | 0.492 | Unannotated | NA | NA |
| metab_579 | Unannotated feature | pos | 123.7268 | 2.065 | 3.518 | 3.678 | 0.160 | 0.171 | 0.343 | 0.492 | Unannotated | NA | NA |
| metab_1697 | Unannotated feature | pos | 340.9293 | 7.285 | 4.620 | 4.606 | -0.014 | -0.171 | 0.343 | 0.492 | Unannotated | NA | NA |
| metab_5875 | Unannotated feature | neg | 674.7308 | 0.570 | 4.875 | 4.848 | -0.027 | -0.171 | 0.343 | 0.492 | Unannotated | NA | NA |
| metab_5290 | Unannotated feature | neg | 999.8024 | 0.554 | 5.360 | 5.351 | -0.009 | -0.171 | 0.343 | 0.492 | Unannotated | NA | NA |
| metab_2177 | Unannotated feature | pos | 868.7073 | 0.567 | 4.739 | 4.695 | -0.045 | -0.171 | 0.343 | 0.492 | Unannotated | NA | NA |
| metab_10667 | Unannotated feature | neg | 704.5998 | 0.582 | 5.005 | 4.960 | -0.045 | -0.171 | 0.343 | 0.492 | Unannotated | NA | NA |
| metab_10969 | Unannotated feature | neg | 156.8944 | 0.509 | 4.891 | 4.896 | 0.005 | 0.171 | 0.343 | 0.492 | Unannotated | NA | NA |
| metab_9816 | Xanthosine | neg | 283.0689 | 2.523 | 4.017 | 4.055 | 0.038 | 0.171 | 0.343 | 0.492 | Strong putative | NA | NA |
| metab_6563 | Unannotated feature | neg | 203.0825 | 4.656 | 4.238 | 4.210 | -0.028 | -0.171 | 0.343 | 0.492 | Unannotated | NA | NA |
| metab_9069 | Unannotated feature | neg | 218.8172 | 4.529 | 4.393 | 4.360 | -0.033 | -0.171 | 0.343 | 0.492 | Unannotated | NA | NA |
| metab_7438 | Unannotated feature | neg | 299.7768 | 7.109 | 4.736 | 4.688 | -0.048 | -0.171 | 0.343 | 0.492 | Unannotated | NA | NA |
| metab_7822 | Unannotated feature | neg | 262.9122 | 7.156 | 5.007 | 4.993 | -0.014 | -0.171 | 0.343 | 0.492 | Unannotated | NA | NA |
| metab_6618 | Unannotated feature | neg | 173.0564 | 0.841 | 5.193 | 5.231 | 0.038 | 0.171 | 0.343 | 0.492 | Unannotated | NA | NA |
| metab_3784 | Unannotated feature | pos | 603.7910 | 3.677 | 6.592 | 6.671 | 0.080 | 0.171 | 0.343 | 0.492 | Unannotated | NA | NA |
| metab_11334 | Unannotated feature | neg | 309.8571 | 7.170 | 4.485 | 4.515 | 0.030 | 0.171 | 0.343 | 0.492 | Unannotated | NA | NA |
| metab_5069 | Unannotated feature | pos | 140.0319 | 0.854 | 4.927 | 4.929 | 0.003 | 0.171 | 0.343 | 0.492 | Unannotated | NA | NA |
| metab_8308 | Precocene II | neg | 265.1087 | 5.859 | 3.993 | 4.095 | 0.102 | 0.171 | 0.343 | 0.492 | Tentative | Organoheterocyclic compounds | Benzopyrans |
| metab_188 | Unannotated feature | pos | 585.8389 | 0.567 | 4.510 | 4.452 | -0.058 | -0.171 | 0.343 | 0.492 | Unannotated | NA | NA |
| metab_4544 | Unannotated feature | pos | 89.0602 | 0.512 | 4.568 | 4.560 | -0.008 | -0.171 | 0.343 | 0.492 | Unannotated | NA | NA |
| metab_10945 | Unannotated feature | neg | 294.9539 | 0.530 | 4.583 | 4.588 | 0.005 | 0.171 | 0.343 | 0.492 | Unannotated | NA | NA |
| metab_4156 | Unannotated feature | pos | 231.1452 | 0.660 | 5.105 | 5.135 | 0.030 | 0.171 | 0.343 | 0.492 | Unannotated | NA | NA |
| metab_4498 | Unannotated feature | pos | 231.0839 | 0.551 | 5.880 | 5.868 | -0.011 | -0.171 | 0.343 | 0.492 | Unannotated | NA | NA |
| metab_6558 | Unannotated feature | neg | 200.5840 | 4.643 | 4.206 | 4.129 | -0.077 | -0.171 | 0.343 | 0.492 | Unannotated | NA | NA |
| metab_8773 | Unannotated feature | neg | 125.0156 | 5.538 | 4.281 | 4.310 | 0.029 | 0.171 | 0.343 | 0.492 | Unannotated | NA | NA |
| metab_7923 | Unannotated feature | neg | 277.8751 | 7.109 | 4.660 | 4.658 | -0.002 | 0.171 | 0.343 | 0.492 | Unannotated | NA | NA |
| metab_9186 | Unannotated feature | neg | 368.8621 | 0.676 | 4.672 | 4.341 | -0.330 | -0.171 | 0.343 | 0.492 | Unannotated | NA | NA |
| metab_2252 | Unannotated feature | pos | 163.0727 | 6.203 | 4.269 | 4.292 | 0.023 | 0.171 | 0.343 | 0.492 | Unannotated | NA | NA |
| metab_4220 | Unannotated feature | pos | 281.9438 | 0.614 | 3.743 | 3.845 | 0.102 | 0.171 | 0.343 | 0.492 | Unannotated | NA | NA |
| metab_2059 | Unannotated feature | pos | 481.3503 | 7.215 | 5.394 | 5.372 | -0.021 | -0.171 | 0.343 | 0.492 | Unannotated | NA | NA |
| metab_3039 | Unannotated feature | pos | 189.1235 | 4.155 | 4.176 | 4.066 | -0.111 | -0.171 | 0.343 | 0.492 | Moderate putative | NA | NA |
| metab_2367 | Unannotated feature | pos | 550.4060 | 5.964 | 6.071 | 6.108 | 0.037 | 0.171 | 0.343 | 0.492 | Unannotated | NA | NA |
| metab_3010 | Unannotated feature | pos | 102.3761 | 4.309 | 4.875 | 4.892 | 0.017 | 0.171 | 0.343 | 0.492 | Unannotated | NA | NA |
| metab_3910 | Unannotated feature | pos | 241.0795 | 0.861 | 4.321 | 4.233 | -0.087 | -0.171 | 0.343 | 0.492 | Unannotated | NA | NA |
| metab_3899 | Unannotated feature | pos | 397.0227 | 0.861 | 4.808 | 4.756 | -0.052 | -0.171 | 0.343 | 0.492 | Unannotated | NA | NA |
| metab_6708 | Unannotated feature | neg | 346.9334 | 0.841 | 4.404 | 4.229 | -0.176 | -0.171 | 0.343 | 0.492 | Unannotated | NA | NA |
| metab_228 | Unannotated feature | pos | 441.0745 | 0.636 | 4.917 | 4.773 | -0.144 | -0.171 | 0.343 | 0.492 | Unannotated | NA | NA |
| metab_4390 | Unannotated feature | pos | 551.8456 | 0.567 | 4.466 | 4.408 | -0.058 | -0.171 | 0.343 | 0.492 | Unannotated | NA | NA |
| metab_4361 | Unannotated feature | pos | 722.8121 | 0.567 | 4.457 | 4.411 | -0.046 | -0.171 | 0.343 | 0.492 | Unannotated | NA | NA |
| metab_4351 | Unannotated feature | pos | 794.8138 | 0.567 | 4.427 | 4.524 | 0.097 | 0.171 | 0.343 | 0.492 | Unannotated | NA | NA |
| metab_11 | Unannotated feature | pos | 272.7947 | 0.591 | 5.671 | 5.616 | -0.055 | -0.171 | 0.343 | 0.492 | Unannotated | NA | NA |
| metab_10411 | Unannotated feature | neg | 179.8415 | 0.692 | 6.226 | 6.242 | 0.016 | 0.171 | 0.343 | 0.492 | Unannotated | NA | NA |
| metab_10975 | Unannotated feature | neg | 472.7642 | 0.582 | 5.789 | 5.757 | -0.032 | -0.171 | 0.343 | 0.492 | Unannotated | NA | NA |
| metab_11156 | Unannotated feature | neg | 119.9069 | 5.779 | 4.785 | 4.729 | -0.056 | -0.171 | 0.343 | 0.492 | Unannotated | NA | NA |
| metab_6186 | Unannotated feature | neg | 343.9635 | 3.290 | 4.558 | 4.547 | -0.011 | -0.171 | 0.343 | 0.492 | Unannotated | NA | NA |
| metab_4091 | Unannotated feature | pos | 99.0557 | 0.729 | 3.745 | 3.601 | -0.143 | -0.171 | 0.343 | 0.492 | Unannotated | NA | NA |
| metab_710 | Benzoylnorecgonine | pos | 317.1495 | 2.560 | 4.687 | 4.483 | -0.204 | -0.171 | 0.343 | 0.492 | Tentative | NA | NA |
| metab_6803 | Unannotated feature | neg | 271.0309 | 5.571 | 3.577 | 3.789 | 0.212 | 0.171 | 0.343 | 0.492 | Unannotated | NA | NA |
| metab_6816 | Unannotated feature | neg | 255.0547 | 5.587 | 4.398 | 4.401 | 0.003 | 0.171 | 0.343 | 0.492 | Unannotated | NA | NA |
| metab_49 | Unannotated feature | pos | 125.9865 | 0.025 | 6.069 | 6.075 | 0.006 | 0.166 | 0.355 | 0.503 | Unannotated | NA | NA |
| metab_11406 | Catechol | neg | 109.0287 | 1.847 | 3.624 | 3.698 | 0.074 | 0.166 | 0.355 | 0.503 | Strong putative | NA | NA |
| metab_6812 | Unannotated feature | neg | 397.8861 | 5.579 | 5.452 | 5.371 | -0.081 | -0.166 | 0.355 | 0.503 | Unannotated | NA | NA |
| metab_7589 | Unannotated feature | neg | 248.9222 | 7.213 | 4.560 | 4.562 | 0.001 | 0.166 | 0.355 | 0.503 | Unannotated | NA | NA |
| metab_11354 | Unannotated feature | neg | 466.8171 | 0.016 | 4.666 | 4.642 | -0.025 | -0.166 | 0.355 | 0.503 | Unannotated | NA | NA |
| metab_7588 | Unannotated feature | neg | 163.0254 | 2.119 | 3.981 | 4.019 | 0.038 | 0.166 | 0.355 | 0.503 | Unannotated | NA | NA |
| metab_554 | Unannotated feature | pos | 279.1592 | 2.026 | 4.789 | 4.788 | -0.001 | -0.166 | 0.355 | 0.503 | Moderate putative | NA | NA |
| metab_3647 | Unannotated feature | pos | 162.0584 | 1.956 | 5.121 | 5.285 | 0.164 | 0.166 | 0.355 | 0.503 | Strong putative | NA | NA |
| metab_994 | N(2)-phenylacetyl-L-glutaminate | pos | 247.1077 | 4.493 | 4.847 | 4.775 | -0.072 | -0.166 | 0.355 | 0.503 | Tentative | NA | NA |
| metab_5363 | Unannotated feature | neg | 633.5949 | 0.587 | 5.185 | 5.119 | -0.066 | -0.166 | 0.355 | 0.503 | Unannotated | NA | NA |
| metab_8550 | Unannotated feature | neg | 477.1629 | 5.691 | 4.257 | 4.014 | -0.243 | -0.166 | 0.355 | 0.503 | Unannotated | NA | NA |
| metab_6858 | Unannotated feature | neg | 287.6124 | 5.627 | 4.232 | 4.156 | -0.077 | -0.166 | 0.355 | 0.503 | Unannotated | NA | NA |
| metab_4334 | Unannotated feature | pos | 533.7850 | 0.575 | 3.813 | 3.804 | -0.010 | -0.166 | 0.355 | 0.503 | Unannotated | NA | NA |
| metab_4823 | Unannotated feature | pos | 140.9914 | 7.324 | 5.024 | 5.026 | 0.002 | 0.166 | 0.355 | 0.503 | Unannotated | NA | NA |
| metab_4885 | Unannotated feature | pos | 230.0812 | 2.058 | 4.261 | 4.305 | 0.044 | 0.166 | 0.355 | 0.503 | Unannotated | NA | NA |
| metab_822 | L-Felinine | pos | 190.0898 | 3.151 | 3.928 | 3.706 | -0.221 | -0.166 | 0.355 | 0.503 | Tentative | NA | NA |
| metab_11361 | Unannotated feature | neg | 212.8846 | 0.039 | 5.926 | 5.920 | -0.006 | -0.166 | 0.355 | 0.503 | Unannotated | NA | NA |
| metab_11306 | Unannotated feature | neg | 580.6501 | 0.587 | 6.175 | 6.163 | -0.012 | -0.166 | 0.355 | 0.503 | Unannotated | NA | NA |
| metab_968 | Unannotated feature | pos | 129.0548 | 1.437 | 5.770 | 5.774 | 0.004 | 0.166 | 0.355 | 0.503 | Unannotated | NA | NA |
| metab_10068 | Unannotated feature | neg | 146.0453 | 1.334 | 3.871 | 3.781 | -0.090 | -0.166 | 0.355 | 0.503 | Unannotated | NA | NA |
| metab_3502 | Glabrin A | pos | 158.0812 | 2.195 | 5.361 | 5.356 | -0.005 | 0.166 | 0.355 | 0.503 | Tentative | NA | NA |
| metab_9151 | 2-[4-(sulfooxy)phenyl]acetic acid | neg | 230.9970 | 4.274 | 4.100 | 4.328 | 0.228 | 0.166 | 0.355 | 0.503 | Tentative | NA | NA |
| metab_10944 | Unannotated feature | neg | 324.9088 | 0.530 | 3.989 | 3.936 | -0.053 | -0.166 | 0.355 | 0.503 | Unannotated | NA | NA |
| metab_9089 | Unannotated feature | neg | 165.0553 | 4.473 | 3.905 | 3.909 | 0.003 | -0.166 | 0.355 | 0.503 | Unannotated | NA | NA |
| metab_1009 | (R)-Pantolactone | pos | 274.2012 | 4.555 | 4.814 | 5.000 | 0.186 | 0.166 | 0.355 | 0.503 | Tentative | NA | NA |
| metab_6132 | Unannotated feature | neg | 570.0456 | 3.201 | 4.192 | 4.153 | -0.039 | -0.166 | 0.355 | 0.503 | Unannotated | NA | NA |
| metab_2717 | Unannotated feature | pos | 198.1853 | 5.740 | 3.716 | 3.690 | -0.025 | -0.166 | 0.355 | 0.503 | Unannotated | NA | NA |
| metab_10499 | Unannotated feature | neg | 214.9748 | 0.636 | 4.198 | 4.357 | 0.159 | 0.166 | 0.355 | 0.503 | Unannotated | NA | NA |
| metab_1220 | Unannotated feature | pos | 186.2218 | 5.793 | 4.332 | 4.336 | 0.004 | 0.166 | 0.355 | 0.503 | Unannotated | NA | NA |
| metab_2030 | Unannotated feature | pos | 514.4830 | 7.269 | 6.847 | 6.847 | 0.001 | -0.166 | 0.355 | 0.503 | Unannotated | NA | NA |
| metab_7382 | Unannotated feature | neg | 159.8689 | 6.993 | 4.022 | 4.040 | 0.018 | 0.166 | 0.355 | 0.503 | Unannotated | NA | NA |
| metab_8225 | Unannotated feature | neg | 157.3338 | 5.930 | 3.519 | 3.491 | -0.029 | -0.166 | 0.355 | 0.503 | Unannotated | NA | NA |
| metab_3444 | Glycyl-leucine | pos | 189.1233 | 2.327 | 3.361 | 2.995 | -0.366 | -0.166 | 0.355 | 0.503 | Strong putative | NA | NA |
| metab_10993 | Unannotated feature | neg | 241.8876 | 0.079 | 4.469 | 4.448 | -0.021 | -0.166 | 0.355 | 0.503 | Unannotated | NA | NA |
| metab_2138 | 2-Acetyl-1-alkyl-sn-glycero-3-phosphocholine | pos | 572.3688 | 6.691 | 5.146 | 5.223 | 0.078 | 0.166 | 0.355 | 0.503 | Tentative | NA | NA |
| metab_733 | Unannotated feature | pos | 125.0155 | 2.637 | 4.971 | 4.977 | 0.006 | 0.166 | 0.355 | 0.503 | Unannotated | NA | NA |
| metab_10640 | Unannotated feature | neg | 575.6356 | 0.590 | 5.036 | 5.000 | -0.036 | -0.166 | 0.355 | 0.503 | Unannotated | NA | NA |
| metab_3323 | Unannotated feature | pos | 141.0102 | 2.644 | 6.193 | 6.203 | 0.009 | 0.166 | 0.355 | 0.503 | Unannotated | NA | NA |
| metab_3806 | Hypoxanthine | pos | 137.0459 | 1.344 | 6.620 | 6.561 | -0.059 | -0.166 | 0.355 | 0.503 | Strong putative | NA | NA |
| metab_5536 | Unannotated feature | neg | 129.9751 | 0.802 | 3.204 | 3.365 | 0.161 | 0.166 | 0.355 | 0.503 | Unannotated | NA | NA |
| metab_7099 | 2-Hydroxy-4-[(1R)-1-hydroxy-8-methyl-6-oxononyl]-3-methyl-2H-furan-5-one | neg | 567.3190 | 5.811 | 5.257 | 5.464 | 0.207 | 0.166 | 0.355 | 0.503 | Tentative | NA | NA |
| metab_2133 | DG(20:5(5Z,8Z,11Z,14Z,16E)-OH(18R)/i-21:0/0:0) | pos | 764.5739 | 6.737 | 6.040 | 5.989 | -0.051 | -0.166 | 0.355 | 0.503 | Tentative | NA | NA |
| metab_392 | Unannotated feature | pos | 147.0442 | 1.025 | 4.857 | 4.823 | -0.034 | -0.166 | 0.355 | 0.503 | Moderate putative | NA | NA |
| metab_2232 | Unannotated feature | pos | 393.2975 | 6.273 | 5.471 | 5.521 | 0.050 | 0.166 | 0.355 | 0.503 | Unannotated | NA | NA |
| metab_3193 | 3h-Indole-3-propanoic acid,a-amino- | pos | 246.1237 | 3.290 | 4.655 | 4.522 | -0.133 | -0.166 | 0.355 | 0.503 | Tentative | NA | NA |
| metab_9426 | Unannotated feature | neg | 180.0663 | 3.412 | 3.690 | 3.722 | 0.032 | 0.166 | 0.355 | 0.503 | Unannotated | NA | NA |
| metab_4427 | Unannotated feature | pos | 596.8666 | 0.559 | 4.270 | 4.194 | -0.076 | -0.166 | 0.355 | 0.503 | Unannotated | NA | NA |
| metab_1972 | Unannotated feature | pos | 872.8312 | 7.293 | 4.533 | 4.427 | -0.106 | -0.166 | 0.355 | 0.503 | Unannotated | NA | NA |
| metab_7963 | Unannotated feature | neg | 195.9996 | 7.101 | 4.985 | 4.979 | -0.006 | -0.166 | 0.355 | 0.503 | Unannotated | NA | NA |
| metab_7993 | Unannotated feature | neg | 99.0080 | 6.993 | 3.457 | 3.390 | -0.067 | -0.166 | 0.355 | 0.503 | Unannotated | NA | NA |
| metab_10845 | Unannotated feature | neg | 659.8648 | 0.554 | 5.681 | 5.705 | 0.025 | 0.166 | 0.355 | 0.503 | Unannotated | NA | NA |
| metab_10034 | Unannotated feature | neg | 116.5808 | 1.649 | 5.467 | 5.459 | -0.008 | -0.166 | 0.355 | 0.503 | Unannotated | NA | NA |
| metab_8986 | Unannotated feature | neg | 89.9249 | 4.798 | 3.554 | 3.577 | 0.022 | 0.166 | 0.355 | 0.503 | Unannotated | NA | NA |
| metab_101 | Unannotated feature | pos | 651.8663 | 0.534 | 5.076 | 5.072 | -0.004 | -0.166 | 0.355 | 0.503 | Unannotated | NA | NA |
| metab_10399 | Unannotated feature | neg | 159.8782 | 0.707 | 4.376 | 4.430 | 0.054 | 0.166 | 0.355 | 0.503 | Unannotated | NA | NA |
| metab_3663 | 3-Amino-3-(4-hydroxyphenyl)propanoate | pos | 223.1076 | 1.949 | 5.061 | 4.991 | -0.070 | -0.166 | 0.355 | 0.503 | Tentative | NA | NA |
| metab_7668 | Unannotated feature | neg | 212.8846 | 7.269 | 5.725 | 5.714 | -0.011 | -0.166 | 0.355 | 0.503 | Unannotated | NA | NA |
| metab_208 | Unannotated feature | pos | 782.4421 | 0.606 | 5.334 | 5.272 | -0.062 | -0.166 | 0.355 | 0.503 | Unannotated | NA | NA |
| metab_1651 | 2-Ethylsuberic acid | pos | 203.1278 | 7.207 | 4.847 | 4.869 | 0.023 | 0.166 | 0.355 | 0.503 | Tentative | NA | NA |
| metab_1654 | RU-0211 | pos | 391.2299 | 7.207 | 5.777 | 5.773 | -0.005 | -0.166 | 0.355 | 0.503 | Tentative | NA | NA |
| metab_897 | Unannotated feature | pos | 675.8174 | 3.746 | 5.860 | 5.873 | 0.012 | 0.166 | 0.355 | 0.503 | Unannotated | NA | NA |
| metab_3651 | L-2-Aminoadipate | pos | 144.0656 | 1.956 | 5.464 | 5.519 | 0.054 | 0.166 | 0.355 | 0.503 | Tentative | NA | NA |
| metab_3791 | Unannotated feature | pos | 90.9482 | 1.554 | 5.038 | 5.034 | -0.004 | -0.166 | 0.355 | 0.503 | Unannotated | NA | NA |
| metab_7543 | Unannotated feature | neg | 275.8803 | 7.173 | 3.875 | 3.841 | -0.034 | -0.166 | 0.355 | 0.503 | Unannotated | NA | NA |
| metab_7638 | Unannotated feature | neg | 278.8653 | 7.961 | 5.926 | 5.941 | 0.015 | 0.166 | 0.355 | 0.503 | Unannotated | NA | NA |
| metab_11375 | Unannotated feature | neg | 88.9873 | 0.481 | 4.930 | 4.946 | 0.016 | 0.166 | 0.355 | 0.503 | Unannotated | NA | NA |
| metab_555 | Unannotated feature | pos | 109.0845 | 2.034 | 3.461 | 3.487 | 0.026 | 0.166 | 0.355 | 0.503 | Unannotated | NA | NA |
| metab_11087 | Unannotated feature | neg | 681.6330 | 0.578 | 4.639 | 4.566 | -0.073 | -0.166 | 0.355 | 0.503 | Unannotated | NA | NA |
| metab_8432 | Unannotated feature | neg | 194.9241 | 5.771 | 4.478 | 4.536 | 0.058 | 0.166 | 0.355 | 0.503 | Unannotated | NA | NA |
| metab_1483 | Unannotated feature | pos | 226.1591 | 6.041 | 4.115 | 4.062 | -0.053 | -0.166 | 0.355 | 0.503 | Unannotated | NA | NA |
| metab_10714 | Unannotated feature | neg | 530.7230 | 0.578 | 5.755 | 5.724 | -0.030 | -0.166 | 0.355 | 0.503 | Unannotated | NA | NA |
| metab_1416 | N-Docosahexaenoyl Lysine | pos | 470.3510 | 5.941 | 5.972 | 6.023 | 0.051 | 0.166 | 0.355 | 0.503 | Tentative | NA | NA |
| metab_2543 | Unannotated feature | pos | 595.4225 | 5.871 | 6.026 | 6.082 | 0.056 | 0.166 | 0.355 | 0.503 | Unannotated | NA | NA |
| metab_3810 | Unannotated feature | pos | 102.5359 | 1.398 | 3.310 | 3.375 | 0.065 | 0.166 | 0.355 | 0.503 | Unannotated | NA | NA |
| metab_8892 | Unannotated feature | neg | 229.9208 | 5.176 | 5.658 | 5.639 | -0.019 | -0.166 | 0.355 | 0.503 | Unannotated | NA | NA |
| metab_4023 | Unannotated feature | pos | 170.1176 | 0.800 | 3.482 | 3.376 | -0.106 | -0.162 | 0.368 | 0.517 | Unannotated | NA | NA |
| metab_7504 | Unannotated feature | neg | 464.8795 | 7.153 | 5.132 | 5.117 | -0.015 | -0.162 | 0.368 | 0.517 | Unannotated | NA | NA |
| metab_1831 | Unannotated feature | pos | 119.0858 | 7.975 | 4.325 | 4.316 | -0.008 | -0.162 | 0.368 | 0.517 | Unannotated | NA | NA |
| metab_10049 | Unannotated feature | neg | 765.4454 | 0.613 | 5.101 | 5.025 | -0.076 | -0.162 | 0.368 | 0.517 | Unannotated | NA | NA |
| metab_10041 | Unannotated feature | neg | 213.8732 | 1.542 | 5.093 | 5.126 | 0.033 | 0.162 | 0.368 | 0.517 | Unannotated | NA | NA |
| metab_7787 | Unannotated feature | neg | 196.9217 | 7.170 | 4.413 | 4.401 | -0.012 | -0.162 | 0.368 | 0.517 | Unannotated | NA | NA |
| metab_5955 | Unannotated feature | neg | 142.0504 | 2.716 | 4.530 | 4.232 | -0.298 | -0.162 | 0.368 | 0.517 | Unannotated | NA | NA |
| metab_4311 | Unannotated feature | pos | 896.5782 | 0.575 | 4.481 | 4.424 | -0.057 | -0.162 | 0.368 | 0.517 | Unannotated | NA | NA |
| metab_2385 | Unannotated feature | pos | 574.8961 | 5.949 | 4.505 | 4.577 | 0.072 | 0.162 | 0.368 | 0.517 | Unannotated | NA | NA |
| metab_1605 | Unannotated feature | pos | 794.0181 | 6.752 | 5.685 | 5.519 | -0.166 | -0.162 | 0.368 | 0.517 | Unannotated | NA | NA |
| metab_2399 | 4-Methylbenzyl alcohol | pos | 245.1538 | 5.941 | 4.592 | 4.584 | -0.009 | 0.162 | 0.368 | 0.517 | Tentative | NA | NA |
| metab_1578 | 4-Cholesten-7alpha,12alpha-diol-3-one | pos | 449.3601 | 6.420 | 7.581 | 7.608 | 0.027 | 0.162 | 0.368 | 0.517 | Tentative | NA | NA |
| metab_8450 | Unannotated feature | neg | 481.2093 | 5.755 | 4.035 | 4.256 | 0.221 | 0.162 | 0.368 | 0.517 | Unannotated | NA | NA |
| metab_11305 | Unannotated feature | neg | 219.8455 | 7.189 | 5.129 | 5.124 | -0.004 | -0.162 | 0.368 | 0.517 | Unannotated | NA | NA |
| metab_11060 | P-CHLOROPHENYLALANINE | neg | 198.0325 | 3.224 | 7.017 | 7.005 | -0.012 | -0.162 | 0.368 | 0.517 | Tentative | NA | NA |
| metab_9897 | Unannotated feature | neg | 324.8589 | 2.197 | 4.556 | 4.581 | 0.025 | 0.162 | 0.368 | 0.517 | Unannotated | NA | NA |
| metab_11071 | Unannotated feature | neg | 300.0398 | 5.579 | 5.109 | 5.192 | 0.083 | 0.162 | 0.368 | 0.517 | Unannotated | NA | NA |
| metab_3322 | Unannotated feature | pos | 155.0452 | 2.644 | 6.933 | 6.940 | 0.007 | 0.162 | 0.368 | 0.517 | Unannotated | NA | NA |
| metab_139 | Unannotated feature | pos | 642.8621 | 0.551 | 5.550 | 5.534 | -0.016 | -0.162 | 0.368 | 0.517 | Unannotated | NA | NA |
| metab_11126 | Unannotated feature | neg | 368.9795 | 3.710 | 4.261 | 4.743 | 0.482 | 0.162 | 0.368 | 0.517 | Unannotated | NA | NA |
| metab_6310 | Unannotated feature | neg | 90.5272 | 3.718 | 3.509 | 3.483 | -0.026 | -0.162 | 0.368 | 0.517 | Unannotated | NA | NA |
| metab_8289 | Unannotated feature | neg | 236.1056 | 5.875 | 4.262 | 4.283 | 0.021 | 0.162 | 0.368 | 0.517 | Unannotated | NA | NA |
| metab_10078 | Unannotated feature | neg | 296.8908 | 1.294 | 4.618 | 4.599 | -0.019 | -0.162 | 0.368 | 0.517 | Unannotated | NA | NA |
| metab_6567 | Unannotated feature | neg | 381.0770 | 4.679 | 4.693 | 4.543 | -0.150 | -0.162 | 0.368 | 0.517 | Unannotated | NA | NA |
| metab_9081 | Fostedil | neg | 406.0919 | 4.505 | 5.101 | 5.038 | -0.063 | -0.162 | 0.368 | 0.517 | Tentative | NA | NA |
| metab_9154 | Unannotated feature | neg | 139.0395 | 4.267 | 4.374 | 4.012 | -0.362 | -0.162 | 0.368 | 0.517 | Unannotated | NA | NA |
| metab_8964 | Unannotated feature | neg | 141.0201 | 4.901 | 3.612 | 3.573 | -0.040 | -0.162 | 0.368 | 0.517 | Unannotated | NA | NA |
| metab_2882 | N-Methyl-2-pyrrolidinone | pos | 100.0761 | 2.103 | 6.237 | 6.072 | -0.166 | -0.162 | 0.368 | 0.517 | Moderate putative | NA | NA |
| metab_2281 | Unannotated feature | pos | 416.3371 | 6.126 | 4.446 | 4.363 | -0.083 | -0.162 | 0.368 | 0.517 | Unannotated | NA | NA |
| metab_8326 | Unannotated feature | neg | 505.3028 | 5.834 | 3.660 | 3.331 | -0.329 | -0.162 | 0.368 | 0.517 | Unannotated | NA | NA |
| metab_4440 | Unannotated feature | pos | 158.4143 | 0.559 | 4.288 | 4.325 | 0.036 | 0.162 | 0.368 | 0.517 | Unannotated | NA | NA |
| metab_2747 | Tetradecanoic acid | pos | 246.2428 | 5.703 | 6.072 | 6.087 | 0.015 | 0.162 | 0.368 | 0.517 | Tentative | NA | NA |
| metab_2881 | Unannotated feature | pos | 156.0389 | 5.122 | 4.938 | 4.932 | -0.006 | 0.162 | 0.368 | 0.517 | Unannotated | NA | NA |
| metab_7699 | Unannotated feature | neg | 173.9258 | 7.213 | 5.208 | 5.223 | 0.015 | 0.162 | 0.368 | 0.517 | Unannotated | NA | NA |
| metab_7700 | Unannotated feature | neg | 160.9790 | 7.213 | 3.192 | 3.351 | 0.159 | 0.162 | 0.368 | 0.517 | Unannotated | NA | NA |
| metab_2934 | Unannotated feature | pos | 204.1054 | 4.808 | 4.282 | 4.179 | -0.103 | -0.162 | 0.368 | 0.517 | Unannotated | NA | NA |
| metab_5481 | Unannotated feature | neg | 507.8767 | 0.530 | 4.477 | 4.433 | -0.045 | -0.162 | 0.368 | 0.517 | Unannotated | NA | NA |
| metab_10684 | Unannotated feature | neg | 464.7323 | 0.582 | 5.677 | 5.663 | -0.014 | -0.162 | 0.368 | 0.517 | Unannotated | NA | NA |
| metab_1434 | Unannotated feature | pos | 72.0815 | 5.964 | 3.271 | 3.305 | 0.033 | 0.162 | 0.368 | 0.517 | Unannotated | NA | NA |
| metab_802 | Unannotated feature | pos | 371.2276 | 3.027 | 3.811 | 4.096 | 0.285 | 0.162 | 0.368 | 0.517 | Unannotated | NA | NA |
| metab_355 | Uracil | pos | 113.0348 | 0.861 | 4.200 | 4.190 | -0.010 | -0.162 | 0.368 | 0.517 | Strong putative | NA | NA |
| metab_7094 | Unannotated feature | neg | 251.1292 | 5.811 | 4.682 | 4.740 | 0.059 | 0.162 | 0.368 | 0.517 | Unannotated | NA | NA |
| metab_107 | Unannotated feature | pos | 677.9177 | 0.538 | 4.560 | 4.467 | -0.093 | -0.162 | 0.368 | 0.517 | Unannotated | NA | NA |
| metab_5735 | Unannotated feature | neg | 180.8993 | 0.023 | 6.312 | 6.302 | -0.011 | -0.162 | 0.368 | 0.517 | Unannotated | NA | NA |
| metab_8991 | Unannotated feature | neg | 178.0143 | 4.782 | 3.357 | 3.514 | 0.156 | 0.162 | 0.368 | 0.517 | Unannotated | NA | NA |
| metab_1901 | Unannotated feature | pos | 156.0333 | 7.331 | 4.288 | 4.255 | -0.033 | -0.162 | 0.368 | 0.517 | Unannotated | NA | NA |
| metab_7089 | Unannotated feature | neg | 329.2340 | 5.803 | 4.191 | 4.306 | 0.115 | 0.162 | 0.368 | 0.517 | Unannotated | NA | NA |
| metab_8399 | Indole-5,6-quinone | neg | 192.0314 | 5.795 | 4.048 | 3.939 | -0.110 | -0.162 | 0.368 | 0.517 | Tentative | NA | NA |
| metab_105 | Unannotated feature | pos | 516.9981 | 0.538 | 5.104 | 5.059 | -0.045 | -0.158 | 0.381 | 0.530 | Unannotated | NA | NA |
| metab_8271 | Unannotated feature | neg | 179.1438 | 5.890 | 3.943 | 4.114 | 0.171 | 0.158 | 0.381 | 0.530 | Unannotated | NA | NA |
| metab_2428 | Unannotated feature | pos | 836.7988 | 0.559 | 4.487 | 4.547 | 0.060 | 0.158 | 0.381 | 0.530 | Unannotated | NA | NA |
| metab_5584 | Unannotated feature | neg | 124.0145 | 0.865 | 4.932 | 4.914 | -0.019 | -0.158 | 0.381 | 0.530 | Unannotated | NA | NA |
| metab_5295 | Unannotated feature | neg | 180.3050 | 0.562 | 4.080 | 4.057 | -0.023 | -0.158 | 0.381 | 0.530 | Unannotated | NA | NA |
| metab_2462 | Unannotated feature | pos | 577.7433 | 5.910 | 4.430 | 4.452 | 0.022 | 0.158 | 0.381 | 0.530 | Unannotated | NA | NA |
| metab_4682 | Unannotated feature | pos | 448.3063 | 5.871 | 4.729 | 4.648 | -0.081 | -0.158 | 0.381 | 0.530 | Unannotated | NA | NA |
| metab_6139 | Vanillate | neg | 167.0346 | 3.217 | 3.144 | 3.172 | 0.029 | 0.158 | 0.381 | 0.530 | Tentative | NA | NA |
| metab_3053 | Luteolinidin | pos | 271.0600 | 4.070 | 4.557 | 4.592 | 0.035 | 0.158 | 0.381 | 0.530 | Moderate putative | NA | NA |
| metab_3277 | 6-Acetamidohexanoic acid | pos | 174.1126 | 2.568 | 3.626 | 3.753 | 0.127 | 0.158 | 0.381 | 0.530 | Tentative | NA | NA |
| metab_6730 | Unannotated feature | neg | 194.0470 | 5.383 | 4.864 | 4.706 | -0.158 | -0.158 | 0.381 | 0.530 | Unannotated | NA | NA |
| metab_6604 | Melibiitol | neg | 381.0771 | 4.861 | 4.801 | 4.638 | -0.163 | -0.158 | 0.381 | 0.530 | Tentative | NA | NA |
| metab_8837 | Unannotated feature | neg | 532.8672 | 7.148 | 5.149 | 5.122 | -0.027 | -0.158 | 0.381 | 0.530 | Unannotated | NA | NA |
| metab_10409 | Unannotated feature | neg | 199.8047 | 0.692 | 7.805 | 7.805 | 0.000 | -0.158 | 0.381 | 0.530 | Unannotated | NA | NA |
| metab_1997 | Unannotated feature | pos | 155.1292 | 7.293 | 4.481 | 4.486 | 0.005 | 0.158 | 0.381 | 0.530 | Unannotated | NA | NA |
| metab_406 | Unannotated feature | pos | 239.1618 | 1.188 | 4.502 | 4.574 | 0.072 | 0.158 | 0.381 | 0.530 | Unannotated | NA | NA |
| metab_5169 | Unannotated feature | neg | 159.0294 | 0.515 | 3.425 | 3.480 | 0.055 | 0.158 | 0.381 | 0.530 | Unannotated | NA | NA |
| metab_6716 | Unannotated feature | neg | 243.0359 | 5.346 | 4.620 | 4.657 | 0.037 | 0.158 | 0.381 | 0.530 | Unannotated | NA | NA |
| metab_11162 | Unannotated feature | neg | 206.8244 | 5.787 | 4.950 | 4.915 | -0.036 | -0.158 | 0.381 | 0.530 | Unannotated | NA | NA |
| metab_10617 | Unannotated feature | neg | 166.8601 | 0.598 | 4.530 | 4.563 | 0.033 | 0.158 | 0.381 | 0.530 | Unannotated | NA | NA |
| metab_1642 | Unannotated feature | pos | 870.7748 | 7.184 | 4.984 | 5.003 | 0.018 | 0.158 | 0.381 | 0.530 | Unannotated | NA | NA |
| metab_10955 | Unannotated feature | neg | 257.9194 | 0.522 | 3.580 | 3.556 | -0.024 | -0.158 | 0.381 | 0.530 | Unannotated | NA | NA |
| metab_2062 | Unannotated feature | pos | 429.3846 | 7.215 | 6.064 | 6.054 | -0.010 | -0.158 | 0.381 | 0.530 | Unannotated | NA | NA |
| metab_4535 | Unannotated feature | pos | 311.9290 | 0.534 | 4.701 | 4.700 | -0.001 | -0.158 | 0.381 | 0.530 | Unannotated | NA | NA |
| metab_5898 | Unannotated feature | neg | 620.2009 | 2.510 | 4.841 | 4.808 | -0.034 | -0.158 | 0.381 | 0.530 | Unannotated | NA | NA |
| metab_10030 | Xanthine | neg | 151.0256 | 1.731 | 5.274 | 5.193 | -0.081 | -0.158 | 0.381 | 0.530 | Strong putative | NA | NA |
| metab_7820 | Unannotated feature | neg | 325.9070 | 7.156 | 4.245 | 4.226 | -0.019 | -0.158 | 0.381 | 0.530 | Unannotated | NA | NA |
| metab_7489 | Unannotated feature | neg | 928.8373 | 7.140 | 5.326 | 5.306 | -0.020 | -0.158 | 0.381 | 0.530 | Unannotated | NA | NA |
| metab_5843 | Unannotated feature | neg | 307.8566 | 0.013 | 6.419 | 6.432 | 0.013 | 0.158 | 0.381 | 0.530 | Unannotated | NA | NA |
| metab_238 | Unannotated feature | pos | 241.1030 | 0.644 | 4.872 | 4.931 | 0.059 | 0.158 | 0.381 | 0.530 | Unannotated | NA | NA |
| metab_7804 | Unannotated feature | neg | 308.9947 | 2.537 | 5.685 | 5.659 | -0.025 | -0.158 | 0.381 | 0.530 | Unannotated | NA | NA |
| metab_9862 | Unannotated feature | neg | 226.9093 | 2.431 | 5.436 | 5.461 | 0.025 | 0.158 | 0.381 | 0.530 | Unannotated | NA | NA |
| metab_4591 | Unannotated feature | pos | 149.0962 | 0.380 | 3.991 | 3.989 | -0.001 | 0.158 | 0.381 | 0.530 | Unannotated | NA | NA |
| metab_1570 | Unannotated feature | pos | 710.6786 | 0.575 | 4.965 | 4.969 | 0.004 | -0.158 | 0.381 | 0.530 | Unannotated | NA | NA |
| metab_10822 | Unannotated feature | neg | 837.8209 | 0.554 | 5.135 | 5.210 | 0.076 | 0.158 | 0.381 | 0.530 | Unannotated | NA | NA |
| metab_3495 | Unannotated feature | pos | 170.0812 | 2.202 | 5.011 | 5.059 | 0.047 | 0.158 | 0.381 | 0.530 | Unannotated | NA | NA |
| metab_7917 | Unannotated feature | neg | 134.9868 | 7.117 | 5.280 | 5.274 | -0.006 | -0.158 | 0.381 | 0.530 | Unannotated | NA | NA |
| metab_5705 | Unannotated feature | neg | 229.9021 | 1.350 | 5.203 | 5.458 | 0.255 | 0.158 | 0.381 | 0.530 | Unannotated | NA | NA |
| metab_7592 | Unannotated feature | neg | 269.9069 | 7.229 | 4.087 | 4.118 | 0.031 | 0.158 | 0.381 | 0.530 | Unannotated | NA | NA |
| metab_3325 | Xi-5-Isopropyl-2(5H)-furanone | pos | 91.0547 | 2.644 | 6.264 | 6.269 | 0.005 | 0.158 | 0.381 | 0.530 | Tentative | NA | NA |
| metab_5354 | Unannotated feature | neg | 672.6034 | 0.582 | 4.919 | 4.938 | 0.019 | 0.158 | 0.381 | 0.530 | Unannotated | NA | NA |
| metab_7815 | Unannotated feature | neg | 371.9905 | 2.565 | 4.985 | 4.957 | -0.028 | -0.158 | 0.381 | 0.530 | Unannotated | NA | NA |
| metab_2623 | Unannotated feature | pos | 525.0337 | 5.832 | 4.619 | 4.539 | -0.080 | -0.158 | 0.381 | 0.530 | Unannotated | NA | NA |
| metab_3127 | Unannotated feature | pos | 84.1315 | 3.692 | 3.469 | 3.514 | 0.045 | 0.158 | 0.381 | 0.530 | Unannotated | NA | NA |
| metab_578 | Unannotated feature | pos | 112.0808 | 2.065 | 5.338 | 5.349 | 0.011 | 0.158 | 0.381 | 0.530 | Unannotated | NA | NA |
| metab_9024 | Unannotated feature | neg | 195.0661 | 4.703 | 2.861 | 2.909 | 0.048 | 0.158 | 0.381 | 0.530 | Unannotated | NA | NA |
| metab_7712 | Unannotated feature | neg | 101.2124 | 7.213 | 3.289 | 3.355 | 0.065 | 0.158 | 0.381 | 0.530 | Unannotated | NA | NA |
| metab_9768 | Glycylproline | neg | 171.0771 | 2.652 | 3.440 | 3.477 | 0.037 | 0.154 | 0.394 | 0.543 | Strong putative | NA | NA |
| metab_2154 | (S)-4-[(S)-2-((S)-1-Formyl-4-guanidino-butylcarbamoyl)-pyrrolidin-1-yl]-4-oxo-3-(2-propyl-pentanoylamino)-butyric acid methyl ester | pos | 493.3141 | 6.528 | 6.240 | 6.226 | -0.014 | -0.154 | 0.394 | 0.543 | Tentative | NA | NA |
| metab_1111 | L-Methionine S-oxide | pos | 166.0533 | 0.644 | 5.139 | 5.228 | 0.089 | 0.154 | 0.394 | 0.543 | Strong putative | NA | NA |
| metab_5544 | Unannotated feature | neg | 144.9668 | 0.818 | 4.227 | 4.158 | -0.069 | -0.154 | 0.394 | 0.543 | Unannotated | NA | NA |
| metab_6777 | Unannotated feature | neg | 246.0752 | 5.532 | 5.835 | 5.780 | -0.055 | -0.154 | 0.394 | 0.543 | Unannotated | NA | NA |
| metab_10095 | Unannotated feature | neg | 241.7936 | 1.214 | 5.656 | 5.660 | 0.004 | 0.154 | 0.394 | 0.543 | Unannotated | NA | NA |
| metab_10496 | Unannotated feature | neg | 217.9120 | 0.636 | 4.772 | 4.696 | -0.076 | -0.154 | 0.394 | 0.543 | Unannotated | NA | NA |
| metab_10843 | Unannotated feature | neg | 667.8514 | 0.554 | 5.069 | 5.101 | 0.032 | 0.154 | 0.394 | 0.543 | Unannotated | NA | NA |
| metab_10227 | Unannotated feature | neg | 174.0405 | 0.865 | 4.558 | 4.615 | 0.057 | 0.154 | 0.394 | 0.543 | Unannotated | NA | NA |
| metab_6607 | Unannotated feature | neg | 218.5276 | 4.885 | 3.622 | 3.489 | -0.133 | -0.154 | 0.394 | 0.543 | Unannotated | NA | NA |
| metab_7875 | Unannotated feature | neg | 562.8995 | 7.140 | 4.533 | 4.504 | -0.030 | -0.154 | 0.394 | 0.543 | Unannotated | NA | NA |
| metab_2090 | Unannotated feature | pos | 485.4308 | 7.184 | 5.479 | 5.480 | 0.001 | -0.154 | 0.394 | 0.543 | Unannotated | NA | NA |
| metab_2562 | Unannotated feature | pos | 522.3732 | 5.863 | 5.794 | 5.861 | 0.067 | 0.154 | 0.394 | 0.543 | Unannotated | NA | NA |
| metab_3772 | Unannotated feature | pos | 82.5376 | 1.701 | 3.642 | 3.619 | -0.024 | -0.154 | 0.394 | 0.543 | Unannotated | NA | NA |
| metab_1733 | Unannotated feature | pos | 89.0088 | 7.324 | 3.708 | 3.712 | 0.004 | 0.154 | 0.394 | 0.543 | Unannotated | NA | NA |
| metab_1313 | Unannotated feature | pos | 624.4425 | 5.879 | 6.192 | 6.240 | 0.048 | 0.154 | 0.394 | 0.543 | Unannotated | NA | NA |
| metab_8635 | Unannotated feature | neg | 685.8665 | 7.140 | 4.413 | 4.382 | -0.030 | -0.154 | 0.394 | 0.543 | Unannotated | NA | NA |
| metab_6692 | Unannotated feature | neg | 144.0449 | 5.239 | 5.133 | 5.060 | -0.073 | -0.154 | 0.394 | 0.543 | Unannotated | NA | NA |
| metab_7562 | Unannotated feature | neg | 221.9029 | 7.189 | 4.223 | 4.238 | 0.015 | 0.154 | 0.394 | 0.543 | Unannotated | NA | NA |
| metab_8604 | Unannotated feature | neg | 277.1086 | 5.667 | 3.543 | 3.690 | 0.147 | 0.154 | 0.394 | 0.543 | Unannotated | NA | NA |
| metab_6378 | 3,4-Dihydroxyphenylacetate | neg | 167.0346 | 3.997 | 3.270 | 3.279 | 0.009 | 0.154 | 0.394 | 0.543 | Strong putative | Benzenoids | Phenols |
| metab_9095 | Unannotated feature | neg | 188.0350 | 4.441 | 3.668 | 3.988 | 0.320 | 0.154 | 0.394 | 0.543 | Unannotated | NA | NA |
| metab_9182 | M1G-dR | neg | 338.0669 | 4.187 | 4.636 | 4.623 | -0.013 | 0.154 | 0.394 | 0.543 | Tentative | NA | NA |
| metab_1867 | Unannotated feature | pos | 103.0757 | 7.455 | 4.298 | 4.275 | -0.023 | -0.154 | 0.394 | 0.543 | Unannotated | NA | NA |
| metab_1844 | Unannotated feature | pos | 128.0194 | 7.936 | 4.103 | 4.102 | -0.001 | 0.154 | 0.394 | 0.543 | Unannotated | NA | NA |
| metab_1213 | Unannotated feature | pos | 364.2316 | 5.778 | 4.493 | 4.513 | 0.019 | 0.154 | 0.394 | 0.543 | Unannotated | NA | NA |
| metab_6350 | Unannotated feature | neg | 213.8732 | 3.843 | 4.540 | 4.529 | -0.011 | -0.154 | 0.394 | 0.543 | Unannotated | NA | NA |
| metab_2883 | Unannotated feature | pos | 119.0732 | 5.122 | 4.196 | 4.144 | -0.052 | 0.154 | 0.394 | 0.543 | Unannotated | NA | NA |
| metab_4183 | Unannotated feature | pos | 614.9500 | 0.636 | 4.852 | 4.752 | -0.100 | -0.154 | 0.394 | 0.543 | Unannotated | NA | NA |
| metab_5379 | Unannotated feature | neg | 408.7246 | 0.598 | 4.524 | 4.528 | 0.004 | 0.154 | 0.394 | 0.543 | Unannotated | NA | NA |
| metab_2397 | Unannotated feature | pos | 419.3163 | 5.941 | 4.675 | 4.746 | 0.071 | 0.154 | 0.394 | 0.543 | Unannotated | NA | NA |
| metab_2386 | Unannotated feature | pos | 572.9205 | 5.949 | 5.716 | 5.768 | 0.052 | 0.154 | 0.394 | 0.543 | Unannotated | NA | NA |
| metab_2021 | Unannotated feature | pos | 425.1344 | 7.277 | 4.895 | 4.897 | 0.002 | 0.154 | 0.394 | 0.543 | Unannotated | NA | NA |
| metab_872 | Unannotated feature | pos | 167.0129 | 7.324 | 6.505 | 6.496 | -0.009 | -0.154 | 0.394 | 0.543 | Unannotated | NA | NA |
| metab_5456 | Unannotated feature | neg | 151.8736 | 0.668 | 4.631 | 4.620 | -0.010 | -0.154 | 0.394 | 0.543 | Unannotated | NA | NA |
| metab_8120 | Unannotated feature | neg | 100.9142 | 6.180 | 5.033 | 5.048 | 0.015 | 0.154 | 0.394 | 0.543 | Unannotated | NA | NA |
| metab_7523 | Unannotated feature | neg | 478.8307 | 7.164 | 4.501 | 4.473 | -0.028 | -0.154 | 0.394 | 0.543 | Unannotated | NA | NA |
| metab_4566 | Dibutyl phthalate | pos | 279.1590 | 0.465 | 5.705 | 5.696 | -0.009 | -0.154 | 0.394 | 0.543 | Strong putative | NA | NA |
| metab_3614 | Coniferyl aldehyde | pos | 179.0703 | 1.988 | 4.242 | 4.209 | -0.033 | -0.154 | 0.394 | 0.543 | Strong putative | NA | NA |
| metab_6980 | Unannotated feature | neg | 424.1988 | 5.707 | 5.101 | 5.021 | -0.080 | -0.154 | 0.394 | 0.543 | Unannotated | NA | NA |
| metab_3713 | Unannotated feature | pos | 128.0195 | 1.886 | 4.206 | 4.182 | -0.024 | -0.154 | 0.394 | 0.543 | Unannotated | NA | NA |
| metab_2813 | Unannotated feature | pos | 141.1135 | 5.573 | 4.401 | 4.441 | 0.040 | 0.154 | 0.394 | 0.543 | Unannotated | NA | NA |
| metab_3278 | 3-Phenyl-2-propenenitrile | pos | 130.0652 | 2.904 | 4.240 | 4.284 | 0.044 | 0.154 | 0.394 | 0.543 | Tentative | NA | NA |
| metab_2074 | Unannotated feature | pos | 463.3392 | 7.200 | 6.679 | 6.664 | -0.015 | -0.154 | 0.394 | 0.543 | Unannotated | NA | NA |
| metab_10121 | Unannotated feature | neg | 162.8421 | 1.039 | 4.112 | 4.179 | 0.067 | 0.154 | 0.394 | 0.543 | Unannotated | NA | NA |
| metab_7861 | Unannotated feature | neg | 847.8498 | 7.140 | 4.178 | 4.131 | -0.047 | -0.154 | 0.394 | 0.543 | Unannotated | NA | NA |
| metab_3420 | Unannotated feature | pos | 109.1015 | 2.405 | 3.558 | 3.453 | -0.105 | -0.154 | 0.394 | 0.543 | Unannotated | NA | NA |
| metab_6177 | Unannotated feature | neg | 547.9529 | 3.266 | 4.589 | 4.664 | 0.075 | 0.154 | 0.394 | 0.543 | Unannotated | NA | NA |
| metab_1286 | Unannotated feature | pos | 124.1061 | 2.073 | 6.544 | 6.543 | -0.001 | 0.154 | 0.394 | 0.543 | Unannotated | NA | NA |
| metab_3637 | Seryllysine | pos | 198.1238 | 1.956 | 3.867 | 3.958 | 0.091 | 0.154 | 0.394 | 0.543 | Tentative | NA | NA |
| metab_4004 | Unannotated feature | pos | 213.0984 | 0.815 | 3.217 | 3.166 | -0.051 | -0.154 | 0.394 | 0.543 | Unannotated | NA | NA |
| metab_5699 | Unannotated feature | neg | 456.9576 | 1.302 | 4.803 | 4.721 | -0.082 | -0.154 | 0.394 | 0.543 | Unannotated | NA | NA |
| metab_6934 | Unannotated feature | neg | 266.0521 | 5.683 | 2.947 | 3.187 | 0.240 | 0.154 | 0.394 | 0.543 | Unannotated | NA | NA |
| metab_2029 | Unannotated feature | pos | 654.7162 | 0.575 | 5.670 | 5.659 | -0.011 | -0.154 | 0.394 | 0.543 | Unannotated | NA | NA |
| metab_5782 | Unannotated feature | neg | 572.2811 | 2.021 | 4.457 | 4.578 | 0.121 | 0.154 | 0.394 | 0.543 | Unannotated | NA | NA |
| metab_1401 | Unannotated feature | pos | 638.4580 | 5.925 | 6.270 | 6.313 | 0.043 | 0.154 | 0.394 | 0.543 | Unannotated | NA | NA |
| metab_5548 | Unannotated feature | neg | 348.7638 | 0.818 | 4.992 | 4.861 | -0.131 | -0.154 | 0.394 | 0.543 | Unannotated | NA | NA |
| metab_9710 | Unannotated feature | neg | 148.0762 | 2.860 | 4.232 | 4.202 | -0.030 | -0.154 | 0.394 | 0.543 | Unannotated | NA | NA |
| metab_6185 | Unannotated feature | neg | 252.0212 | 3.290 | 4.217 | 4.022 | -0.195 | -0.154 | 0.394 | 0.543 | Unannotated | NA | NA |
| metab_423 | Unannotated feature | pos | 85.3009 | 1.390 | 3.483 | 3.504 | 0.020 | 0.149 | 0.407 | 0.556 | Unannotated | NA | NA |
| metab_5165 | Unannotated feature | neg | 120.9541 | 0.509 | 3.278 | 3.348 | 0.070 | 0.149 | 0.407 | 0.556 | Unannotated | NA | NA |
| metab_652 | Unannotated feature | pos | 160.0791 | 2.319 | 3.139 | 3.481 | 0.342 | 0.149 | 0.407 | 0.556 | Unannotated | NA | NA |
| metab_2784 | 3-hydroxydecanoyl carnitine | pos | 332.2430 | 5.642 | 5.265 | 5.557 | 0.292 | 0.149 | 0.407 | 0.556 | Tentative | NA | NA |
| metab_666 | Unannotated feature | pos | 146.1176 | 2.412 | 4.654 | 4.647 | -0.007 | 0.149 | 0.407 | 0.556 | Unannotated | NA | NA |
| metab_10698 | Unannotated feature | neg | 700.6933 | 0.578 | 4.373 | 4.212 | -0.161 | -0.149 | 0.407 | 0.556 | Unannotated | NA | NA |
| metab_1749 | Unannotated feature | pos | 301.1410 | 0.534 | 5.414 | 5.414 | 0.000 | 0.149 | 0.407 | 0.556 | Unannotated | NA | NA |
| metab_7141 | Unannotated feature | neg | 528.7254 | 0.578 | 5.687 | 5.683 | -0.005 | -0.149 | 0.407 | 0.556 | Unannotated | NA | NA |
| metab_3692 | Unannotated feature | pos | 183.1178 | 3.576 | 4.865 | 4.901 | 0.035 | 0.149 | 0.407 | 0.556 | Unannotated | NA | NA |
| metab_1244 | Unannotated feature | pos | 106.0734 | 2.073 | 5.897 | 5.912 | 0.014 | 0.149 | 0.407 | 0.556 | Unannotated | NA | NA |
| metab_2263 | Unannotated feature | pos | 341.2685 | 6.180 | 4.620 | 4.612 | -0.008 | -0.149 | 0.407 | 0.556 | Unannotated | NA | NA |
| metab_8288 | Unannotated feature | neg | 269.1764 | 5.875 | 4.511 | 4.683 | 0.171 | 0.149 | 0.407 | 0.556 | Unannotated | NA | NA |
| metab_2143 | Unannotated feature | pos | 603.2995 | 6.652 | 5.600 | 5.483 | -0.116 | -0.149 | 0.407 | 0.556 | Unannotated | NA | NA |
| metab_1566 | 1-Acyl-sn-glycero-3-phosphocholine | pos | 572.3710 | 6.342 | 5.561 | 5.484 | -0.077 | -0.149 | 0.407 | 0.556 | Tentative | NA | NA |
| metab_1430 | Unannotated feature | pos | 623.4527 | 5.956 | 5.990 | 6.035 | 0.044 | 0.149 | 0.407 | 0.556 | Unannotated | NA | NA |
| metab_10181 | Unannotated feature | neg | 93.1740 | 0.904 | 4.189 | 4.169 | -0.020 | -0.149 | 0.407 | 0.556 | Unannotated | NA | NA |
| metab_73 | Unannotated feature | pos | 109.1015 | 0.434 | 4.182 | 4.187 | 0.005 | 0.149 | 0.407 | 0.556 | Unannotated | NA | NA |
| metab_9009 | Unannotated feature | neg | 220.0615 | 4.718 | 1.359 | 1.218 | -0.140 | -0.149 | 0.407 | 0.556 | Unannotated | NA | NA |
| metab_2723 | Unannotated feature | pos | 440.0546 | 5.733 | 4.436 | 4.384 | -0.052 | -0.149 | 0.407 | 0.556 | Unannotated | NA | NA |
| metab_2465 | Unannotated feature | pos | 527.8766 | 5.910 | 4.906 | 4.936 | 0.030 | 0.149 | 0.407 | 0.556 | Unannotated | NA | NA |
| metab_3091 | Unannotated feature | pos | 573.1766 | 3.854 | 4.431 | 4.403 | -0.028 | -0.149 | 0.407 | 0.556 | Unannotated | NA | NA |
| metab_3086 | Unannotated feature | pos | 209.0809 | 2.436 | 4.625 | 4.569 | -0.056 | -0.149 | 0.407 | 0.556 | Unannotated | NA | NA |
| metab_7833 | Unannotated feature | neg | 534.8624 | 7.153 | 4.536 | 4.573 | 0.036 | 0.149 | 0.407 | 0.556 | Unannotated | NA | NA |
| metab_2196 | Unannotated feature | pos | 517.0903 | 6.303 | 5.921 | 5.934 | 0.013 | 0.149 | 0.407 | 0.556 | Unannotated | NA | NA |
| metab_1726 | Unannotated feature | pos | 204.0544 | 7.316 | 4.280 | 4.246 | -0.034 | -0.149 | 0.407 | 0.556 | Unannotated | NA | NA |
| metab_7847 | Unannotated feature | neg | 676.3074 | 7.148 | 5.169 | 5.157 | -0.012 | -0.149 | 0.407 | 0.556 | Unannotated | NA | NA |
| metab_3026 | Unannotated feature | pos | 155.0703 | 4.217 | 3.992 | 3.483 | -0.509 | -0.149 | 0.407 | 0.556 | Unannotated | NA | NA |
| metab_9881 | Unannotated feature | neg | 285.9097 | 2.342 | 5.366 | 5.357 | -0.009 | -0.149 | 0.407 | 0.556 | Unannotated | NA | NA |
| metab_8180 | Unannotated feature | neg | 526.2946 | 6.000 | 4.458 | 4.700 | 0.242 | 0.149 | 0.407 | 0.556 | Unannotated | NA | NA |
| metab_4723 | Unannotated feature | pos | 542.4265 | 5.995 | 5.773 | 5.774 | 0.001 | -0.149 | 0.407 | 0.556 | Unannotated | NA | NA |
| metab_7336 | Unannotated feature | neg | 133.0136 | 6.397 | 4.723 | 4.743 | 0.020 | 0.149 | 0.407 | 0.556 | Unannotated | NA | NA |
| metab_2947 | Genipic acid | pos | 202.1074 | 2.241 | 4.470 | 4.410 | -0.060 | -0.149 | 0.407 | 0.556 | Tentative | NA | NA |
| metab_7354 | Unannotated feature | neg | 99.0080 | 6.555 | 4.849 | 4.872 | 0.023 | 0.149 | 0.407 | 0.556 | Unannotated | NA | NA |
| metab_4827 | Unannotated feature | pos | 566.3216 | 7.300 | 5.280 | 5.210 | -0.070 | -0.149 | 0.407 | 0.556 | Unannotated | NA | NA |
| metab_4824 | Unannotated feature | pos | 586.3091 | 7.300 | 5.722 | 5.676 | -0.046 | -0.149 | 0.407 | 0.556 | Unannotated | NA | NA |
| metab_4931 | Unannotated feature | pos | 306.9575 | 0.614 | 5.220 | 5.190 | -0.029 | -0.149 | 0.407 | 0.556 | Unannotated | NA | NA |
| metab_6160 | Unannotated feature | neg | 515.9884 | 3.240 | 4.986 | 4.969 | -0.017 | -0.149 | 0.407 | 0.556 | Unannotated | NA | NA |
| metab_4506 | Unannotated feature | pos | 449.0108 | 0.545 | 4.738 | 4.708 | -0.030 | -0.149 | 0.407 | 0.556 | Unannotated | NA | NA |
| metab_3787 | Unannotated feature | pos | 139.0310 | 1.577 | 4.502 | 4.499 | -0.004 | -0.149 | 0.407 | 0.556 | Unannotated | NA | NA |
| metab_10593 | Unannotated feature | neg | 580.5606 | 0.601 | 5.683 | 5.659 | -0.025 | -0.149 | 0.407 | 0.556 | Unannotated | NA | NA |
| metab_8944 | Unannotated feature | neg | 260.0508 | 7.109 | 4.194 | 4.126 | -0.067 | -0.149 | 0.407 | 0.556 | Unannotated | NA | NA |
| metab_2880 | Unannotated feature | pos | 182.0368 | 5.122 | 5.074 | 5.096 | 0.022 | 0.149 | 0.407 | 0.556 | Unannotated | NA | NA |
| metab_10060 | Unannotated feature | neg | 411.3062 | 5.890 | 6.970 | 7.024 | 0.054 | 0.149 | 0.407 | 0.556 | Unannotated | NA | NA |
| metab_8721 | Unannotated feature | neg | 399.8830 | 5.579 | 5.572 | 5.503 | -0.070 | -0.149 | 0.407 | 0.556 | Unannotated | NA | NA |
| metab_7510 | Unannotated feature | neg | 144.8940 | 7.156 | 3.998 | 3.999 | 0.002 | -0.149 | 0.407 | 0.556 | Unannotated | NA | NA |
| metab_11310 | Unannotated feature | neg | 187.8607 | 7.189 | 5.678 | 5.685 | 0.008 | 0.149 | 0.407 | 0.556 | Unannotated | NA | NA |
| metab_7072 | Unannotated feature | neg | 269.9071 | 5.795 | 3.769 | 3.916 | 0.147 | 0.149 | 0.407 | 0.556 | Unannotated | NA | NA |
| metab_2979 | PS(20:4(5Z,8Z,11Z,14Z)/6 keto-PGF1alpha) | pos | 471.7376 | 4.516 | 4.917 | 5.131 | 0.215 | 0.149 | 0.407 | 0.556 | Tentative | NA | NA |
| metab_11064 | Unannotated feature | neg | 370.9508 | 5.543 | 4.542 | 4.552 | 0.010 | 0.149 | 0.407 | 0.556 | Unannotated | NA | NA |
| metab_5963 | Unannotated feature | neg | 442.8948 | 0.570 | 5.862 | 5.833 | -0.029 | -0.149 | 0.407 | 0.556 | Unannotated | NA | NA |
| metab_5968 | Unannotated feature | neg | 90.9328 | 2.748 | 3.613 | 3.651 | 0.038 | 0.149 | 0.407 | 0.556 | Unannotated | NA | NA |
| metab_223 | Unannotated feature | pos | 282.0560 | 0.621 | 4.391 | 4.342 | -0.049 | -0.149 | 0.407 | 0.556 | Unannotated | NA | NA |
| metab_206 | Unannotated feature | pos | 448.6677 | 0.606 | 5.194 | 5.133 | -0.061 | -0.149 | 0.407 | 0.556 | Unannotated | NA | NA |
| metab_4364 | Unannotated feature | pos | 689.8181 | 0.567 | 4.076 | 4.103 | 0.027 | 0.149 | 0.407 | 0.556 | Unannotated | NA | NA |
| metab_6813 | Unannotated feature | neg | 401.8801 | 5.579 | 5.329 | 5.278 | -0.051 | -0.149 | 0.407 | 0.556 | Unannotated | NA | NA |
| metab_1803 | Coumarin | pos | 179.0703 | 7.936 | 5.033 | 5.033 | 0.001 | -0.149 | 0.407 | 0.556 | Moderate putative | NA | NA |
| metab_10962 | Unannotated feature | neg | 147.9733 | 0.515 | 2.761 | 2.774 | 0.013 | 0.149 | 0.407 | 0.556 | Unannotated | NA | NA |
| metab_11070 | Unannotated feature | neg | 141.0202 | 5.579 | 4.594 | 4.728 | 0.134 | 0.149 | 0.407 | 0.556 | Unannotated | NA | NA |
| metab_11056 | Unannotated feature | neg | 169.1027 | 4.798 | 3.823 | 3.884 | 0.061 | 0.149 | 0.407 | 0.556 | Unannotated | NA | NA |
| metab_440 | Unannotated feature | pos | 114.0736 | 1.608 | 5.790 | 5.808 | 0.018 | 0.149 | 0.407 | 0.556 | Unannotated | NA | NA |
| metab_8556 | Unannotated feature | neg | 357.1014 | 5.691 | 3.945 | 4.231 | 0.285 | 0.149 | 0.407 | 0.556 | Unannotated | NA | NA |
| metab_6856 | Unannotated feature | neg | 232.0594 | 5.627 | 5.255 | 5.388 | 0.133 | 0.145 | 0.420 | 0.568 | Unannotated | NA | NA |
| metab_4356 | Unannotated feature | pos | 692.8342 | 0.567 | 5.528 | 5.494 | -0.034 | -0.145 | 0.420 | 0.568 | Unannotated | NA | NA |
| metab_3831 | Unannotated feature | pos | 79.0184 | 1.243 | 3.856 | 3.861 | 0.005 | 0.145 | 0.420 | 0.568 | Unannotated | NA | NA |
| metab_10857 | Unannotated feature | neg | 590.6790 | 0.582 | 5.871 | 5.845 | -0.026 | -0.145 | 0.420 | 0.568 | Unannotated | NA | NA |
| metab_9973 | Unannotated feature | neg | 251.8225 | 2.071 | 4.883 | 4.952 | 0.069 | 0.145 | 0.420 | 0.568 | Unannotated | NA | NA |
| metab_9257 | Unannotated feature | neg | 372.1307 | 3.894 | 4.827 | 4.628 | -0.200 | -0.145 | 0.420 | 0.568 | Unannotated | NA | NA |
| metab_8506 | Unannotated feature | neg | 287.6124 | 5.723 | 4.193 | 3.962 | -0.231 | -0.145 | 0.420 | 0.568 | Unannotated | NA | NA |
| metab_719 | L-Norleucine | pos | 114.0917 | 2.591 | 4.718 | 4.718 | -0.000 | 0.145 | 0.420 | 0.568 | Tentative | NA | NA |
| metab_7530 | Unannotated feature | neg | 165.9930 | 7.170 | 4.794 | 4.779 | -0.015 | -0.145 | 0.420 | 0.568 | Unannotated | NA | NA |
| metab_1168 | Unannotated feature | pos | 202.2166 | 5.717 | 4.565 | 4.603 | 0.038 | 0.145 | 0.420 | 0.568 | Unannotated | NA | NA |
| metab_8599 | Unannotated feature | neg | 190.1396 | 5.675 | 3.828 | 3.799 | -0.028 | -0.145 | 0.420 | 0.568 | Unannotated | NA | NA |
| metab_4886 | Unannotated feature | pos | 258.1197 | 1.949 | 5.095 | 4.965 | -0.130 | -0.145 | 0.420 | 0.568 | Unannotated | NA | NA |
| metab_2334 | Unannotated feature | pos | 94.0656 | 6.003 | 4.365 | 4.370 | 0.005 | 0.145 | 0.420 | 0.568 | Unannotated | NA | NA |
| metab_8764 | Unannotated feature | neg | 183.0029 | 5.543 | 4.248 | 4.256 | 0.008 | 0.145 | 0.420 | 0.568 | Unannotated | NA | NA |
| metab_11054 | MJDBISSN00000001 | neg | 198.0325 | 3.224 | 7.004 | 6.992 | -0.013 | -0.145 | 0.420 | 0.568 | Strong putative | NA | NA |
| metab_2176 | Gymnodimine | pos | 1015.6709 | 6.389 | 6.689 | 6.635 | -0.054 | -0.145 | 0.420 | 0.568 | Tentative | NA | NA |
| metab_11063 | Unannotated feature | neg | 784.6126 | 0.578 | 4.471 | 4.474 | 0.003 | -0.145 | 0.420 | 0.568 | Unannotated | NA | NA |
| metab_1544 | Unannotated feature | pos | 111.0920 | 6.296 | 4.769 | 4.779 | 0.010 | 0.145 | 0.420 | 0.568 | Unannotated | NA | NA |
| metab_1821 | Unannotated feature | pos | 163.0278 | 7.990 | 4.821 | 4.816 | -0.005 | -0.145 | 0.420 | 0.568 | Unannotated | NA | NA |
| metab_4812 | Unannotated feature | pos | 170.0663 | 7.316 | 5.446 | 5.441 | -0.005 | -0.145 | 0.420 | 0.568 | Unannotated | NA | NA |
| metab_5890 | Unannotated feature | neg | 216.9810 | 2.504 | 3.932 | 3.975 | 0.043 | 0.145 | 0.420 | 0.568 | Unannotated | NA | NA |
| metab_2849 | N-(3-Aminopropyl)-N-methylcarbamic acid tert-butyl ester | pos | 171.1493 | 5.306 | 5.068 | 5.074 | 0.007 | 0.145 | 0.420 | 0.568 | Tentative | NA | NA |
| metab_1833 | Unannotated feature | pos | 75.0446 | 7.967 | 3.752 | 3.773 | 0.020 | 0.145 | 0.420 | 0.568 | Unannotated | NA | NA |
| metab_5423 | 3-Hydroxy-3-methylglutarate | neg | 197.0221 | 0.636 | 5.566 | 5.573 | 0.007 | -0.145 | 0.420 | 0.568 | Tentative | NA | NA |
| metab_598 | Unannotated feature | pos | 91.0547 | 2.088 | 5.493 | 5.519 | 0.027 | 0.145 | 0.420 | 0.568 | Unannotated | NA | NA |
| metab_10556 | Unannotated feature | neg | 260.9796 | 0.621 | 5.288 | 5.236 | -0.052 | -0.145 | 0.420 | 0.568 | Unannotated | NA | NA |
| metab_9537 | Unannotated feature | neg | 253.9686 | 3.217 | 4.704 | 4.639 | -0.065 | -0.145 | 0.420 | 0.568 | Unannotated | NA | NA |
| metab_6159 | Unannotated feature | neg | 405.1078 | 3.240 | 4.669 | 4.635 | -0.034 | -0.145 | 0.420 | 0.568 | Unannotated | NA | NA |
| metab_7657 | Unannotated feature | neg | 98.9551 | 7.309 | 5.313 | 5.322 | 0.010 | 0.145 | 0.420 | 0.568 | Unannotated | NA | NA |
| metab_848 | Trp-P-1 | pos | 212.1183 | 3.321 | 5.181 | 5.454 | 0.273 | 0.145 | 0.420 | 0.568 | Tentative | Organoheterocyclic compounds | Indoles and derivatives |
| metab_11227 | Unannotated feature | neg | 499.2926 | 5.811 | 5.363 | 5.397 | 0.034 | 0.145 | 0.420 | 0.568 | Unannotated | NA | NA |
| metab_1655 | Unannotated feature | pos | 475.3237 | 7.207 | 5.331 | 5.323 | -0.008 | -0.145 | 0.420 | 0.568 | Unannotated | NA | NA |
| metab_9772 | Unannotated feature | neg | 141.0187 | 2.636 | 3.823 | 3.848 | 0.025 | 0.145 | 0.420 | 0.568 | Unannotated | NA | NA |
| metab_9776 | Unannotated feature | neg | 398.1689 | 2.613 | 4.055 | 3.691 | -0.364 | -0.145 | 0.420 | 0.568 | Unannotated | NA | NA |
| metab_2725 | Unannotated feature | pos | 257.1746 | 5.733 | 4.123 | 4.105 | -0.019 | -0.145 | 0.420 | 0.568 | Unannotated | NA | NA |
| metab_8798 | 3-Methyl-2-(3-pyridyl)-1-indoleoctanoic acid | neg | 385.1699 | 5.498 | 5.836 | 5.894 | 0.059 | 0.145 | 0.420 | 0.568 | Tentative | NA | NA |
| metab_3875 | Unannotated feature | pos | 103.0546 | 0.900 | 4.732 | 4.687 | -0.045 | -0.145 | 0.420 | 0.568 | Unannotated | NA | NA |
| metab_2412 | Unannotated feature | pos | 470.3683 | 5.933 | 6.094 | 6.097 | 0.004 | -0.145 | 0.420 | 0.568 | Unannotated | NA | NA |
| metab_703 | L-threo-3-Phenylserine | pos | 223.1077 | 2.529 | 5.364 | 5.305 | -0.059 | -0.145 | 0.420 | 0.568 | Tentative | NA | NA |
| metab_6956 | Unannotated feature | neg | 297.8174 | 0.936 | 5.082 | 5.056 | -0.026 | -0.145 | 0.420 | 0.568 | Unannotated | NA | NA |
| metab_1751 | Unannotated feature | pos | 145.0173 | 7.331 | 4.237 | 4.227 | -0.010 | -0.145 | 0.420 | 0.568 | Unannotated | NA | NA |
| metab_8270 | Unannotated feature | neg | 223.1340 | 5.890 | 5.226 | 5.359 | 0.134 | 0.145 | 0.420 | 0.568 | Unannotated | NA | NA |
| metab_3714 | Unannotated feature | pos | 149.0598 | 3.383 | 3.411 | 3.507 | 0.096 | 0.145 | 0.420 | 0.568 | Unannotated | NA | NA |
| metab_6578 | Unannotated feature | neg | 176.8839 | 0.825 | 4.880 | 4.882 | 0.003 | 0.145 | 0.420 | 0.568 | Unannotated | NA | NA |
| metab_11203 | Glycochenodeoxycholate | neg | 448.3077 | 5.890 | 6.207 | 6.249 | 0.042 | 0.145 | 0.420 | 0.568 | Tentative | NA | NA |
| metab_389 | Unannotated feature | pos | 138.0527 | 0.986 | 3.341 | 3.249 | -0.092 | -0.145 | 0.420 | 0.568 | Unannotated | NA | NA |
| metab_9995 | 2,5-Dioxopyrrolidin-1-yl 2-(acetylthio)acetate | neg | 276.0184 | 1.943 | 4.506 | 4.507 | 0.001 | -0.145 | 0.420 | 0.568 | Tentative | NA | NA |
| metab_9784 | Unannotated feature | neg | 204.9811 | 2.589 | 4.315 | 4.664 | 0.349 | 0.145 | 0.420 | 0.568 | Unannotated | NA | NA |
| metab_3885 | Unannotated feature | pos | 162.0138 | 0.877 | 4.606 | 4.629 | 0.023 | 0.145 | 0.420 | 0.568 | Unannotated | NA | NA |
| metab_5166 | Unannotated feature | neg | 187.8885 | 0.509 | 4.828 | 4.832 | 0.004 | 0.145 | 0.420 | 0.568 | Unannotated | NA | NA |
| metab_10856 | Unannotated feature | neg | 191.0559 | 0.723 | 4.641 | 4.808 | 0.167 | 0.145 | 0.420 | 0.568 | Unannotated | NA | NA |
| metab_1990 | Unannotated feature | pos | 459.2482 | 7.293 | 4.797 | 4.792 | -0.005 | -0.145 | 0.420 | 0.568 | Unannotated | NA | NA |
| metab_7866 | Unannotated feature | neg | 753.8541 | 7.140 | 4.313 | 4.158 | -0.155 | -0.145 | 0.420 | 0.568 | Unannotated | NA | NA |
| metab_1494 | Unannotated feature | pos | 658.5098 | 6.072 | 5.146 | 5.070 | -0.076 | -0.145 | 0.420 | 0.568 | Unannotated | NA | NA |
| metab_6748 | Unannotated feature | neg | 107.9931 | 5.490 | 4.257 | 4.280 | 0.022 | 0.145 | 0.420 | 0.568 | Unannotated | NA | NA |
| metab_11039 | Unannotated feature | neg | 354.8496 | 0.582 | 5.592 | 5.558 | -0.033 | -0.145 | 0.420 | 0.568 | Unannotated | NA | NA |
| metab_10072 | Unannotated feature | neg | 707.4864 | 0.613 | 5.485 | 5.484 | -0.000 | -0.145 | 0.420 | 0.568 | Unannotated | NA | NA |
| metab_11175 | Unannotated feature | neg | 624.3406 | 5.811 | 5.776 | 5.633 | -0.143 | -0.145 | 0.420 | 0.568 | Unannotated | NA | NA |
| metab_1530 | Unannotated feature | pos | 523.6205 | 6.203 | 5.871 | 5.895 | 0.024 | 0.145 | 0.420 | 0.568 | Unannotated | NA | NA |
| metab_10070 | Unannotated feature | neg | 423.9381 | 1.302 | 3.908 | 4.028 | 0.120 | 0.145 | 0.420 | 0.568 | Unannotated | NA | NA |
| metab_938 | Unannotated feature | pos | 280.0847 | 4.055 | 5.490 | 5.393 | -0.097 | -0.145 | 0.420 | 0.568 | Unannotated | NA | NA |
| metab_5377 | Unannotated feature | neg | 110.8982 | 0.598 | 5.062 | 5.060 | -0.002 | 0.145 | 0.420 | 0.568 | Unannotated | NA | NA |
| metab_10204 | Unannotated feature | neg | 127.6370 | 0.873 | 3.550 | 3.439 | -0.110 | -0.145 | 0.420 | 0.568 | Unannotated | NA | NA |
| metab_608 | Unannotated feature | pos | 133.0861 | 2.126 | 3.889 | 3.711 | -0.178 | -0.145 | 0.420 | 0.568 | Unannotated | NA | NA |
| metab_9470 | Unannotated feature | neg | 224.0412 | 3.301 | 3.578 | 3.723 | 0.145 | 0.145 | 0.420 | 0.568 | Unannotated | NA | NA |
| metab_6629 | Unannotated feature | neg | 192.9698 | 0.841 | 3.893 | 3.723 | -0.170 | -0.145 | 0.420 | 0.568 | Unannotated | NA | NA |
| metab_8953 | Unannotated feature | neg | 258.9011 | 7.109 | 6.663 | 6.673 | 0.010 | 0.141 | 0.434 | 0.581 | Unannotated | NA | NA |
| metab_5054 | Unannotated feature | pos | 310.1495 | 0.861 | 4.278 | 4.307 | 0.029 | 0.141 | 0.434 | 0.581 | Unannotated | NA | NA |
| metab_2865 | 4-Chlorophenylacetate | pos | 212.0473 | 5.191 | 5.481 | 5.496 | 0.015 | 0.141 | 0.434 | 0.581 | Tentative | NA | NA |
| metab_9578 | Unannotated feature | neg | 175.0140 | 3.176 | 3.187 | 3.017 | -0.171 | -0.141 | 0.434 | 0.581 | Unannotated | NA | NA |
| metab_2137 | Unannotated feature | pos | 954.7560 | 0.567 | 5.124 | 5.064 | -0.060 | -0.141 | 0.434 | 0.581 | Unannotated | NA | NA |
| metab_2970 | alpha-Methyl-m-tyrosine | pos | 178.0863 | 4.555 | 3.757 | 3.764 | 0.007 | 0.141 | 0.434 | 0.581 | Tentative | NA | NA |
| metab_5525 | Unannotated feature | neg | 241.8237 | 0.786 | 4.266 | 4.535 | 0.269 | 0.141 | 0.434 | 0.581 | Unannotated | NA | NA |
| metab_4804 | Unannotated feature | pos | 128.0195 | 0.504 | 5.761 | 5.787 | 0.026 | 0.141 | 0.434 | 0.581 | Unannotated | NA | NA |
| metab_3564 | Unannotated feature | pos | 98.1017 | 2.065 | 4.249 | 4.221 | -0.028 | -0.141 | 0.434 | 0.581 | Unannotated | NA | NA |
| metab_4858 | Unannotated feature | pos | 347.2216 | 5.710 | 4.400 | 4.158 | -0.241 | -0.141 | 0.434 | 0.581 | Unannotated | NA | NA |
| metab_5926 | Unannotated feature | neg | 331.1564 | 2.573 | 4.142 | 4.209 | 0.067 | 0.141 | 0.434 | 0.581 | Unannotated | NA | NA |
| metab_10385 | Unannotated feature | neg | 182.0568 | 0.723 | 4.274 | 4.205 | -0.069 | -0.141 | 0.434 | 0.581 | Unannotated | NA | NA |
| metab_10510 | Unannotated feature | neg | 380.8912 | 0.629 | 4.546 | 4.497 | -0.049 | -0.141 | 0.434 | 0.581 | Unannotated | NA | NA |
| metab_7559 | Unannotated feature | neg | 181.8372 | 7.189 | 4.803 | 4.784 | -0.018 | -0.141 | 0.434 | 0.581 | Unannotated | NA | NA |
| metab_9616 | Unannotated feature | neg | 258.0446 | 3.136 | 4.430 | 4.365 | -0.064 | -0.141 | 0.434 | 0.581 | Unannotated | NA | NA |
| metab_7353 | Unannotated feature | neg | 426.9687 | 6.549 | 4.583 | 4.606 | 0.023 | 0.141 | 0.434 | 0.581 | Unannotated | NA | NA |
| metab_9545 | Unannotated feature | neg | 423.0822 | 3.209 | 4.963 | 4.868 | -0.095 | -0.141 | 0.434 | 0.581 | Unannotated | NA | NA |
| metab_573 | Unannotated feature | pos | 172.7437 | 2.058 | 4.101 | 4.184 | 0.083 | 0.141 | 0.434 | 0.581 | Unannotated | NA | NA |
| metab_6854 | Unannotated feature | neg | 206.0934 | 5.627 | 3.701 | 3.885 | 0.183 | 0.141 | 0.434 | 0.581 | Unannotated | NA | NA |
| metab_9453 | Unannotated feature | neg | 305.9162 | 3.332 | 3.546 | 3.431 | -0.115 | 0.141 | 0.434 | 0.581 | Unannotated | NA | NA |
| metab_6841 | Unannotated feature | neg | 716.6257 | 0.578 | 5.397 | 5.369 | -0.028 | -0.141 | 0.434 | 0.581 | Unannotated | NA | NA |
| metab_9440 | Unannotated feature | neg | 356.9595 | 3.364 | 3.633 | 3.560 | -0.073 | -0.141 | 0.434 | 0.581 | Unannotated | NA | NA |
| metab_10960 | Unannotated feature | neg | 130.0867 | 0.522 | 3.312 | 3.366 | 0.055 | 0.141 | 0.434 | 0.581 | Unannotated | NA | NA |
| metab_7709 | Unannotated feature | neg | 118.8990 | 7.213 | 3.552 | 3.541 | -0.011 | 0.141 | 0.434 | 0.581 | Unannotated | NA | NA |
| metab_6398 | Unannotated feature | neg | 163.0396 | 4.079 | 4.419 | 4.446 | 0.027 | 0.141 | 0.434 | 0.581 | Unannotated | NA | NA |
| metab_2348 | Unannotated feature | pos | 659.4808 | 5.979 | 4.665 | 4.716 | 0.051 | 0.141 | 0.434 | 0.581 | Unannotated | NA | NA |
| metab_4573 | Unannotated feature | pos | 74.0970 | 0.457 | 3.752 | 3.774 | 0.022 | 0.141 | 0.434 | 0.581 | Unannotated | NA | NA |
| metab_4569 | Unannotated feature | pos | 117.0549 | 0.465 | 4.350 | 4.353 | 0.004 | 0.141 | 0.434 | 0.581 | Unannotated | NA | NA |
| metab_7713 | Unannotated feature | neg | 97.9305 | 7.213 | 3.484 | 3.490 | 0.006 | -0.141 | 0.434 | 0.581 | Unannotated | NA | NA |
| metab_4263 | Unannotated feature | pos | 717.5805 | 0.591 | 4.182 | 4.079 | -0.104 | -0.141 | 0.434 | 0.581 | Unannotated | NA | NA |
| metab_7634 | Unannotated feature | neg | 276.8683 | 7.961 | 6.330 | 6.345 | 0.015 | 0.141 | 0.434 | 0.581 | Unannotated | NA | NA |
| metab_9213 | Unannotated feature | neg | 310.9034 | 0.676 | 4.717 | 4.548 | -0.170 | -0.141 | 0.434 | 0.581 | Unannotated | NA | NA |
| metab_2308 | Unannotated feature | pos | 620.8313 | 0.567 | 4.862 | 4.845 | -0.017 | -0.141 | 0.434 | 0.581 | Unannotated | NA | NA |
| metab_5294 | Unannotated feature | neg | 113.9882 | 0.562 | 5.195 | 5.174 | -0.021 | -0.141 | 0.434 | 0.581 | Unannotated | NA | NA |
| metab_5184 | Unannotated feature | neg | 129.9751 | 0.530 | 5.353 | 5.355 | 0.002 | 0.141 | 0.434 | 0.581 | Unannotated | NA | NA |
| metab_7983 | Unannotated feature | neg | 294.9508 | 7.085 | 5.763 | 5.786 | 0.023 | 0.141 | 0.434 | 0.581 | Unannotated | NA | NA |
| metab_5167 | Unannotated feature | neg | 193.9594 | 0.509 | 3.117 | 3.151 | 0.033 | 0.141 | 0.434 | 0.581 | Unannotated | NA | NA |
| metab_5091 | Unannotated feature | pos | 118.0827 | 0.846 | 3.841 | 3.979 | 0.138 | 0.141 | 0.434 | 0.581 | Unannotated | NA | NA |
| metab_1648 | Unannotated feature | pos | 469.2743 | 7.200 | 5.435 | 5.436 | 0.001 | -0.141 | 0.434 | 0.581 | Unannotated | NA | NA |
| metab_7332 | Glycochenodeoxycholate-3-sulfate | neg | 528.2648 | 6.373 | 4.639 | 4.909 | 0.270 | 0.141 | 0.434 | 0.581 | Tentative | NA | NA |
| metab_6588 | Unannotated feature | neg | 267.0120 | 0.825 | 4.270 | 4.320 | 0.051 | 0.141 | 0.434 | 0.581 | Unannotated | NA | NA |
| metab_2010 | Unannotated feature | pos | 352.8975 | 7.285 | 4.876 | 4.892 | 0.016 | 0.141 | 0.434 | 0.581 | Unannotated | NA | NA |
| metab_7759 | Unannotated feature | neg | 126.8803 | 7.181 | 4.818 | 4.793 | -0.025 | -0.141 | 0.434 | 0.581 | Unannotated | NA | NA |
| metab_11308 | Unannotated feature | neg | 209.9190 | 7.197 | 5.281 | 5.284 | 0.003 | 0.141 | 0.434 | 0.581 | Unannotated | NA | NA |
| metab_11165 | Unannotated feature | neg | 266.9689 | 5.795 | 4.872 | 4.884 | 0.013 | 0.141 | 0.434 | 0.581 | Unannotated | NA | NA |
| metab_7805 | Unannotated feature | neg | 472.8345 | 7.164 | 4.742 | 4.741 | -0.001 | -0.141 | 0.434 | 0.581 | Unannotated | NA | NA |
| metab_2128 | Unannotated feature | pos | 487.2796 | 6.776 | 5.719 | 5.706 | -0.013 | -0.141 | 0.434 | 0.581 | Unannotated | NA | NA |
| metab_3185 | Pyridoxine | pos | 152.0707 | 3.344 | 3.596 | 3.649 | 0.053 | 0.141 | 0.434 | 0.581 | Moderate putative | NA | NA |
| metab_9409 | Unannotated feature | neg | 209.9190 | 3.467 | 4.591 | 4.603 | 0.012 | 0.141 | 0.434 | 0.581 | Unannotated | NA | NA |
| metab_8994 | Unannotated feature | neg | 417.8764 | 7.101 | 5.561 | 5.585 | 0.024 | 0.141 | 0.434 | 0.581 | Unannotated | NA | NA |
| metab_6142 | Unannotated feature | neg | 297.9583 | 3.217 | 5.467 | 5.451 | -0.016 | -0.141 | 0.434 | 0.581 | Unannotated | NA | NA |
| metab_10144 | Unannotated feature | neg | 342.7341 | 0.928 | 4.107 | 4.127 | 0.020 | 0.141 | 0.434 | 0.581 | Unannotated | NA | NA |
| metab_2406 | Unannotated feature | pos | 868.8164 | 0.559 | 4.314 | 4.248 | -0.066 | -0.141 | 0.434 | 0.581 | Unannotated | NA | NA |
| metab_11034 | Unannotated feature | neg | 324.8464 | 0.013 | 6.129 | 6.114 | -0.015 | -0.141 | 0.434 | 0.581 | Unannotated | NA | NA |
| metab_4196 | Unannotated feature | pos | 339.9023 | 0.629 | 4.589 | 4.522 | -0.067 | -0.141 | 0.434 | 0.581 | Unannotated | NA | NA |
| metab_1973 | Unannotated feature | pos | 818.3019 | 7.293 | 4.637 | 4.480 | -0.157 | -0.141 | 0.434 | 0.581 | Unannotated | NA | NA |
| metab_6173 | Unannotated feature | neg | 146.9654 | 3.266 | 5.980 | 5.964 | -0.016 | -0.141 | 0.434 | 0.581 | Unannotated | NA | NA |
| metab_7290 | Unannotated feature | neg | 115.9199 | 6.188 | 7.390 | 7.406 | 0.015 | 0.141 | 0.434 | 0.581 | Unannotated | NA | NA |
| metab_8772 | Unannotated feature | neg | 181.0059 | 5.538 | 4.775 | 4.798 | 0.022 | 0.141 | 0.434 | 0.581 | Unannotated | NA | NA |
| metab_1382 | Dihomo-gamma-linolenate | pos | 271.2421 | 5.917 | 4.779 | 4.320 | -0.459 | -0.137 | 0.448 | 0.594 | Tentative | NA | NA |
| metab_5685 | Unannotated feature | neg | 88.0396 | 1.238 | 4.354 | 4.311 | -0.043 | -0.137 | 0.448 | 0.594 | Unannotated | NA | NA |
| metab_2735 | Unannotated feature | pos | 187.1264 | 5.717 | 4.088 | 4.092 | 0.004 | 0.137 | 0.448 | 0.594 | Unannotated | NA | NA |
| metab_10283 | Unannotated feature | neg | 225.8974 | 0.833 | 4.348 | 4.360 | 0.012 | 0.137 | 0.448 | 0.594 | Unannotated | NA | NA |
| metab_2424 | Unannotated feature | pos | 534.8847 | 5.925 | 4.866 | 4.822 | -0.044 | -0.137 | 0.448 | 0.594 | Unannotated | NA | NA |
| metab_7232 | Sterculic acid | neg | 339.2545 | 6.000 | 4.576 | 4.548 | -0.028 | -0.137 | 0.448 | 0.594 | Tentative | NA | NA |
| metab_3602 | Unannotated feature | pos | 122.0603 | 2.026 | 3.498 | 3.527 | 0.030 | 0.137 | 0.448 | 0.594 | Unannotated | NA | NA |
| metab_11389 | Dehydrovomifoliol | neg | 267.1243 | 5.890 | 6.084 | 6.181 | 0.097 | 0.137 | 0.448 | 0.594 | Tentative | NA | NA |
| metab_767 | L-Pipecolate | pos | 171.1130 | 2.849 | 5.925 | 5.840 | -0.085 | -0.137 | 0.448 | 0.594 | Tentative | NA | NA |
| metab_2151 | Unannotated feature | pos | 521.5831 | 6.551 | 5.427 | 5.440 | 0.013 | 0.137 | 0.448 | 0.594 | Unannotated | NA | NA |
| metab_6104 | Hydrochlorothiazide | neg | 295.9579 | 3.168 | 3.880 | 4.168 | 0.287 | 0.137 | 0.448 | 0.594 | Strong putative | Organoheterocyclic compounds | Thiadiazines |
| metab_11307 | Unannotated feature | neg | 191.8548 | 7.189 | 4.692 | 4.707 | 0.015 | 0.137 | 0.448 | 0.594 | Unannotated | NA | NA |
| metab_9848 | Unannotated feature | neg | 129.9357 | 2.477 | 3.910 | 3.948 | 0.038 | 0.137 | 0.448 | 0.594 | Unannotated | NA | NA |
| metab_10102 | Unannotated feature | neg | 170.8704 | 1.158 | 5.147 | 5.172 | 0.025 | 0.137 | 0.448 | 0.594 | Unannotated | NA | NA |
| metab_3924 | 5-Oxoproline | pos | 130.0500 | 0.861 | 6.047 | 6.032 | -0.014 | -0.137 | 0.448 | 0.594 | Strong putative | NA | NA |
| metab_8286 | Unannotated feature | neg | 309.1714 | 5.875 | 4.999 | 4.978 | -0.020 | -0.137 | 0.448 | 0.594 | Unannotated | NA | NA |
| metab_9730 | Unannotated feature | neg | 295.8318 | 2.772 | 5.012 | 5.045 | 0.033 | 0.137 | 0.448 | 0.594 | Unannotated | NA | NA |
| metab_1094 | 3,4,5-Trihydroxypentanoylcarnitine | pos | 276.1442 | 1.902 | 6.500 | 6.516 | 0.016 | 0.137 | 0.448 | 0.594 | Tentative | NA | NA |
| metab_8113 | Unannotated feature | neg | 188.9857 | 6.180 | 5.289 | 5.275 | -0.014 | -0.137 | 0.448 | 0.594 | Unannotated | NA | NA |
| metab_4026 | L-Pipecolate | pos | 112.0760 | 0.800 | 3.972 | 3.999 | 0.027 | 0.137 | 0.448 | 0.594 | Moderate putative | NA | NA |
| metab_3753 | Unannotated feature | pos | 622.7648 | 3.677 | 4.920 | 4.827 | -0.093 | 0.137 | 0.448 | 0.594 | Unannotated | NA | NA |
| metab_7729 | Unannotated feature | neg | 123.8772 | 7.197 | 3.800 | 3.794 | -0.006 | -0.137 | 0.448 | 0.594 | Unannotated | NA | NA |
| metab_11255 | Unannotated feature | neg | 128.9278 | 6.188 | 6.676 | 6.658 | -0.018 | -0.137 | 0.448 | 0.594 | Unannotated | NA | NA |
| metab_4469 | Unannotated feature | pos | 719.7840 | 0.551 | 4.405 | 4.268 | -0.136 | -0.137 | 0.448 | 0.594 | Unannotated | NA | NA |
| metab_10249 | Unannotated feature | neg | 190.0177 | 0.857 | 3.950 | 3.982 | 0.032 | 0.137 | 0.448 | 0.594 | Unannotated | NA | NA |
| metab_10252 | Unannotated feature | neg | 146.0818 | 0.857 | 3.419 | 3.463 | 0.044 | 0.137 | 0.448 | 0.594 | Unannotated | NA | NA |
| metab_4755 | (+/-)-1,4-Nonanediol diacetate | pos | 245.1747 | 6.427 | 6.400 | 6.396 | -0.003 | 0.137 | 0.448 | 0.594 | Tentative | NA | NA |
| metab_11300 | Unannotated feature | neg | 170.8704 | 7.181 | 5.176 | 5.170 | -0.006 | -0.137 | 0.448 | 0.594 | Unannotated | NA | NA |
| metab_700 | Sebacic acid | pos | 185.1173 | 6.427 | 5.870 | 5.873 | 0.002 | 0.137 | 0.448 | 0.594 | Strong putative | NA | NA |
| metab_330 | Unannotated feature | pos | 190.9459 | 0.815 | 3.665 | 3.488 | -0.177 | -0.137 | 0.448 | 0.594 | Unannotated | NA | NA |
| metab_10054 | Unannotated feature | neg | 127.6372 | 1.398 | 3.839 | 3.793 | -0.046 | -0.137 | 0.448 | 0.594 | Unannotated | NA | NA |
| metab_3383 | Unannotated feature | pos | 82.5376 | 2.521 | 3.407 | 3.429 | 0.022 | 0.137 | 0.448 | 0.594 | Unannotated | NA | NA |
| metab_7976 | Unannotated feature | neg | 353.2009 | 7.093 | 4.273 | 4.186 | -0.087 | -0.137 | 0.448 | 0.594 | Unannotated | NA | NA |
| metab_5288 | Unannotated feature | neg | 336.8594 | 0.013 | 6.711 | 6.706 | -0.004 | -0.137 | 0.448 | 0.594 | Unannotated | NA | NA |
| metab_7880 | Unannotated feature | neg | 109.9988 | 2.520 | 4.334 | 4.345 | 0.011 | 0.137 | 0.448 | 0.594 | Unannotated | NA | NA |
| metab_7894 | Unannotated feature | neg | 333.9387 | 7.132 | 4.597 | 4.535 | -0.062 | -0.137 | 0.448 | 0.594 | Unannotated | NA | NA |
| metab_3402 | Unannotated feature | pos | 172.0969 | 2.467 | 3.855 | 3.829 | -0.026 | 0.137 | 0.448 | 0.594 | Moderate putative | NA | NA |
| metab_10126 | Unannotated feature | neg | 199.3456 | 0.991 | 3.540 | 3.404 | -0.136 | -0.137 | 0.448 | 0.594 | Unannotated | NA | NA |
| metab_3275 | Unannotated feature | pos | 175.1079 | 2.911 | 4.340 | 4.326 | -0.015 | -0.137 | 0.448 | 0.594 | Moderate putative | NA | NA |
| metab_3272 | Unannotated feature | pos | 155.0703 | 2.927 | 3.603 | 3.364 | -0.239 | -0.137 | 0.448 | 0.594 | Unannotated | NA | NA |
| metab_9510 | Unannotated feature | neg | 500.9954 | 3.232 | 4.959 | 4.936 | -0.023 | -0.137 | 0.448 | 0.594 | Unannotated | NA | NA |
| metab_7495 | Unannotated feature | neg | 672.8613 | 7.148 | 4.611 | 4.617 | 0.006 | -0.137 | 0.448 | 0.594 | Unannotated | NA | NA |
| metab_2610 | (12Z)-10-Hydroxyoctadecenoylcarnitine | pos | 424.3421 | 5.840 | 6.568 | 6.515 | -0.054 | -0.137 | 0.448 | 0.594 | Tentative | NA | NA |
| metab_3422 | 4-Cresol | pos | 109.0651 | 2.389 | 3.137 | 3.188 | 0.051 | 0.137 | 0.448 | 0.594 | Moderate putative | NA | NA |
| metab_8458 | Unannotated feature | neg | 243.9534 | 5.755 | 5.125 | 5.138 | 0.013 | 0.137 | 0.448 | 0.594 | Unannotated | NA | NA |
| metab_540 | Unannotated feature | pos | 90.5263 | 1.988 | 5.926 | 5.934 | 0.008 | 0.137 | 0.448 | 0.594 | Unannotated | NA | NA |
| metab_11116 | Unannotated feature | neg | 130.0656 | 5.683 | 4.122 | 4.337 | 0.214 | 0.137 | 0.448 | 0.594 | Unannotated | NA | NA |
| metab_2259 | Unannotated feature | pos | 472.3997 | 6.188 | 5.085 | 5.013 | -0.072 | -0.137 | 0.448 | 0.594 | Unannotated | NA | NA |
| metab_5908 | Benzocaine | neg | 164.0713 | 2.530 | 5.752 | 5.739 | -0.013 | -0.137 | 0.448 | 0.594 | Tentative | NA | NA |
| metab_5032 | Unannotated feature | pos | 202.1438 | 0.846 | 3.906 | 4.099 | 0.193 | 0.137 | 0.448 | 0.594 | Unannotated | NA | NA |
| metab_8035 | Unannotated feature | neg | 608.3196 | 6.533 | 6.539 | 6.520 | -0.018 | -0.137 | 0.448 | 0.594 | Unannotated | NA | NA |
| metab_94 | Unannotated feature | pos | 82.9454 | 0.519 | 3.199 | 3.155 | -0.044 | -0.137 | 0.448 | 0.594 | Unannotated | NA | NA |
| metab_8987 | Unannotated feature | neg | 240.1040 | 4.790 | 3.556 | 3.472 | -0.084 | 0.137 | 0.448 | 0.594 | Unannotated | NA | NA |
| metab_3478 | 2-Aminobutan-1-ol | pos | 72.0813 | 2.249 | 3.650 | 3.665 | 0.016 | 0.137 | 0.448 | 0.594 | Tentative | NA | NA |
| metab_9191 | Unannotated feature | neg | 116.0498 | 4.175 | 2.839 | 3.111 | 0.272 | 0.137 | 0.448 | 0.594 | Unannotated | NA | NA |
| metab_2532 | Unannotated feature | pos | 454.3890 | 5.879 | 4.417 | 4.492 | 0.075 | 0.137 | 0.448 | 0.594 | Unannotated | NA | NA |
| metab_3445 | Unannotated feature | pos | 112.1124 | 2.942 | 3.958 | 3.995 | 0.037 | 0.137 | 0.448 | 0.594 | Unannotated | NA | NA |
| metab_7681 | Unannotated feature | neg | 187.8607 | 7.245 | 6.259 | 6.249 | -0.010 | -0.137 | 0.448 | 0.594 | Unannotated | NA | NA |
| metab_10986 | Unannotated feature | neg | 164.9268 | 0.431 | 4.668 | 4.660 | -0.008 | -0.137 | 0.448 | 0.594 | Unannotated | NA | NA |
| metab_1312 | Unannotated feature | pos | 588.4128 | 5.879 | 4.250 | 4.163 | -0.088 | -0.137 | 0.448 | 0.594 | Unannotated | NA | NA |
| metab_7830 | Unannotated feature | neg | 602.8505 | 7.153 | 4.422 | 4.473 | 0.051 | 0.137 | 0.448 | 0.594 | Unannotated | NA | NA |
| metab_2050 | Taxiresinol | pos | 311.1262 | 7.254 | 4.672 | 4.687 | 0.015 | 0.133 | 0.463 | 0.607 | Tentative | NA | NA |
| metab_5717 | Unannotated feature | neg | 239.7985 | 1.422 | 4.187 | 4.144 | -0.043 | -0.133 | 0.463 | 0.607 | Unannotated | NA | NA |
| metab_9062 | Unannotated feature | neg | 121.0288 | 4.574 | 4.840 | 4.825 | -0.015 | -0.133 | 0.463 | 0.607 | Unannotated | NA | NA |
| metab_9180 | Unannotated feature | neg | 601.7780 | 4.193 | 5.768 | 5.829 | 0.061 | 0.133 | 0.463 | 0.607 | Unannotated | NA | NA |
| metab_588 | Unannotated feature | pos | 125.1123 | 2.073 | 7.251 | 7.251 | 0.000 | 0.133 | 0.463 | 0.607 | Unannotated | NA | NA |
| metab_7711 | Unannotated feature | neg | 113.9255 | 7.213 | 3.751 | 3.770 | 0.019 | 0.133 | 0.463 | 0.607 | Unannotated | NA | NA |
| metab_11259 | Unannotated feature | neg | 160.9906 | 6.188 | 6.258 | 6.239 | -0.019 | -0.133 | 0.463 | 0.607 | Unannotated | NA | NA |
| metab_988 | L-Histidine | pos | 156.0769 | 0.854 | 5.994 | 5.996 | 0.002 | -0.133 | 0.463 | 0.607 | Strong putative | NA | NA |
| metab_2580 | Unannotated feature | pos | 639.4477 | 5.855 | 5.795 | 5.779 | -0.016 | -0.133 | 0.463 | 0.607 | Unannotated | NA | NA |
| metab_5832 | Unannotated feature | neg | 181.0364 | 2.167 | 3.651 | 4.028 | 0.378 | 0.133 | 0.463 | 0.607 | Unannotated | NA | NA |
| metab_1465 | Phosphatidylcholine | pos | 780.5534 | 5.995 | 5.854 | 5.880 | 0.026 | 0.133 | 0.463 | 0.607 | Tentative | NA | NA |
| metab_11271 | Unannotated feature | neg | 616.7713 | 0.570 | 5.201 | 5.210 | 0.008 | -0.133 | 0.463 | 0.607 | Unannotated | NA | NA |
| metab_7546 | Unannotated feature | neg | 124.8938 | 7.181 | 4.011 | 3.990 | -0.021 | -0.133 | 0.463 | 0.607 | Unannotated | NA | NA |
| metab_5746 | Unannotated feature | neg | 153.0665 | 1.839 | 5.112 | 5.058 | -0.055 | -0.133 | 0.463 | 0.607 | Unannotated | NA | NA |
| metab_9608 | Unannotated feature | neg | 308.9028 | 3.144 | 4.146 | 4.084 | -0.062 | -0.133 | 0.463 | 0.607 | Unannotated | NA | NA |
| metab_8179 | Penitrem D | neg | 612.3319 | 6.000 | 5.346 | 5.362 | 0.016 | 0.133 | 0.463 | 0.607 | Tentative | NA | NA |
| metab_8148 | 16-Hydroxypalmitate | neg | 271.2283 | 6.102 | 6.068 | 6.120 | 0.053 | 0.133 | 0.463 | 0.607 | Tentative | NA | NA |
| metab_3018 | Unannotated feature | pos | 185.1176 | 2.350 | 3.301 | 3.346 | 0.046 | 0.133 | 0.463 | 0.607 | Unannotated | NA | NA |
| metab_9153 | Unannotated feature | neg | 251.0177 | 4.267 | 4.028 | 3.736 | -0.292 | -0.133 | 0.463 | 0.607 | Unannotated | NA | NA |
| metab_8562 | Unannotated feature | neg | 225.0696 | 5.691 | 5.718 | 5.616 | -0.102 | -0.133 | 0.463 | 0.607 | Unannotated | NA | NA |
| metab_4776 | Unannotated feature | pos | 443.2226 | 7.200 | 5.522 | 5.507 | -0.015 | -0.133 | 0.463 | 0.607 | Unannotated | NA | NA |
| metab_2389 | Unannotated feature | pos | 820.6002 | 5.941 | 6.251 | 6.260 | 0.010 | -0.133 | 0.463 | 0.607 | Unannotated | NA | NA |
| metab_9038 | Unannotated feature | neg | 187.1086 | 4.643 | 3.923 | 3.841 | -0.083 | -0.133 | 0.463 | 0.607 | Unannotated | NA | NA |
| metab_4048 | Unannotated feature | pos | 190.1438 | 0.776 | 3.080 | 3.276 | 0.195 | 0.133 | 0.463 | 0.607 | Unannotated | NA | NA |
| metab_6903 | Unannotated feature | neg | 352.0977 | 5.659 | 3.430 | 3.378 | -0.053 | -0.133 | 0.463 | 0.607 | Unannotated | NA | NA |
| metab_6944 | Unannotated feature | neg | 232.0776 | 5.691 | 3.646 | 3.155 | -0.490 | -0.133 | 0.463 | 0.607 | Unannotated | NA | NA |
| metab_6481 | Am 580 | neg | 747.3703 | 4.355 | 5.257 | 5.115 | -0.142 | -0.133 | 0.463 | 0.607 | Tentative | NA | NA |
| metab_8841 | Ethotoin | neg | 241.0388 | 5.346 | 5.171 | 5.187 | 0.016 | 0.133 | 0.463 | 0.607 | Tentative | NA | NA |
| metab_10255 | Unannotated feature | neg | 154.9737 | 7.164 | 5.566 | 5.563 | -0.002 | -0.133 | 0.463 | 0.607 | Unannotated | NA | NA |
| metab_6780 | Unannotated feature | neg | 344.9276 | 5.538 | 4.446 | 4.462 | 0.016 | 0.133 | 0.463 | 0.607 | Unannotated | NA | NA |
| metab_9488 | Unannotated feature | neg | 142.0868 | 3.266 | 4.581 | 4.474 | -0.107 | -0.133 | 0.463 | 0.607 | Unannotated | NA | NA |
| metab_3699 | Unannotated feature | pos | 94.0455 | 1.910 | 4.895 | 4.880 | -0.015 | -0.133 | 0.463 | 0.607 | Unannotated | NA | NA |
| metab_3290 | L-Serine | pos | 88.0398 | 2.849 | 4.791 | 4.723 | -0.068 | -0.133 | 0.463 | 0.607 | Tentative | NA | NA |
| metab_937 | Unannotated feature | pos | 109.1016 | 4.055 | 4.108 | 4.128 | 0.020 | 0.133 | 0.463 | 0.607 | Unannotated | NA | NA |
| metab_3542 | 2-Phenylacetamide | pos | 118.0654 | 2.081 | 5.573 | 5.586 | 0.013 | 0.133 | 0.463 | 0.607 | Moderate putative | NA | NA |
| metab_7597 | Unannotated feature | neg | 275.9066 | 7.245 | 4.918 | 4.908 | -0.010 | -0.133 | 0.463 | 0.607 | Unannotated | NA | NA |
| metab_10699 | Unannotated feature | neg | 691.6626 | 0.578 | 4.225 | 4.110 | -0.114 | -0.133 | 0.463 | 0.607 | Unannotated | NA | NA |
| metab_5718 | Unannotated feature | neg | 90.9869 | 1.438 | 3.676 | 3.698 | 0.021 | 0.133 | 0.463 | 0.607 | Unannotated | NA | NA |
| metab_4340 | Unannotated feature | pos | 234.8939 | 0.575 | 4.886 | 4.894 | 0.008 | 0.133 | 0.463 | 0.607 | Unannotated | NA | NA |
| metab_8724 | Unannotated feature | neg | 336.9697 | 5.579 | 4.309 | 4.286 | -0.022 | 0.133 | 0.463 | 0.607 | Unannotated | NA | NA |
| metab_5897 | Unannotated feature | neg | 485.0618 | 2.510 | 4.878 | 4.860 | -0.018 | -0.133 | 0.463 | 0.607 | Unannotated | NA | NA |
| metab_5309 | Unannotated feature | neg | 370.8397 | 0.013 | 6.222 | 6.232 | 0.010 | 0.133 | 0.463 | 0.607 | Unannotated | NA | NA |
| metab_9235 | Unannotated feature | neg | 308.9064 | 0.676 | 4.590 | 4.410 | -0.180 | -0.133 | 0.463 | 0.607 | Unannotated | NA | NA |
| metab_2325 | Unannotated feature | pos | 476.2775 | 6.018 | 4.462 | 4.697 | 0.235 | 0.133 | 0.463 | 0.607 | Unannotated | NA | NA |
| metab_7062 | Unannotated feature | neg | 231.9178 | 5.787 | 3.855 | 3.797 | -0.058 | -0.133 | 0.463 | 0.607 | Unannotated | NA | NA |
| metab_269 | (S)-3-Hydroxyisobutyrate | pos | 149.0183 | 0.706 | 4.416 | 4.439 | 0.023 | -0.133 | 0.463 | 0.607 | Tentative | NA | NA |
| metab_754 | Unannotated feature | pos | 125.1123 | 2.764 | 4.860 | 4.849 | -0.011 | -0.133 | 0.463 | 0.607 | Unannotated | NA | NA |
| metab_10655 | Unannotated feature | neg | 203.0671 | 0.699 | 4.501 | 4.615 | 0.113 | 0.133 | 0.463 | 0.607 | Unannotated | NA | NA |
| metab_4933 | Unannotated feature | pos | 177.0247 | 0.614 | 5.163 | 5.137 | -0.026 | -0.133 | 0.463 | 0.607 | Unannotated | NA | NA |
| metab_2867 | 2-Methyl-5-nitroimidazol-1-ylacetic acid | pos | 168.0389 | 5.191 | 4.455 | 4.548 | 0.094 | 0.133 | 0.463 | 0.607 | Tentative | NA | NA |
| metab_5891 | Urate-3-ribonucleoside | neg | 299.0616 | 2.504 | 4.667 | 4.679 | 0.013 | 0.133 | 0.463 | 0.607 | Tentative | NA | NA |
| metab_6293 | Unannotated feature | neg | 224.9089 | 3.650 | 4.113 | 4.150 | 0.037 | 0.133 | 0.463 | 0.607 | Unannotated | NA | NA |
| metab_11205 | Unannotated feature | neg | 413.3126 | 5.898 | 5.351 | 5.380 | 0.029 | 0.133 | 0.463 | 0.607 | Unannotated | NA | NA |
| metab_4423 | Unannotated feature | pos | 166.0419 | 5.191 | 5.100 | 5.100 | 0.000 | 0.133 | 0.463 | 0.607 | Unannotated | NA | NA |
| metab_3054 | Unannotated feature | pos | 125.1124 | 4.070 | 4.723 | 4.717 | -0.006 | 0.133 | 0.463 | 0.607 | Unannotated | NA | NA |
| metab_7973 | Unannotated feature | neg | 86.0063 | 7.101 | 5.994 | 6.002 | 0.008 | 0.133 | 0.463 | 0.607 | Unannotated | NA | NA |
| metab_1428 | Unannotated feature | pos | 448.3377 | 5.956 | 5.603 | 5.625 | 0.022 | 0.133 | 0.463 | 0.607 | Unannotated | NA | NA |
| metab_10691 | Unannotated feature | neg | 244.8771 | 0.582 | 4.088 | 4.207 | 0.119 | 0.133 | 0.463 | 0.607 | Unannotated | NA | NA |
| metab_10133 | Unannotated feature | neg | 339.8983 | 0.960 | 5.012 | 4.956 | -0.056 | -0.133 | 0.463 | 0.607 | Unannotated | NA | NA |
| metab_3424 | Unannotated feature | pos | 247.1288 | 2.389 | 4.819 | 4.840 | 0.021 | 0.133 | 0.463 | 0.607 | Strong putative | NA | NA |
| metab_11095 | Unannotated feature | neg | 608.7397 | 0.578 | 5.484 | 5.462 | -0.022 | -0.133 | 0.463 | 0.607 | Unannotated | NA | NA |
| metab_504 | Glycochenodeoxycholate | pos | 414.3004 | 5.832 | 5.713 | 5.598 | -0.115 | -0.133 | 0.463 | 0.607 | Moderate putative | NA | NA |
| metab_1358 | Unannotated feature | pos | 631.4510 | 5.902 | 6.341 | 6.392 | 0.051 | 0.133 | 0.463 | 0.607 | Unannotated | NA | NA |
| metab_7921 | Unannotated feature | neg | 326.8888 | 7.109 | 4.957 | 4.973 | 0.016 | 0.133 | 0.463 | 0.607 | Unannotated | NA | NA |
| metab_8781 | Unannotated feature | neg | 530.8671 | 7.153 | 4.933 | 4.922 | -0.011 | -0.133 | 0.463 | 0.607 | Unannotated | NA | NA |
| metab_10492 | Unannotated feature | neg | 272.9920 | 0.636 | 5.129 | 5.114 | -0.015 | -0.133 | 0.463 | 0.607 | Unannotated | NA | NA |
| metab_10153 | Unannotated feature | neg | 105.9194 | 0.920 | 3.959 | 3.930 | -0.028 | -0.128 | 0.477 | 0.621 | Unannotated | NA | NA |
| metab_6957 | L-2,3-Dihydrodipicolinate | neg | 168.0298 | 5.699 | 5.554 | 5.531 | -0.022 | -0.128 | 0.477 | 0.621 | Tentative | NA | NA |
| metab_7028 | Unannotated feature | neg | 365.1435 | 5.747 | 3.963 | 4.016 | 0.053 | 0.128 | 0.477 | 0.621 | Unannotated | NA | NA |
| metab_1890 | Unannotated feature | pos | 99.5315 | 7.339 | 4.012 | 3.998 | -0.014 | -0.128 | 0.477 | 0.621 | Unannotated | NA | NA |
| metab_1879 | Unannotated feature | pos | 786.8243 | 0.551 | 5.350 | 5.311 | -0.038 | -0.128 | 0.477 | 0.621 | Unannotated | NA | NA |
| metab_4383 | Unannotated feature | pos | 591.8521 | 0.567 | 4.373 | 4.404 | 0.031 | 0.128 | 0.477 | 0.621 | Unannotated | NA | NA |
| metab_11016 | Unannotated feature | neg | 231.9318 | 0.016 | 6.291 | 6.287 | -0.003 | -0.128 | 0.477 | 0.621 | Unannotated | NA | NA |
| metab_11024 | Unannotated feature | neg | 552.7243 | 0.013 | 4.858 | 4.848 | -0.010 | -0.128 | 0.477 | 0.621 | Unannotated | NA | NA |
| metab_4597 | Unannotated feature | pos | 171.1493 | 0.162 | 4.871 | 4.862 | -0.009 | 0.128 | 0.477 | 0.621 | Unannotated | NA | NA |
| metab_3688 | 2-Amino-2-methyl-1-propanol | pos | 72.0813 | 1.925 | 4.057 | 4.063 | 0.005 | -0.128 | 0.477 | 0.621 | Tentative | NA | NA |
| metab_4624 | Unannotated feature | pos | 283.1078 | 5.619 | 5.102 | 4.953 | -0.149 | -0.128 | 0.477 | 0.621 | Moderate putative | NA | NA |
| metab_8577 | Unannotated feature | neg | 195.1389 | 5.683 | 3.476 | 3.485 | 0.009 | 0.128 | 0.477 | 0.621 | Unannotated | NA | NA |
| metab_1956 | Unannotated feature | pos | 110.0091 | 7.316 | 3.305 | 3.394 | 0.089 | 0.128 | 0.477 | 0.621 | Unannotated | NA | NA |
| metab_704 | 2-(2-Hydroxyethyl)phenyl hydrogen sulfate | pos | 260.0587 | 2.529 | 3.483 | 3.408 | -0.075 | -0.128 | 0.477 | 0.621 | Tentative | NA | NA |
| metab_3593 | rRNA containing N1-methylguanine | pos | 166.0725 | 2.042 | 4.298 | 4.323 | 0.025 | 0.128 | 0.477 | 0.621 | Tentative | NA | NA |
| metab_3649 | 3'-DEOXY-3'-FLUOROTHYMIDINE | pos | 145.0319 | 1.956 | 3.931 | 3.919 | -0.012 | -0.128 | 0.477 | 0.621 | Tentative | NA | NA |
| metab_8576 | Unannotated feature | neg | 221.1184 | 5.683 | 3.848 | 3.835 | -0.013 | 0.128 | 0.477 | 0.621 | Unannotated | NA | NA |
| metab_1300 | Unannotated feature | pos | 587.4079 | 5.871 | 4.751 | 4.675 | -0.076 | -0.128 | 0.477 | 0.621 | Unannotated | NA | NA |
| metab_11316 | Unannotated feature | neg | 189.9209 | 7.213 | 4.355 | 4.332 | -0.023 | -0.128 | 0.477 | 0.621 | Unannotated | NA | NA |
| metab_158 | Unannotated feature | pos | 977.7905 | 0.551 | 5.061 | 5.138 | 0.077 | 0.128 | 0.477 | 0.621 | Unannotated | NA | NA |
| metab_9530 | Unannotated feature | neg | 162.0556 | 3.224 | 5.093 | 5.093 | 0.000 | -0.128 | 0.477 | 0.621 | Unannotated | NA | NA |
| metab_2997 | Xi-2,3-Dihydro-3,5-dihydroxy-6-methyl-4H-pyran-4-one | pos | 123.0443 | 4.401 | 4.235 | 4.126 | -0.108 | -0.128 | 0.477 | 0.621 | Tentative | NA | NA |
| metab_9887 | Unannotated feature | neg | 189.8702 | 2.253 | 4.513 | 4.509 | -0.004 | -0.128 | 0.477 | 0.621 | Unannotated | NA | NA |
| metab_7437 | Unannotated feature | neg | 283.7819 | 7.109 | 5.166 | 5.204 | 0.038 | 0.128 | 0.477 | 0.621 | Unannotated | NA | NA |
| metab_4237 | Unannotated feature | pos | 604.5725 | 0.606 | 5.060 | 5.015 | -0.045 | -0.128 | 0.477 | 0.621 | Unannotated | NA | NA |
| metab_10764 | Unannotated feature | neg | 653.6523 | 0.582 | 4.399 | 4.371 | -0.028 | -0.128 | 0.477 | 0.621 | Unannotated | NA | NA |
| metab_10013 | Unannotated feature | neg | 267.8762 | 1.847 | 3.579 | 3.951 | 0.372 | 0.128 | 0.477 | 0.621 | Unannotated | NA | NA |
| metab_3333 | Unannotated feature | pos | 178.0420 | 2.629 | 3.952 | 3.847 | -0.105 | -0.128 | 0.477 | 0.621 | Unannotated | NA | NA |
| metab_11193 | Unannotated feature | neg | 253.1085 | 5.859 | 4.184 | 4.263 | 0.079 | 0.128 | 0.477 | 0.621 | Unannotated | NA | NA |
| metab_4105 | 2-Aminothiazole | pos | 101.0172 | 0.714 | 4.829 | 4.813 | -0.016 | -0.128 | 0.477 | 0.621 | Tentative | NA | NA |
| metab_8757 | Unannotated feature | neg | 415.8861 | 5.543 | 4.445 | 4.475 | 0.030 | 0.128 | 0.477 | 0.621 | Unannotated | NA | NA |
| metab_3049 | N-Acetyl-DL-Leucine | pos | 174.1126 | 4.093 | 4.566 | 4.504 | -0.061 | -0.128 | 0.477 | 0.621 | Moderate putative | NA | NA |
| metab_6702 | Unannotated feature | neg | 107.9931 | 5.279 | 3.830 | 3.822 | -0.008 | -0.128 | 0.477 | 0.621 | Unannotated | NA | NA |
| metab_3300 | 2-Methylbutyroylcarnitine | pos | 246.1700 | 2.772 | 6.058 | 6.083 | 0.025 | -0.128 | 0.477 | 0.621 | Tentative | NA | NA |
| metab_10497 | Unannotated feature | neg | 216.1530 | 0.636 | 4.712 | 4.689 | -0.023 | -0.128 | 0.477 | 0.621 | Unannotated | NA | NA |
| metab_7796 | Unannotated feature | neg | 143.9595 | 7.170 | 2.841 | 3.016 | 0.175 | 0.128 | 0.477 | 0.621 | Unannotated | NA | NA |
| metab_834 | Unannotated feature | pos | 158.9642 | 0.559 | 6.719 | 6.735 | 0.016 | 0.128 | 0.477 | 0.621 | Unannotated | NA | NA |
| metab_777 | Unannotated feature | pos | 90.5264 | 2.911 | 6.189 | 6.186 | -0.003 | -0.128 | 0.477 | 0.621 | Unannotated | NA | NA |
| metab_622 | Unannotated feature | pos | 194.1175 | 2.179 | 3.984 | 3.991 | 0.006 | -0.128 | 0.477 | 0.621 | Unannotated | NA | NA |
| metab_10165 | Unannotated feature | neg | 218.0332 | 0.912 | 4.457 | 4.335 | -0.122 | -0.128 | 0.477 | 0.621 | Unannotated | NA | NA |
| metab_7685 | Unannotated feature | neg | 206.8595 | 7.237 | 4.898 | 4.914 | 0.016 | 0.128 | 0.477 | 0.621 | Unannotated | NA | NA |
| metab_7727 | Unannotated feature | neg | 433.0995 | 2.520 | 5.496 | 5.500 | 0.004 | -0.128 | 0.477 | 0.621 | Unannotated | NA | NA |
| metab_7585 | Unannotated feature | neg | 171.9314 | 7.213 | 3.677 | 3.699 | 0.022 | 0.128 | 0.477 | 0.621 | Unannotated | NA | NA |
| metab_3165 | Unannotated feature | pos | 245.1860 | 3.476 | 4.729 | 4.810 | 0.081 | 0.128 | 0.477 | 0.621 | Moderate putative | NA | NA |
| metab_7048 | Unannotated feature | neg | 505.2666 | 5.763 | 3.950 | 4.046 | 0.096 | 0.128 | 0.477 | 0.621 | Unannotated | NA | NA |
| metab_8906 | Unannotated feature | neg | 297.1534 | 7.117 | 6.154 | 6.146 | -0.008 | -0.128 | 0.477 | 0.621 | Unannotated | NA | NA |
| metab_10599 | Unannotated feature | neg | 741.4809 | 0.598 | 4.621 | 4.506 | -0.115 | -0.128 | 0.477 | 0.621 | Unannotated | NA | NA |
| metab_7689 | Unannotated feature | neg | 258.9370 | 7.221 | 3.800 | 3.778 | -0.022 | 0.128 | 0.477 | 0.621 | Unannotated | NA | NA |
| metab_1701 | Unannotated feature | pos | 173.0978 | 7.293 | 4.777 | 4.769 | -0.008 | -0.128 | 0.477 | 0.621 | Unannotated | NA | NA |
| metab_6422 | Unannotated feature | neg | 121.0288 | 4.175 | 5.007 | 5.005 | -0.002 | -0.128 | 0.477 | 0.621 | Unannotated | NA | NA |
| metab_6705 | Unannotated feature | neg | 231.0516 | 5.302 | 4.059 | 3.878 | -0.181 | -0.128 | 0.477 | 0.621 | Unannotated | NA | NA |
| metab_6017 | Unannotated feature | neg | 82.9711 | 0.629 | 6.107 | 6.100 | -0.007 | -0.124 | 0.492 | 0.634 | Unannotated | NA | NA |
| metab_3437 | Acetaminophen | pos | 152.0707 | 2.366 | 4.142 | 4.170 | 0.028 | 0.124 | 0.492 | 0.634 | Strong putative | NA | NA |
| metab_10767 | Unannotated feature | neg | 306.9196 | 0.570 | 5.770 | 5.757 | -0.013 | -0.124 | 0.492 | 0.634 | Unannotated | NA | NA |
| metab_5939 | Unannotated feature | neg | 261.8751 | 2.629 | 5.736 | 5.737 | 0.001 | 0.124 | 0.492 | 0.634 | Unannotated | NA | NA |
| metab_1026 | Unannotated feature | pos | 137.0459 | 0.854 | 6.145 | 6.094 | -0.050 | -0.124 | 0.492 | 0.634 | Unannotated | NA | NA |
| metab_8203 | Unannotated feature | neg | 464.1511 | 5.961 | 4.256 | 3.937 | -0.318 | -0.124 | 0.492 | 0.634 | Unannotated | NA | NA |
| metab_4764 | Diacylglycerol | pos | 643.5269 | 6.968 | 6.142 | 6.134 | -0.009 | -0.124 | 0.492 | 0.634 | Tentative | NA | NA |
| metab_1133 | Lobucavir | pos | 283.1513 | 5.634 | 5.642 | 5.645 | 0.003 | 0.124 | 0.492 | 0.634 | Tentative | NA | NA |
| metab_11113 | N-Methylanthranilate | neg | 150.0555 | 5.683 | 4.200 | 4.219 | 0.019 | 0.124 | 0.492 | 0.634 | Strong putative | NA | NA |
| metab_4609 | Unannotated feature | pos | 120.0238 | 0.018 | 4.514 | 4.515 | 0.001 | 0.124 | 0.492 | 0.634 | Unannotated | NA | NA |
| metab_3561 | Putreanine | pos | 126.1157 | 2.065 | 6.204 | 6.207 | 0.003 | 0.124 | 0.492 | 0.634 | Tentative | NA | NA |
| metab_10300 | Unannotated feature | neg | 87.0080 | 0.818 | 4.816 | 4.838 | 0.022 | -0.124 | 0.492 | 0.634 | Unannotated | NA | NA |
| metab_4355 | Unannotated feature | pos | 755.8074 | 0.567 | 4.843 | 4.840 | -0.003 | 0.124 | 0.492 | 0.634 | Unannotated | NA | NA |
| metab_2009 | Unannotated feature | pos | 393.2975 | 7.285 | 6.166 | 6.136 | -0.030 | -0.124 | 0.492 | 0.634 | Unannotated | NA | NA |
| metab_98 | Unannotated feature | pos | 447.9043 | 0.534 | 5.096 | 5.051 | -0.045 | -0.124 | 0.492 | 0.634 | Unannotated | NA | NA |
| metab_1433 | Unannotated feature | pos | 878.6411 | 5.956 | 6.045 | 6.040 | -0.005 | -0.124 | 0.492 | 0.634 | Unannotated | NA | NA |
| metab_7204 | 5-Isopropyl-2-(2-methylpropyl)-2-cyclohexen-1-one | neg | 239.1654 | 5.930 | 4.578 | 4.518 | -0.060 | -0.124 | 0.492 | 0.634 | Tentative | NA | NA |
| metab_7770 | Unannotated feature | neg | 159.0294 | 7.173 | 4.058 | 4.047 | -0.011 | -0.124 | 0.492 | 0.634 | Unannotated | NA | NA |
| metab_2159 | Unannotated feature | pos | 528.4985 | 6.435 | 6.649 | 6.650 | 0.001 | 0.124 | 0.492 | 0.634 | Unannotated | NA | NA |
| metab_5293 | Unannotated feature | neg | 112.9848 | 0.562 | 6.751 | 6.733 | -0.018 | -0.124 | 0.492 | 0.634 | Unannotated | NA | NA |
| metab_10839 | Unannotated feature | neg | 693.8585 | 0.554 | 5.738 | 5.773 | 0.035 | 0.124 | 0.492 | 0.634 | Unannotated | NA | NA |
| metab_6196 | 4-Hydroxy-5-(dihydroxyphenyl)-valeric acid-O-sulphate | neg | 305.0342 | 3.324 | 4.967 | 4.617 | -0.350 | -0.124 | 0.492 | 0.634 | Tentative | NA | NA |
| metab_8003 | Unannotated feature | neg | 401.9262 | 6.931 | 4.883 | 4.949 | 0.067 | 0.124 | 0.492 | 0.634 | Unannotated | NA | NA |
| metab_7774 | Unannotated feature | neg | 102.9750 | 7.173 | 2.986 | 2.966 | -0.020 | -0.124 | 0.492 | 0.634 | Unannotated | NA | NA |
| metab_7524 | Unannotated feature | neg | 542.8227 | 7.164 | 4.604 | 4.586 | -0.018 | -0.124 | 0.492 | 0.634 | Unannotated | NA | NA |
| metab_784 | Unannotated feature | pos | 157.1084 | 7.293 | 4.496 | 4.507 | 0.012 | 0.124 | 0.492 | 0.634 | Unannotated | NA | NA |
| metab_7870 | Unannotated feature | neg | 259.8781 | 2.621 | 6.445 | 6.457 | 0.012 | 0.124 | 0.492 | 0.634 | Unannotated | NA | NA |
| metab_5252 | Unannotated feature | neg | 625.8709 | 0.554 | 5.615 | 5.638 | 0.023 | 0.124 | 0.492 | 0.634 | Unannotated | NA | NA |
| metab_7920 | Unannotated feature | neg | 340.9408 | 7.109 | 6.266 | 6.286 | 0.020 | 0.124 | 0.492 | 0.634 | Unannotated | NA | NA |
| metab_7715 | Unannotated feature | neg | 259.0104 | 7.205 | 4.292 | 4.268 | -0.024 | 0.124 | 0.492 | 0.634 | Unannotated | NA | NA |
| metab_6516 | Unannotated feature | neg | 255.9366 | 4.457 | 4.277 | 4.211 | -0.066 | -0.124 | 0.492 | 0.634 | Unannotated | NA | NA |
| metab_7641 | Unannotated feature | neg | 220.8346 | 2.469 | 4.487 | 4.485 | -0.001 | -0.124 | 0.492 | 0.634 | Unannotated | NA | NA |
| metab_9455 | Unannotated feature | neg | 674.5127 | 0.621 | 5.669 | 5.655 | -0.014 | -0.124 | 0.492 | 0.634 | Unannotated | NA | NA |
| metab_2120 | Unannotated feature | pos | 102.1955 | 6.968 | 3.526 | 3.575 | 0.049 | 0.124 | 0.492 | 0.634 | Unannotated | NA | NA |
| metab_5618 | Unannotated feature | neg | 162.9121 | 0.912 | 4.454 | 4.485 | 0.030 | 0.124 | 0.492 | 0.634 | Unannotated | NA | NA |
| metab_1573 | 1-Acyl-sn-glycero-3-phosphocholine | pos | 522.3556 | 6.381 | 7.592 | 7.603 | 0.011 | -0.124 | 0.492 | 0.634 | Tentative | NA | NA |
| metab_3135 | 1-(2-Furyl)butan-3-one | pos | 103.0546 | 3.646 | 3.692 | 3.677 | -0.014 | 0.124 | 0.492 | 0.634 | Tentative | NA | NA |
| metab_5596 | Unannotated feature | neg | 151.0256 | 0.873 | 4.757 | 4.707 | -0.051 | -0.124 | 0.492 | 0.634 | Unannotated | NA | NA |
| metab_2681 | Unannotated feature | pos | 289.1506 | 1.933 | 5.404 | 5.434 | 0.030 | 0.124 | 0.492 | 0.634 | Unannotated | NA | NA |
| metab_2675 | Unannotated feature | pos | 360.4363 | 5.778 | 4.567 | 4.553 | -0.014 | 0.124 | 0.492 | 0.634 | Unannotated | NA | NA |
| metab_876 | Soyasaponin aa | pos | 539.2697 | 3.591 | 5.669 | 5.657 | -0.011 | 0.124 | 0.492 | 0.634 | Tentative | NA | NA |
| metab_5736 | Unannotated feature | neg | 152.0290 | 1.723 | 3.591 | 3.494 | -0.098 | -0.124 | 0.492 | 0.634 | Unannotated | NA | NA |
| metab_5398 | Unannotated feature | neg | 481.6414 | 0.613 | 4.097 | 4.142 | 0.045 | -0.124 | 0.492 | 0.634 | Unannotated | NA | NA |
| metab_3999 | 2-Pentenoic acid | pos | 83.0496 | 0.823 | 4.556 | 4.562 | 0.006 | 0.124 | 0.492 | 0.634 | Tentative | NA | NA |
| metab_5749 | Unannotated feature | neg | 188.0174 | 1.855 | 3.629 | 3.701 | 0.072 | 0.124 | 0.492 | 0.634 | Unannotated | NA | NA |
| metab_8421 | Unannotated feature | neg | 223.9541 | 5.779 | 3.774 | 3.669 | -0.105 | -0.124 | 0.492 | 0.634 | Unannotated | NA | NA |
| metab_7025 | Unannotated feature | neg | 207.0662 | 5.747 | 4.895 | 4.909 | 0.014 | 0.124 | 0.492 | 0.634 | Unannotated | NA | NA |
| metab_3633 | Unannotated feature | pos | 249.0539 | 1.956 | 3.893 | 3.861 | -0.032 | -0.124 | 0.492 | 0.634 | Unannotated | NA | NA |
| metab_11372 | Methylmalonate | neg | 117.0186 | 0.444 | 4.987 | 5.000 | 0.013 | 0.124 | 0.492 | 0.634 | Moderate putative | NA | NA |
| metab_4930 | Unannotated feature | pos | 260.0740 | 0.614 | 4.858 | 4.832 | -0.026 | -0.124 | 0.492 | 0.634 | Unannotated | NA | NA |
| metab_1167 | Unannotated feature | pos | 103.0757 | 5.717 | 4.948 | 4.941 | -0.007 | -0.124 | 0.492 | 0.634 | Unannotated | NA | NA |
| metab_5009 | Unannotated feature | pos | 203.1139 | 0.807 | 5.151 | 5.211 | 0.060 | 0.124 | 0.492 | 0.634 | Unannotated | NA | NA |
| metab_3769 | beta-L-Aspartylhydroxamate | pos | 113.0349 | 1.724 | 5.161 | 5.148 | -0.013 | -0.124 | 0.492 | 0.634 | Tentative | NA | NA |
| metab_4683 | Unannotated feature | pos | 542.8819 | 5.879 | 5.180 | 5.166 | -0.014 | -0.124 | 0.492 | 0.634 | Unannotated | NA | NA |
| metab_8107 | Unannotated feature | neg | 99.9250 | 6.188 | 6.992 | 7.003 | 0.011 | 0.124 | 0.492 | 0.634 | Unannotated | NA | NA |
| metab_1025 | N-Formimino-L-glutamate | pos | 197.0533 | 0.831 | 5.599 | 5.645 | 0.046 | 0.120 | 0.507 | 0.646 | Tentative | NA | NA |
| metab_1176 | Unannotated feature | pos | 318.2544 | 5.733 | 4.481 | 4.427 | -0.054 | -0.120 | 0.507 | 0.646 | Unannotated | NA | NA |
| metab_11340 | Unannotated feature | neg | 123.9457 | 0.013 | 5.808 | 5.816 | 0.008 | 0.120 | 0.507 | 0.646 | Unannotated | NA | NA |
| metab_7460 | Unannotated feature | neg | 430.9267 | 7.132 | 4.681 | 4.654 | -0.027 | -0.120 | 0.507 | 0.646 | Unannotated | NA | NA |
| metab_1671 | Unannotated feature | pos | 534.3374 | 7.254 | 5.844 | 5.789 | -0.055 | -0.120 | 0.507 | 0.646 | Unannotated | NA | NA |
| metab_3440 | Unannotated feature | pos | 187.1264 | 2.350 | 4.680 | 4.668 | -0.012 | -0.120 | 0.507 | 0.646 | Unannotated | NA | NA |
| metab_5170 | Unannotated feature | neg | 275.9065 | 0.515 | 4.454 | 4.484 | 0.029 | 0.120 | 0.507 | 0.646 | Unannotated | NA | NA |
| metab_1989 | Unannotated feature | pos | 487.2797 | 7.293 | 4.462 | 4.457 | -0.005 | -0.120 | 0.507 | 0.646 | Unannotated | NA | NA |
| metab_8000 | Unannotated feature | neg | 238.9319 | 6.962 | 4.993 | 4.974 | -0.019 | -0.120 | 0.507 | 0.646 | Unannotated | NA | NA |
| metab_11163 | Unannotated feature | neg | 367.0035 | 5.771 | 4.162 | 4.291 | 0.129 | 0.120 | 0.507 | 0.646 | Unannotated | NA | NA |
| metab_1209 | Unannotated feature | pos | 510.8367 | 5.771 | 4.327 | 4.331 | 0.004 | -0.120 | 0.507 | 0.646 | Unannotated | NA | NA |
| metab_6241 | Unannotated feature | neg | 173.0928 | 3.467 | 3.772 | 3.681 | -0.091 | -0.120 | 0.507 | 0.646 | Unannotated | NA | NA |
| metab_691 | Unannotated feature | pos | 459.2498 | 6.296 | 5.745 | 5.816 | 0.071 | 0.120 | 0.507 | 0.646 | Moderate putative | NA | NA |
| metab_8238 | Unannotated feature | neg | 143.9878 | 5.922 | 4.498 | 4.486 | -0.012 | 0.120 | 0.507 | 0.646 | Unannotated | NA | NA |
| metab_3555 | Unannotated feature | pos | 95.0797 | 2.073 | 4.817 | 4.836 | 0.019 | 0.120 | 0.507 | 0.646 | Unannotated | NA | NA |
| metab_1214 | (12E)-10-Hydroxytetradec-12-enoylcarnitine | pos | 386.2900 | 5.778 | 4.576 | 4.891 | 0.315 | 0.120 | 0.507 | 0.646 | Tentative | NA | NA |
| metab_8222 | Formyl-5-hydroxykynurenamine | neg | 253.0837 | 5.930 | 4.214 | 3.934 | -0.280 | -0.120 | 0.507 | 0.646 | Tentative | Organic oxygen compounds | Organooxygen compounds |
| metab_4548 | Unannotated feature | pos | 163.0390 | 0.504 | 4.087 | 4.078 | -0.009 | -0.120 | 0.507 | 0.646 | Unannotated | NA | NA |
| metab_6237 | Unannotated feature | neg | 369.8445 | 3.443 | 4.779 | 4.761 | -0.017 | -0.120 | 0.507 | 0.646 | Unannotated | NA | NA |
| metab_11078 | Unannotated feature | neg | 726.6545 | 0.578 | 4.869 | 4.786 | -0.082 | -0.120 | 0.507 | 0.646 | Unannotated | NA | NA |
| metab_6970 | Unannotated feature | neg | 387.1857 | 5.699 | 5.402 | 5.565 | 0.164 | 0.120 | 0.507 | 0.646 | Unannotated | NA | NA |
| metab_6817 | Unannotated feature | neg | 269.1399 | 5.587 | 3.296 | 3.352 | 0.056 | 0.120 | 0.507 | 0.646 | Unannotated | NA | NA |
| metab_8859 | Unannotated feature | neg | 379.1568 | 7.124 | 5.190 | 5.167 | -0.023 | -0.120 | 0.507 | 0.646 | Unannotated | NA | NA |
| metab_5405 | Unannotated feature | neg | 118.0502 | 0.621 | 4.635 | 4.618 | -0.016 | -0.120 | 0.507 | 0.646 | Unannotated | NA | NA |
| metab_9468 | Unannotated feature | neg | 164.8950 | 3.308 | 4.115 | 4.100 | -0.015 | -0.120 | 0.507 | 0.646 | Unannotated | NA | NA |
| metab_7590 | Unannotated feature | neg | 246.0752 | 7.221 | 3.856 | 3.901 | 0.045 | -0.120 | 0.507 | 0.646 | Unannotated | NA | NA |
| metab_7647 | Unannotated feature | neg | 89.9249 | 7.549 | 3.360 | 3.372 | 0.012 | -0.120 | 0.507 | 0.646 | Unannotated | NA | NA |
| metab_3163 | Unannotated feature | pos | 175.1079 | 3.499 | 4.159 | 4.343 | 0.184 | 0.120 | 0.507 | 0.646 | Unannotated | NA | NA |
| metab_7578 | Unannotated feature | neg | 84.9379 | 7.213 | 3.264 | 3.298 | 0.034 | 0.120 | 0.507 | 0.646 | Unannotated | NA | NA |
| metab_645 | N'-Hydroxymethylnorcotinine | pos | 175.0867 | 2.280 | 3.941 | 3.823 | -0.117 | -0.120 | 0.507 | 0.646 | Tentative | NA | NA |
| metab_6388 | Unannotated feature | neg | 239.0596 | 4.037 | 5.079 | 5.029 | -0.050 | -0.120 | 0.507 | 0.646 | Unannotated | NA | NA |
| metab_7755 | Unannotated feature | neg | 164.8357 | 7.181 | 5.007 | 5.001 | -0.006 | -0.120 | 0.507 | 0.646 | Unannotated | NA | NA |
| metab_11046 | Unannotated feature | neg | 162.9329 | 0.013 | 4.156 | 4.179 | 0.022 | 0.120 | 0.507 | 0.646 | Unannotated | NA | NA |
| metab_5425 | Unannotated feature | neg | 214.1681 | 0.636 | 5.392 | 5.376 | -0.015 | -0.120 | 0.507 | 0.646 | Unannotated | NA | NA |
| metab_11040 | Unannotated feature | neg | 199.8866 | 0.013 | 5.658 | 5.658 | -0.000 | -0.120 | 0.507 | 0.646 | Unannotated | NA | NA |
| metab_7041 | Nitrosobenzene | neg | 152.0348 | 5.763 | 5.535 | 5.551 | 0.016 | 0.120 | 0.507 | 0.646 | Tentative | NA | NA |
| metab_7725 | Unannotated feature | neg | 170.9844 | 7.197 | 3.707 | 3.731 | 0.024 | 0.120 | 0.507 | 0.646 | Unannotated | NA | NA |
| metab_8880 | Unannotated feature | neg | 142.0234 | 5.215 | 4.011 | 3.947 | -0.064 | -0.120 | 0.507 | 0.646 | Unannotated | NA | NA |
| metab_10562 | 3-Ureidoisobutyrate | neg | 145.0613 | 0.621 | 5.868 | 5.863 | -0.004 | -0.120 | 0.507 | 0.646 | Tentative | NA | NA |
| metab_10046 | Unannotated feature | neg | 313.9505 | 1.478 | 4.490 | 4.468 | -0.022 | -0.120 | 0.507 | 0.646 | Unannotated | NA | NA |
| metab_5874 | Unannotated feature | neg | 119.9068 | 2.453 | 4.273 | 4.267 | -0.006 | -0.120 | 0.507 | 0.646 | Unannotated | NA | NA |
| metab_6450 | Unannotated feature | neg | 673.8047 | 4.262 | 5.300 | 5.280 | -0.020 | 0.120 | 0.507 | 0.646 | Unannotated | NA | NA |
| metab_7927 | Unannotated feature | neg | 242.9061 | 7.109 | 4.860 | 4.891 | 0.031 | 0.120 | 0.507 | 0.646 | Unannotated | NA | NA |
| metab_4120 | Unannotated feature | pos | 169.0359 | 0.854 | 6.259 | 6.302 | 0.042 | -0.120 | 0.507 | 0.646 | Unannotated | NA | NA |
| metab_10741 | Unannotated feature | neg | 657.7770 | 0.570 | 4.208 | 4.140 | -0.069 | -0.120 | 0.507 | 0.646 | Unannotated | NA | NA |
| metab_5800 | Unannotated feature | neg | 230.1148 | 2.111 | 4.592 | 4.624 | 0.031 | 0.120 | 0.507 | 0.646 | Unannotated | NA | NA |
| metab_2879 | Ticlopidine | pos | 228.0422 | 5.122 | 5.562 | 5.584 | 0.022 | 0.120 | 0.507 | 0.646 | Moderate putative | NA | NA |
| metab_10037 | Unannotated feature | neg | 251.8225 | 1.577 | 5.807 | 5.793 | -0.014 | -0.120 | 0.507 | 0.646 | Unannotated | NA | NA |
| metab_7508 | Unannotated feature | neg | 98.9060 | 7.156 | 4.682 | 4.669 | -0.013 | -0.120 | 0.507 | 0.646 | Unannotated | NA | NA |
| metab_7922 | Unannotated feature | neg | 288.1404 | 7.109 | 4.654 | 4.670 | 0.016 | 0.120 | 0.507 | 0.646 | Unannotated | NA | NA |
| metab_10000 | Unannotated feature | neg | 216.8538 | 1.935 | 6.353 | 6.337 | -0.016 | -0.120 | 0.507 | 0.646 | Unannotated | NA | NA |
| metab_7939 | Unannotated feature | neg | 288.9746 | 0.841 | 4.644 | 4.477 | -0.166 | -0.120 | 0.507 | 0.646 | Unannotated | NA | NA |
| metab_7096 | Unannotated feature | neg | 329.8603 | 1.294 | 5.168 | 5.125 | -0.043 | -0.120 | 0.507 | 0.646 | Unannotated | NA | NA |
| metab_92 | Unannotated feature | pos | 165.0609 | 0.504 | 4.627 | 4.617 | -0.010 | -0.120 | 0.507 | 0.646 | Unannotated | NA | NA |
| metab_4714 | Unannotated feature | pos | 864.6258 | 5.941 | 6.113 | 6.130 | 0.017 | 0.120 | 0.507 | 0.646 | Unannotated | NA | NA |
| metab_4687 | Unannotated feature | pos | 604.7895 | 0.567 | 5.223 | 5.217 | -0.006 | -0.120 | 0.507 | 0.646 | Unannotated | NA | NA |
| metab_10676 | Unannotated feature | neg | 585.6646 | 0.582 | 4.724 | 4.666 | -0.058 | -0.120 | 0.507 | 0.646 | Unannotated | NA | NA |
| metab_4282 | Unannotated feature | pos | 720.7068 | 0.575 | 4.854 | 4.804 | -0.050 | -0.120 | 0.507 | 0.646 | Unannotated | NA | NA |
| metab_4882 | Unannotated feature | pos | 105.0672 | 2.065 | 5.458 | 5.462 | 0.005 | 0.120 | 0.507 | 0.646 | Unannotated | NA | NA |
| metab_5995 | Unannotated feature | neg | 188.0562 | 2.867 | 3.754 | 3.677 | -0.077 | -0.120 | 0.507 | 0.646 | Unannotated | NA | NA |
| metab_7400 | Unannotated feature | neg | 100.2004 | 7.101 | 3.663 | 3.741 | 0.078 | 0.120 | 0.507 | 0.646 | Unannotated | NA | NA |
| metab_11289 | Unannotated feature | neg | 636.3510 | 6.714 | 6.368 | 6.328 | -0.040 | -0.120 | 0.507 | 0.646 | Unannotated | NA | NA |
| metab_8111 | (S)-2-Hydroxystearate | neg | 299.2596 | 6.180 | 5.509 | 5.425 | -0.085 | -0.120 | 0.507 | 0.646 | Tentative | NA | NA |
| metab_6174 | Unannotated feature | neg | 169.0979 | 3.266 | 4.962 | 4.875 | -0.086 | -0.120 | 0.507 | 0.646 | Unannotated | NA | NA |
| metab_6796 | Unannotated feature | neg | 243.0336 | 5.555 | 4.979 | 4.857 | -0.122 | -0.120 | 0.507 | 0.646 | Unannotated | NA | NA |
| metab_2078 | Octanoic acid | pos | 287.2216 | 7.200 | 5.595 | 5.611 | 0.016 | 0.120 | 0.507 | 0.646 | Tentative | NA | NA |
| metab_6568 | Unannotated feature | neg | 89.9249 | 4.695 | 3.459 | 3.465 | 0.006 | -0.120 | 0.507 | 0.646 | Unannotated | NA | NA |
| metab_7305 | Unannotated feature | neg | 200.8587 | 6.203 | 6.613 | 6.596 | -0.017 | -0.120 | 0.507 | 0.646 | Unannotated | NA | NA |
| metab_8370 | 11-Hydroxy-9-tridecenoic acid | neg | 273.1713 | 5.811 | 3.884 | 4.057 | 0.174 | 0.120 | 0.507 | 0.646 | Tentative | NA | NA |
| metab_1486 | 1-Acyl-sn-glycero-3-phosphocholine | pos | 568.3399 | 6.057 | 6.973 | 6.936 | -0.037 | -0.120 | 0.507 | 0.646 | Tentative | NA | NA |
| metab_4928 | Unannotated feature | pos | 175.1191 | 0.614 | 6.229 | 6.230 | 0.000 | 0.120 | 0.507 | 0.646 | Unannotated | NA | NA |
| metab_8033 | Unannotated feature | neg | 524.3369 | 6.549 | 4.921 | 4.799 | -0.122 | -0.120 | 0.507 | 0.646 | Unannotated | NA | NA |
| metab_96 | Unannotated feature | pos | 120.9875 | 0.527 | 3.239 | 3.221 | -0.018 | -0.120 | 0.507 | 0.646 | Unannotated | NA | NA |
| metab_6622 | Unannotated feature | neg | 187.0973 | 4.961 | 3.928 | 3.702 | -0.226 | -0.120 | 0.507 | 0.646 | Unannotated | NA | NA |
| metab_4942 | Unannotated feature | pos | 140.0683 | 0.636 | 5.293 | 5.324 | 0.031 | 0.120 | 0.507 | 0.646 | Unannotated | NA | NA |
| metab_3790 | Unannotated feature | pos | 147.0223 | 1.562 | 3.774 | 3.729 | -0.045 | -0.120 | 0.507 | 0.646 | Unannotated | NA | NA |
| metab_7756 | Unannotated feature | neg | 162.8386 | 7.181 | 5.520 | 5.511 | -0.008 | -0.120 | 0.507 | 0.646 | Unannotated | NA | NA |
| metab_4211 | Unannotated feature | pos | 248.0741 | 0.621 | 4.645 | 4.560 | -0.086 | -0.120 | 0.507 | 0.646 | Unannotated | NA | NA |
| metab_11378 | Unannotated feature | neg | 190.8881 | 0.505 | 4.985 | 4.992 | 0.007 | 0.116 | 0.522 | 0.659 | Unannotated | NA | NA |
| metab_10301 | Unannotated feature | neg | 288.8592 | 0.810 | 4.270 | 4.058 | -0.212 | -0.116 | 0.522 | 0.659 | Unannotated | NA | NA |
| metab_4567 | Stizolamine | pos | 180.0867 | 0.465 | 5.525 | 5.532 | 0.007 | 0.116 | 0.522 | 0.659 | Tentative | NA | NA |
| metab_1932 | Unannotated feature | pos | 137.9985 | 7.324 | 4.420 | 4.412 | -0.008 | 0.116 | 0.522 | 0.659 | Unannotated | NA | NA |
| metab_4599 | Unannotated feature | pos | 131.9744 | 0.069 | 5.404 | 5.403 | -0.001 | -0.116 | 0.522 | 0.659 | Unannotated | NA | NA |
| metab_1745 | Unannotated feature | pos | 279.6469 | 7.324 | 4.613 | 4.599 | -0.014 | -0.116 | 0.522 | 0.659 | Unannotated | NA | NA |
| metab_7119 | Unannotated feature | neg | 556.3269 | 5.834 | 5.556 | 5.568 | 0.012 | 0.116 | 0.522 | 0.659 | Unannotated | NA | NA |
| metab_549 | (+/-)-6-Hydroxy-3-oxo-alpha-ionol | pos | 207.1380 | 2.011 | 4.432 | 4.438 | 0.006 | -0.116 | 0.522 | 0.659 | Tentative | NA | NA |
| metab_1072 | L-4-Chlorotryptophan | pos | 239.0582 | 5.191 | 6.111 | 6.122 | 0.011 | 0.116 | 0.522 | 0.659 | Tentative | NA | NA |
| metab_7447 | Unannotated feature | neg | 269.1317 | 7.124 | 5.542 | 5.550 | 0.008 | 0.116 | 0.522 | 0.659 | Unannotated | NA | NA |
| metab_7459 | Unannotated feature | neg | 401.9262 | 7.132 | 4.496 | 4.481 | -0.015 | -0.116 | 0.522 | 0.659 | Unannotated | NA | NA |
| metab_8012 | Phosphatidate | neg | 585.3534 | 6.751 | 5.975 | 5.968 | -0.007 | -0.116 | 0.522 | 0.659 | Tentative | NA | NA |
| metab_8086 | Unannotated feature | neg | 126.9333 | 6.196 | 5.165 | 5.146 | -0.019 | -0.116 | 0.522 | 0.659 | Unannotated | NA | NA |
| metab_11313 | Unannotated feature | neg | 201.9060 | 7.213 | 4.526 | 4.524 | -0.002 | -0.116 | 0.522 | 0.659 | Unannotated | NA | NA |
| metab_5062 | Unannotated feature | pos | 198.0373 | 0.861 | 4.742 | 4.748 | 0.006 | 0.116 | 0.522 | 0.659 | Unannotated | NA | NA |
| metab_3655 | Unannotated feature | pos | 115.0392 | 1.956 | 4.290 | 4.285 | -0.006 | -0.116 | 0.522 | 0.659 | Unannotated | NA | NA |
| metab_2342 | Unannotated feature | pos | 892.6557 | 5.987 | 5.450 | 5.459 | 0.008 | -0.116 | 0.522 | 0.659 | Unannotated | NA | NA |
| metab_9872 | 1-Methyluric acid | neg | 181.0364 | 2.417 | 4.437 | 4.625 | 0.188 | 0.116 | 0.522 | 0.659 | Moderate putative | NA | NA |
| metab_8128 | N-[(3a,5b,7b)-7-hydroxy-24-oxo-3-(sulfooxy)cholan-24-yl]-Glycine | neg | 510.2536 | 6.157 | 4.538 | 4.308 | -0.230 | -0.116 | 0.522 | 0.659 | Tentative | NA | NA |
| metab_8594 | Unannotated feature | neg | 452.9238 | 7.132 | 6.244 | 6.228 | -0.016 | -0.116 | 0.522 | 0.659 | Unannotated | NA | NA |
| metab_9732 | Unannotated feature | neg | 114.0271 | 2.764 | 3.099 | 3.006 | -0.093 | -0.116 | 0.522 | 0.659 | Unannotated | NA | NA |
| metab_8102 | Unannotated feature | neg | 160.9789 | 6.188 | 5.939 | 5.912 | -0.027 | -0.116 | 0.522 | 0.659 | Unannotated | NA | NA |
| metab_5942 | Unannotated feature | neg | 114.0553 | 2.652 | 3.704 | 3.718 | 0.014 | 0.116 | 0.522 | 0.659 | Unannotated | NA | NA |
| metab_3525 | Unannotated feature | pos | 156.0575 | 3.097 | 4.708 | 4.694 | -0.014 | -0.116 | 0.522 | 0.659 | Unannotated | NA | NA |
| metab_7335 | Unannotated feature | neg | 557.3221 | 6.392 | 6.507 | 6.536 | 0.029 | 0.116 | 0.522 | 0.659 | Unannotated | NA | NA |
| metab_9809 | Unannotated feature | neg | 210.0764 | 2.530 | 3.358 | 3.449 | 0.091 | 0.116 | 0.522 | 0.659 | Unannotated | NA | NA |
| metab_10438 | Unannotated feature | neg | 164.8357 | 0.668 | 6.317 | 6.331 | 0.015 | 0.116 | 0.522 | 0.659 | Unannotated | NA | NA |
| metab_11094 | Unannotated feature | neg | 610.7368 | 0.578 | 5.116 | 5.106 | -0.009 | -0.116 | 0.522 | 0.659 | Unannotated | NA | NA |
| metab_1275 | Unannotated feature | pos | 471.3401 | 5.855 | 5.639 | 5.611 | -0.029 | -0.116 | 0.522 | 0.659 | Unannotated | NA | NA |
| metab_6200 | Unannotated feature | neg | 429.9069 | 0.652 | 4.866 | 4.878 | 0.012 | -0.116 | 0.522 | 0.659 | Unannotated | NA | NA |
| metab_6167 | Unannotated feature | neg | 252.9603 | 3.255 | 4.740 | 4.703 | -0.038 | -0.116 | 0.522 | 0.659 | Unannotated | NA | NA |
| metab_1071 | Imetit | pos | 193.0529 | 5.191 | 5.377 | 5.405 | 0.028 | 0.116 | 0.522 | 0.659 | Tentative | NA | NA |
| metab_2650 | Unannotated feature | pos | 232.1292 | 1.917 | 5.228 | 5.329 | 0.100 | 0.116 | 0.522 | 0.659 | Strong putative | NA | NA |
| metab_10513 | Unannotated feature | neg | 346.7284 | 0.629 | 5.062 | 5.094 | 0.032 | 0.116 | 0.522 | 0.659 | Unannotated | NA | NA |
| metab_3056 | Unannotated feature | pos | 282.0817 | 4.055 | 4.901 | 4.786 | -0.115 | -0.116 | 0.522 | 0.659 | Unannotated | NA | NA |
| metab_7092 | Unannotated feature | neg | 183.1024 | 5.811 | 4.649 | 4.621 | -0.028 | 0.116 | 0.522 | 0.659 | Unannotated | NA | NA |
| metab_97 | Unannotated feature | pos | 152.0320 | 0.534 | 2.806 | 2.690 | -0.116 | -0.116 | 0.522 | 0.659 | Unannotated | NA | NA |
| metab_10757 | Unannotated feature | neg | 555.7955 | 0.570 | 4.200 | 4.160 | -0.040 | -0.116 | 0.522 | 0.659 | Unannotated | NA | NA |
| metab_7469 | Unannotated feature | neg | 114.9891 | 7.140 | 4.287 | 4.280 | -0.007 | -0.116 | 0.522 | 0.659 | Unannotated | NA | NA |
| metab_5255 | Unannotated feature | neg | 207.9185 | 0.013 | 6.889 | 6.895 | 0.006 | 0.116 | 0.522 | 0.659 | Unannotated | NA | NA |
| metab_7806 | Unannotated feature | neg | 457.2373 | 7.164 | 4.166 | 4.133 | -0.033 | -0.116 | 0.522 | 0.659 | Unannotated | NA | NA |
| metab_893 | Unannotated feature | pos | 580.9767 | 3.707 | 4.088 | 4.440 | 0.352 | 0.116 | 0.522 | 0.659 | Unannotated | NA | NA |
| metab_7873 | Unannotated feature | neg | 651.8726 | 7.140 | 4.141 | 4.028 | -0.113 | -0.116 | 0.522 | 0.659 | Unannotated | NA | NA |
| metab_2853 | Unannotated feature | pos | 95.0496 | 2.088 | 5.686 | 5.711 | 0.024 | 0.116 | 0.522 | 0.659 | Unannotated | NA | NA |
| metab_10592 | Unannotated feature | neg | 161.9935 | 0.605 | 4.123 | 4.106 | -0.016 | 0.116 | 0.522 | 0.659 | Unannotated | NA | NA |
| metab_282 | Thiazoline | pos | 88.0221 | 0.737 | 4.226 | 4.139 | -0.087 | 0.116 | 0.522 | 0.659 | Tentative | NA | NA |
| metab_7104 | Unannotated feature | neg | 255.2333 | 5.819 | 5.424 | 5.436 | 0.012 | 0.116 | 0.522 | 0.659 | Unannotated | NA | NA |
| metab_7854 | Taurochenodeoxycholate-3-sulfate | neg | 288.6201 | 7.148 | 3.690 | 3.801 | 0.111 | 0.116 | 0.522 | 0.659 | Tentative | NA | NA |
| metab_7705 | Unannotated feature | neg | 270.0363 | 2.510 | 4.853 | 4.828 | -0.025 | -0.116 | 0.522 | 0.659 | Unannotated | NA | NA |
| metab_2801 | Unannotated feature | pos | 238.1414 | 5.596 | 4.714 | 4.688 | -0.026 | 0.116 | 0.522 | 0.659 | Unannotated | NA | NA |
| metab_2806 | 2-Azaspiro[4.5]decan-3-one | pos | 171.1493 | 5.588 | 4.261 | 4.283 | 0.022 | 0.116 | 0.522 | 0.659 | Tentative | NA | NA |
| metab_3950 | Unannotated feature | pos | 137.4882 | 0.854 | 3.632 | 3.674 | 0.042 | -0.116 | 0.522 | 0.659 | Unannotated | NA | NA |
| metab_9489 | Unannotated feature | neg | 569.9188 | 3.260 | 4.821 | 4.885 | 0.064 | 0.116 | 0.522 | 0.659 | Unannotated | NA | NA |
| metab_2055 | Euscaphic acid | pos | 521.3811 | 7.223 | 5.266 | 5.249 | -0.017 | -0.116 | 0.522 | 0.659 | Tentative | NA | NA |
| metab_10160 | Unannotated feature | neg | 190.9282 | 7.170 | 6.676 | 6.684 | 0.008 | 0.116 | 0.522 | 0.659 | Unannotated | NA | NA |
| metab_2818 | Aspirin | pos | 163.0390 | 5.558 | 4.882 | 4.894 | 0.012 | 0.116 | 0.522 | 0.659 | Moderate putative | NA | NA |
| metab_5703 | Acetylglycine | neg | 116.0346 | 1.342 | 4.758 | 4.798 | 0.040 | 0.116 | 0.522 | 0.659 | Tentative | NA | NA |
| metab_1378 | Unannotated feature | pos | 807.0630 | 5.910 | 4.992 | 4.948 | -0.045 | -0.116 | 0.522 | 0.659 | Unannotated | NA | NA |
| metab_9424 | Unannotated feature | neg | 251.0597 | 3.412 | 3.780 | 3.867 | 0.088 | 0.116 | 0.522 | 0.659 | Unannotated | NA | NA |
| metab_5629 | Unannotated feature | neg | 108.9012 | 0.920 | 4.510 | 4.529 | 0.019 | 0.116 | 0.522 | 0.659 | Unannotated | NA | NA |
| metab_6557 | Unannotated feature | neg | 89.9249 | 4.643 | 3.034 | 3.080 | 0.046 | 0.116 | 0.522 | 0.659 | Unannotated | NA | NA |
| metab_11033 | Unannotated feature | neg | 351.8549 | 0.013 | 4.908 | 4.894 | -0.013 | -0.116 | 0.522 | 0.659 | Unannotated | NA | NA |
| metab_2094 | Unannotated feature | pos | 120.0658 | 7.161 | 3.330 | 3.421 | 0.092 | 0.112 | 0.538 | 0.672 | Unannotated | NA | NA |
| metab_6389 | Unannotated feature | neg | 243.1717 | 4.037 | 4.027 | 4.093 | 0.067 | 0.112 | 0.538 | 0.672 | Unannotated | NA | NA |
| metab_6416 | Unannotated feature | neg | 209.9490 | 4.161 | 5.947 | 5.958 | 0.011 | 0.112 | 0.538 | 0.672 | Unannotated | NA | NA |
| metab_7196 | Unannotated feature | neg | 248.9787 | 0.865 | 4.090 | 4.282 | 0.192 | 0.112 | 0.538 | 0.672 | Unannotated | NA | NA |
| metab_5517 | Unannotated feature | neg | 184.0725 | 0.770 | 4.362 | 4.483 | 0.121 | 0.112 | 0.538 | 0.672 | Unannotated | NA | NA |
| metab_3742 | Hawkinsin | pos | 324.1125 | 1.794 | 4.044 | 3.816 | -0.228 | -0.112 | 0.538 | 0.672 | Tentative | NA | NA |
| metab_4235 | L-Alanine | pos | 90.0554 | 0.614 | 5.772 | 5.762 | -0.010 | -0.112 | 0.538 | 0.672 | Tentative | NA | NA |
| metab_10131 | Unannotated feature | neg | 177.9053 | 0.975 | 5.032 | 5.013 | -0.018 | -0.112 | 0.538 | 0.672 | Unannotated | NA | NA |
| metab_3747 | Formycin b | pos | 291.0699 | 1.778 | 4.701 | 4.904 | 0.203 | 0.112 | 0.538 | 0.672 | Tentative | NA | NA |
| metab_10804 | Unannotated feature | neg | 113.0069 | 0.562 | 3.918 | 3.888 | -0.030 | -0.112 | 0.538 | 0.672 | Unannotated | NA | NA |
| metab_9722 | Unannotated feature | neg | 109.0399 | 2.804 | 3.571 | 3.466 | -0.105 | -0.112 | 0.538 | 0.672 | Unannotated | NA | NA |
| metab_1557 | Unannotated feature | pos | 361.2735 | 6.303 | 4.170 | 4.138 | -0.032 | -0.112 | 0.538 | 0.672 | Unannotated | NA | NA |
| metab_4312 | Unannotated feature | pos | 830.5889 | 0.575 | 5.058 | 5.052 | -0.005 | -0.112 | 0.538 | 0.672 | Unannotated | NA | NA |
| metab_3084 | Unannotated feature | pos | 335.2225 | 3.885 | 4.038 | 3.920 | -0.119 | -0.112 | 0.538 | 0.672 | Unannotated | NA | NA |
| metab_10550 | Unannotated feature | neg | 402.6900 | 0.621 | 5.607 | 5.587 | -0.020 | -0.112 | 0.538 | 0.672 | Unannotated | NA | NA |
| metab_1099 | Unannotated feature | pos | 234.2064 | 5.459 | 4.201 | 4.143 | -0.057 | -0.112 | 0.538 | 0.672 | Unannotated | NA | NA |
| metab_10392 | Unannotated feature | neg | 223.8858 | 0.715 | 4.788 | 4.783 | -0.004 | 0.112 | 0.538 | 0.672 | Unannotated | NA | NA |
| metab_1131 | Unannotated feature | pos | 126.1280 | 5.634 | 3.930 | 3.913 | -0.017 | -0.112 | 0.538 | 0.672 | Unannotated | NA | NA |
| metab_544 | Unannotated feature | pos | 183.0514 | 2.003 | 4.110 | 4.255 | 0.145 | 0.112 | 0.538 | 0.672 | Unannotated | NA | NA |
| metab_4377 | Unannotated feature | pos | 621.8302 | 0.567 | 3.781 | 3.908 | 0.127 | 0.112 | 0.538 | 0.672 | Unannotated | NA | NA |
| metab_1132 | (4S,8R)-8,9-Dihydroxy-p-menth-1(6)-en-2-one | pos | 207.0988 | 5.634 | 4.725 | 4.725 | -0.001 | 0.112 | 0.538 | 0.672 | Tentative | NA | NA |
| metab_7818 | Unannotated feature | neg | 506.3262 | 7.156 | 4.608 | 4.614 | 0.006 | -0.112 | 0.538 | 0.672 | Unannotated | NA | NA |
| metab_10304 | Unannotated feature | neg | 235.8643 | 0.802 | 4.056 | 3.985 | -0.071 | -0.112 | 0.538 | 0.672 | Unannotated | NA | NA |
| metab_7321 | Budralazine | neg | 539.2902 | 6.297 | 4.183 | 4.059 | -0.124 | 0.112 | 0.538 | 0.672 | Tentative | NA | NA |
| metab_2946 | Unannotated feature | pos | 84.1228 | 4.739 | 3.740 | 3.816 | 0.076 | 0.112 | 0.538 | 0.672 | Unannotated | NA | NA |
| metab_9033 | Unannotated feature | neg | 757.8317 | 4.651 | 4.965 | 5.098 | 0.133 | 0.112 | 0.538 | 0.672 | Unannotated | NA | NA |
| metab_6152 | Unannotated feature | neg | 219.8909 | 3.232 | 3.553 | 3.202 | -0.351 | -0.112 | 0.538 | 0.672 | Unannotated | NA | NA |
| metab_2450 | Unannotated feature | pos | 561.8740 | 0.559 | 4.830 | 4.847 | 0.017 | 0.112 | 0.538 | 0.672 | Unannotated | NA | NA |
| metab_2147 | Unannotated feature | pos | 515.0912 | 6.590 | 5.528 | 5.595 | 0.067 | 0.112 | 0.538 | 0.672 | Unannotated | NA | NA |
| metab_3285 | Unannotated feature | pos | 226.1596 | 2.865 | 4.087 | 4.147 | 0.060 | 0.112 | 0.538 | 0.672 | Unannotated | NA | NA |
| metab_5393 | Unannotated feature | neg | 104.0345 | 0.613 | 4.017 | 3.973 | -0.043 | -0.112 | 0.538 | 0.672 | Unannotated | NA | NA |
| metab_11246 | Unannotated feature | neg | 356.8467 | 0.578 | 5.635 | 5.612 | -0.023 | -0.112 | 0.538 | 0.672 | Unannotated | NA | NA |
| metab_9526 | Unannotated feature | neg | 197.2684 | 3.224 | 4.984 | 4.952 | -0.032 | -0.112 | 0.538 | 0.672 | Unannotated | NA | NA |
| metab_1598 | Unannotated feature | pos | 798.7219 | 0.567 | 5.047 | 5.025 | -0.022 | -0.112 | 0.538 | 0.672 | Unannotated | NA | NA |
| metab_2431 | (Z)-3-Oxo-2-(2-pentenyl)-1-cyclopenteneacetic acid | pos | 209.1173 | 5.925 | 4.358 | 4.489 | 0.131 | 0.112 | 0.538 | 0.672 | Tentative | NA | NA |
| metab_10942 | Unannotated feature | neg | 393.8945 | 0.530 | 3.976 | 3.977 | 0.001 | -0.112 | 0.538 | 0.672 | Unannotated | NA | NA |
| metab_5356 | Unannotated feature | neg | 820.5165 | 0.582 | 4.608 | 4.654 | 0.045 | 0.112 | 0.538 | 0.672 | Unannotated | NA | NA |
| metab_4622 | Unannotated feature | pos | 198.1489 | 5.596 | 4.857 | 4.871 | 0.014 | 0.112 | 0.538 | 0.672 | Unannotated | NA | NA |
| metab_483 | Cytosine | pos | 112.0508 | 1.832 | 4.197 | 4.133 | -0.064 | -0.112 | 0.538 | 0.672 | Moderate putative | NA | NA |
| metab_2427 | Unannotated feature | pos | 441.3297 | 5.925 | 5.710 | 5.775 | 0.065 | 0.112 | 0.538 | 0.672 | Unannotated | NA | NA |
| metab_2396 | Unannotated feature | pos | 490.8582 | 5.941 | 4.298 | 4.395 | 0.097 | 0.112 | 0.538 | 0.672 | Unannotated | NA | NA |
| metab_4742 | Unannotated feature | pos | 357.2999 | 6.211 | 5.418 | 5.378 | -0.040 | -0.112 | 0.538 | 0.672 | Unannotated | NA | NA |
| metab_7819 | Unannotated feature | neg | 330.8997 | 7.156 | 4.972 | 4.962 | -0.010 | -0.112 | 0.538 | 0.672 | Unannotated | NA | NA |
| metab_3002 | Unannotated feature | pos | 170.0368 | 4.378 | 3.850 | 3.759 | -0.091 | -0.112 | 0.538 | 0.672 | Unannotated | NA | NA |
| metab_7071 | Unannotated feature | neg | 227.8945 | 5.795 | 4.981 | 4.922 | -0.059 | -0.112 | 0.538 | 0.672 | Unannotated | NA | NA |
| metab_6120 | Unannotated feature | neg | 247.0138 | 3.192 | 3.700 | 3.674 | -0.026 | -0.112 | 0.538 | 0.672 | Unannotated | NA | NA |
| metab_11149 | Unannotated feature | neg | 411.1858 | 5.715 | 4.858 | 4.945 | 0.088 | 0.112 | 0.538 | 0.672 | Unannotated | NA | NA |
| metab_7599 | Unannotated feature | neg | 90.5272 | 7.253 | 3.002 | 2.976 | -0.026 | -0.112 | 0.538 | 0.672 | Unannotated | NA | NA |
| metab_9187 | Unannotated feature | neg | 249.0884 | 4.181 | 3.199 | 3.389 | 0.190 | 0.112 | 0.538 | 0.672 | Unannotated | NA | NA |
| metab_3447 | Unannotated feature | pos | 90.5263 | 2.311 | 5.698 | 5.702 | 0.004 | 0.112 | 0.538 | 0.672 | Unannotated | NA | NA |
| metab_5134 | Indole | pos | 118.0654 | 2.644 | 7.036 | 7.033 | -0.003 | -0.112 | 0.538 | 0.672 | Strong putative | NA | NA |
| metab_7955 | Unannotated feature | neg | 100.9328 | 7.109 | 7.587 | 7.595 | 0.008 | 0.112 | 0.538 | 0.672 | Unannotated | NA | NA |
| metab_1996 | Unannotated feature | pos | 507.1378 | 7.285 | 4.758 | 4.746 | -0.012 | -0.112 | 0.538 | 0.672 | Unannotated | NA | NA |
| metab_11382 | 4-Nitrophenol | neg | 138.0191 | 5.563 | 4.782 | 4.789 | 0.007 | 0.112 | 0.538 | 0.672 | Moderate putative | NA | NA |
| metab_5550 | Unannotated feature | neg | 232.8459 | 0.825 | 4.654 | 4.578 | -0.076 | -0.112 | 0.538 | 0.672 | Unannotated | NA | NA |
| metab_7171 | Unannotated feature | neg | 194.8281 | 5.898 | 4.133 | 4.153 | 0.019 | 0.112 | 0.538 | 0.672 | Unannotated | NA | NA |
| metab_5207 | Unannotated feature | neg | 911.3243 | 0.539 | 4.519 | 4.475 | -0.044 | -0.112 | 0.538 | 0.672 | Unannotated | NA | NA |
| metab_395 | 2-O-(6-Phospho-alpha-mannosyl)-D-glycerate | pos | 331.0398 | 1.056 | 3.204 | 2.987 | -0.217 | -0.112 | 0.538 | 0.672 | Tentative | NA | NA |
| metab_4902 | Unannotated feature | pos | 154.0863 | 0.426 | 5.359 | 5.362 | 0.003 | -0.112 | 0.538 | 0.672 | Unannotated | NA | NA |
| metab_8059 | Unannotated feature | neg | 116.9821 | 6.360 | 3.440 | 3.463 | 0.023 | 0.112 | 0.538 | 0.672 | Unannotated | NA | NA |
| metab_5081 | Unannotated feature | pos | 194.6189 | 1.243 | 4.812 | 4.892 | 0.080 | 0.112 | 0.538 | 0.672 | Moderate putative | NA | NA |
| metab_4898 | Unannotated feature | pos | 90.9482 | 0.473 | 5.604 | 5.606 | 0.002 | 0.112 | 0.538 | 0.672 | Unannotated | NA | NA |
| metab_10998 | Unannotated feature | neg | 186.9338 | 0.039 | 4.487 | 4.492 | 0.006 | 0.112 | 0.538 | 0.672 | Unannotated | NA | NA |
| metab_6581 | (4As,5aS,6S,12aS)-1,6,10,11-tetrahydroxy-6-methyl-3,12-dioxo-4a,5,5a,12a-tetrahydro-4H-tetracene-2-carboxamide | neg | 406.0919 | 4.774 | 5.439 | 5.378 | -0.061 | -0.107 | 0.554 | 0.686 | Tentative | NA | NA |
| metab_4749 | Unannotated feature | pos | 225.1458 | 6.303 | 5.021 | 5.017 | -0.004 | -0.107 | 0.554 | 0.686 | Unannotated | NA | NA |
| metab_4740 | Unannotated feature | pos | 362.9263 | 0.559 | 6.607 | 6.590 | -0.018 | -0.107 | 0.554 | 0.686 | Unannotated | NA | NA |
| metab_4792 | 1-Acyl-sn-glycero-3-phosphocholine | pos | 532.3372 | 7.293 | 4.782 | 4.825 | 0.043 | -0.107 | 0.554 | 0.686 | Moderate putative | NA | NA |
| metab_7877 | Unannotated feature | neg | 367.9071 | 7.140 | 4.865 | 4.877 | 0.012 | 0.107 | 0.554 | 0.686 | Unannotated | NA | NA |
| metab_4678 | Unannotated feature | pos | 381.3153 | 5.871 | 4.153 | 4.139 | -0.014 | -0.107 | 0.554 | 0.686 | Unannotated | NA | NA |
| metab_9873 | Unannotated feature | neg | 269.9070 | 2.393 | 5.439 | 5.462 | 0.023 | 0.107 | 0.554 | 0.686 | Unannotated | NA | NA |
| metab_5347 | Unannotated feature | neg | 511.7726 | 0.578 | 4.234 | 4.158 | -0.076 | -0.107 | 0.554 | 0.686 | Unannotated | NA | NA |
| metab_6596 | Unannotated feature | neg | 221.0932 | 4.838 | 3.181 | 3.472 | 0.291 | 0.107 | 0.554 | 0.686 | Unannotated | NA | NA |
| metab_11007 | Unannotated feature | neg | 203.9367 | 0.019 | 5.079 | 5.088 | 0.009 | 0.107 | 0.554 | 0.686 | Unannotated | NA | NA |
| metab_8355 | Unannotated feature | neg | 371.1907 | 5.819 | 4.287 | 4.270 | -0.017 | -0.107 | 0.554 | 0.686 | Unannotated | NA | NA |
| metab_2531 | Unannotated feature | pos | 573.4085 | 5.879 | 6.138 | 6.164 | 0.026 | 0.107 | 0.554 | 0.686 | Unannotated | NA | NA |
| metab_9034 | Unannotated feature | neg | 548.9576 | 3.260 | 5.352 | 5.345 | -0.008 | -0.107 | 0.554 | 0.686 | Unannotated | NA | NA |
| metab_4550 | Unannotated feature | pos | 149.0294 | 0.504 | 4.199 | 4.249 | 0.050 | 0.107 | 0.554 | 0.686 | Unannotated | NA | NA |
| metab_10687 | Unannotated feature | neg | 108.9012 | 0.590 | 5.233 | 5.255 | 0.022 | 0.107 | 0.554 | 0.686 | Unannotated | NA | NA |
| metab_11121 | Unannotated feature | neg | 379.1959 | 5.691 | 4.037 | 4.043 | 0.006 | 0.107 | 0.554 | 0.686 | Unannotated | NA | NA |
| metab_10552 | Unannotated feature | neg | 269.5770 | 0.621 | 4.088 | 4.008 | -0.080 | -0.107 | 0.554 | 0.686 | Unannotated | NA | NA |
| metab_10282 | Unannotated feature | neg | 231.0156 | 0.833 | 4.802 | 4.702 | -0.099 | -0.107 | 0.554 | 0.686 | Unannotated | NA | NA |
| metab_2468 | Unannotated feature | pos | 850.6088 | 5.902 | 5.808 | 5.818 | 0.010 | -0.107 | 0.554 | 0.686 | Unannotated | NA | NA |
| metab_5352 | Unannotated feature | neg | 492.7135 | 0.582 | 4.973 | 5.001 | 0.027 | -0.107 | 0.554 | 0.686 | Unannotated | NA | NA |
| metab_4538 | Unannotated feature | pos | 204.9571 | 0.527 | 4.856 | 4.867 | 0.011 | 0.107 | 0.554 | 0.686 | Unannotated | NA | NA |
| metab_10828 | Unannotated feature | neg | 795.8396 | 0.554 | 5.714 | 5.741 | 0.027 | -0.107 | 0.554 | 0.686 | Unannotated | NA | NA |
| metab_10830 | Unannotated feature | neg | 769.8337 | 0.554 | 5.163 | 5.186 | 0.023 | 0.107 | 0.554 | 0.686 | Unannotated | NA | NA |
| metab_8559 | Taurochenodeoxycholate-7-sulfate | neg | 288.6202 | 5.691 | 4.505 | 4.583 | 0.078 | -0.107 | 0.554 | 0.686 | Tentative | NA | NA |
| metab_10766 | Unannotated feature | neg | 376.9041 | 0.570 | 5.082 | 5.065 | -0.017 | -0.107 | 0.554 | 0.686 | Unannotated | NA | NA |
| metab_6529 | Unannotated feature | neg | 186.1133 | 4.529 | 4.279 | 4.308 | 0.029 | 0.107 | 0.554 | 0.686 | Unannotated | NA | NA |
| metab_6495 | N-Acetyl-L-leucine | neg | 172.0975 | 4.379 | 4.422 | 4.385 | -0.037 | -0.107 | 0.554 | 0.686 | Moderate putative | NA | NA |
| metab_5710 | Unannotated feature | neg | 136.0339 | 1.382 | 3.556 | 3.478 | -0.078 | -0.107 | 0.554 | 0.686 | Unannotated | NA | NA |
| metab_6538 | Unannotated feature | neg | 265.0859 | 4.574 | 3.900 | 3.602 | -0.298 | -0.107 | 0.554 | 0.686 | Unannotated | NA | NA |
| metab_1387 | Unannotated feature | pos | 589.9014 | 5.917 | 4.986 | 4.997 | 0.011 | 0.107 | 0.554 | 0.686 | Unannotated | NA | NA |
| metab_7602 | Unannotated feature | neg | 324.8466 | 7.253 | 4.692 | 4.688 | -0.005 | -0.107 | 0.554 | 0.686 | Unannotated | NA | NA |
| metab_6541 | Unannotated feature | neg | 359.9373 | 4.574 | 4.323 | 4.392 | 0.069 | 0.107 | 0.554 | 0.686 | Unannotated | NA | NA |
| metab_3111 | Unannotated feature | pos | 768.8488 | 3.785 | 5.300 | 5.400 | 0.100 | 0.107 | 0.554 | 0.686 | Unannotated | NA | NA |
| metab_743 | Unannotated feature | pos | 222.0292 | 2.644 | 5.686 | 5.727 | 0.041 | 0.107 | 0.554 | 0.686 | Unannotated | NA | NA |
| metab_9170 | Unannotated feature | neg | 320.9214 | 4.235 | 4.553 | 4.630 | 0.076 | 0.107 | 0.554 | 0.686 | Unannotated | NA | NA |
| metab_1658 | Unannotated feature | pos | 297.2033 | 7.215 | 5.085 | 5.079 | -0.006 | -0.107 | 0.554 | 0.686 | Unannotated | NA | NA |
| metab_10997 | Unannotated feature | neg | 188.8945 | 0.047 | 5.568 | 5.567 | -0.001 | -0.107 | 0.554 | 0.686 | Unannotated | NA | NA |
| metab_161 | Unannotated feature | pos | 132.0033 | 0.559 | 4.362 | 4.384 | 0.022 | 0.107 | 0.554 | 0.686 | Unannotated | NA | NA |
| metab_7396 | Unannotated feature | neg | 421.2275 | 7.093 | 4.514 | 4.487 | -0.027 | -0.107 | 0.554 | 0.686 | Unannotated | NA | NA |
| metab_1347 | Unannotated feature | pos | 894.6341 | 5.894 | 5.399 | 5.397 | -0.002 | -0.107 | 0.554 | 0.686 | Unannotated | NA | NA |
| metab_363 | Unannotated feature | pos | 405.0265 | 0.861 | 4.669 | 4.730 | 0.061 | 0.107 | 0.554 | 0.686 | Unannotated | NA | NA |
| metab_7865 | Unannotated feature | neg | 766.8612 | 7.140 | 4.844 | 4.834 | -0.010 | -0.107 | 0.554 | 0.686 | Unannotated | NA | NA |
| metab_6537 | Unannotated feature | neg | 196.3389 | 4.574 | 3.307 | 3.264 | -0.043 | -0.107 | 0.554 | 0.686 | Unannotated | NA | NA |
| metab_10744 | Unannotated feature | neg | 638.8262 | 0.570 | 5.118 | 5.124 | 0.006 | -0.107 | 0.554 | 0.686 | Unannotated | NA | NA |
| metab_5064 | 2,4-Thiazolidinedicarboxylic acid, 2-methyl- | pos | 192.0325 | 0.854 | 5.223 | 5.167 | -0.056 | -0.107 | 0.554 | 0.686 | Tentative | NA | NA |
| metab_10925 | Unannotated feature | neg | 754.8536 | 0.534 | 3.956 | 3.824 | -0.132 | -0.107 | 0.554 | 0.686 | Unannotated | NA | NA |
| metab_838 | Unannotated feature | pos | 171.0992 | 3.259 | 3.802 | 3.851 | 0.049 | 0.107 | 0.554 | 0.686 | Unannotated | NA | NA |
| metab_10653 | Unannotated feature | neg | 549.6734 | 0.587 | 4.554 | 4.480 | -0.073 | -0.107 | 0.554 | 0.686 | Unannotated | NA | NA |
| metab_1135 | Unannotated feature | pos | 200.1646 | 5.649 | 5.612 | 5.606 | -0.006 | 0.107 | 0.554 | 0.686 | Unannotated | NA | NA |
| metab_10056 | Unannotated feature | neg | 245.7879 | 1.382 | 4.450 | 4.485 | 0.035 | 0.107 | 0.554 | 0.686 | Unannotated | NA | NA |
| metab_1809 | Unannotated feature | pos | 145.0173 | 7.959 | 4.233 | 4.223 | -0.010 | 0.107 | 0.554 | 0.686 | Unannotated | NA | NA |
| metab_1812 | Unannotated feature | pos | 120.9811 | 7.975 | 4.500 | 4.498 | -0.002 | 0.107 | 0.554 | 0.686 | Unannotated | NA | NA |
| metab_8993 | Unannotated feature | neg | 408.0886 | 4.774 | 4.757 | 4.817 | 0.060 | -0.107 | 0.554 | 0.686 | Unannotated | NA | NA |
| metab_722 | Unannotated feature | pos | 267.1339 | 2.591 | 4.322 | 4.221 | -0.101 | 0.107 | 0.554 | 0.686 | Strong putative | NA | NA |
| metab_6301 | Unannotated feature | neg | 90.9869 | 3.694 | 4.326 | 4.297 | -0.030 | -0.107 | 0.554 | 0.686 | Unannotated | NA | NA |
| metab_4020 | Unannotated feature | pos | 102.3761 | 0.807 | 2.934 | 2.999 | 0.065 | 0.107 | 0.554 | 0.686 | Unannotated | NA | NA |
| metab_9670 | Unannotated feature | neg | 241.9117 | 2.953 | 5.605 | 5.611 | 0.006 | 0.107 | 0.554 | 0.686 | Unannotated | NA | NA |
| metab_2052 | Agavoside A | pos | 631.3292 | 7.231 | 5.576 | 5.517 | -0.059 | -0.107 | 0.554 | 0.686 | Tentative | NA | NA |
| metab_2134 | DG(15:0/20:4(5Z,8Z,11Z,14Z)-OH(16R)/0:0) | pos | 663.4525 | 6.737 | 5.693 | 5.664 | -0.028 | 0.107 | 0.554 | 0.686 | Tentative | NA | NA |
| metab_7683 | Unannotated feature | neg | 501.1271 | 2.498 | 4.590 | 4.569 | -0.021 | -0.107 | 0.554 | 0.686 | Unannotated | NA | NA |
| metab_8728 | Unannotated feature | neg | 212.0298 | 5.579 | 4.489 | 4.515 | 0.026 | 0.107 | 0.554 | 0.686 | Unannotated | NA | NA |
| metab_4213 | Unannotated feature | pos | 142.0476 | 0.621 | 5.053 | 5.037 | -0.016 | -0.107 | 0.554 | 0.686 | Unannotated | NA | NA |
| metab_3854 | Unannotated feature | pos | 121.4900 | 1.033 | 3.815 | 3.812 | -0.002 | 0.107 | 0.554 | 0.686 | Unannotated | NA | NA |
| metab_5505 | Unannotated feature | neg | 280.1043 | 0.747 | 4.675 | 4.640 | -0.036 | -0.103 | 0.570 | 0.699 | Unannotated | NA | NA |
| metab_5459 | Unannotated feature | neg | 162.8386 | 0.668 | 6.794 | 6.830 | 0.036 | 0.103 | 0.570 | 0.699 | Unannotated | NA | NA |
| metab_9343 | Unannotated feature | neg | 258.0082 | 3.650 | 3.272 | 3.095 | -0.177 | -0.103 | 0.570 | 0.699 | Unannotated | NA | NA |
| metab_2019 | Unannotated feature | pos | 641.2671 | 7.277 | 3.961 | 3.955 | -0.006 | 0.103 | 0.570 | 0.699 | Unannotated | NA | NA |
| metab_10681 | Unannotated feature | neg | 496.7295 | 0.582 | 4.421 | 4.363 | -0.058 | -0.103 | 0.570 | 0.699 | Unannotated | NA | NA |
| metab_11302 | Unannotated feature | neg | 328.8307 | 7.181 | 4.777 | 4.796 | 0.019 | 0.103 | 0.570 | 0.699 | Unannotated | NA | NA |
| metab_3427 | 5-[(2R)-2-Aminopropyl]-2-hydroxybenzoic acid | pos | 196.0969 | 2.389 | 4.008 | 4.037 | 0.029 | 0.103 | 0.570 | 0.699 | Tentative | NA | NA |
| metab_2169 | Unannotated feature | pos | 458.4202 | 6.420 | 6.155 | 6.136 | -0.020 | -0.103 | 0.570 | 0.699 | Unannotated | NA | NA |
| metab_6600 | Unannotated feature | neg | 195.0663 | 4.853 | 2.577 | 2.797 | 0.220 | 0.103 | 0.570 | 0.699 | Unannotated | NA | NA |
| metab_942 | Unannotated feature | pos | 199.1126 | 4.070 | 4.759 | 4.776 | 0.017 | 0.103 | 0.570 | 0.699 | Unannotated | NA | NA |
| metab_6158 | Unannotated feature | neg | 347.9648 | 0.652 | 4.731 | 4.778 | 0.047 | 0.103 | 0.570 | 0.699 | Unannotated | NA | NA |
| metab_2479 | Unannotated feature | pos | 425.8085 | 5.902 | 4.071 | 4.164 | 0.092 | 0.103 | 0.570 | 0.699 | Unannotated | NA | NA |
| metab_2513 | Unannotated feature | pos | 675.4764 | 5.886 | 6.169 | 6.170 | 0.001 | -0.103 | 0.570 | 0.699 | Unannotated | NA | NA |
| metab_2149 | Unannotated feature | pos | 765.9874 | 6.567 | 6.327 | 6.326 | -0.000 | -0.103 | 0.570 | 0.699 | Unannotated | NA | NA |
| metab_4542 | Unannotated feature | pos | 124.0736 | 0.519 | 3.551 | 3.577 | 0.025 | 0.103 | 0.570 | 0.699 | Unannotated | NA | NA |
| metab_4536 | Unannotated feature | pos | 243.9417 | 0.534 | 5.061 | 5.084 | 0.023 | 0.103 | 0.570 | 0.699 | Unannotated | NA | NA |
| metab_1524 | Unannotated feature | pos | 486.4158 | 6.188 | 4.890 | 4.991 | 0.101 | 0.103 | 0.570 | 0.699 | Unannotated | NA | NA |
| metab_5785 | Unannotated feature | neg | 174.8636 | 2.047 | 4.022 | 4.013 | -0.009 | -0.103 | 0.570 | 0.699 | Unannotated | NA | NA |
| metab_1264 | Unannotated feature | pos | 493.3532 | 5.848 | 5.593 | 5.621 | 0.027 | -0.103 | 0.570 | 0.699 | Unannotated | NA | NA |
| metab_5412 | Unannotated feature | neg | 802.4136 | 0.621 | 4.880 | 4.923 | 0.043 | 0.103 | 0.570 | 0.699 | Unannotated | NA | NA |
| metab_2033 | Unannotated feature | pos | 272.9420 | 7.269 | 4.780 | 4.800 | 0.020 | 0.103 | 0.570 | 0.699 | Unannotated | NA | NA |
| metab_4169 | N-(2-Hydroxypropyl)valine | pos | 193.1547 | 0.644 | 5.423 | 5.417 | -0.006 | 0.103 | 0.570 | 0.699 | Tentative | NA | NA |
| metab_5543 | Unannotated feature | neg | 290.8051 | 0.810 | 4.807 | 4.766 | -0.041 | -0.103 | 0.570 | 0.699 | Unannotated | NA | NA |
| metab_3059 | Unannotated feature | pos | 172.1333 | 4.047 | 5.135 | 5.135 | 0.000 | 0.103 | 0.570 | 0.699 | Unannotated | NA | NA |
| metab_10074 | Unannotated feature | neg | 442.0097 | 1.294 | 3.679 | 3.685 | 0.006 | -0.103 | 0.570 | 0.699 | Unannotated | NA | NA |
| metab_10784 | Unannotated feature | neg | 722.8351 | 0.562 | 4.571 | 4.540 | -0.032 | 0.103 | 0.570 | 0.699 | Unannotated | NA | NA |
| metab_4223 | Unannotated feature | pos | 207.0144 | 0.614 | 4.684 | 4.763 | 0.079 | 0.103 | 0.570 | 0.699 | Unannotated | NA | NA |
| metab_10076 | Unannotated feature | neg | 327.8633 | 1.294 | 5.183 | 5.137 | -0.046 | -0.103 | 0.570 | 0.699 | Unannotated | NA | NA |
| metab_7736 | Unannotated feature | neg | 189.8693 | 7.189 | 5.512 | 5.510 | -0.002 | -0.103 | 0.570 | 0.699 | Unannotated | NA | NA |
| metab_4365 | Unannotated feature | pos | 687.8201 | 0.567 | 4.516 | 4.552 | 0.036 | 0.103 | 0.570 | 0.699 | Unannotated | NA | NA |
| metab_4376 | Unannotated feature | pos | 624.8471 | 0.567 | 5.484 | 5.495 | 0.011 | -0.103 | 0.570 | 0.699 | Unannotated | NA | NA |
| metab_4381 | Unannotated feature | pos | 601.7724 | 0.567 | 4.301 | 4.326 | 0.026 | 0.103 | 0.570 | 0.699 | Unannotated | NA | NA |
| metab_2166 | Unannotated feature | pos | 828.8092 | 0.567 | 5.282 | 5.279 | -0.003 | -0.103 | 0.570 | 0.699 | Unannotated | NA | NA |
| metab_7800 | Unannotated feature | neg | 84.9843 | 7.170 | 3.132 | 3.121 | -0.011 | -0.103 | 0.570 | 0.699 | Unannotated | NA | NA |
| metab_11048 | Unannotated feature | neg | 138.9063 | 0.013 | 5.010 | 5.017 | 0.007 | 0.103 | 0.570 | 0.699 | Unannotated | NA | NA |
| metab_4014 | Unannotated feature | pos | 204.0867 | 0.807 | 3.528 | 3.542 | 0.014 | 0.103 | 0.570 | 0.699 | Unannotated | NA | NA |
| metab_4082 | Thioglycolate | pos | 134.0272 | 0.737 | 5.102 | 4.992 | -0.110 | 0.103 | 0.570 | 0.699 | Tentative | NA | NA |
| metab_8737 | Unannotated feature | neg | 309.1005 | 5.571 | 3.592 | 3.721 | 0.128 | 0.103 | 0.570 | 0.699 | Unannotated | NA | NA |
| metab_2146 | Unannotated feature | pos | 916.6366 | 0.567 | 4.666 | 4.719 | 0.052 | 0.103 | 0.570 | 0.699 | Unannotated | NA | NA |
| metab_7722 | Unannotated feature | neg | 218.8764 | 7.197 | 4.161 | 4.157 | -0.004 | -0.103 | 0.570 | 0.699 | Unannotated | NA | NA |
| metab_4124 | Pyrrole | pos | 70.0657 | 0.698 | 5.238 | 5.230 | -0.008 | 0.103 | 0.570 | 0.699 | Tentative | NA | NA |
| metab_2670 | Unannotated feature | pos | 244.2636 | 5.786 | 4.871 | 4.870 | -0.001 | 0.103 | 0.570 | 0.699 | Unannotated | NA | NA |
| metab_2380 | Unannotated feature | pos | 157.1014 | 5.956 | 3.726 | 3.826 | 0.100 | 0.103 | 0.570 | 0.699 | Unannotated | NA | NA |
| metab_4878 | Unannotated feature | pos | 172.1210 | 2.073 | 6.655 | 6.663 | 0.008 | 0.103 | 0.570 | 0.699 | Unannotated | NA | NA |
| metab_3331 | Unannotated feature | pos | 89.0391 | 2.637 | 4.826 | 4.838 | 0.012 | 0.103 | 0.570 | 0.699 | Unannotated | NA | NA |
| metab_9544 | Unannotated feature | neg | 332.9477 | 0.636 | 5.429 | 5.439 | 0.010 | -0.103 | 0.570 | 0.699 | Unannotated | NA | NA |
| metab_10521 | Unannotated feature | neg | 176.0092 | 0.629 | 4.165 | 4.119 | -0.046 | -0.103 | 0.570 | 0.699 | Unannotated | NA | NA |
| metab_2249 | PC(16:1(9E)/0:0) | pos | 516.3066 | 6.203 | 5.466 | 5.396 | -0.069 | -0.103 | 0.570 | 0.699 | Tentative | NA | NA |
| metab_5203 | Unannotated feature | neg | 707.3619 | 0.539 | 4.705 | 4.704 | -0.001 | 0.103 | 0.570 | 0.699 | Unannotated | NA | NA |
| metab_5223 | Unannotated feature | neg | 144.9427 | 0.013 | 5.579 | 5.581 | 0.002 | 0.103 | 0.570 | 0.699 | Unannotated | NA | NA |
| metab_3365 | Unannotated feature | pos | 83.6589 | 2.560 | 3.256 | 3.271 | 0.015 | 0.103 | 0.570 | 0.699 | Unannotated | NA | NA |
| metab_3393 | Unannotated feature | pos | 120.0238 | 2.482 | 4.047 | 4.039 | -0.008 | -0.103 | 0.570 | 0.699 | Unannotated | NA | NA |
| metab_10484 | Unannotated feature | neg | 283.0207 | 0.636 | 4.444 | 4.379 | -0.066 | -0.103 | 0.570 | 0.699 | Unannotated | NA | NA |
| metab_10720 | Unannotated feature | neg | 422.8374 | 0.578 | 5.306 | 5.293 | -0.013 | -0.103 | 0.570 | 0.699 | Unannotated | NA | NA |
| metab_812 | Unannotated feature | pos | 94.0456 | 3.081 | 3.906 | 3.928 | 0.022 | 0.103 | 0.570 | 0.699 | Unannotated | NA | NA |
| metab_5152 | Prenol | pos | 104.1074 | 7.293 | 4.839 | 4.887 | 0.048 | 0.103 | 0.570 | 0.699 | Tentative | NA | NA |
| metab_3052 | Unannotated feature | pos | 153.1071 | 4.078 | 3.977 | 3.932 | -0.045 | 0.103 | 0.570 | 0.699 | Unannotated | NA | NA |
| metab_6998 | Unannotated feature | neg | 132.8996 | 5.731 | 4.486 | 4.496 | 0.010 | 0.103 | 0.570 | 0.699 | Unannotated | NA | NA |
| metab_639 | Phenylalanylhydroxyproline | pos | 279.1340 | 2.249 | 4.065 | 4.243 | 0.178 | 0.103 | 0.570 | 0.699 | Tentative | NA | NA |
| metab_9377 | 2,5-Dihydroxybenzoate | neg | 153.0188 | 3.546 | 4.742 | 4.722 | -0.020 | -0.103 | 0.570 | 0.699 | Tentative | NA | NA |
| metab_6545 | Unannotated feature | neg | 131.0438 | 4.597 | 4.458 | 4.521 | 0.063 | 0.103 | 0.570 | 0.699 | Unannotated | NA | NA |
| metab_7639 | Unannotated feature | neg | 269.9070 | 7.961 | 5.921 | 5.927 | 0.006 | 0.103 | 0.570 | 0.699 | Unannotated | NA | NA |
| metab_6895 | UK-47265 | neg | 383.0451 | 5.651 | 3.690 | 3.558 | -0.132 | 0.103 | 0.570 | 0.699 | Tentative | NA | NA |
| metab_3098 | DL-2-Aminooctanoic acid | pos | 160.1333 | 3.846 | 5.095 | 5.035 | -0.060 | -0.103 | 0.570 | 0.699 | Strong putative | NA | NA |
| metab_9469 | Unannotated feature | neg | 345.9637 | 3.301 | 4.521 | 4.506 | -0.015 | -0.099 | 0.586 | 0.713 | Unannotated | NA | NA |
| metab_9487 | Methyl salicylate | neg | 151.0395 | 3.266 | 3.773 | 3.786 | 0.013 | 0.099 | 0.586 | 0.713 | Strong putative | NA | NA |
| metab_3823 | Unannotated feature | pos | 169.6419 | 1.274 | 4.759 | 4.771 | 0.012 | 0.099 | 0.586 | 0.713 | Unannotated | NA | NA |
| metab_1370 | Unannotated feature | pos | 682.4840 | 5.910 | 6.324 | 6.314 | -0.010 | -0.099 | 0.586 | 0.713 | Unannotated | NA | NA |
| metab_8899 | Unannotated feature | neg | 93.0338 | 5.139 | 3.594 | 3.620 | 0.026 | 0.099 | 0.586 | 0.713 | Unannotated | NA | NA |
| metab_5198 | Unannotated feature | neg | 865.8273 | 0.534 | 3.999 | 3.938 | -0.061 | -0.099 | 0.586 | 0.713 | Unannotated | NA | NA |
| metab_1271 | Unannotated feature | pos | 284.3313 | 5.855 | 4.330 | 4.391 | 0.061 | 0.099 | 0.586 | 0.713 | Unannotated | NA | NA |
| metab_10589 | Unannotated feature | neg | 129.9751 | 0.613 | 3.597 | 3.484 | -0.113 | -0.099 | 0.586 | 0.713 | Unannotated | NA | NA |
| metab_7982 | Unannotated feature | neg | 309.1747 | 7.085 | 4.843 | 4.811 | -0.032 | -0.099 | 0.586 | 0.713 | Unannotated | NA | NA |
| metab_10671 | Unannotated feature | neg | 643.6237 | 0.582 | 4.709 | 4.717 | 0.008 | -0.099 | 0.586 | 0.713 | Unannotated | NA | NA |
| metab_8028 | Unannotated feature | neg | 160.0260 | 2.890 | 4.725 | 4.731 | 0.006 | 0.099 | 0.586 | 0.713 | Unannotated | NA | NA |
| metab_752 | Eupatilin | pos | 408.1055 | 2.749 | 5.406 | 5.393 | -0.012 | -0.099 | 0.586 | 0.713 | Tentative | NA | NA |
| metab_11288 | Unannotated feature | neg | 568.3633 | 6.697 | 7.026 | 7.006 | -0.020 | -0.099 | 0.586 | 0.713 | Unannotated | NA | NA |
| metab_7450 | AB-Chminaca | neg | 393.1726 | 7.124 | 5.206 | 5.193 | -0.012 | -0.099 | 0.586 | 0.713 | Tentative | NA | NA |
| metab_3574 | Unannotated feature | pos | 432.2803 | 3.205 | 4.847 | 4.879 | 0.031 | -0.099 | 0.586 | 0.713 | Unannotated | NA | NA |
| metab_10866 | Unannotated feature | neg | 535.7377 | 0.582 | 4.861 | 4.912 | 0.051 | 0.099 | 0.586 | 0.713 | Unannotated | NA | NA |
| metab_10970 | Unannotated feature | neg | 338.8897 | 0.505 | 4.088 | 4.159 | 0.072 | 0.099 | 0.586 | 0.713 | Unannotated | NA | NA |
| metab_8258 | Unannotated feature | neg | 367.0496 | 5.898 | 4.324 | 4.450 | 0.126 | 0.099 | 0.586 | 0.713 | Unannotated | NA | NA |
| metab_327 | Unannotated feature | pos | 752.7904 | 0.567 | 5.309 | 5.292 | -0.017 | -0.099 | 0.586 | 0.713 | Unannotated | NA | NA |
| metab_9054 | Unannotated feature | neg | 130.0404 | 4.597 | 5.632 | 5.707 | 0.075 | 0.099 | 0.586 | 0.713 | Unannotated | NA | NA |
| metab_274 | Unannotated feature | pos | 123.0919 | 0.721 | 3.723 | 3.756 | 0.032 | 0.099 | 0.586 | 0.713 | Unannotated | NA | NA |
| metab_7529 | Unannotated feature | neg | 152.9780 | 7.170 | 4.391 | 4.381 | -0.010 | -0.099 | 0.586 | 0.713 | Unannotated | NA | NA |
| metab_5358 | Unannotated feature | neg | 148.9492 | 0.587 | 3.677 | 3.626 | -0.051 | 0.099 | 0.586 | 0.713 | Unannotated | NA | NA |
| metab_5355 | Unannotated feature | neg | 677.6175 | 0.582 | 4.445 | 4.423 | -0.022 | -0.099 | 0.586 | 0.713 | Unannotated | NA | NA |
| metab_1723 | Unannotated feature | pos | 94.0455 | 7.316 | 4.698 | 4.694 | -0.003 | -0.099 | 0.586 | 0.713 | Unannotated | NA | NA |
| metab_1592 | 1-Acyl-sn-glycero-3-phosphocholine | pos | 566.3215 | 6.567 | 6.100 | 6.097 | -0.003 | -0.099 | 0.586 | 0.713 | Tentative | NA | NA |
| metab_1411 | Unannotated feature | pos | 512.8714 | 5.933 | 4.624 | 4.601 | -0.023 | -0.099 | 0.586 | 0.713 | Unannotated | NA | NA |
| metab_5865 | Unannotated feature | neg | 560.8096 | 0.570 | 5.508 | 5.508 | -0.000 | -0.099 | 0.586 | 0.713 | Unannotated | NA | NA |
| metab_114 | Unannotated feature | pos | 171.0630 | 0.541 | 4.077 | 4.077 | -0.000 | 0.099 | 0.586 | 0.713 | Unannotated | NA | NA |
| metab_4697 | Unannotated feature | pos | 556.8598 | 0.567 | 5.563 | 5.576 | 0.013 | -0.099 | 0.586 | 0.713 | Unannotated | NA | NA |
| metab_8419 | Unannotated feature | neg | 307.8565 | 5.779 | 4.291 | 4.333 | 0.042 | 0.099 | 0.586 | 0.713 | Unannotated | NA | NA |
| metab_8328 | Glycocholate | neg | 464.3025 | 5.834 | 5.373 | 5.441 | 0.068 | 0.099 | 0.586 | 0.713 | Moderate putative | NA | NA |
| metab_8933 | Unannotated feature | neg | 194.0469 | 4.985 | 4.029 | 4.010 | -0.020 | -0.099 | 0.586 | 0.713 | Unannotated | NA | NA |
| metab_503 | Unannotated feature | pos | 98.5124 | 1.910 | 5.198 | 5.203 | 0.005 | -0.099 | 0.586 | 0.713 | Unannotated | NA | NA |
| metab_8546 | Unannotated feature | neg | 238.0777 | 5.699 | 6.308 | 6.241 | -0.067 | -0.099 | 0.586 | 0.713 | Unannotated | NA | NA |
| metab_2540 | Cardiolipin | pos | 668.4694 | 5.871 | 6.062 | 6.063 | 0.001 | -0.099 | 0.586 | 0.713 | Tentative | NA | NA |
| metab_8566 | Unannotated feature | neg | 146.9654 | 5.691 | 6.654 | 6.659 | 0.005 | 0.099 | 0.586 | 0.713 | Unannotated | NA | NA |
| metab_4224 | Unannotated feature | pos | 191.9775 | 0.614 | 4.163 | 4.160 | -0.003 | -0.099 | 0.586 | 0.713 | Unannotated | NA | NA |
| metab_7746 | Unannotated feature | neg | 330.8284 | 7.181 | 4.499 | 4.523 | 0.024 | 0.099 | 0.586 | 0.713 | Unannotated | NA | NA |
| metab_9742 | Unannotated feature | neg | 209.9190 | 2.740 | 5.313 | 5.324 | 0.011 | 0.099 | 0.586 | 0.713 | Unannotated | NA | NA |
| metab_10710 | Unannotated feature | neg | 593.6964 | 0.578 | 4.306 | 4.288 | -0.018 | 0.099 | 0.586 | 0.713 | Unannotated | NA | NA |
| metab_8483 | Unannotated feature | neg | 218.9969 | 3.224 | 6.041 | 5.838 | -0.203 | 0.099 | 0.586 | 0.713 | Unannotated | NA | NA |
| metab_4410 | Unannotated feature | pos | 829.8101 | 0.559 | 4.443 | 4.463 | 0.020 | 0.099 | 0.586 | 0.713 | Unannotated | NA | NA |
| metab_9329 | Unannotated feature | neg | 233.0127 | 3.702 | 4.773 | 4.360 | -0.414 | 0.099 | 0.586 | 0.713 | Unannotated | NA | NA |
| metab_257 | Unannotated feature | pos | 105.0743 | 0.683 | 3.843 | 3.937 | 0.094 | 0.099 | 0.586 | 0.713 | Unannotated | NA | NA |
| metab_7659 | Unannotated feature | neg | 181.9374 | 7.301 | 5.346 | 5.343 | -0.004 | -0.099 | 0.586 | 0.713 | Unannotated | NA | NA |
| metab_2860 | Unannotated feature | pos | 243.0613 | 5.221 | 3.954 | 3.971 | 0.016 | 0.099 | 0.586 | 0.713 | Unannotated | NA | NA |
| metab_11373 | Unannotated feature | neg | 143.0344 | 0.469 | 4.043 | 4.037 | -0.006 | -0.099 | 0.586 | 0.713 | Unannotated | NA | NA |
| metab_9792 | Unannotated feature | neg | 170.9023 | 2.557 | 4.552 | 4.549 | -0.003 | -0.099 | 0.586 | 0.713 | Unannotated | NA | NA |
| metab_8118 | Unannotated feature | neg | 151.0256 | 1.994 | 4.343 | 4.241 | -0.102 | -0.095 | 0.602 | 0.726 | Unannotated | NA | NA |
| metab_3374 | Xi-4-Hydroxy-4-methyl-2-cyclohexen-1-one | pos | 109.0651 | 2.529 | 3.071 | 3.051 | -0.020 | 0.095 | 0.602 | 0.726 | Tentative | NA | NA |
| metab_2212 | 17-hydroxyprogesterone caproate | pos | 483.2485 | 6.296 | 5.435 | 5.447 | 0.013 | -0.095 | 0.602 | 0.726 | Tentative | NA | NA |
| metab_4904 | Unannotated feature | pos | 163.0278 | 0.449 | 5.729 | 5.726 | -0.003 | -0.095 | 0.602 | 0.726 | Unannotated | NA | NA |
| metab_4917 | Unannotated feature | pos | 446.6706 | 0.598 | 5.357 | 5.327 | -0.030 | -0.095 | 0.602 | 0.726 | Unannotated | NA | NA |
| metab_5045 | Unannotated feature | pos | 388.2301 | 0.861 | 4.549 | 4.628 | 0.079 | 0.095 | 0.602 | 0.726 | Unannotated | NA | NA |
| metab_2320 | Unannotated feature | pos | 664.3455 | 6.026 | 4.621 | 4.685 | 0.064 | -0.095 | 0.602 | 0.726 | Unannotated | NA | NA |
| metab_1662 | Unannotated feature | pos | 641.5114 | 7.215 | 5.195 | 5.207 | 0.013 | 0.095 | 0.602 | 0.726 | Unannotated | NA | NA |
| metab_2282 | 1-Acyl-sn-glycero-3-phosphocholine | pos | 544.3393 | 6.118 | 7.809 | 7.805 | -0.003 | -0.095 | 0.602 | 0.726 | Tentative | NA | NA |
| metab_3486 | Unannotated feature | pos | 124.0759 | 2.234 | 3.640 | 3.625 | -0.015 | 0.095 | 0.602 | 0.726 | Unannotated | NA | NA |
| metab_7615 | Unannotated feature | neg | 233.9106 | 7.301 | 5.694 | 5.700 | 0.006 | 0.095 | 0.602 | 0.726 | Unannotated | NA | NA |
| metab_7483 | Unannotated feature | neg | 758.8682 | 7.140 | 5.304 | 5.300 | -0.004 | 0.095 | 0.602 | 0.726 | Unannotated | NA | NA |
| metab_5904 | Unannotated feature | neg | 85.0036 | 2.523 | 4.230 | 4.252 | 0.022 | 0.095 | 0.602 | 0.726 | Unannotated | NA | NA |
| metab_3128 | Unannotated feature | pos | 270.1546 | 2.389 | 3.854 | 3.843 | -0.012 | -0.095 | 0.602 | 0.726 | Unannotated | NA | NA |
| metab_7623 | Unannotated feature | neg | 196.5664 | 7.517 | 5.202 | 5.190 | -0.012 | -0.095 | 0.602 | 0.726 | Unannotated | NA | NA |
| metab_9049 | Unannotated feature | neg | 260.0546 | 4.620 | 3.621 | 3.545 | -0.076 | -0.095 | 0.602 | 0.726 | Unannotated | NA | NA |
| metab_2195 | 2-Pyrrolidineacetic acid | pos | 162.1125 | 6.311 | 4.788 | 4.770 | -0.018 | -0.095 | 0.602 | 0.726 | Tentative | NA | NA |
| metab_475 | Unannotated feature | pos | 85.8776 | 1.809 | 4.623 | 4.636 | 0.013 | 0.095 | 0.602 | 0.726 | Unannotated | NA | NA |
| metab_486 | N-Acetylimidazole | pos | 128.0820 | 1.855 | 3.542 | 3.383 | -0.159 | 0.095 | 0.602 | 0.726 | Tentative | NA | NA |
| metab_4681 | Unannotated feature | pos | 646.9569 | 5.871 | 5.759 | 5.754 | -0.005 | -0.095 | 0.602 | 0.726 | Unannotated | NA | NA |
| metab_491 | Unannotated feature | pos | 70.0132 | 1.879 | 3.986 | 4.001 | 0.015 | 0.095 | 0.602 | 0.726 | Unannotated | NA | NA |
| metab_9521 | Emtricitabine | neg | 268.0177 | 3.224 | 5.055 | 5.030 | -0.025 | -0.095 | 0.602 | 0.726 | Tentative | NA | NA |
| metab_783 | Statine | pos | 158.1176 | 2.942 | 4.984 | 5.009 | 0.025 | 0.095 | 0.602 | 0.726 | Tentative | NA | NA |
| metab_4739 | Unannotated feature | pos | 269.2263 | 6.172 | 5.327 | 5.350 | 0.023 | 0.095 | 0.602 | 0.726 | Unannotated | NA | NA |
| metab_4699 | Unannotated feature | pos | 549.8897 | 5.910 | 5.355 | 5.312 | -0.043 | -0.095 | 0.602 | 0.726 | Unannotated | NA | NA |
| metab_10663 | Unannotated feature | neg | 834.5399 | 0.582 | 4.507 | 4.532 | 0.025 | 0.095 | 0.602 | 0.726 | Unannotated | NA | NA |
| metab_6029 | Sesamol | neg | 183.0296 | 2.961 | 3.854 | 3.935 | 0.081 | 0.095 | 0.602 | 0.726 | Tentative | NA | NA |
| metab_7845 | Unannotated feature | neg | 772.3261 | 7.148 | 4.520 | 4.515 | -0.005 | -0.095 | 0.602 | 0.726 | Unannotated | NA | NA |
| metab_9341 | Phenylpyruvate | neg | 163.0396 | 3.666 | 3.719 | 3.741 | 0.022 | 0.095 | 0.602 | 0.726 | Strong putative | NA | NA |
| metab_7888 | Unannotated feature | neg | 787.8474 | 7.132 | 4.282 | 4.234 | -0.048 | -0.095 | 0.602 | 0.726 | Unannotated | NA | NA |
| metab_3536 | Unannotated feature | pos | 79.0548 | 2.088 | 4.579 | 4.556 | -0.024 | -0.095 | 0.602 | 0.726 | Unannotated | NA | NA |
| metab_10530 | Unannotated feature | neg | 644.6186 | 0.621 | 4.854 | 4.736 | -0.119 | -0.095 | 0.602 | 0.726 | Unannotated | NA | NA |
| metab_1981 | Unannotated feature | pos | 654.2965 | 7.293 | 5.167 | 5.144 | -0.023 | -0.095 | 0.602 | 0.726 | Unannotated | NA | NA |
| metab_10494 | Aciclovir | neg | 260.0547 | 0.636 | 4.284 | 4.248 | -0.036 | -0.095 | 0.602 | 0.726 | Tentative | NA | NA |
| metab_3307 | Unannotated feature | pos | 125.1075 | 2.749 | 4.909 | 4.797 | -0.112 | -0.095 | 0.602 | 0.726 | Unannotated | NA | NA |
| metab_7772 | Unannotated feature | neg | 136.9092 | 7.173 | 4.894 | 4.905 | 0.011 | 0.095 | 0.602 | 0.726 | Unannotated | NA | NA |
| metab_11244 | Unannotated feature | neg | 480.7960 | 0.578 | 5.593 | 5.579 | -0.014 | -0.095 | 0.602 | 0.726 | Unannotated | NA | NA |
| metab_233 | Unannotated feature | pos | 104.9926 | 0.644 | 4.624 | 4.589 | -0.035 | -0.095 | 0.602 | 0.726 | Unannotated | NA | NA |
| metab_4405 | Unannotated feature | pos | 896.7966 | 0.559 | 5.066 | 5.084 | 0.018 | -0.095 | 0.602 | 0.726 | Unannotated | NA | NA |
| metab_9057 | Unannotated feature | neg | 196.0916 | 4.582 | 3.962 | 3.814 | -0.148 | -0.095 | 0.602 | 0.726 | Unannotated | NA | NA |
| metab_9720 | Unannotated feature | neg | 161.0100 | 2.812 | 4.294 | 4.293 | -0.002 | 0.095 | 0.602 | 0.726 | Unannotated | NA | NA |
| metab_8427 | Unannotated feature | neg | 413.2184 | 5.771 | 4.711 | 4.630 | -0.082 | -0.095 | 0.602 | 0.726 | Unannotated | NA | NA |
| metab_8609 | Unannotated feature | neg | 113.9938 | 5.667 | 4.620 | 4.625 | 0.005 | 0.095 | 0.602 | 0.726 | Unannotated | NA | NA |
| metab_9351 | Unannotated feature | neg | 224.9088 | 3.602 | 3.993 | 4.000 | 0.007 | 0.095 | 0.602 | 0.726 | Unannotated | NA | NA |
| metab_9827 | Unannotated feature | neg | 603.2110 | 2.510 | 4.495 | 4.463 | -0.032 | -0.095 | 0.602 | 0.726 | Unannotated | NA | NA |
| metab_8435 | Unannotated feature | neg | 335.0449 | 3.217 | 3.371 | 3.124 | -0.247 | -0.095 | 0.602 | 0.726 | Unannotated | NA | NA |
| metab_10248 | Unannotated feature | neg | 217.0829 | 0.857 | 3.788 | 3.739 | -0.049 | -0.095 | 0.602 | 0.726 | Unannotated | NA | NA |
| metab_8615 | Unannotated feature | neg | 316.9484 | 7.140 | 6.468 | 6.462 | -0.006 | -0.095 | 0.602 | 0.726 | Unannotated | NA | NA |
| metab_2051 | Unannotated feature | pos | 452.3695 | 7.238 | 5.448 | 5.439 | -0.009 | -0.095 | 0.602 | 0.726 | Unannotated | NA | NA |
| metab_1177 | Unannotated feature | pos | 438.0576 | 5.733 | 4.429 | 4.393 | -0.036 | -0.095 | 0.602 | 0.726 | Unannotated | NA | NA |
| metab_1245 | Unannotated feature | pos | 258.2792 | 5.832 | 4.787 | 4.802 | 0.014 | 0.095 | 0.602 | 0.726 | Unannotated | NA | NA |
| metab_2617 | Unannotated feature | pos | 165.1639 | 5.840 | 4.107 | 4.099 | -0.007 | 0.095 | 0.602 | 0.726 | Unannotated | NA | NA |
| metab_2654 | O-(17-Carboxyheptadecanoyl)carnitine | pos | 458.3476 | 5.793 | 4.755 | 4.700 | -0.054 | -0.095 | 0.602 | 0.726 | Tentative | NA | NA |
| metab_4166 | Ritipenem | pos | 271.0399 | 0.644 | 5.151 | 5.082 | -0.069 | -0.095 | 0.602 | 0.726 | Tentative | NA | NA |
| metab_8731 | Unannotated feature | neg | 166.0425 | 5.579 | 4.854 | 4.868 | 0.014 | 0.095 | 0.602 | 0.726 | Unannotated | NA | NA |
| metab_2132 | Unannotated feature | pos | 521.0841 | 6.745 | 5.316 | 5.321 | 0.005 | -0.095 | 0.602 | 0.726 | Unannotated | NA | NA |
| metab_3209 | 1-O-Isopentyl-3-O-octadec-2-enoyl glycerol | pos | 427.3782 | 6.420 | 8.061 | 8.055 | -0.006 | 0.095 | 0.602 | 0.726 | Tentative | NA | NA |
| metab_7687 | Unannotated feature | neg | 259.9341 | 7.229 | 4.347 | 4.362 | 0.015 | 0.095 | 0.602 | 0.726 | Unannotated | NA | NA |
| metab_7686 | Unannotated feature | neg | 287.9080 | 7.229 | 4.206 | 4.208 | 0.002 | 0.095 | 0.602 | 0.726 | Unannotated | NA | NA |
| metab_5750 | Unannotated feature | neg | 259.9993 | 1.855 | 3.783 | 4.314 | 0.530 | 0.095 | 0.602 | 0.726 | Unannotated | NA | NA |
| metab_6881 | Unannotated feature | neg | 171.1023 | 5.643 | 4.656 | 4.315 | -0.341 | -0.095 | 0.602 | 0.726 | Unannotated | NA | NA |
| metab_2866 | 6-Methylnicotinamide | pos | 195.0499 | 5.191 | 4.848 | 4.831 | -0.016 | 0.095 | 0.602 | 0.726 | Tentative | NA | NA |
| metab_4212 | Unannotated feature | pos | 148.0736 | 0.621 | 3.245 | 3.320 | 0.076 | 0.095 | 0.602 | 0.726 | Unannotated | NA | NA |
| metab_2728 | O-Decanoyl-L-carnitine | pos | 316.2482 | 5.725 | 6.419 | 6.445 | 0.026 | -0.095 | 0.602 | 0.726 | Strong putative | NA | NA |
| metab_9629 | Unannotated feature | neg | 118.0403 | 3.112 | 2.494 | 2.957 | 0.463 | 0.095 | 0.602 | 0.726 | Unannotated | NA | NA |
| metab_2750 | Unannotated feature | pos | 147.0224 | 1.956 | 4.202 | 4.169 | -0.033 | -0.095 | 0.602 | 0.726 | Unannotated | NA | NA |
| metab_3843 | Unannotated feature | pos | 225.1332 | 1.142 | 4.255 | 4.251 | -0.004 | -0.095 | 0.602 | 0.726 | Unannotated | NA | NA |
| metab_6806 | Unannotated feature | neg | 168.0395 | 5.579 | 4.331 | 4.352 | 0.022 | 0.095 | 0.602 | 0.726 | Unannotated | NA | NA |
| metab_33 | Unannotated feature | pos | 211.0713 | 1.956 | 3.637 | 3.614 | -0.022 | -0.091 | 0.619 | 0.740 | Unannotated | NA | NA |
| metab_7021 | (3a,5b,7a,12a)-24-[(carboxymethyl)amino]-1,12-dihydroxy-24-oxocholan-3-yl-b-D-Glucopyranosiduronic acid | neg | 622.3247 | 5.739 | 4.755 | 4.725 | -0.030 | -0.091 | 0.619 | 0.740 | Tentative | NA | NA |
| metab_6634 | Unannotated feature | neg | 163.0396 | 5.001 | 4.135 | 4.116 | -0.019 | -0.091 | 0.619 | 0.740 | Unannotated | NA | NA |
| metab_1635 | Unannotated feature | pos | 88.5694 | 7.130 | 3.170 | 3.215 | 0.044 | 0.091 | 0.619 | 0.740 | Unannotated | NA | NA |
| metab_2002 | Unannotated feature | pos | 1042.7997 | 7.285 | 4.741 | 4.671 | -0.070 | -0.091 | 0.619 | 0.740 | Unannotated | NA | NA |
| metab_1718 | Unannotated feature | pos | 189.0972 | 0.504 | 5.387 | 5.405 | 0.018 | -0.091 | 0.619 | 0.740 | Unannotated | NA | NA |
| metab_3336 | Unannotated feature | pos | 157.0361 | 2.622 | 3.811 | 3.772 | -0.039 | 0.091 | 0.619 | 0.740 | Unannotated | NA | NA |
| metab_11301 | Unannotated feature | neg | 184.8959 | 7.181 | 4.423 | 4.421 | -0.002 | -0.091 | 0.619 | 0.740 | Unannotated | NA | NA |
| metab_7565 | Unannotated feature | neg | 129.8666 | 7.197 | 4.145 | 4.144 | -0.001 | -0.091 | 0.619 | 0.740 | Unannotated | NA | NA |
| metab_5937 | Unannotated feature | neg | 326.1252 | 2.613 | 4.440 | 4.454 | 0.014 | 0.091 | 0.619 | 0.740 | Unannotated | NA | NA |
| metab_7595 | Unannotated feature | neg | 180.9186 | 7.245 | 4.133 | 4.135 | 0.002 | -0.091 | 0.619 | 0.740 | Unannotated | NA | NA |
| metab_9053 | Unannotated feature | neg | 181.0058 | 3.224 | 5.417 | 5.414 | -0.003 | -0.091 | 0.619 | 0.740 | Unannotated | NA | NA |
| metab_7579 | Unannotated feature | neg | 100.6543 | 7.213 | 3.448 | 3.427 | -0.021 | 0.091 | 0.619 | 0.740 | Unannotated | NA | NA |
| metab_1395 | Unannotated feature | pos | 177.0871 | 0.667 | 4.865 | 4.876 | 0.011 | 0.091 | 0.619 | 0.740 | Unannotated | NA | NA |
| metab_9171 | Unannotated feature | neg | 243.0601 | 4.235 | 3.746 | 3.614 | -0.133 | -0.091 | 0.619 | 0.740 | Unannotated | NA | NA |
| metab_7516 | Unannotated feature | neg | 128.9743 | 7.164 | 5.004 | 4.998 | -0.006 | -0.091 | 0.619 | 0.740 | Unannotated | NA | NA |
| metab_7418 | Unannotated feature | neg | 135.9703 | 7.109 | 4.962 | 4.961 | -0.002 | -0.091 | 0.619 | 0.740 | Unannotated | NA | NA |
| metab_9659 | Unannotated feature | neg | 172.8673 | 3.001 | 4.743 | 4.765 | 0.023 | 0.091 | 0.619 | 0.740 | Unannotated | NA | NA |
| metab_1663 | 2-Hydroxypropyl octanoate | pos | 225.1459 | 7.223 | 4.665 | 4.670 | 0.005 | 0.091 | 0.619 | 0.740 | Tentative | NA | NA |
| metab_5095 | Unannotated feature | pos | 120.9811 | 1.554 | 5.090 | 5.070 | -0.020 | -0.091 | 0.619 | 0.740 | Unannotated | NA | NA |
| metab_2235 | 2-Chlorooctadecanoic acid | pos | 341.2221 | 6.257 | 5.610 | 5.614 | 0.004 | 0.091 | 0.619 | 0.740 | Tentative | NA | NA |
| metab_5389 | Unannotated feature | neg | 578.5635 | 0.601 | 6.007 | 5.991 | -0.016 | -0.091 | 0.619 | 0.740 | Unannotated | NA | NA |
| metab_6055 | (S)-Mandelate | neg | 151.0395 | 3.072 | 4.035 | 4.066 | 0.030 | 0.091 | 0.619 | 0.740 | Strong putative | NA | NA |
| metab_7394 | Unannotated feature | neg | 311.9411 | 7.093 | 5.023 | 5.000 | -0.023 | -0.091 | 0.619 | 0.740 | Unannotated | NA | NA |
| metab_6138 | Unannotated feature | neg | 98.9384 | 3.217 | 4.621 | 4.616 | -0.005 | -0.091 | 0.619 | 0.740 | Unannotated | NA | NA |
| metab_3807 | Unannotated feature | pos | 480.2129 | 3.838 | 5.441 | 5.347 | -0.094 | -0.091 | 0.619 | 0.740 | Unannotated | NA | NA |
| metab_7710 | Unannotated feature | neg | 116.9278 | 7.213 | 6.248 | 6.255 | 0.007 | 0.091 | 0.619 | 0.740 | Unannotated | NA | NA |
| metab_7706 | Unannotated feature | neg | 132.8995 | 7.213 | 3.361 | 3.328 | -0.033 | -0.091 | 0.619 | 0.740 | Unannotated | NA | NA |
| metab_9588 | Unannotated feature | neg | 174.0153 | 3.168 | 3.562 | 3.394 | -0.168 | -0.091 | 0.619 | 0.740 | Unannotated | NA | NA |
| metab_3763 | Unannotated feature | pos | 608.3851 | 3.761 | 4.822 | 4.690 | -0.132 | -0.091 | 0.619 | 0.740 | Unannotated | NA | NA |
| metab_7658 | Unannotated feature | neg | 90.9869 | 7.309 | 3.877 | 3.861 | -0.016 | -0.091 | 0.619 | 0.740 | Unannotated | NA | NA |
| metab_973 | Tetracenomycin F1 | pos | 408.1055 | 4.309 | 5.310 | 5.423 | 0.113 | -0.091 | 0.619 | 0.740 | Tentative | NA | NA |
| metab_5337 | Unannotated feature | neg | 676.7274 | 0.570 | 5.313 | 5.310 | -0.003 | -0.091 | 0.619 | 0.740 | Unannotated | NA | NA |
| metab_8780 | Unannotated feature | neg | 329.9558 | 5.763 | 5.111 | 5.045 | -0.066 | -0.091 | 0.619 | 0.740 | Unannotated | NA | NA |
| metab_8761 | Unannotated feature | neg | 311.0059 | 5.543 | 5.178 | 5.127 | -0.051 | -0.091 | 0.619 | 0.740 | Unannotated | NA | NA |
| metab_402 | Unannotated feature | pos | 177.0871 | 1.157 | 4.252 | 4.314 | 0.062 | 0.091 | 0.619 | 0.740 | Unannotated | NA | NA |
| metab_8494 | Unannotated feature | neg | 196.8533 | 5.731 | 4.309 | 4.297 | -0.012 | -0.091 | 0.619 | 0.740 | Unannotated | NA | NA |
| metab_4348 | Unannotated feature | pos | 760.8215 | 0.567 | 5.602 | 5.594 | -0.008 | -0.091 | 0.619 | 0.740 | Unannotated | NA | NA |
| metab_1835 | Unannotated feature | pos | 143.0340 | 7.959 | 4.651 | 4.661 | 0.010 | 0.091 | 0.619 | 0.740 | Unannotated | NA | NA |
| metab_7291 | Unannotated feature | neg | 132.9227 | 6.188 | 7.196 | 7.199 | 0.003 | 0.091 | 0.619 | 0.740 | Unannotated | NA | NA |
| metab_7013 | Unannotated feature | neg | 244.8849 | 5.739 | 3.780 | 3.807 | 0.027 | 0.091 | 0.619 | 0.740 | Unannotated | NA | NA |
| metab_3095 | Unannotated feature | pos | 539.2696 | 3.846 | 5.139 | 5.126 | -0.012 | 0.091 | 0.619 | 0.740 | Unannotated | NA | NA |
| metab_9328 | Unannotated feature | neg | 89.5869 | 3.710 | 3.054 | 3.108 | 0.054 | 0.091 | 0.619 | 0.740 | Unannotated | NA | NA |
| metab_4685 | Unannotated feature | pos | 447.8216 | 5.886 | 4.839 | 4.897 | 0.058 | 0.091 | 0.619 | 0.740 | Unannotated | NA | NA |
| metab_4577 | Unannotated feature | pos | 129.0183 | 0.449 | 4.367 | 4.372 | 0.004 | 0.091 | 0.619 | 0.740 | Unannotated | NA | NA |
| metab_8378 | Unannotated feature | neg | 378.7793 | 5.803 | 4.992 | 4.981 | -0.011 | 0.091 | 0.619 | 0.740 | Unannotated | NA | NA |
| metab_9805 | Unannotated feature | neg | 615.1695 | 2.530 | 4.937 | 4.882 | -0.055 | -0.091 | 0.619 | 0.740 | Unannotated | NA | NA |
| metab_8410 | Unannotated feature | neg | 242.8878 | 5.787 | 4.784 | 4.777 | -0.007 | -0.091 | 0.619 | 0.740 | Unannotated | NA | NA |
| metab_458 | LL-2,6-Diaminoheptanedioate | pos | 155.0816 | 1.740 | 6.515 | 6.448 | -0.067 | -0.091 | 0.619 | 0.740 | Moderate putative | NA | NA |
| metab_1066 | 1-Pentanol | pos | 218.2115 | 5.130 | 5.435 | 5.468 | 0.033 | 0.091 | 0.619 | 0.740 | Tentative | NA | NA |
| metab_2522 | Unannotated feature | pos | 469.8348 | 5.886 | 5.029 | 4.954 | -0.075 | -0.091 | 0.619 | 0.740 | Unannotated | NA | NA |
| metab_2365 | Unannotated feature | pos | 652.9750 | 5.964 | 5.205 | 5.237 | 0.032 | 0.091 | 0.619 | 0.740 | Unannotated | NA | NA |
| metab_8730 | Unannotated feature | neg | 210.0327 | 5.579 | 5.035 | 5.047 | 0.012 | 0.091 | 0.619 | 0.740 | Unannotated | NA | NA |
| metab_8674 | Unannotated feature | neg | 258.9163 | 7.164 | 5.711 | 5.709 | -0.002 | 0.091 | 0.619 | 0.740 | Unannotated | NA | NA |
| metab_4934 | Unannotated feature | pos | 219.0266 | 0.621 | 6.159 | 6.144 | -0.016 | -0.091 | 0.619 | 0.740 | Unannotated | NA | NA |
| metab_5202 | Unannotated feature | neg | 350.0134 | 5.763 | 5.677 | 5.730 | 0.053 | 0.091 | 0.619 | 0.740 | Unannotated | NA | NA |
| metab_9877 | Unannotated feature | neg | 369.8445 | 2.371 | 5.720 | 5.738 | 0.018 | 0.091 | 0.619 | 0.740 | Unannotated | NA | NA |
| metab_9699 | (S)-Dihydroorotate | neg | 203.0322 | 2.890 | 5.084 | 5.087 | 0.002 | 0.091 | 0.619 | 0.740 | Tentative | NA | NA |
| metab_456 | 2-Amino-4-methylpyridine | pos | 109.0764 | 1.740 | 5.699 | 5.632 | -0.067 | -0.091 | 0.619 | 0.740 | Tentative | NA | NA |
| metab_10818 | Unannotated feature | neg | 871.8147 | 0.554 | 5.144 | 5.133 | -0.011 | 0.091 | 0.619 | 0.740 | Unannotated | NA | NA |
| metab_3557 | Unannotated feature | pos | 83.0799 | 2.073 | 3.496 | 3.485 | -0.011 | -0.086 | 0.636 | 0.752 | Unannotated | NA | NA |
| metab_680 | Unannotated feature | pos | 670.4675 | 6.288 | 5.390 | 5.460 | 0.069 | 0.086 | 0.636 | 0.752 | Unannotated | NA | NA |
| metab_2091 | 1-Aminocyclopentanecarboxylate | pos | 162.1125 | 7.184 | 4.528 | 4.502 | -0.026 | -0.086 | 0.636 | 0.752 | Tentative | NA | NA |
| metab_3607 | Unannotated feature | pos | 206.1387 | 2.011 | 3.802 | 3.779 | -0.023 | -0.086 | 0.636 | 0.752 | Unannotated | NA | NA |
| metab_7991 | Unannotated feature | neg | 682.3395 | 7.015 | 4.709 | 4.661 | -0.048 | -0.086 | 0.636 | 0.752 | Unannotated | NA | NA |
| metab_7945 | Unannotated feature | neg | 139.0064 | 7.109 | 5.426 | 5.427 | 0.001 | -0.086 | 0.636 | 0.752 | Unannotated | NA | NA |
| metab_1871 | Unannotated feature | pos | 111.0808 | 7.424 | 3.685 | 3.728 | 0.044 | 0.086 | 0.636 | 0.752 | Unannotated | NA | NA |
| metab_10014 | Unannotated feature | neg | 263.8825 | 1.847 | 4.351 | 4.480 | 0.129 | 0.086 | 0.636 | 0.752 | Unannotated | NA | NA |
| metab_1790 | Unannotated feature | pos | 99.5316 | 7.656 | 5.052 | 5.052 | 0.000 | 0.086 | 0.636 | 0.752 | Unannotated | NA | NA |
| metab_7208 | Unannotated feature | neg | 297.2076 | 5.930 | 3.447 | 3.745 | 0.298 | 0.086 | 0.636 | 0.752 | Unannotated | NA | NA |
| metab_7312 | Unannotated feature | neg | 100.2001 | 6.258 | 3.040 | 3.121 | 0.082 | -0.086 | 0.636 | 0.752 | Unannotated | NA | NA |
| metab_4011 | Unannotated feature | pos | 87.0810 | 0.815 | 3.632 | 3.627 | -0.005 | -0.086 | 0.636 | 0.752 | Unannotated | NA | NA |
| metab_5353 | Unannotated feature | neg | 602.7046 | 0.582 | 4.798 | 4.832 | 0.035 | 0.086 | 0.636 | 0.752 | Unannotated | NA | NA |
| metab_7808 | Unannotated feature | neg | 257.9195 | 7.164 | 4.373 | 4.381 | 0.008 | 0.086 | 0.636 | 0.752 | Unannotated | NA | NA |
| metab_5344 | Unannotated feature | neg | 102.9560 | 0.578 | 6.245 | 6.233 | -0.013 | -0.086 | 0.636 | 0.752 | Unannotated | NA | NA |
| metab_5332 | Unannotated feature | neg | 588.7903 | 0.570 | 4.610 | 4.519 | -0.091 | -0.086 | 0.636 | 0.752 | Unannotated | NA | NA |
| metab_11043 | Unannotated feature | neg | 190.2047 | 0.013 | 4.925 | 4.909 | -0.016 | -0.086 | 0.636 | 0.752 | Unannotated | NA | NA |
| metab_8918 | Unannotated feature | neg | 236.0930 | 5.077 | 4.382 | 4.419 | 0.037 | 0.086 | 0.636 | 0.752 | Unannotated | NA | NA |
| metab_928 | Unannotated feature | pos | 150.0584 | 0.854 | 6.125 | 6.149 | 0.025 | 0.086 | 0.636 | 0.752 | Unannotated | NA | NA |
| metab_8960 | Unannotated feature | neg | 266.9688 | 4.909 | 5.585 | 5.607 | 0.022 | 0.086 | 0.636 | 0.752 | Unannotated | NA | NA |
| metab_3983 | Unannotated feature | pos | 160.6921 | 0.831 | 4.143 | 4.152 | 0.010 | 0.086 | 0.636 | 0.752 | Unannotated | NA | NA |
| metab_3858 | Unannotated feature | pos | 169.0974 | 0.994 | 3.745 | 3.870 | 0.125 | 0.086 | 0.636 | 0.752 | Unannotated | NA | NA |
| metab_3837 | Urocanate | pos | 139.0503 | 1.188 | 4.225 | 4.175 | -0.050 | -0.086 | 0.636 | 0.752 | Strong putative | NA | NA |
| metab_7751 | Unannotated feature | neg | 172.8675 | 7.181 | 4.963 | 4.945 | -0.018 | -0.086 | 0.636 | 0.752 | Unannotated | NA | NA |
| metab_7748 | Unannotated feature | neg | 202.9412 | 7.181 | 4.516 | 4.543 | 0.027 | 0.086 | 0.636 | 0.752 | Unannotated | NA | NA |
| metab_8080 | Unannotated feature | neg | 202.0300 | 6.211 | 4.484 | 4.440 | -0.043 | -0.086 | 0.636 | 0.752 | Unannotated | NA | NA |
| metab_437 | Unannotated feature | pos | 232.1292 | 1.577 | 4.511 | 4.599 | 0.088 | 0.086 | 0.636 | 0.752 | Unannotated | NA | NA |
| metab_8096 | Unannotated feature | neg | 369.1748 | 6.188 | 6.091 | 6.042 | -0.050 | -0.086 | 0.636 | 0.752 | Unannotated | NA | NA |
| metab_4707 | Unannotated feature | pos | 514.3940 | 5.925 | 6.187 | 6.188 | 0.001 | -0.086 | 0.636 | 0.752 | Unannotated | NA | NA |
| metab_4634 | Unannotated feature | pos | 420.8849 | 0.567 | 5.885 | 5.888 | 0.002 | -0.086 | 0.636 | 0.752 | Unannotated | NA | NA |
| metab_8382 | Unannotated feature | neg | 221.8301 | 5.803 | 3.945 | 3.889 | -0.056 | -0.086 | 0.636 | 0.752 | Unannotated | NA | NA |
| metab_4627 | Unannotated feature | pos | 391.2299 | 5.626 | 6.195 | 6.180 | -0.014 | -0.086 | 0.636 | 0.752 | Unannotated | NA | NA |
| metab_8404 | Unannotated feature | neg | 413.2014 | 5.787 | 5.894 | 5.840 | -0.054 | -0.086 | 0.636 | 0.752 | Unannotated | NA | NA |
| metab_6755 | Unannotated feature | neg | 470.1476 | 5.498 | 3.785 | 3.804 | 0.019 | 0.086 | 0.636 | 0.752 | Unannotated | NA | NA |
| metab_3289 | Unannotated feature | pos | 144.1020 | 2.849 | 5.350 | 5.284 | -0.066 | -0.086 | 0.636 | 0.752 | Unannotated | NA | NA |
| metab_9492 | Unannotated feature | neg | 248.9222 | 3.260 | 4.672 | 4.668 | -0.004 | -0.086 | 0.636 | 0.752 | Unannotated | NA | NA |
| metab_8051 | Unannotated feature | neg | 255.2333 | 6.403 | 5.321 | 5.312 | -0.009 | 0.086 | 0.636 | 0.752 | Unannotated | NA | NA |
| metab_599 | Styrene oxide | pos | 121.0650 | 2.088 | 5.393 | 5.400 | 0.006 | 0.086 | 0.636 | 0.752 | Tentative | NA | NA |
| metab_8625 | Unannotated feature | neg | 724.8745 | 7.140 | 6.000 | 5.987 | -0.014 | -0.086 | 0.636 | 0.752 | Unannotated | NA | NA |
| metab_2584 | Unannotated feature | pos | 535.8742 | 5.855 | 4.618 | 4.632 | 0.015 | -0.086 | 0.636 | 0.752 | Unannotated | NA | NA |
| metab_874 | Unannotated feature | pos | 182.1115 | 3.584 | 3.931 | 3.888 | -0.043 | 0.086 | 0.636 | 0.752 | Unannotated | NA | NA |
| metab_4335 | Unannotated feature | pos | 100.0247 | 0.504 | 4.448 | 4.444 | -0.004 | -0.086 | 0.636 | 0.752 | Unannotated | NA | NA |
| metab_6189 | Unannotated feature | neg | 465.8793 | 0.652 | 4.431 | 4.566 | 0.135 | 0.086 | 0.636 | 0.752 | Unannotated | NA | NA |
| metab_4744 | Unannotated feature | pos | 371.3155 | 6.265 | 5.422 | 5.431 | 0.009 | 0.086 | 0.636 | 0.752 | Unannotated | NA | NA |
| metab_7116 | Neomenthol-glucuronide | neg | 331.1769 | 5.834 | 4.137 | 4.223 | 0.085 | 0.086 | 0.636 | 0.752 | Tentative | NA | NA |
| metab_1805 | Unannotated feature | pos | 170.0663 | 7.944 | 5.228 | 5.225 | -0.002 | 0.086 | 0.636 | 0.752 | Unannotated | NA | NA |
| metab_5431 | Unannotated feature | neg | 215.0901 | 0.644 | 4.289 | 4.261 | -0.028 | -0.086 | 0.636 | 0.752 | Unannotated | NA | NA |
| metab_10298 | Unannotated feature | neg | 211.8308 | 0.818 | 4.733 | 4.738 | 0.005 | 0.086 | 0.636 | 0.752 | Unannotated | NA | NA |
| metab_9649 | Unannotated feature | neg | 274.8731 | 3.057 | 4.322 | 4.280 | -0.042 | -0.086 | 0.636 | 0.752 | Unannotated | NA | NA |
| metab_751 | N-(3-Amino-3-oxopropyl)-L-valine | pos | 171.1131 | 2.749 | 5.603 | 5.505 | -0.098 | -0.086 | 0.636 | 0.752 | Tentative | NA | NA |
| metab_7485 | Unannotated feature | neg | 793.3641 | 7.140 | 4.467 | 4.484 | 0.017 | 0.086 | 0.636 | 0.752 | Unannotated | NA | NA |
| metab_10169 | Unannotated feature | neg | 94.6704 | 0.912 | 3.782 | 3.798 | 0.016 | 0.086 | 0.636 | 0.752 | Unannotated | NA | NA |
| metab_682 | Unannotated feature | pos | 229.1547 | 2.451 | 4.246 | 4.263 | 0.017 | 0.086 | 0.636 | 0.752 | Unannotated | NA | NA |
| metab_3418 | Unannotated feature | pos | 195.1129 | 2.405 | 5.579 | 5.557 | -0.022 | 0.086 | 0.636 | 0.752 | Unannotated | NA | NA |
| metab_1353 | Unannotated feature | pos | 412.3266 | 5.902 | 5.428 | 5.429 | 0.000 | -0.086 | 0.636 | 0.752 | Unannotated | NA | NA |
| metab_10752 | Unannotated feature | neg | 583.7763 | 0.570 | 4.007 | 3.986 | -0.021 | -0.086 | 0.636 | 0.752 | Unannotated | NA | NA |
| metab_5740 | Unannotated feature | neg | 117.9780 | 1.817 | 2.725 | 2.712 | -0.014 | -0.086 | 0.636 | 0.752 | Unannotated | NA | NA |
| metab_1695 | Unannotated feature | pos | 293.1698 | 7.285 | 3.914 | 3.841 | -0.073 | -0.086 | 0.636 | 0.752 | Unannotated | NA | NA |
| metab_3037 | Unannotated feature | pos | 202.0863 | 2.381 | 4.421 | 4.944 | 0.523 | 0.086 | 0.636 | 0.752 | Unannotated | NA | NA |
| metab_11002 | Unannotated feature | neg | 240.9109 | 0.023 | 5.005 | 4.999 | -0.006 | -0.086 | 0.636 | 0.752 | Unannotated | NA | NA |
| metab_10222 | Unannotated feature | neg | 230.0168 | 0.865 | 4.677 | 4.659 | -0.018 | -0.086 | 0.636 | 0.752 | Unannotated | NA | NA |
| metab_1708 | Unannotated feature | pos | 612.3245 | 7.293 | 4.700 | 4.658 | -0.042 | -0.086 | 0.636 | 0.752 | Unannotated | NA | NA |
| metab_818 | Unannotated feature | pos | 252.1755 | 3.136 | 5.040 | 5.033 | -0.008 | -0.086 | 0.636 | 0.752 | Unannotated | NA | NA |
| metab_7442 | Unannotated feature | neg | 212.9107 | 7.117 | 5.007 | 4.997 | -0.009 | -0.086 | 0.636 | 0.752 | Unannotated | NA | NA |
| metab_5880 | Unannotated feature | neg | 476.9596 | 2.469 | 4.686 | 4.716 | 0.030 | 0.086 | 0.636 | 0.752 | Unannotated | NA | NA |
| metab_10177 | Unannotated feature | neg | 282.7783 | 0.904 | 4.301 | 4.324 | 0.024 | 0.086 | 0.636 | 0.752 | Unannotated | NA | NA |
| metab_779 | Tyrosyl-Proline | pos | 243.1128 | 2.911 | 3.790 | 3.761 | -0.028 | 0.086 | 0.636 | 0.752 | Tentative | NA | NA |
| metab_10461 | Folpet | neg | 339.8983 | 0.652 | 4.929 | 4.943 | 0.014 | 0.086 | 0.636 | 0.752 | Tentative | NA | NA |
| metab_9828 | Unannotated feature | neg | 441.1396 | 2.510 | 4.437 | 4.400 | -0.037 | -0.086 | 0.636 | 0.752 | Unannotated | NA | NA |
| metab_9847 | Unannotated feature | neg | 241.9203 | 2.477 | 3.656 | 3.698 | 0.043 | -0.086 | 0.636 | 0.752 | Unannotated | NA | NA |
| metab_62 | Unannotated feature | pos | 142.9595 | 0.084 | 3.535 | 3.550 | 0.014 | 0.086 | 0.636 | 0.752 | Unannotated | NA | NA |
| metab_39 | N-Feruloylglycine | pos | 269.1131 | 1.964 | 4.322 | 4.180 | -0.142 | -0.086 | 0.636 | 0.752 | Tentative | NA | NA |
| metab_64 | Unannotated feature | pos | 139.1118 | 0.170 | 3.862 | 3.860 | -0.002 | -0.086 | 0.636 | 0.752 | Unannotated | NA | NA |
| metab_11171 | Unannotated feature | neg | 429.1776 | 5.803 | 4.509 | 4.618 | 0.109 | 0.082 | 0.653 | 0.765 | Unannotated | NA | NA |
| metab_2265 | Unannotated feature | pos | 102.6982 | 6.180 | 3.352 | 3.452 | 0.100 | 0.082 | 0.653 | 0.765 | Unannotated | NA | NA |
| metab_4111 | 4-Trimethylammoniobutanoate | pos | 146.1176 | 0.706 | 5.917 | 5.921 | 0.004 | 0.082 | 0.653 | 0.765 | Tentative | NA | NA |
| metab_2153 | 1-Acyl-sn-glycero-3-phosphocholine | pos | 548.3713 | 6.536 | 5.427 | 5.456 | 0.029 | -0.082 | 0.653 | 0.765 | Tentative | NA | NA |
| metab_10083 | Unannotated feature | neg | 235.0087 | 1.294 | 5.211 | 5.197 | -0.014 | -0.082 | 0.653 | 0.765 | Unannotated | NA | NA |
| metab_10793 | Unannotated feature | neg | 654.8490 | 0.562 | 4.411 | 4.397 | -0.014 | 0.082 | 0.653 | 0.765 | Unannotated | NA | NA |
| metab_10028 | Unannotated feature | neg | 118.9321 | 1.762 | 5.408 | 5.415 | 0.007 | 0.082 | 0.653 | 0.765 | Unannotated | NA | NA |
| metab_5225 | Unannotated feature | neg | 1012.7981 | 0.543 | 4.844 | 4.801 | -0.042 | -0.082 | 0.653 | 0.765 | Unannotated | NA | NA |
| metab_10042 | Unannotated feature | neg | 145.0361 | 1.510 | 4.057 | 4.056 | -0.001 | 0.082 | 0.653 | 0.765 | Unannotated | NA | NA |
| metab_6438 | Unannotated feature | neg | 158.0818 | 4.235 | 5.715 | 5.657 | -0.058 | -0.082 | 0.653 | 0.765 | Unannotated | NA | NA |
| metab_7874 | Unannotated feature | neg | 622.8931 | 7.140 | 5.074 | 5.095 | 0.021 | 0.082 | 0.653 | 0.765 | Unannotated | NA | NA |
| metab_7707 | Unannotated feature | neg | 121.9039 | 7.213 | 3.789 | 3.801 | 0.011 | 0.082 | 0.653 | 0.765 | Unannotated | NA | NA |
| metab_7386 | Unannotated feature | neg | 265.1484 | 7.070 | 5.950 | 5.952 | 0.002 | 0.082 | 0.653 | 0.765 | Moderate putative | NA | NA |
| metab_7621 | Unannotated feature | neg | 145.0501 | 2.119 | 4.353 | 4.379 | 0.026 | 0.082 | 0.653 | 0.765 | Unannotated | NA | NA |
| metab_800 | Fortimicin A | pos | 388.2539 | 3.019 | 4.788 | 4.778 | -0.009 | -0.082 | 0.653 | 0.765 | Tentative | NA | NA |
| metab_5871 | Unannotated feature | neg | 92.0498 | 2.445 | 3.601 | 3.680 | 0.078 | -0.082 | 0.653 | 0.765 | Unannotated | NA | NA |
| metab_5844 | Unannotated feature | neg | 204.8637 | 2.253 | 4.753 | 4.770 | 0.017 | 0.082 | 0.653 | 0.765 | Unannotated | NA | NA |
| metab_3460 | 3,4-Dihydroxyphenylpropanoate | pos | 165.0547 | 2.288 | 4.479 | 4.456 | -0.023 | -0.082 | 0.653 | 0.765 | Tentative | NA | NA |
| metab_7486 | Unannotated feature | neg | 834.8484 | 7.140 | 4.581 | 4.543 | -0.039 | -0.082 | 0.653 | 0.765 | Unannotated | NA | NA |
| metab_1680 | Unannotated feature | pos | 429.3187 | 7.261 | 4.373 | 4.357 | -0.017 | -0.082 | 0.653 | 0.765 | Unannotated | NA | NA |
| metab_3532 | Unannotated feature | pos | 148.0757 | 2.088 | 3.660 | 3.650 | -0.009 | -0.082 | 0.653 | 0.765 | Unannotated | NA | NA |
| metab_3521 | Glycyl-L-Proline | pos | 173.0922 | 2.126 | 5.043 | 5.002 | -0.041 | -0.082 | 0.653 | 0.765 | Moderate putative | NA | NA |
| metab_3465 | 5-(3-Pyridyl)-2-hydroxytetrahydrofuran | pos | 148.0758 | 2.280 | 4.023 | 3.940 | -0.082 | -0.082 | 0.653 | 0.765 | Tentative | NA | NA |
| metab_3611 | Unannotated feature | pos | 74.0970 | 2.011 | 4.460 | 4.459 | -0.001 | -0.082 | 0.653 | 0.765 | Unannotated | NA | NA |
| metab_5747 | Unannotated feature | neg | 171.0271 | 1.847 | 4.315 | 4.446 | 0.131 | 0.082 | 0.653 | 0.765 | Unannotated | NA | NA |
| metab_1693 | Unannotated feature | pos | 164.0918 | 7.285 | 4.191 | 4.171 | -0.019 | 0.082 | 0.653 | 0.765 | Unannotated | NA | NA |
| metab_7574 | Unannotated feature | neg | 146.9654 | 7.205 | 6.142 | 6.137 | -0.005 | -0.082 | 0.653 | 0.765 | Unannotated | NA | NA |
| metab_183 | Unannotated feature | pos | 964.7848 | 0.559 | 4.866 | 4.783 | -0.083 | -0.082 | 0.653 | 0.765 | Unannotated | NA | NA |
| metab_175 | Unannotated feature | pos | 768.8108 | 0.559 | 4.219 | 4.249 | 0.030 | 0.082 | 0.653 | 0.765 | Unannotated | NA | NA |
| metab_3399 | Unannotated feature | pos | 125.1075 | 2.849 | 5.054 | 5.028 | -0.026 | -0.082 | 0.653 | 0.765 | Unannotated | NA | NA |
| metab_10980 | Unannotated feature | neg | 247.8927 | 0.481 | 4.604 | 4.595 | -0.009 | -0.082 | 0.653 | 0.765 | Unannotated | NA | NA |
| metab_9444 | Unannotated feature | neg | 241.0469 | 3.710 | 4.367 | 4.532 | 0.166 | 0.082 | 0.653 | 0.765 | Unannotated | NA | NA |
| metab_10200 | Unannotated feature | neg | 251.9990 | 0.873 | 4.903 | 4.884 | -0.019 | -0.082 | 0.653 | 0.765 | Unannotated | NA | NA |
| metab_1340 | Unannotated feature | pos | 653.4633 | 5.894 | 6.248 | 6.288 | 0.040 | 0.082 | 0.653 | 0.765 | Unannotated | NA | NA |
| metab_9423 | Unannotated feature | neg | 364.9414 | 3.412 | 3.717 | 3.392 | -0.325 | -0.082 | 0.653 | 0.765 | Unannotated | NA | NA |
| metab_7093 | Unannotated feature | neg | 229.0542 | 5.811 | 4.078 | 3.818 | -0.260 | -0.082 | 0.653 | 0.765 | Unannotated | NA | NA |
| metab_1731 | Unannotated feature | pos | 311.1622 | 7.316 | 4.390 | 4.458 | 0.068 | 0.082 | 0.653 | 0.765 | Unannotated | NA | NA |
| metab_6960 | Unannotated feature | neg | 237.0699 | 5.699 | 3.888 | 3.937 | 0.049 | 0.082 | 0.653 | 0.765 | Unannotated | NA | NA |
| metab_8949 | Unannotated feature | neg | 131.0368 | 4.953 | 2.006 | 2.105 | 0.099 | 0.082 | 0.653 | 0.765 | Unannotated | NA | NA |
| metab_948 | Unannotated feature | pos | 323.2173 | 4.117 | 4.478 | 4.481 | 0.003 | -0.082 | 0.653 | 0.765 | Unannotated | NA | NA |
| metab_933 | Unannotated feature | pos | 357.2354 | 3.985 | 4.322 | 4.239 | -0.083 | -0.082 | 0.653 | 0.765 | Unannotated | NA | NA |
| metab_3853 | Unannotated feature | pos | 225.8811 | 1.033 | 4.646 | 4.645 | -0.001 | -0.082 | 0.653 | 0.765 | Unannotated | NA | NA |
| metab_7778 | Unannotated feature | neg | 408.8465 | 7.170 | 4.795 | 4.802 | 0.007 | 0.082 | 0.653 | 0.765 | Unannotated | NA | NA |
| metab_4741 | Unannotated feature | pos | 1013.6556 | 6.196 | 5.279 | 5.299 | 0.020 | 0.082 | 0.653 | 0.765 | Unannotated | NA | NA |
| metab_4702 | Unannotated feature | pos | 412.2846 | 5.863 | 5.346 | 5.410 | 0.064 | 0.082 | 0.653 | 0.765 | Moderate putative | NA | NA |
| metab_8523 | Unannotated feature | neg | 526.2498 | 5.707 | 4.965 | 4.947 | -0.019 | -0.082 | 0.653 | 0.765 | Unannotated | NA | NA |
| metab_4897 | Unannotated feature | pos | 186.0560 | 1.910 | 3.494 | 3.462 | -0.032 | -0.082 | 0.653 | 0.765 | Unannotated | NA | NA |
| metab_869 | Unannotated feature | pos | 210.1286 | 3.576 | 5.533 | 5.539 | 0.006 | 0.082 | 0.653 | 0.765 | Unannotated | NA | NA |
| metab_494 | Unannotated feature | pos | 419.2612 | 5.832 | 6.107 | 6.114 | 0.007 | -0.082 | 0.653 | 0.765 | Unannotated | NA | NA |
| metab_463 | Unannotated feature | pos | 290.2690 | 5.786 | 6.189 | 6.214 | 0.026 | 0.082 | 0.653 | 0.765 | Unannotated | NA | NA |
| metab_4529 | Unannotated feature | pos | 787.8409 | 0.534 | 5.048 | 5.007 | -0.042 | -0.082 | 0.653 | 0.765 | Unannotated | NA | NA |
| metab_4581 | Unannotated feature | pos | 137.0962 | 0.442 | 4.620 | 4.634 | 0.014 | 0.082 | 0.653 | 0.765 | Unannotated | NA | NA |
| metab_10939 | Unannotated feature | neg | 430.9290 | 0.530 | 4.398 | 4.487 | 0.090 | 0.082 | 0.653 | 0.765 | Unannotated | NA | NA |
| metab_4434 | Unannotated feature | pos | 214.0444 | 5.191 | 4.988 | 4.950 | -0.038 | 0.082 | 0.653 | 0.765 | Unannotated | NA | NA |
| metab_8400 | Unannotated feature | neg | 159.1022 | 5.795 | 4.781 | 4.812 | 0.031 | 0.082 | 0.653 | 0.765 | Unannotated | NA | NA |
| metab_2498 | Unannotated feature | pos | 498.8558 | 5.894 | 5.116 | 5.116 | -0.001 | -0.082 | 0.653 | 0.765 | Unannotated | NA | NA |
| metab_2459 | Unannotated feature | pos | 626.7740 | 5.910 | 4.024 | 4.089 | 0.065 | 0.082 | 0.653 | 0.765 | Unannotated | NA | NA |
| metab_9898 | Unannotated feature | neg | 287.7764 | 2.189 | 4.563 | 4.568 | 0.005 | 0.082 | 0.653 | 0.765 | Unannotated | NA | NA |
| metab_4938 | Unannotated feature | pos | 203.8508 | 0.629 | 5.606 | 5.593 | -0.012 | -0.082 | 0.653 | 0.765 | Unannotated | NA | NA |
| metab_3274 | Unannotated feature | pos | 204.1244 | 2.911 | 2.861 | 2.686 | -0.175 | -0.082 | 0.653 | 0.765 | Strong putative | NA | NA |
| metab_4584 | Unannotated feature | pos | 116.9665 | 0.434 | 4.227 | 4.218 | -0.009 | 0.082 | 0.653 | 0.765 | Unannotated | NA | NA |
| metab_7044 | Unannotated feature | neg | 540.7519 | 0.578 | 5.502 | 5.529 | 0.027 | 0.082 | 0.653 | 0.765 | Unannotated | NA | NA |
| metab_7039 | Unannotated feature | neg | 606.3300 | 5.755 | 4.145 | 4.259 | 0.114 | -0.082 | 0.653 | 0.765 | Unannotated | NA | NA |
| metab_9483 | Toluate | neg | 135.0445 | 3.274 | 4.322 | 4.367 | 0.044 | 0.078 | 0.670 | 0.779 | Strong putative | NA | NA |
| metab_2471 | Unannotated feature | pos | 679.8090 | 5.902 | 3.872 | 3.928 | 0.056 | -0.078 | 0.670 | 0.779 | Unannotated | NA | NA |
| metab_4431 | Unannotated feature | pos | 528.8794 | 0.559 | 3.786 | 3.831 | 0.046 | 0.078 | 0.670 | 0.779 | Unannotated | NA | NA |
| metab_5879 | Unannotated feature | neg | 132.0197 | 2.469 | 4.246 | 4.307 | 0.061 | 0.078 | 0.670 | 0.779 | Unannotated | NA | NA |
| metab_106 | Unannotated feature | pos | 584.9856 | 0.538 | 4.781 | 4.795 | 0.015 | -0.078 | 0.670 | 0.779 | Unannotated | NA | NA |
| metab_1113 | Unannotated feature | pos | 170.1540 | 5.581 | 3.715 | 3.709 | -0.006 | 0.078 | 0.670 | 0.779 | Unannotated | NA | NA |
| metab_9517 | Unannotated feature | neg | 124.0159 | 3.232 | 4.424 | 4.251 | -0.174 | 0.078 | 0.670 | 0.779 | Unannotated | NA | NA |
| metab_3397 | Unannotated feature | pos | 81.5211 | 2.475 | 5.552 | 5.545 | -0.007 | -0.078 | 0.670 | 0.779 | Unannotated | NA | NA |
| metab_7694 | Unannotated feature | neg | 314.0262 | 2.492 | 5.645 | 5.656 | 0.012 | 0.078 | 0.670 | 0.779 | Unannotated | NA | NA |
| metab_3643 | Unannotated feature | pos | 174.0817 | 1.956 | 4.504 | 4.515 | 0.011 | 0.078 | 0.670 | 0.779 | Unannotated | NA | NA |
| metab_4757 | Unannotated feature | pos | 1013.6537 | 6.544 | 6.508 | 6.540 | 0.032 | -0.078 | 0.670 | 0.779 | Unannotated | NA | NA |
| metab_11252 | Unannotated feature | neg | 144.9228 | 6.180 | 7.010 | 7.010 | -0.001 | -0.078 | 0.670 | 0.779 | Unannotated | NA | NA |
| metab_11293 | Unannotated feature | neg | 406.8473 | 7.170 | 5.123 | 5.121 | -0.002 | -0.078 | 0.670 | 0.779 | Unannotated | NA | NA |
| metab_3630 | Unannotated feature | pos | 476.3066 | 3.360 | 5.016 | 4.880 | -0.135 | -0.078 | 0.670 | 0.779 | Unannotated | NA | NA |
| metab_10145 | Unannotated feature | neg | 286.7726 | 0.928 | 4.554 | 4.512 | -0.042 | -0.078 | 0.670 | 0.779 | Unannotated | NA | NA |
| metab_2168 | Unannotated feature | pos | 470.4204 | 6.420 | 6.586 | 6.565 | -0.021 | -0.078 | 0.670 | 0.779 | Unannotated | NA | NA |
| metab_234 | Unannotated feature | pos | 117.7343 | 0.644 | 4.702 | 4.679 | -0.023 | -0.078 | 0.670 | 0.779 | Unannotated | NA | NA |
| metab_5076 | (+/-)-2-Hydroxy-4-(methylthio)butanoic acid | pos | 133.0319 | 0.854 | 5.281 | 5.258 | -0.023 | -0.078 | 0.670 | 0.779 | Tentative | NA | NA |
| metab_2330 | Unannotated feature | pos | 329.2319 | 6.010 | 4.390 | 4.446 | 0.055 | -0.078 | 0.670 | 0.779 | Unannotated | NA | NA |
| metab_5075 | Unannotated feature | pos | 138.0494 | 0.854 | 4.950 | 4.911 | -0.039 | -0.078 | 0.670 | 0.779 | Unannotated | NA | NA |
| metab_4168 | Unannotated feature | pos | 225.0345 | 0.644 | 5.217 | 5.215 | -0.002 | 0.078 | 0.670 | 0.779 | Unannotated | NA | NA |
| metab_1589 | Unannotated feature | pos | 455.4092 | 6.551 | 5.390 | 5.370 | -0.019 | -0.078 | 0.670 | 0.779 | Unannotated | NA | NA |
| metab_2864 | Aethallymal | pos | 241.0552 | 5.191 | 5.752 | 5.740 | -0.012 | 0.078 | 0.670 | 0.779 | Tentative | NA | NA |
| metab_8732 | Unannotated feature | neg | 143.0708 | 5.579 | 3.885 | 3.884 | -0.001 | -0.078 | 0.670 | 0.779 | Unannotated | NA | NA |
| metab_10578 | Unannotated feature | neg | 587.5750 | 0.613 | 4.363 | 4.335 | -0.028 | -0.078 | 0.670 | 0.779 | Unannotated | NA | NA |
| metab_4875 | Unannotated feature | pos | 189.0693 | 2.179 | 4.682 | 4.695 | 0.013 | 0.078 | 0.670 | 0.779 | Unannotated | NA | NA |
| metab_7743 | Unannotated feature | neg | 158.8463 | 7.189 | 4.281 | 4.267 | -0.014 | -0.078 | 0.670 | 0.779 | Unannotated | NA | NA |
| metab_1217 | (9S,10S)-9,10-Dihydroxyoctadecanoate | pos | 334.2952 | 5.786 | 4.883 | 4.918 | 0.035 | -0.078 | 0.670 | 0.779 | Tentative | NA | NA |
| metab_2366 | Unannotated feature | pos | 601.4401 | 5.964 | 6.138 | 6.142 | 0.004 | -0.078 | 0.670 | 0.779 | Unannotated | NA | NA |
| metab_6182 | Unannotated feature | neg | 124.0397 | 3.282 | 4.341 | 4.334 | -0.007 | -0.078 | 0.670 | 0.779 | Unannotated | NA | NA |
| metab_4258 | Unannotated feature | pos | 81.1491 | 0.598 | 4.012 | 4.051 | 0.038 | 0.078 | 0.670 | 0.779 | Unannotated | NA | NA |
| metab_5965 | Unannotated feature | neg | 244.8849 | 2.740 | 5.630 | 5.621 | -0.009 | -0.078 | 0.670 | 0.779 | Unannotated | NA | NA |
| metab_4909 | Unannotated feature | pos | 665.6345 | 0.583 | 4.438 | 4.499 | 0.060 | -0.078 | 0.670 | 0.779 | Unannotated | NA | NA |
| metab_8862 | Unannotated feature | neg | 201.0226 | 5.263 | 3.260 | 3.048 | -0.212 | -0.078 | 0.670 | 0.779 | Unannotated | NA | NA |
| metab_1848 | Unannotated feature | pos | 158.0489 | 7.874 | 4.375 | 4.372 | -0.003 | -0.078 | 0.670 | 0.779 | Unannotated | NA | NA |
| metab_10043 | Unannotated feature | neg | 172.8674 | 1.502 | 4.958 | 4.970 | 0.013 | 0.078 | 0.670 | 0.779 | Unannotated | NA | NA |
| metab_3949 | Unannotated feature | pos | 365.2033 | 0.846 | 3.979 | 4.105 | 0.126 | 0.078 | 0.670 | 0.779 | Unannotated | NA | NA |
| metab_3874 | Unannotated feature | pos | 177.0612 | 0.900 | 3.857 | 3.794 | -0.063 | -0.078 | 0.670 | 0.779 | Unannotated | NA | NA |
| metab_7289 | Unannotated feature | neg | 84.9379 | 6.188 | 5.701 | 5.708 | 0.007 | 0.078 | 0.670 | 0.779 | Unannotated | NA | NA |
| metab_10549 | Unannotated feature | neg | 404.6871 | 0.621 | 5.176 | 5.116 | -0.059 | -0.078 | 0.670 | 0.779 | Unannotated | NA | NA |
| metab_4050 | Histidylglycine | pos | 195.0878 | 0.768 | 4.751 | 4.742 | -0.009 | -0.078 | 0.670 | 0.779 | Tentative | NA | NA |
| metab_7839 | Unannotated feature | neg | 398.8870 | 7.153 | 5.073 | 5.087 | 0.014 | 0.078 | 0.670 | 0.779 | Unannotated | NA | NA |
| metab_1761 | Unannotated feature | pos | 272.9443 | 0.527 | 5.342 | 5.333 | -0.009 | -0.078 | 0.670 | 0.779 | Unannotated | NA | NA |
| metab_7876 | Unannotated feature | neg | 498.9147 | 7.140 | 4.336 | 4.323 | -0.013 | -0.078 | 0.670 | 0.779 | Unannotated | NA | NA |
| metab_5267 | Unannotated feature | neg | 727.8523 | 0.554 | 5.820 | 5.817 | -0.003 | -0.078 | 0.670 | 0.779 | Unannotated | NA | NA |
| metab_7944 | Unannotated feature | neg | 141.0200 | 7.109 | 4.091 | 4.129 | 0.038 | 0.078 | 0.670 | 0.779 | Unannotated | NA | NA |
| metab_6086 | Unannotated feature | neg | 331.9278 | 3.144 | 3.980 | 3.958 | -0.022 | -0.078 | 0.670 | 0.779 | Unannotated | NA | NA |
| metab_1616 | Unannotated feature | pos | 237.1458 | 7.007 | 5.023 | 5.027 | 0.004 | 0.078 | 0.670 | 0.779 | Unannotated | NA | NA |
| metab_5896 | Unannotated feature | neg | 402.9468 | 2.510 | 4.698 | 4.696 | -0.001 | -0.078 | 0.670 | 0.779 | Unannotated | NA | NA |
| metab_6663 | Unannotated feature | neg | 157.0865 | 5.123 | 4.322 | 4.325 | 0.003 | 0.078 | 0.670 | 0.779 | Unannotated | NA | NA |
| metab_9971 | Unannotated feature | neg | 189.8700 | 2.087 | 4.813 | 4.801 | -0.012 | -0.078 | 0.670 | 0.779 | Unannotated | NA | NA |
| metab_380 | Unannotated feature | pos | 149.9404 | 0.908 | 4.128 | 4.133 | 0.005 | -0.078 | 0.670 | 0.779 | Unannotated | NA | NA |
| metab_3313 | Unannotated feature | pos | 202.0443 | 2.644 | 7.997 | 7.997 | -0.000 | 0.078 | 0.670 | 0.779 | Unannotated | NA | NA |
| metab_3148 | Unannotated feature | pos | 164.1232 | 3.584 | 4.588 | 4.600 | 0.012 | 0.078 | 0.670 | 0.779 | Unannotated | NA | NA |
| metab_11332 | Unannotated feature | neg | 362.8761 | 7.170 | 5.180 | 5.190 | 0.010 | 0.078 | 0.670 | 0.779 | Unannotated | NA | NA |
| metab_5458 | Unannotated feature | neg | 158.8462 | 0.668 | 5.623 | 5.631 | 0.008 | 0.074 | 0.687 | 0.792 | Unannotated | NA | NA |
| metab_9173 | Unannotated feature | neg | 109.0288 | 4.227 | 4.192 | 4.178 | -0.013 | -0.074 | 0.687 | 0.792 | Unannotated | NA | NA |
| metab_4693 | Unannotated feature | pos | 520.8691 | 5.894 | 5.173 | 5.200 | 0.026 | -0.074 | 0.687 | 0.792 | Unannotated | NA | NA |
| metab_8531 | 9-Anthroic acid | neg | 259.0186 | 5.707 | 2.897 | 2.881 | -0.016 | 0.074 | 0.687 | 0.792 | Tentative | NA | NA |
| metab_9770 | Coformycin | neg | 627.2394 | 2.644 | 4.342 | 4.359 | 0.018 | -0.074 | 0.687 | 0.792 | Tentative | NA | NA |
| metab_7662 | Unannotated feature | neg | 103.9194 | 7.301 | 5.656 | 5.666 | 0.010 | 0.074 | 0.687 | 0.792 | Unannotated | NA | NA |
| metab_1653 | Unannotated feature | pos | 165.0547 | 0.387 | 4.917 | 4.923 | 0.006 | 0.074 | 0.687 | 0.792 | Unannotated | NA | NA |
| metab_9120 | Unannotated feature | neg | 109.9332 | 3.224 | 3.958 | 3.937 | -0.021 | -0.074 | 0.687 | 0.792 | Unannotated | NA | NA |
| metab_3042 | (R)-Mandelate | pos | 194.0813 | 4.124 | 4.781 | 4.811 | 0.029 | 0.074 | 0.687 | 0.792 | Tentative | NA | NA |
| metab_5351 | Unannotated feature | neg | 488.7381 | 0.582 | 4.732 | 4.761 | 0.029 | 0.074 | 0.687 | 0.792 | Unannotated | NA | NA |
| metab_5117 | alpha-Muricholate | pos | 426.3222 | 5.949 | 4.292 | 4.280 | -0.013 | -0.074 | 0.687 | 0.792 | Strong putative | NA | NA |
| metab_6433 | Unannotated feature | neg | 221.8426 | 4.199 | 4.361 | 4.354 | -0.007 | -0.074 | 0.687 | 0.792 | Unannotated | NA | NA |
| metab_6145 | Unannotated feature | neg | 327.9912 | 3.224 | 5.395 | 5.392 | -0.003 | -0.074 | 0.687 | 0.792 | Unannotated | NA | NA |
| metab_7978 | Unannotated feature | neg | 258.8721 | 7.093 | 5.144 | 5.145 | 0.001 | -0.074 | 0.687 | 0.792 | Unannotated | NA | NA |
| metab_10059 | Unannotated feature | neg | 92.0246 | 1.382 | 3.004 | 2.815 | -0.189 | -0.074 | 0.687 | 0.792 | Unannotated | NA | NA |
| metab_5426 | Unannotated feature | neg | 215.0230 | 0.636 | 3.432 | 3.459 | 0.027 | 0.074 | 0.687 | 0.792 | Unannotated | NA | NA |
| metab_6114 | Unannotated feature | neg | 278.9688 | 3.184 | 3.912 | 3.785 | -0.127 | -0.074 | 0.687 | 0.792 | Unannotated | NA | NA |
| metab_5373 | Unannotated feature | neg | 97.1842 | 0.594 | 3.379 | 3.395 | 0.016 | 0.074 | 0.687 | 0.792 | Unannotated | NA | NA |
| metab_8016 | Unannotated feature | neg | 157.0113 | 6.694 | 5.434 | 5.423 | -0.011 | -0.074 | 0.687 | 0.792 | Unannotated | NA | NA |
| metab_8078 | Unannotated feature | neg | 345.9095 | 6.234 | 5.044 | 4.963 | -0.081 | -0.074 | 0.687 | 0.792 | Unannotated | NA | NA |
| metab_1291 | Unannotated feature | pos | 544.8891 | 5.863 | 5.435 | 5.441 | 0.006 | -0.074 | 0.687 | 0.792 | Unannotated | NA | NA |
| metab_4871 | Pantothenate | pos | 220.1180 | 2.226 | 5.754 | 5.779 | 0.026 | -0.074 | 0.687 | 0.792 | Strong putative | NA | NA |
| metab_1590 | Unannotated feature | pos | 200.0473 | 2.637 | 8.467 | 8.471 | 0.004 | 0.074 | 0.687 | 0.792 | Unannotated | NA | NA |
| metab_8925 | Unannotated feature | neg | 146.9654 | 7.117 | 6.574 | 6.583 | 0.009 | 0.074 | 0.687 | 0.792 | Unannotated | NA | NA |
| metab_1832 | 5-Hydroxypentanoate | pos | 83.0497 | 7.975 | 4.453 | 4.460 | 0.007 | 0.074 | 0.687 | 0.792 | Tentative | NA | NA |
| metab_4226 | Unannotated feature | pos | 105.0037 | 0.504 | 5.548 | 5.588 | 0.039 | 0.074 | 0.687 | 0.792 | Unannotated | NA | NA |
| metab_9622 | Unannotated feature | neg | 223.0725 | 3.128 | 3.793 | 3.781 | -0.012 | -0.074 | 0.687 | 0.792 | Unannotated | NA | NA |
| metab_9902 | Unannotated feature | neg | 130.0503 | 2.167 | 4.392 | 4.363 | -0.029 | -0.074 | 0.687 | 0.792 | Unannotated | NA | NA |
| metab_971 | N-Acetyl-L-phenylalanine | pos | 208.0969 | 4.294 | 4.491 | 4.442 | -0.049 | 0.074 | 0.687 | 0.792 | Strong putative | NA | NA |
| metab_4778 | Unannotated feature | pos | 245.1747 | 7.200 | 6.190 | 6.193 | 0.003 | 0.074 | 0.687 | 0.792 | Unannotated | NA | NA |
| metab_9837 | Unannotated feature | neg | 118.0583 | 2.504 | 3.588 | 3.682 | 0.093 | 0.074 | 0.687 | 0.792 | Unannotated | NA | NA |
| metab_2391 | Unannotated feature | pos | 645.4665 | 5.941 | 6.191 | 6.207 | 0.017 | 0.074 | 0.687 | 0.792 | Unannotated | NA | NA |
| metab_4754 | Unannotated feature | pos | 287.2216 | 6.427 | 5.764 | 5.760 | -0.004 | 0.074 | 0.687 | 0.792 | Unannotated | NA | NA |
| metab_5726 | Unannotated feature | neg | 136.8938 | 1.548 | 4.952 | 4.967 | 0.015 | 0.074 | 0.687 | 0.792 | Unannotated | NA | NA |
| metab_3221 | Unannotated feature | pos | 125.9865 | 2.591 | 6.047 | 6.041 | -0.006 | -0.074 | 0.687 | 0.792 | Unannotated | NA | NA |
| metab_1063 | Unannotated feature | pos | 360.1376 | 0.737 | 5.201 | 5.134 | -0.067 | -0.074 | 0.687 | 0.792 | Unannotated | NA | NA |
| metab_1508 | Unannotated feature | pos | 540.8632 | 6.126 | 5.133 | 5.236 | 0.103 | 0.074 | 0.687 | 0.792 | Unannotated | NA | NA |
| metab_6424 | Unannotated feature | neg | 365.9218 | 4.175 | 4.240 | 4.281 | 0.041 | 0.074 | 0.687 | 0.792 | Unannotated | NA | NA |
| metab_2006 | Unannotated feature | pos | 453.1659 | 7.285 | 4.699 | 4.685 | -0.013 | 0.074 | 0.687 | 0.792 | Unannotated | NA | NA |
| metab_1086 | (2R,3R)-pinobanksin | pos | 254.0578 | 5.298 | 4.746 | 4.788 | 0.042 | 0.074 | 0.687 | 0.792 | Tentative | NA | NA |
| metab_9804 | Unannotated feature | neg | 147.0446 | 2.537 | 3.997 | 3.991 | -0.006 | -0.074 | 0.687 | 0.792 | Unannotated | NA | NA |
| metab_3551 | Unannotated feature | pos | 125.0902 | 2.073 | 4.847 | 4.855 | 0.008 | 0.074 | 0.687 | 0.792 | Unannotated | NA | NA |
| metab_11312 | Unannotated feature | neg | 165.8940 | 7.213 | 4.704 | 4.701 | -0.003 | -0.074 | 0.687 | 0.792 | Unannotated | NA | NA |
| metab_10660 | Unannotated feature | neg | 164.9091 | 0.587 | 4.660 | 4.634 | -0.026 | -0.074 | 0.687 | 0.792 | Unannotated | NA | NA |
| metab_6688 | Unannotated feature | neg | 189.0121 | 5.223 | 2.812 | 2.853 | 0.041 | 0.074 | 0.687 | 0.792 | Unannotated | NA | NA |
| metab_6674 | Unannotated feature | neg | 270.0180 | 5.154 | 4.572 | 4.598 | 0.026 | -0.074 | 0.687 | 0.792 | Unannotated | NA | NA |
| metab_1717 | Unannotated feature | pos | 79.9903 | 7.300 | 3.762 | 3.789 | 0.027 | 0.074 | 0.687 | 0.792 | Unannotated | NA | NA |
| metab_2669 | Sterebin E | pos | 356.2793 | 5.786 | 5.273 | 5.230 | -0.043 | -0.074 | 0.687 | 0.792 | Tentative | NA | NA |
| metab_58 | Unannotated feature | pos | 201.0083 | 0.025 | 5.452 | 5.452 | -0.001 | 0.074 | 0.687 | 0.792 | Unannotated | NA | NA |
| metab_10018 | Unannotated feature | neg | 343.9955 | 1.831 | 4.570 | 4.528 | -0.042 | -0.074 | 0.687 | 0.792 | Unannotated | NA | NA |
| metab_6170 | Unannotated feature | neg | 489.8630 | 0.652 | 4.088 | 4.125 | 0.037 | 0.074 | 0.687 | 0.792 | Unannotated | NA | NA |
| metab_6902 | Unannotated feature | neg | 286.1157 | 5.659 | 3.606 | 3.634 | 0.028 | 0.074 | 0.687 | 0.792 | Unannotated | NA | NA |
| metab_2649 | Hydroxytetradecadienyl-l-carnitine | pos | 370.2952 | 5.809 | 6.011 | 6.013 | 0.002 | -0.074 | 0.687 | 0.792 | Tentative | NA | NA |
| metab_10690 | Unannotated feature | neg | 292.8532 | 0.582 | 4.357 | 4.310 | -0.048 | -0.074 | 0.687 | 0.792 | Unannotated | NA | NA |
| metab_7885 | Unannotated feature | neg | 112.9848 | 7.140 | 6.594 | 6.601 | 0.007 | -0.074 | 0.687 | 0.792 | Unannotated | NA | NA |
| metab_2 | Unannotated feature | pos | 208.0693 | 0.768 | 5.146 | 5.077 | -0.069 | -0.074 | 0.687 | 0.792 | Unannotated | NA | NA |
| metab_5855 | Unannotated feature | neg | 386.8348 | 2.379 | 4.526 | 4.556 | 0.030 | 0.074 | 0.687 | 0.792 | Unannotated | NA | NA |
| metab_9139 | 1,2-Benzisoxazole | neg | 164.0348 | 4.306 | 3.394 | 3.286 | -0.108 | -0.074 | 0.687 | 0.792 | Tentative | NA | NA |
| metab_2066 | Unannotated feature | pos | 107.0859 | 7.215 | 3.659 | 3.666 | 0.007 | 0.074 | 0.687 | 0.792 | Unannotated | NA | NA |
| metab_9243 | Unannotated feature | neg | 278.8416 | 3.491 | 4.762 | 4.781 | 0.019 | -0.074 | 0.687 | 0.792 | Unannotated | NA | NA |
| metab_190 | Unannotated feature | pos | 590.8531 | 0.567 | 4.631 | 4.656 | 0.025 | 0.074 | 0.687 | 0.792 | Unannotated | NA | NA |
| metab_5611 | Unannotated feature | neg | 197.8587 | 0.904 | 3.983 | 4.075 | 0.091 | 0.069 | 0.705 | 0.807 | Unannotated | NA | NA |
| metab_6954 | Unannotated feature | neg | 94.0039 | 5.699 | 5.241 | 5.234 | -0.007 | -0.069 | 0.705 | 0.807 | Unannotated | NA | NA |
| metab_2507 | Unannotated feature | pos | 938.6590 | 5.886 | 4.654 | 4.710 | 0.056 | -0.069 | 0.705 | 0.807 | Unannotated | NA | NA |
| metab_4428 | Unannotated feature | pos | 582.8626 | 0.559 | 5.252 | 5.281 | 0.029 | 0.069 | 0.705 | 0.807 | Unannotated | NA | NA |
| metab_5816 | Unannotated feature | neg | 188.9126 | 0.128 | 5.824 | 5.822 | -0.002 | -0.069 | 0.705 | 0.807 | Unannotated | NA | NA |
| metab_9433 | Unannotated feature | neg | 179.0542 | 3.710 | 5.252 | 5.345 | 0.093 | 0.069 | 0.705 | 0.807 | Unannotated | NA | NA |
| metab_7473 | Unannotated feature | neg | 248.9607 | 7.140 | 6.164 | 6.165 | 0.001 | -0.069 | 0.705 | 0.807 | Unannotated | NA | NA |
| metab_1085 | Unannotated feature | pos | 94.0456 | 5.290 | 4.549 | 4.542 | -0.007 | -0.069 | 0.705 | 0.807 | Unannotated | NA | NA |
| metab_1301 | Unannotated feature | pos | 597.8990 | 5.871 | 4.335 | 4.344 | 0.009 | -0.069 | 0.705 | 0.807 | Unannotated | NA | NA |
| metab_1319 | Unannotated feature | pos | 97.0621 | 2.065 | 5.165 | 5.161 | -0.004 | 0.069 | 0.705 | 0.807 | Unannotated | NA | NA |
| metab_1322 | Unannotated feature | pos | 602.4269 | 5.886 | 6.040 | 6.042 | 0.002 | -0.069 | 0.705 | 0.807 | Unannotated | NA | NA |
| metab_1102 | Unannotated feature | pos | 147.0224 | 0.714 | 5.928 | 5.971 | 0.043 | -0.069 | 0.705 | 0.807 | Unannotated | NA | NA |
| metab_2730 | Unannotated feature | pos | 166.0725 | 1.956 | 4.055 | 4.038 | -0.018 | -0.069 | 0.705 | 0.807 | Unannotated | NA | NA |
| metab_7791 | Unannotated feature | neg | 174.9558 | 7.170 | 6.199 | 6.187 | -0.012 | -0.069 | 0.705 | 0.807 | Unannotated | NA | NA |
| metab_5094 | Triacetate | pos | 145.0496 | 1.538 | 6.887 | 6.893 | 0.006 | 0.069 | 0.705 | 0.807 | Tentative | NA | NA |
| metab_2172 | MG(0:0/18:1(11Z)/0:0) | pos | 357.2998 | 6.420 | 5.668 | 5.660 | -0.008 | 0.069 | 0.705 | 0.807 | Tentative | NA | NA |
| metab_7716 | Unannotated feature | neg | 800.4174 | 0.613 | 5.705 | 5.699 | -0.007 | 0.069 | 0.705 | 0.807 | Unannotated | NA | NA |
| metab_8050 | Unannotated feature | neg | 480.3105 | 6.403 | 6.964 | 6.981 | 0.017 | 0.069 | 0.705 | 0.807 | Unannotated | NA | NA |
| metab_4916 | Unannotated feature | pos | 154.8805 | 0.598 | 4.651 | 4.656 | 0.006 | 0.069 | 0.705 | 0.807 | Unannotated | NA | NA |
| metab_5376 | Unannotated feature | neg | 92.9107 | 0.598 | 4.173 | 4.174 | 0.001 | -0.069 | 0.705 | 0.807 | Unannotated | NA | NA |
| metab_2809 | Unannotated feature | pos | 290.2226 | 5.581 | 4.185 | 4.133 | -0.052 | -0.069 | 0.705 | 0.807 | Unannotated | NA | NA |
| metab_9607 | Unannotated feature | neg | 377.1622 | 3.851 | 4.461 | 4.480 | 0.018 | -0.069 | 0.705 | 0.807 | Unannotated | NA | NA |
| metab_4903 | 5-Deoxy-D-ribose | pos | 99.0445 | 0.457 | 5.574 | 5.572 | -0.003 | 0.069 | 0.705 | 0.807 | Tentative | NA | NA |
| metab_6205 | Unannotated feature | neg | 219.8455 | 3.340 | 3.977 | 3.978 | 0.000 | -0.069 | 0.705 | 0.807 | Unannotated | NA | NA |
| metab_3799 | Unannotated feature | pos | 74.0970 | 1.515 | 4.758 | 4.742 | -0.016 | -0.069 | 0.705 | 0.807 | Unannotated | NA | NA |
| metab_643 | Unannotated feature | pos | 119.0494 | 2.280 | 3.431 | 3.337 | -0.094 | -0.069 | 0.705 | 0.807 | Unannotated | NA | NA |
| metab_1290 | Unannotated feature | pos | 449.3272 | 5.863 | 5.510 | 5.525 | 0.014 | 0.069 | 0.705 | 0.807 | Unannotated | NA | NA |
| metab_11151 | Unannotated feature | neg | 253.1085 | 5.755 | 4.997 | 4.758 | -0.240 | 0.069 | 0.705 | 0.807 | Unannotated | NA | NA |
| metab_7741 | Unannotated feature | neg | 166.8327 | 7.189 | 3.968 | 3.958 | -0.009 | -0.069 | 0.705 | 0.807 | Unannotated | NA | NA |
| metab_7721 | Unannotated feature | neg | 229.9210 | 7.197 | 4.851 | 4.858 | 0.006 | 0.069 | 0.705 | 0.807 | Unannotated | NA | NA |
| metab_1841 | Unannotated feature | pos | 113.0600 | 7.944 | 4.686 | 4.679 | -0.007 | -0.069 | 0.705 | 0.807 | Unannotated | NA | NA |
| metab_8608 | Unannotated feature | neg | 155.0709 | 5.667 | 4.184 | 4.155 | -0.029 | -0.069 | 0.705 | 0.807 | Unannotated | NA | NA |
| metab_4252 | Unannotated feature | pos | 454.7022 | 0.598 | 4.439 | 4.471 | 0.032 | -0.069 | 0.705 | 0.807 | Unannotated | NA | NA |
| metab_1065 | Unannotated feature | pos | 230.0393 | 5.122 | 5.054 | 5.056 | 0.002 | 0.069 | 0.705 | 0.807 | Unannotated | NA | NA |
| metab_492 | Unannotated feature | pos | 124.0395 | 1.886 | 4.297 | 4.305 | 0.009 | 0.069 | 0.705 | 0.807 | Unannotated | NA | NA |
| metab_471 | 6-O-Methylguanine | pos | 166.0725 | 1.801 | 4.117 | 4.108 | -0.009 | -0.069 | 0.705 | 0.807 | Moderate putative | NA | NA |
| metab_7401 | Unannotated feature | neg | 101.9406 | 7.101 | 4.434 | 4.439 | 0.005 | -0.069 | 0.705 | 0.807 | Unannotated | NA | NA |
| metab_5921 | Unannotated feature | neg | 173.0452 | 2.549 | 3.975 | 3.980 | 0.005 | 0.069 | 0.705 | 0.807 | Unannotated | NA | NA |
| metab_4644 | Unannotated feature | pos | 614.8191 | 0.567 | 5.415 | 5.417 | 0.002 | -0.069 | 0.705 | 0.807 | Unannotated | NA | NA |
| metab_4772 | Unannotated feature | pos | 458.4203 | 6.296 | 6.154 | 6.109 | -0.045 | -0.069 | 0.705 | 0.807 | Unannotated | NA | NA |
| metab_468 | Unannotated feature | pos | 136.0142 | 1.794 | 3.654 | 3.623 | -0.031 | 0.069 | 0.705 | 0.807 | Unannotated | NA | NA |
| metab_9542 | Unannotated feature | neg | 82.9533 | 3.217 | 4.102 | 4.128 | 0.026 | -0.069 | 0.705 | 0.807 | Unannotated | NA | NA |
| metab_11008 | Unannotated feature | neg | 136.8625 | 0.019 | 4.465 | 4.470 | 0.005 | -0.069 | 0.705 | 0.807 | Unannotated | NA | NA |
| metab_4128 | Unannotated feature | pos | 148.0345 | 0.691 | 4.186 | 4.251 | 0.064 | -0.069 | 0.705 | 0.807 | Unannotated | NA | NA |
| metab_7426 | Unannotated feature | neg | 198.8642 | 7.109 | 5.557 | 5.547 | -0.009 | -0.069 | 0.705 | 0.807 | Unannotated | NA | NA |
| metab_9333 | Isopyridoxal | neg | 166.0505 | 3.694 | 3.238 | 3.225 | -0.013 | 0.069 | 0.705 | 0.807 | Tentative | NA | NA |
| metab_299 | Unannotated feature | pos | 84.0894 | 0.760 | 2.778 | 2.748 | -0.030 | -0.069 | 0.705 | 0.807 | Unannotated | NA | NA |
| metab_11358 | Unannotated feature | neg | 360.8111 | 0.019 | 4.866 | 4.864 | -0.002 | -0.069 | 0.705 | 0.807 | Unannotated | NA | NA |
| metab_7310 | Unannotated feature | neg | 248.9221 | 6.234 | 4.837 | 4.849 | 0.012 | 0.069 | 0.705 | 0.807 | Unannotated | NA | NA |
| metab_7929 | Unannotated feature | neg | 680.5033 | 0.621 | 5.832 | 5.840 | 0.007 | -0.069 | 0.705 | 0.807 | Unannotated | NA | NA |
| metab_10232 | Unannotated feature | neg | 167.0599 | 0.865 | 4.196 | 4.157 | -0.039 | -0.069 | 0.705 | 0.807 | Unannotated | NA | NA |
| metab_1992 | Unannotated feature | pos | 408.9169 | 7.293 | 4.482 | 4.482 | 0.001 | 0.069 | 0.705 | 0.807 | Unannotated | NA | NA |
| metab_11362 | Unannotated feature | neg | 216.8770 | 0.047 | 6.650 | 6.652 | 0.002 | -0.069 | 0.705 | 0.807 | Unannotated | NA | NA |
| metab_6815 | THIARABINE | neg | 240.0436 | 5.587 | 5.361 | 5.349 | -0.012 | -0.069 | 0.705 | 0.807 | Tentative | NA | NA |
| metab_1638 | Unannotated feature | pos | 634.4523 | 7.176 | 5.335 | 5.360 | 0.025 | 0.065 | 0.722 | 0.820 | Unannotated | NA | NA |
| metab_7443 | Unannotated feature | neg | 411.0945 | 7.117 | 4.702 | 4.699 | -0.003 | 0.065 | 0.722 | 0.820 | Unannotated | NA | NA |
| metab_1977 | Unannotated feature | pos | 838.6200 | 0.575 | 5.234 | 5.255 | 0.021 | -0.065 | 0.722 | 0.820 | Unannotated | NA | NA |
| metab_1646 | (6S)-Tetrahydro-L-biopterin | pos | 283.1513 | 7.200 | 5.494 | 5.505 | 0.011 | 0.065 | 0.722 | 0.820 | Tentative | NA | NA |
| metab_11370 | Unannotated feature | neg | 201.9322 | 0.390 | 4.908 | 4.910 | 0.002 | -0.065 | 0.722 | 0.820 | Unannotated | NA | NA |
| metab_332 | Unannotated feature | pos | 116.0835 | 0.823 | 4.234 | 4.250 | 0.016 | 0.065 | 0.722 | 0.820 | Unannotated | NA | NA |
| metab_1647 | Unannotated feature | pos | 458.4202 | 7.200 | 6.375 | 6.382 | 0.007 | 0.065 | 0.722 | 0.820 | Unannotated | NA | NA |
| metab_2228 | Menaquinone | pos | 419.2613 | 6.288 | 6.319 | 6.299 | -0.020 | -0.065 | 0.722 | 0.820 | Tentative | NA | NA |
| metab_7864 | Unannotated feature | neg | 792.8621 | 7.140 | 5.755 | 5.736 | -0.020 | -0.065 | 0.722 | 0.820 | Unannotated | NA | NA |
| metab_7868 | Unannotated feature | neg | 719.8605 | 7.140 | 4.364 | 4.328 | -0.036 | -0.065 | 0.722 | 0.820 | Unannotated | NA | NA |
| metab_292 | Unannotated feature | pos | 80.7484 | 0.753 | 3.267 | 3.289 | 0.022 | 0.065 | 0.722 | 0.820 | Unannotated | NA | NA |
| metab_7838 | Unannotated feature | neg | 452.2794 | 7.153 | 4.284 | 4.382 | 0.098 | 0.065 | 0.722 | 0.820 | Unannotated | NA | NA |
| metab_11049 | Unannotated feature | neg | 106.9430 | 0.013 | 5.286 | 5.283 | -0.003 | -0.065 | 0.722 | 0.820 | Unannotated | NA | NA |
| metab_6540 | Unannotated feature | neg | 357.9404 | 4.574 | 4.383 | 4.486 | 0.104 | 0.065 | 0.722 | 0.820 | Unannotated | NA | NA |
| metab_6551 | Unannotated feature | neg | 277.0445 | 4.627 | 3.578 | 3.469 | -0.109 | -0.065 | 0.722 | 0.820 | Unannotated | NA | NA |
| metab_4045 | Unannotated feature | pos | 241.0795 | 0.776 | 3.725 | 3.749 | 0.023 | 0.065 | 0.722 | 0.820 | Unannotated | NA | NA |
| metab_6154 | Unannotated feature | neg | 481.0261 | 3.232 | 4.867 | 4.855 | -0.013 | -0.065 | 0.722 | 0.820 | Unannotated | NA | NA |
| metab_1846 | Diisobutyl phthalate | pos | 279.1590 | 7.882 | 5.535 | 5.524 | -0.011 | -0.065 | 0.722 | 0.820 | Strong putative | NA | NA |
| metab_5080 | D-Erythrose | pos | 85.0289 | 0.854 | 5.495 | 5.512 | 0.017 | 0.065 | 0.722 | 0.820 | Tentative | NA | NA |
| metab_7798 | Unannotated feature | neg | 91.9204 | 7.170 | 3.920 | 3.928 | 0.008 | -0.065 | 0.722 | 0.820 | Unannotated | NA | NA |
| metab_7327 | Taurocholate | neg | 514.2854 | 6.339 | 4.782 | 4.833 | 0.051 | 0.065 | 0.722 | 0.820 | Tentative | NA | NA |
| metab_1674 | (3S,5R,6R,7E)-3,5,6-Trihydroxy-7-megastigmen-9-one | pos | 207.1380 | 0.411 | 5.502 | 5.502 | 0.000 | -0.065 | 0.722 | 0.820 | Tentative | NA | NA |
| metab_8813 | Unannotated feature | neg | 88.9873 | 5.457 | 4.681 | 4.685 | 0.004 | 0.065 | 0.722 | 0.820 | Unannotated | NA | NA |
| metab_1909 | Unannotated feature | pos | 333.9743 | 7.324 | 4.394 | 4.394 | 0.000 | -0.065 | 0.722 | 0.820 | Unannotated | NA | NA |
| metab_8733 | Unannotated feature | neg | 114.0090 | 5.579 | 3.538 | 3.583 | 0.045 | 0.065 | 0.722 | 0.820 | Unannotated | NA | NA |
| metab_7813 | Unannotated feature | neg | 156.9292 | 7.164 | 2.967 | 2.938 | -0.030 | -0.065 | 0.722 | 0.820 | Unannotated | NA | NA |
| metab_8714 | Unannotated feature | neg | 474.8350 | 7.164 | 5.011 | 5.007 | -0.004 | -0.065 | 0.722 | 0.820 | Unannotated | NA | NA |
| metab_5035 | Unannotated feature | pos | 257.9782 | 0.846 | 4.386 | 4.421 | 0.035 | -0.065 | 0.722 | 0.820 | Unannotated | NA | NA |
| metab_5173 | Unannotated feature | neg | 134.8645 | 0.522 | 5.501 | 5.498 | -0.004 | 0.065 | 0.722 | 0.820 | Unannotated | NA | NA |
| metab_7971 | Unannotated feature | neg | 101.2123 | 7.101 | 5.585 | 5.594 | 0.009 | 0.065 | 0.722 | 0.820 | Unannotated | NA | NA |
| metab_8082 | Unannotated feature | neg | 115.9413 | 6.203 | 4.343 | 4.329 | -0.013 | 0.065 | 0.722 | 0.820 | Unannotated | NA | NA |
| metab_10066 | Unannotated feature | neg | 83.0243 | 1.358 | 3.967 | 4.060 | 0.093 | 0.065 | 0.722 | 0.820 | Unannotated | NA | NA |
| metab_8565 | Unannotated feature | neg | 171.9806 | 5.691 | 4.672 | 4.681 | 0.009 | 0.065 | 0.722 | 0.820 | Unannotated | NA | NA |
| metab_217 | Unannotated feature | pos | 784.4394 | 0.614 | 5.015 | 5.009 | -0.006 | -0.065 | 0.722 | 0.820 | Unannotated | NA | NA |
| metab_8495 | Unannotated feature | neg | 184.9156 | 5.731 | 4.591 | 4.622 | 0.031 | 0.065 | 0.722 | 0.820 | Unannotated | NA | NA |
| metab_8545 | Unannotated feature | neg | 265.9508 | 5.699 | 3.501 | 3.476 | -0.025 | -0.065 | 0.722 | 0.820 | Unannotated | NA | NA |
| metab_11147 | Unannotated feature | neg | 437.0556 | 5.739 | 5.908 | 6.008 | 0.100 | -0.065 | 0.722 | 0.820 | Unannotated | NA | NA |
| metab_873 | Unannotated feature | pos | 137.1122 | 3.584 | 4.207 | 4.222 | 0.015 | 0.065 | 0.722 | 0.820 | Unannotated | NA | NA |
| metab_4239 | Unannotated feature | pos | 494.6465 | 0.606 | 4.579 | 4.599 | 0.020 | 0.065 | 0.722 | 0.820 | Unannotated | NA | NA |
| metab_11059 | Unannotated feature | neg | 228.0248 | 5.538 | 6.088 | 6.083 | -0.005 | 0.065 | 0.722 | 0.820 | Unannotated | NA | NA |
| metab_5395 | Unannotated feature | neg | 131.0456 | 0.613 | 4.188 | 4.170 | -0.018 | -0.065 | 0.722 | 0.820 | Unannotated | NA | NA |
| metab_9634 | Unannotated feature | neg | 193.8154 | 3.096 | 5.536 | 5.533 | -0.003 | -0.065 | 0.722 | 0.820 | Unannotated | NA | NA |
| metab_3567 | Unannotated feature | pos | 96.0558 | 2.065 | 4.499 | 4.486 | -0.013 | 0.065 | 0.722 | 0.820 | Unannotated | NA | NA |
| metab_7603 | Unannotated feature | neg | 129.9590 | 7.261 | 4.261 | 4.260 | -0.000 | 0.065 | 0.722 | 0.820 | Unannotated | NA | NA |
| metab_4688 | Unannotated feature | pos | 482.3607 | 5.886 | 6.179 | 6.192 | 0.013 | 0.065 | 0.722 | 0.820 | Unannotated | NA | NA |
| metab_10725 | Unannotated feature | neg | 103.2439 | 0.578 | 4.156 | 4.139 | -0.017 | -0.065 | 0.722 | 0.820 | Unannotated | NA | NA |
| metab_4658 | Unannotated feature | pos | 388.3058 | 5.809 | 4.692 | 4.767 | 0.075 | -0.065 | 0.722 | 0.820 | Unannotated | NA | NA |
| metab_4763 | 1-Acyl-sn-glycero-3-phosphocholine | pos | 508.3764 | 6.876 | 5.968 | 6.010 | 0.042 | -0.065 | 0.722 | 0.820 | Tentative | NA | NA |
| metab_3438 | Unannotated feature | pos | 187.1442 | 2.358 | 4.017 | 4.086 | 0.069 | 0.065 | 0.722 | 0.820 | Strong putative | NA | NA |
| metab_3207 | 7-Epi-12-hydroxyjasmonic acid | pos | 244.1544 | 3.190 | 4.865 | 4.827 | -0.038 | -0.065 | 0.722 | 0.820 | Tentative | NA | NA |
| metab_7490 | Unannotated feature | neg | 996.8245 | 7.140 | 5.016 | 5.021 | 0.005 | -0.065 | 0.722 | 0.820 | Unannotated | NA | NA |
| metab_7479 | Unannotated feature | neg | 656.8869 | 7.140 | 6.112 | 6.100 | -0.012 | -0.065 | 0.722 | 0.820 | Unannotated | NA | NA |
| metab_6715 | Unannotated feature | neg | 223.0460 | 5.346 | 3.961 | 3.999 | 0.038 | 0.065 | 0.722 | 0.820 | Unannotated | NA | NA |
| metab_11397 | (S)-Malate | neg | 133.0136 | 0.841 | 4.907 | 4.864 | -0.043 | -0.065 | 0.722 | 0.820 | Strong putative | NA | NA |
| metab_6955 | Unannotated feature | neg | 130.9916 | 5.699 | 4.072 | 4.116 | 0.044 | 0.065 | 0.722 | 0.820 | Unannotated | NA | NA |
| metab_8443 | Unannotated feature | neg | 288.0133 | 5.763 | 3.896 | 3.893 | -0.003 | -0.065 | 0.722 | 0.820 | Unannotated | NA | NA |
| metab_1687 | Unannotated feature | pos | 515.4242 | 7.269 | 5.178 | 5.178 | 0.000 | -0.065 | 0.722 | 0.820 | Unannotated | NA | NA |
| metab_515 | Unannotated feature | pos | 466.3166 | 5.863 | 4.685 | 4.771 | 0.086 | 0.065 | 0.722 | 0.820 | Unannotated | NA | NA |
| metab_3043 | L-Adrenaline | pos | 148.0758 | 4.124 | 4.196 | 4.153 | -0.043 | 0.065 | 0.722 | 0.820 | Tentative | NA | NA |
| metab_4539 | Unannotated feature | pos | 175.9543 | 0.527 | 4.254 | 4.249 | -0.004 | 0.065 | 0.722 | 0.820 | Unannotated | NA | NA |
| metab_4137 | Unannotated feature | pos | 260.9738 | 0.675 | 4.002 | 4.079 | 0.077 | 0.061 | 0.740 | 0.833 | Unannotated | NA | NA |
| metab_2648 | Unannotated feature | pos | 390.3577 | 5.809 | 4.320 | 4.368 | 0.048 | 0.061 | 0.740 | 0.833 | Unannotated | NA | NA |
| metab_341 | Unannotated feature | pos | 159.5773 | 0.831 | 4.199 | 4.215 | 0.016 | 0.061 | 0.740 | 0.833 | Unannotated | NA | NA |
| metab_7938 | Unannotated feature | neg | 161.9131 | 7.109 | 5.121 | 5.124 | 0.003 | 0.061 | 0.740 | 0.833 | Unannotated | NA | NA |
| metab_9557 | Unannotated feature | neg | 362.0832 | 3.198 | 5.023 | 4.999 | -0.024 | -0.061 | 0.740 | 0.833 | Unannotated | NA | NA |
| metab_7626 | Unannotated feature | neg | 91.2421 | 7.541 | 5.555 | 5.561 | 0.006 | 0.061 | 0.740 | 0.833 | Unannotated | NA | NA |
| metab_2922 | Unannotated feature | pos | 155.0453 | 4.892 | 3.701 | 3.737 | 0.036 | 0.061 | 0.740 | 0.833 | Unannotated | NA | NA |
| metab_4751 | Unannotated feature | pos | 226.9515 | 0.559 | 7.049 | 7.041 | -0.008 | -0.061 | 0.740 | 0.833 | Unannotated | NA | NA |
| metab_7648 | Unannotated feature | neg | 136.9091 | 7.527 | 4.031 | 4.028 | -0.003 | -0.061 | 0.740 | 0.833 | Unannotated | NA | NA |
| metab_5956 | Unannotated feature | neg | 261.8514 | 2.716 | 6.230 | 6.219 | -0.011 | -0.061 | 0.740 | 0.833 | Unannotated | NA | NA |
| metab_5588 | Unannotated feature | neg | 167.6158 | 0.865 | 5.122 | 5.140 | 0.019 | -0.061 | 0.740 | 0.833 | Unannotated | NA | NA |
| metab_5593 | Unannotated feature | neg | 89.0785 | 0.873 | 3.609 | 3.608 | -0.001 | 0.061 | 0.740 | 0.833 | Unannotated | NA | NA |
| metab_2362 | Unannotated feature | pos | 862.8020 | 0.559 | 4.155 | 4.266 | 0.110 | 0.061 | 0.740 | 0.833 | Unannotated | NA | NA |
| metab_1840 | Unannotated feature | pos | 200.9723 | 7.944 | 4.909 | 4.911 | 0.003 | 0.061 | 0.740 | 0.833 | Unannotated | NA | NA |
| metab_962 | Unannotated feature | pos | 759.8435 | 4.224 | 5.122 | 5.257 | 0.134 | 0.061 | 0.740 | 0.833 | Unannotated | NA | NA |
| metab_11073 | Unannotated feature | neg | 239.0409 | 5.579 | 5.174 | 5.183 | 0.009 | 0.061 | 0.740 | 0.833 | Unannotated | NA | NA |
| metab_7761 | Unannotated feature | neg | 120.9102 | 7.181 | 3.138 | 3.118 | -0.020 | -0.061 | 0.740 | 0.833 | Unannotated | NA | NA |
| metab_1967 | Unannotated feature | pos | 242.9255 | 7.300 | 4.608 | 4.609 | 0.000 | 0.061 | 0.740 | 0.833 | Unannotated | NA | NA |
| metab_6034 | Unannotated feature | neg | 91.0292 | 2.993 | 4.330 | 4.392 | 0.062 | 0.061 | 0.740 | 0.833 | Unannotated | NA | NA |
| metab_2298 | Unannotated feature | pos | 269.2588 | 6.080 | 4.017 | 3.963 | -0.054 | -0.061 | 0.740 | 0.833 | Unannotated | NA | NA |
| metab_591 | Unannotated feature | pos | 152.0319 | 0.861 | 5.733 | 5.739 | 0.006 | 0.061 | 0.740 | 0.833 | Unannotated | NA | NA |
| metab_5396 | Unannotated feature | neg | 138.8746 | 0.613 | 3.087 | 3.202 | 0.115 | -0.061 | 0.740 | 0.833 | Unannotated | NA | NA |
| metab_10666 | Unannotated feature | neg | 608.6306 | 0.587 | 4.718 | 4.716 | -0.002 | -0.061 | 0.740 | 0.833 | Unannotated | NA | NA |
| metab_2345 | LysoPE(0:0/18:2(9Z,12Z)) | pos | 500.2772 | 5.987 | 4.871 | 4.833 | -0.038 | 0.061 | 0.740 | 0.833 | Tentative | NA | NA |
| metab_9857 | Unannotated feature | neg | 164.0349 | 2.445 | 4.468 | 4.549 | 0.081 | -0.061 | 0.740 | 0.833 | Unannotated | NA | NA |
| metab_2652 | p-Menth-8-ene-1,2-diol | pos | 358.2950 | 5.801 | 5.287 | 5.396 | 0.109 | 0.061 | 0.740 | 0.833 | Tentative | NA | NA |
| metab_4039 | Unannotated feature | pos | 121.4900 | 0.784 | 4.674 | 4.681 | 0.007 | 0.061 | 0.740 | 0.833 | Unannotated | NA | NA |
| metab_7900 | Unannotated feature | neg | 447.1446 | 7.124 | 4.224 | 4.200 | -0.024 | -0.061 | 0.740 | 0.833 | Unannotated | NA | NA |
| metab_6788 | Unannotated feature | neg | 313.0029 | 5.543 | 4.637 | 4.581 | -0.056 | -0.061 | 0.740 | 0.833 | Unannotated | NA | NA |
| metab_3249 | Methylgeranate | pos | 214.1550 | 3.019 | 3.360 | 3.176 | -0.184 | -0.061 | 0.740 | 0.833 | Tentative | NA | NA |
| metab_1440 | Unannotated feature | pos | 477.3595 | 5.964 | 5.726 | 5.761 | 0.035 | 0.061 | 0.740 | 0.833 | Unannotated | NA | NA |
| metab_11185 | Unannotated feature | neg | 374.2457 | 5.842 | 5.299 | 5.299 | 0.000 | 0.061 | 0.740 | 0.833 | Unannotated | NA | NA |
| metab_5788 | Unannotated feature | neg | 90.9328 | 2.071 | 3.613 | 3.616 | 0.003 | 0.061 | 0.740 | 0.833 | Unannotated | NA | NA |
| metab_1738 | Unannotated feature | pos | 408.9192 | 0.534 | 5.157 | 5.064 | -0.093 | -0.061 | 0.740 | 0.833 | Unannotated | NA | NA |
| metab_7077 | Unannotated feature | neg | 373.8557 | 5.795 | 4.405 | 4.466 | 0.061 | 0.061 | 0.740 | 0.833 | Unannotated | NA | NA |
| metab_9829 | Unannotated feature | neg | 237.0907 | 2.510 | 4.425 | 4.439 | 0.013 | 0.061 | 0.740 | 0.833 | Unannotated | NA | NA |
| metab_2077 | 9,10,13-Trihydroxystearic acid | pos | 315.2529 | 7.200 | 6.015 | 6.008 | -0.008 | -0.061 | 0.740 | 0.833 | Tentative | NA | NA |
| metab_10723 | Unannotated feature | neg | 172.9409 | 0.578 | 5.012 | 4.991 | -0.021 | -0.061 | 0.740 | 0.833 | Unannotated | NA | NA |
| metab_3464 | 4-Hydroxy-2-quinolone | pos | 194.0812 | 2.280 | 4.452 | 4.438 | -0.014 | -0.061 | 0.740 | 0.833 | Tentative | NA | NA |
| metab_9842 | Unannotated feature | neg | 568.5805 | 0.621 | 5.498 | 5.515 | 0.017 | 0.061 | 0.740 | 0.833 | Unannotated | NA | NA |
| metab_2056 | Unannotated feature | pos | 435.3081 | 7.223 | 5.016 | 5.026 | 0.009 | -0.061 | 0.740 | 0.833 | Unannotated | NA | NA |
| metab_1114 | L-Octanoylcarnitine | pos | 288.2169 | 5.581 | 6.304 | 6.304 | 0.000 | -0.061 | 0.740 | 0.833 | Strong putative | NA | NA |
| metab_4673 | Unannotated feature | pos | 291.1492 | 5.863 | 2.968 | 3.015 | 0.047 | 0.061 | 0.740 | 0.833 | Unannotated | NA | NA |
| metab_10994 | Unannotated feature | neg | 214.8799 | 0.055 | 7.147 | 7.149 | 0.002 | -0.061 | 0.740 | 0.833 | Unannotated | NA | NA |
| metab_1601 | Diacylglycerol | pos | 641.5115 | 6.706 | 5.986 | 5.991 | 0.005 | -0.061 | 0.740 | 0.833 | Tentative | NA | NA |
| metab_8310 | Unannotated feature | neg | 118.9321 | 5.859 | 4.548 | 4.553 | 0.005 | 0.061 | 0.740 | 0.833 | Unannotated | NA | NA |
| metab_10697 | Unannotated feature | neg | 694.5713 | 0.587 | 5.186 | 5.227 | 0.041 | 0.061 | 0.740 | 0.833 | Unannotated | NA | NA |
| metab_1683 | Unannotated feature | pos | 367.1525 | 7.269 | 4.232 | 4.324 | 0.092 | 0.061 | 0.740 | 0.833 | Unannotated | NA | NA |
| metab_4527 | Unannotated feature | pos | 413.2661 | 0.538 | 5.224 | 5.222 | -0.003 | 0.061 | 0.740 | 0.833 | Unannotated | NA | NA |
| metab_3412 | Unannotated feature | pos | 182.0926 | 2.436 | 4.142 | 4.189 | 0.047 | 0.061 | 0.740 | 0.833 | Unannotated | NA | NA |
| metab_10407 | Unannotated feature | neg | 203.7990 | 0.692 | 6.010 | 6.027 | 0.017 | -0.061 | 0.740 | 0.833 | Unannotated | NA | NA |
| metab_1511 | Unannotated feature | pos | 413.3258 | 6.141 | 5.025 | 5.031 | 0.005 | 0.061 | 0.740 | 0.833 | Unannotated | NA | NA |
| metab_11170 | Unannotated feature | neg | 321.9239 | 5.763 | 4.418 | 4.403 | -0.015 | -0.061 | 0.740 | 0.833 | Unannotated | NA | NA |
| metab_2826 | Unannotated feature | pos | 279.1009 | 2.065 | 4.619 | 4.689 | 0.069 | 0.061 | 0.740 | 0.833 | Unannotated | NA | NA |
| metab_10534 | Unannotated feature | neg | 572.5727 | 0.621 | 4.646 | 4.587 | -0.059 | -0.061 | 0.740 | 0.833 | Unannotated | NA | NA |
| metab_2286 | Unannotated feature | pos | 732.7324 | 0.567 | 5.022 | 5.031 | 0.009 | -0.061 | 0.740 | 0.833 | Unannotated | NA | NA |
| metab_7825 | Unannotated feature | neg | 142.8961 | 7.156 | 4.199 | 4.167 | -0.033 | -0.061 | 0.740 | 0.833 | Unannotated | NA | NA |
| metab_6598 | Unannotated feature | neg | 218.8172 | 0.833 | 6.026 | 6.074 | 0.048 | 0.061 | 0.740 | 0.833 | Unannotated | NA | NA |
| metab_270 | Furfural | pos | 97.0288 | 0.714 | 4.987 | 4.986 | -0.001 | 0.061 | 0.740 | 0.833 | Moderate putative | NA | NA |
| metab_3276 | 4-Amino-1-butanol | pos | 72.0813 | 2.911 | 4.022 | 4.000 | -0.022 | -0.061 | 0.740 | 0.833 | Tentative | NA | NA |
| metab_3058 | Unannotated feature | pos | 193.1007 | 2.405 | 4.114 | 4.116 | 0.003 | 0.061 | 0.740 | 0.833 | Unannotated | NA | NA |
| metab_5348 | Unannotated feature | neg | 560.7008 | 0.578 | 4.661 | 4.771 | 0.110 | 0.057 | 0.758 | 0.846 | Unannotated | NA | NA |
| metab_7949 | Unannotated feature | neg | 118.0039 | 7.109 | 5.142 | 5.143 | 0.001 | 0.057 | 0.758 | 0.846 | Unannotated | NA | NA |
| metab_7432 | Unannotated feature | neg | 229.9012 | 7.109 | 4.913 | 4.922 | 0.009 | -0.057 | 0.758 | 0.846 | Unannotated | NA | NA |
| metab_2193 | Unannotated feature | pos | 446.7491 | 6.311 | 3.951 | 4.103 | 0.152 | -0.057 | 0.758 | 0.846 | Unannotated | NA | NA |
| metab_9357 | Unannotated feature | neg | 116.0247 | 3.578 | 3.889 | 3.703 | -0.186 | -0.057 | 0.758 | 0.846 | Unannotated | NA | NA |
| metab_9330 | Unannotated feature | neg | 181.0712 | 3.702 | 3.774 | 3.756 | -0.018 | -0.057 | 0.758 | 0.846 | Unannotated | NA | NA |
| metab_7814 | Unannotated feature | neg | 145.9412 | 7.164 | 4.108 | 4.093 | -0.014 | -0.057 | 0.758 | 0.846 | Unannotated | NA | NA |
| metab_8996 | Unannotated feature | neg | 206.0822 | 4.774 | 4.686 | 4.704 | 0.018 | 0.057 | 0.758 | 0.846 | Unannotated | NA | NA |
| metab_9043 | Unannotated feature | neg | 291.1357 | 3.260 | 3.840 | 4.008 | 0.168 | -0.057 | 0.758 | 0.846 | Unannotated | NA | NA |
| metab_3615 | Unannotated feature | pos | 95.0443 | 1.988 | 3.613 | 3.633 | 0.020 | -0.057 | 0.758 | 0.846 | Unannotated | NA | NA |
| metab_8193 | 9,10-DHOME | neg | 313.2391 | 5.985 | 4.889 | 4.837 | -0.051 | -0.057 | 0.758 | 0.846 | Tentative | NA | NA |
| metab_8536 | Unannotated feature | neg | 160.9906 | 7.109 | 5.956 | 5.954 | -0.002 | -0.057 | 0.758 | 0.846 | Unannotated | NA | NA |
| metab_8580 | Unannotated feature | neg | 145.0865 | 5.683 | 4.142 | 4.104 | -0.038 | 0.057 | 0.758 | 0.846 | Unannotated | NA | NA |
| metab_8467 | Unannotated feature | neg | 228.1607 | 5.747 | 4.179 | 4.196 | 0.017 | 0.057 | 0.758 | 0.846 | Unannotated | NA | NA |
| metab_2130 | Unannotated feature | pos | 614.3402 | 6.760 | 6.433 | 6.511 | 0.078 | 0.057 | 0.758 | 0.846 | Unannotated | NA | NA |
| metab_10778 | Unannotated feature | neg | 858.8087 | 0.562 | 4.590 | 4.613 | 0.023 | 0.057 | 0.758 | 0.846 | Unannotated | NA | NA |
| metab_8896 | Unannotated feature | neg | 232.0595 | 5.139 | 4.981 | 4.900 | -0.080 | 0.057 | 0.758 | 0.846 | Unannotated | NA | NA |
| metab_2144 | Unannotated feature | pos | 148.0969 | 6.606 | 5.075 | 5.086 | 0.011 | 0.057 | 0.758 | 0.846 | Unannotated | NA | NA |
| metab_2353 | Unannotated feature | pos | 936.6821 | 5.972 | 5.637 | 5.620 | -0.018 | 0.057 | 0.758 | 0.846 | Unannotated | NA | NA |
| metab_2119 | Unannotated feature | pos | 323.2944 | 6.976 | 4.551 | 4.543 | -0.009 | -0.057 | 0.758 | 0.846 | Unannotated | NA | NA |
| metab_10134 | Unannotated feature | neg | 295.8204 | 0.960 | 5.255 | 5.261 | 0.006 | 0.057 | 0.758 | 0.846 | Unannotated | NA | NA |
| metab_5489 | Unannotated feature | neg | 362.9416 | 0.530 | 5.019 | 4.987 | -0.033 | 0.057 | 0.758 | 0.846 | Unannotated | NA | NA |
| metab_2868 | Unannotated feature | pos | 131.0731 | 5.191 | 3.611 | 3.551 | -0.060 | 0.057 | 0.758 | 0.846 | Unannotated | NA | NA |
| metab_7767 | Unannotated feature | neg | 182.8964 | 7.173 | 4.972 | 4.972 | -0.000 | -0.057 | 0.758 | 0.846 | Unannotated | NA | NA |
| metab_10108 | Unannotated feature | neg | 566.5841 | 0.613 | 5.765 | 5.760 | -0.005 | -0.057 | 0.758 | 0.846 | Unannotated | NA | NA |
| metab_10924 | Unannotated feature | neg | 797.8401 | 0.534 | 4.441 | 4.424 | -0.017 | 0.057 | 0.758 | 0.846 | Unannotated | NA | NA |
| metab_5182 | Unannotated feature | neg | 588.6818 | 0.582 | 5.878 | 5.870 | -0.009 | -0.057 | 0.758 | 0.846 | Unannotated | NA | NA |
| metab_11058 | Unannotated feature | neg | 169.1027 | 4.167 | 4.419 | 4.413 | -0.006 | -0.057 | 0.758 | 0.846 | Unannotated | NA | NA |
| metab_935 | Unannotated feature | pos | 111.0808 | 4.009 | 3.458 | 3.407 | -0.050 | -0.057 | 0.758 | 0.846 | Unannotated | NA | NA |
| metab_7376 | Unannotated feature | neg | 265.9511 | 6.939 | 5.575 | 5.601 | 0.026 | 0.057 | 0.758 | 0.846 | Unannotated | NA | NA |
| metab_7365 | Unannotated feature | neg | 704.3383 | 6.690 | 5.221 | 5.151 | -0.070 | -0.057 | 0.758 | 0.846 | Unannotated | NA | NA |
| metab_3731 | Unannotated feature | pos | 86.0945 | 1.817 | 3.346 | 3.383 | 0.037 | 0.057 | 0.758 | 0.846 | Unannotated | NA | NA |
| metab_7347 | Unannotated feature | neg | 128.0347 | 6.497 | 5.147 | 5.154 | 0.007 | 0.057 | 0.758 | 0.846 | Unannotated | NA | NA |
| metab_3995 | 1,2,3-Trihydroxybenzene | pos | 127.0392 | 0.823 | 5.432 | 5.433 | 0.001 | -0.057 | 0.758 | 0.846 | Strong putative | NA | NA |
| metab_10320 | Unannotated feature | neg | 222.8112 | 0.786 | 5.548 | 5.577 | 0.029 | 0.057 | 0.758 | 0.846 | Unannotated | NA | NA |
| metab_10281 | Unannotated feature | neg | 292.8020 | 0.833 | 4.601 | 4.601 | 0.000 | -0.057 | 0.758 | 0.846 | Unannotated | NA | NA |
| metab_10278 | Unannotated feature | neg | 90.7646 | 0.841 | 4.188 | 4.169 | -0.019 | 0.057 | 0.758 | 0.846 | Unannotated | NA | NA |
| metab_8809 | Unannotated feature | neg | 394.8914 | 7.153 | 5.529 | 5.523 | -0.006 | -0.057 | 0.758 | 0.846 | Unannotated | NA | NA |
| metab_3966 | Unannotated feature | pos | 140.0683 | 0.846 | 5.516 | 5.520 | 0.004 | -0.057 | 0.758 | 0.846 | Unannotated | NA | NA |
| metab_4027 | Unannotated feature | pos | 223.1176 | 0.792 | 4.233 | 4.247 | 0.014 | 0.057 | 0.758 | 0.846 | Unannotated | NA | NA |
| metab_7907 | Unannotated feature | neg | 493.1344 | 7.117 | 4.615 | 4.698 | 0.083 | 0.057 | 0.758 | 0.846 | Unannotated | NA | NA |
| metab_7124 | Unannotated feature | neg | 397.1698 | 5.842 | 4.704 | 4.838 | 0.134 | 0.057 | 0.758 | 0.846 | Unannotated | NA | NA |
| metab_6439 | Unannotated feature | neg | 318.9244 | 4.235 | 4.628 | 4.669 | 0.040 | 0.057 | 0.758 | 0.846 | Unannotated | NA | NA |
| metab_10509 | Unannotated feature | neg | 344.7313 | 0.629 | 5.595 | 5.582 | -0.014 | -0.057 | 0.758 | 0.846 | Unannotated | NA | NA |
| metab_5758 | Unannotated feature | neg | 111.0192 | 1.900 | 4.142 | 4.132 | -0.010 | -0.057 | 0.758 | 0.846 | Unannotated | NA | NA |
| metab_8231 | Unannotated feature | neg | 283.7819 | 5.922 | 4.612 | 4.651 | 0.040 | 0.057 | 0.758 | 0.846 | Unannotated | NA | NA |
| metab_10724 | Unannotated feature | neg | 170.9439 | 0.578 | 5.503 | 5.497 | -0.006 | 0.057 | 0.758 | 0.846 | Unannotated | NA | NA |
| metab_4841 | Unannotated feature | pos | 828.5507 | 7.285 | 4.792 | 5.077 | 0.285 | -0.057 | 0.758 | 0.846 | Unannotated | NA | NA |
| metab_2420 | Unannotated feature | pos | 711.5045 | 5.925 | 6.007 | 6.011 | 0.003 | -0.057 | 0.758 | 0.846 | Unannotated | NA | NA |
| metab_9258 | Unannotated feature | neg | 155.0358 | 3.894 | 4.347 | 4.467 | 0.119 | 0.057 | 0.758 | 0.846 | Unannotated | NA | NA |
| metab_9259 | Unannotated feature | neg | 100.9328 | 6.188 | 7.885 | 7.879 | -0.005 | 0.057 | 0.758 | 0.846 | Unannotated | NA | NA |
| metab_2443 | Unannotated feature | pos | 463.3432 | 5.917 | 6.101 | 6.115 | 0.014 | -0.057 | 0.758 | 0.846 | Unannotated | NA | NA |
| metab_4660 | Unannotated feature | pos | 396.3108 | 5.817 | 5.237 | 5.209 | -0.027 | -0.057 | 0.758 | 0.846 | Unannotated | NA | NA |
| metab_5793 | Unannotated feature | neg | 92.0246 | 2.111 | 3.411 | 3.315 | -0.096 | -0.057 | 0.758 | 0.846 | Unannotated | NA | NA |
| metab_5836 | Unannotated feature | neg | 174.8636 | 2.189 | 4.217 | 4.220 | 0.003 | 0.057 | 0.758 | 0.846 | Unannotated | NA | NA |
| metab_10754 | Unannotated feature | neg | 656.6698 | 0.582 | 5.377 | 5.380 | 0.004 | 0.057 | 0.758 | 0.846 | Unannotated | NA | NA |
| metab_7491 | Unannotated feature | neg | 123.9013 | 7.148 | 3.876 | 3.874 | -0.002 | -0.057 | 0.758 | 0.846 | Unannotated | NA | NA |
| metab_3168 | 4-Chloro-L-phenylalanine | pos | 241.0738 | 3.468 | 6.478 | 6.467 | -0.010 | 0.057 | 0.758 | 0.846 | Tentative | NA | NA |
| metab_2025 | Unannotated feature | pos | 87.0670 | 7.277 | 3.499 | 3.477 | -0.022 | 0.057 | 0.758 | 0.846 | Unannotated | NA | NA |
| metab_6414 | Unannotated feature | neg | 169.0516 | 4.153 | 3.679 | 3.729 | 0.050 | 0.057 | 0.758 | 0.846 | Unannotated | NA | NA |
| metab_11321 | Unannotated feature | neg | 163.8969 | 7.213 | 5.216 | 5.210 | -0.006 | -0.057 | 0.758 | 0.846 | Unannotated | NA | NA |
| metab_9504 | Unannotated feature | neg | 567.9214 | 3.240 | 5.096 | 5.151 | 0.056 | 0.057 | 0.758 | 0.846 | Unannotated | NA | NA |
| metab_1276 | Cinnamic acid | pos | 166.0863 | 2.081 | 7.663 | 7.662 | -0.001 | -0.053 | 0.776 | 0.859 | Tentative | NA | NA |
| metab_6790 | Unannotated feature | neg | 413.8891 | 5.543 | 4.525 | 4.549 | 0.024 | 0.053 | 0.776 | 0.859 | Unannotated | NA | NA |
| metab_3140 | Skatole | pos | 132.0809 | 3.638 | 4.759 | 4.747 | -0.012 | 0.053 | 0.776 | 0.859 | Strong putative | NA | NA |
| metab_7533 | Unannotated feature | neg | 278.8416 | 2.302 | 5.821 | 5.823 | 0.002 | -0.053 | 0.776 | 0.859 | Unannotated | NA | NA |
| metab_11280 | Unannotated feature | neg | 296.8908 | 0.570 | 5.882 | 5.887 | 0.005 | -0.053 | 0.776 | 0.859 | Unannotated | NA | NA |
| metab_1062 | Unannotated feature | pos | 174.5249 | 5.122 | 3.697 | 3.638 | -0.059 | -0.053 | 0.776 | 0.859 | Unannotated | NA | NA |
| metab_1032 | 4-Hydroxyquinoline | pos | 146.0601 | 4.785 | 4.808 | 4.800 | -0.008 | -0.053 | 0.776 | 0.859 | Strong putative | NA | NA |
| metab_5411 | Unannotated feature | neg | 692.4835 | 0.621 | 4.944 | 4.966 | 0.021 | 0.053 | 0.776 | 0.859 | Unannotated | NA | NA |
| metab_7732 | Unannotated feature | neg | 205.9194 | 7.189 | 3.366 | 3.466 | 0.100 | 0.053 | 0.776 | 0.859 | Unannotated | NA | NA |
| metab_7730 | Unannotated feature | neg | 257.8196 | 7.189 | 4.827 | 4.819 | -0.008 | 0.053 | 0.776 | 0.859 | Unannotated | NA | NA |
| metab_5594 | Unannotated feature | neg | 172.8675 | 0.039 | 5.268 | 5.276 | 0.008 | -0.053 | 0.776 | 0.859 | Unannotated | NA | NA |
| metab_7635 | Unannotated feature | neg | 303.8873 | 7.969 | 5.676 | 5.677 | 0.001 | 0.053 | 0.776 | 0.859 | Unannotated | NA | NA |
| metab_160 | Unannotated feature | pos | 1045.7778 | 0.551 | 5.012 | 4.911 | -0.102 | -0.053 | 0.776 | 0.859 | Unannotated | NA | NA |
| metab_11292 | Unannotated feature | neg | 192.9286 | 7.170 | 5.933 | 5.935 | 0.002 | -0.053 | 0.776 | 0.859 | Unannotated | NA | NA |
| metab_1039 | Lumichrome | pos | 243.0878 | 4.869 | 3.879 | 3.653 | -0.227 | -0.053 | 0.776 | 0.859 | Moderate putative | NA | NA |
| metab_10651 | Unannotated feature | neg | 538.6652 | 0.587 | 5.056 | 5.043 | -0.014 | -0.053 | 0.776 | 0.859 | Unannotated | NA | NA |
| metab_10010 | Unannotated feature | neg | 173.0928 | 1.863 | 4.437 | 4.488 | 0.051 | -0.053 | 0.776 | 0.859 | Unannotated | NA | NA |
| metab_10005 | Unannotated feature | neg | 200.0562 | 1.886 | 2.584 | 2.524 | -0.059 | -0.053 | 0.776 | 0.859 | Unannotated | NA | NA |
| metab_7937 | Unannotated feature | neg | 174.9336 | 7.109 | 5.030 | 5.034 | 0.004 | -0.053 | 0.776 | 0.859 | Unannotated | NA | NA |
| metab_9993 | Unannotated feature | neg | 270.8100 | 1.951 | 4.473 | 4.476 | 0.002 | -0.053 | 0.776 | 0.859 | Unannotated | NA | NA |
| metab_10930 | Unannotated feature | neg | 532.7200 | 0.582 | 5.567 | 5.555 | -0.012 | -0.053 | 0.776 | 0.859 | Unannotated | NA | NA |
| metab_8046 | 3beta-Hydroxypregn-5-en-20-one sulfate | neg | 395.1905 | 6.453 | 4.927 | 4.922 | -0.004 | -0.053 | 0.776 | 0.859 | Moderate putative | NA | NA |
| metab_5098 | Unannotated feature | pos | 172.0969 | 1.515 | 4.765 | 4.733 | -0.032 | 0.053 | 0.776 | 0.859 | Unannotated | NA | NA |
| metab_10531 | Unannotated feature | neg | 632.5280 | 0.621 | 4.578 | 4.588 | 0.010 | 0.053 | 0.776 | 0.859 | Unannotated | NA | NA |
| metab_1410 | Unannotated feature | pos | 483.8506 | 5.933 | 4.925 | 5.010 | 0.086 | 0.053 | 0.776 | 0.859 | Unannotated | NA | NA |
| metab_5266 | Unannotated feature | neg | 190.9281 | 0.013 | 7.057 | 7.056 | -0.000 | 0.053 | 0.776 | 0.859 | Unannotated | NA | NA |
| metab_7886 | Unannotated feature | neg | 112.9627 | 7.140 | 3.588 | 3.594 | 0.006 | 0.053 | 0.776 | 0.859 | Unannotated | NA | NA |
| metab_7862 | Unannotated feature | neg | 826.8560 | 7.140 | 5.148 | 5.157 | 0.010 | -0.053 | 0.776 | 0.859 | Unannotated | NA | NA |
| metab_7913 | Unannotated feature | neg | 293.1798 | 7.117 | 5.472 | 5.470 | -0.002 | -0.053 | 0.776 | 0.859 | Unannotated | NA | NA |
| metab_10044 | Unannotated feature | neg | 184.9158 | 1.494 | 5.310 | 5.299 | -0.011 | -0.053 | 0.776 | 0.859 | Unannotated | NA | NA |
| metab_6006 | Unannotated feature | neg | 738.4626 | 0.621 | 5.351 | 5.298 | -0.053 | -0.053 | 0.776 | 0.859 | Unannotated | NA | NA |
| metab_7735 | Unannotated feature | neg | 191.8661 | 7.189 | 4.924 | 4.933 | 0.010 | 0.053 | 0.776 | 0.859 | Unannotated | NA | NA |
| metab_8117 | Unannotated feature | neg | 736.4658 | 0.613 | 5.635 | 5.640 | 0.005 | -0.053 | 0.776 | 0.859 | Unannotated | NA | NA |
| metab_1451 | Unannotated feature | pos | 703.5063 | 5.972 | 5.111 | 5.146 | 0.035 | 0.053 | 0.776 | 0.859 | Unannotated | NA | NA |
| metab_11159 | Butanedioic acid, octenyl- | neg | 515.2873 | 5.771 | 4.985 | 4.801 | -0.184 | -0.053 | 0.776 | 0.859 | Tentative | NA | NA |
| metab_3552 | Unannotated feature | pos | 124.0839 | 2.073 | 4.728 | 4.735 | 0.007 | 0.053 | 0.776 | 0.859 | Unannotated | NA | NA |
| metab_6491 | Unannotated feature | neg | 183.8480 | 0.786 | 4.375 | 4.537 | 0.162 | 0.053 | 0.776 | 0.859 | Unannotated | NA | NA |
| metab_2729 | 3-(L-Menthoxy)-2-methylpropane-1,2-diol | pos | 262.2377 | 5.725 | 4.844 | 4.857 | 0.013 | 0.053 | 0.776 | 0.859 | Tentative | NA | NA |
| metab_6673 | Unannotated feature | neg | 272.0151 | 5.147 | 3.874 | 3.926 | 0.052 | -0.053 | 0.776 | 0.859 | Unannotated | NA | NA |
| metab_1608 | Unannotated feature | pos | 549.8842 | 6.776 | 5.031 | 5.026 | -0.005 | 0.053 | 0.776 | 0.859 | Unannotated | NA | NA |
| metab_10595 | Unannotated feature | neg | 94.9411 | 0.601 | 3.821 | 3.814 | -0.008 | -0.053 | 0.776 | 0.859 | Unannotated | NA | NA |
| metab_7108 | Unannotated feature | neg | 447.3529 | 5.819 | 5.157 | 5.167 | 0.010 | 0.053 | 0.776 | 0.859 | Unannotated | NA | NA |
| metab_11369 | Unannotated feature | neg | 449.1102 | 5.611 | 4.325 | 4.297 | -0.028 | -0.053 | 0.776 | 0.859 | Unannotated | NA | NA |
| metab_9339 | Unannotated feature | neg | 209.9190 | 3.674 | 4.642 | 4.668 | 0.026 | 0.053 | 0.776 | 0.859 | Unannotated | NA | NA |
| metab_3467 | Unannotated feature | pos | 123.0443 | 2.280 | 4.295 | 4.273 | -0.022 | 0.053 | 0.776 | 0.859 | Unannotated | NA | NA |
| metab_9518 | Unannotated feature | neg | 419.0557 | 3.224 | 4.541 | 4.567 | 0.026 | 0.053 | 0.776 | 0.859 | Unannotated | NA | NA |
| metab_8638 | 1-Methyl-5-(4-benzoyl)pyrrole-2-acetic acid 2-(theophylline-7-yl)ethyl ester | neg | 462.1781 | 5.643 | 4.527 | 4.813 | 0.286 | 0.053 | 0.776 | 0.859 | Tentative | NA | NA |
| metab_596 | Unannotated feature | pos | 167.0897 | 2.081 | 6.639 | 6.642 | 0.003 | -0.053 | 0.776 | 0.859 | Unannotated | NA | NA |
| metab_1507 | Unannotated feature | pos | 223.2057 | 6.126 | 4.690 | 4.684 | -0.006 | 0.053 | 0.776 | 0.859 | Unannotated | NA | NA |
| metab_1077 | Unannotated feature | pos | 264.0398 | 5.221 | 4.152 | 4.153 | 0.001 | 0.053 | 0.776 | 0.859 | Unannotated | NA | NA |
| metab_4349 | Unannotated feature | pos | 785.7861 | 0.567 | 4.305 | 4.188 | -0.117 | -0.053 | 0.776 | 0.859 | Unannotated | NA | NA |
| metab_7131 | LysoPC(0:0/18:0) | neg | 568.3633 | 5.851 | 6.308 | 6.310 | 0.002 | -0.053 | 0.776 | 0.859 | Tentative | NA | NA |
| metab_4176 | Enterolactone | pos | 299.1279 | 4.601 | 2.368 | 2.407 | 0.039 | 0.053 | 0.776 | 0.859 | Moderate putative | NA | NA |
| metab_2587 | L-Palmitoylcarnitine | pos | 400.3422 | 5.855 | 6.323 | 6.288 | -0.035 | -0.053 | 0.776 | 0.859 | Strong putative | NA | NA |
| metab_10971 | Unannotated feature | neg | 304.9093 | 0.505 | 4.511 | 4.538 | 0.027 | 0.053 | 0.776 | 0.859 | Unannotated | NA | NA |
| metab_10633 | Unannotated feature | neg | 138.9203 | 0.594 | 3.604 | 3.528 | -0.076 | -0.053 | 0.776 | 0.859 | Unannotated | NA | NA |
| metab_8256 | Unannotated feature | neg | 413.6118 | 5.898 | 4.060 | 4.079 | 0.019 | 0.053 | 0.776 | 0.859 | Unannotated | NA | NA |
| metab_4616 | MJDBISSP00000009 | pos | 171.1176 | 2.065 | 7.677 | 7.681 | 0.004 | 0.053 | 0.776 | 0.859 | Strong putative | NA | NA |
| metab_8385 | Unannotated feature | neg | 157.0866 | 5.803 | 4.806 | 4.783 | -0.023 | -0.053 | 0.776 | 0.859 | Unannotated | NA | NA |
| metab_9853 | Unannotated feature | neg | 170.8704 | 2.461 | 4.197 | 4.185 | -0.012 | -0.053 | 0.776 | 0.859 | Unannotated | NA | NA |
| metab_6184 | Unannotated feature | neg | 558.9867 | 3.282 | 4.575 | 4.573 | -0.002 | -0.053 | 0.776 | 0.859 | Unannotated | NA | NA |
| metab_6187 | Unannotated feature | neg | 223.0460 | 3.301 | 4.002 | 4.035 | 0.033 | -0.053 | 0.776 | 0.859 | Unannotated | NA | NA |
| metab_6357 | Unannotated feature | neg | 189.8703 | 3.888 | 4.211 | 4.226 | 0.016 | 0.053 | 0.776 | 0.859 | Unannotated | NA | NA |
| metab_6360 | Unannotated feature | neg | 470.7671 | 0.582 | 5.454 | 5.441 | -0.013 | 0.053 | 0.776 | 0.859 | Unannotated | NA | NA |
| metab_3851 | Unannotated feature | pos | 219.0716 | 1.064 | 4.289 | 4.486 | 0.197 | 0.053 | 0.776 | 0.859 | Unannotated | NA | NA |
| metab_7317 | Unannotated feature | neg | 136.8910 | 6.281 | 6.013 | 6.032 | 0.019 | -0.048 | 0.794 | 0.873 | Unannotated | NA | NA |
| metab_2242 | Unannotated feature | pos | 751.7938 | 0.567 | 4.628 | 4.643 | 0.015 | 0.048 | 0.794 | 0.873 | Unannotated | NA | NA |
| metab_5114 | 7-Methylguanine | pos | 166.0725 | 1.468 | 5.321 | 5.309 | -0.012 | 0.048 | 0.794 | 0.873 | Strong putative | NA | NA |
| metab_7738 | Unannotated feature | neg | 161.9132 | 2.523 | 5.781 | 5.781 | -0.000 | 0.048 | 0.794 | 0.873 | Unannotated | NA | NA |
| metab_7283 | Unannotated feature | neg | 156.9805 | 6.180 | 6.985 | 6.993 | 0.008 | 0.048 | 0.794 | 0.873 | Unannotated | NA | NA |
| metab_912 | Unannotated feature | pos | 114.1280 | 3.846 | 4.467 | 4.408 | -0.059 | -0.048 | 0.794 | 0.873 | Unannotated | NA | NA |
| metab_7867 | Unannotated feature | neg | 732.8617 | 7.140 | 4.184 | 4.186 | 0.002 | -0.048 | 0.794 | 0.873 | Unannotated | NA | NA |
| metab_5316 | Unannotated feature | neg | 824.8176 | 0.562 | 4.433 | 4.432 | -0.001 | -0.048 | 0.794 | 0.873 | Unannotated | NA | NA |
| metab_7779 | Unannotated feature | neg | 404.8469 | 7.170 | 4.573 | 4.569 | -0.004 | 0.048 | 0.794 | 0.873 | Unannotated | NA | NA |
| metab_7391 | Unannotated feature | neg | 143.9878 | 7.093 | 3.637 | 3.739 | 0.102 | 0.048 | 0.794 | 0.873 | Unannotated | NA | NA |
| metab_7355 | Unannotated feature | neg | 722.2972 | 6.572 | 5.192 | 5.193 | 0.001 | 0.048 | 0.794 | 0.873 | Unannotated | NA | NA |
| metab_5461 | Unannotated feature | neg | 192.9883 | 0.668 | 5.253 | 5.240 | -0.013 | 0.048 | 0.794 | 0.873 | Unannotated | NA | NA |
| metab_7863 | Unannotated feature | neg | 813.8560 | 7.140 | 4.192 | 4.255 | 0.063 | 0.048 | 0.794 | 0.873 | Unannotated | NA | NA |
| metab_1254 | Unannotated feature | pos | 170.1115 | 2.073 | 6.143 | 6.152 | 0.009 | 0.048 | 0.794 | 0.873 | Unannotated | NA | NA |
| metab_1696 | Unannotated feature | pos | 151.0354 | 0.504 | 5.178 | 5.188 | 0.010 | 0.048 | 0.794 | 0.873 | Unannotated | NA | NA |
| metab_5969 | Unannotated feature | neg | 309.8570 | 2.748 | 5.390 | 5.411 | 0.021 | 0.048 | 0.794 | 0.873 | Unannotated | NA | NA |
| metab_1691 | Unannotated feature | pos | 643.5265 | 7.277 | 4.436 | 4.421 | -0.015 | -0.048 | 0.794 | 0.873 | Unannotated | NA | NA |
| metab_5769 | Unannotated feature | neg | 204.8637 | 1.965 | 4.996 | 5.008 | 0.011 | 0.048 | 0.794 | 0.873 | Unannotated | NA | NA |
| metab_10168 | Unannotated feature | neg | 160.9150 | 0.912 | 4.744 | 4.781 | 0.036 | 0.048 | 0.794 | 0.873 | Unannotated | NA | NA |
| metab_7534 | Unannotated feature | neg | 410.8430 | 7.170 | 4.533 | 4.520 | -0.013 | -0.048 | 0.794 | 0.873 | Unannotated | NA | NA |
| metab_3268 | Gamma-Glu-leu | pos | 261.1445 | 2.934 | 5.683 | 5.674 | -0.009 | -0.048 | 0.794 | 0.873 | Strong putative | NA | NA |
| metab_3208 | Unannotated feature | pos | 85.3009 | 3.182 | 3.826 | 3.874 | 0.048 | 0.048 | 0.794 | 0.873 | Unannotated | NA | NA |
| metab_5878 | Unannotated feature | neg | 236.8529 | 2.461 | 3.804 | 3.852 | 0.048 | 0.048 | 0.794 | 0.873 | Unannotated | NA | NA |
| metab_1277 | Unannotated feature | pos | 544.3876 | 5.855 | 5.766 | 5.761 | -0.004 | -0.048 | 0.794 | 0.873 | Unannotated | NA | NA |
| metab_5929 | Unannotated feature | neg | 204.8637 | 2.581 | 4.966 | 4.972 | 0.006 | 0.048 | 0.794 | 0.873 | Unannotated | NA | NA |
| metab_5620 | Unannotated feature | neg | 214.8363 | 0.912 | 4.671 | 4.672 | 0.001 | -0.048 | 0.794 | 0.873 | Unannotated | NA | NA |
| metab_5547 | Unannotated feature | neg | 237.8614 | 0.818 | 4.023 | 4.020 | -0.003 | 0.048 | 0.794 | 0.873 | Unannotated | NA | NA |
| metab_10707 | Unannotated feature | neg | 594.6942 | 0.578 | 4.675 | 4.682 | 0.006 | -0.048 | 0.794 | 0.873 | Unannotated | NA | NA |
| metab_7679 | Unannotated feature | neg | 238.0777 | 7.245 | 4.444 | 4.465 | 0.021 | 0.048 | 0.794 | 0.873 | Unannotated | NA | NA |
| metab_8313 | Unannotated feature | neg | 270.2082 | 5.851 | 3.792 | 3.844 | 0.052 | 0.048 | 0.794 | 0.873 | Unannotated | NA | NA |
| metab_4600 | Unannotated feature | pos | 102.0343 | 0.061 | 5.148 | 5.161 | 0.013 | 0.048 | 0.794 | 0.873 | Unannotated | NA | NA |
| metab_8684 | Unannotated feature | neg | 331.9528 | 5.763 | 5.090 | 5.078 | -0.012 | -0.048 | 0.794 | 0.873 | Unannotated | NA | NA |
| metab_8690 | Unannotated feature | neg | 390.1237 | 5.611 | 4.196 | 4.281 | 0.085 | 0.048 | 0.794 | 0.873 | Unannotated | NA | NA |
| metab_4345 | Unannotated feature | pos | 858.6781 | 0.567 | 4.842 | 4.838 | -0.004 | -0.048 | 0.794 | 0.873 | Unannotated | NA | NA |
| metab_8412 | Unannotated feature | neg | 225.1861 | 5.787 | 3.604 | 3.661 | 0.056 | 0.048 | 0.794 | 0.873 | Unannotated | NA | NA |
| metab_11325 | Unannotated feature | neg | 134.8940 | 7.213 | 4.975 | 4.974 | -0.002 | -0.048 | 0.794 | 0.873 | Unannotated | NA | NA |
| metab_10738 | Unannotated feature | neg | 676.8358 | 0.570 | 3.962 | 4.031 | 0.069 | 0.048 | 0.794 | 0.873 | Unannotated | NA | NA |
| metab_4398 | Unannotated feature | pos | 177.0611 | 0.567 | 5.712 | 5.693 | -0.019 | -0.048 | 0.794 | 0.873 | Unannotated | NA | NA |
| metab_8462 | 1,2,10-Trihydroxydihydro-trans-linalyl oxide 7-O-beta-D-glucopyranoside | neg | 427.1811 | 5.747 | 4.972 | 5.027 | 0.056 | 0.048 | 0.794 | 0.873 | Tentative | NA | NA |
| metab_9852 | Unannotated feature | neg | 165.8940 | 2.469 | 4.051 | 4.049 | -0.002 | -0.048 | 0.794 | 0.873 | Unannotated | NA | NA |
| metab_4621 | MJDBISSP00000001 | pos | 200.0473 | 2.644 | 8.472 | 8.476 | 0.005 | 0.048 | 0.794 | 0.873 | Strong putative | NA | NA |
| metab_7224 | Unannotated feature | neg | 227.9050 | 1.847 | 5.984 | 6.064 | 0.081 | 0.048 | 0.794 | 0.873 | Unannotated | NA | NA |
| metab_2377 | Unannotated feature | pos | 674.9878 | 5.956 | 5.223 | 5.236 | 0.013 | 0.048 | 0.794 | 0.873 | Unannotated | NA | NA |
| metab_1579 | Unannotated feature | pos | 465.3343 | 6.420 | 4.712 | 4.714 | 0.002 | 0.048 | 0.794 | 0.873 | Unannotated | NA | NA |
| metab_2698 | 12-Hydroxydodecanoylcarnitine | pos | 342.2639 | 5.756 | 5.910 | 5.954 | 0.043 | -0.048 | 0.794 | 0.873 | Tentative | NA | NA |
| metab_8790 | Unannotated feature | neg | 500.2795 | 7.153 | 4.774 | 4.743 | -0.031 | 0.048 | 0.794 | 0.873 | Unannotated | NA | NA |
| metab_10429 | Unannotated feature | neg | 194.9854 | 0.676 | 4.627 | 4.644 | 0.018 | 0.048 | 0.794 | 0.873 | Unannotated | NA | NA |
| metab_1611 | Unannotated feature | pos | 407.2767 | 6.922 | 4.998 | 4.967 | -0.031 | -0.048 | 0.794 | 0.873 | Unannotated | NA | NA |
| metab_3452 | Morpholine | pos | 88.0762 | 2.303 | 4.659 | 4.682 | 0.023 | 0.048 | 0.794 | 0.873 | Moderate putative | NA | NA |
| metab_2910 | Unannotated feature | pos | 183.1017 | 2.148 | 4.266 | 4.147 | -0.119 | -0.048 | 0.794 | 0.873 | Unannotated | NA | NA |
| metab_9060 | Unannotated feature | neg | 197.0978 | 4.574 | 5.887 | 5.815 | -0.072 | -0.048 | 0.794 | 0.873 | Unannotated | NA | NA |
| metab_3664 | Acrylamide-sodium acrylate resin | pos | 215.1391 | 1.949 | 3.919 | 4.008 | 0.090 | 0.048 | 0.794 | 0.873 | Tentative | NA | NA |
| metab_6548 | Unannotated feature | neg | 291.9925 | 4.620 | 3.545 | 3.567 | 0.022 | -0.048 | 0.794 | 0.873 | Unannotated | NA | NA |
| metab_10958 | Unannotated feature | neg | 136.8625 | 0.522 | 4.963 | 4.969 | 0.006 | 0.048 | 0.794 | 0.873 | Unannotated | NA | NA |
| metab_3124 | Unannotated feature | pos | 160.0969 | 3.700 | 4.490 | 4.468 | -0.022 | 0.048 | 0.794 | 0.873 | Unannotated | NA | NA |
| metab_11407 | Sulfoglycolithocholate | neg | 512.2697 | 6.572 | 5.647 | 5.759 | 0.113 | 0.044 | 0.813 | 0.886 | Tentative | NA | NA |
| metab_11374 | Unannotated feature | neg | 96.9688 | 0.476 | 4.566 | 4.567 | 0.001 | -0.044 | 0.813 | 0.886 | Unannotated | NA | NA |
| metab_11360 | Unannotated feature | neg | 226.0276 | 5.538 | 6.587 | 6.573 | -0.014 | 0.044 | 0.813 | 0.886 | Unannotated | NA | NA |
| metab_1699 | Unannotated feature | pos | 447.9039 | 7.285 | 4.837 | 4.837 | 0.001 | 0.044 | 0.813 | 0.886 | Unannotated | NA | NA |
| metab_1735 | Unannotated feature | pos | 100.9572 | 7.324 | 3.726 | 3.723 | -0.002 | -0.044 | 0.813 | 0.886 | Unannotated | NA | NA |
| metab_10185 | Unannotated feature | neg | 173.9936 | 0.896 | 4.043 | 4.018 | -0.025 | 0.044 | 0.813 | 0.886 | Unannotated | NA | NA |
| metab_1829 | Unannotated feature | pos | 144.0656 | 7.975 | 4.355 | 4.376 | 0.021 | 0.044 | 0.813 | 0.886 | Unannotated | NA | NA |
| metab_1822 | Unannotated feature | pos | 102.9706 | 7.990 | 4.948 | 4.945 | -0.003 | 0.044 | 0.813 | 0.886 | Unannotated | NA | NA |
| metab_10036 | Unannotated feature | neg | 253.8513 | 1.593 | 4.317 | 4.407 | 0.090 | 0.044 | 0.813 | 0.886 | Unannotated | NA | NA |
| metab_10480 | Unannotated feature | neg | 215.0327 | 0.644 | 7.451 | 7.459 | 0.009 | -0.044 | 0.813 | 0.886 | Unannotated | NA | NA |
| metab_2770 | Unannotated feature | pos | 116.0531 | 1.956 | 4.043 | 4.064 | 0.021 | 0.044 | 0.813 | 0.886 | Unannotated | NA | NA |
| metab_2685 | alpha-Terpinyl acetate | pos | 214.1802 | 5.771 | 4.536 | 4.560 | 0.024 | 0.044 | 0.813 | 0.886 | Tentative | NA | NA |
| metab_9781 | Unannotated feature | neg | 236.8529 | 2.597 | 4.309 | 4.307 | -0.002 | -0.044 | 0.813 | 0.886 | Unannotated | NA | NA |
| metab_2524 | Piperine | pos | 286.1438 | 5.886 | 5.019 | 4.859 | -0.160 | -0.044 | 0.813 | 0.886 | Strong putative | NA | NA |
| metab_2340 | Unannotated feature | pos | 576.8086 | 0.567 | 4.704 | 4.728 | 0.024 | 0.044 | 0.813 | 0.886 | Unannotated | NA | NA |
| metab_9515 | 2-METHOXYRESORCINOL | neg | 139.0394 | 3.232 | 4.046 | 3.894 | -0.152 | 0.044 | 0.813 | 0.886 | Moderate putative | NA | NA |
| metab_9004 | Unannotated feature | neg | 495.9906 | 3.240 | 5.940 | 5.943 | 0.003 | -0.044 | 0.813 | 0.886 | Unannotated | NA | NA |
| metab_11032 | Unannotated feature | neg | 368.8445 | 0.013 | 4.581 | 4.586 | 0.005 | 0.044 | 0.813 | 0.886 | Unannotated | NA | NA |
| metab_8878 | Unannotated feature | neg | 425.1102 | 7.117 | 4.373 | 4.282 | -0.091 | -0.044 | 0.813 | 0.886 | Unannotated | NA | NA |
| metab_8817 | Unannotated feature | neg | 666.8424 | 7.148 | 4.804 | 4.814 | 0.010 | 0.044 | 0.813 | 0.886 | Unannotated | NA | NA |
| metab_4112 | (R)-3-Amino-2-methylpropanoate | pos | 126.0528 | 0.706 | 4.050 | 4.152 | 0.102 | 0.044 | 0.813 | 0.886 | Tentative | NA | NA |
| metab_8752 | Unannotated feature | neg | 299.9196 | 7.156 | 5.324 | 5.324 | -0.000 | 0.044 | 0.813 | 0.886 | Unannotated | NA | NA |
| metab_600 | Unannotated feature | pos | 163.1118 | 2.088 | 5.305 | 5.306 | 0.001 | -0.044 | 0.813 | 0.886 | Unannotated | NA | NA |
| metab_4217 | 1-Aminocyclopropane-1-carboxylate | pos | 102.0554 | 0.621 | 4.726 | 4.727 | 0.001 | -0.044 | 0.813 | 0.886 | Moderate putative | NA | NA |
| metab_8554 | Unannotated feature | neg | 358.2245 | 5.691 | 3.699 | 3.876 | 0.177 | 0.044 | 0.813 | 0.886 | Unannotated | NA | NA |
| metab_4255 | Unannotated feature | pos | 274.7918 | 0.598 | 5.213 | 5.189 | -0.024 | -0.044 | 0.813 | 0.886 | Unannotated | NA | NA |
| metab_10806 | Unannotated feature | neg | 84.9898 | 0.562 | 3.202 | 3.182 | -0.020 | -0.044 | 0.813 | 0.886 | Unannotated | NA | NA |
| metab_9206 | Unannotated feature | neg | 370.9532 | 4.115 | 4.222 | 4.290 | 0.068 | 0.044 | 0.813 | 0.886 | Unannotated | NA | NA |
| metab_3136 | ROSAMICIN | pos | 564.3589 | 3.638 | 4.987 | 4.880 | -0.107 | -0.044 | 0.813 | 0.886 | Tentative | NA | NA |
| metab_3150 | Unannotated feature | pos | 136.1060 | 3.584 | 4.360 | 4.338 | -0.022 | 0.044 | 0.813 | 0.886 | Unannotated | NA | NA |
| metab_3151 | Unannotated feature | pos | 109.1016 | 3.584 | 3.483 | 3.502 | 0.019 | -0.044 | 0.813 | 0.886 | Unannotated | NA | NA |
| metab_4586 | Unannotated feature | pos | 225.1333 | 0.418 | 5.614 | 5.620 | 0.007 | 0.044 | 0.813 | 0.886 | Unannotated | NA | NA |
| metab_7564 | Unannotated feature | neg | 259.8166 | 7.189 | 4.414 | 4.408 | -0.006 | -0.044 | 0.813 | 0.886 | Unannotated | NA | NA |
| metab_7380 | Unannotated feature | neg | 316.9484 | 6.983 | 5.763 | 5.788 | 0.024 | 0.044 | 0.813 | 0.886 | Unannotated | NA | NA |
| metab_5894 | Unannotated feature | neg | 490.0933 | 2.504 | 4.448 | 4.464 | 0.016 | 0.044 | 0.813 | 0.886 | Unannotated | NA | NA |
| metab_277 | Unannotated feature | pos | 213.0611 | 0.721 | 3.668 | 3.749 | 0.080 | 0.044 | 0.813 | 0.886 | Unannotated | NA | NA |
| metab_11239 | Unannotated feature | neg | 606.7428 | 0.578 | 5.173 | 5.191 | 0.018 | 0.044 | 0.813 | 0.886 | Unannotated | NA | NA |
| metab_252 | Threonate | pos | 181.0083 | 0.667 | 5.115 | 5.169 | 0.054 | 0.044 | 0.813 | 0.886 | Tentative | NA | NA |
| metab_11294 | Unannotated feature | neg | 490.7859 | 7.170 | 4.326 | 4.341 | 0.015 | -0.044 | 0.813 | 0.886 | Unannotated | NA | NA |
| metab_6025 | Unannotated feature | neg | 94.0039 | 2.953 | 4.972 | 4.968 | -0.005 | -0.044 | 0.813 | 0.886 | Unannotated | NA | NA |
| metab_84 | Unannotated feature | pos | 118.9429 | 0.473 | 4.597 | 4.599 | 0.002 | 0.044 | 0.813 | 0.886 | Unannotated | NA | NA |
| metab_6787 | Unannotated feature | neg | 296.0126 | 5.543 | 4.472 | 4.405 | -0.068 | -0.044 | 0.813 | 0.886 | Unannotated | NA | NA |
| metab_6591 | 3-(3-Hydroxyphenyl)propanoic acid | neg | 211.0611 | 4.814 | 3.824 | 3.778 | -0.046 | 0.044 | 0.813 | 0.886 | Strong putative | NA | NA |
| metab_11338 | Unannotated feature | neg | 102.9480 | 0.013 | 5.735 | 5.736 | 0.001 | 0.044 | 0.813 | 0.886 | Unannotated | NA | NA |
| metab_11327 | Unannotated feature | neg | 174.8642 | 7.181 | 4.339 | 4.346 | 0.007 | 0.044 | 0.813 | 0.886 | Unannotated | NA | NA |
| metab_5888 | Unannotated feature | neg | 625.2320 | 2.498 | 4.184 | 4.190 | 0.006 | -0.044 | 0.813 | 0.886 | Unannotated | NA | NA |
| metab_11328 | Unannotated feature | neg | 600.7079 | 0.582 | 5.561 | 5.559 | -0.002 | 0.044 | 0.813 | 0.886 | Unannotated | NA | NA |
| metab_10771 | Unannotated feature | neg | 238.9319 | 0.570 | 5.686 | 5.696 | 0.010 | 0.044 | 0.813 | 0.886 | Unannotated | NA | NA |
| metab_5474 | Unannotated feature | neg | 197.8077 | 0.692 | 8.100 | 8.112 | 0.012 | -0.044 | 0.813 | 0.886 | Unannotated | NA | NA |
| metab_7846 | Unannotated feature | neg | 704.3386 | 7.148 | 4.837 | 4.853 | 0.017 | 0.044 | 0.813 | 0.886 | Unannotated | NA | NA |
| metab_5371 | Unannotated feature | neg | 658.6670 | 0.578 | 5.606 | 5.624 | 0.018 | -0.044 | 0.813 | 0.886 | Unannotated | NA | NA |
| metab_5349 | Unannotated feature | neg | 136.9092 | 0.016 | 5.351 | 5.359 | 0.009 | -0.044 | 0.813 | 0.886 | Unannotated | NA | NA |
| metab_4854 | Unannotated feature | pos | 126.1280 | 5.793 | 3.988 | 3.994 | 0.006 | -0.044 | 0.813 | 0.886 | Unannotated | NA | NA |
| metab_4924 | Unannotated feature | pos | 506.6265 | 0.606 | 5.033 | 5.052 | 0.019 | 0.044 | 0.813 | 0.886 | Unannotated | NA | NA |
| metab_5577 | Unannotated feature | neg | 186.8974 | 0.036 | 6.202 | 6.200 | -0.002 | -0.044 | 0.813 | 0.886 | Unannotated | NA | NA |
| metab_5635 | Unannotated feature | neg | 92.6813 | 0.928 | 4.114 | 4.112 | -0.002 | -0.044 | 0.813 | 0.886 | Unannotated | NA | NA |
| metab_5164 | Unannotated feature | neg | 321.8994 | 0.505 | 4.227 | 4.257 | 0.029 | 0.044 | 0.813 | 0.886 | Unannotated | NA | NA |
| metab_2889 | Unannotated feature | pos | 323.2174 | 5.053 | 5.316 | 5.323 | 0.007 | 0.040 | 0.831 | 0.899 | Unannotated | NA | NA |
| metab_9843 | Unannotated feature | neg | 317.0343 | 2.492 | 4.683 | 4.689 | 0.006 | 0.040 | 0.831 | 0.899 | Unannotated | NA | NA |
| metab_3304 | Beta-Hydroxy-gamma-trimethylaminobutyric acid | pos | 144.1020 | 2.749 | 5.061 | 5.026 | -0.035 | -0.040 | 0.831 | 0.899 | Tentative | NA | NA |
| metab_3671 | Unannotated feature | pos | 520.3328 | 3.507 | 5.035 | 4.913 | -0.122 | 0.040 | 0.831 | 0.899 | Unannotated | NA | NA |
| metab_3620 | 6-Lactoyl-5,6,7,8-tetrahydropterin | pos | 272.1353 | 1.964 | 5.347 | 5.286 | -0.061 | 0.040 | 0.831 | 0.899 | Tentative | NA | NA |
| metab_2626 | Tetracosahexaenoic acid | pos | 339.2684 | 5.832 | 4.870 | 4.842 | -0.029 | -0.040 | 0.831 | 0.899 | Tentative | NA | NA |
| metab_1762 | Unannotated feature | pos | 130.4921 | 7.339 | 4.718 | 4.733 | 0.015 | -0.040 | 0.831 | 0.899 | Unannotated | NA | NA |
| metab_8726 | Unannotated feature | neg | 305.0315 | 5.579 | 4.605 | 4.498 | -0.107 | 0.040 | 0.831 | 0.899 | Unannotated | NA | NA |
| metab_8716 | Unannotated feature | neg | 242.0406 | 5.587 | 4.784 | 4.801 | 0.017 | 0.040 | 0.831 | 0.899 | Unannotated | NA | NA |
| metab_8725 | Unannotated feature | neg | 319.9667 | 5.579 | 4.286 | 4.326 | 0.041 | 0.040 | 0.831 | 0.899 | Unannotated | NA | NA |
| metab_8564 | Unannotated feature | neg | 325.1848 | 7.117 | 6.639 | 6.639 | 0.000 | -0.040 | 0.831 | 0.899 | Unannotated | NA | NA |
| metab_8486 | Unannotated feature | neg | 167.0359 | 5.739 | 4.535 | 4.444 | -0.092 | -0.040 | 0.831 | 0.899 | Unannotated | NA | NA |
| metab_3345 | Unannotated feature | pos | 212.1441 | 2.599 | 5.544 | 5.512 | -0.032 | 0.040 | 0.831 | 0.899 | Unannotated | NA | NA |
| metab_10999 | Unannotated feature | neg | 103.9194 | 0.039 | 6.053 | 6.061 | 0.009 | 0.040 | 0.831 | 0.899 | Unannotated | NA | NA |
| metab_9209 | Unannotated feature | neg | 195.0594 | 4.099 | 4.164 | 4.182 | 0.018 | -0.040 | 0.831 | 0.899 | Unannotated | NA | NA |
| metab_3105 | Isoleucylproline | pos | 211.1442 | 3.831 | 4.914 | 4.934 | 0.021 | 0.040 | 0.831 | 0.899 | Tentative | NA | NA |
| metab_9503 | Unannotated feature | neg | 161.9131 | 3.247 | 5.649 | 5.651 | 0.002 | -0.040 | 0.831 | 0.899 | Unannotated | NA | NA |
| metab_3116 | 2'-Fluoro-2',3'-dideoxyinosine | pos | 255.0895 | 3.754 | 4.796 | 4.878 | 0.082 | 0.040 | 0.831 | 0.899 | Tentative | NA | NA |
| metab_476 | Unannotated feature | pos | 86.3170 | 1.809 | 4.685 | 4.692 | 0.006 | 0.040 | 0.831 | 0.899 | Unannotated | NA | NA |
| metab_418 | Unannotated feature | pos | 223.1294 | 1.297 | 3.615 | 3.548 | -0.067 | 0.040 | 0.831 | 0.899 | Unannotated | NA | NA |
| metab_5189 | Unannotated feature | neg | 566.9042 | 0.530 | 4.450 | 4.529 | 0.079 | 0.040 | 0.831 | 0.899 | Unannotated | NA | NA |
| metab_4700 | Unannotated feature | pos | 476.8427 | 5.910 | 5.126 | 5.132 | 0.006 | 0.040 | 0.831 | 0.899 | Unannotated | NA | NA |
| metab_479 | Unannotated feature | pos | 132.6840 | 1.809 | 4.052 | 4.097 | 0.045 | 0.040 | 0.831 | 0.899 | Unannotated | NA | NA |
| metab_7292 | Unannotated feature | neg | 158.9749 | 6.188 | 8.356 | 8.359 | 0.003 | 0.040 | 0.831 | 0.899 | Unannotated | NA | NA |
| metab_7477 | Unannotated feature | neg | 630.8871 | 7.140 | 4.987 | 4.986 | -0.001 | -0.040 | 0.831 | 0.899 | Unannotated | NA | NA |
| metab_7359 | LysoPE(0:0/20:0) | neg | 554.3475 | 6.622 | 5.426 | 5.355 | -0.072 | -0.040 | 0.831 | 0.899 | Tentative | NA | NA |
| metab_6076 | Unannotated feature | neg | 180.0663 | 3.136 | 4.108 | 4.227 | 0.119 | 0.040 | 0.831 | 0.899 | Moderate putative | NA | NA |
| metab_6130 | 6-Aminomethyl-3-methyl-4H-1,2,4-benzothiadiazine 1,1-dioxide | neg | 270.0544 | 3.201 | 4.684 | 4.708 | 0.025 | 0.040 | 0.831 | 0.899 | Tentative | NA | NA |
| metab_7008 | Unannotated feature | neg | 147.9264 | 5.739 | 3.235 | 3.263 | 0.028 | 0.040 | 0.831 | 0.899 | Unannotated | NA | NA |
| metab_6406 | Unannotated feature | neg | 181.1028 | 4.115 | 4.525 | 4.506 | -0.019 | 0.040 | 0.831 | 0.899 | Unannotated | NA | NA |
| metab_8052 | Unannotated feature | neg | 1035.6648 | 6.392 | 5.584 | 5.567 | -0.018 | 0.040 | 0.831 | 0.899 | Unannotated | NA | NA |
| metab_8420 | Unannotated feature | neg | 259.8782 | 5.779 | 3.832 | 3.893 | 0.061 | 0.040 | 0.831 | 0.899 | Unannotated | NA | NA |
| metab_7646 | Unannotated feature | neg | 106.9429 | 7.665 | 5.569 | 5.569 | 0.000 | 0.040 | 0.831 | 0.899 | Unannotated | NA | NA |
| metab_7726 | Unannotated feature | neg | 161.8566 | 7.197 | 4.080 | 4.106 | 0.026 | -0.040 | 0.831 | 0.899 | Unannotated | NA | NA |
| metab_5522 | Unannotated feature | neg | 185.8451 | 0.786 | 3.736 | 3.892 | 0.156 | 0.040 | 0.831 | 0.899 | Unannotated | NA | NA |
| metab_10789 | Unannotated feature | neg | 688.8427 | 0.562 | 4.545 | 4.571 | 0.026 | -0.040 | 0.831 | 0.899 | Unannotated | NA | NA |
| metab_7480 | Unannotated feature | neg | 698.8743 | 7.140 | 4.978 | 4.941 | -0.037 | -0.040 | 0.831 | 0.899 | Unannotated | NA | NA |
| metab_5791 | Unannotated feature | neg | 378.7792 | 2.087 | 5.026 | 5.045 | 0.019 | 0.040 | 0.831 | 0.899 | Unannotated | NA | NA |
| metab_7545 | Unannotated feature | neg | 118.9071 | 7.181 | 3.179 | 3.179 | -0.000 | -0.040 | 0.831 | 0.899 | Unannotated | NA | NA |
| metab_8436 | Unannotated feature | neg | 118.9236 | 5.771 | 4.857 | 4.862 | 0.005 | -0.040 | 0.831 | 0.899 | Unannotated | NA | NA |
| metab_8376 | Cyasterone | neg | 565.3036 | 5.803 | 4.879 | 5.032 | 0.153 | 0.040 | 0.831 | 0.899 | Tentative | NA | NA |
| metab_8349 | Unannotated feature | neg | 613.3608 | 5.819 | 5.158 | 5.172 | 0.013 | 0.040 | 0.831 | 0.899 | Unannotated | NA | NA |
| metab_5529 | Unannotated feature | neg | 216.8219 | 0.794 | 4.731 | 4.784 | 0.053 | 0.040 | 0.831 | 0.899 | Unannotated | NA | NA |
| metab_7837 | Unannotated feature | neg | 142.9284 | 2.530 | 4.772 | 4.796 | 0.024 | 0.040 | 0.831 | 0.899 | Unannotated | NA | NA |
| metab_7708 | Unannotated feature | neg | 118.9321 | 7.213 | 3.935 | 3.958 | 0.023 | 0.040 | 0.831 | 0.899 | Unannotated | NA | NA |
| metab_2517 | Unannotated feature | pos | 602.9310 | 5.886 | 5.809 | 5.824 | 0.015 | 0.040 | 0.831 | 0.899 | Unannotated | NA | NA |
| metab_10973 | Unannotated feature | neg | 259.8780 | 0.505 | 4.326 | 4.367 | 0.041 | 0.040 | 0.831 | 0.899 | Unannotated | NA | NA |
| metab_9914 | Unannotated feature | neg | 287.1367 | 2.119 | 4.937 | 4.962 | 0.026 | 0.040 | 0.831 | 0.899 | Unannotated | NA | NA |
| metab_9841 | Unannotated feature | neg | 320.9877 | 2.492 | 4.310 | 4.284 | -0.027 | -0.040 | 0.831 | 0.899 | Unannotated | NA | NA |
| metab_9812 | Unannotated feature | neg | 128.9279 | 2.530 | 4.593 | 4.595 | 0.002 | 0.040 | 0.831 | 0.899 | Unannotated | NA | NA |
| metab_8876 | 3-Carbamoyl-2-phenylpropionaldehyde | neg | 230.0209 | 5.223 | 4.045 | 3.940 | -0.105 | 0.040 | 0.831 | 0.899 | Tentative | NA | NA |
| metab_8793 | Unannotated feature | neg | 173.9653 | 5.514 | 4.716 | 4.692 | -0.024 | -0.040 | 0.831 | 0.899 | Unannotated | NA | NA |
| metab_4129 | Unannotated feature | pos | 133.1051 | 0.691 | 3.542 | 3.542 | -0.001 | -0.040 | 0.831 | 0.899 | Unannotated | NA | NA |
| metab_8811 | Unannotated feature | neg | 209.9490 | 5.470 | 6.135 | 6.128 | -0.007 | -0.040 | 0.831 | 0.899 | Unannotated | NA | NA |
| metab_1582 | Unannotated feature | pos | 447.4139 | 6.427 | 4.579 | 4.566 | -0.013 | 0.040 | 0.831 | 0.899 | Unannotated | NA | NA |
| metab_2182 | 5alpha-Cyprinol | pos | 435.3448 | 6.365 | 4.544 | 4.496 | -0.048 | -0.040 | 0.831 | 0.899 | Tentative | NA | NA |
| metab_10045 | Unannotated feature | neg | 441.6820 | 1.486 | 5.026 | 5.032 | 0.006 | -0.040 | 0.831 | 0.899 | Unannotated | NA | NA |
| metab_2150 | Unannotated feature | pos | 477.3917 | 6.559 | 6.005 | 6.014 | 0.009 | 0.040 | 0.831 | 0.899 | Unannotated | NA | NA |
| metab_2171 | Unannotated feature | pos | 430.3881 | 6.420 | 5.140 | 5.094 | -0.047 | -0.040 | 0.831 | 0.899 | Unannotated | NA | NA |
| metab_9740 | Unannotated feature | neg | 276.8683 | 2.740 | 6.200 | 6.199 | -0.001 | 0.040 | 0.831 | 0.899 | Unannotated | NA | NA |
| metab_2873 | Unannotated feature | pos | 208.0430 | 5.160 | 4.206 | 4.179 | -0.026 | -0.040 | 0.831 | 0.899 | Unannotated | NA | NA |
| metab_10898 | Unannotated feature | neg | 586.6846 | 0.582 | 5.458 | 5.438 | -0.020 | 0.040 | 0.831 | 0.899 | Unannotated | NA | NA |
| metab_925 | Unannotated feature | pos | 102.8247 | 3.931 | 3.351 | 3.377 | 0.025 | 0.040 | 0.831 | 0.899 | Unannotated | NA | NA |
| metab_1685 | Unannotated feature | pos | 97.0288 | 0.465 | 5.727 | 5.720 | -0.008 | 0.040 | 0.831 | 0.899 | Unannotated | NA | NA |
| metab_2069 | Unannotated feature | pos | 186.1126 | 7.207 | 3.604 | 3.578 | -0.026 | -0.040 | 0.831 | 0.899 | Unannotated | NA | NA |
| metab_10410 | Unannotated feature | neg | 195.8107 | 0.692 | 7.988 | 7.998 | 0.011 | -0.040 | 0.831 | 0.899 | Unannotated | NA | NA |
| metab_795 | Unannotated feature | pos | 280.1544 | 2.989 | 4.925 | 5.012 | 0.087 | 0.036 | 0.850 | 0.910 | Unannotated | NA | NA |
| metab_4368 | Unannotated feature | pos | 669.7595 | 0.567 | 4.203 | 4.329 | 0.126 | 0.036 | 0.850 | 0.910 | Unannotated | NA | NA |
| metab_3113 | Unannotated feature | pos | 102.3761 | 3.769 | 4.221 | 4.209 | -0.012 | 0.036 | 0.850 | 0.910 | Unannotated | NA | NA |
| metab_9531 | Unannotated feature | neg | 113.9255 | 3.224 | 3.834 | 3.770 | -0.064 | -0.036 | 0.850 | 0.910 | Unannotated | NA | NA |
| metab_3167 | Creatinine aspartate | pos | 243.0709 | 3.468 | 5.972 | 5.978 | 0.006 | 0.036 | 0.850 | 0.910 | Tentative | NA | NA |
| metab_7143 | Unannotated feature | neg | 144.9229 | 5.867 | 6.484 | 6.486 | 0.002 | 0.036 | 0.850 | 0.910 | Unannotated | NA | NA |
| metab_7070 | Unannotated feature | neg | 129.9590 | 5.795 | 3.621 | 3.640 | 0.019 | 0.036 | 0.850 | 0.910 | Unannotated | NA | NA |
| metab_7551 | Unannotated feature | neg | 168.8752 | 7.181 | 3.879 | 3.881 | 0.002 | 0.036 | 0.850 | 0.910 | Unannotated | NA | NA |
| metab_6407 | Unannotated feature | neg | 368.9562 | 4.115 | 4.151 | 4.220 | 0.069 | 0.036 | 0.850 | 0.910 | Unannotated | NA | NA |
| metab_11318 | Unannotated feature | neg | 184.9156 | 7.213 | 4.486 | 4.485 | -0.001 | 0.036 | 0.850 | 0.910 | Unannotated | NA | NA |
| metab_104 | Unannotated feature | pos | 313.0358 | 0.538 | 5.115 | 5.140 | 0.025 | -0.036 | 0.850 | 0.910 | Unannotated | NA | NA |
| metab_8104 | Unannotated feature | neg | 117.9356 | 6.188 | 6.243 | 6.245 | 0.002 | 0.036 | 0.850 | 0.910 | Unannotated | NA | NA |
| metab_5854 | Unannotated feature | neg | 908.7842 | 0.562 | 4.701 | 4.724 | 0.023 | 0.036 | 0.850 | 0.910 | Unannotated | NA | NA |
| metab_5728 | Unannotated feature | neg | 227.8947 | 1.577 | 4.798 | 4.815 | 0.018 | -0.036 | 0.850 | 0.910 | Unannotated | NA | NA |
| metab_5928 | Unannotated feature | neg | 129.9357 | 2.581 | 4.120 | 4.138 | 0.018 | -0.036 | 0.850 | 0.910 | Unannotated | NA | NA |
| metab_5882 | Unannotated feature | neg | 385.9567 | 2.477 | 4.823 | 4.833 | 0.011 | 0.036 | 0.850 | 0.910 | Unannotated | NA | NA |
| metab_10716 | Unannotated feature | neg | 505.7598 | 0.578 | 3.778 | 3.779 | 0.001 | 0.036 | 0.850 | 0.910 | Unannotated | NA | NA |
| metab_7550 | Unannotated feature | neg | 164.9268 | 7.181 | 4.643 | 4.666 | 0.023 | 0.036 | 0.850 | 0.910 | Unannotated | NA | NA |
| metab_10703 | Unannotated feature | neg | 628.6888 | 0.578 | 4.498 | 4.477 | -0.022 | 0.036 | 0.850 | 0.910 | Unannotated | NA | NA |
| metab_7583 | Unannotated feature | neg | 118.9236 | 7.213 | 4.345 | 4.349 | 0.004 | -0.036 | 0.850 | 0.910 | Unannotated | NA | NA |
| metab_1366 | LysoPE(0:0/22:5(7Z,10Z,13Z,16Z,19Z)) | pos | 560.3346 | 5.910 | 5.035 | 4.964 | -0.071 | -0.036 | 0.850 | 0.910 | Tentative | NA | NA |
| metab_8021 | Unannotated feature | neg | 100.3772 | 6.650 | 2.686 | 2.682 | -0.004 | -0.036 | 0.850 | 0.910 | Unannotated | NA | NA |
| metab_5194 | Unannotated feature | neg | 598.8545 | 0.534 | 5.140 | 5.153 | 0.013 | -0.036 | 0.850 | 0.910 | Unannotated | NA | NA |
| metab_4718 | Unannotated feature | pos | 352.8975 | 0.567 | 5.687 | 5.698 | 0.011 | -0.036 | 0.850 | 0.910 | Unannotated | NA | NA |
| metab_4601 | Unannotated feature | pos | 284.2944 | 0.035 | 4.687 | 4.667 | -0.020 | -0.036 | 0.850 | 0.910 | Unannotated | NA | NA |
| metab_4587 | Unannotated feature | pos | 141.0547 | 0.411 | 4.323 | 4.313 | -0.010 | 0.036 | 0.850 | 0.910 | Unannotated | NA | NA |
| metab_8431 | Unannotated feature | neg | 244.7986 | 5.771 | 3.512 | 3.571 | 0.059 | 0.036 | 0.850 | 0.910 | Unannotated | NA | NA |
| metab_781 | PG(22:6(5Z,7Z,10Z,13Z,16Z,19Z)-OH(4)/20:3(8Z,11Z,14Z)) | pos | 453.2454 | 2.927 | 5.722 | 5.716 | -0.006 | 0.036 | 0.850 | 0.910 | Tentative | NA | NA |
| metab_3295 | Unannotated feature | pos | 399.0872 | 2.637 | 5.777 | 5.784 | 0.007 | 0.036 | 0.850 | 0.910 | Unannotated | NA | NA |
| metab_683 | 3-(4-Hydroxyphenyl)pyruvate | pos | 163.0391 | 2.467 | 3.752 | 3.699 | -0.052 | -0.036 | 0.850 | 0.910 | Tentative | NA | NA |
| metab_3324 | Unannotated feature | pos | 117.0576 | 2.644 | 6.181 | 6.187 | 0.006 | -0.036 | 0.850 | 0.910 | Unannotated | NA | NA |
| metab_11088 | Unannotated feature | neg | 666.6986 | 0.578 | 5.219 | 5.246 | 0.027 | 0.036 | 0.850 | 0.910 | Unannotated | NA | NA |
| metab_8468 | Unannotated feature | neg | 197.1546 | 5.747 | 3.340 | 3.295 | -0.045 | -0.036 | 0.850 | 0.910 | Unannotated | NA | NA |
| metab_4343 | Unannotated feature | pos | 688.8186 | 0.567 | 4.480 | 4.492 | 0.012 | 0.036 | 0.850 | 0.910 | Unannotated | NA | NA |
| metab_590 | Dehydrospermidine | pos | 166.1306 | 2.073 | 4.665 | 4.662 | -0.003 | 0.036 | 0.850 | 0.910 | Tentative | NA | NA |
| metab_11062 | Unannotated feature | neg | 225.0959 | 5.543 | 4.591 | 4.551 | -0.040 | -0.036 | 0.850 | 0.910 | Unannotated | NA | NA |
| metab_4384 | Unannotated feature | pos | 590.7435 | 0.567 | 4.380 | 4.384 | 0.004 | -0.036 | 0.850 | 0.910 | Unannotated | NA | NA |
| metab_2706 | 2-Propenyl hexanoate | pos | 330.2638 | 5.748 | 5.183 | 5.314 | 0.132 | 0.036 | 0.850 | 0.910 | Tentative | NA | NA |
| metab_2709 | Unannotated feature | pos | 159.0765 | 1.956 | 5.634 | 5.605 | -0.029 | -0.036 | 0.850 | 0.910 | Unannotated | NA | NA |
| metab_1660 | Tirofiban | pos | 441.2432 | 7.215 | 5.866 | 5.871 | 0.006 | -0.036 | 0.850 | 0.910 | Tentative | NA | NA |
| metab_2705 | O-(13-Carboxytridecanoyl)carnitine | pos | 366.2638 | 5.748 | 4.819 | 4.914 | 0.095 | 0.036 | 0.850 | 0.910 | Tentative | NA | NA |
| metab_2231 | Unannotated feature | pos | 310.3104 | 6.288 | 4.098 | 4.071 | -0.027 | -0.036 | 0.850 | 0.910 | Unannotated | NA | NA |
| metab_2253 | Unannotated feature | pos | 750.7939 | 0.567 | 5.315 | 5.306 | -0.010 | -0.036 | 0.850 | 0.910 | Unannotated | NA | NA |
| metab_9787 | Unannotated feature | neg | 471.9280 | 2.573 | 4.957 | 4.968 | 0.010 | -0.036 | 0.850 | 0.910 | Unannotated | NA | NA |
| metab_2046 | Unannotated feature | pos | 89.0690 | 7.261 | 4.326 | 4.334 | 0.008 | -0.036 | 0.850 | 0.910 | Unannotated | NA | NA |
| metab_2023 | Unannotated feature | pos | 206.8940 | 7.277 | 3.399 | 3.409 | 0.010 | 0.036 | 0.850 | 0.910 | Unannotated | NA | NA |
| metab_1969 | Unannotated feature | pos | 142.1170 | 7.300 | 4.613 | 4.620 | 0.008 | 0.036 | 0.850 | 0.910 | Unannotated | NA | NA |
| metab_1851 | Unannotated feature | pos | 171.1493 | 7.796 | 4.329 | 4.333 | 0.004 | 0.036 | 0.850 | 0.910 | Unannotated | NA | NA |
| metab_1813 | Unannotated feature | pos | 143.9969 | 7.975 | 5.448 | 5.448 | -0.000 | -0.036 | 0.850 | 0.910 | Unannotated | NA | NA |
| metab_2131 | Unannotated feature | pos | 808.0336 | 6.745 | 4.678 | 4.608 | -0.070 | -0.036 | 0.850 | 0.910 | Unannotated | NA | NA |
| metab_2026 | Unannotated feature | pos | 82.9454 | 7.277 | 3.884 | 3.912 | 0.027 | 0.036 | 0.850 | 0.910 | Unannotated | NA | NA |
| metab_10035 | Unannotated feature | neg | 93.0085 | 1.601 | 4.997 | 4.927 | -0.070 | -0.036 | 0.850 | 0.910 | Unannotated | NA | NA |
| metab_2215 | Unannotated feature | pos | 391.2298 | 6.296 | 6.137 | 6.123 | -0.014 | -0.036 | 0.850 | 0.910 | Strong putative | NA | NA |
| metab_1174 | Sterebin A | pos | 328.2482 | 5.725 | 5.794 | 5.764 | -0.029 | -0.036 | 0.850 | 0.910 | Tentative | NA | NA |
| metab_4287 | Unannotated feature | pos | 790.6903 | 0.575 | 4.923 | 4.935 | 0.012 | -0.036 | 0.850 | 0.910 | Unannotated | NA | NA |
| metab_1571 | Unannotated feature | pos | 413.3624 | 6.373 | 4.461 | 4.472 | 0.012 | 0.036 | 0.850 | 0.910 | Unannotated | NA | NA |
| metab_2408 | Unannotated feature | pos | 618.9225 | 5.933 | 4.301 | 4.320 | 0.019 | 0.036 | 0.850 | 0.910 | Unannotated | NA | NA |
| metab_10546 | Unannotated feature | neg | 706.4875 | 0.613 | 5.254 | 5.213 | -0.041 | -0.036 | 0.850 | 0.910 | Unannotated | NA | NA |
| metab_5265 | Unannotated feature | neg | 403.0126 | 0.865 | 5.126 | 5.110 | -0.016 | -0.036 | 0.850 | 0.910 | Unannotated | NA | NA |
| metab_7764 | Unannotated feature | neg | 256.9209 | 7.173 | 3.656 | 3.690 | 0.034 | -0.036 | 0.850 | 0.910 | Unannotated | NA | NA |
| metab_5090 | Unannotated feature | pos | 104.0458 | 1.491 | 4.525 | 4.526 | 0.001 | 0.036 | 0.850 | 0.910 | Unannotated | NA | NA |
| metab_4708 | Unannotated feature | pos | 538.7992 | 0.567 | 5.517 | 5.524 | 0.007 | 0.036 | 0.850 | 0.910 | Unannotated | NA | NA |
| metab_7749 | Unannotated feature | neg | 598.1794 | 2.523 | 4.732 | 4.696 | -0.036 | -0.036 | 0.850 | 0.910 | Unannotated | NA | NA |
| metab_1269 | Unannotated feature | pos | 588.9152 | 5.848 | 5.189 | 5.202 | 0.013 | 0.036 | 0.850 | 0.910 | Unannotated | NA | NA |
| metab_5415 | Unannotated feature | neg | 209.0222 | 0.629 | 4.435 | 4.460 | 0.025 | 0.036 | 0.850 | 0.910 | Unannotated | NA | NA |
| metab_11241 | Unannotated feature | neg | 603.7257 | 0.578 | 4.661 | 4.708 | 0.047 | -0.036 | 0.850 | 0.910 | Unannotated | NA | NA |
| metab_7828 | Unannotated feature | neg | 102.9013 | 7.156 | 3.761 | 3.776 | 0.015 | 0.036 | 0.850 | 0.910 | Unannotated | NA | NA |
| metab_11138 | Unannotated feature | neg | 521.1859 | 5.715 | 4.713 | 4.661 | -0.052 | -0.036 | 0.850 | 0.910 | Unannotated | NA | NA |
| metab_8388 | beta-D-Glucuronoside | neg | 513.2718 | 5.795 | 4.633 | 4.595 | -0.039 | 0.036 | 0.850 | 0.910 | Tentative | Lipids and lipid-like molecules | Steroids and steroid derivatives |
| metab_8962 | N6-(Delta2-Isopentenyl)-adenine | neg | 202.1084 | 4.901 | 4.251 | 4.193 | -0.059 | -0.032 | 0.868 | 0.924 | Strong putative | NA | NA |
| metab_3139 | Metyrosine | pos | 178.0863 | 3.638 | 4.995 | 4.955 | -0.040 | 0.032 | 0.868 | 0.924 | Tentative | NA | NA |
| metab_1643 | Unannotated feature | pos | 387.2351 | 7.192 | 4.959 | 4.959 | 0.001 | 0.032 | 0.868 | 0.924 | Unannotated | NA | NA |
| metab_10978 | Unannotated feature | neg | 311.8707 | 0.501 | 3.712 | 3.726 | 0.015 | -0.032 | 0.868 | 0.924 | Unannotated | NA | NA |
| metab_9190 | Unannotated feature | neg | 132.0812 | 4.175 | 4.859 | 4.875 | 0.016 | 0.032 | 0.868 | 0.924 | Unannotated | NA | NA |
| metab_3519 | Unannotated feature | pos | 109.1015 | 2.141 | 3.451 | 3.461 | 0.011 | 0.032 | 0.868 | 0.924 | Unannotated | NA | NA |
| metab_9047 | Unannotated feature | neg | 352.9089 | 4.627 | 4.495 | 4.497 | 0.002 | -0.032 | 0.868 | 0.924 | Unannotated | NA | NA |
| metab_9527 | Unannotated feature | neg | 183.0030 | 3.224 | 4.880 | 4.881 | 0.001 | -0.032 | 0.868 | 0.924 | Unannotated | NA | NA |
| metab_3183 | 2-Hexyl-1,3-dioxolane-4-methanol | pos | 153.1275 | 3.360 | 3.465 | 3.572 | 0.107 | -0.032 | 0.868 | 0.924 | Tentative | NA | NA |
| metab_3621 | Unannotated feature | pos | 143.0340 | 1.964 | 4.526 | 4.521 | -0.006 | 0.032 | 0.868 | 0.924 | Unannotated | NA | NA |
| metab_2369 | 11-Dehydrocorticosterone | pos | 362.2359 | 5.964 | 3.826 | 4.099 | 0.273 | 0.032 | 0.868 | 0.924 | Tentative | NA | NA |
| metab_10927 | Unannotated feature | neg | 716.8439 | 0.534 | 4.270 | 4.307 | 0.037 | -0.032 | 0.868 | 0.924 | Unannotated | NA | NA |
| metab_9966 | Unannotated feature | neg | 199.0723 | 2.095 | 3.334 | 3.313 | -0.020 | -0.032 | 0.868 | 0.924 | Unannotated | NA | NA |
| metab_1669 | Unannotated feature | pos | 507.3293 | 7.246 | 5.034 | 5.047 | 0.013 | 0.032 | 0.868 | 0.924 | Unannotated | NA | NA |
| metab_2582 | Unannotated feature | pos | 566.4004 | 5.855 | 5.805 | 5.828 | 0.023 | 0.032 | 0.868 | 0.924 | Unannotated | NA | NA |
| metab_9756 | Unannotated feature | neg | 121.0400 | 2.700 | 3.406 | 3.398 | -0.008 | -0.032 | 0.868 | 0.924 | Unannotated | NA | NA |
| metab_2419 | Unannotated feature | pos | 762.5585 | 5.925 | 6.218 | 6.247 | 0.029 | -0.032 | 0.868 | 0.924 | Unannotated | NA | NA |
| metab_9833 | Unannotated feature | neg | 394.1412 | 2.504 | 4.209 | 4.241 | 0.032 | -0.032 | 0.868 | 0.924 | Unannotated | NA | NA |
| metab_10427 | Unannotated feature | neg | 250.9474 | 0.676 | 4.397 | 4.444 | 0.047 | 0.032 | 0.868 | 0.924 | Unannotated | NA | NA |
| metab_10233 | Unannotated feature | neg | 166.9808 | 0.865 | 4.298 | 4.276 | -0.022 | -0.032 | 0.868 | 0.924 | Unannotated | NA | NA |
| metab_1694 | Unannotated feature | pos | 184.0945 | 7.285 | 4.257 | 4.262 | 0.005 | 0.032 | 0.868 | 0.924 | Unannotated | NA | NA |
| metab_2099 | Unannotated feature | pos | 266.2437 | 7.099 | 4.252 | 4.218 | -0.034 | -0.032 | 0.868 | 0.924 | Unannotated | NA | NA |
| metab_7776 | Unannotated feature | neg | 488.7878 | 7.170 | 4.449 | 4.469 | 0.019 | -0.032 | 0.868 | 0.924 | Unannotated | NA | NA |
| metab_5360 | Unannotated feature | neg | 146.9380 | 0.016 | 6.414 | 6.407 | -0.007 | -0.032 | 0.868 | 0.924 | Unannotated | NA | NA |
| metab_7882 | Unannotated feature | neg | 134.0466 | 7.140 | 3.942 | 3.949 | 0.006 | -0.032 | 0.868 | 0.924 | Unannotated | NA | NA |
| metab_4698 | 5 | pos | 558.4177 | 5.910 | 6.323 | 6.348 | 0.025 | 0.032 | 0.868 | 0.924 | Tentative | NA | NA |
| metab_4502 | Unannotated feature | pos | 105.0427 | 0.551 | 4.773 | 4.776 | 0.003 | 0.032 | 0.868 | 0.924 | Unannotated | NA | NA |
| metab_4653 | Unannotated feature | pos | 216.1959 | 5.748 | 4.787 | 4.776 | -0.011 | 0.032 | 0.868 | 0.924 | Unannotated | NA | NA |
| metab_5072 | Unannotated feature | pos | 309.0580 | 1.095 | 5.282 | 5.290 | 0.008 | -0.032 | 0.868 | 0.924 | Unannotated | NA | NA |
| metab_11143 | Unannotated feature | neg | 469.0855 | 5.723 | 4.043 | 4.078 | 0.034 | 0.032 | 0.868 | 0.924 | Unannotated | NA | NA |
| metab_5185 | Unannotated feature | neg | 197.9630 | 0.530 | 4.802 | 4.782 | -0.019 | -0.032 | 0.868 | 0.924 | Unannotated | NA | NA |
| metab_358 | Unannotated feature | pos | 155.0427 | 0.861 | 4.414 | 4.436 | 0.022 | -0.032 | 0.868 | 0.924 | Unannotated | NA | NA |
| metab_7619 | Unannotated feature | neg | 223.0460 | 7.339 | 4.612 | 4.612 | -0.001 | -0.032 | 0.868 | 0.924 | Unannotated | NA | NA |
| metab_192 | Unannotated feature | pos | 625.8482 | 0.567 | 4.747 | 4.740 | -0.006 | -0.032 | 0.868 | 0.924 | Unannotated | NA | NA |
| metab_5422 | Unannotated feature | neg | 162.2686 | 0.636 | 4.618 | 4.634 | 0.016 | -0.032 | 0.868 | 0.924 | Unannotated | NA | NA |
| metab_7684 | Unannotated feature | neg | 247.9628 | 7.237 | 4.178 | 4.169 | -0.009 | -0.032 | 0.868 | 0.924 | Unannotated | NA | NA |
| metab_266 | Unannotated feature | pos | 117.0742 | 0.698 | 5.769 | 5.746 | -0.023 | -0.032 | 0.868 | 0.924 | Unannotated | NA | NA |
| metab_7842 | Unannotated feature | neg | 248.0803 | 7.153 | 5.108 | 5.133 | 0.025 | 0.032 | 0.868 | 0.924 | Unannotated | NA | NA |
| metab_11298 | Unannotated feature | neg | 334.8642 | 7.173 | 4.214 | 4.200 | -0.014 | -0.032 | 0.868 | 0.924 | Unannotated | NA | NA |
| metab_7408 | Unannotated feature | neg | 274.8962 | 7.101 | 4.358 | 4.368 | 0.010 | 0.032 | 0.868 | 0.924 | Unannotated | NA | NA |
| metab_5721 | Unannotated feature | neg | 439.6851 | 1.478 | 4.905 | 4.888 | -0.017 | -0.032 | 0.868 | 0.924 | Unannotated | NA | NA |
| metab_7571 | Unannotated feature | neg | 231.9180 | 7.197 | 4.900 | 4.902 | 0.002 | -0.032 | 0.868 | 0.924 | Unannotated | NA | NA |
| metab_7475 | Unannotated feature | neg | 384.9361 | 7.140 | 6.181 | 6.183 | 0.002 | -0.032 | 0.868 | 0.924 | Unannotated | NA | NA |
| metab_7474 | Unannotated feature | neg | 362.9403 | 7.140 | 4.793 | 4.811 | 0.018 | 0.032 | 0.868 | 0.924 | Unannotated | NA | NA |
| metab_5730 | Unannotated feature | neg | 255.8483 | 1.593 | 4.528 | 4.607 | 0.078 | 0.032 | 0.868 | 0.924 | Unannotated | NA | NA |
| metab_5722 | Unannotated feature | neg | 181.0132 | 1.494 | 3.897 | 3.852 | -0.045 | -0.032 | 0.868 | 0.924 | Unannotated | NA | NA |
| metab_11278 | 1-Acyl-sn-glycero-3-phosphocholine | neg | 540.3319 | 6.392 | 7.542 | 7.539 | -0.004 | 0.032 | 0.868 | 0.924 | Tentative | NA | NA |
| metab_5852 | Unannotated feature | neg | 89.9249 | 2.379 | 4.346 | 4.353 | 0.007 | 0.032 | 0.868 | 0.924 | Unannotated | NA | NA |
| metab_6675 | Unannotated feature | neg | 195.0660 | 5.162 | 3.163 | 3.072 | -0.091 | 0.032 | 0.868 | 0.924 | Unannotated | NA | NA |
| metab_6423 | 1,2,3,4-Tetrahydroisoquinoline-3-carboxylic acid | neg | 176.0714 | 4.175 | 4.501 | 4.476 | -0.025 | 0.032 | 0.868 | 0.924 | Tentative | NA | NA |
| metab_7316 | Unannotated feature | neg | 134.8940 | 6.281 | 6.774 | 6.798 | 0.023 | 0.032 | 0.868 | 0.924 | Unannotated | NA | NA |
| metab_7042 | Unannotated feature | neg | 223.1705 | 5.763 | 3.696 | 3.721 | 0.024 | 0.032 | 0.868 | 0.924 | Unannotated | NA | NA |
| metab_7050 | Unannotated feature | neg | 226.9093 | 5.771 | 4.697 | 4.739 | 0.042 | 0.032 | 0.868 | 0.924 | Unannotated | NA | NA |
| metab_6791 | Unannotated feature | neg | 475.0455 | 5.543 | 3.912 | 3.681 | -0.231 | -0.032 | 0.868 | 0.924 | Unannotated | NA | NA |
| metab_11350 | Unannotated feature | neg | 237.0439 | 5.579 | 5.717 | 5.731 | 0.014 | 0.027 | 0.887 | 0.936 | Unannotated | NA | NA |
| metab_11376 | Unannotated feature | neg | 211.9145 | 0.491 | 4.050 | 4.048 | -0.002 | 0.027 | 0.887 | 0.936 | Unannotated | NA | NA |
| metab_52 | Unannotated feature | pos | 81.0340 | 0.018 | 3.868 | 3.860 | -0.008 | 0.027 | 0.887 | 0.936 | Unannotated | NA | NA |
| metab_6805 | Unannotated feature | neg | 155.0359 | 5.579 | 4.348 | 4.377 | 0.029 | 0.027 | 0.887 | 0.936 | Unannotated | NA | NA |
| metab_6582 | Unannotated feature | neg | 439.0713 | 4.774 | 5.388 | 5.461 | 0.073 | 0.027 | 0.887 | 0.936 | Unannotated | NA | NA |
| metab_6549 | Unannotated feature | neg | 412.8084 | 0.582 | 5.418 | 5.407 | -0.011 | 0.027 | 0.887 | 0.936 | Unannotated | NA | NA |
| metab_7931 | Unannotated feature | neg | 222.8409 | 7.109 | 5.515 | 5.523 | 0.007 | 0.027 | 0.887 | 0.936 | Unannotated | NA | NA |
| metab_1118 | Unannotated feature | pos | 177.1023 | 1.956 | 4.948 | 4.953 | 0.005 | 0.027 | 0.887 | 0.936 | Unannotated | NA | NA |
| metab_8077 | LysoPC(20:3(5Z,8Z,11Z)/0:0) | neg | 590.3470 | 6.234 | 4.352 | 4.196 | -0.156 | -0.027 | 0.887 | 0.936 | Tentative | NA | NA |
| metab_4734 | Unannotated feature | pos | 528.3087 | 6.072 | 5.485 | 5.489 | 0.004 | -0.027 | 0.887 | 0.936 | Unannotated | NA | NA |
| metab_10421 | Unannotated feature | neg | 177.8444 | 0.684 | 6.215 | 6.231 | 0.016 | 0.027 | 0.887 | 0.936 | Unannotated | NA | NA |
| metab_7989 | Unannotated feature | neg | 218.9312 | 7.046 | 3.809 | 3.854 | 0.046 | -0.027 | 0.887 | 0.936 | Unannotated | NA | NA |
| metab_5136 | Arecaidine | pos | 169.0974 | 0.721 | 4.840 | 4.883 | 0.043 | 0.027 | 0.887 | 0.936 | Tentative | NA | NA |
| metab_5127 | 7,8-Dihydrobiopterin | pos | 272.1353 | 2.506 | 5.149 | 5.031 | -0.117 | -0.027 | 0.887 | 0.936 | Tentative | NA | NA |
| metab_8172 | Unannotated feature | neg | 295.2283 | 6.024 | 4.544 | 4.563 | 0.018 | -0.027 | 0.887 | 0.936 | Unannotated | NA | NA |
| metab_1042 | 13(S)-HPOT | pos | 333.2017 | 4.884 | 5.377 | 5.371 | -0.006 | -0.027 | 0.887 | 0.936 | Tentative | NA | NA |
| metab_5314 | Unannotated feature | neg | 790.8211 | 0.562 | 4.518 | 4.504 | -0.013 | 0.027 | 0.887 | 0.936 | Unannotated | NA | NA |
| metab_7871 | Unannotated feature | neg | 690.8806 | 7.140 | 5.317 | 5.308 | -0.010 | -0.027 | 0.887 | 0.936 | Unannotated | NA | NA |
| metab_8425 | Unannotated feature | neg | 217.0982 | 3.217 | 3.712 | 3.629 | -0.083 | -0.027 | 0.887 | 0.936 | Unannotated | NA | NA |
| metab_4371 | Unannotated feature | pos | 658.8402 | 0.567 | 4.751 | 4.735 | -0.016 | 0.027 | 0.887 | 0.936 | Unannotated | NA | NA |
| metab_11190 | 10-hydroxy-2E-decenoic acid | neg | 185.1180 | 5.859 | 5.216 | 5.247 | 0.031 | 0.027 | 0.887 | 0.936 | Moderate putative | NA | NA |
| metab_7765 | Unannotated feature | neg | 239.9496 | 7.173 | 4.826 | 4.838 | 0.012 | -0.027 | 0.887 | 0.936 | Unannotated | NA | NA |
| metab_1905 | Unannotated feature | pos | 126.1279 | 7.331 | 4.551 | 4.544 | -0.007 | -0.027 | 0.887 | 0.936 | Unannotated | NA | NA |
| metab_1876 | Unannotated feature | pos | 84.1315 | 7.378 | 4.479 | 4.474 | -0.005 | -0.027 | 0.887 | 0.936 | Unannotated | NA | NA |
| metab_11324 | Unannotated feature | neg | 142.9284 | 7.213 | 3.882 | 3.892 | 0.009 | 0.027 | 0.887 | 0.936 | Unannotated | NA | NA |
| metab_1817 | Unannotated feature | pos | 115.0393 | 7.983 | 4.579 | 4.581 | 0.001 | -0.027 | 0.887 | 0.936 | Unannotated | NA | NA |
| metab_10336 | Unannotated feature | neg | 242.0316 | 0.770 | 4.459 | 4.348 | -0.111 | -0.027 | 0.887 | 0.936 | Unannotated | NA | NA |
| metab_10321 | Unannotated feature | neg | 220.8143 | 0.786 | 6.017 | 6.053 | 0.035 | 0.027 | 0.887 | 0.936 | Unannotated | NA | NA |
| metab_6191 | Unannotated feature | neg | 405.9516 | 3.308 | 4.617 | 4.642 | 0.025 | -0.027 | 0.887 | 0.936 | Unannotated | NA | NA |
| metab_6246 | Unannotated feature | neg | 196.5665 | 3.483 | 4.402 | 4.416 | 0.014 | -0.027 | 0.887 | 0.936 | Unannotated | NA | NA |
| metab_1793 | Unannotated feature | pos | 170.0663 | 0.504 | 5.683 | 5.713 | 0.030 | -0.027 | 0.887 | 0.936 | Unannotated | NA | NA |
| metab_10439 | Unannotated feature | neg | 160.8415 | 0.668 | 6.815 | 6.838 | 0.023 | 0.027 | 0.887 | 0.936 | Unannotated | NA | NA |
| metab_5228 | Unannotated feature | neg | 382.8658 | 0.546 | 4.697 | 4.693 | -0.004 | -0.027 | 0.887 | 0.936 | Unannotated | NA | NA |
| metab_5532 | Unannotated feature | neg | 228.9030 | 0.794 | 4.365 | 4.353 | -0.012 | -0.027 | 0.887 | 0.936 | Unannotated | NA | NA |
| metab_7632 | Unannotated feature | neg | 286.8971 | 7.932 | 5.576 | 5.583 | 0.007 | -0.027 | 0.887 | 0.936 | Unannotated | NA | NA |
| metab_2456 | Unannotated feature | pos | 704.5139 | 5.910 | 6.102 | 6.129 | 0.027 | -0.027 | 0.887 | 0.936 | Unannotated | NA | NA |
| metab_7673 | Unannotated feature | neg | 148.9901 | 7.261 | 4.389 | 4.387 | -0.002 | -0.027 | 0.887 | 0.936 | Unannotated | NA | NA |
| metab_7177 | Unannotated feature | neg | 410.3000 | 5.898 | 5.053 | 5.047 | -0.006 | -0.027 | 0.887 | 0.936 | Unannotated | NA | NA |
| metab_7163 | Unannotated feature | neg | 160.6421 | 5.890 | 3.427 | 3.527 | 0.100 | 0.027 | 0.887 | 0.936 | Unannotated | NA | NA |
| metab_6420 | Unannotated feature | neg | 241.0881 | 4.167 | 4.519 | 4.531 | 0.012 | 0.027 | 0.887 | 0.936 | Unannotated | NA | NA |
| metab_5856 | Unannotated feature | neg | 295.8318 | 2.385 | 5.196 | 5.191 | -0.006 | 0.027 | 0.887 | 0.936 | Unannotated | NA | NA |
| metab_10664 | Unannotated feature | neg | 764.5549 | 0.582 | 4.604 | 4.671 | 0.067 | 0.027 | 0.887 | 0.936 | Unannotated | NA | NA |
| metab_10616 | Unannotated feature | neg | 224.8193 | 0.598 | 5.057 | 5.025 | -0.032 | -0.027 | 0.887 | 0.936 | Unannotated | NA | NA |
| metab_10631 | Unannotated feature | neg | 292.8073 | 0.594 | 5.073 | 5.043 | -0.030 | 0.027 | 0.887 | 0.936 | Unannotated | NA | NA |
| metab_2564 | Unannotated feature | pos | 452.3734 | 5.863 | 4.752 | 4.781 | 0.030 | 0.027 | 0.887 | 0.936 | Unannotated | NA | NA |
| metab_3112 | 4-Hydroxyheptanoylcarnitine | pos | 272.1855 | 3.769 | 4.227 | 4.197 | -0.030 | -0.027 | 0.887 | 0.936 | Tentative | NA | NA |
| metab_7439 | Unannotated feature | neg | 328.9313 | 7.109 | 4.864 | 4.898 | 0.034 | 0.027 | 0.887 | 0.936 | Unannotated | NA | NA |
| metab_5656 | Unannotated feature | neg | 305.9185 | 1.015 | 4.668 | 4.689 | 0.022 | -0.027 | 0.887 | 0.936 | Unannotated | NA | NA |
| metab_7702 | Unannotated feature | neg | 159.9187 | 7.213 | 2.765 | 2.705 | -0.060 | -0.027 | 0.887 | 0.936 | Unannotated | NA | NA |
| metab_5958 | Unannotated feature | neg | 352.8543 | 2.724 | 6.328 | 6.311 | -0.017 | -0.027 | 0.887 | 0.936 | Unannotated | NA | NA |
| metab_4122 | 3H-Dopamine | pos | 156.1020 | 3.677 | 3.154 | 3.423 | 0.269 | 0.027 | 0.887 | 0.936 | Tentative | NA | NA |
| metab_11052 | Unannotated feature | neg | 92.9192 | 0.013 | 5.020 | 5.022 | 0.002 | 0.027 | 0.887 | 0.936 | Unannotated | NA | NA |
| metab_9996 | Unannotated feature | neg | 437.9438 | 1.935 | 4.638 | 4.624 | -0.013 | -0.027 | 0.887 | 0.936 | Unannotated | NA | NA |
| metab_7717 | Unannotated feature | neg | 438.1306 | 2.504 | 5.198 | 5.216 | 0.018 | -0.027 | 0.887 | 0.936 | Unannotated | NA | NA |
| metab_7392 | Unannotated feature | neg | 198.8319 | 7.093 | 4.937 | 4.935 | -0.002 | -0.027 | 0.887 | 0.936 | Unannotated | NA | NA |
| metab_6065 | Aminomethyl fluorescein | neg | 406.0918 | 3.104 | 4.397 | 4.470 | 0.074 | -0.027 | 0.887 | 0.936 | Tentative | NA | NA |
| metab_5432 | Unannotated feature | neg | 305.9185 | 0.644 | 4.633 | 4.633 | -0.000 | 0.027 | 0.887 | 0.936 | Unannotated | NA | NA |
| metab_2188 | Unannotated feature | pos | 823.7927 | 0.567 | 4.862 | 4.852 | -0.010 | 0.027 | 0.887 | 0.936 | Unannotated | NA | NA |
| metab_882 | Nicotyrine | pos | 159.0918 | 3.638 | 4.713 | 4.638 | -0.074 | 0.027 | 0.887 | 0.936 | Tentative | NA | NA |
| metab_8704 | Unannotated feature | neg | 90.9328 | 7.170 | 4.699 | 4.713 | 0.014 | -0.027 | 0.887 | 0.936 | Unannotated | NA | NA |
| metab_911 | 3-(3,4-Dihydroxyphenyl)lactate | pos | 240.0866 | 3.838 | 3.644 | 3.733 | 0.089 | -0.027 | 0.887 | 0.936 | Tentative | NA | NA |
| metab_875 | Unannotated feature | pos | 163.1168 | 3.591 | 3.711 | 3.597 | -0.114 | 0.027 | 0.887 | 0.936 | Unannotated | NA | NA |
| metab_1399 | Unannotated feature | pos | 538.8675 | 5.925 | 4.800 | 4.929 | 0.130 | 0.027 | 0.887 | 0.936 | Unannotated | NA | NA |
| metab_1308 | Unannotated feature | pos | 171.1176 | 2.065 | 7.681 | 7.683 | 0.002 | 0.027 | 0.887 | 0.936 | Unannotated | NA | NA |
| metab_7063 | Unannotated feature | neg | 242.1764 | 5.787 | 5.838 | 5.832 | -0.006 | -0.027 | 0.887 | 0.936 | Unannotated | NA | NA |
| metab_1228 | Unannotated feature | pos | 286.2378 | 5.801 | 4.533 | 4.525 | -0.008 | -0.027 | 0.887 | 0.936 | Unannotated | NA | NA |
| metab_7075 | Unannotated feature | neg | 339.8753 | 5.795 | 4.330 | 4.320 | -0.009 | 0.027 | 0.887 | 0.936 | Unannotated | NA | NA |
| metab_10241 | Unannotated feature | neg | 282.7783 | 0.598 | 5.804 | 5.787 | -0.017 | 0.023 | 0.906 | 0.947 | Unannotated | NA | NA |
| metab_5380 | Unannotated feature | neg | 466.6833 | 0.598 | 4.784 | 4.801 | 0.017 | 0.023 | 0.906 | 0.947 | Unannotated | NA | NA |
| metab_7784 | Unannotated feature | neg | 232.8916 | 7.170 | 3.254 | 3.281 | 0.027 | -0.023 | 0.906 | 0.947 | Unannotated | NA | NA |
| metab_7910 | Unannotated feature | neg | 345.9095 | 7.117 | 5.402 | 5.410 | 0.007 | 0.023 | 0.906 | 0.947 | Unannotated | NA | NA |
| metab_4388 | Unannotated feature | pos | 558.8570 | 0.567 | 4.987 | 4.994 | 0.007 | -0.023 | 0.906 | 0.947 | Unannotated | NA | NA |
| metab_7233 | Unannotated feature | neg | 754.7437 | 0.570 | 5.367 | 5.374 | 0.007 | -0.023 | 0.906 | 0.947 | Unannotated | NA | NA |
| metab_6271 | Unannotated feature | neg | 704.8189 | 0.570 | 5.738 | 5.738 | 0.000 | -0.023 | 0.906 | 0.947 | Unannotated | NA | NA |
| metab_10426 | Unannotated feature | neg | 259.0104 | 4.782 | 6.321 | 6.344 | 0.022 | 0.023 | 0.906 | 0.947 | Unannotated | NA | NA |
| metab_7909 | Unannotated feature | neg | 424.1784 | 7.117 | 4.722 | 4.675 | -0.047 | -0.023 | 0.906 | 0.947 | Unannotated | NA | NA |
| metab_7896 | Unannotated feature | neg | 265.9510 | 7.132 | 4.367 | 4.367 | -0.000 | -0.023 | 0.906 | 0.947 | Unannotated | NA | NA |
| metab_7869 | Unannotated feature | neg | 711.8744 | 7.140 | 4.276 | 4.335 | 0.058 | 0.023 | 0.906 | 0.947 | Unannotated | NA | NA |
| metab_7940 | Unannotated feature | neg | 160.5329 | 7.109 | 4.653 | 4.664 | 0.011 | 0.023 | 0.906 | 0.947 | Unannotated | NA | NA |
| metab_10683 | Unannotated feature | neg | 627.6906 | 0.578 | 3.950 | 3.986 | 0.036 | -0.023 | 0.906 | 0.947 | Unannotated | NA | NA |
| metab_5013 | Unannotated feature | pos | 179.0703 | 0.800 | 4.416 | 4.408 | -0.008 | 0.023 | 0.906 | 0.947 | Unannotated | NA | NA |
| metab_8079 | LysoPA(18:2(9Z,12Z)/0:0) | neg | 433.2367 | 6.226 | 5.491 | 5.419 | -0.072 | -0.023 | 0.906 | 0.947 | Tentative | NA | NA |
| metab_10537 | Unannotated feature | neg | 556.7645 | 0.621 | 4.662 | 4.646 | -0.016 | 0.023 | 0.906 | 0.947 | Unannotated | NA | NA |
| metab_7952 | Unannotated feature | neg | 116.9277 | 7.109 | 6.609 | 6.605 | -0.005 | 0.023 | 0.906 | 0.947 | Unannotated | NA | NA |
| metab_1593 | Unannotated feature | pos | 778.9944 | 6.567 | 5.949 | 5.941 | -0.008 | 0.023 | 0.906 | 0.947 | Unannotated | NA | NA |
| metab_6569 | Halleridone | neg | 153.0552 | 4.695 | 3.646 | 3.231 | -0.414 | -0.023 | 0.906 | 0.947 | Tentative | NA | NA |
| metab_6904 | 1-(2-Methyl-4-methoxyphenyl)-4-((2-hydroxyethyl)amino)-6-trifluoromethoxy-2,3-dihydropyrrolo(3,2-c)quinoline | neg | 468.1320 | 5.659 | 4.057 | 3.988 | -0.069 | -0.023 | 0.906 | 0.947 | Tentative | NA | NA |
| metab_8032 | Unannotated feature | neg | 113.3158 | 6.568 | 3.986 | 3.981 | -0.004 | 0.023 | 0.906 | 0.947 | Unannotated | NA | NA |
| metab_4839 | Unannotated feature | pos | 86.0605 | 7.293 | 3.950 | 3.953 | 0.002 | -0.023 | 0.906 | 0.947 | Unannotated | NA | NA |
| metab_4779 | Unannotated feature | pos | 407.2768 | 7.223 | 5.125 | 5.204 | 0.080 | 0.023 | 0.906 | 0.947 | Unannotated | NA | NA |
| metab_8009 | Unannotated feature | neg | 101.6505 | 6.871 | 2.960 | 2.907 | -0.053 | -0.023 | 0.906 | 0.947 | Unannotated | NA | NA |
| metab_1810 | Unannotated feature | pos | 146.0530 | 7.967 | 4.954 | 4.941 | -0.014 | -0.023 | 0.906 | 0.947 | Unannotated | NA | NA |
| metab_1850 | Unannotated feature | pos | 113.0236 | 7.812 | 4.226 | 4.224 | -0.002 | 0.023 | 0.906 | 0.947 | Unannotated | NA | NA |
| metab_11326 | Unannotated feature | neg | 185.8654 | 7.189 | 4.603 | 4.618 | 0.015 | -0.023 | 0.906 | 0.947 | Unannotated | NA | NA |
| metab_577 | Unannotated feature | pos | 97.0954 | 2.065 | 4.336 | 4.343 | 0.007 | 0.023 | 0.906 | 0.947 | Unannotated | NA | NA |
| metab_11231 | Unannotated feature | neg | 177.0402 | 5.651 | 4.474 | 4.497 | 0.023 | 0.023 | 0.906 | 0.947 | Unannotated | NA | NA |
| metab_3712 | Unannotated feature | pos | 184.1333 | 1.886 | 3.829 | 3.797 | -0.032 | -0.023 | 0.906 | 0.947 | Unannotated | NA | NA |
| metab_3645 | Unannotated feature | pos | 173.1286 | 1.956 | 4.318 | 4.277 | -0.041 | -0.023 | 0.906 | 0.947 | Unannotated | NA | NA |
| metab_856 | Unannotated feature | pos | 84.1317 | 3.414 | 3.578 | 3.559 | -0.020 | -0.023 | 0.906 | 0.947 | Unannotated | NA | NA |
| metab_2233 | Unannotated feature | pos | 279.1776 | 6.265 | 3.685 | 3.644 | -0.041 | 0.023 | 0.906 | 0.947 | Unannotated | NA | NA |
| metab_10015 | Unannotated feature | neg | 229.9021 | 1.847 | 5.743 | 5.793 | 0.050 | 0.023 | 0.906 | 0.947 | Unannotated | NA | NA |
| metab_3828 | Unannotated feature | pos | 74.0970 | 1.251 | 4.191 | 4.182 | -0.010 | -0.023 | 0.906 | 0.947 | Unannotated | NA | NA |
| metab_3961 | MCPA | pos | 218.0594 | 0.846 | 4.153 | 4.034 | -0.118 | -0.023 | 0.906 | 0.947 | Tentative | NA | NA |
| metab_4042 | Unannotated feature | pos | 103.4008 | 0.784 | 3.494 | 3.501 | 0.008 | 0.023 | 0.906 | 0.947 | Unannotated | NA | NA |
| metab_7799 | Unannotated feature | neg | 89.9249 | 7.170 | 4.485 | 4.487 | 0.002 | 0.023 | 0.906 | 0.947 | Unannotated | NA | NA |
| metab_8848 | Unannotated feature | neg | 588.8991 | 7.140 | 6.272 | 6.258 | -0.014 | 0.023 | 0.906 | 0.947 | Unannotated | NA | NA |
| metab_4333 | Unannotated feature | pos | 561.7657 | 0.575 | 4.031 | 4.059 | 0.028 | 0.023 | 0.906 | 0.947 | Unannotated | NA | NA |
| metab_551 | Unannotated feature | pos | 101.8086 | 2.026 | 3.954 | 3.947 | -0.007 | 0.023 | 0.906 | 0.947 | Unannotated | NA | NA |
| metab_7411 | Unannotated feature | neg | 100.6545 | 7.109 | 5.137 | 5.135 | -0.002 | -0.023 | 0.906 | 0.947 | Unannotated | NA | NA |
| metab_4199 | Unannotated feature | pos | 270.1185 | 0.629 | 3.514 | 3.563 | 0.049 | -0.023 | 0.906 | 0.947 | Unannotated | NA | NA |
| metab_4202 | Unannotated feature | pos | 202.2591 | 0.629 | 5.582 | 5.607 | 0.025 | -0.023 | 0.906 | 0.947 | Unannotated | NA | NA |
| metab_7794 | Unannotated feature | neg | 150.9905 | 7.170 | 3.553 | 3.542 | -0.011 | -0.023 | 0.906 | 0.947 | Unannotated | NA | NA |
| metab_5655 | Unannotated feature | neg | 437.6882 | 1.007 | 5.521 | 5.532 | 0.011 | -0.023 | 0.906 | 0.947 | Unannotated | NA | NA |
| metab_9893 | Unannotated feature | neg | 116.0247 | 2.237 | 4.158 | 4.139 | -0.019 | -0.023 | 0.906 | 0.947 | Unannotated | NA | NA |
| metab_2347 | Unannotated feature | pos | 681.9955 | 5.979 | 4.535 | 4.534 | -0.001 | 0.023 | 0.906 | 0.947 | Unannotated | NA | NA |
| metab_9819 | Unannotated feature | neg | 106.9429 | 2.523 | 5.470 | 5.466 | -0.003 | -0.023 | 0.906 | 0.947 | Unannotated | NA | NA |
| metab_3588 | Unannotated feature | pos | 130.1592 | 2.050 | 4.622 | 4.610 | -0.012 | -0.023 | 0.906 | 0.947 | Unannotated | NA | NA |
| metab_676 | 3-Methoxyanthranilate | pos | 168.0656 | 2.444 | 3.853 | 3.843 | -0.010 | -0.023 | 0.906 | 0.947 | Moderate putative | NA | NA |
| metab_3634 | Unannotated feature | pos | 227.1390 | 1.956 | 3.605 | 3.332 | -0.273 | -0.023 | 0.906 | 0.947 | Unannotated | NA | NA |
| metab_2343 | Unannotated feature | pos | 675.4785 | 5.987 | 5.203 | 5.200 | -0.003 | -0.023 | 0.906 | 0.947 | Unannotated | NA | NA |
| metab_9114 | Unannotated feature | neg | 731.3186 | 4.371 | 5.758 | 5.781 | 0.023 | -0.023 | 0.906 | 0.947 | Unannotated | NA | NA |
| metab_11269 | Unannotated feature | neg | 684.7598 | 0.570 | 5.045 | 5.030 | -0.015 | -0.023 | 0.906 | 0.947 | Unannotated | NA | NA |
| metab_5723 | Unannotated feature | neg | 208.8249 | 1.494 | 4.986 | 5.003 | 0.017 | -0.023 | 0.906 | 0.947 | Unannotated | NA | NA |
| metab_8115 | Unannotated feature | neg | 145.9306 | 6.180 | 5.587 | 5.589 | 0.002 | 0.023 | 0.906 | 0.947 | Unannotated | NA | NA |
| metab_7980 | Unannotated feature | neg | 178.8149 | 7.093 | 4.694 | 4.687 | -0.007 | 0.023 | 0.906 | 0.947 | Unannotated | NA | NA |
| metab_8601 | Unannotated feature | neg | 147.4572 | 5.675 | 4.267 | 4.237 | -0.030 | -0.023 | 0.906 | 0.947 | Unannotated | NA | NA |
| metab_7979 | Unannotated feature | neg | 200.8297 | 7.093 | 4.659 | 4.643 | -0.015 | 0.023 | 0.906 | 0.947 | Unannotated | NA | NA |
| metab_1729 | Unannotated feature | pos | 340.9318 | 0.527 | 5.183 | 5.118 | -0.065 | -0.023 | 0.906 | 0.947 | Unannotated | NA | NA |
| metab_5 | Glycylvaline | pos | 175.1079 | 1.747 | 5.480 | 5.499 | 0.019 | -0.023 | 0.906 | 0.947 | Strong putative | NA | NA |
| metab_3144 | Adipate semialdehyde | pos | 172.0969 | 3.599 | 3.651 | 3.480 | -0.171 | -0.023 | 0.906 | 0.947 | Tentative | NA | NA |
| metab_2810 | Hexyl glucoside | pos | 229.1435 | 5.581 | 3.938 | 3.958 | 0.020 | -0.023 | 0.906 | 0.947 | Tentative | NA | NA |
| metab_7525 | Unannotated feature | neg | 622.7658 | 7.164 | 4.303 | 4.313 | 0.009 | 0.023 | 0.906 | 0.947 | Unannotated | NA | NA |
| metab_3067 | Unannotated feature | pos | 140.0384 | 3.993 | 4.341 | 4.330 | -0.011 | 0.023 | 0.906 | 0.947 | Unannotated | NA | NA |
| metab_7087 | Unannotated feature | neg | 236.9808 | 5.803 | 3.569 | 3.606 | 0.037 | 0.023 | 0.906 | 0.947 | Unannotated | NA | NA |
| metab_7056 | Unannotated feature | neg | 221.9561 | 5.779 | 3.646 | 3.591 | -0.056 | -0.023 | 0.906 | 0.947 | Unannotated | NA | NA |
| metab_7088 | Unannotated feature | neg | 241.9467 | 5.803 | 4.215 | 4.161 | -0.054 | -0.019 | 0.924 | 0.959 | Unannotated | NA | NA |
| metab_1469 | Unannotated feature | pos | 766.5310 | 6.003 | 5.678 | 5.683 | 0.005 | -0.019 | 0.924 | 0.959 | Unannotated | NA | NA |
| metab_6403 | 3-Methylsalicylate | neg | 151.0395 | 4.093 | 4.353 | 4.357 | 0.004 | 0.019 | 0.924 | 0.959 | Moderate putative | NA | NA |
| metab_6426 | (3R)-beta-Leucine | neg | 130.0867 | 4.187 | 4.801 | 4.724 | -0.077 | -0.019 | 0.924 | 0.959 | Strong putative | NA | NA |
| metab_2920 | Unannotated feature | pos | 201.0507 | 4.892 | 4.001 | 3.884 | -0.118 | 0.019 | 0.924 | 0.959 | Unannotated | NA | NA |
| metab_3166 | Unannotated feature | pos | 188.1434 | 3.476 | 4.430 | 4.418 | -0.012 | -0.019 | 0.924 | 0.959 | Unannotated | NA | NA |
| metab_5849 | Unannotated feature | neg | 90.0090 | 2.350 | 4.729 | 4.728 | -0.000 | 0.019 | 0.924 | 0.959 | Unannotated | NA | NA |
| metab_10672 | Unannotated feature | neg | 618.6592 | 0.582 | 4.221 | 4.240 | 0.019 | -0.019 | 0.924 | 0.959 | Unannotated | NA | NA |
| metab_3410 | Unannotated feature | pos | 162.0706 | 2.444 | 4.108 | 4.047 | -0.061 | -0.019 | 0.924 | 0.959 | Unannotated | NA | NA |
| metab_728 | trans-1,2-Dihydrobenzene-1,2-diol | pos | 77.0392 | 2.622 | 3.624 | 3.597 | -0.026 | -0.019 | 0.924 | 0.959 | Tentative | NA | NA |
| metab_3423 | Unannotated feature | pos | 275.1100 | 2.389 | 3.969 | 4.012 | 0.044 | -0.019 | 0.924 | 0.959 | Unannotated | NA | NA |
| metab_9207 | Unannotated feature | neg | 208.1139 | 4.115 | 5.004 | 4.982 | -0.022 | 0.019 | 0.924 | 0.959 | Unannotated | NA | NA |
| metab_9499 | Unannotated feature | neg | 543.9936 | 3.247 | 5.116 | 5.117 | 0.001 | -0.019 | 0.924 | 0.959 | Unannotated | NA | NA |
| metab_9528 | Unannotated feature | neg | 174.2490 | 3.224 | 3.970 | 3.825 | -0.145 | -0.019 | 0.924 | 0.959 | Unannotated | NA | NA |
| metab_140 | Unannotated feature | pos | 650.8498 | 0.551 | 5.243 | 5.235 | -0.008 | 0.019 | 0.924 | 0.959 | Unannotated | NA | NA |
| metab_9507 | Unannotated feature | neg | 405.9952 | 3.240 | 4.624 | 4.626 | 0.003 | 0.019 | 0.924 | 0.959 | Unannotated | NA | NA |
| metab_9858 | Unannotated feature | neg | 388.8745 | 7.170 | 4.916 | 4.905 | -0.011 | 0.019 | 0.924 | 0.959 | Unannotated | NA | NA |
| metab_9832 | Unannotated feature | neg | 510.0617 | 2.504 | 4.936 | 4.910 | -0.026 | -0.019 | 0.924 | 0.959 | Unannotated | NA | NA |
| metab_10951 | Unannotated feature | neg | 343.8943 | 0.522 | 4.166 | 4.184 | 0.018 | 0.019 | 0.924 | 0.959 | Unannotated | NA | NA |
| metab_3384 | Cirsilineol | pos | 408.1055 | 2.514 | 4.709 | 4.844 | 0.135 | 0.019 | 0.924 | 0.959 | Tentative | NA | NA |
| metab_2430 | Unannotated feature | pos | 215.1795 | 5.925 | 3.846 | 3.861 | 0.015 | -0.019 | 0.924 | 0.959 | Unannotated | NA | NA |
| metab_10946 | Unannotated feature | neg | 246.9451 | 0.530 | 3.961 | 3.961 | -0.001 | 0.019 | 0.924 | 0.959 | Unannotated | NA | NA |
| metab_4246 | 2,3-Dihydroxypropanamide | pos | 150.0139 | 0.606 | 4.964 | 4.970 | 0.006 | 0.019 | 0.924 | 0.959 | Tentative | NA | NA |
| metab_2264 | Unannotated feature | pos | 717.7981 | 0.567 | 4.483 | 4.493 | 0.010 | 0.019 | 0.924 | 0.959 | Unannotated | NA | NA |
| metab_2243 | Unannotated feature | pos | 438.2979 | 6.226 | 4.767 | 4.775 | 0.009 | 0.019 | 0.924 | 0.959 | Unannotated | NA | NA |
| metab_7704 | Unannotated feature | neg | 136.8913 | 7.213 | 4.399 | 4.404 | 0.005 | 0.019 | 0.924 | 0.959 | Unannotated | NA | NA |
| metab_7731 | Unannotated feature | neg | 248.8875 | 7.189 | 4.455 | 4.458 | 0.004 | 0.019 | 0.924 | 0.959 | Unannotated | NA | NA |
| metab_7744 | Unannotated feature | neg | 108.8989 | 7.189 | 3.814 | 3.804 | -0.010 | 0.019 | 0.924 | 0.959 | Unannotated | NA | NA |
| metab_5733 | Unannotated feature | neg | 261.8514 | 1.693 | 6.234 | 6.234 | -0.000 | -0.019 | 0.924 | 0.959 | Unannotated | NA | NA |
| metab_3540 | Unannotated feature | pos | 121.0844 | 2.081 | 6.257 | 6.268 | 0.011 | 0.019 | 0.924 | 0.959 | Unannotated | NA | NA |
| metab_9084 | Unannotated feature | neg | 342.9558 | 3.282 | 5.762 | 5.776 | 0.014 | -0.019 | 0.924 | 0.959 | Unannotated | NA | NA |
| metab_9050 | N-Formyl-dl-phenylalanine | neg | 192.0664 | 4.612 | 5.756 | 5.782 | 0.026 | -0.019 | 0.924 | 0.959 | Tentative | NA | NA |
| metab_4037 | Unannotated feature | pos | 156.1020 | 0.784 | 3.306 | 3.258 | -0.048 | -0.019 | 0.924 | 0.959 | Unannotated | NA | NA |
| metab_11047 | Unannotated feature | neg | 153.8994 | 0.013 | 5.112 | 5.113 | 0.001 | -0.019 | 0.924 | 0.959 | Unannotated | NA | NA |
| metab_8884 | Unannotated feature | neg | 141.0201 | 5.207 | 4.955 | 4.909 | -0.046 | -0.019 | 0.924 | 0.959 | Unannotated | NA | NA |
| metab_3841 | Unannotated feature | pos | 120.9810 | 1.165 | 4.625 | 4.645 | 0.021 | 0.019 | 0.924 | 0.959 | Unannotated | NA | NA |
| metab_7672 | Unannotated feature | neg | 377.0220 | 2.492 | 4.953 | 4.933 | -0.021 | -0.019 | 0.924 | 0.959 | Unannotated | NA | NA |
| metab_7781 | Unannotated feature | neg | 342.8554 | 7.170 | 5.114 | 5.106 | -0.008 | -0.019 | 0.924 | 0.959 | Unannotated | NA | NA |
| metab_8713 | Unannotated feature | neg | 269.9070 | 5.587 | 4.075 | 3.996 | -0.079 | 0.019 | 0.924 | 0.959 | Unannotated | NA | NA |
| metab_4206 | Unannotated feature | pos | 126.0222 | 0.629 | 3.576 | 3.638 | 0.062 | -0.019 | 0.924 | 0.959 | Unannotated | NA | NA |
| metab_884 | Tryptophan | pos | 205.0972 | 3.638 | 5.569 | 5.513 | -0.056 | 0.019 | 0.924 | 0.959 | Tentative | NA | NA |
| metab_2197 | 1-Acyl-sn-glycero-3-phosphocholine | pos | 504.3061 | 6.303 | 5.178 | 5.211 | 0.033 | -0.019 | 0.924 | 0.959 | Tentative | NA | NA |
| metab_8496 | Unannotated feature | neg | 150.8890 | 5.731 | 3.811 | 3.853 | 0.043 | 0.019 | 0.924 | 0.959 | Unannotated | NA | NA |
| metab_2158 | Unannotated feature | pos | 107.0859 | 6.451 | 3.946 | 3.921 | -0.024 | -0.019 | 0.924 | 0.959 | Unannotated | NA | NA |
| metab_5960 | Unannotated feature | neg | 194.9241 | 2.732 | 5.189 | 5.185 | -0.004 | -0.019 | 0.924 | 0.959 | Unannotated | NA | NA |
| metab_906 | Unannotated feature | pos | 194.0813 | 3.823 | 3.107 | 3.139 | 0.032 | 0.019 | 0.924 | 0.959 | Moderate putative | NA | NA |
| metab_11119 | TRICIRIBINE PHOSPHATE | neg | 421.0608 | 5.683 | 4.010 | 4.245 | 0.235 | 0.019 | 0.924 | 0.959 | Tentative | NA | NA |
| metab_10522 | Unannotated feature | neg | 80.9740 | 0.629 | 6.243 | 6.240 | -0.003 | -0.019 | 0.924 | 0.959 | Unannotated | NA | NA |
| metab_4532 | Unannotated feature | pos | 612.8816 | 0.534 | 5.007 | 5.032 | 0.025 | 0.019 | 0.924 | 0.959 | Unannotated | NA | NA |
| metab_6155 | Unannotated feature | neg | 654.0299 | 3.232 | 4.794 | 4.888 | 0.094 | 0.019 | 0.924 | 0.959 | Unannotated | NA | NA |
| metab_1911 | Unannotated feature | pos | 731.8421 | 0.559 | 5.086 | 5.095 | 0.008 | -0.019 | 0.924 | 0.959 | Unannotated | NA | NA |
| metab_6356 | Unannotated feature | neg | 188.0926 | 3.874 | 4.086 | 4.111 | 0.025 | 0.019 | 0.924 | 0.959 | Unannotated | NA | NA |
| metab_6221 | Unannotated feature | neg | 371.9481 | 0.652 | 4.844 | 4.801 | -0.043 | -0.019 | 0.924 | 0.959 | Unannotated | NA | NA |
| metab_1676 | Unannotated feature | pos | 277.9219 | 7.261 | 4.696 | 4.731 | 0.035 | 0.019 | 0.924 | 0.959 | Unannotated | NA | NA |
| metab_5191 | Unannotated feature | neg | 396.8921 | 0.534 | 4.711 | 4.730 | 0.019 | -0.019 | 0.924 | 0.959 | Unannotated | NA | NA |
| metab_8505 | Unannotated feature | neg | 327.6526 | 5.723 | 3.475 | 3.512 | 0.037 | -0.019 | 0.924 | 0.959 | Unannotated | NA | NA |
| metab_1945 | Unannotated feature | pos | 81.5211 | 7.324 | 5.030 | 5.025 | -0.005 | -0.019 | 0.924 | 0.959 | Unannotated | NA | NA |
| metab_8465 | Unannotated feature | neg | 695.0314 | 3.224 | 5.627 | 5.623 | -0.004 | -0.019 | 0.924 | 0.959 | Unannotated | NA | NA |
| metab_8491 | Unannotated feature | neg | 714.0190 | 3.240 | 5.696 | 5.736 | 0.040 | 0.019 | 0.924 | 0.959 | Unannotated | NA | NA |
| metab_8456 | Unannotated feature | neg | 697.0288 | 3.224 | 5.684 | 5.695 | 0.011 | 0.019 | 0.924 | 0.959 | Unannotated | NA | NA |
| metab_4590 | Unannotated feature | pos | 171.1492 | 0.380 | 4.594 | 4.591 | -0.003 | -0.019 | 0.924 | 0.959 | Unannotated | NA | NA |
| metab_10149 | Unannotated feature | neg | 318.9243 | 4.425 | 4.837 | 4.834 | -0.003 | -0.019 | 0.924 | 0.959 | Unannotated | NA | NA |
| metab_4346 | Unannotated feature | pos | 856.6813 | 0.567 | 4.337 | 4.327 | -0.011 | -0.019 | 0.924 | 0.959 | Unannotated | NA | NA |
| metab_8532 | 2-(4-Methoxyphenyl)ethyl hydrogen sulfate | neg | 231.0334 | 5.707 | 3.520 | 3.146 | -0.374 | -0.019 | 0.924 | 0.959 | Tentative | NA | NA |
| metab_6820 | Unannotated feature | neg | 361.9095 | 5.587 | 3.930 | 3.954 | 0.024 | 0.019 | 0.924 | 0.959 | Unannotated | NA | NA |
| metab_4901 | Unannotated feature | pos | 200.0408 | 0.606 | 5.527 | 5.539 | 0.012 | -0.019 | 0.924 | 0.959 | Unannotated | NA | NA |
| metab_1587 | Unannotated feature | pos | 1026.6591 | 6.513 | 5.079 | 5.280 | 0.201 | 0.019 | 0.924 | 0.959 | Unannotated | NA | NA |
| metab_11344 | Unannotated feature | neg | 191.6557 | 0.013 | 4.905 | 4.904 | -0.002 | -0.019 | 0.924 | 0.959 | Unannotated | NA | NA |
| metab_1617 | (1'R)-Nepetalic acid | pos | 207.0989 | 7.022 | 4.726 | 4.723 | -0.003 | 0.019 | 0.924 | 0.959 | Tentative | NA | NA |
| metab_559 | Unannotated feature | pos | 137.0599 | 2.042 | 4.464 | 4.457 | -0.007 | -0.015 | 0.943 | 0.970 | Unannotated | NA | NA |
| metab_3584 | Asparaginyl-Valine | pos | 196.1081 | 2.050 | 4.448 | 4.473 | 0.025 | 0.015 | 0.943 | 0.970 | Tentative | NA | NA |
| metab_11014 | Unannotated feature | neg | 316.8023 | 0.016 | 4.342 | 4.351 | 0.009 | -0.015 | 0.943 | 0.970 | Unannotated | NA | NA |
| metab_3969 | Unannotated feature | pos | 196.1081 | 0.839 | 4.821 | 4.835 | 0.013 | 0.015 | 0.943 | 0.970 | Unannotated | NA | NA |
| metab_2173 | 9,10,18-Trihydroxystearate | pos | 315.2529 | 6.420 | 6.061 | 6.058 | -0.003 | 0.015 | 0.943 | 0.970 | Tentative | NA | NA |
| metab_8763 | Unannotated feature | neg | 228.9747 | 5.543 | 3.736 | 3.669 | -0.067 | 0.015 | 0.943 | 0.970 | Unannotated | NA | NA |
| metab_8762 | Glycerophosphoserine | neg | 294.0157 | 5.543 | 5.041 | 4.990 | -0.051 | -0.015 | 0.943 | 0.970 | Tentative | NA | NA |
| metab_5346 | Unannotated feature | neg | 501.7437 | 0.578 | 4.602 | 4.597 | -0.005 | -0.015 | 0.943 | 0.970 | Unannotated | NA | NA |
| metab_9019 | Thienamycin | neg | 253.0626 | 4.710 | 3.016 | 2.891 | -0.125 | -0.015 | 0.943 | 0.970 | Tentative | NA | NA |
| metab_1574 | Unannotated feature | pos | 338.3416 | 6.396 | 5.317 | 5.309 | -0.009 | 0.015 | 0.943 | 0.970 | Strong putative | NA | NA |
| metab_7117 | Unannotated feature | neg | 469.7821 | 5.834 | 5.060 | 5.041 | -0.018 | 0.015 | 0.943 | 0.970 | Unannotated | NA | NA |
| metab_11243 | Unannotated feature | neg | 482.7930 | 0.578 | 5.645 | 5.649 | 0.004 | 0.015 | 0.943 | 0.970 | Unannotated | NA | NA |
| metab_2068 | Unannotated feature | pos | 359.2040 | 7.207 | 5.503 | 5.504 | 0.000 | 0.015 | 0.943 | 0.970 | Unannotated | NA | NA |
| metab_2142 | (R)-2-(4-(Tert-butoxycarbonyl)morpholin-3-yl)acetic acid | pos | 228.1206 | 6.660 | 4.973 | 4.978 | 0.006 | 0.015 | 0.943 | 0.970 | Tentative | NA | NA |
| metab_8329 | Unannotated feature | neg | 446.2922 | 5.834 | 4.538 | 4.539 | 0.000 | -0.015 | 0.943 | 0.970 | Unannotated | NA | NA |
| metab_1688 | Unannotated feature | pos | 175.9543 | 7.277 | 4.764 | 4.757 | -0.007 | -0.015 | 0.943 | 0.970 | Unannotated | NA | NA |
| metab_8444 | Unannotated feature | neg | 228.9048 | 5.763 | 4.555 | 4.505 | -0.050 | -0.015 | 0.943 | 0.970 | Unannotated | NA | NA |
| metab_4579 | Unannotated feature | pos | 155.0703 | 0.442 | 4.617 | 4.615 | -0.002 | -0.015 | 0.943 | 0.970 | Unannotated | NA | NA |
| metab_6197 | N-(3-(3-Hydroxy-4-methoxyphenyl)propyl)-L-alpha-aspartyl-L-phenylalanine | neg | 465.1636 | 3.324 | 4.229 | 4.132 | -0.097 | 0.015 | 0.943 | 0.970 | Tentative | NA | NA |
| metab_4516 | Unannotated feature | pos | 245.0484 | 0.541 | 4.697 | 4.719 | 0.022 | 0.015 | 0.943 | 0.970 | Unannotated | NA | NA |
| metab_6126 | Dehydromonocrotaline | neg | 360.0863 | 3.198 | 5.605 | 5.613 | 0.008 | -0.015 | 0.943 | 0.970 | Tentative | NA | NA |
| metab_1153 | Unannotated feature | pos | 85.0846 | 5.680 | 6.459 | 6.450 | -0.009 | 0.015 | 0.943 | 0.970 | Unannotated | NA | NA |
| metab_5180 | Unannotated feature | neg | 367.9074 | 0.522 | 4.145 | 4.163 | 0.018 | -0.015 | 0.943 | 0.970 | Unannotated | NA | NA |
| metab_4769 | Unannotated feature | pos | 566.3217 | 6.296 | 6.237 | 6.175 | -0.062 | -0.015 | 0.943 | 0.970 | Unannotated | NA | NA |
| metab_4765 | 1-O-Hexadecyl-lyso-sn-glycero-3-phosphocholine | pos | 482.3606 | 6.937 | 5.936 | 5.977 | 0.041 | -0.015 | 0.943 | 0.970 | Tentative | NA | NA |
| metab_425 | Unannotated feature | pos | 241.0311 | 1.414 | 4.347 | 4.396 | 0.049 | 0.015 | 0.943 | 0.970 | Unannotated | NA | NA |
| metab_8001 | Unannotated feature | neg | 333.9388 | 6.955 | 5.527 | 5.526 | -0.001 | -0.015 | 0.943 | 0.970 | Unannotated | NA | NA |
| metab_1954 | Unannotated feature | pos | 151.0354 | 7.316 | 5.290 | 5.293 | 0.003 | 0.015 | 0.943 | 0.970 | Unannotated | NA | NA |
| metab_10308 | Unannotated feature | neg | 126.8803 | 0.802 | 3.564 | 3.568 | 0.004 | -0.015 | 0.943 | 0.970 | Unannotated | NA | NA |
| metab_2014 | Unannotated feature | pos | 171.1241 | 7.285 | 4.649 | 4.635 | -0.014 | 0.015 | 0.943 | 0.970 | Unannotated | NA | NA |
| metab_1934 | Unannotated feature | pos | 140.9618 | 7.324 | 3.961 | 3.954 | -0.006 | 0.015 | 0.943 | 0.970 | Unannotated | NA | NA |
| metab_11090 | Unannotated feature | neg | 598.7107 | 0.578 | 5.620 | 5.625 | 0.005 | 0.015 | 0.943 | 0.970 | Unannotated | NA | NA |
| metab_1994 | Unannotated feature | pos | 226.9515 | 7.293 | 6.366 | 6.355 | -0.011 | -0.015 | 0.943 | 0.970 | Unannotated | NA | NA |
| metab_10234 | 5-Hydroxyisourate | neg | 165.0049 | 0.865 | 4.319 | 4.296 | -0.022 | -0.015 | 0.943 | 0.970 | Tentative | NA | NA |
| metab_4589 | Unannotated feature | pos | 81.0340 | 0.403 | 4.421 | 4.417 | -0.004 | -0.015 | 0.943 | 0.970 | Unannotated | NA | NA |
| metab_542 | Unannotated feature | pos | 121.1014 | 1.995 | 3.854 | 3.840 | -0.014 | -0.015 | 0.943 | 0.970 | Unannotated | NA | NA |
| metab_5419 | Unannotated feature | neg | 286.7725 | 0.629 | 5.720 | 5.709 | -0.011 | 0.015 | 0.943 | 0.970 | Unannotated | NA | NA |
| metab_11035 | Unannotated feature | neg | 334.8443 | 0.013 | 4.997 | 4.998 | 0.001 | 0.015 | 0.943 | 0.970 | Unannotated | NA | NA |
| metab_2310 | Unannotated feature | pos | 640.3456 | 6.049 | 4.644 | 4.678 | 0.034 | 0.015 | 0.943 | 0.970 | Unannotated | NA | NA |
| metab_7357 | Unannotated feature | neg | 676.3072 | 6.581 | 5.732 | 5.728 | -0.005 | -0.015 | 0.943 | 0.970 | Unannotated | NA | NA |
| metab_10544 | Unannotated feature | neg | 440.8470 | 0.621 | 4.092 | 4.111 | 0.019 | 0.015 | 0.943 | 0.970 | Unannotated | NA | NA |
| metab_9447 | Unannotated feature | neg | 228.8211 | 3.348 | 4.366 | 4.372 | 0.006 | 0.015 | 0.943 | 0.970 | Unannotated | NA | NA |
| metab_9478 | Unannotated feature | neg | 85.0035 | 3.282 | 4.315 | 4.337 | 0.023 | -0.015 | 0.943 | 0.970 | Unannotated | NA | NA |
| metab_9875 | Unannotated feature | neg | 352.8543 | 2.379 | 6.208 | 6.215 | 0.006 | 0.015 | 0.943 | 0.970 | Unannotated | NA | NA |
| metab_10956 | Unannotated feature | neg | 187.9341 | 0.522 | 2.838 | 2.831 | -0.007 | -0.015 | 0.943 | 0.970 | Unannotated | NA | NA |
| metab_793 | Unannotated feature | pos | 105.0427 | 7.277 | 6.376 | 6.372 | -0.005 | 0.015 | 0.943 | 0.970 | Unannotated | NA | NA |
| metab_9295 | Unannotated feature | neg | 118.0040 | 3.773 | 4.311 | 4.301 | -0.010 | 0.015 | 0.943 | 0.970 | Unannotated | NA | NA |
| metab_5876 | Unannotated feature | neg | 259.8782 | 2.453 | 5.224 | 5.237 | 0.013 | -0.015 | 0.943 | 0.970 | Unannotated | NA | NA |
| metab_3134 | Unannotated feature | pos | 175.1079 | 3.661 | 4.497 | 4.506 | 0.009 | 0.015 | 0.943 | 0.970 | Unannotated | NA | NA |
| metab_7487 | Unannotated feature | neg | 860.8497 | 7.140 | 5.551 | 5.558 | 0.007 | 0.015 | 0.943 | 0.970 | Unannotated | NA | NA |
| metab_7636 | Unannotated feature | neg | 244.8849 | 7.997 | 5.404 | 5.409 | 0.005 | -0.015 | 0.943 | 0.970 | Unannotated | NA | NA |
| metab_10805 | Unannotated feature | neg | 112.9628 | 0.562 | 3.928 | 3.917 | -0.011 | -0.015 | 0.943 | 0.970 | Unannotated | NA | NA |
| metab_6798 | 2-Styrenesulfonic acid | neg | 229.0179 | 5.563 | 4.242 | 3.995 | -0.247 | -0.015 | 0.943 | 0.970 | Tentative | NA | NA |
| metab_3050 | Unannotated feature | pos | 379.2487 | 4.086 | 4.302 | 4.227 | -0.074 | -0.015 | 0.943 | 0.970 | Unannotated | NA | NA |
| metab_7514 | Unannotated feature | neg | 393.8946 | 7.156 | 4.356 | 4.373 | 0.017 | -0.015 | 0.943 | 0.970 | Unannotated | NA | NA |
| metab_7173 | Unannotated feature | neg | 224.0929 | 5.898 | 4.563 | 4.550 | -0.013 | -0.015 | 0.943 | 0.970 | Unannotated | NA | NA |
| metab_10706 | Unannotated feature | neg | 574.7460 | 0.578 | 4.501 | 4.457 | -0.044 | -0.015 | 0.943 | 0.970 | Unannotated | NA | NA |
| metab_6425 | Unannotated feature | neg | 363.9250 | 4.181 | 4.213 | 4.305 | 0.092 | 0.015 | 0.943 | 0.970 | Unannotated | NA | NA |
| metab_1550 | 3-(6-Aminopurin-9-yl)-5-(hydroxymethyl)cyclopentane-1,2-diol | pos | 283.1513 | 6.296 | 5.855 | 5.848 | -0.007 | -0.015 | 0.943 | 0.970 | Tentative | NA | NA |
| metab_10124 | Unannotated feature | neg | 443.6791 | 0.999 | 5.720 | 5.723 | 0.003 | -0.015 | 0.943 | 0.970 | Unannotated | NA | NA |
| metab_10685 | Unannotated feature | neg | 104.6579 | 0.582 | 3.181 | 3.206 | 0.024 | 0.015 | 0.943 | 0.970 | Unannotated | NA | NA |
| metab_10712 | Unannotated feature | neg | 564.7171 | 0.578 | 4.242 | 4.305 | 0.063 | 0.015 | 0.943 | 0.970 | Unannotated | NA | NA |
| metab_8624 | Unannotated feature | neg | 383.1543 | 5.651 | 5.890 | 5.888 | -0.002 | -0.015 | 0.943 | 0.970 | Unannotated | NA | NA |
| metab_5011 | Unannotated feature | pos | 116.0709 | 0.698 | 7.017 | 6.982 | -0.035 | 0.015 | 0.943 | 0.970 | Moderate putative | NA | NA |
| metab_1572 | Unannotated feature | pos | 1041.6847 | 6.373 | 5.851 | 5.923 | 0.072 | 0.015 | 0.943 | 0.970 | Unannotated | NA | NA |
| metab_5913 | Unannotated feature | neg | 480.0305 | 2.537 | 4.215 | 4.222 | 0.006 | -0.011 | 0.962 | 0.980 | Unannotated | NA | NA |
| metab_2163 | Unannotated feature | pos | 514.4830 | 6.420 | 6.781 | 6.779 | -0.002 | 0.011 | 0.962 | 0.980 | Unannotated | NA | NA |
| metab_943 | Unannotated feature | pos | 124.1061 | 4.078 | 3.774 | 3.727 | -0.047 | 0.011 | 0.962 | 0.980 | Unannotated | NA | NA |
| metab_4007 | 2-Amino-9-[2-(phosphanylmethoxy)ethyl]-1H-purin-6-one | pos | 222.0550 | 4.224 | 4.626 | 4.717 | 0.091 | -0.011 | 0.962 | 0.980 | Tentative | NA | NA |
| metab_195 | Unannotated feature | pos | 654.8246 | 0.567 | 4.410 | 4.469 | 0.060 | -0.011 | 0.962 | 0.980 | Unannotated | NA | NA |
| metab_3653 | Unannotated feature | pos | 130.1592 | 1.956 | 4.743 | 4.752 | 0.009 | 0.011 | 0.962 | 0.980 | Unannotated | NA | NA |
| metab_2276 | Unannotated feature | pos | 127.1120 | 6.149 | 4.028 | 4.046 | 0.017 | -0.011 | 0.962 | 0.980 | Unannotated | NA | NA |
| metab_2297 | Unannotated feature | pos | 712.7840 | 0.567 | 4.662 | 4.631 | -0.030 | -0.011 | 0.962 | 0.980 | Unannotated | NA | NA |
| metab_7385 | Unannotated feature | neg | 224.9685 | 7.038 | 4.003 | 3.953 | -0.051 | -0.011 | 0.962 | 0.980 | Unannotated | NA | NA |
| metab_7383 | Unannotated feature | neg | 362.9385 | 7.015 | 5.172 | 5.208 | 0.036 | 0.011 | 0.962 | 0.980 | Unannotated | NA | NA |
| metab_5404 | Unannotated feature | neg | 78.9181 | 0.621 | 3.926 | 3.974 | 0.048 | -0.011 | 0.962 | 0.980 | Unannotated | NA | NA |
| metab_7542 | Unannotated feature | neg | 241.9467 | 7.173 | 4.348 | 4.361 | 0.013 | 0.011 | 0.962 | 0.980 | Unannotated | NA | NA |
| metab_9098 | Unannotated feature | neg | 320.9214 | 4.425 | 4.780 | 4.793 | 0.013 | -0.011 | 0.962 | 0.980 | Unannotated | NA | NA |
| metab_11283 | Unannotated feature | neg | 364.8784 | 0.570 | 5.681 | 5.689 | 0.008 | 0.011 | 0.962 | 0.980 | Unannotated | NA | NA |
| metab_7829 | Unannotated feature | neg | 632.3198 | 7.153 | 4.577 | 4.591 | 0.014 | 0.011 | 0.962 | 0.980 | Unannotated | NA | NA |
| metab_7691 | Unannotated feature | neg | 257.8570 | 7.213 | 3.954 | 3.975 | 0.021 | 0.011 | 0.962 | 0.980 | Unannotated | NA | NA |
| metab_7850 | Unannotated feature | neg | 634.3351 | 7.148 | 4.388 | 4.416 | 0.027 | 0.011 | 0.962 | 0.980 | Unannotated | NA | NA |
| metab_4231 | Unannotated feature | pos | 128.0320 | 0.614 | 3.989 | 4.017 | 0.029 | -0.011 | 0.962 | 0.980 | Unannotated | NA | NA |
| metab_8799 | Unannotated feature | neg | 462.8794 | 7.153 | 5.013 | 5.023 | 0.010 | -0.011 | 0.962 | 0.980 | Unannotated | NA | NA |
| metab_858 | Unannotated feature | pos | 170.0663 | 3.453 | 4.547 | 4.547 | 0.000 | 0.011 | 0.962 | 0.980 | Unannotated | NA | NA |
| metab_9021 | Unannotated feature | neg | 208.0629 | 4.710 | 4.437 | 4.586 | 0.149 | 0.011 | 0.962 | 0.980 | Unannotated | NA | NA |
| metab_3335 | Unannotated feature | pos | 207.1127 | 2.622 | 4.679 | 4.670 | -0.009 | 0.011 | 0.962 | 0.980 | Unannotated | NA | NA |
| metab_9747 | Unannotated feature | neg | 369.8445 | 2.724 | 5.678 | 5.686 | 0.009 | -0.011 | 0.962 | 0.980 | Unannotated | NA | NA |
| metab_9830 | Unannotated feature | neg | 214.8593 | 2.510 | 4.276 | 4.296 | 0.020 | 0.011 | 0.962 | 0.980 | Unannotated | NA | NA |
| metab_9861 | Resorcinol | neg | 109.0287 | 2.437 | 3.922 | 3.895 | -0.027 | -0.011 | 0.962 | 0.980 | Moderate putative | NA | NA |
| metab_2338 | Unannotated feature | pos | 564.4214 | 5.995 | 4.579 | 4.505 | -0.074 | -0.011 | 0.962 | 0.980 | Unannotated | NA | NA |
| metab_3305 | Unannotated feature | pos | 449.3602 | 7.200 | 8.530 | 8.508 | -0.022 | -0.011 | 0.962 | 0.980 | Moderate putative | NA | NA |
| metab_9751 | Unannotated feature | neg | 259.8565 | 2.716 | 4.815 | 4.816 | 0.001 | 0.011 | 0.962 | 0.980 | Unannotated | NA | NA |
| metab_9761 | Unannotated feature | neg | 187.0544 | 2.692 | 3.465 | 3.563 | 0.098 | 0.011 | 0.962 | 0.980 | Unannotated | NA | NA |
| metab_2581 | Unannotated feature | pos | 619.9120 | 5.855 | 4.587 | 4.597 | 0.010 | -0.011 | 0.962 | 0.980 | Unannotated | NA | NA |
| metab_2985 | Unannotated feature | pos | 84.1227 | 4.493 | 3.203 | 3.281 | 0.078 | -0.011 | 0.962 | 0.980 | Unannotated | NA | NA |
| metab_2846 | Unannotated feature | pos | 119.0733 | 2.088 | 5.212 | 5.232 | 0.020 | 0.011 | 0.962 | 0.980 | Unannotated | NA | NA |
| metab_7507 | Unannotated feature | neg | 668.8428 | 7.153 | 4.996 | 4.987 | -0.009 | 0.011 | 0.962 | 0.980 | Unannotated | NA | NA |
| metab_7476 | Unannotated feature | neg | 520.9112 | 7.140 | 6.233 | 6.223 | -0.010 | -0.011 | 0.962 | 0.980 | Unannotated | NA | NA |
| metab_11345 | Unannotated feature | neg | 353.8495 | 0.013 | 6.293 | 6.295 | 0.002 | 0.011 | 0.962 | 0.980 | Unannotated | NA | NA |
| metab_1368 | Unannotated feature | pos | 611.9145 | 5.910 | 4.622 | 4.658 | 0.035 | 0.011 | 0.962 | 0.980 | Unannotated | NA | NA |
| metab_11392 | Hexadecanedioate | neg | 285.2076 | 5.930 | 4.389 | 4.417 | 0.028 | -0.011 | 0.962 | 0.980 | Tentative | NA | NA |
| metab_1265 | Unannotated feature | pos | 120.0810 | 2.081 | 7.330 | 7.334 | 0.004 | 0.011 | 0.962 | 0.980 | Unannotated | NA | NA |
| metab_1383 | Unannotated feature | pos | 454.8293 | 5.917 | 4.851 | 4.814 | -0.037 | -0.011 | 0.962 | 0.980 | Unannotated | NA | NA |
| metab_10711 | Unannotated feature | neg | 569.7312 | 0.578 | 4.656 | 4.636 | -0.020 | -0.011 | 0.962 | 0.980 | Unannotated | NA | NA |
| metab_6464 | Unannotated feature | neg | 766.8369 | 4.282 | 4.856 | 4.840 | -0.016 | 0.011 | 0.962 | 0.980 | Unannotated | NA | NA |
| metab_5765 | Unannotated feature | neg | 568.8420 | 0.570 | 5.575 | 5.583 | 0.008 | 0.011 | 0.962 | 0.980 | Unannotated | NA | NA |
| metab_3472 | Unannotated feature | pos | 155.0816 | 2.257 | 4.958 | 4.919 | -0.040 | 0.011 | 0.962 | 0.980 | Unannotated | NA | NA |
| metab_9189 | Unannotated feature | neg | 203.0825 | 4.175 | 4.949 | 4.935 | -0.014 | -0.011 | 0.962 | 0.980 | Unannotated | NA | NA |
| metab_7498 | Unannotated feature | neg | 128.9589 | 7.153 | 5.250 | 5.250 | 0.001 | -0.011 | 0.962 | 0.980 | Unannotated | NA | NA |
| metab_9128 | Unannotated feature | neg | 850.0639 | 3.217 | 5.597 | 5.604 | 0.007 | -0.011 | 0.962 | 0.980 | Unannotated | NA | NA |
| metab_3587 | Unannotated feature | pos | 169.0358 | 2.050 | 3.249 | 3.216 | -0.034 | 0.011 | 0.962 | 0.980 | Unannotated | NA | NA |
| metab_3813 | Unannotated feature | pos | 110.0352 | 1.359 | 3.128 | 3.098 | -0.030 | -0.011 | 0.962 | 0.980 | Unannotated | NA | NA |
| metab_3762 | (S)-5-Amino-3-oxohexanoic acid | pos | 128.0708 | 1.740 | 6.085 | 6.062 | -0.024 | 0.011 | 0.962 | 0.980 | Tentative | NA | NA |
| metab_3801 | Unannotated feature | pos | 130.0249 | 1.484 | 4.569 | 4.617 | 0.048 | 0.011 | 0.962 | 0.980 | Unannotated | NA | NA |
| metab_2329 | Unannotated feature | pos | 653.8258 | 0.567 | 4.741 | 4.740 | -0.001 | -0.011 | 0.962 | 0.980 | Unannotated | NA | NA |
| metab_8517 | Unannotated feature | neg | 102.9370 | 7.109 | 4.909 | 4.910 | 0.001 | -0.011 | 0.962 | 0.980 | Unannotated | NA | NA |
| metab_4393 | Unannotated feature | pos | 522.8662 | 0.567 | 4.438 | 4.460 | 0.023 | 0.011 | 0.962 | 0.980 | Unannotated | NA | NA |
| metab_529 | Serylasparagine | pos | 184.0717 | 1.956 | 3.496 | 3.593 | 0.097 | -0.011 | 0.962 | 0.980 | Tentative | NA | NA |
| metab_4656 | Unannotated feature | pos | 368.2796 | 5.793 | 6.081 | 6.192 | 0.111 | -0.011 | 0.962 | 0.980 | Unannotated | NA | NA |
| metab_8584 | Unannotated feature | neg | 461.1603 | 7.124 | 4.551 | 4.552 | 0.001 | -0.011 | 0.962 | 0.980 | Unannotated | NA | NA |
| metab_5366 | Unannotated feature | neg | 544.6586 | 0.590 | 4.477 | 4.585 | 0.108 | 0.011 | 0.962 | 0.980 | Unannotated | NA | NA |
| metab_2013 | Unannotated feature | pos | 216.9227 | 7.285 | 5.145 | 5.143 | -0.001 | -0.011 | 0.962 | 0.980 | Unannotated | NA | NA |
| metab_5099 | Unannotated feature | pos | 136.0428 | 0.768 | 5.061 | 5.011 | -0.050 | -0.011 | 0.962 | 0.980 | Unannotated | NA | NA |
| metab_8223 | Unannotated feature | neg | 204.9402 | 5.930 | 4.668 | 4.651 | -0.017 | 0.011 | 0.962 | 0.980 | Unannotated | NA | NA |
| metab_1710 | Unannotated feature | pos | 668.8692 | 7.293 | 4.376 | 4.397 | 0.021 | -0.011 | 0.962 | 0.980 | Unannotated | NA | NA |
| metab_1748 | Unannotated feature | pos | 97.9917 | 7.331 | 5.520 | 5.515 | -0.005 | 0.011 | 0.962 | 0.980 | Unannotated | NA | NA |
| metab_5275 | Unannotated feature | neg | 803.8272 | 0.554 | 5.215 | 5.214 | -0.001 | 0.011 | 0.962 | 0.980 | Unannotated | NA | NA |
| metab_7849 | Unannotated feature | neg | 636.3511 | 7.148 | 5.101 | 5.092 | -0.009 | -0.011 | 0.962 | 0.980 | Unannotated | NA | NA |
| metab_5270 | Unannotated feature | neg | 761.8460 | 0.554 | 5.796 | 5.786 | -0.009 | 0.011 | 0.962 | 0.980 | Unannotated | NA | NA |
| metab_6166 | Unannotated feature | neg | 142.0656 | 3.255 | 4.835 | 4.872 | 0.037 | -0.011 | 0.962 | 0.980 | Unannotated | NA | NA |
| metab_4433 | Unannotated feature | pos | 514.8751 | 0.559 | 5.278 | 5.260 | -0.019 | -0.011 | 0.962 | 0.980 | Unannotated | NA | NA |
| metab_1998 | Unannotated feature | pos | 101.1077 | 7.293 | 3.515 | 3.520 | 0.005 | 0.011 | 0.962 | 0.980 | Unannotated | NA | NA |
| metab_2076 | Unannotated feature | pos | 447.2923 | 7.200 | 5.745 | 5.743 | -0.002 | 0.011 | 0.962 | 0.980 | Unannotated | NA | NA |
| metab_1948 | Unannotated feature | pos | 317.6598 | 7.316 | 4.464 | 4.451 | -0.013 | 0.011 | 0.962 | 0.980 | Unannotated | NA | NA |
| metab_10604 | Unannotated feature | neg | 586.5958 | 0.598 | 5.434 | 5.436 | 0.002 | -0.011 | 0.962 | 0.980 | Unannotated | NA | NA |
| metab_10553 | Unannotated feature | neg | 288.7697 | 0.621 | 5.145 | 5.144 | -0.001 | -0.011 | 0.962 | 0.980 | Unannotated | NA | NA |
| metab_454 | Unannotated feature | pos | 134.1089 | 1.724 | 3.809 | 3.825 | 0.016 | -0.011 | 0.962 | 0.980 | Unannotated | NA | NA |
| metab_7941 | Unannotated feature | neg | 157.9004 | 7.109 | 4.124 | 4.140 | 0.016 | -0.011 | 0.962 | 0.980 | Unannotated | NA | NA |
| metab_6807 | Unannotated feature | neg | 307.0287 | 5.579 | 4.183 | 4.153 | -0.030 | 0.011 | 0.962 | 0.980 | Unannotated | NA | NA |
| metab_10695 | Unannotated feature | neg | 102.6692 | 0.582 | 4.045 | 4.087 | 0.042 | -0.011 | 0.962 | 0.980 | Unannotated | NA | NA |
| metab_1585 | Unannotated feature | pos | 494.3027 | 6.451 | 4.387 | 4.270 | -0.118 | 0.011 | 0.962 | 0.980 | Unannotated | NA | NA |
| metab_11356 | Unannotated feature | neg | 155.8965 | 0.019 | 4.616 | 4.605 | -0.010 | 0.006 | 0.981 | 0.991 | Unannotated | NA | NA |
| metab_6811 | Unannotated feature | neg | 306.9392 | 0.873 | 5.175 | 5.187 | 0.012 | -0.006 | 0.981 | 0.991 | Unannotated | NA | NA |
| metab_7009 | 2-Hydroxyphenylacetate | neg | 151.0396 | 5.739 | 3.738 | 3.729 | -0.009 | -0.006 | 0.981 | 0.991 | Strong putative | NA | NA |
| metab_6808 | Unannotated feature | neg | 324.0188 | 5.579 | 4.182 | 4.192 | 0.010 | -0.006 | 0.981 | 0.991 | Unannotated | NA | NA |
| metab_1238 | Unannotated feature | pos | 199.1807 | 5.824 | 3.463 | 3.487 | 0.024 | 0.006 | 0.981 | 0.991 | Unannotated | NA | NA |
| metab_414 | Unannotated feature | pos | 128.0195 | 1.259 | 4.720 | 4.735 | 0.015 | -0.006 | 0.981 | 0.991 | Unannotated | NA | NA |
| metab_10769 | Unannotated feature | neg | 298.8878 | 0.570 | 5.583 | 5.589 | 0.006 | -0.006 | 0.981 | 0.991 | Unannotated | NA | NA |
| metab_10755 | Unannotated feature | neg | 579.7602 | 0.570 | 4.403 | 4.419 | 0.016 | -0.006 | 0.981 | 0.991 | Unannotated | NA | NA |
| metab_4956 | L-Threonine | pos | 120.0658 | 0.621 | 5.948 | 5.938 | -0.010 | 0.006 | 0.981 | 0.991 | Strong putative | NA | NA |
| metab_5124 | D-Galactose | pos | 198.0972 | 0.356 | 5.271 | 5.272 | 0.001 | 0.006 | 0.981 | 0.991 | Moderate putative | NA | NA |
| metab_4661 | Unannotated feature | pos | 355.2631 | 5.817 | 4.887 | 4.916 | 0.030 | -0.006 | 0.981 | 0.991 | Unannotated | NA | NA |
| metab_11155 | Unannotated feature | neg | 225.0768 | 5.771 | 3.967 | 3.989 | 0.021 | 0.006 | 0.981 | 0.991 | Unannotated | NA | NA |
| metab_4694 | Unannotated feature | pos | 177.1275 | 5.894 | 5.394 | 5.402 | 0.008 | 0.006 | 0.981 | 0.991 | Unannotated | NA | NA |
| metab_8221 | 9(S)-HPOT | neg | 309.2077 | 5.930 | 5.167 | 5.141 | -0.026 | -0.006 | 0.981 | 0.991 | Moderate putative | NA | NA |
| metab_8433 | Unannotated feature | neg | 189.9209 | 5.771 | 4.889 | 4.890 | 0.001 | 0.006 | 0.981 | 0.991 | Unannotated | NA | NA |
| metab_8441 | Unannotated feature | neg | 293.9790 | 5.763 | 4.730 | 4.779 | 0.049 | 0.006 | 0.981 | 0.991 | Unannotated | NA | NA |
| metab_4294 | Unannotated feature | pos | 723.5934 | 0.583 | 4.471 | 4.413 | -0.058 | -0.006 | 0.981 | 0.991 | Unannotated | NA | NA |
| metab_7788 | Unannotated feature | neg | 191.6557 | 7.170 | 3.733 | 3.707 | -0.026 | -0.006 | 0.981 | 0.991 | Unannotated | NA | NA |
| metab_5345 | Unannotated feature | neg | 484.7901 | 0.578 | 5.055 | 5.065 | 0.010 | 0.006 | 0.981 | 0.991 | Unannotated | NA | NA |
| metab_7852 | Unannotated feature | neg | 435.8949 | 7.148 | 4.685 | 4.696 | 0.011 | 0.006 | 0.981 | 0.991 | Unannotated | NA | NA |
| metab_3922 | Unannotated feature | pos | 152.0067 | 0.861 | 3.419 | 3.306 | -0.113 | 0.006 | 0.981 | 0.991 | Unannotated | NA | NA |
| metab_3849 | N-Acetylornithine | pos | 175.1078 | 1.087 | 5.012 | 5.006 | -0.006 | 0.006 | 0.981 | 0.991 | Tentative | NA | NA |
| metab_5473 | Unannotated feature | neg | 193.8154 | 0.692 | 6.605 | 6.618 | 0.013 | -0.006 | 0.981 | 0.991 | Unannotated | NA | NA |
| metab_11053 | MJDBISSN00000009 | neg | 169.1027 | 2.504 | 5.735 | 5.743 | 0.008 | 0.006 | 0.981 | 0.991 | Strong putative | NA | NA |
| metab_4396 | Unannotated feature | pos | 354.8944 | 0.567 | 5.099 | 5.106 | 0.007 | -0.006 | 0.981 | 0.991 | Unannotated | NA | NA |
| metab_534 | Unannotated feature | pos | 303.1452 | 1.956 | 4.146 | 4.119 | -0.028 | -0.006 | 0.981 | 0.991 | Unannotated | NA | NA |
| metab_8459 | Unannotated feature | neg | 207.9187 | 5.755 | 4.567 | 4.551 | -0.016 | -0.006 | 0.981 | 0.991 | Unannotated | NA | NA |
| metab_8509 | Unannotated feature | neg | 188.9329 | 5.723 | 3.813 | 3.856 | 0.043 | 0.006 | 0.981 | 0.991 | Unannotated | NA | NA |
| metab_5244 | Unannotated feature | neg | 240.8677 | 0.013 | 5.747 | 5.746 | -0.001 | -0.006 | 0.981 | 0.991 | Unannotated | NA | NA |
| metab_8426 | Unannotated feature | neg | 462.2873 | 5.771 | 3.574 | 3.725 | 0.150 | -0.006 | 0.981 | 0.991 | Unannotated | NA | NA |
| metab_8898 | Unannotated feature | neg | 311.1692 | 7.117 | 6.513 | 6.519 | 0.006 | 0.006 | 0.981 | 0.991 | Unannotated | NA | NA |
| metab_5421 | Unannotated feature | neg | 160.2819 | 0.636 | 4.561 | 4.550 | -0.011 | -0.006 | 0.981 | 0.991 | Unannotated | NA | NA |
| metab_7500 | Unannotated feature | neg | 261.8514 | 2.205 | 6.423 | 6.424 | 0.001 | -0.006 | 0.981 | 0.991 | Unannotated | NA | NA |
| metab_5779 | Unannotated feature | neg | 268.8129 | 2.002 | 5.324 | 5.328 | 0.004 | -0.006 | 0.981 | 0.991 | Unannotated | NA | NA |
| metab_5729 | Unannotated feature | neg | 297.7338 | 1.577 | 4.476 | 4.451 | -0.025 | 0.006 | 0.981 | 0.991 | Unannotated | NA | NA |
| metab_9739 | Unannotated feature | neg | 278.8416 | 2.740 | 5.638 | 5.651 | 0.013 | -0.006 | 0.981 | 0.991 | Unannotated | NA | NA |
| metab_9773 | Unannotated feature | neg | 235.8441 | 2.629 | 4.147 | 4.152 | 0.005 | -0.006 | 0.981 | 0.991 | Unannotated | NA | NA |
| metab_2349 | Unannotated feature | pos | 557.4136 | 5.979 | 5.640 | 5.636 | -0.003 | -0.006 | 0.981 | 0.991 | Unannotated | NA | NA |
| metab_9896 | Unannotated feature | neg | 342.8255 | 2.205 | 4.978 | 4.981 | 0.003 | 0.006 | 0.981 | 0.991 | Unannotated | NA | NA |
| metab_2719 | Unannotated feature | pos | 205.0818 | 0.691 | 5.003 | 5.035 | 0.033 | 0.006 | 0.981 | 0.991 | Moderate putative | NA | NA |
| metab_10071 | Unannotated feature | neg | 309.8972 | 1.302 | 4.470 | 4.493 | 0.023 | -0.006 | 0.981 | 0.991 | Unannotated | NA | NA |
| metab_2161 | Unannotated feature | pos | 500.4674 | 6.427 | 4.866 | 4.863 | -0.003 | -0.006 | 0.981 | 0.991 | Unannotated | NA | NA |
| metab_10085 | Unannotated feature | neg | 166.9810 | 1.294 | 4.064 | 4.129 | 0.065 | -0.006 | 0.981 | 0.991 | Unannotated | NA | NA |
| metab_2093 | Unannotated feature | pos | 643.5266 | 7.168 | 5.276 | 5.266 | -0.010 | 0.006 | 0.981 | 0.991 | Unannotated | NA | NA |
| metab_7381 | Unannotated feature | neg | 104.9531 | 6.987 | 5.055 | 5.074 | 0.020 | 0.006 | 0.981 | 0.991 | Unannotated | NA | NA |
| metab_10916 | Unannotated feature | neg | 775.3496 | 0.539 | 4.781 | 4.768 | -0.014 | -0.006 | 0.981 | 0.991 | Unannotated | NA | NA |
| metab_7379 | Abscisic aldehyde | neg | 283.1110 | 6.976 | 3.130 | 3.156 | 0.026 | -0.006 | 0.981 | 0.991 | Tentative | NA | NA |
| metab_5976 | Unannotated feature | neg | 250.9213 | 2.780 | 5.233 | 5.245 | 0.012 | 0.006 | 0.981 | 0.991 | Unannotated | NA | NA |
| metab_3715 | Unannotated feature | pos | 79.0184 | 1.886 | 4.210 | 4.215 | 0.005 | -0.006 | 0.981 | 0.991 | Unannotated | NA | NA |
| metab_3703 | Unannotated feature | pos | 169.0357 | 1.266 | 6.787 | 6.807 | 0.020 | -0.006 | 0.981 | 0.991 | Unannotated | NA | NA |
| metab_9075 | Unannotated feature | neg | 339.2005 | 5.859 | 5.682 | 5.693 | 0.011 | 0.006 | 0.981 | 0.991 | Unannotated | NA | NA |
| metab_5727 | Unannotated feature | neg | 323.9792 | 1.563 | 5.318 | 5.342 | 0.024 | 0.006 | 0.981 | 0.991 | Unannotated | NA | NA |
| metab_3473 | Unannotated feature | pos | 98.5124 | 2.257 | 4.454 | 4.467 | 0.012 | -0.006 | 0.981 | 0.991 | Unannotated | NA | NA |
| metab_9208 | Unannotated feature | neg | 137.1126 | 4.115 | 4.976 | 4.926 | -0.050 | 0.006 | 0.981 | 0.991 | Unannotated | NA | NA |
| metab_5619 | Unannotated feature | neg | 195.8610 | 0.912 | 4.322 | 4.304 | -0.019 | -0.006 | 0.981 | 0.991 | Unannotated | NA | NA |
| metab_9215 | Unannotated feature | neg | 144.0198 | 4.079 | 4.139 | 4.148 | 0.010 | 0.006 | 0.981 | 0.991 | Unannotated | NA | NA |
| metab_9135 | Unannotated feature | neg | 119.0244 | 4.322 | 3.864 | 3.806 | -0.058 | -0.006 | 0.981 | 0.991 | Unannotated | NA | NA |
| metab_3757 | Unannotated feature | pos | 154.5517 | 1.747 | 3.603 | 3.677 | 0.074 | -0.006 | 0.981 | 0.991 | Unannotated | NA | NA |
| metab_3562 | Unannotated feature | pos | 113.0842 | 2.065 | 3.625 | 3.675 | 0.050 | 0.006 | 0.981 | 0.991 | Unannotated | NA | NA |
| metab_10508 | Unannotated feature | neg | 498.8059 | 0.629 | 4.574 | 4.526 | -0.049 | 0.006 | 0.981 | 0.991 | Unannotated | NA | NA |
| metab_7331 | Unannotated feature | neg | 654.3096 | 6.368 | 6.517 | 6.517 | 0.001 | -0.006 | 0.981 | 0.991 | Unannotated | NA | NA |
| metab_7326 | 1-Palmitoylglycerol 3-phosphate | neg | 409.2368 | 6.335 | 4.824 | 4.801 | -0.023 | -0.006 | 0.981 | 0.991 | Tentative | NA | NA |
| metab_1076 | Unannotated feature | pos | 244.0549 | 5.221 | 4.593 | 4.632 | 0.039 | 0.006 | 0.981 | 0.991 | Unannotated | NA | NA |
| metab_1855 | Unannotated feature | pos | 162.0706 | 7.710 | 4.973 | 4.973 | -0.000 | 0.006 | 0.981 | 0.991 | Unannotated | NA | NA |
| metab_7149 | Furanofukinin | neg | 293.1765 | 5.875 | 5.102 | 5.110 | 0.007 | -0.006 | 0.981 | 0.991 | Tentative | NA | NA |
| metab_1786 | Unannotated feature | pos | 111.5222 | 7.532 | 3.676 | 3.690 | 0.014 | 0.006 | 0.981 | 0.991 | Unannotated | NA | NA |
| metab_1615 | Unannotated feature | pos | 102.1836 | 6.999 | 2.975 | 3.011 | 0.036 | -0.006 | 0.981 | 0.991 | Unannotated | NA | NA |
| metab_1541 | Unannotated feature | pos | 677.4499 | 6.280 | 4.101 | 3.981 | -0.119 | 0.006 | 0.981 | 0.991 | Unannotated | NA | NA |
| metab_6961 | Unannotated feature | neg | 245.0674 | 5.699 | 4.858 | 4.856 | -0.002 | 0.006 | 0.981 | 0.991 | Unannotated | NA | NA |
| metab_10815 | Unannotated feature | neg | 905.8086 | 0.554 | 5.063 | 5.119 | 0.057 | 0.006 | 0.981 | 0.991 | Unannotated | NA | NA |
| metab_1462 | Unannotated feature | pos | 484.8681 | 5.995 | 4.489 | 4.529 | 0.040 | -0.006 | 0.981 | 0.991 | Unannotated | NA | NA |
| metab_4010 | Unannotated feature | pos | 112.0373 | 0.815 | 4.313 | 4.332 | 0.019 | 0.002 | 1.000 | 1.000 | Unannotated | NA | NA |
| metab_4005 | Unannotated feature | pos | 200.1281 | 0.815 | 3.846 | 3.849 | 0.004 | 0.002 | 1.000 | 1.000 | Unannotated | NA | NA |
| metab_4053 | Unannotated feature | pos | 119.0164 | 0.768 | 5.075 | 5.133 | 0.058 | -0.002 | 1.000 | 1.000 | Unannotated | NA | NA |
| metab_4038 | Unannotated feature | pos | 120.7566 | 0.784 | 4.659 | 4.673 | 0.013 | -0.002 | 1.000 | 1.000 | Unannotated | NA | NA |
| metab_7848 | Unannotated feature | neg | 496.0958 | 2.523 | 5.010 | 5.006 | -0.004 | 0.002 | 1.000 | 1.000 | Unannotated | NA | NA |
| metab_541 | Unannotated feature | pos | 139.0310 | 1.988 | 3.664 | 3.710 | 0.047 | -0.002 | 1.000 | 1.000 | Unannotated | NA | NA |
| metab_8473 | Unannotated feature | neg | 266.0207 | 3.224 | 5.740 | 5.735 | -0.005 | -0.002 | 1.000 | 1.000 | Unannotated | NA | NA |
| metab_8548 | Unannotated feature | neg | 180.9728 | 5.699 | 4.934 | 4.934 | -0.001 | 0.002 | 1.000 | 1.000 | Unannotated | NA | NA |
| metab_4327 | Unannotated feature | pos | 630.7514 | 0.575 | 4.267 | 4.248 | -0.019 | -0.002 | 1.000 | 1.000 | Unannotated | NA | NA |
| metab_7948 | Unannotated feature | neg | 126.9333 | 7.109 | 4.676 | 4.680 | 0.004 | 0.002 | 1.000 | 1.000 | Unannotated | NA | NA |
| metab_7932 | Unannotated feature | neg | 217.9010 | 7.109 | 3.954 | 3.989 | 0.035 | 0.002 | 1.000 | 1.000 | Unannotated | NA | NA |
| metab_422 | Unannotated feature | pos | 137.4884 | 1.375 | 4.024 | 4.006 | -0.018 | 0.002 | 1.000 | 1.000 | Unannotated | NA | NA |
| metab_10715 | Unannotated feature | neg | 526.7059 | 0.578 | 4.813 | 4.815 | 0.002 | 0.002 | 1.000 | 1.000 | Unannotated | NA | NA |
| metab_7816 | Unannotated feature | neg | 108.9349 | 7.164 | 3.453 | 3.463 | 0.010 | 0.002 | 1.000 | 1.000 | Unannotated | NA | NA |
| metab_4230 | Isoputreanine | pos | 161.1286 | 0.614 | 4.008 | 4.031 | 0.022 | 0.002 | 1.000 | 1.000 | Tentative | NA | NA |
| metab_8643 | Unannotated feature | neg | 604.8731 | 7.148 | 4.855 | 4.876 | 0.021 | 0.002 | 1.000 | 1.000 | Unannotated | NA | NA |
| metab_4110 | Unannotated feature | pos | 152.5845 | 0.706 | 3.643 | 3.609 | -0.034 | -0.002 | 1.000 | 1.000 | Unannotated | NA | NA |
| metab_7904 | Unannotated feature | neg | 238.0776 | 7.124 | 4.961 | 4.952 | -0.009 | 0.002 | 1.000 | 1.000 | Unannotated | NA | NA |
| metab_5291 | Unannotated feature | neg | 1007.7892 | 0.554 | 4.667 | 4.613 | -0.055 | -0.002 | 1.000 | 1.000 | Unannotated | NA | NA |
| metab_342 | Methacholine | pos | 160.1333 | 0.831 | 6.207 | 6.168 | -0.038 | 0.002 | 1.000 | 1.000 | Moderate putative | NA | NA |
| metab_8202 | LysoPE(22:6(4Z,7Z,10Z,13Z,16Z,19Z)/0:0) | neg | 524.2793 | 5.961 | 6.019 | 5.968 | -0.052 | -0.002 | 1.000 | 1.000 | Tentative | NA | NA |
| metab_4664 | Unannotated feature | pos | 283.1521 | 5.840 | 5.512 | 5.491 | -0.021 | -0.002 | 1.000 | 1.000 | Unannotated | NA | NA |
| metab_7563 | Unannotated feature | neg | 255.8226 | 7.189 | 4.840 | 4.850 | 0.010 | -0.002 | 1.000 | 1.000 | Unannotated | NA | NA |
| metab_3775 | Unannotated feature | pos | 70.0132 | 1.686 | 3.957 | 3.959 | 0.003 | 0.002 | 1.000 | 1.000 | Unannotated | NA | NA |
| metab_658 | Unannotated feature | pos | 786.6001 | 6.088 | 6.291 | 6.249 | -0.042 | -0.002 | 1.000 | 1.000 | Unannotated | NA | NA |
| metab_9498 | Unannotated feature | neg | 124.9833 | 3.255 | 4.296 | 4.336 | 0.039 | -0.002 | 1.000 | 1.000 | Unannotated | NA | NA |
| metab_9418 | Unannotated feature | neg | 182.0456 | 3.436 | 3.618 | 3.616 | -0.002 | -0.002 | 1.000 | 1.000 | Unannotated | NA | NA |
| metab_3321 | Feruloylputrescine | pos | 247.1442 | 2.666 | 6.142 | 6.188 | 0.047 | 0.002 | 1.000 | 1.000 | Tentative | NA | NA |
| metab_780 | Adenine | pos | 136.0619 | 2.919 | 4.041 | 4.055 | 0.015 | 0.002 | 1.000 | 1.000 | Strong putative | NA | NA |
| metab_10989 | Unannotated feature | neg | 205.9029 | 0.263 | 5.272 | 5.271 | -0.001 | 0.002 | 1.000 | 1.000 | Unannotated | NA | NA |
| metab_3349 | Unannotated feature | pos | 166.0863 | 2.749 | 5.498 | 5.518 | 0.020 | -0.002 | 1.000 | 1.000 | Unannotated | NA | NA |
| metab_11004 | Unannotated feature | neg | 341.8366 | 0.019 | 5.749 | 5.743 | -0.006 | -0.002 | 1.000 | 1.000 | Unannotated | NA | NA |
| metab_9183 | Unannotated feature | neg | 173.0928 | 4.187 | 4.401 | 4.406 | 0.005 | -0.002 | 1.000 | 1.000 | Unannotated | NA | NA |
| metab_7675 | Unannotated feature | neg | 231.8950 | 7.253 | 5.076 | 5.086 | 0.009 | -0.002 | 1.000 | 1.000 | Unannotated | NA | NA |
| metab_7660 | Unannotated feature | neg | 164.9471 | 7.301 | 4.715 | 4.721 | 0.007 | -0.002 | 1.000 | 1.000 | Unannotated | NA | NA |
| metab_200 | Unannotated feature | pos | 330.7533 | 0.598 | 5.003 | 4.938 | -0.065 | 0.002 | 1.000 | 1.000 | Unannotated | NA | NA |
| metab_5436 | Unannotated feature | neg | 152.0478 | 0.652 | 3.628 | 3.561 | -0.066 | 0.002 | 1.000 | 1.000 | Unannotated | NA | NA |
| metab_221 | Unannotated feature | pos | 133.0609 | 0.621 | 4.436 | 4.440 | 0.004 | -0.002 | 1.000 | 1.000 | Unannotated | NA | NA |
| metab_3612 | Unannotated feature | pos | 81.5211 | 2.003 | 5.759 | 5.762 | 0.003 | -0.002 | 1.000 | 1.000 | Unannotated | NA | NA |
| metab_3609 | Unannotated feature | pos | 156.0575 | 2.011 | 3.885 | 3.870 | -0.015 | -0.002 | 1.000 | 1.000 | Unannotated | NA | NA |
| metab_5903 | Unannotated feature | neg | 474.7612 | 0.582 | 5.417 | 5.414 | -0.004 | 0.002 | 1.000 | 1.000 | Unannotated | NA | NA |
| metab_915 | Unannotated feature | pos | 565.1906 | 3.854 | 5.184 | 5.228 | 0.044 | 0.002 | 1.000 | 1.000 | Unannotated | NA | NA |
| metab_2208 | R-Salbutamol | pos | 520.3401 | 6.296 | 8.122 | 8.125 | 0.003 | -0.002 | 1.000 | 1.000 | Tentative | NA | NA |
| metab_10006 | Unannotated feature | neg | 119.9068 | 1.886 | 4.690 | 4.699 | 0.009 | -0.002 | 1.000 | 1.000 | Unannotated | NA | NA |
| metab_9941 | Unannotated feature | neg | 102.0553 | 2.119 | 3.542 | 3.501 | -0.041 | 0.002 | 1.000 | 1.000 | Unannotated | NA | NA |
| metab_432 | Unannotated feature | pos | 111.0444 | 1.546 | 5.206 | 5.212 | 0.006 | -0.002 | 1.000 | 1.000 | Unannotated | NA | NA |
| metab_7956 | Unannotated feature | neg | 99.6509 | 7.109 | 3.782 | 3.789 | 0.006 | 0.002 | 1.000 | 1.000 | Unannotated | NA | NA |
| metab_7987 | Unannotated feature | neg | 299.9905 | 0.778 | 4.020 | 3.947 | -0.073 | -0.002 | 1.000 | 1.000 | Unannotated | NA | NA |
| metab_5179 | Unannotated feature | neg | 331.9232 | 0.522 | 3.387 | 3.377 | -0.010 | 0.002 | 1.000 | 1.000 | Unannotated | NA | NA |
| metab_4710 | Unannotated feature | pos | 689.4917 | 5.933 | 6.436 | 6.442 | 0.005 | 0.002 | 1.000 | 1.000 | Unannotated | NA | NA |
| metab_436 | Unannotated feature | pos | 143.0340 | 1.577 | 4.848 | 4.852 | 0.004 | -0.002 | 1.000 | 1.000 | Unannotated | NA | NA |
| metab_443 | Unannotated feature | pos | 110.0352 | 1.647 | 3.252 | 3.316 | 0.064 | 0.002 | 1.000 | 1.000 | Unannotated | NA | NA |
| metab_461 | Unannotated feature | pos | 204.9794 | 1.747 | 3.501 | 3.804 | 0.303 | -0.002 | 1.000 | 1.000 | Unannotated | NA | NA |
| metab_4511 | Unannotated feature | pos | 82.0269 | 0.545 | 4.428 | 4.412 | -0.016 | 0.002 | 1.000 | 1.000 | Unannotated | NA | NA |
| metab_8360 | Unannotated feature | neg | 173.1180 | 5.819 | 4.611 | 4.613 | 0.003 | 0.002 | 1.000 | 1.000 | Unannotated | NA | NA |
| metab_7001 | Unannotated feature | neg | 194.8554 | 5.731 | 3.781 | 3.803 | 0.023 | -0.002 | 1.000 | 1.000 | Unannotated | NA | NA |
| metab_10709 | Unannotated feature | neg | 596.7138 | 0.578 | 5.163 | 5.176 | 0.014 | -0.002 | 1.000 | 1.000 | Unannotated | NA | NA |
| metab_10266 | Unannotated feature | neg | 584.5991 | 0.594 | 5.560 | 5.561 | 0.001 | 0.002 | 1.000 | 1.000 | Unannotated | NA | NA |
| metab_2005 | Unannotated feature | pos | 493.1220 | 7.285 | 4.638 | 4.612 | -0.026 | -0.002 | 1.000 | 1.000 | Unannotated | NA | NA |
| metab_2067 | Unannotated feature | pos | 446.9955 | 7.207 | 4.546 | 4.537 | -0.009 | -0.002 | 1.000 | 1.000 | Unannotated | NA | NA |
| metab_2155 | Unannotated feature | pos | 886.7682 | 0.567 | 5.100 | 5.132 | 0.032 | -0.002 | 1.000 | 1.000 | Unannotated | NA | NA |
| metab_11333 | Unannotated feature | neg | 338.8597 | 7.170 | 5.586 | 5.591 | 0.005 | 0.002 | 1.000 | 1.000 | Unannotated | NA | NA |
| metab_6057 | Unannotated feature | neg | 217.0298 | 0.636 | 6.913 | 6.922 | 0.009 | -0.002 | 1.000 | 1.000 | Unannotated | NA | NA |
| metab_5959 | Unannotated feature | neg | 378.7793 | 2.724 | 5.189 | 5.184 | -0.005 | -0.002 | 1.000 | 1.000 | Unannotated | NA | NA |
| metab_10903 | Unannotated feature | neg | 742.9711 | 0.543 | 4.371 | 4.365 | -0.006 | -0.002 | 1.000 | 1.000 | Unannotated | NA | NA |
| metab_9782 | Unannotated feature | neg | 159.8782 | 2.597 | 5.100 | 5.103 | 0.003 | 0.002 | 1.000 | 1.000 | Unannotated | NA | NA |
| metab_7484 | Unannotated feature | neg | 779.8629 | 7.140 | 4.322 | 4.326 | 0.005 | 0.002 | 1.000 | 1.000 | Unannotated | NA | NA |
| metab_10968 | Unannotated feature | neg | 165.9395 | 0.509 | 3.872 | 3.872 | -0.000 | -0.002 | 1.000 | 1.000 | Unannotated | NA | NA |
| metab_2332 | Unannotated feature | pos | 513.3871 | 6.003 | 4.664 | 4.687 | 0.023 | 0.002 | 1.000 | 1.000 | Unannotated | NA | NA |
| metab_6093 | Unannotated feature | neg | 224.0413 | 3.160 | 3.290 | 3.211 | -0.079 | -0.002 | 1.000 | 1.000 | Unannotated | NA | NA |
| metab_10254 | Unannotated feature | neg | 101.0236 | 0.857 | 4.722 | 4.727 | 0.004 | 0.002 | 1.000 | 1.000 | Unannotated | NA | NA |
| metab_10372 | Unannotated feature | neg | 160.0610 | 0.731 | 3.911 | 3.856 | -0.055 | -0.002 | 1.000 | 1.000 | Unannotated | NA | NA |
| metab_6262 | Unannotated feature | neg | 297.7338 | 3.522 | 3.990 | 3.994 | 0.005 | 0.002 | 1.000 | 1.000 | Unannotated | NA | NA |
| metab_1555 | Unannotated feature | pos | 206.1388 | 6.303 | 5.177 | 5.180 | 0.003 | 0.002 | 1.000 | 1.000 | Moderate putative | NA | NA |
| metab_10615 | Unannotated feature | neg | 236.8453 | 0.598 | 4.697 | 4.677 | -0.019 | 0.002 | 1.000 | 1.000 | Unannotated | NA | NA |
| metab_1580 | Unannotated feature | pos | 662.7480 | 0.575 | 5.334 | 5.342 | 0.008 | -0.002 | 1.000 | 1.000 | Unannotated | NA | NA |
| metab_10564 | Unannotated feature | neg | 80.9160 | 0.621 | 4.083 | 4.147 | 0.064 | -0.002 | 1.000 | 1.000 | Unannotated | NA | NA |
| metab_1665 | Unannotated feature | pos | 435.3446 | 7.223 | 4.915 | 4.898 | -0.018 | 0.002 | 1.000 | 1.000 | Unannotated | NA | NA |
| metab_6552 | Unannotated feature | neg | 354.9060 | 4.627 | 4.462 | 4.456 | -0.006 | 0.002 | 1.000 | 1.000 | Unannotated | NA | NA |
| metab_10632 | Unannotated feature | neg | 234.8482 | 0.594 | 4.633 | 4.630 | -0.003 | 0.002 | 1.000 | 1.000 | Unannotated | NA | NA |
| metab_80 | Unannotated feature | pos | 130.9792 | 0.465 | 3.978 | 3.970 | -0.008 | 0.002 | 1.000 | 1.000 | Unannotated | NA | NA |
| metab_10680 | Unannotated feature | neg | 497.7280 | 0.582 | 4.654 | 4.651 | -0.003 | -0.002 | 1.000 | 1.000 | Unannotated | NA | NA |
| metab_11404 | Phenylacetylglutamine | neg | 309.1099 | 2.796 | 4.427 | 4.405 | -0.022 | 0.002 | 1.000 | 1.000 | Tentative | NA | NA |

Values are medians of log10-transformed, sum-normalized feature abundances. Median difference was calculated as stages 3-4 minus stages 1-2; it is not a log2 fold change. P values were obtained using the two-sided Mann-Whitney U test. BH-FDR denotes the Benjamini-Hochberg false-discovery rate. RT, retention time.
