# Supplementary material for: Paired metabolomics identify associations between fecal and serum metabolites in cardiovascular-kidney-metabolic syndrome
Source: Front Endocrinol (Lausanne). 2026 Oct 27;17:1982623. doi: 10.3389/fendo.2026.1982623 (PMC13747780; doi:10.3389/fendo.2026.1982623)
Supplement: Supplementary file 3 [file Table3.docx]

**Supplementary Table S2. Full fecal metabolomics results**

| **Feature ID** | **Putative annotation** | **Ion** | **m/z** | **RT** | **Median 1-2** | **Median 3-4** | **Median difference** | **Cliff's delta** | **P value** | **BH-FDR** | **Confidence** | **Superclass** | **Class** |
| --- | --- | --- | --- | --- | --- | --- | --- | --- | --- | --- | --- | --- | --- |
| metab_4802 | Unannotated feature | pos | 143.0342 | 7.997 | 4.405 | 4.264 | -0.141 | -0.798 | 7.529E-06 | 0.037 | Unannotated | NA | NA |
| metab_699 | Unannotated feature | pos | 675.4170 | 5.922 | 3.260 | 5.840 | 2.580 | 0.789 | 9.390E-06 | 0.037 | Unannotated | NA | NA |
| metab_17126 | Unannotated feature | neg | 73.0284 | 7.827 | 3.845 | 3.514 | -0.331 | -0.789 | 9.390E-06 | 0.037 | Unannotated | NA | NA |
| metab_2639 | Unannotated feature | pos | 768.4701 | 5.906 | 4.441 | 6.143 | 1.702 | 0.777 | 1.302E-05 | 0.038 | Unannotated | NA | NA |
| metab_2724 | Turicine | pos | 124.0761 | 2.179 | 3.962 | 4.245 | 0.283 | 0.768 | 1.616E-05 | 0.038 | Tentative | Organic acids and derivatives | Carboxylic acids and derivatives |
| metab_4243 | Unannotated feature | pos | 645.3971 | 5.929 | 1.407 | 5.706 | 4.299 | 0.752 | 2.470E-05 | 0.049 | Unannotated | NA | NA |
| metab_5969 | Unannotated feature | pos | 623.3816 | 5.914 | 3.407 | 5.851 | 2.444 | 0.735 | 3.744E-05 | 0.063 | Unannotated | NA | NA |
| metab_4214 | Unannotated feature | pos | 784.8137 | 5.914 | 0.295 | 4.771 | 4.476 | 0.718 | 5.626E-05 | 0.073 | Unannotated | NA | NA |
| metab_2690 | Unannotated feature | pos | 701.4343 | 5.914 | 4.594 | 6.598 | 2.004 | 0.718 | 5.626E-05 | 0.073 | Unannotated | NA | NA |
| metab_10008 | 1-Methyladenine | neg | 194.0679 | 2.836 | 2.661 | 4.236 | 1.575 | 0.705 | 7.593E-05 | 0.073 | Tentative | Organoheterocyclic compounds | Imidazopyrimidines |
| metab_6008 | Unannotated feature | pos | 632.3886 | 5.906 | 2.316 | 5.616 | 3.300 | 0.701 | 8.382E-05 | 0.073 | Unannotated | NA | NA |
| metab_1483 | Unannotated feature | pos | 641.3951 | 5.914 | 4.177 | 6.094 | 1.918 | 0.697 | 9.249E-05 | 0.073 | Unannotated | NA | NA |
| metab_5907 | Unannotated feature | pos | 750.4615 | 5.922 | 4.115 | 5.315 | 1.200 | 0.697 | 9.249E-05 | 0.073 | Unannotated | NA | NA |
| metab_685 | Unannotated feature | pos | 716.4405 | 5.906 | 4.484 | 6.109 | 1.625 | 0.693 | 1.020E-04 | 0.073 | Unannotated | NA | NA |
| metab_6549 | Unannotated feature | pos | 625.7315 | 5.526 | 0.292 | 4.939 | 4.647 | 0.688 | 1.124E-04 | 0.073 | Unannotated | NA | NA |
| metab_5887 | Unannotated feature | pos | 683.4190 | 5.929 | 2.382 | 5.381 | 2.999 | 0.684 | 1.238E-04 | 0.073 | Unannotated | NA | NA |
| metab_1489 | Unannotated feature | pos | 670.4106 | 5.898 | 4.295 | 6.280 | 1.985 | 0.680 | 1.363E-04 | 0.073 | Unannotated | NA | NA |
| metab_682 | Unannotated feature | pos | 604.3706 | 5.906 | 3.956 | 5.802 | 1.846 | 0.680 | 1.363E-04 | 0.073 | Unannotated | NA | NA |
| metab_5890 | Unannotated feature | pos | 663.4082 | 5.929 | 3.956 | 5.436 | 1.480 | 0.680 | 1.363E-04 | 0.073 | Unannotated | NA | NA |
| metab_5917 | Unannotated feature | pos | 667.4089 | 5.922 | 2.950 | 5.497 | 2.547 | 0.680 | 1.363E-04 | 0.073 | Unannotated | NA | NA |
| metab_677 | Unannotated feature | pos | 642.8951 | 5.898 | 2.257 | 5.535 | 3.278 | 0.676 | 1.500E-04 | 0.073 | Unannotated | NA | NA |
| metab_13198 | Unannotated feature | neg | 90.9705 | 0.524 | 2.323 | 3.262 | 0.939 | 0.672 | 1.650E-04 | 0.073 | Unannotated | NA | NA |
| metab_2650 | Unannotated feature | pos | 696.9294 | 5.906 | 2.583 | 5.446 | 2.863 | 0.667 | 1.814E-04 | 0.073 | Unannotated | NA | NA |
| metab_2545 | Unannotated feature | pos | 824.5026 | 5.835 | 4.248 | 5.079 | 0.830 | 0.663 | 1.993E-04 | 0.073 | Unannotated | NA | NA |
| metab_2232 | Unannotated feature | pos | 294.1452 | 3.073 | 4.928 | 4.178 | -0.750 | -0.663 | 1.993E-04 | 0.073 | Unannotated | NA | NA |
| metab_6005 | Unannotated feature | pos | 648.3972 | 5.906 | 2.520 | 5.986 | 3.466 | 0.663 | 1.993E-04 | 0.073 | Unannotated | NA | NA |
| metab_1462 | Unannotated feature | pos | 238.1420 | 5.976 | 5.032 | 4.433 | -0.599 | -0.663 | 1.993E-04 | 0.073 | Unannotated | NA | NA |
| metab_6622 | Unannotated feature | pos | 552.3541 | 5.409 | 0.952 | 4.964 | 4.012 | 0.663 | 1.993E-04 | 0.073 | Unannotated | NA | NA |
| metab_2774 | Unannotated feature | pos | 568.6814 | 5.434 | 3.793 | 5.162 | 1.368 | 0.659 | 2.188E-04 | 0.073 | Unannotated | NA | NA |
| metab_1569 | Unannotated feature | pos | 568.8557 | 5.239 | 3.825 | 4.946 | 1.121 | 0.659 | 2.188E-04 | 0.073 | Unannotated | NA | NA |
| metab_4251 | Unannotated feature | pos | 647.3023 | 5.936 | 1.147 | 4.876 | 3.729 | 0.659 | 2.188E-04 | 0.073 | Unannotated | NA | NA |
| metab_978 | Unannotated feature | pos | 140.0933 | 7.941 | 5.580 | 5.223 | -0.356 | -0.659 | 2.188E-04 | 0.073 | Unannotated | NA | NA |
| metab_11124 | Gamma-Glutamylalanine | neg | 217.0826 | 0.714 | 5.785 | 5.378 | -0.407 | -0.659 | 2.188E-04 | 0.073 | Tentative | Organic acids and derivatives | Carboxylic acids and derivatives |
| metab_17111 | Unannotated feature | neg | 141.0186 | 7.853 | 4.519 | 4.403 | -0.116 | -0.659 | 2.188E-04 | 0.073 | Unannotated | NA | NA |
| metab_5884 | Unannotated feature | pos | 855.5206 | 5.929 | 2.198 | 5.450 | 3.252 | 0.655 | 2.401E-04 | 0.073 | Unannotated | NA | NA |
| metab_3069 | N-hydroxy-L-tryptophan | pos | 262.1192 | 3.347 | 4.352 | 4.000 | -0.352 | -0.655 | 2.401E-04 | 0.073 | Tentative | Organoheterocyclic compounds | Indoles and derivatives |
| metab_4949 | Unannotated feature | pos | 485.4329 | 7.602 | 5.706 | 5.620 | -0.086 | -0.655 | 2.401E-04 | 0.073 | Unannotated | NA | NA |
| metab_6642 | Unannotated feature | pos | 537.6784 | 5.381 | 2.262 | 4.927 | 2.666 | 0.655 | 2.401E-04 | 0.073 | Unannotated | NA | NA |
| metab_2248 | Unannotated feature | pos | 568.6814 | 5.597 | 3.388 | 5.407 | 2.020 | 0.655 | 2.401E-04 | 0.073 | Unannotated | NA | NA |
| metab_14208 | Unannotated feature | neg | 221.0929 | 2.951 | 5.238 | 4.627 | -0.612 | -0.651 | 2.634E-04 | 0.074 | Unannotated | NA | NA |
| metab_1622 | PE(20:3(8Z,11Z,14Z)-2OH(5,6)/22:4(7Z,10Z,13Z,16Z)) | pos | 436.7762 | 4.914 | 3.923 | 5.404 | 1.480 | 0.651 | 2.634E-04 | 0.074 | Unannotated | NA | NA |
| metab_8263 | Lysylvaline | pos | 246.1816 | 2.217 | 2.740 | 4.058 | 1.319 | 0.651 | 2.634E-04 | 0.074 | Unannotated | Organic acids and derivatives | Carboxylic acids and derivatives |
| metab_695 | Unannotated feature | pos | 591.3609 | 5.922 | 3.813 | 5.559 | 1.745 | 0.646 | 2.888E-04 | 0.079 | Unannotated | NA | NA |
| metab_548 | Unannotated feature | pos | 539.3302 | 5.550 | 0.639 | 5.151 | 4.511 | 0.642 | 3.164E-04 | 0.080 | Unannotated | NA | NA |
| metab_714 | Unannotated feature | pos | 742.4529 | 5.944 | 3.671 | 5.605 | 1.934 | 0.642 | 3.164E-04 | 0.080 | Unannotated | NA | NA |
| metab_11524 | Unannotated feature | neg | 503.8694 | 0.502 | 3.645 | 4.740 | 1.095 | 0.642 | 3.164E-04 | 0.080 | Unannotated | NA | NA |
| metab_1481 | Unannotated feature | pos | 698.4311 | 5.914 | 3.269 | 5.827 | 2.557 | 0.638 | 3.465E-04 | 0.080 | Unannotated | NA | NA |
| metab_10358 | Glycyl-L-Tyrosine | neg | 237.0882 | 2.183 | 5.395 | 5.007 | -0.387 | -0.638 | 3.465E-04 | 0.080 | Strong putative | Organic acids and derivatives | Carboxylic acids and derivatives |
| metab_6041 | Unannotated feature | pos | 696.4324 | 5.890 | 4.297 | 5.883 | 1.586 | 0.638 | 3.465E-04 | 0.080 | Unannotated | NA | NA |
| metab_4161 | Unannotated feature | pos | 674.4163 | 5.898 | 3.637 | 5.643 | 2.005 | 0.634 | 3.793E-04 | 0.080 | Unannotated | NA | NA |
| metab_16612 | Gamma-Glutaminyl-4-hydroxybenzene | neg | 237.0880 | 1.923 | 5.211 | 4.745 | -0.466 | -0.634 | 3.793E-04 | 0.080 | Unannotated | Organic acids and derivatives | Carboxylic acids and derivatives |
| metab_16863 | Unannotated feature | neg | 233.0669 | 7.737 | 4.256 | 4.874 | 0.618 | 0.634 | 3.793E-04 | 0.080 | Unannotated | NA | NA |
| metab_9714 | Unannotated feature | neg | 221.0929 | 3.112 | 5.583 | 5.032 | -0.551 | -0.634 | 3.793E-04 | 0.080 | Unannotated | NA | NA |
| metab_6530 | Unannotated feature | pos | 537.6787 | 5.550 | 3.726 | 5.523 | 1.797 | 0.634 | 3.793E-04 | 0.080 | Unannotated | NA | NA |
| metab_4203 | Unannotated feature | pos | 635.3879 | 5.914 | 3.659 | 5.837 | 2.178 | 0.634 | 3.793E-04 | 0.080 | Unannotated | NA | NA |
| metab_8387 | L-Dopa | pos | 239.1032 | 1.952 | 5.086 | 4.819 | -0.267 | -0.634 | 3.793E-04 | 0.080 | Tentative | Organic acids and derivatives | Carboxylic acids and derivatives |
| metab_4193 | Unannotated feature | pos | 582.3564 | 5.914 | 3.132 | 5.439 | 2.307 | 0.629 | 4.149E-04 | 0.083 | Unannotated | NA | NA |
| metab_1490 | Unannotated feature | pos | 680.4171 | 5.890 | 3.468 | 5.407 | 1.939 | 0.629 | 4.149E-04 | 0.083 | Unannotated | NA | NA |
| metab_9323 | Unannotated feature | neg | 223.1086 | 3.581 | 4.188 | 3.232 | -0.956 | -0.629 | 4.149E-04 | 0.083 | Unannotated | NA | NA |
| metab_11588 | Unannotated feature | neg | 211.9525 | 0.453 | 4.354 | 4.205 | -0.149 | -0.625 | 4.537E-04 | 0.085 | Unannotated | NA | NA |
| metab_5990 | Unannotated feature | pos | 780.4769 | 5.906 | 4.222 | 5.782 | 1.560 | 0.625 | 4.537E-04 | 0.085 | Unannotated | NA | NA |
| metab_4224 | Unannotated feature | pos | 636.3908 | 5.922 | 3.880 | 5.599 | 1.719 | 0.625 | 4.537E-04 | 0.085 | Unannotated | NA | NA |
| metab_9915 | Unannotated feature | neg | 250.1046 | 2.938 | 4.877 | 4.316 | -0.561 | -0.625 | 4.537E-04 | 0.085 | Unannotated | NA | NA |
| metab_2173 | Ganglioside GM2 (d18:0/25:0) | pos | 753.4744 | 5.488 | 2.575 | 5.213 | 2.638 | 0.621 | 4.958E-04 | 0.088 | Unannotated | Lipids and lipid-like molecules | Sphingolipids |
| metab_290 | Unannotated feature | pos | 209.0336 | 0.485 | 3.548 | 4.583 | 1.036 | 0.621 | 4.958E-04 | 0.088 | Unannotated | NA | NA |
| metab_5959 | Unannotated feature | pos | 659.4067 | 5.914 | 0.857 | 5.308 | 4.451 | 0.621 | 4.958E-04 | 0.088 | Unannotated | NA | NA |
| metab_6581 | Unannotated feature | pos | 493.6519 | 5.474 | 0.190 | 5.324 | 5.135 | 0.621 | 4.958E-04 | 0.088 | Unannotated | NA | NA |
| metab_15647 | Gossypetin | neg | 317.0305 | 5.583 | 3.009 | 5.100 | 2.091 | 0.617 | 5.415E-04 | 0.090 | Strong putative | NA | NA |
| metab_5966 | Unannotated feature | pos | 633.3901 | 5.914 | 3.437 | 5.493 | 2.056 | 0.617 | 5.415E-04 | 0.090 | Unannotated | NA | NA |
| metab_6577 | Unannotated feature | pos | 596.3803 | 5.481 | 1.831 | 5.117 | 3.286 | 0.617 | 5.415E-04 | 0.090 | Unannotated | NA | NA |
| metab_13145 | Unannotated feature | neg | 192.9583 | 0.460 | 6.173 | 6.077 | -0.096 | -0.617 | 5.415E-04 | 0.090 | Unannotated | NA | NA |
| metab_9732 | Phenylalanyl-Gamma-glutamate | neg | 292.1307 | 3.096 | 4.739 | 4.323 | -0.416 | -0.613 | 5.912E-04 | 0.090 | Unannotated | Organic acids and derivatives | Carboxylic acids and derivatives |
| metab_2599 | Unannotated feature | pos | 731.4612 | 5.470 | 2.046 | 5.270 | 3.224 | 0.613 | 5.912E-04 | 0.090 | Unannotated | NA | NA |
| metab_4159 | Unannotated feature | pos | 651.4010 | 5.898 | 1.300 | 5.589 | 4.289 | 0.613 | 5.912E-04 | 0.090 | Unannotated | NA | NA |
| metab_1525 | Unannotated feature | pos | 812.5066 | 5.434 | 1.010 | 4.355 | 3.345 | 0.613 | 5.912E-04 | 0.090 | Unannotated | NA | NA |
| metab_692 | Unannotated feature | pos | 613.3778 | 5.914 | 4.061 | 5.671 | 1.610 | 0.613 | 5.912E-04 | 0.090 | Unannotated | NA | NA |
| metab_1480 | Unannotated feature | pos | 767.4678 | 5.914 | 3.557 | 5.586 | 2.029 | 0.613 | 5.912E-04 | 0.090 | Unannotated | NA | NA |
| metab_13423 | 1,3-Benzodioxole-5,6-diamine | neg | 133.0414 | 0.799 | 4.500 | 3.946 | -0.554 | -0.613 | 5.912E-04 | 0.090 | Unannotated | Organoheterocyclic compounds | Benzodioxoles |
| metab_1838 | Unannotated feature | pos | 216.1458 | 0.744 | 3.392 | 4.528 | 1.136 | 0.608 | 6.450E-04 | 0.091 | Unannotated | NA | NA |
| metab_6756 | Unannotated feature | pos | 577.3689 | 5.239 | 1.110 | 5.471 | 4.361 | 0.608 | 6.450E-04 | 0.091 | Unannotated | NA | NA |
| metab_19092 | Unannotated feature | neg | 431.2117 | 5.401 | 4.097 | 5.112 | 1.015 | 0.608 | 6.450E-04 | 0.091 | Unannotated | NA | NA |
| metab_5745 | Unannotated feature | pos | 161.0124 | 0.477 | 3.410 | 4.193 | 0.783 | 0.608 | 6.450E-04 | 0.091 | Unannotated | NA | NA |
| metab_674 | PA(22:6(5Z,8E,10Z,13Z,15E,19Z)-2OH(7S, 17S)/15:0) | pos | 739.4553 | 5.890 | 4.058 | 5.753 | 1.696 | 0.608 | 6.450E-04 | 0.091 | Unannotated | NA | NA |
| metab_4219 | Unannotated feature | pos | 588.8605 | 5.922 | 3.802 | 5.083 | 1.282 | 0.608 | 6.450E-04 | 0.091 | Unannotated | NA | NA |
| metab_6273 | Unannotated feature | pos | 629.3920 | 5.794 | 3.720 | 5.890 | 2.170 | 0.604 | 7.034E-04 | 0.091 | Unannotated | NA | NA |
| metab_19878 | Malic Acid | neg | 133.0136 | 0.791 | 7.579 | 7.143 | -0.436 | -0.604 | 7.034E-04 | 0.091 | Strong putative | Organic acids and derivatives | Hydroxy acids and derivatives |
| metab_17196 | Unannotated feature | neg | 300.1402 | 7.737 | 5.307 | 5.510 | 0.203 | 0.604 | 7.034E-04 | 0.091 | Unannotated | NA | NA |
| metab_16444 | Unannotated feature | neg | 810.5527 | 6.959 | 5.520 | 4.373 | -1.146 | -0.604 | 7.034E-04 | 0.091 | Unannotated | NA | NA |
| metab_13790 | Unannotated feature | neg | 227.9304 | 0.449 | 4.572 | 4.467 | -0.105 | -0.600 | 7.667E-04 | 0.091 | Unannotated | NA | NA |
| metab_672 | Unannotated feature | pos | 341.1479 | 5.890 | 5.557 | 4.457 | -1.099 | -0.600 | 7.667E-04 | 0.091 | Unannotated | NA | NA |
| metab_8430 | Ile Arg | pos | 288.2034 | 1.944 | 5.500 | 5.049 | -0.450 | -0.600 | 7.667E-04 | 0.091 | Moderate putative | NA | NA |
| metab_14388 | Eltoprazine | neg | 265.1196 | 3.177 | 5.219 | 4.778 | -0.441 | -0.600 | 7.667E-04 | 0.091 | Unannotated | Organoheterocyclic compounds | Diazinanes |
| metab_2723 | Unannotated feature | pos | 676.9143 | 5.914 | 3.971 | 5.730 | 1.759 | 0.600 | 7.667E-04 | 0.091 | Unannotated | NA | NA |
| metab_15662 | Unannotated feature | neg | 431.2119 | 5.607 | 4.601 | 5.470 | 0.868 | 0.600 | 7.667E-04 | 0.091 | Unannotated | NA | NA |
| metab_4139 | Unannotated feature | pos | 599.3658 | 5.882 | 4.725 | 5.598 | 0.873 | 0.596 | 8.352E-04 | 0.091 | Unannotated | NA | NA |
| metab_11604 | Unannotated feature | neg | 236.9486 | 0.443 | 4.219 | 4.081 | -0.138 | -0.596 | 8.352E-04 | 0.091 | Unannotated | NA | NA |
| metab_13927 | Unannotated feature | neg | 435.8853 | 0.485 | 4.772 | 5.506 | 0.734 | 0.596 | 8.352E-04 | 0.091 | Unannotated | NA | NA |
| metab_4247 | Unannotated feature | pos | 682.3156 | 5.929 | 2.079 | 4.640 | 2.562 | 0.596 | 8.352E-04 | 0.091 | Unannotated | NA | NA |
| metab_5845 | Unannotated feature | pos | 687.3229 | 5.960 | 4.135 | 4.964 | 0.829 | 0.596 | 8.352E-04 | 0.091 | Unannotated | NA | NA |
| metab_3752 | Unannotated feature | pos | 478.9765 | 5.434 | 1.344 | 5.249 | 3.905 | 0.596 | 8.352E-04 | 0.091 | Unannotated | NA | NA |
| metab_2194 | Unannotated feature | pos | 612.7072 | 5.494 | 2.388 | 4.509 | 2.121 | 0.596 | 8.352E-04 | 0.091 | Unannotated | NA | NA |
| metab_1584 | Unannotated feature | pos | 546.8424 | 5.175 | 3.604 | 5.038 | 1.434 | 0.596 | 8.352E-04 | 0.091 | Unannotated | NA | NA |
| metab_687 | Unannotated feature | pos | 758.4635 | 5.906 | 4.274 | 6.049 | 1.776 | 0.596 | 8.352E-04 | 0.091 | Unannotated | NA | NA |
| metab_308 | Unannotated feature | pos | 120.5551 | 0.485 | 3.008 | 3.992 | 0.984 | 0.596 | 8.352E-04 | 0.091 | Unannotated | NA | NA |
| metab_6083 | Unannotated feature | pos | 752.4663 | 5.854 | 4.046 | 4.714 | 0.669 | 0.596 | 8.352E-04 | 0.091 | Unannotated | NA | NA |
| metab_13132 | Unannotated feature | neg | 156.9623 | 0.453 | 5.047 | 4.932 | -0.115 | -0.596 | 8.352E-04 | 0.091 | Unannotated | NA | NA |
| metab_7097 | Unannotated feature | pos | 138.9917 | 0.485 | 5.091 | 5.285 | 0.193 | 0.596 | 8.352E-04 | 0.091 | Unannotated | NA | NA |
| metab_4153 | Unannotated feature | pos | 785.4846 | 5.890 | 3.604 | 5.290 | 1.686 | 0.596 | 8.352E-04 | 0.091 | Unannotated | NA | NA |
| metab_4046 | Unannotated feature | pos | 700.9352 | 5.825 | 3.111 | 5.151 | 2.040 | 0.596 | 8.352E-04 | 0.091 | Unannotated | NA | NA |
| metab_4306 | Unannotated feature | pos | 643.4087 | 5.355 | 3.505 | 5.528 | 2.023 | 0.592 | 9.094E-04 | 0.095 | Unannotated | NA | NA |
| metab_4169 | Unannotated feature | pos | 608.7067 | 5.906 | 3.475 | 5.314 | 1.839 | 0.592 | 9.094E-04 | 0.095 | Unannotated | NA | NA |
| metab_6507 | Unannotated feature | pos | 552.3538 | 5.578 | 3.676 | 5.592 | 1.916 | 0.592 | 9.094E-04 | 0.095 | Unannotated | NA | NA |
| metab_6810 | Unannotated feature | pos | 434.2583 | 5.165 | 5.088 | 0.941 | -4.146 | -0.592 | 9.094E-04 | 0.095 | Unannotated | NA | NA |
| metab_16966 | Unannotated feature | neg | 159.0292 | 7.897 | 3.969 | 3.816 | -0.153 | -0.587 | 9.896E-04 | 0.097 | Unannotated | NA | NA |
| metab_385 | Unannotated feature | pos | 284.2955 | 0.068 | 3.950 | 3.796 | -0.154 | -0.587 | 9.896E-04 | 0.097 | Unannotated | NA | NA |
| metab_5949 | Unannotated feature | pos | 760.4676 | 5.914 | 4.081 | 4.645 | 0.564 | 0.587 | 9.896E-04 | 0.097 | Unannotated | NA | NA |
| metab_3779 | Unannotated feature | pos | 523.0030 | 5.519 | 2.652 | 5.354 | 2.703 | 0.587 | 9.896E-04 | 0.097 | Unannotated | NA | NA |
| metab_12939 | Unannotated feature | neg | 322.1148 | 0.738 | 4.504 | 5.142 | 0.638 | 0.587 | 9.896E-04 | 0.097 | Unannotated | NA | NA |
| metab_2574 | Unannotated feature | pos | 668.4113 | 5.851 | 4.077 | 6.073 | 1.996 | 0.587 | 9.896E-04 | 0.097 | Unannotated | NA | NA |
| metab_4205 | Unannotated feature | pos | 654.9013 | 5.914 | 4.022 | 5.789 | 1.766 | 0.587 | 9.896E-04 | 0.097 | Unannotated | NA | NA |
| metab_1473 | Unannotated feature | pos | 661.4039 | 5.922 | 3.116 | 5.537 | 2.421 | 0.587 | 9.896E-04 | 0.097 | Unannotated | NA | NA |
| metab_1427 | Unannotated feature | pos | 369.2262 | 6.040 | 5.791 | 5.243 | -0.548 | -0.583 | 0.001 | 0.100 | Unannotated | NA | NA |
| metab_8276 | N-Hydroxy-L-tyrosine | pos | 239.1031 | 2.165 | 5.307 | 4.931 | -0.376 | -0.583 | 0.001 | 0.100 | Tentative | NA | NA |
| metab_5871 | Unannotated feature | pos | 721.4431 | 5.936 | 0.605 | 5.027 | 4.422 | 0.583 | 0.001 | 0.100 | Unannotated | NA | NA |
| metab_11070 | Tyrosine-tyrosinate | neg | 365.1100 | 0.730 | 3.534 | 4.236 | 0.702 | 0.583 | 0.001 | 0.100 | Unannotated | Organic acids and derivatives | Carboxylic acids and derivatives |
| metab_17118 | Unannotated feature | neg | 159.0293 | 7.835 | 4.168 | 4.043 | -0.125 | -0.583 | 0.001 | 0.100 | Unannotated | NA | NA |
| metab_11598 | Unannotated feature | neg | 165.9392 | 0.449 | 5.257 | 5.158 | -0.099 | -0.583 | 0.001 | 0.100 | Unannotated | NA | NA |
| metab_17141 | Unannotated feature | neg | 154.0503 | 7.792 | 4.455 | 4.355 | -0.101 | -0.579 | 0.001 | 0.103 | Unannotated | NA | NA |
| metab_13671 | Unannotated feature | neg | 286.1887 | 1.685 | 4.499 | 3.861 | -0.638 | -0.579 | 0.001 | 0.103 | Unannotated | NA | NA |
| metab_4208 | Unannotated feature | pos | 714.4373 | 5.914 | 4.277 | 5.876 | 1.598 | 0.579 | 0.001 | 0.103 | Unannotated | NA | NA |
| metab_8011 | Gamma-Glutamyl-2-aminobutyrate | pos | 196.0834 | 2.827 | 2.850 | 3.755 | 0.904 | 0.579 | 0.001 | 0.103 | Unannotated | Organic acids and derivatives | Carboxylic acids and derivatives |
| metab_4204 | Unannotated feature | pos | 651.4956 | 5.914 | 2.357 | 4.769 | 2.412 | 0.579 | 0.001 | 0.103 | Unannotated | NA | NA |
| metab_5930 | Unannotated feature | pos | 611.3746 | 5.922 | 3.920 | 4.900 | 0.980 | 0.579 | 0.001 | 0.103 | Unannotated | NA | NA |
| metab_9346 | Unannotated feature | neg | 367.1039 | 3.552 | 3.600 | 4.188 | 0.588 | 0.579 | 0.001 | 0.103 | Unannotated | NA | NA |
| metab_5286 | Unannotated feature | pos | 482.4216 | 6.598 | 5.143 | 4.013 | -1.130 | -0.575 | 0.001 | 0.103 | Unannotated | NA | NA |
| metab_7836 | Gly Phe | pos | 223.1081 | 3.093 | 5.508 | 5.041 | -0.467 | -0.575 | 0.001 | 0.103 | Moderate putative | NA | NA |
| metab_6664 | Unannotated feature | pos | 523.0032 | 5.355 | 0.483 | 4.938 | 4.455 | 0.575 | 0.001 | 0.103 | Unannotated | NA | NA |
| metab_6165 | Unannotated feature | pos | 614.3792 | 5.831 | 2.927 | 4.970 | 2.042 | 0.575 | 0.001 | 0.103 | Unannotated | NA | NA |
| metab_9672 | N-Methylserotonin | neg | 235.1087 | 3.162 | 5.034 | 4.609 | -0.425 | -0.575 | 0.001 | 0.103 | Tentative | NA | NA |
| metab_11569 | Unannotated feature | neg | 278.9442 | 0.464 | 4.949 | 4.812 | -0.137 | -0.575 | 0.001 | 0.103 | Unannotated | NA | NA |
| metab_6896 | Unannotated feature | pos | 555.3557 | 5.028 | 3.603 | 5.245 | 1.641 | 0.575 | 0.001 | 0.103 | Unannotated | NA | NA |
| metab_1660 | Unannotated feature | pos | 370.7366 | 4.503 | 3.804 | 4.905 | 1.101 | 0.575 | 0.001 | 0.103 | Unannotated | NA | NA |
| metab_6020 | Unannotated feature | pos | 724.3385 | 5.898 | 2.321 | 4.500 | 2.180 | 0.575 | 0.001 | 0.103 | Unannotated | NA | NA |
| metab_14891 | Unannotated feature | neg | 243.1714 | 4.079 | 5.626 | 5.187 | -0.439 | -0.575 | 0.001 | 0.103 | Unannotated | NA | NA |
| metab_667 | Unannotated feature | pos | 762.4693 | 5.874 | 1.716 | 5.344 | 3.628 | 0.575 | 0.001 | 0.103 | Unannotated | NA | NA |
| metab_2638 | Hydroxyl frovatriptan | pos | 294.1817 | 1.864 | 4.629 | 3.883 | -0.746 | -0.575 | 0.001 | 0.103 | Unannotated | Organoheterocyclic compounds | Indoles and derivatives |
| metab_313 | Unannotated feature | pos | 99.5316 | 0.485 | 3.835 | 4.268 | 0.433 | 0.571 | 0.001 | 0.106 | Unannotated | NA | NA |
| metab_11597 | Unannotated feature | neg | 182.9295 | 0.449 | 5.296 | 5.212 | -0.084 | -0.571 | 0.001 | 0.106 | Unannotated | NA | NA |
| metab_5220 | LysoPA(16:0/0:0) | pos | 433.2335 | 6.718 | 5.693 | 5.314 | -0.380 | -0.571 | 0.001 | 0.106 | Tentative | Lipids and lipid-like molecules | Glycerophospholipids |
| metab_8082 | Unannotated feature | pos | 405.1993 | 2.747 | 4.334 | 2.394 | -1.940 | -0.571 | 0.001 | 0.106 | Unannotated | NA | NA |
| metab_8233 | Unannotated feature | pos | 112.0398 | 2.388 | 2.777 | 3.157 | 0.380 | 0.571 | 0.001 | 0.106 | Unannotated | NA | NA |
| metab_3863 | Unannotated feature | pos | 625.7310 | 5.693 | 2.089 | 5.333 | 3.244 | 0.571 | 0.001 | 0.106 | Unannotated | NA | NA |
| metab_14788 | Unannotated feature | neg | 243.1714 | 3.882 | 6.065 | 5.784 | -0.281 | -0.571 | 0.001 | 0.106 | Unannotated | NA | NA |
| metab_619 | Unannotated feature | pos | 652.4070 | 5.799 | 4.449 | 6.361 | 1.912 | 0.571 | 0.001 | 0.106 | Unannotated | NA | NA |
| metab_6176 | Unannotated feature | pos | 620.8824 | 5.825 | 2.088 | 5.125 | 3.037 | 0.566 | 0.001 | 0.107 | Unannotated | NA | NA |
| metab_8507 | Isoleucyl-Threonine | pos | 233.1499 | 1.658 | 6.137 | 5.649 | -0.489 | -0.566 | 0.001 | 0.107 | Unannotated | Organic acids and derivatives | Carboxylic acids and derivatives |
| metab_6792 | Unannotated feature | pos | 555.3558 | 5.181 | 2.665 | 5.496 | 2.831 | 0.566 | 0.001 | 0.107 | Unannotated | NA | NA |
| metab_680 | Unannotated feature | pos | 232.9285 | 0.493 | 4.468 | 5.363 | 0.895 | 0.566 | 0.001 | 0.107 | Unannotated | NA | NA |
| metab_694 | Unannotated feature | pos | 503.3077 | 5.922 | 1.842 | 5.360 | 3.517 | 0.566 | 0.001 | 0.107 | Unannotated | NA | NA |
| metab_3509 | 9'-Carboxy-gamma-tocotrienol | pos | 355.2238 | 4.814 | 4.347 | 3.096 | -1.251 | -0.566 | 0.001 | 0.107 | Unannotated | Lipids and lipid-like molecules | Prenol lipids |
| metab_7856 | Ser Phe | pos | 253.1186 | 3.047 | 5.089 | 4.682 | -0.408 | -0.566 | 0.001 | 0.107 | Strong putative | NA | NA |
| metab_8462 | Unannotated feature | pos | 115.0395 | 1.852 | 4.885 | 4.716 | -0.169 | -0.566 | 0.001 | 0.107 | Unannotated | NA | NA |
| metab_8454 | Unannotated feature | pos | 147.1130 | 1.936 | 4.000 | 3.702 | -0.298 | -0.562 | 0.002 | 0.107 | Unannotated | NA | NA |
| metab_4424 | Unannotated feature | pos | 804.4921 | 5.297 | 3.870 | 6.404 | 2.534 | 0.562 | 0.002 | 0.107 | Unannotated | NA | NA |
| metab_5736 | Unannotated feature | pos | 74.9759 | 0.485 | 3.392 | 4.076 | 0.684 | 0.562 | 0.002 | 0.107 | Unannotated | NA | NA |
| metab_683 | Unannotated feature | pos | 637.3929 | 5.906 | 4.198 | 5.876 | 1.677 | 0.562 | 0.002 | 0.107 | Unannotated | NA | NA |
| metab_11577 | Unannotated feature | neg | 164.9633 | 0.460 | 4.721 | 4.617 | -0.104 | -0.562 | 0.002 | 0.107 | Unannotated | NA | NA |
| metab_5916 | Unannotated feature | pos | 673.5157 | 5.922 | 2.550 | 5.078 | 2.528 | 0.562 | 0.002 | 0.107 | Unannotated | NA | NA |
| metab_5305 | Unannotated feature | pos | 117.0228 | 0.485 | 4.962 | 5.176 | 0.214 | 0.562 | 0.002 | 0.107 | Unannotated | NA | NA |
| metab_3141 | Phenylalanylasparagine | pos | 262.1191 | 3.543 | 5.000 | 4.638 | -0.362 | -0.562 | 0.002 | 0.107 | Unannotated | Organic acids and derivatives | Carboxylic acids and derivatives |
| metab_304 | Unannotated feature | pos | 135.0331 | 0.485 | 4.752 | 4.919 | 0.168 | 0.562 | 0.002 | 0.107 | Unannotated | NA | NA |
| metab_1593 | Unannotated feature | pos | 524.8291 | 5.159 | 1.667 | 4.947 | 3.280 | 0.562 | 0.002 | 0.107 | Unannotated | NA | NA |
| metab_499 | Unannotated feature | pos | 577.3691 | 5.064 | 4.035 | 5.304 | 1.269 | 0.562 | 0.002 | 0.107 | Unannotated | NA | NA |
| metab_17562 | Unannotated feature | neg | 113.9255 | 7.315 | 5.903 | 5.787 | -0.116 | -0.562 | 0.002 | 0.107 | Unannotated | NA | NA |
| metab_5996 | Unannotated feature | pos | 711.4365 | 5.906 | 3.951 | 5.541 | 1.590 | 0.562 | 0.002 | 0.107 | Unannotated | NA | NA |
| metab_17307 | Unannotated feature | neg | 126.9043 | 7.707 | 4.363 | 4.289 | -0.074 | -0.562 | 0.002 | 0.107 | Unannotated | NA | NA |
| metab_18510 | Unannotated feature | neg | 251.1036 | 3.067 | 5.196 | 4.732 | -0.465 | -0.562 | 0.002 | 0.107 | Unannotated | NA | NA |
| metab_6127 | Unannotated feature | pos | 625.3857 | 5.841 | 3.279 | 4.685 | 1.405 | 0.562 | 0.002 | 0.107 | Unannotated | NA | NA |
| metab_6567 | Unannotated feature | pos | 508.3273 | 5.494 | 1.623 | 5.408 | 3.785 | 0.562 | 0.002 | 0.107 | Unannotated | NA | NA |
| metab_8689 | Muscimol | pos | 292.1033 | 0.784 | 5.116 | 5.358 | 0.242 | 0.558 | 0.002 | 0.110 | Tentative | NA | NA |
| metab_2504 | Aspartylglycosamine | pos | 300.1194 | 0.784 | 3.269 | 4.273 | 1.005 | 0.558 | 0.002 | 0.110 | Tentative | Organic oxygen compounds | Organooxygen compounds |
| metab_4662 | DG(16:0/20:3(5Z,8Z,11Z)/0:0) | pos | 663.4936 | 7.427 | 5.648 | 5.175 | -0.473 | -0.558 | 0.002 | 0.110 | Tentative | Lipids and lipid-like molecules | Glycerolipids |
| metab_3720 | Unannotated feature | pos | 524.6549 | 5.355 | 3.759 | 4.732 | 0.972 | 0.558 | 0.002 | 0.110 | Unannotated | NA | NA |
| metab_2353 | Unannotated feature | pos | 79.0183 | 0.485 | 4.041 | 4.334 | 0.293 | 0.558 | 0.002 | 0.110 | Unannotated | NA | NA |
| metab_17133 | Unannotated feature | neg | 105.0185 | 7.810 | 4.407 | 4.309 | -0.098 | -0.558 | 0.002 | 0.110 | Unannotated | NA | NA |
| metab_13147 | Unannotated feature | neg | 452.8757 | 0.460 | 3.150 | 4.525 | 1.375 | 0.558 | 0.002 | 0.110 | Unannotated | NA | NA |
| metab_13319 | N2-Acetyl-L-ornithine | neg | 173.0926 | 0.667 | 5.516 | 5.250 | -0.266 | -0.558 | 0.002 | 0.110 | Moderate putative | Organic acids and derivatives | Carboxylic acids and derivatives |
| metab_16323 | Unannotated feature | neg | 230.1145 | 1.512 | 5.446 | 4.881 | -0.565 | -0.558 | 0.002 | 0.110 | Unannotated | NA | NA |
| metab_282 | PE(22:6(4Z,7Z,10Z,13Z,16Z,19Z)/16:1(9Z)) | pos | 392.7497 | 4.598 | 3.909 | 4.956 | 1.047 | 0.558 | 0.002 | 0.110 | Tentative | Lipids and lipid-like molecules | Glycerophospholipids |
| metab_6044 | Unannotated feature | pos | 346.9092 | 0.501 | 0.108 | 4.631 | 4.523 | 0.558 | 0.002 | 0.110 | Unannotated | NA | NA |
| metab_6144 | Unannotated feature | pos | 695.4288 | 5.835 | 4.201 | 4.842 | 0.641 | 0.554 | 0.002 | 0.112 | Unannotated | NA | NA |
| metab_12983 | Unannotated feature | neg | 132.9857 | 0.791 | 4.430 | 4.024 | -0.406 | -0.554 | 0.002 | 0.112 | Unannotated | NA | NA |
| metab_4259 | PE-NMe2(22:6(4Z,7Z,10Z,13Z,16Z,19Z)/22:6(4Z,7Z,10Z,13Z,16Z,19Z)) | pos | 902.5140 | 5.936 | 2.362 | 4.470 | 2.108 | 0.554 | 0.002 | 0.112 | Tentative | Lipids and lipid-like molecules | Glycerophospholipids |
| metab_4168 | Unannotated feature | pos | 588.3607 | 5.906 | 3.943 | 4.879 | 0.935 | 0.554 | 0.002 | 0.112 | Unannotated | NA | NA |
| metab_12227 | Unannotated feature | neg | 301.0354 | 6.899 | 4.772 | 6.073 | 1.301 | 0.554 | 0.002 | 0.112 | Unannotated | NA | NA |
| metab_2129 | Unannotated feature | pos | 539.3302 | 5.381 | 2.036 | 4.869 | 2.834 | 0.554 | 0.002 | 0.112 | Unannotated | NA | NA |
| metab_6029 | Unannotated feature | pos | 638.4863 | 5.898 | 3.878 | 4.962 | 1.083 | 0.554 | 0.002 | 0.112 | Unannotated | NA | NA |
| metab_4252 | Unannotated feature | pos | 554.0060 | 5.409 | 1.927 | 4.663 | 2.736 | 0.554 | 0.002 | 0.112 | Unannotated | NA | NA |
| metab_296 | Unannotated feature | pos | 190.0026 | 0.485 | 3.548 | 4.300 | 0.752 | 0.554 | 0.002 | 0.112 | Unannotated | NA | NA |
| metab_306 | Unannotated feature | pos | 129.9795 | 0.485 | 3.537 | 4.177 | 0.640 | 0.554 | 0.002 | 0.112 | Unannotated | NA | NA |
| metab_13142 | Unannotated feature | neg | 297.8950 | 0.457 | 3.850 | 4.524 | 0.673 | 0.554 | 0.002 | 0.112 | Unannotated | NA | NA |
| metab_4222 | Unannotated feature | pos | 617.3773 | 5.922 | 1.400 | 5.566 | 4.166 | 0.549 | 0.002 | 0.112 | Unannotated | NA | NA |
| metab_1086 | Unannotated feature | pos | 677.5118 | 6.678 | 6.075 | 5.586 | -0.489 | -0.549 | 0.002 | 0.112 | Unannotated | NA | NA |
| metab_10945 | Unannotated feature | neg | 133.0090 | 0.799 | 3.863 | 3.312 | -0.551 | -0.549 | 0.002 | 0.112 | Unannotated | NA | NA |
| metab_13627 | Glutaminylvaline | neg | 244.1303 | 1.551 | 5.808 | 5.297 | -0.510 | -0.549 | 0.002 | 0.112 | Unannotated | Organic acids and derivatives | Carboxylic acids and derivatives |
| metab_5989 | Unannotated feature | pos | 811.4943 | 5.906 | 2.698 | 5.241 | 2.543 | 0.549 | 0.002 | 0.112 | Unannotated | NA | NA |
| metab_4166 | Unannotated feature | pos | 200.1649 | 6.040 | 6.001 | 5.761 | -0.240 | -0.549 | 0.002 | 0.112 | Unannotated | NA | NA |
| metab_5914 | Unannotated feature | pos | 679.4149 | 5.922 | 3.888 | 5.681 | 1.793 | 0.549 | 0.002 | 0.112 | Unannotated | NA | NA |
| metab_534 | Unannotated feature | pos | 519.9831 | 5.386 | 3.207 | 4.404 | 1.196 | 0.549 | 0.002 | 0.112 | Unannotated | NA | NA |
| metab_9512 | Valylisoleucine | neg | 229.1556 | 3.334 | 5.641 | 5.102 | -0.539 | -0.549 | 0.002 | 0.112 | Unannotated | Organic acids and derivatives | Carboxylic acids and derivatives |
| metab_12561 | Unannotated feature | neg | 333.1375 | 6.162 | 4.587 | 5.299 | 0.712 | 0.549 | 0.002 | 0.112 | Unannotated | NA | NA |
| metab_5391 | Unannotated feature | pos | 209.1539 | 6.454 | 4.860 | 4.716 | -0.144 | -0.549 | 0.002 | 0.112 | Unannotated | NA | NA |
| metab_464 | PE(20:4(6E,8Z,11Z,14Z)-OH(5S)/20:3(5Z,8Z,11Z)) | pos | 414.7629 | 4.832 | 3.917 | 5.422 | 1.506 | 0.549 | 0.002 | 0.112 | Unannotated | NA | NA |
| metab_6931 | Unannotated feature | pos | 467.3027 | 4.975 | 4.383 | 5.687 | 1.304 | 0.549 | 0.002 | 0.112 | Unannotated | NA | NA |
| metab_311 | Unannotated feature | pos | 109.5573 | 0.485 | 3.621 | 4.057 | 0.437 | 0.549 | 0.002 | 0.112 | Unannotated | NA | NA |
| metab_13176 | Unannotated feature | neg | 548.8535 | 0.492 | 4.234 | 5.086 | 0.852 | 0.545 | 0.002 | 0.112 | Unannotated | NA | NA |
| metab_7596 | Rosmaricine | pos | 368.1826 | 3.553 | 4.316 | 2.365 | -1.951 | -0.545 | 0.002 | 0.112 | Unannotated | Lipids and lipid-like molecules | Prenol lipids |
| metab_6069 | Unannotated feature | pos | 652.3080 | 5.870 | 2.487 | 4.532 | 2.046 | 0.545 | 0.002 | 0.112 | Unannotated | NA | NA |
| metab_1928 | Fumarycarnitine | pos | 242.1027 | 0.728 | 4.425 | 5.283 | 0.858 | 0.545 | 0.002 | 0.112 | Unannotated | Lipids and lipid-like molecules | Fatty Acyls |
| metab_708 | Unannotated feature | pos | 790.4853 | 5.936 | 4.130 | 5.426 | 1.296 | 0.545 | 0.002 | 0.112 | Unannotated | NA | NA |
| metab_15070 | Unannotated feature | neg | 239.0559 | 4.480 | 4.371 | 4.723 | 0.351 | 0.545 | 0.002 | 0.112 | Unannotated | NA | NA |
| metab_2042 | Unannotated feature | pos | 148.9760 | 0.485 | 5.051 | 5.256 | 0.205 | 0.545 | 0.002 | 0.112 | Unannotated | NA | NA |
| metab_5926 | Unannotated feature | pos | 638.0574 | 5.922 | 3.771 | 4.954 | 1.183 | 0.545 | 0.002 | 0.112 | Unannotated | NA | NA |
| metab_634 | Unannotated feature | pos | 583.2308 | 5.820 | 2.111 | 4.315 | 2.204 | 0.545 | 0.002 | 0.112 | Unannotated | NA | NA |
| metab_18823 | Unannotated feature | neg | 281.1033 | 5.758 | 4.563 | 5.136 | 0.573 | 0.545 | 0.002 | 0.112 | Unannotated | NA | NA |
| metab_6989 | Unannotated feature | pos | 489.3161 | 4.870 | 3.569 | 5.272 | 1.704 | 0.545 | 0.002 | 0.112 | Unannotated | NA | NA |
| metab_3247 | Gly Tyr Leu | pos | 352.1876 | 3.854 | 4.091 | 3.063 | -1.028 | -0.545 | 0.002 | 0.112 | Strong putative | NA | NA |
| metab_370 | Unannotated feature | pos | 199.1697 | 0.268 | 4.629 | 4.716 | 0.087 | 0.545 | 0.002 | 0.112 | Unannotated | NA | NA |
| metab_4682 | DG(18:1n7/0:0/18:2n6) | pos | 677.5112 | 7.515 | 5.500 | 4.899 | -0.601 | -0.545 | 0.002 | 0.112 | Unannotated | Lipids and lipid-like molecules | Glycerolipids |
| metab_4131 | Unannotated feature | pos | 682.0834 | 5.870 | 2.501 | 5.118 | 2.616 | 0.545 | 0.002 | 0.112 | Unannotated | NA | NA |
| metab_5418 | Unannotated feature | pos | 115.0395 | 2.126 | 5.204 | 5.035 | -0.169 | -0.545 | 0.002 | 0.112 | Unannotated | NA | NA |
| metab_12913 | Unannotated feature | neg | 484.1682 | 0.730 | 2.588 | 4.211 | 1.623 | 0.545 | 0.002 | 0.112 | Unannotated | NA | NA |
| metab_12922 | Unannotated feature | neg | 305.1357 | 0.738 | 5.242 | 4.795 | -0.447 | -0.545 | 0.002 | 0.112 | Unannotated | NA | NA |
| metab_6002 | Unannotated feature | pos | 665.4088 | 5.906 | 4.122 | 5.737 | 1.615 | 0.545 | 0.002 | 0.112 | Unannotated | NA | NA |
| metab_5963 | Unannotated feature | pos | 652.7330 | 5.914 | 1.754 | 5.101 | 3.347 | 0.545 | 0.002 | 0.112 | Unannotated | NA | NA |
| metab_5997 | Unannotated feature | pos | 699.4301 | 5.906 | 2.755 | 5.064 | 2.308 | 0.545 | 0.002 | 0.112 | Unannotated | NA | NA |
| metab_4197 | Unannotated feature | pos | 619.3805 | 5.914 | 0.053 | 5.175 | 5.121 | 0.545 | 0.002 | 0.112 | Unannotated | NA | NA |
| metab_7663 | Unannotated feature | pos | 357.2141 | 3.439 | 4.713 | 3.666 | -1.047 | -0.541 | 0.002 | 0.112 | Unannotated | NA | NA |
| metab_13836 | Unannotated feature | neg | 294.1100 | 2.166 | 4.860 | 4.090 | -0.770 | -0.541 | 0.002 | 0.112 | Unannotated | NA | NA |
| metab_3613 | Unannotated feature | pos | 511.3293 | 5.084 | 3.729 | 5.419 | 1.690 | 0.541 | 0.002 | 0.112 | Unannotated | NA | NA |
| metab_1373 | Unannotated feature | pos | 400.3585 | 6.127 | 4.746 | 2.937 | -1.808 | -0.541 | 0.002 | 0.112 | Unannotated | NA | NA |
| metab_4354 | Unannotated feature | pos | 753.4744 | 5.332 | 2.408 | 4.772 | 2.365 | 0.541 | 0.002 | 0.112 | Unannotated | NA | NA |
| metab_7091 | Unannotated feature | pos | 423.2764 | 4.673 | 4.098 | 5.389 | 1.290 | 0.541 | 0.002 | 0.112 | Unannotated | NA | NA |
| metab_17005 | Unannotated feature | neg | 124.9833 | 7.997 | 3.895 | 3.758 | -0.137 | -0.541 | 0.002 | 0.112 | Unannotated | NA | NA |
| metab_13862 | Unannotated feature | neg | 422.9186 | 0.464 | 5.139 | 4.902 | -0.236 | -0.541 | 0.002 | 0.112 | Unannotated | NA | NA |
| metab_678 | Unannotated feature | pos | 765.4683 | 5.898 | 3.440 | 5.475 | 2.036 | 0.541 | 0.002 | 0.112 | Unannotated | NA | NA |
| metab_1609 | Unannotated feature | pos | 489.3160 | 5.028 | 3.971 | 5.695 | 1.724 | 0.541 | 0.002 | 0.112 | Unannotated | NA | NA |
| metab_6048 | Unannotated feature | pos | 345.2390 | 5.890 | 4.882 | 4.332 | -0.549 | -0.541 | 0.002 | 0.112 | Unannotated | NA | NA |
| metab_5297 | 1-Palmitoyl Lysophosphatidic Acid | pos | 433.2338 | 6.574 | 5.230 | 4.770 | -0.461 | -0.541 | 0.002 | 0.112 | Moderate putative | NA | NA |
| metab_10964 | Unannotated feature | neg | 133.4612 | 0.791 | 4.330 | 3.589 | -0.741 | -0.541 | 0.002 | 0.112 | Unannotated | NA | NA |
| metab_6675 | Unannotated feature | pos | 434.9500 | 5.347 | 0.283 | 4.934 | 4.650 | 0.541 | 0.002 | 0.112 | Unannotated | NA | NA |
| metab_6705 | Unannotated feature | pos | 545.3310 | 5.297 | 3.431 | 4.709 | 1.278 | 0.541 | 0.002 | 0.112 | Unannotated | NA | NA |
| metab_6168 | (+/-)-Metalaxyl | pos | 343.1651 | 5.831 | 6.208 | 5.805 | -0.403 | -0.541 | 0.002 | 0.112 | Tentative | Organic acids and derivatives | Carboxylic acids and derivatives |
| metab_17762 | Unannotated feature | neg | 258.1460 | 2.766 | 6.443 | 5.998 | -0.446 | -0.541 | 0.002 | 0.112 | Unannotated | NA | NA |
| metab_6595 | Unannotated feature | pos | 581.7048 | 5.451 | 1.395 | 5.115 | 3.720 | 0.541 | 0.002 | 0.112 | Unannotated | NA | NA |
| metab_7194 | PA(PGF1alpha/18:1(11Z)) | pos | 399.2475 | 4.448 | 4.016 | 5.297 | 1.281 | 0.537 | 0.003 | 0.116 | Unannotated | NA | NA |
| metab_13162 | Unannotated feature | neg | 549.8655 | 0.485 | 5.010 | 5.523 | 0.513 | 0.537 | 0.003 | 0.116 | Unannotated | NA | NA |
| metab_13405 | Gamma-Aminobutyric acid glutamate | neg | 231.0984 | 0.777 | 5.445 | 5.043 | -0.403 | -0.537 | 0.003 | 0.116 | Unannotated | Organic acids and derivatives | Carboxylic acids and derivatives |
| metab_6231 | Unannotated feature | pos | 282.9032 | 0.531 | 3.231 | 4.288 | 1.057 | 0.537 | 0.003 | 0.116 | Unannotated | NA | NA |
| metab_3633 | Unannotated feature | pos | 533.3425 | 5.142 | 3.693 | 5.477 | 1.784 | 0.537 | 0.003 | 0.116 | Unannotated | NA | NA |
| metab_5902 | (2R,3S,4R,5R,8R,10R,11R,13S,14R)-11-[(2S,3R,4S,6R)-4-(Dimethylamino)-3-hydroxy-6-methyloxan-2-yl]oxy-2-ethyl-3,4,10-trihydroxy-13-[(2R,4R,5S,6S)-5-hydroxy-4-methoxy-4,6-dimethyloxan-2-yl]oxy-3,5,6,8,10,12,14-heptamethyl-1-oxa-6-azacyclopentadecan-15-one | pos | 397.2441 | 5.929 | 2.716 | 4.754 | 2.038 | 0.537 | 0.003 | 0.116 | Unannotated | Organic oxygen compounds | Organooxygen compounds |
| metab_3818 | Unannotated feature | pos | 517.8331 | 5.597 | 3.115 | 4.378 | 1.263 | 0.537 | 0.003 | 0.116 | Unannotated | NA | NA |
| metab_4073 | Unannotated feature | pos | 700.4304 | 5.835 | 4.151 | 4.695 | 0.544 | 0.537 | 0.003 | 0.116 | Unannotated | NA | NA |
| metab_15012 | Unannotated feature | neg | 277.1560 | 4.354 | 5.510 | 5.117 | -0.393 | -0.537 | 0.003 | 0.116 | Unannotated | NA | NA |
| metab_312 | Unannotated feature | pos | 102.0343 | 0.485 | 4.437 | 4.665 | 0.228 | 0.537 | 0.003 | 0.116 | Unannotated | NA | NA |
| metab_3913 | Unannotated feature | pos | 198.0878 | 1.936 | 4.822 | 4.070 | -0.751 | -0.537 | 0.003 | 0.116 | Unannotated | NA | NA |
| metab_6927 | Unannotated feature | pos | 509.3133 | 4.980 | 2.132 | 5.244 | 3.112 | 0.537 | 0.003 | 0.116 | Unannotated | NA | NA |
| metab_6105 | Unannotated feature | pos | 777.4768 | 5.847 | 3.722 | 4.616 | 0.894 | 0.537 | 0.003 | 0.116 | Unannotated | NA | NA |
| metab_2288 | Unannotated feature | pos | 612.7074 | 5.650 | 3.697 | 4.948 | 1.250 | 0.533 | 0.003 | 0.120 | Unannotated | NA | NA |
| metab_6131 | Unannotated feature | pos | 578.3552 | 5.841 | 3.223 | 4.871 | 1.648 | 0.533 | 0.003 | 0.120 | Unannotated | NA | NA |
| metab_2540 | Unannotated feature | pos | 233.1136 | 1.018 | 4.698 | 4.159 | -0.539 | -0.533 | 0.003 | 0.120 | Unannotated | NA | NA |
| metab_4257 | Unannotated feature | pos | 749.4584 | 5.936 | 4.182 | 5.161 | 0.979 | 0.533 | 0.003 | 0.120 | Unannotated | NA | NA |
| metab_3708 | Unannotated feature | pos | 493.6520 | 5.307 | 3.322 | 4.825 | 1.502 | 0.533 | 0.003 | 0.120 | Unannotated | NA | NA |
| metab_11131 | Gamma-Glutamylglutamine | neg | 274.1046 | 0.706 | 5.796 | 5.398 | -0.398 | -0.533 | 0.003 | 0.120 | Moderate putative | Organic acids and derivatives | Carboxylic acids and derivatives |
| metab_1327 | Unannotated feature | pos | 793.5023 | 6.199 | 5.813 | 4.968 | -0.845 | -0.533 | 0.003 | 0.120 | Unannotated | NA | NA |
| metab_6958 | Unannotated feature | pos | 511.3293 | 4.931 | 3.623 | 5.245 | 1.622 | 0.533 | 0.003 | 0.120 | Unannotated | NA | NA |
| metab_17561 | Unannotated feature | neg | 132.9227 | 7.315 | 6.653 | 6.483 | -0.170 | -0.533 | 0.003 | 0.120 | Unannotated | NA | NA |
| metab_2331 | Unannotated feature | pos | 145.0498 | 0.405 | 5.514 | 5.393 | -0.121 | -0.533 | 0.003 | 0.120 | Unannotated | NA | NA |
| metab_2860 | Indole-3-acetylglutamic acid | pos | 269.0927 | 2.780 | 4.475 | 5.265 | 0.789 | 0.533 | 0.003 | 0.120 | Unannotated | Organic acids and derivatives | Carboxylic acids and derivatives |
| metab_2358 | Unannotated feature | pos | 171.9919 | 0.485 | 4.770 | 4.982 | 0.212 | 0.528 | 0.003 | 0.123 | Unannotated | NA | NA |
| metab_7107 | Unannotated feature | pos | 443.2740 | 4.620 | 3.518 | 5.367 | 1.848 | 0.528 | 0.003 | 0.123 | Unannotated | NA | NA |
| metab_18927 | Unannotated feature | neg | 422.1104 | 5.618 | 4.658 | 3.510 | -1.147 | -0.528 | 0.003 | 0.123 | Unannotated | NA | NA |
| metab_8286 | Lys Phe | pos | 294.1818 | 2.126 | 5.051 | 4.455 | -0.596 | -0.528 | 0.003 | 0.123 | Moderate putative | NA | NA |
| metab_4406 | Unannotated feature | pos | 826.5047 | 5.307 | 3.698 | 6.286 | 2.587 | 0.528 | 0.003 | 0.123 | Unannotated | NA | NA |
| metab_1632 | PE(22:4(7Z,10Z,13Z,16Z)/20:3(8Z,11Z,14Z)-2OH(5,6)) | pos | 436.7762 | 4.745 | 2.914 | 4.743 | 1.829 | 0.528 | 0.003 | 0.123 | Unannotated | NA | NA |
| metab_3654 | Unannotated feature | pos | 498.3119 | 5.193 | 3.850 | 5.975 | 2.125 | 0.528 | 0.003 | 0.123 | Unannotated | NA | NA |
| metab_130 | Unannotated feature | pos | 235.2020 | 0.625 | 4.334 | 3.016 | -1.318 | -0.528 | 0.003 | 0.123 | Unannotated | NA | NA |
| metab_6734 | Unannotated feature | pos | 599.3820 | 5.260 | 3.737 | 5.564 | 1.827 | 0.528 | 0.003 | 0.123 | Unannotated | NA | NA |
| metab_6829 | Unannotated feature | pos | 468.9612 | 5.128 | 3.689 | 5.519 | 1.830 | 0.528 | 0.003 | 0.123 | Unannotated | NA | NA |
| metab_501 | Unannotated feature | pos | 502.8158 | 5.084 | 3.784 | 5.104 | 1.320 | 0.528 | 0.003 | 0.123 | Unannotated | NA | NA |
| metab_13133 | Unannotated feature | neg | 177.9721 | 0.453 | 4.164 | 4.037 | -0.127 | -0.528 | 0.003 | 0.123 | Unannotated | NA | NA |
| metab_8042 | Ac-Pro-Gly-Pro-OH | pos | 329.1825 | 2.780 | 4.844 | 4.537 | -0.307 | -0.528 | 0.003 | 0.123 | Unannotated | Organic acids and derivatives | Carboxylic acids and derivatives |
| metab_6736 | Unannotated feature | pos | 527.6635 | 5.260 | 4.441 | 6.462 | 2.021 | 0.528 | 0.003 | 0.123 | Unannotated | NA | NA |
| metab_2267 | Unannotated feature | pos | 70.0131 | 0.493 | 3.792 | 4.089 | 0.297 | 0.528 | 0.003 | 0.123 | Unannotated | NA | NA |
| metab_12906 | Unannotated feature | neg | 275.0886 | 0.730 | 5.790 | 5.570 | -0.220 | -0.528 | 0.003 | 0.123 | Unannotated | NA | NA |
| metab_2081 | Unannotated feature | pos | 260.1610 | 2.755 | 6.273 | 5.908 | -0.365 | -0.524 | 0.003 | 0.124 | Unannotated | NA | NA |
| metab_15463 | Unannotated feature | neg | 103.0029 | 5.204 | 3.253 | 2.967 | -0.286 | -0.524 | 0.003 | 0.124 | Unannotated | NA | NA |
| metab_1192 | Methionyl-Arginine | pos | 369.1664 | 2.740 | 4.493 | 5.201 | 0.708 | 0.524 | 0.003 | 0.124 | Unannotated | Organic acids and derivatives | Carboxylic acids and derivatives |
| metab_9780 | Unannotated feature | neg | 363.1204 | 3.964 | 5.969 | 5.694 | -0.275 | -0.524 | 0.003 | 0.124 | Unannotated | NA | NA |
| metab_11591 | Unannotated feature | neg | 167.9364 | 0.453 | 4.795 | 4.711 | -0.085 | -0.524 | 0.003 | 0.124 | Unannotated | NA | NA |
| metab_3776 | Unannotated feature | pos | 611.0559 | 5.505 | 3.736 | 5.138 | 1.403 | 0.524 | 0.003 | 0.124 | Unannotated | NA | NA |
| metab_2269 | Unannotated feature | pos | 583.3566 | 5.626 | 3.665 | 5.241 | 1.576 | 0.524 | 0.003 | 0.124 | Unannotated | NA | NA |
| metab_1429 | Unannotated feature | pos | 413.2130 | 6.040 | 6.004 | 5.746 | -0.257 | -0.524 | 0.003 | 0.124 | Unannotated | NA | NA |
| metab_12927 | Unannotated feature | neg | 410.1312 | 0.738 | 4.284 | 4.909 | 0.625 | 0.524 | 0.003 | 0.124 | Unannotated | NA | NA |
| metab_4127 | Unannotated feature | pos | 755.4615 | 5.862 | 3.918 | 5.456 | 1.538 | 0.524 | 0.003 | 0.124 | Unannotated | NA | NA |
| metab_13935 | Guanadrel | neg | 258.1461 | 2.579 | 5.649 | 5.139 | -0.510 | -0.524 | 0.003 | 0.124 | Tentative | Organic oxygen compounds | Organooxygen compounds |
| metab_4175 | Unannotated feature | pos | 686.4232 | 5.906 | 2.679 | 5.310 | 2.631 | 0.524 | 0.003 | 0.124 | Unannotated | NA | NA |
| metab_3441 | Unannotated feature | pos | 401.2632 | 4.586 | 4.320 | 5.285 | 0.965 | 0.524 | 0.003 | 0.124 | Unannotated | NA | NA |
| metab_18523 | Unannotated feature | neg | 571.2546 | 6.035 | 5.582 | 4.720 | -0.861 | -0.524 | 0.003 | 0.124 | Unannotated | NA | NA |
| metab_14193 | Unannotated feature | neg | 292.1306 | 2.935 | 4.473 | 4.041 | -0.432 | -0.524 | 0.003 | 0.124 | Unannotated | NA | NA |
| metab_9032 | Unannotated feature | neg | 257.1298 | 4.031 | 4.944 | 4.560 | -0.385 | -0.524 | 0.003 | 0.124 | Unannotated | NA | NA |
| metab_4229 | Unannotated feature | pos | 683.0185 | 5.922 | 2.575 | 4.953 | 2.378 | 0.524 | 0.003 | 0.124 | Unannotated | NA | NA |
| metab_288 | Unannotated feature | pos | 319.0558 | 0.485 | 4.169 | 4.866 | 0.697 | 0.524 | 0.003 | 0.124 | Unannotated | NA | NA |
| metab_5920 | Unannotated feature | pos | 658.4052 | 5.922 | 2.239 | 5.172 | 2.932 | 0.524 | 0.003 | 0.124 | Unannotated | NA | NA |
| metab_2751 | Unannotated feature | pos | 156.0657 | 2.376 | 4.427 | 4.538 | 0.111 | 0.524 | 0.003 | 0.124 | Unannotated | NA | NA |
| metab_14324 | 2-Hydroxy-imipramine glucuronide | neg | 471.2108 | 3.078 | 4.614 | 3.840 | -0.774 | -0.524 | 0.003 | 0.124 | Unannotated | Lipids and lipid-like molecules | Steroids and steroid derivatives |
| metab_246 | Unannotated feature | pos | 279.1705 | 4.564 | 6.112 | 5.632 | -0.480 | -0.520 | 0.004 | 0.128 | Unannotated | NA | NA |
| metab_6450 | Unannotated feature | pos | 611.0556 | 5.650 | 3.352 | 5.478 | 2.126 | 0.520 | 0.004 | 0.128 | Unannotated | NA | NA |
| metab_6007 | Unannotated feature | pos | 637.8936 | 5.906 | 3.078 | 5.150 | 2.071 | 0.520 | 0.004 | 0.128 | Unannotated | NA | NA |
| metab_470 | Unannotated feature | pos | 392.7496 | 4.766 | 4.598 | 5.363 | 0.765 | 0.520 | 0.004 | 0.128 | Unannotated | NA | NA |
| metab_4941 | Unannotated feature | pos | 940.8221 | 7.610 | 4.253 | 4.575 | 0.323 | 0.520 | 0.004 | 0.128 | Unannotated | NA | NA |
| metab_9596 | L-gamma-Glutamyl-beta-phenyl-beta-L-alanine | neg | 293.1145 | 3.231 | 5.540 | 5.079 | -0.461 | -0.520 | 0.004 | 0.128 | Unannotated | Organic acids and derivatives | Carboxylic acids and derivatives |
| metab_362 | PE(20:3(5Z,8Z,11Z)/20:4(5E,8Z,12Z,14Z)-OH(11R)) | pos | 414.7630 | 4.673 | 3.241 | 4.995 | 1.754 | 0.520 | 0.004 | 0.128 | Unannotated | NA | NA |
| metab_11126 | Glyceric Acid | neg | 105.0185 | 0.714 | 5.695 | 5.468 | -0.228 | -0.520 | 0.004 | 0.128 | Strong putative | Organic oxygen compounds | Organooxygen compounds |
| metab_17379 | Unannotated feature | neg | 394.8910 | 7.666 | 5.488 | 5.363 | -0.125 | -0.520 | 0.004 | 0.128 | Unannotated | NA | NA |
| metab_37 | 1-Pyrroline-2-carboxylic acid | pos | 96.0449 | 0.704 | 4.050 | 4.302 | 0.252 | 0.520 | 0.004 | 0.128 | Tentative | NA | NA |
| metab_7803 | Thr Phe | pos | 267.1337 | 3.166 | 5.005 | 4.334 | -0.670 | -0.520 | 0.004 | 0.128 | Unannotated | NA | NA |
| metab_4184 | Lexacalcitol | pos | 505.3261 | 5.914 | 1.140 | 4.066 | 2.926 | 0.520 | 0.004 | 0.128 | Unannotated | Lipids and lipid-like molecules | Steroids and steroid derivatives |
| metab_495 | Unannotated feature | pos | 568.8557 | 5.068 | 3.543 | 3.932 | 0.389 | 0.520 | 0.004 | 0.128 | Unannotated | NA | NA |
| metab_14684 | Unannotated feature | neg | 706.8142 | 0.516 | 4.965 | 5.286 | 0.321 | 0.520 | 0.004 | 0.128 | Unannotated | NA | NA |
| metab_3519 | SM(d18:1/PGF2alpha) | pos | 423.2763 | 4.844 | 4.759 | 5.723 | 0.964 | 0.516 | 0.004 | 0.129 | Unannotated | NA | NA |
| metab_18436 | Unannotated feature | neg | 258.0480 | 3.033 | 6.200 | 5.572 | -0.628 | -0.516 | 0.004 | 0.129 | Unannotated | NA | NA |
| metab_12968 | Unannotated feature | neg | 437.1262 | 0.777 | 2.933 | 4.956 | 2.023 | 0.516 | 0.004 | 0.129 | Unannotated | NA | NA |
| metab_19508 | Unannotated feature | neg | 123.0808 | 4.846 | 4.138 | 3.446 | -0.692 | -0.516 | 0.004 | 0.129 | Unannotated | NA | NA |
| metab_2351 | Unannotated feature | pos | 293.9987 | 0.469 | 3.894 | 4.347 | 0.453 | 0.516 | 0.004 | 0.129 | Unannotated | NA | NA |
| metab_3624 | Unannotated feature | pos | 270.1704 | 5.128 | 4.237 | 3.482 | -0.755 | -0.516 | 0.004 | 0.129 | Unannotated | NA | NA |
| metab_5961 | Unannotated feature | pos | 654.8010 | 5.914 | 2.765 | 4.925 | 2.160 | 0.516 | 0.004 | 0.129 | Unannotated | NA | NA |
| metab_7225 | Unannotated feature | pos | 373.2456 | 4.375 | 4.840 | 4.368 | -0.473 | -0.516 | 0.004 | 0.129 | Unannotated | NA | NA |
| metab_5909 | Unannotated feature | pos | 713.4385 | 5.922 | 3.032 | 4.981 | 1.949 | 0.516 | 0.004 | 0.129 | Unannotated | NA | NA |
| metab_2183 | Unannotated feature | pos | 496.9757 | 5.505 | 3.313 | 4.565 | 1.252 | 0.516 | 0.004 | 0.129 | Unannotated | NA | NA |
| metab_1452 | Unannotated feature | pos | 466.2926 | 6.000 | 5.516 | 4.949 | -0.567 | -0.516 | 0.004 | 0.129 | Unannotated | NA | NA |
| metab_2304 | Unannotated feature | pos | 146.9616 | 0.029 | 3.742 | 3.854 | 0.113 | 0.516 | 0.004 | 0.129 | Unannotated | NA | NA |
| metab_16847 | Unannotated feature | neg | 295.1117 | 7.730 | 4.539 | 4.967 | 0.429 | 0.516 | 0.004 | 0.129 | Unannotated | NA | NA |
| metab_373 | Unannotated feature | pos | 362.2230 | 4.676 | 3.494 | 4.928 | 1.434 | 0.516 | 0.004 | 0.129 | Unannotated | NA | NA |
| metab_15567 | Unannotated feature | neg | 400.1623 | 5.427 | 5.045 | 5.886 | 0.841 | 0.516 | 0.004 | 0.129 | Unannotated | NA | NA |
| metab_7538 | Sinapic Acid | pos | 225.0761 | 3.681 | 0.120 | 2.807 | 2.687 | 0.516 | 0.004 | 0.129 | Strong putative | NA | NA |
| metab_690 | Unannotated feature | pos | 616.8781 | 5.914 | 2.153 | 4.803 | 2.649 | 0.516 | 0.004 | 0.129 | Unannotated | NA | NA |
| metab_11241 | N-NITROSOMORPHOLINE | neg | 161.0561 | 0.636 | 4.497 | 3.617 | -0.879 | -0.516 | 0.004 | 0.129 | Tentative | Organoheterocyclic compounds | Oxazinanes |
| metab_4188 | Unannotated feature | pos | 560.3432 | 5.914 | 3.253 | 5.177 | 1.924 | 0.516 | 0.004 | 0.129 | Unannotated | NA | NA |
| metab_13148 | Unannotated feature | neg | 270.9434 | 0.464 | 5.223 | 5.041 | -0.182 | -0.516 | 0.004 | 0.129 | Unannotated | NA | NA |
| metab_668 | Unannotated feature | pos | 727.5539 | 5.874 | 3.173 | 4.598 | 1.424 | 0.516 | 0.004 | 0.129 | Unannotated | NA | NA |
| metab_18586 | Anthracene-9-carboxylic acid | neg | 259.0188 | 6.003 | 3.821 | 2.027 | -1.794 | -0.516 | 0.004 | 0.129 | Tentative | Benzenoids | Anthracenes |
| metab_4228 | Unannotated feature | pos | 678.4159 | 5.922 | 3.080 | 5.459 | 2.379 | 0.516 | 0.004 | 0.129 | Unannotated | NA | NA |
| metab_8363 | Unannotated feature | pos | 373.7071 | 1.952 | 4.443 | 2.746 | -1.697 | -0.512 | 0.004 | 0.132 | Unannotated | NA | NA |
| metab_4398 | Unannotated feature | pos | 621.3953 | 5.313 | 2.285 | 5.515 | 3.230 | 0.512 | 0.004 | 0.132 | Unannotated | NA | NA |
| metab_1620 | Unannotated feature | pos | 664.4123 | 4.923 | 3.805 | 5.606 | 1.801 | 0.512 | 0.004 | 0.132 | Unannotated | NA | NA |
| metab_11545 | Unannotated feature | neg | 307.9244 | 0.489 | 4.519 | 5.238 | 0.719 | 0.512 | 0.004 | 0.132 | Unannotated | NA | NA |
| metab_11498 | Unannotated feature | neg | 632.8139 | 0.512 | 4.188 | 4.513 | 0.325 | 0.512 | 0.004 | 0.132 | Unannotated | NA | NA |
| metab_8188 | Gln Ile | pos | 260.1609 | 2.556 | 5.578 | 5.002 | -0.576 | -0.512 | 0.004 | 0.132 | Strong putative | NA | NA |
| metab_2227 | Unannotated feature | pos | 554.0059 | 5.573 | 3.777 | 5.248 | 1.471 | 0.512 | 0.004 | 0.132 | Unannotated | NA | NA |
| metab_13492 | Epsilon-Deoxy-fructosyl-lysine | neg | 305.1358 | 1.029 | 5.168 | 4.133 | -1.035 | -0.512 | 0.004 | 0.132 | Unannotated | Organic acids and derivatives | Carboxylic acids and derivatives |
| metab_5982 | Unannotated feature | pos | 564.6793 | 5.914 | 3.300 | 4.489 | 1.190 | 0.512 | 0.004 | 0.132 | Unannotated | NA | NA |
| metab_3712 | Unannotated feature | pos | 557.0146 | 5.332 | 4.169 | 6.332 | 2.163 | 0.512 | 0.004 | 0.132 | Unannotated | NA | NA |
| metab_10928 | Unannotated feature | neg | 217.9915 | 0.810 | 5.435 | 4.833 | -0.602 | -0.512 | 0.004 | 0.132 | Unannotated | NA | NA |
| metab_6630 | Unannotated feature | pos | 464.3010 | 5.396 | 2.192 | 5.078 | 2.886 | 0.512 | 0.004 | 0.132 | Unannotated | NA | NA |
| metab_12606 | Unannotated feature | neg | 423.2876 | 6.068 | 4.670 | 4.004 | -0.666 | -0.512 | 0.004 | 0.132 | Unannotated | NA | NA |
| metab_2485 | Unannotated feature | pos | 673.2240 | 5.806 | 3.741 | 4.302 | 0.561 | 0.512 | 0.004 | 0.132 | Unannotated | NA | NA |
| metab_3599 | Unannotated feature | pos | 650.3985 | 5.060 | 5.354 | 6.537 | 1.183 | 0.512 | 0.004 | 0.132 | Unannotated | NA | NA |
| metab_4680 | Unannotated feature | pos | 363.2319 | 7.515 | 4.691 | 4.425 | -0.266 | -0.512 | 0.004 | 0.132 | Unannotated | NA | NA |
| metab_2681 | Unannotated feature | pos | 221.1386 | 5.922 | 2.720 | 4.889 | 2.169 | 0.512 | 0.004 | 0.132 | Unannotated | NA | NA |
| metab_5859 | Unannotated feature | pos | 741.4490 | 5.944 | 2.217 | 4.543 | 2.325 | 0.512 | 0.004 | 0.132 | Unannotated | NA | NA |
| metab_11933 | Unannotated feature | neg | 567.3011 | 6.115 | 6.588 | 6.987 | 0.399 | 0.512 | 0.004 | 0.132 | Unannotated | NA | NA |
| metab_14019 | Unannotated feature | neg | 374.1575 | 2.754 | 5.789 | 5.331 | -0.458 | -0.512 | 0.004 | 0.132 | Unannotated | NA | NA |
| metab_4743 | Unannotated feature | pos | 158.1178 | 7.700 | 5.138 | 5.187 | 0.048 | 0.507 | 0.004 | 0.132 | Unannotated | NA | NA |
| metab_765 | Unannotated feature | pos | 229.1802 | 6.119 | 4.426 | 3.884 | -0.542 | -0.507 | 0.004 | 0.132 | Unannotated | NA | NA |
| metab_4108 | Unannotated feature | pos | 689.4246 | 5.851 | 3.999 | 5.470 | 1.471 | 0.507 | 0.004 | 0.132 | Unannotated | NA | NA |
| metab_9682 | Unannotated feature | neg | 357.1165 | 3.074 | 4.610 | 5.378 | 0.768 | 0.507 | 0.004 | 0.132 | Unannotated | NA | NA |
| metab_13487 | Alanylproline | neg | 231.0984 | 1.021 | 5.290 | 4.930 | -0.361 | -0.507 | 0.004 | 0.132 | Unannotated | Organic acids and derivatives | Carboxylic acids and derivatives |
| metab_9897 | Baikiain | neg | 253.1195 | 2.956 | 5.670 | 5.418 | -0.252 | -0.507 | 0.004 | 0.132 | Unannotated | Organic acids and derivatives | Carboxylic acids and derivatives |
| metab_1586 | Unannotated feature | pos | 538.3289 | 5.175 | 3.033 | 4.415 | 1.382 | 0.507 | 0.004 | 0.132 | Unannotated | NA | NA |
| metab_4196 | Unannotated feature | pos | 607.3729 | 5.914 | 3.947 | 5.291 | 1.344 | 0.507 | 0.004 | 0.132 | Unannotated | NA | NA |
| metab_1579 | Unannotated feature | pos | 560.3413 | 5.213 | 0.773 | 4.647 | 3.874 | 0.507 | 0.004 | 0.132 | Unannotated | NA | NA |
| metab_3085 | Asp Ala Phe | pos | 352.1512 | 3.384 | 4.703 | 4.197 | -0.506 | -0.507 | 0.004 | 0.132 | Moderate putative | NA | NA |
| metab_18308 | Unannotated feature | neg | 195.0505 | 0.651 | 6.720 | 6.502 | -0.218 | -0.507 | 0.004 | 0.132 | Unannotated | NA | NA |
| metab_4947 | Unannotated feature | pos | 155.1293 | 7.610 | 4.551 | 4.457 | -0.094 | -0.507 | 0.004 | 0.132 | Unannotated | NA | NA |
| metab_10953 | Unannotated feature | neg | 278.1247 | 0.791 | 4.330 | 5.050 | 0.720 | 0.507 | 0.004 | 0.132 | Unannotated | NA | NA |
| metab_2660 | Unannotated feature | pos | 682.9190 | 5.914 | 1.464 | 5.004 | 3.539 | 0.507 | 0.004 | 0.132 | Unannotated | NA | NA |
| metab_6685 | Unannotated feature | pos | 571.6901 | 5.332 | 2.654 | 6.243 | 3.589 | 0.507 | 0.004 | 0.132 | Unannotated | NA | NA |
| metab_9325 | Secologanin | neg | 369.1196 | 3.577 | 1.087 | 3.558 | 2.471 | 0.507 | 0.004 | 0.132 | Tentative | Lipids and lipid-like molecules | Prenol lipids |
| metab_228 | Lys Gly | pos | 204.1347 | 0.561 | 4.718 | 3.668 | -1.050 | -0.507 | 0.004 | 0.132 | Moderate putative | NA | NA |
| metab_3465 | SM(d16:2(4E,8Z)/20:5(6E,8Z,11Z,14Z,17Z)-OH(5)) | pos | 379.2499 | 4.681 | 4.834 | 5.692 | 0.858 | 0.507 | 0.004 | 0.132 | Unannotated | NA | NA |
| metab_2151 | Unannotated feature | pos | 687.4348 | 5.414 | 3.987 | 5.272 | 1.285 | 0.507 | 0.004 | 0.132 | Unannotated | NA | NA |
| metab_13075 | Unannotated feature | neg | 312.9170 | 0.460 | 5.682 | 5.572 | -0.110 | -0.507 | 0.004 | 0.132 | Unannotated | NA | NA |
| metab_2928 | Unannotated feature | pos | 230.1867 | 2.907 | 3.440 | 4.004 | 0.564 | 0.507 | 0.004 | 0.132 | Unannotated | NA | NA |
| metab_486 | Unannotated feature | pos | 608.8494 | 4.984 | 4.341 | 5.361 | 1.021 | 0.507 | 0.004 | 0.132 | Unannotated | NA | NA |
| metab_17858 | PE(16:0/0:0) | neg | 452.2790 | 6.726 | 7.730 | 7.461 | -0.269 | -0.507 | 0.004 | 0.132 | Strong putative | Lipids and lipid-like molecules | Glycerophospholipids |
| metab_531 | Unannotated feature | pos | 774.4759 | 5.260 | 3.666 | 5.473 | 1.806 | 0.507 | 0.004 | 0.132 | Unannotated | NA | NA |
| metab_7242 | L-Norleucine | pos | 132.1021 | 0.688 | 6.396 | 5.854 | -0.542 | -0.507 | 0.004 | 0.132 | Tentative | Organic acids and derivatives | Carboxylic acids and derivatives |
| metab_1221 | Unannotated feature | pos | 872.5452 | 4.914 | 3.411 | 4.509 | 1.098 | 0.507 | 0.004 | 0.132 | Unannotated | NA | NA |
| metab_6050 | Unannotated feature | pos | 694.4271 | 5.882 | 3.938 | 5.465 | 1.527 | 0.507 | 0.004 | 0.132 | Unannotated | NA | NA |
| metab_17185 | Unannotated feature | neg | 255.0803 | 7.741 | 1.981 | 3.727 | 1.746 | 0.507 | 0.004 | 0.132 | Unannotated | NA | NA |
| metab_4573 | Unannotated feature | pos | 740.4662 | 4.681 | 4.730 | 5.744 | 1.014 | 0.503 | 0.005 | 0.134 | Unannotated | NA | NA |
| metab_1821 | Unannotated feature | pos | 736.4349 | 4.524 | 4.259 | 5.565 | 1.306 | 0.503 | 0.005 | 0.134 | Unannotated | NA | NA |
| metab_4290 | Unannotated feature | pos | 665.4216 | 5.372 | 3.428 | 5.471 | 2.043 | 0.503 | 0.005 | 0.134 | Unannotated | NA | NA |
| metab_3562 | Unannotated feature | pos | 481.3188 | 4.980 | 0.698 | 4.559 | 3.861 | 0.503 | 0.005 | 0.134 | Unannotated | NA | NA |
| metab_7685 | Unannotated feature | pos | 372.2602 | 3.400 | 3.428 | 4.821 | 1.393 | 0.503 | 0.005 | 0.134 | Unannotated | NA | NA |
| metab_3986 | Vulgaxanthin I | pos | 357.1427 | 5.806 | 5.252 | 4.452 | -0.801 | -0.503 | 0.005 | 0.134 | Tentative | Organic acids and derivatives | Carboxylic acids and derivatives |
| metab_4650 | Unannotated feature | pos | 740.4663 | 4.503 | 3.658 | 5.135 | 1.476 | 0.503 | 0.005 | 0.134 | Unannotated | NA | NA |
| metab_2329 | Unannotated feature | pos | 219.0193 | 0.385 | 4.380 | 4.435 | 0.055 | 0.503 | 0.005 | 0.134 | Unannotated | NA | NA |
| metab_5191 | Unannotated feature | pos | 191.0229 | 0.485 | 4.928 | 5.078 | 0.150 | 0.503 | 0.005 | 0.134 | Unannotated | NA | NA |
| metab_9569 | Unannotated feature | neg | 281.1141 | 3.254 | 4.740 | 4.427 | -0.313 | -0.503 | 0.005 | 0.134 | Unannotated | NA | NA |
| metab_6686 | Unannotated feature | pos | 508.3276 | 5.332 | 3.143 | 4.864 | 1.721 | 0.503 | 0.005 | 0.134 | Unannotated | NA | NA |
| metab_307 | Unannotated feature | pos | 124.9741 | 0.485 | 3.693 | 4.274 | 0.581 | 0.503 | 0.005 | 0.134 | Unannotated | NA | NA |
| metab_546 | Unannotated feature | pos | 526.3267 | 5.556 | 3.761 | 4.490 | 0.729 | 0.503 | 0.005 | 0.134 | Unannotated | NA | NA |
| metab_13293 | Unannotated feature | neg | 353.0736 | 0.791 | 5.481 | 5.139 | -0.342 | -0.503 | 0.005 | 0.134 | Unannotated | NA | NA |
| metab_4320 | Unannotated feature | pos | 588.0168 | 5.355 | 3.981 | 5.756 | 1.774 | 0.503 | 0.005 | 0.134 | Unannotated | NA | NA |
| metab_6025 | Unannotated feature | pos | 677.4160 | 5.898 | 4.018 | 5.110 | 1.092 | 0.503 | 0.005 | 0.134 | Unannotated | NA | NA |
| metab_2613 | Arg Leu | pos | 288.2034 | 1.685 | 5.688 | 5.108 | -0.580 | -0.503 | 0.005 | 0.134 | Moderate putative | NA | NA |
| metab_17076 | Unannotated feature | neg | 285.9102 | 7.905 | 4.831 | 4.754 | -0.077 | -0.503 | 0.005 | 0.134 | Unannotated | NA | NA |
| metab_2671 | Unannotated feature | pos | 717.5478 | 5.914 | 3.846 | 4.761 | 0.916 | 0.503 | 0.005 | 0.134 | Unannotated | NA | NA |
| metab_5881 | Dihydro-beta-erythroidine | pos | 276.1601 | 5.936 | 4.332 | 3.251 | -1.081 | -0.503 | 0.005 | 0.134 | Tentative | Organoheterocyclic compounds | Indoles and derivatives |
| metab_6071 | Unannotated feature | pos | 255.9445 | 0.493 | 3.388 | 4.367 | 0.980 | 0.503 | 0.005 | 0.134 | Unannotated | NA | NA |
| metab_12751 | Gamma-Glutamylleucine | neg | 259.1301 | 1.952 | 6.061 | 5.763 | -0.298 | -0.503 | 0.005 | 0.134 | Unannotated | Organic acids and derivatives | Carboxylic acids and derivatives |
| metab_2712 | Unannotated feature | pos | 824.5012 | 5.929 | 3.498 | 5.062 | 1.564 | 0.503 | 0.005 | 0.134 | Unannotated | NA | NA |
| metab_5712 | Unannotated feature | pos | 406.3537 | 6.103 | 5.019 | 4.855 | -0.164 | -0.499 | 0.005 | 0.138 | Unannotated | NA | NA |
| metab_234 | Unannotated feature | pos | 190.0369 | 0.553 | 2.779 | 3.334 | 0.554 | 0.499 | 0.005 | 0.138 | Unannotated | NA | NA |
| metab_10910 | Unannotated feature | neg | 274.8987 | 0.858 | 4.668 | 3.112 | -1.556 | -0.499 | 0.005 | 0.138 | Unannotated | NA | NA |
| metab_19101 | Unannotated feature | neg | 422.1102 | 5.386 | 4.490 | 3.514 | -0.976 | -0.499 | 0.005 | 0.138 | Unannotated | NA | NA |
| metab_10109 | Ala-Leu | neg | 201.1240 | 2.766 | 5.785 | 5.284 | -0.502 | -0.499 | 0.005 | 0.138 | Strong putative | Organic acids and derivatives | Carboxylic acids and derivatives |
| metab_4137 | Unannotated feature | pos | 775.4786 | 5.874 | 2.307 | 4.832 | 2.524 | 0.499 | 0.005 | 0.138 | Unannotated | NA | NA |
| metab_11499 | Unannotated feature | neg | 586.8194 | 0.512 | 4.573 | 5.116 | 0.543 | 0.499 | 0.005 | 0.138 | Unannotated | NA | NA |
| metab_1933 | Unannotated feature | pos | 314.1242 | 1.936 | 4.299 | 4.858 | 0.559 | 0.499 | 0.005 | 0.138 | Unannotated | NA | NA |
| metab_6190 | Unannotated feature | pos | 612.8848 | 5.820 | 2.957 | 5.212 | 2.255 | 0.499 | 0.005 | 0.138 | Unannotated | NA | NA |
| metab_14601 | Unannotated feature | neg | 422.1580 | 3.505 | 4.662 | 3.667 | -0.995 | -0.499 | 0.005 | 0.138 | Unannotated | NA | NA |
| metab_660 | Unannotated feature | pos | 678.5201 | 5.854 | 3.911 | 4.730 | 0.818 | 0.499 | 0.005 | 0.138 | Unannotated | NA | NA |
| metab_4234 | Unannotated feature | pos | 746.4585 | 5.922 | 3.353 | 4.975 | 1.621 | 0.499 | 0.005 | 0.138 | Unannotated | NA | NA |
| metab_5947 | Unannotated feature | pos | 779.4767 | 5.914 | 1.772 | 4.681 | 2.909 | 0.499 | 0.005 | 0.138 | Unannotated | NA | NA |
| metab_1598 | Unannotated feature | pos | 704.9178 | 5.142 | 2.155 | 4.316 | 2.161 | 0.499 | 0.005 | 0.138 | Unannotated | NA | NA |
| metab_14164 | Unannotated feature | neg | 360.1055 | 2.903 | 4.078 | 5.057 | 0.979 | 0.499 | 0.005 | 0.138 | Unannotated | NA | NA |
| metab_539 | Unannotated feature | pos | 578.6849 | 5.505 | 2.154 | 4.251 | 2.097 | 0.499 | 0.005 | 0.138 | Unannotated | NA | NA |
| metab_17432 | Unannotated feature | neg | 619.2476 | 2.649 | 0.803 | 3.763 | 2.960 | 0.499 | 0.005 | 0.138 | Unannotated | NA | NA |
| metab_13851 | (2E)-3-[3-(sulfooxy)phenyl]prop-2-enoic acid | neg | 224.9882 | 2.229 | 3.925 | 2.712 | -1.213 | -0.499 | 0.005 | 0.138 | Unannotated | Phenylpropanoids and polyketides | Cinnamic acids and derivatives |
| metab_10837 | Hydroxypropionylcarnitine | neg | 278.1247 | 1.041 | 4.106 | 4.611 | 0.506 | 0.499 | 0.005 | 0.138 | Unannotated | Lipids and lipid-like molecules | Fatty Acyls |
| metab_8643 | Carboxyethyllysine | pos | 219.1343 | 1.026 | 5.153 | 4.878 | -0.275 | -0.495 | 0.006 | 0.140 | Unannotated | Organic acids and derivatives | Carboxylic acids and derivatives |
| metab_5870 | Unannotated feature | pos | 745.4591 | 5.936 | 0.382 | 4.788 | 4.406 | 0.495 | 0.006 | 0.140 | Unannotated | NA | NA |
| metab_16458 | 3-Hydroxymonoethylglycinexylidide | neg | 489.2721 | 6.997 | 5.373 | 5.640 | 0.268 | 0.495 | 0.006 | 0.140 | Tentative | NA | NA |
| metab_18563 | Unannotated feature | neg | 233.0777 | 0.667 | 5.805 | 5.537 | -0.268 | -0.495 | 0.006 | 0.140 | Unannotated | NA | NA |
| metab_2766 | Unannotated feature | pos | 258.1340 | 2.439 | 2.880 | 3.615 | 0.735 | 0.495 | 0.006 | 0.140 | Unannotated | NA | NA |
| metab_3507 | Unannotated feature | pos | 467.3027 | 4.810 | 3.799 | 5.283 | 1.484 | 0.495 | 0.006 | 0.140 | Unannotated | NA | NA |
| metab_2343 | Unannotated feature | pos | 627.3805 | 5.682 | 3.428 | 4.868 | 1.440 | 0.495 | 0.006 | 0.140 | Unannotated | NA | NA |
| metab_6897 | Unannotated feature | pos | 483.6362 | 5.028 | 3.578 | 5.365 | 1.786 | 0.495 | 0.006 | 0.140 | Unannotated | NA | NA |
| metab_17610 | 2-Hexadec-7-enyloctadecanedioic acid | neg | 557.4588 | 7.194 | 7.385 | 6.957 | -0.428 | -0.495 | 0.006 | 0.140 | Unannotated | Lipids and lipid-like molecules | Fatty Acyls |
| metab_11396 | Tributyrylglycerol | neg | 301.1659 | 5.185 | 4.461 | 5.534 | 1.073 | 0.495 | 0.006 | 0.140 | Tentative | NA | NA |
| metab_10571 | Unannotated feature | neg | 201.1240 | 1.749 | 4.947 | 4.626 | -0.321 | -0.495 | 0.006 | 0.140 | Unannotated | NA | NA |
| metab_2019 | 2-(acetylamino)-1,5-anhydro-2-deoxy-3-O-b-D-galactopyranosyl-D-arabino-Hex-1-enitol | pos | 366.1404 | 0.720 | 3.408 | 4.345 | 0.937 | 0.495 | 0.006 | 0.140 | Unannotated | Organic oxygen compounds | Organooxygen compounds |
| metab_9644 | Unannotated feature | neg | 364.1610 | 3.192 | 4.275 | 3.623 | -0.652 | -0.495 | 0.006 | 0.140 | Unannotated | NA | NA |
| metab_6490 | Unannotated feature | pos | 567.0297 | 5.597 | 3.427 | 5.613 | 2.186 | 0.495 | 0.006 | 0.140 | Unannotated | NA | NA |
| metab_10263 | Asparaginylisoleucine | neg | 244.1303 | 2.531 | 5.554 | 4.863 | -0.691 | -0.495 | 0.006 | 0.140 | Unannotated | Organic acids and derivatives | Carboxylic acids and derivatives |
| metab_6160 | Unannotated feature | pos | 707.9381 | 5.831 | 1.460 | 4.281 | 2.821 | 0.495 | 0.006 | 0.140 | Unannotated | NA | NA |
| metab_10191 | Serylleucine | neg | 217.1191 | 2.659 | 6.066 | 5.556 | -0.509 | -0.495 | 0.006 | 0.140 | Unannotated | Organic acids and derivatives | Carboxylic acids and derivatives |
| metab_6484 | Unannotated feature | pos | 581.7051 | 5.600 | 3.838 | 5.577 | 1.739 | 0.495 | 0.006 | 0.140 | Unannotated | NA | NA |
| metab_7073 | Unannotated feature | pos | 393.2653 | 4.704 | 0.040 | 4.750 | 4.710 | 0.495 | 0.006 | 0.140 | Unannotated | NA | NA |
| metab_9184 | Unannotated feature | neg | 376.1529 | 3.925 | 5.266 | 4.695 | -0.571 | -0.495 | 0.006 | 0.140 | Unannotated | NA | NA |
| metab_4283 | Unannotated feature | pos | 775.4664 | 5.960 | 3.407 | 4.468 | 1.061 | 0.495 | 0.006 | 0.140 | Unannotated | NA | NA |
| metab_13301 | Molinate | neg | 295.1038 | 0.722 | 4.150 | 5.028 | 0.879 | 0.495 | 0.006 | 0.140 | Tentative | Organic nitrogen compounds | Organonitrogen compounds |
| metab_13146 | Unannotated feature | neg | 395.8983 | 0.460 | 4.779 | 4.508 | -0.271 | -0.495 | 0.006 | 0.140 | Unannotated | NA | NA |
| metab_684 | Unannotated feature | pos | 647.9963 | 5.906 | 3.739 | 4.312 | 0.573 | 0.495 | 0.006 | 0.140 | Unannotated | NA | NA |
| metab_19749 | Unannotated feature | neg | 263.0778 | 4.506 | 3.049 | 4.201 | 1.152 | 0.495 | 0.006 | 0.140 | Unannotated | NA | NA |
| metab_6652 | Unannotated feature | pos | 595.0338 | 5.369 | 2.806 | 4.848 | 2.041 | 0.495 | 0.006 | 0.140 | Unannotated | NA | NA |
| metab_6825 | Unannotated feature | pos | 694.4249 | 5.136 | 5.099 | 6.690 | 1.591 | 0.495 | 0.006 | 0.140 | Unannotated | NA | NA |
| metab_14147 | L-Leucyl-L-Alanine | neg | 201.1240 | 2.885 | 5.564 | 5.121 | -0.444 | -0.495 | 0.006 | 0.140 | Unannotated | Organic acids and derivatives | Carboxylic acids and derivatives |
| metab_626 | Unannotated feature | pos | 586.8638 | 5.811 | 2.692 | 4.976 | 2.284 | 0.495 | 0.006 | 0.140 | Unannotated | NA | NA |
| metab_4230 | Unannotated feature | pos | 709.4481 | 5.430 | 3.627 | 5.235 | 1.608 | 0.491 | 0.006 | 0.144 | Unannotated | NA | NA |
| metab_2171 | Unannotated feature | pos | 360.2137 | 2.928 | 5.403 | 5.097 | -0.306 | -0.491 | 0.006 | 0.144 | Unannotated | NA | NA |
| metab_4253 | Unannotated feature | pos | 667.9109 | 5.936 | 2.331 | 5.036 | 2.705 | 0.491 | 0.006 | 0.144 | Unannotated | NA | NA |
| metab_4021 | Unannotated feature | pos | 608.3820 | 5.814 | 3.460 | 5.548 | 2.088 | 0.491 | 0.006 | 0.144 | Unannotated | NA | NA |
| metab_505 | Unannotated feature | pos | 599.3820 | 5.102 | 3.642 | 5.213 | 1.572 | 0.491 | 0.006 | 0.144 | Unannotated | NA | NA |
| metab_3727 | Unannotated feature | pos | 449.6255 | 5.381 | 2.769 | 5.014 | 2.245 | 0.491 | 0.006 | 0.144 | Unannotated | NA | NA |
| metab_11487 | Unannotated feature | neg | 118.9656 | 0.520 | 4.130 | 4.587 | 0.457 | 0.491 | 0.006 | 0.144 | Unannotated | NA | NA |
| metab_453 | Unannotated feature | pos | 200.0477 | 5.187 | 4.215 | 4.108 | -0.107 | -0.491 | 0.006 | 0.144 | Unannotated | NA | NA |
| metab_7675 | Unannotated feature | pos | 344.2287 | 3.421 | 4.238 | 4.902 | 0.664 | 0.491 | 0.006 | 0.144 | Unannotated | NA | NA |
| metab_5892 | Unannotated feature | pos | 647.3968 | 5.929 | 3.050 | 5.221 | 2.172 | 0.491 | 0.006 | 0.144 | Unannotated | NA | NA |
| metab_7165 | Unannotated feature | pos | 379.2499 | 4.503 | 4.261 | 5.237 | 0.977 | 0.491 | 0.006 | 0.144 | Unannotated | NA | NA |
| metab_4698 | Unannotated feature | pos | 648.3824 | 4.111 | 3.690 | 5.305 | 1.614 | 0.491 | 0.006 | 0.144 | Unannotated | NA | NA |
| metab_11565 | Unannotated feature | neg | 466.8778 | 0.464 | 4.901 | 4.597 | -0.304 | -0.491 | 0.006 | 0.144 | Unannotated | NA | NA |
| metab_617 | Unannotated feature | pos | 728.4554 | 5.794 | 3.243 | 5.011 | 1.768 | 0.491 | 0.006 | 0.144 | Unannotated | NA | NA |
| metab_19096 | Dihydrolipoate | neg | 243.0299 | 5.394 | 5.843 | 4.591 | -1.252 | -0.491 | 0.006 | 0.144 | Tentative | Lipids and lipid-like molecules | Fatty Acyls |
| metab_6534 | Unannotated feature | pos | 640.4066 | 5.544 | 3.424 | 4.949 | 1.525 | 0.491 | 0.006 | 0.144 | Unannotated | NA | NA |
| metab_7938 | (1S,2S,4R)-p-Menth-8-ene-1,2,10-triol 2-glucoside | pos | 393.1490 | 3.388 | 5.098 | 4.778 | -0.320 | -0.491 | 0.006 | 0.144 | Unannotated | NA | NA |
| metab_8235 | Unannotated feature | pos | 192.0658 | 2.373 | 3.023 | 3.645 | 0.622 | 0.491 | 0.006 | 0.144 | Unannotated | NA | NA |
| metab_662 | Unannotated feature | pos | 623.2892 | 5.862 | 3.971 | 4.871 | 0.901 | 0.491 | 0.006 | 0.144 | Unannotated | NA | NA |
| metab_1778 | Unannotated feature | pos | 314.1393 | 3.268 | 2.393 | 4.313 | 1.920 | 0.491 | 0.006 | 0.144 | Unannotated | NA | NA |
| metab_16387 | LysoPA(0:0/18:2(9Z,12Z)) | neg | 433.2367 | 6.797 | 4.818 | 3.477 | -1.341 | -0.486 | 0.006 | 0.146 | Unannotated | Lipids and lipid-like molecules | Glycerophospholipids |
| metab_3154 | Caffeine | pos | 195.0881 | 3.582 | 4.910 | 3.234 | -1.677 | -0.486 | 0.006 | 0.146 | Strong putative | Organoheterocyclic compounds | Imidazopyrimidines |
| metab_17301 | Unannotated feature | neg | 116.9278 | 7.710 | 6.486 | 6.402 | -0.084 | -0.486 | 0.006 | 0.146 | Unannotated | NA | NA |
| metab_13997 | Unannotated feature | neg | 618.8616 | 0.489 | 5.033 | 5.480 | 0.447 | 0.486 | 0.006 | 0.146 | Unannotated | NA | NA |
| metab_10288 | Unannotated feature | neg | 280.1151 | 2.442 | 4.375 | 3.130 | -1.245 | -0.486 | 0.006 | 0.146 | Unannotated | NA | NA |
| metab_5283 | Unannotated feature | pos | 456.4059 | 6.606 | 5.345 | 4.539 | -0.806 | -0.486 | 0.006 | 0.146 | Unannotated | NA | NA |
| metab_18004 | Unannotated feature | neg | 195.0519 | 2.880 | 6.488 | 5.098 | -1.390 | -0.486 | 0.006 | 0.146 | Unannotated | NA | NA |
| metab_4285 | Mdp-gdp | pos | 544.3378 | 5.969 | 0.231 | 4.668 | 4.437 | 0.486 | 0.006 | 0.146 | Unannotated | Organic acids and derivatives | Carboxylic acids and derivatives |
| metab_3500 | Unannotated feature | pos | 246.1453 | 2.724 | 5.894 | 5.596 | -0.298 | -0.486 | 0.006 | 0.146 | Unannotated | NA | NA |
| metab_756 | Unannotated feature | pos | 383.3278 | 6.080 | 5.678 | 5.045 | -0.634 | -0.486 | 0.006 | 0.146 | Unannotated | NA | NA |
| metab_4531 | Unannotated feature | pos | 621.3953 | 5.151 | 3.694 | 5.045 | 1.351 | 0.486 | 0.006 | 0.146 | Unannotated | NA | NA |
| metab_11514 | Unannotated feature | neg | 502.8570 | 0.507 | 4.763 | 5.035 | 0.272 | 0.486 | 0.006 | 0.146 | Unannotated | NA | NA |
| metab_1802 | Unannotated feature | pos | 692.4086 | 4.422 | 3.977 | 5.757 | 1.780 | 0.486 | 0.006 | 0.146 | Unannotated | NA | NA |
| metab_15044 | Unannotated feature | neg | 195.0294 | 4.409 | 4.880 | 5.502 | 0.622 | 0.486 | 0.006 | 0.146 | Unannotated | NA | NA |
| metab_9795 | Unannotated feature | neg | 393.1888 | 3.041 | 4.792 | 4.241 | -0.552 | -0.486 | 0.006 | 0.146 | Unannotated | NA | NA |
| metab_466 | Ala Val Ile | pos | 302.2080 | 4.794 | 5.559 | 5.048 | -0.511 | -0.486 | 0.006 | 0.146 | Moderate putative | NA | NA |
| metab_2014 | Glutamylmethionine | pos | 279.1014 | 1.944 | 4.657 | 4.209 | -0.448 | -0.486 | 0.006 | 0.146 | Unannotated | NA | NA |
| metab_4593 | PE(22:6(4Z,7Z,10Z,13E,15E,19Z)-OH(17)/18:3(9Z,12Z,15Z)) | pos | 412.7472 | 4.717 | 3.840 | 5.491 | 1.651 | 0.486 | 0.006 | 0.146 | Unannotated | NA | NA |
| metab_2283 | Unannotated feature | pos | 231.1707 | 3.319 | 5.934 | 5.616 | -0.319 | -0.486 | 0.006 | 0.146 | Unannotated | NA | NA |
| metab_5161 | Unannotated feature | pos | 216.0871 | 0.728 | 4.692 | 5.122 | 0.430 | 0.486 | 0.006 | 0.146 | Unannotated | NA | NA |
| metab_3725 | Unannotated feature | pos | 586.3657 | 5.372 | 4.017 | 6.139 | 2.122 | 0.486 | 0.006 | 0.146 | Unannotated | NA | NA |
| metab_6078 | Unannotated feature | pos | 666.4104 | 5.862 | 4.159 | 5.225 | 1.066 | 0.486 | 0.006 | 0.146 | Unannotated | NA | NA |
| metab_12889 | Unannotated feature | neg | 456.1369 | 0.722 | 3.814 | 4.687 | 0.873 | 0.486 | 0.006 | 0.146 | Unannotated | NA | NA |
| metab_13984 | De-O-methylsimmondsin | neg | 406.1365 | 2.687 | 4.888 | 5.405 | 0.517 | 0.486 | 0.006 | 0.146 | Unannotated | Organic oxygen compounds | Organooxygen compounds |
| metab_16069 | Stachyflin | neg | 430.2273 | 6.209 | 5.790 | 5.394 | -0.396 | -0.486 | 0.006 | 0.146 | Unannotated | NA | NA |
| metab_297 | Unannotated feature | pos | 186.0441 | 0.485 | 6.230 | 6.355 | 0.125 | 0.486 | 0.006 | 0.146 | Unannotated | NA | NA |
| metab_18539 | Unannotated feature | neg | 399.1012 | 3.070 | 2.244 | 4.635 | 2.391 | 0.486 | 0.006 | 0.146 | Unannotated | NA | NA |
| metab_2595 | Asparaginyl-Leucine | pos | 228.1347 | 1.517 | 4.097 | 3.430 | -0.667 | -0.486 | 0.006 | 0.146 | Unannotated | Organic acids and derivatives | Carboxylic acids and derivatives |
| metab_19573 | Unannotated feature | neg | 330.2038 | 4.749 | 5.411 | 4.915 | -0.496 | -0.486 | 0.006 | 0.146 | Unannotated | NA | NA |
| metab_17490 | Unannotated feature | neg | 941.2186 | 7.514 | 4.881 | 5.272 | 0.391 | 0.486 | 0.006 | 0.146 | Unannotated | NA | NA |
| metab_13801 | Unannotated feature | neg | 184.9267 | 0.449 | 4.793 | 4.663 | -0.130 | -0.482 | 0.007 | 0.148 | Unannotated | NA | NA |
| metab_10615 | Glutamylisoleucine | neg | 259.1301 | 1.685 | 5.755 | 5.483 | -0.271 | -0.482 | 0.007 | 0.148 | Unannotated | Organic acids and derivatives | Carboxylic acids and derivatives |
| metab_5231 | Unannotated feature | pos | 163.0280 | 0.485 | 6.443 | 6.589 | 0.146 | 0.482 | 0.007 | 0.148 | Unannotated | NA | NA |
| metab_8069 | Unannotated feature | pos | 148.0607 | 2.762 | 3.628 | 3.322 | -0.307 | -0.482 | 0.007 | 0.148 | Unannotated | NA | NA |
| metab_18056 | 18-Hydroxy-11-dehydrotetrahydrocorticosterone | neg | 345.2104 | 6.436 | 5.810 | 5.361 | -0.449 | -0.482 | 0.007 | 0.148 | Unannotated | Lipids and lipid-like molecules | Steroids and steroid derivatives |
| metab_11566 | Unannotated feature | neg | 393.9006 | 0.464 | 4.946 | 4.686 | -0.260 | -0.482 | 0.007 | 0.148 | Unannotated | NA | NA |
| metab_19247 | Unannotated feature | neg | 364.1520 | 3.379 | 4.700 | 4.261 | -0.439 | -0.482 | 0.007 | 0.148 | Unannotated | NA | NA |
| metab_6317 | Unannotated feature | pos | 536.3334 | 5.782 | 3.776 | 4.118 | 0.341 | 0.482 | 0.007 | 0.148 | Unannotated | NA | NA |
| metab_6012 | Unannotated feature | pos | 601.3699 | 5.906 | 3.924 | 5.293 | 1.369 | 0.482 | 0.007 | 0.148 | Unannotated | NA | NA |
| metab_15982 | (+/-)-Rollipyrrole | neg | 621.2947 | 6.083 | 5.655 | 5.133 | -0.523 | -0.482 | 0.007 | 0.148 | Unannotated | Organoheterocyclic compounds | Pyrrolines |
| metab_620 | Unannotated feature | pos | 655.8170 | 5.799 | 3.677 | 5.025 | 1.348 | 0.482 | 0.007 | 0.148 | Unannotated | NA | NA |
| metab_5726 | Unannotated feature | pos | 251.9517 | 0.469 | 4.038 | 4.577 | 0.539 | 0.482 | 0.007 | 0.148 | Unannotated | NA | NA |
| metab_10611 | Unannotated feature | neg | 287.1361 | 1.685 | 4.083 | 3.119 | -0.964 | -0.482 | 0.007 | 0.148 | Unannotated | NA | NA |
| metab_2147 | Unannotated feature | pos | 364.1014 | 0.633 | 2.473 | 4.395 | 1.922 | 0.482 | 0.007 | 0.148 | Unannotated | NA | NA |
| metab_2097 | Unannotated feature | pos | 782.4784 | 5.252 | 4.801 | 6.558 | 1.757 | 0.482 | 0.007 | 0.148 | Unannotated | NA | NA |
| metab_754 | Unannotated feature | pos | 141.0548 | 6.080 | 2.994 | 4.684 | 1.689 | 0.482 | 0.007 | 0.148 | Unannotated | NA | NA |
| metab_758 | 3-Isopropenylpentanedioic acid | pos | 155.0705 | 6.087 | 3.944 | 5.707 | 1.763 | 0.482 | 0.007 | 0.148 | Unannotated | Lipids and lipid-like molecules | Fatty Acyls |
| metab_4101 | Unannotated feature | pos | 747.4620 | 5.847 | 1.914 | 4.511 | 2.598 | 0.482 | 0.007 | 0.148 | Unannotated | NA | NA |
| metab_4133 | Unannotated feature | pos | 744.4588 | 5.870 | 0.880 | 4.959 | 4.080 | 0.482 | 0.007 | 0.148 | Unannotated | NA | NA |
| metab_10151 | Tryptophyl-Phenylalanine | neg | 372.1306 | 2.737 | 4.514 | 5.171 | 0.658 | 0.482 | 0.007 | 0.148 | Unannotated | Organic acids and derivatives | Carboxylic acids and derivatives |
| metab_314 | Unannotated feature | pos | 91.0365 | 0.485 | 2.853 | 3.535 | 0.682 | 0.482 | 0.007 | 0.148 | Unannotated | NA | NA |
| metab_7178 | Asn Ile Ile | pos | 359.2298 | 4.479 | 5.810 | 5.210 | -0.600 | -0.482 | 0.007 | 0.148 | Moderate putative | NA | NA |
| metab_17437 | Unannotated feature | neg | 643.8870 | 7.617 | 5.207 | 5.402 | 0.196 | 0.482 | 0.007 | 0.148 | Unannotated | NA | NA |
| metab_5880 | Unannotated feature | pos | 688.4233 | 5.929 | 3.703 | 5.555 | 1.852 | 0.482 | 0.007 | 0.148 | Unannotated | NA | NA |
| metab_18833 | Unannotated feature | neg | 293.1510 | 3.207 | 5.171 | 4.891 | -0.280 | -0.482 | 0.007 | 0.148 | Unannotated | NA | NA |
| metab_10897 | Unannotated feature | neg | 233.9569 | 0.889 | 4.349 | 3.130 | -1.219 | -0.482 | 0.007 | 0.148 | Unannotated | NA | NA |
| metab_5995 | Unannotated feature | pos | 722.4437 | 5.906 | 4.170 | 5.350 | 1.180 | 0.482 | 0.007 | 0.148 | Unannotated | NA | NA |
| metab_7984 | Unannotated feature | pos | 255.1343 | 2.859 | 5.188 | 5.039 | -0.148 | -0.482 | 0.007 | 0.148 | Unannotated | NA | NA |
| metab_3433 | PA(TXB2/20:0) | pos | 421.2607 | 4.537 | 3.358 | 5.315 | 1.957 | 0.482 | 0.007 | 0.148 | Unannotated | NA | NA |
| metab_6009 | Unannotated feature | pos | 618.3806 | 5.906 | 3.271 | 5.136 | 1.864 | 0.482 | 0.007 | 0.148 | Unannotated | NA | NA |
| metab_14850 | Unannotated feature | neg | 350.1726 | 3.995 | 4.782 | 4.074 | -0.708 | -0.482 | 0.007 | 0.148 | Unannotated | NA | NA |
| metab_6758 | Unannotated feature | pos | 512.9876 | 5.239 | 3.734 | 6.153 | 2.419 | 0.482 | 0.007 | 0.148 | Unannotated | NA | NA |
| metab_13780 | Unannotated feature | neg | 173.9525 | 0.449 | 4.459 | 4.358 | -0.100 | -0.478 | 0.007 | 0.148 | Unannotated | NA | NA |
| metab_11880 | (+/-)-3-Hydroxynonanoic acid | neg | 347.2442 | 6.018 | 5.939 | 5.682 | -0.257 | -0.478 | 0.007 | 0.148 | Unannotated | NA | NA |
| metab_2430 | Unannotated feature | pos | 170.0580 | 0.625 | 4.977 | 3.689 | -1.288 | -0.478 | 0.007 | 0.148 | Unannotated | NA | NA |
| metab_8321 | Unannotated feature | pos | 108.0449 | 2.021 | 3.701 | 3.899 | 0.198 | 0.478 | 0.007 | 0.148 | Unannotated | NA | NA |
| metab_13102 | Unannotated feature | neg | 125.8725 | 0.100 | 4.866 | 4.995 | 0.129 | 0.478 | 0.007 | 0.148 | Unannotated | NA | NA |
| metab_1448 | Unannotated feature | pos | 345.1798 | 6.008 | 5.073 | 4.756 | -0.316 | -0.478 | 0.007 | 0.148 | Unannotated | NA | NA |
| metab_3308 | Leu Ile | pos | 245.1864 | 4.084 | 5.835 | 5.526 | -0.309 | -0.478 | 0.007 | 0.148 | Moderate putative | NA | NA |
| metab_6366 | Unannotated feature | pos | 517.8331 | 5.757 | 2.541 | 4.605 | 2.064 | 0.478 | 0.007 | 0.148 | Unannotated | NA | NA |
| metab_1726 | Unannotated feature | pos | 120.9814 | 0.485 | 6.350 | 6.515 | 0.165 | 0.478 | 0.007 | 0.148 | Unannotated | NA | NA |
| metab_627 | Unannotated feature | pos | 97.9918 | 0.485 | 4.913 | 5.077 | 0.163 | 0.478 | 0.007 | 0.148 | Unannotated | NA | NA |
| metab_285 | Unannotated feature | pos | 205.0751 | 0.485 | 5.748 | 5.891 | 0.142 | 0.478 | 0.007 | 0.148 | Unannotated | NA | NA |
| metab_4684 | DG(14:0/22:4(7Z,10Z,13Z,16Z)/0:0) | pos | 639.4972 | 7.522 | 5.181 | 5.057 | -0.124 | -0.478 | 0.007 | 0.148 | Tentative | NA | NA |
| metab_2901 | Niazirin | pos | 262.1079 | 2.853 | 3.167 | 4.360 | 1.193 | 0.478 | 0.007 | 0.148 | Unannotated | Organic oxygen compounds | Organooxygen compounds |
| metab_8717 | 2,3-Dihydro-3-hydroxy-6-methoxy-2,2-dimethyl-4H-1-benzopyran-4-one | pos | 240.1234 | 0.768 | 3.510 | 4.485 | 0.975 | 0.478 | 0.007 | 0.148 | Unannotated | Organoheterocyclic compounds | Benzopyrans |
| metab_15989 | Lumirubin | neg | 629.2631 | 6.091 | 4.217 | 3.077 | -1.140 | -0.478 | 0.007 | 0.148 | Unannotated | Organoheterocyclic compounds | Tetrapyrroles and derivatives |
| metab_9246 | Ser-Tyr | neg | 267.0988 | 3.697 | 4.604 | 4.129 | -0.476 | -0.478 | 0.007 | 0.148 | Unannotated | Organic acids and derivatives | Carboxylic acids and derivatives |
| metab_5345 | Unannotated feature | pos | 454.3903 | 6.526 | 5.675 | 5.365 | -0.310 | -0.478 | 0.007 | 0.148 | Unannotated | NA | NA |
| metab_10091 | Unannotated feature | neg | 307.1263 | 2.771 | 5.216 | 4.624 | -0.592 | -0.478 | 0.007 | 0.148 | Unannotated | NA | NA |
| metab_7358 | Unannotated feature | pos | 416.2306 | 4.059 | 4.536 | 3.272 | -1.265 | -0.478 | 0.007 | 0.148 | Unannotated | NA | NA |
| metab_5716 | Unannotated feature | pos | 113.0602 | 6.103 | 2.980 | 3.732 | 0.752 | 0.478 | 0.007 | 0.148 | Unannotated | NA | NA |
| metab_5970 | Unannotated feature | pos | 619.2859 | 5.914 | 3.942 | 4.527 | 0.586 | 0.478 | 0.007 | 0.148 | Unannotated | NA | NA |
| metab_8965 | Unannotated feature | neg | 277.1559 | 4.146 | 5.425 | 4.981 | -0.444 | -0.478 | 0.007 | 0.148 | Unannotated | NA | NA |
| metab_7196 | Unannotated feature | pos | 413.2631 | 4.444 | 2.643 | 4.292 | 1.649 | 0.478 | 0.007 | 0.148 | Unannotated | NA | NA |
| metab_6138 | Unannotated feature | pos | 726.4465 | 5.835 | 4.106 | 5.129 | 1.022 | 0.478 | 0.007 | 0.148 | Unannotated | NA | NA |
| metab_5974 | Unannotated feature | pos | 606.3722 | 5.914 | 2.248 | 4.803 | 2.555 | 0.478 | 0.007 | 0.148 | Unannotated | NA | NA |
| metab_4058 | Unannotated feature | pos | 711.4357 | 5.831 | 4.087 | 5.114 | 1.027 | 0.478 | 0.007 | 0.148 | Unannotated | NA | NA |
| metab_4031 | Unannotated feature | pos | 606.0388 | 5.820 | 2.745 | 4.377 | 1.632 | 0.478 | 0.007 | 0.148 | Unannotated | NA | NA |
| metab_6627 | Unannotated feature | pos | 602.6921 | 5.402 | 3.336 | 5.202 | 1.866 | 0.478 | 0.007 | 0.148 | Unannotated | NA | NA |
| metab_2228 | Unannotated feature | pos | 171.0533 | 0.593 | 4.571 | 3.909 | -0.662 | -0.478 | 0.007 | 0.148 | Unannotated | NA | NA |
| metab_3831 | Unannotated feature | pos | 539.8464 | 5.629 | 3.384 | 4.491 | 1.107 | 0.478 | 0.007 | 0.148 | Unannotated | NA | NA |
| metab_12568 | Unannotated feature | neg | 257.0820 | 6.154 | 5.973 | 5.200 | -0.772 | -0.478 | 0.007 | 0.148 | Unannotated | NA | NA |
| metab_315 | Unannotated feature | pos | 90.5263 | 0.485 | 4.848 | 5.056 | 0.208 | 0.478 | 0.007 | 0.148 | Unannotated | NA | NA |
| metab_3488 | SM(d18:2(4E,14Z)/PGJ2) | pos | 401.2631 | 4.766 | 4.926 | 5.699 | 0.773 | 0.478 | 0.007 | 0.148 | Unannotated | NA | NA |
| metab_8259 | Delta-Valerolactone | pos | 101.0602 | 2.227 | 5.407 | 4.945 | -0.462 | -0.478 | 0.007 | 0.148 | Tentative | NA | NA |
| metab_9555 | Nordihydrocapsaicin | neg | 314.1726 | 3.281 | 5.595 | 5.180 | -0.415 | -0.478 | 0.007 | 0.148 | Tentative | Benzenoids | Phenols |
| metab_454 | Unannotated feature | pos | 200.0477 | 5.621 | 4.326 | 4.134 | -0.192 | -0.478 | 0.007 | 0.148 | Unannotated | NA | NA |
| metab_6930 | Unannotated feature | pos | 533.3425 | 4.975 | 3.108 | 5.132 | 2.025 | 0.478 | 0.007 | 0.148 | Unannotated | NA | NA |
| metab_13874 | Alanyl-Tyrosine | neg | 251.1037 | 2.329 | 4.937 | 4.611 | -0.326 | -0.478 | 0.007 | 0.148 | Unannotated | NA | NA |
| metab_7164 | Unannotated feature | pos | 393.2655 | 4.503 | 3.451 | 4.218 | 0.768 | 0.478 | 0.007 | 0.148 | Unannotated | NA | NA |
| metab_2087 | Unannotated feature | pos | 154.0501 | 2.762 | 5.302 | 5.542 | 0.241 | 0.478 | 0.007 | 0.148 | Unannotated | NA | NA |
| metab_1014 | PA(18:1(9Z)-O(12,13)/i-18:0) | pos | 734.5341 | 7.100 | 5.443 | 4.916 | -0.527 | -0.474 | 0.008 | 0.152 | Unannotated | NA | NA |
| metab_7065 | PA(20:0/TXB2) | pos | 421.2606 | 4.717 | 4.084 | 5.773 | 1.689 | 0.474 | 0.008 | 0.152 | Unannotated | NA | NA |
| metab_1608 | Unannotated feature | pos | 630.8625 | 5.028 | 4.113 | 5.701 | 1.588 | 0.474 | 0.008 | 0.152 | Unannotated | NA | NA |
| metab_5053 | Unannotated feature | pos | 604.4408 | 7.156 | 5.046 | 4.111 | -0.935 | -0.474 | 0.008 | 0.152 | Unannotated | NA | NA |
| metab_7972 | Unannotated feature | pos | 237.6500 | 2.871 | 3.764 | 2.551 | -1.213 | -0.474 | 0.008 | 0.152 | Unannotated | NA | NA |
| metab_3931 | Unannotated feature | pos | 539.8462 | 5.770 | 3.849 | 4.991 | 1.143 | 0.474 | 0.008 | 0.152 | Unannotated | NA | NA |
| metab_441 | Unannotated feature | pos | 176.9848 | 0.013 | 3.770 | 3.867 | 0.096 | 0.474 | 0.008 | 0.152 | Unannotated | NA | NA |
| metab_2112 | Unannotated feature | pos | 268.1549 | 2.732 | 4.159 | 4.902 | 0.743 | 0.474 | 0.008 | 0.152 | Unannotated | NA | NA |
| metab_3428 | PA(P-16:0/PGE1) | pos | 377.2343 | 4.521 | 4.038 | 5.305 | 1.267 | 0.474 | 0.008 | 0.152 | Unannotated | NA | NA |
| metab_10007 | 6-(1-Hydroxyethyl)-2,2-dimethyl-2H-1-benzopyran | neg | 239.0825 | 2.836 | 4.755 | 5.139 | 0.384 | 0.474 | 0.008 | 0.152 | Unannotated | Organoheterocyclic compounds | Benzopyrans |
| metab_9017 | Deoxypyridinoline | neg | 457.1947 | 3.817 | 3.620 | 2.323 | -1.297 | -0.474 | 0.008 | 0.152 | Unannotated | Organic acids and derivatives | Carboxylic acids and derivatives |
| metab_5951 | Unannotated feature | pos | 730.4500 | 5.914 | 3.962 | 5.292 | 1.330 | 0.474 | 0.008 | 0.152 | Unannotated | NA | NA |
| metab_497 | Unannotated feature | pos | 652.8760 | 5.068 | 4.423 | 5.943 | 1.521 | 0.474 | 0.008 | 0.152 | Unannotated | NA | NA |
| metab_4240 | Unannotated feature | pos | 529.3235 | 5.929 | 1.217 | 5.007 | 3.790 | 0.474 | 0.008 | 0.152 | Unannotated | NA | NA |
| metab_1791 | Unannotated feature | pos | 696.4399 | 4.406 | 4.590 | 5.584 | 0.994 | 0.474 | 0.008 | 0.152 | Unannotated | NA | NA |
| metab_5986 | Unannotated feature | pos | 538.3300 | 5.914 | 3.260 | 5.291 | 2.032 | 0.474 | 0.008 | 0.152 | Unannotated | NA | NA |
| metab_16949 | Unannotated feature | neg | 280.0830 | 1.958 | 3.878 | 4.677 | 0.799 | 0.474 | 0.008 | 0.152 | Unannotated | NA | NA |
| metab_651 | Unannotated feature | pos | 582.8577 | 5.841 | 4.152 | 5.193 | 1.040 | 0.474 | 0.008 | 0.152 | Unannotated | NA | NA |
| metab_13754 | Alanylleucine | neg | 201.1240 | 1.952 | 5.359 | 5.034 | -0.325 | -0.474 | 0.008 | 0.152 | Unannotated | Organic acids and derivatives | Carboxylic acids and derivatives |
| metab_14227 | Unannotated feature | neg | 456.8605 | 0.520 | 5.404 | 5.864 | 0.460 | 0.474 | 0.008 | 0.152 | Unannotated | NA | NA |
| metab_15932 | Dodecyl-beta-D-maltoside | neg | 491.2867 | 6.029 | 5.833 | 5.432 | -0.402 | -0.474 | 0.008 | 0.152 | Unannotated | Lipids and lipid-like molecules | Fatty Acyls |
| metab_1610 | Unannotated feature | pos | 480.8027 | 5.028 | 3.566 | 5.073 | 1.507 | 0.474 | 0.008 | 0.152 | Unannotated | NA | NA |
| metab_14634 | Unannotated feature | neg | 366.1672 | 3.566 | 4.268 | 3.628 | -0.640 | -0.474 | 0.008 | 0.152 | Unannotated | NA | NA |
| metab_6757 | Unannotated feature | pos | 514.6397 | 5.239 | 5.162 | 5.863 | 0.701 | 0.474 | 0.008 | 0.152 | Unannotated | NA | NA |
| metab_6607 | Unannotated feature | pos | 567.0295 | 5.434 | 3.547 | 5.028 | 1.480 | 0.474 | 0.008 | 0.152 | Unannotated | NA | NA |
| metab_4086 | Unannotated feature | pos | 650.4972 | 5.841 | 2.907 | 5.183 | 2.276 | 0.469 | 0.009 | 0.155 | Unannotated | NA | NA |
| metab_5893 | Unannotated feature | pos | 645.7966 | 5.929 | 3.250 | 4.855 | 1.605 | 0.469 | 0.009 | 0.155 | Unannotated | NA | NA |
| metab_11561 | Unannotated feature | neg | 352.8969 | 0.467 | 5.246 | 5.092 | -0.154 | -0.469 | 0.009 | 0.155 | Unannotated | NA | NA |
| metab_15089 | Unannotated feature | neg | 357.2148 | 4.510 | 5.842 | 5.485 | -0.357 | -0.469 | 0.009 | 0.155 | Unannotated | NA | NA |
| metab_2024 | Unannotated feature | pos | 376.1725 | 2.457 | 4.876 | 4.409 | -0.467 | -0.469 | 0.009 | 0.155 | Unannotated | NA | NA |
| metab_2651 | Unannotated feature | pos | 205.1081 | 1.944 | 3.706 | 3.135 | -0.571 | -0.469 | 0.009 | 0.155 | Unannotated | NA | NA |
| metab_6237 | Unannotated feature | pos | 593.7138 | 5.811 | 4.451 | 4.917 | 0.467 | 0.469 | 0.009 | 0.155 | Unannotated | NA | NA |
| metab_16817 | Unannotated feature | neg | 280.1244 | 7.720 | 5.196 | 5.532 | 0.336 | 0.469 | 0.009 | 0.155 | Unannotated | NA | NA |
| metab_14056 | Unannotated feature | neg | 358.1739 | 2.780 | 4.876 | 4.011 | -0.865 | -0.469 | 0.009 | 0.155 | Unannotated | NA | NA |
| metab_4522 | Unannotated feature | pos | 738.4514 | 5.175 | 4.919 | 6.880 | 1.961 | 0.469 | 0.009 | 0.155 | Unannotated | NA | NA |
| metab_10561 | 5,7-DICHLOROKYNURENIC ACID | neg | 255.9580 | 1.850 | 4.697 | 3.793 | -0.904 | -0.469 | 0.009 | 0.155 | Moderate putative | Organoheterocyclic compounds | Quinolines and derivatives |
| metab_5707 | Unannotated feature | pos | 125.9867 | 0.485 | 6.281 | 6.419 | 0.138 | 0.469 | 0.009 | 0.155 | Unannotated | NA | NA |
| metab_17162 | Unannotated feature | neg | 143.9383 | 7.765 | 3.645 | 3.472 | -0.173 | -0.469 | 0.009 | 0.155 | Unannotated | NA | NA |
| metab_13274 | Unannotated feature | neg | 115.0030 | 0.799 | 6.335 | 6.042 | -0.293 | -0.469 | 0.009 | 0.155 | Unannotated | NA | NA |
| metab_4269 | Unannotated feature | pos | 724.5543 | 5.944 | 3.924 | 4.446 | 0.522 | 0.469 | 0.009 | 0.155 | Unannotated | NA | NA |
| metab_14621 | Unannotated feature | neg | 394.1625 | 3.544 | 4.703 | 4.087 | -0.616 | -0.469 | 0.009 | 0.155 | Unannotated | NA | NA |
| metab_676 | Unannotated feature | pos | 595.8640 | 5.898 | 3.019 | 4.820 | 1.801 | 0.469 | 0.009 | 0.155 | Unannotated | NA | NA |
| metab_15043 | Unannotated feature | neg | 300.1931 | 4.405 | 5.770 | 5.375 | -0.396 | -0.469 | 0.009 | 0.155 | Unannotated | NA | NA |
| metab_1484 | Unannotated feature | pos | 676.6132 | 5.906 | 3.787 | 4.282 | 0.495 | 0.469 | 0.009 | 0.155 | Unannotated | NA | NA |
| metab_6715 | Unannotated feature | pos | 542.3391 | 5.283 | 4.510 | 6.419 | 1.908 | 0.469 | 0.009 | 0.155 | Unannotated | NA | NA |
| metab_4207 | Unannotated feature | pos | 702.7635 | 5.914 | 2.774 | 4.204 | 1.431 | 0.469 | 0.009 | 0.155 | Unannotated | NA | NA |
| metab_6939 | Unannotated feature | pos | 163.1119 | 4.965 | 2.815 | 3.264 | 0.449 | 0.469 | 0.009 | 0.155 | Unannotated | NA | NA |
| metab_13580 | Unannotated feature | neg | 115.0030 | 1.308 | 5.852 | 5.572 | -0.281 | -0.469 | 0.009 | 0.155 | Unannotated | NA | NA |
| metab_18588 | Unannotated feature | neg | 597.2751 | 5.999 | 4.572 | 5.343 | 0.771 | 0.469 | 0.009 | 0.155 | Unannotated | NA | NA |
| metab_11042 | N-Ribosylhistidine | neg | 332.1101 | 0.738 | 5.045 | 4.859 | -0.186 | -0.469 | 0.009 | 0.155 | Unannotated | Organic acids and derivatives | Carboxylic acids and derivatives |
| metab_18685 | 2,3-Dihydro-6-methyl-1H-pyrrolizine-5-carboxaldehyde | neg | 148.0760 | 5.918 | 4.625 | 4.182 | -0.443 | -0.469 | 0.009 | 0.155 | Unannotated | Organoheterocyclic compounds | Pyrrolizines |
| metab_8005 | Cristacarpin | pos | 319.1296 | 2.835 | 4.408 | 4.862 | 0.454 | 0.469 | 0.009 | 0.155 | Tentative | Lipids and lipid-like molecules | Fatty Acyls |
| metab_2149 | Unannotated feature | pos | 299.1243 | 2.880 | 4.432 | 5.206 | 0.774 | 0.469 | 0.009 | 0.155 | Unannotated | NA | NA |
| metab_9195 | Unannotated feature | neg | 366.1669 | 3.773 | 4.882 | 3.773 | -1.109 | -0.469 | 0.009 | 0.155 | Unannotated | NA | NA |
| metab_6842 | Unannotated feature | pos | 528.3308 | 5.106 | 3.106 | 4.990 | 1.884 | 0.469 | 0.009 | 0.155 | Unannotated | NA | NA |
| metab_10032 | Unannotated feature | neg | 221.0929 | 2.813 | 4.593 | 4.159 | -0.433 | -0.469 | 0.009 | 0.155 | Unannotated | NA | NA |
| metab_9556 | 2-Amino-3-phosphonopropionic acid | neg | 168.0058 | 3.281 | 2.805 | 2.507 | -0.298 | -0.469 | 0.009 | 0.155 | Tentative | NA | NA |
| metab_9105 | Metoclopramide | neg | 280.1192 | 3.925 | 3.929 | 4.463 | 0.534 | 0.469 | 0.009 | 0.155 | Tentative | Benzenoids | Phenol ethers |
| metab_2808 | Histidyltryptophan | pos | 306.1344 | 2.687 | 4.014 | 4.868 | 0.854 | 0.469 | 0.009 | 0.155 | Unannotated | Organic acids and derivatives | Carboxylic acids and derivatives |
| metab_706 | Unannotated feature | pos | 634.4861 | 5.936 | 4.042 | 5.330 | 1.287 | 0.469 | 0.009 | 0.155 | Unannotated | NA | NA |
| metab_6874 | Unannotated feature | pos | 553.3366 | 5.068 | 3.339 | 4.582 | 1.244 | 0.465 | 0.009 | 0.158 | Unannotated | NA | NA |
| metab_2801 | Ser-Leu | pos | 219.1344 | 2.643 | 6.105 | 5.553 | -0.552 | -0.465 | 0.009 | 0.158 | Unannotated | NA | NA |
| metab_6777 | Unannotated feature | pos | 499.9636 | 5.213 | 3.598 | 5.782 | 2.184 | 0.465 | 0.009 | 0.158 | Unannotated | NA | NA |
| metab_18638 | Valylleucine | neg | 229.1556 | 3.085 | 6.290 | 5.889 | -0.401 | -0.465 | 0.009 | 0.158 | Unannotated | Organic acids and derivatives | Carboxylic acids and derivatives |
| metab_1506 | Unannotated feature | pos | 627.3823 | 5.519 | 0.778 | 4.448 | 3.670 | 0.465 | 0.009 | 0.158 | Unannotated | NA | NA |
| metab_14463 | Cilomilast | neg | 388.1734 | 3.288 | 5.795 | 5.482 | -0.313 | -0.465 | 0.009 | 0.158 | Unannotated | NA | NA |
| metab_474 | Gly Ile Phe | pos | 336.1925 | 4.923 | 5.235 | 4.684 | -0.550 | -0.465 | 0.009 | 0.158 | Moderate putative | NA | NA |
| metab_4018 | Unannotated feature | pos | 579.6923 | 5.814 | 4.145 | 4.815 | 0.669 | 0.465 | 0.009 | 0.158 | Unannotated | NA | NA |
| metab_15822 | Unannotated feature | neg | 231.9314 | 5.896 | 3.050 | 3.785 | 0.735 | 0.465 | 0.009 | 0.158 | Unannotated | NA | NA |
| metab_14628 | Unannotated feature | neg | 722.7889 | 0.520 | 4.860 | 5.422 | 0.563 | 0.465 | 0.009 | 0.158 | Unannotated | NA | NA |
| metab_6965 | PG(20:4(7E,9E,11Z,13E)-3OH(5S,6R,15S)/i-21:0) | pos | 445.2895 | 4.914 | 4.833 | 5.646 | 0.812 | 0.465 | 0.009 | 0.158 | Unannotated | NA | NA |
| metab_5967 | Unannotated feature | pos | 631.4783 | 5.914 | 2.203 | 4.150 | 1.947 | 0.465 | 0.009 | 0.158 | Unannotated | NA | NA |
| metab_11144 | Glutaminylalanine | neg | 216.0986 | 0.691 | 4.641 | 4.300 | -0.341 | -0.465 | 0.009 | 0.158 | Unannotated | Organic acids and derivatives | Carboxylic acids and derivatives |
| metab_7076 | PG(i-21:0/TXB2) | pos | 465.2872 | 4.699 | 3.563 | 5.286 | 1.723 | 0.465 | 0.009 | 0.158 | Unannotated | NA | NA |
| metab_14083 | Unannotated feature | neg | 327.1677 | 2.806 | 4.971 | 4.166 | -0.805 | -0.465 | 0.009 | 0.158 | Unannotated | NA | NA |
| metab_6755 | Unannotated feature | pos | 760.4648 | 5.239 | 3.975 | 6.511 | 2.536 | 0.465 | 0.009 | 0.158 | Unannotated | NA | NA |
| metab_5269 | Unannotated feature | pos | 314.2877 | 6.638 | 3.856 | 3.238 | -0.617 | -0.465 | 0.009 | 0.158 | Unannotated | NA | NA |
| metab_8339 | (R)-Lipoic acid | pos | 229.0347 | 1.984 | 3.591 | 3.030 | -0.561 | -0.465 | 0.009 | 0.158 | Tentative | Organoheterocyclic compounds | Dithiolanes |
| metab_10582 | Unannotated feature | neg | 406.1363 | 1.695 | 0.880 | 3.808 | 2.928 | 0.465 | 0.009 | 0.158 | Unannotated | NA | NA |
| metab_16476 | Unannotated feature | neg | 100.9256 | 7.057 | 3.766 | 3.572 | -0.194 | -0.465 | 0.009 | 0.158 | Unannotated | NA | NA |
| metab_8278 | Unannotated feature | pos | 322.1882 | 2.150 | 4.363 | 3.489 | -0.873 | -0.465 | 0.009 | 0.158 | Unannotated | NA | NA |
| metab_12133 | 2-ISOPRENYL-3-HYDROXY-5-METHYL-a-PYRONE | neg | 193.0865 | 5.865 | 5.606 | 4.633 | -0.973 | -0.465 | 0.009 | 0.158 | Moderate putative | NA | NA |
| metab_652 | Unannotated feature | pos | 607.4630 | 5.847 | 3.924 | 4.315 | 0.391 | 0.465 | 0.009 | 0.158 | Unannotated | NA | NA |
| metab_649 | Unannotated feature | pos | 685.5234 | 5.841 | 4.334 | 4.939 | 0.605 | 0.465 | 0.009 | 0.158 | Unannotated | NA | NA |
| metab_679 | Unannotated feature | pos | 707.9361 | 5.898 | 2.237 | 5.229 | 2.991 | 0.465 | 0.009 | 0.158 | Unannotated | NA | NA |
| metab_6089 | Unannotated feature | pos | 647.3018 | 5.854 | 0.810 | 4.760 | 3.950 | 0.465 | 0.009 | 0.158 | Unannotated | NA | NA |
| metab_2889 | Tyr Ile Ser | pos | 382.1978 | 2.827 | 4.271 | 3.450 | -0.821 | -0.465 | 0.009 | 0.158 | Moderate putative | NA | NA |
| metab_13917 | Unannotated feature | neg | 307.1263 | 2.535 | 4.859 | 3.885 | -0.974 | -0.465 | 0.009 | 0.158 | Unannotated | NA | NA |
| metab_14165 | Unannotated feature | neg | 372.1894 | 2.903 | 5.105 | 4.219 | -0.885 | -0.465 | 0.009 | 0.158 | Unannotated | NA | NA |
| metab_6903 | CL(8:0/i-12:0/18:2(9Z,11Z)/18:2(9Z,11Z)) | pos | 628.3852 | 5.020 | 5.377 | 6.400 | 1.023 | 0.465 | 0.009 | 0.158 | Unannotated | Lipids and lipid-like molecules | Glycerophospholipids |
| metab_8527 | Lys Leu | pos | 260.1973 | 1.435 | 6.245 | 5.760 | -0.486 | -0.461 | 0.010 | 0.160 | Moderate putative | NA | NA |
| metab_6461 | Unannotated feature | pos | 596.3798 | 5.639 | 3.862 | 5.839 | 1.977 | 0.461 | 0.010 | 0.160 | Unannotated | NA | NA |
| metab_6242 | Unannotated feature | pos | 555.8411 | 5.811 | 2.690 | 4.503 | 1.813 | 0.461 | 0.010 | 0.160 | Unannotated | NA | NA |
| metab_4172 | Unannotated feature | pos | 639.3923 | 5.906 | 3.915 | 5.302 | 1.387 | 0.461 | 0.010 | 0.160 | Unannotated | NA | NA |
| metab_4183 | Unannotated feature | pos | 353.2176 | 5.914 | 0.698 | 5.010 | 4.312 | 0.461 | 0.010 | 0.160 | Unannotated | NA | NA |
| metab_13923 | ALA-ILE | neg | 201.1240 | 2.554 | 5.417 | 4.994 | -0.423 | -0.461 | 0.010 | 0.160 | Strong putative | Organic acids and derivatives | Carboxylic acids and derivatives |
| metab_9537 | Unannotated feature | neg | 209.0928 | 3.303 | 4.509 | 3.774 | -0.735 | -0.461 | 0.010 | 0.160 | Unannotated | NA | NA |
| metab_8111 | Spectinomycin | pos | 355.1507 | 2.732 | 4.002 | 4.562 | 0.559 | 0.461 | 0.010 | 0.160 | Tentative | Organoheterocyclic compounds | Dioxanes |
| metab_6909 | Glu Ile Phe | pos | 408.2142 | 5.013 | 5.208 | 3.928 | -1.280 | -0.461 | 0.010 | 0.160 | Moderate putative | NA | NA |
| metab_658 | Unannotated feature | pos | 666.1565 | 5.851 | 3.195 | 4.549 | 1.354 | 0.461 | 0.010 | 0.160 | Unannotated | NA | NA |
| metab_1931 | Unannotated feature | pos | 198.0765 | 0.728 | 3.587 | 4.431 | 0.844 | 0.461 | 0.010 | 0.160 | Unannotated | NA | NA |
| metab_16881 | Unannotated feature | neg | 254.0724 | 7.741 | 3.950 | 4.302 | 0.352 | 0.461 | 0.010 | 0.160 | Unannotated | NA | NA |
| metab_3665 | Unannotated feature | pos | 506.9799 | 5.219 | 3.632 | 5.355 | 1.723 | 0.461 | 0.010 | 0.160 | Unannotated | NA | NA |
| metab_1812 | PE(18:4(6Z,9Z,12Z,15Z)/20:5(5Z,8Z,11Z,14Z,17Z)) | pos | 780.4611 | 4.448 | 2.899 | 5.109 | 2.210 | 0.461 | 0.010 | 0.160 | Tentative | Lipids and lipid-like molecules | Glycerophospholipids |
| metab_1381 | Unannotated feature | pos | 419.2620 | 6.111 | 6.064 | 5.929 | -0.136 | -0.461 | 0.010 | 0.160 | Unannotated | NA | NA |
| metab_12794 | Unannotated feature | neg | 501.3591 | 6.868 | 4.631 | 5.750 | 1.118 | 0.461 | 0.010 | 0.160 | Unannotated | NA | NA |
| metab_2125 | Unannotated feature | pos | 292.1186 | 2.835 | 3.838 | 4.825 | 0.988 | 0.461 | 0.010 | 0.160 | Unannotated | NA | NA |
| metab_2106 | Unannotated feature | pos | 192.1022 | 2.795 | 4.081 | 4.654 | 0.573 | 0.461 | 0.010 | 0.160 | Unannotated | NA | NA |
| metab_6180 | Unannotated feature | pos | 718.4447 | 5.820 | 3.341 | 4.662 | 1.320 | 0.461 | 0.010 | 0.160 | Unannotated | NA | NA |
| metab_5354 | PGP(i-22:0/PGF1alpha) | pos | 494.3025 | 5.084 | 0.037 | 4.195 | 4.158 | 0.461 | 0.010 | 0.160 | Unannotated | NA | NA |
| metab_3692 | Unannotated feature | pos | 478.9765 | 5.275 | 1.135 | 4.763 | 3.628 | 0.461 | 0.010 | 0.160 | Unannotated | NA | NA |
| metab_946 | Unannotated feature | pos | 699.4943 | 7.586 | 5.733 | 5.307 | -0.426 | -0.461 | 0.010 | 0.160 | Unannotated | NA | NA |
| metab_18264 | Unannotated feature | neg | 285.0887 | 2.975 | 4.391 | 4.987 | 0.596 | 0.461 | 0.010 | 0.160 | Unannotated | NA | NA |
| metab_5722 | Dolasetron | pos | 325.1532 | 6.087 | 5.742 | 5.132 | -0.610 | -0.461 | 0.010 | 0.160 | Tentative | Organoheterocyclic compounds | Indoles and derivatives |
| metab_1951 | Unannotated feature | pos | 451.1810 | 1.952 | 2.435 | 4.180 | 1.745 | 0.461 | 0.010 | 0.160 | Unannotated | NA | NA |
| metab_7208 | Unannotated feature | pos | 357.2367 | 4.406 | 4.383 | 5.311 | 0.928 | 0.461 | 0.010 | 0.160 | Unannotated | NA | NA |
| metab_11594 | Unannotated feature | neg | 329.9072 | 0.449 | 4.991 | 4.876 | -0.115 | -0.461 | 0.010 | 0.160 | Unannotated | NA | NA |
| metab_9708 | Aesculin | neg | 339.0727 | 3.120 | 3.474 | 4.278 | 0.804 | 0.461 | 0.010 | 0.160 | Tentative | Phenylpropanoids and polyketides | Coumarins and derivatives |
| metab_776 | Unannotated feature | pos | 398.2423 | 6.159 | 5.654 | 5.509 | -0.145 | -0.461 | 0.010 | 0.160 | Unannotated | NA | NA |
| metab_5960 | Unannotated feature | pos | 656.5011 | 5.914 | 3.637 | 4.512 | 0.875 | 0.461 | 0.010 | 0.160 | Unannotated | NA | NA |
| metab_11484 | Unannotated feature | neg | 180.9208 | 0.520 | 4.582 | 4.265 | -0.317 | -0.461 | 0.010 | 0.160 | Unannotated | NA | NA |
| metab_19774 | 6-Phosphogluconic acid | neg | 275.0201 | 4.469 | 5.278 | 4.053 | -1.225 | -0.461 | 0.010 | 0.160 | Tentative | Organic oxygen compounds | Organooxygen compounds |
| metab_703 | Unannotated feature | pos | 459.2813 | 5.929 | 2.030 | 4.914 | 2.885 | 0.461 | 0.010 | 0.160 | Unannotated | NA | NA |
| metab_9288 | Diacylglycerol kinase inhibitor i | neg | 504.1790 | 3.628 | 5.275 | 3.854 | -1.421 | -0.461 | 0.010 | 0.160 | Unannotated | Benzenoids | Benzene and substituted derivatives |
| metab_1494 | Unannotated feature | pos | 664.6220 | 5.806 | 3.168 | 5.056 | 1.887 | 0.461 | 0.010 | 0.160 | Unannotated | NA | NA |
| metab_6891 | Unannotated feature | pos | 716.4378 | 5.034 | 3.875 | 6.056 | 2.180 | 0.461 | 0.010 | 0.160 | Unannotated | NA | NA |
| metab_15431 | Gentisein | neg | 243.0299 | 5.140 | 5.341 | 4.261 | -1.080 | -0.461 | 0.010 | 0.160 | Tentative | Organoheterocyclic compounds | Benzopyrans |
| metab_19672 | Unannotated feature | neg | 300.1931 | 4.603 | 5.914 | 5.644 | -0.269 | -0.461 | 0.010 | 0.160 | Unannotated | NA | NA |
| metab_1758 | Unannotated feature | pos | 648.3824 | 4.301 | 4.433 | 5.761 | 1.328 | 0.461 | 0.010 | 0.160 | Unannotated | NA | NA |
| metab_7012 | Leu Glu Phe | pos | 408.2142 | 4.814 | 5.236 | 4.375 | -0.861 | -0.461 | 0.010 | 0.160 | Moderate putative | NA | NA |
| metab_9744 | Beta-L-Fucose | neg | 209.0673 | 3.085 | 4.966 | 4.550 | -0.416 | -0.457 | 0.010 | 0.163 | Tentative | Organic oxygen compounds | Organooxygen compounds |
| metab_14691 | Unannotated feature | neg | 326.2089 | 3.685 | 5.102 | 4.772 | -0.330 | -0.457 | 0.010 | 0.163 | Unannotated | NA | NA |
| metab_10559 | Unannotated feature | neg | 216.0123 | 1.854 | 4.704 | 4.152 | -0.552 | -0.457 | 0.010 | 0.163 | Unannotated | NA | NA |
| metab_18788 | Unannotated feature | neg | 350.1362 | 3.184 | 4.869 | 4.585 | -0.284 | -0.457 | 0.010 | 0.163 | Unannotated | NA | NA |
| metab_12793 | Unannotated feature | neg | 445.2005 | 5.261 | 5.418 | 5.694 | 0.276 | 0.457 | 0.010 | 0.163 | Unannotated | NA | NA |
| metab_1578 | Unannotated feature | pos | 740.9285 | 5.205 | 3.140 | 6.008 | 2.868 | 0.457 | 0.010 | 0.163 | Unannotated | NA | NA |
| metab_14288 | Unannotated feature | neg | 241.0353 | 3.037 | 1.971 | 3.873 | 1.902 | 0.457 | 0.010 | 0.163 | Unannotated | NA | NA |
| metab_10069 | Unannotated feature | neg | 315.1677 | 2.780 | 5.743 | 5.338 | -0.405 | -0.457 | 0.010 | 0.163 | Unannotated | NA | NA |
| metab_16568 | Unannotated feature | neg | 116.9278 | 7.310 | 7.127 | 7.045 | -0.082 | -0.457 | 0.010 | 0.163 | Unannotated | NA | NA |
| metab_16974 | Unannotated feature | neg | 157.8624 | 7.914 | 4.878 | 4.755 | -0.123 | -0.457 | 0.010 | 0.163 | Unannotated | NA | NA |
| metab_14743 | Unannotated feature | neg | 343.1990 | 3.777 | 5.848 | 5.419 | -0.429 | -0.457 | 0.010 | 0.163 | Unannotated | NA | NA |
| metab_18416 | Unannotated feature | neg | 431.2119 | 6.115 | 6.095 | 6.342 | 0.248 | 0.457 | 0.010 | 0.163 | Unannotated | NA | NA |
| metab_633 | Unannotated feature | pos | 616.3827 | 5.814 | 4.073 | 5.060 | 0.986 | 0.457 | 0.010 | 0.163 | Unannotated | NA | NA |
| metab_19634 | (E)-8-Hydroxy-2-octene-4,6-diynoic acid | neg | 195.0294 | 4.660 | 5.259 | 5.811 | 0.553 | 0.457 | 0.010 | 0.163 | Unannotated | Lipids and lipid-like molecules | Fatty Acyls |
| metab_4363 | Unannotated feature | pos | 573.3419 | 5.332 | 3.756 | 5.991 | 2.235 | 0.457 | 0.010 | 0.163 | Unannotated | NA | NA |
| metab_6725 | Unannotated feature | pos | 784.9545 | 5.267 | 0.867 | 5.252 | 4.385 | 0.457 | 0.010 | 0.163 | Unannotated | NA | NA |
| metab_2791 | Unannotated feature | pos | 367.2103 | 6.008 | 6.033 | 5.757 | -0.276 | -0.457 | 0.010 | 0.163 | Unannotated | NA | NA |
| metab_8922 | Unannotated feature | neg | 371.2304 | 4.231 | 4.941 | 4.490 | -0.451 | -0.457 | 0.010 | 0.163 | Unannotated | NA | NA |
| metab_7580 | Unannotated feature | pos | 316.6850 | 3.586 | 4.491 | 3.637 | -0.855 | -0.457 | 0.010 | 0.163 | Unannotated | NA | NA |
| metab_14261 | Unannotated feature | neg | 227.0559 | 3.007 | 4.711 | 5.165 | 0.454 | 0.457 | 0.010 | 0.163 | Unannotated | NA | NA |
| metab_14804 | Unannotated feature | neg | 349.1732 | 3.909 | 5.062 | 4.659 | -0.403 | -0.457 | 0.010 | 0.163 | Unannotated | NA | NA |
| metab_10177 | Unannotated feature | neg | 379.1842 | 2.698 | 4.053 | 3.548 | -0.505 | -0.457 | 0.010 | 0.163 | Unannotated | NA | NA |
| metab_14790 | Unannotated feature | neg | 306.1673 | 3.882 | 4.165 | 3.290 | -0.875 | -0.457 | 0.010 | 0.163 | Unannotated | NA | NA |
| metab_5964 | Unannotated feature | pos | 641.2929 | 5.914 | 4.234 | 4.823 | 0.589 | 0.457 | 0.010 | 0.163 | Unannotated | NA | NA |
| metab_7592 | Ile Leu | pos | 245.1864 | 3.569 | 6.693 | 6.354 | -0.339 | -0.457 | 0.010 | 0.163 | Moderate putative | NA | NA |
| metab_8869 | Unannotated feature | neg | 357.2148 | 4.319 | 5.642 | 5.207 | -0.435 | -0.457 | 0.010 | 0.163 | Unannotated | NA | NA |
| metab_9273 | Oxydemeton-methyl | neg | 291.0153 | 3.655 | 4.568 | 3.069 | -1.499 | -0.457 | 0.010 | 0.163 | Tentative | Organosulfur compounds | Sulfoxides |
| metab_6619 | Unannotated feature | pos | 615.7160 | 5.414 | 3.631 | 5.566 | 1.934 | 0.457 | 0.010 | 0.163 | Unannotated | NA | NA |
| metab_17570 | Unannotated feature | neg | 99.9250 | 7.310 | 6.736 | 6.649 | -0.088 | -0.457 | 0.010 | 0.163 | Unannotated | NA | NA |
| metab_6158 | 2-Amino-3-methylbenzoate | pos | 303.1344 | 5.835 | 5.830 | 5.564 | -0.266 | -0.457 | 0.010 | 0.163 | Tentative | Benzenoids | Benzene and substituted derivatives |
| metab_6202 | Unannotated feature | pos | 631.0592 | 5.814 | 4.058 | 4.669 | 0.612 | 0.457 | 0.010 | 0.163 | Unannotated | NA | NA |
| metab_3172 | [3-[2-Aminoethoxy(hydroxy)phosphoryl]oxy-2-hydroxypropyl] hexadecanoate | pos | 498.2580 | 6.718 | 6.241 | 5.983 | -0.258 | -0.457 | 0.010 | 0.163 | Unannotated | Lipids and lipid-like molecules | Glycerophospholipids |
| metab_16683 | Unannotated feature | neg | 609.8923 | 7.617 | 4.223 | 4.645 | 0.422 | 0.457 | 0.010 | 0.163 | Unannotated | NA | NA |
| metab_16571 | Unannotated feature | neg | 115.9200 | 7.315 | 7.344 | 7.251 | -0.093 | -0.457 | 0.010 | 0.163 | Unannotated | NA | NA |
| metab_17064 | Unannotated feature | neg | 159.8594 | 7.939 | 4.631 | 4.487 | -0.144 | -0.457 | 0.010 | 0.163 | Unannotated | NA | NA |
| metab_10509 | Unannotated feature | neg | 376.1255 | 1.947 | 1.609 | 3.722 | 2.113 | 0.457 | 0.010 | 0.163 | Unannotated | NA | NA |
| metab_519 | Unannotated feature | pos | 707.9246 | 5.169 | 3.272 | 6.168 | 2.896 | 0.457 | 0.010 | 0.163 | Unannotated | NA | NA |
| metab_2628 | Unannotated feature | pos | 583.3567 | 5.451 | 2.850 | 4.739 | 1.889 | 0.457 | 0.010 | 0.163 | Unannotated | NA | NA |
| metab_8404 | Methyl 5-[2-(pyrrolidine-1-carbonyl)phenyl]-1,3-oxazole-4-carboxylate | pos | 301.1188 | 3.859 | 3.611 | 4.644 | 1.033 | 0.453 | 0.011 | 0.166 | Moderate putative | NA | NA |
| metab_5507 | Unannotated feature | pos | 398.2423 | 6.335 | 6.041 | 5.880 | -0.162 | -0.453 | 0.011 | 0.166 | Unannotated | NA | NA |
| metab_625 | Unannotated feature | pos | 346.1425 | 5.811 | 3.727 | 4.243 | 0.516 | 0.453 | 0.011 | 0.166 | Unannotated | NA | NA |
| metab_9151 | Unannotated feature | neg | 316.1880 | 3.849 | 5.606 | 5.363 | -0.243 | -0.453 | 0.011 | 0.166 | Unannotated | NA | NA |
| metab_11572 | Unannotated feature | neg | 369.8874 | 0.460 | 4.470 | 4.373 | -0.097 | -0.453 | 0.011 | 0.166 | Unannotated | NA | NA |
| metab_195 | Unannotated feature | pos | 248.1609 | 0.593 | 4.291 | 3.047 | -1.243 | -0.453 | 0.011 | 0.166 | Unannotated | NA | NA |
| metab_9685 | Unannotated feature | neg | 346.1624 | 3.141 | 5.817 | 5.371 | -0.446 | -0.453 | 0.011 | 0.166 | Unannotated | NA | NA |
| metab_19447 | Gly Leu Phe | neg | 334.1776 | 4.945 | 5.120 | 4.671 | -0.449 | -0.453 | 0.011 | 0.166 | Strong putative | NA | NA |
| metab_10051 | TRIETHYLENE GLYCOL | neg | 345.1780 | 2.789 | 5.621 | 5.019 | -0.602 | -0.453 | 0.011 | 0.166 | Unannotated | Organic oxygen compounds | Organooxygen compounds |
| metab_5933 | Unannotated feature | pos | 482.2999 | 5.457 | 2.905 | 4.451 | 1.546 | 0.453 | 0.011 | 0.166 | Unannotated | NA | NA |
| metab_11579 | Unannotated feature | neg | 311.9208 | 0.457 | 4.167 | 4.023 | -0.144 | -0.453 | 0.011 | 0.166 | Unannotated | NA | NA |
| metab_17992 | (3beta,17alpha,23S)-17,23-Epoxy-3,29-dihydroxy-27-norlanost-8-en-24-one | neg | 503.3384 | 6.522 | 6.232 | 6.499 | 0.267 | 0.453 | 0.011 | 0.166 | Unannotated | NA | NA |
| metab_12424 | Unannotated feature | neg | 104.9282 | 7.730 | 4.806 | 4.744 | -0.062 | -0.453 | 0.011 | 0.166 | Unannotated | NA | NA |
| metab_17864 | Unannotated feature | neg | 537.2568 | 6.719 | 5.363 | 4.738 | -0.625 | -0.453 | 0.011 | 0.166 | Unannotated | NA | NA |
| metab_10025 | 4-Coumaroylputrescine | neg | 279.1352 | 2.817 | 5.206 | 4.894 | -0.312 | -0.453 | 0.011 | 0.166 | Unannotated | Phenylpropanoids and polyketides | Cinnamic acids and derivatives |
| metab_7654 | Unannotated feature | pos | 316.1874 | 3.455 | 5.270 | 4.909 | -0.360 | -0.453 | 0.011 | 0.166 | Unannotated | NA | NA |
| metab_3881 | Unannotated feature | pos | 655.0818 | 5.725 | 3.910 | 5.096 | 1.186 | 0.453 | 0.011 | 0.166 | Unannotated | NA | NA |
| metab_8047 | Unannotated feature | pos | 359.2298 | 3.491 | 5.628 | 5.307 | -0.320 | -0.453 | 0.011 | 0.166 | Unannotated | NA | NA |
| metab_5946 | Unannotated feature | pos | 799.4875 | 5.914 | 3.667 | 4.625 | 0.958 | 0.453 | 0.011 | 0.166 | Unannotated | NA | NA |
| metab_6837 | Simonin IV | pos | 672.4118 | 5.113 | 5.208 | 6.649 | 1.441 | 0.453 | 0.011 | 0.166 | Unannotated | Lipids and lipid-like molecules | Fatty Acyls |
| metab_17218 | Unannotated feature | neg | 309.1452 | 7.730 | 4.033 | 4.325 | 0.292 | 0.453 | 0.011 | 0.166 | Unannotated | NA | NA |
| metab_9046 | Unannotated feature | neg | 229.0716 | 4.008 | 2.855 | 3.847 | 0.993 | 0.453 | 0.011 | 0.166 | Unannotated | NA | NA |
| metab_11130 | N-Acetyl-6-O-L-fucosyl-D-glucosamine | neg | 412.1472 | 0.706 | 3.659 | 4.649 | 0.991 | 0.453 | 0.011 | 0.166 | Unannotated | Lipids and lipid-like molecules | Fatty Acyls |
| metab_3588 | Boviquinone 4 | pos | 476.2760 | 6.710 | 7.438 | 7.184 | -0.254 | -0.453 | 0.011 | 0.166 | Unannotated | Lipids and lipid-like molecules | Prenol lipids |
| metab_9080 | Unannotated feature | neg | 277.1559 | 3.952 | 4.900 | 4.359 | -0.541 | -0.453 | 0.011 | 0.166 | Unannotated | NA | NA |
| metab_2467 | N,n'-Dimethylarginine | pos | 203.1506 | 0.688 | 5.479 | 4.797 | -0.682 | -0.453 | 0.011 | 0.166 | Unannotated | NA | NA |
| metab_8822 | Methylprednisolone acetate | pos | 381.2027 | 5.952 | 4.224 | 2.854 | -1.370 | -0.453 | 0.011 | 0.166 | Tentative | Lipids and lipid-like molecules | Steroids and steroid derivatives |
| metab_1649 | 9-Oxo-10-acridineacetic acid | pos | 285.1240 | 4.586 | 0.042 | 4.582 | 4.540 | 0.453 | 0.011 | 0.166 | Unannotated | Organoheterocyclic compounds | Quinolines and derivatives |
| metab_8767 | 3-Hydroxy-Hexanoyl-DL-Homoserine Lactone | pos | 233.1499 | 0.728 | 4.469 | 3.974 | -0.496 | -0.453 | 0.011 | 0.166 | Moderate putative | NA | NA |
| metab_7674 | Unannotated feature | pos | 336.1925 | 3.421 | 4.476 | 4.084 | -0.392 | -0.453 | 0.011 | 0.166 | Unannotated | NA | NA |
| metab_16829 | Unannotated feature | neg | 301.2656 | 7.726 | 3.263 | 3.927 | 0.664 | 0.453 | 0.011 | 0.166 | Unannotated | NA | NA |
| metab_13738 | Unannotated feature | neg | 345.1782 | 1.947 | 4.471 | 3.971 | -0.500 | -0.453 | 0.011 | 0.166 | Unannotated | NA | NA |
| metab_558 | 4-hydroxy-2-nonenal-[Cys-Gly] conjugate | pos | 335.1608 | 5.600 | 5.263 | 4.879 | -0.384 | -0.453 | 0.011 | 0.166 | Unannotated | Organic acids and derivatives | Carboxylic acids and derivatives |
| metab_2652 | Triterpenoid | pos | 288.1558 | 1.944 | 4.412 | 3.946 | -0.466 | -0.453 | 0.011 | 0.166 | Tentative | Lipids and lipid-like molecules | Prenol lipids |
| metab_17135 | Unannotated feature | neg | 254.9170 | 7.802 | 4.479 | 4.416 | -0.063 | -0.453 | 0.011 | 0.166 | Unannotated | NA | NA |
| metab_17422 | Unannotated feature | neg | 991.8109 | 7.633 | 4.956 | 5.276 | 0.319 | 0.453 | 0.011 | 0.166 | Unannotated | NA | NA |
| metab_4700 | From Dolabella auricularia (sea hare) | pos | 767.4825 | 7.586 | 5.296 | 4.262 | -1.034 | -0.453 | 0.011 | 0.166 | Tentative | NA | NA |
| metab_13715 | 3-Mercaptopyruvic acid | neg | 238.9676 | 1.864 | 4.762 | 4.219 | -0.543 | -0.448 | 0.012 | 0.170 | Tentative | Organic acids and derivatives | Keto acids and derivatives |
| metab_18491 | 6-Hydroxymelatonin | neg | 293.1146 | 3.063 | 5.342 | 5.032 | -0.310 | -0.448 | 0.012 | 0.170 | Tentative | Organoheterocyclic compounds | Indoles and derivatives |
| metab_4220 | Unannotated feature | pos | 467.6250 | 5.434 | 3.036 | 4.128 | 1.092 | 0.448 | 0.012 | 0.170 | Unannotated | NA | NA |
| metab_8636 | Valaciclovir | pos | 347.1458 | 1.034 | 3.655 | 2.929 | -0.726 | -0.448 | 0.012 | 0.170 | Tentative | Organic acids and derivatives | Carboxylic acids and derivatives |
| metab_8068 | Unannotated feature | pos | 223.6344 | 2.762 | 3.855 | 3.039 | -0.816 | -0.448 | 0.012 | 0.170 | Unannotated | NA | NA |
| metab_12525 | C75 | neg | 567.3179 | 6.287 | 5.560 | 5.835 | 0.275 | 0.448 | 0.012 | 0.170 | Moderate putative | NA | NA |
| metab_14192 | Leucyl-Valine | neg | 229.1556 | 2.935 | 6.260 | 5.818 | -0.442 | -0.448 | 0.012 | 0.170 | Unannotated | Organic acids and derivatives | Carboxylic acids and derivatives |
| metab_1012 | Unannotated feature | pos | 601.4217 | 7.124 | 5.019 | 4.057 | -0.962 | -0.448 | 0.012 | 0.170 | Unannotated | NA | NA |
| metab_13783 | Unannotated feature | neg | 251.1034 | 1.981 | 4.735 | 4.182 | -0.553 | -0.448 | 0.012 | 0.170 | Unannotated | NA | NA |
| metab_3530 | PG(a-21:0/TXB2) | pos | 465.2872 | 4.874 | 3.883 | 5.619 | 1.736 | 0.448 | 0.012 | 0.170 | Unannotated | NA | NA |
| metab_10317 | Unannotated feature | neg | 103.0029 | 2.329 | 4.133 | 4.075 | -0.059 | -0.448 | 0.012 | 0.170 | Unannotated | NA | NA |
| metab_18418 | Unannotated feature | neg | 195.0519 | 3.037 | 6.921 | 5.575 | -1.346 | -0.448 | 0.012 | 0.170 | Unannotated | NA | NA |
| metab_7102 | SM(d16:2(4E,8Z)/20:4(6E,8Z,11Z,13E)-2OH(5S,15S)) | pos | 399.2475 | 4.630 | 4.158 | 5.673 | 1.515 | 0.448 | 0.012 | 0.170 | Unannotated | NA | NA |
| metab_8346 | Ile Asp Asp | pos | 362.1564 | 1.960 | 4.202 | 3.944 | -0.257 | -0.448 | 0.012 | 0.170 | Moderate putative | NA | NA |
| metab_1776 | Unannotated feature | pos | 343.1984 | 3.275 | 5.225 | 4.785 | -0.439 | -0.448 | 0.012 | 0.170 | Unannotated | NA | NA |
| metab_647 | Unannotated feature | pos | 625.4802 | 5.841 | 4.045 | 5.020 | 0.975 | 0.448 | 0.012 | 0.170 | Unannotated | NA | NA |
| metab_1181 | Unannotated feature | pos | 203.1395 | 2.732 | 6.091 | 5.885 | -0.206 | -0.448 | 0.012 | 0.170 | Unannotated | NA | NA |
| metab_2176 | Unannotated feature | pos | 317.1139 | 2.941 | 3.167 | 4.382 | 1.216 | 0.448 | 0.012 | 0.170 | Unannotated | NA | NA |
| metab_4033 | Unannotated feature | pos | 630.8909 | 5.820 | 3.342 | 4.499 | 1.157 | 0.448 | 0.012 | 0.170 | Unannotated | NA | NA |
| metab_10961 | Unannotated feature | neg | 168.0660 | 0.791 | 3.133 | 3.664 | 0.531 | 0.448 | 0.012 | 0.170 | Unannotated | NA | NA |
| metab_8525 | N-alpha-Acetyl-L-lysine | pos | 189.1236 | 1.469 | 6.011 | 5.579 | -0.431 | -0.448 | 0.012 | 0.170 | Tentative | Organic acids and derivatives | Carboxylic acids and derivatives |
| metab_4124 | Unannotated feature | pos | 646.8997 | 5.862 | 2.923 | 4.205 | 1.282 | 0.448 | 0.012 | 0.170 | Unannotated | NA | NA |
| metab_16269 | Unannotated feature | neg | 272.9932 | 1.418 | 5.406 | 5.246 | -0.160 | -0.448 | 0.012 | 0.170 | Unannotated | NA | NA |
| metab_14382 | Phenylalanyl-prolyl-arginine | neg | 453.2002 | 3.162 | 5.780 | 5.032 | -0.748 | -0.448 | 0.012 | 0.170 | Unannotated | Organic acids and derivatives | Carboxylic acids and derivatives |
| metab_2635 | Unannotated feature | pos | 189.0074 | 1.844 | 3.687 | 2.945 | -0.742 | -0.448 | 0.012 | 0.170 | Unannotated | NA | NA |
| metab_5993 | Unannotated feature | pos | 277.0086 | 0.493 | 5.413 | 5.594 | 0.181 | 0.448 | 0.012 | 0.170 | Unannotated | NA | NA |
| metab_67 | Methylguanidine | pos | 74.0718 | 0.665 | 2.670 | 2.973 | 0.303 | 0.448 | 0.012 | 0.170 | Strong putative | Organic nitrogen compounds | Organonitrogen compounds |
| metab_13890 | Serylisoleucine | neg | 217.1191 | 2.432 | 5.515 | 5.063 | -0.452 | -0.448 | 0.012 | 0.170 | Unannotated | Organic acids and derivatives | Carboxylic acids and derivatives |
| metab_1443 | Unannotated feature | pos | 393.2759 | 6.008 | 5.245 | 4.734 | -0.512 | -0.448 | 0.012 | 0.170 | Unannotated | NA | NA |
| metab_14640 | Leucylleucine | neg | 243.1714 | 3.573 | 6.032 | 5.702 | -0.329 | -0.448 | 0.012 | 0.170 | Tentative | Organic acids and derivatives | Carboxylic acids and derivatives |
| metab_13848 | Unannotated feature | neg | 248.0403 | 2.211 | 3.577 | 4.468 | 0.891 | 0.448 | 0.012 | 0.170 | Unannotated | NA | NA |
| metab_12757 | Unannotated feature | neg | 406.1364 | 1.947 | 4.062 | 4.990 | 0.928 | 0.448 | 0.012 | 0.170 | Unannotated | NA | NA |
| metab_836 | Auda | pos | 823.5714 | 6.470 | 6.192 | 5.511 | -0.681 | -0.448 | 0.012 | 0.170 | Unannotated | Lipids and lipid-like molecules | Fatty Acyls |
| metab_11559 | Unannotated feature | neg | 491.9134 | 0.472 | 5.086 | 4.712 | -0.374 | -0.448 | 0.012 | 0.170 | Unannotated | NA | NA |
| metab_11064 | Gly-pro-arg-pro | neg | 460.2061 | 0.730 | 4.877 | 4.224 | -0.653 | -0.448 | 0.012 | 0.170 | Unannotated | Organic acids and derivatives | Carboxylic acids and derivatives |
| metab_1889 | Unannotated feature | pos | 205.1186 | 1.360 | 5.934 | 5.632 | -0.302 | -0.448 | 0.012 | 0.170 | Unannotated | NA | NA |
| metab_14721 | Unannotated feature | neg | 250.8939 | 0.516 | 4.070 | 2.514 | -1.557 | -0.448 | 0.012 | 0.170 | Unannotated | NA | NA |
| metab_12132 | Unannotated feature | neg | 844.5735 | 6.577 | 5.651 | 5.199 | -0.452 | -0.448 | 0.012 | 0.170 | Unannotated | NA | NA |
| metab_9913 | Unannotated feature | neg | 424.0851 | 2.938 | 4.347 | 3.354 | -0.994 | -0.444 | 0.013 | 0.174 | Unannotated | NA | NA |
| metab_11501 | Unannotated feature | neg | 430.8639 | 0.512 | 4.248 | 3.588 | -0.660 | -0.444 | 0.013 | 0.174 | Unannotated | NA | NA |
| metab_8268 | 4-Methylbenzoic acid | pos | 178.0866 | 2.205 | 3.033 | 3.831 | 0.798 | 0.444 | 0.013 | 0.174 | Tentative | Benzenoids | Benzene and substituted derivatives |
| metab_6795 | Unannotated feature | pos | 483.6365 | 5.175 | 4.031 | 5.793 | 1.762 | 0.444 | 0.013 | 0.174 | Unannotated | NA | NA |
| metab_8006 | Unannotated feature | pos | 195.0293 | 2.835 | 3.925 | 4.585 | 0.660 | 0.444 | 0.013 | 0.174 | Unannotated | NA | NA |
| metab_3614 | Unannotated feature | pos | 512.9874 | 5.095 | 3.732 | 5.633 | 1.900 | 0.444 | 0.013 | 0.174 | Unannotated | NA | NA |
| metab_6665 | Unannotated feature | pos | 385.2209 | 5.355 | 5.126 | 4.700 | -0.426 | -0.444 | 0.013 | 0.174 | Unannotated | NA | NA |
| metab_4838 | Unannotated feature | pos | 284.2954 | 7.756 | 5.211 | 5.070 | -0.141 | -0.444 | 0.013 | 0.174 | Unannotated | NA | NA |
| metab_3484 | PG(a-21:0/20:4(7E,9E,11Z,13E)-3OH(5S,6R,15S)) | pos | 445.2895 | 4.750 | 4.330 | 5.329 | 0.999 | 0.444 | 0.013 | 0.174 | Unannotated | NA | NA |
| metab_2119 | Epsilon-(gamma-Glutamyl)lysine | pos | 317.1825 | 2.819 | 5.681 | 5.196 | -0.485 | -0.444 | 0.013 | 0.174 | Unannotated | NA | NA |
| metab_3397 | Ser Leu Ile | pos | 332.2187 | 4.389 | 5.408 | 4.681 | -0.727 | -0.444 | 0.013 | 0.174 | Strong putative | NA | NA |
| metab_1738 | Unannotated feature | pos | 403.2715 | 3.654 | 3.369 | 1.863 | -1.505 | -0.444 | 0.013 | 0.174 | Unannotated | NA | NA |
| metab_7094 | Ile Thr Ile | pos | 346.2344 | 4.660 | 5.195 | 4.476 | -0.719 | -0.444 | 0.013 | 0.174 | Moderate putative | NA | NA |
| metab_4202 | Unannotated feature | pos | 632.7879 | 5.914 | 2.893 | 4.335 | 1.442 | 0.444 | 0.013 | 0.174 | Unannotated | NA | NA |
| metab_1724 | Unannotated feature | pos | 382.1973 | 3.817 | 4.604 | 4.175 | -0.428 | -0.444 | 0.013 | 0.174 | Unannotated | NA | NA |
| metab_1626 | Unannotated feature | pos | 366.2033 | 4.900 | 4.857 | 4.475 | -0.382 | -0.444 | 0.013 | 0.174 | Unannotated | NA | NA |
| metab_10629 | Unannotated feature | neg | 369.0290 | 1.680 | 3.644 | 2.760 | -0.884 | -0.444 | 0.013 | 0.174 | Unannotated | NA | NA |
| metab_663 | Unannotated feature | pos | 677.3139 | 5.862 | 4.053 | 4.635 | 0.581 | 0.444 | 0.013 | 0.174 | Unannotated | NA | NA |
| metab_18536 | Unannotated feature | neg | 487.2546 | 6.029 | 3.464 | 2.897 | -0.567 | -0.444 | 0.013 | 0.174 | Unannotated | NA | NA |
| metab_4165 | Unannotated feature | pos | 84.3058 | 5.906 | 3.484 | 3.073 | -0.411 | -0.444 | 0.013 | 0.174 | Unannotated | NA | NA |
| metab_1843 | Unannotated feature | pos | 696.4401 | 4.592 | 4.897 | 5.813 | 0.915 | 0.444 | 0.013 | 0.174 | Unannotated | NA | NA |
| metab_8110 | Dehydromonocrotaline | pos | 356.1712 | 2.732 | 3.453 | 4.683 | 1.231 | 0.444 | 0.013 | 0.174 | Unannotated | Organoheterocyclic compounds | Pyrrolizines |
| metab_5793 | Myristic acid | pos | 246.2432 | 6.016 | 4.385 | 4.121 | -0.263 | -0.444 | 0.013 | 0.174 | Tentative | Lipids and lipid-like molecules | Fatty Acyls |
| metab_4233 | Unannotated feature | pos | 726.1115 | 5.922 | 3.955 | 4.553 | 0.598 | 0.444 | 0.013 | 0.174 | Unannotated | NA | NA |
| metab_12953 | 3-(4,5-Dimethylthiazol-2-yl)-2,5-diphenyl-2,3-dihydro-1H-tetrazole | neg | 380.1204 | 0.770 | 4.236 | 4.954 | 0.718 | 0.444 | 0.013 | 0.174 | Unannotated | Organoheterocyclic compounds | Azoles |
| metab_63 | Methyl oxindole-3-acetate | pos | 428.1776 | 0.665 | 2.008 | 4.253 | 2.244 | 0.444 | 0.013 | 0.174 | Unannotated | Organoheterocyclic compounds | Indoles and derivatives |
| metab_13126 | Unannotated feature | neg | 209.9487 | 0.449 | 7.262 | 7.213 | -0.049 | -0.444 | 0.013 | 0.174 | Unannotated | NA | NA |
| metab_5923 | Unannotated feature | pos | 650.2995 | 5.922 | 4.088 | 5.006 | 0.919 | 0.444 | 0.013 | 0.174 | Unannotated | NA | NA |
| metab_1074 | Unannotated feature | pos | 313.2740 | 6.718 | 6.098 | 5.863 | -0.236 | -0.444 | 0.013 | 0.174 | Unannotated | NA | NA |
| metab_4070 | Unannotated feature | pos | 667.9144 | 5.835 | 2.125 | 4.885 | 2.760 | 0.444 | 0.013 | 0.174 | Unannotated | NA | NA |
| metab_4602 | PE(20:5(5Z,8Z,11Z,14Z,17Z)/18:4(6Z,9Z,12Z,15Z)) | pos | 780.4612 | 4.636 | 3.080 | 5.499 | 2.419 | 0.444 | 0.013 | 0.174 | Tentative | Lipids and lipid-like molecules | Glycerophospholipids |
| metab_14046 | Unannotated feature | neg | 239.1037 | 2.776 | 4.620 | 4.298 | -0.322 | -0.444 | 0.013 | 0.174 | Unannotated | NA | NA |
| metab_7425 | N-gamma-L-Glutamyl-D-alanine | pos | 219.0979 | 0.712 | 5.802 | 5.524 | -0.278 | -0.444 | 0.013 | 0.174 | Unannotated | Organic acids and derivatives | Carboxylic acids and derivatives |
| metab_6115 | Unannotated feature | pos | 529.3232 | 5.847 | 3.299 | 4.944 | 1.645 | 0.444 | 0.013 | 0.174 | Unannotated | NA | NA |
| metab_12634 | Unannotated feature | neg | 214.1444 | 5.991 | 5.743 | 5.585 | -0.158 | -0.444 | 0.013 | 0.174 | Unannotated | NA | NA |
| metab_11568 | Unannotated feature | neg | 281.9175 | 0.464 | 5.809 | 5.687 | -0.122 | -0.444 | 0.013 | 0.174 | Unannotated | NA | NA |
| metab_1539 | Unannotated feature | pos | 517.3267 | 5.355 | 3.573 | 4.396 | 0.823 | 0.440 | 0.014 | 0.179 | Unannotated | NA | NA |
| metab_17442 | Unannotated feature | neg | 787.8487 | 7.611 | 5.810 | 5.982 | 0.172 | 0.440 | 0.014 | 0.179 | Unannotated | NA | NA |
| metab_6028 | Unannotated feature | pos | 659.3079 | 5.898 | 3.742 | 4.610 | 0.868 | 0.440 | 0.014 | 0.179 | Unannotated | NA | NA |
| metab_6774 | Unannotated feature | pos | 501.3041 | 5.219 | 2.576 | 4.534 | 1.958 | 0.440 | 0.014 | 0.179 | Unannotated | NA | NA |
| metab_14441 | 1,3,7-Trimethyluric Acid | neg | 209.0674 | 3.254 | 5.116 | 4.730 | -0.386 | -0.440 | 0.014 | 0.179 | Strong putative | Organoheterocyclic compounds | Imidazopyrimidines |
| metab_6251 | Unannotated feature | pos | 583.8730 | 5.806 | 4.210 | 4.689 | 0.479 | 0.440 | 0.014 | 0.179 | Unannotated | NA | NA |
| metab_4897 | Unannotated feature | pos | 126.1281 | 7.700 | 3.476 | 3.623 | 0.146 | 0.440 | 0.014 | 0.179 | Unannotated | NA | NA |
| metab_10822 | N-Acetyl-L-Alanine | neg | 130.0503 | 1.061 | 5.408 | 5.133 | -0.275 | -0.440 | 0.014 | 0.179 | Moderate putative | Organic acids and derivatives | Carboxylic acids and derivatives |
| metab_14353 | Undeca-5,7,9-trienedioylcarnitine | neg | 374.1576 | 3.123 | 5.653 | 5.055 | -0.598 | -0.440 | 0.014 | 0.179 | Unannotated | Lipids and lipid-like molecules | Fatty Acyls |
| metab_16185 | Unannotated feature | neg | 177.9672 | 0.619 | 5.013 | 4.779 | -0.234 | -0.440 | 0.014 | 0.179 | Unannotated | NA | NA |
| metab_2300 | Unannotated feature | pos | 328.9804 | 0.016 | 4.449 | 4.525 | 0.077 | 0.440 | 0.014 | 0.179 | Unannotated | NA | NA |
| metab_316 | Unannotated feature | pos | 81.5210 | 0.485 | 4.418 | 4.626 | 0.208 | 0.440 | 0.014 | 0.179 | Unannotated | NA | NA |
| metab_6424 | Unannotated feature | pos | 594.8794 | 5.700 | 4.155 | 4.932 | 0.777 | 0.440 | 0.014 | 0.179 | Unannotated | NA | NA |
| metab_2245 | Unannotated feature | pos | 211.1080 | 3.113 | 4.883 | 4.145 | -0.738 | -0.440 | 0.014 | 0.179 | Unannotated | NA | NA |
| metab_17478 | Unannotated feature | neg | 843.8458 | 7.575 | 4.955 | 5.377 | 0.422 | 0.440 | 0.014 | 0.179 | Unannotated | NA | NA |
| metab_1600 | Unannotated feature | pos | 696.9022 | 5.136 | 4.115 | 6.191 | 2.075 | 0.440 | 0.014 | 0.179 | Unannotated | NA | NA |
| metab_5463 | Dihomo-gamma-linolenoylethanolamide | pos | 367.3327 | 6.382 | 5.099 | 4.439 | -0.659 | -0.440 | 0.014 | 0.179 | Tentative | NA | NA |
| metab_6413 | Unannotated feature | pos | 640.4054 | 5.711 | 3.901 | 5.287 | 1.386 | 0.440 | 0.014 | 0.179 | Unannotated | NA | NA |
| metab_17812 | PE(17:0/0:0) | neg | 466.2944 | 6.836 | 6.085 | 5.939 | -0.145 | -0.440 | 0.014 | 0.179 | Strong putative | Lipids and lipid-like molecules | Glycerophospholipids |
| metab_18694 | Unannotated feature | neg | 233.0668 | 5.909 | 4.995 | 5.559 | 0.564 | 0.440 | 0.014 | 0.179 | Unannotated | NA | NA |
| metab_4783 | Unannotated feature | pos | 98.5125 | 7.818 | 4.709 | 4.628 | -0.081 | -0.440 | 0.014 | 0.179 | Unannotated | NA | NA |
| metab_17626 | Unannotated feature | neg | 380.1468 | 2.766 | 5.464 | 5.094 | -0.370 | -0.440 | 0.014 | 0.179 | Unannotated | NA | NA |
| metab_1897 | Unannotated feature | pos | 178.0899 | 1.396 | 5.400 | 4.247 | -1.152 | -0.440 | 0.014 | 0.179 | Unannotated | NA | NA |
| metab_9668 | 1-Octanesulfonic acid | neg | 387.1893 | 3.165 | 5.451 | 5.066 | -0.385 | -0.440 | 0.014 | 0.179 | Unannotated | NA | NA |
| metab_4171 | Unannotated feature | pos | 629.9861 | 5.906 | 1.150 | 4.528 | 3.378 | 0.440 | 0.014 | 0.179 | Unannotated | NA | NA |
| metab_14020 | Unannotated feature | neg | 444.2113 | 2.758 | 4.878 | 4.089 | -0.789 | -0.440 | 0.014 | 0.179 | Unannotated | NA | NA |
| metab_2864 | N6-Acetyl-L-lysine | pos | 189.1237 | 2.788 | 6.529 | 6.297 | -0.232 | -0.440 | 0.014 | 0.179 | Tentative | NA | NA |
| metab_16906 | Unannotated feature | neg | 147.0655 | 7.765 | 4.672 | 4.473 | -0.199 | -0.440 | 0.014 | 0.179 | Unannotated | NA | NA |
| metab_5992 | Unannotated feature | pos | 754.4641 | 5.906 | 3.858 | 4.296 | 0.438 | 0.440 | 0.014 | 0.179 | Unannotated | NA | NA |
| metab_6235 | Unannotated feature | pos | 601.8754 | 5.811 | 4.067 | 4.844 | 0.777 | 0.440 | 0.014 | 0.179 | Unannotated | NA | NA |
| metab_4242 | Unannotated feature | pos | 603.3695 | 5.929 | 2.014 | 5.327 | 3.313 | 0.440 | 0.014 | 0.179 | Unannotated | NA | NA |
| metab_10649 | Unannotated feature | neg | 205.0651 | 1.650 | 3.739 | 1.925 | -1.813 | -0.440 | 0.014 | 0.179 | Unannotated | NA | NA |
| metab_16388 | Unannotated feature | neg | 217.1191 | 1.647 | 6.304 | 5.934 | -0.370 | -0.440 | 0.014 | 0.179 | Unannotated | NA | NA |
| metab_7134 | Unannotated feature | pos | 348.7233 | 4.575 | 4.255 | 5.155 | 0.900 | 0.440 | 0.014 | 0.179 | Unannotated | NA | NA |
| metab_8401 | Unannotated feature | pos | 200.1216 | 1.952 | 3.734 | 0.420 | -3.314 | -0.440 | 0.014 | 0.179 | Unannotated | NA | NA |
| metab_2453 | Unannotated feature | pos | 622.7114 | 5.537 | 1.887 | 3.834 | 1.947 | 0.440 | 0.014 | 0.179 | Unannotated | NA | NA |
| metab_7019 | Unannotated feature | pos | 443.2739 | 4.798 | 4.253 | 5.768 | 1.515 | 0.440 | 0.014 | 0.179 | Unannotated | NA | NA |
| metab_17589 | Unannotated feature | neg | 379.1569 | 7.259 | 3.429 | 3.732 | 0.303 | 0.436 | 0.015 | 0.182 | Unannotated | NA | NA |
| metab_7245 | Asn Ile Leu | pos | 359.2298 | 4.314 | 5.457 | 5.012 | -0.446 | -0.436 | 0.015 | 0.182 | Moderate putative | NA | NA |
| metab_16633 | Unannotated feature | neg | 653.5154 | 7.517 | 5.885 | 5.410 | -0.475 | -0.436 | 0.015 | 0.182 | Unannotated | NA | NA |
| metab_1734 | Unannotated feature | pos | 345.2141 | 3.775 | 5.746 | 5.375 | -0.372 | -0.436 | 0.015 | 0.182 | Unannotated | NA | NA |
| metab_2773 | Metyrosine | pos | 178.0866 | 2.490 | 3.503 | 4.177 | 0.674 | 0.436 | 0.015 | 0.182 | Tentative | Phenylpropanoids and polyketides | Phenylpropanoic acids |
| metab_19278 | Unannotated feature | neg | 373.1289 | 5.169 | 4.901 | 4.571 | -0.330 | -0.436 | 0.015 | 0.182 | Unannotated | NA | NA |
| metab_3563 | Unannotated feature | pos | 606.3720 | 4.980 | 4.856 | 6.164 | 1.308 | 0.436 | 0.015 | 0.182 | Unannotated | NA | NA |
| metab_19533 | Unannotated feature | neg | 300.1931 | 4.803 | 5.659 | 5.237 | -0.422 | -0.436 | 0.015 | 0.182 | Unannotated | NA | NA |
| metab_654 | Unannotated feature | pos | 673.9149 | 5.847 | 3.228 | 4.503 | 1.275 | 0.436 | 0.015 | 0.182 | Unannotated | NA | NA |
| metab_4672 | Unannotated feature | pos | 692.4088 | 4.239 | 3.838 | 5.284 | 1.445 | 0.436 | 0.015 | 0.182 | Unannotated | NA | NA |
| metab_13929 | Unannotated feature | neg | 321.1420 | 2.572 | 4.474 | 3.535 | -0.939 | -0.436 | 0.015 | 0.182 | Unannotated | NA | NA |
| metab_6304 | Unannotated feature | pos | 562.1104 | 5.787 | 3.969 | 4.669 | 0.700 | 0.436 | 0.015 | 0.182 | Unannotated | NA | NA |
| metab_15020 | Unannotated feature | neg | 371.2305 | 4.378 | 5.148 | 4.767 | -0.381 | -0.436 | 0.015 | 0.182 | Unannotated | NA | NA |
| metab_16437 | Unannotated feature | neg | 360.1416 | 1.680 | 5.110 | 4.928 | -0.182 | -0.436 | 0.015 | 0.182 | Unannotated | NA | NA |
| metab_19217 | 3-Methyl-2-(3-pyridyl)-1-indoleoctanoic acid | neg | 385.1697 | 5.238 | 5.349 | 6.009 | 0.661 | 0.436 | 0.015 | 0.182 | Unannotated | Organoheterocyclic compounds | Indoles and derivatives |
| metab_10589 | Unannotated feature | neg | 357.1415 | 1.691 | 4.156 | 3.769 | -0.387 | -0.436 | 0.015 | 0.182 | Unannotated | NA | NA |
| metab_7026 | 7,8-Dihydro-2'-deoxyguanosine | pos | 252.1082 | 0.657 | 4.255 | 4.668 | 0.413 | 0.436 | 0.015 | 0.182 | Unannotated | NA | NA |
| metab_14240 | Unannotated feature | neg | 343.1991 | 2.981 | 5.088 | 4.432 | -0.656 | -0.436 | 0.015 | 0.182 | Unannotated | NA | NA |
| metab_13499 | Unannotated feature | neg | 305.0185 | 1.041 | 4.630 | 3.263 | -1.367 | -0.436 | 0.015 | 0.182 | Unannotated | NA | NA |
| metab_6585 | Unannotated feature | pos | 630.3914 | 5.470 | 3.955 | 5.205 | 1.251 | 0.436 | 0.015 | 0.182 | Unannotated | NA | NA |
| metab_14712 | Unannotated feature | neg | 654.8039 | 0.516 | 5.042 | 5.578 | 0.536 | 0.436 | 0.015 | 0.182 | Unannotated | NA | NA |
| metab_11485 | Unannotated feature | neg | 146.9606 | 0.520 | 5.368 | 5.539 | 0.172 | 0.436 | 0.015 | 0.182 | Unannotated | NA | NA |
| metab_8070 | Unannotated feature | pos | 141.1025 | 2.762 | 3.534 | 2.705 | -0.829 | -0.436 | 0.015 | 0.182 | Unannotated | NA | NA |
| metab_5576 | Unannotated feature | pos | 242.1504 | 2.747 | 5.365 | 5.057 | -0.307 | -0.436 | 0.015 | 0.182 | Unannotated | NA | NA |
| metab_14975 | Unannotated feature | neg | 461.1861 | 4.274 | 3.646 | 4.562 | 0.916 | 0.436 | 0.015 | 0.182 | Unannotated | NA | NA |
| metab_11352 | Unannotated feature | neg | 202.1193 | 0.582 | 4.103 | 3.216 | -0.888 | -0.436 | 0.015 | 0.182 | Unannotated | NA | NA |
| metab_3445 | Gly Leu Leu | pos | 302.2080 | 4.598 | 5.816 | 5.463 | -0.353 | -0.436 | 0.015 | 0.182 | Moderate putative | NA | NA |
| metab_10150 | Unannotated feature | neg | 520.1895 | 2.737 | 3.788 | 5.283 | 1.495 | 0.436 | 0.015 | 0.182 | Unannotated | NA | NA |
| metab_3517 | Unannotated feature | pos | 437.2918 | 4.838 | 3.001 | 4.744 | 1.743 | 0.436 | 0.015 | 0.182 | Unannotated | NA | NA |
| metab_6902 | Unannotated feature | pos | 186.1492 | 5.028 | 4.058 | 3.754 | -0.304 | -0.436 | 0.015 | 0.182 | Unannotated | NA | NA |
| metab_6148 | Unannotated feature | pos | 651.2032 | 5.835 | 4.149 | 4.852 | 0.703 | 0.436 | 0.015 | 0.182 | Unannotated | NA | NA |
| metab_7956 | Ser Gly Ile | pos | 276.1558 | 2.897 | 4.494 | 3.979 | -0.516 | -0.436 | 0.015 | 0.182 | Moderate putative | NA | NA |
| metab_17785 | Unannotated feature | neg | 462.2997 | 6.868 | 6.280 | 5.686 | -0.594 | -0.436 | 0.015 | 0.182 | Unannotated | NA | NA |
| metab_4845 | 3-Amino-2-methoxynonadec-5-en-4-ol | pos | 310.3112 | 7.732 | 5.580 | 5.450 | -0.130 | -0.436 | 0.015 | 0.182 | Unannotated | NA | NA |
| metab_15541 | Unannotated feature | neg | 356.2560 | 5.355 | 5.076 | 4.410 | -0.666 | -0.436 | 0.015 | 0.182 | Unannotated | NA | NA |
| metab_9215 | Unannotated feature | neg | 376.1542 | 3.745 | 4.988 | 4.202 | -0.786 | -0.436 | 0.015 | 0.182 | Unannotated | NA | NA |
| metab_1474 | Unannotated feature | pos | 643.4902 | 5.922 | 3.225 | 4.847 | 1.622 | 0.436 | 0.015 | 0.182 | Unannotated | NA | NA |
| metab_719 | Unannotated feature | pos | 681.5188 | 5.952 | 3.894 | 4.829 | 0.935 | 0.436 | 0.015 | 0.182 | Unannotated | NA | NA |
| metab_9289 | Tyrosyl-Isoleucine | neg | 293.1510 | 3.628 | 5.543 | 5.245 | -0.299 | -0.436 | 0.015 | 0.182 | Unannotated | Organic acids and derivatives | Carboxylic acids and derivatives |
| metab_19825 | Unannotated feature | neg | 330.2039 | 4.398 | 5.580 | 5.018 | -0.562 | -0.436 | 0.015 | 0.182 | Unannotated | NA | NA |
| metab_3544 | Unannotated feature | pos | 487.3002 | 4.931 | 3.557 | 5.528 | 1.971 | 0.436 | 0.015 | 0.182 | Unannotated | NA | NA |
| metab_8650 | Penicillin K | pos | 307.1506 | 1.018 | 4.560 | 3.936 | -0.624 | -0.436 | 0.015 | 0.182 | Tentative | Organic acids and derivatives | Carboxylic acids and derivatives |
| metab_2843 | Unannotated feature | pos | 208.0973 | 2.755 | 4.006 | 4.620 | 0.614 | 0.436 | 0.015 | 0.182 | Unannotated | NA | NA |
| metab_13150 | Unannotated feature | neg | 436.8984 | 0.464 | 5.156 | 5.467 | 0.311 | 0.436 | 0.015 | 0.182 | Unannotated | NA | NA |
| metab_2716 | Unannotated feature | pos | 297.0821 | 2.126 | 5.550 | 5.344 | -0.207 | -0.432 | 0.016 | 0.185 | Unannotated | NA | NA |
| metab_3188 | Propafenone | pos | 359.2298 | 3.694 | 5.344 | 4.827 | -0.517 | -0.432 | 0.016 | 0.185 | Tentative | Phenylpropanoids and polyketides | Linear 1,3-diarylpropanoids |
| metab_18458 | Unannotated feature | neg | 415.2170 | 6.083 | 5.800 | 6.159 | 0.359 | 0.432 | 0.016 | 0.185 | Unannotated | NA | NA |
| metab_14523 | Unannotated feature | neg | 164.9342 | 0.507 | 5.262 | 5.006 | -0.256 | -0.432 | 0.016 | 0.185 | Unannotated | NA | NA |
| metab_5076 | Unannotated feature | pos | 441.3574 | 7.100 | 4.592 | 4.385 | -0.207 | -0.432 | 0.016 | 0.185 | Unannotated | NA | NA |
| metab_9071 | 1,5-Naphthalene diisocyanate | neg | 246.9918 | 3.971 | 2.910 | 4.189 | 1.280 | 0.432 | 0.016 | 0.185 | Tentative | Benzenoids | Naphthalenes |
| metab_7508 | Unannotated feature | pos | 352.1875 | 3.729 | 4.616 | 4.004 | -0.612 | -0.432 | 0.016 | 0.185 | Unannotated | NA | NA |
| metab_11564 | Unannotated feature | neg | 580.8588 | 0.464 | 4.658 | 4.103 | -0.555 | -0.432 | 0.016 | 0.185 | Unannotated | NA | NA |
| metab_18133 | Unannotated feature | neg | 521.2584 | 6.365 | 4.693 | 5.345 | 0.652 | 0.432 | 0.016 | 0.185 | Unannotated | NA | NA |
| metab_13938 | Unannotated feature | neg | 588.1112 | 2.579 | 5.304 | 4.407 | -0.897 | -0.432 | 0.016 | 0.185 | Unannotated | NA | NA |
| metab_1669 | Unannotated feature | pos | 326.7101 | 4.472 | 4.287 | 5.086 | 0.799 | 0.432 | 0.016 | 0.185 | Unannotated | NA | NA |
| metab_6306 | Unannotated feature | pos | 546.8478 | 5.787 | 3.692 | 4.351 | 0.658 | 0.432 | 0.016 | 0.185 | Unannotated | NA | NA |
| metab_1769 | Unannotated feature | pos | 736.4349 | 4.339 | 2.316 | 5.169 | 2.853 | 0.432 | 0.016 | 0.185 | Unannotated | NA | NA |
| metab_974 | Unannotated feature | pos | 174.0764 | 7.864 | 5.114 | 5.009 | -0.105 | -0.432 | 0.016 | 0.185 | Unannotated | NA | NA |
| metab_18926 | Unannotated feature | neg | 157.0863 | 5.621 | 3.833 | 3.239 | -0.594 | -0.432 | 0.016 | 0.185 | Unannotated | NA | NA |
| metab_4932 | Unannotated feature | pos | 315.1558 | 0.712 | 4.568 | 5.130 | 0.562 | 0.432 | 0.016 | 0.185 | Unannotated | NA | NA |
| metab_5010 | Unannotated feature | pos | 652.4136 | 4.475 | 5.466 | 6.151 | 0.685 | 0.432 | 0.016 | 0.185 | Unannotated | NA | NA |
| metab_5030 | Unannotated feature | pos | 603.4374 | 7.276 | 6.219 | 5.443 | -0.777 | -0.432 | 0.016 | 0.185 | Unannotated | NA | NA |
| metab_17567 | Unannotated feature | neg | 117.9356 | 7.310 | 4.920 | 4.820 | -0.100 | -0.432 | 0.016 | 0.185 | Unannotated | NA | NA |
| metab_6727 | Val Phe Ile | pos | 378.2398 | 5.267 | 5.057 | 4.258 | -0.800 | -0.432 | 0.016 | 0.185 | Moderate putative | NA | NA |
| metab_5928 | Unannotated feature | pos | 627.3842 | 5.922 | 4.156 | 5.662 | 1.506 | 0.432 | 0.016 | 0.185 | Unannotated | NA | NA |
| metab_2233 | Unannotated feature | pos | 302.1240 | 3.087 | 2.673 | 4.100 | 1.427 | 0.432 | 0.016 | 0.185 | Unannotated | NA | NA |
| metab_17958 | Unannotated feature | neg | 368.0990 | 2.762 | 3.794 | 4.615 | 0.822 | 0.432 | 0.016 | 0.185 | Unannotated | NA | NA |
| metab_13125 | Unannotated feature | neg | 155.9105 | 0.449 | 3.572 | 3.502 | -0.070 | -0.432 | 0.016 | 0.185 | Unannotated | NA | NA |
| metab_7948 | 4-Hydroxy-L-Proline | pos | 132.0658 | 2.907 | 3.591 | 3.386 | -0.205 | -0.432 | 0.016 | 0.185 | Moderate putative | NA | NA |
| metab_6667 | Ile Leu Ile | pos | 358.2709 | 5.355 | 5.237 | 4.589 | -0.648 | -0.432 | 0.016 | 0.185 | Moderate putative | NA | NA |
| metab_16747 | Unannotated feature | neg | 663.8479 | 7.670 | 4.165 | 4.458 | 0.293 | 0.432 | 0.016 | 0.185 | Unannotated | NA | NA |
| metab_7741 | Unannotated feature | pos | 100.1126 | 3.312 | 3.400 | 3.605 | 0.205 | 0.432 | 0.016 | 0.185 | Unannotated | NA | NA |
| metab_11531 | Unannotated feature | neg | 174.9555 | 0.502 | 6.638 | 6.830 | 0.193 | 0.432 | 0.016 | 0.185 | Unannotated | NA | NA |
| metab_17420 | Unannotated feature | neg | 651.8733 | 7.637 | 5.332 | 5.514 | 0.182 | 0.432 | 0.016 | 0.185 | Unannotated | NA | NA |
| metab_2714 | Alpha-Fluoro-beta-ureidopropionic acid | pos | 189.0074 | 2.116 | 4.526 | 4.159 | -0.367 | -0.432 | 0.016 | 0.185 | Tentative | Organic acids and derivatives | Carboxylic acids and derivatives |
| metab_9540 | Unannotated feature | neg | 355.1992 | 3.298 | 4.606 | 3.679 | -0.927 | -0.432 | 0.016 | 0.185 | Unannotated | NA | NA |
| metab_13149 | Unannotated feature | neg | 279.9203 | 0.464 | 6.035 | 5.911 | -0.124 | -0.432 | 0.016 | 0.185 | Unannotated | NA | NA |
| metab_1421 | Unannotated feature | pos | 652.4136 | 4.298 | 5.106 | 5.886 | 0.779 | 0.432 | 0.016 | 0.185 | Unannotated | NA | NA |
| metab_6471 | Unannotated feature | pos | 540.0972 | 5.626 | 3.746 | 4.623 | 0.876 | 0.432 | 0.016 | 0.185 | Unannotated | NA | NA |
| metab_2086 | Unannotated feature | pos | 687.4346 | 5.239 | 3.336 | 4.818 | 1.481 | 0.432 | 0.016 | 0.185 | Unannotated | NA | NA |
| metab_15853 | Octopine | neg | 281.1032 | 5.930 | 5.011 | 5.406 | 0.395 | 0.432 | 0.016 | 0.185 | Tentative | Organic acids and derivatives | Carboxylic acids and derivatives |
| metab_3010 | Beta-Funaltrexamine | pos | 455.2150 | 3.140 | 5.404 | 3.577 | -1.828 | -0.432 | 0.016 | 0.185 | Tentative | Benzenoids | Phenanthrenes and derivatives |
| metab_3828 | 7-Hydroxygliclazide | pos | 340.1334 | 5.626 | 3.376 | 2.903 | -0.473 | -0.432 | 0.016 | 0.185 | Unannotated | Benzenoids | Benzene and substituted derivatives |
| metab_18655 | Unannotated feature | neg | 294.9824 | 5.940 | 5.073 | 4.797 | -0.276 | -0.432 | 0.016 | 0.185 | Unannotated | NA | NA |
| metab_6034 | Unannotated feature | pos | 535.3282 | 5.898 | 3.828 | 4.563 | 0.735 | 0.432 | 0.016 | 0.185 | Unannotated | NA | NA |
| metab_14713 | Unannotated feature | neg | 316.8842 | 0.516 | 4.691 | 4.186 | -0.506 | -0.432 | 0.016 | 0.185 | Unannotated | NA | NA |
| metab_7465 | Ser Val Leu | pos | 318.2030 | 3.835 | 5.534 | 5.285 | -0.249 | -0.432 | 0.016 | 0.185 | Moderate putative | NA | NA |
| metab_13640 | Gamma-Glutamylvaline | neg | 245.1143 | 1.647 | 6.612 | 6.306 | -0.306 | -0.432 | 0.016 | 0.185 | Unannotated | Organic acids and derivatives | Carboxylic acids and derivatives |
| metab_102 | Aspartyl-Lysine | pos | 262.1402 | 0.641 | 4.400 | 3.903 | -0.497 | -0.432 | 0.016 | 0.185 | Unannotated | NA | NA |
| metab_6775 | Unannotated feature | pos | 449.6256 | 5.219 | 3.396 | 4.506 | 1.109 | 0.432 | 0.016 | 0.185 | Unannotated | NA | NA |
| metab_4836 | Unannotated feature | pos | 256.2640 | 7.764 | 5.891 | 5.825 | -0.066 | -0.432 | 0.016 | 0.185 | Unannotated | NA | NA |
| metab_4957 | Unannotated feature | pos | 787.8431 | 7.586 | 5.609 | 5.677 | 0.068 | 0.427 | 0.017 | 0.189 | Unannotated | NA | NA |
| metab_9052 | Unannotated feature | neg | 371.2305 | 3.988 | 4.414 | 3.237 | -1.178 | -0.427 | 0.017 | 0.189 | Unannotated | NA | NA |
| metab_352 | Unannotated feature | pos | 144.9613 | 0.393 | 3.941 | 3.989 | 0.048 | 0.427 | 0.017 | 0.189 | Unannotated | NA | NA |
| metab_3677 | Unannotated feature | pos | 464.3010 | 5.247 | 3.553 | 4.598 | 1.045 | 0.427 | 0.017 | 0.189 | Unannotated | NA | NA |
| metab_5190 | Unannotated feature | pos | 784.4924 | 4.766 | 3.670 | 5.078 | 1.408 | 0.427 | 0.017 | 0.189 | Unannotated | NA | NA |
| metab_2346 | Unannotated feature | pos | 320.0685 | 0.457 | 4.696 | 4.446 | -0.250 | -0.427 | 0.017 | 0.189 | Unannotated | NA | NA |
| metab_7064 | Unannotated feature | pos | 435.2762 | 4.717 | 3.456 | 5.073 | 1.617 | 0.427 | 0.017 | 0.189 | Unannotated | NA | NA |
| metab_11877 | 3-Carboxy-4-methyl-5-propyl-2-furanpropionic acid | neg | 239.0920 | 6.018 | 4.680 | 4.240 | -0.439 | -0.427 | 0.017 | 0.189 | Unannotated | Lipids and lipid-like molecules | Fatty Acyls |
| metab_12387 | 5-[(3As,4S,6aR)-2-oxo-1,3,3a,4,6,6a-hexahydrothieno[3,4-d]imidazol-4-yl]-N'-propylpentanehydrazide | neg | 321.1357 | 6.201 | 4.407 | 5.144 | 0.737 | 0.427 | 0.017 | 0.189 | Unannotated | Organoheterocyclic compounds | Thiolanes |
| metab_8129 | Unannotated feature | pos | 366.2147 | 2.724 | 4.992 | 4.469 | -0.523 | -0.427 | 0.017 | 0.189 | Unannotated | NA | NA |
| metab_530 | Unannotated feature | pos | 590.8687 | 5.260 | 3.661 | 4.550 | 0.889 | 0.427 | 0.017 | 0.189 | Unannotated | NA | NA |
| metab_8122 | Unannotated feature | pos | 260.0922 | 2.732 | 3.593 | 4.301 | 0.708 | 0.427 | 0.017 | 0.189 | Unannotated | NA | NA |
| metab_3339 | Unannotated feature | pos | 302.2080 | 4.198 | 4.837 | 4.149 | -0.688 | -0.427 | 0.017 | 0.189 | Unannotated | NA | NA |
| metab_14397 | Unannotated feature | neg | 291.1354 | 3.192 | 3.770 | 3.360 | -0.410 | -0.427 | 0.017 | 0.189 | Unannotated | NA | NA |
| metab_18574 | Unannotated feature | neg | 234.1134 | 6.007 | 5.076 | 4.667 | -0.409 | -0.427 | 0.017 | 0.189 | Unannotated | NA | NA |
| metab_19709 | Unannotated feature | neg | 330.2038 | 4.542 | 5.854 | 5.347 | -0.507 | -0.427 | 0.017 | 0.189 | Unannotated | NA | NA |
| metab_6586 | Unannotated feature | pos | 517.3204 | 5.470 | 2.516 | 4.964 | 2.449 | 0.427 | 0.017 | 0.189 | Unannotated | NA | NA |
| metab_7206 | Unannotated feature | pos | 348.7233 | 4.406 | 3.441 | 4.776 | 1.335 | 0.427 | 0.017 | 0.189 | Unannotated | NA | NA |
| metab_3729 | Unannotated feature | pos | 601.0405 | 5.386 | 4.558 | 5.935 | 1.377 | 0.427 | 0.017 | 0.189 | Unannotated | NA | NA |
| metab_13313 | Unannotated feature | neg | 205.0826 | 0.651 | 4.941 | 4.386 | -0.556 | -0.427 | 0.017 | 0.189 | Unannotated | NA | NA |
| metab_333 | {[5-(5-Nitro-2-furyl)-1,3,4-oxadiazol-2-YL]thio}acetic acid | pos | 235.9741 | 0.457 | 4.831 | 4.622 | -0.210 | -0.427 | 0.017 | 0.189 | Unannotated | NA | NA |
| metab_1974 | Lys Ile Ala | pos | 331.2348 | 1.952 | 5.273 | 4.913 | -0.360 | -0.427 | 0.017 | 0.189 | Unannotated | NA | NA |
| metab_10062 | 3-Hydroxyanthranilic Acid | neg | 152.0347 | 2.785 | 5.343 | 5.516 | 0.173 | 0.427 | 0.017 | 0.189 | Strong putative | Benzenoids | Benzene and substituted derivatives |
| metab_5988 | Unannotated feature | pos | 265.1648 | 5.914 | 2.684 | 5.046 | 2.362 | 0.427 | 0.017 | 0.189 | Unannotated | NA | NA |
| metab_10613 | Unannotated feature | neg | 274.1411 | 1.685 | 5.293 | 4.909 | -0.384 | -0.427 | 0.017 | 0.189 | Unannotated | NA | NA |
| metab_16559 | Unannotated feature | neg | 579.4409 | 7.297 | 6.992 | 6.602 | -0.390 | -0.427 | 0.017 | 0.189 | Unannotated | NA | NA |
| metab_8118 | Pyrrhoxanthinol | pos | 286.1766 | 2.732 | 4.899 | 4.602 | -0.298 | -0.427 | 0.017 | 0.189 | Unannotated | Lipids and lipid-like molecules | Prenol lipids |
| metab_1639 | Unannotated feature | pos | 368.2188 | 4.717 | 4.702 | 4.150 | -0.552 | -0.427 | 0.017 | 0.189 | Unannotated | NA | NA |
| metab_17440 | Unannotated feature | neg | 881.8442 | 7.611 | 5.473 | 5.670 | 0.197 | 0.427 | 0.017 | 0.189 | Unannotated | NA | NA |
| metab_3238 | Phe Ile Thr | pos | 380.2188 | 3.823 | 4.640 | 4.089 | -0.551 | -0.427 | 0.017 | 0.189 | Moderate putative | NA | NA |
| metab_13916 | Deca-3,5,7-trienedioylcarnitine | neg | 360.1418 | 2.531 | 5.389 | 4.789 | -0.600 | -0.427 | 0.017 | 0.189 | Unannotated | Lipids and lipid-like molecules | Fatty Acyls |
| metab_7183 | Unannotated feature | pos | 335.2234 | 4.475 | 4.569 | 5.272 | 0.703 | 0.427 | 0.017 | 0.189 | Unannotated | NA | NA |
| metab_2031 | Methylisopelletierine | pos | 173.1651 | 2.523 | 5.963 | 5.225 | -0.738 | -0.427 | 0.017 | 0.189 | Tentative | Organoheterocyclic compounds | Piperidines |
| metab_12231 | Unannotated feature | neg | 655.5320 | 6.915 | 6.176 | 5.910 | -0.266 | -0.427 | 0.017 | 0.189 | Unannotated | NA | NA |
| metab_19620 | Unannotated feature | neg | 357.2148 | 4.677 | 5.550 | 5.125 | -0.424 | -0.427 | 0.017 | 0.189 | Unannotated | NA | NA |
| metab_5681 | N-Myristoyl Threonine | pos | 393.2756 | 6.135 | 5.499 | 4.911 | -0.588 | -0.427 | 0.017 | 0.189 | Unannotated | NA | NA |
| metab_327 | Unannotated feature | pos | 278.0210 | 0.469 | 5.163 | 5.022 | -0.141 | -0.427 | 0.017 | 0.189 | Unannotated | NA | NA |
| metab_6390 | Unannotated feature | pos | 638.9056 | 5.732 | 3.840 | 4.574 | 0.734 | 0.427 | 0.017 | 0.189 | Unannotated | NA | NA |
| metab_12846 | Desciclovir | neg | 190.0716 | 0.683 | 5.299 | 5.466 | 0.168 | 0.427 | 0.017 | 0.189 | Unannotated | NA | NA |
| metab_6847 | Unannotated feature | pos | 507.3117 | 5.102 | 3.668 | 4.380 | 0.712 | 0.427 | 0.017 | 0.189 | Unannotated | NA | NA |
| metab_3981 | Unannotated feature | pos | 574.3559 | 5.799 | 3.628 | 4.827 | 1.199 | 0.427 | 0.017 | 0.189 | Unannotated | NA | NA |
| metab_14087 | Telotristat | neg | 545.1306 | 2.809 | 5.624 | 5.014 | -0.610 | -0.427 | 0.017 | 0.189 | Unannotated | Organic acids and derivatives | Carboxylic acids and derivatives |
| metab_10130 | Unannotated feature | neg | 219.0772 | 2.758 | 4.013 | 3.613 | -0.401 | -0.427 | 0.017 | 0.189 | Unannotated | NA | NA |
| metab_8596 | Val Glu | pos | 247.1293 | 1.151 | 5.660 | 5.493 | -0.167 | -0.427 | 0.017 | 0.189 | Strong putative | NA | NA |
| metab_35 | Unannotated feature | pos | 140.0708 | 0.704 | 4.261 | 4.604 | 0.343 | 0.427 | 0.017 | 0.189 | Unannotated | NA | NA |
| metab_18542 | Phenylalanylglutamine | neg | 645.2919 | 6.022 | 4.925 | 3.931 | -0.994 | -0.423 | 0.018 | 0.193 | Unannotated | Organic acids and derivatives | Carboxylic acids and derivatives |
| metab_4212 | Unannotated feature | pos | 748.4601 | 5.914 | 3.208 | 4.676 | 1.468 | 0.423 | 0.018 | 0.193 | Unannotated | NA | NA |
| metab_13375 | Unannotated feature | neg | 240.0877 | 0.738 | 2.276 | 4.473 | 2.197 | 0.423 | 0.018 | 0.193 | Unannotated | NA | NA |
| metab_19581 | Unannotated feature | neg | 334.1777 | 4.740 | 4.018 | 3.463 | -0.555 | -0.423 | 0.018 | 0.193 | Unannotated | NA | NA |
| metab_7044 | Unannotated feature | pos | 487.3002 | 4.766 | 3.462 | 5.002 | 1.540 | 0.423 | 0.018 | 0.193 | Unannotated | NA | NA |
| metab_7046 | Unannotated feature | pos | 316.2237 | 4.763 | 5.399 | 5.039 | -0.360 | -0.423 | 0.018 | 0.193 | Unannotated | NA | NA |
| metab_18073 | Unannotated feature | neg | 178.0505 | 2.915 | 3.474 | 4.059 | 0.585 | 0.423 | 0.018 | 0.193 | Unannotated | NA | NA |
| metab_1884 | Glu Leu Lys | pos | 389.2403 | 1.294 | 5.586 | 5.022 | -0.565 | -0.423 | 0.018 | 0.193 | Strong putative | NA | NA |
| metab_5912 | Unannotated feature | pos | 702.9851 | 5.922 | 3.076 | 4.526 | 1.450 | 0.423 | 0.018 | 0.193 | Unannotated | NA | NA |
| metab_9375 | Unannotated feature | neg | 357.2149 | 3.505 | 5.518 | 4.974 | -0.544 | -0.423 | 0.018 | 0.193 | Unannotated | NA | NA |
| metab_10467 | Unannotated feature | neg | 140.0347 | 1.958 | 3.854 | 3.749 | -0.105 | -0.423 | 0.018 | 0.193 | Unannotated | NA | NA |
| metab_527 | Unannotated feature | pos | 751.9510 | 5.239 | 3.424 | 5.805 | 2.381 | 0.423 | 0.018 | 0.193 | Unannotated | NA | NA |
| metab_12330 | Unannotated feature | neg | 559.4735 | 7.379 | 6.736 | 6.049 | -0.686 | -0.423 | 0.018 | 0.193 | Unannotated | NA | NA |
| metab_16670 | Unannotated feature | neg | 1005.3187 | 7.607 | 5.681 | 5.984 | 0.303 | 0.423 | 0.018 | 0.193 | Unannotated | NA | NA |
| metab_1548 | Unannotated feature | pos | 490.6318 | 5.332 | 3.684 | 4.445 | 0.761 | 0.423 | 0.018 | 0.193 | Unannotated | NA | NA |
| metab_7117 | Unannotated feature | pos | 357.2367 | 4.603 | 4.801 | 5.535 | 0.734 | 0.423 | 0.018 | 0.193 | Unannotated | NA | NA |
| metab_3155 | Asn Ile Val | pos | 345.2141 | 3.582 | 5.750 | 5.346 | -0.404 | -0.423 | 0.018 | 0.193 | Moderate putative | NA | NA |
| metab_9320 | Unannotated feature | neg | 250.0721 | 3.983 | 4.347 | 4.921 | 0.574 | 0.423 | 0.018 | 0.193 | Unannotated | NA | NA |
| metab_9465 | Unannotated feature | neg | 350.1361 | 3.391 | 5.302 | 5.109 | -0.193 | -0.423 | 0.018 | 0.193 | Unannotated | NA | NA |
| metab_4036 | Unannotated feature | pos | 652.9043 | 5.820 | 4.053 | 4.549 | 0.496 | 0.423 | 0.018 | 0.193 | Unannotated | NA | NA |
| metab_6010 | Unannotated feature | pos | 612.3755 | 5.906 | 3.827 | 5.063 | 1.236 | 0.423 | 0.018 | 0.193 | Unannotated | NA | NA |
| metab_18653 | Unannotated feature | neg | 230.9860 | 5.944 | 5.271 | 5.110 | -0.161 | -0.423 | 0.018 | 0.193 | Unannotated | NA | NA |
| metab_12827 | Unannotated feature | neg | 175.0718 | 0.659 | 5.605 | 5.279 | -0.326 | -0.423 | 0.018 | 0.193 | Unannotated | NA | NA |
| metab_17198 | Unannotated feature | neg | 266.0532 | 7.737 | 3.039 | 4.267 | 1.228 | 0.423 | 0.018 | 0.193 | Unannotated | NA | NA |
| metab_13582 | Unannotated feature | neg | 629.1946 | 1.952 | 4.568 | 4.167 | -0.401 | -0.423 | 0.018 | 0.193 | Unannotated | NA | NA |
| metab_4258 | Unannotated feature | pos | 776.2249 | 5.936 | 2.264 | 4.393 | 2.129 | 0.423 | 0.018 | 0.193 | Unannotated | NA | NA |
| metab_601 | Unannotated feature | pos | 524.6548 | 5.537 | 3.732 | 5.115 | 1.383 | 0.423 | 0.018 | 0.193 | Unannotated | NA | NA |
| metab_16680 | Unannotated feature | neg | 983.8253 | 7.611 | 5.060 | 5.249 | 0.189 | 0.423 | 0.018 | 0.193 | Unannotated | NA | NA |
| metab_2226 | Unannotated feature | pos | 375.1884 | 3.058 | 5.667 | 5.253 | -0.414 | -0.423 | 0.018 | 0.193 | Unannotated | NA | NA |
| metab_14372 | Unannotated feature | neg | 484.2425 | 3.149 | 4.310 | 3.380 | -0.930 | -0.423 | 0.018 | 0.193 | Unannotated | NA | NA |
| metab_14435 | Unannotated feature | neg | 300.1931 | 3.242 | 4.592 | 3.950 | -0.643 | -0.423 | 0.018 | 0.193 | Unannotated | NA | NA |
| metab_9982 | Delta-Hydroxylysylnorleucine | neg | 272.1619 | 2.865 | 5.121 | 4.769 | -0.352 | -0.423 | 0.018 | 0.193 | Unannotated | Organic acids and derivatives | Carboxylic acids and derivatives |
| metab_3542 | Unannotated feature | pos | 652.8757 | 4.919 | 2.810 | 5.295 | 2.485 | 0.423 | 0.018 | 0.193 | Unannotated | NA | NA |
| metab_16550 | Unannotated feature | neg | 602.2841 | 7.246 | 5.025 | 5.400 | 0.375 | 0.423 | 0.018 | 0.193 | Unannotated | NA | NA |
| metab_9758 | O-Demethylmetoprolol | neg | 274.1411 | 3.078 | 4.888 | 4.314 | -0.574 | -0.423 | 0.018 | 0.193 | Unannotated | Benzenoids | Phenols |
| metab_89 | Unannotated feature | pos | 151.6257 | 0.649 | 5.375 | 4.468 | -0.907 | -0.423 | 0.018 | 0.193 | Unannotated | NA | NA |
| metab_1502 | Unannotated feature | pos | 620.5952 | 5.794 | 3.917 | 5.107 | 1.190 | 0.423 | 0.018 | 0.193 | Unannotated | NA | NA |
| metab_6236 | Unannotated feature | pos | 600.7154 | 5.811 | 4.071 | 4.902 | 0.830 | 0.423 | 0.018 | 0.193 | Unannotated | NA | NA |
| metab_12940 | Unannotated feature | neg | 293.0882 | 0.738 | 5.181 | 5.618 | 0.438 | 0.423 | 0.018 | 0.193 | Unannotated | NA | NA |
| metab_4216 | Unannotated feature | pos | 243.1517 | 5.922 | 0.070 | 4.745 | 4.674 | 0.423 | 0.018 | 0.193 | Unannotated | NA | NA |
| metab_689 | Unannotated feature | pos | 594.0299 | 5.914 | 4.001 | 4.896 | 0.895 | 0.423 | 0.018 | 0.193 | Unannotated | NA | NA |
| metab_7253 | Unannotated feature | pos | 335.2234 | 4.298 | 3.880 | 4.823 | 0.943 | 0.423 | 0.018 | 0.193 | Unannotated | NA | NA |
| metab_2431 | L-erythro-4-Hydroxyarginine | pos | 232.1408 | 0.625 | 4.443 | 3.192 | -1.251 | -0.423 | 0.018 | 0.193 | Tentative | Organic acids and derivatives | Carboxylic acids and derivatives |
| metab_13137 | Unannotated feature | neg | 323.9292 | 0.453 | 4.839 | 4.770 | -0.068 | -0.423 | 0.018 | 0.193 | Unannotated | NA | NA |
| metab_17239 | Unannotated feature | neg | 89.9248 | 7.730 | 4.470 | 4.390 | -0.080 | -0.423 | 0.018 | 0.193 | Unannotated | NA | NA |
| metab_1486 | Unannotated feature | pos | 629.7844 | 5.906 | 3.276 | 4.510 | 1.234 | 0.419 | 0.019 | 0.196 | Unannotated | NA | NA |
| metab_1902 | Unannotated feature | pos | 486.1833 | 0.728 | 3.960 | 4.692 | 0.732 | 0.419 | 0.019 | 0.196 | Unannotated | NA | NA |
| metab_1839 | Unannotated feature | pos | 220.1184 | 1.026 | 3.742 | 4.275 | 0.533 | 0.419 | 0.019 | 0.196 | Unannotated | NA | NA |
| metab_13712 | Unannotated feature | neg | 131.0343 | 1.854 | 6.324 | 6.078 | -0.245 | -0.419 | 0.019 | 0.196 | Unannotated | NA | NA |
| metab_15502 | Unannotated feature | neg | 414.1235 | 5.276 | 3.971 | 3.174 | -0.797 | -0.419 | 0.019 | 0.196 | Unannotated | NA | NA |
| metab_13448 | Benzyl-6-hydroxy-2-cyclohexene-on-oyl | neg | 245.0819 | 4.983 | 3.817 | 4.523 | 0.706 | 0.419 | 0.019 | 0.196 | Unannotated | Benzenoids | Benzene and substituted derivatives |
| metab_11231 | Unannotated feature | neg | 304.1044 | 0.636 | 3.900 | 4.713 | 0.813 | 0.419 | 0.019 | 0.196 | Unannotated | NA | NA |
| metab_5976 | Unannotated feature | pos | 591.1953 | 5.914 | 3.885 | 4.147 | 0.262 | 0.419 | 0.019 | 0.196 | Unannotated | NA | NA |
| metab_8793 | Alpha-Aminobutyric acid | pos | 86.0605 | 7.874 | 4.533 | 4.653 | 0.120 | 0.419 | 0.019 | 0.196 | Tentative | NA | NA |
| metab_2581 | Unannotated feature | pos | 255.1343 | 1.266 | 4.912 | 4.612 | -0.300 | -0.419 | 0.019 | 0.196 | Unannotated | NA | NA |
| metab_13480 | (2Z)-2-Morpholin-4-yliminoacetonitrile | neg | 184.0722 | 1.000 | 5.176 | 4.729 | -0.447 | -0.419 | 0.019 | 0.196 | Unannotated | Organoheterocyclic compounds | Oxazinanes |
| metab_502 | Unannotated feature | pos | 453.9533 | 5.095 | 3.583 | 5.170 | 1.587 | 0.419 | 0.019 | 0.196 | Unannotated | NA | NA |
| metab_7503 | Unannotated feature | pos | 350.1454 | 0.720 | 3.263 | 4.655 | 1.392 | 0.419 | 0.019 | 0.196 | Unannotated | NA | NA |
| metab_16579 | Unannotated feature | neg | 174.0013 | 7.332 | 4.731 | 4.921 | 0.189 | 0.419 | 0.019 | 0.196 | Unannotated | NA | NA |
| metab_14740 | Unannotated feature | neg | 752.8077 | 0.512 | 4.314 | 4.948 | 0.635 | 0.419 | 0.019 | 0.196 | Unannotated | NA | NA |
| metab_7406 | Unannotated feature | pos | 407.2664 | 3.961 | 4.464 | 3.503 | -0.961 | -0.419 | 0.019 | 0.196 | Unannotated | NA | NA |
| metab_7541 | Unannotated feature | pos | 265.1550 | 3.674 | 5.322 | 4.915 | -0.407 | -0.419 | 0.019 | 0.196 | Unannotated | NA | NA |
| metab_2004 | Unannotated feature | pos | 281.0505 | 2.232 | 3.167 | 2.784 | -0.383 | -0.419 | 0.019 | 0.196 | Unannotated | NA | NA |
| metab_15003 | Unannotated feature | neg | 762.7571 | 0.538 | 4.211 | 4.728 | 0.517 | 0.419 | 0.019 | 0.196 | Unannotated | NA | NA |
| metab_4112 | Unannotated feature | pos | 846.5158 | 5.851 | 4.152 | 4.435 | 0.283 | 0.419 | 0.019 | 0.196 | Unannotated | NA | NA |
| metab_19408 | Unannotated feature | neg | 335.1357 | 3.210 | 4.331 | 3.792 | -0.539 | -0.419 | 0.019 | 0.196 | Unannotated | NA | NA |
| metab_8520 | Morph | pos | 246.1452 | 1.535 | 5.856 | 5.458 | -0.398 | -0.419 | 0.019 | 0.196 | Tentative | Alkaloids and derivatives | Morphinans |
| metab_9440 | Methyl cinnamate | neg | 207.0658 | 3.422 | 4.986 | 4.598 | -0.388 | -0.419 | 0.019 | 0.196 | Tentative | Phenylpropanoids and polyketides | Cinnamic acids and derivatives |
| metab_1384 | Unannotated feature | pos | 213.1125 | 6.111 | 3.286 | 5.080 | 1.794 | 0.419 | 0.019 | 0.196 | Unannotated | NA | NA |
| metab_10041 | Unannotated feature | neg | 143.1183 | 2.806 | 4.698 | 4.266 | -0.432 | -0.419 | 0.019 | 0.196 | Unannotated | NA | NA |
| metab_10645 | Unannotated feature | neg | 282.0103 | 1.656 | 5.595 | 5.485 | -0.110 | -0.419 | 0.019 | 0.196 | Unannotated | NA | NA |
| metab_16679 | Unannotated feature | neg | 855.8366 | 7.611 | 5.531 | 5.786 | 0.255 | 0.419 | 0.019 | 0.196 | Unannotated | NA | NA |
| metab_12801 | Unannotated feature | neg | 217.0476 | 0.644 | 4.172 | 4.789 | 0.617 | 0.419 | 0.019 | 0.196 | Unannotated | NA | NA |
| metab_7811 | Matairesinol | pos | 376.1725 | 3.135 | 5.028 | 4.187 | -0.841 | -0.419 | 0.019 | 0.196 | Tentative | NA | NA |
| metab_10195 | Unannotated feature | neg | 429.1999 | 2.656 | 4.487 | 3.738 | -0.749 | -0.419 | 0.019 | 0.196 | Unannotated | NA | NA |
| metab_1476 | Unannotated feature | pos | 450.2761 | 5.922 | 3.028 | 4.422 | 1.393 | 0.419 | 0.019 | 0.196 | Unannotated | NA | NA |
| metab_670 | Unannotated feature | pos | 653.9042 | 5.851 | 1.838 | 4.492 | 2.654 | 0.419 | 0.019 | 0.196 | Unannotated | NA | NA |
| metab_8944 | Unannotated feature | neg | 300.1931 | 4.199 | 5.070 | 4.597 | -0.473 | -0.419 | 0.019 | 0.196 | Unannotated | NA | NA |
| metab_3459 | Leu Glu Leu | pos | 374.2296 | 4.673 | 6.043 | 5.513 | -0.530 | -0.419 | 0.019 | 0.196 | Moderate putative | NA | NA |
| metab_7355 | Unannotated feature | pos | 354.2088 | 4.063 | 3.866 | 0.675 | -3.190 | -0.419 | 0.019 | 0.196 | Unannotated | NA | NA |
| metab_12759 | Unannotated feature | neg | 274.1411 | 1.947 | 5.685 | 5.389 | -0.296 | -0.419 | 0.019 | 0.196 | Unannotated | NA | NA |
| metab_7590 | Unannotated feature | pos | 382.1977 | 3.569 | 4.613 | 3.871 | -0.742 | -0.419 | 0.019 | 0.196 | Unannotated | NA | NA |
| metab_6247 | Ganglioside GD1b (d18:0/22:0) | pos | 647.0116 | 5.806 | 2.991 | 4.803 | 1.812 | 0.419 | 0.019 | 0.196 | Unannotated | Lipids and lipid-like molecules | Sphingolipids |
| metab_16700 | Unannotated feature | neg | 979.3126 | 7.633 | 5.252 | 5.601 | 0.349 | 0.419 | 0.019 | 0.196 | Unannotated | NA | NA |
| metab_2142 | Unannotated feature | pos | 356.1211 | 2.864 | 1.756 | 3.137 | 1.381 | 0.419 | 0.019 | 0.196 | Unannotated | NA | NA |
| metab_13967 | Unannotated feature | neg | 760.8372 | 0.485 | 4.639 | 5.019 | 0.380 | 0.419 | 0.019 | 0.196 | Unannotated | NA | NA |
| metab_4288 | Unannotated feature | pos | 675.3256 | 5.969 | 4.436 | 5.007 | 0.571 | 0.419 | 0.019 | 0.196 | Unannotated | NA | NA |
| metab_8440 | Unannotated feature | pos | 187.6238 | 1.944 | 4.348 | 3.420 | -0.928 | -0.419 | 0.019 | 0.196 | Unannotated | NA | NA |
| metab_8746 | (2S,3R,4S,5R,6R)-6-Ethyloxane-2,3,4,5-tetrol | pos | 220.1183 | 0.736 | 4.354 | 4.814 | 0.459 | 0.419 | 0.019 | 0.196 | Unannotated | NA | NA |
| metab_8194 | Asn Leu | pos | 246.1453 | 2.513 | 5.471 | 4.946 | -0.524 | -0.419 | 0.019 | 0.196 | Strong putative | NA | NA |
| metab_4621 | Unannotated feature | pos | 317.1504 | 4.579 | 3.276 | 3.995 | 0.720 | 0.419 | 0.019 | 0.196 | Unannotated | NA | NA |
| metab_3412 | Unannotated feature | pos | 382.2208 | 4.448 | 2.575 | 4.168 | 1.593 | 0.419 | 0.019 | 0.196 | Unannotated | NA | NA |
| metab_14502 | Unannotated feature | neg | 154.0867 | 3.354 | 3.609 | 2.923 | -0.687 | -0.419 | 0.019 | 0.196 | Unannotated | NA | NA |
| metab_9554 | Unannotated feature | neg | 341.1834 | 3.281 | 5.311 | 4.903 | -0.409 | -0.419 | 0.019 | 0.196 | Unannotated | NA | NA |
| metab_9307 | Unannotated feature | neg | 343.1990 | 3.600 | 5.820 | 5.291 | -0.529 | -0.419 | 0.019 | 0.196 | Unannotated | NA | NA |
| metab_17425 | Unannotated feature | neg | 889.8301 | 7.625 | 5.396 | 5.708 | 0.313 | 0.419 | 0.019 | 0.196 | Unannotated | NA | NA |
| metab_10137 | Unannotated feature | neg | 372.2145 | 4.469 | 5.832 | 5.253 | -0.580 | -0.419 | 0.019 | 0.196 | Unannotated | NA | NA |
| metab_1497 | Unannotated feature | pos | 647.2118 | 5.799 | 3.635 | 5.153 | 1.518 | 0.419 | 0.019 | 0.196 | Unannotated | NA | NA |
| metab_1498 | Unannotated feature | pos | 638.2066 | 5.799 | 3.452 | 5.107 | 1.656 | 0.419 | 0.019 | 0.196 | Unannotated | NA | NA |
| metab_2907 | Unannotated feature | pos | 204.1426 | 2.868 | 4.418 | 3.882 | -0.535 | -0.419 | 0.019 | 0.196 | Unannotated | NA | NA |
| metab_5604 | PA(22:6(4Z,7Z,10Z,13E,15E,19Z)-OH(17)/22:5(7Z,10Z,13Z,16Z,19Z)) | pos | 828.5189 | 4.838 | 3.612 | 4.633 | 1.020 | 0.419 | 0.019 | 0.196 | Unannotated | NA | NA |
| metab_104 | Ala Val Leu | pos | 302.2080 | 4.399 | 5.699 | 5.341 | -0.358 | -0.419 | 0.019 | 0.196 | Moderate putative | NA | NA |
| metab_3218 | Unannotated feature | pos | 336.1922 | 3.761 | 4.707 | 4.040 | -0.667 | -0.415 | 0.020 | 0.200 | Unannotated | NA | NA |
| metab_8602 | Leucylhydroxyproline | pos | 245.1500 | 1.126 | 5.773 | 5.193 | -0.579 | -0.415 | 0.020 | 0.200 | Unannotated | Organic acids and derivatives | Carboxylic acids and derivatives |
| metab_2769 | Dihydroxyphenyl-valerolactone | pos | 250.1078 | 2.452 | 3.102 | 3.984 | 0.882 | 0.415 | 0.020 | 0.200 | Unannotated | Organoheterocyclic compounds | Lactones |
| metab_2391 | Unannotated feature | pos | 204.0182 | 0.561 | 3.078 | 3.949 | 0.871 | 0.415 | 0.020 | 0.200 | Unannotated | NA | NA |
| metab_12837 | Unannotated feature | neg | 295.0608 | 0.675 | 3.988 | 4.446 | 0.458 | 0.415 | 0.020 | 0.200 | Unannotated | NA | NA |
| metab_7104 | Unannotated feature | pos | 382.2208 | 4.625 | 3.543 | 4.665 | 1.123 | 0.415 | 0.020 | 0.200 | Unannotated | NA | NA |
| metab_4782 | Unannotated feature | pos | 97.1017 | 7.810 | 4.506 | 4.561 | 0.056 | 0.415 | 0.020 | 0.200 | Unannotated | NA | NA |
| metab_2729 | Unannotated feature | pos | 119.0708 | 2.205 | 3.524 | 2.225 | -1.300 | -0.415 | 0.020 | 0.200 | Unannotated | NA | NA |
| metab_4690 | Unannotated feature | pos | 604.3561 | 4.166 | 4.782 | 5.665 | 0.884 | 0.415 | 0.020 | 0.200 | Unannotated | NA | NA |
| metab_8701 | Unannotated feature | pos | 636.4188 | 4.179 | 4.707 | 5.119 | 0.413 | 0.415 | 0.020 | 0.200 | Unannotated | NA | NA |
| metab_6017 | Unannotated feature | pos | 568.3483 | 5.906 | 2.106 | 4.099 | 1.993 | 0.415 | 0.020 | 0.200 | Unannotated | NA | NA |
| metab_9421 | Unannotated feature | neg | 342.1674 | 3.446 | 5.580 | 5.334 | -0.246 | -0.415 | 0.020 | 0.200 | Unannotated | NA | NA |
| metab_6021 | Unannotated feature | pos | 686.2232 | 5.898 | 2.237 | 4.189 | 1.952 | 0.415 | 0.020 | 0.200 | Unannotated | NA | NA |
| metab_2150 | Unannotated feature | pos | 216.9510 | 0.493 | 5.935 | 6.115 | 0.180 | 0.415 | 0.020 | 0.200 | Unannotated | NA | NA |
| metab_19626 | Unannotated feature | neg | 344.2194 | 4.667 | 5.116 | 4.532 | -0.584 | -0.415 | 0.020 | 0.200 | Unannotated | NA | NA |
| metab_5624 | 3-O-Demethylfortimicin A | pos | 414.2320 | 6.199 | 5.162 | 4.721 | -0.442 | -0.415 | 0.020 | 0.200 | Unannotated | Organic oxygen compounds | Organooxygen compounds |
| metab_2971 | Val Ser Val | pos | 304.1871 | 3.021 | 5.344 | 4.704 | -0.640 | -0.415 | 0.020 | 0.200 | Moderate putative | NA | NA |
| metab_630 | Unannotated feature | pos | 717.6547 | 5.811 | 3.035 | 4.292 | 1.258 | 0.415 | 0.020 | 0.200 | Unannotated | NA | NA |
| metab_16248 | 6-Ethylchenodeoxycholic acid | neg | 419.3172 | 6.522 | 5.242 | 4.810 | -0.432 | -0.415 | 0.020 | 0.200 | Tentative | NA | NA |
| metab_9944 | Unannotated feature | neg | 274.1410 | 2.906 | 5.131 | 4.216 | -0.915 | -0.415 | 0.020 | 0.200 | Unannotated | NA | NA |
| metab_6101 | Unannotated feature | pos | 634.3900 | 5.851 | 4.297 | 5.224 | 0.927 | 0.415 | 0.020 | 0.200 | Unannotated | NA | NA |
| metab_14853 | Unannotated feature | neg | 340.2245 | 4.004 | 4.922 | 3.426 | -1.497 | -0.415 | 0.020 | 0.200 | Unannotated | NA | NA |
| metab_10042 | Threonylleucine | neg | 231.1348 | 2.802 | 6.211 | 5.749 | -0.463 | -0.415 | 0.020 | 0.200 | Unannotated | Organic acids and derivatives | Carboxylic acids and derivatives |
| metab_16678 | Unannotated feature | neg | 794.8667 | 7.611 | 5.498 | 5.617 | 0.120 | 0.415 | 0.020 | 0.200 | Unannotated | NA | NA |
| metab_5685 | Unannotated feature | pos | 612.8821 | 5.297 | 3.246 | 4.654 | 1.409 | 0.415 | 0.020 | 0.200 | Unannotated | NA | NA |
| metab_11563 | Unannotated feature | neg | 254.8985 | 0.467 | 4.482 | 4.357 | -0.124 | -0.415 | 0.020 | 0.200 | Unannotated | NA | NA |
| metab_7553 | Thr Val Val | pos | 318.2030 | 3.647 | 5.584 | 5.203 | -0.382 | -0.415 | 0.020 | 0.200 | Moderate putative | NA | NA |
| metab_12756 | Unannotated feature | neg | 408.1525 | 1.947 | 4.138 | 4.579 | 0.442 | 0.415 | 0.020 | 0.200 | Unannotated | NA | NA |
| metab_16692 | Unannotated feature | neg | 1004.8123 | 7.621 | 5.235 | 5.539 | 0.303 | 0.415 | 0.020 | 0.200 | Unannotated | NA | NA |
| metab_5666 | Unannotated feature | pos | 401.3385 | 6.151 | 5.772 | 5.302 | -0.470 | -0.415 | 0.020 | 0.200 | Unannotated | NA | NA |
| metab_1621 | Unannotated feature | pos | 502.8158 | 4.923 | 1.473 | 4.449 | 2.976 | 0.415 | 0.020 | 0.200 | Unannotated | NA | NA |
| metab_431 | Unannotated feature | pos | 131.9746 | 0.016 | 5.446 | 5.492 | 0.046 | 0.415 | 0.020 | 0.200 | Unannotated | NA | NA |
| metab_2561 | Unannotated feature | pos | 306.1190 | 1.126 | 3.081 | 4.439 | 1.357 | 0.415 | 0.020 | 0.200 | Unannotated | NA | NA |
| metab_11861 | Arginyl-Gamma-glutamate | neg | 301.1659 | 4.959 | 5.096 | 5.463 | 0.368 | 0.415 | 0.020 | 0.200 | Unannotated | Organic acids and derivatives | Carboxylic acids and derivatives |
| metab_2923 | N-(3-acetamidopropyl)pyrrolidin-2-one | pos | 185.1288 | 2.897 | 5.176 | 5.520 | 0.344 | 0.415 | 0.020 | 0.200 | Unannotated | NA | NA |
| metab_11073 | 5,9:7,10a-Dimethano-10aH-[1,3]dioxocino[6,5-d]pyrimidine-4,7,10,11,12-pentol, octahydro-12-(hydroxymethyl)-2-imino-,(4R,4aR,5R,7S,9S,10S,10aR,11S,12S)- | neg | 318.0944 | 0.730 | 4.644 | 3.982 | -0.662 | -0.415 | 0.020 | 0.200 | Unannotated | Organoheterocyclic compounds | Diazanaphthalenes |
| metab_7942 | Lucyoside N | pos | 326.2082 | 2.907 | 5.100 | 4.600 | -0.500 | -0.415 | 0.020 | 0.200 | Unannotated | NA | NA |
| metab_380 | Unannotated feature | pos | 906.2608 | 0.093 | 6.276 | 6.353 | 0.078 | 0.415 | 0.020 | 0.200 | Unannotated | NA | NA |
| metab_14360 | Unannotated feature | neg | 341.1833 | 3.131 | 4.836 | 4.459 | -0.377 | -0.415 | 0.020 | 0.200 | Unannotated | NA | NA |
| metab_945 | Unannotated feature | pos | 627.5339 | 7.538 | 5.941 | 5.999 | 0.059 | 0.415 | 0.020 | 0.200 | Unannotated | NA | NA |
| metab_5251 | Unannotated feature | pos | 268.2638 | 6.662 | 4.684 | 4.499 | -0.185 | -0.415 | 0.020 | 0.200 | Unannotated | NA | NA |
| metab_15961 | Unannotated feature | neg | 359.2707 | 6.068 | 5.394 | 4.783 | -0.611 | -0.415 | 0.020 | 0.200 | Unannotated | NA | NA |
| metab_4729 | Unannotated feature | pos | 218.9201 | 7.658 | 4.517 | 4.607 | 0.090 | 0.415 | 0.020 | 0.200 | Unannotated | NA | NA |
| metab_4723 | Unannotated feature | pos | 556.8623 | 7.642 | 5.198 | 5.277 | 0.079 | 0.415 | 0.020 | 0.200 | Unannotated | NA | NA |
| metab_9218 | Unannotated feature | neg | 422.1944 | 3.741 | 4.785 | 3.574 | -1.211 | -0.415 | 0.020 | 0.200 | Unannotated | NA | NA |
| metab_13411 | Unannotated feature | neg | 324.0942 | 0.784 | 2.779 | 3.337 | 0.558 | 0.415 | 0.020 | 0.200 | Unannotated | NA | NA |
| metab_6547 | Unannotated feature | pos | 319.1297 | 5.532 | 5.128 | 4.738 | -0.390 | -0.415 | 0.020 | 0.200 | Unannotated | NA | NA |
| metab_14680 | Unannotated feature | neg | 316.1880 | 3.655 | 5.775 | 5.401 | -0.374 | -0.415 | 0.020 | 0.200 | Unannotated | NA | NA |
| metab_6503 | Unannotated feature | pos | 506.8266 | 5.583 | 3.226 | 4.354 | 1.129 | 0.415 | 0.020 | 0.200 | Unannotated | NA | NA |
| metab_728 | LysoPC(20:3(5Z,8Z,11Z)/0:0) | pos | 546.3555 | 5.984 | 4.593 | 3.808 | -0.785 | -0.415 | 0.020 | 0.200 | Unannotated | Lipids and lipid-like molecules | Glycerophospholipids |
| metab_9271 | Unannotated feature | neg | 379.1843 | 3.655 | 5.160 | 4.413 | -0.747 | -0.415 | 0.020 | 0.200 | Unannotated | NA | NA |
| metab_9981 | Unannotated feature | neg | 407.1580 | 2.865 | 4.021 | 3.708 | -0.313 | -0.411 | 0.022 | 0.206 | Unannotated | NA | NA |
| metab_14866 | Unannotated feature | neg | 616.8383 | 0.502 | 5.112 | 5.406 | 0.294 | 0.411 | 0.022 | 0.206 | Unannotated | NA | NA |
| metab_8025 | 4-Amino-3-hydroxybutanoylcarnitine | pos | 304.1871 | 2.803 | 5.497 | 5.090 | -0.407 | -0.411 | 0.022 | 0.206 | Unannotated | Lipids and lipid-like molecules | Fatty Acyls |
| metab_1819 | N-lactoyl-Tyrosine | pos | 286.1294 | 0.768 | 4.446 | 5.295 | 0.849 | 0.411 | 0.022 | 0.206 | Unannotated | NA | NA |
| metab_14563 | Unannotated feature | neg | 459.2108 | 3.453 | 4.340 | 3.234 | -1.107 | -0.411 | 0.022 | 0.206 | Unannotated | NA | NA |
| metab_13639 | Unannotated feature | neg | 373.2097 | 1.635 | 5.576 | 5.243 | -0.333 | -0.411 | 0.022 | 0.206 | Unannotated | NA | NA |
| metab_7527 | 2'-Deoxymugineic acid | pos | 269.1138 | 3.704 | 4.254 | 3.587 | -0.668 | -0.411 | 0.022 | 0.206 | Tentative | Organic acids and derivatives | Carboxylic acids and derivatives |
| metab_3343 | Glu Ile Leu | pos | 374.2296 | 4.212 | 4.932 | 3.933 | -0.999 | -0.411 | 0.022 | 0.206 | Moderate putative | NA | NA |
| metab_2157 | Unannotated feature | pos | 126.0917 | 2.900 | 4.343 | 4.519 | 0.176 | 0.411 | 0.022 | 0.206 | Unannotated | NA | NA |
| metab_18590 | Unannotated feature | neg | 364.1518 | 3.096 | 4.944 | 4.528 | -0.416 | -0.411 | 0.022 | 0.206 | Unannotated | NA | NA |
| metab_16802 | Unannotated feature | neg | 132.8994 | 7.714 | 3.297 | 3.172 | -0.125 | -0.411 | 0.022 | 0.206 | Unannotated | NA | NA |
| metab_17273 | Unannotated feature | neg | 331.1222 | 7.720 | 3.328 | 3.571 | 0.242 | 0.411 | 0.022 | 0.206 | Unannotated | NA | NA |
| metab_17882 | Unannotated feature | neg | 297.2072 | 6.695 | 5.003 | 4.413 | -0.590 | -0.411 | 0.022 | 0.206 | Unannotated | NA | NA |
| metab_18575 | Didox | neg | 168.0295 | 6.007 | 5.447 | 5.314 | -0.133 | -0.411 | 0.022 | 0.206 | Unannotated | Benzenoids | Benzene and substituted derivatives |
| metab_1903 | Asp Ile Lys | pos | 188.1158 | 1.642 | 5.343 | 4.963 | -0.380 | -0.411 | 0.022 | 0.206 | Moderate putative | NA | NA |
| metab_1567 | Unannotated feature | pos | 461.2804 | 5.247 | 3.550 | 3.679 | 0.129 | 0.411 | 0.022 | 0.206 | Unannotated | NA | NA |
| metab_6341 | Unannotated feature | pos | 540.0972 | 5.770 | 2.657 | 4.778 | 2.121 | 0.411 | 0.022 | 0.206 | Unannotated | NA | NA |
| metab_528 | Unannotated feature | pos | 370.6167 | 5.247 | 4.341 | 4.112 | -0.229 | -0.411 | 0.022 | 0.206 | Unannotated | NA | NA |
| metab_2759 | Ser Leu | pos | 219.1343 | 2.419 | 5.616 | 5.045 | -0.571 | -0.411 | 0.022 | 0.206 | Strong putative | NA | NA |
| metab_2527 | Unannotated feature | pos | 638.7285 | 5.825 | 3.466 | 4.812 | 1.346 | 0.411 | 0.022 | 0.206 | Unannotated | NA | NA |
| metab_6589 | Unannotated feature | pos | 645.0664 | 5.457 | 3.773 | 4.728 | 0.955 | 0.411 | 0.022 | 0.206 | Unannotated | NA | NA |
| metab_19476 | Unannotated feature | neg | 364.1884 | 4.899 | 4.981 | 4.549 | -0.431 | -0.411 | 0.022 | 0.206 | Unannotated | NA | NA |
| metab_4091 | Unannotated feature | pos | 736.9504 | 5.841 | 2.874 | 4.510 | 1.635 | 0.411 | 0.022 | 0.206 | Unannotated | NA | NA |
| metab_1989 | Unannotated feature | pos | 212.0234 | 2.155 | 5.113 | 4.792 | -0.321 | -0.411 | 0.022 | 0.206 | Unannotated | NA | NA |
| metab_4138 | Unannotated feature | pos | 570.3528 | 5.882 | 2.340 | 4.163 | 1.822 | 0.411 | 0.022 | 0.206 | Unannotated | NA | NA |
| metab_3492 | Unannotated feature | pos | 415.2786 | 4.774 | 3.480 | 4.709 | 1.229 | 0.411 | 0.022 | 0.206 | Unannotated | NA | NA |
| metab_6112 | Unannotated feature | pos | 637.8940 | 5.847 | 4.238 | 4.568 | 0.330 | 0.411 | 0.022 | 0.206 | Unannotated | NA | NA |
| metab_17004 | Unannotated feature | neg | 238.9676 | 2.141 | 5.741 | 5.455 | -0.287 | -0.411 | 0.022 | 0.206 | Unannotated | NA | NA |
| metab_9726 | Lactucin | neg | 321.0982 | 3.100 | 3.586 | 4.457 | 0.871 | 0.411 | 0.022 | 0.206 | Tentative | Organoheterocyclic compounds | Lactones |
| metab_19014 | Unannotated feature | neg | 305.0781 | 3.234 | 4.304 | 4.693 | 0.388 | 0.411 | 0.022 | 0.206 | Unannotated | NA | NA |
| metab_19069 | Unannotated feature | neg | 175.0970 | 5.435 | 3.908 | 3.391 | -0.517 | -0.411 | 0.022 | 0.206 | Unannotated | NA | NA |
| metab_1786 | Unannotated feature | pos | 389.2403 | 0.768 | 4.876 | 4.183 | -0.693 | -0.411 | 0.022 | 0.206 | Unannotated | NA | NA |
| metab_14070 | Unannotated feature | neg | 402.1048 | 2.789 | 3.384 | 4.503 | 1.119 | 0.411 | 0.022 | 0.206 | Unannotated | NA | NA |
| metab_7235 | PA(PGE1/P-16:0) | pos | 377.2343 | 4.350 | 4.403 | 5.087 | 0.684 | 0.411 | 0.022 | 0.206 | Unannotated | NA | NA |
| metab_13601 | Leucyl-Lysine | neg | 258.1824 | 1.438 | 4.623 | 3.860 | -0.762 | -0.411 | 0.022 | 0.206 | Unannotated | NA | NA |
| metab_6750 | 5-HEPE | pos | 382.2346 | 5.247 | 4.667 | 3.491 | -1.176 | -0.411 | 0.022 | 0.206 | Unannotated | Lipids and lipid-like molecules | Fatty Acyls |
| metab_10731 | Unannotated feature | neg | 233.1029 | 1.288 | 3.571 | 4.120 | 0.549 | 0.411 | 0.022 | 0.206 | Unannotated | NA | NA |
| metab_5998 | Unannotated feature | pos | 693.3175 | 5.906 | 3.201 | 4.690 | 1.489 | 0.411 | 0.022 | 0.206 | Unannotated | NA | NA |
| metab_2057 | Unannotated feature | pos | 203.0918 | 2.716 | 2.836 | 4.321 | 1.484 | 0.406 | 0.023 | 0.211 | Unannotated | NA | NA |
| metab_6230 | Unannotated feature | pos | 634.8951 | 5.332 | 3.561 | 4.433 | 0.872 | 0.406 | 0.023 | 0.211 | Unannotated | NA | NA |
| metab_17576 | DG(12:0/0:0/PGF1alpha) | neg | 647.4285 | 7.297 | 6.023 | 5.501 | -0.522 | -0.406 | 0.023 | 0.211 | Unannotated | NA | NA |
| metab_7862 | N-Methylethanolaminium phosphate | pos | 197.0675 | 3.035 | 5.037 | 2.059 | -2.979 | -0.406 | 0.023 | 0.211 | Tentative | Organic acids and derivatives | Organic phosphoric acids and derivatives |
| metab_12692 | Unannotated feature | neg | 459.2159 | 5.398 | 2.503 | 3.910 | 1.406 | 0.406 | 0.023 | 0.211 | Unannotated | NA | NA |
| metab_3598 | Unannotated feature | pos | 498.3117 | 5.060 | 3.550 | 5.573 | 2.024 | 0.406 | 0.023 | 0.211 | Unannotated | NA | NA |
| metab_4991 | Unannotated feature | pos | 414.1616 | 0.728 | 3.863 | 4.717 | 0.854 | 0.406 | 0.023 | 0.211 | Unannotated | NA | NA |
| metab_11061 | Imidazoleacetic acid riboside | neg | 561.1689 | 0.730 | 1.517 | 4.682 | 3.165 | 0.406 | 0.023 | 0.211 | Tentative | Nucleosides, nucleotides, and analogues | Imidazole ribonucleosides and ribonucleotides |
| metab_17042 | Unannotated feature | neg | 145.9491 | 7.989 | 4.022 | 3.926 | -0.096 | -0.406 | 0.023 | 0.211 | Unannotated | NA | NA |
| metab_18405 | Unannotated feature | neg | 228.1602 | 6.130 | 5.062 | 4.814 | -0.247 | -0.406 | 0.023 | 0.211 | Unannotated | NA | NA |
| metab_16296 | Tricarballylic Acid | neg | 175.0242 | 1.471 | 7.288 | 6.831 | -0.458 | -0.406 | 0.023 | 0.211 | Moderate putative | Organic acids and derivatives | Carboxylic acids and derivatives |
| metab_6525 | Unannotated feature | pos | 495.8198 | 5.563 | 2.980 | 4.371 | 1.391 | 0.406 | 0.023 | 0.211 | Unannotated | NA | NA |
| metab_11012 | Unannotated feature | neg | 140.0823 | 0.770 | 4.615 | 4.072 | -0.542 | -0.406 | 0.023 | 0.211 | Unannotated | NA | NA |
| metab_1677 | Unannotated feature | pos | 337.7010 | 4.301 | 3.231 | 4.004 | 0.773 | 0.406 | 0.023 | 0.211 | Unannotated | NA | NA |
| metab_10345 | Unannotated feature | neg | 360.1418 | 2.222 | 4.879 | 4.280 | -0.599 | -0.406 | 0.023 | 0.211 | Unannotated | NA | NA |
| metab_7991 | Ala Leu Ala | pos | 274.1766 | 2.849 | 5.203 | 4.930 | -0.272 | -0.406 | 0.023 | 0.211 | Moderate putative | NA | NA |
| metab_10252 | Unannotated feature | neg | 279.0989 | 2.554 | 4.516 | 4.101 | -0.416 | -0.406 | 0.023 | 0.211 | Unannotated | NA | NA |
| metab_4614 | Unannotated feature | pos | 571.3985 | 7.100 | 4.027 | 4.353 | 0.326 | 0.406 | 0.023 | 0.211 | Unannotated | NA | NA |
| metab_8840 | Sinapyl alcohol | neg | 209.0815 | 4.837 | 6.103 | 5.637 | -0.466 | -0.406 | 0.023 | 0.211 | Tentative | Benzenoids | Phenols |
| metab_10560 | Unannotated feature | neg | 87.0442 | 1.854 | 4.723 | 4.444 | -0.279 | -0.406 | 0.023 | 0.211 | Unannotated | NA | NA |
| metab_17453 | Unannotated feature | neg | 331.1626 | 1.952 | 5.192 | 4.623 | -0.569 | -0.406 | 0.023 | 0.211 | Unannotated | NA | NA |
| metab_13418 | Unannotated feature | neg | 229.1192 | 0.791 | 4.079 | 3.477 | -0.602 | -0.406 | 0.023 | 0.211 | Unannotated | NA | NA |
| metab_18761 | Unannotated feature | neg | 214.1444 | 5.834 | 5.214 | 5.045 | -0.169 | -0.406 | 0.023 | 0.211 | Unannotated | NA | NA |
| metab_4890 | Unannotated feature | pos | 82.5376 | 7.708 | 3.672 | 3.546 | -0.126 | -0.406 | 0.023 | 0.211 | Unannotated | NA | NA |
| metab_17060 | Unannotated feature | neg | 243.0485 | 2.183 | 5.422 | 5.791 | 0.369 | 0.406 | 0.023 | 0.211 | Unannotated | NA | NA |
| metab_18582 | Sulindac | neg | 401.0883 | 6.003 | 5.486 | 5.272 | -0.214 | -0.406 | 0.023 | 0.211 | Tentative | NA | NA |
| metab_10076 | Unannotated feature | neg | 332.1832 | 2.776 | 5.613 | 5.178 | -0.435 | -0.406 | 0.023 | 0.211 | Unannotated | NA | NA |
| metab_5746 | 5b-Cholestane-3a,7a,12a,23R,25-pentol | pos | 417.3338 | 6.064 | 5.419 | 4.843 | -0.577 | -0.406 | 0.023 | 0.211 | Tentative | Lipids and lipid-like molecules | Fatty Acyls |
| metab_7119 | Unannotated feature | pos | 415.2788 | 4.598 | 3.501 | 4.229 | 0.728 | 0.406 | 0.023 | 0.211 | Unannotated | NA | NA |
| metab_2676 | Unannotated feature | pos | 245.1500 | 1.968 | 4.628 | 4.206 | -0.421 | -0.406 | 0.023 | 0.211 | Unannotated | NA | NA |
| metab_5968 | Unannotated feature | pos | 627.2909 | 5.914 | 3.944 | 4.815 | 0.871 | 0.406 | 0.023 | 0.211 | Unannotated | NA | NA |
| metab_11091 | Unannotated feature | neg | 108.0447 | 0.730 | 3.729 | 4.239 | 0.509 | 0.406 | 0.023 | 0.211 | Unannotated | NA | NA |
| metab_10189 | Unannotated feature | neg | 572.1162 | 2.659 | 5.500 | 4.859 | -0.642 | -0.406 | 0.023 | 0.211 | Unannotated | NA | NA |
| metab_17834 | Unannotated feature | neg | 520.2662 | 6.766 | 6.227 | 6.050 | -0.177 | -0.406 | 0.023 | 0.211 | Unannotated | NA | NA |
| metab_1503 | Unannotated feature | pos | 611.7900 | 5.794 | 3.411 | 5.354 | 1.943 | 0.406 | 0.023 | 0.211 | Unannotated | NA | NA |
| metab_4299 | Unannotated feature | pos | 143.9971 | 0.485 | 6.472 | 6.636 | 0.163 | 0.406 | 0.023 | 0.211 | Unannotated | NA | NA |
| metab_14540 | Unannotated feature | neg | 526.8428 | 0.524 | 5.214 | 5.746 | 0.532 | 0.406 | 0.023 | 0.211 | Unannotated | NA | NA |
| metab_6889 | Unannotated feature | pos | 503.3325 | 5.040 | 3.715 | 4.320 | 0.606 | 0.406 | 0.023 | 0.211 | Unannotated | NA | NA |
| metab_19136 | Unannotated feature | neg | 286.1662 | 5.343 | 5.187 | 4.943 | -0.244 | -0.406 | 0.023 | 0.211 | Unannotated | NA | NA |
| metab_691 | Unannotated feature | pos | 258.9978 | 0.493 | 5.413 | 5.573 | 0.160 | 0.406 | 0.023 | 0.211 | Unannotated | NA | NA |
| metab_17469 | Unannotated feature | neg | 775.8576 | 7.590 | 5.072 | 5.519 | 0.447 | 0.406 | 0.023 | 0.211 | Unannotated | NA | NA |
| metab_6353 | Unannotated feature | pos | 542.3398 | 5.761 | 0.069 | 4.386 | 4.317 | 0.406 | 0.023 | 0.211 | Unannotated | NA | NA |
| metab_16494 | Unannotated feature | neg | 384.1924 | 7.080 | 4.282 | 3.912 | -0.370 | -0.406 | 0.023 | 0.211 | Unannotated | NA | NA |
| metab_3092 | Glu Leu Val | pos | 360.2140 | 3.400 | 5.013 | 4.431 | -0.582 | -0.406 | 0.023 | 0.211 | Moderate putative | NA | NA |
| metab_2317 | Unannotated feature | pos | 199.1697 | 0.156 | 4.567 | 4.624 | 0.057 | 0.406 | 0.023 | 0.211 | Unannotated | NA | NA |
| metab_17498 | Unannotated feature | neg | 754.2458 | 7.499 | 4.073 | 4.591 | 0.518 | 0.406 | 0.023 | 0.211 | Unannotated | NA | NA |
| metab_13954 | Unannotated feature | neg | 153.0380 | 2.637 | 3.409 | 3.767 | 0.358 | 0.406 | 0.023 | 0.211 | Unannotated | NA | NA |
| metab_14095 | Unannotated feature | neg | 351.1529 | 2.817 | 5.430 | 4.979 | -0.451 | -0.406 | 0.023 | 0.211 | Unannotated | NA | NA |
| metab_1479 | Unannotated feature | pos | 820.5016 | 5.914 | 4.118 | 4.644 | 0.526 | 0.406 | 0.023 | 0.211 | Unannotated | NA | NA |
| metab_19759 | Unannotated feature | neg | 420.2111 | 4.493 | 4.998 | 4.428 | -0.570 | -0.402 | 0.024 | 0.216 | Unannotated | NA | NA |
| metab_2915 | Quinoline-4,8-diol | pos | 194.0816 | 2.884 | 3.793 | 4.296 | 0.503 | 0.402 | 0.024 | 0.216 | Tentative | NA | NA |
| metab_4190 | Unannotated feature | pos | 573.3503 | 5.914 | 3.645 | 5.270 | 1.625 | 0.402 | 0.024 | 0.216 | Unannotated | NA | NA |
| metab_1582 | Unannotated feature | pos | 492.3041 | 5.187 | 2.803 | 5.301 | 2.499 | 0.402 | 0.024 | 0.216 | Unannotated | NA | NA |
| metab_1478 | Unannotated feature | pos | 362.2546 | 5.922 | 4.612 | 4.186 | -0.426 | -0.402 | 0.024 | 0.216 | Unannotated | NA | NA |
| metab_5359 | Unannotated feature | pos | 429.3781 | 6.510 | 5.514 | 5.293 | -0.220 | -0.402 | 0.024 | 0.216 | Unannotated | NA | NA |
| metab_13811 | Unannotated feature | neg | 296.9103 | 0.453 | 4.797 | 4.732 | -0.065 | -0.402 | 0.024 | 0.216 | Unannotated | NA | NA |
| metab_17586 | Unannotated feature | neg | 737.2561 | 7.259 | 5.008 | 5.603 | 0.595 | 0.402 | 0.024 | 0.216 | Unannotated | NA | NA |
| metab_14263 | Phenylglyoxylic acid | neg | 195.0295 | 3.011 | 4.116 | 4.610 | 0.494 | 0.402 | 0.024 | 0.216 | Tentative | Benzenoids | Benzene and substituted derivatives |
| metab_17405 | Unannotated feature | neg | 761.8416 | 7.648 | 4.182 | 4.586 | 0.404 | 0.402 | 0.024 | 0.216 | Unannotated | NA | NA |
| metab_17623 | Unannotated feature | neg | 100.9256 | 7.148 | 3.926 | 3.801 | -0.125 | -0.402 | 0.024 | 0.216 | Unannotated | NA | NA |
| metab_13842 | Unannotated feature | neg | 306.9394 | 0.460 | 6.997 | 6.942 | -0.055 | -0.402 | 0.024 | 0.216 | Unannotated | NA | NA |
| metab_7190 | Unannotated feature | pos | 398.9067 | 0.477 | 4.720 | 5.006 | 0.286 | 0.402 | 0.024 | 0.216 | Unannotated | NA | NA |
| metab_10024 | Unannotated feature | neg | 302.1724 | 2.817 | 5.694 | 5.284 | -0.410 | -0.402 | 0.024 | 0.216 | Unannotated | NA | NA |
| metab_6252 | Unannotated feature | pos | 572.3571 | 5.806 | 3.958 | 4.826 | 0.868 | 0.402 | 0.024 | 0.216 | Unannotated | NA | NA |
| metab_16629 | Unannotated feature | neg | 447.9161 | 7.510 | 4.423 | 4.924 | 0.502 | 0.402 | 0.024 | 0.216 | Unannotated | NA | NA |
| metab_14619 | Unannotated feature | neg | 169.9911 | 3.544 | 3.436 | 2.652 | -0.784 | -0.402 | 0.024 | 0.216 | Unannotated | NA | NA |
| metab_10442 | Unannotated feature | neg | 387.1892 | 1.965 | 4.214 | 3.651 | -0.563 | -0.402 | 0.024 | 0.216 | Unannotated | NA | NA |
| metab_2023 | Unannotated feature | pos | 516.3147 | 5.159 | 4.218 | 5.038 | 0.821 | 0.402 | 0.024 | 0.216 | Unannotated | NA | NA |
| metab_6920 | Unannotated feature | pos | 261.1450 | 1.936 | 5.977 | 5.799 | -0.177 | -0.402 | 0.024 | 0.216 | Unannotated | NA | NA |
| metab_9928 | Unannotated feature | neg | 243.0509 | 2.928 | 4.492 | 4.991 | 0.499 | 0.402 | 0.024 | 0.216 | Unannotated | NA | NA |
| metab_7481 | Unannotated feature | pos | 277.1653 | 3.791 | 4.562 | 4.036 | -0.525 | -0.402 | 0.024 | 0.216 | Unannotated | NA | NA |
| metab_337 | Unannotated feature | pos | 187.0567 | 0.451 | 4.373 | 4.254 | -0.119 | -0.402 | 0.024 | 0.216 | Unannotated | NA | NA |
| metab_3730 | Unannotated feature | pos | 846.5879 | 6.566 | 5.674 | 5.090 | -0.584 | -0.402 | 0.024 | 0.216 | Unannotated | NA | NA |
| metab_5788 | Unannotated feature | pos | 345.2390 | 6.016 | 5.363 | 5.175 | -0.188 | -0.402 | 0.024 | 0.216 | Unannotated | NA | NA |
| metab_19575 | 17-beta-estradiol 3-sulfate-17-(beta-D-glucuronide) | neg | 263.0777 | 4.749 | 4.706 | 5.364 | 0.658 | 0.402 | 0.024 | 0.216 | Tentative | Lipids and lipid-like molecules | Steroids and steroid derivatives |
| metab_611 | Unannotated feature | pos | 602.9849 | 5.791 | 3.516 | 4.944 | 1.428 | 0.402 | 0.024 | 0.216 | Unannotated | NA | NA |
| metab_5921 | Unannotated feature | pos | 653.7323 | 5.922 | 0.070 | 4.127 | 4.057 | 0.402 | 0.024 | 0.216 | Unannotated | NA | NA |
| metab_12544 | Unannotated feature | neg | 369.1743 | 6.217 | 6.237 | 6.588 | 0.351 | 0.402 | 0.024 | 0.216 | Unannotated | NA | NA |
| metab_6079 | Unannotated feature | pos | 661.9079 | 5.862 | 2.001 | 4.870 | 2.868 | 0.402 | 0.024 | 0.216 | Unannotated | NA | NA |
| metab_13170 | Unannotated feature | neg | 147.9731 | 0.489 | 4.885 | 4.841 | -0.045 | -0.402 | 0.024 | 0.216 | Unannotated | NA | NA |
| metab_18361 | Unannotated feature | neg | 357.0620 | 6.162 | 4.760 | 4.426 | -0.334 | -0.402 | 0.024 | 0.216 | Unannotated | NA | NA |
| metab_13596 | [(2S)-6-Amino-1-methoxy-1-oxohexan-2-yl]carbamic acid | neg | 203.1032 | 1.397 | 5.642 | 5.188 | -0.454 | -0.402 | 0.024 | 0.216 | Unannotated | Organic acids and derivatives | Carboxylic acids and derivatives |
| metab_17361 | Unannotated feature | neg | 702.8793 | 7.684 | 4.745 | 4.909 | 0.164 | 0.402 | 0.024 | 0.216 | Unannotated | NA | NA |
| metab_5635 | Unannotated feature | pos | 159.0191 | 0.016 | 4.388 | 4.439 | 0.051 | 0.402 | 0.024 | 0.216 | Unannotated | NA | NA |
| metab_14325 | Phenyl(piperidin-4-yl)methanone | neg | 234.1134 | 3.082 | 2.682 | 2.301 | -0.381 | -0.402 | 0.024 | 0.216 | Unannotated | Organic oxygen compounds | Organooxygen compounds |
| metab_11606 | Unannotated feature | neg | 247.9115 | 0.433 | 6.215 | 6.189 | -0.026 | -0.402 | 0.024 | 0.216 | Unannotated | NA | NA |
| metab_12290 | Unannotated feature | neg | 555.4431 | 7.139 | 6.806 | 6.228 | -0.579 | -0.402 | 0.024 | 0.216 | Unannotated | NA | NA |
| metab_2593 | Homocitrulline | pos | 172.1082 | 1.474 | 3.468 | 4.116 | 0.648 | 0.402 | 0.024 | 0.216 | Tentative | Organic acids and derivatives | Carboxylic acids and derivatives |
| metab_2265 | Phenylalanyl-Alanine | pos | 237.1237 | 3.159 | 5.057 | 4.667 | -0.390 | -0.402 | 0.024 | 0.216 | Unannotated | NA | NA |
| metab_2731 | Unannotated feature | pos | 182.0294 | 2.213 | 4.096 | 4.190 | 0.094 | 0.402 | 0.024 | 0.216 | Unannotated | NA | NA |
| metab_6272 | Unannotated feature | pos | 630.5928 | 5.794 | 3.463 | 4.551 | 1.089 | 0.402 | 0.024 | 0.216 | Unannotated | NA | NA |
| metab_5172 | Unannotated feature | pos | 258.2704 | 6.829 | 4.645 | 4.502 | -0.142 | -0.402 | 0.024 | 0.216 | Unannotated | NA | NA |
| metab_2100 | Thr Ile | pos | 233.1500 | 2.780 | 6.300 | 5.920 | -0.380 | -0.402 | 0.024 | 0.216 | Strong putative | NA | NA |
| metab_11281 | Beta-Alanyl-L-lysine | neg | 216.1351 | 0.619 | 4.418 | 3.251 | -1.167 | -0.402 | 0.024 | 0.216 | Tentative | Organic acids and derivatives | Peptidomimetics |
| metab_11510 | Unannotated feature | neg | 136.9393 | 0.512 | 3.842 | 3.422 | -0.420 | -0.402 | 0.024 | 0.216 | Unannotated | NA | NA |
| metab_18929 | Unannotated feature | neg | 226.0273 | 5.614 | 3.418 | 3.046 | -0.372 | -0.402 | 0.024 | 0.216 | Unannotated | NA | NA |
| metab_7973 | Unannotated feature | pos | 129.1026 | 2.871 | 4.155 | 3.686 | -0.469 | -0.402 | 0.024 | 0.216 | Unannotated | NA | NA |
| metab_890 | LysoPC(15:0/0:0) | pos | 504.3073 | 6.965 | 5.157 | 4.522 | -0.635 | -0.402 | 0.024 | 0.216 | Tentative | Lipids and lipid-like molecules | Glycerophospholipids |
| metab_5374 | Unannotated feature | pos | 361.2455 | 1.952 | 5.059 | 4.433 | -0.626 | -0.402 | 0.024 | 0.216 | Unannotated | NA | NA |
| metab_13333 | N-Acetyl-b-glucosaminylamine | neg | 219.0983 | 0.691 | 5.182 | 4.811 | -0.370 | -0.398 | 0.026 | 0.221 | Tentative | NA | NA |
| metab_1298 | N-Palmitoyl Lysine | pos | 385.3442 | 6.247 | 6.016 | 5.609 | -0.407 | -0.398 | 0.026 | 0.221 | Unannotated | NA | NA |
| metab_1304 | Unannotated feature | pos | 167.0454 | 2.928 | 3.794 | 4.629 | 0.835 | 0.398 | 0.026 | 0.221 | Unannotated | NA | NA |
| metab_8113 | N-(1,5-dimethyl-3-oxo-2-phenylpyrazol-4-yl)-1,3-dimethylpyrazole-4-carboxamide | pos | 326.1608 | 2.732 | 4.221 | 4.977 | 0.756 | 0.398 | 0.026 | 0.221 | Moderate putative | NA | NA |
| metab_10663 | Unannotated feature | neg | 129.1026 | 1.594 | 4.468 | 3.732 | -0.736 | -0.398 | 0.026 | 0.221 | Unannotated | NA | NA |
| metab_9030 | Unannotated feature | neg | 345.1091 | 4.031 | 5.574 | 5.384 | -0.190 | -0.398 | 0.026 | 0.221 | Unannotated | NA | NA |
| metab_19520 | Dodec-2-enedioylcarnitine | neg | 406.1992 | 4.825 | 5.217 | 4.894 | -0.323 | -0.398 | 0.026 | 0.221 | Unannotated | Lipids and lipid-like molecules | Fatty Acyls |
| metab_504 | Unannotated feature | pos | 674.8892 | 5.102 | 4.174 | 6.021 | 1.846 | 0.398 | 0.026 | 0.221 | Unannotated | NA | NA |
| metab_14612 | DIPROTIN B | neg | 326.2089 | 3.529 | 5.330 | 4.664 | -0.667 | -0.398 | 0.026 | 0.221 | Unannotated | NA | NA |
| metab_1602 | Unannotated feature | pos | 288.1923 | 3.694 | 6.179 | 5.855 | -0.324 | -0.398 | 0.026 | 0.221 | Unannotated | NA | NA |
| metab_2563 | Unannotated feature | pos | 661.2182 | 5.806 | 0.754 | 4.579 | 3.824 | 0.398 | 0.026 | 0.221 | Unannotated | NA | NA |
| metab_5801 | Methadone | pos | 292.2063 | 6.008 | 4.572 | 4.405 | -0.167 | -0.398 | 0.026 | 0.221 | Moderate putative | NA | NA |
| metab_14121 | Allantoic acid | neg | 211.0245 | 2.852 | 5.585 | 5.987 | 0.402 | 0.398 | 0.026 | 0.221 | Tentative | Organic acids and derivatives | Carboxylic acids and derivatives |
| metab_1946 | Unannotated feature | pos | 516.3155 | 4.975 | 3.441 | 4.134 | 0.693 | 0.398 | 0.026 | 0.221 | Unannotated | NA | NA |
| metab_645 | Unannotated feature | pos | 652.6110 | 5.811 | 0.716 | 4.474 | 3.757 | 0.398 | 0.026 | 0.221 | Unannotated | NA | NA |
| metab_19451 | Unannotated feature | neg | 348.1932 | 4.936 | 4.473 | 4.207 | -0.266 | -0.398 | 0.026 | 0.221 | Unannotated | NA | NA |
| metab_14025 | Unannotated feature | neg | 554.1422 | 2.762 | 6.132 | 5.530 | -0.602 | -0.398 | 0.026 | 0.221 | Unannotated | NA | NA |
| metab_8690 | Fructosyl valine | pos | 280.1395 | 0.784 | 4.657 | 5.071 | 0.414 | 0.398 | 0.026 | 0.221 | Unannotated | Organic acids and derivatives | Carboxylic acids and derivatives |
| metab_8648 | Unannotated feature | pos | 115.0395 | 1.026 | 4.522 | 4.428 | -0.094 | -0.398 | 0.026 | 0.221 | Unannotated | NA | NA |
| metab_2126 | Unannotated feature | pos | 371.1927 | 2.835 | 3.990 | 4.592 | 0.602 | 0.398 | 0.026 | 0.221 | Unannotated | NA | NA |
| metab_10378 | CAY10577 | neg | 255.9580 | 2.137 | 5.665 | 5.390 | -0.275 | -0.398 | 0.026 | 0.221 | Moderate putative | NA | NA |
| metab_2841 | O-6-deoxy-a-L-galactopyranosyl-(1->2)-O-b-D-galactopyranosyl-(1->3)-2-(acetylamino)-1,5-anhydro-2-deoxy-D-arabino-Hex-1-enitol | pos | 556.1573 | 2.747 | 5.455 | 4.617 | -0.837 | -0.398 | 0.026 | 0.221 | Unannotated | Organic oxygen compounds | Organooxygen compounds |
| metab_6207 | Unannotated feature | pos | 609.2133 | 5.814 | 3.564 | 4.267 | 0.703 | 0.398 | 0.026 | 0.221 | Unannotated | NA | NA |
| metab_18765 | Radalbuvir | neg | 542.2615 | 5.825 | 4.637 | 4.856 | 0.219 | 0.398 | 0.026 | 0.221 | Unannotated | NA | NA |
| metab_2266 | Unannotated feature | pos | 86.0969 | 0.493 | 6.311 | 5.626 | -0.685 | -0.398 | 0.026 | 0.221 | Unannotated | NA | NA |
| metab_11062 | D-Galactopyranosyl-(1->3)-D-galactopyranosyl-(1->3)-L-arabinose | neg | 519.1578 | 0.730 | 4.753 | 5.199 | 0.446 | 0.398 | 0.026 | 0.221 | Unannotated | NA | NA |
| metab_4024 | Unannotated feature | pos | 637.5739 | 5.814 | 1.270 | 4.293 | 3.024 | 0.398 | 0.026 | 0.221 | Unannotated | NA | NA |
| metab_12375 | 3-(2,5-dimethoxyphenyl)propanoic acid | neg | 209.0815 | 4.600 | 5.180 | 4.931 | -0.248 | -0.398 | 0.026 | 0.221 | Unannotated | NA | NA |
| metab_16668 | Unannotated feature | neg | 949.8314 | 7.607 | 5.073 | 5.407 | 0.334 | 0.398 | 0.026 | 0.221 | Unannotated | NA | NA |
| metab_7311 | Gluten exorphin C | pos | 632.3874 | 4.166 | 3.736 | 4.988 | 1.253 | 0.398 | 0.026 | 0.221 | Unannotated | Organic acids and derivatives | Carboxylic acids and derivatives |
| metab_7042 | Unannotated feature | pos | 143.0342 | 4.774 | 4.564 | 4.419 | -0.145 | -0.398 | 0.026 | 0.221 | Unannotated | NA | NA |
| metab_15749 | Unannotated feature | neg | 126.9043 | 5.767 | 4.187 | 3.932 | -0.254 | -0.398 | 0.026 | 0.221 | Unannotated | NA | NA |
| metab_3402 | Unannotated feature | pos | 355.2211 | 4.417 | 4.668 | 5.048 | 0.381 | 0.398 | 0.026 | 0.221 | Unannotated | NA | NA |
| metab_14748 | Unannotated feature | neg | 406.1964 | 3.780 | 5.418 | 5.132 | -0.286 | -0.398 | 0.026 | 0.221 | Unannotated | NA | NA |
| metab_14677 | Olopatadine | neg | 318.1493 | 3.647 | 4.561 | 4.055 | -0.506 | -0.398 | 0.026 | 0.221 | Tentative | Organoheterocyclic compounds | Benzoxepines |
| metab_11980 | Unannotated feature | neg | 510.2540 | 6.201 | 5.160 | 5.499 | 0.339 | 0.398 | 0.026 | 0.221 | Unannotated | NA | NA |
| metab_13793 | Unannotated feature | neg | 215.1398 | 2.004 | 5.746 | 5.438 | -0.308 | -0.398 | 0.026 | 0.221 | Unannotated | NA | NA |
| metab_3626 | Unannotated feature | pos | 470.6127 | 5.128 | 4.222 | 5.137 | 0.914 | 0.398 | 0.026 | 0.221 | Unannotated | NA | NA |
| metab_1400 | Unannotated feature | pos | 530.3605 | 6.080 | 4.720 | 4.170 | -0.551 | -0.398 | 0.026 | 0.221 | Unannotated | NA | NA |
| metab_2962 | ROSIN | pos | 314.1604 | 2.984 | 2.963 | 4.081 | 1.117 | 0.398 | 0.026 | 0.221 | Unannotated | Lipids and lipid-like molecules | Fatty Acyls |
| metab_10577 | Unannotated feature | neg | 357.1164 | 3.298 | 4.947 | 5.633 | 0.686 | 0.398 | 0.026 | 0.221 | Unannotated | NA | NA |
| metab_5617 | 17-Epimethyltestosterone | pos | 344.2591 | 6.207 | 4.989 | 4.042 | -0.947 | -0.398 | 0.026 | 0.221 | Tentative | Lipids and lipid-like molecules | Steroids and steroid derivatives |
| metab_7672 | Methohexital | pos | 295.1658 | 3.426 | 5.977 | 5.724 | -0.252 | -0.398 | 0.026 | 0.221 | Tentative | NA | NA |
| metab_2159 | 6-Hydroxy-5-methoxy-1h-indole-2-carboxylic acid | pos | 208.0608 | 2.907 | 5.515 | 5.850 | 0.335 | 0.398 | 0.026 | 0.221 | Unannotated | NA | NA |
| metab_5410 | Unannotated feature | pos | 348.1775 | 1.952 | 5.652 | 5.365 | -0.287 | -0.398 | 0.026 | 0.221 | Unannotated | NA | NA |
| metab_811 | Unannotated feature | pos | 256.9829 | 0.585 | 4.392 | 3.597 | -0.795 | -0.398 | 0.026 | 0.221 | Unannotated | NA | NA |
| metab_14999 | Unannotated feature | neg | 344.2195 | 4.328 | 5.026 | 4.192 | -0.834 | -0.398 | 0.026 | 0.221 | Unannotated | NA | NA |
| metab_6356 | Unannotated feature | pos | 699.1084 | 5.757 | 3.882 | 4.732 | 0.850 | 0.398 | 0.026 | 0.221 | Unannotated | NA | NA |
| metab_11562 | Unannotated feature | neg | 379.9093 | 0.467 | 4.891 | 4.568 | -0.324 | -0.398 | 0.026 | 0.221 | Unannotated | NA | NA |
| metab_16155 | Unannotated feature | neg | 314.0734 | 1.245 | 4.259 | 4.769 | 0.510 | 0.398 | 0.026 | 0.221 | Unannotated | NA | NA |
| metab_6244 | Unannotated feature | pos | 664.8229 | 5.806 | 3.361 | 4.733 | 1.372 | 0.398 | 0.026 | 0.221 | Unannotated | NA | NA |
| metab_11067 | 5-[(3S)-1,2-Dithiolan-3-yl]pentanoylcarnitine | neg | 394.1362 | 0.730 | 3.646 | 4.452 | 0.806 | 0.394 | 0.028 | 0.228 | Unannotated | Lipids and lipid-like molecules | Fatty Acyls |
| metab_13711 | Unannotated feature | neg | 324.0573 | 1.850 | 4.663 | 4.308 | -0.355 | -0.394 | 0.028 | 0.228 | Unannotated | NA | NA |
| metab_8250 | Unannotated feature | pos | 408.2139 | 3.678 | 4.971 | 4.455 | -0.516 | -0.394 | 0.028 | 0.228 | Unannotated | NA | NA |
| metab_1356 | Unannotated feature | pos | 100.0762 | 2.590 | 7.231 | 6.284 | -0.947 | -0.394 | 0.028 | 0.228 | Unannotated | NA | NA |
| metab_11788 | Unannotated feature | neg | 337.1087 | 5.803 | 4.613 | 3.865 | -0.748 | -0.394 | 0.028 | 0.228 | Unannotated | NA | NA |
| metab_16814 | Unannotated feature | neg | 272.0714 | 7.720 | 2.942 | 3.554 | 0.612 | 0.394 | 0.028 | 0.228 | Unannotated | NA | NA |
| metab_10652 | Unannotated feature | neg | 280.1152 | 1.647 | 5.011 | 3.888 | -1.124 | -0.394 | 0.028 | 0.228 | Unannotated | NA | NA |
| metab_7867 | Gly Val Val | pos | 274.1765 | 3.021 | 5.318 | 4.963 | -0.355 | -0.394 | 0.028 | 0.228 | Moderate putative | NA | NA |
| metab_4225 | Unannotated feature | pos | 651.2054 | 5.922 | 0.847 | 4.425 | 3.578 | 0.394 | 0.028 | 0.228 | Unannotated | NA | NA |
| metab_3200 | Unannotated feature | pos | 256.6635 | 3.725 | 4.361 | 3.717 | -0.643 | -0.394 | 0.028 | 0.228 | Unannotated | NA | NA |
| metab_8571 | Unannotated feature | pos | 375.2246 | 1.211 | 4.969 | 4.276 | -0.694 | -0.394 | 0.028 | 0.228 | Unannotated | NA | NA |
| metab_9423 | Unannotated feature | neg | 293.1510 | 3.439 | 5.551 | 5.080 | -0.471 | -0.394 | 0.028 | 0.228 | Unannotated | NA | NA |
| metab_14202 | Kaempferol 3-rhamnoside 4'-xyloside | neg | 545.1306 | 2.942 | 6.062 | 5.546 | -0.516 | -0.394 | 0.028 | 0.228 | Unannotated | Phenylpropanoids and polyketides | Flavonoids |
| metab_1437 | Unannotated feature | pos | 383.2021 | 6.024 | 5.099 | 4.158 | -0.941 | -0.394 | 0.028 | 0.228 | Unannotated | NA | NA |
| metab_8495 | Vomifoliol | pos | 247.1293 | 1.685 | 6.211 | 6.112 | -0.098 | -0.394 | 0.028 | 0.228 | Tentative | NA | NA |
| metab_3565 | Unannotated feature | pos | 642.9022 | 4.988 | 2.145 | 4.427 | 2.282 | 0.394 | 0.028 | 0.228 | Unannotated | NA | NA |
| metab_2732 | Unannotated feature | pos | 136.0808 | 2.217 | 2.624 | 3.018 | 0.394 | 0.394 | 0.028 | 0.228 | Unannotated | NA | NA |
| metab_1472 | Unannotated feature | pos | 550.0035 | 5.929 | 3.741 | 4.137 | 0.397 | 0.394 | 0.028 | 0.228 | Unannotated | NA | NA |
| metab_1323 | 7-Dehydrodesmosterol | pos | 383.3279 | 6.207 | 6.259 | 5.828 | -0.432 | -0.394 | 0.028 | 0.228 | Tentative | Lipids and lipid-like molecules | Steroids and steroid derivatives |
| metab_9348 | Unannotated feature | neg | 350.1728 | 3.548 | 4.612 | 4.072 | -0.540 | -0.394 | 0.028 | 0.228 | Unannotated | NA | NA |
| metab_15061 | Unannotated feature | neg | 344.2194 | 4.454 | 5.233 | 4.777 | -0.456 | -0.394 | 0.028 | 0.228 | Unannotated | NA | NA |
| metab_6233 | Unannotated feature | pos | 624.7854 | 5.811 | 3.915 | 4.129 | 0.214 | 0.394 | 0.028 | 0.228 | Unannotated | NA | NA |
| metab_12709 | Unannotated feature | neg | 599.2912 | 5.264 | 4.114 | 4.722 | 0.608 | 0.394 | 0.028 | 0.228 | Unannotated | NA | NA |
| metab_7643 | His Leu Leu | pos | 382.2460 | 3.470 | 5.416 | 4.922 | -0.494 | -0.394 | 0.028 | 0.228 | Strong putative | NA | NA |
| metab_544 | Unannotated feature | pos | 364.2240 | 5.532 | 4.927 | 4.327 | -0.600 | -0.394 | 0.028 | 0.228 | Unannotated | NA | NA |
| metab_6155 | Unannotated feature | pos | 567.3478 | 5.835 | 3.838 | 4.211 | 0.372 | 0.394 | 0.028 | 0.228 | Unannotated | NA | NA |
| metab_6253 | CDP-DG(a-21:0/i-24:0) | pos | 568.8609 | 5.806 | 3.843 | 4.410 | 0.567 | 0.394 | 0.028 | 0.228 | Tentative | Lipids and lipid-like molecules | Glycerophospholipids |
| metab_665 | Unannotated feature | pos | 665.3083 | 5.870 | 4.029 | 4.423 | 0.394 | 0.394 | 0.028 | 0.228 | Unannotated | NA | NA |
| metab_12204 | Pollenin A | neg | 301.0355 | 6.007 | 4.420 | 5.452 | 1.033 | 0.394 | 0.028 | 0.228 | Tentative | Phenylpropanoids and polyketides | Flavonoids |
| metab_5629 | Unannotated feature | pos | 157.9742 | 0.016 | 5.189 | 5.228 | 0.039 | 0.394 | 0.028 | 0.228 | Unannotated | NA | NA |
| metab_7597 | Leu Thr Val | pos | 332.2188 | 3.553 | 5.002 | 4.296 | -0.706 | -0.394 | 0.028 | 0.228 | Unannotated | NA | NA |
| metab_1382 | Unannotated feature | pos | 362.3272 | 6.111 | 5.950 | 5.857 | -0.093 | -0.394 | 0.028 | 0.228 | Unannotated | NA | NA |
| metab_8810 | Gallocatechin | pos | 307.0817 | 2.819 | 4.532 | 3.061 | -1.471 | -0.394 | 0.028 | 0.228 | Strong putative | Phenylpropanoids and polyketides | Flavonoids |
| metab_2134 | 5-Methyl-2-furaldehyde | pos | 111.0445 | 2.853 | 4.469 | 4.705 | 0.236 | 0.394 | 0.028 | 0.228 | Moderate putative | NA | NA |
| metab_17015 | Unannotated feature | neg | 199.0218 | 2.155 | 5.262 | 4.962 | -0.300 | -0.394 | 0.028 | 0.228 | Unannotated | NA | NA |
| metab_12404 | Unannotated feature | neg | 482.2535 | 7.697 | 4.349 | 3.990 | -0.359 | -0.394 | 0.028 | 0.228 | Unannotated | NA | NA |
| metab_12485 | Unannotated feature | neg | 315.1604 | 6.404 | 3.571 | 3.710 | 0.139 | 0.394 | 0.028 | 0.228 | Unannotated | NA | NA |
| metab_17028 | Unannotated feature | neg | 168.9454 | 7.997 | 3.241 | 3.046 | -0.194 | -0.394 | 0.028 | 0.228 | Unannotated | NA | NA |
| metab_18988 | Desmethyl Erlotinib | neg | 414.1236 | 5.542 | 4.477 | 3.300 | -1.177 | -0.394 | 0.028 | 0.228 | Unannotated | NA | NA |
| metab_1732 | Unannotated feature | pos | 346.1982 | 3.781 | 5.704 | 5.369 | -0.334 | -0.394 | 0.028 | 0.228 | Unannotated | NA | NA |
| metab_5765 | Unannotated feature | pos | 359.2050 | 6.040 | 5.230 | 4.949 | -0.281 | -0.394 | 0.028 | 0.228 | Unannotated | NA | NA |
| metab_738 | Unannotated feature | pos | 359.2683 | 6.032 | 4.954 | 4.551 | -0.404 | -0.389 | 0.029 | 0.234 | Unannotated | NA | NA |
| metab_13648 | Unannotated feature | neg | 305.9268 | 0.489 | 6.356 | 6.429 | 0.073 | 0.389 | 0.029 | 0.234 | Unannotated | NA | NA |
| metab_1630 | Unannotated feature | pos | 584.3589 | 4.886 | 5.167 | 5.952 | 0.785 | 0.389 | 0.029 | 0.234 | Unannotated | NA | NA |
| metab_6720 | Unannotated feature | pos | 530.6554 | 5.275 | 2.993 | 4.731 | 1.739 | 0.389 | 0.029 | 0.234 | Unannotated | NA | NA |
| metab_11914 | Unannotated feature | neg | 375.2654 | 6.076 | 5.071 | 4.527 | -0.544 | -0.389 | 0.029 | 0.234 | Unannotated | NA | NA |
| metab_2476 | Unannotated feature | pos | 598.0321 | 5.481 | 3.867 | 4.724 | 0.857 | 0.389 | 0.029 | 0.234 | Unannotated | NA | NA |
| metab_656 | Unannotated feature | pos | 657.7383 | 5.851 | 1.192 | 4.319 | 3.127 | 0.389 | 0.029 | 0.234 | Unannotated | NA | NA |
| metab_359 | Unannotated feature | pos | 179.0005 | 0.368 | 4.267 | 4.305 | 0.038 | 0.389 | 0.029 | 0.234 | Unannotated | NA | NA |
| metab_14863 | Unannotated feature | neg | 320.1618 | 4.031 | 4.853 | 4.415 | -0.438 | -0.389 | 0.029 | 0.234 | Unannotated | NA | NA |
| metab_8543 | Unannotated feature | pos | 142.0978 | 1.321 | 4.402 | 2.685 | -1.717 | -0.389 | 0.029 | 0.234 | Unannotated | NA | NA |
| metab_11593 | Unannotated feature | neg | 374.9274 | 0.449 | 3.658 | 3.440 | -0.218 | -0.389 | 0.029 | 0.234 | Unannotated | NA | NA |
| metab_9756 | Leucyl-Tryptophan | neg | 354.1199 | 3.078 | 4.180 | 4.933 | 0.753 | 0.389 | 0.029 | 0.234 | Unannotated | Organic acids and derivatives | Carboxylic acids and derivatives |
| metab_10940 | Glutamylvaline | neg | 245.1143 | 0.799 | 5.608 | 5.340 | -0.268 | -0.389 | 0.029 | 0.234 | Unannotated | Organic acids and derivatives | Carboxylic acids and derivatives |
| metab_10311 | Unannotated feature | neg | 215.1398 | 2.339 | 6.061 | 5.747 | -0.314 | -0.389 | 0.029 | 0.234 | Unannotated | NA | NA |
| metab_14096 | Unannotated feature | neg | 438.1527 | 2.817 | 4.380 | 3.746 | -0.634 | -0.389 | 0.029 | 0.234 | Unannotated | NA | NA |
| metab_16707 | Unannotated feature | neg | 945.3194 | 7.637 | 4.986 | 5.230 | 0.244 | 0.389 | 0.029 | 0.234 | Unannotated | NA | NA |
| metab_18752 | Unannotated feature | neg | 234.1133 | 5.847 | 4.792 | 4.465 | -0.327 | -0.389 | 0.029 | 0.234 | Unannotated | NA | NA |
| metab_5856 | Unannotated feature | pos | 177.0050 | 0.016 | 4.349 | 4.392 | 0.044 | 0.389 | 0.029 | 0.234 | Unannotated | NA | NA |
| metab_9347 | Unannotated feature | neg | 330.2039 | 3.552 | 5.132 | 4.525 | -0.607 | -0.389 | 0.029 | 0.234 | Unannotated | NA | NA |
| metab_700 | Unannotated feature | pos | 672.5134 | 5.922 | 3.901 | 4.891 | 0.990 | 0.389 | 0.029 | 0.234 | Unannotated | NA | NA |
| metab_18506 | MG(14:1(9Z)/0:0/0:0) | neg | 345.2285 | 6.046 | 6.519 | 6.310 | -0.210 | -0.389 | 0.029 | 0.234 | Unannotated | Lipids and lipid-like molecules | Glycerolipids |
| metab_7283 | Licoricone | pos | 347.1246 | 4.232 | 5.712 | 5.482 | -0.230 | -0.389 | 0.029 | 0.234 | Tentative | NA | NA |
| metab_15498 | Unannotated feature | neg | 127.0758 | 5.272 | 4.388 | 4.338 | -0.050 | -0.389 | 0.029 | 0.234 | Unannotated | NA | NA |
| metab_1275 | Unannotated feature | pos | 297.2040 | 6.303 | 5.262 | 5.166 | -0.097 | -0.389 | 0.029 | 0.234 | Unannotated | NA | NA |
| metab_9161 | Unannotated feature | neg | 340.2245 | 3.834 | 4.623 | 3.507 | -1.116 | -0.389 | 0.029 | 0.234 | Unannotated | NA | NA |
| metab_16663 | Unannotated feature | neg | 719.8612 | 7.607 | 5.761 | 5.929 | 0.168 | 0.389 | 0.029 | 0.234 | Unannotated | NA | NA |
| metab_1926 | Glutamylglutamic acid | pos | 277.1035 | 0.728 | 5.291 | 5.084 | -0.207 | -0.389 | 0.029 | 0.234 | Tentative | NA | NA |
| metab_6238 | Unannotated feature | pos | 587.0333 | 5.811 | 3.961 | 4.115 | 0.155 | 0.389 | 0.029 | 0.234 | Unannotated | NA | NA |
| metab_10382 | Unannotated feature | neg | 131.0343 | 2.137 | 6.778 | 6.517 | -0.261 | -0.389 | 0.029 | 0.234 | Unannotated | NA | NA |
| metab_13345 | N-(Hydroxymethyl)acrylamide | neg | 247.0935 | 0.714 | 5.730 | 5.427 | -0.303 | -0.389 | 0.029 | 0.234 | Tentative | Organic acids and derivatives | Carboxylic acids and derivatives |
| metab_13372 | N-Acetylmuramoyl-Ala | neg | 399.1153 | 0.730 | 5.030 | 5.803 | 0.773 | 0.389 | 0.029 | 0.234 | Tentative | Organic oxygen compounds | Organooxygen compounds |
| metab_1517 | Unannotated feature | pos | 617.3675 | 5.451 | 3.706 | 4.830 | 1.124 | 0.389 | 0.029 | 0.234 | Unannotated | NA | NA |
| metab_11121 | Unannotated feature | neg | 286.1669 | 5.017 | 4.506 | 4.052 | -0.454 | -0.389 | 0.029 | 0.234 | Unannotated | NA | NA |
| metab_6147 | Unannotated feature | pos | 659.5034 | 5.835 | 3.052 | 4.378 | 1.326 | 0.389 | 0.029 | 0.234 | Unannotated | NA | NA |
| metab_8797 | Beta-Zearalenol | pos | 362.1932 | 2.770 | 5.689 | 5.374 | -0.315 | -0.389 | 0.029 | 0.234 | Tentative | NA | NA |
| metab_14525 | Leucyl-leucine | neg | 243.1714 | 3.387 | 5.396 | 5.049 | -0.347 | -0.389 | 0.029 | 0.234 | Tentative | Organic acids and derivatives | Carboxylic acids and derivatives |
| metab_4794 | Aminoacetone | pos | 74.0606 | 7.980 | 4.549 | 4.465 | -0.084 | -0.389 | 0.029 | 0.234 | Tentative | NA | NA |
| metab_6195 | Unannotated feature | pos | 335.6497 | 5.820 | 5.359 | 4.221 | -1.138 | -0.389 | 0.029 | 0.234 | Unannotated | NA | NA |
| metab_18273 | Unannotated feature | neg | 415.2167 | 6.232 | 6.081 | 6.377 | 0.296 | 0.389 | 0.029 | 0.234 | Unannotated | NA | NA |
| metab_17663 | Unannotated feature | neg | 515.4113 | 7.057 | 4.724 | 4.048 | -0.676 | -0.389 | 0.029 | 0.234 | Unannotated | NA | NA |
| metab_2278 | Unannotated feature | pos | 137.9712 | 0.446 | 3.697 | 3.919 | 0.221 | 0.389 | 0.029 | 0.234 | Unannotated | NA | NA |
| metab_2620 | Unannotated feature | pos | 705.7669 | 5.898 | 1.269 | 4.625 | 3.356 | 0.389 | 0.029 | 0.234 | Unannotated | NA | NA |
| metab_6853 | Unannotated feature | pos | 514.9734 | 5.095 | 4.417 | 5.114 | 0.697 | 0.389 | 0.029 | 0.234 | Unannotated | NA | NA |
| metab_5929 | Levobunolol | pos | 621.3301 | 5.922 | 5.555 | 3.703 | -1.852 | -0.389 | 0.029 | 0.234 | Tentative | Benzenoids | Tetralins |
| metab_8405 | Unannotated feature | pos | 194.6317 | 1.952 | 3.638 | 3.026 | -0.612 | -0.389 | 0.029 | 0.234 | Unannotated | NA | NA |
| metab_16278 | Unannotated feature | neg | 236.9519 | 1.438 | 4.820 | 4.357 | -0.463 | -0.389 | 0.029 | 0.234 | Unannotated | NA | NA |
| metab_7756 | Goshonoside F4 | pos | 316.1873 | 3.287 | 5.135 | 4.900 | -0.235 | -0.389 | 0.029 | 0.234 | Unannotated | Lipids and lipid-like molecules | Prenol lipids |
| metab_6065 | Unannotated feature | pos | 746.4605 | 5.870 | 2.841 | 4.898 | 2.057 | 0.389 | 0.029 | 0.234 | Unannotated | NA | NA |
| metab_11489 | Unannotated feature | neg | 638.8272 | 0.516 | 5.246 | 5.566 | 0.320 | 0.389 | 0.029 | 0.234 | Unannotated | NA | NA |
| metab_1785 | Unannotated feature | pos | 374.2408 | 0.768 | 4.968 | 3.920 | -1.048 | -0.389 | 0.029 | 0.234 | Unannotated | NA | NA |
| metab_15027 | Trimethaphan | neg | 400.1370 | 4.390 | 4.261 | 2.988 | -1.273 | -0.389 | 0.029 | 0.234 | Tentative | Organoheterocyclic compounds | Thienoimidazolidines |
| metab_8428 | Dansylcadaverine | pos | 318.1666 | 1.944 | 5.320 | 5.077 | -0.243 | -0.389 | 0.029 | 0.234 | Unannotated | NA | NA |
| metab_12971 | Unannotated feature | neg | 175.0606 | 0.784 | 5.120 | 5.307 | 0.187 | 0.389 | 0.029 | 0.234 | Unannotated | NA | NA |
| metab_2982 | Asn Phe | pos | 280.1297 | 3.052 | 5.040 | 4.726 | -0.314 | -0.385 | 0.031 | 0.239 | Strong putative | NA | NA |
| metab_8787 | 3-Hydroxybutyric acid | pos | 146.0815 | 0.641 | 5.097 | 5.294 | 0.197 | 0.385 | 0.031 | 0.239 | Tentative | NA | NA |
| metab_2018 | Unannotated feature | pos | 170.0815 | 2.422 | 4.009 | 4.152 | 0.143 | 0.385 | 0.031 | 0.239 | Unannotated | NA | NA |
| metab_16229 | 9,10-Epoxystearic acid | neg | 343.2491 | 6.475 | 4.115 | 4.618 | 0.503 | 0.385 | 0.031 | 0.239 | Tentative | NA | NA |
| metab_5927 | Unannotated feature | pos | 634.3893 | 5.922 | 1.654 | 5.259 | 3.605 | 0.385 | 0.031 | 0.239 | Unannotated | NA | NA |
| metab_4109 | Unannotated feature | pos | 318.3008 | 6.111 | 6.411 | 6.330 | -0.081 | -0.385 | 0.031 | 0.239 | Unannotated | NA | NA |
| metab_3377 | Ile Phe | pos | 279.1705 | 4.332 | 5.763 | 5.311 | -0.451 | -0.385 | 0.031 | 0.239 | Moderate putative | NA | NA |
| metab_12758 | Unannotated feature | neg | 329.2199 | 1.947 | 4.341 | 3.772 | -0.569 | -0.385 | 0.031 | 0.239 | Unannotated | NA | NA |
| metab_5841 | Unannotated feature | pos | 267.1384 | 5.969 | 4.415 | 3.500 | -0.914 | -0.385 | 0.031 | 0.239 | Unannotated | NA | NA |
| metab_9257 | Unannotated feature | neg | 404.1358 | 3.952 | 4.577 | 3.999 | -0.578 | -0.385 | 0.031 | 0.239 | Unannotated | NA | NA |
| metab_2363 | Imiprothrin | pos | 319.1658 | 5.732 | 6.830 | 6.591 | -0.238 | -0.385 | 0.031 | 0.239 | Unannotated | NA | NA |
| metab_7342 | Unannotated feature | pos | 132.0629 | 4.088 | 3.114 | 3.291 | 0.178 | 0.385 | 0.031 | 0.239 | Unannotated | NA | NA |
| metab_7635 | Unannotated feature | pos | 302.2079 | 3.481 | 4.535 | 3.493 | -1.042 | -0.385 | 0.031 | 0.239 | Unannotated | NA | NA |
| metab_12970 | Unannotated feature | neg | 457.1210 | 0.777 | 5.334 | 5.735 | 0.400 | 0.385 | 0.031 | 0.239 | Unannotated | NA | NA |
| metab_1611 | PE(20:4(8Z,11Z,14Z,17Z)-2OH(5S,6R)/DiMe(13,5)) | pos | 458.7893 | 4.971 | 4.588 | 5.207 | 0.619 | 0.385 | 0.031 | 0.239 | Unannotated | NA | NA |
| metab_592 | Unannotated feature | pos | 558.9587 | 5.764 | 3.810 | 4.583 | 0.773 | 0.385 | 0.031 | 0.239 | Unannotated | NA | NA |
| metab_12967 | Unannotated feature | neg | 374.1094 | 0.777 | 3.844 | 4.342 | 0.498 | 0.385 | 0.031 | 0.239 | Unannotated | NA | NA |
| metab_7290 | Gln Ile Ile | pos | 373.2456 | 4.223 | 4.946 | 4.473 | -0.473 | -0.385 | 0.031 | 0.239 | Strong putative | NA | NA |
| metab_16630 | Unannotated feature | neg | 459.8849 | 7.510 | 4.006 | 4.896 | 0.890 | 0.385 | 0.031 | 0.239 | Unannotated | NA | NA |
| metab_13130 | Unannotated feature | neg | 339.9072 | 0.449 | 4.356 | 4.483 | 0.127 | 0.385 | 0.031 | 0.239 | Unannotated | NA | NA |
| metab_14904 | Unannotated feature | neg | 522.8524 | 0.496 | 4.305 | 4.717 | 0.411 | 0.385 | 0.031 | 0.239 | Unannotated | NA | NA |
| metab_7573 | Glu Ile Val | pos | 360.2139 | 3.607 | 5.614 | 5.033 | -0.581 | -0.385 | 0.031 | 0.239 | Moderate putative | NA | NA |
| metab_6451 | CDP-DG(i-24:0/i-20:0) | pos | 561.8593 | 5.650 | 3.870 | 4.554 | 0.685 | 0.385 | 0.031 | 0.239 | Tentative | Lipids and lipid-like molecules | Glycerophospholipids |
| metab_2003 | Unannotated feature | pos | 173.1651 | 2.268 | 5.599 | 4.932 | -0.666 | -0.385 | 0.031 | 0.239 | Unannotated | NA | NA |
| metab_12557 | Unannotated feature | neg | 511.2382 | 6.162 | 4.718 | 5.095 | 0.377 | 0.385 | 0.031 | 0.239 | Unannotated | NA | NA |
| metab_632 | Unannotated feature | pos | 623.0629 | 5.814 | 4.248 | 4.717 | 0.469 | 0.385 | 0.031 | 0.239 | Unannotated | NA | NA |
| metab_3160 | Unannotated feature | pos | 373.2820 | 3.618 | 5.108 | 4.432 | -0.676 | -0.385 | 0.031 | 0.239 | Unannotated | NA | NA |
| metab_16718 | Unannotated feature | neg | 547.1213 | 1.947 | 4.443 | 4.200 | -0.243 | -0.385 | 0.031 | 0.239 | Unannotated | NA | NA |
| metab_10854 | 5-Hydroxy-p-mentha-6,8-dien-2-one | neg | 165.0915 | 4.841 | 4.570 | 3.177 | -1.393 | -0.385 | 0.031 | 0.239 | Unannotated | NA | NA |
| metab_14369 | Unannotated feature | neg | 332.1831 | 3.144 | 4.605 | 3.903 | -0.701 | -0.385 | 0.031 | 0.239 | Unannotated | NA | NA |
| metab_18631 | (+/-)-2,6-Dimethyl-5-heptenal | neg | 139.1121 | 5.958 | 4.327 | 3.849 | -0.478 | -0.385 | 0.031 | 0.239 | Unannotated | Organic oxygen compounds | Organooxygen compounds |
| metab_12836 | Unannotated feature | neg | 203.0669 | 0.675 | 5.653 | 5.479 | -0.174 | -0.385 | 0.031 | 0.239 | Unannotated | NA | NA |
| metab_4035 | Unannotated feature | pos | 643.0651 | 5.820 | 2.615 | 4.473 | 1.858 | 0.385 | 0.031 | 0.239 | Unannotated | NA | NA |
| metab_4006 | Unannotated feature | pos | 605.8792 | 5.811 | 1.836 | 4.557 | 2.721 | 0.385 | 0.031 | 0.239 | Unannotated | NA | NA |
| metab_5167 | Unannotated feature | pos | 537.5368 | 6.837 | 5.059 | 4.773 | -0.286 | -0.385 | 0.031 | 0.239 | Unannotated | NA | NA |
| metab_16698 | Unannotated feature | neg | 842.8325 | 7.633 | 4.729 | 5.036 | 0.308 | 0.385 | 0.031 | 0.239 | Unannotated | NA | NA |
| metab_12838 | Unannotated feature | neg | 327.0938 | 0.675 | 4.855 | 5.082 | 0.227 | 0.385 | 0.031 | 0.239 | Unannotated | NA | NA |
| metab_13294 | N(6),O(2)-Dimethyladenosine | neg | 340.1254 | 0.625 | 3.837 | 4.912 | 1.075 | 0.385 | 0.031 | 0.239 | Unannotated | Nucleosides, nucleotides, and analogues | Purine nucleosides |
| metab_18732 | Unannotated feature | neg | 447.2067 | 5.876 | 5.299 | 5.602 | 0.303 | 0.385 | 0.031 | 0.239 | Unannotated | NA | NA |
| metab_16011 | Unannotated feature | neg | 257.1661 | 6.130 | 6.179 | 5.918 | -0.262 | -0.385 | 0.031 | 0.239 | Unannotated | NA | NA |
| metab_13123 | Unannotated feature | neg | 244.9207 | 0.443 | 4.159 | 4.083 | -0.076 | -0.385 | 0.031 | 0.239 | Unannotated | NA | NA |
| metab_14553 | Unannotated feature | neg | 341.1834 | 3.446 | 5.099 | 4.713 | -0.386 | -0.385 | 0.031 | 0.239 | Unannotated | NA | NA |
| metab_9727 | Unannotated feature | neg | 314.1338 | 3.100 | 4.406 | 3.877 | -0.529 | -0.385 | 0.031 | 0.239 | Unannotated | NA | NA |
| metab_11837 | Unannotated feature | neg | 227.0347 | 5.940 | 5.239 | 4.866 | -0.373 | -0.385 | 0.031 | 0.239 | Unannotated | NA | NA |
| metab_14211 | Phaseic acid | neg | 315.0996 | 2.956 | 4.260 | 4.871 | 0.610 | 0.385 | 0.031 | 0.239 | Tentative | Lipids and lipid-like molecules | Prenol lipids |
| metab_13620 | Unannotated feature | neg | 543.1582 | 1.952 | 4.522 | 3.906 | -0.616 | -0.385 | 0.031 | 0.239 | Unannotated | NA | NA |
| metab_11836 | Unannotated feature | neg | 599.2910 | 5.937 | 5.223 | 5.616 | 0.394 | 0.385 | 0.031 | 0.239 | Unannotated | NA | NA |
| metab_283 | Unannotated feature | pos | 104.1268 | 0.501 | 4.410 | 3.736 | -0.674 | -0.385 | 0.031 | 0.239 | Unannotated | NA | NA |
| metab_1720 | Unannotated feature | pos | 302.2078 | 3.882 | 5.533 | 5.347 | -0.187 | -0.385 | 0.031 | 0.239 | Unannotated | NA | NA |
| metab_14042 | Unannotated feature | neg | 421.2101 | 2.771 | 4.582 | 3.414 | -1.167 | -0.385 | 0.031 | 0.239 | Unannotated | NA | NA |
| metab_3079 | Ile Ile | pos | 245.1865 | 3.375 | 5.780 | 5.348 | -0.432 | -0.385 | 0.031 | 0.239 | Moderate putative | NA | NA |
| metab_14570 | Unannotated feature | neg | 328.1882 | 3.461 | 5.534 | 5.221 | -0.313 | -0.385 | 0.031 | 0.239 | Unannotated | NA | NA |
| metab_14152 | Unannotated feature | neg | 366.1309 | 2.890 | 5.045 | 4.687 | -0.357 | -0.385 | 0.031 | 0.239 | Unannotated | NA | NA |
| metab_11158 | Unannotated feature | neg | 470.1524 | 0.675 | 4.254 | 4.821 | 0.566 | 0.385 | 0.031 | 0.239 | Unannotated | NA | NA |
| metab_17071 | Unannotated feature | neg | 231.9775 | 2.155 | 5.433 | 5.125 | -0.308 | -0.385 | 0.031 | 0.239 | Unannotated | NA | NA |
| metab_17066 | Unannotated feature | neg | 129.8666 | 7.926 | 4.027 | 4.156 | 0.130 | 0.385 | 0.031 | 0.239 | Unannotated | NA | NA |
| metab_7889 | Unannotated feature | pos | 265.1551 | 3.357 | 5.835 | 5.484 | -0.351 | -0.385 | 0.031 | 0.239 | Unannotated | NA | NA |
| metab_8270 | Alpha-[3-(Nitrosoamino)propyl]-3-pyridinemethanol | pos | 213.1350 | 2.193 | 2.222 | 2.944 | 0.722 | 0.385 | 0.031 | 0.239 | Tentative | NA | NA |
| metab_16659 | Unannotated feature | neg | 971.3252 | 7.600 | 6.001 | 6.180 | 0.179 | 0.381 | 0.033 | 0.243 | Unannotated | NA | NA |
| metab_17065 | Unannotated feature | neg | 123.9457 | 7.934 | 4.420 | 4.218 | -0.201 | -0.381 | 0.033 | 0.243 | Unannotated | NA | NA |
| metab_7085 | PG(20:4(6Z,8E,10E,14Z)-2OH(5S,12R)/i-21:0) | pos | 437.2920 | 4.676 | 2.795 | 4.394 | 1.600 | 0.381 | 0.033 | 0.243 | Unannotated | NA | NA |
| metab_12056 | Unannotated feature | neg | 513.2200 | 6.381 | 3.900 | 5.914 | 2.014 | 0.381 | 0.033 | 0.243 | Unannotated | NA | NA |
| metab_9459 | 5-Methoxydimethyltryptamine | neg | 263.1403 | 3.400 | 5.163 | 4.773 | -0.390 | -0.381 | 0.033 | 0.243 | Tentative | NA | NA |
| metab_9112 | Unannotated feature | neg | 379.1842 | 3.863 | 5.348 | 4.971 | -0.377 | -0.381 | 0.033 | 0.243 | Unannotated | NA | NA |
| metab_2491 | Val Arg | pos | 274.1878 | 0.752 | 5.160 | 4.474 | -0.686 | -0.381 | 0.033 | 0.243 | Moderate putative | NA | NA |
| metab_17451 | Unannotated feature | neg | 1019.4865 | 7.607 | 4.232 | 4.333 | 0.101 | 0.381 | 0.033 | 0.243 | Unannotated | NA | NA |
| metab_16677 | Unannotated feature | neg | 779.8628 | 7.611 | 5.701 | 5.862 | 0.161 | 0.381 | 0.033 | 0.243 | Unannotated | NA | NA |
| metab_19731 | Unannotated feature | neg | 465.1419 | 4.518 | 2.325 | 3.006 | 0.680 | 0.381 | 0.033 | 0.243 | Unannotated | NA | NA |
| metab_7461 | Unannotated feature | pos | 272.1136 | 0.720 | 4.434 | 5.304 | 0.870 | 0.381 | 0.033 | 0.243 | Unannotated | NA | NA |
| metab_8541 | Herierin IV | pos | 212.0921 | 1.333 | 4.441 | 5.034 | 0.593 | 0.381 | 0.033 | 0.243 | Unannotated | Organoheterocyclic compounds | Pyrans |
| metab_3905 | P-Hydroxyphenyl-phenylhydantoin | pos | 301.1188 | 5.757 | 5.485 | 4.978 | -0.507 | -0.381 | 0.033 | 0.243 | Unannotated | Organoheterocyclic compounds | Azolidines |
| metab_14049 | Unannotated feature | neg | 330.1200 | 2.776 | 3.725 | 4.825 | 1.100 | 0.381 | 0.033 | 0.243 | Unannotated | NA | NA |
| metab_3937 | Unannotated feature | pos | 535.8413 | 5.778 | 3.751 | 4.289 | 0.538 | 0.381 | 0.033 | 0.243 | Unannotated | NA | NA |
| metab_10329 | Unannotated feature | neg | 358.0417 | 2.281 | 4.389 | 3.782 | -0.607 | -0.381 | 0.033 | 0.243 | Unannotated | NA | NA |
| metab_12848 | Unannotated feature | neg | 416.1076 | 0.683 | 5.284 | 4.906 | -0.379 | -0.381 | 0.033 | 0.243 | Unannotated | NA | NA |
| metab_9240 | Unannotated feature | neg | 349.1734 | 3.701 | 5.309 | 5.047 | -0.261 | -0.381 | 0.033 | 0.243 | Unannotated | NA | NA |
| metab_9875 | Unannotated feature | neg | 232.1189 | 2.972 | 2.746 | 3.328 | 0.582 | 0.381 | 0.033 | 0.243 | Unannotated | NA | NA |
| metab_4816 | Unannotated feature | pos | 101.0715 | 7.911 | 3.832 | 3.993 | 0.161 | 0.381 | 0.033 | 0.243 | Unannotated | NA | NA |
| metab_8660 | Glycyl-Glutamine | pos | 186.0875 | 0.989 | 5.431 | 4.977 | -0.455 | -0.381 | 0.033 | 0.243 | Unannotated | Organic acids and derivatives | Carboxylic acids and derivatives |
| metab_1068 | PE(18:1/0:0) | pos | 502.2917 | 6.726 | 6.120 | 5.694 | -0.425 | -0.381 | 0.033 | 0.243 | Moderate putative | NA | NA |
| metab_339 | Unannotated feature | pos | 145.0099 | 0.451 | 4.119 | 4.017 | -0.102 | -0.381 | 0.033 | 0.243 | Unannotated | NA | NA |
| metab_10962 | L-2-Amino-3-(1-pyrazolyl)propanoic acid | neg | 154.0615 | 0.791 | 3.881 | 3.454 | -0.427 | -0.381 | 0.033 | 0.243 | Unannotated | Organic acids and derivatives | Carboxylic acids and derivatives |
| metab_1655 | Unannotated feature | pos | 316.2238 | 4.550 | 5.524 | 5.224 | -0.301 | -0.381 | 0.033 | 0.243 | Unannotated | NA | NA |
| metab_3972 | Unannotated feature | pos | 561.8592 | 5.794 | 3.978 | 4.771 | 0.793 | 0.381 | 0.033 | 0.243 | Unannotated | NA | NA |
| metab_2537 | Unannotated feature | pos | 649.2999 | 5.835 | 3.144 | 4.841 | 1.697 | 0.381 | 0.033 | 0.243 | Unannotated | NA | NA |
| metab_4342 | (2'E,4'Z,7'Z,8E)-Colnelenic acid | pos | 310.2363 | 6.119 | 4.870 | 4.533 | -0.338 | -0.381 | 0.033 | 0.243 | Tentative | Lipids and lipid-like molecules | Fatty Acyls |
| metab_11584 | Unannotated feature | neg | 311.8832 | 0.453 | 3.813 | 4.137 | 0.324 | 0.381 | 0.033 | 0.243 | Unannotated | NA | NA |
| metab_17826 | Prolyl-Valine | neg | 259.1300 | 2.817 | 6.544 | 6.162 | -0.382 | -0.381 | 0.033 | 0.243 | Unannotated | Organic acids and derivatives | Carboxylic acids and derivatives |
| metab_2090 | Unannotated feature | pos | 334.1980 | 2.762 | 5.361 | 4.903 | -0.458 | -0.381 | 0.033 | 0.243 | Unannotated | NA | NA |
| metab_4798 | Unannotated feature | pos | 280.1185 | 3.569 | 2.973 | 3.711 | 0.738 | 0.381 | 0.033 | 0.243 | Unannotated | NA | NA |
| metab_1342 | 1,25-Dihydroxy-16-ene-vitamin D3 | pos | 397.3078 | 6.167 | 5.863 | 5.560 | -0.303 | -0.381 | 0.033 | 0.243 | Unannotated | NA | NA |
| metab_18477 | Unannotated feature | neg | 318.1673 | 3.055 | 5.159 | 4.770 | -0.389 | -0.381 | 0.033 | 0.243 | Unannotated | NA | NA |
| metab_16312 | Unannotated feature | neg | 241.2171 | 6.648 | 5.515 | 5.123 | -0.392 | -0.381 | 0.033 | 0.243 | Unannotated | NA | NA |
| metab_14778 | Unannotated feature | neg | 245.0489 | 3.852 | 4.144 | 3.322 | -0.823 | -0.381 | 0.033 | 0.243 | Unannotated | NA | NA |
| metab_12390 | Unannotated feature | neg | 936.8253 | 7.666 | 5.813 | 5.952 | 0.139 | 0.381 | 0.033 | 0.243 | Unannotated | NA | NA |
| metab_597 | Unannotated feature | pos | 576.5689 | 5.778 | 3.811 | 4.802 | 0.991 | 0.381 | 0.033 | 0.243 | Unannotated | NA | NA |
| metab_4065 | Unannotated feature | pos | 571.3531 | 5.835 | 3.208 | 4.333 | 1.125 | 0.381 | 0.033 | 0.243 | Unannotated | NA | NA |
| metab_4250 | Unannotated feature | pos | 625.4820 | 5.936 | 3.320 | 4.540 | 1.220 | 0.381 | 0.033 | 0.243 | Unannotated | NA | NA |
| metab_11793 | Unannotated feature | neg | 475.2105 | 5.813 | 3.710 | 4.037 | 0.327 | 0.381 | 0.033 | 0.243 | Unannotated | NA | NA |
| metab_4052 | Unannotated feature | pos | 637.3962 | 5.831 | 4.303 | 4.959 | 0.656 | 0.381 | 0.033 | 0.243 | Unannotated | NA | NA |
| metab_5755 | Delta4-Dafachronic acid | pos | 415.3182 | 6.048 | 5.061 | 4.613 | -0.448 | -0.381 | 0.033 | 0.243 | Moderate putative | NA | NA |
| metab_9063 | 4-Amino-2-methylenebutanoic acid | neg | 344.1831 | 3.983 | 5.766 | 5.446 | -0.320 | -0.381 | 0.033 | 0.243 | Unannotated | Organic acids and derivatives | Carboxylic acids and derivatives |
| metab_6088 | Unannotated feature | pos | 656.2049 | 5.854 | 3.385 | 4.282 | 0.897 | 0.381 | 0.033 | 0.243 | Unannotated | NA | NA |
| metab_18186 | (24E)-3alpha-Acetoxy-15alpha-hydroxy-23-oxo-7,9(11),24-lanostatrien-26-oic acid | neg | 507.3149 | 6.310 | 6.512 | 6.140 | -0.372 | -0.381 | 0.033 | 0.243 | Unannotated | NA | NA |
| metab_1803 | Unannotated feature | pos | 247.1293 | 0.784 | 5.531 | 5.355 | -0.176 | -0.381 | 0.033 | 0.243 | Unannotated | NA | NA |
| metab_320 | Unannotated feature | pos | 434.7606 | 4.620 | 3.340 | 4.605 | 1.265 | 0.381 | 0.033 | 0.243 | Unannotated | NA | NA |
| metab_16379 | Unannotated feature | neg | 588.2545 | 6.774 | 5.199 | 4.858 | -0.341 | -0.381 | 0.033 | 0.243 | Unannotated | NA | NA |
| metab_18055 | Unannotated feature | neg | 313.9803 | 2.906 | 4.368 | 5.019 | 0.651 | 0.381 | 0.033 | 0.243 | Unannotated | NA | NA |
| metab_17408 | Unannotated feature | neg | 774.8440 | 7.645 | 4.784 | 4.987 | 0.203 | 0.381 | 0.033 | 0.243 | Unannotated | NA | NA |
| metab_4936 | Unannotated feature | pos | 880.8283 | 7.618 | 5.026 | 5.249 | 0.223 | 0.381 | 0.033 | 0.243 | Unannotated | NA | NA |
| metab_18030 | Unannotated feature | neg | 297.1093 | 2.903 | 4.874 | 5.529 | 0.655 | 0.381 | 0.033 | 0.243 | Unannotated | NA | NA |
| metab_17470 | Unannotated feature | neg | 580.8676 | 7.590 | 5.443 | 5.612 | 0.169 | 0.381 | 0.033 | 0.243 | Unannotated | NA | NA |
| metab_3686 | Unannotated feature | pos | 523.6396 | 5.260 | 3.772 | 4.190 | 0.419 | 0.381 | 0.033 | 0.243 | Unannotated | NA | NA |
| metab_14349 | Unannotated feature | neg | 316.1516 | 3.120 | 5.811 | 5.506 | -0.305 | -0.381 | 0.033 | 0.243 | Unannotated | NA | NA |
| metab_10063 | Unannotated feature | neg | 108.0448 | 2.785 | 4.199 | 4.472 | 0.273 | 0.381 | 0.033 | 0.243 | Unannotated | NA | NA |
| metab_11560 | Unannotated feature | neg | 322.9413 | 0.472 | 4.823 | 4.746 | -0.077 | -0.381 | 0.033 | 0.243 | Unannotated | NA | NA |
| metab_9399 | Unannotated feature | neg | 380.2309 | 3.476 | 4.924 | 4.171 | -0.753 | -0.381 | 0.033 | 0.243 | Unannotated | NA | NA |
| metab_12337 | Unannotated feature | neg | 639.4971 | 7.420 | 5.744 | 5.491 | -0.253 | -0.381 | 0.033 | 0.243 | Unannotated | NA | NA |
| metab_10416 | Unannotated feature | neg | 243.9079 | 2.016 | 4.005 | 3.810 | -0.196 | -0.381 | 0.033 | 0.243 | Unannotated | NA | NA |
| metab_9553 | Unannotated feature | neg | 351.1530 | 3.281 | 4.496 | 3.973 | -0.523 | -0.381 | 0.033 | 0.243 | Unannotated | NA | NA |
| metab_1873 | Unannotated feature | pos | 374.2409 | 1.211 | 4.879 | 3.320 | -1.559 | -0.381 | 0.033 | 0.243 | Unannotated | NA | NA |
| metab_17435 | Unannotated feature | neg | 923.8241 | 7.617 | 5.484 | 5.725 | 0.240 | 0.381 | 0.033 | 0.243 | Unannotated | NA | NA |
| metab_8949 | 3-O-Caffeoyl-4-O-methylquinic acid | neg | 367.1038 | 4.183 | 3.455 | 3.903 | 0.448 | 0.381 | 0.033 | 0.243 | Unannotated | NA | NA |
| metab_22 | Cytarabine | pos | 276.1194 | 0.712 | 5.728 | 5.501 | -0.227 | -0.381 | 0.033 | 0.243 | Tentative | Nucleosides, nucleotides, and analogues | Pyrimidine nucleosides |
| metab_1924 | PGP(18:1(12Z)-O(9S,10R)/i-20:0) | pos | 450.2759 | 4.971 | 0.002 | 4.365 | 4.363 | 0.381 | 0.033 | 0.243 | Unannotated | NA | NA |
| metab_14886 | Unannotated feature | neg | 408.8729 | 0.502 | 4.770 | 5.034 | 0.264 | 0.381 | 0.033 | 0.243 | Unannotated | NA | NA |
| metab_16200 | Unannotated feature | neg | 497.2225 | 6.412 | 3.999 | 5.765 | 1.766 | 0.377 | 0.035 | 0.250 | Unannotated | NA | NA |
| metab_9029 | Gartanin | neg | 377.1359 | 4.031 | 5.794 | 5.593 | -0.200 | -0.377 | 0.035 | 0.250 | Tentative | Organoheterocyclic compounds | Benzopyrans |
| metab_19481 | Unannotated feature | neg | 213.0224 | 3.480 | 4.739 | 4.657 | -0.082 | -0.377 | 0.035 | 0.250 | Unannotated | NA | NA |
| metab_19509 | 3,4-Methylene suberic acid | neg | 243.0874 | 4.841 | 0.918 | 3.421 | 2.503 | 0.377 | 0.035 | 0.250 | Unannotated | Lipids and lipid-like molecules | Fatty Acyls |
| metab_6059 | Unannotated feature | pos | 706.5412 | 5.874 | 3.387 | 4.245 | 0.858 | 0.377 | 0.035 | 0.250 | Unannotated | NA | NA |
| metab_5096 | PA(22:5(7Z,10Z,13Z,16Z,19Z)/22:6(4Z,7Z,10Z,13E,15E,19Z)-OH(17)) | pos | 828.5191 | 4.676 | 3.535 | 4.305 | 0.770 | 0.377 | 0.035 | 0.250 | Unannotated | NA | NA |
| metab_13702 | Unannotated feature | neg | 243.9081 | 1.737 | 3.999 | 3.642 | -0.357 | -0.377 | 0.035 | 0.250 | Unannotated | NA | NA |
| metab_1820 | Unannotated feature | pos | 219.1343 | 0.768 | 5.479 | 5.253 | -0.226 | -0.377 | 0.035 | 0.250 | Unannotated | NA | NA |
| metab_637 | Unannotated feature | pos | 198.1493 | 5.825 | 4.295 | 3.732 | -0.563 | -0.377 | 0.035 | 0.250 | Unannotated | NA | NA |
| metab_1618 | Unannotated feature | pos | 478.7867 | 4.931 | 3.158 | 4.381 | 1.224 | 0.377 | 0.035 | 0.250 | Unannotated | NA | NA |
| metab_7498 | Isoleucyl-Glutamate | pos | 225.1238 | 3.756 | 4.953 | 4.693 | -0.260 | -0.377 | 0.035 | 0.250 | Unannotated | NA | NA |
| metab_17661 | Unannotated feature | neg | 316.1516 | 2.771 | 6.263 | 6.076 | -0.187 | -0.377 | 0.035 | 0.250 | Unannotated | NA | NA |
| metab_6137 | Unannotated feature | pos | 756.4657 | 5.835 | 3.664 | 4.570 | 0.906 | 0.377 | 0.035 | 0.250 | Unannotated | NA | NA |
| metab_13736 | Unannotated feature | neg | 316.1517 | 1.947 | 5.528 | 5.314 | -0.215 | -0.377 | 0.035 | 0.250 | Unannotated | NA | NA |
| metab_653 | Unannotated feature | pos | 691.6229 | 5.847 | 4.183 | 4.588 | 0.405 | 0.377 | 0.035 | 0.250 | Unannotated | NA | NA |
| metab_12599 | Unannotated feature | neg | 242.1760 | 6.076 | 5.982 | 5.857 | -0.125 | -0.377 | 0.035 | 0.250 | Unannotated | NA | NA |
| metab_1759 | Unannotated feature | pos | 330.2031 | 3.444 | 5.655 | 5.473 | -0.182 | -0.377 | 0.035 | 0.250 | Unannotated | NA | NA |
| metab_14700 | Unannotated feature | neg | 286.1774 | 3.701 | 6.046 | 5.769 | -0.278 | -0.377 | 0.035 | 0.250 | Unannotated | NA | NA |
| metab_2552 | Mimosine | pos | 231.0979 | 1.065 | 5.708 | 5.412 | -0.295 | -0.377 | 0.035 | 0.250 | Tentative | NA | NA |
| metab_5627 | Unannotated feature | pos | 189.0913 | 6.199 | 4.170 | 4.068 | -0.101 | -0.377 | 0.035 | 0.250 | Unannotated | NA | NA |
| metab_3560 | Aniracetam | pos | 202.0866 | 4.975 | 3.082 | 3.944 | 0.863 | 0.377 | 0.035 | 0.250 | Tentative | Benzenoids | Benzene and substituted derivatives |
| metab_3837 | Unannotated feature | pos | 551.1037 | 5.645 | 4.146 | 4.483 | 0.337 | 0.377 | 0.035 | 0.250 | Unannotated | NA | NA |
| metab_7785 | Unannotated feature | pos | 163.0756 | 3.291 | 3.596 | 2.898 | -0.697 | -0.377 | 0.035 | 0.250 | Unannotated | NA | NA |
| metab_16686 | Unannotated feature | neg | 915.8381 | 7.617 | 5.389 | 5.648 | 0.260 | 0.377 | 0.035 | 0.250 | Unannotated | NA | NA |
| metab_8372 | Ala Ile Asn | pos | 317.1826 | 1.952 | 4.874 | 4.367 | -0.507 | -0.377 | 0.035 | 0.250 | Unannotated | NA | NA |
| metab_1440 | Ecklonialactone A | pos | 291.1960 | 6.024 | 5.435 | 5.190 | -0.245 | -0.377 | 0.035 | 0.250 | Unannotated | NA | NA |
| metab_7840 | Ala Val Val | pos | 288.1922 | 3.083 | 4.864 | 4.319 | -0.546 | -0.377 | 0.035 | 0.250 | Moderate putative | NA | NA |
| metab_11581 | Unannotated feature | neg | 186.9729 | 0.457 | 3.700 | 3.546 | -0.153 | -0.377 | 0.035 | 0.250 | Unannotated | NA | NA |
| metab_15808 | Gaboxadol | neg | 339.1329 | 5.880 | 4.962 | 3.987 | -0.975 | -0.377 | 0.035 | 0.250 | Tentative | NA | NA |
| metab_5944 | Unannotated feature | pos | 133.0862 | 5.922 | 4.035 | 4.521 | 0.485 | 0.377 | 0.035 | 0.250 | Unannotated | NA | NA |
| metab_10821 | Citric Acid | neg | 191.0192 | 1.061 | 6.565 | 6.091 | -0.474 | -0.377 | 0.035 | 0.250 | Strong putative | Organic acids and derivatives | Carboxylic acids and derivatives |
| metab_4129 | Unannotated feature | pos | 622.1314 | 5.870 | 2.877 | 4.178 | 1.301 | 0.377 | 0.035 | 0.250 | Unannotated | NA | NA |
| metab_7195 | Thr Ile Leu | pos | 346.2345 | 4.448 | 5.096 | 4.552 | -0.544 | -0.377 | 0.035 | 0.250 | Moderate putative | NA | NA |
| metab_10680 | Unannotated feature | neg | 188.1116 | 1.508 | 4.413 | 3.416 | -0.997 | -0.377 | 0.035 | 0.250 | Unannotated | NA | NA |
| metab_6311 | Unannotated feature | pos | 586.1626 | 5.782 | 3.589 | 4.557 | 0.968 | 0.377 | 0.035 | 0.250 | Unannotated | NA | NA |
| metab_1560 | Unannotated feature | pos | 360.2289 | 5.283 | 4.570 | 3.516 | -1.054 | -0.377 | 0.035 | 0.250 | Unannotated | NA | NA |
| metab_24 | PA(10:0/PGF1alpha) | pos | 682.4245 | 4.314 | 1.492 | 4.170 | 2.678 | 0.377 | 0.035 | 0.250 | Unannotated | NA | NA |
| metab_16982 | Unannotated feature | neg | 147.0656 | 2.137 | 5.842 | 5.552 | -0.290 | -0.377 | 0.035 | 0.250 | Unannotated | NA | NA |
| metab_4244 | Unannotated feature | pos | 653.4996 | 5.929 | 3.715 | 4.179 | 0.464 | 0.377 | 0.035 | 0.250 | Unannotated | NA | NA |
| metab_6990 | 2-O-alpha-D-Galactopyranosyl-1-deoxynojirimycin | pos | 308.1347 | 0.649 | 4.779 | 5.248 | 0.469 | 0.377 | 0.035 | 0.250 | Unannotated | Organic oxygen compounds | Organooxygen compounds |
| metab_9468 | Unannotated feature | neg | 358.1154 | 3.387 | 3.436 | 4.799 | 1.363 | 0.377 | 0.035 | 0.250 | Unannotated | NA | NA |
| metab_14027 | Unannotated feature | neg | 288.9367 | 0.496 | 6.856 | 6.924 | 0.068 | 0.377 | 0.035 | 0.250 | Unannotated | NA | NA |
| metab_14289 | 5-Hydroxylysinonorleucine | neg | 272.1618 | 3.037 | 5.341 | 4.965 | -0.376 | -0.377 | 0.035 | 0.250 | Unannotated | Organic acids and derivatives | Carboxylic acids and derivatives |
| metab_2084 | Unannotated feature | pos | 137.0600 | 2.762 | 4.098 | 4.261 | 0.162 | 0.377 | 0.035 | 0.250 | Unannotated | NA | NA |
| metab_11162 | 6-Amino-9H-purine-9-propanoic acid | neg | 252.0742 | 0.675 | 2.420 | 3.376 | 0.956 | 0.377 | 0.035 | 0.250 | Unannotated | NA | NA |
| metab_2819 | 7-Methylinosine | pos | 301.1401 | 2.716 | 4.268 | 4.988 | 0.719 | 0.377 | 0.035 | 0.250 | Tentative | NA | NA |
| metab_13973 | Threonylisoleucine | neg | 231.1349 | 2.659 | 5.433 | 5.150 | -0.282 | -0.377 | 0.035 | 0.250 | Unannotated | Organic acids and derivatives | Carboxylic acids and derivatives |
| metab_13825 | Unannotated feature | neg | 216.0289 | 2.122 | 2.915 | 3.594 | 0.679 | 0.377 | 0.035 | 0.250 | Unannotated | NA | NA |
| metab_17446 | Unannotated feature | neg | 685.8666 | 7.611 | 5.670 | 5.801 | 0.130 | 0.377 | 0.035 | 0.250 | Unannotated | NA | NA |
| metab_18373 | P-(1,1-Dimethylheptyl)phenol | neg | 255.1506 | 6.154 | 5.865 | 5.469 | -0.395 | -0.373 | 0.037 | 0.254 | Unannotated | Benzenoids | Benzene and substituted derivatives |
| metab_704 | Unannotated feature | pos | 629.8876 | 5.929 | 3.887 | 4.352 | 0.465 | 0.373 | 0.037 | 0.254 | Unannotated | NA | NA |
| metab_2468 | Unannotated feature | pos | 591.3200 | 5.799 | 8.481 | 7.858 | -0.624 | -0.373 | 0.037 | 0.254 | Unannotated | NA | NA |
| metab_8191 | Ala Ile | pos | 203.1394 | 2.528 | 5.500 | 5.155 | -0.345 | -0.373 | 0.037 | 0.254 | Strong putative | NA | NA |
| metab_7156 | Unannotated feature | pos | 313.1553 | 4.524 | 4.211 | 3.626 | -0.585 | -0.373 | 0.037 | 0.254 | Unannotated | NA | NA |
| metab_13852 | Unannotated feature | neg | 322.9170 | 0.460 | 5.924 | 5.980 | 0.056 | 0.373 | 0.037 | 0.254 | Unannotated | NA | NA |
| metab_16 | Unannotated feature | pos | 154.0501 | 0.720 | 4.110 | 4.347 | 0.237 | 0.373 | 0.037 | 0.254 | Unannotated | NA | NA |
| metab_3907 | Unannotated feature | pos | 518.0841 | 5.757 | 3.782 | 4.497 | 0.715 | 0.373 | 0.037 | 0.254 | Unannotated | NA | NA |
| metab_9187 | DL-Allylglycine | neg | 344.1831 | 3.784 | 5.813 | 5.477 | -0.337 | -0.373 | 0.037 | 0.254 | Unannotated | Organic acids and derivatives | Carboxylic acids and derivatives |
| metab_15254 | Unannotated feature | neg | 378.2042 | 4.810 | 4.448 | 3.604 | -0.844 | -0.373 | 0.037 | 0.254 | Unannotated | NA | NA |
| metab_9733 | Unannotated feature | neg | 185.1654 | 3.096 | 4.004 | 2.818 | -1.186 | -0.373 | 0.037 | 0.254 | Unannotated | NA | NA |
| metab_14153 | (3R,4S)-1-(4-Fluorophenyl)-4-[(3S)-3-(4-fluorophenyl)-3-hydroxypropyl]-3-(4-hydroxyphenyl)azetidin-2-one | neg | 408.1422 | 2.890 | 5.278 | 4.859 | -0.419 | -0.373 | 0.037 | 0.254 | Unannotated | Organoheterocyclic compounds | Lactams |
| metab_7939 | Unannotated feature | pos | 385.2568 | 2.915 | 4.603 | 2.856 | -1.747 | -0.373 | 0.037 | 0.254 | Unannotated | NA | NA |
| metab_16573 | Unannotated feature | neg | 299.0207 | 1.722 | 5.190 | 5.128 | -0.062 | -0.373 | 0.037 | 0.254 | Unannotated | NA | NA |
| metab_6817 | Unannotated feature | pos | 208.1911 | 5.151 | 4.991 | 4.850 | -0.141 | -0.373 | 0.037 | 0.254 | Unannotated | NA | NA |
| metab_2643 | 2-Acetylfuran | pos | 111.0446 | 1.936 | 5.074 | 5.193 | 0.119 | 0.373 | 0.037 | 0.254 | Unannotated | NA | NA |
| metab_3325 | Lys Ile Phe | pos | 407.2663 | 4.139 | 4.228 | 3.402 | -0.826 | -0.373 | 0.037 | 0.254 | Unannotated | NA | NA |
| metab_6305 | Unannotated feature | pos | 550.8529 | 5.787 | 3.342 | 4.788 | 1.446 | 0.373 | 0.037 | 0.254 | Unannotated | NA | NA |
| metab_7050 | Unannotated feature | pos | 459.3051 | 4.750 | 1.288 | 4.146 | 2.859 | 0.373 | 0.037 | 0.254 | Unannotated | NA | NA |
| metab_4645 | Unannotated feature | pos | 458.3840 | 7.260 | 4.486 | 4.211 | -0.275 | -0.373 | 0.037 | 0.254 | Unannotated | NA | NA |
| metab_1344 | Fluocortolone Pivalate | pos | 425.2525 | 6.167 | 5.304 | 4.921 | -0.383 | -0.373 | 0.037 | 0.254 | Unannotated | NA | NA |
| metab_19445 | N-formylanthranilic acid | neg | 164.0347 | 4.949 | 4.570 | 4.394 | -0.176 | -0.373 | 0.037 | 0.254 | Moderate putative | Benzenoids | Benzene and substituted derivatives |
| metab_18160 | Cyclic 3-Hydroxymelatonin | neg | 293.1147 | 2.758 | 5.590 | 5.323 | -0.267 | -0.373 | 0.037 | 0.254 | Unannotated | Organoheterocyclic compounds | Indoles and derivatives |
| metab_1594 | Unannotated feature | pos | 804.4902 | 5.147 | 3.747 | 5.373 | 1.627 | 0.373 | 0.037 | 0.254 | Unannotated | NA | NA |
| metab_7214 | Unannotated feature | pos | 336.1927 | 4.394 | 3.821 | 2.266 | -1.555 | -0.373 | 0.037 | 0.254 | Unannotated | NA | NA |
| metab_16604 | Unannotated feature | neg | 147.0656 | 1.882 | 5.695 | 5.444 | -0.251 | -0.373 | 0.037 | 0.254 | Unannotated | NA | NA |
| metab_7662 | Unannotated feature | pos | 373.2820 | 3.439 | 5.239 | 4.740 | -0.499 | -0.373 | 0.037 | 0.254 | Unannotated | NA | NA |
| metab_11776 | 2-(p-Acetamidophenyl)-2-ethylglutarimide | neg | 255.1141 | 5.753 | 5.893 | 5.432 | -0.461 | -0.373 | 0.037 | 0.254 | Unannotated | Organoheterocyclic compounds | Piperidines |
| metab_14133 | Unannotated feature | neg | 357.2513 | 2.865 | 4.864 | 4.352 | -0.512 | -0.373 | 0.037 | 0.254 | Unannotated | NA | NA |
| metab_14294 | Unannotated feature | neg | 335.1357 | 3.041 | 4.468 | 3.926 | -0.542 | -0.373 | 0.037 | 0.254 | Unannotated | NA | NA |
| metab_17441 | Unannotated feature | neg | 813.8571 | 7.611 | 5.663 | 5.784 | 0.121 | 0.373 | 0.037 | 0.254 | Unannotated | NA | NA |
| metab_6199 | Unannotated feature | pos | 645.0774 | 5.814 | 3.830 | 4.325 | 0.496 | 0.373 | 0.037 | 0.254 | Unannotated | NA | NA |
| metab_2848 | Val Ile Asp | pos | 346.1982 | 2.762 | 5.699 | 5.459 | -0.240 | -0.373 | 0.037 | 0.254 | Moderate putative | NA | NA |
| metab_13939 | Cytidine, 2'-deoxy-2',2'-difluoro-N-(1-oxo-2-propylpentyl)- | neg | 424.1474 | 2.583 | 4.899 | 5.517 | 0.618 | 0.373 | 0.037 | 0.254 | Unannotated | Organoheterocyclic compounds | Diazines |
| metab_18947 | Unannotated feature | neg | 294.1198 | 5.586 | 4.349 | 3.069 | -1.280 | -0.373 | 0.037 | 0.254 | Unannotated | NA | NA |
| metab_2046 | Eriodictyol 7-(6-trans-p-coumaroylglucoside) | pos | 299.0825 | 2.665 | 4.874 | 3.933 | -0.941 | -0.373 | 0.037 | 0.254 | Unannotated | Phenylpropanoids and polyketides | Flavonoids |
| metab_4041 | Unannotated feature | pos | 618.8829 | 5.825 | 2.870 | 4.416 | 1.546 | 0.373 | 0.037 | 0.254 | Unannotated | NA | NA |
| metab_325 | Lysyllysine | pos | 138.1077 | 0.477 | 4.427 | 3.320 | -1.107 | -0.373 | 0.037 | 0.254 | Unannotated | Organic acids and derivatives | Carboxylic acids and derivatives |
| metab_9856 | Unannotated feature | neg | 330.1675 | 2.984 | 5.230 | 4.870 | -0.360 | -0.373 | 0.037 | 0.254 | Unannotated | NA | NA |
| metab_6226 | Unannotated feature | pos | 682.2320 | 5.811 | 3.812 | 4.610 | 0.798 | 0.373 | 0.037 | 0.254 | Unannotated | NA | NA |
| metab_8544 | Histidinyl-Leucine | pos | 269.1612 | 1.309 | 5.860 | 5.471 | -0.389 | -0.373 | 0.037 | 0.254 | Tentative | NA | NA |
| metab_11011 | Glutaminylglycine | neg | 184.0722 | 0.770 | 5.265 | 4.872 | -0.393 | -0.373 | 0.037 | 0.254 | Unannotated | Organic acids and derivatives | Carboxylic acids and derivatives |
| metab_19015 | 6-Hydroxy-4-methoxy-3-(3-methyl-2-butenyl)-2-(2-phenylethenyl)benzoic acid | neg | 359.1250 | 5.512 | 5.133 | 4.802 | -0.330 | -0.373 | 0.037 | 0.254 | Tentative | Phenylpropanoids and polyketides | Stilbenes |
| metab_1704 | Unannotated feature | pos | 560.3299 | 4.016 | 4.724 | 5.383 | 0.659 | 0.373 | 0.037 | 0.254 | Unannotated | NA | NA |
| metab_3508 | Unannotated feature | pos | 481.3183 | 4.810 | 3.683 | 4.002 | 0.320 | 0.373 | 0.037 | 0.254 | Unannotated | NA | NA |
| metab_19109 | Unannotated feature | neg | 613.2701 | 5.374 | 3.620 | 4.115 | 0.495 | 0.373 | 0.037 | 0.254 | Unannotated | NA | NA |
| metab_2933 | Ile Val | pos | 231.1707 | 2.915 | 6.557 | 6.247 | -0.311 | -0.373 | 0.037 | 0.254 | Moderate putative | NA | NA |
| metab_13366 | Unannotated feature | neg | 270.0987 | 0.730 | 0.527 | 4.520 | 3.993 | 0.373 | 0.037 | 0.254 | Unannotated | NA | NA |
| metab_13365 | Fructose lactate | neg | 267.0726 | 0.730 | 5.565 | 5.677 | 0.112 | 0.373 | 0.037 | 0.254 | Unannotated | NA | NA |
| metab_17936 | 4-ene-Valproic acid | neg | 283.1915 | 6.624 | 5.062 | 4.836 | -0.226 | -0.373 | 0.037 | 0.254 | Tentative | Lipids and lipid-like molecules | Fatty Acyls |
| metab_4142 | Unannotated feature | pos | 711.9371 | 5.882 | 3.006 | 4.554 | 1.548 | 0.373 | 0.037 | 0.254 | Unannotated | NA | NA |
| metab_11119 | Unannotated feature | neg | 365.1204 | 0.714 | 4.543 | 4.921 | 0.378 | 0.373 | 0.037 | 0.254 | Unannotated | NA | NA |
| metab_15935 | Unannotated feature | neg | 357.0620 | 6.035 | 4.557 | 4.410 | -0.147 | -0.373 | 0.037 | 0.254 | Unannotated | NA | NA |
| metab_6357 | Unannotated feature | pos | 683.1833 | 5.757 | 0.109 | 4.354 | 4.245 | 0.373 | 0.037 | 0.254 | Unannotated | NA | NA |
| metab_14310 | N-Jasmonoylisoleucine | neg | 344.1831 | 3.063 | 5.713 | 5.100 | -0.614 | -0.373 | 0.037 | 0.254 | Tentative | NA | NA |
| metab_6093 | Unannotated feature | pos | 705.5418 | 5.851 | 4.088 | 4.644 | 0.556 | 0.373 | 0.037 | 0.254 | Unannotated | NA | NA |
| metab_3457 | Unannotated feature | pos | 413.2631 | 4.630 | 3.115 | 4.833 | 1.717 | 0.373 | 0.037 | 0.254 | Unannotated | NA | NA |
| metab_4727 | Unannotated feature | pos | 352.8982 | 7.650 | 5.124 | 5.217 | 0.092 | 0.373 | 0.037 | 0.254 | Unannotated | NA | NA |
| metab_7763 | LysoPC(P-16:0/0:0) | pos | 251.6657 | 3.279 | 4.358 | 3.200 | -1.158 | -0.373 | 0.037 | 0.254 | Unannotated | Lipids and lipid-like molecules | Glycerophospholipids |
| metab_5309 | Lincomycin | pos | 448.2489 | 6.558 | 5.815 | 5.695 | -0.120 | -0.373 | 0.037 | 0.254 | Tentative | Organic acids and derivatives | Carboxylic acids and derivatives |
| metab_1519 | Unannotated feature | pos | 389.1694 | 5.457 | 4.236 | 3.096 | -1.140 | -0.373 | 0.037 | 0.254 | Unannotated | NA | NA |
| metab_7896 | Gln Val Val | pos | 345.2140 | 2.965 | 4.976 | 4.439 | -0.537 | -0.373 | 0.037 | 0.254 | Moderate putative | NA | NA |
| metab_16490 | Unannotated feature | neg | 135.9703 | 7.080 | 5.095 | 4.986 | -0.109 | -0.373 | 0.037 | 0.254 | Unannotated | NA | NA |
| metab_2900 | Unannotated feature | pos | 377.2808 | 6.151 | 6.323 | 5.900 | -0.423 | -0.373 | 0.037 | 0.254 | Unannotated | NA | NA |
| metab_14016 | Unannotated feature | neg | 406.9119 | 0.492 | 4.608 | 4.838 | 0.230 | 0.373 | 0.037 | 0.254 | Unannotated | NA | NA |
| metab_11993 | Unannotated feature | neg | 583.3116 | 6.217 | 5.249 | 5.638 | 0.389 | 0.373 | 0.037 | 0.254 | Unannotated | NA | NA |
| metab_18037 | Unannotated feature | neg | 346.1624 | 2.903 | 5.770 | 5.411 | -0.358 | -0.373 | 0.037 | 0.254 | Unannotated | NA | NA |
| metab_19887 | Nicotinic Acid | neg | 122.0241 | 1.061 | 6.207 | 6.063 | -0.145 | -0.373 | 0.037 | 0.254 | Strong putative | Organoheterocyclic compounds | Pyridines and derivatives |
| metab_3055 | Vanillactic acid | pos | 254.1028 | 3.324 | 3.954 | 4.351 | 0.396 | 0.373 | 0.037 | 0.254 | Unannotated | NA | NA |
| metab_6502 | Unannotated feature | pos | 669.7579 | 5.583 | 3.612 | 4.311 | 0.699 | 0.373 | 0.037 | 0.254 | Unannotated | NA | NA |
| metab_11971 | Unannotated feature | neg | 477.2264 | 5.480 | 4.797 | 5.168 | 0.371 | 0.368 | 0.039 | 0.261 | Unannotated | NA | NA |
| metab_11981 | Unannotated feature | neg | 375.2652 | 6.201 | 5.445 | 5.076 | -0.369 | -0.368 | 0.039 | 0.261 | Unannotated | NA | NA |
| metab_4958 | Unannotated feature | pos | 719.8553 | 7.586 | 5.760 | 5.865 | 0.106 | 0.368 | 0.039 | 0.261 | Unannotated | NA | NA |
| metab_3605 | Unannotated feature | pos | 468.2710 | 5.068 | 2.123 | 3.572 | 1.449 | 0.368 | 0.039 | 0.261 | Unannotated | NA | NA |
| metab_17571 | Unannotated feature | neg | 84.9377 | 7.310 | 5.113 | 5.267 | 0.154 | 0.368 | 0.039 | 0.261 | Unannotated | NA | NA |
| metab_8007 | Unannotated feature | pos | 359.2662 | 2.827 | 5.755 | 5.422 | -0.333 | -0.368 | 0.039 | 0.261 | Unannotated | NA | NA |
| metab_8010 | 5'-N-Ethylcarboxamidoadenosine | pos | 273.1085 | 2.827 | 3.469 | 3.970 | 0.501 | 0.368 | 0.039 | 0.261 | Unannotated | NA | NA |
| metab_9125 | Unannotated feature | neg | 300.1930 | 3.886 | 5.481 | 5.226 | -0.255 | -0.368 | 0.039 | 0.261 | Unannotated | NA | NA |
| metab_6831 | Unannotated feature | pos | 686.9270 | 5.121 | 3.879 | 4.671 | 0.792 | 0.368 | 0.039 | 0.261 | Unannotated | NA | NA |
| metab_8112 | Unannotated feature | pos | 316.1874 | 2.732 | 4.992 | 4.818 | -0.174 | -0.368 | 0.039 | 0.261 | Unannotated | NA | NA |
| metab_4658 | Unannotated feature | pos | 653.3243 | 7.403 | 5.822 | 5.943 | 0.121 | 0.368 | 0.039 | 0.261 | Unannotated | NA | NA |
| metab_9635 | Unannotated feature | neg | 295.1149 | 3.203 | 4.224 | 4.915 | 0.690 | 0.368 | 0.039 | 0.261 | Unannotated | NA | NA |
| metab_15371 | Ancistrocladine | neg | 406.1992 | 5.026 | 5.424 | 4.854 | -0.570 | -0.368 | 0.039 | 0.261 | Tentative | Organoheterocyclic compounds | Isoquinolines and derivatives |
| metab_2333 | Unannotated feature | pos | 157.0184 | 0.410 | 4.716 | 4.757 | 0.041 | 0.368 | 0.039 | 0.261 | Unannotated | NA | NA |
| metab_4708 | Unannotated feature | pos | 906.8287 | 7.610 | 6.133 | 6.261 | 0.128 | 0.368 | 0.039 | 0.261 | Unannotated | NA | NA |
| metab_15492 | LF | neg | 277.1558 | 5.257 | 4.594 | 3.868 | -0.726 | -0.368 | 0.039 | 0.261 | Unannotated | NA | NA |
| metab_1616 | Unannotated feature | pos | 586.8362 | 4.931 | 3.720 | 5.032 | 1.312 | 0.368 | 0.039 | 0.261 | Unannotated | NA | NA |
| metab_8299 | Val Lys Val | pos | 345.2506 | 2.070 | 5.281 | 4.756 | -0.525 | -0.368 | 0.039 | 0.261 | Unannotated | NA | NA |
| metab_3848 | Unannotated feature | pos | 572.8661 | 5.671 | 3.318 | 4.645 | 1.328 | 0.368 | 0.039 | 0.261 | Unannotated | NA | NA |
| metab_8522 | Physoperuvine | pos | 159.1495 | 1.510 | 7.133 | 6.160 | -0.973 | -0.368 | 0.039 | 0.261 | Tentative | Alkaloids and derivatives | Tropane alkaloids |
| metab_3870 | Unannotated feature | pos | 607.1242 | 5.711 | 3.954 | 4.199 | 0.245 | 0.368 | 0.039 | 0.261 | Unannotated | NA | NA |
| metab_13153 | Unannotated feature | neg | 212.9373 | 0.467 | 3.623 | 3.485 | -0.138 | -0.368 | 0.039 | 0.261 | Unannotated | NA | NA |
| metab_10466 | Unannotated feature | neg | 196.0611 | 1.958 | 4.654 | 4.755 | 0.101 | 0.368 | 0.039 | 0.261 | Unannotated | NA | NA |
| metab_8222 | Unannotated feature | pos | 98.5125 | 2.430 | 3.514 | 3.803 | 0.288 | 0.368 | 0.039 | 0.261 | Unannotated | NA | NA |
| metab_416 | Unannotated feature | pos | 174.0215 | 0.016 | 5.426 | 5.458 | 0.033 | 0.368 | 0.039 | 0.261 | Unannotated | NA | NA |
| metab_1492 | Unannotated feature | pos | 335.1608 | 5.811 | 5.386 | 4.904 | -0.482 | -0.368 | 0.039 | 0.261 | Unannotated | NA | NA |
| metab_11475 | Unannotated feature | neg | 86.9928 | 0.524 | 3.445 | 3.258 | -0.187 | -0.368 | 0.039 | 0.261 | Unannotated | NA | NA |
| metab_17673 | Unannotated feature | neg | 126.9043 | 7.044 | 5.509 | 5.447 | -0.062 | -0.368 | 0.039 | 0.261 | Unannotated | NA | NA |
| metab_17686 | Unannotated feature | neg | 553.4275 | 7.019 | 5.591 | 4.536 | -1.055 | -0.368 | 0.039 | 0.261 | Unannotated | NA | NA |
| metab_4559 | N-Linoleoyl Valine | pos | 362.3037 | 6.853 | 4.735 | 5.047 | 0.312 | 0.368 | 0.039 | 0.261 | Unannotated | NA | NA |
| metab_3275 | Val Asp Leu | pos | 346.1981 | 3.974 | 5.551 | 5.194 | -0.357 | -0.368 | 0.039 | 0.261 | Moderate putative | NA | NA |
| metab_822 | Cysteinyl-Lysine | pos | 499.2411 | 6.398 | 4.932 | 5.909 | 0.977 | 0.368 | 0.039 | 0.261 | Unannotated | Organic acids and derivatives | Carboxylic acids and derivatives |
| metab_1717 | Unannotated feature | pos | 403.2714 | 3.920 | 3.898 | 2.128 | -1.771 | -0.368 | 0.039 | 0.261 | Unannotated | NA | NA |
| metab_17429 | Unannotated feature | neg | 868.8378 | 7.621 | 5.753 | 5.849 | 0.096 | 0.368 | 0.039 | 0.261 | Unannotated | NA | NA |
| metab_650 | Unannotated feature | pos | 687.2140 | 5.841 | 2.773 | 4.077 | 1.304 | 0.368 | 0.039 | 0.261 | Unannotated | NA | NA |
| metab_10143 | Unannotated feature | neg | 394.1623 | 2.746 | 5.069 | 4.887 | -0.182 | -0.368 | 0.039 | 0.261 | Unannotated | NA | NA |
| metab_2132 | Unannotated feature | pos | 194.1180 | 2.840 | 4.098 | 4.580 | 0.481 | 0.368 | 0.039 | 0.261 | Unannotated | NA | NA |
| metab_13530 | Glutaric acid | neg | 309.0833 | 1.096 | 3.809 | 4.289 | 0.480 | 0.368 | 0.039 | 0.261 | Tentative | NA | NA |
| metab_10046 | Unannotated feature | neg | 318.1673 | 2.799 | 5.661 | 5.244 | -0.417 | -0.368 | 0.039 | 0.261 | Unannotated | NA | NA |
| metab_14307 | Unannotated feature | neg | 227.1399 | 3.059 | 6.302 | 6.163 | -0.139 | -0.368 | 0.039 | 0.261 | Unannotated | NA | NA |
| metab_1967 | Unannotated feature | pos | 317.2191 | 1.952 | 4.935 | 4.390 | -0.545 | -0.368 | 0.039 | 0.261 | Unannotated | NA | NA |
| metab_7524 | Unannotated feature | pos | 276.1584 | 3.712 | 4.844 | 4.434 | -0.411 | -0.368 | 0.039 | 0.261 | Unannotated | NA | NA |
| metab_6264 | Unannotated feature | pos | 635.0028 | 5.799 | 1.365 | 4.197 | 2.832 | 0.368 | 0.039 | 0.261 | Unannotated | NA | NA |
| metab_2709 | Unannotated feature | pos | 139.0755 | 2.084 | 3.111 | 3.490 | 0.379 | 0.368 | 0.039 | 0.261 | Unannotated | NA | NA |
| metab_12149 | Unannotated feature | neg | 571.2907 | 6.640 | 6.340 | 5.963 | -0.377 | -0.368 | 0.039 | 0.261 | Unannotated | NA | NA |
| metab_7177 | Centhaquine | pos | 680.4447 | 4.479 | 3.972 | 4.972 | 1.000 | 0.368 | 0.039 | 0.261 | Unannotated | Organoheterocyclic compounds | Diazinanes |
| metab_7913 | Cibaric acid | pos | 369.1665 | 2.947 | 2.665 | 3.915 | 1.250 | 0.368 | 0.039 | 0.261 | Unannotated | Lipids and lipid-like molecules | Fatty Acyls |
| metab_5835 | Anabsin | pos | 545.3142 | 5.969 | 5.042 | 4.152 | -0.890 | -0.368 | 0.039 | 0.261 | Unannotated | Lipids and lipid-like molecules | Prenol lipids |
| metab_4097 | Unannotated feature | pos | 694.3264 | 5.847 | 3.871 | 4.307 | 0.436 | 0.368 | 0.039 | 0.261 | Unannotated | NA | NA |
| metab_10139 | Unannotated feature | neg | 270.1462 | 2.750 | 4.660 | 4.309 | -0.351 | -0.368 | 0.039 | 0.261 | Unannotated | NA | NA |
| metab_6657 | 2',3'-Didehydro-2',3'-dideoxycytidine | pos | 436.1979 | 5.362 | 5.078 | 4.381 | -0.698 | -0.368 | 0.039 | 0.261 | Unannotated | NA | NA |
| metab_1635 | DL-m-Tyrosine | pos | 164.0708 | 4.733 | 5.943 | 5.784 | -0.159 | -0.368 | 0.039 | 0.261 | Strong putative | NA | NA |
| metab_18147 | Unannotated feature | neg | 721.4310 | 6.342 | 5.506 | 4.021 | -1.486 | -0.368 | 0.039 | 0.261 | Unannotated | NA | NA |
| metab_18085 | Propofol | neg | 223.1336 | 6.404 | 4.443 | 4.937 | 0.495 | 0.368 | 0.039 | 0.261 | Tentative | NA | NA |
| metab_8377 | Unannotated feature | pos | 274.1768 | 1.952 | 4.316 | 3.794 | -0.521 | -0.368 | 0.039 | 0.261 | Unannotated | NA | NA |
| metab_8171 | Agar | pos | 319.1371 | 2.640 | 0.000 | 3.724 | 3.724 | 0.364 | 0.042 | 0.269 | Tentative | Organic oxygen compounds | Organooxygen compounds |
| metab_7821 | Unannotated feature | pos | 194.1180 | 1.936 | 5.003 | 4.761 | -0.241 | -0.364 | 0.042 | 0.269 | Unannotated | NA | NA |
| metab_17597 | Unannotated feature | neg | 342.1206 | 2.762 | 5.022 | 5.339 | 0.317 | 0.364 | 0.042 | 0.269 | Unannotated | NA | NA |
| metab_14878 | Unannotated feature | neg | 392.1835 | 4.052 | 5.143 | 4.667 | -0.476 | -0.364 | 0.042 | 0.269 | Unannotated | NA | NA |
| metab_3574 | Cyclocalopin C1 | pos | 338.1606 | 5.013 | 4.082 | 3.465 | -0.617 | -0.364 | 0.042 | 0.269 | Unannotated | Organoheterocyclic compounds | Furopyrans |
| metab_9254 | Unannotated feature | neg | 300.1931 | 3.688 | 5.253 | 5.007 | -0.246 | -0.364 | 0.042 | 0.269 | Unannotated | NA | NA |
| metab_5571 | PA(20:4(5Z,7E,11Z,14Z)-OH(9)/8:0) | pos | 639.3052 | 6.255 | 6.209 | 5.799 | -0.410 | -0.364 | 0.042 | 0.269 | Unannotated | NA | NA |
| metab_4829 | Unannotated feature | pos | 144.1021 | 7.788 | 4.824 | 4.884 | 0.061 | 0.364 | 0.042 | 0.269 | Unannotated | NA | NA |
| metab_9504 | Unannotated feature | neg | 407.1580 | 3.338 | 4.575 | 3.649 | -0.926 | -0.364 | 0.042 | 0.269 | Unannotated | NA | NA |
| metab_13155 | Unannotated feature | neg | 489.9163 | 0.472 | 5.340 | 4.983 | -0.357 | -0.364 | 0.042 | 0.269 | Unannotated | NA | NA |
| metab_13182 | Unannotated feature | neg | 178.9483 | 0.502 | 5.151 | 5.257 | 0.105 | 0.364 | 0.042 | 0.269 | Unannotated | NA | NA |
| metab_11596 | Unannotated feature | neg | 218.9456 | 0.449 | 4.310 | 4.541 | 0.231 | 0.364 | 0.042 | 0.269 | Unannotated | NA | NA |
| metab_8347 | Unannotated feature | pos | 346.1618 | 1.960 | 4.989 | 4.687 | -0.302 | -0.364 | 0.042 | 0.269 | Unannotated | NA | NA |
| metab_747 | Dibekacin | pos | 434.2583 | 6.056 | 5.271 | 4.799 | -0.472 | -0.364 | 0.042 | 0.269 | Unannotated | Organic oxygen compounds | Organooxygen compounds |
| metab_7608 | Pro Glu Ile | pos | 358.1982 | 3.533 | 5.747 | 5.493 | -0.254 | -0.364 | 0.042 | 0.269 | Moderate putative | NA | NA |
| metab_7379 | Unannotated feature | pos | 342.2396 | 4.006 | 5.199 | 4.068 | -1.131 | -0.364 | 0.042 | 0.269 | Unannotated | NA | NA |
| metab_16897 | Unannotated feature | neg | 286.0938 | 1.958 | 4.750 | 5.130 | 0.380 | 0.364 | 0.042 | 0.269 | Unannotated | NA | NA |
| metab_1380 | Unannotated feature | pos | 605.2988 | 6.111 | 6.394 | 6.116 | -0.277 | -0.364 | 0.042 | 0.269 | Unannotated | NA | NA |
| metab_2257 | Unannotated feature | pos | 332.1823 | 3.129 | 5.490 | 4.814 | -0.676 | -0.364 | 0.042 | 0.269 | Unannotated | NA | NA |
| metab_11464 | Unannotated feature | neg | 624.7843 | 0.524 | 4.747 | 5.128 | 0.381 | 0.364 | 0.042 | 0.269 | Unannotated | NA | NA |
| metab_3746 | Histidinol | pos | 321.1452 | 5.425 | 5.897 | 5.638 | -0.259 | -0.364 | 0.042 | 0.269 | Tentative | Organic nitrogen compounds | Organonitrogen compounds |
| metab_2767 | Unannotated feature | pos | 786.2549 | 2.439 | 5.464 | 5.605 | 0.141 | 0.364 | 0.042 | 0.269 | Unannotated | NA | NA |
| metab_3220 | Delta-Valerolactam | pos | 100.0762 | 2.268 | 6.857 | 5.879 | -0.978 | -0.364 | 0.042 | 0.269 | Unannotated | NA | NA |
| metab_16784 | Unannotated feature | neg | 718.8570 | 7.697 | 4.438 | 4.659 | 0.221 | 0.364 | 0.042 | 0.269 | Unannotated | NA | NA |
| metab_1361 | Melitracen | pos | 292.2063 | 6.143 | 5.532 | 5.301 | -0.230 | -0.364 | 0.042 | 0.269 | Unannotated | NA | NA |
| metab_6835 | Unannotated feature | pos | 455.9375 | 5.121 | 3.543 | 4.233 | 0.691 | 0.364 | 0.042 | 0.269 | Unannotated | NA | NA |
| metab_16693 | Unannotated feature | neg | 617.8785 | 7.625 | 4.473 | 4.735 | 0.262 | 0.364 | 0.042 | 0.269 | Unannotated | NA | NA |
| metab_4888 | Unannotated feature | pos | 114.5588 | 7.708 | 3.272 | 3.447 | 0.175 | 0.364 | 0.042 | 0.269 | Unannotated | NA | NA |
| metab_10864 | Unannotated feature | neg | 140.0823 | 1.000 | 4.550 | 3.951 | -0.598 | -0.364 | 0.042 | 0.269 | Unannotated | NA | NA |
| metab_1948 | Unannotated feature | pos | 230.1029 | 1.952 | 5.011 | 5.339 | 0.328 | 0.364 | 0.042 | 0.269 | Unannotated | NA | NA |
| metab_11792 | Quercetin 3-sulfate | neg | 380.9926 | 5.808 | 1.745 | 4.178 | 2.432 | 0.364 | 0.042 | 0.269 | Tentative | Phenylpropanoids and polyketides | Flavonoids |
| metab_5619 | Unannotated feature | pos | 149.9406 | 0.016 | 4.544 | 4.576 | 0.032 | 0.364 | 0.042 | 0.269 | Unannotated | NA | NA |
| metab_6100 | Unannotated feature | pos | 650.1985 | 5.851 | 1.658 | 4.071 | 2.413 | 0.364 | 0.042 | 0.269 | Unannotated | NA | NA |
| metab_17115 | Unannotated feature | neg | 84.9502 | 7.840 | 3.545 | 3.443 | -0.102 | -0.364 | 0.042 | 0.269 | Unannotated | NA | NA |
| metab_3900 | Unannotated feature | pos | 529.0909 | 5.754 | 4.048 | 4.810 | 0.762 | 0.364 | 0.042 | 0.269 | Unannotated | NA | NA |
| metab_4170 | Unannotated feature | pos | 610.7752 | 5.906 | 3.199 | 4.138 | 0.938 | 0.364 | 0.042 | 0.269 | Unannotated | NA | NA |
| metab_6302 | Unannotated feature | pos | 563.0982 | 5.787 | 4.072 | 4.389 | 0.317 | 0.364 | 0.042 | 0.269 | Unannotated | NA | NA |
| metab_8769 | Unannotated feature | pos | 160.1334 | 0.728 | 7.184 | 6.809 | -0.376 | -0.364 | 0.042 | 0.269 | Unannotated | NA | NA |
| metab_18199 | Unannotated feature | neg | 359.1576 | 2.951 | 5.151 | 4.655 | -0.496 | -0.364 | 0.042 | 0.269 | Unannotated | NA | NA |
| metab_6770 | Unannotated feature | pos | 665.4216 | 5.219 | 3.865 | 4.753 | 0.888 | 0.364 | 0.042 | 0.269 | Unannotated | NA | NA |
| metab_14283 | N-Malonyltryptophan | neg | 335.0888 | 3.028 | 4.413 | 4.875 | 0.462 | 0.364 | 0.042 | 0.269 | Unannotated | NA | NA |
| metab_2032 | Unannotated feature | pos | 122.0580 | 2.528 | 4.833 | 3.351 | -1.483 | -0.364 | 0.042 | 0.269 | Unannotated | NA | NA |
| metab_467 | PS(MonoMe(13,5)/DiMe(11,5)) | pos | 480.8026 | 4.870 | 3.349 | 4.495 | 1.146 | 0.364 | 0.042 | 0.269 | Unannotated | Lipids and lipid-like molecules | Glycerophospholipids |
| metab_10003 | Unannotated feature | neg | 215.1399 | 2.841 | 4.030 | 3.093 | -0.937 | -0.364 | 0.042 | 0.269 | Unannotated | NA | NA |
| metab_4116 | Unannotated feature | pos | 640.8961 | 5.854 | 4.055 | 4.748 | 0.693 | 0.364 | 0.042 | 0.269 | Unannotated | NA | NA |
| metab_1925 | (2S,2'S)-Pyrosaccharopine | pos | 291.1556 | 1.706 | 4.769 | 4.559 | -0.210 | -0.364 | 0.042 | 0.269 | Unannotated | Organic acids and derivatives | Carboxylic acids and derivatives |
| metab_9507 | Unannotated feature | neg | 298.1775 | 3.334 | 4.492 | 3.942 | -0.550 | -0.364 | 0.042 | 0.269 | Unannotated | NA | NA |
| metab_17530 | Unannotated feature | neg | 340.9404 | 7.417 | 5.833 | 5.945 | 0.112 | 0.364 | 0.042 | 0.269 | Unannotated | NA | NA |
| metab_16623 | Unannotated feature | neg | 805.2440 | 7.499 | 5.792 | 5.990 | 0.198 | 0.364 | 0.042 | 0.269 | Unannotated | NA | NA |
| metab_16661 | Unannotated feature | neg | 937.3321 | 7.603 | 6.098 | 6.214 | 0.117 | 0.364 | 0.042 | 0.269 | Unannotated | NA | NA |
| metab_14068 | Unannotated feature | neg | 470.9032 | 0.502 | 5.971 | 6.164 | 0.193 | 0.364 | 0.042 | 0.269 | Unannotated | NA | NA |
| metab_16032 | Unannotated feature | neg | 511.2920 | 6.154 | 4.017 | 4.326 | 0.309 | 0.364 | 0.042 | 0.269 | Unannotated | NA | NA |
| metab_4972 | Unannotated feature | pos | 721.3117 | 7.546 | 5.727 | 5.828 | 0.101 | 0.360 | 0.044 | 0.274 | Unannotated | NA | NA |
| metab_16660 | Unannotated feature | neg | 862.8541 | 7.603 | 4.943 | 5.144 | 0.200 | 0.360 | 0.044 | 0.274 | Unannotated | NA | NA |
| metab_11100 | Unannotated feature | neg | 299.1363 | 0.722 | 4.588 | 4.357 | -0.231 | -0.360 | 0.044 | 0.274 | Unannotated | NA | NA |
| metab_15804 | 4-Nonylphenol | neg | 257.1298 | 5.871 | 6.179 | 5.924 | -0.255 | -0.360 | 0.044 | 0.274 | Tentative | Benzenoids | Phenols |
| metab_6245 | Unannotated feature | pos | 656.0173 | 5.806 | 4.012 | 4.926 | 0.914 | 0.360 | 0.044 | 0.274 | Unannotated | NA | NA |
| metab_14039 | Unannotated feature | neg | 321.1421 | 2.771 | 5.303 | 4.843 | -0.459 | -0.360 | 0.044 | 0.274 | Unannotated | NA | NA |
| metab_16714 | Unannotated feature | neg | 714.8458 | 7.648 | 6.121 | 6.205 | 0.084 | 0.360 | 0.044 | 0.274 | Unannotated | NA | NA |
| metab_6798 | Unannotated feature | pos | 358.2709 | 5.175 | 4.743 | 3.802 | -0.941 | -0.360 | 0.044 | 0.274 | Unannotated | NA | NA |
| metab_16684 | Unannotated feature | neg | 821.8427 | 7.617 | 5.746 | 5.909 | 0.163 | 0.360 | 0.044 | 0.274 | Unannotated | NA | NA |
| metab_4706 | Unannotated feature | pos | 634.8783 | 7.610 | 6.492 | 6.558 | 0.066 | 0.360 | 0.044 | 0.274 | Unannotated | NA | NA |
| metab_16056 | MG(0:0/14:1(9Z)/0:0) | neg | 345.2283 | 6.193 | 6.139 | 5.816 | -0.323 | -0.360 | 0.044 | 0.274 | Unannotated | NA | NA |
| metab_3002 | Unannotated feature | pos | 201.1598 | 3.135 | 4.689 | 4.160 | -0.529 | -0.360 | 0.044 | 0.274 | Unannotated | NA | NA |
| metab_16799 | Unannotated feature | neg | 316.1532 | 7.710 | 4.827 | 4.937 | 0.110 | 0.360 | 0.044 | 0.274 | Unannotated | NA | NA |
| metab_12890 | SA-beta-Gal | neg | 545.1735 | 0.722 | 3.196 | 4.633 | 1.437 | 0.360 | 0.044 | 0.274 | Unannotated | Organic oxygen compounds | Organooxygen compounds |
| metab_5910 | Unannotated feature | pos | 707.5389 | 5.922 | 1.609 | 4.383 | 2.774 | 0.360 | 0.044 | 0.274 | Unannotated | NA | NA |
| metab_4787 | Unannotated feature | pos | 388.2552 | 3.607 | 5.292 | 5.662 | 0.370 | 0.360 | 0.044 | 0.274 | Unannotated | NA | NA |
| metab_4751 | Unannotated feature | pos | 244.1763 | 7.708 | 4.187 | 4.264 | 0.077 | 0.360 | 0.044 | 0.274 | Unannotated | NA | NA |
| metab_7781 | Ile Leu Gly | pos | 302.2079 | 3.233 | 4.811 | 3.771 | -1.040 | -0.360 | 0.044 | 0.274 | Strong putative | NA | NA |
| metab_1189 | Unannotated feature | pos | 414.3587 | 6.478 | 5.016 | 4.422 | -0.594 | -0.360 | 0.044 | 0.274 | Unannotated | NA | NA |
| metab_15427 | Therafectin | neg | 286.1663 | 5.132 | 4.742 | 4.501 | -0.241 | -0.360 | 0.044 | 0.274 | Unannotated | NA | NA |
| metab_14770 | Dodec-9-enedioylcarnitine | neg | 408.1785 | 3.834 | 4.811 | 3.775 | -1.036 | -0.360 | 0.044 | 0.274 | Unannotated | NA | NA |
| metab_8115 | Unannotated feature | pos | 315.1349 | 3.527 | 2.735 | 4.423 | 1.688 | 0.360 | 0.044 | 0.274 | Unannotated | NA | NA |
| metab_11544 | Unannotated feature | neg | 532.8757 | 0.489 | 5.930 | 6.038 | 0.108 | 0.360 | 0.044 | 0.274 | Unannotated | NA | NA |
| metab_1868 | Unannotated feature | pos | 331.2348 | 1.176 | 4.212 | 3.482 | -0.729 | -0.360 | 0.044 | 0.274 | Unannotated | NA | NA |
| metab_6303 | Unannotated feature | pos | 564.3571 | 5.787 | 3.885 | 4.718 | 0.834 | 0.360 | 0.044 | 0.274 | Unannotated | NA | NA |
| metab_17619 | Unannotated feature | neg | 242.0670 | 2.766 | 4.656 | 5.071 | 0.416 | 0.360 | 0.044 | 0.274 | Unannotated | NA | NA |
| metab_17103 | Unannotated feature | neg | 236.9723 | 2.141 | 4.783 | 4.031 | -0.753 | -0.360 | 0.044 | 0.274 | Unannotated | NA | NA |
| metab_6659 | Unannotated feature | pos | 323.1608 | 4.733 | 6.476 | 6.126 | -0.349 | -0.360 | 0.044 | 0.274 | Unannotated | NA | NA |
| metab_4022 | Unannotated feature | pos | 616.5522 | 5.814 | 4.112 | 4.592 | 0.480 | 0.360 | 0.044 | 0.274 | Unannotated | NA | NA |
| metab_8155 | Unannotated feature | pos | 139.0756 | 2.703 | 3.235 | 3.673 | 0.438 | 0.360 | 0.044 | 0.274 | Unannotated | NA | NA |
| metab_18452 | Unannotated feature | neg | 301.1518 | 3.028 | 5.012 | 4.620 | -0.392 | -0.360 | 0.044 | 0.274 | Unannotated | NA | NA |
| metab_17928 | Unannotated feature | neg | 483.2959 | 6.632 | 4.652 | 3.866 | -0.786 | -0.360 | 0.044 | 0.274 | Unannotated | NA | NA |
| metab_10152 | N-(2-Furoyl)glycine | neg | 168.0296 | 2.737 | 3.590 | 3.794 | 0.203 | 0.360 | 0.044 | 0.274 | Moderate putative | NA | NA |
| metab_635 | Unannotated feature | pos | 613.7115 | 5.820 | 4.062 | 4.407 | 0.345 | 0.360 | 0.044 | 0.274 | Unannotated | NA | NA |
| metab_606 | Unannotated feature | pos | 594.1798 | 5.787 | 2.733 | 4.657 | 1.924 | 0.360 | 0.044 | 0.274 | Unannotated | NA | NA |
| metab_10240 | Unannotated feature | neg | 323.9667 | 2.575 | 5.060 | 4.943 | -0.117 | -0.360 | 0.044 | 0.274 | Unannotated | NA | NA |
| metab_13020 | Unannotated feature | neg | 131.0456 | 0.628 | 4.814 | 4.460 | -0.354 | -0.360 | 0.044 | 0.274 | Unannotated | NA | NA |
| metab_13958 | Unannotated feature | neg | 682.2435 | 2.637 | 3.679 | 5.008 | 1.329 | 0.360 | 0.044 | 0.274 | Unannotated | NA | NA |
| metab_4278 | Unannotated feature | pos | 492.2122 | 5.960 | 4.653 | 4.248 | -0.404 | -0.360 | 0.044 | 0.274 | Unannotated | NA | NA |
| metab_10356 | Unannotated feature | neg | 230.9357 | 2.186 | 4.184 | 3.676 | -0.508 | -0.360 | 0.044 | 0.274 | Unannotated | NA | NA |
| metab_9972 | Unannotated feature | neg | 258.0482 | 2.880 | 5.738 | 4.352 | -1.386 | -0.360 | 0.044 | 0.274 | Unannotated | NA | NA |
| metab_2396 | Unannotated feature | pos | 218.1503 | 0.569 | 4.481 | 3.620 | -0.861 | -0.360 | 0.044 | 0.274 | Unannotated | NA | NA |
| metab_17572 | Unannotated feature | neg | 710.5382 | 7.305 | 6.057 | 5.676 | -0.381 | -0.360 | 0.044 | 0.274 | Unannotated | NA | NA |
| metab_17049 | Unannotated feature | neg | 216.0123 | 2.186 | 5.550 | 5.293 | -0.257 | -0.360 | 0.044 | 0.274 | Unannotated | NA | NA |
| metab_6712 | Unannotated feature | pos | 796.4882 | 5.283 | 3.217 | 4.977 | 1.760 | 0.360 | 0.044 | 0.274 | Unannotated | NA | NA |
| metab_13971 | Unannotated feature | neg | 143.1183 | 2.659 | 4.228 | 3.788 | -0.439 | -0.360 | 0.044 | 0.274 | Unannotated | NA | NA |
| metab_9519 | 3',5-Dihydroxy-4',7-dimethoxyflavanone | neg | 315.0880 | 4.123 | 3.126 | 4.498 | 1.372 | 0.360 | 0.044 | 0.274 | Unannotated | NA | NA |
| metab_3117 | Unannotated feature | pos | 344.1826 | 3.477 | 5.734 | 5.362 | -0.372 | -0.360 | 0.044 | 0.274 | Unannotated | NA | NA |
| metab_8253 | Unannotated feature | pos | 122.0580 | 2.268 | 4.774 | 3.394 | -1.380 | -0.360 | 0.044 | 0.274 | Unannotated | NA | NA |
| metab_9265 | Unannotated feature | neg | 380.2310 | 3.666 | 4.560 | 3.629 | -0.931 | -0.360 | 0.044 | 0.274 | Unannotated | NA | NA |
| metab_1551 | Unannotated feature | pos | 319.1297 | 5.324 | 4.789 | 4.360 | -0.430 | -0.360 | 0.044 | 0.274 | Unannotated | NA | NA |
| metab_3808 | Unannotated feature | pos | 507.0775 | 5.578 | 3.773 | 4.271 | 0.498 | 0.360 | 0.044 | 0.274 | Unannotated | NA | NA |
| metab_17339 | Unannotated feature | neg | 633.3155 | 7.692 | 5.083 | 5.154 | 0.070 | 0.360 | 0.044 | 0.274 | Unannotated | NA | NA |
| metab_9451 | Unannotated feature | neg | 344.2194 | 3.407 | 5.075 | 4.571 | -0.505 | -0.360 | 0.044 | 0.274 | Unannotated | NA | NA |
| metab_14637 | Unannotated feature | neg | 306.1674 | 3.570 | 3.935 | 3.351 | -0.584 | -0.360 | 0.044 | 0.274 | Unannotated | NA | NA |
| metab_697 | Unannotated feature | pos | 640.4916 | 5.922 | 2.582 | 4.440 | 1.858 | 0.360 | 0.044 | 0.274 | Unannotated | NA | NA |
| metab_6173 | Unannotated feature | pos | 656.9121 | 5.825 | 3.943 | 4.316 | 0.373 | 0.360 | 0.044 | 0.274 | Unannotated | NA | NA |
| metab_9796 | (2S)-2-Amino-6-[(3-formylpiperidin-1-yl)amino]hexanoic acid | neg | 302.1723 | 3.041 | 5.502 | 5.137 | -0.365 | -0.360 | 0.044 | 0.274 | Unannotated | NA | NA |
| metab_6175 | Unannotated feature | pos | 628.0522 | 5.825 | 2.890 | 4.055 | 1.164 | 0.360 | 0.044 | 0.274 | Unannotated | NA | NA |
| metab_2861 | 5-Hydroxy-N-formylkynurenine | pos | 285.1086 | 2.780 | 4.239 | 4.590 | 0.351 | 0.360 | 0.044 | 0.274 | Tentative | Organic oxygen compounds | Organooxygen compounds |
| metab_3207 | Unannotated feature | pos | 417.2356 | 3.734 | 4.354 | 3.612 | -0.743 | -0.360 | 0.044 | 0.274 | Unannotated | NA | NA |
| metab_19211 | Unannotated feature | neg | 179.0707 | 5.249 | 3.269 | 2.717 | -0.552 | -0.360 | 0.044 | 0.274 | Unannotated | NA | NA |
| metab_12474 | Unannotated feature | neg | 192.9584 | 7.997 | 5.189 | 5.147 | -0.042 | -0.360 | 0.044 | 0.274 | Unannotated | NA | NA |
| metab_9683 | Unannotated feature | neg | 320.1619 | 4.228 | 4.961 | 4.574 | -0.387 | -0.360 | 0.044 | 0.274 | Unannotated | NA | NA |
| metab_13963 | Unannotated feature | neg | 611.1391 | 2.646 | 5.603 | 4.909 | -0.693 | -0.360 | 0.044 | 0.274 | Unannotated | NA | NA |
| metab_5706 | Unannotated feature | pos | 250.1781 | 6.111 | 5.787 | 5.699 | -0.088 | -0.360 | 0.044 | 0.274 | Unannotated | NA | NA |
| metab_9565 | S-Farnesyl cysteine | neg | 360.1782 | 3.258 | 5.536 | 5.037 | -0.499 | -0.360 | 0.044 | 0.274 | Tentative | NA | NA |
| metab_13918 | Unannotated feature | neg | 318.1674 | 2.535 | 4.809 | 4.363 | -0.446 | -0.356 | 0.047 | 0.281 | Unannotated | NA | NA |
| metab_7631 | Nafenopin | pos | 333.1456 | 3.491 | 4.326 | 3.821 | -0.505 | -0.356 | 0.047 | 0.281 | Tentative | NA | NA |
| metab_6240 | Unannotated feature | pos | 579.8647 | 5.811 | 3.927 | 4.689 | 0.762 | 0.356 | 0.047 | 0.281 | Unannotated | NA | NA |
| metab_1945 | Unannotated feature | pos | 209.1286 | 1.952 | 5.234 | 5.090 | -0.144 | -0.356 | 0.047 | 0.281 | Unannotated | NA | NA |
| metab_641 | Unannotated feature | pos | 615.7256 | 5.814 | 1.819 | 3.979 | 2.159 | 0.356 | 0.047 | 0.281 | Unannotated | NA | NA |
| metab_10065 | Unannotated feature | neg | 378.1644 | 2.780 | 4.889 | 4.485 | -0.404 | -0.356 | 0.047 | 0.281 | Unannotated | NA | NA |
| metab_10052 | (2R,3S,5R)-5-(4,5-Dihydroimidazo[2,1-f]purin-3-yl)-2-(hydroxymethyl)oxolan-3-ol | neg | 276.1090 | 2.789 | 3.757 | 4.231 | 0.474 | 0.356 | 0.047 | 0.281 | Unannotated | NA | NA |
| metab_17941 | Unannotated feature | neg | 211.0470 | 2.841 | 4.292 | 0.393 | -3.899 | -0.356 | 0.047 | 0.281 | Unannotated | NA | NA |
| metab_8102 | Unannotated feature | pos | 179.1287 | 2.740 | 4.247 | 3.284 | -0.963 | -0.356 | 0.047 | 0.281 | Unannotated | NA | NA |
| metab_19178 | Unannotated feature | neg | 421.1738 | 3.358 | 4.627 | 3.984 | -0.643 | -0.356 | 0.047 | 0.281 | Unannotated | NA | NA |
| metab_6976 | Permetin A | pos | 562.3456 | 4.896 | 4.168 | 5.340 | 1.172 | 0.356 | 0.047 | 0.281 | Unannotated | Organic acids and derivatives | Peptidomimetics |
| metab_7731 | Tyrosyl-Leucine | pos | 312.1923 | 3.319 | 3.560 | 3.939 | 0.379 | 0.356 | 0.047 | 0.281 | Unannotated | Organic acids and derivatives | Carboxylic acids and derivatives |
| metab_10090 | Unannotated feature | neg | 328.1045 | 2.771 | 4.179 | 4.468 | 0.288 | 0.356 | 0.047 | 0.281 | Unannotated | NA | NA |
| metab_13546 | Unannotated feature | neg | 277.0501 | 1.154 | 4.248 | 4.996 | 0.749 | 0.356 | 0.047 | 0.281 | Unannotated | NA | NA |
| metab_494 | Unannotated feature | pos | 436.2455 | 5.068 | 4.895 | 4.437 | -0.458 | -0.356 | 0.047 | 0.281 | Unannotated | NA | NA |
| metab_18810 | 8-Azaspiro[4.5]decane-7,9-dione | neg | 148.0760 | 5.774 | 4.631 | 4.340 | -0.291 | -0.356 | 0.047 | 0.281 | Unannotated | Organoheterocyclic compounds | Azaspirodecane derivatives |
| metab_8720 | Unannotated feature | pos | 195.1237 | 0.768 | 4.403 | 3.685 | -0.717 | -0.356 | 0.047 | 0.281 | Unannotated | NA | NA |
| metab_19849 | Aspartic Acid | neg | 132.0296 | 0.636 | 6.508 | 6.291 | -0.217 | -0.356 | 0.047 | 0.281 | Strong putative | NA | NA |
| metab_3056 | Lys Ile Leu | pos | 373.2820 | 3.324 | 5.064 | 4.476 | -0.588 | -0.356 | 0.047 | 0.281 | Unannotated | NA | NA |
| metab_18859 | Unannotated feature | neg | 378.1671 | 3.215 | 5.054 | 4.793 | -0.260 | -0.356 | 0.047 | 0.281 | Unannotated | NA | NA |
| metab_1004 | Tildipirosin | pos | 734.5345 | 7.292 | 5.629 | 4.517 | -1.112 | -0.356 | 0.047 | 0.281 | Unannotated | Organic oxygen compounds | Organooxygen compounds |
| metab_2898 | Unannotated feature | pos | 180.1365 | 2.853 | 3.779 | 3.295 | -0.484 | -0.356 | 0.047 | 0.281 | Unannotated | NA | NA |
| metab_11862 | Unannotated feature | neg | 587.2894 | 5.991 | 6.301 | 5.874 | -0.427 | -0.356 | 0.047 | 0.281 | Unannotated | NA | NA |
| metab_6431 | Unannotated feature | pos | 583.8728 | 5.686 | 3.175 | 4.658 | 1.483 | 0.356 | 0.047 | 0.281 | Unannotated | NA | NA |
| metab_2323 | Unannotated feature | pos | 219.0193 | 0.332 | 4.129 | 4.181 | 0.053 | 0.356 | 0.047 | 0.281 | Unannotated | NA | NA |
| metab_10142 | Unannotated feature | neg | 503.1997 | 2.746 | 4.701 | 5.550 | 0.848 | 0.356 | 0.047 | 0.281 | Unannotated | NA | NA |
| metab_10457 | Unannotated feature | neg | 288.1094 | 1.958 | 3.095 | 4.340 | 1.245 | 0.356 | 0.047 | 0.281 | Unannotated | NA | NA |
| metab_17735 | Unannotated feature | neg | 403.1844 | 2.766 | 4.901 | 4.253 | -0.648 | -0.356 | 0.047 | 0.281 | Unannotated | NA | NA |
| metab_6019 | Unannotated feature | pos | 756.4642 | 5.898 | 2.754 | 4.737 | 1.983 | 0.356 | 0.047 | 0.281 | Unannotated | NA | NA |
| metab_10485 | Unannotated feature | neg | 255.1352 | 1.952 | 4.162 | 3.565 | -0.597 | -0.356 | 0.047 | 0.281 | Unannotated | NA | NA |
| metab_2757 | Unannotated feature | pos | 860.2735 | 2.411 | 5.353 | 5.474 | 0.121 | 0.356 | 0.047 | 0.281 | Unannotated | NA | NA |
| metab_7618 | Ginsenoside M7cd | pos | 328.2238 | 3.517 | 5.133 | 4.742 | -0.391 | -0.356 | 0.047 | 0.281 | Unannotated | Lipids and lipid-like molecules | Prenol lipids |
| metab_18312 | Unannotated feature | neg | 359.2705 | 6.209 | 5.665 | 5.246 | -0.419 | -0.356 | 0.047 | 0.281 | Unannotated | NA | NA |
| metab_16395 | Unannotated feature | neg | 260.0454 | 1.650 | 4.782 | 4.680 | -0.101 | -0.356 | 0.047 | 0.281 | Unannotated | NA | NA |
| metab_9314 | (S)-Abscisic acid | neg | 299.1038 | 3.589 | 3.144 | 3.979 | 0.836 | 0.356 | 0.047 | 0.281 | Tentative | Lipids and lipid-like molecules | Prenol lipids |
| metab_3806 | Anandamide | pos | 392.2554 | 5.578 | 5.085 | 4.314 | -0.771 | -0.356 | 0.047 | 0.281 | Tentative | Organic nitrogen compounds | Organonitrogen compounds |
| metab_14323 | Unannotated feature | neg | 336.1571 | 3.078 | 4.982 | 4.730 | -0.252 | -0.356 | 0.047 | 0.281 | Unannotated | NA | NA |
| metab_7683 | Ile Leu Thr | pos | 346.2344 | 3.404 | 5.046 | 4.641 | -0.405 | -0.356 | 0.047 | 0.281 | Strong putative | NA | NA |
| metab_1386 | (4Z,7Z,10Z,13Z,16Z)-19,20-Dihydroxydocosa-4,7,10,13,16-pentaenoylcarnitine | pos | 544.3068 | 6.103 | 4.772 | 4.368 | -0.404 | -0.356 | 0.047 | 0.281 | Unannotated | Lipids and lipid-like molecules | Fatty Acyls |
| metab_1375 | Unannotated feature | pos | 204.1234 | 2.724 | 4.749 | 5.014 | 0.264 | 0.356 | 0.047 | 0.281 | Unannotated | NA | NA |
| metab_8406 | Methyldopa | pos | 194.0816 | 1.952 | 5.038 | 5.269 | 0.231 | 0.356 | 0.047 | 0.281 | Tentative | NA | NA |
| metab_8531 | Policapram | pos | 98.0969 | 1.396 | 4.059 | 4.184 | 0.125 | 0.356 | 0.047 | 0.281 | Tentative | NA | NA |
| metab_1679 | Unannotated feature | pos | 512.3207 | 4.295 | 4.834 | 3.940 | -0.894 | -0.356 | 0.047 | 0.281 | Unannotated | NA | NA |
| metab_1768 | Unannotated feature | pos | 482.3101 | 3.404 | 4.877 | 3.591 | -1.286 | -0.356 | 0.047 | 0.281 | Unannotated | NA | NA |
| metab_13305 | 2-C-Methyl-1,4-erythrono-D-lactone | neg | 113.0237 | 0.644 | 5.904 | 5.737 | -0.167 | -0.356 | 0.047 | 0.281 | Unannotated | Organoheterocyclic compounds | Lactones |
| metab_2535 | Unannotated feature | pos | 146.1541 | 0.997 | 4.824 | 4.312 | -0.512 | -0.356 | 0.047 | 0.281 | Unannotated | NA | NA |
| metab_9735 | Unannotated feature | neg | 373.1734 | 3.090 | 6.382 | 6.019 | -0.363 | -0.356 | 0.047 | 0.281 | Unannotated | NA | NA |
| metab_13903 | Threonylphenylalanine | neg | 265.1196 | 2.494 | 4.757 | 4.344 | -0.413 | -0.356 | 0.047 | 0.281 | Unannotated | NA | NA |
| metab_3469 | Unannotated feature | pos | 456.7737 | 4.699 | 2.486 | 4.534 | 2.048 | 0.356 | 0.047 | 0.281 | Unannotated | NA | NA |
| metab_8359 | Linustatin | pos | 410.1678 | 1.952 | 3.894 | 4.636 | 0.742 | 0.356 | 0.047 | 0.281 | Tentative | NA | NA |
| metab_17402 | Unannotated feature | neg | 850.8215 | 7.648 | 5.518 | 5.693 | 0.176 | 0.356 | 0.047 | 0.281 | Unannotated | NA | NA |
| metab_18236 | Unannotated feature | neg | 565.2850 | 6.263 | 6.264 | 6.576 | 0.312 | 0.356 | 0.047 | 0.281 | Unannotated | NA | NA |
| metab_17484 | Unannotated feature | neg | 721.5033 | 7.534 | 5.647 | 5.309 | -0.338 | -0.356 | 0.047 | 0.281 | Unannotated | NA | NA |
| metab_4992 | Unannotated feature | pos | 387.2354 | 7.482 | 4.787 | 4.675 | -0.112 | -0.356 | 0.047 | 0.281 | Unannotated | NA | NA |
| metab_354 | Unannotated feature | pos | 238.0502 | 0.385 | 5.147 | 5.187 | 0.040 | 0.356 | 0.047 | 0.281 | Unannotated | NA | NA |
| metab_7249 | Imidazole Lactic Acid | pos | 174.0876 | 0.688 | 4.290 | 4.849 | 0.559 | 0.356 | 0.047 | 0.281 | Strong putative | Organoheterocyclic compounds | Azoles |
| metab_2484 | D-Alanyl-l-alanine | pos | 125.0713 | 0.736 | 3.792 | 4.158 | 0.367 | 0.356 | 0.047 | 0.281 | Tentative | NA | NA |
| metab_14150 | Unannotated feature | neg | 270.1462 | 2.890 | 4.831 | 4.166 | -0.665 | -0.356 | 0.047 | 0.281 | Unannotated | NA | NA |
| metab_2970 | Unannotated feature | pos | 287.1970 | 3.021 | 3.200 | 2.317 | -0.883 | -0.356 | 0.047 | 0.281 | Unannotated | NA | NA |
| metab_1081 | Unannotated feature | pos | 202.1190 | 1.944 | 5.284 | 4.548 | -0.736 | -0.356 | 0.047 | 0.281 | Unannotated | NA | NA |
| metab_301 | Unannotated feature | pos | 339.1559 | 4.598 | 4.837 | 4.378 | -0.459 | -0.356 | 0.047 | 0.281 | Unannotated | NA | NA |
| metab_14374 | Epsilon-caprolactam | neg | 158.0816 | 3.153 | 5.946 | 5.872 | -0.075 | -0.356 | 0.047 | 0.281 | Tentative | NA | NA |
| metab_2433 | (2R,3S,4R,5R,8R,9S,10R,11R)-5-Amino-1,2,3,4,8,9,10,11-octahydroxydodecane-6,7-dione | pos | 380.0962 | 0.625 | 3.493 | 4.427 | 0.934 | 0.356 | 0.047 | 0.281 | Unannotated | Organic oxygen compounds | Organooxygen compounds |
| metab_2420 | Unannotated feature | pos | 176.0111 | 0.617 | 3.487 | 3.875 | 0.388 | 0.356 | 0.047 | 0.281 | Unannotated | NA | NA |
| metab_3612 | N-Arachidonoyl Proline | pos | 424.2834 | 6.678 | 5.940 | 5.695 | -0.246 | -0.352 | 0.049 | 0.286 | Unannotated | NA | NA |
| metab_17557 | Unannotated feature | neg | 102.9484 | 7.335 | 4.824 | 4.896 | 0.072 | 0.352 | 0.049 | 0.286 | Unannotated | NA | NA |
| metab_2416 | Unannotated feature | pos | 598.0319 | 5.639 | 3.500 | 5.003 | 1.503 | 0.352 | 0.049 | 0.286 | Unannotated | NA | NA |
| metab_2477 | Artabsin | pos | 271.1294 | 0.712 | 4.212 | 4.614 | 0.403 | 0.352 | 0.049 | 0.286 | Tentative | NA | NA |
| metab_353 | Unannotated feature | pos | 860.2735 | 0.390 | 5.779 | 5.826 | 0.047 | 0.352 | 0.049 | 0.286 | Unannotated | NA | NA |
| metab_19428 | Unannotated feature | neg | 336.1094 | 4.970 | 2.925 | 3.359 | 0.435 | 0.352 | 0.049 | 0.286 | Unannotated | NA | NA |
| metab_17428 | Unannotated feature | neg | 970.8208 | 7.621 | 5.699 | 5.891 | 0.192 | 0.352 | 0.049 | 0.286 | Unannotated | NA | NA |
| metab_7451 | Trp Val | pos | 304.1661 | 3.862 | 4.716 | 4.319 | -0.397 | -0.352 | 0.049 | 0.286 | Unannotated | NA | NA |
| metab_1737 | Unannotated feature | pos | 534.3141 | 4.123 | 4.030 | 4.429 | 0.399 | 0.352 | 0.049 | 0.286 | Unannotated | NA | NA |
| metab_13651 | Unannotated feature | neg | 83.0129 | 1.675 | 3.370 | 3.046 | -0.324 | -0.352 | 0.049 | 0.286 | Unannotated | NA | NA |
| metab_4576 | Unannotated feature | pos | 413.3248 | 6.925 | 5.123 | 4.894 | -0.229 | -0.352 | 0.049 | 0.286 | Unannotated | NA | NA |
| metab_8397 | Valylvaline | pos | 217.1551 | 1.952 | 6.137 | 5.987 | -0.150 | -0.352 | 0.049 | 0.286 | Unannotated | NA | NA |
| metab_6011 | Unannotated feature | pos | 608.8752 | 5.906 | 3.386 | 3.831 | 0.445 | 0.352 | 0.049 | 0.286 | Unannotated | NA | NA |
| metab_8370 | Unannotated feature | pos | 320.1822 | 1.952 | 4.521 | 4.153 | -0.367 | -0.352 | 0.049 | 0.286 | Unannotated | NA | NA |
| metab_16932 | Unannotated feature | neg | 186.9293 | 7.819 | 4.713 | 4.621 | -0.092 | -0.352 | 0.049 | 0.286 | Unannotated | NA | NA |
| metab_6370 | Unannotated feature | pos | 684.4311 | 5.754 | 4.018 | 5.089 | 1.071 | 0.352 | 0.049 | 0.286 | Unannotated | NA | NA |
| metab_284 | Unannotated feature | pos | 302.9366 | 0.493 | 4.430 | 4.549 | 0.119 | 0.352 | 0.049 | 0.286 | Unannotated | NA | NA |
| metab_6905 | Unannotated feature | pos | 382.2347 | 5.020 | 4.541 | 3.496 | -1.044 | -0.352 | 0.049 | 0.286 | Unannotated | NA | NA |
| metab_3291 | Unannotated feature | pos | 394.1983 | 4.027 | 4.468 | 4.171 | -0.297 | -0.352 | 0.049 | 0.286 | Unannotated | NA | NA |
| metab_1818 | Unannotated feature | pos | 358.1617 | 0.768 | 4.502 | 3.887 | -0.615 | -0.352 | 0.049 | 0.286 | Unannotated | NA | NA |
| metab_8414 | 3-Amino-5-hydroxybenzoic acid | pos | 154.0501 | 1.952 | 4.873 | 5.273 | 0.400 | 0.352 | 0.049 | 0.286 | Tentative | NA | NA |
| metab_5268 | Ribostamycin | pos | 419.2180 | 6.638 | 5.349 | 5.097 | -0.252 | -0.352 | 0.049 | 0.286 | Tentative | Organic oxygen compounds | Organooxygen compounds |
| metab_642 | Unannotated feature | pos | 691.0380 | 5.811 | 2.515 | 4.835 | 2.321 | 0.352 | 0.049 | 0.286 | Unannotated | NA | NA |
| metab_8918 | Nootkatol | neg | 257.1297 | 4.239 | 5.270 | 5.022 | -0.248 | -0.352 | 0.049 | 0.286 | Tentative | Lipids and lipid-like molecules | Prenol lipids |
| metab_17452 | Unannotated feature | neg | 962.8313 | 7.607 | 6.320 | 6.464 | 0.144 | 0.352 | 0.049 | 0.286 | Unannotated | NA | NA |
| metab_13633 | Glycylvaline | neg | 173.0926 | 1.594 | 6.153 | 5.777 | -0.377 | -0.352 | 0.049 | 0.286 | Strong putative | NA | NA |
| metab_15233 | Unannotated feature | neg | 351.0991 | 4.781 | 1.656 | 3.629 | 1.973 | 0.352 | 0.049 | 0.286 | Unannotated | NA | NA |
| metab_3811 | 2-(Methylamino)benzoic acid | pos | 303.1345 | 5.593 | 5.896 | 5.689 | -0.207 | -0.352 | 0.049 | 0.286 | Tentative | Benzenoids | Benzene and substituted derivatives |
| metab_14452 | Unannotated feature | neg | 220.0976 | 3.271 | 4.105 | 3.732 | -0.373 | -0.352 | 0.049 | 0.286 | Unannotated | NA | NA |
| metab_2279 | Glu Phe | pos | 295.1293 | 3.228 | 5.430 | 5.020 | -0.409 | -0.352 | 0.049 | 0.286 | Moderate putative | NA | NA |
| metab_2685 | Unannotated feature | pos | 150.0916 | 1.998 | 4.380 | 4.604 | 0.224 | 0.352 | 0.049 | 0.286 | Unannotated | NA | NA |
| metab_17391 | Unannotated feature | neg | 689.8505 | 7.660 | 5.252 | 5.335 | 0.083 | 0.352 | 0.049 | 0.286 | Unannotated | NA | NA |
| metab_8152 | Unannotated feature | pos | 312.1923 | 3.613 | 3.250 | 3.783 | 0.533 | 0.352 | 0.049 | 0.286 | Unannotated | NA | NA |
| metab_8473 | (2-Ethyl-2,4,4-trimethyl-1,3-oxazolidin-3-yl)oxidanyl | pos | 142.1229 | 1.698 | 5.558 | 4.837 | -0.721 | -0.352 | 0.049 | 0.286 | Unannotated | Organoheterocyclic compounds | Azolidines |
| metab_3894 | Unannotated feature | pos | 650.1635 | 5.741 | 4.107 | 4.712 | 0.605 | 0.352 | 0.049 | 0.286 | Unannotated | NA | NA |
| metab_2163 | Naloxazone | pos | 374.2045 | 2.897 | 5.364 | 4.994 | -0.370 | -0.352 | 0.049 | 0.286 | Unannotated | NA | NA |
| metab_2139 | Unannotated feature | pos | 678.9212 | 5.402 | 2.211 | 4.020 | 1.808 | 0.352 | 0.049 | 0.286 | Unannotated | NA | NA |
| metab_9862 | Unannotated feature | neg | 314.2089 | 4.328 | 4.503 | 4.175 | -0.328 | -0.352 | 0.049 | 0.286 | Unannotated | NA | NA |
| metab_9885 | Unannotated feature | neg | 298.1775 | 2.964 | 3.655 | 3.070 | -0.585 | -0.352 | 0.049 | 0.286 | Unannotated | NA | NA |
| metab_9868 | N-Formyl-methionyl-leucyl-phenyl-alanine | neg | 474.1476 | 2.975 | 2.361 | 3.608 | 1.247 | 0.352 | 0.049 | 0.286 | Unannotated | NA | NA |
| metab_4707 | Unannotated feature | pos | 838.8413 | 7.610 | 6.339 | 6.428 | 0.089 | 0.352 | 0.049 | 0.286 | Unannotated | NA | NA |
| metab_10718 | Leonurine | neg | 356.1468 | 1.336 | 5.135 | 4.848 | -0.288 | -0.352 | 0.049 | 0.286 | Tentative | Benzenoids | Benzene and substituted derivatives |
| metab_8698 | Phenethylamine glucuronide | pos | 330.1555 | 0.776 | 3.620 | 4.685 | 1.065 | 0.352 | 0.049 | 0.286 | Tentative | Organic oxygen compounds | Organooxygen compounds |
| metab_6054 | Asi-222 | pos | 484.3031 | 5.882 | 6.205 | 6.042 | -0.163 | -0.352 | 0.049 | 0.286 | Unannotated | NA | NA |
| metab_15596 | Germacrone | neg | 255.1143 | 5.496 | 5.649 | 5.266 | -0.384 | -0.352 | 0.049 | 0.286 | Tentative | Lipids and lipid-like molecules | Prenol lipids |
| metab_16560 | Unannotated feature | neg | 715.4163 | 7.297 | 5.484 | 4.802 | -0.682 | -0.352 | 0.049 | 0.286 | Unannotated | NA | NA |
| metab_14800 | Unannotated feature | neg | 356.9218 | 0.512 | 6.087 | 6.226 | 0.139 | 0.352 | 0.049 | 0.286 | Unannotated | NA | NA |
| metab_7131 | Unannotated feature | pos | 359.7142 | 4.579 | 3.328 | 4.604 | 1.276 | 0.352 | 0.049 | 0.286 | Unannotated | NA | NA |
| metab_9211 | Unannotated feature | neg | 482.1728 | 2.972 | 2.809 | 3.678 | 0.869 | 0.352 | 0.049 | 0.286 | Unannotated | NA | NA |
| metab_10119 | Unannotated feature | neg | 346.1624 | 2.762 | 6.065 | 5.728 | -0.337 | -0.352 | 0.049 | 0.286 | Unannotated | NA | NA |
| metab_3947 | Unannotated feature | pos | 552.0914 | 5.782 | 2.075 | 4.415 | 2.341 | 0.352 | 0.049 | 0.286 | Unannotated | NA | NA |
| metab_10094 | Unannotated feature | neg | 245.0667 | 2.771 | 4.500 | 4.701 | 0.201 | 0.352 | 0.049 | 0.286 | Unannotated | NA | NA |
| metab_332 | Unannotated feature | pos | 301.0372 | 0.457 | 4.464 | 4.350 | -0.114 | -0.352 | 0.049 | 0.286 | Unannotated | NA | NA |
| metab_344 | Unannotated feature | pos | 786.2549 | 0.420 | 5.842 | 5.871 | 0.029 | 0.352 | 0.049 | 0.286 | Unannotated | NA | NA |
| metab_13690 | Unannotated feature | neg | 249.9069 | 0.208 | 7.057 | 7.039 | -0.018 | -0.352 | 0.049 | 0.286 | Unannotated | NA | NA |
| metab_11583 | Unannotated feature | neg | 325.9273 | 0.453 | 3.886 | 3.673 | -0.213 | -0.352 | 0.049 | 0.286 | Unannotated | NA | NA |
| metab_16988 | Unannotated feature | neg | 154.9459 | 7.985 | 4.608 | 4.565 | -0.043 | -0.352 | 0.049 | 0.286 | Unannotated | NA | NA |
| metab_10646 | N2-Succinyl-L-ornithine | neg | 231.0984 | 1.656 | 5.057 | 4.807 | -0.250 | -0.352 | 0.049 | 0.286 | Tentative | Organic acids and derivatives | Carboxylic acids and derivatives |
| metab_4681 | Unannotated feature | pos | 608.3875 | 4.179 | 5.433 | 5.876 | 0.442 | 0.352 | 0.049 | 0.286 | Unannotated | NA | NA |
| metab_7701 | Unannotated feature | pos | 246.6449 | 3.371 | 3.747 | 2.578 | -1.168 | -0.352 | 0.049 | 0.286 | Unannotated | NA | NA |
| metab_578 | Unannotated feature | pos | 339.2399 | 5.615 | 2.758 | 3.445 | 0.687 | 0.352 | 0.049 | 0.286 | Unannotated | NA | NA |
| metab_10164 | Amidinophenylpyruvic acid | neg | 411.1303 | 2.714 | 4.187 | 5.078 | 0.891 | 0.352 | 0.049 | 0.286 | Unannotated | Benzenoids | Benzene and substituted derivatives |
| metab_7959 | 2,8-Quinolinediol | pos | 162.0552 | 2.897 | 4.862 | 5.193 | 0.331 | 0.352 | 0.049 | 0.286 | Strong putative | NA | NA |
| metab_5083 | Unannotated feature | pos | 312.3264 | 7.084 | 5.136 | 4.948 | -0.187 | -0.352 | 0.049 | 0.286 | Unannotated | NA | NA |
| metab_10860 | 2-Amino-4-[(2-hydroxy-1-oxopropyl)amino]butanoic acid | neg | 171.0769 | 1.004 | 4.340 | 4.248 | -0.092 | -0.352 | 0.049 | 0.286 | Unannotated | Organic acids and derivatives | Carboxylic acids and derivatives |
| metab_10905 | Picolinic Acid | neg | 122.0241 | 0.868 | 5.635 | 5.480 | -0.154 | -0.352 | 0.049 | 0.286 | Moderate putative | Organoheterocyclic compounds | Pyridines and derivatives |
| metab_13558 | Unannotated feature | neg | 187.0243 | 1.205 | 4.373 | 4.534 | 0.161 | 0.352 | 0.049 | 0.286 | Unannotated | NA | NA |
| metab_933 | Unannotated feature | pos | 165.9880 | 0.609 | 4.206 | 4.500 | 0.294 | 0.352 | 0.049 | 0.286 | Unannotated | NA | NA |
| metab_3057 | [(2S,3R)-2-Amino-3-hydroxybutanoyl] (2S)-pyrrolidine-2-carboxylate | pos | 455.2153 | 3.324 | 4.975 | 3.986 | -0.989 | -0.352 | 0.049 | 0.286 | Unannotated | Organic acids and derivatives | Carboxylic acids and derivatives |
| metab_7493 | Pentostatin | pos | 301.1509 | 0.720 | 4.934 | 4.834 | -0.100 | -0.352 | 0.049 | 0.286 | Tentative | Organoheterocyclic compounds | Imidazodiazepines |
| metab_19743 | Unannotated feature | neg | 377.1359 | 4.510 | 4.723 | 4.489 | -0.233 | -0.352 | 0.049 | 0.286 | Unannotated | NA | NA |
| metab_2636 | Unannotated feature | pos | 297.0821 | 1.844 | 4.927 | 4.756 | -0.171 | -0.352 | 0.049 | 0.286 | Unannotated | NA | NA |
| metab_8705 | Unannotated feature | pos | 279.1705 | 4.171 | 5.877 | 5.471 | -0.405 | -0.352 | 0.049 | 0.286 | Unannotated | NA | NA |
| metab_14107 | Unannotated feature | neg | 668.8458 | 0.507 | 5.085 | 5.362 | 0.277 | 0.352 | 0.049 | 0.286 | Unannotated | NA | NA |
| metab_2677 | Unannotated feature | pos | 280.1295 | 1.968 | 3.996 | 3.409 | -0.586 | -0.352 | 0.049 | 0.286 | Unannotated | NA | NA |
| metab_8712 | Ala Val Lys | pos | 317.2190 | 0.768 | 5.025 | 4.257 | -0.768 | -0.352 | 0.049 | 0.286 | Moderate putative | NA | NA |
| metab_2514 | D-Methionine (S)-S-oxide | pos | 148.0428 | 0.846 | 3.131 | 3.636 | 0.505 | 0.347 | 0.052 | 0.291 | Unannotated | NA | NA |
| metab_11459 | Unannotated feature | neg | 258.9175 | 0.527 | 5.393 | 5.829 | 0.436 | 0.347 | 0.052 | 0.291 | Unannotated | NA | NA |
| metab_6170 | Unannotated feature | pos | 697.7609 | 5.825 | 2.321 | 3.738 | 1.417 | 0.347 | 0.052 | 0.291 | Unannotated | NA | NA |
| metab_6610 | 21-Deoxycortisone | pos | 367.1873 | 5.434 | 4.789 | 3.555 | -1.235 | -0.347 | 0.052 | 0.291 | Tentative | NA | NA |
| metab_6946 | Val Ile Ile | pos | 344.2552 | 4.950 | 5.457 | 5.242 | -0.215 | -0.347 | 0.052 | 0.291 | Strong putative | NA | NA |
| metab_2505 | Unannotated feature | pos | 118.1102 | 0.800 | 4.241 | 3.933 | -0.308 | -0.347 | 0.052 | 0.291 | Unannotated | NA | NA |
| metab_251 | Unannotated feature | pos | 366.8639 | 0.531 | 4.340 | 4.994 | 0.655 | 0.347 | 0.052 | 0.291 | Unannotated | NA | NA |
| metab_265 | Unannotated feature | pos | 395.2778 | 6.590 | 5.748 | 5.975 | 0.226 | 0.347 | 0.052 | 0.291 | Unannotated | NA | NA |
| metab_11801 | Unannotated feature | neg | 647.2656 | 5.839 | 5.125 | 3.628 | -1.497 | -0.347 | 0.052 | 0.291 | Unannotated | NA | NA |
| metab_11799 | Unannotated feature | neg | 652.3007 | 5.834 | 6.854 | 6.085 | -0.769 | -0.347 | 0.052 | 0.291 | Unannotated | NA | NA |
| metab_6099 | Unannotated feature | pos | 645.9949 | 5.851 | 2.554 | 4.279 | 1.726 | 0.347 | 0.052 | 0.291 | Unannotated | NA | NA |
| metab_14188 | Unannotated feature | neg | 297.1438 | 2.931 | 4.730 | 4.426 | -0.304 | -0.347 | 0.052 | 0.291 | Unannotated | NA | NA |
| metab_9722 | Unannotated feature | neg | 297.1438 | 3.105 | 4.810 | 4.577 | -0.233 | -0.347 | 0.052 | 0.291 | Unannotated | NA | NA |
| metab_7047 | Unannotated feature | pos | 220.1006 | 4.763 | 4.482 | 3.758 | -0.725 | -0.347 | 0.052 | 0.291 | Unannotated | NA | NA |
| metab_10393 | Unannotated feature | neg | 243.9079 | 2.074 | 3.894 | 3.706 | -0.188 | -0.347 | 0.052 | 0.291 | Unannotated | NA | NA |
| metab_3498 | 1-(4-Hydroxy-3-methoxyphenyl)-3-decanone | pos | 317.1504 | 4.783 | 3.867 | 4.500 | 0.633 | 0.347 | 0.052 | 0.291 | Tentative | Benzenoids | Phenols |
| metab_7595 | Beta-D-Galactopyranosyl-(1->4)-2-amino-2-deoxy-beta-D-glucopyranosyl-(1->6)-D-mannose | pos | 468.1726 | 0.728 | 4.538 | 5.087 | 0.550 | 0.347 | 0.052 | 0.291 | Unannotated | Organic oxygen compounds | Organooxygen compounds |
| metab_13135 | Unannotated feature | neg | 216.9368 | 0.453 | 3.754 | 3.636 | -0.117 | -0.347 | 0.052 | 0.291 | Unannotated | NA | NA |
| metab_7614 | Unannotated feature | pos | 212.0557 | 0.736 | 3.324 | 4.075 | 0.750 | 0.347 | 0.052 | 0.291 | Unannotated | NA | NA |
| metab_5769 | Naldemedine | pos | 603.2831 | 6.032 | 5.289 | 4.829 | -0.460 | -0.347 | 0.052 | 0.291 | Unannotated | Alkaloids and derivatives | Morphinans |
| metab_2888 | Pro Gln Val | pos | 343.1983 | 2.827 | 4.800 | 4.266 | -0.533 | -0.347 | 0.052 | 0.291 | Moderate putative | NA | NA |
| metab_2568 | Methylmalonic acid | pos | 101.0238 | 1.176 | 3.114 | 2.523 | -0.592 | -0.347 | 0.052 | 0.291 | Tentative | Organic acids and derivatives | Carboxylic acids and derivatives |
| metab_12307 | Unannotated feature | neg | 355.3219 | 6.899 | 5.556 | 5.950 | 0.395 | 0.347 | 0.052 | 0.291 | Unannotated | NA | NA |
| metab_5477 | Xi-4-Hydroxy-4-methyl-2-cyclohexen-1-one | pos | 91.0547 | 6.375 | 5.206 | 5.138 | -0.068 | -0.347 | 0.052 | 0.291 | Unannotated | NA | NA |
| metab_7258 | Unannotated feature | pos | 162.0762 | 0.688 | 5.626 | 5.672 | 0.046 | 0.347 | 0.052 | 0.291 | Unannotated | NA | NA |
| metab_8747 | Unannotated feature | pos | 208.1183 | 0.736 | 3.546 | 4.082 | 0.536 | 0.347 | 0.052 | 0.291 | Unannotated | NA | NA |
| metab_16333 | DG(PGD1/0:0/2:0) | neg | 505.2593 | 6.671 | 5.745 | 6.110 | 0.365 | 0.347 | 0.052 | 0.291 | Unannotated | NA | NA |
| metab_17491 | Unannotated feature | neg | 873.2314 | 7.510 | 5.447 | 5.595 | 0.148 | 0.347 | 0.052 | 0.291 | Unannotated | NA | NA |
| metab_1842 | Unannotated feature | pos | 290.1350 | 1.034 | 5.354 | 5.503 | 0.149 | 0.347 | 0.052 | 0.291 | Unannotated | NA | NA |
| metab_12520 | 3-Hydroxy-2-Octylpentanedioic Acid | neg | 259.1550 | 6.295 | 4.845 | 5.118 | 0.273 | 0.347 | 0.052 | 0.291 | Moderate putative | NA | NA |
| metab_1254 | Unannotated feature | pos | 287.1243 | 2.732 | 4.530 | 4.815 | 0.285 | 0.347 | 0.052 | 0.291 | Unannotated | NA | NA |
| metab_1910 | Unannotated feature | pos | 298.1655 | 1.685 | 2.110 | 4.617 | 2.507 | 0.347 | 0.052 | 0.291 | Unannotated | NA | NA |
| metab_11391 | Unannotated feature | neg | 233.9931 | 0.552 | 3.868 | 4.660 | 0.791 | 0.347 | 0.052 | 0.291 | Unannotated | NA | NA |
| metab_12488 | Unannotated feature | neg | 507.3337 | 6.397 | 5.184 | 5.354 | 0.171 | 0.347 | 0.052 | 0.291 | Unannotated | NA | NA |
| metab_8180 | Unannotated feature | pos | 401.2883 | 3.627 | 4.988 | 4.313 | -0.676 | -0.347 | 0.052 | 0.291 | Unannotated | NA | NA |
| metab_16845 | Unannotated feature | neg | 232.0591 | 7.730 | 5.442 | 5.791 | 0.349 | 0.347 | 0.052 | 0.291 | Unannotated | NA | NA |
| metab_6395 | Unannotated feature | pos | 507.0772 | 5.732 | 3.933 | 4.374 | 0.441 | 0.347 | 0.052 | 0.291 | Unannotated | NA | NA |
| metab_8696 | Unannotated feature | pos | 118.0829 | 0.784 | 4.304 | 4.091 | -0.213 | -0.347 | 0.052 | 0.291 | Unannotated | NA | NA |
| metab_16647 | Unannotated feature | neg | 161.9860 | 1.947 | 5.047 | 4.883 | -0.164 | -0.347 | 0.052 | 0.291 | Unannotated | NA | NA |
| metab_19807 | Unannotated feature | neg | 507.2106 | 4.413 | 4.715 | 3.732 | -0.983 | -0.347 | 0.052 | 0.291 | Unannotated | NA | NA |
| metab_5006 | Unannotated feature | pos | 585.3369 | 7.411 | 5.393 | 5.541 | 0.148 | 0.347 | 0.052 | 0.291 | Unannotated | NA | NA |
| metab_4882 | Unannotated feature | pos | 484.2039 | 0.696 | 5.579 | 5.713 | 0.134 | 0.347 | 0.052 | 0.291 | Unannotated | NA | NA |
| metab_1992 | Isodityrosine | pos | 361.1404 | 2.150 | 4.494 | 4.140 | -0.354 | -0.347 | 0.052 | 0.291 | Unannotated | NA | NA |
| metab_6879 | Unannotated feature | pos | 664.4119 | 5.060 | 3.894 | 4.991 | 1.097 | 0.347 | 0.052 | 0.291 | Unannotated | NA | NA |
| metab_8382 | 4-Aminophenylmannoside | pos | 254.1025 | 1.952 | 4.551 | 5.010 | 0.459 | 0.347 | 0.052 | 0.291 | Unannotated | NA | NA |
| metab_6287 | Unannotated feature | pos | 603.1851 | 5.791 | 3.607 | 4.685 | 1.078 | 0.347 | 0.052 | 0.291 | Unannotated | NA | NA |
| metab_19280 | Unannotated feature | neg | 356.2560 | 5.164 | 4.522 | 3.507 | -1.015 | -0.347 | 0.052 | 0.291 | Unannotated | NA | NA |
| metab_18197 | Unannotated feature | neg | 281.1394 | 6.303 | 1.364 | 4.526 | 3.161 | 0.347 | 0.052 | 0.291 | Unannotated | NA | NA |
| metab_13870 | Unannotated feature | neg | 144.0660 | 2.317 | 5.365 | 5.258 | -0.107 | -0.347 | 0.052 | 0.291 | Unannotated | NA | NA |
| metab_3324 | 6-[2,3-Dihydroxy-1-(hydroxymethyl)propyl]-1,2-dihydro-7-hydroxy-9-methoxy-cyclopenta[c][1]benzopyran-3,4-dione | pos | 315.0873 | 4.132 | 4.169 | 2.582 | -1.587 | -0.347 | 0.052 | 0.291 | Tentative | Phenylpropanoids and polyketides | Coumarins and derivatives |
| metab_11044 | Hydroxyprolyl-Gamma-glutamate | neg | 304.1149 | 0.738 | 5.251 | 4.912 | -0.340 | -0.347 | 0.052 | 0.291 | Unannotated | Organic acids and derivatives | Carboxylic acids and derivatives |
| metab_3623 | Unannotated feature | pos | 782.9786 | 5.121 | 3.878 | 5.156 | 1.279 | 0.347 | 0.052 | 0.291 | Unannotated | NA | NA |
| metab_12598 | Unannotated feature | neg | 667.2971 | 6.076 | 4.682 | 4.049 | -0.633 | -0.347 | 0.052 | 0.291 | Unannotated | NA | NA |
| metab_12603 | Unannotated feature | neg | 583.2961 | 6.068 | 6.345 | 6.415 | 0.070 | 0.347 | 0.052 | 0.291 | Unannotated | NA | NA |
| metab_6824 | Unannotated feature | pos | 348.1280 | 0.633 | 3.522 | 4.215 | 0.692 | 0.347 | 0.052 | 0.291 | Unannotated | NA | NA |
| metab_16563 | Unannotated feature | neg | 246.0983 | 1.685 | 4.004 | 4.457 | 0.453 | 0.347 | 0.052 | 0.291 | Unannotated | NA | NA |
| metab_8027 | Glutamylleucine | pos | 243.1344 | 2.803 | 6.036 | 5.828 | -0.208 | -0.347 | 0.052 | 0.291 | Unannotated | NA | NA |
| metab_3020 | Unannotated feature | pos | 187.1809 | 3.193 | 5.177 | 3.654 | -1.523 | -0.347 | 0.052 | 0.291 | Unannotated | NA | NA |
| metab_8077 | Unannotated feature | pos | 244.6579 | 2.755 | 4.526 | 3.877 | -0.650 | -0.347 | 0.052 | 0.291 | Unannotated | NA | NA |
| metab_6458 | Unannotated feature | pos | 550.8530 | 5.645 | 3.865 | 4.725 | 0.860 | 0.347 | 0.052 | 0.291 | Unannotated | NA | NA |
| metab_17282 | Unannotated feature | neg | 134.8939 | 7.720 | 5.170 | 5.133 | -0.038 | -0.347 | 0.052 | 0.291 | Unannotated | NA | NA |
| metab_5676 | Unannotated feature | pos | 246.2431 | 6.143 | 4.876 | 4.768 | -0.108 | -0.347 | 0.052 | 0.291 | Unannotated | NA | NA |
| metab_17468 | Unannotated feature | neg | 1013.8132 | 7.590 | 5.304 | 5.566 | 0.262 | 0.347 | 0.052 | 0.291 | Unannotated | NA | NA |
| metab_1999 | Unannotated feature | pos | 223.6344 | 1.952 | 4.176 | 3.570 | -0.605 | -0.347 | 0.052 | 0.291 | Unannotated | NA | NA |
| metab_1300 | Unannotated feature | pos | 362.3271 | 6.247 | 6.449 | 6.337 | -0.112 | -0.347 | 0.052 | 0.291 | Unannotated | NA | NA |
| metab_14720 | Unannotated feature | neg | 393.2000 | 3.733 | 4.694 | 3.494 | -1.200 | -0.347 | 0.052 | 0.291 | Unannotated | NA | NA |
| metab_3103 | Ser Leu Val | pos | 318.2029 | 3.426 | 4.607 | 3.552 | -1.055 | -0.347 | 0.052 | 0.291 | Unannotated | NA | NA |
| metab_16719 | Unannotated feature | neg | 620.8474 | 7.652 | 4.496 | 4.603 | 0.107 | 0.347 | 0.052 | 0.291 | Unannotated | NA | NA |
| metab_13536 | Unannotated feature | neg | 714.7619 | 0.533 | 4.593 | 4.892 | 0.299 | 0.347 | 0.052 | 0.291 | Unannotated | NA | NA |
| metab_11570 | Unannotated feature | neg | 536.8974 | 0.460 | 4.678 | 4.527 | -0.151 | -0.347 | 0.052 | 0.291 | Unannotated | NA | NA |
| metab_4792 | Unannotated feature | pos | 124.0397 | 7.971 | 4.138 | 4.085 | -0.052 | -0.347 | 0.052 | 0.291 | Unannotated | NA | NA |
| metab_16600 | Unannotated feature | neg | 653.5163 | 7.417 | 5.768 | 5.064 | -0.704 | -0.347 | 0.052 | 0.291 | Unannotated | NA | NA |
| metab_7963 | Unannotated feature | pos | 230.1584 | 2.892 | 5.344 | 5.243 | -0.101 | -0.347 | 0.052 | 0.291 | Unannotated | NA | NA |
| metab_2054 | Unannotated feature | pos | 565.1688 | 2.703 | 4.880 | 3.721 | -1.158 | -0.347 | 0.052 | 0.291 | Unannotated | NA | NA |
| metab_9947 | Adrenochrome o-semiquinone | neg | 162.0554 | 2.906 | 5.044 | 5.484 | 0.441 | 0.347 | 0.052 | 0.291 | Unannotated | NA | NA |
| metab_9942 | Unannotated feature | neg | 330.9692 | 2.906 | 3.339 | 3.780 | 0.441 | 0.347 | 0.052 | 0.291 | Unannotated | NA | NA |
| metab_11983 | Unannotated feature | neg | 300.1218 | 6.201 | 5.728 | 5.337 | -0.391 | -0.347 | 0.052 | 0.291 | Unannotated | NA | NA |
| metab_9160 | Unannotated feature | neg | 378.2040 | 3.834 | 4.533 | 3.668 | -0.865 | -0.347 | 0.052 | 0.291 | Unannotated | NA | NA |
| metab_16656 | Unannotated feature | neg | 597.3940 | 7.590 | 4.708 | 4.916 | 0.208 | 0.347 | 0.052 | 0.291 | Unannotated | NA | NA |
| metab_21 | Unannotated feature | pos | 295.0963 | 0.712 | 3.516 | 4.073 | 0.557 | 0.343 | 0.055 | 0.296 | Unannotated | NA | NA |
| metab_14032 | Unannotated feature | neg | 396.1410 | 2.766 | 5.231 | 5.009 | -0.222 | -0.343 | 0.055 | 0.296 | Unannotated | NA | NA |
| metab_2910 | Unannotated feature | pos | 208.6473 | 2.871 | 3.655 | 2.950 | -0.705 | -0.343 | 0.055 | 0.296 | Unannotated | NA | NA |
| metab_137 | Unannotated feature | pos | 154.0840 | 0.625 | 3.917 | 2.983 | -0.934 | -0.343 | 0.055 | 0.296 | Unannotated | NA | NA |
| metab_14040 | Nor-ketotifen | neg | 330.0702 | 2.771 | 3.753 | 4.250 | 0.497 | 0.343 | 0.055 | 0.296 | Unannotated | Organoheterocyclic compounds | Cycloheptathiophenes |
| metab_7572 | Unannotated feature | pos | 416.2867 | 3.607 | 4.582 | 4.903 | 0.320 | 0.343 | 0.055 | 0.296 | Unannotated | NA | NA |
| metab_15879 | Unannotated feature | neg | 727.3782 | 5.964 | 4.844 | 4.141 | -0.703 | -0.343 | 0.055 | 0.296 | Unannotated | NA | NA |
| metab_3216 | Monocrotaline | pos | 290.1393 | 3.756 | 2.091 | 2.824 | 0.732 | 0.343 | 0.055 | 0.296 | Tentative | Organoheterocyclic compounds | Pyrrolizines |
| metab_13645 | Unannotated feature | neg | 256.0308 | 1.656 | 5.581 | 5.449 | -0.131 | -0.343 | 0.055 | 0.296 | Unannotated | NA | NA |
| metab_10175 | Unannotated feature | neg | 665.2534 | 2.698 | 1.470 | 5.227 | 3.756 | 0.343 | 0.055 | 0.296 | Unannotated | NA | NA |
| metab_1915 | Unannotated feature | pos | 216.0870 | 1.692 | 4.196 | 4.599 | 0.404 | 0.343 | 0.055 | 0.296 | Unannotated | NA | NA |
| metab_10131 | Unannotated feature | neg | 423.1535 | 2.754 | 5.166 | 4.725 | -0.440 | -0.343 | 0.055 | 0.296 | Unannotated | NA | NA |
| metab_15315 | Unannotated feature | neg | 400.1730 | 4.910 | 4.240 | 4.931 | 0.691 | 0.343 | 0.055 | 0.296 | Unannotated | NA | NA |
| metab_1319 | Unannotated feature | pos | 623.3100 | 6.215 | 5.879 | 5.038 | -0.842 | -0.343 | 0.055 | 0.296 | Unannotated | NA | NA |
| metab_4409 | Unannotated feature | pos | 304.3004 | 6.311 | 4.078 | 3.637 | -0.440 | -0.343 | 0.055 | 0.296 | Unannotated | NA | NA |
| metab_15116 | Unannotated feature | neg | 314.2089 | 4.559 | 5.242 | 5.005 | -0.236 | -0.343 | 0.055 | 0.296 | Unannotated | NA | NA |
| metab_6393 | Unannotated feature | pos | 628.1501 | 5.732 | 4.061 | 4.789 | 0.728 | 0.343 | 0.055 | 0.296 | Unannotated | NA | NA |
| metab_17963 | Sphingosine 1-phosphate | neg | 424.2475 | 6.561 | 6.356 | 6.081 | -0.274 | -0.343 | 0.055 | 0.296 | Tentative | Lipids and lipid-like molecules | Sphingolipids |
| metab_18156 | Atorvastatin | neg | 557.2467 | 6.334 | 2.141 | 4.087 | 1.946 | 0.343 | 0.055 | 0.296 | Strong putative | Organoheterocyclic compounds | Pyrroles |
| metab_5012 | DG(8:0/a-13:0/0:0) | pos | 459.3098 | 7.363 | 4.533 | 4.243 | -0.289 | -0.343 | 0.055 | 0.296 | Tentative | NA | NA |
| metab_13989 | 6''-p-Coumaroylprunin | neg | 579.1490 | 2.698 | 5.534 | 4.835 | -0.699 | -0.343 | 0.055 | 0.296 | Unannotated | NA | NA |
| metab_12549 | Unannotated feature | neg | 373.1693 | 6.193 | 5.964 | 6.239 | 0.275 | 0.343 | 0.055 | 0.296 | Unannotated | NA | NA |
| metab_17354 | Unannotated feature | neg | 527.8725 | 7.687 | 4.625 | 4.766 | 0.141 | 0.343 | 0.055 | 0.296 | Unannotated | NA | NA |
| metab_4876 | Unannotated feature | pos | 99.5316 | 7.724 | 3.785 | 3.571 | -0.214 | -0.343 | 0.055 | 0.296 | Unannotated | NA | NA |
| metab_10664 | Unannotated feature | neg | 216.9091 | 1.584 | 3.859 | 3.682 | -0.177 | -0.343 | 0.055 | 0.296 | Unannotated | NA | NA |
| metab_14747 | Unannotated feature | neg | 390.1705 | 3.780 | 4.580 | 3.732 | -0.848 | -0.343 | 0.055 | 0.296 | Unannotated | NA | NA |
| metab_5728 | Piperettine | pos | 623.3099 | 6.080 | 5.949 | 5.155 | -0.794 | -0.343 | 0.055 | 0.296 | Unannotated | Organoheterocyclic compounds | Benzodioxoles |
| metab_4338 | Unannotated feature | pos | 565.3971 | 6.111 | 4.052 | 3.826 | -0.226 | -0.343 | 0.055 | 0.296 | Unannotated | NA | NA |
| metab_418 | Unannotated feature | pos | 172.0261 | 0.016 | 4.016 | 4.095 | 0.078 | 0.343 | 0.055 | 0.296 | Unannotated | NA | NA |
| metab_7335 | L-Rhamnulose | pos | 206.1026 | 0.704 | 5.257 | 5.426 | 0.169 | 0.343 | 0.055 | 0.296 | Tentative | NA | NA |
| metab_11428 | Unannotated feature | neg | 384.8605 | 0.538 | 5.321 | 5.141 | -0.180 | -0.343 | 0.055 | 0.296 | Unannotated | NA | NA |
| metab_1640 | Unannotated feature | pos | 418.2350 | 4.714 | 4.329 | 3.662 | -0.667 | -0.343 | 0.055 | 0.296 | Unannotated | NA | NA |
| metab_6163 | Unannotated feature | pos | 641.4933 | 5.831 | 3.190 | 4.459 | 1.268 | 0.343 | 0.055 | 0.296 | Unannotated | NA | NA |
| metab_9552 | Phosphorylcholine | neg | 229.0715 | 3.284 | 5.004 | 5.212 | 0.208 | 0.343 | 0.055 | 0.296 | Tentative | Organic nitrogen compounds | Organonitrogen compounds |
| metab_12873 | Unannotated feature | neg | 276.1092 | 0.714 | 4.680 | 5.292 | 0.612 | 0.343 | 0.055 | 0.296 | Unannotated | NA | NA |
| metab_2623 | Salidroside | pos | 342.1552 | 1.692 | 3.001 | 4.074 | 1.072 | 0.343 | 0.055 | 0.296 | Tentative | Organic oxygen compounds | Organooxygen compounds |
| metab_7697 | 4-Nitro-o-phenylenediamine | pos | 195.0881 | 3.375 | 4.372 | 2.703 | -1.670 | -0.343 | 0.055 | 0.296 | Tentative | Benzenoids | Benzene and substituted derivatives |
| metab_6108 | Unannotated feature | pos | 732.1172 | 5.847 | 4.101 | 4.282 | 0.181 | 0.343 | 0.055 | 0.296 | Unannotated | NA | NA |
| metab_6314 | Unannotated feature | pos | 576.3742 | 5.782 | 4.065 | 4.345 | 0.281 | 0.343 | 0.055 | 0.296 | Unannotated | NA | NA |
| metab_1526 | Unannotated feature | pos | 370.6167 | 5.425 | 4.772 | 4.616 | -0.156 | -0.343 | 0.055 | 0.296 | Unannotated | NA | NA |
| metab_6747 | Diethyl 1,4-dihydro-2,6-dimethyl-3,5-pyridinedicarboxylate | pos | 524.2999 | 5.247 | 3.556 | 4.489 | 0.933 | 0.343 | 0.055 | 0.296 | Unannotated | Organoheterocyclic compounds | Pyridines and derivatives |
| metab_7792 | N-Formyl-dl-phenylalanine | pos | 211.1082 | 3.291 | 5.073 | 4.835 | -0.239 | -0.343 | 0.055 | 0.296 | Unannotated | NA | NA |
| metab_4072 | Unannotated feature | pos | 682.9188 | 5.835 | 3.692 | 4.203 | 0.511 | 0.343 | 0.055 | 0.296 | Unannotated | NA | NA |
| metab_4071 | Unannotated feature | pos | 676.3178 | 5.835 | 3.632 | 4.136 | 0.505 | 0.343 | 0.055 | 0.296 | Unannotated | NA | NA |
| metab_1781 | Unannotated feature | pos | 186.0875 | 0.768 | 5.748 | 5.398 | -0.350 | -0.343 | 0.055 | 0.296 | Unannotated | NA | NA |
| metab_13295 | Unannotated feature | neg | 71.0128 | 0.636 | 4.658 | 4.425 | -0.232 | -0.343 | 0.055 | 0.296 | Unannotated | NA | NA |
| metab_8290 | Unannotated feature | pos | 87.0446 | 2.112 | 4.672 | 4.494 | -0.178 | -0.343 | 0.055 | 0.296 | Unannotated | NA | NA |
| metab_4596 | Unannotated feature | pos | 344.3528 | 7.005 | 4.683 | 4.384 | -0.298 | -0.343 | 0.055 | 0.296 | Unannotated | NA | NA |
| metab_16147 | Unannotated feature | neg | 556.3577 | 6.310 | 4.303 | 3.460 | -0.842 | -0.343 | 0.055 | 0.296 | Unannotated | NA | NA |
| metab_6537 | Unannotated feature | pos | 797.5008 | 5.541 | 3.109 | 3.975 | 0.867 | 0.343 | 0.055 | 0.296 | Unannotated | NA | NA |
| metab_14366 | Unannotated feature | neg | 372.2145 | 3.141 | 5.262 | 4.970 | -0.292 | -0.343 | 0.055 | 0.296 | Unannotated | NA | NA |
| metab_1764 | Propanidid | pos | 382.1621 | 3.421 | 4.602 | 4.181 | -0.421 | -0.343 | 0.055 | 0.296 | Unannotated | Benzenoids | Phenol ethers |
| metab_4381 | Unannotated feature | pos | 709.4475 | 5.267 | 4.157 | 4.814 | 0.657 | 0.343 | 0.055 | 0.296 | Unannotated | NA | NA |
| metab_17417 | Unannotated feature | neg | 876.8246 | 7.637 | 5.856 | 5.963 | 0.107 | 0.343 | 0.055 | 0.296 | Unannotated | NA | NA |
| metab_11746 | Unannotated feature | neg | 434.1464 | 5.645 | 3.437 | 4.751 | 1.314 | 0.343 | 0.055 | 0.296 | Unannotated | NA | NA |
| metab_4155 | Unannotated feature | pos | 569.3507 | 5.898 | 3.691 | 3.837 | 0.146 | 0.343 | 0.055 | 0.296 | Unannotated | NA | NA |
| metab_10182 | Betanin | neg | 595.1441 | 2.676 | 5.658 | 5.185 | -0.473 | -0.343 | 0.055 | 0.296 | Tentative | NA | NA |
| metab_19417 | Unannotated feature | neg | 261.0440 | 3.461 | 4.408 | 3.373 | -1.035 | -0.343 | 0.055 | 0.296 | Unannotated | NA | NA |
| metab_18237 | Unannotated feature | neg | 539.3063 | 6.263 | 6.045 | 5.636 | -0.409 | -0.343 | 0.055 | 0.296 | Unannotated | NA | NA |
| metab_18251 | Unannotated feature | neg | 395.1900 | 6.256 | 5.298 | 6.005 | 0.708 | 0.343 | 0.055 | 0.296 | Unannotated | NA | NA |
| metab_2449 | Unannotated feature | pos | 362.0963 | 0.649 | 2.513 | 3.900 | 1.387 | 0.343 | 0.055 | 0.296 | Unannotated | NA | NA |
| metab_10696 | Unannotated feature | neg | 306.0582 | 1.438 | 6.492 | 5.978 | -0.514 | -0.343 | 0.055 | 0.296 | Unannotated | NA | NA |
| metab_1336 | Unannotated feature | pos | 350.1970 | 2.976 | 3.567 | 4.061 | 0.494 | 0.343 | 0.055 | 0.296 | Unannotated | NA | NA |
| metab_4669 | Unannotated feature | pos | 357.2615 | 7.466 | 4.105 | 4.323 | 0.218 | 0.343 | 0.055 | 0.296 | Unannotated | NA | NA |
| metab_6676 | Dihydrocapsiate | pos | 309.2066 | 5.347 | 5.196 | 4.733 | -0.464 | -0.343 | 0.055 | 0.296 | Tentative | NA | NA |
| metab_6332 | Unannotated feature | pos | 552.3424 | 5.778 | 4.012 | 4.573 | 0.560 | 0.343 | 0.055 | 0.296 | Unannotated | NA | NA |
| metab_7935 | 7-Aminoheptanoic acid | pos | 329.1825 | 2.923 | 4.246 | 3.811 | -0.435 | -0.343 | 0.055 | 0.296 | Unannotated | Lipids and lipid-like molecules | Fatty Acyls |
| metab_503 | Unannotated feature | pos | 307.1658 | 5.102 | 6.104 | 5.795 | -0.310 | -0.343 | 0.055 | 0.296 | Unannotated | NA | NA |
| metab_11146 | Unannotated feature | neg | 175.0242 | 0.691 | 5.851 | 5.734 | -0.117 | -0.343 | 0.055 | 0.296 | Unannotated | NA | NA |
| metab_17447 | Unannotated feature | neg | 657.3887 | 7.611 | 5.714 | 5.804 | 0.090 | 0.343 | 0.055 | 0.296 | Unannotated | NA | NA |
| metab_381 | Unannotated feature | pos | 182.0294 | 0.085 | 4.555 | 4.613 | 0.058 | 0.343 | 0.055 | 0.296 | Unannotated | NA | NA |
| metab_2013 | Unannotated feature | pos | 346.1979 | 1.944 | 5.243 | 5.036 | -0.207 | -0.343 | 0.055 | 0.296 | Unannotated | NA | NA |
| metab_12948 | Unannotated feature | neg | 297.0832 | 0.730 | 5.454 | 5.648 | 0.194 | 0.343 | 0.055 | 0.296 | Unannotated | NA | NA |
| metab_19134 | Unannotated feature | neg | 475.2105 | 5.343 | 4.286 | 5.020 | 0.735 | 0.343 | 0.055 | 0.296 | Unannotated | NA | NA |
| metab_3175 | His Ile Ile | pos | 382.2460 | 3.647 | 5.048 | 4.730 | -0.319 | -0.343 | 0.055 | 0.296 | Strong putative | NA | NA |
| metab_9217 | Unannotated feature | neg | 330.2038 | 3.745 | 5.401 | 4.894 | -0.507 | -0.343 | 0.055 | 0.296 | Unannotated | NA | NA |
| metab_10232 | Benzoyl meso-tartaric acid | neg | 253.0355 | 2.586 | 3.936 | 3.571 | -0.366 | -0.343 | 0.055 | 0.296 | Unannotated | Benzenoids | Benzene and substituted derivatives |
| metab_10225 | 2,4,5-Trihydroxyphenylalanine | neg | 258.0621 | 2.602 | 4.907 | 5.176 | 0.269 | 0.343 | 0.055 | 0.296 | Unannotated | NA | NA |
| metab_6384 | Phenylalanyl-prolyl-arginine nitrile | pos | 364.2239 | 5.741 | 4.405 | 3.893 | -0.512 | -0.339 | 0.058 | 0.304 | Unannotated | Organic acids and derivatives | Carboxylic acids and derivatives |
| metab_9800 | Unannotated feature | neg | 376.1550 | 3.037 | 4.402 | 3.674 | -0.729 | -0.339 | 0.058 | 0.304 | Unannotated | NA | NA |
| metab_2230 | Unannotated feature | pos | 231.1706 | 3.077 | 6.532 | 6.403 | -0.129 | -0.339 | 0.058 | 0.304 | Unannotated | NA | NA |
| metab_19254 | Gamma-Glutamylglutamic acid | neg | 311.0675 | 5.192 | 1.948 | 3.391 | 1.444 | 0.339 | 0.058 | 0.304 | Tentative | Organic acids and derivatives | Carboxylic acids and derivatives |
| metab_1377 | Unannotated feature | pos | 631.3120 | 6.119 | 6.002 | 5.760 | -0.242 | -0.339 | 0.058 | 0.304 | Unannotated | NA | NA |
| metab_13051 | Unannotated feature | neg | 154.0615 | 0.989 | 4.471 | 4.254 | -0.217 | -0.339 | 0.058 | 0.304 | Unannotated | NA | NA |
| metab_13105 | Unannotated feature | neg | 204.9213 | 0.134 | 5.308 | 5.284 | -0.024 | -0.339 | 0.058 | 0.304 | Unannotated | NA | NA |
| metab_9209 | Feruloylquinic Acid | neg | 367.1038 | 3.754 | 3.547 | 4.108 | 0.561 | 0.339 | 0.058 | 0.304 | Moderate putative | NA | NA |
| metab_2873 | Unannotated feature | pos | 291.1749 | 2.803 | 3.966 | 3.531 | -0.436 | -0.339 | 0.058 | 0.304 | Unannotated | NA | NA |
| metab_15885 | Sagopilone | neg | 542.2627 | 5.975 | 5.664 | 5.897 | 0.234 | 0.339 | 0.058 | 0.304 | Unannotated | NA | NA |
| metab_971 | 6,10,14-Trimethyl-5,9,13-pentadecatrien-2-one | pos | 280.2641 | 7.772 | 6.055 | 5.943 | -0.112 | -0.339 | 0.058 | 0.304 | Unannotated | NA | NA |
| metab_3745 | 17alpha-Trenbolone | pos | 315.1344 | 5.418 | 2.188 | 3.167 | 0.978 | 0.339 | 0.058 | 0.304 | Unannotated | Lipids and lipid-like molecules | Steroids and steroid derivatives |
| metab_14459 | Unannotated feature | neg | 230.0491 | 3.284 | 3.811 | 3.229 | -0.581 | -0.339 | 0.058 | 0.304 | Unannotated | NA | NA |
| metab_14437 | Unannotated feature | neg | 213.0224 | 3.247 | 4.306 | 4.199 | -0.107 | -0.339 | 0.058 | 0.304 | Unannotated | NA | NA |
| metab_7727 | Unannotated feature | pos | 374.2197 | 3.324 | 3.529 | 2.842 | -0.687 | -0.339 | 0.058 | 0.304 | Unannotated | NA | NA |
| metab_13817 | Unannotated feature | neg | 387.1893 | 2.070 | 4.515 | 3.808 | -0.707 | -0.339 | 0.058 | 0.304 | Unannotated | NA | NA |
| metab_9485 | Unannotated feature | neg | 223.1086 | 3.371 | 3.894 | 3.408 | -0.485 | -0.339 | 0.058 | 0.304 | Unannotated | NA | NA |
| metab_10618 | Unannotated feature | neg | 216.0874 | 1.685 | 5.206 | 5.637 | 0.431 | 0.339 | 0.058 | 0.304 | Unannotated | NA | NA |
| metab_5397 | Solasodine | pos | 396.3268 | 6.446 | 4.522 | 3.187 | -1.335 | -0.339 | 0.058 | 0.304 | Tentative | NA | NA |
| metab_6297 | Unannotated feature | pos | 602.7847 | 5.787 | 4.172 | 4.399 | 0.227 | 0.339 | 0.058 | 0.304 | Unannotated | NA | NA |
| metab_940 | Unannotated feature | pos | 520.4373 | 7.522 | 5.205 | 5.278 | 0.073 | 0.339 | 0.058 | 0.304 | Unannotated | NA | NA |
| metab_11201 | Sulfoacetic acid | neg | 138.9699 | 0.651 | 3.743 | 2.856 | -0.887 | -0.339 | 0.058 | 0.304 | Tentative | NA | NA |
| metab_2210 | Unannotated feature | pos | 187.1808 | 3.021 | 4.917 | 3.393 | -1.525 | -0.339 | 0.058 | 0.304 | Unannotated | NA | NA |
| metab_18713 | (R)-Boschniakine | neg | 206.0818 | 5.896 | 3.891 | 3.313 | -0.578 | -0.339 | 0.058 | 0.304 | Tentative | Organoheterocyclic compounds | Pyridines and derivatives |
| metab_9356 | Val-pro-pro | neg | 356.1832 | 3.541 | 5.801 | 5.512 | -0.288 | -0.339 | 0.058 | 0.304 | Unannotated | Organic acids and derivatives | Carboxylic acids and derivatives |
| metab_336 | Unannotated feature | pos | 254.9834 | 0.451 | 4.512 | 4.431 | -0.082 | -0.339 | 0.058 | 0.304 | Unannotated | NA | NA |
| metab_6466 | Unannotated feature | pos | 570.3538 | 5.633 | 2.926 | 3.884 | 0.958 | 0.339 | 0.058 | 0.304 | Unannotated | NA | NA |
| metab_1132 | Unannotated feature | pos | 324.1297 | 0.649 | 4.388 | 4.636 | 0.249 | 0.339 | 0.058 | 0.304 | Unannotated | NA | NA |
| metab_13884 | Allobarbital | neg | 253.0831 | 2.379 | 4.869 | 5.251 | 0.382 | 0.339 | 0.058 | 0.304 | Unannotated | Organoheterocyclic compounds | Diazines |
| metab_7917 | Alpha-Methyltryptamine | pos | 175.1233 | 2.941 | 4.042 | 2.746 | -1.296 | -0.339 | 0.058 | 0.304 | Tentative | NA | NA |
| metab_9881 | Unannotated feature | neg | 542.2833 | 4.343 | 4.439 | 3.550 | -0.888 | -0.339 | 0.058 | 0.304 | Unannotated | NA | NA |
| metab_12711 | Unannotated feature | neg | 347.1143 | 5.233 | 4.139 | 4.923 | 0.784 | 0.339 | 0.058 | 0.304 | Unannotated | NA | NA |
| metab_361 | Linolenic Acid | pos | 296.2588 | 6.534 | 6.256 | 6.148 | -0.108 | -0.339 | 0.058 | 0.304 | Moderate putative | NA | NA |
| metab_5886 | Unannotated feature | pos | 709.5444 | 5.929 | 3.962 | 4.147 | 0.185 | 0.339 | 0.058 | 0.304 | Unannotated | NA | NA |
| metab_2073 | Unannotated feature | pos | 224.0921 | 2.740 | 4.764 | 4.985 | 0.221 | 0.339 | 0.058 | 0.304 | Unannotated | NA | NA |
| metab_14631 | Unannotated feature | neg | 390.1709 | 3.562 | 4.451 | 3.794 | -0.657 | -0.339 | 0.058 | 0.304 | Unannotated | NA | NA |
| metab_6188 | Unannotated feature | pos | 626.8848 | 5.820 | 2.984 | 4.108 | 1.125 | 0.339 | 0.058 | 0.304 | Unannotated | NA | NA |
| metab_9448 | 5-Caffeoylquinic acid | neg | 353.0885 | 3.410 | 4.100 | 4.571 | 0.470 | 0.339 | 0.058 | 0.304 | Tentative | Organic oxygen compounds | Organooxygen compounds |
| metab_3515 | Unannotated feature | pos | 620.3866 | 4.832 | 3.723 | 4.794 | 1.071 | 0.339 | 0.058 | 0.304 | Unannotated | NA | NA |
| metab_2604 | Unannotated feature | pos | 160.1527 | 1.654 | 5.656 | 4.327 | -1.328 | -0.339 | 0.058 | 0.304 | Unannotated | NA | NA |
| metab_16027 | Nitrosobenzene | neg | 152.0346 | 6.154 | 4.835 | 4.684 | -0.152 | -0.339 | 0.058 | 0.304 | Tentative | Benzenoids | Benzene and substituted derivatives |
| metab_8443 | Unannotated feature | pos | 173.1162 | 1.944 | 4.622 | 4.098 | -0.524 | -0.339 | 0.058 | 0.304 | Unannotated | NA | NA |
| metab_17438 | Unannotated feature | neg | 1030.8183 | 7.611 | 5.921 | 6.089 | 0.168 | 0.339 | 0.058 | 0.304 | Unannotated | NA | NA |
| metab_8239 | Unannotated feature | pos | 307.2387 | 3.716 | 4.641 | 4.067 | -0.574 | -0.339 | 0.058 | 0.304 | Unannotated | NA | NA |
| metab_10951 | Unannotated feature | neg | 352.1255 | 0.791 | 0.585 | 4.055 | 3.470 | 0.339 | 0.058 | 0.304 | Unannotated | NA | NA |
| metab_614 | Unannotated feature | pos | 613.1824 | 5.794 | 3.032 | 4.014 | 0.983 | 0.339 | 0.058 | 0.304 | Unannotated | NA | NA |
| metab_6263 | Unannotated feature | pos | 648.0020 | 5.799 | 4.140 | 4.743 | 0.603 | 0.339 | 0.058 | 0.304 | Unannotated | NA | NA |
| metab_14758 | 20-COOH-leukotriene E4 | neg | 504.1789 | 3.822 | 5.549 | 3.905 | -1.644 | -0.339 | 0.058 | 0.304 | Unannotated | NA | NA |
| metab_2789 | Glutaconic acid | pos | 278.0889 | 2.584 | 2.958 | 4.251 | 1.293 | 0.339 | 0.058 | 0.304 | Tentative | Organic acids and derivatives | Carboxylic acids and derivatives |
| metab_7576 | Vindoline | pos | 474.2603 | 3.596 | 4.844 | 3.874 | -0.969 | -0.339 | 0.058 | 0.304 | Tentative | NA | NA |
| metab_4238 | Unannotated feature | pos | 294.5157 | 5.929 | 3.449 | 3.559 | 0.110 | 0.339 | 0.058 | 0.304 | Unannotated | NA | NA |
| metab_334 | Unannotated feature | pos | 354.9121 | 0.451 | 4.657 | 4.500 | -0.157 | -0.339 | 0.058 | 0.304 | Unannotated | NA | NA |
| metab_20 | Unannotated feature | pos | 288.1085 | 0.712 | 4.505 | 5.076 | 0.570 | 0.339 | 0.058 | 0.304 | Unannotated | NA | NA |
| metab_2071 | Riddelliine | pos | 332.1501 | 2.732 | 3.887 | 4.959 | 1.073 | 0.339 | 0.058 | 0.304 | Unannotated | NA | NA |
| metab_8973 | Unannotated feature | neg | 357.2148 | 4.123 | 4.984 | 4.496 | -0.488 | -0.339 | 0.058 | 0.304 | Unannotated | NA | NA |
| metab_16669 | Unannotated feature | neg | 996.8251 | 7.607 | 6.672 | 6.742 | 0.070 | 0.339 | 0.058 | 0.304 | Unannotated | NA | NA |
| metab_15017 | 9-(2,3-dihydroxypropoxy)-9-Oxononanoic Acid | neg | 261.1345 | 4.371 | 4.428 | 4.796 | 0.368 | 0.339 | 0.058 | 0.304 | Unannotated | NA | NA |
| metab_10192 | Glycylleucine | neg | 187.1083 | 2.659 | 5.828 | 5.584 | -0.244 | -0.339 | 0.058 | 0.304 | Tentative | Organic acids and derivatives | Carboxylic acids and derivatives |
| metab_19890 | Phenyllactic acid | neg | 147.0444 | 5.355 | 3.941 | 3.778 | -0.162 | -0.339 | 0.058 | 0.304 | Tentative | NA | NA |
| metab_12950 | Unannotated feature | neg | 218.1031 | 0.770 | 3.878 | 4.406 | 0.528 | 0.339 | 0.058 | 0.304 | Unannotated | NA | NA |
| metab_10510 | Unannotated feature | neg | 359.1577 | 1.947 | 5.622 | 5.280 | -0.342 | -0.339 | 0.058 | 0.304 | Unannotated | NA | NA |
| metab_14706 | 6-[5-(4-Carbamimidoylphenyl)-2-furyl]pyridine-3-carboxamidine | neg | 326.0996 | 3.709 | 3.366 | 4.041 | 0.675 | 0.335 | 0.061 | 0.311 | Unannotated | NA | NA |
| metab_9148 | Unannotated feature | neg | 318.1483 | 3.852 | 4.467 | 3.725 | -0.741 | -0.335 | 0.061 | 0.311 | Unannotated | NA | NA |
| metab_19883 | Methylnoradrenaline | neg | 164.0711 | 2.579 | 6.267 | 6.087 | -0.180 | -0.335 | 0.061 | 0.311 | Tentative | NA | NA |
| metab_4067 | Unannotated feature | pos | 649.4994 | 5.835 | 3.784 | 4.547 | 0.763 | 0.335 | 0.061 | 0.311 | Unannotated | NA | NA |
| metab_8713 | Unannotated feature | pos | 359.2299 | 4.111 | 4.994 | 4.362 | -0.632 | -0.335 | 0.061 | 0.311 | Unannotated | NA | NA |
| metab_8368 | Unannotated feature | pos | 345.2250 | 1.952 | 4.699 | 4.322 | -0.377 | -0.335 | 0.061 | 0.311 | Unannotated | NA | NA |
| metab_5943 | Azimexon | pos | 177.1124 | 5.922 | 4.378 | 4.526 | 0.148 | 0.335 | 0.061 | 0.311 | Unannotated | Organic acids and derivatives | Carboxylic acids and derivatives |
| metab_17430 | Unannotated feature | neg | 753.8546 | 7.621 | 5.835 | 5.967 | 0.132 | 0.335 | 0.061 | 0.311 | Unannotated | NA | NA |
| metab_8360 | Endoxifen | pos | 418.1720 | 1.952 | 1.375 | 3.188 | 1.813 | 0.335 | 0.061 | 0.311 | Tentative | Phenylpropanoids and polyketides | Stilbenes |
| metab_8423 | 3,4-Dihydroxyphenylglycol | pos | 382.1504 | 1.944 | 3.964 | 4.582 | 0.618 | 0.335 | 0.061 | 0.311 | Tentative | Benzenoids | Phenols |
| metab_4095 | Unannotated feature | pos | 619.8825 | 5.847 | 3.919 | 4.079 | 0.160 | 0.335 | 0.061 | 0.311 | Unannotated | NA | NA |
| metab_6139 | Unannotated feature | pos | 720.9400 | 5.835 | 4.177 | 4.434 | 0.258 | 0.335 | 0.061 | 0.311 | Unannotated | NA | NA |
| metab_7693 | Unannotated feature | pos | 286.1766 | 3.384 | 5.327 | 4.979 | -0.348 | -0.335 | 0.061 | 0.311 | Unannotated | NA | NA |
| metab_8234 | Unannotated feature | pos | 237.0874 | 2.376 | 3.240 | 3.910 | 0.670 | 0.335 | 0.061 | 0.311 | Unannotated | NA | NA |
| metab_18082 | Diosbulbin D | neg | 343.1157 | 2.915 | 4.035 | 4.458 | 0.423 | 0.335 | 0.061 | 0.311 | Unannotated | NA | NA |
| metab_4001 | Unannotated feature | pos | 572.1892 | 5.811 | 3.746 | 4.370 | 0.624 | 0.335 | 0.061 | 0.311 | Unannotated | NA | NA |
| metab_4910 | Unannotated feature | pos | 247.1404 | 0.688 | 5.956 | 5.434 | -0.522 | -0.335 | 0.061 | 0.311 | Unannotated | NA | NA |
| metab_11252 | Unannotated feature | neg | 324.1304 | 0.628 | 1.755 | 4.369 | 2.613 | 0.335 | 0.061 | 0.311 | Unannotated | NA | NA |
| metab_5568 | Unannotated feature | pos | 338.1607 | 2.747 | 4.709 | 5.043 | 0.335 | 0.335 | 0.061 | 0.311 | Unannotated | NA | NA |
| metab_10320 | Unannotated feature | neg | 466.1212 | 2.309 | 2.884 | 2.025 | -0.859 | -0.335 | 0.061 | 0.311 | Unannotated | NA | NA |
| metab_543 | Unannotated feature | pos | 511.6513 | 5.519 | 3.543 | 4.239 | 0.696 | 0.335 | 0.061 | 0.311 | Unannotated | NA | NA |
| metab_4149 | Unannotated feature | pos | 379.2965 | 6.080 | 6.792 | 6.447 | -0.345 | -0.335 | 0.061 | 0.311 | Unannotated | NA | NA |
| metab_12298 | Unannotated feature | neg | 715.4162 | 7.194 | 5.184 | 4.372 | -0.811 | -0.335 | 0.061 | 0.311 | Unannotated | NA | NA |
| metab_7203 | Unannotated feature | pos | 346.7077 | 4.417 | 1.510 | 4.242 | 2.732 | 0.335 | 0.061 | 0.311 | Unannotated | NA | NA |
| metab_8599 | Unannotated feature | pos | 588.3606 | 4.037 | 3.723 | 4.939 | 1.216 | 0.335 | 0.061 | 0.311 | Unannotated | NA | NA |
| metab_16709 | Unannotated feature | neg | 843.3381 | 7.641 | 5.596 | 5.720 | 0.124 | 0.335 | 0.061 | 0.311 | Unannotated | NA | NA |
| metab_2686 | 2-Pyrazinoylguanidine | pos | 148.0607 | 2.003 | 4.479 | 4.279 | -0.200 | -0.335 | 0.061 | 0.311 | Unannotated | NA | NA |
| metab_4921 | Unannotated feature | pos | 608.3875 | 4.355 | 5.837 | 6.289 | 0.452 | 0.335 | 0.061 | 0.311 | Unannotated | NA | NA |
| metab_8265 | Unannotated feature | pos | 135.0745 | 2.217 | 4.279 | 4.386 | 0.107 | 0.335 | 0.061 | 0.311 | Unannotated | NA | NA |
| metab_18380 | Unannotated feature | neg | 527.2689 | 6.146 | 6.303 | 6.462 | 0.159 | 0.335 | 0.061 | 0.311 | Unannotated | NA | NA |
| metab_16688 | Unannotated feature | neg | 1038.8074 | 7.617 | 5.656 | 5.833 | 0.178 | 0.335 | 0.061 | 0.311 | Unannotated | NA | NA |
| metab_13522 | 2-Furoic Acid | neg | 111.0081 | 1.071 | 4.904 | 4.423 | -0.481 | -0.335 | 0.061 | 0.311 | Moderate putative | Organoheterocyclic compounds | Furans |
| metab_7968 | Unannotated feature | pos | 401.2045 | 2.875 | 4.087 | 3.722 | -0.364 | -0.335 | 0.061 | 0.311 | Unannotated | NA | NA |
| metab_12777 | Unannotated feature | neg | 133.0328 | 0.628 | 5.137 | 4.840 | -0.297 | -0.335 | 0.061 | 0.311 | Unannotated | NA | NA |
| metab_14656 | Unannotated feature | neg | 691.2113 | 3.592 | 4.704 | 1.290 | -3.414 | -0.335 | 0.061 | 0.311 | Unannotated | NA | NA |
| metab_10419 | Unannotated feature | neg | 400.1371 | 4.613 | 4.444 | 3.595 | -0.849 | -0.335 | 0.061 | 0.311 | Unannotated | NA | NA |
| metab_6459 | 5-(10,13-Nonadecadienyl)-1,3-benzenediol | pos | 395.2915 | 5.645 | 4.777 | 4.042 | -0.735 | -0.335 | 0.061 | 0.311 | Unannotated | NA | NA |
| metab_409 | Unannotated feature | pos | 200.0009 | 0.016 | 4.821 | 4.842 | 0.021 | 0.335 | 0.061 | 0.311 | Unannotated | NA | NA |
| metab_5366 | Unannotated feature | pos | 312.3630 | 6.502 | 5.400 | 5.241 | -0.159 | -0.335 | 0.061 | 0.311 | Unannotated | NA | NA |
| metab_4334 | Resolvin D1 | pos | 359.2195 | 6.103 | 4.284 | 3.131 | -1.153 | -0.335 | 0.061 | 0.311 | Moderate putative | NA | NA |
| metab_15751 | (E)-10-Hydroxy-8-decenoic acid | neg | 185.1178 | 5.771 | 4.069 | 3.775 | -0.294 | -0.335 | 0.061 | 0.311 | Unannotated | Organic acids and derivatives | Hydroxy acids and derivatives |
| metab_7484 | Unannotated feature | pos | 278.1240 | 0.720 | 5.107 | 5.343 | 0.235 | 0.335 | 0.061 | 0.311 | Unannotated | NA | NA |
| metab_3278 | Inuline | pos | 604.3560 | 3.987 | 4.118 | 5.101 | 0.983 | 0.335 | 0.061 | 0.311 | Strong putative | Lipids and lipid-like molecules | Prenol lipids |
| metab_3271 | Phe Ile | pos | 279.1705 | 3.953 | 5.269 | 4.887 | -0.382 | -0.335 | 0.061 | 0.311 | Strong putative | NA | NA |
| metab_1896 | Unannotated feature | pos | 101.0238 | 1.435 | 3.678 | 3.262 | -0.416 | -0.335 | 0.061 | 0.311 | Unannotated | NA | NA |
| metab_17574 | Unannotated feature | neg | 778.5268 | 7.301 | 4.983 | 4.389 | -0.594 | -0.335 | 0.061 | 0.311 | Unannotated | NA | NA |
| metab_8020 | Santamarin | pos | 287.1032 | 2.811 | 3.128 | 4.271 | 1.143 | 0.335 | 0.061 | 0.311 | Tentative | Lipids and lipid-like molecules | Prenol lipids |
| metab_6883 | Unannotated feature | pos | 752.4642 | 5.052 | 3.899 | 4.898 | 0.999 | 0.335 | 0.061 | 0.311 | Unannotated | NA | NA |
| metab_7904 | (Z)-3-(1-Formyl-1-propenyl)pentanedioic acid | pos | 165.0549 | 2.960 | 2.901 | 3.678 | 0.777 | 0.335 | 0.061 | 0.311 | Unannotated | NA | NA |
| metab_9497 | Sesamol | neg | 183.0293 | 3.354 | 5.102 | 4.737 | -0.365 | -0.335 | 0.061 | 0.311 | Tentative | NA | NA |
| metab_2483 | Unannotated feature | pos | 130.6024 | 0.728 | 4.239 | 3.541 | -0.698 | -0.335 | 0.061 | 0.311 | Unannotated | NA | NA |
| metab_13608 | Unannotated feature | neg | 260.0026 | 1.471 | 5.115 | 4.300 | -0.815 | -0.335 | 0.061 | 0.311 | Unannotated | NA | NA |
| metab_6149 | Unannotated feature | pos | 646.8998 | 5.835 | 1.373 | 4.060 | 2.687 | 0.335 | 0.061 | 0.311 | Unannotated | NA | NA |
| metab_10926 | 2-(2-Hydroxyethyl)-4-oxohexanedioic acid | neg | 185.0450 | 0.815 | 5.240 | 5.403 | 0.163 | 0.335 | 0.061 | 0.311 | Unannotated | NA | NA |
| metab_15499 | Hygrine | neg | 186.1130 | 5.272 | 5.954 | 5.713 | -0.241 | -0.335 | 0.061 | 0.311 | Tentative | NA | NA |
| metab_13159 | Unannotated feature | neg | 360.9412 | 0.481 | 4.813 | 4.439 | -0.374 | -0.335 | 0.061 | 0.311 | Unannotated | NA | NA |
| metab_6265 | Unannotated feature | pos | 638.0052 | 5.799 | 4.191 | 4.655 | 0.464 | 0.335 | 0.061 | 0.311 | Unannotated | NA | NA |
| metab_16701 | Unannotated feature | neg | 142.0251 | 7.637 | 4.209 | 4.108 | -0.100 | -0.335 | 0.061 | 0.311 | Unannotated | NA | NA |
| metab_7441 | Amastatin | pos | 516.3032 | 3.882 | 4.781 | 4.949 | 0.168 | 0.335 | 0.061 | 0.311 | Tentative | Organic acids and derivatives | Peptidomimetics |
| metab_462 | Pterosin J | pos | 285.1240 | 4.783 | 2.067 | 4.123 | 2.057 | 0.335 | 0.061 | 0.311 | Unannotated | Benzenoids | Indanes |
| metab_7343 | Unannotated feature | pos | 346.1874 | 4.084 | 4.840 | 4.382 | -0.459 | -0.335 | 0.061 | 0.311 | Unannotated | NA | NA |
| metab_7748 | Alongside | pos | 242.1391 | 3.295 | 2.778 | 3.097 | 0.319 | 0.335 | 0.061 | 0.311 | Unannotated | Phenylpropanoids and polyketides | Macrolides and analogues |
| metab_12929 | Unannotated feature | neg | 473.1526 | 0.738 | 4.695 | 5.139 | 0.444 | 0.335 | 0.061 | 0.311 | Unannotated | NA | NA |
| metab_4903 | Unannotated feature | pos | 276.1449 | 0.704 | 4.101 | 5.377 | 1.276 | 0.335 | 0.061 | 0.311 | Unannotated | NA | NA |
| metab_14562 | Hemiphloin | neg | 433.1147 | 3.453 | 5.455 | 5.023 | -0.432 | -0.335 | 0.061 | 0.311 | Strong putative | NA | NA |
| metab_5147 | (E)-6,10-Dimethyl-9-methylene-5-undecen-2-one | pos | 226.2168 | 6.877 | 5.204 | 5.119 | -0.086 | -0.335 | 0.061 | 0.311 | Unannotated | NA | NA |
| metab_6409 | Unannotated feature | pos | 617.1435 | 5.717 | 2.896 | 4.991 | 2.095 | 0.335 | 0.061 | 0.311 | Unannotated | NA | NA |
| metab_12701 | Unannotated feature | neg | 361.2236 | 5.351 | 6.686 | 6.353 | -0.333 | -0.335 | 0.061 | 0.311 | Unannotated | NA | NA |
| metab_11174 | N-Methyl-1-deoxynojirimycin | neg | 222.0980 | 0.667 | 4.338 | 4.759 | 0.421 | 0.335 | 0.061 | 0.311 | Unannotated | NA | NA |
| metab_17313 | Unannotated feature | neg | 382.1567 | 7.702 | 5.816 | 5.869 | 0.053 | 0.335 | 0.061 | 0.311 | Unannotated | NA | NA |
| metab_10669 | Unannotated feature | neg | 259.1454 | 4.740 | 6.009 | 5.762 | -0.246 | -0.335 | 0.061 | 0.311 | Unannotated | NA | NA |
| metab_752 | Estreptoquinasa | pos | 395.2916 | 6.072 | 6.154 | 5.851 | -0.303 | -0.335 | 0.061 | 0.311 | Unannotated | NA | NA |
| metab_18125 | Unannotated feature | neg | 291.0238 | 2.906 | 5.001 | 5.348 | 0.348 | 0.335 | 0.061 | 0.311 | Unannotated | NA | NA |
| metab_11046 | Unannotated feature | neg | 279.0725 | 0.738 | 4.786 | 5.231 | 0.445 | 0.331 | 0.065 | 0.319 | Unannotated | NA | NA |
| metab_10033 | Cyclokievitone | neg | 335.0891 | 2.809 | 4.231 | 4.748 | 0.517 | 0.331 | 0.065 | 0.319 | Tentative | NA | NA |
| metab_8628 | Unannotated feature | pos | 125.0430 | 1.049 | 5.280 | 5.144 | -0.136 | -0.331 | 0.065 | 0.319 | Unannotated | NA | NA |
| metab_2721 | Unannotated feature | pos | 127.0288 | 2.168 | 3.113 | 2.531 | -0.583 | -0.331 | 0.065 | 0.319 | Unannotated | NA | NA |
| metab_17813 | Unannotated feature | neg | 391.2626 | 6.836 | 4.116 | 5.606 | 1.490 | 0.331 | 0.065 | 0.319 | Unannotated | NA | NA |
| metab_4092 | Unannotated feature | pos | 748.4611 | 5.841 | 3.480 | 4.526 | 1.046 | 0.331 | 0.065 | 0.319 | Unannotated | NA | NA |
| metab_9871 | 2-Pyrocatechuic Acid | neg | 153.0187 | 2.975 | 5.293 | 5.486 | 0.192 | 0.331 | 0.065 | 0.319 | Strong putative | Benzenoids | Benzene and substituted derivatives |
| metab_4051 | Unannotated feature | pos | 628.8909 | 5.831 | 3.949 | 4.447 | 0.498 | 0.331 | 0.065 | 0.319 | Unannotated | NA | NA |
| metab_6751 | Unannotated feature | pos | 359.1587 | 5.247 | 5.379 | 4.967 | -0.412 | -0.331 | 0.065 | 0.319 | Unannotated | NA | NA |
| metab_6670 | Leu Phe | pos | 279.1705 | 5.355 | 5.327 | 4.837 | -0.490 | -0.331 | 0.065 | 0.319 | Unannotated | NA | NA |
| metab_12271 | Unannotated feature | neg | 549.4536 | 7.068 | 5.512 | 4.813 | -0.699 | -0.331 | 0.065 | 0.319 | Unannotated | NA | NA |
| metab_1828 | Unannotated feature | pos | 300.1450 | 0.760 | 4.214 | 4.849 | 0.635 | 0.331 | 0.065 | 0.319 | Unannotated | NA | NA |
| metab_18106 | Unannotated feature | neg | 250.0721 | 2.915 | 3.886 | 4.179 | 0.293 | 0.331 | 0.065 | 0.319 | Unannotated | NA | NA |
| metab_9158 | Unannotated feature | neg | 379.1039 | 3.889 | 3.918 | 3.200 | -0.718 | -0.331 | 0.065 | 0.319 | Unannotated | NA | NA |
| metab_8096 | Asp Ile Thr | pos | 348.1775 | 2.740 | 5.634 | 5.379 | -0.255 | -0.331 | 0.065 | 0.319 | Moderate putative | NA | NA |
| metab_9224 | Unannotated feature | neg | 274.9870 | 3.728 | 2.835 | 3.955 | 1.119 | 0.331 | 0.065 | 0.319 | Unannotated | NA | NA |
| metab_4563 | 4-(2,6,6-Trimethyl-1-cyclohexen-1-yl)-2-butanone | pos | 212.2012 | 6.869 | 5.152 | 5.053 | -0.099 | -0.331 | 0.065 | 0.319 | Tentative | NA | NA |
| metab_9919 | Unannotated feature | neg | 336.1571 | 2.935 | 4.005 | 3.645 | -0.360 | -0.331 | 0.065 | 0.319 | Unannotated | NA | NA |
| metab_9376 | Unannotated feature | neg | 267.0987 | 3.505 | 4.247 | 3.985 | -0.262 | -0.331 | 0.065 | 0.319 | Unannotated | NA | NA |
| metab_17471 | Unannotated feature | neg | 877.8391 | 7.584 | 6.218 | 6.285 | 0.068 | 0.331 | 0.065 | 0.319 | Unannotated | NA | NA |
| metab_7944 | 4-Amino-1-[(1S,2S,4S)-4,5-bis(hydroxymethyl)-2-bicyclo[3.1.0]hexanyl]pyrimidin-2-one | pos | 293.1611 | 2.907 | 4.116 | 3.636 | -0.480 | -0.331 | 0.065 | 0.319 | Unannotated | Nucleosides, nucleotides, and analogues | Nucleoside and nucleotide analogues |
| metab_19858 | D-Glucuronic Acid | neg | 193.0349 | 0.651 | 6.420 | 6.253 | -0.167 | -0.331 | 0.065 | 0.319 | Strong putative | NA | NA |
| metab_949 | Unannotated feature | pos | 498.9027 | 7.610 | 6.803 | 6.857 | 0.054 | 0.331 | 0.065 | 0.319 | Unannotated | NA | NA |
| metab_18495 | N-[(3a,5b,7b)-7-hydroxy-24-oxo-3-(sulfooxy)cholan-24-yl]-Glycine | neg | 510.2540 | 6.052 | 5.169 | 5.478 | 0.309 | 0.331 | 0.065 | 0.319 | Unannotated | NA | NA |
| metab_346 | Unannotated feature | pos | 143.0341 | 0.417 | 4.675 | 4.654 | -0.021 | -0.331 | 0.065 | 0.319 | Unannotated | NA | NA |
| metab_4896 | Unannotated feature | pos | 168.0508 | 7.700 | 4.216 | 4.275 | 0.059 | 0.331 | 0.065 | 0.319 | Unannotated | NA | NA |
| metab_8774 | Unannotated feature | pos | 146.1540 | 0.728 | 5.068 | 4.837 | -0.231 | -0.331 | 0.065 | 0.319 | Unannotated | NA | NA |
| metab_12831 | Unannotated feature | neg | 250.0933 | 0.651 | 5.239 | 5.436 | 0.197 | 0.331 | 0.065 | 0.319 | Unannotated | NA | NA |
| metab_11522 | Unannotated feature | neg | 564.8310 | 0.502 | 3.338 | 3.848 | 0.511 | 0.331 | 0.065 | 0.319 | Unannotated | NA | NA |
| metab_19287 | 5,7-Dihydroxy-3',4'-dimethoxy-8-(3-hydroxy-3-methylbutyl)-isoflavone 7-glucoside | neg | 280.0969 | 5.156 | 3.209 | 3.678 | 0.469 | 0.331 | 0.065 | 0.319 | Unannotated | NA | NA |
| metab_17578 | Unannotated feature | neg | 323.0785 | 2.758 | 3.811 | 4.253 | 0.442 | 0.331 | 0.065 | 0.319 | Unannotated | NA | NA |
| metab_7962 | ((2R,3S)-3-Amino-2-hydroxy-2-(1H-imidazol-4-ylmethyl)-5-methyl)-5-methylhexanoic Acid | pos | 259.1769 | 2.892 | 4.507 | 4.300 | -0.207 | -0.331 | 0.065 | 0.319 | Unannotated | Organic acids and derivatives | Carboxylic acids and derivatives |
| metab_1116 | DG(TXB2/i-13:0/0:0) | pos | 679.4197 | 6.638 | 5.443 | 5.003 | -0.440 | -0.331 | 0.065 | 0.319 | Unannotated | NA | NA |
| metab_9286 | Dodec-8-enedioylcarnitine | neg | 408.1785 | 3.631 | 4.975 | 4.336 | -0.639 | -0.331 | 0.065 | 0.319 | Unannotated | Lipids and lipid-like molecules | Fatty Acyls |
| metab_16006 | Unannotated feature | neg | 421.2446 | 6.123 | 3.889 | 4.034 | 0.145 | 0.331 | 0.065 | 0.319 | Unannotated | NA | NA |
| metab_8337 | Leu Asp Asn | pos | 361.1727 | 1.984 | 4.973 | 4.668 | -0.305 | -0.331 | 0.065 | 0.319 | Unannotated | NA | NA |
| metab_16823 | Unannotated feature | neg | 217.1119 | 7.726 | 3.749 | 3.852 | 0.103 | 0.331 | 0.065 | 0.319 | Unannotated | NA | NA |
| metab_13857 | Unannotated feature | neg | 216.0289 | 2.244 | 2.834 | 3.244 | 0.410 | 0.331 | 0.065 | 0.319 | Unannotated | NA | NA |
| metab_2506 | Unannotated feature | pos | 232.0821 | 0.807 | 3.606 | 4.044 | 0.438 | 0.331 | 0.065 | 0.319 | Unannotated | NA | NA |
| metab_970 | Unannotated feature | pos | 119.1071 | 7.756 | 5.920 | 5.970 | 0.050 | 0.331 | 0.065 | 0.319 | Unannotated | NA | NA |
| metab_3824 | Unannotated feature | pos | 528.8401 | 5.607 | 3.765 | 4.234 | 0.469 | 0.331 | 0.065 | 0.319 | Unannotated | NA | NA |
| metab_15212 | Unannotated feature | neg | 393.1999 | 4.754 | 4.426 | 3.743 | -0.683 | -0.331 | 0.065 | 0.319 | Unannotated | NA | NA |
| metab_4873 | Unannotated feature | pos | 122.5475 | 7.724 | 5.930 | 5.960 | 0.030 | 0.331 | 0.065 | 0.319 | Unannotated | NA | NA |
| metab_9292 | 2-Chloro-1-[(2S,3S,5S,10S,13S)-3-hydroxy-10,13-dimethyl-2-morpholino-2,3,4,5,6,7,8,9,11,12,14,15,16,17-tetradecahydro-1H-cyclopenta[a]phenanthren-17-yl]ethanone | neg | 472.2430 | 3.620 | 4.786 | 3.742 | -1.044 | -0.331 | 0.065 | 0.319 | Unannotated | Lipids and lipid-like molecules | Steroids and steroid derivatives |
| metab_7357 | Unannotated feature | pos | 352.1611 | 0.704 | 3.159 | 4.536 | 1.376 | 0.331 | 0.065 | 0.319 | Unannotated | NA | NA |
| metab_18292 | Unannotated feature | neg | 483.2061 | 6.217 | 5.108 | 5.283 | 0.175 | 0.331 | 0.065 | 0.319 | Unannotated | NA | NA |
| metab_14255 | Unannotated feature | neg | 351.1529 | 2.994 | 5.206 | 4.749 | -0.457 | -0.331 | 0.065 | 0.319 | Unannotated | NA | NA |
| metab_13641 | Unannotated feature | neg | 283.0296 | 1.647 | 5.436 | 5.307 | -0.129 | -0.331 | 0.065 | 0.319 | Unannotated | NA | NA |
| metab_6375 | Unannotated feature | pos | 287.0145 | 0.577 | 4.088 | 0.022 | -4.066 | -0.331 | 0.065 | 0.319 | Unannotated | NA | NA |
| metab_2348 | Unannotated feature | pos | 95.0194 | 0.469 | 3.825 | 3.643 | -0.182 | -0.331 | 0.065 | 0.319 | Unannotated | NA | NA |
| metab_6299 | Unannotated feature | pos | 586.3666 | 5.787 | 4.021 | 4.834 | 0.814 | 0.331 | 0.065 | 0.319 | Unannotated | NA | NA |
| metab_12453 | Unannotated feature | neg | 485.3273 | 6.506 | 5.845 | 6.380 | 0.535 | 0.331 | 0.065 | 0.319 | Unannotated | NA | NA |
| metab_16730 | Unannotated feature | neg | 796.3436 | 7.660 | 4.762 | 4.992 | 0.230 | 0.331 | 0.065 | 0.319 | Unannotated | NA | NA |
| metab_15335 | 3-Oxopentanoic acid | neg | 231.0872 | 4.955 | 3.838 | 4.401 | 0.564 | 0.331 | 0.065 | 0.319 | Tentative | Organic acids and derivatives | Keto acids and derivatives |
| metab_9208 | Norhygrine | neg | 172.0973 | 3.759 | 5.548 | 5.459 | -0.089 | -0.331 | 0.065 | 0.319 | Unannotated | NA | NA |
| metab_9634 | Unannotated feature | neg | 408.1783 | 3.203 | 5.187 | 4.868 | -0.319 | -0.331 | 0.065 | 0.319 | Unannotated | NA | NA |
| metab_2289 | Unannotated feature | pos | 861.2742 | 0.006 | 3.780 | 4.302 | 0.522 | 0.331 | 0.065 | 0.319 | Unannotated | NA | NA |
| metab_9693 | Unannotated feature | neg | 288.0607 | 3.131 | 4.410 | 3.985 | -0.425 | -0.331 | 0.065 | 0.319 | Unannotated | NA | NA |
| metab_3735 | Unannotated feature | pos | 753.7983 | 5.390 | 3.964 | 4.525 | 0.562 | 0.331 | 0.065 | 0.319 | Unannotated | NA | NA |
| metab_18459 | Unannotated feature | neg | 410.2895 | 6.083 | 1.734 | 0.888 | -0.846 | -0.331 | 0.065 | 0.319 | Unannotated | NA | NA |
| metab_4697 | Unannotated feature | pos | 651.8683 | 7.578 | 5.763 | 5.829 | 0.066 | 0.331 | 0.065 | 0.319 | Unannotated | NA | NA |
| metab_14177 | Unannotated feature | neg | 381.1162 | 2.915 | 3.939 | 4.694 | 0.755 | 0.331 | 0.065 | 0.319 | Unannotated | NA | NA |
| metab_3753 | 2-Methoxyestrone | pos | 639.3055 | 5.438 | 6.048 | 5.459 | -0.589 | -0.331 | 0.065 | 0.319 | Tentative | NA | NA |
| metab_14155 | Unannotated feature | neg | 401.2050 | 2.896 | 4.374 | 4.023 | -0.352 | -0.331 | 0.065 | 0.319 | Unannotated | NA | NA |
| metab_2884 | Val His Ile | pos | 368.2303 | 2.823 | 5.227 | 4.573 | -0.654 | -0.331 | 0.065 | 0.319 | Moderate putative | NA | NA |
| metab_12100 | Unannotated feature | neg | 309.2072 | 6.498 | 5.804 | 5.961 | 0.157 | 0.331 | 0.065 | 0.319 | Unannotated | NA | NA |
| metab_2981 | Unannotated feature | pos | 254.1027 | 3.052 | 4.811 | 5.120 | 0.309 | 0.331 | 0.065 | 0.319 | Unannotated | NA | NA |
| metab_16048 | Unannotated feature | neg | 524.3138 | 6.177 | 5.928 | 5.756 | -0.172 | -0.326 | 0.068 | 0.326 | Unannotated | NA | NA |
| metab_6015 | Unannotated feature | pos | 187.9644 | 0.013 | 4.588 | 4.627 | 0.039 | 0.326 | 0.068 | 0.326 | Unannotated | NA | NA |
| metab_9679 | Unannotated feature | neg | 256.0906 | 3.149 | 1.501 | 2.900 | 1.399 | 0.326 | 0.068 | 0.326 | Unannotated | NA | NA |
| metab_781 | Unannotated feature | pos | 395.2916 | 6.191 | 6.531 | 6.211 | -0.320 | -0.326 | 0.068 | 0.326 | Unannotated | NA | NA |
| metab_16403 | Unannotated feature | neg | 284.0258 | 1.665 | 6.681 | 6.566 | -0.116 | -0.326 | 0.068 | 0.326 | Unannotated | NA | NA |
| metab_8564 | Unannotated feature | pos | 245.0773 | 1.239 | 5.046 | 4.334 | -0.713 | -0.326 | 0.068 | 0.326 | Unannotated | NA | NA |
| metab_13672 | 3-Hydroxybutyrylcarnitine | neg | 292.1404 | 1.685 | 5.113 | 5.431 | 0.318 | 0.326 | 0.068 | 0.326 | Unannotated | NA | NA |
| metab_13704 | Unannotated feature | neg | 303.0130 | 1.749 | 4.044 | 3.809 | -0.235 | -0.326 | 0.068 | 0.326 | Unannotated | NA | NA |
| metab_18748 | Unannotated feature | neg | 214.0619 | 3.165 | 4.952 | 4.800 | -0.152 | -0.326 | 0.068 | 0.326 | Unannotated | NA | NA |
| metab_12800 | Unannotated feature | neg | 213.0071 | 0.644 | 6.408 | 5.983 | -0.425 | -0.326 | 0.068 | 0.326 | Unannotated | NA | NA |
| metab_2203 | 3-Hydroxytamoxifen (Droloxifene) | pos | 432.1915 | 3.002 | 4.387 | 3.561 | -0.826 | -0.326 | 0.068 | 0.326 | Tentative | Phenylpropanoids and polyketides | Stilbenes |
| metab_2166 | Unannotated feature | pos | 387.2725 | 2.907 | 5.391 | 5.217 | -0.175 | -0.326 | 0.068 | 0.326 | Unannotated | NA | NA |
| metab_12376 | Unannotated feature | neg | 459.2160 | 5.148 | 3.720 | 4.611 | 0.890 | 0.326 | 0.068 | 0.326 | Unannotated | NA | NA |
| metab_19495 | Unannotated feature | neg | 391.1992 | 4.865 | 5.180 | 4.693 | -0.487 | -0.326 | 0.068 | 0.326 | Unannotated | NA | NA |
| metab_8943 | Unannotated feature | neg | 330.2038 | 4.199 | 4.693 | 4.292 | -0.400 | -0.326 | 0.068 | 0.326 | Unannotated | NA | NA |
| metab_4625 | Unannotated feature | pos | 175.9545 | 7.140 | 4.287 | 4.721 | 0.434 | 0.326 | 0.068 | 0.326 | Unannotated | NA | NA |
| metab_4369 | Calcitriol | pos | 399.3242 | 6.207 | 5.739 | 5.541 | -0.198 | -0.326 | 0.068 | 0.326 | Tentative | NA | NA |
| metab_7534 | Ala Leu Val | pos | 302.2080 | 3.689 | 5.232 | 5.013 | -0.218 | -0.326 | 0.068 | 0.326 | Moderate putative | NA | NA |
| metab_17745 | Unannotated feature | neg | 599.3216 | 6.915 | 5.785 | 5.251 | -0.534 | -0.326 | 0.068 | 0.326 | Unannotated | NA | NA |
| metab_8019 | Gln Leu Ala | pos | 331.1983 | 2.811 | 5.467 | 5.246 | -0.221 | -0.326 | 0.068 | 0.326 | Moderate putative | NA | NA |
| metab_6867 | Val Leu Phe | pos | 378.2398 | 5.076 | 5.166 | 4.634 | -0.533 | -0.326 | 0.068 | 0.326 | Moderate putative | NA | NA |
| metab_6274 | Unannotated feature | pos | 625.9961 | 5.794 | 2.708 | 4.098 | 1.389 | 0.326 | 0.068 | 0.326 | Unannotated | NA | NA |
| metab_14214 | Geniposidic acid | neg | 355.1042 | 2.956 | 4.364 | 4.854 | 0.491 | 0.326 | 0.068 | 0.326 | Tentative | NA | NA |
| metab_19040 | Unannotated feature | neg | 452.1206 | 5.475 | 4.836 | 3.684 | -1.152 | -0.326 | 0.068 | 0.326 | Unannotated | NA | NA |
| metab_18565 | Unannotated feature | neg | 565.3238 | 6.007 | 5.708 | 5.046 | -0.663 | -0.326 | 0.068 | 0.326 | Unannotated | NA | NA |
| metab_19232 | Unannotated feature | neg | 245.0670 | 5.227 | 3.194 | 3.944 | 0.750 | 0.326 | 0.068 | 0.326 | Unannotated | NA | NA |
| metab_12467 | Unannotated feature | neg | 82.9531 | 7.978 | 4.903 | 4.841 | -0.062 | -0.326 | 0.068 | 0.326 | Unannotated | NA | NA |
| metab_954 | Unannotated feature | pos | 676.8657 | 7.626 | 5.406 | 5.553 | 0.147 | 0.326 | 0.068 | 0.326 | Unannotated | NA | NA |
| metab_3676 | NH-DVal(NMe)-Val-OMe | pos | 245.1864 | 5.247 | 5.279 | 4.857 | -0.422 | -0.326 | 0.068 | 0.326 | Moderate putative | NA | NA |
| metab_14614 | Unannotated feature | neg | 126.0012 | 3.541 | 3.329 | 2.435 | -0.894 | -0.326 | 0.068 | 0.326 | Unannotated | NA | NA |
| metab_8516 | Neferine | pos | 625.3292 | 3.979 | 2.039 | 2.989 | 0.951 | 0.326 | 0.068 | 0.326 | Unannotated | Organoheterocyclic compounds | Isoquinolines and derivatives |
| metab_12402 | Unannotated feature | neg | 189.9205 | 7.697 | 5.261 | 5.193 | -0.067 | -0.326 | 0.068 | 0.326 | Unannotated | NA | NA |
| metab_19387 | Unannotated feature | neg | 191.0556 | 0.675 | 6.047 | 6.159 | 0.112 | 0.326 | 0.068 | 0.326 | Unannotated | NA | NA |
| metab_998 | Unannotated feature | pos | 81.0316 | 7.610 | 4.726 | 4.782 | 0.056 | 0.326 | 0.068 | 0.326 | Unannotated | NA | NA |
| metab_16882 | Unannotated feature | neg | 262.0700 | 7.741 | 3.983 | 4.402 | 0.419 | 0.326 | 0.068 | 0.326 | Unannotated | NA | NA |
| metab_2624 | Unannotated feature | pos | 140.0344 | 1.698 | 4.462 | 4.614 | 0.152 | 0.326 | 0.068 | 0.326 | Unannotated | NA | NA |
| metab_8123 | Unannotated feature | pos | 236.6422 | 2.732 | 3.671 | 3.153 | -0.518 | -0.326 | 0.068 | 0.326 | Unannotated | NA | NA |
| metab_1283 | Unannotated feature | pos | 238.0714 | 2.871 | 5.333 | 5.496 | 0.163 | 0.326 | 0.068 | 0.326 | Unannotated | NA | NA |
| metab_6571 | Unannotated feature | pos | 872.5454 | 4.738 | 3.394 | 4.115 | 0.721 | 0.326 | 0.068 | 0.326 | Unannotated | NA | NA |
| metab_10651 | Unannotated feature | neg | 299.0207 | 1.647 | 5.199 | 5.019 | -0.179 | -0.326 | 0.068 | 0.326 | Unannotated | NA | NA |
| metab_12570 | Unannotated feature | neg | 389.1644 | 6.130 | 5.207 | 5.561 | 0.353 | 0.326 | 0.068 | 0.326 | Unannotated | NA | NA |
| metab_8637 | Fructosylvaline | pos | 280.1396 | 1.034 | 4.216 | 4.613 | 0.397 | 0.326 | 0.068 | 0.326 | Unannotated | Organic acids and derivatives | Carboxylic acids and derivatives |
| metab_7354 | Unannotated feature | pos | 137.0963 | 4.068 | 3.013 | 3.438 | 0.426 | 0.326 | 0.068 | 0.326 | Unannotated | NA | NA |
| metab_11590 | Unannotated feature | neg | 195.9386 | 0.453 | 4.205 | 4.294 | 0.089 | 0.326 | 0.068 | 0.326 | Unannotated | NA | NA |
| metab_9213 | Unannotated feature | neg | 250.0721 | 3.754 | 4.500 | 4.868 | 0.368 | 0.326 | 0.068 | 0.326 | Unannotated | NA | NA |
| metab_8655 | 3-Carbamoyl-2-phenylpropionic acid | pos | 210.0764 | 1.010 | 3.934 | 4.190 | 0.255 | 0.326 | 0.068 | 0.326 | Tentative | NA | NA |
| metab_8453 | Unannotated feature | pos | 183.1132 | 1.936 | 4.861 | 5.129 | 0.268 | 0.326 | 0.068 | 0.326 | Unannotated | NA | NA |
| metab_4085 | Unannotated feature | pos | 639.4843 | 5.841 | 2.573 | 4.581 | 2.008 | 0.326 | 0.068 | 0.326 | Unannotated | NA | NA |
| metab_19122 | Unannotated feature | neg | 277.1558 | 5.355 | 5.384 | 4.934 | -0.449 | -0.326 | 0.068 | 0.326 | Unannotated | NA | NA |
| metab_4705 | Unannotated feature | pos | 566.8904 | 7.610 | 6.561 | 6.623 | 0.062 | 0.326 | 0.068 | 0.326 | Unannotated | NA | NA |
| metab_12994 | Unannotated feature | neg | 452.1419 | 0.791 | 5.092 | 5.412 | 0.321 | 0.326 | 0.068 | 0.326 | Unannotated | NA | NA |
| metab_2092 | Unannotated feature | pos | 281.1501 | 2.747 | 5.739 | 5.643 | -0.096 | -0.326 | 0.068 | 0.326 | Unannotated | NA | NA |
| metab_9943 | H-Arg(NO2)-Obzl | neg | 290.1248 | 2.906 | 4.563 | 5.075 | 0.512 | 0.326 | 0.068 | 0.326 | Unannotated | NA | NA |
| metab_6970 | Unannotated feature | pos | 459.3053 | 4.906 | 2.952 | 4.653 | 1.701 | 0.326 | 0.068 | 0.326 | Unannotated | NA | NA |
| metab_8424 | Unannotated feature | pos | 339.1559 | 1.944 | 3.396 | 4.420 | 1.024 | 0.326 | 0.068 | 0.326 | Unannotated | NA | NA |
| metab_13185 | Unannotated feature | neg | 556.8429 | 0.502 | 4.218 | 4.535 | 0.317 | 0.326 | 0.068 | 0.326 | Unannotated | NA | NA |
| metab_6494 | Unannotated feature | pos | 519.3224 | 5.593 | 2.092 | 3.721 | 1.629 | 0.326 | 0.068 | 0.326 | Unannotated | NA | NA |
| metab_4995 | Isoquinoline, 1,2,3,4-tetrahydro-2-(4-amino-6-((4-phenyl-1-piperazinyl)methyl)-s-triazin-2-yl)- | pos | 419.2628 | 7.474 | 5.779 | 5.738 | -0.041 | -0.326 | 0.068 | 0.326 | Unannotated | NA | NA |
| metab_7048 | Val Ile Leu | pos | 344.2552 | 4.757 | 5.346 | 5.050 | -0.296 | -0.326 | 0.068 | 0.326 | Moderate putative | NA | NA |
| metab_3980 | Unannotated feature | pos | 379.2964 | 6.207 | 7.222 | 6.963 | -0.259 | -0.326 | 0.068 | 0.326 | Unannotated | NA | NA |
| metab_9400 | (S)-3-[(Cyanophenylmethyl)amino]-3-oxopropanoic acid | neg | 263.0675 | 3.476 | 4.261 | 4.939 | 0.678 | 0.326 | 0.068 | 0.326 | Unannotated | Benzenoids | Benzene and substituted derivatives |
| metab_11601 | Unannotated feature | neg | 346.8972 | 0.443 | 4.361 | 4.288 | -0.073 | -0.326 | 0.068 | 0.326 | Unannotated | NA | NA |
| metab_4959 | Unannotated feature | pos | 515.8933 | 7.586 | 5.871 | 5.929 | 0.058 | 0.326 | 0.068 | 0.326 | Unannotated | NA | NA |
| metab_4373 | MG(PGE2/0:0/0:0) | pos | 427.2679 | 6.215 | 5.906 | 5.939 | 0.033 | -0.326 | 0.068 | 0.326 | Unannotated | NA | NA |
| metab_4558 | Unannotated feature | pos | 434.7605 | 4.798 | 4.184 | 5.320 | 1.136 | 0.326 | 0.068 | 0.326 | Unannotated | NA | NA |
| metab_19832 | 2,6-Dimethoxy-1,4-benzoquinone | neg | 167.0343 | 4.393 | 4.670 | 4.889 | 0.219 | 0.326 | 0.068 | 0.326 | Tentative | Organic oxygen compounds | Organooxygen compounds |
| metab_5756 | Unannotated feature | pos | 656.9083 | 5.362 | 3.205 | 4.303 | 1.097 | 0.326 | 0.068 | 0.326 | Unannotated | NA | NA |
| metab_1368 | Unannotated feature | pos | 368.1898 | 6.135 | 5.324 | 4.634 | -0.690 | -0.326 | 0.068 | 0.326 | Unannotated | NA | NA |
| metab_16601 | SM(d17:2(4E,8Z)/16:0) | neg | 721.5037 | 7.417 | 5.133 | 4.382 | -0.750 | -0.326 | 0.068 | 0.326 | Unannotated | Organic nitrogen compounds | Organonitrogen compounds |
| metab_7804 | Adrenochrome | pos | 180.0657 | 3.159 | 2.946 | 3.945 | 0.999 | 0.326 | 0.068 | 0.326 | Moderate putative | Organoheterocyclic compounds | Indoles and derivatives |
| metab_1833 | Unannotated feature | pos | 412.7473 | 4.533 | 3.423 | 4.974 | 1.550 | 0.326 | 0.068 | 0.326 | Unannotated | NA | NA |
| metab_1860 | Unannotated feature | pos | 318.1297 | 1.139 | 5.201 | 5.409 | 0.208 | 0.326 | 0.068 | 0.326 | Unannotated | NA | NA |
| metab_5438 | Unannotated feature | pos | 472.2888 | 5.044 | 4.401 | 4.706 | 0.305 | 0.322 | 0.072 | 0.335 | Unannotated | NA | NA |
| metab_7964 | Rotenone | pos | 412.1728 | 2.884 | 3.772 | 3.394 | -0.378 | -0.322 | 0.072 | 0.335 | Tentative | Phenylpropanoids and polyketides | Isoflavonoids |
| metab_10931 | Unannotated feature | neg | 96.9593 | 0.810 | 7.017 | 6.814 | -0.203 | -0.322 | 0.072 | 0.335 | Unannotated | NA | NA |
| metab_10952 | Sarmentosin | neg | 320.0991 | 0.791 | 4.510 | 4.735 | 0.225 | 0.322 | 0.072 | 0.335 | Tentative | NA | NA |
| metab_11622 | Unannotated feature | neg | 359.1256 | 5.290 | 5.098 | 4.936 | -0.161 | -0.322 | 0.072 | 0.335 | Unannotated | NA | NA |
| metab_16657 | Unannotated feature | neg | 665.3817 | 7.596 | 6.145 | 6.212 | 0.067 | 0.322 | 0.072 | 0.335 | Unannotated | NA | NA |
| metab_17377 | Unannotated feature | neg | 470.9166 | 7.666 | 4.508 | 4.658 | 0.150 | 0.322 | 0.072 | 0.335 | Unannotated | NA | NA |
| metab_10628 | Beta-D-Xylopyranosyl-(1->5)-alpha-L-arabinofuranosyl-(1->5)-L-arabinose | neg | 413.1312 | 1.680 | 3.477 | 3.999 | 0.522 | 0.322 | 0.072 | 0.335 | Unannotated | Organic oxygen compounds | Organooxygen compounds |
| metab_12870 | Unannotated feature | neg | 225.0072 | 0.714 | 6.222 | 5.953 | -0.269 | -0.322 | 0.072 | 0.335 | Unannotated | NA | NA |
| metab_8511 | Unannotated feature | pos | 131.6623 | 1.642 | 3.426 | 3.247 | -0.179 | -0.322 | 0.072 | 0.335 | Unannotated | NA | NA |
| metab_10586 | Unannotated feature | neg | 462.1477 | 1.691 | 2.467 | 4.121 | 1.655 | 0.322 | 0.072 | 0.335 | Unannotated | NA | NA |
| metab_2295 | Unannotated feature | pos | 172.0057 | 0.016 | 4.175 | 4.213 | 0.039 | 0.322 | 0.072 | 0.335 | Unannotated | NA | NA |
| metab_12919 | Unannotated feature | neg | 239.0230 | 0.738 | 4.782 | 4.503 | -0.279 | -0.322 | 0.072 | 0.335 | Unannotated | NA | NA |
| metab_14792 | Aucubin | neg | 327.1089 | 3.886 | 3.277 | 3.604 | 0.327 | 0.322 | 0.072 | 0.335 | Tentative | NA | NA |
| metab_5469 | Chavicol | pos | 117.0703 | 6.382 | 4.940 | 4.789 | -0.151 | -0.322 | 0.072 | 0.335 | Tentative | NA | NA |
| metab_17650 | Unannotated feature | neg | 283.2642 | 7.080 | 5.114 | 4.926 | -0.188 | -0.322 | 0.072 | 0.335 | Unannotated | NA | NA |
| metab_5123 | Unannotated feature | pos | 296.2952 | 6.957 | 3.976 | 3.653 | -0.323 | -0.322 | 0.072 | 0.335 | Unannotated | NA | NA |
| metab_2871 | Asp Leu | pos | 247.1293 | 2.803 | 5.632 | 5.445 | -0.187 | -0.322 | 0.072 | 0.335 | Moderate putative | NA | NA |
| metab_10443 | 2,2'-Bityrosine | neg | 381.1049 | 1.965 | 4.977 | 4.335 | -0.642 | -0.322 | 0.072 | 0.335 | Unannotated | Organic acids and derivatives | Carboxylic acids and derivatives |
| metab_16360 | Unannotated feature | neg | 275.1686 | 6.734 | 4.729 | 4.304 | -0.425 | -0.322 | 0.072 | 0.335 | Unannotated | NA | NA |
| metab_15952 | Unannotated feature | neg | 158.0452 | 1.061 | 5.731 | 5.592 | -0.139 | -0.322 | 0.072 | 0.335 | Unannotated | NA | NA |
| metab_7857 | Purpurogallin | pos | 238.0713 | 3.047 | 5.335 | 5.506 | 0.171 | 0.322 | 0.072 | 0.335 | Tentative | NA | NA |
| metab_7193 | Ile Glu Leu | pos | 374.2296 | 4.457 | 5.939 | 5.389 | -0.550 | -0.322 | 0.072 | 0.335 | Moderate putative | NA | NA |
| metab_10843 | Unannotated feature | neg | 275.1252 | 1.033 | 4.833 | 4.493 | -0.341 | -0.322 | 0.072 | 0.335 | Unannotated | NA | NA |
| metab_14235 | Unannotated feature | neg | 408.1771 | 2.972 | 5.092 | 4.693 | -0.399 | -0.322 | 0.072 | 0.335 | Unannotated | NA | NA |
| metab_3979 | Unannotated feature | pos | 557.8529 | 5.799 | 3.913 | 4.330 | 0.417 | 0.322 | 0.072 | 0.335 | Unannotated | NA | NA |
| metab_13598 | Unannotated feature | neg | 241.9421 | 1.411 | 5.520 | 5.233 | -0.287 | -0.322 | 0.072 | 0.335 | Unannotated | NA | NA |
| metab_1018 | Unannotated feature | pos | 494.3024 | 4.919 | 2.980 | 3.640 | 0.660 | 0.322 | 0.072 | 0.335 | Unannotated | NA | NA |
| metab_763 | Unannotated feature | pos | 589.3037 | 6.103 | 7.031 | 6.758 | -0.274 | -0.322 | 0.072 | 0.335 | Unannotated | NA | NA |
| metab_383 | Dimethylethanolamine | pos | 90.0918 | 0.079 | 3.811 | 3.904 | 0.093 | 0.322 | 0.072 | 0.335 | Tentative | NA | NA |
| metab_11736 | Unannotated feature | neg | 385.1696 | 5.586 | 6.307 | 6.748 | 0.440 | 0.322 | 0.072 | 0.335 | Unannotated | NA | NA |
| metab_16935 | Unannotated feature | neg | 178.8146 | 7.822 | 4.998 | 4.955 | -0.043 | -0.322 | 0.072 | 0.335 | Unannotated | NA | NA |
| metab_17086 | Unannotated feature | neg | 297.8694 | 7.893 | 4.683 | 4.746 | 0.063 | 0.322 | 0.072 | 0.335 | Unannotated | NA | NA |
| metab_10299 | Unannotated feature | neg | 305.0342 | 2.409 | 3.532 | 2.158 | -1.374 | -0.322 | 0.072 | 0.335 | Unannotated | NA | NA |
| metab_8425 | Unannotated feature | pos | 345.1816 | 1.944 | 4.085 | 3.691 | -0.394 | -0.322 | 0.072 | 0.335 | Unannotated | NA | NA |
| metab_10075 | 1-(3-Methyl-2-butenoyl)-6-apiosylglucose | neg | 415.1260 | 2.776 | 4.127 | 4.556 | 0.429 | 0.322 | 0.072 | 0.335 | Unannotated | Lipids and lipid-like molecules | Fatty Acyls |
| metab_12291 | Unannotated feature | neg | 577.4253 | 7.162 | 6.229 | 5.703 | -0.525 | -0.322 | 0.072 | 0.335 | Unannotated | NA | NA |
| metab_4106 | Unannotated feature | pos | 597.4610 | 5.851 | 4.112 | 4.645 | 0.533 | 0.322 | 0.072 | 0.335 | Unannotated | NA | NA |
| metab_13537 | N6-Methyl-2'-deoxyadenosine | neg | 246.0982 | 1.119 | 3.412 | 4.135 | 0.723 | 0.322 | 0.072 | 0.335 | Tentative | NA | NA |
| metab_1028 | Unannotated feature | pos | 284.2949 | 7.005 | 6.911 | 6.805 | -0.105 | -0.322 | 0.072 | 0.335 | Unannotated | NA | NA |
| metab_10688 | Unannotated feature | neg | 275.9677 | 1.471 | 4.722 | 3.317 | -1.406 | -0.322 | 0.072 | 0.335 | Unannotated | NA | NA |
| metab_1019 | Unannotated feature | pos | 198.0975 | 0.641 | 5.408 | 4.958 | -0.450 | -0.322 | 0.072 | 0.335 | Unannotated | NA | NA |
| metab_16301 | PE(16:1/0:0) | neg | 450.2628 | 6.624 | 5.361 | 5.194 | -0.167 | -0.322 | 0.072 | 0.335 | Moderate putative | NA | NA |
| metab_18831 | Unannotated feature | neg | 459.2158 | 5.748 | 5.492 | 5.726 | 0.234 | 0.322 | 0.072 | 0.335 | Unannotated | NA | NA |
| metab_19209 | Unannotated feature | neg | 239.0092 | 3.371 | 4.186 | 2.869 | -1.317 | -0.322 | 0.072 | 0.335 | Unannotated | NA | NA |
| metab_10322 | Unannotated feature | neg | 332.1468 | 2.298 | 5.383 | 5.080 | -0.302 | -0.322 | 0.072 | 0.335 | Unannotated | NA | NA |
| metab_8100 | Unannotated feature | pos | 252.0869 | 2.740 | 3.146 | 3.830 | 0.684 | 0.322 | 0.072 | 0.335 | Unannotated | NA | NA |
| metab_4028 | Unannotated feature | pos | 607.3146 | 6.167 | 6.521 | 6.336 | -0.185 | -0.322 | 0.072 | 0.335 | Unannotated | NA | NA |
| metab_213 | Unannotated feature | pos | 209.0092 | 0.585 | 3.908 | 3.672 | -0.236 | -0.322 | 0.072 | 0.335 | Unannotated | NA | NA |
| metab_3070 | Pro Glu Leu | pos | 358.1981 | 3.347 | 5.557 | 5.384 | -0.173 | -0.322 | 0.072 | 0.335 | Moderate putative | NA | NA |
| metab_7847 | Glu Val Val | pos | 346.1980 | 3.058 | 5.126 | 4.124 | -1.001 | -0.322 | 0.072 | 0.335 | Moderate putative | NA | NA |
| metab_13579 | Unannotated feature | neg | 387.2256 | 1.288 | 5.447 | 5.042 | -0.405 | -0.322 | 0.072 | 0.335 | Unannotated | NA | NA |
| metab_12363 | Unannotated feature | neg | 809.8522 | 7.578 | 6.392 | 6.472 | 0.080 | 0.322 | 0.072 | 0.335 | Unannotated | NA | NA |
| metab_586 | Unannotated feature | pos | 90.9482 | 0.016 | 5.231 | 5.273 | 0.042 | 0.322 | 0.072 | 0.335 | Unannotated | NA | NA |
| metab_10110 | 3,6-Dihydroxycyclohepta-2,4,6-trien-1-one | neg | 183.0293 | 2.766 | 4.616 | 4.807 | 0.190 | 0.322 | 0.072 | 0.335 | Unannotated | NA | NA |
| metab_13737 | Unannotated feature | neg | 318.1673 | 1.947 | 4.434 | 4.144 | -0.290 | -0.322 | 0.072 | 0.335 | Unannotated | NA | NA |
| metab_14058 | Unannotated feature | neg | 399.1894 | 2.780 | 4.136 | 3.264 | -0.872 | -0.322 | 0.072 | 0.335 | Unannotated | NA | NA |
| metab_1414 | Unannotated feature | pos | 409.2212 | 6.064 | 5.143 | 4.546 | -0.597 | -0.322 | 0.072 | 0.335 | Unannotated | NA | NA |
| metab_6051 | Unannotated feature | pos | 133.0974 | 0.501 | 5.243 | 4.929 | -0.314 | -0.322 | 0.072 | 0.335 | Unannotated | NA | NA |
| metab_17200 | Unannotated feature | neg | 247.0827 | 7.737 | 4.386 | 4.669 | 0.283 | 0.322 | 0.072 | 0.335 | Unannotated | NA | NA |
| metab_12007 | Unannotated feature | neg | 361.1684 | 6.256 | 4.572 | 4.799 | 0.227 | 0.322 | 0.072 | 0.335 | Unannotated | NA | NA |
| metab_12651 | Unannotated feature | neg | 229.1079 | 5.944 | 5.212 | 4.981 | -0.231 | -0.322 | 0.072 | 0.335 | Unannotated | NA | NA |
| metab_11217 | Unannotated feature | neg | 303.1813 | 5.065 | 4.246 | 4.902 | 0.656 | 0.318 | 0.076 | 0.345 | Unannotated | NA | NA |
| metab_8963 | Unannotated feature | neg | 223.1086 | 3.773 | 4.351 | 4.250 | -0.101 | -0.318 | 0.076 | 0.345 | Unannotated | NA | NA |
| metab_6636 | Unannotated feature | pos | 208.1911 | 5.390 | 5.345 | 5.250 | -0.095 | -0.318 | 0.076 | 0.345 | Unannotated | NA | NA |
| metab_249 | Unannotated feature | pos | 450.8258 | 0.531 | 3.982 | 4.804 | 0.822 | 0.318 | 0.076 | 0.345 | Unannotated | NA | NA |
| metab_121 | N-Methyl-L-Histidine | pos | 170.0926 | 0.633 | 4.344 | 4.210 | -0.134 | -0.318 | 0.076 | 0.345 | Moderate putative | NA | NA |
| metab_12579 | Unannotated feature | neg | 297.2071 | 6.115 | 4.160 | 3.719 | -0.441 | -0.318 | 0.076 | 0.345 | Unannotated | NA | NA |
| metab_3152 | Unannotated feature | pos | 286.1766 | 3.574 | 5.463 | 5.111 | -0.352 | -0.318 | 0.076 | 0.345 | Unannotated | NA | NA |
| metab_4930 | Unannotated feature | pos | 106.0867 | 7.634 | 4.164 | 4.147 | -0.017 | -0.318 | 0.076 | 0.345 | Unannotated | NA | NA |
| metab_10324 | Unannotated feature | neg | 278.1359 | 2.298 | 4.069 | 2.787 | -1.282 | -0.318 | 0.076 | 0.345 | Unannotated | NA | NA |
| metab_4914 | Unannotated feature | pos | 216.9230 | 7.658 | 5.398 | 5.468 | 0.070 | 0.318 | 0.076 | 0.345 | Unannotated | NA | NA |
| metab_515 | Unannotated feature | pos | 597.3606 | 5.159 | 2.448 | 3.662 | 1.214 | 0.318 | 0.076 | 0.345 | Unannotated | NA | NA |
| metab_13581 | Unannotated feature | neg | 267.1465 | 1.308 | 4.342 | 3.697 | -0.645 | -0.318 | 0.076 | 0.345 | Unannotated | NA | NA |
| metab_698 | Unannotated feature | pos | 650.1987 | 5.922 | 3.471 | 4.440 | 0.969 | 0.318 | 0.076 | 0.345 | Unannotated | NA | NA |
| metab_8458 | Unannotated feature | pos | 231.1343 | 1.928 | 4.540 | 4.338 | -0.202 | -0.318 | 0.076 | 0.345 | Unannotated | NA | NA |
| metab_16733 | Unannotated feature | neg | 512.8794 | 7.663 | 5.498 | 5.575 | 0.078 | 0.318 | 0.076 | 0.345 | Unannotated | NA | NA |
| metab_12754 | Unannotated feature | neg | 617.5123 | 6.766 | 6.667 | 6.437 | -0.230 | -0.318 | 0.076 | 0.345 | Unannotated | NA | NA |
| metab_7909 | Unannotated feature | pos | 275.2009 | 6.382 | 5.574 | 5.447 | -0.127 | -0.318 | 0.076 | 0.345 | Unannotated | NA | NA |
| metab_12749 | Unannotated feature | neg | 570.1373 | 2.737 | 5.817 | 5.350 | -0.467 | -0.318 | 0.076 | 0.345 | Unannotated | NA | NA |
| metab_16673 | Unannotated feature | neg | 622.8933 | 7.611 | 6.206 | 6.309 | 0.102 | 0.318 | 0.076 | 0.345 | Unannotated | NA | NA |
| metab_14468 | Unannotated feature | neg | 372.2144 | 3.303 | 5.004 | 4.688 | -0.317 | -0.318 | 0.076 | 0.345 | Unannotated | NA | NA |
| metab_4955 | Unannotated feature | pos | 923.8179 | 7.586 | 5.397 | 5.525 | 0.128 | 0.318 | 0.076 | 0.345 | Unannotated | NA | NA |
| metab_18599 | 1-Ipomeanol | neg | 395.1713 | 5.991 | 4.349 | 3.920 | -0.428 | -0.318 | 0.076 | 0.345 | Unannotated | NA | NA |
| metab_16653 | Unannotated feature | neg | 815.8580 | 7.575 | 4.225 | 4.597 | 0.372 | 0.318 | 0.076 | 0.345 | Unannotated | NA | NA |
| metab_17466 | Unannotated feature | neg | 869.3448 | 7.600 | 6.236 | 6.383 | 0.148 | 0.318 | 0.076 | 0.345 | Unannotated | NA | NA |
| metab_4715 | Unannotated feature | pos | 948.8151 | 7.618 | 5.075 | 5.201 | 0.126 | 0.318 | 0.076 | 0.345 | Unannotated | NA | NA |
| metab_2123 | Unannotated feature | pos | 236.0921 | 2.732 | 4.698 | 4.941 | 0.243 | 0.318 | 0.076 | 0.345 | Unannotated | NA | NA |
| metab_605 | Unannotated feature | pos | 179.0005 | 0.016 | 5.407 | 5.457 | 0.050 | 0.318 | 0.076 | 0.345 | Unannotated | NA | NA |
| metab_2127 | Unannotated feature | pos | 222.0877 | 2.840 | 4.205 | 2.743 | -1.462 | -0.318 | 0.076 | 0.345 | Unannotated | NA | NA |
| metab_11361 | D-Sorbitol | neg | 181.0712 | 0.577 | 4.897 | 5.144 | 0.247 | 0.318 | 0.076 | 0.345 | Strong putative | NA | NA |
| metab_2424 | Unannotated feature | pos | 262.0691 | 0.617 | 2.820 | 3.404 | 0.584 | 0.318 | 0.076 | 0.345 | Unannotated | NA | NA |
| metab_18860 | Deoxycorticosterone sulfate | neg | 455.1751 | 5.710 | 3.449 | 3.957 | 0.509 | 0.318 | 0.076 | 0.345 | Unannotated | NA | NA |
| metab_4944 | Unannotated feature | pos | 770.8534 | 7.610 | 6.372 | 6.444 | 0.072 | 0.318 | 0.076 | 0.345 | Unannotated | NA | NA |
| metab_3951 | Unannotated feature | pos | 585.5750 | 5.782 | 3.855 | 4.149 | 0.294 | 0.318 | 0.076 | 0.345 | Unannotated | NA | NA |
| metab_11602 | Unannotated feature | neg | 318.8736 | 0.443 | 3.885 | 3.800 | -0.085 | -0.318 | 0.076 | 0.345 | Unannotated | NA | NA |
| metab_1729 | Unannotated feature | pos | 394.2347 | 3.808 | 4.648 | 3.428 | -1.220 | -0.318 | 0.076 | 0.345 | Unannotated | NA | NA |
| metab_7585 | 5-hydroxypropafenone | pos | 375.2246 | 0.728 | 5.226 | 4.957 | -0.269 | -0.318 | 0.076 | 0.345 | Unannotated | Phenylpropanoids and polyketides | Linear 1,3-diarylpropanoids |
| metab_5240 | Unannotated feature | pos | 228.2325 | 6.678 | 5.197 | 5.123 | -0.075 | -0.318 | 0.076 | 0.345 | Unannotated | NA | NA |
| metab_14629 | Unannotated feature | neg | 393.2003 | 3.559 | 4.421 | 3.595 | -0.826 | -0.318 | 0.076 | 0.345 | Unannotated | NA | NA |
| metab_8554 | Pyridoxine | pos | 170.0814 | 1.266 | 4.336 | 4.586 | 0.250 | 0.318 | 0.076 | 0.345 | Strong putative | Organoheterocyclic compounds | Pyridines and derivatives |
| metab_17724 | Unannotated feature | neg | 393.2319 | 6.959 | 4.785 | 4.032 | -0.753 | -0.318 | 0.076 | 0.345 | Unannotated | NA | NA |
| metab_10234 | Unannotated feature | neg | 454.0747 | 2.579 | 4.903 | 4.259 | -0.644 | -0.318 | 0.076 | 0.345 | Unannotated | NA | NA |
| metab_19228 | Valeric acid | neg | 203.1284 | 5.233 | 5.377 | 5.038 | -0.339 | -0.318 | 0.076 | 0.345 | Tentative | Lipids and lipid-like molecules | Fatty Acyls |
| metab_8410 | Unannotated feature | pos | 184.6185 | 1.952 | 4.171 | 3.383 | -0.788 | -0.318 | 0.076 | 0.345 | Unannotated | NA | NA |
| metab_15154 | 4-Hydroxy-2-butenoic acid gamma-lactone | neg | 167.0343 | 4.639 | 5.007 | 5.208 | 0.201 | 0.318 | 0.076 | 0.345 | Tentative | Organoheterocyclic compounds | Dihydrofurans |
| metab_16320 | Myristoylcarnitine | neg | 408.2525 | 6.656 | 5.131 | 4.744 | -0.387 | -0.318 | 0.076 | 0.345 | Unannotated | Lipids and lipid-like molecules | Fatty Acyls |
| metab_8559 | Unannotated feature | pos | 256.1184 | 1.252 | 4.527 | 5.041 | 0.514 | 0.318 | 0.076 | 0.345 | Unannotated | NA | NA |
| metab_16713 | Unannotated feature | neg | 707.3628 | 7.648 | 5.448 | 5.562 | 0.113 | 0.318 | 0.076 | 0.345 | Unannotated | NA | NA |
| metab_456 | Hexyl benzoate | pos | 171.1172 | 6.390 | 5.221 | 5.052 | -0.170 | -0.318 | 0.076 | 0.345 | Tentative | NA | NA |
| metab_2760 | Leu Thr Asp | pos | 348.1774 | 2.422 | 5.035 | 4.753 | -0.282 | -0.318 | 0.076 | 0.345 | Moderate putative | NA | NA |
| metab_10289 | Unannotated feature | neg | 143.0707 | 2.442 | 3.821 | 3.285 | -0.536 | -0.318 | 0.076 | 0.345 | Unannotated | NA | NA |
| metab_12645 | Telmisartan | neg | 513.2307 | 5.964 | 4.715 | 3.959 | -0.757 | -0.318 | 0.076 | 0.345 | Strong putative | NA | NA |
| metab_12610 | Unannotated feature | neg | 447.1820 | 6.035 | 4.470 | 3.915 | -0.555 | -0.318 | 0.076 | 0.345 | Unannotated | NA | NA |
| metab_6373 | Unannotated feature | pos | 649.9126 | 5.749 | 2.433 | 4.810 | 2.377 | 0.318 | 0.076 | 0.345 | Unannotated | NA | NA |
| metab_4778 | Unannotated feature | pos | 432.2818 | 3.607 | 5.094 | 5.702 | 0.608 | 0.318 | 0.076 | 0.345 | Unannotated | NA | NA |
| metab_18279 | Dihydroxyphaseic acid | neg | 621.2945 | 6.224 | 5.563 | 4.733 | -0.830 | -0.318 | 0.076 | 0.345 | Unannotated | Lipids and lipid-like molecules | Prenol lipids |
| metab_15080 | Unannotated feature | neg | 91.2560 | 4.498 | 3.343 | 3.218 | -0.126 | -0.318 | 0.076 | 0.345 | Unannotated | NA | NA |
| metab_3352 | Gly Val Phe | pos | 322.1768 | 4.239 | 4.280 | 3.557 | -0.723 | -0.318 | 0.076 | 0.345 | Moderate putative | NA | NA |
| metab_8179 | Unannotated feature | pos | 265.1551 | 2.603 | 5.597 | 4.673 | -0.925 | -0.318 | 0.076 | 0.345 | Unannotated | NA | NA |
| metab_3858 | Unannotated feature | pos | 584.1238 | 5.686 | 3.989 | 4.392 | 0.403 | 0.318 | 0.076 | 0.345 | Unannotated | NA | NA |
| metab_5742 | Orcinol | pos | 125.0601 | 6.072 | 2.697 | 3.611 | 0.914 | 0.318 | 0.076 | 0.345 | Tentative | NA | NA |
| metab_17416 | Unannotated feature | neg | 944.8119 | 7.637 | 5.887 | 6.050 | 0.163 | 0.318 | 0.076 | 0.345 | Unannotated | NA | NA |
| metab_5952 | Unannotated feature | pos | 153.9561 | 0.402 | 4.977 | 5.010 | 0.033 | 0.318 | 0.076 | 0.345 | Unannotated | NA | NA |
| metab_8757 | Unannotated feature | pos | 347.2296 | 0.728 | 5.260 | 4.597 | -0.664 | -0.318 | 0.076 | 0.345 | Unannotated | NA | NA |
| metab_16708 | Unannotated feature | neg | 706.8567 | 7.641 | 4.504 | 4.737 | 0.233 | 0.318 | 0.076 | 0.345 | Unannotated | NA | NA |
| metab_4973 | Unannotated feature | pos | 175.9545 | 7.546 | 5.209 | 5.255 | 0.046 | 0.314 | 0.080 | 0.355 | Unannotated | NA | NA |
| metab_8048 | Unannotated feature | pos | 180.0636 | 2.780 | 2.882 | 3.501 | 0.619 | 0.314 | 0.080 | 0.355 | Unannotated | NA | NA |
| metab_4013 | Unannotated feature | pos | 554.3428 | 5.814 | 4.136 | 4.307 | 0.170 | 0.314 | 0.080 | 0.355 | Unannotated | NA | NA |
| metab_12845 | Unannotated feature | neg | 194.9964 | 0.683 | 6.494 | 6.138 | -0.356 | -0.314 | 0.080 | 0.355 | Unannotated | NA | NA |
| metab_16715 | Unannotated feature | neg | 911.3258 | 7.648 | 5.597 | 5.741 | 0.144 | 0.314 | 0.080 | 0.355 | Unannotated | NA | NA |
| metab_9550 | Unannotated feature | neg | 339.0728 | 3.284 | 3.205 | 3.685 | 0.480 | 0.314 | 0.080 | 0.355 | Unannotated | NA | NA |
| metab_4726 | Kaempferol 3-glucosyl-(1->2)-(6''-acetylgalactoside)-7-glucoside | pos | 832.2426 | 7.642 | 5.261 | 5.332 | 0.071 | 0.314 | 0.080 | 0.355 | Unannotated | NA | NA |
| metab_17384 | Unannotated feature | neg | 749.3449 | 7.663 | 4.076 | 4.260 | 0.185 | 0.314 | 0.080 | 0.355 | Unannotated | NA | NA |
| metab_10705 | Unannotated feature | neg | 136.0761 | 4.759 | 5.623 | 5.511 | -0.112 | -0.314 | 0.080 | 0.355 | Unannotated | NA | NA |
| metab_11730 | Unannotated feature | neg | 279.0908 | 5.583 | 4.485 | 4.918 | 0.433 | 0.314 | 0.080 | 0.355 | Unannotated | NA | NA |
| metab_7960 | Unannotated feature | pos | 298.1767 | 3.410 | 4.416 | 4.068 | -0.348 | -0.314 | 0.080 | 0.355 | Unannotated | NA | NA |
| metab_1752 | Unannotated feature | pos | 341.2304 | 3.559 | 4.579 | 3.990 | -0.589 | -0.314 | 0.080 | 0.355 | Unannotated | NA | NA |
| metab_15023 | Trinexapac-ethyl | neg | 251.0925 | 4.382 | 1.565 | 3.065 | 1.500 | 0.314 | 0.080 | 0.355 | Tentative | NA | NA |
| metab_4718 | Unannotated feature | pos | 786.8281 | 7.634 | 4.717 | 4.913 | 0.195 | 0.314 | 0.080 | 0.355 | Unannotated | NA | NA |
| metab_7910 | Unannotated feature | pos | 280.1185 | 3.371 | 2.672 | 3.594 | 0.923 | 0.314 | 0.080 | 0.355 | Unannotated | NA | NA |
| metab_2656 | Unannotated feature | pos | 156.0657 | 1.952 | 5.060 | 5.130 | 0.070 | 0.314 | 0.080 | 0.355 | Unannotated | NA | NA |
| metab_5038 | Unannotated feature | pos | 465.3536 | 7.244 | 5.255 | 5.224 | -0.031 | -0.314 | 0.080 | 0.355 | Unannotated | NA | NA |
| metab_9101 | Unannotated feature | neg | 378.1676 | 3.930 | 4.612 | 4.098 | -0.514 | -0.314 | 0.080 | 0.355 | Unannotated | NA | NA |
| metab_14024 | Unannotated feature | neg | 474.2218 | 2.762 | 4.751 | 3.815 | -0.936 | -0.314 | 0.080 | 0.355 | Unannotated | NA | NA |
| metab_12245 | Unannotated feature | neg | 571.2903 | 6.959 | 6.392 | 6.318 | -0.074 | -0.314 | 0.080 | 0.355 | Unannotated | NA | NA |
| metab_13128 | Unannotated feature | neg | 312.8963 | 0.449 | 3.710 | 3.448 | -0.262 | -0.314 | 0.080 | 0.355 | Unannotated | NA | NA |
| metab_849 | Unannotated feature | pos | 223.1695 | 6.550 | 6.241 | 6.197 | -0.044 | -0.314 | 0.080 | 0.355 | Unannotated | NA | NA |
| metab_13988 | Unannotated feature | neg | 460.1952 | 2.698 | 4.481 | 3.790 | -0.691 | -0.314 | 0.080 | 0.355 | Unannotated | NA | NA |
| metab_3025 | Unannotated feature | pos | 375.1774 | 1.952 | 5.237 | 5.132 | -0.105 | -0.314 | 0.080 | 0.355 | Unannotated | NA | NA |
| metab_355 | Unannotated feature | pos | 142.0866 | 0.385 | 4.554 | 4.588 | 0.034 | 0.314 | 0.080 | 0.355 | Unannotated | NA | NA |
| metab_19725 | Unannotated feature | neg | 259.1454 | 4.522 | 5.543 | 5.298 | -0.245 | -0.314 | 0.080 | 0.355 | Unannotated | NA | NA |
| metab_7011 | Unannotated feature | pos | 509.3131 | 4.820 | 3.642 | 4.931 | 1.289 | 0.314 | 0.080 | 0.355 | Unannotated | NA | NA |
| metab_15336 | Unannotated feature | neg | 303.1717 | 4.955 | 2.373 | 2.915 | 0.542 | 0.314 | 0.080 | 0.355 | Unannotated | NA | NA |
| metab_6572 | Unannotated feature | pos | 134.0191 | 0.609 | 3.866 | 3.129 | -0.736 | -0.314 | 0.080 | 0.355 | Unannotated | NA | NA |
| metab_17127 | Unannotated feature | neg | 174.8643 | 7.822 | 3.799 | 3.901 | 0.102 | 0.314 | 0.080 | 0.355 | Unannotated | NA | NA |
| metab_2220 | Unannotated feature | pos | 259.1769 | 3.047 | 5.118 | 4.270 | -0.849 | -0.314 | 0.080 | 0.355 | Unannotated | NA | NA |
| metab_5955 | Unannotated feature | pos | 665.2066 | 5.914 | 3.350 | 4.287 | 0.937 | 0.314 | 0.080 | 0.355 | Unannotated | NA | NA |
| metab_13982 | Dethiobiotin | neg | 259.1301 | 2.676 | 5.810 | 5.700 | -0.110 | -0.314 | 0.080 | 0.355 | Tentative | Lipids and lipid-like molecules | Fatty Acyls |
| metab_8170 | Thr Leu | pos | 233.1500 | 2.643 | 5.618 | 5.262 | -0.355 | -0.314 | 0.080 | 0.355 | Strong putative | NA | NA |
| metab_14595 | Unannotated feature | neg | 470.1578 | 3.502 | 4.163 | 3.745 | -0.418 | -0.314 | 0.080 | 0.355 | Unannotated | NA | NA |
| metab_1613 | Unannotated feature | pos | 209.0812 | 3.689 | 5.211 | 4.990 | -0.221 | -0.314 | 0.080 | 0.355 | Unannotated | NA | NA |
| metab_5940 | Unannotated feature | pos | 287.1778 | 5.922 | 2.657 | 4.028 | 1.371 | 0.314 | 0.080 | 0.355 | Unannotated | NA | NA |
| metab_2750 | Unannotated feature | pos | 99.5316 | 2.367 | 4.781 | 4.909 | 0.128 | 0.314 | 0.080 | 0.355 | Unannotated | NA | NA |
| metab_8026 | N2-Ethyl-2'-deoxyguanosine | pos | 260.1134 | 2.803 | 3.531 | 4.260 | 0.728 | 0.314 | 0.080 | 0.355 | Unannotated | NA | NA |
| metab_18640 | Unannotated feature | neg | 94.0038 | 5.952 | 4.885 | 4.767 | -0.118 | -0.314 | 0.080 | 0.355 | Unannotated | NA | NA |
| metab_17332 | Unannotated feature | neg | 294.0300 | 2.579 | 5.688 | 5.479 | -0.209 | -0.314 | 0.080 | 0.355 | Unannotated | NA | NA |
| metab_4117 | Unannotated feature | pos | 667.5079 | 5.854 | 4.086 | 4.549 | 0.462 | 0.314 | 0.080 | 0.355 | Unannotated | NA | NA |
| metab_4209 | Unannotated feature | pos | 718.9427 | 5.914 | 4.117 | 4.496 | 0.379 | 0.314 | 0.080 | 0.355 | Unannotated | NA | NA |
| metab_5260 | Unannotated feature | pos | 501.3929 | 6.646 | 5.176 | 5.550 | 0.374 | 0.314 | 0.080 | 0.355 | Unannotated | NA | NA |
| metab_16574 | Unannotated feature | neg | 160.9902 | 7.320 | 5.672 | 5.721 | 0.049 | 0.314 | 0.080 | 0.355 | Unannotated | NA | NA |
| metab_7581 | Unannotated feature | pos | 277.1657 | 3.586 | 3.363 | 2.675 | -0.687 | -0.314 | 0.080 | 0.355 | Unannotated | NA | NA |
| metab_7704 | Unannotated feature | pos | 240.1234 | 3.366 | 4.163 | 4.358 | 0.195 | 0.314 | 0.080 | 0.355 | Unannotated | NA | NA |
| metab_4695 | Unannotated feature | pos | 447.9052 | 7.570 | 5.926 | 5.999 | 0.073 | 0.314 | 0.080 | 0.355 | Unannotated | NA | NA |
| metab_14392 | Epsilon-(Hexanoyl)lysine | neg | 279.1463 | 3.184 | 3.710 | 3.015 | -0.695 | -0.314 | 0.080 | 0.355 | Unannotated | NA | NA |
| metab_14383 | Unannotated feature | neg | 363.1100 | 3.169 | 4.055 | 4.618 | 0.563 | 0.314 | 0.080 | 0.355 | Unannotated | NA | NA |
| metab_12258 | Unannotated feature | neg | 349.2363 | 7.004 | 5.983 | 6.113 | 0.130 | 0.314 | 0.080 | 0.355 | Unannotated | NA | NA |
| metab_15741 | L-Targinine | neg | 223.0972 | 5.753 | 3.671 | 4.329 | 0.658 | 0.314 | 0.080 | 0.355 | Tentative | Organic acids and derivatives | Carboxylic acids and derivatives |
| metab_18940 | Unannotated feature | neg | 613.2719 | 5.595 | 4.484 | 4.628 | 0.144 | 0.314 | 0.080 | 0.355 | Unannotated | NA | NA |
| metab_10235 | Fenquizone | neg | 318.0102 | 2.579 | 6.076 | 5.912 | -0.164 | -0.314 | 0.080 | 0.355 | Unannotated | NA | NA |
| metab_10588 | Unannotated feature | neg | 169.0500 | 4.701 | 3.399 | 4.034 | 0.634 | 0.314 | 0.080 | 0.355 | Unannotated | NA | NA |
| metab_4699 | Unannotated feature | pos | 583.8809 | 7.586 | 5.839 | 5.885 | 0.046 | 0.314 | 0.080 | 0.355 | Unannotated | NA | NA |
| metab_5962 | Unannotated feature | pos | 139.9880 | 0.016 | 5.424 | 5.457 | 0.033 | 0.314 | 0.080 | 0.355 | Unannotated | NA | NA |
| metab_2313 | Kaempferol 3-[2''-glucosyl-6''-acetyl-galactoside] 7-glucoside | pos | 832.2435 | 0.085 | 6.609 | 6.651 | 0.041 | 0.314 | 0.080 | 0.355 | Unannotated | NA | NA |
| metab_18537 | Unannotated feature | neg | 429.1961 | 6.029 | 6.177 | 6.378 | 0.202 | 0.314 | 0.080 | 0.355 | Unannotated | NA | NA |
| metab_19741 | Unannotated feature | neg | 137.0601 | 4.515 | 4.139 | 4.019 | -0.121 | -0.309 | 0.084 | 0.361 | Unannotated | NA | NA |
| metab_1048 | 4-Dodecylmorpholine | pos | 256.2637 | 6.829 | 7.156 | 7.105 | -0.051 | -0.309 | 0.084 | 0.361 | Unannotated | NA | NA |
| metab_5379 | Unannotated feature | pos | 441.2820 | 6.470 | 5.510 | 5.144 | -0.366 | -0.309 | 0.084 | 0.361 | Unannotated | NA | NA |
| metab_11585 | Unannotated feature | neg | 302.8882 | 0.453 | 3.933 | 3.831 | -0.103 | -0.309 | 0.084 | 0.361 | Unannotated | NA | NA |
| metab_10219 | Unannotated feature | neg | 375.1090 | 4.522 | 4.849 | 5.333 | 0.484 | 0.309 | 0.084 | 0.361 | Unannotated | NA | NA |
| metab_4246 | Unannotated feature | pos | 668.0086 | 5.929 | 1.549 | 4.479 | 2.930 | 0.309 | 0.084 | 0.361 | Unannotated | NA | NA |
| metab_18149 | para-hydroxyatorvastatin | neg | 555.2311 | 6.342 | 4.549 | 5.158 | 0.609 | 0.309 | 0.084 | 0.361 | Unannotated | Organoheterocyclic compounds | Pyrroles |
| metab_10292 | Unannotated feature | neg | 246.0256 | 2.432 | 3.053 | 3.731 | 0.678 | 0.309 | 0.084 | 0.361 | Unannotated | NA | NA |
| metab_10654 | Unannotated feature | neg | 229.0352 | 1.640 | 4.569 | 5.020 | 0.451 | 0.309 | 0.084 | 0.361 | Unannotated | NA | NA |
| metab_10121 | Dimethylpropiothetin | neg | 267.0754 | 2.762 | 5.559 | 5.858 | 0.299 | 0.309 | 0.084 | 0.361 | Tentative | Organic acids and derivatives | Carboxylic acids and derivatives |
| metab_6255 | Unannotated feature | pos | 550.1739 | 5.806 | 3.110 | 3.580 | 0.470 | 0.309 | 0.084 | 0.361 | Unannotated | NA | NA |
| metab_4347 | CL(16:1(9Z)/16:1(9Z)/22:6(4Z,7Z,10Z,13Z,16Z,19Z)/20:4(5Z,8Z,11Z,14Z)) | pos | 746.4668 | 5.340 | 4.069 | 4.543 | 0.474 | 0.309 | 0.084 | 0.361 | Tentative | Lipids and lipid-like molecules | Glycerophospholipids |
| metab_6521 | Unannotated feature | pos | 656.7332 | 5.563 | 2.717 | 3.970 | 1.252 | 0.309 | 0.084 | 0.361 | Unannotated | NA | NA |
| metab_10178 | Unannotated feature | neg | 136.0761 | 4.510 | 5.227 | 5.098 | -0.129 | -0.309 | 0.084 | 0.361 | Unannotated | NA | NA |
| metab_10632 | Unannotated feature | neg | 274.0573 | 1.680 | 3.834 | 3.304 | -0.530 | -0.309 | 0.084 | 0.361 | Unannotated | NA | NA |
| metab_14760 | Unannotated feature | neg | 554.8646 | 0.512 | 5.783 | 6.186 | 0.403 | 0.309 | 0.084 | 0.361 | Unannotated | NA | NA |
| metab_6269 | Unannotated feature | pos | 584.1240 | 5.799 | 4.001 | 4.075 | 0.074 | 0.309 | 0.084 | 0.361 | Unannotated | NA | NA |
| metab_13346 | 3-beta-D-galactosyl-sn-glycerol | neg | 253.0929 | 0.714 | 4.945 | 5.219 | 0.275 | 0.309 | 0.084 | 0.361 | Tentative | Lipids and lipid-like molecules | Glycerolipids |
| metab_2105 | Unannotated feature | pos | 143.1182 | 2.795 | 5.055 | 4.914 | -0.140 | -0.309 | 0.084 | 0.361 | Unannotated | NA | NA |
| metab_2102 | Unannotated feature | pos | 206.1180 | 2.747 | 4.570 | 4.984 | 0.414 | 0.309 | 0.084 | 0.361 | Unannotated | NA | NA |
| metab_13007 | Unannotated feature | neg | 194.9270 | 0.815 | 5.964 | 5.669 | -0.295 | -0.309 | 0.084 | 0.361 | Unannotated | NA | NA |
| metab_10647 | Unannotated feature | neg | 318.0061 | 1.650 | 5.629 | 5.486 | -0.143 | -0.309 | 0.084 | 0.361 | Unannotated | NA | NA |
| metab_3997 | Matesaponin 4 | pos | 633.2944 | 5.806 | 5.298 | 5.082 | -0.216 | -0.309 | 0.084 | 0.361 | Unannotated | NA | NA |
| metab_2111 | Unannotated feature | pos | 272.1859 | 2.732 | 4.379 | 3.376 | -1.002 | -0.309 | 0.084 | 0.361 | Unannotated | NA | NA |
| metab_16825 | Unannotated feature | neg | 245.0671 | 7.726 | 3.536 | 4.053 | 0.517 | 0.309 | 0.084 | 0.361 | Unannotated | NA | NA |
| metab_14731 | Unannotated feature | neg | 248.8963 | 0.516 | 4.937 | 4.566 | -0.371 | -0.309 | 0.084 | 0.361 | Unannotated | NA | NA |
| metab_18412 | Unannotated feature | neg | 589.3412 | 6.115 | 4.397 | 4.738 | 0.341 | 0.309 | 0.084 | 0.361 | Unannotated | NA | NA |
| metab_17013 | Unannotated feature | neg | 259.8778 | 7.997 | 4.205 | 4.167 | -0.039 | -0.309 | 0.084 | 0.361 | Unannotated | NA | NA |
| metab_1998 | Unannotated feature | pos | 170.1116 | 2.223 | 5.268 | 5.314 | 0.047 | 0.309 | 0.084 | 0.361 | Unannotated | NA | NA |
| metab_17886 | Unannotated feature | neg | 225.0402 | 2.841 | 4.399 | 4.615 | 0.216 | 0.309 | 0.084 | 0.361 | Unannotated | NA | NA |
| metab_19371 | Unannotated feature | neg | 145.9491 | 5.044 | 4.055 | 3.926 | -0.130 | -0.309 | 0.084 | 0.361 | Unannotated | NA | NA |
| metab_455 | MJDBISSP00000002 | pos | 403.3620 | 6.438 | 7.353 | 7.296 | -0.057 | -0.309 | 0.084 | 0.361 | Moderate putative | NA | NA |
| metab_5780 | Unannotated feature | pos | 223.9887 | 0.026 | 4.661 | 4.684 | 0.023 | 0.309 | 0.084 | 0.361 | Unannotated | NA | NA |
| metab_9940 | Unannotated feature | neg | 385.1735 | 2.906 | 3.865 | 3.545 | -0.321 | -0.309 | 0.084 | 0.361 | Unannotated | NA | NA |
| metab_3711 | Unannotated feature | pos | 660.3947 | 5.324 | 4.242 | 5.411 | 1.168 | 0.309 | 0.084 | 0.361 | Unannotated | NA | NA |
| metab_13740 | Unannotated feature | neg | 230.9212 | 0.075 | 5.991 | 5.955 | -0.035 | -0.309 | 0.084 | 0.361 | Unannotated | NA | NA |
| metab_2445 | Unannotated feature | pos | 132.1133 | 0.649 | 4.726 | 4.542 | -0.184 | -0.309 | 0.084 | 0.361 | Unannotated | NA | NA |
| metab_17622 | Unannotated feature | neg | 346.1639 | 7.148 | 4.098 | 4.331 | 0.233 | 0.309 | 0.084 | 0.361 | Unannotated | NA | NA |
| metab_5500 | Nafazatrom | pos | 301.1551 | 6.343 | 6.286 | 6.037 | -0.249 | -0.309 | 0.084 | 0.361 | Unannotated | Benzenoids | Naphthalenes |
| metab_11728 | Unannotated feature | neg | 538.2068 | 5.567 | 2.902 | 3.748 | 0.847 | 0.309 | 0.084 | 0.361 | Unannotated | NA | NA |
| metab_996 | Tris(2-carboxyethyl)phosphine | pos | 314.0755 | 0.641 | 4.876 | 4.396 | -0.479 | -0.309 | 0.084 | 0.361 | Unannotated | Organic acids and derivatives | Carboxylic acids and derivatives |
| metab_4923 | Phosphono [2,3,4-trihydroxy-5,6-bis[[hydroxy(phosphonooxy)phosphoryl]oxy]cyclohexyl] hydrogen phosphate | pos | 624.8500 | 7.642 | 4.998 | 5.179 | 0.181 | 0.309 | 0.084 | 0.361 | Unannotated | Organic oxygen compounds | Organooxygen compounds |
| metab_17489 | Unannotated feature | neg | 486.9039 | 7.523 | 5.106 | 5.207 | 0.101 | 0.309 | 0.084 | 0.361 | Unannotated | NA | NA |
| metab_7255 | Unannotated feature | pos | 326.7101 | 4.295 | 3.326 | 4.225 | 0.899 | 0.309 | 0.084 | 0.361 | Unannotated | NA | NA |
| metab_7702 | Unannotated feature | pos | 396.1777 | 3.366 | 4.210 | 2.879 | -1.331 | -0.309 | 0.084 | 0.361 | Unannotated | NA | NA |
| metab_19507 | Unannotated feature | neg | 334.0054 | 4.846 | 4.243 | 1.963 | -2.279 | -0.309 | 0.084 | 0.361 | Unannotated | NA | NA |
| metab_12690 | Unannotated feature | neg | 395.2077 | 5.431 | 1.823 | 3.537 | 1.714 | 0.309 | 0.084 | 0.361 | Unannotated | NA | NA |
| metab_16652 | Unannotated feature | neg | 707.8705 | 7.575 | 4.915 | 5.108 | 0.192 | 0.309 | 0.084 | 0.361 | Unannotated | NA | NA |
| metab_15234 | Unannotated feature | neg | 189.9758 | 0.558 | 4.526 | 3.835 | -0.691 | -0.309 | 0.084 | 0.361 | Unannotated | NA | NA |
| metab_8514 | Ile Ser | pos | 219.1343 | 1.622 | 6.169 | 5.762 | -0.407 | -0.309 | 0.084 | 0.361 | Moderate putative | NA | NA |
| metab_3803 | Unannotated feature | pos | 655.0818 | 5.563 | 2.709 | 4.703 | 1.995 | 0.309 | 0.084 | 0.361 | Unannotated | NA | NA |
| metab_4996 | Unannotated feature | pos | 311.1832 | 7.474 | 4.880 | 4.799 | -0.081 | -0.309 | 0.084 | 0.361 | Unannotated | NA | NA |
| metab_16003 | Unannotated feature | neg | 280.9831 | 6.123 | 4.336 | 3.937 | -0.399 | -0.309 | 0.084 | 0.361 | Unannotated | NA | NA |
| metab_618 | Unannotated feature | pos | 608.5853 | 5.799 | 3.880 | 4.124 | 0.244 | 0.309 | 0.084 | 0.361 | Unannotated | NA | NA |
| metab_13694 | Gallic Acid | neg | 169.0137 | 1.699 | 4.888 | 4.662 | -0.226 | -0.309 | 0.084 | 0.361 | Strong putative | NA | NA |
| metab_19332 | Unannotated feature | neg | 156.9748 | 5.095 | 5.435 | 5.308 | -0.127 | -0.309 | 0.084 | 0.361 | Unannotated | NA | NA |
| metab_2179 | Phe Ile Lys | pos | 204.1367 | 2.951 | 2.958 | 2.190 | -0.768 | -0.309 | 0.084 | 0.361 | Moderate putative | NA | NA |
| metab_3864 | Unannotated feature | pos | 261.1852 | 6.382 | 5.535 | 5.454 | -0.081 | -0.309 | 0.084 | 0.361 | Unannotated | NA | NA |
| metab_553 | Unannotated feature | pos | 228.1961 | 6.111 | 6.034 | 5.986 | -0.048 | -0.309 | 0.084 | 0.361 | Unannotated | NA | NA |
| metab_18283 | Unannotated feature | neg | 329.1834 | 2.984 | 5.630 | 5.526 | -0.104 | -0.309 | 0.084 | 0.361 | Unannotated | NA | NA |
| metab_3876 | Unannotated feature | pos | 495.8197 | 5.721 | 3.856 | 4.429 | 0.572 | 0.309 | 0.084 | 0.361 | Unannotated | NA | NA |
| metab_3891 | Unannotated feature | pos | 669.7572 | 5.738 | 4.183 | 5.067 | 0.885 | 0.309 | 0.084 | 0.361 | Unannotated | NA | NA |
| metab_2158 | Unannotated feature | pos | 357.2506 | 2.900 | 5.249 | 4.287 | -0.962 | -0.309 | 0.084 | 0.361 | Unannotated | NA | NA |
| metab_11495 | Unannotated feature | neg | 114.9879 | 0.516 | 4.940 | 4.798 | -0.142 | -0.309 | 0.084 | 0.361 | Unannotated | NA | NA |
| metab_595 | Unannotated feature | pos | 159.9695 | 0.016 | 6.256 | 6.276 | 0.020 | 0.309 | 0.084 | 0.361 | Unannotated | NA | NA |
| metab_3423 | Unannotated feature | pos | 143.0342 | 4.503 | 4.130 | 4.030 | -0.100 | -0.309 | 0.084 | 0.361 | Unannotated | NA | NA |
| metab_18379 | Unannotated feature | neg | 502.2165 | 2.750 | 4.659 | 3.802 | -0.857 | -0.309 | 0.084 | 0.361 | Unannotated | NA | NA |
| metab_17439 | Unannotated feature | neg | 1017.8179 | 7.611 | 4.669 | 4.932 | 0.262 | 0.309 | 0.084 | 0.361 | Unannotated | NA | NA |
| metab_1496 | Unannotated feature | pos | 655.6164 | 5.806 | 4.196 | 4.730 | 0.534 | 0.309 | 0.084 | 0.361 | Unannotated | NA | NA |
| metab_11366 | Descyclopropyl Abacavir | neg | 291.1201 | 0.571 | 3.640 | 4.017 | 0.377 | 0.309 | 0.084 | 0.361 | Unannotated | Nucleosides, nucleotides, and analogues | Nucleoside and nucleotide analogues |
| metab_16753 | Unannotated feature | neg | 571.2903 | 7.684 | 5.162 | 5.016 | -0.146 | -0.309 | 0.084 | 0.361 | Unannotated | NA | NA |
| metab_3698 | Unannotated feature | pos | 604.3683 | 5.291 | 1.705 | 4.669 | 2.964 | 0.309 | 0.084 | 0.361 | Unannotated | NA | NA |
| metab_18952 | (1R,2R,4R)-1,8-Epoxy-p-menthane-2,4-diol | neg | 231.1236 | 5.583 | 5.430 | 5.247 | -0.183 | -0.309 | 0.084 | 0.361 | Unannotated | Lipids and lipid-like molecules | Prenol lipids |
| metab_5301 | Unannotated feature | pos | 285.2428 | 6.558 | 4.293 | 3.657 | -0.636 | -0.309 | 0.084 | 0.361 | Unannotated | NA | NA |
| metab_16920 | Unannotated feature | neg | 146.9382 | 7.785 | 5.087 | 5.008 | -0.078 | -0.309 | 0.084 | 0.361 | Unannotated | NA | NA |
| metab_11751 | 2-Benzylsuccinic Acid | neg | 207.0658 | 3.925 | 5.511 | 5.319 | -0.192 | -0.309 | 0.084 | 0.361 | Strong putative | NA | NA |
| metab_2701 | Unannotated feature | pos | 716.2729 | 5.922 | 4.345 | 4.917 | 0.571 | 0.309 | 0.084 | 0.361 | Unannotated | NA | NA |
| metab_13932 | Unannotated feature | neg | 315.9946 | 2.575 | 4.775 | 4.492 | -0.284 | -0.309 | 0.084 | 0.361 | Unannotated | NA | NA |
| metab_19563 | Unannotated feature | neg | 137.0601 | 4.762 | 4.710 | 4.573 | -0.137 | -0.309 | 0.084 | 0.361 | Unannotated | NA | NA |
| metab_9219 | Unannotated feature | neg | 311.1250 | 3.741 | 3.532 | 4.092 | 0.560 | 0.309 | 0.084 | 0.361 | Unannotated | NA | NA |
| metab_4850 | Unannotated feature | pos | 119.5473 | 7.732 | 3.905 | 3.958 | 0.053 | 0.309 | 0.084 | 0.361 | Unannotated | NA | NA |
| metab_17324 | LysoPC(17:0/0:0) | neg | 508.3426 | 7.697 | 4.829 | 4.377 | -0.452 | -0.309 | 0.084 | 0.361 | Tentative | NA | NA |
| metab_3206 | Val Thr Ile | pos | 332.2187 | 3.734 | 5.615 | 5.351 | -0.263 | -0.309 | 0.084 | 0.361 | Unannotated | NA | NA |
| metab_16902 | Unannotated feature | neg | 326.9038 | 7.760 | 5.899 | 5.864 | -0.035 | -0.309 | 0.084 | 0.361 | Unannotated | NA | NA |
| metab_5687 | N-Dodecylsarcosinate | pos | 290.2694 | 6.135 | 4.863 | 4.709 | -0.154 | -0.309 | 0.084 | 0.361 | Unannotated | NA | NA |
| metab_18358 | Unannotated feature | neg | 371.1902 | 6.162 | 6.245 | 6.547 | 0.301 | 0.309 | 0.084 | 0.361 | Unannotated | NA | NA |
| metab_4541 | Unannotated feature | pos | 211.0970 | 4.828 | 4.405 | 4.132 | -0.273 | -0.309 | 0.084 | 0.361 | Unannotated | NA | NA |
| metab_11004 | Unannotated feature | neg | 137.0349 | 0.777 | 5.168 | 4.965 | -0.203 | -0.309 | 0.084 | 0.361 | Unannotated | NA | NA |
| metab_8766 | Histidylvaline | pos | 255.1452 | 0.728 | 4.948 | 4.441 | -0.508 | -0.309 | 0.084 | 0.361 | Unannotated | NA | NA |
| metab_2669 | 1-Nitrohydroxyphenyl-N-benzoylalanine | pos | 166.0502 | 1.960 | 3.909 | 4.072 | 0.163 | 0.309 | 0.084 | 0.361 | Unannotated | NA | NA |
| metab_1996 | N-OCTANOYL-L-HOMOSERINE LACTONE | pos | 228.1598 | 1.952 | 2.611 | 3.017 | 0.407 | 0.305 | 0.088 | 0.370 | Tentative | NA | NA |
| metab_10653 | Unannotated feature | neg | 190.0174 | 1.647 | 4.645 | 4.197 | -0.448 | -0.305 | 0.088 | 0.370 | Unannotated | NA | NA |
| metab_9998 | Unannotated feature | neg | 812.2973 | 2.845 | 4.006 | 3.658 | -0.347 | -0.305 | 0.088 | 0.370 | Unannotated | NA | NA |
| metab_9997 | Picolinamide | neg | 243.0874 | 2.848 | 4.144 | 4.548 | 0.404 | 0.305 | 0.088 | 0.370 | Tentative | NA | NA |
| metab_15059 | Unannotated feature | neg | 120.9541 | 0.547 | 4.954 | 4.818 | -0.135 | -0.305 | 0.088 | 0.370 | Unannotated | NA | NA |
| metab_16667 | Unannotated feature | neg | 894.8443 | 7.607 | 6.538 | 6.612 | 0.074 | 0.305 | 0.088 | 0.370 | Unannotated | NA | NA |
| metab_15248 | Isoketocamphoric acid | neg | 215.0921 | 4.803 | 4.864 | 5.197 | 0.333 | 0.305 | 0.088 | 0.370 | Unannotated | NA | NA |
| metab_4062 | Unannotated feature | pos | 463.2831 | 5.835 | 1.383 | 3.716 | 2.333 | 0.305 | 0.088 | 0.370 | Unannotated | NA | NA |
| metab_540 | Unannotated feature | pos | 593.3598 | 5.509 | 3.575 | 4.327 | 0.752 | 0.305 | 0.088 | 0.370 | Unannotated | NA | NA |
| metab_5771 | N-Linoleoyl Phenylalanine | pos | 450.2975 | 6.032 | 5.697 | 5.569 | -0.128 | -0.305 | 0.088 | 0.370 | Unannotated | NA | NA |
| metab_4066 | Unannotated feature | pos | 631.8904 | 5.835 | 3.777 | 4.351 | 0.574 | 0.305 | 0.088 | 0.370 | Unannotated | NA | NA |
| metab_6617 | 6-Hydroxygliclazide | pos | 340.1332 | 5.425 | 4.638 | 4.471 | -0.167 | -0.305 | 0.088 | 0.370 | Unannotated | NA | NA |
| metab_8083 | Unannotated feature | pos | 392.2190 | 3.517 | 4.845 | 4.325 | -0.519 | -0.305 | 0.088 | 0.370 | Unannotated | NA | NA |
| metab_9761 | 6,11-Dihydroxy-3-methyl-3-(4-methyl-3-pentenyl)-3H,7H-pyrano[2,3-c]xanthen-7-one | neg | 377.1360 | 4.239 | 6.152 | 6.031 | -0.121 | -0.305 | 0.088 | 0.370 | Unannotated | Organoheterocyclic compounds | Benzopyrans |
| metab_3903 | Unannotated feature | pos | 661.1697 | 5.754 | 4.163 | 4.520 | 0.357 | 0.305 | 0.088 | 0.370 | Unannotated | NA | NA |
| metab_9827 | Unannotated feature | neg | 335.1577 | 3.017 | 4.346 | 3.592 | -0.754 | -0.305 | 0.088 | 0.370 | Unannotated | NA | NA |
| metab_16655 | Unannotated feature | neg | 605.8895 | 7.584 | 6.679 | 6.742 | 0.063 | 0.305 | 0.088 | 0.370 | Unannotated | NA | NA |
| metab_3912 | Unannotated feature | pos | 660.9189 | 5.757 | 3.450 | 4.486 | 1.036 | 0.305 | 0.088 | 0.370 | Unannotated | NA | NA |
| metab_13642 | Erosnin | neg | 301.0160 | 1.647 | 6.334 | 6.293 | -0.041 | -0.305 | 0.088 | 0.370 | Unannotated | NA | NA |
| metab_6427 | Unannotated feature | pos | 595.1309 | 5.693 | 3.396 | 3.964 | 0.567 | 0.305 | 0.088 | 0.370 | Unannotated | NA | NA |
| metab_11097 | Unannotated feature | neg | 248.0928 | 5.000 | 5.875 | 5.462 | -0.413 | -0.305 | 0.088 | 0.370 | Unannotated | NA | NA |
| metab_11106 | Coumarinate | neg | 385.0934 | 5.007 | 5.348 | 4.684 | -0.664 | -0.305 | 0.088 | 0.370 | Tentative | Phenylpropanoids and polyketides | Cinnamic acids and derivatives |
| metab_5380 | N-Acetylsphinganine | pos | 344.3164 | 6.470 | 5.034 | 4.611 | -0.423 | -0.305 | 0.088 | 0.370 | Unannotated | NA | NA |
| metab_7482 | 5-Acetamidovalerate | pos | 360.2138 | 3.791 | 6.128 | 5.698 | -0.430 | -0.305 | 0.088 | 0.370 | Tentative | NA | NA |
| metab_10991 | Unannotated feature | neg | 345.1418 | 0.777 | 4.587 | 4.296 | -0.291 | -0.305 | 0.088 | 0.370 | Unannotated | NA | NA |
| metab_12024 | Unannotated feature | neg | 445.1993 | 5.519 | 6.235 | 6.360 | 0.124 | 0.305 | 0.088 | 0.370 | Unannotated | NA | NA |
| metab_3082 | Faropenem | pos | 303.0982 | 3.380 | 2.698 | 3.230 | 0.532 | 0.305 | 0.088 | 0.370 | Tentative | Organic acids and derivatives | Carboxylic acids and derivatives |
| metab_4249 | Unannotated feature | pos | 551.3556 | 5.936 | 2.766 | 4.801 | 2.036 | 0.305 | 0.088 | 0.370 | Unannotated | NA | NA |
| metab_912 | Unannotated feature | pos | 784.4924 | 4.586 | 3.656 | 4.514 | 0.858 | 0.305 | 0.088 | 0.370 | Unannotated | NA | NA |
| metab_11313 | Unannotated feature | neg | 373.1386 | 5.127 | 5.062 | 4.936 | -0.127 | -0.305 | 0.088 | 0.370 | Unannotated | NA | NA |
| metab_500 | Unannotated feature | pos | 762.9414 | 5.084 | 2.967 | 4.696 | 1.729 | 0.305 | 0.088 | 0.370 | Unannotated | NA | NA |
| metab_6278 | Unannotated feature | pos | 611.9906 | 5.794 | 4.183 | 5.044 | 0.861 | 0.305 | 0.088 | 0.370 | Unannotated | NA | NA |
| metab_14773 | Unannotated feature | neg | 160.9119 | 3.842 | 3.442 | 2.951 | -0.491 | -0.305 | 0.088 | 0.370 | Unannotated | NA | NA |
| metab_8168 | Deacetylasperulosidic acid | pos | 408.1514 | 2.651 | 3.178 | 4.292 | 1.114 | 0.305 | 0.088 | 0.370 | Unannotated | Lipids and lipid-like molecules | Prenol lipids |
| metab_19438 | Unannotated feature | neg | 342.2402 | 4.955 | 5.336 | 5.141 | -0.195 | -0.305 | 0.088 | 0.370 | Unannotated | NA | NA |
| metab_19401 | 5-Oxo-prolyl-glycyl-arginine-4-nitroanilide | neg | 461.1863 | 5.011 | 4.408 | 4.842 | 0.434 | 0.305 | 0.088 | 0.370 | Unannotated | Organic acids and derivatives | Carboxylic acids and derivatives |
| metab_8362 | Lys Glu Ile | pos | 389.2404 | 1.952 | 5.242 | 4.689 | -0.552 | -0.305 | 0.088 | 0.370 | Moderate putative | NA | NA |
| metab_16315 | PE(15:0/0:0) | neg | 438.2631 | 6.648 | 7.473 | 7.189 | -0.284 | -0.305 | 0.088 | 0.370 | Strong putative | NA | NA |
| metab_3427 | 9-Oxo-nonanoic acid | pos | 214.1442 | 4.521 | 5.410 | 5.254 | -0.157 | -0.305 | 0.088 | 0.370 | Tentative | NA | NA |
| metab_9392 | Unannotated feature | neg | 300.1931 | 3.487 | 4.041 | 3.601 | -0.440 | -0.305 | 0.088 | 0.370 | Unannotated | NA | NA |
| metab_2598 | Tyramine | pos | 138.0915 | 1.622 | 5.320 | 4.932 | -0.389 | -0.305 | 0.088 | 0.370 | Moderate putative | NA | NA |
| metab_14917 | Nalbuphine | neg | 378.1675 | 4.146 | 5.039 | 4.917 | -0.122 | -0.305 | 0.088 | 0.370 | Tentative | Benzenoids | Phenanthrenes and derivatives |
| metab_3487 | Coumaryl acetate | pos | 210.1128 | 4.766 | 6.239 | 5.662 | -0.577 | -0.305 | 0.088 | 0.370 | Tentative | Benzenoids | Benzene and substituted derivatives |
| metab_9409 | Noroxyhydrastinine | neg | 236.0563 | 3.461 | 4.899 | 5.191 | 0.292 | 0.305 | 0.088 | 0.370 | Unannotated | Organoheterocyclic compounds | Isoquinolines and derivatives |
| metab_15910 | Unannotated feature | neg | 185.0813 | 6.013 | 4.530 | 4.311 | -0.218 | -0.305 | 0.088 | 0.370 | Unannotated | NA | NA |
| metab_2621 | D-1-[(3-Carboxypropyl)amino]-1-deoxyfructose | pos | 230.1028 | 1.692 | 4.514 | 5.445 | 0.931 | 0.305 | 0.088 | 0.370 | Unannotated | Organic acids and derivatives | Carboxylic acids and derivatives |
| metab_6335 | Unannotated feature | pos | 713.7840 | 5.770 | 4.287 | 4.491 | 0.205 | 0.305 | 0.088 | 0.370 | Unannotated | NA | NA |
| metab_12852 | Unannotated feature | neg | 434.2203 | 6.318 | 5.579 | 5.778 | 0.198 | 0.305 | 0.088 | 0.370 | Unannotated | NA | NA |
| metab_18826 | Unannotated feature | neg | 225.1493 | 5.758 | 2.870 | 3.452 | 0.582 | 0.305 | 0.088 | 0.370 | Unannotated | NA | NA |
| metab_19007 | Ethyl glucoside | neg | 461.1860 | 5.522 | 4.424 | 4.956 | 0.532 | 0.305 | 0.088 | 0.370 | Unannotated | Organic oxygen compounds | Organooxygen compounds |
| metab_6162 | Unannotated feature | pos | 646.4960 | 5.831 | 3.628 | 3.966 | 0.339 | 0.305 | 0.088 | 0.370 | Unannotated | NA | NA |
| metab_12876 | Unannotated feature | neg | 420.1100 | 0.714 | 4.819 | 5.226 | 0.408 | 0.305 | 0.088 | 0.370 | Unannotated | NA | NA |
| metab_13668 | Unannotated feature | neg | 219.0772 | 1.685 | 3.123 | 1.965 | -1.158 | -0.305 | 0.088 | 0.370 | Unannotated | NA | NA |
| metab_19191 | N-(5-Amino-2-methylphenyl)-4-(3-pyridyl)-2-pyrimidineamine | neg | 322.1309 | 5.276 | 3.305 | 2.504 | -0.801 | -0.305 | 0.088 | 0.370 | Unannotated | NA | NA |
| metab_567 | Unannotated feature | pos | 633.2938 | 5.639 | 5.018 | 4.291 | -0.728 | -0.305 | 0.088 | 0.370 | Unannotated | NA | NA |
| metab_13655 | Unannotated feature | neg | 241.0353 | 1.680 | 3.429 | 3.977 | 0.548 | 0.305 | 0.088 | 0.370 | Unannotated | NA | NA |
| metab_4872 | Unannotated feature | pos | 131.0551 | 7.724 | 4.185 | 4.239 | 0.054 | 0.305 | 0.088 | 0.370 | Unannotated | NA | NA |
| metab_17376 | Unannotated feature | neg | 538.9049 | 7.666 | 4.420 | 4.636 | 0.216 | 0.305 | 0.088 | 0.370 | Unannotated | NA | NA |
| metab_3349 | Unannotated feature | pos | 355.2212 | 4.232 | 4.567 | 4.922 | 0.355 | 0.305 | 0.088 | 0.370 | Unannotated | NA | NA |
| metab_6481 | Unannotated feature | pos | 529.0910 | 5.607 | 3.767 | 4.304 | 0.537 | 0.305 | 0.088 | 0.370 | Unannotated | NA | NA |
| metab_16627 | Unannotated feature | neg | 350.9285 | 7.510 | 5.635 | 5.656 | 0.021 | 0.305 | 0.088 | 0.370 | Unannotated | NA | NA |
| metab_16540 | Unannotated feature | neg | 383.1899 | 7.212 | 6.265 | 6.193 | -0.072 | -0.305 | 0.088 | 0.370 | Unannotated | NA | NA |
| metab_117 | Unannotated feature | pos | 219.0270 | 0.633 | 6.622 | 6.376 | -0.245 | -0.305 | 0.088 | 0.370 | Unannotated | NA | NA |
| metab_15491 | Unannotated feature | neg | 415.1807 | 5.253 | 0.002 | 3.449 | 3.447 | 0.305 | 0.088 | 0.370 | Unannotated | NA | NA |
| metab_253 | Unannotated feature | pos | 336.8445 | 0.531 | 4.730 | 5.293 | 0.562 | 0.305 | 0.088 | 0.370 | Unannotated | NA | NA |
| metab_15881 | Unannotated feature | neg | 333.1822 | 5.971 | 4.706 | 4.063 | -0.643 | -0.305 | 0.088 | 0.370 | Unannotated | NA | NA |
| metab_14705 | 5-Methoxytryptophol | neg | 236.0927 | 3.709 | 5.224 | 5.027 | -0.197 | -0.305 | 0.088 | 0.370 | Unannotated | Organoheterocyclic compounds | Indoles and derivatives |
| metab_11821 | Mycorradicin | neg | 293.1034 | 5.901 | 4.770 | 4.947 | 0.176 | 0.305 | 0.088 | 0.370 | Tentative | NA | NA |
| metab_19544 | 3-Ethylphenol | neg | 121.0652 | 4.785 | 5.407 | 4.948 | -0.459 | -0.305 | 0.088 | 0.370 | Moderate putative | NA | NA |
| metab_10585 | Unannotated feature | neg | 125.0238 | 1.695 | 4.803 | 4.713 | -0.090 | -0.305 | 0.088 | 0.370 | Unannotated | NA | NA |
| metab_279 | Unannotated feature | pos | 348.9424 | 0.501 | 4.328 | 4.563 | 0.235 | 0.305 | 0.088 | 0.370 | Unannotated | NA | NA |
| metab_19592 | Unannotated feature | neg | 202.0481 | 4.726 | 3.288 | 2.750 | -0.538 | -0.305 | 0.088 | 0.370 | Unannotated | NA | NA |
| metab_16637 | Unannotated feature | neg | 130.9784 | 7.543 | 4.064 | 3.984 | -0.080 | -0.305 | 0.088 | 0.370 | Unannotated | NA | NA |
| metab_50 | Flumethasone | pos | 375.1769 | 0.680 | 3.568 | 4.612 | 1.044 | 0.305 | 0.088 | 0.370 | Tentative | Lipids and lipid-like molecules | Steroids and steroid derivatives |
| metab_98 | Unannotated feature | pos | 317.1714 | 0.641 | 3.700 | 4.597 | 0.897 | 0.301 | 0.093 | 0.378 | Unannotated | NA | NA |
| metab_12101 | Unannotated feature | neg | 589.3049 | 5.834 | 7.818 | 7.279 | -0.539 | -0.301 | 0.093 | 0.378 | Unannotated | NA | NA |
| metab_9099 | Harmalan | neg | 183.0922 | 3.934 | 4.258 | 3.757 | -0.502 | -0.301 | 0.093 | 0.378 | Unannotated | NA | NA |
| metab_9185 | Unannotated feature | neg | 311.1593 | 3.787 | 3.929 | 3.473 | -0.456 | -0.301 | 0.093 | 0.378 | Unannotated | NA | NA |
| metab_10482 | 2-Hydroxy-4,7-dimethoxy-2H-1,4-benzoxazin-3(4H)-one | neg | 270.0623 | 1.952 | 4.078 | 4.512 | 0.435 | 0.301 | 0.093 | 0.378 | Unannotated | NA | NA |
| metab_4533 | Isophorone | pos | 156.1385 | 6.749 | 4.875 | 4.741 | -0.134 | -0.301 | 0.093 | 0.378 | Moderate putative | NA | NA |
| metab_12118 | Unannotated feature | neg | 507.3146 | 6.545 | 7.219 | 6.893 | -0.326 | -0.301 | 0.093 | 0.378 | Unannotated | NA | NA |
| metab_11552 | Unannotated feature | neg | 283.1299 | 3.938 | 4.502 | 4.793 | 0.291 | 0.301 | 0.093 | 0.378 | Unannotated | NA | NA |
| metab_14624 | Fasoracetam | neg | 241.1192 | 3.548 | 4.935 | 4.681 | -0.254 | -0.301 | 0.093 | 0.378 | Tentative | NA | NA |
| metab_1606 | Unannotated feature | pos | 918.5084 | 5.076 | 3.302 | 3.871 | 0.569 | 0.301 | 0.093 | 0.378 | Unannotated | NA | NA |
| metab_16865 | Unannotated feature | neg | 240.0748 | 7.737 | 4.670 | 4.828 | 0.159 | 0.301 | 0.093 | 0.378 | Unannotated | NA | NA |
| metab_570 | Unannotated feature | pos | 528.3008 | 5.700 | 3.723 | 4.996 | 1.273 | 0.301 | 0.093 | 0.378 | Unannotated | NA | NA |
| metab_19330 | Bz-Arg-OEt | neg | 343.1204 | 5.095 | 4.623 | 5.216 | 0.593 | 0.301 | 0.093 | 0.378 | Tentative | NA | NA |
| metab_1634 | Unannotated feature | pos | 432.2817 | 3.786 | 5.574 | 6.018 | 0.444 | 0.301 | 0.093 | 0.378 | Unannotated | NA | NA |
| metab_638 | Unannotated feature | pos | 72.0813 | 0.485 | 5.365 | 5.070 | -0.295 | -0.301 | 0.093 | 0.378 | Unannotated | NA | NA |
| metab_16000 | Unannotated feature | neg | 432.2431 | 6.115 | 5.338 | 4.714 | -0.624 | -0.301 | 0.093 | 0.378 | Unannotated | NA | NA |
| metab_4636 | Unannotated feature | pos | 472.4373 | 7.196 | 7.061 | 7.036 | -0.025 | -0.301 | 0.093 | 0.378 | Unannotated | NA | NA |
| metab_4642 | Unannotated feature | pos | 323.1609 | 4.516 | 6.032 | 5.754 | -0.278 | -0.301 | 0.093 | 0.378 | Unannotated | NA | NA |
| metab_8349 | Unannotated feature | pos | 264.1446 | 1.960 | 4.542 | 4.415 | -0.127 | -0.301 | 0.093 | 0.378 | Unannotated | NA | NA |
| metab_8350 | L-3-Hydroxykynurenine | pos | 257.1136 | 1.960 | 4.935 | 5.117 | 0.182 | 0.301 | 0.093 | 0.378 | Tentative | NA | NA |
| metab_1700 | Unannotated feature | pos | 302.1506 | 4.016 | 6.068 | 5.750 | -0.319 | -0.301 | 0.093 | 0.378 | Unannotated | NA | NA |
| metab_1715 | Unannotated feature | pos | 564.3611 | 4.054 | 5.650 | 6.010 | 0.361 | 0.301 | 0.093 | 0.378 | Unannotated | NA | NA |
| metab_506 | Leu Trp | pos | 362.1427 | 5.113 | 4.915 | 3.710 | -1.205 | -0.301 | 0.093 | 0.378 | Moderate putative | NA | NA |
| metab_19353 | Unannotated feature | neg | 391.1982 | 5.065 | 5.220 | 4.914 | -0.306 | -0.301 | 0.093 | 0.378 | Unannotated | NA | NA |
| metab_4435 | Unannotated feature | pos | 552.3372 | 6.359 | 5.007 | 4.461 | -0.546 | -0.301 | 0.093 | 0.378 | Unannotated | NA | NA |
| metab_3889 | Unannotated feature | pos | 639.1567 | 5.738 | 3.744 | 4.685 | 0.942 | 0.301 | 0.093 | 0.378 | Unannotated | NA | NA |
| metab_13400 | Unannotated feature | neg | 134.0464 | 0.777 | 3.774 | 4.053 | 0.279 | 0.301 | 0.093 | 0.378 | Unannotated | NA | NA |
| metab_7946 | Unannotated feature | pos | 230.0428 | 2.907 | 4.206 | 4.729 | 0.522 | 0.301 | 0.093 | 0.378 | Unannotated | NA | NA |
| metab_7450 | Latrunculin B | pos | 378.1767 | 0.712 | 3.998 | 4.940 | 0.942 | 0.301 | 0.093 | 0.378 | Strong putative | Phenylpropanoids and polyketides | Macrolides and analogues |
| metab_4198 | Sinapoyl aldehyde | pos | 209.0813 | 3.416 | 4.940 | 4.779 | -0.161 | -0.301 | 0.093 | 0.378 | Moderate putative | NA | NA |
| metab_18057 | Unannotated feature | neg | 257.0778 | 6.436 | 5.710 | 5.646 | -0.064 | -0.301 | 0.093 | 0.378 | Unannotated | NA | NA |
| metab_1782 | Unannotated feature | pos | 245.1500 | 0.768 | 5.484 | 5.196 | -0.288 | -0.301 | 0.093 | 0.378 | Unannotated | NA | NA |
| metab_1284 | Unannotated feature | pos | 441.2445 | 6.271 | 6.277 | 6.143 | -0.134 | -0.301 | 0.093 | 0.378 | Unannotated | NA | NA |
| metab_11537 | Unannotated feature | neg | 261.9542 | 0.496 | 5.382 | 5.322 | -0.059 | -0.301 | 0.093 | 0.378 | Unannotated | NA | NA |
| metab_12436 | Unannotated feature | neg | 520.8337 | 7.730 | 4.588 | 4.655 | 0.067 | 0.301 | 0.093 | 0.378 | Unannotated | NA | NA |
| metab_16228 | Unannotated feature | neg | 236.9685 | 1.347 | 4.096 | 3.033 | -1.063 | -0.301 | 0.093 | 0.378 | Unannotated | NA | NA |
| metab_7428 | 1H-Indol-3-amine | pos | 165.1025 | 3.910 | 3.830 | 2.909 | -0.920 | -0.301 | 0.093 | 0.378 | Tentative | Organoheterocyclic compounds | Indoles and derivatives |
| metab_17027 | Unannotated feature | neg | 294.9735 | 2.158 | 4.531 | 3.830 | -0.702 | -0.301 | 0.093 | 0.378 | Unannotated | NA | NA |
| metab_3982 | Unannotated feature | pos | 617.1903 | 5.799 | 4.018 | 4.111 | 0.093 | 0.301 | 0.093 | 0.378 | Unannotated | NA | NA |
| metab_2128 | Unannotated feature | pos | 283.2022 | 2.840 | 0.746 | 3.416 | 2.670 | 0.301 | 0.093 | 0.378 | Unannotated | NA | NA |
| metab_16227 | 10Z-Pentadecenoic acid | neg | 285.2071 | 6.475 | 5.405 | 5.546 | 0.141 | 0.301 | 0.093 | 0.378 | Unannotated | Lipids and lipid-like molecules | Fatty Acyls |
| metab_4965 | Unannotated feature | pos | 379.9171 | 7.562 | 5.747 | 5.801 | 0.054 | 0.301 | 0.093 | 0.378 | Unannotated | NA | NA |
| metab_1244 | Unannotated feature | pos | 714.4961 | 6.375 | 6.442 | 6.107 | -0.335 | -0.301 | 0.093 | 0.378 | Unannotated | NA | NA |
| metab_12910 | 2-O-a-D-Galactopyranuronosyl-L-rhamnose | neg | 339.0945 | 0.730 | 5.674 | 5.887 | 0.212 | 0.301 | 0.093 | 0.378 | Unannotated | NA | NA |
| metab_6095 | Unannotated feature | pos | 680.3098 | 5.851 | 3.270 | 4.058 | 0.788 | 0.301 | 0.093 | 0.378 | Unannotated | NA | NA |
| metab_5626 | Unannotated feature | pos | 254.2482 | 6.199 | 4.342 | 3.830 | -0.512 | -0.301 | 0.093 | 0.378 | Unannotated | NA | NA |
| metab_8917 | Unannotated feature | neg | 345.1092 | 4.239 | 5.822 | 5.694 | -0.128 | -0.301 | 0.093 | 0.378 | Unannotated | NA | NA |
| metab_12786 | Unannotated feature | neg | 201.0069 | 0.636 | 3.745 | 4.198 | 0.452 | 0.301 | 0.093 | 0.378 | Unannotated | NA | NA |
| metab_12176 | Unannotated feature | neg | 441.2535 | 6.719 | 5.288 | 5.357 | 0.069 | 0.301 | 0.093 | 0.378 | Unannotated | NA | NA |
| metab_8024 | Unannotated feature | pos | 360.1890 | 2.803 | 4.634 | 3.982 | -0.652 | -0.301 | 0.093 | 0.378 | Unannotated | NA | NA |
| metab_15308 | Unannotated feature | neg | 269.0382 | 4.896 | 2.356 | 2.154 | -0.202 | -0.301 | 0.093 | 0.378 | Unannotated | NA | NA |
| metab_15965 | 13,14-Dihydro PGF-1a | neg | 403.2706 | 6.076 | 5.795 | 5.750 | -0.045 | -0.301 | 0.093 | 0.378 | Unannotated | Lipids and lipid-like molecules | Fatty Acyls |
| metab_12601 | Unannotated feature | neg | 923.4708 | 6.068 | 3.730 | 4.389 | 0.659 | 0.301 | 0.093 | 0.378 | Unannotated | NA | NA |
| metab_12311 | Unannotated feature | neg | 573.4540 | 6.892 | 7.390 | 7.081 | -0.309 | -0.301 | 0.093 | 0.378 | Unannotated | NA | NA |
| metab_18892 | Unannotated feature | neg | 315.1134 | 5.671 | 4.551 | 5.018 | 0.467 | 0.301 | 0.093 | 0.378 | Unannotated | NA | NA |
| metab_9637 | Unannotated feature | neg | 333.0622 | 4.199 | 3.778 | 3.148 | -0.630 | -0.301 | 0.093 | 0.378 | Unannotated | NA | NA |
| metab_7326 | Unannotated feature | pos | 130.1362 | 0.704 | 3.241 | 3.831 | 0.590 | 0.301 | 0.093 | 0.378 | Unannotated | NA | NA |
| metab_5009 | Unannotated feature | pos | 453.3345 | 7.387 | 4.513 | 3.828 | -0.685 | -0.301 | 0.093 | 0.378 | Unannotated | NA | NA |
| metab_7000 | Unannotated feature | pos | 177.0872 | 0.657 | 4.942 | 4.641 | -0.301 | -0.301 | 0.093 | 0.378 | Unannotated | NA | NA |
| metab_13646 | Unannotated feature | neg | 277.0361 | 1.660 | 4.419 | 4.294 | -0.125 | -0.301 | 0.093 | 0.378 | Unannotated | NA | NA |
| metab_17655 | Unannotated feature | neg | 485.2117 | 7.068 | 3.827 | 4.722 | 0.895 | 0.301 | 0.093 | 0.378 | Unannotated | NA | NA |
| metab_3773 | Unannotated feature | pos | 403.3620 | 6.446 | 7.366 | 7.300 | -0.067 | -0.301 | 0.093 | 0.378 | Unannotated | NA | NA |
| metab_8569 | Unannotated feature | pos | 330.1555 | 1.217 | 1.981 | 4.207 | 2.226 | 0.301 | 0.093 | 0.378 | Unannotated | NA | NA |
| metab_9690 | DHMBOA hexose | neg | 386.1100 | 3.131 | 2.353 | 3.460 | 1.107 | 0.301 | 0.093 | 0.378 | Unannotated | Organic oxygen compounds | Organooxygen compounds |
| metab_11343 | Unannotated feature | neg | 309.0233 | 0.588 | 5.069 | 4.772 | -0.297 | -0.301 | 0.093 | 0.378 | Unannotated | NA | NA |
| metab_4808 | Unannotated feature | pos | 293.9770 | 7.980 | 4.495 | 4.547 | 0.052 | 0.301 | 0.093 | 0.378 | Unannotated | NA | NA |
| metab_17113 | Unannotated feature | neg | 163.9281 | 7.840 | 5.099 | 5.055 | -0.044 | -0.301 | 0.093 | 0.378 | Unannotated | NA | NA |
| metab_17100 | Unannotated feature | neg | 167.8361 | 7.870 | 4.326 | 4.423 | 0.097 | 0.301 | 0.093 | 0.378 | Unannotated | NA | NA |
| metab_8743 | Unannotated feature | pos | 295.0719 | 4.232 | 4.262 | 4.638 | 0.376 | 0.301 | 0.093 | 0.378 | Unannotated | NA | NA |
| metab_9278 | Unannotated feature | neg | 248.0963 | 3.647 | 2.576 | 2.924 | 0.348 | 0.301 | 0.093 | 0.378 | Unannotated | NA | NA |
| metab_5595 | Unannotated feature | pos | 130.9794 | 0.016 | 4.790 | 4.837 | 0.046 | 0.301 | 0.093 | 0.378 | Unannotated | NA | NA |
| metab_5590 | Unannotated feature | pos | 111.9689 | 0.016 | 4.757 | 4.789 | 0.032 | 0.301 | 0.093 | 0.378 | Unannotated | NA | NA |
| metab_8801 | Cortisone | pos | 383.1822 | 3.439 | 4.012 | 4.477 | 0.465 | 0.301 | 0.093 | 0.378 | Tentative | NA | NA |
| metab_17373 | Unannotated feature | neg | 825.8265 | 7.666 | 4.617 | 4.795 | 0.178 | 0.301 | 0.093 | 0.378 | Unannotated | NA | NA |
| metab_13992 | Neoacrimarine K | neg | 540.1695 | 2.703 | 3.688 | 4.182 | 0.494 | 0.301 | 0.093 | 0.378 | Unannotated | Organoheterocyclic compounds | Quinolines and derivatives |
| metab_8901 | Suberylglycine | neg | 230.1030 | 4.266 | 4.647 | 4.363 | -0.283 | -0.301 | 0.093 | 0.378 | Unannotated | Organic acids and derivatives | Carboxylic acids and derivatives |
| metab_9253 | Niazirinin | neg | 366.1199 | 3.688 | 2.932 | 3.726 | 0.794 | 0.301 | 0.093 | 0.378 | Unannotated | Organic oxygen compounds | Organooxygen compounds |
| metab_9203 | Unannotated feature | neg | 307.0937 | 3.766 | 3.405 | 4.114 | 0.708 | 0.301 | 0.093 | 0.378 | Unannotated | NA | NA |
| metab_4178 | Unannotated feature | pos | 721.3325 | 5.906 | 1.341 | 4.166 | 2.825 | 0.297 | 0.097 | 0.387 | Unannotated | NA | NA |
| metab_9968 | Aspartyl-Threonine | neg | 279.0843 | 2.885 | 4.996 | 5.136 | 0.140 | 0.297 | 0.097 | 0.387 | Unannotated | NA | NA |
| metab_2069 | Unannotated feature | pos | 293.1503 | 2.732 | 4.625 | 4.783 | 0.158 | 0.297 | 0.097 | 0.387 | Unannotated | NA | NA |
| metab_6221 | Unannotated feature | pos | 252.8828 | 0.531 | 4.167 | 4.796 | 0.629 | 0.297 | 0.097 | 0.387 | Unannotated | NA | NA |
| metab_4460 | Unannotated feature | pos | 284.3316 | 6.438 | 4.864 | 4.633 | -0.232 | -0.297 | 0.097 | 0.387 | Unannotated | NA | NA |
| metab_2101 | Unannotated feature | pos | 332.1825 | 2.780 | 5.912 | 5.789 | -0.123 | -0.297 | 0.097 | 0.387 | Unannotated | NA | NA |
| metab_10942 | Unannotated feature | neg | 197.0675 | 0.799 | 5.254 | 4.550 | -0.704 | -0.297 | 0.097 | 0.387 | Unannotated | NA | NA |
| metab_17130 | Unannotated feature | neg | 158.9777 | 7.819 | 5.572 | 5.553 | -0.018 | -0.297 | 0.097 | 0.387 | Unannotated | NA | NA |
| metab_2872 | Unannotated feature | pos | 279.2073 | 2.803 | 4.400 | 3.848 | -0.551 | -0.297 | 0.097 | 0.387 | Unannotated | NA | NA |
| metab_12247 | Unannotated feature | neg | 663.4236 | 6.974 | 5.333 | 4.952 | -0.381 | -0.297 | 0.097 | 0.387 | Unannotated | NA | NA |
| metab_2749 | Val Val | pos | 217.1551 | 2.355 | 6.397 | 6.105 | -0.293 | -0.297 | 0.097 | 0.387 | Strong putative | NA | NA |
| metab_17128 | Unannotated feature | neg | 161.8839 | 7.822 | 3.963 | 3.893 | -0.070 | -0.297 | 0.097 | 0.387 | Unannotated | NA | NA |
| metab_2607 | Prostratin | pos | 432.2393 | 1.664 | 4.606 | 4.171 | -0.435 | -0.297 | 0.097 | 0.387 | Tentative | NA | NA |
| metab_17418 | Unannotated feature | neg | 775.3505 | 7.637 | 5.601 | 5.738 | 0.137 | 0.297 | 0.097 | 0.387 | Unannotated | NA | NA |
| metab_13913 | Unannotated feature | neg | 332.1832 | 2.524 | 4.209 | 3.799 | -0.410 | -0.297 | 0.097 | 0.387 | Unannotated | NA | NA |
| metab_9499 | Unannotated feature | neg | 306.0990 | 3.349 | 3.562 | 4.123 | 0.561 | 0.297 | 0.097 | 0.387 | Unannotated | NA | NA |
| metab_17912 | Cohibin C | neg | 621.5088 | 6.648 | 5.124 | 5.743 | 0.619 | 0.297 | 0.097 | 0.387 | Unannotated | NA | NA |
| metab_13551 | D-erythro-L-galacto-Nonulose | neg | 251.0773 | 1.179 | 5.472 | 5.756 | 0.284 | 0.297 | 0.097 | 0.387 | Unannotated | NA | NA |
| metab_6933 | Unannotated feature | pos | 686.4248 | 4.971 | 2.331 | 4.834 | 2.504 | 0.297 | 0.097 | 0.387 | Unannotated | NA | NA |
| metab_3940 | N-Stearoyl Asparagine | pos | 381.3129 | 6.247 | 6.506 | 6.250 | -0.256 | -0.297 | 0.097 | 0.387 | Unannotated | NA | NA |
| metab_11175 | Unannotated feature | neg | 214.0718 | 0.667 | 5.118 | 5.340 | 0.222 | 0.297 | 0.097 | 0.387 | Unannotated | NA | NA |
| metab_15718 | Unannotated feature | neg | 425.9735 | 0.596 | 3.492 | 4.101 | 0.609 | 0.297 | 0.097 | 0.387 | Unannotated | NA | NA |
| metab_3908 | Unannotated feature | pos | 530.0785 | 5.757 | 3.800 | 4.488 | 0.688 | 0.297 | 0.097 | 0.387 | Unannotated | NA | NA |
| metab_8457 | Nivalenol | pos | 330.1557 | 1.928 | 3.656 | 4.600 | 0.944 | 0.297 | 0.097 | 0.387 | Tentative | Lipids and lipid-like molecules | Prenol lipids |
| metab_10154 | Unannotated feature | neg | 368.1465 | 2.732 | 4.736 | 4.607 | -0.129 | -0.297 | 0.097 | 0.387 | Unannotated | NA | NA |
| metab_18211 | 9,10,13-Trihydroxystearic acid | neg | 331.2492 | 6.287 | 5.928 | 5.533 | -0.395 | -0.297 | 0.097 | 0.387 | Unannotated | NA | NA |
| metab_13430 | Unannotated feature | neg | 116.0710 | 0.804 | 5.540 | 5.435 | -0.105 | -0.297 | 0.097 | 0.387 | Unannotated | NA | NA |
| metab_4893 | Unannotated feature | pos | 225.1453 | 7.700 | 4.717 | 4.781 | 0.064 | 0.297 | 0.097 | 0.387 | Unannotated | NA | NA |
| metab_7443 | Unannotated feature | pos | 287.1246 | 0.712 | 4.612 | 4.902 | 0.290 | 0.297 | 0.097 | 0.387 | Unannotated | NA | NA |
| metab_12746 | Unannotated feature | neg | 611.1846 | 2.746 | 5.279 | 4.239 | -1.039 | -0.297 | 0.097 | 0.387 | Unannotated | NA | NA |
| metab_14651 | Unannotated feature | neg | 284.1617 | 3.589 | 5.498 | 5.051 | -0.447 | -0.297 | 0.097 | 0.387 | Unannotated | NA | NA |
| metab_4622 | Unannotated feature | pos | 207.0992 | 7.132 | 4.669 | 4.582 | -0.088 | -0.297 | 0.097 | 0.387 | Unannotated | NA | NA |
| metab_4634 | Ent-kaur-16-en-19-oate | pos | 625.4199 | 7.156 | 4.829 | 4.238 | -0.591 | -0.297 | 0.097 | 0.387 | Tentative | Lipids and lipid-like molecules | Prenol lipids |
| metab_4613 | Unannotated feature | pos | 325.1765 | 4.676 | 6.346 | 6.235 | -0.111 | -0.297 | 0.097 | 0.387 | Unannotated | NA | NA |
| metab_1728 | Unannotated feature | pos | 349.1766 | 3.808 | 4.893 | 4.564 | -0.329 | -0.297 | 0.097 | 0.387 | Unannotated | NA | NA |
| metab_13195 | Unannotated feature | neg | 593.3358 | 6.217 | 6.793 | 7.043 | 0.250 | 0.297 | 0.097 | 0.387 | Unannotated | NA | NA |
| metab_4266 | Unannotated feature | pos | 627.4762 | 5.944 | 3.583 | 4.052 | 0.469 | 0.297 | 0.097 | 0.387 | Unannotated | NA | NA |
| metab_15137 | Thalidomide | neg | 293.0348 | 4.600 | 2.875 | 2.729 | -0.146 | -0.297 | 0.097 | 0.387 | Tentative | Organoheterocyclic compounds | Isoindoles and derivatives |
| metab_577 | Unannotated feature | pos | 136.9534 | 0.016 | 5.496 | 5.523 | 0.027 | 0.297 | 0.097 | 0.387 | Unannotated | NA | NA |
| metab_1328 | LysoPC(P-18:0/0:0) | pos | 549.4022 | 6.199 | 5.129 | 4.797 | -0.333 | -0.297 | 0.097 | 0.387 | Tentative | NA | NA |
| metab_1663 | Ile Ile Val | pos | 344.2553 | 4.510 | 4.883 | 4.383 | -0.500 | -0.297 | 0.097 | 0.387 | Moderate putative | NA | NA |
| metab_1659 | Unannotated feature | pos | 351.1941 | 4.533 | 3.452 | 4.237 | 0.784 | 0.297 | 0.097 | 0.387 | Unannotated | NA | NA |
| metab_593 | Unannotated feature | pos | 714.2626 | 5.761 | 2.166 | 4.104 | 1.938 | 0.297 | 0.097 | 0.387 | Unannotated | NA | NA |
| metab_8287 | Unannotated feature | pos | 155.0029 | 2.126 | 4.228 | 4.275 | 0.047 | 0.297 | 0.097 | 0.387 | Unannotated | NA | NA |
| metab_4939 | Unannotated feature | pos | 540.8908 | 7.618 | 5.811 | 5.895 | 0.084 | 0.297 | 0.097 | 0.387 | Unannotated | NA | NA |
| metab_8218 | Ile Phe Asp | pos | 394.1983 | 3.689 | 4.644 | 4.344 | -0.300 | -0.297 | 0.097 | 0.387 | Strong putative | NA | NA |
| metab_10438 | 3,4-Dihydroxy-2-methoxy-4-methyl-3-[2-methyl-3-(3-methyl-but-2-enyl)-oxiranyl]-cyclohexanone | neg | 319.1512 | 1.969 | 3.217 | 1.652 | -1.566 | -0.297 | 0.097 | 0.387 | Unannotated | Organic oxygen compounds | Organooxygen compounds |
| metab_10461 | Unannotated feature | neg | 271.0465 | 1.958 | 3.833 | 4.464 | 0.631 | 0.297 | 0.097 | 0.387 | Unannotated | NA | NA |
| metab_11870 | Unannotated feature | neg | 653.3398 | 6.007 | 5.184 | 4.554 | -0.629 | -0.297 | 0.097 | 0.387 | Unannotated | NA | NA |
| metab_138 | Diethanolamine | pos | 106.0867 | 0.625 | 2.967 | 3.475 | 0.508 | 0.297 | 0.097 | 0.387 | Moderate putative | NA | NA |
| metab_19874 | Isocitrate | neg | 191.0192 | 0.784 | 6.185 | 5.852 | -0.333 | -0.297 | 0.097 | 0.387 | Strong putative | Organic acids and derivatives | Carboxylic acids and derivatives |
| metab_12573 | Unannotated feature | neg | 287.1865 | 6.123 | 4.812 | 5.115 | 0.304 | 0.297 | 0.097 | 0.387 | Unannotated | NA | NA |
| metab_2853 | Unannotated feature | pos | 273.1924 | 2.770 | 4.430 | 3.609 | -0.821 | -0.297 | 0.097 | 0.387 | Unannotated | NA | NA |
| metab_15611 | 3,5-Dihydroxy-6,7-megastigmadien-9-one | neg | 269.1396 | 5.522 | 5.106 | 5.256 | 0.150 | 0.297 | 0.097 | 0.387 | Unannotated | NA | NA |
| metab_17352 | Unannotated feature | neg | 349.0894 | 2.602 | 3.792 | 4.531 | 0.739 | 0.297 | 0.097 | 0.387 | Unannotated | NA | NA |
| metab_9381 | Unannotated feature | neg | 298.1774 | 3.502 | 4.820 | 4.277 | -0.543 | -0.297 | 0.097 | 0.387 | Unannotated | NA | NA |
| metab_16465 | Unannotated feature | neg | 87.0025 | 7.019 | 7.305 | 7.280 | -0.025 | -0.297 | 0.097 | 0.387 | Unannotated | NA | NA |
| metab_14303 | Unannotated feature | neg | 378.1660 | 3.055 | 5.026 | 4.732 | -0.294 | -0.297 | 0.097 | 0.387 | Unannotated | NA | NA |
| metab_10858 | Unannotated feature | neg | 146.9960 | 1.011 | 3.706 | 3.425 | -0.280 | -0.297 | 0.097 | 0.387 | Unannotated | NA | NA |
| metab_3617 | LysoPE(15:0/0:0) | pos | 462.2602 | 6.646 | 7.186 | 7.004 | -0.182 | -0.297 | 0.097 | 0.387 | Strong putative | Lipids and lipid-like molecules | Glycerophospholipids |
| metab_5541 | Fluocortolone caproate | pos | 457.2787 | 6.287 | 5.744 | 5.673 | -0.070 | -0.297 | 0.097 | 0.387 | Unannotated | NA | NA |
| metab_6415 | Unannotated feature | pos | 607.3745 | 5.711 | 3.977 | 4.113 | 0.136 | 0.297 | 0.097 | 0.387 | Unannotated | NA | NA |
| metab_18656 | Unannotated feature | neg | 180.9888 | 5.940 | 3.893 | 3.761 | -0.132 | -0.297 | 0.097 | 0.387 | Unannotated | NA | NA |
| metab_17371 | Unannotated feature | neg | 334.9419 | 7.670 | 4.586 | 4.665 | 0.079 | 0.297 | 0.097 | 0.387 | Unannotated | NA | NA |
| metab_9295 | Tetrahydrofurfuryl butyrate | neg | 153.0914 | 3.620 | 2.571 | 3.624 | 1.052 | 0.297 | 0.097 | 0.387 | Unannotated | Lipids and lipid-like molecules | Fatty Acyls |
| metab_7054 | Indeloxazine | pos | 463.2565 | 4.738 | 3.752 | 4.324 | 0.572 | 0.297 | 0.097 | 0.387 | Tentative | NA | NA |
| metab_11153 | Unannotated feature | neg | 230.1145 | 0.683 | 4.652 | 4.277 | -0.375 | -0.297 | 0.097 | 0.387 | Unannotated | NA | NA |
| metab_16775 | Unannotated feature | neg | 145.9304 | 7.697 | 5.135 | 5.086 | -0.049 | -0.297 | 0.097 | 0.387 | Unannotated | NA | NA |
| metab_11463 | Unannotated feature | neg | 676.7948 | 0.524 | 4.941 | 5.106 | 0.165 | 0.297 | 0.097 | 0.387 | Unannotated | NA | NA |
| metab_802 | 7-Ketodeoxycholic Acid | pos | 389.2690 | 6.279 | 6.503 | 6.420 | -0.083 | -0.297 | 0.097 | 0.387 | Strong putative | NA | NA |
| metab_12362 | Unannotated feature | neg | 741.8648 | 7.584 | 6.589 | 6.645 | 0.055 | 0.297 | 0.097 | 0.387 | Unannotated | NA | NA |
| metab_17454 | Unannotated feature | neg | 928.8378 | 7.607 | 6.888 | 6.947 | 0.058 | 0.297 | 0.097 | 0.387 | Unannotated | NA | NA |
| metab_16635 | Unannotated feature | neg | 789.4915 | 7.527 | 4.933 | 4.196 | -0.737 | -0.297 | 0.097 | 0.387 | Unannotated | NA | NA |
| metab_955 | Unannotated feature | pos | 488.8743 | 7.642 | 5.325 | 5.372 | 0.046 | 0.297 | 0.097 | 0.387 | Unannotated | NA | NA |
| metab_6426 | Leu Thr Ser Lys Tyr | pos | 611.3457 | 5.693 | 3.920 | 6.221 | 2.300 | 0.297 | 0.097 | 0.387 | Moderate putative | NA | NA |
| metab_9774 | 5-Phenyl-1,3-oxazinane-2,4-dione | neg | 236.0563 | 3.059 | 5.560 | 5.814 | 0.254 | 0.297 | 0.097 | 0.387 | Tentative | Benzenoids | Benzene and substituted derivatives |
| metab_5975 | Unannotated feature | pos | 598.5327 | 5.914 | 3.851 | 3.912 | 0.061 | 0.297 | 0.097 | 0.387 | Unannotated | NA | NA |
| metab_16270 | Unannotated feature | neg | 545.3267 | 6.553 | 3.538 | 3.977 | 0.439 | 0.293 | 0.102 | 0.394 | Unannotated | NA | NA |
| metab_4034 | Unannotated feature | pos | 638.5652 | 5.820 | 0.049 | 4.067 | 4.018 | 0.293 | 0.102 | 0.394 | Unannotated | NA | NA |
| metab_2109 | Unannotated feature | pos | 480.1895 | 2.788 | 4.526 | 4.915 | 0.389 | 0.293 | 0.102 | 0.394 | Unannotated | NA | NA |
| metab_7626 | Eugenin | pos | 207.0655 | 3.502 | 4.182 | 3.109 | -1.073 | -0.293 | 0.102 | 0.394 | Tentative | Organoheterocyclic compounds | Benzopyrans |
| metab_15215 | Unannotated feature | neg | 342.2402 | 4.762 | 5.150 | 4.897 | -0.252 | -0.293 | 0.102 | 0.394 | Unannotated | NA | NA |
| metab_4937 | Unannotated feature | pos | 744.8531 | 7.618 | 5.438 | 5.509 | 0.071 | 0.293 | 0.102 | 0.394 | Unannotated | NA | NA |
| metab_1555 | Unannotated feature | pos | 337.1765 | 5.321 | 5.869 | 5.706 | -0.163 | -0.293 | 0.102 | 0.394 | Unannotated | NA | NA |
| metab_1505 | Unannotated feature | pos | 567.7637 | 5.787 | 4.118 | 4.642 | 0.524 | 0.293 | 0.102 | 0.394 | Unannotated | NA | NA |
| metab_14697 | 6-Methoxymellein | neg | 207.0658 | 3.697 | 5.126 | 5.046 | -0.080 | -0.293 | 0.102 | 0.394 | Tentative | Organoheterocyclic compounds | Benzopyrans |
| metab_15098 | Unannotated feature | neg | 806.7502 | 0.520 | 4.349 | 4.811 | 0.462 | 0.293 | 0.102 | 0.394 | Unannotated | NA | NA |
| metab_14076 | 8-Methoxykynurenate | neg | 264.0516 | 2.802 | 4.737 | 5.032 | 0.295 | 0.293 | 0.102 | 0.394 | Tentative | NA | NA |
| metab_19299 | Unannotated feature | neg | 175.0970 | 5.148 | 3.950 | 3.806 | -0.144 | -0.293 | 0.102 | 0.394 | Unannotated | NA | NA |
| metab_18274 | Unannotated feature | neg | 280.0829 | 2.754 | 4.125 | 4.532 | 0.407 | 0.293 | 0.102 | 0.394 | Unannotated | NA | NA |
| metab_4422 | Unannotated feature | pos | 477.3808 | 6.335 | 4.675 | 4.131 | -0.544 | -0.293 | 0.102 | 0.394 | Unannotated | NA | NA |
| metab_16365 | PE(18:1(9Z)/0:0) | neg | 478.2946 | 6.734 | 6.738 | 6.534 | -0.204 | -0.293 | 0.102 | 0.394 | Strong putative | NA | NA |
| metab_9117 | Unannotated feature | neg | 210.0768 | 3.897 | 4.103 | 4.175 | 0.072 | 0.293 | 0.102 | 0.394 | Unannotated | NA | NA |
| metab_16907 | Unannotated feature | neg | 178.9572 | 7.765 | 4.805 | 4.767 | -0.038 | -0.293 | 0.102 | 0.394 | Unannotated | NA | NA |
| metab_1918 | Unannotated feature | pos | 331.2348 | 1.692 | 4.859 | 4.403 | -0.456 | -0.293 | 0.102 | 0.394 | Unannotated | NA | NA |
| metab_1980 | Unannotated feature | pos | 126.0917 | 2.070 | 4.544 | 4.343 | -0.201 | -0.293 | 0.102 | 0.394 | Unannotated | NA | NA |
| metab_2051 | Lys Val | pos | 246.1816 | 0.641 | 6.223 | 5.952 | -0.271 | -0.293 | 0.102 | 0.394 | Moderate putative | NA | NA |
| metab_4069 | Unannotated feature | pos | 655.5040 | 5.835 | 3.960 | 4.141 | 0.181 | 0.293 | 0.102 | 0.394 | Unannotated | NA | NA |
| metab_6288 | Unannotated feature | pos | 599.5798 | 5.791 | 1.064 | 4.245 | 3.182 | 0.293 | 0.102 | 0.394 | Unannotated | NA | NA |
| metab_489 | Unannotated feature | pos | 337.1765 | 5.034 | 5.526 | 5.313 | -0.213 | -0.293 | 0.102 | 0.394 | Unannotated | NA | NA |
| metab_888 | Unannotated feature | pos | 385.1790 | 6.909 | 5.518 | 5.718 | 0.200 | 0.293 | 0.102 | 0.394 | Unannotated | NA | NA |
| metab_7753 | (R)-Laudanidine | pos | 344.1845 | 3.291 | 5.183 | 4.756 | -0.427 | -0.293 | 0.102 | 0.394 | Tentative | NA | NA |
| metab_7746 | Ser Val Ile | pos | 318.2030 | 3.295 | 4.509 | 3.878 | -0.631 | -0.293 | 0.102 | 0.394 | Unannotated | NA | NA |
| metab_1310 | Unannotated feature | pos | 456.3330 | 6.231 | 4.517 | 4.754 | 0.237 | 0.293 | 0.102 | 0.394 | Unannotated | NA | NA |
| metab_18439 | Unannotated feature | neg | 485.2763 | 6.099 | 4.624 | 4.251 | -0.372 | -0.293 | 0.102 | 0.394 | Unannotated | NA | NA |
| metab_14954 | Pelargonic acid | neg | 203.1284 | 4.235 | 4.677 | 4.824 | 0.147 | 0.293 | 0.102 | 0.394 | Tentative | NA | NA |
| metab_13332 | Aminofructose 6-phosphate | neg | 295.9944 | 0.644 | 4.019 | 2.541 | -1.477 | -0.293 | 0.102 | 0.394 | Tentative | Organic oxygen compounds | Organooxygen compounds |
| metab_6266 | Unannotated feature | pos | 629.8019 | 5.799 | 3.823 | 4.129 | 0.306 | 0.293 | 0.102 | 0.394 | Unannotated | NA | NA |
| metab_6380 | Unannotated feature | pos | 629.8911 | 5.741 | 3.664 | 3.837 | 0.173 | 0.293 | 0.102 | 0.394 | Unannotated | NA | NA |
| metab_10332 | Unannotated feature | neg | 177.0221 | 2.267 | 4.793 | 4.513 | -0.280 | -0.293 | 0.102 | 0.394 | Unannotated | NA | NA |
| metab_5355 | P-Hydroxyfelbamate | pos | 287.1244 | 1.952 | 5.246 | 5.422 | 0.176 | 0.293 | 0.102 | 0.394 | Tentative | NA | NA |
| metab_542 | Unannotated feature | pos | 409.1632 | 6.127 | 6.669 | 6.597 | -0.071 | -0.293 | 0.102 | 0.394 | Unannotated | NA | NA |
| metab_4336 | Unannotated feature | pos | 271.2273 | 6.111 | 4.651 | 4.604 | -0.047 | -0.293 | 0.102 | 0.394 | Unannotated | NA | NA |
| metab_4284 | Unannotated feature | pos | 468.3082 | 5.969 | 6.629 | 6.322 | -0.307 | -0.293 | 0.102 | 0.394 | Unannotated | NA | NA |
| metab_12382 | Unannotated feature | neg | 782.8335 | 7.645 | 6.226 | 6.284 | 0.058 | 0.293 | 0.102 | 0.394 | Unannotated | NA | NA |
| metab_13619 | Unannotated feature | neg | 321.1459 | 4.749 | 6.563 | 6.389 | -0.174 | -0.293 | 0.102 | 0.394 | Unannotated | NA | NA |
| metab_4971 | Unannotated feature | pos | 102.1094 | 7.554 | 4.016 | 3.981 | -0.035 | -0.293 | 0.102 | 0.394 | Unannotated | NA | NA |
| metab_14876 | 4-Hydroxyphenylpyruvic acid | neg | 225.0402 | 4.052 | 4.656 | 4.875 | 0.219 | 0.293 | 0.102 | 0.394 | Tentative | NA | NA |
| metab_4733 | Unannotated feature | pos | 259.9161 | 7.666 | 4.256 | 4.319 | 0.063 | 0.293 | 0.102 | 0.394 | Unannotated | NA | NA |
| metab_11470 | Unannotated feature | neg | 302.8389 | 0.524 | 4.331 | 3.807 | -0.525 | -0.293 | 0.102 | 0.394 | Unannotated | NA | NA |
| metab_18499 | (6E)-8-hydroxygeraniol | neg | 215.1285 | 6.052 | 5.147 | 4.955 | -0.192 | -0.293 | 0.102 | 0.394 | Tentative | NA | NA |
| metab_3578 | Unannotated feature | pos | 188.1284 | 5.020 | 4.913 | 4.828 | -0.086 | -0.293 | 0.102 | 0.394 | Unannotated | NA | NA |
| metab_10836 | (2S,3S,4R,5R)-5-(6-Aminopurin-9-yl)-N-cyclopropyl-3,4-dihydroxyoxolane-2-carboxamide | neg | 319.1150 | 1.041 | 4.313 | 4.601 | 0.288 | 0.293 | 0.102 | 0.394 | Unannotated | NA | NA |
| metab_5918 | Unannotated feature | pos | 663.5068 | 5.922 | 3.094 | 4.716 | 1.622 | 0.293 | 0.102 | 0.394 | Unannotated | NA | NA |
| metab_13794 | Unannotated feature | neg | 455.1168 | 2.007 | 3.357 | 4.847 | 1.490 | 0.293 | 0.102 | 0.394 | Unannotated | NA | NA |
| metab_3636 | Domoic acid | pos | 312.1448 | 5.147 | 5.194 | 4.361 | -0.833 | -0.293 | 0.102 | 0.394 | Tentative | Organic acids and derivatives | Carboxylic acids and derivatives |
| metab_16162 | Testolactone | neg | 345.1710 | 6.365 | 4.366 | 4.748 | 0.382 | 0.293 | 0.102 | 0.394 | Tentative | NA | NA |
| metab_7270 | 4-Formyl-3-(formylmethyl)-4-hexenoic acid | pos | 217.1072 | 4.262 | 3.888 | 4.022 | 0.134 | 0.293 | 0.102 | 0.394 | Unannotated | NA | NA |
| metab_364 | Unannotated feature | pos | 229.0554 | 0.348 | 5.195 | 5.214 | 0.018 | 0.293 | 0.102 | 0.394 | Unannotated | NA | NA |
| metab_11266 | Unannotated feature | neg | 227.9446 | 0.625 | 4.213 | 3.919 | -0.294 | -0.293 | 0.102 | 0.394 | Unannotated | NA | NA |
| metab_5021 | 28-Homobrassinolide | pos | 517.3489 | 7.307 | 7.051 | 7.101 | 0.050 | 0.293 | 0.102 | 0.394 | Unannotated | NA | NA |
| metab_10826 | 2-Amino-3-oxoadipate | neg | 174.0402 | 1.054 | 6.462 | 6.255 | -0.207 | -0.293 | 0.102 | 0.394 | Tentative | NA | NA |
| metab_12855 | Unannotated feature | neg | 370.1360 | 0.636 | 4.177 | 4.716 | 0.539 | 0.293 | 0.102 | 0.394 | Unannotated | NA | NA |
| metab_411 | Unannotated feature | pos | 191.9876 | 0.016 | 4.467 | 4.480 | 0.013 | 0.293 | 0.102 | 0.394 | Unannotated | NA | NA |
| metab_12898 | Unannotated feature | neg | 177.0398 | 0.730 | 6.629 | 6.470 | -0.158 | -0.293 | 0.102 | 0.394 | Unannotated | NA | NA |
| metab_2293 | Unannotated feature | pos | 158.0114 | 0.016 | 3.996 | 4.033 | 0.037 | 0.293 | 0.102 | 0.394 | Unannotated | NA | NA |
| metab_16769 | Unannotated feature | neg | 346.1640 | 7.692 | 5.817 | 5.868 | 0.052 | 0.293 | 0.102 | 0.394 | Unannotated | NA | NA |
| metab_15060 | Homoarecoline | neg | 214.1081 | 4.454 | 5.072 | 4.958 | -0.114 | -0.293 | 0.102 | 0.394 | Unannotated | NA | NA |
| metab_7360 | Unannotated feature | pos | 368.2190 | 4.054 | 4.835 | 4.265 | -0.570 | -0.293 | 0.102 | 0.394 | Unannotated | NA | NA |
| metab_9742 | Unannotated feature | neg | 308.1252 | 3.085 | 4.374 | 4.144 | -0.230 | -0.293 | 0.102 | 0.394 | Unannotated | NA | NA |
| metab_7361 | M-Hydroxybenzoylecgonine | pos | 323.1609 | 4.054 | 5.679 | 5.407 | -0.273 | -0.293 | 0.102 | 0.394 | Unannotated | NA | NA |
| metab_11674 | Unannotated feature | neg | 617.3020 | 5.339 | 4.369 | 4.678 | 0.309 | 0.293 | 0.102 | 0.394 | Unannotated | NA | NA |
| metab_7329 | Unannotated feature | pos | 321.1453 | 4.120 | 4.198 | 4.448 | 0.250 | 0.293 | 0.102 | 0.394 | Unannotated | NA | NA |
| metab_16797 | Unannotated feature | neg | 108.0447 | 1.965 | 4.668 | 4.824 | 0.156 | 0.293 | 0.102 | 0.394 | Unannotated | NA | NA |
| metab_7281 | Unannotated feature | pos | 126.1280 | 4.239 | 2.980 | 3.134 | 0.153 | 0.293 | 0.102 | 0.394 | Unannotated | NA | NA |
| metab_15305 | Unannotated feature | neg | 376.2247 | 4.884 | 4.063 | 3.229 | -0.834 | -0.293 | 0.102 | 0.394 | Unannotated | NA | NA |
| metab_13924 | Unannotated feature | neg | 360.1781 | 2.554 | 4.860 | 4.514 | -0.346 | -0.293 | 0.102 | 0.394 | Unannotated | NA | NA |
| metab_15894 | 7-Hydroxy-5-methoxy-4-methyl-3-(4-methylpiperazin-1-yl)-2H-chromen-2-one | neg | 349.1404 | 5.991 | 3.708 | 4.749 | 1.041 | 0.293 | 0.102 | 0.394 | Unannotated | NA | NA |
| metab_13893 | Unannotated feature | neg | 384.9241 | 0.467 | 5.243 | 5.159 | -0.085 | -0.293 | 0.102 | 0.394 | Unannotated | NA | NA |
| metab_5511 | Unannotated feature | pos | 254.2482 | 6.335 | 4.787 | 4.174 | -0.613 | -0.293 | 0.102 | 0.394 | Unannotated | NA | NA |
| metab_13820 | Unannotated feature | neg | 321.1460 | 4.518 | 6.253 | 6.052 | -0.200 | -0.293 | 0.102 | 0.394 | Unannotated | NA | NA |
| metab_17392 | Unannotated feature | neg | 639.3756 | 7.660 | 4.379 | 4.653 | 0.274 | 0.293 | 0.102 | 0.394 | Unannotated | NA | NA |
| metab_2753 | Unannotated feature | pos | 222.0812 | 2.393 | 4.331 | 4.406 | 0.076 | 0.293 | 0.102 | 0.394 | Unannotated | NA | NA |
| metab_13943 | Unannotated feature | neg | 213.1242 | 2.599 | 5.369 | 5.223 | -0.146 | -0.293 | 0.102 | 0.394 | Unannotated | NA | NA |
| metab_14269 | Unannotated feature | neg | 392.1803 | 3.014 | 4.672 | 4.097 | -0.575 | -0.293 | 0.102 | 0.394 | Unannotated | NA | NA |
| metab_6833 | Unannotated feature | pos | 564.3611 | 4.229 | 6.048 | 6.353 | 0.305 | 0.293 | 0.102 | 0.394 | Unannotated | NA | NA |
| metab_17388 | Unannotated feature | neg | 830.3378 | 7.660 | 4.136 | 4.558 | 0.422 | 0.293 | 0.102 | 0.394 | Unannotated | NA | NA |
| metab_345 | Unannotated feature | pos | 324.2177 | 0.417 | 5.487 | 5.528 | 0.041 | 0.293 | 0.102 | 0.394 | Unannotated | NA | NA |
| metab_16690 | Unannotated feature | neg | 766.8599 | 7.621 | 6.687 | 6.731 | 0.044 | 0.293 | 0.102 | 0.394 | Unannotated | NA | NA |
| metab_2662 | 1,2-Cyclohexanedicarboxylic acid, 4-methyl- | pos | 204.1235 | 1.952 | 4.855 | 5.090 | 0.235 | 0.293 | 0.102 | 0.394 | Unannotated | NA | NA |
| metab_3410 | Miroestrol | pos | 376.1729 | 2.732 | 5.521 | 5.458 | -0.063 | -0.293 | 0.102 | 0.394 | Tentative | NA | NA |
| metab_12789 | Unannotated feature | neg | 266.0048 | 0.636 | 4.240 | 4.178 | -0.062 | -0.293 | 0.102 | 0.394 | Unannotated | NA | NA |
| metab_17323 | Unannotated feature | neg | 583.8935 | 7.697 | 4.536 | 4.654 | 0.118 | 0.293 | 0.102 | 0.394 | Unannotated | NA | NA |
| metab_18962 | Acetylpterosin C | neg | 597.2755 | 5.571 | 4.128 | 5.237 | 1.110 | 0.293 | 0.102 | 0.394 | Unannotated | Benzenoids | Indanes |
| metab_3267 | Actinodaphnine | pos | 329.1502 | 3.937 | 4.408 | 3.968 | -0.440 | -0.293 | 0.102 | 0.394 | Tentative | NA | NA |
| metab_2825 | Unannotated feature | pos | 382.1502 | 2.724 | 3.368 | 3.961 | 0.593 | 0.293 | 0.102 | 0.394 | Unannotated | NA | NA |
| metab_11184 | 2',3'-Didehydro-2',3'-dideoxyguanosine | neg | 294.0835 | 0.659 | 4.134 | 4.438 | 0.305 | 0.293 | 0.102 | 0.394 | Unannotated | Organoheterocyclic compounds | Imidazopyrimidines |
| metab_18759 | Ganolucidic acid D | neg | 545.3145 | 5.834 | 6.167 | 5.400 | -0.767 | -0.293 | 0.102 | 0.394 | Unannotated | Organoheterocyclic compounds | Imidolactams |
| metab_18729 | Unannotated feature | neg | 380.1465 | 5.880 | 5.805 | 5.473 | -0.332 | -0.293 | 0.102 | 0.394 | Unannotated | NA | NA |
| metab_14064 | Bursopoietin | neg | 360.1782 | 2.785 | 5.940 | 5.654 | -0.286 | -0.293 | 0.102 | 0.394 | Unannotated | Organic acids and derivatives | Carboxylic acids and derivatives |
| metab_12016 | Unannotated feature | neg | 563.2694 | 6.271 | 5.257 | 5.679 | 0.422 | 0.293 | 0.102 | 0.394 | Unannotated | NA | NA |
| metab_19785 | Unannotated feature | neg | 280.1192 | 3.688 | 3.879 | 4.488 | 0.609 | 0.293 | 0.102 | 0.394 | Unannotated | NA | NA |
| metab_5046 | 2-Amino-3-methyl-1-butanol | pos | 145.1338 | 0.768 | 6.773 | 6.238 | -0.534 | -0.293 | 0.102 | 0.394 | Unannotated | Organic nitrogen compounds | Organonitrogen compounds |
| metab_11888 | Unannotated feature | neg | 277.1082 | 6.035 | 4.157 | 4.578 | 0.420 | 0.288 | 0.107 | 0.402 | Unannotated | NA | NA |
| metab_15653 | Unannotated feature | neg | 760.7562 | 0.527 | 4.652 | 5.112 | 0.460 | 0.288 | 0.107 | 0.402 | Unannotated | NA | NA |
| metab_14094 | Unannotated feature | neg | 329.1834 | 2.817 | 5.511 | 5.272 | -0.239 | -0.288 | 0.107 | 0.402 | Unannotated | NA | NA |
| metab_17227 | Unannotated feature | neg | 246.0749 | 7.730 | 5.123 | 5.387 | 0.264 | 0.288 | 0.107 | 0.402 | Unannotated | NA | NA |
| metab_14995 | Unannotated feature | neg | 420.2111 | 4.315 | 4.417 | 3.890 | -0.528 | -0.288 | 0.107 | 0.402 | Unannotated | NA | NA |
| metab_7767 | Unannotated feature | pos | 331.2235 | 3.275 | 4.760 | 3.733 | -1.027 | -0.288 | 0.107 | 0.402 | Unannotated | NA | NA |
| metab_12584 | Rislenemdaz | neg | 357.1748 | 6.099 | 4.848 | 5.236 | 0.389 | 0.288 | 0.107 | 0.402 | Unannotated | Benzenoids | Benzene and substituted derivatives |
| metab_1039 | Unannotated feature | pos | 209.1286 | 1.685 | 5.011 | 4.714 | -0.297 | -0.288 | 0.107 | 0.402 | Unannotated | NA | NA |
| metab_16697 | Unannotated feature | neg | 902.8342 | 7.625 | 6.466 | 6.542 | 0.076 | 0.288 | 0.107 | 0.402 | Unannotated | NA | NA |
| metab_4860 | Unannotated feature | pos | 208.0398 | 7.724 | 5.933 | 5.972 | 0.039 | 0.288 | 0.107 | 0.402 | Unannotated | NA | NA |
| metab_10977 | 2-Quinoxalinecarboxylic acid | neg | 209.0122 | 0.784 | 5.033 | 4.829 | -0.204 | -0.288 | 0.107 | 0.402 | Tentative | Organoheterocyclic compounds | Diazanaphthalenes |
| metab_10844 | Unannotated feature | neg | 235.0823 | 1.033 | 4.730 | 5.039 | 0.310 | 0.288 | 0.107 | 0.402 | Unannotated | NA | NA |
| metab_19159 | Unannotated feature | neg | 298.1046 | 3.346 | 4.609 | 5.080 | 0.471 | 0.288 | 0.107 | 0.402 | Unannotated | NA | NA |
| metab_19168 | Unannotated feature | neg | 245.0125 | 3.354 | 5.552 | 4.227 | -1.325 | -0.288 | 0.107 | 0.402 | Unannotated | NA | NA |
| metab_12565 | Unannotated feature | neg | 569.3332 | 6.154 | 6.215 | 6.474 | 0.259 | 0.288 | 0.107 | 0.402 | Unannotated | NA | NA |
| metab_18750 | 2,3-Dihydroxypropyl 4-phenylbutanoate | neg | 283.1188 | 5.847 | 5.707 | 5.915 | 0.208 | 0.288 | 0.107 | 0.402 | Unannotated | Lipids and lipid-like molecules | Glycerolipids |
| metab_18803 | P-Coumaraldehyde | neg | 147.0444 | 5.783 | 3.131 | 2.744 | -0.388 | -0.288 | 0.107 | 0.402 | Strong putative | NA | NA |
| metab_16720 | Unannotated feature | neg | 762.3489 | 7.652 | 4.786 | 4.947 | 0.160 | 0.288 | 0.107 | 0.402 | Unannotated | NA | NA |
| metab_7926 | Nixylic acid | pos | 243.1130 | 2.937 | 3.405 | 3.711 | 0.305 | 0.288 | 0.107 | 0.402 | Moderate putative | NA | NA |
| metab_4812 | Unannotated feature | pos | 684.2049 | 7.964 | 5.069 | 5.095 | 0.025 | 0.288 | 0.107 | 0.402 | Unannotated | NA | NA |
| metab_1521 | Unannotated feature | pos | 388.2552 | 3.429 | 5.042 | 5.332 | 0.290 | 0.288 | 0.107 | 0.402 | Unannotated | NA | NA |
| metab_590 | Unannotated feature | pos | 550.1526 | 5.764 | 4.238 | 4.483 | 0.244 | 0.288 | 0.107 | 0.402 | Unannotated | NA | NA |
| metab_15090 | Unannotated feature | neg | 124.0761 | 4.515 | 3.073 | 2.680 | -0.393 | -0.288 | 0.107 | 0.402 | Unannotated | NA | NA |
| metab_11142 | Unannotated feature | neg | 232.8832 | 0.698 | 4.274 | 3.851 | -0.424 | -0.288 | 0.107 | 0.402 | Unannotated | NA | NA |
| metab_4670 | Unannotated feature | pos | 463.3036 | 7.466 | 5.401 | 5.345 | -0.056 | -0.288 | 0.107 | 0.402 | Unannotated | NA | NA |
| metab_1529 | Unannotated feature | pos | 303.1346 | 5.425 | 5.732 | 5.499 | -0.233 | -0.288 | 0.107 | 0.402 | Unannotated | NA | NA |
| metab_16665 | Unannotated feature | neg | 758.8686 | 7.607 | 6.783 | 6.814 | 0.032 | 0.288 | 0.107 | 0.402 | Unannotated | NA | NA |
| metab_7622 | Unannotated feature | pos | 408.2136 | 3.506 | 4.475 | 3.891 | -0.584 | -0.288 | 0.107 | 0.402 | Unannotated | NA | NA |
| metab_10694 | Unannotated feature | neg | 280.9578 | 1.445 | 4.255 | 3.665 | -0.591 | -0.288 | 0.107 | 0.402 | Unannotated | NA | NA |
| metab_14802 | Vignafuran | neg | 315.0880 | 3.905 | 3.004 | 3.823 | 0.819 | 0.288 | 0.107 | 0.402 | Tentative | NA | NA |
| metab_1222 | P-HPEA-EDA | pos | 368.1462 | 2.732 | 5.102 | 4.980 | -0.122 | -0.288 | 0.107 | 0.402 | Unannotated | NA | NA |
| metab_10871 | Unannotated feature | neg | 126.9044 | 0.985 | 3.008 | 2.661 | -0.347 | -0.288 | 0.107 | 0.402 | Unannotated | NA | NA |
| metab_3856 | Cholylmethionine | pos | 504.3164 | 6.367 | 4.763 | 4.581 | -0.182 | -0.288 | 0.107 | 0.402 | Unannotated | NA | NA |
| metab_15535 | Unannotated feature | neg | 658.3802 | 5.343 | 4.762 | 5.778 | 1.016 | 0.288 | 0.107 | 0.402 | Unannotated | NA | NA |
| metab_12401 | Unannotated feature | neg | 568.3636 | 7.692 | 5.678 | 5.355 | -0.323 | -0.288 | 0.107 | 0.402 | Unannotated | NA | NA |
| metab_13746 | Sorbic Acid | neg | 111.0444 | 1.952 | 4.388 | 4.475 | 0.086 | 0.288 | 0.107 | 0.402 | Moderate putative | NA | NA |
| metab_17056 | Unannotated feature | neg | 107.9931 | 7.978 | 4.440 | 4.370 | -0.070 | -0.288 | 0.107 | 0.402 | Unannotated | NA | NA |
| metab_5002 | Unannotated feature | pos | 458.3843 | 7.458 | 4.959 | 4.863 | -0.097 | -0.288 | 0.107 | 0.402 | Unannotated | NA | NA |
| metab_17641 | Unannotated feature | neg | 708.5225 | 7.101 | 5.137 | 4.247 | -0.891 | -0.288 | 0.107 | 0.402 | Unannotated | NA | NA |
| metab_17054 | Unannotated feature | neg | 144.9417 | 7.985 | 4.243 | 4.199 | -0.044 | -0.288 | 0.107 | 0.402 | Unannotated | NA | NA |
| metab_16634 | Unannotated feature | neg | 418.9164 | 7.527 | 5.588 | 5.649 | 0.061 | 0.288 | 0.107 | 0.402 | Unannotated | NA | NA |
| metab_5805 | Lactose-lysine | pos | 471.2154 | 6.000 | 3.130 | 2.892 | -0.238 | -0.288 | 0.107 | 0.402 | Unannotated | NA | NA |
| metab_2541 | Pseudouridine | pos | 245.0773 | 1.018 | 4.589 | 4.290 | -0.299 | -0.288 | 0.107 | 0.402 | Moderate putative | Nucleosides, nucleotides, and analogues | Nucleoside and nucleotide analogues |
| metab_5535 | Macrophorin D | pos | 505.2784 | 6.295 | 5.301 | 5.034 | -0.266 | -0.288 | 0.107 | 0.402 | Unannotated | Lipids and lipid-like molecules | Fatty Acyls |
| metab_17477 | Unannotated feature | neg | 673.8771 | 7.578 | 6.593 | 6.641 | 0.048 | 0.288 | 0.107 | 0.402 | Unannotated | NA | NA |
| metab_13796 | Unannotated feature | neg | 177.0221 | 2.019 | 4.196 | 3.631 | -0.565 | -0.288 | 0.107 | 0.402 | Unannotated | NA | NA |
| metab_6141 | Unannotated feature | pos | 713.4394 | 5.835 | 4.208 | 4.463 | 0.255 | 0.288 | 0.107 | 0.402 | Unannotated | NA | NA |
| metab_13760 | Unannotated feature | neg | 232.9166 | 0.402 | 6.721 | 6.694 | -0.027 | -0.288 | 0.107 | 0.402 | Unannotated | NA | NA |
| metab_376 | Unannotated feature | pos | 74.0969 | 0.131 | 3.280 | 3.342 | 0.062 | 0.288 | 0.107 | 0.402 | Unannotated | NA | NA |
| metab_9457 | Unannotated feature | neg | 123.0319 | 3.403 | 3.731 | 3.505 | -0.225 | -0.288 | 0.107 | 0.402 | Unannotated | NA | NA |
| metab_952 | Unannotated feature | pos | 1042.8023 | 7.626 | 5.548 | 5.610 | 0.062 | 0.288 | 0.107 | 0.402 | Unannotated | NA | NA |
| metab_10095 | Protocatechuic acid 3-O-sulfate | neg | 232.9760 | 2.771 | 3.186 | 3.794 | 0.608 | 0.288 | 0.107 | 0.402 | Unannotated | Organic acids and derivatives | Organic sulfuric acids and derivatives |
| metab_15566 | Unannotated feature | neg | 281.9696 | 0.577 | 4.617 | 3.819 | -0.799 | -0.288 | 0.107 | 0.402 | Unannotated | NA | NA |
| metab_17030 | Unannotated feature | neg | 128.9466 | 7.997 | 4.137 | 4.079 | -0.058 | -0.288 | 0.107 | 0.402 | Unannotated | NA | NA |
| metab_3965 | Unannotated feature | pos | 551.1038 | 5.791 | 4.129 | 4.582 | 0.453 | 0.288 | 0.107 | 0.402 | Unannotated | NA | NA |
| metab_15558 | 2-(7'-methylthio)heptylmalate | neg | 297.0770 | 5.417 | 4.566 | 3.270 | -1.296 | -0.288 | 0.107 | 0.402 | Tentative | Organic acids and derivatives | Hydroxy acids and derivatives |
| metab_1898 | P-Tolualdehyde | pos | 121.0653 | 1.474 | 5.988 | 5.632 | -0.356 | -0.288 | 0.107 | 0.402 | Strong putative | NA | NA |
| metab_13374 | Beta-D-galactosyl | neg | 223.0821 | 0.738 | 4.711 | 5.046 | 0.335 | 0.288 | 0.107 | 0.402 | Unannotated | Organic oxygen compounds | Organooxygen compounds |
| metab_7752 | Leu Leu Glu | pos | 374.2295 | 3.291 | 5.106 | 4.547 | -0.559 | -0.288 | 0.107 | 0.402 | Unannotated | NA | NA |
| metab_10327 | L-Phenylalanine | neg | 164.0711 | 2.298 | 5.900 | 5.773 | -0.127 | -0.288 | 0.107 | 0.402 | Strong putative | NA | NA |
| metab_569 | Unannotated feature | pos | 439.1656 | 6.087 | 5.513 | 5.357 | -0.155 | -0.288 | 0.107 | 0.402 | Unannotated | NA | NA |
| metab_19313 | Unannotated feature | neg | 198.1131 | 5.127 | 3.621 | 3.173 | -0.448 | -0.288 | 0.107 | 0.402 | Unannotated | NA | NA |
| metab_6364 | Unannotated feature | pos | 528.8397 | 5.757 | 4.028 | 4.801 | 0.773 | 0.288 | 0.107 | 0.402 | Unannotated | NA | NA |
| metab_14609 | 3-Isovalidene-3alpha,4-dihydrophthalide | neg | 249.1133 | 3.525 | 2.892 | 2.464 | -0.428 | -0.288 | 0.107 | 0.402 | Unannotated | Organoheterocyclic compounds | Isobenzofurans |
| metab_948 | Unannotated feature | pos | 362.9271 | 7.610 | 6.984 | 7.019 | 0.035 | 0.288 | 0.107 | 0.402 | Unannotated | NA | NA |
| metab_6081 | Unannotated feature | pos | 145.1702 | 0.516 | 5.617 | 4.970 | -0.647 | -0.288 | 0.107 | 0.402 | Unannotated | NA | NA |
| metab_7146 | Unannotated feature | pos | 129.0663 | 0.665 | 4.131 | 4.553 | 0.422 | 0.288 | 0.107 | 0.402 | Unannotated | NA | NA |
| metab_2914 | Unannotated feature | pos | 196.1210 | 2.880 | 3.645 | 2.968 | -0.677 | -0.288 | 0.107 | 0.402 | Unannotated | NA | NA |
| metab_16030 | Unannotated feature | neg | 491.2871 | 6.154 | 6.113 | 5.910 | -0.202 | -0.288 | 0.107 | 0.402 | Unannotated | NA | NA |
| metab_9127 | Unannotated feature | neg | 311.1594 | 3.882 | 3.987 | 3.612 | -0.375 | -0.288 | 0.107 | 0.402 | Unannotated | NA | NA |
| metab_17201 | Unannotated feature | neg | 239.0851 | 7.737 | 4.823 | 5.092 | 0.269 | 0.288 | 0.107 | 0.402 | Unannotated | NA | NA |
| metab_16533 | Unannotated feature | neg | 283.2644 | 7.194 | 5.398 | 5.179 | -0.219 | -0.288 | 0.107 | 0.402 | Unannotated | NA | NA |
| metab_13994 | Unannotated feature | neg | 232.1189 | 2.707 | 3.224 | 3.659 | 0.435 | 0.288 | 0.107 | 0.402 | Unannotated | NA | NA |
| metab_2814 | Unannotated feature | pos | 276.1559 | 2.703 | 4.562 | 4.371 | -0.191 | -0.288 | 0.107 | 0.402 | Unannotated | NA | NA |
| metab_12071 | Unannotated feature | neg | 424.2476 | 6.412 | 5.804 | 5.564 | -0.240 | -0.288 | 0.107 | 0.402 | Unannotated | NA | NA |
| metab_15652 | Unannotated feature | neg | 134.0605 | 5.595 | 3.984 | 3.430 | -0.554 | -0.288 | 0.107 | 0.402 | Unannotated | NA | NA |
| metab_174 | Unannotated feature | pos | 323.2559 | 6.670 | 5.247 | 5.455 | 0.208 | 0.288 | 0.107 | 0.402 | Unannotated | NA | NA |
| metab_12225 | Unannotated feature | neg | 362.2370 | 6.892 | 5.379 | 4.404 | -0.975 | -0.288 | 0.107 | 0.402 | Unannotated | NA | NA |
| metab_3005 | Ile Leu Glu | pos | 374.2295 | 3.135 | 4.907 | 4.655 | -0.251 | -0.288 | 0.107 | 0.402 | Strong putative | NA | NA |
| metab_15488 | Unannotated feature | neg | 134.0604 | 5.242 | 4.387 | 4.179 | -0.208 | -0.288 | 0.107 | 0.402 | Unannotated | NA | NA |
| metab_13980 | Unannotated feature | neg | 499.2044 | 2.667 | 2.582 | 3.516 | 0.934 | 0.288 | 0.107 | 0.402 | Unannotated | NA | NA |
| metab_13936 | Unannotated feature | neg | 335.0005 | 2.579 | 5.665 | 5.554 | -0.111 | -0.288 | 0.107 | 0.402 | Unannotated | NA | NA |
| metab_12604 | Unannotated feature | neg | 461.2314 | 6.068 | 5.562 | 5.931 | 0.369 | 0.288 | 0.107 | 0.402 | Unannotated | NA | NA |
| metab_17400 | Unannotated feature | neg | 552.8589 | 7.652 | 4.099 | 4.419 | 0.321 | 0.288 | 0.107 | 0.402 | Unannotated | NA | NA |
| metab_17436 | Unannotated feature | neg | 847.8507 | 7.617 | 5.565 | 5.741 | 0.176 | 0.288 | 0.107 | 0.402 | Unannotated | NA | NA |
| metab_3494 | Unannotated feature | pos | 392.2188 | 4.778 | 5.098 | 4.335 | -0.763 | -0.288 | 0.107 | 0.402 | Unannotated | NA | NA |
| metab_8659 | LL-2,6-Diaminopimelic Acid | pos | 173.0924 | 0.997 | 5.357 | 5.181 | -0.176 | -0.288 | 0.107 | 0.402 | Moderate putative | NA | NA |
| metab_15094 | Unannotated feature | neg | 342.2401 | 4.518 | 4.569 | 4.158 | -0.412 | -0.284 | 0.112 | 0.412 | Unannotated | NA | NA |
| metab_11500 | Unannotated feature | neg | 473.8504 | 0.512 | 3.645 | 4.402 | 0.757 | 0.284 | 0.112 | 0.412 | Unannotated | NA | NA |
| metab_4649 | Unannotated feature | pos | 470.4209 | 7.307 | 6.491 | 6.550 | 0.059 | 0.284 | 0.112 | 0.412 | Unannotated | NA | NA |
| metab_14669 | Unannotated feature | neg | 370.1625 | 3.624 | 4.574 | 4.060 | -0.513 | -0.284 | 0.112 | 0.412 | Unannotated | NA | NA |
| metab_644 | Unannotated feature | pos | 606.4680 | 5.835 | 2.709 | 4.383 | 1.674 | 0.284 | 0.112 | 0.412 | Unannotated | NA | NA |
| metab_659 | Unannotated feature | pos | 132.9949 | 0.016 | 4.989 | 5.010 | 0.021 | 0.284 | 0.112 | 0.412 | Unannotated | NA | NA |
| metab_6400 | Unannotated feature | pos | 616.8926 | 5.725 | 4.099 | 4.823 | 0.724 | 0.284 | 0.112 | 0.412 | Unannotated | NA | NA |
| metab_13027 | Unannotated feature | neg | 146.9823 | 0.858 | 2.644 | 3.015 | 0.371 | 0.284 | 0.112 | 0.412 | Unannotated | NA | NA |
| metab_16901 | Unannotated feature | neg | 188.8934 | 7.760 | 3.596 | 3.514 | -0.082 | -0.284 | 0.112 | 0.412 | Unannotated | NA | NA |
| metab_4920 | Unannotated feature | pos | 650.8520 | 7.642 | 5.072 | 5.113 | 0.041 | 0.284 | 0.112 | 0.412 | Unannotated | NA | NA |
| metab_9833 | Unannotated feature | neg | 239.0923 | 4.298 | 5.550 | 5.810 | 0.260 | 0.284 | 0.112 | 0.412 | Unannotated | NA | NA |
| metab_12658 | Unannotated feature | neg | 287.1136 | 5.918 | 2.532 | 3.699 | 1.167 | 0.284 | 0.112 | 0.412 | Unannotated | NA | NA |
| metab_7903 | Unannotated feature | pos | 279.2072 | 2.960 | 4.778 | 4.479 | -0.299 | -0.284 | 0.112 | 0.412 | Unannotated | NA | NA |
| metab_13543 | Unannotated feature | neg | 173.0449 | 1.944 | 5.411 | 5.525 | 0.114 | 0.284 | 0.112 | 0.412 | Unannotated | NA | NA |
| metab_4048 | Unannotated feature | pos | 711.9406 | 5.825 | 2.471 | 4.081 | 1.610 | 0.284 | 0.112 | 0.412 | Unannotated | NA | NA |
| metab_10000 | Unannotated feature | neg | 380.0992 | 2.845 | 4.480 | 4.895 | 0.414 | 0.284 | 0.112 | 0.412 | Unannotated | NA | NA |
| metab_9983 | (2R)-2-Hydroxy-2-methylbutanenitrile | neg | 243.1350 | 2.865 | 4.397 | 3.998 | -0.398 | -0.284 | 0.112 | 0.412 | Tentative | NA | NA |
| metab_18046 | Unannotated feature | neg | 318.1196 | 2.903 | 4.585 | 5.131 | 0.546 | 0.284 | 0.112 | 0.412 | Unannotated | NA | NA |
| metab_10236 | Unannotated feature | neg | 290.0153 | 2.579 | 4.844 | 4.612 | -0.232 | -0.284 | 0.112 | 0.412 | Unannotated | NA | NA |
| metab_1190 | Unannotated feature | pos | 333.2044 | 6.478 | 4.325 | 4.523 | 0.198 | 0.284 | 0.112 | 0.412 | Unannotated | NA | NA |
| metab_10714 | N-Methylporphyroxine | neg | 384.1418 | 4.749 | 5.970 | 5.766 | -0.204 | -0.284 | 0.112 | 0.412 | Unannotated | Alkaloids and derivatives | Rhoeadine alkaloids |
| metab_8105 | Unannotated feature | pos | 157.1339 | 2.740 | 3.968 | 3.580 | -0.387 | -0.284 | 0.112 | 0.412 | Unannotated | NA | NA |
| metab_18518 | Unannotated feature | neg | 295.1115 | 6.041 | 5.004 | 5.250 | 0.246 | 0.284 | 0.112 | 0.412 | Unannotated | NA | NA |
| metab_16666 | Unannotated feature | neg | 826.8568 | 7.607 | 6.704 | 6.762 | 0.058 | 0.284 | 0.112 | 0.412 | Unannotated | NA | NA |
| metab_16672 | Unannotated feature | neg | 562.8993 | 7.611 | 5.803 | 5.910 | 0.107 | 0.284 | 0.112 | 0.412 | Unannotated | NA | NA |
| metab_4766 | Unannotated feature | pos | 313.9617 | 7.732 | 4.370 | 4.278 | -0.092 | -0.284 | 0.112 | 0.412 | Unannotated | NA | NA |
| metab_1219 | Unannotated feature | pos | 240.2689 | 6.406 | 4.337 | 5.086 | 0.750 | 0.284 | 0.112 | 0.412 | Unannotated | NA | NA |
| metab_10768 | Unannotated feature | neg | 501.1476 | 0.730 | 4.761 | 5.076 | 0.315 | 0.284 | 0.112 | 0.412 | Unannotated | NA | NA |
| metab_8058 | Unannotated feature | pos | 403.2715 | 3.496 | 2.828 | 2.005 | -0.823 | -0.284 | 0.112 | 0.412 | Unannotated | NA | NA |
| metab_3035 | Ile Ile Asp | pos | 360.2138 | 3.257 | 5.167 | 4.536 | -0.631 | -0.284 | 0.112 | 0.412 | Moderate putative | NA | NA |
| metab_11876 | Unannotated feature | neg | 533.2261 | 6.013 | 3.843 | 4.820 | 0.978 | 0.284 | 0.112 | 0.412 | Unannotated | NA | NA |
| metab_11859 | Unannotated feature | neg | 283.0591 | 5.991 | 4.022 | 3.485 | -0.538 | -0.284 | 0.112 | 0.412 | Unannotated | NA | NA |
| metab_18775 | Unannotated feature | neg | 631.2792 | 5.813 | 4.906 | 3.857 | -1.049 | -0.284 | 0.112 | 0.412 | Unannotated | NA | NA |
| metab_12778 | L-Glutamine | neg | 145.0613 | 0.628 | 5.929 | 5.819 | -0.109 | -0.284 | 0.112 | 0.412 | Tentative | NA | NA |
| metab_16689 | Unannotated feature | neg | 698.8732 | 7.621 | 6.671 | 6.731 | 0.060 | 0.284 | 0.112 | 0.412 | Unannotated | NA | NA |
| metab_16710 | Unannotated feature | neg | 1012.7996 | 7.641 | 5.470 | 5.544 | 0.074 | 0.284 | 0.112 | 0.412 | Unannotated | NA | NA |
| metab_16711 | Unannotated feature | neg | 877.3318 | 7.645 | 5.288 | 5.485 | 0.197 | 0.284 | 0.112 | 0.412 | Unannotated | NA | NA |
| metab_16459 | Unannotated feature | neg | 146.0452 | 7.004 | 5.011 | 4.929 | -0.082 | -0.284 | 0.112 | 0.412 | Unannotated | NA | NA |
| metab_18632 | Unannotated feature | neg | 637.2651 | 5.952 | 3.606 | 2.242 | -1.365 | -0.284 | 0.112 | 0.412 | Unannotated | NA | NA |
| metab_6476 | (+)-cis-Khellactone | pos | 304.1185 | 5.621 | 5.342 | 5.188 | -0.154 | -0.284 | 0.112 | 0.412 | Unannotated | Phenylpropanoids and polyketides | Coumarins and derivatives |
| metab_14835 | Unannotated feature | neg | 242.0305 | 3.975 | 4.434 | 3.952 | -0.482 | -0.284 | 0.112 | 0.412 | Unannotated | NA | NA |
| metab_11226 | Unannotated feature | neg | 102.0552 | 0.644 | 5.804 | 5.665 | -0.139 | -0.284 | 0.112 | 0.412 | Unannotated | NA | NA |
| metab_5804 | (2S)-2-Amino-N-[(1S)-7,7-dimethyl-1-[[4-(2-methylphenyl)piperazin-1-yl]sulfonylmethyl]-2-bicyclo[2.2.1]heptanyl]-4-methylsulfonylbutanamide | pos | 587.2884 | 6.000 | 5.119 | 4.458 | -0.661 | -0.284 | 0.112 | 0.412 | Unannotated | NA | NA |
| metab_17467 | Unannotated feature | neg | 801.3571 | 7.596 | 6.337 | 6.430 | 0.093 | 0.284 | 0.112 | 0.412 | Unannotated | NA | NA |
| metab_11754 | Unannotated feature | neg | 401.1981 | 5.671 | 3.991 | 4.391 | 0.400 | 0.284 | 0.112 | 0.412 | Unannotated | NA | NA |
| metab_2165 | Unannotated feature | pos | 173.1652 | 2.915 | 4.573 | 3.683 | -0.890 | -0.284 | 0.112 | 0.412 | Unannotated | NA | NA |
| metab_17690 | Unannotated feature | neg | 86.0061 | 7.019 | 6.604 | 6.568 | -0.036 | -0.284 | 0.112 | 0.412 | Unannotated | NA | NA |
| metab_2262 | Unannotated feature | pos | 295.1657 | 3.113 | 5.549 | 5.386 | -0.164 | -0.284 | 0.112 | 0.412 | Unannotated | NA | NA |
| metab_7870 | Unannotated feature | pos | 78.4258 | 3.007 | 3.306 | 3.459 | 0.153 | 0.284 | 0.112 | 0.412 | Unannotated | NA | NA |
| metab_8557 | Unannotated feature | pos | 270.1340 | 4.011 | 4.658 | 4.295 | -0.363 | -0.284 | 0.112 | 0.412 | Unannotated | NA | NA |
| metab_9765 | Deca-4,6,8-trienedioylcarnitine | neg | 360.1416 | 3.067 | 4.535 | 4.234 | -0.301 | -0.284 | 0.112 | 0.412 | Unannotated | NA | NA |
| metab_3877 | Unannotated feature | pos | 508.0648 | 5.721 | 4.083 | 4.349 | 0.266 | 0.284 | 0.112 | 0.412 | Unannotated | NA | NA |
| metab_6945 | Unannotated feature | pos | 674.8889 | 4.950 | 3.967 | 5.162 | 1.195 | 0.284 | 0.112 | 0.412 | Unannotated | NA | NA |
| metab_9889 | Unannotated feature | neg | 300.1932 | 2.960 | 5.075 | 4.809 | -0.266 | -0.284 | 0.112 | 0.412 | Unannotated | NA | NA |
| metab_8385 | 2-Methyl-3-oxopropanoic acid | pos | 246.0977 | 1.952 | 4.241 | 4.570 | 0.329 | 0.284 | 0.112 | 0.412 | Tentative | NA | NA |
| metab_10359 | Unannotated feature | neg | 348.1667 | 4.592 | 4.320 | 4.850 | 0.530 | 0.284 | 0.112 | 0.412 | Unannotated | NA | NA |
| metab_4434 | Unannotated feature | pos | 509.3105 | 6.359 | 5.673 | 5.376 | -0.297 | -0.284 | 0.112 | 0.412 | Unannotated | NA | NA |
| metab_5130 | Unannotated feature | pos | 148.7118 | 6.917 | 3.376 | 3.789 | 0.413 | 0.284 | 0.112 | 0.412 | Unannotated | NA | NA |
| metab_5112 | Unannotated feature | pos | 441.3577 | 6.989 | 4.916 | 4.866 | -0.050 | -0.284 | 0.112 | 0.412 | Unannotated | NA | NA |
| metab_16502 | Unannotated feature | neg | 464.2849 | 7.097 | 4.832 | 4.420 | -0.412 | -0.284 | 0.112 | 0.412 | Unannotated | NA | NA |
| metab_15199 | Unannotated feature | neg | 486.2580 | 4.726 | 4.612 | 3.596 | -1.016 | -0.284 | 0.112 | 0.412 | Unannotated | NA | NA |
| metab_1841 | Unannotated feature | pos | 274.1878 | 1.026 | 4.916 | 4.552 | -0.365 | -0.284 | 0.112 | 0.412 | Unannotated | NA | NA |
| metab_5364 | Yucalexin P15 | pos | 353.1716 | 1.952 | 4.250 | 4.851 | 0.602 | 0.284 | 0.112 | 0.412 | Unannotated | NA | NA |
| metab_2596 | N2-Acetylornithine | pos | 175.1080 | 1.566 | 6.173 | 5.929 | -0.245 | -0.284 | 0.112 | 0.412 | Tentative | NA | NA |
| metab_3315 | Deoxycholylasparagine | pos | 548.3659 | 4.094 | 5.116 | 5.569 | 0.453 | 0.284 | 0.112 | 0.412 | Unannotated | NA | NA |
| metab_6818 | Unannotated feature | pos | 660.3948 | 5.147 | 3.387 | 4.897 | 1.510 | 0.284 | 0.112 | 0.412 | Unannotated | NA | NA |
| metab_17342 | Unannotated feature | neg | 180.9304 | 0.603 | 5.137 | 4.997 | -0.140 | -0.284 | 0.112 | 0.412 | Unannotated | NA | NA |
| metab_15409 | Unannotated feature | neg | 343.9791 | 0.566 | 3.825 | 4.389 | 0.564 | 0.284 | 0.112 | 0.412 | Unannotated | NA | NA |
| metab_11774 | Unannotated feature | neg | 523.2213 | 5.748 | 3.486 | 4.144 | 0.658 | 0.284 | 0.112 | 0.412 | Unannotated | NA | NA |
| metab_14170 | Unannotated feature | neg | 251.0965 | 2.906 | 3.515 | 3.083 | -0.432 | -0.284 | 0.112 | 0.412 | Unannotated | NA | NA |
| metab_3506 | (5E)-3-Hydroxyhept-5-enoylcarnitine | pos | 288.1810 | 4.810 | 4.410 | 3.837 | -0.573 | -0.284 | 0.112 | 0.412 | Unannotated | NA | NA |
| metab_15383 | Unannotated feature | neg | 214.9530 | 5.044 | 3.727 | 3.582 | -0.145 | -0.284 | 0.112 | 0.412 | Unannotated | NA | NA |
| metab_2728 | Unannotated feature | pos | 170.0816 | 2.199 | 3.919 | 4.003 | 0.084 | 0.284 | 0.112 | 0.412 | Unannotated | NA | NA |
| metab_2974 | (2S)-dihydrotricetin | pos | 326.0383 | 3.027 | 4.881 | 5.005 | 0.124 | 0.284 | 0.112 | 0.412 | Unannotated | NA | NA |
| metab_3181 | 9-Fluoroprednisolone | pos | 442.1986 | 3.678 | 4.654 | 3.463 | -1.191 | -0.284 | 0.112 | 0.412 | Tentative | NA | NA |
| metab_319 | Caffeic acid 3-O-sulfate | pos | 304.9696 | 0.477 | 4.776 | 4.409 | -0.366 | -0.284 | 0.112 | 0.412 | Unannotated | NA | NA |
| metab_6766 | (E,E)-Futoamide | pos | 625.3250 | 5.223 | 4.697 | 4.114 | -0.583 | -0.280 | 0.118 | 0.419 | Unannotated | Organoheterocyclic compounds | Benzodioxoles |
| metab_14135 | Unannotated feature | neg | 265.0832 | 2.869 | 3.824 | 4.237 | 0.412 | 0.280 | 0.118 | 0.419 | Unannotated | NA | NA |
| metab_19635 | Unannotated feature | neg | 245.0125 | 3.581 | 5.377 | 4.845 | -0.531 | -0.280 | 0.118 | 0.419 | Unannotated | NA | NA |
| metab_16552 | LysoPA(0:0/16:0) | neg | 391.2262 | 7.259 | 5.589 | 5.435 | -0.154 | -0.280 | 0.118 | 0.419 | Unannotated | NA | NA |
| metab_6491 | Unannotated feature | pos | 518.0840 | 5.597 | 2.297 | 4.374 | 2.077 | 0.280 | 0.118 | 0.419 | Unannotated | NA | NA |
| metab_424 | Unannotated feature | pos | 140.9959 | 0.016 | 4.648 | 4.679 | 0.031 | 0.280 | 0.118 | 0.419 | Unannotated | NA | NA |
| metab_13594 | Unannotated feature | neg | 224.9518 | 1.384 | 4.837 | 4.669 | -0.168 | -0.280 | 0.118 | 0.419 | Unannotated | NA | NA |
| metab_7789 | Unannotated feature | pos | 326.0383 | 3.200 | 5.235 | 5.513 | 0.278 | 0.280 | 0.118 | 0.419 | Unannotated | NA | NA |
| metab_2255 | N-Acetyl-L-glutamic acid | pos | 379.1370 | 3.129 | 4.443 | 4.655 | 0.212 | 0.280 | 0.118 | 0.419 | Tentative | Organic acids and derivatives | Carboxylic acids and derivatives |
| metab_5860 | Unannotated feature | pos | 690.4253 | 5.944 | 3.995 | 4.754 | 0.758 | 0.280 | 0.118 | 0.419 | Unannotated | NA | NA |
| metab_6544 | Unannotated feature | pos | 486.3034 | 5.537 | 3.593 | 3.641 | 0.047 | 0.280 | 0.118 | 0.419 | Unannotated | NA | NA |
| metab_17217 | Unannotated feature | neg | 314.1196 | 7.730 | 4.775 | 4.999 | 0.224 | 0.280 | 0.118 | 0.419 | Unannotated | NA | NA |
| metab_16695 | Unannotated feature | neg | 834.8476 | 7.625 | 6.626 | 6.678 | 0.051 | 0.280 | 0.118 | 0.419 | Unannotated | NA | NA |
| metab_5152 | Unannotated feature | pos | 260.1497 | 0.728 | 4.455 | 4.878 | 0.423 | 0.280 | 0.118 | 0.419 | Unannotated | NA | NA |
| metab_10989 | Arginylphenylalanine | neg | 356.1469 | 0.777 | 4.536 | 4.093 | -0.443 | -0.280 | 0.118 | 0.419 | Unannotated | Organic acids and derivatives | Carboxylic acids and derivatives |
| metab_8926 | Unannotated feature | neg | 322.0935 | 4.228 | 3.073 | 3.880 | 0.807 | 0.280 | 0.118 | 0.419 | Unannotated | NA | NA |
| metab_3297 | 2-Methylbenzoic acid | pos | 119.0496 | 4.051 | 4.083 | 3.070 | -1.012 | -0.280 | 0.118 | 0.419 | Tentative | Benzenoids | Benzene and substituted derivatives |
| metab_3667 | Unannotated feature | pos | 242.1137 | 1.952 | 4.937 | 5.161 | 0.224 | 0.280 | 0.118 | 0.419 | Unannotated | NA | NA |
| metab_14381 | Acidissiminol | neg | 430.1780 | 3.162 | 4.858 | 3.423 | -1.435 | -0.280 | 0.118 | 0.419 | Unannotated | NA | NA |
| metab_15857 | Unannotated feature | neg | 448.2826 | 5.937 | 4.586 | 4.421 | -0.165 | -0.280 | 0.118 | 0.419 | Unannotated | NA | NA |
| metab_15858 | Cyclopropanecarboxamide, 1,2,2,3,3-pentamethyl- | neg | 200.1287 | 5.944 | 5.680 | 5.511 | -0.169 | -0.280 | 0.118 | 0.419 | Unannotated | Organic acids and derivatives | Carboxylic acids and derivatives |
| metab_14477 | Unannotated feature | neg | 395.1780 | 3.318 | 4.225 | 3.654 | -0.571 | -0.280 | 0.118 | 0.419 | Unannotated | NA | NA |
| metab_11609 | Unannotated feature | neg | 289.8707 | 0.388 | 4.579 | 4.532 | -0.047 | -0.280 | 0.118 | 0.419 | Unannotated | NA | NA |
| metab_2148 | Unannotated feature | pos | 371.1825 | 2.880 | 3.523 | 4.634 | 1.111 | 0.280 | 0.118 | 0.419 | Unannotated | NA | NA |
| metab_7997 | Unannotated feature | pos | 306.1345 | 2.840 | 3.578 | 4.064 | 0.486 | 0.280 | 0.118 | 0.419 | Unannotated | NA | NA |
| metab_4805 | Unannotated feature | pos | 245.1033 | 7.987 | 4.956 | 4.993 | 0.037 | 0.280 | 0.118 | 0.419 | Unannotated | NA | NA |
| metab_6849 | (R)-Apiumetin glucoside | pos | 371.1130 | 5.102 | 4.683 | 4.527 | -0.156 | -0.280 | 0.118 | 0.419 | Unannotated | NA | NA |
| metab_14860 | Unannotated feature | neg | 326.1725 | 4.017 | 4.730 | 3.923 | -0.807 | -0.280 | 0.118 | 0.419 | Unannotated | NA | NA |
| metab_18793 | Unannotated feature | neg | 313.1289 | 5.789 | 3.296 | 3.619 | 0.323 | 0.280 | 0.118 | 0.419 | Unannotated | NA | NA |
| metab_17479 | Unannotated feature | neg | 537.9014 | 7.575 | 6.753 | 6.800 | 0.047 | 0.280 | 0.118 | 0.419 | Unannotated | NA | NA |
| metab_2547 | Thiomorpholine 3-carboxylate | pos | 148.0429 | 1.049 | 2.744 | 3.155 | 0.412 | 0.280 | 0.118 | 0.419 | Tentative | NA | NA |
| metab_17445 | Unannotated feature | neg | 745.8692 | 7.611 | 5.781 | 5.838 | 0.058 | 0.280 | 0.118 | 0.419 | Unannotated | NA | NA |
| metab_18469 | Estriol-16-Glucuronide | neg | 445.1908 | 6.076 | 6.593 | 6.737 | 0.144 | 0.280 | 0.118 | 0.419 | Tentative | NA | NA |
| metab_11749 | Isoamericanin A | neg | 363.0650 | 5.567 | 3.035 | 3.553 | 0.518 | 0.280 | 0.118 | 0.419 | Unannotated | Organoheterocyclic compounds | Benzodioxanes |
| metab_17334 | Unannotated feature | neg | 160.9176 | 7.697 | 4.170 | 4.160 | -0.010 | -0.280 | 0.118 | 0.419 | Unannotated | NA | NA |
| metab_13004 | Unannotated feature | neg | 397.2064 | 6.209 | 6.509 | 6.766 | 0.257 | 0.280 | 0.118 | 0.419 | Unannotated | NA | NA |
| metab_12972 | Unannotated feature | neg | 217.1190 | 0.784 | 4.828 | 4.650 | -0.179 | -0.280 | 0.118 | 0.419 | Unannotated | NA | NA |
| metab_7610 | Unannotated feature | pos | 266.1755 | 3.533 | 4.987 | 4.878 | -0.109 | -0.280 | 0.118 | 0.419 | Unannotated | NA | NA |
| metab_17994 | Diisononyl phthalate | neg | 463.3073 | 6.522 | 6.018 | 5.819 | -0.199 | -0.280 | 0.118 | 0.419 | Tentative | Benzenoids | Benzene and substituted derivatives |
| metab_13863 | Unannotated feature | neg | 276.1090 | 2.274 | 3.109 | 3.836 | 0.727 | 0.280 | 0.118 | 0.419 | Unannotated | NA | NA |
| metab_9533 | Unannotated feature | neg | 290.1152 | 3.306 | 3.930 | 2.948 | -0.982 | -0.280 | 0.118 | 0.419 | Unannotated | NA | NA |
| metab_1852 | Cyclohexanecarboxamide | pos | 145.1338 | 1.069 | 6.831 | 6.300 | -0.530 | -0.280 | 0.118 | 0.419 | Unannotated | Organic acids and derivatives | Carboxylic acids and derivatives |
| metab_11717 | Unannotated feature | neg | 380.1466 | 5.530 | 5.628 | 5.383 | -0.245 | -0.280 | 0.118 | 0.419 | Unannotated | NA | NA |
| metab_2475 | Glu-Thr | pos | 249.1085 | 0.712 | 5.414 | 5.234 | -0.180 | -0.280 | 0.118 | 0.419 | Strong putative | NA | NA |
| metab_2314 | Aspirin | pos | 163.0391 | 0.098 | 5.569 | 5.595 | 0.026 | 0.280 | 0.118 | 0.419 | Tentative | NA | NA |
| metab_734 | Unannotated feature | pos | 659.3300 | 6.000 | 4.818 | 5.143 | 0.325 | 0.280 | 0.118 | 0.419 | Unannotated | NA | NA |
| metab_17053 | Unannotated feature | neg | 164.9469 | 7.985 | 5.391 | 5.350 | -0.041 | -0.280 | 0.118 | 0.419 | Unannotated | NA | NA |
| metab_9658 | Unannotated feature | neg | 312.1934 | 3.180 | 4.622 | 4.477 | -0.145 | -0.280 | 0.118 | 0.419 | Unannotated | NA | NA |
| metab_18520 | Unannotated feature | neg | 222.0769 | 3.067 | 5.456 | 5.191 | -0.266 | -0.280 | 0.118 | 0.419 | Unannotated | NA | NA |
| metab_8120 | Unannotated feature | pos | 272.1610 | 2.732 | 5.147 | 5.013 | -0.134 | -0.280 | 0.118 | 0.419 | Unannotated | NA | NA |
| metab_5417 | Leukotriene B4 ethanolamide | pos | 421.3076 | 6.414 | 4.512 | 4.953 | 0.441 | 0.280 | 0.118 | 0.419 | Unannotated | NA | NA |
| metab_6973 | Ile Val Phe | pos | 378.2396 | 4.900 | 4.244 | 3.700 | -0.545 | -0.280 | 0.118 | 0.419 | Moderate putative | NA | NA |
| metab_10015 | Unannotated feature | neg | 281.0992 | 2.826 | 6.633 | 6.523 | -0.110 | -0.280 | 0.118 | 0.419 | Unannotated | NA | NA |
| metab_6552 | 17alpha-Methylestradiol | pos | 611.3461 | 5.519 | 4.251 | 6.044 | 1.792 | 0.280 | 0.118 | 0.419 | Tentative | NA | NA |
| metab_4712 | Unannotated feature | pos | 608.8787 | 7.618 | 5.855 | 5.935 | 0.080 | 0.280 | 0.118 | 0.419 | Unannotated | NA | NA |
| metab_10701 | Unannotated feature | neg | 174.9773 | 1.411 | 3.864 | 3.282 | -0.583 | -0.280 | 0.118 | 0.419 | Unannotated | NA | NA |
| metab_18564 | Unannotated feature | neg | 172.9908 | 3.082 | 4.859 | 4.146 | -0.713 | -0.280 | 0.118 | 0.419 | Unannotated | NA | NA |
| metab_10764 | Unannotated feature | neg | 452.1419 | 1.179 | 5.407 | 5.691 | 0.285 | 0.280 | 0.118 | 0.419 | Unannotated | NA | NA |
| metab_8089 | Ascorbalamic acid | pos | 228.0507 | 2.747 | 4.274 | 4.607 | 0.332 | 0.280 | 0.118 | 0.419 | Unannotated | NA | NA |
| metab_12504 | Unannotated feature | neg | 357.2649 | 6.357 | 5.156 | 5.308 | 0.152 | 0.280 | 0.118 | 0.419 | Unannotated | NA | NA |
| metab_793 | Unannotated feature | pos | 250.1781 | 6.247 | 6.169 | 6.067 | -0.101 | -0.280 | 0.118 | 0.419 | Unannotated | NA | NA |
| metab_7680 | Unannotated feature | pos | 233.1136 | 0.752 | 5.298 | 5.119 | -0.180 | -0.280 | 0.118 | 0.419 | Unannotated | NA | NA |
| metab_6430 | Unannotated feature | pos | 585.3627 | 5.686 | 3.901 | 4.507 | 0.606 | 0.280 | 0.118 | 0.419 | Unannotated | NA | NA |
| metab_16722 | Unannotated feature | neg | 128.9589 | 7.656 | 5.671 | 5.636 | -0.035 | -0.280 | 0.118 | 0.419 | Unannotated | NA | NA |
| metab_13775 | Unannotated feature | neg | 202.1080 | 1.969 | 4.800 | 5.056 | 0.256 | 0.280 | 0.118 | 0.419 | Unannotated | NA | NA |
| metab_3559 | Unannotated feature | pos | 620.8894 | 4.971 | 3.779 | 4.598 | 0.819 | 0.280 | 0.118 | 0.419 | Unannotated | NA | NA |
| metab_5017 | Unannotated feature | pos | 534.3394 | 7.315 | 6.212 | 6.224 | 0.012 | 0.280 | 0.118 | 0.419 | Unannotated | NA | NA |
| metab_16621 | Unannotated feature | neg | 618.2708 | 7.495 | 4.553 | 4.887 | 0.334 | 0.280 | 0.118 | 0.419 | Unannotated | NA | NA |
| metab_17173 | Unannotated feature | neg | 184.8956 | 7.745 | 4.076 | 4.009 | -0.067 | -0.280 | 0.118 | 0.419 | Unannotated | NA | NA |
| metab_5000 | 2-Hydroxypropyl octanoate | pos | 225.1462 | 7.466 | 4.447 | 4.356 | -0.090 | -0.280 | 0.118 | 0.419 | Unannotated | NA | NA |
| metab_5062 | Unannotated feature | pos | 148.9936 | 7.140 | 4.515 | 4.788 | 0.273 | 0.280 | 0.118 | 0.419 | Unannotated | NA | NA |
| metab_19857 | D-Galactose | neg | 225.0614 | 0.577 | 6.330 | 6.056 | -0.273 | -0.280 | 0.118 | 0.419 | Moderate putative | Organic oxygen compounds | Organooxygen compounds |
| metab_9026 | Unannotated feature | neg | 207.0506 | 0.722 | 6.395 | 6.290 | -0.105 | -0.280 | 0.118 | 0.419 | Unannotated | NA | NA |
| metab_7794 | Unannotated feature | pos | 155.0260 | 3.193 | 3.990 | 3.874 | -0.116 | -0.280 | 0.118 | 0.419 | Unannotated | NA | NA |
| metab_14052 | Unannotated feature | neg | 199.0223 | 2.780 | 3.835 | 4.138 | 0.303 | 0.280 | 0.118 | 0.419 | Unannotated | NA | NA |
| metab_3097 | Unannotated feature | pos | 213.1237 | 3.416 | 4.441 | 4.159 | -0.283 | -0.280 | 0.118 | 0.419 | Unannotated | NA | NA |
| metab_11938 | Unannotated feature | neg | 251.1285 | 6.130 | 5.251 | 5.534 | 0.284 | 0.280 | 0.118 | 0.419 | Unannotated | NA | NA |
| metab_16631 | Unannotated feature | neg | 737.2562 | 7.510 | 6.143 | 6.322 | 0.179 | 0.280 | 0.118 | 0.419 | Unannotated | NA | NA |
| metab_11276 | Unannotated feature | neg | 245.9161 | 0.619 | 4.547 | 4.039 | -0.508 | -0.280 | 0.118 | 0.419 | Unannotated | NA | NA |
| metab_12628 | Unannotated feature | neg | 526.2489 | 6.003 | 5.178 | 5.500 | 0.322 | 0.280 | 0.118 | 0.419 | Unannotated | NA | NA |
| metab_10319 | Unannotated feature | neg | 351.9906 | 2.313 | 5.566 | 5.410 | -0.157 | -0.280 | 0.118 | 0.419 | Unannotated | NA | NA |
| metab_7488 | Unannotated feature | pos | 504.3396 | 3.775 | 4.495 | 5.080 | 0.585 | 0.280 | 0.118 | 0.419 | Unannotated | NA | NA |
| metab_18216 | Unannotated feature | neg | 473.3277 | 6.279 | 4.287 | 4.780 | 0.493 | 0.280 | 0.118 | 0.419 | Unannotated | NA | NA |
| metab_1748 | Unannotated feature | pos | 214.1554 | 3.586 | 4.598 | 3.412 | -1.187 | -0.280 | 0.118 | 0.419 | Unannotated | NA | NA |
| metab_13302 | Unannotated feature | neg | 284.0990 | 0.636 | 5.204 | 4.993 | -0.211 | -0.280 | 0.118 | 0.419 | Unannotated | NA | NA |
| metab_19008 | Unannotated feature | neg | 263.0960 | 5.522 | 3.792 | 4.637 | 0.846 | 0.280 | 0.118 | 0.419 | Unannotated | NA | NA |
| metab_14943 | Unannotated feature | neg | 472.9250 | 0.485 | 6.120 | 5.843 | -0.277 | -0.280 | 0.118 | 0.419 | Unannotated | NA | NA |
| metab_16955 | Unannotated feature | neg | 203.9224 | 7.866 | 4.562 | 4.628 | 0.065 | 0.280 | 0.118 | 0.419 | Unannotated | NA | NA |
| metab_8289 | Unannotated feature | pos | 187.1445 | 2.116 | 4.167 | 3.702 | -0.465 | -0.280 | 0.118 | 0.419 | Unannotated | NA | NA |
| metab_1556 | Unannotated feature | pos | 468.2509 | 5.313 | 6.224 | 5.756 | -0.468 | -0.280 | 0.118 | 0.419 | Unannotated | NA | NA |
| metab_8327 | Prostaglandin C1 | pos | 359.2186 | 2.003 | 4.372 | 4.781 | 0.408 | 0.280 | 0.118 | 0.419 | Tentative | Lipids and lipid-like molecules | Fatty Acyls |
| metab_6326 | Penitrem B | pos | 625.3614 | 5.778 | 4.694 | 6.056 | 1.362 | 0.280 | 0.118 | 0.419 | Tentative | Organoheterocyclic compounds | Naphthopyrans |
| metab_18364 | Isoleucylhydroxyproline | neg | 243.1350 | 3.011 | 5.186 | 4.910 | -0.276 | -0.280 | 0.118 | 0.419 | Unannotated | Organic acids and derivatives | Carboxylic acids and derivatives |
| metab_1454 | Unannotated feature | pos | 400.3586 | 6.000 | 5.566 | 5.468 | -0.098 | -0.280 | 0.118 | 0.419 | Unannotated | NA | NA |
| metab_1475 | Unannotated feature | pos | 474.2612 | 5.922 | 3.065 | 4.058 | 0.992 | 0.280 | 0.118 | 0.419 | Unannotated | NA | NA |
| metab_10481 | Unannotated feature | neg | 274.0935 | 1.952 | 5.272 | 5.424 | 0.152 | 0.280 | 0.118 | 0.419 | Unannotated | NA | NA |
| metab_7851 | Phenylalanylglutamic acid | pos | 277.1187 | 3.052 | 4.605 | 4.264 | -0.341 | -0.280 | 0.118 | 0.419 | Unannotated | NA | NA |
| metab_14906 | Dimethylmethoxy chromanol | neg | 253.1082 | 4.123 | 3.703 | 4.151 | 0.448 | 0.276 | 0.124 | 0.428 | Unannotated | NA | NA |
| metab_3952 | Unannotated feature | pos | 586.5657 | 5.782 | 2.303 | 4.163 | 1.860 | 0.276 | 0.124 | 0.428 | Unannotated | NA | NA |
| metab_12278 | Unannotated feature | neg | 115.0029 | 7.004 | 5.071 | 4.895 | -0.176 | -0.276 | 0.124 | 0.428 | Unannotated | NA | NA |
| metab_11769 | Unannotated feature | neg | 317.1508 | 5.734 | 7.173 | 7.030 | -0.144 | -0.276 | 0.124 | 0.428 | Unannotated | NA | NA |
| metab_12266 | Unannotated feature | neg | 366.2264 | 7.044 | 5.770 | 5.964 | 0.194 | 0.276 | 0.124 | 0.428 | Unannotated | NA | NA |
| metab_4054 | Unannotated feature | pos | 656.3064 | 5.831 | 3.380 | 4.300 | 0.920 | 0.276 | 0.124 | 0.428 | Unannotated | NA | NA |
| metab_14224 | Unannotated feature | neg | 287.0226 | 2.964 | 3.298 | 1.416 | -1.882 | -0.276 | 0.124 | 0.428 | Unannotated | NA | NA |
| metab_4869 | Unannotated feature | pos | 154.0052 | 7.724 | 3.702 | 3.626 | -0.076 | -0.276 | 0.124 | 0.428 | Unannotated | NA | NA |
| metab_10996 | Unannotated feature | neg | 260.1141 | 0.777 | 4.545 | 4.241 | -0.304 | -0.276 | 0.124 | 0.428 | Unannotated | NA | NA |
| metab_18725 | Unannotated feature | neg | 390.1676 | 5.884 | 2.568 | 3.455 | 0.888 | 0.276 | 0.124 | 0.428 | Unannotated | NA | NA |
| metab_12783 | Unannotated feature | neg | 88.0394 | 0.636 | 5.174 | 5.082 | -0.092 | -0.276 | 0.124 | 0.428 | Unannotated | NA | NA |
| metab_14805 | Dichlorophenyl-bis-triazolylpropanol | neg | 383.0450 | 3.909 | 4.997 | 4.443 | -0.554 | -0.276 | 0.124 | 0.428 | Tentative | Benzenoids | Benzene and substituted derivatives |
| metab_7980 | 1,7-Dimethyluric Acid | pos | 197.0674 | 2.864 | 4.596 | 2.827 | -1.769 | -0.276 | 0.124 | 0.428 | Strong putative | Organoheterocyclic compounds | Imidazopyrimidines |
| metab_18625 | 3-Isobutyl-1-methylxanthine | neg | 443.2203 | 5.964 | 5.785 | 6.002 | 0.217 | 0.276 | 0.124 | 0.428 | Tentative | NA | NA |
| metab_11885 | Unannotated feature | neg | 296.1186 | 6.029 | 5.506 | 5.028 | -0.478 | -0.276 | 0.124 | 0.428 | Unannotated | NA | NA |
| metab_7893 | Tocopheronic acid | pos | 312.1813 | 2.970 | 3.503 | 3.720 | 0.217 | 0.276 | 0.124 | 0.428 | Unannotated | Lipids and lipid-like molecules | Prenol lipids |
| metab_7890 | Unannotated feature | pos | 286.1765 | 2.976 | 4.767 | 4.486 | -0.281 | -0.276 | 0.124 | 0.428 | Unannotated | NA | NA |
| metab_9186 | Unannotated feature | neg | 186.0766 | 3.787 | 4.321 | 4.255 | -0.067 | -0.276 | 0.124 | 0.428 | Unannotated | NA | NA |
| metab_9210 | Unannotated feature | neg | 281.0547 | 3.754 | 4.401 | 3.894 | -0.507 | -0.276 | 0.124 | 0.428 | Unannotated | NA | NA |
| metab_17279 | Unannotated feature | neg | 257.0923 | 7.720 | 5.270 | 5.346 | 0.076 | 0.276 | 0.124 | 0.428 | Unannotated | NA | NA |
| metab_9201 | 1,2,3,4-Tetrahydro-b-carboline-1,3-dicarboxylic acid | neg | 259.0726 | 3.769 | 6.329 | 6.051 | -0.279 | -0.276 | 0.124 | 0.428 | Unannotated | Alkaloids and derivatives | Harmala alkaloids |
| metab_2925 | Diethylstilbestrol | pos | 291.1345 | 2.897 | 3.497 | 4.480 | 0.983 | 0.276 | 0.124 | 0.428 | Tentative | NA | NA |
| metab_980 | Unannotated feature | pos | 131.5338 | 7.732 | 5.661 | 5.686 | 0.025 | 0.276 | 0.124 | 0.428 | Unannotated | NA | NA |
| metab_3833 | Falcarindiol | pos | 305.1501 | 5.633 | 4.969 | 4.873 | -0.096 | -0.276 | 0.124 | 0.428 | Tentative | Lipids and lipid-like molecules | Fatty Acyls |
| metab_8524 | 3-HYDROXYPHENETHYLAMINE HCL | pos | 138.0915 | 1.474 | 5.177 | 4.813 | -0.363 | -0.276 | 0.124 | 0.428 | Moderate putative | NA | NA |
| metab_2205 | (2S)-2-[[2-(5-methoxyindol-1-yl)acetyl]amino]-4-methylpentanoic acid | pos | 319.1659 | 5.526 | 6.251 | 6.091 | -0.160 | -0.276 | 0.124 | 0.428 | Unannotated | NA | NA |
| metab_13595 | Unannotated feature | neg | 360.0209 | 1.393 | 4.877 | 4.465 | -0.412 | -0.276 | 0.124 | 0.428 | Unannotated | NA | NA |
| metab_9060 | Unannotated feature | neg | 179.0707 | 3.988 | 4.335 | 3.878 | -0.457 | -0.276 | 0.124 | 0.428 | Unannotated | NA | NA |
| metab_13643 | Unannotated feature | neg | 289.9823 | 1.650 | 5.783 | 5.702 | -0.081 | -0.276 | 0.124 | 0.428 | Unannotated | NA | NA |
| metab_17035 | Unannotated feature | neg | 187.8730 | 7.993 | 4.346 | 4.296 | -0.050 | -0.276 | 0.124 | 0.428 | Unannotated | NA | NA |
| metab_986 | Unannotated feature | pos | 202.1292 | 7.708 | 4.710 | 4.737 | 0.027 | 0.276 | 0.124 | 0.428 | Unannotated | NA | NA |
| metab_10138 | 5-Hydroxyoct-2-enedioylcarnitine | neg | 366.1309 | 2.750 | 5.414 | 5.284 | -0.130 | -0.276 | 0.124 | 0.428 | Unannotated | Lipids and lipid-like molecules | Fatty Acyls |
| metab_18110 | Unannotated feature | neg | 483.3327 | 6.381 | 5.745 | 5.767 | 0.023 | 0.276 | 0.124 | 0.428 | Unannotated | NA | NA |
| metab_13821 | Unannotated feature | neg | 287.9336 | 0.453 | 4.555 | 4.506 | -0.049 | -0.276 | 0.124 | 0.428 | Unannotated | NA | NA |
| metab_8598 | S-Allyl-L-cysteine | pos | 162.0585 | 1.147 | 5.472 | 5.271 | -0.202 | -0.276 | 0.124 | 0.428 | Moderate putative | NA | NA |
| metab_13094 | Unannotated feature | neg | 206.9169 | 0.038 | 5.875 | 5.837 | -0.039 | -0.276 | 0.124 | 0.428 | Unannotated | NA | NA |
| metab_16848 | 2,4,6-Trihydroxybenzoic acid | neg | 169.0137 | 1.992 | 5.084 | 4.845 | -0.239 | -0.276 | 0.124 | 0.428 | Unannotated | NA | NA |
| metab_8134 | Leucyl-Glutamate | pos | 225.1238 | 3.574 | 4.647 | 4.540 | -0.108 | -0.276 | 0.124 | 0.428 | Unannotated | NA | NA |
| metab_19245 | Carbofuran | neg | 220.0976 | 5.209 | 4.951 | 4.833 | -0.118 | -0.276 | 0.124 | 0.428 | Tentative | Organoheterocyclic compounds | Coumarans |
| metab_14244 | Unannotated feature | neg | 159.9360 | 2.984 | 3.392 | 3.049 | -0.343 | -0.276 | 0.124 | 0.428 | Unannotated | NA | NA |
| metab_9319 | Unannotated feature | neg | 254.0539 | 3.585 | 3.594 | 4.099 | 0.504 | 0.276 | 0.124 | 0.428 | Unannotated | NA | NA |
| metab_10665 | Unannotated feature | neg | 216.0333 | 1.577 | 3.846 | 3.658 | -0.188 | -0.276 | 0.124 | 0.428 | Unannotated | NA | NA |
| metab_17343 | Unannotated feature | neg | 311.0203 | 2.569 | 5.267 | 5.073 | -0.194 | -0.276 | 0.124 | 0.428 | Unannotated | NA | NA |
| metab_8142 | Unannotated feature | pos | 193.1320 | 2.724 | 3.956 | 3.549 | -0.407 | -0.276 | 0.124 | 0.428 | Unannotated | NA | NA |
| metab_1411 | Cinaciguat | pos | 629.2967 | 6.064 | 5.796 | 5.569 | -0.227 | -0.276 | 0.124 | 0.428 | Unannotated | NA | NA |
| metab_4568 | Stearamide | pos | 325.3217 | 6.893 | 4.652 | 4.549 | -0.103 | -0.276 | 0.124 | 0.428 | Strong putative | NA | NA |
| metab_7010 | Unannotated feature | pos | 540.3324 | 4.820 | 3.811 | 4.916 | 1.105 | 0.276 | 0.124 | 0.428 | Unannotated | NA | NA |
| metab_18549 | Rutacultin | neg | 319.1212 | 6.022 | 4.559 | 4.865 | 0.306 | 0.276 | 0.124 | 0.428 | Unannotated | NA | NA |
| metab_10016 | Unannotated feature | neg | 146.0816 | 2.826 | 4.998 | 4.898 | -0.100 | -0.276 | 0.124 | 0.428 | Unannotated | NA | NA |
| metab_16282 | Unannotated feature | neg | 297.2436 | 6.592 | 6.938 | 7.029 | 0.091 | 0.276 | 0.124 | 0.428 | Unannotated | NA | NA |
| metab_10055 | Unannotated feature | neg | 437.1690 | 2.785 | 4.310 | 3.902 | -0.408 | -0.276 | 0.124 | 0.428 | Unannotated | NA | NA |
| metab_17155 | Unannotated feature | neg | 102.9480 | 7.773 | 5.043 | 5.021 | -0.022 | -0.276 | 0.124 | 0.428 | Unannotated | NA | NA |
| metab_1208 | Unannotated feature | pos | 303.2323 | 6.430 | 5.354 | 4.708 | -0.645 | -0.276 | 0.124 | 0.428 | Unannotated | NA | NA |
| metab_16773 | Unannotated feature | neg | 472.9173 | 7.692 | 4.308 | 4.394 | 0.086 | 0.276 | 0.124 | 0.428 | Unannotated | NA | NA |
| metab_5889 | Unannotated feature | pos | 671.5057 | 5.929 | 3.557 | 3.983 | 0.426 | 0.276 | 0.124 | 0.428 | Unannotated | NA | NA |
| metab_16230 | Emodin | neg | 269.0456 | 6.483 | 3.522 | 4.555 | 1.033 | 0.276 | 0.124 | 0.428 | Strong putative | NA | NA |
| metab_7058 | CL(8:0/11:0/12:0/a-15:0) | pos | 562.3455 | 4.729 | 4.172 | 5.076 | 0.904 | 0.276 | 0.124 | 0.428 | Unannotated | Lipids and lipid-like molecules | Glycerophospholipids |
| metab_2312 | Unannotated feature | pos | 684.2049 | 0.082 | 6.112 | 6.122 | 0.010 | 0.276 | 0.124 | 0.428 | Unannotated | NA | NA |
| metab_17573 | LysoPI(0:0/18:0) | neg | 599.3218 | 7.305 | 5.685 | 5.313 | -0.372 | -0.276 | 0.124 | 0.428 | Unannotated | Lipids and lipid-like molecules | Glycerophospholipids |
| metab_11592 | Unannotated feature | neg | 140.9174 | 0.453 | 3.581 | 3.527 | -0.054 | -0.276 | 0.124 | 0.428 | Unannotated | NA | NA |
| metab_19591 | 8-Hydroxy-5,6-octadienoic acid | neg | 201.0764 | 3.566 | 5.373 | 5.599 | 0.227 | 0.276 | 0.124 | 0.428 | Unannotated | NA | NA |
| metab_6729 | Unannotated feature | pos | 266.1391 | 5.267 | 3.705 | 3.467 | -0.238 | -0.276 | 0.124 | 0.428 | Unannotated | NA | NA |
| metab_607 | Unannotated feature | pos | 719.2178 | 5.787 | 3.667 | 4.275 | 0.608 | 0.276 | 0.124 | 0.428 | Unannotated | NA | NA |
| metab_14918 | 3h-Indole-3-propanoic acid,a-amino- | neg | 239.0593 | 4.150 | 4.954 | 5.204 | 0.250 | 0.276 | 0.124 | 0.428 | Unannotated | NA | NA |
| metab_8605 | Unannotated feature | pos | 316.1508 | 1.118 | 3.703 | 4.333 | 0.629 | 0.276 | 0.124 | 0.428 | Unannotated | NA | NA |
| metab_3646 | Unannotated feature | pos | 551.3383 | 5.169 | 1.697 | 4.640 | 2.943 | 0.276 | 0.124 | 0.428 | Unannotated | NA | NA |
| metab_9597 | Unannotated feature | neg | 439.1254 | 4.180 | 4.199 | 4.033 | -0.166 | -0.276 | 0.124 | 0.428 | Unannotated | NA | NA |
| metab_11573 | Unannotated feature | neg | 234.9348 | 0.460 | 4.104 | 3.952 | -0.151 | -0.276 | 0.124 | 0.428 | Unannotated | NA | NA |
| metab_8309 | Glycyl-D-proline | pos | 155.0817 | 2.049 | 5.059 | 4.858 | -0.202 | -0.276 | 0.124 | 0.428 | Unannotated | NA | NA |
| metab_550 | Unannotated feature | pos | 436.1980 | 5.573 | 5.135 | 4.465 | -0.670 | -0.276 | 0.124 | 0.428 | Unannotated | NA | NA |
| metab_10395 | Unannotated feature | neg | 264.1203 | 2.058 | 4.507 | 4.202 | -0.305 | -0.276 | 0.124 | 0.428 | Unannotated | NA | NA |
| metab_580 | Unannotated feature | pos | 333.1452 | 5.749 | 5.154 | 4.443 | -0.711 | -0.276 | 0.124 | 0.428 | Unannotated | NA | NA |
| metab_15341 | Unannotated feature | neg | 232.0590 | 4.966 | 6.682 | 6.942 | 0.260 | 0.276 | 0.124 | 0.428 | Unannotated | NA | NA |
| metab_13868 | Unannotated feature | neg | 323.9666 | 2.298 | 5.337 | 5.157 | -0.181 | -0.276 | 0.124 | 0.428 | Unannotated | NA | NA |
| metab_17455 | Unannotated feature | neg | 860.8503 | 7.607 | 7.054 | 7.113 | 0.058 | 0.276 | 0.124 | 0.428 | Unannotated | NA | NA |
| metab_4456 | Unannotated feature | pos | 476.3082 | 3.775 | 5.530 | 5.811 | 0.281 | 0.276 | 0.124 | 0.428 | Unannotated | NA | NA |
| metab_2916 | 5'-Deoxyadenosine | pos | 274.0927 | 2.884 | 1.542 | 3.725 | 2.183 | 0.276 | 0.124 | 0.428 | Tentative | Nucleosides, nucleotides, and analogues | 5''-deoxyribonucleosides |
| metab_14168 | Unannotated feature | neg | 83.9956 | 2.906 | 5.589 | 5.556 | -0.033 | -0.276 | 0.124 | 0.428 | Unannotated | NA | NA |
| metab_14172 | 7-Amino-4-(trifluoromethyl)coumarin | neg | 274.0336 | 2.906 | 4.674 | 5.063 | 0.389 | 0.276 | 0.124 | 0.428 | Unannotated | NA | NA |
| metab_12639 | Unannotated feature | neg | 457.2002 | 5.975 | 5.051 | 5.288 | 0.237 | 0.276 | 0.124 | 0.428 | Unannotated | NA | NA |
| metab_16588 | Unannotated feature | neg | 225.0352 | 7.358 | 3.269 | 3.616 | 0.347 | 0.276 | 0.124 | 0.428 | Unannotated | NA | NA |
| metab_17220 | Unannotated feature | neg | 303.1091 | 7.730 | 4.315 | 4.559 | 0.244 | 0.276 | 0.124 | 0.428 | Unannotated | NA | NA |
| metab_3023 | Unannotated feature | pos | 265.1551 | 3.200 | 5.270 | 4.908 | -0.362 | -0.276 | 0.124 | 0.428 | Unannotated | NA | NA |
| metab_9072 | Unannotated feature | neg | 311.1247 | 3.967 | 3.076 | 4.317 | 1.240 | 0.276 | 0.124 | 0.428 | Unannotated | NA | NA |
| metab_11936 | Unannotated feature | neg | 413.2012 | 6.123 | 6.357 | 6.573 | 0.216 | 0.272 | 0.129 | 0.438 | Unannotated | NA | NA |
| metab_19700 | Unannotated feature | neg | 276.0336 | 4.563 | 4.481 | 4.685 | 0.204 | 0.272 | 0.129 | 0.438 | Unannotated | NA | NA |
| metab_12677 | Unannotated feature | neg | 461.1861 | 5.748 | 5.599 | 5.809 | 0.210 | 0.272 | 0.129 | 0.438 | Unannotated | NA | NA |
| metab_18919 | Alpha-Bisabolol oxide B | neg | 275.1404 | 5.632 | 5.548 | 5.491 | -0.056 | -0.272 | 0.129 | 0.438 | Tentative | Organoheterocyclic compounds | Tetrahydrofurans |
| metab_7015 | 3-Pyridinecarboximidamide, N-(2-hydroxy-3-(1-piperidinyl)propoxy)- | pos | 261.1714 | 4.810 | 2.617 | 1.801 | -0.816 | -0.272 | 0.129 | 0.438 | Unannotated | Organoheterocyclic compounds | Pyridines and derivatives |
| metab_5830 | Unannotated feature | pos | 152.9952 | 0.016 | 4.343 | 4.389 | 0.046 | 0.272 | 0.129 | 0.438 | Unannotated | NA | NA |
| metab_2413 | Unannotated feature | pos | 264.8449 | 0.601 | 4.928 | 4.521 | -0.406 | -0.272 | 0.129 | 0.438 | Unannotated | NA | NA |
| metab_10261 | Unannotated feature | neg | 287.1866 | 4.542 | 5.811 | 5.717 | -0.094 | -0.272 | 0.129 | 0.438 | Unannotated | NA | NA |
| metab_4360 | Xi-2,3-Dihydro-2-oxo-1H-indole-3-acetic acid | pos | 224.0919 | 6.167 | 3.163 | 3.421 | 0.258 | 0.272 | 0.129 | 0.438 | Unannotated | Organoheterocyclic compounds | Indoles and derivatives |
| metab_4322 | Dehydrozingerone | pos | 210.1128 | 6.080 | 4.810 | 4.297 | -0.514 | -0.272 | 0.129 | 0.438 | Tentative | Phenylpropanoids and polyketides | Cinnamic acids and derivatives |
| metab_4321 | Unannotated feature | pos | 144.1385 | 6.072 | 4.373 | 4.323 | -0.050 | -0.272 | 0.129 | 0.438 | Unannotated | NA | NA |
| metab_7510 | Gamma-Glutamyl-S-methylcysteine | pos | 229.0646 | 3.729 | 4.284 | 4.046 | -0.238 | -0.272 | 0.129 | 0.438 | Unannotated | NA | NA |
| metab_13186 | Corticrocin | neg | 245.0819 | 4.749 | 1.745 | 2.934 | 1.189 | 0.272 | 0.129 | 0.438 | Unannotated | Lipids and lipid-like molecules | Fatty Acyls |
| metab_1672 | Unannotated feature | pos | 476.3082 | 3.943 | 6.158 | 6.204 | 0.046 | 0.272 | 0.129 | 0.438 | Unannotated | NA | NA |
| metab_4410 | 1,2-Cyclohexanediol, 1-methyl-4-(1-methylethenyl)- | pos | 358.2950 | 6.311 | 5.265 | 5.084 | -0.181 | -0.272 | 0.129 | 0.438 | Tentative | NA | NA |
| metab_16335 | Unannotated feature | neg | 483.2962 | 6.679 | 4.338 | 3.748 | -0.590 | -0.272 | 0.129 | 0.438 | Unannotated | NA | NA |
| metab_13131 | Unannotated feature | neg | 137.9443 | 0.453 | 3.824 | 3.767 | -0.057 | -0.272 | 0.129 | 0.438 | Unannotated | NA | NA |
| metab_1575 | Unannotated feature | pos | 270.1704 | 5.219 | 4.582 | 4.019 | -0.563 | -0.272 | 0.129 | 0.438 | Unannotated | NA | NA |
| metab_1488 | Unannotated feature | pos | 201.1601 | 3.319 | 5.768 | 5.396 | -0.372 | -0.272 | 0.129 | 0.438 | Unannotated | NA | NA |
| metab_10417 | 1-Methylpseudouridine | neg | 239.0673 | 2.012 | 5.017 | 5.174 | 0.157 | 0.272 | 0.129 | 0.438 | Moderate putative | NA | NA |
| metab_4528 | 11-Aminoundecanoic Acid | pos | 184.1699 | 6.741 | 5.221 | 5.118 | -0.103 | -0.272 | 0.129 | 0.438 | Moderate putative | NA | NA |
| metab_14717 | Unannotated feature | neg | 169.0765 | 3.728 | 3.551 | 2.975 | -0.576 | -0.272 | 0.129 | 0.438 | Unannotated | NA | NA |
| metab_16912 | Unannotated feature | neg | 130.9785 | 7.773 | 4.503 | 4.485 | -0.018 | -0.272 | 0.129 | 0.438 | Unannotated | NA | NA |
| metab_8695 | 5-Aminopentanoic acid | pos | 118.0868 | 0.784 | 7.454 | 7.416 | -0.038 | -0.272 | 0.129 | 0.438 | Strong putative | NA | NA |
| metab_5549 | 1-Phenylethanol | pos | 105.0704 | 6.287 | 5.496 | 5.459 | -0.037 | -0.272 | 0.129 | 0.438 | Tentative | NA | NA |
| metab_15191 | Unannotated feature | neg | 239.0559 | 4.714 | 4.639 | 4.878 | 0.239 | 0.272 | 0.129 | 0.438 | Unannotated | NA | NA |
| metab_18184 | Unannotated feature | neg | 173.1176 | 6.318 | 4.913 | 4.690 | -0.223 | -0.272 | 0.129 | 0.438 | Unannotated | NA | NA |
| metab_2489 | Shanzhiside | pos | 434.1669 | 0.736 | 3.636 | 4.590 | 0.954 | 0.272 | 0.129 | 0.438 | Tentative | Lipids and lipid-like molecules | Prenol lipids |
| metab_13831 | Unannotated feature | neg | 328.8737 | 0.443 | 3.274 | 3.417 | 0.143 | 0.272 | 0.129 | 0.438 | Unannotated | NA | NA |
| metab_5506 | 11-(5-Butyl-3,4-dimethylfuran-2-yl)undecanoylcarnitine | pos | 444.3490 | 6.335 | 3.870 | 3.619 | -0.251 | -0.272 | 0.129 | 0.438 | Unannotated | NA | NA |
| metab_9578 | Unannotated feature | neg | 344.2195 | 3.242 | 4.912 | 4.589 | -0.323 | -0.272 | 0.129 | 0.438 | Unannotated | NA | NA |
| metab_4632 | Unannotated feature | pos | 520.3346 | 3.920 | 5.478 | 5.958 | 0.480 | 0.272 | 0.129 | 0.438 | Unannotated | NA | NA |
| metab_8206 | Unannotated feature | pos | 97.0622 | 2.495 | 3.737 | 3.950 | 0.214 | 0.272 | 0.129 | 0.438 | Unannotated | NA | NA |
| metab_8192 | Unannotated feature | pos | 102.9708 | 2.528 | 3.485 | 3.547 | 0.063 | 0.272 | 0.129 | 0.438 | Unannotated | NA | NA |
| metab_4620 | Unannotated feature | pos | 311.1831 | 7.124 | 4.938 | 4.887 | -0.051 | -0.272 | 0.129 | 0.438 | Unannotated | NA | NA |
| metab_612 | Unannotated feature | pos | 494.2958 | 5.794 | 4.649 | 5.000 | 0.351 | 0.272 | 0.129 | 0.438 | Unannotated | NA | NA |
| metab_17338 | Unannotated feature | neg | 101.9405 | 7.697 | 4.642 | 4.593 | -0.049 | -0.272 | 0.129 | 0.438 | Unannotated | NA | NA |
| metab_17404 | Unannotated feature | neg | 809.3446 | 7.648 | 5.525 | 5.546 | 0.021 | 0.272 | 0.129 | 0.438 | Unannotated | NA | NA |
| metab_17401 | Unannotated feature | neg | 918.8096 | 7.648 | 4.985 | 5.266 | 0.281 | 0.272 | 0.129 | 0.438 | Unannotated | NA | NA |
| metab_2659 | Unannotated feature | pos | 180.1386 | 1.952 | 4.921 | 4.411 | -0.509 | -0.272 | 0.129 | 0.438 | Unannotated | NA | NA |
| metab_12952 | Unannotated feature | neg | 253.0025 | 0.730 | 4.948 | 4.555 | -0.393 | -0.272 | 0.129 | 0.438 | Unannotated | NA | NA |
| metab_1268 | Unannotated feature | pos | 313.2378 | 6.319 | 5.209 | 5.035 | -0.174 | -0.272 | 0.129 | 0.438 | Unannotated | NA | NA |
| metab_2048 | Unannotated feature | pos | 581.1638 | 2.676 | 4.660 | 3.112 | -1.548 | -0.272 | 0.129 | 0.438 | Unannotated | NA | NA |
| metab_13427 | Unannotated feature | neg | 217.0714 | 0.799 | 4.195 | 4.714 | 0.520 | 0.272 | 0.129 | 0.438 | Unannotated | NA | NA |
| metab_19383 | Sinapine | neg | 347.1144 | 5.029 | 4.299 | 4.662 | 0.363 | 0.272 | 0.129 | 0.438 | Tentative | NA | NA |
| metab_4701 | Unannotated feature | pos | 855.8301 | 7.586 | 5.567 | 5.655 | 0.088 | 0.272 | 0.129 | 0.438 | Unannotated | NA | NA |
| metab_19259 | Unannotated feature | neg | 303.1717 | 5.188 | 2.650 | 3.321 | 0.671 | 0.272 | 0.129 | 0.438 | Unannotated | NA | NA |
| metab_5249 | Glycochenodeoxycholic Acid | pos | 432.3150 | 6.662 | 4.110 | 4.488 | 0.379 | 0.272 | 0.129 | 0.438 | Moderate putative | NA | NA |
| metab_18529 | 2-Butenedioic acid (2E)-, 1,4-diethyl ester | neg | 389.1478 | 3.074 | 4.593 | 4.308 | -0.285 | -0.272 | 0.129 | 0.438 | Unannotated | NA | NA |
| metab_6452 | Unannotated feature | pos | 552.0918 | 5.650 | 4.177 | 4.328 | 0.150 | 0.272 | 0.129 | 0.438 | Unannotated | NA | NA |
| metab_5901 | Unannotated feature | pos | 201.0605 | 0.372 | 5.663 | 5.681 | 0.017 | 0.272 | 0.129 | 0.438 | Unannotated | NA | NA |
| metab_12468 | Unannotated feature | neg | 127.8695 | 7.910 | 5.251 | 5.307 | 0.056 | 0.272 | 0.129 | 0.438 | Unannotated | NA | NA |
| metab_16430 | Unannotated feature | neg | 229.1444 | 6.922 | 3.920 | 3.633 | -0.286 | -0.272 | 0.129 | 0.438 | Unannotated | NA | NA |
| metab_3249 | Statine | pos | 158.1178 | 3.859 | 4.579 | 4.964 | 0.385 | 0.272 | 0.129 | 0.438 | Unannotated | NA | NA |
| metab_2887 | Urea glutamate | pos | 245.0884 | 2.827 | 3.878 | 2.635 | -1.242 | -0.272 | 0.129 | 0.438 | Unannotated | Organic acids and derivatives | Carboxylic acids and derivatives |
| metab_12269 | DG(15:0/18:3(9Z,12Z,15Z)/0:0) | neg | 575.4692 | 7.057 | 6.981 | 6.836 | -0.145 | -0.272 | 0.129 | 0.438 | Tentative | NA | NA |
| metab_8470 | Prostaglandin E2 | pos | 375.2139 | 1.702 | 5.174 | 4.942 | -0.232 | -0.272 | 0.129 | 0.438 | Tentative | NA | NA |
| metab_7385 | Unannotated feature | pos | 100.0762 | 0.712 | 5.674 | 5.230 | -0.445 | -0.272 | 0.129 | 0.438 | Unannotated | NA | NA |
| metab_5445 | MG(0:0/20:3(11Z,14Z,17Z)/0:0) | pos | 783.5786 | 6.390 | 6.242 | 5.931 | -0.311 | -0.272 | 0.129 | 0.438 | Unannotated | Lipids and lipid-like molecules | Endocannabinoids |
| metab_2122 | Unannotated feature | pos | 247.1810 | 2.732 | 2.780 | 2.991 | 0.210 | 0.272 | 0.129 | 0.438 | Unannotated | NA | NA |
| metab_17617 | Unannotated feature | neg | 643.5287 | 7.171 | 5.634 | 5.417 | -0.217 | -0.272 | 0.129 | 0.438 | Unannotated | NA | NA |
| metab_1146 | Unannotated feature | pos | 397.3107 | 6.566 | 5.676 | 5.484 | -0.192 | -0.272 | 0.129 | 0.438 | Unannotated | NA | NA |
| metab_8008 | Unannotated feature | pos | 433.3421 | 6.550 | 6.250 | 6.124 | -0.126 | -0.272 | 0.129 | 0.438 | Unannotated | NA | NA |
| metab_14829 | Unannotated feature | neg | 540.9042 | 0.507 | 5.199 | 5.073 | -0.126 | -0.272 | 0.129 | 0.438 | Unannotated | NA | NA |
| metab_15787 | Scytalone | neg | 193.0501 | 5.834 | 4.592 | 4.429 | -0.164 | -0.272 | 0.129 | 0.438 | Strong putative | NA | NA |
| metab_16477 | Unannotated feature | neg | 430.3335 | 7.057 | 4.286 | 3.701 | -0.586 | -0.272 | 0.129 | 0.438 | Unannotated | NA | NA |
| metab_7966 | Unannotated feature | pos | 240.1234 | 2.880 | 3.699 | 3.913 | 0.214 | 0.272 | 0.129 | 0.438 | Unannotated | NA | NA |
| metab_5150 | Unannotated feature | pos | 627.5339 | 6.869 | 6.902 | 7.031 | 0.129 | 0.272 | 0.129 | 0.438 | Unannotated | NA | NA |
| metab_5142 | Unannotated feature | pos | 316.1402 | 0.728 | 4.678 | 4.879 | 0.201 | 0.272 | 0.129 | 0.438 | Unannotated | NA | NA |
| metab_5876 | Unannotated feature | pos | 637.1896 | 5.936 | 3.549 | 4.000 | 0.451 | 0.272 | 0.129 | 0.438 | Unannotated | NA | NA |
| metab_7921 | Unannotated feature | pos | 99.5316 | 2.941 | 3.309 | 3.376 | 0.067 | 0.272 | 0.129 | 0.438 | Unannotated | NA | NA |
| metab_4931 | Unannotated feature | pos | 1016.8033 | 7.626 | 4.387 | 4.663 | 0.276 | 0.272 | 0.129 | 0.438 | Unannotated | NA | NA |
| metab_13678 | Nitrendipine | neg | 381.1047 | 1.685 | 4.766 | 4.448 | -0.318 | -0.272 | 0.129 | 0.438 | Tentative | NA | NA |
| metab_410 | Unannotated feature | pos | 193.0525 | 0.016 | 4.205 | 4.245 | 0.040 | 0.272 | 0.129 | 0.438 | Unannotated | NA | NA |
| metab_2223 | Unannotated feature | pos | 318.2030 | 3.052 | 4.562 | 4.138 | -0.424 | -0.272 | 0.129 | 0.438 | Unannotated | NA | NA |
| metab_6955 | Unannotated feature | pos | 107.9673 | 4.938 | 4.026 | 3.734 | -0.291 | -0.272 | 0.129 | 0.438 | Unannotated | NA | NA |
| metab_8523 | Unannotated feature | pos | 204.0635 | 1.506 | 5.288 | 5.152 | -0.136 | -0.272 | 0.129 | 0.438 | Unannotated | NA | NA |
| metab_12674 | Glucosamine, N-carbamoyl-(6CI) | neg | 221.0793 | 5.774 | 3.438 | 4.090 | 0.651 | 0.272 | 0.129 | 0.438 | Unannotated | NA | NA |
| metab_16658 | Unannotated feature | neg | 733.3695 | 7.600 | 6.410 | 6.463 | 0.053 | 0.272 | 0.129 | 0.438 | Unannotated | NA | NA |
| metab_5721 | Penicillin F | pos | 345.1451 | 6.087 | 4.712 | 4.542 | -0.170 | -0.272 | 0.129 | 0.438 | Tentative | NA | NA |
| metab_17238 | Unannotated feature | neg | 91.9202 | 7.730 | 4.078 | 4.023 | -0.055 | -0.272 | 0.129 | 0.438 | Unannotated | NA | NA |
| metab_28 | Unannotated feature | pos | 144.1022 | 0.712 | 5.843 | 5.651 | -0.193 | -0.267 | 0.136 | 0.445 | Unannotated | NA | NA |
| metab_14098 | Unannotated feature | neg | 460.8800 | 0.507 | 4.324 | 4.008 | -0.316 | -0.267 | 0.136 | 0.445 | Unannotated | NA | NA |
| metab_17256 | Unannotated feature | neg | 288.1220 | 7.726 | 4.546 | 4.866 | 0.321 | 0.267 | 0.136 | 0.445 | Unannotated | NA | NA |
| metab_12178 | Unannotated feature | neg | 257.2122 | 6.726 | 6.141 | 6.100 | -0.041 | -0.267 | 0.136 | 0.445 | Unannotated | NA | NA |
| metab_12171 | Unannotated feature | neg | 295.1300 | 3.418 | 5.555 | 5.110 | -0.445 | -0.267 | 0.136 | 0.445 | Unannotated | NA | NA |
| metab_11846 | Unannotated feature | neg | 475.2103 | 5.952 | 4.212 | 4.489 | 0.278 | 0.267 | 0.136 | 0.445 | Unannotated | NA | NA |
| metab_11866 | Unannotated feature | neg | 269.1395 | 6.003 | 5.696 | 5.838 | 0.142 | 0.267 | 0.136 | 0.445 | Unannotated | NA | NA |
| metab_16626 | Unannotated feature | neg | 686.2584 | 7.503 | 4.043 | 4.433 | 0.390 | 0.267 | 0.136 | 0.445 | Unannotated | NA | NA |
| metab_12680 | Unannotated feature | neg | 341.1036 | 5.675 | 5.008 | 4.869 | -0.138 | -0.267 | 0.136 | 0.445 | Unannotated | NA | NA |
| metab_11223 | 3-Mercapto-3-methylbutyl formate | neg | 147.0485 | 0.644 | 5.933 | 5.807 | -0.126 | -0.267 | 0.136 | 0.445 | Unannotated | NA | NA |
| metab_18981 | Dihydro-O-methylsterigmatocystin | neg | 385.0933 | 5.550 | 4.855 | 4.040 | -0.815 | -0.267 | 0.136 | 0.445 | Tentative | NA | NA |
| metab_19016 | Unannotated feature | neg | 217.1442 | 5.512 | 4.084 | 3.919 | -0.166 | -0.267 | 0.136 | 0.445 | Unannotated | NA | NA |
| metab_19877 | L-Glutamate | neg | 146.0452 | 0.644 | 7.155 | 7.044 | -0.110 | -0.267 | 0.136 | 0.445 | Strong putative | NA | NA |
| metab_2303 | Unannotated feature | pos | 116.5310 | 0.023 | 3.515 | 3.596 | 0.081 | 0.267 | 0.136 | 0.445 | Unannotated | NA | NA |
| metab_2272 | Unannotated feature | pos | 314.1755 | 3.200 | 4.435 | 4.627 | 0.192 | 0.267 | 0.136 | 0.445 | Unannotated | NA | NA |
| metab_430 | Unannotated feature | pos | 114.9716 | 0.016 | 5.432 | 5.467 | 0.035 | 0.267 | 0.136 | 0.445 | Unannotated | NA | NA |
| metab_3873 | Unannotated feature | pos | 496.0707 | 5.717 | 4.059 | 4.119 | 0.059 | 0.267 | 0.136 | 0.445 | Unannotated | NA | NA |
| metab_3862 | Unannotated feature | pos | 596.1185 | 5.693 | 3.785 | 3.817 | 0.032 | 0.267 | 0.136 | 0.445 | Unannotated | NA | NA |
| metab_3850 | Unannotated feature | pos | 335.6498 | 5.677 | 5.425 | 4.327 | -1.098 | -0.267 | 0.136 | 0.445 | Unannotated | NA | NA |
| metab_5458 | Unannotated feature | pos | 259.1293 | 1.952 | 5.693 | 5.622 | -0.071 | -0.267 | 0.136 | 0.445 | Unannotated | NA | NA |
| metab_17029 | Unannotated feature | neg | 160.9121 | 7.997 | 4.413 | 4.383 | -0.030 | -0.267 | 0.136 | 0.445 | Unannotated | NA | NA |
| metab_12374 | Unannotated feature | neg | 389.2472 | 6.750 | 5.075 | 5.726 | 0.651 | 0.267 | 0.136 | 0.445 | Unannotated | NA | NA |
| metab_7318 | Asp Val Phe | pos | 380.1827 | 4.143 | 4.775 | 4.297 | -0.478 | -0.267 | 0.136 | 0.445 | Moderate putative | NA | NA |
| metab_9648 | P-Toluenesulfonic Acid | neg | 171.0115 | 3.192 | 4.967 | 4.949 | -0.019 | -0.267 | 0.136 | 0.445 | Moderate putative | NA | NA |
| metab_17853 | Unannotated feature | neg | 441.3010 | 6.734 | 5.221 | 5.436 | 0.216 | 0.267 | 0.136 | 0.445 | Unannotated | NA | NA |
| metab_4084 | Unannotated feature | pos | 615.4672 | 5.841 | 3.477 | 4.336 | 0.859 | 0.267 | 0.136 | 0.445 | Unannotated | NA | NA |
| metab_9985 | Unannotated feature | neg | 486.2581 | 3.941 | 5.121 | 4.865 | -0.256 | -0.267 | 0.136 | 0.445 | Unannotated | NA | NA |
| metab_13447 | N-acetylaspartate | neg | 174.0402 | 0.854 | 6.407 | 6.125 | -0.283 | -0.267 | 0.136 | 0.445 | Moderate putative | NA | NA |
| metab_6988 | Unannotated feature | pos | 642.3979 | 4.870 | 4.484 | 5.075 | 0.591 | 0.267 | 0.136 | 0.445 | Unannotated | NA | NA |
| metab_5390 | 13-Hydroxyoctadecanoylcarnitine | pos | 426.3587 | 6.454 | 6.085 | 5.594 | -0.491 | -0.267 | 0.136 | 0.445 | Unannotated | NA | NA |
| metab_10108 | Unannotated feature | neg | 269.0821 | 4.473 | 4.824 | 4.714 | -0.110 | -0.267 | 0.136 | 0.445 | Unannotated | NA | NA |
| metab_1943 | 2-Hydroxynicotinic acid | pos | 140.0344 | 1.952 | 4.819 | 4.945 | 0.126 | 0.267 | 0.136 | 0.445 | Unannotated | NA | NA |
| metab_1935 | Unannotated feature | pos | 353.1714 | 4.984 | 5.240 | 4.926 | -0.313 | -0.267 | 0.136 | 0.445 | Unannotated | NA | NA |
| metab_4167 | Unannotated feature | pos | 576.3529 | 5.906 | 3.621 | 4.132 | 0.511 | 0.267 | 0.136 | 0.445 | Unannotated | NA | NA |
| metab_18092 | Acetoxolone | neg | 493.3366 | 6.397 | 4.654 | 4.279 | -0.375 | -0.267 | 0.136 | 0.445 | Tentative | NA | NA |
| metab_18076 | 1-(8Z,11Z,14Z-eicosatrienoyl)-glycero-3-phosphate | neg | 505.2587 | 6.412 | 4.925 | 4.916 | -0.009 | 0.267 | 0.136 | 0.445 | Unannotated | NA | NA |
| metab_1877 | Unannotated feature | pos | 142.0866 | 1.239 | 4.818 | 4.975 | 0.157 | 0.267 | 0.136 | 0.445 | Unannotated | NA | NA |
| metab_15192 | Eugenyl glucoside | neg | 347.1144 | 4.714 | 4.347 | 4.789 | 0.442 | 0.267 | 0.136 | 0.445 | Unannotated | Organic oxygen compounds | Organooxygen compounds |
| metab_5381 | Unannotated feature | pos | 359.2412 | 1.952 | 5.011 | 4.880 | -0.131 | -0.267 | 0.136 | 0.445 | Unannotated | NA | NA |
| metab_13336 | Unannotated feature | neg | 227.1147 | 0.698 | 4.554 | 4.351 | -0.202 | -0.267 | 0.136 | 0.445 | Unannotated | NA | NA |
| metab_1846 | Unannotated feature | pos | 124.0398 | 1.049 | 6.611 | 6.562 | -0.049 | -0.267 | 0.136 | 0.445 | Unannotated | NA | NA |
| metab_5517 | 6',7'-Dihydroxybergamottin | pos | 390.1883 | 2.740 | 5.343 | 5.252 | -0.091 | -0.267 | 0.136 | 0.445 | Tentative | NA | NA |
| metab_8616 | 2-Amino-4-oxo-6-(1',2',3'-trihydroxypropyl)-diquinoid-7,8-dihydroxypterin | pos | 254.0888 | 1.065 | 3.227 | 3.556 | 0.329 | 0.267 | 0.136 | 0.445 | Tentative | NA | NA |
| metab_13283 | DL-2-hydroxy stearic acid | neg | 299.2592 | 6.695 | 7.751 | 8.044 | 0.293 | 0.267 | 0.136 | 0.445 | Moderate putative | NA | NA |
| metab_15168 | Unannotated feature | neg | 332.1219 | 4.663 | 4.347 | 4.744 | 0.397 | 0.267 | 0.136 | 0.445 | Unannotated | NA | NA |
| metab_16981 | Unannotated feature | neg | 164.9047 | 7.961 | 3.237 | 3.357 | 0.120 | 0.267 | 0.136 | 0.445 | Unannotated | NA | NA |
| metab_18249 | Unannotated feature | neg | 321.0731 | 2.975 | 4.036 | 4.460 | 0.424 | 0.267 | 0.136 | 0.445 | Unannotated | NA | NA |
| metab_4429 | Palmitoleic acid | pos | 272.2588 | 6.351 | 5.035 | 4.864 | -0.171 | -0.267 | 0.136 | 0.445 | Tentative | NA | NA |
| metab_13161 | Unannotated feature | neg | 358.9447 | 0.485 | 4.784 | 4.299 | -0.485 | -0.267 | 0.136 | 0.445 | Unannotated | NA | NA |
| metab_13160 | Unannotated feature | neg | 305.1510 | 5.110 | 6.396 | 6.246 | -0.150 | -0.267 | 0.136 | 0.445 | Unannotated | NA | NA |
| metab_15924 | Unannotated feature | neg | 621.2686 | 6.022 | 3.346 | 2.833 | -0.513 | -0.267 | 0.136 | 0.445 | Unannotated | NA | NA |
| metab_8280 | Unannotated feature | pos | 135.0331 | 2.142 | 4.218 | 4.170 | -0.048 | -0.267 | 0.136 | 0.445 | Unannotated | NA | NA |
| metab_16941 | Unannotated feature | neg | 180.8124 | 7.830 | 4.525 | 4.479 | -0.045 | -0.267 | 0.136 | 0.445 | Unannotated | NA | NA |
| metab_10464 | Unannotated feature | neg | 201.0400 | 1.958 | 4.883 | 5.096 | 0.214 | 0.267 | 0.136 | 0.445 | Unannotated | NA | NA |
| metab_2646 | Unannotated feature | pos | 238.1552 | 1.936 | 3.305 | 2.910 | -0.395 | -0.267 | 0.136 | 0.445 | Unannotated | NA | NA |
| metab_17433 | Unannotated feature | neg | 113.0067 | 7.621 | 4.999 | 4.963 | -0.036 | -0.267 | 0.136 | 0.445 | Unannotated | NA | NA |
| metab_17431 | Unannotated feature | neg | 630.8866 | 7.621 | 6.470 | 6.524 | 0.054 | 0.267 | 0.136 | 0.445 | Unannotated | NA | NA |
| metab_3493 | Ethyl 3-aminobenzoate | pos | 198.1129 | 4.778 | 4.167 | 3.823 | -0.344 | -0.267 | 0.136 | 0.445 | Tentative | Benzenoids | Benzene and substituted derivatives |
| metab_5566 | Unannotated feature | pos | 334.2958 | 6.263 | 4.412 | 3.954 | -0.457 | -0.267 | 0.136 | 0.445 | Unannotated | NA | NA |
| metab_3534 | Unannotated feature | pos | 531.3255 | 4.883 | 3.527 | 4.654 | 1.127 | 0.267 | 0.136 | 0.445 | Unannotated | NA | NA |
| metab_13091 | Unannotated feature | neg | 221.8827 | 0.036 | 6.334 | 6.310 | -0.024 | -0.267 | 0.136 | 0.445 | Unannotated | NA | NA |
| metab_11528 | Unannotated feature | neg | 373.9125 | 0.502 | 4.473 | 4.618 | 0.145 | 0.267 | 0.136 | 0.445 | Unannotated | NA | NA |
| metab_15085 | 3h-Adrenaline | neg | 230.1032 | 4.510 | 4.446 | 4.240 | -0.205 | -0.267 | 0.136 | 0.445 | Unannotated | Organic oxygen compounds | Organooxygen compounds |
| metab_17139 | Unannotated feature | neg | 174.0014 | 7.802 | 4.061 | 4.109 | 0.049 | 0.267 | 0.136 | 0.445 | Unannotated | NA | NA |
| metab_2780 | Prolyl-Threonine | pos | 199.1081 | 2.528 | 4.659 | 4.901 | 0.243 | 0.267 | 0.136 | 0.445 | Unannotated | NA | NA |
| metab_2740 | Unannotated feature | pos | 100.1126 | 2.276 | 2.549 | 3.095 | 0.546 | 0.267 | 0.136 | 0.445 | Unannotated | NA | NA |
| metab_19546 | 3,4-Methyleneazelaic acid | neg | 257.1032 | 3.537 | 4.758 | 5.096 | 0.338 | 0.267 | 0.136 | 0.445 | Unannotated | NA | NA |
| metab_5589 | Unannotated feature | pos | 123.1172 | 6.247 | 3.807 | 3.944 | 0.137 | 0.267 | 0.136 | 0.445 | Unannotated | NA | NA |
| metab_19530 | Unannotated feature | neg | 274.1299 | 4.807 | 2.399 | 3.557 | 1.158 | 0.267 | 0.136 | 0.445 | Unannotated | NA | NA |
| metab_8745 | N2-Succinyl-L-glutamic acid 5-semialdehyde | pos | 264.1083 | 0.736 | 4.765 | 5.010 | 0.245 | 0.267 | 0.136 | 0.445 | Tentative | NA | NA |
| metab_1347 | Unannotated feature | pos | 187.1445 | 2.941 | 6.054 | 5.961 | -0.093 | -0.267 | 0.136 | 0.445 | Unannotated | NA | NA |
| metab_4691 | Unannotated feature | pos | 311.9294 | 7.554 | 5.708 | 5.756 | 0.047 | 0.267 | 0.136 | 0.445 | Unannotated | NA | NA |
| metab_8135 | Unannotated feature | pos | 279.1693 | 2.724 | 3.353 | 3.897 | 0.544 | 0.267 | 0.136 | 0.445 | Unannotated | NA | NA |
| metab_11533 | Unannotated feature | neg | 621.8221 | 0.496 | 3.671 | 3.868 | 0.197 | 0.267 | 0.136 | 0.445 | Unannotated | NA | NA |
| metab_12954 | Unannotated feature | neg | 433.1187 | 6.240 | 4.266 | 4.936 | 0.669 | 0.267 | 0.136 | 0.445 | Unannotated | NA | NA |
| metab_4703 | Unannotated feature | pos | 991.8052 | 7.594 | 5.117 | 5.316 | 0.199 | 0.267 | 0.136 | 0.445 | Unannotated | NA | NA |
| metab_8099 | 1'-Acetoxyeugenol acetate | pos | 282.1343 | 2.740 | 3.281 | 3.990 | 0.709 | 0.267 | 0.136 | 0.445 | Tentative | Benzenoids | Phenol esters |
| metab_4719 | Unannotated feature | pos | 520.3347 | 4.094 | 5.934 | 6.399 | 0.465 | 0.267 | 0.136 | 0.445 | Unannotated | NA | NA |
| metab_12447 | Unannotated feature | neg | 258.9161 | 7.777 | 5.991 | 5.965 | -0.026 | -0.267 | 0.136 | 0.445 | Unannotated | NA | NA |
| metab_11472 | Unannotated feature | neg | 245.0629 | 0.524 | 4.769 | 4.253 | -0.516 | -0.267 | 0.136 | 0.445 | Unannotated | NA | NA |
| metab_1243 | Unannotated feature | pos | 237.1237 | 2.795 | 5.151 | 5.050 | -0.101 | -0.267 | 0.136 | 0.445 | Unannotated | NA | NA |
| metab_12849 | Unannotated feature | neg | 462.0934 | 0.683 | 2.490 | 3.900 | 1.410 | 0.267 | 0.136 | 0.445 | Unannotated | NA | NA |
| metab_5897 | Unannotated feature | pos | 594.8649 | 5.929 | 3.886 | 3.934 | 0.048 | 0.267 | 0.136 | 0.445 | Unannotated | NA | NA |
| metab_809 | Unannotated feature | pos | 429.2624 | 6.311 | 6.686 | 6.466 | -0.220 | -0.267 | 0.136 | 0.445 | Unannotated | NA | NA |
| metab_5213 | D-(+)-dihydrocarvone | pos | 170.1541 | 6.734 | 5.065 | 4.942 | -0.123 | -0.267 | 0.136 | 0.445 | Tentative | NA | NA |
| metab_10802 | Gamma-Glutamylthreonine | neg | 229.0827 | 1.096 | 5.409 | 5.263 | -0.146 | -0.267 | 0.136 | 0.445 | Unannotated | NA | NA |
| metab_8881 | Emtricitabine | neg | 292.0400 | 4.298 | 0.495 | 0.238 | -0.257 | -0.267 | 0.136 | 0.445 | Tentative | NA | NA |
| metab_5618 | Unannotated feature | pos | 286.2381 | 6.207 | 4.216 | 4.091 | -0.125 | -0.267 | 0.136 | 0.445 | Unannotated | NA | NA |
| metab_12261 | PS(16:0/18:0) | neg | 762.5364 | 7.019 | 6.007 | 5.786 | -0.222 | -0.267 | 0.136 | 0.445 | Tentative | NA | NA |
| metab_9245 | Unannotated feature | neg | 340.0548 | 3.697 | 3.244 | 3.539 | 0.295 | 0.267 | 0.136 | 0.445 | Unannotated | NA | NA |
| metab_3264 | 4-Hydroxy-alprenolol | pos | 548.3660 | 3.923 | 4.592 | 5.143 | 0.550 | 0.267 | 0.136 | 0.445 | Unannotated | Benzenoids | Phenols |
| metab_2838 | Unannotated feature | pos | 291.1346 | 2.747 | 3.609 | 4.323 | 0.714 | 0.267 | 0.136 | 0.445 | Unannotated | NA | NA |
| metab_14831 | Unannotated feature | neg | 311.1171 | 3.956 | 2.398 | 3.162 | 0.764 | 0.267 | 0.136 | 0.445 | Unannotated | NA | NA |
| metab_6479 | Unannotated feature | pos | 699.1087 | 5.607 | 2.470 | 4.220 | 1.750 | 0.267 | 0.136 | 0.445 | Unannotated | NA | NA |
| metab_7698 | Non-5-enedioylcarnitine | pos | 312.1818 | 3.371 | 2.176 | 3.557 | 1.382 | 0.267 | 0.136 | 0.445 | Unannotated | NA | NA |
| metab_18736 | Unannotated feature | neg | 542.2469 | 5.871 | 2.742 | 3.521 | 0.778 | 0.267 | 0.136 | 0.445 | Unannotated | NA | NA |
| metab_4881 | Unannotated feature | pos | 196.0408 | 7.716 | 4.049 | 4.128 | 0.079 | 0.267 | 0.136 | 0.445 | Unannotated | NA | NA |
| metab_5131 | 23S,25,26-Trihydroxyvitamin D3 | pos | 455.3129 | 6.909 | 5.015 | 4.886 | -0.129 | -0.267 | 0.136 | 0.445 | Unannotated | NA | NA |
| metab_864 | LysoPE(P-16:0/0:0) | pos | 438.2991 | 6.654 | 5.534 | 5.388 | -0.146 | -0.267 | 0.136 | 0.445 | Unannotated | NA | NA |
| metab_1027 | Geranylcitronellol | pos | 310.3108 | 7.005 | 7.159 | 7.068 | -0.090 | -0.267 | 0.136 | 0.445 | Unannotated | NA | NA |
| metab_997 | Unannotated feature | pos | 226.9518 | 7.610 | 7.388 | 7.415 | 0.027 | 0.267 | 0.136 | 0.445 | Unannotated | NA | NA |
| metab_4946 | Unannotated feature | pos | 420.8858 | 7.610 | 5.373 | 5.435 | 0.062 | 0.267 | 0.136 | 0.445 | Unannotated | NA | NA |
| metab_18839 | Unannotated feature | neg | 402.1290 | 5.739 | 5.050 | 4.872 | -0.178 | -0.267 | 0.136 | 0.445 | Unannotated | NA | NA |
| metab_16651 | Unannotated feature | neg | 634.8891 | 7.575 | 5.575 | 5.644 | 0.068 | 0.267 | 0.136 | 0.445 | Unannotated | NA | NA |
| metab_8950 | Unannotated feature | neg | 283.1299 | 4.183 | 3.577 | 3.928 | 0.352 | 0.267 | 0.136 | 0.445 | Unannotated | NA | NA |
| metab_14003 | Unannotated feature | neg | 563.1537 | 2.725 | 5.253 | 4.839 | -0.414 | -0.267 | 0.136 | 0.445 | Unannotated | NA | NA |
| metab_19633 | Unannotated feature | neg | 391.1993 | 4.660 | 4.547 | 3.758 | -0.789 | -0.267 | 0.136 | 0.445 | Unannotated | NA | NA |
| metab_16648 | Unannotated feature | neg | 566.9013 | 7.562 | 5.743 | 5.802 | 0.059 | 0.267 | 0.136 | 0.445 | Unannotated | NA | NA |
| metab_7018 | Unannotated feature | pos | 457.2893 | 4.802 | 3.694 | 4.915 | 1.221 | 0.267 | 0.136 | 0.445 | Unannotated | NA | NA |
| metab_19870 | Hexanal | neg | 145.0863 | 5.853 | 4.259 | 4.025 | -0.233 | -0.263 | 0.142 | 0.456 | Tentative | NA | NA |
| metab_11 | Unannotated feature | pos | 227.1393 | 0.720 | 4.439 | 4.721 | 0.282 | 0.263 | 0.142 | 0.456 | Unannotated | NA | NA |
| metab_15485 | Unannotated feature | neg | 500.2196 | 5.233 | 3.716 | 4.284 | 0.568 | 0.263 | 0.142 | 0.456 | Unannotated | NA | NA |
| metab_11635 | Unannotated feature | neg | 124.9833 | 0.075 | 5.007 | 4.997 | -0.010 | -0.263 | 0.142 | 0.456 | Unannotated | NA | NA |
| metab_18822 | Unannotated feature | neg | 364.1617 | 5.758 | 4.244 | 4.536 | 0.292 | 0.263 | 0.142 | 0.456 | Unannotated | NA | NA |
| metab_11092 | 2-Pyridone | neg | 94.0290 | 0.730 | 4.253 | 4.471 | 0.218 | 0.263 | 0.142 | 0.456 | Moderate putative | NA | NA |
| metab_14982 | Unannotated feature | neg | 426.8410 | 0.538 | 4.994 | 5.481 | 0.488 | 0.263 | 0.142 | 0.456 | Unannotated | NA | NA |
| metab_7924 | Unannotated feature | pos | 300.1923 | 2.937 | 4.512 | 4.322 | -0.190 | -0.263 | 0.142 | 0.456 | Unannotated | NA | NA |
| metab_18639 | Unannotated feature | neg | 238.1083 | 5.952 | 5.375 | 5.023 | -0.353 | -0.263 | 0.142 | 0.456 | Unannotated | NA | NA |
| metab_7882 | Phenylbutyrylglutamine | pos | 293.1502 | 3.347 | 4.751 | 4.879 | 0.127 | 0.263 | 0.142 | 0.456 | Unannotated | NA | NA |
| metab_4913 | Unannotated feature | pos | 463.8793 | 7.658 | 4.405 | 4.456 | 0.052 | 0.263 | 0.142 | 0.456 | Unannotated | NA | NA |
| metab_7858 | (+)-trans-Carveol glucoside | pos | 332.2080 | 3.040 | 4.807 | 4.566 | -0.241 | -0.263 | 0.142 | 0.456 | Unannotated | Lipids and lipid-like molecules | Prenol lipids |
| metab_1171 | Unannotated feature | pos | 285.1813 | 2.724 | 3.903 | 4.575 | 0.672 | 0.263 | 0.142 | 0.456 | Unannotated | NA | NA |
| metab_15767 | Unannotated feature | neg | 288.1276 | 5.797 | 3.739 | 3.243 | -0.495 | -0.263 | 0.142 | 0.456 | Unannotated | NA | NA |
| metab_1173 | Unannotated feature | pos | 338.3171 | 6.518 | 4.771 | 4.036 | -0.735 | -0.263 | 0.142 | 0.456 | Unannotated | NA | NA |
| metab_7200 | 2-Propylglutaric acid | pos | 216.1235 | 4.435 | 3.527 | 3.265 | -0.262 | -0.263 | 0.142 | 0.456 | Tentative | NA | NA |
| metab_1643 | Unannotated feature | pos | 560.3298 | 3.842 | 4.005 | 4.805 | 0.800 | 0.263 | 0.142 | 0.456 | Unannotated | NA | NA |
| metab_4737 | Glycodeoxycholic Acid | pos | 414.3014 | 7.681 | 4.163 | 3.824 | -0.339 | -0.263 | 0.142 | 0.456 | Tentative | NA | NA |
| metab_10453 | Unannotated feature | neg | 436.1471 | 1.958 | 2.815 | 3.560 | 0.745 | 0.263 | 0.142 | 0.456 | Unannotated | NA | NA |
| metab_13119 | Unannotated feature | neg | 202.8969 | 0.370 | 5.194 | 5.163 | -0.030 | -0.263 | 0.142 | 0.456 | Unannotated | NA | NA |
| metab_4611 | Unannotated feature | pos | 833.6291 | 7.092 | 2.925 | 4.288 | 1.364 | 0.263 | 0.142 | 0.456 | Unannotated | NA | NA |
| metab_14723 | Unannotated feature | neg | 486.2580 | 3.741 | 4.735 | 4.425 | -0.310 | -0.263 | 0.142 | 0.456 | Unannotated | NA | NA |
| metab_4713 | Unannotated feature | pos | 736.8592 | 7.618 | 4.806 | 4.945 | 0.139 | 0.263 | 0.142 | 0.456 | Unannotated | NA | NA |
| metab_10527 | Unannotated feature | neg | 201.0876 | 1.947 | 4.669 | 4.557 | -0.111 | -0.263 | 0.142 | 0.456 | Unannotated | NA | NA |
| metab_18449 | Unannotated feature | neg | 397.2228 | 6.091 | 5.970 | 5.761 | -0.209 | -0.263 | 0.142 | 0.456 | Unannotated | NA | NA |
| metab_18476 | Unannotated feature | neg | 245.0432 | 0.659 | 5.450 | 5.280 | -0.170 | -0.263 | 0.142 | 0.456 | Unannotated | NA | NA |
| metab_4874 | Unannotated feature | pos | 110.5420 | 7.724 | 5.211 | 5.262 | 0.050 | 0.263 | 0.142 | 0.456 | Unannotated | NA | NA |
| metab_12894 | Unannotated feature | neg | 241.0023 | 0.722 | 5.141 | 4.927 | -0.214 | -0.263 | 0.142 | 0.456 | Unannotated | NA | NA |
| metab_13002 | Unannotated feature | neg | 200.0308 | 0.777 | 4.325 | 3.923 | -0.402 | -0.263 | 0.142 | 0.456 | Unannotated | NA | NA |
| metab_1370 | Unannotated feature | pos | 272.2587 | 6.135 | 3.948 | 3.703 | -0.245 | -0.263 | 0.142 | 0.456 | Unannotated | NA | NA |
| metab_10323 | Unannotated feature | neg | 315.9946 | 2.298 | 5.012 | 4.826 | -0.185 | -0.263 | 0.142 | 0.456 | Unannotated | NA | NA |
| metab_3194 | Unannotated feature | pos | 392.2190 | 3.704 | 4.527 | 3.695 | -0.831 | -0.263 | 0.142 | 0.456 | Unannotated | NA | NA |
| metab_6980 | 24-Oxo-1alpha,23,25-trihydroxyvitamin D3 | pos | 485.2663 | 4.886 | 2.830 | 3.404 | 0.574 | 0.263 | 0.142 | 0.456 | Unannotated | Lipids and lipid-like molecules | Steroids and steroid derivatives |
| metab_4147 | Unannotated feature | pos | 603.7701 | 5.890 | 2.837 | 3.859 | 1.022 | 0.263 | 0.142 | 0.456 | Unannotated | NA | NA |
| metab_4003 | Unannotated feature | pos | 587.2001 | 5.811 | 1.045 | 3.939 | 2.894 | 0.263 | 0.142 | 0.456 | Unannotated | NA | NA |
| metab_8501 | Unannotated feature | pos | 173.1161 | 1.685 | 4.127 | 3.159 | -0.968 | -0.263 | 0.142 | 0.456 | Unannotated | NA | NA |
| metab_17034 | Unannotated feature | neg | 224.9211 | 7.993 | 4.676 | 4.643 | -0.033 | -0.263 | 0.142 | 0.456 | Unannotated | NA | NA |
| metab_17073 | Unannotated feature | neg | 156.9979 | 7.914 | 4.494 | 4.448 | -0.047 | -0.263 | 0.142 | 0.456 | Unannotated | NA | NA |
| metab_17041 | Unannotated feature | neg | 179.8980 | 7.989 | 4.217 | 4.162 | -0.055 | -0.263 | 0.142 | 0.456 | Unannotated | NA | NA |
| metab_3230 | Unannotated feature | pos | 119.0496 | 3.802 | 3.690 | 2.726 | -0.964 | -0.263 | 0.142 | 0.456 | Unannotated | NA | NA |
| metab_9650 | Melilotoside | neg | 307.0830 | 3.188 | 3.947 | 4.257 | 0.309 | 0.263 | 0.142 | 0.456 | Tentative | NA | NA |
| metab_9712 | Unannotated feature | neg | 261.1283 | 3.116 | 4.544 | 4.372 | -0.172 | -0.263 | 0.142 | 0.456 | Unannotated | NA | NA |
| metab_9686 | Unannotated feature | neg | 116.0499 | 3.141 | 4.387 | 4.170 | -0.218 | -0.263 | 0.142 | 0.456 | Unannotated | NA | NA |
| metab_8915 | Unannotated feature | neg | 172.0398 | 4.243 | 3.401 | 3.596 | 0.196 | 0.263 | 0.142 | 0.456 | Unannotated | NA | NA |
| metab_9824 | 4-O-alpha-D-Galactopyranosylcalystegine B2 | neg | 318.1197 | 3.021 | 4.263 | 4.725 | 0.462 | 0.263 | 0.142 | 0.456 | Unannotated | Organic oxygen compounds | Organooxygen compounds |
| metab_13660 | Rosuvastatin 5 S-lactone | neg | 444.1382 | 1.680 | 3.568 | 4.401 | 0.833 | 0.263 | 0.142 | 0.456 | Unannotated | Organoheterocyclic compounds | Diazines |
| metab_2219 | Unannotated feature | pos | 207.1131 | 3.047 | 3.240 | 3.820 | 0.580 | 0.263 | 0.142 | 0.456 | Unannotated | NA | NA |
| metab_17699 | Unannotated feature | neg | 231.9314 | 7.004 | 5.700 | 5.622 | -0.078 | -0.263 | 0.142 | 0.456 | Unannotated | NA | NA |
| metab_14145 | Folinic acid | neg | 472.1599 | 2.880 | 3.343 | 3.774 | 0.431 | 0.263 | 0.142 | 0.456 | Tentative | Organoheterocyclic compounds | Pteridines and derivatives |
| metab_15540 | Unannotated feature | neg | 135.0444 | 5.355 | 5.009 | 4.947 | -0.063 | -0.263 | 0.142 | 0.456 | Unannotated | NA | NA |
| metab_1801 | Dimethyl (R)-pyrrolidine-1,2-dicarboxylate | pos | 188.0920 | 0.784 | 5.295 | 5.510 | 0.215 | 0.263 | 0.142 | 0.456 | Moderate putative | NA | NA |
| metab_6178 | Unannotated feature | pos | 553.3428 | 5.825 | 4.021 | 4.124 | 0.103 | 0.263 | 0.142 | 0.456 | Unannotated | NA | NA |
| metab_10064 | Unannotated feature | neg | 461.1901 | 2.780 | 4.122 | 3.623 | -0.499 | -0.263 | 0.142 | 0.456 | Unannotated | NA | NA |
| metab_16998 | Unannotated feature | neg | 212.9153 | 7.989 | 5.131 | 5.090 | -0.041 | -0.263 | 0.142 | 0.456 | Unannotated | NA | NA |
| metab_9936 | Unannotated feature | neg | 355.2357 | 2.915 | 3.529 | 3.209 | -0.320 | -0.263 | 0.142 | 0.456 | Unannotated | NA | NA |
| metab_18183 | Unannotated feature | neg | 355.1586 | 6.318 | 5.373 | 5.422 | 0.049 | 0.263 | 0.142 | 0.456 | Unannotated | NA | NA |
| metab_4313 | Aflatoxin B1 dialcohol | pos | 313.1077 | 5.362 | 4.960 | 4.326 | -0.634 | -0.263 | 0.142 | 0.456 | Unannotated | NA | NA |
| metab_8371 | Unannotated feature | pos | 318.1303 | 1.952 | 4.905 | 5.100 | 0.195 | 0.263 | 0.142 | 0.456 | Unannotated | NA | NA |
| metab_19405 | Unannotated feature | neg | 233.0567 | 5.007 | 3.393 | 4.217 | 0.825 | 0.263 | 0.142 | 0.456 | Unannotated | NA | NA |
| metab_15620 | Unannotated feature | neg | 363.2394 | 5.533 | 4.642 | 3.984 | -0.659 | -0.263 | 0.142 | 0.456 | Unannotated | NA | NA |
| metab_15606 | Unannotated feature | neg | 123.0808 | 5.515 | 3.722 | 3.542 | -0.180 | -0.263 | 0.142 | 0.456 | Unannotated | NA | NA |
| metab_13252 | Unannotated feature | neg | 325.1621 | 0.582 | 4.262 | 3.496 | -0.766 | -0.263 | 0.142 | 0.456 | Unannotated | NA | NA |
| metab_1706 | 2-Hydroxyestrone | pos | 331.1296 | 3.979 | 4.961 | 5.204 | 0.243 | 0.263 | 0.142 | 0.456 | Tentative | NA | NA |
| metab_19194 | Unannotated feature | neg | 264.1243 | 5.272 | 4.854 | 4.500 | -0.354 | -0.263 | 0.142 | 0.456 | Unannotated | NA | NA |
| metab_12174 | Unannotated feature | neg | 552.3366 | 6.711 | 5.394 | 5.247 | -0.148 | -0.263 | 0.142 | 0.456 | Unannotated | NA | NA |
| metab_244 | Unannotated feature | pos | 310.8381 | 0.537 | 4.272 | 4.063 | -0.209 | -0.263 | 0.142 | 0.456 | Unannotated | NA | NA |
| metab_2832 | Unannotated feature | pos | 347.1244 | 6.048 | 2.986 | 3.161 | 0.175 | 0.263 | 0.142 | 0.456 | Unannotated | NA | NA |
| metab_12523 | Unannotated feature | neg | 569.3324 | 6.287 | 6.026 | 6.177 | 0.151 | 0.263 | 0.142 | 0.456 | Unannotated | NA | NA |
| metab_7485 | Mirogabalin | pos | 460.3130 | 3.786 | 4.981 | 5.266 | 0.285 | 0.263 | 0.142 | 0.456 | Unannotated | Organic acids and derivatives | Carboxylic acids and derivatives |
| metab_295 | Unannotated feature | pos | 190.9889 | 0.485 | 4.516 | 4.202 | -0.314 | -0.263 | 0.142 | 0.456 | Unannotated | NA | NA |
| metab_7231 | (2E,6E,8E)-Decatrienoylcarnitine | pos | 636.4186 | 4.355 | 4.508 | 5.251 | 0.744 | 0.263 | 0.142 | 0.456 | Unannotated | Lipids and lipid-like molecules | Fatty Acyls |
| metab_7557 | Isoleucyl-thiazolidine | pos | 446.2613 | 3.638 | 4.153 | 4.490 | 0.337 | 0.263 | 0.142 | 0.456 | Unannotated | NA | NA |
| metab_15218 | 5'-Methoxy-O-desmethylangolensin | neg | 333.0987 | 4.767 | 4.756 | 4.485 | -0.271 | -0.263 | 0.142 | 0.456 | Unannotated | NA | NA |
| metab_13956 | Unannotated feature | neg | 730.8820 | 0.485 | 5.500 | 5.013 | -0.487 | -0.263 | 0.142 | 0.456 | Unannotated | NA | NA |
| metab_17365 | Unannotated feature | neg | 595.8605 | 7.684 | 4.700 | 4.780 | 0.079 | 0.263 | 0.142 | 0.456 | Unannotated | NA | NA |
| metab_2870 | Unannotated feature | pos | 224.0558 | 2.803 | 3.884 | 4.261 | 0.376 | 0.263 | 0.142 | 0.456 | Unannotated | NA | NA |
| metab_3088 | Bufuralol | pos | 306.1453 | 3.393 | 4.084 | 3.551 | -0.533 | -0.263 | 0.142 | 0.456 | Tentative | NA | NA |
| metab_2961 | Alpha-Terpineol propanoate | pos | 228.1961 | 6.263 | 6.419 | 6.403 | -0.016 | -0.263 | 0.142 | 0.456 | Unannotated | NA | NA |
| metab_11865 | Unannotated feature | neg | 387.1852 | 5.999 | 6.252 | 6.585 | 0.333 | 0.263 | 0.142 | 0.456 | Unannotated | NA | NA |
| metab_12147 | Unannotated feature | neg | 471.3484 | 6.640 | 6.004 | 6.185 | 0.181 | 0.263 | 0.142 | 0.456 | Unannotated | NA | NA |
| metab_783 | Unannotated feature | pos | 460.2708 | 6.199 | 5.725 | 5.564 | -0.161 | -0.263 | 0.142 | 0.456 | Unannotated | NA | NA |
| metab_8240 | Aminocaproic Acid | pos | 173.1287 | 2.337 | 5.590 | 4.957 | -0.633 | -0.259 | 0.148 | 0.466 | Moderate putative | NA | NA |
| metab_8328 | Tyrosyl-Alanine | pos | 253.1188 | 2.003 | 5.149 | 5.043 | -0.105 | -0.259 | 0.148 | 0.466 | Unannotated | NA | NA |
| metab_10495 | 2-Methyladipic acid | neg | 159.0656 | 1.952 | 5.980 | 5.724 | -0.255 | -0.259 | 0.148 | 0.466 | Unannotated | Lipids and lipid-like molecules | Fatty Acyls |
| metab_1500 | Unannotated feature | pos | 620.9969 | 5.794 | 4.149 | 4.467 | 0.318 | 0.259 | 0.148 | 0.466 | Unannotated | NA | NA |
| metab_1592 | Unannotated feature | pos | 366.1669 | 3.586 | 4.802 | 4.591 | -0.211 | -0.259 | 0.148 | 0.466 | Unannotated | NA | NA |
| metab_16836 | Unannotated feature | neg | 543.8409 | 7.726 | 4.599 | 4.734 | 0.136 | 0.259 | 0.148 | 0.466 | Unannotated | NA | NA |
| metab_18355 | Unannotated feature | neg | 263.0958 | 6.170 | 2.382 | 3.552 | 1.170 | 0.259 | 0.148 | 0.466 | Unannotated | NA | NA |
| metab_1612 | Xi-Linalool 3-[rhamnosyl-(1->6)-glucoside] | pos | 463.2566 | 4.975 | 3.357 | 3.815 | 0.458 | 0.259 | 0.148 | 0.466 | Unannotated | NA | NA |
| metab_10612 | 3-[4-methoxy-3-(sulfooxy)phenyl]propanoic acid | neg | 275.0235 | 1.685 | 4.320 | 3.758 | -0.562 | -0.259 | 0.148 | 0.466 | Unannotated | NA | NA |
| metab_7978 | Petasitenine | pos | 346.1657 | 2.864 | 3.188 | 3.713 | 0.526 | 0.259 | 0.148 | 0.466 | Tentative | NA | NA |
| metab_10742 | Tuliposide A | neg | 277.0932 | 1.245 | 5.160 | 5.576 | 0.416 | 0.259 | 0.148 | 0.466 | Tentative | NA | NA |
| metab_8074 | Methylimidazole acetaldehyde | pos | 290.1618 | 2.755 | 3.191 | 3.404 | 0.212 | 0.259 | 0.148 | 0.466 | Tentative | NA | NA |
| metab_4753 | Unannotated feature | pos | 109.5221 | 7.716 | 4.713 | 4.746 | 0.034 | 0.259 | 0.148 | 0.466 | Unannotated | NA | NA |
| metab_1495 | Unannotated feature | pos | 615.9811 | 5.806 | 3.615 | 3.708 | 0.093 | 0.259 | 0.148 | 0.466 | Unannotated | NA | NA |
| metab_16675 | Unannotated feature | neg | 677.8811 | 7.611 | 5.548 | 5.666 | 0.118 | 0.259 | 0.148 | 0.466 | Unannotated | NA | NA |
| metab_18808 | Unannotated feature | neg | 400.1730 | 5.774 | 4.259 | 4.999 | 0.740 | 0.259 | 0.148 | 0.466 | Unannotated | NA | NA |
| metab_17969 | Unannotated feature | neg | 521.2949 | 6.553 | 6.295 | 5.745 | -0.550 | -0.259 | 0.148 | 0.466 | Unannotated | NA | NA |
| metab_13565 | Unannotated feature | neg | 233.0665 | 1.947 | 5.447 | 5.184 | -0.263 | -0.259 | 0.148 | 0.466 | Unannotated | NA | NA |
| metab_2058 | Unannotated feature | pos | 134.0450 | 0.641 | 5.634 | 5.478 | -0.156 | -0.259 | 0.148 | 0.466 | Unannotated | NA | NA |
| metab_9899 | (+/-)-Tryptophan | neg | 203.0822 | 2.956 | 5.784 | 5.530 | -0.254 | -0.259 | 0.148 | 0.466 | Tentative | NA | NA |
| metab_1890 | Prolyl-Alanine | pos | 219.1343 | 1.370 | 5.952 | 5.758 | -0.194 | -0.259 | 0.148 | 0.466 | Unannotated | NA | NA |
| metab_10112 | Unannotated feature | neg | 139.0394 | 2.766 | 4.368 | 4.485 | 0.117 | 0.259 | 0.148 | 0.466 | Unannotated | NA | NA |
| metab_10127 | 2-(6'-methylthio)hexylmalate | neg | 523.1694 | 2.754 | 5.037 | 3.934 | -1.102 | -0.259 | 0.148 | 0.466 | Tentative | NA | NA |
| metab_10180 | Unannotated feature | neg | 308.0259 | 2.690 | 4.193 | 3.962 | -0.231 | -0.259 | 0.148 | 0.466 | Unannotated | NA | NA |
| metab_11555 | Unannotated feature | neg | 558.9108 | 0.485 | 4.222 | 3.507 | -0.714 | -0.259 | 0.148 | 0.466 | Unannotated | NA | NA |
| metab_12438 | Unannotated feature | neg | 191.9458 | 7.726 | 6.632 | 6.608 | -0.024 | -0.259 | 0.148 | 0.466 | Unannotated | NA | NA |
| metab_12596 | Unannotated feature | neg | 727.3777 | 6.076 | 5.108 | 4.535 | -0.573 | -0.259 | 0.148 | 0.466 | Unannotated | NA | NA |
| metab_19337 | Unannotated feature | neg | 269.0418 | 3.407 | 5.389 | 5.139 | -0.250 | -0.259 | 0.148 | 0.466 | Unannotated | NA | NA |
| metab_19364 | Unannotated feature | neg | 243.1714 | 5.052 | 4.277 | 3.925 | -0.352 | -0.259 | 0.148 | 0.466 | Unannotated | NA | NA |
| metab_10367 | Unannotated feature | neg | 310.0111 | 2.151 | 3.662 | 3.813 | 0.150 | 0.259 | 0.148 | 0.466 | Unannotated | NA | NA |
| metab_18304 | 1-a,24R,25-Trihydroxyvitamin D2 | neg | 489.3229 | 6.209 | 4.433 | 4.870 | 0.437 | 0.259 | 0.148 | 0.466 | Unannotated | Lipids and lipid-like molecules | Steroids and steroid derivatives |
| metab_5872 | Unannotated feature | pos | 159.0134 | 0.325 | 5.701 | 5.732 | 0.031 | 0.259 | 0.148 | 0.466 | Unannotated | NA | NA |
| metab_10697 | Unannotated feature | neg | 73.0285 | 1.433 | 4.883 | 4.872 | -0.012 | -0.259 | 0.148 | 0.466 | Unannotated | NA | NA |
| metab_329 | Unannotated feature | pos | 443.0987 | 4.648 | 3.065 | 2.863 | -0.202 | -0.259 | 0.148 | 0.466 | Unannotated | NA | NA |
| metab_15033 | Unannotated feature | neg | 250.1085 | 4.398 | 5.147 | 5.031 | -0.116 | -0.259 | 0.148 | 0.466 | Unannotated | NA | NA |
| metab_6654 | D-Phenyllactic acid | pos | 149.0600 | 5.369 | 3.433 | 2.761 | -0.672 | -0.259 | 0.148 | 0.466 | Tentative | NA | NA |
| metab_12072 | 3-(4-Hydroxy-3-methoxyphenyl)-2-methyllactic acid | neg | 225.0766 | 3.215 | 5.506 | 5.303 | -0.203 | -0.259 | 0.148 | 0.466 | Unannotated | Phenylpropanoids and polyketides | Phenylpropanoic acids |
| metab_19862 | Dodecanedioic Acid | neg | 229.1442 | 6.115 | 5.915 | 5.674 | -0.240 | -0.259 | 0.148 | 0.466 | Strong putative | Lipids and lipid-like molecules | Fatty Acyls |
| metab_7141 | Unannotated feature | pos | 142.1229 | 4.571 | 4.652 | 4.610 | -0.041 | -0.259 | 0.148 | 0.466 | Unannotated | NA | NA |
| metab_5 | Unannotated feature | pos | 303.2032 | 0.720 | 4.715 | 4.336 | -0.379 | -0.259 | 0.148 | 0.466 | Unannotated | NA | NA |
| metab_18601 | Clofop | neg | 329.0007 | 5.991 | 3.636 | 3.676 | 0.040 | 0.259 | 0.148 | 0.466 | Tentative | Benzenoids | Benzene and substituted derivatives |
| metab_18902 | L-Aspartyl-L-Phenylalanine | neg | 279.0988 | 3.227 | 5.318 | 5.062 | -0.255 | -0.259 | 0.148 | 0.466 | Moderate putative | NA | NA |
| metab_1197 | Unannotated feature | pos | 457.2783 | 6.462 | 6.429 | 6.293 | -0.136 | -0.259 | 0.148 | 0.466 | Unannotated | NA | NA |
| metab_10881 | Unannotated feature | neg | 270.0102 | 0.952 | 5.684 | 5.505 | -0.179 | -0.259 | 0.148 | 0.466 | Unannotated | NA | NA |
| metab_7874 | Unannotated feature | pos | 91.0547 | 3.002 | 4.814 | 4.834 | 0.019 | 0.259 | 0.148 | 0.466 | Unannotated | NA | NA |
| metab_7865 | Undecanoic acid | pos | 225.1237 | 3.027 | 4.524 | 4.689 | 0.165 | 0.259 | 0.148 | 0.466 | Tentative | Lipids and lipid-like molecules | Fatty Acyls |
| metab_6873 | Unannotated feature | pos | 323.0747 | 0.641 | 3.177 | 3.766 | 0.589 | 0.259 | 0.148 | 0.466 | Unannotated | NA | NA |
| metab_11371 | 3-Hydroxy-L-proline | neg | 321.1307 | 0.566 | 4.129 | 4.572 | 0.442 | 0.259 | 0.148 | 0.466 | Tentative | Organic acids and derivatives | Carboxylic acids and derivatives |
| metab_17800 | Unannotated feature | neg | 256.0830 | 2.809 | 5.475 | 5.765 | 0.291 | 0.259 | 0.148 | 0.466 | Unannotated | NA | NA |
| metab_2495 | L-Furosine | pos | 255.1345 | 0.768 | 4.575 | 4.446 | -0.129 | -0.259 | 0.148 | 0.466 | Unannotated | NA | NA |
| metab_9630 | Unannotated feature | neg | 103.0029 | 3.210 | 4.337 | 4.284 | -0.053 | -0.259 | 0.148 | 0.466 | Unannotated | NA | NA |
| metab_17773 | Unannotated feature | neg | 381.2110 | 6.892 | 4.563 | 5.880 | 1.317 | 0.259 | 0.148 | 0.466 | Unannotated | NA | NA |
| metab_8617 | Unannotated feature | pos | 178.1077 | 1.065 | 3.522 | 3.643 | 0.121 | 0.259 | 0.148 | 0.466 | Unannotated | NA | NA |
| metab_3531 | Unannotated feature | pos | 456.7737 | 4.878 | 3.415 | 4.922 | 1.507 | 0.259 | 0.148 | 0.466 | Unannotated | NA | NA |
| metab_4200 | Unannotated feature | pos | 623.4729 | 5.914 | 3.821 | 4.238 | 0.417 | 0.259 | 0.148 | 0.466 | Unannotated | NA | NA |
| metab_14516 | Unannotated feature | neg | 235.0610 | 3.379 | 3.042 | 3.608 | 0.566 | 0.259 | 0.148 | 0.466 | Unannotated | NA | NA |
| metab_14511 | Unannotated feature | neg | 314.2089 | 3.367 | 5.034 | 4.748 | -0.286 | -0.259 | 0.148 | 0.466 | Unannotated | NA | NA |
| metab_4191 | Ile Ile Arg Ser Ser | pos | 575.3541 | 5.914 | 2.272 | 4.403 | 2.131 | 0.259 | 0.148 | 0.466 | Strong putative | NA | NA |
| metab_3880 | Unannotated feature | pos | 627.8990 | 5.725 | 3.969 | 4.857 | 0.888 | 0.259 | 0.148 | 0.466 | Unannotated | NA | NA |
| metab_14359 | Unannotated feature | neg | 284.1144 | 3.131 | 3.660 | 4.350 | 0.689 | 0.259 | 0.148 | 0.466 | Unannotated | NA | NA |
| metab_17125 | Unannotated feature | neg | 335.0005 | 2.295 | 5.869 | 5.707 | -0.162 | -0.259 | 0.148 | 0.466 | Unannotated | NA | NA |
| metab_2028 | Unannotated feature | pos | 134.0273 | 0.712 | 3.935 | 4.319 | 0.383 | 0.259 | 0.148 | 0.466 | Unannotated | NA | NA |
| metab_11525 | Unannotated feature | neg | 454.9276 | 0.502 | 5.342 | 5.182 | -0.160 | -0.259 | 0.148 | 0.466 | Unannotated | NA | NA |
| metab_13156 | Unannotated feature | neg | 519.9088 | 0.472 | 5.622 | 5.379 | -0.243 | -0.259 | 0.148 | 0.466 | Unannotated | NA | NA |
| metab_11571 | Unannotated feature | neg | 420.9204 | 0.460 | 5.522 | 5.482 | -0.040 | -0.259 | 0.148 | 0.466 | Unannotated | NA | NA |
| metab_17225 | Unannotated feature | neg | 258.0556 | 7.730 | 3.799 | 4.395 | 0.596 | 0.259 | 0.148 | 0.466 | Unannotated | NA | NA |
| metab_14057 | Unannotated feature | neg | 294.8928 | 0.502 | 4.952 | 5.117 | 0.164 | 0.259 | 0.148 | 0.466 | Unannotated | NA | NA |
| metab_17306 | Unannotated feature | neg | 175.9680 | 7.707 | 5.688 | 5.647 | -0.040 | -0.259 | 0.148 | 0.466 | Unannotated | NA | NA |
| metab_14048 | L-beta-aspartyl-L-phenylalanine | neg | 279.0990 | 2.776 | 4.634 | 4.437 | -0.197 | -0.259 | 0.148 | 0.466 | Unannotated | NA | NA |
| metab_13859 | Unannotated feature | neg | 243.1462 | 2.248 | 4.463 | 4.003 | -0.460 | -0.259 | 0.148 | 0.466 | Unannotated | NA | NA |
| metab_8978 | 2,3-Dihydroxybenzoic acid | neg | 153.0187 | 4.118 | 5.676 | 5.422 | -0.254 | -0.259 | 0.148 | 0.466 | Strong putative | NA | NA |
| metab_11466 | Unannotated feature | neg | 524.8454 | 0.524 | 5.595 | 5.840 | 0.245 | 0.259 | 0.148 | 0.466 | Unannotated | NA | NA |
| metab_7445 | Unannotated feature | pos | 282.1342 | 3.882 | 4.407 | 4.616 | 0.210 | 0.259 | 0.148 | 0.466 | Unannotated | NA | NA |
| metab_2851 | Unannotated feature | pos | 266.1731 | 6.064 | 5.802 | 5.743 | -0.059 | -0.259 | 0.148 | 0.466 | Unannotated | NA | NA |
| metab_6136 | Unannotated feature | pos | 785.1431 | 5.835 | 2.495 | 2.590 | 0.095 | 0.259 | 0.148 | 0.466 | Unannotated | NA | NA |
| metab_17228 | Unannotated feature | neg | 238.0773 | 7.730 | 5.249 | 5.448 | 0.199 | 0.259 | 0.148 | 0.466 | Unannotated | NA | NA |
| metab_17456 | Unannotated feature | neg | 792.8624 | 7.607 | 7.245 | 7.300 | 0.055 | 0.259 | 0.148 | 0.466 | Unannotated | NA | NA |
| metab_19193 | Unannotated feature | neg | 453.1461 | 5.272 | 3.375 | 4.152 | 0.778 | 0.259 | 0.148 | 0.466 | Unannotated | NA | NA |
| metab_6574 | Semilepidinoside A | pos | 301.1189 | 5.488 | 5.391 | 5.021 | -0.370 | -0.259 | 0.148 | 0.466 | Unannotated | NA | NA |
| metab_11823 | Unannotated feature | neg | 203.1284 | 5.905 | 4.907 | 4.480 | -0.427 | -0.259 | 0.148 | 0.466 | Unannotated | NA | NA |
| metab_7348 | Unannotated feature | pos | 158.1178 | 4.084 | 4.979 | 5.374 | 0.395 | 0.259 | 0.148 | 0.466 | Unannotated | NA | NA |
| metab_7619 | Unannotated feature | pos | 215.0819 | 3.517 | 3.973 | 3.827 | -0.146 | -0.259 | 0.148 | 0.466 | Unannotated | NA | NA |
| metab_11618 | Unannotated feature | neg | 159.9360 | 0.213 | 5.371 | 5.357 | -0.014 | -0.259 | 0.148 | 0.466 | Unannotated | NA | NA |
| metab_11878 | Unannotated feature | neg | 385.1696 | 6.018 | 6.251 | 6.552 | 0.300 | 0.259 | 0.148 | 0.466 | Unannotated | NA | NA |
| metab_7402 | Unannotated feature | pos | 337.1766 | 3.970 | 4.877 | 4.643 | -0.234 | -0.255 | 0.155 | 0.475 | Unannotated | NA | NA |
| metab_7328 | Fenoldopam | pos | 347.1142 | 4.123 | 3.695 | 3.268 | -0.426 | -0.255 | 0.155 | 0.475 | Tentative | Organoheterocyclic compounds | Benzazepines |
| metab_16261 | Doxercalciferol | neg | 457.3329 | 6.538 | 5.233 | 5.525 | 0.292 | 0.255 | 0.155 | 0.475 | Tentative | NA | NA |
| metab_7420 | Val Ile Val | pos | 330.2395 | 3.930 | 5.239 | 4.790 | -0.449 | -0.255 | 0.155 | 0.475 | Strong putative | NA | NA |
| metab_7520 | Unannotated feature | pos | 320.1758 | 3.716 | 3.451 | 2.901 | -0.550 | -0.255 | 0.155 | 0.475 | Unannotated | NA | NA |
| metab_2682 | 3-hydroxypentadecanoic acid | pos | 297.1815 | 1.984 | 3.681 | 4.298 | 0.617 | 0.255 | 0.155 | 0.475 | Unannotated | Lipids and lipid-like molecules | Fatty Acyls |
| metab_13902 | Unannotated feature | neg | 344.1831 | 2.484 | 5.341 | 5.002 | -0.340 | -0.255 | 0.155 | 0.475 | Unannotated | NA | NA |
| metab_11308 | Unannotated feature | neg | 122.9253 | 0.607 | 3.819 | 3.738 | -0.081 | -0.255 | 0.155 | 0.475 | Unannotated | NA | NA |
| metab_9291 | Unannotated feature | neg | 136.0761 | 3.624 | 4.142 | 3.845 | -0.297 | -0.255 | 0.155 | 0.475 | Unannotated | NA | NA |
| metab_2883 | 3,4-Dihydroxyphenylvaleric acid | pos | 228.1235 | 2.823 | 3.533 | 4.049 | 0.516 | 0.255 | 0.155 | 0.475 | Unannotated | NA | NA |
| metab_17321 | Unannotated feature | neg | 466.1213 | 2.569 | 3.840 | 3.488 | -0.351 | -0.255 | 0.155 | 0.475 | Unannotated | NA | NA |
| metab_14158 | Salicyluric Acid | neg | 194.0454 | 2.899 | 4.244 | 4.514 | 0.270 | 0.255 | 0.155 | 0.475 | Moderate putative | NA | NA |
| metab_15848 | Unannotated feature | neg | 191.0191 | 5.927 | 4.248 | 4.610 | 0.362 | 0.255 | 0.155 | 0.475 | Unannotated | NA | NA |
| metab_17566 | Unannotated feature | neg | 142.9800 | 7.310 | 4.891 | 4.956 | 0.065 | 0.255 | 0.155 | 0.475 | Unannotated | NA | NA |
| metab_15852 | Unannotated feature | neg | 231.9315 | 5.930 | 3.336 | 3.817 | 0.480 | 0.255 | 0.155 | 0.475 | Unannotated | NA | NA |
| metab_13763 | Unannotated feature | neg | 374.1098 | 1.958 | 4.109 | 4.723 | 0.614 | 0.255 | 0.155 | 0.475 | Unannotated | NA | NA |
| metab_8825 | Norlaudanosoline | pos | 305.1502 | 5.434 | 4.858 | 4.540 | -0.318 | -0.255 | 0.155 | 0.475 | Tentative | Organoheterocyclic compounds | Isoquinolines and derivatives |
| metab_17147 | Unannotated feature | neg | 318.0102 | 2.306 | 6.012 | 5.864 | -0.148 | -0.255 | 0.155 | 0.475 | Unannotated | NA | NA |
| metab_3310 | Unannotated feature | pos | 124.0761 | 4.091 | 3.672 | 3.554 | -0.118 | -0.255 | 0.155 | 0.475 | Unannotated | NA | NA |
| metab_8740 | Patulin | pos | 172.0606 | 0.744 | 4.463 | 4.756 | 0.293 | 0.255 | 0.155 | 0.475 | Tentative | NA | NA |
| metab_17638 | Tetrahydrodeoxycortisol | neg | 699.4832 | 7.111 | 5.694 | 5.201 | -0.493 | -0.255 | 0.155 | 0.475 | Tentative | NA | NA |
| metab_6150 | Unannotated feature | pos | 637.4869 | 5.835 | 3.919 | 4.151 | 0.232 | 0.255 | 0.155 | 0.475 | Unannotated | NA | NA |
| metab_17604 | Unannotated feature | neg | 449.1313 | 2.762 | 5.622 | 5.377 | -0.244 | -0.255 | 0.155 | 0.475 | Unannotated | NA | NA |
| metab_2328 | Unannotated feature | pos | 116.9667 | 0.385 | 5.925 | 5.964 | 0.039 | 0.255 | 0.155 | 0.475 | Unannotated | NA | NA |
| metab_11422 | Unannotated feature | neg | 485.1675 | 5.192 | 4.638 | 4.756 | 0.118 | 0.255 | 0.155 | 0.475 | Unannotated | NA | NA |
| metab_11400 | Unannotated feature | neg | 308.8415 | 0.547 | 4.384 | 4.018 | -0.366 | -0.255 | 0.155 | 0.475 | Unannotated | NA | NA |
| metab_6024 | 3-Pyridinamine | pos | 112.0874 | 0.501 | 5.237 | 4.889 | -0.348 | -0.255 | 0.155 | 0.475 | Moderate putative | NA | NA |
| metab_740 | Unannotated feature | pos | 677.3406 | 6.032 | 5.200 | 4.893 | -0.306 | -0.255 | 0.155 | 0.475 | Unannotated | NA | NA |
| metab_8888 | Unannotated feature | neg | 215.0922 | 4.286 | 5.544 | 5.664 | 0.120 | 0.255 | 0.155 | 0.475 | Unannotated | NA | NA |
| metab_15700 | Curcumin | neg | 403.0981 | 5.683 | 4.548 | 4.201 | -0.347 | -0.255 | 0.155 | 0.475 | Tentative | NA | NA |
| metab_19248 | Cis-Caffeic acid | neg | 179.0344 | 5.204 | 4.784 | 4.686 | -0.099 | -0.255 | 0.155 | 0.475 | Tentative | NA | NA |
| metab_9840 | Unannotated feature | neg | 399.1053 | 3.007 | 3.481 | 4.072 | 0.591 | 0.255 | 0.155 | 0.475 | Unannotated | NA | NA |
| metab_14356 | L-Tryptophan | neg | 203.0822 | 3.131 | 6.225 | 6.013 | -0.212 | -0.255 | 0.155 | 0.475 | Strong putative | NA | NA |
| metab_8570 | 1-(beta-D-Ribofuranosyl)-1,4-dihydronicotinamide | pos | 221.0927 | 1.217 | 3.114 | 3.449 | 0.335 | 0.255 | 0.155 | 0.475 | Tentative | NA | NA |
| metab_13304 | Unannotated feature | neg | 95.0130 | 0.644 | 3.947 | 3.569 | -0.379 | -0.255 | 0.155 | 0.475 | Unannotated | NA | NA |
| metab_951 | Unannotated feature | pos | 294.9393 | 7.610 | 6.513 | 6.546 | 0.033 | 0.255 | 0.155 | 0.475 | Unannotated | NA | NA |
| metab_12612 | Unannotated feature | neg | 339.1087 | 6.035 | 5.174 | 4.532 | -0.642 | -0.255 | 0.155 | 0.475 | Unannotated | NA | NA |
| metab_10238 | Unannotated feature | neg | 351.9906 | 2.575 | 5.231 | 5.169 | -0.062 | -0.255 | 0.155 | 0.475 | Unannotated | NA | NA |
| metab_1850 | Unannotated feature | pos | 286.0974 | 1.062 | 5.090 | 4.791 | -0.299 | -0.255 | 0.155 | 0.475 | Unannotated | NA | NA |
| metab_1701 | Unannotated feature | pos | 331.0934 | 4.006 | 3.805 | 4.417 | 0.612 | 0.255 | 0.155 | 0.475 | Unannotated | NA | NA |
| metab_6822 | Unannotated feature | pos | 279.1704 | 5.142 | 4.679 | 3.774 | -0.905 | -0.255 | 0.155 | 0.475 | Unannotated | NA | NA |
| metab_19293 | Unannotated feature | neg | 277.1558 | 5.152 | 4.690 | 4.370 | -0.320 | -0.255 | 0.155 | 0.475 | Unannotated | NA | NA |
| metab_1698 | Gly Phe Val | pos | 322.1769 | 4.016 | 4.666 | 4.383 | -0.283 | -0.255 | 0.155 | 0.475 | Moderate putative | NA | NA |
| metab_640 | Unannotated feature | pos | 678.8260 | 5.814 | 3.338 | 3.430 | 0.092 | 0.255 | 0.155 | 0.475 | Unannotated | NA | NA |
| metab_4308 | Irbesartan | pos | 429.2410 | 6.032 | 5.477 | 5.209 | -0.268 | -0.255 | 0.155 | 0.475 | Strong putative | NA | NA |
| metab_8354 | O-Succinyl-L-homoserine | pos | 184.0608 | 1.960 | 5.220 | 5.315 | 0.095 | 0.255 | 0.155 | 0.475 | Tentative | NA | NA |
| metab_8433 | Unannotated feature | pos | 255.1344 | 1.944 | 4.690 | 4.601 | -0.090 | -0.255 | 0.155 | 0.475 | Unannotated | NA | NA |
| metab_2053 | Unannotated feature | pos | 303.1345 | 5.199 | 5.347 | 5.044 | -0.303 | -0.255 | 0.155 | 0.475 | Unannotated | NA | NA |
| metab_17698 | Unannotated feature | neg | 255.2329 | 7.004 | 5.971 | 5.877 | -0.094 | -0.255 | 0.155 | 0.475 | Unannotated | NA | NA |
| metab_17700 | Unannotated feature | neg | 462.2694 | 6.997 | 6.108 | 5.887 | -0.221 | -0.255 | 0.155 | 0.475 | Unannotated | NA | NA |
| metab_3945 | Unannotated feature | pos | 299.1395 | 6.223 | 5.455 | 4.794 | -0.661 | -0.255 | 0.155 | 0.475 | Unannotated | NA | NA |
| metab_3869 | Unannotated feature | pos | 497.3100 | 5.711 | 3.927 | 4.319 | 0.392 | 0.255 | 0.155 | 0.475 | Unannotated | NA | NA |
| metab_10011 | Unannotated feature | neg | 308.0259 | 2.831 | 4.933 | 4.812 | -0.122 | -0.255 | 0.155 | 0.475 | Unannotated | NA | NA |
| metab_13407 | GHB-Gluc | neg | 619.1747 | 0.777 | 4.261 | 4.799 | 0.538 | 0.255 | 0.155 | 0.475 | Unannotated | Lipids and lipid-like molecules | Saccharolipids |
| metab_18049 | Dehydroepiandrosterone sulfate | neg | 367.1585 | 6.444 | 5.706 | 6.036 | 0.330 | 0.255 | 0.155 | 0.475 | Tentative | NA | NA |
| metab_8716 | 2-Hydroxypyridine | pos | 254.0888 | 0.768 | 1.651 | 2.378 | 0.727 | 0.255 | 0.155 | 0.475 | Tentative | NA | NA |
| metab_9841 | Unannotated feature | neg | 207.0330 | 3.007 | 4.856 | 5.071 | 0.214 | 0.255 | 0.155 | 0.475 | Unannotated | NA | NA |
| metab_17752 | Unannotated feature | neg | 246.0439 | 2.793 | 5.957 | 5.805 | -0.151 | -0.255 | 0.155 | 0.475 | Unannotated | NA | NA |
| metab_8751 | Unannotated feature | pos | 81.0340 | 0.736 | 3.832 | 4.111 | 0.280 | 0.255 | 0.155 | 0.475 | Unannotated | NA | NA |
| metab_17109 | Unannotated feature | neg | 197.9019 | 7.858 | 3.456 | 3.628 | 0.171 | 0.255 | 0.155 | 0.475 | Unannotated | NA | NA |
| metab_6950 | Indolelactic Acid | pos | 206.0816 | 4.943 | 3.336 | 3.701 | 0.365 | 0.255 | 0.155 | 0.475 | Strong putative | NA | NA |
| metab_16871 | Unannotated feature | neg | 379.9217 | 7.737 | 3.389 | 3.512 | 0.123 | 0.255 | 0.155 | 0.475 | Unannotated | NA | NA |
| metab_8229 | Unannotated feature | pos | 324.2178 | 2.411 | 4.739 | 4.873 | 0.135 | 0.255 | 0.155 | 0.475 | Unannotated | NA | NA |
| metab_16942 | Unannotated feature | neg | 98.0239 | 7.835 | 4.301 | 4.271 | -0.029 | -0.255 | 0.155 | 0.475 | Unannotated | NA | NA |
| metab_15951 | Taurohyocholate | neg | 514.2854 | 6.052 | 5.683 | 5.544 | -0.139 | -0.255 | 0.155 | 0.475 | Tentative | NA | NA |
| metab_7854 | Dobutamine | pos | 324.1561 | 3.047 | 4.463 | 4.299 | -0.164 | -0.255 | 0.155 | 0.475 | Tentative | Benzenoids | Phenols |
| metab_10929 | Unannotated feature | neg | 377.0930 | 4.515 | 3.958 | 4.224 | 0.266 | 0.255 | 0.155 | 0.475 | Unannotated | NA | NA |
| metab_18594 | Unannotated feature | neg | 603.2637 | 5.991 | 4.006 | 3.686 | -0.319 | -0.255 | 0.155 | 0.475 | Unannotated | NA | NA |
| metab_1167 | 12-oxo-PDA | pos | 293.2098 | 6.526 | 5.567 | 5.430 | -0.137 | -0.255 | 0.155 | 0.475 | Tentative | NA | NA |
| metab_7218 | Desaminotyrosine | pos | 184.0972 | 4.394 | 5.106 | 4.991 | -0.115 | -0.255 | 0.155 | 0.475 | Tentative | Phenylpropanoids and polyketides | Phenylpropanoic acids |
| metab_18751 | 2,3-Dihydroxypropyl octanoate | neg | 239.1287 | 5.847 | 4.684 | 4.964 | 0.280 | 0.255 | 0.155 | 0.475 | Unannotated | NA | NA |
| metab_18908 | 2-Hydroxypropyl 2-isopropyl-5-methylcyclohexyl carbonate | neg | 303.1814 | 5.642 | 4.478 | 4.620 | 0.142 | 0.255 | 0.155 | 0.475 | Unannotated | NA | NA |
| metab_7691 | Metanephrine | pos | 395.2187 | 3.388 | 3.288 | 3.904 | 0.616 | 0.255 | 0.155 | 0.475 | Tentative | Benzenoids | Phenols |
| metab_5230 | Arctiol | pos | 254.2481 | 6.702 | 6.019 | 5.950 | -0.069 | -0.255 | 0.155 | 0.475 | Unannotated | NA | NA |
| metab_348 | Unannotated feature | pos | 934.2923 | 0.405 | 5.379 | 5.448 | 0.069 | 0.255 | 0.155 | 0.475 | Unannotated | NA | NA |
| metab_19521 | Unannotated feature | neg | 344.0505 | 3.512 | 4.253 | 3.649 | -0.604 | -0.255 | 0.155 | 0.475 | Unannotated | NA | NA |
| metab_1378 | Oleoyl glycine | pos | 381.3125 | 6.119 | 6.159 | 5.896 | -0.263 | -0.255 | 0.155 | 0.475 | Unannotated | NA | NA |
| metab_15969 | Unannotated feature | neg | 456.8945 | 6.076 | 3.905 | 4.565 | 0.661 | 0.255 | 0.155 | 0.475 | Unannotated | NA | NA |
| metab_7958 | Umbelliferone | pos | 204.0658 | 2.897 | 2.036 | 2.166 | 0.130 | 0.255 | 0.155 | 0.475 | Tentative | Phenylpropanoids and polyketides | Coumarins and derivatives |
| metab_16676 | Unannotated feature | neg | 711.8750 | 7.611 | 5.690 | 5.757 | 0.067 | 0.255 | 0.155 | 0.475 | Unannotated | NA | NA |
| metab_7965 | Unannotated feature | pos | 309.1580 | 2.884 | 2.847 | 3.125 | 0.278 | 0.255 | 0.155 | 0.475 | Unannotated | NA | NA |
| metab_7977 | S-Prenyl-L-cysteine | pos | 190.0897 | 2.868 | 3.603 | 3.663 | 0.060 | 0.255 | 0.155 | 0.475 | Tentative | NA | NA |
| metab_16694 | Unannotated feature | neg | 732.8621 | 7.625 | 5.855 | 5.970 | 0.115 | 0.255 | 0.155 | 0.475 | Unannotated | NA | NA |
| metab_4870 | Unannotated feature | pos | 132.0629 | 7.724 | 4.714 | 4.743 | 0.030 | 0.255 | 0.155 | 0.475 | Unannotated | NA | NA |
| metab_1249 | Unannotated feature | pos | 268.2639 | 6.367 | 4.889 | 4.469 | -0.420 | -0.255 | 0.155 | 0.475 | Unannotated | NA | NA |
| metab_10599 | Indoleacetaldehyde | neg | 204.0662 | 4.710 | 4.875 | 5.053 | 0.178 | 0.255 | 0.155 | 0.475 | Tentative | NA | NA |
| metab_10515 | 6-[(2R,4S,5R)-4-Hydroxy-5-(hydroxymethyl)oxolan-2-yl]-7,8-dihydroimidazo[1,2-c]pyrimidin-5-one | neg | 298.1045 | 1.947 | 4.838 | 4.976 | 0.138 | 0.255 | 0.155 | 0.475 | Unannotated | Organic oxygen compounds | Organooxygen compounds |
| metab_4800 | Unannotated feature | pos | 284.1342 | 7.991 | 5.084 | 5.116 | 0.031 | 0.255 | 0.155 | 0.475 | Unannotated | NA | NA |
| metab_1628 | Unannotated feature | pos | 553.8321 | 4.892 | 3.580 | 4.329 | 0.749 | 0.255 | 0.155 | 0.475 | Unannotated | NA | NA |
| metab_4780 | Unannotated feature | pos | 84.0791 | 7.788 | 4.569 | 4.635 | 0.066 | 0.255 | 0.155 | 0.475 | Unannotated | NA | NA |
| metab_8037 | (S)-corytuberine | pos | 328.1551 | 3.477 | 4.825 | 4.397 | -0.428 | -0.255 | 0.155 | 0.475 | Tentative | Alkaloids and derivatives | Aporphines |
| metab_1531 | Unannotated feature | pos | 372.1875 | 3.444 | 3.478 | 4.061 | 0.583 | 0.255 | 0.155 | 0.475 | Unannotated | NA | NA |
| metab_8190 | (+/-)-erythro-Isoleucine | pos | 173.1287 | 2.543 | 5.787 | 5.265 | -0.522 | -0.255 | 0.155 | 0.475 | Tentative | Organic acids and derivatives | Carboxylic acids and derivatives |
| metab_12035 | Unannotated feature | neg | 652.3006 | 5.678 | 6.711 | 6.225 | -0.486 | -0.255 | 0.155 | 0.475 | Unannotated | NA | NA |
| metab_1109 | Unannotated feature | pos | 643.4917 | 6.646 | 3.794 | 4.436 | 0.642 | 0.255 | 0.155 | 0.475 | Unannotated | NA | NA |
| metab_412 | Unannotated feature | pos | 172.9567 | 0.016 | 4.868 | 4.879 | 0.011 | 0.255 | 0.155 | 0.475 | Unannotated | NA | NA |
| metab_2341 | Unannotated feature | pos | 168.0256 | 0.451 | 3.853 | 3.746 | -0.107 | -0.251 | 0.162 | 0.484 | Unannotated | NA | NA |
| metab_3285 | 4-Hydroxy-2,6,6-trimethyl-3-oxo-1,4-cyclohexadiene-1-carboxaldehyde | pos | 163.0755 | 4.006 | 4.836 | 4.127 | -0.709 | -0.251 | 0.162 | 0.484 | Unannotated | Organic oxygen compounds | Organooxygen compounds |
| metab_14246 | Unannotated feature | neg | 197.0926 | 2.984 | 4.081 | 3.613 | -0.468 | -0.251 | 0.162 | 0.484 | Unannotated | NA | NA |
| metab_13709 | Unannotated feature | neg | 159.9361 | 1.830 | 3.345 | 3.101 | -0.244 | -0.251 | 0.162 | 0.484 | Unannotated | NA | NA |
| metab_17214 | Unannotated feature | neg | 322.1168 | 7.730 | 3.882 | 4.323 | 0.440 | 0.251 | 0.162 | 0.484 | Unannotated | NA | NA |
| metab_14130 | Unannotated feature | neg | 289.0392 | 2.862 | 4.168 | 3.730 | -0.438 | -0.251 | 0.162 | 0.484 | Unannotated | NA | NA |
| metab_17167 | Unannotated feature | neg | 294.0300 | 2.298 | 5.583 | 5.425 | -0.158 | -0.251 | 0.162 | 0.484 | Unannotated | NA | NA |
| metab_3240 | Glaucine | pos | 356.1865 | 3.835 | 4.457 | 2.636 | -1.820 | -0.251 | 0.162 | 0.484 | Moderate putative | NA | NA |
| metab_818 | Unannotated feature | pos | 460.2707 | 6.343 | 6.227 | 6.165 | -0.062 | -0.251 | 0.162 | 0.484 | Unannotated | NA | NA |
| metab_15691 | Unannotated feature | neg | 468.1159 | 5.668 | 1.978 | 3.421 | 1.443 | 0.251 | 0.162 | 0.484 | Unannotated | NA | NA |
| metab_8953 | Unannotated feature | neg | 344.0504 | 3.769 | 4.782 | 4.438 | -0.344 | -0.251 | 0.162 | 0.484 | Unannotated | NA | NA |
| metab_17458 | Unannotated feature | neg | 690.8810 | 7.607 | 6.721 | 6.784 | 0.063 | 0.251 | 0.162 | 0.484 | Unannotated | NA | NA |
| metab_11626 | Unannotated feature | neg | 154.9459 | 0.142 | 5.259 | 5.245 | -0.014 | -0.251 | 0.162 | 0.484 | Unannotated | NA | NA |
| metab_16233 | Unannotated feature | neg | 467.2479 | 6.483 | 6.129 | 5.944 | -0.185 | -0.251 | 0.162 | 0.484 | Unannotated | NA | NA |
| metab_287 | Arctic acid | pos | 286.9590 | 0.485 | 5.496 | 5.180 | -0.316 | -0.251 | 0.162 | 0.484 | Unannotated | NA | NA |
| metab_19730 | 2,4-Dimethylphenol | neg | 121.0652 | 4.522 | 5.043 | 4.483 | -0.559 | -0.251 | 0.162 | 0.484 | Moderate putative | NA | NA |
| metab_16271 | 2-Decaprenyl-6-methoxyphenol | neg | 841.6219 | 6.553 | 5.624 | 4.938 | -0.686 | -0.251 | 0.162 | 0.484 | Unannotated | NA | NA |
| metab_7545 | Unannotated feature | pos | 217.1551 | 3.670 | 4.724 | 4.453 | -0.271 | -0.251 | 0.162 | 0.484 | Unannotated | NA | NA |
| metab_15127 | Unannotated feature | neg | 229.1554 | 4.582 | 4.762 | 4.307 | -0.455 | -0.251 | 0.162 | 0.484 | Unannotated | NA | NA |
| metab_7500 | Gamma-Aminobutyric acid-betaxanthin | pos | 261.0875 | 3.751 | 5.608 | 5.313 | -0.295 | -0.251 | 0.162 | 0.484 | Unannotated | NA | NA |
| metab_434 | Unannotated feature | pos | 113.9642 | 0.016 | 5.813 | 5.837 | 0.025 | 0.251 | 0.162 | 0.484 | Unannotated | NA | NA |
| metab_16179 | 4-[2-Acetylamino-2-(1-biphenyl-4-ylmethyl-2-oxo-azepan-3-ylcarbamoyl)-ethyl]-2-formyl-benzoic acid | neg | 576.2164 | 6.381 | 2.883 | 4.252 | 1.369 | 0.251 | 0.162 | 0.484 | Unannotated | Organic acids and derivatives | Carboxylic acids and derivatives |
| metab_9428 | Unannotated feature | neg | 284.1618 | 3.434 | 5.322 | 4.985 | -0.337 | -0.251 | 0.162 | 0.484 | Unannotated | NA | NA |
| metab_11934 | Unannotated feature | neg | 587.2893 | 6.115 | 6.439 | 6.070 | -0.369 | -0.251 | 0.162 | 0.484 | Unannotated | NA | NA |
| metab_13974 | Unannotated feature | neg | 316.1880 | 2.659 | 5.408 | 5.115 | -0.293 | -0.251 | 0.162 | 0.484 | Unannotated | NA | NA |
| metab_2706 | Unannotated feature | pos | 243.1075 | 2.075 | 5.877 | 5.903 | 0.027 | 0.251 | 0.162 | 0.484 | Unannotated | NA | NA |
| metab_6124 | Unannotated feature | pos | 655.7358 | 5.841 | 0.064 | 2.344 | 2.280 | 0.251 | 0.162 | 0.484 | Unannotated | NA | NA |
| metab_17374 | Unannotated feature | neg | 757.8378 | 7.666 | 4.952 | 5.108 | 0.156 | 0.251 | 0.162 | 0.484 | Unannotated | NA | NA |
| metab_9230 | Unannotated feature | neg | 390.2042 | 3.716 | 4.381 | 3.286 | -1.095 | -0.251 | 0.162 | 0.484 | Unannotated | NA | NA |
| metab_2942 | Unannotated feature | pos | 232.2023 | 2.937 | 4.795 | 4.553 | -0.242 | -0.251 | 0.162 | 0.484 | Unannotated | NA | NA |
| metab_14074 | Unannotated feature | neg | 340.1406 | 2.799 | 3.935 | 4.359 | 0.423 | 0.251 | 0.162 | 0.484 | Unannotated | NA | NA |
| metab_11621 | Unannotated feature | neg | 219.8871 | 0.199 | 5.180 | 5.147 | -0.033 | -0.251 | 0.162 | 0.484 | Unannotated | NA | NA |
| metab_11206 | Unannotated feature | neg | 362.1064 | 0.644 | 4.419 | 4.670 | 0.251 | 0.251 | 0.162 | 0.484 | Unannotated | NA | NA |
| metab_11213 | 5-Chloro-2'-deoxyuridine | neg | 243.0180 | 0.644 | 5.814 | 5.608 | -0.206 | -0.251 | 0.162 | 0.484 | Unannotated | Nucleosides, nucleotides, and analogues | Pyrimidine nucleosides |
| metab_15576 | 4-Dimethylaminocinnamaldehyde | neg | 220.0976 | 5.450 | 5.334 | 5.245 | -0.089 | -0.251 | 0.162 | 0.484 | Unannotated | Phenylpropanoids and polyketides | Cinnamaldehydes |
| metab_11238 | Unannotated feature | neg | 226.0648 | 0.636 | 5.276 | 5.172 | -0.104 | -0.251 | 0.162 | 0.484 | Unannotated | NA | NA |
| metab_12602 | Unannotated feature | neg | 605.2999 | 6.068 | 6.388 | 6.203 | -0.185 | -0.251 | 0.162 | 0.484 | Unannotated | NA | NA |
| metab_512 | Palitantin, (+)- | pos | 237.1489 | 5.113 | 3.122 | 3.557 | 0.435 | 0.251 | 0.162 | 0.484 | Unannotated | NA | NA |
| metab_1753 | Unannotated feature | pos | 321.1818 | 3.559 | 4.777 | 4.568 | -0.209 | -0.251 | 0.162 | 0.484 | Unannotated | NA | NA |
| metab_12555 | Unannotated feature | neg | 599.2530 | 6.162 | 4.839 | 4.275 | -0.564 | -0.251 | 0.162 | 0.484 | Unannotated | NA | NA |
| metab_6781 | Unannotated feature | pos | 303.1557 | 0.625 | 5.337 | 5.629 | 0.292 | 0.251 | 0.162 | 0.484 | Unannotated | NA | NA |
| metab_6614 | Unannotated feature | pos | 172.0009 | 0.617 | 4.376 | 4.095 | -0.281 | -0.251 | 0.162 | 0.484 | Unannotated | NA | NA |
| metab_526 | Unannotated feature | pos | 455.1609 | 5.223 | 3.606 | 3.086 | -0.519 | -0.251 | 0.162 | 0.484 | Unannotated | NA | NA |
| metab_18185 | Unannotated feature | neg | 511.2736 | 6.310 | 6.621 | 6.723 | 0.102 | 0.251 | 0.162 | 0.484 | Unannotated | NA | NA |
| metab_4294 | 7-(3-((3-Acetylphenoxy)methyl)-1,5-dimethyl-1H-pyrazol-4-yl)-3-(3-(naphthalen-1-yloxy)propyl)-1H-indole-2-carboxylic acid | pos | 605.2791 | 5.976 | 4.734 | 4.633 | -0.100 | -0.251 | 0.162 | 0.484 | Unannotated | NA | NA |
| metab_18098 | Unannotated feature | neg | 336.0043 | 2.920 | 3.119 | 3.764 | 0.646 | 0.251 | 0.162 | 0.484 | Unannotated | NA | NA |
| metab_8343 | Unannotated feature | pos | 330.2031 | 1.968 | 4.193 | 4.073 | -0.120 | -0.251 | 0.162 | 0.484 | Unannotated | NA | NA |
| metab_16953 | Unannotated feature | neg | 166.8326 | 7.861 | 4.354 | 4.386 | 0.033 | 0.251 | 0.162 | 0.484 | Unannotated | NA | NA |
| metab_12647 | Unannotated feature | neg | 272.1294 | 5.964 | 4.421 | 3.908 | -0.513 | -0.251 | 0.162 | 0.484 | Unannotated | NA | NA |
| metab_11199 | 2-Hydroxy-L-methionine | neg | 164.0381 | 0.651 | 5.249 | 5.042 | -0.207 | -0.251 | 0.162 | 0.484 | Unannotated | NA | NA |
| metab_17841 | Unannotated feature | neg | 77.9549 | 6.758 | 3.087 | 3.712 | 0.625 | 0.251 | 0.162 | 0.484 | Unannotated | NA | NA |
| metab_13299 | Unannotated feature | neg | 221.9753 | 0.636 | 5.004 | 4.951 | -0.053 | -0.251 | 0.162 | 0.484 | Unannotated | NA | NA |
| metab_13576 | Unannotated feature | neg | 301.1045 | 1.278 | 4.479 | 4.235 | -0.244 | -0.251 | 0.162 | 0.484 | Unannotated | NA | NA |
| metab_2121 | Unannotated feature | pos | 216.1023 | 2.811 | 4.477 | 4.855 | 0.379 | 0.251 | 0.162 | 0.484 | Unannotated | NA | NA |
| metab_17463 | Unannotated feature | neg | 830.3373 | 7.603 | 4.252 | 4.426 | 0.175 | 0.251 | 0.162 | 0.484 | Unannotated | NA | NA |
| metab_2182 | 2-Methylindole | pos | 132.0811 | 2.941 | 4.655 | 4.474 | -0.181 | -0.251 | 0.162 | 0.484 | Strong putative | NA | NA |
| metab_17069 | Unannotated feature | neg | 177.8442 | 7.918 | 4.934 | 4.922 | -0.012 | -0.251 | 0.162 | 0.484 | Unannotated | NA | NA |
| metab_14455 | (R)-5-Hydroxy-2-(4-hydroxyphenyl)-7-(((2S,3R,4S,5S,6R)-3,4,5-trihydroxy-6-(hydroxymethyl)tetrahydro-2H-pyran-2-yl)oxy)chroman-4-one | neg | 433.1146 | 3.276 | 5.018 | 4.490 | -0.528 | -0.251 | 0.162 | 0.484 | Unannotated | NA | NA |
| metab_3709 | Unannotated feature | pos | 84.3058 | 5.313 | 3.142 | 2.964 | -0.178 | -0.251 | 0.162 | 0.484 | Unannotated | NA | NA |
| metab_8542 | Integerrimine | pos | 358.1617 | 1.321 | 4.645 | 4.118 | -0.527 | -0.251 | 0.162 | 0.484 | Tentative | Phenylpropanoids and polyketides | Macrolides and analogues |
| metab_14471 | 3-Methyl-1-(2,4,6-trihydroxyphenyl)-1-butanone | neg | 255.0875 | 3.306 | 4.778 | 5.174 | 0.396 | 0.251 | 0.162 | 0.484 | Tentative | NA | NA |
| metab_14515 | Unannotated feature | neg | 196.0611 | 3.379 | 5.272 | 5.432 | 0.160 | 0.251 | 0.162 | 0.484 | Unannotated | NA | NA |
| metab_1987 | Unannotated feature | pos | 224.1034 | 2.133 | 3.986 | 3.654 | -0.331 | -0.251 | 0.162 | 0.484 | Unannotated | NA | NA |
| metab_6211 | Unannotated feature | pos | 338.8424 | 0.531 | 3.397 | 4.490 | 1.093 | 0.251 | 0.162 | 0.484 | Unannotated | NA | NA |
| metab_17089 | Unannotated feature | neg | 156.9748 | 7.888 | 4.744 | 4.733 | -0.011 | -0.251 | 0.162 | 0.484 | Unannotated | NA | NA |
| metab_2251 | Unannotated feature | pos | 409.1880 | 3.125 | 5.158 | 4.721 | -0.437 | -0.251 | 0.162 | 0.484 | Unannotated | NA | NA |
| metab_2811 | Unannotated feature | pos | 547.3307 | 6.016 | 5.671 | 6.039 | 0.368 | 0.251 | 0.162 | 0.484 | Unannotated | NA | NA |
| metab_6913 | Unannotated feature | pos | 164.0920 | 0.649 | 4.381 | 4.566 | 0.185 | 0.251 | 0.162 | 0.484 | Unannotated | NA | NA |
| metab_16654 | Unannotated feature | neg | 287.1361 | 1.947 | 4.241 | 3.807 | -0.434 | -0.251 | 0.162 | 0.484 | Unannotated | NA | NA |
| metab_6919 | Unannotated feature | pos | 708.4385 | 4.988 | 2.700 | 4.869 | 2.169 | 0.251 | 0.162 | 0.484 | Unannotated | NA | NA |
| metab_4809 | Unannotated feature | pos | 293.1027 | 3.533 | 1.944 | 3.260 | 1.316 | 0.251 | 0.162 | 0.484 | Unannotated | NA | NA |
| metab_12999 | Unannotated feature | neg | 281.0342 | 0.777 | 3.918 | 4.370 | 0.452 | 0.251 | 0.162 | 0.484 | Unannotated | NA | NA |
| metab_16741 | Unannotated feature | neg | 417.9002 | 7.666 | 5.089 | 5.147 | 0.058 | 0.251 | 0.162 | 0.484 | Unannotated | NA | NA |
| metab_10403 | Unannotated feature | neg | 248.1141 | 2.043 | 2.892 | 3.998 | 1.106 | 0.251 | 0.162 | 0.484 | Unannotated | NA | NA |
| metab_8257 | Unannotated feature | pos | 126.1159 | 2.227 | 5.192 | 5.238 | 0.046 | 0.251 | 0.162 | 0.484 | Unannotated | NA | NA |
| metab_1432 | Calystegine C1 | pos | 192.0869 | 0.665 | 5.159 | 5.274 | 0.115 | 0.251 | 0.162 | 0.484 | Unannotated | NA | NA |
| metab_1354 | Unannotated feature | pos | 162.1127 | 6.151 | 4.483 | 4.329 | -0.155 | -0.251 | 0.162 | 0.484 | Unannotated | NA | NA |
| metab_16736 | Unannotated feature | neg | 129.9751 | 7.666 | 6.287 | 6.248 | -0.038 | -0.251 | 0.162 | 0.484 | Unannotated | NA | NA |
| metab_5085 | Unannotated feature | pos | 425.2491 | 7.068 | 4.688 | 4.557 | -0.131 | -0.251 | 0.162 | 0.484 | Unannotated | NA | NA |
| metab_14811 | Unannotated feature | neg | 608.8077 | 0.524 | 5.312 | 5.890 | 0.578 | 0.251 | 0.162 | 0.484 | Unannotated | NA | NA |
| metab_15422 | Unannotated feature | neg | 266.1400 | 5.110 | 3.776 | 3.545 | -0.231 | -0.251 | 0.162 | 0.484 | Unannotated | NA | NA |
| metab_7169 | L-Threo-3-Phenylserine | pos | 164.0708 | 4.503 | 5.289 | 5.175 | -0.114 | -0.251 | 0.162 | 0.484 | Tentative | NA | NA |
| metab_10794 | Hypoxanthine | neg | 135.0305 | 1.119 | 6.931 | 6.770 | -0.161 | -0.251 | 0.162 | 0.484 | Strong putative | NA | NA |
| metab_12874 | Unannotated feature | neg | 203.1033 | 0.714 | 5.066 | 4.947 | -0.120 | -0.251 | 0.162 | 0.484 | Unannotated | NA | NA |
| metab_10872 | Unannotated feature | neg | 141.0186 | 0.981 | 3.503 | 3.587 | 0.084 | 0.251 | 0.162 | 0.484 | Unannotated | NA | NA |
| metab_1133 | Unannotated feature | pos | 851.6394 | 6.574 | 5.312 | 4.407 | -0.905 | -0.251 | 0.162 | 0.484 | Unannotated | NA | NA |
| metab_12814 | Unannotated feature | neg | 379.1014 | 0.651 | 4.956 | 4.740 | -0.216 | -0.251 | 0.162 | 0.484 | Unannotated | NA | NA |
| metab_1155 | PE(14:0/0:0) | pos | 426.2628 | 6.558 | 5.736 | 5.508 | -0.229 | -0.251 | 0.162 | 0.484 | Moderate putative | NA | NA |
| metab_16521 | Unannotated feature | neg | 216.0333 | 1.691 | 4.760 | 4.629 | -0.131 | -0.251 | 0.162 | 0.484 | Unannotated | NA | NA |
| metab_18737 | Unannotated feature | neg | 231.1599 | 5.871 | 4.359 | 3.940 | -0.419 | -0.251 | 0.162 | 0.484 | Unannotated | NA | NA |
| metab_1051 | Unannotated feature | pos | 317.2070 | 6.821 | 5.532 | 5.310 | -0.222 | -0.251 | 0.162 | 0.484 | Unannotated | NA | NA |
| metab_11135 | Unannotated feature | neg | 100.0396 | 0.706 | 4.460 | 4.320 | -0.140 | -0.251 | 0.162 | 0.484 | Unannotated | NA | NA |
| metab_18880 | Pentalenolactone O | neg | 293.1033 | 5.683 | 4.231 | 4.453 | 0.222 | 0.251 | 0.162 | 0.484 | Moderate putative | NA | NA |
| metab_7692 | Gentisate aldehyde | pos | 180.0658 | 3.388 | 4.810 | 5.023 | 0.214 | 0.251 | 0.162 | 0.484 | Tentative | NA | NA |
| metab_19582 | Unannotated feature | neg | 141.0187 | 4.740 | 3.465 | 3.337 | -0.128 | -0.251 | 0.162 | 0.484 | Unannotated | NA | NA |
| metab_7833 | Unannotated feature | pos | 100.1126 | 3.099 | 3.410 | 3.541 | 0.131 | 0.251 | 0.162 | 0.484 | Unannotated | NA | NA |
| metab_11735 | Propoxate | neg | 257.1298 | 5.603 | 6.316 | 6.280 | -0.035 | -0.251 | 0.162 | 0.484 | Unannotated | Organoheterocyclic compounds | Azoles |
| metab_11639 | Unannotated feature | neg | 176.8973 | 0.073 | 5.172 | 5.158 | -0.014 | -0.251 | 0.162 | 0.484 | Unannotated | NA | NA |
| metab_19463 | Unannotated feature | neg | 152.0710 | 4.919 | 4.605 | 4.488 | -0.117 | -0.251 | 0.162 | 0.484 | Unannotated | NA | NA |
| metab_5452 | Tetranor-12(S)-HETE | pos | 249.1852 | 6.390 | 5.407 | 5.273 | -0.133 | -0.251 | 0.162 | 0.484 | Moderate putative | NA | NA |
| metab_81 | Unannotated feature | pos | 381.0804 | 0.649 | 6.589 | 6.255 | -0.334 | -0.251 | 0.162 | 0.484 | Unannotated | NA | NA |
| metab_16086 | Unannotated feature | neg | 359.1530 | 6.224 | 4.281 | 4.760 | 0.479 | 0.251 | 0.162 | 0.484 | Unannotated | NA | NA |
| metab_12066 | Unannotated feature | neg | 783.5801 | 6.397 | 7.419 | 7.152 | -0.267 | -0.246 | 0.169 | 0.496 | Unannotated | NA | NA |
| metab_12221 | Unannotated feature | neg | 313.2749 | 6.884 | 4.824 | 4.631 | -0.192 | -0.246 | 0.169 | 0.496 | Unannotated | NA | NA |
| metab_19601 | L-Tyrosine ethyl ester | neg | 208.0975 | 4.714 | 4.933 | 4.541 | -0.391 | -0.246 | 0.169 | 0.496 | Unannotated | Organic acids and derivatives | Carboxylic acids and derivatives |
| metab_5485 | Unannotated feature | pos | 199.1445 | 1.952 | 3.951 | 4.131 | 0.180 | 0.246 | 0.169 | 0.496 | Unannotated | NA | NA |
| metab_7263 | Unannotated feature | pos | 125.0965 | 4.285 | 3.305 | 3.513 | 0.207 | 0.246 | 0.169 | 0.496 | Unannotated | NA | NA |
| metab_7227 | Ac-Tyr-OEt | pos | 252.1235 | 4.375 | 4.294 | 4.077 | -0.217 | -0.246 | 0.169 | 0.496 | Tentative | NA | NA |
| metab_16150 | 9-Hydroxy-10-O-D-glucuronoside-12Z-octadecenoate | neg | 489.2712 | 6.318 | 3.471 | 3.763 | 0.292 | 0.246 | 0.169 | 0.496 | Unannotated | Lipids and lipid-like molecules | Saccharolipids |
| metab_16104 | Unannotated feature | neg | 637.2893 | 6.240 | 5.377 | 3.751 | -1.626 | -0.246 | 0.169 | 0.496 | Unannotated | NA | NA |
| metab_11159 | 6-methylthiohexyldesulfoglucosinolate | neg | 368.1203 | 0.675 | 3.782 | 4.428 | 0.646 | 0.246 | 0.169 | 0.496 | Unannotated | Organic oxygen compounds | Organooxygen compounds |
| metab_18843 | Unannotated feature | neg | 380.1467 | 5.734 | 6.079 | 5.959 | -0.121 | -0.246 | 0.169 | 0.496 | Unannotated | NA | NA |
| metab_18867 | Eriodictyol | neg | 287.0563 | 5.698 | 3.429 | 4.131 | 0.702 | 0.246 | 0.169 | 0.496 | Moderate putative | NA | NA |
| metab_10990 | Unannotated feature | neg | 350.1097 | 0.777 | 5.326 | 5.647 | 0.321 | 0.246 | 0.169 | 0.496 | Unannotated | NA | NA |
| metab_12847 | Unannotated feature | neg | 217.0296 | 0.644 | 6.394 | 6.205 | -0.189 | -0.246 | 0.169 | 0.496 | Unannotated | NA | NA |
| metab_1144 | Unannotated feature | pos | 385.3438 | 6.566 | 6.419 | 6.298 | -0.121 | -0.246 | 0.169 | 0.496 | Unannotated | NA | NA |
| metab_12877 | Unannotated feature | neg | 418.1130 | 0.714 | 5.088 | 5.339 | 0.251 | 0.246 | 0.169 | 0.496 | Unannotated | NA | NA |
| metab_10797 | 2-Aminomuconic acid semialdehyde | neg | 186.0403 | 1.100 | 6.434 | 6.655 | 0.221 | 0.246 | 0.169 | 0.496 | Tentative | NA | NA |
| metab_6438 | 3-(2,4-Dimethyl-5-(2-oxo-1,2-dihydroindol-3-ylidenemethyl)-1H-pyrrol-3-yl)propionic acid | pos | 343.1655 | 5.677 | 5.940 | 5.462 | -0.478 | -0.246 | 0.169 | 0.496 | Unannotated | NA | NA |
| metab_1258 | Unannotated feature | pos | 341.2482 | 6.343 | 4.551 | 4.666 | 0.114 | 0.246 | 0.169 | 0.496 | Unannotated | NA | NA |
| metab_1424 | Unannotated feature | pos | 613.3613 | 6.040 | 3.256 | 4.784 | 1.528 | 0.246 | 0.169 | 0.496 | Unannotated | NA | NA |
| metab_13134 | Unannotated feature | neg | 202.9680 | 0.453 | 3.429 | 3.295 | -0.134 | -0.246 | 0.169 | 0.496 | Unannotated | NA | NA |
| metab_13188 | Unannotated feature | neg | 216.9687 | 0.507 | 3.835 | 3.432 | -0.403 | -0.246 | 0.169 | 0.496 | Unannotated | NA | NA |
| metab_891 | Unannotated feature | pos | 128.0113 | 0.617 | 3.794 | 3.750 | -0.044 | -0.246 | 0.169 | 0.496 | Unannotated | NA | NA |
| metab_914 | Unannotated feature | pos | 391.2309 | 7.148 | 5.462 | 5.432 | -0.030 | -0.246 | 0.169 | 0.496 | Unannotated | NA | NA |
| metab_11215 | Unannotated feature | neg | 215.0325 | 0.644 | 6.895 | 6.710 | -0.185 | -0.246 | 0.169 | 0.496 | Unannotated | NA | NA |
| metab_13264 | Threonolactone | neg | 295.0674 | 0.738 | 5.468 | 5.660 | 0.192 | 0.246 | 0.169 | 0.496 | Unannotated | NA | NA |
| metab_10247 | Unannotated feature | neg | 196.0723 | 2.569 | 3.588 | 2.891 | -0.697 | -0.246 | 0.169 | 0.496 | Unannotated | NA | NA |
| metab_1863 | Unannotated feature | pos | 227.1281 | 4.625 | 5.808 | 5.511 | -0.297 | -0.246 | 0.169 | 0.496 | Unannotated | NA | NA |
| metab_18091 | Unannotated feature | neg | 352.1043 | 2.920 | 3.803 | 4.360 | 0.557 | 0.246 | 0.169 | 0.496 | Unannotated | NA | NA |
| metab_13389 | Unannotated feature | neg | 358.0904 | 0.762 | 3.622 | 3.963 | 0.341 | 0.246 | 0.169 | 0.496 | Unannotated | NA | NA |
| metab_17993 | Unannotated feature | neg | 236.0563 | 2.890 | 6.032 | 6.074 | 0.043 | 0.246 | 0.169 | 0.496 | Unannotated | NA | NA |
| metab_2012 | Unannotated feature | pos | 163.1331 | 5.147 | 5.740 | 5.690 | -0.050 | -0.246 | 0.169 | 0.496 | Unannotated | NA | NA |
| metab_13497 | (3R,4R)-3-Amino-4-Hydroxypentanoic Acid | neg | 132.0660 | 1.041 | 4.379 | 4.571 | 0.192 | 0.246 | 0.169 | 0.496 | Unannotated | NA | NA |
| metab_17860 | Unannotated feature | neg | 240.0513 | 2.817 | 6.039 | 6.280 | 0.241 | 0.246 | 0.169 | 0.496 | Unannotated | NA | NA |
| metab_9896 | Unannotated feature | neg | 339.0730 | 2.956 | 3.299 | 3.417 | 0.119 | 0.246 | 0.169 | 0.496 | Unannotated | NA | NA |
| metab_2160 | Unannotated feature | pos | 181.0723 | 2.907 | 4.348 | 3.743 | -0.605 | -0.246 | 0.169 | 0.496 | Unannotated | NA | NA |
| metab_17758 | Unannotated feature | neg | 107.9931 | 6.907 | 4.944 | 4.861 | -0.083 | -0.246 | 0.169 | 0.496 | Unannotated | NA | NA |
| metab_2332 | Unannotated feature | pos | 634.7309 | 5.711 | 4.168 | 4.543 | 0.375 | 0.246 | 0.169 | 0.496 | Unannotated | NA | NA |
| metab_2446 | Methionine Sulfoxide | pos | 166.0535 | 0.649 | 5.257 | 5.195 | -0.062 | -0.246 | 0.169 | 0.496 | Strong putative | NA | NA |
| metab_2450 | 2-(Ethylamino)ethanol | pos | 90.0918 | 0.657 | 4.229 | 4.385 | 0.156 | 0.246 | 0.169 | 0.496 | Moderate putative | NA | NA |
| metab_17485 | Unannotated feature | neg | 430.9258 | 7.534 | 6.067 | 6.096 | 0.029 | 0.246 | 0.169 | 0.496 | Unannotated | NA | NA |
| metab_9432 | Unannotated feature | neg | 276.0549 | 3.429 | 3.879 | 3.144 | -0.735 | -0.246 | 0.169 | 0.496 | Unannotated | NA | NA |
| metab_759 | Unannotated feature | pos | 333.1815 | 6.087 | 5.424 | 5.238 | -0.187 | -0.246 | 0.169 | 0.496 | Unannotated | NA | NA |
| metab_9405 | Unannotated feature | neg | 264.0798 | 3.469 | 1.998 | 3.050 | 1.052 | 0.246 | 0.169 | 0.496 | Unannotated | NA | NA |
| metab_13969 | Unannotated feature | neg | 334.0589 | 2.656 | 3.957 | 3.411 | -0.546 | -0.246 | 0.169 | 0.496 | Unannotated | NA | NA |
| metab_9188 | Unannotated feature | neg | 161.0601 | 3.784 | 3.824 | 3.200 | -0.624 | -0.246 | 0.169 | 0.496 | Unannotated | NA | NA |
| metab_9144 | Unannotated feature | neg | 350.1618 | 3.860 | 3.187 | 2.908 | -0.278 | -0.246 | 0.169 | 0.496 | Unannotated | NA | NA |
| metab_17209 | Unannotated feature | neg | 380.8459 | 7.730 | 4.223 | 4.320 | 0.097 | 0.246 | 0.169 | 0.496 | Unannotated | NA | NA |
| metab_3159 | N,N,O-Tridesmethyltramadol | pos | 460.3130 | 3.613 | 4.350 | 4.979 | 0.628 | 0.246 | 0.169 | 0.496 | Unannotated | Benzenoids | Benzene and substituted derivatives |
| metab_3196 | Unannotated feature | pos | 266.1755 | 3.712 | 4.061 | 3.879 | -0.182 | -0.246 | 0.169 | 0.496 | Unannotated | NA | NA |
| metab_17132 | Unannotated feature | neg | 136.8622 | 7.814 | 5.828 | 5.813 | -0.015 | -0.246 | 0.169 | 0.496 | Unannotated | NA | NA |
| metab_8463 | Unannotated feature | pos | 212.0234 | 1.844 | 3.248 | 2.819 | -0.429 | -0.246 | 0.169 | 0.496 | Unannotated | NA | NA |
| metab_4019 | Unannotated feature | pos | 699.2870 | 6.175 | 5.510 | 4.512 | -0.998 | -0.246 | 0.169 | 0.496 | Unannotated | NA | NA |
| metab_4185 | Unannotated feature | pos | 551.8385 | 5.914 | 3.909 | 3.999 | 0.090 | 0.246 | 0.169 | 0.496 | Unannotated | NA | NA |
| metab_19392 | Unannotated feature | neg | 387.1857 | 5.022 | 0.896 | 2.842 | 1.947 | 0.246 | 0.169 | 0.496 | Unannotated | NA | NA |
| metab_19338 | Sulforidazine | neg | 383.1285 | 5.087 | 4.278 | 4.614 | 0.336 | 0.246 | 0.169 | 0.496 | Unannotated | NA | NA |
| metab_16963 | Unannotated feature | neg | 204.9241 | 7.874 | 5.254 | 5.221 | -0.033 | -0.246 | 0.169 | 0.496 | Unannotated | NA | NA |
| metab_16915 | Unannotated feature | neg | 164.9342 | 0.566 | 4.512 | 3.832 | -0.681 | -0.246 | 0.169 | 0.496 | Unannotated | NA | NA |
| metab_16840 | Unannotated feature | neg | 663.2937 | 7.726 | 4.423 | 4.545 | 0.122 | 0.246 | 0.169 | 0.496 | Unannotated | NA | NA |
| metab_14763 | Unannotated feature | neg | 271.0474 | 3.826 | 3.541 | 4.121 | 0.580 | 0.246 | 0.169 | 0.496 | Unannotated | NA | NA |
| metab_16803 | Unannotated feature | neg | 179.8917 | 7.714 | 3.642 | 3.490 | -0.152 | -0.246 | 0.169 | 0.496 | Unannotated | NA | NA |
| metab_8054 | Unannotated feature | pos | 253.1551 | 2.774 | 4.783 | 4.733 | -0.051 | -0.246 | 0.169 | 0.496 | Unannotated | NA | NA |
| metab_16662 | Unannotated feature | neg | 656.8869 | 7.607 | 7.479 | 7.522 | 0.043 | 0.246 | 0.169 | 0.496 | Unannotated | NA | NA |
| metab_7877 | 2-Phenylacetamide | pos | 118.0656 | 3.002 | 5.638 | 5.675 | 0.037 | 0.246 | 0.169 | 0.496 | Moderate putative | NA | NA |
| metab_5004 | Unannotated feature | pos | 204.1234 | 0.736 | 4.377 | 4.737 | 0.361 | 0.246 | 0.169 | 0.496 | Unannotated | NA | NA |
| metab_5016 | Unannotated feature | pos | 943.7200 | 7.315 | 5.111 | 5.413 | 0.301 | 0.246 | 0.169 | 0.496 | Unannotated | NA | NA |
| metab_7798 | Unannotated feature | pos | 91.0547 | 3.179 | 5.269 | 5.290 | 0.021 | 0.246 | 0.169 | 0.496 | Unannotated | NA | NA |
| metab_7720 | Unannotated feature | pos | 124.0761 | 0.768 | 2.780 | 3.014 | 0.233 | 0.246 | 0.169 | 0.496 | Unannotated | NA | NA |
| metab_16498 | N-Stearoyl Isoleucine | neg | 396.3488 | 7.090 | 4.483 | 4.493 | 0.010 | -0.246 | 0.169 | 0.496 | Unannotated | NA | NA |
| metab_15032 | Unannotated feature | neg | 306.8441 | 0.542 | 4.510 | 4.206 | -0.303 | -0.246 | 0.169 | 0.496 | Unannotated | NA | NA |
| metab_16428 | Unannotated feature | neg | 153.0308 | 1.680 | 4.156 | 4.440 | 0.284 | 0.246 | 0.169 | 0.496 | Unannotated | NA | NA |
| metab_386 | Unannotated feature | pos | 112.0762 | 0.068 | 5.053 | 5.085 | 0.032 | 0.246 | 0.169 | 0.496 | Unannotated | NA | NA |
| metab_12364 | Unannotated feature | neg | 588.8991 | 7.596 | 7.689 | 7.727 | 0.037 | 0.246 | 0.169 | 0.496 | Unannotated | NA | NA |
| metab_16457 | Unannotated feature | neg | 166.0220 | 6.997 | 4.339 | 4.157 | -0.182 | -0.246 | 0.169 | 0.496 | Unannotated | NA | NA |
| metab_5332 | Unannotated feature | pos | 767.5838 | 6.534 | 6.082 | 5.431 | -0.651 | -0.246 | 0.169 | 0.496 | Unannotated | NA | NA |
| metab_15134 | Unannotated feature | neg | 285.1710 | 4.596 | 5.653 | 5.925 | 0.272 | 0.246 | 0.169 | 0.496 | Unannotated | NA | NA |
| metab_7525 | Nalidixic Acid | pos | 215.0820 | 3.712 | 4.626 | 4.393 | -0.233 | -0.246 | 0.169 | 0.496 | Tentative | NA | NA |
| metab_7155 | 2-Phenylethyl beta-D-glucopyranoside | pos | 249.1124 | 4.528 | 3.160 | 3.229 | 0.069 | -0.246 | 0.169 | 0.496 | Unannotated | Organic oxygen compounds | Organooxygen compounds |
| metab_6432 | Unannotated feature | pos | 484.8126 | 5.686 | 3.781 | 3.789 | 0.008 | 0.242 | 0.177 | 0.504 | Unannotated | NA | NA |
| metab_6886 | Unannotated feature | pos | 265.1121 | 0.641 | 5.606 | 5.938 | 0.331 | 0.242 | 0.177 | 0.504 | Unannotated | NA | NA |
| metab_12450 | Unannotated feature | neg | 190.9279 | 7.792 | 6.602 | 6.575 | -0.027 | -0.242 | 0.177 | 0.504 | Unannotated | NA | NA |
| metab_9584 | Unannotated feature | neg | 225.0437 | 3.239 | 3.538 | 4.028 | 0.491 | 0.242 | 0.177 | 0.504 | Unannotated | NA | NA |
| metab_1773 | Thr Val Ala Lys | pos | 382.2459 | 3.283 | 5.067 | 4.359 | -0.708 | -0.242 | 0.177 | 0.504 | Unannotated | NA | NA |
| metab_16596 | Unannotated feature | neg | 199.0219 | 1.861 | 4.204 | 3.982 | -0.222 | -0.242 | 0.177 | 0.504 | Unannotated | NA | NA |
| metab_5073 | Phorbol 12-myristate 13-acetate | pos | 639.3861 | 7.108 | 2.648 | 2.791 | 0.143 | 0.242 | 0.177 | 0.504 | Tentative | NA | NA |
| metab_5058 | Unannotated feature | pos | 362.9271 | 7.148 | 3.973 | 4.726 | 0.752 | 0.242 | 0.177 | 0.504 | Unannotated | NA | NA |
| metab_1535 | Unannotated feature | pos | 374.2450 | 5.369 | 4.253 | 4.068 | -0.185 | -0.242 | 0.177 | 0.504 | Unannotated | NA | NA |
| metab_12576 | Unannotated feature | neg | 545.2799 | 6.115 | 5.648 | 5.855 | 0.207 | 0.242 | 0.177 | 0.504 | Unannotated | NA | NA |
| metab_19077 | Doisynoestrol | neg | 319.1302 | 5.423 | 6.373 | 6.304 | -0.070 | -0.242 | 0.177 | 0.504 | Tentative | Lipids and lipid-like molecules | Steroids and steroid derivatives |
| metab_19075 | Alpha-Bisabolol oxide A | neg | 275.1406 | 5.427 | 5.543 | 5.478 | -0.065 | -0.242 | 0.177 | 0.504 | Tentative | NA | NA |
| metab_4759 | Unannotated feature | pos | 227.0707 | 7.724 | 6.183 | 6.196 | 0.013 | 0.242 | 0.177 | 0.504 | Unannotated | NA | NA |
| metab_14207 | 3-Ethoxypropanoic Acid | neg | 117.0550 | 2.951 | 6.017 | 5.952 | -0.065 | -0.242 | 0.177 | 0.504 | Moderate putative | NA | NA |
| metab_5294 | Unannotated feature | pos | 558.4386 | 6.582 | 5.717 | 5.622 | -0.095 | -0.242 | 0.177 | 0.504 | Unannotated | NA | NA |
| metab_11270 | Unannotated feature | neg | 141.0663 | 0.625 | 2.929 | 3.602 | 0.673 | 0.242 | 0.177 | 0.504 | Unannotated | NA | NA |
| metab_2722 | Unannotated feature | pos | 299.1338 | 2.168 | 5.411 | 5.348 | -0.064 | -0.242 | 0.177 | 0.504 | Unannotated | NA | NA |
| metab_9357 | Unannotated feature | neg | 154.0867 | 3.541 | 3.950 | 3.503 | -0.447 | -0.242 | 0.177 | 0.504 | Unannotated | NA | NA |
| metab_2684 | Unannotated feature | pos | 182.1292 | 1.994 | 5.300 | 4.657 | -0.644 | -0.242 | 0.177 | 0.504 | Unannotated | NA | NA |
| metab_729 | Unannotated feature | pos | 589.3041 | 5.984 | 7.021 | 6.573 | -0.447 | -0.242 | 0.177 | 0.504 | Unannotated | NA | NA |
| metab_14951 | Unannotated feature | neg | 353.1359 | 4.231 | 5.372 | 5.263 | -0.109 | -0.242 | 0.177 | 0.504 | Unannotated | NA | NA |
| metab_14264 | Unannotated feature | neg | 330.2038 | 3.011 | 5.569 | 5.418 | -0.151 | -0.242 | 0.177 | 0.504 | Unannotated | NA | NA |
| metab_12905 | Histidine glutamate | neg | 281.0880 | 0.730 | 5.500 | 5.632 | 0.132 | 0.242 | 0.177 | 0.504 | Unannotated | NA | NA |
| metab_10066 | Unannotated feature | neg | 336.1204 | 2.780 | 4.758 | 4.589 | -0.169 | -0.242 | 0.177 | 0.504 | Unannotated | NA | NA |
| metab_1975 | Unannotated feature | pos | 300.1452 | 1.952 | 4.580 | 4.992 | 0.412 | 0.242 | 0.177 | 0.504 | Unannotated | NA | NA |
| metab_4711 | Unannotated feature | pos | 472.9030 | 7.618 | 5.657 | 5.728 | 0.072 | 0.242 | 0.177 | 0.504 | Unannotated | NA | NA |
| metab_12587 | Unannotated feature | neg | 299.1196 | 6.099 | 5.633 | 5.496 | -0.138 | -0.242 | 0.177 | 0.504 | Unannotated | NA | NA |
| metab_463 | PE(DiMe(13,5)/20:4(8Z,11Z,14Z,17Z)-2OH(5S,6R)) | pos | 458.7894 | 4.810 | 4.022 | 4.439 | 0.418 | 0.242 | 0.177 | 0.504 | Unannotated | NA | NA |
| metab_8166 | Unannotated feature | pos | 262.1484 | 2.661 | 4.428 | 3.844 | -0.585 | -0.242 | 0.177 | 0.504 | Unannotated | NA | NA |
| metab_9701 | 5-(4-Carboxybutylperoxy)pentanoic acid | neg | 233.1029 | 4.246 | 3.766 | 4.346 | 0.580 | 0.242 | 0.177 | 0.504 | Unannotated | Organic acids and derivatives | Carboxylic acids and derivatives |
| metab_4714 | Unannotated feature | pos | 804.8467 | 7.618 | 4.663 | 4.805 | 0.142 | 0.242 | 0.177 | 0.504 | Unannotated | NA | NA |
| metab_4639 | Unannotated feature | pos | 446.6065 | 7.212 | 4.714 | 4.643 | -0.072 | -0.242 | 0.177 | 0.504 | Unannotated | NA | NA |
| metab_11627 | Unannotated feature | neg | 293.8686 | 0.132 | 4.906 | 4.888 | -0.018 | -0.242 | 0.177 | 0.504 | Unannotated | NA | NA |
| metab_3243 | Unannotated feature | pos | 379.2824 | 6.877 | 6.685 | 6.740 | 0.055 | 0.242 | 0.177 | 0.504 | Unannotated | NA | NA |
| metab_6193 | Unannotated feature | pos | 584.0250 | 5.820 | 3.081 | 3.971 | 0.890 | 0.242 | 0.177 | 0.504 | Unannotated | NA | NA |
| metab_6154 | Unannotated feature | pos | 575.4407 | 5.835 | 3.680 | 3.974 | 0.294 | 0.242 | 0.177 | 0.504 | Unannotated | NA | NA |
| metab_8711 | N-Succinyl-L,L-2,6-diaminopimelate | pos | 332.1450 | 0.768 | 3.967 | 4.040 | 0.073 | 0.242 | 0.177 | 0.504 | Tentative | NA | NA |
| metab_1201 | Unannotated feature | pos | 517.3891 | 6.438 | 5.591 | 5.789 | 0.198 | 0.242 | 0.177 | 0.504 | Unannotated | NA | NA |
| metab_2857 | Unannotated feature | pos | 385.2568 | 2.774 | 3.516 | 2.792 | -0.724 | -0.242 | 0.177 | 0.504 | Unannotated | NA | NA |
| metab_9276 | Enalapril | neg | 397.1722 | 3.647 | 2.570 | 3.257 | 0.687 | 0.242 | 0.177 | 0.504 | Tentative | Organic acids and derivatives | Carboxylic acids and derivatives |
| metab_12723 | Unannotated feature | neg | 326.1039 | 5.087 | 2.397 | 3.456 | 1.059 | 0.242 | 0.177 | 0.504 | Unannotated | NA | NA |
| metab_4525 | N-Lauroyl Isoleucine | pos | 377.2779 | 6.726 | 2.221 | 2.701 | 0.480 | 0.242 | 0.177 | 0.504 | Unannotated | Organic acids and derivatives | Carboxylic acids and derivatives |
| metab_8193 | Val Glu Asp | pos | 362.1567 | 2.518 | 4.119 | 3.415 | -0.704 | -0.242 | 0.177 | 0.504 | Moderate putative | NA | NA |
| metab_15194 | Unannotated feature | neg | 300.8989 | 0.552 | 5.321 | 5.130 | -0.191 | -0.242 | 0.177 | 0.504 | Unannotated | NA | NA |
| metab_12741 | Unannotated feature | neg | 134.0604 | 3.647 | 3.970 | 3.519 | -0.450 | -0.242 | 0.177 | 0.504 | Unannotated | NA | NA |
| metab_8305 | Unannotated feature | pos | 159.0134 | 2.065 | 4.042 | 4.089 | 0.048 | 0.242 | 0.177 | 0.504 | Unannotated | NA | NA |
| metab_17299 | Unannotated feature | neg | 150.9463 | 7.710 | 4.117 | 4.065 | -0.052 | -0.242 | 0.177 | 0.504 | Unannotated | NA | NA |
| metab_2895 | Unannotated feature | pos | 246.1129 | 2.845 | 3.009 | 3.536 | 0.526 | 0.242 | 0.177 | 0.504 | Unannotated | NA | NA |
| metab_12888 | Prolylglycine | neg | 389.1686 | 0.722 | 4.724 | 4.360 | -0.364 | -0.242 | 0.177 | 0.504 | Unannotated | NA | NA |
| metab_8715 | 5-[1-Carboxy-2-(trimethylazaniumyl)ethoxy]-5-oxopentanoate | pos | 262.1290 | 0.768 | 4.852 | 5.040 | 0.187 | 0.242 | 0.177 | 0.504 | Unannotated | Organic acids and derivatives | Carboxylic acids and derivatives |
| metab_3510 | Unannotated feature | pos | 184.0285 | 7.724 | 5.179 | 5.185 | 0.006 | 0.242 | 0.177 | 0.504 | Unannotated | NA | NA |
| metab_14194 | 3-(7'-methylthio)heptylmalate | neg | 321.0988 | 2.935 | 3.519 | 3.947 | 0.428 | 0.242 | 0.177 | 0.504 | Tentative | NA | NA |
| metab_12857 | Unannotated feature | neg | 360.1072 | 0.636 | 3.440 | 4.300 | 0.860 | 0.242 | 0.177 | 0.504 | Unannotated | NA | NA |
| metab_1170 | Unannotated feature | pos | 487.3042 | 6.518 | 4.750 | 4.475 | -0.276 | -0.242 | 0.177 | 0.504 | Unannotated | NA | NA |
| metab_14357 | 1-Methyladenosine | neg | 262.0940 | 3.131 | 3.605 | 4.075 | 0.470 | 0.242 | 0.177 | 0.504 | Tentative | NA | NA |
| metab_2221 | Unannotated feature | pos | 247.1444 | 3.047 | 3.543 | 4.343 | 0.800 | 0.242 | 0.177 | 0.504 | Unannotated | NA | NA |
| metab_18244 | Unannotated feature | neg | 588.3765 | 6.256 | 4.641 | 4.379 | -0.262 | -0.242 | 0.177 | 0.504 | Unannotated | NA | NA |
| metab_17457 | Unannotated feature | neg | 725.8779 | 7.607 | 6.363 | 6.439 | 0.076 | 0.242 | 0.177 | 0.504 | Unannotated | NA | NA |
| metab_13078 | Unannotated feature | neg | 175.0718 | 1.046 | 5.058 | 5.235 | 0.177 | 0.242 | 0.177 | 0.504 | Unannotated | NA | NA |
| metab_17310 | Unannotated feature | neg | 307.9816 | 2.575 | 4.554 | 4.395 | -0.159 | -0.242 | 0.177 | 0.504 | Unannotated | NA | NA |
| metab_15274 | 2-Propyl-2,4-pentadienoic acid | neg | 185.0814 | 4.837 | 4.412 | 4.481 | 0.069 | 0.242 | 0.177 | 0.504 | Tentative | Lipids and lipid-like molecules | Fatty Acyls |
| metab_11068 | Indole-3-acetaldehyde | neg | 204.0662 | 4.992 | 5.300 | 5.525 | 0.225 | 0.242 | 0.177 | 0.504 | Moderate putative | NA | NA |
| metab_4467 | Unannotated feature | pos | 439.3629 | 6.454 | 4.994 | 4.585 | -0.408 | -0.242 | 0.177 | 0.504 | Unannotated | NA | NA |
| metab_4464 | Unannotated feature | pos | 186.1855 | 6.454 | 3.806 | 3.731 | -0.074 | -0.242 | 0.177 | 0.504 | Unannotated | NA | NA |
| metab_8332 | 4-Hydroxyequilenin | pos | 247.1115 | 1.994 | 3.985 | 4.228 | 0.244 | 0.242 | 0.177 | 0.504 | Unannotated | Lipids and lipid-like molecules | Steroids and steroid derivatives |
| metab_16237 | N-Acetylaspartylglutamic Acid | neg | 303.0834 | 1.354 | 4.597 | 5.161 | 0.564 | 0.242 | 0.177 | 0.504 | Strong putative | NA | NA |
| metab_8649 | Hydroxylated lecithin | pos | 113.0351 | 1.026 | 6.348 | 6.268 | -0.080 | -0.242 | 0.177 | 0.504 | Tentative | NA | NA |
| metab_7749 | Verbenalol | pos | 209.0811 | 3.233 | 4.117 | 3.825 | -0.292 | -0.242 | 0.177 | 0.504 | Unannotated | Lipids and lipid-like molecules | Prenol lipids |
| metab_11486 | Unannotated feature | neg | 415.1371 | 5.227 | 4.168 | 3.961 | -0.206 | -0.242 | 0.177 | 0.504 | Unannotated | NA | NA |
| metab_11507 | Unannotated feature | neg | 259.9303 | 0.512 | 4.875 | 4.988 | 0.113 | 0.242 | 0.177 | 0.504 | Unannotated | NA | NA |
| metab_1878 | Unannotated feature | pos | 254.1027 | 1.231 | 4.758 | 5.059 | 0.300 | 0.242 | 0.177 | 0.504 | Unannotated | NA | NA |
| metab_4883 | Unannotated feature | pos | 111.5197 | 7.716 | 4.752 | 4.739 | -0.013 | -0.242 | 0.177 | 0.504 | Unannotated | NA | NA |
| metab_4943 | 4-Pentenoic acid | pos | 101.0602 | 0.720 | 5.857 | 5.421 | -0.435 | -0.242 | 0.177 | 0.504 | Unannotated | NA | NA |
| metab_2327 | Unannotated feature | pos | 161.9698 | 0.380 | 5.301 | 5.335 | 0.034 | 0.242 | 0.177 | 0.504 | Unannotated | NA | NA |
| metab_15024 | Unannotated feature | neg | 220.0612 | 4.386 | 3.175 | 3.805 | 0.630 | 0.242 | 0.177 | 0.504 | Unannotated | NA | NA |
| metab_12478 | Unannotated feature | neg | 125.8725 | 7.910 | 5.355 | 5.395 | 0.040 | 0.242 | 0.177 | 0.504 | Unannotated | NA | NA |
| metab_18786 | Foliandrin | neg | 621.3313 | 5.793 | 5.065 | 5.625 | 0.561 | 0.242 | 0.177 | 0.504 | Unannotated | NA | NA |
| metab_9852 | 3,4,5,6-Tetrahydrohippuric acid | neg | 228.0876 | 2.994 | 4.895 | 5.007 | 0.112 | 0.242 | 0.177 | 0.504 | Unannotated | NA | NA |
| metab_11703 | Unannotated feature | neg | 540.2226 | 5.480 | 4.353 | 4.546 | 0.193 | 0.242 | 0.177 | 0.504 | Unannotated | NA | NA |
| metab_9825 | 4-Vinylcyclohexene dioxide | neg | 185.0814 | 3.021 | 4.650 | 4.790 | 0.141 | 0.242 | 0.177 | 0.504 | Tentative | NA | NA |
| metab_17932 | Unannotated feature | neg | 329.0885 | 2.858 | 4.253 | 4.425 | 0.172 | 0.242 | 0.177 | 0.504 | Unannotated | NA | NA |
| metab_5680 | Alfaprostolum | pos | 429.2625 | 6.135 | 5.695 | 5.216 | -0.479 | -0.242 | 0.177 | 0.504 | Unannotated | NA | NA |
| metab_19603 | Cetiedil | neg | 386.1572 | 4.710 | 4.447 | 5.300 | 0.853 | 0.242 | 0.177 | 0.504 | Tentative | NA | NA |
| metab_13022 | Unannotated feature | neg | 111.0193 | 0.850 | 6.551 | 6.537 | -0.013 | -0.242 | 0.177 | 0.504 | Unannotated | NA | NA |
| metab_17772 | Unannotated feature | neg | 211.0834 | 2.802 | 4.714 | 4.294 | -0.421 | -0.242 | 0.177 | 0.504 | Unannotated | NA | NA |
| metab_13606 | Unannotated feature | neg | 246.9772 | 1.460 | 3.424 | 2.964 | -0.461 | -0.242 | 0.177 | 0.504 | Unannotated | NA | NA |
| metab_8398 | Unannotated feature | pos | 211.1446 | 1.952 | 3.917 | 3.728 | -0.190 | -0.242 | 0.177 | 0.504 | Unannotated | NA | NA |
| metab_3932 | Unannotated feature | pos | 541.3385 | 5.770 | 4.024 | 4.273 | 0.249 | 0.242 | 0.177 | 0.504 | Unannotated | NA | NA |
| metab_11742 | Unannotated feature | neg | 297.0770 | 5.632 | 4.940 | 4.024 | -0.915 | -0.242 | 0.177 | 0.504 | Unannotated | NA | NA |
| metab_1110 | Unannotated feature | pos | 471.3461 | 6.646 | 6.624 | 6.709 | 0.085 | 0.242 | 0.177 | 0.504 | Unannotated | NA | NA |
| metab_12270 | Vaccenic Acid | neg | 327.2543 | 7.004 | 5.095 | 5.648 | 0.553 | 0.242 | 0.177 | 0.504 | Tentative | NA | NA |
| metab_10968 | L-quinate | neg | 441.1258 | 0.784 | 1.715 | 3.867 | 2.152 | 0.242 | 0.177 | 0.504 | Tentative | NA | NA |
| metab_3939 | Unannotated feature | pos | 559.1592 | 5.778 | 3.811 | 3.851 | 0.040 | 0.242 | 0.177 | 0.504 | Unannotated | NA | NA |
| metab_335 | Unannotated feature | pos | 297.0305 | 0.451 | 4.617 | 4.588 | -0.029 | -0.242 | 0.177 | 0.504 | Unannotated | NA | NA |
| metab_19289 | Unannotated feature | neg | 658.3803 | 5.152 | 4.411 | 5.430 | 1.020 | 0.242 | 0.177 | 0.504 | Unannotated | NA | NA |
| metab_3769 | Unannotated feature | pos | 468.2508 | 5.494 | 6.455 | 6.226 | -0.228 | -0.242 | 0.177 | 0.504 | Unannotated | NA | NA |
| metab_3762 | Prostaglandin A2 | pos | 379.1887 | 5.470 | 3.761 | 4.156 | 0.395 | 0.242 | 0.177 | 0.504 | Tentative | Lipids and lipid-like molecules | Fatty Acyls |
| metab_5653 | Unannotated feature | pos | 197.0376 | 0.016 | 4.999 | 5.034 | 0.034 | 0.242 | 0.177 | 0.504 | Unannotated | NA | NA |
| metab_10644 | 2-Acetamido-2-deoxy-6-O-a-L-fucopyranosyl-D-glucose | neg | 412.1484 | 1.660 | 3.406 | 2.867 | -0.539 | -0.242 | 0.177 | 0.504 | Unannotated | Lipids and lipid-like molecules | Fatty Acyls |
| metab_6612 | Unannotated feature | pos | 270.1704 | 5.434 | 4.646 | 4.333 | -0.313 | -0.242 | 0.177 | 0.504 | Unannotated | NA | NA |
| metab_13464 | Unannotated feature | neg | 217.9616 | 1.445 | 5.119 | 4.694 | -0.425 | -0.242 | 0.177 | 0.504 | Unannotated | NA | NA |
| metab_8843 | 20R-Camptothecin | neg | 385.0604 | 3.705 | 5.021 | 5.262 | 0.241 | 0.242 | 0.177 | 0.504 | Unannotated | NA | NA |
| metab_12106 | Unannotated feature | neg | 549.2896 | 6.514 | 6.630 | 6.781 | 0.151 | 0.242 | 0.177 | 0.504 | Unannotated | NA | NA |
| metab_13493 | Unannotated feature | neg | 201.0389 | 1.033 | 4.939 | 5.154 | 0.215 | 0.242 | 0.177 | 0.504 | Unannotated | NA | NA |
| metab_19888 | Oxalic acid | neg | 88.9872 | 0.142 | 6.256 | 6.242 | -0.013 | -0.242 | 0.177 | 0.504 | Strong putative | NA | NA |
| metab_19860 | Dehydrovomifoliol | neg | 267.1238 | 6.256 | 5.031 | 5.150 | 0.119 | 0.242 | 0.177 | 0.504 | Tentative | NA | NA |
| metab_14257 | Unannotated feature | neg | 261.1345 | 2.999 | 4.649 | 4.877 | 0.228 | 0.238 | 0.185 | 0.515 | Unannotated | NA | NA |
| metab_1771 | Unannotated feature | pos | 390.1883 | 3.291 | 5.325 | 4.972 | -0.352 | -0.238 | 0.185 | 0.515 | Unannotated | NA | NA |
| metab_12077 | 2-[[3-Cyclohexyl-1-[2-[3-(diaminomethylideneamino)propylcarbamoyl]piperidin-1-yl]-1-oxopropan-2-yl]amino]acetic acid | neg | 437.2912 | 6.420 | 7.537 | 7.403 | -0.135 | -0.238 | 0.185 | 0.515 | Unannotated | NA | NA |
| metab_17952 | Unannotated feature | neg | 451.3430 | 6.577 | 3.777 | 4.320 | 0.543 | 0.238 | 0.185 | 0.515 | Unannotated | NA | NA |
| metab_19556 | Unannotated feature | neg | 461.1861 | 4.771 | 4.270 | 5.109 | 0.839 | 0.238 | 0.185 | 0.515 | Unannotated | NA | NA |
| metab_3108 | Cumi-101 | pos | 374.2199 | 3.444 | 4.144 | 3.525 | -0.619 | -0.238 | 0.185 | 0.515 | Unannotated | NA | NA |
| metab_16088 | Unannotated feature | neg | 418.2141 | 6.224 | 5.310 | 5.612 | 0.302 | 0.238 | 0.185 | 0.515 | Unannotated | NA | NA |
| metab_10885 | Unannotated feature | neg | 275.9664 | 0.931 | 5.123 | 4.885 | -0.239 | -0.238 | 0.185 | 0.515 | Unannotated | NA | NA |
| metab_6354 | Unannotated feature | pos | 535.5897 | 5.761 | 3.745 | 3.882 | 0.137 | 0.238 | 0.185 | 0.515 | Unannotated | NA | NA |
| metab_17102 | Unannotated feature | neg | 119.9463 | 7.870 | 5.909 | 5.871 | -0.038 | -0.238 | 0.185 | 0.515 | Unannotated | NA | NA |
| metab_9651 | Unannotated feature | neg | 242.0128 | 3.188 | 4.245 | 3.967 | -0.278 | -0.238 | 0.185 | 0.515 | Unannotated | NA | NA |
| metab_9418 | Isomaltoside | neg | 353.1458 | 3.453 | 3.368 | 3.777 | 0.409 | 0.238 | 0.185 | 0.515 | Unannotated | Organic oxygen compounds | Organooxygen compounds |
| metab_10435 | Unannotated feature | neg | 387.2256 | 1.976 | 4.200 | 3.468 | -0.731 | -0.238 | 0.185 | 0.515 | Unannotated | NA | NA |
| metab_19309 | Unannotated feature | neg | 378.1563 | 5.132 | 4.117 | 3.460 | -0.656 | -0.238 | 0.185 | 0.515 | Unannotated | NA | NA |
| metab_7542 | 5-Nitro-2-(3-phenylpropylamino)benzoic acid | pos | 333.1454 | 3.670 | 4.795 | 4.695 | -0.100 | -0.238 | 0.185 | 0.515 | Tentative | NA | NA |
| metab_893 | DG(15:0/18:4(6Z,9Z,12Z,15Z)/0:0) | pos | 597.4505 | 6.973 | 6.905 | 6.809 | -0.096 | -0.238 | 0.185 | 0.515 | Tentative | NA | NA |
| metab_1969 | Unannotated feature | pos | 97.0653 | 2.012 | 4.581 | 4.594 | 0.013 | 0.238 | 0.185 | 0.515 | Unannotated | NA | NA |
| metab_1972 | Unannotated feature | pos | 341.1354 | 1.952 | 3.422 | 4.167 | 0.745 | 0.238 | 0.185 | 0.515 | Unannotated | NA | NA |
| metab_14421 | Unannotated feature | neg | 239.0490 | 3.223 | 1.560 | 2.040 | 0.480 | 0.238 | 0.185 | 0.515 | Unannotated | NA | NA |
| metab_16508 | Unannotated feature | neg | 113.0067 | 7.114 | 3.273 | 2.791 | -0.483 | -0.238 | 0.185 | 0.515 | Unannotated | NA | NA |
| metab_5029 | Glycylproline | pos | 173.0924 | 0.760 | 5.749 | 5.746 | -0.003 | -0.238 | 0.185 | 0.515 | Moderate putative | NA | NA |
| metab_11625 | Unannotated feature | neg | 213.9106 | 0.142 | 6.224 | 6.213 | -0.011 | -0.238 | 0.185 | 0.515 | Unannotated | NA | NA |
| metab_4626 | Unannotated feature | pos | 294.9393 | 7.140 | 4.715 | 4.989 | 0.274 | 0.238 | 0.185 | 0.515 | Unannotated | NA | NA |
| metab_17680 | Sterculate | neg | 631.4970 | 7.031 | 6.993 | 6.669 | -0.324 | -0.238 | 0.185 | 0.515 | Tentative | Lipids and lipid-like molecules | Fatty Acyls |
| metab_2736 | Unannotated feature | pos | 107.0798 | 2.227 | 4.900 | 4.934 | 0.034 | 0.238 | 0.185 | 0.515 | Unannotated | NA | NA |
| metab_13122 | Unannotated feature | neg | 343.8690 | 0.424 | 3.853 | 3.894 | 0.041 | 0.238 | 0.185 | 0.515 | Unannotated | NA | NA |
| metab_1965 | Unannotated feature | pos | 304.1508 | 1.952 | 5.444 | 5.326 | -0.118 | -0.238 | 0.185 | 0.515 | Unannotated | NA | NA |
| metab_4674 | Unannotated feature | pos | 727.4611 | 7.474 | 4.704 | 4.475 | -0.229 | -0.238 | 0.185 | 0.515 | Unannotated | NA | NA |
| metab_12679 | Unannotated feature | neg | 679.2745 | 5.675 | 5.019 | 4.738 | -0.281 | -0.238 | 0.185 | 0.515 | Unannotated | NA | NA |
| metab_935 | Unannotated feature | pos | 739.5541 | 7.482 | 5.642 | 4.797 | -0.846 | -0.238 | 0.185 | 0.515 | Unannotated | NA | NA |
| metab_11613 | Unannotated feature | neg | 282.8805 | 0.346 | 4.215 | 4.151 | -0.064 | -0.238 | 0.185 | 0.515 | Unannotated | NA | NA |
| metab_9315 | N5-(4-Methoxybenzyl)glutamine | neg | 265.1196 | 3.589 | 2.974 | 3.309 | 0.336 | 0.238 | 0.185 | 0.515 | Unannotated | NA | NA |
| metab_16880 | Unannotated feature | neg | 248.0791 | 7.741 | 4.869 | 4.939 | 0.070 | 0.238 | 0.185 | 0.515 | Unannotated | NA | NA |
| metab_14658 | Unannotated feature | neg | 304.1306 | 3.608 | 4.219 | 3.388 | -0.831 | -0.238 | 0.185 | 0.515 | Unannotated | NA | NA |
| metab_19570 | Unannotated feature | neg | 134.0604 | 4.754 | 3.943 | 3.693 | -0.250 | -0.238 | 0.185 | 0.515 | Unannotated | NA | NA |
| metab_13369 | Unannotated feature | neg | 455.2657 | 6.217 | 6.811 | 6.753 | -0.058 | -0.238 | 0.185 | 0.515 | Unannotated | NA | NA |
| metab_8450 | Butanoyl (2S)-2-amino-5-(diaminomethylideneamino)pentanoate | pos | 245.1612 | 1.936 | 4.959 | 4.306 | -0.653 | -0.238 | 0.185 | 0.515 | Unannotated | Organic acids and derivatives | Carboxylic acids and derivatives |
| metab_13985 | Unannotated feature | neg | 294.0445 | 2.690 | 4.467 | 4.185 | -0.282 | -0.238 | 0.185 | 0.515 | Unannotated | NA | NA |
| metab_9223 | Unannotated feature | neg | 284.1508 | 3.733 | 4.356 | 2.275 | -2.081 | -0.238 | 0.185 | 0.515 | Unannotated | NA | NA |
| metab_4500 | DG(16:0/6 keto-PGF1alpha/0:0) | pos | 705.4926 | 6.614 | 5.165 | 4.663 | -0.503 | -0.238 | 0.185 | 0.515 | Unannotated | NA | NA |
| metab_1783 | Unannotated feature | pos | 296.0995 | 0.768 | 4.506 | 4.581 | 0.075 | 0.238 | 0.185 | 0.515 | Unannotated | NA | NA |
| metab_19288 | Unannotated feature | neg | 147.0444 | 5.156 | 4.162 | 3.970 | -0.191 | -0.238 | 0.185 | 0.515 | Unannotated | NA | NA |
| metab_18609 | Unannotated feature | neg | 272.0930 | 5.983 | 5.372 | 5.113 | -0.258 | -0.238 | 0.185 | 0.515 | Unannotated | NA | NA |
| metab_3212 | Unannotated feature | pos | 170.0604 | 3.751 | 4.595 | 4.045 | -0.550 | -0.238 | 0.185 | 0.515 | Unannotated | NA | NA |
| metab_17150 | Unannotated feature | neg | 88.0395 | 7.777 | 4.748 | 4.687 | -0.061 | -0.238 | 0.185 | 0.515 | Unannotated | NA | NA |
| metab_5446 | Unannotated feature | pos | 537.3416 | 6.390 | 6.129 | 5.963 | -0.166 | -0.238 | 0.185 | 0.515 | Unannotated | NA | NA |
| metab_5453 | Unannotated feature | pos | 235.1696 | 6.390 | 4.526 | 4.297 | -0.229 | -0.238 | 0.185 | 0.515 | Unannotated | NA | NA |
| metab_7368 | Gancaonin X | pos | 356.1866 | 4.037 | 4.906 | 4.143 | -0.763 | -0.238 | 0.185 | 0.515 | Unannotated | NA | NA |
| metab_10528 | Unannotated feature | neg | 337.1410 | 4.592 | 5.385 | 5.587 | 0.202 | 0.238 | 0.185 | 0.515 | Unannotated | NA | NA |
| metab_609 | Unannotated feature | pos | 569.1552 | 5.787 | 3.906 | 3.954 | 0.048 | 0.238 | 0.185 | 0.515 | Unannotated | NA | NA |
| metab_655 | Amphibine H | pos | 570.3115 | 5.851 | 4.268 | 5.364 | 1.096 | 0.238 | 0.185 | 0.515 | Unannotated | Organic acids and derivatives | Carboxylic acids and derivatives |
| metab_5533 | Unannotated feature | pos | 398.1446 | 2.747 | 4.678 | 4.810 | 0.132 | 0.238 | 0.185 | 0.515 | Unannotated | NA | NA |
| metab_12304 | 4-Hydroxyvalsartan | neg | 450.2154 | 6.123 | 5.376 | 5.721 | 0.345 | 0.238 | 0.185 | 0.515 | Unannotated | NA | NA |
| metab_17807 | Unannotated feature | neg | 281.2394 | 6.845 | 2.846 | 3.211 | 0.365 | 0.238 | 0.185 | 0.515 | Unannotated | NA | NA |
| metab_9157 | Unannotated feature | neg | 311.1176 | 3.837 | 3.678 | 4.185 | 0.507 | 0.238 | 0.185 | 0.515 | Unannotated | NA | NA |
| metab_3031 | Ile Ile Thr | pos | 346.2344 | 3.228 | 4.622 | 4.062 | -0.560 | -0.238 | 0.185 | 0.515 | Strong putative | NA | NA |
| metab_16990 | Unannotated feature | neg | 181.9169 | 7.985 | 5.602 | 5.579 | -0.024 | -0.238 | 0.185 | 0.515 | Unannotated | NA | NA |
| metab_11084 | 1-Methylhistidine | neg | 214.0830 | 0.730 | 4.751 | 4.300 | -0.451 | -0.238 | 0.185 | 0.515 | Tentative | NA | NA |
| metab_14573 | Unannotated feature | neg | 348.0664 | 3.465 | 2.024 | 3.246 | 1.222 | 0.238 | 0.185 | 0.515 | Unannotated | NA | NA |
| metab_10365 | Unannotated feature | neg | 291.8769 | 2.158 | 4.656 | 4.313 | -0.343 | -0.238 | 0.185 | 0.515 | Unannotated | NA | NA |
| metab_1627 | Inproquone | pos | 307.1658 | 4.896 | 6.150 | 5.817 | -0.333 | -0.238 | 0.185 | 0.515 | Unannotated | NA | NA |
| metab_1876 | Unannotated feature | pos | 403.1357 | 1.217 | 4.552 | 4.731 | 0.179 | 0.238 | 0.185 | 0.515 | Unannotated | NA | NA |
| metab_13534 | Unannotated feature | neg | 171.0073 | 1.119 | 3.973 | 3.513 | -0.460 | -0.238 | 0.185 | 0.515 | Unannotated | NA | NA |
| metab_2371 | Unannotated feature | pos | 315.1236 | 5.671 | 4.259 | 5.029 | 0.771 | 0.238 | 0.185 | 0.515 | Unannotated | NA | NA |
| metab_2068 | Unannotated feature | pos | 288.1925 | 2.732 | 5.370 | 5.239 | -0.131 | -0.238 | 0.185 | 0.515 | Unannotated | NA | NA |
| metab_17850 | Unannotated feature | neg | 218.1032 | 2.826 | 7.066 | 6.973 | -0.093 | -0.238 | 0.185 | 0.515 | Unannotated | NA | NA |
| metab_382 | Unannotated feature | pos | 444.4061 | 0.082 | 5.400 | 5.364 | -0.035 | -0.238 | 0.185 | 0.515 | Unannotated | NA | NA |
| metab_377 | Unannotated feature | pos | 393.0877 | 0.123 | 5.547 | 5.590 | 0.043 | 0.238 | 0.185 | 0.515 | Unannotated | NA | NA |
| metab_12760 | Unannotated feature | neg | 268.1193 | 1.947 | 4.299 | 4.415 | 0.117 | 0.238 | 0.185 | 0.515 | Unannotated | NA | NA |
| metab_16978 | Unannotated feature | neg | 72.9921 | 7.934 | 4.171 | 4.138 | -0.033 | -0.238 | 0.185 | 0.515 | Unannotated | NA | NA |
| metab_18062 | Unannotated feature | neg | 447.1156 | 2.906 | 5.327 | 4.542 | -0.785 | -0.238 | 0.185 | 0.515 | Unannotated | NA | NA |
| metab_3012 | Unannotated feature | pos | 99.5316 | 3.156 | 3.135 | 2.878 | -0.257 | -0.238 | 0.185 | 0.515 | Unannotated | NA | NA |
| metab_8988 | Glutarate semialdehyde | neg | 231.0873 | 4.100 | 4.833 | 5.068 | 0.235 | 0.238 | 0.185 | 0.515 | Tentative | Lipids and lipid-like molecules | Fatty Acyls |
| metab_3176 | N-Methoxysuccinyl-Ala-Ala-Pro-Val | pos | 488.2720 | 3.661 | 4.165 | 3.764 | -0.401 | -0.238 | 0.185 | 0.515 | Unannotated | NA | NA |
| metab_5655 | Unannotated feature | pos | 227.1257 | 6.167 | 5.426 | 5.316 | -0.111 | -0.238 | 0.185 | 0.515 | Unannotated | NA | NA |
| metab_7129 | Coniferaldehyde | pos | 211.0970 | 4.586 | 3.429 | 3.315 | -0.114 | -0.238 | 0.185 | 0.515 | Tentative | NA | NA |
| metab_5692 | Unannotated feature | pos | 396.2735 | 6.127 | 4.869 | 4.454 | -0.415 | -0.238 | 0.185 | 0.515 | Unannotated | NA | NA |
| metab_2055 | Obtustyrene | pos | 285.0876 | 2.708 | 3.487 | 3.864 | 0.377 | 0.238 | 0.185 | 0.515 | Unannotated | Phenylpropanoids and polyketides | Linear 1,3-diarylpropanoids |
| metab_16638 | Unannotated feature | neg | 498.9135 | 7.546 | 5.856 | 5.889 | 0.033 | 0.238 | 0.185 | 0.515 | Unannotated | NA | NA |
| metab_15729 | Unannotated feature | neg | 266.9239 | 5.730 | 4.158 | 3.417 | -0.742 | -0.238 | 0.185 | 0.515 | Unannotated | NA | NA |
| metab_13486 | Unannotated feature | neg | 209.0122 | 1.021 | 4.806 | 4.620 | -0.185 | -0.238 | 0.185 | 0.515 | Unannotated | NA | NA |
| metab_10733 | 8-Hydroxyguanine | neg | 166.0364 | 1.278 | 4.077 | 4.537 | 0.460 | 0.238 | 0.185 | 0.515 | Tentative | NA | NA |
| metab_3262 | Unannotated feature | pos | 266.1755 | 3.902 | 4.580 | 4.374 | -0.206 | -0.238 | 0.185 | 0.515 | Unannotated | NA | NA |
| metab_18552 | Unannotated feature | neg | 493.3013 | 6.018 | 4.216 | 3.941 | -0.276 | -0.238 | 0.185 | 0.515 | Unannotated | NA | NA |
| metab_8808 | Fludrocortisone acetate | pos | 486.2248 | 5.811 | 5.227 | 4.932 | -0.295 | -0.238 | 0.185 | 0.515 | Tentative | NA | NA |
| metab_8814 | Indicine | pos | 621.3304 | 5.782 | 6.122 | 5.489 | -0.633 | -0.238 | 0.185 | 0.515 | Tentative | NA | NA |
| metab_14245 | Cinnamyl alcohol | neg | 179.0708 | 2.984 | 3.496 | 3.169 | -0.327 | -0.238 | 0.185 | 0.515 | Tentative | NA | NA |
| metab_19333 | Gein | neg | 493.1490 | 5.091 | 3.963 | 4.637 | 0.674 | 0.238 | 0.185 | 0.515 | Tentative | NA | NA |
| metab_744 | 6Z-8-Hydroxygeraniol 8-O-glucoside | pos | 377.1536 | 6.048 | 4.085 | 3.562 | -0.524 | -0.238 | 0.185 | 0.515 | Unannotated | NA | NA |
| metab_7845 | Unannotated feature | pos | 337.1402 | 3.307 | 5.529 | 5.365 | -0.164 | -0.234 | 0.193 | 0.526 | Unannotated | NA | NA |
| metab_7843 | Tryptophyl-Glutamate | pos | 366.1668 | 3.066 | 4.207 | 3.600 | -0.607 | -0.234 | 0.193 | 0.526 | Unannotated | Organic acids and derivatives | Carboxylic acids and derivatives |
| metab_12448 | Unannotated feature | neg | 537.3261 | 6.514 | 7.577 | 7.264 | -0.313 | -0.234 | 0.193 | 0.526 | Unannotated | NA | NA |
| metab_12418 | Unannotated feature | neg | 173.9256 | 7.726 | 5.850 | 5.841 | -0.009 | -0.234 | 0.193 | 0.526 | Unannotated | NA | NA |
| metab_8824 | N-Acetylneuraminic Acid | pos | 310.1139 | 0.680 | 4.809 | 5.165 | 0.356 | 0.234 | 0.193 | 0.526 | Strong putative | NA | NA |
| metab_6855 | 11-Hydroxy-9-tridecenoic acid | pos | 273.1450 | 0.641 | 4.849 | 5.106 | 0.258 | 0.234 | 0.193 | 0.526 | Unannotated | NA | NA |
| metab_16447 | Unannotated feature | neg | 148.9491 | 0.547 | 6.190 | 5.951 | -0.239 | -0.234 | 0.193 | 0.526 | Unannotated | NA | NA |
| metab_4806 | Unannotated feature | pos | 264.1283 | 7.983 | 5.360 | 5.406 | 0.046 | 0.234 | 0.193 | 0.526 | Unannotated | NA | NA |
| metab_10755 | Unannotated feature | neg | 274.0934 | 1.219 | 4.880 | 5.102 | 0.223 | 0.234 | 0.193 | 0.526 | Unannotated | NA | NA |
| metab_6472 | Unannotated feature | pos | 530.0787 | 5.626 | 3.810 | 3.852 | 0.042 | 0.234 | 0.193 | 0.526 | Unannotated | NA | NA |
| metab_12752 | Inosine,2',3'-dideoxy- | neg | 257.0671 | 1.952 | 4.722 | 4.960 | 0.238 | 0.234 | 0.193 | 0.526 | Unannotated | NA | NA |
| metab_4372 | Cardanolmonoene | pos | 325.2532 | 6.215 | 4.813 | 4.382 | -0.431 | -0.234 | 0.193 | 0.526 | Unannotated | NA | NA |
| metab_8574 | Unannotated feature | pos | 159.0281 | 1.204 | 4.525 | 4.307 | -0.218 | -0.234 | 0.193 | 0.526 | Unannotated | NA | NA |
| metab_16054 | Unannotated feature | neg | 529.3054 | 6.185 | 3.316 | 3.645 | 0.329 | 0.234 | 0.193 | 0.526 | Unannotated | NA | NA |
| metab_11977 | Unannotated feature | neg | 462.2871 | 6.177 | 4.844 | 4.640 | -0.204 | -0.234 | 0.193 | 0.526 | Unannotated | NA | NA |
| metab_2022 | Unannotated feature | pos | 259.0930 | 0.720 | 4.807 | 4.631 | -0.176 | -0.234 | 0.193 | 0.526 | Unannotated | NA | NA |
| metab_14497 | Nevirapine | neg | 311.1143 | 3.342 | 4.923 | 4.627 | -0.296 | -0.234 | 0.193 | 0.526 | Tentative | NA | NA |
| metab_12433 | Unannotated feature | neg | 224.0615 | 7.737 | 5.703 | 5.842 | 0.139 | 0.234 | 0.193 | 0.526 | Unannotated | NA | NA |
| metab_16933 | Unannotated feature | neg | 159.8861 | 7.822 | 4.377 | 4.355 | -0.022 | -0.234 | 0.193 | 0.526 | Unannotated | NA | NA |
| metab_13792 | Zearalanone | neg | 341.1358 | 2.000 | 4.574 | 4.105 | -0.469 | -0.234 | 0.193 | 0.526 | Tentative | NA | NA |
| metab_5155 | Guaiol | pos | 240.2324 | 6.861 | 5.079 | 4.964 | -0.114 | -0.234 | 0.193 | 0.526 | Tentative | NA | NA |
| metab_10344 | Unannotated feature | neg | 363.0730 | 2.222 | 4.235 | 3.918 | -0.317 | -0.234 | 0.193 | 0.526 | Unannotated | NA | NA |
| metab_2214 | Unannotated feature | pos | 283.1294 | 3.035 | 4.934 | 5.032 | 0.098 | 0.234 | 0.193 | 0.526 | Unannotated | NA | NA |
| metab_1858 | [(2R,3S,4R,5R)-3,4,5,6-Tetrahydroxy-1-oxohexan-2-yl] 2-hydroxypropanoate | pos | 270.1188 | 1.122 | 3.369 | 3.674 | 0.305 | 0.234 | 0.193 | 0.526 | Unannotated | NA | NA |
| metab_596 | Unannotated feature | pos | 642.0575 | 5.537 | 3.640 | 3.825 | 0.185 | 0.234 | 0.193 | 0.526 | Unannotated | NA | NA |
| metab_4879 | Unannotated feature | pos | 274.1932 | 7.716 | 4.431 | 4.342 | -0.089 | -0.234 | 0.193 | 0.526 | Unannotated | NA | NA |
| metab_9505 | Sporol | neg | 287.1250 | 3.338 | 4.580 | 4.823 | 0.242 | 0.234 | 0.193 | 0.526 | Tentative | NA | NA |
| metab_4340 | (R)-1-Octen-3-ol | pos | 274.2745 | 6.119 | 6.606 | 6.560 | -0.047 | -0.234 | 0.193 | 0.526 | Tentative | NA | NA |
| metab_10294 | Unannotated feature | neg | 393.2002 | 4.554 | 5.216 | 4.907 | -0.309 | -0.234 | 0.193 | 0.526 | Unannotated | NA | NA |
| metab_12733 | Unannotated feature | neg | 391.2858 | 6.530 | 8.097 | 7.933 | -0.164 | -0.234 | 0.193 | 0.526 | Unannotated | NA | NA |
| metab_8485 | Unannotated feature | pos | 201.1237 | 1.692 | 4.649 | 4.565 | -0.084 | -0.234 | 0.193 | 0.526 | Unannotated | NA | NA |
| metab_2994 | Benzamide | pos | 243.1124 | 3.099 | 3.973 | 4.163 | 0.191 | 0.234 | 0.193 | 0.526 | Tentative | Benzenoids | Benzene and substituted derivatives |
| metab_12750 | Unannotated feature | neg | 275.0234 | 1.952 | 4.512 | 4.175 | -0.337 | -0.234 | 0.193 | 0.526 | Unannotated | NA | NA |
| metab_19452 | Unannotated feature | neg | 313.0503 | 3.484 | 4.095 | 3.781 | -0.314 | -0.234 | 0.193 | 0.526 | Unannotated | NA | NA |
| metab_7457 | Zaleplon | pos | 323.1609 | 3.854 | 5.328 | 5.052 | -0.276 | -0.234 | 0.193 | 0.526 | Moderate putative | NA | NA |
| metab_11656 | Unannotated feature | neg | 325.0721 | 5.304 | 4.068 | 4.694 | 0.627 | 0.234 | 0.193 | 0.526 | Unannotated | NA | NA |
| metab_15993 | Unannotated feature | neg | 474.2432 | 6.107 | 5.254 | 5.025 | -0.229 | -0.234 | 0.193 | 0.526 | Unannotated | NA | NA |
| metab_18648 | Glabrin A | neg | 220.0823 | 0.667 | 5.585 | 5.737 | 0.152 | 0.234 | 0.193 | 0.526 | Unannotated | NA | NA |
| metab_14319 | Unannotated feature | neg | 329.0248 | 0.533 | 3.435 | 3.172 | -0.263 | -0.234 | 0.193 | 0.526 | Unannotated | NA | NA |
| metab_9228 | Unannotated feature | neg | 286.1410 | 3.721 | 1.318 | 2.445 | 1.127 | 0.234 | 0.193 | 0.526 | Unannotated | NA | NA |
| metab_9979 | Unannotated feature | neg | 260.1141 | 2.869 | 3.972 | 4.330 | 0.357 | 0.234 | 0.193 | 0.526 | Unannotated | NA | NA |
| metab_17085 | Unannotated feature | neg | 233.9103 | 7.897 | 5.062 | 5.038 | -0.024 | -0.234 | 0.193 | 0.526 | Unannotated | NA | NA |
| metab_8693 | Glycyl-Threonine | pos | 177.0873 | 0.784 | 4.312 | 4.529 | 0.217 | 0.234 | 0.193 | 0.526 | Unannotated | NA | NA |
| metab_8474 | Unannotated feature | pos | 504.3394 | 3.948 | 5.036 | 5.432 | 0.396 | 0.234 | 0.193 | 0.526 | Unannotated | NA | NA |
| metab_12274 | Unannotated feature | neg | 528.1822 | 6.091 | 4.189 | 4.484 | 0.294 | 0.234 | 0.193 | 0.526 | Unannotated | NA | NA |
| metab_17232 | Unannotated feature | neg | 192.9283 | 7.730 | 6.078 | 6.055 | -0.022 | -0.234 | 0.193 | 0.526 | Unannotated | NA | NA |
| metab_17233 | Unannotated feature | neg | 164.9267 | 7.730 | 5.664 | 5.625 | -0.039 | -0.234 | 0.193 | 0.526 | Unannotated | NA | NA |
| metab_9035 | N-Acetyl-DL-Leucine | neg | 172.0973 | 4.020 | 5.722 | 5.703 | -0.019 | -0.234 | 0.193 | 0.526 | Moderate putative | NA | NA |
| metab_7373 | Cis-Sinapic acid | pos | 207.0656 | 4.027 | 4.846 | 3.465 | -1.381 | -0.234 | 0.193 | 0.526 | Unannotated | Phenylpropanoids and polyketides | Cinnamic acids and derivatives |
| metab_14589 | Unannotated feature | neg | 413.2051 | 3.495 | 5.089 | 4.696 | -0.393 | -0.234 | 0.193 | 0.526 | Unannotated | NA | NA |
| metab_12735 | Unannotated feature | neg | 238.0833 | 4.417 | 3.842 | 3.594 | -0.248 | -0.234 | 0.193 | 0.526 | Unannotated | NA | NA |
| metab_10530 | Calystegine B2 | neg | 156.0660 | 1.947 | 4.639 | 4.728 | 0.089 | 0.234 | 0.193 | 0.526 | Tentative | NA | NA |
| metab_9670 | Unannotated feature | neg | 137.0601 | 3.165 | 5.355 | 4.594 | -0.760 | -0.234 | 0.193 | 0.526 | Unannotated | NA | NA |
| metab_14826 | Ile-Val-Val | neg | 328.2246 | 3.941 | 4.813 | 4.660 | -0.154 | -0.234 | 0.193 | 0.526 | Unannotated | Organic acids and derivatives | Carboxylic acids and derivatives |
| metab_2322 | Unannotated feature | pos | 243.1075 | 0.325 | 6.436 | 6.461 | 0.024 | 0.234 | 0.193 | 0.526 | Unannotated | NA | NA |
| metab_18444 | Unannotated feature | neg | 365.0189 | 3.028 | 1.581 | 3.265 | 1.685 | 0.234 | 0.193 | 0.526 | Unannotated | NA | NA |
| metab_4038 | Unannotated feature | pos | 319.1911 | 6.151 | 4.869 | 4.576 | -0.293 | -0.234 | 0.193 | 0.526 | Unannotated | NA | NA |
| metab_11195 | Unannotated feature | neg | 187.0188 | 0.651 | 5.494 | 5.447 | -0.047 | -0.234 | 0.193 | 0.526 | Unannotated | NA | NA |
| metab_16822 | Unannotated feature | neg | 174.9555 | 7.726 | 6.986 | 6.962 | -0.024 | -0.234 | 0.193 | 0.526 | Unannotated | NA | NA |
| metab_10476 | Unannotated feature | neg | 392.0994 | 4.634 | 6.032 | 6.225 | 0.193 | 0.234 | 0.193 | 0.526 | Unannotated | NA | NA |
| metab_2044 | Glycyl-leucine | pos | 189.1237 | 2.643 | 5.740 | 5.514 | -0.226 | -0.234 | 0.193 | 0.526 | Strong putative | NA | NA |
| metab_15129 | Unannotated feature | neg | 390.8062 | 0.547 | 4.800 | 4.634 | -0.166 | -0.234 | 0.193 | 0.526 | Unannotated | NA | NA |
| metab_18354 | Unannotated feature | neg | 381.1163 | 3.011 | 4.366 | 4.637 | 0.271 | 0.234 | 0.193 | 0.526 | Unannotated | NA | NA |
| metab_968 | Unannotated feature | pos | 201.9761 | 7.732 | 5.013 | 4.953 | -0.060 | -0.234 | 0.193 | 0.526 | Unannotated | NA | NA |
| metab_1712 | Unannotated feature | pos | 293.1024 | 3.937 | 3.531 | 2.778 | -0.753 | -0.234 | 0.193 | 0.526 | Unannotated | NA | NA |
| metab_4974 | N-(3-Oxobutanoyl)-DL-Homoserine Lactone | pos | 218.1027 | 0.728 | 5.910 | 6.023 | 0.113 | 0.234 | 0.193 | 0.526 | Moderate putative | NA | NA |
| metab_7728 | 10-Nitrolinoleic acid | pos | 370.1983 | 3.324 | 3.749 | 3.289 | -0.460 | -0.234 | 0.193 | 0.526 | Tentative | NA | NA |
| metab_14896 | Unannotated feature | neg | 288.0882 | 4.095 | 4.423 | 4.827 | 0.403 | 0.234 | 0.193 | 0.526 | Unannotated | NA | NA |
| metab_16392 | Teneligliptin | neg | 407.2026 | 6.821 | 4.384 | 3.939 | -0.445 | -0.234 | 0.193 | 0.526 | Unannotated | Organic acids and derivatives | Carboxylic acids and derivatives |
| metab_514 | Unannotated feature | pos | 612.8820 | 5.147 | 2.918 | 3.759 | 0.841 | 0.234 | 0.193 | 0.526 | Unannotated | NA | NA |
| metab_7555 | Docebenone | pos | 349.1766 | 3.642 | 4.523 | 4.406 | -0.116 | -0.234 | 0.193 | 0.526 | Tentative | Lipids and lipid-like molecules | Fatty Acyls |
| metab_10887 | Unannotated feature | neg | 260.8985 | 0.917 | 4.807 | 4.291 | -0.517 | -0.234 | 0.193 | 0.526 | Unannotated | NA | NA |
| metab_18280 | Unannotated feature | neg | 508.2320 | 6.224 | 4.529 | 4.669 | 0.140 | 0.234 | 0.193 | 0.526 | Unannotated | NA | NA |
| metab_1128 | Unannotated feature | pos | 854.5542 | 6.606 | 4.966 | 4.565 | -0.401 | -0.234 | 0.193 | 0.526 | Unannotated | NA | NA |
| metab_169 | Unannotated feature | pos | 320.2356 | 0.601 | 4.328 | 4.224 | -0.104 | -0.234 | 0.193 | 0.526 | Unannotated | NA | NA |
| metab_19041 | Unannotated feature | neg | 322.1411 | 3.303 | 4.601 | 4.383 | -0.217 | -0.234 | 0.193 | 0.526 | Unannotated | NA | NA |
| metab_18019 | Unannotated feature | neg | 477.3590 | 6.491 | 5.026 | 5.456 | 0.430 | 0.234 | 0.193 | 0.526 | Unannotated | NA | NA |
| metab_4717 | Unannotated feature | pos | 582.8648 | 7.634 | 5.071 | 5.174 | 0.103 | 0.234 | 0.193 | 0.526 | Unannotated | NA | NA |
| metab_4740 | PC(18:0/0:0) | pos | 524.3730 | 7.689 | 5.385 | 4.978 | -0.408 | -0.234 | 0.193 | 0.526 | Strong putative | NA | NA |
| metab_16787 | Unannotated feature | neg | 187.0821 | 7.702 | 3.924 | 3.866 | -0.058 | -0.234 | 0.193 | 0.526 | Unannotated | NA | NA |
| metab_17104 | Unannotated feature | neg | 201.9256 | 7.866 | 4.837 | 4.854 | 0.017 | 0.234 | 0.193 | 0.526 | Unannotated | NA | NA |
| metab_913 | Unannotated feature | pos | 193.9830 | 0.609 | 4.315 | 3.873 | -0.442 | -0.234 | 0.193 | 0.526 | Unannotated | NA | NA |
| metab_4667 | Unannotated feature | pos | 459.3098 | 7.458 | 4.756 | 4.669 | -0.086 | -0.234 | 0.193 | 0.526 | Unannotated | NA | NA |
| metab_5324 | Unannotated feature | pos | 317.2092 | 6.550 | 5.253 | 5.539 | 0.286 | 0.234 | 0.193 | 0.526 | Unannotated | NA | NA |
| metab_9689 | Sobetirome | neg | 349.1416 | 3.134 | 4.116 | 3.911 | -0.204 | -0.234 | 0.193 | 0.526 | Tentative | NA | NA |
| metab_2291 | Unannotated feature | pos | 125.9727 | 0.013 | 3.800 | 3.815 | 0.015 | 0.234 | 0.193 | 0.526 | Unannotated | NA | NA |
| metab_16325 | Phthalic Acid | neg | 165.0186 | 6.663 | 6.165 | 6.123 | -0.042 | -0.234 | 0.193 | 0.526 | Strong putative | NA | NA |
| metab_1964 | Unannotated feature | pos | 95.0609 | 1.990 | 3.859 | 3.503 | -0.357 | -0.234 | 0.193 | 0.526 | Unannotated | NA | NA |
| metab_18931 | Gibberellin A32 | neg | 359.1147 | 5.611 | 5.672 | 5.460 | -0.212 | -0.234 | 0.193 | 0.526 | Unannotated | NA | NA |
| metab_3127 | Fusaric Acid | pos | 197.1289 | 3.511 | 4.827 | 4.710 | -0.118 | -0.234 | 0.193 | 0.526 | Moderate putative | NA | NA |
| metab_13439 | Unannotated feature | neg | 197.9112 | 0.823 | 5.052 | 4.719 | -0.333 | -0.234 | 0.193 | 0.526 | Unannotated | NA | NA |
| metab_10054 | 3,4-Dihydroxyphenylacetaldehyde | neg | 197.0451 | 2.789 | 4.887 | 5.002 | 0.115 | 0.234 | 0.193 | 0.526 | Tentative | NA | NA |
| metab_6790 | Unannotated feature | pos | 120.1023 | 0.633 | 2.738 | 3.242 | 0.504 | 0.234 | 0.193 | 0.526 | Unannotated | NA | NA |
| metab_1982 | Unannotated feature | pos | 288.1926 | 1.952 | 4.397 | 4.582 | 0.184 | 0.234 | 0.193 | 0.526 | Unannotated | NA | NA |
| metab_17426 | Unannotated feature | neg | 664.8744 | 7.625 | 5.602 | 5.714 | 0.111 | 0.234 | 0.193 | 0.526 | Unannotated | NA | NA |
| metab_1206 | 25-Hydroxyvitamin D3 3-sulfate ester | pos | 481.2965 | 6.430 | 5.515 | 5.443 | -0.072 | -0.234 | 0.193 | 0.526 | Unannotated | NA | NA |
| metab_795 | Phytosphingosine | pos | 318.3009 | 6.255 | 6.935 | 6.839 | -0.096 | -0.229 | 0.201 | 0.535 | Tentative | NA | NA |
| metab_13414 | Unannotated feature | neg | 175.0718 | 0.791 | 4.937 | 5.152 | 0.215 | 0.229 | 0.201 | 0.535 | Unannotated | NA | NA |
| metab_3541 | Sclareol oxide | pos | 280.2640 | 6.749 | 7.473 | 7.406 | -0.067 | -0.229 | 0.201 | 0.535 | Tentative | NA | NA |
| metab_1420 | Isodeoxycholic acid | pos | 807.5771 | 6.526 | 7.936 | 7.823 | -0.113 | -0.229 | 0.201 | 0.535 | Tentative | NA | NA |
| metab_16947 | Unannotated feature | neg | 226.8010 | 7.840 | 4.459 | 4.404 | -0.055 | -0.229 | 0.201 | 0.535 | Unannotated | NA | NA |
| metab_11066 | Unannotated feature | neg | 401.1312 | 0.730 | 4.095 | 4.447 | 0.352 | 0.229 | 0.201 | 0.535 | Unannotated | NA | NA |
| metab_4477 | N-Linoleoyl Lysine | pos | 409.3438 | 6.510 | 6.001 | 5.913 | -0.088 | -0.229 | 0.201 | 0.535 | Unannotated | NA | NA |
| metab_8336 | Unannotated feature | pos | 195.0657 | 1.990 | 4.587 | 4.734 | 0.147 | 0.229 | 0.201 | 0.535 | Unannotated | NA | NA |
| metab_2444 | L-Prolinamide | pos | 115.0871 | 0.649 | 4.539 | 4.475 | -0.064 | -0.229 | 0.201 | 0.535 | Strong putative | NA | NA |
| metab_2066 | Unannotated feature | pos | 313.1766 | 2.724 | 4.358 | 4.689 | 0.332 | 0.229 | 0.201 | 0.535 | Unannotated | NA | NA |
| metab_9951 | Unannotated feature | neg | 340.1881 | 2.903 | 4.889 | 4.702 | -0.187 | -0.229 | 0.201 | 0.535 | Unannotated | NA | NA |
| metab_9616 | Unannotated feature | neg | 293.1510 | 4.183 | 4.367 | 3.664 | -0.703 | -0.229 | 0.201 | 0.535 | Unannotated | NA | NA |
| metab_1872 | Unannotated feature | pos | 296.1133 | 1.211 | 3.754 | 4.738 | 0.984 | 0.229 | 0.201 | 0.535 | Unannotated | NA | NA |
| metab_1947 | Unannotated feature | pos | 224.1285 | 1.952 | 4.444 | 4.793 | 0.350 | 0.229 | 0.201 | 0.535 | Unannotated | NA | NA |
| metab_19504 | Unannotated feature | neg | 236.0927 | 3.509 | 4.893 | 4.805 | -0.088 | -0.229 | 0.201 | 0.535 | Unannotated | NA | NA |
| metab_4942 | Unannotated feature | pos | 872.8332 | 7.610 | 4.428 | 4.700 | 0.272 | 0.229 | 0.201 | 0.535 | Unannotated | NA | NA |
| metab_15346 | (-)-Wikstromol | neg | 373.1305 | 4.983 | 5.029 | 4.868 | -0.160 | -0.229 | 0.201 | 0.535 | Tentative | NA | NA |
| metab_12325 | Unannotated feature | neg | 273.2341 | 6.868 | 5.604 | 5.510 | -0.094 | -0.229 | 0.201 | 0.535 | Unannotated | NA | NA |
| metab_4346 | Antibiotic SB 202742 | pos | 335.2372 | 6.135 | 4.366 | 2.818 | -1.548 | -0.229 | 0.201 | 0.535 | Unannotated | NA | NA |
| metab_10159 | Nikkomycin | neg | 530.1310 | 2.725 | 5.656 | 5.190 | -0.466 | -0.229 | 0.201 | 0.535 | Unannotated | Lipids and lipid-like molecules | Steroids and steroid derivatives |
| metab_9385 | Unannotated feature | neg | 290.1149 | 3.495 | 4.613 | 3.968 | -0.645 | -0.229 | 0.201 | 0.535 | Unannotated | NA | NA |
| metab_16364 | 11'-Carboxy-alpha-chromanol | neg | 417.3013 | 6.734 | 5.858 | 6.043 | 0.185 | 0.229 | 0.201 | 0.535 | Unannotated | NA | NA |
| metab_12129 | Unannotated feature | neg | 265.1479 | 6.592 | 7.130 | 7.255 | 0.125 | 0.229 | 0.201 | 0.535 | Unannotated | NA | NA |
| metab_72 | Emedastine | pos | 347.1820 | 0.657 | 3.916 | 4.557 | 0.640 | 0.229 | 0.201 | 0.535 | Tentative | Organoheterocyclic compounds | Benzimidazoles |
| metab_6823 | Unannotated feature | pos | 139.1119 | 5.142 | 4.120 | 3.992 | -0.127 | -0.229 | 0.201 | 0.535 | Unannotated | NA | NA |
| metab_4392 | Cinncassiol D1 | pos | 353.2311 | 6.255 | 5.933 | 5.879 | -0.054 | -0.229 | 0.201 | 0.535 | Tentative | NA | NA |
| metab_16186 | 4-Hydroxy-3-methoxy-2,10-bisaboladien-9-one | neg | 797.5596 | 6.389 | 5.638 | 4.903 | -0.735 | -0.229 | 0.201 | 0.535 | Unannotated | NA | NA |
| metab_1907 | Unannotated feature | pos | 370.1728 | 0.728 | 4.987 | 5.148 | 0.161 | 0.229 | 0.201 | 0.535 | Unannotated | NA | NA |
| metab_18102 | Unannotated feature | neg | 393.2919 | 6.389 | 6.240 | 6.100 | -0.140 | -0.229 | 0.201 | 0.535 | Unannotated | NA | NA |
| metab_12932 | Unannotated feature | neg | 302.1360 | 0.745 | 5.168 | 5.005 | -0.164 | -0.229 | 0.201 | 0.535 | Unannotated | NA | NA |
| metab_12103 | 16-B1-phytoprostane | neg | 307.1917 | 6.506 | 5.266 | 5.664 | 0.398 | 0.229 | 0.201 | 0.535 | Unannotated | Lipids and lipid-like molecules | Fatty Acyls |
| metab_12120 | Unannotated feature | neg | 393.2918 | 6.553 | 6.583 | 6.397 | -0.186 | -0.229 | 0.201 | 0.535 | Unannotated | NA | NA |
| metab_2834 | Unannotated feature | pos | 183.1132 | 2.740 | 4.510 | 4.434 | -0.076 | -0.229 | 0.201 | 0.535 | Unannotated | NA | NA |
| metab_5972 | Unannotated feature | pos | 610.4697 | 5.914 | 3.563 | 3.736 | 0.173 | 0.229 | 0.201 | 0.535 | Unannotated | NA | NA |
| metab_15850 | 3-Cyclopentylpropionic acid | neg | 343.2128 | 5.927 | 6.854 | 6.801 | -0.052 | -0.229 | 0.201 | 0.535 | Unannotated | NA | NA |
| metab_5979 | Unannotated feature | pos | 576.5200 | 5.914 | 3.775 | 4.002 | 0.227 | 0.229 | 0.201 | 0.535 | Unannotated | NA | NA |
| metab_5609 | Meproscillarin | pos | 577.3366 | 6.215 | 5.150 | 4.975 | -0.175 | -0.229 | 0.201 | 0.535 | Unannotated | NA | NA |
| metab_3840 | Kanzonol J | pos | 486.2247 | 5.650 | 4.495 | 3.858 | -0.637 | -0.229 | 0.201 | 0.535 | Unannotated | Phenylpropanoids and polyketides | Isoflavonoids |
| metab_7202 | 9-(4-Fluoro-3-(hydroxymethyl)butyl)guanine | pos | 256.1183 | 4.430 | 3.396 | 4.098 | 0.702 | 0.229 | 0.201 | 0.535 | Unannotated | NA | NA |
| metab_16152 | CD 1790 | neg | 454.2044 | 6.326 | 5.060 | 6.011 | 0.952 | 0.229 | 0.201 | 0.535 | Unannotated | Organoheterocyclic compounds | Imidazopyrimidines |
| metab_16287 | Unannotated feature | neg | 214.9230 | 1.411 | 3.613 | 3.261 | -0.353 | -0.229 | 0.201 | 0.535 | Unannotated | NA | NA |
| metab_1002 | Unannotated feature | pos | 660.5263 | 7.276 | 5.232 | 5.101 | -0.131 | -0.229 | 0.201 | 0.535 | Unannotated | NA | NA |
| metab_4482 | Unannotated feature | pos | 163.1483 | 6.534 | 4.527 | 4.377 | -0.150 | -0.229 | 0.201 | 0.535 | Unannotated | NA | NA |
| metab_1252 | 13-Nor-6-eremophilene-8,11-dione | pos | 221.1539 | 6.367 | 5.905 | 5.726 | -0.179 | -0.229 | 0.201 | 0.535 | Unannotated | NA | NA |
| metab_12188 | Unannotated feature | neg | 379.1950 | 6.750 | 4.871 | 5.390 | 0.518 | 0.229 | 0.201 | 0.535 | Unannotated | NA | NA |
| metab_9140 | Unannotated feature | neg | 185.1078 | 3.867 | 3.941 | 3.777 | -0.164 | -0.229 | 0.201 | 0.535 | Unannotated | NA | NA |
| metab_3016 | Unannotated feature | pos | 319.1909 | 6.327 | 5.031 | 4.977 | -0.055 | -0.229 | 0.201 | 0.535 | Unannotated | NA | NA |
| metab_9043 | Unannotated feature | neg | 353.1358 | 4.013 | 4.416 | 4.243 | -0.173 | -0.229 | 0.201 | 0.535 | Unannotated | NA | NA |
| metab_2906 | Unannotated feature | pos | 324.1451 | 2.864 | 4.171 | 4.718 | 0.547 | 0.229 | 0.201 | 0.535 | Unannotated | NA | NA |
| metab_11016 | Unannotated feature | neg | 382.1363 | 0.753 | 4.261 | 4.434 | 0.173 | 0.229 | 0.201 | 0.535 | Unannotated | NA | NA |
| metab_9810 | Unannotated feature | neg | 336.1206 | 3.028 | 3.967 | 3.724 | -0.243 | -0.229 | 0.201 | 0.535 | Unannotated | NA | NA |
| metab_9789 | Unannotated feature | neg | 339.0451 | 3.045 | 4.438 | 4.272 | -0.167 | -0.229 | 0.201 | 0.535 | Unannotated | NA | NA |
| metab_4263 | Unannotated feature | pos | 515.9799 | 5.402 | 1.154 | 2.821 | 1.667 | 0.229 | 0.201 | 0.535 | Unannotated | NA | NA |
| metab_11744 | Ferulic Acid | neg | 193.0501 | 5.642 | 4.740 | 4.640 | -0.099 | -0.229 | 0.201 | 0.535 | Strong putative | NA | NA |
| metab_1939 | Arginylproline | pos | 294.1553 | 1.944 | 5.126 | 5.526 | 0.400 | 0.229 | 0.201 | 0.535 | Unannotated | NA | NA |
| metab_11660 | P-CHLOROPHENYLALANINE | neg | 198.0323 | 3.025 | 6.515 | 6.470 | -0.045 | -0.229 | 0.201 | 0.535 | Moderate putative | NA | NA |
| metab_2229 | Unannotated feature | pos | 181.0722 | 3.070 | 4.947 | 4.527 | -0.420 | -0.229 | 0.201 | 0.535 | Unannotated | NA | NA |
| metab_3587 | Pantothenic Acid | pos | 220.1184 | 2.811 | 6.800 | 6.705 | -0.095 | -0.229 | 0.201 | 0.535 | Strong putative | NA | NA |
| metab_415 | Unannotated feature | pos | 174.9537 | 0.016 | 4.227 | 4.264 | 0.037 | 0.229 | 0.201 | 0.535 | Unannotated | NA | NA |
| metab_10254 | Unannotated feature | neg | 477.1736 | 2.542 | 3.658 | 4.792 | 1.135 | 0.229 | 0.201 | 0.535 | Unannotated | NA | NA |
| metab_5211 | Unannotated feature | pos | 535.3964 | 6.734 | 5.686 | 5.880 | 0.195 | 0.229 | 0.201 | 0.535 | Unannotated | NA | NA |
| metab_7689 | 2-(2-methoxyethoxy)-N-[(7-oxoazepan-2-yl)methyl]acetamide | pos | 259.1657 | 3.393 | 4.304 | 3.893 | -0.411 | -0.229 | 0.201 | 0.535 | Unannotated | NA | NA |
| metab_18234 | Unannotated feature | neg | 923.3286 | 2.968 | 3.200 | 3.749 | 0.549 | 0.229 | 0.201 | 0.535 | Unannotated | NA | NA |
| metab_5208 | Unannotated feature | pos | 377.2670 | 6.741 | 5.905 | 6.181 | 0.277 | 0.229 | 0.201 | 0.535 | Unannotated | NA | NA |
| metab_9180 | 3-Feruloylquinic acid | neg | 349.0933 | 3.795 | 4.355 | 3.631 | -0.724 | -0.229 | 0.201 | 0.535 | Tentative | NA | NA |
| metab_2992 | Unannotated feature | pos | 242.1390 | 3.093 | 2.027 | 2.326 | 0.298 | 0.229 | 0.201 | 0.535 | Unannotated | NA | NA |
| metab_11741 | 9-Hydroxy-risperidone | neg | 447.1791 | 5.627 | 5.256 | 4.676 | -0.580 | -0.229 | 0.201 | 0.535 | Unannotated | NA | NA |
| metab_16936 | Unannotated feature | neg | 121.9433 | 7.827 | 5.412 | 5.377 | -0.035 | -0.229 | 0.201 | 0.535 | Unannotated | NA | NA |
| metab_3011 | Benzoylnorecgonine | pos | 293.1501 | 3.150 | 4.038 | 3.678 | -0.360 | -0.229 | 0.201 | 0.535 | Unannotated | NA | NA |
| metab_17554 | Unannotated feature | neg | 649.5041 | 7.347 | 4.903 | 4.206 | -0.697 | -0.229 | 0.201 | 0.535 | Unannotated | NA | NA |
| metab_15529 | N1-trans-Feruloylagmatine | neg | 287.1502 | 5.334 | 5.140 | 5.327 | 0.186 | 0.229 | 0.201 | 0.535 | Tentative | NA | NA |
| metab_5592 | Unannotated feature | pos | 403.3217 | 6.239 | 4.870 | 4.944 | 0.074 | 0.229 | 0.201 | 0.535 | Unannotated | NA | NA |
| metab_10190 | Unannotated feature | neg | 294.1309 | 2.659 | 4.491 | 4.160 | -0.331 | -0.229 | 0.201 | 0.535 | Unannotated | NA | NA |
| metab_5338 | Shyobunyl acetate | pos | 229.1953 | 6.534 | 4.366 | 4.183 | -0.183 | -0.229 | 0.201 | 0.535 | Unannotated | NA | NA |
| metab_2238 | Unannotated feature | pos | 353.1714 | 3.093 | 3.660 | 4.282 | 0.622 | 0.229 | 0.201 | 0.535 | Unannotated | NA | NA |
| metab_17503 | Unannotated feature | neg | 375.2309 | 7.490 | 4.584 | 3.833 | -0.751 | -0.229 | 0.201 | 0.535 | Unannotated | NA | NA |
| metab_17499 | Unannotated feature | neg | 669.2684 | 7.499 | 6.460 | 6.561 | 0.101 | 0.229 | 0.201 | 0.535 | Unannotated | NA | NA |
| metab_16267 | Unannotated feature | neg | 395.1898 | 6.553 | 5.615 | 5.858 | 0.243 | 0.229 | 0.201 | 0.535 | Unannotated | NA | NA |
| metab_12631 | Unannotated feature | neg | 267.1238 | 6.003 | 4.960 | 5.167 | 0.207 | 0.229 | 0.201 | 0.535 | Unannotated | NA | NA |
| metab_2049 | Unannotated feature | pos | 112.0511 | 0.665 | 4.248 | 4.388 | 0.141 | 0.229 | 0.201 | 0.535 | Unannotated | NA | NA |
| metab_8015 | Val Ile Glu | pos | 360.2138 | 2.819 | 5.290 | 4.879 | -0.411 | -0.229 | 0.201 | 0.535 | Moderate putative | NA | NA |
| metab_16454 | Unannotated feature | neg | 236.0563 | 6.993 | 5.522 | 5.475 | -0.047 | -0.229 | 0.201 | 0.535 | Unannotated | NA | NA |
| metab_13398 | Unannotated feature | neg | 386.0949 | 0.770 | 5.593 | 5.755 | 0.163 | 0.229 | 0.201 | 0.535 | Unannotated | NA | NA |
| metab_10631 | Unannotated feature | neg | 290.0633 | 1.680 | 6.632 | 6.415 | -0.217 | -0.229 | 0.201 | 0.535 | Unannotated | NA | NA |
| metab_16798 | Unannotated feature | neg | 187.9777 | 7.710 | 4.729 | 4.688 | -0.041 | -0.229 | 0.201 | 0.535 | Unannotated | NA | NA |
| metab_13904 | Unannotated feature | neg | 702.8859 | 0.481 | 4.781 | 4.101 | -0.680 | -0.229 | 0.201 | 0.535 | Unannotated | NA | NA |
| metab_15057 | 2-Nonene-1,4-diol | neg | 203.1284 | 4.446 | 5.170 | 5.237 | 0.067 | 0.229 | 0.201 | 0.535 | Unannotated | NA | NA |
| metab_11607 | Unannotated feature | neg | 145.9490 | 0.417 | 5.067 | 5.048 | -0.019 | -0.229 | 0.201 | 0.535 | Unannotated | NA | NA |
| metab_12421 | Unannotated feature | neg | 289.1291 | 7.726 | 3.359 | 3.932 | 0.573 | 0.229 | 0.201 | 0.535 | Unannotated | NA | NA |
| metab_15097 | Unannotated feature | neg | 134.0604 | 4.522 | 3.203 | 2.795 | -0.408 | -0.229 | 0.201 | 0.535 | Unannotated | NA | NA |
| metab_9322 | Tyrosylhydroxyproline | neg | 293.1146 | 3.581 | 5.498 | 5.458 | -0.039 | -0.229 | 0.201 | 0.535 | Unannotated | NA | NA |
| metab_18368 | Unannotated feature | neg | 446.2918 | 6.154 | 3.799 | 3.623 | -0.175 | -0.229 | 0.201 | 0.535 | Unannotated | NA | NA |
| metab_992 | Unannotated feature | pos | 111.0396 | 7.724 | 5.780 | 5.820 | 0.041 | 0.229 | 0.201 | 0.535 | Unannotated | NA | NA |
| metab_7740 | N-Methylphenylalanine | pos | 197.1289 | 3.312 | 4.703 | 4.652 | -0.052 | -0.229 | 0.201 | 0.535 | Moderate putative | NA | NA |
| metab_17496 | Unannotated feature | neg | 162.9816 | 7.503 | 5.130 | 5.101 | -0.029 | -0.229 | 0.201 | 0.535 | Unannotated | NA | NA |
| metab_16650 | Unannotated feature | neg | 469.9136 | 7.575 | 6.790 | 6.802 | 0.012 | 0.229 | 0.201 | 0.535 | Unannotated | NA | NA |
| metab_1205 | Unannotated feature | pos | 573.3435 | 6.430 | 5.197 | 5.596 | 0.400 | 0.229 | 0.201 | 0.535 | Unannotated | NA | NA |
| metab_2666 | Unannotated feature | pos | 398.1453 | 1.952 | 3.294 | 3.806 | 0.513 | 0.229 | 0.201 | 0.535 | Unannotated | NA | NA |
| metab_14970 | Unannotated feature | neg | 245.1394 | 4.266 | 4.593 | 4.772 | 0.179 | 0.229 | 0.201 | 0.535 | Unannotated | NA | NA |
| metab_6365 | Unannotated feature | pos | 519.0719 | 5.757 | 4.237 | 4.309 | 0.072 | 0.229 | 0.201 | 0.535 | Unannotated | NA | NA |
| metab_10463 | Unannotated feature | neg | 232.0285 | 1.958 | 3.207 | 2.938 | -0.269 | -0.229 | 0.201 | 0.535 | Unannotated | NA | NA |
| metab_9926 | S-Glutaryldihydrolipoamide | neg | 366.1061 | 2.928 | 5.053 | 4.909 | -0.144 | -0.229 | 0.201 | 0.535 | Tentative | NA | NA |
| metab_16076 | Unannotated feature | neg | 242.1760 | 6.217 | 6.271 | 6.184 | -0.087 | -0.229 | 0.201 | 0.535 | Unannotated | NA | NA |
| metab_17870 | Unannotated feature | neg | 422.2683 | 6.711 | 5.926 | 5.568 | -0.358 | -0.229 | 0.201 | 0.535 | Unannotated | NA | NA |
| metab_6488 | Unannotated feature | pos | 684.4330 | 5.597 | 4.082 | 4.474 | 0.392 | 0.229 | 0.201 | 0.535 | Unannotated | NA | NA |
| metab_10019 | Unannotated feature | neg | 140.0346 | 2.822 | 3.808 | 3.667 | -0.142 | -0.229 | 0.201 | 0.535 | Unannotated | NA | NA |
| metab_13419 | Unannotated feature | neg | 248.1140 | 0.791 | 3.255 | 3.862 | 0.607 | 0.229 | 0.201 | 0.535 | Unannotated | NA | NA |
| metab_15758 | Unannotated feature | neg | 128.9589 | 0.603 | 6.980 | 6.851 | -0.130 | -0.229 | 0.201 | 0.535 | Unannotated | NA | NA |
| metab_10785 | Unannotated feature | neg | 222.9916 | 1.135 | 4.247 | 4.666 | 0.419 | 0.229 | 0.201 | 0.535 | Unannotated | NA | NA |
| metab_12059 | Unannotated feature | neg | 493.3182 | 6.381 | 7.042 | 6.908 | -0.134 | -0.229 | 0.201 | 0.535 | Unannotated | NA | NA |
| metab_11955 | Unannotated feature | neg | 615.3179 | 6.146 | 4.014 | 4.542 | 0.528 | 0.225 | 0.209 | 0.544 | Unannotated | NA | NA |
| metab_12946 | Unannotated feature | neg | 139.0506 | 0.738 | 4.719 | 4.506 | -0.214 | -0.225 | 0.209 | 0.544 | Unannotated | NA | NA |
| metab_19732 | Litebamine | neg | 384.1419 | 4.518 | 5.558 | 5.419 | -0.139 | -0.225 | 0.209 | 0.544 | Unannotated | Benzenoids | Phenanthrenes and derivatives |
| metab_15697 | Physalolactone B | neg | 545.3146 | 5.675 | 5.888 | 5.296 | -0.592 | -0.225 | 0.209 | 0.544 | Unannotated | Lipids and lipid-like molecules | Steroids and steroid derivatives |
| metab_16618 | Unannotated feature | neg | 726.5545 | 7.485 | 5.788 | 5.830 | 0.042 | 0.225 | 0.209 | 0.544 | Unannotated | NA | NA |
| metab_8817 | Isonicotinic acid | pos | 124.0397 | 0.854 | 6.322 | 6.291 | -0.031 | -0.225 | 0.209 | 0.544 | Strong putative | NA | NA |
| metab_17138 | Unannotated feature | neg | 311.0202 | 2.298 | 5.231 | 5.097 | -0.134 | -0.225 | 0.209 | 0.544 | Unannotated | NA | NA |
| metab_6870 | Unannotated feature | pos | 477.9603 | 5.073 | 2.521 | 3.773 | 1.252 | 0.225 | 0.209 | 0.544 | Unannotated | NA | NA |
| metab_7606 | N-Acetylvanilalanine | pos | 254.1028 | 3.538 | 4.031 | 4.376 | 0.345 | 0.225 | 0.209 | 0.544 | Unannotated | Organic acids and derivatives | Carboxylic acids and derivatives |
| metab_7607 | Edulitine | pos | 170.0604 | 3.538 | 3.656 | 2.961 | -0.695 | -0.225 | 0.209 | 0.544 | Unannotated | NA | NA |
| metab_13701 | Tranexamic Acid | neg | 202.1080 | 1.726 | 4.546 | 4.690 | 0.144 | 0.225 | 0.209 | 0.544 | Tentative | NA | NA |
| metab_13708 | Unannotated feature | neg | 241.8989 | 0.073 | 6.862 | 6.834 | -0.028 | -0.225 | 0.209 | 0.544 | Unannotated | NA | NA |
| metab_2320 | Unannotated feature | pos | 221.0479 | 0.263 | 4.228 | 4.251 | 0.023 | 0.225 | 0.209 | 0.544 | Unannotated | NA | NA |
| metab_13937 | Unannotated feature | neg | 586.9064 | 0.485 | 6.230 | 5.891 | -0.339 | -0.225 | 0.209 | 0.544 | Unannotated | NA | NA |
| metab_9324 | (4Z,7Z)-9-[(1S,2R,3R)-3-Hydroxy-2-[(1E,3S,5Z)-3-hydroxyocta-1,5-dien-1-yl]-5-oxocyclopentyl]nona-4,7-dienoylcarnitine | neg | 554.2852 | 3.577 | 4.817 | 3.744 | -1.072 | -0.225 | 0.209 | 0.544 | Unannotated | Lipids and lipid-like molecules | Fatty Acyls |
| metab_8084 | Unannotated feature | pos | 293.1139 | 2.747 | 3.812 | 4.065 | 0.253 | 0.225 | 0.209 | 0.544 | Unannotated | NA | NA |
| metab_16355 | Unannotated feature | neg | 76.2821 | 6.726 | 1.548 | 3.240 | 1.692 | 0.225 | 0.209 | 0.544 | Unannotated | NA | NA |
| metab_449 | Unannotated feature | pos | 208.0972 | 4.774 | 4.846 | 4.728 | -0.117 | -0.225 | 0.209 | 0.544 | Unannotated | NA | NA |
| metab_10259 | Captopril-cysteine disulfide | neg | 317.0630 | 2.535 | 4.284 | 4.752 | 0.468 | 0.225 | 0.209 | 0.544 | Unannotated | Organic acids and derivatives | Carboxylic acids and derivatives |
| metab_2642 | Unannotated feature | pos | 236.0922 | 1.928 | 3.159 | 3.750 | 0.591 | 0.225 | 0.209 | 0.544 | Unannotated | NA | NA |
| metab_4797 | Unannotated feature | pos | 201.0087 | 7.991 | 5.182 | 5.168 | -0.013 | -0.225 | 0.209 | 0.544 | Unannotated | NA | NA |
| metab_894 | Neurotensin 11-13 | pos | 471.2546 | 6.989 | 5.824 | 5.791 | -0.033 | -0.225 | 0.209 | 0.544 | Unannotated | NA | NA |
| metab_16838 | Unannotated feature | neg | 588.8214 | 7.726 | 4.660 | 4.672 | 0.013 | 0.225 | 0.209 | 0.544 | Unannotated | NA | NA |
| metab_422 | Unannotated feature | pos | 158.0266 | 0.016 | 4.321 | 4.340 | 0.019 | 0.225 | 0.209 | 0.544 | Unannotated | NA | NA |
| metab_17368 | Unannotated feature | neg | 554.8918 | 7.675 | 4.999 | 5.032 | 0.033 | 0.225 | 0.209 | 0.544 | Unannotated | NA | NA |
| metab_1447 | Unannotated feature | pos | 307.2021 | 6.008 | 5.266 | 5.087 | -0.178 | -0.225 | 0.209 | 0.544 | Unannotated | NA | NA |
| metab_15161 | Unannotated feature | neg | 249.0790 | 4.653 | 3.871 | 4.382 | 0.512 | 0.225 | 0.209 | 0.544 | Unannotated | NA | NA |
| metab_16895 | Unannotated feature | neg | 338.8595 | 7.745 | 4.871 | 4.844 | -0.028 | -0.225 | 0.209 | 0.544 | Unannotated | NA | NA |
| metab_8202 | Unannotated feature | pos | 107.0798 | 2.500 | 5.337 | 5.415 | 0.078 | 0.225 | 0.209 | 0.544 | Unannotated | NA | NA |
| metab_7459 | Unannotated feature | pos | 206.0851 | 3.846 | 4.083 | 3.680 | -0.402 | -0.225 | 0.209 | 0.544 | Unannotated | NA | NA |
| metab_17302 | Unannotated feature | neg | 488.8937 | 7.707 | 4.271 | 4.345 | 0.074 | 0.225 | 0.209 | 0.544 | Unannotated | NA | NA |
| metab_8273 | Unannotated feature | pos | 257.0867 | 2.179 | 5.159 | 5.085 | -0.074 | -0.225 | 0.209 | 0.544 | Unannotated | NA | NA |
| metab_1774 | Unannotated feature | pos | 362.1930 | 3.283 | 5.280 | 5.014 | -0.266 | -0.225 | 0.209 | 0.544 | Unannotated | NA | NA |
| metab_1798 | Unannotated feature | pos | 119.0900 | 0.784 | 6.222 | 6.186 | -0.036 | -0.225 | 0.209 | 0.544 | Unannotated | NA | NA |
| metab_7464 | Unannotated feature | pos | 276.1585 | 3.839 | 4.107 | 3.922 | -0.184 | -0.225 | 0.209 | 0.544 | Unannotated | NA | NA |
| metab_13683 | Parachlorophenol | neg | 126.9965 | 1.691 | 4.364 | 4.226 | -0.138 | -0.225 | 0.209 | 0.544 | Tentative | Benzenoids | Phenols |
| metab_12389 | Unannotated feature | neg | 327.1809 | 6.561 | 5.735 | 5.780 | 0.044 | 0.225 | 0.209 | 0.544 | Unannotated | NA | NA |
| metab_11652 | Unannotated feature | neg | 170.9209 | 0.025 | 4.800 | 4.763 | -0.038 | -0.225 | 0.209 | 0.544 | Unannotated | NA | NA |
| metab_406 | Marinobufagenin | pos | 439.1876 | 4.704 | 3.343 | 3.610 | 0.266 | 0.225 | 0.209 | 0.544 | Tentative | NA | NA |
| metab_5443 | (1R,7S,13S,15S)-2,15-Dihydroxy-7-methyl-6-oxabicyclo[11.3.0]hexadeca-3,11-dien-5-one | pos | 245.1539 | 6.398 | 5.198 | 4.974 | -0.224 | -0.225 | 0.209 | 0.544 | Unannotated | NA | NA |
| metab_10532 | 3-Hydroxypicolinic acid | neg | 138.0190 | 1.947 | 5.461 | 5.698 | 0.237 | 0.225 | 0.209 | 0.544 | Strong putative | NA | NA |
| metab_10537 | Unannotated feature | neg | 335.0005 | 1.944 | 4.609 | 4.349 | -0.259 | -0.225 | 0.209 | 0.544 | Unannotated | NA | NA |
| metab_1030 | Unannotated feature | pos | 383.3135 | 6.997 | 4.977 | 4.600 | -0.377 | -0.225 | 0.209 | 0.544 | Unannotated | NA | NA |
| metab_13324 | Unannotated feature | neg | 315.0813 | 0.644 | 4.934 | 5.286 | 0.352 | 0.225 | 0.209 | 0.544 | Unannotated | NA | NA |
| metab_2956 | Unannotated feature | pos | 251.1756 | 2.965 | 4.127 | 3.248 | -0.879 | -0.225 | 0.209 | 0.544 | Unannotated | NA | NA |
| metab_2959 | Dulcin | pos | 181.0974 | 2.976 | 4.006 | 3.592 | -0.414 | -0.225 | 0.209 | 0.544 | Moderate putative | NA | NA |
| metab_5472 | Lithocholate 3-O-glucuronide | pos | 535.3261 | 6.375 | 6.369 | 6.277 | -0.092 | -0.225 | 0.209 | 0.544 | Tentative | NA | NA |
| metab_15035 | Noroxymorphone | neg | 332.1148 | 4.398 | 3.803 | 4.210 | 0.407 | 0.225 | 0.209 | 0.544 | Unannotated | NA | NA |
| metab_11546 | Unannotated feature | neg | 378.0833 | 5.257 | 3.298 | 4.129 | 0.831 | 0.225 | 0.209 | 0.544 | Unannotated | NA | NA |
| metab_1683 | Unannotated feature | pos | 592.3924 | 4.239 | 5.117 | 5.441 | 0.324 | 0.225 | 0.209 | 0.544 | Unannotated | NA | NA |
| metab_2352 | Unannotated feature | pos | 656.7329 | 5.732 | 3.223 | 4.680 | 1.457 | 0.225 | 0.209 | 0.544 | Unannotated | NA | NA |
| metab_16209 | (S)-10,16-Dihydroxyhexadecanoic acid | neg | 287.2228 | 6.436 | 5.022 | 5.256 | 0.233 | 0.225 | 0.209 | 0.544 | Tentative | Lipids and lipid-like molecules | Fatty Acyls |
| metab_372 | Unannotated feature | pos | 283.1593 | 0.201 | 6.256 | 6.276 | 0.020 | 0.225 | 0.209 | 0.544 | Unannotated | NA | NA |
| metab_1393 | Unannotated feature | pos | 395.1975 | 6.095 | 5.131 | 4.836 | -0.295 | -0.225 | 0.209 | 0.544 | Unannotated | NA | NA |
| metab_14532 | Unannotated feature | neg | 325.1321 | 3.400 | 3.701 | 3.404 | -0.297 | -0.225 | 0.209 | 0.544 | Unannotated | NA | NA |
| metab_10987 | Quinaprilat | neg | 445.1573 | 0.777 | 4.308 | 4.959 | 0.651 | 0.225 | 0.209 | 0.544 | Tentative | NA | NA |
| metab_17263 | Unannotated feature | neg | 176.9601 | 7.726 | 5.011 | 4.998 | -0.013 | -0.225 | 0.209 | 0.544 | Unannotated | NA | NA |
| metab_12286 | Unannotated feature | neg | 585.2730 | 6.907 | 5.007 | 4.497 | -0.510 | -0.225 | 0.209 | 0.544 | Unannotated | NA | NA |
| metab_8491 | Unannotated feature | pos | 295.1586 | 1.685 | 2.553 | 3.284 | 0.731 | 0.225 | 0.209 | 0.544 | Unannotated | NA | NA |
| metab_2131 | Unannotated feature | pos | 203.1758 | 2.845 | 3.809 | 3.260 | -0.549 | -0.225 | 0.209 | 0.544 | Unannotated | NA | NA |
| metab_1351 | Unannotated feature | pos | 430.2265 | 6.151 | 4.802 | 4.604 | -0.199 | -0.225 | 0.209 | 0.544 | Unannotated | NA | NA |
| metab_10614 | Unannotated feature | neg | 262.0869 | 1.685 | 3.273 | 2.062 | -1.211 | -0.225 | 0.209 | 0.544 | Unannotated | NA | NA |
| metab_10627 | Unannotated feature | neg | 108.0447 | 1.685 | 4.399 | 4.562 | 0.164 | 0.225 | 0.209 | 0.544 | Unannotated | NA | NA |
| metab_5158 | Unannotated feature | pos | 408.3090 | 6.853 | 6.159 | 6.256 | 0.097 | 0.225 | 0.209 | 0.544 | Unannotated | NA | NA |
| metab_5159 | Unannotated feature | pos | 284.2860 | 6.853 | 6.138 | 6.069 | -0.069 | -0.225 | 0.209 | 0.544 | Unannotated | NA | NA |
| metab_4945 | (3b,9R)-5-Megastigmene-3,9-diol 9-[apiosyl-(1->6)-glucoside] | pos | 471.2550 | 7.610 | 5.218 | 5.176 | -0.041 | -0.225 | 0.209 | 0.544 | Unannotated | NA | NA |
| metab_13830 | Unannotated feature | neg | 320.0738 | 2.137 | 5.657 | 5.489 | -0.168 | -0.225 | 0.209 | 0.544 | Unannotated | NA | NA |
| metab_15433 | Unannotated feature | neg | 205.1077 | 5.144 | 4.055 | 3.586 | -0.470 | -0.225 | 0.209 | 0.544 | Unannotated | NA | NA |
| metab_14101 | Tyrosyl-Proline | neg | 277.1196 | 2.826 | 5.695 | 5.589 | -0.106 | -0.225 | 0.209 | 0.544 | Unannotated | NA | NA |
| metab_3136 | 2'-Fluorothymidine | pos | 261.0874 | 3.538 | 5.276 | 5.007 | -0.269 | -0.225 | 0.209 | 0.544 | Unannotated | NA | NA |
| metab_8532 | Unannotated feature | pos | 91.0547 | 1.392 | 5.618 | 5.502 | -0.115 | -0.225 | 0.209 | 0.544 | Unannotated | NA | NA |
| metab_17055 | Unannotated feature | neg | 154.9181 | 7.981 | 4.497 | 4.478 | -0.019 | -0.225 | 0.209 | 0.544 | Unannotated | NA | NA |
| metab_17059 | Unannotated feature | neg | 184.9929 | 7.970 | 3.280 | 3.350 | 0.070 | 0.225 | 0.209 | 0.544 | Unannotated | NA | NA |
| metab_3805 | Methyltrienolone | pos | 607.3146 | 5.573 | 6.163 | 5.627 | -0.536 | -0.225 | 0.209 | 0.544 | Tentative | NA | NA |
| metab_3185 | Unannotated feature | pos | 282.1342 | 3.689 | 4.283 | 4.332 | 0.049 | 0.225 | 0.209 | 0.544 | Unannotated | NA | NA |
| metab_14375 | Metaxalone | neg | 258.0521 | 3.153 | 5.924 | 5.705 | -0.219 | -0.225 | 0.209 | 0.544 | Tentative | NA | NA |
| metab_11829 | Unannotated feature | neg | 255.1236 | 5.918 | 4.181 | 4.386 | 0.205 | 0.225 | 0.209 | 0.544 | Unannotated | NA | NA |
| metab_788 | Unannotated feature | pos | 273.1853 | 6.231 | 5.171 | 5.071 | -0.100 | -0.225 | 0.209 | 0.544 | Unannotated | NA | NA |
| metab_9580 | Unannotated feature | neg | 233.0124 | 3.242 | 2.605 | 2.057 | -0.548 | -0.225 | 0.209 | 0.544 | Unannotated | NA | NA |
| metab_2465 | 2-Amino-2,3,6,7-tetrahydro-1H-pteridin-4-one | pos | 168.0869 | 0.688 | 4.400 | 4.334 | -0.066 | -0.225 | 0.209 | 0.544 | Unannotated | NA | NA |
| metab_792 | Unannotated feature | pos | 172.1698 | 6.247 | 4.862 | 4.824 | -0.038 | -0.225 | 0.209 | 0.544 | Unannotated | NA | NA |
| metab_5144 | Unannotated feature | pos | 475.3402 | 6.877 | 4.873 | 4.879 | 0.006 | 0.225 | 0.209 | 0.544 | Unannotated | NA | NA |
| metab_17152 | Unannotated feature | neg | 272.8990 | 7.773 | 4.019 | 3.984 | -0.034 | -0.225 | 0.209 | 0.544 | Unannotated | NA | NA |
| metab_17068 | Unannotated feature | neg | 186.9330 | 7.918 | 3.428 | 3.165 | -0.263 | -0.225 | 0.209 | 0.544 | Unannotated | NA | NA |
| metab_8600 | 3,4-Dihydroxy-2-hydroxymethyl-1-pyrrolidinepropanamide | pos | 187.1080 | 1.139 | 6.078 | 5.872 | -0.206 | -0.225 | 0.209 | 0.544 | Unannotated | NA | NA |
| metab_17168 | Unannotated feature | neg | 313.2389 | 7.750 | 4.482 | 4.576 | 0.094 | 0.225 | 0.209 | 0.544 | Unannotated | NA | NA |
| metab_5749 | (5Z,7S,8E,10Z,13Z,15E,17S,19Z)-7,17-Dihydroxydocosa-5,8,10,13,15,19-hexaenoylcarnitine | pos | 542.2913 | 6.056 | 5.225 | 4.682 | -0.543 | -0.225 | 0.209 | 0.544 | Unannotated | NA | NA |
| metab_5807 | Unannotated feature | pos | 221.0475 | 0.016 | 5.987 | 6.003 | 0.016 | 0.225 | 0.209 | 0.544 | Unannotated | NA | NA |
| metab_5815 | Unannotated feature | pos | 156.9978 | 0.082 | 5.238 | 5.271 | 0.033 | 0.225 | 0.209 | 0.544 | Unannotated | NA | NA |
| metab_8921 | N-Tert-Butyl-3-(4-(2-methoxyphenyl)-piperazin-1-yl)-2-phenylpropanamide | neg | 440.2551 | 4.231 | 4.658 | 3.879 | -0.779 | -0.225 | 0.209 | 0.544 | Unannotated | Organoheterocyclic compounds | Diazinanes |
| metab_10693 | Flumazenil acid | neg | 310.0414 | 1.457 | 5.113 | 4.757 | -0.357 | -0.225 | 0.209 | 0.544 | Unannotated | NA | NA |
| metab_209 | Unannotated feature | pos | 302.9885 | 0.585 | 4.633 | 4.029 | -0.604 | -0.225 | 0.209 | 0.544 | Unannotated | NA | NA |
| metab_3253 | Unannotated feature | pos | 143.0732 | 3.868 | 4.309 | 4.067 | -0.241 | -0.225 | 0.209 | 0.544 | Unannotated | NA | NA |
| metab_8898 | Unannotated feature | neg | 243.1350 | 4.270 | 3.483 | 3.085 | -0.397 | -0.225 | 0.209 | 0.544 | Unannotated | NA | NA |
| metab_3429 | (+/-)-(Z)-2-(5-Tetradecenyl)cyclobutanone | pos | 282.2795 | 6.853 | 8.233 | 8.214 | -0.018 | -0.225 | 0.209 | 0.544 | Unannotated | NA | NA |
| metab_3400 | Unannotated feature | pos | 637.3705 | 7.108 | 5.142 | 5.141 | -0.002 | -0.225 | 0.209 | 0.544 | Unannotated | NA | NA |
| metab_19755 | Unannotated feature | neg | 230.1475 | 4.498 | 3.580 | 2.386 | -1.194 | -0.225 | 0.209 | 0.544 | Unannotated | NA | NA |
| metab_184 | Unannotated feature | pos | 114.8933 | 0.601 | 4.666 | 4.597 | -0.069 | -0.225 | 0.209 | 0.544 | Unannotated | NA | NA |
| metab_12451 | Unannotated feature | neg | 138.8594 | 7.814 | 5.103 | 5.073 | -0.029 | -0.225 | 0.209 | 0.544 | Unannotated | NA | NA |
| metab_6399 | Unannotated feature | pos | 629.3895 | 5.725 | 3.938 | 4.367 | 0.429 | 0.225 | 0.209 | 0.544 | Unannotated | NA | NA |
| metab_6467 | (E)-4-[[(1R)-2-[[(2R)-2-(2-Adamantyloxycarbonylamino)-3-(1H-indol-3-yl)-2-methylpropanoyl]amino]-1-phenylethyl]amino]-4-oxobut-2-enoic acid | pos | 613.3016 | 5.629 | 6.659 | 6.030 | -0.629 | -0.225 | 0.209 | 0.544 | Unannotated | Organoheterocyclic compounds | Indoles and derivatives |
| metab_8802 | D-TYROSINE | pos | 164.0707 | 5.239 | 5.936 | 5.828 | -0.108 | -0.225 | 0.209 | 0.544 | Moderate putative | Organic acids and derivatives | Carboxylic acids and derivatives |
| metab_6754 | (S)-7-(3-Amino-1-pyrrolidinyl)-1-cyclopropyl-6-fluoro-1,4-dihydro-4-oxo-1,8-naphthyridine-3-carboxylic acid | pos | 333.1342 | 4.262 | 4.426 | 4.526 | 0.100 | 0.225 | 0.209 | 0.544 | Unannotated | NA | NA |
| metab_1986 | Unannotated feature | pos | 270.1343 | 1.952 | 4.435 | 4.965 | 0.531 | 0.225 | 0.209 | 0.544 | Unannotated | NA | NA |
| metab_3348 | Unannotated feature | pos | 453.3345 | 7.260 | 4.903 | 4.712 | -0.191 | -0.221 | 0.218 | 0.552 | Unannotated | NA | NA |
| metab_3372 | 4-hydroxy Nonenal Alkyne | pos | 153.0913 | 4.311 | 3.440 | 3.812 | 0.372 | 0.221 | 0.218 | 0.552 | Unannotated | NA | NA |
| metab_10760 | Unannotated feature | neg | 73.0285 | 1.209 | 4.353 | 4.277 | -0.075 | -0.221 | 0.218 | 0.552 | Unannotated | NA | NA |
| metab_18578 | Unannotated feature | neg | 570.3383 | 6.003 | 3.991 | 3.367 | -0.625 | -0.221 | 0.218 | 0.552 | Unannotated | NA | NA |
| metab_17119 | Unannotated feature | neg | 195.8940 | 7.830 | 3.990 | 4.092 | 0.102 | 0.221 | 0.218 | 0.552 | Unannotated | NA | NA |
| metab_1856 | Methylene bisacrylamide | pos | 155.0817 | 1.034 | 5.361 | 5.092 | -0.269 | -0.221 | 0.218 | 0.552 | Unannotated | NA | NA |
| metab_11683 | Unannotated feature | neg | 289.1295 | 5.401 | 4.098 | 4.122 | 0.023 | 0.221 | 0.218 | 0.552 | Unannotated | NA | NA |
| metab_11901 | Unannotated feature | neg | 399.1486 | 6.052 | 5.302 | 5.629 | 0.328 | 0.221 | 0.218 | 0.552 | Unannotated | NA | NA |
| metab_19267 | Unannotated feature | neg | 100.0396 | 5.181 | 2.348 | 2.238 | -0.110 | -0.221 | 0.218 | 0.552 | Unannotated | NA | NA |
| metab_5151 | Unannotated feature | pos | 447.3079 | 6.869 | 4.706 | 4.756 | 0.050 | 0.221 | 0.218 | 0.552 | Unannotated | NA | NA |
| metab_11465 | Unannotated feature | neg | 592.8331 | 0.524 | 5.354 | 5.361 | 0.007 | 0.221 | 0.218 | 0.552 | Unannotated | NA | NA |
| metab_19236 | Eurycomanol | neg | 409.1517 | 3.379 | 4.529 | 4.323 | -0.206 | -0.221 | 0.218 | 0.552 | Unannotated | NA | NA |
| metab_16649 | Unannotated feature | neg | 401.9260 | 7.570 | 6.750 | 6.769 | 0.019 | 0.221 | 0.218 | 0.552 | Unannotated | NA | NA |
| metab_10331 | Unannotated feature | neg | 495.1712 | 2.274 | 6.264 | 6.133 | -0.131 | -0.221 | 0.218 | 0.552 | Unannotated | NA | NA |
| metab_10315 | 3-Hydroxydodecanedioic acid | neg | 291.1451 | 4.563 | 4.575 | 4.720 | 0.145 | 0.221 | 0.218 | 0.552 | Unannotated | NA | NA |
| metab_13255 | Unannotated feature | neg | 386.8009 | 0.588 | 4.296 | 3.875 | -0.422 | -0.221 | 0.218 | 0.552 | Unannotated | NA | NA |
| metab_7891 | (2R,3S)-5-oxo-3-phenylmorpholine-2-carboxylic acid | pos | 222.0763 | 2.976 | 4.373 | 4.800 | 0.427 | 0.221 | 0.218 | 0.552 | Unannotated | NA | NA |
| metab_12432 | Unannotated feature | neg | 209.9186 | 7.730 | 5.720 | 5.695 | -0.026 | -0.221 | 0.218 | 0.552 | Unannotated | NA | NA |
| metab_16474 | Unannotated feature | neg | 382.3330 | 7.053 | 5.090 | 4.660 | -0.431 | -0.221 | 0.218 | 0.552 | Unannotated | NA | NA |
| metab_715 | Unannotated feature | pos | 703.9325 | 5.944 | 3.251 | 4.289 | 1.039 | 0.221 | 0.218 | 0.552 | Unannotated | NA | NA |
| metab_14299 | Unannotated feature | neg | 223.1086 | 3.045 | 3.657 | 4.033 | 0.376 | 0.221 | 0.218 | 0.552 | Unannotated | NA | NA |
| metab_16918 | Unannotated feature | neg | 188.9326 | 7.781 | 5.432 | 5.401 | -0.032 | -0.221 | 0.218 | 0.552 | Unannotated | NA | NA |
| metab_4885 | 5-(Hydroxymethyl)piperidine-3,4-diol | pos | 192.0621 | 7.708 | 4.922 | 4.949 | 0.027 | 0.221 | 0.218 | 0.552 | Unannotated | NA | NA |
| metab_2336 | Unannotated feature | pos | 177.9926 | 0.420 | 4.551 | 4.563 | 0.012 | 0.221 | 0.218 | 0.552 | Unannotated | NA | NA |
| metab_10512 | Unannotated feature | neg | 330.1308 | 1.947 | 4.954 | 4.844 | -0.110 | -0.221 | 0.218 | 0.552 | Unannotated | NA | NA |
| metab_7859 | L-alpha-Amino-1H-pyrrole-1-hexanoic acid | pos | 229.1551 | 3.040 | 6.745 | 6.661 | -0.084 | -0.221 | 0.218 | 0.552 | Unannotated | NA | NA |
| metab_8902 | Unannotated feature | neg | 465.1268 | 4.262 | 3.918 | 4.478 | 0.560 | 0.221 | 0.218 | 0.552 | Unannotated | NA | NA |
| metab_3261 | PA(16:0/14:1(9Z)) | pos | 619.4327 | 6.957 | 5.251 | 5.022 | -0.229 | -0.221 | 0.218 | 0.552 | Tentative | NA | NA |
| metab_11090 | Unannotated feature | neg | 113.0349 | 0.730 | 4.088 | 3.745 | -0.343 | -0.221 | 0.218 | 0.552 | Unannotated | NA | NA |
| metab_7397 | Unannotated feature | pos | 295.1657 | 3.987 | 4.314 | 4.064 | -0.250 | -0.221 | 0.218 | 0.552 | Unannotated | NA | NA |
| metab_8380 | Unannotated feature | pos | 260.1134 | 1.952 | 4.300 | 4.570 | 0.270 | 0.221 | 0.218 | 0.552 | Unannotated | NA | NA |
| metab_12328 | AS 1-5 | neg | 714.5537 | 7.358 | 5.180 | 4.145 | -1.035 | -0.221 | 0.218 | 0.552 | Unannotated | NA | NA |
| metab_17261 | Unannotated feature | neg | 178.9484 | 7.726 | 5.290 | 5.266 | -0.024 | -0.221 | 0.218 | 0.552 | Unannotated | NA | NA |
| metab_13397 | 2-(1,2,3,4-Tetrahydroxybutyl)-6-(2,3,4-trihydroxybutyl)pyrazine | neg | 349.1257 | 0.770 | 4.419 | 4.683 | 0.263 | 0.221 | 0.218 | 0.552 | Unannotated | Organoheterocyclic compounds | Diazines |
| metab_16924 | Unannotated feature | neg | 401.8528 | 7.788 | 4.557 | 4.528 | -0.029 | -0.221 | 0.218 | 0.552 | Unannotated | NA | NA |
| metab_4391 | Unannotated feature | pos | 320.3071 | 6.255 | 4.851 | 4.737 | -0.115 | -0.221 | 0.218 | 0.552 | Unannotated | NA | NA |
| metab_15531 | Unannotated feature | neg | 357.1380 | 5.339 | 3.406 | 3.747 | 0.341 | 0.221 | 0.218 | 0.552 | Unannotated | NA | NA |
| metab_3596 | Leu Leu | pos | 245.1864 | 5.060 | 4.777 | 4.310 | -0.467 | -0.221 | 0.218 | 0.552 | Moderate putative | NA | NA |
| metab_16991 | Unannotated feature | neg | 193.8151 | 7.985 | 5.645 | 5.607 | -0.038 | -0.221 | 0.218 | 0.552 | Unannotated | NA | NA |
| metab_2062 | Unannotated feature | pos | 310.1294 | 2.724 | 5.624 | 5.806 | 0.182 | 0.221 | 0.218 | 0.552 | Unannotated | NA | NA |
| metab_18289 | Unannotated feature | neg | 524.2692 | 6.217 | 3.165 | 3.287 | 0.122 | 0.221 | 0.218 | 0.552 | Unannotated | NA | NA |
| metab_19110 | Unannotated feature | neg | 266.1036 | 3.334 | 5.314 | 5.483 | 0.169 | 0.221 | 0.218 | 0.552 | Unannotated | NA | NA |
| metab_17975 | Unannotated feature | neg | 439.2476 | 6.545 | 4.656 | 4.423 | -0.233 | -0.221 | 0.218 | 0.552 | Unannotated | NA | NA |
| metab_4796 | Unannotated feature | pos | 182.0294 | 7.983 | 4.669 | 4.737 | 0.068 | 0.221 | 0.218 | 0.552 | Unannotated | NA | NA |
| metab_18456 | Unannotated feature | neg | 500.2697 | 6.083 | 5.244 | 5.093 | -0.151 | -0.221 | 0.218 | 0.552 | Unannotated | NA | NA |
| metab_8974 | Unannotated feature | neg | 328.2246 | 4.123 | 5.279 | 5.129 | -0.149 | -0.221 | 0.218 | 0.552 | Unannotated | NA | NA |
| metab_1065 | Unannotated feature | pos | 378.2987 | 6.765 | 4.408 | 3.577 | -0.830 | -0.221 | 0.218 | 0.552 | Unannotated | NA | NA |
| metab_5701 | Geldanamycin | pos | 583.2570 | 6.111 | 5.155 | 4.690 | -0.465 | -0.221 | 0.218 | 0.552 | Tentative | NA | NA |
| metab_11413 | Unannotated feature | neg | 615.2857 | 5.188 | 4.484 | 4.657 | 0.173 | 0.221 | 0.218 | 0.552 | Unannotated | NA | NA |
| metab_12483 | Unannotated feature | neg | 551.3057 | 6.404 | 6.718 | 6.982 | 0.264 | 0.221 | 0.218 | 0.552 | Unannotated | NA | NA |
| metab_4884 | Unannotated feature | pos | 238.9078 | 7.708 | 4.153 | 4.214 | 0.061 | 0.221 | 0.218 | 0.552 | Unannotated | NA | NA |
| metab_14862 | Prephenate | neg | 271.0475 | 4.031 | 3.703 | 4.281 | 0.578 | 0.221 | 0.218 | 0.552 | Tentative | NA | NA |
| metab_17148 | Unannotated feature | neg | 437.2918 | 7.777 | 4.774 | 4.701 | -0.073 | -0.221 | 0.218 | 0.552 | Unannotated | NA | NA |
| metab_11839 | Unannotated feature | neg | 549.2360 | 5.944 | 4.639 | 4.933 | 0.293 | 0.221 | 0.218 | 0.552 | Unannotated | NA | NA |
| metab_2531 | Unannotated feature | pos | 91.0579 | 0.961 | 2.588 | 2.992 | 0.405 | 0.221 | 0.218 | 0.552 | Unannotated | NA | NA |
| metab_12463 | Unannotated feature | neg | 203.8439 | 7.910 | 5.280 | 5.237 | -0.043 | -0.221 | 0.218 | 0.552 | Unannotated | NA | NA |
| metab_1070 | Unannotated feature | pos | 471.3816 | 6.718 | 3.755 | 4.629 | 0.875 | 0.221 | 0.218 | 0.552 | Unannotated | NA | NA |
| metab_18390 | Unannotated feature | neg | 565.3235 | 6.138 | 5.929 | 5.401 | -0.528 | -0.221 | 0.218 | 0.552 | Unannotated | NA | NA |
| metab_260 | Unannotated feature | pos | 213.0014 | 0.531 | 4.290 | 4.453 | 0.164 | 0.221 | 0.218 | 0.552 | Unannotated | NA | NA |
| metab_10692 | 6-Aminonaphthalene-2-sulfonic acid | neg | 222.0228 | 1.460 | 4.829 | 5.507 | 0.678 | 0.221 | 0.218 | 0.552 | Unannotated | NA | NA |
| metab_16699 | Unannotated feature | neg | 910.8193 | 7.633 | 4.406 | 4.725 | 0.319 | 0.221 | 0.218 | 0.552 | Unannotated | NA | NA |
| metab_12166 | Unannotated feature | neg | 367.2467 | 6.687 | 7.023 | 7.092 | 0.069 | 0.221 | 0.218 | 0.552 | Unannotated | NA | NA |
| metab_14198 | Unannotated feature | neg | 264.0046 | 2.938 | 3.947 | 4.316 | 0.369 | 0.221 | 0.218 | 0.552 | Unannotated | NA | NA |
| metab_9766 | Unannotated feature | neg | 309.1191 | 3.067 | 2.749 | 3.429 | 0.680 | 0.221 | 0.218 | 0.552 | Unannotated | NA | NA |
| metab_9406 | (R)-Shinanolone | neg | 237.0767 | 3.465 | 3.172 | 3.635 | 0.463 | 0.221 | 0.218 | 0.552 | Unannotated | NA | NA |
| metab_8427 | Unannotated feature | pos | 337.1479 | 1.944 | 3.010 | 3.654 | 0.644 | 0.221 | 0.218 | 0.552 | Unannotated | NA | NA |
| metab_7463 | Glutaminylphenylalanine | pos | 326.1717 | 3.839 | 4.896 | 4.720 | -0.177 | -0.221 | 0.218 | 0.552 | Unannotated | Organic acids and derivatives | Carboxylic acids and derivatives |
| metab_13379 | Unannotated feature | neg | 373.2097 | 0.738 | 4.530 | 4.235 | -0.295 | -0.221 | 0.218 | 0.552 | Unannotated | NA | NA |
| metab_461 | Unannotated feature | pos | 353.1715 | 4.778 | 4.629 | 4.382 | -0.247 | -0.221 | 0.218 | 0.552 | Unannotated | NA | NA |
| metab_2237 | 4-Hydroxyretinoic acid | pos | 339.1921 | 5.566 | 4.425 | 5.818 | 1.393 | 0.221 | 0.218 | 0.552 | Tentative | NA | NA |
| metab_10207 | Unannotated feature | neg | 265.0834 | 2.642 | 4.219 | 4.536 | 0.317 | 0.221 | 0.218 | 0.552 | Unannotated | NA | NA |
| metab_18987 | Unannotated feature | neg | 259.1454 | 5.546 | 5.278 | 5.184 | -0.094 | -0.221 | 0.218 | 0.552 | Unannotated | NA | NA |
| metab_7515 | Prostaglandin I2 | pos | 391.1873 | 3.725 | 4.760 | 4.424 | -0.335 | -0.221 | 0.218 | 0.552 | Tentative | NA | NA |
| metab_5331 | 1-(2,6,6-Trimethyl-2-cyclohexen-1-yl)-1,6-heptadien-3-one | pos | 215.1797 | 6.542 | 4.872 | 4.784 | -0.088 | -0.221 | 0.218 | 0.552 | Unannotated | NA | NA |
| metab_18678 | Unannotated feature | neg | 249.0881 | 3.131 | 4.911 | 4.889 | -0.023 | -0.221 | 0.218 | 0.552 | Unannotated | NA | NA |
| metab_17670 | Unannotated feature | neg | 373.0782 | 2.771 | 4.018 | 3.863 | -0.154 | -0.221 | 0.218 | 0.552 | Unannotated | NA | NA |
| metab_5114 | Azitromycin | pos | 729.5291 | 6.981 | 5.814 | 4.890 | -0.924 | -0.221 | 0.218 | 0.552 | Unannotated | NA | NA |
| metab_17229 | Unannotated feature | neg | 231.0512 | 7.730 | 4.845 | 4.967 | 0.121 | 0.221 | 0.218 | 0.552 | Unannotated | NA | NA |
| metab_2756 | Unannotated feature | pos | 250.0761 | 2.407 | 4.358 | 4.435 | 0.077 | 0.221 | 0.218 | 0.552 | Unannotated | NA | NA |
| metab_7822 | Unannotated feature | pos | 415.2125 | 6.199 | 6.930 | 6.829 | -0.101 | -0.221 | 0.218 | 0.552 | Unannotated | NA | NA |
| metab_11992 | Unannotated feature | neg | 317.1509 | 5.530 | 6.548 | 6.404 | -0.144 | -0.221 | 0.218 | 0.552 | Unannotated | NA | NA |
| metab_1251 | 2,3-Dimethyl-2-cyclohexen-1-one | pos | 107.0860 | 6.367 | 4.969 | 4.948 | -0.020 | -0.221 | 0.218 | 0.552 | Unannotated | NA | NA |
| metab_17910 | (R)-2-Hydroxystearic Acid | neg | 335.2359 | 6.656 | 5.434 | 5.626 | 0.192 | 0.221 | 0.218 | 0.552 | Unannotated | NA | NA |
| metab_2733 | Unannotated feature | pos | 97.0622 | 2.227 | 3.607 | 3.668 | 0.061 | 0.221 | 0.218 | 0.552 | Unannotated | NA | NA |
| metab_14474 | Unannotated feature | neg | 428.1497 | 3.314 | 3.798 | 3.452 | -0.347 | -0.221 | 0.218 | 0.552 | Unannotated | NA | NA |
| metab_18949 | Unannotated feature | neg | 451.1270 | 3.247 | 4.480 | 4.111 | -0.369 | -0.221 | 0.218 | 0.552 | Unannotated | NA | NA |
| metab_7217 | Leu Val | pos | 231.1707 | 4.394 | 5.073 | 4.897 | -0.176 | -0.221 | 0.218 | 0.552 | Moderate putative | NA | NA |
| metab_5547 | 3-Methylbenzaldehyde | pos | 121.0653 | 6.287 | 5.290 | 5.224 | -0.066 | -0.221 | 0.218 | 0.552 | Tentative | NA | NA |
| metab_8182 | Erinapyrone B | pos | 107.0497 | 2.590 | 5.315 | 5.179 | -0.136 | -0.221 | 0.218 | 0.552 | Unannotated | NA | NA |
| metab_722 | Unannotated feature | pos | 699.3301 | 5.969 | 2.126 | 4.702 | 2.576 | 0.221 | 0.218 | 0.552 | Unannotated | NA | NA |
| metab_5064 | Unannotated feature | pos | 318.1301 | 0.768 | 5.029 | 5.116 | 0.087 | 0.221 | 0.218 | 0.552 | Unannotated | NA | NA |
| metab_12914 | B-D-fructosyl-a-D-(6-O-(E))-feruloylglucoside | neg | 471.1366 | 0.730 | 4.754 | 5.068 | 0.314 | 0.221 | 0.218 | 0.552 | Unannotated | NA | NA |
| metab_17001 | Unannotated feature | neg | 142.9459 | 7.993 | 4.787 | 4.766 | -0.021 | -0.221 | 0.218 | 0.552 | Unannotated | NA | NA |
| metab_14692 | Unannotated feature | neg | 383.0449 | 3.688 | 4.711 | 4.058 | -0.653 | -0.221 | 0.218 | 0.552 | Unannotated | NA | NA |
| metab_5318 | Unannotated feature | pos | 497.3251 | 6.550 | 5.285 | 5.484 | 0.199 | 0.221 | 0.218 | 0.552 | Unannotated | NA | NA |
| metab_3895 | Unannotated feature | pos | 491.2996 | 6.359 | 6.337 | 6.152 | -0.185 | -0.221 | 0.218 | 0.552 | Unannotated | NA | NA |
| metab_5288 | Eicosapentaenoic acid | pos | 320.2565 | 6.598 | 6.574 | 6.431 | -0.143 | -0.221 | 0.218 | 0.552 | Tentative | NA | NA |
| metab_18652 | 4',6-Dihydroxyflavone | neg | 253.0506 | 5.944 | 5.296 | 5.055 | -0.241 | -0.221 | 0.218 | 0.552 | Moderate putative | NA | NA |
| metab_8513 | Asp Val | pos | 233.1136 | 1.622 | 5.089 | 4.900 | -0.189 | -0.221 | 0.218 | 0.552 | Strong putative | NA | NA |
| metab_16182 | Deoxycholic Acid | neg | 391.2858 | 6.389 | 7.768 | 7.671 | -0.097 | -0.221 | 0.218 | 0.552 | Moderate putative | NA | NA |
| metab_18342 | Ala Thr Val Glu | neg | 417.1960 | 6.177 | 5.563 | 5.691 | 0.129 | 0.221 | 0.218 | 0.552 | Moderate putative | NA | NA |
| metab_19053 | Unannotated feature | neg | 153.0187 | 3.310 | 5.184 | 5.413 | 0.229 | 0.221 | 0.218 | 0.552 | Unannotated | NA | NA |
| metab_14401 | Unannotated feature | neg | 194.0454 | 3.199 | 4.207 | 4.395 | 0.188 | 0.221 | 0.218 | 0.552 | Unannotated | NA | NA |
| metab_17007 | Unannotated feature | neg | 162.9392 | 7.997 | 4.928 | 4.897 | -0.031 | -0.221 | 0.218 | 0.552 | Unannotated | NA | NA |
| metab_10449 | Glutarimide | neg | 271.0935 | 1.965 | 4.607 | 4.888 | 0.281 | 0.221 | 0.218 | 0.552 | Tentative | Organoheterocyclic compounds | Pyridines and derivatives |
| metab_10230 | Unannotated feature | neg | 351.1316 | 2.590 | 4.400 | 4.283 | -0.117 | -0.221 | 0.218 | 0.552 | Unannotated | NA | NA |
| metab_17837 | Unannotated feature | neg | 389.2592 | 6.766 | 4.647 | 4.968 | 0.321 | 0.221 | 0.218 | 0.552 | Unannotated | NA | NA |
| metab_3841 | Mesobilirubinogen | pos | 625.3616 | 5.653 | 1.296 | 5.634 | 4.338 | 0.221 | 0.218 | 0.552 | Tentative | NA | NA |
| metab_3060 | 3-Hydroxychavicol 1-glucoside | pos | 277.1077 | 3.331 | 2.644 | 2.399 | -0.245 | -0.221 | 0.218 | 0.552 | Unannotated | NA | NA |
| metab_4716 | Unannotated feature | pos | 760.8253 | 7.626 | 4.575 | 4.696 | 0.120 | 0.221 | 0.218 | 0.552 | Unannotated | NA | NA |
| metab_3822 | Cilengitide | pos | 621.3304 | 5.600 | 5.674 | 5.271 | -0.403 | -0.221 | 0.218 | 0.552 | Unannotated | NA | NA |
| metab_16358 | Unannotated feature | neg | 509.2896 | 6.726 | 6.517 | 6.388 | -0.128 | -0.221 | 0.218 | 0.552 | Unannotated | NA | NA |
| metab_8247 | Benzaldehyde | pos | 107.0497 | 2.296 | 5.085 | 4.967 | -0.118 | -0.221 | 0.218 | 0.552 | Tentative | NA | NA |
| metab_12409 | Unannotated feature | neg | 549.8575 | 7.707 | 4.434 | 4.566 | 0.133 | 0.221 | 0.218 | 0.552 | Unannotated | NA | NA |
| metab_14520 | Unannotated feature | neg | 95.0494 | 3.382 | 3.782 | 2.840 | -0.942 | -0.221 | 0.218 | 0.552 | Unannotated | NA | NA |
| metab_17419 | Unannotated feature | neg | 740.8488 | 7.637 | 6.257 | 6.335 | 0.078 | 0.221 | 0.218 | 0.552 | Unannotated | NA | NA |
| metab_1159 | 3b-Hydroxy-5-cholenoic acid | pos | 339.2684 | 6.542 | 5.793 | 5.711 | -0.082 | -0.221 | 0.218 | 0.552 | Unannotated | NA | NA |
| metab_3784 | 4-Hydroxy-5-phenyltetrahydro-1,3-oxazin-2-one | pos | 194.0816 | 5.532 | 4.322 | 4.035 | -0.287 | -0.221 | 0.218 | 0.552 | Tentative | Benzenoids | Benzene and substituted derivatives |
| metab_18614 | Niazimin | neg | 364.1407 | 5.975 | 5.174 | 4.787 | -0.386 | -0.221 | 0.218 | 0.552 | Unannotated | Organic oxygen compounds | Organooxygen compounds |
| metab_17057 | Unannotated feature | neg | 231.9432 | 7.974 | 5.467 | 5.431 | -0.036 | -0.221 | 0.218 | 0.552 | Unannotated | NA | NA |
| metab_17010 | Unannotated feature | neg | 197.9018 | 7.997 | 4.279 | 4.253 | -0.025 | -0.221 | 0.218 | 0.552 | Unannotated | NA | NA |
| metab_17011 | Unannotated feature | neg | 222.8876 | 7.997 | 4.473 | 4.448 | -0.026 | -0.221 | 0.218 | 0.552 | Unannotated | NA | NA |
| metab_16251 | Unannotated feature | neg | 586.3434 | 6.522 | 5.680 | 5.509 | -0.171 | -0.217 | 0.227 | 0.562 | Unannotated | NA | NA |
| metab_10376 | Unannotated feature | neg | 293.8740 | 2.137 | 4.511 | 4.221 | -0.290 | -0.217 | 0.227 | 0.562 | Unannotated | NA | NA |
| metab_12472 | Unannotated feature | neg | 162.9078 | 7.989 | 4.797 | 4.757 | -0.040 | -0.217 | 0.227 | 0.562 | Unannotated | NA | NA |
| metab_1719 | Indole-3-propionic acid | pos | 231.1133 | 3.898 | 6.557 | 6.458 | -0.099 | -0.217 | 0.227 | 0.562 | Unannotated | NA | NA |
| metab_7950 | 3-O-alpha-D-Glucopyranuronosyl-D-xylose | pos | 368.1212 | 2.900 | 4.258 | 3.796 | -0.462 | -0.217 | 0.227 | 0.562 | Unannotated | NA | NA |
| metab_9522 | Unannotated feature | neg | 181.0136 | 3.321 | 4.946 | 5.113 | 0.167 | 0.217 | 0.227 | 0.562 | Unannotated | NA | NA |
| metab_2099 | Unannotated feature | pos | 229.1188 | 2.747 | 4.972 | 5.037 | 0.065 | 0.217 | 0.227 | 0.562 | Unannotated | NA | NA |
| metab_9938 | Unannotated feature | neg | 281.0670 | 3.948 | 3.725 | 4.355 | 0.630 | 0.217 | 0.227 | 0.562 | Unannotated | NA | NA |
| metab_545 | Unannotated feature | pos | 337.1764 | 5.550 | 5.630 | 5.602 | -0.028 | -0.217 | 0.227 | 0.562 | Unannotated | NA | NA |
| metab_5246 | Unannotated feature | pos | 441.3738 | 6.662 | 4.997 | 4.790 | -0.207 | -0.217 | 0.227 | 0.562 | Unannotated | NA | NA |
| metab_16755 | Unannotated feature | neg | 634.8909 | 7.684 | 5.237 | 5.324 | 0.087 | 0.217 | 0.227 | 0.562 | Unannotated | NA | NA |
| metab_2321 | Unannotated feature | pos | 595.3506 | 5.705 | 6.589 | 7.901 | 1.312 | 0.217 | 0.227 | 0.562 | Unannotated | NA | NA |
| metab_3671 | Citrusinine I | pos | 319.1296 | 5.231 | 5.009 | 4.842 | -0.167 | -0.217 | 0.227 | 0.562 | Unannotated | NA | NA |
| metab_12224 | Unannotated feature | neg | 283.1188 | 5.999 | 5.975 | 6.223 | 0.248 | 0.217 | 0.227 | 0.562 | Unannotated | NA | NA |
| metab_2640 | 4,5-Dihydro-4-((1-piperazinyl)acetyl)-7-(3-(trifluoromethyl)phenyl)pyrazolo(1,5-a)pyrimidine-3-carbonitrile | pos | 434.1905 | 1.893 | 5.989 | 5.918 | -0.072 | -0.217 | 0.227 | 0.562 | Unannotated | NA | NA |
| metab_17314 | Unannotated feature | neg | 340.9457 | 7.702 | 3.794 | 3.907 | 0.113 | 0.217 | 0.227 | 0.562 | Unannotated | NA | NA |
| metab_13983 | Didanosine | neg | 235.0830 | 2.679 | 2.933 | 3.262 | 0.329 | 0.217 | 0.227 | 0.562 | Tentative | NA | NA |
| metab_17025 | Unannotated feature | neg | 180.9119 | 7.997 | 4.083 | 4.052 | -0.031 | -0.217 | 0.227 | 0.562 | Unannotated | NA | NA |
| metab_18733 | 4'-Hydroxy-R-phenprocoumon | neg | 341.1036 | 5.876 | 4.543 | 4.015 | -0.528 | -0.217 | 0.227 | 0.562 | Unannotated | NA | NA |
| metab_10997 | Zalcitabine | neg | 256.0940 | 0.777 | 4.813 | 4.617 | -0.196 | -0.217 | 0.227 | 0.562 | Tentative | NA | NA |
| metab_1949 | Gln Asn Met | pos | 392.1565 | 1.952 | 3.630 | 3.995 | 0.364 | 0.217 | 0.227 | 0.562 | Moderate putative | NA | NA |
| metab_14361 | Unannotated feature | neg | 357.0213 | 3.131 | 5.313 | 5.109 | -0.204 | -0.217 | 0.227 | 0.562 | Unannotated | NA | NA |
| metab_17318 | Unannotated feature | neg | 173.0300 | 7.702 | 4.767 | 4.713 | -0.054 | -0.217 | 0.227 | 0.562 | Unannotated | NA | NA |
| metab_17708 | Unannotated feature | neg | 396.3489 | 6.993 | 4.496 | 4.227 | -0.269 | -0.217 | 0.227 | 0.562 | Unannotated | NA | NA |
| metab_4855 | Unannotated feature | pos | 333.9754 | 7.724 | 4.517 | 4.487 | -0.030 | -0.217 | 0.227 | 0.562 | Unannotated | NA | NA |
| metab_17153 | Unannotated feature | neg | 165.0408 | 7.773 | 4.468 | 4.436 | -0.032 | -0.217 | 0.227 | 0.562 | Unannotated | NA | NA |
| metab_12354 | Unannotated feature | neg | 539.2690 | 6.162 | 5.025 | 5.360 | 0.334 | 0.217 | 0.227 | 0.562 | Unannotated | NA | NA |
| metab_12358 | Unannotated feature | neg | 546.2823 | 6.766 | 4.836 | 4.389 | -0.447 | -0.217 | 0.227 | 0.562 | Unannotated | NA | NA |
| metab_12345 | Unannotated feature | neg | 271.2280 | 6.860 | 7.600 | 7.440 | -0.160 | -0.217 | 0.227 | 0.562 | Unannotated | NA | NA |
| metab_4940 | Unannotated feature | pos | 170.0790 | 7.618 | 3.726 | 3.763 | 0.037 | 0.217 | 0.227 | 0.562 | Unannotated | NA | NA |
| metab_16923 | Unannotated feature | neg | 275.9062 | 7.788 | 5.657 | 5.638 | -0.020 | -0.217 | 0.227 | 0.562 | Unannotated | NA | NA |
| metab_3223 | Dextrorphan O-glucuronide | pos | 398.1934 | 3.781 | 4.715 | 4.574 | -0.141 | -0.217 | 0.227 | 0.562 | Unannotated | NA | NA |
| metab_3504 | Isovalerylcarnitine | pos | 246.1705 | 4.810 | 3.714 | 3.528 | -0.186 | -0.217 | 0.227 | 0.562 | Tentative | NA | NA |
| metab_17720 | Hydroxydesmthyl doxepin glucuronide | neg | 516.1849 | 2.766 | 3.809 | 4.337 | 0.528 | 0.217 | 0.227 | 0.562 | Unannotated | Organoheterocyclic compounds | Benzoxepines |
| metab_9438 | 4-p-Coumaroylquinic acid | neg | 337.0936 | 3.422 | 4.256 | 4.583 | 0.327 | 0.217 | 0.227 | 0.562 | Tentative | NA | NA |
| metab_5740 | Unannotated feature | pos | 395.2562 | 6.072 | 3.735 | 3.402 | -0.333 | -0.217 | 0.227 | 0.562 | Unannotated | NA | NA |
| metab_9475 | Glutamylphenylalanine | neg | 293.1145 | 3.379 | 5.224 | 5.174 | -0.050 | -0.217 | 0.227 | 0.562 | Unannotated | NA | NA |
| metab_9165 | 2-Hydroxy-3-(4-hydroxyphenyl)propenoic acid | neg | 225.0402 | 3.829 | 4.209 | 4.433 | 0.223 | 0.217 | 0.227 | 0.562 | Tentative | NA | NA |
| metab_12309 | Unannotated feature | neg | 279.2330 | 6.899 | 6.606 | 6.650 | 0.044 | 0.217 | 0.227 | 0.562 | Unannotated | NA | NA |
| metab_18526 | Unannotated feature | neg | 462.2869 | 6.035 | 3.851 | 3.598 | -0.252 | -0.217 | 0.227 | 0.562 | Unannotated | NA | NA |
| metab_1746 | 3-Succinoylpyridine | pos | 162.0552 | 3.602 | 5.061 | 5.204 | 0.143 | 0.217 | 0.227 | 0.562 | Tentative | NA | NA |
| metab_7742 | MG(0:0/10:0/0:0) | pos | 285.1451 | 3.307 | 2.565 | 1.668 | -0.897 | -0.217 | 0.227 | 0.562 | Unannotated | NA | NA |
| metab_8320 | Unannotated feature | pos | 171.9922 | 2.021 | 3.987 | 3.831 | -0.156 | -0.217 | 0.227 | 0.562 | Unannotated | NA | NA |
| metab_11509 | 5-(Methoxycarbonyl)thiophene-2-carboxylic acid | neg | 206.9723 | 0.512 | 3.616 | 3.714 | 0.098 | 0.217 | 0.227 | 0.562 | Unannotated | Organoheterocyclic compounds | Thiophenes |
| metab_1703 | 5-Hydroxyindoleacetic acid | pos | 192.0659 | 3.997 | 5.637 | 5.580 | -0.057 | 0.217 | 0.227 | 0.562 | Strong putative | NA | NA |
| metab_11085 | (E)-indol-3-ylacetaldoxime | neg | 211.0279 | 0.730 | 5.302 | 5.032 | -0.270 | -0.217 | 0.227 | 0.562 | Tentative | Organoheterocyclic compounds | Indoles and derivatives |
| metab_14268 | Unannotated feature | neg | 262.0392 | 3.014 | 4.449 | 4.356 | -0.093 | -0.217 | 0.227 | 0.562 | Unannotated | NA | NA |
| metab_14277 | Unannotated feature | neg | 200.0293 | 3.025 | 6.032 | 5.986 | -0.046 | -0.217 | 0.227 | 0.562 | Unannotated | NA | NA |
| metab_6301 | Unannotated feature | pos | 152.1107 | 0.493 | 3.312 | 2.690 | -0.623 | -0.217 | 0.227 | 0.562 | Unannotated | NA | NA |
| metab_171 | Unannotated feature | pos | 266.1602 | 0.601 | 3.667 | 4.212 | 0.545 | 0.217 | 0.227 | 0.562 | Unannotated | NA | NA |
| metab_395 | Unannotated feature | pos | 195.1384 | 4.708 | 4.346 | 3.515 | -0.831 | -0.217 | 0.227 | 0.562 | Unannotated | NA | NA |
| metab_1412 | PA(18:1(12Z)-2OH(9,10)/10:0) | pos | 661.3465 | 6.056 | 3.667 | 4.556 | 0.890 | 0.217 | 0.227 | 0.562 | Unannotated | NA | NA |
| metab_9803 | L-beta-aspartyl-L-threonine | neg | 279.0850 | 3.037 | 4.973 | 5.036 | 0.063 | 0.217 | 0.227 | 0.562 | Unannotated | NA | NA |
| metab_8333 | Dihydrozeatin-7-N-glucoside | pos | 416.2154 | 1.990 | 4.425 | 3.788 | -0.636 | -0.217 | 0.227 | 0.562 | Unannotated | NA | NA |
| metab_2041 | Unannotated feature | pos | 143.1182 | 2.636 | 4.514 | 4.444 | -0.070 | -0.217 | 0.227 | 0.562 | Unannotated | NA | NA |
| metab_15882 | Unannotated feature | neg | 118.9301 | 0.607 | 5.940 | 5.861 | -0.079 | -0.217 | 0.227 | 0.562 | Unannotated | NA | NA |
| metab_7868 | Unannotated feature | pos | 94.1662 | 3.017 | 3.292 | 3.490 | 0.199 | 0.217 | 0.227 | 0.562 | Unannotated | NA | NA |
| metab_6922 | Unannotated feature | pos | 162.1126 | 0.649 | 5.732 | 5.598 | -0.134 | -0.217 | 0.227 | 0.562 | Unannotated | NA | NA |
| metab_9745 | Unannotated feature | neg | 181.0137 | 3.085 | 4.453 | 4.769 | 0.317 | 0.217 | 0.227 | 0.562 | Unannotated | NA | NA |
| metab_8355 | Unannotated feature | pos | 174.1492 | 1.960 | 4.443 | 4.227 | -0.216 | -0.217 | 0.227 | 0.562 | Unannotated | NA | NA |
| metab_7380 | Unannotated feature | pos | 310.1767 | 4.006 | 4.148 | 3.847 | -0.301 | -0.217 | 0.227 | 0.562 | Unannotated | NA | NA |
| metab_19450 | Syringic acid | neg | 179.0344 | 4.941 | 4.833 | 4.940 | 0.108 | 0.217 | 0.227 | 0.562 | Tentative | NA | NA |
| metab_1329 | Unannotated feature | pos | 542.2913 | 6.199 | 5.641 | 5.324 | -0.317 | -0.217 | 0.227 | 0.562 | Unannotated | NA | NA |
| metab_1543 | 2-(6,7-Dimethyl-9-oxoxanthen-4-yl)acetic acid | pos | 315.1234 | 5.355 | 3.766 | 4.508 | 0.743 | 0.217 | 0.227 | 0.562 | Unannotated | NA | NA |
| metab_17043 | Unannotated feature | neg | 293.9144 | 7.985 | 4.648 | 4.596 | -0.052 | -0.217 | 0.227 | 0.562 | Unannotated | NA | NA |
| metab_16338 | Unannotated feature | neg | 301.2652 | 6.695 | 5.822 | 6.013 | 0.191 | 0.217 | 0.227 | 0.562 | Unannotated | NA | NA |
| metab_13706 | Unannotated feature | neg | 303.0132 | 1.785 | 3.749 | 3.598 | -0.151 | -0.217 | 0.227 | 0.562 | Unannotated | NA | NA |
| metab_2152 | Unannotated feature | pos | 216.1599 | 2.892 | 3.395 | 3.177 | -0.218 | -0.217 | 0.227 | 0.562 | Unannotated | NA | NA |
| metab_8961 | Undecanedioic acid | neg | 261.1344 | 4.150 | 4.754 | 4.956 | 0.202 | 0.217 | 0.227 | 0.562 | Unannotated | NA | NA |
| metab_8419 | Uracil | pos | 113.0351 | 1.952 | 5.721 | 5.630 | -0.090 | -0.217 | 0.227 | 0.562 | Strong putative | NA | NA |
| metab_3072 | Unannotated feature | pos | 423.3630 | 6.438 | 5.923 | 5.670 | -0.253 | -0.217 | 0.227 | 0.562 | Unannotated | NA | NA |
| metab_2110 | Unannotated feature | pos | 265.1551 | 2.732 | 4.755 | 4.588 | -0.166 | -0.217 | 0.227 | 0.562 | Unannotated | NA | NA |
| metab_1083 | Unannotated feature | pos | 507.2717 | 6.686 | 5.021 | 5.272 | 0.251 | 0.217 | 0.227 | 0.562 | Unannotated | NA | NA |
| metab_11149 | Unannotated feature | neg | 326.1004 | 0.683 | 2.634 | 2.865 | 0.231 | 0.217 | 0.227 | 0.562 | Unannotated | NA | NA |
| metab_15208 | Unannotated feature | neg | 310.1380 | 4.740 | 4.201 | 4.151 | -0.051 | -0.217 | 0.227 | 0.562 | Unannotated | NA | NA |
| metab_10981 | Unannotated feature | neg | 111.0081 | 0.784 | 4.449 | 4.043 | -0.405 | -0.217 | 0.227 | 0.562 | Unannotated | NA | NA |
| metab_13118 | 2'-Hydroxy-4,4',6'-trimethoxychalcone | neg | 313.1086 | 5.355 | 5.782 | 6.049 | 0.267 | 0.217 | 0.227 | 0.562 | Unannotated | NA | NA |
| metab_15206 | Melatonin glucuronide | neg | 389.1341 | 4.735 | 5.151 | 4.971 | -0.180 | -0.217 | 0.227 | 0.562 | Unannotated | Nucleosides, nucleotides, and analogues | Nucleoside and nucleotide analogues |
| metab_17356 | Unannotated feature | neg | 486.9041 | 7.687 | 5.113 | 5.129 | 0.016 | 0.217 | 0.227 | 0.562 | Unannotated | NA | NA |
| metab_19437 | Unannotated feature | neg | 468.2473 | 4.955 | 5.217 | 4.979 | -0.238 | -0.217 | 0.227 | 0.562 | Unannotated | NA | NA |
| metab_17210 | Unannotated feature | neg | 359.8458 | 7.730 | 3.928 | 3.823 | -0.105 | -0.217 | 0.227 | 0.562 | Unannotated | NA | NA |
| metab_5573 | Scymnol | pos | 433.3323 | 6.255 | 5.618 | 5.372 | -0.246 | -0.217 | 0.227 | 0.562 | Tentative | NA | NA |
| metab_16811 | Unannotated feature | neg | 136.8913 | 7.720 | 4.703 | 4.685 | -0.017 | -0.217 | 0.227 | 0.562 | Unannotated | NA | NA |
| metab_11244 | Unannotated feature | neg | 110.9846 | 0.636 | 5.263 | 4.779 | -0.484 | -0.217 | 0.227 | 0.562 | Unannotated | NA | NA |
| metab_18328 | Unannotated feature | neg | 281.0893 | 2.999 | 5.130 | 5.411 | 0.281 | 0.217 | 0.227 | 0.562 | Unannotated | NA | NA |
| metab_14791 | Unannotated feature | neg | 239.0589 | 3.886 | 4.962 | 5.054 | 0.091 | 0.217 | 0.227 | 0.562 | Unannotated | NA | NA |
| metab_351 | Furapyrimidone | pos | 271.1025 | 0.402 | 6.316 | 6.337 | 0.021 | 0.217 | 0.227 | 0.562 | Unannotated | NA | NA |
| metab_5524 | Didemnin A | pos | 472.2894 | 4.870 | 3.517 | 3.816 | 0.299 | 0.217 | 0.227 | 0.562 | Tentative | NA | NA |
| metab_12239 | Unannotated feature | neg | 409.2367 | 6.953 | 5.315 | 4.956 | -0.359 | -0.217 | 0.227 | 0.562 | Unannotated | NA | NA |
| metab_8174 | Acrylamide-sodium acrylate resin | pos | 215.1394 | 2.630 | 5.154 | 5.034 | -0.119 | -0.217 | 0.227 | 0.562 | Unannotated | NA | NA |
| metab_4652 | Unannotated feature | pos | 457.2779 | 7.315 | 6.397 | 6.382 | -0.014 | -0.217 | 0.227 | 0.562 | Unannotated | NA | NA |
| metab_18966 | Unannotated feature | neg | 241.0540 | 5.571 | 4.993 | 4.937 | -0.056 | -0.217 | 0.227 | 0.562 | Unannotated | NA | NA |
| metab_14733 | Unannotated feature | neg | 399.1891 | 3.754 | 4.622 | 3.749 | -0.873 | -0.217 | 0.227 | 0.562 | Unannotated | NA | NA |
| metab_9848 | Histidylproline | neg | 233.1029 | 2.999 | 5.063 | 5.224 | 0.161 | 0.217 | 0.227 | 0.562 | Unannotated | NA | NA |
| metab_0 | 3-Oxocholic acid | pos | 371.2588 | 6.359 | 6.920 | 6.608 | -0.311 | -0.217 | 0.227 | 0.562 | Unannotated | NA | NA |
| metab_16856 | Unannotated feature | neg | 323.1249 | 7.730 | 4.925 | 5.039 | 0.114 | 0.217 | 0.227 | 0.562 | Unannotated | NA | NA |
| metab_11619 | Unannotated feature | neg | 215.9262 | 0.208 | 6.913 | 6.888 | -0.024 | -0.217 | 0.227 | 0.562 | Unannotated | NA | NA |
| metab_3028 | Unannotated feature | pos | 78.4258 | 3.223 | 3.761 | 3.846 | 0.085 | 0.217 | 0.227 | 0.562 | Unannotated | NA | NA |
| metab_1345 | Unannotated feature | pos | 412.3068 | 6.167 | 5.346 | 5.189 | -0.157 | -0.217 | 0.227 | 0.562 | Unannotated | NA | NA |
| metab_1849 | 2-Pyrimidine Acetic Acid | pos | 139.0504 | 1.049 | 5.980 | 6.046 | 0.066 | -0.217 | 0.227 | 0.562 | Moderate putative | NA | NA |
| metab_14060 | Unannotated feature | neg | 153.0551 | 2.785 | 4.855 | 4.955 | 0.100 | 0.217 | 0.227 | 0.562 | Unannotated | NA | NA |
| metab_8162 | Val Leu Ser | pos | 318.2030 | 2.684 | 5.203 | 5.011 | -0.192 | -0.217 | 0.227 | 0.562 | Moderate putative | NA | NA |
| metab_2380 | Unannotated feature | pos | 159.9920 | 0.545 | 2.471 | 3.017 | 0.546 | 0.217 | 0.227 | 0.562 | Unannotated | NA | NA |
| metab_9610 | Urothion | neg | 324.0234 | 3.219 | 5.638 | 5.730 | 0.092 | 0.217 | 0.227 | 0.562 | Unannotated | NA | NA |
| metab_13209 | Unannotated feature | neg | 92.9590 | 0.542 | 3.914 | 3.439 | -0.475 | -0.217 | 0.227 | 0.562 | Unannotated | NA | NA |
| metab_6743 | Unannotated feature | pos | 270.9622 | 0.625 | 4.320 | 4.084 | -0.236 | -0.217 | 0.227 | 0.562 | Unannotated | NA | NA |
| metab_15679 | 1-(1-Methylindol-5-yl)-3-(3-methyl-1,2-thiazol-5-yl)urea | neg | 285.0794 | 5.653 | 4.313 | 4.587 | 0.274 | 0.217 | 0.227 | 0.562 | Unannotated | Organoheterocyclic compounds | Indoles and derivatives |
| metab_6714 | Unannotated feature | pos | 200.1762 | 0.625 | 4.430 | 4.078 | -0.352 | -0.217 | 0.227 | 0.562 | Unannotated | NA | NA |
| metab_11906 | Unannotated feature | neg | 441.2040 | 6.060 | 5.399 | 5.567 | 0.168 | 0.217 | 0.227 | 0.562 | Unannotated | NA | NA |
| metab_8849 | Methyl (3b,11x)-3-Hydroxy-8-oxo-6-eremophilen-12-oate | neg | 301.1405 | 4.363 | 4.384 | 4.744 | 0.360 | 0.217 | 0.227 | 0.562 | Unannotated | NA | NA |
| metab_14254 | Unannotated feature | neg | 321.0481 | 2.994 | 5.077 | 4.947 | -0.131 | -0.217 | 0.227 | 0.562 | Unannotated | NA | NA |
| metab_1997 | Unannotated feature | pos | 111.0748 | 2.217 | 4.020 | 4.059 | 0.040 | 0.217 | 0.227 | 0.562 | Unannotated | NA | NA |
| metab_14241 | Unannotated feature | neg | 416.1205 | 2.981 | 3.609 | 3.965 | 0.356 | 0.213 | 0.236 | 0.570 | Unannotated | NA | NA |
| metab_8838 | Xanthohumol | pos | 387.1773 | 3.487 | 3.587 | 4.241 | 0.653 | 0.213 | 0.236 | 0.570 | Tentative | NA | NA |
| metab_8834 | Styrene | pos | 105.0704 | 2.859 | 4.767 | 4.507 | -0.260 | -0.213 | 0.236 | 0.570 | Strong putative | NA | NA |
| metab_10316 | Unannotated feature | neg | 202.1080 | 2.329 | 4.019 | 4.648 | 0.630 | 0.213 | 0.236 | 0.570 | Unannotated | NA | NA |
| metab_64 | N1,N12-Diacetylspermine | pos | 287.2447 | 0.665 | 5.860 | 5.065 | -0.795 | -0.213 | 0.236 | 0.570 | Moderate putative | Organic acids and derivatives | Carboximidic acids and derivatives |
| metab_19834 | Deferiprone | neg | 337.1410 | 4.390 | 4.997 | 4.864 | -0.134 | -0.213 | 0.236 | 0.570 | Unannotated | NA | NA |
| metab_6796 | 2,3-Dihydroxypropanamide | pos | 147.0766 | 0.633 | 5.634 | 5.417 | -0.217 | -0.213 | 0.236 | 0.570 | Unannotated | NA | NA |
| metab_3265 | N(2)-phenylacetyl-L-glutaminate | pos | 229.0976 | 3.930 | 5.234 | 4.982 | -0.252 | -0.213 | 0.236 | 0.570 | Tentative | NA | NA |
| metab_6441 | Unannotated feature | pos | 336.6575 | 5.671 | 3.673 | 4.080 | 0.407 | 0.213 | 0.236 | 0.570 | Unannotated | NA | NA |
| metab_178 | Unannotated feature | pos | 209.1288 | 0.601 | 3.458 | 3.978 | 0.519 | 0.213 | 0.236 | 0.570 | Unannotated | NA | NA |
| metab_11995 | Unannotated feature | neg | 621.3272 | 6.224 | 3.546 | 2.120 | -1.427 | -0.213 | 0.236 | 0.570 | Unannotated | NA | NA |
| metab_59 | Unannotated feature | pos | 233.0429 | 0.672 | 3.334 | 3.746 | 0.412 | 0.213 | 0.236 | 0.570 | Unannotated | NA | NA |
| metab_6748 | Unannotated feature | pos | 468.2703 | 5.247 | 4.603 | 4.170 | -0.433 | -0.213 | 0.236 | 0.570 | Unannotated | NA | NA |
| metab_1461 | Unannotated feature | pos | 481.2807 | 5.976 | 4.167 | 3.977 | -0.190 | -0.213 | 0.236 | 0.570 | Unannotated | NA | NA |
| metab_4643 | Unannotated feature | pos | 727.4618 | 7.252 | 4.400 | 4.477 | 0.077 | 0.213 | 0.236 | 0.570 | Unannotated | NA | NA |
| metab_5350 | Unannotated feature | pos | 845.5537 | 6.518 | 4.755 | 4.329 | -0.426 | -0.213 | 0.236 | 0.570 | Unannotated | NA | NA |
| metab_14054 | Unannotated feature | neg | 277.0390 | 2.780 | 3.506 | 3.100 | -0.406 | -0.213 | 0.236 | 0.570 | Unannotated | NA | NA |
| metab_17020 | Unannotated feature | neg | 243.7552 | 7.997 | 4.768 | 4.737 | -0.030 | -0.213 | 0.236 | 0.570 | Unannotated | NA | NA |
| metab_11617 | Unannotated feature | neg | 135.9202 | 0.252 | 3.327 | 3.275 | -0.052 | -0.213 | 0.236 | 0.570 | Unannotated | NA | NA |
| metab_432 | Unannotated feature | pos | 129.9954 | 0.016 | 3.161 | 3.251 | 0.090 | 0.213 | 0.236 | 0.570 | Unannotated | NA | NA |
| metab_16309 | Prostaglandin D1 Alcohol | neg | 339.2539 | 6.640 | 4.431 | 4.456 | 0.025 | 0.213 | 0.236 | 0.570 | Strong putative | NA | NA |
| metab_7522 | Sarecycline | pos | 470.1883 | 0.720 | 4.145 | 4.683 | 0.538 | 0.213 | 0.236 | 0.570 | Unannotated | Phenylpropanoids and polyketides | Tetracyclines |
| metab_15391 | Unannotated feature | neg | 134.0604 | 5.052 | 4.094 | 4.014 | -0.079 | -0.213 | 0.236 | 0.570 | Unannotated | NA | NA |
| metab_11756 | Unannotated feature | neg | 673.2575 | 5.678 | 4.920 | 3.877 | -1.043 | -0.213 | 0.236 | 0.570 | Unannotated | NA | NA |
| metab_11210 | L-Cysteine | neg | 301.0531 | 0.644 | 2.985 | 3.848 | 0.863 | 0.213 | 0.236 | 0.570 | Tentative | NA | NA |
| metab_5329 | (1(10)E,4a,5E)-1(10),5-Germacradiene-12-acetoxy-4,11-diol | pos | 261.1852 | 6.542 | 4.790 | 4.573 | -0.217 | -0.213 | 0.236 | 0.570 | Unannotated | NA | NA |
| metab_9073 | (S,E)-Zearalenone | neg | 317.1395 | 3.964 | 4.489 | 4.366 | -0.122 | -0.213 | 0.236 | 0.570 | Tentative | Phenylpropanoids and polyketides | Macrolides and analogues |
| metab_10120 | Saxitoxin | neg | 334.1051 | 2.762 | 3.595 | 3.919 | 0.325 | 0.213 | 0.236 | 0.570 | Tentative | Phenylpropanoids and polyketides | Saxitoxins, gonyautoxins, and derivatives |
| metab_16843 | Unannotated feature | neg | 166.9237 | 7.730 | 5.357 | 5.337 | -0.020 | -0.213 | 0.236 | 0.570 | Unannotated | NA | NA |
| metab_967 | Unannotated feature | pos | 156.9909 | 7.732 | 4.483 | 4.444 | -0.040 | -0.213 | 0.236 | 0.570 | Unannotated | NA | NA |
| metab_1837 | Unannotated feature | pos | 176.0556 | 1.026 | 4.975 | 4.687 | -0.288 | -0.213 | 0.236 | 0.570 | Unannotated | NA | NA |
| metab_16263 | Unannotated feature | neg | 325.1482 | 6.545 | 5.191 | 5.152 | -0.039 | -0.213 | 0.236 | 0.570 | Unannotated | NA | NA |
| metab_13098 | Unannotated feature | neg | 249.8489 | 0.051 | 5.836 | 5.804 | -0.032 | -0.213 | 0.236 | 0.570 | Unannotated | NA | NA |
| metab_7549 | Unannotated feature | pos | 452.2302 | 6.111 | 5.667 | 5.844 | 0.176 | 0.213 | 0.236 | 0.570 | Unannotated | NA | NA |
| metab_11612 | Unannotated feature | neg | 197.9111 | 0.370 | 4.172 | 4.157 | -0.015 | -0.213 | 0.236 | 0.570 | Unannotated | NA | NA |
| metab_5643 | Unannotated feature | pos | 186.9813 | 0.016 | 5.432 | 5.435 | 0.002 | 0.213 | 0.236 | 0.570 | Unannotated | NA | NA |
| metab_5599 | HOMATROPINE | pos | 293.1863 | 6.231 | 4.008 | 4.852 | 0.844 | 0.213 | 0.236 | 0.570 | Tentative | NA | NA |
| metab_12978 | Sipoglitazar | neg | 462.1478 | 0.784 | 3.784 | 4.504 | 0.720 | 0.213 | 0.236 | 0.570 | Unannotated | Benzenoids | Phenol ethers |
| metab_14404 | Unannotated feature | neg | 266.0283 | 3.203 | 3.513 | 3.120 | -0.393 | -0.213 | 0.236 | 0.570 | Unannotated | NA | NA |
| metab_16352 | Unannotated feature | neg | 215.9913 | 0.619 | 4.714 | 4.456 | -0.258 | -0.213 | 0.236 | 0.570 | Unannotated | NA | NA |
| metab_2697 | Etiracetam | pos | 203.1394 | 2.039 | 5.802 | 5.771 | -0.030 | -0.213 | 0.236 | 0.570 | Tentative | NA | NA |
| metab_9340 | Unannotated feature | neg | 187.9311 | 3.562 | 3.965 | 3.858 | -0.107 | -0.213 | 0.236 | 0.570 | Unannotated | NA | NA |
| metab_19343 | Unannotated feature | neg | 145.0864 | 5.076 | 4.360 | 4.271 | -0.089 | -0.213 | 0.236 | 0.570 | Unannotated | NA | NA |
| metab_2311 | Unannotated feature | pos | 226.0724 | 0.072 | 5.190 | 5.227 | 0.037 | 0.213 | 0.236 | 0.570 | Unannotated | NA | NA |
| metab_15434 | Unannotated feature | neg | 229.9983 | 0.571 | 4.552 | 4.947 | 0.395 | 0.213 | 0.236 | 0.570 | Unannotated | NA | NA |
| metab_9013 | 3-Carboxy-4-Methyl-5-Propyl-2-Furanpropanoic Acid | neg | 239.0923 | 4.052 | 4.965 | 5.103 | 0.138 | 0.213 | 0.236 | 0.570 | Moderate putative | NA | NA |
| metab_11642 | Unannotated feature | neg | 260.8984 | 0.036 | 5.897 | 5.880 | -0.017 | -0.213 | 0.236 | 0.570 | Unannotated | NA | NA |
| metab_5385 | Unannotated feature | pos | 163.0728 | 6.462 | 4.370 | 4.188 | -0.182 | -0.213 | 0.236 | 0.570 | Unannotated | NA | NA |
| metab_2840 | Unannotated feature | pos | 344.1824 | 2.747 | 5.075 | 4.978 | -0.097 | -0.213 | 0.236 | 0.570 | Unannotated | NA | NA |
| metab_16140 | Unannotated feature | neg | 190.9480 | 6.303 | 4.453 | 3.934 | -0.519 | -0.213 | 0.236 | 0.570 | Unannotated | NA | NA |
| metab_19568 | Unannotated feature | neg | 219.0886 | 4.759 | 3.522 | 3.018 | -0.503 | -0.213 | 0.236 | 0.570 | Unannotated | NA | NA |
| metab_7628 | Nitrolinoleic acid | pos | 370.1983 | 3.496 | 4.484 | 4.042 | -0.441 | -0.213 | 0.236 | 0.570 | Tentative | NA | NA |
| metab_19320 | Unannotated feature | neg | 368.1467 | 5.114 | 4.326 | 3.766 | -0.559 | -0.213 | 0.236 | 0.570 | Unannotated | NA | NA |
| metab_8553 | Unannotated feature | pos | 377.1309 | 1.266 | 4.605 | 4.659 | 0.054 | 0.213 | 0.236 | 0.570 | Unannotated | NA | NA |
| metab_2078 | Unannotated feature | pos | 247.0575 | 2.747 | 3.797 | 3.658 | -0.139 | -0.213 | 0.236 | 0.570 | Unannotated | NA | NA |
| metab_9914 | Unannotated feature | neg | 314.1255 | 2.938 | 4.674 | 4.864 | 0.190 | 0.213 | 0.236 | 0.570 | Unannotated | NA | NA |
| metab_827 | Unannotated feature | pos | 272.2952 | 6.422 | 4.715 | 4.901 | 0.186 | 0.213 | 0.236 | 0.570 | Unannotated | NA | NA |
| metab_12200 | Unannotated feature | neg | 370.2214 | 6.797 | 4.585 | 4.946 | 0.361 | 0.213 | 0.236 | 0.570 | Unannotated | NA | NA |
| metab_5678 | Unannotated feature | pos | 309.9494 | 0.016 | 5.507 | 5.521 | 0.014 | 0.213 | 0.236 | 0.570 | Unannotated | NA | NA |
| metab_5658 | Unannotated feature | pos | 515.2833 | 6.159 | 4.940 | 4.652 | -0.287 | -0.213 | 0.236 | 0.570 | Unannotated | NA | NA |
| metab_1654 | Unannotated feature | pos | 172.1334 | 4.571 | 6.015 | 5.978 | -0.037 | -0.213 | 0.236 | 0.570 | Unannotated | NA | NA |
| metab_2658 | Coumarin | pos | 179.0705 | 1.952 | 3.667 | 3.799 | 0.132 | 0.213 | 0.236 | 0.570 | Strong putative | NA | NA |
| metab_10243 | Unannotated feature | neg | 437.0844 | 2.572 | 5.013 | 4.735 | -0.278 | -0.213 | 0.236 | 0.570 | Unannotated | NA | NA |
| metab_3699 | 4-Ethylphenol | pos | 140.1072 | 5.297 | 4.832 | 4.521 | -0.312 | -0.213 | 0.236 | 0.570 | Tentative | Benzenoids | Phenols |
| metab_3600 | Unannotated feature | pos | 729.9375 | 5.064 | 3.994 | 4.917 | 0.923 | 0.213 | 0.236 | 0.570 | Unannotated | NA | NA |
| metab_7455 | Butyl 4-aminobenzoate | pos | 158.0967 | 3.859 | 5.662 | 5.562 | -0.100 | -0.213 | 0.236 | 0.570 | Tentative | NA | NA |
| metab_19462 | (1S,2S,4S,5R)-1,8-Epoxy-p-menthane-2,5-diol | neg | 231.1236 | 4.923 | 4.446 | 4.765 | 0.319 | 0.213 | 0.236 | 0.570 | Unannotated | NA | NA |
| metab_5209 | Unannotated feature | pos | 199.0830 | 1.041 | 4.454 | 3.728 | -0.725 | -0.213 | 0.236 | 0.570 | Unannotated | NA | NA |
| metab_17165 | Unannotated feature | neg | 148.9695 | 7.760 | 5.135 | 5.113 | -0.022 | -0.213 | 0.236 | 0.570 | Unannotated | NA | NA |
| metab_1162 | Unannotated feature | pos | 175.1484 | 6.534 | 4.682 | 4.502 | -0.180 | -0.213 | 0.236 | 0.570 | Unannotated | NA | NA |
| metab_3537 | Unannotated feature | pos | 105.0429 | 4.906 | 3.808 | 3.755 | -0.052 | -0.213 | 0.236 | 0.570 | Unannotated | NA | NA |
| metab_13284 | Unannotated feature | neg | 252.9636 | 0.804 | 5.026 | 4.952 | -0.074 | -0.213 | 0.236 | 0.570 | Unannotated | NA | NA |
| metab_4871 | Unannotated feature | pos | 365.1562 | 0.696 | 3.946 | 4.227 | 0.281 | 0.213 | 0.236 | 0.570 | Unannotated | NA | NA |
| metab_807 | Unannotated feature | pos | 489.2838 | 6.303 | 6.039 | 5.847 | -0.192 | -0.213 | 0.236 | 0.570 | Unannotated | NA | NA |
| metab_4864 | Unannotated feature | pos | 185.0581 | 7.724 | 3.801 | 3.756 | -0.045 | -0.213 | 0.236 | 0.570 | Unannotated | NA | NA |
| metab_16706 | Unannotated feature | neg | 558.1802 | 1.947 | 3.628 | 4.141 | 0.513 | 0.213 | 0.236 | 0.570 | Unannotated | NA | NA |
| metab_10703 | Glucaron | neg | 257.0280 | 1.404 | 5.001 | 4.962 | -0.039 | -0.213 | 0.236 | 0.570 | Unannotated | NA | NA |
| metab_12369 | Unannotated feature | neg | 520.9110 | 7.596 | 7.725 | 7.750 | 0.026 | 0.213 | 0.236 | 0.570 | Unannotated | NA | NA |
| metab_11103 | 5-Amino-2-[(2-amino-3-carboxypropanoyl)oxyamino]pentanoic acid | neg | 244.0938 | 0.722 | 4.932 | 4.356 | -0.575 | -0.213 | 0.236 | 0.570 | Unannotated | NA | NA |
| metab_8300 | Unannotated feature | pos | 159.9694 | 2.070 | 5.876 | 5.952 | 0.076 | 0.213 | 0.236 | 0.570 | Unannotated | NA | NA |
| metab_13395 | Unannotated feature | neg | 188.0018 | 0.770 | 4.169 | 4.094 | -0.075 | -0.213 | 0.236 | 0.570 | Unannotated | NA | NA |
| metab_17782 | Nepsilon-Acetyl-L-lysine | neg | 187.1083 | 2.802 | 6.598 | 6.508 | -0.090 | -0.213 | 0.236 | 0.570 | Tentative | NA | NA |
| metab_17774 | Unannotated feature | neg | 84.9502 | 6.892 | 4.725 | 4.717 | -0.008 | -0.213 | 0.236 | 0.570 | Unannotated | NA | NA |
| metab_5201 | Unannotated feature | pos | 239.9669 | 0.485 | 5.543 | 5.748 | 0.206 | 0.213 | 0.236 | 0.570 | Unannotated | NA | NA |
| metab_15854 | Unannotated feature | neg | 120.9271 | 0.607 | 5.488 | 5.404 | -0.083 | -0.213 | 0.236 | 0.570 | Unannotated | NA | NA |
| metab_15908 | Unannotated feature | neg | 542.2453 | 6.003 | 4.404 | 4.606 | 0.203 | 0.213 | 0.236 | 0.570 | Unannotated | NA | NA |
| metab_17094 | Unannotated feature | neg | 191.8545 | 7.874 | 5.240 | 5.310 | 0.071 | 0.213 | 0.236 | 0.570 | Unannotated | NA | NA |
| metab_3449 | Oxyglutinosone | pos | 269.1752 | 4.608 | 4.882 | 5.073 | 0.191 | 0.213 | 0.236 | 0.570 | Unannotated | NA | NA |
| metab_7695 | Unannotated feature | pos | 307.1770 | 3.375 | 4.699 | 4.057 | -0.642 | -0.213 | 0.236 | 0.570 | Unannotated | NA | NA |
| metab_669 | Unannotated feature | pos | 138.9956 | 0.016 | 4.943 | 4.965 | 0.022 | 0.213 | 0.236 | 0.570 | Unannotated | NA | NA |
| metab_17888 | Unannotated feature | neg | 339.2652 | 6.687 | 3.829 | 4.007 | 0.177 | 0.213 | 0.236 | 0.570 | Unannotated | NA | NA |
| metab_3244 | Anthralin | pos | 244.0973 | 3.842 | 4.960 | 5.147 | 0.187 | 0.213 | 0.236 | 0.570 | Tentative | NA | NA |
| metab_17114 | Unannotated feature | neg | 274.0936 | 2.259 | 5.241 | 5.496 | 0.256 | 0.213 | 0.236 | 0.570 | Unannotated | NA | NA |
| metab_2037 | Unannotated feature | pos | 120.0813 | 2.590 | 7.202 | 7.076 | -0.126 | -0.213 | 0.236 | 0.570 | Unannotated | NA | NA |
| metab_5026 | Unannotated feature | pos | 97.1016 | 7.307 | 4.147 | 4.175 | 0.028 | 0.213 | 0.236 | 0.570 | Unannotated | NA | NA |
| metab_13088 | Unannotated feature | neg | 172.9365 | 0.036 | 6.074 | 6.045 | -0.029 | -0.213 | 0.236 | 0.570 | Unannotated | NA | NA |
| metab_14645 | Gentisic acid | neg | 153.0187 | 3.585 | 5.291 | 5.532 | 0.241 | 0.213 | 0.236 | 0.570 | Strong putative | NA | NA |
| metab_12380 | Unannotated feature | neg | 808.8367 | 7.637 | 6.128 | 6.207 | 0.080 | 0.213 | 0.236 | 0.570 | Unannotated | NA | NA |
| metab_11055 | 2-Keto-3-Deoxy-D-Mannooctanoic Acid | neg | 219.0508 | 0.738 | 5.641 | 5.745 | 0.104 | 0.213 | 0.236 | 0.570 | Unannotated | NA | NA |
| metab_8376 | Unannotated feature | pos | 251.0643 | 1.952 | 4.456 | 4.233 | -0.223 | -0.213 | 0.236 | 0.570 | Unannotated | NA | NA |
| metab_11704 | Unannotated feature | neg | 479.1720 | 5.484 | 3.836 | 3.499 | -0.337 | -0.213 | 0.236 | 0.570 | Unannotated | NA | NA |
| metab_1751 | Unannotated feature | pos | 391.1874 | 3.553 | 4.697 | 4.413 | -0.284 | -0.213 | 0.236 | 0.570 | Unannotated | NA | NA |
| metab_11511 | Unannotated feature | neg | 477.2265 | 5.238 | 3.645 | 3.813 | 0.168 | 0.213 | 0.236 | 0.570 | Unannotated | NA | NA |
| metab_13253 | (3S)-3,6-Diaminohexanoate | neg | 145.0976 | 0.588 | 5.295 | 5.163 | -0.132 | -0.213 | 0.236 | 0.570 | Tentative | NA | NA |
| metab_14591 | Tetranor-PGFM | neg | 365.1359 | 3.498 | 5.301 | 5.779 | 0.478 | 0.213 | 0.236 | 0.570 | Unannotated | NA | NA |
| metab_7349 | 4-{[(5,5,8,8-Tetramethyl-5,6,7,8-tetrahydronaphthalen-2-yl)carbonyl]amino}benzoic acid | pos | 334.1770 | 4.078 | 3.791 | 3.527 | -0.263 | -0.213 | 0.236 | 0.570 | Tentative | Benzenoids | Benzene and substituted derivatives |
| metab_2217 | Unannotated feature | pos | 380.1348 | 3.035 | 4.258 | 4.508 | 0.250 | 0.213 | 0.236 | 0.570 | Unannotated | NA | NA |
| metab_7346 | Unannotated feature | pos | 130.1090 | 0.704 | 6.077 | 5.902 | -0.174 | -0.213 | 0.236 | 0.570 | Unannotated | NA | NA |
| metab_11679 | Unannotated feature | neg | 457.2019 | 5.362 | 4.732 | 5.287 | 0.555 | 0.213 | 0.236 | 0.570 | Unannotated | NA | NA |
| metab_16960 | Unannotated feature | neg | 259.0936 | 1.958 | 4.743 | 4.990 | 0.247 | 0.213 | 0.236 | 0.570 | Unannotated | NA | NA |
| metab_2969 | Unannotated feature | pos | 353.2481 | 6.271 | 6.236 | 5.914 | -0.322 | -0.213 | 0.236 | 0.570 | Unannotated | NA | NA |
| metab_17289 | Unannotated feature | neg | 346.0645 | 2.417 | 4.308 | 4.529 | 0.221 | 0.213 | 0.236 | 0.570 | Unannotated | NA | NA |
| metab_12904 | Unannotated feature | neg | 255.0181 | 0.730 | 4.287 | 3.867 | -0.420 | -0.213 | 0.236 | 0.570 | Unannotated | NA | NA |
| metab_6318 | Unannotated feature | pos | 466.2927 | 5.782 | 5.173 | 4.955 | -0.218 | -0.213 | 0.236 | 0.570 | Unannotated | NA | NA |
| metab_6270 | Unannotated feature | pos | 574.1047 | 5.799 | 3.907 | 3.909 | 0.002 | 0.213 | 0.236 | 0.570 | Unannotated | NA | NA |
| metab_9996 | Unannotated feature | neg | 200.0383 | 2.852 | 3.770 | 3.396 | -0.375 | -0.213 | 0.236 | 0.570 | Unannotated | NA | NA |
| metab_13429 | N-Acetyllactosamine | neg | 364.1255 | 0.799 | 4.633 | 4.838 | 0.205 | 0.213 | 0.236 | 0.570 | Tentative | Organic oxygen compounds | Organooxygen compounds |
| metab_1240 | Unannotated feature | pos | 247.1696 | 6.382 | 5.516 | 5.389 | -0.128 | -0.213 | 0.236 | 0.570 | Unannotated | NA | NA |
| metab_10030 | Unannotated feature | neg | 478.1744 | 2.813 | 3.719 | 4.896 | 1.177 | 0.208 | 0.246 | 0.581 | Unannotated | NA | NA |
| metab_14236 | 2-Keto-6-acetamidocaproate | neg | 168.0660 | 2.975 | 4.835 | 4.718 | -0.117 | -0.208 | 0.246 | 0.581 | Tentative | NA | NA |
| metab_2027 | Unannotated feature | pos | 111.0748 | 2.495 | 4.280 | 4.326 | 0.046 | 0.208 | 0.246 | 0.581 | Unannotated | NA | NA |
| metab_7345 | Bisnoryangonin | pos | 272.0923 | 4.084 | 3.125 | 3.898 | 0.773 | 0.208 | 0.246 | 0.581 | Tentative | NA | NA |
| metab_9189 | Unannotated feature | neg | 349.1411 | 3.780 | 4.890 | 5.298 | 0.408 | 0.208 | 0.246 | 0.581 | Unannotated | NA | NA |
| metab_16951 | Unannotated feature | neg | 249.9782 | 7.858 | 5.733 | 5.705 | -0.028 | -0.208 | 0.246 | 0.581 | Unannotated | NA | NA |
| metab_16958 | Unannotated feature | neg | 191.8659 | 7.870 | 4.271 | 4.312 | 0.041 | 0.208 | 0.246 | 0.581 | Unannotated | NA | NA |
| metab_4420 | (10Z)-12-Hydroxyhexadecenoylcarnitine | pos | 396.3119 | 6.335 | 5.424 | 5.243 | -0.181 | -0.208 | 0.246 | 0.581 | Unannotated | NA | NA |
| metab_10333 | 2'-Deoxy-2'-methylenecytidine | neg | 260.0639 | 2.259 | 3.754 | 2.500 | -1.253 | -0.208 | 0.246 | 0.581 | Unannotated | NA | NA |
| metab_18257 | Unannotated feature | neg | 443.0107 | 2.975 | 3.299 | 3.726 | 0.427 | 0.208 | 0.246 | 0.581 | Unannotated | NA | NA |
| metab_18528 | Unannotated feature | neg | 365.1618 | 6.035 | 3.875 | 4.043 | 0.168 | 0.208 | 0.246 | 0.581 | Unannotated | NA | NA |
| metab_15011 | Unannotated feature | neg | 150.9463 | 0.542 | 5.713 | 5.423 | -0.290 | -0.208 | 0.246 | 0.581 | Unannotated | NA | NA |
| metab_7284 | 1-Hydroxyisoquinoline | pos | 146.0603 | 4.232 | 5.661 | 5.617 | -0.044 | 0.208 | 0.246 | 0.581 | Strong putative | NA | NA |
| metab_11651 | Unannotated feature | neg | 289.8707 | 0.033 | 4.222 | 4.159 | -0.063 | -0.208 | 0.246 | 0.581 | Unannotated | NA | NA |
| metab_2941 | Unannotated feature | pos | 226.1805 | 6.215 | 6.173 | 6.081 | -0.093 | -0.208 | 0.246 | 0.581 | Unannotated | NA | NA |
| metab_17277 | Unannotated feature | neg | 294.1041 | 7.720 | 2.993 | 3.068 | 0.075 | 0.208 | 0.246 | 0.581 | Unannotated | NA | NA |
| metab_18501 | Gabazine | neg | 573.2421 | 6.046 | 3.735 | 4.813 | 1.078 | 0.208 | 0.246 | 0.581 | Tentative | Organoheterocyclic compounds | Diazines |
| metab_13597 | Succinic Acid | neg | 117.0186 | 1.404 | 6.928 | 6.897 | -0.031 | -0.208 | 0.246 | 0.581 | Strong putative | Organic acids and derivatives | Carboxylic acids and derivatives |
| metab_17330 | Unannotated feature | neg | 325.1587 | 7.697 | 5.101 | 5.154 | 0.053 | 0.208 | 0.246 | 0.581 | Unannotated | NA | NA |
| metab_14300 | Unannotated feature | neg | 324.0234 | 3.045 | 5.259 | 5.312 | 0.053 | 0.208 | 0.246 | 0.581 | Unannotated | NA | NA |
| metab_17242 | Unannotated feature | neg | 570.8481 | 7.726 | 4.577 | 4.636 | 0.059 | 0.208 | 0.246 | 0.581 | Unannotated | NA | NA |
| metab_12400 | Unannotated feature | neg | 326.1880 | 7.692 | 3.815 | 3.931 | 0.116 | 0.208 | 0.246 | 0.581 | Unannotated | NA | NA |
| metab_9269 | 3-Hydroxybenzoic Acid | neg | 137.0237 | 3.658 | 5.240 | 5.136 | -0.103 | -0.208 | 0.246 | 0.581 | Strong putative | NA | NA |
| metab_11456 | Unannotated feature | neg | 348.8962 | 0.527 | 5.090 | 4.938 | -0.152 | -0.208 | 0.246 | 0.581 | Unannotated | NA | NA |
| metab_17765 | Capric acid | neg | 209.0943 | 6.899 | 5.977 | 5.940 | -0.037 | -0.208 | 0.246 | 0.581 | Tentative | NA | NA |
| metab_18836 | Unannotated feature | neg | 271.0503 | 5.743 | 4.069 | 3.524 | -0.545 | -0.208 | 0.246 | 0.581 | Unannotated | NA | NA |
| metab_1011 | Unannotated feature | pos | 797.5197 | 7.092 | 4.204 | 4.430 | 0.226 | 0.208 | 0.246 | 0.581 | Unannotated | NA | NA |
| metab_9238 | 1-O-Sinapoyl-Beta-D-Glucose | neg | 385.1146 | 3.701 | 5.624 | 5.238 | -0.386 | -0.208 | 0.246 | 0.581 | Strong putative | Phenylpropanoids and polyketides | Cinnamic acids and derivatives |
| metab_5431 | Unannotated feature | pos | 469.2760 | 6.406 | 5.991 | 5.907 | -0.083 | -0.208 | 0.246 | 0.581 | Unannotated | NA | NA |
| metab_1191 | Unannotated feature | pos | 316.3215 | 6.478 | 6.003 | 6.246 | 0.243 | 0.208 | 0.246 | 0.581 | Unannotated | NA | NA |
| metab_18568 | Unannotated feature | neg | 553.3393 | 6.007 | 4.972 | 4.837 | -0.135 | -0.208 | 0.246 | 0.581 | Unannotated | NA | NA |
| metab_4906 | Vitamin D3 | pos | 385.3476 | 7.681 | 5.167 | 4.921 | -0.246 | -0.208 | 0.246 | 0.581 | Tentative | Lipids and lipid-like molecules | Steroids and steroid derivatives |
| metab_7532 | (E)-2-Hydroxy-N-desmethyldoxepin | pos | 314.1758 | 3.694 | 3.394 | 3.236 | -0.158 | -0.208 | 0.246 | 0.581 | Unannotated | Organoheterocyclic compounds | Benzoxepines |
| metab_1263 | Unannotated feature | pos | 281.1077 | 2.732 | 4.041 | 4.324 | 0.283 | 0.208 | 0.246 | 0.581 | Unannotated | NA | NA |
| metab_8181 | Unannotated feature | pos | 121.0846 | 2.590 | 6.116 | 5.984 | -0.132 | -0.208 | 0.246 | 0.581 | Unannotated | NA | NA |
| metab_16831 | Unannotated feature | neg | 387.8773 | 7.726 | 4.148 | 4.143 | -0.004 | 0.208 | 0.246 | 0.581 | Unannotated | NA | NA |
| metab_4831 | Beta-D-Glucopyranuronic acid | pos | 347.0955 | 0.672 | 4.343 | 4.917 | 0.574 | 0.208 | 0.246 | 0.581 | Tentative | NA | NA |
| metab_1637 | Unannotated feature | pos | 291.1708 | 4.729 | 4.808 | 4.596 | -0.211 | -0.208 | 0.246 | 0.581 | Unannotated | NA | NA |
| metab_14154 | Unannotated feature | neg | 311.1342 | 2.896 | 2.888 | 2.590 | -0.297 | -0.208 | 0.246 | 0.581 | Unannotated | NA | NA |
| metab_924 | 1-Deoxynojirimycin | pos | 196.1185 | 0.625 | 3.894 | 4.459 | 0.565 | 0.208 | 0.246 | 0.581 | Tentative | Organoheterocyclic compounds | Piperidines |
| metab_3925 | Unannotated feature | pos | 541.0851 | 5.764 | 3.808 | 4.391 | 0.583 | 0.208 | 0.246 | 0.581 | Unannotated | NA | NA |
| metab_3923 | Pregnan-20-one, 17-(acetyloxy)-3-hydroxy-6-methyl-, (3a,5b,6a)- | pos | 391.2852 | 6.255 | 6.688 | 6.464 | -0.224 | -0.208 | 0.246 | 0.581 | Strong putative | NA | NA |
| metab_14443 | Unannotated feature | neg | 299.0532 | 3.254 | 3.698 | 3.952 | 0.254 | 0.208 | 0.246 | 0.581 | Unannotated | NA | NA |
| metab_8499 | 3-Hydroxyoctanoic acid | pos | 202.1441 | 1.685 | 3.465 | 3.528 | 0.063 | 0.208 | 0.246 | 0.581 | Tentative | NA | NA |
| metab_8130 | (15S)-Prostaglandin A2 | pos | 357.2029 | 2.724 | 3.687 | 4.128 | 0.441 | 0.208 | 0.246 | 0.581 | Tentative | NA | NA |
| metab_5551 | Unannotated feature | pos | 256.1184 | 2.755 | 4.797 | 5.069 | 0.272 | 0.208 | 0.246 | 0.581 | Unannotated | NA | NA |
| metab_13856 | Unannotated feature | neg | 105.0185 | 2.244 | 3.361 | 3.335 | -0.027 | -0.208 | 0.246 | 0.581 | Unannotated | NA | NA |
| metab_1690 | Unannotated feature | pos | 158.0491 | 4.103 | 5.470 | 5.497 | 0.027 | 0.208 | 0.246 | 0.581 | Unannotated | NA | NA |
| metab_12700 | Unannotated feature | neg | 376.1042 | 5.359 | 6.101 | 6.279 | 0.178 | 0.208 | 0.246 | 0.581 | Unannotated | NA | NA |
| metab_16999 | Unannotated feature | neg | 222.8296 | 7.989 | 5.855 | 5.806 | -0.049 | -0.208 | 0.246 | 0.581 | Unannotated | NA | NA |
| metab_4598 | Unannotated feature | pos | 683.4357 | 7.005 | 4.948 | 5.064 | 0.117 | 0.208 | 0.246 | 0.581 | Unannotated | NA | NA |
| metab_963 | Unannotated feature | pos | 230.1242 | 7.700 | 5.383 | 5.408 | 0.025 | 0.208 | 0.246 | 0.581 | Unannotated | NA | NA |
| metab_8230 | Unannotated feature | pos | 350.1451 | 2.398 | 3.687 | 3.391 | -0.296 | -0.208 | 0.246 | 0.581 | Unannotated | NA | NA |
| metab_13957 | Unannotated feature | neg | 417.1418 | 2.637 | 3.111 | 3.665 | 0.554 | 0.208 | 0.246 | 0.581 | Unannotated | NA | NA |
| metab_18346 | Unannotated feature | neg | 328.1882 | 3.007 | 4.647 | 4.190 | -0.456 | -0.208 | 0.246 | 0.581 | Unannotated | NA | NA |
| metab_7469 | Unannotated feature | pos | 302.1975 | 3.823 | 3.949 | 3.662 | -0.287 | -0.208 | 0.246 | 0.581 | Unannotated | NA | NA |
| metab_3842 | Unannotated feature | pos | 359.2877 | 6.390 | 6.519 | 6.414 | -0.105 | -0.208 | 0.246 | 0.581 | Unannotated | NA | NA |
| metab_7556 | Acetaminophen | pos | 303.1345 | 3.642 | 4.569 | 4.905 | 0.335 | 0.208 | 0.246 | 0.581 | Tentative | NA | NA |
| metab_12985 | Unannotated feature | neg | 155.0343 | 0.791 | 5.417 | 5.564 | 0.148 | 0.208 | 0.246 | 0.581 | Unannotated | NA | NA |
| metab_19377 | Unannotated feature | neg | 136.0761 | 5.038 | 5.334 | 5.126 | -0.208 | -0.208 | 0.246 | 0.581 | Unannotated | NA | NA |
| metab_10609 | Fructosyl-lysine | neg | 289.1408 | 1.685 | 5.285 | 5.099 | -0.186 | -0.208 | 0.246 | 0.581 | Tentative | Organic oxygen compounds | Organooxygen compounds |
| metab_3887 | Unannotated feature | pos | 223.1695 | 6.367 | 5.930 | 5.878 | -0.052 | -0.208 | 0.246 | 0.581 | Unannotated | NA | NA |
| metab_3843 | Unannotated feature | pos | 84.3059 | 5.656 | 3.201 | 3.066 | -0.135 | -0.208 | 0.246 | 0.581 | Unannotated | NA | NA |
| metab_18118 | Unannotated feature | neg | 269.1758 | 6.381 | 4.839 | 4.840 | 0.001 | 0.208 | 0.246 | 0.581 | Unannotated | NA | NA |
| metab_10648 | Unannotated feature | neg | 247.1550 | 4.726 | 5.268 | 4.946 | -0.322 | -0.208 | 0.246 | 0.581 | Unannotated | NA | NA |
| metab_13698 | Unannotated feature | neg | 271.0935 | 1.706 | 3.908 | 4.300 | 0.392 | 0.208 | 0.246 | 0.581 | Unannotated | NA | NA |
| metab_12420 | Unannotated feature | neg | 194.9238 | 7.726 | 5.703 | 5.691 | -0.012 | -0.208 | 0.246 | 0.581 | Unannotated | NA | NA |
| metab_1572 | Fluvoxamino acid | pos | 301.1189 | 5.231 | 5.039 | 4.902 | -0.138 | -0.208 | 0.246 | 0.581 | Unannotated | NA | NA |
| metab_19085 | Unannotated feature | neg | 246.0748 | 5.413 | 6.136 | 6.473 | 0.337 | 0.208 | 0.246 | 0.581 | Unannotated | NA | NA |
| metab_10104 | Unannotated feature | neg | 367.1144 | 2.766 | 3.761 | 3.957 | 0.196 | 0.208 | 0.246 | 0.581 | Unannotated | NA | NA |
| metab_2093 | 7-Benzylidenenaltrexone | pos | 394.1840 | 2.770 | 3.793 | 3.975 | 0.182 | 0.208 | 0.246 | 0.581 | Tentative | NA | NA |
| metab_16426 | Unannotated feature | neg | 379.1579 | 6.915 | 6.163 | 6.314 | 0.151 | 0.208 | 0.246 | 0.581 | Unannotated | NA | NA |
| metab_16771 | Unannotated feature | neg | 404.9297 | 7.692 | 4.708 | 4.750 | 0.041 | 0.208 | 0.246 | 0.581 | Unannotated | NA | NA |
| metab_7080 | Unannotated feature | pos | 346.2236 | 4.687 | 2.883 | 2.088 | -0.796 | -0.208 | 0.246 | 0.581 | Unannotated | NA | NA |
| metab_19630 | Plicatic acid | neg | 403.1040 | 4.663 | 5.047 | 4.784 | -0.263 | -0.208 | 0.246 | 0.581 | Tentative | Lignans, neolignans and related compounds | Aryltetralin lignans |
| metab_8576 | 2-Deoxystreptamine | pos | 145.0974 | 1.196 | 3.306 | 3.506 | 0.200 | 0.208 | 0.246 | 0.581 | Tentative | Organic oxygen compounds | Organooxygen compounds |
| metab_12219 | Unannotated feature | neg | 492.3102 | 6.868 | 4.312 | 3.096 | -1.216 | -0.208 | 0.246 | 0.581 | Unannotated | NA | NA |
| metab_17450 | Unannotated feature | neg | 229.9549 | 7.611 | 4.347 | 4.202 | -0.145 | -0.208 | 0.246 | 0.581 | Unannotated | NA | NA |
| metab_14616 | Unannotated feature | neg | 390.2042 | 3.541 | 4.095 | 3.814 | -0.281 | -0.208 | 0.246 | 0.581 | Unannotated | NA | NA |
| metab_11975 | Unannotated feature | neg | 479.2653 | 6.193 | 5.999 | 5.888 | -0.111 | -0.208 | 0.246 | 0.581 | Unannotated | NA | NA |
| metab_6598 | Unannotated feature | pos | 135.0807 | 5.451 | 2.668 | 3.089 | 0.421 | 0.208 | 0.246 | 0.581 | Unannotated | NA | NA |
| metab_7667 | Unannotated feature | pos | 294.1185 | 3.436 | 4.891 | 4.780 | -0.111 | -0.208 | 0.246 | 0.581 | Unannotated | NA | NA |
| metab_1288 | Unannotated feature | pos | 230.2481 | 6.271 | 5.789 | 5.744 | -0.046 | -0.208 | 0.246 | 0.581 | Unannotated | NA | NA |
| metab_8975 | Unannotated feature | neg | 182.0817 | 4.123 | 5.169 | 5.108 | -0.061 | -0.208 | 0.246 | 0.581 | Unannotated | NA | NA |
| metab_2007 | Cinnamic acid | pos | 166.0866 | 2.296 | 7.385 | 7.279 | -0.106 | -0.208 | 0.246 | 0.581 | Tentative | NA | NA |
| metab_6772 | Unannotated feature | pos | 366.1163 | 0.625 | 4.034 | 4.514 | 0.480 | 0.208 | 0.246 | 0.581 | Unannotated | NA | NA |
| metab_9669 | Unannotated feature | neg | 368.0991 | 3.165 | 4.384 | 3.955 | -0.429 | -0.208 | 0.246 | 0.581 | Unannotated | NA | NA |
| metab_8584 | Unannotated feature | pos | 138.0493 | 1.176 | 5.777 | 5.667 | -0.110 | -0.208 | 0.246 | 0.581 | Unannotated | NA | NA |
| metab_10305 | 4-Hydroxy-3-nitrophenylacetaldehyde | neg | 162.0190 | 2.379 | 3.877 | 3.895 | 0.018 | -0.208 | 0.246 | 0.581 | Unannotated | NA | NA |
| metab_12449 | Unannotated feature | neg | 229.1442 | 6.263 | 6.301 | 6.211 | -0.090 | -0.208 | 0.246 | 0.581 | Unannotated | NA | NA |
| metab_797 | Unannotated feature | pos | 423.2745 | 6.263 | 5.620 | 5.488 | -0.133 | -0.208 | 0.246 | 0.581 | Unannotated | NA | NA |
| metab_14927 | Unannotated feature | neg | 250.1086 | 4.169 | 4.774 | 4.679 | -0.095 | -0.208 | 0.246 | 0.581 | Unannotated | NA | NA |
| metab_17756 | Unannotated feature | neg | 325.2749 | 6.907 | 5.392 | 5.220 | -0.172 | -0.208 | 0.246 | 0.581 | Unannotated | NA | NA |
| metab_200 | Unannotated feature | pos | 200.0687 | 0.593 | 4.662 | 4.503 | -0.159 | -0.208 | 0.246 | 0.581 | Unannotated | NA | NA |
| metab_17067 | Unannotated feature | neg | 247.8923 | 7.921 | 4.760 | 4.800 | 0.040 | 0.208 | 0.246 | 0.581 | Unannotated | NA | NA |
| metab_13175 | Unannotated feature | neg | 293.9438 | 0.492 | 5.060 | 4.992 | -0.068 | -0.208 | 0.246 | 0.581 | Unannotated | NA | NA |
| metab_2704 | Unannotated feature | pos | 271.1024 | 2.065 | 5.879 | 5.922 | 0.042 | 0.208 | 0.246 | 0.581 | Unannotated | NA | NA |
| metab_15104 | Unannotated feature | neg | 389.1342 | 4.530 | 4.564 | 4.416 | -0.149 | -0.208 | 0.246 | 0.581 | Unannotated | NA | NA |
| metab_2319 | Unannotated feature | pos | 285.1547 | 0.201 | 7.095 | 7.114 | 0.019 | 0.208 | 0.246 | 0.581 | Unannotated | NA | NA |
| metab_16079 | Unannotated feature | neg | 391.2494 | 6.217 | 4.550 | 4.958 | 0.409 | 0.208 | 0.246 | 0.581 | Unannotated | NA | NA |
| metab_5789 | Unannotated feature | pos | 176.9596 | 0.019 | 4.730 | 4.754 | 0.024 | 0.208 | 0.246 | 0.581 | Unannotated | NA | NA |
| metab_8727 | Pent-1-en-2-ol | pos | 128.1074 | 0.768 | 5.625 | 5.046 | -0.578 | -0.208 | 0.246 | 0.581 | Unannotated | Organic oxygen compounds | Organooxygen compounds |
| metab_9627 | Unannotated feature | neg | 198.9879 | 3.210 | 4.123 | 3.837 | -0.287 | -0.208 | 0.246 | 0.581 | Unannotated | NA | NA |
| metab_3518 | Unannotated feature | pos | 107.9673 | 4.844 | 3.568 | 3.195 | -0.373 | -0.208 | 0.246 | 0.581 | Unannotated | NA | NA |
| metab_8061 | Sabeluzole | pos | 448.2060 | 2.762 | 5.082 | 4.838 | -0.244 | -0.208 | 0.246 | 0.581 | Unannotated | NA | NA |
| metab_8055 | Unannotated feature | pos | 168.0488 | 2.774 | 3.436 | 3.867 | 0.431 | 0.208 | 0.246 | 0.581 | Unannotated | NA | NA |
| metab_14776 | Unannotated feature | neg | 283.0937 | 3.849 | 3.807 | 4.170 | 0.363 | 0.208 | 0.246 | 0.581 | Unannotated | NA | NA |
| metab_8438 | Unannotated feature | pos | 215.1394 | 1.944 | 4.974 | 4.878 | -0.096 | -0.204 | 0.255 | 0.590 | Unannotated | NA | NA |
| metab_11010 | L-3-Aminodihydro-2(3H)-furanone | neg | 201.0877 | 0.770 | 4.847 | 4.680 | -0.167 | -0.204 | 0.255 | 0.590 | Tentative | NA | NA |
| metab_4059 | Unannotated feature | pos | 738.4553 | 5.831 | 4.223 | 4.266 | 0.043 | 0.204 | 0.255 | 0.590 | Unannotated | NA | NA |
| metab_9724 | Unannotated feature | neg | 187.9311 | 3.105 | 4.100 | 3.861 | -0.239 | -0.204 | 0.255 | 0.590 | Unannotated | NA | NA |
| metab_16922 | Unannotated feature | neg | 343.8940 | 7.785 | 5.516 | 5.483 | -0.032 | -0.204 | 0.255 | 0.590 | Unannotated | NA | NA |
| metab_13108 | Unannotated feature | neg | 173.9650 | 0.142 | 5.673 | 5.661 | -0.012 | -0.204 | 0.255 | 0.590 | Unannotated | NA | NA |
| metab_3411 | Unannotated feature | pos | 158.9643 | 7.602 | 6.728 | 6.773 | 0.045 | 0.204 | 0.255 | 0.590 | Unannotated | NA | NA |
| metab_7864 | Unannotated feature | pos | 387.1771 | 3.295 | 3.074 | 3.849 | 0.776 | 0.204 | 0.255 | 0.590 | Unannotated | NA | NA |
| metab_14899 | Quaternary ammonium chloride combination | neg | 229.0980 | 4.103 | 5.451 | 5.402 | -0.048 | -0.204 | 0.255 | 0.590 | Tentative | NA | NA |
| metab_12469 | Unannotated feature | neg | 208.9360 | 7.985 | 6.265 | 6.240 | -0.024 | -0.204 | 0.255 | 0.590 | Unannotated | NA | NA |
| metab_9136 | Benzidine | neg | 229.0981 | 3.872 | 6.013 | 5.926 | -0.087 | -0.204 | 0.255 | 0.590 | Tentative | NA | NA |
| metab_8872 | Unannotated feature | neg | 295.0857 | 4.315 | 3.293 | 4.001 | 0.708 | 0.204 | 0.255 | 0.590 | Unannotated | NA | NA |
| metab_8870 | Unannotated feature | neg | 288.0880 | 4.319 | 4.961 | 5.220 | 0.259 | 0.204 | 0.255 | 0.590 | Unannotated | NA | NA |
| metab_2204 | Zanthodioline | pos | 306.1342 | 3.002 | 4.058 | 4.258 | 0.201 | 0.204 | 0.255 | 0.590 | Unannotated | NA | NA |
| metab_6918 | 2-Ethylphenol | pos | 140.1072 | 4.995 | 4.516 | 4.227 | -0.289 | -0.204 | 0.255 | 0.590 | Tentative | Benzenoids | Phenols |
| metab_3299 | Arg-Thr-Lys-Arg | pos | 592.3924 | 4.054 | 4.597 | 5.084 | 0.487 | 0.204 | 0.255 | 0.590 | Unannotated | Organic acids and derivatives | Carboxylic acids and derivatives |
| metab_1238 | Unannotated feature | pos | 115.0547 | 6.382 | 4.843 | 4.662 | -0.181 | -0.204 | 0.255 | 0.590 | Unannotated | NA | NA |
| metab_11896 | Unannotated feature | neg | 579.2652 | 6.041 | 4.016 | 4.579 | 0.563 | 0.204 | 0.255 | 0.590 | Unannotated | NA | NA |
| metab_13077 | Unannotated feature | neg | 197.0675 | 1.046 | 5.498 | 5.259 | -0.239 | -0.204 | 0.255 | 0.590 | Unannotated | NA | NA |
| metab_1743 | Fenamic acid | pos | 231.1133 | 3.627 | 6.164 | 6.051 | -0.112 | -0.204 | 0.255 | 0.590 | Moderate putative | NA | NA |
| metab_17257 | Unannotated feature | neg | 260.9164 | 7.726 | 5.890 | 5.873 | -0.017 | -0.204 | 0.255 | 0.590 | Unannotated | NA | NA |
| metab_12614 | Unannotated feature | neg | 270.1137 | 6.035 | 4.843 | 4.302 | -0.540 | -0.204 | 0.255 | 0.590 | Unannotated | NA | NA |
| metab_4676 | (4S,8R)-8,9-Dihydroxy-p-menth-1(6)-en-2-one | pos | 207.0992 | 7.490 | 4.575 | 4.556 | -0.019 | -0.204 | 0.255 | 0.590 | Unannotated | NA | NA |
| metab_8464 | Unannotated feature | pos | 182.0676 | 1.840 | 4.989 | 5.178 | 0.189 | 0.204 | 0.255 | 0.590 | Unannotated | NA | NA |
| metab_9518 | Unannotated feature | neg | 317.1255 | 3.326 | 4.236 | 3.985 | -0.251 | -0.204 | 0.255 | 0.590 | Unannotated | NA | NA |
| metab_9912 | 1-(4-Aminophenyl)-7,8-dimethoxy-3,5-dihydro-2,3-benzodiazepin-4-one | neg | 356.1257 | 2.942 | 3.593 | 4.018 | 0.425 | 0.204 | 0.255 | 0.590 | Unannotated | NA | NA |
| metab_8471 | 5-Hydroxykynurenine | pos | 257.1136 | 1.698 | 4.463 | 4.588 | 0.125 | 0.204 | 0.255 | 0.590 | Tentative | NA | NA |
| metab_1087 | Unannotated feature | pos | 488.2760 | 6.678 | 4.958 | 4.553 | -0.405 | -0.204 | 0.255 | 0.590 | Unannotated | NA | NA |
| metab_426 | Unannotated feature | pos | 142.9620 | 0.016 | 4.647 | 4.667 | 0.020 | 0.204 | 0.255 | 0.590 | Unannotated | NA | NA |
| metab_10684 | Unannotated feature | neg | 367.9856 | 1.489 | 5.025 | 4.970 | -0.055 | -0.204 | 0.255 | 0.590 | Unannotated | NA | NA |
| metab_3964 | Glutaminyllysine | pos | 587.2882 | 6.215 | 5.511 | 5.303 | -0.208 | -0.204 | 0.255 | 0.590 | Unannotated | NA | NA |
| metab_9836 | Unannotated feature | neg | 301.0676 | 3.011 | 4.523 | 4.721 | 0.198 | 0.204 | 0.255 | 0.590 | Unannotated | NA | NA |
| metab_14203 | 2,6-Dimethoxyphenol | neg | 153.0551 | 2.946 | 4.631 | 4.768 | 0.137 | 0.204 | 0.255 | 0.590 | Tentative | NA | NA |
| metab_15958 | Glycocholenate sulfate | neg | 524.2273 | 6.060 | 4.461 | 4.857 | 0.396 | 0.204 | 0.255 | 0.590 | Unannotated | NA | NA |
| metab_19705 | Unannotated feature | neg | 319.1169 | 4.547 | 1.574 | 3.390 | 1.816 | 0.204 | 0.255 | 0.590 | Unannotated | NA | NA |
| metab_11407 | Unannotated feature | neg | 122.9513 | 0.547 | 4.535 | 4.237 | -0.298 | -0.204 | 0.255 | 0.590 | Unannotated | NA | NA |
| metab_1693 | (R)-2-Benzylsuccinate | pos | 209.0812 | 3.917 | 5.370 | 5.090 | -0.281 | -0.204 | 0.255 | 0.590 | Tentative | Phenylpropanoids and polyketides | Phenylpropanoic acids |
| metab_5542 | (3S,7E,9S)-9-Hydroxy-4,7-megastigmadien-3-one 9-glucoside | pos | 409.1631 | 6.287 | 6.870 | 6.733 | -0.137 | -0.204 | 0.255 | 0.590 | Unannotated | NA | NA |
| metab_9546 | Isopropyl apiosylglucoside | neg | 353.1456 | 3.288 | 3.030 | 3.516 | 0.487 | 0.204 | 0.255 | 0.590 | Unannotated | Organic oxygen compounds | Organooxygen compounds |
| metab_11526 | Unannotated feature | neg | 426.9319 | 0.502 | 3.850 | 3.624 | -0.226 | -0.204 | 0.255 | 0.590 | Unannotated | NA | NA |
| metab_11539 | Unannotated feature | neg | 273.1253 | 5.246 | 4.959 | 4.959 | -0.000 | -0.204 | 0.255 | 0.590 | Unannotated | NA | NA |
| metab_5171 | Unannotated feature | pos | 173.1287 | 0.728 | 4.229 | 3.909 | -0.320 | -0.204 | 0.255 | 0.590 | Unannotated | NA | NA |
| metab_8729 | Unannotated feature | pos | 129.0551 | 0.768 | 4.942 | 4.998 | 0.057 | 0.204 | 0.255 | 0.590 | Unannotated | NA | NA |
| metab_6159 | Unannotated feature | pos | 737.4546 | 5.831 | 2.743 | 4.153 | 1.410 | 0.204 | 0.255 | 0.590 | Unannotated | NA | NA |
| metab_17083 | Unannotated feature | neg | 122.8935 | 7.905 | 5.052 | 5.006 | -0.045 | -0.204 | 0.255 | 0.590 | Unannotated | NA | NA |
| metab_8224 | Porphobilinogen | pos | 209.0925 | 2.422 | 3.785 | 4.022 | 0.237 | 0.204 | 0.255 | 0.590 | Tentative | NA | NA |
| metab_18747 | Crocetin | neg | 349.1406 | 5.847 | 4.030 | 4.552 | 0.522 | 0.204 | 0.255 | 0.590 | Tentative | Lipids and lipid-like molecules | Prenol lipids |
| metab_18742 | Glu-Ile-Leu-Asp-Val | neg | 568.2965 | 5.853 | 4.209 | 4.970 | 0.761 | 0.204 | 0.255 | 0.590 | Unannotated | NA | NA |
| metab_11623 | Unannotated feature | neg | 207.8943 | 0.181 | 5.616 | 5.600 | -0.016 | -0.204 | 0.255 | 0.590 | Unannotated | NA | NA |
| metab_10593 | Unannotated feature | neg | 210.0880 | 1.691 | 4.358 | 4.306 | -0.052 | -0.204 | 0.255 | 0.590 | Unannotated | NA | NA |
| metab_1366 | Unannotated feature | pos | 412.2858 | 6.135 | 5.382 | 5.059 | -0.322 | -0.204 | 0.255 | 0.590 | Unannotated | NA | NA |
| metab_14051 | Unannotated feature | neg | 114.0917 | 2.780 | 3.029 | 2.462 | -0.567 | -0.204 | 0.255 | 0.590 | Unannotated | NA | NA |
| metab_3968 | Unannotated feature | pos | 760.2258 | 5.791 | 4.054 | 4.320 | 0.266 | 0.204 | 0.255 | 0.590 | Unannotated | NA | NA |
| metab_16846 | Unannotated feature | neg | 279.6143 | 7.730 | 4.225 | 4.372 | 0.147 | 0.204 | 0.255 | 0.590 | Unannotated | NA | NA |
| metab_9854 | Unannotated feature | neg | 447.1155 | 2.990 | 5.077 | 4.090 | -0.986 | -0.204 | 0.255 | 0.590 | Unannotated | NA | NA |
| metab_12287 | N-Oleoyl Phenylalanine | neg | 428.3176 | 6.907 | 6.014 | 5.745 | -0.269 | -0.204 | 0.255 | 0.590 | Strong putative | NA | NA |
| metab_1938 | Unannotated feature | pos | 309.1451 | 1.944 | 4.471 | 4.591 | 0.120 | 0.204 | 0.255 | 0.590 | Unannotated | NA | NA |
| metab_984 | Unannotated feature | pos | 123.0407 | 7.708 | 5.011 | 5.054 | 0.043 | 0.204 | 0.255 | 0.590 | Unannotated | NA | NA |
| metab_5454 | Unannotated feature | pos | 801.5892 | 6.382 | 6.126 | 5.433 | -0.693 | -0.204 | 0.255 | 0.590 | Unannotated | NA | NA |
| metab_19301 | (1beta,2beta,5beta)-p-Menth-3-ene-1,2,5-triol | neg | 231.1236 | 5.144 | 4.633 | 4.947 | 0.315 | 0.204 | 0.255 | 0.590 | Unannotated | NA | NA |
| metab_7711 | Imidaprilat | pos | 378.1672 | 3.347 | 4.347 | 3.843 | -0.504 | -0.204 | 0.255 | 0.590 | Tentative | Organic acids and derivatives | Carboxylic acids and derivatives |
| metab_16556 | Unannotated feature | neg | 182.0453 | 1.702 | 6.216 | 6.350 | 0.133 | 0.204 | 0.255 | 0.590 | Unannotated | NA | NA |
| metab_11902 | (+/-)-3',4'-Methylenedioxy-5,7-dimethylepicatechin | neg | 365.0800 | 5.156 | 4.477 | 4.172 | -0.305 | -0.204 | 0.255 | 0.590 | Unannotated | NA | NA |
| metab_19845 | 9,10-Epoxyoctadecanoic acid | neg | 349.2149 | 6.459 | 3.809 | 4.343 | 0.534 | 0.204 | 0.255 | 0.590 | Tentative | NA | NA |
| metab_6513 | 1-Hydroxycarvedilol | pos | 423.1927 | 5.573 | 4.255 | 4.797 | 0.542 | 0.204 | 0.255 | 0.590 | Unannotated | NA | NA |
| metab_13 | Isobutyryl carnitine | pos | 214.1442 | 4.306 | 5.276 | 5.135 | -0.141 | -0.204 | 0.255 | 0.590 | Unannotated | NA | NA |
| metab_8306 | Unannotated feature | pos | 120.0661 | 2.061 | 4.294 | 4.239 | -0.055 | -0.204 | 0.255 | 0.590 | Unannotated | NA | NA |
| metab_1883 | Unannotated feature | pos | 168.1134 | 1.211 | 5.677 | 5.414 | -0.263 | -0.204 | 0.255 | 0.590 | Unannotated | NA | NA |
| metab_10163 | 1-Methylinosine | neg | 317.0632 | 2.718 | 5.081 | 5.260 | 0.178 | 0.204 | 0.255 | 0.590 | Unannotated | Nucleosides, nucleotides, and analogues | Purine nucleosides |
| metab_19471 | N-acetyl-S-(3-oxo-3-carboxy-n-propyl)cysteine | neg | 244.0285 | 3.484 | 4.822 | 4.050 | -0.771 | -0.204 | 0.255 | 0.590 | Unannotated | NA | NA |
| metab_7454 | N-phenylanthranilic acid | pos | 214.0866 | 3.859 | 5.615 | 5.538 | -0.078 | -0.204 | 0.255 | 0.590 | Tentative | NA | NA |
| metab_16929 | Unannotated feature | neg | 230.9557 | 7.806 | 4.483 | 4.482 | -0.001 | 0.204 | 0.255 | 0.590 | Unannotated | NA | NA |
| metab_1161 | Unannotated feature | pos | 210.1129 | 2.680 | 5.161 | 5.287 | 0.126 | 0.204 | 0.255 | 0.590 | Unannotated | NA | NA |
| metab_18443 | 9,10,13-TriHOME | neg | 329.2335 | 6.099 | 6.151 | 5.916 | -0.235 | -0.204 | 0.255 | 0.590 | Tentative | NA | NA |
| metab_17048 | Unannotated feature | neg | 199.8045 | 7.985 | 6.713 | 6.672 | -0.041 | -0.204 | 0.255 | 0.590 | Unannotated | NA | NA |
| metab_19698 | Unannotated feature | neg | 377.2049 | 4.563 | 4.040 | 3.485 | -0.555 | -0.204 | 0.255 | 0.590 | Unannotated | NA | NA |
| metab_1152 | (E)-3-(2-Hydroxyphenyl)-2-propenal | pos | 166.0866 | 2.590 | 7.714 | 7.570 | -0.143 | -0.204 | 0.255 | 0.590 | Unannotated | NA | NA |
| metab_12995 | Unannotated feature | neg | 413.1311 | 0.777 | 4.793 | 4.937 | 0.144 | 0.204 | 0.255 | 0.590 | Unannotated | NA | NA |
| metab_4664 | Unannotated feature | pos | 465.3545 | 7.450 | 4.902 | 4.864 | -0.039 | -0.204 | 0.255 | 0.590 | Unannotated | NA | NA |
| metab_2298 | Unannotated feature | pos | 318.8873 | 0.016 | 4.612 | 4.631 | 0.019 | 0.204 | 0.255 | 0.590 | Unannotated | NA | NA |
| metab_1847 | Unannotated feature | pos | 150.0586 | 1.049 | 7.098 | 6.949 | -0.149 | -0.204 | 0.255 | 0.590 | Unannotated | NA | NA |
| metab_19050 | Unannotated feature | neg | 259.9338 | 5.457 | 6.329 | 6.236 | -0.093 | -0.204 | 0.255 | 0.590 | Unannotated | NA | NA |
| metab_2135 | Unannotated feature | pos | 299.1611 | 2.724 | 4.621 | 4.831 | 0.211 | 0.204 | 0.255 | 0.590 | Unannotated | NA | NA |
| metab_14458 | Unannotated feature | neg | 384.2146 | 3.281 | 4.553 | 4.548 | -0.006 | -0.204 | 0.255 | 0.590 | Unannotated | NA | NA |
| metab_15974 | 2-Ethyl-2-heptenal | neg | 139.1121 | 6.083 | 4.259 | 3.566 | -0.692 | -0.204 | 0.255 | 0.590 | Unannotated | NA | NA |
| metab_4823 | Unannotated feature | pos | 111.9849 | 7.854 | 3.475 | 3.449 | -0.026 | -0.204 | 0.255 | 0.590 | Unannotated | NA | NA |
| metab_2529 | Unannotated feature | pos | 131.0705 | 0.949 | 3.155 | 3.204 | 0.048 | 0.204 | 0.255 | 0.590 | Unannotated | NA | NA |
| metab_2538 | Unannotated feature | pos | 130.0977 | 1.018 | 4.475 | 3.743 | -0.733 | -0.204 | 0.255 | 0.590 | Unannotated | NA | NA |
| metab_16040 | (6R,8Z)-6-Hydroxy-3-oxotetradecenoic acid | neg | 255.1598 | 6.177 | 5.215 | 5.422 | 0.208 | 0.204 | 0.255 | 0.590 | Unannotated | NA | NA |
| metab_3583 | Beta-Tyrosine | pos | 164.0708 | 5.028 | 5.422 | 5.368 | -0.054 | -0.204 | 0.255 | 0.590 | Tentative | NA | NA |
| metab_12228 | Unannotated feature | neg | 417.3384 | 6.899 | 5.624 | 5.716 | 0.092 | 0.204 | 0.255 | 0.590 | Unannotated | NA | NA |
| metab_8067 | Unannotated feature | pos | 541.3405 | 3.502 | 4.985 | 4.625 | -0.360 | -0.204 | 0.255 | 0.590 | Unannotated | NA | NA |
| metab_16776 | 4-Pyridoxic Acid | neg | 182.0453 | 1.965 | 6.277 | 6.327 | 0.050 | 0.204 | 0.255 | 0.590 | Strong putative | NA | NA |
| metab_509 | Unannotated feature | pos | 465.3563 | 6.885 | 7.394 | 7.437 | 0.043 | 0.204 | 0.255 | 0.590 | Unannotated | NA | NA |
| metab_16762 | Unannotated feature | neg | 566.9032 | 7.687 | 5.486 | 5.488 | 0.002 | 0.204 | 0.255 | 0.590 | Unannotated | NA | NA |
| metab_1561 | Unannotated feature | pos | 297.1452 | 3.410 | 5.830 | 5.443 | -0.387 | -0.204 | 0.255 | 0.590 | Unannotated | NA | NA |
| metab_1528 | Unannotated feature | pos | 304.1186 | 5.425 | 5.278 | 5.126 | -0.152 | -0.204 | 0.255 | 0.590 | Unannotated | NA | NA |
| metab_19100 | Dodecatrienoic acid | neg | 239.1288 | 5.390 | 5.001 | 4.881 | -0.120 | -0.204 | 0.255 | 0.590 | Unannotated | Lipids and lipid-like molecules | Fatty Acyls |
| metab_16723 | Unannotated feature | neg | 376.9039 | 7.656 | 5.377 | 5.445 | 0.068 | 0.204 | 0.255 | 0.590 | Unannotated | NA | NA |
| metab_13720 | Unannotated feature | neg | 186.8969 | 0.079 | 5.674 | 5.664 | -0.010 | -0.204 | 0.255 | 0.590 | Unannotated | NA | NA |
| metab_3104 | Cis-3-Hexenyl salicylate | pos | 238.1442 | 3.436 | 2.772 | 3.240 | 0.468 | 0.204 | 0.255 | 0.590 | Unannotated | NA | NA |
| metab_12981 | Unannotated feature | neg | 87.0078 | 0.791 | 5.590 | 5.501 | -0.089 | -0.204 | 0.255 | 0.590 | Unannotated | NA | NA |
| metab_160 | Unannotated feature | pos | 189.0162 | 0.609 | 5.671 | 5.591 | -0.079 | -0.204 | 0.255 | 0.590 | Unannotated | NA | NA |
| metab_15577 | Ethylmethylacetic acid | neg | 203.1284 | 5.453 | 4.630 | 4.308 | -0.322 | -0.204 | 0.255 | 0.590 | Tentative | Lipids and lipid-like molecules | Fatty Acyls |
| metab_2569 | Unannotated feature | pos | 137.0460 | 1.176 | 7.084 | 6.993 | -0.090 | -0.204 | 0.255 | 0.590 | Unannotated | NA | NA |
| metab_14807 | 7-Methyl-1,4,5-naphthalenetriol 4-[xylosyl-(1->6)-glucoside] | neg | 529.1575 | 3.917 | 5.994 | 4.986 | -1.009 | -0.204 | 0.255 | 0.590 | Unannotated | Organic oxygen compounds | Organooxygen compounds |
| metab_3191 | Pro-Pro-Pro | pos | 310.1762 | 3.701 | 4.524 | 4.407 | -0.117 | -0.204 | 0.255 | 0.590 | Unannotated | NA | NA |
| metab_10433 | Unannotated feature | neg | 114.0553 | 1.981 | 4.473 | 4.375 | -0.098 | -0.204 | 0.255 | 0.590 | Unannotated | NA | NA |
| metab_10179 | Unannotated feature | neg | 296.1000 | 2.698 | 4.295 | 4.098 | -0.197 | -0.204 | 0.255 | 0.590 | Unannotated | NA | NA |
| metab_11670 | Unannotated feature | neg | 266.1400 | 5.314 | 4.201 | 4.037 | -0.164 | -0.204 | 0.255 | 0.590 | Unannotated | NA | NA |
| metab_201 | Unannotated feature | pos | 192.9105 | 0.593 | 4.195 | 3.963 | -0.232 | -0.204 | 0.255 | 0.590 | Unannotated | NA | NA |
| metab_11082 | 2-Aminobicyclo[3.1.0]hexane-2,6-dicarboxylic acid | neg | 230.0668 | 0.730 | 5.433 | 5.746 | 0.314 | 0.204 | 0.255 | 0.590 | Unannotated | NA | NA |
| metab_2360 | Unannotated feature | pos | 330.9317 | 0.493 | 4.601 | 4.726 | 0.125 | 0.204 | 0.255 | 0.590 | Unannotated | NA | NA |
| metab_18225 | Unannotated feature | neg | 385.0242 | 2.964 | 4.154 | 4.401 | 0.247 | 0.204 | 0.255 | 0.590 | Unannotated | NA | NA |
| metab_13767 | Unannotated feature | neg | 253.1195 | 1.965 | 3.692 | 3.660 | -0.032 | -0.204 | 0.255 | 0.590 | Unannotated | NA | NA |
| metab_19093 | Marmesin rhamnoside | neg | 373.1296 | 5.401 | 4.386 | 4.200 | -0.186 | -0.204 | 0.255 | 0.590 | Unannotated | NA | NA |
| metab_2571 | Unannotated feature | pos | 228.0870 | 1.189 | 4.167 | 4.483 | 0.316 | 0.204 | 0.255 | 0.590 | Unannotated | NA | NA |
| metab_11976 | Unannotated feature | neg | 605.2997 | 6.193 | 6.234 | 5.885 | -0.349 | -0.204 | 0.255 | 0.590 | Unannotated | NA | NA |
| metab_1988 | Unannotated feature | pos | 182.0676 | 2.142 | 5.731 | 5.868 | 0.137 | 0.204 | 0.255 | 0.590 | Unannotated | NA | NA |
| metab_736 | Unannotated feature | pos | 513.3012 | 6.024 | 4.546 | 4.290 | -0.256 | -0.200 | 0.265 | 0.601 | Unannotated | NA | NA |
| metab_19243 | Aziridyl benzoquinone | neg | 319.1303 | 5.209 | 5.692 | 5.554 | -0.138 | -0.200 | 0.265 | 0.601 | Tentative | NA | NA |
| metab_9586 | Unannotated feature | neg | 290.1039 | 4.172 | 4.334 | 4.161 | -0.173 | -0.200 | 0.265 | 0.601 | Unannotated | NA | NA |
| metab_391 | Unannotated feature | pos | 155.9749 | 0.023 | 4.418 | 4.460 | 0.042 | 0.200 | 0.265 | 0.601 | Unannotated | NA | NA |
| metab_13310 | Difructose anhydride III | neg | 323.0988 | 0.730 | 6.343 | 6.524 | 0.181 | 0.200 | 0.265 | 0.601 | Unannotated | NA | NA |
| metab_5467 | Phenylpropionylglycine | pos | 240.1234 | 1.952 | 5.507 | 5.655 | 0.148 | 0.200 | 0.265 | 0.601 | Moderate putative | NA | NA |
| metab_4444 | Unannotated feature | pos | 398.3274 | 6.382 | 5.758 | 5.714 | -0.044 | -0.200 | 0.265 | 0.601 | Unannotated | NA | NA |
| metab_12040 | Acoric acid | neg | 267.1602 | 6.326 | 4.517 | 4.748 | 0.231 | 0.200 | 0.265 | 0.601 | Unannotated | NA | NA |
| metab_27 | Unannotated feature | pos | 159.1132 | 0.712 | 4.567 | 4.503 | -0.065 | -0.200 | 0.265 | 0.601 | Unannotated | NA | NA |
| metab_10321 | Unannotated feature | neg | 454.0747 | 2.298 | 4.631 | 4.052 | -0.579 | -0.200 | 0.265 | 0.601 | Unannotated | NA | NA |
| metab_5219 | 22-Acetylpriverogenin B | pos | 481.3682 | 6.718 | 4.716 | 4.805 | 0.089 | 0.200 | 0.265 | 0.601 | Unannotated | NA | NA |
| metab_13692 | Unannotated feature | neg | 195.0771 | 1.695 | 3.885 | 3.541 | -0.344 | -0.200 | 0.265 | 0.601 | Unannotated | NA | NA |
| metab_7649 | C18-Sphingosine 1-phosphate D-erythro-Sphingosine-1-phosphate | pos | 402.2347 | 3.461 | 3.656 | 3.864 | 0.208 | 0.200 | 0.265 | 0.601 | Unannotated | NA | NA |
| metab_17427 | Unannotated feature | neg | 494.9117 | 7.625 | 5.567 | 5.653 | 0.087 | 0.200 | 0.265 | 0.601 | Unannotated | NA | NA |
| metab_3163 | Genipin | pos | 191.0706 | 3.622 | 4.139 | 4.006 | -0.133 | -0.200 | 0.265 | 0.601 | Tentative | Lipids and lipid-like molecules | Prenol lipids |
| metab_12965 | Unannotated feature | neg | 293.0340 | 0.777 | 3.465 | 3.926 | 0.462 | 0.200 | 0.265 | 0.601 | Unannotated | NA | NA |
| metab_1291 | Unannotated feature | pos | 199.1485 | 6.263 | 4.661 | 4.487 | -0.174 | -0.200 | 0.265 | 0.601 | Unannotated | NA | NA |
| metab_10282 | Unannotated feature | neg | 387.1891 | 2.465 | 4.930 | 4.700 | -0.230 | -0.200 | 0.265 | 0.601 | Unannotated | NA | NA |
| metab_2617 | D-Digitoxose | pos | 190.1076 | 1.692 | 4.964 | 4.987 | 0.023 | 0.200 | 0.265 | 0.601 | Tentative | NA | NA |
| metab_18245 | Unannotated feature | neg | 573.3113 | 6.256 | 5.164 | 4.929 | -0.236 | -0.200 | 0.265 | 0.601 | Unannotated | NA | NA |
| metab_7673 | Unannotated feature | pos | 254.1392 | 3.426 | 3.443 | 3.563 | 0.119 | -0.200 | 0.265 | 0.601 | Unannotated | NA | NA |
| metab_2060 | Unannotated feature | pos | 195.1132 | 2.724 | 4.403 | 4.581 | 0.179 | 0.200 | 0.265 | 0.601 | Unannotated | NA | NA |
| metab_4803 | Unannotated feature | pos | 283.1594 | 7.991 | 5.121 | 5.158 | 0.037 | 0.200 | 0.265 | 0.601 | Unannotated | NA | NA |
| metab_10102 | Unannotated feature | neg | 398.1098 | 2.766 | 3.821 | 4.155 | 0.334 | 0.200 | 0.265 | 0.601 | Unannotated | NA | NA |
| metab_14417 | Fructose citrate | neg | 387.0194 | 3.219 | 4.850 | 4.956 | 0.106 | 0.200 | 0.265 | 0.601 | Unannotated | NA | NA |
| metab_10870 | Unannotated feature | neg | 139.0506 | 0.989 | 4.683 | 4.641 | -0.042 | -0.200 | 0.265 | 0.601 | Unannotated | NA | NA |
| metab_2299 | Unannotated feature | pos | 591.3200 | 5.677 | 8.178 | 7.699 | -0.479 | -0.200 | 0.265 | 0.601 | Unannotated | NA | NA |
| metab_15425 | Unannotated feature | neg | 317.1299 | 5.122 | 3.751 | 4.009 | 0.258 | 0.200 | 0.265 | 0.601 | Unannotated | NA | NA |
| metab_14116 | N-Phenyldiethanolamine | neg | 226.1083 | 2.845 | 2.936 | 3.897 | 0.961 | 0.200 | 0.265 | 0.601 | Unannotated | NA | NA |
| metab_13729 | Unannotated feature | neg | 214.8797 | 0.096 | 6.904 | 6.889 | -0.015 | -0.200 | 0.265 | 0.601 | Unannotated | NA | NA |
| metab_13734 | (R)-3-hydroxybutyrylcarnitine | neg | 292.1404 | 1.947 | 5.186 | 5.228 | 0.042 | 0.200 | 0.265 | 0.601 | Unannotated | NA | NA |
| metab_10272 | Unannotated feature | neg | 295.1301 | 3.247 | 5.116 | 5.049 | -0.067 | -0.200 | 0.265 | 0.601 | Unannotated | NA | NA |
| metab_12864 | Unannotated feature | neg | 332.0758 | 0.706 | 4.291 | 4.630 | 0.339 | 0.200 | 0.265 | 0.601 | Unannotated | NA | NA |
| metab_16004 | Unannotated feature | neg | 692.7692 | 0.524 | 5.167 | 5.382 | 0.215 | 0.200 | 0.265 | 0.601 | Unannotated | NA | NA |
| metab_12152 | Unannotated feature | neg | 589.3050 | 5.678 | 7.544 | 7.087 | -0.457 | -0.200 | 0.265 | 0.601 | Unannotated | NA | NA |
| metab_3814 | Unannotated feature | pos | 172.1698 | 6.398 | 5.548 | 5.500 | -0.048 | -0.200 | 0.265 | 0.601 | Unannotated | NA | NA |
| metab_10092 | Unannotated feature | neg | 303.0184 | 2.771 | 4.100 | 3.409 | -0.692 | -0.200 | 0.265 | 0.601 | Unannotated | NA | NA |
| metab_2315 | Unannotated feature | pos | 762.2209 | 0.098 | 4.747 | 4.787 | 0.039 | 0.200 | 0.265 | 0.601 | Unannotated | NA | NA |
| metab_9660 | Unannotated feature | neg | 252.0728 | 3.177 | 3.940 | 4.250 | 0.310 | 0.200 | 0.265 | 0.601 | Unannotated | NA | NA |
| metab_14402 | 7,8-Dihydropteroic acid | neg | 335.0888 | 3.199 | 3.387 | 3.992 | 0.605 | 0.200 | 0.265 | 0.601 | Tentative | Organoheterocyclic compounds | Pteridines and derivatives |
| metab_1321 | Unannotated feature | pos | 198.1856 | 6.215 | 4.756 | 4.733 | -0.023 | -0.200 | 0.265 | 0.601 | Unannotated | NA | NA |
| metab_19619 | (S)-Homostachydrine | neg | 202.1081 | 4.681 | 3.604 | 3.170 | -0.434 | -0.200 | 0.265 | 0.601 | Tentative | NA | NA |
| metab_11802 | Unannotated feature | neg | 371.1074 | 4.515 | 4.459 | 3.993 | -0.466 | -0.200 | 0.265 | 0.601 | Unannotated | NA | NA |
| metab_19389 | (+/-)-Pelletierine | neg | 186.1130 | 5.026 | 5.804 | 5.821 | 0.017 | -0.200 | 0.265 | 0.601 | Tentative | NA | NA |
| metab_11235 | Unannotated feature | neg | 281.9785 | 0.636 | 3.682 | 3.413 | -0.269 | -0.200 | 0.265 | 0.601 | Unannotated | NA | NA |
| metab_3899 | Unannotated feature | pos | 651.4011 | 5.749 | 3.999 | 4.320 | 0.321 | 0.200 | 0.265 | 0.601 | Unannotated | NA | NA |
| metab_10474 | Unannotated feature | neg | 335.0449 | 1.952 | 3.180 | 2.435 | -0.745 | -0.200 | 0.265 | 0.601 | Unannotated | NA | NA |
| metab_10959 | N-(3-(Dimethylamino)propyl)acrylamide | neg | 201.1241 | 0.791 | 4.375 | 4.176 | -0.199 | -0.200 | 0.265 | 0.601 | Unannotated | NA | NA |
| metab_14735 | Unannotated feature | neg | 260.8986 | 3.762 | 3.980 | 3.749 | -0.232 | -0.200 | 0.265 | 0.601 | Unannotated | NA | NA |
| metab_10377 | Unannotated feature | neg | 260.8985 | 2.137 | 5.588 | 5.377 | -0.211 | -0.200 | 0.265 | 0.601 | Unannotated | NA | NA |
| metab_7025 | Piperdial | pos | 251.1645 | 4.794 | 3.117 | 3.649 | 0.532 | 0.200 | 0.265 | 0.601 | Tentative | NA | NA |
| metab_14312 | 2,3-Hexanedione | neg | 113.0601 | 3.067 | 2.453 | 2.986 | 0.533 | 0.200 | 0.265 | 0.601 | Moderate putative | NA | NA |
| metab_15139 | Unannotated feature | neg | 164.9267 | 0.552 | 5.413 | 5.494 | 0.081 | 0.200 | 0.265 | 0.601 | Unannotated | NA | NA |
| metab_14050 | Enoxacin | neg | 357.0768 | 2.776 | 6.048 | 5.241 | -0.806 | -0.200 | 0.265 | 0.601 | Tentative | NA | NA |
| metab_14071 | Protocatechuic Acid | neg | 153.0187 | 2.793 | 5.129 | 5.296 | 0.167 | 0.200 | 0.265 | 0.601 | Strong putative | NA | NA |
| metab_3045 | Aloesol 7-glucoside | pos | 379.1371 | 3.287 | 4.850 | 5.149 | 0.299 | 0.200 | 0.265 | 0.601 | Unannotated | Organic oxygen compounds | Organooxygen compounds |
| metab_8497 | Unannotated feature | pos | 217.1550 | 1.685 | 5.333 | 5.149 | -0.185 | -0.200 | 0.265 | 0.601 | Unannotated | NA | NA |
| metab_2777 | Unannotated feature | pos | 154.0913 | 2.500 | 4.169 | 4.215 | 0.046 | 0.200 | 0.265 | 0.601 | Unannotated | NA | NA |
| metab_13665 | 3-Hydroxy-2-methylpyridine-4,5-dicarboxylate | neg | 196.0247 | 1.685 | 3.736 | 4.008 | 0.272 | 0.200 | 0.265 | 0.601 | Tentative | NA | NA |
| metab_10186 | Unannotated feature | neg | 255.0628 | 2.663 | 2.293 | 2.913 | 0.620 | 0.200 | 0.265 | 0.601 | Unannotated | NA | NA |
| metab_10215 | Unannotated feature | neg | 203.0015 | 2.629 | 3.779 | 3.878 | 0.099 | 0.200 | 0.265 | 0.601 | Unannotated | NA | NA |
| metab_13818 | Unannotated feature | neg | 226.9178 | 2.079 | 5.333 | 5.245 | -0.088 | -0.200 | 0.265 | 0.601 | Unannotated | NA | NA |
| metab_3473 | Unannotated feature | pos | 111.0207 | 4.704 | 4.280 | 4.251 | -0.029 | -0.200 | 0.265 | 0.601 | Unannotated | NA | NA |
| metab_1194 | Unannotated feature | pos | 470.3850 | 6.470 | 5.229 | 5.076 | -0.154 | -0.200 | 0.265 | 0.601 | Unannotated | NA | NA |
| metab_202 | Unannotated feature | pos | 373.1225 | 4.516 | 4.588 | 4.474 | -0.113 | -0.200 | 0.265 | 0.601 | Unannotated | NA | NA |
| metab_6995 | Unannotated feature | pos | 576.3608 | 4.861 | 1.978 | 4.041 | 2.063 | 0.200 | 0.265 | 0.601 | Unannotated | NA | NA |
| metab_6023 | Unannotated feature | pos | 690.9252 | 5.898 | 3.995 | 4.050 | 0.056 | 0.200 | 0.265 | 0.601 | Unannotated | NA | NA |
| metab_8246 | Unannotated feature | pos | 121.0846 | 2.296 | 5.785 | 5.667 | -0.118 | -0.200 | 0.265 | 0.601 | Unannotated | NA | NA |
| metab_982 | Unannotated feature | pos | 225.0299 | 7.724 | 5.835 | 5.846 | 0.011 | 0.200 | 0.265 | 0.601 | Unannotated | NA | NA |
| metab_12717 | Unannotated feature | neg | 421.1780 | 5.173 | 3.477 | 4.295 | 0.818 | 0.200 | 0.265 | 0.601 | Unannotated | NA | NA |
| metab_11138 | 4-Hydroxy-5-(phenyl)-valeric acid-O-sulphate | neg | 273.0442 | 5.007 | 0.171 | 3.561 | 3.390 | 0.200 | 0.265 | 0.601 | Unannotated | NA | NA |
| metab_17353 | Unannotated feature | neg | 540.9052 | 7.687 | 3.944 | 4.135 | 0.191 | 0.200 | 0.265 | 0.601 | Unannotated | NA | NA |
| metab_14512 | 3-(Azetidin-2-ylmethoxy)-5-iodopyridine | neg | 334.9893 | 3.371 | 4.791 | 3.308 | -1.484 | -0.200 | 0.265 | 0.601 | Unannotated | Organic oxygen compounds | Organooxygen compounds |
| metab_4241 | Unannotated feature | pos | 163.1331 | 5.430 | 6.278 | 6.218 | -0.060 | -0.200 | 0.265 | 0.601 | Unannotated | NA | NA |
| metab_4951 | Morpholine-4-carboxamide | pos | 113.0714 | 7.602 | 4.648 | 4.629 | -0.019 | -0.200 | 0.265 | 0.601 | Unannotated | NA | NA |
| metab_4953 | 6-O-Desmethyldonepezil | pos | 410.1669 | 0.720 | 4.639 | 4.796 | 0.156 | 0.200 | 0.265 | 0.601 | Unannotated | NA | NA |
| metab_19224 | Unannotated feature | neg | 317.1145 | 5.233 | 5.875 | 5.720 | -0.155 | -0.200 | 0.265 | 0.601 | Unannotated | NA | NA |
| metab_1273 | Unannotated feature | pos | 320.1824 | 2.724 | 5.412 | 5.287 | -0.125 | -0.200 | 0.265 | 0.601 | Unannotated | NA | NA |
| metab_19227 | Methionyl-Histidine | neg | 307.0829 | 3.379 | 5.100 | 5.224 | 0.124 | 0.200 | 0.265 | 0.601 | Unannotated | Organic acids and derivatives | Carboxylic acids and derivatives |
| metab_13197 | Unannotated feature | neg | 790.7755 | 0.520 | 4.086 | 4.189 | 0.103 | 0.200 | 0.265 | 0.601 | Unannotated | NA | NA |
| metab_17488 | Unannotated feature | neg | 362.9381 | 7.527 | 6.034 | 6.027 | -0.008 | 0.200 | 0.265 | 0.601 | Unannotated | NA | NA |
| metab_18723 | Absinthin | neg | 533.2264 | 5.884 | 4.164 | 4.746 | 0.582 | 0.200 | 0.265 | 0.601 | Tentative | NA | NA |
| metab_11646 | Unannotated feature | neg | 216.8767 | 0.036 | 6.328 | 6.313 | -0.015 | -0.200 | 0.265 | 0.601 | Unannotated | NA | NA |
| metab_7437 | Unannotated feature | pos | 324.1922 | 3.898 | 4.254 | 4.567 | 0.313 | 0.200 | 0.265 | 0.601 | Unannotated | NA | NA |
| metab_4510 | Unannotated feature | pos | 536.3715 | 6.670 | 4.580 | 4.821 | 0.241 | 0.200 | 0.265 | 0.601 | Unannotated | NA | NA |
| metab_2909 | Unannotated feature | pos | 429.2412 | 6.167 | 6.495 | 6.423 | -0.073 | -0.200 | 0.265 | 0.601 | Unannotated | NA | NA |
| metab_16989 | Unannotated feature | neg | 172.9365 | 7.985 | 5.950 | 5.931 | -0.020 | -0.200 | 0.265 | 0.601 | Unannotated | NA | NA |
| metab_9177 | Unannotated feature | neg | 363.0989 | 3.879 | 3.822 | 4.133 | 0.311 | 0.200 | 0.265 | 0.601 | Unannotated | NA | NA |
| metab_1900 | Unannotated feature | pos | 212.0534 | 1.365 | 4.884 | 4.969 | 0.085 | 0.200 | 0.265 | 0.601 | Unannotated | NA | NA |
| metab_1007 | Unannotated feature | pos | 204.0095 | 0.641 | 4.683 | 4.448 | -0.235 | -0.200 | 0.265 | 0.601 | Unannotated | NA | NA |
| metab_5419 | Secalciferol | pos | 417.3373 | 6.414 | 5.974 | 5.881 | -0.093 | -0.200 | 0.265 | 0.601 | Tentative | NA | NA |
| metab_17710 | Unannotated feature | neg | 78.9178 | 6.993 | 3.179 | 3.157 | -0.022 | -0.200 | 0.265 | 0.601 | Unannotated | NA | NA |
| metab_5493 | Unannotated feature | pos | 199.1081 | 1.952 | 4.875 | 4.849 | -0.026 | -0.200 | 0.265 | 0.601 | Unannotated | NA | NA |
| metab_8845 | Unannotated feature | neg | 145.9491 | 4.378 | 4.021 | 3.879 | -0.143 | -0.200 | 0.265 | 0.601 | Unannotated | NA | NA |
| metab_8765 | (1r,2s)-2-Aminocyclopentanecarboxylic acid | pos | 259.1656 | 0.728 | 2.708 | 1.804 | -0.904 | -0.200 | 0.265 | 0.601 | Unannotated | Organic acids and derivatives | Carboxylic acids and derivatives |
| metab_8770 | Glutaminylthreonine | pos | 212.1033 | 0.728 | 4.960 | 4.649 | -0.312 | -0.200 | 0.265 | 0.601 | Unannotated | NA | NA |
| metab_17129 | Unannotated feature | neg | 152.0823 | 7.822 | 4.263 | 4.358 | 0.095 | 0.200 | 0.265 | 0.601 | Unannotated | NA | NA |
| metab_11488 | Unannotated feature | neg | 622.8526 | 0.516 | 5.068 | 5.217 | 0.149 | 0.200 | 0.265 | 0.601 | Unannotated | NA | NA |
| metab_7791 | Unannotated feature | pos | 154.0362 | 3.200 | 4.153 | 4.083 | -0.070 | -0.200 | 0.265 | 0.601 | Unannotated | NA | NA |
| metab_8367 | 4-O-(Indole-3-acetyl)-D-glucopyranose | pos | 355.1507 | 1.952 | 4.202 | 4.289 | 0.087 | 0.200 | 0.265 | 0.601 | Tentative | NA | NA |
| metab_7412 | Ketorolac | pos | 273.1239 | 3.948 | 3.479 | 4.037 | 0.558 | 0.200 | 0.265 | 0.601 | Tentative | NA | NA |
| metab_5444 | Dehydrocurdione | pos | 199.1485 | 6.398 | 4.953 | 4.770 | -0.183 | -0.200 | 0.265 | 0.601 | Tentative | NA | NA |
| metab_5479 | Cucurbitacin C | pos | 561.3416 | 6.367 | 6.086 | 5.908 | -0.178 | -0.200 | 0.265 | 0.601 | Tentative | NA | NA |
| metab_14206 | Unannotated feature | neg | 359.0811 | 2.946 | 3.873 | 3.351 | -0.522 | -0.200 | 0.265 | 0.601 | Unannotated | NA | NA |
| metab_10059 | Unannotated feature | neg | 280.0677 | 2.785 | 3.772 | 3.822 | 0.049 | 0.200 | 0.265 | 0.601 | Unannotated | NA | NA |
| metab_19778 | 11b-Hydroxyprogesterone | neg | 353.1722 | 4.466 | 4.987 | 4.759 | -0.228 | -0.196 | 0.276 | 0.611 | Tentative | NA | NA |
| metab_19882 | Mesaconic Acid | neg | 129.0187 | 0.893 | 5.377 | 5.273 | -0.104 | -0.196 | 0.276 | 0.611 | Moderate putative | NA | NA |
| metab_3280 | 2-Hydroxyquinoline | pos | 146.0603 | 3.997 | 5.264 | 5.224 | -0.039 | 0.196 | 0.276 | 0.611 | Strong putative | NA | NA |
| metab_14217 | Aspartyl-Leucine | neg | 227.1035 | 2.960 | 5.396 | 5.248 | -0.147 | -0.196 | 0.276 | 0.611 | Unannotated | NA | NA |
| metab_11841 | Unannotated feature | neg | 305.1511 | 4.906 | 6.180 | 5.937 | -0.243 | -0.196 | 0.276 | 0.611 | Unannotated | NA | NA |
| metab_11882 | Unannotated feature | neg | 385.1331 | 6.022 | 4.371 | 4.533 | 0.162 | 0.196 | 0.276 | 0.611 | Unannotated | NA | NA |
| metab_17991 | Unannotated feature | neg | 543.3356 | 6.522 | 6.068 | 6.116 | 0.048 | 0.196 | 0.276 | 0.611 | Unannotated | NA | NA |
| metab_2009 | Unannotated feature | pos | 120.0813 | 2.296 | 6.892 | 6.782 | -0.110 | -0.196 | 0.276 | 0.611 | Unannotated | NA | NA |
| metab_2077 | Unannotated feature | pos | 208.6473 | 2.747 | 3.870 | 3.474 | -0.396 | -0.196 | 0.276 | 0.611 | Unannotated | NA | NA |
| metab_17854 | N-Linoleoyl Methionine | neg | 410.2741 | 6.734 | 4.084 | 4.753 | 0.669 | 0.196 | 0.276 | 0.611 | Unannotated | NA | NA |
| metab_3475 | P-Cymene | pos | 135.1171 | 4.714 | 4.459 | 3.933 | -0.526 | -0.196 | 0.276 | 0.611 | Tentative | Lipids and lipid-like molecules | Prenol lipids |
| metab_2630 | N-Lactoylleucine | pos | 204.1235 | 1.740 | 4.241 | 4.397 | 0.157 | 0.196 | 0.276 | 0.611 | Unannotated | NA | NA |
| metab_4811 | Unannotated feature | pos | 242.0353 | 7.971 | 4.739 | 4.678 | -0.061 | -0.196 | 0.276 | 0.611 | Unannotated | NA | NA |
| metab_2580 | Unannotated feature | pos | 160.1083 | 1.266 | 3.554 | 4.154 | 0.600 | 0.196 | 0.276 | 0.611 | Unannotated | NA | NA |
| metab_9436 | Unannotated feature | neg | 200.0196 | 3.426 | 3.293 | 3.592 | 0.299 | 0.196 | 0.276 | 0.611 | Unannotated | NA | NA |
| metab_15469 | Daucol | neg | 275.1410 | 5.209 | 4.832 | 4.713 | -0.120 | -0.196 | 0.276 | 0.611 | Tentative | NA | NA |
| metab_12539 | Unannotated feature | neg | 403.2705 | 6.217 | 6.547 | 6.386 | -0.161 | -0.196 | 0.276 | 0.611 | Unannotated | NA | NA |
| metab_7995 | (3S)-3-hydroxycyclocitral | pos | 186.1493 | 2.845 | 3.219 | 3.432 | 0.213 | 0.196 | 0.276 | 0.611 | Tentative | NA | NA |
| metab_7975 | Unannotated feature | pos | 432.2467 | 2.868 | 4.161 | 3.349 | -0.812 | -0.196 | 0.276 | 0.611 | Unannotated | NA | NA |
| metab_2136 | Unannotated feature | pos | 348.1776 | 2.853 | 5.147 | 4.973 | -0.174 | -0.196 | 0.276 | 0.611 | Unannotated | NA | NA |
| metab_17181 | Unannotated feature | neg | 324.1328 | 7.741 | 4.818 | 4.856 | 0.038 | 0.196 | 0.276 | 0.611 | Unannotated | NA | NA |
| metab_14169 | Unannotated feature | neg | 187.9311 | 2.906 | 3.770 | 3.522 | -0.249 | -0.196 | 0.276 | 0.611 | Unannotated | NA | NA |
| metab_17406 | Unannotated feature | neg | 646.8585 | 7.648 | 5.744 | 5.757 | 0.012 | 0.196 | 0.276 | 0.611 | Unannotated | NA | NA |
| metab_2536 | Unannotated feature | pos | 269.1137 | 0.720 | 4.832 | 5.001 | 0.169 | 0.196 | 0.276 | 0.611 | Unannotated | NA | NA |
| metab_16115 | Unannotated feature | neg | 399.1852 | 6.263 | 6.078 | 6.174 | 0.095 | 0.196 | 0.276 | 0.611 | Unannotated | NA | NA |
| metab_14313 | Unannotated feature | neg | 274.0394 | 3.067 | 4.006 | 3.595 | -0.412 | -0.196 | 0.276 | 0.611 | Unannotated | NA | NA |
| metab_8633 | Unannotated feature | pos | 203.1394 | 1.041 | 5.505 | 5.347 | -0.158 | -0.196 | 0.276 | 0.611 | Unannotated | NA | NA |
| metab_1127 | Cholylasparagine | pos | 555.3674 | 6.606 | 5.781 | 5.966 | 0.185 | 0.196 | 0.276 | 0.611 | Unannotated | NA | NA |
| metab_12303 | Unannotated feature | neg | 391.2263 | 6.899 | 5.083 | 4.792 | -0.291 | -0.196 | 0.276 | 0.611 | Unannotated | NA | NA |
| metab_12966 | Unannotated feature | neg | 351.0880 | 0.777 | 4.534 | 4.782 | 0.248 | 0.196 | 0.276 | 0.611 | Unannotated | NA | NA |
| metab_9723 | Unannotated feature | neg | 220.0613 | 3.105 | 4.037 | 4.253 | 0.215 | 0.196 | 0.276 | 0.611 | Unannotated | NA | NA |
| metab_15249 | Unannotated feature | neg | 285.1710 | 4.803 | 5.553 | 5.638 | 0.085 | 0.196 | 0.276 | 0.611 | Unannotated | NA | NA |
| metab_12975 | Aspartyl-Proline | neg | 229.0828 | 0.784 | 5.292 | 5.253 | -0.039 | -0.196 | 0.276 | 0.611 | Unannotated | NA | NA |
| metab_18413 | N-[(3a,5b,7a,12a)-3,7-dihydroxy-24-oxo-12-(sulfooxy)cholan-24-yl]-Glycine | neg | 526.2494 | 6.115 | 4.748 | 4.998 | 0.250 | 0.196 | 0.276 | 0.611 | Unannotated | NA | NA |
| metab_3879 | Unannotated feature | pos | 508.3166 | 5.725 | 4.039 | 4.509 | 0.470 | 0.196 | 0.276 | 0.611 | Unannotated | NA | NA |
| metab_3885 | Unannotated feature | pos | 273.1603 | 5.738 | 4.295 | 3.844 | -0.450 | -0.196 | 0.276 | 0.611 | Unannotated | NA | NA |
| metab_9097 | Unannotated feature | neg | 287.1772 | 3.934 | 4.404 | 4.410 | 0.007 | -0.196 | 0.276 | 0.611 | Unannotated | NA | NA |
| metab_9074 | Unannotated feature | neg | 349.1411 | 3.964 | 4.834 | 5.362 | 0.528 | 0.196 | 0.276 | 0.611 | Unannotated | NA | NA |
| metab_10954 | Unannotated feature | neg | 251.0232 | 0.791 | 4.566 | 4.273 | -0.292 | -0.196 | 0.276 | 0.611 | Unannotated | NA | NA |
| metab_9317 | Unannotated feature | neg | 422.1943 | 3.585 | 4.449 | 4.171 | -0.278 | -0.196 | 0.276 | 0.611 | Unannotated | NA | NA |
| metab_4899 | Unannotated feature | pos | 204.9572 | 7.695 | 4.205 | 4.250 | 0.045 | 0.196 | 0.276 | 0.611 | Unannotated | NA | NA |
| metab_12629 | Unannotated feature | neg | 489.2716 | 6.003 | 4.956 | 4.643 | -0.313 | -0.196 | 0.276 | 0.611 | Unannotated | NA | NA |
| metab_4655 | Unannotated feature | pos | 617.5136 | 7.355 | 5.215 | 5.158 | -0.057 | -0.196 | 0.276 | 0.611 | Unannotated | NA | NA |
| metab_19513 | Unannotated feature | neg | 311.1142 | 3.509 | 5.517 | 5.347 | -0.170 | -0.196 | 0.276 | 0.611 | Unannotated | NA | NA |
| metab_16218 | Unannotated feature | neg | 353.2002 | 6.451 | 5.319 | 5.303 | -0.016 | -0.196 | 0.276 | 0.611 | Unannotated | NA | NA |
| metab_5466 | Unannotated feature | pos | 229.1953 | 6.382 | 4.067 | 3.956 | -0.111 | -0.196 | 0.276 | 0.611 | Unannotated | NA | NA |
| metab_8163 | Unannotated feature | pos | 158.0967 | 3.627 | 5.207 | 5.086 | -0.121 | -0.196 | 0.276 | 0.611 | Unannotated | NA | NA |
| metab_14848 | 1-(2,4,5-Trimethoxyphenyl)-1,2-propanedione | neg | 283.0827 | 3.995 | 4.547 | 4.660 | 0.113 | 0.196 | 0.276 | 0.611 | Unannotated | NA | NA |
| metab_8437 | Unannotated feature | pos | 217.1188 | 1.944 | 4.498 | 4.393 | -0.105 | -0.196 | 0.276 | 0.611 | Unannotated | NA | NA |
| metab_16262 | Hyodeoxycholic Acid | neg | 783.5802 | 6.538 | 7.588 | 7.461 | -0.126 | -0.196 | 0.276 | 0.611 | Strong putative | NA | NA |
| metab_13114 | Unannotated feature | neg | 82.9531 | 0.247 | 4.968 | 4.960 | -0.008 | -0.196 | 0.276 | 0.611 | Unannotated | NA | NA |
| metab_4757 | Unannotated feature | pos | 90.5263 | 7.724 | 4.835 | 4.801 | -0.034 | -0.196 | 0.276 | 0.611 | Unannotated | NA | NA |
| metab_18721 | Unannotated feature | neg | 333.0410 | 3.149 | 4.907 | 4.709 | -0.198 | -0.196 | 0.276 | 0.611 | Unannotated | NA | NA |
| metab_13657 | Unannotated feature | neg | 277.0679 | 1.680 | 4.246 | 4.170 | -0.076 | -0.196 | 0.276 | 0.611 | Unannotated | NA | NA |
| metab_13364 | Semidehydroascorbic acid | neg | 221.0300 | 0.730 | 4.363 | 4.283 | -0.080 | -0.196 | 0.276 | 0.611 | Tentative | NA | NA |
| metab_1869 | Unannotated feature | pos | 224.0921 | 1.196 | 5.235 | 5.488 | 0.253 | 0.196 | 0.276 | 0.611 | Unannotated | NA | NA |
| metab_14728 | L-histidinol-phosphate | neg | 242.0303 | 3.754 | 3.883 | 3.482 | -0.401 | -0.196 | 0.276 | 0.611 | Tentative | NA | NA |
| metab_18968 | Unannotated feature | neg | 263.1403 | 3.239 | 4.871 | 4.676 | -0.195 | -0.196 | 0.276 | 0.611 | Unannotated | NA | NA |
| metab_18628 | Delapril diacid | neg | 459.1703 | 5.958 | 5.817 | 5.346 | -0.471 | -0.196 | 0.276 | 0.611 | Unannotated | NA | NA |
| metab_18629 | Unannotated feature | neg | 412.1815 | 5.958 | 4.883 | 4.591 | -0.293 | -0.196 | 0.276 | 0.611 | Unannotated | NA | NA |
| metab_12961 | Unannotated feature | neg | 243.0622 | 0.777 | 5.615 | 5.526 | -0.089 | -0.196 | 0.276 | 0.611 | Unannotated | NA | NA |
| metab_9688 | Unannotated feature | neg | 377.1223 | 3.138 | 4.300 | 4.614 | 0.314 | 0.196 | 0.276 | 0.611 | Unannotated | NA | NA |
| metab_2234 | Unannotated feature | pos | 325.1764 | 3.087 | 5.241 | 5.151 | -0.090 | -0.196 | 0.276 | 0.611 | Unannotated | NA | NA |
| metab_16266 | 9(S)-HODE | neg | 295.2278 | 6.553 | 5.921 | 6.118 | 0.197 | 0.196 | 0.276 | 0.611 | Moderate putative | NA | NA |
| metab_14646 | Quetiapine | neg | 364.1514 | 3.585 | 5.146 | 5.036 | -0.111 | -0.196 | 0.276 | 0.611 | Tentative | Organoheterocyclic compounds | Benzothiazepines |
| metab_16939 | Unannotated feature | neg | 207.9182 | 7.827 | 6.121 | 6.103 | -0.018 | -0.196 | 0.276 | 0.611 | Unannotated | NA | NA |
| metab_15050 | Unannotated feature | neg | 136.9317 | 0.547 | 4.036 | 4.157 | 0.120 | 0.196 | 0.276 | 0.611 | Unannotated | NA | NA |
| metab_1807 | Unannotated feature | pos | 274.0927 | 0.800 | 6.046 | 6.228 | 0.182 | 0.196 | 0.276 | 0.611 | Unannotated | NA | NA |
| metab_1435 | 3-[4-(3-Trifluoromethyl-phenyl)-piperazin-1-yl]-dihydrofuran-2-one | pos | 315.1346 | 6.032 | 5.061 | 4.673 | -0.388 | -0.196 | 0.276 | 0.611 | Unannotated | Organic acids and derivatives | Carboxylic acids and derivatives |
| metab_11535 | Unannotated feature | neg | 487.0402 | 0.496 | 5.076 | 4.701 | -0.375 | -0.196 | 0.276 | 0.611 | Unannotated | NA | NA |
| metab_8340 | Unannotated feature | pos | 159.0767 | 1.984 | 5.183 | 5.124 | -0.059 | -0.196 | 0.276 | 0.611 | Unannotated | NA | NA |
| metab_1054 | Unannotated feature | pos | 525.3397 | 6.813 | 5.801 | 5.693 | -0.107 | -0.196 | 0.276 | 0.611 | Unannotated | NA | NA |
| metab_12455 | Unannotated feature | neg | 183.9139 | 7.861 | 5.270 | 5.253 | -0.017 | -0.196 | 0.276 | 0.611 | Unannotated | NA | NA |
| metab_7703 | Glutamyltryptophan | pos | 366.1679 | 3.366 | 4.527 | 4.449 | -0.078 | -0.196 | 0.276 | 0.611 | Unannotated | Organic acids and derivatives | Carboxylic acids and derivatives |
| metab_987 | Unannotated feature | pos | 139.0181 | 7.724 | 5.991 | 6.039 | 0.048 | 0.196 | 0.276 | 0.611 | Unannotated | NA | NA |
| metab_2939 | Unannotated feature | pos | 192.1386 | 2.937 | 4.272 | 4.193 | -0.080 | -0.196 | 0.276 | 0.611 | Unannotated | NA | NA |
| metab_19271 | Unannotated feature | neg | 380.1355 | 5.176 | 4.236 | 4.708 | 0.472 | 0.196 | 0.276 | 0.611 | Unannotated | NA | NA |
| metab_767 | Unannotated feature | pos | 303.1708 | 6.127 | 6.854 | 6.620 | -0.234 | -0.196 | 0.276 | 0.611 | Unannotated | NA | NA |
| metab_11065 | Unannotated feature | neg | 453.1191 | 0.730 | 3.441 | 2.986 | -0.455 | -0.196 | 0.276 | 0.611 | Unannotated | NA | NA |
| metab_1415 | Unannotated feature | pos | 631.2800 | 6.056 | 4.145 | 4.074 | -0.071 | -0.196 | 0.276 | 0.611 | Unannotated | NA | NA |
| metab_1806 | Unannotated feature | pos | 241.1550 | 0.776 | 4.499 | 4.607 | 0.108 | 0.196 | 0.276 | 0.611 | Unannotated | NA | NA |
| metab_2000 | 3-Hydroxyphenylpyruvic acid | pos | 198.0765 | 1.952 | 4.260 | 4.350 | 0.090 | 0.196 | 0.276 | 0.611 | Unannotated | NA | NA |
| metab_15785 | Unannotated feature | neg | 273.1572 | 0.603 | 4.058 | 3.852 | -0.207 | -0.196 | 0.276 | 0.611 | Unannotated | NA | NA |
| metab_6885 | Protopine | pos | 336.1239 | 5.052 | 2.529 | 3.793 | 1.264 | 0.196 | 0.276 | 0.611 | Tentative | NA | NA |
| metab_12125 | Unannotated feature | neg | 717.4784 | 6.561 | 5.356 | 5.235 | -0.121 | -0.196 | 0.276 | 0.611 | Unannotated | NA | NA |
| metab_17905 | Unannotated feature | neg | 172.9908 | 2.845 | 4.327 | 3.440 | -0.887 | -0.196 | 0.276 | 0.611 | Unannotated | NA | NA |
| metab_1271 | Unannotated feature | pos | 448.2243 | 6.311 | 2.395 | 1.613 | -0.781 | -0.196 | 0.276 | 0.611 | Unannotated | NA | NA |
| metab_14213 | Unannotated feature | neg | 343.0609 | 2.956 | 5.214 | 4.904 | -0.310 | -0.196 | 0.276 | 0.611 | Unannotated | NA | NA |
| metab_8792 | Allysine | pos | 187.1080 | 0.776 | 5.742 | 5.628 | -0.114 | -0.196 | 0.276 | 0.611 | Tentative | NA | NA |
| metab_19852 | Caffeic Acid | neg | 179.0344 | 5.450 | 4.933 | 4.835 | -0.098 | -0.196 | 0.276 | 0.611 | Strong putative | NA | NA |
| metab_8815 | Indole | pos | 118.0656 | 3.179 | 6.162 | 6.162 | 0.000 | 0.196 | 0.276 | 0.611 | Strong putative | NA | NA |
| metab_15628 | Proxyphylline | neg | 475.2104 | 5.546 | 4.474 | 5.379 | 0.905 | 0.196 | 0.276 | 0.611 | Unannotated | NA | NA |
| metab_12054 | Unannotated feature | neg | 523.3094 | 6.373 | 7.103 | 7.088 | -0.015 | -0.196 | 0.276 | 0.611 | Unannotated | NA | NA |
| metab_19784 | Unannotated feature | neg | 316.1669 | 4.454 | 4.391 | 4.315 | -0.076 | -0.196 | 0.276 | 0.611 | Unannotated | NA | NA |
| metab_19841 | 3-O-Methylquercetin | neg | 315.0515 | 5.546 | 2.169 | 3.440 | 1.271 | 0.196 | 0.276 | 0.611 | Strong putative | Phenylpropanoids and polyketides | Flavonoids |
| metab_1645 | Unannotated feature | pos | 181.1226 | 4.630 | 4.351 | 4.080 | -0.271 | -0.196 | 0.276 | 0.611 | Unannotated | NA | NA |
| metab_2846 | Unannotated feature | pos | 239.1759 | 2.762 | 4.265 | 4.428 | 0.162 | 0.196 | 0.276 | 0.611 | Unannotated | NA | NA |
| metab_972 | Unannotated feature | pos | 224.0122 | 7.772 | 5.521 | 5.563 | 0.042 | 0.196 | 0.276 | 0.611 | Unannotated | NA | NA |
| metab_4964 | Unannotated feature | pos | 89.1096 | 7.570 | 3.907 | 3.851 | -0.056 | -0.196 | 0.276 | 0.611 | Unannotated | NA | NA |
| metab_8407 | Unannotated feature | pos | 191.1393 | 1.952 | 4.265 | 4.184 | -0.081 | -0.196 | 0.276 | 0.611 | Unannotated | NA | NA |
| metab_16342 | Unannotated feature | neg | 155.0455 | 1.364 | 5.673 | 5.631 | -0.043 | -0.196 | 0.276 | 0.611 | Unannotated | NA | NA |
| metab_1041 | Unannotated feature | pos | 569.4190 | 6.853 | 5.421 | 4.794 | -0.627 | -0.196 | 0.276 | 0.611 | Unannotated | NA | NA |
| metab_5276 | Unannotated feature | pos | 273.1085 | 1.252 | 4.541 | 4.597 | 0.057 | 0.196 | 0.276 | 0.611 | Unannotated | NA | NA |
| metab_14901 | Sulotroban | neg | 316.0653 | 4.107 | 4.192 | 4.787 | 0.595 | 0.196 | 0.276 | 0.611 | Unannotated | NA | NA |
| metab_2292 | Unannotated feature | pos | 98.5125 | 0.016 | 4.637 | 4.645 | 0.008 | 0.196 | 0.276 | 0.611 | Unannotated | NA | NA |
| metab_13828 | 8-Hydroxy-7-methylguanine | neg | 180.0522 | 2.137 | 6.079 | 6.267 | 0.188 | 0.196 | 0.276 | 0.611 | Unannotated | NA | NA |
| metab_12477 | Unannotated feature | neg | 265.9096 | 7.989 | 4.072 | 4.018 | -0.054 | -0.196 | 0.276 | 0.611 | Unannotated | NA | NA |
| metab_17333 | Unannotated feature | neg | 268.9544 | 7.697 | 4.414 | 4.320 | -0.094 | -0.196 | 0.276 | 0.611 | Unannotated | NA | NA |
| metab_3657 | Azvudine | pos | 304.1186 | 5.199 | 4.420 | 4.357 | -0.064 | -0.192 | 0.286 | 0.622 | Unannotated | NA | NA |
| metab_14166 | Unannotated feature | neg | 399.1894 | 2.903 | 4.501 | 4.209 | -0.291 | -0.192 | 0.286 | 0.622 | Unannotated | NA | NA |
| metab_10834 | Unannotated feature | neg | 93.0449 | 1.046 | 4.391 | 4.313 | -0.078 | -0.192 | 0.286 | 0.622 | Unannotated | NA | NA |
| metab_3235 | Unannotated feature | pos | 322.1767 | 3.813 | 4.469 | 4.302 | -0.166 | -0.192 | 0.286 | 0.622 | Unannotated | NA | NA |
| metab_17087 | Unannotated feature | neg | 261.8130 | 7.888 | 4.171 | 4.229 | 0.058 | 0.192 | 0.286 | 0.622 | Unannotated | NA | NA |
| metab_2673 | Unannotated feature | pos | 232.0820 | 1.960 | 2.996 | 3.488 | 0.493 | 0.192 | 0.286 | 0.622 | Unannotated | NA | NA |
| metab_11454 | Unannotated feature | neg | 562.8134 | 0.527 | 5.110 | 5.221 | 0.111 | 0.192 | 0.286 | 0.622 | Unannotated | NA | NA |
| metab_11445 | Unannotated feature | neg | 400.8527 | 0.533 | 5.142 | 5.031 | -0.111 | -0.192 | 0.286 | 0.622 | Unannotated | NA | NA |
| metab_815 | Unannotated feature | pos | 346.3323 | 6.335 | 4.580 | 4.511 | -0.069 | -0.192 | 0.286 | 0.622 | Unannotated | NA | NA |
| metab_11442 | Unannotated feature | neg | 494.8260 | 0.533 | 5.021 | 5.410 | 0.390 | 0.192 | 0.286 | 0.622 | Unannotated | NA | NA |
| metab_4846 | Unannotated feature | pos | 203.9716 | 7.732 | 3.798 | 3.704 | -0.093 | -0.192 | 0.286 | 0.622 | Unannotated | NA | NA |
| metab_273 | Unannotated feature | pos | 317.0514 | 0.509 | 3.435 | 3.802 | 0.367 | 0.192 | 0.286 | 0.622 | Unannotated | NA | NA |
| metab_343 | Unannotated feature | pos | 155.0029 | 0.424 | 4.529 | 4.559 | 0.029 | 0.192 | 0.286 | 0.622 | Unannotated | NA | NA |
| metab_12282 | Unannotated feature | neg | 495.3119 | 7.114 | 6.462 | 6.286 | -0.176 | -0.192 | 0.286 | 0.622 | Unannotated | NA | NA |
| metab_7181 | Unannotated feature | pos | 525.1234 | 0.672 | 5.095 | 4.428 | -0.666 | -0.192 | 0.286 | 0.622 | Unannotated | NA | NA |
| metab_8663 | Unannotated feature | pos | 133.0611 | 0.961 | 3.162 | 3.115 | -0.048 | -0.192 | 0.286 | 0.622 | Unannotated | NA | NA |
| metab_14161 | Unannotated feature | neg | 217.0173 | 2.903 | 3.916 | 3.641 | -0.275 | -0.192 | 0.286 | 0.622 | Unannotated | NA | NA |
| metab_90 | 3-epi-Fagomine | pos | 148.0968 | 0.649 | 4.841 | 4.827 | -0.014 | -0.192 | 0.286 | 0.622 | Unannotated | NA | NA |
| metab_19891 | Phloretin | neg | 255.0663 | 5.600 | 4.206 | 4.397 | 0.192 | -0.192 | 0.286 | 0.622 | Tentative | NA | NA |
| metab_17116 | Unannotated feature | neg | 271.9074 | 7.835 | 3.724 | 3.623 | -0.101 | -0.192 | 0.286 | 0.622 | Unannotated | NA | NA |
| metab_12774 | Unannotated feature | neg | 205.9624 | 0.625 | 4.661 | 4.440 | -0.221 | -0.192 | 0.286 | 0.622 | Unannotated | NA | NA |
| metab_4007 | Unannotated feature | pos | 615.5588 | 5.811 | 3.958 | 3.959 | 0.000 | 0.192 | 0.286 | 0.622 | Unannotated | NA | NA |
| metab_3958 | Unannotated feature | pos | 585.1740 | 5.787 | 4.011 | 4.102 | 0.091 | 0.192 | 0.286 | 0.622 | Unannotated | NA | NA |
| metab_7418 | Unannotated feature | pos | 315.0860 | 3.934 | 4.069 | 2.578 | -1.490 | -0.192 | 0.286 | 0.622 | Unannotated | NA | NA |
| metab_11663 | Unannotated feature | neg | 169.1025 | 7.765 | 5.044 | 5.016 | -0.028 | -0.192 | 0.286 | 0.622 | Unannotated | NA | NA |
| metab_4927 | Unannotated feature | pos | 854.8157 | 7.634 | 4.837 | 4.869 | 0.032 | 0.192 | 0.286 | 0.622 | Unannotated | NA | NA |
| metab_11649 | Unannotated feature | neg | 189.9267 | 0.036 | 5.957 | 5.934 | -0.023 | -0.192 | 0.286 | 0.622 | Unannotated | NA | NA |
| metab_13151 | Unannotated feature | neg | 231.0637 | 5.334 | 4.194 | 3.862 | -0.333 | -0.192 | 0.286 | 0.622 | Unannotated | NA | NA |
| metab_930 | Unannotated feature | pos | 447.2938 | 7.435 | 5.855 | 5.829 | -0.026 | -0.192 | 0.286 | 0.622 | Unannotated | NA | NA |
| metab_8177 | Unannotated feature | pos | 119.0735 | 2.614 | 4.169 | 4.081 | -0.088 | -0.192 | 0.286 | 0.622 | Unannotated | NA | NA |
| metab_4892 | Unannotated feature | pos | 306.8955 | 7.700 | 4.287 | 4.399 | 0.112 | 0.192 | 0.286 | 0.622 | Unannotated | NA | NA |
| metab_12816 | Unannotated feature | neg | 139.0064 | 0.659 | 5.524 | 5.274 | -0.251 | -0.192 | 0.286 | 0.622 | Unannotated | NA | NA |
| metab_13659 | Unannotated feature | neg | 390.1411 | 1.680 | 2.453 | 3.612 | 1.159 | 0.192 | 0.286 | 0.622 | Unannotated | NA | NA |
| metab_15265 | Cimiracemate A | neg | 403.1041 | 4.825 | 5.200 | 5.005 | -0.195 | -0.192 | 0.286 | 0.622 | Unannotated | Phenylpropanoids and polyketides | Cinnamic acids and derivatives |
| metab_10920 | Unannotated feature | neg | 238.1084 | 4.869 | 2.969 | 3.552 | 0.584 | 0.192 | 0.286 | 0.622 | Unannotated | NA | NA |
| metab_2162 | Unannotated feature | pos | 418.2139 | 5.474 | 5.940 | 5.678 | -0.262 | -0.192 | 0.286 | 0.622 | Unannotated | NA | NA |
| metab_4685 | Unannotated feature | pos | 903.7647 | 7.522 | 5.441 | 5.410 | -0.030 | -0.192 | 0.286 | 0.622 | Unannotated | NA | NA |
| metab_4677 | Unannotated feature | pos | 128.1437 | 7.498 | 4.374 | 4.345 | -0.029 | -0.192 | 0.286 | 0.622 | Unannotated | NA | NA |
| metab_10491 | Unannotated feature | neg | 204.9920 | 1.952 | 4.756 | 4.847 | 0.092 | -0.192 | 0.286 | 0.622 | Unannotated | NA | NA |
| metab_2743 | Unannotated feature | pos | 657.3150 | 5.952 | 5.426 | 5.718 | 0.293 | 0.192 | 0.286 | 0.622 | Unannotated | NA | NA |
| metab_9327 | Unannotated feature | neg | 311.1595 | 3.577 | 4.482 | 4.221 | -0.262 | -0.192 | 0.286 | 0.622 | Unannotated | NA | NA |
| metab_2737 | Unannotated feature | pos | 125.1126 | 2.227 | 6.320 | 6.357 | 0.037 | 0.192 | 0.286 | 0.622 | Unannotated | NA | NA |
| metab_4721 | Methenamine | pos | 141.1136 | 7.642 | 4.441 | 4.485 | 0.044 | 0.192 | 0.286 | 0.622 | Moderate putative | NA | NA |
| metab_12608 | Unannotated feature | neg | 589.3740 | 6.475 | 4.842 | 4.806 | -0.036 | -0.192 | 0.286 | 0.622 | Unannotated | NA | NA |
| metab_7260 | Oxycodone | pos | 316.1550 | 4.290 | 2.780 | 3.128 | 0.349 | 0.192 | 0.286 | 0.622 | Moderate putative | NA | NA |
| metab_18061 | 10-Hydroxydecanoic Acid | neg | 187.1333 | 6.428 | 4.322 | 4.465 | 0.142 | 0.192 | 0.286 | 0.622 | Moderate putative | NA | NA |
| metab_14108 | Unannotated feature | neg | 188.0640 | 2.836 | 3.505 | 3.880 | 0.375 | 0.192 | 0.286 | 0.622 | Unannotated | NA | NA |
| metab_14102 | 1-[(3R,4R,5R)-3-Fluoro-3,4-dihydroxy-5-(1-hydroxyethyl)oxolan-2-yl]pyrimidine-2,4-dione | neg | 321.0728 | 2.826 | 4.371 | 4.629 | 0.259 | 0.192 | 0.286 | 0.622 | Unannotated | NA | NA |
| metab_5488 | Lidoflazine | pos | 533.3103 | 6.359 | 6.030 | 5.953 | -0.077 | -0.192 | 0.286 | 0.622 | Unannotated | NA | NA |
| metab_8548 | N6-Methyladenosine | pos | 282.1201 | 1.294 | 3.928 | 4.422 | 0.494 | 0.192 | 0.286 | 0.622 | Strong putative | NA | NA |
| metab_16051 | Losartan | neg | 421.1556 | 6.185 | 4.163 | 4.445 | 0.282 | 0.192 | 0.286 | 0.622 | Strong putative | NA | NA |
| metab_12944 | Unannotated feature | neg | 544.1898 | 0.762 | 4.178 | 4.629 | 0.451 | 0.192 | 0.286 | 0.622 | Unannotated | NA | NA |
| metab_18573 | Unannotated feature | neg | 443.1570 | 3.085 | 3.078 | 3.765 | 0.687 | 0.192 | 0.286 | 0.622 | Unannotated | NA | NA |
| metab_4804 | Unannotated feature | pos | 226.0722 | 7.991 | 5.066 | 5.093 | 0.027 | 0.192 | 0.286 | 0.622 | Unannotated | NA | NA |
| metab_12542 | Unannotated feature | neg | 429.2498 | 6.217 | 6.312 | 6.188 | -0.125 | -0.192 | 0.286 | 0.622 | Unannotated | NA | NA |
| metab_8023 | Dihydroconiferyl alcohol | pos | 387.1774 | 2.803 | 4.013 | 4.290 | 0.278 | 0.192 | 0.286 | 0.622 | Tentative | Benzenoids | Phenols |
| metab_845 | Unannotated feature | pos | 318.2409 | 6.534 | 6.723 | 6.598 | -0.125 | -0.192 | 0.286 | 0.622 | Unannotated | NA | NA |
| metab_2614 | Tyrosyl-Glutamate | pos | 311.1243 | 1.685 | 4.207 | 3.942 | -0.266 | -0.192 | 0.286 | 0.622 | Unannotated | NA | NA |
| metab_4810 | Unannotated feature | pos | 760.2225 | 7.971 | 5.486 | 5.513 | 0.027 | 0.192 | 0.286 | 0.622 | Unannotated | NA | NA |
| metab_14812 | Unannotated feature | neg | 346.9027 | 0.512 | 4.318 | 4.017 | -0.301 | -0.192 | 0.286 | 0.622 | Unannotated | NA | NA |
| metab_5662 | Unannotated feature | pos | 202.0165 | 0.016 | 6.533 | 6.542 | 0.009 | 0.192 | 0.286 | 0.622 | Unannotated | NA | NA |
| metab_12911 | Unannotated feature | neg | 603.1796 | 0.730 | 4.705 | 4.909 | 0.204 | 0.192 | 0.286 | 0.622 | Unannotated | NA | NA |
| metab_14343 | Unannotated feature | neg | 144.0660 | 3.112 | 4.268 | 4.190 | -0.078 | -0.192 | 0.286 | 0.622 | Unannotated | NA | NA |
| metab_10825 | Vanillyl mandelic acid | neg | 323.0676 | 4.818 | 5.148 | 5.389 | 0.242 | 0.192 | 0.286 | 0.622 | Unannotated | NA | NA |
| metab_1308 | Unannotated feature | pos | 342.2645 | 6.231 | 4.557 | 4.127 | -0.429 | -0.192 | 0.286 | 0.622 | Unannotated | NA | NA |
| metab_8686 | 2-Deoxy-2,3-dehydro-n-acetyl-neuraminic acid | pos | 314.0853 | 0.807 | 4.843 | 5.051 | 0.208 | 0.192 | 0.286 | 0.622 | Unannotated | NA | NA |
| metab_17157 | Unannotated feature | neg | 272.0047 | 2.302 | 4.077 | 3.986 | -0.091 | -0.192 | 0.286 | 0.622 | Unannotated | NA | NA |
| metab_2488 | Lactosamine | pos | 342.1404 | 0.736 | 5.368 | 5.519 | 0.151 | 0.192 | 0.286 | 0.622 | Tentative | NA | NA |
| metab_5206 | Unannotated feature | pos | 561.5363 | 6.741 | 5.825 | 5.748 | -0.077 | -0.192 | 0.286 | 0.622 | Unannotated | NA | NA |
| metab_2973 | Abeado | pos | 314.1755 | 3.027 | 4.535 | 4.652 | 0.116 | 0.192 | 0.286 | 0.622 | Unannotated | NA | NA |
| metab_10554 | Unannotated feature | neg | 145.9490 | 1.886 | 3.512 | 3.402 | -0.110 | -0.192 | 0.286 | 0.622 | Unannotated | NA | NA |
| metab_16357 | Unannotated feature | neg | 495.3150 | 6.726 | 6.271 | 6.132 | -0.139 | -0.192 | 0.286 | 0.622 | Unannotated | NA | NA |
| metab_7589 | 5-amino-2-(1,3-dioxooctahydro-2H-4,7-methanoisoindol-2-yl)-5-oxopentanoic acid | pos | 295.1295 | 3.569 | 5.391 | 5.310 | -0.081 | -0.192 | 0.286 | 0.622 | Unannotated | NA | NA |
| metab_16353 | Unannotated feature | neg | 589.4491 | 6.719 | 6.457 | 6.160 | -0.297 | -0.192 | 0.286 | 0.622 | Unannotated | NA | NA |
| metab_12426 | Unannotated feature | neg | 211.9141 | 7.730 | 5.219 | 5.192 | -0.027 | -0.192 | 0.286 | 0.622 | Unannotated | NA | NA |
| metab_10539 | Unannotated feature | neg | 233.1030 | 1.944 | 4.436 | 4.649 | 0.213 | 0.192 | 0.286 | 0.622 | Unannotated | NA | NA |
| metab_4603 | DG(15:0/18:3(6Z,9Z,12Z)/0:0) | pos | 599.4661 | 7.044 | 6.593 | 6.485 | -0.108 | -0.192 | 0.286 | 0.622 | Tentative | NA | NA |
| metab_17251 | Unannotated feature | neg | 342.1315 | 7.726 | 4.674 | 4.897 | 0.223 | 0.192 | 0.286 | 0.622 | Unannotated | NA | NA |
| metab_2246 | Unannotated feature | pos | 271.1656 | 3.120 | 4.595 | 4.240 | -0.355 | -0.192 | 0.286 | 0.622 | Unannotated | NA | NA |
| metab_19394 | Unannotated feature | neg | 330.1646 | 5.022 | 2.731 | 3.672 | 0.941 | 0.192 | 0.286 | 0.622 | Unannotated | NA | NA |
| metab_4929 | Unannotated feature | pos | 514.8771 | 7.634 | 5.247 | 5.307 | 0.060 | 0.192 | 0.286 | 0.622 | Unannotated | NA | NA |
| metab_18906 | Unannotated feature | neg | 231.1599 | 5.645 | 4.500 | 4.150 | -0.350 | -0.192 | 0.286 | 0.622 | Unannotated | NA | NA |
| metab_17493 | Unannotated feature | neg | 294.9503 | 7.510 | 5.876 | 5.882 | 0.006 | 0.192 | 0.286 | 0.622 | Unannotated | NA | NA |
| metab_7497 | [(1S,2S,4R,5S)-9-Methyl-3-oxa-9-azatricyclo[3.3.1.02,4]nonan-7-yl] (2S)-3-hydroxy-2-phenylpropanoate | pos | 321.1815 | 3.756 | 4.674 | 4.499 | -0.175 | -0.192 | 0.286 | 0.622 | Unannotated | NA | NA |
| metab_18782 | Unannotated feature | neg | 320.1507 | 5.803 | 3.869 | 3.615 | -0.254 | -0.192 | 0.286 | 0.622 | Unannotated | NA | NA |
| metab_13085 | Unannotated feature | neg | 179.8980 | 0.022 | 5.223 | 5.200 | -0.023 | -0.192 | 0.286 | 0.622 | Unannotated | NA | NA |
| metab_2407 | Unannotated feature | pos | 112.8962 | 0.601 | 5.173 | 5.106 | -0.067 | -0.192 | 0.286 | 0.622 | Unannotated | NA | NA |
| metab_7796 | 4-Chlorophenyl | pos | 154.0421 | 3.179 | 7.341 | 7.367 | 0.026 | 0.192 | 0.286 | 0.622 | Unannotated | NA | NA |
| metab_5063 | Unannotated feature | pos | 128.1437 | 7.140 | 3.313 | 3.569 | 0.256 | 0.192 | 0.286 | 0.622 | Unannotated | NA | NA |
| metab_4532 | Ganoderic acid H | pos | 537.2875 | 6.741 | 6.642 | 6.486 | -0.155 | -0.192 | 0.286 | 0.622 | Unannotated | Lipids and lipid-like molecules | Prenol lipids |
| metab_16903 | Unannotated feature | neg | 411.8820 | 7.760 | 5.560 | 5.550 | -0.010 | -0.192 | 0.286 | 0.622 | Unannotated | NA | NA |
| metab_16909 | Unannotated feature | neg | 384.8623 | 7.765 | 4.913 | 4.868 | -0.044 | -0.192 | 0.286 | 0.622 | Unannotated | NA | NA |
| metab_7709 | Unannotated feature | pos | 266.1755 | 3.352 | 4.557 | 4.456 | -0.100 | -0.192 | 0.286 | 0.622 | Unannotated | NA | NA |
| metab_7734 | Unannotated feature | pos | 295.1170 | 3.316 | 3.755 | 3.204 | -0.551 | -0.192 | 0.286 | 0.622 | Unannotated | NA | NA |
| metab_8319 | Unannotated feature | pos | 156.9978 | 2.025 | 4.209 | 4.262 | 0.053 | 0.192 | 0.286 | 0.622 | Unannotated | NA | NA |
| metab_7640 | Glycohyocholic Acid | pos | 488.3000 | 6.135 | 4.976 | 4.514 | -0.463 | -0.192 | 0.286 | 0.622 | Unannotated | NA | NA |
| metab_4402 | Aplaviroc | pos | 595.3509 | 6.287 | 6.779 | 6.982 | 0.203 | 0.192 | 0.286 | 0.622 | Unannotated | NA | NA |
| metab_2955 | Unannotated feature | pos | 234.1163 | 2.965 | 4.772 | 4.009 | -0.762 | -0.192 | 0.286 | 0.622 | Unannotated | NA | NA |
| metab_1754 | Unannotated feature | pos | 328.1905 | 3.553 | 5.184 | 4.998 | -0.186 | -0.192 | 0.286 | 0.622 | Unannotated | NA | NA |
| metab_7755 | Scoparone | pos | 207.0656 | 3.291 | 3.885 | 3.019 | -0.866 | -0.192 | 0.286 | 0.622 | Tentative | Phenylpropanoids and polyketides | Coumarins and derivatives |
| metab_1822 | Unannotated feature | pos | 176.1284 | 0.768 | 4.617 | 4.717 | 0.100 | 0.192 | 0.286 | 0.622 | Unannotated | NA | NA |
| metab_15796 | Unannotated feature | neg | 333.1460 | 5.847 | 5.573 | 5.503 | -0.070 | -0.192 | 0.286 | 0.622 | Unannotated | NA | NA |
| metab_13504 | Unannotated feature | neg | 137.0349 | 1.051 | 5.517 | 5.417 | -0.100 | -0.192 | 0.286 | 0.622 | Unannotated | NA | NA |
| metab_6834 | Unannotated feature | pos | 132.0658 | 0.641 | 4.851 | 4.692 | -0.159 | -0.192 | 0.286 | 0.622 | Unannotated | NA | NA |
| metab_17922 | 4-Hydroxy-5-(3',5'-dihydroxyphenyl)-valeric acid-O-methyl-O-glucuronide | neg | 479.1419 | 2.858 | 5.592 | 5.122 | -0.470 | -0.192 | 0.286 | 0.622 | Unannotated | NA | NA |
| metab_13440 | Uridine | neg | 243.0622 | 1.226 | 6.533 | 6.444 | -0.090 | -0.192 | 0.286 | 0.622 | Strong putative | NA | NA |
| metab_2867 | Unannotated feature | pos | 188.0921 | 2.795 | 4.142 | 4.178 | 0.036 | 0.192 | 0.286 | 0.622 | Unannotated | NA | NA |
| metab_16527 | Demethylphylloquinone | neg | 473.2834 | 7.167 | 5.866 | 6.009 | 0.144 | 0.192 | 0.286 | 0.622 | Tentative | NA | NA |
| metab_3241 | LysoPS(18:1(9Z)/0:0) | pos | 488.2733 | 3.835 | 5.287 | 5.016 | -0.272 | -0.192 | 0.286 | 0.622 | Unannotated | NA | NA |
| metab_17146 | Unannotated feature | neg | 209.0663 | 7.781 | 5.144 | 5.086 | -0.058 | -0.192 | 0.286 | 0.622 | Unannotated | NA | NA |
| metab_3329 | Unannotated feature | pos | 468.3583 | 6.933 | 5.721 | 6.051 | 0.330 | 0.192 | 0.286 | 0.622 | Unannotated | NA | NA |
| metab_26 | 1-Pyrroline-4-hydroxy-2-carboxylate | pos | 171.0766 | 0.712 | 4.945 | 5.002 | 0.058 | 0.192 | 0.286 | 0.622 | Tentative | NA | NA |
| metab_11927 | Unannotated feature | neg | 377.1246 | 6.107 | 4.294 | 4.193 | -0.101 | -0.187 | 0.297 | 0.632 | Unannotated | NA | NA |
| metab_3311 | Unannotated feature | pos | 655.4926 | 7.124 | 7.205 | 7.158 | -0.048 | -0.187 | 0.297 | 0.632 | Unannotated | NA | NA |
| metab_3309 | Unannotated feature | pos | 324.1923 | 4.084 | 3.519 | 4.675 | 1.156 | 0.187 | 0.297 | 0.632 | Unannotated | NA | NA |
| metab_18299 | Unannotated feature | neg | 246.0983 | 2.754 | 5.425 | 5.443 | 0.017 | 0.187 | 0.297 | 0.632 | Unannotated | NA | NA |
| metab_8733 | 2-(3-Hydroxy-1H-indol-2-yl)acetic acid | pos | 192.0659 | 4.232 | 5.943 | 5.895 | -0.049 | 0.187 | 0.297 | 0.632 | Unannotated | NA | NA |
| metab_14265 | Unannotated feature | neg | 344.0846 | 3.011 | 4.058 | 4.214 | 0.156 | 0.187 | 0.297 | 0.632 | Unannotated | NA | NA |
| metab_15814 | Perindopril | neg | 403.1977 | 5.884 | 5.392 | 5.342 | -0.050 | 0.187 | 0.297 | 0.632 | Tentative | NA | NA |
| metab_11897 | Unannotated feature | neg | 467.2119 | 6.046 | 6.297 | 6.190 | -0.107 | -0.187 | 0.297 | 0.632 | Unannotated | NA | NA |
| metab_11108 | Unannotated feature | neg | 200.0924 | 0.722 | 3.375 | 3.777 | 0.402 | 0.187 | 0.297 | 0.632 | Unannotated | NA | NA |
| metab_5129 | Unannotated feature | pos | 619.5283 | 6.917 | 4.139 | 4.221 | 0.082 | 0.187 | 0.297 | 0.632 | Unannotated | NA | NA |
| metab_16542 | SM(d18:0/5-iso PGF2VI) | neg | 809.5188 | 7.212 | 4.718 | 5.305 | 0.587 | 0.187 | 0.297 | 0.632 | Unannotated | NA | NA |
| metab_16535 | Sterculic acid | neg | 633.5112 | 7.199 | 6.081 | 6.025 | -0.056 | -0.187 | 0.297 | 0.632 | Tentative | Lipids and lipid-like molecules | Fatty Acyls |
| metab_16516 | 1-Stearoylglycerophosphoglycerol | neg | 493.2947 | 7.132 | 6.953 | 6.915 | -0.038 | -0.187 | 0.297 | 0.632 | Unannotated | NA | NA |
| metab_5089 | 3h-Phenylalanine | pos | 209.1287 | 0.768 | 3.946 | 4.237 | 0.291 | 0.187 | 0.297 | 0.632 | Unannotated | NA | NA |
| metab_13765 | 3-(1H-Pyrrol-2-yl)propanoic Acid | neg | 138.0554 | 1.965 | 5.237 | 5.297 | 0.061 | 0.187 | 0.297 | 0.632 | Unannotated | NA | NA |
| metab_16938 | Unannotated feature | neg | 285.1094 | 1.958 | 5.039 | 5.257 | 0.218 | 0.187 | 0.297 | 0.632 | Unannotated | NA | NA |
| metab_17308 | Unannotated feature | neg | 667.8600 | 7.702 | 4.208 | 4.321 | 0.113 | 0.187 | 0.297 | 0.632 | Unannotated | NA | NA |
| metab_13996 | Unannotated feature | neg | 304.0789 | 2.714 | 6.483 | 6.437 | -0.047 | -0.187 | 0.297 | 0.632 | Unannotated | NA | NA |
| metab_13101 | Unannotated feature | neg | 122.8934 | 0.096 | 5.185 | 5.169 | -0.017 | -0.187 | 0.297 | 0.632 | Unannotated | NA | NA |
| metab_13100 | Unannotated feature | neg | 185.8651 | 0.075 | 5.298 | 5.282 | -0.016 | -0.187 | 0.297 | 0.632 | Unannotated | NA | NA |
| metab_16586 | Unannotated feature | neg | 475.2895 | 7.354 | 3.691 | 4.986 | 1.294 | 0.187 | 0.297 | 0.632 | Unannotated | NA | NA |
| metab_7846 | Unannotated feature | pos | 679.8179 | 3.058 | 4.993 | 5.065 | 0.072 | 0.187 | 0.297 | 0.632 | Unannotated | NA | NA |
| metab_5025 | Unannotated feature | pos | 111.1173 | 7.307 | 4.051 | 4.012 | -0.039 | -0.187 | 0.297 | 0.632 | Unannotated | NA | NA |
| metab_14018 | Unannotated feature | neg | 163.0254 | 2.750 | 4.572 | 4.508 | -0.064 | -0.187 | 0.297 | 0.632 | Unannotated | NA | NA |
| metab_16930 | Unannotated feature | neg | 90.7508 | 7.814 | 3.470 | 3.404 | -0.066 | -0.187 | 0.297 | 0.632 | Unannotated | NA | NA |
| metab_1799 | Unannotated feature | pos | 200.1034 | 0.784 | 4.241 | 3.318 | -0.922 | -0.187 | 0.297 | 0.632 | Unannotated | NA | NA |
| metab_13290 | Unannotated feature | neg | 238.0932 | 0.625 | 3.463 | 3.841 | 0.378 | 0.187 | 0.297 | 0.632 | Unannotated | NA | NA |
| metab_1826 | Unannotated feature | pos | 203.1030 | 0.997 | 5.164 | 5.110 | -0.055 | -0.187 | 0.297 | 0.632 | Unannotated | NA | NA |
| metab_7700 | Unannotated feature | pos | 452.1774 | 0.760 | 3.885 | 4.790 | 0.905 | 0.187 | 0.297 | 0.632 | Unannotated | NA | NA |
| metab_14560 | 4-Hydroxybenzoic acid glucoside | neg | 281.0695 | 3.453 | 4.269 | 4.503 | 0.234 | 0.187 | 0.297 | 0.632 | Unannotated | NA | NA |
| metab_18153 | Citronellyl acetate | neg | 243.1599 | 6.342 | 5.035 | 5.141 | 0.107 | 0.187 | 0.297 | 0.632 | Tentative | NA | NA |
| metab_18190 | 8-Deoxy-11,13-dihydroxygrosheimin | neg | 619.2787 | 6.303 | 5.360 | 4.271 | -1.089 | -0.187 | 0.297 | 0.632 | Unannotated | NA | NA |
| metab_18771 | (R)-Bitalin A | neg | 263.0925 | 5.822 | 3.036 | 3.613 | 0.577 | 0.187 | 0.297 | 0.632 | Unannotated | NA | NA |
| metab_11050 | Isopentenyladenine-9-N-glucoside | neg | 364.1617 | 4.962 | 3.775 | 4.282 | 0.507 | 0.187 | 0.297 | 0.632 | Unannotated | NA | NA |
| metab_14557 | Unannotated feature | neg | 370.1625 | 3.450 | 4.444 | 4.266 | -0.178 | -0.187 | 0.297 | 0.632 | Unannotated | NA | NA |
| metab_1398 | Unannotated feature | pos | 381.2022 | 6.087 | 3.451 | 3.071 | -0.380 | -0.187 | 0.297 | 0.632 | Unannotated | NA | NA |
| metab_17258 | Unannotated feature | neg | 259.9338 | 7.726 | 6.058 | 6.041 | -0.016 | -0.187 | 0.297 | 0.632 | Unannotated | NA | NA |
| metab_2318 | Unannotated feature | pos | 74.0969 | 0.181 | 3.517 | 3.549 | 0.032 | 0.187 | 0.297 | 0.632 | Unannotated | NA | NA |
| metab_8394 | Aminocarb | pos | 226.1554 | 1.952 | 5.250 | 4.868 | -0.382 | -0.187 | 0.297 | 0.632 | Tentative | Benzenoids | Benzene and substituted derivatives |
| metab_12471 | Unannotated feature | neg | 241.7579 | 7.985 | 5.206 | 5.190 | -0.016 | -0.187 | 0.297 | 0.632 | Unannotated | NA | NA |
| metab_19216 | Unannotated feature | neg | 403.1494 | 5.238 | 3.943 | 4.147 | 0.205 | 0.187 | 0.297 | 0.632 | Unannotated | NA | NA |
| metab_16868 | Unannotated feature | neg | 392.1208 | 2.007 | 4.591 | 5.117 | 0.526 | 0.187 | 0.297 | 0.632 | Unannotated | NA | NA |
| metab_4548 | Solanocardinol | pos | 495.3547 | 6.813 | 4.210 | 4.301 | 0.091 | 0.187 | 0.297 | 0.632 | Unannotated | NA | NA |
| metab_3000 | Unannotated feature | pos | 251.1757 | 3.125 | 3.845 | 3.113 | -0.732 | -0.187 | 0.297 | 0.632 | Unannotated | NA | NA |
| metab_12780 | Unannotated feature | neg | 307.1514 | 0.628 | 4.631 | 4.331 | -0.300 | -0.187 | 0.297 | 0.632 | Unannotated | NA | NA |
| metab_11620 | Unannotated feature | neg | 112.9556 | 0.208 | 4.857 | 4.846 | -0.011 | -0.187 | 0.297 | 0.632 | Unannotated | NA | NA |
| metab_16292 | Unannotated feature | neg | 350.1257 | 6.609 | 4.688 | 4.845 | 0.157 | 0.187 | 0.297 | 0.632 | Unannotated | NA | NA |
| metab_2296 | Unannotated feature | pos | 188.9788 | 0.016 | 4.389 | 4.402 | 0.013 | 0.187 | 0.297 | 0.632 | Unannotated | NA | NA |
| metab_16272 | Unannotated feature | neg | 391.2620 | 6.561 | 4.046 | 3.393 | -0.653 | -0.187 | 0.297 | 0.632 | Unannotated | NA | NA |
| metab_2276 | Unannotated feature | pos | 156.0390 | 3.208 | 6.845 | 6.872 | 0.027 | 0.187 | 0.297 | 0.632 | Unannotated | NA | NA |
| metab_4081 | 4-Oxododecanedioic Acid | pos | 262.1653 | 5.841 | 4.105 | 3.909 | -0.196 | -0.187 | 0.297 | 0.632 | Strong putative | NA | NA |
| metab_3975 | Unannotated feature | pos | 629.2696 | 5.794 | 5.505 | 5.004 | -0.501 | -0.187 | 0.297 | 0.632 | Unannotated | NA | NA |
| metab_1864 | Unannotated feature | pos | 156.0657 | 1.169 | 5.454 | 5.569 | 0.115 | 0.187 | 0.297 | 0.632 | Unannotated | NA | NA |
| metab_1851 | Unannotated feature | pos | 104.0534 | 1.049 | 5.462 | 5.303 | -0.160 | -0.187 | 0.297 | 0.632 | Unannotated | NA | NA |
| metab_7392 | Nicotinuric acid | pos | 378.1384 | 3.991 | 3.656 | 3.682 | 0.026 | 0.187 | 0.297 | 0.632 | Tentative | Organic acids and derivatives | Carboxylic acids and derivatives |
| metab_17465 | Unannotated feature | neg | 100.9043 | 7.603 | 3.436 | 3.354 | -0.082 | -0.187 | 0.297 | 0.632 | Unannotated | NA | NA |
| metab_2490 | Unannotated feature | pos | 131.0536 | 0.744 | 3.509 | 3.379 | -0.130 | -0.187 | 0.297 | 0.632 | Unannotated | NA | NA |
| metab_4894 | Arkofix | pos | 211.0931 | 7.700 | 5.599 | 5.627 | 0.028 | 0.187 | 0.297 | 0.632 | Unannotated | NA | NA |
| metab_11205 | Unannotated feature | neg | 203.1284 | 4.959 | 4.990 | 4.865 | -0.125 | -0.187 | 0.297 | 0.632 | Unannotated | NA | NA |
| metab_12620 | Unannotated feature | neg | 585.2738 | 6.007 | 5.143 | 4.537 | -0.606 | -0.187 | 0.297 | 0.632 | Unannotated | NA | NA |
| metab_9102 | Unannotated feature | neg | 229.1191 | 3.930 | 3.794 | 3.290 | -0.504 | -0.187 | 0.297 | 0.632 | Unannotated | NA | NA |
| metab_17230 | Unannotated feature | neg | 205.9979 | 7.730 | 4.540 | 4.498 | -0.042 | -0.187 | 0.297 | 0.632 | Unannotated | NA | NA |
| metab_1325 | Unannotated feature | pos | 375.2807 | 6.239 | 5.217 | 4.663 | -0.554 | -0.187 | 0.297 | 0.632 | Unannotated | NA | NA |
| metab_18714 | Unannotated feature | neg | 364.1729 | 5.892 | 2.673 | 3.440 | 0.767 | 0.187 | 0.297 | 0.632 | Unannotated | NA | NA |
| metab_15257 | Unannotated feature | neg | 410.1464 | 4.814 | 3.944 | 3.653 | -0.291 | -0.187 | 0.297 | 0.632 | Unannotated | NA | NA |
| metab_7316 | L-Proline, 1-acetyl-4-hydroxy-, cis- | pos | 138.0551 | 0.704 | 5.691 | 5.626 | -0.065 | -0.187 | 0.297 | 0.632 | Unannotated | NA | NA |
| metab_2195 | Unannotated feature | pos | 390.8525 | 0.593 | 3.377 | 3.704 | 0.327 | 0.187 | 0.297 | 0.632 | Unannotated | NA | NA |
| metab_1914 | Unannotated feature | pos | 185.0924 | 1.692 | 4.958 | 5.080 | 0.122 | 0.187 | 0.297 | 0.632 | Unannotated | NA | NA |
| metab_17786 | Unannotated feature | neg | 397.2359 | 6.868 | 3.677 | 3.834 | 0.157 | 0.187 | 0.297 | 0.632 | Unannotated | NA | NA |
| metab_371 | Unannotated feature | pos | 98.0969 | 0.206 | 3.813 | 3.822 | 0.009 | 0.187 | 0.297 | 0.632 | Unannotated | NA | NA |
| metab_12317 | Unannotated feature | neg | 571.4379 | 6.876 | 6.369 | 6.269 | -0.101 | -0.187 | 0.297 | 0.632 | Unannotated | NA | NA |
| metab_11693 | Unannotated feature | neg | 428.1684 | 5.446 | 3.554 | 4.036 | 0.482 | 0.187 | 0.297 | 0.632 | Unannotated | NA | NA |
| metab_9353 | Unannotated feature | neg | 314.2090 | 3.544 | 5.200 | 4.953 | -0.247 | -0.187 | 0.297 | 0.632 | Unannotated | NA | NA |
| metab_8128 | Unannotated feature | pos | 208.1336 | 2.732 | 4.121 | 4.640 | 0.519 | 0.187 | 0.297 | 0.632 | Unannotated | NA | NA |
| metab_18365 | Unannotated feature | neg | 537.3444 | 6.154 | 6.026 | 5.886 | -0.140 | -0.187 | 0.297 | 0.632 | Unannotated | NA | NA |
| metab_17396 | Unannotated feature | neg | 314.9326 | 7.656 | 4.147 | 4.303 | 0.156 | 0.187 | 0.297 | 0.632 | Unannotated | NA | NA |
| metab_5565 | 2-Cyclohexen-1-one, 3-(15-hydroxypentadecyl)-2,4,4-trimethyl- | pos | 403.2963 | 6.263 | 4.109 | 3.825 | -0.284 | -0.187 | 0.297 | 0.632 | Unannotated | NA | NA |
| metab_9867 | Unannotated feature | neg | 242.9435 | 2.981 | 3.950 | 3.862 | -0.088 | -0.187 | 0.297 | 0.632 | Unannotated | NA | NA |
| metab_13616 | Unannotated feature | neg | 331.9896 | 1.512 | 4.498 | 4.445 | -0.053 | -0.187 | 0.297 | 0.632 | Unannotated | NA | NA |
| metab_14391 | Ascladiol | neg | 137.0238 | 3.184 | 5.008 | 5.055 | 0.046 | 0.187 | 0.297 | 0.632 | Unannotated | NA | NA |
| metab_18471 | Unannotated feature | neg | 369.1746 | 6.076 | 6.284 | 6.395 | 0.111 | 0.187 | 0.297 | 0.632 | Unannotated | NA | NA |
| metab_10852 | Unannotated feature | neg | 153.0298 | 1.021 | 4.413 | 4.240 | -0.174 | -0.187 | 0.297 | 0.632 | Unannotated | NA | NA |
| metab_3705 | 27-Nor-5b-cholestane-3a,7a,12a,24,25-pentol | pos | 483.3095 | 6.590 | 5.858 | 6.002 | 0.143 | 0.187 | 0.297 | 0.632 | Unannotated | NA | NA |
| metab_8056 | Unannotated feature | pos | 154.0865 | 2.774 | 4.053 | 4.060 | 0.007 | 0.187 | 0.297 | 0.632 | Unannotated | NA | NA |
| metab_13928 | Unannotated feature | neg | 200.0924 | 2.565 | 4.582 | 4.598 | 0.016 | 0.187 | 0.297 | 0.632 | Unannotated | NA | NA |
| metab_4857 | Unannotated feature | pos | 258.1382 | 7.724 | 4.287 | 4.366 | 0.079 | 0.187 | 0.297 | 0.632 | Unannotated | NA | NA |
| metab_1615 | Unannotated feature | pos | 268.1188 | 4.938 | 5.437 | 5.381 | -0.056 | -0.187 | 0.297 | 0.632 | Unannotated | NA | NA |
| metab_17480 | Unannotated feature | neg | 333.9384 | 7.558 | 6.768 | 6.760 | -0.008 | 0.187 | 0.297 | 0.632 | Unannotated | NA | NA |
| metab_18255 | Unannotated feature | neg | 416.2478 | 6.248 | 4.522 | 4.649 | 0.127 | 0.187 | 0.297 | 0.632 | Unannotated | NA | NA |
| metab_11794 | Unannotated feature | neg | 255.0664 | 5.813 | 4.992 | 4.894 | -0.098 | -0.187 | 0.297 | 0.632 | Unannotated | NA | NA |
| metab_277 | Unannotated feature | pos | 112.1126 | 0.509 | 4.737 | 4.704 | -0.033 | -0.187 | 0.297 | 0.632 | Unannotated | NA | NA |
| metab_15379 | Unannotated feature | neg | 232.0311 | 0.566 | 4.770 | 5.089 | 0.319 | 0.187 | 0.297 | 0.632 | Unannotated | NA | NA |
| metab_11764 | Gentamicin A sulfate | neg | 489.2149 | 5.706 | 3.234 | 4.036 | 0.802 | 0.187 | 0.297 | 0.632 | Unannotated | NA | NA |
| metab_3601 | Unannotated feature | pos | 182.0293 | 5.068 | 3.940 | 3.757 | -0.183 | -0.187 | 0.297 | 0.632 | Unannotated | NA | NA |
| metab_10841 | Unannotated feature | neg | 300.0728 | 1.037 | 4.016 | 4.431 | 0.416 | 0.187 | 0.297 | 0.632 | Unannotated | NA | NA |
| metab_8903 | Unannotated feature | neg | 439.1363 | 4.258 | 4.147 | 3.912 | -0.235 | -0.187 | 0.297 | 0.632 | Unannotated | NA | NA |
| metab_8658 | Zingerone | pos | 258.1089 | 1.002 | 4.271 | 3.583 | -0.688 | -0.187 | 0.297 | 0.632 | Tentative | Benzenoids | Phenols |
| metab_12197 | Unannotated feature | neg | 293.1793 | 6.782 | 6.745 | 6.865 | 0.119 | 0.187 | 0.297 | 0.632 | Unannotated | NA | NA |
| metab_1520 | Unannotated feature | pos | 144.0658 | 1.952 | 5.676 | 5.738 | 0.061 | 0.187 | 0.297 | 0.632 | Unannotated | NA | NA |
| metab_2618 | Unannotated feature | pos | 198.1606 | 1.692 | 3.179 | 3.480 | 0.301 | 0.187 | 0.297 | 0.632 | Unannotated | NA | NA |
| metab_8032 | Unannotated feature | pos | 299.1712 | 2.795 | 3.465 | 3.206 | -0.259 | -0.187 | 0.297 | 0.632 | Unannotated | NA | NA |
| metab_11406 | Unannotated feature | neg | 166.9237 | 0.547 | 5.428 | 5.401 | -0.027 | 0.187 | 0.297 | 0.632 | Unannotated | NA | NA |
| metab_12217 | Unannotated feature | neg | 469.2580 | 6.860 | 6.126 | 6.358 | 0.233 | 0.187 | 0.297 | 0.632 | Unannotated | NA | NA |
| metab_15467 | Unannotated feature | neg | 243.1601 | 5.209 | 3.174 | 3.979 | 0.806 | 0.187 | 0.297 | 0.632 | Unannotated | NA | NA |
| metab_5644 | 9alpha-(3-Methylbutanoyloxy)-4S-hydroxy-10(14)-oplopen-3-one | pos | 301.2167 | 6.175 | 5.009 | 4.777 | -0.232 | -0.187 | 0.297 | 0.632 | Unannotated | NA | NA |
| metab_7072 | 1-(9-ethyl-5,6,7,9-tetrahydroimidazo[1,2-a][1,4]diazepin-8-yl)-2-methoxyethanone | pos | 282.1190 | 0.657 | 4.508 | 4.702 | 0.194 | 0.187 | 0.297 | 0.632 | Unannotated | NA | NA |
| metab_16055 | 6-(4-sulfophenyl)decanoic Acid | neg | 327.1267 | 6.193 | 4.815 | 4.973 | 0.157 | 0.187 | 0.297 | 0.632 | Unannotated | NA | NA |
| metab_7093 | Phenylalanyltyrosine | pos | 329.1480 | 4.673 | 4.388 | 3.904 | -0.484 | -0.187 | 0.297 | 0.632 | Unannotated | NA | NA |
| metab_15873 | Oxyresveratrol (cis-) | neg | 349.1309 | 5.958 | 4.695 | 5.014 | 0.319 | 0.187 | 0.297 | 0.632 | Unannotated | NA | NA |
| metab_3437 | Unannotated feature | pos | 217.1915 | 4.571 | 5.706 | 5.650 | -0.056 | -0.187 | 0.297 | 0.632 | Unannotated | NA | NA |
| metab_8786 | 2-keto-D-Gluconic acid | pos | 233.0062 | 0.641 | 4.540 | 4.273 | -0.268 | -0.187 | 0.297 | 0.632 | Moderate putative | NA | NA |
| metab_69 | Unannotated feature | pos | 339.1559 | 4.375 | 4.496 | 4.344 | -0.152 | -0.187 | 0.297 | 0.632 | Unannotated | NA | NA |
| metab_19822 | 2-Methoxy-5-methylaniline | neg | 182.0817 | 4.401 | 5.375 | 5.304 | -0.071 | -0.187 | 0.297 | 0.632 | Tentative | Benzenoids | Phenol ethers |
| metab_19871 | Homogentisic Acid | neg | 213.0401 | 2.817 | 4.408 | 4.735 | 0.327 | 0.183 | 0.308 | 0.638 | Moderate putative | NA | NA |
| metab_1229 | 4,7,10,13-Hexadecatetraenoic acid | pos | 215.1797 | 6.390 | 5.404 | 5.371 | -0.032 | -0.183 | 0.308 | 0.638 | Unannotated | NA | NA |
| metab_15725 | Moracin C | neg | 355.1191 | 5.722 | 3.617 | 3.737 | 0.120 | -0.183 | 0.308 | 0.638 | Unannotated | NA | NA |
| metab_15599 | Unannotated feature | neg | 422.1771 | 5.501 | 4.886 | 5.246 | 0.361 | 0.183 | 0.308 | 0.638 | Unannotated | NA | NA |
| metab_11364 | Unannotated feature | neg | 383.1721 | 5.164 | 4.577 | 4.232 | -0.345 | -0.183 | 0.308 | 0.638 | Unannotated | NA | NA |
| metab_4822 | Unannotated feature | pos | 146.9805 | 7.864 | 4.777 | 4.816 | 0.039 | 0.183 | 0.308 | 0.638 | Unannotated | NA | NA |
| metab_14820 | Unannotated feature | neg | 285.1710 | 3.934 | 6.468 | 6.409 | -0.059 | -0.183 | 0.308 | 0.638 | Unannotated | NA | NA |
| metab_14808 | Unannotated feature | neg | 321.9045 | 3.921 | 3.698 | 3.367 | -0.331 | -0.183 | 0.308 | 0.638 | Unannotated | NA | NA |
| metab_13869 | Unannotated feature | neg | 290.0153 | 2.313 | 4.817 | 4.599 | -0.218 | -0.183 | 0.308 | 0.638 | Unannotated | NA | NA |
| metab_4769 | Unannotated feature | pos | 79.0758 | 7.740 | 5.092 | 5.125 | 0.033 | 0.183 | 0.308 | 0.638 | Unannotated | NA | NA |
| metab_11318 | Unannotated feature | neg | 358.8441 | 0.603 | 3.711 | 3.557 | -0.154 | -0.183 | 0.308 | 0.638 | Unannotated | NA | NA |
| metab_11314 | Unannotated feature | neg | 538.7390 | 0.603 | 4.143 | 4.595 | 0.451 | 0.183 | 0.308 | 0.638 | Unannotated | NA | NA |
| metab_6974 | Danatrol | pos | 355.2346 | 4.900 | 2.755 | 2.416 | -0.339 | -0.183 | 0.308 | 0.638 | Tentative | NA | NA |
| metab_15920 | Unannotated feature | neg | 511.2388 | 6.018 | 4.390 | 4.442 | 0.052 | 0.183 | 0.308 | 0.638 | Unannotated | NA | NA |
| metab_7032 | Osmaronin | pos | 224.0921 | 4.783 | 3.456 | 3.753 | 0.297 | 0.183 | 0.308 | 0.638 | Unannotated | Lipids and lipid-like molecules | Fatty Acyls |
| metab_7013 | Unannotated feature | pos | 203.0343 | 4.814 | 3.952 | 3.744 | -0.208 | -0.183 | 0.308 | 0.638 | Unannotated | NA | NA |
| metab_15946 | Tilsuprost | neg | 428.2118 | 6.046 | 5.294 | 5.009 | -0.285 | -0.183 | 0.308 | 0.638 | Unannotated | NA | NA |
| metab_1182 | Unannotated feature | pos | 300.3265 | 6.502 | 3.790 | 4.694 | 0.903 | 0.183 | 0.308 | 0.638 | Unannotated | NA | NA |
| metab_8852 | Unannotated feature | neg | 391.2207 | 4.354 | 4.452 | 4.232 | -0.219 | -0.183 | 0.308 | 0.638 | Unannotated | NA | NA |
| metab_16057 | Unannotated feature | neg | 467.2118 | 6.193 | 6.047 | 6.258 | 0.210 | 0.183 | 0.308 | 0.638 | Unannotated | NA | NA |
| metab_5823 | Unannotated feature | pos | 184.9858 | 0.016 | 5.733 | 5.755 | 0.022 | 0.183 | 0.308 | 0.638 | Unannotated | NA | NA |
| metab_12155 | Unannotated feature | neg | 397.2059 | 6.663 | 4.649 | 4.996 | 0.346 | 0.183 | 0.308 | 0.638 | Unannotated | NA | NA |
| metab_15536 | Unannotated feature | neg | 401.0884 | 5.347 | 4.254 | 3.560 | -0.693 | -0.183 | 0.308 | 0.638 | Unannotated | NA | NA |
| metab_12139 | Unannotated feature | neg | 383.3282 | 6.569 | 5.253 | 5.077 | -0.176 | -0.183 | 0.308 | 0.638 | Unannotated | NA | NA |
| metab_6329 | Unannotated feature | pos | 465.3356 | 0.561 | 5.362 | 5.314 | -0.048 | -0.183 | 0.308 | 0.638 | Unannotated | NA | NA |
| metab_6259 | Unannotated feature | pos | 388.8550 | 0.524 | 4.860 | 4.718 | -0.143 | -0.183 | 0.308 | 0.638 | Unannotated | NA | NA |
| metab_2674 | Unannotated feature | pos | 259.2021 | 1.960 | 3.360 | 2.558 | -0.803 | -0.183 | 0.308 | 0.638 | Unannotated | NA | NA |
| metab_865 | Unannotated feature | pos | 579.4244 | 6.678 | 5.312 | 5.668 | 0.356 | 0.183 | 0.308 | 0.638 | Unannotated | NA | NA |
| metab_12508 | Unannotated feature | neg | 377.2336 | 6.350 | 5.820 | 5.912 | 0.092 | 0.183 | 0.308 | 0.638 | Unannotated | NA | NA |
| metab_823 | Sphingosine | pos | 300.2901 | 6.406 | 6.808 | 6.560 | -0.248 | -0.183 | 0.308 | 0.638 | Strong putative | NA | NA |
| metab_9470 | Unannotated feature | neg | 376.1522 | 3.379 | 4.301 | 4.091 | -0.210 | -0.183 | 0.308 | 0.638 | Unannotated | NA | NA |
| metab_12609 | Unannotated feature | neg | 673.3462 | 6.052 | 4.409 | 4.965 | 0.556 | 0.183 | 0.308 | 0.638 | Unannotated | NA | NA |
| metab_13116 | Unannotated feature | neg | 204.8924 | 0.263 | 6.335 | 6.323 | -0.012 | -0.183 | 0.308 | 0.638 | Unannotated | NA | NA |
| metab_881 | Unannotated feature | pos | 209.9569 | 0.609 | 4.797 | 4.687 | -0.110 | -0.183 | 0.308 | 0.638 | Unannotated | NA | NA |
| metab_4741 | Unannotated feature | pos | 94.0656 | 7.695 | 3.246 | 3.241 | -0.005 | -0.183 | 0.308 | 0.638 | Unannotated | NA | NA |
| metab_8103 | Unannotated feature | pos | 413.3753 | 6.686 | 5.554 | 5.240 | -0.315 | -0.183 | 0.308 | 0.638 | Unannotated | NA | NA |
| metab_10505 | Oxytetracycline | neg | 441.1261 | 1.947 | 4.332 | 3.800 | -0.532 | -0.183 | 0.308 | 0.638 | Tentative | Phenylpropanoids and polyketides | Tetracyclines |
| metab_2727 | Acetophenazine | pos | 434.1905 | 2.187 | 6.844 | 6.712 | -0.132 | -0.183 | 0.308 | 0.638 | Tentative | NA | NA |
| metab_4926 | Unannotated feature | pos | 134.0192 | 7.642 | 3.327 | 3.193 | -0.134 | -0.183 | 0.308 | 0.638 | Unannotated | NA | NA |
| metab_4922 | Trans-4-Aminocyclohexanecarboxylic acid | pos | 161.1286 | 0.712 | 5.239 | 4.786 | -0.453 | -0.183 | 0.308 | 0.638 | Unannotated | NA | NA |
| metab_1671 | Unannotated feature | pos | 472.2822 | 4.411 | 4.665 | 4.076 | -0.589 | -0.183 | 0.308 | 0.638 | Unannotated | NA | NA |
| metab_9511 | N-[(2R,3R,4S,6R)-4,6-Dihydroxy-6-methyl-2-[(1R,2R)-1,2,3-trihydroxypropyl]oxan-3-yl]acetamide | neg | 260.1140 | 3.334 | 3.509 | 3.817 | 0.308 | 0.183 | 0.308 | 0.638 | Unannotated | NA | NA |
| metab_17078 | Unannotated feature | neg | 140.9854 | 7.910 | 4.685 | 4.663 | -0.022 | -0.183 | 0.308 | 0.638 | Unannotated | NA | NA |
| metab_17075 | Unannotated feature | neg | 207.8364 | 7.910 | 6.495 | 6.458 | -0.037 | -0.183 | 0.308 | 0.638 | Unannotated | NA | NA |
| metab_17160 | Unannotated feature | neg | 229.1444 | 7.765 | 4.113 | 4.012 | -0.101 | -0.183 | 0.308 | 0.638 | Unannotated | NA | NA |
| metab_14345 | Unannotated feature | neg | 259.9338 | 3.112 | 4.126 | 3.897 | -0.229 | -0.183 | 0.308 | 0.638 | Unannotated | NA | NA |
| metab_7133 | Unannotated feature | pos | 124.1125 | 4.579 | 3.390 | 3.308 | -0.082 | -0.183 | 0.308 | 0.638 | Unannotated | NA | NA |
| metab_10107 | 5-Hydroxyferulic acid | neg | 255.0512 | 2.766 | 5.072 | 5.086 | 0.014 | 0.183 | 0.308 | 0.638 | Tentative | NA | NA |
| metab_12834 | Unannotated feature | neg | 244.1667 | 0.644 | 4.689 | 4.198 | -0.491 | -0.183 | 0.308 | 0.638 | Unannotated | NA | NA |
| metab_17188 | Unannotated feature | neg | 231.9315 | 7.741 | 6.044 | 6.023 | -0.021 | -0.183 | 0.308 | 0.638 | Unannotated | NA | NA |
| metab_17186 | Spongothymidine | neg | 257.0780 | 2.137 | 5.409 | 5.431 | 0.022 | -0.183 | 0.308 | 0.638 | Tentative | NA | NA |
| metab_1141 | PC(14:1(9Z)/PGF2alpha) | pos | 865.5357 | 6.566 | 5.324 | 5.477 | 0.152 | 0.183 | 0.308 | 0.638 | Unannotated | NA | NA |
| metab_7197 | L-2-Amino-3-methylenehexanoic acid | pos | 126.0917 | 4.444 | 4.784 | 4.671 | -0.113 | -0.183 | 0.308 | 0.638 | Unannotated | NA | NA |
| metab_16107 | Unannotated feature | neg | 414.2326 | 6.248 | 5.819 | 5.583 | -0.236 | -0.183 | 0.308 | 0.638 | Unannotated | NA | NA |
| metab_3627 | Androstenedione | pos | 611.3461 | 5.128 | 3.506 | 4.596 | 1.090 | 0.183 | 0.308 | 0.638 | Tentative | NA | NA |
| metab_8187 | Unannotated feature | pos | 358.1406 | 3.661 | 4.071 | 4.396 | 0.325 | 0.183 | 0.308 | 0.638 | Unannotated | NA | NA |
| metab_18351 | Unannotated feature | neg | 385.1329 | 6.170 | 4.517 | 4.963 | 0.445 | 0.183 | 0.308 | 0.638 | Unannotated | NA | NA |
| metab_1442 | Unannotated feature | pos | 200.0478 | 3.208 | 7.986 | 8.007 | 0.021 | 0.183 | 0.308 | 0.638 | Unannotated | NA | NA |
| metab_17363 | Unannotated feature | neg | 205.0350 | 1.958 | 5.296 | 5.173 | -0.122 | -0.183 | 0.308 | 0.638 | Unannotated | NA | NA |
| metab_14732 | Acerosin | neg | 359.0775 | 3.754 | 4.417 | 4.807 | 0.390 | 0.183 | 0.308 | 0.638 | Tentative | NA | NA |
| metab_16643 | Unannotated feature | neg | 1009.2068 | 7.549 | 4.327 | 4.604 | 0.277 | 0.183 | 0.308 | 0.638 | Unannotated | NA | NA |
| metab_17511 | Unannotated feature | neg | 616.4631 | 7.473 | 3.832 | 4.666 | 0.834 | 0.183 | 0.308 | 0.638 | Unannotated | NA | NA |
| metab_1723 | Unannotated feature | pos | 349.1403 | 3.817 | 5.140 | 5.344 | 0.204 | 0.183 | 0.308 | 0.638 | Unannotated | NA | NA |
| metab_19218 | Unannotated feature | neg | 312.1933 | 3.375 | 4.886 | 4.793 | -0.093 | -0.183 | 0.308 | 0.638 | Unannotated | NA | NA |
| metab_3807 | Unannotated feature | pos | 355.2639 | 6.398 | 7.617 | 7.266 | -0.352 | -0.183 | 0.308 | 0.638 | Unannotated | NA | NA |
| metab_3789 | Unannotated feature | pos | 251.2009 | 6.406 | 5.216 | 5.105 | -0.110 | -0.183 | 0.308 | 0.638 | Unannotated | NA | NA |
| metab_18669 | Unannotated feature | neg | 391.2712 | 5.927 | 3.885 | 4.459 | 0.574 | 0.183 | 0.308 | 0.638 | Unannotated | NA | NA |
| metab_12253 | Unannotated feature | neg | 623.4922 | 6.993 | 5.522 | 5.592 | 0.069 | 0.183 | 0.308 | 0.638 | Unannotated | NA | NA |
| metab_15381 | Isoleucyl-Phenylalanine | neg | 259.1455 | 5.041 | 5.257 | 4.996 | -0.261 | -0.183 | 0.308 | 0.638 | Unannotated | Organic acids and derivatives | Carboxylic acids and derivatives |
| metab_9873 | Unannotated feature | neg | 93.0337 | 2.975 | 3.498 | 3.416 | -0.083 | -0.183 | 0.308 | 0.638 | Unannotated | NA | NA |
| metab_10496 | Unannotated feature | neg | 152.0711 | 1.952 | 3.464 | 3.716 | 0.252 | 0.183 | 0.308 | 0.638 | Unannotated | NA | NA |
| metab_13110 | Unannotated feature | neg | 180.9409 | 0.169 | 5.240 | 5.233 | -0.008 | -0.183 | 0.308 | 0.638 | Unannotated | NA | NA |
| metab_9574 | Unannotated feature | neg | 373.1734 | 3.247 | 5.243 | 5.126 | -0.117 | -0.183 | 0.308 | 0.638 | Unannotated | NA | NA |
| metab_1917 | Methionyl-Threonine | pos | 289.0606 | 1.692 | 3.089 | 3.484 | 0.396 | 0.183 | 0.308 | 0.638 | Unannotated | NA | NA |
| metab_379 | Unannotated feature | pos | 297.0831 | 0.098 | 4.702 | 4.686 | -0.016 | -0.183 | 0.308 | 0.638 | Unannotated | NA | NA |
| metab_8539 | METAMITRON | pos | 235.1193 | 1.348 | 4.865 | 4.158 | -0.708 | -0.183 | 0.308 | 0.638 | Tentative | NA | NA |
| metab_8533 | Unannotated feature | pos | 283.0660 | 1.383 | 3.939 | 3.217 | -0.722 | -0.183 | 0.308 | 0.638 | Unannotated | NA | NA |
| metab_9009 | 4-Hydroxyphenylacetaldehyde | neg | 331.1192 | 4.056 | 5.125 | 5.222 | 0.097 | 0.183 | 0.308 | 0.638 | Tentative | NA | NA |
| metab_1390 | Unannotated feature | pos | 293.1863 | 6.103 | 5.164 | 5.402 | 0.238 | 0.183 | 0.308 | 0.638 | Unannotated | NA | NA |
| metab_12998 | Unannotated feature | neg | 496.1790 | 0.799 | 3.289 | 4.271 | 0.982 | 0.183 | 0.308 | 0.638 | Unannotated | NA | NA |
| metab_12829 | Unannotated feature | neg | 308.0991 | 0.667 | 6.473 | 6.533 | 0.060 | 0.183 | 0.308 | 0.638 | Unannotated | NA | NA |
| metab_3086 | 10-Hydroxy-2-oxabicyclo[6.2.2]dodeca-1(10),8,11-trien-3-one | pos | 210.1129 | 3.393 | 5.381 | 5.490 | 0.110 | 0.183 | 0.308 | 0.638 | Unannotated | NA | NA |
| metab_17203 | Unannotated feature | neg | 201.9057 | 7.737 | 5.082 | 5.058 | -0.024 | -0.183 | 0.308 | 0.638 | Unannotated | NA | NA |
| metab_14085 | Beta-Glucogallin | neg | 313.0571 | 2.809 | 3.694 | 3.814 | 0.119 | 0.183 | 0.308 | 0.638 | Tentative | NA | NA |
| metab_18425 | Unannotated feature | neg | 442.1361 | 3.037 | 3.403 | 3.744 | 0.340 | 0.183 | 0.308 | 0.638 | Unannotated | NA | NA |
| metab_1114 | Unannotated feature | pos | 172.1334 | 6.646 | 5.099 | 4.989 | -0.109 | -0.183 | 0.308 | 0.638 | Unannotated | NA | NA |
| metab_10162 | Unannotated feature | neg | 366.0063 | 2.718 | 4.471 | 4.039 | -0.432 | -0.183 | 0.308 | 0.638 | Unannotated | NA | NA |
| metab_1008 | Unannotated feature | pos | 603.4974 | 7.276 | 4.226 | 3.212 | -1.014 | -0.183 | 0.308 | 0.638 | Unannotated | NA | NA |
| metab_16569 | Unannotated feature | neg | 182.9880 | 7.310 | 6.193 | 6.130 | -0.063 | -0.183 | 0.308 | 0.638 | Unannotated | NA | NA |
| metab_14910 | Unannotated feature | neg | 321.1469 | 4.130 | 5.632 | 5.562 | -0.070 | -0.183 | 0.308 | 0.638 | Unannotated | NA | NA |
| metab_1742 | Unannotated feature | pos | 402.2347 | 3.638 | 4.549 | 4.602 | 0.052 | 0.183 | 0.308 | 0.638 | Unannotated | NA | NA |
| metab_13213 | Unannotated feature | neg | 140.9174 | 0.552 | 4.627 | 4.426 | -0.201 | -0.183 | 0.308 | 0.638 | Unannotated | NA | NA |
| metab_19514 | Isoferulic Acid | neg | 193.0501 | 4.837 | 4.983 | 4.752 | -0.231 | -0.183 | 0.308 | 0.638 | Strong putative | Phenylpropanoids and polyketides | Cinnamic acids and derivatives |
| metab_11655 | 5-Hydroxy-7-(hydroxymethyl)-2-Methyl-2-(5-Oxooxolan-2-yl)-3H-Chromen-4-one | neg | 291.0878 | 3.960 | 4.786 | 4.951 | 0.165 | 0.183 | 0.308 | 0.638 | Moderate putative | NA | NA |
| metab_10971 | 3-(3-Fluorophenyl)-5,5-dimethyl-4-(4-methylsulfonylphenyl)furan-2-one | neg | 341.0647 | 0.784 | 4.306 | 4.457 | 0.151 | 0.183 | 0.308 | 0.638 | Unannotated | NA | NA |
| metab_8508 | Aspartyl-Isoleucine | pos | 229.1187 | 1.654 | 5.566 | 5.573 | 0.007 | -0.183 | 0.308 | 0.638 | Unannotated | NA | NA |
| metab_3075 | 4-Quinolinecarboxylic Acid | pos | 174.0553 | 3.366 | 3.713 | 4.088 | 0.375 | 0.183 | 0.308 | 0.638 | Moderate putative | NA | NA |
| metab_14066 | Unannotated feature | neg | 200.0924 | 2.789 | 4.341 | 4.400 | 0.059 | 0.183 | 0.308 | 0.638 | Unannotated | NA | NA |
| metab_17696 | Unannotated feature | neg | 465.1256 | 2.776 | 3.619 | 3.433 | -0.186 | -0.183 | 0.308 | 0.638 | Unannotated | NA | NA |
| metab_13673 | Unannotated feature | neg | 298.1046 | 1.685 | 4.804 | 4.894 | 0.090 | 0.183 | 0.308 | 0.638 | Unannotated | NA | NA |
| metab_5428 | Unannotated feature | pos | 342.1559 | 1.952 | 5.310 | 5.445 | 0.136 | 0.183 | 0.308 | 0.638 | Unannotated | NA | NA |
| metab_5395 | Aspartyl-L-proline | pos | 213.0874 | 2.021 | 5.395 | 5.486 | 0.091 | 0.183 | 0.308 | 0.638 | Unannotated | NA | NA |
| metab_12408 | Unannotated feature | neg | 299.1864 | 6.232 | 5.582 | 5.656 | 0.074 | 0.183 | 0.308 | 0.638 | Unannotated | NA | NA |
| metab_16940 | Unannotated feature | neg | 147.0291 | 7.830 | 4.611 | 4.614 | 0.003 | -0.183 | 0.308 | 0.638 | Unannotated | NA | NA |
| metab_17275 | Unannotated feature | neg | 314.9242 | 7.720 | 3.836 | 3.890 | 0.054 | 0.183 | 0.308 | 0.638 | Unannotated | NA | NA |
| metab_8308 | Unannotated feature | pos | 143.0706 | 2.055 | 4.407 | 4.255 | -0.152 | -0.183 | 0.308 | 0.638 | Unannotated | NA | NA |
| metab_7733 | Unannotated feature | pos | 239.1294 | 3.319 | 1.474 | 1.075 | -0.399 | -0.183 | 0.308 | 0.638 | Unannotated | NA | NA |
| metab_18196 | 5-Hydroxy-4-methoxybisabola-2,10-dien-9-one | neg | 311.1864 | 6.303 | 5.394 | 5.511 | 0.118 | 0.183 | 0.308 | 0.638 | Unannotated | NA | NA |
| metab_7782 | Phenylephrine | pos | 168.1021 | 3.233 | 3.037 | 3.296 | 0.258 | 0.183 | 0.308 | 0.638 | Moderate putative | NA | NA |
| metab_12405 | Unannotated feature | neg | 226.9652 | 7.702 | 6.041 | 6.022 | -0.019 | -0.183 | 0.308 | 0.638 | Unannotated | NA | NA |
| metab_2281 | 3-[3-(2-methylphenyl)-1,2-oxazol-4-yl]propanoic acid | pos | 249.1237 | 3.242 | 5.114 | 4.730 | -0.383 | -0.183 | 0.308 | 0.638 | Moderate putative | NA | NA |
| metab_2243 | Unannotated feature | pos | 174.8962 | 0.585 | 4.156 | 3.682 | -0.474 | -0.183 | 0.308 | 0.638 | Unannotated | NA | NA |
| metab_5376 | Unannotated feature | pos | 252.2326 | 6.486 | 3.900 | 3.861 | -0.039 | -0.183 | 0.308 | 0.638 | Unannotated | NA | NA |
| metab_4068 | Unannotated feature | pos | 513.3012 | 6.135 | 4.772 | 4.430 | -0.342 | -0.183 | 0.308 | 0.638 | Unannotated | NA | NA |
| metab_3019 | Unannotated feature | pos | 141.0103 | 3.193 | 5.449 | 5.475 | 0.026 | 0.183 | 0.308 | 0.638 | Unannotated | NA | NA |
| metab_18399 | 5-[[Pyridin-3-yl-[3-(trifluoromethyl)phenyl]methylidene]amino]oxypentanoic acid | neg | 401.0883 | 6.130 | 5.442 | 5.377 | -0.064 | -0.183 | 0.308 | 0.638 | Unannotated | NA | NA |
| metab_17002 | Unannotated feature | neg | 198.9072 | 7.993 | 5.128 | 5.092 | -0.036 | -0.183 | 0.308 | 0.638 | Unannotated | NA | NA |
| metab_16313 | Unannotated feature | neg | 398.0945 | 1.485 | 4.096 | 4.407 | 0.311 | 0.183 | 0.308 | 0.638 | Unannotated | NA | NA |
| metab_9700 | Indole-3-acetyl-beta-4-D-glucose | neg | 382.1149 | 3.123 | 3.098 | 3.781 | 0.684 | 0.183 | 0.308 | 0.638 | Unannotated | Organoheterocyclic compounds | Indoles and derivatives |
| metab_1848 | Unannotated feature | pos | 262.1289 | 1.049 | 4.476 | 4.742 | 0.265 | 0.183 | 0.308 | 0.638 | Unannotated | NA | NA |
| metab_615 | Unannotated feature | pos | 626.1974 | 5.794 | 3.878 | 3.921 | 0.043 | 0.183 | 0.308 | 0.638 | Unannotated | NA | NA |
| metab_13717 | Unannotated feature | neg | 243.9080 | 1.871 | 3.888 | 3.608 | -0.280 | -0.183 | 0.308 | 0.638 | Unannotated | NA | NA |
| metab_574 | Esculeogenin A | pos | 448.3433 | 5.732 | 4.668 | 5.213 | 0.545 | 0.183 | 0.308 | 0.638 | Unannotated | NA | NA |
| metab_11536 | Unannotated feature | neg | 289.9491 | 0.496 | 7.130 | 7.095 | -0.036 | -0.183 | 0.308 | 0.638 | Unannotated | NA | NA |
| metab_1409 | Unannotated feature | pos | 229.1437 | 6.072 | 3.651 | 3.306 | -0.345 | -0.183 | 0.308 | 0.638 | Unannotated | NA | NA |
| metab_1404 | Unannotated feature | pos | 317.1502 | 6.080 | 6.105 | 5.881 | -0.224 | -0.183 | 0.308 | 0.638 | Unannotated | NA | NA |
| metab_14001 | Unannotated feature | neg | 355.0768 | 2.718 | 3.981 | 3.944 | -0.036 | -0.183 | 0.308 | 0.638 | Unannotated | NA | NA |
| metab_17260 | Unannotated feature | neg | 218.9820 | 7.726 | 3.052 | 3.215 | 0.163 | 0.183 | 0.308 | 0.638 | Unannotated | NA | NA |
| metab_13072 | Unannotated feature | neg | 368.8866 | 0.538 | 4.828 | 4.420 | -0.408 | -0.183 | 0.308 | 0.638 | Unannotated | NA | NA |
| metab_7583 | (R)-Glabridin | pos | 342.1709 | 3.578 | 5.697 | 5.237 | -0.460 | -0.183 | 0.308 | 0.638 | Tentative | NA | NA |
| metab_15093 | Unannotated feature | neg | 263.1404 | 4.518 | 4.440 | 4.041 | -0.399 | -0.183 | 0.308 | 0.638 | Unannotated | NA | NA |
| metab_9657 | Unannotated feature | neg | 281.0670 | 4.207 | 4.116 | 4.493 | 0.377 | 0.183 | 0.308 | 0.638 | Unannotated | NA | NA |
| metab_9678 | Unannotated feature | neg | 321.0481 | 3.153 | 5.581 | 5.341 | -0.240 | -0.183 | 0.308 | 0.638 | Unannotated | NA | NA |
| metab_19303 | Unannotated feature | neg | 468.2473 | 5.140 | 5.575 | 5.275 | -0.300 | -0.183 | 0.308 | 0.638 | Unannotated | NA | NA |
| metab_16950 | Unannotated feature | neg | 92.9271 | 7.858 | 4.678 | 4.672 | -0.006 | -0.183 | 0.308 | 0.638 | Unannotated | NA | NA |
| metab_16972 | Unannotated feature | neg | 209.8334 | 7.910 | 5.961 | 5.940 | -0.021 | -0.183 | 0.308 | 0.638 | Unannotated | NA | NA |
| metab_15068 | Unannotated feature | neg | 234.9082 | 0.547 | 5.377 | 5.229 | -0.147 | -0.183 | 0.308 | 0.638 | Unannotated | NA | NA |
| metab_13695 | Unannotated feature | neg | 232.0098 | 1.699 | 4.200 | 4.093 | -0.107 | -0.183 | 0.308 | 0.638 | Unannotated | NA | NA |
| metab_19285 | Unannotated feature | neg | 417.1424 | 2.750 | 3.917 | 4.313 | 0.396 | 0.183 | 0.308 | 0.638 | Unannotated | NA | NA |
| metab_12766 | Unannotated feature | neg | 547.1543 | 1.944 | 3.692 | 3.757 | 0.065 | -0.183 | 0.308 | 0.638 | Unannotated | NA | NA |
| metab_14534 | Unannotated feature | neg | 183.1133 | 3.403 | 3.772 | 3.196 | -0.576 | -0.183 | 0.308 | 0.638 | Unannotated | NA | NA |
| metab_132 | Unannotated feature | pos | 191.9826 | 0.625 | 3.041 | 3.868 | 0.828 | 0.183 | 0.308 | 0.638 | Unannotated | NA | NA |
| metab_2002 | Unannotated feature | pos | 186.1492 | 1.952 | 3.948 | 4.375 | 0.427 | 0.183 | 0.308 | 0.638 | Unannotated | NA | NA |
| metab_8836 | Triethylenemelamine | pos | 237.1461 | 7.482 | 5.022 | 4.972 | -0.050 | -0.183 | 0.308 | 0.638 | Tentative | NA | NA |
| metab_11969 | Unannotated feature | neg | 557.2466 | 6.177 | 2.959 | 4.364 | 1.406 | 0.183 | 0.308 | 0.638 | Unannotated | NA | NA |
| metab_58 | Pro His | pos | 253.1299 | 0.672 | 5.275 | 5.024 | -0.251 | -0.183 | 0.308 | 0.638 | Strong putative | NA | NA |
| metab_1510 | Unannotated feature | pos | 176.0921 | 0.672 | 5.901 | 6.008 | 0.107 | 0.183 | 0.308 | 0.638 | Unannotated | NA | NA |
| metab_17120 | Unannotated feature | neg | 157.8712 | 7.830 | 4.106 | 4.082 | -0.025 | -0.183 | 0.308 | 0.638 | Unannotated | NA | NA |
| metab_9973 | Unannotated feature | neg | 113.9938 | 2.880 | 5.084 | 5.034 | -0.050 | -0.183 | 0.308 | 0.638 | Unannotated | NA | NA |
| metab_10005 | Unannotated feature | neg | 382.1012 | 2.836 | 4.631 | 4.305 | -0.326 | -0.183 | 0.308 | 0.638 | Unannotated | NA | NA |
| metab_9995 | Unannotated feature | neg | 242.9436 | 2.852 | 4.122 | 3.855 | -0.268 | -0.183 | 0.308 | 0.638 | Unannotated | NA | NA |
| metab_8780 | Firuglipel | pos | 432.1723 | 0.720 | 4.956 | 4.327 | -0.629 | -0.183 | 0.308 | 0.638 | Unannotated | NA | NA |
| metab_3371 | Unannotated feature | pos | 526.3003 | 4.290 | 5.031 | 4.393 | -0.638 | -0.183 | 0.308 | 0.638 | Unannotated | NA | NA |
| metab_1231 | 13(S)-HOTrE | pos | 277.2166 | 6.390 | 6.349 | 6.247 | -0.102 | -0.183 | 0.308 | 0.638 | Moderate putative | NA | NA |
| metab_14222 | (R)-2-Hydroxy-7,8-dimethoxy-2H-1,4-benzoxazin-3(4H)-one 2-glucoside | neg | 386.1100 | 2.960 | 3.529 | 3.844 | 0.316 | 0.183 | 0.308 | 0.638 | Unannotated | Organic oxygen compounds | Organooxygen compounds |
| metab_14278 | Unannotated feature | neg | 268.0175 | 3.025 | 4.998 | 4.927 | -0.071 | -0.179 | 0.320 | 0.648 | Unannotated | NA | NA |
| metab_3409 | Unannotated feature | pos | 436.2353 | 6.295 | 6.002 | 6.218 | 0.216 | 0.179 | 0.320 | 0.648 | Unannotated | NA | NA |
| metab_3408 | Isoleucyl-Tryptophan | pos | 318.1820 | 4.444 | 4.911 | 4.789 | -0.121 | -0.179 | 0.320 | 0.648 | Unannotated | Organic acids and derivatives | Carboxylic acids and derivatives |
| metab_12909 | Idarubicinone | neg | 367.0820 | 0.730 | 5.393 | 5.105 | -0.288 | -0.179 | 0.320 | 0.648 | Unannotated | Benzenoids | Naphthacenes |
| metab_12082 | Americanin A | neg | 327.0881 | 5.762 | 4.996 | 4.694 | -0.302 | -0.179 | 0.320 | 0.648 | Unannotated | NA | NA |
| metab_15684 | Lurasidone | neg | 513.2354 | 5.656 | 3.386 | 3.449 | 0.063 | 0.179 | 0.320 | 0.648 | Unannotated | Organoheterocyclic compounds | Diazinanes |
| metab_2565 | Pyroglutamic Acid | pos | 130.0503 | 1.151 | 6.094 | 6.130 | 0.036 | 0.179 | 0.320 | 0.648 | Moderate putative | NA | NA |
| metab_9429 | L-2,3-Dihydrodipicolinate | neg | 168.0296 | 3.434 | 4.350 | 4.290 | -0.059 | -0.179 | 0.320 | 0.648 | Tentative | NA | NA |
| metab_5253 | Unannotated feature | pos | 465.3367 | 6.654 | 4.383 | 4.208 | -0.175 | -0.179 | 0.320 | 0.648 | Unannotated | NA | NA |
| metab_1824 | Methylimidazoleacetic acid | pos | 141.0660 | 0.981 | 5.276 | 5.379 | 0.103 | -0.179 | 0.320 | 0.648 | Tentative | NA | NA |
| metab_16398 | Unannotated feature | neg | 190.9480 | 6.845 | 5.599 | 5.577 | -0.022 | -0.179 | 0.320 | 0.648 | Unannotated | NA | NA |
| metab_10993 | Unannotated feature | neg | 247.1551 | 4.932 | 5.464 | 5.252 | -0.212 | -0.179 | 0.320 | 0.648 | Unannotated | NA | NA |
| metab_12764 | Unannotated feature | neg | 126.0554 | 1.947 | 4.391 | 4.239 | -0.152 | -0.179 | 0.320 | 0.648 | Unannotated | NA | NA |
| metab_14221 | N-Succinimidyl 3-(2-pyridyldithio)propionate | neg | 357.0213 | 2.960 | 4.833 | 4.689 | -0.144 | -0.179 | 0.320 | 0.648 | Unannotated | NA | NA |
| metab_14522 | 1,8-Naphthyridine, 2-fluoro- | neg | 341.0884 | 3.382 | 3.201 | 4.231 | 1.030 | 0.179 | 0.320 | 0.648 | Unannotated | NA | NA |
| metab_17651 | Unannotated feature | neg | 229.0828 | 2.771 | 4.329 | 4.748 | 0.419 | 0.179 | 0.320 | 0.648 | Unannotated | NA | NA |
| metab_10222 | Dihydroprudomenin | neg | 539.1424 | 2.606 | 5.469 | 5.124 | -0.344 | -0.179 | 0.320 | 0.648 | Unannotated | NA | NA |
| metab_5339 | Neocnidilide | pos | 195.1383 | 6.534 | 4.843 | 4.513 | -0.330 | -0.179 | 0.320 | 0.648 | Moderate putative | NA | NA |
| metab_5295 | Unannotated feature | pos | 411.3595 | 6.582 | 6.315 | 6.084 | -0.231 | -0.179 | 0.320 | 0.648 | Unannotated | NA | NA |
| metab_10581 | 5-Amino-2-Naphthalenesulfonic Acid | neg | 222.0228 | 1.699 | 5.248 | 5.506 | 0.259 | 0.179 | 0.320 | 0.648 | Moderate putative | NA | NA |
| metab_17254 | Unannotated feature | neg | 297.1272 | 7.726 | 3.369 | 3.791 | 0.422 | 0.179 | 0.320 | 0.648 | Unannotated | NA | NA |
| metab_10594 | Unannotated feature | neg | 204.9921 | 1.691 | 4.342 | 4.401 | 0.059 | -0.179 | 0.320 | 0.648 | Unannotated | NA | NA |
| metab_12773 | Unannotated feature | neg | 108.9012 | 0.625 | 5.404 | 5.293 | -0.111 | -0.179 | 0.320 | 0.648 | Unannotated | NA | NA |
| metab_15186 | 5-(2-Furanyl)-1,2,3,4,5,6-hexahydro-7H-cyclopenta[b]pyridin-7-one | neg | 248.0928 | 4.701 | 4.972 | 4.652 | -0.320 | -0.179 | 0.320 | 0.648 | Unannotated | NA | NA |
| metab_11553 | Unannotated feature | neg | 259.1454 | 5.261 | 5.688 | 5.720 | 0.032 | -0.179 | 0.320 | 0.648 | Unannotated | NA | NA |
| metab_14993 | Benzyl acetate | neg | 195.0658 | 4.315 | 4.428 | 4.562 | 0.134 | 0.179 | 0.320 | 0.648 | Tentative | NA | NA |
| metab_3017 | Unannotated feature | pos | 314.2079 | 3.170 | 5.326 | 5.202 | -0.124 | -0.179 | 0.320 | 0.648 | Unannotated | NA | NA |
| metab_459 | Unannotated feature | pos | 171.1179 | 2.223 | 6.906 | 6.939 | 0.033 | 0.179 | 0.320 | 0.648 | Unannotated | NA | NA |
| metab_13787 | Unannotated feature | neg | 183.0293 | 1.992 | 4.545 | 4.524 | -0.021 | 0.179 | 0.320 | 0.648 | Unannotated | NA | NA |
| metab_18868 | Unannotated feature | neg | 523.9941 | 3.219 | 4.800 | 4.610 | -0.190 | -0.179 | 0.320 | 0.648 | Unannotated | NA | NA |
| metab_16145 | Unannotated feature | neg | 615.3173 | 6.303 | 4.288 | 4.638 | 0.350 | 0.179 | 0.320 | 0.648 | Unannotated | NA | NA |
| metab_12340 | Unannotated feature | neg | 493.3365 | 7.448 | 6.665 | 6.534 | -0.131 | -0.179 | 0.320 | 0.648 | Unannotated | NA | NA |
| metab_11678 | Unannotated feature | neg | 421.1778 | 5.355 | 3.937 | 4.462 | 0.525 | 0.179 | 0.320 | 0.648 | Unannotated | NA | NA |
| metab_9589 | Flumioxazin | neg | 399.1013 | 3.234 | 3.093 | 3.204 | 0.111 | 0.179 | 0.320 | 0.648 | Tentative | Organoheterocyclic compounds | Benzoxazines |
| metab_7819 | Matricin | pos | 348.1780 | 3.120 | 5.531 | 5.346 | -0.185 | -0.179 | 0.320 | 0.648 | Tentative | NA | NA |
| metab_17298 | Unannotated feature | neg | 255.2330 | 7.710 | 3.785 | 3.690 | -0.095 | -0.179 | 0.320 | 0.648 | Unannotated | NA | NA |
| metab_13350 | 2-[(1S,2S,4R)-2-Hydroxy-7,7-dimethyl-1-(spiro[indene-1,4'-piperidine]-1'-ylsulfonylmethyl)-2-bicyclo[2.2.1]heptanyl]acetic acid | neg | 480.1847 | 0.714 | 4.165 | 4.483 | 0.318 | 0.179 | 0.320 | 0.648 | Unannotated | NA | NA |
| metab_15258 | Unannotated feature | neg | 255.1238 | 4.818 | 3.964 | 4.569 | 0.605 | 0.179 | 0.320 | 0.648 | Unannotated | NA | NA |
| metab_979 | Unannotated feature | pos | 267.1261 | 7.983 | 5.640 | 5.677 | 0.037 | 0.179 | 0.320 | 0.648 | Unannotated | NA | NA |
| metab_12790 | Unannotated feature | neg | 261.0281 | 0.636 | 4.099 | 4.496 | 0.397 | 0.179 | 0.320 | 0.648 | Unannotated | NA | NA |
| metab_11733 | Unannotated feature | neg | 303.1088 | 5.595 | 4.449 | 4.697 | 0.248 | 0.179 | 0.320 | 0.648 | Unannotated | NA | NA |
| metab_12319 | Unannotated feature | neg | 158.9749 | 7.310 | 8.410 | 8.376 | -0.034 | -0.179 | 0.320 | 0.648 | Unannotated | NA | NA |
| metab_5448 | Unannotated feature | pos | 267.1336 | 1.952 | 4.326 | 4.572 | 0.245 | 0.179 | 0.320 | 0.648 | Unannotated | NA | NA |
| metab_10956 | Unannotated feature | neg | 305.0700 | 3.461 | 6.136 | 5.891 | -0.245 | -0.179 | 0.320 | 0.648 | Unannotated | NA | NA |
| metab_1090 | Unannotated feature | pos | 194.1397 | 1.944 | 4.896 | 4.642 | -0.254 | -0.179 | 0.320 | 0.648 | Unannotated | NA | NA |
| metab_9808 | Unannotated feature | neg | 357.9279 | 3.028 | 4.484 | 4.147 | -0.337 | -0.179 | 0.320 | 0.648 | Unannotated | NA | NA |
| metab_5468 | Jasmolone | pos | 145.1014 | 6.382 | 5.153 | 5.029 | -0.124 | -0.179 | 0.320 | 0.648 | Unannotated | NA | NA |
| metab_1853 | Unannotated feature | pos | 175.1444 | 1.041 | 4.895 | 4.488 | -0.407 | -0.179 | 0.320 | 0.648 | Unannotated | NA | NA |
| metab_17733 | Unannotated feature | neg | 475.1755 | 6.946 | 4.854 | 5.222 | 0.368 | 0.179 | 0.320 | 0.648 | Unannotated | NA | NA |
| metab_5435 | Unannotated feature | pos | 252.2689 | 6.406 | 5.259 | 5.116 | -0.143 | -0.179 | 0.320 | 0.648 | Unannotated | NA | NA |
| metab_18695 | Unannotated feature | neg | 149.0271 | 3.082 | 5.261 | 5.372 | 0.111 | 0.179 | 0.320 | 0.648 | Unannotated | NA | NA |
| metab_1365 | 4-Hydroxy-(3',4'-dihydroxyphenyl)-valeric acid | pos | 209.0811 | 3.035 | 3.362 | 3.215 | -0.147 | -0.179 | 0.320 | 0.648 | Unannotated | Organic acids and derivatives | Hydroxy acids and derivatives |
| metab_14444 | Unannotated feature | neg | 333.0102 | 3.254 | 4.456 | 3.984 | -0.472 | -0.179 | 0.320 | 0.648 | Unannotated | NA | NA |
| metab_5416 | 3alpha,7alpha,12alpha-trihydroxy-5beta-cholestanate | pos | 468.3696 | 6.414 | 5.417 | 4.766 | -0.650 | -0.179 | 0.320 | 0.648 | Tentative | Lipids and lipid-like molecules | Steroids and steroid derivatives |
| metab_2920 | 12a-Hydroxy-3-oxocholadienic acid | pos | 387.2533 | 6.175 | 5.664 | 5.540 | -0.124 | -0.179 | 0.320 | 0.648 | Unannotated | NA | NA |
| metab_10524 | Unannotated feature | neg | 226.0355 | 1.947 | 4.108 | 4.240 | 0.133 | 0.179 | 0.320 | 0.648 | Unannotated | NA | NA |
| metab_11071 | Unannotated feature | neg | 361.0910 | 0.730 | 4.601 | 4.742 | 0.140 | 0.179 | 0.320 | 0.648 | Unannotated | NA | NA |
| metab_7730 | Urocanic Acid | pos | 139.0504 | 0.768 | 5.883 | 5.899 | 0.016 | -0.179 | 0.320 | 0.648 | Strong putative | NA | NA |
| metab_2381 | Unannotated feature | pos | 461.2307 | 5.757 | 4.111 | 4.765 | 0.654 | 0.179 | 0.320 | 0.648 | Unannotated | NA | NA |
| metab_13272 | Unannotated feature | neg | 260.8510 | 0.614 | 4.339 | 4.109 | -0.230 | -0.179 | 0.320 | 0.648 | Unannotated | NA | NA |
| metab_7784 | Estriol | pos | 311.1606 | 3.223 | 3.594 | 4.360 | 0.766 | 0.179 | 0.320 | 0.648 | Tentative | NA | NA |
| metab_720 | Unannotated feature | pos | 579.3541 | 5.969 | 5.178 | 5.529 | 0.351 | 0.179 | 0.320 | 0.648 | Unannotated | NA | NA |
| metab_5384 | Unannotated feature | pos | 316.2253 | 6.462 | 5.157 | 4.916 | -0.242 | -0.179 | 0.320 | 0.648 | Unannotated | NA | NA |
| metab_16620 | Unannotated feature | neg | 258.9008 | 7.495 | 5.284 | 5.293 | 0.009 | 0.179 | 0.320 | 0.648 | Unannotated | NA | NA |
| metab_18637 | Unannotated feature | neg | 265.0731 | 5.952 | 3.100 | 3.198 | 0.098 | 0.179 | 0.320 | 0.648 | Unannotated | NA | NA |
| metab_17050 | Unannotated feature | neg | 195.8107 | 7.985 | 6.935 | 6.903 | -0.032 | -0.179 | 0.320 | 0.648 | Unannotated | NA | NA |
| metab_14390 | Unannotated feature | neg | 694.7673 | 0.527 | 4.709 | 5.165 | 0.456 | 0.179 | 0.320 | 0.648 | Unannotated | NA | NA |
| metab_13563 | Unannotated feature | neg | 247.0395 | 1.214 | 4.516 | 4.292 | -0.224 | -0.179 | 0.320 | 0.648 | Unannotated | NA | NA |
| metab_15462 | Unannotated feature | neg | 398.1721 | 5.201 | 3.466 | 3.188 | -0.278 | -0.179 | 0.320 | 0.648 | Unannotated | NA | NA |
| metab_8631 | Citramalic acid | pos | 314.1102 | 1.041 | 1.636 | 3.451 | 1.815 | 0.179 | 0.320 | 0.648 | Tentative | NA | NA |
| metab_14292 | Unannotated feature | neg | 271.1301 | 3.041 | 3.782 | 3.478 | -0.303 | -0.179 | 0.320 | 0.648 | Unannotated | NA | NA |
| metab_19628 | Unannotated feature | neg | 243.0187 | 4.667 | 3.875 | 4.177 | 0.303 | 0.179 | 0.320 | 0.648 | Unannotated | NA | NA |
| metab_10829 | Unannotated feature | neg | 242.1145 | 1.051 | 4.293 | 4.088 | -0.205 | -0.179 | 0.320 | 0.648 | Unannotated | NA | NA |
| metab_15486 | Arecoline | neg | 136.0761 | 5.238 | 5.480 | 5.454 | -0.025 | -0.179 | 0.320 | 0.648 | Tentative | Alkaloids and derivatives | NA |
| metab_11232 | Unannotated feature | neg | 298.1146 | 0.636 | 5.582 | 5.366 | -0.216 | -0.179 | 0.320 | 0.648 | Unannotated | NA | NA |
| metab_17933 | Unannotated feature | neg | 666.0613 | 6.624 | 5.214 | 4.791 | -0.423 | -0.179 | 0.320 | 0.648 | Unannotated | NA | NA |
| metab_255 | Unannotated feature | pos | 306.8905 | 0.531 | 4.477 | 4.185 | -0.292 | -0.179 | 0.320 | 0.648 | Unannotated | NA | NA |
| metab_9946 | Unannotated feature | neg | 202.1081 | 2.906 | 4.696 | 4.864 | 0.168 | 0.179 | 0.320 | 0.648 | Unannotated | NA | NA |
| metab_9962 | Unannotated feature | neg | 222.1134 | 2.896 | 2.296 | 3.014 | 0.718 | 0.179 | 0.320 | 0.648 | Unannotated | NA | NA |
| metab_16071 | Unannotated feature | neg | 253.9476 | 0.614 | 4.928 | 4.801 | -0.127 | -0.179 | 0.320 | 0.648 | Unannotated | NA | NA |
| metab_19827 | Unannotated feature | neg | 229.1555 | 4.398 | 4.822 | 4.577 | -0.245 | -0.179 | 0.320 | 0.648 | Unannotated | NA | NA |
| metab_6597 | Unannotated feature | pos | 461.9494 | 5.451 | 3.569 | 3.613 | 0.043 | 0.179 | 0.320 | 0.648 | Unannotated | NA | NA |
| metab_3330 | (-)-Epinephrine | pos | 184.0971 | 4.175 | 4.539 | 4.524 | -0.015 | -0.179 | 0.320 | 0.648 | Moderate putative | NA | NA |
| metab_15590 | Unannotated feature | neg | 345.1557 | 5.487 | 4.567 | 4.783 | 0.216 | 0.179 | 0.320 | 0.648 | Unannotated | NA | NA |
| metab_11393 | Unannotated feature | neg | 142.9146 | 0.552 | 3.749 | 3.383 | -0.366 | -0.179 | 0.320 | 0.648 | Unannotated | NA | NA |
| metab_10801 | Isovalerylglutamic acid | neg | 276.1090 | 1.096 | 3.666 | 4.594 | 0.928 | 0.179 | 0.320 | 0.648 | Unannotated | NA | NA |
| metab_8730 | GUVACINE | pos | 128.0710 | 0.768 | 5.705 | 5.753 | 0.049 | 0.179 | 0.320 | 0.648 | Tentative | NA | NA |
| metab_8868 | Unannotated feature | neg | 394.1149 | 4.319 | 5.022 | 5.458 | 0.436 | 0.179 | 0.320 | 0.648 | Unannotated | NA | NA |
| metab_7020 | Prolylphenylalanine | pos | 280.1659 | 4.798 | 4.990 | 4.765 | -0.225 | -0.179 | 0.320 | 0.648 | Unannotated | NA | NA |
| metab_15564 | Trigraecum | neg | 313.0723 | 5.427 | 3.975 | 4.377 | 0.402 | 0.179 | 0.320 | 0.648 | Unannotated | NA | NA |
| metab_1175 | Unannotated feature | pos | 571.3618 | 6.510 | 5.611 | 5.785 | 0.174 | 0.179 | 0.320 | 0.648 | Unannotated | NA | NA |
| metab_12537 | Unannotated feature | neg | 477.2497 | 6.224 | 5.592 | 5.329 | -0.263 | -0.179 | 0.320 | 0.648 | Unannotated | NA | NA |
| metab_8076 | Desmosflavone | pos | 314.1393 | 3.496 | 3.340 | 3.864 | 0.525 | 0.179 | 0.320 | 0.648 | Unannotated | NA | NA |
| metab_8066 | Methionyl-Proline | pos | 247.1115 | 2.762 | 5.620 | 5.507 | -0.113 | -0.179 | 0.320 | 0.648 | Unannotated | NA | NA |
| metab_879 | Unannotated feature | pos | 371.1635 | 6.829 | 5.061 | 5.241 | 0.180 | 0.179 | 0.320 | 0.648 | Unannotated | NA | NA |
| metab_10079 | Phenylglucuronide | neg | 269.0671 | 2.776 | 5.010 | 5.138 | 0.128 | 0.179 | 0.320 | 0.648 | Unannotated | NA | NA |
| metab_4761 | Unannotated feature | pos | 341.9054 | 7.724 | 4.590 | 4.636 | 0.046 | 0.179 | 0.320 | 0.648 | Unannotated | NA | NA |
| metab_9393 | Unannotated feature | neg | 428.1500 | 3.484 | 3.983 | 3.484 | -0.499 | -0.179 | 0.320 | 0.648 | Unannotated | NA | NA |
| metab_13591 | Unannotated feature | neg | 293.0341 | 1.958 | 4.413 | 4.169 | -0.244 | -0.179 | 0.320 | 0.648 | Unannotated | NA | NA |
| metab_9891 | Unannotated feature | neg | 242.9435 | 2.960 | 4.109 | 3.852 | -0.257 | -0.179 | 0.320 | 0.648 | Unannotated | NA | NA |
| metab_1731 | (2R,4S)-4-Carbamimidamido-3-acetamido-2-((1R,2R)-2-hydroxy-1-methoxy-3-(octanoyloxy)propyl)-3,4-dihydro-2H-pyran-6-carboxylic acid | pos | 490.2875 | 3.799 | 4.114 | 4.043 | -0.070 | 0.179 | 0.320 | 0.648 | Unannotated | NA | NA |
| metab_4948 | Unannotated feature | pos | 146.9805 | 7.610 | 4.893 | 4.934 | 0.041 | 0.179 | 0.320 | 0.648 | Unannotated | NA | NA |
| metab_18903 | Alpha-Artemether | neg | 333.1461 | 5.649 | 5.305 | 5.226 | -0.079 | -0.179 | 0.320 | 0.648 | Unannotated | Lipids and lipid-like molecules | Prenol lipids |
| metab_8551 | Mytilitol | pos | 236.1132 | 1.274 | 3.598 | 3.989 | 0.391 | 0.179 | 0.320 | 0.648 | Unannotated | NA | NA |
| metab_994 | Unannotated feature | pos | 183.0981 | 7.708 | 5.309 | 5.336 | 0.027 | 0.179 | 0.320 | 0.648 | Unannotated | NA | NA |
| metab_15393 | Gibberellin A29 | neg | 347.1505 | 5.057 | 5.542 | 5.399 | -0.143 | -0.179 | 0.320 | 0.648 | Tentative | NA | NA |
| metab_17815 | Unannotated feature | neg | 564.3737 | 6.821 | 5.342 | 5.268 | -0.074 | -0.179 | 0.320 | 0.648 | Unannotated | NA | NA |
| metab_19032 | 20-Hydroxy-leukotriene B4 | neg | 373.2032 | 5.487 | 2.284 | 1.917 | -0.368 | -0.179 | 0.320 | 0.648 | Tentative | NA | NA |
| metab_10477 | Unannotated feature | neg | 319.1147 | 1.952 | 4.349 | 4.657 | 0.308 | 0.179 | 0.320 | 0.648 | Unannotated | NA | NA |
| metab_17383 | Unannotated feature | neg | 893.8129 | 7.663 | 4.123 | 4.360 | 0.237 | 0.179 | 0.320 | 0.648 | Unannotated | NA | NA |
| metab_4928 | Unannotated feature | pos | 718.8394 | 7.634 | 5.091 | 5.148 | 0.057 | 0.179 | 0.320 | 0.648 | Unannotated | NA | NA |
| metab_2525 | Unannotated feature | pos | 257.0858 | 0.910 | 4.420 | 4.489 | 0.069 | 0.179 | 0.320 | 0.648 | Unannotated | NA | NA |
| metab_18256 | Unannotated feature | neg | 415.1799 | 6.248 | 5.647 | 5.830 | 0.183 | 0.179 | 0.320 | 0.648 | Unannotated | NA | NA |
| metab_2831 | Unannotated feature | pos | 194.0330 | 0.609 | 4.761 | 4.509 | -0.252 | -0.179 | 0.320 | 0.648 | Unannotated | NA | NA |
| metab_9570 | N-Acetyl-L-Tyrosine | neg | 222.0769 | 3.254 | 5.416 | 5.319 | -0.098 | -0.179 | 0.320 | 0.648 | Strong putative | NA | NA |
| metab_17808 | Unannotated feature | neg | 188.9509 | 6.845 | 5.064 | 4.977 | -0.087 | -0.179 | 0.320 | 0.648 | Unannotated | NA | NA |
| metab_2277 | Enokipodin D | pos | 280.1547 | 3.223 | 3.349 | 3.817 | 0.468 | 0.175 | 0.331 | 0.660 | Unannotated | NA | NA |
| metab_2268 | Unannotated feature | pos | 202.0445 | 3.179 | 7.499 | 7.520 | 0.022 | 0.175 | 0.331 | 0.660 | Unannotated | NA | NA |
| metab_16265 | Capsidiol | neg | 531.3693 | 6.545 | 5.841 | 6.021 | 0.179 | 0.175 | 0.331 | 0.660 | Tentative | NA | NA |
| metab_17659 | Unannotated feature | neg | 591.4405 | 7.061 | 2.879 | 2.795 | -0.084 | -0.175 | 0.331 | 0.660 | Unannotated | NA | NA |
| metab_16505 | Unannotated feature | neg | 778.5280 | 7.101 | 5.178 | 4.661 | -0.517 | -0.175 | 0.331 | 0.660 | Unannotated | NA | NA |
| metab_16957 | Unannotated feature | neg | 164.8355 | 7.870 | 5.560 | 5.580 | 0.019 | 0.175 | 0.331 | 0.660 | Unannotated | NA | NA |
| metab_581 | Unannotated feature | pos | 229.1438 | 5.615 | 3.812 | 4.340 | 0.528 | 0.175 | 0.331 | 0.660 | Unannotated | NA | NA |
| metab_4050 | Unannotated feature | pos | 599.7056 | 5.831 | 2.888 | 2.932 | 0.043 | 0.175 | 0.331 | 0.660 | Unannotated | NA | NA |
| metab_17000 | Unannotated feature | neg | 229.9784 | 7.989 | 4.485 | 4.463 | -0.022 | -0.175 | 0.331 | 0.660 | Unannotated | NA | NA |
| metab_9698 | Unannotated feature | neg | 374.0115 | 3.127 | 4.860 | 4.704 | -0.157 | -0.175 | 0.331 | 0.660 | Unannotated | NA | NA |
| metab_13043 | Unannotated feature | neg | 516.8195 | 0.596 | 4.683 | 4.892 | 0.208 | 0.175 | 0.331 | 0.660 | Unannotated | NA | NA |
| metab_19790 | Unannotated feature | neg | 403.1040 | 4.446 | 4.312 | 4.203 | -0.109 | -0.175 | 0.331 | 0.660 | Unannotated | NA | NA |
| metab_13099 | Unannotated feature | neg | 189.8576 | 0.073 | 6.394 | 6.379 | -0.015 | -0.175 | 0.331 | 0.660 | Unannotated | NA | NA |
| metab_7230 | Val Val Ile | pos | 330.2395 | 4.359 | 5.334 | 5.355 | 0.020 | -0.175 | 0.331 | 0.660 | Moderate putative | NA | NA |
| metab_18051 | Unannotated feature | neg | 295.1915 | 6.444 | 4.451 | 4.496 | 0.045 | 0.175 | 0.331 | 0.660 | Unannotated | NA | NA |
| metab_11737 | Unannotated feature | neg | 147.0444 | 5.571 | 4.643 | 4.603 | -0.040 | -0.175 | 0.331 | 0.660 | Unannotated | NA | NA |
| metab_11726 | Unannotated feature | neg | 390.0839 | 5.571 | 4.195 | 4.920 | 0.725 | 0.175 | 0.331 | 0.660 | Unannotated | NA | NA |
| metab_3893 | Unannotated feature | pos | 506.8265 | 5.741 | 4.172 | 4.455 | 0.283 | 0.175 | 0.331 | 0.660 | Unannotated | NA | NA |
| metab_17047 | Unannotated feature | neg | 201.8015 | 7.985 | 6.011 | 5.977 | -0.034 | -0.175 | 0.331 | 0.660 | Unannotated | NA | NA |
| metab_11700 | Gibberellin A8 | neg | 345.1347 | 5.470 | 5.404 | 5.220 | -0.184 | -0.175 | 0.331 | 0.660 | Tentative | NA | NA |
| metab_14407 | 8-methylthiooctyldesulfoglucosinolate | neg | 378.1412 | 3.207 | 5.199 | 5.159 | -0.041 | -0.175 | 0.331 | 0.660 | Unannotated | Organic oxygen compounds | Organooxygen compounds |
| metab_1340 | Formiminoglutamic acid | pos | 110.0205 | 6.175 | 5.171 | 5.184 | 0.013 | -0.175 | 0.331 | 0.660 | Tentative | NA | NA |
| metab_5087 | Unannotated feature | pos | 503.3725 | 7.060 | 5.962 | 6.014 | 0.052 | 0.175 | 0.331 | 0.660 | Unannotated | NA | NA |
| metab_7815 | Unannotated feature | pos | 404.1591 | 3.312 | 3.492 | 3.864 | 0.371 | 0.175 | 0.331 | 0.660 | Unannotated | NA | NA |
| metab_4527 | Capsicum annuum Fluorescent chlorophyll catabolite | pos | 593.2777 | 6.726 | 5.998 | 5.631 | -0.366 | -0.175 | 0.331 | 0.660 | Unannotated | NA | NA |
| metab_8279 | Unannotated feature | pos | 223.0642 | 2.150 | 4.560 | 4.585 | 0.025 | 0.175 | 0.331 | 0.660 | Unannotated | NA | NA |
| metab_15332 | Unannotated feature | neg | 305.9729 | 0.558 | 3.551 | 3.796 | 0.245 | -0.175 | 0.331 | 0.660 | Unannotated | NA | NA |
| metab_15259 | Unannotated feature | neg | 353.1715 | 4.818 | 2.562 | 3.447 | 0.885 | 0.175 | 0.331 | 0.660 | Unannotated | NA | NA |
| metab_7797 | Unannotated feature | pos | 119.0734 | 3.179 | 5.806 | 5.820 | 0.014 | 0.175 | 0.331 | 0.660 | Unannotated | NA | NA |
| metab_4490 | Contignasterol | pos | 473.3254 | 6.550 | 6.449 | 6.341 | -0.108 | -0.175 | 0.331 | 0.660 | Tentative | NA | NA |
| metab_16944 | Unannotated feature | neg | 256.9553 | 7.835 | 4.620 | 4.623 | 0.003 | 0.175 | 0.331 | 0.660 | Unannotated | NA | NA |
| metab_14485 | Unannotated feature | neg | 263.0321 | 3.331 | 3.681 | 4.456 | 0.775 | 0.175 | 0.331 | 0.660 | Unannotated | NA | NA |
| metab_3949 | Unannotated feature | pos | 573.1644 | 5.782 | 3.913 | 3.960 | 0.048 | 0.175 | 0.331 | 0.660 | Unannotated | NA | NA |
| metab_9116 | Unannotated feature | neg | 340.0547 | 3.897 | 4.067 | 4.502 | 0.435 | 0.175 | 0.331 | 0.660 | Unannotated | NA | NA |
| metab_15204 | Unannotated feature | neg | 232.9108 | 0.547 | 5.715 | 5.581 | -0.134 | -0.175 | 0.331 | 0.660 | Unannotated | NA | NA |
| metab_14989 | Unannotated feature | neg | 230.1396 | 4.311 | 5.010 | 4.960 | -0.050 | -0.175 | 0.331 | 0.660 | Unannotated | NA | NA |
| metab_9767 | Unannotated feature | neg | 255.0510 | 3.067 | 4.759 | 4.877 | 0.118 | 0.175 | 0.331 | 0.660 | Unannotated | NA | NA |
| metab_13686 | Inosine | neg | 267.0738 | 1.691 | 5.743 | 5.602 | -0.142 | -0.175 | 0.331 | 0.660 | Strong putative | Nucleosides, nucleotides, and analogues | Purine nucleosides |
| metab_11680 | Unannotated feature | neg | 238.1084 | 5.369 | 4.012 | 4.175 | 0.163 | -0.175 | 0.331 | 0.660 | Unannotated | NA | NA |
| metab_1862 | (2,5-Dioxopyrrolidin-1-yl) 5-(2-hydroxyethoxy)-4-oxopentanoate | pos | 274.0927 | 1.151 | 6.282 | 6.534 | 0.252 | 0.175 | 0.331 | 0.660 | Unannotated | NA | NA |
| metab_13321 | Unannotated feature | neg | 198.0767 | 0.667 | 4.342 | 4.633 | 0.291 | 0.175 | 0.331 | 0.660 | Unannotated | NA | NA |
| metab_11968 | Unannotated feature | neg | 636.2379 | 6.177 | 0.284 | 3.485 | 3.202 | 0.175 | 0.331 | 0.660 | Unannotated | NA | NA |
| metab_15801 | PA(8:0/22:6(4Z,8Z,10Z,13Z,16Z,19Z)-OH(7)) | neg | 661.2898 | 5.865 | 4.400 | 4.313 | -0.087 | -0.175 | 0.331 | 0.660 | Unannotated | NA | NA |
| metab_1242 | Unannotated feature | pos | 135.1171 | 6.382 | 5.354 | 5.305 | -0.049 | -0.175 | 0.331 | 0.660 | Unannotated | NA | NA |
| metab_7609 | Unannotated feature | pos | 276.1585 | 3.533 | 4.547 | 4.218 | -0.330 | -0.175 | 0.331 | 0.660 | Unannotated | NA | NA |
| metab_16987 | Unannotated feature | neg | 234.8556 | 7.981 | 5.102 | 5.081 | -0.021 | -0.175 | 0.331 | 0.660 | Unannotated | NA | NA |
| metab_16948 | Unannotated feature | neg | 141.9118 | 7.853 | 4.136 | 4.076 | -0.060 | -0.175 | 0.331 | 0.660 | Unannotated | NA | NA |
| metab_1078 | Unannotated feature | pos | 535.3044 | 6.702 | 5.615 | 5.870 | 0.256 | 0.175 | 0.331 | 0.660 | Unannotated | NA | NA |
| metab_4396 | Unannotated feature | pos | 444.2950 | 6.263 | 4.704 | 4.156 | -0.549 | -0.175 | 0.331 | 0.660 | Unannotated | NA | NA |
| metab_11574 | Unannotated feature | neg | 251.9255 | 0.460 | 3.502 | 3.406 | -0.096 | -0.175 | 0.331 | 0.660 | Unannotated | NA | NA |
| metab_18187 | Unannotated feature | neg | 325.1480 | 6.310 | 5.276 | 5.143 | -0.133 | -0.175 | 0.331 | 0.660 | Unannotated | NA | NA |
| metab_16439 | Unannotated feature | neg | 549.3655 | 6.953 | 4.448 | 4.916 | 0.467 | 0.175 | 0.331 | 0.660 | Unannotated | NA | NA |
| metab_167 | Unannotated feature | pos | 116.8914 | 0.609 | 3.555 | 3.340 | -0.215 | -0.175 | 0.331 | 0.660 | Unannotated | NA | NA |
| metab_16970 | Unannotated feature | neg | 205.8394 | 7.910 | 6.501 | 6.482 | -0.019 | -0.175 | 0.331 | 0.660 | Unannotated | NA | NA |
| metab_14281 | Isopropylmaleic acid | neg | 157.0500 | 3.028 | 4.503 | 4.508 | 0.005 | 0.175 | 0.331 | 0.660 | Tentative | NA | NA |
| metab_12110 | Unannotated feature | neg | 591.3200 | 6.522 | 5.609 | 5.776 | 0.167 | 0.175 | 0.331 | 0.660 | Unannotated | NA | NA |
| metab_1527 | Enprostil | pos | 433.2244 | 5.434 | 4.864 | 4.221 | -0.644 | -0.175 | 0.331 | 0.660 | Unannotated | NA | NA |
| metab_16725 | Unannotated feature | neg | 442.8947 | 7.660 | 5.671 | 5.687 | 0.016 | 0.175 | 0.331 | 0.660 | Unannotated | NA | NA |
| metab_10558 | Unannotated feature | neg | 318.9941 | 4.527 | 4.692 | 3.920 | -0.772 | -0.175 | 0.331 | 0.660 | Unannotated | NA | NA |
| metab_9198 | Unannotated feature | neg | 189.1127 | 3.773 | 4.108 | 4.292 | 0.184 | 0.175 | 0.331 | 0.660 | Unannotated | NA | NA |
| metab_443 | Unannotated feature | pos | 119.5360 | 0.013 | 3.544 | 3.591 | 0.047 | 0.175 | 0.331 | 0.660 | Unannotated | NA | NA |
| metab_5285 | Unannotated feature | pos | 480.3101 | 6.598 | 4.999 | 4.739 | -0.260 | -0.175 | 0.331 | 0.660 | Unannotated | NA | NA |
| metab_646 | Unannotated feature | pos | 466.2346 | 5.841 | 5.061 | 4.538 | -0.523 | -0.175 | 0.331 | 0.660 | Unannotated | NA | NA |
| metab_599 | Unannotated feature | pos | 367.2107 | 5.782 | 5.321 | 5.179 | -0.143 | -0.175 | 0.331 | 0.660 | Unannotated | NA | NA |
| metab_12441 | Unannotated feature | neg | 553.3215 | 6.263 | 7.202 | 6.986 | -0.216 | -0.175 | 0.331 | 0.660 | Unannotated | NA | NA |
| metab_18024 | Unannotated feature | neg | 369.2648 | 6.483 | 4.692 | 4.704 | 0.012 | 0.175 | 0.331 | 0.660 | Unannotated | NA | NA |
| metab_9901 | Combretastatin | neg | 333.1351 | 4.359 | 4.092 | 4.696 | 0.605 | 0.175 | 0.331 | 0.660 | Unannotated | NA | NA |
| metab_18045 | Unannotated feature | neg | 184.0610 | 0.644 | 4.832 | 5.249 | 0.417 | 0.175 | 0.331 | 0.660 | Unannotated | NA | NA |
| metab_18624 | Unannotated feature | neg | 466.2928 | 5.964 | 6.166 | 6.056 | -0.110 | -0.175 | 0.331 | 0.660 | Unannotated | NA | NA |
| metab_10922 | Dihydroferulic acid 4-O-glucuronide | neg | 353.0841 | 0.819 | 5.492 | 5.518 | 0.026 | 0.175 | 0.331 | 0.660 | Unannotated | NA | NA |
| metab_11478 | Unannotated feature | neg | 576.8590 | 0.520 | 4.613 | 4.790 | 0.177 | 0.175 | 0.331 | 0.660 | Unannotated | NA | NA |
| metab_9892 | Unannotated feature | neg | 171.0115 | 2.960 | 4.339 | 4.315 | -0.024 | -0.175 | 0.331 | 0.660 | Unannotated | NA | NA |
| metab_18960 | Menthol glucuronide | neg | 331.1764 | 5.576 | 4.780 | 4.997 | 0.217 | 0.175 | 0.331 | 0.660 | Unannotated | NA | NA |
| metab_16844 | Unannotated feature | neg | 195.9387 | 7.730 | 4.686 | 4.663 | -0.023 | -0.175 | 0.331 | 0.660 | Unannotated | NA | NA |
| metab_5018 | Unannotated feature | pos | 452.1419 | 7.315 | 4.946 | 4.874 | -0.072 | -0.175 | 0.331 | 0.660 | Unannotated | NA | NA |
| metab_965 | Unannotated feature | pos | 314.9442 | 7.724 | 5.077 | 5.061 | -0.016 | -0.175 | 0.331 | 0.660 | Unannotated | NA | NA |
| metab_2668 | 5-Methylcytosine | pos | 126.0666 | 1.960 | 3.662 | 3.497 | -0.165 | -0.175 | 0.331 | 0.660 | Moderate putative | NA | NA |
| metab_4739 | Unannotated feature | pos | 97.1016 | 7.689 | 3.721 | 3.794 | 0.073 | 0.175 | 0.331 | 0.660 | Unannotated | NA | NA |
| metab_13914 | Unannotated feature | neg | 304.0790 | 2.531 | 6.331 | 6.312 | -0.019 | -0.175 | 0.331 | 0.660 | Unannotated | NA | NA |
| metab_2543 | 7-Aminomethyl-7-carbaguanine | pos | 180.0882 | 1.026 | 3.832 | 3.940 | 0.107 | 0.175 | 0.331 | 0.660 | Tentative | NA | NA |
| metab_18980 | Unannotated feature | neg | 406.1151 | 5.550 | 4.054 | 3.925 | -0.128 | -0.175 | 0.331 | 0.660 | Unannotated | NA | NA |
| metab_2726 | N-gamma-Glutamylcysteine ethyl ester | pos | 279.1014 | 2.187 | 5.180 | 5.148 | -0.032 | -0.175 | 0.331 | 0.660 | Unannotated | NA | NA |
| metab_4889 | Unannotated feature | pos | 103.5610 | 7.708 | 3.812 | 3.862 | 0.050 | 0.175 | 0.331 | 0.660 | Unannotated | NA | NA |
| metab_12481 | Unannotated feature | neg | 351.0851 | 5.355 | 4.673 | 4.269 | -0.404 | -0.175 | 0.331 | 0.660 | Unannotated | NA | NA |
| metab_3526 | L-Valine, N-(2-hydroxy-3-butenyl)- | pos | 188.1284 | 4.870 | 4.404 | 4.313 | -0.091 | -0.175 | 0.331 | 0.660 | Unannotated | NA | NA |
| metab_2076 | Unannotated feature | pos | 350.1722 | 2.740 | 4.740 | 4.561 | -0.179 | -0.175 | 0.331 | 0.660 | Unannotated | NA | NA |
| metab_17051 | Unannotated feature | neg | 190.9041 | 7.985 | 4.860 | 4.835 | -0.026 | -0.175 | 0.331 | 0.660 | Unannotated | NA | NA |
| metab_2141 | Unannotated feature | pos | 224.1285 | 2.864 | 4.710 | 4.838 | 0.128 | 0.175 | 0.331 | 0.660 | Unannotated | NA | NA |
| metab_1295 | Unannotated feature | pos | 338.2565 | 6.255 | 4.856 | 4.711 | -0.145 | -0.175 | 0.331 | 0.660 | Unannotated | NA | NA |
| metab_1260 | Cucurbitacin I | pos | 559.2623 | 6.327 | 5.155 | 5.221 | 0.067 | 0.175 | 0.331 | 0.660 | Tentative | Lipids and lipid-like molecules | Steroids and steroid derivatives |
| metab_16597 | Unannotated feature | neg | 185.9106 | 7.409 | 5.242 | 5.265 | 0.023 | 0.175 | 0.331 | 0.660 | Unannotated | NA | NA |
| metab_2457 | 5-Hydroxypentanoic acid | pos | 160.0972 | 0.665 | 5.559 | 5.775 | 0.216 | 0.175 | 0.331 | 0.660 | Tentative | NA | NA |
| metab_17801 | Unannotated feature | neg | 393.2591 | 6.853 | 4.883 | 5.134 | 0.252 | 0.175 | 0.331 | 0.660 | Unannotated | NA | NA |
| metab_16889 | Unannotated feature | neg | 172.8298 | 7.745 | 4.715 | 4.751 | 0.035 | 0.175 | 0.331 | 0.660 | Unannotated | NA | NA |
| metab_16896 | Unannotated feature | neg | 406.8476 | 7.745 | 4.259 | 4.310 | 0.051 | 0.175 | 0.331 | 0.660 | Unannotated | NA | NA |
| metab_2143 | Histidinal | pos | 122.0717 | 0.641 | 4.462 | 4.679 | 0.217 | 0.175 | 0.331 | 0.660 | Tentative | NA | NA |
| metab_9559 | Gamma-Glu-leu | neg | 259.1300 | 3.276 | 5.917 | 5.800 | -0.117 | -0.175 | 0.331 | 0.660 | Strong putative | NA | NA |
| metab_5704 | Unannotated feature | pos | 410.2911 | 6.111 | 4.357 | 4.117 | -0.240 | -0.175 | 0.331 | 0.660 | Unannotated | NA | NA |
| metab_19699 | Unannotated feature | neg | 269.0418 | 3.620 | 5.667 | 5.522 | -0.144 | -0.175 | 0.331 | 0.660 | Unannotated | NA | NA |
| metab_13413 | Quinolinic Acid | neg | 166.0141 | 0.791 | 4.138 | 4.508 | 0.370 | 0.175 | 0.331 | 0.660 | Moderate putative | Organoheterocyclic compounds | Pyridines and derivatives |
| metab_12168 | Unannotated feature | neg | 642.4061 | 6.656 | 5.865 | 5.401 | -0.463 | -0.175 | 0.331 | 0.660 | Unannotated | NA | NA |
| metab_15953 | Melatonin | neg | 523.2559 | 6.052 | 3.939 | 3.489 | -0.451 | -0.175 | 0.331 | 0.660 | Tentative | NA | NA |
| metab_8044 | Glucitol-lysine | pos | 275.1605 | 2.780 | 4.086 | 3.966 | -0.120 | -0.175 | 0.331 | 0.660 | Unannotated | NA | NA |
| metab_12497 | Unannotated feature | neg | 446.1414 | 6.373 | 0.083 | 6.084 | 6.001 | 0.175 | 0.331 | 0.660 | Unannotated | NA | NA |
| metab_16761 | Unannotated feature | neg | 529.8697 | 7.687 | 3.807 | 3.931 | 0.124 | 0.175 | 0.331 | 0.660 | Unannotated | NA | NA |
| metab_4976 | Unannotated feature | pos | 590.4279 | 7.538 | 5.079 | 5.118 | 0.039 | 0.171 | 0.343 | 0.671 | Unannotated | NA | NA |
| metab_17974 | Methyl n-acetylanthranilate | neg | 238.0720 | 2.880 | 5.290 | 5.491 | 0.201 | 0.171 | 0.343 | 0.671 | Tentative | NA | NA |
| metab_2124 | Unannotated feature | pos | 252.1599 | 2.835 | 3.603 | 3.860 | 0.256 | 0.171 | 0.343 | 0.671 | Unannotated | NA | NA |
| metab_5415 | Cholylvaline | pos | 472.3439 | 6.414 | 5.331 | 4.917 | -0.414 | -0.171 | 0.343 | 0.671 | Unannotated | NA | NA |
| metab_3373 | Unannotated feature | pos | 316.2235 | 4.314 | 4.188 | 3.793 | -0.395 | -0.171 | 0.343 | 0.671 | Unannotated | NA | NA |
| metab_1232 | Unannotated feature | pos | 330.1667 | 2.732 | 5.600 | 5.564 | -0.036 | -0.171 | 0.343 | 0.671 | Unannotated | NA | NA |
| metab_1228 | Unannotated feature | pos | 799.5744 | 6.390 | 6.161 | 5.807 | -0.354 | -0.171 | 0.343 | 0.671 | Unannotated | NA | NA |
| metab_10757 | Unannotated feature | neg | 199.0244 | 1.214 | 2.771 | 3.271 | 0.501 | 0.171 | 0.343 | 0.671 | Unannotated | NA | NA |
| metab_15220 | 3,5,5-Trimethyl-2-cyclohexen-1-one | neg | 183.1020 | 4.771 | 3.123 | 3.252 | 0.129 | 0.171 | 0.343 | 0.671 | Tentative | NA | NA |
| metab_18029 | Unannotated feature | neg | 383.2808 | 6.475 | 4.548 | 4.753 | 0.205 | 0.171 | 0.343 | 0.671 | Unannotated | NA | NA |
| metab_8736 | Unannotated feature | pos | 171.1494 | 0.760 | 4.040 | 3.954 | -0.086 | 0.171 | 0.343 | 0.671 | Unannotated | NA | NA |
| metab_12899 | Unannotated feature | neg | 205.0348 | 0.730 | 5.234 | 5.339 | 0.105 | 0.171 | 0.343 | 0.671 | Unannotated | NA | NA |
| metab_8001 | Unannotated feature | pos | 195.0656 | 2.840 | 4.602 | 4.652 | 0.049 | 0.171 | 0.343 | 0.671 | Unannotated | NA | NA |
| metab_6611 | PA(a-13:0/5-iso PGF2VI) | pos | 339.2056 | 5.434 | 3.604 | 3.574 | -0.031 | -0.171 | 0.343 | 0.671 | Unannotated | NA | NA |
| metab_6504 | LEUCOGENENOL | pos | 348.1450 | 5.583 | 4.569 | 4.098 | -0.470 | -0.171 | 0.343 | 0.671 | Unannotated | Organoheterocyclic compounds | Oxazines |
| metab_12092 | Unannotated feature | neg | 215.1648 | 6.467 | 5.048 | 5.189 | 0.141 | 0.171 | 0.343 | 0.671 | Unannotated | NA | NA |
| metab_10704 | Unannotated feature | neg | 262.0195 | 1.384 | 3.790 | 3.898 | 0.107 | 0.171 | 0.343 | 0.671 | Unannotated | NA | NA |
| metab_17145 | Unannotated feature | neg | 142.9955 | 7.785 | 4.204 | 4.122 | -0.083 | -0.171 | 0.343 | 0.671 | Unannotated | NA | NA |
| metab_16728 | Unannotated feature | neg | 282.0846 | 1.952 | 5.608 | 5.485 | -0.123 | -0.171 | 0.343 | 0.671 | Unannotated | NA | NA |
| metab_11494 | Unannotated feature | neg | 285.1135 | 5.233 | 4.544 | 4.066 | -0.478 | -0.171 | 0.343 | 0.671 | Unannotated | NA | NA |
| metab_13618 | L-Tyrosine | neg | 180.0660 | 1.526 | 6.694 | 6.546 | -0.148 | -0.171 | 0.343 | 0.671 | Strong putative | NA | NA |
| metab_2170 | Unannotated feature | pos | 361.1728 | 2.928 | 5.060 | 4.971 | -0.089 | -0.171 | 0.343 | 0.671 | Unannotated | NA | NA |
| metab_11666 | MJDBISSN00000001 | neg | 198.0323 | 3.210 | 6.918 | 6.818 | -0.100 | -0.171 | 0.343 | 0.671 | Strong putative | NA | NA |
| metab_17182 | Unannotated feature | neg | 308.1201 | 7.741 | 4.987 | 5.101 | 0.114 | 0.171 | 0.343 | 0.671 | Unannotated | NA | NA |
| metab_2161 | Unannotated feature | pos | 309.1814 | 2.907 | 4.391 | 4.778 | 0.387 | 0.171 | 0.343 | 0.671 | Unannotated | NA | NA |
| metab_17781 | Unannotated feature | neg | 96.9687 | 0.625 | 5.965 | 5.395 | -0.570 | -0.171 | 0.343 | 0.671 | Unannotated | NA | NA |
| metab_3471 | Unannotated feature | pos | 200.0962 | 7.724 | 6.158 | 6.184 | 0.027 | 0.171 | 0.343 | 0.671 | Unannotated | NA | NA |
| metab_1875 | Balenine | pos | 263.1105 | 1.217 | 3.532 | 3.561 | 0.028 | 0.171 | 0.343 | 0.671 | Unannotated | NA | NA |
| metab_12095 | Unannotated feature | neg | 378.2331 | 6.459 | 4.178 | 4.358 | 0.180 | 0.171 | 0.343 | 0.671 | Unannotated | NA | NA |
| metab_12065 | Unannotated feature | neg | 611.3811 | 6.397 | 4.479 | 4.602 | 0.123 | 0.171 | 0.343 | 0.671 | Unannotated | NA | NA |
| metab_8500 | Unannotated feature | pos | 180.1387 | 1.685 | 4.591 | 4.151 | -0.440 | -0.171 | 0.343 | 0.671 | Unannotated | NA | NA |
| metab_9083 | Unannotated feature | neg | 261.1342 | 3.948 | 3.958 | 4.470 | 0.513 | 0.171 | 0.343 | 0.671 | Unannotated | NA | NA |
| metab_1315 | Unannotated feature | pos | 288.1236 | 2.937 | 4.290 | 4.562 | 0.272 | 0.171 | 0.343 | 0.671 | Unannotated | NA | NA |
| metab_17191 | Unannotated feature | neg | 524.8504 | 7.737 | 4.599 | 4.635 | 0.036 | 0.171 | 0.343 | 0.671 | Unannotated | NA | NA |
| metab_8108 | Demethoxycurcumin | pos | 371.1459 | 2.732 | 5.368 | 5.054 | -0.314 | -0.171 | 0.343 | 0.671 | Tentative | NA | NA |
| metab_11245 | Unannotated feature | neg | 80.9739 | 0.636 | 4.628 | 4.564 | -0.064 | -0.171 | 0.343 | 0.671 | Unannotated | NA | NA |
| metab_8565 | Dihydrouracil | pos | 147.0766 | 1.239 | 4.077 | 3.976 | -0.102 | -0.171 | 0.343 | 0.671 | Tentative | NA | NA |
| metab_17653 | Unannotated feature | neg | 134.8939 | 7.080 | 4.907 | 4.917 | 0.010 | -0.171 | 0.343 | 0.671 | Unannotated | NA | NA |
| metab_887 | Unannotated feature | pos | 519.3665 | 6.893 | 6.181 | 6.200 | 0.019 | 0.171 | 0.343 | 0.671 | Unannotated | NA | NA |
| metab_4745 | Unannotated feature | pos | 288.9226 | 7.700 | 5.215 | 5.237 | 0.021 | 0.171 | 0.343 | 0.671 | Unannotated | NA | NA |
| metab_14772 | Peperinic acid | neg | 227.0923 | 3.837 | 4.196 | 4.326 | 0.130 | 0.171 | 0.343 | 0.671 | Unannotated | NA | NA |
| metab_9628 | Unannotated feature | neg | 181.0057 | 3.210 | 4.442 | 4.428 | -0.014 | -0.171 | 0.343 | 0.671 | Unannotated | NA | NA |
| metab_17202 | Unannotated feature | neg | 234.9117 | 7.737 | 4.374 | 4.333 | -0.040 | -0.171 | 0.343 | 0.671 | Unannotated | NA | NA |
| metab_13722 | N5-(3,4-Dioxo-1,5-cyclohexadien-1-yl)-L-glutamine | neg | 287.0461 | 1.919 | 3.702 | 3.660 | -0.041 | 0.171 | 0.343 | 0.671 | Unannotated | NA | NA |
| metab_9711 | (2-oxo-2,3-Dihydro-1H-Indol-3-yl)acetic Acid | neg | 190.0504 | 4.246 | 6.421 | 6.489 | 0.067 | 0.171 | 0.343 | 0.671 | Strong putative | NA | NA |
| metab_14122 | Unannotated feature | neg | 187.9311 | 2.858 | 3.961 | 3.650 | -0.311 | -0.171 | 0.343 | 0.671 | Unannotated | NA | NA |
| metab_17219 | Unannotated feature | neg | 308.1378 | 7.730 | 5.956 | 6.050 | 0.094 | 0.171 | 0.343 | 0.671 | Unannotated | NA | NA |
| metab_17074 | Unannotated feature | neg | 211.8305 | 7.910 | 4.887 | 4.865 | -0.022 | -0.171 | 0.343 | 0.671 | Unannotated | NA | NA |
| metab_19721 | Unannotated feature | neg | 223.0140 | 4.527 | 4.233 | 3.714 | -0.519 | -0.171 | 0.343 | 0.671 | Unannotated | NA | NA |
| metab_12165 | Unannotated feature | neg | 432.2794 | 6.695 | 5.483 | 5.608 | 0.125 | 0.171 | 0.343 | 0.671 | Unannotated | NA | NA |
| metab_8085 | Asparaginyl-Lysine | pos | 274.1583 | 2.747 | 3.618 | 3.404 | -0.213 | -0.171 | 0.343 | 0.671 | Unannotated | NA | NA |
| metab_6061 | Unannotated feature | pos | 146.1655 | 0.509 | 6.125 | 6.140 | 0.014 | -0.171 | 0.343 | 0.671 | Unannotated | NA | NA |
| metab_10187 | Unannotated feature | neg | 228.0362 | 2.663 | 5.271 | 5.355 | 0.083 | 0.171 | 0.343 | 0.671 | Unannotated | NA | NA |
| metab_12113 | Unannotated feature | neg | 535.3107 | 6.530 | 7.165 | 7.287 | 0.123 | 0.171 | 0.343 | 0.671 | Unannotated | NA | NA |
| metab_16485 | 12-hydroxyicosanoic acid | neg | 327.2907 | 7.068 | 5.701 | 5.560 | -0.140 | -0.171 | 0.343 | 0.671 | Unannotated | NA | NA |
| metab_15832 | Unannotated feature | neg | 543.2821 | 5.905 | 2.744 | 3.078 | 0.335 | 0.171 | 0.343 | 0.671 | Unannotated | NA | NA |
| metab_17172 | Unannotated feature | neg | 198.9219 | 7.745 | 4.425 | 4.386 | -0.039 | -0.171 | 0.343 | 0.671 | Unannotated | NA | NA |
| metab_12135 | Unannotated feature | neg | 570.3650 | 6.601 | 5.524 | 4.758 | -0.766 | -0.171 | 0.343 | 0.671 | Unannotated | NA | NA |
| metab_1287 | Lauryldiethanolamine | pos | 274.2745 | 6.271 | 7.120 | 7.070 | -0.050 | -0.171 | 0.343 | 0.671 | Strong putative | NA | NA |
| metab_1307 | 3-ketosphingosine | pos | 298.2745 | 6.239 | 5.544 | 5.200 | -0.344 | -0.171 | 0.343 | 0.671 | Moderate putative | NA | NA |
| metab_8638 | 6-Aminonicotinamide | pos | 275.1243 | 1.034 | 4.858 | 4.950 | 0.093 | 0.171 | 0.343 | 0.671 | Unannotated | NA | NA |
| metab_14367 | 2-Amino-3-cyclohexylpropanoic acid | neg | 216.1238 | 3.144 | 5.064 | 4.987 | -0.077 | -0.171 | 0.343 | 0.671 | Unannotated | Organic acids and derivatives | Carboxylic acids and derivatives |
| metab_1164 | Unannotated feature | pos | 527.3360 | 6.526 | 5.869 | 6.184 | 0.314 | 0.171 | 0.343 | 0.671 | Unannotated | NA | NA |
| metab_3198 | Indoleacrylic acid | pos | 229.0976 | 3.720 | 4.820 | 4.607 | -0.212 | -0.171 | 0.343 | 0.671 | Unannotated | NA | NA |
| metab_1358 | Unannotated feature | pos | 407.2802 | 6.143 | 6.021 | 5.697 | -0.324 | -0.171 | 0.343 | 0.671 | Unannotated | NA | NA |
| metab_16290 | Unannotated feature | neg | 409.3440 | 6.601 | 5.001 | 5.022 | 0.021 | -0.171 | 0.343 | 0.671 | Unannotated | NA | NA |
| metab_16331 | Unannotated feature | neg | 296.9733 | 1.404 | 4.856 | 4.899 | 0.044 | 0.171 | 0.343 | 0.671 | Unannotated | NA | NA |
| metab_7561 | Trandolapril-d5 Diketopiperazine | pos | 430.2662 | 3.634 | 4.181 | 4.437 | 0.256 | 0.171 | 0.343 | 0.671 | Unannotated | NA | NA |
| metab_9193 | L-phenylalanyl-L-proline | neg | 261.1248 | 3.777 | 6.084 | 5.981 | -0.103 | -0.171 | 0.343 | 0.671 | Unannotated | NA | NA |
| metab_10525 | Unannotated feature | neg | 216.0334 | 1.947 | 4.634 | 4.393 | -0.240 | -0.171 | 0.343 | 0.671 | Unannotated | NA | NA |
| metab_1736 | Coixinden B | pos | 252.1235 | 3.734 | 4.437 | 4.560 | 0.123 | 0.171 | 0.343 | 0.671 | Tentative | NA | NA |
| metab_14696 | Unannotated feature | neg | 426.1415 | 3.693 | 3.655 | 4.090 | 0.435 | 0.171 | 0.343 | 0.671 | Unannotated | NA | NA |
| metab_16391 | AB-Chminaca | neg | 393.1723 | 6.821 | 6.037 | 6.183 | 0.146 | 0.171 | 0.343 | 0.671 | Unannotated | NA | NA |
| metab_17348 | Unannotated feature | neg | 336.9420 | 7.692 | 4.734 | 4.743 | 0.009 | 0.171 | 0.343 | 0.671 | Unannotated | NA | NA |
| metab_16443 | Unannotated feature | neg | 577.3756 | 6.959 | 5.347 | 5.077 | -0.270 | -0.171 | 0.343 | 0.671 | Unannotated | NA | NA |
| metab_16393 | Unannotated feature | neg | 183.0111 | 6.836 | 5.301 | 5.277 | -0.024 | -0.171 | 0.343 | 0.671 | Unannotated | NA | NA |
| metab_5647 | 2-Pentyl-3-phenyl-2-propenal | pos | 443.2341 | 6.167 | 4.487 | 4.057 | -0.430 | -0.171 | 0.343 | 0.671 | Tentative | NA | NA |
| metab_15444 | Unannotated feature | neg | 379.1766 | 5.156 | 3.886 | 3.895 | 0.009 | 0.171 | 0.343 | 0.671 | Unannotated | NA | NA |
| metab_2502 | Cyclo(his-pro) | pos | 235.1192 | 0.784 | 4.174 | 3.626 | -0.548 | -0.171 | 0.343 | 0.671 | Unannotated | NA | NA |
| metab_12327 | Unannotated feature | neg | 777.5518 | 7.362 | 5.016 | 4.558 | -0.458 | -0.171 | 0.343 | 0.671 | Unannotated | NA | NA |
| metab_2866 | Unannotated feature | pos | 146.1178 | 2.795 | 4.517 | 4.249 | -0.268 | -0.171 | 0.343 | 0.671 | Unannotated | NA | NA |
| metab_8421 | 5-Methyl-3(2H)-furanone | pos | 81.0340 | 1.952 | 3.975 | 3.955 | -0.020 | -0.171 | 0.343 | 0.671 | Unannotated | NA | NA |
| metab_9345 | Unannotated feature | neg | 108.0210 | 3.556 | 2.940 | 3.503 | 0.562 | 0.171 | 0.343 | 0.671 | Unannotated | NA | NA |
| metab_14638 | Unannotated feature | neg | 660.8209 | 0.520 | 4.610 | 4.815 | 0.204 | 0.171 | 0.343 | 0.671 | Unannotated | NA | NA |
| metab_12437 | Unannotated feature | neg | 262.9119 | 7.726 | 5.484 | 5.459 | -0.024 | -0.171 | 0.343 | 0.671 | Unannotated | NA | NA |
| metab_7688 | Gibberellin A24 | pos | 311.1608 | 3.396 | 3.830 | 4.192 | 0.362 | 0.171 | 0.343 | 0.671 | Tentative | NA | NA |
| metab_2603 | Toluene | pos | 93.0704 | 1.642 | 4.048 | 3.968 | -0.080 | -0.171 | 0.343 | 0.671 | Tentative | NA | NA |
| metab_11105 | Unannotated feature | neg | 234.0440 | 0.722 | 4.587 | 4.618 | 0.031 | -0.171 | 0.343 | 0.671 | Unannotated | NA | NA |
| metab_11925 | Unannotated feature | neg | 581.2801 | 6.099 | 6.294 | 6.464 | 0.171 | 0.171 | 0.343 | 0.671 | Unannotated | NA | NA |
| metab_11945 | Unannotated feature | neg | 549.3282 | 6.130 | 3.661 | 3.642 | -0.019 | -0.171 | 0.343 | 0.671 | Unannotated | NA | NA |
| metab_6556 | Unannotated feature | pos | 266.8424 | 0.601 | 4.819 | 4.620 | -0.200 | -0.171 | 0.343 | 0.671 | Unannotated | NA | NA |
| metab_11997 | Unannotated feature | neg | 483.2967 | 6.232 | 6.884 | 6.814 | -0.070 | -0.171 | 0.343 | 0.671 | Unannotated | NA | NA |
| metab_10037 | Unannotated feature | neg | 345.1006 | 4.439 | 4.960 | 5.178 | 0.218 | 0.171 | 0.343 | 0.671 | Unannotated | NA | NA |
| metab_12000 | Unannotated feature | neg | 551.3055 | 6.240 | 6.723 | 6.804 | 0.081 | 0.171 | 0.343 | 0.671 | Unannotated | NA | NA |
| metab_1966 | Unannotated feature | pos | 290.1393 | 1.994 | 4.292 | 4.387 | 0.096 | 0.171 | 0.343 | 0.671 | Unannotated | NA | NA |
| metab_17943 | Unannotated feature | neg | 323.2592 | 6.601 | 4.760 | 4.784 | 0.024 | -0.171 | 0.343 | 0.671 | Unannotated | NA | NA |
| metab_1035 | Unannotated feature | pos | 555.4033 | 6.885 | 3.301 | 4.003 | 0.702 | 0.171 | 0.343 | 0.671 | Unannotated | NA | NA |
| metab_16996 | Unannotated feature | neg | 283.9142 | 7.985 | 5.038 | 5.010 | -0.027 | -0.171 | 0.343 | 0.671 | Unannotated | NA | NA |
| metab_8391 | Unannotated feature | pos | 229.0824 | 1.952 | 4.337 | 4.277 | -0.060 | -0.171 | 0.343 | 0.671 | Unannotated | NA | NA |
| metab_14538 | Phlorizin | neg | 435.1295 | 3.414 | 4.160 | 3.613 | -0.546 | -0.171 | 0.343 | 0.671 | Tentative | NA | NA |
| metab_2466 | Unannotated feature | pos | 199.0483 | 0.688 | 4.123 | 3.735 | -0.388 | -0.171 | 0.343 | 0.671 | Unannotated | NA | NA |
| metab_15450 | Unannotated feature | neg | 426.1890 | 5.169 | 4.510 | 4.411 | -0.099 | -0.171 | 0.343 | 0.671 | Unannotated | NA | NA |
| metab_922 | Enantio-PAF C-16 | pos | 546.3546 | 7.252 | 5.104 | 4.488 | -0.616 | -0.171 | 0.343 | 0.671 | Moderate putative | NA | NA |
| metab_12310 | Unannotated feature | neg | 207.0658 | 6.899 | 6.290 | 6.296 | 0.006 | -0.171 | 0.343 | 0.671 | Unannotated | NA | NA |
| metab_10979 | L-rhamnofuranose | neg | 163.0605 | 0.784 | 5.357 | 5.101 | -0.255 | -0.171 | 0.343 | 0.671 | Tentative | NA | NA |
| metab_12771 | Unannotated feature | neg | 110.8983 | 0.625 | 5.233 | 5.110 | -0.123 | -0.171 | 0.343 | 0.671 | Unannotated | NA | NA |
| metab_8183 | Unannotated feature | pos | 91.0547 | 2.590 | 4.864 | 4.767 | -0.097 | -0.171 | 0.343 | 0.671 | Unannotated | NA | NA |
| metab_17246 | Unannotated feature | neg | 434.8726 | 7.726 | 4.381 | 4.449 | 0.068 | 0.171 | 0.343 | 0.671 | Unannotated | NA | NA |
| metab_13092 | Unannotated feature | neg | 226.8237 | 0.036 | 5.889 | 5.871 | -0.018 | -0.171 | 0.343 | 0.671 | Unannotated | NA | NA |
| metab_5346 | Unannotated feature | pos | 383.2050 | 6.526 | 5.392 | 5.309 | -0.083 | -0.171 | 0.343 | 0.671 | Unannotated | NA | NA |
| metab_12425 | Unannotated feature | neg | 148.9383 | 7.730 | 4.817 | 4.806 | -0.012 | -0.171 | 0.343 | 0.671 | Unannotated | NA | NA |
| metab_13771 | Unannotated feature | neg | 205.8393 | 0.443 | 6.245 | 6.222 | -0.023 | -0.171 | 0.343 | 0.671 | Unannotated | NA | NA |
| metab_17262 | Unannotated feature | neg | 187.9413 | 7.726 | 4.728 | 4.717 | -0.011 | -0.171 | 0.343 | 0.671 | Unannotated | NA | NA |
| metab_7126 | Unannotated feature | pos | 262.1038 | 0.665 | 5.071 | 4.845 | -0.226 | -0.171 | 0.343 | 0.671 | Unannotated | NA | NA |
| metab_11638 | Unannotated feature | neg | 196.9203 | 0.073 | 6.301 | 6.277 | -0.023 | -0.166 | 0.355 | 0.683 | Unannotated | NA | NA |
| metab_5387 | Unannotated feature | pos | 359.1458 | 1.952 | 4.445 | 4.831 | 0.386 | 0.166 | 0.355 | 0.683 | Unannotated | NA | NA |
| metab_17164 | Unannotated feature | neg | 204.9565 | 7.760 | 4.933 | 4.906 | -0.028 | -0.166 | 0.355 | 0.683 | Unannotated | NA | NA |
| metab_1272 | Unannotated feature | pos | 412.3067 | 6.303 | 5.558 | 5.436 | -0.123 | -0.166 | 0.355 | 0.683 | Unannotated | NA | NA |
| metab_4799 | Unannotated feature | pos | 268.1203 | 7.991 | 5.115 | 5.164 | 0.049 | 0.166 | 0.355 | 0.683 | Unannotated | NA | NA |
| metab_11474 | Unannotated feature | neg | 102.9879 | 0.524 | 5.315 | 5.290 | -0.025 | -0.166 | 0.355 | 0.683 | Unannotated | NA | NA |
| metab_15743 | 7,4'-Dihydroxyflavone | neg | 253.0506 | 5.758 | 5.043 | 4.780 | -0.264 | -0.166 | 0.355 | 0.683 | Tentative | NA | NA |
| metab_8753 | Unannotated feature | pos | 232.1295 | 0.728 | 4.084 | 3.662 | -0.422 | -0.166 | 0.355 | 0.683 | Unannotated | NA | NA |
| metab_17121 | Unannotated feature | neg | 224.8033 | 7.827 | 4.753 | 4.739 | -0.014 | -0.166 | 0.355 | 0.683 | Unannotated | NA | NA |
| metab_8731 | 2-Hydroxy-3-(1H-imidazol-5-yl)propanoic acid | pos | 121.0401 | 0.768 | 4.080 | 4.153 | 0.073 | -0.166 | 0.355 | 0.683 | Tentative | NA | NA |
| metab_13392 | Unannotated feature | neg | 333.8646 | 0.628 | 4.736 | 4.623 | -0.113 | -0.166 | 0.355 | 0.683 | Unannotated | NA | NA |
| metab_13380 | Cellulose, microcrystalline | neg | 415.1467 | 0.738 | 4.207 | 4.255 | 0.048 | 0.166 | 0.355 | 0.683 | Unannotated | NA | NA |
| metab_19125 | Unannotated feature | neg | 337.1772 | 5.351 | 3.697 | 4.639 | 0.941 | 0.166 | 0.355 | 0.683 | Unannotated | NA | NA |
| metab_5351 | Ginsenoside F1 | pos | 661.4302 | 6.518 | 5.063 | 4.738 | -0.324 | -0.166 | 0.355 | 0.683 | Tentative | NA | NA |
| metab_15272 | Unannotated feature | neg | 327.8947 | 4.833 | 4.802 | 4.646 | -0.156 | -0.166 | 0.355 | 0.683 | Unannotated | NA | NA |
| metab_10944 | L-2-Amino-4-methylenepentanedioic acid | neg | 158.0452 | 0.799 | 5.320 | 5.304 | -0.017 | -0.166 | 0.355 | 0.683 | Tentative | NA | NA |
| metab_661 | Unannotated feature | pos | 663.3047 | 5.854 | 5.162 | 5.547 | 0.386 | 0.166 | 0.355 | 0.683 | Unannotated | NA | NA |
| metab_1107 | Unannotated feature | pos | 472.4373 | 6.654 | 6.291 | 6.210 | -0.081 | -0.166 | 0.355 | 0.683 | Unannotated | NA | NA |
| metab_1789 | Unannotated feature | pos | 203.1394 | 0.776 | 5.510 | 5.377 | -0.133 | -0.166 | 0.355 | 0.683 | Unannotated | NA | NA |
| metab_7569 | 3,5-Dimethyl-3'-isopropyl-L-thyronine | pos | 326.1718 | 3.618 | 4.699 | 4.811 | 0.112 | -0.166 | 0.355 | 0.683 | Unannotated | Organic acids and derivatives | Carboxylic acids and derivatives |
| metab_3785 | Ameltolide | pos | 273.1603 | 5.532 | 4.298 | 4.045 | -0.254 | -0.166 | 0.355 | 0.683 | Tentative | Benzenoids | Benzene and substituted derivatives |
| metab_457 | Unannotated feature | pos | 200.0478 | 3.179 | 7.959 | 7.999 | 0.040 | 0.166 | 0.355 | 0.683 | Unannotated | NA | NA |
| metab_4724 | Unannotated feature | pos | 639.4972 | 7.642 | 4.179 | 4.042 | -0.137 | -0.166 | 0.355 | 0.683 | Unannotated | NA | NA |
| metab_11243 | Taurine | neg | 124.0067 | 0.636 | 5.007 | 4.439 | -0.568 | -0.166 | 0.355 | 0.683 | Strong putative | NA | NA |
| metab_17184 | Unannotated feature | neg | 279.9021 | 7.741 | 4.703 | 4.659 | -0.044 | -0.166 | 0.355 | 0.683 | Unannotated | NA | NA |
| metab_8972 | BEFLOXATONE | neg | 394.1148 | 4.123 | 4.481 | 4.887 | 0.407 | 0.166 | 0.355 | 0.683 | Unannotated | NA | NA |
| metab_2324 | Linatine | pos | 242.1111 | 0.348 | 5.249 | 5.268 | 0.019 | 0.166 | 0.355 | 0.683 | Tentative | NA | NA |
| metab_1811 | Unannotated feature | pos | 166.0726 | 0.776 | 4.928 | 4.898 | -0.030 | 0.166 | 0.355 | 0.683 | Unannotated | NA | NA |
| metab_1148 | Unannotated feature | pos | 829.5591 | 6.558 | 6.669 | 6.284 | -0.384 | -0.166 | 0.355 | 0.683 | Unannotated | NA | NA |
| metab_8577 | Unannotated feature | pos | 347.1246 | 4.022 | 5.949 | 5.834 | -0.115 | -0.166 | 0.355 | 0.683 | Unannotated | NA | NA |
| metab_9768 | Unannotated feature | neg | 155.0707 | 3.067 | 3.403 | 3.565 | 0.162 | 0.166 | 0.355 | 0.683 | Unannotated | NA | NA |
| metab_9692 | Unannotated feature | neg | 350.0312 | 3.131 | 4.609 | 4.453 | -0.156 | -0.166 | 0.355 | 0.683 | Unannotated | NA | NA |
| metab_17732 | Unannotated feature | neg | 543.1633 | 6.946 | 4.392 | 4.865 | 0.472 | 0.166 | 0.355 | 0.683 | Unannotated | NA | NA |
| metab_18115 | Unannotated feature | neg | 361.2055 | 6.381 | 5.011 | 4.564 | -0.447 | -0.166 | 0.355 | 0.683 | Unannotated | NA | NA |
| metab_15200 | Unannotated feature | neg | 211.1132 | 4.730 | 4.657 | 4.609 | -0.048 | -0.166 | 0.355 | 0.683 | Unannotated | NA | NA |
| metab_1865 | Unannotated feature | pos | 359.1655 | 1.169 | 4.197 | 4.045 | -0.152 | -0.166 | 0.355 | 0.683 | Unannotated | NA | NA |
| metab_14175 | Unannotated feature | neg | 410.1579 | 2.910 | 3.779 | 3.556 | -0.223 | -0.166 | 0.355 | 0.683 | Unannotated | NA | NA |
| metab_3576 | Unannotated feature | pos | 561.4146 | 6.710 | 6.349 | 6.464 | 0.114 | 0.166 | 0.355 | 0.683 | Unannotated | NA | NA |
| metab_3723 | Unannotated feature | pos | 84.3058 | 5.369 | 3.152 | 3.048 | -0.104 | -0.166 | 0.355 | 0.683 | Unannotated | NA | NA |
| metab_18634 | Unannotated feature | neg | 443.1753 | 5.952 | 5.917 | 5.935 | 0.018 | 0.166 | 0.355 | 0.683 | Unannotated | NA | NA |
| metab_6327 | N5-Acetyl-N2-gamma-L-glutamyl-L-ornithine | pos | 607.2932 | 5.778 | 3.318 | 2.679 | -0.638 | -0.166 | 0.355 | 0.683 | Unannotated | NA | NA |
| metab_13622 | Unannotated feature | neg | 310.0251 | 1.526 | 5.395 | 5.246 | -0.149 | -0.166 | 0.355 | 0.683 | Unannotated | NA | NA |
| metab_15252 | Unannotated feature | neg | 276.0337 | 4.810 | 3.438 | 3.640 | 0.202 | 0.166 | 0.355 | 0.683 | Unannotated | NA | NA |
| metab_8929 | Unannotated feature | neg | 214.1082 | 4.220 | 4.205 | 4.049 | -0.156 | -0.166 | 0.355 | 0.683 | Unannotated | NA | NA |
| metab_17830 | Unannotated feature | neg | 378.3013 | 6.782 | 4.907 | 5.397 | 0.490 | 0.166 | 0.355 | 0.683 | Unannotated | NA | NA |
| metab_2095 | Unannotated feature | pos | 240.1235 | 2.747 | 4.940 | 5.162 | 0.222 | 0.166 | 0.355 | 0.683 | Unannotated | NA | NA |
| metab_8737 | Adenine | pos | 136.0620 | 0.760 | 4.581 | 4.691 | 0.110 | 0.166 | 0.355 | 0.683 | Strong putative | NA | NA |
| metab_12900 | Unannotated feature | neg | 216.0874 | 0.730 | 4.675 | 4.823 | 0.148 | 0.166 | 0.355 | 0.683 | Unannotated | NA | NA |
| metab_8794 | Alpha-Muricholic Acid | pos | 373.2745 | 6.398 | 7.567 | 7.424 | -0.143 | -0.166 | 0.355 | 0.683 | Moderate putative | NA | NA |
| metab_16249 | Unannotated feature | neg | 453.3226 | 6.522 | 6.069 | 6.405 | 0.336 | 0.166 | 0.355 | 0.683 | Unannotated | NA | NA |
| metab_13613 | Unannotated feature | neg | 436.0850 | 1.489 | 5.338 | 5.129 | -0.209 | -0.166 | 0.355 | 0.683 | Unannotated | NA | NA |
| metab_1930 | Unannotated feature | pos | 216.0982 | 0.728 | 4.960 | 4.749 | -0.211 | -0.166 | 0.355 | 0.683 | Unannotated | NA | NA |
| metab_17997 | Unannotated feature | neg | 328.2493 | 6.522 | 4.761 | 4.737 | -0.024 | -0.166 | 0.355 | 0.683 | Unannotated | NA | NA |
| metab_17810 | DG(15:0/14:1(9Z)/0:0) | neg | 545.4222 | 6.836 | 5.791 | 5.358 | -0.432 | -0.166 | 0.355 | 0.683 | Tentative | NA | NA |
| metab_10004 | 3,4-dihydroxyphenylacetic Acid | neg | 167.0344 | 2.841 | 5.419 | 5.647 | 0.228 | 0.166 | 0.355 | 0.683 | Strong putative | NA | NA |
| metab_7795 | Unannotated feature | pos | 102.0469 | 3.185 | 3.745 | 3.675 | -0.070 | -0.166 | 0.355 | 0.683 | Unannotated | NA | NA |
| metab_16642 | Unannotated feature | neg | 714.5015 | 7.549 | 5.358 | 5.185 | -0.174 | -0.166 | 0.355 | 0.683 | Unannotated | NA | NA |
| metab_1944 | Unannotated feature | pos | 216.1598 | 1.952 | 3.355 | 3.785 | 0.429 | 0.166 | 0.355 | 0.683 | Unannotated | NA | NA |
| metab_11951 | Unannotated feature | neg | 329.1035 | 5.435 | 6.020 | 5.660 | -0.360 | -0.166 | 0.355 | 0.683 | Unannotated | NA | NA |
| metab_1923 | Unannotated feature | pos | 257.1500 | 0.728 | 4.640 | 4.916 | 0.277 | 0.166 | 0.355 | 0.683 | Unannotated | NA | NA |
| metab_19640 | Valproic acid glucuronide | neg | 319.1387 | 4.653 | 4.475 | 4.535 | 0.060 | 0.166 | 0.355 | 0.683 | Unannotated | NA | NA |
| metab_476 | Unannotated feature | pos | 598.3737 | 4.931 | 4.733 | 4.913 | 0.179 | 0.166 | 0.355 | 0.683 | Unannotated | NA | NA |
| metab_9313 | Strigol | neg | 391.1383 | 3.589 | 4.621 | 4.472 | -0.149 | -0.166 | 0.355 | 0.683 | Tentative | Lipids and lipid-like molecules | Prenol lipids |
| metab_16839 | Unannotated feature | neg | 656.8090 | 7.726 | 4.299 | 4.406 | 0.107 | 0.166 | 0.355 | 0.683 | Unannotated | NA | NA |
| metab_944 | Unannotated feature | pos | 783.2346 | 7.538 | 5.145 | 5.277 | 0.132 | 0.166 | 0.355 | 0.683 | Unannotated | NA | NA |
| metab_15105 | Unannotated feature | neg | 230.1397 | 4.533 | 5.358 | 5.372 | 0.014 | -0.166 | 0.355 | 0.683 | Unannotated | NA | NA |
| metab_14496 | Unannotated feature | neg | 141.0550 | 3.342 | 3.658 | 3.561 | -0.097 | -0.166 | 0.355 | 0.683 | Unannotated | NA | NA |
| metab_16356 | Unannotated feature | neg | 378.2321 | 6.726 | 4.221 | 3.692 | -0.529 | -0.166 | 0.355 | 0.683 | Unannotated | NA | NA |
| metab_10563 | Unannotated feature | neg | 180.0521 | 1.843 | 5.435 | 5.640 | 0.205 | 0.166 | 0.355 | 0.683 | Unannotated | NA | NA |
| metab_5489 | Tetradecanoylcarnitine | pos | 372.3116 | 6.359 | 4.223 | 4.197 | -0.026 | -0.166 | 0.355 | 0.683 | Unannotated | NA | NA |
| metab_9606 | Visnagin | neg | 275.0565 | 3.223 | 4.043 | 3.834 | -0.209 | -0.166 | 0.355 | 0.683 | Tentative | NA | NA |
| metab_2377 | Unannotated feature | pos | 312.8356 | 0.537 | 3.991 | 3.648 | -0.343 | -0.166 | 0.355 | 0.683 | Unannotated | NA | NA |
| metab_7038 | Unannotated feature | pos | 518.3190 | 4.774 | 3.651 | 4.176 | 0.525 | 0.166 | 0.355 | 0.683 | Unannotated | NA | NA |
| metab_7016 | Unannotated feature | pos | 198.0531 | 0.657 | 3.965 | 4.208 | 0.242 | 0.166 | 0.355 | 0.683 | Unannotated | NA | NA |
| metab_11532 | Unannotated feature | neg | 670.8677 | 0.496 | 4.440 | 3.946 | -0.494 | -0.166 | 0.355 | 0.683 | Unannotated | NA | NA |
| metab_16813 | Unannotated feature | neg | 242.9436 | 7.720 | 6.546 | 6.549 | 0.002 | -0.166 | 0.355 | 0.683 | Unannotated | NA | NA |
| metab_12428 | Unannotated feature | neg | 276.9240 | 7.730 | 5.641 | 5.616 | -0.025 | -0.166 | 0.355 | 0.683 | Unannotated | NA | NA |
| metab_12356 | Unannotated feature | neg | 467.2788 | 6.797 | 6.718 | 6.640 | -0.078 | -0.166 | 0.355 | 0.683 | Unannotated | NA | NA |
| metab_16424 | Unannotated feature | neg | 283.2642 | 6.915 | 4.801 | 4.325 | -0.475 | -0.166 | 0.355 | 0.683 | Unannotated | NA | NA |
| metab_19526 | (E)-2-Methyl-2-buten-1-ol O-beta-D-Glucopyranoside | neg | 247.1187 | 4.818 | 5.870 | 5.783 | -0.087 | -0.166 | 0.355 | 0.683 | Unannotated | Lipids and lipid-like molecules | Fatty Acyls |
| metab_7632 | L-arginino-succinate | pos | 307.1505 | 0.736 | 5.256 | 4.988 | -0.268 | -0.166 | 0.355 | 0.683 | Unannotated | NA | NA |
| metab_16435 | Unannotated feature | neg | 424.1783 | 6.946 | 4.872 | 5.051 | 0.179 | 0.166 | 0.355 | 0.683 | Unannotated | NA | NA |
| metab_18773 | Unannotated feature | neg | 303.0632 | 3.173 | 4.675 | 4.607 | -0.068 | -0.166 | 0.355 | 0.683 | Unannotated | NA | NA |
| metab_1040 | Unannotated feature | pos | 308.2952 | 6.877 | 4.690 | 4.630 | -0.060 | -0.166 | 0.355 | 0.683 | Unannotated | NA | NA |
| metab_9528 | Beta-D-Glucopyranosyl anthranilate | neg | 280.0829 | 3.314 | 4.516 | 4.422 | -0.094 | -0.166 | 0.355 | 0.683 | Unannotated | NA | NA |
| metab_2524 | Unannotated feature | pos | 131.9746 | 0.910 | 3.336 | 3.511 | 0.175 | 0.166 | 0.355 | 0.683 | Unannotated | NA | NA |
| metab_2785 | Trans-1,2-Dihydrobenzene-1,2-diol | pos | 95.0497 | 2.560 | 5.361 | 5.263 | -0.098 | -0.166 | 0.355 | 0.683 | Tentative | NA | NA |
| metab_16168 | Succinic acid, sulfo-, 1,4-dioctyl ester | neg | 403.2156 | 6.373 | 4.983 | 5.168 | 0.185 | 0.166 | 0.355 | 0.683 | Unannotated | NA | NA |
| metab_11170 | Unannotated feature | neg | 317.0643 | 0.667 | 5.362 | 4.977 | -0.385 | -0.166 | 0.355 | 0.683 | Unannotated | NA | NA |
| metab_1066 | Unannotated feature | pos | 376.3193 | 6.741 | 5.485 | 5.634 | 0.149 | 0.166 | 0.355 | 0.683 | Unannotated | NA | NA |
| metab_2793 | Unannotated feature | pos | 102.0469 | 2.614 | 4.298 | 4.210 | -0.088 | -0.166 | 0.355 | 0.683 | Unannotated | NA | NA |
| metab_2800 | Unannotated feature | pos | 381.1887 | 6.024 | 4.779 | 4.227 | -0.552 | -0.166 | 0.355 | 0.683 | Unannotated | NA | NA |
| metab_10475 | Unannotated feature | neg | 223.0609 | 3.303 | 5.391 | 5.311 | -0.081 | -0.166 | 0.355 | 0.683 | Unannotated | NA | NA |
| metab_11101 | Unannotated feature | neg | 291.0675 | 0.722 | 4.767 | 5.043 | 0.276 | 0.166 | 0.355 | 0.683 | Unannotated | NA | NA |
| metab_15019 | Unannotated feature | neg | 328.2246 | 4.378 | 5.214 | 5.138 | -0.077 | -0.166 | 0.355 | 0.683 | Unannotated | NA | NA |
| metab_2648 | Unannotated feature | pos | 342.1661 | 1.936 | 3.976 | 3.911 | -0.065 | -0.166 | 0.355 | 0.683 | Unannotated | NA | NA |
| metab_1636 | Unannotated feature | pos | 136.0759 | 4.733 | 5.456 | 5.414 | -0.041 | -0.166 | 0.355 | 0.683 | Unannotated | NA | NA |
| metab_2573 | Unannotated feature | pos | 758.2238 | 1.211 | 5.390 | 5.478 | 0.088 | 0.166 | 0.355 | 0.683 | Unannotated | NA | NA |
| metab_11122 | Unannotated feature | neg | 252.0990 | 0.714 | 4.030 | 4.263 | 0.233 | 0.166 | 0.355 | 0.683 | Unannotated | NA | NA |
| metab_8292 | Mycotoxin T 2 | pos | 383.1822 | 3.734 | 4.635 | 4.883 | 0.248 | 0.166 | 0.355 | 0.683 | Unannotated | NA | NA |
| metab_18340 | Unannotated feature | neg | 453.2331 | 6.177 | 6.069 | 6.332 | 0.263 | 0.166 | 0.355 | 0.683 | Unannotated | NA | NA |
| metab_9363 | Unannotated feature | neg | 317.1259 | 3.525 | 3.761 | 3.385 | -0.376 | -0.166 | 0.355 | 0.683 | Unannotated | NA | NA |
| metab_798 | Unannotated feature | pos | 815.5689 | 6.263 | 4.861 | 4.733 | -0.128 | -0.166 | 0.355 | 0.683 | Unannotated | NA | NA |
| metab_16664 | Unannotated feature | neg | 343.1147 | 1.947 | 4.594 | 4.811 | 0.217 | 0.166 | 0.355 | 0.683 | Unannotated | NA | NA |
| metab_17902 | LysoPE(16:0/0:0) | neg | 434.2687 | 6.663 | 5.407 | 5.157 | -0.250 | -0.166 | 0.355 | 0.683 | Unannotated | NA | NA |
| metab_2040 | L-Pyridosine | pos | 255.1344 | 0.672 | 4.972 | 4.947 | -0.025 | -0.166 | 0.355 | 0.683 | Unannotated | NA | NA |
| metab_10471 | Unannotated feature | neg | 342.0834 | 1.952 | 3.382 | 3.950 | 0.568 | 0.166 | 0.355 | 0.683 | Unannotated | NA | NA |
| metab_17935 | Unannotated feature | neg | 316.9363 | 6.624 | 5.066 | 4.998 | -0.068 | -0.166 | 0.355 | 0.683 | Unannotated | NA | NA |
| metab_11926 | Unannotated feature | neg | 271.1916 | 6.107 | 4.059 | 4.376 | 0.317 | 0.166 | 0.355 | 0.683 | Unannotated | NA | NA |
| metab_12058 | Unannotated feature | neg | 497.2592 | 6.381 | 5.326 | 5.393 | 0.066 | 0.162 | 0.368 | 0.693 | Unannotated | NA | NA |
| metab_13415 | Unannotated feature | neg | 96.9213 | 0.625 | 4.104 | 4.053 | -0.051 | -0.162 | 0.368 | 0.693 | Unannotated | NA | NA |
| metab_2067 | Unannotated feature | pos | 238.1443 | 2.732 | 4.205 | 4.199 | -0.006 | 0.162 | 0.368 | 0.693 | Unannotated | NA | NA |
| metab_12466 | Unannotated feature | neg | 151.0605 | 7.953 | 5.024 | 5.007 | -0.018 | -0.162 | 0.368 | 0.693 | Unannotated | NA | NA |
| metab_40 | Unannotated feature | pos | 272.1861 | 4.344 | 4.930 | 4.920 | -0.010 | -0.162 | 0.368 | 0.693 | Unannotated | NA | NA |
| metab_51 | Unannotated feature | pos | 243.1344 | 0.680 | 5.170 | 5.324 | 0.154 | 0.162 | 0.368 | 0.693 | Unannotated | NA | NA |
| metab_17 | N-Acetylputrescine | pos | 131.1182 | 0.720 | 5.926 | 5.472 | -0.454 | -0.162 | 0.368 | 0.693 | Moderate putative | NA | NA |
| metab_1994 | Tyr-Gly | pos | 256.1296 | 1.952 | 4.063 | 3.917 | -0.145 | -0.162 | 0.368 | 0.693 | Unannotated | NA | NA |
| metab_11996 | Unannotated feature | neg | 281.1759 | 6.232 | 4.701 | 4.836 | 0.135 | 0.162 | 0.368 | 0.693 | Unannotated | NA | NA |
| metab_15047 | Unannotated feature | neg | 385.1706 | 4.413 | 3.756 | 4.039 | 0.284 | 0.162 | 0.368 | 0.693 | Unannotated | NA | NA |
| metab_7636 | Apronal | pos | 217.1550 | 3.481 | 4.551 | 4.385 | -0.167 | -0.162 | 0.368 | 0.693 | Unannotated | NA | NA |
| metab_1940 | Unannotated feature | pos | 387.2725 | 1.944 | 3.976 | 2.916 | -1.060 | -0.162 | 0.368 | 0.693 | Unannotated | NA | NA |
| metab_2155 | Tyrphostin A25 | pos | 185.0326 | 0.625 | 4.964 | 5.047 | 0.084 | -0.162 | 0.368 | 0.693 | Unannotated | NA | NA |
| metab_3317 | 7-Epi-12-hydroxyjasmonic acid | pos | 209.1176 | 4.111 | 4.181 | 4.498 | 0.318 | 0.162 | 0.368 | 0.693 | Unannotated | NA | NA |
| metab_5424 | Unannotated feature | pos | 328.2850 | 6.414 | 4.873 | 4.590 | -0.283 | -0.162 | 0.368 | 0.693 | Unannotated | NA | NA |
| metab_10767 | Unannotated feature | neg | 245.0125 | 1.179 | 4.106 | 3.764 | -0.343 | -0.162 | 0.368 | 0.693 | Unannotated | NA | NA |
| metab_3425 | Unannotated feature | pos | 124.1125 | 4.510 | 3.686 | 3.484 | -0.202 | -0.162 | 0.368 | 0.693 | Unannotated | NA | NA |
| metab_11923 | 4-Hydroxy-3-prenylbenzoic acid glucoside | neg | 349.1297 | 6.099 | 4.097 | 3.885 | -0.212 | -0.162 | 0.368 | 0.693 | Unannotated | NA | NA |
| metab_1893 | Unannotated feature | pos | 215.1030 | 1.396 | 6.034 | 5.901 | -0.133 | -0.162 | 0.368 | 0.693 | Unannotated | NA | NA |
| metab_18034 | Unannotated feature | neg | 269.1312 | 6.467 | 6.803 | 6.768 | -0.035 | -0.162 | 0.368 | 0.693 | Unannotated | NA | NA |
| metab_18093 | Unannotated feature | neg | 475.3069 | 6.397 | 6.079 | 6.257 | 0.179 | 0.162 | 0.368 | 0.693 | Unannotated | NA | NA |
| metab_5612 | 3-Hydroxy-7,12-Diketocholanoic Acid | pos | 405.2641 | 6.215 | 5.805 | 5.774 | -0.031 | -0.162 | 0.368 | 0.693 | Moderate putative | NA | NA |
| metab_18330 | Unannotated feature | neg | 495.2131 | 6.193 | 3.866 | 4.236 | 0.370 | 0.162 | 0.368 | 0.693 | Unannotated | NA | NA |
| metab_18870 | Unannotated feature | neg | 526.2856 | 5.692 | 4.617 | 5.037 | 0.419 | 0.162 | 0.368 | 0.693 | Unannotated | NA | NA |
| metab_4538 | Unannotated feature | pos | 483.3821 | 6.773 | 6.484 | 6.706 | 0.222 | 0.162 | 0.368 | 0.693 | Unannotated | NA | NA |
| metab_13905 | Unannotated feature | neg | 126.9043 | 2.508 | 4.049 | 4.034 | -0.015 | -0.162 | 0.368 | 0.693 | Unannotated | NA | NA |
| metab_17081 | Unannotated feature | neg | 432.1754 | 2.222 | 5.548 | 5.368 | -0.180 | -0.162 | 0.368 | 0.693 | Unannotated | NA | NA |
| metab_11631 | Unannotated feature | neg | 212.8843 | 0.096 | 5.570 | 5.559 | -0.011 | -0.162 | 0.368 | 0.693 | Unannotated | NA | NA |
| metab_11633 | Unannotated feature | neg | 191.8546 | 0.075 | 5.558 | 5.547 | -0.011 | -0.162 | 0.368 | 0.693 | Unannotated | NA | NA |
| metab_11634 | Unannotated feature | neg | 187.8606 | 0.075 | 6.598 | 6.591 | -0.006 | -0.162 | 0.368 | 0.693 | Unannotated | NA | NA |
| metab_5352 | 2-Hydroxymyristoylcarnitine | pos | 388.3066 | 6.518 | 4.224 | 4.508 | 0.284 | 0.162 | 0.368 | 0.693 | Unannotated | NA | NA |
| metab_10706 | Netivudine | neg | 303.0599 | 1.372 | 4.867 | 5.038 | 0.171 | 0.162 | 0.368 | 0.693 | Unannotated | NA | NA |
| metab_10707 | Unannotated feature | neg | 350.9954 | 1.369 | 5.753 | 5.684 | -0.069 | -0.162 | 0.368 | 0.693 | Unannotated | NA | NA |
| metab_19126 | Unannotated feature | neg | 332.1412 | 5.351 | 3.882 | 3.789 | -0.093 | 0.162 | 0.368 | 0.693 | Unannotated | NA | NA |
| metab_8029 | Phe Gly | pos | 223.1081 | 2.803 | 5.035 | 5.003 | -0.032 | -0.162 | 0.368 | 0.693 | Moderate putative | NA | NA |
| metab_4863 | Unannotated feature | pos | 167.0131 | 7.724 | 6.684 | 6.719 | 0.036 | 0.162 | 0.368 | 0.693 | Unannotated | NA | NA |
| metab_347 | Unannotated feature | pos | 74.0969 | 0.413 | 3.336 | 3.357 | 0.022 | 0.162 | 0.368 | 0.693 | Unannotated | NA | NA |
| metab_17385 | Unannotated feature | neg | 621.8631 | 7.663 | 5.438 | 5.446 | 0.008 | 0.162 | 0.368 | 0.693 | Unannotated | NA | NA |
| metab_10472 | Beta-D-Xylopyranosyl-(1->5)-alpha-L-arabinofuranosyl-(1->3)-L-arabinose | neg | 413.1316 | 1.952 | 4.130 | 4.322 | 0.192 | 0.162 | 0.368 | 0.693 | Unannotated | NA | NA |
| metab_1468 | Unannotated feature | pos | 196.0972 | 5.936 | 4.750 | 4.817 | 0.068 | -0.162 | 0.368 | 0.693 | Unannotated | NA | NA |
| metab_375 | Unannotated feature | pos | 99.0809 | 0.173 | 3.371 | 3.422 | 0.050 | 0.162 | 0.368 | 0.693 | Unannotated | NA | NA |
| metab_12284 | DG(5-iso PGF2VI/0:0/2:0) | neg | 487.2546 | 6.091 | 4.123 | 3.997 | -0.125 | -0.162 | 0.368 | 0.693 | Unannotated | NA | NA |
| metab_18282 | Unannotated feature | neg | 437.2551 | 6.224 | 6.323 | 6.217 | -0.106 | -0.162 | 0.368 | 0.693 | Unannotated | NA | NA |
| metab_11783 | Unannotated feature | neg | 385.1695 | 5.783 | 6.055 | 6.332 | 0.277 | 0.162 | 0.368 | 0.693 | Unannotated | NA | NA |
| metab_5743 | 2'-O-Methylglabridin | pos | 699.2871 | 6.064 | 4.787 | 4.317 | -0.470 | -0.162 | 0.368 | 0.693 | Unannotated | NA | NA |
| metab_2196 | Unannotated feature | pos | 245.1500 | 2.994 | 4.980 | 4.872 | -0.108 | -0.162 | 0.368 | 0.693 | Unannotated | NA | NA |
| metab_15547 | Unannotated feature | neg | 262.0699 | 5.386 | 5.764 | 5.756 | -0.008 | 0.162 | 0.368 | 0.693 | Unannotated | NA | NA |
| metab_7381 | 1-(D-3-Mercapto-2-methyl-1-oxopropyl)-L-proline | pos | 281.0926 | 4.002 | 3.685 | 3.848 | 0.164 | 0.162 | 0.368 | 0.693 | Unannotated | NA | NA |
| metab_17158 | Unannotated feature | neg | 144.0660 | 7.769 | 4.451 | 4.412 | -0.039 | -0.162 | 0.368 | 0.693 | Unannotated | NA | NA |
| metab_14212 | Unannotated feature | neg | 333.0410 | 2.956 | 4.299 | 4.072 | -0.227 | -0.162 | 0.368 | 0.693 | Unannotated | NA | NA |
| metab_19121 | Unannotated feature | neg | 392.0994 | 5.355 | 5.381 | 5.047 | -0.334 | -0.162 | 0.368 | 0.693 | Unannotated | NA | NA |
| metab_15509 | Unannotated feature | neg | 255.0804 | 5.295 | 4.096 | 4.179 | 0.083 | 0.162 | 0.368 | 0.693 | Unannotated | NA | NA |
| metab_8331 | Unannotated feature | pos | 345.2136 | 1.994 | 4.401 | 4.386 | -0.016 | -0.162 | 0.368 | 0.693 | Unannotated | NA | NA |
| metab_11576 | Unannotated feature | neg | 199.8494 | 0.460 | 4.055 | 3.518 | -0.537 | -0.162 | 0.368 | 0.693 | Unannotated | NA | NA |
| metab_19311 | Unannotated feature | neg | 389.0918 | 5.127 | 4.484 | 4.295 | -0.189 | -0.162 | 0.368 | 0.693 | Unannotated | NA | NA |
| metab_19318 | 5-(2-Methylpropyl)tetrahydro-2-oxo-3-furancarboxylic acid | neg | 185.0814 | 5.118 | 4.903 | 4.954 | 0.051 | 0.162 | 0.368 | 0.693 | Unannotated | NA | NA |
| metab_5532 | (S)-N-Methylcoclaurine | pos | 621.2939 | 6.295 | 5.108 | 4.439 | -0.669 | -0.162 | 0.368 | 0.693 | Tentative | NA | NA |
| metab_1449 | Unannotated feature | pos | 297.1451 | 3.238 | 5.351 | 5.003 | -0.348 | -0.162 | 0.368 | 0.693 | Unannotated | NA | NA |
| metab_13112 | Unannotated feature | neg | 289.8707 | 0.204 | 4.616 | 4.590 | -0.027 | -0.162 | 0.368 | 0.693 | Unannotated | NA | NA |
| metab_14163 | Unannotated feature | neg | 335.1255 | 2.903 | 5.163 | 5.166 | 0.003 | -0.162 | 0.368 | 0.693 | Unannotated | NA | NA |
| metab_8936 | Menthyl pyrrolidone carboxylate | neg | 353.1718 | 4.207 | 5.006 | 4.874 | -0.132 | -0.162 | 0.368 | 0.693 | Tentative | NA | NA |
| metab_12575 | Unannotated feature | neg | 585.2736 | 6.115 | 4.989 | 4.796 | -0.193 | -0.162 | 0.368 | 0.693 | Unannotated | NA | NA |
| metab_11319 | Unannotated feature | neg | 340.8647 | 0.603 | 4.186 | 3.771 | -0.415 | -0.162 | 0.368 | 0.693 | Unannotated | NA | NA |
| metab_880 | Unannotated feature | pos | 461.3250 | 6.845 | 6.562 | 6.589 | 0.028 | 0.162 | 0.368 | 0.693 | Unannotated | NA | NA |
| metab_8073 | Unannotated feature | pos | 359.2298 | 2.755 | 4.129 | 3.589 | -0.540 | -0.162 | 0.368 | 0.693 | Unannotated | NA | NA |
| metab_2213 | Unannotated feature | pos | 260.8978 | 0.593 | 4.241 | 3.946 | -0.294 | -0.162 | 0.368 | 0.693 | Unannotated | NA | NA |
| metab_13342 | Glycylhydroxyproline | neg | 169.0612 | 0.714 | 4.394 | 4.392 | -0.003 | 0.162 | 0.368 | 0.693 | Unannotated | Organic acids and derivatives | Carboxylic acids and derivatives |
| metab_17695 | Unannotated feature | neg | 237.9735 | 0.619 | 4.967 | 4.802 | -0.165 | -0.162 | 0.368 | 0.693 | Unannotated | NA | NA |
| metab_19662 | RepSox | neg | 332.1145 | 4.621 | 3.984 | 4.270 | 0.286 | 0.162 | 0.368 | 0.693 | Unannotated | NA | NA |
| metab_14960 | Unannotated feature | neg | 258.0384 | 4.250 | 4.970 | 5.014 | 0.045 | 0.162 | 0.368 | 0.693 | Unannotated | NA | NA |
| metab_9820 | Lamivudine sulfoxide | neg | 266.0204 | 3.025 | 5.621 | 5.528 | -0.093 | -0.162 | 0.368 | 0.693 | Unannotated | NA | NA |
| metab_16968 | Unannotated feature | neg | 216.8766 | 7.902 | 5.664 | 5.714 | 0.051 | 0.162 | 0.368 | 0.693 | Unannotated | NA | NA |
| metab_18756 | 3-[4-(sulfooxy)phenyl]propanoic acid | neg | 245.0125 | 3.169 | 4.849 | 4.334 | -0.515 | -0.162 | 0.368 | 0.693 | Unannotated | Organic acids and derivatives | Organic sulfuric acids and derivatives |
| metab_2908 | Unannotated feature | pos | 100.0418 | 2.871 | 3.585 | 3.476 | -0.109 | -0.162 | 0.368 | 0.693 | Unannotated | NA | NA |
| metab_10531 | Diethyl hydrogen phosphate | neg | 153.0315 | 1.947 | 4.525 | 4.676 | 0.151 | 0.162 | 0.368 | 0.693 | Strong putative | NA | NA |
| metab_9194 | Unannotated feature | neg | 525.1626 | 3.925 | 3.668 | 3.329 | -0.339 | -0.162 | 0.368 | 0.693 | Unannotated | NA | NA |
| metab_12698 | Unannotated feature | neg | 303.1089 | 5.366 | 4.011 | 4.379 | 0.367 | 0.162 | 0.368 | 0.693 | Unannotated | NA | NA |
| metab_12775 | Unannotated feature | neg | 587.2886 | 6.813 | 4.805 | 4.481 | -0.324 | -0.162 | 0.368 | 0.693 | Unannotated | NA | NA |
| metab_17692 | Unannotated feature | neg | 591.3918 | 7.012 | 6.184 | 6.050 | -0.134 | -0.162 | 0.368 | 0.693 | Unannotated | NA | NA |
| metab_8611 | Unannotated feature | pos | 124.0761 | 1.118 | 3.619 | 3.588 | -0.031 | 0.162 | 0.368 | 0.693 | Unannotated | NA | NA |
| metab_3190 | Unannotated feature | pos | 254.1391 | 3.701 | 4.177 | 3.901 | -0.275 | -0.162 | 0.368 | 0.693 | Unannotated | NA | NA |
| metab_895 | Unannotated feature | pos | 769.4885 | 6.997 | 5.020 | 5.127 | 0.107 | 0.162 | 0.368 | 0.693 | Unannotated | NA | NA |
| metab_906 | Unannotated feature | pos | 717.5057 | 7.108 | 4.604 | 5.167 | 0.564 | 0.162 | 0.368 | 0.693 | Unannotated | NA | NA |
| metab_883 | Unannotated feature | pos | 325.2120 | 6.861 | 1.677 | 3.750 | 2.073 | 0.162 | 0.368 | 0.693 | Unannotated | NA | NA |
| metab_19051 | Unannotated feature | neg | 197.0814 | 5.457 | 4.222 | 4.378 | 0.156 | 0.162 | 0.368 | 0.693 | Unannotated | NA | NA |
| metab_4110 | Unannotated feature | pos | 701.7670 | 5.851 | 0.873 | 4.026 | 3.153 | 0.162 | 0.368 | 0.693 | Unannotated | NA | NA |
| metab_10544 | Unannotated feature | neg | 98.0239 | 1.944 | 3.798 | 3.806 | 0.008 | -0.162 | 0.368 | 0.693 | Unannotated | NA | NA |
| metab_19002 | Unannotated feature | neg | 288.0404 | 5.526 | 4.149 | 3.928 | -0.220 | -0.162 | 0.368 | 0.693 | Unannotated | NA | NA |
| metab_2447 | Unannotated feature | pos | 173.0424 | 0.649 | 5.343 | 5.175 | -0.168 | -0.162 | 0.368 | 0.693 | Unannotated | NA | NA |
| metab_19590 | Undecylenic acid | neg | 229.1444 | 4.726 | 5.633 | 5.578 | -0.055 | -0.162 | 0.368 | 0.693 | Tentative | NA | NA |
| metab_13742 | Unannotated feature | neg | 466.1575 | 1.947 | 3.001 | 3.391 | 0.390 | 0.162 | 0.368 | 0.693 | Unannotated | NA | NA |
| metab_7565 | Carbazole | pos | 168.0811 | 3.627 | 3.821 | 3.436 | -0.385 | -0.162 | 0.368 | 0.693 | Strong putative | NA | NA |
| metab_16192 | Unannotated feature | neg | 179.0014 | 1.304 | 5.438 | 5.311 | -0.127 | -0.162 | 0.368 | 0.693 | Unannotated | NA | NA |
| metab_16215 | Muricin A | neg | 641.4645 | 6.444 | 5.617 | 5.254 | -0.364 | -0.162 | 0.368 | 0.693 | Unannotated | NA | NA |
| metab_1800 | Unannotated feature | pos | 208.0640 | 0.784 | 3.767 | 3.996 | 0.229 | 0.162 | 0.368 | 0.693 | Unannotated | NA | NA |
| metab_2367 | Temazepam | pos | 301.0737 | 0.509 | 4.534 | 4.381 | -0.154 | -0.162 | 0.368 | 0.693 | Moderate putative | NA | NA |
| metab_15940 | Unannotated feature | neg | 413.2183 | 6.041 | 5.452 | 5.249 | -0.203 | -0.162 | 0.368 | 0.693 | Unannotated | NA | NA |
| metab_1101 | Unannotated feature | pos | 174.1240 | 1.944 | 5.117 | 5.426 | 0.309 | 0.162 | 0.368 | 0.693 | Unannotated | NA | NA |
| metab_1391 | Stallimycin | pos | 482.2300 | 6.095 | 5.156 | 4.906 | -0.249 | -0.162 | 0.368 | 0.693 | Unannotated | NA | NA |
| metab_1348 | Unannotated feature | pos | 358.2955 | 6.167 | 4.535 | 4.210 | -0.325 | -0.162 | 0.368 | 0.693 | Unannotated | NA | NA |
| metab_16235 | Unannotated feature | neg | 350.8970 | 6.491 | 5.361 | 5.397 | 0.037 | 0.162 | 0.368 | 0.693 | Unannotated | NA | NA |
| metab_11538 | Unannotated feature | neg | 158.9779 | 0.496 | 7.321 | 7.307 | -0.014 | -0.162 | 0.368 | 0.693 | Unannotated | NA | NA |
| metab_16468 | Unannotated feature | neg | 200.0712 | 7.037 | 3.815 | 3.653 | -0.162 | -0.162 | 0.368 | 0.693 | Unannotated | NA | NA |
| metab_2325 | Unannotated feature | pos | 280.0974 | 0.360 | 5.496 | 5.516 | 0.020 | 0.162 | 0.368 | 0.693 | Unannotated | NA | NA |
| metab_10312 | Unannotated feature | neg | 244.0324 | 2.336 | 5.299 | 5.498 | 0.198 | 0.162 | 0.368 | 0.693 | Unannotated | NA | NA |
| metab_17565 | Unannotated feature | neg | 156.9805 | 7.310 | 7.031 | 7.022 | -0.009 | -0.162 | 0.368 | 0.693 | Unannotated | NA | NA |
| metab_5492 | Unannotated feature | pos | 369.3484 | 6.351 | 5.164 | 5.275 | 0.111 | 0.162 | 0.368 | 0.693 | Unannotated | NA | NA |
| metab_12808 | Unannotated feature | neg | 397.0541 | 0.644 | 3.602 | 4.516 | 0.914 | 0.162 | 0.368 | 0.693 | Unannotated | NA | NA |
| metab_5497 | Unannotated feature | pos | 421.2577 | 6.343 | 5.413 | 5.229 | -0.183 | -0.162 | 0.368 | 0.693 | Unannotated | NA | NA |
| metab_13028 | Unannotated feature | neg | 375.0663 | 0.858 | 5.129 | 5.342 | 0.213 | 0.162 | 0.368 | 0.693 | Unannotated | NA | NA |
| metab_3036 | Unannotated feature | pos | 433.3320 | 6.406 | 6.361 | 6.243 | -0.118 | -0.162 | 0.368 | 0.693 | Unannotated | NA | NA |
| metab_3039 | 11-Dehydrocorticosterone | pos | 386.2294 | 3.268 | 4.975 | 4.888 | -0.086 | -0.162 | 0.368 | 0.693 | Tentative | NA | NA |
| metab_12653 | Unannotated feature | neg | 442.1906 | 5.937 | 4.808 | 4.791 | -0.017 | -0.162 | 0.368 | 0.693 | Unannotated | NA | NA |
| metab_10114 | Unannotated feature | neg | 477.1260 | 2.762 | 5.022 | 4.620 | -0.402 | -0.162 | 0.368 | 0.693 | Unannotated | NA | NA |
| metab_11636 | Unannotated feature | neg | 222.8296 | 0.073 | 6.370 | 6.352 | -0.018 | -0.162 | 0.368 | 0.693 | Unannotated | NA | NA |
| metab_19190 | Cocaine-alcohol | neg | 364.1407 | 5.276 | 4.406 | 4.150 | -0.257 | -0.162 | 0.368 | 0.693 | Unannotated | NA | NA |
| metab_15693 | Cladosporin | neg | 313.1085 | 5.671 | 6.238 | 6.451 | 0.213 | 0.162 | 0.368 | 0.693 | Unannotated | NA | NA |
| metab_5198 | Unannotated feature | pos | 341.2671 | 6.757 | 4.949 | 4.974 | 0.025 | 0.162 | 0.368 | 0.693 | Unannotated | NA | NA |
| metab_9394 | (+)-8-Acetoxycarvone | neg | 253.1083 | 3.484 | 4.142 | 4.428 | 0.287 | 0.162 | 0.368 | 0.693 | Unannotated | Lipids and lipid-like molecules | Prenol lipids |
| metab_17407 | Unannotated feature | neg | 264.9344 | 7.648 | 5.702 | 5.692 | -0.010 | -0.162 | 0.368 | 0.693 | Unannotated | NA | NA |
| metab_12255 | Unannotated feature | neg | 315.2176 | 6.997 | 4.015 | 3.599 | -0.416 | -0.162 | 0.368 | 0.693 | Unannotated | NA | NA |
| metab_15613 | Unannotated feature | neg | 277.0670 | 5.526 | 3.916 | 3.573 | -0.343 | -0.162 | 0.368 | 0.693 | Unannotated | NA | NA |
| metab_11970 | Unannotated feature | neg | 303.1350 | 6.185 | 5.296 | 5.166 | -0.129 | -0.158 | 0.381 | 0.701 | Unannotated | NA | NA |
| metab_11455 | Unannotated feature | neg | 478.8516 | 0.527 | 5.337 | 5.437 | 0.100 | 0.158 | 0.381 | 0.701 | Unannotated | NA | NA |
| metab_2006 | Unannotated feature | pos | 86.0606 | 1.952 | 4.624 | 4.697 | 0.073 | 0.158 | 0.381 | 0.701 | Unannotated | NA | NA |
| metab_19525 | Dioscoretine | neg | 286.1665 | 4.818 | 3.484 | 3.574 | 0.090 | -0.158 | 0.381 | 0.701 | Unannotated | NA | NA |
| metab_19572 | 3,4-Dihydro-2H-1-benzopyran-2-one | neg | 341.1040 | 4.749 | 5.068 | 4.491 | -0.577 | -0.158 | 0.381 | 0.701 | Tentative | Phenylpropanoids and polyketides | 3,4-dihydrocoumarins |
| metab_13115 | Unannotated feature | neg | 160.9120 | 0.259 | 4.749 | 4.722 | -0.027 | -0.158 | 0.381 | 0.701 | Unannotated | NA | NA |
| metab_2664 | Unannotated feature | pos | 217.1914 | 1.952 | 3.664 | 3.177 | -0.487 | -0.158 | 0.381 | 0.701 | Unannotated | NA | NA |
| metab_5205 | Unannotated feature | pos | 87.8450 | 6.749 | 3.183 | 3.269 | 0.086 | 0.158 | 0.381 | 0.701 | Unannotated | NA | NA |
| metab_8241 | Hypoglycin B | pos | 253.1188 | 2.324 | 5.092 | 4.985 | -0.107 | -0.158 | 0.381 | 0.701 | Tentative | NA | NA |
| metab_14690 | Unannotated feature | neg | 316.1040 | 3.681 | 4.100 | 4.497 | 0.397 | 0.158 | 0.381 | 0.701 | Unannotated | NA | NA |
| metab_8275 | D-Galactosamine | pos | 180.0882 | 2.168 | 3.202 | 3.174 | -0.027 | 0.158 | 0.381 | 0.701 | Tentative | NA | NA |
| metab_15688 | (4S,7S,12Br)-6-oxo-7-[[(2S)-3-phenyl-2-sulfanylpropanoyl]amino]-2,3,4,7,8,12b-hexahydro-1H-pyrido[2,1-a][2]benzazepine-4-carboxylic acid | neg | 437.1506 | 5.664 | 4.526 | 4.900 | 0.373 | 0.158 | 0.381 | 0.701 | Unannotated | Organic acids and derivatives | Carboxylic acids and derivatives |
| metab_4854 | Unannotated feature | pos | 350.8934 | 7.724 | 4.585 | 4.588 | 0.003 | -0.158 | 0.381 | 0.701 | Unannotated | NA | NA |
| metab_5080 | Unannotated feature | pos | 175.1444 | 0.776 | 4.926 | 4.130 | -0.796 | -0.158 | 0.381 | 0.701 | Unannotated | NA | NA |
| metab_2026 | Unannotated feature | pos | 304.1399 | 0.712 | 5.143 | 5.268 | 0.125 | 0.158 | 0.381 | 0.701 | Unannotated | NA | NA |
| metab_17918 | Unannotated feature | neg | 523.2406 | 6.640 | 5.006 | 4.889 | -0.117 | -0.158 | 0.381 | 0.701 | Unannotated | NA | NA |
| metab_17907 | Unannotated feature | neg | 615.4969 | 6.656 | 5.146 | 4.702 | -0.443 | -0.158 | 0.381 | 0.701 | Unannotated | NA | NA |
| metab_11645 | Unannotated feature | neg | 224.9086 | 0.036 | 4.665 | 4.595 | -0.070 | -0.158 | 0.381 | 0.701 | Unannotated | NA | NA |
| metab_5394 | Unannotated feature | pos | 437.3275 | 6.446 | 4.634 | 4.963 | 0.329 | 0.158 | 0.381 | 0.701 | Unannotated | NA | NA |
| metab_14274 | Unannotated feature | neg | 183.0026 | 3.021 | 3.874 | 3.897 | 0.023 | -0.158 | 0.381 | 0.701 | Unannotated | NA | NA |
| metab_9905 | Unannotated feature | neg | 248.1252 | 2.951 | 4.266 | 3.551 | -0.715 | -0.158 | 0.381 | 0.701 | Unannotated | NA | NA |
| metab_2089 | Unannotated feature | pos | 381.1662 | 2.755 | 3.710 | 4.013 | 0.303 | 0.158 | 0.381 | 0.701 | Unannotated | NA | NA |
| metab_6799 | Glutaminylhistidine | pos | 325.1633 | 5.175 | 3.056 | 3.072 | 0.017 | 0.158 | 0.381 | 0.701 | Unannotated | NA | NA |
| metab_11847 | Unannotated feature | neg | 379.1874 | 5.952 | 5.490 | 5.284 | -0.206 | -0.158 | 0.381 | 0.701 | Unannotated | NA | NA |
| metab_9130 | Unannotated feature | neg | 215.1286 | 3.882 | 3.951 | 4.033 | 0.082 | 0.158 | 0.381 | 0.701 | Unannotated | NA | NA |
| metab_11027 | Unannotated feature | neg | 227.1034 | 0.745 | 3.554 | 3.713 | 0.159 | 0.158 | 0.381 | 0.701 | Unannotated | NA | NA |
| metab_14481 | Unannotated feature | neg | 239.1036 | 3.326 | 4.583 | 4.483 | -0.100 | -0.158 | 0.381 | 0.701 | Unannotated | NA | NA |
| metab_12796 | Unannotated feature | neg | 408.9657 | 0.636 | 3.676 | 4.253 | 0.577 | 0.158 | 0.381 | 0.701 | Unannotated | NA | NA |
| metab_8175 | Hydroxyphenylacetylglycine | pos | 210.0765 | 2.630 | 4.602 | 4.742 | 0.139 | 0.158 | 0.381 | 0.701 | Tentative | NA | NA |
| metab_17545 | Unannotated feature | neg | 477.3051 | 7.379 | 5.869 | 6.178 | 0.309 | 0.158 | 0.381 | 0.701 | Unannotated | NA | NA |
| metab_2335 | Unannotated feature | pos | 201.0087 | 0.417 | 5.830 | 5.850 | 0.020 | 0.158 | 0.381 | 0.701 | Unannotated | NA | NA |
| metab_15284 | Unannotated feature | neg | 373.0231 | 0.558 | 4.079 | 4.069 | -0.009 | 0.158 | 0.381 | 0.701 | Unannotated | NA | NA |
| metab_1346 | Unannotated feature | pos | 377.2315 | 6.167 | 5.898 | 5.713 | -0.185 | -0.158 | 0.381 | 0.701 | Unannotated | NA | NA |
| metab_17235 | Unannotated feature | neg | 130.9651 | 7.730 | 2.967 | 2.921 | -0.046 | -0.158 | 0.381 | 0.701 | Unannotated | NA | NA |
| metab_9077 | Unannotated feature | neg | 429.1595 | 3.960 | 3.528 | 3.166 | -0.362 | -0.158 | 0.381 | 0.701 | Unannotated | NA | NA |
| metab_8487 | 4-Hydroxystachydrine | pos | 142.0866 | 1.692 | 4.293 | 4.375 | 0.082 | 0.158 | 0.381 | 0.701 | Tentative | NA | NA |
| metab_8550 | Unannotated feature | pos | 231.1455 | 1.286 | 5.181 | 4.955 | -0.225 | -0.158 | 0.381 | 0.701 | Unannotated | NA | NA |
| metab_11222 | Unannotated feature | neg | 157.0612 | 0.644 | 4.219 | 4.272 | 0.053 | 0.158 | 0.381 | 0.701 | Unannotated | NA | NA |
| metab_11221 | Alanylalanine | neg | 159.0768 | 0.644 | 5.619 | 5.466 | -0.153 | -0.158 | 0.381 | 0.701 | Strong putative | Organic acids and derivatives | Carboxylic acids and derivatives |
| metab_7790 | Unannotated feature | pos | 248.1478 | 3.200 | 3.198 | 3.386 | 0.188 | 0.158 | 0.381 | 0.701 | Unannotated | NA | NA |
| metab_9619 | Unannotated feature | neg | 183.0027 | 3.215 | 4.485 | 4.398 | -0.087 | -0.158 | 0.381 | 0.701 | Unannotated | NA | NA |
| metab_1790 | 5'-Amino-5'-deoxyadenosine | pos | 231.0979 | 0.776 | 5.536 | 5.512 | -0.024 | -0.158 | 0.381 | 0.701 | Unannotated | NA | NA |
| metab_1784 | Ligstroside-aglycone | pos | 327.1191 | 0.768 | 4.303 | 4.319 | 0.016 | 0.158 | 0.381 | 0.701 | Unannotated | Lipids and lipid-like molecules | Prenol lipids |
| metab_2350 | Unannotated feature | pos | 158.0120 | 0.469 | 4.313 | 4.282 | -0.031 | -0.158 | 0.381 | 0.701 | Unannotated | NA | NA |
| metab_9633 | 5-Dihydroxyhept-6-enoic acid | neg | 205.0714 | 3.207 | 4.141 | 3.889 | -0.253 | -0.158 | 0.381 | 0.701 | Unannotated | NA | NA |
| metab_18410 | Unannotated feature | neg | 344.9811 | 3.028 | 5.056 | 5.020 | -0.036 | -0.158 | 0.381 | 0.701 | Unannotated | NA | NA |
| metab_5483 | 4-Propylphenol | pos | 119.0860 | 6.367 | 5.788 | 5.741 | -0.047 | -0.158 | 0.381 | 0.701 | Tentative | NA | NA |
| metab_5306 | Unannotated feature | pos | 571.3280 | 6.558 | 3.711 | 4.076 | 0.364 | 0.158 | 0.381 | 0.701 | Unannotated | NA | NA |
| metab_14415 | Unannotated feature | neg | 175.0970 | 3.219 | 4.202 | 4.350 | 0.148 | 0.158 | 0.381 | 0.701 | Unannotated | NA | NA |
| metab_252 | Unannotated feature | pos | 326.9822 | 0.531 | 3.721 | 3.820 | 0.100 | 0.158 | 0.381 | 0.701 | Unannotated | NA | NA |
| metab_1069 | Unannotated feature | pos | 441.2981 | 6.726 | 5.791 | 5.736 | -0.056 | -0.158 | 0.381 | 0.701 | Unannotated | NA | NA |
| metab_15458 | Unannotated feature | neg | 200.1287 | 5.192 | 4.346 | 4.312 | -0.034 | -0.158 | 0.381 | 0.701 | Unannotated | NA | NA |
| metab_10326 | Unannotated feature | neg | 335.1405 | 4.574 | 3.816 | 4.389 | 0.572 | 0.158 | 0.381 | 0.701 | Unannotated | NA | NA |
| metab_10325 | Unannotated feature | neg | 278.0522 | 2.298 | 4.512 | 4.400 | -0.112 | -0.158 | 0.381 | 0.701 | Unannotated | NA | NA |
| metab_13228 | Unannotated feature | neg | 515.1263 | 0.777 | 4.120 | 4.563 | 0.443 | 0.158 | 0.381 | 0.701 | Unannotated | NA | NA |
| metab_13220 | Peroxysimulenoline | neg | 340.1557 | 3.592 | 4.617 | 4.200 | -0.417 | -0.158 | 0.381 | 0.701 | Unannotated | NA | NA |
| metab_8143 | Xymedon | pos | 186.1241 | 2.724 | 3.600 | 3.123 | -0.477 | -0.158 | 0.381 | 0.701 | Unannotated | Lipids and lipid-like molecules | Steroids and steroid derivatives |
| metab_384 | Unannotated feature | pos | 298.1291 | 4.699 | 4.707 | 3.991 | -0.715 | -0.158 | 0.381 | 0.701 | Unannotated | NA | NA |
| metab_8417 | 2-Amino-4-ethoxy-3-hydroxybutanoic acid | pos | 146.0814 | 1.952 | 5.118 | 5.072 | -0.047 | -0.158 | 0.381 | 0.701 | Unannotated | NA | NA |
| metab_10835 | Unannotated feature | neg | 345.1004 | 4.701 | 5.139 | 5.353 | 0.214 | 0.158 | 0.381 | 0.701 | Unannotated | NA | NA |
| metab_12861 | 4-O-alpha-D-Galactopyranuronosyl-D-galacturonic acid | neg | 351.0574 | 0.698 | 5.256 | 4.939 | -0.318 | -0.158 | 0.381 | 0.701 | Tentative | NA | NA |
| metab_16815 | Unannotated feature | neg | 452.8488 | 0.542 | 3.953 | 3.879 | -0.074 | -0.158 | 0.381 | 0.701 | Unannotated | NA | NA |
| metab_1314 | Unannotated feature | pos | 416.2476 | 6.223 | 5.953 | 5.833 | -0.121 | -0.158 | 0.381 | 0.701 | Unannotated | NA | NA |
| metab_10916 | Unannotated feature | neg | 277.0389 | 0.838 | 3.653 | 3.575 | -0.078 | 0.158 | 0.381 | 0.701 | Unannotated | NA | NA |
| metab_18603 | N-Docosahexaenoyl Aspartic acid | neg | 480.2147 | 5.983 | 4.755 | 4.594 | -0.161 | -0.158 | 0.381 | 0.701 | Unannotated | NA | NA |
| metab_17687 | LysoPG(18:1(9Z)/0:0) | neg | 509.2895 | 7.019 | 6.957 | 6.913 | -0.044 | -0.158 | 0.381 | 0.701 | Unannotated | NA | NA |
| metab_2235 | Unannotated feature | pos | 264.1598 | 3.087 | 4.485 | 4.149 | -0.335 | -0.158 | 0.381 | 0.701 | Unannotated | NA | NA |
| metab_15859 | Unannotated feature | neg | 238.0772 | 5.944 | 5.949 | 6.130 | 0.181 | 0.158 | 0.381 | 0.701 | Unannotated | NA | NA |
| metab_1082 | Unannotated feature | pos | 507.3669 | 6.694 | 5.814 | 6.068 | 0.254 | 0.158 | 0.381 | 0.701 | Unannotated | NA | NA |
| metab_8212 | Unannotated feature | pos | 184.0033 | 2.465 | 4.733 | 4.758 | 0.024 | 0.158 | 0.381 | 0.701 | Unannotated | NA | NA |
| metab_8211 | Phe Asp | pos | 281.1138 | 2.473 | 4.306 | 4.061 | -0.245 | -0.158 | 0.381 | 0.701 | Moderate putative | NA | NA |
| metab_18937 | Artemorin | neg | 293.1398 | 5.600 | 3.352 | 3.688 | 0.337 | -0.158 | 0.381 | 0.701 | Tentative | NA | NA |
| metab_4600 | Hexadecyl ferulate | pos | 441.2987 | 7.013 | 5.801 | 5.879 | 0.078 | 0.158 | 0.381 | 0.701 | Unannotated | NA | NA |
| metab_16867 | Unannotated feature | neg | 264.9094 | 7.737 | 4.077 | 4.023 | -0.054 | -0.158 | 0.381 | 0.701 | Unannotated | NA | NA |
| metab_4572 | Unannotated feature | pos | 270.2794 | 6.917 | 4.138 | 3.941 | -0.196 | -0.158 | 0.381 | 0.701 | Unannotated | NA | NA |
| metab_8221 | Dyphylline | pos | 237.0986 | 2.430 | 2.503 | 2.911 | 0.408 | 0.158 | 0.381 | 0.701 | Tentative | NA | NA |
| metab_9227 | Unannotated feature | neg | 348.1669 | 3.934 | 5.695 | 5.677 | -0.018 | -0.158 | 0.381 | 0.701 | Unannotated | NA | NA |
| metab_7873 | Unannotated feature | pos | 119.0734 | 3.007 | 5.401 | 5.389 | -0.012 | 0.158 | 0.381 | 0.701 | Unannotated | NA | NA |
| metab_12571 | Unannotated feature | neg | 363.1456 | 6.130 | 5.397 | 5.234 | -0.163 | -0.158 | 0.381 | 0.701 | Unannotated | NA | NA |
| metab_11277 | Unannotated feature | neg | 241.0924 | 0.619 | 4.063 | 4.360 | 0.297 | 0.158 | 0.381 | 0.701 | Unannotated | NA | NA |
| metab_17072 | Unannotated feature | neg | 235.9260 | 7.914 | 6.072 | 6.044 | -0.027 | -0.158 | 0.381 | 0.701 | Unannotated | NA | NA |
| metab_4380 | (E,E)-Piperlonguminine | pos | 291.1708 | 6.231 | 4.521 | 4.428 | -0.093 | -0.158 | 0.381 | 0.701 | Unannotated | Organoheterocyclic compounds | Benzodioxoles |
| metab_14493 | Proazulene | neg | 327.1196 | 3.338 | 3.283 | 3.783 | 0.500 | 0.158 | 0.381 | 0.701 | Tentative | NA | NA |
| metab_17370 | Unannotated feature | neg | 154.9902 | 7.675 | 4.491 | 4.457 | -0.034 | -0.158 | 0.381 | 0.701 | Unannotated | NA | NA |
| metab_564 | 1-(3-methylbutyl)-3-[2-[1-(4-methylphenyl)sulfonylpiperidin-4-yl]ethyl]urea | pos | 418.2137 | 5.653 | 5.852 | 5.834 | -0.019 | -0.158 | 0.381 | 0.701 | Moderate putative | NA | NA |
| metab_16959 | Unannotated feature | neg | 265.8512 | 7.870 | 5.107 | 5.164 | 0.056 | 0.158 | 0.381 | 0.701 | Unannotated | NA | NA |
| metab_4407 | Tenuazonic acid | pos | 180.1021 | 6.303 | 6.123 | 6.289 | 0.166 | 0.158 | 0.381 | 0.701 | Tentative | NA | NA |
| metab_17540 | Unannotated feature | neg | 760.5217 | 7.395 | 6.698 | 6.613 | -0.085 | -0.158 | 0.381 | 0.701 | Unannotated | NA | NA |
| metab_10352 | Unannotated feature | neg | 244.9980 | 2.193 | 2.811 | 3.616 | 0.806 | 0.158 | 0.381 | 0.701 | Unannotated | NA | NA |
| metab_12850 | Unannotated feature | neg | 149.0084 | 0.691 | 3.920 | 3.051 | -0.869 | -0.158 | 0.381 | 0.701 | Unannotated | NA | NA |
| metab_3533 | Unannotated feature | pos | 485.3613 | 6.757 | 7.500 | 7.584 | 0.084 | 0.158 | 0.381 | 0.701 | Unannotated | NA | NA |
| metab_8892 | Unannotated feature | neg | 392.1835 | 4.279 | 4.717 | 4.485 | -0.232 | -0.158 | 0.381 | 0.701 | Unannotated | NA | NA |
| metab_10682 | Unannotated feature | neg | 339.9615 | 1.497 | 5.230 | 5.156 | -0.073 | -0.158 | 0.381 | 0.701 | Unannotated | NA | NA |
| metab_10674 | Unannotated feature | neg | 238.0247 | 1.526 | 4.112 | 4.113 | 0.001 | -0.158 | 0.381 | 0.701 | Unannotated | NA | NA |
| metab_17154 | Unannotated feature | neg | 146.9653 | 7.773 | 7.097 | 7.087 | -0.010 | -0.158 | 0.381 | 0.701 | Unannotated | NA | NA |
| metab_414 | Unannotated feature | pos | 181.9776 | 0.016 | 3.877 | 3.837 | -0.040 | -0.158 | 0.381 | 0.701 | Unannotated | NA | NA |
| metab_16259 | Unannotated feature | neg | 201.9965 | 1.407 | 5.487 | 5.364 | -0.123 | -0.158 | 0.381 | 0.701 | Unannotated | NA | NA |
| metab_8912 | Unannotated feature | neg | 297.9839 | 4.246 | 4.661 | 4.716 | 0.055 | 0.158 | 0.381 | 0.701 | Unannotated | NA | NA |
| metab_17747 | Adb-chminaca, (+/-)- | neg | 407.1882 | 6.915 | 6.332 | 6.439 | 0.107 | 0.158 | 0.381 | 0.701 | Unannotated | NA | NA |
| metab_16853 | Unannotated feature | neg | 312.8582 | 7.730 | 4.418 | 4.458 | 0.040 | 0.158 | 0.381 | 0.701 | Unannotated | NA | NA |
| metab_7163 | Unannotated feature | pos | 183.0850 | 4.510 | 3.001 | 2.946 | -0.055 | -0.158 | 0.381 | 0.701 | Unannotated | NA | NA |
| metab_16058 | Unannotated feature | neg | 539.3240 | 6.193 | 5.257 | 5.042 | -0.215 | -0.158 | 0.381 | 0.701 | Unannotated | NA | NA |
| metab_2481 | Furfural | pos | 97.0289 | 0.728 | 4.584 | 4.592 | 0.008 | 0.158 | 0.381 | 0.701 | Moderate putative | NA | NA |
| metab_13835 | Unannotated feature | neg | 276.9241 | 2.166 | 3.781 | 3.845 | 0.063 | 0.158 | 0.381 | 0.701 | Unannotated | NA | NA |
| metab_2778 | 1-[(5-Amino-5-carboxypentyl)amino]-1-deoxyfructose | pos | 273.1450 | 2.500 | 4.433 | 4.546 | 0.113 | 0.158 | 0.381 | 0.701 | Unannotated | NA | NA |
| metab_7210 | Moringyne | pos | 354.1552 | 4.399 | 4.102 | 4.053 | -0.048 | -0.158 | 0.381 | 0.701 | Unannotated | Organic oxygen compounds | Organooxygen compounds |
| metab_820 | Unannotated feature | pos | 276.8718 | 0.593 | 4.844 | 4.553 | -0.290 | -0.158 | 0.381 | 0.701 | Unannotated | NA | NA |
| metab_8004 | Unannotated feature | pos | 334.1878 | 2.835 | 3.791 | 3.852 | 0.061 | 0.158 | 0.381 | 0.701 | Unannotated | NA | NA |
| metab_8828 | Panaxynol | pos | 227.1797 | 6.390 | 4.588 | 4.406 | -0.181 | -0.158 | 0.381 | 0.701 | Tentative | NA | NA |
| metab_1247 | Unannotated feature | pos | 397.2725 | 6.367 | 4.535 | 4.776 | 0.241 | 0.158 | 0.381 | 0.701 | Unannotated | NA | NA |
| metab_3288 | Dihydrogenistein | pos | 305.1026 | 4.027 | 1.334 | 1.066 | -0.269 | -0.158 | 0.381 | 0.701 | Tentative | Phenylpropanoids and polyketides | Isoflavonoids |
| metab_18560 | Unannotated feature | neg | 309.1275 | 6.013 | 5.499 | 5.627 | 0.129 | 0.158 | 0.381 | 0.701 | Unannotated | NA | NA |
| metab_19543 | Aceteugenol | neg | 251.0924 | 4.785 | 4.113 | 4.256 | 0.143 | 0.158 | 0.381 | 0.701 | Tentative | NA | NA |
| metab_17337 | Unannotated feature | neg | 143.9877 | 7.697 | 4.321 | 4.299 | -0.022 | 0.158 | 0.381 | 0.701 | Unannotated | NA | NA |
| metab_9404 | 2-(Arabinosylamino)-3-(glucosylamino)propanenitrile | neg | 360.1417 | 3.469 | 4.254 | 3.999 | -0.254 | -0.158 | 0.381 | 0.701 | Unannotated | Organic oxygen compounds | Organooxygen compounds |
| metab_18281 | 2,6-Diamino-5-hydroxyhexanoic acid | neg | 485.2924 | 6.224 | 3.976 | 3.750 | -0.226 | -0.158 | 0.381 | 0.701 | Tentative | NA | NA |
| metab_18300 | Unannotated feature | neg | 183.1384 | 6.217 | 4.467 | 4.052 | -0.415 | -0.158 | 0.381 | 0.701 | Unannotated | NA | NA |
| metab_1614 | Unannotated feature | pos | 271.1521 | 4.938 | 3.830 | 3.724 | -0.106 | -0.158 | 0.381 | 0.701 | Unannotated | NA | NA |
| metab_18849 | Eudesmic acid | neg | 211.0608 | 3.210 | 5.181 | 5.240 | 0.059 | 0.158 | 0.381 | 0.701 | Moderate putative | Benzenoids | Benzene and substituted derivatives |
| metab_12722 | Unannotated feature | neg | 469.2583 | 6.561 | 5.907 | 6.084 | 0.177 | 0.158 | 0.381 | 0.701 | Unannotated | NA | NA |
| metab_9368 | Unannotated feature | neg | 223.0608 | 3.517 | 5.469 | 5.487 | 0.018 | -0.158 | 0.381 | 0.701 | Unannotated | NA | NA |
| metab_7616 | 4-Acetoxy-4-androstene-3,17-dione | pos | 184.0972 | 3.527 | 4.426 | 4.534 | 0.108 | 0.158 | 0.381 | 0.701 | Unannotated | NA | NA |
| metab_19527 | Verimol D | neg | 347.1504 | 4.814 | 5.238 | 4.963 | -0.275 | -0.158 | 0.381 | 0.701 | Unannotated | Phenylpropanoids and polyketides | Stilbenes |
| metab_1010 | DG(20:4(6E,8Z,11Z,13E)-2OH(5S,15S)/0:0/16:0) | pos | 613.4821 | 7.124 | 5.008 | 4.842 | -0.167 | -0.158 | 0.381 | 0.701 | Unannotated | NA | NA |
| metab_18834 | Unannotated feature | neg | 359.1718 | 5.748 | 4.844 | 5.032 | 0.188 | 0.158 | 0.381 | 0.701 | Unannotated | NA | NA |
| metab_1214 | Deoxyspergualin | pos | 426.2627 | 6.414 | 5.247 | 5.076 | -0.171 | -0.158 | 0.381 | 0.701 | Unannotated | NA | NA |
| metab_3335 | Unannotated feature | pos | 231.1707 | 4.194 | 4.011 | 3.682 | -0.330 | -0.158 | 0.381 | 0.701 | Unannotated | NA | NA |
| metab_19750 | Unannotated feature | neg | 215.0922 | 4.506 | 4.906 | 4.938 | 0.032 | 0.158 | 0.381 | 0.701 | Unannotated | NA | NA |
| metab_9878 | Unannotated feature | neg | 269.1145 | 2.968 | 4.983 | 5.135 | 0.152 | 0.158 | 0.381 | 0.701 | Unannotated | NA | NA |
| metab_10023 | Unannotated feature | neg | 344.1358 | 2.817 | 3.989 | 4.205 | 0.216 | 0.158 | 0.381 | 0.701 | Unannotated | NA | NA |
| metab_42 | Pro Arg | pos | 272.1721 | 0.696 | 5.550 | 5.397 | -0.153 | -0.158 | 0.381 | 0.701 | Unannotated | NA | NA |
| metab_6535 | Hemorphin-4 | pos | 607.2903 | 5.544 | 3.471 | 3.158 | -0.312 | -0.158 | 0.381 | 0.701 | Unannotated | NA | NA |
| metab_12048 | Unannotated feature | neg | 451.2167 | 6.357 | 5.782 | 5.922 | 0.141 | 0.158 | 0.381 | 0.701 | Unannotated | NA | NA |
| metab_14854 | Desmethyldeschlorobenzoyl Indomethacin | neg | 190.0504 | 4.008 | 6.041 | 6.070 | 0.029 | 0.158 | 0.381 | 0.701 | Moderate putative | NA | NA |
| metab_13403 | Ureidosuccinic Acid | neg | 175.0354 | 0.777 | 4.847 | 4.469 | -0.378 | -0.158 | 0.381 | 0.701 | Strong putative | NA | NA |
| metab_13490 | L-trans-alpha-Amino-2-carboxycyclopropaneacetic acid | neg | 377.1207 | 1.026 | 4.238 | 3.713 | -0.525 | -0.158 | 0.381 | 0.701 | Unannotated | NA | NA |
| metab_13541 | Unannotated feature | neg | 355.1252 | 1.127 | 3.955 | 3.559 | -0.396 | -0.158 | 0.381 | 0.701 | Unannotated | NA | NA |
| metab_17964 | Unannotated feature | neg | 427.2635 | 6.561 | 6.675 | 6.649 | -0.026 | -0.158 | 0.381 | 0.701 | Unannotated | NA | NA |
| metab_19872 | Hydroquinone | neg | 109.0288 | 2.984 | 4.521 | 4.570 | 0.049 | 0.154 | 0.394 | 0.714 | Strong putative | NA | NA |
| metab_12871 | Unannotated feature | neg | 236.0774 | 0.714 | 5.428 | 5.469 | 0.041 | 0.154 | 0.394 | 0.714 | Unannotated | NA | NA |
| metab_3334 | Ser Ile Leu | pos | 332.2186 | 4.190 | 4.264 | 4.070 | -0.194 | -0.154 | 0.394 | 0.714 | Moderate putative | NA | NA |
| metab_8674 | Unannotated feature | pos | 137.0271 | 0.854 | 4.405 | 4.478 | 0.072 | 0.154 | 0.394 | 0.714 | Unannotated | NA | NA |
| metab_12928 | 4-Hydroxymethyl Ambrisentan | neg | 429.1259 | 0.738 | 5.117 | 5.042 | -0.075 | -0.154 | 0.394 | 0.714 | Unannotated | NA | NA |
| metab_15793 | 2-Phenylethanol glucuronide | neg | 297.0981 | 5.842 | 5.155 | 5.219 | 0.064 | 0.154 | 0.394 | 0.714 | Tentative | NA | NA |
| metab_16704 | Unannotated feature | neg | 672.8609 | 7.637 | 6.255 | 6.326 | 0.071 | 0.154 | 0.394 | 0.714 | Unannotated | NA | NA |
| metab_12529 | Avocadyne | neg | 329.2334 | 6.263 | 6.755 | 6.690 | -0.065 | -0.154 | 0.394 | 0.714 | Unannotated | NA | NA |
| metab_4821 | Unannotated feature | pos | 200.9961 | 7.867 | 5.021 | 5.043 | 0.022 | 0.154 | 0.394 | 0.714 | Unannotated | NA | NA |
| metab_6321 | Unannotated feature | pos | 265.0234 | 0.561 | 4.676 | 4.703 | 0.027 | 0.154 | 0.394 | 0.714 | Unannotated | NA | NA |
| metab_11338 | 5-Methoxy-3-(1,2,3,6-tetrahydropyridin-4-yl)-1H-indole | neg | 263.0974 | 0.596 | 4.600 | 4.761 | 0.161 | 0.154 | 0.394 | 0.714 | Unannotated | NA | NA |
| metab_18054 | Unannotated feature | neg | 492.2586 | 6.436 | 5.736 | 5.941 | 0.205 | 0.154 | 0.394 | 0.714 | Unannotated | NA | NA |
| metab_13328 | Unannotated feature | neg | 226.0485 | 0.683 | 3.389 | 3.602 | 0.213 | 0.154 | 0.394 | 0.714 | Unannotated | NA | NA |
| metab_19141 | Coumatetralyl | neg | 337.1061 | 5.339 | 4.969 | 4.980 | 0.012 | 0.154 | 0.394 | 0.714 | Tentative | NA | NA |
| metab_19208 | Unannotated feature | neg | 171.0656 | 5.257 | 5.348 | 5.302 | -0.046 | -0.154 | 0.394 | 0.714 | Unannotated | NA | NA |
| metab_16768 | Unannotated feature | neg | 340.2036 | 7.692 | 3.310 | 3.514 | 0.204 | 0.154 | 0.394 | 0.714 | Unannotated | NA | NA |
| metab_854 | Unannotated feature | pos | 771.4891 | 6.558 | 5.882 | 5.797 | -0.085 | -0.154 | 0.394 | 0.714 | Unannotated | NA | NA |
| metab_5135 | Unannotated feature | pos | 591.5839 | 6.901 | 4.883 | 4.685 | -0.198 | -0.154 | 0.394 | 0.714 | Unannotated | NA | NA |
| metab_7377 | 2-Hydroxy-4-[(1R)-1-hydroxy-8-methyl-6-oxononyl]-3-methyl-2H-furan-5-one | pos | 302.1969 | 4.016 | 4.223 | 3.921 | -0.302 | -0.154 | 0.394 | 0.714 | Unannotated | NA | NA |
| metab_7371 | 2-Polyprenyl-3-methyl-5-hydroxy-6-methoxy-1,4-benzoquinone | pos | 349.1403 | 4.032 | 5.133 | 5.350 | 0.217 | 0.154 | 0.394 | 0.714 | Tentative | NA | NA |
| metab_405 | Unannotated feature | pos | 219.0521 | 0.016 | 4.348 | 4.356 | 0.009 | 0.154 | 0.394 | 0.714 | Unannotated | NA | NA |
| metab_11657 | Unannotated feature | neg | 146.9379 | 0.022 | 4.814 | 4.792 | -0.022 | -0.154 | 0.394 | 0.714 | Unannotated | NA | NA |
| metab_15203 | Unannotated feature | neg | 289.1564 | 4.735 | 2.594 | 3.079 | 0.484 | 0.154 | 0.394 | 0.714 | Unannotated | NA | NA |
| metab_7769 | Unannotated feature | pos | 330.2030 | 3.268 | 5.096 | 5.062 | -0.034 | -0.154 | 0.394 | 0.714 | Unannotated | NA | NA |
| metab_7779 | Valylhydroxyproline | pos | 213.1237 | 3.238 | 3.538 | 3.283 | -0.255 | -0.154 | 0.394 | 0.714 | Unannotated | NA | NA |
| metab_5088 | Unannotated feature | pos | 481.2841 | 7.060 | 5.366 | 5.353 | -0.013 | -0.154 | 0.394 | 0.714 | Unannotated | NA | NA |
| metab_12160 | Unannotated feature | neg | 455.3381 | 6.663 | 5.670 | 6.005 | 0.335 | 0.154 | 0.394 | 0.714 | Unannotated | NA | NA |
| metab_2337 | Unannotated feature | pos | 111.1173 | 0.424 | 3.775 | 3.814 | 0.039 | 0.154 | 0.394 | 0.714 | Unannotated | NA | NA |
| metab_897 | Unannotated feature | pos | 507.2708 | 7.005 | 5.466 | 5.309 | -0.157 | -0.154 | 0.394 | 0.714 | Unannotated | NA | NA |
| metab_16820 | Unannotated feature | neg | 644.8527 | 7.720 | 4.976 | 5.016 | 0.040 | 0.154 | 0.394 | 0.714 | Unannotated | NA | NA |
| metab_16464 | Unannotated feature | neg | 170.0453 | 1.685 | 4.816 | 4.683 | -0.133 | -0.154 | 0.394 | 0.714 | Unannotated | NA | NA |
| metab_16509 | Sorbitan palmitate | neg | 437.2685 | 7.114 | 4.489 | 3.997 | -0.492 | -0.154 | 0.394 | 0.714 | Unannotated | NA | NA |
| metab_9614 | Unannotated feature | neg | 349.9558 | 3.215 | 4.639 | 4.536 | -0.104 | -0.154 | 0.394 | 0.714 | Unannotated | NA | NA |
| metab_18179 | Unannotated feature | neg | 457.2813 | 6.318 | 6.369 | 6.262 | -0.108 | -0.154 | 0.394 | 0.714 | Unannotated | NA | NA |
| metab_12992 | Unannotated feature | neg | 509.3124 | 6.310 | 7.116 | 7.021 | -0.095 | -0.154 | 0.394 | 0.714 | Unannotated | NA | NA |
| metab_12812 | Unannotated feature | neg | 287.1363 | 0.651 | 4.870 | 4.620 | -0.250 | -0.154 | 0.394 | 0.714 | Unannotated | NA | NA |
| metab_18589 | Unannotated feature | neg | 498.2256 | 5.999 | 4.851 | 4.718 | -0.133 | -0.154 | 0.394 | 0.714 | Unannotated | NA | NA |
| metab_8604 | Leucyl-Arginine | pos | 144.6054 | 1.122 | 3.108 | 3.052 | -0.056 | 0.154 | 0.394 | 0.714 | Unannotated | NA | NA |
| metab_13222 | Creatinine nitrite nitrate | neg | 200.0047 | 0.558 | 4.969 | 4.778 | -0.191 | -0.154 | 0.394 | 0.714 | Unannotated | NA | NA |
| metab_908 | Unannotated feature | pos | 539.2733 | 7.132 | 5.200 | 5.258 | 0.058 | -0.154 | 0.394 | 0.714 | Unannotated | NA | NA |
| metab_19003 | Unannotated feature | neg | 271.0504 | 5.526 | 3.896 | 3.644 | -0.252 | -0.154 | 0.394 | 0.714 | Unannotated | NA | NA |
| metab_4673 | Unannotated feature | pos | 458.4217 | 7.474 | 5.229 | 5.162 | -0.067 | -0.154 | 0.394 | 0.714 | Unannotated | NA | NA |
| metab_4635 | Unannotated feature | pos | 449.1879 | 7.180 | 4.519 | 4.380 | -0.139 | -0.154 | 0.394 | 0.714 | Unannotated | NA | NA |
| metab_17600 | Unannotated feature | neg | 465.3051 | 7.216 | 7.908 | 7.827 | -0.081 | -0.154 | 0.394 | 0.714 | Unannotated | NA | NA |
| metab_9541 | Unannotated feature | neg | 264.0879 | 3.298 | 5.112 | 5.002 | -0.110 | -0.154 | 0.394 | 0.714 | Unannotated | NA | NA |
| metab_16851 | Unannotated feature | neg | 307.1300 | 7.730 | 5.312 | 5.429 | 0.117 | 0.154 | 0.394 | 0.714 | Unannotated | NA | NA |
| metab_12654 | Unannotated feature | neg | 231.1236 | 5.937 | 5.040 | 5.081 | 0.041 | -0.154 | 0.394 | 0.714 | Unannotated | NA | NA |
| metab_2443 | 2-Pyrrolidinone | pos | 86.0605 | 0.649 | 4.588 | 4.380 | -0.208 | -0.154 | 0.394 | 0.714 | Strong putative | NA | NA |
| metab_13168 | Unannotated feature | neg | 233.0431 | 4.785 | 5.820 | 5.529 | -0.291 | -0.154 | 0.394 | 0.714 | Unannotated | NA | NA |
| metab_16911 | Unannotated feature | neg | 323.9097 | 7.769 | 5.042 | 5.030 | -0.013 | -0.154 | 0.394 | 0.714 | Unannotated | NA | NA |
| metab_8249 | Adipate semialdehyde | pos | 95.0497 | 2.288 | 5.205 | 5.139 | -0.066 | -0.154 | 0.394 | 0.714 | Tentative | NA | NA |
| metab_8245 | Unannotated feature | pos | 91.0547 | 2.299 | 4.673 | 4.628 | -0.045 | -0.154 | 0.394 | 0.714 | Unannotated | NA | NA |
| metab_5243 | Polyoxyethylene (600) monoricinoleate | pos | 341.3055 | 6.670 | 5.699 | 5.753 | 0.054 | 0.154 | 0.394 | 0.714 | Unannotated | NA | NA |
| metab_16441 | Cer(d18:0/20:4) | neg | 632.5305 | 6.953 | 5.546 | 5.337 | -0.209 | -0.154 | 0.394 | 0.714 | Unannotated | NA | NA |
| metab_19567 | Unannotated feature | neg | 157.0500 | 3.210 | 5.060 | 5.201 | 0.141 | 0.154 | 0.394 | 0.714 | Unannotated | NA | NA |
| metab_278 | Unannotated feature | pos | 416.9176 | 0.501 | 4.102 | 4.327 | 0.224 | 0.154 | 0.394 | 0.714 | Unannotated | NA | NA |
| metab_3077 | Unannotated feature | pos | 295.1295 | 3.371 | 5.045 | 5.036 | -0.009 | -0.154 | 0.394 | 0.714 | Unannotated | NA | NA |
| metab_16214 | Unannotated feature | neg | 485.3108 | 6.444 | 3.813 | 4.474 | 0.662 | 0.154 | 0.394 | 0.714 | Unannotated | NA | NA |
| metab_15275 | Unannotated feature | neg | 316.0013 | 0.558 | 4.657 | 5.061 | 0.403 | 0.154 | 0.394 | 0.714 | Unannotated | NA | NA |
| metab_1587 | Unannotated feature | pos | 397.2134 | 5.175 | 4.163 | 4.340 | 0.177 | 0.154 | 0.394 | 0.714 | Unannotated | NA | NA |
| metab_12706 | Unannotated feature | neg | 301.1449 | 5.280 | 5.667 | 5.604 | -0.063 | -0.154 | 0.394 | 0.714 | Unannotated | NA | NA |
| metab_13140 | Unannotated feature | neg | 232.8585 | 7.993 | 5.545 | 5.511 | -0.034 | -0.154 | 0.394 | 0.714 | Unannotated | NA | NA |
| metab_17448 | Unannotated feature | neg | 384.9358 | 7.611 | 7.754 | 7.772 | 0.018 | 0.154 | 0.394 | 0.714 | Unannotated | NA | NA |
| metab_4478 | Unannotated feature | pos | 533.4329 | 6.518 | 5.203 | 5.425 | 0.222 | 0.154 | 0.394 | 0.714 | Unannotated | NA | NA |
| metab_4442 | Unannotated feature | pos | 481.3535 | 6.375 | 5.410 | 5.254 | -0.156 | -0.154 | 0.394 | 0.714 | Unannotated | NA | NA |
| metab_8316 | Serotonin | pos | 177.1026 | 2.032 | 3.832 | 3.662 | -0.170 | -0.154 | 0.394 | 0.714 | Strong putative | NA | NA |
| metab_8342 | Unannotated feature | pos | 132.0659 | 1.976 | 4.425 | 4.405 | -0.021 | -0.154 | 0.394 | 0.714 | Unannotated | NA | NA |
| metab_12795 | Unannotated feature | neg | 344.8953 | 0.636 | 3.811 | 3.838 | 0.027 | -0.154 | 0.394 | 0.714 | Unannotated | NA | NA |
| metab_3847 | Pentenylbenzene | pos | 147.1170 | 6.390 | 5.156 | 5.101 | -0.056 | -0.154 | 0.394 | 0.714 | Unannotated | NA | NA |
| metab_10943 | Unannotated feature | neg | 170.0452 | 0.799 | 5.115 | 5.261 | 0.146 | 0.154 | 0.394 | 0.714 | Unannotated | NA | NA |
| metab_18722 | Unannotated feature | neg | 649.2875 | 5.884 | 4.630 | 4.171 | -0.460 | -0.154 | 0.394 | 0.714 | Unannotated | NA | NA |
| metab_17344 | Unannotated feature | neg | 461.8825 | 7.692 | 3.975 | 4.049 | 0.073 | 0.154 | 0.394 | 0.714 | Unannotated | NA | NA |
| metab_4426 | Unannotated feature | pos | 158.1542 | 6.343 | 4.749 | 4.751 | 0.002 | -0.154 | 0.394 | 0.714 | Unannotated | NA | NA |
| metab_4394 | Withanolide B | pos | 499.2405 | 6.255 | 4.122 | 5.072 | 0.950 | 0.154 | 0.394 | 0.714 | Tentative | Lipids and lipid-like molecules | Prenol lipids |
| metab_7302 | Unannotated feature | pos | 295.1658 | 4.194 | 4.458 | 4.317 | -0.141 | -0.154 | 0.394 | 0.714 | Unannotated | NA | NA |
| metab_2786 | Unannotated feature | pos | 411.1850 | 2.560 | 3.629 | 3.853 | 0.224 | 0.154 | 0.394 | 0.714 | Unannotated | NA | NA |
| metab_17293 | Unannotated feature | neg | 599.8724 | 7.710 | 4.931 | 4.972 | 0.041 | 0.154 | 0.394 | 0.714 | Unannotated | NA | NA |
| metab_13082 | Unannotated feature | neg | 241.7579 | 0.027 | 5.729 | 5.696 | -0.032 | -0.154 | 0.394 | 0.714 | Unannotated | NA | NA |
| metab_2904 | Phaseollinisoflavan | pos | 289.1255 | 2.859 | 2.626 | 3.315 | 0.689 | 0.154 | 0.394 | 0.714 | Tentative | NA | NA |
| metab_4267 | Unannotated feature | pos | 646.3029 | 5.944 | 2.933 | 3.828 | 0.896 | 0.154 | 0.394 | 0.714 | Unannotated | NA | NA |
| metab_8392 | Unannotated feature | pos | 228.1234 | 1.952 | 4.127 | 4.285 | 0.158 | 0.154 | 0.394 | 0.714 | Unannotated | NA | NA |
| metab_11006 | Xi-3-Hydroxy-5-phenylpentanoic acid O-beta-D-Glucopyranoside | neg | 377.1208 | 0.770 | 4.077 | 4.319 | 0.242 | 0.154 | 0.394 | 0.714 | Unannotated | NA | NA |
| metab_1058 | Unannotated feature | pos | 405.3459 | 6.789 | 5.272 | 5.197 | -0.075 | -0.154 | 0.394 | 0.714 | Unannotated | NA | NA |
| metab_13979 | Unannotated feature | neg | 657.1963 | 2.663 | 4.322 | 3.532 | -0.790 | -0.154 | 0.394 | 0.714 | Unannotated | NA | NA |
| metab_2822 | 1-Methylhypoxanthine | pos | 151.0618 | 2.724 | 4.050 | 4.247 | 0.196 | 0.154 | 0.394 | 0.714 | Unannotated | NA | NA |
| metab_7295 | 1-Aminocyclohexanecarboxylic acid | pos | 126.0917 | 4.212 | 4.292 | 4.351 | 0.059 | -0.154 | 0.394 | 0.714 | Unannotated | NA | NA |
| metab_7267 | Val Glu Phe | pos | 394.1985 | 4.268 | 4.522 | 4.146 | -0.375 | -0.154 | 0.394 | 0.714 | Moderate putative | NA | NA |
| metab_12419 | Unannotated feature | neg | 435.1346 | 6.248 | 1.876 | 4.219 | 2.343 | 0.154 | 0.394 | 0.714 | Unannotated | NA | NA |
| metab_14503 | Xanthurenic Acid | neg | 204.0298 | 3.354 | 4.903 | 5.065 | 0.162 | 0.154 | 0.394 | 0.714 | Strong putative | NA | NA |
| metab_12787 | Unannotated feature | neg | 219.9781 | 0.636 | 5.580 | 5.687 | 0.107 | -0.154 | 0.394 | 0.714 | Unannotated | NA | NA |
| metab_12781 | Isoleucine glutamate | neg | 321.1310 | 0.628 | 4.776 | 4.903 | 0.127 | 0.154 | 0.394 | 0.714 | Unannotated | NA | NA |
| metab_11608 | Unannotated feature | neg | 195.8685 | 0.405 | 5.302 | 5.280 | -0.022 | -0.154 | 0.394 | 0.714 | Unannotated | NA | NA |
| metab_13472 | Unannotated feature | neg | 242.1762 | 0.968 | 4.637 | 4.586 | -0.052 | -0.154 | 0.394 | 0.714 | Unannotated | NA | NA |
| metab_7456 | Unannotated feature | pos | 368.2190 | 3.854 | 4.040 | 3.519 | -0.521 | -0.154 | 0.394 | 0.714 | Unannotated | NA | NA |
| metab_16289 | Unannotated feature | neg | 365.2314 | 6.601 | 4.972 | 5.221 | 0.249 | 0.154 | 0.394 | 0.714 | Unannotated | NA | NA |
| metab_8775 | Imidazolepropionic acid | pos | 123.0557 | 0.728 | 4.762 | 4.766 | 0.004 | -0.154 | 0.394 | 0.714 | Tentative | NA | NA |
| metab_17099 | Unannotated feature | neg | 169.8334 | 7.870 | 4.502 | 4.553 | 0.051 | 0.154 | 0.394 | 0.714 | Unannotated | NA | NA |
| metab_7955 | Pilsicainide | pos | 295.1768 | 2.897 | 3.512 | 3.296 | -0.217 | -0.154 | 0.394 | 0.714 | Unannotated | NA | NA |
| metab_14849 | Unannotated feature | neg | 243.9559 | 0.507 | 5.086 | 5.089 | 0.002 | -0.154 | 0.394 | 0.714 | Unannotated | NA | NA |
| metab_4880 | Unannotated feature | pos | 237.9252 | 7.716 | 4.203 | 4.164 | -0.040 | 0.154 | 0.394 | 0.714 | Unannotated | NA | NA |
| metab_13396 | Unannotated feature | neg | 239.0673 | 0.770 | 4.138 | 4.414 | 0.276 | 0.154 | 0.394 | 0.714 | Unannotated | NA | NA |
| metab_13483 | Unannotated feature | neg | 272.8772 | 0.603 | 4.999 | 4.761 | -0.238 | -0.154 | 0.394 | 0.714 | Unannotated | NA | NA |
| metab_17887 | Unannotated feature | neg | 348.2215 | 6.687 | 4.079 | 3.891 | -0.187 | -0.149 | 0.407 | 0.724 | Unannotated | NA | NA |
| metab_17899 | 3-Hydroxylinoleoylcarnitine | neg | 476.2785 | 6.663 | 6.773 | 6.804 | 0.030 | -0.149 | 0.407 | 0.724 | Unannotated | NA | NA |
| metab_19796 | Unannotated feature | neg | 440.1836 | 3.688 | 4.556 | 3.792 | -0.764 | -0.149 | 0.407 | 0.724 | Unannotated | NA | NA |
| metab_34 | (R)-2-Hydroxy-2H-1,4-benzoxazin-3(4H)-one | pos | 183.0766 | 0.704 | 4.178 | 3.756 | -0.422 | -0.149 | 0.407 | 0.724 | Tentative | NA | NA |
| metab_13998 | Diacetoxyscirpenol | neg | 387.1416 | 2.714 | 6.120 | 6.042 | -0.079 | -0.149 | 0.407 | 0.724 | Tentative | NA | NA |
| metab_1405 | Unannotated feature | pos | 291.1708 | 6.080 | 5.281 | 5.249 | -0.032 | -0.149 | 0.407 | 0.724 | Unannotated | NA | NA |
| metab_19361 | {4-[4-(6-Carbamoyl-3,5-dimethylpyrazin-2-yl)phenyl]cyclohexyl}acetic acid | neg | 402.1563 | 5.057 | 4.831 | 4.555 | -0.276 | -0.149 | 0.407 | 0.724 | Unannotated | NA | NA |
| metab_13093 | Unannotated feature | neg | 252.9262 | 0.036 | 5.782 | 5.767 | -0.015 | -0.149 | 0.407 | 0.724 | Unannotated | NA | NA |
| metab_17265 | Unannotated feature | neg | 165.8938 | 7.726 | 4.459 | 4.451 | -0.008 | -0.149 | 0.407 | 0.724 | Unannotated | NA | NA |
| metab_18372 | 3b-Hydroxy-6b-methoxy-7(11)-eremophilen-12,8a-olide | neg | 325.1660 | 6.154 | 5.015 | 4.912 | -0.103 | 0.149 | 0.407 | 0.724 | Unannotated | NA | NA |
| metab_9887 | Unannotated feature | neg | 374.0114 | 2.960 | 4.321 | 4.095 | -0.226 | -0.149 | 0.407 | 0.724 | Unannotated | NA | NA |
| metab_10115 | Unannotated feature | neg | 377.1096 | 2.762 | 3.798 | 3.479 | -0.319 | -0.149 | 0.407 | 0.724 | Unannotated | NA | NA |
| metab_5077 | Unannotated feature | pos | 164.0920 | 7.100 | 3.998 | 4.003 | 0.005 | 0.149 | 0.407 | 0.724 | Unannotated | NA | NA |
| metab_16594 | Unannotated feature | neg | 692.9980 | 7.395 | 6.892 | 6.969 | 0.077 | 0.149 | 0.407 | 0.724 | Unannotated | NA | NA |
| metab_11383 | Unannotated feature | neg | 215.9824 | 0.558 | 3.302 | 3.865 | 0.562 | 0.149 | 0.407 | 0.724 | Unannotated | NA | NA |
| metab_19240 | Unannotated feature | neg | 430.1990 | 5.209 | 3.870 | 4.243 | 0.374 | 0.149 | 0.407 | 0.724 | Unannotated | NA | NA |
| metab_13573 | Nopalinic acid | neg | 243.0986 | 1.267 | 5.768 | 5.802 | 0.033 | -0.149 | 0.407 | 0.724 | Tentative | NA | NA |
| metab_8734 | Unannotated feature | pos | 306.1379 | 0.760 | 3.081 | 3.225 | 0.144 | 0.149 | 0.407 | 0.724 | Unannotated | NA | NA |
| metab_5490 | Dhv-PGE2 | pos | 361.2368 | 6.359 | 5.428 | 5.128 | -0.300 | -0.149 | 0.407 | 0.724 | Unannotated | NA | NA |
| metab_17022 | Unannotated feature | neg | 205.8973 | 7.997 | 4.987 | 4.967 | -0.020 | -0.149 | 0.407 | 0.724 | Unannotated | NA | NA |
| metab_12415 | Unannotated feature | neg | 429.2975 | 6.530 | 6.196 | 6.077 | -0.120 | -0.149 | 0.407 | 0.724 | Unannotated | NA | NA |
| metab_12417 | Unannotated feature | neg | 594.8391 | 7.720 | 5.028 | 5.065 | 0.037 | 0.149 | 0.407 | 0.724 | Unannotated | NA | NA |
| metab_11589 | 3-benzyl-4-hydroxy-5-(4-hydroxyphenyl)furan-2(5H)-one | neg | 327.0879 | 5.280 | 5.993 | 5.822 | -0.171 | -0.149 | 0.407 | 0.724 | Moderate putative | NA | NA |
| metab_5509 | Unannotated feature | pos | 142.0866 | 1.952 | 5.054 | 5.051 | -0.002 | 0.149 | 0.407 | 0.724 | Unannotated | NA | NA |
| metab_18376 | Milbemycin A4 | neg | 587.3272 | 6.146 | 5.009 | 5.202 | 0.193 | 0.149 | 0.407 | 0.724 | Unannotated | NA | NA |
| metab_16986 | Unannotated feature | neg | 239.7604 | 7.978 | 4.662 | 4.649 | -0.013 | -0.149 | 0.407 | 0.724 | Unannotated | NA | NA |
| metab_18802 | Unannotated feature | neg | 238.1083 | 5.783 | 5.127 | 5.103 | -0.024 | -0.149 | 0.407 | 0.724 | Unannotated | NA | NA |
| metab_16742 | Unannotated feature | neg | 485.8877 | 7.666 | 5.289 | 5.326 | 0.037 | 0.149 | 0.407 | 0.724 | Unannotated | NA | NA |
| metab_13661 | 6-Hydroxynicotinic Acid | neg | 138.0190 | 1.685 | 5.558 | 5.633 | 0.076 | 0.149 | 0.407 | 0.724 | Strong putative | NA | NA |
| metab_13360 | Unannotated feature | neg | 97.0286 | 0.730 | 4.429 | 4.498 | 0.069 | 0.149 | 0.407 | 0.724 | Unannotated | NA | NA |
| metab_4784 | Unannotated feature | pos | 156.0108 | 7.824 | 5.044 | 5.033 | -0.011 | -0.149 | 0.407 | 0.724 | Unannotated | NA | NA |
| metab_19274 | 10-(2,3-Dihydroxypropoxy)-10-oxodecanoic acid | neg | 275.1499 | 5.173 | 4.799 | 5.018 | 0.219 | 0.149 | 0.407 | 0.724 | Unannotated | NA | NA |
| metab_9822 | Unannotated feature | neg | 370.9585 | 3.021 | 4.805 | 4.796 | -0.010 | -0.149 | 0.407 | 0.724 | Unannotated | NA | NA |
| metab_15870 | Unannotated feature | neg | 240.0747 | 5.958 | 5.423 | 5.540 | 0.117 | 0.149 | 0.407 | 0.724 | Unannotated | NA | NA |
| metab_2954 | Pyridoxal | pos | 168.0658 | 2.965 | 3.995 | 4.000 | 0.006 | 0.149 | 0.407 | 0.724 | Strong putative | NA | NA |
| metab_15298 | Unannotated feature | neg | 205.1077 | 4.876 | 3.824 | 3.186 | -0.638 | -0.149 | 0.407 | 0.724 | Unannotated | NA | NA |
| metab_12324 | Unannotated feature | neg | 607.3153 | 6.123 | 6.580 | 6.456 | -0.124 | -0.149 | 0.407 | 0.724 | Unannotated | NA | NA |
| metab_16973 | Unannotated feature | neg | 212.8843 | 7.910 | 5.260 | 5.276 | 0.015 | 0.149 | 0.407 | 0.724 | Unannotated | NA | NA |
| metab_5244 | 2-Methylhexadecanoic acid | pos | 253.2529 | 6.670 | 4.449 | 4.649 | 0.201 | 0.149 | 0.407 | 0.724 | Unannotated | NA | NA |
| metab_19329 | Unannotated feature | neg | 149.0601 | 5.098 | 2.893 | 3.433 | 0.541 | 0.149 | 0.407 | 0.724 | Unannotated | NA | NA |
| metab_7233 | Unannotated feature | pos | 492.1127 | 0.680 | 2.824 | 3.537 | 0.713 | 0.149 | 0.407 | 0.724 | Unannotated | NA | NA |
| metab_13106 | Unannotated feature | neg | 121.9433 | 0.142 | 5.096 | 5.076 | -0.019 | -0.149 | 0.407 | 0.724 | Unannotated | NA | NA |
| metab_10660 | Unannotated feature | neg | 174.0766 | 1.604 | 5.423 | 5.623 | 0.200 | 0.149 | 0.407 | 0.724 | Unannotated | NA | NA |
| metab_7475 | Unannotated feature | pos | 263.1032 | 3.808 | 4.072 | 4.343 | 0.271 | 0.149 | 0.407 | 0.724 | Unannotated | NA | NA |
| metab_18608 | Unannotated feature | neg | 297.0986 | 5.983 | 5.389 | 5.493 | 0.104 | 0.149 | 0.407 | 0.724 | Unannotated | NA | NA |
| metab_14363 | ACEXAMIC ACID | neg | 172.0973 | 3.134 | 5.019 | 4.827 | -0.193 | -0.149 | 0.407 | 0.724 | Moderate putative | NA | NA |
| metab_7394 | Unannotated feature | pos | 203.0343 | 3.991 | 4.068 | 4.042 | -0.027 | -0.149 | 0.407 | 0.724 | Unannotated | NA | NA |
| metab_10672 | 4-Amino-1,3-dihydroxy-9,10-dioxo-9,10-dihydroanthracene-2-sulfonic acid | neg | 334.0053 | 1.526 | 5.931 | 5.908 | -0.023 | -0.149 | 0.407 | 0.724 | Unannotated | NA | NA |
| metab_4764 | Unannotated feature | pos | 199.0178 | 7.732 | 4.698 | 4.678 | -0.020 | -0.149 | 0.407 | 0.724 | Unannotated | NA | NA |
| metab_855 | Unannotated feature | pos | 397.2936 | 6.574 | 3.983 | 3.808 | -0.175 | -0.149 | 0.407 | 0.724 | Unannotated | NA | NA |
| metab_12578 | Unannotated feature | neg | 385.2233 | 6.115 | 6.404 | 6.182 | -0.222 | -0.149 | 0.407 | 0.724 | Unannotated | NA | NA |
| metab_3004 | Isopilocarpinic acid | pos | 268.1660 | 3.135 | 3.844 | 4.171 | 0.326 | 0.149 | 0.407 | 0.724 | Unannotated | NA | NA |
| metab_9129 | Lumiflavin | neg | 255.0885 | 3.882 | 5.454 | 5.581 | 0.127 | 0.149 | 0.407 | 0.724 | Unannotated | NA | NA |
| metab_14445 | Unannotated feature | neg | 784.1527 | 3.254 | 5.339 | 5.291 | -0.047 | -0.149 | 0.407 | 0.724 | Unannotated | NA | NA |
| metab_3882 | Unannotated feature | pos | 280.2639 | 6.367 | 6.613 | 6.473 | -0.140 | -0.149 | 0.407 | 0.724 | Unannotated | NA | NA |
| metab_1369 | (2E,4E)-5-[2-Methyl-2-(1,1,4,4-tetramethyl-1,2,3,4-tetrahydronaphthalene-6-yl)cyclopropyl]-3-methyl-2,4-pentadienoic acid | pos | 353.2479 | 6.135 | 5.658 | 5.638 | -0.020 | -0.149 | 0.407 | 0.724 | Unannotated | NA | NA |
| metab_16380 | Icos-19-ene-1,2,4-Triol | neg | 327.2905 | 6.782 | 5.715 | 5.763 | 0.047 | 0.149 | 0.407 | 0.724 | Unannotated | NA | NA |
| metab_11075 | Unannotated feature | neg | 290.1248 | 0.730 | 4.049 | 4.637 | 0.589 | 0.149 | 0.407 | 0.724 | Unannotated | NA | NA |
| metab_16945 | Unannotated feature | neg | 337.8771 | 7.835 | 5.215 | 5.194 | -0.021 | -0.149 | 0.407 | 0.724 | Unannotated | NA | NA |
| metab_275 | Unannotated feature | pos | 413.0215 | 0.509 | 3.583 | 4.398 | 0.815 | 0.149 | 0.407 | 0.724 | Unannotated | NA | NA |
| metab_13719 | 3-(3-hydroxyphenyl)propanoic Acid | neg | 165.0551 | 4.522 | 6.605 | 6.540 | -0.065 | -0.149 | 0.407 | 0.724 | Strong putative | NA | NA |
| metab_1797 | Unannotated feature | pos | 110.0718 | 0.784 | 3.755 | 3.585 | -0.169 | -0.149 | 0.407 | 0.724 | Unannotated | NA | NA |
| metab_16788 | Unannotated feature | neg | 272.9590 | 7.702 | 4.630 | 4.611 | -0.019 | -0.149 | 0.407 | 0.724 | Unannotated | NA | NA |
| metab_4747 | Unannotated feature | pos | 73.5323 | 7.708 | 3.749 | 3.770 | 0.020 | 0.149 | 0.407 | 0.724 | Unannotated | NA | NA |
| metab_16546 | Unannotated feature | neg | 758.5066 | 7.230 | 5.939 | 5.837 | -0.101 | -0.149 | 0.407 | 0.724 | Unannotated | NA | NA |
| metab_14972 | Sativan | neg | 331.1192 | 4.270 | 5.558 | 5.429 | -0.129 | 0.149 | 0.407 | 0.724 | Tentative | NA | NA |
| metab_7759 | Gingerol | pos | 339.1560 | 3.283 | 4.098 | 4.092 | -0.006 | 0.149 | 0.407 | 0.724 | Tentative | NA | NA |
| metab_15925 | Unannotated feature | neg | 671.2764 | 6.022 | 4.599 | 4.149 | -0.450 | -0.149 | 0.407 | 0.724 | Unannotated | NA | NA |
| metab_6987 | (1R,2R,3S,1'R)-Nepetalinic acid | pos | 165.0913 | 4.874 | 3.150 | 2.969 | -0.181 | -0.149 | 0.407 | 0.724 | Unannotated | NA | NA |
| metab_12955 | Unannotated feature | neg | 193.0713 | 0.730 | 5.425 | 5.601 | 0.176 | 0.149 | 0.407 | 0.724 | Unannotated | NA | NA |
| metab_357 | Unannotated feature | pos | 815.2158 | 0.375 | 6.090 | 6.094 | 0.004 | 0.149 | 0.407 | 0.724 | Unannotated | NA | NA |
| metab_15389 | Unannotated feature | neg | 532.7932 | 0.533 | 5.005 | 5.232 | 0.227 | 0.149 | 0.407 | 0.724 | Unannotated | NA | NA |
| metab_10484 | Unannotated feature | neg | 262.1298 | 1.952 | 4.238 | 4.117 | -0.120 | -0.149 | 0.407 | 0.724 | Unannotated | NA | NA |
| metab_13143 | Unannotated feature | neg | 417.8921 | 0.457 | 4.237 | 4.227 | -0.010 | 0.149 | 0.407 | 0.724 | Unannotated | NA | NA |
| metab_4491 | Proscillaridin | pos | 553.2818 | 6.550 | 5.097 | 5.101 | 0.004 | -0.149 | 0.407 | 0.724 | Tentative | NA | NA |
| metab_16928 | Unannotated feature | neg | 161.0813 | 7.806 | 4.069 | 4.040 | -0.029 | -0.149 | 0.407 | 0.724 | Unannotated | NA | NA |
| metab_16812 | Unannotated feature | neg | 189.0076 | 7.720 | 5.641 | 5.621 | -0.019 | -0.149 | 0.407 | 0.724 | Unannotated | NA | NA |
| metab_5840 | Unannotated feature | pos | 256.2639 | 0.141 | 6.091 | 6.074 | -0.017 | -0.149 | 0.407 | 0.724 | Unannotated | NA | NA |
| metab_10202 | Unannotated feature | neg | 208.0614 | 2.649 | 4.822 | 4.913 | 0.091 | 0.149 | 0.407 | 0.724 | Unannotated | NA | NA |
| metab_19021 | Unannotated feature | neg | 466.2355 | 5.501 | 5.526 | 5.174 | -0.352 | -0.149 | 0.407 | 0.724 | Unannotated | NA | NA |
| metab_11267 | Unannotated feature | neg | 225.0988 | 0.625 | 4.071 | 3.992 | -0.080 | -0.149 | 0.407 | 0.724 | Unannotated | NA | NA |
| metab_2294 | Unannotated feature | pos | 160.9898 | 0.016 | 5.235 | 5.254 | 0.019 | 0.149 | 0.407 | 0.724 | Unannotated | NA | NA |
| metab_9647 | Nonate | neg | 233.1029 | 3.192 | 5.165 | 5.297 | 0.133 | 0.149 | 0.407 | 0.724 | Unannotated | NA | NA |
| metab_13282 | Unannotated feature | neg | 243.9186 | 0.619 | 4.752 | 4.418 | -0.334 | -0.149 | 0.407 | 0.724 | Unannotated | NA | NA |
| metab_2665 | Unannotated feature | pos | 230.1390 | 1.952 | 4.556 | 4.453 | -0.103 | -0.149 | 0.407 | 0.724 | Unannotated | NA | NA |
| metab_2680 | 2-Amino-4-(phosphonomethyl)hept-3-enoic acid | pos | 238.0827 | 1.984 | 3.689 | 2.794 | -0.895 | -0.149 | 0.407 | 0.724 | Unannotated | NA | NA |
| metab_17031 | Unannotated feature | neg | 113.9547 | 7.997 | 3.400 | 3.309 | -0.091 | -0.149 | 0.407 | 0.724 | Unannotated | NA | NA |
| metab_16203 | Unannotated feature | neg | 484.1338 | 6.420 | 4.006 | 5.175 | 1.169 | 0.149 | 0.407 | 0.724 | Unannotated | NA | NA |
| metab_10857 | Unannotated feature | neg | 201.0878 | 1.015 | 4.818 | 4.724 | -0.094 | -0.149 | 0.407 | 0.724 | Unannotated | NA | NA |
| metab_17061 | Unannotated feature | neg | 212.9880 | 7.965 | 5.207 | 5.206 | -0.001 | -0.149 | 0.407 | 0.724 | Unannotated | NA | NA |
| metab_3781 | Unannotated feature | pos | 84.3057 | 5.526 | 3.112 | 3.222 | 0.110 | 0.149 | 0.407 | 0.724 | Unannotated | NA | NA |
| metab_1312 | Unannotated feature | pos | 406.3536 | 6.231 | 5.619 | 5.618 | -0.001 | -0.149 | 0.407 | 0.724 | Unannotated | NA | NA |
| metab_3114 | Unannotated feature | pos | 423.2214 | 3.465 | 3.841 | 3.400 | -0.440 | -0.149 | 0.407 | 0.724 | Unannotated | NA | NA |
| metab_7051 | Unannotated feature | pos | 564.8231 | 4.738 | 1.136 | 3.769 | 2.634 | 0.149 | 0.407 | 0.724 | Unannotated | NA | NA |
| metab_16029 | Glyceryl palmitoleate | neg | 373.2599 | 6.154 | 5.389 | 5.512 | 0.123 | 0.149 | 0.407 | 0.724 | Unannotated | NA | NA |
| metab_12203 | Unannotated feature | neg | 352.2857 | 6.805 | 4.885 | 5.315 | 0.430 | 0.149 | 0.407 | 0.724 | Unannotated | NA | NA |
| metab_2500 | 2-Aminoheptanedioic acid | pos | 217.1187 | 0.784 | 5.425 | 5.375 | -0.049 | -0.149 | 0.407 | 0.724 | Unannotated | NA | NA |
| metab_13203 | Unannotated feature | neg | 173.0300 | 0.533 | 3.937 | 3.768 | -0.170 | -0.149 | 0.407 | 0.724 | Unannotated | NA | NA |
| metab_13749 | N,N'-METHYLENEBISACRYLAMIDE | neg | 153.0663 | 1.952 | 4.356 | 4.384 | 0.028 | -0.149 | 0.407 | 0.724 | Unannotated | NA | NA |
| metab_2419 | Zearalenone | pos | 319.1513 | 0.609 | 5.387 | 5.470 | 0.083 | 0.149 | 0.407 | 0.724 | Tentative | NA | NA |
| metab_17541 | Unannotated feature | neg | 728.4800 | 7.395 | 4.955 | 4.385 | -0.570 | -0.149 | 0.407 | 0.724 | Unannotated | NA | NA |
| metab_17544 | Unannotated feature | neg | 394.1263 | 2.746 | 4.970 | 5.198 | 0.228 | 0.149 | 0.407 | 0.724 | Unannotated | NA | NA |
| metab_12676 | (4R,5S,7R,11x)-11,12-Dihydroxy-1(10)-spirovetiven-2-one 12-glucoside | neg | 413.2184 | 5.758 | 4.837 | 5.114 | 0.277 | 0.149 | 0.407 | 0.724 | Unannotated | NA | NA |
| metab_8153 | Unannotated feature | pos | 292.1663 | 2.703 | 3.423 | 3.055 | -0.368 | -0.149 | 0.407 | 0.724 | Unannotated | NA | NA |
| metab_12652 | Unannotated feature | neg | 539.3415 | 6.577 | 6.985 | 7.029 | 0.044 | 0.149 | 0.407 | 0.724 | Unannotated | NA | NA |
| metab_18446 | Unannotated feature | neg | 272.1293 | 6.099 | 4.356 | 4.874 | 0.518 | 0.149 | 0.407 | 0.724 | Unannotated | NA | NA |
| metab_1153 | Unannotated feature | pos | 529.3518 | 6.558 | 6.475 | 6.595 | 0.119 | 0.149 | 0.407 | 0.724 | Unannotated | NA | NA |
| metab_2528 | Unannotated feature | pos | 266.0872 | 0.943 | 3.837 | 4.230 | 0.392 | 0.149 | 0.407 | 0.724 | Unannotated | NA | NA |
| metab_15397 | 2,3-Bis(3-hydroxybenzyl)butane-1,4-diol | neg | 301.1449 | 5.061 | 5.089 | 4.941 | -0.148 | -0.149 | 0.407 | 0.724 | Tentative | NA | NA |
| metab_12712 | Unannotated feature | neg | 555.2855 | 6.561 | 4.803 | 4.115 | -0.688 | -0.149 | 0.407 | 0.724 | Unannotated | NA | NA |
| metab_1740 | Unannotated feature | pos | 416.2140 | 3.642 | 3.742 | 3.851 | 0.110 | 0.149 | 0.407 | 0.724 | Unannotated | NA | NA |
| metab_5702 | DG(2:0/6 keto-PGF1alpha/0:0) | pos | 519.3176 | 6.111 | 4.195 | 3.889 | -0.305 | -0.149 | 0.407 | 0.724 | Unannotated | NA | NA |
| metab_8200 | Pinolidoxin | pos | 321.1664 | 2.500 | 4.575 | 4.820 | 0.245 | 0.149 | 0.407 | 0.724 | Tentative | NA | NA |
| metab_1722 | 1h-Benzo[g]indole | pos | 168.0810 | 3.877 | 4.614 | 4.503 | -0.110 | -0.149 | 0.407 | 0.724 | Unannotated | NA | NA |
| metab_1733 | Unannotated feature | pos | 316.1870 | 3.781 | 4.276 | 4.116 | -0.160 | -0.149 | 0.407 | 0.724 | Unannotated | NA | NA |
| metab_2056 | (+/-)-(E)-Ethyl-2-[(E)-hydroxyimino]-5-nitro-3-hexeneamide | pos | 198.0857 | 0.641 | 5.241 | 5.286 | 0.045 | 0.149 | 0.407 | 0.724 | Unannotated | NA | NA |
| metab_9956 | Unannotated feature | neg | 141.0550 | 2.903 | 4.136 | 4.086 | -0.050 | -0.149 | 0.407 | 0.724 | Unannotated | NA | NA |
| metab_19189 | Unannotated feature | neg | 227.0559 | 3.362 | 5.755 | 5.482 | -0.273 | -0.149 | 0.407 | 0.724 | Unannotated | NA | NA |
| metab_17986 | Boldenone | neg | 331.1915 | 6.530 | 5.339 | 5.467 | 0.128 | 0.149 | 0.407 | 0.724 | Tentative | NA | NA |
| metab_2021 | Asparaginylhydroxyproline | pos | 246.1089 | 0.720 | 5.715 | 5.397 | -0.318 | -0.149 | 0.407 | 0.724 | Unannotated | Organic acids and derivatives | Carboxylic acids and derivatives |
| metab_11948 | Unannotated feature | neg | 591.3201 | 6.138 | 6.101 | 6.014 | -0.087 | -0.149 | 0.407 | 0.724 | Unannotated | NA | NA |
| metab_18571 | 3-Oxohexanoic acid | neg | 259.1187 | 6.007 | 5.072 | 5.127 | 0.055 | 0.149 | 0.407 | 0.724 | Tentative | Organic acids and derivatives | Keto acids and derivatives |
| metab_8656 | FAPy-adenine | pos | 136.0621 | 1.010 | 4.657 | 4.753 | 0.097 | 0.149 | 0.407 | 0.724 | Tentative | NA | NA |
| metab_8844 | Unannotated feature | neg | 319.1304 | 4.378 | 5.204 | 5.465 | 0.261 | 0.149 | 0.407 | 0.724 | Unannotated | NA | NA |
| metab_12925 | Unannotated feature | neg | 371.0658 | 0.738 | 4.902 | 4.404 | -0.498 | -0.149 | 0.407 | 0.724 | Unannotated | NA | NA |
| metab_13434 | Unannotated feature | neg | 684.7449 | 0.603 | 4.129 | 4.411 | 0.281 | 0.149 | 0.407 | 0.724 | Unannotated | NA | NA |
| metab_17945 | Unannotated feature | neg | 517.2614 | 6.592 | 4.081 | 4.643 | 0.562 | 0.149 | 0.407 | 0.724 | Unannotated | NA | NA |
| metab_11963 | Unannotated feature | neg | 339.1638 | 6.170 | 4.984 | 4.912 | -0.073 | -0.145 | 0.420 | 0.734 | Unannotated | NA | NA |
| metab_19863 | EPIGALLOCATECHIN | neg | 305.0672 | 2.848 | 4.223 | 4.368 | 0.145 | 0.145 | 0.420 | 0.734 | Strong putative | NA | NA |
| metab_19792 | 4-Hydroxybenzyl alcohol | neg | 169.0500 | 4.446 | 3.398 | 3.828 | 0.429 | 0.145 | 0.420 | 0.734 | Tentative | Benzenoids | Benzene and substituted derivatives |
| metab_15686 | Unannotated feature | neg | 267.1603 | 5.664 | 4.317 | 4.843 | 0.527 | 0.145 | 0.420 | 0.734 | Unannotated | NA | NA |
| metab_6647 | 5'-Hydroxycarvedilol | pos | 423.1928 | 5.377 | 3.947 | 4.647 | 0.700 | 0.145 | 0.420 | 0.734 | Unannotated | NA | NA |
| metab_10749 | Coformycin | neg | 283.1049 | 1.230 | 4.831 | 5.036 | 0.205 | 0.145 | 0.420 | 0.734 | Tentative | NA | NA |
| metab_8830 | Phenol | pos | 136.0759 | 1.365 | 6.724 | 6.593 | -0.131 | -0.145 | 0.420 | 0.734 | Strong putative | NA | NA |
| metab_11629 | Unannotated feature | neg | 265.9091 | 0.104 | 5.504 | 5.480 | -0.024 | -0.145 | 0.420 | 0.734 | Unannotated | NA | NA |
| metab_19421 | Unannotated feature | neg | 395.1979 | 4.983 | 3.233 | 3.659 | 0.426 | 0.145 | 0.420 | 0.734 | Unannotated | NA | NA |
| metab_17098 | Unannotated feature | neg | 217.8653 | 7.870 | 5.779 | 5.735 | -0.044 | -0.145 | 0.420 | 0.734 | Unannotated | NA | NA |
| metab_12022 | Unannotated feature | neg | 481.2813 | 6.279 | 6.666 | 6.602 | -0.064 | -0.145 | 0.420 | 0.734 | Unannotated | NA | NA |
| metab_1233 | Cyperolone | pos | 201.1641 | 6.390 | 5.286 | 5.121 | -0.165 | -0.145 | 0.420 | 0.734 | Tentative | NA | NA |
| metab_12891 | Unannotated feature | neg | 407.2808 | 6.248 | 7.350 | 7.261 | -0.089 | -0.145 | 0.420 | 0.734 | Unannotated | NA | NA |
| metab_12902 | Unannotated feature | neg | 527.3226 | 6.240 | 6.445 | 6.389 | -0.057 | -0.145 | 0.420 | 0.734 | Unannotated | NA | NA |
| metab_1211 | Unannotated feature | pos | 330.3372 | 6.422 | 5.743 | 5.667 | -0.076 | -0.145 | 0.420 | 0.734 | Unannotated | NA | NA |
| metab_85 | Oxoadipic acid | pos | 178.0712 | 0.649 | 4.267 | 4.457 | 0.190 | 0.145 | 0.420 | 0.734 | Tentative | NA | NA |
| metab_4982 | Unannotated feature | pos | 546.4019 | 7.522 | 4.306 | 4.428 | 0.122 | 0.145 | 0.420 | 0.734 | Unannotated | NA | NA |
| metab_14877 | Unannotated feature | neg | 584.8841 | 0.502 | 5.002 | 4.810 | -0.192 | -0.145 | 0.420 | 0.734 | Unannotated | NA | NA |
| metab_4925 | Unannotated feature | pos | 378.9011 | 7.642 | 5.422 | 5.448 | 0.027 | 0.145 | 0.420 | 0.734 | Unannotated | NA | NA |
| metab_13533 | Unannotated feature | neg | 191.0015 | 1.115 | 5.328 | 5.230 | -0.098 | -0.145 | 0.420 | 0.734 | Unannotated | NA | NA |
| metab_16031 | 1-O-all-trans-retinoyl-beta-glucuronic Acid | neg | 497.2168 | 6.154 | 4.173 | 4.039 | -0.134 | -0.145 | 0.420 | 0.734 | Tentative | NA | NA |
| metab_16859 | Unannotated feature | neg | 244.0324 | 1.992 | 4.768 | 4.950 | 0.182 | 0.145 | 0.420 | 0.734 | Unannotated | NA | NA |
| metab_16857 | Unannotated feature | neg | 342.8552 | 7.730 | 4.770 | 4.745 | -0.025 | -0.145 | 0.420 | 0.734 | Unannotated | NA | NA |
| metab_10413 | (2S,3'S)-alpha-Amino-2-carboxy-5-oxo-1-pyrrolidinebutanoic acid | neg | 211.0721 | 2.026 | 5.700 | 5.779 | 0.080 | 0.145 | 0.420 | 0.734 | Unannotated | NA | NA |
| metab_16073 | Unannotated feature | neg | 573.3473 | 6.209 | 4.539 | 4.680 | 0.141 | 0.145 | 0.420 | 0.734 | Unannotated | NA | NA |
| metab_12243 | Unannotated feature | neg | 359.1147 | 5.390 | 5.304 | 5.278 | -0.026 | -0.145 | 0.420 | 0.734 | Unannotated | NA | NA |
| metab_9493 | Unannotated feature | neg | 421.0679 | 0.753 | 5.168 | 5.036 | -0.131 | -0.145 | 0.420 | 0.734 | Unannotated | NA | NA |
| metab_9460 | 4,6-O-Ethylidene-alpha-D-glucose | neg | 205.0714 | 3.400 | 4.119 | 3.915 | -0.204 | -0.145 | 0.420 | 0.734 | Unannotated | NA | NA |
| metab_13233 | Unannotated feature | neg | 196.0097 | 0.566 | 3.772 | 3.602 | -0.169 | -0.145 | 0.420 | 0.734 | Unannotated | NA | NA |
| metab_14724 | Unannotated feature | neg | 329.0615 | 3.745 | 3.481 | 3.894 | 0.413 | 0.145 | 0.420 | 0.734 | Unannotated | NA | NA |
| metab_18955 | Unannotated feature | neg | 405.1644 | 5.579 | 3.769 | 4.149 | 0.380 | 0.145 | 0.420 | 0.734 | Unannotated | NA | NA |
| metab_9520 | Unannotated feature | neg | 216.1238 | 3.326 | 5.388 | 5.325 | -0.063 | -0.145 | 0.420 | 0.734 | Unannotated | NA | NA |
| metab_17487 | Unannotated feature | neg | 384.1420 | 1.952 | 4.893 | 4.739 | -0.154 | -0.145 | 0.420 | 0.734 | Unannotated | NA | NA |
| metab_12154 | Unannotated feature | neg | 467.3188 | 6.656 | 5.700 | 5.930 | 0.231 | 0.145 | 0.420 | 0.734 | Unannotated | NA | NA |
| metab_19688 | Unannotated feature | neg | 209.0452 | 4.592 | 6.230 | 6.218 | -0.011 | -0.145 | 0.420 | 0.734 | Unannotated | NA | NA |
| metab_19687 | Unannotated feature | neg | 255.1239 | 4.592 | 4.216 | 4.566 | 0.350 | 0.145 | 0.420 | 0.734 | Unannotated | NA | NA |
| metab_17192 | Unannotated feature | neg | 473.8531 | 7.737 | 4.315 | 4.243 | -0.072 | -0.145 | 0.420 | 0.734 | Unannotated | NA | NA |
| metab_14104 | Fluoromisonidazole | neg | 234.0518 | 2.831 | 2.735 | 1.792 | -0.944 | -0.145 | 0.420 | 0.734 | Unannotated | NA | NA |
| metab_12991 | Unannotated feature | neg | 619.2105 | 0.791 | 3.125 | 3.504 | 0.379 | 0.145 | 0.420 | 0.734 | Unannotated | NA | NA |
| metab_1311 | Unannotated feature | pos | 442.2113 | 6.231 | 5.321 | 5.163 | -0.158 | -0.145 | 0.420 | 0.734 | Unannotated | NA | NA |
| metab_12841 | Stercobilinogen | neg | 595.3512 | 6.318 | 6.220 | 6.356 | 0.136 | 0.145 | 0.420 | 0.734 | Tentative | NA | NA |
| metab_3083 | Unannotated feature | pos | 413.2673 | 6.446 | 7.351 | 7.335 | -0.016 | -0.145 | 0.420 | 0.734 | Unannotated | NA | NA |
| metab_14125 | Unannotated feature | neg | 159.9360 | 2.862 | 3.876 | 3.343 | -0.533 | -0.145 | 0.420 | 0.734 | Unannotated | NA | NA |
| metab_14123 | Unannotated feature | neg | 496.1321 | 2.858 | 4.713 | 3.456 | -1.257 | -0.145 | 0.420 | 0.734 | Unannotated | NA | NA |
| metab_18432 | Unannotated feature | neg | 437.1505 | 6.107 | 4.281 | 4.620 | 0.339 | 0.145 | 0.420 | 0.734 | Unannotated | NA | NA |
| metab_16337 | Ascorbyl palmitate | neg | 435.2345 | 6.687 | 5.753 | 5.916 | 0.163 | 0.145 | 0.420 | 0.734 | Unannotated | NA | NA |
| metab_5302 | (1R,4R,5S)-1-[(1S)-1-Hydroxy-2-methylpropyl]-4-propyl-6-oxa-2-azabicyclo[3.2.0]heptane-3,7-dione | pos | 483.2706 | 6.566 | 5.762 | 5.605 | -0.157 | -0.145 | 0.420 | 0.734 | Unannotated | NA | NA |
| metab_19433 | 4-Hydroxycinnamoylagmatine | neg | 275.1502 | 4.962 | 4.167 | 4.378 | 0.211 | 0.145 | 0.420 | 0.734 | Tentative | NA | NA |
| metab_17039 | Unannotated feature | neg | 224.8267 | 7.989 | 5.976 | 5.949 | -0.027 | -0.145 | 0.420 | 0.734 | Unannotated | NA | NA |
| metab_17033 | Unannotated feature | neg | 239.9034 | 7.993 | 4.932 | 4.904 | -0.028 | -0.145 | 0.420 | 0.734 | Unannotated | NA | NA |
| metab_398 | Unannotated feature | pos | 336.8033 | 0.016 | 4.210 | 4.136 | -0.073 | -0.145 | 0.420 | 0.734 | Unannotated | NA | NA |
| metab_15283 | Phenacetyl-carnitine | neg | 261.1345 | 4.854 | 4.995 | 4.967 | -0.029 | -0.145 | 0.420 | 0.734 | Unannotated | NA | NA |
| metab_13920 | Unannotated feature | neg | 366.0063 | 2.542 | 4.394 | 4.546 | 0.152 | -0.145 | 0.420 | 0.734 | Unannotated | NA | NA |
| metab_1817 | Unannotated feature | pos | 150.0586 | 0.870 | 6.932 | 6.770 | -0.162 | -0.145 | 0.420 | 0.734 | Unannotated | NA | NA |
| metab_4732 | Unannotated feature | pos | 223.9888 | 7.666 | 4.593 | 4.565 | -0.028 | -0.145 | 0.420 | 0.734 | Unannotated | NA | NA |
| metab_9721 | Unannotated feature | neg | 319.0967 | 3.105 | 4.117 | 4.352 | 0.234 | 0.145 | 0.420 | 0.734 | Unannotated | NA | NA |
| metab_10209 | Unannotated feature | neg | 233.0430 | 4.522 | 5.340 | 5.126 | -0.214 | -0.145 | 0.420 | 0.734 | Unannotated | NA | NA |
| metab_16484 | Unannotated feature | neg | 142.0251 | 7.068 | 4.675 | 4.673 | -0.002 | -0.145 | 0.420 | 0.734 | Unannotated | NA | NA |
| metab_702 | Unannotated feature | pos | 169.0461 | 0.485 | 5.331 | 5.436 | 0.105 | 0.145 | 0.420 | 0.734 | Unannotated | NA | NA |
| metab_5160 | Unannotated feature | pos | 266.1730 | 6.853 | 5.221 | 5.239 | 0.018 | 0.145 | 0.420 | 0.734 | Unannotated | NA | NA |
| metab_8125 | Unannotated feature | pos | 444.2456 | 3.481 | 4.069 | 4.272 | 0.203 | 0.145 | 0.420 | 0.734 | Unannotated | NA | NA |
| metab_1563 | Unannotated feature | pos | 510.2902 | 5.267 | 5.046 | 4.908 | -0.137 | -0.145 | 0.420 | 0.734 | Unannotated | NA | NA |
| metab_11547 | Unannotated feature | neg | 160.9818 | 0.489 | 5.349 | 5.345 | -0.004 | -0.145 | 0.420 | 0.734 | Unannotated | NA | NA |
| metab_4506 | Unannotated feature | pos | 727.4219 | 6.630 | 4.285 | 4.334 | 0.048 | -0.145 | 0.420 | 0.734 | Unannotated | NA | NA |
| metab_18817 | Unannotated feature | neg | 200.1287 | 5.767 | 5.224 | 5.233 | 0.009 | -0.145 | 0.420 | 0.734 | Unannotated | NA | NA |
| metab_18343 | Unannotated feature | neg | 401.2549 | 6.177 | 5.818 | 5.659 | -0.159 | -0.145 | 0.420 | 0.734 | Unannotated | NA | NA |
| metab_9444 | Unannotated feature | neg | 227.1035 | 3.418 | 5.414 | 5.370 | -0.044 | -0.145 | 0.420 | 0.734 | Unannotated | NA | NA |
| metab_2764 | 7-(2-Hydroxyethyl)guanine | pos | 178.0725 | 2.439 | 2.378 | 2.935 | 0.557 | 0.145 | 0.420 | 0.734 | Unannotated | NA | NA |
| metab_1465 | Chrysophanol | pos | 255.0656 | 5.936 | 4.475 | 4.233 | -0.242 | -0.145 | 0.420 | 0.734 | Strong putative | NA | NA |
| metab_5569 | Gliocladic Acid | pos | 237.1483 | 6.263 | 5.258 | 5.239 | -0.019 | -0.145 | 0.420 | 0.734 | Unannotated | NA | NA |
| metab_9362 | Unannotated feature | neg | 233.1029 | 3.529 | 4.832 | 4.961 | 0.129 | 0.145 | 0.420 | 0.734 | Unannotated | NA | NA |
| metab_7824 | 5-Acetylamino-6-formylamino-3-methyluracil | pos | 227.0794 | 3.120 | 4.669 | 4.542 | -0.126 | -0.145 | 0.420 | 0.734 | Tentative | NA | NA |
| metab_14795 | Ethyl 3-oxohexanoate | neg | 203.0920 | 3.889 | 4.996 | 5.032 | 0.037 | -0.145 | 0.420 | 0.734 | Tentative | NA | NA |
| metab_11271 | L-Serine | neg | 104.0345 | 0.625 | 5.737 | 5.719 | -0.018 | -0.145 | 0.420 | 0.734 | Strong putative | NA | NA |
| metab_211 | KAPA | pos | 170.1178 | 4.521 | 5.465 | 5.386 | -0.079 | -0.145 | 0.420 | 0.734 | Tentative | NA | NA |
| metab_11860 | Unannotated feature | neg | 239.1287 | 5.991 | 4.992 | 5.132 | 0.139 | 0.145 | 0.420 | 0.734 | Unannotated | NA | NA |
| metab_13292 | Emorfazone | neg | 284.1254 | 0.625 | 4.029 | 3.720 | -0.309 | -0.145 | 0.420 | 0.734 | Unannotated | NA | NA |
| metab_1029 | 1-Aminocyclopropanecarboxylic Acid | pos | 84.0449 | 0.641 | 5.898 | 5.826 | -0.072 | -0.145 | 0.420 | 0.734 | Tentative | NA | NA |
| metab_10603 | Clethodim | neg | 358.1257 | 1.685 | 4.665 | 4.590 | -0.075 | -0.145 | 0.420 | 0.734 | Tentative | NA | NA |
| metab_10592 | Unannotated feature | neg | 246.0620 | 1.691 | 5.788 | 5.726 | -0.062 | -0.145 | 0.420 | 0.734 | Unannotated | NA | NA |
| metab_8486 | L-Histidinol | pos | 174.1240 | 1.692 | 4.564 | 4.801 | 0.237 | 0.145 | 0.420 | 0.734 | Tentative | NA | NA |
| metab_10933 | Unannotated feature | neg | 335.0499 | 0.804 | 3.079 | 3.384 | 0.305 | 0.145 | 0.420 | 0.734 | Unannotated | NA | NA |
| metab_16300 | Unannotated feature | neg | 337.2056 | 6.624 | 5.313 | 5.297 | -0.016 | -0.145 | 0.420 | 0.734 | Unannotated | NA | NA |
| metab_3046 | 3-Oxododecanoic acid | pos | 256.1912 | 3.291 | 3.766 | 3.308 | -0.458 | 0.145 | 0.420 | 0.734 | Tentative | NA | NA |
| metab_5154 | Unannotated feature | pos | 394.3304 | 6.861 | 4.395 | 4.463 | 0.068 | 0.145 | 0.420 | 0.734 | Unannotated | NA | NA |
| metab_5143 | DG(18:1(12Z)-2OH(9,10)/0:0/i-19:0) | pos | 713.5331 | 6.877 | 4.275 | 4.474 | 0.199 | 0.145 | 0.420 | 0.734 | Unannotated | NA | NA |
| metab_6445 | Unannotated feature | pos | 241.9654 | 0.593 | 3.871 | 3.763 | -0.108 | -0.145 | 0.420 | 0.734 | Unannotated | NA | NA |
| metab_7422 | Biotin | pos | 245.0959 | 3.930 | 4.861 | 4.899 | 0.037 | 0.145 | 0.420 | 0.734 | Strong putative | NA | NA |
| metab_12859 | Unannotated feature | neg | 283.0784 | 0.698 | 6.255 | 6.158 | -0.097 | -0.145 | 0.420 | 0.734 | Unannotated | NA | NA |
| metab_3540 | N-Lactoylvaline | pos | 154.0865 | 4.914 | 4.407 | 4.383 | -0.024 | -0.145 | 0.420 | 0.734 | Unannotated | NA | NA |
| metab_5370 | All-trans-18-Hydroxyretinoic acid | pos | 349.2361 | 6.494 | 4.852 | 4.935 | 0.083 | 0.145 | 0.420 | 0.734 | Tentative | Lipids and lipid-like molecules | Prenol lipids |
| metab_18384 | ALPINETIN METHYL ETHER | neg | 283.0985 | 6.146 | 4.427 | 4.473 | 0.046 | 0.145 | 0.420 | 0.734 | Moderate putative | NA | NA |
| metab_9258 | SCHRADAN | neg | 331.1298 | 3.685 | 4.788 | 4.718 | -0.070 | -0.145 | 0.420 | 0.734 | Tentative | NA | NA |
| metab_7250 | Unannotated feature | pos | 197.0377 | 4.306 | 3.944 | 4.030 | 0.086 | 0.145 | 0.420 | 0.734 | Unannotated | NA | NA |
| metab_19317 | Unannotated feature | neg | 231.0512 | 5.122 | 6.142 | 6.210 | 0.068 | 0.145 | 0.420 | 0.734 | Unannotated | NA | NA |
| metab_5518 | Unannotated feature | pos | 94.0656 | 6.327 | 3.062 | 3.169 | 0.108 | 0.145 | 0.420 | 0.734 | Unannotated | NA | NA |
| metab_1422 | Unannotated feature | pos | 202.0714 | 0.665 | 3.978 | 4.194 | 0.216 | 0.145 | 0.420 | 0.734 | Unannotated | NA | NA |
| metab_17280 | Unannotated feature | neg | 279.0568 | 2.539 | 4.988 | 4.989 | 0.001 | -0.145 | 0.420 | 0.734 | Unannotated | NA | NA |
| metab_17305 | Unannotated feature | neg | 216.9369 | 7.707 | 4.262 | 4.218 | -0.044 | -0.145 | 0.420 | 0.734 | Unannotated | NA | NA |
| metab_6361 | Cfp-aaf-pab | pos | 621.2937 | 5.757 | 3.274 | 2.834 | -0.440 | -0.145 | 0.420 | 0.734 | Unannotated | NA | NA |
| metab_11476 | Unannotated feature | neg | 605.2999 | 5.221 | 5.123 | 3.851 | -1.273 | -0.145 | 0.420 | 0.734 | Unannotated | NA | NA |
| metab_766 | 1-(2-Furyl)butan-3-one | pos | 121.0653 | 6.127 | 4.910 | 4.862 | -0.048 | -0.145 | 0.420 | 0.734 | Unannotated | NA | NA |
| metab_16746 | Unannotated feature | neg | 435.8945 | 7.670 | 4.732 | 4.810 | 0.079 | 0.145 | 0.420 | 0.734 | Unannotated | NA | NA |
| metab_17788 | Unannotated feature | neg | 360.2677 | 6.868 | 4.804 | 4.354 | -0.450 | -0.145 | 0.420 | 0.734 | Unannotated | NA | NA |
| metab_10173 | 20-Hydroxy-leukotriene E4 | neg | 492.1849 | 2.703 | 4.009 | 4.425 | 0.415 | 0.145 | 0.420 | 0.734 | Tentative | NA | NA |
| metab_13603 | 2,4-Dihydroxy-2H-1,4-benzoxazin-3(4H)-one | neg | 162.0190 | 1.453 | 3.311 | 3.599 | 0.288 | 0.145 | 0.420 | 0.734 | Tentative | NA | NA |
| metab_13688 | Unannotated feature | neg | 345.0807 | 1.691 | 4.647 | 4.514 | -0.133 | -0.145 | 0.420 | 0.734 | Unannotated | NA | NA |
| metab_9809 | Unannotated feature | neg | 351.9713 | 3.028 | 5.788 | 5.733 | -0.055 | -0.145 | 0.420 | 0.734 | Unannotated | NA | NA |
| metab_1071 | Unannotated feature | pos | 226.0940 | 1.944 | 5.260 | 5.046 | -0.214 | -0.145 | 0.420 | 0.734 | Unannotated | NA | NA |
| metab_10564 | Unannotated feature | neg | 321.9511 | 1.822 | 4.276 | 3.831 | -0.445 | -0.145 | 0.420 | 0.734 | Unannotated | NA | NA |
| metab_7280 | Pentanoic acid, 5-(dipentylamino)-5-oxo-4-((3-quinolinylcarbonyl)amino)-, (R)- | pos | 442.2711 | 4.239 | 4.513 | 4.382 | -0.130 | -0.145 | 0.420 | 0.734 | Unannotated | Organic acids and derivatives | Carboxylic acids and derivatives |
| metab_469 | Allodeoxycholic acid | pos | 357.2794 | 6.382 | 7.020 | 7.091 | 0.071 | -0.145 | 0.420 | 0.734 | Tentative | NA | NA |
| metab_8461 | Unannotated feature | pos | 182.0294 | 1.869 | 3.863 | 3.896 | 0.033 | 0.145 | 0.420 | 0.734 | Unannotated | NA | NA |
| metab_14488 | Unannotated feature | neg | 274.0473 | 3.334 | 3.661 | 3.624 | -0.038 | -0.145 | 0.420 | 0.734 | Unannotated | NA | NA |
| metab_390 | Unannotated feature | pos | 178.9911 | 0.023 | 4.704 | 4.737 | 0.034 | 0.145 | 0.420 | 0.734 | Unannotated | NA | NA |
| metab_14215 | Unannotated feature | neg | 130.9829 | 0.520 | 6.196 | 6.174 | -0.023 | -0.145 | 0.420 | 0.734 | Unannotated | NA | NA |
| metab_8899 | Caprylic acid | neg | 189.1127 | 4.270 | 3.846 | 3.879 | 0.032 | -0.145 | 0.420 | 0.734 | Tentative | NA | NA |
| metab_15748 | Unannotated feature | neg | 548.7681 | 0.596 | 5.026 | 5.193 | 0.167 | 0.145 | 0.420 | 0.734 | Unannotated | NA | NA |
| metab_19752 | Unannotated feature | neg | 386.1572 | 4.502 | 4.029 | 4.905 | 0.877 | 0.145 | 0.420 | 0.734 | Unannotated | NA | NA |
| metab_11378 | Unannotated feature | neg | 217.9980 | 0.563 | 4.419 | 4.453 | 0.033 | 0.145 | 0.420 | 0.734 | Unannotated | NA | NA |
| metab_12527 | Unannotated feature | neg | 507.3522 | 6.287 | 4.746 | 5.005 | 0.259 | 0.145 | 0.420 | 0.734 | Unannotated | NA | NA |
| metab_2113 | Unannotated feature | pos | 236.0670 | 2.811 | 4.691 | 4.363 | -0.328 | -0.145 | 0.420 | 0.734 | Unannotated | NA | NA |
| metab_6888 | Unannotated feature | pos | 625.3251 | 5.040 | 4.317 | 4.124 | -0.193 | -0.145 | 0.420 | 0.734 | Unannotated | NA | NA |
| metab_12342 | Unannotated feature | neg | 303.2330 | 6.860 | 5.987 | 5.812 | -0.175 | -0.145 | 0.420 | 0.734 | Unannotated | NA | NA |
| metab_12336 | Unannotated feature | neg | 412.2891 | 6.860 | 5.319 | 5.368 | 0.049 | 0.145 | 0.420 | 0.734 | Unannotated | NA | NA |
| metab_9176 | Homopisatin | neg | 299.0926 | 3.817 | 2.926 | 3.029 | 0.103 | 0.145 | 0.420 | 0.734 | Tentative | Phenylpropanoids and polyketides | Isoflavonoids |
| metab_10553 | Unannotated feature | neg | 187.9311 | 1.903 | 3.642 | 3.429 | -0.213 | -0.145 | 0.420 | 0.734 | Unannotated | NA | NA |
| metab_16985 | Unannotated feature | neg | 207.9305 | 7.974 | 5.409 | 5.389 | -0.020 | -0.145 | 0.420 | 0.734 | Unannotated | NA | NA |
| metab_487 | Unannotated feature | pos | 437.1947 | 6.359 | 7.632 | 7.490 | -0.142 | -0.145 | 0.420 | 0.734 | Unannotated | NA | NA |
| metab_19776 | Unannotated feature | neg | 305.0703 | 3.685 | 6.188 | 6.141 | -0.047 | -0.145 | 0.420 | 0.734 | Unannotated | NA | NA |
| metab_15595 | Unannotated feature | neg | 170.8326 | 5.496 | 5.135 | 5.010 | -0.125 | -0.145 | 0.420 | 0.734 | Unannotated | NA | NA |
| metab_19873 | Irinotecan | neg | 631.2787 | 6.099 | 4.289 | 4.053 | -0.236 | -0.141 | 0.434 | 0.744 | Tentative | NA | NA |
| metab_19865 | Epicatechin | neg | 289.0721 | 3.677 | 4.526 | 4.032 | -0.494 | -0.141 | 0.434 | 0.744 | Strong putative | NA | NA |
| metab_4844 | Unannotated feature | pos | 316.9396 | 7.732 | 4.352 | 4.364 | 0.013 | -0.141 | 0.434 | 0.744 | Unannotated | NA | NA |
| metab_11443 | Unannotated feature | neg | 468.8418 | 0.533 | 3.460 | 3.338 | -0.122 | -0.141 | 0.434 | 0.744 | Unannotated | NA | NA |
| metab_5071 | Unannotated feature | pos | 575.4679 | 7.116 | 5.559 | 5.499 | -0.059 | -0.141 | 0.434 | 0.744 | Unannotated | NA | NA |
| metab_17746 | Unannotated feature | neg | 491.2792 | 6.915 | 5.061 | 5.305 | 0.244 | -0.141 | 0.434 | 0.744 | Unannotated | NA | NA |
| metab_11380 | Erianin | neg | 317.1398 | 5.176 | 5.086 | 5.294 | 0.207 | 0.141 | 0.434 | 0.744 | Unannotated | NA | NA |
| metab_13355 | (2R)-1-O-beta-D-Galactopyranosylglycerol | neg | 289.0697 | 0.722 | 4.955 | 5.065 | 0.110 | 0.141 | 0.434 | 0.744 | Unannotated | NA | NA |
| metab_13351 | 3,4,5-trihydroxy-6-{3-[(E)-2-(4-hydroxyphenyl)ethenyl]-5-{[3,4,5-trihydroxy-6-(hydroxymethyl)oxan-2-yl]oxy}phenoxy}oxane-2-carboxylic acid | neg | 601.1351 | 0.714 | 5.233 | 4.999 | -0.234 | -0.141 | 0.434 | 0.744 | Unannotated | NA | NA |
| metab_13348 | Mabuterol | neg | 331.0806 | 0.714 | 4.168 | 4.188 | 0.020 | 0.141 | 0.434 | 0.744 | Unannotated | NA | NA |
| metab_6506 | Unannotated feature | pos | 171.1494 | 0.601 | 5.343 | 5.187 | -0.157 | -0.141 | 0.434 | 0.744 | Unannotated | NA | NA |
| metab_19139 | Unannotated feature | neg | 268.0174 | 3.227 | 5.412 | 5.339 | -0.073 | -0.141 | 0.434 | 0.744 | Unannotated | NA | NA |
| metab_15752 | Unannotated feature | neg | 323.6223 | 5.771 | 4.608 | 5.041 | 0.434 | 0.141 | 0.434 | 0.744 | Unannotated | NA | NA |
| metab_12630 | Unannotated feature | neg | 495.2790 | 6.530 | 6.759 | 6.880 | 0.121 | 0.141 | 0.434 | 0.744 | Unannotated | NA | NA |
| metab_4605 | Unannotated feature | pos | 505.3877 | 7.052 | 5.968 | 6.019 | 0.052 | 0.141 | 0.434 | 0.744 | Unannotated | NA | NA |
| metab_4659 | Unannotated feature | pos | 463.3762 | 7.411 | 5.864 | 5.829 | -0.034 | -0.141 | 0.434 | 0.744 | Unannotated | NA | NA |
| metab_2212 | Unannotated feature | pos | 200.0478 | 3.002 | 7.589 | 7.576 | -0.013 | 0.141 | 0.434 | 0.744 | Unannotated | NA | NA |
| metab_6907 | Unannotated feature | pos | 319.1297 | 5.020 | 4.562 | 4.397 | -0.164 | -0.141 | 0.434 | 0.744 | Unannotated | NA | NA |
| metab_6929 | 12S-HHT | pos | 319.1659 | 4.980 | 5.630 | 5.562 | -0.068 | -0.141 | 0.434 | 0.744 | Tentative | NA | NA |
| metab_11881 | Unannotated feature | neg | 271.1552 | 6.022 | 6.031 | 6.133 | 0.102 | 0.141 | 0.434 | 0.744 | Unannotated | NA | NA |
| metab_11224 | Unannotated feature | neg | 145.5328 | 0.644 | 3.561 | 3.378 | -0.183 | -0.141 | 0.434 | 0.744 | Unannotated | NA | NA |
| metab_16480 | Probucol | neg | 515.3037 | 7.061 | 4.198 | 4.513 | 0.315 | 0.141 | 0.434 | 0.744 | Strong putative | NA | NA |
| metab_8133 | Unannotated feature | pos | 282.1924 | 2.724 | 4.127 | 3.659 | -0.468 | -0.141 | 0.434 | 0.744 | Unannotated | NA | NA |
| metab_664 | Stercobilin | pos | 595.3503 | 5.870 | 6.740 | 7.500 | 0.760 | 0.141 | 0.434 | 0.744 | Strong putative | NA | NA |
| metab_886 | Unannotated feature | pos | 959.5725 | 6.885 | 5.824 | 5.829 | 0.005 | 0.141 | 0.434 | 0.744 | Unannotated | NA | NA |
| metab_4742 | Unannotated feature | pos | 94.0455 | 7.700 | 5.571 | 5.588 | 0.017 | 0.141 | 0.434 | 0.744 | Unannotated | NA | NA |
| metab_8121 | Unannotated feature | pos | 258.2181 | 2.732 | 4.478 | 4.391 | -0.088 | -0.141 | 0.434 | 0.744 | Unannotated | NA | NA |
| metab_8095 | PE(DiMe(9,3)/22:6(4Z,7Z,11E,13Z,15E,19Z)-2OH(10S,17)) | pos | 286.1647 | 2.740 | 3.660 | 3.885 | 0.225 | 0.141 | 0.434 | 0.744 | Unannotated | NA | NA |
| metab_4755 | Unannotated feature | pos | 300.0248 | 7.716 | 4.821 | 4.810 | -0.011 | -0.141 | 0.434 | 0.744 | Unannotated | NA | NA |
| metab_5156 | Unannotated feature | pos | 87.8450 | 6.861 | 4.205 | 4.271 | 0.066 | 0.141 | 0.434 | 0.744 | Unannotated | NA | NA |
| metab_12551 | Menthol-glucoronide | neg | 315.1813 | 6.170 | 5.588 | 5.601 | 0.013 | 0.141 | 0.434 | 0.744 | Unannotated | NA | NA |
| metab_16765 | Unannotated feature | neg | 197.9627 | 7.692 | 5.607 | 5.584 | -0.023 | -0.141 | 0.434 | 0.744 | Unannotated | NA | NA |
| metab_10992 | Glycylprolylhydroxyproline | neg | 330.1305 | 0.777 | 4.468 | 4.354 | -0.114 | -0.141 | 0.434 | 0.744 | Unannotated | NA | NA |
| metab_14514 | 4-Hydroxypyridine | neg | 94.0290 | 3.379 | 4.921 | 4.959 | 0.038 | -0.141 | 0.434 | 0.744 | Moderate putative | NA | NA |
| metab_12430 | Unannotated feature | neg | 347.1866 | 6.263 | 5.363 | 5.672 | 0.309 | 0.141 | 0.434 | 0.744 | Unannotated | NA | NA |
| metab_7582 | N-Acetyl-7-O-acetylneuraminic acid | pos | 393.1528 | 3.582 | 5.026 | 4.853 | -0.174 | -0.141 | 0.434 | 0.744 | Tentative | NA | NA |
| metab_8468 | Unannotated feature | pos | 132.5443 | 1.726 | 3.142 | 3.291 | 0.148 | 0.141 | 0.434 | 0.744 | Unannotated | NA | NA |
| metab_15464 | Unannotated feature | neg | 153.0914 | 5.204 | 2.602 | 3.209 | 0.607 | 0.141 | 0.434 | 0.744 | Unannotated | NA | NA |
| metab_7571 | Myricanone | pos | 398.1934 | 3.602 | 4.342 | 4.213 | -0.128 | -0.141 | 0.434 | 0.744 | Unannotated | NA | NA |
| metab_1149 | Unannotated feature | pos | 815.5150 | 6.558 | 5.424 | 5.267 | -0.157 | -0.141 | 0.434 | 0.744 | Unannotated | NA | NA |
| metab_11842 | 3-Methylazelaic acid | neg | 201.1127 | 5.952 | 6.033 | 5.709 | -0.323 | -0.141 | 0.434 | 0.744 | Unannotated | NA | NA |
| metab_16014 | (E)-2-Glucosyl-3,4',5-trihydroxystilbene | neg | 435.1282 | 1.108 | 3.791 | 4.335 | 0.544 | 0.141 | 0.434 | 0.744 | Unannotated | NA | NA |
| metab_18623 | Unannotated feature | neg | 239.0924 | 3.109 | 4.397 | 4.551 | 0.154 | 0.141 | 0.434 | 0.744 | Unannotated | NA | NA |
| metab_14387 | 3,4-Methylenedioxybenzaldehyde | neg | 195.0294 | 3.177 | 4.659 | 4.766 | 0.108 | 0.141 | 0.434 | 0.744 | Tentative | NA | NA |
| metab_13212 | Unannotated feature | neg | 361.0937 | 3.658 | 4.617 | 4.967 | 0.350 | 0.141 | 0.434 | 0.744 | Unannotated | NA | NA |
| metab_5847 | Unannotated feature | pos | 154.0865 | 0.190 | 4.940 | 4.963 | 0.023 | 0.141 | 0.434 | 0.744 | Unannotated | NA | NA |
| metab_13773 | Unannotated feature | neg | 372.1416 | 1.965 | 4.527 | 4.461 | -0.065 | -0.141 | 0.434 | 0.744 | Unannotated | NA | NA |
| metab_1747 | Di-Hydroxymelatonin | pos | 247.1081 | 3.596 | 5.667 | 5.523 | -0.143 | -0.141 | 0.434 | 0.744 | Unannotated | NA | NA |
| metab_10257 | Unannotated feature | neg | 237.0902 | 2.539 | 4.696 | 4.615 | -0.081 | -0.141 | 0.434 | 0.744 | Unannotated | NA | NA |
| metab_225 | Alanyl-Aspartic acid | pos | 249.0458 | 0.561 | 5.694 | 5.630 | -0.064 | -0.141 | 0.434 | 0.744 | Unannotated | NA | NA |
| metab_2316 | Unannotated feature | pos | 154.0865 | 0.123 | 3.878 | 3.884 | 0.005 | 0.141 | 0.434 | 0.744 | Unannotated | NA | NA |
| metab_9659 | 3-Pyridinol | neg | 94.0290 | 3.180 | 4.605 | 4.717 | 0.112 | -0.141 | 0.434 | 0.744 | Moderate putative | NA | NA |
| metab_8373 | Unannotated feature | pos | 315.1648 | 1.952 | 4.103 | 4.173 | 0.069 | -0.141 | 0.434 | 0.744 | Unannotated | NA | NA |
| metab_14533 | 2-Methyl-2H-1,3-benzoxazin-4(3H)-one | neg | 162.0554 | 3.403 | 4.786 | 4.666 | -0.120 | -0.141 | 0.434 | 0.744 | Unannotated | NA | NA |
| metab_8402 | Unannotated feature | pos | 193.1340 | 1.952 | 4.041 | 3.918 | -0.122 | -0.141 | 0.434 | 0.744 | Unannotated | NA | NA |
| metab_221 | Unannotated feature | pos | 466.2449 | 4.537 | 4.058 | 4.600 | 0.542 | 0.141 | 0.434 | 0.744 | Unannotated | NA | NA |
| metab_16992 | Unannotated feature | neg | 197.8076 | 7.985 | 7.037 | 7.018 | -0.019 | -0.141 | 0.434 | 0.744 | Unannotated | NA | NA |
| metab_16931 | Unannotated feature | neg | 123.9013 | 7.819 | 3.859 | 3.819 | -0.040 | -0.141 | 0.434 | 0.744 | Unannotated | NA | NA |
| metab_1037 | Unannotated feature | pos | 595.4351 | 6.877 | 6.251 | 6.121 | -0.130 | -0.141 | 0.434 | 0.744 | Unannotated | NA | NA |
| metab_16914 | Unannotated feature | neg | 200.0293 | 7.773 | 4.933 | 4.871 | -0.062 | -0.141 | 0.434 | 0.744 | Unannotated | NA | NA |
| metab_14668 | Unannotated feature | neg | 440.8831 | 0.520 | 6.389 | 6.512 | 0.123 | 0.141 | 0.434 | 0.744 | Unannotated | NA | NA |
| metab_14657 | 6-Hydroxy-3,4-dihydro-2(1H)-quinolinone | neg | 162.0554 | 3.608 | 5.188 | 5.045 | -0.143 | -0.141 | 0.434 | 0.744 | Unannotated | NA | NA |
| metab_12444 | Unannotated feature | neg | 505.3543 | 6.522 | 6.741 | 6.737 | -0.004 | 0.141 | 0.434 | 0.744 | Unannotated | NA | NA |
| metab_16900 | Unannotated feature | neg | 299.9195 | 7.755 | 5.201 | 5.192 | -0.009 | -0.141 | 0.434 | 0.744 | Unannotated | NA | NA |
| metab_10859 | Unannotated feature | neg | 271.9523 | 1.004 | 3.817 | 4.381 | 0.564 | 0.141 | 0.434 | 0.744 | Unannotated | NA | NA |
| metab_1619 | Unannotated feature | pos | 312.1448 | 4.931 | 4.790 | 4.297 | -0.493 | -0.141 | 0.434 | 0.744 | Unannotated | NA | NA |
| metab_13845 | Unannotated feature | neg | 252.9263 | 2.190 | 5.196 | 5.106 | -0.091 | -0.141 | 0.434 | 0.744 | Unannotated | NA | NA |
| metab_17414 | Unannotated feature | neg | 619.1747 | 1.952 | 3.832 | 4.248 | 0.416 | 0.141 | 0.434 | 0.744 | Unannotated | NA | NA |
| metab_2993 | Unannotated feature | pos | 284.1861 | 3.093 | 3.204 | 3.336 | 0.131 | 0.141 | 0.434 | 0.744 | Unannotated | NA | NA |
| metab_9205 | 2-Mercapto-3-furan-2-ylpropenoic acid | neg | 399.0205 | 3.762 | 1.706 | 1.701 | -0.005 | -0.141 | 0.434 | 0.744 | Unannotated | NA | NA |
| metab_18341 | Unannotated feature | neg | 428.2120 | 6.177 | 4.884 | 4.570 | -0.313 | -0.141 | 0.434 | 0.744 | Unannotated | NA | NA |
| metab_18337 | Unannotated feature | neg | 187.0607 | 3.007 | 5.753 | 5.737 | -0.017 | 0.141 | 0.434 | 0.744 | Unannotated | NA | NA |
| metab_11748 | Unannotated feature | neg | 623.3465 | 5.653 | 5.181 | 5.620 | 0.440 | 0.141 | 0.434 | 0.744 | Unannotated | NA | NA |
| metab_349 | Unannotated feature | pos | 118.9635 | 0.405 | 4.653 | 4.672 | 0.019 | 0.141 | 0.434 | 0.744 | Unannotated | NA | NA |
| metab_19551 | N-Acetyl-D-phenylalanine | neg | 206.0819 | 4.777 | 5.541 | 5.471 | -0.070 | -0.141 | 0.434 | 0.744 | Strong putative | NA | NA |
| metab_2696 | Unannotated feature | pos | 224.0559 | 2.035 | 3.345 | 3.162 | -0.183 | -0.141 | 0.434 | 0.744 | Unannotated | NA | NA |
| metab_7223 | 5-Deoxydiplosporin | pos | 191.1070 | 4.389 | 4.688 | 4.401 | -0.288 | -0.141 | 0.434 | 0.744 | Unannotated | NA | NA |
| metab_7222 | 3-Hydroxyphenyl-valeric acid | pos | 227.1281 | 4.389 | 5.140 | 5.121 | -0.020 | -0.141 | 0.434 | 0.744 | Unannotated | NA | NA |
| metab_5562 | Unannotated feature | pos | 271.2272 | 6.271 | 5.161 | 5.179 | 0.018 | -0.141 | 0.434 | 0.744 | Unannotated | NA | NA |
| metab_2788 | Unannotated feature | pos | 170.1116 | 2.571 | 5.450 | 5.494 | 0.044 | 0.141 | 0.434 | 0.744 | Unannotated | NA | NA |
| metab_2114 | Unannotated feature | pos | 285.0875 | 2.811 | 4.073 | 4.522 | 0.449 | 0.141 | 0.434 | 0.744 | Unannotated | NA | NA |
| metab_10099 | Unannotated feature | neg | 87.0442 | 2.771 | 5.086 | 4.989 | -0.097 | -0.141 | 0.434 | 0.744 | Unannotated | NA | NA |
| metab_13578 | Unannotated feature | neg | 289.1042 | 1.288 | 4.654 | 4.945 | 0.291 | 0.141 | 0.434 | 0.744 | Unannotated | NA | NA |
| metab_4950 | Unannotated feature | pos | 124.0737 | 7.602 | 4.855 | 4.882 | 0.027 | 0.141 | 0.434 | 0.744 | Unannotated | NA | NA |
| metab_7871 | Chlorobenzene | pos | 154.0420 | 3.007 | 6.944 | 6.940 | -0.004 | 0.141 | 0.434 | 0.744 | Tentative | NA | NA |
| metab_14900 | Unannotated feature | neg | 363.1104 | 4.103 | 4.421 | 4.374 | -0.047 | -0.141 | 0.434 | 0.744 | Unannotated | NA | NA |
| metab_31 | Unannotated feature | pos | 434.2414 | 4.336 | 3.628 | 3.936 | 0.308 | 0.141 | 0.434 | 0.744 | Unannotated | NA | NA |
| metab_14885 | Unannotated feature | neg | 169.0864 | 4.064 | 3.475 | 3.488 | 0.014 | 0.141 | 0.434 | 0.744 | Unannotated | NA | NA |
| metab_6805 | Unannotated feature | pos | 205.1550 | 0.633 | 4.293 | 3.983 | -0.310 | -0.141 | 0.434 | 0.744 | Unannotated | NA | NA |
| metab_12256 | Unannotated feature | neg | 281.2487 | 7.004 | 6.599 | 6.638 | 0.039 | 0.141 | 0.434 | 0.744 | Unannotated | NA | NA |
| metab_11772 | Unannotated feature | neg | 495.1848 | 1.933 | 5.533 | 5.670 | 0.137 | 0.141 | 0.434 | 0.744 | Unannotated | NA | NA |
| metab_5584 | Adrenic acid | pos | 355.2638 | 6.247 | 7.003 | 6.671 | -0.332 | -0.141 | 0.434 | 0.744 | Tentative | NA | NA |
| metab_7172 | (S)-(-)-Perillyl alcohol | pos | 135.1171 | 4.495 | 3.939 | 3.670 | -0.268 | -0.141 | 0.434 | 0.744 | Moderate putative | Lipids and lipid-like molecules | Prenol lipids |
| metab_16124 | Unannotated feature | neg | 287.2228 | 6.279 | 4.752 | 5.033 | 0.282 | 0.141 | 0.434 | 0.744 | Unannotated | NA | NA |
| metab_19625 | 3'-thiacytidine | neg | 266.0205 | 3.207 | 5.999 | 5.938 | -0.061 | -0.141 | 0.434 | 0.744 | Unannotated | NA | NA |
| metab_18319 | Unannotated feature | neg | 230.1033 | 2.754 | 5.058 | 5.107 | 0.049 | 0.141 | 0.434 | 0.744 | Unannotated | NA | NA |
| metab_118 | Unannotated feature | pos | 188.0148 | 0.633 | 3.924 | 3.907 | -0.017 | -0.141 | 0.434 | 0.744 | Unannotated | NA | NA |
| metab_9508 | Ethyl (S)-3-hydroxybutyrate glucoside | neg | 275.1135 | 3.334 | 3.569 | 4.338 | 0.769 | 0.141 | 0.434 | 0.744 | Unannotated | NA | NA |
| metab_2510 | Unannotated feature | pos | 71.0497 | 0.823 | 3.550 | 3.557 | 0.007 | 0.141 | 0.434 | 0.744 | Unannotated | NA | NA |
| metab_15414 | (z)-3-Hexenyl Beta-d-Glucopyranoside | neg | 261.1345 | 5.095 | 5.572 | 5.479 | -0.093 | -0.141 | 0.434 | 0.744 | Unannotated | NA | NA |
| metab_3346 | 16-Ketoestradiol | pos | 350.1719 | 4.226 | 4.421 | 4.492 | 0.071 | 0.141 | 0.434 | 0.744 | Tentative | NA | NA |
| metab_8826 | P-Aminobenzoic acid | pos | 170.0814 | 2.811 | 4.918 | 4.944 | 0.026 | 0.141 | 0.434 | 0.744 | Strong putative | NA | NA |
| metab_3281 | Unannotated feature | pos | 647.4872 | 6.997 | 6.148 | 6.130 | -0.018 | 0.141 | 0.434 | 0.744 | Unannotated | NA | NA |
| metab_7363 | Valyltryptophan | pos | 304.1665 | 4.047 | 4.012 | 3.417 | -0.595 | -0.141 | 0.434 | 0.744 | Unannotated | NA | NA |
| metab_13034 | Unannotated feature | neg | 487.2378 | 6.162 | 6.961 | 7.054 | 0.093 | 0.141 | 0.434 | 0.744 | Unannotated | NA | NA |
| metab_14029 | Unannotated feature | neg | 167.0820 | 2.766 | 3.313 | 2.983 | -0.330 | -0.141 | 0.434 | 0.744 | Unannotated | NA | NA |
| metab_10601 | Unannotated feature | neg | 390.0718 | 1.685 | 4.949 | 4.845 | -0.104 | -0.141 | 0.434 | 0.744 | Unannotated | NA | NA |
| metab_9120 | Unannotated feature | neg | 319.0497 | 2.951 | 4.061 | 4.329 | 0.269 | 0.141 | 0.434 | 0.744 | Unannotated | NA | NA |
| metab_17216 | Unannotated feature | neg | 315.1276 | 7.730 | 5.831 | 5.889 | 0.058 | 0.141 | 0.434 | 0.744 | Unannotated | NA | NA |
| metab_18417 | Unannotated feature | neg | 423.2035 | 6.115 | 5.132 | 5.020 | -0.111 | -0.141 | 0.434 | 0.744 | Unannotated | NA | NA |
| metab_15246 | Unannotated feature | neg | 390.2041 | 4.800 | 4.591 | 4.071 | -0.520 | -0.141 | 0.434 | 0.744 | Unannotated | NA | NA |
| metab_10597 | Unannotated feature | neg | 495.1845 | 1.685 | 4.799 | 5.362 | 0.564 | 0.141 | 0.434 | 0.744 | Unannotated | NA | NA |
| metab_1691 | 4-(Phenylamino)benzoic acid | pos | 231.1132 | 4.094 | 6.064 | 6.023 | -0.041 | -0.141 | 0.434 | 0.744 | Tentative | NA | NA |
| metab_17082 | Unannotated feature | neg | 266.9686 | 7.905 | 4.823 | 4.815 | -0.008 | -0.141 | 0.434 | 0.744 | Unannotated | NA | NA |
| metab_5401 | Palmitoylcarnitine | pos | 400.3431 | 6.438 | 6.188 | 6.046 | -0.142 | -0.141 | 0.434 | 0.744 | Strong putative | NA | NA |
| metab_2515 | N-Acetyl-DL-Aspartic Acid | pos | 176.0556 | 0.854 | 4.738 | 4.591 | -0.147 | -0.141 | 0.434 | 0.744 | Moderate putative | NA | NA |
| metab_10342 | Unannotated feature | neg | 340.0318 | 2.238 | 5.266 | 5.275 | 0.009 | 0.141 | 0.434 | 0.744 | Unannotated | NA | NA |
| metab_5686 | Unannotated feature | pos | 306.9203 | 0.016 | 4.991 | 5.005 | 0.014 | 0.141 | 0.434 | 0.744 | Unannotated | NA | NA |
| metab_1212 | Unannotated feature | pos | 821.6084 | 6.414 | 5.720 | 5.508 | -0.212 | -0.141 | 0.434 | 0.744 | Unannotated | NA | NA |
| metab_7449 | Unannotated feature | pos | 158.0492 | 3.868 | 4.998 | 5.050 | 0.052 | 0.141 | 0.434 | 0.744 | Unannotated | NA | NA |
| metab_17096 | Unannotated feature | neg | 94.9242 | 7.874 | 4.511 | 4.505 | -0.006 | -0.141 | 0.434 | 0.744 | Unannotated | NA | NA |
| metab_15160 | Phthalide | neg | 179.0344 | 4.653 | 4.731 | 4.933 | 0.202 | 0.141 | 0.434 | 0.744 | Tentative | NA | NA |
| metab_7477 | Unannotated feature | pos | 201.1238 | 0.720 | 4.292 | 4.632 | 0.340 | 0.141 | 0.434 | 0.744 | Unannotated | NA | NA |
| metab_11614 | Unannotated feature | neg | 278.8740 | 0.311 | 5.060 | 5.045 | -0.015 | -0.141 | 0.434 | 0.744 | Unannotated | NA | NA |
| metab_15175 | Unannotated feature | neg | 283.9668 | 0.552 | 4.478 | 4.442 | -0.037 | -0.141 | 0.434 | 0.744 | Unannotated | NA | NA |
| metab_14344 | 1,2-Cyclohexanediol | neg | 161.0813 | 3.112 | 4.818 | 4.639 | -0.179 | -0.141 | 0.434 | 0.744 | Unannotated | NA | NA |
| metab_14695 | 3-Oxovalproic acid | neg | 203.0921 | 3.693 | 4.675 | 4.827 | 0.151 | 0.137 | 0.448 | 0.754 | Tentative | NA | NA |
| metab_958 | Unannotated feature | pos | 242.9258 | 7.650 | 5.893 | 5.919 | 0.026 | 0.137 | 0.448 | 0.754 | Unannotated | NA | NA |
| metab_14378 | Unannotated feature | neg | 95.0494 | 3.162 | 2.848 | 2.704 | -0.144 | -0.137 | 0.448 | 0.754 | Unannotated | NA | NA |
| metab_1126 | Unannotated feature | pos | 431.3344 | 6.614 | 3.813 | 3.916 | 0.103 | 0.137 | 0.448 | 0.754 | Unannotated | NA | NA |
| metab_5911 | Unannotated feature | pos | 140.0708 | 0.375 | 5.042 | 5.056 | 0.014 | -0.137 | 0.448 | 0.754 | Unannotated | NA | NA |
| metab_7041 | Oxymorphone | pos | 319.1658 | 4.774 | 5.117 | 5.004 | -0.113 | -0.137 | 0.448 | 0.754 | Tentative | NA | NA |
| metab_13799 | Hydralazine pyruvate hydrazone | neg | 275.0778 | 2.023 | 4.635 | 4.692 | 0.057 | 0.137 | 0.448 | 0.754 | Unannotated | NA | NA |
| metab_9566 | Unannotated feature | neg | 348.1668 | 4.138 | 5.944 | 5.869 | -0.075 | -0.137 | 0.448 | 0.754 | Unannotated | NA | NA |
| metab_16322 | Unannotated feature | neg | 437.3641 | 6.656 | 5.222 | 5.426 | 0.204 | 0.137 | 0.448 | 0.754 | Unannotated | NA | NA |
| metab_11007 | Unannotated feature | neg | 358.1625 | 0.770 | 4.436 | 4.225 | -0.212 | -0.137 | 0.448 | 0.754 | Unannotated | NA | NA |
| metab_17023 | Unannotated feature | neg | 195.9056 | 7.997 | 4.635 | 4.601 | -0.034 | -0.137 | 0.448 | 0.754 | Unannotated | NA | NA |
| metab_11595 | Unannotated feature | neg | 260.9464 | 0.449 | 4.057 | 4.035 | -0.022 | -0.137 | 0.448 | 0.754 | Unannotated | NA | NA |
| metab_1100 | Unannotated feature | pos | 436.3409 | 6.662 | 5.785 | 5.696 | -0.089 | -0.137 | 0.448 | 0.754 | Unannotated | NA | NA |
| metab_14409 | 3-(2-Hydroxy-3-methoxyphenyl)-2-propenoic acid | neg | 239.0560 | 3.210 | 4.943 | 5.028 | 0.086 | 0.137 | 0.448 | 0.754 | Unannotated | NA | NA |
| metab_1104 | Unannotated feature | pos | 579.2620 | 6.654 | 5.198 | 4.737 | -0.462 | -0.137 | 0.448 | 0.754 | Unannotated | NA | NA |
| metab_18642 | Unannotated feature | neg | 441.1591 | 5.947 | 4.479 | 4.621 | 0.142 | 0.137 | 0.448 | 0.754 | Unannotated | NA | NA |
| metab_8535 | (2S,3S,5R)-5-(6-Aminopurin-9-yl)-2-(2H-oxet-2-yl)oxolan-3-ol | pos | 240.0871 | 3.997 | 4.965 | 4.796 | -0.169 | -0.137 | 0.448 | 0.754 | Unannotated | NA | NA |
| metab_18753 | Rothin B | neg | 587.2894 | 5.842 | 5.777 | 5.332 | -0.445 | -0.137 | 0.448 | 0.754 | Unannotated | NA | NA |
| metab_2362 | Unannotated feature | pos | 404.8291 | 0.531 | 4.728 | 4.955 | 0.227 | 0.137 | 0.448 | 0.754 | Unannotated | NA | NA |
| metab_9622 | Unannotated feature | neg | 387.9489 | 3.210 | 4.466 | 4.179 | -0.287 | -0.137 | 0.448 | 0.754 | Unannotated | NA | NA |
| metab_4397 | Unannotated feature | pos | 475.3409 | 6.271 | 4.568 | 4.365 | -0.204 | -0.137 | 0.448 | 0.754 | Unannotated | NA | NA |
| metab_11567 | (2S,3R,4S,5S,6R)-2-[3-Hydroxy-5-[2-(4-hydroxyphenyl)ethenyl]phenoxy]-6-(hydroxymethyl)oxane-3,4,5-triol | neg | 389.1248 | 5.272 | 5.757 | 5.628 | -0.129 | -0.137 | 0.448 | 0.754 | Unannotated | NA | NA |
| metab_16389 | Unannotated feature | neg | 410.1624 | 6.805 | 4.831 | 5.126 | 0.295 | 0.137 | 0.448 | 0.754 | Unannotated | NA | NA |
| metab_4450 | Vamonolide | pos | 473.2889 | 6.406 | 5.896 | 5.832 | -0.065 | -0.137 | 0.448 | 0.754 | Unannotated | NA | NA |
| metab_5180 | Unannotated feature | pos | 223.0249 | 0.585 | 5.652 | 5.526 | -0.126 | -0.137 | 0.448 | 0.754 | Unannotated | NA | NA |
| metab_995 | Unannotated feature | pos | 151.0355 | 7.700 | 5.927 | 5.955 | 0.028 | 0.137 | 0.448 | 0.754 | Unannotated | NA | NA |
| metab_16432 | Unannotated feature | neg | 82.9709 | 6.937 | 5.013 | 5.005 | -0.008 | -0.137 | 0.448 | 0.754 | Unannotated | NA | NA |
| metab_1792 | Unannotated feature | pos | 248.1495 | 0.776 | 2.706 | 3.862 | 1.156 | 0.137 | 0.448 | 0.754 | Unannotated | NA | NA |
| metab_2339 | Unannotated feature | pos | 140.9959 | 0.435 | 4.611 | 4.607 | -0.004 | 0.137 | 0.448 | 0.754 | Unannotated | NA | NA |
| metab_10237 | Unannotated feature | neg | 179.0344 | 2.579 | 4.017 | 4.170 | 0.153 | 0.137 | 0.448 | 0.754 | Unannotated | NA | NA |
| metab_1829 | (6S)-Tetrahydro-L-biopterin | pos | 305.1350 | 1.010 | 5.081 | 5.177 | 0.096 | 0.137 | 0.448 | 0.754 | Unannotated | NA | NA |
| metab_8381 | Carprazidil | pos | 256.0820 | 1.952 | 5.187 | 5.202 | 0.015 | 0.137 | 0.448 | 0.754 | Unannotated | NA | NA |
| metab_4390 | Unannotated feature | pos | 109.1017 | 6.255 | 4.722 | 4.750 | 0.028 | 0.137 | 0.448 | 0.754 | Unannotated | NA | NA |
| metab_953 | Unannotated feature | pos | 222.0201 | 0.633 | 4.848 | 4.589 | -0.258 | -0.137 | 0.448 | 0.754 | Unannotated | NA | NA |
| metab_4772 | Unannotated feature | pos | 126.1281 | 7.756 | 3.910 | 3.922 | 0.012 | 0.137 | 0.448 | 0.754 | Unannotated | NA | NA |
| metab_12560 | Unannotated feature | neg | 465.1954 | 6.162 | 5.473 | 5.622 | 0.149 | 0.137 | 0.448 | 0.754 | Unannotated | NA | NA |
| metab_11335 | Unannotated feature | neg | 432.8579 | 0.596 | 4.800 | 4.851 | 0.051 | 0.137 | 0.448 | 0.754 | Unannotated | NA | NA |
| metab_5020 | Unannotated feature | pos | 403.2312 | 0.728 | 4.396 | 3.647 | -0.750 | -0.137 | 0.448 | 0.754 | Unannotated | NA | NA |
| metab_6486 | PGP(18:1(9Z)-O(12,13)/i-20:0) | pos | 916.5714 | 4.802 | 2.791 | 3.736 | 0.945 | 0.137 | 0.448 | 0.754 | Unannotated | NA | NA |
| metab_12128 | Unannotated feature | neg | 433.2963 | 6.585 | 6.089 | 6.209 | 0.121 | 0.137 | 0.448 | 0.754 | Unannotated | NA | NA |
| metab_9821 | 3-Iodothyroacetic acid | neg | 368.9615 | 3.021 | 5.405 | 5.385 | -0.020 | -0.137 | 0.448 | 0.754 | Unannotated | NA | NA |
| metab_15736 | Unannotated feature | neg | 424.1918 | 5.739 | 3.274 | 3.965 | 0.692 | 0.137 | 0.448 | 0.754 | Unannotated | NA | NA |
| metab_9816 | Unannotated feature | neg | 505.0069 | 3.025 | 4.469 | 4.442 | -0.028 | -0.137 | 0.448 | 0.754 | Unannotated | NA | NA |
| metab_2174 | Unannotated feature | pos | 304.1509 | 2.937 | 5.156 | 5.071 | -0.085 | -0.137 | 0.448 | 0.754 | Unannotated | NA | NA |
| metab_19867 | Estrone | neg | 291.1354 | 3.349 | 3.939 | 3.724 | -0.215 | -0.137 | 0.448 | 0.754 | Tentative | NA | NA |
| metab_12532 | Unannotated feature | neg | 470.2761 | 6.248 | 5.422 | 5.373 | -0.049 | -0.137 | 0.448 | 0.754 | Unannotated | NA | NA |
| metab_8009 | Diphenylhydantoic acid | pos | 303.1346 | 3.455 | 4.007 | 4.447 | 0.440 | 0.137 | 0.448 | 0.754 | Unannotated | NA | NA |
| metab_4819 | Dihydrokainic acid | pos | 216.1235 | 4.212 | 4.019 | 3.883 | -0.136 | -0.137 | 0.448 | 0.754 | Strong putative | NA | NA |
| metab_5055 | DG(PGE2/0:0/18:0) | pos | 657.5076 | 7.148 | 7.062 | 7.047 | -0.015 | -0.137 | 0.448 | 0.754 | Unannotated | NA | NA |
| metab_2208 | Unannotated feature | pos | 202.0446 | 3.007 | 7.098 | 7.088 | -0.010 | 0.137 | 0.448 | 0.754 | Unannotated | NA | NA |
| metab_12486 | 9(S)-HpODE | neg | 311.2228 | 6.404 | 6.283 | 6.322 | 0.039 | 0.137 | 0.448 | 0.754 | Strong putative | NA | NA |
| metab_4866 | Unannotated feature | pos | 181.0147 | 7.724 | 5.403 | 5.435 | 0.031 | 0.137 | 0.448 | 0.754 | Unannotated | NA | NA |
| metab_810 | Ricinoleic acid | pos | 321.2405 | 6.614 | 6.924 | 7.048 | 0.123 | 0.137 | 0.448 | 0.754 | Tentative | NA | NA |
| metab_19719 | N-Acetyl-L-phenylalanine | neg | 206.0819 | 4.527 | 5.288 | 5.258 | -0.030 | -0.137 | 0.448 | 0.754 | Strong putative | NA | NA |
| metab_12452 | Unannotated feature | neg | 178.9772 | 7.827 | 5.607 | 5.592 | -0.015 | -0.137 | 0.448 | 0.754 | Unannotated | NA | NA |
| metab_16472 | Unannotated feature | neg | 265.0412 | 1.685 | 4.835 | 4.955 | 0.120 | -0.137 | 0.448 | 0.754 | Unannotated | NA | NA |
| metab_16794 | Unannotated feature | neg | 257.9190 | 7.707 | 5.009 | 4.981 | -0.028 | -0.137 | 0.448 | 0.754 | Unannotated | NA | NA |
| metab_14798 | Haloperidol | neg | 356.1256 | 3.901 | 5.001 | 5.037 | 0.035 | 0.137 | 0.448 | 0.754 | Tentative | NA | NA |
| metab_11890 | Unannotated feature | neg | 375.2758 | 6.035 | 4.745 | 4.630 | -0.115 | -0.137 | 0.448 | 0.754 | Unannotated | NA | NA |
| metab_15843 | Unannotated feature | neg | 606.3317 | 5.918 | 4.328 | 4.161 | -0.167 | -0.137 | 0.448 | 0.754 | Unannotated | NA | NA |
| metab_17712 | Unannotated feature | neg | 283.0939 | 2.771 | 5.186 | 5.464 | 0.279 | 0.137 | 0.448 | 0.754 | Unannotated | NA | NA |
| metab_18990 | Unannotated feature | neg | 160.8413 | 5.542 | 4.613 | 4.569 | -0.044 | -0.137 | 0.448 | 0.754 | Unannotated | NA | NA |
| metab_909 | Unannotated feature | pos | 643.5284 | 7.132 | 7.228 | 7.249 | 0.021 | 0.137 | 0.448 | 0.754 | Unannotated | NA | NA |
| metab_10765 | Gibberellic Acid | neg | 391.1366 | 1.179 | 3.722 | 4.329 | 0.607 | 0.137 | 0.448 | 0.754 | Moderate putative | NA | NA |
| metab_18570 | Unannotated feature | neg | 223.1337 | 6.007 | 4.197 | 4.436 | 0.239 | 0.137 | 0.448 | 0.754 | Unannotated | NA | NA |
| metab_1172 | Unannotated feature | pos | 455.2790 | 6.518 | 4.463 | 4.650 | 0.187 | 0.137 | 0.448 | 0.754 | Unannotated | NA | NA |
| metab_12348 | Unannotated feature | neg | 601.2806 | 7.499 | 6.651 | 6.664 | 0.013 | 0.137 | 0.448 | 0.754 | Unannotated | NA | NA |
| metab_15362 | Hymenoxon | neg | 317.1144 | 5.022 | 5.387 | 5.340 | -0.047 | -0.137 | 0.448 | 0.754 | Tentative | NA | NA |
| metab_13044 | Unannotated feature | neg | 464.8058 | 0.596 | 5.253 | 5.439 | 0.186 | 0.137 | 0.448 | 0.754 | Unannotated | NA | NA |
| metab_13059 | Unannotated feature | neg | 337.1146 | 1.015 | 4.312 | 4.373 | 0.060 | 0.137 | 0.448 | 0.754 | Unannotated | NA | NA |
| metab_13062 | N2-gamma-Glutamylglutamine | neg | 256.0940 | 1.021 | 4.900 | 4.707 | -0.193 | -0.137 | 0.448 | 0.754 | Tentative | NA | NA |
| metab_11668 | MJDBISSN00000009 | neg | 169.1025 | 2.579 | 5.788 | 5.703 | -0.085 | -0.137 | 0.448 | 0.754 | Strong putative | NA | NA |
| metab_17231 | Unannotated feature | neg | 196.9957 | 7.730 | 3.942 | 4.008 | 0.066 | 0.137 | 0.448 | 0.754 | Unannotated | NA | NA |
| metab_13035 | Unannotated feature | neg | 298.8807 | 0.603 | 5.045 | 5.117 | 0.072 | 0.137 | 0.448 | 0.754 | Unannotated | NA | NA |
| metab_11685 | Unannotated feature | neg | 426.1891 | 5.409 | 4.820 | 4.784 | -0.036 | -0.137 | 0.448 | 0.754 | Unannotated | NA | NA |
| metab_7332 | Unannotated feature | pos | 272.1861 | 4.111 | 4.690 | 4.582 | -0.109 | -0.137 | 0.448 | 0.754 | Unannotated | NA | NA |
| metab_9104 | Unannotated feature | neg | 394.1148 | 3.925 | 3.440 | 3.450 | 0.010 | 0.137 | 0.448 | 0.754 | Unannotated | NA | NA |
| metab_3232 | Unannotated feature | pos | 669.4717 | 6.853 | 6.479 | 6.370 | -0.110 | -0.137 | 0.448 | 0.754 | Unannotated | NA | NA |
| metab_18435 | Unannotated feature | neg | 134.0968 | 6.107 | 3.815 | 3.503 | -0.311 | -0.137 | 0.448 | 0.754 | Unannotated | NA | NA |
| metab_17177 | Unannotated feature | neg | 170.8326 | 7.745 | 5.050 | 5.050 | -0.000 | 0.137 | 0.448 | 0.754 | Unannotated | NA | NA |
| metab_3122 | Unannotated feature | pos | 394.1357 | 1.994 | 3.913 | 4.406 | 0.493 | 0.137 | 0.448 | 0.754 | Unannotated | NA | NA |
| metab_18433 | Unannotated feature | neg | 308.1374 | 6.107 | 6.438 | 6.574 | 0.136 | 0.137 | 0.448 | 0.754 | Unannotated | NA | NA |
| metab_1241 | Unannotated feature | pos | 243.2111 | 6.382 | 5.133 | 5.085 | -0.048 | -0.137 | 0.448 | 0.754 | Unannotated | NA | NA |
| metab_14190 | Unannotated feature | neg | 332.8585 | 0.516 | 4.588 | 4.446 | -0.142 | -0.137 | 0.448 | 0.754 | Unannotated | NA | NA |
| metab_407 | Unannotated feature | pos | 206.9954 | 0.016 | 3.995 | 4.007 | 0.012 | 0.137 | 0.448 | 0.754 | Unannotated | NA | NA |
| metab_7409 | Unannotated feature | pos | 141.9588 | 3.957 | 4.011 | 3.941 | -0.070 | -0.137 | 0.448 | 0.754 | Unannotated | NA | NA |
| metab_5363 | Unannotated feature | pos | 383.3422 | 6.502 | 3.248 | 2.555 | -0.693 | -0.137 | 0.448 | 0.754 | Unannotated | NA | NA |
| metab_8651 | 2',3'-Dideoxyuridine | pos | 230.1139 | 1.018 | 5.029 | 4.953 | -0.076 | -0.137 | 0.448 | 0.754 | Unannotated | NA | NA |
| metab_14279 | Unannotated feature | neg | 361.9715 | 3.025 | 4.193 | 3.947 | -0.246 | -0.137 | 0.448 | 0.754 | Unannotated | NA | NA |
| metab_9463 | Unannotated feature | neg | 170.0817 | 4.092 | 3.758 | 4.315 | 0.557 | 0.137 | 0.448 | 0.754 | Unannotated | NA | NA |
| metab_9490 | 4-Methylorcinol | neg | 137.0601 | 3.362 | 5.878 | 5.021 | -0.857 | -0.137 | 0.448 | 0.754 | Unannotated | NA | NA |
| metab_17508 | PE(22:4(7Z,10Z,13Z,16Z)/P-18:0) | neg | 760.5574 | 7.481 | 5.292 | 5.120 | -0.173 | -0.137 | 0.448 | 0.754 | Unannotated | NA | NA |
| metab_2470 | N2-Methyl-L-lysine | pos | 143.1182 | 0.704 | 4.399 | 4.115 | -0.284 | -0.137 | 0.448 | 0.754 | Unannotated | NA | NA |
| metab_11827 | Unannotated feature | neg | 352.0967 | 5.909 | 4.154 | 4.125 | -0.029 | -0.137 | 0.448 | 0.754 | Unannotated | NA | NA |
| metab_11817 | Unannotated feature | neg | 501.2150 | 5.888 | 5.794 | 5.768 | -0.026 | -0.137 | 0.448 | 0.754 | Unannotated | NA | NA |
| metab_7108 | Unannotated feature | pos | 264.1193 | 0.665 | 4.648 | 4.628 | -0.019 | 0.137 | 0.448 | 0.754 | Unannotated | NA | NA |
| metab_3355 | 5-Hydroxyindoleacetate | pos | 174.0553 | 4.250 | 4.162 | 4.141 | -0.020 | 0.137 | 0.448 | 0.754 | Strong putative | NA | NA |
| metab_10734 | Glycitin | neg | 445.1149 | 4.740 | 3.585 | 4.077 | 0.492 | 0.137 | 0.448 | 0.754 | Tentative | NA | NA |
| metab_14327 | Risdiplam | neg | 436.1697 | 3.082 | 4.438 | 4.010 | -0.429 | -0.137 | 0.448 | 0.754 | Unannotated | NA | NA |
| metab_5557 | Unannotated feature | pos | 382.2961 | 6.271 | 4.609 | 4.801 | 0.193 | 0.137 | 0.448 | 0.754 | Unannotated | NA | NA |
| metab_10501 | Glycerol lactate pyruvate | neg | 513.1472 | 1.947 | 5.633 | 5.510 | -0.123 | -0.137 | 0.448 | 0.754 | Unannotated | NA | NA |
| metab_2745 | Unannotated feature | pos | 79.0183 | 2.303 | 3.497 | 3.459 | -0.038 | 0.137 | 0.448 | 0.754 | Unannotated | NA | NA |
| metab_16181 | Traumatic Acid | neg | 227.1285 | 6.389 | 5.241 | 5.175 | -0.065 | -0.137 | 0.448 | 0.754 | Strong putative | Lipids and lipid-like molecules | Fatty Acyls |
| metab_16135 | Unannotated feature | neg | 508.7889 | 0.603 | 4.155 | 3.788 | -0.368 | -0.137 | 0.448 | 0.754 | Unannotated | NA | NA |
| metab_1499 | Pyroglutamyl-glutamyl-proline amide | pos | 372.1875 | 3.275 | 3.270 | 3.087 | -0.184 | -0.137 | 0.448 | 0.754 | Unannotated | NA | NA |
| metab_324 | Unannotated feature | pos | 177.0429 | 0.477 | 4.419 | 4.327 | -0.091 | -0.137 | 0.448 | 0.754 | Unannotated | NA | NA |
| metab_7157 | Unannotated feature | pos | 233.1864 | 4.524 | 5.038 | 5.072 | 0.034 | -0.137 | 0.448 | 0.754 | Unannotated | NA | NA |
| metab_5661 | Unannotated feature | pos | 158.1542 | 6.159 | 4.256 | 4.314 | 0.058 | -0.137 | 0.448 | 0.754 | Unannotated | NA | NA |
| metab_5672 | Nafoxidine | pos | 408.2291 | 6.143 | 4.362 | 3.655 | -0.707 | -0.137 | 0.448 | 0.754 | Tentative | NA | NA |
| metab_2592 | Unannotated feature | pos | 447.2152 | 5.481 | 3.844 | 4.222 | 0.378 | 0.137 | 0.448 | 0.754 | Unannotated | NA | NA |
| metab_14037 | Unannotated feature | neg | 264.0991 | 2.771 | 0.316 | 3.426 | 3.110 | 0.137 | 0.448 | 0.754 | Unannotated | NA | NA |
| metab_11800 | Sapidolide A | neg | 265.1059 | 5.839 | 4.758 | 4.817 | 0.059 | -0.137 | 0.448 | 0.754 | Unannotated | NA | NA |
| metab_17284 | Unannotated feature | neg | 611.8287 | 7.714 | 4.187 | 4.218 | 0.031 | -0.137 | 0.448 | 0.754 | Unannotated | NA | NA |
| metab_17347 | Unannotated feature | neg | 342.8731 | 7.692 | 4.000 | 4.035 | 0.035 | 0.137 | 0.448 | 0.754 | Unannotated | NA | NA |
| metab_2708 | Unannotated feature | pos | 220.0396 | 2.079 | 5.398 | 5.404 | 0.005 | 0.137 | 0.448 | 0.754 | Unannotated | NA | NA |
| metab_13457 | Unannotated feature | neg | 534.7920 | 0.603 | 4.511 | 4.612 | 0.101 | 0.137 | 0.448 | 0.754 | Unannotated | NA | NA |
| metab_5583 | Unannotated feature | pos | 180.9902 | 0.013 | 5.645 | 5.657 | 0.012 | 0.137 | 0.448 | 0.754 | Unannotated | NA | NA |
| metab_7872 | 8,9-Dihydroxy-1,5,6,10B-tetrahydropyrrolo[2,1-A]isoquinolin-3(2H)-one | pos | 237.1238 | 3.347 | 4.341 | 4.240 | -0.101 | -0.137 | 0.448 | 0.754 | Unannotated | NA | NA |
| metab_4904 | Unannotated feature | pos | 95.0860 | 7.689 | 3.861 | 3.808 | -0.053 | -0.137 | 0.448 | 0.754 | Unannotated | NA | NA |
| metab_7916 | Ephedrine | pos | 148.1123 | 2.941 | 4.043 | 4.056 | 0.013 | -0.137 | 0.448 | 0.754 | Moderate putative | NA | NA |
| metab_16671 | Unannotated feature | neg | 139.8962 | 7.611 | 4.353 | 4.342 | -0.011 | 0.137 | 0.448 | 0.754 | Unannotated | NA | NA |
| metab_19231 | 2-Methoxy-4-vinylphenol | neg | 299.1291 | 5.227 | 4.763 | 5.334 | 0.571 | 0.137 | 0.448 | 0.754 | Tentative | NA | NA |
| metab_13370 | Unannotated feature | neg | 359.1202 | 0.636 | 4.007 | 4.233 | 0.226 | 0.137 | 0.448 | 0.754 | Unannotated | NA | NA |
| metab_1960 | Unannotated feature | pos | 398.2047 | 1.960 | 4.588 | 4.603 | 0.015 | -0.137 | 0.448 | 0.754 | Unannotated | NA | NA |
| metab_6591 | Delapril | pos | 470.2666 | 5.457 | 5.170 | 5.424 | 0.254 | 0.137 | 0.448 | 0.754 | Unannotated | NA | NA |
| metab_6806 | (9Z,11E,13S,15Z)-13-Hydroxyoctadeca-9,11,15-trienoic acid | pos | 333.1815 | 5.169 | 2.206 | 2.704 | 0.498 | 0.137 | 0.448 | 0.754 | Tentative | Lipids and lipid-like molecules | Fatty Acyls |
| metab_9986 | Unannotated feature | neg | 299.0346 | 2.862 | 3.098 | 3.670 | 0.573 | 0.137 | 0.448 | 0.754 | Unannotated | NA | NA |
| metab_10053 | Unannotated feature | neg | 255.9580 | 2.789 | 3.322 | 3.404 | 0.081 | -0.137 | 0.448 | 0.754 | Unannotated | NA | NA |
| metab_13433 | N-Acetyl-a-neuraminic acid | neg | 290.0885 | 0.804 | 6.955 | 7.179 | 0.224 | 0.133 | 0.463 | 0.765 | Tentative | NA | NA |
| metab_2050 | Unannotated feature | pos | 202.1918 | 0.649 | 5.154 | 4.774 | -0.380 | -0.133 | 0.463 | 0.765 | Unannotated | NA | NA |
| metab_13512 | Unannotated feature | neg | 89.0235 | 1.061 | 6.005 | 6.206 | 0.201 | 0.133 | 0.463 | 0.765 | Unannotated | NA | NA |
| metab_10096 | 4-Allylpyrocatechol sulfate | neg | 229.0175 | 2.771 | 3.722 | 4.050 | 0.328 | 0.133 | 0.463 | 0.765 | Unannotated | NA | NA |
| metab_17867 | Unannotated feature | neg | 462.2694 | 6.711 | 5.289 | 4.516 | -0.774 | -0.133 | 0.463 | 0.765 | Unannotated | NA | NA |
| metab_2074 | Unannotated feature | pos | 281.1865 | 2.740 | 3.578 | 4.124 | 0.546 | 0.133 | 0.463 | 0.765 | Unannotated | NA | NA |
| metab_4886 | Unannotated feature | pos | 181.9885 | 7.708 | 4.793 | 4.828 | 0.035 | 0.133 | 0.463 | 0.765 | Unannotated | NA | NA |
| metab_4962 | Unannotated feature | pos | 497.3069 | 7.578 | 5.570 | 5.526 | -0.045 | -0.133 | 0.463 | 0.765 | Unannotated | NA | NA |
| metab_2005 | Unannotated feature | pos | 245.1612 | 2.280 | 5.485 | 5.113 | -0.372 | -0.133 | 0.463 | 0.765 | Unannotated | NA | NA |
| metab_13426 | Unannotated feature | neg | 191.0015 | 0.799 | 5.011 | 4.882 | -0.129 | -0.133 | 0.463 | 0.765 | Unannotated | NA | NA |
| metab_5580 | Diflucortolone valerate | pos | 479.2629 | 6.247 | 5.135 | 4.885 | -0.250 | -0.133 | 0.463 | 0.765 | Unannotated | NA | NA |
| metab_18324 | Unannotated feature | neg | 298.1448 | 6.201 | 4.858 | 4.669 | -0.188 | -0.133 | 0.463 | 0.765 | Unannotated | NA | NA |
| metab_18339 | Unannotated feature | neg | 490.2220 | 6.177 | 4.885 | 5.041 | 0.156 | 0.133 | 0.463 | 0.765 | Unannotated | NA | NA |
| metab_9367 | Tamoxifen-ol | neg | 379.1467 | 3.517 | 4.701 | 4.587 | -0.114 | -0.133 | 0.463 | 0.765 | Unannotated | NA | NA |
| metab_2705 | Unannotated feature | pos | 298.1515 | 2.070 | 5.491 | 5.355 | -0.136 | -0.133 | 0.463 | 0.765 | Unannotated | NA | NA |
| metab_13940 | Unannotated feature | neg | 442.1160 | 2.586 | 4.518 | 4.328 | -0.189 | -0.133 | 0.463 | 0.765 | Unannotated | NA | NA |
| metab_16143 | Unannotated feature | neg | 556.2208 | 6.303 | 4.083 | 4.711 | 0.628 | 0.133 | 0.463 | 0.765 | Unannotated | NA | NA |
| metab_19555 | Unannotated feature | neg | 200.0293 | 3.210 | 6.417 | 6.383 | -0.034 | -0.133 | 0.463 | 0.765 | Unannotated | NA | NA |
| metab_12305 | Unannotated feature | neg | 577.4844 | 7.216 | 6.193 | 6.158 | -0.036 | -0.133 | 0.463 | 0.765 | Unannotated | NA | NA |
| metab_17393 | Unannotated feature | neg | 205.0172 | 1.958 | 4.333 | 4.452 | 0.118 | 0.133 | 0.463 | 0.765 | Unannotated | NA | NA |
| metab_10508 | Unannotated feature | neg | 227.0536 | 4.660 | 4.823 | 4.900 | 0.077 | 0.133 | 0.463 | 0.765 | Unannotated | NA | NA |
| metab_13121 | Unannotated feature | neg | 190.9041 | 0.413 | 4.976 | 4.960 | -0.016 | -0.133 | 0.463 | 0.765 | Unannotated | NA | NA |
| metab_16164 | Unannotated feature | neg | 556.3325 | 6.365 | 6.465 | 6.386 | -0.079 | -0.133 | 0.463 | 0.765 | Unannotated | NA | NA |
| metab_11720 | Unannotated feature | neg | 335.1617 | 5.546 | 5.622 | 5.712 | 0.090 | 0.133 | 0.463 | 0.765 | Unannotated | NA | NA |
| metab_16142 | Unannotated feature | neg | 459.2964 | 6.303 | 5.543 | 5.345 | -0.198 | -0.133 | 0.463 | 0.765 | Unannotated | NA | NA |
| metab_2852 | 3-Oxopentanoylcarnitine | pos | 260.1499 | 2.770 | 2.958 | 3.047 | 0.088 | 0.133 | 0.463 | 0.765 | Unannotated | NA | NA |
| metab_13087 | Unannotated feature | neg | 160.8413 | 0.036 | 5.031 | 4.981 | -0.050 | -0.133 | 0.463 | 0.765 | Unannotated | NA | NA |
| metab_18404 | Unannotated feature | neg | 279.0988 | 3.028 | 4.425 | 4.402 | -0.023 | -0.133 | 0.463 | 0.765 | Unannotated | NA | NA |
| metab_17271 | Unannotated feature | neg | 328.9041 | 7.720 | 5.921 | 5.916 | -0.006 | -0.133 | 0.463 | 0.765 | Unannotated | NA | NA |
| metab_13846 | Unannotated feature | neg | 195.8685 | 2.193 | 2.878 | 3.143 | 0.265 | 0.133 | 0.463 | 0.765 | Unannotated | NA | NA |
| metab_2558 | 4-Hydroxyquinazoline | pos | 169.0359 | 1.118 | 4.756 | 5.022 | 0.266 | 0.133 | 0.463 | 0.765 | Unannotated | NA | NA |
| metab_11784 | Unannotated feature | neg | 343.0854 | 5.789 | 4.587 | 5.010 | 0.423 | 0.133 | 0.463 | 0.765 | Unannotated | NA | NA |
| metab_2589 | Unannotated feature | pos | 182.0453 | 1.341 | 4.801 | 4.636 | -0.165 | -0.133 | 0.463 | 0.765 | Unannotated | NA | NA |
| metab_13901 | Unannotated feature | neg | 188.0923 | 2.476 | 5.043 | 5.298 | 0.255 | 0.133 | 0.463 | 0.765 | Unannotated | NA | NA |
| metab_1401 | Unannotated feature | pos | 447.2521 | 6.080 | 5.090 | 4.904 | -0.186 | -0.133 | 0.463 | 0.765 | Unannotated | NA | NA |
| metab_12350 | Hovenidulcigenin A | neg | 525.3263 | 6.853 | 7.004 | 7.104 | 0.100 | 0.133 | 0.463 | 0.765 | Unannotated | NA | NA |
| metab_17236 | Unannotated feature | neg | 666.7659 | 0.538 | 4.160 | 4.202 | 0.041 | -0.133 | 0.463 | 0.765 | Unannotated | NA | NA |
| metab_7337 | 8-Isoquinoline methanamine (hydrochloride) | pos | 158.0967 | 4.094 | 5.063 | 4.990 | -0.073 | -0.133 | 0.463 | 0.765 | Moderate putative | NA | NA |
| metab_18429 | Notoginsenoside R10 | neg | 553.3397 | 6.107 | 4.463 | 4.382 | -0.081 | -0.133 | 0.463 | 0.765 | Unannotated | NA | NA |
| metab_9056 | Unannotated feature | neg | 285.0731 | 3.988 | 3.687 | 3.971 | 0.284 | -0.133 | 0.463 | 0.765 | Unannotated | NA | NA |
| metab_17211 | Unannotated feature | neg | 344.9117 | 7.730 | 5.465 | 5.428 | -0.036 | -0.133 | 0.463 | 0.765 | Unannotated | NA | NA |
| metab_3112 | Unannotated feature | pos | 615.3167 | 6.510 | 6.055 | 6.360 | 0.305 | 0.133 | 0.463 | 0.765 | Unannotated | NA | NA |
| metab_3115 | Asp Asp Leu | pos | 362.1567 | 3.470 | 4.012 | 3.862 | -0.151 | -0.133 | 0.463 | 0.765 | Strong putative | NA | NA |
| metab_3170 | Cromakalim | pos | 304.1663 | 3.642 | 3.907 | 3.988 | 0.080 | -0.133 | 0.463 | 0.765 | Tentative | NA | NA |
| metab_7427 | 2,5-Dihydroxybenzaldehyde | pos | 139.0392 | 3.923 | 1.880 | 1.711 | -0.169 | -0.133 | 0.463 | 0.765 | Tentative | NA | NA |
| metab_14181 | Unannotated feature | neg | 262.9302 | 0.516 | 6.291 | 6.211 | -0.080 | -0.133 | 0.463 | 0.765 | Unannotated | NA | NA |
| metab_18538 | Unannotated feature | neg | 383.1537 | 6.029 | 5.499 | 5.589 | 0.090 | 0.133 | 0.463 | 0.765 | Unannotated | NA | NA |
| metab_12915 | L-2-Amino-3-(oxalylamino)propanoic acid | neg | 157.0248 | 0.738 | 4.254 | 4.223 | -0.031 | -0.133 | 0.463 | 0.765 | Tentative | NA | NA |
| metab_12193 | Unannotated feature | neg | 619.2576 | 5.922 | 4.806 | 4.862 | 0.056 | 0.133 | 0.463 | 0.765 | Unannotated | NA | NA |
| metab_19613 | Unannotated feature | neg | 436.2213 | 4.691 | 4.692 | 4.388 | -0.304 | -0.133 | 0.463 | 0.765 | Unannotated | NA | NA |
| metab_10380 | Unannotated feature | neg | 217.1078 | 4.596 | 5.286 | 5.363 | 0.077 | 0.133 | 0.463 | 0.765 | Unannotated | NA | NA |
| metab_11637 | Unannotated feature | neg | 224.8267 | 0.073 | 6.370 | 6.338 | -0.032 | -0.133 | 0.463 | 0.765 | Unannotated | NA | NA |
| metab_19458 | Unannotated feature | neg | 379.1766 | 4.927 | 4.690 | 4.280 | -0.410 | -0.133 | 0.463 | 0.765 | Unannotated | NA | NA |
| metab_5439 | Unannotated feature | pos | 298.1295 | 1.952 | 5.203 | 5.241 | 0.038 | 0.133 | 0.463 | 0.765 | Unannotated | NA | NA |
| metab_10779 | N6-(L-1,3-Dicarboxypropyl)-L-lysine | neg | 257.1144 | 1.149 | 5.504 | 5.436 | -0.069 | -0.133 | 0.463 | 0.765 | Moderate putative | NA | NA |
| metab_12875 | Unannotated feature | neg | 387.0610 | 0.714 | 6.151 | 6.029 | -0.122 | -0.133 | 0.463 | 0.765 | Unannotated | NA | NA |
| metab_12885 | Unannotated feature | neg | 235.0457 | 0.722 | 5.500 | 5.485 | -0.015 | -0.133 | 0.463 | 0.765 | Unannotated | NA | NA |
| metab_12862 | Unannotated feature | neg | 791.4982 | 7.012 | 5.133 | 5.139 | 0.005 | 0.133 | 0.463 | 0.765 | Unannotated | NA | NA |
| metab_8583 | Unannotated feature | pos | 182.1038 | 1.176 | 4.972 | 4.794 | -0.178 | -0.133 | 0.463 | 0.765 | Unannotated | NA | NA |
| metab_18657 | Unannotated feature | neg | 264.0880 | 3.120 | 4.782 | 5.012 | 0.230 | 0.133 | 0.463 | 0.765 | Unannotated | NA | NA |
| metab_13250 | Unannotated feature | neg | 230.0069 | 0.582 | 5.251 | 5.170 | -0.082 | -0.133 | 0.463 | 0.765 | Unannotated | NA | NA |
| metab_13210 | Unannotated feature | neg | 188.0044 | 0.547 | 4.395 | 4.530 | 0.135 | 0.133 | 0.463 | 0.765 | Unannotated | NA | NA |
| metab_1750 | N-(2-Hydroxypropyl)valine | pos | 158.1178 | 3.574 | 4.936 | 5.017 | 0.081 | 0.133 | 0.463 | 0.765 | Unannotated | NA | NA |
| metab_2438 | 4-Methyl-5-Thiazoleethanol | pos | 144.0480 | 0.641 | 4.061 | 4.390 | 0.328 | 0.133 | 0.463 | 0.765 | Strong putative | NA | NA |
| metab_13798 | 3-Dehydroshikimate | neg | 171.0293 | 2.023 | 4.158 | 4.307 | 0.149 | 0.133 | 0.463 | 0.765 | Moderate putative | NA | NA |
| metab_14419 | Unannotated feature | neg | 496.8236 | 0.527 | 4.896 | 5.154 | 0.258 | 0.133 | 0.463 | 0.765 | Unannotated | NA | NA |
| metab_8545 | 4-Hydroxybenzaldehyde | pos | 123.0445 | 1.309 | 6.264 | 6.133 | -0.130 | -0.133 | 0.463 | 0.765 | Strong putative | NA | NA |
| metab_447 | Unannotated feature | pos | 324.2179 | 0.006 | 4.102 | 4.107 | 0.005 | 0.133 | 0.463 | 0.765 | Unannotated | NA | NA |
| metab_15146 | Unannotated feature | neg | 361.1409 | 4.617 | 5.531 | 5.615 | 0.083 | 0.133 | 0.463 | 0.765 | Unannotated | NA | NA |
| metab_8509 | Unannotated feature | pos | 367.1718 | 1.650 | 4.076 | 4.088 | 0.012 | -0.133 | 0.463 | 0.765 | Unannotated | NA | NA |
| metab_1095 | Unannotated feature | pos | 505.3517 | 6.662 | 6.772 | 6.854 | 0.082 | 0.133 | 0.463 | 0.765 | Unannotated | NA | NA |
| metab_18679 | Unannotated feature | neg | 503.2513 | 5.922 | 4.610 | 4.237 | -0.373 | -0.133 | 0.463 | 0.765 | Unannotated | NA | NA |
| metab_14460 | Deca-2,5,8-trienedioylcarnitine | neg | 360.1417 | 3.284 | 4.320 | 4.215 | -0.105 | -0.133 | 0.463 | 0.765 | Unannotated | NA | NA |
| metab_12779 | Unannotated feature | neg | 193.9802 | 0.628 | 3.999 | 3.937 | -0.062 | -0.133 | 0.463 | 0.765 | Unannotated | NA | NA |
| metab_18777 | Unannotated feature | neg | 364.1406 | 5.813 | 4.819 | 4.712 | -0.106 | -0.133 | 0.463 | 0.765 | Unannotated | NA | NA |
| metab_11875 | Unannotated feature | neg | 401.2181 | 6.013 | 6.208 | 6.048 | -0.160 | -0.133 | 0.463 | 0.765 | Unannotated | NA | NA |
| metab_204 | Unannotated feature | pos | 108.9492 | 0.593 | 2.901 | 2.607 | -0.295 | -0.133 | 0.463 | 0.765 | Unannotated | NA | NA |
| metab_9709 | Unannotated feature | neg | 235.0725 | 3.120 | 4.452 | 4.363 | -0.090 | 0.133 | 0.463 | 0.765 | Unannotated | NA | NA |
| metab_9677 | Unannotated feature | neg | 351.1095 | 3.153 | 3.470 | 3.258 | -0.213 | -0.133 | 0.463 | 0.765 | Unannotated | NA | NA |
| metab_9663 | Unannotated feature | neg | 280.9942 | 3.173 | 3.461 | 3.342 | -0.119 | -0.133 | 0.463 | 0.765 | Unannotated | NA | NA |
| metab_573 | Unannotated feature | pos | 317.1505 | 5.725 | 4.822 | 5.049 | 0.227 | 0.133 | 0.463 | 0.765 | Unannotated | NA | NA |
| metab_5271 | 3b,7a-Dihydroxy-5b-cholanoic acid | pos | 434.3254 | 6.630 | 4.153 | 4.347 | 0.194 | 0.133 | 0.463 | 0.765 | Tentative | Lipids and lipid-like molecules | Steroids and steroid derivatives |
| metab_4448 | Progestoral | pos | 313.2169 | 6.406 | 4.689 | 4.322 | -0.367 | -0.133 | 0.463 | 0.765 | Tentative | NA | NA |
| metab_16913 | Unannotated feature | neg | 152.9779 | 7.773 | 4.961 | 4.953 | -0.008 | -0.133 | 0.463 | 0.765 | Unannotated | NA | NA |
| metab_4518 | Unannotated feature | pos | 619.3117 | 6.694 | 5.089 | 4.698 | -0.391 | -0.133 | 0.463 | 0.765 | Unannotated | NA | NA |
| metab_18877 | Unannotated feature | neg | 231.0872 | 3.223 | 5.266 | 5.278 | 0.012 | 0.133 | 0.463 | 0.765 | Unannotated | NA | NA |
| metab_950 | Lucidone C | pos | 437.2859 | 7.610 | 5.844 | 5.820 | -0.024 | -0.133 | 0.463 | 0.765 | Unannotated | NA | NA |
| metab_8172 | Methoxamine | pos | 253.1551 | 2.640 | 4.790 | 4.650 | -0.140 | -0.133 | 0.463 | 0.765 | Tentative | NA | NA |
| metab_925 | Unannotated feature | pos | 491.2757 | 7.132 | 5.453 | 5.457 | 0.004 | -0.133 | 0.463 | 0.765 | Unannotated | NA | NA |
| metab_17749 | Unannotated feature | neg | 353.2152 | 6.915 | 5.598 | 5.631 | 0.033 | 0.133 | 0.463 | 0.765 | Unannotated | NA | NA |
| metab_13652 | Unannotated feature | neg | 125.0350 | 1.675 | 5.508 | 5.437 | -0.071 | -0.133 | 0.463 | 0.765 | Unannotated | NA | NA |
| metab_19744 | Unannotated feature | neg | 327.8946 | 4.510 | 4.692 | 4.581 | -0.110 | -0.133 | 0.463 | 0.765 | Unannotated | NA | NA |
| metab_2209 | Unannotated feature | pos | 156.0390 | 3.007 | 6.443 | 6.437 | -0.006 | 0.133 | 0.463 | 0.765 | Unannotated | NA | NA |
| metab_13325 | Unannotated feature | neg | 119.0343 | 0.683 | 4.741 | 4.697 | -0.044 | -0.133 | 0.463 | 0.765 | Unannotated | NA | NA |
| metab_13326 | Unannotated feature | neg | 137.0349 | 0.683 | 4.488 | 4.454 | -0.035 | -0.133 | 0.463 | 0.765 | Unannotated | NA | NA |
| metab_13335 | 3-Hydroxy-4-aminopyridine | neg | 155.0455 | 0.698 | 4.326 | 4.324 | -0.002 | 0.133 | 0.463 | 0.765 | Unannotated | NA | NA |
| metab_9778 | A-L-Arabinofuranosyl-(1->3)-b-D-xylopyranosyl-(1->4)-D-xylose | neg | 827.2706 | 0.730 | 4.825 | 4.690 | -0.135 | 0.133 | 0.463 | 0.765 | Unannotated | NA | NA |
| metab_14774 | Unannotated feature | neg | 254.1035 | 3.842 | 4.497 | 4.700 | 0.203 | 0.133 | 0.463 | 0.765 | Unannotated | NA | NA |
| metab_16805 | Unannotated feature | neg | 350.1292 | 7.714 | 4.803 | 4.685 | -0.117 | 0.133 | 0.463 | 0.765 | Unannotated | NA | NA |
| metab_16486 | Unannotated feature | neg | 381.1742 | 7.068 | 6.729 | 6.863 | 0.134 | 0.133 | 0.463 | 0.765 | Unannotated | NA | NA |
| metab_15000 | Unannotated feature | neg | 192.0661 | 4.332 | 4.353 | 4.268 | -0.085 | -0.133 | 0.463 | 0.765 | Unannotated | NA | NA |
| metab_19296 | Naltrexone | neg | 340.1558 | 3.403 | 3.654 | 3.393 | -0.261 | -0.133 | 0.463 | 0.765 | Tentative | NA | NA |
| metab_5162 | Unannotated feature | pos | 250.1780 | 6.853 | 5.416 | 5.459 | 0.043 | 0.133 | 0.463 | 0.765 | Unannotated | NA | NA |
| metab_19052 | Unannotated feature | neg | 153.0914 | 5.457 | 4.026 | 4.291 | 0.265 | 0.133 | 0.463 | 0.765 | Unannotated | NA | NA |
| metab_7786 | Unannotated feature | pos | 94.1662 | 3.214 | 3.932 | 4.017 | 0.085 | 0.133 | 0.463 | 0.765 | Unannotated | NA | NA |
| metab_4777 | Unannotated feature | pos | 195.0423 | 7.772 | 3.971 | 3.919 | -0.052 | -0.133 | 0.463 | 0.765 | Unannotated | NA | NA |
| metab_11337 | Unannotated feature | neg | 380.8443 | 0.596 | 5.043 | 5.408 | 0.365 | 0.133 | 0.463 | 0.765 | Unannotated | NA | NA |
| metab_11367 | Unannotated feature | neg | 287.9537 | 0.571 | 4.603 | 4.358 | -0.244 | -0.133 | 0.463 | 0.765 | Unannotated | NA | NA |
| metab_16758 | Unannotated feature | neg | 258.9009 | 7.687 | 4.056 | 4.120 | 0.064 | 0.133 | 0.463 | 0.765 | Unannotated | NA | NA |
| metab_15754 | Unannotated feature | neg | 231.1236 | 5.783 | 4.489 | 4.445 | -0.045 | -0.133 | 0.463 | 0.765 | Unannotated | NA | NA |
| metab_9828 | Unannotated feature | neg | 285.0620 | 3.017 | 4.704 | 4.748 | 0.044 | 0.133 | 0.463 | 0.765 | Unannotated | NA | NA |
| metab_10157 | Homovanillin | neg | 331.1193 | 4.498 | 5.592 | 5.544 | -0.048 | 0.133 | 0.463 | 0.765 | Tentative | NA | NA |
| metab_770 | Unannotated feature | pos | 202.0201 | 0.553 | 4.309 | 4.261 | -0.048 | -0.133 | 0.463 | 0.765 | Unannotated | NA | NA |
| metab_11480 | Unannotated feature | neg | 508.8707 | 0.520 | 5.647 | 5.665 | 0.018 | 0.133 | 0.463 | 0.765 | Unannotated | NA | NA |
| metab_7849 | Unannotated feature | pos | 301.1763 | 3.052 | 3.620 | 4.397 | 0.777 | 0.133 | 0.463 | 0.765 | Unannotated | NA | NA |
| metab_12543 | Unannotated feature | neg | 863.5367 | 6.389 | 5.961 | 5.672 | -0.290 | -0.133 | 0.463 | 0.765 | Unannotated | NA | NA |
| metab_4832 | Unannotated feature | pos | 238.0642 | 7.772 | 4.612 | 4.576 | -0.036 | -0.133 | 0.463 | 0.765 | Unannotated | NA | NA |
| metab_1984 | Unannotated feature | pos | 786.2550 | 2.104 | 6.052 | 6.061 | 0.010 | 0.133 | 0.463 | 0.765 | Unannotated | NA | NA |
| metab_17946 | P-Menthane-3,8-diol | neg | 343.2854 | 6.592 | 5.050 | 5.031 | -0.019 | 0.133 | 0.463 | 0.765 | Tentative | NA | NA |
| metab_6697 | Unannotated feature | pos | 158.0217 | 0.625 | 3.969 | 3.881 | -0.088 | -0.133 | 0.463 | 0.765 | Unannotated | NA | NA |
| metab_5357 | Unannotated feature | pos | 611.2862 | 6.510 | 5.689 | 5.574 | -0.115 | -0.128 | 0.477 | 0.777 | Unannotated | NA | NA |
| metab_181 | Unannotated feature | pos | 181.9619 | 0.601 | 4.841 | 4.796 | -0.046 | -0.128 | 0.477 | 0.777 | Unannotated | NA | NA |
| metab_8271 | Unannotated feature | pos | 346.1980 | 2.187 | 4.078 | 3.753 | -0.325 | -0.128 | 0.477 | 0.777 | Unannotated | NA | NA |
| metab_12713 | 4'-Methoxy-7-O-Beta-D-Glucopyranosyl-8,3'-Dihydroxyflavanone | neg | 485.1101 | 5.221 | 4.286 | 4.019 | -0.267 | -0.128 | 0.477 | 0.777 | Strong putative | NA | NA |
| metab_1638 | Unannotated feature | pos | 202.0866 | 4.724 | 4.797 | 4.712 | -0.086 | -0.128 | 0.477 | 0.777 | Unannotated | NA | NA |
| metab_18397 | Unannotated feature | neg | 353.9682 | 3.025 | 5.286 | 5.259 | -0.027 | -0.128 | 0.477 | 0.777 | Unannotated | NA | NA |
| metab_1762 | Unannotated feature | pos | 386.2294 | 3.439 | 5.399 | 5.388 | -0.011 | -0.128 | 0.477 | 0.777 | Unannotated | NA | NA |
| metab_1246 | Unannotated feature | pos | 325.2531 | 6.375 | 5.141 | 5.122 | -0.018 | -0.128 | 0.477 | 0.777 | Unannotated | NA | NA |
| metab_1871 | Unannotated feature | pos | 256.1296 | 1.204 | 4.696 | 4.906 | 0.210 | 0.128 | 0.477 | 0.777 | Unannotated | NA | NA |
| metab_15171 | Ethyl (3R,5Z)-3-hydroxy-5-octenoate | neg | 231.1236 | 4.667 | 4.221 | 4.201 | -0.020 | 0.128 | 0.477 | 0.777 | Unannotated | NA | NA |
| metab_1763 | Unannotated feature | pos | 342.1709 | 3.439 | 5.393 | 5.116 | -0.277 | -0.128 | 0.477 | 0.777 | Unannotated | NA | NA |
| metab_1932 | Unannotated feature | pos | 180.0879 | 0.728 | 4.108 | 4.272 | 0.164 | 0.128 | 0.477 | 0.777 | Unannotated | NA | NA |
| metab_18130 | Unannotated feature | neg | 353.2335 | 6.373 | 5.883 | 5.943 | 0.060 | 0.128 | 0.477 | 0.777 | Unannotated | NA | NA |
| metab_10584 | Unannotated feature | neg | 262.1298 | 1.695 | 4.016 | 3.672 | -0.344 | -0.128 | 0.477 | 0.777 | Unannotated | NA | NA |
| metab_15256 | Unannotated feature | neg | 117.0339 | 4.814 | 3.895 | 3.690 | -0.205 | -0.128 | 0.477 | 0.777 | Unannotated | NA | NA |
| metab_1195 | Unannotated feature | pos | 297.2038 | 6.470 | 5.744 | 5.730 | -0.013 | -0.128 | 0.477 | 0.777 | Unannotated | NA | NA |
| metab_6338 | Unannotated feature | pos | 555.7535 | 5.770 | 3.716 | 3.712 | -0.004 | 0.128 | 0.477 | 0.777 | Unannotated | NA | NA |
| metab_10605 | Unannotated feature | neg | 335.0005 | 1.685 | 4.181 | 4.104 | -0.077 | -0.128 | 0.477 | 0.777 | Unannotated | NA | NA |
| metab_264 | Unannotated feature | pos | 253.0439 | 0.524 | 4.359 | 4.238 | -0.121 | -0.128 | 0.477 | 0.777 | Unannotated | NA | NA |
| metab_8303 | Unannotated feature | pos | 789.5658 | 6.949 | 4.828 | 5.164 | 0.336 | 0.128 | 0.477 | 0.777 | Unannotated | NA | NA |
| metab_10557 | Unannotated feature | neg | 285.0606 | 1.861 | 3.209 | 3.793 | 0.584 | 0.128 | 0.477 | 0.777 | Unannotated | NA | NA |
| metab_1157 | Unannotated feature | pos | 289.2529 | 6.550 | 4.428 | 4.164 | -0.263 | -0.128 | 0.477 | 0.777 | Unannotated | NA | NA |
| metab_13069 | Fluocinonide | neg | 531.1582 | 1.029 | 5.278 | 4.745 | -0.533 | -0.128 | 0.477 | 0.777 | Tentative | NA | NA |
| metab_18613 | Unannotated feature | neg | 385.1870 | 5.975 | 4.786 | 4.606 | -0.181 | -0.128 | 0.477 | 0.777 | Unannotated | NA | NA |
| metab_2347 | Unannotated feature | pos | 171.0435 | 0.461 | 4.292 | 4.258 | -0.035 | -0.128 | 0.477 | 0.777 | Unannotated | NA | NA |
| metab_18649 | Unannotated feature | neg | 429.1556 | 3.120 | 4.712 | 4.603 | -0.110 | -0.128 | 0.477 | 0.777 | Unannotated | NA | NA |
| metab_2250 | Unannotated feature | pos | 492.2091 | 3.120 | 3.642 | 3.391 | -0.251 | -0.128 | 0.477 | 0.777 | Unannotated | NA | NA |
| metab_18794 | Aminonitropyrene | neg | 307.0727 | 5.789 | 4.853 | 5.232 | 0.379 | 0.128 | 0.477 | 0.777 | Unannotated | NA | NA |
| metab_18730 | Unannotated feature | neg | 287.1250 | 3.157 | 4.571 | 4.751 | 0.180 | 0.128 | 0.477 | 0.777 | Unannotated | NA | NA |
| metab_17500 | Unannotated feature | neg | 603.2958 | 7.499 | 4.287 | 4.491 | 0.204 | 0.128 | 0.477 | 0.777 | Unannotated | NA | NA |
| metab_17535 | Unannotated feature | neg | 602.4477 | 7.409 | 4.243 | 5.113 | 0.870 | 0.128 | 0.477 | 0.777 | Unannotated | NA | NA |
| metab_15671 | Unannotated feature | neg | 392.9853 | 0.588 | 4.018 | 3.884 | -0.134 | -0.128 | 0.477 | 0.777 | Unannotated | NA | NA |
| metab_2422 | Unannotated feature | pos | 197.9757 | 0.617 | 3.479 | 3.411 | -0.068 | -0.128 | 0.477 | 0.777 | Unannotated | NA | NA |
| metab_5257 | Buprenorphine | pos | 468.3076 | 6.654 | 5.913 | 5.837 | -0.075 | -0.128 | 0.477 | 0.777 | Moderate putative | NA | NA |
| metab_5236 | Unannotated feature | pos | 372.9999 | 6.694 | 4.013 | 4.165 | 0.152 | 0.128 | 0.477 | 0.777 | Unannotated | NA | NA |
| metab_14582 | Unannotated feature | neg | 322.0937 | 3.487 | 4.407 | 4.705 | 0.298 | 0.128 | 0.477 | 0.777 | Unannotated | NA | NA |
| metab_19600 | Unannotated feature | neg | 399.0398 | 3.566 | 3.051 | 2.683 | -0.367 | -0.128 | 0.477 | 0.777 | Unannotated | NA | NA |
| metab_12150 | Unannotated feature | neg | 767.5849 | 6.648 | 6.087 | 5.706 | -0.381 | -0.128 | 0.477 | 0.777 | Unannotated | NA | NA |
| metab_5189 | Cholic Acid | pos | 373.2745 | 6.239 | 6.851 | 6.509 | -0.342 | -0.128 | 0.477 | 0.777 | Strong putative | NA | NA |
| metab_2619 | Pilocarpine | pos | 226.1553 | 1.692 | 5.164 | 5.094 | -0.070 | -0.128 | 0.477 | 0.777 | Strong putative | NA | NA |
| metab_10056 | Unannotated feature | neg | 397.1874 | 4.451 | 5.851 | 5.669 | -0.182 | -0.128 | 0.477 | 0.777 | Unannotated | NA | NA |
| metab_2518 | Unannotated feature | pos | 104.0534 | 0.862 | 5.271 | 5.106 | -0.165 | -0.128 | 0.477 | 0.777 | Unannotated | NA | NA |
| metab_17505 | Glyuranolide | neg | 533.2928 | 7.485 | 7.389 | 7.377 | -0.012 | 0.128 | 0.477 | 0.777 | Unannotated | NA | NA |
| metab_2441 | Ptaquiloside | pos | 363.1770 | 0.641 | 4.630 | 4.766 | 0.136 | 0.128 | 0.477 | 0.777 | Tentative | Organic oxygen compounds | Organooxygen compounds |
| metab_10002 | Unannotated feature | neg | 227.0537 | 4.405 | 4.476 | 4.518 | 0.042 | 0.128 | 0.477 | 0.777 | Unannotated | NA | NA |
| metab_17403 | Unannotated feature | neg | 125.0238 | 1.958 | 5.256 | 5.220 | -0.035 | -0.128 | 0.477 | 0.777 | Unannotated | NA | NA |
| metab_13500 | Unannotated feature | neg | 413.1310 | 1.041 | 4.258 | 4.320 | 0.062 | 0.128 | 0.477 | 0.777 | Unannotated | NA | NA |
| metab_15888 | DG(20:5(5Z,8Z,11Z,14Z,16E)-OH(18R)/0:0/2:0) | neg | 479.2659 | 5.983 | 4.884 | 4.797 | -0.086 | -0.128 | 0.477 | 0.777 | Unannotated | NA | NA |
| metab_11868 | Unannotated feature | neg | 403.2347 | 6.007 | 5.798 | 5.647 | -0.152 | -0.128 | 0.477 | 0.777 | Unannotated | NA | NA |
| metab_6972 | 3-O-Methylniveusin A | pos | 391.1724 | 0.649 | 4.711 | 4.765 | 0.055 | 0.128 | 0.477 | 0.777 | Unannotated | NA | NA |
| metab_15574 | Unannotated feature | neg | 162.8384 | 5.450 | 4.885 | 4.905 | 0.020 | -0.128 | 0.477 | 0.777 | Unannotated | NA | NA |
| metab_5153 | Unannotated feature | pos | 585.5344 | 6.861 | 5.377 | 5.275 | -0.102 | -0.128 | 0.477 | 0.777 | Unannotated | NA | NA |
| metab_5086 | Unannotated feature | pos | 543.4034 | 7.060 | 5.534 | 5.538 | 0.004 | 0.128 | 0.477 | 0.777 | Unannotated | NA | NA |
| metab_5109 | Unannotated feature | pos | 497.3066 | 6.997 | 6.003 | 5.977 | -0.026 | -0.128 | 0.477 | 0.777 | Unannotated | NA | NA |
| metab_8519 | Unannotated feature | pos | 87.1003 | 1.543 | 5.550 | 5.465 | -0.084 | -0.128 | 0.477 | 0.777 | Unannotated | NA | NA |
| metab_2905 | 4-[(S)-Amino(carboxy)methyl]-3-methylbenzoic acid | pos | 192.0657 | 2.864 | 4.763 | 4.777 | 0.014 | -0.128 | 0.477 | 0.777 | Unannotated | NA | NA |
| metab_907 | Unannotated feature | pos | 435.3455 | 7.116 | 6.439 | 6.416 | -0.023 | -0.128 | 0.477 | 0.777 | Unannotated | NA | NA |
| metab_17346 | Unannotated feature | neg | 350.9286 | 7.692 | 5.557 | 5.546 | -0.011 | -0.128 | 0.477 | 0.777 | Unannotated | NA | NA |
| metab_6784 | Unannotated feature | pos | 258.1529 | 5.199 | 4.727 | 4.515 | -0.211 | -0.128 | 0.477 | 0.777 | Unannotated | NA | NA |
| metab_18941 | Ethyl 15-methoxy-12-oxo-2,4,11-triazatetracyclo[11.4.0.0^{2,6}.0^{7,11}]heptadeca-1(13),3,5,14,16-pentaene-5-carboxylate | neg | 376.1042 | 5.595 | 6.577 | 6.725 | 0.148 | 0.128 | 0.477 | 0.777 | Unannotated | NA | NA |
| metab_7668 | Kynurenic Acid | pos | 190.0502 | 3.432 | 5.765 | 5.795 | 0.030 | 0.128 | 0.477 | 0.777 | Strong putative | NA | NA |
| metab_7661 | Unannotated feature | pos | 646.2573 | 0.728 | 3.705 | 3.854 | 0.149 | 0.128 | 0.477 | 0.777 | Unannotated | NA | NA |
| metab_17264 | Unannotated feature | neg | 171.9312 | 7.726 | 3.973 | 3.935 | -0.038 | -0.128 | 0.477 | 0.777 | Unannotated | NA | NA |
| metab_13676 | Secologanate | neg | 355.1001 | 1.685 | 6.273 | 6.303 | 0.030 | -0.128 | 0.477 | 0.777 | Tentative | NA | NA |
| metab_12427 | Unannotated feature | neg | 277.9066 | 7.730 | 5.558 | 5.526 | -0.032 | -0.128 | 0.477 | 0.777 | Unannotated | NA | NA |
| metab_394 | Unannotated feature | pos | 197.0144 | 0.019 | 4.582 | 4.597 | 0.014 | 0.128 | 0.477 | 0.777 | Unannotated | NA | NA |
| metab_16625 | Unannotated feature | neg | 199.8505 | 7.503 | 3.756 | 3.842 | 0.085 | 0.128 | 0.477 | 0.777 | Unannotated | NA | NA |
| metab_14350 | Unannotated feature | neg | 248.9605 | 0.527 | 4.220 | 3.998 | -0.221 | -0.128 | 0.477 | 0.777 | Unannotated | NA | NA |
| metab_17171 | Unannotated feature | neg | 266.9101 | 7.745 | 3.631 | 3.577 | -0.054 | -0.128 | 0.477 | 0.777 | Unannotated | NA | NA |
| metab_19063 | Unannotated feature | neg | 267.1604 | 5.441 | 4.046 | 4.557 | 0.511 | 0.128 | 0.477 | 0.777 | Unannotated | NA | NA |
| metab_3063 | Unannotated feature | pos | 839.5672 | 6.430 | 7.156 | 7.034 | -0.122 | -0.128 | 0.477 | 0.777 | Unannotated | NA | NA |
| metab_9664 | Hydroxy Tyrosol -Acetate | neg | 270.0986 | 3.173 | 4.829 | 4.952 | 0.123 | 0.128 | 0.477 | 0.777 | Unannotated | NA | NA |
| metab_17142 | Unannotated feature | neg | 336.8588 | 7.788 | 4.527 | 4.516 | -0.011 | -0.128 | 0.477 | 0.777 | Unannotated | NA | NA |
| metab_3290 | Unannotated feature | pos | 789.5660 | 7.036 | 6.000 | 6.083 | 0.083 | 0.128 | 0.477 | 0.777 | Unannotated | NA | NA |
| metab_17092 | Unannotated feature | neg | 247.9115 | 2.144 | 4.704 | 4.170 | -0.534 | -0.128 | 0.477 | 0.777 | Unannotated | NA | NA |
| metab_12361 | Unannotated feature | neg | 415.3224 | 6.758 | 5.560 | 5.645 | 0.084 | 0.128 | 0.477 | 0.777 | Unannotated | NA | NA |
| metab_3380 | (Z)-6-(2-Methoxyvinyl)-7-methyl-2H-1-benzopyran-2-one | pos | 249.1108 | 4.339 | 4.214 | 4.201 | -0.013 | -0.128 | 0.477 | 0.777 | Unannotated | NA | NA |
| metab_15392 | Unannotated feature | neg | 353.1717 | 5.052 | 3.926 | 4.612 | 0.687 | 0.128 | 0.477 | 0.777 | Unannotated | NA | NA |
| metab_15525 | Unannotated feature | neg | 383.1284 | 5.330 | 5.099 | 5.224 | 0.124 | 0.128 | 0.477 | 0.777 | Unannotated | NA | NA |
| metab_19127 | Unannotated feature | neg | 149.0601 | 5.351 | 3.090 | 3.526 | 0.435 | 0.128 | 0.477 | 0.777 | Unannotated | NA | NA |
| metab_14997 | Cerasinone | neg | 375.1090 | 4.324 | 5.354 | 5.483 | 0.129 | 0.128 | 0.477 | 0.777 | Unannotated | NA | NA |
| metab_9551 | 4,4'-Diphenylmethane diisocyanate | neg | 287.0234 | 3.284 | 3.972 | 3.334 | -0.639 | -0.128 | 0.477 | 0.777 | Tentative | Benzenoids | Benzene and substituted derivatives |
| metab_408 | Unannotated feature | pos | 200.0212 | 0.016 | 5.282 | 5.292 | 0.010 | 0.128 | 0.477 | 0.777 | Unannotated | NA | NA |
| metab_401 | Unannotated feature | pos | 203.9715 | 0.016 | 4.482 | 4.494 | 0.012 | 0.128 | 0.477 | 0.777 | Unannotated | NA | NA |
| metab_4858 | Unannotated feature | pos | 239.1072 | 7.724 | 4.337 | 4.337 | -0.000 | 0.128 | 0.477 | 0.777 | Unannotated | NA | NA |
| metab_11694 | Unannotated feature | neg | 365.1722 | 5.441 | 5.091 | 4.815 | -0.276 | -0.128 | 0.477 | 0.777 | Unannotated | NA | NA |
| metab_3971 | 7alpha-hydroxy-3-oxochol-4-en-24-oic Acid | pos | 411.2514 | 6.215 | 5.203 | 4.972 | -0.231 | -0.128 | 0.477 | 0.777 | Unannotated | NA | NA |
| metab_3834 | Unannotated feature | pos | 186.0166 | 0.633 | 5.608 | 5.575 | -0.034 | -0.128 | 0.477 | 0.777 | Unannotated | NA | NA |
| metab_13881 | Unannotated feature | neg | 112.9556 | 2.375 | 4.393 | 4.328 | -0.065 | -0.128 | 0.477 | 0.777 | Unannotated | NA | NA |
| metab_13877 | 1-Methyluric acid | neg | 181.0362 | 2.336 | 6.104 | 6.372 | 0.268 | 0.128 | 0.477 | 0.777 | Moderate putative | NA | NA |
| metab_9419 | Gamma-Glutamylisoleucine | neg | 259.1300 | 3.453 | 5.759 | 5.697 | -0.063 | -0.128 | 0.477 | 0.777 | Unannotated | NA | NA |
| metab_9410 | Unannotated feature | neg | 384.2146 | 3.457 | 5.001 | 5.023 | 0.022 | -0.128 | 0.477 | 0.777 | Unannotated | NA | NA |
| metab_11740 | Methoxyhydroxymandelic Acid | neg | 179.0344 | 3.975 | 5.491 | 5.313 | -0.178 | -0.128 | 0.477 | 0.777 | Unannotated | NA | NA |
| metab_19212 | Unannotated feature | neg | 167.0707 | 5.246 | 2.907 | 3.167 | 0.260 | 0.128 | 0.477 | 0.777 | Unannotated | NA | NA |
| metab_779 | Unannotated feature | pos | 518.8105 | 0.524 | 4.783 | 5.122 | 0.339 | 0.128 | 0.477 | 0.777 | Unannotated | NA | NA |
| metab_9301 | Unannotated feature | neg | 273.1248 | 3.612 | 3.764 | 3.448 | -0.317 | -0.128 | 0.477 | 0.777 | Unannotated | NA | NA |
| metab_13965 | Unannotated feature | neg | 235.1186 | 2.649 | 3.909 | 3.907 | -0.002 | 0.128 | 0.477 | 0.777 | Unannotated | NA | NA |
| metab_9365 | Unannotated feature | neg | 332.0726 | 3.521 | 3.756 | 3.619 | -0.137 | -0.128 | 0.477 | 0.777 | Unannotated | NA | NA |
| metab_421 | Unannotated feature | pos | 158.9490 | 0.016 | 3.897 | 3.904 | 0.006 | 0.128 | 0.477 | 0.777 | Unannotated | NA | NA |
| metab_8884 | Unannotated feature | neg | 399.1080 | 4.291 | 4.262 | 4.149 | -0.114 | -0.128 | 0.477 | 0.777 | Unannotated | NA | NA |
| metab_4418 | 2-Lysophosphatidylcholine | pos | 524.3723 | 6.327 | 6.241 | 6.082 | -0.159 | -0.128 | 0.477 | 0.777 | Tentative | Lipids and lipid-like molecules | Glycerophospholipids |
| metab_14078 | Unannotated feature | neg | 672.8667 | 0.502 | 4.701 | 4.498 | -0.203 | -0.128 | 0.477 | 0.777 | Unannotated | NA | NA |
| metab_9061 | Unannotated feature | neg | 107.0495 | 3.988 | 5.786 | 5.488 | -0.298 | -0.128 | 0.477 | 0.777 | Unannotated | NA | NA |
| metab_14120 | Unannotated feature | neg | 198.0226 | 2.848 | 3.129 | 3.448 | 0.319 | 0.128 | 0.477 | 0.777 | Unannotated | NA | NA |
| metab_12209 | Unannotated feature | neg | 339.2146 | 6.828 | 5.489 | 5.429 | -0.059 | -0.128 | 0.477 | 0.777 | Unannotated | NA | NA |
| metab_537 | Dehydroglyasperin D | pos | 333.1453 | 5.499 | 4.215 | 3.733 | -0.482 | -0.128 | 0.477 | 0.777 | Unannotated | Phenylpropanoids and polyketides | Isoflavonoids |
| metab_16855 | Rescinnamine | neg | 316.1353 | 7.730 | 5.985 | 5.960 | -0.025 | 0.128 | 0.477 | 0.777 | Tentative | NA | NA |
| metab_11830 | Unannotated feature | neg | 545.2657 | 5.918 | 4.703 | 4.751 | 0.048 | 0.128 | 0.477 | 0.777 | Unannotated | NA | NA |
| metab_19345 | Unannotated feature | neg | 373.0933 | 5.072 | 3.572 | 4.134 | 0.562 | 0.128 | 0.477 | 0.777 | Unannotated | NA | NA |
| metab_14113 | Unannotated feature | neg | 335.1548 | 2.841 | 4.491 | 4.075 | -0.416 | -0.128 | 0.477 | 0.777 | Unannotated | NA | NA |
| metab_4496 | THYMOQUINONE | pos | 165.0913 | 6.598 | 5.542 | 5.606 | 0.064 | 0.128 | 0.477 | 0.777 | Moderate putative | NA | NA |
| metab_12731 | Unannotated feature | neg | 377.1257 | 4.454 | 4.592 | 4.640 | 0.048 | -0.128 | 0.477 | 0.777 | Unannotated | NA | NA |
| metab_103 | Unannotated feature | pos | 260.0535 | 0.641 | 6.095 | 5.848 | -0.247 | -0.128 | 0.477 | 0.777 | Unannotated | NA | NA |
| metab_7053 | Dihydroorotic Acid | pos | 358.1020 | 0.657 | 5.523 | 5.369 | -0.154 | -0.128 | 0.477 | 0.777 | Moderate putative | NA | NA |
| metab_92 | Gamma-Aminobutyric Acid | pos | 104.0712 | 0.649 | 6.021 | 5.724 | -0.297 | -0.128 | 0.477 | 0.777 | Strong putative | NA | NA |
| metab_8088 | MG(0:0/20:5(5Z,8Z,11Z,14Z,17Z)/0:0) | pos | 211.1190 | 2.747 | 3.513 | 2.904 | -0.609 | -0.124 | 0.492 | 0.787 | Unannotated | NA | NA |
| metab_5442 | D-erythro-Sphingosine C-17 | pos | 286.2744 | 6.398 | 5.596 | 5.422 | -0.174 | -0.124 | 0.492 | 0.787 | Moderate putative | NA | NA |
| metab_5447 | 3-keto Fusidic acid | pos | 563.3572 | 6.390 | 6.289 | 6.207 | -0.082 | -0.124 | 0.492 | 0.787 | Unannotated | NA | NA |
| metab_15448 | Unannotated feature | neg | 338.1460 | 5.169 | 3.681 | 4.130 | 0.449 | 0.124 | 0.492 | 0.787 | Unannotated | NA | NA |
| metab_18317 | Unannotated feature | neg | 541.2849 | 6.201 | 4.792 | 4.957 | 0.165 | 0.124 | 0.492 | 0.787 | Unannotated | NA | NA |
| metab_12687 | Unannotated feature | neg | 223.0972 | 5.484 | 4.176 | 4.417 | 0.240 | 0.124 | 0.492 | 0.787 | Unannotated | NA | NA |
| metab_10849 | 7-Methyl-2-(2-furyl)-1,8-naphthyridine-4(1H)-one | neg | 263.0233 | 1.021 | 3.453 | 4.004 | 0.551 | 0.124 | 0.492 | 0.787 | Unannotated | NA | NA |
| metab_11163 | Unannotated feature | neg | 351.1565 | 4.997 | 5.287 | 5.172 | -0.115 | -0.124 | 0.492 | 0.787 | Unannotated | NA | NA |
| metab_1912 | (S)-Isosclerone | pos | 196.0972 | 1.685 | 5.013 | 5.106 | 0.093 | 0.124 | 0.492 | 0.787 | Unannotated | NA | NA |
| metab_18038 | Lysylarginine | neg | 283.1915 | 6.459 | 5.137 | 5.217 | 0.080 | 0.124 | 0.492 | 0.787 | Unannotated | Organic acids and derivatives | Carboxylic acids and derivatives |
| metab_10659 | Unannotated feature | neg | 343.0842 | 4.722 | 5.003 | 5.270 | 0.267 | 0.124 | 0.492 | 0.787 | Unannotated | NA | NA |
| metab_10690 | 2-O-Methyl-L-fucose | neg | 159.0656 | 1.467 | 5.673 | 5.606 | -0.067 | -0.124 | 0.492 | 0.787 | Unannotated | NA | NA |
| metab_12973 | Unannotated feature | neg | 231.0509 | 0.784 | 5.643 | 5.816 | 0.173 | 0.124 | 0.492 | 0.787 | Unannotated | NA | NA |
| metab_1941 | N1-Methyl-4-pyridone-3-carboxamide | pos | 153.0661 | 1.952 | 5.268 | 5.330 | 0.062 | 0.124 | 0.492 | 0.787 | Tentative | NA | NA |
| metab_1830 | (1r,3r)-1-Aminocyclopentane-1,3-dicarboxylic acid | pos | 174.0764 | 0.760 | 5.994 | 6.093 | 0.099 | 0.124 | 0.492 | 0.787 | Unannotated | NA | NA |
| metab_15179 | Unannotated feature | neg | 305.1513 | 4.681 | 5.457 | 5.396 | -0.061 | -0.124 | 0.492 | 0.787 | Unannotated | NA | NA |
| metab_18566 | Unannotated feature | neg | 597.2522 | 6.007 | 3.082 | 3.494 | 0.412 | 0.124 | 0.492 | 0.787 | Unannotated | NA | NA |
| metab_13023 | Unannotated feature | neg | 327.8807 | 0.619 | 4.174 | 3.881 | -0.293 | -0.124 | 0.492 | 0.787 | Unannotated | NA | NA |
| metab_15162 | Unannotated feature | neg | 266.9991 | 4.653 | 3.608 | 3.808 | 0.200 | 0.124 | 0.492 | 0.787 | Unannotated | NA | NA |
| metab_7887 | Unannotated feature | pos | 307.1660 | 2.976 | 3.756 | 3.888 | 0.132 | 0.124 | 0.492 | 0.787 | Unannotated | NA | NA |
| metab_14873 | N-Palmitoyl Cysteine | neg | 299.1503 | 4.044 | 5.540 | 5.654 | 0.113 | 0.124 | 0.492 | 0.787 | Unannotated | NA | NA |
| metab_16866 | Unannotated feature | neg | 252.5727 | 7.737 | 4.153 | 4.137 | -0.016 | -0.124 | 0.492 | 0.787 | Unannotated | NA | NA |
| metab_16870 | Unannotated feature | neg | 318.8749 | 7.737 | 4.881 | 4.848 | -0.033 | -0.124 | 0.492 | 0.787 | Unannotated | NA | NA |
| metab_4384 | Physapubenolide | pos | 573.2415 | 6.239 | 2.240 | 4.777 | 2.537 | 0.124 | 0.492 | 0.787 | Unannotated | Lipids and lipid-like molecules | Steroids and steroid derivatives |
| metab_9079 | Unannotated feature | neg | 245.1394 | 3.956 | 4.738 | 4.745 | 0.007 | 0.124 | 0.492 | 0.787 | Unannotated | NA | NA |
| metab_9119 | 1-(2,6-Dihydroxy-4-methoxyphenyl)-3-phenyl-1-propanone | neg | 317.1035 | 3.889 | 3.117 | 3.306 | 0.189 | 0.124 | 0.492 | 0.787 | Tentative | NA | NA |
| metab_11778 | Unannotated feature | neg | 384.0729 | 5.767 | 2.312 | 2.759 | 0.447 | 0.124 | 0.492 | 0.787 | Unannotated | NA | NA |
| metab_12241 | Unannotated feature | neg | 516.3068 | 6.959 | 4.788 | 4.870 | 0.082 | 0.124 | 0.492 | 0.787 | Unannotated | NA | NA |
| metab_10541 | Unannotated feature | neg | 144.1023 | 1.944 | 3.352 | 3.224 | -0.128 | -0.124 | 0.492 | 0.787 | Unannotated | NA | NA |
| metab_8369 | (2E,4E,7R)-2,7-Dimethyl-2,4-octadiene-1,8-diol 8-O-b-D-glucopyranoside | pos | 315.1782 | 1.952 | 2.352 | 2.117 | -0.235 | -0.124 | 0.492 | 0.787 | Unannotated | Lipids and lipid-like molecules | Fatty Acyls |
| metab_12096 | Unannotated feature | neg | 455.2478 | 6.428 | 7.045 | 6.989 | -0.056 | -0.124 | 0.492 | 0.787 | Unannotated | NA | NA |
| metab_10420 | Ilicifolinoside A | neg | 309.1191 | 2.004 | 4.222 | 3.968 | -0.254 | -0.124 | 0.492 | 0.787 | Unannotated | NA | NA |
| metab_17894 | Unannotated feature | neg | 644.4215 | 6.663 | 5.279 | 5.325 | 0.046 | -0.124 | 0.492 | 0.787 | Unannotated | NA | NA |
| metab_2036 | Sucrose | pos | 360.1512 | 0.696 | 5.034 | 4.991 | -0.043 | -0.124 | 0.492 | 0.787 | Moderate putative | NA | NA |
| metab_11259 | Unannotated feature | neg | 287.1866 | 5.083 | 4.573 | 4.901 | 0.328 | 0.124 | 0.492 | 0.787 | Unannotated | NA | NA |
| metab_1158 | Unannotated feature | pos | 557.3831 | 6.542 | 6.693 | 6.785 | 0.093 | 0.124 | 0.492 | 0.787 | Unannotated | NA | NA |
| metab_14914 | Unannotated feature | neg | 285.1710 | 4.138 | 6.798 | 6.727 | -0.072 | -0.124 | 0.492 | 0.787 | Unannotated | NA | NA |
| metab_19298 | Unannotated feature | neg | 312.1456 | 5.148 | 4.049 | 4.355 | 0.306 | 0.124 | 0.492 | 0.787 | Unannotated | NA | NA |
| metab_10337 | Cis-Isoferulic acid | neg | 193.0501 | 4.588 | 4.538 | 4.369 | -0.168 | -0.124 | 0.492 | 0.787 | Unannotated | Phenylpropanoids and polyketides | Cinnamic acids and derivatives |
| metab_10339 | Unannotated feature | neg | 234.0982 | 2.244 | 2.830 | 3.447 | 0.618 | 0.124 | 0.492 | 0.787 | Unannotated | NA | NA |
| metab_10494 | Unannotated feature | neg | 183.0769 | 1.952 | 3.827 | 3.874 | 0.048 | -0.124 | 0.492 | 0.787 | Unannotated | NA | NA |
| metab_2156 | Unannotated feature | pos | 288.1923 | 2.897 | 4.912 | 4.947 | 0.035 | -0.124 | 0.492 | 0.787 | Unannotated | NA | NA |
| metab_17753 | Unannotated feature | neg | 588.4683 | 6.907 | 6.539 | 6.489 | -0.051 | -0.124 | 0.492 | 0.787 | Unannotated | NA | NA |
| metab_11285 | L-Histidine | neg | 154.0615 | 0.619 | 5.623 | 5.483 | -0.140 | -0.124 | 0.492 | 0.787 | Strong putative | NA | NA |
| metab_15887 | Unannotated feature | neg | 273.1708 | 5.983 | 6.265 | 6.144 | -0.120 | -0.124 | 0.492 | 0.787 | Unannotated | NA | NA |
| metab_13236 | DIMP | neg | 353.0255 | 0.571 | 4.344 | 4.334 | -0.010 | -0.124 | 0.492 | 0.787 | Tentative | NA | NA |
| metab_17657 | Unannotated feature | neg | 379.1795 | 7.068 | 4.227 | 4.499 | 0.272 | 0.124 | 0.492 | 0.787 | Unannotated | NA | NA |
| metab_17628 | Sorbitan stearate | neg | 467.2789 | 7.129 | 6.943 | 6.920 | -0.022 | -0.124 | 0.492 | 0.787 | Unannotated | NA | NA |
| metab_17634 | Unannotated feature | neg | 591.3918 | 7.114 | 6.448 | 6.327 | -0.121 | -0.124 | 0.492 | 0.787 | Unannotated | NA | NA |
| metab_1118 | Unannotated feature | pos | 365.2790 | 6.638 | 3.902 | 4.121 | 0.219 | 0.124 | 0.492 | 0.787 | Unannotated | NA | NA |
| metab_14028 | Unannotated feature | neg | 111.0444 | 2.766 | 4.465 | 4.491 | 0.027 | 0.124 | 0.492 | 0.787 | Unannotated | NA | NA |
| metab_8893 | Unannotated feature | neg | 294.0986 | 4.279 | 3.707 | 4.221 | 0.513 | 0.124 | 0.492 | 0.787 | Unannotated | NA | NA |
| metab_16816 | Unannotated feature | neg | 401.1209 | 1.958 | 4.488 | 4.450 | -0.038 | 0.124 | 0.492 | 0.787 | Unannotated | NA | NA |
| metab_4696 | Unannotated feature | pos | 578.5133 | 7.578 | 5.278 | 5.443 | 0.165 | 0.124 | 0.492 | 0.787 | Unannotated | NA | NA |
| metab_5596 | Unannotated feature | pos | 544.3070 | 6.231 | 5.049 | 4.802 | -0.247 | -0.124 | 0.492 | 0.787 | Unannotated | NA | NA |
| metab_420 | Unannotated feature | pos | 159.5037 | 0.016 | 4.097 | 4.138 | 0.041 | 0.124 | 0.492 | 0.787 | Unannotated | NA | NA |
| metab_15844 | Pregnenolone-3,21-disulfate | neg | 245.0669 | 5.922 | 4.759 | 5.011 | 0.252 | 0.124 | 0.492 | 0.787 | Unannotated | NA | NA |
| metab_10307 | Unannotated feature | neg | 127.0394 | 2.368 | 4.090 | 4.217 | 0.127 | 0.124 | 0.492 | 0.787 | Unannotated | NA | NA |
| metab_10269 | Unannotated feature | neg | 87.0442 | 2.520 | 4.529 | 4.436 | -0.093 | -0.124 | 0.492 | 0.787 | Unannotated | NA | NA |
| metab_17688 | Unannotated feature | neg | 198.0767 | 2.776 | 6.108 | 5.995 | -0.113 | -0.124 | 0.492 | 0.787 | Unannotated | NA | NA |
| metab_5465 | Carotol | pos | 240.2325 | 6.382 | 4.656 | 4.487 | -0.169 | -0.124 | 0.492 | 0.787 | Tentative | NA | NA |
| metab_13509 | 2'-Deoxyuridine | neg | 273.0733 | 1.680 | 6.350 | 6.440 | 0.090 | -0.124 | 0.492 | 0.787 | Moderate putative | NA | NA |
| metab_9975 | Unannotated feature | neg | 473.1676 | 3.149 | 4.232 | 4.233 | 0.001 | -0.124 | 0.492 | 0.787 | Unannotated | NA | NA |
| metab_9988 | Unannotated feature | neg | 220.0724 | 2.862 | 3.137 | 2.803 | -0.334 | -0.124 | 0.492 | 0.787 | Unannotated | NA | NA |
| metab_12465 | Unannotated feature | neg | 179.8401 | 7.910 | 5.488 | 5.473 | -0.015 | -0.124 | 0.492 | 0.787 | Unannotated | NA | NA |
| metab_9935 | Unannotated feature | neg | 215.0017 | 2.920 | 3.569 | 3.638 | 0.069 | -0.124 | 0.492 | 0.787 | Unannotated | NA | NA |
| metab_2517 | 2-Amino-7-[(2R)-2,3-dihydroxypropanoyl]-5,6,7,8-tetrahydro-3H-pteridin-4-one | pos | 238.0922 | 0.854 | 5.288 | 5.297 | 0.009 | 0.124 | 0.492 | 0.787 | Unannotated | NA | NA |
| metab_10045 | Unannotated feature | neg | 392.1470 | 2.799 | 4.534 | 4.373 | -0.161 | -0.124 | 0.492 | 0.787 | Unannotated | NA | NA |
| metab_9304 | Unannotated feature | neg | 161.0813 | 3.612 | 4.986 | 4.882 | -0.104 | -0.124 | 0.492 | 0.787 | Unannotated | NA | NA |
| metab_13990 | Unannotated feature | neg | 335.1545 | 2.703 | 3.617 | 3.311 | -0.306 | -0.124 | 0.492 | 0.787 | Unannotated | NA | NA |
| metab_19264 | Unannotated feature | neg | 261.1706 | 5.185 | 3.164 | 2.897 | -0.267 | -0.124 | 0.492 | 0.787 | Unannotated | NA | NA |
| metab_780 | Unannotated feature | pos | 315.0980 | 6.191 | 3.734 | 3.786 | 0.052 | 0.124 | 0.492 | 0.787 | Unannotated | NA | NA |
| metab_4411 | Unannotated feature | pos | 531.2946 | 6.311 | 4.981 | 4.799 | -0.182 | -0.124 | 0.492 | 0.787 | Unannotated | NA | NA |
| metab_3959 | Urobilin | pos | 623.3456 | 5.787 | 5.407 | 5.569 | 0.163 | 0.124 | 0.492 | 0.787 | Tentative | NA | NA |
| metab_13588 | Unannotated feature | neg | 108.0196 | 1.354 | 4.893 | 4.879 | -0.014 | -0.124 | 0.492 | 0.787 | Unannotated | NA | NA |
| metab_9895 | Unannotated feature | neg | 350.0311 | 2.956 | 3.817 | 3.589 | -0.228 | -0.124 | 0.492 | 0.787 | Unannotated | NA | NA |
| metab_5075 | Unannotated feature | pos | 653.4765 | 7.100 | 6.083 | 6.098 | 0.014 | -0.124 | 0.492 | 0.787 | Unannotated | NA | NA |
| metab_19540 | Unannotated feature | neg | 239.1036 | 3.533 | 4.914 | 4.905 | -0.009 | -0.124 | 0.492 | 0.787 | Unannotated | NA | NA |
| metab_13417 | Unannotated feature | neg | 188.0923 | 0.791 | 4.638 | 4.476 | -0.162 | -0.124 | 0.492 | 0.787 | Unannotated | NA | NA |
| metab_2951 | Unannotated feature | pos | 245.1539 | 6.255 | 5.015 | 4.734 | -0.281 | -0.124 | 0.492 | 0.787 | Unannotated | NA | NA |
| metab_15364 | Unannotated feature | neg | 369.1668 | 5.022 | 3.322 | 3.871 | 0.549 | 0.124 | 0.492 | 0.787 | Unannotated | NA | NA |
| metab_15643 | Unannotated feature | neg | 373.1297 | 5.579 | 3.589 | 3.867 | 0.278 | 0.124 | 0.492 | 0.787 | Unannotated | NA | NA |
| metab_13557 | Unannotated feature | neg | 357.1314 | 1.202 | 4.373 | 4.284 | -0.088 | -0.124 | 0.492 | 0.787 | Unannotated | NA | NA |
| metab_13559 | Deoxyuridine | neg | 273.0733 | 1.947 | 6.496 | 6.612 | 0.116 | -0.124 | 0.492 | 0.787 | Strong putative | NA | NA |
| metab_13572 | Unannotated feature | neg | 262.0836 | 1.263 | 3.967 | 4.619 | 0.652 | 0.124 | 0.492 | 0.787 | Unannotated | NA | NA |
| metab_17319 | Unannotated feature | neg | 144.9228 | 7.702 | 6.873 | 6.855 | -0.018 | -0.124 | 0.492 | 0.787 | Unannotated | NA | NA |
| metab_4079 | Unannotated feature | pos | 515.2457 | 6.080 | 5.877 | 6.197 | 0.320 | 0.124 | 0.492 | 0.787 | Unannotated | NA | NA |
| metab_14247 | 2-(2-Acetyl-3-Hydroxy-5-methoxyphenyl)acetic Acid | neg | 223.0609 | 2.984 | 5.465 | 5.429 | -0.036 | -0.124 | 0.492 | 0.787 | Unannotated | NA | NA |
| metab_16782 | Unannotated feature | neg | 515.9058 | 7.697 | 4.811 | 4.805 | -0.006 | 0.124 | 0.492 | 0.787 | Unannotated | NA | NA |
| metab_16702 | Unannotated feature | neg | 228.9939 | 7.637 | 4.301 | 4.359 | 0.057 | 0.124 | 0.492 | 0.787 | Unannotated | NA | NA |
| metab_9542 | Unannotated feature | neg | 428.1323 | 3.295 | 4.633 | 4.579 | -0.054 | -0.124 | 0.492 | 0.787 | Unannotated | NA | NA |
| metab_3007 | 3-Pyridinebutanoic acid | pos | 198.1131 | 3.140 | 3.788 | 3.654 | -0.134 | -0.124 | 0.492 | 0.787 | Unannotated | NA | NA |
| metab_3024 | Unannotated feature | pos | 241.0740 | 3.208 | 4.366 | 4.405 | 0.039 | 0.124 | 0.492 | 0.787 | Unannotated | NA | NA |
| metab_15998 | Unannotated feature | neg | 301.1566 | 6.115 | 5.053 | 4.656 | -0.397 | -0.124 | 0.492 | 0.787 | Unannotated | NA | NA |
| metab_8561 | Unannotated feature | pos | 79.0183 | 1.252 | 3.282 | 3.326 | 0.044 | 0.124 | 0.492 | 0.787 | Unannotated | NA | NA |
| metab_15384 | 4,11,13,15-Tetrahydroridentin B | neg | 303.1359 | 5.044 | 5.349 | 5.413 | 0.064 | -0.124 | 0.492 | 0.787 | Unannotated | Lipids and lipid-like molecules | Prenol lipids |
| metab_2988 | Unannotated feature | pos | 615.3173 | 6.295 | 6.823 | 6.384 | -0.440 | -0.124 | 0.492 | 0.787 | Unannotated | NA | NA |
| metab_14986 | 2,2,3,3-Tetramethylcyclopropanecarboxamide | neg | 186.1130 | 4.303 | 5.459 | 5.432 | -0.026 | -0.124 | 0.492 | 0.787 | Unannotated | NA | NA |
| metab_6844 | Unannotated feature | pos | 148.0605 | 0.641 | 6.810 | 6.874 | 0.065 | -0.124 | 0.492 | 0.787 | Unannotated | NA | NA |
| metab_17046 | Hexathiepane | neg | 226.8238 | 7.985 | 5.464 | 5.432 | -0.031 | -0.124 | 0.492 | 0.787 | Unannotated | NA | NA |
| metab_14991 | Unannotated feature | neg | 524.2851 | 4.311 | 4.881 | 4.522 | -0.359 | -0.124 | 0.492 | 0.787 | Unannotated | NA | NA |
| metab_19128 | Unannotated feature | neg | 287.1614 | 3.234 | 4.982 | 4.892 | -0.091 | -0.124 | 0.492 | 0.787 | Unannotated | NA | NA |
| metab_7710 | (1R,2R)-2-Amino-1-(4-nitrophenyl)propane-1,3-diol | pos | 442.1932 | 0.760 | 5.397 | 5.552 | 0.155 | 0.124 | 0.492 | 0.787 | Unannotated | NA | NA |
| metab_9762 | Unannotated feature | neg | 252.9827 | 3.074 | 4.257 | 4.468 | 0.211 | 0.124 | 0.492 | 0.787 | Unannotated | NA | NA |
| metab_9764 | Unannotated feature | neg | 250.0721 | 3.070 | 4.183 | 4.405 | 0.222 | 0.124 | 0.492 | 0.787 | Unannotated | NA | NA |
| metab_3074 | Unannotated feature | pos | 314.2080 | 3.362 | 5.546 | 5.501 | -0.045 | -0.124 | 0.492 | 0.787 | Unannotated | NA | NA |
| metab_3080 | Norgalanthamine | pos | 274.1442 | 3.375 | 3.055 | 3.300 | 0.245 | 0.124 | 0.492 | 0.787 | Tentative | NA | NA |
| metab_19062 | Unannotated feature | neg | 325.0075 | 3.314 | 5.654 | 5.847 | 0.193 | 0.124 | 0.492 | 0.787 | Unannotated | NA | NA |
| metab_17166 | Unannotated feature | neg | 154.9735 | 7.755 | 5.410 | 5.398 | -0.012 | -0.124 | 0.492 | 0.787 | Unannotated | NA | NA |
| metab_3342 | Mertiatide | pos | 281.0926 | 4.207 | 4.517 | 4.535 | 0.018 | 0.124 | 0.492 | 0.787 | Unannotated | NA | NA |
| metab_12371 | Unannotated feature | neg | 452.9235 | 7.611 | 8.065 | 8.076 | 0.011 | 0.124 | 0.492 | 0.787 | Unannotated | NA | NA |
| metab_19160 | N-Eicosapentaenoyl Glutamic acid | neg | 466.2355 | 5.317 | 4.962 | 4.281 | -0.681 | -0.124 | 0.492 | 0.787 | Unannotated | NA | NA |
| metab_3452 | Unannotated feature | pos | 364.2251 | 4.615 | 3.949 | 4.369 | 0.420 | 0.124 | 0.492 | 0.787 | Unannotated | NA | NA |
| metab_13747 | Unannotated feature | neg | 144.0659 | 1.952 | 5.260 | 5.216 | -0.044 | -0.124 | 0.492 | 0.787 | Unannotated | NA | NA |
| metab_3124 | DL-Alanyl-DL-leucine | pos | 185.1288 | 3.502 | 3.629 | 4.165 | 0.537 | 0.124 | 0.492 | 0.787 | Unannotated | NA | NA |
| metab_3144 | Unannotated feature | pos | 203.1758 | 3.549 | 4.091 | 4.114 | 0.023 | -0.124 | 0.492 | 0.787 | Unannotated | NA | NA |
| metab_3151 | Unannotated feature | pos | 419.2766 | 6.622 | 5.980 | 5.891 | -0.089 | -0.124 | 0.492 | 0.787 | Unannotated | NA | NA |
| metab_3234 | Unannotated feature | pos | 417.1667 | 3.808 | 3.446 | 3.983 | 0.537 | 0.124 | 0.492 | 0.787 | Unannotated | NA | NA |
| metab_11957 | Unannotated feature | neg | 467.2655 | 6.154 | 5.874 | 5.714 | -0.160 | -0.124 | 0.492 | 0.787 | Unannotated | NA | NA |
| metab_11961 | Unannotated feature | neg | 637.2906 | 5.441 | 5.790 | 5.658 | -0.132 | -0.124 | 0.492 | 0.787 | Unannotated | NA | NA |
| metab_110 | Trimethylamine N-oxide | pos | 76.0762 | 0.641 | 3.057 | 3.054 | -0.003 | -0.124 | 0.492 | 0.787 | Strong putative | NA | NA |
| metab_18419 | Unannotated feature | neg | 349.1671 | 6.115 | 4.963 | 4.946 | -0.018 | -0.124 | 0.492 | 0.787 | Unannotated | NA | NA |
| metab_15198 | Unannotated feature | neg | 377.1359 | 4.726 | 4.468 | 4.227 | -0.241 | -0.124 | 0.492 | 0.787 | Unannotated | NA | NA |
| metab_8131 | Unannotated feature | pos | 299.1972 | 2.724 | 4.269 | 4.459 | 0.190 | 0.124 | 0.492 | 0.787 | Unannotated | NA | NA |
| metab_194 | Unannotated feature | pos | 274.8738 | 0.593 | 5.393 | 5.353 | -0.040 | -0.124 | 0.492 | 0.787 | Unannotated | NA | NA |
| metab_11988 | 1-Hydroxyacorenone | neg | 499.3086 | 6.217 | 5.939 | 6.070 | 0.131 | 0.124 | 0.492 | 0.787 | Unannotated | NA | NA |
| metab_8098 | Unannotated feature | pos | 309.1816 | 2.740 | 4.210 | 4.277 | 0.067 | 0.120 | 0.507 | 0.797 | Unannotated | NA | NA |
| metab_16242 | (9S,10S)-10-hydroxy-9-(phosphonooxy)octadecanoate | neg | 377.2089 | 6.506 | 4.371 | 3.933 | -0.438 | -0.120 | 0.507 | 0.797 | Tentative | NA | NA |
| metab_16326 | Unannotated feature | neg | 309.1741 | 6.663 | 6.556 | 6.530 | -0.026 | -0.120 | 0.507 | 0.797 | Unannotated | NA | NA |
| metab_8256 | Unannotated feature | pos | 141.0524 | 2.232 | 4.266 | 4.362 | 0.096 | 0.120 | 0.507 | 0.797 | Unannotated | NA | NA |
| metab_5371 | Unannotated feature | pos | 491.3345 | 6.486 | 4.986 | 4.929 | -0.057 | 0.120 | 0.507 | 0.797 | Unannotated | NA | NA |
| metab_16283 | Unannotated feature | neg | 361.2387 | 6.592 | 5.094 | 5.009 | -0.085 | -0.120 | 0.507 | 0.797 | Unannotated | NA | NA |
| metab_5323 | Unannotated feature | pos | 351.2523 | 6.550 | 5.738 | 5.900 | 0.162 | 0.120 | 0.507 | 0.797 | Unannotated | NA | NA |
| metab_14704 | Unannotated feature | neg | 354.1651 | 3.705 | 3.796 | 3.667 | -0.129 | -0.120 | 0.507 | 0.797 | Unannotated | NA | NA |
| metab_5473 | DG(22:6(4Z,7Z,10Z,13E,15E,19Z)-OH(17)/0:0/2:0) | pos | 493.3158 | 6.375 | 6.066 | 6.081 | 0.015 | -0.120 | 0.507 | 0.797 | Unannotated | NA | NA |
| metab_19114 | Unannotated feature | neg | 458.1889 | 5.366 | 0.987 | 3.291 | 2.304 | 0.120 | 0.507 | 0.797 | Unannotated | NA | NA |
| metab_11095 | Raffinose | neg | 549.1687 | 0.722 | 5.130 | 5.165 | 0.035 | 0.120 | 0.507 | 0.797 | Strong putative | NA | NA |
| metab_10937 | Uric Acid | neg | 167.0204 | 0.804 | 6.090 | 6.096 | 0.006 | 0.120 | 0.507 | 0.797 | Strong putative | NA | NA |
| metab_1673 | Unannotated feature | pos | 363.1559 | 4.399 | 4.046 | 4.476 | 0.430 | 0.120 | 0.507 | 0.797 | Unannotated | NA | NA |
| metab_1279 | Unannotated feature | pos | 175.1120 | 6.295 | 4.484 | 4.268 | -0.217 | -0.120 | 0.507 | 0.797 | Unannotated | NA | NA |
| metab_11167 | Unannotated feature | neg | 73.0284 | 0.675 | 4.531 | 4.483 | -0.049 | -0.120 | 0.507 | 0.797 | Unannotated | NA | NA |
| metab_1727 | Unannotated feature | pos | 269.1753 | 4.129 | 5.898 | 5.780 | -0.118 | -0.120 | 0.507 | 0.797 | Unannotated | NA | NA |
| metab_18071 | Unannotated feature | neg | 386.1565 | 6.420 | 3.631 | 4.890 | 1.259 | 0.120 | 0.507 | 0.797 | Unannotated | NA | NA |
| metab_12959 | Unannotated feature | neg | 210.0880 | 0.777 | 4.071 | 4.018 | -0.053 | -0.120 | 0.507 | 0.797 | Unannotated | NA | NA |
| metab_1793 | Hydroxyprolylhydroxyproline | pos | 245.1136 | 0.776 | 5.434 | 5.507 | 0.072 | 0.120 | 0.507 | 0.797 | Unannotated | NA | NA |
| metab_1832 | Unannotated feature | pos | 264.8733 | 0.016 | 5.174 | 5.202 | 0.027 | 0.120 | 0.507 | 0.797 | Unannotated | NA | NA |
| metab_10949 | Astringin | neg | 405.1157 | 0.791 | 5.214 | 5.070 | -0.145 | -0.120 | 0.507 | 0.797 | Tentative | NA | NA |
| metab_3823 | Unannotated feature | pos | 817.5852 | 6.390 | 6.475 | 6.414 | -0.061 | -0.120 | 0.507 | 0.797 | Unannotated | NA | NA |
| metab_3672 | Prostaglandin B2 | pos | 379.1886 | 5.231 | 3.878 | 4.148 | 0.270 | 0.120 | 0.507 | 0.797 | Tentative | NA | NA |
| metab_9571 | 1-(2-Furanylmethyl)-1H-pyrrole | neg | 192.0661 | 3.254 | 4.499 | 4.448 | -0.051 | -0.120 | 0.507 | 0.797 | Unannotated | NA | NA |
| metab_7712 | Ile Ile Ala | pos | 316.2238 | 3.347 | 5.332 | 5.235 | -0.097 | -0.120 | 0.507 | 0.797 | Moderate putative | NA | NA |
| metab_13687 | Unannotated feature | neg | 330.0696 | 1.691 | 4.966 | 4.831 | -0.135 | -0.120 | 0.507 | 0.797 | Unannotated | NA | NA |
| metab_12406 | Unannotated feature | neg | 532.8673 | 7.702 | 5.079 | 5.156 | 0.077 | 0.120 | 0.507 | 0.797 | Unannotated | NA | NA |
| metab_15172 | Unannotated feature | neg | 256.0649 | 4.667 | 4.049 | 4.098 | 0.049 | -0.120 | 0.507 | 0.797 | Unannotated | NA | NA |
| metab_1185 | Unannotated feature | pos | 310.3109 | 6.494 | 5.557 | 5.456 | -0.100 | -0.120 | 0.507 | 0.797 | Unannotated | NA | NA |
| metab_1922 | Unannotated feature | pos | 451.1811 | 1.698 | 4.343 | 4.660 | 0.317 | 0.120 | 0.507 | 0.797 | Unannotated | NA | NA |
| metab_9007 | Unannotated feature | neg | 233.1029 | 3.762 | 5.049 | 5.173 | 0.124 | 0.120 | 0.507 | 0.797 | Unannotated | NA | NA |
| metab_11812 | Trans-Resveratrol 3-O-glucuronide | neg | 385.0935 | 4.749 | 5.732 | 5.013 | -0.720 | -0.120 | 0.507 | 0.797 | Unannotated | Phenylpropanoids and polyketides | Stilbenes |
| metab_14176 | 5-Methylthioribose | neg | 179.0376 | 2.915 | 3.599 | 3.522 | -0.078 | 0.120 | 0.507 | 0.797 | Tentative | NA | NA |
| metab_1662 | 3,11,12-Trihydroxy-1(10)-spirovetiven-2-one | pos | 269.1752 | 3.930 | 5.533 | 5.493 | -0.040 | -0.120 | 0.507 | 0.797 | Unannotated | Lipids and lipid-like molecules | Prenol lipids |
| metab_5035 | Unannotated feature | pos | 447.2938 | 7.252 | 5.373 | 5.337 | -0.037 | 0.120 | 0.507 | 0.797 | Unannotated | NA | NA |
| metab_5032 | Saponin H | pos | 683.4355 | 7.268 | 4.966 | 4.917 | -0.049 | -0.120 | 0.507 | 0.797 | Unannotated | NA | NA |
| metab_7180 | Unannotated feature | pos | 253.1419 | 4.479 | 4.495 | 4.072 | -0.422 | -0.120 | 0.507 | 0.797 | Unannotated | NA | NA |
| metab_2976 | 5-Hydroxybenzofuran-2(3H)-one | pos | 192.0656 | 3.040 | 4.994 | 4.958 | -0.036 | -0.120 | 0.507 | 0.797 | Unannotated | NA | NA |
| metab_17079 | Unannotated feature | neg | 124.8905 | 7.910 | 4.443 | 4.396 | -0.046 | -0.120 | 0.507 | 0.797 | Unannotated | NA | NA |
| metab_16202 | Unannotated feature | neg | 452.2482 | 6.420 | 3.272 | 3.106 | -0.166 | -0.120 | 0.507 | 0.797 | Unannotated | NA | NA |
| metab_14655 | 20-Carboxyleukotriene B4 | neg | 403.1520 | 3.592 | 3.058 | 3.191 | 0.133 | 0.120 | 0.507 | 0.797 | Tentative | NA | NA |
| metab_5273 | Unannotated feature | pos | 252.2326 | 6.630 | 4.702 | 4.587 | -0.114 | -0.120 | 0.507 | 0.797 | Unannotated | NA | NA |
| metab_1136 | Ganoderiol F | pos | 487.3770 | 6.574 | 5.010 | 5.123 | 0.113 | 0.120 | 0.507 | 0.797 | Tentative | NA | NA |
| metab_10619 | Unannotated feature | neg | 213.1241 | 1.685 | 3.781 | 3.668 | -0.113 | -0.120 | 0.507 | 0.797 | Unannotated | NA | NA |
| metab_13071 | D-Erythroascorbic acid 1'-a-D-glucoside | neg | 353.0751 | 1.033 | 3.765 | 3.845 | 0.080 | 0.120 | 0.507 | 0.797 | Unannotated | NA | NA |
| metab_13045 | Unannotated feature | neg | 439.2704 | 6.162 | 6.852 | 6.808 | -0.044 | -0.120 | 0.507 | 0.797 | Unannotated | NA | NA |
| metab_13056 | Unannotated feature | neg | 392.8035 | 0.542 | 4.734 | 4.611 | -0.123 | -0.120 | 0.507 | 0.797 | Unannotated | NA | NA |
| metab_2039 | Unannotated feature | pos | 363.0696 | 0.672 | 5.605 | 5.630 | 0.025 | 0.120 | 0.507 | 0.797 | Unannotated | NA | NA |
| metab_18597 | Pentamorphone | neg | 405.1592 | 5.991 | 4.691 | 4.850 | 0.159 | 0.120 | 0.507 | 0.797 | Unannotated | NA | NA |
| metab_2094 | 5-[(2R)-2-Aminopropyl]-2-hydroxybenzoic acid | pos | 196.0973 | 2.774 | 5.085 | 5.069 | -0.016 | 0.120 | 0.507 | 0.797 | Unannotated | NA | NA |
| metab_12210 | Unannotated feature | neg | 523.3436 | 6.836 | 6.177 | 6.263 | 0.086 | 0.120 | 0.507 | 0.797 | Unannotated | NA | NA |
| metab_19391 | Unannotated feature | neg | 427.1806 | 5.022 | 3.255 | 3.720 | 0.465 | 0.120 | 0.507 | 0.797 | Unannotated | NA | NA |
| metab_14913 | Unannotated feature | neg | 415.1292 | 4.134 | 4.364 | 4.305 | -0.059 | -0.120 | 0.507 | 0.797 | Unannotated | NA | NA |
| metab_12158 | Unannotated feature | neg | 515.3748 | 6.663 | 5.780 | 5.950 | 0.170 | 0.120 | 0.507 | 0.797 | Unannotated | NA | NA |
| metab_11543 | Unannotated feature | neg | 605.8441 | 0.489 | 2.677 | 2.623 | -0.054 | 0.120 | 0.507 | 0.797 | Unannotated | NA | NA |
| metab_11558 | Unannotated feature | neg | 635.3106 | 5.267 | 4.181 | 5.260 | 1.079 | 0.120 | 0.507 | 0.797 | Unannotated | NA | NA |
| metab_12423 | Unannotated feature | neg | 322.8869 | 7.726 | 5.788 | 5.784 | -0.004 | -0.120 | 0.507 | 0.797 | Unannotated | NA | NA |
| metab_903 | Unannotated feature | pos | 488.3119 | 7.068 | 5.463 | 5.300 | -0.162 | -0.120 | 0.507 | 0.797 | Unannotated | NA | NA |
| metab_18967 | 2-[4,7,10-Tris(carboxymethyl)-6-[4-[3-(2,5-dioxopyrrol-1-yl)propanoylamino]butyl]-1,4,7,10-tetrazacyclododec-1-yl]acetic acid | neg | 647.2721 | 5.567 | 5.372 | 4.968 | -0.404 | -0.120 | 0.507 | 0.797 | Unannotated | NA | NA |
| metab_2922 | Sumiki's acid | pos | 125.0237 | 2.897 | 3.645 | 3.350 | -0.296 | -0.120 | 0.507 | 0.797 | Tentative | Organoheterocyclic compounds | Furans |
| metab_17291 | Unannotated feature | neg | 288.9366 | 7.714 | 5.465 | 5.470 | 0.005 | -0.120 | 0.507 | 0.797 | Unannotated | NA | NA |
| metab_749 | Deoxycholylproline | pos | 490.3542 | 6.064 | 5.903 | 5.875 | -0.028 | 0.120 | 0.507 | 0.797 | Unannotated | NA | NA |
| metab_4469 | Unannotated feature | pos | 330.3009 | 6.470 | 5.001 | 4.959 | -0.043 | -0.120 | 0.507 | 0.797 | Unannotated | NA | NA |
| metab_9202 | Unannotated feature | neg | 281.1064 | 3.917 | 4.136 | 4.265 | 0.129 | 0.120 | 0.507 | 0.797 | Unannotated | NA | NA |
| metab_10351 | Unannotated feature | neg | 281.1257 | 2.197 | 2.811 | 2.841 | 0.030 | 0.120 | 0.507 | 0.797 | Unannotated | NA | NA |
| metab_2199 | Dehydrooreadone | pos | 252.1598 | 2.997 | 3.526 | 3.307 | -0.220 | 0.120 | 0.507 | 0.797 | Unannotated | NA | NA |
| metab_11260 | Unannotated feature | neg | 135.9202 | 0.628 | 4.764 | 4.708 | -0.057 | -0.120 | 0.507 | 0.797 | Unannotated | NA | NA |
| metab_11284 | Unannotated feature | neg | 156.0272 | 0.619 | 3.176 | 3.620 | 0.444 | 0.120 | 0.507 | 0.797 | Unannotated | NA | NA |
| metab_18913 | Unannotated feature | neg | 287.0886 | 2.531 | 6.515 | 6.542 | 0.028 | -0.120 | 0.507 | 0.797 | Unannotated | NA | NA |
| metab_11530 | Unannotated feature | neg | 333.1459 | 5.246 | 5.028 | 4.794 | -0.234 | -0.120 | 0.507 | 0.797 | Unannotated | NA | NA |
| metab_4098 | Unannotated feature | pos | 242.0000 | 0.485 | 6.350 | 6.242 | -0.108 | -0.120 | 0.507 | 0.797 | Unannotated | NA | NA |
| metab_427 | Unannotated feature | pos | 141.9589 | 0.016 | 6.258 | 6.259 | 0.001 | 0.120 | 0.507 | 0.797 | Unannotated | NA | NA |
| metab_19415 | Unannotated feature | neg | 197.8518 | 4.997 | 4.206 | 4.137 | -0.069 | -0.120 | 0.507 | 0.797 | Unannotated | NA | NA |
| metab_12183 | 6-Ketoestriol | neg | 301.1447 | 5.971 | 5.694 | 5.548 | -0.146 | -0.120 | 0.507 | 0.797 | Unannotated | NA | NA |
| metab_1134 | Gingerglycolipid B | pos | 701.3743 | 6.574 | 4.808 | 4.693 | -0.114 | -0.120 | 0.507 | 0.797 | Unannotated | NA | NA |
| metab_11390 | Unannotated feature | neg | 278.9082 | 0.552 | 5.711 | 5.738 | 0.027 | 0.120 | 0.507 | 0.797 | Unannotated | NA | NA |
| metab_18799 | Pravastatin lactone | neg | 441.2049 | 5.783 | 3.718 | 3.985 | 0.266 | 0.120 | 0.507 | 0.797 | Unannotated | NA | NA |
| metab_10429 | 1-Octen-3-yl glucoside | neg | 311.1504 | 4.621 | 4.844 | 4.932 | 0.088 | 0.120 | 0.507 | 0.797 | Unannotated | NA | NA |
| metab_10462 | Unannotated feature | neg | 254.1147 | 1.958 | 3.161 | 3.337 | 0.176 | 0.120 | 0.507 | 0.797 | Unannotated | NA | NA |
| metab_14226 | Trans-Grandmarin | neg | 291.0879 | 3.562 | 4.411 | 4.684 | 0.273 | 0.120 | 0.507 | 0.797 | Unannotated | NA | NA |
| metab_11848 | Unannotated feature | neg | 285.1136 | 5.964 | 5.248 | 5.187 | -0.060 | -0.120 | 0.507 | 0.797 | Unannotated | NA | NA |
| metab_9341 | 3'-O-methyl-(-)-epicatechin | neg | 349.0934 | 3.999 | 4.925 | 4.640 | -0.285 | -0.120 | 0.507 | 0.797 | Unannotated | NA | NA |
| metab_6151 | Unannotated feature | pos | 337.0056 | 0.531 | 4.320 | 4.360 | 0.041 | -0.120 | 0.507 | 0.797 | Unannotated | NA | NA |
| metab_2471 | L-Homocitrulline | pos | 190.1189 | 0.704 | 4.627 | 4.604 | -0.022 | -0.120 | 0.507 | 0.797 | Strong putative | NA | NA |
| metab_10295 | Unannotated feature | neg | 164.9267 | 2.417 | 4.274 | 4.249 | -0.025 | -0.120 | 0.507 | 0.797 | Unannotated | NA | NA |
| metab_2437 | Unannotated feature | pos | 236.0411 | 0.633 | 4.298 | 4.583 | 0.285 | 0.120 | 0.507 | 0.797 | Unannotated | NA | NA |
| metab_2297 | Unannotated feature | pos | 199.9758 | 0.016 | 5.617 | 5.634 | 0.017 | 0.120 | 0.507 | 0.797 | Unannotated | NA | NA |
| metab_12582 | Unannotated feature | neg | 484.2418 | 6.099 | 5.288 | 5.136 | -0.152 | -0.120 | 0.507 | 0.797 | Unannotated | NA | NA |
| metab_9388 | Unannotated feature | neg | 287.1610 | 3.491 | 3.521 | 3.059 | -0.462 | -0.120 | 0.507 | 0.797 | Unannotated | NA | NA |
| metab_11691 | Unannotated feature | neg | 259.1817 | 5.420 | 3.911 | 4.938 | 1.026 | 0.120 | 0.507 | 0.797 | Unannotated | NA | NA |
| metab_19188 | Unannotated feature | neg | 287.0886 | 2.714 | 6.787 | 6.824 | 0.038 | -0.120 | 0.507 | 0.797 | Unannotated | NA | NA |
| metab_17331 | Unannotated feature | neg | 294.9533 | 7.697 | 5.889 | 5.877 | -0.011 | -0.120 | 0.507 | 0.797 | Unannotated | NA | NA |
| metab_8452 | 7-Methylhypoxanthine | pos | 151.0617 | 1.936 | 4.755 | 4.893 | 0.138 | 0.120 | 0.507 | 0.797 | Unannotated | NA | NA |
| metab_5095 | Unannotated feature | pos | 533.2862 | 7.036 | 5.238 | 5.083 | -0.155 | -0.120 | 0.507 | 0.797 | Unannotated | NA | NA |
| metab_6981 | Unannotated feature | pos | 383.0948 | 0.649 | 3.640 | 3.673 | 0.033 | 0.120 | 0.507 | 0.797 | Unannotated | NA | NA |
| metab_13460 | Unannotated feature | neg | 232.0073 | 0.910 | 3.304 | 3.761 | 0.457 | 0.120 | 0.507 | 0.797 | Unannotated | NA | NA |
| metab_2924 | 2-(3-Carboxy-3-(methylammonio)propyl)-L-histidine | pos | 236.1284 | 2.897 | 3.547 | 3.496 | -0.051 | -0.120 | 0.507 | 0.797 | Tentative | NA | NA |
| metab_2458 | Vidarabine phosphate | pos | 348.0700 | 0.665 | 4.807 | 4.830 | 0.023 | 0.120 | 0.507 | 0.797 | Unannotated | NA | NA |
| metab_13349 | Unannotated feature | neg | 439.0799 | 0.714 | 5.733 | 5.166 | -0.567 | -0.120 | 0.507 | 0.797 | Unannotated | NA | NA |
| metab_13363 | Unannotated feature | neg | 200.0672 | 0.730 | 3.956 | 3.199 | -0.758 | -0.120 | 0.507 | 0.797 | Unannotated | NA | NA |
| metab_4310 | Alpha-Terpineol acetate | pos | 214.1806 | 6.040 | 4.557 | 4.558 | 0.002 | -0.120 | 0.507 | 0.797 | Tentative | NA | NA |
| metab_4382 | Unannotated feature | pos | 300.1997 | 6.239 | 4.685 | 4.904 | 0.219 | 0.120 | 0.507 | 0.797 | Unannotated | NA | NA |
| metab_11724 | Unannotated feature | neg | 287.1502 | 5.567 | 5.589 | 5.608 | 0.019 | 0.120 | 0.507 | 0.797 | Unannotated | NA | NA |
| metab_13873 | Unannotated feature | neg | 514.8819 | 0.467 | 4.442 | 4.357 | -0.085 | -0.120 | 0.507 | 0.797 | Unannotated | NA | NA |
| metab_15937 | Unannotated feature | neg | 490.2218 | 6.035 | 4.685 | 4.717 | 0.032 | 0.120 | 0.507 | 0.797 | Unannotated | NA | NA |
| metab_13409 | Unannotated feature | neg | 257.9345 | 0.628 | 5.590 | 5.530 | -0.060 | -0.120 | 0.507 | 0.797 | Unannotated | NA | NA |
| metab_15624 | Unannotated feature | neg | 294.0574 | 5.542 | 4.372 | 4.189 | -0.183 | -0.120 | 0.507 | 0.797 | Unannotated | NA | NA |
| metab_11879 | Unannotated feature | neg | 463.1777 | 6.018 | 4.376 | 4.555 | 0.180 | 0.120 | 0.507 | 0.797 | Unannotated | NA | NA |
| metab_11895 | Unannotated feature | neg | 459.2407 | 6.041 | 4.968 | 5.113 | 0.145 | 0.120 | 0.507 | 0.797 | Unannotated | NA | NA |
| metab_19597 | Unannotated feature | neg | 338.1725 | 4.718 | 3.757 | 3.885 | 0.129 | 0.120 | 0.507 | 0.797 | Unannotated | NA | NA |
| metab_274 | Unannotated feature | pos | 319.1560 | 4.592 | 3.709 | 3.408 | -0.301 | -0.120 | 0.507 | 0.797 | Unannotated | NA | NA |
| metab_5232 | Unannotated feature | pos | 533.3828 | 6.694 | 6.606 | 6.718 | 0.113 | 0.120 | 0.507 | 0.797 | Unannotated | NA | NA |
| metab_5259 | Unannotated feature | pos | 182.9856 | 0.016 | 6.765 | 6.763 | -0.002 | 0.120 | 0.507 | 0.797 | Unannotated | NA | NA |
| metab_15115 | Unannotated feature | neg | 227.0382 | 4.559 | 4.797 | 4.812 | 0.015 | 0.120 | 0.507 | 0.797 | Unannotated | NA | NA |
| metab_5460 | Unannotated feature | pos | 579.3518 | 6.382 | 5.208 | 5.081 | -0.127 | -0.120 | 0.507 | 0.797 | Unannotated | NA | NA |
| metab_19762 | Isohomovanillic acid | neg | 163.0395 | 4.488 | 4.997 | 5.044 | 0.046 | 0.120 | 0.507 | 0.797 | Unannotated | Benzenoids | Phenols |
| metab_15424 | Unannotated feature | neg | 216.0181 | 0.571 | 4.595 | 4.416 | -0.178 | -0.120 | 0.507 | 0.797 | Unannotated | NA | NA |
| metab_14090 | Zeatin | neg | 437.2151 | 2.813 | 4.722 | 4.673 | -0.049 | -0.120 | 0.507 | 0.797 | Moderate putative | Organoheterocyclic compounds | Imidazopyrimidines |
| metab_4516 | Unannotated feature | pos | 409.2942 | 6.694 | 4.908 | 4.922 | 0.013 | -0.120 | 0.507 | 0.797 | Unannotated | NA | NA |
| metab_18194 | Unannotated feature | neg | 427.1802 | 6.303 | 4.817 | 4.935 | 0.119 | -0.120 | 0.507 | 0.797 | Unannotated | NA | NA |
| metab_7466 | Unannotated feature | pos | 291.1742 | 3.826 | 4.416 | 4.210 | -0.206 | -0.120 | 0.507 | 0.797 | Unannotated | NA | NA |
| metab_6500 | Valnemulin | pos | 609.3299 | 5.589 | 5.629 | 5.814 | 0.185 | 0.120 | 0.507 | 0.797 | Tentative | NA | NA |
| metab_1647 | Cinncassiol C3 | pos | 383.2071 | 4.620 | 3.746 | 4.180 | 0.435 | 0.120 | 0.507 | 0.797 | Tentative | NA | NA |
| metab_12344 | VALSARTAN | neg | 434.2205 | 6.154 | 5.061 | 5.181 | 0.120 | 0.120 | 0.507 | 0.797 | Strong putative | NA | NA |
| metab_7826 | 3-Methylindole | pos | 132.0811 | 3.113 | 4.960 | 4.797 | -0.163 | -0.120 | 0.507 | 0.797 | Moderate putative | NA | NA |
| metab_9615 | Unannotated feature | neg | 323.9766 | 3.215 | 4.467 | 4.395 | -0.072 | -0.120 | 0.507 | 0.797 | Unannotated | NA | NA |
| metab_11965 | Unannotated feature | neg | 595.3513 | 6.170 | 5.203 | 5.700 | 0.497 | 0.120 | 0.507 | 0.797 | Unannotated | NA | NA |
| metab_15756 | Unannotated feature | neg | 404.1695 | 5.783 | 3.485 | 3.687 | 0.203 | 0.120 | 0.507 | 0.797 | Unannotated | NA | NA |
| metab_18427 | Unannotated feature | neg | 653.3409 | 6.107 | 5.275 | 4.874 | -0.401 | -0.120 | 0.507 | 0.797 | Unannotated | NA | NA |
| metab_1385 | Unannotated feature | pos | 338.2331 | 6.103 | 4.483 | 4.473 | -0.010 | -0.120 | 0.507 | 0.797 | Unannotated | NA | NA |
| metab_8165 | Unannotated feature | pos | 94.0456 | 2.671 | 4.200 | 4.218 | 0.019 | 0.120 | 0.507 | 0.797 | Unannotated | NA | NA |
| metab_14714 | Unannotated feature | neg | 245.1394 | 3.725 | 4.235 | 4.313 | 0.078 | 0.116 | 0.522 | 0.804 | Unannotated | NA | NA |
| metab_7408 | 2-(5-Hydroxy-1H-indol-2-yl)acetic acid | pos | 224.0921 | 3.957 | 4.077 | 4.543 | 0.466 | 0.116 | 0.522 | 0.804 | Unannotated | NA | NA |
| metab_12743 | Unannotated feature | neg | 781.5642 | 6.514 | 6.725 | 6.648 | -0.077 | -0.116 | 0.522 | 0.804 | Unannotated | NA | NA |
| metab_1341 | Unannotated feature | pos | 613.3613 | 6.167 | 4.273 | 5.432 | 1.159 | 0.116 | 0.522 | 0.804 | Unannotated | NA | NA |
| metab_9611 | Unannotated feature | neg | 283.0106 | 3.219 | 5.399 | 5.362 | -0.037 | -0.116 | 0.522 | 0.804 | Unannotated | NA | NA |
| metab_13769 | Unannotated feature | neg | 289.1042 | 1.965 | 5.199 | 5.233 | 0.034 | 0.116 | 0.522 | 0.804 | Unannotated | NA | NA |
| metab_13731 | Unannotated feature | neg | 200.0560 | 1.947 | 5.777 | 5.881 | 0.104 | 0.116 | 0.522 | 0.804 | Unannotated | NA | NA |
| metab_13829 | Unannotated feature | neg | 221.8827 | 2.137 | 5.665 | 5.671 | 0.005 | -0.116 | 0.522 | 0.804 | Unannotated | NA | NA |
| metab_16221 | Unannotated feature | neg | 395.2804 | 6.467 | 4.792 | 5.014 | 0.222 | 0.116 | 0.522 | 0.804 | Unannotated | NA | NA |
| metab_18192 | Unannotated feature | neg | 568.3630 | 6.303 | 5.525 | 5.338 | -0.187 | -0.116 | 0.522 | 0.804 | Unannotated | NA | NA |
| metab_1749 | Unannotated feature | pos | 383.1823 | 3.574 | 5.095 | 5.035 | -0.060 | 0.116 | 0.522 | 0.804 | Unannotated | NA | NA |
| metab_8998 | Unannotated feature | neg | 217.1078 | 4.083 | 5.139 | 5.225 | 0.086 | 0.116 | 0.522 | 0.804 | Unannotated | NA | NA |
| metab_4495 | Unannotated feature | pos | 473.3639 | 6.582 | 4.220 | 4.616 | 0.396 | 0.116 | 0.522 | 0.804 | Unannotated | NA | NA |
| metab_79 | Creatinine | pos | 114.0667 | 0.657 | 5.095 | 4.843 | -0.252 | -0.116 | 0.522 | 0.804 | Moderate putative | Organic acids and derivatives | Carboxylic acids and derivatives |
| metab_12009 | Unannotated feature | neg | 411.2393 | 6.256 | 6.708 | 6.554 | -0.155 | -0.116 | 0.522 | 0.804 | Unannotated | NA | NA |
| metab_5553 | Unannotated feature | pos | 290.2694 | 6.279 | 5.221 | 5.148 | -0.073 | -0.116 | 0.522 | 0.804 | Unannotated | NA | NA |
| metab_14579 | Remifentanil | neg | 397.1722 | 3.476 | 2.334 | 2.961 | 0.628 | 0.116 | 0.522 | 0.804 | Tentative | NA | NA |
| metab_19661 | Unannotated feature | neg | 277.1294 | 4.625 | 4.911 | 4.719 | -0.192 | -0.116 | 0.522 | 0.804 | Unannotated | NA | NA |
| metab_8379 | Unannotated feature | pos | 269.0886 | 1.952 | 5.166 | 5.086 | -0.080 | -0.116 | 0.522 | 0.804 | Unannotated | NA | NA |
| metab_18830 | Unannotated feature | neg | 503.2519 | 5.748 | 3.878 | 3.598 | -0.280 | -0.116 | 0.522 | 0.804 | Unannotated | NA | NA |
| metab_10122 | Unannotated feature | neg | 249.0622 | 2.762 | 3.971 | 4.080 | 0.110 | 0.116 | 0.522 | 0.804 | Unannotated | NA | NA |
| metab_5973 | Unannotated feature | pos | 347.9472 | 0.013 | 5.337 | 5.345 | 0.008 | 0.116 | 0.522 | 0.804 | Unannotated | NA | NA |
| metab_3557 | Unannotated feature | pos | 542.3234 | 6.726 | 5.786 | 5.830 | 0.044 | 0.116 | 0.522 | 0.804 | Unannotated | NA | NA |
| metab_13995 | Unannotated feature | neg | 105.0185 | 2.714 | 3.629 | 3.602 | -0.027 | -0.116 | 0.522 | 0.804 | Unannotated | NA | NA |
| metab_2512 | Unannotated feature | pos | 186.0441 | 0.831 | 4.235 | 4.295 | 0.060 | 0.116 | 0.522 | 0.804 | Unannotated | NA | NA |
| metab_10170 | Darodipine | neg | 408.0978 | 2.707 | 3.331 | 3.423 | 0.091 | -0.116 | 0.522 | 0.804 | Unannotated | NA | NA |
| metab_983 | Unannotated feature | pos | 218.1069 | 7.724 | 5.112 | 5.125 | 0.014 | 0.116 | 0.522 | 0.804 | Unannotated | NA | NA |
| metab_17381 | Unannotated feature | neg | 265.9507 | 7.666 | 5.961 | 5.937 | -0.025 | -0.116 | 0.522 | 0.804 | Unannotated | NA | NA |
| metab_2720 | Unannotated feature | pos | 934.2925 | 2.160 | 5.400 | 5.408 | 0.008 | 0.116 | 0.522 | 0.804 | Unannotated | NA | NA |
| metab_2601 | Unannotated feature | pos | 250.0761 | 1.622 | 4.446 | 4.668 | 0.223 | 0.116 | 0.522 | 0.804 | Unannotated | NA | NA |
| metab_6107 | Unannotated feature | pos | 288.9191 | 0.524 | 3.859 | 4.065 | 0.205 | -0.116 | 0.522 | 0.804 | Unannotated | NA | NA |
| metab_15918 | Unannotated feature | neg | 380.8442 | 0.527 | 5.765 | 5.933 | 0.168 | 0.116 | 0.522 | 0.804 | Unannotated | NA | NA |
| metab_8374 | Vidarabine | pos | 250.0940 | 1.952 | 3.850 | 4.124 | 0.274 | 0.116 | 0.522 | 0.804 | Tentative | NA | NA |
| metab_16491 | Cyclocalopin A | neg | 317.0992 | 1.685 | 5.499 | 5.375 | -0.123 | -0.116 | 0.522 | 0.804 | Unannotated | NA | NA |
| metab_5098 | Cucurbitacin D | pos | 555.2689 | 7.028 | 4.688 | 4.859 | 0.171 | 0.116 | 0.522 | 0.804 | Tentative | NA | NA |
| metab_16577 | Unannotated feature | neg | 158.9386 | 7.324 | 5.322 | 5.323 | 0.001 | -0.116 | 0.522 | 0.804 | Unannotated | NA | NA |
| metab_17300 | Unannotated feature | neg | 735.3082 | 2.569 | 3.552 | 4.102 | 0.550 | 0.116 | 0.522 | 0.804 | Unannotated | NA | NA |
| metab_2302 | Unannotated feature | pos | 243.0198 | 0.019 | 4.708 | 4.727 | 0.019 | 0.116 | 0.522 | 0.804 | Unannotated | NA | NA |
| metab_17689 | Unannotated feature | neg | 372.2910 | 7.019 | 4.142 | 4.972 | 0.829 | 0.116 | 0.522 | 0.804 | Unannotated | NA | NA |
| metab_10268 | Unannotated feature | neg | 214.9871 | 2.520 | 4.143 | 4.036 | -0.107 | -0.116 | 0.522 | 0.804 | Unannotated | NA | NA |
| metab_10221 | Unannotated feature | neg | 247.1122 | 2.613 | 4.036 | 3.943 | -0.094 | -0.116 | 0.522 | 0.804 | Unannotated | NA | NA |
| metab_17580 | Unannotated feature | neg | 381.3378 | 7.279 | 5.995 | 5.957 | -0.038 | -0.116 | 0.522 | 0.804 | Unannotated | NA | NA |
| metab_8785 | 2-Hydroxycinnamic acid | pos | 182.0816 | 1.309 | 7.627 | 7.481 | -0.146 | -0.116 | 0.522 | 0.804 | Strong putative | NA | NA |
| metab_8781 | Palatinose | pos | 384.1511 | 0.720 | 3.763 | 3.884 | 0.121 | 0.116 | 0.522 | 0.804 | Tentative | NA | NA |
| metab_5572 | Biliverdin | pos | 583.2569 | 6.255 | 5.703 | 5.509 | -0.194 | -0.116 | 0.522 | 0.804 | Strong putative | NA | NA |
| metab_14928 | 2-n-Propyl-4-oxopentanoic acid | neg | 203.0920 | 4.172 | 4.869 | 4.927 | 0.058 | -0.116 | 0.522 | 0.804 | Tentative | NA | NA |
| metab_16791 | Unannotated feature | neg | 650.8696 | 7.702 | 5.110 | 5.161 | 0.051 | 0.116 | 0.522 | 0.804 | Unannotated | NA | NA |
| metab_8806 | Estradiol valerate | pos | 389.2692 | 6.127 | 5.640 | 5.438 | -0.201 | -0.116 | 0.522 | 0.804 | Tentative | NA | NA |
| metab_16095 | 17-Hydroxyandrostane-3-glucuronide | neg | 449.2551 | 6.232 | 5.808 | 5.760 | -0.047 | -0.116 | 0.522 | 0.804 | Tentative | NA | NA |
| metab_17668 | Unannotated feature | neg | 196.9956 | 7.053 | 3.108 | 3.555 | 0.447 | 0.116 | 0.522 | 0.804 | Unannotated | NA | NA |
| metab_17662 | Unannotated feature | neg | 577.3759 | 7.057 | 5.535 | 5.219 | -0.316 | -0.116 | 0.522 | 0.804 | Unannotated | NA | NA |
| metab_10373 | Unannotated feature | neg | 231.9434 | 2.141 | 3.657 | 3.543 | -0.114 | -0.116 | 0.522 | 0.804 | Unannotated | NA | NA |
| metab_13117 | Unannotated feature | neg | 295.8642 | 0.304 | 4.437 | 4.451 | 0.014 | 0.116 | 0.522 | 0.804 | Unannotated | NA | NA |
| metab_18785 | Unannotated feature | neg | 623.3465 | 5.797 | 2.512 | 5.850 | 3.338 | 0.116 | 0.522 | 0.804 | Unannotated | NA | NA |
| metab_11435 | Unannotated feature | neg | 386.8581 | 0.538 | 4.912 | 4.916 | 0.004 | -0.116 | 0.522 | 0.804 | Unannotated | NA | NA |
| metab_17021 | Unannotated feature | neg | 207.8943 | 7.997 | 4.406 | 4.407 | 0.001 | -0.116 | 0.522 | 0.804 | Unannotated | NA | NA |
| metab_8683 | Unannotated feature | pos | 158.0128 | 0.815 | 4.422 | 4.468 | 0.046 | 0.116 | 0.522 | 0.804 | Unannotated | NA | NA |
| metab_8665 | Unannotated feature | pos | 332.0615 | 0.954 | 2.912 | 3.386 | 0.474 | 0.116 | 0.522 | 0.804 | Unannotated | NA | NA |
| metab_8728 | Pipecolic Acid | pos | 130.0866 | 0.768 | 6.537 | 6.532 | -0.004 | -0.116 | 0.522 | 0.804 | Moderate putative | NA | NA |
| metab_14259 | Unannotated feature | neg | 159.0768 | 3.003 | 3.109 | 2.949 | -0.161 | -0.116 | 0.522 | 0.804 | Unannotated | NA | NA |
| metab_12439 | Unannotated feature | neg | 480.8872 | 7.720 | 4.929 | 5.002 | 0.073 | 0.116 | 0.522 | 0.804 | Unannotated | NA | NA |
| metab_2816 | Unannotated feature | pos | 288.1558 | 2.708 | 3.984 | 4.042 | 0.058 | 0.116 | 0.522 | 0.804 | Unannotated | NA | NA |
| metab_2771 | Val Val Glu | pos | 346.1982 | 2.465 | 4.549 | 4.509 | -0.040 | -0.116 | 0.522 | 0.804 | Moderate putative | NA | NA |
| metab_13644 | N-(6-Cyano-3-hydroxy-2,2-dimethyl-3,4-dihydrochromen-4-yl)-N-methylethanesulfonamide | neg | 369.1152 | 1.650 | 5.006 | 4.876 | -0.130 | -0.116 | 0.522 | 0.804 | Tentative | NA | NA |
| metab_15021 | Unannotated feature | neg | 236.9059 | 0.542 | 4.026 | 3.984 | -0.041 | -0.116 | 0.522 | 0.804 | Unannotated | NA | NA |
| metab_18630 | Unannotated feature | neg | 437.1060 | 3.112 | 3.465 | 3.587 | 0.123 | 0.116 | 0.522 | 0.804 | Unannotated | NA | NA |
| metab_2218 | Unannotated feature | pos | 258.8998 | 0.593 | 5.017 | 4.886 | -0.131 | -0.116 | 0.522 | 0.804 | Unannotated | NA | NA |
| metab_13063 | Unannotated feature | neg | 330.8994 | 7.720 | 5.630 | 5.615 | -0.016 | -0.116 | 0.522 | 0.804 | Unannotated | NA | NA |
| metab_2344 | Unannotated feature | pos | 258.9907 | 0.451 | 4.787 | 4.745 | -0.042 | -0.116 | 0.522 | 0.804 | Unannotated | NA | NA |
| metab_1094 | Unannotated feature | pos | 497.3620 | 6.670 | 5.945 | 6.045 | 0.100 | 0.116 | 0.522 | 0.804 | Unannotated | NA | NA |
| metab_4443 | Tetracosahexaenoic acid, n-3 | pos | 339.2687 | 6.382 | 5.455 | 5.519 | 0.064 | -0.116 | 0.522 | 0.804 | Unannotated | NA | NA |
| metab_12281 | Unannotated feature | neg | 674.5462 | 7.111 | 6.229 | 6.103 | -0.126 | -0.116 | 0.522 | 0.804 | Unannotated | NA | NA |
| metab_11755 | Unannotated feature | neg | 697.2392 | 5.671 | 4.572 | 3.770 | -0.801 | -0.116 | 0.522 | 0.804 | Unannotated | NA | NA |
| metab_5688 | N,N-Dimethyldodecylamine-N-oxide | pos | 230.2482 | 6.135 | 4.951 | 4.976 | 0.025 | -0.116 | 0.522 | 0.804 | Moderate putative | NA | NA |
| metab_9838 | Unannotated feature | neg | 198.9879 | 3.011 | 3.380 | 3.054 | -0.326 | -0.116 | 0.522 | 0.804 | Unannotated | NA | NA |
| metab_9819 | Unannotated feature | neg | 327.9909 | 3.025 | 5.361 | 5.281 | -0.080 | -0.116 | 0.522 | 0.804 | Unannotated | NA | NA |
| metab_3044 | Unannotated feature | pos | 708.5793 | 6.406 | 5.865 | 5.673 | -0.192 | -0.116 | 0.522 | 0.804 | Unannotated | NA | NA |
| metab_3041 | 4-O-Methylgalactinol | pos | 374.1639 | 3.275 | 3.189 | 3.581 | 0.392 | 0.116 | 0.522 | 0.804 | Unannotated | Organic oxygen compounds | Organooxygen compounds |
| metab_18979 | Eupatilin | neg | 343.0843 | 5.554 | 4.835 | 4.989 | 0.155 | 0.116 | 0.522 | 0.804 | Tentative | NA | NA |
| metab_12416 | Unannotated feature | neg | 440.8897 | 7.720 | 5.772 | 5.779 | 0.007 | -0.116 | 0.522 | 0.804 | Unannotated | NA | NA |
| metab_3014 | Carbocysteine-lysine | pos | 325.1512 | 3.156 | 2.065 | 3.800 | 1.734 | 0.116 | 0.522 | 0.804 | Unannotated | Organic acids and derivatives | Carboxylic acids and derivatives |
| metab_8022 | Unannotated feature | pos | 190.1440 | 2.811 | 3.263 | 3.175 | -0.087 | -0.116 | 0.522 | 0.804 | Unannotated | NA | NA |
| metab_14036 | Unannotated feature | neg | 234.0440 | 2.771 | 3.878 | 4.088 | 0.209 | 0.116 | 0.522 | 0.804 | Unannotated | NA | NA |
| metab_7831 | Unannotated feature | pos | 218.1391 | 3.312 | 5.541 | 5.510 | -0.030 | -0.116 | 0.522 | 0.804 | Unannotated | NA | NA |
| metab_9226 | Furanone A | neg | 167.0343 | 3.728 | 4.103 | 4.070 | -0.033 | 0.116 | 0.522 | 0.804 | Tentative | NA | NA |
| metab_9214 | Unannotated feature | neg | 107.0495 | 3.754 | 5.086 | 4.705 | -0.381 | -0.116 | 0.522 | 0.804 | Unannotated | NA | NA |
| metab_17885 | PE(17:1/0:0) | neg | 464.2789 | 6.687 | 5.215 | 4.854 | -0.361 | -0.116 | 0.522 | 0.804 | Moderate putative | NA | NA |
| metab_17915 | Unannotated feature | neg | 481.3537 | 6.648 | 6.404 | 6.584 | 0.180 | 0.116 | 0.522 | 0.804 | Unannotated | NA | NA |
| metab_10427 | Unannotated feature | neg | 232.0098 | 1.985 | 4.556 | 4.582 | 0.026 | -0.116 | 0.522 | 0.804 | Unannotated | NA | NA |
| metab_13041 | Unannotated feature | neg | 603.8107 | 0.596 | 3.939 | 4.350 | 0.411 | 0.116 | 0.522 | 0.804 | Unannotated | NA | NA |
| metab_14676 | Unannotated feature | neg | 343.8920 | 0.520 | 4.740 | 5.035 | 0.296 | 0.116 | 0.522 | 0.804 | Unannotated | NA | NA |
| metab_12063 | Unannotated feature | neg | 411.3057 | 6.397 | 6.864 | 6.836 | -0.028 | 0.116 | 0.522 | 0.804 | Unannotated | NA | NA |
| metab_256 | S-4-Hydroxymephenytoin | pos | 235.1081 | 4.571 | 5.264 | 5.427 | 0.163 | 0.116 | 0.522 | 0.804 | Unannotated | NA | NA |
| metab_16376 | 3,4-Dimethyl-5-pentyl-2-furantridecanoic acid | neg | 399.2861 | 6.766 | 5.435 | 5.468 | 0.034 | 0.116 | 0.522 | 0.804 | Unannotated | NA | NA |
| metab_16372 | Unannotated feature | neg | 483.3149 | 6.750 | 6.417 | 6.577 | 0.160 | 0.116 | 0.522 | 0.804 | Unannotated | NA | NA |
| metab_5505 | Unannotated feature | pos | 497.3074 | 6.335 | 4.462 | 4.280 | -0.183 | -0.116 | 0.522 | 0.804 | Unannotated | NA | NA |
| metab_14782 | Unannotated feature | neg | 215.0152 | 3.860 | 3.087 | 3.025 | -0.063 | -0.116 | 0.522 | 0.804 | Unannotated | NA | NA |
| metab_8064 | 1-Tert-butyl 4-ethyl 5-oxoazepane-1,4-dicarboxylate | pos | 327.1921 | 2.762 | 3.472 | 4.120 | 0.648 | 0.116 | 0.522 | 0.804 | Unannotated | NA | NA |
| metab_8365 | Unannotated feature | pos | 244.2024 | 1.952 | 5.623 | 5.509 | -0.114 | -0.116 | 0.522 | 0.804 | Unannotated | NA | NA |
| metab_14038 | Unannotated feature | neg | 503.0179 | 0.496 | 4.433 | 4.771 | 0.338 | 0.116 | 0.522 | 0.804 | Unannotated | NA | NA |
| metab_12156 | Unannotated feature | neg | 463.3438 | 6.663 | 6.166 | 6.322 | 0.156 | 0.116 | 0.522 | 0.804 | Unannotated | NA | NA |
| metab_15007 | Heliotridine | neg | 355.1878 | 4.340 | 3.939 | 4.652 | 0.713 | 0.116 | 0.522 | 0.804 | Tentative | NA | NA |
| metab_3593 | Loliolide | pos | 197.1176 | 5.044 | 4.248 | 3.998 | -0.250 | -0.116 | 0.522 | 0.804 | Strong putative | NA | NA |
| metab_5039 | 4,4'-Dioctyldiphenylamine | pos | 394.3479 | 7.236 | 4.766 | 4.765 | -0.001 | 0.116 | 0.522 | 0.804 | Strong putative | NA | NA |
| metab_15385 | 4-oxo-Retinoic acid | neg | 335.1618 | 5.044 | 5.514 | 5.415 | -0.100 | -0.116 | 0.522 | 0.804 | Tentative | Lipids and lipid-like molecules | Prenol lipids |
| metab_374 | Unannotated feature | pos | 299.0624 | 0.185 | 4.632 | 4.615 | -0.017 | -0.116 | 0.522 | 0.804 | Unannotated | NA | NA |
| metab_14370 | 4-Butyloxazole | neg | 170.0817 | 3.149 | 3.252 | 3.557 | 0.305 | 0.116 | 0.522 | 0.804 | Unannotated | NA | NA |
| metab_8021 | Lysylisoleucine | pos | 242.1868 | 2.811 | 4.287 | 4.003 | -0.283 | -0.116 | 0.522 | 0.804 | Unannotated | Organic acids and derivatives | Carboxylic acids and derivatives |
| metab_14819 | Unannotated feature | neg | 175.0970 | 3.934 | 4.073 | 4.049 | -0.024 | -0.116 | 0.522 | 0.804 | Unannotated | NA | NA |
| metab_14347 | Betalamic acid | neg | 210.0404 | 3.120 | 5.041 | 5.125 | 0.084 | 0.116 | 0.522 | 0.804 | Tentative | NA | NA |
| metab_8607 | Unannotated feature | pos | 223.1081 | 1.118 | 3.682 | 3.429 | -0.253 | -0.116 | 0.522 | 0.804 | Unannotated | NA | NA |
| metab_9128 | Unannotated feature | neg | 303.1448 | 3.882 | 5.063 | 5.253 | 0.189 | 0.116 | 0.522 | 0.804 | Unannotated | NA | NA |
| metab_4393 | 5,20-DiHETE | pos | 359.2199 | 6.255 | 4.872 | 4.314 | -0.558 | -0.116 | 0.522 | 0.804 | Unannotated | NA | NA |
| metab_1887 | Benzyl formate | pos | 119.0496 | 1.302 | 5.971 | 5.941 | -0.031 | -0.116 | 0.522 | 0.804 | Tentative | NA | NA |
| metab_18095 | Terpestacin | neg | 401.2667 | 6.397 | 3.958 | 3.863 | -0.095 | -0.116 | 0.522 | 0.804 | Moderate putative | NA | NA |
| metab_6454 | Unannotated feature | pos | 181.1225 | 5.650 | 3.678 | 3.775 | 0.096 | 0.116 | 0.522 | 0.804 | Unannotated | NA | NA |
| metab_7513 | D-4-Hydroxyphenylglycine | pos | 168.0658 | 3.729 | 3.994 | 3.922 | -0.072 | -0.116 | 0.522 | 0.804 | Strong putative | NA | NA |
| metab_15237 | Heptenophos | neg | 295.0137 | 4.785 | 4.685 | 4.250 | -0.435 | -0.116 | 0.522 | 0.804 | Tentative | NA | NA |
| metab_15288 | Unannotated feature | neg | 256.1554 | 4.861 | 3.253 | 3.283 | 0.030 | 0.116 | 0.522 | 0.804 | Unannotated | NA | NA |
| metab_15141 | (+)-taxifolin | neg | 303.0512 | 4.603 | 3.131 | 3.888 | 0.757 | 0.116 | 0.522 | 0.804 | Tentative | NA | NA |
| metab_17170 | Unannotated feature | neg | 474.8352 | 7.745 | 4.351 | 4.427 | 0.076 | 0.116 | 0.522 | 0.804 | Unannotated | NA | NA |
| metab_19009 | Unannotated feature | neg | 475.2365 | 5.519 | 3.071 | 3.799 | 0.728 | 0.116 | 0.522 | 0.804 | Unannotated | NA | NA |
| metab_13700 | Unannotated feature | neg | 188.9071 | 0.346 | 6.180 | 6.154 | -0.026 | -0.116 | 0.522 | 0.804 | Unannotated | NA | NA |
| metab_7705 | Unannotated feature | pos | 167.0818 | 3.362 | 3.429 | 3.490 | 0.061 | 0.116 | 0.522 | 0.804 | Unannotated | NA | NA |
| metab_3284 | Unannotated feature | pos | 239.1394 | 4.002 | 5.057 | 5.017 | -0.040 | -0.116 | 0.522 | 0.804 | Unannotated | NA | NA |
| metab_17106 | Unannotated feature | neg | 171.8308 | 7.861 | 3.642 | 3.731 | 0.088 | 0.116 | 0.522 | 0.804 | Unannotated | NA | NA |
| metab_15987 | Unannotated feature | neg | 547.2940 | 6.091 | 5.098 | 5.017 | -0.082 | -0.116 | 0.522 | 0.804 | Unannotated | NA | NA |
| metab_7718 | 6-Hydroxyetodolac | pos | 321.1817 | 3.340 | 2.359 | 2.616 | 0.257 | 0.116 | 0.522 | 0.804 | Unannotated | NA | NA |
| metab_7717 | Unannotated feature | pos | 202.0165 | 3.343 | 4.321 | 4.373 | 0.052 | 0.116 | 0.522 | 0.804 | Unannotated | NA | NA |
| metab_15356 | Unannotated feature | neg | 348.1662 | 5.014 | 4.064 | 4.170 | 0.106 | -0.116 | 0.522 | 0.804 | Unannotated | NA | NA |
| metab_13000 | Unannotated feature | neg | 260.0777 | 0.804 | 6.127 | 6.261 | 0.134 | 0.116 | 0.522 | 0.804 | Unannotated | NA | NA |
| metab_18120 | Cholyltryptophan | neg | 593.3600 | 6.373 | 4.244 | 4.457 | 0.213 | 0.116 | 0.522 | 0.804 | Unannotated | NA | NA |
| metab_18105 | Unannotated feature | neg | 585.4019 | 6.381 | 5.814 | 5.647 | -0.167 | -0.116 | 0.522 | 0.804 | Unannotated | NA | NA |
| metab_15770 | Unannotated feature | neg | 390.0836 | 5.797 | 4.125 | 3.461 | -0.664 | -0.116 | 0.522 | 0.804 | Unannotated | NA | NA |
| metab_18158 | Cetilistat | neg | 446.2918 | 6.334 | 5.071 | 4.964 | -0.107 | -0.116 | 0.522 | 0.804 | Unannotated | NA | NA |
| metab_18195 | Unannotated feature | neg | 390.2303 | 6.303 | 3.510 | 3.559 | 0.049 | 0.116 | 0.522 | 0.804 | Unannotated | NA | NA |
| metab_10789 | Unannotated feature | neg | 225.0112 | 4.807 | 3.830 | 3.101 | -0.729 | -0.116 | 0.522 | 0.804 | Unannotated | NA | NA |
| metab_12842 | Unannotated feature | neg | 139.0506 | 0.675 | 4.771 | 4.563 | -0.208 | -0.116 | 0.522 | 0.804 | Unannotated | NA | NA |
| metab_3208 | Unannotated feature | pos | 278.1744 | 3.740 | 3.548 | 3.460 | -0.088 | -0.116 | 0.522 | 0.804 | Unannotated | NA | NA |
| metab_18239 | Unannotated feature | neg | 339.2176 | 6.263 | 5.130 | 5.141 | 0.011 | 0.116 | 0.522 | 0.804 | Unannotated | NA | NA |
| metab_1689 | Unannotated feature | pos | 330.2395 | 4.103 | 5.515 | 5.490 | -0.026 | -0.116 | 0.522 | 0.804 | Unannotated | NA | NA |
| metab_18077 | 3,17,20-Trihydroxypregnane | neg | 381.2649 | 6.412 | 5.651 | 5.680 | 0.029 | 0.116 | 0.522 | 0.804 | Unannotated | NA | NA |
| metab_11193 | Unannotated feature | neg | 258.0564 | 0.651 | 6.246 | 6.010 | -0.236 | -0.116 | 0.522 | 0.804 | Unannotated | NA | NA |
| metab_11191 | Unannotated feature | neg | 281.9697 | 0.651 | 4.633 | 4.380 | -0.253 | -0.116 | 0.522 | 0.804 | Unannotated | NA | NA |
| metab_11173 | 5,7-Dihydroxyflavone | neg | 235.0398 | 0.667 | 4.814 | 4.470 | -0.344 | -0.116 | 0.522 | 0.804 | Tentative | Phenylpropanoids and polyketides | Flavonoids |
| metab_12907 | Unannotated feature | neg | 333.0956 | 0.730 | 5.239 | 5.229 | -0.010 | 0.116 | 0.522 | 0.804 | Unannotated | NA | NA |
| metab_12815 | Unannotated feature | neg | 343.1129 | 0.651 | 4.506 | 4.518 | 0.012 | -0.116 | 0.522 | 0.804 | Unannotated | NA | NA |
| metab_12684 | (4R,5S,7R,11S)-11,12-Dihydroxy-1(10)-spirovetiven-2-one 11-glucoside | neg | 413.2188 | 5.561 | 4.411 | 4.773 | 0.362 | 0.116 | 0.522 | 0.804 | Unannotated | NA | NA |
| metab_18441 | Unannotated feature | neg | 375.1475 | 6.099 | 4.184 | 4.279 | 0.095 | 0.116 | 0.522 | 0.804 | Unannotated | NA | NA |
| metab_10994 | Unannotated feature | neg | 314.0999 | 0.777 | 3.776 | 3.724 | -0.052 | -0.116 | 0.522 | 0.804 | Unannotated | NA | NA |
| metab_12788 | Unannotated feature | neg | 268.1041 | 0.636 | 4.884 | 4.919 | 0.035 | -0.116 | 0.522 | 0.804 | Unannotated | NA | NA |
| metab_11986 | Unannotated feature | neg | 469.2273 | 6.209 | 7.086 | 7.232 | 0.146 | 0.116 | 0.522 | 0.804 | Unannotated | NA | NA |
| metab_16302 | Unannotated feature | neg | 553.4118 | 6.624 | 5.864 | 5.874 | 0.010 | 0.116 | 0.522 | 0.804 | Unannotated | NA | NA |
| metab_5365 | Unannotated feature | pos | 358.3686 | 6.502 | 5.706 | 5.670 | -0.036 | -0.116 | 0.522 | 0.804 | Unannotated | NA | NA |
| metab_12045 | Unannotated feature | neg | 656.3318 | 5.710 | 4.615 | 6.273 | 1.658 | 0.116 | 0.522 | 0.804 | Unannotated | NA | NA |
| metab_19753 | 3,4-Dihydro-1H-2-benzopyran-1-one | neg | 341.1041 | 4.498 | 4.975 | 3.885 | -1.090 | -0.116 | 0.522 | 0.804 | Unannotated | Organoheterocyclic compounds | Benzopyrans |
| metab_14633 | Unannotated feature | neg | 354.0836 | 3.566 | 3.134 | 3.323 | 0.189 | 0.116 | 0.522 | 0.804 | Unannotated | NA | NA |
| metab_8282 | Unannotated feature | pos | 860.2737 | 2.138 | 6.013 | 6.007 | -0.006 | 0.116 | 0.522 | 0.804 | Unannotated | NA | NA |
| metab_14767 | C75 trans | neg | 299.1503 | 3.834 | 5.155 | 5.296 | 0.141 | 0.116 | 0.522 | 0.804 | Unannotated | NA | NA |
| metab_19851 | Brassinolide | neg | 501.3222 | 6.459 | 6.479 | 6.545 | 0.066 | 0.112 | 0.538 | 0.813 | Tentative | NA | NA |
| metab_11973 | Unannotated feature | neg | 413.2551 | 6.177 | 6.567 | 6.545 | -0.022 | -0.112 | 0.538 | 0.813 | Unannotated | NA | NA |
| metab_18421 | (+/-)-2-Hydroxypiperitone | neg | 213.1128 | 6.115 | 4.930 | 4.800 | -0.130 | -0.112 | 0.538 | 0.813 | Tentative | NA | NA |
| metab_15690 | Unannotated feature | neg | 435.8867 | 0.588 | 4.938 | 5.138 | 0.200 | 0.112 | 0.538 | 0.813 | Unannotated | NA | NA |
| metab_1514 | Unannotated feature | pos | 524.3000 | 5.474 | 4.782 | 4.413 | -0.369 | -0.112 | 0.538 | 0.813 | Unannotated | NA | NA |
| metab_13658 | Unannotated feature | neg | 197.8076 | 0.036 | 7.497 | 7.478 | -0.019 | -0.112 | 0.538 | 0.813 | Unannotated | NA | NA |
| metab_13548 | Unannotated feature | neg | 242.1762 | 1.164 | 3.707 | 3.664 | -0.042 | -0.112 | 0.538 | 0.813 | Unannotated | NA | NA |
| metab_2869 | Unannotated feature | pos | 218.1026 | 2.803 | 4.529 | 4.585 | 0.056 | 0.112 | 0.538 | 0.813 | Unannotated | NA | NA |
| metab_2807 | Unannotated feature | pos | 265.0800 | 2.687 | 4.639 | 4.675 | 0.035 | -0.112 | 0.538 | 0.813 | Unannotated | NA | NA |
| metab_18964 | Ononin | neg | 411.1070 | 5.571 | 5.304 | 5.276 | -0.028 | -0.112 | 0.538 | 0.813 | Tentative | NA | NA |
| metab_14400 | Unannotated feature | neg | 646.7753 | 0.527 | 4.760 | 5.232 | 0.472 | 0.112 | 0.538 | 0.813 | Unannotated | NA | NA |
| metab_15608 | Unannotated feature | neg | 449.9323 | 0.582 | 4.018 | 4.468 | 0.450 | 0.112 | 0.538 | 0.813 | Unannotated | NA | NA |
| metab_17360 | Unannotated feature | neg | 170.9436 | 7.687 | 4.839 | 4.843 | 0.004 | -0.112 | 0.538 | 0.813 | Unannotated | NA | NA |
| metab_917 | Cer(d18:2(4E,14Z)/20:3(6,8,11)-OH(5)) | pos | 602.5131 | 7.148 | 5.026 | 4.603 | -0.422 | -0.112 | 0.538 | 0.813 | Unannotated | NA | NA |
| metab_3091 | Unannotated feature | pos | 299.1609 | 3.400 | 3.790 | 3.861 | 0.071 | 0.112 | 0.538 | 0.813 | Unannotated | NA | NA |
| metab_13437 | Unannotated feature | neg | 292.0936 | 0.819 | 4.057 | 4.074 | 0.018 | 0.112 | 0.538 | 0.813 | Unannotated | NA | NA |
| metab_18871 | Unannotated feature | neg | 416.1988 | 5.692 | 5.052 | 5.259 | 0.207 | 0.112 | 0.538 | 0.813 | Unannotated | NA | NA |
| metab_18934 | Apomine | neg | 607.3152 | 5.607 | 4.794 | 5.153 | 0.359 | 0.112 | 0.538 | 0.813 | Unannotated | NA | NA |
| metab_18889 | 22-Deoxoisocucurbitacin D | neg | 547.3304 | 5.671 | 4.858 | 5.071 | 0.212 | 0.112 | 0.538 | 0.813 | Unannotated | NA | NA |
| metab_5103 | Alisol A | pos | 491.3721 | 7.005 | 6.193 | 6.201 | 0.008 | 0.112 | 0.538 | 0.813 | Tentative | NA | NA |
| metab_14440 | 3-Hydroxypropyl methacrylate | neg | 189.0763 | 3.254 | 5.016 | 5.035 | 0.019 | 0.112 | 0.538 | 0.813 | Unannotated | NA | NA |
| metab_9760 | Tifluadom | neg | 374.1137 | 3.074 | 4.445 | 4.470 | 0.025 | -0.112 | 0.538 | 0.813 | Tentative | Organoheterocyclic compounds | Benzodiazepines |
| metab_1296 | Unannotated feature | pos | 309.2581 | 6.255 | 5.561 | 5.203 | -0.357 | -0.112 | 0.538 | 0.813 | Unannotated | NA | NA |
| metab_19339 | Unannotated feature | neg | 294.1350 | 5.087 | 4.056 | 4.101 | 0.045 | -0.112 | 0.538 | 0.813 | Unannotated | NA | NA |
| metab_17449 | Unannotated feature | neg | 316.9480 | 7.611 | 7.867 | 7.853 | -0.014 | 0.112 | 0.538 | 0.813 | Unannotated | NA | NA |
| metab_7613 | Unannotated feature | pos | 291.1638 | 3.527 | 3.003 | 2.923 | -0.080 | -0.112 | 0.538 | 0.813 | Unannotated | NA | NA |
| metab_15911 | 4-Heptyloxyphenol | neg | 253.1445 | 6.013 | 4.700 | 4.802 | 0.102 | 0.112 | 0.538 | 0.813 | Tentative | NA | NA |
| metab_13732 | Valylproline | neg | 213.1241 | 1.947 | 4.200 | 4.048 | -0.151 | -0.112 | 0.538 | 0.813 | Unannotated | NA | NA |
| metab_5715 | Unannotated feature | pos | 300.1997 | 6.103 | 4.020 | 4.218 | 0.198 | 0.112 | 0.538 | 0.813 | Unannotated | NA | NA |
| metab_5720 | 5-[(1S,2S,3S,5R)-3,5-Dihydroxy-2-[(E,3R)-3-hydroxyoct-1-enyl]cyclopentyl]-4-oxopentanoic acid | pos | 325.2009 | 6.087 | 5.085 | 4.858 | -0.226 | -0.112 | 0.538 | 0.813 | Unannotated | NA | NA |
| metab_8400 | Unannotated feature | pos | 201.1238 | 1.952 | 4.990 | 4.861 | -0.129 | -0.112 | 0.538 | 0.813 | Unannotated | NA | NA |
| metab_10072 | N-VINYL-2-PYRROLIDONE | neg | 156.0660 | 2.780 | 5.036 | 5.037 | 0.000 | -0.112 | 0.538 | 0.813 | Tentative | NA | NA |
| metab_1031 | Unannotated feature | pos | 621.5077 | 6.997 | 4.410 | 4.175 | -0.235 | -0.112 | 0.538 | 0.813 | Unannotated | NA | NA |
| metab_17482 | Unannotated feature | neg | 265.9507 | 7.553 | 6.321 | 6.314 | -0.008 | 0.112 | 0.538 | 0.813 | Unannotated | NA | NA |
| metab_14412 | 4-Methylbenzyl alcohol | neg | 167.0707 | 3.215 | 3.255 | 3.190 | -0.065 | 0.112 | 0.538 | 0.813 | Tentative | Benzenoids | Benzene and substituted derivatives |
| metab_16566 | Unannotated feature | neg | 98.9383 | 7.310 | 6.507 | 6.490 | -0.017 | -0.112 | 0.538 | 0.813 | Unannotated | NA | NA |
| metab_5094 | Unannotated feature | pos | 647.3934 | 7.036 | 4.561 | 4.368 | -0.193 | -0.112 | 0.538 | 0.813 | Unannotated | NA | NA |
| metab_12398 | Unannotated feature | neg | 399.2217 | 6.209 | 6.495 | 6.574 | 0.079 | 0.112 | 0.538 | 0.813 | Unannotated | NA | NA |
| metab_19024 | Unannotated feature | neg | 465.1957 | 5.496 | 3.508 | 3.636 | 0.129 | 0.112 | 0.538 | 0.813 | Unannotated | NA | NA |
| metab_4627 | Unannotated feature | pos | 419.2628 | 7.140 | 5.812 | 5.798 | -0.014 | -0.112 | 0.538 | 0.813 | Unannotated | NA | NA |
| metab_12817 | Unannotated feature | neg | 256.0595 | 0.651 | 6.788 | 6.535 | -0.253 | -0.112 | 0.538 | 0.813 | Unannotated | NA | NA |
| metab_1694 | Unannotated feature | pos | 321.0955 | 4.068 | 4.411 | 3.863 | -0.548 | -0.112 | 0.538 | 0.813 | Unannotated | NA | NA |
| metab_3229 | Unannotated feature | pos | 202.0165 | 3.799 | 3.810 | 3.801 | -0.009 | -0.112 | 0.538 | 0.813 | Unannotated | NA | NA |
| metab_17518 | Unannotated feature | neg | 318.0835 | 1.952 | 4.229 | 4.451 | 0.222 | 0.112 | 0.538 | 0.813 | Unannotated | NA | NA |
| metab_858 | Ginsenoside Rh7 | pos | 601.4089 | 6.614 | 5.179 | 5.587 | 0.408 | 0.112 | 0.538 | 0.813 | Unannotated | NA | NA |
| metab_15976 | Propargite | neg | 387.1042 | 6.083 | 5.862 | 5.985 | 0.123 | -0.112 | 0.538 | 0.813 | Tentative | NA | NA |
| metab_8412 | S-Methylthiocitrulline | pos | 170.0750 | 1.952 | 3.943 | 4.170 | 0.226 | 0.112 | 0.538 | 0.813 | Unannotated | NA | NA |
| metab_5139 | Ganoderic acid F | pos | 535.2720 | 6.885 | 6.301 | 6.424 | 0.123 | -0.112 | 0.538 | 0.813 | Unannotated | NA | NA |
| metab_12162 | Unannotated feature | neg | 749.5741 | 6.671 | 5.151 | 6.053 | 0.902 | 0.112 | 0.538 | 0.813 | Unannotated | NA | NA |
| metab_15610 | Unannotated feature | neg | 526.2857 | 5.519 | 3.866 | 5.088 | 1.222 | 0.112 | 0.538 | 0.813 | Unannotated | NA | NA |
| metab_7558 | 4-Amino-5-hydroxymethyl-2-methylpyrimidine | pos | 181.1080 | 0.728 | 4.180 | 4.122 | -0.058 | -0.112 | 0.538 | 0.813 | Moderate putative | Organoheterocyclic compounds | Diazines |
| metab_2404 | Unannotated feature | pos | 122.9250 | 0.593 | 5.984 | 5.929 | -0.055 | -0.112 | 0.538 | 0.813 | Unannotated | NA | NA |
| metab_17556 | Unannotated feature | neg | 491.3367 | 7.335 | 4.173 | 4.727 | 0.554 | 0.112 | 0.538 | 0.813 | Unannotated | NA | NA |
| metab_5433 | 9,10-Epoxy-18-hydroxy-octadecanoic acid | pos | 279.2323 | 6.406 | 6.171 | 6.124 | -0.047 | -0.112 | 0.538 | 0.813 | Tentative | NA | NA |
| metab_8804 | Dihydrobiopterin | pos | 303.1194 | 0.792 | 4.721 | 4.620 | -0.101 | 0.112 | 0.538 | 0.813 | Tentative | NA | NA |
| metab_4746 | Unannotated feature | pos | 263.1396 | 3.661 | 6.690 | 6.625 | -0.065 | -0.112 | 0.538 | 0.813 | Unannotated | NA | NA |
| metab_19566 | Unannotated feature | neg | 155.0013 | 0.651 | 4.711 | 4.691 | -0.021 | 0.112 | 0.538 | 0.813 | Unannotated | NA | NA |
| metab_14524 | Unannotated feature | neg | 327.0664 | 0.524 | 4.610 | 4.550 | -0.060 | -0.112 | 0.538 | 0.813 | Unannotated | NA | NA |
| metab_5188 | Unannotated feature | pos | 540.5366 | 6.781 | 5.921 | 5.893 | -0.027 | 0.112 | 0.538 | 0.813 | Unannotated | NA | NA |
| metab_11402 | 3,4-Dichloroisocoumarin | neg | 234.9349 | 0.547 | 3.830 | 3.926 | 0.096 | -0.112 | 0.538 | 0.813 | Unannotated | NA | NA |
| metab_8639 | Unannotated feature | pos | 248.1135 | 1.034 | 4.462 | 4.448 | -0.014 | -0.112 | 0.538 | 0.813 | Unannotated | NA | NA |
| metab_16738 | Unannotated feature | neg | 367.9068 | 7.666 | 5.138 | 5.148 | 0.010 | 0.112 | 0.538 | 0.813 | Unannotated | NA | NA |
| metab_4827 | Unannotated feature | pos | 84.3059 | 7.803 | 3.498 | 3.461 | -0.037 | -0.112 | 0.538 | 0.813 | Unannotated | NA | NA |
| metab_14322 | Unannotated feature | neg | 192.0661 | 3.078 | 4.610 | 4.820 | 0.210 | 0.112 | 0.538 | 0.813 | Unannotated | NA | NA |
| metab_9548 | Unannotated feature | neg | 195.0692 | 3.288 | 3.428 | 3.781 | 0.354 | 0.112 | 0.538 | 0.813 | Unannotated | NA | NA |
| metab_7236 | Unannotated feature | pos | 274.2018 | 4.350 | 4.639 | 4.503 | -0.136 | -0.112 | 0.538 | 0.813 | Unannotated | NA | NA |
| metab_1057 | Unannotated feature | pos | 443.3149 | 6.789 | 4.730 | 4.825 | 0.095 | 0.112 | 0.538 | 0.813 | Unannotated | NA | NA |
| metab_10336 | Unannotated feature | neg | 316.0515 | 2.248 | 4.297 | 4.381 | 0.084 | 0.112 | 0.538 | 0.813 | Unannotated | NA | NA |
| metab_13263 | Unannotated feature | neg | 372.8129 | 0.603 | 4.475 | 4.939 | 0.464 | 0.112 | 0.538 | 0.813 | Unannotated | NA | NA |
| metab_1061 | Tetrahydrodeoxycorticosterone | pos | 335.2566 | 6.789 | 5.265 | 5.540 | 0.276 | 0.112 | 0.538 | 0.813 | Moderate putative | NA | NA |
| metab_16792 | Unannotated feature | neg | 148.9490 | 7.707 | 4.566 | 4.562 | -0.004 | -0.112 | 0.538 | 0.813 | Unannotated | NA | NA |
| metab_10793 | Unannotated feature | neg | 168.0237 | 1.119 | 5.111 | 5.233 | 0.123 | 0.112 | 0.538 | 0.813 | Unannotated | NA | NA |
| metab_18138 | Corchorifatty acid F | neg | 655.4439 | 6.357 | 4.681 | 4.341 | -0.340 | -0.112 | 0.538 | 0.813 | Unannotated | NA | NA |
| metab_14109 | 3'-Deoxythymidine | neg | 271.0934 | 2.836 | 4.816 | 4.711 | -0.105 | -0.112 | 0.538 | 0.813 | Unannotated | NA | NA |
| metab_18218 | Unannotated feature | neg | 415.2706 | 6.279 | 6.097 | 6.000 | -0.097 | -0.112 | 0.538 | 0.813 | Unannotated | NA | NA |
| metab_10387 | Unannotated feature | neg | 200.9041 | 2.107 | 3.114 | 3.281 | 0.167 | 0.112 | 0.538 | 0.813 | Unannotated | NA | NA |
| metab_12558 | Unannotated feature | neg | 498.2909 | 6.162 | 6.215 | 6.276 | 0.061 | -0.112 | 0.538 | 0.813 | Unannotated | NA | NA |
| metab_15001 | Acetyl-DL-Leucine | neg | 172.0973 | 4.335 | 6.025 | 5.913 | -0.112 | -0.112 | 0.538 | 0.813 | Strong putative | NA | NA |
| metab_10415 | Unannotated feature | neg | 326.9954 | 2.019 | 4.131 | 4.042 | -0.090 | -0.112 | 0.538 | 0.813 | Unannotated | NA | NA |
| metab_10425 | 3-Methyluric acid | neg | 181.0362 | 1.992 | 5.788 | 5.941 | 0.153 | 0.112 | 0.538 | 0.813 | Unannotated | NA | NA |
| metab_19644 | Unannotated feature | neg | 397.1257 | 3.165 | 4.515 | 4.685 | 0.170 | 0.112 | 0.538 | 0.813 | Unannotated | NA | NA |
| metab_12119 | Unannotated feature | neg | 474.2623 | 6.545 | 5.667 | 5.131 | -0.536 | -0.112 | 0.538 | 0.813 | Unannotated | NA | NA |
| metab_12121 | Unannotated feature | neg | 507.2744 | 6.553 | 5.870 | 6.026 | 0.156 | 0.112 | 0.538 | 0.813 | Unannotated | NA | NA |
| metab_5233 | Unannotated feature | pos | 515.3722 | 6.694 | 6.343 | 6.368 | 0.025 | 0.112 | 0.538 | 0.813 | Unannotated | NA | NA |
| metab_6812 | Unannotated feature | pos | 189.1349 | 0.633 | 3.866 | 3.695 | -0.170 | -0.112 | 0.538 | 0.813 | Unannotated | NA | NA |
| metab_18166 | Unannotated feature | neg | 534.2518 | 6.326 | 3.903 | 4.231 | 0.328 | 0.112 | 0.538 | 0.813 | Unannotated | NA | NA |
| metab_508 | Unannotated feature | pos | 309.1815 | 5.121 | 3.013 | 4.856 | 1.844 | 0.112 | 0.538 | 0.813 | Unannotated | NA | NA |
| metab_14140 | Unannotated feature | neg | 457.0220 | 0.512 | 4.439 | 4.445 | 0.006 | 0.112 | 0.538 | 0.813 | Unannotated | NA | NA |
| metab_8704 | Unannotated feature | pos | 153.0660 | 0.776 | 2.983 | 3.114 | 0.131 | 0.112 | 0.538 | 0.813 | Unannotated | NA | NA |
| metab_16729 | Unannotated feature | neg | 756.8234 | 7.660 | 4.447 | 4.462 | 0.015 | 0.112 | 0.538 | 0.813 | Unannotated | NA | NA |
| metab_14272 | Unannotated feature | neg | 285.1458 | 3.017 | 4.154 | 4.020 | -0.134 | -0.112 | 0.538 | 0.813 | Unannotated | NA | NA |
| metab_13910 | Unannotated feature | neg | 131.0343 | 2.520 | 6.139 | 6.082 | -0.057 | -0.112 | 0.538 | 0.813 | Unannotated | NA | NA |
| metab_18621 | Unannotated feature | neg | 397.1871 | 5.971 | 5.402 | 5.005 | -0.397 | -0.112 | 0.538 | 0.813 | Unannotated | NA | NA |
| metab_17721 | SM(d19:0/20:4(8Z,11Z,14Z,17Z)-2OH(5S,6R)) | neg | 833.5560 | 6.959 | 4.948 | 4.221 | -0.727 | -0.112 | 0.538 | 0.813 | Unannotated | NA | NA |
| metab_8783 | 17a,20a-Dihydroxycholesterol | pos | 383.3310 | 6.574 | 5.559 | 5.518 | -0.040 | -0.112 | 0.538 | 0.813 | Tentative | NA | NA |
| metab_13961 | Unannotated feature | neg | 234.0244 | 2.646 | 2.798 | 2.653 | -0.145 | -0.112 | 0.538 | 0.813 | Unannotated | NA | NA |
| metab_1143 | Unannotated feature | pos | 203.0530 | 0.633 | 5.833 | 5.464 | -0.368 | -0.112 | 0.538 | 0.813 | Unannotated | NA | NA |
| metab_16156 | Unannotated feature | neg | 487.2710 | 6.342 | 4.985 | 5.168 | 0.183 | -0.112 | 0.538 | 0.813 | Unannotated | NA | NA |
| metab_12308 | Unannotated feature | neg | 339.2001 | 6.899 | 8.006 | 8.009 | 0.004 | 0.112 | 0.538 | 0.813 | Unannotated | NA | NA |
| metab_814 | Unannotated feature | pos | 597.3664 | 6.327 | 5.474 | 6.826 | 1.352 | 0.112 | 0.538 | 0.813 | Unannotated | NA | NA |
| metab_13947 | Unannotated feature | neg | 284.0890 | 2.606 | 1.651 | 1.824 | 0.173 | 0.112 | 0.538 | 0.813 | Unannotated | NA | NA |
| metab_4094 | Unannotated feature | pos | 587.1662 | 5.847 | 2.482 | 3.867 | 1.384 | 0.112 | 0.538 | 0.813 | Unannotated | NA | NA |
| metab_6470 | 3beta-Hydroxy-17-(1H-imidazol-1-yl)androsta-5,16-diene | pos | 383.2051 | 5.629 | 4.521 | 4.319 | -0.201 | -0.112 | 0.538 | 0.813 | Unannotated | NA | NA |
| metab_11176 | L-Glutamic gamma-semialdehyde | neg | 176.0558 | 0.667 | 5.411 | 5.527 | 0.116 | 0.112 | 0.538 | 0.813 | Tentative | NA | NA |
| metab_16121 | Cepanone | neg | 209.1542 | 6.279 | 4.449 | 4.569 | 0.119 | 0.112 | 0.538 | 0.813 | Unannotated | NA | NA |
| metab_13057 | Unannotated feature | neg | 768.7024 | 0.538 | 4.632 | 4.573 | -0.059 | -0.112 | 0.538 | 0.813 | Unannotated | NA | NA |
| metab_1990 | Loureirin B | pos | 334.1657 | 5.113 | 5.437 | 5.447 | 0.010 | 0.112 | 0.538 | 0.813 | Unannotated | NA | NA |
| metab_8329 | Phenacetin | pos | 180.1022 | 2.003 | 5.080 | 5.162 | 0.082 | 0.112 | 0.538 | 0.813 | Tentative | NA | NA |
| metab_11913 | Sapienic acid | neg | 291.1714 | 6.076 | 3.818 | 3.284 | -0.533 | -0.112 | 0.538 | 0.813 | Tentative | NA | NA |
| metab_15135 | Norajmaline | neg | 349.1302 | 4.596 | 4.026 | 4.062 | 0.036 | 0.112 | 0.538 | 0.813 | Tentative | NA | NA |
| metab_6669 | Unannotated feature | pos | 151.9513 | 0.601 | 4.553 | 4.543 | -0.010 | 0.112 | 0.538 | 0.813 | Unannotated | NA | NA |
| metab_4187 | Unannotated feature | pos | 402.1834 | 6.016 | 5.264 | 5.355 | 0.091 | 0.112 | 0.538 | 0.813 | Unannotated | NA | NA |
| metab_4770 | Unannotated feature | pos | 90.0287 | 7.740 | 3.020 | 2.902 | -0.118 | -0.112 | 0.538 | 0.813 | Unannotated | NA | NA |
| metab_17942 | Unannotated feature | neg | 521.3501 | 6.601 | 3.534 | 3.701 | 0.167 | 0.112 | 0.538 | 0.813 | Unannotated | NA | NA |
| metab_8315 | Val Val Asp | pos | 332.1824 | 2.032 | 4.966 | 4.894 | -0.072 | -0.112 | 0.538 | 0.813 | Strong putative | NA | NA |
| metab_739 | Unannotated feature | pos | 394.8753 | 0.524 | 3.455 | 3.332 | -0.123 | -0.112 | 0.538 | 0.813 | Unannotated | NA | NA |
| metab_19251 | Combretastatin A4 | neg | 361.1299 | 5.201 | 5.147 | 5.054 | -0.093 | -0.112 | 0.538 | 0.813 | Tentative | NA | NA |
| metab_18025 | Unannotated feature | neg | 348.9000 | 6.483 | 5.151 | 5.101 | -0.051 | -0.112 | 0.538 | 0.813 | Unannotated | NA | NA |
| metab_17961 | Unannotated feature | neg | 499.3646 | 6.561 | 5.300 | 5.401 | 0.101 | 0.112 | 0.538 | 0.813 | Unannotated | NA | NA |
| metab_7533 | Isopenicillin N | pos | 377.1465 | 3.689 | 5.752 | 5.767 | 0.015 | 0.112 | 0.538 | 0.813 | Tentative | NA | NA |
| metab_5552 | Unannotated feature | pos | 419.3165 | 6.279 | 4.807 | 4.889 | 0.082 | 0.112 | 0.538 | 0.813 | Unannotated | NA | NA |
| metab_12198 | Unannotated feature | neg | 425.3643 | 6.782 | 6.310 | 6.591 | 0.280 | 0.112 | 0.538 | 0.813 | Unannotated | NA | NA |
| metab_10702 | Succinic Anhydride | neg | 99.0079 | 1.411 | 5.024 | 5.014 | -0.010 | -0.112 | 0.538 | 0.813 | Moderate putative | Organic acids and derivatives | Carboxylic acids and derivatives |
| metab_16980 | Unannotated feature | neg | 149.0084 | 7.944 | 3.476 | 3.389 | -0.087 | -0.112 | 0.538 | 0.813 | Unannotated | NA | NA |
| metab_14073 | Unannotated feature | neg | 269.1144 | 2.799 | 4.856 | 4.822 | -0.034 | -0.112 | 0.538 | 0.813 | Unannotated | NA | NA |
| metab_753 | Unannotated feature | pos | 549.3454 | 6.072 | 5.917 | 6.146 | 0.228 | -0.112 | 0.538 | 0.813 | Unannotated | NA | NA |
| metab_14920 | Sudan II | neg | 311.0929 | 4.154 | 2.387 | 3.105 | 0.718 | 0.112 | 0.538 | 0.813 | Tentative | NA | NA |
| metab_248 | Unannotated feature | pos | 472.8170 | 0.531 | 4.531 | 4.575 | 0.044 | -0.112 | 0.538 | 0.813 | Unannotated | NA | NA |
| metab_19627 | Unannotated feature | neg | 277.1650 | 4.667 | 4.128 | 3.959 | -0.169 | -0.112 | 0.538 | 0.813 | Unannotated | NA | NA |
| metab_14679 | Unannotated feature | neg | 97.0650 | 3.651 | 2.309 | 3.057 | 0.749 | 0.112 | 0.538 | 0.813 | Unannotated | NA | NA |
| metab_8244 | Cyclohexa-2,4-dienylmethanol | pos | 93.0704 | 2.299 | 4.396 | 4.330 | -0.067 | -0.112 | 0.538 | 0.813 | Unannotated | NA | NA |
| metab_16327 | Unannotated feature | neg | 341.1959 | 6.663 | 5.762 | 5.776 | 0.014 | 0.112 | 0.538 | 0.813 | Unannotated | NA | NA |
| metab_616 | Unannotated feature | pos | 219.0067 | 0.019 | 5.026 | 5.039 | 0.013 | 0.112 | 0.538 | 0.813 | Unannotated | NA | NA |
| metab_19284 | Unannotated feature | neg | 310.1300 | 5.156 | 4.600 | 4.509 | -0.092 | -0.112 | 0.538 | 0.813 | Unannotated | NA | NA |
| metab_4436 | Unannotated feature | pos | 330.2469 | 6.367 | 4.640 | 4.640 | 0.000 | -0.112 | 0.538 | 0.813 | Unannotated | NA | NA |
| metab_14013 | Sanfetrinem | neg | 326.1251 | 2.742 | 5.119 | 5.316 | 0.197 | 0.112 | 0.538 | 0.813 | Unannotated | NA | NA |
| metab_16191 | Unannotated feature | neg | 803.5988 | 6.397 | 5.811 | 5.810 | -0.001 | -0.112 | 0.538 | 0.813 | Unannotated | NA | NA |
| metab_16260 | Unannotated feature | neg | 385.1694 | 6.538 | 4.729 | 4.694 | -0.035 | 0.112 | 0.538 | 0.813 | Unannotated | NA | NA |
| metab_8900 | Unannotated feature | neg | 333.0993 | 4.266 | 4.690 | 4.606 | -0.084 | -0.112 | 0.538 | 0.813 | Unannotated | NA | NA |
| metab_13629 | Unannotated feature | neg | 230.0127 | 1.566 | 4.857 | 4.728 | -0.129 | -0.112 | 0.538 | 0.813 | Unannotated | NA | NA |
| metab_2902 | O-Toluidine | pos | 108.0813 | 2.859 | 4.944 | 4.949 | 0.006 | 0.112 | 0.538 | 0.813 | Strong putative | NA | NA |
| metab_5817 | Melagatran | pos | 452.2305 | 5.984 | 3.775 | 3.637 | -0.138 | -0.112 | 0.538 | 0.813 | Tentative | NA | NA |
| metab_8119 | Unannotated feature | pos | 269.1864 | 2.732 | 4.975 | 5.053 | 0.079 | 0.107 | 0.554 | 0.823 | Unannotated | NA | NA |
| metab_8104 | Unannotated feature | pos | 316.2238 | 3.527 | 5.377 | 5.302 | -0.075 | -0.107 | 0.554 | 0.823 | Unannotated | NA | NA |
| metab_5475 | Unannotated feature | pos | 447.2741 | 6.375 | 5.912 | 5.805 | -0.107 | -0.107 | 0.554 | 0.823 | Unannotated | NA | NA |
| metab_1357 | Unannotated feature | pos | 436.2355 | 6.143 | 4.525 | 4.695 | 0.170 | 0.107 | 0.554 | 0.823 | Unannotated | NA | NA |
| metab_4427 | Unannotated feature | pos | 502.3195 | 6.343 | 5.506 | 5.487 | -0.019 | -0.107 | 0.554 | 0.823 | Unannotated | NA | NA |
| metab_18042 | Unannotated feature | neg | 465.2337 | 6.451 | 5.816 | 5.624 | -0.192 | -0.107 | 0.554 | 0.823 | Unannotated | NA | NA |
| metab_5292 | PE(18:3(9Z,12Z,15Z)/16:0) | pos | 678.4817 | 6.582 | 5.570 | 5.690 | 0.120 | 0.107 | 0.554 | 0.823 | Tentative | NA | NA |
| metab_12459 | Unannotated feature | neg | 463.2708 | 6.256 | 6.330 | 6.268 | -0.063 | -0.107 | 0.554 | 0.823 | Unannotated | NA | NA |
| metab_2828 | Celgosivir | pos | 301.1764 | 2.732 | 3.412 | 3.918 | 0.506 | 0.107 | 0.554 | 0.823 | Unannotated | NA | NA |
| metab_13553 | Rose oxide (cis) | neg | 419.1317 | 1.179 | 5.623 | 5.662 | 0.039 | 0.107 | 0.554 | 0.823 | Tentative | NA | NA |
| metab_13662 | Unannotated feature | neg | 144.1023 | 1.685 | 3.252 | 2.936 | -0.316 | -0.107 | 0.554 | 0.823 | Unannotated | NA | NA |
| metab_12768 | Unannotated feature | neg | 172.0246 | 1.944 | 4.418 | 4.402 | -0.016 | -0.107 | 0.554 | 0.823 | Unannotated | NA | NA |
| metab_15 | Unannotated feature | pos | 185.0924 | 0.720 | 4.101 | 4.333 | 0.232 | 0.107 | 0.554 | 0.823 | Unannotated | NA | NA |
| metab_12061 | Azelaic Acid | neg | 187.0970 | 5.753 | 6.657 | 6.594 | -0.064 | -0.107 | 0.554 | 0.823 | Strong putative | NA | NA |
| metab_12055 | Unannotated feature | neg | 387.2541 | 6.381 | 5.847 | 5.929 | 0.082 | 0.107 | 0.554 | 0.823 | Unannotated | NA | NA |
| metab_7648 | Unannotated feature | pos | 202.0165 | 3.465 | 4.109 | 4.133 | 0.025 | -0.107 | 0.554 | 0.823 | Unannotated | NA | NA |
| metab_2763 | Unannotated feature | pos | 90.5263 | 2.439 | 5.789 | 5.810 | 0.021 | 0.107 | 0.554 | 0.823 | Unannotated | NA | NA |
| metab_9959 | D-Leucic acid | neg | 113.0601 | 2.899 | 3.182 | 3.176 | -0.006 | 0.107 | 0.554 | 0.823 | Tentative | NA | NA |
| metab_898 | Unannotated feature | pos | 469.2759 | 7.044 | 6.765 | 6.740 | -0.025 | -0.107 | 0.554 | 0.823 | Unannotated | NA | NA |
| metab_12062 | Unannotated feature | neg | 837.5522 | 6.389 | 6.049 | 5.564 | -0.485 | -0.107 | 0.554 | 0.823 | Unannotated | NA | NA |
| metab_11211 | Unannotated feature | neg | 259.0132 | 0.644 | 4.538 | 4.358 | -0.180 | -0.107 | 0.554 | 0.823 | Unannotated | NA | NA |
| metab_1236 | N-di-Demethyl roxithromycin | pos | 845.4837 | 6.382 | 5.625 | 5.391 | -0.235 | -0.107 | 0.554 | 0.823 | Unannotated | NA | NA |
| metab_5040 | Unannotated feature | pos | 449.3321 | 7.220 | 5.316 | 5.244 | -0.072 | -0.107 | 0.554 | 0.823 | Unannotated | NA | NA |
| metab_11491 | Unannotated feature | neg | 492.8958 | 0.516 | 5.099 | 5.029 | -0.069 | -0.107 | 0.554 | 0.823 | Unannotated | NA | NA |
| metab_18882 | Unannotated feature | neg | 417.1595 | 5.678 | 5.285 | 5.489 | 0.204 | 0.107 | 0.554 | 0.823 | Unannotated | NA | NA |
| metab_13494 | Unannotated feature | neg | 213.0514 | 1.037 | 4.092 | 3.978 | -0.115 | -0.107 | 0.554 | 0.823 | Unannotated | NA | NA |
| metab_11099 | Unannotated feature | neg | 301.1044 | 0.722 | 4.814 | 4.484 | -0.330 | -0.107 | 0.554 | 0.823 | Unannotated | NA | NA |
| metab_1450 | Pyridin-3-ylmethyl N-[(2S,4S,5S)-4-hydroxy-5-[[(2S)-3-methyl-2-[[methyl(1,3-thiazol-4-ylmethyl)carbamoyl]amino]butanoyl]amino]-1,6-diphenylhexan-2-yl]carbamate | pos | 655.3001 | 6.000 | 5.660 | 5.869 | 0.209 | 0.107 | 0.554 | 0.823 | Unannotated | NA | NA |
| metab_18369 | Unannotated feature | neg | 379.1771 | 6.154 | 4.783 | 4.642 | -0.141 | -0.107 | 0.554 | 0.823 | Unannotated | NA | NA |
| metab_11040 | 8-Azido-cyclic AMP | neg | 369.0503 | 0.738 | 5.113 | 4.935 | -0.178 | -0.107 | 0.554 | 0.823 | Unannotated | NA | NA |
| metab_3061 | Unannotated feature | pos | 200.1285 | 3.335 | 3.733 | 4.052 | 0.319 | 0.107 | 0.554 | 0.823 | Unannotated | NA | NA |
| metab_17222 | Unannotated feature | neg | 296.8816 | 7.730 | 3.618 | 3.568 | -0.051 | -0.107 | 0.554 | 0.823 | Unannotated | NA | NA |
| metab_16128 | Unannotated feature | neg | 465.2477 | 6.279 | 4.796 | 4.792 | -0.004 | 0.107 | 0.554 | 0.823 | Unannotated | NA | NA |
| metab_4952 | Unannotated feature | pos | 105.0428 | 7.602 | 6.440 | 6.471 | 0.031 | 0.107 | 0.554 | 0.823 | Unannotated | NA | NA |
| metab_12229 | Unannotated feature | neg | 377.2729 | 6.915 | 6.008 | 6.128 | 0.120 | 0.107 | 0.554 | 0.823 | Unannotated | NA | NA |
| metab_10650 | Unannotated feature | neg | 202.0177 | 1.650 | 3.969 | 4.090 | 0.121 | -0.107 | 0.554 | 0.823 | Unannotated | NA | NA |
| metab_10633 | Unannotated feature | neg | 263.0233 | 1.680 | 4.822 | 4.771 | -0.052 | -0.107 | 0.554 | 0.823 | Unannotated | NA | NA |
| metab_2627 | Unannotated feature | pos | 206.8863 | 0.593 | 5.634 | 5.515 | -0.119 | -0.107 | 0.554 | 0.823 | Unannotated | NA | NA |
| metab_973 | Unannotated feature | pos | 132.0354 | 7.732 | 4.383 | 4.400 | 0.017 | 0.107 | 0.554 | 0.823 | Unannotated | NA | NA |
| metab_4875 | Unannotated feature | pos | 104.0308 | 7.724 | 4.372 | 4.358 | -0.014 | -0.107 | 0.554 | 0.823 | Unannotated | NA | NA |
| metab_19409 | Amrinone | neg | 373.1385 | 5.003 | 4.100 | 4.069 | -0.030 | -0.107 | 0.554 | 0.823 | Tentative | NA | NA |
| metab_1289 | Unannotated feature | pos | 375.2154 | 6.263 | 4.795 | 4.826 | 0.032 | -0.107 | 0.554 | 0.823 | Unannotated | NA | NA |
| metab_15929 | Unannotated feature | neg | 331.1555 | 6.029 | 3.874 | 3.876 | 0.003 | -0.107 | 0.554 | 0.823 | Unannotated | NA | NA |
| metab_1025 | DG(18:4(6Z,9Z,12Z,15Z)/16:1(9Z)/0:0) | pos | 619.4916 | 7.028 | 6.115 | 6.094 | -0.021 | -0.107 | 0.554 | 0.823 | Tentative | NA | NA |
| metab_17413 | Unannotated feature | neg | 536.8851 | 7.641 | 6.291 | 6.339 | 0.047 | 0.107 | 0.554 | 0.823 | Unannotated | NA | NA |
| metab_10941 | Unannotated feature | neg | 204.0331 | 0.799 | 4.246 | 3.737 | -0.508 | -0.107 | 0.554 | 0.823 | Unannotated | NA | NA |
| metab_10893 | Unannotated feature | neg | 310.0051 | 0.897 | 3.127 | 3.223 | 0.095 | 0.107 | 0.554 | 0.823 | Unannotated | NA | NA |
| metab_12189 | Unannotated feature | neg | 439.3798 | 6.750 | 6.093 | 6.228 | 0.136 | 0.107 | 0.554 | 0.823 | Unannotated | NA | NA |
| metab_11225 | D-Ornithine | neg | 131.0819 | 0.644 | 5.893 | 5.840 | -0.053 | -0.107 | 0.554 | 0.823 | Tentative | NA | NA |
| metab_5671 | PA(20:3(6,8,11)-OH(5)/8:0) | pos | 641.3208 | 6.143 | 5.435 | 5.422 | -0.014 | -0.107 | 0.554 | 0.823 | Unannotated | NA | NA |
| metab_10596 | Unannotated feature | neg | 116.0169 | 1.691 | 3.824 | 3.585 | -0.239 | -0.107 | 0.554 | 0.823 | Unannotated | NA | NA |
| metab_4771 | Unannotated feature | pos | 312.9438 | 7.740 | 4.886 | 4.879 | -0.006 | -0.107 | 0.554 | 0.823 | Unannotated | NA | NA |
| metab_419 | Unannotated feature | pos | 161.0145 | 0.016 | 3.539 | 3.553 | 0.014 | 0.107 | 0.554 | 0.823 | Unannotated | NA | NA |
| metab_11838 | Unannotated feature | neg | 319.1663 | 5.944 | 5.665 | 6.346 | 0.681 | -0.107 | 0.554 | 0.823 | Unannotated | NA | NA |
| metab_10160 | Unannotated feature | neg | 381.1161 | 2.725 | 4.922 | 4.984 | 0.062 | 0.107 | 0.554 | 0.823 | Unannotated | NA | NA |
| metab_11648 | Unannotated feature | neg | 195.8107 | 0.036 | 7.379 | 7.361 | -0.019 | -0.107 | 0.554 | 0.823 | Unannotated | NA | NA |
| metab_11647 | Unannotated feature | neg | 199.8045 | 0.036 | 7.156 | 7.140 | -0.016 | -0.107 | 0.554 | 0.823 | Unannotated | NA | NA |
| metab_17101 | Unannotated feature | neg | 159.8780 | 7.870 | 5.344 | 5.345 | 0.002 | 0.107 | 0.554 | 0.823 | Unannotated | NA | NA |
| metab_19037 | Unannotated feature | neg | 337.1772 | 5.480 | 4.475 | 5.168 | 0.693 | 0.107 | 0.554 | 0.823 | Unannotated | NA | NA |
| metab_882 | Unannotated feature | pos | 304.2614 | 6.853 | 6.940 | 6.960 | 0.020 | -0.107 | 0.554 | 0.823 | Unannotated | NA | NA |
| metab_10262 | Unannotated feature | neg | 349.0160 | 2.531 | 4.731 | 4.783 | 0.052 | -0.107 | 0.554 | 0.823 | Unannotated | NA | NA |
| metab_17537 | Unannotated feature | neg | 299.1251 | 2.746 | 5.000 | 5.114 | 0.115 | 0.107 | 0.554 | 0.823 | Unannotated | NA | NA |
| metab_2508 | Unannotated feature | pos | 242.0429 | 0.815 | 4.421 | 2.585 | -1.836 | -0.107 | 0.554 | 0.823 | Unannotated | NA | NA |
| metab_10303 | 2-(Malonylamino)benzoic acid | neg | 222.0406 | 2.379 | 4.810 | 4.838 | 0.028 | -0.107 | 0.554 | 0.823 | Tentative | NA | NA |
| metab_7033 | Unannotated feature | pos | 194.1180 | 4.783 | 4.596 | 4.707 | 0.111 | -0.107 | 0.554 | 0.823 | Unannotated | NA | NA |
| metab_8431 | 4-amino-4-deoxychorismate | pos | 243.0982 | 1.944 | 3.526 | 3.804 | 0.277 | 0.107 | 0.554 | 0.823 | Tentative | NA | NA |
| metab_19149 | Tiglic acid | neg | 99.0443 | 3.223 | 4.700 | 4.628 | -0.072 | -0.107 | 0.554 | 0.823 | Moderate putative | NA | NA |
| metab_12206 | Unannotated feature | neg | 623.4178 | 6.821 | 5.489 | 5.828 | 0.339 | 0.107 | 0.554 | 0.823 | Unannotated | NA | NA |
| metab_4174 | Gliadins | pos | 664.3343 | 5.906 | 4.010 | 4.226 | 0.215 | 0.107 | 0.554 | 0.823 | Unannotated | NA | NA |
| metab_15286 | Unannotated feature | neg | 189.0763 | 4.857 | 4.880 | 4.725 | -0.155 | 0.107 | 0.554 | 0.823 | Unannotated | NA | NA |
| metab_6422 | Unannotated feature | pos | 337.6653 | 5.705 | 5.022 | 4.631 | -0.391 | -0.107 | 0.554 | 0.823 | Unannotated | NA | NA |
| metab_13048 | Unannotated feature | neg | 364.8701 | 0.596 | 5.255 | 5.398 | 0.143 | 0.107 | 0.554 | 0.823 | Unannotated | NA | NA |
| metab_8184 | Unannotated feature | pos | 93.0704 | 2.571 | 4.554 | 4.518 | -0.037 | -0.107 | 0.554 | 0.823 | Unannotated | NA | NA |
| metab_16298 | Unannotated feature | neg | 314.9390 | 6.624 | 5.197 | 5.114 | -0.083 | -0.107 | 0.554 | 0.823 | Unannotated | NA | NA |
| metab_12840 | Unannotated feature | neg | 196.9922 | 0.675 | 5.103 | 4.800 | -0.304 | -0.107 | 0.554 | 0.823 | Unannotated | NA | NA |
| metab_3321 | Nona-3,5,7-trienedioylcarnitine | pos | 367.1873 | 1.952 | 3.919 | 3.943 | 0.024 | 0.107 | 0.554 | 0.823 | Unannotated | Lipids and lipid-like molecules | Fatty Acyls |
| metab_17577 | Unannotated feature | neg | 505.3367 | 7.288 | 4.517 | 4.482 | -0.035 | -0.107 | 0.554 | 0.823 | Unannotated | NA | NA |
| metab_19210 | N-(3-Amino-2-hydroxy-3-oxopropyl)-L-valine | neg | 467.2346 | 5.253 | 3.640 | 3.134 | -0.507 | -0.107 | 0.554 | 0.823 | Unannotated | NA | NA |
| metab_7823 | Unannotated feature | pos | 421.1695 | 3.312 | 3.985 | 3.796 | -0.190 | -0.107 | 0.554 | 0.823 | Unannotated | NA | NA |
| metab_16703 | Unannotated feature | neg | 604.8731 | 7.637 | 6.321 | 6.367 | 0.046 | 0.107 | 0.554 | 0.823 | Unannotated | NA | NA |
| metab_4801 | Unannotated feature | pos | 199.0448 | 7.997 | 3.517 | 3.556 | 0.039 | 0.107 | 0.554 | 0.823 | Unannotated | NA | NA |
| metab_8837 | Urobilinogen | pos | 623.3456 | 5.485 | 2.442 | 3.811 | 1.369 | 0.107 | 0.554 | 0.823 | Tentative | NA | NA |
| metab_8931 | Dihydrophaseic acid | neg | 281.1397 | 4.216 | 4.427 | 4.738 | 0.311 | 0.107 | 0.554 | 0.823 | Tentative | NA | NA |
| metab_8925 | Unannotated feature | neg | 280.9983 | 3.754 | 4.567 | 4.076 | -0.491 | -0.107 | 0.554 | 0.823 | Unannotated | NA | NA |
| metab_17914 | Unannotated feature | neg | 507.3694 | 6.648 | 6.756 | 6.830 | 0.074 | 0.107 | 0.554 | 0.823 | Unannotated | NA | NA |
| metab_10568 | Unannotated feature | neg | 267.0936 | 1.765 | 5.001 | 5.029 | 0.029 | -0.107 | 0.554 | 0.823 | Unannotated | NA | NA |
| metab_17879 | Unannotated feature | neg | 215.0558 | 2.766 | 5.828 | 5.874 | 0.047 | 0.107 | 0.554 | 0.823 | Unannotated | NA | NA |
| metab_4357 | Paromomycin I | pos | 633.3274 | 6.159 | 4.203 | 5.140 | 0.937 | 0.107 | 0.554 | 0.823 | Tentative | NA | NA |
| metab_14869 | Unannotated feature | neg | 356.1254 | 4.039 | 4.163 | 4.438 | 0.275 | 0.107 | 0.554 | 0.823 | Unannotated | NA | NA |
| metab_14867 | Unannotated feature | neg | 305.1064 | 4.036 | 4.278 | 4.472 | 0.194 | 0.107 | 0.554 | 0.823 | Unannotated | NA | NA |
| metab_4752 | Unannotated feature | pos | 305.9129 | 7.708 | 4.656 | 4.582 | -0.073 | -0.107 | 0.554 | 0.823 | Unannotated | NA | NA |
| metab_1224 | Unannotated feature | pos | 312.1600 | 6.398 | 4.256 | 4.214 | -0.041 | -0.107 | 0.554 | 0.823 | Unannotated | NA | NA |
| metab_16879 | Unannotated feature | neg | 232.0772 | 7.741 | 4.298 | 4.288 | -0.010 | 0.107 | 0.554 | 0.823 | Unannotated | NA | NA |
| metab_4566 | Unannotated feature | pos | 369.2983 | 6.885 | 5.593 | 5.446 | -0.147 | -0.107 | 0.554 | 0.823 | Unannotated | NA | NA |
| metab_10761 | Unannotated feature | neg | 230.0668 | 1.205 | 6.201 | 6.328 | 0.127 | 0.107 | 0.554 | 0.823 | Unannotated | NA | NA |
| metab_1253 | Unannotated feature | pos | 409.2571 | 6.359 | 5.404 | 5.085 | -0.319 | -0.107 | 0.554 | 0.823 | Unannotated | NA | NA |
| metab_286 | Alcophosphamide | pos | 261.0311 | 0.485 | 6.543 | 6.458 | -0.085 | -0.107 | 0.554 | 0.823 | Tentative | NA | NA |
| metab_11373 | Unannotated feature | neg | 240.9690 | 0.566 | 4.426 | 4.044 | -0.381 | -0.107 | 0.554 | 0.823 | Unannotated | NA | NA |
| metab_2402 | Unannotated feature | pos | 79.9902 | 0.593 | 4.417 | 4.390 | -0.027 | -0.107 | 0.554 | 0.823 | Unannotated | NA | NA |
| metab_17609 | Unannotated feature | neg | 160.9786 | 7.199 | 6.063 | 6.060 | -0.002 | -0.107 | 0.554 | 0.823 | Unannotated | NA | NA |
| metab_7215 | N-alpha-Acetyl-L-citrulline | pos | 259.1404 | 0.680 | 5.149 | 5.113 | -0.036 | -0.107 | 0.554 | 0.823 | Tentative | NA | NA |
| metab_12663 | Unannotated feature | neg | 654.3160 | 5.834 | 5.971 | 5.844 | -0.128 | -0.107 | 0.554 | 0.823 | Unannotated | NA | NA |
| metab_18191 | 5-Pyridoxolactone | neg | 210.0404 | 2.956 | 5.029 | 5.203 | 0.174 | 0.107 | 0.554 | 0.823 | Tentative | NA | NA |
| metab_10739 | N-(gamma-Glutamyl)ethanolamine | neg | 171.0769 | 1.258 | 4.146 | 4.051 | -0.095 | -0.107 | 0.554 | 0.823 | Unannotated | NA | NA |
| metab_18161 | Unannotated feature | neg | 346.1637 | 6.334 | 5.828 | 5.869 | 0.041 | 0.107 | 0.554 | 0.823 | Unannotated | NA | NA |
| metab_13950 | Unannotated feature | neg | 174.0224 | 2.617 | 3.675 | 3.752 | 0.077 | -0.107 | 0.554 | 0.823 | Unannotated | NA | NA |
| metab_14789 | Unannotated feature | neg | 362.8775 | 0.512 | 4.645 | 4.544 | -0.101 | -0.107 | 0.554 | 0.823 | Unannotated | NA | NA |
| metab_15008 | Unannotated feature | neg | 288.1088 | 4.347 | 4.532 | 4.537 | 0.005 | -0.107 | 0.554 | 0.823 | Unannotated | NA | NA |
| metab_5347 | Ent-16b,19-Kauranediol 19-acetate | pos | 366.2991 | 6.526 | 4.943 | 4.908 | -0.035 | -0.107 | 0.554 | 0.823 | Unannotated | NA | NA |
| metab_11937 | Unannotated feature | neg | 356.1760 | 6.123 | 4.341 | 4.273 | -0.068 | 0.107 | 0.554 | 0.823 | Unannotated | NA | NA |
| metab_10465 | Unannotated feature | neg | 201.0764 | 1.958 | 4.477 | 4.416 | -0.061 | 0.107 | 0.554 | 0.823 | Unannotated | NA | NA |
| metab_16759 | Unannotated feature | neg | 391.8974 | 7.687 | 4.745 | 4.747 | 0.001 | 0.107 | 0.554 | 0.823 | Unannotated | NA | NA |
| metab_5274 | Unannotated feature | pos | 577.4089 | 6.622 | 5.974 | 6.060 | 0.086 | 0.107 | 0.554 | 0.823 | Unannotated | NA | NA |
| metab_6523 | Unannotated feature | pos | 236.0089 | 0.601 | 3.446 | 2.471 | -0.975 | -0.107 | 0.554 | 0.823 | Unannotated | NA | NA |
| metab_2029 | Unannotated feature | pos | 460.2037 | 0.704 | 5.190 | 4.477 | -0.713 | -0.107 | 0.554 | 0.823 | Unannotated | NA | NA |
| metab_9382 | Tetramethylchromanol glucoside | neg | 403.1730 | 3.498 | 5.292 | 5.408 | 0.116 | 0.107 | 0.554 | 0.823 | Unannotated | NA | NA |
| metab_13926 | Unannotated feature | neg | 361.0936 | 3.872 | 5.124 | 5.529 | 0.405 | 0.107 | 0.554 | 0.823 | Unannotated | NA | NA |
| metab_17019 | Unannotated feature | neg | 249.9060 | 7.997 | 5.420 | 5.395 | -0.024 | -0.107 | 0.554 | 0.823 | Unannotated | NA | NA |
| metab_17018 | Unannotated feature | neg | 260.8985 | 7.997 | 4.378 | 4.350 | -0.028 | -0.107 | 0.554 | 0.823 | Unannotated | NA | NA |
| metab_2104 | Unannotated feature | pos | 256.0934 | 2.740 | 4.345 | 4.488 | 0.143 | 0.107 | 0.554 | 0.823 | Unannotated | NA | NA |
| metab_12621 | Unannotated feature | neg | 451.1297 | 6.007 | 3.981 | 3.708 | -0.273 | -0.107 | 0.554 | 0.823 | Unannotated | NA | NA |
| metab_3757 | Unannotated feature | pos | 516.2545 | 6.470 | 5.710 | 5.612 | -0.098 | 0.107 | 0.554 | 0.823 | Unannotated | NA | NA |
| metab_2231 | Unannotated feature | pos | 115.0759 | 3.077 | 4.034 | 4.373 | 0.338 | -0.107 | 0.554 | 0.823 | Unannotated | NA | NA |
| metab_2225 | 3-(6-methyl-2-oxo-1H-quinolin-3-yl)propanoic acid | pos | 249.1237 | 3.058 | 4.787 | 4.764 | -0.023 | -0.107 | 0.554 | 0.823 | Moderate putative | NA | NA |
| metab_12742 | Unannotated feature | neg | 349.9761 | 3.021 | 4.813 | 4.606 | -0.207 | -0.107 | 0.554 | 0.823 | Unannotated | NA | NA |
| metab_2940 | 2',3'-Dideoxyadenosine | pos | 218.1026 | 2.937 | 4.641 | 4.762 | 0.121 | 0.107 | 0.554 | 0.823 | Unannotated | NA | NA |
| metab_17297 | Unannotated feature | neg | 356.9240 | 7.710 | 5.090 | 5.101 | 0.011 | -0.107 | 0.554 | 0.823 | Unannotated | NA | NA |
| metab_19005 | Unannotated feature | neg | 283.0687 | 2.421 | 5.887 | 5.985 | 0.098 | 0.107 | 0.554 | 0.823 | Unannotated | NA | NA |
| metab_12031 | Unannotated feature | neg | 441.2862 | 6.295 | 6.571 | 6.556 | -0.015 | -0.107 | 0.554 | 0.823 | Unannotated | NA | NA |
| metab_19838 | 1-Pyrroline-5-carboxylic acid | neg | 285.1090 | 2.771 | 4.296 | 4.398 | 0.101 | 0.107 | 0.554 | 0.823 | Tentative | NA | NA |
| metab_12008 | MG(0:0/20:3(5Z,8Z,11Z)/0:0) | neg | 819.5942 | 6.256 | 4.821 | 4.669 | -0.152 | -0.107 | 0.554 | 0.823 | Unannotated | NA | NA |
| metab_19843 | 4-Hydroxyphenylacetic Acid | neg | 151.0394 | 3.754 | 5.139 | 4.930 | -0.209 | -0.107 | 0.554 | 0.823 | Moderate putative | NA | NA |
| metab_19804 | Unannotated feature | neg | 175.0969 | 4.424 | 3.335 | 3.729 | 0.393 | -0.103 | 0.570 | 0.830 | Unannotated | NA | NA |
| metab_19868 | Genistein | neg | 269.0457 | 6.123 | 4.869 | 4.481 | -0.388 | -0.103 | 0.570 | 0.830 | Strong putative | NA | NA |
| metab_14699 | Cis-Ferulic acid | neg | 239.0559 | 3.701 | 4.961 | 4.974 | 0.013 | 0.103 | 0.570 | 0.830 | Unannotated | NA | NA |
| metab_4432 | Unannotated feature | pos | 281.1387 | 6.359 | 5.502 | 5.429 | -0.073 | -0.103 | 0.570 | 0.830 | Unannotated | NA | NA |
| metab_19866 | Estradiol | neg | 317.1759 | 6.326 | 3.983 | 4.698 | 0.715 | 0.103 | 0.570 | 0.830 | Tentative | NA | NA |
| metab_25 | Unannotated feature | pos | 190.1440 | 0.712 | 3.749 | 4.222 | 0.473 | 0.103 | 0.570 | 0.830 | Unannotated | NA | NA |
| metab_12001 | Unannotated feature | neg | 451.2707 | 6.240 | 6.502 | 6.432 | -0.070 | -0.103 | 0.570 | 0.830 | Unannotated | NA | NA |
| metab_11984 | Unannotated feature | neg | 215.1284 | 6.201 | 5.372 | 5.293 | -0.078 | -0.103 | 0.570 | 0.830 | Unannotated | NA | NA |
| metab_15578 | Unannotated feature | neg | 379.1765 | 5.453 | 3.768 | 3.981 | 0.213 | 0.103 | 0.570 | 0.830 | Unannotated | NA | NA |
| metab_12244 | Unannotated feature | neg | 808.4879 | 6.959 | 5.595 | 5.410 | -0.185 | -0.103 | 0.570 | 0.830 | Unannotated | NA | NA |
| metab_541 | Glu Pro Arg | pos | 401.2159 | 5.515 | 3.947 | 4.175 | 0.227 | 0.103 | 0.570 | 0.830 | Moderate putative | NA | NA |
| metab_1899 | Unannotated feature | pos | 241.1550 | 1.510 | 5.918 | 5.663 | -0.254 | -0.103 | 0.570 | 0.830 | Unannotated | NA | NA |
| metab_1929 | Unannotated feature | pos | 226.0690 | 0.728 | 5.894 | 5.878 | -0.016 | 0.103 | 0.570 | 0.830 | Unannotated | NA | NA |
| metab_4415 | 4-ethylamino-6-isopropylamino-1,3,5-triazin-2-ol | pos | 412.2856 | 6.327 | 4.328 | 4.038 | -0.290 | -0.103 | 0.570 | 0.830 | Tentative | NA | NA |
| metab_9135 | Leucopelargonidin | neg | 335.0778 | 3.875 | 3.887 | 3.903 | 0.016 | -0.103 | 0.570 | 0.830 | Tentative | NA | NA |
| metab_14053 | Unannotated feature | neg | 238.9677 | 2.780 | 4.389 | 4.385 | -0.004 | -0.103 | 0.570 | 0.830 | Unannotated | NA | NA |
| metab_19683 | Unannotated feature | neg | 290.1248 | 4.592 | 4.617 | 4.212 | -0.404 | -0.103 | 0.570 | 0.830 | Unannotated | NA | NA |
| metab_10726 | 3-Oxoadipic acid | neg | 205.0349 | 1.308 | 5.657 | 5.537 | -0.120 | -0.103 | 0.570 | 0.830 | Tentative | NA | NA |
| metab_15552 | Unannotated feature | neg | 289.1116 | 5.409 | 4.136 | 4.501 | 0.365 | 0.103 | 0.570 | 0.830 | Unannotated | NA | NA |
| metab_15549 | Unannotated feature | neg | 321.2029 | 5.394 | 3.766 | 3.811 | 0.046 | -0.103 | 0.570 | 0.830 | Unannotated | NA | NA |
| metab_17798 | Unannotated feature | neg | 479.2823 | 6.853 | 5.185 | 5.037 | -0.148 | -0.103 | 0.570 | 0.830 | Unannotated | NA | NA |
| metab_17748 | Unannotated feature | neg | 388.2531 | 6.915 | 5.121 | 5.192 | 0.071 | -0.103 | 0.570 | 0.830 | Unannotated | NA | NA |
| metab_844 | Unannotated feature | pos | 427.2467 | 6.526 | 4.124 | 4.008 | -0.115 | -0.103 | 0.570 | 0.830 | Unannotated | NA | NA |
| metab_9379 | Acetyl-L-tyrosine | neg | 222.0770 | 3.975 | 4.668 | 4.465 | -0.202 | -0.103 | 0.570 | 0.830 | Moderate putative | NA | NA |
| metab_3960 | 3-Methyldioxyindole | pos | 196.0972 | 5.791 | 4.510 | 4.426 | -0.084 | -0.103 | 0.570 | 0.830 | Tentative | Organoheterocyclic compounds | Indoles and derivatives |
| metab_1163 | Unannotated feature | pos | 613.3018 | 6.526 | 6.841 | 6.674 | -0.167 | -0.103 | 0.570 | 0.830 | Unannotated | NA | NA |
| metab_18591 | Unannotated feature | neg | 322.1168 | 5.999 | 5.045 | 4.976 | -0.069 | -0.103 | 0.570 | 0.830 | Unannotated | NA | NA |
| metab_12592 | Unannotated feature | neg | 444.2611 | 6.091 | 4.633 | 4.093 | -0.541 | -0.103 | 0.570 | 0.830 | Unannotated | NA | NA |
| metab_17766 | Terephthalic Acid | neg | 165.0186 | 6.899 | 5.781 | 5.737 | -0.044 | -0.103 | 0.570 | 0.830 | Strong putative | NA | NA |
| metab_11898 | Unannotated feature | neg | 555.2639 | 6.046 | 4.690 | 4.505 | -0.186 | 0.103 | 0.570 | 0.830 | Unannotated | NA | NA |
| metab_2606 | L-Isoleucine | pos | 132.1022 | 1.664 | 8.187 | 8.072 | -0.115 | -0.103 | 0.570 | 0.830 | Moderate putative | NA | NA |
| metab_17205 | Unannotated feature | neg | 168.0409 | 2.442 | 4.198 | 4.193 | -0.005 | 0.103 | 0.570 | 0.830 | Unannotated | NA | NA |
| metab_1562 | Unannotated feature | pos | 918.5088 | 5.267 | 3.768 | 4.204 | 0.436 | 0.103 | 0.570 | 0.830 | Unannotated | NA | NA |
| metab_12461 | Unannotated feature | neg | 215.8682 | 7.888 | 6.054 | 6.043 | -0.011 | -0.103 | 0.570 | 0.830 | Unannotated | NA | NA |
| metab_18541 | Unannotated feature | neg | 673.2931 | 6.022 | 3.758 | 4.202 | 0.444 | 0.103 | 0.570 | 0.830 | Unannotated | NA | NA |
| metab_1916 | Unannotated feature | pos | 251.0643 | 1.692 | 4.246 | 4.394 | 0.148 | -0.103 | 0.570 | 0.830 | Unannotated | NA | NA |
| metab_13141 | Unannotated feature | neg | 195.8547 | 0.457 | 4.249 | 4.068 | -0.182 | -0.103 | 0.570 | 0.830 | Unannotated | NA | NA |
| metab_11317 | Unannotated feature | neg | 374.8109 | 0.603 | 3.838 | 3.794 | -0.044 | -0.103 | 0.570 | 0.830 | Unannotated | NA | NA |
| metab_13103 | Unannotated feature | neg | 159.8780 | 0.108 | 5.418 | 5.403 | -0.015 | -0.103 | 0.570 | 0.830 | Unannotated | NA | NA |
| metab_10480 | Unannotated feature | neg | 300.0729 | 1.952 | 3.479 | 3.907 | 0.428 | 0.103 | 0.570 | 0.830 | Unannotated | NA | NA |
| metab_14248 | Unannotated feature | neg | 242.9400 | 0.524 | 6.765 | 6.736 | -0.029 | -0.103 | 0.570 | 0.830 | Unannotated | NA | NA |
| metab_16925 | Unannotated feature | neg | 340.8998 | 7.797 | 4.559 | 4.528 | -0.031 | -0.103 | 0.570 | 0.830 | Unannotated | NA | NA |
| metab_3741 | 6-Hydroxymelatonin glucuronide | pos | 457.1843 | 5.409 | 4.855 | 4.379 | -0.476 | -0.103 | 0.570 | 0.830 | Unannotated | NA | NA |
| metab_15829 | Unannotated feature | neg | 415.1412 | 5.901 | 4.165 | 4.055 | -0.110 | -0.103 | 0.570 | 0.830 | Unannotated | NA | NA |
| metab_18081 | Ganolucidic acid A | neg | 481.2998 | 6.404 | 7.062 | 7.018 | -0.044 | -0.103 | 0.570 | 0.830 | Unannotated | NA | NA |
| metab_19426 | Unannotated feature | neg | 327.9909 | 3.210 | 5.763 | 5.690 | -0.073 | -0.103 | 0.570 | 0.830 | Unannotated | NA | NA |
| metab_451 | MJDBISSP00000001 | pos | 200.0478 | 3.007 | 7.567 | 7.552 | -0.014 | 0.103 | 0.570 | 0.830 | Strong putative | NA | NA |
| metab_17962 | Unannotated feature | neg | 483.2429 | 6.561 | 6.272 | 6.094 | -0.178 | -0.103 | 0.570 | 0.830 | Unannotated | NA | NA |
| metab_13441 | 2,3-Butanediol apiosylglucoside | neg | 419.1317 | 0.829 | 4.935 | 4.836 | -0.099 | 0.103 | 0.570 | 0.830 | Unannotated | NA | NA |
| metab_2584 | Gamma-Glutamylarginine | pos | 268.1407 | 1.274 | 4.339 | 4.215 | -0.124 | -0.103 | 0.570 | 0.830 | Unannotated | NA | NA |
| metab_9791 | Beta-D-ribosylnicotinate | neg | 254.0671 | 3.045 | 6.332 | 6.343 | 0.010 | -0.103 | 0.570 | 0.830 | Tentative | NA | NA |
| metab_479 | 4-Hydroxypropofol | pos | 411.2518 | 6.367 | 6.017 | 5.862 | -0.154 | -0.103 | 0.570 | 0.830 | Unannotated | NA | NA |
| metab_18450 | Unannotated feature | neg | 443.1752 | 6.091 | 5.571 | 5.530 | -0.040 | 0.103 | 0.570 | 0.830 | Unannotated | NA | NA |
| metab_9695 | Unannotated feature | neg | 229.1192 | 3.131 | 4.032 | 4.201 | 0.169 | 0.103 | 0.570 | 0.830 | Unannotated | NA | NA |
| metab_9694 | Aminoglutethimide | neg | 277.1198 | 3.131 | 3.412 | 3.637 | 0.225 | 0.103 | 0.570 | 0.830 | Tentative | NA | NA |
| metab_18828 | Alpha-Muramic acid | neg | 250.0931 | 5.753 | 4.732 | 4.613 | -0.118 | -0.103 | 0.570 | 0.830 | Unannotated | NA | NA |
| metab_8521 | Piperidine | pos | 86.0970 | 1.521 | 7.195 | 7.092 | -0.103 | -0.103 | 0.570 | 0.830 | Moderate putative | NA | NA |
| metab_1880 | Gamma-Glutamylhistidine | pos | 285.1197 | 1.259 | 5.621 | 5.661 | 0.040 | 0.103 | 0.570 | 0.830 | Unannotated | NA | NA |
| metab_9411 | S-(2-Methylbutanoyl)-dihydrolipoamide | neg | 326.0994 | 3.457 | 3.833 | 4.133 | 0.300 | 0.103 | 0.570 | 0.830 | Tentative | NA | NA |
| metab_7908 | 3-Methylsuberic acid | pos | 230.1391 | 2.951 | 3.701 | 3.965 | 0.263 | 0.103 | 0.570 | 0.830 | Unannotated | NA | NA |
| metab_7879 | Unannotated feature | pos | 206.0451 | 2.997 | 4.040 | 3.918 | -0.122 | 0.103 | 0.570 | 0.830 | Unannotated | NA | NA |
| metab_12053 | Unannotated feature | neg | 385.2058 | 6.373 | 5.519 | 5.598 | 0.079 | 0.103 | 0.570 | 0.830 | Unannotated | NA | NA |
| metab_19718 | Allylisopropylacetamide | neg | 186.1130 | 4.530 | 5.779 | 5.717 | -0.062 | -0.103 | 0.570 | 0.830 | Unannotated | NA | NA |
| metab_19702 | Unannotated feature | neg | 407.1354 | 4.554 | 4.088 | 4.019 | -0.068 | -0.103 | 0.570 | 0.830 | Unannotated | NA | NA |
| metab_13898 | 7-Methylxanthine | neg | 165.0412 | 2.472 | 5.554 | 5.507 | -0.047 | -0.103 | 0.570 | 0.830 | Tentative | Organoheterocyclic compounds | Imidazopyrimidines |
| metab_13889 | Unannotated feature | neg | 208.0610 | 2.430 | 4.427 | 4.365 | -0.061 | 0.103 | 0.570 | 0.830 | Unannotated | NA | NA |
| metab_6478 | Unannotated feature | pos | 96.9223 | 0.601 | 3.332 | 3.143 | -0.190 | -0.103 | 0.570 | 0.830 | Unannotated | NA | NA |
| metab_9371 | 4-Allylpyrocatechol | neg | 195.0658 | 3.512 | 4.407 | 4.525 | 0.119 | 0.103 | 0.570 | 0.830 | Strong putative | NA | NA |
| metab_19222 | Batatasin I | neg | 329.1036 | 5.233 | 5.848 | 5.718 | -0.130 | -0.103 | 0.570 | 0.830 | Tentative | NA | NA |
| metab_19215 | 2-Naphthylaminomethyl-gamma-aminobutyric acid | neg | 303.1357 | 5.242 | 5.695 | 5.696 | 0.001 | -0.103 | 0.570 | 0.830 | Unannotated | Organic acids and derivatives | Carboxylic acids and derivatives |
| metab_1120 | Unannotated feature | pos | 641.3839 | 6.614 | 5.030 | 5.074 | 0.044 | 0.103 | 0.570 | 0.830 | Unannotated | NA | NA |
| metab_13167 | Unannotated feature | neg | 746.8602 | 0.485 | 4.907 | 4.749 | -0.158 | -0.103 | 0.570 | 0.830 | Unannotated | NA | NA |
| metab_17671 | 5alpha-Androstane-3beta,17beta-diol | neg | 629.4809 | 7.044 | 5.993 | 5.749 | -0.244 | -0.103 | 0.570 | 0.830 | Tentative | NA | NA |
| metab_1981 | Unannotated feature | pos | 271.0818 | 1.952 | 4.731 | 4.538 | -0.193 | -0.103 | 0.570 | 0.830 | Unannotated | NA | NA |
| metab_17875 | Unannotated feature | neg | 529.3057 | 6.703 | 5.328 | 5.237 | -0.091 | 0.103 | 0.570 | 0.830 | Unannotated | NA | NA |
| metab_9294 | Unannotated feature | neg | 245.0932 | 3.620 | 5.316 | 5.372 | 0.056 | -0.103 | 0.570 | 0.830 | Unannotated | NA | NA |
| metab_13723 | Unannotated feature | neg | 378.0858 | 1.919 | 3.168 | 3.562 | 0.395 | 0.103 | 0.570 | 0.830 | Unannotated | NA | NA |
| metab_13431 | Unannotated feature | neg | 179.0014 | 0.804 | 4.593 | 4.544 | -0.049 | -0.103 | 0.570 | 0.830 | Unannotated | NA | NA |
| metab_2661 | Unannotated feature | pos | 190.1441 | 1.952 | 4.371 | 4.255 | -0.115 | 0.103 | 0.570 | 0.830 | Unannotated | NA | NA |
| metab_10918 | 3-Methylcrotonylglycine | neg | 202.0717 | 0.829 | 7.021 | 6.484 | -0.537 | -0.103 | 0.570 | 0.830 | Tentative | NA | NA |
| metab_16872 | Unannotated feature | neg | 408.8470 | 7.737 | 4.371 | 4.347 | -0.024 | -0.103 | 0.570 | 0.830 | Unannotated | NA | NA |
| metab_13753 | 2-Oxo-4-methylthiobutanoic acid | neg | 193.0171 | 1.952 | 4.113 | 4.290 | 0.177 | 0.103 | 0.570 | 0.830 | Tentative | NA | NA |
| metab_2631 | 1-[3-(Trifluoromethyl)phenyl]piperazine | pos | 275.0757 | 1.774 | 4.669 | 4.159 | -0.510 | -0.103 | 0.570 | 0.830 | Unannotated | NA | NA |
| metab_12185 | Unannotated feature | neg | 321.2105 | 6.742 | 5.555 | 5.582 | 0.027 | 0.103 | 0.570 | 0.830 | Unannotated | NA | NA |
| metab_12175 | Unannotated feature | neg | 697.5401 | 6.711 | 4.081 | 4.291 | 0.209 | 0.103 | 0.570 | 0.830 | Unannotated | NA | NA |
| metab_4298 | Unannotated feature | pos | 447.2931 | 6.390 | 6.185 | 6.116 | -0.069 | -0.103 | 0.570 | 0.830 | Unannotated | NA | NA |
| metab_16772 | Unannotated feature | neg | 430.9274 | 7.692 | 5.732 | 5.744 | 0.011 | 0.103 | 0.570 | 0.830 | Unannotated | NA | NA |
| metab_4551 | DG(16:0/PGF2alpha/0:0) | pos | 689.4978 | 6.821 | 6.266 | 6.004 | -0.262 | -0.103 | 0.570 | 0.830 | Unannotated | NA | NA |
| metab_10780 | Unannotated feature | neg | 184.0973 | 4.749 | 5.449 | 5.269 | -0.180 | -0.103 | 0.570 | 0.830 | Unannotated | NA | NA |
| metab_18142 | Unannotated feature | neg | 355.2492 | 6.357 | 5.666 | 5.836 | 0.170 | 0.103 | 0.570 | 0.830 | Unannotated | NA | NA |
| metab_1745 | 5-Hydroxy-6-methoxy-1h-indole-2-carboxylic acid | pos | 208.0608 | 3.602 | 5.163 | 5.061 | -0.102 | -0.103 | 0.570 | 0.830 | Unannotated | NA | NA |
| metab_6037 | L-Pipecolic acid | pos | 147.1130 | 0.501 | 6.553 | 6.463 | -0.090 | -0.103 | 0.570 | 0.830 | Moderate putative | NA | NA |
| metab_1215 | Unannotated feature | pos | 443.3165 | 6.406 | 5.325 | 5.304 | -0.022 | -0.103 | 0.570 | 0.830 | Unannotated | NA | NA |
| metab_11690 | Pentaleno(1,6a-c)pyran-5-carboxylic acid, 1-(chloromethyl)-1,2,4,4a,6a,7-hexahydro-1-hydroxy-7,8-dimethyl-2-oxo-, (1S-(1alpha,4aalpha,6abeta,7alpha,9aR*))- | neg | 311.0675 | 5.420 | 2.752 | 2.521 | -0.231 | 0.103 | 0.570 | 0.830 | Unannotated | Lipids and lipid-like molecules | Prenol lipids |
| metab_11689 | Unannotated feature | neg | 607.3156 | 5.420 | 4.640 | 4.874 | 0.234 | 0.103 | 0.570 | 0.830 | Unannotated | NA | NA |
| metab_17122 | Unannotated feature | neg | 155.8735 | 7.827 | 4.667 | 4.650 | -0.017 | -0.103 | 0.570 | 0.830 | Unannotated | NA | NA |
| metab_12617 | Capsianoside IV | neg | 643.3366 | 6.007 | 4.907 | 5.339 | 0.433 | 0.103 | 0.570 | 0.830 | Unannotated | NA | NA |
| metab_746 | Unannotated feature | pos | 315.0980 | 6.048 | 3.775 | 3.743 | -0.033 | 0.103 | 0.570 | 0.830 | Unannotated | NA | NA |
| metab_8324 | Unannotated feature | pos | 273.1450 | 2.012 | 4.441 | 4.993 | 0.552 | 0.103 | 0.570 | 0.830 | Unannotated | NA | NA |
| metab_8296 | Unannotated feature | pos | 285.0836 | 2.079 | 4.017 | 3.343 | -0.674 | -0.103 | 0.570 | 0.830 | Unannotated | NA | NA |
| metab_13368 | Dihydro-3-coumaric acid | neg | 165.0551 | 4.781 | 6.908 | 6.781 | -0.128 | -0.103 | 0.570 | 0.830 | Strong putative | NA | NA |
| metab_10214 | Unannotated feature | neg | 295.0250 | 2.629 | 2.722 | 2.786 | 0.063 | -0.103 | 0.570 | 0.830 | Unannotated | NA | NA |
| metab_10058 | Gamma-Glutamyltyrosine | neg | 309.1097 | 2.785 | 5.179 | 5.281 | 0.102 | -0.103 | 0.570 | 0.830 | Strong putative | NA | NA |
| metab_3338 | 4-Nitrosodiphenylamine | pos | 199.0870 | 4.198 | 2.141 | 2.192 | 0.051 | 0.103 | 0.570 | 0.830 | Moderate putative | NA | NA |
| metab_832 | 5-[(Z)-14-(3,5-dihydroxyphenyl)tetradec-10-enyl]benzene-1,3-Diol | pos | 454.2940 | 6.446 | 6.346 | 6.097 | -0.248 | -0.103 | 0.570 | 0.830 | Moderate putative | NA | NA |
| metab_429 | Unannotated feature | pos | 108.0233 | 0.016 | 3.765 | 3.753 | -0.012 | -0.103 | 0.570 | 0.830 | Unannotated | NA | NA |
| metab_5204 | Unannotated feature | pos | 302.2457 | 6.749 | 6.086 | 6.043 | -0.043 | -0.103 | 0.570 | 0.830 | Unannotated | NA | NA |
| metab_5192 | Unannotated feature | pos | 459.3095 | 6.781 | 6.728 | 6.766 | 0.038 | 0.103 | 0.570 | 0.830 | Unannotated | NA | NA |
| metab_19399 | Unannotated feature | neg | 344.9810 | 3.210 | 5.431 | 5.360 | -0.071 | -0.103 | 0.570 | 0.830 | Unannotated | NA | NA |
| metab_19376 | Homovanillic Acid | neg | 181.0500 | 3.434 | 6.089 | 5.827 | -0.261 | -0.103 | 0.570 | 0.830 | Strong putative | Benzenoids | Phenols |
| metab_15236 | Phenacyl | neg | 119.0495 | 4.785 | 5.338 | 5.151 | -0.187 | -0.103 | 0.570 | 0.830 | Unannotated | NA | NA |
| metab_1198 | Unannotated feature | pos | 444.3699 | 6.462 | 5.083 | 4.938 | -0.144 | -0.103 | 0.570 | 0.830 | Unannotated | NA | NA |
| metab_12392 | Unannotated feature | neg | 476.2641 | 6.561 | 7.067 | 7.024 | -0.042 | -0.103 | 0.570 | 0.830 | Unannotated | NA | NA |
| metab_3227 | 1-Nitro-5,6-dihydroxy-dihydronaphthalene | pos | 240.0871 | 3.795 | 4.487 | 4.155 | -0.332 | -0.103 | 0.570 | 0.830 | Tentative | NA | NA |
| metab_18301 | Hericene A | neg | 593.3603 | 6.209 | 3.225 | 3.921 | 0.696 | 0.103 | 0.570 | 0.830 | Unannotated | NA | NA |
| metab_18297 | Unannotated feature | neg | 368.1902 | 6.217 | 4.603 | 4.601 | -0.001 | -0.103 | 0.570 | 0.830 | Unannotated | NA | NA |
| metab_18240 | Unannotated feature | neg | 319.1042 | 2.754 | 4.255 | 4.426 | 0.171 | 0.103 | 0.570 | 0.830 | Unannotated | NA | NA |
| metab_17144 | Unannotated feature | neg | 255.9221 | 7.785 | 4.601 | 4.565 | -0.036 | -0.103 | 0.570 | 0.830 | Unannotated | NA | NA |
| metab_17107 | Unannotated feature | neg | 162.8384 | 7.861 | 6.136 | 6.137 | 0.000 | 0.103 | 0.570 | 0.830 | Unannotated | NA | NA |
| metab_3386 | (-)-Jasmonic acid | pos | 211.1332 | 4.355 | 5.006 | 5.012 | 0.006 | 0.103 | 0.570 | 0.830 | Moderate putative | NA | NA |
| metab_16591 | Unannotated feature | neg | 198.8315 | 7.387 | 3.284 | 3.283 | -0.001 | 0.103 | 0.570 | 0.830 | Unannotated | NA | NA |
| metab_14469 | Unannotated feature | neg | 161.0813 | 3.306 | 5.041 | 4.970 | -0.071 | -0.103 | 0.570 | 0.830 | Unannotated | NA | NA |
| metab_8547 | Unannotated feature | pos | 90.5263 | 1.302 | 5.311 | 5.348 | 0.036 | 0.103 | 0.570 | 0.830 | Unannotated | NA | NA |
| metab_17917 | DG(18:3(9Z,12Z,15Z)/14:1(9Z)/0:0) | neg | 605.4434 | 6.640 | 5.822 | 5.403 | -0.419 | -0.103 | 0.570 | 0.830 | Tentative | NA | NA |
| metab_19682 | 2-S-glutathionyl acetate | neg | 386.0633 | 4.592 | 4.438 | 3.841 | -0.597 | -0.103 | 0.570 | 0.830 | Tentative | NA | NA |
| metab_11912 | 7-Hydroxy-2',4',5'-trimethoxyisoflavan | neg | 315.1243 | 5.122 | 6.203 | 6.209 | 0.006 | 0.103 | 0.570 | 0.830 | Unannotated | NA | NA |
| metab_5263 | Momordicoside L | pos | 599.3927 | 6.638 | 4.550 | 4.773 | 0.222 | 0.103 | 0.570 | 0.830 | Unannotated | NA | NA |
| metab_17924 | 4-Decan-4-Ylbenzenesulfonic Acid | neg | 297.1530 | 6.640 | 6.751 | 6.725 | -0.027 | -0.103 | 0.570 | 0.830 | Moderate putative | NA | NA |
| metab_2070 | Unannotated feature | pos | 476.2359 | 2.732 | 4.384 | 4.129 | -0.255 | -0.103 | 0.570 | 0.830 | Unannotated | NA | NA |
| metab_18465 | 3-Carboxy-4-methyl-5-ethyl-2-furanpropionic acid | neg | 225.0766 | 3.050 | 4.951 | 4.978 | 0.027 | -0.103 | 0.570 | 0.830 | Unannotated | Lipids and lipid-like molecules | Fatty Acyls |
| metab_1267 | Unannotated feature | pos | 438.3222 | 6.319 | 4.806 | 4.958 | 0.152 | 0.103 | 0.570 | 0.830 | Unannotated | NA | NA |
| metab_8204 | N-Acetylmuramate | pos | 276.1083 | 2.495 | 4.936 | 5.242 | 0.306 | 0.103 | 0.570 | 0.830 | Strong putative | NA | NA |
| metab_12844 | Unannotated feature | neg | 110.9751 | 0.675 | 4.470 | 4.504 | 0.033 | 0.103 | 0.570 | 0.830 | Unannotated | NA | NA |
| metab_2436 | Unannotated feature | pos | 154.0266 | 0.633 | 4.253 | 4.507 | 0.253 | 0.103 | 0.570 | 0.830 | Unannotated | NA | NA |
| metab_13279 | Unannotated feature | neg | 155.0649 | 0.619 | 4.278 | 4.175 | -0.104 | -0.103 | 0.570 | 0.830 | Unannotated | NA | NA |
| metab_17502 | Unannotated feature | neg | 160.9175 | 7.499 | 4.449 | 4.486 | 0.037 | 0.103 | 0.570 | 0.830 | Unannotated | NA | NA |
| metab_6128 | Unannotated feature | pos | 593.3350 | 5.664 | 7.392 | 7.573 | 0.181 | 0.103 | 0.570 | 0.830 | Unannotated | NA | NA |
| metab_7306 | Naloxone | pos | 360.1816 | 4.184 | 4.398 | 4.403 | 0.005 | 0.103 | 0.570 | 0.830 | Tentative | NA | NA |
| metab_15334 | 2-succinylbenzoate | neg | 221.0452 | 4.952 | 2.772 | 2.773 | 0.002 | -0.103 | 0.570 | 0.830 | Tentative | NA | NA |
| metab_16497 | Unannotated feature | neg | 739.5144 | 7.084 | 6.213 | 6.071 | -0.141 | -0.103 | 0.570 | 0.830 | Unannotated | NA | NA |
| metab_3625 | Unannotated feature | pos | 559.3985 | 6.670 | 6.645 | 6.686 | 0.041 | 0.103 | 0.570 | 0.830 | Unannotated | NA | NA |
| metab_19170 | Unannotated feature | neg | 567.3168 | 5.304 | 4.737 | 4.860 | 0.123 | 0.103 | 0.570 | 0.830 | Unannotated | NA | NA |
| metab_19162 | Unannotated feature | neg | 280.1038 | 5.317 | 3.815 | 3.971 | 0.156 | 0.103 | 0.570 | 0.830 | Unannotated | NA | NA |
| metab_15291 | Unannotated feature | neg | 391.1408 | 4.861 | 4.274 | 4.221 | -0.053 | -0.103 | 0.570 | 0.830 | Unannotated | NA | NA |
| metab_2835 | Unannotated feature | pos | 239.1395 | 2.740 | 4.459 | 4.751 | 0.292 | 0.103 | 0.570 | 0.830 | Unannotated | NA | NA |
| metab_11492 | Unannotated feature | neg | 379.9311 | 0.516 | 3.247 | 3.282 | 0.035 | -0.103 | 0.570 | 0.830 | Unannotated | NA | NA |
| metab_13669 | Unannotated feature | neg | 164.8354 | 0.075 | 5.454 | 5.450 | -0.003 | -0.103 | 0.570 | 0.830 | Unannotated | NA | NA |
| metab_9696 | 1,2-Dihydroxy-3,4-epoxy-1,2,3,4-tetrahydronaphthalene | neg | 223.0610 | 3.131 | 4.954 | 4.953 | -0.001 | -0.103 | 0.570 | 0.830 | Tentative | Benzenoids | Tetralins |
| metab_5007 | Unannotated feature | pos | 455.4100 | 7.411 | 5.492 | 5.521 | 0.028 | 0.103 | 0.570 | 0.830 | Unannotated | NA | NA |
| metab_8897 | Unannotated feature | neg | 356.1255 | 4.270 | 4.476 | 4.567 | 0.090 | 0.103 | 0.570 | 0.830 | Unannotated | NA | NA |
| metab_4604 | Unannotated feature | pos | 441.2442 | 7.052 | 6.983 | 6.959 | -0.025 | -0.103 | 0.570 | 0.830 | Unannotated | NA | NA |
| metab_840 | Unannotated feature | pos | 589.3728 | 6.510 | 5.359 | 5.695 | 0.336 | 0.103 | 0.570 | 0.830 | Unannotated | NA | NA |
| metab_6940 | 4-Hydroxyquinoline | pos | 146.0603 | 4.965 | 4.887 | 5.083 | 0.197 | 0.103 | 0.570 | 0.830 | Strong putative | NA | NA |
| metab_10407 | Deoxyguanosine | neg | 266.0897 | 2.036 | 5.999 | 5.855 | -0.143 | -0.103 | 0.570 | 0.830 | Strong putative | NA | NA |
| metab_19864 | Enalaprilat | neg | 347.1614 | 5.748 | 5.510 | 5.542 | 0.032 | -0.103 | 0.570 | 0.830 | Tentative | Organic acids and derivatives | Carboxylic acids and derivatives |
| metab_12018 | Unannotated feature | neg | 607.2967 | 6.271 | 4.541 | 4.661 | 0.119 | 0.103 | 0.570 | 0.830 | Unannotated | NA | NA |
| metab_47 | Unannotated feature | pos | 117.0745 | 0.688 | 5.352 | 5.425 | 0.073 | 0.103 | 0.570 | 0.830 | Unannotated | NA | NA |
| metab_17268 | Unannotated feature | neg | 528.8459 | 7.720 | 4.601 | 4.497 | -0.104 | -0.103 | 0.570 | 0.830 | Unannotated | NA | NA |
| metab_9886 | Unannotated feature | neg | 141.0186 | 2.964 | 3.672 | 3.751 | 0.079 | 0.103 | 0.570 | 0.830 | Unannotated | NA | NA |
| metab_2890 | Unannotated feature | pos | 449.3277 | 6.143 | 4.893 | 4.518 | -0.375 | -0.103 | 0.570 | 0.830 | Unannotated | NA | NA |
| metab_17315 | Unannotated feature | neg | 284.9247 | 7.702 | 4.328 | 4.271 | -0.057 | -0.103 | 0.570 | 0.830 | Unannotated | NA | NA |
| metab_8248 | Unannotated feature | pos | 163.1120 | 2.293 | 3.965 | 4.023 | 0.058 | -0.103 | 0.570 | 0.830 | Unannotated | NA | NA |
| metab_8274 | Phe Thr | pos | 267.1337 | 2.174 | 3.865 | 3.808 | -0.057 | -0.103 | 0.570 | 0.830 | Unannotated | NA | NA |
| metab_393 | Unannotated feature | pos | 220.0271 | 0.019 | 4.078 | 4.085 | 0.007 | 0.099 | 0.586 | 0.839 | Unannotated | NA | NA |
| metab_13623 | Unannotated feature | neg | 419.0951 | 1.526 | 5.410 | 5.245 | -0.165 | -0.099 | 0.586 | 0.839 | Unannotated | NA | NA |
| metab_12736 | Parasorbic acid | neg | 269.1033 | 3.917 | 5.220 | 5.146 | -0.073 | -0.099 | 0.586 | 0.839 | Tentative | NA | NA |
| metab_13885 | Unannotated feature | neg | 200.9042 | 2.383 | 3.296 | 3.409 | 0.113 | 0.099 | 0.586 | 0.839 | Unannotated | NA | NA |
| metab_14199 | Unannotated feature | neg | 395.9047 | 0.516 | 5.073 | 5.086 | 0.012 | 0.099 | 0.586 | 0.839 | Unannotated | NA | NA |
| metab_5028 | Unannotated feature | pos | 142.1593 | 7.276 | 3.889 | 3.854 | -0.034 | -0.099 | 0.586 | 0.839 | Unannotated | NA | NA |
| metab_14424 | Unannotated feature | neg | 353.9681 | 3.227 | 5.526 | 5.481 | -0.045 | -0.099 | 0.586 | 0.839 | Unannotated | NA | NA |
| metab_9954 | Unannotated feature | neg | 242.1762 | 2.903 | 3.706 | 3.680 | -0.026 | -0.099 | 0.586 | 0.839 | Unannotated | NA | NA |
| metab_17355 | Unannotated feature | neg | 498.9152 | 7.687 | 5.619 | 5.623 | 0.004 | 0.099 | 0.586 | 0.839 | Unannotated | NA | NA |
| metab_17358 | Unannotated feature | neg | 240.9286 | 7.687 | 5.353 | 5.386 | 0.033 | 0.099 | 0.586 | 0.839 | Unannotated | NA | NA |
| metab_15361 | Unannotated feature | neg | 273.1253 | 5.022 | 4.473 | 4.445 | -0.028 | -0.099 | 0.586 | 0.839 | Unannotated | NA | NA |
| metab_7049 | Romucosine C | pos | 338.1394 | 4.757 | 3.946 | 3.455 | -0.491 | -0.099 | 0.586 | 0.839 | Tentative | NA | NA |
| metab_12395 | Unannotated feature | neg | 449.3274 | 6.561 | 6.623 | 6.614 | -0.008 | -0.099 | 0.586 | 0.839 | Unannotated | NA | NA |
| metab_874 | Unannotated feature | pos | 377.2783 | 6.797 | 1.850 | 2.255 | 0.405 | 0.099 | 0.586 | 0.839 | Unannotated | NA | NA |
| metab_13362 | Unannotated feature | neg | 136.9907 | 0.730 | 4.651 | 4.257 | -0.394 | -0.099 | 0.586 | 0.839 | Unannotated | NA | NA |
| metab_10181 | Unannotated feature | neg | 285.0557 | 2.690 | 4.102 | 4.435 | 0.333 | 0.099 | 0.586 | 0.839 | Unannotated | NA | NA |
| metab_14491 | 2-Hydroxy-3-morpholinopropanesulfonic Acid | neg | 246.0425 | 3.338 | 5.416 | 5.219 | -0.197 | -0.099 | 0.586 | 0.839 | Unannotated | NA | NA |
| metab_12164 | 7-Methyl-cholic acid | neg | 403.2854 | 6.687 | 6.521 | 6.557 | 0.036 | 0.099 | 0.586 | 0.839 | Unannotated | NA | NA |
| metab_9549 | Unannotated feature | neg | 377.1223 | 3.284 | 4.892 | 4.815 | -0.077 | 0.099 | 0.586 | 0.839 | Unannotated | NA | NA |
| metab_15096 | Unannotated feature | neg | 119.0495 | 4.522 | 4.901 | 4.742 | -0.159 | -0.099 | 0.586 | 0.839 | Unannotated | NA | NA |
| metab_2513 | Unannotated feature | pos | 210.0609 | 0.839 | 4.432 | 4.306 | -0.126 | -0.099 | 0.586 | 0.839 | Unannotated | NA | NA |
| metab_15616 | Unannotated feature | neg | 411.8802 | 0.582 | 3.248 | 2.921 | -0.327 | 0.099 | 0.586 | 0.839 | Unannotated | NA | NA |
| metab_16418 | Unannotated feature | neg | 428.3383 | 6.892 | 3.579 | 2.915 | -0.664 | -0.099 | 0.586 | 0.839 | Unannotated | NA | NA |
| metab_17920 | LysoSM(d18:1) | neg | 446.3280 | 6.640 | 5.328 | 5.427 | 0.099 | 0.099 | 0.586 | 0.839 | Tentative | NA | NA |
| metab_15931 | 8-iso-15-keto-PGE2 | neg | 395.2092 | 6.029 | 5.782 | 5.740 | -0.042 | -0.099 | 0.586 | 0.839 | Unannotated | NA | NA |
| metab_17178 | Unannotated feature | neg | 136.8714 | 7.745 | 4.490 | 4.503 | 0.013 | 0.099 | 0.586 | 0.839 | Unannotated | NA | NA |
| metab_3217 | Ptelatoside B | pos | 393.1528 | 3.756 | 5.334 | 5.258 | -0.075 | -0.099 | 0.586 | 0.839 | Unannotated | NA | NA |
| metab_12792 | Unannotated feature | neg | 331.8674 | 0.636 | 4.849 | 4.676 | -0.173 | -0.099 | 0.586 | 0.839 | Unannotated | NA | NA |
| metab_3204 | 5-Methoxytryptophan | pos | 217.0976 | 3.729 | 5.815 | 5.756 | -0.059 | -0.099 | 0.586 | 0.839 | Unannotated | NA | NA |
| metab_12360 | Unannotated feature | neg | 565.3160 | 6.758 | 4.006 | 3.701 | -0.305 | -0.099 | 0.586 | 0.839 | Unannotated | NA | NA |
| metab_5605 | Unannotated feature | pos | 97.9692 | 0.016 | 4.514 | 4.539 | 0.025 | 0.099 | 0.586 | 0.839 | Unannotated | NA | NA |
| metab_8820 | Melphalan | pos | 287.0700 | 2.795 | 3.678 | 3.827 | 0.148 | -0.099 | 0.586 | 0.839 | Tentative | NA | NA |
| metab_5210 | Unannotated feature | pos | 128.1073 | 6.741 | 5.083 | 5.029 | -0.055 | -0.099 | 0.586 | 0.839 | Unannotated | NA | NA |
| metab_4842 | Unannotated feature | pos | 867.2404 | 0.672 | 3.819 | 4.312 | 0.493 | 0.099 | 0.586 | 0.839 | Unannotated | NA | NA |
| metab_2435 | Unannotated feature | pos | 461.2994 | 5.787 | 3.485 | 3.766 | 0.281 | 0.099 | 0.586 | 0.839 | Unannotated | NA | NA |
| metab_1009 | Unannotated feature | pos | 463.3036 | 7.132 | 5.358 | 5.340 | -0.017 | -0.099 | 0.586 | 0.839 | Unannotated | NA | NA |
| metab_17190 | Unannotated feature | neg | 170.9841 | 7.741 | 5.222 | 5.216 | -0.006 | -0.099 | 0.586 | 0.839 | Unannotated | NA | NA |
| metab_2579 | 3-(2-Hydroxyethyl)histidine | pos | 200.1034 | 1.259 | 5.184 | 5.007 | -0.177 | -0.099 | 0.586 | 0.839 | Unannotated | NA | NA |
| metab_10067 | Unannotated feature | neg | 351.9713 | 3.215 | 6.053 | 6.014 | -0.038 | -0.099 | 0.586 | 0.839 | Unannotated | NA | NA |
| metab_11850 | Unannotated feature | neg | 619.2795 | 5.964 | 4.670 | 4.380 | -0.290 | -0.099 | 0.586 | 0.839 | Unannotated | NA | NA |
| metab_14282 | Unannotated feature | neg | 283.0106 | 3.028 | 5.005 | 5.007 | 0.002 | -0.099 | 0.586 | 0.839 | Unannotated | NA | NA |
| metab_12285 | Unannotated feature | neg | 651.5215 | 6.993 | 5.635 | 5.606 | -0.029 | -0.099 | 0.586 | 0.839 | Unannotated | NA | NA |
| metab_16344 | Unannotated feature | neg | 342.2649 | 6.703 | 6.048 | 6.036 | -0.012 | 0.099 | 0.586 | 0.839 | Unannotated | NA | NA |
| metab_16351 | Unannotated feature | neg | 513.3584 | 6.719 | 4.545 | 4.888 | 0.343 | 0.099 | 0.586 | 0.839 | Unannotated | NA | NA |
| metab_12611 | Unannotated feature | neg | 427.2266 | 6.035 | 6.028 | 5.990 | -0.037 | -0.099 | 0.586 | 0.839 | Unannotated | NA | NA |
| metab_11677 | Unannotated feature | neg | 350.1459 | 5.339 | 4.606 | 4.811 | 0.205 | 0.099 | 0.586 | 0.839 | Unannotated | NA | NA |
| metab_17607 | Unannotated feature | neg | 619.4231 | 7.199 | 4.604 | 4.682 | 0.078 | -0.099 | 0.586 | 0.839 | Unannotated | NA | NA |
| metab_10328 | Unannotated feature | neg | 179.0379 | 2.292 | 4.541 | 4.793 | 0.252 | 0.099 | 0.586 | 0.839 | Unannotated | NA | NA |
| metab_11777 | Unannotated feature | neg | 605.2777 | 5.762 | 3.929 | 3.670 | -0.259 | -0.099 | 0.586 | 0.839 | Unannotated | NA | NA |
| metab_4199 | Unannotated feature | pos | 472.2456 | 6.008 | 5.661 | 6.192 | 0.530 | 0.099 | 0.586 | 0.839 | Unannotated | NA | NA |
| metab_363 | Unannotated feature | pos | 519.1405 | 0.356 | 5.319 | 5.321 | 0.002 | -0.099 | 0.586 | 0.839 | Unannotated | NA | NA |
| metab_19058 | Unannotated feature | neg | 415.1372 | 5.446 | 4.502 | 4.195 | -0.307 | -0.099 | 0.586 | 0.839 | Unannotated | NA | NA |
| metab_19010 | Prostaglandin E2 methyl ester | neg | 401.2122 | 5.519 | 5.007 | 4.886 | -0.120 | -0.099 | 0.586 | 0.839 | Unannotated | NA | NA |
| metab_16834 | Unannotated feature | neg | 460.8582 | 7.726 | 4.913 | 4.958 | 0.045 | 0.099 | 0.586 | 0.839 | Unannotated | NA | NA |
| metab_16837 | Unannotated feature | neg | 429.1272 | 1.981 | 4.653 | 4.679 | 0.026 | 0.099 | 0.586 | 0.839 | Unannotated | NA | NA |
| metab_8955 | Homocamptothecin | neg | 397.0970 | 4.169 | 4.177 | 3.827 | -0.350 | -0.099 | 0.586 | 0.839 | Unannotated | Organoheterocyclic compounds | Quinolines and derivatives |
| metab_10900 | Unannotated feature | neg | 89.0235 | 0.882 | 5.719 | 5.860 | 0.141 | 0.099 | 0.586 | 0.839 | Unannotated | NA | NA |
| metab_13025 | Unannotated feature | neg | 399.1486 | 6.193 | 5.473 | 5.610 | 0.137 | 0.099 | 0.586 | 0.839 | Unannotated | NA | NA |
| metab_17635 | Unannotated feature | neg | 431.1206 | 2.766 | 4.437 | 4.320 | -0.117 | -0.099 | 0.586 | 0.839 | Unannotated | NA | NA |
| metab_1111 | Unannotated feature | pos | 188.0920 | 1.692 | 5.008 | 4.919 | -0.089 | 0.099 | 0.586 | 0.839 | Unannotated | NA | NA |
| metab_11350 | Gibberellin A28 | neg | 375.1437 | 5.152 | 4.736 | 4.459 | -0.277 | -0.099 | 0.586 | 0.839 | Unannotated | NA | NA |
| metab_7747 | Diethofencarb | pos | 268.1548 | 3.295 | 3.841 | 3.816 | -0.025 | 0.099 | 0.586 | 0.839 | Tentative | NA | NA |
| metab_14613 | Unannotated feature | neg | 249.9970 | 3.533 | 3.858 | 4.010 | 0.152 | 0.099 | 0.586 | 0.839 | Unannotated | NA | NA |
| metab_16199 | Unannotated feature | neg | 387.2212 | 6.412 | 4.723 | 4.866 | 0.144 | 0.099 | 0.586 | 0.839 | Unannotated | NA | NA |
| metab_17006 | Unannotated feature | neg | 159.9361 | 7.997 | 4.455 | 4.441 | -0.014 | -0.099 | 0.586 | 0.839 | Unannotated | NA | NA |
| metab_6469 | Unannotated feature | pos | 345.8903 | 0.593 | 4.697 | 4.645 | -0.051 | -0.099 | 0.586 | 0.839 | Unannotated | NA | NA |
| metab_18162 | Unannotated feature | neg | 339.1636 | 6.334 | 6.004 | 5.918 | -0.086 | -0.099 | 0.586 | 0.839 | Unannotated | NA | NA |
| metab_3516 | Unannotated feature | pos | 125.9866 | 7.724 | 5.710 | 5.726 | 0.015 | 0.099 | 0.586 | 0.839 | Unannotated | NA | NA |
| metab_203 | Unannotated feature | pos | 124.9231 | 0.593 | 5.152 | 5.119 | -0.033 | -0.099 | 0.586 | 0.839 | Unannotated | NA | NA |
| metab_240 | N1-Acetylspermidine | pos | 188.1761 | 0.545 | 5.795 | 5.764 | -0.031 | -0.099 | 0.586 | 0.839 | Strong putative | Organic acids and derivatives | Carboximidic acids and derivatives |
| metab_16842 | Unannotated feature | neg | 161.0125 | 7.730 | 4.016 | 4.043 | 0.026 | -0.099 | 0.586 | 0.839 | Unannotated | NA | NA |
| metab_1881 | Unannotated feature | pos | 190.0713 | 1.231 | 5.949 | 6.016 | 0.067 | 0.099 | 0.586 | 0.839 | Unannotated | NA | NA |
| metab_5082 | Unannotated feature | pos | 675.5179 | 7.084 | 6.345 | 6.277 | -0.067 | -0.099 | 0.586 | 0.839 | Unannotated | NA | NA |
| metab_10100 | Unannotated feature | neg | 426.1528 | 2.766 | 3.859 | 3.779 | -0.079 | 0.099 | 0.586 | 0.839 | Unannotated | NA | NA |
| metab_2520 | Trachelogenin | pos | 353.1384 | 0.870 | 4.059 | 4.220 | 0.161 | 0.099 | 0.586 | 0.839 | Tentative | Lignans, neolignans and related compounds | Furanoid lignans |
| metab_17481 | Unannotated feature | neg | 403.1479 | 1.952 | 4.120 | 4.090 | -0.030 | 0.099 | 0.586 | 0.839 | Unannotated | NA | NA |
| metab_1302 | Unannotated feature | pos | 97.1016 | 6.247 | 3.852 | 3.790 | -0.063 | -0.099 | 0.586 | 0.839 | Unannotated | NA | NA |
| metab_482 | (2R,4S)-1-(Tert-butoxycarbonyl)-4-phenylpyrrolidine-2-carboxylic acid | pos | 309.1814 | 4.965 | 3.427 | 4.605 | 1.178 | 0.099 | 0.586 | 0.839 | Unannotated | Organic acids and derivatives | Carboxylic acids and derivatives |
| metab_11824 | Unannotated feature | neg | 406.2090 | 5.905 | 6.135 | 6.022 | -0.113 | -0.099 | 0.586 | 0.839 | Unannotated | NA | NA |
| metab_404 | Unannotated feature | pos | 267.9024 | 0.016 | 4.175 | 4.154 | -0.021 | -0.099 | 0.586 | 0.839 | Unannotated | NA | NA |
| metab_17243 | Unannotated feature | neg | 525.8675 | 7.726 | 4.965 | 5.034 | 0.069 | 0.099 | 0.586 | 0.839 | Unannotated | NA | NA |
| metab_7063 | Adelmidrol | pos | 587.3408 | 4.720 | 4.400 | 4.743 | 0.344 | 0.099 | 0.586 | 0.839 | Unannotated | NA | NA |
| metab_15820 | Unannotated feature | neg | 396.1416 | 5.892 | 4.414 | 3.783 | -0.631 | -0.099 | 0.586 | 0.839 | Unannotated | NA | NA |
| metab_13978 | Cyclopentenyl cytosine | neg | 537.1953 | 2.663 | 4.447 | 4.615 | 0.169 | 0.099 | 0.586 | 0.839 | Unannotated | NA | NA |
| metab_445 | Unannotated feature | pos | 787.2557 | 0.006 | 4.053 | 4.181 | 0.129 | 0.099 | 0.586 | 0.839 | Unannotated | NA | NA |
| metab_1226 | Unannotated feature | pos | 577.3366 | 6.390 | 5.861 | 5.580 | -0.281 | -0.099 | 0.586 | 0.839 | Unannotated | NA | NA |
| metab_1861 | Unannotated feature | pos | 218.0663 | 1.155 | 5.113 | 5.106 | -0.007 | 0.099 | 0.586 | 0.839 | Unannotated | NA | NA |
| metab_12943 | Unannotated feature | neg | 377.1995 | 6.248 | 6.077 | 6.006 | -0.071 | -0.099 | 0.586 | 0.839 | Unannotated | NA | NA |
| metab_9427 | Citpressine II | neg | 360.1098 | 3.434 | 3.843 | 4.389 | 0.546 | 0.099 | 0.586 | 0.839 | Unannotated | NA | NA |
| metab_13861 | Unannotated feature | neg | 231.9434 | 2.255 | 3.868 | 3.807 | -0.061 | -0.099 | 0.586 | 0.839 | Unannotated | NA | NA |
| metab_2309 | Unannotated feature | pos | 141.9789 | 0.068 | 3.939 | 3.902 | -0.038 | -0.099 | 0.586 | 0.839 | Unannotated | NA | NA |
| metab_7929 | Unannotated feature | pos | 401.1929 | 3.366 | 4.564 | 4.672 | 0.108 | 0.099 | 0.586 | 0.839 | Unannotated | NA | NA |
| metab_14326 | Unannotated feature | neg | 311.1252 | 3.082 | 4.161 | 4.057 | -0.104 | -0.099 | 0.586 | 0.839 | Unannotated | NA | NA |
| metab_14308 | Unannotated feature | neg | 410.8640 | 0.533 | 6.024 | 6.243 | 0.219 | 0.099 | 0.586 | 0.839 | Unannotated | NA | NA |
| metab_10478 | Lagociclovir | neg | 314.0887 | 1.952 | 4.864 | 4.978 | 0.114 | 0.099 | 0.586 | 0.839 | Unannotated | NA | NA |
| metab_13104 | Unannotated feature | neg | 161.8751 | 0.132 | 5.210 | 5.201 | -0.010 | -0.099 | 0.586 | 0.839 | Unannotated | NA | NA |
| metab_1125 | Pubescenol | pos | 507.3305 | 6.614 | 6.722 | 6.708 | -0.015 | -0.099 | 0.586 | 0.839 | Unannotated | NA | NA |
| metab_14926 | Unannotated feature | neg | 427.1638 | 4.165 | 4.454 | 4.282 | -0.172 | -0.099 | 0.586 | 0.839 | Unannotated | NA | NA |
| metab_7981 | Unannotated feature | pos | 291.0869 | 3.432 | 3.915 | 3.648 | -0.268 | -0.099 | 0.586 | 0.839 | Unannotated | NA | NA |
| metab_16149 | Unannotated feature | neg | 281.0359 | 1.241 | 4.478 | 4.796 | 0.318 | -0.099 | 0.586 | 0.839 | Unannotated | NA | NA |
| metab_1650 | Butanedioic acid, octenyl- | pos | 251.1262 | 4.586 | 4.208 | 3.855 | -0.353 | -0.099 | 0.586 | 0.839 | Unannotated | Lipids and lipid-like molecules | Fatty Acyls |
| metab_11116 | Unannotated feature | neg | 453.1026 | 0.714 | 4.324 | 4.411 | 0.087 | -0.099 | 0.586 | 0.839 | Unannotated | NA | NA |
| metab_12720 | Unannotated feature | neg | 378.1198 | 5.122 | 5.402 | 5.437 | 0.034 | 0.099 | 0.586 | 0.839 | Unannotated | NA | NA |
| metab_3073 | Unannotated feature | pos | 328.1905 | 3.357 | 4.380 | 4.476 | 0.096 | -0.099 | 0.586 | 0.839 | Unannotated | NA | NA |
| metab_2698 | Alanylisoleucine | pos | 185.1288 | 2.044 | 4.879 | 4.661 | -0.218 | -0.099 | 0.586 | 0.839 | Unannotated | NA | NA |
| metab_12492 | Unannotated feature | neg | 513.2895 | 6.334 | 7.207 | 7.110 | -0.098 | -0.099 | 0.586 | 0.839 | Unannotated | NA | NA |
| metab_12498 | Unannotated feature | neg | 453.2326 | 6.373 | 6.712 | 6.985 | 0.273 | 0.099 | 0.586 | 0.839 | Unannotated | NA | NA |
| metab_16500 | Unannotated feature | neg | 129.9751 | 7.097 | 7.078 | 7.117 | 0.039 | 0.099 | 0.586 | 0.839 | Unannotated | NA | NA |
| metab_12146 | Unannotated feature | neg | 415.2821 | 6.640 | 6.793 | 6.644 | -0.149 | -0.099 | 0.586 | 0.839 | Unannotated | NA | NA |
| metab_10468 | Unannotated feature | neg | 519.1731 | 4.634 | 4.223 | 4.582 | 0.358 | 0.099 | 0.586 | 0.839 | Unannotated | NA | NA |
| metab_17763 | Unannotated feature | neg | 324.2545 | 6.899 | 4.818 | 4.887 | 0.069 | 0.099 | 0.586 | 0.839 | Unannotated | NA | NA |
| metab_17793 | Unannotated feature | neg | 392.3174 | 6.860 | 5.721 | 5.854 | 0.134 | 0.099 | 0.586 | 0.839 | Unannotated | NA | NA |
| metab_17044 | Unannotated feature | neg | 266.9235 | 7.985 | 4.633 | 4.605 | -0.028 | -0.099 | 0.586 | 0.839 | Unannotated | NA | NA |
| metab_13086 | Unannotated feature | neg | 242.1761 | 0.031 | 5.298 | 5.286 | -0.012 | -0.099 | 0.586 | 0.839 | Unannotated | NA | NA |
| metab_13090 | Unannotated feature | neg | 201.8015 | 0.036 | 6.454 | 6.433 | -0.021 | -0.099 | 0.586 | 0.839 | Unannotated | NA | NA |
| metab_13095 | Allocholic acid | neg | 407.2807 | 6.404 | 7.411 | 7.008 | -0.402 | -0.099 | 0.586 | 0.839 | Moderate putative | NA | NA |
| metab_11698 | Unannotated feature | neg | 635.3105 | 5.457 | 4.419 | 5.493 | 1.074 | 0.099 | 0.586 | 0.839 | Unannotated | NA | NA |
| metab_12917 | Unannotated feature | neg | 217.0464 | 0.738 | 4.684 | 4.840 | 0.156 | 0.099 | 0.586 | 0.839 | Unannotated | NA | NA |
| metab_9121 | Unannotated feature | neg | 115.0757 | 3.879 | 3.504 | 3.776 | 0.271 | 0.099 | 0.586 | 0.839 | Unannotated | NA | NA |
| metab_18000 | DG(PGF1alpha/0:0/13:0) | neg | 625.4692 | 6.514 | 5.210 | 4.995 | -0.215 | -0.099 | 0.586 | 0.839 | Unannotated | NA | NA |
| metab_560 | Unannotated feature | pos | 470.2664 | 5.629 | 4.998 | 6.201 | 1.203 | 0.099 | 0.586 | 0.839 | Unannotated | NA | NA |
| metab_4492 | Phorbol-12-monomyristate | pos | 575.3941 | 6.550 | 6.300 | 6.330 | 0.031 | 0.099 | 0.586 | 0.839 | Unannotated | NA | NA |
| metab_15412 | Unannotated feature | neg | 366.1390 | 5.079 | 3.889 | 3.626 | -0.263 | -0.099 | 0.586 | 0.839 | Unannotated | NA | NA |
| metab_7123 | Dopamine quinone | pos | 134.0603 | 4.598 | 5.306 | 4.874 | -0.432 | -0.099 | 0.586 | 0.839 | Tentative | NA | NA |
| metab_11978 | Unannotated feature | neg | 253.1348 | 6.201 | 4.232 | 3.949 | -0.283 | -0.099 | 0.586 | 0.839 | Unannotated | NA | NA |
| metab_12748 | Unannotated feature | neg | 647.2063 | 2.742 | 4.417 | 4.335 | -0.082 | -0.099 | 0.586 | 0.839 | Unannotated | NA | NA |
| metab_15720 | 2-(2-Aminoethoxymethyl)-4-(2-chlorophenyl)-6-methyl-1,4-dihydropyridine-3,5-dicarboxylic acid O3-ethyl ester O5-methyl esterbenzenesulfonic acid | neg | 453.1458 | 5.714 | 4.534 | 4.808 | 0.274 | 0.099 | 0.586 | 0.839 | Unannotated | NA | NA |
| metab_8924 | Unannotated feature | neg | 379.1765 | 4.228 | 3.753 | 3.958 | 0.205 | -0.099 | 0.586 | 0.839 | Unannotated | NA | NA |
| metab_15530 | Unannotated feature | neg | 365.9703 | 0.577 | 4.034 | 4.408 | 0.374 | 0.099 | 0.586 | 0.839 | Unannotated | NA | NA |
| metab_12057 | Ganolucidic acid C | neg | 499.3101 | 6.381 | 6.904 | 6.985 | 0.082 | 0.099 | 0.586 | 0.839 | Unannotated | NA | NA |
| metab_6498 | Unannotated feature | pos | 323.2183 | 5.593 | 4.568 | 4.532 | -0.036 | -0.099 | 0.586 | 0.839 | Unannotated | NA | NA |
| metab_19897 | Ubiquinone-1 | neg | 295.1184 | 4.359 | 4.764 | 4.723 | -0.042 | 0.099 | 0.586 | 0.839 | Tentative | NA | NA |
| metab_5461 | Unannotated feature | pos | 479.3167 | 6.382 | 4.343 | 4.293 | -0.051 | 0.099 | 0.586 | 0.839 | Unannotated | NA | NA |
| metab_19769 | Unannotated feature | neg | 211.1133 | 4.477 | 4.532 | 4.471 | -0.062 | -0.099 | 0.586 | 0.839 | Unannotated | NA | NA |
| metab_1545 | Unannotated feature | pos | 335.1985 | 5.340 | 4.450 | 4.570 | 0.120 | 0.099 | 0.586 | 0.839 | Unannotated | NA | NA |
| metab_18453 | Unannotated feature | neg | 314.1197 | 6.091 | 5.523 | 5.410 | -0.113 | -0.099 | 0.586 | 0.839 | Unannotated | NA | NA |
| metab_11982 | Unannotated feature | neg | 187.0971 | 5.522 | 6.403 | 6.351 | -0.052 | -0.099 | 0.586 | 0.839 | Unannotated | NA | NA |
| metab_1416 | Unannotated feature | pos | 433.1984 | 6.056 | 4.403 | 4.578 | 0.175 | 0.099 | 0.586 | 0.839 | Unannotated | NA | NA |
| metab_12004 | Unannotated feature | neg | 473.1842 | 6.248 | 3.703 | 3.795 | 0.091 | 0.095 | 0.602 | 0.848 | Unannotated | NA | NA |
| metab_136 | Unannotated feature | pos | 161.0296 | 0.625 | 4.001 | 4.015 | 0.014 | 0.095 | 0.602 | 0.848 | Unannotated | NA | NA |
| metab_15037 | Unannotated feature | neg | 266.9991 | 4.401 | 4.003 | 4.063 | 0.060 | 0.095 | 0.602 | 0.848 | Unannotated | NA | NA |
| metab_9877 | Unannotated feature | neg | 81.0336 | 2.972 | 3.086 | 3.387 | 0.301 | 0.095 | 0.602 | 0.848 | Unannotated | NA | NA |
| metab_17259 | Unannotated feature | neg | 321.0464 | 2.524 | 4.714 | 4.538 | -0.176 | -0.095 | 0.602 | 0.848 | Unannotated | NA | NA |
| metab_5008 | Unannotated feature | pos | 736.5361 | 7.395 | 6.258 | 6.313 | 0.056 | -0.095 | 0.602 | 0.848 | Unannotated | NA | NA |
| metab_8588 | Unannotated feature | pos | 258.2068 | 4.037 | 4.165 | 4.330 | 0.166 | 0.095 | 0.602 | 0.848 | Unannotated | NA | NA |
| metab_8558 | Unannotated feature | pos | 102.0343 | 1.259 | 5.566 | 5.588 | 0.023 | 0.095 | 0.602 | 0.848 | Unannotated | NA | NA |
| metab_14416 | Unannotated feature | neg | 285.0076 | 3.219 | 4.880 | 4.806 | -0.074 | -0.095 | 0.602 | 0.848 | Unannotated | NA | NA |
| metab_18272 | Deflazacort | neg | 440.2117 | 6.232 | 3.691 | 3.979 | 0.287 | 0.095 | 0.602 | 0.848 | Unannotated | NA | NA |
| metab_18267 | Unannotated feature | neg | 382.1563 | 6.240 | 4.920 | 4.732 | -0.188 | -0.095 | 0.602 | 0.848 | Unannotated | NA | NA |
| metab_10846 | Unannotated feature | neg | 311.1503 | 4.829 | 5.304 | 5.365 | 0.061 | 0.095 | 0.602 | 0.848 | Unannotated | NA | NA |
| metab_8967 | Unannotated feature | neg | 287.1765 | 4.138 | 4.456 | 4.451 | -0.004 | -0.095 | 0.602 | 0.848 | Unannotated | NA | NA |
| metab_18556 | Unannotated feature | neg | 308.1197 | 6.018 | 5.525 | 5.468 | -0.057 | -0.095 | 0.602 | 0.848 | Unannotated | NA | NA |
| metab_16800 | Unannotated feature | neg | 424.9120 | 7.710 | 5.157 | 5.124 | -0.033 | -0.095 | 0.602 | 0.848 | Unannotated | NA | NA |
| metab_8856 | Unannotated feature | neg | 225.1130 | 4.350 | 5.648 | 5.704 | 0.056 | -0.095 | 0.602 | 0.848 | Unannotated | NA | NA |
| metab_4709 | 3,5,7-Trimethylepicatechin | pos | 333.1341 | 4.054 | 3.792 | 3.840 | 0.048 | 0.095 | 0.602 | 0.848 | Unannotated | NA | NA |
| metab_7949 | 4-(2-Aminophenyl)-2,4-dioxobutanoic acid | pos | 208.0608 | 3.400 | 4.826 | 4.773 | -0.052 | -0.095 | 0.602 | 0.848 | Tentative | NA | NA |
| metab_1269 | Unannotated feature | pos | 617.3324 | 6.311 | 6.107 | 6.287 | 0.180 | 0.095 | 0.602 | 0.848 | Unannotated | NA | NA |
| metab_12697 | (Z)-Resveratrol 4'-glucoside | neg | 389.1247 | 5.366 | 6.083 | 5.987 | -0.097 | -0.095 | 0.602 | 0.848 | Unannotated | NA | NA |
| metab_12435 | Unannotated feature | neg | 226.9090 | 7.737 | 5.543 | 5.537 | -0.006 | -0.095 | 0.602 | 0.848 | Unannotated | NA | NA |
| metab_2815 | Unannotated feature | pos | 81.0341 | 2.708 | 3.375 | 3.540 | 0.165 | 0.095 | 0.602 | 0.848 | Unannotated | NA | NA |
| metab_2978 | Unannotated feature | pos | 485.2693 | 6.255 | 5.920 | 5.781 | -0.140 | -0.095 | 0.602 | 0.848 | Unannotated | NA | NA |
| metab_15603 | Unannotated feature | neg | 91.0542 | 5.512 | 3.653 | 3.633 | -0.020 | -0.095 | 0.602 | 0.848 | Unannotated | NA | NA |
| metab_7285 | Carbetamide | pos | 237.1238 | 4.232 | 4.066 | 4.142 | 0.076 | 0.095 | 0.602 | 0.848 | Tentative | NA | NA |
| metab_15365 | Unannotated feature | neg | 403.1041 | 5.022 | 4.826 | 4.698 | -0.129 | -0.095 | 0.602 | 0.848 | Unannotated | NA | NA |
| metab_1788 | Castanospermine | pos | 190.1077 | 0.776 | 5.424 | 5.361 | -0.063 | 0.095 | 0.602 | 0.848 | Moderate putative | NA | NA |
| metab_14937 | Unannotated feature | neg | 361.1298 | 4.195 | 4.718 | 4.780 | 0.062 | -0.095 | 0.602 | 0.848 | Unannotated | NA | NA |
| metab_5611 | Unannotated feature | pos | 139.9635 | 0.016 | 5.306 | 5.308 | 0.003 | 0.095 | 0.602 | 0.848 | Unannotated | NA | NA |
| metab_4595 | Unannotated feature | pos | 452.3704 | 6.997 | 6.267 | 6.304 | 0.037 | 0.095 | 0.602 | 0.848 | Unannotated | NA | NA |
| metab_16841 | Unannotated feature | neg | 121.9039 | 7.730 | 3.413 | 3.469 | 0.056 | 0.095 | 0.602 | 0.848 | Unannotated | NA | NA |
| metab_10769 | Unannotated feature | neg | 358.1257 | 3.418 | 4.869 | 4.831 | -0.038 | 0.095 | 0.602 | 0.848 | Unannotated | NA | NA |
| metab_12854 | Unannotated feature | neg | 132.0658 | 0.644 | 4.394 | 4.468 | 0.074 | 0.095 | 0.602 | 0.848 | Unannotated | NA | NA |
| metab_10808 | Unannotated feature | neg | 318.9727 | 1.071 | 4.882 | 5.062 | 0.180 | 0.095 | 0.602 | 0.848 | Unannotated | NA | NA |
| metab_13529 | N2-Acetyl,n6-methyllysine | neg | 201.1242 | 1.092 | 5.092 | 4.925 | -0.168 | -0.095 | 0.602 | 0.848 | Unannotated | NA | NA |
| metab_4909 | Unannotated feature | pos | 243.0198 | 7.666 | 4.594 | 4.522 | -0.072 | -0.095 | 0.602 | 0.848 | Unannotated | NA | NA |
| metab_17245 | Unannotated feature | neg | 458.8627 | 7.726 | 5.427 | 5.464 | 0.037 | 0.095 | 0.602 | 0.848 | Unannotated | NA | NA |
| metab_12672 | Unannotated feature | neg | 351.2542 | 6.617 | 5.661 | 5.693 | 0.032 | 0.095 | 0.602 | 0.848 | Unannotated | NA | NA |
| metab_4515 | Unannotated feature | pos | 366.2622 | 6.694 | 4.917 | 4.712 | -0.205 | 0.095 | 0.602 | 0.848 | Unannotated | NA | NA |
| metab_9067 | Unannotated feature | neg | 427.2193 | 3.837 | 3.744 | 3.977 | 0.233 | 0.095 | 0.602 | 0.848 | Unannotated | NA | NA |
| metab_18174 | Unannotated feature | neg | 341.2334 | 6.326 | 4.703 | 4.795 | 0.092 | 0.095 | 0.602 | 0.848 | Unannotated | NA | NA |
| metab_12083 | Unannotated feature | neg | 311.1325 | 6.444 | 5.301 | 5.255 | -0.047 | -0.095 | 0.602 | 0.848 | Unannotated | NA | NA |
| metab_12088 | Unannotated feature | neg | 465.3218 | 6.451 | 6.906 | 6.823 | -0.083 | -0.095 | 0.602 | 0.848 | Unannotated | NA | NA |
| metab_10725 | Unannotated feature | neg | 260.0453 | 1.308 | 3.841 | 3.385 | -0.456 | -0.095 | 0.602 | 0.848 | Unannotated | NA | NA |
| metab_7480 | Unannotated feature | pos | 215.1758 | 3.795 | 4.605 | 4.170 | -0.434 | -0.095 | 0.602 | 0.848 | Unannotated | NA | NA |
| metab_12230 | Unannotated feature | neg | 430.3542 | 6.915 | 4.364 | 5.064 | 0.700 | 0.095 | 0.602 | 0.848 | Unannotated | NA | NA |
| metab_19429 | Unannotated feature | neg | 295.1219 | 4.970 | 4.600 | 4.637 | 0.037 | 0.095 | 0.602 | 0.848 | Unannotated | NA | NA |
| metab_5588 | (4E,7E,10E,13E)-Hexadeca-4,7,10,13-tetraenoic acid | pos | 213.1641 | 6.247 | 4.566 | 4.224 | -0.342 | -0.095 | 0.602 | 0.848 | Unannotated | NA | NA |
| metab_1203 | Unannotated feature | pos | 342.1550 | 2.740 | 5.062 | 5.250 | 0.187 | 0.095 | 0.602 | 0.848 | Unannotated | NA | NA |
| metab_12641 | Unannotated feature | neg | 805.5623 | 6.545 | 7.361 | 7.130 | -0.231 | -0.095 | 0.602 | 0.848 | Unannotated | NA | NA |
| metab_18530 | Unannotated feature | neg | 317.1970 | 6.035 | 5.354 | 5.246 | -0.109 | -0.095 | 0.602 | 0.848 | Unannotated | NA | NA |
| metab_3008 | Glycocholic Acid | pos | 488.3000 | 6.311 | 5.124 | 4.994 | -0.130 | -0.095 | 0.602 | 0.848 | Moderate putative | NA | NA |
| metab_2746 | Tetraazacyclododecanetetraacetic acid | pos | 369.1760 | 2.329 | 2.991 | 3.451 | 0.460 | 0.095 | 0.602 | 0.848 | Unannotated | NA | NA |
| metab_2710 | Unannotated feature | pos | 115.9705 | 2.088 | 4.738 | 4.793 | 0.055 | 0.095 | 0.602 | 0.848 | Unannotated | NA | NA |
| metab_5543 | Unannotated feature | pos | 295.1658 | 2.755 | 5.330 | 5.396 | 0.067 | 0.095 | 0.602 | 0.848 | Unannotated | NA | NA |
| metab_5070 | Unannotated feature | pos | 217.1049 | 7.124 | 3.659 | 3.801 | 0.142 | 0.095 | 0.602 | 0.848 | Unannotated | NA | NA |
| metab_1952 | Unannotated feature | pos | 434.1675 | 1.952 | 4.260 | 3.772 | -0.488 | -0.095 | 0.602 | 0.848 | Unannotated | NA | NA |
| metab_10625 | Unannotated feature | neg | 160.0067 | 1.685 | 4.700 | 4.444 | -0.255 | -0.095 | 0.602 | 0.848 | Unannotated | NA | NA |
| metab_16952 | Unannotated feature | neg | 160.8414 | 7.861 | 6.154 | 6.155 | 0.001 | 0.095 | 0.602 | 0.848 | Unannotated | NA | NA |
| metab_786 | Unannotated feature | pos | 450.2396 | 6.223 | 4.178 | 3.936 | -0.242 | -0.095 | 0.602 | 0.848 | Unannotated | NA | NA |
| metab_6250 | Unannotated feature | pos | 158.1655 | 0.553 | 4.468 | 4.051 | -0.417 | -0.095 | 0.602 | 0.848 | Unannotated | NA | NA |
| metab_11921 | Unannotated feature | neg | 289.1560 | 6.099 | 5.082 | 4.991 | -0.091 | -0.095 | 0.602 | 0.848 | Unannotated | NA | NA |
| metab_17988 | Unannotated feature | neg | 614.3738 | 6.522 | 5.608 | 5.213 | -0.396 | -0.095 | 0.602 | 0.848 | Unannotated | NA | NA |
| metab_16743 | Unannotated feature | neg | 553.8754 | 7.666 | 5.448 | 5.440 | -0.008 | 0.095 | 0.602 | 0.848 | Unannotated | NA | NA |
| metab_16394 | Dodecylbenzenesulfonic Acid | neg | 325.1844 | 6.836 | 7.883 | 7.884 | 0.001 | 0.095 | 0.602 | 0.848 | Strong putative | NA | NA |
| metab_1983 | Unannotated feature | pos | 281.1500 | 1.952 | 4.561 | 4.574 | 0.013 | 0.095 | 0.602 | 0.848 | Unannotated | NA | NA |
| metab_9236 | Unannotated feature | neg | 311.1241 | 3.945 | 4.807 | 4.912 | 0.106 | 0.095 | 0.602 | 0.848 | Unannotated | NA | NA |
| metab_16108 | Unannotated feature | neg | 481.2645 | 6.248 | 4.629 | 4.838 | 0.210 | -0.095 | 0.602 | 0.848 | Unannotated | NA | NA |
| metab_16036 | Unannotated feature | neg | 289.1293 | 6.170 | 3.683 | 3.664 | -0.019 | -0.095 | 0.602 | 0.848 | Unannotated | NA | NA |
| metab_9308 | Unannotated feature | neg | 101.0600 | 3.596 | 4.160 | 4.279 | 0.120 | 0.095 | 0.602 | 0.848 | Unannotated | NA | NA |
| metab_10034 | Unannotated feature | neg | 212.0561 | 2.809 | 4.195 | 4.169 | -0.027 | -0.095 | 0.602 | 0.848 | Unannotated | NA | NA |
| metab_17424 | Unannotated feature | neg | 301.0937 | 2.656 | 4.072 | 4.049 | -0.024 | 0.095 | 0.602 | 0.848 | Unannotated | NA | NA |
| metab_7634 | Unannotated feature | pos | 194.1544 | 3.491 | 4.344 | 4.242 | -0.102 | -0.095 | 0.602 | 0.848 | Unannotated | NA | NA |
| metab_2562 | Unannotated feature | pos | 208.0643 | 1.139 | 4.215 | 4.057 | -0.158 | -0.095 | 0.602 | 0.848 | Unannotated | NA | NA |
| metab_12177 | Unannotated feature | neg | 665.5012 | 6.719 | 5.306 | 5.007 | -0.299 | -0.095 | 0.602 | 0.848 | Unannotated | NA | NA |
| metab_1154 | Unannotated feature | pos | 459.3596 | 6.558 | 4.656 | 4.621 | -0.035 | -0.095 | 0.602 | 0.848 | Unannotated | NA | NA |
| metab_11249 | Unannotated feature | neg | 271.0614 | 5.076 | 4.890 | 4.789 | -0.101 | 0.095 | 0.602 | 0.848 | Unannotated | NA | NA |
| metab_9338 | Unannotated feature | neg | 141.0550 | 3.566 | 3.563 | 3.373 | -0.190 | -0.095 | 0.602 | 0.848 | Unannotated | NA | NA |
| metab_9337 | Unannotated feature | neg | 189.1127 | 3.566 | 3.810 | 4.092 | 0.282 | 0.095 | 0.602 | 0.848 | Unannotated | NA | NA |
| metab_14786 | Phenmedipham | neg | 345.1091 | 3.867 | 5.685 | 5.537 | -0.148 | -0.095 | 0.602 | 0.848 | Tentative | NA | NA |
| metab_2224 | Unannotated feature | pos | 174.9383 | 0.593 | 3.774 | 3.680 | -0.094 | -0.095 | 0.602 | 0.848 | Unannotated | NA | NA |
| metab_2133 | Atenolol | pos | 267.1708 | 2.849 | 4.664 | 4.832 | 0.168 | 0.095 | 0.602 | 0.848 | Moderate putative | NA | NA |
| metab_11699 | Unannotated feature | neg | 343.1404 | 5.461 | 3.719 | 3.981 | 0.262 | 0.095 | 0.602 | 0.848 | Unannotated | NA | NA |
| metab_6928 | Alpha-AMINO-3-HYDROXY-5-METHYL-4-ISOXAZOLEPROPIONIC ACID | pos | 204.0982 | 0.649 | 4.850 | 4.960 | 0.109 | 0.095 | 0.602 | 0.848 | Tentative | NA | NA |
| metab_6906 | Salutaridinol | pos | 352.1551 | 5.020 | 3.635 | 3.496 | -0.139 | -0.095 | 0.602 | 0.848 | Tentative | NA | NA |
| metab_3829 | Unannotated feature | pos | 309.2581 | 6.390 | 6.371 | 6.355 | -0.015 | -0.095 | 0.602 | 0.848 | Unannotated | NA | NA |
| metab_13089 | Unannotated feature | neg | 193.8152 | 0.036 | 6.087 | 6.065 | -0.022 | -0.095 | 0.602 | 0.848 | Unannotated | NA | NA |
| metab_2107 | Unannotated feature | pos | 237.1601 | 2.795 | 4.944 | 4.793 | -0.151 | -0.095 | 0.602 | 0.848 | Unannotated | NA | NA |
| metab_11630 | Unannotated feature | neg | 239.9035 | 0.096 | 5.465 | 5.456 | -0.008 | -0.095 | 0.602 | 0.848 | Unannotated | NA | NA |
| metab_12511 | Unannotated feature | neg | 411.2758 | 6.334 | 5.522 | 5.534 | 0.012 | 0.095 | 0.602 | 0.848 | Unannotated | NA | NA |
| metab_12499 | Unannotated feature | neg | 379.2495 | 6.373 | 6.067 | 6.128 | 0.061 | 0.095 | 0.602 | 0.848 | Unannotated | NA | NA |
| metab_15617 | Unannotated feature | neg | 553.3397 | 5.530 | 3.336 | 3.648 | 0.312 | 0.095 | 0.602 | 0.848 | Unannotated | NA | NA |
| metab_16042 | Unannotated feature | neg | 445.1573 | 1.119 | 5.523 | 5.735 | 0.212 | 0.095 | 0.602 | 0.848 | Unannotated | NA | NA |
| metab_3111 | Unannotated feature | pos | 160.0760 | 3.461 | 4.026 | 3.867 | -0.159 | -0.095 | 0.602 | 0.848 | Unannotated | NA | NA |
| metab_13714 | Unannotated feature | neg | 116.0427 | 1.864 | 3.897 | 4.212 | 0.315 | 0.095 | 0.602 | 0.848 | Unannotated | NA | NA |
| metab_13750 | Unannotated feature | neg | 208.9361 | 0.349 | 6.471 | 6.471 | -0.000 | -0.095 | 0.602 | 0.848 | Unannotated | NA | NA |
| metab_11640 | Unannotated feature | neg | 179.8406 | 0.046 | 4.537 | 4.512 | -0.025 | -0.095 | 0.602 | 0.848 | Unannotated | NA | NA |
| metab_8396 | Cis-4-Decenedioic acid | pos | 218.1391 | 1.952 | 3.926 | 4.173 | 0.247 | 0.095 | 0.602 | 0.848 | Unannotated | NA | NA |
| metab_5084 | Erucamide | pos | 338.3422 | 7.076 | 6.358 | 6.367 | 0.009 | 0.095 | 0.602 | 0.848 | Moderate putative | NA | NA |
| metab_19084 | Unannotated feature | neg | 387.2394 | 5.413 | 5.437 | 5.018 | -0.419 | -0.095 | 0.602 | 0.848 | Unannotated | NA | NA |
| metab_3192 | Unannotated feature | pos | 421.2932 | 6.765 | 6.102 | 6.120 | 0.017 | 0.095 | 0.602 | 0.848 | Unannotated | NA | NA |
| metab_5464 | Cocamidopropyl Betaine | pos | 343.2960 | 6.382 | 5.044 | 4.807 | -0.237 | -0.095 | 0.602 | 0.848 | Strong putative | NA | NA |
| metab_12124 | Unannotated feature | neg | 579.4277 | 6.561 | 5.177 | 5.257 | 0.080 | 0.095 | 0.602 | 0.848 | Unannotated | NA | NA |
| metab_10455 | Unannotated feature | neg | 380.0523 | 1.958 | 2.929 | 1.691 | -1.238 | -0.095 | 0.602 | 0.848 | Unannotated | NA | NA |
| metab_10440 | Unannotated feature | neg | 135.0114 | 1.969 | 2.833 | 2.917 | 0.084 | -0.095 | 0.602 | 0.848 | Unannotated | NA | NA |
| metab_1088 | Unannotated feature | pos | 465.3188 | 6.678 | 4.039 | 3.941 | -0.098 | -0.095 | 0.602 | 0.848 | Unannotated | NA | NA |
| metab_13191 | Unannotated feature | neg | 408.9345 | 0.512 | 4.434 | 4.684 | 0.250 | 0.095 | 0.602 | 0.848 | Unannotated | NA | NA |
| metab_2306 | Unannotated feature | pos | 97.0527 | 0.039 | 4.565 | 4.585 | 0.020 | 0.095 | 0.602 | 0.848 | Unannotated | NA | NA |
| metab_3566 | METHOPRENE (S) | pos | 333.2405 | 6.718 | 5.053 | 4.411 | -0.641 | -0.095 | 0.602 | 0.848 | Moderate putative | NA | NA |
| metab_9461 | Unannotated feature | neg | 454.1971 | 3.396 | 4.129 | 4.328 | 0.199 | 0.095 | 0.602 | 0.848 | Unannotated | NA | NA |
| metab_17063 | Unannotated feature | neg | 255.8398 | 7.948 | 5.024 | 5.023 | -0.001 | -0.095 | 0.602 | 0.848 | Unannotated | NA | NA |
| metab_3659 | Unannotated feature | pos | 427.3586 | 6.646 | 5.962 | 5.987 | 0.025 | 0.095 | 0.602 | 0.848 | Unannotated | NA | NA |
| metab_1059 | Unannotated feature | pos | 349.2733 | 6.789 | 4.528 | 4.465 | -0.063 | 0.095 | 0.602 | 0.848 | Unannotated | NA | NA |
| metab_2399 | Unannotated feature | pos | 253.9916 | 0.569 | 4.279 | 4.398 | 0.119 | -0.095 | 0.602 | 0.848 | Unannotated | NA | NA |
| metab_10355 | Uralenneoside | neg | 285.0596 | 2.186 | 4.107 | 4.088 | -0.019 | -0.095 | 0.602 | 0.848 | Unannotated | NA | NA |
| metab_7036 | Unannotated feature | pos | 293.0638 | 0.657 | 5.050 | 5.102 | 0.052 | 0.095 | 0.602 | 0.848 | Unannotated | NA | NA |
| metab_19119 | Unannotated feature | neg | 473.1675 | 3.292 | 4.111 | 3.900 | -0.211 | -0.095 | 0.602 | 0.848 | Unannotated | NA | NA |
| metab_17131 | Unannotated feature | neg | 172.8673 | 7.814 | 5.234 | 5.218 | -0.016 | 0.095 | 0.602 | 0.848 | Unannotated | NA | NA |
| metab_889 | Unannotated feature | pos | 611.2860 | 6.933 | 5.331 | 4.491 | -0.840 | -0.095 | 0.602 | 0.848 | Unannotated | NA | NA |
| metab_10133 | Imidazole-4-acetaldehyde | neg | 265.0942 | 2.754 | 3.908 | 3.906 | -0.003 | 0.095 | 0.602 | 0.848 | Tentative | NA | NA |
| metab_15665 | Resokaempherol | neg | 269.0457 | 5.611 | 5.481 | 5.333 | -0.147 | -0.095 | 0.602 | 0.848 | Strong putative | NA | NA |
| metab_6191 | Unannotated feature | pos | 488.7911 | 0.531 | 4.341 | 4.784 | 0.443 | 0.095 | 0.602 | 0.848 | Unannotated | NA | NA |
| metab_428 | Unannotated feature | pos | 433.2381 | 4.757 | 3.737 | 2.929 | -0.808 | -0.095 | 0.602 | 0.848 | Unannotated | NA | NA |
| metab_17558 | Unannotated feature | neg | 271.1302 | 2.754 | 4.854 | 4.789 | -0.065 | -0.095 | 0.602 | 0.848 | Unannotated | NA | NA |
| metab_18779 | Unannotated feature | neg | 412.1733 | 5.808 | 3.421 | 3.797 | 0.376 | 0.095 | 0.602 | 0.848 | Unannotated | NA | NA |
| metab_18754 | Unannotated feature | neg | 420.2295 | 5.842 | 4.545 | 4.362 | -0.183 | -0.095 | 0.602 | 0.848 | Unannotated | NA | NA |
| metab_18806 | Unannotated feature | neg | 227.0559 | 3.192 | 5.424 | 5.546 | 0.122 | 0.095 | 0.602 | 0.848 | Unannotated | NA | NA |
| metab_18795 | Ethyl (2E,6Z)-dodecadienoate | neg | 269.1759 | 5.789 | 4.121 | 4.518 | 0.396 | 0.095 | 0.602 | 0.848 | Unannotated | NA | NA |
| metab_7341 | 4-Fluoro-L-phenylalanine | pos | 216.1023 | 4.088 | 3.791 | 4.096 | 0.305 | 0.095 | 0.602 | 0.848 | Tentative | NA | NA |
| metab_7324 | Aspulvinone E | pos | 297.0759 | 4.129 | 3.893 | 3.637 | -0.256 | -0.095 | 0.602 | 0.848 | Moderate putative | NA | NA |
| metab_15329 | Cannabidiolic acid | neg | 393.1830 | 4.941 | 4.968 | 4.651 | -0.318 | -0.095 | 0.602 | 0.848 | Tentative | NA | NA |
| metab_8451 | Unannotated feature | pos | 229.0136 | 1.936 | 4.022 | 3.769 | -0.253 | -0.095 | 0.602 | 0.848 | Unannotated | NA | NA |
| metab_13853 | Unannotated feature | neg | 323.0417 | 2.238 | 5.456 | 5.449 | -0.007 | 0.095 | 0.602 | 0.848 | Unannotated | NA | NA |
| metab_12359 | Unannotated feature | neg | 353.2812 | 6.766 | 3.518 | 3.427 | -0.091 | -0.095 | 0.602 | 0.848 | Unannotated | NA | NA |
| metab_10276 | Unannotated feature | neg | 231.9774 | 2.508 | 4.145 | 4.014 | -0.131 | -0.095 | 0.602 | 0.848 | Unannotated | NA | NA |
| metab_18434 | Unannotated feature | neg | 279.6141 | 6.107 | 4.434 | 4.479 | 0.045 | 0.095 | 0.602 | 0.848 | Unannotated | NA | NA |
| metab_10873 | Unannotated feature | neg | 295.0136 | 4.522 | 4.641 | 4.325 | -0.316 | -0.095 | 0.602 | 0.848 | Unannotated | NA | NA |
| metab_18451 | Lovastatin acid | neg | 403.2493 | 6.091 | 5.239 | 5.014 | -0.225 | -0.095 | 0.602 | 0.848 | Tentative | NA | NA |
| metab_18437 | Unannotated feature | neg | 553.2851 | 6.099 | 4.964 | 4.865 | -0.100 | 0.095 | 0.602 | 0.848 | Unannotated | NA | NA |
| metab_10998 | Unannotated feature | neg | 253.0388 | 0.777 | 4.277 | 4.337 | 0.060 | 0.095 | 0.602 | 0.848 | Unannotated | NA | NA |
| metab_10973 | Unannotated feature | neg | 323.0423 | 0.784 | 4.030 | 4.283 | 0.254 | 0.095 | 0.602 | 0.848 | Unannotated | NA | NA |
| metab_1652 | Unannotated feature | pos | 460.2403 | 3.817 | 3.806 | 3.666 | -0.141 | -0.095 | 0.602 | 0.848 | Unannotated | NA | NA |
| metab_12755 | Unannotated feature | neg | 208.0724 | 1.952 | 4.128 | 4.388 | 0.260 | 0.095 | 0.602 | 0.848 | Unannotated | NA | NA |
| metab_18366 | Unannotated feature | neg | 535.3288 | 6.154 | 5.838 | 5.687 | -0.150 | -0.095 | 0.602 | 0.848 | Unannotated | NA | NA |
| metab_8906 | N-(4-Benzyloxybenzyl)acetohydroxamic acid | neg | 316.1193 | 4.254 | 3.283 | 3.788 | 0.505 | 0.095 | 0.602 | 0.848 | Unannotated | Benzenoids | Phenol ethers |
| metab_87 | Unannotated feature | pos | 443.0988 | 4.375 | 2.877 | 2.841 | -0.036 | 0.091 | 0.619 | 0.857 | Unannotated | NA | NA |
| metab_95 | N-(2-propan-2-yl-[1,2,4]triazolo[1,5-a]pyridin-8-yl)cyclopropanecarboxamide | pos | 262.1654 | 4.394 | 4.194 | 4.260 | 0.066 | 0.091 | 0.619 | 0.857 | Moderate putative | NA | NA |
| metab_7136 | 3-Carboxypropyl trimethylammonium | pos | 146.1178 | 0.672 | 6.387 | 6.555 | 0.168 | -0.091 | 0.619 | 0.857 | Moderate putative | NA | NA |
| metab_11972 | Unannotated feature | neg | 397.1691 | 6.185 | 6.139 | 6.237 | 0.098 | 0.091 | 0.619 | 0.857 | Unannotated | NA | NA |
| metab_2464 | Unannotated feature | pos | 132.1385 | 0.688 | 3.910 | 4.337 | 0.427 | 0.091 | 0.619 | 0.857 | Unannotated | NA | NA |
| metab_1024 | Unannotated feature | pos | 673.5029 | 7.028 | 6.900 | 6.867 | -0.033 | -0.091 | 0.619 | 0.857 | Unannotated | NA | NA |
| metab_8426 | 7,8-Dimethyl-10-[(2R,3R,4S)-2,3,4,5-tetrahydroxypentyl]benzo[g]pteridine-2,4-dione | pos | 377.1466 | 3.882 | 6.076 | 6.114 | 0.039 | 0.091 | 0.619 | 0.857 | Unannotated | NA | NA |
| metab_5124 | Unannotated feature | pos | 433.3776 | 6.949 | 4.572 | 4.753 | 0.181 | 0.091 | 0.619 | 0.857 | Unannotated | NA | NA |
| metab_5140 | N-(3-Methyl-2-pyridyl)-3-phenylsuccinimide | pos | 299.1393 | 6.885 | 4.744 | 4.867 | 0.122 | -0.091 | 0.619 | 0.857 | Unannotated | NA | NA |
| metab_5182 | Estradiol cypionate | pos | 414.2991 | 6.797 | 5.213 | 5.367 | 0.154 | 0.091 | 0.619 | 0.857 | Tentative | NA | NA |
| metab_16779 | Unannotated feature | neg | 325.9066 | 7.697 | 5.067 | 5.033 | -0.034 | -0.091 | 0.619 | 0.857 | Unannotated | NA | NA |
| metab_11516 | Unannotated feature | neg | 340.9465 | 0.507 | 5.685 | 5.724 | 0.039 | -0.091 | 0.619 | 0.857 | Unannotated | NA | NA |
| metab_11517 | Unannotated feature | neg | 244.9638 | 0.507 | 5.783 | 5.773 | -0.011 | -0.091 | 0.619 | 0.857 | Unannotated | NA | NA |
| metab_4730 | Bedoradrine | pos | 446.2610 | 3.808 | 4.982 | 5.068 | 0.086 | 0.091 | 0.619 | 0.857 | Unannotated | NA | NA |
| metab_11155 | Aceglutamide | neg | 187.0719 | 0.683 | 4.727 | 4.571 | -0.156 | -0.091 | 0.619 | 0.857 | Moderate putative | NA | NA |
| metab_10880 | Unannotated feature | neg | 287.0003 | 0.956 | 5.138 | 5.065 | -0.073 | -0.091 | 0.619 | 0.857 | Unannotated | NA | NA |
| metab_11822 | Unannotated feature | neg | 265.9986 | 4.837 | 4.346 | 3.616 | -0.730 | -0.091 | 0.619 | 0.857 | Unannotated | NA | NA |
| metab_8959 | Heliannone C | neg | 331.0832 | 4.154 | 4.299 | 4.216 | -0.083 | -0.091 | 0.619 | 0.857 | Unannotated | Phenylpropanoids and polyketides | Flavonoids |
| metab_16685 | Unannotated feature | neg | 412.1486 | 1.947 | 4.651 | 4.836 | 0.185 | 0.091 | 0.619 | 0.857 | Unannotated | NA | NA |
| metab_3462 | Unannotated feature | pos | 246.1018 | 7.724 | 5.901 | 5.926 | 0.025 | 0.091 | 0.619 | 0.857 | Unannotated | NA | NA |
| metab_17630 | Unannotated feature | neg | 369.1742 | 7.129 | 4.290 | 4.399 | 0.109 | 0.091 | 0.619 | 0.857 | Unannotated | NA | NA |
| metab_2374 | Unannotated feature | pos | 779.1530 | 0.531 | 5.258 | 5.239 | -0.019 | 0.091 | 0.619 | 0.857 | Unannotated | NA | NA |
| metab_15865 | Unannotated feature | neg | 413.2015 | 5.947 | 6.121 | 6.252 | 0.131 | 0.091 | 0.619 | 0.857 | Unannotated | NA | NA |
| metab_15277 | Caroverine | neg | 402.1555 | 4.837 | 4.330 | 4.204 | -0.126 | -0.091 | 0.619 | 0.857 | Unannotated | NA | NA |
| metab_10265 | Unannotated feature | neg | 216.0123 | 2.524 | 4.692 | 4.640 | -0.052 | -0.091 | 0.619 | 0.857 | Unannotated | NA | NA |
| metab_11672 | Unannotated feature | neg | 438.1988 | 5.322 | 3.840 | 3.704 | -0.136 | 0.091 | 0.619 | 0.857 | Unannotated | NA | NA |
| metab_1670 | Unannotated feature | pos | 354.1708 | 4.417 | 3.511 | 3.539 | 0.028 | 0.091 | 0.619 | 0.857 | Unannotated | NA | NA |
| metab_16852 | Unannotated feature | neg | 309.1277 | 7.730 | 4.870 | 5.027 | 0.157 | 0.091 | 0.619 | 0.857 | Unannotated | NA | NA |
| metab_14935 | Unannotated feature | neg | 175.0970 | 4.191 | 3.949 | 3.763 | -0.185 | -0.091 | 0.619 | 0.857 | Unannotated | NA | NA |
| metab_17267 | Unannotated feature | neg | 593.8554 | 7.720 | 4.704 | 4.716 | 0.012 | 0.091 | 0.619 | 0.857 | Unannotated | NA | NA |
| metab_18959 | Unannotated feature | neg | 229.1192 | 3.247 | 4.160 | 4.316 | 0.155 | -0.091 | 0.619 | 0.857 | Unannotated | NA | NA |
| metab_9782 | Unannotated feature | neg | 285.0077 | 3.055 | 3.548 | 3.395 | -0.154 | -0.091 | 0.619 | 0.857 | Unannotated | NA | NA |
| metab_11508 | Unannotated feature | neg | 261.9278 | 0.512 | 3.799 | 3.926 | 0.127 | -0.091 | 0.619 | 0.857 | Unannotated | NA | NA |
| metab_831 | Unannotated feature | pos | 544.7316 | 0.593 | 4.590 | 4.417 | -0.173 | -0.091 | 0.619 | 0.857 | Unannotated | NA | NA |
| metab_2366 | Unannotated feature | pos | 266.9278 | 0.509 | 3.290 | 3.580 | 0.290 | 0.091 | 0.619 | 0.857 | Unannotated | NA | NA |
| metab_2382 | Unannotated feature | pos | 189.1793 | 0.545 | 4.636 | 4.666 | 0.031 | -0.091 | 0.619 | 0.857 | Unannotated | NA | NA |
| metab_289 | Unannotated feature | pos | 303.0782 | 0.485 | 6.050 | 5.978 | -0.072 | -0.091 | 0.619 | 0.857 | Unannotated | NA | NA |
| metab_16858 | Unannotated feature | neg | 345.8942 | 7.730 | 5.354 | 5.335 | -0.019 | -0.091 | 0.619 | 0.857 | Unannotated | NA | NA |
| metab_17244 | Unannotated feature | neg | 471.2431 | 7.726 | 4.868 | 4.894 | 0.026 | 0.091 | 0.619 | 0.857 | Unannotated | NA | NA |
| metab_9797 | Unannotated feature | neg | 276.0996 | 3.041 | 4.250 | 4.287 | 0.037 | 0.091 | 0.619 | 0.857 | Unannotated | NA | NA |
| metab_11582 | Unannotated feature | neg | 170.8326 | 0.457 | 4.374 | 4.235 | -0.139 | 0.091 | 0.619 | 0.857 | Unannotated | NA | NA |
| metab_17253 | Unannotated feature | neg | 304.9145 | 7.726 | 5.911 | 5.910 | -0.001 | -0.091 | 0.619 | 0.857 | Unannotated | NA | NA |
| metab_3027 | Unannotated feature | pos | 94.8096 | 3.219 | 3.709 | 3.768 | 0.059 | 0.091 | 0.619 | 0.857 | Unannotated | NA | NA |
| metab_4934 | Unannotated feature | pos | 301.1416 | 7.626 | 5.058 | 5.036 | -0.022 | -0.091 | 0.619 | 0.857 | Unannotated | NA | NA |
| metab_12456 | Unannotated feature | neg | 185.8651 | 7.870 | 5.528 | 5.540 | 0.012 | 0.091 | 0.619 | 0.857 | Unannotated | NA | NA |
| metab_4750 | Unannotated feature | pos | 220.9350 | 7.708 | 5.173 | 5.191 | 0.018 | 0.091 | 0.619 | 0.857 | Unannotated | NA | NA |
| metab_7490 | Unannotated feature | pos | 307.1658 | 3.775 | 4.104 | 4.054 | -0.050 | 0.091 | 0.619 | 0.857 | Unannotated | NA | NA |
| metab_15159 | Unannotated feature | neg | 115.0757 | 4.653 | 5.278 | 5.330 | 0.052 | 0.091 | 0.619 | 0.857 | Unannotated | NA | NA |
| metab_196 | Unannotated feature | pos | 225.0230 | 0.593 | 4.235 | 4.120 | -0.116 | -0.091 | 0.619 | 0.857 | Unannotated | NA | NA |
| metab_11940 | 3-Dehydrocholic Acid | neg | 405.2650 | 6.130 | 6.505 | 6.471 | -0.033 | -0.091 | 0.619 | 0.857 | Moderate putative | NA | NA |
| metab_4523 | Unannotated feature | pos | 512.5051 | 6.710 | 6.562 | 6.551 | -0.011 | 0.091 | 0.619 | 0.857 | Unannotated | NA | NA |
| metab_16937 | Unannotated feature | neg | 128.8774 | 7.827 | 4.601 | 4.587 | -0.015 | 0.091 | 0.619 | 0.857 | Unannotated | NA | NA |
| metab_14855 | 2,4-Dimethylpimelic acid | neg | 233.1029 | 4.008 | 4.524 | 4.580 | 0.056 | 0.091 | 0.619 | 0.857 | Unannotated | Lipids and lipid-like molecules | Fatty Acyls |
| metab_3683 | Tyrosine methylester | pos | 228.1234 | 5.260 | 3.375 | 2.963 | -0.412 | -0.091 | 0.619 | 0.857 | Tentative | NA | NA |
| metab_17052 | Unannotated feature | neg | 176.8364 | 7.985 | 4.502 | 4.476 | -0.026 | -0.091 | 0.619 | 0.857 | Unannotated | NA | NA |
| metab_13841 | Unannotated feature | neg | 115.0394 | 2.183 | 5.972 | 6.177 | 0.205 | 0.091 | 0.619 | 0.857 | Unannotated | NA | NA |
| metab_10379 | Unannotated feature | neg | 247.1089 | 3.239 | 5.275 | 5.208 | -0.067 | -0.091 | 0.619 | 0.857 | Unannotated | NA | NA |
| metab_2252 | Unannotated feature | pos | 143.0732 | 3.129 | 4.480 | 4.405 | -0.075 | 0.091 | 0.619 | 0.857 | Unannotated | NA | NA |
| metab_18691 | Unannotated feature | neg | 323.6223 | 5.909 | 5.191 | 5.440 | 0.249 | 0.091 | 0.619 | 0.857 | Unannotated | NA | NA |
| metab_8777 | 4-Methylpyrimidine | pos | 95.0609 | 0.728 | 4.544 | 4.463 | -0.081 | -0.091 | 0.619 | 0.857 | Moderate putative | NA | NA |
| metab_16756 | Unannotated feature | neg | 317.0992 | 1.958 | 5.272 | 5.215 | -0.057 | -0.091 | 0.619 | 0.857 | Unannotated | NA | NA |
| metab_10483 | Unannotated feature | neg | 265.0411 | 1.952 | 4.760 | 4.869 | 0.110 | -0.091 | 0.619 | 0.857 | Unannotated | NA | NA |
| metab_11265 | Unannotated feature | neg | 386.1576 | 5.087 | 4.523 | 5.216 | 0.693 | 0.091 | 0.619 | 0.857 | Unannotated | NA | NA |
| metab_11286 | Unannotated feature | neg | 437.0920 | 5.095 | 5.018 | 4.276 | -0.743 | -0.091 | 0.619 | 0.857 | Unannotated | NA | NA |
| metab_8269 | Unannotated feature | pos | 193.1000 | 2.199 | 4.244 | 4.293 | 0.049 | 0.091 | 0.619 | 0.857 | Unannotated | NA | NA |
| metab_604 | DG(15:0/18:0/0:0) | pos | 605.5131 | 7.450 | 4.220 | 3.997 | -0.223 | -0.091 | 0.619 | 0.857 | Tentative | NA | NA |
| metab_9153 | Unannotated feature | neg | 324.1568 | 3.845 | 3.904 | 3.604 | -0.299 | -0.091 | 0.619 | 0.857 | Unannotated | NA | NA |
| metab_9162 | Unannotated feature | neg | 300.1823 | 3.834 | 2.588 | 2.297 | -0.291 | -0.091 | 0.619 | 0.857 | Unannotated | NA | NA |
| metab_1234 | Unannotated feature | pos | 187.1484 | 6.390 | 5.255 | 5.164 | -0.091 | -0.091 | 0.619 | 0.857 | Unannotated | NA | NA |
| metab_1237 | Unannotated feature | pos | 528.2179 | 6.375 | 4.143 | 4.505 | 0.363 | 0.091 | 0.619 | 0.857 | Unannotated | NA | NA |
| metab_12681 | Unannotated feature | neg | 654.3160 | 5.671 | 5.883 | 6.080 | 0.196 | 0.091 | 0.619 | 0.857 | Unannotated | NA | NA |
| metab_1123 | Unannotated feature | pos | 322.2731 | 6.622 | 5.137 | 4.995 | -0.142 | -0.091 | 0.619 | 0.857 | Unannotated | NA | NA |
| metab_12112 | Unannotated feature | neg | 457.2002 | 5.834 | 5.283 | 5.674 | 0.391 | 0.091 | 0.619 | 0.857 | Unannotated | NA | NA |
| metab_19621 | Vanillin | neg | 303.0879 | 4.677 | 3.236 | 3.785 | 0.549 | 0.091 | 0.619 | 0.857 | Tentative | NA | NA |
| metab_870 | Unannotated feature | pos | 613.4456 | 6.734 | 6.527 | 6.476 | -0.051 | -0.091 | 0.619 | 0.857 | Unannotated | NA | NA |
| metab_2501 | Aplysiatoxin, 17-debromo- | pos | 593.3351 | 5.814 | 7.596 | 7.586 | -0.010 | -0.091 | 0.619 | 0.857 | Unannotated | NA | NA |
| metab_8537 | Unannotated feature | pos | 371.1457 | 1.348 | 4.501 | 4.566 | 0.065 | 0.091 | 0.619 | 0.857 | Unannotated | NA | NA |
| metab_8540 | Glycyl-Lysine | pos | 186.1239 | 1.341 | 4.471 | 4.509 | 0.038 | 0.091 | 0.619 | 0.857 | Unannotated | NA | NA |
| metab_600 | Unannotated feature | pos | 309.0875 | 5.782 | 4.467 | 4.877 | 0.410 | 0.091 | 0.619 | 0.857 | Unannotated | NA | NA |
| metab_12964 | Unannotated feature | neg | 365.1971 | 6.240 | 6.431 | 6.357 | -0.074 | -0.091 | 0.619 | 0.857 | Unannotated | NA | NA |
| metab_8479 | Unannotated feature | pos | 257.1500 | 1.692 | 4.789 | 4.655 | -0.135 | -0.091 | 0.619 | 0.857 | Unannotated | NA | NA |
| metab_7990 | Unannotated feature | pos | 124.0761 | 2.853 | 3.568 | 3.623 | 0.055 | 0.091 | 0.619 | 0.857 | Unannotated | NA | NA |
| metab_7999 | Unannotated feature | pos | 261.1450 | 3.444 | 5.539 | 5.593 | 0.054 | -0.091 | 0.619 | 0.857 | Unannotated | NA | NA |
| metab_5510 | (4E,14Z)-2-Aminooctadeca-4,14-diene-1,3-diol | pos | 330.3009 | 6.335 | 4.696 | 4.411 | -0.285 | -0.091 | 0.619 | 0.857 | Unannotated | NA | NA |
| metab_9665 | 5-Hydroxyconiferyl alcohol | neg | 241.0717 | 3.173 | 4.461 | 4.538 | 0.078 | 0.091 | 0.619 | 0.857 | Tentative | NA | NA |
| metab_2739 | Unannotated feature | pos | 102.5360 | 2.240 | 3.622 | 3.593 | -0.029 | 0.091 | 0.619 | 0.857 | Unannotated | NA | NA |
| metab_18938 | Unannotated feature | neg | 261.1344 | 5.600 | 4.287 | 4.173 | -0.114 | -0.091 | 0.619 | 0.857 | Unannotated | NA | NA |
| metab_5099 | Carbocyclic 3-deazaadenosine | pos | 529.2523 | 7.021 | 4.822 | 4.543 | -0.279 | 0.091 | 0.619 | 0.857 | Unannotated | NA | NA |
| metab_10061 | Unannotated feature | neg | 155.0707 | 2.785 | 3.541 | 3.478 | -0.063 | -0.091 | 0.619 | 0.857 | Unannotated | NA | NA |
| metab_10093 | Osmundalin | neg | 271.0830 | 2.771 | 5.082 | 5.124 | 0.043 | 0.091 | 0.619 | 0.857 | Unannotated | NA | NA |
| metab_11452 | Unannotated feature | neg | 159.0143 | 0.533 | 3.508 | 3.663 | 0.155 | 0.091 | 0.619 | 0.857 | Unannotated | NA | NA |
| metab_17829 | 5-(14-Nonadecenyl)-1,3-benzenediol | neg | 419.3161 | 6.782 | 4.814 | 5.258 | 0.444 | 0.091 | 0.619 | 0.857 | Unannotated | NA | NA |
| metab_9389 | Unannotated feature | neg | 221.0452 | 4.049 | 5.490 | 5.382 | -0.107 | -0.091 | 0.619 | 0.857 | Unannotated | NA | NA |
| metab_10543 | 2-Methoxy-1,3-benzenediol | neg | 139.0394 | 1.944 | 4.199 | 4.358 | 0.159 | 0.091 | 0.619 | 0.857 | Unannotated | NA | NA |
| metab_2206 | Unannotated feature | pos | 284.0001 | 3.007 | 4.267 | 4.139 | -0.129 | -0.091 | 0.619 | 0.857 | Unannotated | NA | NA |
| metab_17761 | Unannotated feature | neg | 380.3173 | 6.899 | 5.748 | 5.566 | -0.182 | -0.091 | 0.619 | 0.857 | Unannotated | NA | NA |
| metab_8338 | Tetrahydroharmine | pos | 239.1143 | 1.984 | 4.735 | 4.925 | 0.190 | 0.091 | 0.619 | 0.857 | Tentative | NA | NA |
| metab_5278 | Unannotated feature | pos | 737.5322 | 6.614 | 4.750 | 4.700 | -0.051 | 0.091 | 0.619 | 0.857 | Unannotated | NA | NA |
| metab_14602 | Cis-4-Hydroxycyclohexylacetic acid | neg | 203.0921 | 3.509 | 4.941 | 4.972 | 0.031 | 0.091 | 0.619 | 0.857 | Unannotated | NA | NA |
| metab_16204 | Unannotated feature | neg | 511.1528 | 6.420 | 3.000 | 4.688 | 1.687 | 0.091 | 0.619 | 0.857 | Unannotated | NA | NA |
| metab_12312 | Unannotated feature | neg | 402.3019 | 6.884 | 5.414 | 5.458 | 0.044 | -0.091 | 0.619 | 0.857 | Unannotated | NA | NA |
| metab_13972 | Unannotated feature | neg | 199.0066 | 2.659 | 5.125 | 5.080 | -0.045 | -0.091 | 0.619 | 0.857 | Unannotated | NA | NA |
| metab_1145 | Unannotated feature | pos | 475.3404 | 6.566 | 5.994 | 5.948 | -0.046 | -0.091 | 0.619 | 0.857 | Unannotated | NA | NA |
| metab_19488 | Unannotated feature | neg | 364.0866 | 3.502 | 4.171 | 4.094 | -0.077 | -0.091 | 0.619 | 0.857 | Unannotated | NA | NA |
| metab_8031 | 5-Hydroxymethyl-2-furancarboxaldehyde | pos | 168.0658 | 2.803 | 4.321 | 4.359 | 0.038 | 0.091 | 0.619 | 0.857 | Tentative | NA | NA |
| metab_18896 | Unannotated feature | neg | 309.1192 | 3.227 | 3.784 | 3.790 | 0.006 | 0.091 | 0.619 | 0.857 | Unannotated | NA | NA |
| metab_17947 | Unannotated feature | neg | 250.1448 | 6.592 | 5.224 | 5.051 | -0.173 | -0.091 | 0.619 | 0.857 | Unannotated | NA | NA |
| metab_13011 | Unannotated feature | neg | 317.1469 | 0.628 | 4.872 | 4.925 | 0.053 | -0.091 | 0.619 | 0.857 | Unannotated | NA | NA |
| metab_6292 | Unannotated feature | pos | 484.2834 | 5.791 | 3.527 | 3.378 | -0.148 | -0.091 | 0.619 | 0.857 | Unannotated | NA | NA |
| metab_6703 | Unannotated feature | pos | 115.0371 | 0.625 | 3.085 | 3.587 | 0.502 | 0.091 | 0.619 | 0.857 | Unannotated | NA | NA |
| metab_773 | Unannotated feature | pos | 611.3457 | 6.151 | 5.586 | 6.356 | 0.770 | 0.091 | 0.619 | 0.857 | Unannotated | NA | NA |
| metab_775 | Unannotated feature | pos | 180.1021 | 6.159 | 6.488 | 6.673 | 0.185 | 0.091 | 0.619 | 0.857 | Unannotated | NA | NA |
| metab_16033 | Unannotated feature | neg | 167.0204 | 1.119 | 6.503 | 6.478 | -0.024 | 0.091 | 0.619 | 0.857 | Unannotated | NA | NA |
| metab_731 | Unannotated feature | pos | 456.8429 | 0.531 | 4.267 | 4.101 | -0.166 | -0.091 | 0.619 | 0.857 | Unannotated | NA | NA |
| metab_19256 | Unannotated feature | neg | 368.9615 | 3.210 | 5.688 | 5.659 | -0.029 | -0.091 | 0.619 | 0.857 | Unannotated | NA | NA |
| metab_13449 | Unannotated feature | neg | 674.7138 | 0.603 | 3.652 | 3.762 | 0.109 | 0.091 | 0.619 | 0.857 | Unannotated | NA | NA |
| metab_7199 | N-formimidoyl-glutamic acid | pos | 175.0717 | 0.680 | 4.846 | 5.139 | 0.293 | 0.091 | 0.619 | 0.857 | Tentative | NA | NA |
| metab_4260 | Norethynodrel | pos | 321.1814 | 5.944 | 5.359 | 5.864 | 0.505 | -0.091 | 0.619 | 0.857 | Tentative | NA | NA |
| metab_4296 | Osthenol | pos | 272.1286 | 5.984 | 4.243 | 3.878 | -0.365 | -0.091 | 0.619 | 0.857 | Tentative | NA | NA |
| metab_4305 | Cinncassiol D4 2-glucoside | pos | 515.2842 | 6.024 | 4.560 | 4.030 | -0.530 | -0.091 | 0.619 | 0.857 | Unannotated | NA | NA |
| metab_17892 | Unannotated feature | neg | 598.3800 | 6.671 | 4.302 | 4.627 | 0.325 | -0.091 | 0.619 | 0.857 | Unannotated | NA | NA |
| metab_13042 | Unannotated feature | neg | 522.7648 | 0.596 | 4.553 | 4.732 | 0.179 | 0.091 | 0.619 | 0.857 | Unannotated | NA | NA |
| metab_12618 | Unannotated feature | neg | 641.3213 | 6.007 | 5.054 | 5.373 | 0.319 | 0.091 | 0.619 | 0.857 | Unannotated | NA | NA |
| metab_15806 | 6-(alpha-D-Glucosaminyl)-1D-myo-inositol | neg | 376.1041 | 5.876 | 5.791 | 5.913 | 0.122 | -0.091 | 0.619 | 0.857 | Tentative | Organic oxygen compounds | Organooxygen compounds |
| metab_1413 | Unannotated feature | pos | 452.3130 | 6.064 | 6.272 | 6.278 | 0.006 | -0.091 | 0.619 | 0.857 | Unannotated | NA | NA |
| metab_17274 | Unannotated feature | neg | 327.9215 | 7.720 | 5.990 | 5.978 | -0.012 | -0.091 | 0.619 | 0.857 | Unannotated | NA | NA |
| metab_2972 | Unannotated feature | pos | 156.1022 | 3.027 | 4.776 | 4.575 | -0.202 | -0.091 | 0.619 | 0.857 | Unannotated | NA | NA |
| metab_19894 | Pyrocatechol | neg | 109.0288 | 3.414 | 4.966 | 4.902 | -0.063 | -0.091 | 0.619 | 0.857 | Strong putative | NA | NA |
| metab_12014 | Unannotated feature | neg | 349.2021 | 6.271 | 5.668 | 5.577 | -0.091 | 0.091 | 0.619 | 0.857 | Unannotated | NA | NA |
| metab_11058 | Unannotated feature | neg | 125.0002 | 0.738 | 4.425 | 4.434 | 0.008 | -0.091 | 0.619 | 0.857 | Unannotated | NA | NA |
| metab_19837 | (+/-)-Catechin | neg | 289.0721 | 3.882 | 4.783 | 4.549 | -0.234 | -0.091 | 0.619 | 0.857 | Strong putative | NA | NA |
| metab_12826 | Unannotated feature | neg | 461.0481 | 0.659 | 4.311 | 3.925 | -0.386 | -0.091 | 0.619 | 0.857 | Unannotated | NA | NA |
| metab_14660 | 2-Ketohexanoic acid | neg | 175.0606 | 3.612 | 5.904 | 5.827 | -0.076 | 0.091 | 0.619 | 0.857 | Tentative | NA | NA |
| metab_16222 | Unannotated feature | neg | 519.2788 | 6.467 | 5.160 | 5.058 | -0.102 | -0.091 | 0.619 | 0.857 | Unannotated | NA | NA |
| metab_16639 | Unannotated feature | neg | 94.0290 | 1.947 | 5.037 | 5.127 | 0.090 | 0.091 | 0.619 | 0.857 | Unannotated | NA | NA |
| metab_388 | Unannotated feature | pos | 102.0344 | 0.039 | 5.046 | 5.052 | 0.007 | 0.091 | 0.619 | 0.857 | Unannotated | NA | NA |
| metab_13593 | Valylaspartic acid | neg | 269.0530 | 1.369 | 4.702 | 4.752 | 0.050 | -0.091 | 0.619 | 0.857 | Unannotated | NA | NA |
| metab_9876 | Unannotated feature | neg | 193.0502 | 2.972 | 2.786 | 2.436 | -0.350 | -0.091 | 0.619 | 0.857 | Unannotated | NA | NA |
| metab_15949 | Unannotated feature | neg | 280.6220 | 6.052 | 4.637 | 4.068 | -0.569 | -0.086 | 0.636 | 0.866 | Unannotated | NA | NA |
| metab_13600 | Clavulanate | neg | 198.0403 | 1.422 | 5.478 | 5.648 | 0.170 | 0.086 | 0.636 | 0.866 | Tentative | NA | NA |
| metab_12440 | Unannotated feature | neg | 390.8749 | 7.720 | 5.726 | 5.701 | -0.026 | -0.086 | 0.636 | 0.866 | Unannotated | NA | NA |
| metab_396 | Unannotated feature | pos | 138.4802 | 0.019 | 4.356 | 4.370 | 0.014 | 0.086 | 0.636 | 0.866 | Unannotated | NA | NA |
| metab_368 | Unannotated feature | pos | 429.0900 | 0.276 | 5.602 | 5.613 | 0.011 | -0.086 | 0.636 | 0.866 | Unannotated | NA | NA |
| metab_5405 | Unannotated feature | pos | 296.2953 | 6.438 | 4.057 | 4.042 | -0.015 | -0.086 | 0.636 | 0.866 | Unannotated | NA | NA |
| metab_16310 | DG(14:1(9Z)/18:4(6Z,9Z,12Z,15Z)/0:0) | neg | 603.4275 | 6.640 | 4.970 | 4.521 | -0.449 | -0.086 | 0.636 | 0.866 | Tentative | NA | NA |
| metab_10923 | 4'-Azidocytidine | neg | 265.0679 | 0.819 | 5.736 | 5.207 | -0.528 | -0.086 | 0.636 | 0.866 | Unannotated | NA | NA |
| metab_16876 | Unannotated feature | neg | 171.9804 | 7.741 | 5.879 | 5.870 | -0.009 | -0.086 | 0.636 | 0.866 | Unannotated | NA | NA |
| metab_11048 | 5-phosphonooxy-L-lysine | neg | 277.0383 | 0.738 | 3.429 | 3.183 | -0.246 | -0.086 | 0.636 | 0.866 | Tentative | NA | NA |
| metab_18392 | Unannotated feature | neg | 426.1964 | 6.138 | 5.404 | 5.340 | -0.064 | -0.086 | 0.636 | 0.866 | Unannotated | NA | NA |
| metab_18382 | 12-Hydroxy-13-O-D-glucuronoside-octadec-9Z-enoate | neg | 489.2717 | 6.146 | 4.806 | 4.740 | -0.067 | 0.086 | 0.636 | 0.866 | Unannotated | NA | NA |
| metab_1394 | Capsianoside I | pos | 643.3360 | 6.087 | 4.581 | 4.728 | 0.146 | 0.086 | 0.636 | 0.866 | Unannotated | NA | NA |
| metab_11140 | Decarbamoylsaxitoxin | neg | 277.1041 | 0.698 | 3.529 | 3.993 | 0.464 | 0.086 | 0.636 | 0.866 | Tentative | NA | NA |
| metab_18371 | 3-Methoxyanthranilate | neg | 166.0503 | 3.003 | 4.998 | 4.850 | -0.147 | -0.086 | 0.636 | 0.866 | Tentative | NA | NA |
| metab_18329 | (3R,5R)-7-[2-(4-Fluorophenyl)-3-phenyl-4-(phenylcarbamoyl)-5-propan-2-ylpyrrol-1-yl]-3-hydroperoxy-5-hydroxyheptanoic acid | neg | 555.2310 | 6.193 | 5.140 | 5.255 | 0.115 | 0.086 | 0.636 | 0.866 | Unannotated | NA | NA |
| metab_19764 | 1,4-Cyclohexanedicarboxylic Acid | neg | 171.0656 | 4.484 | 5.069 | 5.084 | 0.015 | 0.086 | 0.636 | 0.866 | Moderate putative | NA | NA |
| metab_16740 | Unannotated feature | neg | 402.9294 | 7.666 | 4.602 | 4.635 | 0.034 | 0.086 | 0.636 | 0.866 | Unannotated | NA | NA |
| metab_4781 | Unannotated feature | pos | 110.0205 | 7.796 | 6.056 | 6.068 | 0.012 | 0.086 | 0.636 | 0.866 | Unannotated | NA | NA |
| metab_16390 | Unannotated feature | neg | 387.2870 | 6.821 | 5.179 | 5.511 | 0.332 | 0.086 | 0.636 | 0.866 | Unannotated | NA | NA |
| metab_5254 | 3-O-(beta-D-Glucopyranosyl)-soyasapogenol B | pos | 585.4142 | 6.654 | 5.713 | 5.672 | -0.041 | -0.086 | 0.636 | 0.866 | Unannotated | NA | NA |
| metab_6541 | Unannotated feature | pos | 134.0715 | 5.541 | 3.991 | 4.031 | 0.040 | 0.086 | 0.636 | 0.866 | Unannotated | NA | NA |
| metab_13977 | Unannotated feature | neg | 500.9207 | 0.485 | 6.744 | 6.748 | 0.004 | -0.086 | 0.636 | 0.866 | Unannotated | NA | NA |
| metab_13467 | Unannotated feature | neg | 268.0147 | 0.943 | 4.077 | 3.908 | -0.169 | -0.086 | 0.636 | 0.866 | Unannotated | NA | NA |
| metab_5891 | Unannotated feature | pos | 223.0641 | 0.308 | 5.435 | 5.455 | 0.020 | 0.086 | 0.636 | 0.866 | Unannotated | NA | NA |
| metab_12263 | Cer(d18:0/18:1(12Z)-O(9S,10R)) | neg | 624.5221 | 7.025 | 5.297 | 5.168 | -0.129 | -0.086 | 0.636 | 0.866 | Unannotated | NA | NA |
| metab_16994 | Unannotated feature | neg | 241.8989 | 7.985 | 6.095 | 6.087 | -0.008 | -0.086 | 0.636 | 0.866 | Unannotated | NA | NA |
| metab_13036 | Unannotated feature | neg | 286.8541 | 0.603 | 5.479 | 5.459 | -0.020 | -0.086 | 0.636 | 0.866 | Unannotated | NA | NA |
| metab_11915 | Unannotated feature | neg | 445.2364 | 6.076 | 5.216 | 5.302 | 0.086 | 0.086 | 0.636 | 0.866 | Unannotated | NA | NA |
| metab_16370 | LysoPC(0:0/18:1(9Z)) | neg | 566.3477 | 6.742 | 4.832 | 4.407 | -0.425 | -0.086 | 0.636 | 0.866 | Unannotated | NA | NA |
| metab_16363 | Unannotated feature | neg | 397.2265 | 6.734 | 5.899 | 5.859 | -0.040 | -0.086 | 0.636 | 0.866 | Unannotated | NA | NA |
| metab_11715 | Unannotated feature | neg | 371.0710 | 5.526 | 3.952 | 3.927 | -0.025 | -0.086 | 0.636 | 0.866 | Unannotated | NA | NA |
| metab_4865 | Unannotated feature | pos | 182.0225 | 7.724 | 5.198 | 5.209 | 0.011 | 0.086 | 0.636 | 0.866 | Unannotated | NA | NA |
| metab_8619 | Unannotated feature | pos | 151.0390 | 1.062 | 4.104 | 4.248 | 0.143 | 0.086 | 0.636 | 0.866 | Unannotated | NA | NA |
| metab_8591 | 4-(Glutamylamino) butanoate | pos | 197.0925 | 1.161 | 4.175 | 4.414 | 0.238 | 0.086 | 0.636 | 0.866 | Tentative | NA | NA |
| metab_4980 | Cyclohexane | pos | 102.1282 | 7.530 | 7.628 | 7.643 | 0.014 | 0.086 | 0.636 | 0.866 | Tentative | NA | NA |
| metab_16176 | Unannotated feature | neg | 405.3012 | 6.381 | 5.090 | 5.075 | -0.014 | -0.086 | 0.636 | 0.866 | Unannotated | NA | NA |
| metab_9326 | 1-O-Caffeoylglucose | neg | 341.0883 | 3.577 | 3.261 | 3.810 | 0.549 | 0.086 | 0.636 | 0.866 | Tentative | NA | NA |
| metab_13941 | Unannotated feature | neg | 218.0126 | 2.590 | 4.205 | 4.578 | 0.373 | 0.086 | 0.636 | 0.866 | Unannotated | NA | NA |
| metab_16171 | Ecdysone | neg | 463.3071 | 6.373 | 6.324 | 6.365 | 0.041 | 0.086 | 0.636 | 0.866 | Tentative | NA | NA |
| metab_17799 | Unannotated feature | neg | 431.3026 | 6.853 | 3.552 | 3.570 | 0.018 | 0.086 | 0.636 | 0.866 | Unannotated | NA | NA |
| metab_2187 | Unannotated feature | pos | 351.1558 | 2.970 | 4.718 | 4.638 | -0.079 | -0.086 | 0.636 | 0.866 | Unannotated | NA | NA |
| metab_17884 | Unannotated feature | neg | 483.2742 | 6.687 | 6.595 | 6.566 | -0.030 | -0.086 | 0.636 | 0.866 | Unannotated | NA | NA |
| metab_13408 | Unannotated feature | neg | 126.0189 | 0.784 | 4.002 | 4.067 | 0.065 | 0.086 | 0.636 | 0.866 | Unannotated | NA | NA |
| metab_19025 | 3-(3,4-Dihydroxyphenyl)-N-[2-(3,4-dihydroxyphenyl)ethyl]prop-2-enamide | neg | 360.1098 | 3.281 | 4.205 | 4.182 | -0.024 | 0.086 | 0.636 | 0.866 | Unannotated | NA | NA |
| metab_9645 | Epiheterodendrin | neg | 306.1198 | 3.192 | 3.962 | 4.168 | 0.205 | 0.086 | 0.636 | 0.866 | Unannotated | NA | NA |
| metab_8490 | 2-Hydroxynevirapine | pos | 300.1453 | 1.685 | 4.358 | 4.480 | 0.121 | 0.086 | 0.636 | 0.866 | Tentative | NA | NA |
| metab_15378 | Medicarpin | neg | 315.0883 | 5.038 | 3.756 | 3.710 | -0.046 | 0.086 | 0.636 | 0.866 | Tentative | NA | NA |
| metab_11709 | Unannotated feature | neg | 482.1879 | 5.491 | 4.497 | 4.129 | -0.368 | -0.086 | 0.636 | 0.866 | Unannotated | NA | NA |
| metab_18022 | Unannotated feature | neg | 149.0270 | 2.903 | 5.085 | 5.057 | -0.027 | 0.086 | 0.636 | 0.866 | Unannotated | NA | NA |
| metab_18009 | Dimethisterone | neg | 385.2391 | 6.506 | 4.103 | 4.362 | 0.259 | 0.086 | 0.636 | 0.866 | Tentative | NA | NA |
| metab_1177 | Unannotated feature | pos | 408.3117 | 6.510 | 6.351 | 6.327 | -0.024 | -0.086 | 0.636 | 0.866 | Unannotated | NA | NA |
| metab_5093 | Unannotated feature | pos | 507.3665 | 7.036 | 5.631 | 5.656 | 0.025 | 0.086 | 0.636 | 0.866 | Unannotated | NA | NA |
| metab_16570 | Unannotated feature | neg | 527.6481 | 7.310 | 4.579 | 4.559 | -0.020 | 0.086 | 0.636 | 0.866 | Unannotated | NA | NA |
| metab_17461 | Unannotated feature | neg | 112.9632 | 7.607 | 4.912 | 4.879 | -0.033 | -0.086 | 0.636 | 0.866 | Unannotated | NA | NA |
| metab_2479 | N-Nitroso-3-hydroxypyrrolidine | pos | 158.0927 | 0.720 | 3.750 | 3.705 | -0.045 | -0.086 | 0.636 | 0.866 | Unannotated | NA | NA |
| metab_13373 | Unannotated feature | neg | 181.0170 | 0.738 | 4.752 | 4.754 | 0.002 | -0.086 | 0.636 | 0.866 | Unannotated | NA | NA |
| metab_10201 | O-Desmethyl-lacosamide | neg | 281.1141 | 2.649 | 4.215 | 4.195 | -0.020 | -0.086 | 0.636 | 0.866 | Unannotated | NA | NA |
| metab_13353 | N-Acetylhistidine | neg | 196.0722 | 0.722 | 4.520 | 4.601 | 0.082 | -0.086 | 0.636 | 0.866 | Strong putative | NA | NA |
| metab_3182 | Unannotated feature | pos | 364.2828 | 6.734 | 5.199 | 4.730 | -0.470 | -0.086 | 0.636 | 0.866 | Unannotated | NA | NA |
| metab_17176 | Unannotated feature | neg | 174.8281 | 7.745 | 4.640 | 4.665 | 0.025 | 0.086 | 0.636 | 0.866 | Unannotated | NA | NA |
| metab_12648 | Unannotated feature | neg | 362.1148 | 5.952 | 5.083 | 5.099 | 0.016 | -0.086 | 0.636 | 0.866 | Unannotated | NA | NA |
| metab_1239 | Unannotated feature | pos | 149.1327 | 6.382 | 4.986 | 5.055 | 0.069 | -0.086 | 0.636 | 0.866 | Unannotated | NA | NA |
| metab_10670 | Unannotated feature | neg | 344.0052 | 1.530 | 5.000 | 4.834 | -0.167 | -0.086 | 0.636 | 0.866 | Unannotated | NA | NA |
| metab_18035 | Unannotated feature | neg | 528.3086 | 6.459 | 4.758 | 4.867 | 0.108 | 0.086 | 0.636 | 0.866 | Unannotated | NA | NA |
| metab_18068 | Unannotated feature | neg | 477.3227 | 6.420 | 6.564 | 6.668 | 0.104 | 0.086 | 0.636 | 0.866 | Unannotated | NA | NA |
| metab_14031 | Unannotated feature | neg | 361.1142 | 2.766 | 4.563 | 4.437 | -0.126 | -0.086 | 0.636 | 0.866 | Unannotated | NA | NA |
| metab_16330 | Unannotated feature | neg | 624.3953 | 6.663 | 5.651 | 5.741 | 0.090 | -0.086 | 0.636 | 0.866 | Unannotated | NA | NA |
| metab_5287 | Unannotated feature | pos | 352.2830 | 6.598 | 4.914 | 4.913 | -0.001 | 0.086 | 0.636 | 0.866 | Unannotated | NA | NA |
| metab_964 | N-[2-(3,5-Dioxopiperazino)propyl]-N-(2-amino-2-oxoethyl)glycine | pos | 319.1616 | 0.633 | 5.682 | 5.743 | 0.061 | 0.086 | 0.636 | 0.866 | Unannotated | NA | NA |
| metab_10473 | Unannotated feature | neg | 345.0807 | 1.952 | 5.010 | 4.876 | -0.135 | -0.086 | 0.636 | 0.866 | Unannotated | NA | NA |
| metab_16732 | Unannotated feature | neg | 374.9068 | 7.663 | 5.562 | 5.572 | 0.010 | 0.086 | 0.636 | 0.866 | Unannotated | NA | NA |
| metab_14237 | Unannotated feature | neg | 644.7778 | 0.520 | 3.846 | 3.997 | 0.151 | 0.086 | 0.636 | 0.866 | Unannotated | NA | NA |
| metab_17036 | Unannotated feature | neg | 251.8460 | 7.989 | 5.032 | 4.983 | -0.049 | -0.086 | 0.636 | 0.866 | Unannotated | NA | NA |
| metab_12548 | Unannotated feature | neg | 327.2178 | 6.209 | 6.787 | 6.651 | -0.136 | -0.086 | 0.636 | 0.866 | Unannotated | NA | NA |
| metab_4597 | Unannotated feature | pos | 393.2983 | 7.005 | 7.400 | 7.443 | 0.043 | 0.086 | 0.636 | 0.866 | Unannotated | NA | NA |
| metab_12220 | Unannotated feature | neg | 459.3486 | 6.876 | 6.676 | 6.731 | 0.055 | 0.086 | 0.636 | 0.866 | Unannotated | NA | NA |
| metab_12076 | Unannotated feature | neg | 479.2481 | 6.420 | 5.727 | 5.601 | -0.126 | -0.086 | 0.636 | 0.866 | Unannotated | NA | NA |
| metab_18127 | MG(0:0/20:4(5Z,8Z,11Z,14Z)/0:0) | neg | 423.2757 | 6.373 | 6.739 | 6.570 | -0.169 | -0.086 | 0.636 | 0.866 | Tentative | Lipids and lipid-like molecules | Endocannabinoids |
| metab_1892 | Unannotated feature | pos | 143.0705 | 1.378 | 3.140 | 3.436 | 0.296 | 0.086 | 0.636 | 0.866 | Unannotated | NA | NA |
| metab_7502 | Unannotated feature | pos | 141.9588 | 3.751 | 4.104 | 4.089 | -0.015 | -0.086 | 0.636 | 0.866 | Unannotated | NA | NA |
| metab_436 | Unannotated feature | pos | 99.5127 | 0.016 | 4.113 | 4.126 | 0.013 | 0.086 | 0.636 | 0.866 | Unannotated | NA | NA |
| metab_4762 | Unannotated feature | pos | 97.9919 | 7.732 | 5.170 | 5.181 | 0.011 | 0.086 | 0.636 | 0.866 | Unannotated | NA | NA |
| metab_13539 | Unannotated feature | neg | 203.0188 | 1.127 | 4.531 | 4.403 | -0.128 | -0.086 | 0.636 | 0.866 | Unannotated | NA | NA |
| metab_2776 | Unannotated feature | pos | 106.0736 | 2.500 | 4.655 | 4.642 | -0.013 | 0.086 | 0.636 | 0.866 | Unannotated | NA | NA |
| metab_2758 | Pretyrosine | pos | 210.0765 | 2.415 | 4.186 | 4.342 | 0.156 | 0.086 | 0.636 | 0.866 | Tentative | NA | NA |
| metab_9966 | Unannotated feature | neg | 329.1036 | 4.393 | 5.513 | 5.809 | 0.297 | 0.086 | 0.636 | 0.866 | Unannotated | NA | NA |
| metab_9916 | 2-[3-(sulfooxy)phenyl]acetic acid | neg | 230.9967 | 2.938 | 5.382 | 5.481 | 0.099 | 0.086 | 0.636 | 0.866 | Unannotated | NA | NA |
| metab_15604 | 13(S)-Hydroperoxylinolenic acid | neg | 347.1614 | 5.512 | 5.238 | 5.211 | -0.027 | -0.086 | 0.636 | 0.866 | Tentative | Lipids and lipid-like molecules | Fatty Acyls |
| metab_12671 | (S)-Oleuropeic acid | neg | 367.2129 | 5.774 | 5.805 | 5.660 | -0.144 | -0.086 | 0.636 | 0.866 | Unannotated | NA | NA |
| metab_1834 | Unannotated feature | pos | 146.0926 | 0.760 | 4.502 | 4.443 | -0.058 | -0.086 | 0.636 | 0.866 | Unannotated | NA | NA |
| metab_3675 | Unannotated feature | pos | 682.4100 | 6.638 | 3.323 | 3.985 | 0.661 | 0.086 | 0.636 | 0.866 | Unannotated | NA | NA |
| metab_16448 | Unannotated feature | neg | 94.0290 | 1.685 | 4.854 | 4.947 | 0.093 | 0.086 | 0.636 | 0.866 | Unannotated | NA | NA |
| metab_291 | Unannotated feature | pos | 218.9840 | 0.485 | 6.442 | 6.361 | -0.080 | -0.086 | 0.636 | 0.866 | Unannotated | NA | NA |
| metab_1049 | Unannotated feature | pos | 257.1500 | 1.944 | 4.975 | 5.039 | 0.064 | 0.086 | 0.636 | 0.866 | Unannotated | NA | NA |
| metab_7800 | 4-[(2,4-Dihydroxy-3,3-dimethylbutanoyl)amino]butanoic acid | pos | 234.1339 | 3.295 | 5.212 | 5.097 | -0.115 | -0.086 | 0.636 | 0.866 | Unannotated | Organic acids and derivatives | Carboxylic acids and derivatives |
| metab_7788 | DG(2:0/PGF2alpha/0:0) | pos | 247.1445 | 3.208 | 3.585 | 3.924 | 0.339 | 0.086 | 0.636 | 0.866 | Unannotated | NA | NA |
| metab_7777 | (2S)-3-Hydroxy-1-[(2S)-pyrrolidine-2-carbonyl]pyrrolidine-2-carboxylic acid | pos | 261.1449 | 3.275 | 5.608 | 5.643 | 0.035 | -0.086 | 0.636 | 0.866 | Unannotated | NA | NA |
| metab_14955 | Unannotated feature | neg | 347.0778 | 4.235 | 3.928 | 4.227 | 0.298 | 0.086 | 0.636 | 0.866 | Unannotated | NA | NA |
| metab_15014 | Unannotated feature | neg | 333.1462 | 4.359 | 3.803 | 4.030 | 0.227 | 0.086 | 0.636 | 0.866 | Unannotated | NA | NA |
| metab_9617 | Unannotated feature | neg | 317.9621 | 3.215 | 4.325 | 4.130 | -0.195 | -0.086 | 0.636 | 0.866 | Unannotated | NA | NA |
| metab_9560 | Unannotated feature | neg | 445.1684 | 3.271 | 3.027 | 3.072 | 0.045 | -0.086 | 0.636 | 0.866 | Unannotated | NA | NA |
| metab_16024 | Unannotated feature | neg | 391.1796 | 6.146 | 6.285 | 6.306 | 0.021 | 0.086 | 0.636 | 0.866 | Unannotated | NA | NA |
| metab_18910 | Ethyl 2-furanacrylate | neg | 391.1399 | 5.638 | 4.147 | 3.919 | -0.228 | -0.086 | 0.636 | 0.866 | Unannotated | NA | NA |
| metab_9779 | Unannotated feature | neg | 339.0395 | 0.738 | 4.189 | 3.877 | -0.312 | -0.086 | 0.636 | 0.866 | Unannotated | NA | NA |
| metab_15311 | Unannotated feature | neg | 256.0650 | 4.903 | 4.534 | 4.556 | 0.022 | -0.086 | 0.636 | 0.866 | Unannotated | NA | NA |
| metab_9902 | Unannotated feature | neg | 175.0970 | 2.956 | 4.398 | 4.611 | 0.213 | 0.086 | 0.636 | 0.866 | Unannotated | NA | NA |
| metab_12882 | Unannotated feature | neg | 557.3166 | 6.295 | 5.807 | 5.793 | -0.014 | -0.086 | 0.636 | 0.866 | Unannotated | NA | NA |
| metab_16087 | Pirmenol | neg | 375.1848 | 6.224 | 6.555 | 6.441 | -0.114 | -0.086 | 0.636 | 0.866 | Unannotated | NA | NA |
| metab_5621 | Unannotated feature | pos | 281.1387 | 6.199 | 4.943 | 4.903 | -0.041 | -0.086 | 0.636 | 0.866 | Unannotated | NA | NA |
| metab_16884 | Unannotated feature | neg | 320.8866 | 7.741 | 5.681 | 5.654 | -0.027 | -0.086 | 0.636 | 0.866 | Unannotated | NA | NA |
| metab_12851 | Unannotated feature | neg | 212.0618 | 0.644 | 4.670 | 4.846 | 0.176 | 0.086 | 0.636 | 0.866 | Unannotated | NA | NA |
| metab_17143 | Unannotated feature | neg | 136.9091 | 7.788 | 4.885 | 4.887 | 0.002 | -0.086 | 0.636 | 0.866 | Unannotated | NA | NA |
| metab_13807 | Unannotated feature | neg | 330.1675 | 2.036 | 5.135 | 5.092 | -0.042 | -0.086 | 0.636 | 0.866 | Unannotated | NA | NA |
| metab_11653 | Unannotated feature | neg | 192.9493 | 0.022 | 3.836 | 3.812 | -0.025 | -0.086 | 0.636 | 0.866 | Unannotated | NA | NA |
| metab_10134 | 5-Hydroxy-1-methylpyrrolidin-2-one | neg | 229.1192 | 2.754 | 4.177 | 4.249 | 0.072 | -0.086 | 0.636 | 0.866 | Unannotated | NA | NA |
| metab_15678 | Unannotated feature | neg | 603.3226 | 5.649 | 3.686 | 3.813 | 0.127 | 0.086 | 0.636 | 0.866 | Unannotated | NA | NA |
| metab_15279 | Unannotated feature | neg | 247.0556 | 4.850 | 1.102 | 1.327 | 0.225 | -0.086 | 0.636 | 0.866 | Unannotated | NA | NA |
| metab_17095 | Unannotated feature | neg | 187.8606 | 7.874 | 6.653 | 6.656 | 0.003 | 0.086 | 0.636 | 0.866 | Unannotated | NA | NA |
| metab_8626 | Adenosine | pos | 268.1044 | 1.049 | 3.489 | 3.206 | -0.282 | -0.086 | 0.636 | 0.866 | Strong putative | NA | NA |
| metab_16833 | Unannotated feature | neg | 457.8802 | 7.726 | 5.084 | 5.094 | 0.010 | 0.086 | 0.636 | 0.866 | Unannotated | NA | NA |
| metab_460 | Paullinic acid | pos | 328.3215 | 6.470 | 5.546 | 5.482 | -0.064 | -0.086 | 0.636 | 0.866 | Tentative | NA | NA |
| metab_10804 | Unannotated feature | neg | 233.0302 | 1.088 | 3.386 | 3.340 | -0.046 | 0.086 | 0.636 | 0.866 | Unannotated | NA | NA |
| metab_12703 | Unannotated feature | neg | 423.1700 | 5.334 | 4.003 | 4.156 | 0.153 | 0.086 | 0.636 | 0.866 | Unannotated | NA | NA |
| metab_16102 | Unannotated feature | neg | 403.2497 | 6.240 | 5.371 | 5.314 | -0.057 | -0.086 | 0.636 | 0.866 | Unannotated | NA | NA |
| metab_19404 | Isomaltose | neg | 341.1065 | 5.007 | 3.591 | 3.998 | 0.407 | 0.086 | 0.636 | 0.866 | Tentative | Organic oxygen compounds | Organooxygen compounds |
| metab_12694 | Unannotated feature | neg | 315.1243 | 5.339 | 6.597 | 6.601 | 0.004 | 0.086 | 0.636 | 0.866 | Unannotated | NA | NA |
| metab_12693 | Unannotated feature | neg | 491.2053 | 5.369 | 4.545 | 4.243 | -0.302 | -0.086 | 0.636 | 0.866 | Unannotated | NA | NA |
| metab_8158 | Unannotated feature | pos | 99.0446 | 2.695 | 4.225 | 4.190 | -0.034 | 0.086 | 0.636 | 0.866 | Unannotated | NA | NA |
| metab_14509 | Unannotated feature | neg | 229.1080 | 3.367 | 5.042 | 5.147 | 0.105 | 0.086 | 0.636 | 0.866 | Unannotated | NA | NA |
| metab_19165 | Unannotated feature | neg | 507.1632 | 5.308 | 4.382 | 3.799 | -0.583 | -0.086 | 0.636 | 0.866 | Unannotated | NA | NA |
| metab_12339 | Unannotated feature | neg | 465.3051 | 7.417 | 7.999 | 7.911 | -0.088 | -0.086 | 0.636 | 0.866 | Unannotated | NA | NA |
| metab_3307 | Unannotated feature | pos | 203.0344 | 4.084 | 4.137 | 4.027 | -0.110 | -0.086 | 0.636 | 0.866 | Unannotated | NA | NA |
| metab_15400 | Unannotated feature | neg | 303.9391 | 0.566 | 3.791 | 3.183 | -0.607 | -0.086 | 0.636 | 0.866 | Unannotated | NA | NA |
| metab_15742 | Unannotated feature | neg | 125.0965 | 5.758 | 4.954 | 4.899 | -0.056 | -0.086 | 0.636 | 0.866 | Unannotated | NA | NA |
| metab_14187 | Kinetin riboside | neg | 392.1207 | 2.928 | 4.785 | 4.928 | 0.143 | 0.086 | 0.636 | 0.866 | Unannotated | NA | NA |
| metab_113 | N2-Fructopyranosylarginine | pos | 337.1724 | 0.633 | 4.149 | 4.215 | 0.066 | 0.086 | 0.636 | 0.866 | Unannotated | NA | NA |
| metab_142 | L-Glycine | pos | 76.0398 | 0.625 | 4.576 | 4.621 | 0.045 | 0.086 | 0.636 | 0.866 | Tentative | NA | NA |
| metab_11979 | Unannotated feature | neg | 301.1292 | 6.201 | 5.897 | 5.985 | 0.088 | 0.082 | 0.653 | 0.875 | Unannotated | NA | NA |
| metab_19812 | Unannotated feature | neg | 243.0187 | 4.409 | 3.313 | 3.631 | 0.318 | 0.082 | 0.653 | 0.875 | Unannotated | NA | NA |
| metab_7900 | 5-hydroxy-coniferaldehyde | pos | 195.0656 | 3.347 | 4.649 | 4.594 | -0.054 | -0.082 | 0.653 | 0.875 | Unannotated | NA | NA |
| metab_7899 | 3-(3,4-Dihydroxyphenyl)lactic acid | pos | 240.0869 | 2.965 | 4.180 | 4.250 | 0.069 | 0.082 | 0.653 | 0.875 | Tentative | NA | NA |
| metab_14111 | Unannotated feature | neg | 725.9463 | 2.836 | 4.275 | 4.031 | -0.244 | -0.082 | 0.653 | 0.875 | Unannotated | NA | NA |
| metab_7919 | Unannotated feature | pos | 174.1128 | 2.941 | 4.174 | 4.146 | -0.028 | -0.082 | 0.653 | 0.875 | Unannotated | NA | NA |
| metab_14092 | 1h,8h-Pyrano[3,4-c]pyran-1,8-dione | neg | 209.0087 | 2.817 | 3.487 | 3.744 | 0.257 | 0.082 | 0.653 | 0.875 | Unannotated | NA | NA |
| metab_9034 | Unannotated feature | neg | 458.0041 | 4.028 | 6.973 | 7.162 | 0.189 | 0.082 | 0.653 | 0.875 | Unannotated | NA | NA |
| metab_1804 | Dihydromaleimide beta-D-glucoside | pos | 294.1189 | 0.784 | 4.844 | 5.003 | 0.159 | 0.082 | 0.653 | 0.875 | Unannotated | NA | NA |
| metab_1266 | 4alpha,5alpha-Epoxy-11-eudesmen-3a-ol | pos | 254.2119 | 6.327 | 4.379 | 4.292 | -0.086 | -0.082 | 0.653 | 0.875 | Unannotated | Lipids and lipid-like molecules | Prenol lipids |
| metab_8186 | Unannotated feature | pos | 125.1126 | 2.560 | 6.737 | 6.757 | 0.021 | 0.082 | 0.653 | 0.875 | Unannotated | NA | NA |
| metab_8157 | Unannotated feature | pos | 362.1572 | 2.695 | 3.833 | 3.966 | 0.133 | 0.082 | 0.653 | 0.875 | Unannotated | NA | NA |
| metab_8220 | Alanylglutamine | pos | 182.0928 | 2.434 | 4.328 | 4.351 | 0.023 | 0.082 | 0.653 | 0.875 | Unannotated | NA | NA |
| metab_5383 | Corticosterone | pos | 347.2203 | 6.462 | 4.141 | 4.013 | -0.128 | -0.082 | 0.653 | 0.875 | Strong putative | NA | NA |
| metab_12803 | Unannotated feature | neg | 250.9940 | 0.644 | 4.339 | 4.106 | -0.233 | -0.082 | 0.653 | 0.875 | Unannotated | NA | NA |
| metab_12941 | Unannotated feature | neg | 144.0295 | 0.738 | 4.778 | 4.928 | 0.149 | 0.082 | 0.653 | 0.875 | Unannotated | NA | NA |
| metab_7518 | 3,4-Dihydroxybenzaldehyde | pos | 139.0392 | 3.725 | 2.771 | 2.595 | -0.176 | -0.082 | 0.653 | 0.875 | Moderate putative | NA | NA |
| metab_7479 | 2beta,9xi-Dihydroxy-8-oxo-1(10),4,11(13)-germacratrien-12,6alpha-olide | pos | 296.1498 | 3.799 | 3.129 | 3.417 | 0.287 | 0.082 | 0.653 | 0.875 | Unannotated | NA | NA |
| metab_18089 | DG(20:4(6E,8Z,11Z,13E)-2OH(5S,15S)/i-12:0/0:0) | neg | 637.4332 | 6.397 | 5.282 | 4.810 | -0.472 | -0.082 | 0.653 | 0.875 | Unannotated | NA | NA |
| metab_11183 | Galactinol | neg | 377.0861 | 0.659 | 6.527 | 6.395 | -0.132 | -0.082 | 0.653 | 0.875 | Strong putative | NA | NA |
| metab_12662 | Unannotated feature | neg | 459.2735 | 6.569 | 7.338 | 7.329 | -0.009 | -0.082 | 0.653 | 0.875 | Unannotated | NA | NA |
| metab_1816 | Unannotated feature | pos | 361.1983 | 0.768 | 4.066 | 4.086 | 0.021 | 0.082 | 0.653 | 0.875 | Unannotated | NA | NA |
| metab_5378 | DHAP(18:0) | pos | 478.2939 | 6.470 | 4.463 | 4.500 | 0.037 | 0.082 | 0.653 | 0.875 | Tentative | NA | NA |
| metab_193 | Unannotated feature | pos | 280.8190 | 0.593 | 4.386 | 4.442 | 0.055 | -0.082 | 0.653 | 0.875 | Unannotated | NA | NA |
| metab_14630 | Unannotated feature | neg | 212.0383 | 3.562 | 3.938 | 3.786 | -0.152 | -0.082 | 0.653 | 0.875 | Unannotated | NA | NA |
| metab_9197 | Unannotated feature | neg | 249.9970 | 3.773 | 4.487 | 4.545 | 0.058 | 0.082 | 0.653 | 0.875 | Unannotated | NA | NA |
| metab_9166 | (1R,6R)-6-hydroxy-2-succinylcyclohexa-2,4-diene-1-carboxylate | neg | 221.0452 | 3.829 | 5.093 | 4.936 | -0.157 | -0.082 | 0.653 | 0.875 | Tentative | NA | NA |
| metab_4475 | Unannotated feature | pos | 827.5435 | 6.502 | 0.903 | 2.995 | 2.092 | -0.082 | 0.653 | 0.875 | Unannotated | NA | NA |
| metab_11768 | 4-Hydroxy-2',4',6'-Trimethoxychalcone | neg | 349.0850 | 5.683 | 4.131 | 4.704 | 0.573 | 0.082 | 0.653 | 0.875 | Strong putative | NA | NA |
| metab_785 | Unannotated feature | pos | 393.2262 | 6.207 | 5.345 | 5.173 | -0.172 | -0.082 | 0.653 | 0.875 | Unannotated | NA | NA |
| metab_4779 | Biguanide | pos | 84.0674 | 7.780 | 4.871 | 4.899 | 0.028 | 0.082 | 0.653 | 0.875 | Tentative | NA | NA |
| metab_4767 | Unannotated feature | pos | 410.1933 | 3.642 | 4.710 | 4.412 | -0.298 | -0.082 | 0.653 | 0.875 | Unannotated | NA | NA |
| metab_448 | Unannotated feature | pos | 199.0450 | 0.006 | 3.511 | 3.493 | -0.019 | 0.082 | 0.653 | 0.875 | Unannotated | NA | NA |
| metab_1963 | Unannotated feature | pos | 240.0871 | 1.952 | 5.456 | 5.593 | 0.137 | 0.082 | 0.653 | 0.875 | Unannotated | NA | NA |
| metab_1956 | Unannotated feature | pos | 350.1820 | 1.960 | 3.675 | 3.944 | 0.269 | 0.082 | 0.653 | 0.875 | Unannotated | NA | NA |
| metab_13040 | Unannotated feature | neg | 616.7558 | 0.596 | 4.970 | 4.972 | 0.002 | 0.082 | 0.653 | 0.875 | Unannotated | NA | NA |
| metab_17972 | Unannotated feature | neg | 337.1845 | 6.553 | 3.775 | 3.858 | 0.083 | 0.082 | 0.653 | 0.875 | Unannotated | NA | NA |
| metab_12649 | Unannotated feature | neg | 335.0879 | 5.952 | 5.222 | 5.322 | 0.101 | -0.082 | 0.653 | 0.875 | Unannotated | NA | NA |
| metab_12644 | Unannotated feature | neg | 437.1508 | 5.971 | 3.952 | 3.971 | 0.019 | -0.082 | 0.653 | 0.875 | Unannotated | NA | NA |
| metab_18532 | 5,6-Dihydro-5-hydroxy-6-methyl-2H-pyran-2-one | neg | 273.0770 | 6.035 | 5.071 | 4.850 | -0.221 | -0.082 | 0.653 | 0.875 | Tentative | NA | NA |
| metab_18005 | 3-Dehydroteasterone | neg | 491.3384 | 6.514 | 6.039 | 6.095 | 0.056 | 0.082 | 0.653 | 0.875 | Tentative | NA | NA |
| metab_12947 | Unannotated feature | neg | 310.1148 | 0.730 | 3.949 | 4.201 | 0.252 | 0.082 | 0.653 | 0.875 | Unannotated | NA | NA |
| metab_4327 | Phenylalanylphenylalanine | pos | 295.1447 | 6.087 | 4.805 | 4.626 | -0.178 | -0.082 | 0.653 | 0.875 | Unannotated | NA | NA |
| metab_17026 | Unannotated feature | neg | 214.0205 | 0.571 | 5.510 | 5.356 | -0.154 | -0.082 | 0.653 | 0.875 | Unannotated | NA | NA |
| metab_17024 | Unannotated feature | neg | 186.9116 | 7.997 | 3.525 | 3.572 | 0.047 | -0.082 | 0.653 | 0.875 | Unannotated | NA | NA |
| metab_13942 | Unannotated feature | neg | 383.1104 | 2.594 | 4.493 | 4.726 | 0.232 | 0.082 | 0.653 | 0.875 | Unannotated | NA | NA |
| metab_15357 | Unannotated feature | neg | 381.1018 | 5.014 | 3.469 | 3.432 | -0.037 | -0.082 | 0.653 | 0.875 | Unannotated | NA | NA |
| metab_14305 | Unannotated feature | neg | 174.0224 | 3.059 | 3.446 | 3.528 | 0.081 | -0.082 | 0.653 | 0.875 | Unannotated | NA | NA |
| metab_16748 | Unannotated feature | neg | 234.0441 | 1.958 | 4.132 | 4.094 | -0.038 | -0.082 | 0.653 | 0.875 | Unannotated | NA | NA |
| metab_13068 | Unannotated feature | neg | 484.7974 | 0.538 | 5.283 | 5.151 | -0.132 | -0.082 | 0.653 | 0.875 | Unannotated | NA | NA |
| metab_10579 | Unannotated feature | neg | 344.0994 | 1.702 | 4.098 | 4.138 | 0.039 | 0.082 | 0.653 | 0.875 | Unannotated | NA | NA |
| metab_15242 | P-Hydroxyphenethyl trans-ferulate | neg | 359.1139 | 4.796 | 5.023 | 5.410 | 0.387 | 0.082 | 0.653 | 0.875 | Unannotated | Phenylpropanoids and polyketides | Cinnamic acids and derivatives |
| metab_7364 | Unannotated feature | pos | 224.1341 | 4.042 | 2.257 | 3.350 | 1.093 | 0.082 | 0.653 | 0.875 | Unannotated | NA | NA |
| metab_15151 | Halofenozide | neg | 365.0800 | 4.630 | 4.269 | 4.400 | 0.131 | 0.082 | 0.653 | 0.875 | Strong putative | NA | NA |
| metab_15117 | 3,4-DHPEA-EA | neg | 359.1142 | 4.559 | 4.540 | 4.777 | 0.237 | 0.082 | 0.653 | 0.875 | Unannotated | NA | NA |
| metab_16367 | Unannotated feature | neg | 537.4168 | 6.734 | 6.145 | 6.199 | 0.054 | 0.082 | 0.653 | 0.875 | Unannotated | NA | NA |
| metab_9401 | Unannotated feature | neg | 320.1618 | 3.472 | 3.685 | 3.618 | -0.066 | -0.082 | 0.653 | 0.875 | Unannotated | NA | NA |
| metab_830 | 12-Hydroxyheptadecanoylcarnitine | pos | 412.3432 | 6.430 | 4.860 | 5.009 | 0.149 | 0.082 | 0.653 | 0.875 | Unannotated | NA | NA |
| metab_7812 | Unannotated feature | pos | 281.1501 | 3.135 | 4.477 | 4.456 | -0.021 | -0.082 | 0.653 | 0.875 | Unannotated | NA | NA |
| metab_4820 | Unannotated feature | pos | 327.2022 | 3.410 | 4.419 | 4.416 | -0.004 | 0.082 | 0.653 | 0.875 | Unannotated | NA | NA |
| metab_4834 | Unannotated feature | pos | 180.9901 | 7.772 | 5.384 | 5.404 | 0.020 | 0.082 | 0.653 | 0.875 | Unannotated | NA | NA |
| metab_4833 | Unannotated feature | pos | 197.0376 | 7.772 | 6.086 | 6.098 | 0.012 | 0.082 | 0.653 | 0.875 | Unannotated | NA | NA |
| metab_3455 | Unannotated feature | pos | 102.0345 | 7.724 | 6.435 | 6.452 | 0.016 | 0.082 | 0.653 | 0.875 | Unannotated | NA | NA |
| metab_9523 | Unannotated feature | neg | 108.0210 | 3.321 | 2.728 | 2.600 | -0.128 | -0.082 | 0.653 | 0.875 | Unannotated | NA | NA |
| metab_10398 | Unannotated feature | neg | 204.8924 | 2.054 | 5.374 | 5.368 | -0.006 | -0.082 | 0.653 | 0.875 | Unannotated | NA | NA |
| metab_13124 | Unannotated feature | neg | 257.8193 | 0.443 | 4.791 | 4.788 | -0.003 | 0.082 | 0.653 | 0.875 | Unannotated | NA | NA |
| metab_2239 | Unannotated feature | pos | 342.8615 | 0.585 | 4.352 | 4.364 | 0.011 | -0.082 | 0.653 | 0.875 | Unannotated | NA | NA |
| metab_17789 | Docosapentaenoic acid (22n-3) | neg | 329.2486 | 6.868 | 5.396 | 5.020 | -0.376 | -0.082 | 0.653 | 0.875 | Tentative | NA | NA |
| metab_10497 | Unannotated feature | neg | 125.0350 | 1.952 | 5.909 | 5.901 | -0.008 | -0.082 | 0.653 | 0.875 | Unannotated | NA | NA |
| metab_11230 | Unannotated feature | neg | 309.1309 | 0.636 | 3.873 | 3.950 | 0.077 | 0.082 | 0.653 | 0.875 | Unannotated | NA | NA |
| metab_17927 | Unannotated feature | neg | 529.3523 | 6.632 | 5.638 | 5.798 | 0.159 | 0.082 | 0.653 | 0.875 | Unannotated | NA | NA |
| metab_12338 | Unannotated feature | neg | 764.5513 | 7.424 | 4.668 | 4.818 | 0.150 | 0.082 | 0.653 | 0.875 | Unannotated | NA | NA |
| metab_12368 | Unannotated feature | neg | 112.9849 | 7.607 | 8.115 | 8.105 | -0.010 | -0.082 | 0.653 | 0.875 | Unannotated | NA | NA |
| metab_3384 | 2-Hydroxy-2,6,6-trimethylcyclohexanone | pos | 139.1119 | 7.307 | 6.676 | 6.685 | 0.009 | 0.082 | 0.653 | 0.875 | Unannotated | NA | NA |
| metab_3376 | Unannotated feature | pos | 174.1128 | 4.332 | 5.159 | 5.234 | 0.075 | 0.082 | 0.653 | 0.875 | Unannotated | NA | NA |
| metab_9572 | Unannotated feature | neg | 290.0342 | 3.250 | 3.263 | 3.486 | 0.223 | 0.082 | 0.653 | 0.875 | Unannotated | NA | NA |
| metab_7723 | Lapachol | pos | 284.1287 | 3.331 | 3.821 | 3.742 | -0.080 | -0.082 | 0.653 | 0.875 | Tentative | NA | NA |
| metab_869 | LysoPC(18:1(9Z)/0:0) | pos | 522.3566 | 6.718 | 5.658 | 5.676 | 0.018 | 0.082 | 0.653 | 0.875 | Unannotated | NA | NA |
| metab_11643 | Unannotated feature | neg | 415.2024 | 5.295 | 4.822 | 5.158 | 0.336 | 0.082 | 0.653 | 0.875 | Unannotated | NA | NA |
| metab_19044 | Unannotated feature | neg | 325.1294 | 5.470 | 3.666 | 3.611 | -0.055 | -0.082 | 0.653 | 0.875 | Unannotated | NA | NA |
| metab_4977 | Unannotated feature | pos | 272.9417 | 7.538 | 4.875 | 4.901 | 0.026 | 0.082 | 0.653 | 0.875 | Unannotated | NA | NA |
| metab_13261 | Unannotated feature | neg | 130.9570 | 0.603 | 5.550 | 5.563 | 0.014 | -0.082 | 0.653 | 0.875 | Unannotated | NA | NA |
| metab_11344 | Unannotated feature | neg | 302.8391 | 0.588 | 4.762 | 4.354 | -0.407 | -0.082 | 0.653 | 0.875 | Unannotated | NA | NA |
| metab_11341 | Unannotated feature | neg | 491.2057 | 5.140 | 3.529 | 3.707 | 0.178 | 0.082 | 0.653 | 0.875 | Unannotated | NA | NA |
| metab_1091 | Unannotated feature | pos | 629.4404 | 6.670 | 6.117 | 6.076 | -0.041 | -0.082 | 0.653 | 0.875 | Unannotated | NA | NA |
| metab_11331 | Unannotated feature | neg | 128.9325 | 0.603 | 3.171 | 3.116 | -0.055 | -0.082 | 0.653 | 0.875 | Unannotated | NA | NA |
| metab_2273 | Gibberellin A59 | pos | 424.1375 | 3.208 | 4.448 | 4.614 | 0.166 | -0.082 | 0.653 | 0.875 | Unannotated | NA | NA |
| metab_2307 | Unannotated feature | pos | 83.0497 | 0.055 | 3.061 | 3.073 | 0.012 | -0.082 | 0.653 | 0.875 | Unannotated | NA | NA |
| metab_13113 | Unannotated feature | neg | 181.9169 | 0.208 | 5.714 | 5.699 | -0.015 | -0.082 | 0.653 | 0.875 | Unannotated | NA | NA |
| metab_5228 | Unannotated feature | pos | 533.2865 | 6.702 | 4.377 | 4.105 | -0.273 | 0.082 | 0.653 | 0.875 | Unannotated | NA | NA |
| metab_10450 | Tatridin B | neg | 309.1348 | 4.630 | 4.387 | 4.088 | -0.300 | -0.082 | 0.653 | 0.875 | Unannotated | NA | NA |
| metab_11605 | Unannotated feature | neg | 209.8335 | 0.443 | 4.469 | 4.385 | -0.084 | -0.082 | 0.653 | 0.875 | Unannotated | NA | NA |
| metab_9656 | Unannotated feature | neg | 442.1376 | 3.180 | 3.300 | 4.008 | 0.708 | 0.082 | 0.653 | 0.875 | Unannotated | NA | NA |
| metab_7967 | Unannotated feature | pos | 142.0865 | 2.880 | 4.939 | 4.992 | 0.053 | 0.082 | 0.653 | 0.875 | Unannotated | NA | NA |
| metab_5033 | Fagomine | pos | 148.0969 | 7.268 | 4.024 | 3.962 | -0.063 | -0.082 | 0.653 | 0.875 | Tentative | NA | NA |
| metab_16674 | Unannotated feature | neg | 449.1311 | 1.947 | 4.286 | 4.136 | -0.150 | -0.082 | 0.653 | 0.875 | Unannotated | NA | NA |
| metab_17248 | Unannotated feature | neg | 228.0373 | 2.517 | 4.914 | 4.938 | 0.024 | 0.082 | 0.653 | 0.875 | Unannotated | NA | NA |
| metab_19004 | Unannotated feature | neg | 125.0965 | 5.526 | 4.582 | 4.530 | -0.051 | -0.082 | 0.653 | 0.875 | Unannotated | NA | NA |
| metab_12442 | Unannotated feature | neg | 310.9314 | 7.720 | 6.348 | 6.332 | -0.016 | -0.082 | 0.653 | 0.875 | Unannotated | NA | NA |
| metab_18954 | Bisindolylmaleimide i | neg | 457.1862 | 5.579 | 4.387 | 5.336 | 0.949 | 0.082 | 0.653 | 0.875 | Tentative | NA | NA |
| metab_15034 | 1,2-dibutyrin | neg | 277.1294 | 4.398 | 4.837 | 4.721 | -0.116 | -0.082 | 0.653 | 0.875 | Unannotated | NA | NA |
| metab_9798 | Unannotated feature | neg | 202.1080 | 3.041 | 4.283 | 4.594 | 0.310 | 0.082 | 0.653 | 0.875 | Unannotated | NA | NA |
| metab_8582 | Unannotated feature | pos | 162.5471 | 1.176 | 3.799 | 3.146 | -0.653 | -0.082 | 0.653 | 0.875 | Unannotated | NA | NA |
| metab_9842 | Unannotated feature | neg | 242.0128 | 3.003 | 3.228 | 3.291 | 0.063 | -0.082 | 0.653 | 0.875 | Unannotated | NA | NA |
| metab_5811 | Imperialine | pos | 430.3327 | 5.992 | 5.279 | 5.850 | 0.571 | 0.082 | 0.653 | 0.875 | Tentative | NA | NA |
| metab_10297 | Unannotated feature | neg | 230.9212 | 2.417 | 4.691 | 4.676 | -0.015 | -0.082 | 0.653 | 0.875 | Unannotated | NA | NA |
| metab_10270 | Unannotated feature | neg | 328.9979 | 2.517 | 4.373 | 4.132 | -0.241 | -0.082 | 0.653 | 0.875 | Unannotated | NA | NA |
| metab_6157 | (Z)-Narceine imide | pos | 459.2151 | 5.835 | 4.352 | 4.539 | 0.187 | 0.082 | 0.653 | 0.875 | Unannotated | NA | NA |
| metab_1045 | Unannotated feature | pos | 639.4973 | 6.845 | 6.358 | 6.266 | -0.092 | -0.082 | 0.653 | 0.875 | Unannotated | NA | NA |
| metab_18763 | Unannotated feature | neg | 208.0610 | 3.169 | 4.509 | 4.594 | 0.085 | 0.082 | 0.653 | 0.875 | Unannotated | NA | NA |
| metab_14530 | Sherry lactone | neg | 175.0606 | 3.396 | 5.575 | 5.463 | -0.111 | 0.082 | 0.653 | 0.875 | Unannotated | NA | NA |
| metab_2338 | Unannotated feature | pos | 139.1120 | 0.429 | 4.737 | 4.709 | -0.028 | -0.082 | 0.653 | 0.875 | Unannotated | NA | NA |
| metab_10128 | Unannotated feature | neg | 445.1148 | 4.469 | 3.729 | 4.243 | 0.514 | 0.082 | 0.653 | 0.875 | Unannotated | NA | NA |
| metab_17519 | Unannotated feature | neg | 794.5116 | 7.448 | 4.606 | 4.404 | -0.202 | 0.082 | 0.653 | 0.875 | Unannotated | NA | NA |
| metab_10166 | Unannotated feature | neg | 279.0567 | 2.714 | 5.040 | 5.183 | 0.143 | -0.082 | 0.653 | 0.875 | Unannotated | NA | NA |
| metab_15669 | (1S,2R,4R,8S)-p-Menthane-2,8,9-triol 9-glucoside | neg | 349.1863 | 5.627 | 3.075 | 3.513 | 0.438 | 0.082 | 0.653 | 0.875 | Unannotated | NA | NA |
| metab_8447 | Unannotated feature | pos | 254.1390 | 3.923 | 4.089 | 4.203 | 0.114 | -0.082 | 0.653 | 0.875 | Unannotated | NA | NA |
| metab_5126 | Docosahexaenoylethanolamine | pos | 354.2772 | 6.941 | 3.972 | 3.989 | 0.018 | 0.082 | 0.653 | 0.875 | Unannotated | NA | NA |
| metab_17552 | Unannotated feature | neg | 463.1107 | 2.750 | 2.139 | 1.891 | -0.248 | -0.082 | 0.653 | 0.875 | Unannotated | NA | NA |
| metab_11504 | Unannotated feature | neg | 180.9119 | 0.512 | 5.374 | 5.376 | 0.002 | 0.082 | 0.653 | 0.875 | Unannotated | NA | NA |
| metab_937 | Unannotated feature | pos | 634.4537 | 7.506 | 5.093 | 5.062 | -0.031 | -0.082 | 0.653 | 0.875 | Unannotated | NA | NA |
| metab_2830 | Unannotated feature | pos | 125.0714 | 2.740 | 4.008 | 4.075 | 0.067 | -0.082 | 0.653 | 0.875 | Unannotated | NA | NA |
| metab_17350 | Unannotated feature | neg | 269.1316 | 7.692 | 3.714 | 3.871 | 0.157 | 0.082 | 0.653 | 0.875 | Unannotated | NA | NA |
| metab_13527 | Unannotated feature | neg | 346.8923 | 0.538 | 4.802 | 4.765 | -0.037 | 0.082 | 0.653 | 0.875 | Unannotated | NA | NA |
| metab_12457 | Unannotated feature | neg | 161.8751 | 7.874 | 5.295 | 5.298 | 0.004 | 0.082 | 0.653 | 0.875 | Unannotated | NA | NA |
| metab_11457 | 2,3-Dihydro-2,3-dihydroxy-4-(4-hydroxyphenyl)-1H-phenalen-1-one | neg | 343.0404 | 0.527 | 5.025 | 5.042 | 0.017 | -0.082 | 0.653 | 0.875 | Unannotated | NA | NA |
| metab_10012 | Unannotated feature | neg | 244.0438 | 4.413 | 4.795 | 4.817 | 0.022 | 0.082 | 0.653 | 0.875 | Unannotated | NA | NA |
| metab_5067 | Unannotated feature | pos | 323.2950 | 7.132 | 5.265 | 5.297 | 0.032 | -0.082 | 0.653 | 0.875 | Unannotated | NA | NA |
| metab_2679 | Unannotated feature | pos | 225.1601 | 1.984 | 3.858 | 3.746 | -0.112 | 0.082 | 0.653 | 0.875 | Unannotated | NA | NA |
| metab_2645 | Unannotated feature | pos | 216.1235 | 1.936 | 3.937 | 4.063 | 0.126 | 0.082 | 0.653 | 0.875 | Unannotated | NA | NA |
| metab_15923 | Unannotated feature | neg | 489.2398 | 6.022 | 4.875 | 5.025 | 0.150 | 0.082 | 0.653 | 0.875 | Unannotated | NA | NA |
| metab_7621 | 1,1-Dimethylbiguanide | pos | 168.0658 | 3.511 | 3.819 | 3.884 | 0.065 | 0.082 | 0.653 | 0.875 | Tentative | NA | NA |
| metab_2570 | Unannotated feature | pos | 79.0183 | 1.189 | 3.531 | 3.629 | 0.098 | 0.082 | 0.653 | 0.875 | Unannotated | NA | NA |
| metab_12512 | Unannotated feature | neg | 393.2287 | 6.326 | 6.112 | 6.063 | -0.049 | -0.082 | 0.653 | 0.875 | Unannotated | NA | NA |
| metab_16615 | Unannotated feature | neg | 550.2832 | 7.473 | 6.254 | 6.308 | 0.054 | 0.082 | 0.653 | 0.875 | Unannotated | NA | NA |
| metab_16828 | Unannotated feature | neg | 296.1195 | 7.726 | 3.899 | 4.189 | 0.290 | 0.082 | 0.653 | 0.875 | Unannotated | NA | NA |
| metab_11831 | (2S)-5-Amino-2-[(2-carboxy-2-oxoethyl)amino]-5-oxopentanoic acid | neg | 277.0695 | 4.865 | 3.729 | 4.371 | 0.642 | 0.082 | 0.653 | 0.875 | Unannotated | Organic acids and derivatives | Carboxylic acids and derivatives |
| metab_481 | Unannotated feature | pos | 433.2380 | 4.943 | 3.869 | 3.436 | -0.433 | -0.082 | 0.653 | 0.875 | Unannotated | NA | NA |
| metab_8841 | Unannotated feature | neg | 369.0088 | 0.636 | 5.712 | 5.440 | -0.272 | -0.082 | 0.653 | 0.875 | Unannotated | NA | NA |
| metab_8927 | Unannotated feature | neg | 352.1308 | 4.224 | 4.552 | 4.766 | 0.214 | 0.082 | 0.653 | 0.875 | Unannotated | NA | NA |
| metab_12802 | Unannotated feature | neg | 246.9973 | 0.644 | 4.740 | 4.903 | 0.164 | 0.082 | 0.653 | 0.875 | Unannotated | NA | NA |
| metab_12821 | 1-Methyladenosin | neg | 326.1096 | 0.659 | 5.510 | 5.568 | 0.058 | 0.082 | 0.653 | 0.875 | Unannotated | Nucleosides, nucleotides, and analogues | Purine nucleosides |
| metab_11015 | Unannotated feature | neg | 423.1631 | 0.753 | 4.025 | 4.529 | 0.504 | 0.082 | 0.653 | 0.875 | Unannotated | NA | NA |
| metab_1425 | ortho-hydroxyatorvastatin | pos | 575.2572 | 6.040 | 3.542 | 4.062 | 0.520 | 0.082 | 0.653 | 0.875 | Unannotated | Organoheterocyclic compounds | Pyrroles |
| metab_15196 | Unannotated feature | neg | 434.1850 | 4.714 | 2.874 | 3.479 | 0.605 | 0.082 | 0.653 | 0.875 | Unannotated | NA | NA |
| metab_6563 | Unannotated feature | pos | 218.2119 | 5.505 | 4.013 | 3.955 | -0.058 | -0.082 | 0.653 | 0.875 | Unannotated | NA | NA |
| metab_14755 | Unannotated feature | neg | 273.1248 | 3.803 | 4.288 | 3.978 | -0.310 | -0.078 | 0.670 | 0.881 | Unannotated | NA | NA |
| metab_7419 | Unannotated feature | pos | 309.1681 | 3.934 | 3.552 | 2.812 | -0.740 | -0.078 | 0.670 | 0.881 | Unannotated | NA | NA |
| metab_1446 | Unannotated feature | pos | 399.2289 | 6.008 | 4.180 | 4.730 | 0.550 | 0.078 | 0.670 | 0.881 | Unannotated | NA | NA |
| metab_11022 | 7-Hydroxy-3-(3-hydroxy-4-methoxybenzyl)-5-methoxy-4-chromanone | neg | 329.1036 | 4.949 | 5.194 | 5.344 | 0.150 | -0.078 | 0.670 | 0.881 | Unannotated | Phenylpropanoids and polyketides | Homoisoflavonoids |
| metab_11038 | Unannotated feature | neg | 443.1569 | 3.234 | 3.572 | 4.194 | 0.622 | 0.078 | 0.670 | 0.881 | Unannotated | NA | NA |
| metab_1372 | Unannotated feature | pos | 605.2784 | 6.127 | 4.898 | 4.855 | -0.042 | -0.078 | 0.670 | 0.881 | Unannotated | NA | NA |
| metab_1658 | Unannotated feature | pos | 223.0602 | 4.537 | 4.579 | 4.610 | 0.031 | 0.078 | 0.670 | 0.881 | Unannotated | NA | NA |
| metab_18316 | Leu-Arg-Asn-Arg | neg | 556.3329 | 6.201 | 5.617 | 5.600 | -0.017 | -0.078 | 0.670 | 0.881 | Unannotated | NA | NA |
| metab_11098 | 7-Methylguanosine | neg | 343.1146 | 0.722 | 4.418 | 4.441 | 0.023 | -0.078 | 0.670 | 0.881 | Unannotated | NA | NA |
| metab_12799 | Unannotated feature | neg | 192.0872 | 0.644 | 4.036 | 3.966 | -0.070 | -0.078 | 0.670 | 0.881 | Unannotated | NA | NA |
| metab_19397 | (3Z,6Z)-3,6-Nonadienal | neg | 183.1021 | 5.022 | 3.974 | 3.870 | -0.104 | 0.078 | 0.670 | 0.881 | Tentative | NA | NA |
| metab_11832 | Cyclocreatine | neg | 285.1344 | 5.927 | 5.308 | 5.388 | 0.080 | 0.078 | 0.670 | 0.881 | Unannotated | NA | NA |
| metab_4607 | LysoPC(16:0/0:0) | pos | 496.3408 | 7.060 | 6.165 | 6.070 | -0.095 | -0.078 | 0.670 | 0.881 | Tentative | NA | NA |
| metab_14185 | 6-(2-Hydroxyethoxy)-6-oxohexanoic acid | neg | 189.0763 | 2.928 | 5.438 | 5.422 | -0.016 | 0.078 | 0.670 | 0.881 | Unannotated | NA | NA |
| metab_4686 | Unannotated feature | pos | 715.2470 | 7.530 | 5.265 | 5.327 | 0.063 | 0.078 | 0.670 | 0.881 | Unannotated | NA | NA |
| metab_16819 | Unannotated feature | neg | 526.8503 | 7.720 | 5.238 | 5.252 | 0.014 | 0.078 | 0.670 | 0.881 | Unannotated | NA | NA |
| metab_16821 | Unannotated feature | neg | 119.9068 | 7.726 | 4.279 | 4.315 | 0.035 | 0.078 | 0.670 | 0.881 | Unannotated | NA | NA |
| metab_11991 | Unannotated feature | neg | 527.1482 | 6.217 | 3.668 | 3.782 | 0.114 | 0.078 | 0.670 | 0.881 | Unannotated | NA | NA |
| metab_93 | Unannotated feature | pos | 87.0445 | 0.649 | 4.519 | 4.434 | -0.086 | -0.078 | 0.670 | 0.881 | Unannotated | NA | NA |
| metab_15455 | Unannotated feature | neg | 91.0542 | 5.188 | 2.881 | 3.111 | 0.230 | -0.078 | 0.670 | 0.881 | Unannotated | NA | NA |
| metab_11077 | Unannotated feature | neg | 414.1528 | 4.997 | 3.682 | 3.708 | 0.027 | -0.078 | 0.670 | 0.881 | Unannotated | NA | NA |
| metab_16558 | Unannotated feature | neg | 621.4386 | 7.282 | 5.204 | 5.093 | -0.110 | -0.078 | 0.670 | 0.881 | Unannotated | NA | NA |
| metab_10048 | Unannotated feature | neg | 217.0715 | 2.799 | 5.132 | 5.104 | -0.028 | -0.078 | 0.670 | 0.881 | Unannotated | NA | NA |
| metab_12505 | Unannotated feature | neg | 383.2440 | 6.357 | 5.880 | 5.772 | -0.108 | -0.078 | 0.670 | 0.881 | Unannotated | NA | NA |
| metab_12516 | Unannotated feature | neg | 397.2599 | 6.318 | 6.260 | 6.226 | -0.033 | -0.078 | 0.670 | 0.881 | Unannotated | NA | NA |
| metab_18874 | Carthamoside A1 | neg | 395.1714 | 5.688 | 4.055 | 4.212 | 0.157 | 0.078 | 0.670 | 0.881 | Unannotated | NA | NA |
| metab_985 | Unannotated feature | pos | 198.0806 | 7.724 | 5.112 | 5.092 | -0.019 | -0.078 | 0.670 | 0.881 | Unannotated | NA | NA |
| metab_15905 | Unannotated feature | neg | 389.2551 | 6.003 | 5.390 | 5.454 | 0.064 | 0.078 | 0.670 | 0.881 | Unannotated | NA | NA |
| metab_15045 | Unannotated feature | neg | 283.9894 | 4.409 | 3.849 | 3.857 | 0.009 | 0.078 | 0.670 | 0.881 | Unannotated | NA | NA |
| metab_2602 | Unannotated feature | pos | 158.9617 | 1.629 | 5.389 | 5.416 | 0.027 | 0.078 | 0.670 | 0.881 | Unannotated | NA | NA |
| metab_15493 | 2-(4-Methylphenoxy)-7-(4-chlorophenyl)heptanoic acid | neg | 381.1018 | 5.257 | 4.461 | 4.761 | 0.300 | 0.078 | 0.670 | 0.881 | Unannotated | NA | NA |
| metab_15565 | Unannotated feature | neg | 776.7308 | 0.527 | 4.361 | 4.919 | 0.558 | 0.078 | 0.670 | 0.881 | Unannotated | NA | NA |
| metab_2707 | Unannotated feature | pos | 184.9859 | 2.079 | 5.320 | 5.347 | 0.027 | 0.078 | 0.670 | 0.881 | Unannotated | NA | NA |
| metab_18879 | Carteolol | neg | 337.1771 | 5.683 | 5.081 | 5.699 | 0.618 | 0.078 | 0.670 | 0.881 | Tentative | NA | NA |
| metab_9911 | N',N'-Diphenylhydrazinecarbohydrazide | neg | 241.1081 | 4.340 | 5.521 | 5.787 | 0.266 | 0.078 | 0.670 | 0.881 | Unannotated | NA | NA |
| metab_13571 | Unannotated feature | neg | 177.0762 | 1.255 | 5.050 | 5.052 | 0.002 | 0.078 | 0.670 | 0.881 | Unannotated | NA | NA |
| metab_17311 | Unannotated feature | neg | 582.8821 | 7.702 | 5.403 | 5.398 | -0.004 | 0.078 | 0.670 | 0.881 | Unannotated | NA | NA |
| metab_2415 | Unannotated feature | pos | 183.9601 | 0.609 | 3.351 | 3.469 | 0.118 | -0.078 | 0.670 | 0.881 | Unannotated | NA | NA |
| metab_1000 | Unannotated feature | pos | 638.5733 | 7.482 | 4.771 | 4.855 | 0.084 | 0.078 | 0.670 | 0.881 | Unannotated | NA | NA |
| metab_19483 | Gibberellin A29-catabolite | neg | 345.1346 | 4.884 | 5.312 | 5.068 | -0.243 | -0.078 | 0.670 | 0.881 | Tentative | NA | NA |
| metab_2455 | 6-[3-(2-carboxyethyl)-5-hydroxyphenoxy]-3,4,5-trihydroxyoxane-2-carboxylic acid | pos | 422.1072 | 0.657 | 4.939 | 5.064 | 0.126 | 0.078 | 0.670 | 0.881 | Unannotated | NA | NA |
| metab_2463 | Unannotated feature | pos | 70.0656 | 0.688 | 4.568 | 4.671 | 0.103 | 0.078 | 0.670 | 0.881 | Unannotated | NA | NA |
| metab_10199 | Unannotated feature | neg | 441.1233 | 4.515 | 4.253 | 3.475 | -0.779 | -0.078 | 0.670 | 0.881 | Unannotated | NA | NA |
| metab_2498 | Isoeugenitol | pos | 224.0921 | 0.776 | 4.748 | 4.478 | -0.269 | 0.078 | 0.670 | 0.881 | Unannotated | NA | NA |
| metab_17464 | Unannotated feature | neg | 416.1204 | 1.952 | 4.246 | 4.210 | -0.036 | 0.078 | 0.670 | 0.881 | Unannotated | NA | NA |
| metab_13385 | Unannotated feature | neg | 392.9520 | 0.628 | 4.714 | 4.702 | -0.012 | -0.078 | 0.670 | 0.881 | Unannotated | NA | NA |
| metab_13204 | Unannotated feature | neg | 434.9787 | 3.817 | 5.428 | 5.684 | 0.256 | 0.078 | 0.670 | 0.881 | Unannotated | NA | NA |
| metab_17654 | LysoPC(18:1(11Z)/0:0) | neg | 566.3478 | 7.068 | 5.089 | 5.207 | 0.118 | 0.078 | 0.670 | 0.881 | Tentative | NA | NA |
| metab_12167 | Unannotated feature | neg | 373.2750 | 6.663 | 6.205 | 6.767 | 0.562 | 0.078 | 0.670 | 0.881 | Unannotated | NA | NA |
| metab_12137 | Capsicoside B2 | neg | 739.4306 | 6.577 | 4.221 | 3.864 | -0.357 | -0.078 | 0.670 | 0.881 | Unannotated | NA | NA |
| metab_19577 | Phenylpyruvic Acid | neg | 163.0395 | 4.749 | 5.254 | 5.352 | 0.098 | 0.078 | 0.670 | 0.881 | Strong putative | Benzenoids | Benzene and substituted derivatives |
| metab_5216 | Unannotated feature | pos | 412.2832 | 6.726 | 3.277 | 3.617 | 0.340 | 0.078 | 0.670 | 0.881 | Unannotated | NA | NA |
| metab_5223 | Unannotated feature | pos | 531.2588 | 6.710 | 4.960 | 4.940 | -0.020 | 0.078 | 0.670 | 0.881 | Unannotated | NA | NA |
| metab_17627 | Mupirocin | neg | 535.2666 | 7.129 | 5.285 | 5.064 | -0.220 | -0.078 | 0.670 | 0.881 | Tentative | NA | NA |
| metab_15851 | Unannotated feature | neg | 103.0028 | 5.930 | 3.854 | 3.982 | 0.128 | 0.078 | 0.670 | 0.881 | Unannotated | NA | NA |
| metab_17272 | Unannotated feature | neg | 413.8822 | 7.720 | 5.406 | 5.407 | 0.001 | -0.078 | 0.670 | 0.881 | Unannotated | NA | NA |
| metab_8562 | Threonylproline | pos | 217.1187 | 1.245 | 6.164 | 6.136 | -0.028 | -0.078 | 0.670 | 0.881 | Unannotated | NA | NA |
| metab_16632 | Unannotated feature | neg | 226.9626 | 7.517 | 6.475 | 6.481 | 0.007 | 0.078 | 0.670 | 0.881 | Unannotated | NA | NA |
| metab_9813 | Unannotated feature | neg | 285.0076 | 3.028 | 4.192 | 4.077 | -0.115 | -0.078 | 0.670 | 0.881 | Unannotated | NA | NA |
| metab_2964 | 6-Ketoprostaglandin E1 | pos | 391.2086 | 2.984 | 4.718 | 4.803 | 0.086 | 0.078 | 0.670 | 0.881 | Tentative | NA | NA |
| metab_7639 | (5S,6R)-Methyl 5,6,7-trihydroxyheptanoate | pos | 234.1340 | 3.477 | 5.338 | 5.238 | -0.100 | -0.078 | 0.670 | 0.881 | Unannotated | Lipids and lipid-like molecules | Fatty Acyls |
| metab_7679 | Cycloheximide | pos | 323.1972 | 3.410 | 3.493 | 4.159 | 0.666 | 0.078 | 0.670 | 0.881 | Tentative | Organoheterocyclic compounds | Piperidines |
| metab_18942 | Unannotated feature | neg | 513.1420 | 3.242 | 4.025 | 4.202 | 0.176 | -0.078 | 0.670 | 0.881 | Unannotated | NA | NA |
| metab_15369 | Unannotated feature | neg | 244.1554 | 5.026 | 4.120 | 4.122 | 0.002 | -0.078 | 0.670 | 0.881 | Unannotated | NA | NA |
| metab_12443 | Unannotated feature | neg | 479.3383 | 6.530 | 6.777 | 6.877 | 0.099 | 0.078 | 0.670 | 0.881 | Unannotated | NA | NA |
| metab_12445 | Unannotated feature | neg | 180.8614 | 7.755 | 4.930 | 4.916 | -0.014 | -0.078 | 0.670 | 0.881 | Unannotated | NA | NA |
| metab_17247 | Unannotated feature | neg | 415.8776 | 7.726 | 4.766 | 4.780 | 0.014 | -0.078 | 0.670 | 0.881 | Unannotated | NA | NA |
| metab_4891 | Unannotated feature | pos | 356.9104 | 7.700 | 4.246 | 4.231 | -0.015 | 0.078 | 0.670 | 0.881 | Unannotated | NA | NA |
| metab_4967 | Unannotated feature | pos | 544.8933 | 7.554 | 4.880 | 4.921 | 0.041 | 0.078 | 0.670 | 0.881 | Unannotated | NA | NA |
| metab_5525 | Unannotated feature | pos | 283.1296 | 2.747 | 5.260 | 5.196 | -0.063 | -0.078 | 0.670 | 0.881 | Unannotated | NA | NA |
| metab_17223 | Unannotated feature | neg | 294.8855 | 7.730 | 4.450 | 4.481 | 0.031 | 0.078 | 0.670 | 0.881 | Unannotated | NA | NA |
| metab_17199 | Unannotated feature | neg | 252.8837 | 7.737 | 4.193 | 4.228 | 0.035 | -0.078 | 0.670 | 0.881 | Unannotated | NA | NA |
| metab_17208 | Unannotated feature | neg | 407.8652 | 7.730 | 4.913 | 4.936 | 0.023 | 0.078 | 0.670 | 0.881 | Unannotated | NA | NA |
| metab_5168 | Unannotated feature | pos | 525.3929 | 6.829 | 4.506 | 4.525 | 0.020 | 0.078 | 0.670 | 0.881 | Unannotated | NA | NA |
| metab_5178 | 7b-Hydroxy-3-oxo-5b-cholanoic acid | pos | 435.3090 | 6.813 | 6.902 | 6.897 | -0.005 | 0.078 | 0.670 | 0.881 | Unannotated | NA | NA |
| metab_12115 | Unannotated feature | neg | 568.3321 | 6.530 | 5.713 | 5.598 | -0.116 | -0.078 | 0.670 | 0.881 | Unannotated | NA | NA |
| metab_13177 | (-)-Catechin gallate | neg | 441.0839 | 4.701 | 4.459 | 4.914 | 0.455 | 0.078 | 0.670 | 0.881 | Strong putative | NA | NA |
| metab_10310 | Unannotated feature | neg | 314.0999 | 2.339 | 2.863 | 2.205 | -0.658 | -0.078 | 0.670 | 0.881 | Unannotated | NA | NA |
| metab_13248 | Unannotated feature | neg | 186.0378 | 0.582 | 5.105 | 5.028 | -0.077 | 0.078 | 0.670 | 0.881 | Unannotated | NA | NA |
| metab_4969 | Unannotated feature | pos | 408.9172 | 7.554 | 5.130 | 5.112 | -0.018 | 0.078 | 0.670 | 0.881 | Unannotated | NA | NA |
| metab_8594 | N-Methylolpentamethylmelamine | pos | 249.1449 | 1.155 | 4.693 | 4.866 | 0.174 | -0.078 | 0.670 | 0.881 | Unannotated | NA | NA |
| metab_19038 | 3-[[5-Methyl-2-(1-methylethyl)cyclohexyl]oxy]-1,2-propanediol | neg | 275.1865 | 5.480 | 5.156 | 5.002 | -0.154 | 0.078 | 0.670 | 0.881 | Unannotated | Lipids and lipid-like molecules | Prenol lipids |
| metab_19046 | N-Arachidonoyl Glutamic acid | neg | 468.2512 | 5.465 | 3.712 | 4.489 | 0.777 | 0.078 | 0.670 | 0.881 | Unannotated | NA | NA |
| metab_848 | Unannotated feature | pos | 573.3779 | 6.542 | 6.114 | 6.225 | 0.111 | 0.078 | 0.670 | 0.881 | Unannotated | NA | NA |
| metab_7829 | Unannotated feature | pos | 174.1128 | 3.109 | 4.550 | 4.468 | -0.081 | -0.078 | 0.670 | 0.881 | Unannotated | NA | NA |
| metab_16241 | Unannotated feature | neg | 239.0671 | 6.498 | 4.663 | 4.560 | -0.103 | -0.078 | 0.670 | 0.881 | Unannotated | NA | NA |
| metab_10538 | Unannotated feature | neg | 244.0438 | 4.670 | 5.205 | 5.283 | 0.079 | 0.078 | 0.670 | 0.881 | Unannotated | NA | NA |
| metab_13066 | Unannotated feature | neg | 568.7596 | 0.538 | 4.532 | 4.837 | 0.305 | 0.078 | 0.670 | 0.881 | Unannotated | NA | NA |
| metab_2198 | Unannotated feature | pos | 333.8899 | 0.593 | 3.999 | 4.003 | 0.003 | -0.078 | 0.670 | 0.881 | Unannotated | NA | NA |
| metab_17764 | Unannotated feature | neg | 281.2485 | 6.899 | 5.991 | 6.014 | 0.023 | 0.078 | 0.670 | 0.881 | Unannotated | NA | NA |
| metab_2117 | Unannotated feature | pos | 283.1654 | 2.819 | 4.909 | 4.935 | 0.026 | -0.078 | 0.670 | 0.881 | Unannotated | NA | NA |
| metab_17701 | Unannotated feature | neg | 379.2689 | 6.997 | 5.557 | 5.604 | 0.047 | -0.078 | 0.670 | 0.881 | Unannotated | NA | NA |
| metab_12597 | Unannotated feature | neg | 541.3229 | 6.483 | 6.937 | 6.971 | 0.034 | 0.078 | 0.670 | 0.881 | Unannotated | NA | NA |
| metab_11279 | Unannotated feature | neg | 229.0231 | 0.619 | 4.532 | 4.367 | -0.165 | -0.078 | 0.670 | 0.881 | Unannotated | NA | NA |
| metab_3436 | Unannotated feature | pos | 611.2859 | 6.813 | 4.723 | 4.329 | -0.394 | -0.078 | 0.670 | 0.881 | Unannotated | NA | NA |
| metab_16777 | Unannotated feature | neg | 265.1481 | 7.697 | 4.331 | 4.371 | 0.040 | 0.078 | 0.670 | 0.881 | Unannotated | NA | NA |
| metab_14981 | Unannotated feature | neg | 260.8985 | 4.286 | 4.534 | 4.414 | -0.120 | -0.078 | 0.670 | 0.881 | Unannotated | NA | NA |
| metab_15350 | Unannotated feature | neg | 124.9907 | 0.566 | 4.113 | 4.105 | -0.008 | -0.078 | 0.670 | 0.881 | Unannotated | NA | NA |
| metab_3738 | Unannotated feature | pos | 500.2608 | 6.566 | 6.650 | 6.643 | -0.007 | -0.078 | 0.670 | 0.881 | Unannotated | NA | NA |
| metab_3501 | Unannotated feature | pos | 186.0441 | 7.724 | 6.346 | 6.376 | 0.031 | 0.078 | 0.670 | 0.881 | Unannotated | NA | NA |
| metab_3524 | Unannotated feature | pos | 193.9741 | 7.716 | 5.287 | 5.287 | 0.000 | -0.078 | 0.670 | 0.881 | Unannotated | NA | NA |
| metab_13864 | L-erythro-5-(1-Hydroxyethyl)-2(5H)-furanone | neg | 173.0450 | 2.277 | 5.254 | 5.346 | 0.092 | 0.078 | 0.670 | 0.881 | Unannotated | NA | NA |
| metab_17921 | Pregnanediol | neg | 365.2697 | 6.640 | 5.618 | 5.560 | -0.057 | -0.078 | 0.670 | 0.881 | Tentative | NA | NA |
| metab_15122 | Histidylglutamine | neg | 304.1040 | 4.566 | 5.293 | 5.634 | 0.341 | 0.078 | 0.670 | 0.881 | Unannotated | NA | NA |
| metab_15813 | Unannotated feature | neg | 383.1541 | 5.884 | 5.716 | 5.795 | 0.079 | 0.078 | 0.670 | 0.881 | Unannotated | NA | NA |
| metab_18581 | Unannotated feature | neg | 263.0321 | 3.090 | 4.502 | 4.792 | 0.290 | 0.078 | 0.670 | 0.881 | Unannotated | NA | NA |
| metab_6599 | Unannotated feature | pos | 637.3256 | 5.445 | 4.965 | 6.286 | 1.321 | 0.078 | 0.670 | 0.881 | Unannotated | NA | NA |
| metab_6618 | Thr Phe Leu Thr Gln | pos | 609.3301 | 5.418 | 5.230 | 5.485 | 0.255 | 0.078 | 0.670 | 0.881 | Moderate putative | NA | NA |
| metab_13021 | Unannotated feature | neg | 303.0239 | 0.843 | 3.717 | 4.316 | 0.599 | 0.078 | 0.670 | 0.881 | Unannotated | NA | NA |
| metab_13029 | Unannotated feature | neg | 668.7715 | 0.603 | 3.107 | 3.703 | 0.597 | 0.078 | 0.670 | 0.881 | Unannotated | NA | NA |
| metab_17908 | Unannotated feature | neg | 453.2634 | 6.656 | 5.587 | 5.875 | 0.288 | 0.078 | 0.670 | 0.881 | Unannotated | NA | NA |
| metab_8703 | Miglitol | pos | 172.0970 | 0.776 | 4.875 | 4.860 | -0.016 | 0.078 | 0.670 | 0.881 | Tentative | NA | NA |
| metab_16757 | Unannotated feature | neg | 238.9316 | 7.687 | 5.128 | 5.111 | -0.017 | -0.078 | 0.670 | 0.881 | Unannotated | NA | NA |
| metab_13925 | Isopropyl tartaric acid | neg | 173.0450 | 2.561 | 5.213 | 5.241 | 0.028 | 0.078 | 0.670 | 0.881 | Unannotated | NA | NA |
| metab_17012 | Unannotated feature | neg | 249.8488 | 7.997 | 5.240 | 5.203 | -0.037 | -0.078 | 0.670 | 0.881 | Unannotated | NA | NA |
| metab_9312 | Unannotated feature | neg | 230.0281 | 3.592 | 3.497 | 3.572 | 0.075 | 0.078 | 0.670 | 0.881 | Unannotated | NA | NA |
| metab_9237 | Unannotated feature | neg | 225.0766 | 3.705 | 4.847 | 4.723 | -0.124 | -0.078 | 0.670 | 0.881 | Unannotated | NA | NA |
| metab_4309 | Unannotated feature | pos | 443.3167 | 6.032 | 3.304 | 3.302 | -0.002 | -0.078 | 0.670 | 0.881 | Unannotated | NA | NA |
| metab_4371 | Unannotated feature | pos | 334.1657 | 5.332 | 5.759 | 5.682 | -0.077 | 0.078 | 0.670 | 0.881 | Unannotated | NA | NA |
| metab_12316 | Unannotated feature | neg | 775.5346 | 7.292 | 5.060 | 5.236 | 0.177 | 0.078 | 0.670 | 0.881 | Unannotated | NA | NA |
| metab_18058 | Unannotated feature | neg | 533.3594 | 6.428 | 4.691 | 4.625 | -0.066 | -0.078 | 0.670 | 0.881 | Unannotated | NA | NA |
| metab_12093 | 5(S)-Hydroperoxyeicosatetraenoic acid | neg | 335.2230 | 6.467 | 5.236 | 5.145 | -0.092 | -0.078 | 0.670 | 0.881 | Tentative | NA | NA |
| metab_12102 | Octadecyl fumarate | neg | 389.2701 | 6.483 | 7.206 | 7.164 | -0.042 | -0.078 | 0.670 | 0.881 | Unannotated | NA | NA |
| metab_12977 | Unannotated feature | neg | 347.1099 | 0.784 | 5.097 | 5.080 | -0.016 | 0.078 | 0.670 | 0.881 | Unannotated | NA | NA |
| metab_12962 | 2,3-Dimethoxyphenol sulfate | neg | 279.0182 | 0.777 | 4.594 | 4.448 | -0.146 | -0.078 | 0.670 | 0.881 | Unannotated | Organic acids and derivatives | Organic sulfuric acids and derivatives |
| metab_10590 | Unannotated feature | neg | 330.1308 | 1.691 | 4.596 | 4.607 | 0.012 | -0.078 | 0.670 | 0.881 | Unannotated | NA | NA |
| metab_10607 | Unannotated feature | neg | 307.0498 | 1.685 | 3.257 | 3.076 | -0.181 | -0.078 | 0.670 | 0.881 | Unannotated | NA | NA |
| metab_12318 | Unannotated feature | neg | 128.9278 | 7.310 | 6.520 | 6.516 | -0.003 | 0.078 | 0.670 | 0.881 | Unannotated | NA | NA |
| metab_12201 | Unannotated feature | neg | 509.3306 | 6.797 | 5.900 | 5.968 | 0.068 | 0.078 | 0.670 | 0.881 | Unannotated | NA | NA |
| metab_16962 | Unannotated feature | neg | 189.8576 | 7.874 | 6.442 | 6.453 | 0.012 | 0.078 | 0.670 | 0.881 | Unannotated | NA | NA |
| metab_5700 | Unannotated feature | pos | 591.3482 | 6.111 | 4.084 | 4.141 | 0.057 | -0.078 | 0.670 | 0.881 | Unannotated | NA | NA |
| metab_16034 | Unannotated feature | neg | 214.1444 | 6.162 | 4.905 | 4.993 | 0.088 | 0.078 | 0.670 | 0.881 | Unannotated | NA | NA |
| metab_563 | (Z)-3-Oxo-2-(2-pentenyl)-1-cyclopenteneacetic acid | pos | 209.1176 | 5.650 | 4.194 | 4.288 | 0.094 | 0.078 | 0.670 | 0.881 | Unannotated | NA | NA |
| metab_4439 | Unannotated feature | pos | 559.3257 | 6.367 | 5.137 | 5.180 | 0.043 | -0.078 | 0.670 | 0.881 | Unannotated | NA | NA |
| metab_9092 | Unannotated feature | neg | 379.1407 | 3.938 | 3.884 | 4.185 | 0.301 | 0.078 | 0.670 | 0.881 | Unannotated | NA | NA |
| metab_11942 | Unannotated feature | neg | 551.2696 | 6.130 | 5.414 | 5.468 | 0.054 | -0.078 | 0.670 | 0.881 | Unannotated | NA | NA |
| metab_5358 | Polypodine B | pos | 497.3077 | 6.510 | 5.795 | 5.737 | -0.058 | -0.078 | 0.670 | 0.881 | Tentative | NA | NA |
| metab_12682 | Unannotated feature | neg | 591.3204 | 5.671 | 6.763 | 6.877 | 0.113 | 0.078 | 0.670 | 0.881 | Unannotated | NA | NA |
| metab_11189 | Unannotated feature | neg | 406.0787 | 4.952 | 3.430 | 3.748 | 0.317 | 0.078 | 0.670 | 0.881 | Unannotated | NA | NA |
| metab_18078 | Unannotated feature | neg | 236.0926 | 6.412 | 4.768 | 4.775 | 0.007 | 0.078 | 0.670 | 0.881 | Unannotated | NA | NA |
| metab_18090 | Unannotated feature | neg | 609.3656 | 6.397 | 5.095 | 5.041 | -0.054 | 0.078 | 0.670 | 0.881 | Unannotated | NA | NA |
| metab_1708 | Unannotated feature | pos | 420.2255 | 3.974 | 4.259 | 4.290 | 0.031 | 0.078 | 0.670 | 0.881 | Unannotated | NA | NA |
| metab_8160 | Unannotated feature | pos | 311.1336 | 2.687 | 2.912 | 2.816 | -0.096 | 0.078 | 0.670 | 0.881 | Unannotated | NA | NA |
| metab_14742 | Unannotated feature | neg | 313.1044 | 3.777 | 2.799 | 2.823 | 0.025 | 0.078 | 0.670 | 0.881 | Unannotated | NA | NA |
| metab_8197 | Linoleoyl Ethanolamide | pos | 346.2721 | 6.710 | 6.281 | 6.180 | -0.101 | -0.078 | 0.670 | 0.881 | Moderate putative | NA | NA |
| metab_18472 | (-)-trans-Carveol glucoside | neg | 359.1717 | 6.076 | 5.036 | 4.989 | -0.048 | -0.078 | 0.670 | 0.881 | Unannotated | NA | NA |
| metab_18200 | Pregnanediol 3-O-glucuronide | neg | 477.2864 | 6.295 | 5.488 | 5.519 | 0.031 | -0.078 | 0.670 | 0.881 | Tentative | Lipids and lipid-like molecules | Steroids and steroid derivatives |
| metab_18206 | Unannotated feature | neg | 289.0390 | 2.758 | 4.538 | 4.304 | -0.235 | -0.078 | 0.670 | 0.881 | Unannotated | NA | NA |
| metab_12869 | Unannotated feature | neg | 143.0343 | 0.714 | 4.849 | 4.781 | -0.067 | -0.078 | 0.670 | 0.881 | Unannotated | NA | NA |
| metab_12879 | Unannotated feature | neg | 541.1369 | 0.714 | 4.970 | 5.106 | 0.136 | 0.078 | 0.670 | 0.881 | Unannotated | NA | NA |
| metab_9152 | Unannotated feature | neg | 210.0591 | 3.849 | 3.834 | 3.783 | -0.051 | -0.078 | 0.670 | 0.881 | Unannotated | NA | NA |
| metab_14047 | Unannotated feature | neg | 616.9987 | 0.496 | 4.073 | 4.589 | 0.516 | 0.078 | 0.670 | 0.881 | Unannotated | NA | NA |
| metab_16801 | Unannotated feature | neg | 466.8750 | 7.710 | 4.763 | 4.780 | 0.017 | 0.078 | 0.670 | 0.881 | Unannotated | NA | NA |
| metab_4758 | Unannotated feature | pos | 111.5498 | 7.724 | 4.922 | 4.902 | -0.020 | -0.078 | 0.670 | 0.881 | Unannotated | NA | NA |
| metab_14861 | Unannotated feature | neg | 460.0011 | 4.028 | 6.962 | 7.158 | 0.196 | 0.078 | 0.670 | 0.881 | Unannotated | NA | NA |
| metab_11791 | Unannotated feature | neg | 415.1043 | 4.644 | 4.009 | 4.178 | 0.169 | 0.078 | 0.670 | 0.881 | Unannotated | NA | NA |
| metab_19370 | Unannotated feature | neg | 264.1243 | 5.044 | 4.637 | 4.323 | -0.314 | -0.078 | 0.670 | 0.881 | Unannotated | NA | NA |
| metab_4488 | Unannotated feature | pos | 228.2325 | 6.550 | 4.613 | 4.643 | 0.030 | -0.078 | 0.670 | 0.881 | Unannotated | NA | NA |
| metab_4549 | Solutol HS 15 | pos | 367.2823 | 6.821 | 4.392 | 4.097 | -0.295 | -0.078 | 0.670 | 0.881 | Tentative | NA | NA |
| metab_4581 | Unannotated feature | pos | 395.3134 | 6.957 | 5.318 | 5.367 | 0.049 | 0.078 | 0.670 | 0.881 | Unannotated | NA | NA |
| metab_15465 | Unannotated feature | neg | 256.1555 | 5.204 | 4.314 | 4.224 | -0.090 | -0.078 | 0.670 | 0.881 | Unannotated | NA | NA |
| metab_7130 | Norendoxifen | pos | 392.2189 | 4.579 | 4.445 | 4.467 | 0.022 | -0.078 | 0.670 | 0.881 | Unannotated | NA | NA |
| metab_19893 | Prostaglandin J2 | neg | 379.2130 | 6.232 | 6.093 | 6.132 | 0.039 | 0.078 | 0.670 | 0.881 | Tentative | NA | NA |
| metab_19886 | N-Epsilon-Acetyl-L-Lysine | neg | 187.1083 | 0.714 | 5.188 | 5.174 | -0.013 | -0.074 | 0.687 | 0.888 | Strong putative | NA | NA |
| metab_19861 | Deoxycholic acid glycine conjugate | neg | 448.3073 | 6.263 | 5.826 | 5.844 | 0.018 | -0.074 | 0.687 | 0.888 | Tentative | NA | NA |
| metab_19896 | Salicylic Acid | neg | 137.0237 | 5.475 | 4.851 | 4.924 | 0.072 | 0.074 | 0.687 | 0.888 | Moderate putative | NA | NA |
| metab_8408 | 2,3-dihydroxy-3-methylvalerate | pos | 190.1077 | 1.952 | 5.216 | 5.214 | -0.002 | 0.074 | 0.687 | 0.888 | Tentative | NA | NA |
| metab_5136 | DG(18:1(12Z)-2OH(9,10)/0:0/i-16:0) | pos | 671.4874 | 6.893 | 6.857 | 6.801 | -0.056 | -0.074 | 0.687 | 0.888 | Unannotated | NA | NA |
| metab_16567 | Unannotated feature | neg | 100.9328 | 7.310 | 7.914 | 7.912 | -0.003 | -0.074 | 0.687 | 0.888 | Unannotated | NA | NA |
| metab_16548 | Unannotated feature | neg | 285.1094 | 1.702 | 4.978 | 4.958 | -0.020 | 0.074 | 0.687 | 0.888 | Unannotated | NA | NA |
| metab_5079 | Unannotated feature | pos | 529.3882 | 7.092 | 5.897 | 5.954 | 0.057 | 0.074 | 0.687 | 0.888 | Unannotated | NA | NA |
| metab_14842 | 3-Hydroxyphenylacetic Acid | neg | 151.0394 | 3.988 | 5.423 | 5.324 | -0.099 | -0.074 | 0.687 | 0.888 | Strong putative | NA | NA |
| metab_11721 | Unannotated feature | neg | 419.1860 | 5.550 | 3.893 | 4.005 | 0.112 | 0.074 | 0.687 | 0.888 | Unannotated | NA | NA |
| metab_760 | Unannotated feature | pos | 335.0318 | 0.553 | 4.323 | 4.135 | -0.188 | -0.074 | 0.687 | 0.888 | Unannotated | NA | NA |
| metab_556 | Unannotated feature | pos | 465.3224 | 5.573 | 2.174 | 3.768 | 1.594 | 0.074 | 0.687 | 0.888 | Unannotated | NA | NA |
| metab_12199 | Unannotated feature | neg | 269.2123 | 6.774 | 5.776 | 5.817 | 0.041 | 0.074 | 0.687 | 0.888 | Unannotated | NA | NA |
| metab_12157 | Unannotated feature | neg | 535.4012 | 6.663 | 6.590 | 6.584 | -0.005 | 0.074 | 0.687 | 0.888 | Unannotated | NA | NA |
| metab_19587 | (2E,4E)-Hexa-2,4-dienedioylcarnitine | neg | 266.1035 | 4.730 | 3.378 | 3.389 | 0.011 | -0.074 | 0.687 | 0.888 | Unannotated | NA | NA |
| metab_326 | Unannotated feature | pos | 117.0401 | 0.477 | 4.361 | 4.313 | -0.048 | -0.074 | 0.687 | 0.888 | Unannotated | NA | NA |
| metab_11580 | Unannotated feature | neg | 197.8516 | 0.457 | 4.353 | 4.143 | -0.210 | -0.074 | 0.687 | 0.888 | Unannotated | NA | NA |
| metab_11611 | Unannotated feature | neg | 315.0881 | 5.280 | 4.720 | 4.732 | 0.012 | 0.074 | 0.687 | 0.888 | Unannotated | NA | NA |
| metab_12399 | Unannotated feature | neg | 551.3964 | 6.530 | 6.478 | 6.508 | 0.030 | 0.074 | 0.687 | 0.888 | Unannotated | NA | NA |
| metab_12407 | Unannotated feature | neg | 600.8551 | 7.702 | 4.726 | 4.802 | 0.075 | 0.074 | 0.687 | 0.888 | Unannotated | NA | NA |
| metab_19030 | 4',6-Diamidino-2-phenylindole | neg | 322.1310 | 5.491 | 3.258 | 3.309 | 0.050 | 0.074 | 0.687 | 0.888 | Unannotated | Organoheterocyclic compounds | Indoles and derivatives |
| metab_19099 | Cyclohexylsulfamate | neg | 178.0538 | 3.292 | 5.599 | 5.996 | 0.397 | 0.074 | 0.687 | 0.888 | Moderate putative | NA | NA |
| metab_12373 | Unannotated feature | neg | 312.2544 | 6.758 | 4.638 | 4.956 | 0.317 | 0.074 | 0.687 | 0.888 | Unannotated | NA | NA |
| metab_15539 | Unannotated feature | neg | 381.9445 | 0.577 | 4.793 | 5.020 | 0.226 | 0.074 | 0.687 | 0.888 | Unannotated | NA | NA |
| metab_806 | Unannotated feature | pos | 352.1775 | 6.303 | 3.333 | 3.825 | 0.492 | 0.074 | 0.687 | 0.888 | Unannotated | NA | NA |
| metab_808 | Unannotated feature | pos | 207.1746 | 6.311 | 4.322 | 4.294 | -0.027 | -0.074 | 0.687 | 0.888 | Unannotated | NA | NA |
| metab_8609 | Unannotated feature | pos | 345.2384 | 6.678 | 6.359 | 6.601 | 0.242 | 0.074 | 0.687 | 0.888 | Unannotated | NA | NA |
| metab_8697 | 1,3-Butadiene | pos | 72.0813 | 0.784 | 6.320 | 6.216 | -0.104 | -0.074 | 0.687 | 0.888 | Tentative | NA | NA |
| metab_14270 | Unannotated feature | neg | 193.0534 | 3.017 | 4.322 | 4.380 | 0.058 | 0.074 | 0.687 | 0.888 | Unannotated | NA | NA |
| metab_8788 | 4-Hydroxybutyric acid | pos | 250.1289 | 1.034 | 3.663 | 3.903 | 0.240 | 0.074 | 0.687 | 0.888 | Tentative | NA | NA |
| metab_4749 | Unannotated feature | pos | 110.0091 | 7.708 | 3.757 | 3.753 | -0.004 | 0.074 | 0.687 | 0.888 | Unannotated | NA | NA |
| metab_16785 | Unannotated feature | neg | 130.9829 | 7.702 | 4.119 | 4.109 | -0.009 | -0.074 | 0.687 | 0.888 | Unannotated | NA | NA |
| metab_18835 | Unannotated feature | neg | 294.0577 | 5.748 | 4.434 | 4.489 | 0.056 | 0.074 | 0.687 | 0.888 | Unannotated | NA | NA |
| metab_981 | Unannotated feature | pos | 260.1539 | 7.724 | 5.203 | 5.226 | 0.023 | 0.074 | 0.687 | 0.888 | Unannotated | NA | NA |
| metab_12475 | Unannotated feature | neg | 220.8343 | 7.997 | 4.724 | 4.722 | -0.002 | -0.074 | 0.687 | 0.888 | Unannotated | NA | NA |
| metab_18932 | Hexyl glucoside | neg | 245.1394 | 5.611 | 5.821 | 5.919 | 0.098 | 0.074 | 0.687 | 0.888 | Unannotated | NA | NA |
| metab_18885 | 1-(3-Pyridinyl)-1,4-butanediol | neg | 379.1874 | 5.675 | 3.789 | 3.785 | -0.004 | 0.074 | 0.687 | 0.888 | Tentative | NA | NA |
| metab_18924 | Unannotated feature | neg | 293.0649 | 3.239 | 4.776 | 4.650 | -0.126 | -0.074 | 0.687 | 0.888 | Unannotated | NA | NA |
| metab_11520 | Unannotated feature | neg | 365.1721 | 5.238 | 4.828 | 4.768 | -0.060 | -0.074 | 0.687 | 0.888 | Unannotated | NA | NA |
| metab_11529 | Unannotated feature | neg | 272.9592 | 0.502 | 7.436 | 7.433 | -0.003 | -0.074 | 0.687 | 0.888 | Unannotated | NA | NA |
| metab_7268 | Reticulol | pos | 223.0602 | 4.268 | 4.092 | 4.060 | -0.032 | -0.074 | 0.687 | 0.888 | Unannotated | NA | NA |
| metab_15592 | Unannotated feature | neg | 267.9633 | 0.582 | 5.838 | 5.774 | -0.063 | -0.074 | 0.687 | 0.888 | Unannotated | NA | NA |
| metab_15313 | 4-Hydroxycyclohexylcarboxylic acid | neg | 333.1548 | 4.906 | 4.768 | 4.734 | -0.033 | 0.074 | 0.687 | 0.888 | Unannotated | NA | NA |
| metab_8883 | Unannotated feature | neg | 443.1755 | 4.291 | 2.395 | 2.923 | 0.527 | 0.074 | 0.687 | 0.888 | Unannotated | NA | NA |
| metab_4656 | DG(PGE1/0:0/18:0) | pos | 659.5230 | 7.355 | 6.411 | 6.390 | -0.021 | -0.074 | 0.687 | 0.888 | Unannotated | NA | NA |
| metab_8919 | Clidinium | neg | 397.1874 | 4.235 | 5.357 | 5.188 | -0.169 | -0.074 | 0.687 | 0.888 | Tentative | NA | NA |
| metab_8942 | Unannotated feature | neg | 132.0740 | 4.203 | 4.286 | 4.293 | 0.007 | -0.074 | 0.687 | 0.888 | Unannotated | NA | NA |
| metab_8954 | Unannotated feature | neg | 281.1066 | 4.172 | 4.037 | 4.227 | 0.190 | 0.074 | 0.687 | 0.888 | Unannotated | NA | NA |
| metab_4601 | Tafluprost free acid | pos | 443.2240 | 7.036 | 5.707 | 5.719 | 0.012 | -0.074 | 0.687 | 0.888 | Unannotated | NA | NA |
| metab_16891 | Unannotated feature | neg | 184.8571 | 7.745 | 3.772 | 3.764 | -0.008 | 0.074 | 0.687 | 0.888 | Unannotated | NA | NA |
| metab_1130 | Unannotated feature | pos | 540.3082 | 6.598 | 5.288 | 5.286 | -0.002 | -0.074 | 0.687 | 0.888 | Unannotated | NA | NA |
| metab_11299 | Unannotated feature | neg | 173.1038 | 0.614 | 4.440 | 4.695 | 0.255 | -0.074 | 0.687 | 0.888 | Unannotated | NA | NA |
| metab_1099 | DIHYDROCELASTROL | pos | 485.3258 | 6.662 | 6.533 | 6.573 | 0.040 | -0.074 | 0.687 | 0.888 | Strong putative | NA | NA |
| metab_1064 | Unannotated feature | pos | 469.3301 | 6.765 | 6.280 | 6.389 | 0.109 | 0.074 | 0.687 | 0.888 | Unannotated | NA | NA |
| metab_1047 | Unannotated feature | pos | 322.2722 | 6.845 | 4.830 | 5.266 | 0.436 | 0.074 | 0.687 | 0.888 | Unannotated | NA | NA |
| metab_1006 | Glucocerebrosides | pos | 736.5358 | 7.268 | 5.458 | 5.757 | 0.298 | -0.074 | 0.687 | 0.888 | Strong putative | NA | NA |
| metab_15618 | Unannotated feature | neg | 232.1011 | 5.533 | 4.240 | 4.355 | 0.116 | 0.074 | 0.687 | 0.888 | Unannotated | NA | NA |
| metab_4550 | Unannotated feature | pos | 235.1081 | 4.802 | 5.793 | 5.765 | -0.028 | -0.074 | 0.687 | 0.888 | Unannotated | NA | NA |
| metab_4557 | Unannotated feature | pos | 228.1961 | 6.853 | 4.978 | 4.923 | -0.054 | 0.074 | 0.687 | 0.888 | Unannotated | NA | NA |
| metab_16888 | Unannotated feature | neg | 168.8356 | 7.745 | 5.161 | 5.171 | 0.011 | 0.074 | 0.687 | 0.888 | Unannotated | NA | NA |
| metab_14128 | 3,4-Dihydroxyphenylpropanoate | neg | 181.0501 | 3.647 | 5.549 | 5.555 | 0.006 | 0.074 | 0.687 | 0.888 | Strong putative | NA | NA |
| metab_14093 | Dihydrocaffeic acid 3-sulfate | neg | 261.0076 | 2.817 | 5.370 | 5.371 | 0.001 | 0.074 | 0.687 | 0.888 | Unannotated | NA | NA |
| metab_4451 | Unannotated feature | pos | 284.2861 | 6.414 | 2.487 | 1.968 | -0.518 | -0.074 | 0.687 | 0.888 | Unannotated | NA | NA |
| metab_14004 | Unannotated feature | neg | 215.0381 | 2.729 | 3.812 | 3.866 | 0.054 | -0.074 | 0.687 | 0.888 | Unannotated | NA | NA |
| metab_16965 | Unannotated feature | neg | 259.8162 | 7.888 | 5.497 | 5.492 | -0.004 | 0.074 | 0.687 | 0.888 | Unannotated | NA | NA |
| metab_16979 | Unannotated feature | neg | 245.8948 | 7.934 | 5.699 | 5.699 | 0.000 | 0.074 | 0.687 | 0.888 | Unannotated | NA | NA |
| metab_9216 | Unannotated feature | neg | 354.0835 | 3.745 | 3.225 | 3.354 | 0.129 | 0.074 | 0.687 | 0.888 | Unannotated | NA | NA |
| metab_16091 | Unannotated feature | neg | 200.0561 | 1.145 | 6.395 | 6.331 | -0.064 | 0.074 | 0.687 | 0.888 | Unannotated | NA | NA |
| metab_5699 | Unannotated feature | pos | 226.9753 | 0.019 | 4.545 | 4.557 | 0.012 | 0.074 | 0.687 | 0.888 | Unannotated | NA | NA |
| metab_13960 | Unannotated feature | neg | 197.0087 | 2.646 | 4.358 | 4.393 | 0.035 | 0.074 | 0.687 | 0.888 | Unannotated | NA | NA |
| metab_13948 | Unannotated feature | neg | 630.8792 | 0.485 | 5.664 | 5.880 | 0.216 | 0.074 | 0.687 | 0.888 | Unannotated | NA | NA |
| metab_4089 | Unannotated feature | pos | 485.2686 | 6.119 | 5.840 | 5.623 | -0.216 | -0.074 | 0.687 | 0.888 | Unannotated | NA | NA |
| metab_3904 | Unannotated feature | pos | 699.3584 | 6.335 | 4.538 | 4.349 | -0.189 | -0.074 | 0.687 | 0.888 | Unannotated | NA | NA |
| metab_13883 | Unannotated feature | neg | 403.9295 | 0.472 | 6.626 | 6.654 | 0.029 | -0.074 | 0.687 | 0.888 | Unannotated | NA | NA |
| metab_3767 | Spisulosine | pos | 286.3108 | 6.462 | 5.612 | 5.653 | 0.041 | 0.074 | 0.687 | 0.888 | Unannotated | NA | NA |
| metab_17045 | Unannotated feature | neg | 252.9163 | 7.985 | 5.431 | 5.404 | -0.027 | -0.074 | 0.687 | 0.888 | Unannotated | NA | NA |
| metab_17058 | Unannotated feature | neg | 163.8462 | 7.974 | 4.392 | 4.399 | 0.006 | -0.074 | 0.687 | 0.888 | Unannotated | NA | NA |
| metab_17077 | Unannotated feature | neg | 181.8369 | 7.910 | 5.309 | 5.294 | -0.015 | -0.074 | 0.687 | 0.888 | Unannotated | NA | NA |
| metab_11081 | Neuraminic acid | neg | 248.0776 | 0.730 | 5.178 | 5.299 | 0.121 | 0.074 | 0.687 | 0.888 | Tentative | NA | NA |
| metab_1360 | Unannotated feature | pos | 387.2261 | 6.143 | 5.342 | 5.437 | 0.095 | 0.074 | 0.687 | 0.888 | Unannotated | NA | NA |
| metab_18485 | Unannotated feature | neg | 429.1590 | 6.060 | 3.868 | 4.102 | 0.234 | 0.074 | 0.687 | 0.888 | Unannotated | NA | NA |
| metab_1265 | Unannotated feature | pos | 257.1903 | 6.327 | 3.954 | 3.984 | 0.029 | 0.074 | 0.687 | 0.888 | Unannotated | NA | NA |
| metab_1270 | Unannotated feature | pos | 470.2663 | 6.311 | 5.675 | 5.476 | -0.199 | -0.074 | 0.687 | 0.888 | Unannotated | NA | NA |
| metab_11192 | D-glycero-L-galacto-Octulose | neg | 277.0330 | 0.651 | 4.537 | 4.875 | 0.338 | 0.074 | 0.687 | 0.888 | Unannotated | NA | NA |
| metab_1216 | Unannotated feature | pos | 431.3163 | 6.406 | 5.919 | 5.974 | 0.055 | 0.074 | 0.687 | 0.888 | Unannotated | NA | NA |
| metab_17080 | Unannotated feature | neg | 299.8665 | 7.905 | 4.653 | 4.649 | -0.004 | 0.074 | 0.687 | 0.888 | Unannotated | NA | NA |
| metab_13822 | Unannotated feature | neg | 190.0365 | 2.089 | 2.912 | 3.073 | 0.161 | 0.074 | 0.687 | 0.888 | Unannotated | NA | NA |
| metab_17105 | Unannotated feature | neg | 195.8547 | 7.861 | 4.944 | 4.952 | 0.008 | -0.074 | 0.687 | 0.888 | Unannotated | NA | NA |
| metab_3365 | 1,3,5-Trihydroxy-10-methylacridone | pos | 275.1031 | 4.276 | 4.428 | 4.534 | 0.106 | -0.074 | 0.687 | 0.888 | Unannotated | NA | NA |
| metab_3366 | Unannotated feature | pos | 773.5706 | 7.276 | 4.819 | 4.978 | 0.159 | 0.074 | 0.687 | 0.888 | Unannotated | NA | NA |
| metab_3269 | Unannotated feature | pos | 244.0608 | 3.953 | 4.365 | 4.383 | 0.018 | -0.074 | 0.687 | 0.888 | Unannotated | NA | NA |
| metab_3270 | Unannotated feature | pos | 685.4374 | 6.989 | 7.089 | 7.060 | -0.029 | -0.074 | 0.687 | 0.888 | Unannotated | NA | NA |
| metab_17136 | (+/-)-Enterolactone | neg | 297.1133 | 5.944 | 6.455 | 6.477 | 0.022 | -0.074 | 0.687 | 0.888 | Strong putative | NA | NA |
| metab_17140 | Unannotated feature | neg | 166.0584 | 7.792 | 4.004 | 3.957 | -0.047 | -0.074 | 0.687 | 0.888 | Unannotated | NA | NA |
| metab_7721 | Unannotated feature | pos | 309.1815 | 3.335 | 4.333 | 4.397 | 0.064 | 0.074 | 0.687 | 0.888 | Unannotated | NA | NA |
| metab_7738 | Cadabicine | pos | 468.2508 | 6.135 | 5.444 | 5.503 | 0.059 | 0.074 | 0.687 | 0.888 | Unannotated | Organic oxygen compounds | Organooxygen compounds |
| metab_5718 | Unannotated feature | pos | 163.0453 | 0.469 | 4.311 | 4.216 | -0.095 | -0.074 | 0.687 | 0.888 | Unannotated | NA | NA |
| metab_1808 | Unannotated feature | pos | 134.9968 | 0.807 | 4.085 | 4.253 | 0.168 | 0.074 | 0.687 | 0.888 | Unannotated | NA | NA |
| metab_18246 | Unannotated feature | neg | 527.3052 | 6.256 | 4.820 | 5.007 | 0.187 | 0.074 | 0.687 | 0.888 | Unannotated | NA | NA |
| metab_18263 | Unannotated feature | neg | 509.2760 | 6.240 | 4.332 | 4.378 | 0.046 | 0.074 | 0.687 | 0.888 | Unannotated | NA | NA |
| metab_12822 | Unannotated feature | neg | 379.0831 | 0.659 | 5.999 | 5.857 | -0.143 | -0.074 | 0.687 | 0.888 | Unannotated | NA | NA |
| metab_10946 | Acetylglycine | neg | 116.0346 | 0.799 | 4.778 | 4.712 | -0.065 | -0.074 | 0.687 | 0.888 | Moderate putative | NA | NA |
| metab_1549 | Unannotated feature | pos | 611.3460 | 5.324 | 4.949 | 5.019 | 0.070 | 0.074 | 0.687 | 0.888 | Unannotated | NA | NA |
| metab_12804 | Unannotated feature | neg | 453.3381 | 6.884 | 5.037 | 5.243 | 0.205 | 0.074 | 0.687 | 0.888 | Unannotated | NA | NA |
| metab_10960 | Acrylamide | neg | 187.0719 | 0.791 | 5.341 | 5.337 | -0.004 | -0.074 | 0.687 | 0.888 | Tentative | NA | NA |
| metab_11001 | 5-Hydroxymethyluracil | neg | 187.0355 | 0.777 | 4.651 | 4.640 | -0.012 | 0.074 | 0.687 | 0.888 | Tentative | NA | NA |
| metab_6505 | Unannotated feature | pos | 94.0455 | 5.583 | 4.719 | 4.701 | -0.018 | -0.074 | 0.687 | 0.888 | Unannotated | NA | NA |
| metab_17206 | Unannotated feature | neg | 476.8343 | 7.730 | 4.493 | 4.497 | 0.003 | -0.074 | 0.687 | 0.888 | Unannotated | NA | NA |
| metab_17215 | Unannotated feature | neg | 330.0696 | 2.465 | 5.709 | 5.935 | 0.226 | 0.074 | 0.687 | 0.888 | Unannotated | NA | NA |
| metab_9703 | Orotidine | neg | 325.0075 | 3.123 | 5.327 | 5.496 | 0.169 | 0.074 | 0.687 | 0.888 | Tentative | NA | NA |
| metab_9704 | Unannotated feature | neg | 230.0302 | 3.123 | 4.816 | 4.731 | -0.085 | -0.074 | 0.687 | 0.888 | Unannotated | NA | NA |
| metab_2960 | Phenol glucuronide | pos | 271.0816 | 2.984 | 3.727 | 3.745 | 0.018 | -0.074 | 0.687 | 0.888 | Unannotated | NA | NA |
| metab_7665 | Uridine, 2'-deoxy-2'-fluoro-2'-methyl-, (2'R)- | pos | 243.0769 | 3.439 | 3.751 | 3.558 | -0.193 | -0.074 | 0.687 | 0.888 | Unannotated | NA | NA |
| metab_15944 | Unannotated feature | neg | 202.0717 | 1.061 | 7.321 | 7.165 | -0.156 | -0.074 | 0.687 | 0.888 | Unannotated | NA | NA |
| metab_9814 | Valylphenylalanine | neg | 245.1312 | 3.028 | 3.848 | 4.261 | 0.413 | 0.074 | 0.687 | 0.888 | Unannotated | NA | NA |
| metab_17290 | Unannotated feature | neg | 349.1213 | 7.714 | 4.556 | 4.669 | 0.113 | 0.074 | 0.687 | 0.888 | Unannotated | NA | NA |
| metab_13614 | Unannotated feature | neg | 321.9522 | 1.497 | 4.420 | 4.204 | -0.215 | -0.074 | 0.687 | 0.888 | Unannotated | NA | NA |
| metab_13574 | Allopurinol riboside | neg | 267.0738 | 1.952 | 6.199 | 6.144 | -0.056 | -0.074 | 0.687 | 0.888 | Unannotated | Nucleosides, nucleotides, and analogues | Pyrazolo[3,4-d]pyrimidine glycosides |
| metab_17296 | Unannotated feature | neg | 398.8870 | 7.710 | 5.355 | 5.384 | 0.029 | 0.074 | 0.687 | 0.888 | Unannotated | NA | NA |
| metab_17312 | Unannotated feature | neg | 530.8676 | 7.702 | 4.893 | 4.877 | -0.016 | -0.074 | 0.687 | 0.888 | Unannotated | NA | NA |
| metab_2854 | Lotaustralin | pos | 226.1078 | 2.774 | 5.013 | 5.129 | 0.116 | 0.074 | 0.687 | 0.888 | Tentative | NA | NA |
| metab_13532 | N-Methacryloyl-L-glutamic acid | neg | 260.0777 | 1.108 | 6.501 | 6.588 | 0.087 | 0.074 | 0.687 | 0.888 | Unannotated | NA | NA |
| metab_2647 | Vephylline | pos | 276.1448 | 1.936 | 4.793 | 4.746 | -0.048 | 0.074 | 0.687 | 0.888 | Unannotated | NA | NA |
| metab_17459 | Unannotated feature | neg | 493.3520 | 7.607 | 2.950 | 2.583 | -0.367 | -0.074 | 0.687 | 0.888 | Unannotated | NA | NA |
| metab_7605 | Unannotated feature | pos | 271.1022 | 3.538 | 4.740 | 4.747 | 0.008 | 0.074 | 0.687 | 0.888 | Unannotated | NA | NA |
| metab_7630 | Pantoyllactone glucoside | pos | 310.1480 | 3.496 | 4.889 | 4.742 | -0.146 | -0.074 | 0.687 | 0.888 | Unannotated | NA | NA |
| metab_15039 | 4-Hydroxy-4-(3-pyridyl)-butanoic acid | neg | 361.1403 | 4.401 | 4.978 | 5.276 | 0.298 | 0.074 | 0.687 | 0.888 | Tentative | NA | NA |
| metab_5922 | Unannotated feature | pos | 148.9772 | 0.016 | 4.356 | 4.360 | 0.004 | 0.074 | 0.687 | 0.888 | Unannotated | NA | NA |
| metab_17940 | Tavulin | neg | 527.2613 | 6.601 | 6.168 | 6.081 | -0.086 | -0.074 | 0.687 | 0.888 | Unannotated | NA | NA |
| metab_10616 | Unannotated feature | neg | 226.0355 | 1.685 | 3.697 | 3.742 | 0.044 | 0.074 | 0.687 | 0.888 | Unannotated | NA | NA |
| metab_12974 | Unannotated feature | neg | 550.4854 | 7.460 | 4.911 | 4.192 | -0.719 | -0.074 | 0.687 | 0.888 | Unannotated | NA | NA |
| metab_12933 | Unannotated feature | neg | 712.5397 | 7.282 | 6.038 | 6.114 | 0.076 | -0.074 | 0.687 | 0.888 | Unannotated | NA | NA |
| metab_10710 | Unannotated feature | neg | 288.9994 | 1.354 | 4.764 | 5.050 | 0.286 | 0.074 | 0.687 | 0.888 | Unannotated | NA | NA |
| metab_18104 | Mosin C | neg | 639.4487 | 6.381 | 5.459 | 5.110 | -0.349 | -0.074 | 0.687 | 0.888 | Tentative | NA | NA |
| metab_10740 | Unannotated feature | neg | 395.0961 | 1.255 | 4.211 | 3.812 | -0.399 | -0.074 | 0.687 | 0.888 | Unannotated | NA | NA |
| metab_2577 | Cbhcy | pos | 218.0850 | 1.245 | 4.093 | 3.883 | -0.210 | 0.074 | 0.687 | 0.888 | Unannotated | NA | NA |
| metab_2494 | Unannotated feature | pos | 253.1551 | 0.768 | 4.351 | 3.977 | -0.375 | -0.074 | 0.687 | 0.888 | Unannotated | NA | NA |
| metab_2530 | Unannotated feature | pos | 371.1023 | 0.954 | 5.736 | 5.746 | 0.010 | 0.074 | 0.687 | 0.888 | Unannotated | NA | NA |
| metab_13315 | Unannotated feature | neg | 392.0636 | 0.659 | 4.117 | 4.121 | 0.004 | 0.074 | 0.687 | 0.888 | Unannotated | NA | NA |
| metab_17531 | Unannotated feature | neg | 243.9530 | 7.417 | 6.390 | 6.410 | 0.020 | 0.074 | 0.687 | 0.888 | Unannotated | NA | NA |
| metab_17492 | Unannotated feature | neg | 467.3200 | 7.510 | 6.298 | 6.628 | 0.330 | 0.074 | 0.687 | 0.888 | Unannotated | NA | NA |
| metab_2459 | Unannotated feature | pos | 309.1814 | 5.485 | 4.693 | 5.421 | 0.728 | 0.074 | 0.687 | 0.888 | Unannotated | NA | NA |
| metab_17569 | Unannotated feature | neg | 102.9370 | 7.310 | 4.785 | 4.780 | -0.004 | -0.074 | 0.687 | 0.888 | Unannotated | NA | NA |
| metab_17703 | DG(15:0/20:3(5Z,8Z,11Z)/0:0) | neg | 649.5063 | 6.993 | 6.122 | 6.197 | 0.076 | -0.074 | 0.687 | 0.888 | Tentative | NA | NA |
| metab_13111 | Unannotated feature | neg | 188.8886 | 0.185 | 3.421 | 3.467 | 0.046 | 0.074 | 0.687 | 0.888 | Unannotated | NA | NA |
| metab_17739 | Unannotated feature | neg | 305.2487 | 6.929 | 5.774 | 5.227 | -0.546 | -0.074 | 0.687 | 0.888 | Unannotated | NA | NA |
| metab_17857 | Unannotated feature | neg | 559.4003 | 6.726 | 5.362 | 5.153 | -0.210 | 0.074 | 0.687 | 0.888 | Unannotated | NA | NA |
| metab_15126 | N-Acetylleucine | neg | 172.0973 | 4.582 | 5.777 | 5.801 | 0.024 | -0.074 | 0.687 | 0.888 | Strong putative | NA | NA |
| metab_5481 | Unannotated feature | pos | 289.2166 | 6.367 | 4.350 | 4.311 | -0.039 | 0.074 | 0.687 | 0.888 | Unannotated | NA | NA |
| metab_16256 | Unannotated feature | neg | 293.1793 | 6.530 | 6.070 | 6.129 | 0.060 | 0.074 | 0.687 | 0.888 | Unannotated | NA | NA |
| metab_8140 | 5-Hydroxyvalproic acid | pos | 202.1442 | 2.724 | 4.023 | 3.832 | -0.191 | -0.074 | 0.687 | 0.888 | Tentative | NA | NA |
| metab_16303 | DG(20:2(11Z,14Z)/20:4(8Z,11Z,14Z,17Z)/0:0) | neg | 713.5350 | 6.624 | 5.422 | 5.608 | 0.185 | 0.074 | 0.687 | 0.888 | Tentative | NA | NA |
| metab_16362 | Unannotated feature | neg | 552.7849 | 0.542 | 4.627 | 4.451 | -0.176 | -0.074 | 0.687 | 0.888 | Unannotated | NA | NA |
| metab_16328 | 7alpha-Hydroxy-3-oxo-4-cholestenoate | neg | 429.2975 | 6.663 | 6.692 | 6.674 | -0.018 | -0.074 | 0.687 | 0.888 | Tentative | NA | NA |
| metab_8277 | Unannotated feature | pos | 324.2177 | 2.155 | 5.175 | 5.131 | -0.044 | -0.074 | 0.687 | 0.888 | Unannotated | NA | NA |
| metab_5486 | Unannotated feature | pos | 165.1277 | 6.367 | 3.706 | 3.721 | 0.015 | 0.074 | 0.687 | 0.888 | Unannotated | NA | NA |
| metab_356 | Unannotated feature | pos | 270.1063 | 0.380 | 5.132 | 5.140 | 0.008 | 0.074 | 0.687 | 0.888 | Unannotated | NA | NA |
| metab_300 | Unannotated feature | pos | 149.0544 | 0.485 | 4.638 | 4.598 | -0.040 | -0.074 | 0.687 | 0.888 | Unannotated | NA | NA |
| metab_19583 | Bruceine D | neg | 409.1512 | 3.548 | 3.957 | 3.630 | -0.327 | 0.074 | 0.687 | 0.888 | Tentative | NA | NA |
| metab_19632 | Unannotated feature | neg | 283.9894 | 4.663 | 3.972 | 4.128 | 0.156 | 0.074 | 0.687 | 0.888 | Unannotated | NA | NA |
| metab_267 | Unannotated feature | pos | 317.1156 | 0.524 | 4.505 | 4.375 | -0.129 | -0.074 | 0.687 | 0.888 | Unannotated | NA | NA |
| metab_19701 | Unannotated feature | neg | 422.1127 | 4.559 | 3.332 | 3.757 | 0.425 | -0.074 | 0.687 | 0.888 | Unannotated | NA | NA |
| metab_15532 | Unannotated feature | neg | 197.1178 | 5.343 | 3.772 | 4.014 | 0.241 | 0.074 | 0.687 | 0.888 | Unannotated | NA | NA |
| metab_19840 | 3-Hydroxybutanoic Acid | neg | 103.0393 | 1.675 | 5.642 | 5.562 | -0.080 | -0.069 | 0.705 | 0.897 | Moderate putative | NA | NA |
| metab_7061 | N-heptanoyl-homoserine lactone | pos | 214.1442 | 4.724 | 4.617 | 4.245 | -0.371 | -0.069 | 0.705 | 0.897 | Tentative | NA | NA |
| metab_7098 | Unannotated feature | pos | 187.0580 | 0.665 | 3.822 | 4.169 | 0.347 | 0.069 | 0.705 | 0.897 | Unannotated | NA | NA |
| metab_19806 | Unannotated feature | neg | 322.0937 | 3.688 | 4.581 | 4.702 | 0.121 | 0.069 | 0.705 | 0.897 | Unannotated | NA | NA |
| metab_19745 | Cis-p-Menthane-1,7,8-triol | neg | 233.1392 | 4.510 | 2.997 | 3.049 | 0.052 | -0.069 | 0.705 | 0.897 | Unannotated | NA | NA |
| metab_19638 | Unannotated feature | neg | 141.0550 | 4.656 | 3.954 | 3.999 | 0.045 | 0.069 | 0.705 | 0.897 | Unannotated | NA | NA |
| metab_11894 | Unannotated feature | neg | 421.1556 | 6.041 | 4.054 | 4.026 | -0.029 | -0.069 | 0.705 | 0.897 | Unannotated | NA | NA |
| metab_11887 | Unannotated feature | neg | 522.2114 | 6.013 | 4.937 | 4.977 | 0.040 | 0.069 | 0.705 | 0.897 | Unannotated | NA | NA |
| metab_331 | Unannotated feature | pos | 172.0457 | 0.461 | 4.149 | 4.115 | -0.034 | -0.069 | 0.705 | 0.897 | Unannotated | NA | NA |
| metab_8060 | Lysylproline | pos | 244.1660 | 2.770 | 4.256 | 4.266 | 0.009 | -0.069 | 0.705 | 0.897 | Unannotated | Organic acids and derivatives | Carboxylic acids and derivatives |
| metab_8304 | Iprofenin | pos | 273.1238 | 3.734 | 3.768 | 4.223 | 0.455 | 0.069 | 0.705 | 0.897 | Unannotated | NA | NA |
| metab_5280 | Glucosylsphingosine | pos | 444.3335 | 6.614 | 5.782 | 5.814 | 0.032 | 0.069 | 0.705 | 0.897 | Tentative | NA | NA |
| metab_8255 | 4-Hydroxyproline galactoside | pos | 276.1083 | 2.232 | 4.777 | 4.998 | 0.222 | 0.069 | 0.705 | 0.897 | Unannotated | NA | NA |
| metab_5300 | Unannotated feature | pos | 148.0970 | 6.574 | 5.100 | 5.104 | 0.004 | 0.069 | 0.705 | 0.897 | Unannotated | NA | NA |
| metab_16273 | N-Oleoyl Aspartic acid | neg | 396.2757 | 6.569 | 4.498 | 4.225 | -0.273 | 0.069 | 0.705 | 0.897 | Unannotated | NA | NA |
| metab_5362 | Unannotated feature | pos | 567.3885 | 6.502 | 4.974 | 4.964 | -0.010 | -0.069 | 0.705 | 0.897 | Unannotated | NA | NA |
| metab_8196 | Unannotated feature | pos | 182.0526 | 2.513 | 3.603 | 3.983 | 0.380 | 0.069 | 0.705 | 0.897 | Unannotated | NA | NA |
| metab_6453 | Unannotated feature | pos | 262.1653 | 5.650 | 4.375 | 4.527 | 0.152 | 0.069 | 0.705 | 0.897 | Unannotated | NA | NA |
| metab_10578 | Unannotated feature | neg | 245.1029 | 4.527 | 4.489 | 4.349 | -0.140 | 0.069 | 0.705 | 0.897 | Unannotated | NA | NA |
| metab_17876 | Unannotated feature | neg | 483.3696 | 6.703 | 5.248 | 5.466 | 0.219 | 0.069 | 0.705 | 0.897 | Unannotated | NA | NA |
| metab_13047 | Unannotated feature | neg | 349.0206 | 0.952 | 4.004 | 3.723 | -0.281 | 0.069 | 0.705 | 0.897 | Unannotated | NA | NA |
| metab_13070 | Glucose pyruvate | neg | 247.0457 | 1.033 | 4.650 | 4.856 | 0.206 | 0.069 | 0.705 | 0.897 | Unannotated | NA | NA |
| metab_2082 | N-lactoyl-phenylalanine | pos | 279.1346 | 2.755 | 6.187 | 6.171 | -0.016 | -0.069 | 0.705 | 0.897 | Unannotated | NA | NA |
| metab_17706 | Unannotated feature | neg | 163.0064 | 2.780 | 4.449 | 4.560 | 0.111 | 0.069 | 0.705 | 0.897 | Unannotated | NA | NA |
| metab_10430 | 2,3-Butanediol glucoside | neg | 297.1194 | 1.981 | 2.762 | 3.118 | 0.356 | 0.069 | 0.705 | 0.897 | Unannotated | NA | NA |
| metab_10388 | Prolylhydroxyproline | neg | 273.1094 | 2.100 | 4.670 | 5.019 | 0.348 | 0.069 | 0.705 | 0.897 | Unannotated | NA | NA |
| metab_2308 | Unannotated feature | pos | 97.9919 | 0.068 | 4.722 | 4.733 | 0.011 | 0.069 | 0.705 | 0.897 | Unannotated | NA | NA |
| metab_2326 | Unannotated feature | pos | 299.0623 | 0.372 | 4.605 | 4.604 | -0.001 | 0.069 | 0.705 | 0.897 | Unannotated | NA | NA |
| metab_13223 | Unannotated feature | neg | 202.0202 | 0.558 | 5.077 | 5.160 | 0.083 | 0.069 | 0.705 | 0.897 | Unannotated | NA | NA |
| metab_15111 | Unannotated feature | neg | 373.1775 | 4.542 | 3.549 | 3.773 | 0.224 | 0.069 | 0.705 | 0.897 | Unannotated | NA | NA |
| metab_17529 | Unannotated feature | neg | 258.8717 | 7.417 | 4.953 | 4.932 | -0.021 | -0.069 | 0.705 | 0.897 | Unannotated | NA | NA |
| metab_10168 | Unannotated feature | neg | 299.0866 | 4.502 | 3.622 | 3.076 | -0.546 | -0.069 | 0.705 | 0.897 | Unannotated | NA | NA |
| metab_10126 | Pinoresinol | neg | 357.1308 | 2.758 | 4.784 | 4.865 | 0.081 | 0.069 | 0.705 | 0.897 | Moderate putative | NA | NA |
| metab_10125 | Unannotated feature | neg | 562.1208 | 2.758 | 4.664 | 4.681 | 0.017 | -0.069 | 0.705 | 0.897 | Unannotated | NA | NA |
| metab_10103 | Unannotated feature | neg | 396.1295 | 2.766 | 5.516 | 5.494 | -0.022 | -0.069 | 0.705 | 0.897 | Unannotated | NA | NA |
| metab_10077 | 3',4'-Dihydrodiol | neg | 321.0654 | 2.776 | 4.450 | 4.737 | 0.287 | 0.069 | 0.705 | 0.897 | Unannotated | NA | NA |
| metab_18182 | Unannotated feature | neg | 413.2914 | 6.318 | 3.866 | 3.811 | -0.054 | -0.069 | 0.705 | 0.897 | Unannotated | NA | NA |
| metab_15773 | Kamolonol | neg | 419.1861 | 5.808 | 4.733 | 4.502 | -0.231 | -0.069 | 0.705 | 0.897 | Unannotated | NA | NA |
| metab_1874 | 3-Quinolinecarboxylic acid, 7-((3R)-3-(1-aminocyclopropyl)-1-pyrrolidinyl)-1-((1R,2S)-2-fluorocyclopropyl)-1,4-dihydro-8-methoxy-4-oxo- | pos | 402.1836 | 4.774 | 4.272 | 4.183 | -0.089 | -0.069 | 0.705 | 0.897 | Unannotated | NA | NA |
| metab_10699 | Unannotated feature | neg | 185.0068 | 1.422 | 5.152 | 5.141 | -0.011 | -0.069 | 0.705 | 0.897 | Unannotated | NA | NA |
| metab_18003 | LysoPC(0:0/18:2(9Z,12Z)) | neg | 564.3328 | 6.514 | 4.231 | 4.475 | 0.243 | 0.069 | 0.705 | 0.897 | Unannotated | NA | NA |
| metab_17981 | DG(5-iso PGF2VI/a-13:0/0:0) | neg | 577.4118 | 6.530 | 4.868 | 5.032 | 0.164 | 0.069 | 0.705 | 0.897 | Unannotated | NA | NA |
| metab_12976 | Unannotated feature | neg | 337.1146 | 0.784 | 4.504 | 4.606 | 0.101 | 0.069 | 0.705 | 0.897 | Unannotated | NA | NA |
| metab_10617 | Unannotated feature | neg | 224.0926 | 1.685 | 4.220 | 4.525 | 0.305 | 0.069 | 0.705 | 0.897 | Unannotated | NA | NA |
| metab_17982 | Unannotated feature | neg | 525.3802 | 6.530 | 6.203 | 6.334 | 0.131 | 0.069 | 0.705 | 0.897 | Unannotated | NA | NA |
| metab_2748 | Unannotated feature | pos | 79.0183 | 2.355 | 3.237 | 3.263 | 0.026 | 0.069 | 0.705 | 0.897 | Unannotated | NA | NA |
| metab_2702 | Unannotated feature | pos | 163.0280 | 2.058 | 5.299 | 5.323 | 0.023 | 0.069 | 0.705 | 0.897 | Unannotated | NA | NA |
| metab_13547 | Unannotated feature | neg | 337.9996 | 1.158 | 3.813 | 3.455 | -0.357 | -0.069 | 0.705 | 0.897 | Unannotated | NA | NA |
| metab_9849 | Unannotated feature | neg | 122.0605 | 2.999 | 3.454 | 3.324 | -0.130 | -0.069 | 0.705 | 0.897 | Unannotated | NA | NA |
| metab_17269 | Unannotated feature | neg | 508.8772 | 7.720 | 5.421 | 5.455 | 0.034 | 0.069 | 0.705 | 0.897 | Unannotated | NA | NA |
| metab_5839 | Procyanidin dimer B7 | pos | 408.1664 | 5.355 | 5.564 | 5.515 | -0.049 | -0.069 | 0.705 | 0.897 | Unannotated | NA | NA |
| metab_5798 | Unannotated feature | pos | 287.8894 | 0.016 | 5.765 | 5.765 | 0.000 | 0.069 | 0.705 | 0.897 | Unannotated | NA | NA |
| metab_17241 | Unannotated feature | neg | 638.8358 | 7.726 | 4.482 | 4.459 | -0.023 | 0.069 | 0.705 | 0.897 | Unannotated | NA | NA |
| metab_17189 | Unannotated feature | neg | 225.0691 | 7.741 | 5.357 | 5.399 | 0.042 | 0.069 | 0.705 | 0.897 | Unannotated | NA | NA |
| metab_9624 | Unannotated feature | neg | 312.0136 | 3.210 | 4.146 | 4.154 | 0.007 | -0.069 | 0.705 | 0.897 | Unannotated | NA | NA |
| metab_9623 | Unannotated feature | neg | 329.9881 | 3.210 | 4.969 | 4.867 | -0.102 | -0.069 | 0.705 | 0.897 | Unannotated | NA | NA |
| metab_7458 | Unannotated feature | pos | 233.1191 | 3.854 | 3.674 | 3.565 | -0.109 | -0.069 | 0.705 | 0.897 | Unannotated | NA | NA |
| metab_15698 | Unannotated feature | neg | 342.0114 | 0.588 | 4.188 | 4.420 | 0.232 | 0.069 | 0.705 | 0.897 | Unannotated | NA | NA |
| metab_12807 | Ginnalin B | neg | 353.0259 | 0.644 | 4.793 | 4.786 | -0.007 | 0.069 | 0.705 | 0.897 | Unannotated | NA | NA |
| metab_1581 | Unannotated feature | pos | 280.1550 | 3.569 | 3.918 | 4.220 | 0.302 | 0.069 | 0.705 | 0.897 | Unannotated | NA | NA |
| metab_18275 | Panaxytriol | neg | 323.1864 | 6.232 | 5.528 | 5.602 | 0.075 | 0.069 | 0.705 | 0.897 | Tentative | NA | NA |
| metab_18215 | Unannotated feature | neg | 641.1959 | 2.754 | 4.119 | 3.533 | -0.586 | -0.069 | 0.705 | 0.897 | Unannotated | NA | NA |
| metab_13772 | Unannotated feature | neg | 344.1467 | 1.965 | 5.097 | 5.059 | -0.038 | -0.069 | 0.705 | 0.897 | Unannotated | NA | NA |
| metab_9599 | Unannotated feature | neg | 421.0533 | 3.227 | 3.908 | 3.940 | 0.032 | 0.069 | 0.705 | 0.897 | Unannotated | NA | NA |
| metab_17108 | Unannotated feature | neg | 158.8461 | 7.861 | 4.834 | 4.830 | -0.004 | 0.069 | 0.705 | 0.897 | Unannotated | NA | NA |
| metab_13804 | Unannotated feature | neg | 167.0820 | 2.036 | 3.988 | 3.974 | -0.014 | 0.069 | 0.705 | 0.897 | Unannotated | NA | NA |
| metab_15656 | 4-Methylpentanal | neg | 145.0864 | 5.600 | 2.602 | 3.088 | 0.486 | 0.069 | 0.705 | 0.897 | Tentative | NA | NA |
| metab_6687 | Unannotated feature | pos | 106.0504 | 0.625 | 5.283 | 5.181 | -0.102 | -0.069 | 0.705 | 0.897 | Unannotated | NA | NA |
| metab_7367 | Dimboa glucoside | pos | 406.1331 | 0.704 | 3.037 | 3.872 | 0.835 | 0.069 | 0.705 | 0.897 | Unannotated | Organic oxygen compounds | Organooxygen compounds |
| metab_11220 | Unannotated feature | neg | 203.9916 | 0.644 | 4.433 | 4.262 | -0.171 | -0.069 | 0.705 | 0.897 | Unannotated | NA | NA |
| metab_11219 | Unannotated feature | neg | 204.0041 | 0.644 | 4.889 | 4.878 | -0.010 | -0.069 | 0.705 | 0.897 | Unannotated | NA | NA |
| metab_18461 | Unannotated feature | neg | 315.1270 | 6.083 | 6.660 | 6.567 | -0.093 | 0.069 | 0.705 | 0.897 | Unannotated | NA | NA |
| metab_12695 | ENOXOLONE | neg | 469.3325 | 6.648 | 6.226 | 6.570 | 0.344 | 0.069 | 0.705 | 0.897 | Tentative | NA | NA |
| metab_18440 | Unannotated feature | neg | 422.1861 | 6.099 | 3.890 | 4.081 | 0.191 | -0.069 | 0.705 | 0.897 | Unannotated | NA | NA |
| metab_18438 | Unannotated feature | neg | 557.2800 | 6.099 | 4.856 | 4.841 | -0.014 | 0.069 | 0.705 | 0.897 | Unannotated | NA | NA |
| metab_3701 | Unannotated feature | pos | 417.2178 | 5.297 | 4.129 | 4.425 | 0.296 | 0.069 | 0.705 | 0.897 | Unannotated | NA | NA |
| metab_3649 | Unannotated feature | pos | 627.4248 | 6.654 | 5.797 | 5.430 | -0.367 | 0.069 | 0.705 | 0.897 | Unannotated | NA | NA |
| metab_3835 | Unannotated feature | pos | 403.2967 | 6.390 | 4.483 | 4.934 | 0.451 | 0.069 | 0.705 | 0.897 | Unannotated | NA | NA |
| metab_17008 | Unannotated feature | neg | 178.8334 | 7.997 | 4.453 | 4.466 | 0.013 | 0.069 | 0.705 | 0.897 | Unannotated | NA | NA |
| metab_4099 | Unannotated feature | pos | 609.3303 | 6.119 | 6.725 | 6.737 | 0.012 | -0.069 | 0.705 | 0.897 | Unannotated | NA | NA |
| metab_9287 | Unannotated feature | neg | 307.0857 | 3.631 | 5.183 | 5.264 | 0.081 | 0.069 | 0.705 | 0.897 | Unannotated | NA | NA |
| metab_16964 | Unannotated feature | neg | 334.9118 | 7.884 | 5.104 | 5.097 | -0.007 | -0.069 | 0.705 | 0.897 | Unannotated | NA | NA |
| metab_9232 | UK-121,265 | neg | 410.1100 | 3.709 | 4.654 | 4.697 | 0.042 | 0.069 | 0.705 | 0.897 | Unannotated | NA | NA |
| metab_9212 | Unannotated feature | neg | 408.0945 | 3.921 | 5.055 | 4.830 | -0.225 | -0.069 | 0.705 | 0.897 | Unannotated | NA | NA |
| metab_9207 | Unannotated feature | neg | 391.1380 | 3.759 | 4.990 | 4.929 | -0.061 | -0.069 | 0.705 | 0.897 | Unannotated | NA | NA |
| metab_14149 | Unannotated feature | neg | 560.8178 | 0.512 | 4.627 | 4.532 | -0.095 | 0.069 | 0.705 | 0.897 | Unannotated | NA | NA |
| metab_11418 | Unannotated feature | neg | 316.8729 | 0.542 | 5.382 | 5.341 | -0.041 | -0.069 | 0.705 | 0.897 | Unannotated | NA | NA |
| metab_18738 | Agelin | neg | 437.1506 | 5.865 | 4.386 | 4.630 | 0.244 | 0.069 | 0.705 | 0.897 | Unannotated | NA | NA |
| metab_1112 | 3-Methyl-5-pentyl-2-furannonanoic acid | pos | 331.2247 | 6.630 | 4.290 | 4.278 | -0.012 | 0.069 | 0.705 | 0.897 | Unannotated | NA | NA |
| metab_11306 | Unannotated feature | neg | 296.8905 | 0.607 | 4.365 | 4.226 | -0.140 | 0.069 | 0.705 | 0.897 | Unannotated | NA | NA |
| metab_1124 | Unannotated feature | pos | 551.3572 | 6.614 | 6.789 | 6.788 | -0.001 | 0.069 | 0.705 | 0.897 | Unannotated | NA | NA |
| metab_1122 | Unannotated feature | pos | 226.0939 | 1.685 | 4.881 | 4.727 | -0.154 | -0.069 | 0.705 | 0.897 | Unannotated | NA | NA |
| metab_11280 | Unannotated feature | neg | 213.0490 | 0.619 | 4.864 | 4.794 | -0.070 | -0.069 | 0.705 | 0.897 | Unannotated | NA | NA |
| metab_18611 | Retaspimycin | neg | 568.2971 | 5.975 | 5.515 | 5.756 | 0.242 | 0.069 | 0.705 | 0.897 | Unannotated | Phenylpropanoids and polyketides | Macrolactams |
| metab_11227 | Oxindanac | neg | 327.0880 | 5.065 | 5.597 | 5.353 | -0.243 | -0.069 | 0.705 | 0.897 | Unannotated | NA | NA |
| metab_16861 | Unannotated feature | neg | 412.8996 | 7.730 | 5.284 | 5.282 | -0.002 | 0.069 | 0.705 | 0.897 | Unannotated | NA | NA |
| metab_7925 | Pro Lys | pos | 244.1659 | 2.937 | 3.940 | 3.551 | -0.389 | 0.069 | 0.705 | 0.897 | Moderate putative | NA | NA |
| metab_7892 | Pyridoxine (Vitamin B6) | pos | 170.0814 | 2.976 | 4.097 | 4.138 | 0.041 | 0.069 | 0.705 | 0.897 | Unannotated | NA | NA |
| metab_16849 | Unannotated feature | neg | 300.1222 | 7.730 | 5.048 | 4.956 | -0.093 | -0.069 | 0.705 | 0.897 | Unannotated | NA | NA |
| metab_16875 | Unannotated feature | neg | 140.8865 | 7.741 | 3.424 | 3.471 | 0.047 | -0.069 | 0.705 | 0.897 | Unannotated | NA | NA |
| metab_14157 | Unannotated feature | neg | 167.0707 | 2.899 | 3.716 | 3.611 | -0.105 | -0.069 | 0.705 | 0.897 | Unannotated | NA | NA |
| metab_8916 | Unannotated feature | neg | 283.0397 | 3.754 | 3.960 | 4.206 | 0.246 | 0.069 | 0.705 | 0.897 | Unannotated | NA | NA |
| metab_4688 | Unannotated feature | pos | 528.5005 | 7.538 | 6.768 | 6.747 | -0.020 | -0.069 | 0.705 | 0.897 | Unannotated | NA | NA |
| metab_8886 | Unannotated feature | neg | 233.0852 | 3.728 | 4.145 | 4.211 | 0.066 | -0.069 | 0.705 | 0.897 | Unannotated | NA | NA |
| metab_926 | Unannotated feature | pos | 644.5322 | 7.331 | 5.811 | 5.879 | 0.068 | -0.069 | 0.705 | 0.897 | Unannotated | NA | NA |
| metab_927 | Unannotated feature | pos | 287.2219 | 7.307 | 6.166 | 6.166 | 0.000 | 0.069 | 0.705 | 0.897 | Unannotated | NA | NA |
| metab_12487 | Unannotated feature | neg | 569.4068 | 6.397 | 5.945 | 5.940 | -0.004 | -0.069 | 0.705 | 0.897 | Unannotated | NA | NA |
| metab_18856 | Unannotated feature | neg | 375.1452 | 5.714 | 3.518 | 3.494 | -0.024 | 0.069 | 0.705 | 0.897 | Unannotated | NA | NA |
| metab_12503 | Unannotated feature | neg | 583.3863 | 6.350 | 5.842 | 5.895 | 0.053 | -0.069 | 0.705 | 0.897 | Unannotated | NA | NA |
| metab_8816 | Indole-3-Carboxaldehyde | pos | 146.0602 | 5.223 | 5.259 | 5.422 | 0.163 | 0.069 | 0.705 | 0.897 | Strong putative | NA | NA |
| metab_7953 | Delta-Hexalactone | pos | 115.0759 | 2.900 | 3.601 | 3.884 | 0.283 | 0.069 | 0.705 | 0.897 | Moderate putative | NA | NA |
| metab_16101 | Unannotated feature | neg | 278.8224 | 0.614 | 4.679 | 4.623 | -0.056 | -0.069 | 0.705 | 0.897 | Unannotated | NA | NA |
| metab_5578 | Unannotated feature | pos | 201.1641 | 6.255 | 4.245 | 4.249 | 0.004 | 0.069 | 0.705 | 0.897 | Unannotated | NA | NA |
| metab_5567 | Unannotated feature | pos | 250.1124 | 6.263 | 5.219 | 5.166 | -0.053 | -0.069 | 0.705 | 0.897 | Unannotated | NA | NA |
| metab_4776 | Unannotated feature | pos | 180.0222 | 7.772 | 3.869 | 3.892 | 0.022 | -0.069 | 0.705 | 0.897 | Unannotated | NA | NA |
| metab_8759 | (3R)-3,4-Dihydroxy-3-(hydroxymethyl)butanenitrile 4-glucoside | pos | 335.1458 | 0.728 | 4.506 | 4.507 | 0.002 | 0.069 | 0.705 | 0.897 | Unannotated | NA | NA |
| metab_4788 | Unannotated feature | pos | 99.5316 | 7.880 | 3.174 | 3.215 | 0.041 | 0.069 | 0.705 | 0.897 | Unannotated | NA | NA |
| metab_16712 | Unannotated feature | neg | 400.9097 | 7.648 | 6.041 | 6.042 | 0.001 | -0.069 | 0.705 | 0.897 | Unannotated | NA | NA |
| metab_4978 | Unannotated feature | pos | 514.4840 | 7.530 | 6.897 | 6.877 | -0.021 | -0.069 | 0.705 | 0.897 | Unannotated | NA | NA |
| metab_6863 | Unannotated feature | pos | 290.0755 | 0.641 | 3.734 | 3.143 | -0.591 | 0.069 | 0.705 | 0.897 | Unannotated | NA | NA |
| metab_6819 | 4-Aminohex-5-ynoic acid | pos | 169.0974 | 0.633 | 4.937 | 5.040 | 0.103 | 0.069 | 0.705 | 0.897 | Unannotated | NA | NA |
| metab_11671 | Unannotated feature | neg | 209.0452 | 5.317 | 5.531 | 5.469 | -0.062 | -0.069 | 0.705 | 0.897 | Unannotated | NA | NA |
| metab_867 | Unannotated feature | pos | 326.3058 | 6.702 | 4.891 | 4.683 | -0.208 | -0.069 | 0.705 | 0.897 | Unannotated | NA | NA |
| metab_11650 | Unannotated feature | neg | 162.8383 | 0.036 | 5.925 | 5.905 | -0.020 | -0.069 | 0.705 | 0.897 | Unannotated | NA | NA |
| metab_19017 | Unannotated feature | neg | 425.1649 | 5.506 | 3.987 | 3.979 | -0.008 | -0.069 | 0.705 | 0.897 | Unannotated | NA | NA |
| metab_7192 | Unannotated feature | pos | 203.0343 | 4.460 | 4.002 | 3.970 | -0.032 | 0.069 | 0.705 | 0.897 | Unannotated | NA | NA |
| metab_7152 | Unannotated feature | pos | 375.1924 | 4.533 | 4.290 | 4.267 | -0.023 | -0.069 | 0.705 | 0.897 | Unannotated | NA | NA |
| metab_358 | Unannotated feature | pos | 75.0446 | 0.375 | 4.593 | 4.613 | 0.019 | 0.069 | 0.705 | 0.897 | Unannotated | NA | NA |
| metab_11843 | Unannotated feature | neg | 360.1093 | 5.952 | 6.764 | 6.742 | -0.022 | -0.069 | 0.705 | 0.897 | Unannotated | NA | NA |
| metab_19342 | Egonol | neg | 371.1140 | 5.076 | 2.294 | 2.997 | 0.703 | 0.069 | 0.705 | 0.897 | Unannotated | NA | NA |
| metab_498 | Unannotated feature | pos | 547.3300 | 6.231 | 5.126 | 5.103 | -0.023 | 0.069 | 0.705 | 0.897 | Unannotated | NA | NA |
| metab_721 | Unannotated feature | pos | 156.0143 | 0.493 | 5.518 | 5.440 | -0.078 | -0.069 | 0.705 | 0.897 | Unannotated | NA | NA |
| metab_12283 | Unannotated feature | neg | 659.3795 | 7.117 | 5.645 | 5.373 | -0.272 | -0.069 | 0.705 | 0.897 | Unannotated | NA | NA |
| metab_19192 | Unannotated feature | neg | 603.3225 | 5.272 | 3.164 | 3.405 | 0.240 | -0.069 | 0.705 | 0.897 | Unannotated | NA | NA |
| metab_8059 | Glucosylisomaltol | pos | 271.0818 | 2.770 | 4.264 | 4.470 | 0.206 | 0.069 | 0.705 | 0.897 | Unannotated | NA | NA |
| metab_16137 | 2-(4,4-Difluoro-1-piperidinyl)-6-methoxy-N-[1-(1-methylethyl)-4-piperidinyl]-7-[3-(1-pyrrolidinyl)propoxy]-4-quinazolinamine | neg | 591.3444 | 6.295 | 4.289 | 4.234 | -0.055 | -0.069 | 0.705 | 0.897 | Unannotated | NA | NA |
| metab_5014 | Unannotated feature | pos | 638.5735 | 7.355 | 4.563 | 4.628 | 0.064 | -0.069 | 0.705 | 0.897 | Unannotated | NA | NA |
| metab_8575 | Unannotated feature | pos | 245.0854 | 1.196 | 3.069 | 3.335 | 0.266 | 0.069 | 0.705 | 0.897 | Unannotated | NA | NA |
| metab_14413 | Unannotated feature | neg | 320.1465 | 3.215 | 3.078 | 3.292 | 0.213 | 0.069 | 0.705 | 0.897 | Unannotated | NA | NA |
| metab_14439 | Unannotated feature | neg | 412.8615 | 0.527 | 5.815 | 5.867 | 0.052 | 0.069 | 0.705 | 0.897 | Unannotated | NA | NA |
| metab_14467 | Unannotated feature | neg | 394.8895 | 0.527 | 6.014 | 6.028 | 0.014 | 0.069 | 0.705 | 0.897 | Unannotated | NA | NA |
| metab_14504 | Unannotated feature | neg | 744.7826 | 0.524 | 3.891 | 4.089 | 0.198 | 0.069 | 0.705 | 0.897 | Unannotated | NA | NA |
| metab_5222 | Unannotated feature | pos | 541.3359 | 6.710 | 4.212 | 4.859 | 0.648 | 0.069 | 0.705 | 0.897 | Unannotated | NA | NA |
| metab_16396 | Unannotated feature | neg | 439.2478 | 6.836 | 5.705 | 5.855 | 0.149 | -0.069 | 0.705 | 0.897 | Unannotated | NA | NA |
| metab_12027 | Unannotated feature | neg | 591.3202 | 6.287 | 6.682 | 6.559 | -0.123 | -0.069 | 0.705 | 0.897 | Unannotated | NA | NA |
| metab_19824 | 12-Oxo-20-trihydroxy-leukotriene B4 | neg | 381.1924 | 4.398 | 4.899 | 5.005 | 0.106 | 0.069 | 0.705 | 0.897 | Unannotated | NA | NA |
| metab_11999 | Unannotated feature | neg | 239.1287 | 6.232 | 4.851 | 4.948 | 0.097 | 0.065 | 0.722 | 0.904 | Unannotated | NA | NA |
| metab_17838 | Unannotated feature | neg | 365.2312 | 6.766 | 4.733 | 4.608 | -0.125 | 0.065 | 0.722 | 0.904 | Unannotated | NA | NA |
| metab_2202 | Leu Val Ala | pos | 302.2080 | 3.002 | 5.626 | 5.573 | -0.053 | -0.065 | 0.722 | 0.904 | Moderate putative | NA | NA |
| metab_10402 | Unannotated feature | neg | 334.0777 | 2.043 | 3.718 | 3.531 | -0.188 | -0.065 | 0.722 | 0.904 | Unannotated | NA | NA |
| metab_13139 | Unannotated feature | neg | 172.8298 | 0.457 | 4.121 | 3.926 | -0.195 | 0.065 | 0.722 | 0.904 | Unannotated | NA | NA |
| metab_15495 | Unannotated feature | neg | 339.0093 | 0.566 | 3.964 | 3.513 | -0.451 | -0.065 | 0.722 | 0.904 | Unannotated | NA | NA |
| metab_392 | Unannotated feature | pos | 271.9170 | 0.019 | 4.402 | 4.375 | -0.026 | -0.065 | 0.722 | 0.904 | Unannotated | NA | NA |
| metab_323 | Unannotated feature | pos | 187.0818 | 0.477 | 5.137 | 5.096 | -0.041 | -0.065 | 0.722 | 0.904 | Unannotated | NA | NA |
| metab_4375 | Unannotated feature | pos | 483.2539 | 6.215 | 4.956 | 4.766 | -0.190 | -0.065 | 0.722 | 0.904 | Unannotated | NA | NA |
| metab_14041 | Unannotated feature | neg | 380.0651 | 2.771 | 4.102 | 4.189 | 0.087 | -0.065 | 0.722 | 0.904 | Unannotated | NA | NA |
| metab_10389 | Unannotated feature | neg | 147.0115 | 2.097 | 3.888 | 3.898 | 0.010 | 0.065 | 0.722 | 0.904 | Unannotated | NA | NA |
| metab_10013 | Unannotated feature | neg | 276.0618 | 2.831 | 4.048 | 4.049 | 0.002 | -0.065 | 0.722 | 0.904 | Unannotated | NA | NA |
| metab_13455 | Unannotated feature | neg | 301.9823 | 0.893 | 4.986 | 5.057 | 0.070 | -0.065 | 0.722 | 0.904 | Unannotated | NA | NA |
| metab_2768 | MALEAMIC ACID | pos | 272.0883 | 2.443 | 3.045 | 3.311 | 0.267 | 0.065 | 0.722 | 0.904 | Tentative | NA | NA |
| metab_13462 | Unannotated feature | neg | 326.9953 | 0.917 | 3.938 | 2.342 | -1.596 | -0.065 | 0.722 | 0.904 | Unannotated | NA | NA |
| metab_49 | L-Proline | pos | 116.0711 | 0.688 | 6.664 | 6.743 | 0.078 | 0.065 | 0.722 | 0.904 | Strong putative | NA | NA |
| metab_2691 | Unannotated feature | pos | 79.0759 | 2.021 | 4.046 | 4.078 | 0.032 | 0.065 | 0.722 | 0.904 | Unannotated | NA | NA |
| metab_13475 | Unannotated feature | neg | 348.8963 | 0.603 | 5.357 | 5.338 | -0.018 | -0.065 | 0.722 | 0.904 | Unannotated | NA | NA |
| metab_18381 | Unannotated feature | neg | 499.3283 | 6.146 | 4.921 | 5.074 | 0.153 | -0.065 | 0.722 | 0.904 | Unannotated | NA | NA |
| metab_8036 | Biocytin | pos | 390.2149 | 2.788 | 3.674 | 3.641 | -0.033 | 0.065 | 0.722 | 0.904 | Tentative | NA | NA |
| metab_12753 | Prolyl-Asparagine | neg | 228.0987 | 1.952 | 5.076 | 4.969 | -0.107 | -0.065 | 0.722 | 0.904 | Unannotated | NA | NA |
| metab_1458 | Ruscogenin | pos | 448.3433 | 5.992 | 5.415 | 5.543 | 0.128 | -0.065 | 0.722 | 0.904 | Tentative | NA | NA |
| metab_18492 | Unannotated feature | neg | 239.0852 | 6.060 | 5.605 | 5.415 | -0.190 | -0.065 | 0.722 | 0.904 | Unannotated | NA | NA |
| metab_14851 | Unannotated feature | neg | 304.1151 | 3.999 | 3.471 | 3.501 | 0.030 | 0.065 | 0.722 | 0.904 | Unannotated | NA | NA |
| metab_5262 | Goshonoside F3 | pos | 677.3756 | 6.638 | 5.884 | 5.760 | -0.124 | -0.065 | 0.722 | 0.904 | Unannotated | NA | NA |
| metab_14600 | 2-Propylthiazolo[4,5-c]quinolin-4-amine | neg | 288.0811 | 3.505 | 2.715 | 2.919 | 0.204 | 0.065 | 0.722 | 0.904 | Unannotated | NA | NA |
| metab_10550 | Unannotated feature | neg | 335.0618 | 1.923 | 4.573 | 4.547 | -0.026 | 0.065 | 0.722 | 0.904 | Unannotated | NA | NA |
| metab_13067 | Unannotated feature | neg | 498.8422 | 0.538 | 4.392 | 4.551 | 0.160 | 0.065 | 0.722 | 0.904 | Unannotated | NA | NA |
| metab_17861 | 2-Hydroxymyristic Acid | neg | 243.1964 | 6.726 | 5.623 | 5.617 | -0.006 | -0.065 | 0.722 | 0.904 | Moderate putative | NA | NA |
| metab_4140 | Morpholin-4-yl-[2-(piperidin-3-ylmethoxy)phenyl]methanone | pos | 305.1863 | 6.095 | 5.847 | 6.413 | 0.567 | 0.065 | 0.722 | 0.904 | Strong putative | NA | NA |
| metab_4156 | Unannotated feature | pos | 608.3465 | 5.898 | 4.366 | 4.416 | 0.050 | -0.065 | 0.722 | 0.904 | Unannotated | NA | NA |
| metab_4239 | Unannotated feature | pos | 494.2953 | 5.929 | 4.604 | 4.824 | 0.220 | 0.065 | 0.722 | 0.904 | Unannotated | NA | NA |
| metab_9965 | Unannotated feature | neg | 285.1458 | 2.890 | 4.265 | 4.176 | -0.089 | -0.065 | 0.722 | 0.904 | Unannotated | NA | NA |
| metab_4984 | Unannotated feature | pos | 455.4102 | 7.522 | 5.610 | 5.600 | -0.010 | -0.065 | 0.722 | 0.904 | Unannotated | NA | NA |
| metab_8610 | 2-hydroxydesipramine | pos | 305.1614 | 4.068 | 4.138 | 4.233 | 0.095 | -0.065 | 0.722 | 0.904 | Unannotated | NA | NA |
| metab_2874 | 2-O-p-Coumaroylhydroxycitric acid | pos | 355.0643 | 2.803 | 4.266 | 4.203 | -0.063 | -0.065 | 0.722 | 0.904 | Unannotated | NA | NA |
| metab_2879 | Unannotated feature | pos | 593.3349 | 6.135 | 6.693 | 6.847 | 0.154 | -0.065 | 0.722 | 0.904 | Unannotated | NA | NA |
| metab_16127 | Unannotated feature | neg | 187.9961 | 0.614 | 5.230 | 5.247 | 0.017 | -0.065 | 0.722 | 0.904 | Unannotated | NA | NA |
| metab_14872 | Unannotated feature | neg | 133.0652 | 4.044 | 3.986 | 4.000 | 0.014 | -0.065 | 0.722 | 0.904 | Unannotated | NA | NA |
| metab_12637 | Unannotated feature | neg | 359.2080 | 5.983 | 6.478 | 6.398 | -0.079 | -0.065 | 0.722 | 0.904 | Unannotated | NA | NA |
| metab_11207 | Unannotated feature | neg | 339.1412 | 0.644 | 5.498 | 5.574 | 0.076 | 0.065 | 0.722 | 0.904 | Unannotated | NA | NA |
| metab_1250 | Unannotated feature | pos | 237.1852 | 6.367 | 3.939 | 3.904 | -0.036 | -0.065 | 0.722 | 0.904 | Unannotated | NA | NA |
| metab_4637 | Unannotated feature | pos | 875.7339 | 7.196 | 7.908 | 7.922 | 0.014 | 0.065 | 0.722 | 0.904 | Unannotated | NA | NA |
| metab_16383 | 2,6,10,10-Tetramethyl-1-oxaspiro[4.5]decan-6-ol | neg | 469.3534 | 6.782 | 2.690 | 2.827 | 0.137 | -0.065 | 0.722 | 0.904 | Unannotated | NA | NA |
| metab_8297 | Unannotated feature | pos | 200.9728 | 2.079 | 4.476 | 4.427 | -0.049 | -0.065 | 0.722 | 0.904 | Unannotated | NA | NA |
| metab_8314 | Unannotated feature | pos | 138.9917 | 2.039 | 4.015 | 4.004 | -0.011 | 0.065 | 0.722 | 0.904 | Unannotated | NA | NA |
| metab_13061 | Ectoine | neg | 187.0719 | 1.021 | 5.151 | 5.214 | 0.063 | 0.065 | 0.722 | 0.904 | Tentative | NA | NA |
| metab_10572 | Unannotated feature | neg | 220.0283 | 1.737 | 3.404 | 3.831 | 0.427 | 0.065 | 0.722 | 0.904 | Unannotated | NA | NA |
| metab_13052 | Unannotated feature | neg | 218.9820 | 0.992 | 3.553 | 3.786 | 0.233 | 0.065 | 0.722 | 0.904 | Unannotated | NA | NA |
| metab_13120 | Unannotated feature | neg | 197.8892 | 0.388 | 3.873 | 3.884 | 0.011 | -0.065 | 0.722 | 0.904 | Unannotated | NA | NA |
| metab_8352 | Unannotated feature | pos | 209.0925 | 1.960 | 4.006 | 4.006 | -0.000 | -0.065 | 0.722 | 0.904 | Unannotated | NA | NA |
| metab_15680 | Unannotated feature | neg | 519.8484 | 0.588 | 4.362 | 4.803 | 0.441 | 0.065 | 0.722 | 0.904 | Unannotated | NA | NA |
| metab_4687 | Unannotated feature | pos | 103.1315 | 7.538 | 6.367 | 6.371 | 0.005 | 0.065 | 0.722 | 0.904 | Unannotated | NA | NA |
| metab_16804 | Unannotated feature | neg | 305.9267 | 7.714 | 4.834 | 4.851 | 0.017 | -0.065 | 0.722 | 0.904 | Unannotated | NA | NA |
| metab_9398 | Unannotated feature | neg | 480.9666 | 4.024 | 4.871 | 5.142 | 0.271 | 0.065 | 0.722 | 0.904 | Unannotated | NA | NA |
| metab_9316 | (S)-2-Aceto-2-hydroxybutanoic acid | neg | 145.0500 | 3.589 | 5.833 | 5.857 | 0.024 | 0.065 | 0.722 | 0.904 | Tentative | NA | NA |
| metab_17003 | Unannotated feature | neg | 345.8849 | 7.993 | 4.474 | 4.499 | 0.025 | 0.065 | 0.722 | 0.904 | Unannotated | NA | NA |
| metab_9331 | Esculetin | neg | 177.0187 | 3.573 | 3.530 | 3.603 | 0.074 | 0.065 | 0.722 | 0.904 | Strong putative | NA | NA |
| metab_15664 | Unannotated feature | neg | 351.9250 | 0.588 | 5.000 | 5.272 | 0.272 | 0.065 | 0.722 | 0.904 | Unannotated | NA | NA |
| metab_6560 | Unannotated feature | pos | 510.2907 | 5.505 | 3.874 | 3.750 | -0.124 | 0.065 | 0.722 | 0.904 | Unannotated | NA | NA |
| metab_1570 | Isoelemicin | pos | 191.1070 | 3.543 | 3.489 | 3.904 | 0.415 | 0.065 | 0.722 | 0.904 | Tentative | NA | NA |
| metab_16599 | Unannotated feature | neg | 621.4387 | 7.413 | 5.070 | 4.992 | -0.077 | -0.065 | 0.722 | 0.904 | Unannotated | NA | NA |
| metab_8482 | Exo,exo-1,8-Epoxy-p-menthane-2,6-diol | pos | 228.1599 | 1.692 | 1.484 | 1.761 | 0.276 | 0.065 | 0.722 | 0.904 | Tentative | NA | NA |
| metab_8504 | Unannotated feature | pos | 601.4806 | 7.124 | 5.836 | 5.557 | -0.279 | -0.065 | 0.722 | 0.904 | Unannotated | NA | NA |
| metab_2943 | Unannotated feature | pos | 143.0732 | 2.941 | 3.923 | 3.695 | -0.228 | -0.065 | 0.722 | 0.904 | Unannotated | NA | NA |
| metab_1137 | Benzenepropanoic acid, beta-((2-carboxyethyl)thio)-2-(8-phenyloctyl)-, (betaS)- | pos | 425.2157 | 6.574 | 5.797 | 5.737 | -0.060 | -0.065 | 0.722 | 0.904 | Unannotated | NA | NA |
| metab_13254 | Galactosyl 4-hydroxyproline | neg | 292.1039 | 0.777 | 5.584 | 5.614 | 0.029 | -0.065 | 0.722 | 0.904 | Unannotated | NA | NA |
| metab_13258 | Unannotated feature | neg | 229.0594 | 0.596 | 4.592 | 4.570 | -0.022 | -0.065 | 0.722 | 0.904 | Unannotated | NA | NA |
| metab_14671 | (1xi,3xi)-1,2,3,4-Tetrahydro-1-methyl-beta-carboline-3-carboxylic acid | neg | 229.0981 | 3.631 | 5.587 | 5.613 | 0.026 | -0.065 | 0.722 | 0.904 | Unannotated | NA | NA |
| metab_10620 | Unannotated feature | neg | 201.0400 | 1.685 | 4.528 | 4.565 | 0.037 | 0.065 | 0.722 | 0.904 | Unannotated | NA | NA |
| metab_10621 | Unannotated feature | neg | 296.1141 | 4.710 | 4.764 | 4.625 | -0.139 | -0.065 | 0.722 | 0.904 | Unannotated | NA | NA |
| metab_19366 | Unannotated feature | neg | 255.0511 | 3.439 | 4.465 | 4.518 | 0.052 | 0.065 | 0.722 | 0.904 | Unannotated | NA | NA |
| metab_6562 | Unannotated feature | pos | 213.0752 | 0.601 | 3.986 | 4.235 | 0.249 | -0.065 | 0.722 | 0.904 | Unannotated | NA | NA |
| metab_15708 | Unannotated feature | neg | 382.8425 | 0.596 | 4.942 | 5.088 | 0.145 | 0.065 | 0.722 | 0.904 | Unannotated | NA | NA |
| metab_4849 | Unannotated feature | pos | 142.9914 | 7.732 | 4.954 | 4.980 | 0.026 | 0.065 | 0.722 | 0.904 | Unannotated | NA | NA |
| metab_8664 | Unannotated feature | pos | 84.9602 | 0.961 | 4.590 | 4.505 | -0.085 | -0.065 | 0.722 | 0.904 | Unannotated | NA | NA |
| metab_9792 | 4-Hydroxy-1-(3-pyridinyl)-1-butanone | neg | 210.0768 | 3.045 | 5.112 | 5.081 | -0.032 | -0.065 | 0.722 | 0.904 | Tentative | NA | NA |
| metab_5730 | Unannotated feature | pos | 420.2293 | 6.080 | 5.025 | 4.890 | -0.134 | -0.065 | 0.722 | 0.904 | Unannotated | NA | NA |
| metab_16008 | Unannotated feature | neg | 544.3400 | 6.123 | 4.260 | 4.002 | -0.258 | -0.065 | 0.722 | 0.904 | Unannotated | NA | NA |
| metab_12553 | Unannotated feature | neg | 495.3334 | 6.373 | 6.943 | 6.906 | -0.037 | -0.065 | 0.722 | 0.904 | Unannotated | NA | NA |
| metab_12552 | Unannotated feature | neg | 307.1662 | 6.170 | 3.258 | 4.177 | 0.919 | 0.065 | 0.722 | 0.904 | Unannotated | NA | NA |
| metab_1106 | Unannotated feature | pos | 525.3389 | 6.654 | 5.835 | 5.848 | 0.013 | 0.065 | 0.722 | 0.904 | Unannotated | NA | NA |
| metab_4916 | Unannotated feature | pos | 493.3667 | 7.650 | 4.912 | 4.919 | 0.008 | 0.065 | 0.722 | 0.904 | Unannotated | NA | NA |
| metab_4841 | Unannotated feature | pos | 190.9796 | 7.740 | 4.754 | 4.771 | 0.017 | 0.065 | 0.722 | 0.904 | Unannotated | NA | NA |
| metab_15201 | Dibutyl malate | neg | 291.1451 | 4.730 | 3.991 | 4.021 | 0.030 | 0.065 | 0.722 | 0.904 | Unannotated | NA | NA |
| metab_8851 | Unannotated feature | neg | 263.1039 | 3.681 | 5.325 | 5.596 | 0.272 | 0.065 | 0.722 | 0.904 | Unannotated | NA | NA |
| metab_7483 | Pheneturide | pos | 239.1394 | 3.791 | 4.927 | 4.974 | 0.047 | 0.065 | 0.722 | 0.904 | Tentative | NA | NA |
| metab_7509 | Unannotated feature | pos | 329.1504 | 3.729 | 3.963 | 3.895 | -0.068 | 0.065 | 0.722 | 0.904 | Unannotated | NA | NA |
| metab_11767 | Unannotated feature | neg | 319.1573 | 5.730 | 5.450 | 5.706 | 0.256 | 0.065 | 0.722 | 0.904 | Unannotated | NA | NA |
| metab_10912 | Unannotated feature | neg | 431.0991 | 4.869 | 3.658 | 3.949 | 0.290 | 0.065 | 0.722 | 0.904 | Unannotated | NA | NA |
| metab_16253 | (9S,10E,12Z)-9-hydroperoxy-10,12-octadecadienoate | neg | 621.4381 | 6.522 | 5.593 | 5.341 | -0.252 | -0.065 | 0.722 | 0.904 | Tentative | NA | NA |
| metab_1681 | Unannotated feature | pos | 274.1079 | 4.254 | 4.185 | 4.365 | 0.180 | 0.065 | 0.722 | 0.904 | Unannotated | NA | NA |
| metab_3202 | Tryptophol | pos | 144.0810 | 3.729 | 5.282 | 5.264 | -0.019 | -0.065 | 0.722 | 0.904 | Moderate putative | NA | NA |
| metab_17156 | Unannotated feature | neg | 391.8976 | 7.769 | 4.635 | 4.583 | -0.052 | -0.065 | 0.722 | 0.904 | Unannotated | NA | NA |
| metab_17161 | Unannotated feature | neg | 181.0712 | 7.765 | 5.031 | 4.975 | -0.056 | -0.065 | 0.722 | 0.904 | Unannotated | NA | NA |
| metab_13766 | Unannotated feature | neg | 162.0223 | 1.965 | 2.844 | 2.650 | -0.193 | -0.065 | 0.722 | 0.904 | Unannotated | NA | NA |
| metab_15015 | Unannotated feature | neg | 227.1287 | 4.363 | 6.164 | 6.184 | 0.020 | 0.065 | 0.722 | 0.904 | Unannotated | NA | NA |
| metab_4815 | Alatanin 2 | pos | 832.2422 | 7.916 | 6.206 | 6.196 | -0.010 | 0.065 | 0.722 | 0.904 | Unannotated | NA | NA |
| metab_9747 | Deoxyloganin | neg | 395.1353 | 3.082 | 4.620 | 4.558 | -0.063 | -0.065 | 0.722 | 0.904 | Tentative | NA | NA |
| metab_11708 | Unannotated feature | neg | 434.9787 | 4.028 | 5.825 | 6.028 | 0.203 | 0.065 | 0.722 | 0.904 | Unannotated | NA | NA |
| metab_5311 | Unannotated feature | pos | 407.2415 | 6.558 | 5.950 | 5.922 | -0.027 | -0.065 | 0.722 | 0.904 | Unannotated | NA | NA |
| metab_16305 | Unannotated feature | neg | 400.1802 | 6.632 | 5.179 | 5.213 | 0.034 | 0.065 | 0.722 | 0.904 | Unannotated | NA | NA |
| metab_18072 | Unannotated feature | neg | 224.0925 | 6.420 | 5.169 | 5.122 | -0.046 | -0.065 | 0.722 | 0.904 | Unannotated | NA | NA |
| metab_10661 | Glycine-betaxanthin | neg | 303.0409 | 1.594 | 3.101 | 2.990 | -0.111 | -0.065 | 0.722 | 0.904 | Tentative | NA | NA |
| metab_10642 | Unannotated feature | neg | 355.1363 | 1.665 | 3.924 | 3.824 | -0.100 | -0.065 | 0.722 | 0.904 | Unannotated | NA | NA |
| metab_5164 | Enkephalinamide-leu | pos | 555.2953 | 6.845 | 4.260 | 4.078 | -0.181 | -0.065 | 0.722 | 0.904 | Unannotated | NA | NA |
| metab_13245 | Unannotated feature | neg | 513.1470 | 0.777 | 4.577 | 4.645 | 0.068 | 0.065 | 0.722 | 0.904 | Unannotated | NA | NA |
| metab_10184 | 1-METHYLXANTHINE | neg | 165.0412 | 2.667 | 5.818 | 5.794 | -0.025 | -0.065 | 0.722 | 0.904 | Moderate putative | Organoheterocyclic compounds | Imidazopyrimidines |
| metab_10129 | Unannotated feature | neg | 230.0127 | 2.758 | 4.203 | 4.268 | 0.065 | 0.065 | 0.722 | 0.904 | Unannotated | NA | NA |
| metab_1765 | Unannotated feature | pos | 313.1494 | 3.421 | 4.468 | 4.454 | -0.014 | -0.065 | 0.722 | 0.904 | Unannotated | NA | NA |
| metab_10763 | Unannotated feature | neg | 366.0107 | 1.186 | 4.394 | 4.230 | -0.165 | -0.065 | 0.722 | 0.904 | Unannotated | NA | NA |
| metab_12476 | Unannotated feature | neg | 328.8947 | 7.997 | 4.968 | 4.963 | -0.005 | -0.065 | 0.722 | 0.904 | Unannotated | NA | NA |
| metab_957 | Unannotated feature | pos | 174.9383 | 7.658 | 4.700 | 4.703 | 0.004 | 0.065 | 0.722 | 0.904 | Unannotated | NA | NA |
| metab_11473 | Unannotated feature | neg | 145.9491 | 0.524 | 5.207 | 5.230 | 0.023 | -0.065 | 0.722 | 0.904 | Unannotated | NA | NA |
| metab_9613 | Unannotated feature | neg | 325.9728 | 3.215 | 4.200 | 4.085 | -0.116 | -0.065 | 0.722 | 0.904 | Unannotated | NA | NA |
| metab_6463 | Unannotated feature | pos | 306.9864 | 0.593 | 4.111 | 4.153 | 0.042 | -0.065 | 0.722 | 0.904 | Unannotated | NA | NA |
| metab_12367 | Unannotated feature | neg | 600.5223 | 7.607 | 5.206 | 5.327 | 0.121 | 0.065 | 0.722 | 0.904 | Unannotated | NA | NA |
| metab_8789 | 4-aminobenzoate | pos | 273.0845 | 1.161 | 5.216 | 4.945 | -0.271 | -0.065 | 0.722 | 0.904 | Tentative | NA | NA |
| metab_3438 | Phenethylacetate | pos | 165.0913 | 4.575 | 4.118 | 3.745 | -0.373 | -0.065 | 0.722 | 0.904 | Moderate putative | NA | NA |
| metab_12331 | Unannotated feature | neg | 758.5438 | 7.387 | 6.034 | 6.096 | 0.062 | -0.065 | 0.722 | 0.904 | Unannotated | NA | NA |
| metab_10752 | Betonicine | neg | 204.0872 | 1.222 | 5.406 | 5.444 | 0.037 | -0.065 | 0.722 | 0.904 | Tentative | NA | NA |
| metab_10246 | Unannotated feature | neg | 244.0827 | 2.569 | 4.247 | 4.230 | -0.016 | 0.065 | 0.722 | 0.904 | Unannotated | NA | NA |
| metab_10249 | Unannotated feature | neg | 274.0936 | 2.561 | 5.573 | 5.735 | 0.162 | 0.065 | 0.722 | 0.904 | Unannotated | NA | NA |
| metab_2460 | Creatine | pos | 132.0771 | 0.672 | 4.650 | 4.454 | -0.196 | 0.065 | 0.722 | 0.904 | Strong putative | NA | NA |
| metab_17124 | Unannotated feature | neg | 126.8803 | 7.827 | 4.853 | 4.853 | 0.000 | 0.065 | 0.722 | 0.904 | Unannotated | NA | NA |
| metab_4754 | Unannotated feature | pos | 213.0051 | 7.716 | 4.373 | 4.368 | -0.005 | -0.065 | 0.722 | 0.904 | Unannotated | NA | NA |
| metab_899 | Unannotated feature | pos | 413.2126 | 7.052 | 6.934 | 6.929 | -0.006 | -0.065 | 0.722 | 0.904 | Unannotated | NA | NA |
| metab_6090 | Unannotated feature | pos | 127.1234 | 0.516 | 3.598 | 3.556 | -0.042 | 0.065 | 0.722 | 0.904 | Unannotated | NA | NA |
| metab_936 | Unannotated feature | pos | 552.3109 | 7.498 | 6.091 | 5.956 | -0.135 | -0.065 | 0.722 | 0.904 | Unannotated | NA | NA |
| metab_11664 | Unannotated feature | neg | 411.3057 | 6.397 | 6.809 | 6.770 | -0.039 | 0.065 | 0.722 | 0.904 | Unannotated | NA | NA |
| metab_15075 | Unannotated feature | neg | 127.0758 | 4.488 | 4.041 | 4.089 | 0.048 | 0.065 | 0.722 | 0.904 | Unannotated | NA | NA |
| metab_15867 | Unannotated feature | neg | 224.0614 | 5.952 | 6.348 | 6.413 | 0.065 | 0.065 | 0.722 | 0.904 | Unannotated | NA | NA |
| metab_12396 | Unannotated feature | neg | 241.1807 | 6.561 | 4.277 | 3.951 | -0.326 | 0.065 | 0.722 | 0.904 | Unannotated | NA | NA |
| metab_11632 | Unannotated feature | neg | 408.1398 | 5.295 | 3.858 | 4.113 | 0.255 | -0.065 | 0.722 | 0.904 | Unannotated | NA | NA |
| metab_892 | N,N-Dimethylsphingosine | pos | 350.3036 | 6.973 | 5.303 | 5.303 | 0.000 | -0.065 | 0.722 | 0.904 | Tentative | NA | NA |
| metab_13768 | Xanthosine | neg | 283.0687 | 1.965 | 5.740 | 5.483 | -0.258 | -0.065 | 0.722 | 0.904 | Strong putative | NA | NA |
| metab_8 | Thymidine | pos | 260.1245 | 0.720 | 4.805 | 4.769 | -0.035 | -0.065 | 0.722 | 0.904 | Tentative | NA | NA |
| metab_7089 | Unannotated feature | pos | 438.2387 | 4.673 | 4.550 | 4.398 | -0.152 | -0.065 | 0.722 | 0.904 | Unannotated | NA | NA |
| metab_11941 | Unannotated feature | neg | 473.1951 | 5.398 | 5.689 | 5.634 | -0.055 | 0.065 | 0.722 | 0.904 | Unannotated | NA | NA |
| metab_4590 | DG(15:0/22:4(7Z,10Z,13Z,16Z)/0:0) | pos | 653.5125 | 6.981 | 4.116 | 4.264 | 0.149 | -0.065 | 0.722 | 0.904 | Tentative | NA | NA |
| metab_9012 | 3,4,5,6-Tetrahydrophthalimide | neg | 347.1246 | 4.052 | 5.735 | 5.722 | -0.014 | 0.065 | 0.722 | 0.904 | Unannotated | NA | NA |
| metab_11909 | Unannotated feature | neg | 483.2073 | 6.068 | 5.951 | 5.897 | -0.055 | -0.065 | 0.722 | 0.904 | Unannotated | NA | NA |
| metab_11911 | 5-Heptyltetrahydro-2-oxo-3-furancarboxylic acid | neg | 227.1285 | 6.076 | 5.622 | 5.495 | -0.128 | -0.065 | 0.722 | 0.904 | Unannotated | NA | NA |
| metab_2137 | Unannotated feature | pos | 333.1087 | 2.853 | 3.858 | 3.766 | -0.093 | -0.065 | 0.722 | 0.904 | Unannotated | NA | NA |
| metab_17818 | Unannotated feature | neg | 356.2054 | 6.821 | 6.027 | 5.989 | -0.038 | -0.065 | 0.722 | 0.904 | Unannotated | NA | NA |
| metab_12068 | Unannotated feature | neg | 353.1794 | 6.404 | 6.276 | 6.258 | -0.018 | -0.065 | 0.722 | 0.904 | Unannotated | NA | NA |
| metab_247 | Unannotated feature | pos | 853.1716 | 0.531 | 5.247 | 5.164 | -0.083 | -0.065 | 0.722 | 0.904 | Unannotated | NA | NA |
| metab_19655 | Unannotated feature | neg | 183.0293 | 3.596 | 4.851 | 5.047 | 0.196 | 0.065 | 0.722 | 0.904 | Unannotated | NA | NA |
| metab_4485 | PG(i-12:0/i-12:0) | pos | 643.4197 | 6.534 | 5.238 | 4.891 | -0.347 | -0.065 | 0.722 | 0.904 | Tentative | NA | NA |
| metab_16921 | Unannotated feature | neg | 208.0822 | 7.785 | 4.780 | 4.783 | 0.003 | -0.065 | 0.722 | 0.904 | Unannotated | NA | NA |
| metab_14097 | Unannotated feature | neg | 126.0554 | 2.822 | 3.372 | 3.406 | 0.034 | -0.065 | 0.722 | 0.904 | Unannotated | NA | NA |
| metab_14100 | 3-[[(2S)-2,4-Dihydroxy-3,3-dimethylbutanoyl]amino]propanoic acid | neg | 254.0793 | 2.822 | 5.300 | 5.270 | -0.030 | -0.065 | 0.722 | 0.904 | Unannotated | NA | NA |
| metab_11964 | Unannotated feature | neg | 611.3465 | 6.170 | 5.624 | 5.908 | 0.284 | 0.065 | 0.722 | 0.904 | Unannotated | NA | NA |
| metab_116 | Unannotated feature | pos | 242.0541 | 0.633 | 3.845 | 3.669 | -0.176 | -0.065 | 0.722 | 0.904 | Unannotated | NA | NA |
| metab_12052 | Unannotated feature | neg | 593.3357 | 5.710 | 5.925 | 7.222 | 1.298 | 0.065 | 0.722 | 0.904 | Unannotated | NA | NA |
| metab_140 | Unannotated feature | pos | 105.1108 | 0.625 | 5.012 | 4.968 | -0.045 | 0.065 | 0.722 | 0.904 | Unannotated | NA | NA |
| metab_4509 | 6S,9R-Dihydroxy-4,7E-megastigmadien-3-one 9-[apiosyl-(1->6)-glucoside] | pos | 551.2669 | 6.638 | 6.040 | 6.038 | -0.002 | -0.065 | 0.722 | 0.904 | Unannotated | NA | NA |
| metab_19735 | Unannotated feature | neg | 309.1348 | 4.518 | 3.664 | 3.737 | 0.074 | -0.061 | 0.740 | 0.910 | Unannotated | NA | NA |
| metab_108 | Unannotated feature | pos | 160.0801 | 0.641 | 4.361 | 4.143 | -0.219 | -0.061 | 0.740 | 0.910 | Unannotated | NA | NA |
| metab_9085 | Acetamide, 2-(4-(2-((2-hydroxy-3-phenoxypropyl)amino)ethoxy)phenoxy)-N-(2-methoxyethyl)-, (S)- | neg | 399.1892 | 3.945 | 4.444 | 4.102 | -0.343 | -0.061 | 0.740 | 0.910 | Unannotated | NA | NA |
| metab_226 | Unannotated feature | pos | 216.0357 | 0.561 | 4.670 | 4.652 | -0.017 | -0.061 | 0.740 | 0.910 | Unannotated | NA | NA |
| metab_17805 | 4,4-Dimethyl-2-[3-carboxylatopropyl]-2-tridecyloxazolidine 3-oxide | neg | 366.3013 | 6.845 | 4.970 | 4.937 | -0.033 | 0.061 | 0.740 | 0.910 | Unannotated | NA | NA |
| metab_2146 | Unannotated feature | pos | 474.1747 | 2.875 | 3.910 | 3.597 | -0.313 | -0.061 | 0.740 | 0.910 | Unannotated | NA | NA |
| metab_14593 | 4-Hydroxy-5-(dihydroxyphenyl)-valeric acid-O-sulphate | neg | 287.0234 | 3.502 | 3.863 | 4.140 | 0.276 | 0.061 | 0.740 | 0.910 | Unannotated | NA | NA |
| metab_11908 | Unannotated feature | neg | 337.1313 | 6.068 | 4.002 | 4.221 | 0.219 | 0.061 | 0.740 | 0.910 | Unannotated | NA | NA |
| metab_14082 | Unannotated feature | neg | 260.0413 | 2.806 | 4.236 | 4.283 | 0.047 | 0.061 | 0.740 | 0.910 | Unannotated | NA | NA |
| metab_9108 | Unannotated feature | neg | 225.0765 | 3.921 | 5.227 | 5.241 | 0.014 | -0.061 | 0.740 | 0.910 | Unannotated | NA | NA |
| metab_9045 | Unannotated feature | neg | 258.0384 | 4.008 | 4.385 | 4.434 | 0.050 | 0.061 | 0.740 | 0.910 | Unannotated | NA | NA |
| metab_8726 | 8-Oxo-7,8-dihydrodeoxyguanine | pos | 152.0567 | 0.768 | 5.127 | 5.084 | -0.044 | 0.061 | 0.740 | 0.910 | Unannotated | NA | NA |
| metab_19034 | Unannotated feature | neg | 347.0779 | 3.281 | 4.025 | 3.922 | -0.104 | -0.061 | 0.740 | 0.910 | Unannotated | NA | NA |
| metab_12383 | Unannotated feature | neg | 561.4170 | 6.640 | 5.744 | 5.579 | -0.165 | -0.061 | 0.740 | 0.910 | Unannotated | NA | NA |
| metab_12410 | Unannotated feature | neg | 514.8942 | 7.707 | 5.669 | 5.693 | 0.024 | 0.061 | 0.740 | 0.910 | Unannotated | NA | NA |
| metab_850 | 3-Sulfopyruvic acid | pos | 231.9889 | 0.601 | 4.020 | 4.316 | 0.296 | -0.061 | 0.740 | 0.910 | Tentative | NA | NA |
| metab_7540 | Unannotated feature | pos | 150.0773 | 0.728 | 4.059 | 4.074 | 0.014 | 0.061 | 0.740 | 0.910 | Unannotated | NA | NA |
| metab_7539 | Brisoral | pos | 428.0706 | 3.674 | 3.763 | 3.775 | 0.011 | -0.061 | 0.740 | 0.910 | Tentative | NA | NA |
| metab_7531 | Ethinamate | pos | 185.1288 | 3.701 | 3.653 | 4.576 | 0.923 | 0.061 | 0.740 | 0.910 | Tentative | NA | NA |
| metab_15187 | Unannotated feature | neg | 381.0921 | 4.707 | 4.139 | 4.133 | -0.006 | -0.061 | 0.740 | 0.910 | Unannotated | NA | NA |
| metab_12429 | Unannotated feature | neg | 246.8864 | 7.730 | 4.591 | 4.593 | 0.002 | 0.061 | 0.740 | 0.910 | Unannotated | NA | NA |
| metab_15896 | Terbogrel | neg | 450.2156 | 5.991 | 4.779 | 4.575 | -0.204 | -0.061 | 0.740 | 0.910 | Unannotated | NA | NA |
| metab_19000 | 2,3-Dimethoxy-5-methyl-6-(9'-carboxynonyl)-1,4-benzoquinone | neg | 397.1870 | 5.526 | 4.297 | 4.359 | 0.062 | 0.061 | 0.740 | 0.910 | Unannotated | NA | NA |
| metab_13815 | Inosine pyruvate | neg | 319.0666 | 2.058 | 5.536 | 5.540 | 0.005 | 0.061 | 0.740 | 0.910 | Unannotated | NA | NA |
| metab_3368 | Oxindole | pos | 134.0603 | 4.285 | 5.004 | 4.642 | -0.362 | -0.061 | 0.740 | 0.910 | Strong putative | NA | NA |
| metab_12892 | Unannotated feature | neg | 626.5369 | 7.154 | 5.698 | 5.482 | -0.216 | -0.061 | 0.740 | 0.910 | Unannotated | NA | NA |
| metab_10750 | Chlorogenic acid | neg | 375.0662 | 1.222 | 5.289 | 5.263 | -0.025 | 0.061 | 0.740 | 0.910 | Tentative | NA | NA |
| metab_19108 | 1,11-Undecanedicarboxylic acid | neg | 289.1658 | 5.378 | 5.587 | 5.406 | -0.181 | -0.061 | 0.740 | 0.910 | Unannotated | NA | NA |
| metab_12341 | Unannotated feature | neg | 337.2057 | 6.860 | 6.055 | 6.021 | -0.034 | -0.061 | 0.740 | 0.910 | Unannotated | NA | NA |
| metab_3481 | Unannotated feature | pos | 199.0758 | 7.724 | 5.798 | 5.817 | 0.019 | 0.061 | 0.740 | 0.910 | Unannotated | NA | NA |
| metab_13837 | Unannotated feature | neg | 193.8151 | 2.174 | 3.995 | 3.561 | -0.434 | 0.061 | 0.740 | 0.910 | Unannotated | NA | NA |
| metab_8790 | 7-Methylguanine | pos | 166.0726 | 1.217 | 6.119 | 6.076 | -0.043 | 0.061 | 0.740 | 0.910 | Strong putative | NA | NA |
| metab_16774 | Unannotated feature | neg | 142.9283 | 7.697 | 5.306 | 5.312 | 0.007 | -0.061 | 0.740 | 0.910 | Unannotated | NA | NA |
| metab_12357 | Unannotated feature | neg | 337.1847 | 6.797 | 5.182 | 5.217 | 0.034 | 0.061 | 0.740 | 0.910 | Unannotated | NA | NA |
| metab_11695 | Unannotated feature | neg | 417.1596 | 5.435 | 4.956 | 5.303 | 0.347 | 0.061 | 0.740 | 0.910 | Unannotated | NA | NA |
| metab_11692 | Unannotated feature | neg | 496.2099 | 5.435 | 4.000 | 3.625 | -0.375 | -0.061 | 0.740 | 0.910 | Unannotated | NA | NA |
| metab_15766 | (-)-Linalyl acetate | neg | 241.1444 | 5.797 | 4.028 | 3.995 | -0.032 | -0.061 | 0.740 | 0.910 | Tentative | NA | NA |
| metab_15826 | Unannotated feature | neg | 456.7748 | 0.603 | 4.394 | 4.623 | 0.229 | 0.061 | 0.740 | 0.910 | Unannotated | NA | NA |
| metab_7687 | Indole-3-Carboxylic Acid | pos | 162.0552 | 3.400 | 4.538 | 4.802 | 0.264 | 0.061 | 0.740 | 0.910 | Moderate putative | NA | NA |
| metab_18872 | Unannotated feature | neg | 241.0504 | 5.692 | 3.522 | 3.271 | -0.250 | -0.061 | 0.740 | 0.910 | Unannotated | NA | NA |
| metab_18840 | Unannotated feature | neg | 181.0500 | 3.210 | 6.230 | 6.077 | -0.153 | 0.061 | 0.740 | 0.910 | Unannotated | NA | NA |
| metab_11471 | Unannotated feature | neg | 294.9535 | 0.524 | 5.120 | 5.225 | 0.105 | 0.061 | 0.740 | 0.910 | Unannotated | NA | NA |
| metab_18151 | Unannotated feature | neg | 311.0888 | 2.758 | 6.227 | 6.303 | 0.076 | -0.061 | 0.740 | 0.910 | Unannotated | NA | NA |
| metab_1809 | Unannotated feature | pos | 75.0810 | 0.807 | 4.811 | 4.819 | 0.007 | 0.061 | 0.740 | 0.910 | Unannotated | NA | NA |
| metab_10807 | Unannotated feature | neg | 348.1666 | 4.814 | 3.937 | 4.634 | 0.697 | 0.061 | 0.740 | 0.910 | Unannotated | NA | NA |
| metab_8156 | Homomethionine | pos | 128.0540 | 2.699 | 4.425 | 4.511 | 0.086 | 0.061 | 0.740 | 0.910 | Tentative | Organic acids and derivatives | Carboxylic acids and derivatives |
| metab_15155 | Unannotated feature | neg | 393.0658 | 4.639 | 5.218 | 4.657 | -0.561 | -0.061 | 0.740 | 0.910 | Unannotated | NA | NA |
| metab_19180 | 1-Phenyl-1,2-propanedione | neg | 193.0501 | 5.290 | 5.376 | 5.555 | 0.179 | 0.061 | 0.740 | 0.910 | Tentative | NA | NA |
| metab_12314 | Unannotated feature | neg | 473.2763 | 6.130 | 5.528 | 5.447 | -0.081 | -0.061 | 0.740 | 0.910 | Unannotated | NA | NA |
| metab_16461 | PG(16:0/0:0)[U] | neg | 483.2738 | 7.004 | 7.020 | 6.936 | -0.084 | -0.061 | 0.740 | 0.910 | Moderate putative | NA | NA |
| metab_10698 | Unannotated feature | neg | 261.1610 | 4.759 | 5.434 | 5.562 | 0.128 | -0.061 | 0.740 | 0.910 | Unannotated | NA | NA |
| metab_5335 | Unannotated feature | pos | 188.0920 | 1.952 | 5.268 | 5.224 | -0.044 | 0.061 | 0.740 | 0.910 | Unannotated | NA | NA |
| metab_10721 | Unannotated feature | neg | 251.0522 | 1.329 | 4.957 | 5.061 | 0.105 | -0.061 | 0.740 | 0.910 | Unannotated | NA | NA |
| metab_10271 | Unannotated feature | neg | 199.0218 | 2.517 | 4.012 | 3.885 | -0.127 | -0.061 | 0.740 | 0.910 | Unannotated | NA | NA |
| metab_18271 | Unannotated feature | neg | 535.3289 | 6.232 | 6.077 | 6.031 | -0.046 | -0.061 | 0.740 | 0.910 | Unannotated | NA | NA |
| metab_13432 | Unannotated feature | neg | 264.9887 | 0.804 | 3.798 | 4.100 | 0.302 | 0.061 | 0.740 | 0.910 | Unannotated | NA | NA |
| metab_10080 | Unannotated feature | neg | 150.0554 | 2.776 | 4.590 | 4.488 | -0.102 | -0.061 | 0.740 | 0.910 | Unannotated | NA | NA |
| metab_3187 | Unannotated feature | pos | 399.1287 | 3.689 | 4.056 | 4.105 | 0.049 | 0.061 | 0.740 | 0.910 | Unannotated | NA | NA |
| metab_9625 | Unannotated feature | neg | 281.9943 | 3.210 | 4.285 | 4.299 | 0.014 | -0.061 | 0.740 | 0.910 | Unannotated | NA | NA |
| metab_17194 | Unannotated feature | neg | 403.8527 | 7.737 | 4.517 | 4.504 | -0.013 | 0.061 | 0.740 | 0.910 | Unannotated | NA | NA |
| metab_16739 | Unannotated feature | neg | 126.9965 | 1.958 | 4.513 | 4.477 | -0.036 | -0.061 | 0.740 | 0.910 | Unannotated | NA | NA |
| metab_12501 | Unannotated feature | neg | 265.1479 | 6.365 | 6.170 | 6.224 | 0.054 | 0.061 | 0.740 | 0.910 | Unannotated | NA | NA |
| metab_1021 | Unannotated feature | pos | 521.3824 | 7.052 | 6.133 | 6.188 | 0.055 | 0.061 | 0.740 | 0.910 | Unannotated | NA | NA |
| metab_18824 | Unannotated feature | neg | 267.0738 | 2.435 | 6.559 | 6.773 | 0.213 | 0.061 | 0.740 | 0.910 | Unannotated | NA | NA |
| metab_9837 | Unannotated feature | neg | 216.0146 | 3.011 | 4.668 | 4.659 | -0.009 | 0.061 | 0.740 | 0.910 | Unannotated | NA | NA |
| metab_1067 | MG(0:0/20:3(6,8,11)-OH(5)/0:0) | pos | 429.3197 | 6.734 | 6.035 | 6.061 | 0.026 | 0.061 | 0.740 | 0.910 | Unannotated | NA | NA |
| metab_11415 | Unannotated feature | neg | 372.8128 | 0.542 | 4.366 | 4.300 | -0.066 | -0.061 | 0.740 | 0.910 | Unannotated | NA | NA |
| metab_15990 | Benzarone | neg | 311.0929 | 6.099 | 4.280 | 3.910 | -0.371 | -0.061 | 0.740 | 0.910 | Tentative | NA | NA |
| metab_13344 | Unannotated feature | neg | 192.0508 | 0.714 | 3.917 | 4.193 | 0.276 | -0.061 | 0.740 | 0.910 | Unannotated | NA | NA |
| metab_5121 | Unannotated feature | pos | 481.2842 | 6.965 | 5.257 | 5.300 | 0.043 | 0.061 | 0.740 | 0.910 | Unannotated | NA | NA |
| metab_16511 | Unannotated feature | neg | 262.0561 | 1.685 | 3.215 | 3.317 | 0.102 | -0.061 | 0.740 | 0.910 | Unannotated | NA | NA |
| metab_12865 | Unannotated feature | neg | 335.1465 | 0.706 | 4.673 | 4.610 | -0.063 | 0.061 | 0.740 | 0.910 | Unannotated | NA | NA |
| metab_1684 | N-Lauroylglycine | pos | 258.2069 | 4.226 | 4.642 | 4.758 | 0.116 | 0.061 | 0.740 | 0.910 | Unannotated | NA | NA |
| metab_16257 | DG(i-12:0/0:0/18:3(10,12,15)-OH(9)) | neg | 595.4226 | 6.530 | 4.982 | 5.148 | 0.166 | 0.061 | 0.740 | 0.910 | Unannotated | NA | NA |
| metab_14769 | Unannotated feature | neg | 538.8901 | 0.512 | 5.261 | 5.289 | 0.028 | 0.061 | 0.740 | 0.910 | Unannotated | NA | NA |
| metab_12809 | Unannotated feature | neg | 124.9907 | 0.651 | 4.817 | 4.943 | 0.125 | -0.061 | 0.740 | 0.910 | Unannotated | NA | NA |
| metab_11706 | Unannotated feature | neg | 415.2024 | 5.496 | 5.504 | 5.705 | 0.201 | 0.061 | 0.740 | 0.910 | Unannotated | NA | NA |
| metab_799 | Unannotated feature | pos | 303.1707 | 6.271 | 6.260 | 6.308 | 0.048 | 0.061 | 0.740 | 0.910 | Unannotated | NA | NA |
| metab_777 | Unannotated feature | pos | 597.3664 | 6.159 | 4.512 | 6.207 | 1.695 | 0.061 | 0.740 | 0.910 | Unannotated | NA | NA |
| metab_12293 | Unannotated feature | neg | 467.3006 | 7.177 | 4.978 | 4.598 | -0.379 | -0.061 | 0.740 | 0.910 | Unannotated | NA | NA |
| metab_12292 | 3beta-hydroxy-4beta-methyl-5alpha-cholest-7-ene-4alpha-carbaldehyde | neg | 473.3640 | 6.899 | 5.501 | 5.756 | 0.255 | 0.061 | 0.740 | 0.910 | Tentative | NA | NA |
| metab_9482 | Unannotated feature | neg | 339.0844 | 3.371 | 3.040 | 3.716 | 0.676 | 0.061 | 0.740 | 0.910 | Unannotated | NA | NA |
| metab_3718 | Unannotated feature | pos | 339.1921 | 5.355 | 3.719 | 5.215 | 1.496 | 0.061 | 0.740 | 0.910 | Unannotated | NA | NA |
| metab_3641 | L-Metanephrine | pos | 230.1391 | 5.165 | 3.683 | 3.693 | 0.010 | -0.061 | 0.740 | 0.910 | Tentative | NA | NA |
| metab_11782 | Unannotated feature | neg | 333.1352 | 4.574 | 4.371 | 4.569 | 0.198 | 0.061 | 0.740 | 0.910 | Unannotated | NA | NA |
| metab_11781 | Unannotated feature | neg | 382.1623 | 5.778 | 4.584 | 5.176 | 0.592 | 0.061 | 0.740 | 0.910 | Unannotated | NA | NA |
| metab_15713 | Unannotated feature | neg | 345.1558 | 5.702 | 4.071 | 4.037 | -0.034 | 0.061 | 0.740 | 0.910 | Unannotated | NA | NA |
| metab_2936 | N-Nitrosobis(2-oxopropyl)amine | pos | 141.0660 | 2.928 | 3.887 | 3.883 | -0.005 | 0.061 | 0.740 | 0.910 | Unannotated | NA | NA |
| metab_14944 | Unannotated feature | neg | 241.1192 | 4.211 | 4.931 | 4.984 | 0.054 | 0.061 | 0.740 | 0.910 | Unannotated | NA | NA |
| metab_16012 | Unannotated feature | neg | 331.2128 | 6.130 | 4.144 | 4.405 | 0.261 | 0.061 | 0.740 | 0.910 | Unannotated | NA | NA |
| metab_3040 | Unannotated feature | pos | 160.0759 | 3.275 | 3.206 | 3.331 | 0.125 | 0.061 | 0.740 | 0.910 | Unannotated | NA | NA |
| metab_3026 | Unannotated feature | pos | 387.1810 | 6.287 | 6.838 | 6.843 | 0.005 | -0.061 | 0.740 | 0.910 | Unannotated | NA | NA |
| metab_3021 | Valganciclovir, (S)- | pos | 319.1519 | 3.193 | 4.982 | 4.854 | -0.129 | -0.061 | 0.740 | 0.910 | Unannotated | NA | NA |
| metab_6596 | Unannotated feature | pos | 296.0666 | 0.609 | 2.862 | 3.289 | 0.427 | 0.061 | 0.740 | 0.910 | Unannotated | NA | NA |
| metab_1959 | Unannotated feature | pos | 456.2092 | 1.960 | 4.217 | 4.428 | 0.211 | 0.061 | 0.740 | 0.910 | Unannotated | NA | NA |
| metab_1958 | Unannotated feature | pos | 414.1630 | 1.960 | 4.430 | 4.454 | 0.025 | 0.061 | 0.740 | 0.910 | Unannotated | NA | NA |
| metab_17966 | Unannotated feature | neg | 350.0735 | 2.762 | 4.878 | 4.837 | -0.042 | -0.061 | 0.740 | 0.910 | Unannotated | NA | NA |
| metab_14678 | Unannotated feature | neg | 388.1189 | 3.647 | 3.437 | 3.511 | 0.074 | -0.061 | 0.740 | 0.910 | Unannotated | NA | NA |
| metab_18007 | TOFA | neg | 323.2228 | 6.514 | 4.970 | 5.197 | 0.227 | -0.061 | 0.740 | 0.910 | Moderate putative | NA | NA |
| metab_10283 | Unannotated feature | neg | 199.0583 | 4.559 | 5.071 | 4.912 | -0.159 | -0.061 | 0.740 | 0.910 | Unannotated | NA | NA |
| metab_2403 | Unannotated feature | pos | 106.9511 | 0.593 | 4.351 | 4.380 | 0.028 | -0.061 | 0.740 | 0.910 | Unannotated | NA | NA |
| metab_11295 | Beta-D-Glucosamine | neg | 224.0774 | 0.614 | 4.030 | 4.001 | -0.029 | 0.061 | 0.740 | 0.910 | Tentative | NA | NA |
| metab_9870 | Unannotated feature | neg | 198.0401 | 2.975 | 4.713 | 4.700 | -0.013 | -0.061 | 0.740 | 0.910 | Unannotated | NA | NA |
| metab_17294 | Unannotated feature | neg | 492.8996 | 7.710 | 4.993 | 5.026 | 0.033 | 0.061 | 0.740 | 0.910 | Unannotated | NA | NA |
| metab_2539 | Unannotated feature | pos | 149.1077 | 1.018 | 3.785 | 3.210 | -0.575 | -0.061 | 0.740 | 0.910 | Unannotated | NA | NA |
| metab_14490 | 7-[(6-Hydroxy-3,7-dimethyl-2,7-octadienyl)oxy]-2H-1-benzopyran-2-one | neg | 335.1255 | 3.334 | 5.663 | 5.633 | -0.029 | -0.061 | 0.740 | 0.910 | Unannotated | NA | NA |
| metab_18295 | Unannotated feature | neg | 453.2500 | 6.217 | 6.315 | 6.318 | 0.003 | -0.061 | 0.740 | 0.910 | Unannotated | NA | NA |
| metab_10925 | Unannotated feature | neg | 230.0166 | 0.815 | 3.699 | 3.871 | 0.172 | 0.061 | 0.740 | 0.910 | Unannotated | NA | NA |
| metab_12828 | Unannotated feature | neg | 152.9857 | 0.667 | 5.445 | 5.752 | 0.307 | 0.061 | 0.740 | 0.910 | Unannotated | NA | NA |
| metab_12819 | Unannotated feature | neg | 300.0398 | 0.659 | 5.865 | 5.862 | -0.003 | -0.061 | 0.740 | 0.910 | Unannotated | NA | NA |
| metab_17288 | Unannotated feature | neg | 378.9189 | 7.714 | 6.153 | 6.151 | -0.002 | -0.061 | 0.740 | 0.910 | Unannotated | NA | NA |
| metab_12216 | Unannotated feature | neg | 413.3005 | 6.860 | 5.106 | 4.571 | -0.534 | -0.061 | 0.740 | 0.910 | Unannotated | NA | NA |
| metab_3954 | Chenodeoxycholic acid glycine conjugate | pos | 414.3013 | 6.223 | 5.614 | 5.355 | -0.259 | -0.061 | 0.740 | 0.910 | Tentative | NA | NA |
| metab_13912 | Unannotated feature | neg | 357.0221 | 2.520 | 4.946 | 4.872 | -0.075 | -0.061 | 0.740 | 0.910 | Unannotated | NA | NA |
| metab_437 | Unannotated feature | pos | 96.5153 | 0.016 | 3.458 | 3.454 | -0.004 | 0.061 | 0.740 | 0.910 | Unannotated | NA | NA |
| metab_435 | Unannotated feature | pos | 110.0205 | 0.016 | 4.706 | 4.689 | -0.017 | 0.061 | 0.740 | 0.910 | Unannotated | NA | NA |
| metab_7378 | 11,12,13-Trinor-1,3,5-bisabolatrien-10-oic acid | pos | 193.1228 | 4.016 | 3.744 | 3.224 | -0.520 | -0.061 | 0.740 | 0.910 | Unannotated | Lipids and lipid-like molecules | Prenol lipids |
| metab_15625 | Unannotated feature | neg | 394.1515 | 5.542 | 3.124 | 3.350 | 0.226 | 0.061 | 0.740 | 0.910 | Unannotated | NA | NA |
| metab_15668 | Gemfibrozil | neg | 295.1552 | 5.627 | 3.613 | 3.607 | -0.007 | -0.061 | 0.740 | 0.910 | Tentative | NA | NA |
| metab_8378 | Unannotated feature | pos | 274.1404 | 1.952 | 4.287 | 4.410 | 0.122 | 0.061 | 0.740 | 0.910 | Unannotated | NA | NA |
| metab_10576 | Unannotated feature | neg | 172.0246 | 1.706 | 3.693 | 3.771 | 0.078 | -0.061 | 0.740 | 0.910 | Unannotated | NA | NA |
| metab_16366 | Soyasapogenol D | neg | 517.3905 | 6.734 | 6.437 | 6.390 | -0.047 | -0.061 | 0.740 | 0.910 | Tentative | NA | NA |
| metab_13015 | Unannotated feature | neg | 427.2258 | 6.201 | 6.582 | 6.506 | -0.076 | -0.061 | 0.740 | 0.910 | Unannotated | NA | NA |
| metab_4647 | Unannotated feature | pos | 463.3763 | 7.284 | 5.287 | 5.284 | -0.003 | -0.061 | 0.740 | 0.910 | Unannotated | NA | NA |
| metab_8937 | Unannotated feature | neg | 230.1396 | 4.207 | 4.339 | 4.344 | 0.006 | -0.061 | 0.740 | 0.910 | Unannotated | NA | NA |
| metab_1165 | Unannotated feature | pos | 501.3207 | 6.526 | 6.279 | 6.365 | 0.086 | 0.061 | 0.740 | 0.910 | Unannotated | NA | NA |
| metab_5628 | Alpha-Methylstyrene | pos | 119.0860 | 6.199 | 5.188 | 5.177 | -0.011 | -0.061 | 0.740 | 0.910 | Tentative | NA | NA |
| metab_2847 | Unannotated feature | pos | 293.1865 | 2.762 | 4.081 | 3.780 | -0.301 | -0.061 | 0.740 | 0.910 | Unannotated | NA | NA |
| metab_8595 | Unannotated feature | pos | 354.0671 | 1.151 | 5.098 | 5.081 | -0.017 | 0.061 | 0.740 | 0.910 | Unannotated | NA | NA |
| metab_9920 | Unannotated feature | neg | 311.1143 | 2.935 | 4.544 | 4.562 | 0.019 | -0.061 | 0.740 | 0.910 | Unannotated | NA | NA |
| metab_1151 | Unannotated feature | pos | 683.4371 | 6.558 | 6.511 | 6.491 | -0.020 | -0.061 | 0.740 | 0.910 | Unannotated | NA | NA |
| metab_19418 | Unannotated feature | neg | 255.1504 | 4.992 | 4.558 | 4.618 | 0.060 | -0.061 | 0.740 | 0.910 | Unannotated | NA | NA |
| metab_11852 | Unannotated feature | neg | 175.0970 | 4.896 | 4.949 | 4.923 | -0.026 | -0.061 | 0.740 | 0.910 | Unannotated | NA | NA |
| metab_15635 | Unannotated feature | neg | 258.0501 | 0.588 | 5.232 | 5.117 | -0.115 | 0.061 | 0.740 | 0.910 | Unannotated | NA | NA |
| metab_13084 | Decanedioic acid | neg | 201.1127 | 6.091 | 6.262 | 5.958 | -0.304 | -0.061 | 0.740 | 0.910 | Strong putative | NA | NA |
| metab_2011 | Unannotated feature | pos | 361.1405 | 1.944 | 4.925 | 4.770 | -0.155 | -0.061 | 0.740 | 0.910 | Unannotated | NA | NA |
| metab_17925 | DG(22:4(7Z,10Z,13Z,16Z)/22:4(7Z,10Z,13Z,16Z)/0:0) | neg | 765.5690 | 6.632 | 4.183 | 4.857 | 0.674 | 0.061 | 0.740 | 0.910 | Tentative | NA | NA |
| metab_17645 | 1,1,1,3,3-Pentafluoropropane | neg | 154.9902 | 7.097 | 5.605 | 5.611 | 0.006 | 0.061 | 0.740 | 0.910 | Unannotated | NA | NA |
| metab_17643 | 2-Deoxyribonic acid | neg | 131.0343 | 2.771 | 6.360 | 6.360 | -0.001 | -0.061 | 0.740 | 0.910 | Unannotated | NA | NA |
| metab_18490 | Fisetin | neg | 285.0406 | 6.060 | 4.759 | 4.905 | 0.147 | 0.061 | 0.740 | 0.910 | Moderate putative | NA | NA |
| metab_5559 | Unannotated feature | pos | 379.1509 | 2.747 | 4.473 | 4.479 | 0.005 | -0.061 | 0.740 | 0.910 | Unannotated | NA | NA |
| metab_1460 | Unannotated feature | pos | 484.2458 | 5.984 | 5.311 | 5.249 | -0.063 | -0.061 | 0.740 | 0.910 | Unannotated | NA | NA |
| metab_1456 | Unannotated feature | pos | 595.2678 | 5.992 | 4.142 | 4.582 | 0.441 | 0.061 | 0.740 | 0.910 | Unannotated | NA | NA |
| metab_1431 | Glu Lys Leu Thr His | pos | 627.3410 | 6.032 | 5.661 | 5.641 | -0.020 | -0.061 | 0.740 | 0.910 | Moderate putative | NA | NA |
| metab_1410 | (3beta,6beta)-Furanoeremophilane-3,6-diol 6-acetate | pos | 337.1401 | 3.129 | 5.185 | 5.204 | 0.019 | -0.061 | 0.740 | 0.910 | Unannotated | NA | NA |
| metab_1402 | Unannotated feature | pos | 205.0974 | 3.120 | 7.195 | 7.089 | -0.106 | -0.061 | 0.740 | 0.910 | Unannotated | NA | NA |
| metab_2711 | Unannotated feature | pos | 445.1214 | 2.109 | 5.597 | 5.604 | 0.007 | 0.061 | 0.740 | 0.910 | Unannotated | NA | NA |
| metab_15482 | Unannotated feature | neg | 331.1101 | 5.227 | 3.715 | 3.566 | -0.149 | -0.061 | 0.740 | 0.910 | Unannotated | NA | NA |
| metab_15478 | Unannotated feature | neg | 297.9825 | 0.571 | 5.344 | 5.403 | 0.059 | 0.061 | 0.740 | 0.910 | Unannotated | NA | NA |
| metab_2683 | Unannotated feature | pos | 315.1922 | 1.990 | 4.035 | 4.014 | -0.021 | -0.061 | 0.740 | 0.910 | Unannotated | NA | NA |
| metab_14448 | 3-hydroxy-3-(3-hydroxyphenyl)propanoic acid-O-sulphate | neg | 261.0076 | 3.262 | 4.575 | 4.090 | -0.486 | -0.061 | 0.740 | 0.910 | Unannotated | NA | NA |
| metab_1396 | (25R)-3beta-hydroxycholest-5-en-7-one-26-oate | pos | 469.2704 | 6.087 | 5.142 | 5.347 | 0.205 | 0.061 | 0.740 | 0.910 | Unannotated | NA | NA |
| metab_1359 | Unannotated feature | pos | 352.1776 | 6.143 | 3.688 | 3.950 | 0.262 | -0.061 | 0.740 | 0.910 | Unannotated | NA | NA |
| metab_1349 | Unannotated feature | pos | 391.2107 | 6.159 | 4.522 | 4.225 | -0.297 | -0.061 | 0.740 | 0.910 | Unannotated | NA | NA |
| metab_1326 | Unannotated feature | pos | 205.0975 | 2.941 | 6.792 | 6.712 | -0.080 | -0.061 | 0.740 | 0.910 | Unannotated | NA | NA |
| metab_8030 | Unannotated feature | pos | 217.0977 | 2.803 | 4.137 | 4.182 | 0.046 | 0.061 | 0.740 | 0.910 | Unannotated | NA | NA |
| metab_13485 | 8-Hydroxyadenine | neg | 150.0415 | 1.021 | 4.695 | 4.749 | 0.055 | -0.061 | 0.740 | 0.910 | Unannotated | NA | NA |
| metab_2779 | Thymine | pos | 127.0506 | 2.509 | 5.457 | 5.581 | 0.123 | -0.061 | 0.740 | 0.910 | Strong putative | NA | NA |
| metab_16934 | Unannotated feature | neg | 170.8702 | 7.822 | 5.422 | 5.414 | -0.008 | 0.061 | 0.740 | 0.910 | Unannotated | NA | NA |
| metab_4459 | Unannotated feature | pos | 254.2846 | 6.438 | 4.664 | 4.580 | -0.084 | 0.061 | 0.740 | 0.910 | Unannotated | NA | NA |
| metab_19461 | O-Desmethylquinidine | neg | 347.1145 | 3.491 | 5.002 | 4.940 | -0.062 | -0.061 | 0.740 | 0.910 | Unannotated | NA | NA |
| metab_10421 | Histidylthreonine | neg | 237.0992 | 1.997 | 3.977 | 4.314 | 0.337 | 0.061 | 0.740 | 0.910 | Unannotated | NA | NA |
| metab_2178 | N-(N-(3-Carboxyoxirane-2-carbonyl)leucyl)isoamylamine | pos | 332.2185 | 2.951 | 5.688 | 5.705 | 0.017 | -0.061 | 0.740 | 0.910 | Unannotated | NA | NA |
| metab_17819 | Unannotated feature | neg | 389.1572 | 2.806 | 5.646 | 5.549 | -0.097 | -0.061 | 0.740 | 0.910 | Unannotated | NA | NA |
| metab_9051 | Heptyl 4-hydroxybenzoate | neg | 281.1396 | 3.992 | 4.156 | 4.309 | 0.153 | 0.061 | 0.740 | 0.910 | Tentative | NA | NA |
| metab_12002 | Unannotated feature | neg | 515.3048 | 6.240 | 6.722 | 6.681 | -0.041 | -0.061 | 0.740 | 0.910 | Unannotated | NA | NA |
| metab_44 | Unannotated feature | pos | 244.0796 | 0.688 | 5.193 | 5.081 | -0.111 | -0.061 | 0.740 | 0.910 | Unannotated | NA | NA |
| metab_19879 | Maltose | neg | 387.1150 | 0.675 | 6.297 | 6.221 | -0.076 | -0.061 | 0.740 | 0.910 | Moderate putative | NA | NA |
| metab_4553 | Basilimoside | pos | 589.4455 | 6.829 | 5.090 | 4.958 | -0.132 | -0.057 | 0.758 | 0.915 | Unannotated | NA | NA |
| metab_16910 | Unannotated feature | neg | 114.0553 | 7.769 | 4.588 | 4.605 | 0.017 | 0.057 | 0.758 | 0.915 | Unannotated | NA | NA |
| metab_19754 | Phenylalanylleucine | neg | 323.1615 | 4.498 | 4.174 | 5.416 | 1.242 | -0.057 | 0.758 | 0.915 | Unannotated | NA | NA |
| metab_100 | 2-(3-Carboxy-3-aminopropyl)-L-histidine | pos | 289.1508 | 0.641 | 5.505 | 5.500 | -0.005 | -0.057 | 0.758 | 0.915 | Tentative | NA | NA |
| metab_11959 | Unannotated feature | neg | 619.2788 | 6.154 | 5.169 | 4.679 | -0.490 | -0.057 | 0.758 | 0.915 | Unannotated | NA | NA |
| metab_12094 | Unannotated feature | neg | 515.3049 | 6.459 | 7.144 | 7.152 | 0.007 | -0.057 | 0.758 | 0.915 | Unannotated | NA | NA |
| metab_263 | Unannotated feature | pos | 139.9149 | 0.524 | 4.345 | 4.328 | -0.018 | -0.057 | 0.758 | 0.915 | Unannotated | NA | NA |
| metab_19697 | Unannotated feature | neg | 181.0864 | 4.566 | 4.127 | 4.807 | 0.679 | 0.057 | 0.758 | 0.915 | Unannotated | NA | NA |
| metab_12075 | Unannotated feature | neg | 451.3071 | 6.420 | 6.535 | 6.574 | 0.039 | 0.057 | 0.758 | 0.915 | Unannotated | NA | NA |
| metab_2657 | Unannotated feature | pos | 161.1004 | 1.952 | 3.643 | 3.658 | 0.015 | -0.057 | 0.758 | 0.915 | Unannotated | NA | NA |
| metab_10043 | 3-(3,4-Dihydroxyphenyl)prop-2-enoic Acid | neg | 179.0345 | 2.802 | 4.080 | 3.995 | -0.085 | -0.057 | 0.758 | 0.915 | Moderate putative | NA | NA |
| metab_1395 | Unannotated feature | pos | 470.2304 | 6.087 | 5.839 | 5.851 | 0.012 | -0.057 | 0.758 | 0.915 | Unannotated | NA | NA |
| metab_16148 | [(3S,4S,5S,6R)-3,4,5-Trihydroxy-6-(hydroxymethyl)oxan-2-yl] octadec-9-enoate | neg | 443.3020 | 6.318 | 5.969 | 6.024 | 0.055 | -0.057 | 0.758 | 0.915 | Unannotated | NA | NA |
| metab_11128 | Unannotated feature | neg | 249.0381 | 3.647 | 3.883 | 3.484 | -0.398 | -0.057 | 0.758 | 0.915 | Unannotated | NA | NA |
| metab_2812 | L-4-Hydroxyglutamine | pos | 127.0506 | 2.699 | 5.632 | 5.736 | 0.103 | -0.057 | 0.758 | 0.915 | Unannotated | NA | NA |
| metab_2794 | Unannotated feature | pos | 271.0817 | 2.614 | 4.491 | 4.379 | -0.112 | -0.057 | 0.758 | 0.915 | Unannotated | NA | NA |
| metab_9970 | Unannotated feature | neg | 752.2757 | 2.880 | 3.852 | 3.746 | -0.105 | -0.057 | 0.758 | 0.915 | Unannotated | NA | NA |
| metab_5031 | Unannotated feature | pos | 419.2624 | 7.276 | 5.465 | 5.455 | -0.010 | -0.057 | 0.758 | 0.915 | Unannotated | NA | NA |
| metab_18479 | Ubiquinone-2 | neg | 363.1816 | 6.068 | 5.389 | 5.361 | -0.028 | 0.057 | 0.758 | 0.915 | Tentative | NA | NA |
| metab_12708 | Unannotated feature | neg | 371.0777 | 5.267 | 4.054 | 4.335 | 0.281 | 0.057 | 0.758 | 0.915 | Unannotated | NA | NA |
| metab_7961 | Lysyltyrosine | pos | 342.2031 | 2.892 | 4.796 | 4.653 | -0.143 | -0.057 | 0.758 | 0.915 | Unannotated | NA | NA |
| metab_11172 | 5-Amino-1-[(2R,3R,4S,5R)-3,4-dihydroxy-5-(hydroxymethyl)oxolan-2-yl]oxyimidazole-4-carboxamide | neg | 255.0727 | 0.667 | 4.566 | 4.585 | 0.018 | -0.057 | 0.758 | 0.915 | Unannotated | Organic oxygen compounds | Organooxygen compounds |
| metab_1261 | Unannotated feature | pos | 446.3489 | 6.327 | 4.819 | 4.824 | 0.005 | -0.057 | 0.758 | 0.915 | Unannotated | NA | NA |
| metab_1257 | Unannotated feature | pos | 433.2427 | 6.351 | 5.997 | 5.968 | -0.030 | -0.057 | 0.758 | 0.915 | Unannotated | NA | NA |
| metab_10972 | Unannotated feature | neg | 333.1310 | 0.784 | 4.112 | 4.367 | 0.255 | 0.057 | 0.758 | 0.915 | Unannotated | NA | NA |
| metab_12785 | Unannotated feature | neg | 184.0012 | 0.636 | 5.391 | 5.358 | -0.033 | -0.057 | 0.758 | 0.915 | Unannotated | NA | NA |
| metab_8075 | Unannotated feature | pos | 259.1657 | 2.755 | 3.458 | 3.595 | 0.137 | 0.057 | 0.758 | 0.915 | Unannotated | NA | NA |
| metab_2689 | Unannotated feature | pos | 155.0030 | 2.017 | 3.901 | 3.946 | 0.045 | 0.057 | 0.758 | 0.915 | Unannotated | NA | NA |
| metab_18580 | Unannotated feature | neg | 426.1966 | 6.003 | 5.072 | 4.957 | -0.114 | -0.057 | 0.758 | 0.915 | Unannotated | NA | NA |
| metab_12613 | Unannotated feature | neg | 313.1659 | 6.035 | 5.789 | 5.708 | -0.081 | -0.057 | 0.758 | 0.915 | Unannotated | NA | NA |
| metab_14895 | Unannotated feature | neg | 390.0500 | 0.502 | 4.295 | 4.234 | -0.060 | -0.057 | 0.758 | 0.915 | Unannotated | NA | NA |
| metab_9910 | 3-Hydroxybenzyl Alcohol | neg | 123.0445 | 2.946 | 6.056 | 6.056 | -0.000 | -0.057 | 0.758 | 0.915 | Strong putative | NA | NA |
| metab_17329 | Unannotated feature | neg | 352.9123 | 7.697 | 4.497 | 4.476 | -0.021 | -0.057 | 0.758 | 0.915 | Unannotated | NA | NA |
| metab_2878 | Keratan sulfate II (core 2-linked), degradation product 1 | pos | 139.0392 | 2.819 | 2.485 | 2.662 | 0.177 | 0.057 | 0.758 | 0.915 | Unannotated | NA | NA |
| metab_9967 | Unannotated feature | neg | 233.0389 | 2.890 | 3.292 | 3.108 | -0.185 | -0.057 | 0.758 | 0.915 | Unannotated | NA | NA |
| metab_9958 | Unannotated feature | neg | 385.0593 | 4.393 | 4.546 | 4.700 | 0.154 | 0.057 | 0.758 | 0.915 | Unannotated | NA | NA |
| metab_1160 | Unannotated feature | pos | 278.2482 | 6.542 | 5.681 | 5.668 | -0.013 | -0.057 | 0.758 | 0.915 | Unannotated | NA | NA |
| metab_5650 | Tomatidine | pos | 416.3533 | 6.167 | 4.513 | 4.860 | 0.347 | 0.057 | 0.758 | 0.915 | Tentative | NA | NA |
| metab_9826 | 3-(4-Hydroxyphenyl)lactate | neg | 181.0500 | 3.021 | 5.542 | 5.530 | -0.011 | 0.057 | 0.758 | 0.915 | Strong putative | NA | NA |
| metab_5107 | DG(20:4(6E,8Z,11Z,13E)-2OH(5S,15S)/0:0/i-18:0) | pos | 641.5132 | 6.997 | 7.454 | 7.464 | 0.010 | 0.057 | 0.758 | 0.915 | Unannotated | NA | NA |
| metab_10146 | Unannotated feature | neg | 444.1637 | 2.742 | 3.675 | 3.848 | 0.174 | -0.057 | 0.758 | 0.915 | Unannotated | NA | NA |
| metab_1646 | Unannotated feature | pos | 363.1682 | 4.636 | 3.617 | 3.832 | 0.215 | 0.057 | 0.758 | 0.915 | Unannotated | NA | NA |
| metab_12791 | Unannotated feature | neg | 317.0397 | 0.636 | 4.636 | 4.708 | 0.072 | -0.057 | 0.758 | 0.915 | Unannotated | NA | NA |
| metab_16195 | Unannotated feature | neg | 417.3010 | 6.404 | 3.490 | 3.542 | 0.051 | -0.057 | 0.758 | 0.915 | Unannotated | NA | NA |
| metab_5474 | Unannotated feature | pos | 470.3277 | 6.375 | 4.897 | 4.890 | -0.007 | -0.057 | 0.758 | 0.915 | Unannotated | NA | NA |
| metab_4840 | Unannotated feature | pos | 178.9910 | 7.748 | 5.303 | 5.296 | -0.006 | 0.057 | 0.758 | 0.915 | Unannotated | NA | NA |
| metab_1092 | Ret-P-man | pos | 529.2548 | 6.670 | 4.003 | 4.112 | 0.109 | -0.057 | 0.758 | 0.915 | Unannotated | NA | NA |
| metab_16017 | Unannotated feature | neg | 333.1709 | 6.138 | 4.203 | 4.739 | 0.536 | 0.057 | 0.758 | 0.915 | Unannotated | NA | NA |
| metab_9787 | Unannotated feature | neg | 101.0600 | 3.050 | 4.414 | 4.467 | 0.053 | -0.057 | 0.758 | 0.915 | Unannotated | NA | NA |
| metab_13663 | L-Allysine Ethylene Acetal | neg | 188.0923 | 1.685 | 5.245 | 5.382 | 0.137 | 0.057 | 0.758 | 0.915 | Unannotated | NA | NA |
| metab_8675 | Unannotated feature | pos | 130.9821 | 0.854 | 4.694 | 4.662 | -0.031 | 0.057 | 0.758 | 0.915 | Unannotated | NA | NA |
| metab_12538 | Unannotated feature | neg | 455.2485 | 6.224 | 6.474 | 6.348 | -0.126 | -0.057 | 0.758 | 0.915 | Unannotated | NA | NA |
| metab_5773 | Unannotated feature | pos | 119.0558 | 0.477 | 5.146 | 5.096 | -0.049 | -0.057 | 0.758 | 0.915 | Unannotated | NA | NA |
| metab_16023 | Unannotated feature | neg | 220.0086 | 1.112 | 4.887 | 4.918 | 0.031 | -0.057 | 0.758 | 0.915 | Unannotated | NA | NA |
| metab_14992 | Unannotated feature | neg | 382.8422 | 0.538 | 4.629 | 4.787 | 0.158 | 0.057 | 0.758 | 0.915 | Unannotated | NA | NA |
| metab_18209 | 3a,6b,7a,12a-Tetrahydroxy-5b-cholanoic acid | neg | 405.2650 | 6.287 | 6.725 | 6.670 | -0.054 | -0.057 | 0.758 | 0.915 | Tentative | NA | NA |
| metab_10071 | Unannotated feature | neg | 179.0351 | 2.780 | 4.939 | 4.828 | -0.111 | 0.057 | 0.758 | 0.915 | Unannotated | NA | NA |
| metab_3174 | Cyclohexanecarboxylic acid | pos | 298.2019 | 3.647 | 4.721 | 4.356 | -0.365 | -0.057 | 0.758 | 0.915 | Tentative | NA | NA |
| metab_4828 | Unannotated feature | pos | 97.0653 | 7.796 | 4.522 | 4.508 | -0.015 | -0.057 | 0.758 | 0.915 | Unannotated | NA | NA |
| metab_12500 | Unannotated feature | neg | 481.3174 | 6.365 | 6.199 | 6.324 | 0.125 | 0.057 | 0.758 | 0.915 | Unannotated | NA | NA |
| metab_1038 | Unannotated feature | pos | 761.5919 | 6.869 | 6.001 | 6.040 | 0.039 | 0.057 | 0.758 | 0.915 | Unannotated | NA | NA |
| metab_1013 | Unannotated feature | pos | 360.3240 | 7.108 | 4.886 | 4.936 | 0.050 | 0.057 | 0.758 | 0.915 | Unannotated | NA | NA |
| metab_18825 | Unannotated feature | neg | 249.0381 | 3.203 | 4.794 | 4.706 | -0.088 | -0.057 | 0.758 | 0.915 | Unannotated | NA | NA |
| metab_18815 | Unannotated feature | neg | 365.0994 | 3.192 | 5.140 | 5.278 | 0.138 | 0.057 | 0.758 | 0.915 | Unannotated | NA | NA |
| metab_3283 | (2S)-2-Amino-3-[4-[(2S)-2-amino-4-methylsulfanylbutanoyl]oxyphenyl]propanoic acid | pos | 354.1457 | 3.997 | 4.733 | 4.902 | 0.169 | 0.057 | 0.758 | 0.915 | Unannotated | NA | NA |
| metab_9621 | Unannotated feature | neg | 481.0263 | 3.210 | 4.501 | 4.405 | -0.095 | 0.057 | 0.758 | 0.915 | Unannotated | NA | NA |
| metab_16745 | Unannotated feature | neg | 142.9955 | 7.670 | 3.632 | 3.614 | -0.018 | -0.057 | 0.758 | 0.915 | Unannotated | NA | NA |
| metab_18939 | Unannotated feature | neg | 179.0708 | 5.600 | 3.575 | 3.460 | -0.115 | -0.057 | 0.758 | 0.915 | Unannotated | NA | NA |
| metab_8198 | Unannotated feature | pos | 328.1551 | 3.665 | 3.721 | 3.669 | -0.052 | -0.057 | 0.758 | 0.915 | Unannotated | NA | NA |
| metab_18134 | Unannotated feature | neg | 343.1246 | 0.644 | 5.011 | 5.034 | 0.023 | 0.057 | 0.758 | 0.915 | Unannotated | NA | NA |
| metab_1777 | 2-Naphthylamine | pos | 144.0810 | 3.275 | 4.803 | 4.910 | 0.107 | 0.057 | 0.758 | 0.915 | Moderate putative | NA | NA |
| metab_12884 | Unannotated feature | neg | 174.0766 | 0.722 | 4.894 | 4.933 | 0.040 | -0.057 | 0.758 | 0.915 | Unannotated | NA | NA |
| metab_2511 | 2,2,3,3-Tetramethylcyclopropanecarbonylurea | pos | 229.0917 | 0.823 | 4.476 | 4.567 | 0.092 | 0.057 | 0.758 | 0.915 | Unannotated | NA | NA |
| metab_10106 | Unannotated feature | neg | 292.0500 | 2.766 | 4.061 | 4.224 | 0.162 | 0.057 | 0.758 | 0.915 | Unannotated | NA | NA |
| metab_17527 | 4-Azaspiro[bicyclo[2.2.2]octane-2,2'-[1,4]oxazolidine]-5'-one | neg | 181.0977 | 2.742 | 3.997 | 3.730 | -0.266 | -0.057 | 0.758 | 0.915 | Unannotated | NA | NA |
| metab_13367 | Glutaminylproline | neg | 288.1202 | 0.730 | 5.336 | 5.407 | 0.071 | 0.057 | 0.758 | 0.915 | Unannotated | NA | NA |
| metab_15066 | (2S,3S,4S,5R,6R)-3,4,5-Trihydroxy-6-[2-hydroxy-2-(4-hydroxy-3-methoxyphenyl)ethoxy]oxane-2-carboxylic acid | neg | 341.0885 | 4.477 | 1.970 | 2.445 | 0.475 | 0.057 | 0.758 | 0.915 | Unannotated | NA | NA |
| metab_11586 | Unannotated feature | neg | 234.8556 | 0.453 | 3.499 | 3.507 | 0.009 | 0.057 | 0.758 | 0.915 | Unannotated | NA | NA |
| metab_11616 | Unannotated feature | neg | 183.9139 | 0.263 | 5.335 | 5.312 | -0.023 | -0.057 | 0.758 | 0.915 | Unannotated | NA | NA |
| metab_902 | Unannotated feature | pos | 629.4765 | 7.060 | 5.518 | 5.429 | -0.089 | 0.057 | 0.758 | 0.915 | Unannotated | NA | NA |
| metab_13814 | Unannotated feature | neg | 381.0368 | 2.054 | 4.794 | 4.688 | -0.106 | 0.057 | 0.758 | 0.915 | Unannotated | NA | NA |
| metab_3374 | Sebacic acid | pos | 185.1175 | 7.307 | 6.394 | 6.393 | -0.002 | -0.057 | 0.758 | 0.915 | Strong putative | NA | NA |
| metab_10267 | Ribavirin monophosphate | neg | 323.0417 | 2.520 | 5.646 | 5.635 | -0.012 | -0.057 | 0.758 | 0.915 | Unannotated | NA | NA |
| metab_10250 | Unannotated feature | neg | 293.1357 | 4.533 | 4.809 | 4.832 | 0.023 | -0.057 | 0.758 | 0.915 | Unannotated | NA | NA |
| metab_16492 | Unannotated feature | neg | 170.9840 | 7.080 | 5.327 | 5.295 | -0.032 | -0.057 | 0.758 | 0.915 | Unannotated | NA | NA |
| metab_5138 | Unannotated feature | pos | 332.2927 | 6.893 | 5.716 | 5.645 | -0.071 | -0.057 | 0.758 | 0.915 | Unannotated | NA | NA |
| metab_5137 | Unannotated feature | pos | 457.3506 | 6.893 | 5.622 | 5.737 | 0.115 | 0.057 | 0.758 | 0.915 | Unannotated | NA | NA |
| metab_5132 | Asparaginylglutamic acid | pos | 325.1137 | 0.728 | 5.076 | 5.019 | -0.057 | 0.057 | 0.758 | 0.915 | Unannotated | Organic acids and derivatives | Carboxylic acids and derivatives |
| metab_14517 | Meconine | neg | 239.0560 | 3.379 | 5.093 | 5.061 | -0.031 | 0.057 | 0.758 | 0.915 | Unannotated | NA | NA |
| metab_10302 | Ethanethioic acid, S-(2-(((2R)-2-(acetylamino)-3-mercapto-1-oxopropyl)amino)ethyl)ester | neg | 285.0367 | 2.379 | 4.762 | 3.653 | -1.109 | -0.057 | 0.758 | 0.915 | Unannotated | NA | NA |
| metab_1886 | Unannotated feature | pos | 81.5210 | 1.309 | 4.608 | 4.632 | 0.025 | 0.057 | 0.758 | 0.915 | Unannotated | NA | NA |
| metab_10735 | Unannotated feature | neg | 215.1033 | 1.274 | 5.227 | 5.182 | -0.044 | -0.057 | 0.758 | 0.915 | Unannotated | NA | NA |
| metab_11587 | Unannotated feature | neg | 232.8585 | 0.453 | 4.049 | 4.009 | -0.040 | -0.057 | 0.758 | 0.915 | Unannotated | NA | NA |
| metab_873 | Unannotated feature | pos | 217.1953 | 6.773 | 4.077 | 4.037 | -0.041 | -0.057 | 0.758 | 0.915 | Unannotated | NA | NA |
| metab_15871 | 7(14)-Bisabolene-2,3,10,11-tetrol | neg | 271.1916 | 5.958 | 4.646 | 4.572 | -0.075 | -0.057 | 0.758 | 0.915 | Unannotated | NA | NA |
| metab_6117 | Unannotated feature | pos | 420.9677 | 0.524 | 4.610 | 4.738 | 0.128 | -0.057 | 0.758 | 0.915 | Unannotated | NA | NA |
| metab_11681 | Unannotated feature | neg | 375.1433 | 5.374 | 5.282 | 4.887 | -0.394 | -0.057 | 0.758 | 0.915 | Unannotated | NA | NA |
| metab_6447 | Unannotated feature | pos | 315.1346 | 5.656 | 4.627 | 4.117 | -0.510 | -0.057 | 0.758 | 0.915 | Unannotated | NA | NA |
| metab_19161 | Unannotated feature | neg | 366.1344 | 5.317 | 4.158 | 4.500 | 0.341 | 0.057 | 0.758 | 0.915 | Unannotated | NA | NA |
| metab_3454 | 4-Hydroxynonenal | pos | 121.1018 | 4.625 | 4.470 | 4.523 | 0.053 | -0.057 | 0.758 | 0.915 | Tentative | Lipids and lipid-like molecules | Fatty Acyls |
| metab_3440 | 9-(4-Hydroxyphenyl)-2-methoxy-1H-phenalen-1-one | pos | 325.0827 | 4.579 | 4.613 | 4.676 | 0.062 | 0.057 | 0.758 | 0.915 | Unannotated | NA | NA |
| metab_9524 | Prednisone | neg | 403.1733 | 3.318 | 4.830 | 4.827 | -0.003 | -0.057 | 0.758 | 0.915 | Tentative | NA | NA |
| metab_9562 | Unannotated feature | neg | 313.0502 | 3.266 | 3.559 | 3.545 | -0.014 | -0.057 | 0.758 | 0.915 | Unannotated | NA | NA |
| metab_841 | Unannotated feature | pos | 572.7534 | 0.593 | 4.141 | 4.143 | 0.002 | 0.057 | 0.758 | 0.915 | Unannotated | NA | NA |
| metab_15776 | (S)-9-Hydroxy-10-undecenoic acid | neg | 245.1393 | 5.818 | 6.104 | 6.075 | -0.029 | 0.057 | 0.758 | 0.915 | Unannotated | NA | NA |
| metab_2330 | Unannotated feature | pos | 355.0709 | 0.402 | 5.643 | 5.648 | 0.005 | -0.057 | 0.758 | 0.915 | Unannotated | NA | NA |
| metab_16482 | Unannotated feature | neg | 697.4730 | 7.061 | 4.744 | 4.766 | 0.023 | 0.057 | 0.758 | 0.915 | Unannotated | NA | NA |
| metab_10361 | Unannotated feature | neg | 159.8780 | 2.178 | 4.359 | 4.496 | 0.137 | 0.057 | 0.758 | 0.915 | Unannotated | NA | NA |
| metab_5336 | Unannotated feature | pos | 377.2575 | 6.534 | 2.796 | 2.966 | 0.171 | 0.057 | 0.758 | 0.915 | Unannotated | NA | NA |
| metab_11707 | Unannotated feature | neg | 478.1988 | 5.496 | 4.674 | 4.465 | -0.209 | 0.057 | 0.758 | 0.915 | Unannotated | NA | NA |
| metab_11750 | Unannotated feature | neg | 505.1377 | 5.660 | 3.872 | 3.699 | -0.173 | -0.057 | 0.758 | 0.915 | Unannotated | NA | NA |
| metab_11745 | Unannotated feature | neg | 243.1237 | 5.645 | 6.172 | 6.159 | -0.013 | 0.057 | 0.758 | 0.915 | Unannotated | NA | NA |
| metab_12300 | Araliacerebroside | neg | 730.5494 | 7.199 | 4.364 | 4.125 | -0.239 | -0.057 | 0.758 | 0.915 | Unannotated | NA | NA |
| metab_7474 | Carboxyprimaquine | pos | 275.1395 | 3.808 | 4.775 | 4.619 | -0.157 | -0.057 | 0.758 | 0.915 | Moderate putative | NA | NA |
| metab_13907 | Unannotated feature | neg | 255.9579 | 2.514 | 4.319 | 4.316 | -0.003 | -0.057 | 0.758 | 0.915 | Unannotated | NA | NA |
| metab_3685 | Unannotated feature | pos | 463.3037 | 6.606 | 6.413 | 6.433 | 0.020 | 0.057 | 0.758 | 0.915 | Unannotated | NA | NA |
| metab_13844 | Unannotated feature | neg | 226.8238 | 2.190 | 4.583 | 4.565 | -0.018 | 0.057 | 0.758 | 0.915 | Unannotated | NA | NA |
| metab_4722 | Unannotated feature | pos | 310.9133 | 7.642 | 5.154 | 5.151 | -0.003 | 0.057 | 0.758 | 0.915 | Unannotated | NA | NA |
| metab_19281 | Unannotated feature | neg | 248.0775 | 5.161 | 3.419 | 3.419 | -0.000 | 0.057 | 0.758 | 0.915 | Unannotated | NA | NA |
| metab_12235 | Unannotated feature | neg | 587.2889 | 6.929 | 5.373 | 5.146 | -0.226 | -0.057 | 0.758 | 0.915 | Unannotated | NA | NA |
| metab_13003 | Unannotated feature | neg | 120.9958 | 0.810 | 2.932 | 2.889 | -0.043 | -0.057 | 0.758 | 0.915 | Unannotated | NA | NA |
| metab_10606 | Unannotated feature | neg | 317.0630 | 1.685 | 4.554 | 4.940 | 0.386 | -0.057 | 0.758 | 0.915 | Unannotated | NA | NA |
| metab_5293 | Unannotated feature | pos | 132.9985 | 0.485 | 5.790 | 5.700 | -0.089 | -0.057 | 0.758 | 0.915 | Unannotated | NA | NA |
| metab_1936 | Unannotated feature | pos | 233.1089 | 1.936 | 3.577 | 3.747 | 0.170 | -0.057 | 0.758 | 0.915 | Unannotated | NA | NA |
| metab_11804 | Unannotated feature | neg | 547.3304 | 5.842 | 5.259 | 5.266 | 0.007 | 0.057 | 0.758 | 0.915 | Unannotated | NA | NA |
| metab_11811 | Unannotated feature | neg | 656.3316 | 5.871 | 4.879 | 6.096 | 1.217 | 0.057 | 0.758 | 0.915 | Unannotated | NA | NA |
| metab_6731 | Unannotated feature | pos | 637.3255 | 5.260 | 4.613 | 5.861 | 1.248 | 0.057 | 0.758 | 0.915 | Unannotated | NA | NA |
| metab_6707 | Pseudoecgonine | pos | 168.1021 | 5.297 | 2.856 | 3.056 | 0.200 | 0.057 | 0.758 | 0.915 | Tentative | NA | NA |
| metab_450 | MJDBISSP00000009 | pos | 171.1179 | 2.560 | 7.283 | 7.295 | 0.011 | 0.057 | 0.758 | 0.915 | Strong putative | NA | NA |
| metab_13976 | Unannotated feature | neg | 233.0123 | 2.663 | 2.911 | 3.095 | 0.185 | 0.057 | 0.758 | 0.915 | Unannotated | NA | NA |
| metab_9280 | Unannotated feature | neg | 95.0494 | 3.647 | 2.742 | 2.457 | -0.285 | -0.057 | 0.758 | 0.915 | Unannotated | NA | NA |
| metab_17014 | Unannotated feature | neg | 265.8904 | 7.997 | 4.145 | 4.185 | 0.040 | 0.057 | 0.758 | 0.915 | Unannotated | NA | NA |
| metab_4047 | 1-cyclohexyl-3-[2-(4-ethoxyphenoxy)ethyl]urea | pos | 307.2020 | 6.151 | 5.296 | 5.310 | 0.015 | -0.057 | 0.758 | 0.915 | Moderate putative | NA | NA |
| metab_13934 | 3'-Deamino-3'-(2-methoxy-4-morpholinyl)doxorubicin | neg | 648.2036 | 2.575 | 3.756 | 3.613 | -0.143 | 0.057 | 0.758 | 0.915 | Unannotated | NA | NA |
| metab_9306 | Gibberellin A9 | neg | 337.1409 | 3.604 | 4.073 | 4.179 | 0.106 | 0.057 | 0.758 | 0.915 | Tentative | NA | NA |
| metab_16808 | Unannotated feature | neg | 481.8699 | 7.714 | 5.093 | 5.097 | 0.004 | -0.057 | 0.758 | 0.915 | Unannotated | NA | NA |
| metab_12192 | Unannotated feature | neg | 431.3165 | 6.766 | 5.681 | 5.691 | 0.010 | 0.057 | 0.758 | 0.915 | Unannotated | NA | NA |
| metab_11833 | Unannotated feature | neg | 371.1712 | 5.927 | 5.367 | 5.251 | -0.115 | 0.057 | 0.758 | 0.915 | Unannotated | NA | NA |
| metab_15262 | Cis-3-Hexenyl lactate | neg | 217.1078 | 4.825 | 5.267 | 5.324 | 0.057 | 0.057 | 0.758 | 0.915 | Unannotated | NA | NA |
| metab_10369 | Kifunensine | neg | 277.0695 | 4.600 | 3.718 | 3.858 | 0.141 | -0.057 | 0.758 | 0.915 | Unannotated | NA | NA |
| metab_8348 | Aspergillomarasmine A | pos | 272.0885 | 1.960 | 3.696 | 3.761 | 0.065 | 0.057 | 0.758 | 0.915 | Unannotated | Organic acids and derivatives | Carboxylic acids and derivatives |
| metab_10456 | Unannotated feature | neg | 369.1412 | 1.958 | 3.887 | 3.919 | 0.032 | 0.057 | 0.758 | 0.915 | Unannotated | NA | NA |
| metab_8307 | Unannotated feature | pos | 145.0174 | 2.058 | 4.607 | 4.558 | -0.048 | -0.057 | 0.758 | 0.915 | Unannotated | NA | NA |
| metab_9221 | Unannotated feature | neg | 250.1089 | 3.737 | 4.058 | 4.329 | 0.271 | 0.057 | 0.758 | 0.915 | Unannotated | NA | NA |
| metab_8934 | Unannotated feature | neg | 566.1589 | 0.714 | 5.142 | 5.110 | -0.032 | 0.057 | 0.758 | 0.915 | Unannotated | NA | NA |
| metab_425 | Unannotated feature | pos | 146.9805 | 0.016 | 4.231 | 4.233 | 0.002 | 0.057 | 0.758 | 0.915 | Unannotated | NA | NA |
| metab_11840 | Unannotated feature | neg | 370.0683 | 5.947 | 5.226 | 5.428 | 0.202 | -0.057 | 0.758 | 0.915 | Unannotated | NA | NA |
| metab_15294 | ORTHOTHYMOTINIC ACID | neg | 193.0865 | 6.007 | 6.250 | 6.186 | -0.063 | -0.057 | 0.758 | 0.915 | Strong putative | NA | NA |
| metab_12182 | Unannotated feature | neg | 521.3301 | 6.726 | 6.874 | 6.894 | 0.020 | 0.057 | 0.758 | 0.915 | Unannotated | NA | NA |
| metab_4480 | Unannotated feature | pos | 301.1418 | 6.526 | 5.466 | 5.366 | -0.101 | -0.057 | 0.758 | 0.915 | Unannotated | NA | NA |
| metab_10533 | Unannotated feature | neg | 116.0169 | 1.947 | 4.116 | 3.905 | -0.211 | -0.057 | 0.758 | 0.915 | Unannotated | NA | NA |
| metab_5267 | Ganolucidic acid B | pos | 503.3362 | 6.638 | 6.676 | 6.837 | 0.161 | 0.057 | 0.758 | 0.915 | Unannotated | NA | NA |
| metab_2015 | Unannotated feature | pos | 227.1394 | 1.944 | 4.434 | 4.391 | -0.043 | -0.057 | 0.758 | 0.915 | Unannotated | NA | NA |
| metab_17637 | Unannotated feature | neg | 742.5338 | 7.111 | 5.766 | 5.706 | -0.061 | -0.057 | 0.758 | 0.915 | Unannotated | NA | NA |
| metab_17681 | Unannotated feature | neg | 577.2772 | 7.031 | 5.229 | 5.397 | 0.168 | 0.057 | 0.758 | 0.915 | Unannotated | NA | NA |
| metab_14035 | Unannotated feature | neg | 181.0613 | 2.771 | 4.185 | 4.348 | 0.164 | 0.057 | 0.758 | 0.915 | Unannotated | NA | NA |
| metab_9141 | Lactaronecatorin A | neg | 287.1041 | 3.863 | 5.192 | 5.255 | 0.063 | -0.057 | 0.758 | 0.915 | Unannotated | NA | NA |
| metab_9168 | Unannotated feature | neg | 345.0996 | 3.909 | 5.169 | 5.257 | 0.088 | -0.057 | 0.758 | 0.915 | Unannotated | NA | NA |
| metab_16869 | Unannotated feature | neg | 301.1299 | 7.737 | 5.521 | 5.444 | -0.077 | 0.057 | 0.758 | 0.915 | Unannotated | NA | NA |
| metab_8995 | 3-Carboxy-2,3,4,9-tetrahydro-1H-pyrido[3,4-b]indole-1-propanoic acid | neg | 287.1038 | 4.087 | 5.615 | 5.625 | 0.010 | -0.057 | 0.758 | 0.915 | Unannotated | NA | NA |
| metab_8977 | Unannotated feature | neg | 225.1130 | 4.118 | 5.332 | 5.321 | -0.011 | -0.057 | 0.758 | 0.915 | Unannotated | NA | NA |
| metab_19517 | Unannotated feature | neg | 145.0864 | 4.833 | 3.325 | 3.440 | 0.115 | 0.057 | 0.758 | 0.915 | Unannotated | NA | NA |
| metab_397 | Unannotated feature | pos | 116.5247 | 0.019 | 3.234 | 3.206 | -0.028 | -0.057 | 0.758 | 0.915 | Unannotated | NA | NA |
| metab_367 | Unannotated feature | pos | 79.0759 | 0.319 | 5.323 | 5.344 | 0.021 | 0.057 | 0.758 | 0.915 | Unannotated | NA | NA |
| metab_10526 | Isopyridoxal | neg | 212.0561 | 1.947 | 5.189 | 5.148 | -0.041 | 0.057 | 0.758 | 0.915 | Tentative | NA | NA |
| metab_10499 | 2-Hydroxybutyric Acid | neg | 103.0393 | 1.952 | 5.753 | 5.687 | -0.066 | -0.057 | 0.758 | 0.915 | Moderate putative | NA | NA |
| metab_5242 | Unannotated feature | pos | 144.9824 | 0.016 | 4.989 | 4.982 | -0.008 | 0.057 | 0.758 | 0.915 | Unannotated | NA | NA |
| metab_7291 | Tyrosyl-Methionine | pos | 354.1457 | 4.223 | 5.246 | 5.368 | 0.122 | 0.057 | 0.758 | 0.915 | Unannotated | NA | NA |
| metab_11872 | Ethyl glucuronide | neg | 665.2166 | 0.722 | 6.043 | 6.084 | 0.041 | 0.057 | 0.758 | 0.915 | Unannotated | NA | NA |
| metab_12159 | Unannotated feature | neg | 388.2528 | 6.671 | 4.552 | 4.301 | -0.251 | -0.057 | 0.758 | 0.915 | Unannotated | NA | NA |
| metab_7234 | Thymyl acetate | pos | 193.1228 | 4.355 | 4.896 | 4.895 | -0.001 | 0.057 | 0.758 | 0.915 | Tentative | NA | NA |
| metab_7220 | 5-Hydroxy-3-(4-hydroxybenzyl)-7,8-dimethoxy-4-chromanone | pos | 331.1185 | 4.389 | 4.177 | 4.337 | 0.160 | -0.057 | 0.758 | 0.915 | Unannotated | NA | NA |
| metab_11918 | Unannotated feature | neg | 671.3310 | 6.076 | 4.994 | 5.165 | 0.171 | -0.057 | 0.758 | 0.915 | Unannotated | NA | NA |
| metab_11907 | Unannotated feature | neg | 393.1953 | 6.060 | 6.515 | 6.609 | 0.094 | 0.057 | 0.758 | 0.915 | Unannotated | NA | NA |
| metab_14088 | Unannotated feature | neg | 276.9540 | 0.507 | 5.512 | 5.589 | 0.078 | 0.057 | 0.758 | 0.915 | Unannotated | NA | NA |
| metab_14148 | Dimethyl-3-hydroxypyrid-4-one | neg | 184.0610 | 2.890 | 5.865 | 5.883 | 0.018 | 0.057 | 0.758 | 0.915 | Unannotated | NA | NA |
| metab_15477 | Unannotated feature | neg | 486.7942 | 0.527 | 4.787 | 4.836 | 0.050 | 0.057 | 0.758 | 0.915 | Unannotated | NA | NA |
| metab_19818 | Unannotated feature | neg | 115.0757 | 4.405 | 4.884 | 4.886 | 0.002 | 0.057 | 0.758 | 0.915 | Unannotated | NA | NA |
| metab_9 | Unannotated feature | pos | 239.1394 | 0.720 | 4.731 | 4.702 | -0.029 | -0.053 | 0.776 | 0.922 | Unannotated | NA | NA |
| metab_11987 | Unannotated feature | neg | 357.2285 | 6.217 | 5.604 | 5.379 | -0.225 | -0.053 | 0.776 | 0.922 | Unannotated | NA | NA |
| metab_10368 | Unannotated feature | neg | 197.8075 | 2.151 | 5.214 | 5.207 | -0.008 | 0.053 | 0.776 | 0.922 | Unannotated | NA | NA |
| metab_16349 | 9(S)-HOTrE | neg | 587.4326 | 6.711 | 5.545 | 5.290 | -0.255 | -0.053 | 0.776 | 0.922 | Tentative | NA | NA |
| metab_14652 | Unannotated feature | neg | 349.1411 | 3.589 | 3.924 | 4.321 | 0.397 | 0.053 | 0.776 | 0.922 | Unannotated | NA | NA |
| metab_1955 | Unannotated feature | pos | 267.0982 | 1.960 | 4.432 | 4.432 | -0.000 | -0.053 | 0.776 | 0.922 | Unannotated | NA | NA |
| metab_1937 | Unannotated feature | pos | 273.1081 | 1.944 | 4.687 | 4.646 | -0.041 | -0.053 | 0.776 | 0.922 | Unannotated | NA | NA |
| metab_8964 | Unannotated feature | neg | 307.1223 | 4.146 | 3.680 | 3.689 | 0.009 | -0.053 | 0.776 | 0.922 | Unannotated | NA | NA |
| metab_19472 | Pinocembrin | neg | 301.0721 | 4.903 | 4.226 | 4.786 | 0.560 | 0.053 | 0.776 | 0.922 | Tentative | NA | NA |
| metab_403 | Unannotated feature | pos | 276.8972 | 0.016 | 4.718 | 4.691 | -0.027 | -0.053 | 0.776 | 0.922 | Unannotated | NA | NA |
| metab_12184 | Unannotated feature | neg | 509.3854 | 6.711 | 6.484 | 6.492 | 0.008 | 0.053 | 0.776 | 0.922 | Unannotated | NA | NA |
| metab_7298 | 3-(2-Hydroxyphenyl)propanoic acid | pos | 208.0972 | 4.198 | 3.883 | 3.885 | 0.002 | 0.053 | 0.776 | 0.922 | Tentative | NA | NA |
| metab_12131 | Unannotated feature | neg | 519.2412 | 6.592 | 5.098 | 4.960 | -0.138 | 0.053 | 0.776 | 0.922 | Unannotated | NA | NA |
| metab_19531 | Unannotated feature | neg | 387.0398 | 3.521 | 2.772 | 2.993 | 0.220 | 0.053 | 0.776 | 0.922 | Unannotated | NA | NA |
| metab_15514 | Butyl 3-hydroxy-2-methylidenebutanoate | neg | 217.1078 | 5.304 | 5.778 | 5.883 | 0.105 | -0.053 | 0.776 | 0.922 | Unannotated | NA | NA |
| metab_4631 | LysoPE(0:0/22:4(7Z,10Z,13Z,16Z)) | pos | 552.3110 | 7.148 | 4.907 | 4.660 | -0.247 | -0.053 | 0.776 | 0.922 | Unannotated | NA | NA |
| metab_16854 | Unannotated feature | neg | 314.8545 | 7.730 | 3.923 | 3.970 | 0.047 | 0.053 | 0.776 | 0.922 | Unannotated | NA | NA |
| metab_19478 | Unannotated feature | neg | 274.0248 | 4.896 | 3.179 | 3.718 | 0.539 | 0.053 | 0.776 | 0.922 | Unannotated | NA | NA |
| metab_15295 | Unannotated feature | neg | 578.7881 | 0.533 | 5.266 | 5.389 | 0.123 | 0.053 | 0.776 | 0.922 | Unannotated | NA | NA |
| metab_9126 | Rufloxacin | neg | 408.1055 | 3.882 | 4.311 | 4.175 | -0.136 | -0.053 | 0.776 | 0.922 | Tentative | NA | NA |
| metab_14129 | Unannotated feature | neg | 327.9177 | 0.512 | 4.208 | 4.460 | 0.252 | -0.053 | 0.776 | 0.922 | Unannotated | NA | NA |
| metab_13237 | Unannotated feature | neg | 357.9279 | 3.199 | 5.010 | 4.884 | -0.126 | -0.053 | 0.776 | 0.922 | Unannotated | NA | NA |
| metab_5174 | DG(16:0/0:0/PGE2) | pos | 687.4815 | 6.821 | 5.822 | 5.677 | -0.145 | -0.053 | 0.776 | 0.922 | Unannotated | NA | NA |
| metab_5165 | Unannotated feature | pos | 469.3513 | 6.845 | 4.429 | 4.197 | -0.233 | -0.053 | 0.776 | 0.922 | Unannotated | NA | NA |
| metab_16501 | Unannotated feature | neg | 282.0846 | 1.685 | 5.312 | 5.245 | -0.067 | -0.053 | 0.776 | 0.922 | Unannotated | NA | NA |
| metab_10641 | Unannotated feature | neg | 236.0411 | 1.670 | 4.006 | 3.987 | -0.020 | -0.053 | 0.776 | 0.922 | Unannotated | NA | NA |
| metab_18040 | Arginylvaline | neg | 545.3475 | 6.451 | 5.593 | 5.680 | 0.087 | 0.053 | 0.776 | 0.922 | Unannotated | NA | NA |
| metab_16304 | Mono-methyl-adipate | neg | 205.0713 | 1.489 | 5.125 | 5.147 | 0.022 | 0.053 | 0.776 | 0.922 | Unannotated | NA | NA |
| metab_5322 | 5,6-DHET | pos | 361.2353 | 6.550 | 5.230 | 5.225 | -0.005 | 0.053 | 0.776 | 0.922 | Tentative | NA | NA |
| metab_17504 | 11-Oxo-androsterone glucuronide | neg | 515.2009 | 2.725 | 3.292 | 3.376 | 0.084 | -0.053 | 0.776 | 0.922 | Unannotated | Lipids and lipid-like molecules | Glycerolipids |
| metab_6540 | Unannotated feature | pos | 186.1492 | 5.541 | 4.240 | 4.259 | 0.019 | 0.053 | 0.776 | 0.922 | Unannotated | NA | NA |
| metab_12207 | Unannotated feature | neg | 72.9920 | 6.828 | 4.470 | 4.517 | 0.046 | -0.053 | 0.776 | 0.922 | Unannotated | NA | NA |
| metab_12205 | Unannotated feature | neg | 607.3868 | 6.797 | 5.263 | 5.379 | 0.116 | 0.053 | 0.776 | 0.922 | Unannotated | NA | NA |
| metab_12191 | DG(a-15:0/18:3(9,11,15)-OH(13)/0:0) | neg | 591.4644 | 6.758 | 6.681 | 6.824 | 0.143 | -0.053 | 0.776 | 0.922 | Unannotated | NA | NA |
| metab_6763 | Fluvoxamine | pos | 357.1178 | 0.625 | 3.919 | 3.970 | 0.051 | 0.053 | 0.776 | 0.922 | Tentative | NA | NA |
| metab_19422 | Unannotated feature | neg | 381.1673 | 4.983 | 4.053 | 4.252 | 0.199 | 0.053 | 0.776 | 0.922 | Unannotated | NA | NA |
| metab_16830 | Unannotated feature | neg | 347.8897 | 7.726 | 4.690 | 4.744 | 0.054 | 0.053 | 0.776 | 0.922 | Unannotated | NA | NA |
| metab_1888 | Gamma-Glutamylproline | pos | 245.1136 | 1.176 | 6.069 | 6.126 | 0.058 | 0.053 | 0.776 | 0.922 | Unannotated | NA | NA |
| metab_1879 | Unannotated feature | pos | 140.0344 | 1.252 | 4.981 | 5.065 | 0.084 | 0.053 | 0.776 | 0.922 | Unannotated | NA | NA |
| metab_1867 | Unannotated feature | pos | 253.1551 | 1.184 | 4.332 | 4.381 | 0.049 | -0.053 | 0.776 | 0.922 | Unannotated | NA | NA |
| metab_18128 | MG(18:2(9Z,12Z)/0:0/0:0) | neg | 399.2756 | 6.373 | 5.739 | 5.793 | 0.054 | 0.053 | 0.776 | 0.922 | Unannotated | NA | NA |
| metab_10743 | Unannotated feature | neg | 319.1763 | 4.785 | 4.315 | 4.113 | -0.202 | -0.053 | 0.776 | 0.922 | Unannotated | NA | NA |
| metab_16268 | Unannotated feature | neg | 400.8348 | 0.538 | 5.523 | 5.550 | 0.027 | -0.053 | 0.776 | 0.922 | Unannotated | NA | NA |
| metab_10773 | Unannotated feature | neg | 332.0304 | 1.171 | 3.980 | 3.836 | -0.144 | 0.053 | 0.776 | 0.922 | Unannotated | NA | NA |
| metab_10766 | Gamma-Glutamyltryptophan | neg | 354.1059 | 1.179 | 5.006 | 4.833 | -0.174 | -0.053 | 0.776 | 0.922 | Unannotated | NA | NA |
| metab_13839 | Unannotated feature | neg | 231.9434 | 2.178 | 4.013 | 3.926 | -0.088 | 0.053 | 0.776 | 0.922 | Unannotated | NA | NA |
| metab_4731 | Unannotated feature | pos | 489.8942 | 7.658 | 4.148 | 4.106 | -0.042 | 0.053 | 0.776 | 0.922 | Unannotated | NA | NA |
| metab_17093 | Unannotated feature | neg | 231.9175 | 7.874 | 4.653 | 4.611 | -0.041 | -0.053 | 0.776 | 0.922 | Unannotated | NA | NA |
| metab_17088 | Unannotated feature | neg | 214.8797 | 7.888 | 6.554 | 6.570 | 0.016 | 0.053 | 0.776 | 0.922 | Unannotated | NA | NA |
| metab_11787 | Unannotated feature | neg | 493.1513 | 5.797 | 4.059 | 4.120 | 0.061 | -0.053 | 0.776 | 0.922 | Unannotated | NA | NA |
| metab_15193 | Unannotated feature | neg | 427.8541 | 0.520 | 4.863 | 5.019 | 0.156 | 0.053 | 0.776 | 0.922 | Unannotated | NA | NA |
| metab_15716 | 3-hydroxytridecanoic acid | neg | 275.1864 | 5.710 | 4.504 | 4.534 | 0.030 | 0.053 | 0.776 | 0.922 | Unannotated | Lipids and lipid-like molecules | Fatty Acyls |
| metab_19315 | Unannotated feature | neg | 349.1409 | 5.122 | 4.522 | 4.483 | -0.039 | 0.053 | 0.776 | 0.922 | Unannotated | NA | NA |
| metab_561 | Unannotated feature | pos | 397.2131 | 6.103 | 4.710 | 4.907 | 0.197 | 0.053 | 0.776 | 0.922 | Unannotated | NA | NA |
| metab_13394 | N-Acetyl-L-glutamate 5-semialdehyde | neg | 172.0609 | 0.770 | 6.045 | 6.079 | 0.034 | -0.053 | 0.776 | 0.922 | Tentative | NA | NA |
| metab_10165 | Unannotated feature | neg | 349.0160 | 2.714 | 4.863 | 5.044 | 0.182 | -0.053 | 0.776 | 0.922 | Unannotated | NA | NA |
| metab_8126 | Unannotated feature | pos | 225.1238 | 2.732 | 4.510 | 4.625 | 0.114 | 0.053 | 0.776 | 0.922 | Unannotated | NA | NA |
| metab_1744 | Unannotated feature | pos | 156.1022 | 3.582 | 5.515 | 5.635 | 0.120 | -0.053 | 0.776 | 0.922 | Unannotated | NA | NA |
| metab_12830 | DG(PGF2alpha/i-16:0/0:0) | neg | 647.4908 | 6.892 | 6.011 | 6.220 | 0.210 | -0.053 | 0.776 | 0.922 | Unannotated | NA | NA |
| metab_10868 | Unannotated feature | neg | 174.0014 | 0.992 | 3.147 | 3.142 | -0.006 | 0.053 | 0.776 | 0.922 | Unannotated | NA | NA |
| metab_10828 | Unannotated feature | neg | 278.0019 | 1.051 | 4.634 | 4.840 | 0.205 | -0.053 | 0.776 | 0.922 | Unannotated | NA | NA |
| metab_10824 | 1,2,3-Triazine, 4-phenyl- | neg | 202.0629 | 1.054 | 4.011 | 3.941 | -0.070 | 0.053 | 0.776 | 0.922 | Unannotated | NA | NA |
| metab_13436 | Gamma-L-Glutamyl-L-pipecolic acid | neg | 257.1145 | 0.815 | 4.342 | 4.221 | -0.121 | -0.053 | 0.776 | 0.922 | Unannotated | NA | NA |
| metab_12334 | Unannotated feature | neg | 409.1902 | 6.115 | 6.454 | 6.394 | -0.060 | 0.053 | 0.776 | 0.922 | Unannotated | NA | NA |
| metab_13832 | Unannotated feature | neg | 330.0696 | 2.137 | 5.166 | 5.447 | 0.280 | 0.053 | 0.776 | 0.922 | Unannotated | NA | NA |
| metab_13819 | Unannotated feature | neg | 187.9311 | 2.081 | 5.224 | 5.177 | -0.047 | -0.053 | 0.776 | 0.922 | Unannotated | NA | NA |
| metab_17110 | Unannotated feature | neg | 197.8517 | 7.853 | 5.163 | 5.180 | 0.017 | 0.053 | 0.776 | 0.922 | Unannotated | NA | NA |
| metab_9576 | Unannotated feature | neg | 214.0330 | 3.247 | 4.329 | 4.218 | -0.112 | -0.053 | 0.776 | 0.922 | Unannotated | NA | NA |
| metab_9573 | N-(1-Carboxy-3-carboxanilidopropyl)alanylproline | neg | 436.1695 | 3.247 | 3.630 | 3.582 | -0.048 | -0.053 | 0.776 | 0.922 | Unannotated | NA | NA |
| metab_19175 | P-Hydroxyubenimex | neg | 323.1615 | 5.295 | 5.157 | 6.118 | 0.962 | 0.053 | 0.776 | 0.922 | Unannotated | NA | NA |
| metab_19241 | Cannabigerolate | neg | 395.1978 | 5.209 | 3.591 | 3.730 | 0.140 | -0.053 | 0.776 | 0.922 | Tentative | NA | NA |
| metab_12257 | 24-Hydroxytetracosanoic acid | neg | 383.3533 | 7.004 | 5.515 | 5.806 | 0.291 | 0.053 | 0.776 | 0.922 | Unannotated | NA | NA |
| metab_13894 | Unannotated feature | neg | 335.0615 | 2.449 | 4.622 | 5.019 | 0.397 | 0.053 | 0.776 | 0.922 | Unannotated | NA | NA |
| metab_10078 | Unannotated feature | neg | 296.1141 | 4.458 | 4.602 | 4.463 | -0.139 | -0.053 | 0.776 | 0.922 | Unannotated | NA | NA |
| metab_2551 | Lotusine | pos | 353.1385 | 1.062 | 4.815 | 5.145 | 0.330 | 0.053 | 0.776 | 0.922 | Tentative | NA | NA |
| metab_8498 | Unannotated feature | pos | 215.1394 | 1.685 | 4.626 | 4.628 | 0.002 | -0.053 | 0.776 | 0.922 | Unannotated | NA | NA |
| metab_18288 | Unannotated feature | neg | 529.2845 | 6.217 | 6.349 | 6.339 | -0.010 | 0.053 | 0.776 | 0.922 | Unannotated | NA | NA |
| metab_12835 | Unannotated feature | neg | 149.0448 | 0.675 | 5.909 | 5.904 | -0.005 | -0.053 | 0.776 | 0.922 | Unannotated | NA | NA |
| metab_16190 | Unannotated feature | neg | 612.3585 | 6.397 | 5.149 | 5.072 | -0.076 | 0.053 | 0.776 | 0.922 | Unannotated | NA | NA |
| metab_11043 | Unannotated feature | neg | 322.0784 | 0.738 | 3.945 | 4.152 | 0.208 | -0.053 | 0.776 | 0.922 | Unannotated | NA | NA |
| metab_915 | Unannotated feature | pos | 750.5603 | 7.148 | 5.346 | 5.305 | -0.041 | -0.053 | 0.776 | 0.922 | Unannotated | NA | NA |
| metab_15883 | Unannotated feature | neg | 279.6142 | 5.975 | 4.095 | 4.069 | -0.026 | -0.053 | 0.776 | 0.922 | Unannotated | NA | NA |
| metab_14276 | Unannotated feature | neg | 164.9439 | 0.533 | 4.236 | 4.094 | -0.142 | -0.053 | 0.776 | 0.922 | Unannotated | NA | NA |
| metab_8762 | 5-Methyldeoxycytidine | pos | 274.1400 | 0.728 | 4.722 | 4.663 | -0.059 | 0.053 | 0.776 | 0.922 | Tentative | NA | NA |
| metab_8738 | N-a-Acetyl-L-arginine | pos | 217.1298 | 0.752 | 4.729 | 4.467 | -0.262 | -0.053 | 0.776 | 0.922 | Unannotated | NA | NA |
| metab_11599 | Unannotated feature | neg | 157.9076 | 0.449 | 3.291 | 3.295 | 0.003 | -0.053 | 0.776 | 0.922 | Unannotated | NA | NA |
| metab_12413 | Unannotated feature | neg | 184.9153 | 7.697 | 5.111 | 5.108 | -0.004 | 0.053 | 0.776 | 0.922 | Unannotated | NA | NA |
| metab_15100 | Unannotated feature | neg | 181.0136 | 4.522 | 5.006 | 4.844 | -0.162 | -0.053 | 0.776 | 0.922 | Unannotated | NA | NA |
| metab_2713 | Gamma-Glutamyl-beta-aminopropiononitrile | pos | 182.0928 | 2.116 | 3.314 | 3.562 | 0.248 | 0.053 | 0.776 | 0.922 | Tentative | NA | NA |
| metab_2644 | Glycerol alpha-monochlorohydrin | pos | 128.0476 | 1.936 | 3.183 | 3.316 | 0.133 | -0.053 | 0.776 | 0.922 | Tentative | NA | NA |
| metab_1313 | Unannotated feature | pos | 256.2274 | 6.231 | 3.758 | 3.739 | -0.019 | -0.053 | 0.776 | 0.922 | Unannotated | NA | NA |
| metab_16144 | Unannotated feature | neg | 540.7368 | 0.603 | 4.334 | 4.594 | 0.260 | 0.053 | 0.776 | 0.922 | Unannotated | NA | NA |
| metab_16130 | Unannotated feature | neg | 543.2999 | 6.279 | 5.252 | 5.014 | -0.238 | -0.053 | 0.776 | 0.922 | Unannotated | NA | NA |
| metab_18888 | Unannotated feature | neg | 649.2816 | 5.671 | 4.327 | 4.564 | 0.237 | 0.053 | 0.776 | 0.922 | Unannotated | NA | NA |
| metab_15071 | Unannotated feature | neg | 333.0988 | 4.480 | 4.423 | 4.487 | 0.064 | -0.053 | 0.776 | 0.922 | Unannotated | NA | NA |
| metab_939 | Unannotated feature | pos | 204.9543 | 7.530 | 4.930 | 4.911 | -0.019 | -0.053 | 0.776 | 0.922 | Unannotated | NA | NA |
| metab_929 | (+/-)-1,4-Nonanediol diacetate | pos | 245.1750 | 7.307 | 6.804 | 6.805 | 0.001 | 0.053 | 0.776 | 0.922 | Unannotated | NA | NA |
| metab_12446 | Unannotated feature | neg | 633.3821 | 6.514 | 5.366 | 5.537 | 0.171 | 0.053 | 0.776 | 0.922 | Unannotated | NA | NA |
| metab_921 | Unannotated feature | pos | 617.5123 | 7.132 | 6.044 | 5.987 | -0.057 | -0.053 | 0.776 | 0.922 | Unannotated | NA | NA |
| metab_2842 | Unannotated feature | pos | 472.3435 | 6.048 | 5.219 | 5.387 | 0.168 | -0.053 | 0.776 | 0.922 | Unannotated | NA | NA |
| metab_2784 | Guanidine, (4-chloro-3-methoxy-1-oxo-1H-2-benzopyran-7-yl)- | pos | 250.0391 | 2.551 | 4.497 | 4.447 | -0.050 | -0.053 | 0.776 | 0.922 | Unannotated | NA | NA |
| metab_8556 | Unannotated feature | pos | 215.1394 | 1.259 | 4.907 | 4.964 | 0.057 | 0.053 | 0.776 | 0.922 | Unannotated | NA | NA |
| metab_12721 | Chrysaloin | neg | 383.1122 | 5.118 | 4.597 | 4.568 | -0.029 | 0.053 | 0.776 | 0.922 | Unannotated | NA | NA |
| metab_11132 | Beta-N-Acetylglucosamine | neg | 266.0883 | 0.706 | 6.243 | 6.154 | -0.090 | -0.053 | 0.776 | 0.922 | Tentative | Organic oxygen compounds | Organooxygen compounds |
| metab_16103 | Unannotated feature | neg | 483.3329 | 6.240 | 5.625 | 5.667 | 0.042 | 0.053 | 0.776 | 0.922 | Unannotated | NA | NA |
| metab_12507 | Diisooctyl phthalate | neg | 435.2754 | 6.350 | 6.397 | 6.402 | 0.004 | -0.053 | 0.776 | 0.922 | Tentative | NA | NA |
| metab_1023 | Unannotated feature | pos | 693.5076 | 7.028 | 5.745 | 5.690 | -0.055 | 0.053 | 0.776 | 0.922 | Unannotated | NA | NA |
| metab_990 | Unannotated feature | pos | 181.0651 | 7.724 | 6.206 | 6.229 | 0.023 | 0.053 | 0.776 | 0.922 | Unannotated | NA | NA |
| metab_15947 | Unannotated feature | neg | 473.2197 | 6.046 | 4.905 | 4.790 | -0.116 | -0.053 | 0.776 | 0.922 | Unannotated | NA | NA |
| metab_15031 | Brassicanal A | neg | 222.0228 | 4.398 | 4.555 | 4.639 | 0.084 | 0.053 | 0.776 | 0.922 | Tentative | NA | NA |
| metab_3273 | Methylone | pos | 240.1234 | 3.961 | 4.719 | 4.688 | -0.032 | -0.053 | 0.776 | 0.922 | Tentative | NA | NA |
| metab_3246 | Pummeline | pos | 289.1188 | 3.854 | 5.039 | 5.187 | 0.148 | 0.053 | 0.776 | 0.922 | Unannotated | NA | NA |
| metab_3162 | PC(16:0/0:0) | pos | 518.3231 | 6.694 | 6.549 | 6.590 | 0.041 | -0.053 | 0.776 | 0.922 | Strong putative | NA | NA |
| metab_4795 | Unannotated feature | pos | 144.0658 | 7.983 | 4.174 | 4.188 | 0.013 | 0.053 | 0.776 | 0.922 | Unannotated | NA | NA |
| metab_16117 | 1-Dodecanesulfonic acid | neg | 559.3307 | 6.263 | 5.205 | 5.312 | 0.107 | -0.053 | 0.776 | 0.922 | Unannotated | NA | NA |
| metab_8601 | Unannotated feature | pos | 158.0926 | 1.133 | 4.599 | 4.548 | -0.052 | -0.053 | 0.776 | 0.922 | Unannotated | NA | NA |
| metab_16116 | 2,3-Secoporrigenin | neg | 505.2816 | 6.263 | 5.538 | 5.575 | 0.037 | -0.053 | 0.776 | 0.922 | Unannotated | NA | NA |
| metab_12594 | Unannotated feature | neg | 297.1707 | 6.083 | 5.325 | 5.466 | 0.140 | 0.053 | 0.776 | 0.922 | Unannotated | NA | NA |
| metab_11312 | Unannotated feature | neg | 550.7665 | 0.603 | 4.298 | 4.467 | 0.168 | 0.053 | 0.776 | 0.922 | Unannotated | NA | NA |
| metab_12585 | METHYL 4-FORMYLBENZOATE | neg | 327.0879 | 5.571 | 4.918 | 4.951 | 0.033 | -0.053 | 0.776 | 0.922 | Unannotated | NA | NA |
| metab_1135 | 1-(5Z,8Z,11Z,14Z-eicosatetraenoyl)-sn-glycero-3-phosphate | pos | 500.2751 | 6.574 | 5.837 | 5.936 | 0.099 | -0.053 | 0.776 | 0.922 | Unannotated | NA | NA |
| metab_12524 | Unannotated feature | neg | 553.3384 | 6.373 | 6.794 | 6.814 | 0.020 | -0.053 | 0.776 | 0.922 | Unannotated | NA | NA |
| metab_11447 | Unannotated feature | neg | 328.8992 | 0.533 | 5.518 | 5.697 | 0.178 | 0.053 | 0.776 | 0.922 | Unannotated | NA | NA |
| metab_11424 | Unannotated feature | neg | 296.8825 | 0.538 | 5.459 | 5.519 | 0.060 | 0.053 | 0.776 | 0.922 | Unannotated | NA | NA |
| metab_11417 | Unannotated feature | neg | 318.8702 | 0.542 | 5.123 | 5.074 | -0.049 | -0.053 | 0.776 | 0.922 | Unannotated | NA | NA |
| metab_5785 | Unannotated feature | pos | 547.3074 | 6.016 | 3.455 | 3.052 | -0.403 | -0.053 | 0.776 | 0.922 | Unannotated | NA | NA |
| metab_5761 | Pyroglutamyl-prolyl-arginine-4-nitroanilide | pos | 467.2176 | 6.040 | 5.315 | 4.971 | -0.344 | -0.053 | 0.776 | 0.922 | Unannotated | NA | NA |
| metab_12515 | Unannotated feature | neg | 423.3118 | 6.326 | 5.117 | 5.091 | -0.026 | -0.053 | 0.776 | 0.922 | Unannotated | NA | NA |
| metab_14346 | Unannotated feature | neg | 260.1335 | 3.116 | 3.957 | 3.939 | -0.018 | 0.053 | 0.776 | 0.922 | Unannotated | NA | NA |
| metab_3123 | 2-Hydroxyoctadecanoic acid | pos | 323.2565 | 6.526 | 4.448 | 4.339 | -0.109 | 0.053 | 0.776 | 0.922 | Tentative | NA | NA |
| metab_13615 | Unannotated feature | neg | 159.9360 | 1.501 | 3.334 | 3.370 | 0.036 | 0.053 | 0.776 | 0.922 | Unannotated | NA | NA |
| metab_4861 | Unannotated feature | pos | 320.1458 | 0.672 | 6.435 | 6.489 | 0.054 | 0.053 | 0.776 | 0.922 | Unannotated | NA | NA |
| metab_18720 | Unannotated feature | neg | 378.1197 | 5.888 | 4.501 | 4.955 | 0.454 | 0.053 | 0.776 | 0.922 | Unannotated | NA | NA |
| metab_18708 | (1R,2S,5S,6R,9S,10S)-3,6,17,20-Tetrahydroxy-6-(2-hydroxyacetyl)-1,5-dimethylpentacyclo[11.8.0.02,10.05,9.016,21]henicosa-13,16,18,20-tetraen-15-one | neg | 463.1770 | 5.896 | 3.698 | 3.622 | -0.076 | -0.053 | 0.776 | 0.922 | Unannotated | NA | NA |
| metab_18701 | Unannotated feature | neg | 498.1753 | 5.901 | 4.438 | 4.501 | 0.063 | -0.053 | 0.776 | 0.922 | Unannotated | NA | NA |
| metab_1096 | Unannotated feature | pos | 299.2584 | 6.670 | 5.955 | 5.941 | -0.014 | -0.053 | 0.776 | 0.922 | Unannotated | NA | NA |
| metab_54 | Imidazole-4-Acetic Acid | pos | 127.0506 | 0.680 | 4.382 | 4.156 | -0.227 | -0.053 | 0.776 | 0.922 | Moderate putative | NA | NA |
| metab_9033 | Unannotated feature | neg | 461.9980 | 4.028 | 6.470 | 6.664 | 0.194 | 0.053 | 0.776 | 0.922 | Unannotated | NA | NA |
| metab_9031 | 2,9-Dimethyl-2,9-diazatricyclo[10.2.2.25,8]octadeca-5,7,12,14,15,17-hexaene-3,10-diol, 9CI | neg | 333.1348 | 4.031 | 4.562 | 4.750 | 0.189 | 0.053 | 0.776 | 0.922 | Unannotated | NA | NA |
| metab_185 | Genipic acid | pos | 217.1075 | 4.491 | 4.730 | 4.731 | 0.001 | -0.053 | 0.776 | 0.922 | Unannotated | NA | NA |
| metab_19739 | Unannotated feature | neg | 357.0984 | 4.515 | 3.121 | 3.053 | -0.068 | 0.053 | 0.776 | 0.922 | Unannotated | NA | NA |
| metab_10518 | Unannotated feature | neg | 292.1042 | 1.947 | 6.110 | 6.163 | 0.053 | -0.053 | 0.776 | 0.922 | Unannotated | NA | NA |
| metab_13096 | Unannotated feature | neg | 470.2761 | 6.083 | 5.076 | 5.073 | -0.003 | -0.053 | 0.776 | 0.922 | Unannotated | NA | NA |
| metab_17821 | Unannotated feature | neg | 511.3098 | 6.805 | 7.093 | 7.100 | 0.007 | 0.053 | 0.776 | 0.922 | Unannotated | NA | NA |
| metab_15386 | Unannotated feature | neg | 408.0942 | 5.044 | 3.647 | 3.422 | -0.226 | 0.053 | 0.776 | 0.922 | Unannotated | NA | NA |
| metab_14144 | Unannotated feature | neg | 301.0678 | 2.880 | 4.375 | 4.448 | 0.073 | 0.053 | 0.776 | 0.922 | Unannotated | NA | NA |
| metab_9123 | Unannotated feature | neg | 438.1274 | 3.886 | 5.553 | 5.591 | 0.038 | -0.053 | 0.776 | 0.922 | Unannotated | NA | NA |
| metab_4508 | Unannotated feature | pos | 501.3563 | 6.638 | 6.663 | 6.707 | 0.044 | 0.053 | 0.776 | 0.922 | Unannotated | NA | NA |
| metab_224 | Feruloylcholine | pos | 298.1879 | 0.561 | 4.544 | 4.330 | -0.214 | -0.053 | 0.776 | 0.922 | Unannotated | NA | NA |
| metab_7062 | Unannotated feature | pos | 321.0589 | 0.657 | 4.586 | 4.575 | -0.012 | 0.053 | 0.776 | 0.922 | Unannotated | NA | NA |
| metab_186 | Unannotated feature | pos | 86.9091 | 0.601 | 3.760 | 3.721 | -0.038 | -0.053 | 0.776 | 0.922 | Unannotated | NA | NA |
| metab_15453 | Unannotated feature | neg | 257.8193 | 5.185 | 4.179 | 4.379 | 0.200 | -0.053 | 0.776 | 0.922 | Unannotated | NA | NA |
| metab_11998 | Unannotated feature | neg | 445.2362 | 6.232 | 5.815 | 5.846 | 0.031 | 0.053 | 0.776 | 0.922 | Unannotated | NA | NA |
| metab_19895 | Riboflavin | neg | 375.1314 | 3.697 | 5.509 | 5.495 | -0.015 | -0.053 | 0.776 | 0.922 | Strong putative | NA | NA |
| metab_3 | Unannotated feature | pos | 170.1178 | 4.290 | 5.027 | 5.015 | -0.012 | -0.048 | 0.794 | 0.928 | Unannotated | NA | NA |
| metab_7109 | Polyethylene, oxidized | pos | 209.1176 | 4.620 | 4.328 | 4.232 | -0.096 | -0.048 | 0.794 | 0.928 | Unannotated | Organic acids and derivatives | Keto acids and derivatives |
| metab_19658 | Caffeic acid ester | neg | 329.1035 | 4.634 | 5.895 | 5.990 | 0.095 | 0.048 | 0.794 | 0.928 | Tentative | Phenylpropanoids and polyketides | Cinnamic acids and derivatives |
| metab_4569 | DG(O-18:0/2:0/0:0) | pos | 431.3140 | 6.901 | 5.286 | 5.536 | 0.250 | -0.048 | 0.794 | 0.928 | Tentative | NA | NA |
| metab_9016 | Valproic acid | neg | 189.1127 | 4.049 | 4.252 | 4.339 | 0.087 | -0.048 | 0.794 | 0.928 | Tentative | NA | NA |
| metab_19612 | Unannotated feature | neg | 305.1596 | 4.696 | 3.833 | 3.940 | 0.107 | 0.048 | 0.794 | 0.928 | Unannotated | NA | NA |
| metab_17855 | Piperonyl sulfoxide | neg | 323.1688 | 6.734 | 5.203 | 5.229 | 0.026 | 0.048 | 0.794 | 0.928 | Tentative | Organoheterocyclic compounds | Benzodioxoles |
| metab_2108 | Unannotated feature | pos | 447.2152 | 5.260 | 3.627 | 3.791 | 0.164 | -0.048 | 0.794 | 0.928 | Unannotated | NA | NA |
| metab_11958 | Unannotated feature | neg | 436.2273 | 6.154 | 5.274 | 5.226 | -0.048 | -0.048 | 0.794 | 0.928 | Unannotated | NA | NA |
| metab_11962 | Unannotated feature | neg | 371.1529 | 6.170 | 4.705 | 4.558 | -0.147 | -0.048 | 0.794 | 0.928 | Unannotated | NA | NA |
| metab_105 | Unannotated feature | pos | 214.0592 | 0.641 | 6.003 | 6.097 | 0.094 | -0.048 | 0.794 | 0.928 | Unannotated | NA | NA |
| metab_146 | Unannotated feature | pos | 195.9776 | 0.617 | 4.768 | 4.807 | 0.039 | -0.048 | 0.794 | 0.928 | Unannotated | NA | NA |
| metab_9021 | Unannotated feature | neg | 290.1248 | 4.039 | 4.322 | 4.286 | -0.036 | 0.048 | 0.794 | 0.928 | Unannotated | NA | NA |
| metab_9028 | Unannotated feature | neg | 436.9755 | 4.031 | 4.523 | 4.810 | 0.287 | 0.048 | 0.794 | 0.928 | Unannotated | NA | NA |
| metab_9776 | Unannotated feature | neg | 170.9752 | 3.059 | 4.011 | 4.341 | 0.330 | 0.048 | 0.794 | 0.928 | Unannotated | NA | NA |
| metab_8681 | Unannotated feature | pos | 145.0175 | 0.831 | 4.559 | 4.534 | -0.025 | -0.048 | 0.794 | 0.928 | Unannotated | NA | NA |
| metab_1097 | DG(i-13:0/20:3(8Z,11Z,14Z)-2OH(5,6)/0:0) | pos | 631.4561 | 6.662 | 6.178 | 6.316 | 0.138 | -0.048 | 0.794 | 0.928 | Unannotated | NA | NA |
| metab_11329 | Unannotated feature | neg | 180.9725 | 0.603 | 4.330 | 4.490 | 0.161 | 0.048 | 0.794 | 0.928 | Unannotated | NA | NA |
| metab_1102 | Unannotated feature | pos | 433.2936 | 6.662 | 6.255 | 6.244 | -0.011 | 0.048 | 0.794 | 0.928 | Unannotated | NA | NA |
| metab_4902 | Unannotated feature | pos | 272.9448 | 7.689 | 4.887 | 4.878 | -0.009 | 0.048 | 0.794 | 0.928 | Unannotated | NA | NA |
| metab_4839 | Unannotated feature | pos | 192.9767 | 7.748 | 4.216 | 4.235 | 0.018 | 0.048 | 0.794 | 0.928 | Unannotated | NA | NA |
| metab_2932 | Unannotated feature | pos | 575.2572 | 6.183 | 4.905 | 4.874 | -0.031 | 0.048 | 0.794 | 0.928 | Unannotated | NA | NA |
| metab_17276 | Unannotated feature | neg | 306.9099 | 7.720 | 4.626 | 4.614 | -0.012 | -0.048 | 0.794 | 0.928 | Unannotated | NA | NA |
| metab_17285 | Unannotated feature | neg | 531.8846 | 7.714 | 5.277 | 5.295 | 0.018 | 0.048 | 0.794 | 0.928 | Unannotated | NA | NA |
| metab_17193 | Unannotated feature | neg | 409.8607 | 7.737 | 3.946 | 4.011 | 0.065 | 0.048 | 0.794 | 0.928 | Unannotated | NA | NA |
| metab_16025 | Unannotated feature | neg | 547.2761 | 6.146 | 3.998 | 4.094 | 0.096 | 0.048 | 0.794 | 0.928 | Unannotated | NA | NA |
| metab_11379 | Unannotated feature | neg | 443.0106 | 3.822 | 5.977 | 6.170 | 0.192 | 0.048 | 0.794 | 0.928 | Unannotated | NA | NA |
| metab_8669 | Unannotated feature | pos | 153.9981 | 0.897 | 4.415 | 4.499 | 0.084 | 0.048 | 0.794 | 0.928 | Unannotated | NA | NA |
| metab_8677 | Unannotated feature | pos | 223.0602 | 0.846 | 4.521 | 4.478 | -0.043 | -0.048 | 0.794 | 0.928 | Unannotated | NA | NA |
| metab_2996 | 1-Naphthylamine | pos | 144.0810 | 3.109 | 5.387 | 5.441 | 0.053 | 0.048 | 0.794 | 0.928 | Moderate putative | NA | NA |
| metab_13689 | Unannotated feature | neg | 373.1257 | 1.691 | 3.348 | 3.463 | 0.115 | -0.048 | 0.794 | 0.928 | Unannotated | NA | NA |
| metab_16113 | Unannotated feature | neg | 301.1559 | 6.263 | 5.158 | 4.927 | -0.232 | -0.048 | 0.794 | 0.928 | Unannotated | NA | NA |
| metab_18617 | Unannotated feature | neg | 247.0826 | 5.975 | 5.126 | 5.156 | 0.030 | 0.048 | 0.794 | 0.928 | Unannotated | NA | NA |
| metab_11296 | Unannotated feature | neg | 255.0885 | 3.681 | 5.521 | 5.541 | 0.021 | 0.048 | 0.794 | 0.928 | Unannotated | NA | NA |
| metab_18602 | Bitolterol | neg | 506.2164 | 5.983 | 4.757 | 4.645 | -0.112 | 0.048 | 0.794 | 0.928 | Tentative | NA | NA |
| metab_11289 | Unannotated feature | neg | 337.9094 | 0.614 | 4.140 | 4.215 | 0.075 | -0.048 | 0.794 | 0.928 | Unannotated | NA | NA |
| metab_12566 | Unannotated feature | neg | 367.1587 | 6.154 | 6.568 | 6.523 | -0.045 | 0.048 | 0.794 | 0.928 | Unannotated | NA | NA |
| metab_2899 | Unannotated feature | pos | 235.1445 | 2.853 | 3.912 | 4.033 | 0.121 | 0.048 | 0.794 | 0.928 | Unannotated | NA | NA |
| metab_14352 | Unannotated feature | neg | 269.1509 | 3.123 | 2.986 | 3.248 | 0.263 | 0.048 | 0.794 | 0.928 | Unannotated | NA | NA |
| metab_1180 | Unannotated feature | pos | 339.2149 | 6.502 | 4.951 | 4.955 | 0.003 | -0.048 | 0.794 | 0.928 | Unannotated | NA | NA |
| metab_9601 | Unannotated feature | neg | 370.9584 | 3.227 | 5.041 | 4.987 | -0.054 | -0.048 | 0.794 | 0.928 | Unannotated | NA | NA |
| metab_15025 | Plafibride | neg | 354.1198 | 4.386 | 4.027 | 3.901 | -0.126 | -0.048 | 0.794 | 0.928 | Unannotated | NA | NA |
| metab_1020 | Unannotated feature | pos | 518.3231 | 7.060 | 6.576 | 6.393 | -0.183 | -0.048 | 0.794 | 0.928 | Unannotated | NA | NA |
| metab_4830 | Unannotated feature | pos | 153.0112 | 7.780 | 4.955 | 4.966 | 0.011 | 0.048 | 0.794 | 0.928 | Unannotated | NA | NA |
| metab_17237 | Unannotated feature | neg | 249.9068 | 2.413 | 5.565 | 5.553 | -0.012 | -0.048 | 0.794 | 0.928 | Unannotated | NA | NA |
| metab_17213 | Unannotated feature | neg | 339.8771 | 7.730 | 4.965 | 4.965 | 0.001 | -0.048 | 0.794 | 0.928 | Unannotated | NA | NA |
| metab_9706 | Unannotated feature | neg | 428.1321 | 3.120 | 4.266 | 4.335 | 0.068 | -0.048 | 0.794 | 0.928 | Unannotated | NA | NA |
| metab_18559 | Unannotated feature | neg | 355.2237 | 6.013 | 2.680 | 2.746 | 0.066 | -0.048 | 0.794 | 0.928 | Unannotated | NA | NA |
| metab_12659 | Unannotated feature | neg | 601.3068 | 5.914 | 5.242 | 5.269 | 0.027 | -0.048 | 0.794 | 0.928 | Unannotated | NA | NA |
| metab_1225 | Unannotated feature | pos | 288.2901 | 6.398 | 6.366 | 6.414 | 0.048 | 0.048 | 0.794 | 0.928 | Unannotated | NA | NA |
| metab_18511 | 17-Dimethylaminogeldanamycin | neg | 637.3253 | 6.041 | 3.794 | 4.378 | 0.584 | 0.048 | 0.794 | 0.928 | Unannotated | NA | NA |
| metab_18517 | Unannotated feature | neg | 299.1290 | 6.041 | 4.815 | 4.577 | -0.238 | 0.048 | 0.794 | 0.928 | Unannotated | NA | NA |
| metab_7941 | Unannotated feature | pos | 359.1934 | 2.907 | 4.075 | 3.978 | -0.096 | 0.048 | 0.794 | 0.928 | Unannotated | NA | NA |
| metab_1278 | Unannotated feature | pos | 260.2587 | 6.295 | 5.032 | 4.815 | -0.216 | -0.048 | 0.794 | 0.928 | Unannotated | NA | NA |
| metab_8580 | Unannotated feature | pos | 199.1082 | 1.189 | 4.447 | 4.341 | -0.106 | -0.048 | 0.794 | 0.928 | Unannotated | NA | NA |
| metab_2796 | Unannotated feature | pos | 144.0811 | 2.625 | 4.168 | 4.137 | -0.031 | -0.048 | 0.794 | 0.928 | Unannotated | NA | NA |
| metab_13484 | Unannotated feature | neg | 165.0221 | 1.015 | 4.497 | 4.437 | -0.060 | -0.048 | 0.794 | 0.928 | Unannotated | NA | NA |
| metab_18945 | Unannotated feature | neg | 80.9739 | 5.590 | 4.279 | 4.262 | -0.017 | -0.048 | 0.794 | 0.928 | Unannotated | NA | NA |
| metab_11490 | Unannotated feature | neg | 525.0094 | 0.516 | 4.027 | 3.922 | -0.105 | 0.048 | 0.794 | 0.928 | Unannotated | NA | NA |
| metab_5942 | Unannotated feature | pos | 220.0397 | 0.402 | 6.008 | 6.012 | 0.004 | 0.048 | 0.794 | 0.928 | Unannotated | NA | NA |
| metab_7652 | Unannotated feature | pos | 98.9847 | 0.744 | 4.030 | 4.315 | 0.285 | 0.048 | 0.794 | 0.928 | Unannotated | NA | NA |
| metab_7657 | Unannotated feature | pos | 271.1024 | 3.444 | 4.770 | 4.749 | -0.021 | -0.048 | 0.794 | 0.928 | Unannotated | NA | NA |
| metab_960 | Unannotated feature | pos | 228.1209 | 7.610 | 5.007 | 5.017 | 0.010 | -0.048 | 0.794 | 0.928 | Unannotated | NA | NA |
| metab_8684 | 3-Aminopropionaldehyde | pos | 74.0606 | 0.815 | 4.827 | 4.820 | -0.008 | 0.048 | 0.794 | 0.928 | Tentative | NA | NA |
| metab_14290 | Unannotated feature | neg | 300.0550 | 3.037 | 3.186 | 3.581 | 0.395 | 0.048 | 0.794 | 0.928 | Unannotated | NA | NA |
| metab_4791 | Unannotated feature | pos | 169.9776 | 7.941 | 5.007 | 4.995 | -0.012 | -0.048 | 0.794 | 0.928 | Unannotated | NA | NA |
| metab_4793 | Unannotated feature | pos | 472.4377 | 7.971 | 6.052 | 6.029 | -0.023 | -0.048 | 0.794 | 0.928 | Unannotated | NA | NA |
| metab_13562 | Unannotated feature | neg | 296.9733 | 1.209 | 4.569 | 4.593 | 0.024 | 0.048 | 0.794 | 0.928 | Unannotated | NA | NA |
| metab_12726 | Unannotated feature | neg | 418.0789 | 4.903 | 3.545 | 3.714 | 0.169 | 0.048 | 0.794 | 0.928 | Unannotated | NA | NA |
| metab_8000 | Unannotated feature | pos | 233.1288 | 2.840 | 3.930 | 3.952 | 0.022 | 0.048 | 0.794 | 0.928 | Unannotated | NA | NA |
| metab_5519 | Glycyrrhetinic acid | pos | 488.3748 | 6.319 | 3.064 | 3.173 | 0.109 | 0.048 | 0.794 | 0.928 | Tentative | NA | NA |
| metab_1343 | Ganosporelactone B | pos | 559.2623 | 6.167 | 4.094 | 4.265 | 0.171 | 0.048 | 0.794 | 0.928 | Unannotated | Benzenoids | Benzene and substituted derivatives |
| metab_1379 | Unannotated feature | pos | 253.1426 | 6.119 | 4.615 | 4.514 | -0.101 | -0.048 | 0.794 | 0.928 | Unannotated | NA | NA |
| metab_2588 | QUISQUALIC ACID | pos | 154.0249 | 1.333 | 4.658 | 4.624 | -0.033 | -0.048 | 0.794 | 0.928 | Tentative | NA | NA |
| metab_2600 | Unannotated feature | pos | 229.0554 | 1.622 | 4.717 | 4.756 | 0.040 | 0.048 | 0.794 | 0.928 | Unannotated | NA | NA |
| metab_14434 | Unannotated feature | neg | 269.1143 | 3.242 | 3.302 | 3.265 | -0.037 | -0.048 | 0.794 | 0.928 | Unannotated | NA | NA |
| metab_17415 | Unannotated feature | neg | 468.8973 | 7.641 | 6.207 | 6.235 | 0.028 | 0.048 | 0.794 | 0.928 | Unannotated | NA | NA |
| metab_10017 | 2-Methylidene-4-(oxiran-2-yl)butanoic Acid | neg | 123.0445 | 2.826 | 4.849 | 4.836 | -0.013 | 0.048 | 0.794 | 0.928 | Unannotated | NA | NA |
| metab_6200 | Unannotated feature | pos | 406.8267 | 0.531 | 3.952 | 4.382 | 0.430 | 0.048 | 0.794 | 0.928 | Unannotated | NA | NA |
| metab_11669 | Unannotated feature | neg | 169.1025 | 2.244 | 5.396 | 5.347 | -0.049 | -0.048 | 0.794 | 0.928 | Unannotated | NA | NA |
| metab_19013 | 7-Epijasmonic acid | neg | 255.1235 | 5.515 | 5.040 | 4.877 | -0.163 | -0.048 | 0.794 | 0.928 | Tentative | NA | NA |
| metab_14304 | Unannotated feature | neg | 83.0493 | 3.059 | 3.690 | 3.921 | 0.231 | 0.048 | 0.794 | 0.928 | Unannotated | NA | NA |
| metab_8764 | Unannotated feature | pos | 271.0816 | 0.728 | 3.702 | 3.953 | 0.252 | 0.048 | 0.794 | 0.928 | Unannotated | NA | NA |
| metab_4774 | Unannotated feature | pos | 243.0431 | 7.764 | 4.496 | 4.543 | 0.047 | 0.048 | 0.794 | 0.928 | Unannotated | NA | NA |
| metab_17117 | Unannotated feature | neg | 239.9494 | 7.835 | 4.937 | 4.918 | -0.020 | -0.048 | 0.794 | 0.928 | Unannotated | NA | NA |
| metab_9583 | Unannotated feature | neg | 261.1341 | 3.239 | 3.934 | 3.761 | -0.173 | -0.048 | 0.794 | 0.928 | Unannotated | NA | NA |
| metab_8052 | Unannotated feature | pos | 281.1136 | 2.774 | 4.445 | 4.273 | -0.172 | -0.048 | 0.794 | 0.928 | Unannotated | NA | NA |
| metab_16173 | Unannotated feature | neg | 280.0688 | 1.237 | 3.848 | 4.339 | 0.491 | 0.048 | 0.794 | 0.928 | Unannotated | NA | NA |
| metab_12782 | Unannotated feature | neg | 118.0503 | 0.636 | 6.089 | 6.080 | -0.009 | -0.048 | 0.794 | 0.928 | Unannotated | NA | NA |
| metab_8091 | Diaminopimelic acid | pos | 173.0924 | 2.747 | 3.871 | 3.818 | -0.053 | -0.048 | 0.794 | 0.928 | Tentative | NA | NA |
| metab_14764 | Unannotated feature | neg | 133.0652 | 3.829 | 3.643 | 3.750 | 0.107 | -0.048 | 0.794 | 0.928 | Unannotated | NA | NA |
| metab_12806 | Unannotated feature | neg | 319.9260 | 0.644 | 3.945 | 3.927 | -0.017 | -0.048 | 0.794 | 0.928 | Unannotated | NA | NA |
| metab_18287 | Unannotated feature | neg | 575.3265 | 6.217 | 4.766 | 4.753 | -0.013 | -0.048 | 0.794 | 0.928 | Unannotated | NA | NA |
| metab_8505 | Unannotated feature | pos | 582.2831 | 3.970 | 3.844 | 3.715 | -0.129 | -0.048 | 0.794 | 0.928 | Unannotated | NA | NA |
| metab_13399 | Dihydrozeatin-O-glucoside | neg | 418.1476 | 0.770 | 4.215 | 4.115 | -0.101 | -0.048 | 0.794 | 0.928 | Tentative | NA | NA |
| metab_9413 | Unannotated feature | neg | 103.0029 | 3.457 | 3.292 | 3.277 | -0.016 | -0.048 | 0.794 | 0.928 | Unannotated | NA | NA |
| metab_7495 | Unannotated feature | pos | 243.1344 | 3.761 | 4.593 | 4.568 | -0.025 | -0.048 | 0.794 | 0.928 | Unannotated | NA | NA |
| metab_11727 | Unannotated feature | neg | 351.0845 | 5.576 | 4.842 | 4.583 | -0.259 | 0.048 | 0.794 | 0.928 | Unannotated | NA | NA |
| metab_11687 | Unannotated feature | neg | 441.0137 | 4.028 | 6.295 | 6.486 | 0.190 | 0.048 | 0.794 | 0.928 | Unannotated | NA | NA |
| metab_3395 | 3,4-Methylenesebacic acid | pos | 209.1176 | 4.389 | 4.918 | 4.966 | 0.048 | 0.048 | 0.794 | 0.928 | Unannotated | NA | NA |
| metab_12372 | Unannotated feature | neg | 356.2807 | 6.758 | 5.149 | 5.188 | 0.039 | 0.048 | 0.794 | 0.928 | Unannotated | NA | NA |
| metab_16562 | Unannotated feature | neg | 100.9512 | 7.305 | 4.898 | 4.901 | 0.003 | 0.048 | 0.794 | 0.928 | Unannotated | NA | NA |
| metab_16517 | Unannotated feature | neg | 561.2823 | 7.135 | 5.363 | 5.272 | -0.091 | -0.048 | 0.794 | 0.928 | Unannotated | NA | NA |
| metab_16538 | Unannotated feature | neg | 377.0820 | 1.695 | 4.732 | 4.670 | -0.062 | -0.048 | 0.794 | 0.928 | Unannotated | NA | NA |
| metab_16584 | Unannotated feature | neg | 479.3208 | 7.343 | 6.173 | 6.096 | -0.077 | -0.048 | 0.794 | 0.928 | Unannotated | NA | NA |
| metab_17536 | Unannotated feature | neg | 268.9683 | 7.402 | 5.631 | 5.634 | 0.003 | 0.048 | 0.794 | 0.928 | Unannotated | NA | NA |
| metab_8139 | Unannotated feature | pos | 223.1445 | 2.724 | 4.286 | 4.644 | 0.359 | 0.048 | 0.794 | 0.928 | Unannotated | NA | NA |
| metab_8144 | Unannotated feature | pos | 460.2781 | 3.574 | 3.948 | 3.741 | -0.207 | -0.048 | 0.794 | 0.928 | Unannotated | NA | NA |
| metab_12242 | Unannotated feature | neg | 321.2106 | 6.965 | 6.091 | 6.128 | 0.037 | 0.048 | 0.794 | 0.928 | Unannotated | NA | NA |
| metab_12250 | Unannotated feature | neg | 521.3833 | 6.988 | 5.007 | 5.038 | 0.031 | 0.048 | 0.794 | 0.928 | Unannotated | NA | NA |
| metab_19388 | Unannotated feature | neg | 274.0335 | 3.396 | 3.334 | 3.497 | 0.163 | -0.048 | 0.794 | 0.928 | Unannotated | NA | NA |
| metab_11795 | Enterodiol | neg | 301.1447 | 5.808 | 5.264 | 5.336 | 0.072 | -0.048 | 0.794 | 0.928 | Strong putative | NA | NA |
| metab_19294 | Unannotated feature | neg | 210.1132 | 5.152 | 4.627 | 4.698 | 0.071 | -0.048 | 0.794 | 0.928 | Unannotated | NA | NA |
| metab_4735 | 5-Hydroxyproline | pos | 164.0921 | 7.674 | 5.092 | 5.110 | 0.018 | 0.048 | 0.794 | 0.928 | Unannotated | NA | NA |
| metab_9473 | Glutamyllysine | neg | 320.1464 | 3.379 | 3.620 | 3.603 | -0.018 | 0.048 | 0.794 | 0.928 | Unannotated | NA | NA |
| metab_12868 | Unannotated feature | neg | 539.1399 | 0.706 | 5.695 | 5.752 | 0.057 | 0.048 | 0.794 | 0.928 | Unannotated | NA | NA |
| metab_18143 | Unannotated feature | neg | 255.0512 | 2.938 | 5.132 | 5.050 | -0.082 | -0.048 | 0.794 | 0.928 | Unannotated | NA | NA |
| metab_18155 | Homodolicholide | neg | 537.3448 | 6.334 | 5.379 | 5.171 | -0.208 | -0.048 | 0.794 | 0.928 | Unannotated | NA | NA |
| metab_18107 | Unannotated feature | neg | 565.3755 | 6.381 | 6.001 | 6.041 | 0.040 | -0.048 | 0.794 | 0.928 | Unannotated | NA | NA |
| metab_12921 | Unannotated feature | neg | 155.0091 | 0.738 | 6.121 | 6.212 | 0.091 | -0.048 | 0.794 | 0.928 | Unannotated | NA | NA |
| metab_1831 | Nalpha-Acetyl-L-arginine | pos | 217.1298 | 1.018 | 4.224 | 4.097 | -0.126 | -0.048 | 0.794 | 0.928 | Moderate putative | NA | NA |
| metab_10717 | Unannotated feature | neg | 312.9797 | 1.343 | 4.526 | 4.892 | 0.366 | 0.048 | 0.794 | 0.928 | Unannotated | NA | NA |
| metab_2421 | Alanyl-Gamma-glutamate | pos | 182.0927 | 0.617 | 3.271 | 3.668 | 0.397 | 0.048 | 0.794 | 0.928 | Unannotated | NA | NA |
| metab_16826 | Unannotated feature | neg | 145.0612 | 1.981 | 4.008 | 4.018 | 0.010 | -0.048 | 0.794 | 0.928 | Unannotated | NA | NA |
| metab_16832 | Unannotated feature | neg | 392.8702 | 7.726 | 4.965 | 4.974 | 0.009 | -0.048 | 0.794 | 0.928 | Unannotated | NA | NA |
| metab_9241 | Unannotated feature | neg | 321.0996 | 3.701 | 3.357 | 3.606 | 0.249 | 0.048 | 0.794 | 0.928 | Unannotated | NA | NA |
| metab_15264 | Unannotated feature | neg | 270.0084 | 0.558 | 3.968 | 3.772 | -0.197 | -0.048 | 0.794 | 0.928 | Unannotated | NA | NA |
| metab_6765 | Unannotated feature | pos | 659.3582 | 5.223 | 4.634 | 4.618 | -0.016 | -0.048 | 0.794 | 0.928 | Unannotated | NA | NA |
| metab_15287 | Unannotated feature | neg | 275.1139 | 4.857 | 3.955 | 3.713 | -0.242 | -0.048 | 0.794 | 0.928 | Unannotated | NA | NA |
| metab_433 | Unannotated feature | pos | 127.9802 | 0.016 | 3.416 | 3.388 | -0.028 | 0.048 | 0.794 | 0.928 | Unannotated | NA | NA |
| metab_13909 | Unannotated feature | neg | 340.0317 | 2.517 | 5.394 | 5.385 | -0.009 | 0.048 | 0.794 | 0.928 | Unannotated | NA | NA |
| metab_7383 | Unannotated feature | pos | 170.1178 | 4.002 | 3.572 | 3.746 | 0.174 | 0.048 | 0.794 | 0.928 | Unannotated | NA | NA |
| metab_2426 | Unannotated feature | pos | 263.9266 | 0.617 | 3.888 | 4.153 | 0.265 | -0.048 | 0.794 | 0.928 | Unannotated | NA | NA |
| metab_14473 | Pivagabine | neg | 232.1189 | 3.314 | 4.898 | 4.817 | -0.081 | -0.048 | 0.794 | 0.928 | Unannotated | NA | NA |
| metab_10677 | Unannotated feature | neg | 333.1309 | 1.508 | 4.844 | 4.507 | -0.338 | -0.048 | 0.794 | 0.928 | Unannotated | NA | NA |
| metab_18043 | Unannotated feature | neg | 266.1034 | 6.451 | 4.700 | 4.762 | 0.062 | 0.048 | 0.794 | 0.928 | Unannotated | NA | NA |
| metab_18047 | Cervonoyl ethanolamide | neg | 417.2653 | 6.444 | 5.311 | 5.334 | 0.023 | 0.048 | 0.794 | 0.928 | Tentative | NA | NA |
| metab_17603 | DG(PGE2/20:0/0:0) | neg | 741.5292 | 7.212 | 6.510 | 6.444 | -0.066 | 0.048 | 0.794 | 0.928 | Unannotated | NA | NA |
| metab_10278 | Unannotated feature | neg | 147.0114 | 2.476 | 4.605 | 4.591 | -0.014 | 0.048 | 0.794 | 0.928 | Unannotated | NA | NA |
| metab_4481 | Unannotated feature | pos | 375.2515 | 6.526 | 5.608 | 5.587 | -0.021 | -0.048 | 0.794 | 0.928 | Unannotated | NA | NA |
| metab_15629 | 6-keto PGE1 | neg | 367.2130 | 5.550 | 5.526 | 5.361 | -0.165 | -0.048 | 0.794 | 0.928 | Moderate putative | NA | NA |
| metab_15632 | Unannotated feature | neg | 605.2758 | 5.554 | 3.196 | 3.299 | 0.103 | -0.048 | 0.794 | 0.928 | Unannotated | NA | NA |
| metab_15319 | 2-(1-Phenylpropan-2-yl)-1,3,2-dioxazetidine | neg | 224.0926 | 4.927 | 4.405 | 4.404 | -0.000 | -0.048 | 0.794 | 0.928 | Unannotated | NA | NA |
| metab_15323 | Isopentenyladenine | neg | 202.1081 | 4.932 | 3.750 | 3.502 | -0.248 | 0.048 | 0.794 | 0.928 | Moderate putative | NA | NA |
| metab_11853 | Unannotated feature | neg | 303.1717 | 5.975 | 4.309 | 4.308 | -0.001 | -0.048 | 0.794 | 0.928 | Unannotated | NA | NA |
| metab_11854 | Unannotated feature | neg | 375.2029 | 5.975 | 5.592 | 5.402 | -0.190 | -0.048 | 0.794 | 0.928 | Unannotated | NA | NA |
| metab_19416 | Unannotated feature | neg | 319.1756 | 4.992 | 4.696 | 4.780 | 0.085 | -0.048 | 0.794 | 0.928 | Unannotated | NA | NA |
| metab_16993 | Unannotated feature | neg | 268.9785 | 2.137 | 4.587 | 4.717 | 0.130 | 0.048 | 0.794 | 0.928 | Unannotated | NA | NA |
| metab_8932 | Unannotated feature | neg | 181.0137 | 4.216 | 4.785 | 4.654 | -0.131 | -0.048 | 0.794 | 0.928 | Unannotated | NA | NA |
| metab_15390 | Unannotated feature | neg | 248.8962 | 0.566 | 4.425 | 4.278 | -0.147 | -0.048 | 0.794 | 0.928 | Unannotated | NA | NA |
| metab_12114 | Unannotated feature | neg | 589.3044 | 6.530 | 6.369 | 6.568 | 0.199 | -0.048 | 0.794 | 0.928 | Unannotated | NA | NA |
| metab_19542 | Unannotated feature | neg | 317.1512 | 4.785 | 4.002 | 4.192 | 0.191 | 0.048 | 0.794 | 0.928 | Unannotated | NA | NA |
| metab_11874 | Unannotated feature | neg | 351.1916 | 6.013 | 4.468 | 4.458 | -0.010 | -0.048 | 0.794 | 0.928 | Unannotated | NA | NA |
| metab_12134 | 4-Hydroxy-3-(16-methylheptadecyl)-2H-pyran-2-one | neg | 409.2962 | 6.601 | 5.719 | 5.778 | 0.059 | 0.048 | 0.794 | 0.928 | Unannotated | NA | NA |
| metab_15609 | Unannotated feature | neg | 351.1925 | 5.519 | 4.451 | 4.160 | -0.291 | -0.048 | 0.794 | 0.928 | Unannotated | NA | NA |
| metab_19444 | Unannotated feature | neg | 319.1671 | 4.949 | 3.618 | 3.971 | 0.352 | 0.048 | 0.794 | 0.928 | Unannotated | NA | NA |
| metab_9249 | Unannotated feature | neg | 438.1273 | 3.693 | 5.174 | 5.205 | 0.031 | 0.048 | 0.794 | 0.928 | Unannotated | NA | NA |
| metab_1934 | Unannotated feature | pos | 100.0249 | 0.485 | 5.676 | 5.597 | -0.079 | -0.048 | 0.794 | 0.928 | Unannotated | NA | NA |
| metab_8283 | Unannotated feature | pos | 815.2160 | 2.133 | 5.326 | 5.323 | -0.003 | 0.048 | 0.794 | 0.928 | Unannotated | NA | NA |
| metab_1973 | 4-Hydroxy-3-methoxy-cinnamoylglycine | pos | 269.1135 | 2.032 | 5.220 | 5.341 | 0.121 | 0.048 | 0.794 | 0.928 | Tentative | NA | NA |
| metab_16373 | Unannotated feature | neg | 519.4062 | 6.758 | 5.851 | 5.798 | -0.053 | -0.048 | 0.794 | 0.928 | Unannotated | NA | NA |
| metab_2008 | Unannotated feature | pos | 119.0735 | 2.299 | 4.251 | 4.245 | -0.006 | -0.048 | 0.794 | 0.928 | Unannotated | NA | NA |
| metab_13076 | Unannotated feature | neg | 301.9823 | 1.041 | 5.357 | 5.414 | 0.057 | 0.048 | 0.794 | 0.928 | Unannotated | NA | NA |
| metab_5186 | Unannotated feature | pos | 426.2987 | 6.789 | 3.962 | 3.981 | 0.019 | -0.048 | 0.794 | 0.928 | Unannotated | NA | NA |
| metab_2264 | Unannotated feature | pos | 160.0759 | 3.113 | 4.463 | 4.322 | -0.141 | -0.048 | 0.794 | 0.928 | Unannotated | NA | NA |
| metab_13193 | Unannotated feature | neg | 424.9090 | 0.516 | 5.911 | 5.931 | 0.021 | 0.048 | 0.794 | 0.928 | Unannotated | NA | NA |
| metab_13194 | Unannotated feature | neg | 147.9472 | 0.520 | 3.422 | 3.363 | -0.059 | -0.048 | 0.794 | 0.928 | Unannotated | NA | NA |
| metab_5261 | Pregnenolone | pos | 349.2739 | 6.646 | 5.073 | 5.085 | 0.011 | 0.048 | 0.794 | 0.928 | Tentative | Lipids and lipid-like molecules | Steroids and steroid derivatives |
| metab_1 | Unannotated feature | pos | 239.1394 | 1.952 | 5.158 | 5.161 | 0.002 | -0.044 | 0.813 | 0.934 | Unannotated | NA | NA |
| metab_12033 | Unannotated feature | neg | 490.2428 | 6.295 | 4.578 | 4.706 | 0.128 | 0.044 | 0.813 | 0.934 | Unannotated | NA | NA |
| metab_16905 | Unannotated feature | neg | 222.0405 | 2.036 | 4.556 | 4.628 | 0.071 | 0.044 | 0.813 | 0.934 | Unannotated | NA | NA |
| metab_16899 | Unannotated feature | neg | 421.2970 | 7.750 | 4.617 | 4.597 | -0.020 | -0.044 | 0.813 | 0.934 | Unannotated | NA | NA |
| metab_191 | Unannotated feature | pos | 292.8457 | 0.593 | 4.609 | 4.927 | 0.318 | 0.044 | 0.813 | 0.934 | Unannotated | NA | NA |
| metab_19757 | Unannotated feature | neg | 184.0973 | 4.498 | 4.826 | 4.750 | -0.076 | -0.044 | 0.813 | 0.934 | Unannotated | NA | NA |
| metab_11953 | W123 | neg | 305.1870 | 6.146 | 3.960 | 4.026 | 0.066 | 0.044 | 0.813 | 0.934 | Moderate putative | NA | NA |
| metab_19766 | Unannotated feature | neg | 389.1248 | 4.480 | 3.872 | 3.747 | -0.126 | -0.044 | 0.813 | 0.934 | Unannotated | NA | NA |
| metab_12036 | Unannotated feature | neg | 427.2165 | 6.303 | 6.568 | 6.635 | 0.067 | 0.044 | 0.813 | 0.934 | Unannotated | NA | NA |
| metab_11946 | Unannotated feature | neg | 427.1803 | 6.138 | 5.804 | 5.764 | -0.040 | 0.044 | 0.813 | 0.934 | Unannotated | NA | NA |
| metab_19643 | Epsilon-Caprolactone | neg | 159.0656 | 4.653 | 6.691 | 6.756 | 0.065 | 0.044 | 0.813 | 0.934 | Tentative | NA | NA |
| metab_12097 | Unannotated feature | neg | 425.2915 | 6.420 | 6.601 | 6.585 | -0.016 | -0.044 | 0.813 | 0.934 | Unannotated | NA | NA |
| metab_19684 | Dehydroxymethylflazine | neg | 323.0677 | 4.592 | 4.626 | 4.642 | 0.017 | 0.044 | 0.813 | 0.934 | Unannotated | NA | NA |
| metab_7224 | Unannotated feature | pos | 148.0970 | 4.383 | 4.111 | 4.226 | 0.115 | 0.044 | 0.813 | 0.934 | Unannotated | NA | NA |
| metab_7007 | Lysyl-Glycine | pos | 186.1240 | 0.657 | 3.890 | 3.871 | -0.019 | -0.044 | 0.813 | 0.934 | Unannotated | NA | NA |
| metab_11863 | Dihydroisorhamnetin | neg | 299.0562 | 5.999 | 4.487 | 4.287 | -0.200 | 0.044 | 0.813 | 0.934 | Unannotated | NA | NA |
| metab_342 | Unannotated feature | pos | 130.9793 | 0.435 | 4.008 | 4.001 | -0.007 | 0.044 | 0.813 | 0.934 | Unannotated | NA | NA |
| metab_11869 | Unannotated feature | neg | 635.3098 | 6.007 | 4.030 | 3.983 | -0.046 | -0.044 | 0.813 | 0.934 | Unannotated | NA | NA |
| metab_16898 | Unannotated feature | neg | 310.8580 | 7.750 | 4.062 | 4.060 | -0.001 | -0.044 | 0.813 | 0.934 | Unannotated | NA | NA |
| metab_14146 | Unannotated feature | neg | 83.0493 | 2.885 | 2.953 | 3.435 | 0.482 | 0.044 | 0.813 | 0.934 | Unannotated | NA | NA |
| metab_321 | Unannotated feature | pos | 249.1551 | 0.477 | 5.243 | 5.184 | -0.059 | -0.044 | 0.813 | 0.934 | Unannotated | NA | NA |
| metab_360 | Unannotated feature | pos | 503.1090 | 0.360 | 5.477 | 5.481 | 0.004 | -0.044 | 0.813 | 0.934 | Unannotated | NA | NA |
| metab_15582 | Vulgarin | neg | 285.1135 | 5.465 | 5.634 | 5.118 | -0.516 | -0.044 | 0.813 | 0.934 | Tentative | NA | NA |
| metab_15575 | Unannotated feature | neg | 183.1246 | 0.582 | 4.361 | 4.633 | 0.272 | 0.044 | 0.813 | 0.934 | Unannotated | NA | NA |
| metab_6788 | 4-Hydroxyhexanoycarnitine | pos | 258.1704 | 5.193 | 3.805 | 3.304 | -0.501 | -0.044 | 0.813 | 0.934 | Unannotated | NA | NA |
| metab_19449 | Unannotated feature | neg | 392.0994 | 4.941 | 4.925 | 5.265 | 0.341 | -0.044 | 0.813 | 0.934 | Unannotated | NA | NA |
| metab_7313 | Unannotated feature | pos | 203.0343 | 4.166 | 4.154 | 4.146 | -0.009 | 0.044 | 0.813 | 0.934 | Unannotated | NA | NA |
| metab_7307 | Ile Trp | pos | 318.1820 | 4.179 | 3.869 | 3.716 | -0.154 | 0.044 | 0.813 | 0.934 | Strong putative | NA | NA |
| metab_423 | Unannotated feature | pos | 154.0155 | 0.016 | 3.433 | 3.483 | 0.050 | 0.044 | 0.813 | 0.934 | Unannotated | NA | NA |
| metab_6649 | Unannotated feature | pos | 161.0214 | 0.609 | 3.108 | 3.281 | 0.173 | 0.044 | 0.813 | 0.934 | Unannotated | NA | NA |
| metab_19368 | Isoamericanol A | neg | 375.1091 | 5.044 | 4.945 | 5.130 | 0.185 | -0.044 | 0.813 | 0.934 | Unannotated | NA | NA |
| metab_19386 | Unannotated feature | neg | 306.1351 | 5.026 | 4.282 | 4.406 | 0.125 | 0.044 | 0.813 | 0.934 | Unannotated | NA | NA |
| metab_6578 | Unannotated feature | pos | 200.1285 | 5.481 | 3.687 | 4.369 | 0.682 | 0.044 | 0.813 | 0.934 | Unannotated | NA | NA |
| metab_19244 | Jasmonic acid | neg | 255.1236 | 5.209 | 5.095 | 5.062 | -0.034 | -0.044 | 0.813 | 0.934 | Tentative | NA | NA |
| metab_790 | Unannotated feature | pos | 447.2928 | 6.239 | 5.687 | 5.668 | -0.018 | -0.044 | 0.813 | 0.934 | Unannotated | NA | NA |
| metab_19272 | Unannotated feature | neg | 255.8223 | 5.176 | 5.700 | 5.690 | -0.010 | -0.044 | 0.813 | 0.934 | Unannotated | NA | NA |
| metab_15125 | Pro-Pro-Phe | neg | 396.1306 | 4.574 | 4.175 | 4.469 | 0.293 | -0.044 | 0.813 | 0.934 | Unannotated | NA | NA |
| metab_6129 | Unannotated feature | pos | 502.8362 | 0.524 | 4.603 | 4.578 | -0.025 | -0.044 | 0.813 | 0.934 | Unannotated | NA | NA |
| metab_11667 | Unannotated feature | neg | 169.1024 | 3.025 | 3.497 | 3.509 | 0.013 | -0.044 | 0.813 | 0.934 | Unannotated | NA | NA |
| metab_851 | Unannotated feature | pos | 549.3777 | 6.550 | 6.562 | 6.575 | 0.013 | 0.044 | 0.813 | 0.934 | Unannotated | NA | NA |
| metab_19095 | Unannotated feature | neg | 380.1355 | 5.398 | 5.453 | 5.404 | -0.049 | -0.044 | 0.813 | 0.934 | Unannotated | NA | NA |
| metab_15860 | Perilloside A | neg | 359.1719 | 5.944 | 5.489 | 5.487 | -0.002 | 0.044 | 0.813 | 0.934 | Unannotated | NA | NA |
| metab_905 | Unannotated feature | pos | 627.4982 | 7.092 | 3.660 | 3.691 | 0.031 | -0.044 | 0.813 | 0.934 | Unannotated | NA | NA |
| metab_12378 | Unannotated feature | neg | 443.2790 | 6.711 | 5.996 | 6.247 | 0.251 | 0.044 | 0.813 | 0.934 | Unannotated | NA | NA |
| metab_11644 | Unannotated feature | neg | 245.8948 | 0.036 | 5.563 | 5.585 | 0.022 | 0.044 | 0.813 | 0.934 | Unannotated | NA | NA |
| metab_11624 | Unannotated feature | neg | 298.8887 | 0.165 | 4.616 | 4.620 | 0.005 | 0.044 | 0.813 | 0.934 | Unannotated | NA | NA |
| metab_15038 | Unannotated feature | neg | 327.0743 | 4.401 | 3.462 | 3.369 | -0.092 | -0.044 | 0.813 | 0.934 | Unannotated | NA | NA |
| metab_7624 | 6-Methylquinoline | pos | 144.0810 | 3.506 | 4.858 | 4.919 | 0.061 | 0.044 | 0.813 | 0.934 | Moderate putative | NA | NA |
| metab_942 | Unannotated feature | pos | 426.8233 | 0.593 | 4.363 | 4.292 | -0.071 | -0.044 | 0.813 | 0.934 | Unannotated | NA | NA |
| metab_18965 | 2H-1,4-Benzodiazepin-2-one, 1,3-dihydro-7-chloro-5-(2-fluorophenyl)-1-(2-hydroxyethyl)- | neg | 377.0705 | 5.571 | 4.501 | 5.422 | 0.921 | 0.044 | 0.813 | 0.934 | Unannotated | NA | NA |
| metab_16975 | Unannotated feature | neg | 91.2560 | 7.918 | 3.378 | 3.326 | -0.052 | -0.044 | 0.813 | 0.934 | Unannotated | NA | NA |
| metab_9204 | 7-(1-Imidazolyl)heptanoic acid | neg | 241.1193 | 3.766 | 5.334 | 5.401 | 0.067 | -0.044 | 0.813 | 0.934 | Unannotated | NA | NA |
| metab_5826 | Capsiate | pos | 307.1910 | 5.976 | 4.625 | 4.724 | 0.098 | -0.044 | 0.813 | 0.934 | Tentative | NA | NA |
| metab_993 | Unannotated feature | pos | 81.5210 | 7.724 | 5.358 | 5.366 | 0.007 | -0.044 | 0.813 | 0.934 | Unannotated | NA | NA |
| metab_5763 | Unannotated feature | pos | 123.0407 | 0.477 | 4.814 | 4.748 | -0.066 | -0.044 | 0.813 | 0.934 | Unannotated | NA | NA |
| metab_12510 | Unannotated feature | neg | 461.2916 | 6.334 | 5.574 | 5.636 | 0.062 | -0.044 | 0.813 | 0.934 | Unannotated | NA | NA |
| metab_12533 | Unannotated feature | neg | 297.1529 | 6.381 | 6.042 | 6.024 | -0.018 | 0.044 | 0.813 | 0.934 | Unannotated | NA | NA |
| metab_12526 | Unannotated feature | neg | 535.2746 | 6.287 | 5.618 | 5.466 | -0.152 | -0.044 | 0.813 | 0.934 | Unannotated | NA | NA |
| metab_7757 | Unannotated feature | pos | 186.1492 | 3.287 | 3.354 | 3.381 | 0.026 | -0.044 | 0.813 | 0.934 | Unannotated | NA | NA |
| metab_16007 | Unannotated feature | neg | 465.2491 | 6.123 | 4.835 | 4.857 | 0.022 | 0.044 | 0.813 | 0.934 | Unannotated | NA | NA |
| metab_18651 | 5,8-Dihydroxy-3-(4-hydroxybenzyl)-7-methoxy-4-chromanone 8-acetate | neg | 357.1009 | 5.944 | 4.720 | 4.669 | -0.051 | -0.044 | 0.813 | 0.934 | Unannotated | NA | NA |
| metab_12622 | Unannotated feature | neg | 355.1765 | 6.007 | 6.029 | 5.935 | -0.094 | 0.044 | 0.813 | 0.934 | Unannotated | NA | NA |
| metab_1188 | N-hexadecanoyl-L-Homoserine lactone | pos | 362.2672 | 6.486 | 4.682 | 4.413 | -0.269 | -0.044 | 0.813 | 0.934 | Unannotated | NA | NA |
| metab_1256 | (12Z,15Z)-10-Hydroxyoctadeca-12,15-dienoylcarnitine | pos | 440.3383 | 6.351 | 5.499 | 5.554 | 0.056 | 0.044 | 0.813 | 0.934 | Unannotated | NA | NA |
| metab_11196 | 3-Hydroxy-1,4-benzodiazepine | neg | 197.0120 | 0.651 | 5.024 | 4.919 | -0.106 | -0.044 | 0.813 | 0.934 | Unannotated | NA | NA |
| metab_5563 | Unannotated feature | pos | 177.0885 | 6.271 | 5.277 | 5.280 | 0.003 | -0.044 | 0.813 | 0.934 | Unannotated | NA | NA |
| metab_12715 | 4-Ipomeanol | neg | 395.1713 | 5.176 | 4.039 | 4.129 | 0.090 | 0.044 | 0.813 | 0.934 | Unannotated | NA | NA |
| metab_11117 | Unannotated feature | neg | 445.1322 | 0.714 | 4.907 | 4.811 | -0.097 | 0.044 | 0.813 | 0.934 | Unannotated | NA | NA |
| metab_18547 | Unannotated feature | neg | 379.1774 | 6.022 | 4.866 | 4.675 | -0.191 | -0.044 | 0.813 | 0.934 | Unannotated | NA | NA |
| metab_5540 | Unannotated feature | pos | 593.3352 | 6.287 | 7.173 | 7.161 | -0.013 | -0.044 | 0.813 | 0.934 | Unannotated | NA | NA |
| metab_8101 | Pemoline | pos | 209.0925 | 2.740 | 3.874 | 3.818 | -0.055 | 0.044 | 0.813 | 0.934 | Tentative | NA | NA |
| metab_1407 | Unannotated feature | pos | 180.1749 | 6.080 | 3.631 | 3.633 | 0.002 | -0.044 | 0.813 | 0.934 | Unannotated | NA | NA |
| metab_5400 | Unannotated feature | pos | 452.2785 | 6.438 | 3.905 | 3.861 | -0.044 | -0.044 | 0.813 | 0.934 | Unannotated | NA | NA |
| metab_5389 | Unannotated feature | pos | 546.4020 | 6.454 | 5.005 | 4.952 | -0.053 | -0.044 | 0.813 | 0.934 | Unannotated | NA | NA |
| metab_5412 | Unannotated feature | pos | 97.1016 | 6.422 | 3.255 | 3.229 | -0.026 | -0.044 | 0.813 | 0.934 | Unannotated | NA | NA |
| metab_18167 | Unannotated feature | neg | 510.2907 | 6.326 | 4.485 | 5.018 | 0.533 | 0.044 | 0.813 | 0.934 | Unannotated | NA | NA |
| metab_16264 | Unannotated feature | neg | 510.2900 | 6.545 | 5.613 | 5.527 | -0.086 | -0.044 | 0.813 | 0.934 | Unannotated | NA | NA |
| metab_12920 | Lycoperdic acid | neg | 262.0570 | 0.738 | 5.570 | 5.525 | -0.045 | 0.044 | 0.813 | 0.934 | Unannotated | NA | NA |
| metab_8227 | D-Glucamine | pos | 164.0934 | 2.415 | 4.310 | 4.215 | -0.095 | -0.044 | 0.813 | 0.934 | Unannotated | NA | NA |
| metab_16314 | Unannotated feature | neg | 412.3437 | 6.648 | 5.264 | 5.483 | 0.219 | 0.044 | 0.813 | 0.934 | Unannotated | NA | NA |
| metab_18070 | Unannotated feature | neg | 427.3072 | 6.420 | 5.391 | 5.538 | 0.146 | 0.044 | 0.813 | 0.934 | Unannotated | NA | NA |
| metab_12993 | Unannotated feature | neg | 491.2095 | 6.217 | 5.499 | 5.626 | 0.127 | 0.044 | 0.813 | 0.934 | Unannotated | NA | NA |
| metab_12988 | Unannotated feature | neg | 248.0235 | 0.791 | 4.483 | 4.299 | -0.184 | -0.044 | 0.813 | 0.934 | Unannotated | NA | NA |
| metab_1942 | Unannotated feature | pos | 136.0621 | 1.952 | 4.711 | 4.596 | -0.115 | -0.044 | 0.813 | 0.934 | Unannotated | NA | NA |
| metab_9247 | Unannotated feature | neg | 331.0830 | 3.948 | 4.327 | 4.207 | -0.120 | 0.044 | 0.813 | 0.934 | Unannotated | NA | NA |
| metab_4630 | Unannotated feature | pos | 142.1592 | 7.148 | 4.400 | 4.422 | 0.022 | -0.044 | 0.813 | 0.934 | Unannotated | NA | NA |
| metab_14201 | Unannotated feature | neg | 348.0664 | 2.938 | 4.476 | 4.577 | 0.102 | -0.044 | 0.813 | 0.934 | Unannotated | NA | NA |
| metab_9299 | Unannotated feature | neg | 168.9806 | 0.738 | 4.740 | 4.691 | -0.049 | 0.044 | 0.813 | 0.934 | Unannotated | NA | NA |
| metab_17931 | Unannotated feature | neg | 781.5241 | 6.624 | 4.171 | 4.217 | 0.045 | 0.044 | 0.813 | 0.934 | Unannotated | NA | NA |
| metab_13079 | Unannotated feature | neg | 418.1476 | 1.046 | 3.800 | 3.909 | 0.108 | -0.044 | 0.813 | 0.934 | Unannotated | NA | NA |
| metab_13074 | Unannotated feature | neg | 436.8784 | 7.726 | 5.100 | 5.105 | 0.004 | 0.044 | 0.813 | 0.934 | Unannotated | NA | NA |
| metab_13039 | Unannotated feature | neg | 254.9057 | 0.603 | 4.381 | 4.193 | -0.188 | -0.044 | 0.813 | 0.934 | Unannotated | NA | NA |
| metab_8323 | Unannotated feature | pos | 164.0932 | 2.017 | 4.137 | 4.182 | 0.045 | 0.044 | 0.813 | 0.934 | Unannotated | NA | NA |
| metab_13083 | Unannotated feature | neg | 197.9020 | 0.018 | 3.678 | 3.626 | -0.052 | -0.044 | 0.813 | 0.934 | Unannotated | NA | NA |
| metab_2096 | Cappariloside A | pos | 317.1140 | 2.774 | 4.787 | 4.812 | 0.026 | 0.044 | 0.813 | 0.934 | Unannotated | Organic oxygen compounds | Organooxygen compounds |
| metab_10529 | 3-Amino-2,3-dihydrobenzoic acid | neg | 184.0610 | 1.947 | 5.984 | 6.012 | 0.028 | 0.044 | 0.813 | 0.934 | Tentative | NA | NA |
| metab_2144 | Unannotated feature | pos | 182.0294 | 2.871 | 4.601 | 4.578 | -0.023 | 0.044 | 0.813 | 0.934 | Unannotated | NA | NA |
| metab_17777 | Unannotated feature | neg | 381.2316 | 6.884 | 5.651 | 5.671 | 0.020 | 0.044 | 0.813 | 0.934 | Unannotated | NA | NA |
| metab_9277 | Unannotated feature | neg | 287.1249 | 3.647 | 4.309 | 4.299 | -0.009 | 0.044 | 0.813 | 0.934 | Unannotated | NA | NA |
| metab_13931 | Unannotated feature | neg | 286.0030 | 2.575 | 4.174 | 4.416 | 0.243 | 0.044 | 0.813 | 0.934 | Unannotated | NA | NA |
| metab_2191 | Unannotated feature | pos | 248.1145 | 2.984 | 3.599 | 4.152 | 0.553 | 0.044 | 0.813 | 0.934 | Unannotated | NA | NA |
| metab_2172 | Unannotated feature | pos | 104.1075 | 0.625 | 6.364 | 6.290 | -0.074 | 0.044 | 0.813 | 0.934 | Unannotated | NA | NA |
| metab_2167 | Unannotated feature | pos | 337.1402 | 2.897 | 4.886 | 4.942 | 0.056 | -0.044 | 0.813 | 0.934 | Unannotated | NA | NA |
| metab_10493 | Hydroxytyrosol | neg | 199.0608 | 1.952 | 4.809 | 4.893 | 0.083 | 0.044 | 0.813 | 0.934 | Unannotated | NA | NA |
| metab_8341 | Unannotated feature | pos | 375.2246 | 1.976 | 3.617 | 3.496 | -0.121 | 0.044 | 0.813 | 0.934 | Unannotated | NA | NA |
| metab_10357 | Unannotated feature | neg | 213.9106 | 2.186 | 4.401 | 4.307 | -0.093 | -0.044 | 0.813 | 0.934 | Unannotated | NA | NA |
| metab_10375 | Unannotated feature | neg | 325.0047 | 2.137 | 4.048 | 4.186 | 0.138 | -0.044 | 0.813 | 0.934 | Unannotated | NA | NA |
| metab_10340 | Unannotated feature | neg | 357.0221 | 2.241 | 5.015 | 4.958 | -0.057 | 0.044 | 0.813 | 0.934 | Unannotated | NA | NA |
| metab_2357 | Unannotated feature | pos | 142.0716 | 0.485 | 5.065 | 5.004 | -0.061 | -0.044 | 0.813 | 0.934 | Unannotated | NA | NA |
| metab_2387 | Unannotated feature | pos | 158.0301 | 0.561 | 3.955 | 3.921 | -0.034 | -0.044 | 0.813 | 0.934 | Unannotated | NA | NA |
| metab_8439 | Unannotated feature | pos | 206.6134 | 1.944 | 3.038 | 2.905 | -0.134 | -0.044 | 0.813 | 0.934 | Unannotated | NA | NA |
| metab_14513 | Unannotated feature | neg | 728.8091 | 0.524 | 4.129 | 4.068 | -0.061 | 0.044 | 0.813 | 0.934 | Unannotated | NA | NA |
| metab_17587 | Unannotated feature | neg | 621.4753 | 7.259 | 4.234 | 4.309 | 0.075 | -0.044 | 0.813 | 0.934 | Unannotated | NA | NA |
| metab_14476 | Unannotated feature | neg | 332.9224 | 0.527 | 3.937 | 3.788 | -0.149 | -0.044 | 0.813 | 0.934 | Unannotated | NA | NA |
| metab_2473 | N-Alpha-acetyllysine | pos | 189.1236 | 0.712 | 5.877 | 5.886 | 0.008 | -0.044 | 0.813 | 0.934 | Strong putative | NA | NA |
| metab_9397 | Unannotated feature | neg | 647.2059 | 0.738 | 4.368 | 5.108 | 0.740 | 0.044 | 0.813 | 0.934 | Unannotated | NA | NA |
| metab_9354 | Prenyl glucoside | neg | 293.1240 | 3.544 | 2.960 | 3.186 | 0.225 | -0.044 | 0.813 | 0.934 | Tentative | Lipids and lipid-like molecules | Fatty Acyls |
| metab_10241 | Unannotated feature | neg | 289.1408 | 2.575 | 3.560 | 3.441 | -0.119 | -0.044 | 0.813 | 0.934 | Unannotated | NA | NA |
| metab_13341 | Unannotated feature | neg | 174.0878 | 0.644 | 6.179 | 6.113 | -0.067 | -0.044 | 0.813 | 0.934 | Unannotated | NA | NA |
| metab_2532 | Unannotated feature | pos | 220.0396 | 0.961 | 4.931 | 4.855 | -0.076 | -0.044 | 0.813 | 0.934 | Unannotated | NA | NA |
| metab_13422 | Unannotated feature | neg | 132.0661 | 0.799 | 4.249 | 4.153 | -0.097 | -0.044 | 0.813 | 0.934 | Unannotated | NA | NA |
| metab_2575 | N-(3-Hydroxypropyl)valine | pos | 176.1284 | 1.217 | 4.318 | 4.358 | 0.040 | 0.044 | 0.813 | 0.934 | Unannotated | NA | NA |
| metab_2559 | Unannotated feature | pos | 215.0242 | 1.118 | 4.266 | 4.547 | 0.280 | 0.044 | 0.813 | 0.934 | Unannotated | NA | NA |
| metab_2546 | Unannotated feature | pos | 212.1033 | 1.041 | 4.109 | 4.236 | 0.127 | 0.044 | 0.813 | 0.934 | Unannotated | NA | NA |
| metab_5069 | Unannotated feature | pos | 590.4279 | 7.124 | 4.299 | 4.246 | -0.052 | 0.044 | 0.813 | 0.934 | Unannotated | NA | NA |
| metab_5060 | Unannotated feature | pos | 638.5735 | 7.140 | 5.136 | 5.174 | 0.038 | 0.044 | 0.813 | 0.934 | Unannotated | NA | NA |
| metab_2754 | Unannotated feature | pos | 314.1604 | 5.438 | 3.793 | 4.930 | 1.137 | 0.044 | 0.813 | 0.934 | Unannotated | NA | NA |
| metab_8572 | Unannotated feature | pos | 318.1210 | 1.211 | 5.482 | 5.513 | 0.030 | -0.044 | 0.813 | 0.934 | Unannotated | NA | NA |
| metab_2804 | Galactosylglycerol | pos | 237.0986 | 2.655 | 2.463 | 2.510 | 0.047 | 0.044 | 0.813 | 0.934 | Tentative | NA | NA |
| metab_4924 | Unannotated feature | pos | 465.3356 | 7.642 | 6.987 | 7.016 | 0.029 | -0.044 | 0.813 | 0.934 | Unannotated | NA | NA |
| metab_3809 | 5-(3-Pyridyl)-2-hydroxytetrahydrofuran | pos | 148.0759 | 5.583 | 4.177 | 4.150 | -0.026 | -0.044 | 0.813 | 0.934 | Tentative | NA | NA |
| metab_8593 | 2-Amino-4-oxopentanoic acid | pos | 114.0555 | 1.161 | 5.671 | 5.709 | 0.038 | 0.044 | 0.813 | 0.934 | Tentative | NA | NA |
| metab_4979 | Unannotated feature | pos | 142.1593 | 7.530 | 4.859 | 4.853 | -0.005 | -0.044 | 0.813 | 0.934 | Unannotated | NA | NA |
| metab_9955 | Unannotated feature | neg | 149.0601 | 2.903 | 3.872 | 3.820 | -0.052 | -0.044 | 0.813 | 0.934 | Unannotated | NA | NA |
| metab_9880 | Unannotated feature | neg | 531.1578 | 0.738 | 5.745 | 5.919 | 0.174 | -0.044 | 0.813 | 0.934 | Unannotated | NA | NA |
| metab_2882 | Unannotated feature | pos | 170.0665 | 2.823 | 4.652 | 4.704 | 0.051 | -0.044 | 0.813 | 0.934 | Unannotated | NA | NA |
| metab_13617 | Unannotated feature | neg | 383.1229 | 1.523 | 5.293 | 5.305 | 0.012 | -0.044 | 0.813 | 0.934 | Unannotated | NA | NA |
| metab_17283 | Unannotated feature | neg | 129.9357 | 7.720 | 4.880 | 4.917 | 0.037 | 0.044 | 0.813 | 0.934 | Unannotated | NA | NA |
| metab_4856 | Unannotated feature | pos | 294.7066 | 7.724 | 3.650 | 3.745 | 0.094 | 0.044 | 0.813 | 0.934 | Unannotated | NA | NA |
| metab_9839 | Methyl Gallate | neg | 183.0294 | 3.011 | 3.961 | 4.110 | 0.149 | 0.044 | 0.813 | 0.934 | Strong putative | NA | NA |
| metab_9811 | Unannotated feature | neg | 323.9766 | 3.028 | 4.043 | 3.919 | -0.125 | -0.044 | 0.813 | 0.934 | Unannotated | NA | NA |
| metab_9783 | Adipic Acid | neg | 145.0500 | 3.055 | 6.343 | 6.383 | 0.040 | -0.044 | 0.813 | 0.934 | Strong putative | NA | NA |
| metab_2975 | Unannotated feature | pos | 358.1979 | 3.027 | 4.237 | 3.947 | -0.290 | -0.044 | 0.813 | 0.934 | Unannotated | NA | NA |
| metab_4814 | Unannotated feature | pos | 199.9757 | 7.936 | 5.120 | 5.143 | 0.023 | 0.044 | 0.813 | 0.934 | Unannotated | NA | NA |
| metab_4813 | Unannotated feature | pos | 261.0664 | 7.957 | 5.364 | 5.383 | 0.019 | 0.044 | 0.813 | 0.934 | Unannotated | NA | NA |
| metab_13681 | Unannotated feature | neg | 417.0276 | 1.685 | 4.227 | 4.171 | -0.056 | -0.044 | 0.813 | 0.934 | Unannotated | NA | NA |
| metab_9478 | 3-Methyglutaric Acid | neg | 145.0500 | 3.379 | 5.515 | 5.509 | -0.006 | 0.044 | 0.813 | 0.934 | Strong putative | NA | NA |
| metab_9445 | Unannotated feature | neg | 121.0288 | 4.083 | 6.193 | 6.279 | 0.086 | 0.044 | 0.813 | 0.934 | Unannotated | NA | NA |
| metab_9435 | Unannotated feature | neg | 307.0858 | 4.020 | 5.235 | 5.333 | 0.098 | -0.044 | 0.813 | 0.934 | Unannotated | NA | NA |
| metab_13882 | Unannotated feature | neg | 189.9071 | 2.379 | 3.728 | 3.865 | 0.137 | 0.044 | 0.813 | 0.934 | Unannotated | NA | NA |
| metab_17174 | Unannotated feature | neg | 182.8591 | 7.745 | 4.450 | 4.423 | -0.027 | 0.044 | 0.813 | 0.934 | Unannotated | NA | NA |
| metab_3178 | Dimethylstrobochrysin | pos | 314.1395 | 3.670 | 3.337 | 3.453 | 0.116 | 0.044 | 0.813 | 0.934 | Unannotated | NA | NA |
| metab_13757 | Unannotated feature | neg | 268.0941 | 1.952 | 3.450 | 3.477 | 0.028 | 0.044 | 0.813 | 0.934 | Unannotated | NA | NA |
| metab_9600 | Unannotated feature | neg | 385.9519 | 3.227 | 5.206 | 5.144 | -0.062 | -0.044 | 0.813 | 0.934 | Unannotated | NA | NA |
| metab_17134 | Unannotated feature | neg | 206.9723 | 7.806 | 5.423 | 5.442 | 0.019 | 0.044 | 0.813 | 0.934 | Unannotated | NA | NA |
| metab_14243 | Unannotated feature | neg | 126.0554 | 2.984 | 3.246 | 3.379 | 0.133 | -0.044 | 0.813 | 0.934 | Unannotated | NA | NA |
| metab_8778 | Acetoacetic acid | pos | 85.0289 | 0.728 | 4.709 | 4.646 | -0.064 | 0.044 | 0.813 | 0.934 | Tentative | NA | NA |
| metab_3405 | Unannotated feature | pos | 126.1280 | 4.435 | 3.730 | 3.687 | -0.043 | -0.044 | 0.813 | 0.934 | Unannotated | NA | NA |
| metab_4738 | Trans-Isoasarone | pos | 191.1070 | 3.756 | 4.325 | 4.363 | 0.039 | 0.044 | 0.813 | 0.934 | Tentative | NA | NA |
| metab_9538 | Unannotated feature | neg | 345.0991 | 4.123 | 5.479 | 5.542 | 0.063 | -0.044 | 0.813 | 0.934 | Unannotated | NA | NA |
| metab_3603 | Unannotated feature | pos | 587.4298 | 6.686 | 5.976 | 5.921 | -0.055 | 0.044 | 0.813 | 0.934 | Unannotated | NA | NA |
| metab_12029 | Unannotated feature | neg | 654.3160 | 6.287 | 4.906 | 4.599 | -0.307 | -0.044 | 0.813 | 0.934 | Unannotated | NA | NA |
| metab_83 | Citrulline | pos | 176.1032 | 0.649 | 6.301 | 6.339 | 0.038 | -0.044 | 0.813 | 0.934 | Strong putative | NA | NA |
| metab_7082 | Unannotated feature | pos | 244.1659 | 0.657 | 5.636 | 5.591 | -0.045 | -0.044 | 0.813 | 0.934 | Unannotated | NA | NA |
| metab_12043 | Unannotated feature | neg | 581.3706 | 6.326 | 5.583 | 5.773 | 0.190 | 0.044 | 0.813 | 0.934 | Unannotated | NA | NA |
| metab_12042 | Unannotated feature | neg | 589.2371 | 6.326 | 5.048 | 5.658 | 0.610 | 0.044 | 0.813 | 0.934 | Unannotated | NA | NA |
| metab_19830 | Alpha-Ketooctanoic acid | neg | 203.0920 | 4.393 | 5.194 | 5.262 | 0.068 | -0.044 | 0.813 | 0.934 | Unannotated | NA | NA |
| metab_14114 | Unannotated feature | neg | 382.0428 | 2.841 | 4.336 | 4.118 | -0.218 | -0.040 | 0.831 | 0.941 | Unannotated | NA | NA |
| metab_4536 | Unannotated feature | pos | 686.4408 | 6.749 | 6.156 | 6.055 | -0.101 | -0.040 | 0.831 | 0.941 | Unannotated | NA | NA |
| metab_4562 | Unannotated feature | pos | 382.2047 | 6.861 | 3.785 | 3.874 | 0.089 | 0.040 | 0.831 | 0.941 | Unannotated | NA | NA |
| metab_9514 | 3-Methylpimelic acid | neg | 155.0707 | 3.331 | 3.526 | 3.443 | -0.084 | 0.040 | 0.831 | 0.941 | Unannotated | NA | NA |
| metab_12085 | Unannotated feature | neg | 471.3330 | 6.444 | 5.616 | 5.638 | 0.022 | 0.040 | 0.831 | 0.941 | Unannotated | NA | NA |
| metab_11137 | Unannotated feature | neg | 380.1681 | 0.698 | 3.886 | 3.857 | -0.029 | -0.040 | 0.831 | 0.941 | Unannotated | NA | NA |
| metab_11216 | Unannotated feature | neg | 197.0428 | 3.749 | 5.590 | 5.548 | -0.042 | 0.040 | 0.831 | 0.941 | Unannotated | NA | NA |
| metab_1277 | Unannotated feature | pos | 295.1557 | 6.295 | 3.146 | 3.295 | 0.149 | 0.040 | 0.831 | 0.941 | Unannotated | NA | NA |
| metab_1282 | Unannotated feature | pos | 302.2152 | 6.279 | 5.346 | 5.291 | -0.055 | -0.040 | 0.831 | 0.941 | Unannotated | NA | NA |
| metab_1248 | Unannotated feature | pos | 269.1730 | 6.367 | 4.114 | 4.054 | -0.059 | -0.040 | 0.831 | 0.941 | Unannotated | NA | NA |
| metab_4370 | Unannotated feature | pos | 313.1496 | 3.670 | 5.185 | 5.157 | -0.028 | 0.040 | 0.831 | 0.941 | Unannotated | NA | NA |
| metab_12591 | Unannotated feature | neg | 442.1625 | 6.091 | 2.498 | 2.751 | 0.254 | 0.040 | 0.831 | 0.941 | Unannotated | NA | NA |
| metab_12744 | Unannotated feature | neg | 437.1071 | 2.750 | 3.621 | 3.556 | -0.064 | 0.040 | 0.831 | 0.941 | Unannotated | NA | NA |
| metab_1371 | Unannotated feature | pos | 245.1539 | 6.135 | 4.278 | 4.217 | -0.061 | 0.040 | 0.831 | 0.941 | Unannotated | NA | NA |
| metab_1294 | Unannotated feature | pos | 379.2481 | 6.255 | 5.160 | 4.816 | -0.344 | 0.040 | 0.831 | 0.941 | Unannotated | NA | NA |
| metab_12762 | Unannotated feature | neg | 205.0713 | 1.947 | 5.595 | 5.647 | 0.052 | 0.040 | 0.831 | 0.941 | Unannotated | NA | NA |
| metab_10947 | Suberic acid | neg | 173.0813 | 4.896 | 6.139 | 6.198 | 0.059 | 0.040 | 0.831 | 0.941 | Moderate putative | NA | NA |
| metab_14933 | Unannotated feature | neg | 386.9398 | 0.489 | 7.066 | 7.131 | 0.065 | -0.040 | 0.831 | 0.941 | Unannotated | NA | NA |
| metab_16105 | Unannotated feature | neg | 311.1323 | 6.248 | 4.293 | 4.319 | 0.026 | -0.040 | 0.831 | 0.941 | Unannotated | NA | NA |
| metab_12580 | Monic acid | neg | 341.1971 | 6.115 | 6.473 | 6.437 | -0.036 | -0.040 | 0.831 | 0.941 | Unannotated | NA | NA |
| metab_18363 | Unannotated feature | neg | 225.1129 | 6.162 | 5.189 | 5.081 | -0.108 | -0.040 | 0.831 | 0.941 | Unannotated | NA | NA |
| metab_11039 | Columbianetin acetate | neg | 333.0987 | 4.966 | 4.805 | 4.594 | -0.211 | -0.040 | 0.831 | 0.941 | Unannotated | NA | NA |
| metab_12761 | Unannotated feature | neg | 238.0719 | 1.947 | 5.507 | 5.452 | -0.055 | -0.040 | 0.831 | 0.941 | Unannotated | NA | NA |
| metab_18258 | Unannotated feature | neg | 395.2442 | 6.248 | 6.168 | 6.205 | 0.037 | -0.040 | 0.831 | 0.941 | Unannotated | NA | NA |
| metab_1464 | Unannotated feature | pos | 309.0875 | 5.969 | 4.968 | 5.017 | 0.049 | -0.040 | 0.831 | 0.941 | Unannotated | NA | NA |
| metab_18345 | Prostaglandin E1 | neg | 353.2336 | 6.177 | 5.572 | 5.609 | 0.037 | -0.040 | 0.831 | 0.941 | Tentative | Lipids and lipid-like molecules | Fatty Acyls |
| metab_5476 | Unannotated feature | pos | 285.0835 | 2.376 | 3.875 | 3.668 | -0.207 | -0.040 | 0.831 | 0.941 | Unannotated | NA | NA |
| metab_14961 | Unannotated feature | neg | 275.0287 | 4.250 | 3.955 | 3.866 | -0.089 | 0.040 | 0.831 | 0.941 | Unannotated | NA | NA |
| metab_18703 | Unannotated feature | neg | 377.1722 | 5.901 | 4.705 | 4.626 | -0.079 | -0.040 | 0.831 | 0.941 | Unannotated | NA | NA |
| metab_12556 | Unannotated feature | neg | 522.2353 | 6.162 | 4.785 | 4.723 | -0.062 | -0.040 | 0.831 | 0.941 | Unannotated | NA | NA |
| metab_11345 | Unannotated feature | neg | 236.0152 | 0.588 | 4.694 | 4.613 | -0.081 | -0.040 | 0.831 | 0.941 | Unannotated | NA | NA |
| metab_16232 | Unannotated feature | neg | 339.2541 | 6.483 | 4.982 | 5.015 | 0.032 | 0.040 | 0.831 | 0.941 | Unannotated | NA | NA |
| metab_16245 | Unannotated feature | neg | 407.3281 | 6.514 | 4.807 | 4.728 | -0.079 | 0.040 | 0.831 | 0.941 | Unannotated | NA | NA |
| metab_5670 | Unannotated feature | pos | 300.9211 | 0.016 | 4.320 | 4.345 | 0.025 | 0.040 | 0.831 | 0.941 | Unannotated | NA | NA |
| metab_1084 | Unannotated feature | pos | 539.3723 | 6.686 | 5.820 | 5.736 | -0.085 | 0.040 | 0.831 | 0.941 | Unannotated | NA | NA |
| metab_14228 | Lupinic acid | neg | 228.1240 | 2.968 | 2.025 | 2.632 | 0.607 | 0.040 | 0.831 | 0.941 | Tentative | NA | NA |
| metab_18168 | Sphingosylphosphorylcholine | neg | 485.3122 | 6.326 | 6.348 | 6.318 | -0.030 | 0.040 | 0.831 | 0.941 | Tentative | NA | NA |
| metab_18170 | 2'-Aminoacetophenone | neg | 134.0605 | 2.956 | 4.347 | 4.316 | -0.031 | -0.040 | 0.831 | 0.941 | Unannotated | NA | NA |
| metab_1721 | Unannotated feature | pos | 291.1639 | 3.882 | 3.473 | 3.811 | 0.338 | 0.040 | 0.831 | 0.941 | Unannotated | NA | NA |
| metab_18276 | Unannotated feature | neg | 319.1915 | 6.232 | 4.361 | 4.481 | 0.120 | -0.040 | 0.831 | 0.941 | Unannotated | NA | NA |
| metab_5432 | (Z)-5-[(2R,3S,4S)-4-Hydroxy-2-[(E)-3-hydroxyoct-1-enyl]oxan-3-yl]pent-3-enoic acid | pos | 349.1993 | 6.406 | 5.561 | 5.540 | -0.022 | -0.040 | 0.831 | 0.941 | Unannotated | NA | NA |
| metab_1072 | Unannotated feature | pos | 374.3037 | 6.718 | 5.456 | 5.438 | -0.018 | 0.040 | 0.831 | 0.941 | Unannotated | NA | NA |
| metab_1075 | Unannotated feature | pos | 578.5135 | 6.702 | 5.619 | 5.788 | 0.169 | 0.040 | 0.831 | 0.941 | Unannotated | NA | NA |
| metab_5714 | Unannotated feature | pos | 118.0354 | 0.485 | 5.093 | 5.021 | -0.072 | -0.040 | 0.831 | 0.941 | Unannotated | NA | NA |
| metab_14729 | Unannotated feature | neg | 304.9785 | 3.754 | 4.560 | 4.346 | -0.214 | -0.040 | 0.831 | 0.941 | Unannotated | NA | NA |
| metab_12908 | Unannotated feature | neg | 353.1095 | 0.730 | 5.387 | 5.390 | 0.003 | 0.040 | 0.831 | 0.941 | Unannotated | NA | NA |
| metab_8173 | Unannotated feature | pos | 206.0850 | 3.631 | 4.302 | 4.392 | 0.090 | -0.040 | 0.831 | 0.941 | Unannotated | NA | NA |
| metab_8213 | Gamma-Glutamylmethionine | pos | 279.1014 | 2.461 | 4.952 | 4.928 | -0.023 | 0.040 | 0.831 | 0.941 | Strong putative | NA | NA |
| metab_1840 | Unannotated feature | pos | 296.0996 | 1.026 | 5.307 | 5.334 | 0.027 | 0.040 | 0.831 | 0.941 | Unannotated | NA | NA |
| metab_18094 | Unannotated feature | neg | 445.2573 | 6.397 | 5.444 | 5.437 | -0.007 | 0.040 | 0.831 | 0.941 | Unannotated | NA | NA |
| metab_15979 | Lucidenic acid H | neg | 513.2304 | 6.083 | 5.255 | 5.050 | -0.205 | 0.040 | 0.831 | 0.941 | Unannotated | NA | NA |
| metab_15036 | Unannotated feature | neg | 392.0994 | 4.398 | 5.560 | 5.722 | 0.163 | 0.040 | 0.831 | 0.941 | Unannotated | NA | NA |
| metab_8225 | Oxypurinol | pos | 153.0410 | 2.422 | 5.710 | 5.932 | 0.223 | 0.040 | 0.831 | 0.941 | Strong putative | NA | NA |
| metab_12924 | Unannotated feature | neg | 368.1578 | 0.738 | 4.562 | 4.637 | 0.075 | 0.040 | 0.831 | 0.941 | Unannotated | NA | NA |
| metab_12931 | Unannotated feature | neg | 202.0354 | 0.745 | 4.619 | 4.647 | 0.029 | -0.040 | 0.831 | 0.941 | Unannotated | NA | NA |
| metab_12960 | Unannotated feature | neg | 228.0987 | 0.777 | 4.805 | 4.768 | -0.037 | -0.040 | 0.831 | 0.941 | Unannotated | NA | NA |
| metab_5315 | Thr Met | pos | 289.0607 | 1.944 | 4.750 | 4.771 | 0.021 | 0.040 | 0.831 | 0.941 | Moderate putative | NA | NA |
| metab_5303 | Cyprodinil | pos | 473.2419 | 6.566 | 5.515 | 5.389 | -0.127 | -0.040 | 0.831 | 0.941 | Tentative | NA | NA |
| metab_1911 | Grepafloxacin | pos | 382.1506 | 1.685 | 4.792 | 4.885 | 0.092 | 0.040 | 0.831 | 0.941 | Tentative | NA | NA |
| metab_5824 | 3,7-Dihydroxy-12-oxocholanoic acid | pos | 445.2360 | 5.976 | 4.438 | 4.415 | -0.023 | 0.040 | 0.831 | 0.941 | Unannotated | NA | NA |
| metab_15957 | Unannotated feature | neg | 389.2088 | 6.060 | 4.247 | 4.291 | 0.044 | 0.040 | 0.831 | 0.941 | Unannotated | NA | NA |
| metab_15962 | Unannotated feature | neg | 503.2311 | 6.068 | 4.883 | 4.943 | 0.060 | 0.040 | 0.831 | 0.941 | Unannotated | NA | NA |
| metab_7655 | 1,2,10-Trihydroxydihydro-trans-linalyl oxide 7-O-beta-D-glucopyranoside | pos | 400.2184 | 3.451 | 4.255 | 4.272 | 0.017 | -0.040 | 0.831 | 0.941 | Unannotated | NA | NA |
| metab_12473 | Unannotated feature | neg | 316.9214 | 7.993 | 5.156 | 5.164 | 0.008 | -0.040 | 0.831 | 0.941 | Unannotated | NA | NA |
| metab_12462 | Unannotated feature | neg | 188.8941 | 7.910 | 4.717 | 4.730 | 0.013 | 0.040 | 0.831 | 0.941 | Unannotated | NA | NA |
| metab_14197 | Unannotated feature | neg | 165.0663 | 2.938 | 2.710 | 3.054 | 0.344 | 0.040 | 0.831 | 0.941 | Unannotated | NA | NA |
| metab_9605 | 2-[(E)-2-(3-Nitrophenyl)vinyl]quinoline | neg | 297.0666 | 3.223 | 3.905 | 3.951 | 0.047 | 0.040 | 0.831 | 0.941 | Unannotated | NA | NA |
| metab_12116 | Varanic acid | neg | 435.3121 | 6.538 | 5.991 | 6.168 | 0.177 | 0.040 | 0.831 | 0.941 | Unannotated | NA | NA |
| metab_13049 | Unannotated feature | neg | 335.9511 | 0.588 | 4.988 | 5.050 | 0.063 | -0.040 | 0.831 | 0.941 | Unannotated | NA | NA |
| metab_18909 | Unannotated feature | neg | 631.2800 | 5.638 | 4.530 | 4.297 | -0.233 | -0.040 | 0.831 | 0.941 | Unannotated | NA | NA |
| metab_969 | Unannotated feature | pos | 143.9812 | 7.732 | 4.532 | 4.537 | 0.005 | -0.040 | 0.831 | 0.941 | Unannotated | NA | NA |
| metab_976 | Unannotated feature | pos | 182.9856 | 7.849 | 6.589 | 6.598 | 0.009 | 0.040 | 0.831 | 0.941 | Unannotated | NA | NA |
| metab_16399 | Unannotated feature | neg | 285.2435 | 6.845 | 6.483 | 6.397 | -0.086 | -0.040 | 0.831 | 0.941 | Unannotated | NA | NA |
| metab_13033 | Unannotated feature | neg | 454.7771 | 0.603 | 4.402 | 4.752 | 0.350 | 0.040 | 0.831 | 0.941 | Unannotated | NA | NA |
| metab_10548 | Unannotated feature | neg | 131.0707 | 4.554 | 6.474 | 6.385 | -0.090 | -0.040 | 0.831 | 0.941 | Unannotated | NA | NA |
| metab_2059 | Unannotated feature | pos | 723.3333 | 2.716 | 3.496 | 3.908 | 0.412 | -0.040 | 0.831 | 0.941 | Unannotated | NA | NA |
| metab_17842 | Unannotated feature | neg | 285.0552 | 2.817 | 4.860 | 4.880 | 0.020 | 0.040 | 0.831 | 0.941 | Unannotated | NA | NA |
| metab_17843 | Unannotated feature | neg | 313.2748 | 6.758 | 4.558 | 4.473 | -0.085 | -0.040 | 0.831 | 0.941 | Unannotated | NA | NA |
| metab_17869 | Unannotated feature | neg | 323.0381 | 2.831 | 4.935 | 5.171 | 0.236 | 0.040 | 0.831 | 0.941 | Unannotated | NA | NA |
| metab_14026 | Unannotated feature | neg | 291.0878 | 3.745 | 5.227 | 5.152 | -0.075 | 0.040 | 0.831 | 0.941 | Unannotated | NA | NA |
| metab_919 | Unannotated feature | pos | 452.3699 | 7.220 | 6.832 | 6.850 | 0.018 | -0.040 | 0.831 | 0.941 | Unannotated | NA | NA |
| metab_15515 | Unannotated feature | neg | 328.0015 | 0.566 | 4.530 | 4.525 | -0.005 | -0.040 | 0.831 | 0.941 | Unannotated | NA | NA |
| metab_6966 | Aacocf3 | pos | 395.1977 | 4.914 | 5.373 | 5.385 | 0.011 | -0.040 | 0.831 | 0.941 | Tentative | NA | NA |
| metab_17769 | Unannotated feature | neg | 583.2577 | 6.892 | 5.179 | 4.968 | -0.211 | -0.040 | 0.831 | 0.941 | Unannotated | NA | NA |
| metab_17792 | Unannotated feature | neg | 213.0877 | 2.809 | 5.207 | 5.216 | 0.009 | 0.040 | 0.831 | 0.941 | Unannotated | NA | NA |
| metab_10498 | Unannotated feature | neg | 173.0813 | 4.656 | 5.757 | 5.766 | 0.008 | 0.040 | 0.831 | 0.941 | Unannotated | NA | NA |
| metab_18956 | 3'-Hydroxy-4',5',7,8-tetramethoxyflavone | neg | 357.0985 | 5.579 | 4.165 | 4.175 | 0.011 | -0.040 | 0.831 | 0.941 | Unannotated | NA | NA |
| metab_11603 | Unannotated feature | neg | 255.8222 | 0.443 | 4.680 | 4.685 | 0.005 | 0.040 | 0.831 | 0.941 | Unannotated | NA | NA |
| metab_15899 | Unannotated feature | neg | 287.6117 | 5.999 | 4.539 | 4.212 | -0.327 | -0.040 | 0.831 | 0.941 | Unannotated | NA | NA |
| metab_19018 | Unannotated feature | neg | 415.1806 | 5.506 | 4.312 | 4.325 | 0.013 | -0.040 | 0.831 | 0.941 | Unannotated | NA | NA |
| metab_8366 | Unannotated feature | pos | 248.1496 | 1.952 | 3.777 | 3.907 | 0.130 | 0.040 | 0.831 | 0.941 | Unannotated | NA | NA |
| metab_14544 | Ortho-Hydroxyphenylacetic acid | neg | 363.1092 | 3.426 | 4.854 | 4.945 | 0.091 | 0.040 | 0.831 | 0.941 | Tentative | NA | NA |
| metab_16473 | Unannotated feature | neg | 355.1585 | 7.048 | 6.095 | 6.114 | 0.019 | -0.040 | 0.831 | 0.941 | Unannotated | NA | NA |
| metab_8415 | Unannotated feature | pos | 187.1808 | 3.877 | 4.584 | 4.572 | -0.012 | -0.040 | 0.831 | 0.941 | Unannotated | NA | NA |
| metab_2286 | Unannotated feature | pos | 741.1970 | 0.006 | 4.126 | 4.028 | -0.098 | -0.040 | 0.831 | 0.941 | Unannotated | NA | NA |
| metab_2301 | Unannotated feature | pos | 88.9529 | 0.019 | 3.628 | 3.604 | -0.024 | 0.040 | 0.831 | 0.941 | Unannotated | NA | NA |
| metab_875 | Unannotated feature | pos | 463.3404 | 6.797 | 7.855 | 7.875 | 0.020 | 0.040 | 0.831 | 0.941 | Unannotated | NA | NA |
| metab_15544 | Unannotated feature | neg | 241.1234 | 5.369 | 4.332 | 4.275 | -0.057 | 0.040 | 0.831 | 0.941 | Unannotated | NA | NA |
| metab_303 | Unannotated feature | pos | 145.0348 | 0.485 | 5.167 | 5.142 | -0.025 | -0.040 | 0.831 | 0.941 | Unannotated | NA | NA |
| metab_9638 | Phenylacetic Acid | neg | 135.0445 | 3.203 | 4.667 | 4.651 | -0.016 | 0.040 | 0.831 | 0.941 | Strong putative | NA | NA |
| metab_8996 | Tryptophyl-Proline | neg | 300.1357 | 4.083 | 5.480 | 5.413 | -0.067 | 0.040 | 0.831 | 0.941 | Unannotated | NA | NA |
| metab_11659 | Unannotated feature | neg | 197.8892 | 0.013 | 3.449 | 3.401 | -0.047 | -0.040 | 0.831 | 0.941 | Unannotated | NA | NA |
| metab_15909 | Unannotated feature | neg | 444.7482 | 0.607 | 3.856 | 4.111 | 0.256 | -0.040 | 0.831 | 0.941 | Unannotated | NA | NA |
| metab_15148 | Unannotated feature | neg | 218.0153 | 0.552 | 5.003 | 5.109 | 0.106 | -0.040 | 0.831 | 0.941 | Unannotated | NA | NA |
| metab_5166 | N-Stearoyl Valine | pos | 406.3298 | 6.845 | 5.593 | 5.591 | -0.002 | 0.040 | 0.831 | 0.941 | Unannotated | NA | NA |
| metab_13217 | Unannotated feature | neg | 301.9842 | 0.552 | 4.248 | 4.500 | 0.252 | 0.040 | 0.831 | 0.941 | Unannotated | NA | NA |
| metab_10354 | Unannotated feature | neg | 230.9213 | 2.190 | 4.999 | 4.956 | -0.043 | -0.040 | 0.831 | 0.941 | Unannotated | NA | NA |
| metab_839 | Unannotated feature | pos | 339.2514 | 6.510 | 5.790 | 5.764 | -0.025 | 0.040 | 0.831 | 0.941 | Unannotated | NA | NA |
| metab_19151 | Lisofylline | neg | 261.1345 | 5.330 | 5.018 | 5.077 | 0.059 | -0.040 | 0.831 | 0.941 | Unannotated | NA | NA |
| metab_12323 | Unannotated feature | neg | 455.3537 | 6.876 | 6.320 | 6.310 | -0.010 | 0.040 | 0.831 | 0.941 | Unannotated | NA | NA |
| metab_14480 | 3-Oxooctanoic acid | neg | 203.0920 | 3.326 | 4.640 | 4.527 | -0.113 | -0.040 | 0.831 | 0.941 | Unannotated | NA | NA |
| metab_16580 | Unannotated feature | neg | 467.3194 | 7.335 | 6.631 | 6.786 | 0.155 | 0.040 | 0.831 | 0.941 | Unannotated | NA | NA |
| metab_15169 | Voriconazole | neg | 394.1146 | 4.663 | 4.532 | 4.523 | -0.009 | 0.040 | 0.831 | 0.941 | Tentative | NA | NA |
| metab_828 | Unannotated feature | pos | 475.3056 | 6.422 | 6.285 | 6.396 | 0.111 | -0.040 | 0.831 | 0.941 | Unannotated | NA | NA |
| metab_12297 | Unannotated feature | neg | 409.2059 | 7.190 | 4.969 | 4.935 | -0.034 | -0.040 | 0.831 | 0.941 | Unannotated | NA | NA |
| metab_17017 | Unannotated feature | neg | 282.8804 | 7.997 | 4.237 | 4.242 | 0.005 | -0.040 | 0.831 | 0.941 | Unannotated | NA | NA |
| metab_4517 | Armillaramide | pos | 556.5316 | 6.694 | 6.029 | 6.060 | 0.031 | 0.040 | 0.831 | 0.941 | Unannotated | NA | NA |
| metab_11867 | Unannotated feature | neg | 367.1589 | 6.003 | 6.203 | 5.936 | -0.267 | -0.040 | 0.831 | 0.941 | Unannotated | NA | NA |
| metab_7256 | Unannotated feature | pos | 233.1864 | 4.295 | 4.737 | 4.746 | 0.009 | -0.040 | 0.831 | 0.941 | Unannotated | NA | NA |
| metab_7262 | Unannotated feature | pos | 141.9589 | 4.285 | 3.638 | 3.630 | -0.008 | 0.040 | 0.831 | 0.941 | Unannotated | NA | NA |
| metab_15338 | Gibberellin A8-catabolite | neg | 361.1299 | 4.955 | 5.183 | 4.953 | -0.230 | -0.040 | 0.831 | 0.941 | Tentative | NA | NA |
| metab_13406 | 3-(3-Amino-3-carboxypropyl)uridine | neg | 326.0999 | 0.777 | 3.519 | 3.857 | 0.338 | 0.040 | 0.831 | 0.941 | Unannotated | NA | NA |
| metab_9372 | Unannotated feature | neg | 111.0808 | 3.512 | 3.453 | 3.131 | -0.322 | -0.040 | 0.831 | 0.941 | Unannotated | NA | NA |
| metab_19220 | Precocene II | neg | 265.1082 | 5.238 | 3.956 | 4.051 | 0.095 | 0.040 | 0.831 | 0.941 | Tentative | NA | NA |
| metab_19182 | Arbaprostil | neg | 401.2111 | 5.285 | 4.317 | 4.246 | -0.072 | -0.040 | 0.831 | 0.941 | Unannotated | NA | NA |
| metab_5056 | Pro-Ile | pos | 229.1550 | 0.768 | 4.808 | 4.846 | 0.039 | 0.040 | 0.831 | 0.941 | Unannotated | NA | NA |
| metab_10060 | Unannotated feature | neg | 216.0126 | 2.785 | 4.522 | 4.686 | 0.164 | -0.040 | 0.831 | 0.941 | Unannotated | NA | NA |
| metab_16919 | Unannotated feature | neg | 428.8720 | 7.781 | 4.820 | 4.855 | 0.035 | 0.040 | 0.831 | 0.941 | Unannotated | NA | NA |
| metab_16893 | Unannotated feature | neg | 200.9191 | 7.745 | 3.771 | 3.765 | -0.006 | -0.040 | 0.831 | 0.941 | Unannotated | NA | NA |
| metab_11763 | Unannotated feature | neg | 378.1193 | 5.702 | 4.587 | 4.854 | 0.268 | 0.040 | 0.831 | 0.941 | Unannotated | NA | NA |
| metab_11765 | DG(2:0/0:0/20:5(5Z,8Z,11Z,14Z,16E)-OH(18R)) | neg | 479.2660 | 5.722 | 4.169 | 4.079 | -0.090 | -0.040 | 0.831 | 0.941 | Unannotated | NA | NA |
| metab_322 | Unannotated feature | pos | 191.0950 | 0.477 | 5.786 | 5.735 | -0.052 | -0.040 | 0.831 | 0.941 | Unannotated | NA | NA |
| metab_681 | Unannotated feature | pos | 448.2241 | 5.906 | 4.084 | 4.366 | 0.282 | 0.040 | 0.831 | 0.941 | Unannotated | NA | NA |
| metab_8552 | Unannotated feature | pos | 99.5316 | 1.274 | 3.750 | 3.781 | 0.031 | 0.040 | 0.831 | 0.941 | Unannotated | NA | NA |
| metab_10035 | Unannotated feature | neg | 450.1514 | 2.806 | 3.673 | 3.728 | 0.055 | 0.040 | 0.831 | 0.941 | Unannotated | NA | NA |
| metab_10006 | Unannotated feature | neg | 275.0525 | 2.836 | 4.161 | 4.262 | 0.101 | 0.040 | 0.831 | 0.941 | Unannotated | NA | NA |
| metab_3799 | Unannotated feature | pos | 442.3540 | 6.398 | 5.841 | 5.914 | 0.072 | 0.040 | 0.831 | 0.941 | Unannotated | NA | NA |
| metab_17037 | Unannotated feature | neg | 230.9212 | 7.989 | 3.860 | 3.871 | 0.011 | 0.040 | 0.831 | 0.941 | Unannotated | NA | NA |
| metab_8853 | Unannotated feature | neg | 290.1248 | 4.354 | 4.441 | 4.464 | 0.023 | 0.040 | 0.831 | 0.941 | Unannotated | NA | NA |
| metab_14186 | Unannotated feature | neg | 350.0731 | 2.928 | 4.380 | 4.298 | -0.082 | -0.040 | 0.831 | 0.941 | Unannotated | NA | NA |
| metab_4668 | Unannotated feature | pos | 683.4360 | 7.458 | 5.481 | 5.461 | -0.020 | -0.040 | 0.831 | 0.941 | Unannotated | NA | NA |
| metab_13535 | Unannotated feature | neg | 443.0106 | 4.028 | 6.319 | 6.534 | 0.214 | 0.040 | 0.831 | 0.941 | Unannotated | NA | NA |
| metab_19347 | Unannotated feature | neg | 333.1462 | 5.072 | 4.561 | 4.663 | 0.102 | -0.040 | 0.831 | 0.941 | Unannotated | NA | NA |
| metab_11805 | Phaseolic acid | neg | 243.1237 | 5.847 | 6.465 | 6.303 | -0.162 | -0.040 | 0.831 | 0.941 | Tentative | NA | NA |
| metab_11803 | Beta-D-Glucopyranosylurea | neg | 221.0792 | 5.842 | 4.079 | 4.318 | 0.239 | 0.040 | 0.831 | 0.941 | Unannotated | NA | NA |
| metab_4591 | Unannotated feature | pos | 477.3926 | 6.989 | 7.035 | 7.053 | 0.018 | 0.040 | 0.831 | 0.941 | Unannotated | NA | NA |
| metab_2997 | Unannotated feature | pos | 408.2291 | 6.287 | 4.505 | 4.368 | -0.137 | -0.040 | 0.831 | 0.941 | Unannotated | NA | NA |
| metab_7405 | Unannotated feature | pos | 141.0660 | 0.712 | 6.106 | 6.181 | 0.075 | -0.040 | 0.831 | 0.941 | Unannotated | NA | NA |
| metab_9879 | 1-(1-hydroxybutyl)-1,3,4,5,6,7-Hexahydro-2-Benzofuran-4,5,6,7-Tetrol | neg | 241.1081 | 2.968 | 5.372 | 5.099 | -0.274 | -0.040 | 0.831 | 0.941 | Moderate putative | NA | NA |
| metab_17325 | Unannotated feature | neg | 476.9217 | 7.697 | 4.065 | 4.048 | -0.017 | 0.040 | 0.831 | 0.941 | Unannotated | NA | NA |
| metab_17328 | Unannotated feature | neg | 393.8944 | 7.697 | 4.934 | 4.968 | 0.034 | -0.040 | 0.831 | 0.941 | Unannotated | NA | NA |
| metab_13550 | Unannotated feature | neg | 616.7553 | 0.533 | 4.765 | 5.090 | 0.325 | 0.040 | 0.831 | 0.941 | Unannotated | NA | NA |
| metab_4898 | Unannotated feature | pos | 102.5532 | 7.700 | 3.799 | 3.770 | -0.028 | -0.040 | 0.831 | 0.941 | Unannotated | NA | NA |
| metab_510 | Unannotated feature | pos | 463.2682 | 6.231 | 6.022 | 6.206 | 0.185 | 0.040 | 0.831 | 0.941 | Unannotated | NA | NA |
| metab_11858 | Alpha-Carboxy-delta-nonalactone | neg | 199.0971 | 5.991 | 6.135 | 6.175 | 0.040 | 0.040 | 0.831 | 0.941 | Unannotated | NA | NA |
| metab_417 | Unannotated feature | pos | 268.1189 | 4.724 | 5.081 | 5.054 | -0.027 | -0.040 | 0.831 | 0.941 | Unannotated | NA | NA |
| metab_11813 | Unannotated feature | neg | 518.2460 | 5.871 | 4.186 | 3.938 | -0.247 | -0.040 | 0.831 | 0.941 | Unannotated | NA | NA |
| metab_15302 | Unannotated feature | neg | 200.1288 | 4.884 | 3.366 | 3.361 | -0.005 | -0.040 | 0.831 | 0.941 | Unannotated | NA | NA |
| metab_6785 | 10-hydroxy-(2E,8E)-decadien-4-ynoic Acid | pos | 222.1128 | 5.199 | 3.908 | 4.116 | 0.209 | 0.040 | 0.831 | 0.941 | Unannotated | NA | NA |
| metab_8657 | Senkyunolide N | pos | 227.1281 | 4.107 | 4.573 | 4.649 | 0.076 | 0.040 | 0.831 | 0.941 | Unannotated | NA | NA |
| metab_12013 | Dehydrocyanaropicrin | neg | 389.1248 | 5.550 | 6.544 | 6.627 | 0.083 | -0.040 | 0.831 | 0.941 | Unannotated | NA | NA |
| metab_144 | Unannotated feature | pos | 261.9285 | 0.617 | 4.874 | 4.968 | 0.095 | -0.040 | 0.831 | 0.941 | Unannotated | NA | NA |
| metab_11949 | Unannotated feature | neg | 143.0244 | 6.146 | 2.919 | 2.926 | 0.007 | -0.040 | 0.831 | 0.941 | Unannotated | NA | NA |
| metab_4564 | Unannotated feature | pos | 350.3036 | 6.869 | 4.677 | 4.846 | 0.169 | 0.040 | 0.831 | 0.941 | Unannotated | NA | NA |
| metab_17070 | 2,4-Dichloro-6-nitrophenol | neg | 243.8993 | 7.914 | 6.128 | 6.135 | 0.007 | 0.036 | 0.850 | 0.948 | Unannotated | NA | NA |
| metab_78 | Unannotated feature | pos | 380.1828 | 4.379 | 5.198 | 5.331 | 0.133 | 0.036 | 0.850 | 0.948 | Unannotated | NA | NA |
| metab_12181 | Unannotated feature | neg | 385.2712 | 6.734 | 5.604 | 5.744 | 0.140 | 0.036 | 0.850 | 0.948 | Unannotated | NA | NA |
| metab_12153 | Unannotated feature | neg | 622.3796 | 6.648 | 4.376 | 4.556 | 0.181 | -0.036 | 0.850 | 0.948 | Unannotated | NA | NA |
| metab_18463 | Unannotated feature | neg | 515.2696 | 6.076 | 5.857 | 5.897 | 0.039 | -0.036 | 0.850 | 0.948 | Unannotated | NA | NA |
| metab_18470 | Unannotated feature | neg | 442.1911 | 6.076 | 4.381 | 4.506 | 0.125 | -0.036 | 0.850 | 0.948 | Unannotated | NA | NA |
| metab_53 | Unannotated feature | pos | 230.1867 | 0.680 | 5.171 | 4.922 | -0.250 | -0.036 | 0.850 | 0.948 | Unannotated | NA | NA |
| metab_7111 | (S)-5'-Deoxy-5'-(methylsulfinyl)adenosine | pos | 331.1185 | 4.620 | 4.395 | 4.726 | 0.331 | -0.036 | 0.850 | 0.948 | Unannotated | NA | NA |
| metab_13840 | Unannotated feature | neg | 249.9069 | 2.178 | 5.637 | 5.534 | -0.103 | -0.036 | 0.850 | 0.948 | Unannotated | NA | NA |
| metab_12169 | Unannotated feature | neg | 433.3319 | 6.703 | 6.003 | 6.082 | 0.079 | 0.036 | 0.850 | 0.948 | Unannotated | NA | NA |
| metab_2274 | Unannotated feature | pos | 222.0295 | 3.214 | 4.877 | 4.860 | -0.017 | -0.036 | 0.850 | 0.948 | Unannotated | NA | NA |
| metab_18770 | Gardenin B | neg | 403.1007 | 5.822 | 4.111 | 3.890 | -0.221 | -0.036 | 0.850 | 0.948 | Tentative | NA | NA |
| metab_19690 | Unannotated feature | neg | 227.1287 | 4.588 | 5.993 | 5.796 | -0.198 | -0.036 | 0.850 | 0.948 | Unannotated | NA | NA |
| metab_12108 | Unannotated feature | neg | 765.5693 | 6.514 | 3.860 | 4.738 | 0.878 | 0.036 | 0.850 | 0.948 | Unannotated | NA | NA |
| metab_19089 | Rhamnose | neg | 209.0663 | 0.675 | 5.893 | 5.886 | -0.007 | 0.036 | 0.850 | 0.948 | Tentative | NA | NA |
| metab_17183 | Unannotated feature | neg | 293.1325 | 7.741 | 4.230 | 4.324 | 0.094 | -0.036 | 0.850 | 0.948 | Unannotated | NA | NA |
| metab_9641 | 2-Acetyl-4-methylthiazole | neg | 186.0225 | 3.199 | 5.231 | 5.075 | -0.156 | 0.036 | 0.850 | 0.948 | Unannotated | Organic oxygen compounds | Organooxygen compounds |
| metab_3201 | Unannotated feature | pos | 391.3304 | 6.789 | 4.251 | 4.585 | 0.334 | 0.036 | 0.850 | 0.948 | Unannotated | NA | NA |
| metab_19302 | Unannotated feature | neg | 192.0661 | 5.144 | 3.396 | 3.452 | 0.057 | -0.036 | 0.850 | 0.948 | Unannotated | NA | NA |
| metab_15240 | Bisdemethoxycurcumin | neg | 353.1010 | 4.791 | 3.391 | 3.787 | 0.396 | 0.036 | 0.850 | 0.948 | Moderate putative | NA | NA |
| metab_6660 | Unannotated feature | pos | 156.0423 | 0.609 | 4.306 | 4.556 | 0.250 | 0.036 | 0.850 | 0.948 | Unannotated | NA | NA |
| metab_6723 | Unannotated feature | pos | 223.9725 | 0.625 | 5.550 | 5.561 | 0.010 | -0.036 | 0.850 | 0.948 | Unannotated | NA | NA |
| metab_17316 | Unannotated feature | neg | 243.9538 | 7.702 | 5.457 | 5.446 | -0.011 | -0.036 | 0.850 | 0.948 | Unannotated | NA | NA |
| metab_9930 | Unannotated feature | neg | 354.0835 | 3.964 | 3.058 | 3.164 | 0.106 | -0.036 | 0.850 | 0.948 | Unannotated | NA | NA |
| metab_18633 | Unannotated feature | neg | 503.2312 | 5.952 | 4.947 | 4.780 | -0.168 | -0.036 | 0.850 | 0.948 | Unannotated | NA | NA |
| metab_9817 | Unannotated feature | neg | 385.9519 | 3.025 | 4.869 | 4.870 | 0.001 | -0.036 | 0.850 | 0.948 | Unannotated | NA | NA |
| metab_7623 | L-1,2,3,4-Tetrahydro-beta-carboline-3-carboxylic acid | pos | 217.0976 | 3.506 | 5.443 | 5.433 | -0.010 | -0.036 | 0.850 | 0.948 | Unannotated | NA | NA |
| metab_14309 | Unannotated feature | neg | 269.9737 | 3.063 | 3.894 | 3.969 | 0.075 | -0.036 | 0.850 | 0.948 | Unannotated | NA | NA |
| metab_8997 | Unannotated feature | neg | 129.0551 | 3.773 | 6.792 | 6.834 | 0.042 | -0.036 | 0.850 | 0.948 | Unannotated | NA | NA |
| metab_8403 | N-Stearoyl Methionine | pos | 438.2989 | 6.813 | 5.816 | 6.023 | 0.206 | -0.036 | 0.850 | 0.948 | Unannotated | NA | NA |
| metab_1825 | Unannotated feature | pos | 154.0977 | 0.989 | 5.534 | 5.339 | -0.195 | -0.036 | 0.850 | 0.948 | Unannotated | NA | NA |
| metab_11405 | Unannotated feature | neg | 392.0994 | 5.185 | 5.355 | 5.603 | 0.248 | -0.036 | 0.850 | 0.948 | Unannotated | NA | NA |
| metab_4511 | Unannotated feature | pos | 513.3565 | 6.678 | 6.583 | 6.604 | 0.021 | 0.036 | 0.850 | 0.948 | Unannotated | NA | NA |
| metab_11661 | MJDBISSN00000003 | neg | 411.3057 | 6.248 | 6.277 | 6.259 | -0.018 | -0.036 | 0.850 | 0.948 | Unannotated | NA | NA |
| metab_6293 | Unannotated feature | pos | 350.8864 | 0.524 | 5.503 | 5.469 | -0.035 | -0.036 | 0.850 | 0.948 | Unannotated | NA | NA |
| metab_2334 | Unannotated feature | pos | 144.0658 | 0.413 | 4.325 | 4.318 | -0.008 | 0.036 | 0.850 | 0.948 | Unannotated | NA | NA |
| metab_17620 | MG(18:1(12Z)-2OH(9,10)/0:0/0:0) | neg | 423.2526 | 7.162 | 5.296 | 4.909 | -0.387 | -0.036 | 0.850 | 0.948 | Unannotated | NA | NA |
| metab_2369 | Unannotated feature | pos | 268.9610 | 0.516 | 3.447 | 3.811 | 0.364 | 0.036 | 0.850 | 0.948 | Unannotated | NA | NA |
| metab_2989 | 4(1H)-Pyridinone, 2-ethyl-3-hydroxy-1-(2-hydroxyethyl)- | pos | 216.1234 | 3.066 | 4.537 | 4.628 | 0.091 | -0.036 | 0.850 | 0.948 | Unannotated | NA | NA |
| metab_16075 | Unannotated feature | neg | 195.1385 | 6.217 | 4.400 | 4.286 | -0.114 | -0.036 | 0.850 | 0.948 | Unannotated | NA | NA |
| metab_1362 | Unannotated feature | pos | 797.5579 | 6.135 | 2.772 | 2.778 | 0.007 | -0.036 | 0.850 | 0.948 | Unannotated | NA | NA |
| metab_10957 | Unannotated feature | neg | 282.1096 | 4.910 | 2.853 | 3.105 | 0.252 | 0.036 | 0.850 | 0.948 | Unannotated | NA | NA |
| metab_15778 | Unannotated feature | neg | 272.0929 | 5.818 | 4.986 | 4.932 | -0.054 | -0.036 | 0.850 | 0.948 | Unannotated | NA | NA |
| metab_14994 | Unannotated feature | neg | 293.1358 | 4.315 | 4.359 | 4.375 | 0.016 | -0.036 | 0.850 | 0.948 | Unannotated | NA | NA |
| metab_12454 | (9S,10S)-9,10-dihydroxyoctadecanoate | neg | 315.2540 | 6.506 | 6.765 | 6.735 | -0.030 | -0.036 | 0.850 | 0.948 | Tentative | NA | NA |
| metab_11610 | Unannotated feature | neg | 362.8752 | 0.384 | 5.593 | 5.588 | -0.005 | -0.036 | 0.850 | 0.948 | Unannotated | NA | NA |
| metab_6035 | Glucose propionate | pos | 543.1341 | 0.665 | 5.968 | 5.764 | -0.204 | -0.036 | 0.850 | 0.948 | Unannotated | NA | NA |
| metab_6063 | Nuatigenin | pos | 448.3433 | 5.874 | 4.853 | 5.341 | 0.488 | 0.036 | 0.850 | 0.948 | Tentative | NA | NA |
| metab_14719 | 2-Furanmethanol | neg | 195.0658 | 3.733 | 4.793 | 4.811 | 0.018 | 0.036 | 0.850 | 0.948 | Tentative | NA | NA |
| metab_8201 | Unannotated feature | pos | 193.0999 | 2.500 | 3.895 | 3.859 | -0.036 | -0.036 | 0.850 | 0.948 | Unannotated | NA | NA |
| metab_8215 | Unannotated feature | pos | 167.0565 | 2.452 | 4.945 | 5.011 | 0.065 | 0.036 | 0.850 | 0.948 | Unannotated | NA | NA |
| metab_7462 | L-Leucine | pos | 114.0919 | 0.720 | 6.168 | 6.162 | -0.006 | -0.036 | 0.850 | 0.948 | Moderate putative | NA | NA |
| metab_16997 | Unannotated feature | neg | 351.9020 | 7.985 | 4.935 | 4.932 | -0.003 | 0.036 | 0.850 | 0.948 | Unannotated | NA | NA |
| metab_13060 | Unannotated feature | neg | 514.8163 | 0.538 | 4.890 | 4.764 | -0.126 | -0.036 | 0.850 | 0.948 | Unannotated | NA | NA |
| metab_8318 | Unannotated feature | pos | 816.2164 | 2.025 | 4.770 | 4.745 | -0.025 | -0.036 | 0.850 | 0.948 | Unannotated | NA | NA |
| metab_5279 | Unannotated feature | pos | 471.3283 | 6.614 | 5.018 | 5.086 | 0.067 | 0.036 | 0.850 | 0.948 | Unannotated | NA | NA |
| metab_14597 | Unannotated feature | neg | 326.9019 | 0.524 | 6.503 | 6.495 | -0.008 | 0.036 | 0.850 | 0.948 | Unannotated | NA | NA |
| metab_18044 | Unannotated feature | neg | 402.8322 | 0.533 | 5.434 | 5.501 | 0.066 | 0.036 | 0.850 | 0.948 | Unannotated | NA | NA |
| metab_12211 | Unannotated feature | neg | 565.4477 | 6.836 | 5.320 | 5.519 | 0.199 | 0.036 | 0.850 | 0.948 | Unannotated | NA | NA |
| metab_4986 | Unannotated feature | pos | 647.2592 | 7.506 | 5.836 | 5.799 | -0.037 | 0.036 | 0.850 | 0.948 | Unannotated | NA | NA |
| metab_13540 | Unannotated feature | neg | 251.9987 | 1.127 | 4.220 | 4.209 | -0.011 | -0.036 | 0.850 | 0.948 | Unannotated | NA | NA |
| metab_1814 | Unannotated feature | pos | 257.1230 | 0.846 | 5.383 | 5.427 | 0.045 | 0.036 | 0.850 | 0.948 | Unannotated | NA | NA |
| metab_8432 | 2'-Deoxy-2'-fluorocytidine | pos | 263.1140 | 1.944 | 3.871 | 4.134 | 0.263 | 0.036 | 0.850 | 0.948 | Unannotated | NA | NA |
| metab_2385 | Unannotated feature | pos | 363.9095 | 0.553 | 4.244 | 4.117 | -0.127 | -0.036 | 0.850 | 0.948 | Unannotated | NA | NA |
| metab_13257 | Unannotated feature | neg | 128.9855 | 0.596 | 3.192 | 3.275 | 0.084 | -0.036 | 0.850 | 0.948 | Unannotated | NA | NA |
| metab_19102 | Arctiopicrin | neg | 395.1714 | 5.386 | 4.366 | 4.438 | 0.073 | 0.036 | 0.850 | 0.948 | Tentative | NA | NA |
| metab_8455 | (2S)-2-Amino-5-aminooxy-5-oxopentanoic acid | pos | 127.0506 | 1.936 | 6.115 | 6.029 | -0.086 | -0.036 | 0.850 | 0.948 | Unannotated | NA | NA |
| metab_17555 | DG(18:0/15:0/0:0) | neg | 581.5163 | 7.347 | 3.708 | 3.769 | 0.061 | 0.036 | 0.850 | 0.948 | Tentative | Lipids and lipid-like molecules | Glycerolipids |
| metab_15167 | Unannotated feature | neg | 321.9048 | 4.656 | 4.266 | 4.157 | -0.109 | -0.036 | 0.850 | 0.948 | Unannotated | NA | NA |
| metab_6367 | 6alpha-hydroxy-castasterone | pos | 505.3264 | 5.757 | 2.227 | 4.258 | 2.030 | 0.036 | 0.850 | 0.948 | Tentative | Lipids and lipid-like molecules | Steroids and steroid derivatives |
| metab_1079 | Unannotated feature | pos | 407.2777 | 6.702 | 6.779 | 6.779 | -0.000 | -0.036 | 0.850 | 0.948 | Unannotated | NA | NA |
| metab_3756 | Unannotated feature | pos | 204.0871 | 1.065 | 6.735 | 6.440 | -0.295 | 0.036 | 0.850 | 0.948 | Unannotated | NA | NA |
| metab_12656 | Unannotated feature | neg | 382.1623 | 5.930 | 5.097 | 5.323 | 0.225 | 0.036 | 0.850 | 0.948 | Unannotated | NA | NA |
| metab_7186 | Unannotated feature | pos | 339.1922 | 4.472 | 4.261 | 5.030 | 0.769 | 0.036 | 0.850 | 0.948 | Unannotated | NA | NA |
| metab_3820 | Unannotated feature | pos | 484.2828 | 5.600 | 3.296 | 3.485 | 0.189 | -0.036 | 0.850 | 0.948 | Unannotated | NA | NA |
| metab_9231 | Unannotated feature | neg | 175.0970 | 3.713 | 3.665 | 3.620 | -0.045 | -0.036 | 0.850 | 0.948 | Unannotated | NA | NA |
| metab_9243 | Unannotated feature | neg | 216.1238 | 3.701 | 3.872 | 3.990 | 0.118 | -0.036 | 0.850 | 0.948 | Unannotated | NA | NA |
| metab_4789 | Unannotated feature | pos | 154.9904 | 7.895 | 5.626 | 5.643 | 0.016 | 0.036 | 0.850 | 0.948 | Unannotated | NA | NA |
| metab_3093 | Unannotated feature | pos | 565.3727 | 6.478 | 6.022 | 6.128 | 0.106 | 0.036 | 0.850 | 0.948 | Unannotated | NA | NA |
| metab_1003 | Cuminaldehyde | pos | 149.0963 | 7.307 | 5.407 | 5.406 | -0.001 | 0.036 | 0.850 | 0.948 | Moderate putative | NA | NA |
| metab_10709 | 4-(6-Carbamoyl-5-formyl-2H-pyran-4-yl)-5-formyl-2H-pyran-6-carboxamide | neg | 325.0419 | 1.354 | 4.066 | 4.231 | 0.164 | 0.036 | 0.850 | 0.948 | Unannotated | NA | NA |
| metab_1725 | Unannotated feature | pos | 203.0344 | 3.817 | 4.103 | 4.115 | 0.012 | 0.036 | 0.850 | 0.948 | Unannotated | NA | NA |
| metab_1044 | DG(16:0/PGF1alpha/0:0) | pos | 691.5134 | 6.845 | 5.582 | 5.527 | -0.055 | -0.036 | 0.850 | 0.948 | Unannotated | NA | NA |
| metab_18181 | N-[(3s)-2-Oxotetrahydrofuran-3-Yl]butanamide | neg | 216.0875 | 2.956 | 6.136 | 6.185 | 0.049 | 0.036 | 0.850 | 0.948 | Unannotated | NA | NA |
| metab_14618 | Unannotated feature | neg | 259.0786 | 0.524 | 5.727 | 5.802 | 0.074 | -0.036 | 0.850 | 0.948 | Unannotated | NA | NA |
| metab_15967 | Unannotated feature | neg | 370.1021 | 1.067 | 5.362 | 5.274 | -0.089 | -0.036 | 0.850 | 0.948 | Unannotated | NA | NA |
| metab_5865 | Unannotated feature | pos | 741.1970 | 0.305 | 6.342 | 6.368 | 0.026 | 0.036 | 0.850 | 0.948 | Unannotated | NA | NA |
| metab_5866 | (3beta,17alpha,23R)-17,23-Epoxy-3,29-dihydroxy-27-norlanost-8-ene-15,24-dione | pos | 490.3542 | 5.944 | 5.610 | 5.674 | 0.064 | 0.036 | 0.850 | 0.948 | Unannotated | NA | NA |
| metab_15912 | Unannotated feature | neg | 287.1499 | 6.013 | 5.334 | 5.286 | -0.048 | -0.036 | 0.850 | 0.948 | Unannotated | NA | NA |
| metab_15913 | Oxymetazoline | neg | 305.1871 | 6.013 | 1.911 | 2.286 | 0.375 | 0.036 | 0.850 | 0.948 | Tentative | NA | NA |
| metab_1131 | Unannotated feature | pos | 341.2071 | 6.582 | 4.642 | 4.640 | -0.002 | 0.036 | 0.850 | 0.948 | Unannotated | NA | NA |
| metab_1138 | Unannotated feature | pos | 312.2538 | 6.574 | 4.687 | 4.720 | 0.033 | -0.036 | 0.850 | 0.948 | Unannotated | NA | NA |
| metab_18739 | 1-O-Caffeoyl-beta-D-glucose | neg | 341.0885 | 3.157 | 4.693 | 4.715 | 0.022 | 0.036 | 0.850 | 0.948 | Tentative | NA | NA |
| metab_218 | Unannotated feature | pos | 291.9477 | 0.577 | 4.336 | 4.404 | 0.068 | -0.036 | 0.850 | 0.948 | Unannotated | NA | NA |
| metab_13827 | Unannotated feature | neg | 132.9170 | 2.133 | 3.384 | 3.364 | -0.020 | -0.036 | 0.850 | 0.948 | Unannotated | NA | NA |
| metab_3337 | Unannotated feature | pos | 697.5391 | 7.108 | 6.788 | 6.586 | -0.202 | -0.036 | 0.850 | 0.948 | Unannotated | NA | NA |
| metab_17568 | Unannotated feature | neg | 451.1453 | 2.754 | 4.391 | 4.342 | -0.049 | -0.036 | 0.850 | 0.948 | Unannotated | NA | NA |
| metab_9667 | Unannotated feature | neg | 283.0826 | 4.199 | 4.974 | 4.970 | -0.003 | 0.036 | 0.850 | 0.948 | Unannotated | NA | NA |
| metab_16219 | Unannotated feature | neg | 543.3908 | 6.451 | 4.860 | 5.054 | 0.194 | -0.036 | 0.850 | 0.948 | Unannotated | NA | NA |
| metab_10865 | Unannotated feature | neg | 273.9475 | 0.997 | 5.833 | 5.897 | 0.064 | 0.036 | 0.850 | 0.948 | Unannotated | NA | NA |
| metab_17520 | Unannotated feature | neg | 561.3245 | 7.448 | 4.399 | 4.725 | 0.326 | 0.036 | 0.850 | 0.948 | Unannotated | NA | NA |
| metab_751 | Unannotated feature | pos | 198.1857 | 6.072 | 4.261 | 4.252 | -0.009 | -0.036 | 0.850 | 0.948 | Unannotated | NA | NA |
| metab_9408 | Unannotated feature | neg | 311.1170 | 4.052 | 4.721 | 4.865 | 0.144 | 0.036 | 0.850 | 0.948 | Unannotated | NA | NA |
| metab_13858 | Unannotated feature | neg | 299.0612 | 2.244 | 4.562 | 4.540 | -0.021 | -0.036 | 0.850 | 0.948 | Unannotated | NA | NA |
| metab_9759 | Unannotated feature | neg | 214.1081 | 3.078 | 4.305 | 4.362 | 0.057 | -0.036 | 0.850 | 0.948 | Unannotated | NA | NA |
| metab_2655 | Unannotated feature | pos | 151.1233 | 1.952 | 3.717 | 3.784 | 0.067 | -0.036 | 0.850 | 0.948 | Unannotated | NA | NA |
| metab_18854 | Isovaleric acid | neg | 203.1284 | 5.718 | 4.492 | 4.359 | -0.133 | -0.036 | 0.850 | 0.948 | Tentative | NA | NA |
| metab_1908 | Unannotated feature | pos | 276.1083 | 1.685 | 6.358 | 6.304 | -0.054 | -0.036 | 0.850 | 0.948 | Unannotated | NA | NA |
| metab_966 | Unannotated feature | pos | 296.9714 | 7.724 | 5.430 | 5.416 | -0.014 | -0.036 | 0.850 | 0.948 | Unannotated | NA | NA |
| metab_2085 | (+/-)-2-Hydroxy-3-(2-hydroxyphenyl)propanoic acid | pos | 200.0921 | 2.762 | 5.179 | 5.261 | 0.083 | 0.036 | 0.850 | 0.948 | Unannotated | NA | NA |
| metab_17828 | Unannotated feature | neg | 426.3020 | 6.782 | 5.410 | 5.588 | 0.178 | 0.036 | 0.850 | 0.948 | Unannotated | NA | NA |
| metab_13726 | Unannotated feature | neg | 100.0396 | 1.944 | 4.063 | 4.169 | 0.106 | -0.036 | 0.850 | 0.948 | Unannotated | NA | NA |
| metab_804 | Unannotated feature | pos | 425.2912 | 6.287 | 5.124 | 5.248 | 0.125 | -0.036 | 0.850 | 0.948 | Unannotated | NA | NA |
| metab_816 | Unannotated feature | pos | 423.3237 | 6.335 | 5.327 | 5.425 | 0.098 | 0.036 | 0.850 | 0.948 | Unannotated | NA | NA |
| metab_1060 | Unannotated feature | pos | 252.1234 | 1.944 | 4.676 | 4.776 | 0.100 | 0.036 | 0.850 | 0.948 | Unannotated | NA | NA |
| metab_9175 | Unannotated feature | neg | 460.0011 | 3.817 | 6.618 | 6.798 | 0.179 | 0.036 | 0.850 | 0.948 | Unannotated | NA | NA |
| metab_9179 | Unannotated feature | neg | 396.1781 | 3.803 | 4.150 | 4.097 | -0.053 | -0.036 | 0.850 | 0.948 | Unannotated | NA | NA |
| metab_12431 | Unannotated feature | neg | 341.8726 | 7.730 | 4.586 | 4.580 | -0.006 | -0.036 | 0.850 | 0.948 | Unannotated | NA | NA |
| metab_19006 | Unannotated feature | neg | 438.1921 | 3.234 | 4.369 | 4.461 | 0.093 | -0.036 | 0.850 | 0.948 | Unannotated | NA | NA |
| metab_10513 | Unannotated feature | neg | 326.1359 | 1.947 | 3.666 | 3.644 | -0.022 | -0.036 | 0.850 | 0.948 | Unannotated | NA | NA |
| metab_10517 | Unannotated feature | neg | 175.0969 | 4.663 | 4.643 | 4.619 | -0.024 | -0.036 | 0.850 | 0.948 | Unannotated | NA | NA |
| metab_10523 | Unannotated feature | neg | 232.1189 | 1.947 | 3.840 | 3.917 | 0.077 | 0.036 | 0.850 | 0.948 | Unannotated | NA | NA |
| metab_8093 | Unannotated feature | pos | 256.1912 | 3.491 | 4.513 | 4.469 | -0.044 | 0.036 | 0.850 | 0.948 | Unannotated | NA | NA |
| metab_14757 | Unannotated feature | neg | 458.0041 | 3.817 | 6.632 | 6.810 | 0.178 | 0.036 | 0.850 | 0.948 | Unannotated | NA | NA |
| metab_4458 | Unannotated feature | pos | 507.3694 | 6.422 | 5.800 | 5.653 | -0.147 | -0.036 | 0.850 | 0.948 | Unannotated | NA | NA |
| metab_8469 | 3,4-Dihydroxymandelic acid | pos | 202.0713 | 1.706 | 4.374 | 4.381 | 0.008 | 0.036 | 0.850 | 0.948 | Tentative | NA | NA |
| metab_10935 | Glycerol glutamate | neg | 216.0510 | 0.804 | 5.778 | 5.744 | -0.035 | 0.036 | 0.850 | 0.948 | Unannotated | NA | NA |
| metab_18270 | Unannotated feature | neg | 553.3389 | 6.232 | 5.936 | 5.992 | 0.056 | 0.036 | 0.850 | 0.948 | Unannotated | NA | NA |
| metab_10036 | DCMP | neg | 352.0557 | 2.806 | 2.826 | 2.987 | 0.161 | 0.036 | 0.850 | 0.948 | Tentative | Nucleosides, nucleotides, and analogues | Pyrimidine nucleotides |
| metab_4497 | Unannotated feature | pos | 431.3533 | 6.606 | 5.376 | 5.294 | -0.083 | 0.036 | 0.850 | 0.948 | Unannotated | NA | NA |
| metab_4503 | Ginsenoside Rh1 | pos | 603.4249 | 6.622 | 5.573 | 5.486 | -0.088 | 0.036 | 0.850 | 0.948 | Tentative | NA | NA |
| metab_17494 | Unannotated feature | neg | 200.8583 | 7.503 | 5.102 | 5.105 | 0.003 | 0.036 | 0.850 | 0.948 | Unannotated | NA | NA |
| metab_12702 | Unannotated feature | neg | 333.1837 | 5.351 | 4.618 | 4.795 | 0.177 | 0.036 | 0.850 | 0.948 | Unannotated | NA | NA |
| metab_14131 | Unannotated feature | neg | 170.9752 | 2.865 | 3.322 | 3.822 | 0.500 | 0.036 | 0.850 | 0.948 | Unannotated | NA | NA |
| metab_4859 | Unannotated feature | pos | 139.9881 | 7.724 | 5.870 | 5.867 | -0.003 | 0.036 | 0.850 | 0.948 | Unannotated | NA | NA |
| metab_17097 | Unannotated feature | neg | 267.8480 | 7.870 | 5.231 | 5.225 | -0.006 | 0.036 | 0.850 | 0.948 | Unannotated | NA | NA |
| metab_852 | Unannotated feature | pos | 405.2623 | 6.550 | 5.719 | 5.693 | -0.026 | -0.036 | 0.850 | 0.948 | Unannotated | NA | NA |
| metab_853 | 15d PGD2 | pos | 335.2199 | 6.550 | 6.301 | 6.303 | 0.002 | 0.036 | 0.850 | 0.948 | Unannotated | NA | NA |
| metab_11654 | Unannotated feature | neg | 369.1044 | 0.730 | 6.260 | 6.256 | -0.004 | 0.036 | 0.850 | 0.948 | Unannotated | NA | NA |
| metab_2930 | Gynocardin | pos | 345.1315 | 2.907 | 3.604 | 4.071 | 0.467 | 0.036 | 0.850 | 0.948 | Tentative | NA | NA |
| metab_2934 | Isoleucyl-Arginine | pos | 270.1929 | 2.915 | 4.398 | 4.221 | -0.177 | -0.036 | 0.850 | 0.948 | Unannotated | NA | NA |
| metab_8750 | Unannotated feature | pos | 243.1343 | 4.232 | 3.695 | 3.930 | 0.236 | -0.036 | 0.850 | 0.948 | Unannotated | NA | NA |
| metab_16780 | Unannotated feature | neg | 379.9320 | 7.697 | 5.286 | 5.292 | 0.006 | -0.036 | 0.850 | 0.948 | Unannotated | NA | NA |
| metab_17180 | Unannotated feature | neg | 362.8753 | 7.741 | 5.238 | 5.236 | -0.002 | 0.036 | 0.850 | 0.948 | Unannotated | NA | NA |
| metab_3276 | Unannotated feature | pos | 215.1758 | 3.987 | 4.433 | 4.531 | 0.099 | 0.036 | 0.850 | 0.948 | Unannotated | NA | NA |
| metab_13739 | Unannotated feature | neg | 358.1253 | 1.947 | 4.890 | 5.028 | 0.137 | -0.036 | 0.850 | 0.948 | Unannotated | NA | NA |
| metab_1978 | Unannotated feature | pos | 505.1808 | 2.044 | 5.191 | 4.834 | -0.357 | 0.036 | 0.850 | 0.948 | Unannotated | NA | NA |
| metab_12249 | Unannotated feature | neg | 453.2637 | 6.959 | 6.397 | 6.507 | 0.110 | -0.036 | 0.850 | 0.948 | Unannotated | NA | NA |
| metab_7365 | VAPIPROST | pos | 442.2712 | 4.037 | 3.847 | 4.097 | 0.250 | 0.036 | 0.850 | 0.948 | Unannotated | NA | NA |
| metab_101 | Unannotated feature | pos | 279.0482 | 0.641 | 3.813 | 3.608 | -0.204 | -0.036 | 0.850 | 0.948 | Unannotated | NA | NA |
| metab_7923 | Unannotated feature | pos | 332.2079 | 2.937 | 2.652 | 2.553 | -0.099 | -0.032 | 0.868 | 0.954 | Unannotated | NA | NA |
| metab_12194 | Unannotated feature | neg | 341.1794 | 6.774 | 6.467 | 6.483 | 0.016 | 0.032 | 0.868 | 0.954 | Unannotated | NA | NA |
| metab_13031 | Unannotated feature | neg | 484.8717 | 0.603 | 3.401 | 3.517 | 0.117 | 0.032 | 0.868 | 0.954 | Unannotated | NA | NA |
| metab_1976 | Unannotated feature | pos | 138.0493 | 2.049 | 5.667 | 5.681 | 0.014 | 0.032 | 0.868 | 0.954 | Unannotated | NA | NA |
| metab_13053 | Unannotated feature | neg | 385.1871 | 6.091 | 5.342 | 5.268 | -0.074 | 0.032 | 0.868 | 0.954 | Unannotated | NA | NA |
| metab_17149 | Unannotated feature | neg | 160.0609 | 7.777 | 3.693 | 3.670 | -0.023 | -0.032 | 0.868 | 0.954 | Unannotated | NA | NA |
| metab_3211 | Oleoylethanolamide | pos | 348.2878 | 6.821 | 5.875 | 5.792 | -0.084 | -0.032 | 0.868 | 0.954 | Tentative | NA | NA |
| metab_5226 | PA(P-16:0/22:6(4Z,7Z,10Z,12E,16Z,19Z)-OH(14)) | pos | 685.4650 | 6.702 | 5.717 | 5.730 | 0.013 | 0.032 | 0.868 | 0.954 | Unannotated | NA | NA |
| metab_5224 | Unannotated feature | pos | 487.2323 | 6.710 | 4.325 | 4.665 | 0.340 | 0.032 | 0.868 | 0.954 | Unannotated | NA | NA |
| metab_14526 | Unannotated feature | neg | 378.1412 | 3.387 | 4.669 | 4.948 | 0.279 | -0.032 | 0.868 | 0.954 | Unannotated | NA | NA |
| metab_2244 | Unannotated feature | pos | 90.9772 | 0.585 | 3.148 | 3.581 | 0.433 | -0.032 | 0.868 | 0.954 | Unannotated | NA | NA |
| metab_8629 | Unannotated feature | pos | 289.1188 | 4.084 | 5.662 | 5.659 | -0.004 | 0.032 | 0.868 | 0.954 | Unannotated | NA | NA |
| metab_4619 | Unannotated feature | pos | 171.0993 | 7.124 | 3.800 | 3.815 | 0.015 | -0.032 | 0.868 | 0.954 | Unannotated | NA | NA |
| metab_134 | 2-Amino-4-[carbamimidoyl(methyl)amino]butanoic acid | pos | 175.1193 | 0.625 | 5.263 | 5.499 | 0.236 | -0.032 | 0.868 | 0.954 | Unannotated | NA | NA |
| metab_17495 | Unannotated feature | neg | 369.1044 | 1.952 | 4.288 | 4.457 | 0.169 | 0.032 | 0.868 | 0.954 | Unannotated | NA | NA |
| metab_16916 | Unannotated feature | neg | 329.0858 | 2.039 | 5.294 | 5.260 | -0.034 | -0.032 | 0.868 | 0.954 | Unannotated | NA | NA |
| metab_13391 | Unannotated feature | neg | 128.0710 | 0.770 | 4.217 | 4.363 | 0.145 | 0.032 | 0.868 | 0.954 | Unannotated | NA | NA |
| metab_12545 | Unannotated feature | neg | 351.1822 | 6.217 | 5.610 | 5.585 | -0.025 | 0.032 | 0.868 | 0.954 | Unannotated | NA | NA |
| metab_12839 | Unannotated feature | neg | 315.1313 | 0.675 | 4.322 | 4.558 | 0.235 | -0.032 | 0.868 | 0.954 | Unannotated | NA | NA |
| metab_16092 | Unannotated feature | neg | 301.2020 | 6.232 | 5.731 | 5.646 | -0.085 | -0.032 | 0.868 | 0.954 | Unannotated | NA | NA |
| metab_8518 | Unannotated feature | pos | 182.0295 | 1.592 | 5.294 | 5.357 | 0.063 | 0.032 | 0.868 | 0.954 | Unannotated | NA | NA |
| metab_5052 | Unannotated feature | pos | 660.5263 | 7.156 | 5.260 | 5.256 | -0.003 | 0.032 | 0.868 | 0.954 | Unannotated | NA | NA |
| metab_14043 | (Tetrahydropyran-4-yl)-[2-phenyl-5-(1,1-dioxo-thiomorpholin-4-yl)methyl-1H-indol-7-yl]amine | neg | 484.1947 | 2.771 | 3.743 | 4.187 | 0.444 | -0.032 | 0.868 | 0.954 | Unannotated | NA | NA |
| metab_9147 | Chrysophanein | neg | 415.1044 | 3.852 | 4.047 | 4.103 | 0.056 | 0.032 | 0.868 | 0.954 | Tentative | NA | NA |
| metab_1418 | Xanthoplanine | pos | 321.1714 | 6.048 | 4.637 | 4.736 | 0.099 | 0.032 | 0.868 | 0.954 | Unannotated | NA | NA |
| metab_16316 | Unannotated feature | neg | 581.4431 | 6.648 | 4.487 | 4.247 | -0.239 | -0.032 | 0.868 | 0.954 | Unannotated | NA | NA |
| metab_9133 | Unannotated feature | neg | 141.0914 | 3.875 | 4.177 | 4.009 | -0.167 | -0.032 | 0.868 | 0.954 | Unannotated | NA | NA |
| metab_15930 | Unannotated feature | neg | 369.1919 | 6.029 | 5.443 | 5.358 | -0.086 | -0.032 | 0.868 | 0.954 | Unannotated | NA | NA |
| metab_16193 | Rishitin | neg | 221.1543 | 6.404 | 4.764 | 4.842 | 0.078 | 0.032 | 0.868 | 0.954 | Tentative | NA | NA |
| metab_1113 | Unannotated feature | pos | 619.4033 | 6.630 | 5.711 | 5.773 | 0.062 | -0.032 | 0.868 | 0.954 | Unannotated | NA | NA |
| metab_6455 | Unannotated feature | pos | 290.8478 | 0.593 | 5.472 | 5.587 | 0.114 | -0.032 | 0.868 | 0.954 | Unannotated | NA | NA |
| metab_340 | Unannotated feature | pos | 121.9937 | 0.451 | 3.679 | 3.689 | 0.010 | -0.032 | 0.868 | 0.954 | Unannotated | NA | NA |
| metab_17824 | Unannotated feature | neg | 382.2965 | 6.797 | 4.747 | 5.042 | 0.295 | 0.032 | 0.868 | 0.954 | Unannotated | NA | NA |
| metab_962 | Unannotated feature | pos | 170.0665 | 7.700 | 5.681 | 5.683 | 0.002 | 0.032 | 0.868 | 0.954 | Unannotated | NA | NA |
| metab_1909 | Unannotated feature | pos | 272.1244 | 1.685 | 4.610 | 4.654 | 0.045 | 0.032 | 0.868 | 0.954 | Unannotated | NA | NA |
| metab_15827 | Byssochlamic acid | neg | 313.1087 | 5.901 | 5.742 | 6.050 | 0.308 | -0.032 | 0.868 | 0.954 | Tentative | NA | NA |
| metab_4683 | Unannotated feature | pos | 477.3927 | 7.522 | 7.055 | 7.067 | 0.011 | 0.032 | 0.868 | 0.954 | Unannotated | NA | NA |
| metab_18335 | Unannotated feature | neg | 243.1600 | 6.185 | 4.515 | 4.476 | -0.039 | 0.032 | 0.868 | 0.954 | Unannotated | NA | NA |
| metab_18323 | Unannotated feature | neg | 327.1814 | 6.201 | 5.335 | 5.287 | -0.048 | 0.032 | 0.868 | 0.954 | Unannotated | NA | NA |
| metab_4624 | Unannotated feature | pos | 144.1749 | 7.140 | 4.560 | 4.605 | 0.045 | 0.032 | 0.868 | 0.954 | Unannotated | NA | NA |
| metab_14942 | 2-Isopropylmalic acid | neg | 157.0500 | 4.211 | 4.880 | 4.873 | -0.007 | 0.032 | 0.868 | 0.954 | Tentative | NA | NA |
| metab_19714 | Unannotated feature | neg | 197.0975 | 4.537 | 4.340 | 4.293 | -0.047 | -0.032 | 0.868 | 0.954 | Unannotated | NA | NA |
| metab_3393 | 4-Piperidinone, 1-hydroxy-2,2,6,6-tetramethyl- | pos | 172.1334 | 4.375 | 5.426 | 5.471 | 0.045 | 0.032 | 0.868 | 0.954 | Strong putative | NA | NA |
| metab_17409 | Unannotated feature | neg | 332.9221 | 7.645 | 5.974 | 5.963 | -0.010 | -0.032 | 0.868 | 0.954 | Unannotated | NA | NA |
| metab_19275 | Unannotated feature | neg | 197.8518 | 5.173 | 3.435 | 3.313 | -0.122 | -0.032 | 0.868 | 0.954 | Unannotated | NA | NA |
| metab_8678 | Unannotated feature | pos | 144.9977 | 0.839 | 4.236 | 4.260 | 0.024 | 0.032 | 0.868 | 0.954 | Unannotated | NA | NA |
| metab_17062 | Unannotated feature | neg | 200.9041 | 7.953 | 4.416 | 4.401 | -0.015 | -0.032 | 0.868 | 0.954 | Unannotated | NA | NA |
| metab_2616 | 5-Hydroxy-4-pentenoic acid d-lactone | pos | 81.0340 | 1.692 | 3.525 | 3.506 | -0.019 | -0.032 | 0.868 | 0.954 | Unannotated | Organoheterocyclic compounds | Pyrans |
| metab_19200 | Unannotated feature | neg | 237.0403 | 3.346 | 3.903 | 3.984 | 0.081 | 0.032 | 0.868 | 0.954 | Unannotated | NA | NA |
| metab_18238 | Polyporusterone F | neg | 507.3331 | 6.263 | 5.323 | 5.454 | 0.131 | 0.032 | 0.868 | 0.954 | Unannotated | NA | NA |
| metab_16246 | Unannotated feature | neg | 619.4224 | 6.514 | 5.045 | 5.162 | 0.117 | 0.032 | 0.868 | 0.954 | Unannotated | NA | NA |
| metab_8489 | Cndac | pos | 316.1010 | 1.685 | 4.961 | 4.560 | -0.401 | -0.032 | 0.868 | 0.954 | Unannotated | NA | NA |
| metab_12141 | Unannotated feature | neg | 351.1638 | 6.632 | 4.874 | 4.906 | 0.031 | -0.032 | 0.868 | 0.954 | Unannotated | NA | NA |
| metab_12140 | Unannotated feature | neg | 617.3866 | 6.624 | 5.222 | 5.242 | 0.020 | 0.032 | 0.868 | 0.954 | Unannotated | NA | NA |
| metab_9590 | Hematoxylene | neg | 331.0827 | 3.234 | 3.550 | 3.955 | 0.405 | 0.032 | 0.868 | 0.954 | Unannotated | NA | NA |
| metab_18673 | Auberganol | neg | 275.1769 | 5.927 | 4.828 | 5.261 | 0.434 | -0.032 | 0.868 | 0.954 | Unannotated | NA | NA |
| metab_18362 | Unannotated feature | neg | 259.1816 | 6.162 | 5.182 | 5.599 | 0.416 | 0.032 | 0.868 | 0.954 | Unannotated | NA | NA |
| metab_12577 | Unannotated feature | neg | 543.2997 | 6.115 | 4.910 | 4.866 | -0.044 | -0.032 | 0.868 | 0.954 | Unannotated | NA | NA |
| metab_2080 | Unannotated feature | pos | 188.0380 | 2.755 | 4.453 | 4.157 | -0.297 | -0.032 | 0.868 | 0.954 | Unannotated | NA | NA |
| metab_17863 | Unannotated feature | neg | 609.4740 | 6.719 | 5.120 | 5.027 | -0.093 | 0.032 | 0.868 | 0.954 | Unannotated | NA | NA |
| metab_10626 | Unannotated feature | neg | 140.0710 | 1.685 | 3.735 | 3.839 | 0.104 | 0.032 | 0.868 | 0.954 | Unannotated | NA | NA |
| metab_4301 | Ecgonine | pos | 168.1022 | 6.000 | 3.597 | 3.907 | 0.310 | -0.032 | 0.868 | 0.954 | Tentative | NA | NA |
| metab_8217 | Glucose lactate acetate | pos | 291.0705 | 2.439 | 3.847 | 4.477 | 0.631 | 0.032 | 0.868 | 0.954 | Unannotated | NA | NA |
| metab_12856 | Unannotated feature | neg | 160.0609 | 0.698 | 5.125 | 5.161 | 0.036 | 0.032 | 0.868 | 0.954 | Unannotated | NA | NA |
| metab_2140 | Unannotated feature | pos | 250.1442 | 2.724 | 3.725 | 3.991 | 0.266 | 0.032 | 0.868 | 0.954 | Unannotated | NA | NA |
| metab_5250 | Unannotated feature | pos | 147.0504 | 0.485 | 6.992 | 6.952 | -0.041 | -0.032 | 0.868 | 0.954 | Unannotated | NA | NA |
| metab_16429 | Unannotated feature | neg | 509.3132 | 6.915 | 5.320 | 5.332 | 0.012 | -0.032 | 0.868 | 0.954 | Unannotated | NA | NA |
| metab_17832 | Unannotated feature | neg | 283.2279 | 6.782 | 4.564 | 4.618 | 0.054 | -0.032 | 0.868 | 0.954 | Unannotated | NA | NA |
| metab_10790 | 4-Nitroanisole | neg | 458.1173 | 1.119 | 4.863 | 4.737 | -0.127 | 0.032 | 0.868 | 0.954 | Tentative | NA | NA |
| metab_13298 | Unannotated feature | neg | 211.0823 | 0.636 | 3.918 | 4.340 | 0.422 | 0.032 | 0.868 | 0.954 | Unannotated | NA | NA |
| metab_10260 | Unannotated feature | neg | 295.1033 | 2.535 | 4.580 | 4.347 | -0.233 | 0.032 | 0.868 | 0.954 | Unannotated | NA | NA |
| metab_14196 | Unannotated feature | neg | 406.1131 | 2.935 | 3.638 | 3.794 | 0.156 | -0.032 | 0.868 | 0.954 | Unannotated | NA | NA |
| metab_16770 | Unannotated feature | neg | 362.9406 | 7.692 | 6.007 | 6.014 | 0.006 | -0.032 | 0.868 | 0.954 | Unannotated | NA | NA |
| metab_7137 | (3E,5Z)-3,5-Octadien-1-ol | pos | 109.1017 | 4.575 | 5.042 | 5.082 | 0.041 | 0.032 | 0.868 | 0.954 | Unannotated | NA | NA |
| metab_11086 | (2R,4S)-Pyrrolidine-2,4-dicarboxylic acid | neg | 204.0509 | 0.730 | 5.041 | 5.072 | 0.031 | -0.032 | 0.868 | 0.954 | Unannotated | NA | NA |
| metab_16236 | Unannotated feature | neg | 352.8941 | 6.491 | 4.807 | 4.801 | -0.006 | -0.032 | 0.868 | 0.954 | Unannotated | NA | NA |
| metab_17032 | Unannotated feature | neg | 269.9067 | 7.993 | 4.299 | 4.307 | 0.008 | -0.032 | 0.868 | 0.954 | Unannotated | NA | NA |
| metab_9999 | Unannotated feature | neg | 399.1416 | 2.845 | 3.248 | 3.317 | 0.069 | 0.032 | 0.868 | 0.954 | Unannotated | NA | NA |
| metab_9987 | Unannotated feature | neg | 283.0395 | 2.862 | 4.870 | 4.983 | 0.113 | -0.032 | 0.868 | 0.954 | Unannotated | NA | NA |
| metab_13570 | Unannotated feature | neg | 294.9940 | 1.249 | 4.720 | 4.525 | -0.195 | -0.032 | 0.868 | 0.954 | Unannotated | NA | NA |
| metab_2803 | 6-Keto-prostaglandin F1a | pos | 409.1979 | 2.651 | 4.344 | 4.624 | 0.280 | 0.032 | 0.868 | 0.954 | Tentative | NA | NA |
| metab_14487 | Unannotated feature | neg | 244.9376 | 0.527 | 5.756 | 5.752 | -0.005 | 0.032 | 0.868 | 0.954 | Unannotated | NA | NA |
| metab_19174 | Moracin P | neg | 371.1140 | 5.299 | 3.903 | 3.860 | -0.043 | 0.032 | 0.868 | 0.954 | Unannotated | NA | NA |
| metab_19164 | Doramapimod | neg | 526.2857 | 5.308 | 4.076 | 4.626 | 0.550 | 0.032 | 0.868 | 0.954 | Unannotated | NA | NA |
| metab_2472 | Glycyl-Arginine | pos | 214.1302 | 0.704 | 3.004 | 3.210 | 0.206 | 0.032 | 0.868 | 0.954 | Unannotated | NA | NA |
| metab_2388 | Unannotated feature | pos | 255.0656 | 5.754 | 4.222 | 4.446 | 0.223 | 0.032 | 0.868 | 0.954 | Unannotated | NA | NA |
| metab_10248 | Unannotated feature | neg | 163.0064 | 2.565 | 4.116 | 4.206 | 0.090 | 0.032 | 0.868 | 0.954 | Unannotated | NA | NA |
| metab_18298 | Unannotated feature | neg | 280.6219 | 6.217 | 4.115 | 4.094 | -0.022 | 0.032 | 0.868 | 0.954 | Unannotated | NA | NA |
| metab_19374 | Phenylalanylisoleucine | neg | 323.1615 | 5.038 | 5.270 | 5.875 | 0.606 | -0.032 | 0.868 | 0.954 | Unannotated | NA | NA |
| metab_10636 | Unannotated feature | neg | 199.0608 | 1.680 | 4.574 | 4.530 | -0.044 | -0.032 | 0.868 | 0.954 | Unannotated | NA | NA |
| metab_13073 | Unannotated feature | neg | 548.1483 | 1.037 | 5.005 | 5.221 | 0.216 | -0.032 | 0.868 | 0.954 | Unannotated | NA | NA |
| metab_2010 | Unannotated feature | pos | 144.0480 | 2.329 | 5.527 | 5.517 | -0.009 | -0.032 | 0.868 | 0.954 | Unannotated | NA | NA |
| metab_16408 | Unannotated feature | neg | 354.3014 | 6.876 | 5.829 | 5.824 | -0.005 | 0.032 | 0.868 | 0.954 | Unannotated | NA | NA |
| metab_8154 | Unannotated feature | pos | 182.0528 | 2.703 | 4.152 | 4.344 | 0.192 | -0.032 | 0.868 | 0.954 | Unannotated | NA | NA |
| metab_8151 | Unannotated feature | pos | 136.0620 | 2.708 | 3.921 | 3.845 | -0.076 | -0.032 | 0.868 | 0.954 | Unannotated | NA | NA |
| metab_943 | 1-Octene | pos | 130.1593 | 7.554 | 7.039 | 7.029 | -0.010 | -0.032 | 0.868 | 0.954 | Unannotated | NA | NA |
| metab_5299 | Unannotated feature | pos | 105.0038 | 0.485 | 6.749 | 6.704 | -0.045 | -0.032 | 0.868 | 0.954 | Unannotated | NA | NA |
| metab_5291 | PE(16:1(9Z)/P-18:1(9Z)) | pos | 722.5069 | 6.582 | 5.480 | 5.471 | -0.009 | 0.032 | 0.868 | 0.954 | Tentative | NA | NA |
| metab_5328 | Unannotated feature | pos | 531.2722 | 6.542 | 4.532 | 4.369 | -0.163 | 0.032 | 0.868 | 0.954 | Unannotated | NA | NA |
| metab_10713 | Xanthine | neg | 151.0255 | 1.354 | 7.633 | 7.652 | 0.019 | -0.032 | 0.868 | 0.954 | Strong putative | NA | NA |
| metab_16013 | Bornyl acetate | neg | 241.1444 | 6.138 | 4.457 | 4.323 | -0.134 | -0.032 | 0.868 | 0.954 | Tentative | NA | NA |
| metab_15800 | Unannotated feature | neg | 332.9224 | 0.603 | 3.497 | 4.332 | 0.835 | 0.032 | 0.868 | 0.954 | Unannotated | NA | NA |
| metab_5491 | 2,5-Dimethylbenzaldehyde | pos | 135.0807 | 6.359 | 5.483 | 5.500 | 0.016 | -0.032 | 0.868 | 0.954 | Strong putative | NA | NA |
| metab_16208 | Alpha-Methyl-m-tyrosine | neg | 194.0817 | 6.436 | 6.891 | 6.877 | -0.014 | -0.032 | 0.868 | 0.954 | Tentative | NA | NA |
| metab_8033 | Unannotated feature | pos | 222.1492 | 2.795 | 3.068 | 2.881 | -0.186 | -0.032 | 0.868 | 0.954 | Unannotated | NA | NA |
| metab_1306 | Unannotated feature | pos | 359.2889 | 6.239 | 5.931 | 5.881 | -0.049 | -0.032 | 0.868 | 0.954 | Unannotated | NA | NA |
| metab_1316 | Unannotated feature | pos | 395.2558 | 6.223 | 4.731 | 4.776 | 0.045 | 0.032 | 0.868 | 0.954 | Unannotated | NA | NA |
| metab_5613 | Unannotated feature | pos | 369.3485 | 6.215 | 4.341 | 4.270 | -0.071 | 0.032 | 0.868 | 0.954 | Unannotated | NA | NA |
| metab_13630 | Unannotated feature | neg | 254.0671 | 1.952 | 5.908 | 6.025 | 0.117 | 0.032 | 0.868 | 0.954 | Unannotated | NA | NA |
| metab_3001 | Butyric acid | pos | 218.1390 | 3.129 | 4.999 | 4.978 | -0.021 | -0.032 | 0.868 | 0.954 | Tentative | NA | NA |
| metab_8672 | Unannotated feature | pos | 286.0974 | 0.862 | 4.142 | 4.014 | -0.128 | -0.032 | 0.868 | 0.954 | Unannotated | NA | NA |
| metab_400 | Unannotated feature | pos | 328.9162 | 0.016 | 5.509 | 5.507 | -0.001 | 0.032 | 0.868 | 0.954 | Unannotated | NA | NA |
| metab_13109 | Unannotated feature | neg | 290.9299 | 0.149 | 4.136 | 4.155 | 0.019 | 0.032 | 0.868 | 0.954 | Unannotated | NA | NA |
| metab_9275 | Pimelic Acid | neg | 159.0656 | 3.651 | 5.611 | 5.589 | -0.022 | -0.032 | 0.868 | 0.954 | Strong putative | Lipids and lipid-like molecules | Fatty Acyls |
| metab_10338 | Unannotated feature | neg | 197.0087 | 2.248 | 3.542 | 3.053 | -0.489 | -0.032 | 0.868 | 0.954 | Unannotated | NA | NA |
| metab_10304 | Unannotated feature | neg | 241.1081 | 4.566 | 5.970 | 6.140 | 0.170 | 0.032 | 0.868 | 0.954 | Unannotated | NA | NA |
| metab_10296 | Unannotated feature | neg | 242.9435 | 2.417 | 5.426 | 5.332 | -0.094 | -0.032 | 0.868 | 0.954 | Unannotated | NA | NA |
| metab_13205 | Unannotated feature | neg | 222.0367 | 0.533 | 4.145 | 4.188 | 0.042 | -0.032 | 0.868 | 0.954 | Unannotated | NA | NA |
| metab_13196 | Sucralose | neg | 441.0137 | 3.817 | 5.949 | 6.135 | 0.186 | 0.032 | 0.868 | 0.954 | Strong putative | NA | NA |
| metab_11439 | Unannotated feature | neg | 580.7856 | 0.533 | 4.238 | 4.606 | 0.369 | 0.032 | 0.868 | 0.954 | Unannotated | NA | NA |
| metab_5179 | Unannotated feature | pos | 417.3460 | 6.813 | 3.950 | 4.000 | 0.050 | -0.032 | 0.868 | 0.954 | Unannotated | NA | NA |
| metab_1823 | Unannotated feature | pos | 163.0280 | 0.961 | 4.998 | 4.998 | -0.000 | 0.032 | 0.868 | 0.954 | Unannotated | NA | NA |
| metab_10400 | Unannotated feature | neg | 385.1705 | 4.606 | 4.533 | 4.526 | -0.007 | -0.032 | 0.868 | 0.954 | Unannotated | NA | NA |
| metab_12414 | Unannotated feature | neg | 463.8970 | 7.714 | 5.539 | 5.565 | 0.026 | 0.032 | 0.868 | 0.954 | Unannotated | NA | NA |
| metab_19045 | Unannotated feature | neg | 277.1200 | 3.303 | 4.644 | 4.674 | 0.030 | 0.032 | 0.868 | 0.954 | Unannotated | NA | NA |
| metab_1866 | Caffeoylmalic acid | pos | 314.0853 | 1.169 | 5.158 | 4.919 | -0.239 | -0.032 | 0.868 | 0.954 | Unannotated | NA | NA |
| metab_8236 | Unannotated feature | pos | 159.0767 | 2.363 | 4.128 | 4.164 | 0.037 | 0.032 | 0.868 | 0.954 | Unannotated | NA | NA |
| metab_8228 | Unannotated feature | pos | 472.2768 | 3.707 | 4.699 | 4.797 | 0.099 | 0.032 | 0.868 | 0.954 | Unannotated | NA | NA |
| metab_7707 | Uniconazole P | pos | 292.1186 | 3.357 | 3.847 | 3.891 | 0.045 | 0.032 | 0.868 | 0.954 | Tentative | NA | NA |
| metab_5762 | Cyclopamine | pos | 412.3219 | 6.040 | 4.517 | 4.595 | 0.078 | 0.032 | 0.868 | 0.954 | Tentative | Lipids and lipid-like molecules | Steroids and steroid derivatives |
| metab_9607 | Unannotated feature | neg | 255.9915 | 3.223 | 4.342 | 4.167 | -0.175 | -0.032 | 0.868 | 0.954 | Unannotated | NA | NA |
| metab_13778 | Asparagine-betaxanthin | neg | 346.0646 | 1.972 | 3.749 | 3.986 | 0.237 | -0.032 | 0.868 | 0.954 | Unannotated | NA | NA |
| metab_19511 | R-138727 | neg | 386.0636 | 4.837 | 4.652 | 4.512 | -0.140 | 0.032 | 0.868 | 0.954 | Unannotated | NA | NA |
| metab_8606 | Asp Pro Thr | pos | 332.1459 | 1.118 | 3.609 | 3.706 | 0.097 | 0.032 | 0.868 | 0.954 | Moderate putative | NA | NA |
| metab_2967 | Unannotated feature | pos | 94.8096 | 3.017 | 3.482 | 3.474 | -0.009 | 0.032 | 0.868 | 0.954 | Unannotated | NA | NA |
| metab_12729 | Unannotated feature | neg | 209.1291 | 4.498 | 3.089 | 2.797 | -0.292 | -0.032 | 0.868 | 0.954 | Unannotated | NA | NA |
| metab_1178 | Unannotated feature | pos | 301.1548 | 6.510 | 5.449 | 5.659 | 0.211 | 0.032 | 0.868 | 0.954 | Unannotated | NA | NA |
| metab_13584 | Unannotated feature | neg | 126.0302 | 1.332 | 4.260 | 4.271 | 0.011 | -0.032 | 0.868 | 0.954 | Unannotated | NA | NA |
| metab_2802 | Unannotated feature | pos | 167.0565 | 2.651 | 5.091 | 5.163 | 0.072 | 0.032 | 0.868 | 0.954 | Unannotated | NA | NA |
| metab_2863 | Unannotated feature | pos | 160.0607 | 2.788 | 3.800 | 4.103 | 0.303 | 0.032 | 0.868 | 0.954 | Unannotated | NA | NA |
| metab_15722 | Hexylresorcinol | neg | 239.1287 | 5.718 | 4.634 | 4.614 | -0.020 | -0.032 | 0.868 | 0.954 | Unannotated | NA | NA |
| metab_4868 | Unannotated feature | pos | 156.0334 | 7.724 | 4.698 | 4.700 | 0.001 | -0.032 | 0.868 | 0.954 | Unannotated | NA | NA |
| metab_9652 | 2-Hydroxypentanoic Acid | neg | 117.0550 | 3.188 | 5.937 | 5.921 | -0.016 | -0.032 | 0.868 | 0.954 | Strong putative | NA | NA |
| metab_14315 | Unannotated feature | neg | 175.0606 | 3.070 | 4.771 | 4.790 | 0.019 | 0.032 | 0.868 | 0.954 | Unannotated | NA | NA |
| metab_15543 | 6-Methyl-2-methylene-6-octene-1,3,8-triol | neg | 231.1236 | 5.362 | 5.067 | 5.125 | 0.058 | 0.032 | 0.868 | 0.954 | Unannotated | NA | NA |
| metab_12384 | Unannotated feature | neg | 533.3854 | 6.640 | 6.375 | 6.456 | 0.081 | 0.032 | 0.868 | 0.954 | Unannotated | NA | NA |
| metab_293 | Unannotated feature | pos | 207.1081 | 0.485 | 5.922 | 5.893 | -0.029 | -0.032 | 0.868 | 0.954 | Unannotated | NA | NA |
| metab_16796 | Unannotated feature | neg | 145.9411 | 7.710 | 4.173 | 4.165 | -0.008 | -0.032 | 0.868 | 0.954 | Unannotated | NA | NA |
| metab_15224 | Momilactone B | neg | 351.1567 | 4.774 | 4.840 | 4.715 | -0.125 | 0.032 | 0.868 | 0.954 | Unannotated | NA | NA |
| metab_19850 | Benzoate | neg | 301.0722 | 4.653 | 3.413 | 4.265 | 0.852 | 0.032 | 0.868 | 0.954 | Tentative | NA | NA |
| metab_86 | Unannotated feature | pos | 531.3677 | 6.638 | 7.090 | 7.087 | -0.003 | 0.032 | 0.868 | 0.954 | Unannotated | NA | NA |
| metab_3511 | Palmatine | pos | 352.1551 | 4.820 | 3.288 | 3.303 | 0.015 | -0.032 | 0.868 | 0.954 | Strong putative | NA | NA |
| metab_18468 | Unannotated feature | neg | 450.2977 | 6.076 | 5.358 | 5.286 | -0.073 | -0.032 | 0.868 | 0.954 | Unannotated | NA | NA |
| metab_12208 | Petrosaspongiolide m | neg | 441.2631 | 6.828 | 5.303 | 5.436 | 0.133 | 0.032 | 0.868 | 0.954 | Unannotated | NA | NA |
| metab_13637 | 1,3-Cyclohexanedione | neg | 157.0500 | 1.627 | 5.256 | 5.330 | 0.074 | 0.032 | 0.868 | 0.954 | Tentative | NA | NA |
| metab_17281 | Unannotated feature | neg | 203.0597 | 7.720 | 4.950 | 4.946 | -0.004 | -0.032 | 0.868 | 0.954 | Unannotated | NA | NA |
| metab_15991 | Unannotated feature | neg | 420.2305 | 6.099 | 5.222 | 5.251 | 0.029 | -0.032 | 0.868 | 0.954 | Unannotated | NA | NA |
| metab_15473 | Unannotated feature | neg | 416.2198 | 5.209 | 4.061 | 4.047 | -0.013 | 0.032 | 0.868 | 0.954 | Unannotated | NA | NA |
| metab_15451 | 3,4-Methylenedioxyamphetamine | neg | 224.0926 | 5.173 | 4.575 | 4.432 | -0.143 | -0.032 | 0.868 | 0.954 | Tentative | NA | NA |
| metab_7087 | Unannotated feature | pos | 305.1504 | 4.676 | 3.828 | 3.903 | 0.075 | -0.032 | 0.868 | 0.954 | Unannotated | NA | NA |
| metab_14209 | Unannotated feature | neg | 446.8377 | 0.516 | 4.653 | 4.620 | -0.033 | -0.027 | 0.887 | 0.961 | Unannotated | NA | NA |
| metab_8839 | Zeranol | pos | 305.1773 | 6.080 | 4.895 | 5.021 | 0.126 | 0.027 | 0.887 | 0.961 | Tentative | NA | NA |
| metab_149 | Unannotated feature | pos | 180.0036 | 0.617 | 4.132 | 4.065 | -0.067 | -0.027 | 0.887 | 0.961 | Unannotated | NA | NA |
| metab_7920 | 4-Amino-3-phenylbutanoic acid | pos | 144.0810 | 2.941 | 4.864 | 4.760 | -0.103 | -0.027 | 0.887 | 0.961 | Unannotated | NA | NA |
| metab_109 | L-Threonine | pos | 120.0660 | 0.641 | 5.880 | 5.918 | 0.037 | 0.027 | 0.887 | 0.961 | Strong putative | NA | NA |
| metab_7 | Gly Val Thr | pos | 276.1556 | 0.720 | 4.900 | 4.592 | -0.308 | -0.027 | 0.887 | 0.961 | Unannotated | NA | NA |
| metab_15497 | Gibberellin A6 | neg | 345.1346 | 5.264 | 5.458 | 5.437 | -0.022 | -0.027 | 0.887 | 0.961 | Tentative | NA | NA |
| metab_4366 | 1,4-Ipomeadiol | pos | 135.0807 | 6.199 | 4.957 | 4.951 | -0.007 | -0.027 | 0.887 | 0.961 | Unannotated | NA | NA |
| metab_4374 | Unannotated feature | pos | 447.2517 | 6.215 | 4.599 | 4.677 | 0.079 | 0.027 | 0.887 | 0.961 | Unannotated | NA | NA |
| metab_8945 | Unannotated feature | neg | 271.1189 | 3.766 | 4.832 | 5.063 | 0.231 | 0.027 | 0.887 | 0.961 | Unannotated | NA | NA |
| metab_14868 | Unannotated feature | neg | 254.1035 | 4.039 | 4.568 | 4.713 | 0.145 | 0.027 | 0.887 | 0.961 | Unannotated | NA | NA |
| metab_16100 | Unannotated feature | neg | 630.8004 | 0.524 | 5.218 | 5.240 | 0.022 | 0.027 | 0.887 | 0.961 | Unannotated | NA | NA |
| metab_11190 | Unannotated feature | neg | 367.1362 | 0.651 | 5.122 | 5.087 | -0.035 | -0.027 | 0.887 | 0.961 | Unannotated | NA | NA |
| metab_18497 | Posatirelin | neg | 397.2066 | 6.052 | 5.868 | 5.985 | 0.117 | 0.027 | 0.887 | 0.961 | Unannotated | NA | NA |
| metab_8984 | Acetylshikonin | neg | 375.1090 | 4.103 | 4.891 | 5.031 | 0.139 | 0.027 | 0.887 | 0.961 | Tentative | NA | NA |
| metab_15646 | Unannotated feature | neg | 269.9614 | 0.588 | 4.804 | 4.802 | -0.002 | -0.027 | 0.887 | 0.961 | Unannotated | NA | NA |
| metab_16885 | Unannotated feature | neg | 385.8804 | 7.741 | 5.004 | 4.976 | -0.028 | -0.027 | 0.887 | 0.961 | Unannotated | NA | NA |
| metab_3999 | Unannotated feature | pos | 261.1449 | 1.692 | 5.686 | 5.717 | 0.031 | -0.027 | 0.887 | 0.961 | Unannotated | NA | NA |
| metab_884 | Unannotated feature | pos | 711.5185 | 6.861 | 5.444 | 5.228 | -0.216 | 0.027 | 0.887 | 0.961 | Unannotated | NA | NA |
| metab_2222 | Unannotated feature | pos | 208.8843 | 0.593 | 4.827 | 4.839 | 0.012 | -0.027 | 0.887 | 0.961 | Unannotated | NA | NA |
| metab_5218 | Unannotated feature | pos | 189.0975 | 0.485 | 6.768 | 6.710 | -0.058 | -0.027 | 0.887 | 0.961 | Unannotated | NA | NA |
| metab_2282 | Unannotated feature | pos | 367.1509 | 3.245 | 5.823 | 5.895 | 0.072 | 0.027 | 0.887 | 0.961 | Unannotated | NA | NA |
| metab_18796 | 4-Hydroxybenzoic Acid | neg | 137.0237 | 5.789 | 5.137 | 5.186 | 0.048 | 0.027 | 0.887 | 0.961 | Moderate putative | NA | NA |
| metab_294 | Unannotated feature | pos | 193.0561 | 0.485 | 5.513 | 5.474 | -0.039 | -0.027 | 0.887 | 0.961 | Unannotated | NA | NA |
| metab_19646 | Unannotated feature | neg | 339.1563 | 4.648 | 3.953 | 4.308 | 0.355 | 0.027 | 0.887 | 0.961 | Unannotated | NA | NA |
| metab_15856 | (Z)-4-Hydroxy-6-dodecenoic acid lactone | neg | 241.1444 | 5.937 | 4.244 | 4.053 | -0.191 | 0.027 | 0.887 | 0.961 | Tentative | NA | NA |
| metab_12412 | Unannotated feature | neg | 464.8795 | 7.707 | 5.419 | 5.448 | 0.029 | 0.027 | 0.887 | 0.961 | Unannotated | NA | NA |
| metab_2197 | Unannotated feature | pos | 325.1512 | 2.990 | 3.133 | 3.620 | 0.487 | 0.027 | 0.887 | 0.961 | Unannotated | NA | NA |
| metab_9057 | Unannotated feature | neg | 296.0777 | 3.834 | 4.204 | 3.890 | -0.314 | -0.027 | 0.887 | 0.961 | Unannotated | NA | NA |
| metab_16926 | Unannotated feature | neg | 209.9266 | 7.802 | 3.548 | 3.564 | 0.016 | -0.027 | 0.887 | 0.961 | Unannotated | NA | NA |
| metab_14739 | Phthalate | neg | 165.0187 | 3.773 | 6.139 | 6.105 | -0.034 | 0.027 | 0.887 | 0.961 | Moderate putative | NA | NA |
| metab_1836 | Glycerophosphocholine | pos | 290.1349 | 0.744 | 5.711 | 5.742 | 0.031 | 0.027 | 0.887 | 0.961 | Moderate putative | NA | NA |
| metab_18122 | Lithocholic acid glucuronide | neg | 533.3124 | 6.373 | 6.108 | 6.048 | -0.060 | -0.027 | 0.887 | 0.961 | Tentative | NA | NA |
| metab_7563 | 4-Hydroxy-2,2,6,6-tetramethylpiperidine-1-oxyl | pos | 174.1492 | 3.634 | 3.566 | 3.887 | 0.321 | -0.027 | 0.887 | 0.961 | Unannotated | NA | NA |
| metab_7574 | 1-[5-(morpholine-4-carbonyl)-2,3-dihydroindol-1-yl]ethanone | pos | 275.1396 | 3.602 | 4.081 | 4.309 | 0.228 | 0.027 | 0.887 | 0.961 | Moderate putative | NA | NA |
| metab_10300 | Unannotated feature | neg | 247.9115 | 2.405 | 3.748 | 3.645 | -0.103 | -0.027 | 0.887 | 0.961 | Unannotated | NA | NA |
| metab_2349 | Unannotated feature | pos | 129.0229 | 0.469 | 5.133 | 5.110 | -0.023 | -0.027 | 0.887 | 0.961 | Unannotated | NA | NA |
| metab_10348 | Unannotated feature | neg | 247.1186 | 4.592 | 5.419 | 5.549 | 0.129 | 0.027 | 0.887 | 0.961 | Unannotated | NA | NA |
| metab_15817 | Unannotated feature | neg | 470.2304 | 5.888 | 5.164 | 5.749 | 0.585 | 0.027 | 0.887 | 0.961 | Unannotated | NA | NA |
| metab_12912 | Unannotated feature | neg | 393.1830 | 6.248 | 5.531 | 5.646 | 0.115 | 0.027 | 0.887 | 0.961 | Unannotated | NA | NA |
| metab_1034 | Unannotated feature | pos | 408.3457 | 6.901 | 4.368 | 4.318 | -0.051 | -0.027 | 0.887 | 0.961 | Unannotated | NA | NA |
| metab_8243 | Unannotated feature | pos | 250.0391 | 2.299 | 4.539 | 4.502 | -0.037 | 0.027 | 0.887 | 0.961 | Unannotated | NA | NA |
| metab_12460 | Unannotated feature | neg | 186.8971 | 7.893 | 5.374 | 5.363 | -0.011 | -0.027 | 0.887 | 0.961 | Unannotated | NA | NA |
| metab_5984 | Unannotated feature | pos | 170.0665 | 0.485 | 7.028 | 7.016 | -0.013 | -0.027 | 0.887 | 0.961 | Unannotated | NA | NA |
| metab_9349 | Unannotated feature | neg | 297.1092 | 3.548 | 4.156 | 4.128 | -0.029 | -0.027 | 0.887 | 0.961 | Unannotated | NA | NA |
| metab_14703 | Unannotated feature | neg | 229.1192 | 3.705 | 3.953 | 3.870 | -0.083 | -0.027 | 0.887 | 0.961 | Unannotated | NA | NA |
| metab_11365 | Unannotated feature | neg | 282.0085 | 0.571 | 4.434 | 4.443 | 0.010 | -0.027 | 0.887 | 0.961 | Unannotated | NA | NA |
| metab_838 | Unannotated feature | pos | 591.3885 | 6.494 | 5.828 | 5.917 | 0.088 | -0.027 | 0.887 | 0.961 | Unannotated | NA | NA |
| metab_11702 | Unannotated feature | neg | 312.1456 | 5.435 | 5.546 | 5.376 | -0.171 | -0.027 | 0.887 | 0.961 | Unannotated | NA | NA |
| metab_19172 | Hydroxyethyl methacrylic acid | neg | 259.1188 | 5.304 | 5.703 | 5.651 | -0.052 | -0.027 | 0.887 | 0.961 | Tentative | NA | NA |
| metab_8422 | Simmondsin | pos | 408.1878 | 1.944 | 3.379 | 3.559 | 0.180 | 0.027 | 0.887 | 0.961 | Unannotated | Organic oxygen compounds | Organooxygen compounds |
| metab_787 | Unannotated feature | pos | 441.2862 | 6.223 | 4.543 | 4.473 | -0.070 | -0.027 | 0.887 | 0.961 | Unannotated | NA | NA |
| metab_3901 | Mauritine A | pos | 608.3447 | 5.754 | 3.416 | 3.749 | 0.333 | -0.027 | 0.887 | 0.961 | Unannotated | NA | NA |
| metab_16983 | Unannotated feature | neg | 288.8976 | 7.965 | 4.722 | 4.715 | -0.008 | -0.027 | 0.887 | 0.961 | Unannotated | NA | NA |
| metab_17831 | C-2 Ceramide | neg | 340.2856 | 6.782 | 5.309 | 5.324 | 0.014 | 0.027 | 0.887 | 0.961 | Moderate putative | NA | NA |
| metab_2115 | Unannotated feature | pos | 311.1608 | 2.811 | 4.870 | 4.776 | -0.094 | -0.027 | 0.887 | 0.961 | Unannotated | NA | NA |
| metab_5238 | Unannotated feature | pos | 151.0355 | 0.485 | 6.727 | 6.693 | -0.034 | -0.027 | 0.887 | 0.961 | Unannotated | NA | NA |
| metab_17796 | Unannotated feature | neg | 547.3498 | 6.853 | 5.112 | 5.115 | 0.003 | -0.027 | 0.887 | 0.961 | Unannotated | NA | NA |
| metab_16971 | Unannotated feature | neg | 186.9117 | 2.110 | 4.623 | 4.651 | 0.029 | 0.027 | 0.887 | 0.961 | Unannotated | NA | NA |
| metab_18904 | Unannotated feature | neg | 306.1196 | 5.645 | 5.410 | 5.297 | -0.113 | -0.027 | 0.887 | 0.961 | Unannotated | NA | NA |
| metab_12491 | Unannotated feature | neg | 449.2920 | 6.381 | 6.595 | 6.574 | -0.021 | -0.027 | 0.887 | 0.961 | Unannotated | NA | NA |
| metab_10800 | Unannotated feature | neg | 330.1310 | 1.096 | 4.384 | 4.409 | 0.025 | 0.027 | 0.887 | 0.961 | Unannotated | NA | NA |
| metab_1755 | Unannotated feature | pos | 416.2867 | 3.465 | 5.257 | 5.194 | -0.063 | -0.027 | 0.887 | 0.961 | Unannotated | NA | NA |
| metab_19717 | Unannotated feature | neg | 387.9489 | 3.021 | 4.086 | 4.183 | 0.098 | -0.027 | 0.887 | 0.961 | Unannotated | NA | NA |
| metab_205 | Unannotated feature | pos | 84.9117 | 0.593 | 4.210 | 4.195 | -0.015 | 0.027 | 0.887 | 0.961 | Unannotated | NA | NA |
| metab_11920 | Unannotated feature | neg | 335.0871 | 6.083 | 5.310 | 5.438 | 0.128 | 0.027 | 0.887 | 0.961 | Unannotated | NA | NA |
| metab_9691 | Unannotated feature | neg | 293.1243 | 3.971 | 4.109 | 3.899 | -0.210 | 0.027 | 0.887 | 0.961 | Unannotated | NA | NA |
| metab_5455 | Unannotated feature | pos | 200.2012 | 6.390 | 4.714 | 4.705 | -0.009 | -0.027 | 0.887 | 0.961 | Unannotated | NA | NA |
| metab_5404 | Unannotated feature | pos | 253.0936 | 2.049 | 5.959 | 5.907 | -0.052 | -0.027 | 0.887 | 0.961 | Unannotated | NA | NA |
| metab_17512 | Unannotated feature | neg | 330.0696 | 1.952 | 5.231 | 5.158 | -0.073 | -0.027 | 0.887 | 0.961 | Unannotated | NA | NA |
| metab_18069 | Unannotated feature | neg | 470.2764 | 6.420 | 5.652 | 5.519 | -0.133 | -0.027 | 0.887 | 0.961 | Unannotated | NA | NA |
| metab_988 | Unannotated feature | pos | 184.9859 | 7.724 | 6.147 | 6.157 | 0.010 | 0.027 | 0.887 | 0.961 | Unannotated | NA | NA |
| metab_14615 | Isophthalic Acid | neg | 165.0187 | 3.541 | 5.662 | 5.643 | -0.019 | 0.027 | 0.887 | 0.961 | Moderate putative | NA | NA |
| metab_15380 | Unannotated feature | neg | 391.1499 | 5.038 | 3.843 | 3.877 | 0.034 | -0.027 | 0.887 | 0.961 | Unannotated | NA | NA |
| metab_15524 | Unannotated feature | neg | 317.1299 | 5.325 | 4.346 | 4.375 | 0.029 | 0.027 | 0.887 | 0.961 | Unannotated | NA | NA |
| metab_13752 | L-Hypoglycin A | neg | 186.0766 | 1.952 | 5.359 | 5.468 | 0.109 | 0.027 | 0.887 | 0.961 | Tentative | NA | NA |
| metab_14287 | Unannotated feature | neg | 430.8540 | 0.533 | 5.274 | 5.091 | -0.183 | 0.027 | 0.887 | 0.961 | Unannotated | NA | NA |
| metab_19177 | Unannotated feature | neg | 261.1610 | 5.295 | 3.903 | 5.550 | 1.647 | 0.027 | 0.887 | 0.961 | Unannotated | NA | NA |
| metab_4476 | Unannotated feature | pos | 351.2150 | 6.510 | 5.649 | 5.632 | -0.017 | -0.027 | 0.887 | 0.961 | Unannotated | NA | NA |
| metab_5061 | DG(PGD1/16:0/0:0) | pos | 631.4925 | 7.140 | 5.428 | 5.426 | -0.002 | 0.027 | 0.887 | 0.961 | Unannotated | NA | NA |
| metab_14454 | Unannotated feature | neg | 220.0361 | 3.276 | 4.076 | 4.149 | 0.072 | -0.027 | 0.887 | 0.961 | Unannotated | NA | NA |
| metab_14948 | Unannotated feature | neg | 271.9849 | 4.220 | 3.868 | 3.871 | 0.003 | -0.027 | 0.887 | 0.961 | Unannotated | NA | NA |
| metab_1591 | Demethylclomipramine | pos | 283.1334 | 5.165 | 3.881 | 4.032 | 0.151 | 0.027 | 0.887 | 0.961 | Moderate putative | NA | NA |
| metab_1653 | Unannotated feature | pos | 174.1128 | 4.579 | 4.990 | 4.980 | -0.010 | 0.027 | 0.887 | 0.961 | Unannotated | NA | NA |
| metab_12564 | Tetradecanedioic acid | neg | 257.1757 | 6.373 | 6.168 | 6.167 | -0.001 | -0.027 | 0.887 | 0.961 | Strong putative | NA | NA |
| metab_8526 | Unannotated feature | pos | 360.1819 | 3.991 | 4.043 | 4.214 | 0.171 | 0.027 | 0.887 | 0.961 | Unannotated | NA | NA |
| metab_13378 | (S)-2-Hydroxyglutarate | neg | 147.0292 | 1.037 | 6.632 | 6.698 | 0.066 | 0.027 | 0.887 | 0.961 | Tentative | NA | NA |
| metab_9174 | Unannotated feature | neg | 461.9980 | 3.817 | 6.129 | 6.308 | 0.179 | 0.027 | 0.887 | 0.961 | Unannotated | NA | NA |
| metab_4401 | Unannotated feature | pos | 452.3230 | 6.287 | 5.020 | 5.030 | 0.009 | -0.027 | 0.887 | 0.961 | Unannotated | NA | NA |
| metab_16956 | Unannotated feature | neg | 141.8674 | 7.870 | 5.016 | 5.017 | 0.001 | 0.027 | 0.887 | 0.961 | Unannotated | NA | NA |
| metab_12434 | Unannotated feature | neg | 228.9043 | 7.737 | 4.993 | 5.007 | 0.014 | -0.027 | 0.887 | 0.961 | Unannotated | NA | NA |
| metab_16317 | Unannotated feature | neg | 613.4808 | 6.648 | 4.423 | 4.357 | -0.066 | -0.027 | 0.887 | 0.961 | Unannotated | NA | NA |
| metab_14639 | ((4-(4-Amidinophenoxy)butanoyl)aspartyl)valine | neg | 435.1897 | 3.570 | 4.807 | 4.789 | -0.019 | -0.027 | 0.887 | 0.961 | Unannotated | NA | NA |
| metab_8106 | Maltol | pos | 109.0289 | 2.740 | 3.515 | 3.400 | -0.114 | 0.027 | 0.887 | 0.961 | Tentative | NA | NA |
| metab_18350 | Unannotated feature | neg | 416.2328 | 6.170 | 4.794 | 4.598 | -0.197 | -0.027 | 0.887 | 0.961 | Unannotated | NA | NA |
| metab_1121 | Cis-Piceid | pos | 408.1664 | 5.556 | 6.102 | 6.021 | -0.082 | -0.027 | 0.887 | 0.961 | Unannotated | NA | NA |
| metab_9980 | Unannotated feature | neg | 204.0592 | 2.869 | 3.534 | 3.828 | 0.295 | 0.027 | 0.887 | 0.961 | Unannotated | NA | NA |
| metab_9450 | (R)-2,7-Dihydroxy-2H-1,4-benzoxazin-3(4H)-one | neg | 162.0190 | 3.410 | 3.752 | 3.743 | -0.009 | -0.027 | 0.887 | 0.961 | Unannotated | NA | NA |
| metab_9477 | Coniferyl alcohol | neg | 225.0766 | 3.379 | 4.753 | 4.927 | 0.174 | -0.027 | 0.887 | 0.961 | Tentative | NA | NA |
| metab_13892 | Desacetylcephalothin | neg | 375.0066 | 2.442 | 4.354 | 4.972 | 0.618 | 0.027 | 0.887 | 0.961 | Unannotated | NA | NA |
| metab_13867 | Unannotated feature | neg | 199.0065 | 2.298 | 4.351 | 4.335 | -0.017 | -0.027 | 0.887 | 0.961 | Unannotated | NA | NA |
| metab_389 | Unannotated feature | pos | 147.0505 | 0.029 | 3.939 | 3.958 | 0.020 | 0.027 | 0.887 | 0.961 | Unannotated | NA | NA |
| metab_19249 | Unannotated feature | neg | 513.2843 | 5.201 | 4.287 | 4.017 | -0.271 | -0.027 | 0.887 | 0.961 | Unannotated | NA | NA |
| metab_11779 | Unannotated feature | neg | 619.3151 | 5.771 | 5.895 | 5.713 | -0.182 | -0.027 | 0.887 | 0.961 | Unannotated | NA | NA |
| metab_15205 | Unannotated feature | neg | 305.1600 | 4.735 | 3.591 | 3.632 | 0.041 | -0.027 | 0.887 | 0.961 | Unannotated | NA | NA |
| metab_15730 | Oryzalide B | neg | 365.1971 | 5.730 | 5.524 | 5.435 | -0.089 | -0.027 | 0.887 | 0.961 | Unannotated | NA | NA |
| metab_12073 | Unannotated feature | neg | 319.1662 | 5.778 | 5.347 | 6.345 | 0.998 | -0.027 | 0.887 | 0.961 | Unannotated | NA | NA |
| metab_12078 | Unannotated feature | neg | 523.3645 | 6.428 | 6.745 | 6.854 | 0.108 | 0.027 | 0.887 | 0.961 | Unannotated | NA | NA |
| metab_14659 | Unannotated feature | neg | 540.9835 | 0.520 | 4.758 | 5.107 | 0.349 | -0.027 | 0.887 | 0.961 | Unannotated | NA | NA |
| metab_15966 | Unannotated feature | neg | 446.2224 | 6.076 | 3.791 | 2.934 | -0.858 | -0.027 | 0.887 | 0.961 | Unannotated | NA | NA |
| metab_19316 | L-Epicatechol | neg | 289.0722 | 3.422 | 4.990 | 5.053 | 0.063 | 0.027 | 0.887 | 0.961 | Strong putative | NA | NA |
| metab_12238 | N-Oleoyl Leucine | neg | 394.3330 | 6.929 | 5.913 | 5.997 | 0.084 | -0.027 | 0.887 | 0.961 | Moderate putative | NA | NA |
| metab_7452 | Cefprozil | pos | 428.0707 | 3.859 | 4.333 | 4.274 | -0.060 | -0.027 | 0.887 | 0.961 | Tentative | NA | NA |
| metab_2833 | (Z)-[(4-hydroxyphenyl)acetaldehyde oxime] | pos | 169.0974 | 2.740 | 4.227 | 4.263 | 0.036 | 0.027 | 0.887 | 0.961 | Tentative | NA | NA |
| metab_9921 | Unannotated feature | neg | 131.0707 | 4.294 | 5.823 | 5.766 | -0.057 | -0.027 | 0.887 | 0.961 | Unannotated | NA | NA |
| metab_14414 | Unannotated feature | neg | 507.0037 | 3.215 | 5.004 | 4.934 | -0.070 | -0.027 | 0.887 | 0.961 | Unannotated | NA | NA |
| metab_9543 | Unannotated feature | neg | 303.1562 | 3.295 | 3.293 | 3.274 | -0.019 | 0.027 | 0.887 | 0.961 | Unannotated | NA | NA |
| metab_13806 | Unannotated feature | neg | 289.0525 | 2.036 | 4.535 | 4.457 | -0.078 | 0.027 | 0.887 | 0.961 | Unannotated | NA | NA |
| metab_4671 | Unannotated feature | pos | 379.2825 | 7.474 | 5.544 | 5.527 | -0.017 | -0.027 | 0.887 | 0.961 | Unannotated | NA | NA |
| metab_17878 | Unannotated feature | neg | 353.2005 | 6.703 | 6.197 | 6.140 | -0.057 | -0.027 | 0.887 | 0.961 | Unannotated | NA | NA |
| metab_8326 | Unannotated feature | pos | 182.0295 | 2.012 | 4.156 | 4.176 | 0.020 | 0.027 | 0.887 | 0.961 | Unannotated | NA | NA |
| metab_1993 | Unannotated feature | pos | 255.1707 | 1.952 | 4.797 | 4.753 | -0.043 | 0.027 | 0.887 | 0.961 | Unannotated | NA | NA |
| metab_10556 | 5-Dehydroquinic acid | neg | 189.0400 | 1.864 | 4.659 | 4.744 | 0.085 | 0.027 | 0.887 | 0.961 | Strong putative | NA | NA |
| metab_13064 | Unannotated feature | neg | 636.7467 | 0.538 | 4.233 | 4.198 | -0.035 | -0.027 | 0.887 | 0.961 | Unannotated | NA | NA |
| metab_18048 | Unannotated feature | neg | 441.3003 | 6.444 | 1.810 | 1.817 | 0.007 | -0.027 | 0.887 | 0.961 | Unannotated | NA | NA |
| metab_4571 | Unannotated feature | pos | 657.4718 | 6.909 | 2.326 | 2.235 | -0.092 | 0.027 | 0.887 | 0.961 | Unannotated | NA | NA |
| metab_4825 | Unannotated feature | pos | 89.5072 | 7.824 | 4.777 | 4.786 | 0.009 | -0.027 | 0.887 | 0.961 | Unannotated | NA | NA |
| metab_9801 | Unannotated feature | neg | 377.0930 | 4.298 | 3.599 | 3.837 | 0.237 | 0.027 | 0.887 | 0.961 | Unannotated | NA | NA |
| metab_11053 | Unannotated feature | neg | 224.0232 | 0.738 | 4.519 | 4.172 | -0.347 | 0.027 | 0.887 | 0.961 | Unannotated | NA | NA |
| metab_12172 | Unannotated feature | neg | 435.1347 | 5.937 | 3.760 | 4.073 | 0.313 | 0.027 | 0.887 | 0.961 | Unannotated | NA | NA |
| metab_7869 | Unannotated feature | pos | 174.0221 | 3.011 | 3.412 | 3.465 | 0.053 | 0.027 | 0.887 | 0.961 | Unannotated | NA | NA |
| metab_16146 | Octinoxate | neg | 335.1866 | 6.310 | 5.299 | 5.321 | 0.022 | -0.027 | 0.887 | 0.961 | Tentative | NA | NA |
| metab_9969 | Unannotated feature | neg | 146.0533 | 2.885 | 4.347 | 4.470 | 0.123 | 0.027 | 0.887 | 0.961 | Unannotated | NA | NA |
| metab_2963 | 3a-Hydroxydesogestrel | pos | 371.1931 | 2.984 | 4.090 | 4.277 | 0.187 | 0.027 | 0.887 | 0.961 | Unannotated | NA | NA |
| metab_13590 | 2-Mercapto-3-phenylthieno[3,2-d]pyrimidin-4(3H)-one | neg | 280.9843 | 1.354 | 4.870 | 4.865 | -0.005 | 0.027 | 0.887 | 0.961 | Unannotated | NA | NA |
| metab_2845 | Unannotated feature | pos | 205.1551 | 2.762 | 4.383 | 4.414 | 0.032 | -0.027 | 0.887 | 0.961 | Unannotated | NA | NA |
| metab_4773 | Unannotated feature | pos | 108.0233 | 7.764 | 4.357 | 4.360 | 0.003 | -0.027 | 0.887 | 0.961 | Unannotated | NA | NA |
| metab_17163 | Unannotated feature | neg | 479.8697 | 7.760 | 4.904 | 4.892 | -0.012 | -0.027 | 0.887 | 0.961 | Unannotated | NA | NA |
| metab_2020 | Unannotated feature | pos | 409.1980 | 2.430 | 4.269 | 4.036 | -0.233 | 0.027 | 0.887 | 0.961 | Unannotated | NA | NA |
| metab_16361 | 4-(undecan-5-yl)benzene-1-Sulfonic Acid | neg | 311.1688 | 6.734 | 7.524 | 7.517 | -0.007 | 0.027 | 0.887 | 0.961 | Strong putative | NA | NA |
| metab_8310 | 9H-Purine-9-ol | pos | 137.0460 | 2.049 | 6.872 | 6.889 | 0.017 | -0.027 | 0.887 | 0.961 | Unannotated | NA | NA |
| metab_19480 | Hydroxypropionic acid | neg | 179.0555 | 0.667 | 6.523 | 6.577 | 0.054 | 0.027 | 0.887 | 0.961 | Tentative | NA | NA |
| metab_9851 | Desmethyl Fluvoxamine | neg | 341.0888 | 2.994 | 4.409 | 4.462 | 0.053 | 0.027 | 0.887 | 0.961 | Unannotated | NA | NA |
| metab_15316 | Unannotated feature | neg | 434.1872 | 4.910 | 3.386 | 3.342 | -0.044 | 0.027 | 0.887 | 0.961 | Unannotated | NA | NA |
| metab_4554 | Unannotated feature | pos | 693.3260 | 6.829 | 4.662 | 4.749 | 0.088 | 0.027 | 0.887 | 0.961 | Unannotated | NA | NA |
| metab_16904 | Unannotated feature | neg | 128.9743 | 7.765 | 3.902 | 3.906 | 0.003 | 0.027 | 0.887 | 0.961 | Unannotated | NA | NA |
| metab_2 | Unannotated feature | pos | 427.3791 | 7.307 | 8.535 | 8.530 | -0.005 | 0.027 | 0.887 | 0.961 | Unannotated | NA | NA |
| metab_7132 | [3-Oxo-2-(2-penten-1-yl)cyclopentyl]acetic Acid | pos | 211.1332 | 4.579 | 5.136 | 4.764 | -0.372 | -0.027 | 0.887 | 0.961 | Strong putative | NA | NA |
| metab_15468 | Unannotated feature | neg | 495.9254 | 0.571 | 3.889 | 4.157 | 0.269 | 0.027 | 0.887 | 0.961 | Unannotated | NA | NA |
| metab_19821 | Unannotated feature | neg | 321.9048 | 4.401 | 4.307 | 4.270 | -0.036 | -0.027 | 0.887 | 0.961 | Unannotated | NA | NA |
| metab_7105 | (S)-3-Butyl-1(3H)-isobenzofuranone | pos | 191.1070 | 4.625 | 4.430 | 4.566 | 0.136 | -0.027 | 0.887 | 0.961 | Tentative | Organoheterocyclic compounds | Benzofurans |
| metab_14354 | Glutamine-glutamate | neg | 290.0995 | 3.127 | 4.665 | 4.642 | -0.024 | 0.023 | 0.906 | 0.968 | Unannotated | NA | NA |
| metab_3215 | Golotimod | pos | 334.1411 | 3.751 | 3.634 | 3.949 | 0.315 | 0.023 | 0.906 | 0.968 | Unannotated | NA | NA |
| metab_13008 | 2-Hydroxy-2-(2-oxopropyl)butanedioic acid | neg | 189.0399 | 0.815 | 5.418 | 5.379 | -0.039 | 0.023 | 0.906 | 0.968 | Unannotated | NA | NA |
| metab_15483 | Ubenimex | neg | 307.1666 | 5.233 | 3.967 | 5.473 | 1.507 | 0.023 | 0.906 | 0.968 | Tentative | NA | NA |
| metab_12683 | Unannotated feature | neg | 575.3954 | 6.561 | 5.862 | 5.639 | -0.223 | -0.023 | 0.906 | 0.968 | Unannotated | NA | NA |
| metab_1227 | Pentyl 2-furyl ketone | pos | 131.0858 | 6.398 | 5.193 | 5.192 | -0.002 | -0.023 | 0.906 | 0.968 | Tentative | Organic oxygen compounds | Organooxygen compounds |
| metab_14136 | Unannotated feature | neg | 122.0605 | 2.873 | 3.952 | 3.860 | -0.091 | 0.023 | 0.906 | 0.968 | Unannotated | NA | NA |
| metab_4617 | Unannotated feature | pos | 447.3455 | 7.116 | 5.653 | 5.648 | -0.006 | -0.023 | 0.906 | 0.968 | Unannotated | NA | NA |
| metab_10623 | Unannotated feature | neg | 186.0766 | 1.685 | 5.054 | 5.176 | 0.121 | 0.023 | 0.906 | 0.968 | Unannotated | NA | NA |
| metab_17898 | 3-beta-Hydroxy-4-beta-methyl-5-alpha-cholest-7-ene-4-alpha-carboxylate | neg | 489.3592 | 6.663 | 6.562 | 6.571 | 0.009 | -0.023 | 0.906 | 0.968 | Tentative | NA | NA |
| metab_2919 | Chloroethyl nitrosourea | pos | 281.0998 | 2.892 | 4.198 | 4.223 | 0.025 | 0.023 | 0.906 | 0.968 | Unannotated | NA | NA |
| metab_15630 | Unannotated feature | neg | 197.1178 | 5.554 | 4.246 | 4.108 | -0.138 | 0.023 | 0.906 | 0.968 | Unannotated | NA | NA |
| metab_7292 | Unannotated feature | pos | 131.1057 | 0.696 | 4.340 | 4.323 | -0.018 | 0.023 | 0.906 | 0.968 | Unannotated | NA | NA |
| metab_12151 | Unannotated feature | neg | 384.2370 | 6.656 | 5.791 | 6.040 | 0.249 | 0.023 | 0.906 | 0.968 | Unannotated | NA | NA |
| metab_12716 | Unannotated feature | neg | 383.2080 | 5.176 | 5.187 | 5.001 | -0.186 | 0.023 | 0.906 | 0.968 | Unannotated | NA | NA |
| metab_14368 | Unannotated feature | neg | 614.8272 | 0.527 | 4.647 | 4.791 | 0.145 | -0.023 | 0.906 | 0.968 | Unannotated | NA | NA |
| metab_19737 | Unannotated feature | neg | 381.1563 | 3.639 | 4.170 | 4.253 | 0.083 | 0.023 | 0.906 | 0.968 | Unannotated | NA | NA |
| metab_13585 | Unannotated feature | neg | 241.0214 | 1.336 | 4.396 | 4.308 | -0.088 | -0.023 | 0.906 | 0.968 | Unannotated | NA | NA |
| metab_12623 | Unannotated feature | neg | 321.1817 | 6.007 | 5.114 | 5.425 | 0.311 | 0.023 | 0.906 | 0.968 | Unannotated | NA | NA |
| metab_5531 | 2-Propionylpyrrole | pos | 106.0656 | 6.303 | 4.223 | 4.251 | 0.028 | 0.023 | 0.906 | 0.968 | Unannotated | NA | NA |
| metab_11072 | Unannotated feature | neg | 299.0445 | 0.730 | 3.775 | 3.380 | -0.394 | 0.023 | 0.906 | 0.968 | Unannotated | NA | NA |
| metab_11180 | Unannotated feature | neg | 171.0657 | 4.987 | 4.769 | 4.775 | 0.006 | -0.023 | 0.906 | 0.968 | Unannotated | NA | NA |
| metab_11248 | Unannotated feature | neg | 176.0090 | 0.628 | 3.921 | 3.992 | 0.071 | -0.023 | 0.906 | 0.968 | Unannotated | NA | NA |
| metab_1147 | Unannotated feature | pos | 384.2540 | 6.566 | 5.251 | 5.186 | -0.065 | 0.023 | 0.906 | 0.968 | Unannotated | NA | NA |
| metab_11262 | Unannotated feature | neg | 325.9223 | 0.625 | 4.486 | 4.478 | -0.007 | -0.023 | 0.906 | 0.968 | Unannotated | NA | NA |
| metab_17295 | Unannotated feature | neg | 446.9067 | 7.710 | 5.816 | 5.842 | 0.026 | 0.023 | 0.906 | 0.968 | Unannotated | NA | NA |
| metab_13589 | Unannotated feature | neg | 152.0287 | 1.354 | 6.336 | 6.357 | 0.021 | -0.023 | 0.906 | 0.968 | Unannotated | NA | NA |
| metab_17847 | Unannotated feature | neg | 412.2894 | 6.742 | 4.247 | 4.324 | 0.078 | 0.023 | 0.906 | 0.968 | Unannotated | NA | NA |
| metab_7637 | 11,12-Methylenedioxykopsinaline | pos | 416.2142 | 3.477 | 3.265 | 3.005 | -0.260 | 0.023 | 0.906 | 0.968 | Moderate putative | NA | NA |
| metab_5932 | Glutaminylglutamic acid | pos | 592.2564 | 5.922 | 3.768 | 4.010 | 0.241 | 0.023 | 0.906 | 0.968 | Unannotated | NA | NA |
| metab_14567 | Coriandrone D | neg | 397.1510 | 3.457 | 3.711 | 3.344 | -0.367 | -0.023 | 0.906 | 0.968 | Unannotated | NA | NA |
| metab_14576 | Unannotated feature | neg | 365.8859 | 0.524 | 3.017 | 3.271 | 0.254 | -0.023 | 0.906 | 0.968 | Unannotated | NA | NA |
| metab_11527 | Unannotated feature | neg | 392.8921 | 0.502 | 4.907 | 5.073 | 0.166 | 0.023 | 0.906 | 0.968 | Unannotated | NA | NA |
| metab_4975 | Unannotated feature | pos | 88.1126 | 7.546 | 7.461 | 7.459 | -0.002 | -0.023 | 0.906 | 0.968 | Unannotated | NA | NA |
| metab_4981 | Unannotated feature | pos | 619.4924 | 7.522 | 5.142 | 5.146 | 0.004 | -0.023 | 0.906 | 0.968 | Unannotated | NA | NA |
| metab_4985 | Unannotated feature | pos | 164.9207 | 7.515 | 4.658 | 4.551 | -0.107 | 0.023 | 0.906 | 0.968 | Unannotated | NA | NA |
| metab_4594 | Unannotated feature | pos | 332.2927 | 6.997 | 5.819 | 5.880 | 0.061 | -0.023 | 0.906 | 0.968 | Unannotated | NA | NA |
| metab_5607 | 2-Methyl-3-[4-(pyridin-3-ylmethyl)phenyl]prop-2-enoic acid | pos | 295.1446 | 6.223 | 5.313 | 5.118 | -0.195 | -0.023 | 0.906 | 0.968 | Unannotated | NA | NA |
| metab_15522 | Unannotated feature | neg | 288.0690 | 0.577 | 4.387 | 4.654 | 0.267 | 0.023 | 0.906 | 0.968 | Unannotated | NA | NA |
| metab_13713 | Unannotated feature | neg | 231.0703 | 1.854 | 3.240 | 3.354 | 0.114 | -0.023 | 0.906 | 0.968 | Unannotated | NA | NA |
| metab_13730 | Unannotated feature | neg | 142.0503 | 1.947 | 5.324 | 5.382 | 0.057 | -0.023 | 0.906 | 0.968 | Unannotated | NA | NA |
| metab_10486 | Triazolopropionic acid | neg | 252.0627 | 1.952 | 3.233 | 3.587 | 0.354 | -0.023 | 0.906 | 0.968 | Unannotated | NA | NA |
| metab_5325 | 3-Hydroxyoctanedioylcarnitine | pos | 375.2137 | 1.944 | 5.180 | 5.262 | 0.082 | -0.023 | 0.906 | 0.968 | Unannotated | NA | NA |
| metab_9334 | Unannotated feature | neg | 381.1672 | 3.566 | 4.675 | 4.536 | -0.139 | 0.023 | 0.906 | 0.968 | Unannotated | NA | NA |
| metab_11003 | 2-Hydroxyadenine | neg | 150.0415 | 0.777 | 3.736 | 3.667 | -0.069 | 0.023 | 0.906 | 0.968 | Unannotated | NA | NA |
| metab_15704 | Cyclo(Leu-Phe) | neg | 305.1509 | 5.688 | 5.393 | 5.810 | 0.417 | 0.023 | 0.906 | 0.968 | Tentative | NA | NA |
| metab_9991 | Unannotated feature | neg | 179.0375 | 2.858 | 4.704 | 4.727 | 0.022 | -0.023 | 0.906 | 0.968 | Unannotated | NA | NA |
| metab_11089 | N-Acetylhistamine | neg | 152.0822 | 0.730 | 3.898 | 3.504 | -0.393 | 0.023 | 0.906 | 0.968 | Strong putative | NA | NA |
| metab_12732 | Unannotated feature | neg | 401.1248 | 4.451 | 3.228 | 3.303 | 0.075 | -0.023 | 0.906 | 0.968 | Unannotated | NA | NA |
| metab_4736 | 5-Methyl-1H-Benzotriazole | pos | 134.0715 | 7.681 | 4.259 | 4.268 | 0.009 | 0.023 | 0.906 | 0.968 | Strong putative | NA | NA |
| metab_16786 | Unannotated feature | neg | 323.0776 | 1.965 | 3.585 | 3.773 | 0.188 | -0.023 | 0.906 | 0.968 | Unannotated | NA | NA |
| metab_16766 | Unannotated feature | neg | 455.1419 | 1.958 | 4.291 | 4.552 | 0.261 | 0.023 | 0.906 | 0.968 | Unannotated | NA | NA |
| metab_8803 | Dermatan L-iduronate | pos | 473.1781 | 1.239 | 4.472 | 4.016 | -0.456 | 0.023 | 0.906 | 0.968 | Tentative | NA | NA |
| metab_9567 | Unannotated feature | neg | 255.0513 | 3.258 | 4.282 | 4.296 | 0.014 | -0.023 | 0.906 | 0.968 | Unannotated | NA | NA |
| metab_3375 | Unannotated feature | pos | 244.1548 | 4.327 | 4.319 | 4.027 | -0.292 | 0.023 | 0.906 | 0.968 | Unannotated | NA | NA |
| metab_2797 | 3-Methyl-1-phenyl-1-butanone | pos | 163.1120 | 2.630 | 4.116 | 4.134 | 0.018 | 0.023 | 0.906 | 0.968 | Unannotated | NA | NA |
| metab_13513 | Triacetic acid | neg | 189.0399 | 1.061 | 5.696 | 5.761 | 0.065 | 0.023 | 0.906 | 0.968 | Tentative | NA | NA |
| metab_4970 | Unannotated feature | pos | 142.1229 | 7.554 | 5.530 | 5.530 | -0.000 | 0.023 | 0.906 | 0.968 | Unannotated | NA | NA |
| metab_12458 | Unannotated feature | neg | 255.8223 | 7.874 | 6.065 | 6.047 | -0.018 | 0.023 | 0.906 | 0.968 | Unannotated | NA | NA |
| metab_9336 | Unannotated feature | neg | 362.1646 | 3.566 | 3.062 | 3.134 | 0.073 | 0.023 | 0.906 | 0.968 | Unannotated | NA | NA |
| metab_15056 | (+/-)-Sphaerosin | neg | 347.1145 | 4.439 | 4.723 | 4.712 | -0.011 | 0.023 | 0.906 | 0.968 | Unannotated | NA | NA |
| metab_15971 | Unannotated feature | neg | 491.2099 | 6.076 | 5.022 | 4.828 | -0.194 | 0.023 | 0.906 | 0.968 | Unannotated | NA | NA |
| metab_15975 | Unannotated feature | neg | 360.1093 | 6.083 | 6.931 | 7.012 | 0.080 | -0.023 | 0.906 | 0.968 | Unannotated | NA | NA |
| metab_15986 | Unannotated feature | neg | 362.1148 | 6.091 | 5.167 | 5.282 | 0.114 | -0.023 | 0.906 | 0.968 | Unannotated | NA | NA |
| metab_12517 | Unannotated feature | neg | 359.1899 | 6.318 | 6.347 | 6.457 | 0.110 | -0.023 | 0.906 | 0.968 | Unannotated | NA | NA |
| metab_14718 | Paramethadione | neg | 373.1622 | 3.728 | 3.956 | 4.077 | 0.122 | 0.023 | 0.906 | 0.968 | Tentative | Organoheterocyclic compounds | Azolidines |
| metab_896 | Unannotated feature | pos | 371.3163 | 7.005 | 5.817 | 5.812 | -0.005 | -0.023 | 0.906 | 0.968 | Unannotated | NA | NA |
| metab_18971 | Tilisolol | neg | 349.1772 | 5.567 | 4.075 | 4.491 | 0.416 | 0.023 | 0.906 | 0.968 | Unannotated | NA | NA |
| metab_17755 | N-Palmitoyl Leucine | neg | 368.3171 | 6.907 | 5.884 | 5.844 | -0.040 | -0.023 | 0.906 | 0.968 | Unannotated | NA | NA |
| metab_19073 | Unannotated feature | neg | 184.0973 | 5.431 | 3.441 | 3.855 | 0.414 | 0.023 | 0.906 | 0.968 | Unannotated | NA | NA |
| metab_2211 | Unannotated feature | pos | 267.1707 | 3.002 | 4.596 | 4.632 | 0.037 | 0.023 | 0.906 | 0.968 | Unannotated | NA | NA |
| metab_8356 | Unannotated feature | pos | 160.1335 | 1.960 | 4.081 | 4.064 | -0.016 | 0.023 | 0.906 | 0.968 | Unannotated | NA | NA |
| metab_6140 | Unannotated feature | pos | 238.9030 | 0.531 | 4.681 | 4.742 | 0.061 | 0.023 | 0.906 | 0.968 | Unannotated | NA | NA |
| metab_11441 | Unannotated feature | neg | 516.8172 | 0.533 | 5.097 | 5.189 | 0.092 | 0.023 | 0.906 | 0.968 | Unannotated | NA | NA |
| metab_292 | Unannotated feature | pos | 123.0444 | 4.608 | 2.628 | 3.027 | 0.399 | 0.023 | 0.906 | 0.968 | Unannotated | NA | NA |
| metab_254 | Unannotated feature | pos | 322.8647 | 0.531 | 5.094 | 5.218 | 0.124 | 0.023 | 0.906 | 0.968 | Unannotated | NA | NA |
| metab_12123 | Unannotated feature | neg | 593.3356 | 5.871 | 6.021 | 7.303 | 1.282 | 0.023 | 0.906 | 0.968 | Unannotated | NA | NA |
| metab_4760 | Unannotated feature | pos | 316.0025 | 7.724 | 5.235 | 5.225 | -0.010 | 0.023 | 0.906 | 0.968 | Unannotated | NA | NA |
| metab_19080 | Unannotated feature | neg | 285.1092 | 3.318 | 4.813 | 4.808 | -0.004 | -0.023 | 0.906 | 0.968 | Unannotated | NA | NA |
| metab_9441 | Unannotated feature | neg | 134.0604 | 3.422 | 2.800 | 2.816 | 0.016 | -0.023 | 0.906 | 0.968 | Unannotated | NA | NA |
| metab_9453 | Unannotated feature | neg | 335.0778 | 4.083 | 4.334 | 4.601 | 0.267 | -0.023 | 0.906 | 0.968 | Unannotated | NA | NA |
| metab_9167 | Unannotated feature | neg | 121.0288 | 3.829 | 5.850 | 5.998 | 0.148 | 0.023 | 0.906 | 0.968 | Unannotated | NA | NA |
| metab_19703 | Unannotated feature | neg | 216.0486 | 4.554 | 5.048 | 4.993 | -0.055 | -0.023 | 0.906 | 0.968 | Unannotated | NA | NA |
| metab_5689 | Unannotated feature | pos | 637.3249 | 6.127 | 3.551 | 3.554 | 0.003 | -0.023 | 0.906 | 0.968 | Unannotated | NA | NA |
| metab_16037 | Unannotated feature | neg | 393.2656 | 6.170 | 4.638 | 4.664 | 0.025 | 0.023 | 0.906 | 0.968 | Unannotated | NA | NA |
| metab_12583 | Unannotated feature | neg | 387.2394 | 6.099 | 6.125 | 6.030 | -0.095 | -0.023 | 0.906 | 0.968 | Unannotated | NA | NA |
| metab_876 | Unannotated feature | pos | 471.3818 | 6.821 | 4.466 | 4.392 | -0.074 | -0.023 | 0.906 | 0.968 | Unannotated | NA | NA |
| metab_298 | 5-Hydroxy-2-imino-1-methylimidazolidin-4-one | pos | 174.0250 | 0.485 | 5.220 | 5.192 | -0.028 | -0.023 | 0.906 | 0.968 | Unannotated | NA | NA |
| metab_10781 | Unannotated feature | neg | 129.0380 | 1.149 | 5.323 | 5.259 | -0.064 | 0.023 | 0.906 | 0.968 | Unannotated | NA | NA |
| metab_1870 | 8-Amino-7-oxononanoic acid | pos | 229.1551 | 1.196 | 5.298 | 5.254 | -0.043 | -0.023 | 0.906 | 0.968 | Tentative | NA | NA |
| metab_1885 | Unannotated feature | pos | 216.1598 | 4.790 | 4.398 | 4.286 | -0.112 | -0.023 | 0.906 | 0.968 | Unannotated | NA | NA |
| metab_11665 | Unannotated feature | neg | 377.0706 | 5.308 | 4.614 | 5.053 | 0.438 | -0.023 | 0.906 | 0.968 | Unannotated | NA | NA |
| metab_2566 | Saccharopine | pos | 259.1293 | 1.155 | 5.484 | 5.512 | 0.029 | 0.023 | 0.906 | 0.968 | Tentative | NA | NA |
| metab_15761 | S-(Formylmethyl)glutathione | neg | 370.0684 | 5.789 | 5.025 | 5.180 | 0.156 | -0.023 | 0.906 | 0.968 | Tentative | NA | NA |
| metab_2725 | 2,6-Pyridinedicarboxylic Acid | pos | 168.0294 | 2.187 | 3.630 | 3.679 | 0.049 | 0.023 | 0.906 | 0.968 | Moderate putative | NA | NA |
| metab_18314 | Unannotated feature | neg | 297.1268 | 6.209 | 4.421 | 4.463 | 0.042 | -0.023 | 0.906 | 0.968 | Unannotated | NA | NA |
| metab_10999 | Deoxycytidine | neg | 226.0831 | 0.777 | 3.797 | 3.763 | -0.034 | -0.023 | 0.906 | 0.968 | Moderate putative | NA | NA |
| metab_16240 | Unannotated feature | neg | 233.1544 | 6.498 | 3.845 | 4.004 | 0.160 | -0.023 | 0.906 | 0.968 | Unannotated | NA | NA |
| metab_9156 | Unannotated feature | neg | 161.0813 | 3.842 | 5.049 | 5.044 | -0.005 | -0.023 | 0.906 | 0.968 | Unannotated | NA | NA |
| metab_4437 | Unannotated feature | pos | 363.2531 | 6.367 | 4.853 | 4.888 | 0.035 | 0.023 | 0.906 | 0.968 | Unannotated | NA | NA |
| metab_4454 | N-Docosahexaenoyl Asparagine | pos | 443.2892 | 6.414 | 5.791 | 5.694 | -0.097 | -0.023 | 0.906 | 0.968 | Unannotated | NA | NA |
| metab_5068 | Unannotated feature | pos | 393.2621 | 7.132 | 4.882 | 4.851 | -0.031 | -0.023 | 0.906 | 0.968 | Unannotated | NA | NA |
| metab_1624 | Unannotated feature | pos | 293.1026 | 3.734 | 3.112 | 3.548 | 0.435 | -0.023 | 0.906 | 0.968 | Unannotated | NA | NA |
| metab_18293 | Unannotated feature | neg | 235.0223 | 2.984 | 5.115 | 4.921 | -0.194 | 0.023 | 0.906 | 0.968 | Unannotated | NA | NA |
| metab_11348 | Unannotated feature | neg | 465.9063 | 0.582 | 4.362 | 4.746 | 0.384 | 0.023 | 0.906 | 0.968 | Unannotated | NA | NA |
| metab_10896 | Unannotated feature | neg | 278.0019 | 0.889 | 4.281 | 4.298 | 0.017 | -0.023 | 0.906 | 0.968 | Unannotated | NA | NA |
| metab_13269 | Unannotated feature | neg | 110.0716 | 0.614 | 3.080 | 3.257 | 0.178 | 0.023 | 0.906 | 0.968 | Unannotated | NA | NA |
| metab_13276 | Unannotated feature | neg | 471.0768 | 0.614 | 4.355 | 4.478 | 0.123 | -0.023 | 0.906 | 0.968 | Unannotated | NA | NA |
| metab_5122 | N-Acetylmannosamine | pos | 204.0871 | 0.807 | 6.519 | 6.562 | 0.043 | -0.023 | 0.906 | 0.968 | Tentative | NA | NA |
| metab_861 | Unannotated feature | pos | 458.7713 | 0.593 | 4.928 | 4.981 | 0.054 | 0.023 | 0.906 | 0.968 | Unannotated | NA | NA |
| metab_866 | Unannotated feature | pos | 541.3880 | 6.694 | 6.370 | 6.232 | -0.138 | 0.023 | 0.906 | 0.968 | Unannotated | NA | NA |
| metab_16038 | Delta9-Tetrahydrocannabinol hemisuccinate | neg | 459.2402 | 6.170 | 5.285 | 5.049 | -0.236 | -0.023 | 0.906 | 0.968 | Unannotated | NA | NA |
| metab_12534 | Unannotated feature | neg | 411.3057 | 6.248 | 6.323 | 6.277 | -0.046 | -0.023 | 0.906 | 0.968 | Unannotated | NA | NA |
| metab_1050 | Hericene B | pos | 615.4619 | 6.821 | 6.746 | 6.836 | 0.090 | -0.023 | 0.906 | 0.968 | Unannotated | NA | NA |
| metab_10171 | Unannotated feature | neg | 200.9859 | 2.707 | 4.695 | 4.698 | 0.003 | 0.023 | 0.906 | 0.968 | Unannotated | NA | NA |
| metab_5398 | Alpha-Irone | pos | 207.1746 | 6.446 | 3.712 | 3.805 | 0.093 | 0.023 | 0.906 | 0.968 | Moderate putative | NA | NA |
| metab_10839 | Unannotated feature | neg | 181.9945 | 1.041 | 4.442 | 4.278 | -0.165 | -0.023 | 0.906 | 0.968 | Unannotated | NA | NA |
| metab_10818 | Aceneuramic acid | neg | 290.0885 | 1.061 | 7.258 | 7.275 | 0.017 | 0.023 | 0.906 | 0.968 | Tentative | NA | NA |
| metab_10820 | Tetrahydrodipicolinate | neg | 216.0510 | 1.061 | 6.040 | 6.010 | -0.030 | 0.023 | 0.906 | 0.968 | Tentative | NA | NA |
| metab_7772 | Unannotated feature | pos | 679.8181 | 3.261 | 4.703 | 4.661 | -0.041 | 0.023 | 0.906 | 0.968 | Unannotated | NA | NA |
| metab_10233 | Unannotated feature | neg | 215.0381 | 2.583 | 3.872 | 3.981 | 0.108 | 0.023 | 0.906 | 0.968 | Unannotated | NA | NA |
| metab_4678 | Unannotated feature | pos | 579.2721 | 7.498 | 5.868 | 5.788 | -0.079 | -0.023 | 0.906 | 0.968 | Unannotated | NA | NA |
| metab_15840 | Alniditan | neg | 347.2078 | 5.914 | 5.315 | 5.292 | -0.023 | 0.023 | 0.906 | 0.968 | Unannotated | NA | NA |
| metab_17016 | Unannotated feature | neg | 306.9977 | 7.997 | 4.473 | 4.444 | -0.029 | -0.023 | 0.906 | 0.968 | Unannotated | NA | NA |
| metab_8772 | Unannotated feature | pos | 197.1288 | 0.728 | 3.813 | 3.743 | -0.070 | 0.023 | 0.906 | 0.968 | Unannotated | NA | NA |
| metab_10786 | Unannotated feature | neg | 264.9887 | 1.127 | 4.887 | 4.952 | 0.065 | 0.023 | 0.906 | 0.968 | Unannotated | NA | NA |
| metab_12880 | Unannotated feature | neg | 701.1934 | 0.714 | 5.243 | 5.232 | -0.011 | -0.023 | 0.906 | 0.968 | Unannotated | NA | NA |
| metab_18220 | Stearidonic acid | neg | 321.2071 | 6.279 | 4.568 | 4.852 | 0.284 | 0.023 | 0.906 | 0.968 | Tentative | NA | NA |
| metab_12903 | Unannotated feature | neg | 259.0935 | 0.730 | 4.482 | 4.452 | -0.031 | -0.023 | 0.906 | 0.968 | Unannotated | NA | NA |
| metab_5434 | Unannotated feature | pos | 278.2097 | 6.406 | 3.817 | 4.040 | 0.222 | -0.023 | 0.906 | 0.968 | Unannotated | NA | NA |
| metab_16244 | Unannotated feature | neg | 214.0215 | 1.364 | 6.695 | 6.681 | -0.014 | -0.023 | 0.906 | 0.968 | Unannotated | NA | NA |
| metab_19726 | Unannotated feature | neg | 252.0336 | 4.522 | 3.993 | 4.239 | 0.246 | 0.023 | 0.906 | 0.968 | Unannotated | NA | NA |
| metab_19727 | Unannotated feature | neg | 244.0462 | 3.647 | 4.532 | 4.491 | -0.041 | -0.023 | 0.906 | 0.968 | Unannotated | NA | NA |
| metab_17123 | Unannotated feature | neg | 130.9916 | 7.827 | 4.333 | 4.350 | 0.017 | 0.023 | 0.906 | 0.968 | Unannotated | NA | NA |
| metab_9588 | Unannotated feature | neg | 450.1463 | 3.234 | 4.308 | 4.526 | 0.218 | -0.023 | 0.906 | 0.968 | Unannotated | NA | NA |
| metab_2452 | Unannotated feature | pos | 236.9052 | 0.531 | 5.710 | 5.706 | -0.004 | 0.023 | 0.906 | 0.968 | Unannotated | NA | NA |
| metab_4534 | Taccagenin | pos | 479.3357 | 6.749 | 7.413 | 7.426 | 0.014 | 0.023 | 0.906 | 0.968 | Unannotated | NA | NA |
| metab_13705 | 3'-Deoxyinosine | neg | 251.0787 | 1.772 | 6.618 | 6.496 | -0.121 | -0.023 | 0.906 | 0.968 | Unannotated | NA | NA |
| metab_9675 | (R)-3,4-Dihydro-6,8-dihydroxy-3-methyl-1H-2-benzopyran-1-one | neg | 193.0506 | 3.157 | 4.132 | 4.212 | 0.079 | -0.023 | 0.906 | 0.968 | Unannotated | NA | NA |
| metab_9730 | Unannotated feature | neg | 341.0883 | 4.254 | 4.097 | 4.481 | 0.383 | 0.023 | 0.906 | 0.968 | Unannotated | NA | NA |
| metab_9746 | Azeloprazole | neg | 450.1463 | 3.082 | 4.060 | 4.105 | 0.044 | -0.023 | 0.906 | 0.968 | Unannotated | Organoheterocyclic compounds | Benzimidazoles |
| metab_7112 | Unannotated feature | pos | 163.1119 | 4.620 | 4.179 | 4.312 | 0.133 | -0.023 | 0.906 | 0.968 | Unannotated | NA | NA |
| metab_16340 | Unannotated feature | neg | 529.6433 | 6.695 | 6.882 | 6.849 | -0.033 | -0.023 | 0.906 | 0.968 | Unannotated | NA | NA |
| metab_11468 | Unannotated feature | neg | 378.9154 | 0.524 | 5.490 | 5.611 | 0.121 | -0.023 | 0.906 | 0.968 | Unannotated | NA | NA |
| metab_2034 | Unannotated feature | pos | 209.1288 | 2.513 | 4.628 | 4.647 | 0.020 | 0.023 | 0.906 | 0.968 | Unannotated | NA | NA |
| metab_1995 | Unannotated feature | pos | 230.1139 | 1.952 | 5.666 | 5.696 | 0.030 | -0.023 | 0.906 | 0.968 | Unannotated | NA | NA |
| metab_17913 | Unannotated feature | neg | 616.4636 | 6.648 | 5.684 | 5.656 | -0.028 | 0.023 | 0.906 | 0.968 | Unannotated | NA | NA |
| metab_14586 | Unannotated feature | neg | 363.0305 | 0.524 | 3.380 | 3.382 | 0.003 | -0.023 | 0.906 | 0.968 | Unannotated | NA | NA |
| metab_10542 | Unannotated feature | neg | 154.0503 | 1.944 | 5.084 | 5.105 | 0.022 | 0.023 | 0.906 | 0.968 | Unannotated | NA | NA |
| metab_9220 | Neomenthol-glucuronide | neg | 353.1589 | 3.938 | 5.157 | 5.110 | -0.047 | -0.023 | 0.906 | 0.968 | Unannotated | NA | NA |
| metab_13855 | Unannotated feature | neg | 328.9981 | 2.241 | 4.719 | 4.698 | -0.021 | -0.023 | 0.906 | 0.968 | Unannotated | NA | NA |
| metab_8842 | Unannotated feature | neg | 218.0667 | 0.714 | 5.886 | 5.895 | 0.009 | -0.023 | 0.906 | 0.968 | Unannotated | NA | NA |
| metab_12655 | 9-Amino-1,2,3,4-tetrahydroacridin-2-ol | neg | 473.2197 | 5.930 | 4.574 | 4.404 | -0.169 | 0.023 | 0.906 | 0.968 | Unannotated | NA | NA |
| metab_18023 | Unannotated feature | neg | 400.2326 | 6.483 | 4.838 | 4.693 | -0.145 | 0.023 | 0.906 | 0.968 | Unannotated | NA | NA |
| metab_8754 | Gibberellin A4 glucosyl ester | pos | 248.1134 | 0.728 | 4.731 | 4.852 | 0.121 | 0.023 | 0.906 | 0.968 | Unannotated | NA | NA |
| metab_12026 | Unannotated feature | neg | 351.2179 | 6.287 | 6.071 | 6.088 | 0.018 | -0.023 | 0.906 | 0.968 | Unannotated | NA | NA |
| metab_9492 | Myxin | neg | 303.0627 | 3.358 | 4.414 | 4.156 | -0.258 | 0.023 | 0.906 | 0.968 | Unannotated | NA | NA |
| metab_16790 | Unannotated feature | neg | 354.9097 | 7.702 | 3.670 | 3.546 | -0.124 | 0.019 | 0.924 | 0.974 | Unannotated | NA | NA |
| metab_16731 | Unannotated feature | neg | 306.9192 | 7.663 | 5.755 | 5.743 | -0.013 | -0.019 | 0.924 | 0.974 | Unannotated | NA | NA |
| metab_8749 | 2,4-Thiazolidinedicarboxylic acid, 2-methyl- | pos | 192.0328 | 0.736 | 4.226 | 4.081 | -0.146 | -0.019 | 0.924 | 0.974 | Unannotated | NA | NA |
| metab_5800 | Unannotated feature | pos | 309.1690 | 6.008 | 3.563 | 3.978 | 0.415 | 0.019 | 0.924 | 0.974 | Unannotated | NA | NA |
| metab_12464 | Unannotated feature | neg | 257.8192 | 7.914 | 6.038 | 6.015 | -0.024 | -0.019 | 0.924 | 0.974 | Unannotated | NA | NA |
| metab_12012 | Unannotated feature | neg | 471.2428 | 6.263 | 8.093 | 8.063 | -0.030 | 0.019 | 0.924 | 0.974 | Unannotated | NA | NA |
| metab_15417 | Unannotated feature | neg | 361.0120 | 0.566 | 4.423 | 4.254 | -0.169 | -0.019 | 0.924 | 0.974 | Unannotated | NA | NA |
| metab_11168 | Unannotated feature | neg | 80.9642 | 0.675 | 4.083 | 4.138 | 0.055 | -0.019 | 0.924 | 0.974 | Unannotated | NA | NA |
| metab_15533 | Unannotated feature | neg | 291.1273 | 5.343 | 4.572 | 4.757 | 0.185 | -0.019 | 0.924 | 0.974 | Unannotated | NA | NA |
| metab_12699 | Unannotated feature | neg | 389.0919 | 5.362 | 4.461 | 4.715 | 0.254 | 0.019 | 0.924 | 0.974 | Unannotated | NA | NA |
| metab_18508 | Unannotated feature | neg | 303.2178 | 6.046 | 4.820 | 4.695 | -0.125 | 0.019 | 0.924 | 0.974 | Unannotated | NA | NA |
| metab_18445 | Alkhanin | neg | 285.1133 | 6.099 | 4.923 | 5.102 | 0.179 | 0.019 | 0.924 | 0.974 | Unannotated | Lipids and lipid-like molecules | Prenol lipids |
| metab_4628 | Unannotated feature | pos | 475.3248 | 7.140 | 5.001 | 4.983 | -0.018 | 0.019 | 0.924 | 0.974 | Unannotated | NA | NA |
| metab_1920 | Unannotated feature | pos | 229.1187 | 1.698 | 5.639 | 5.643 | 0.004 | 0.019 | 0.924 | 0.974 | Unannotated | NA | NA |
| metab_1970 | Unannotated feature | pos | 223.1445 | 2.012 | 4.260 | 4.147 | -0.113 | 0.019 | 0.924 | 0.974 | Unannotated | NA | NA |
| metab_5275 | Unannotated feature | pos | 551.3926 | 6.622 | 5.216 | 5.314 | 0.098 | 0.019 | 0.924 | 0.974 | Unannotated | NA | NA |
| metab_3049 | Unannotated feature | pos | 444.2455 | 3.295 | 3.500 | 3.556 | 0.056 | 0.019 | 0.924 | 0.974 | Unannotated | NA | NA |
| metab_15347 | 2-Propenamide, 2-cyano-3-(4-hydroxy-3,5-bis(1-methylethyl)phenyl)- | neg | 317.1513 | 4.992 | 4.755 | 4.839 | 0.083 | 0.019 | 0.924 | 0.974 | Unannotated | NA | NA |
| metab_3327 | Alpha-Carboxy-delta-decalactone | pos | 232.1547 | 4.156 | 4.628 | 4.591 | -0.037 | -0.019 | 0.924 | 0.974 | Unannotated | NA | NA |
| metab_19676 | 2-Hydroxyhept-5-enoylcarnitine | neg | 286.1666 | 4.600 | 3.543 | 3.577 | 0.034 | 0.019 | 0.924 | 0.974 | Unannotated | NA | NA |
| metab_18700 | Allixin | neg | 271.1187 | 5.905 | 4.711 | 4.667 | -0.043 | 0.019 | 0.924 | 0.974 | Unannotated | Organoheterocyclic compounds | Pyrans |
| metab_18693 | Unannotated feature | neg | 285.1817 | 5.909 | 2.954 | 2.835 | -0.119 | 0.019 | 0.924 | 0.974 | Unannotated | NA | NA |
| metab_14947 | Unannotated feature | neg | 187.0065 | 4.220 | 5.826 | 5.906 | 0.080 | 0.019 | 0.924 | 0.974 | Unannotated | NA | NA |
| metab_9592 | Unannotated feature | neg | 219.1235 | 3.234 | 4.320 | 4.302 | -0.018 | 0.019 | 0.924 | 0.974 | Unannotated | NA | NA |
| metab_3914 | Ganoderic acid eta | pos | 515.2996 | 6.351 | 5.589 | 5.701 | 0.112 | 0.019 | 0.924 | 0.974 | Unannotated | NA | NA |
| metab_3852 | Unannotated feature | pos | 568.6092 | 5.677 | 3.895 | 3.856 | -0.039 | 0.019 | 0.924 | 0.974 | Unannotated | NA | NA |
| metab_7512 | Glu Asp Lys | pos | 391.1835 | 0.720 | 4.757 | 4.708 | -0.049 | 0.019 | 0.924 | 0.974 | Moderate putative | NA | NA |
| metab_11697 | Unannotated feature | neg | 408.0943 | 4.123 | 5.151 | 5.113 | -0.038 | -0.019 | 0.924 | 0.974 | Unannotated | NA | NA |
| metab_8436 | Ethylmorphine | pos | 314.1758 | 3.882 | 4.852 | 4.899 | 0.047 | 0.019 | 0.924 | 0.974 | Tentative | NA | NA |
| metab_842 | PC(18:2/0:0) | pos | 520.3408 | 6.518 | 6.377 | 6.380 | 0.003 | 0.019 | 0.924 | 0.974 | Strong putative | NA | NA |
| metab_16818 | Unannotated feature | neg | 319.8897 | 7.720 | 4.591 | 4.565 | -0.026 | -0.019 | 0.924 | 0.974 | Unannotated | NA | NA |
| metab_10842 | Adenosine dialdehyde | neg | 246.0619 | 1.037 | 4.898 | 4.986 | 0.088 | 0.019 | 0.924 | 0.974 | Unannotated | NA | NA |
| metab_18250 | Unannotated feature | neg | 421.2455 | 6.256 | 3.987 | 3.966 | -0.021 | -0.019 | 0.924 | 0.974 | Unannotated | NA | NA |
| metab_8132 | (2R)-2-Amino-6-[[(3S,4R,5R)-3,4,5,6-tetrahydroxy-2-oxohexyl]amino]hexanoic acid | pos | 273.1450 | 2.724 | 4.408 | 4.500 | 0.093 | 0.019 | 0.924 | 0.974 | Unannotated | NA | NA |
| metab_5441 | (12Z)-10-Hydroxyoctadecenoylcarnitine | pos | 424.3433 | 6.398 | 5.796 | 5.838 | 0.041 | 0.019 | 0.924 | 0.974 | Unannotated | NA | NA |
| metab_12811 | Unannotated feature | neg | 253.0566 | 0.651 | 5.098 | 5.134 | 0.036 | 0.019 | 0.924 | 0.974 | Unannotated | NA | NA |
| metab_13331 | Unannotated feature | neg | 190.0174 | 0.691 | 3.610 | 3.667 | 0.057 | -0.019 | 0.924 | 0.974 | Unannotated | NA | NA |
| metab_835 | R-Palmitoyl-(2-methyl) Ethanolamide | pos | 314.3059 | 6.462 | 5.611 | 5.730 | 0.119 | -0.019 | 0.924 | 0.974 | Moderate putative | NA | NA |
| metab_868 | Unannotated feature | pos | 515.3202 | 6.702 | 5.923 | 5.891 | -0.031 | 0.019 | 0.924 | 0.974 | Unannotated | NA | NA |
| metab_12530 | Unannotated feature | neg | 623.3094 | 6.248 | 4.915 | 4.829 | -0.086 | -0.019 | 0.924 | 0.974 | Unannotated | NA | NA |
| metab_16018 | Unannotated feature | neg | 366.1748 | 6.138 | 4.345 | 4.406 | 0.060 | -0.019 | 0.924 | 0.974 | Unannotated | NA | NA |
| metab_16005 | Unannotated feature | neg | 265.0679 | 1.100 | 6.217 | 6.161 | -0.056 | -0.019 | 0.924 | 0.974 | Unannotated | NA | NA |
| metab_10350 | Unannotated feature | neg | 166.0139 | 2.208 | 3.766 | 3.770 | 0.004 | 0.019 | 0.924 | 0.974 | Unannotated | NA | NA |
| metab_10284 | Unannotated feature | neg | 235.1186 | 2.465 | 3.318 | 3.619 | 0.301 | 0.019 | 0.924 | 0.974 | Unannotated | NA | NA |
| metab_16503 | DG(18:1(9Z)-O(12,13)/0:0/i-19:0) | neg | 671.5273 | 7.097 | 6.339 | 6.421 | 0.082 | 0.019 | 0.924 | 0.974 | Unannotated | NA | NA |
| metab_11332 | Unannotated feature | neg | 471.2612 | 5.140 | 3.878 | 3.904 | 0.026 | -0.019 | 0.924 | 0.974 | Unannotated | NA | NA |
| metab_12824 | (2S)-3-[3-[(4-Carbamimidoylbenzoyl)amino]propanoylamino]-2-[(4-ethylphenyl)sulfonylamino]propanoic acid | neg | 488.1632 | 0.659 | 5.188 | 5.277 | 0.088 | 0.019 | 0.924 | 0.974 | Unannotated | NA | NA |
| metab_7808 | Unannotated feature | pos | 310.1481 | 3.303 | 4.462 | 4.543 | 0.081 | 0.019 | 0.924 | 0.974 | Unannotated | NA | NA |
| metab_5074 | Unannotated feature | pos | 675.4248 | 7.108 | 6.647 | 6.648 | 0.001 | 0.019 | 0.924 | 0.974 | Unannotated | NA | NA |
| metab_5041 | Unannotated feature | pos | 357.3004 | 7.212 | 6.275 | 6.284 | 0.010 | -0.019 | 0.924 | 0.974 | Unannotated | NA | NA |
| metab_10132 | Unannotated feature | neg | 639.1764 | 2.750 | 4.320 | 4.357 | 0.037 | -0.019 | 0.924 | 0.974 | Unannotated | NA | NA |
| metab_9137 | Unannotated feature | neg | 170.0817 | 3.872 | 3.753 | 3.917 | 0.164 | -0.019 | 0.924 | 0.974 | Unannotated | NA | NA |
| metab_19237 | Buparlisib | neg | 455.1696 | 5.212 | 4.978 | 4.849 | -0.129 | 0.019 | 0.924 | 0.974 | Unannotated | NA | NA |
| metab_13733 | NOTA | neg | 284.1254 | 1.947 | 4.638 | 4.609 | -0.029 | -0.019 | 0.924 | 0.974 | Unannotated | NA | NA |
| metab_2609 | Glutamine hydroxamate | pos | 127.0506 | 1.677 | 5.667 | 5.639 | -0.029 | 0.019 | 0.924 | 0.974 | Unannotated | NA | NA |
| metab_18676 | Unannotated feature | neg | 133.0135 | 5.927 | 4.100 | 4.140 | 0.041 | 0.019 | 0.924 | 0.974 | Unannotated | NA | NA |
| metab_5470 | Unannotated feature | pos | 702.4958 | 6.375 | 5.210 | 5.570 | 0.360 | 0.019 | 0.924 | 0.974 | Unannotated | NA | NA |
| metab_17380 | Unannotated feature | neg | 349.9123 | 7.666 | 4.963 | 4.984 | 0.021 | 0.019 | 0.924 | 0.974 | Unannotated | NA | NA |
| metab_2719 | Unannotated feature | pos | 83.0497 | 2.150 | 4.290 | 4.295 | 0.006 | 0.019 | 0.924 | 0.974 | Unannotated | NA | NA |
| metab_12276 | Unannotated feature | neg | 765.5694 | 7.080 | 5.387 | 5.641 | 0.253 | 0.019 | 0.924 | 0.974 | Unannotated | NA | NA |
| metab_11760 | Unannotated feature | neg | 521.1838 | 5.688 | 3.331 | 2.649 | -0.682 | -0.019 | 0.924 | 0.974 | Unannotated | NA | NA |
| metab_2594 | Galactosylhydroxylysine | pos | 289.1400 | 1.506 | 5.330 | 5.230 | -0.100 | -0.019 | 0.924 | 0.974 | Tentative | NA | NA |
| metab_2590 | (S)-5-Amino-3-oxohexanoate | pos | 291.1556 | 1.409 | 4.074 | 4.131 | 0.057 | -0.019 | 0.924 | 0.974 | Tentative | NA | NA |
| metab_10366 | Unannotated feature | neg | 241.8989 | 2.151 | 4.381 | 4.461 | 0.081 | -0.019 | 0.924 | 0.974 | Unannotated | NA | NA |
| metab_10364 | Unannotated feature | neg | 185.8651 | 2.166 | 3.962 | 4.040 | 0.078 | 0.019 | 0.924 | 0.974 | Unannotated | NA | NA |
| metab_7567 | Ala Glu Lys Asp | pos | 231.6136 | 0.728 | 4.976 | 5.020 | 0.044 | -0.019 | 0.924 | 0.974 | Moderate putative | NA | NA |
| metab_7550 | Nomega-Acetylhistamine | pos | 154.0977 | 0.728 | 5.582 | 5.457 | -0.125 | 0.019 | 0.924 | 0.974 | Strong putative | NA | NA |
| metab_11673 | Unannotated feature | neg | 378.1200 | 5.330 | 5.806 | 5.800 | -0.006 | 0.019 | 0.924 | 0.974 | Unannotated | NA | NA |
| metab_10751 | Aloesone 7-O-glucoside | neg | 375.1090 | 4.791 | 5.206 | 5.319 | 0.113 | 0.019 | 0.924 | 0.974 | Unannotated | Organic oxygen compounds | Organooxygen compounds |
| metab_10746 | 1-Methylguanine | neg | 164.0571 | 1.237 | 5.252 | 4.938 | -0.315 | -0.019 | 0.924 | 0.974 | Moderate putative | NA | NA |
| metab_1891 | Hydroxysimazine | pos | 184.1196 | 1.378 | 4.485 | 4.433 | -0.051 | -0.019 | 0.924 | 0.974 | Unannotated | NA | NA |
| metab_10778 | Unannotated feature | neg | 281.9737 | 1.149 | 4.434 | 4.193 | -0.241 | -0.019 | 0.924 | 0.974 | Unannotated | NA | NA |
| metab_19574 | Unannotated feature | neg | 271.1190 | 3.552 | 4.495 | 4.861 | 0.366 | 0.019 | 0.924 | 0.974 | Unannotated | NA | NA |
| metab_7844 | Unannotated feature | pos | 309.1814 | 3.066 | 4.227 | 3.910 | -0.318 | -0.019 | 0.924 | 0.974 | Unannotated | NA | NA |
| metab_13478 | Unannotated feature | neg | 290.9378 | 0.985 | 4.003 | 4.093 | 0.090 | -0.019 | 0.924 | 0.974 | Unannotated | NA | NA |
| metab_2694 | Zidovudine | pos | 268.1045 | 2.028 | 6.097 | 6.001 | -0.096 | -0.019 | 0.924 | 0.974 | Tentative | NA | NA |
| metab_11770 | Delpazolid | neg | 353.1269 | 5.743 | 4.372 | 4.580 | 0.208 | 0.019 | 0.924 | 0.974 | Unannotated | NA | NA |
| metab_19283 | Valine-betaxanthin | neg | 355.1128 | 5.156 | 4.263 | 4.071 | -0.192 | -0.019 | 0.924 | 0.974 | Unannotated | NA | NA |
| metab_750 | Unannotated feature | pos | 284.9350 | 0.531 | 4.712 | 4.823 | 0.111 | 0.019 | 0.924 | 0.974 | Unannotated | NA | NA |
| metab_733 | 1-(6Z,9Z,12Z,15Z-octadecatetraenoyl)-glycero-3-phosphate | pos | 494.2277 | 6.000 | 4.538 | 4.948 | 0.410 | 0.019 | 0.924 | 0.974 | Unannotated | NA | NA |
| metab_9769 | Unannotated feature | neg | 126.9852 | 3.067 | 3.133 | 3.811 | 0.678 | 0.019 | 0.924 | 0.974 | Unannotated | NA | NA |
| metab_3721 | Unannotated feature | pos | 595.3829 | 6.574 | 6.700 | 6.692 | -0.008 | 0.019 | 0.924 | 0.974 | Unannotated | NA | NA |
| metab_19617 | Cerasidin | neg | 389.1252 | 4.687 | 4.316 | 3.950 | -0.366 | -0.019 | 0.924 | 0.974 | Unannotated | NA | NA |
| metab_12098 | Unannotated feature | neg | 452.2658 | 6.491 | 5.632 | 5.597 | -0.035 | 0.019 | 0.924 | 0.974 | Unannotated | NA | NA |
| metab_18780 | Unannotated feature | neg | 285.1458 | 3.173 | 4.965 | 4.741 | -0.225 | -0.019 | 0.924 | 0.974 | Unannotated | NA | NA |
| metab_5169 | Unannotated feature | pos | 543.4033 | 6.829 | 5.757 | 5.515 | -0.242 | 0.019 | 0.924 | 0.974 | Unannotated | NA | NA |
| metab_14539 | P-Salicylic acid | neg | 137.0237 | 3.418 | 5.507 | 5.466 | -0.040 | -0.019 | 0.924 | 0.974 | Tentative | NA | NA |
| metab_17009 | Unannotated feature | neg | 181.8949 | 7.997 | 3.005 | 3.005 | -0.001 | 0.019 | 0.924 | 0.974 | Unannotated | NA | NA |
| metab_10414 | Unannotated feature | neg | 204.0761 | 2.023 | 3.522 | 3.756 | 0.234 | 0.019 | 0.924 | 0.974 | Unannotated | NA | NA |
| metab_2193 | Unannotated feature | pos | 390.9483 | 0.593 | 4.339 | 4.554 | 0.215 | 0.019 | 0.924 | 0.974 | Unannotated | NA | NA |
| metab_1859 | Unannotated feature | pos | 445.1152 | 1.118 | 5.429 | 5.324 | -0.105 | -0.019 | 0.924 | 0.974 | Unannotated | NA | NA |
| metab_7696 | Tyrosyl-Threonine | pos | 247.1082 | 3.375 | 5.042 | 5.131 | 0.089 | -0.019 | 0.924 | 0.974 | Unannotated | NA | NA |
| metab_3301 | DG(13:0/0:0/20:4(5Z,8Z,11Z,14Z)-OH(18R)) | pos | 573.4509 | 7.028 | 5.178 | 4.983 | -0.195 | 0.019 | 0.924 | 0.974 | Unannotated | NA | NA |
| metab_13518 | Unannotated feature | neg | 296.8826 | 0.596 | 5.935 | 5.914 | -0.021 | 0.019 | 0.924 | 0.974 | Unannotated | NA | NA |
| metab_7429 | 15-Deoxy-d-12,14-PGJ2 | pos | 339.1926 | 3.917 | 3.756 | 3.377 | -0.379 | -0.019 | 0.924 | 0.974 | Tentative | NA | NA |
| metab_12422 | Unannotated feature | neg | 324.8824 | 7.726 | 5.281 | 5.280 | -0.001 | -0.019 | 0.924 | 0.974 | Unannotated | NA | NA |
| metab_7732 | Unannotated feature | pos | 288.1810 | 3.319 | 3.392 | 3.380 | -0.012 | -0.019 | 0.924 | 0.974 | Unannotated | NA | NA |
| metab_18067 | Unannotated feature | neg | 497.3493 | 6.420 | 6.613 | 6.676 | 0.063 | -0.019 | 0.924 | 0.974 | Unannotated | NA | NA |
| metab_12493 | Unannotated feature | neg | 368.1902 | 6.381 | 5.546 | 4.649 | -0.897 | -0.019 | 0.924 | 0.974 | Unannotated | NA | NA |
| metab_13097 | Unannotated feature | neg | 235.9263 | 0.038 | 5.505 | 5.526 | 0.020 | 0.019 | 0.924 | 0.974 | Unannotated | NA | NA |
| metab_17840 | Unannotated feature | neg | 477.3439 | 6.758 | 5.135 | 5.285 | 0.151 | -0.019 | 0.924 | 0.974 | Unannotated | NA | NA |
| metab_15538 | Unannotated feature | neg | 419.1358 | 5.351 | 4.203 | 4.189 | -0.015 | -0.019 | 0.924 | 0.974 | Unannotated | NA | NA |
| metab_4786 | Unannotated feature | pos | 137.9877 | 7.874 | 5.427 | 5.433 | 0.006 | 0.019 | 0.924 | 0.974 | Unannotated | NA | NA |
| metab_19548 | Unannotated feature | neg | 250.0333 | 4.781 | 5.395 | 5.381 | -0.014 | -0.019 | 0.924 | 0.974 | Unannotated | NA | NA |
| metab_17820 | 1-Linoleoyl-sn-Glycero-3-Phosphocholine | neg | 564.3322 | 6.813 | 5.179 | 5.428 | 0.249 | 0.019 | 0.924 | 0.974 | Strong putative | NA | NA |
| metab_12170 | DG(a-13:0/18:3(9,11,15)-OH(13)/0:0) | neg | 563.4326 | 6.703 | 5.816 | 5.568 | -0.249 | -0.019 | 0.924 | 0.974 | Unannotated | NA | NA |
| metab_5502 | Unannotated feature | pos | 196.0973 | 1.952 | 5.301 | 5.320 | 0.019 | 0.019 | 0.924 | 0.974 | Unannotated | NA | NA |
| metab_14781 | D-Linalool 3-(6''-malonylglucoside) | neg | 437.1574 | 3.856 | 4.370 | 4.137 | -0.233 | 0.019 | 0.924 | 0.974 | Unannotated | NA | NA |
| metab_8043 | Unannotated feature | pos | 289.1763 | 2.780 | 1.658 | 1.761 | 0.102 | -0.019 | 0.924 | 0.974 | Unannotated | NA | NA |
| metab_11045 | Glycyl-prolyl-glutamic acid | neg | 300.1203 | 0.738 | 4.874 | 4.874 | 0.000 | -0.019 | 0.924 | 0.974 | Unannotated | NA | NA |
| metab_1293 | Unannotated feature | pos | 480.2088 | 2.897 | 4.288 | 4.385 | 0.098 | 0.019 | 0.924 | 0.974 | Unannotated | NA | NA |
| metab_19519 | Tetranor-PGEM | neg | 309.1347 | 4.829 | 5.149 | 5.106 | -0.043 | -0.019 | 0.924 | 0.974 | Unannotated | NA | NA |
| metab_13560 | Unannotated feature | neg | 381.0726 | 1.205 | 3.674 | 3.531 | -0.142 | 0.019 | 0.924 | 0.974 | Unannotated | NA | NA |
| metab_8592 | Unannotated feature | pos | 223.0601 | 1.161 | 4.952 | 4.883 | -0.069 | 0.019 | 0.924 | 0.974 | Unannotated | NA | NA |
| metab_19359 | CRESOPYRINE | neg | 193.0501 | 5.061 | 4.932 | 5.089 | 0.157 | -0.019 | 0.924 | 0.974 | Moderate putative | NA | NA |
| metab_15195 | Unannotated feature | neg | 398.1576 | 4.714 | 4.356 | 4.660 | 0.304 | 0.019 | 0.924 | 0.974 | Unannotated | NA | NA |
| metab_12984 | Unannotated feature | neg | 145.0136 | 0.791 | 5.517 | 5.490 | -0.026 | 0.019 | 0.924 | 0.974 | Unannotated | NA | NA |
| metab_15964 | Unannotated feature | neg | 316.1350 | 6.076 | 6.614 | 6.539 | -0.075 | 0.019 | 0.924 | 0.974 | Unannotated | NA | NA |
| metab_5825 | Methysergide | pos | 417.2292 | 5.976 | 4.285 | 4.216 | -0.069 | -0.019 | 0.924 | 0.974 | Tentative | NA | NA |
| metab_991 | Unannotated feature | pos | 280.9938 | 7.716 | 4.704 | 4.703 | -0.001 | -0.019 | 0.924 | 0.974 | Unannotated | NA | NA |
| metab_10676 | Unannotated feature | neg | 350.1209 | 1.512 | 4.966 | 4.589 | -0.377 | -0.019 | 0.924 | 0.974 | Unannotated | NA | NA |
| metab_10662 | Unannotated feature | neg | 237.0727 | 1.594 | 4.289 | 4.062 | -0.227 | 0.019 | 0.924 | 0.974 | Unannotated | NA | NA |
| metab_18032 | Unannotated feature | neg | 222.1132 | 6.475 | 4.168 | 4.131 | -0.037 | -0.019 | 0.924 | 0.974 | Unannotated | NA | NA |
| metab_2030 | Unannotated feature | pos | 265.0800 | 2.513 | 4.656 | 4.904 | 0.248 | 0.019 | 0.924 | 0.974 | Unannotated | NA | NA |
| metab_2063 | Unannotated feature | pos | 377.1929 | 2.724 | 4.560 | 4.512 | -0.048 | -0.019 | 0.924 | 0.974 | Unannotated | NA | NA |
| metab_17900 | 24-Epibrassinolide | neg | 461.3279 | 6.663 | 6.559 | 6.609 | 0.050 | 0.019 | 0.924 | 0.974 | Tentative | NA | NA |
| metab_16154 | Unannotated feature | neg | 513.3438 | 6.334 | 6.451 | 6.468 | 0.016 | -0.019 | 0.924 | 0.974 | Unannotated | NA | NA |
| metab_5513 | Unannotated feature | pos | 428.3661 | 6.327 | 3.232 | 3.168 | -0.064 | -0.019 | 0.924 | 0.974 | Unannotated | NA | NA |
| metab_1179 | Unannotated feature | pos | 547.3622 | 6.502 | 6.664 | 6.668 | 0.004 | 0.019 | 0.924 | 0.974 | Unannotated | NA | NA |
| metab_12615 | Unannotated feature | neg | 367.1768 | 6.013 | 5.584 | 5.355 | -0.229 | -0.019 | 0.924 | 0.974 | Unannotated | NA | NA |
| metab_17317 | Unannotated feature | neg | 218.9341 | 7.702 | 3.676 | 3.660 | -0.016 | -0.019 | 0.924 | 0.974 | Unannotated | NA | NA |
| metab_19443 | Unannotated feature | neg | 129.0551 | 3.484 | 6.812 | 6.819 | 0.007 | -0.019 | 0.924 | 0.974 | Unannotated | NA | NA |
| metab_16908 | Unannotated feature | neg | 227.0670 | 7.765 | 3.373 | 3.493 | 0.120 | -0.019 | 0.924 | 0.974 | Unannotated | NA | NA |
| metab_4350 | 3-Amino-4-methoxybenzanilide | pos | 207.0920 | 6.151 | 3.715 | 3.500 | -0.215 | -0.019 | 0.924 | 0.974 | Unannotated | NA | NA |
| metab_4348 | Chenodeoxycholyltryptophan | pos | 617.3326 | 6.143 | 5.641 | 5.724 | 0.083 | 0.019 | 0.924 | 0.974 | Unannotated | NA | NA |
| metab_18525 | Unannotated feature | neg | 465.3216 | 6.035 | 3.484 | 3.765 | 0.281 | 0.019 | 0.924 | 0.974 | Unannotated | NA | NA |
| metab_9853 | Unannotated feature | neg | 199.0583 | 4.298 | 4.407 | 4.291 | -0.115 | 0.019 | 0.924 | 0.974 | Unannotated | NA | NA |
| metab_7315 | (S)-2,3-Dihydro-6-hydroxy-5-(hydroxyacetyl)-2-isopropenylbenzofuran | pos | 252.1234 | 4.156 | 3.770 | 3.886 | 0.117 | -0.019 | 0.924 | 0.974 | Unannotated | NA | NA |
| metab_12251 | Unannotated feature | neg | 667.4265 | 6.915 | 4.735 | 4.563 | -0.171 | 0.019 | 0.924 | 0.974 | Unannotated | NA | NA |
| metab_511 | Unannotated feature | pos | 335.1980 | 5.136 | 4.191 | 4.466 | 0.276 | 0.019 | 0.924 | 0.974 | Unannotated | NA | NA |
| metab_4919 | Unannotated feature | pos | 906.2610 | 7.642 | 4.770 | 4.675 | -0.095 | -0.019 | 0.924 | 0.974 | Unannotated | NA | NA |
| metab_9235 | Unannotated feature | neg | 379.1480 | 3.705 | 4.482 | 4.223 | -0.259 | 0.019 | 0.924 | 0.974 | Unannotated | NA | NA |
| metab_19815 | Unannotated feature | neg | 306.1197 | 4.405 | 3.972 | 4.457 | 0.484 | 0.019 | 0.924 | 0.974 | Unannotated | NA | NA |
| metab_9818 | Unannotated feature | neg | 349.9558 | 3.025 | 4.082 | 4.078 | -0.005 | 0.019 | 0.924 | 0.974 | Unannotated | NA | NA |
| metab_12688 | Unannotated feature | neg | 307.1666 | 5.480 | 4.518 | 5.805 | 1.286 | 0.019 | 0.924 | 0.974 | Unannotated | NA | NA |
| metab_8948 | Unannotated feature | neg | 85.0649 | 4.199 | 2.532 | 2.677 | 0.144 | -0.019 | 0.924 | 0.974 | Unannotated | NA | NA |
| metab_11212 | Milrinone | neg | 248.0211 | 0.644 | 4.750 | 4.730 | -0.020 | -0.019 | 0.924 | 0.974 | Tentative | NA | NA |
| metab_1213 | Unannotated feature | pos | 277.1916 | 2.740 | 5.121 | 5.103 | -0.018 | 0.019 | 0.924 | 0.974 | Unannotated | NA | NA |
| metab_11194 | Unannotated feature | neg | 247.0401 | 0.651 | 4.685 | 4.634 | -0.052 | -0.019 | 0.924 | 0.974 | Unannotated | NA | NA |
| metab_11150 | Unannotated feature | neg | 274.1411 | 0.683 | 3.936 | 3.814 | -0.122 | -0.019 | 0.924 | 0.974 | Unannotated | NA | NA |
| metab_16877 | Unannotated feature | neg | 226.0671 | 7.741 | 3.644 | 3.687 | 0.043 | 0.019 | 0.924 | 0.974 | Unannotated | NA | NA |
| metab_15435 | Neotussilagine | neg | 244.1192 | 5.144 | 3.315 | 3.097 | -0.218 | -0.019 | 0.924 | 0.974 | Unannotated | NA | NA |
| metab_16385 | 13-Hode | neg | 277.2173 | 6.797 | 4.397 | 4.494 | 0.097 | -0.019 | 0.924 | 0.974 | Tentative | NA | NA |
| metab_9620 | Benzofuran | neg | 163.0395 | 3.215 | 4.890 | 4.909 | 0.019 | -0.019 | 0.924 | 0.974 | Tentative | NA | NA |
| metab_9020 | (E)-Zeatin glucoside | neg | 362.1461 | 4.039 | 5.642 | 5.587 | -0.055 | 0.015 | 0.943 | 0.981 | Unannotated | NA | NA |
| metab_19817 | 3-Methyladipic Acid | neg | 159.0656 | 4.405 | 6.271 | 6.289 | 0.018 | 0.015 | 0.943 | 0.981 | Strong putative | NA | NA |
| metab_13849 | Unannotated feature | neg | 231.9434 | 2.215 | 3.837 | 3.765 | -0.072 | -0.015 | 0.943 | 0.981 | Unannotated | NA | NA |
| metab_11956 | Unannotated feature | neg | 359.1901 | 6.154 | 5.586 | 5.697 | 0.111 | 0.015 | 0.943 | 0.981 | Unannotated | NA | NA |
| metab_9517 | (4S,6R)-p-Mentha-1,8-diene-6,7-diol 7-glucoside | neg | 365.1358 | 3.326 | 5.628 | 5.643 | 0.015 | 0.015 | 0.943 | 0.981 | Unannotated | NA | NA |
| metab_7118 | Unannotated feature | pos | 455.1177 | 0.665 | 4.356 | 4.647 | 0.292 | 0.015 | 0.943 | 0.981 | Unannotated | NA | NA |
| metab_12586 | Unannotated feature | neg | 539.3594 | 6.326 | 6.695 | 6.773 | 0.077 | 0.015 | 0.943 | 0.981 | Unannotated | NA | NA |
| metab_2799 | Glutaminylhydroxyproline | pos | 242.1139 | 2.640 | 3.532 | 3.841 | 0.309 | 0.015 | 0.943 | 0.981 | Unannotated | NA | NA |
| metab_4960 | Butylisopropylamine | pos | 116.1438 | 7.586 | 7.018 | 7.018 | -0.000 | -0.015 | 0.943 | 0.981 | Moderate putative | NA | NA |
| metab_591 | Unannotated feature | pos | 202.1441 | 5.770 | 3.909 | 4.144 | 0.234 | -0.015 | 0.943 | 0.981 | Unannotated | NA | NA |
| metab_15421 | 2,4,6(1H,3H,5H)-Pyrimidinetrione, 5-ethyl-5-(3-hydroxy-1-methylbutyl)- | neg | 241.1191 | 5.110 | 4.428 | 4.119 | -0.308 | -0.015 | 0.943 | 0.981 | Unannotated | NA | NA |
| metab_15479 | Unannotated feature | neg | 207.0409 | 5.227 | 3.587 | 4.073 | 0.486 | 0.015 | 0.943 | 0.981 | Unannotated | NA | NA |
| metab_7912 | Ethyl maltol | pos | 123.0445 | 2.951 | 3.796 | 3.788 | -0.008 | -0.015 | 0.943 | 0.981 | Tentative | NA | NA |
| metab_15411 | 2-Methylpropyl 3-hydroxy-2-methylidenebutanoate | neg | 217.1078 | 5.079 | 5.389 | 5.558 | 0.169 | 0.015 | 0.943 | 0.981 | Unannotated | NA | NA |
| metab_18596 | Unannotated feature | neg | 445.2449 | 5.991 | 4.647 | 4.774 | 0.127 | -0.015 | 0.943 | 0.981 | Unannotated | NA | NA |
| metab_1339 | Unannotated feature | pos | 267.1495 | 6.175 | 4.697 | 4.611 | -0.085 | -0.015 | 0.943 | 0.981 | Unannotated | NA | NA |
| metab_10314 | 13E-Tetranor-16-carboxy-LTE4 | neg | 394.1363 | 2.329 | 3.276 | 3.372 | 0.096 | 0.015 | 0.943 | 0.981 | Unannotated | NA | NA |
| metab_13911 | Unannotated feature | neg | 299.0613 | 2.520 | 4.952 | 4.895 | -0.057 | -0.015 | 0.943 | 0.981 | Unannotated | NA | NA |
| metab_15626 | Unannotated feature | neg | 372.0309 | 0.582 | 4.141 | 4.289 | 0.148 | 0.015 | 0.943 | 0.981 | Unannotated | NA | NA |
| metab_19423 | Unannotated feature | neg | 615.2855 | 4.977 | 4.113 | 3.929 | -0.185 | -0.015 | 0.943 | 0.981 | Unannotated | NA | NA |
| metab_18431 | Panax ginseng Tetrapeptide | neg | 480.2146 | 6.107 | 4.835 | 4.888 | 0.053 | -0.015 | 0.943 | 0.981 | Unannotated | NA | NA |
| metab_18391 | Unannotated feature | neg | 485.2221 | 6.138 | 6.701 | 6.712 | 0.011 | 0.015 | 0.943 | 0.981 | Unannotated | NA | NA |
| metab_14752 | Camonagrel | neg | 307.0859 | 3.791 | 5.365 | 5.377 | 0.013 | -0.015 | 0.943 | 0.981 | Unannotated | NA | NA |
| metab_18626 | Flazine | neg | 307.0727 | 5.964 | 5.190 | 5.478 | 0.288 | -0.015 | 0.943 | 0.981 | Unannotated | NA | NA |
| metab_13789 | Unannotated feature | neg | 159.0768 | 1.997 | 3.732 | 3.829 | 0.097 | 0.015 | 0.943 | 0.981 | Unannotated | NA | NA |
| metab_4765 | Unannotated feature | pos | 204.0548 | 7.732 | 4.598 | 4.589 | -0.009 | 0.015 | 0.943 | 0.981 | Unannotated | NA | NA |
| metab_13803 | Unannotated feature | neg | 303.0161 | 2.032 | 3.701 | 3.743 | 0.042 | 0.015 | 0.943 | 0.981 | Unannotated | NA | NA |
| metab_10301 | Unannotated feature | neg | 186.9117 | 2.390 | 4.256 | 4.276 | 0.020 | -0.015 | 0.943 | 0.981 | Unannotated | NA | NA |
| metab_17614 | Unannotated feature | neg | 424.0929 | 2.762 | 4.544 | 4.603 | 0.059 | 0.015 | 0.943 | 0.981 | Unannotated | NA | NA |
| metab_15082 | Disperse Yellow 7 | neg | 361.1305 | 4.502 | 4.409 | 4.562 | 0.153 | -0.015 | 0.943 | 0.981 | Unannotated | NA | NA |
| metab_7305 | Ibuprofen | pos | 251.1032 | 4.190 | 3.709 | 3.686 | -0.023 | 0.015 | 0.943 | 0.981 | Tentative | NA | NA |
| metab_13680 | 2,6-Dichloro-4-nitrophenol | neg | 243.8994 | 0.073 | 6.600 | 6.593 | -0.007 | 0.015 | 0.943 | 0.981 | Unannotated | NA | NA |
| metab_369 | Unannotated feature | pos | 445.1215 | 0.305 | 5.909 | 5.930 | 0.021 | 0.015 | 0.943 | 0.981 | Unannotated | NA | NA |
| metab_387 | Unannotated feature | pos | 90.5263 | 0.063 | 5.176 | 5.178 | 0.003 | 0.015 | 0.943 | 0.981 | Unannotated | NA | NA |
| metab_6334 | Scopolamine | pos | 321.1814 | 5.778 | 5.129 | 5.952 | 0.823 | 0.015 | 0.943 | 0.981 | Tentative | NA | NA |
| metab_5134 | Unannotated feature | pos | 721.3580 | 6.901 | 6.229 | 6.299 | 0.071 | -0.015 | 0.943 | 0.981 | Unannotated | NA | NA |
| metab_15812 | Unannotated feature | neg | 327.1452 | 5.884 | 4.911 | 4.695 | -0.216 | -0.015 | 0.943 | 0.981 | Unannotated | NA | NA |
| metab_4807 | Unannotated feature | pos | 145.0174 | 7.983 | 4.015 | 4.002 | -0.013 | 0.015 | 0.943 | 0.981 | Unannotated | NA | NA |
| metab_4826 | Unannotated feature | pos | 202.0167 | 7.810 | 5.474 | 5.484 | 0.009 | 0.015 | 0.943 | 0.981 | Unannotated | NA | NA |
| metab_9749 | Unannotated feature | neg | 178.0538 | 3.082 | 5.300 | 5.183 | -0.117 | 0.015 | 0.943 | 0.981 | Unannotated | NA | NA |
| metab_402 | Unannotated feature | pos | 303.9047 | 0.016 | 4.065 | 4.037 | -0.028 | -0.015 | 0.943 | 0.981 | Unannotated | NA | NA |
| metab_10167 | Unannotated feature | neg | 288.0728 | 3.215 | 4.818 | 4.852 | 0.034 | -0.015 | 0.943 | 0.981 | Unannotated | NA | NA |
| metab_15771 | Unannotated feature | neg | 551.3240 | 5.797 | 4.634 | 4.759 | 0.126 | -0.015 | 0.943 | 0.981 | Unannotated | NA | NA |
| metab_2305 | Unannotated feature | pos | 81.5211 | 0.033 | 4.206 | 4.210 | 0.004 | -0.015 | 0.943 | 0.981 | Unannotated | NA | NA |
| metab_17632 | Unannotated feature | neg | 194.9880 | 7.120 | 3.210 | 3.271 | 0.061 | -0.015 | 0.943 | 0.981 | Unannotated | NA | NA |
| metab_12385 | Unannotated feature | neg | 444.3124 | 6.640 | 5.420 | 5.448 | 0.028 | -0.015 | 0.943 | 0.981 | Unannotated | NA | NA |
| metab_10274 | Unannotated feature | neg | 316.0513 | 2.514 | 4.800 | 4.739 | -0.061 | 0.015 | 0.943 | 0.981 | Unannotated | NA | NA |
| metab_18266 | Unannotated feature | neg | 420.2307 | 6.240 | 5.605 | 5.633 | 0.028 | -0.015 | 0.943 | 0.981 | Unannotated | NA | NA |
| metab_8613 | Unannotated feature | pos | 158.9770 | 1.089 | 3.780 | 4.048 | 0.268 | -0.015 | 0.943 | 0.981 | Unannotated | NA | NA |
| metab_2958 | Unannotated feature | pos | 167.0566 | 2.976 | 4.013 | 3.968 | -0.045 | 0.015 | 0.943 | 0.981 | Unannotated | NA | NA |
| metab_4895 | Unannotated feature | pos | 172.0640 | 7.700 | 4.600 | 4.625 | 0.025 | -0.015 | 0.943 | 0.981 | Unannotated | NA | NA |
| metab_4918 | Unannotated feature | pos | 79.9902 | 7.650 | 3.919 | 3.925 | 0.006 | 0.015 | 0.943 | 0.981 | Unannotated | NA | NA |
| metab_13561 | Deoxyribose | neg | 133.0499 | 1.209 | 5.969 | 5.891 | -0.079 | 0.015 | 0.943 | 0.981 | Moderate putative | NA | NA |
| metab_15760 | Unannotated feature | neg | 271.1916 | 5.789 | 4.519 | 4.581 | 0.062 | -0.015 | 0.943 | 0.981 | Unannotated | NA | NA |
| metab_7415 | (3R)-3-Hydroxy-N-[(3S)-2-oxooxolan-3-yl]butanamide | pos | 205.1186 | 0.712 | 4.896 | 5.054 | 0.158 | 0.015 | 0.943 | 0.981 | Unannotated | NA | NA |
| metab_11327 | Unannotated feature | neg | 212.9206 | 0.603 | 5.159 | 5.322 | 0.162 | -0.015 | 0.943 | 0.981 | Unannotated | NA | NA |
| metab_18659 | Unannotated feature | neg | 275.1500 | 5.937 | 3.535 | 3.515 | -0.019 | 0.015 | 0.943 | 0.981 | Unannotated | NA | NA |
| metab_14912 | Unannotated feature | neg | 176.9598 | 0.496 | 4.943 | 4.909 | -0.035 | -0.015 | 0.943 | 0.981 | Unannotated | NA | NA |
| metab_12818 | Unannotated feature | neg | 182.9963 | 0.659 | 5.233 | 5.259 | 0.026 | 0.015 | 0.943 | 0.981 | Unannotated | NA | NA |
| metab_1682 | 3-Ketopetromyzonol sulfate | pos | 490.2875 | 3.987 | 4.873 | 4.689 | -0.185 | -0.015 | 0.943 | 0.981 | Unannotated | NA | NA |
| metab_16250 | Unannotated feature | neg | 549.3805 | 6.522 | 6.200 | 6.304 | 0.104 | 0.015 | 0.943 | 0.981 | Unannotated | NA | NA |
| metab_10847 | Dextran | neg | 485.1523 | 1.026 | 4.687 | 4.768 | 0.081 | 0.015 | 0.943 | 0.981 | Tentative | NA | NA |
| metab_12916 | Unannotated feature | neg | 167.0014 | 0.738 | 5.078 | 4.983 | -0.095 | -0.015 | 0.943 | 0.981 | Unannotated | NA | NA |
| metab_1117 | Goyaglycoside c | pos | 645.4354 | 6.638 | 5.844 | 5.896 | 0.051 | -0.015 | 0.943 | 0.981 | Unannotated | NA | NA |
| metab_1073 | Unannotated feature | pos | 611.4299 | 6.710 | 5.960 | 5.839 | -0.121 | 0.015 | 0.943 | 0.981 | Unannotated | NA | NA |
| metab_2605 | Unannotated feature | pos | 132.5446 | 1.658 | 3.480 | 3.528 | 0.048 | 0.015 | 0.943 | 0.981 | Unannotated | NA | NA |
| metab_11759 | Unannotated feature | neg | 396.1418 | 5.683 | 4.484 | 4.408 | -0.076 | 0.015 | 0.943 | 0.981 | Unannotated | NA | NA |
| metab_438 | Unannotated feature | pos | 89.5072 | 0.016 | 4.321 | 4.303 | -0.018 | 0.015 | 0.943 | 0.981 | Unannotated | NA | NA |
| metab_14548 | 2-Hydroxyquinoline-3-carboxylic acid | neg | 188.0347 | 3.434 | 6.100 | 6.092 | -0.008 | -0.015 | 0.943 | 0.981 | Unannotated | NA | NA |
| metab_5225 | Unannotated feature | pos | 190.1076 | 1.057 | 5.507 | 5.504 | -0.003 | 0.015 | 0.943 | 0.981 | Unannotated | NA | NA |
| metab_904 | Unannotated feature | pos | 695.5234 | 7.060 | 6.572 | 6.560 | -0.012 | -0.015 | 0.943 | 0.981 | Unannotated | NA | NA |
| metab_15387 | Unannotated feature | neg | 159.8780 | 5.049 | 5.373 | 5.463 | 0.090 | -0.015 | 0.943 | 0.981 | Unannotated | NA | NA |
| metab_15516 | Garbanzol | neg | 271.0614 | 5.304 | 4.867 | 4.834 | -0.034 | 0.015 | 0.943 | 0.981 | Tentative | NA | NA |
| metab_17112 | Unannotated feature | neg | 227.8942 | 7.848 | 4.748 | 4.745 | -0.002 | -0.015 | 0.943 | 0.981 | Unannotated | NA | NA |
| metab_13809 | Deoxyinosine | neg | 251.0787 | 2.043 | 7.044 | 7.025 | -0.019 | 0.015 | 0.943 | 0.981 | Strong putative | NA | NA |
| metab_741 | (2S,3S,5S,8S,9S,10S,13S,14S,17S)-17-Acetyl-2-(2,2-dimethylmorpholino)-3-hydroxy-10,13-dimethylhexadecahydro-11H-cyclopenta[a]phenanthren-11-one | pos | 446.3278 | 6.040 | 4.248 | 4.337 | 0.089 | -0.015 | 0.943 | 0.981 | Unannotated | NA | NA |
| metab_3780 | DL-DIHYDROSPHINGOSINE | pos | 302.3058 | 6.438 | 6.693 | 6.722 | 0.029 | 0.015 | 0.943 | 0.981 | Unannotated | NA | NA |
| metab_15733 | Unannotated feature | neg | 509.8195 | 0.596 | 3.108 | 3.946 | 0.838 | 0.015 | 0.943 | 0.981 | Unannotated | NA | NA |
| metab_10439 | Unannotated feature | neg | 243.1238 | 4.625 | 6.357 | 6.267 | -0.090 | 0.015 | 0.943 | 0.981 | Unannotated | NA | NA |
| metab_4057 | Unannotated feature | pos | 399.2290 | 6.143 | 4.584 | 4.706 | 0.122 | 0.015 | 0.943 | 0.981 | Unannotated | NA | NA |
| metab_13993 | Unannotated feature | neg | 179.0377 | 2.707 | 4.268 | 4.276 | 0.008 | 0.015 | 0.943 | 0.981 | Unannotated | NA | NA |
| metab_19486 | Unannotated feature | neg | 281.0147 | 4.880 | 3.037 | 3.145 | 0.108 | 0.015 | 0.943 | 0.981 | Unannotated | NA | NA |
| metab_9264 | 2-Methyl-3-hydroxy-5-formylpyridine-4-carboxylate | neg | 162.0190 | 3.673 | 4.239 | 4.208 | -0.031 | 0.015 | 0.943 | 0.981 | Tentative | NA | NA |
| metab_16752 | Unannotated feature | neg | 200.8584 | 7.684 | 4.283 | 4.298 | 0.015 | -0.015 | 0.943 | 0.981 | Unannotated | NA | NA |
| metab_16406 | Unannotated feature | neg | 370.2964 | 6.868 | 4.818 | 4.739 | -0.079 | 0.015 | 0.943 | 0.981 | Unannotated | NA | NA |
| metab_16419 | OR-1855 | neg | 224.0787 | 1.675 | 5.887 | 5.827 | -0.060 | -0.015 | 0.943 | 0.981 | Unannotated | NA | NA |
| metab_17835 | Unannotated feature | neg | 511.4007 | 6.766 | 5.365 | 5.274 | -0.091 | -0.015 | 0.943 | 0.981 | Unannotated | NA | NA |
| metab_920 | Unannotated feature | pos | 621.4473 | 7.140 | 5.333 | 5.403 | 0.070 | -0.015 | 0.943 | 0.981 | Unannotated | NA | NA |
| metab_12218 | Avocadyne 4-acetate | neg | 347.2205 | 6.868 | 5.535 | 5.631 | 0.096 | 0.015 | 0.943 | 0.981 | Strong putative | NA | NA |
| metab_12226 | Unannotated feature | neg | 253.2172 | 6.892 | 4.775 | 4.777 | 0.002 | 0.015 | 0.943 | 0.981 | Unannotated | NA | NA |
| metab_4748 | Unannotated feature | pos | 95.0443 | 7.708 | 4.389 | 4.380 | -0.009 | -0.015 | 0.943 | 0.981 | Unannotated | NA | NA |
| metab_19685 | Unannotated feature | neg | 401.1096 | 3.021 | 3.758 | 3.767 | 0.009 | -0.015 | 0.943 | 0.981 | Unannotated | NA | NA |
| metab_12117 | Unannotated feature | neg | 293.2487 | 6.545 | 5.035 | 5.099 | 0.064 | 0.015 | 0.943 | 0.981 | Unannotated | NA | NA |
| metab_18221 | 3-Hydroxy-6,8-dimethoxy-7(11)-eremophilen-12,8-olide | neg | 291.1604 | 6.279 | 4.650 | 4.490 | -0.160 | 0.015 | 0.943 | 0.981 | Unannotated | NA | NA |
| metab_5344 | Lucerastat | pos | 202.1442 | 1.952 | 4.362 | 4.350 | -0.012 | 0.015 | 0.943 | 0.981 | Unannotated | NA | NA |
| metab_10737 | Unannotated feature | neg | 304.9646 | 1.263 | 5.278 | 5.146 | -0.132 | -0.015 | 0.943 | 0.981 | Unannotated | NA | NA |
| metab_14332 | Unannotated feature | neg | 219.0871 | 3.090 | 3.937 | 3.993 | 0.056 | -0.015 | 0.943 | 0.981 | Unannotated | NA | NA |
| metab_9847 | Unannotated feature | neg | 275.0527 | 2.999 | 4.198 | 4.167 | -0.032 | -0.015 | 0.943 | 0.981 | Unannotated | NA | NA |
| metab_2663 | 2-Methylhippuric acid | pos | 211.1081 | 1.952 | 4.977 | 5.056 | 0.079 | 0.015 | 0.943 | 0.981 | Tentative | NA | NA |
| metab_8312 | Lomefloxacin | pos | 369.1761 | 2.044 | 3.036 | 3.099 | 0.063 | -0.015 | 0.943 | 0.981 | Tentative | NA | NA |
| metab_16995 | Unannotated feature | neg | 253.8429 | 7.985 | 5.092 | 5.098 | 0.005 | -0.015 | 0.943 | 0.981 | Unannotated | NA | NA |
| metab_11469 | Unannotated feature | neg | 362.9414 | 0.524 | 3.934 | 4.246 | 0.312 | -0.015 | 0.943 | 0.981 | Unannotated | NA | NA |
| metab_977 | Unannotated feature | pos | 88.0762 | 7.964 | 5.095 | 5.098 | 0.003 | 0.015 | 0.943 | 0.981 | Unannotated | NA | NA |
| metab_2103 | Unannotated feature | pos | 313.1037 | 2.732 | 5.093 | 5.397 | 0.304 | -0.015 | 0.943 | 0.981 | Unannotated | NA | NA |
| metab_17825 | Docosahexaenoic Acid | neg | 327.2330 | 6.797 | 5.685 | 5.395 | -0.290 | -0.015 | 0.943 | 0.981 | Moderate putative | Lipids and lipid-like molecules | Fatty Acyls |
| metab_14610 | Prallethrin | neg | 321.1460 | 3.525 | 3.951 | 3.782 | -0.169 | 0.015 | 0.943 | 0.981 | Tentative | NA | NA |
| metab_7651 | R-Deprenyl N-Oxide | pos | 221.1653 | 3.461 | 3.434 | 3.894 | 0.460 | -0.015 | 0.943 | 0.981 | Moderate putative | NA | NA |
| metab_11444 | Unannotated feature | neg | 416.8292 | 0.533 | 4.264 | 4.042 | -0.222 | 0.015 | 0.943 | 0.981 | Unannotated | NA | NA |
| metab_18809 | 7-Hydroxy-3',4',5,6-tetramethoxyflavone | neg | 357.1002 | 5.774 | 4.493 | 4.467 | -0.027 | 0.015 | 0.943 | 0.981 | Unannotated | NA | NA |
| metab_12489 | Unannotated feature | neg | 497.2946 | 6.397 | 7.195 | 7.141 | -0.054 | -0.015 | 0.943 | 0.981 | Unannotated | NA | NA |
| metab_12495 | Unannotated feature | neg | 653.4266 | 6.373 | 4.898 | 4.841 | -0.057 | 0.015 | 0.943 | 0.981 | Unannotated | NA | NA |
| metab_10708 | Unannotated feature | neg | 219.0133 | 1.364 | 5.160 | 5.138 | -0.022 | -0.015 | 0.943 | 0.981 | Unannotated | NA | NA |
| metab_1026 | (7Z,9Z,12Z,15Z,18Z,21Z)-Tetracosa-7,9,12,15,18,21-hexaenoylcarnitine | pos | 544.3387 | 7.021 | 6.324 | 6.315 | -0.009 | 0.015 | 0.943 | 0.981 | Unannotated | NA | NA |
| metab_5752 | Unannotated feature | pos | 179.1130 | 0.477 | 3.786 | 3.792 | 0.006 | -0.015 | 0.943 | 0.981 | Unannotated | NA | NA |
| metab_12997 | Unannotated feature | neg | 349.0717 | 0.777 | 4.635 | 4.390 | -0.245 | 0.015 | 0.943 | 0.981 | Unannotated | NA | NA |
| metab_18041 | Physangulide | neg | 543.2564 | 6.451 | 5.415 | 5.117 | -0.298 | -0.015 | 0.943 | 0.981 | Unannotated | NA | NA |
| metab_16329 | Unannotated feature | neg | 481.2998 | 6.663 | 7.560 | 7.627 | 0.066 | -0.015 | 0.943 | 0.981 | Unannotated | NA | NA |
| metab_18911 | Unannotated feature | neg | 209.1177 | 5.638 | 3.059 | 3.092 | 0.033 | -0.015 | 0.943 | 0.981 | Unannotated | NA | NA |
| metab_18918 | Unannotated feature | neg | 381.2030 | 5.632 | 2.778 | 2.848 | 0.071 | 0.015 | 0.943 | 0.981 | Unannotated | NA | NA |
| metab_13019 | Unannotated feature | neg | 191.9831 | 0.628 | 4.427 | 4.545 | 0.118 | 0.015 | 0.943 | 0.981 | Unannotated | NA | NA |
| metab_10029 | Unannotated feature | neg | 519.1730 | 4.409 | 4.031 | 4.074 | 0.043 | 0.015 | 0.943 | 0.981 | Unannotated | NA | NA |
| metab_9111 | P-Tolyl Sulfate | neg | 187.0065 | 3.909 | 5.358 | 5.417 | 0.060 | 0.015 | 0.943 | 0.981 | Moderate putative | NA | NA |
| metab_7448 | Unannotated feature | pos | 188.1437 | 3.872 | 3.204 | 3.086 | -0.118 | 0.015 | 0.943 | 0.981 | Unannotated | NA | NA |
| metab_17349 | Unannotated feature | neg | 297.1531 | 7.692 | 4.122 | 4.113 | -0.009 | 0.015 | 0.943 | 0.981 | Unannotated | NA | NA |
| metab_8549 | Unannotated feature | pos | 150.0778 | 1.291 | 3.923 | 3.790 | -0.133 | -0.015 | 0.943 | 0.981 | Unannotated | NA | NA |
| metab_711 | DG(i-12:0/22:6(5Z,7Z,10Z,13Z,16Z,19Z)-OH(4)/0:0) | pos | 633.4710 | 6.670 | 5.968 | 6.165 | 0.196 | 0.015 | 0.943 | 0.981 | Unannotated | NA | NA |
| metab_959 | Unannotated feature | pos | 157.1087 | 7.650 | 4.711 | 4.721 | 0.009 | -0.015 | 0.943 | 0.981 | Unannotated | NA | NA |
| metab_10591 | Unannotated feature | neg | 303.0504 | 1.691 | 3.657 | 3.721 | 0.064 | 0.015 | 0.943 | 0.981 | Unannotated | NA | NA |
| metab_14648 | Unannotated feature | neg | 624.9458 | 0.520 | 3.817 | 4.297 | 0.480 | 0.015 | 0.943 | 0.981 | Unannotated | NA | NA |
| metab_12519 | Unannotated feature | neg | 257.0921 | 6.318 | 4.027 | 4.111 | 0.084 | 0.015 | 0.943 | 0.981 | Unannotated | NA | NA |
| metab_15548 | Dimefuron | neg | 383.1124 | 5.386 | 4.519 | 4.297 | -0.221 | 0.015 | 0.943 | 0.981 | Strong putative | NA | NA |
| metab_302 | Unannotated feature | pos | 146.0301 | 0.485 | 6.205 | 6.177 | -0.028 | 0.015 | 0.943 | 0.981 | Unannotated | NA | NA |
| metab_12143 | FP-biotin | neg | 591.3205 | 5.825 | 6.947 | 7.042 | 0.095 | -0.015 | 0.943 | 0.981 | Unannotated | Organoheterocyclic compounds | Biotin and derivatives |
| metab_19535 | Unannotated feature | neg | 125.0965 | 4.803 | 3.667 | 3.864 | 0.197 | -0.015 | 0.943 | 0.981 | Unannotated | NA | NA |
| metab_999 | 4-Acetylbutyrate | pos | 148.0970 | 7.586 | 5.602 | 5.609 | 0.008 | -0.015 | 0.943 | 0.981 | Tentative | NA | NA |
| metab_8813 | Hexadecanedioic acid | pos | 325.1766 | 3.517 | 3.425 | 3.731 | 0.306 | 0.015 | 0.943 | 0.981 | Tentative | NA | NA |
| metab_14220 | Unannotated feature | neg | 304.1041 | 2.960 | 4.949 | 4.796 | -0.152 | -0.015 | 0.943 | 0.981 | Unannotated | NA | NA |
| metab_11994 | Unannotated feature | neg | 479.2496 | 6.224 | 5.178 | 5.045 | -0.134 | 0.015 | 0.943 | 0.981 | Unannotated | NA | NA |
| metab_7090 | Piperidine-4-Carboxamide | pos | 129.1026 | 0.665 | 4.176 | 4.216 | 0.040 | -0.015 | 0.943 | 0.981 | Moderate putative | NA | NA |
| metab_8947 | Unannotated feature | neg | 216.0486 | 4.199 | 4.065 | 4.204 | 0.139 | 0.015 | 0.943 | 0.981 | Unannotated | NA | NA |
| metab_7994 | 13'-Carboxy-alpha-tocotrienol | pos | 250.1443 | 2.845 | 3.664 | 3.414 | -0.250 | -0.015 | 0.943 | 0.981 | Unannotated | NA | NA |
| metab_12625 | Unannotated feature | neg | 324.1324 | 6.007 | 5.868 | 5.638 | -0.230 | -0.015 | 0.943 | 0.981 | Unannotated | NA | NA |
| metab_14840 | 3-Hydroxybenzyl alcohol glucoside | neg | 285.0993 | 3.983 | 4.123 | 4.432 | 0.309 | -0.015 | 0.943 | 0.981 | Tentative | NA | NA |
| metab_18395 | Unannotated feature | neg | 635.3098 | 6.130 | 4.176 | 4.279 | 0.103 | -0.015 | 0.943 | 0.981 | Unannotated | NA | NA |
| metab_16197 | Unannotated feature | neg | 521.3490 | 6.404 | 6.610 | 6.718 | 0.107 | 0.015 | 0.943 | 0.981 | Unannotated | NA | NA |
| metab_14887 | 2-Deoxy-2-fluoro-alpha-D-galactopyranose | neg | 163.0394 | 4.068 | 4.643 | 4.629 | -0.014 | -0.015 | 0.943 | 0.981 | Unannotated | NA | NA |
| metab_11924 | Unannotated feature | neg | 391.1447 | 6.099 | 4.329 | 4.463 | 0.134 | -0.015 | 0.943 | 0.981 | Unannotated | NA | NA |
| metab_14844 | Unannotated feature | neg | 241.1192 | 3.988 | 5.033 | 5.055 | 0.022 | 0.015 | 0.943 | 0.981 | Unannotated | NA | NA |
| metab_18558 | Unannotated feature | neg | 331.2129 | 6.013 | 4.225 | 4.131 | -0.094 | 0.015 | 0.943 | 0.981 | Unannotated | NA | NA |
| metab_19856 | D-Arabitol | neg | 151.0605 | 0.582 | 4.039 | 4.073 | 0.035 | 0.015 | 0.943 | 0.981 | Strong putative | NA | NA |
| metab_70 | 4-O-Methyl-a-D-glucosyl-(1->2)-b-D-xylosyl-(1->4)-D-xylose | pos | 490.1781 | 0.657 | 4.442 | 4.517 | 0.075 | -0.015 | 0.943 | 0.981 | Unannotated | NA | NA |
| metab_1202 | Oryzalexin E | pos | 305.2479 | 6.446 | 3.923 | 3.899 | -0.024 | 0.015 | 0.943 | 0.981 | Tentative | Benzenoids | Phenanthrenes and derivatives |
| metab_16850 | Unannotated feature | neg | 302.8994 | 7.730 | 4.654 | 4.667 | 0.013 | 0.015 | 0.943 | 0.981 | Unannotated | NA | NA |
| metab_189 | Unannotated feature | pos | 360.8335 | 0.593 | 3.800 | 3.735 | -0.065 | -0.015 | 0.943 | 0.981 | Unannotated | NA | NA |
| metab_19736 | Unannotated feature | neg | 250.0333 | 4.518 | 4.774 | 4.712 | -0.062 | 0.011 | 0.962 | 0.986 | Unannotated | NA | NA |
| metab_16890 | Unannotated feature | neg | 178.8644 | 7.745 | 4.786 | 4.788 | 0.001 | -0.011 | 0.962 | 0.986 | Unannotated | NA | NA |
| metab_16886 | Unannotated feature | neg | 134.8744 | 7.745 | 4.299 | 4.292 | -0.007 | 0.011 | 0.962 | 0.986 | Unannotated | NA | NA |
| metab_16878 | Unannotated feature | neg | 372.1153 | 2.004 | 3.163 | 3.393 | 0.230 | 0.011 | 0.962 | 0.986 | Unannotated | NA | NA |
| metab_19836 | Unannotated feature | neg | 224.8267 | 4.382 | 5.766 | 5.828 | 0.062 | -0.011 | 0.962 | 0.986 | Unannotated | NA | NA |
| metab_19663 | Unannotated feature | neg | 306.1196 | 4.621 | 4.472 | 4.638 | 0.166 | -0.011 | 0.962 | 0.986 | Unannotated | NA | NA |
| metab_1196 | (6S,8Z)-6-Hydroxy-3-oxotetradecenoic acid | pos | 239.1625 | 6.470 | 4.764 | 4.751 | -0.014 | 0.011 | 0.962 | 0.986 | Unannotated | NA | NA |
| metab_14780 | Unannotated feature | neg | 373.0593 | 0.512 | 5.677 | 5.654 | -0.023 | 0.011 | 0.962 | 0.986 | Unannotated | NA | NA |
| metab_14836 | Ent-7alpha,12beta-Dihydroxy-16-kauren-19,6beta-olide | neg | 353.1717 | 3.975 | 4.279 | 4.494 | 0.215 | -0.011 | 0.962 | 0.986 | Tentative | NA | NA |
| metab_8946 | Unannotated feature | neg | 277.1294 | 4.199 | 3.456 | 3.844 | 0.388 | 0.011 | 0.962 | 0.986 | Unannotated | NA | NA |
| metab_15466 | Unannotated feature | neg | 211.1335 | 5.209 | 3.988 | 4.085 | 0.098 | -0.011 | 0.962 | 0.986 | Unannotated | NA | NA |
| metab_15398 | Delta-Guanidinovaleric acid | neg | 377.2185 | 5.061 | 5.092 | 5.189 | 0.097 | -0.011 | 0.962 | 0.986 | Tentative | NA | NA |
| metab_3434 | Loxapine | pos | 372.0880 | 4.550 | 1.735 | 1.355 | -0.380 | -0.011 | 0.962 | 0.986 | Tentative | NA | NA |
| metab_18816 | Unannotated feature | neg | 232.1011 | 5.767 | 4.127 | 4.326 | 0.199 | -0.011 | 0.962 | 0.986 | Unannotated | NA | NA |
| metab_11919 | Unannotated feature | neg | 418.2143 | 6.083 | 5.288 | 5.340 | 0.052 | -0.011 | 0.962 | 0.986 | Unannotated | NA | NA |
| metab_17175 | Unannotated feature | neg | 267.0738 | 2.137 | 6.050 | 6.316 | 0.266 | 0.011 | 0.962 | 0.986 | Unannotated | NA | NA |
| metab_9618 | 4-Formylaminoantipyrine | neg | 276.0996 | 3.215 | 4.976 | 4.939 | -0.036 | 0.011 | 0.962 | 0.986 | Unannotated | NA | NA |
| metab_3332 | Unannotated feature | pos | 274.2017 | 4.179 | 4.363 | 4.742 | 0.380 | 0.011 | 0.962 | 0.986 | Unannotated | NA | NA |
| metab_12479 | Unannotated feature | neg | 623.4540 | 6.498 | 5.926 | 5.879 | -0.047 | -0.011 | 0.962 | 0.986 | Unannotated | NA | NA |
| metab_10679 | (S)-Bitalin A 12-glucoside | neg | 361.1299 | 4.754 | 4.748 | 4.798 | 0.050 | -0.011 | 0.962 | 0.986 | Unannotated | Organic oxygen compounds | Organooxygen compounds |
| metab_1901 | Unannotated feature | pos | 226.1441 | 1.161 | 3.108 | 3.129 | 0.021 | 0.011 | 0.962 | 0.986 | Unannotated | NA | NA |
| metab_8261 | Unannotated feature | pos | 321.1664 | 2.223 | 4.278 | 4.586 | 0.307 | -0.011 | 0.962 | 0.986 | Unannotated | NA | NA |
| metab_17970 | Unannotated feature | neg | 497.3278 | 6.553 | 3.439 | 3.499 | 0.060 | 0.011 | 0.962 | 0.986 | Unannotated | NA | NA |
| metab_18021 | Unannotated feature | neg | 477.2299 | 6.483 | 5.857 | 5.803 | -0.054 | -0.011 | 0.962 | 0.986 | Unannotated | NA | NA |
| metab_712 | Unannotated feature | pos | 215.0881 | 0.509 | 5.085 | 5.055 | -0.030 | -0.011 | 0.962 | 0.986 | Unannotated | NA | NA |
| metab_7492 | Ipazine | pos | 261.1602 | 3.770 | 4.471 | 4.404 | -0.067 | -0.011 | 0.962 | 0.986 | Tentative | NA | NA |
| metab_18897 | Strobilurin | neg | 463.1771 | 5.664 | 3.858 | 3.921 | 0.063 | -0.011 | 0.962 | 0.986 | Unannotated | NA | NA |
| metab_16319 | Unannotated feature | neg | 328.2494 | 6.656 | 5.849 | 5.794 | -0.055 | -0.011 | 0.962 | 0.986 | Unannotated | NA | NA |
| metab_18124 | MG(0:0/22:6(4Z,7Z,10Z,13Z,16Z,19Z)/0:0) | neg | 447.2754 | 6.373 | 6.098 | 5.895 | -0.203 | -0.011 | 0.962 | 0.986 | Unannotated | NA | NA |
| metab_12918 | Unannotated feature | neg | 234.0618 | 0.738 | 5.451 | 5.519 | 0.068 | 0.011 | 0.962 | 0.986 | Unannotated | NA | NA |
| metab_12514 | Unannotated feature | neg | 483.2443 | 6.357 | 6.461 | 6.441 | -0.021 | 0.011 | 0.962 | 0.986 | Unannotated | NA | NA |
| metab_10640 | Unannotated feature | neg | 273.0441 | 4.726 | 1.489 | 2.355 | 0.867 | -0.011 | 0.962 | 0.986 | Unannotated | NA | NA |
| metab_18766 | Racemetirosine | neg | 449.1939 | 5.825 | 5.251 | 5.200 | -0.050 | 0.011 | 0.962 | 0.986 | Tentative | NA | NA |
| metab_14617 | Unannotated feature | neg | 558.8852 | 0.502 | 5.538 | 5.438 | -0.100 | -0.011 | 0.962 | 0.986 | Unannotated | NA | NA |
| metab_10520 | Acyclovir | neg | 224.0788 | 1.947 | 3.676 | 3.825 | 0.149 | -0.011 | 0.962 | 0.986 | Strong putative | NA | NA |
| metab_10570 | Unannotated feature | neg | 601.1642 | 1.757 | 4.494 | 4.861 | 0.367 | -0.011 | 0.962 | 0.986 | Unannotated | NA | NA |
| metab_10567 | Unannotated feature | neg | 314.0750 | 1.782 | 4.558 | 4.374 | -0.184 | -0.011 | 0.962 | 0.986 | Unannotated | NA | NA |
| metab_8317 | Unannotated feature | pos | 116.0837 | 2.032 | 4.114 | 4.070 | -0.044 | -0.011 | 0.962 | 0.986 | Unannotated | NA | NA |
| metab_8288 | Isoniazid | pos | 275.1243 | 2.116 | 2.904 | 3.729 | 0.825 | -0.011 | 0.962 | 0.986 | Tentative | NA | NA |
| metab_16347 | Unannotated feature | neg | 327.1637 | 6.711 | 5.643 | 5.657 | 0.014 | 0.011 | 0.962 | 0.986 | Unannotated | NA | NA |
| metab_1991 | Unannotated feature | pos | 371.1023 | 2.160 | 6.123 | 6.092 | -0.031 | 0.011 | 0.962 | 0.986 | Unannotated | NA | NA |
| metab_5023 | Floionolic acid | pos | 315.2534 | 7.307 | 6.724 | 6.718 | -0.006 | -0.011 | 0.962 | 0.986 | Tentative | NA | NA |
| metab_16595 | Unannotated feature | neg | 491.3210 | 7.399 | 6.192 | 6.207 | 0.016 | -0.011 | 0.962 | 0.986 | Unannotated | NA | NA |
| metab_10156 | Unannotated feature | neg | 493.1692 | 2.729 | 4.176 | 4.257 | 0.082 | 0.011 | 0.962 | 0.986 | Unannotated | NA | NA |
| metab_6733 | Unannotated feature | pos | 245.9545 | 0.625 | 4.684 | 4.747 | 0.064 | 0.011 | 0.962 | 0.986 | Unannotated | NA | NA |
| metab_11855 | Unannotated feature | neg | 155.1070 | 5.983 | 5.133 | 5.266 | 0.134 | -0.011 | 0.962 | 0.986 | Unannotated | NA | NA |
| metab_14358 | Unannotated feature | neg | 510.9639 | 0.527 | 4.176 | 4.665 | 0.489 | 0.011 | 0.962 | 0.986 | Unannotated | NA | NA |
| metab_4852 | Unannotated feature | pos | 111.0207 | 7.732 | 6.263 | 6.262 | -0.002 | -0.011 | 0.962 | 0.986 | Unannotated | NA | NA |
| metab_9832 | Unannotated feature | neg | 186.0225 | 3.014 | 4.734 | 4.590 | -0.144 | 0.011 | 0.962 | 0.986 | Unannotated | NA | NA |
| metab_9831 | Succinic acid semialdehyde | neg | 203.0557 | 3.014 | 5.600 | 5.584 | -0.015 | 0.011 | 0.962 | 0.986 | Tentative | NA | NA |
| metab_9823 | Unannotated feature | neg | 209.0452 | 4.311 | 5.946 | 5.943 | -0.004 | -0.011 | 0.962 | 0.986 | Unannotated | NA | NA |
| metab_13703 | Unannotated feature | neg | 159.0769 | 1.741 | 3.133 | 3.128 | -0.005 | 0.011 | 0.962 | 0.986 | Unannotated | NA | NA |
| metab_15342 | Unannotated feature | neg | 305.1604 | 4.966 | 4.153 | 4.024 | -0.128 | -0.011 | 0.962 | 0.986 | Unannotated | NA | NA |
| metab_16927 | Unannotated feature | neg | 601.1640 | 2.051 | 5.530 | 5.668 | 0.138 | -0.011 | 0.962 | 0.986 | Unannotated | NA | NA |
| metab_18123 | Unannotated feature | neg | 495.2794 | 6.373 | 6.384 | 6.397 | 0.013 | -0.011 | 0.962 | 0.986 | Unannotated | NA | NA |
| metab_18109 | (3beta,17alpha,23S)-17,23-Epoxy-3,28,29-trihydroxy-27-norlanost-8-en-24-one | neg | 519.3331 | 6.381 | 6.541 | 6.541 | 0.000 | 0.011 | 0.962 | 0.986 | Unannotated | NA | NA |
| metab_5377 | PE(18:2/0:0) | pos | 500.2758 | 6.470 | 4.346 | 4.577 | 0.231 | 0.011 | 0.962 | 0.986 | Moderate putative | NA | NA |
| metab_5343 | Vitamin D3 glucosiduronate | pos | 593.4038 | 6.526 | 5.548 | 5.577 | 0.029 | 0.011 | 0.962 | 0.986 | Unannotated | NA | NA |
| metab_5340 | Unannotated feature | pos | 138.9955 | 6.534 | 4.018 | 3.912 | -0.105 | 0.011 | 0.962 | 0.986 | Unannotated | NA | NA |
| metab_19654 | Unannotated feature | neg | 110.9573 | 0.714 | 4.999 | 4.970 | -0.029 | 0.011 | 0.962 | 0.986 | Unannotated | NA | NA |
| metab_9581 | Unannotated feature | neg | 129.0550 | 3.242 | 6.087 | 6.157 | 0.070 | 0.011 | 0.962 | 0.986 | Unannotated | NA | NA |
| metab_12215 | Unannotated feature | neg | 388.3069 | 6.860 | 5.605 | 5.359 | -0.246 | 0.011 | 0.962 | 0.986 | Unannotated | NA | NA |
| metab_11523 | Unannotated feature | neg | 556.8864 | 0.502 | 5.359 | 5.348 | -0.010 | 0.011 | 0.962 | 0.986 | Unannotated | NA | NA |
| metab_16976 | Unannotated feature | neg | 180.9310 | 7.918 | 3.792 | 3.827 | 0.035 | 0.011 | 0.962 | 0.986 | Unannotated | NA | NA |
| metab_13107 | Unannotated feature | neg | 104.9531 | 7.773 | 5.756 | 5.782 | 0.025 | 0.011 | 0.962 | 0.986 | Unannotated | NA | NA |
| metab_17780 | 18-hydroxyoleate | neg | 593.4797 | 6.876 | 6.318 | 6.429 | 0.111 | 0.011 | 0.962 | 0.986 | Tentative | NA | NA |
| metab_5270 | Ganoderiol H | pos | 491.3741 | 6.630 | 5.550 | 5.491 | -0.059 | 0.011 | 0.962 | 0.986 | Unannotated | NA | NA |
| metab_6894 | Unannotated feature | pos | 113.0715 | 0.649 | 4.973 | 4.928 | -0.044 | -0.011 | 0.962 | 0.986 | Unannotated | NA | NA |
| metab_3102 | Unannotated feature | pos | 699.3586 | 6.494 | 4.578 | 4.409 | -0.169 | -0.011 | 0.962 | 0.986 | Unannotated | NA | NA |
| metab_4790 | Unannotated feature | pos | 102.0555 | 7.911 | 4.502 | 4.481 | -0.021 | -0.011 | 0.962 | 0.986 | Unannotated | NA | NA |
| metab_8694 | 3-Cyanoalanine | pos | 156.0771 | 0.784 | 6.061 | 6.065 | 0.004 | -0.011 | 0.962 | 0.986 | Tentative | NA | NA |
| metab_13756 | Unannotated feature | neg | 244.0826 | 1.952 | 5.902 | 5.921 | 0.019 | 0.011 | 0.962 | 0.986 | Unannotated | NA | NA |
| metab_2695 | 3-Hydroxydesloratadine | pos | 327.1289 | 2.032 | 3.465 | 3.443 | -0.023 | 0.011 | 0.962 | 0.986 | Unannotated | NA | NA |
| metab_2153 | Protocatechuic acid 4-glucoside | pos | 317.0838 | 0.633 | 4.313 | 4.460 | 0.147 | -0.011 | 0.962 | 0.986 | Unannotated | NA | NA |
| metab_9268 | Puerarin | neg | 415.1044 | 3.658 | 3.484 | 3.536 | 0.052 | 0.011 | 0.962 | 0.986 | Strong putative | NA | NA |
| metab_3199 | Dihydrocoumarin | pos | 149.0600 | 3.725 | 4.071 | 4.030 | -0.040 | -0.011 | 0.962 | 0.986 | Tentative | NA | NA |
| metab_9639 | Unannotated feature | neg | 401.1099 | 3.199 | 3.406 | 3.518 | 0.112 | 0.011 | 0.962 | 0.986 | Unannotated | NA | NA |
| metab_4592 | Unannotated feature | pos | 510.3421 | 6.989 | 5.111 | 5.173 | 0.062 | 0.011 | 0.962 | 0.986 | Unannotated | NA | NA |
| metab_4589 | Unannotated feature | pos | 413.2668 | 6.981 | 6.499 | 6.486 | -0.013 | 0.011 | 0.962 | 0.986 | Unannotated | NA | NA |
| metab_6036 | Unannotated feature | pos | 479.2418 | 5.445 | 4.937 | 4.836 | -0.102 | -0.011 | 0.962 | 0.986 | Unannotated | NA | NA |
| metab_18992 | Osajin | neg | 385.1446 | 5.538 | 4.935 | 4.937 | 0.002 | -0.011 | 0.962 | 0.986 | Moderate putative | NA | NA |
| metab_4011 | Unannotated feature | pos | 466.3126 | 5.814 | 3.007 | 3.093 | 0.086 | -0.011 | 0.962 | 0.986 | Unannotated | NA | NA |
| metab_10428 | Unannotated feature | neg | 314.1097 | 1.981 | 2.457 | 2.801 | 0.344 | 0.011 | 0.962 | 0.986 | Unannotated | NA | NA |
| metab_10519 | Forodesine | neg | 287.0775 | 1.947 | 5.332 | 5.313 | -0.019 | -0.011 | 0.962 | 0.986 | Unannotated | NA | NA |
| metab_6487 | Unannotated feature | pos | 460.7694 | 0.593 | 3.175 | 3.992 | 0.817 | 0.011 | 0.962 | 0.986 | Unannotated | NA | NA |
| metab_17357 | Unannotated feature | neg | 281.9247 | 7.687 | 4.143 | 4.135 | -0.008 | 0.011 | 0.962 | 0.986 | Unannotated | NA | NA |
| metab_2775 | Unannotated feature | pos | 288.1924 | 2.490 | 3.854 | 4.149 | 0.295 | 0.011 | 0.962 | 0.986 | Unannotated | NA | NA |
| metab_8861 | Unannotated feature | neg | 181.0864 | 4.340 | 3.404 | 4.181 | 0.777 | 0.011 | 0.962 | 0.986 | Unannotated | NA | NA |
| metab_5019 | Unannotated feature | pos | 875.7340 | 7.307 | 8.114 | 8.117 | 0.002 | -0.011 | 0.962 | 0.986 | Unannotated | NA | NA |
| metab_5015 | P,P-Dioctyldiphenylamine | pos | 394.3478 | 7.347 | 4.988 | 4.911 | -0.078 | 0.011 | 0.962 | 0.986 | Unannotated | NA | NA |
| metab_15777 | Unannotated feature | neg | 222.0494 | 0.603 | 4.538 | 4.380 | -0.159 | 0.011 | 0.962 | 0.986 | Unannotated | NA | NA |
| metab_11790 | (2-((3,5-Di-t-butyl-4-hydroxyphenyl)thio)-1-methylpropoxy)acetic acid | neg | 413.2015 | 5.808 | 5.785 | 5.853 | 0.068 | 0.011 | 0.962 | 0.986 | Unannotated | NA | NA |
| metab_15396 | Unannotated feature | neg | 197.0006 | 5.061 | 3.796 | 3.784 | -0.012 | -0.011 | 0.962 | 0.986 | Unannotated | NA | NA |
| metab_17685 | Unannotated feature | neg | 595.4947 | 7.019 | 5.414 | 5.426 | 0.012 | 0.011 | 0.962 | 0.986 | Unannotated | NA | NA |
| metab_16475 | (24E)-3alpha-Acetoxy-15alpha,22S-dihydroxylanosta-7,9(11),24-trien-26-oic acid | neg | 549.3210 | 7.053 | 4.148 | 4.753 | 0.605 | 0.011 | 0.962 | 0.986 | Unannotated | NA | NA |
| metab_7611 | N-Acetylserotonin | pos | 219.1132 | 3.533 | 3.534 | 3.762 | 0.228 | 0.011 | 0.962 | 0.986 | Strong putative | NA | NA |
| metab_440 | Unannotated feature | pos | 248.9011 | 0.013 | 4.029 | 3.991 | -0.038 | -0.011 | 0.962 | 0.986 | Unannotated | NA | NA |
| metab_10145 | Homoanserine | neg | 253.1306 | 2.746 | 3.019 | 3.672 | 0.653 | 0.011 | 0.962 | 0.986 | Unannotated | NA | NA |
| metab_13420 | Unannotated feature | neg | 300.0838 | 0.791 | 3.454 | 3.543 | 0.089 | -0.011 | 0.962 | 0.986 | Unannotated | NA | NA |
| metab_2576 | Unannotated feature | pos | 154.0442 | 1.245 | 5.506 | 5.514 | 0.008 | 0.011 | 0.962 | 0.986 | Unannotated | NA | NA |
| metab_12288 | Unannotated feature | neg | 479.3593 | 6.899 | 4.944 | 4.936 | -0.008 | 0.011 | 0.962 | 0.986 | Unannotated | NA | NA |
| metab_11739 | Darexaban | neg | 519.2248 | 5.621 | 3.327 | 3.389 | 0.062 | -0.011 | 0.962 | 0.986 | Unannotated | NA | NA |
| metab_2154 | BENAZOLIN | pos | 225.9707 | 0.625 | 4.375 | 4.429 | 0.054 | 0.011 | 0.962 | 0.986 | Tentative | NA | NA |
| metab_16045 | Unannotated feature | neg | 369.2284 | 6.177 | 6.366 | 6.261 | -0.106 | -0.011 | 0.962 | 0.986 | Unannotated | NA | NA |
| metab_12893 | Unannotated feature | neg | 404.1054 | 0.722 | 5.643 | 5.543 | -0.101 | -0.011 | 0.962 | 0.986 | Unannotated | NA | NA |
| metab_1022 | Unannotated feature | pos | 819.6705 | 7.036 | 6.099 | 6.134 | 0.034 | 0.011 | 0.962 | 0.986 | Unannotated | NA | NA |
| metab_12866 | Unannotated feature | neg | 285.1096 | 0.706 | 4.463 | 4.510 | 0.047 | -0.011 | 0.962 | 0.986 | Unannotated | NA | NA |
| metab_18252 | Unannotated feature | neg | 341.1427 | 6.256 | 5.042 | 5.082 | 0.040 | 0.011 | 0.962 | 0.986 | Unannotated | NA | NA |
| metab_2875 | Unannotated feature | pos | 362.1572 | 2.803 | 3.551 | 3.417 | -0.135 | 0.011 | 0.962 | 0.986 | Unannotated | NA | NA |
| metab_2868 | Unannotated feature | pos | 463.2464 | 6.064 | 5.248 | 4.990 | -0.258 | 0.011 | 0.962 | 0.986 | Unannotated | NA | NA |
| metab_15747 | Unannotated feature | neg | 93.0337 | 5.767 | 3.358 | 3.249 | -0.109 | -0.011 | 0.962 | 0.986 | Unannotated | NA | NA |
| metab_19314 | Unannotated feature | neg | 381.2027 | 5.122 | 2.544 | 2.775 | 0.231 | -0.011 | 0.962 | 0.986 | Unannotated | NA | NA |
| metab_4917 | Unannotated feature | pos | 244.9238 | 7.650 | 4.087 | 4.128 | 0.041 | 0.011 | 0.962 | 0.986 | Unannotated | NA | NA |
| metab_17309 | Unannotated feature | neg | 598.8557 | 7.702 | 4.488 | 4.474 | -0.014 | -0.011 | 0.962 | 0.986 | Unannotated | NA | NA |
| metab_2957 | Unannotated feature | pos | 285.1812 | 2.970 | 3.769 | 3.808 | 0.039 | 0.011 | 0.962 | 0.986 | Unannotated | NA | NA |
| metab_16252 | Unannotated feature | neg | 248.9935 | 1.369 | 5.476 | 5.409 | -0.067 | -0.011 | 0.962 | 0.986 | Unannotated | NA | NA |
| metab_10955 | Unannotated feature | neg | 247.0401 | 0.791 | 4.625 | 4.924 | 0.299 | 0.011 | 0.962 | 0.986 | Unannotated | NA | NA |
| metab_10396 | Unannotated feature | neg | 376.0019 | 2.054 | 5.045 | 4.996 | -0.049 | -0.011 | 0.962 | 0.986 | Unannotated | NA | NA |
| metab_2526 | Unannotated feature | pos | 131.0343 | 0.918 | 3.292 | 3.385 | 0.092 | 0.011 | 0.962 | 0.986 | Unannotated | NA | NA |
| metab_8494 | Glutamylproline | pos | 286.1402 | 1.685 | 5.349 | 5.301 | -0.049 | -0.011 | 0.962 | 0.986 | Unannotated | NA | NA |
| metab_13631 | Unannotated feature | neg | 174.0151 | 1.584 | 3.920 | 3.901 | -0.019 | -0.011 | 0.962 | 0.986 | Unannotated | NA | NA |
| metab_13624 | Unannotated feature | neg | 198.0880 | 1.530 | 3.455 | 3.376 | -0.079 | 0.011 | 0.962 | 0.986 | Unannotated | NA | NA |
| metab_8679 | Unannotated feature | pos | 79.0759 | 0.839 | 5.019 | 5.029 | 0.009 | 0.011 | 0.962 | 0.986 | Unannotated | NA | NA |
| metab_17250 | Unannotated feature | neg | 372.9019 | 7.726 | 5.581 | 5.569 | -0.013 | -0.011 | 0.962 | 0.986 | Unannotated | NA | NA |
| metab_3643 | Unannotated feature | pos | 769.5717 | 6.654 | 4.609 | 4.801 | 0.191 | -0.011 | 0.962 | 0.986 | Unannotated | NA | NA |
| metab_3062 | 3,4-Dihydroxyhydrocinnamic acid | pos | 165.0550 | 3.340 | 3.428 | 3.472 | 0.044 | 0.011 | 0.962 | 0.986 | Tentative | NA | NA |
| metab_15189 | Leucylphenylalanine | neg | 323.1615 | 4.710 | 4.899 | 6.116 | 1.217 | -0.011 | 0.962 | 0.986 | Unannotated | NA | NA |
| metab_7584 | Dihydroalprenolol | pos | 315.2035 | 3.578 | 2.973 | 3.020 | 0.047 | 0.011 | 0.962 | 0.986 | Unannotated | NA | NA |
| metab_2356 | Unannotated feature | pos | 128.0197 | 0.485 | 6.961 | 6.941 | -0.020 | -0.011 | 0.962 | 0.986 | Unannotated | NA | NA |
| metab_11323 | Unannotated feature | neg | 239.1288 | 5.136 | 4.549 | 4.651 | 0.102 | -0.011 | 0.962 | 0.986 | Unannotated | NA | NA |
| metab_8796 | Ascorbic acid | pos | 218.0663 | 0.854 | 4.286 | 4.086 | -0.200 | -0.011 | 0.962 | 0.986 | Tentative | NA | NA |
| metab_4744 | Unannotated feature | pos | 200.1136 | 7.700 | 4.108 | 4.111 | 0.002 | 0.011 | 0.962 | 0.986 | Unannotated | NA | NA |
| metab_7191 | Unannotated feature | pos | 365.1061 | 0.672 | 5.582 | 5.747 | 0.165 | 0.011 | 0.962 | 0.986 | Unannotated | NA | NA |
| metab_7143 | 3-Phenylpropyl acetate | pos | 179.1069 | 4.564 | 3.872 | 4.148 | 0.276 | -0.011 | 0.962 | 0.986 | Tentative | Benzenoids | Benzene and substituted derivatives |
| metab_3289 | Unannotated feature | pos | 323.1113 | 4.027 | 3.143 | 3.346 | 0.204 | -0.011 | 0.962 | 0.986 | Unannotated | NA | NA |
| metab_19593 | Lefamulin | neg | 544.2459 | 4.722 | 4.323 | 4.664 | 0.341 | 0.011 | 0.962 | 0.986 | Unannotated | NA | NA |
| metab_12574 | Unannotated feature | neg | 407.2454 | 6.357 | 5.839 | 5.743 | -0.096 | 0.011 | 0.962 | 0.986 | Unannotated | NA | NA |
| metab_5705 | Demethylsuberosin | pos | 272.1285 | 6.111 | 4.309 | 4.140 | -0.170 | -0.011 | 0.962 | 0.986 | Tentative | NA | NA |
| metab_1428 | Unannotated feature | pos | 414.3377 | 6.040 | 5.236 | 5.349 | 0.113 | -0.011 | 0.962 | 0.986 | Unannotated | NA | NA |
| metab_1408 | All-trans-retinoyl B-glucuronide | pos | 459.2393 | 6.072 | 4.902 | 4.890 | -0.012 | -0.011 | 0.962 | 0.986 | Unannotated | NA | NA |
| metab_12767 | Unannotated feature | neg | 243.0986 | 1.944 | 4.430 | 4.418 | -0.013 | -0.011 | 0.962 | 0.986 | Unannotated | NA | NA |
| metab_12763 | Unannotated feature | neg | 188.0923 | 1.947 | 5.368 | 5.387 | 0.019 | -0.011 | 0.962 | 0.986 | Unannotated | NA | NA |
| metab_4835 | Unannotated feature | pos | 138.9956 | 7.772 | 5.433 | 5.436 | 0.003 | 0.011 | 0.962 | 0.986 | Unannotated | NA | NA |
| metab_12329 | Unannotated feature | neg | 772.5603 | 7.374 | 3.693 | 3.872 | 0.179 | -0.011 | 0.962 | 0.986 | Unannotated | NA | NA |
| metab_13306 | Glycineamideribotide | neg | 331.0554 | 0.644 | 5.349 | 5.168 | -0.181 | -0.011 | 0.962 | 0.986 | Tentative | NA | NA |
| metab_13891 | Unannotated feature | neg | 391.9971 | 2.432 | 4.545 | 4.697 | 0.152 | 0.011 | 0.962 | 0.986 | Unannotated | NA | NA |
| metab_9395 | Unannotated feature | neg | 197.0426 | 3.484 | 5.195 | 5.130 | -0.065 | 0.011 | 0.962 | 0.986 | Unannotated | NA | NA |
| metab_9387 | Unannotated feature | neg | 214.0330 | 3.495 | 5.145 | 5.108 | -0.038 | -0.011 | 0.962 | 0.986 | Unannotated | NA | NA |
| metab_13266 | Unannotated feature | neg | 360.7869 | 0.607 | 4.408 | 4.373 | -0.034 | 0.011 | 0.962 | 0.986 | Unannotated | NA | NA |
| metab_834 | 2beta-Hydroxytestosterone | pos | 337.2360 | 6.454 | 6.301 | 6.280 | -0.021 | -0.011 | 0.962 | 0.986 | Tentative | NA | NA |
| metab_1463 | Unannotated feature | pos | 407.1981 | 5.969 | 5.022 | 5.006 | -0.016 | 0.011 | 0.962 | 0.986 | Unannotated | NA | NA |
| metab_18592 | Taurochenodeoxycholate-7-sulfate | neg | 288.6196 | 5.999 | 4.307 | 4.518 | 0.211 | -0.011 | 0.962 | 0.986 | Unannotated | NA | NA |
| metab_12010 | Unannotated feature | neg | 357.1739 | 6.263 | 6.291 | 6.340 | 0.049 | 0.011 | 0.962 | 0.986 | Unannotated | NA | NA |
| metab_14859 | Unannotated feature | neg | 352.1308 | 4.013 | 3.830 | 4.147 | 0.316 | 0.011 | 0.962 | 0.986 | Unannotated | NA | NA |
| metab_7928 | 8-Hydroxyquinoline | pos | 146.0603 | 2.937 | 5.302 | 5.312 | 0.010 | 0.011 | 0.962 | 0.986 | Strong putative | NA | NA |
| metab_12738 | 2-Methyl-4-pentenoic acid | neg | 159.0656 | 3.863 | 5.879 | 5.915 | 0.036 | 0.011 | 0.962 | 0.986 | Unannotated | NA | NA |
| metab_3490 | Unannotated feature | pos | 186.9813 | 7.724 | 5.820 | 5.836 | 0.016 | 0.011 | 0.962 | 0.986 | Unannotated | NA | NA |
| metab_13838 | 3-Dehydroquinic acid | neg | 189.0399 | 2.178 | 5.375 | 5.311 | -0.064 | -0.011 | 0.962 | 0.986 | Tentative | NA | NA |
| metab_12020 | Unannotated feature | neg | 283.1005 | 6.279 | 5.157 | 5.132 | -0.025 | -0.011 | 0.962 | 0.986 | Unannotated | NA | NA |
| metab_12032 | Unannotated feature | neg | 493.2250 | 6.295 | 6.787 | 6.750 | -0.037 | -0.011 | 0.962 | 0.986 | Unannotated | NA | NA |
| metab_3635 | Hexyl 2-methyl-4-pentenoate | pos | 419.3152 | 6.670 | 5.031 | 5.054 | 0.023 | -0.011 | 0.962 | 0.986 | Unannotated | NA | NA |
| metab_3595 | Unannotated feature | pos | 821.5561 | 6.694 | 4.857 | 4.911 | 0.054 | -0.006 | 0.981 | 0.995 | Unannotated | NA | NA |
| metab_12705 | Unannotated feature | neg | 375.2906 | 6.663 | 7.540 | 7.456 | -0.084 | -0.006 | 0.981 | 0.995 | Unannotated | NA | NA |
| metab_1199 | Unannotated feature | pos | 391.2461 | 6.462 | 5.111 | 5.079 | -0.032 | -0.006 | 0.981 | 0.995 | Unannotated | NA | NA |
| metab_1186 | Unannotated feature | pos | 455.3532 | 6.486 | 5.518 | 5.515 | -0.003 | -0.006 | 0.981 | 0.995 | Unannotated | NA | NA |
| metab_11152 | Unannotated feature | neg | 209.0452 | 5.029 | 5.490 | 5.440 | -0.051 | -0.006 | 0.981 | 0.995 | Unannotated | NA | NA |
| metab_1374 | Unannotated feature | pos | 365.2441 | 6.127 | 5.098 | 5.139 | 0.040 | 0.006 | 0.981 | 0.995 | Unannotated | NA | NA |
| metab_11102 | Unannotated feature | neg | 258.0741 | 0.722 | 4.198 | 4.093 | -0.105 | -0.006 | 0.981 | 0.995 | Unannotated | NA | NA |
| metab_16862 | Unannotated feature | neg | 475.8529 | 7.730 | 4.929 | 4.944 | 0.015 | -0.006 | 0.981 | 0.995 | Unannotated | NA | NA |
| metab_7382 | Unannotated feature | pos | 275.1032 | 4.002 | 4.058 | 4.222 | 0.164 | 0.006 | 0.981 | 0.995 | Unannotated | NA | NA |
| metab_11705 | Unannotated feature | neg | 365.1972 | 5.484 | 5.192 | 5.014 | -0.178 | -0.006 | 0.981 | 0.995 | Unannotated | NA | NA |
| metab_12353 | Unannotated feature | neg | 493.2949 | 6.853 | 6.588 | 6.705 | 0.117 | 0.006 | 0.981 | 0.995 | Unannotated | NA | NA |
| metab_2462 | N-(N-(3-Amino-3-carboxypropyl)-3-amino-3-carboxypropyl)azetidine-2-carboxylic acid | pos | 286.1402 | 0.680 | 4.251 | 4.318 | 0.067 | -0.006 | 0.981 | 0.995 | Unannotated | NA | NA |
| metab_19145 | Unannotated feature | neg | 209.1178 | 5.334 | 3.369 | 3.253 | -0.116 | 0.006 | 0.981 | 0.995 | Unannotated | NA | NA |
| metab_19072 | Panipenem | neg | 338.1181 | 5.431 | 3.565 | 3.776 | 0.211 | 0.006 | 0.981 | 0.995 | Unannotated | NA | NA |
| metab_15816 | Unannotated feature | neg | 450.8339 | 0.603 | 5.110 | 5.092 | -0.018 | -0.006 | 0.981 | 0.995 | Unannotated | NA | NA |
| metab_5133 | Unannotated feature | pos | 105.0428 | 6.909 | 6.592 | 6.600 | 0.008 | 0.006 | 0.981 | 0.995 | Unannotated | NA | NA |
| metab_13199 | Unannotated feature | neg | 116.9855 | 0.524 | 3.332 | 3.385 | 0.053 | 0.006 | 0.981 | 0.995 | Unannotated | NA | NA |
| metab_2384 | Unannotated feature | pos | 279.9474 | 0.553 | 4.107 | 4.129 | 0.022 | 0.006 | 0.981 | 0.995 | Unannotated | NA | NA |
| metab_17255 | Unannotated feature | neg | 294.6197 | 7.726 | 4.502 | 4.354 | -0.148 | 0.006 | 0.981 | 0.995 | Unannotated | NA | NA |
| metab_12186 | Unannotated feature | neg | 358.2520 | 6.742 | 5.338 | 5.214 | -0.124 | -0.006 | 0.981 | 0.995 | Unannotated | NA | NA |
| metab_17581 | Unannotated feature | neg | 225.0353 | 7.279 | 3.830 | 3.776 | -0.054 | -0.006 | 0.981 | 0.995 | Unannotated | NA | NA |
| metab_19087 | Ovalicin | neg | 317.1397 | 5.409 | 5.357 | 5.333 | -0.024 | 0.006 | 0.981 | 0.995 | Tentative | NA | NA |
| metab_5119 | Unannotated feature | pos | 617.4768 | 6.973 | 6.422 | 6.460 | 0.038 | 0.006 | 0.981 | 0.995 | Unannotated | NA | NA |
| metab_8492 | Unannotated feature | pos | 288.1926 | 1.685 | 3.139 | 2.376 | -0.763 | -0.006 | 0.981 | 0.995 | Unannotated | NA | NA |
| metab_8483 | Asparaginyl-Proline | pos | 212.1033 | 1.692 | 4.021 | 4.084 | 0.064 | -0.006 | 0.981 | 0.995 | Unannotated | NA | NA |
| metab_15824 | Unannotated feature | neg | 453.1457 | 5.896 | 4.519 | 4.747 | 0.228 | 0.006 | 0.981 | 0.995 | Unannotated | NA | NA |
| metab_7517 | Unannotated feature | pos | 225.0320 | 3.725 | 4.758 | 4.739 | -0.019 | 0.006 | 0.981 | 0.995 | Unannotated | NA | NA |
| metab_17682 | Physapubescin | neg | 551.2617 | 7.031 | 5.232 | 5.159 | -0.073 | -0.006 | 0.981 | 0.995 | Unannotated | NA | NA |
| metab_2260 | 7-Hydroxy-3,3',4',5,6-pentamethoxyflavone | pos | 371.1134 | 5.615 | 4.025 | 3.992 | -0.033 | 0.006 | 0.981 | 0.995 | Unannotated | NA | NA |
| metab_2259 | Unannotated feature | pos | 320.8673 | 0.531 | 5.943 | 5.905 | -0.038 | 0.006 | 0.981 | 0.995 | Unannotated | NA | NA |
| metab_5229 | Unannotated feature | pos | 399.2987 | 6.702 | 4.475 | 4.454 | -0.022 | -0.006 | 0.981 | 0.995 | Unannotated | NA | NA |
| metab_8409 | N-Acetyl-b-D-galactosamine | pos | 186.0764 | 1.952 | 5.687 | 5.665 | -0.021 | 0.006 | 0.981 | 0.995 | Tentative | NA | NA |
| metab_16481 | Unannotated feature | neg | 292.1042 | 1.685 | 6.073 | 6.173 | 0.100 | -0.006 | 0.981 | 0.995 | Unannotated | NA | NA |
| metab_901 | Unannotated feature | pos | 427.0771 | 0.609 | 3.497 | 3.579 | 0.081 | -0.006 | 0.981 | 0.995 | Unannotated | NA | NA |
| metab_7598 | 3-methylheptanedioylcarnitine | pos | 318.1927 | 3.553 | 4.242 | 4.344 | 0.102 | -0.006 | 0.981 | 0.995 | Unannotated | NA | NA |
| metab_2185 | Unannotated feature | pos | 444.7953 | 0.593 | 4.010 | 4.126 | 0.116 | -0.006 | 0.981 | 0.995 | Unannotated | NA | NA |
| metab_13127 | Unannotated feature | neg | 301.9125 | 0.449 | 3.583 | 3.665 | 0.082 | 0.006 | 0.981 | 0.995 | Unannotated | NA | NA |
| metab_6640 | Unannotated feature | pos | 165.9670 | 0.609 | 5.091 | 5.092 | 0.000 | 0.006 | 0.981 | 0.995 | Unannotated | NA | NA |
| metab_10135 | Unannotated feature | neg | 468.1565 | 2.750 | 3.858 | 3.943 | 0.085 | -0.006 | 0.981 | 0.995 | Unannotated | NA | NA |
| metab_803 | Unannotated feature | pos | 437.3028 | 6.279 | 4.781 | 4.657 | -0.123 | 0.006 | 0.981 | 0.995 | Unannotated | NA | NA |
| metab_13435 | Unannotated feature | neg | 262.0393 | 0.810 | 4.175 | 4.319 | 0.145 | -0.006 | 0.981 | 0.995 | Unannotated | NA | NA |
| metab_10050 | Unannotated feature | neg | 237.0439 | 2.793 | 4.835 | 4.953 | 0.118 | -0.006 | 0.981 | 0.995 | Unannotated | NA | NA |
| metab_11743 | Unannotated feature | neg | 289.1657 | 5.627 | 5.676 | 5.546 | -0.130 | 0.006 | 0.981 | 0.995 | Unannotated | NA | NA |
| metab_15163 | Cinncassiol A | neg | 381.1924 | 4.653 | 5.425 | 5.350 | -0.075 | 0.006 | 0.981 | 0.995 | Tentative | NA | NA |
| metab_19078 | Unannotated feature | neg | 603.3226 | 5.417 | 3.949 | 3.873 | -0.076 | -0.006 | 0.981 | 0.995 | Unannotated | NA | NA |
| metab_4210 | Unannotated feature | pos | 323.1968 | 6.000 | 4.623 | 4.693 | 0.070 | 0.006 | 0.981 | 0.995 | Unannotated | NA | NA |
| metab_14316 | Unannotated feature | neg | 245.9932 | 3.070 | 3.401 | 3.740 | 0.339 | 0.006 | 0.981 | 0.995 | Unannotated | NA | NA |
| metab_3169 | 4-Phenylpyridine | pos | 156.0811 | 3.642 | 3.615 | 3.666 | 0.051 | 0.006 | 0.981 | 0.995 | Tentative | NA | NA |
| metab_3140 | Unannotated feature | pos | 639.4094 | 6.590 | 6.706 | 6.645 | -0.061 | 0.006 | 0.981 | 0.995 | Unannotated | NA | NA |
| metab_3133 | Bisnortilidin | pos | 263.1759 | 3.533 | 3.860 | 3.689 | -0.171 | -0.006 | 0.981 | 0.995 | Unannotated | NA | NA |
| metab_11892 | Unannotated feature | neg | 225.0691 | 6.041 | 5.721 | 5.495 | -0.226 | -0.006 | 0.981 | 0.995 | Unannotated | NA | NA |
| metab_12148 | Unannotated feature | neg | 607.4591 | 6.640 | 6.091 | 6.060 | -0.030 | -0.006 | 0.981 | 0.995 | Unannotated | NA | NA |
| metab_318 | Unannotated feature | pos | 263.0104 | 0.477 | 3.922 | 3.976 | 0.054 | 0.006 | 0.981 | 0.995 | Unannotated | NA | NA |
| metab_317 | Unannotated feature | pos | 211.0930 | 0.477 | 5.271 | 5.229 | -0.042 | -0.006 | 0.981 | 0.995 | Unannotated | NA | NA |
| metab_13933 | 1-O-Feruloyl-beta-D-glucose | neg | 337.0896 | 2.575 | 5.276 | 5.339 | 0.063 | -0.006 | 0.981 | 0.995 | Tentative | NA | NA |
| metab_923 | Unannotated feature | pos | 627.5336 | 7.252 | 5.814 | 5.839 | 0.025 | -0.006 | 0.981 | 0.995 | Unannotated | NA | NA |
| metab_16946 | Unannotated feature | neg | 153.8993 | 7.840 | 4.764 | 4.760 | -0.004 | -0.006 | 0.981 | 0.995 | Unannotated | NA | NA |
| metab_16977 | Unannotated feature | neg | 197.9018 | 7.918 | 4.024 | 4.023 | -0.001 | 0.006 | 0.981 | 0.995 | Unannotated | NA | NA |
| metab_10492 | (S)-Methylmalonic acid semialdehyde | neg | 203.0557 | 1.952 | 5.014 | 5.100 | 0.086 | 0.006 | 0.981 | 0.995 | Tentative | NA | NA |
| metab_1985 | Unannotated feature | pos | 355.0709 | 2.104 | 5.280 | 5.265 | -0.015 | -0.006 | 0.981 | 0.995 | Unannotated | NA | NA |
| metab_5022 | Unannotated feature | pos | 449.3615 | 7.307 | 9.423 | 9.439 | 0.016 | -0.006 | 0.981 | 0.995 | Unannotated | NA | NA |
| metab_5105 | Unannotated feature | pos | 347.1465 | 0.728 | 4.725 | 4.674 | -0.051 | -0.006 | 0.981 | 0.995 | Unannotated | NA | NA |
| metab_6718 | Unannotated feature | pos | 347.1588 | 5.283 | 4.145 | 5.351 | 1.207 | -0.006 | 0.981 | 0.995 | Unannotated | NA | NA |
| metab_12142 | Unannotated feature | neg | 331.2314 | 6.632 | 4.412 | 3.923 | -0.489 | 0.006 | 0.981 | 0.995 | Unannotated | NA | NA |
| metab_17234 | Unannotated feature | neg | 138.8894 | 7.730 | 4.035 | 4.033 | -0.002 | 0.006 | 0.981 | 0.995 | Unannotated | NA | NA |
| metab_13081 | Unannotated feature | neg | 598.7788 | 0.533 | 4.911 | 4.804 | -0.107 | -0.006 | 0.981 | 0.995 | Unannotated | NA | NA |
| metab_14588 | 1-Naphthalenesulfonic acid, 3,4-dihydro-3,4-dioxo- | neg | 282.9941 | 3.495 | 4.991 | 4.927 | -0.064 | 0.006 | 0.981 | 0.995 | Unannotated | NA | NA |
| metab_14022 | 1-Carboxyethylisoleucine | neg | 202.1081 | 2.762 | 4.613 | 4.664 | 0.051 | 0.006 | 0.981 | 0.995 | Unannotated | NA | NA |
| metab_17948 | Unannotated feature | neg | 311.1253 | 2.865 | 4.465 | 4.520 | 0.054 | -0.006 | 0.981 | 0.995 | Unannotated | NA | NA |
| metab_12996 | Tripdiolide | neg | 357.1310 | 0.777 | 4.139 | 4.106 | -0.033 | 0.006 | 0.981 | 0.995 | Tentative | NA | NA |
| metab_12496 | Unannotated feature | neg | 541.3756 | 6.373 | 6.198 | 6.228 | 0.031 | 0.006 | 0.981 | 0.995 | Unannotated | NA | NA |
| metab_14426 | Unannotated feature | neg | 419.0557 | 3.227 | 4.405 | 4.461 | 0.057 | 0.006 | 0.981 | 0.995 | Unannotated | NA | NA |
| metab_14410 | Unannotated feature | neg | 361.9715 | 3.210 | 4.603 | 4.640 | 0.037 | 0.006 | 0.981 | 0.995 | Unannotated | NA | NA |
| metab_8546 | Swainsonine | pos | 174.1128 | 4.002 | 4.741 | 4.832 | 0.091 | -0.006 | 0.981 | 0.995 | Moderate putative | NA | NA |
| metab_14450 | 4-Hydroxy-3-methoxybenzenemethanol | neg | 153.0551 | 3.266 | 4.024 | 4.033 | 0.009 | -0.006 | 0.981 | 0.995 | Tentative | NA | NA |
| metab_2625 | 4-Oxo-2-azetidinecarboxylic acid | pos | 272.0883 | 1.698 | 2.771 | 2.735 | -0.036 | 0.006 | 0.981 | 0.995 | Unannotated | NA | NA |
| metab_11761 | Unannotated feature | neg | 501.2142 | 5.692 | 4.703 | 4.677 | -0.026 | 0.006 | 0.981 | 0.995 | Unannotated | NA | NA |
| metab_6515 | Unannotated feature | pos | 189.1600 | 0.601 | 4.828 | 4.813 | -0.015 | -0.006 | 0.981 | 0.995 | Unannotated | NA | NA |
| metab_13488 | Unannotated feature | neg | 349.1257 | 1.021 | 3.953 | 3.687 | -0.265 | 0.006 | 0.981 | 0.995 | Unannotated | NA | NA |
| metab_4328 | Indole-3-Carbinol | pos | 130.0655 | 6.095 | 4.008 | 3.890 | -0.118 | 0.006 | 0.981 | 0.995 | Unannotated | NA | NA |
| metab_11502 | Unannotated feature | neg | 428.1685 | 5.233 | 3.591 | 3.449 | -0.143 | 0.006 | 0.981 | 0.995 | Unannotated | NA | NA |
| metab_989 | Unannotated feature | pos | 141.9836 | 7.724 | 5.505 | 5.508 | 0.003 | -0.006 | 0.981 | 0.995 | Unannotated | NA | NA |
| metab_12111 | Unannotated feature | neg | 767.5848 | 6.522 | 6.125 | 6.111 | -0.014 | -0.006 | 0.981 | 0.995 | Unannotated | NA | NA |
| metab_9603 | Unannotated feature | neg | 101.0600 | 3.227 | 3.895 | 4.051 | 0.156 | 0.006 | 0.981 | 0.995 | Unannotated | NA | NA |
| metab_17159 | Unannotated feature | neg | 486.9042 | 7.765 | 4.140 | 4.150 | 0.010 | -0.006 | 0.981 | 0.995 | Unannotated | NA | NA |
| metab_12963 | Unannotated feature | neg | 305.0883 | 0.777 | 6.115 | 6.146 | 0.031 | -0.006 | 0.981 | 0.995 | Unannotated | NA | NA |
| metab_10637 | Beta-D-Fucose | neg | 163.0606 | 1.680 | 4.822 | 4.869 | 0.047 | 0.006 | 0.981 | 0.995 | Tentative | NA | NA |
| metab_12490 | Unannotated feature | neg | 633.4012 | 6.389 | 4.538 | 4.489 | -0.049 | -0.006 | 0.981 | 0.995 | Unannotated | NA | NA |
| metab_12926 | Unannotated feature | neg | 323.0291 | 0.738 | 4.579 | 4.585 | 0.006 | 0.006 | 0.981 | 0.995 | Unannotated | NA | NA |
| metab_1766 | Descarbonyl-lacosamide | pos | 191.1182 | 3.347 | 3.290 | 3.522 | 0.232 | -0.006 | 0.981 | 0.995 | Unannotated | NA | NA |
| metab_16022 | 20-Trihydroxy-leukotriene-B4 | neg | 383.2078 | 6.146 | 5.987 | 5.883 | -0.104 | 0.006 | 0.981 | 0.995 | Unannotated | NA | NA |
| metab_270 | Unannotated feature | pos | 83.0472 | 0.524 | 3.624 | 3.532 | -0.092 | 0.006 | 0.981 | 0.995 | Unannotated | NA | NA |
| metab_8776 | Cytosine | pos | 112.0511 | 0.728 | 4.463 | 4.430 | -0.033 | -0.006 | 0.981 | 0.995 | Moderate putative | NA | NA |
| metab_13810 | Saxalin | neg | 359.0118 | 2.043 | 5.319 | 5.227 | -0.092 | 0.006 | 0.981 | 0.995 | Unannotated | NA | NA |
| metab_8603 | Unannotated feature | pos | 266.0873 | 1.122 | 5.033 | 5.075 | 0.042 | 0.006 | 0.981 | 0.995 | Unannotated | NA | NA |
| metab_8857 | Unannotated feature | neg | 222.8297 | 4.347 | 5.782 | 5.865 | 0.083 | -0.006 | 0.981 | 0.995 | Unannotated | NA | NA |
| metab_7212 | Dihydro-5-pentyl-2(3H)-furanone | pos | 121.1018 | 4.399 | 4.074 | 4.163 | 0.089 | 0.006 | 0.981 | 0.995 | Tentative | Organoheterocyclic compounds | Lactones |
| metab_1056 | Unannotated feature | pos | 477.3197 | 6.789 | 7.550 | 7.569 | 0.020 | 0.006 | 0.981 | 0.995 | Unannotated | NA | NA |
| metab_14746 | Nicotine glucuronide | neg | 337.1410 | 3.780 | 4.162 | 4.026 | -0.137 | 0.006 | 0.981 | 0.995 | Unannotated | NA | NA |
| metab_18148 | Monensin | neg | 651.4122 | 6.342 | 4.305 | 4.398 | 0.092 | -0.006 | 0.981 | 0.995 | Tentative | NA | NA |
| metab_12554 | Unannotated feature | neg | 637.3249 | 6.162 | 5.088 | 5.178 | 0.090 | 0.006 | 0.981 | 0.995 | Unannotated | NA | NA |
| metab_16094 | Embelin | neg | 339.1813 | 6.232 | 5.831 | 5.822 | -0.010 | -0.006 | 0.981 | 0.995 | Tentative | NA | NA |
| metab_10851 | Unannotated feature | neg | 190.0602 | 1.021 | 3.360 | 3.471 | 0.110 | 0.006 | 0.981 | 0.995 | Unannotated | NA | NA |
| metab_10838 | Unannotated feature | neg | 262.0393 | 1.041 | 4.308 | 4.191 | -0.117 | -0.006 | 0.981 | 0.995 | Unannotated | NA | NA |
| metab_5414 | Milbemycin beta1 | pos | 545.3466 | 6.414 | 6.399 | 6.464 | 0.065 | 0.006 | 0.981 | 0.995 | Unannotated | NA | NA |
| metab_12863 | Unannotated feature | neg | 325.1256 | 0.636 | 5.336 | 5.337 | 0.001 | -0.006 | 0.981 | 0.995 | Unannotated | NA | NA |
| metab_14983 | Unannotated feature | neg | 216.0486 | 4.294 | 4.367 | 4.389 | 0.022 | 0.006 | 0.981 | 0.995 | Unannotated | NA | NA |
| metab_12626 | Unannotated feature | neg | 603.3223 | 6.003 | 5.107 | 5.024 | -0.083 | -0.006 | 0.981 | 0.995 | Unannotated | NA | NA |
| metab_2894 | Isoquinoline | pos | 130.0655 | 2.845 | 3.712 | 3.736 | 0.025 | 0.006 | 0.981 | 0.995 | Tentative | NA | NA |
| metab_4907 | Unannotated feature | pos | 408.9191 | 7.674 | 4.856 | 4.807 | -0.049 | -0.006 | 0.981 | 0.995 | Unannotated | NA | NA |
| metab_14768 | (R)-Pantothenic acid 4'-O-b-D-glucoside | neg | 362.1463 | 3.834 | 5.482 | 5.476 | -0.006 | 0.006 | 0.981 | 0.995 | Unannotated | NA | NA |
| metab_5408 | 9,10-DHOME | pos | 297.2428 | 6.430 | 5.269 | 5.264 | -0.005 | 0.006 | 0.981 | 0.995 | Tentative | NA | NA |
| metab_16217 | Unannotated feature | neg | 187.0023 | 1.336 | 5.135 | 5.110 | -0.025 | -0.006 | 0.981 | 0.995 | Unannotated | NA | NA |
| metab_11002 | Unannotated feature | neg | 544.2474 | 4.927 | 4.815 | 5.654 | 0.839 | -0.006 | 0.981 | 0.995 | Unannotated | NA | NA |
| metab_11000 | Unannotated feature | neg | 195.0328 | 0.777 | 4.525 | 4.321 | -0.205 | 0.006 | 0.981 | 0.995 | Unannotated | NA | NA |
| metab_9558 | (4R,6S)-p-Menth-1-ene-4,6-diol 4-glucoside | neg | 353.1590 | 4.146 | 5.538 | 5.543 | 0.004 | -0.006 | 0.981 | 0.995 | Unannotated | Lipids and lipid-like molecules | Prenol lipids |
| metab_8752 | L-Theanine | pos | 157.0975 | 0.728 | 3.373 | 4.001 | 0.628 | 0.006 | 0.981 | 0.995 | Tentative | NA | NA |
| metab_5480 | Tetradecadienyl-l-carnitine | pos | 386.3270 | 6.367 | 5.027 | 5.046 | 0.020 | 0.006 | 0.981 | 0.995 | Unannotated | NA | NA |
| metab_1657 | Unannotated feature | pos | 338.1394 | 4.542 | 4.376 | 4.165 | -0.211 | 0.006 | 0.981 | 0.995 | Unannotated | NA | NA |
| metab_15490 | Momilactone A | neg | 335.1617 | 5.249 | 6.106 | 6.062 | -0.044 | -0.006 | 0.981 | 0.995 | Tentative | NA | NA |
| metab_11944 | Unannotated feature | neg | 639.3055 | 6.130 | 5.209 | 5.330 | 0.121 | -0.006 | 0.981 | 0.995 | Unannotated | NA | NA |
| metab_12212 | Unannotated feature | neg | 555.2854 | 6.853 | 5.812 | 5.633 | -0.180 | -0.006 | 0.981 | 0.995 | Unannotated | NA | NA |
| metab_648 | Unannotated feature | pos | 345.9517 | 0.016 | 4.425 | 4.424 | -0.002 | -0.006 | 0.981 | 0.995 | Unannotated | NA | NA |
| metab_19336 | Acrimarine I | neg | 546.1898 | 5.087 | 3.745 | 4.371 | 0.627 | -0.006 | 0.981 | 0.995 | Unannotated | NA | NA |
| metab_9872 | Unannotated feature | neg | 304.1041 | 4.340 | 5.552 | 5.435 | -0.117 | 0.006 | 0.981 | 0.995 | Unannotated | NA | NA |
| metab_11187 | Erythrose | neg | 165.0398 | 0.659 | 5.491 | 5.545 | 0.054 | 0.006 | 0.981 | 0.995 | Tentative | NA | NA |
| metab_16183 | Enterolactone | neg | 297.1134 | 6.083 | 6.604 | 6.701 | 0.097 | 0.006 | 0.981 | 0.995 | Strong putative | NA | NA |
| metab_18393 | Unannotated feature | neg | 407.1746 | 6.138 | 5.551 | 5.673 | 0.122 | 0.006 | 0.981 | 0.995 | Unannotated | NA | NA |
| metab_14839 | Unannotated feature | neg | 476.8577 | 0.507 | 4.679 | 4.622 | -0.057 | 0.006 | 0.981 | 0.995 | Unannotated | NA | NA |
| metab_11141 | DI(Hydroxyethyl)ether | neg | 257.1256 | 0.698 | 3.913 | 3.980 | 0.066 | 0.006 | 0.981 | 0.995 | Tentative | NA | NA |
| metab_17090 | Unannotated feature | neg | 218.8759 | 7.884 | 4.422 | 4.439 | 0.016 | -0.006 | 0.981 | 0.995 | Unannotated | NA | NA |
| metab_8850 | Gatifloxacin | neg | 396.1308 | 4.359 | 4.116 | 4.226 | 0.109 | 0.002 | 1.000 | 1.000 | Tentative | NA | NA |
| metab_12060 | Unannotated feature | neg | 389.1999 | 6.389 | 5.076 | 5.226 | 0.150 | -0.002 | 1.000 | 1.000 | Unannotated | NA | NA |
| metab_14903 | Unannotated feature | neg | 546.8390 | 0.524 | 5.214 | 5.267 | 0.053 | 0.002 | 1.000 | 1.000 | Unannotated | NA | NA |
| metab_12640 | Unannotated feature | neg | 269.1760 | 5.975 | 4.819 | 4.911 | 0.092 | -0.002 | 1.000 | 1.000 | Unannotated | NA | NA |
| metab_19889 | Phenylacetylglutamine | neg | 263.1039 | 3.461 | 3.303 | 3.289 | -0.014 | 0.002 | 1.000 | 1.000 | Strong putative | NA | NA |
| metab_11930 | Unannotated feature | neg | 425.2184 | 6.115 | 6.011 | 5.952 | -0.060 | 0.002 | 1.000 | 1.000 | Unannotated | NA | NA |
| metab_587 | DL-o-Tyrosine | pos | 164.0707 | 5.566 | 5.295 | 5.294 | -0.001 | 0.002 | 1.000 | 1.000 | Moderate putative | NA | NA |
| metab_6588 | Unannotated feature | pos | 167.9650 | 0.609 | 3.835 | 3.882 | 0.047 | -0.002 | 1.000 | 1.000 | Unannotated | NA | NA |
| metab_12237 | Unannotated feature | neg | 765.5693 | 6.940 | 5.276 | 5.454 | 0.179 | 0.002 | 1.000 | 1.000 | Unannotated | NA | NA |
| metab_4867 | Unannotated feature | pos | 171.0059 | 7.724 | 4.656 | 4.675 | 0.019 | 0.002 | 1.000 | 1.000 | Unannotated | NA | NA |
| metab_17327 | Unannotated feature | neg | 447.9192 | 7.697 | 4.951 | 4.919 | -0.032 | -0.002 | 1.000 | 1.000 | Unannotated | NA | NA |
| metab_2770 | Unannotated feature | pos | 81.5211 | 2.465 | 5.245 | 5.237 | -0.008 | -0.002 | 1.000 | 1.000 | Unannotated | NA | NA |
| metab_5024 | 6-Hydroxyhexanoic acid | pos | 97.0653 | 7.307 | 5.115 | 5.123 | 0.007 | -0.002 | 1.000 | 1.000 | Tentative | NA | NA |
| metab_4660 | Unannotated feature | pos | 903.7646 | 7.411 | 5.334 | 5.345 | 0.011 | -0.002 | 1.000 | 1.000 | Unannotated | NA | NA |
| metab_3747 | MG(18:1(12Z)-O(9S,10R)/0:0/0:0) | pos | 393.2620 | 6.494 | 6.207 | 6.183 | -0.024 | 0.002 | 1.000 | 1.000 | Unannotated | NA | NA |
| metab_710 | Retinoyl b-glucuronide | pos | 459.2392 | 5.944 | 4.274 | 4.210 | -0.065 | -0.002 | 1.000 | 1.000 | Tentative | Lipids and lipid-like molecules | Prenol lipids |
| metab_718 | Unannotated feature | pos | 570.3115 | 5.960 | 5.438 | 5.428 | -0.010 | -0.002 | 1.000 | 1.000 | Unannotated | NA | NA |
| metab_9984 | Unannotated feature | neg | 200.9859 | 2.865 | 5.176 | 5.126 | -0.050 | -0.002 | 1.000 | 1.000 | Unannotated | NA | NA |
| metab_5066 | Unannotated feature | pos | 546.4028 | 7.132 | 4.449 | 4.361 | -0.089 | 0.002 | 1.000 | 1.000 | Unannotated | NA | NA |
| metab_16943 | Unannotated feature | neg | 197.8893 | 7.835 | 5.820 | 5.822 | 0.002 | 0.002 | 1.000 | 1.000 | Unannotated | NA | NA |
| metab_9976 | Herbarumin II | neg | 243.1237 | 4.401 | 5.489 | 5.739 | 0.251 | 0.002 | 1.000 | 1.000 | Unannotated | NA | NA |
| metab_8536 | 6,8-Dihydroxypurine | pos | 153.0409 | 1.348 | 6.874 | 6.873 | -0.002 | 0.002 | 1.000 | 1.000 | Unannotated | NA | NA |
| metab_8481 | Vanillylmandelic acid | pos | 240.0870 | 1.692 | 5.344 | 5.360 | 0.016 | 0.002 | 1.000 | 1.000 | Tentative | NA | NA |
| metab_16614 | Unannotated feature | neg | 700.4843 | 7.468 | 5.816 | 5.764 | -0.051 | -0.002 | 1.000 | 1.000 | Unannotated | NA | NA |
| metab_19485 | Unannotated feature | neg | 408.1313 | 4.880 | 4.765 | 4.423 | -0.343 | 0.002 | 1.000 | 1.000 | Unannotated | NA | NA |
| metab_4851 | Unannotated feature | pos | 120.5551 | 7.732 | 4.162 | 4.166 | 0.005 | -0.002 | 1.000 | 1.000 | Unannotated | NA | NA |
| metab_4843 | Unannotated feature | pos | 335.0335 | 7.732 | 4.035 | 4.062 | 0.027 | -0.002 | 1.000 | 1.000 | Unannotated | NA | NA |
| metab_19382 | Balofloxacin | neg | 410.1465 | 5.029 | 3.483 | 3.882 | 0.399 | -0.002 | 1.000 | 1.000 | Tentative | NA | NA |
| metab_10088 | Unannotated feature | neg | 343.0847 | 4.462 | 4.986 | 5.109 | 0.123 | 0.002 | 1.000 | 1.000 | Unannotated | NA | NA |
| metab_2578 | Unannotated feature | pos | 230.1867 | 1.252 | 6.253 | 6.231 | -0.022 | -0.002 | 1.000 | 1.000 | Unannotated | NA | NA |
| metab_829 | Unannotated feature | pos | 203.1798 | 6.430 | 4.668 | 4.607 | -0.062 | 0.002 | 1.000 | 1.000 | Unannotated | NA | NA |
| metab_4692 | Unannotated feature | pos | 476.9044 | 7.554 | 4.855 | 4.852 | -0.003 | 0.002 | 1.000 | 1.000 | Unannotated | NA | NA |
| metab_10123 | Lipoamide | neg | 226.0355 | 2.762 | 4.564 | 4.677 | 0.112 | -0.002 | 1.000 | 1.000 | Tentative | NA | NA |
| metab_10111 | Unannotated feature | neg | 161.0813 | 2.766 | 5.022 | 5.140 | 0.118 | 0.002 | 1.000 | 1.000 | Unannotated | NA | NA |
| metab_13402 | Unannotated feature | neg | 159.0292 | 0.777 | 5.152 | 5.206 | 0.054 | -0.002 | 1.000 | 1.000 | Unannotated | NA | NA |
| metab_5113 | Unannotated feature | pos | 839.6390 | 6.981 | 4.474 | 4.507 | 0.033 | 0.002 | 1.000 | 1.000 | Unannotated | NA | NA |
| metab_16513 | Unannotated feature | neg | 153.9939 | 7.129 | 3.647 | 3.664 | 0.017 | 0.002 | 1.000 | 1.000 | Unannotated | NA | NA |
| metab_8446 | Imigliptin | pos | 399.1887 | 1.936 | 3.818 | 4.086 | 0.268 | -0.002 | 1.000 | 1.000 | Unannotated | NA | NA |
| metab_9751 | O-Acetyl-L-homoserine | neg | 160.0609 | 3.082 | 2.887 | 2.818 | -0.068 | 0.002 | 1.000 | 1.000 | Moderate putative | NA | NA |
| metab_13664 | Unannotated feature | neg | 193.0172 | 1.685 | 3.785 | 3.705 | -0.080 | -0.002 | 1.000 | 1.000 | Unannotated | NA | NA |
| metab_4818 | Unannotated feature | pos | 196.0173 | 7.874 | 4.537 | 4.516 | -0.021 | -0.002 | 1.000 | 1.000 | Unannotated | NA | NA |
| metab_9812 | Unannotated feature | neg | 563.1422 | 4.303 | 3.921 | 3.475 | -0.446 | 0.002 | 1.000 | 1.000 | Unannotated | NA | NA |
| metab_7516 | All-trans-5,6-Epoxyretinoic acid | pos | 339.1925 | 3.725 | 3.636 | 3.492 | -0.144 | -0.002 | 1.000 | 1.000 | Tentative | NA | NA |
| metab_11731 | Unannotated feature | neg | 619.3164 | 5.586 | 5.427 | 5.725 | 0.299 | 0.002 | 1.000 | 1.000 | Unannotated | NA | NA |
| metab_8444 | Unannotated feature | pos | 125.0602 | 1.944 | 3.953 | 3.947 | -0.006 | 0.002 | 1.000 | 1.000 | Unannotated | NA | NA |
| metab_19070 | 5'-Hydroxy-3',4',7-trimethoxyflavan | neg | 361.1299 | 5.431 | 4.633 | 4.798 | 0.165 | 0.002 | 1.000 | 1.000 | Unannotated | NA | NA |
| metab_15819 | Unannotated feature | neg | 253.0644 | 5.892 | 4.134 | 3.941 | -0.193 | 0.002 | 1.000 | 1.000 | Unannotated | NA | NA |
| metab_16493 | Unannotated feature | neg | 309.2801 | 7.080 | 4.326 | 3.987 | -0.339 | -0.002 | 1.000 | 1.000 | Unannotated | NA | NA |
| metab_8416 | Guanine | pos | 152.0570 | 1.952 | 5.951 | 5.933 | -0.017 | -0.002 | 1.000 | 1.000 | Strong putative | NA | NA |
| metab_17542 | Unannotated feature | neg | 256.8740 | 7.395 | 5.280 | 5.275 | -0.004 | 0.002 | 1.000 | 1.000 | Unannotated | NA | NA |
| metab_2432 | 3,4-Dihydro-9-hydroxy-[1]benzothieno[2,3-f]-1,4-thiazepin-5(2H)-one | pos | 252.0151 | 0.625 | 5.003 | 5.172 | 0.168 | -0.002 | 1.000 | 1.000 | Unannotated | NA | NA |
| metab_13184 | Unannotated feature | neg | 491.8631 | 0.502 | 3.061 | 3.038 | -0.023 | -0.002 | 1.000 | 1.000 | Unannotated | NA | NA |
| metab_13179 | Unannotated feature | neg | 506.8740 | 0.496 | 4.974 | 5.051 | 0.077 | 0.002 | 1.000 | 1.000 | Unannotated | NA | NA |
| metab_3047 | Unannotated feature | pos | 351.1558 | 3.291 | 3.946 | 4.018 | 0.072 | -0.002 | 1.000 | 1.000 | Unannotated | NA | NA |
| metab_17252 | Unannotated feature | neg | 321.9045 | 7.726 | 5.576 | 5.568 | -0.008 | 0.002 | 1.000 | 1.000 | Unannotated | NA | NA |
| metab_17249 | Unannotated feature | neg | 389.8923 | 7.726 | 5.117 | 5.093 | -0.023 | 0.002 | 1.000 | 1.000 | Unannotated | NA | NA |
| metab_13879 | Unannotated feature | neg | 139.0394 | 2.357 | 4.320 | 4.392 | 0.072 | 0.002 | 1.000 | 1.000 | Unannotated | NA | NA |
| metab_3048 | 2-Methylbutyroylcarnitine | pos | 246.1705 | 3.295 | 2.949 | 3.002 | 0.053 | 0.002 | 1.000 | 1.000 | Moderate putative | Lipids and lipid-like molecules | Fatty Acyls |
| metab_13667 | Unannotated feature | neg | 214.0718 | 1.685 | 4.998 | 5.075 | 0.077 | 0.002 | 1.000 | 1.000 | Unannotated | NA | NA |
| metab_15589 | 5-Hydroxypentanoylcarnitine | neg | 567.3169 | 5.484 | 5.138 | 5.183 | 0.045 | 0.002 | 1.000 | 1.000 | Unannotated | NA | NA |
| metab_19104 | Fenchyl acetate | neg | 241.1444 | 5.386 | 4.856 | 4.883 | 0.027 | -0.002 | 1.000 | 1.000 | Tentative | NA | NA |
| metab_13240 | Unannotated feature | neg | 167.0819 | 0.577 | 3.814 | 3.972 | 0.158 | -0.002 | 1.000 | 1.000 | Unannotated | NA | NA |
| metab_17608 | Unannotated feature | neg | 351.2520 | 7.199 | 4.692 | 4.707 | 0.014 | 0.002 | 1.000 | 1.000 | Unannotated | NA | NA |
| metab_17760 | Unannotated feature | neg | 425.2583 | 6.899 | 5.218 | 5.224 | 0.007 | -0.002 | 1.000 | 1.000 | Unannotated | NA | NA |
| metab_2249 | Unannotated feature | pos | 146.0603 | 3.125 | 5.527 | 5.433 | -0.095 | -0.002 | 1.000 | 1.000 | Unannotated | NA | NA |
| metab_13152 | Unannotated feature | neg | 523.8611 | 0.464 | 3.914 | 3.927 | 0.013 | -0.002 | 1.000 | 1.000 | Unannotated | NA | NA |
| metab_17742 | Unannotated feature | neg | 241.2171 | 6.922 | 4.676 | 4.662 | -0.015 | -0.002 | 1.000 | 1.000 | Unannotated | NA | NA |
| metab_2189 | Unannotated feature | pos | 316.1397 | 2.970 | 3.727 | 4.046 | 0.319 | -0.002 | 1.000 | 1.000 | Unannotated | NA | NA |
| metab_12379 | Unannotated feature | neg | 413.3028 | 6.703 | 4.522 | 4.330 | -0.191 | -0.002 | 1.000 | 1.000 | Unannotated | NA | NA |
| metab_11641 | Unannotated feature | neg | 207.9308 | 0.038 | 5.201 | 5.198 | -0.003 | -0.002 | 1.000 | 1.000 | Unannotated | NA | NA |
| metab_19059 | Unannotated feature | neg | 271.1917 | 5.446 | 4.832 | 4.788 | -0.044 | -0.002 | 1.000 | 1.000 | Unannotated | NA | NA |
| metab_12411 | Unannotated feature | neg | 396.8918 | 7.710 | 5.903 | 5.896 | -0.007 | -0.002 | 1.000 | 1.000 | Unannotated | NA | NA |
| metab_859 | Unannotated feature | pos | 449.2894 | 6.622 | 6.930 | 6.926 | -0.005 | -0.002 | 1.000 | 1.000 | Unannotated | NA | NA |
| metab_9636 | Unannotated feature | neg | 232.1189 | 3.203 | 4.438 | 4.450 | 0.012 | -0.002 | 1.000 | 1.000 | Unannotated | NA | NA |
| metab_9626 | Unannotated feature | neg | 285.0994 | 4.199 | 4.449 | 4.735 | 0.285 | -0.002 | 1.000 | 1.000 | Unannotated | NA | NA |
| metab_3126 | Unannotated feature | pos | 160.1335 | 3.511 | 3.495 | 3.521 | 0.027 | -0.002 | 1.000 | 1.000 | Unannotated | NA | NA |
| metab_15872 | Tryptophyl-Lysine | neg | 331.1765 | 5.958 | 4.474 | 4.215 | -0.259 | 0.002 | 1.000 | 1.000 | Unannotated | NA | NA |
| metab_15941 | Unannotated feature | neg | 522.2358 | 6.041 | 4.112 | 4.288 | 0.176 | 0.002 | 1.000 | 1.000 | Unannotated | NA | NA |
| metab_10444 | Octanedioylcarnitine | neg | 354.1310 | 1.965 | 4.623 | 4.683 | 0.060 | 0.002 | 1.000 | 1.000 | Unannotated | NA | NA |
| metab_4386 | Tanacetol B | pos | 261.1852 | 6.247 | 4.769 | 4.736 | -0.033 | -0.002 | 1.000 | 1.000 | Unannotated | NA | NA |
| metab_16455 | Unannotated feature | neg | 184.0610 | 1.685 | 5.875 | 5.904 | 0.030 | 0.002 | 1.000 | 1.000 | Unannotated | NA | NA |
| metab_16449 | Manglupenone | neg | 473.2834 | 6.974 | 5.965 | 5.992 | 0.027 | -0.002 | 1.000 | 1.000 | Unannotated | NA | NA |
| metab_2130 | 4-Hydroxy-5-(3',4'-dihydroxyphenyl)-valeric acid-O-methyl-O-glucuronide | pos | 452.1777 | 2.840 | 4.943 | 4.945 | 0.001 | -0.002 | 1.000 | 1.000 | Unannotated | NA | NA |
| metab_2164 | Unannotated feature | pos | 474.2211 | 2.907 | 4.259 | 4.313 | 0.054 | 0.002 | 1.000 | 1.000 | Unannotated | NA | NA |
| metab_5266 | Unannotated feature | pos | 212.1397 | 1.239 | 5.339 | 5.325 | -0.014 | 0.002 | 1.000 | 1.000 | Unannotated | NA | NA |
| metab_6947 | Jervine | pos | 470.2624 | 4.943 | 4.499 | 4.223 | -0.276 | 0.002 | 1.000 | 1.000 | Tentative | Lipids and lipid-like molecules | Steroids and steroid derivatives |
| metab_14296 | Unannotated feature | neg | 136.0761 | 3.045 | 2.801 | 2.989 | 0.187 | 0.002 | 1.000 | 1.000 | Unannotated | NA | NA |
| metab_8702 | Unannotated feature | pos | 230.1867 | 0.776 | 5.896 | 5.682 | -0.214 | -0.002 | 1.000 | 1.000 | Unannotated | NA | NA |
| metab_13759 | Pyramid | neg | 170.0453 | 1.958 | 4.822 | 4.753 | -0.069 | -0.002 | 1.000 | 1.000 | Unannotated | NA | NA |
| metab_9169 | 7,8-Dihydroxycoumarin | neg | 177.0187 | 3.826 | 4.147 | 4.158 | 0.011 | -0.002 | 1.000 | 1.000 | Strong putative | NA | NA |
| metab_14566 | Unannotated feature | neg | 427.0024 | 0.524 | 4.679 | 4.799 | 0.120 | 0.002 | 1.000 | 1.000 | Unannotated | NA | NA |
| metab_18842 | Unannotated feature | neg | 487.2382 | 5.734 | 5.871 | 6.067 | 0.196 | 0.002 | 1.000 | 1.000 | Unannotated | NA | NA |
| metab_11467 | Riboflavin (Vitamin B2) | neg | 421.1374 | 3.882 | 5.893 | 5.923 | 0.030 | 0.002 | 1.000 | 1.000 | Strong putative | NA | NA |
| metab_11461 | Unannotated feature | neg | 180.9724 | 0.527 | 4.507 | 4.528 | 0.021 | -0.002 | 1.000 | 1.000 | Unannotated | NA | NA |
| metab_2016 | Unannotated feature | pos | 99.0446 | 1.944 | 5.080 | 5.081 | 0.001 | -0.002 | 1.000 | 1.000 | Unannotated | NA | NA |
| metab_13038 | Unannotated feature | neg | 264.9344 | 0.603 | 4.989 | 5.146 | 0.157 | -0.002 | 1.000 | 1.000 | Unannotated | NA | NA |
| metab_18886 | Unannotated feature | neg | 505.0070 | 3.223 | 5.286 | 5.236 | -0.050 | -0.002 | 1.000 | 1.000 | Unannotated | NA | NA |
| metab_12470 | Unannotated feature | neg | 511.3281 | 6.310 | 6.890 | 6.963 | 0.073 | 0.002 | 1.000 | 1.000 | Unannotated | NA | NA |
| metab_8938 | Unannotated feature | neg | 197.1179 | 4.207 | 3.660 | 3.613 | -0.047 | 0.002 | 1.000 | 1.000 | Unannotated | NA | NA |
| metab_18912 | Unannotated feature | neg | 370.8154 | 0.603 | 5.402 | 5.433 | 0.031 | 0.002 | 1.000 | 1.000 | Unannotated | NA | NA |
| metab_947 | Unannotated feature | pos | 172.0946 | 7.594 | 5.559 | 5.561 | 0.002 | 0.002 | 1.000 | 1.000 | Unannotated | NA | NA |
| metab_18821 | 3-Methoxy-4-hydroxyphenylglycolaldehyde | neg | 163.0394 | 5.762 | 4.927 | 4.919 | -0.008 | -0.002 | 1.000 | 1.000 | Tentative | Benzenoids | Phenols |
| metab_3221 | Unannotated feature | pos | 563.4285 | 6.829 | 5.164 | 5.311 | 0.147 | 0.002 | 1.000 | 1.000 | Unannotated | NA | NA |
| metab_9612 | Unannotated feature | neg | 521.9970 | 3.215 | 4.812 | 4.807 | -0.005 | -0.002 | 1.000 | 1.000 | Unannotated | NA | NA |
| metab_4768 | Unannotated feature | pos | 332.9925 | 7.732 | 4.226 | 4.112 | -0.114 | -0.002 | 1.000 | 1.000 | Unannotated | NA | NA |
| metab_19560 | Unannotated feature | neg | 261.1344 | 4.767 | 4.644 | 4.657 | 0.013 | -0.002 | 1.000 | 1.000 | Unannotated | NA | NA |
| metab_299 | Unannotated feature | pos | 168.0509 | 0.485 | 5.044 | 5.058 | 0.014 | -0.002 | 1.000 | 1.000 | Unannotated | NA | NA |
| metab_1962 | Unannotated feature | pos | 141.0526 | 1.984 | 4.445 | 4.468 | 0.023 | -0.002 | 1.000 | 1.000 | Unannotated | NA | NA |
| metab_11389 | Uracil mustard | neg | 285.9906 | 0.552 | 4.303 | 4.351 | 0.048 | -0.002 | 1.000 | 1.000 | Tentative | NA | NA |
| metab_5304 | Unannotated feature | pos | 313.2374 | 6.566 | 4.782 | 4.793 | 0.011 | 0.002 | 1.000 | 1.000 | Unannotated | NA | NA |
| metab_18052 | Unannotated feature | neg | 210.0767 | 6.444 | 4.497 | 4.508 | 0.010 | 0.002 | 1.000 | 1.000 | Unannotated | NA | NA |
| metab_12951 | Unannotated feature | neg | 265.0569 | 0.770 | 5.188 | 5.150 | -0.038 | 0.002 | 1.000 | 1.000 | Unannotated | NA | NA |
| metab_10747 | Unannotated feature | neg | 372.1052 | 1.233 | 3.910 | 3.613 | -0.297 | 0.002 | 1.000 | 1.000 | Unannotated | NA | NA |
| metab_10738 | Unannotated feature | neg | 201.0149 | 1.263 | 5.101 | 5.081 | -0.021 | -0.002 | 1.000 | 1.000 | Unannotated | NA | NA |
| metab_18050 | Unannotated feature | neg | 255.2329 | 6.444 | 4.536 | 4.521 | -0.016 | -0.002 | 1.000 | 1.000 | Unannotated | NA | NA |
| metab_1882 | Unannotated feature | pos | 184.1196 | 1.211 | 4.001 | 3.832 | -0.169 | 0.002 | 1.000 | 1.000 | Unannotated | NA | NA |
| metab_12936 | Unannotated feature | neg | 511.1260 | 0.738 | 4.254 | 3.884 | -0.370 | -0.002 | 1.000 | 1.000 | Unannotated | NA | NA |
| metab_18172 | Unannotated feature | neg | 375.2183 | 6.326 | 5.101 | 5.035 | -0.067 | -0.002 | 1.000 | 1.000 | Unannotated | NA | NA |
| metab_14702 | Unannotated feature | neg | 654.9646 | 0.516 | 4.423 | 4.723 | 0.301 | 0.002 | 1.000 | 1.000 | Unannotated | NA | NA |
| metab_12087 | Unannotated feature | neg | 313.2385 | 6.444 | 6.815 | 6.826 | 0.011 | -0.002 | 1.000 | 1.000 | Unannotated | NA | NA |
| metab_7017 | (5R,6S)-6-[(1R)-1-Hydroxyethyl]-7-oxo-3-pyridin-3-yl-4-thia-1-azabicyclo[3.2.0]hept-2-ene-2-carboxylic acid | pos | 325.0826 | 4.802 | 5.138 | 5.118 | -0.020 | 0.002 | 1.000 | 1.000 | Unannotated | NA | NA |
| metab_18735 | 1-beta-Hydroxyeuscaphic acid | neg | 549.3458 | 5.871 | 2.830 | 5.616 | 2.787 | -0.002 | 1.000 | 1.000 | Unannotated | NA | NA |
| metab_1016 | DG(18:3(6Z,9Z,12Z)/18:4(6Z,9Z,12Z,15Z)/0:0) | pos | 611.4664 | 7.092 | 4.548 | 4.911 | 0.363 | -0.002 | 1.000 | 1.000 | Tentative | NA | NA |
| metab_18749 | Unannotated feature | neg | 306.1196 | 5.847 | 5.381 | 5.425 | 0.043 | -0.002 | 1.000 | 1.000 | Unannotated | NA | NA |
| metab_12853 | Unannotated feature | neg | 206.0011 | 0.644 | 4.157 | 4.290 | 0.133 | 0.002 | 1.000 | 1.000 | Unannotated | NA | NA |
| metab_10819 | N-Succinyl-2-amino-6-ketopimelate | neg | 270.0598 | 1.061 | 4.869 | 4.855 | -0.014 | 0.002 | 1.000 | 1.000 | Tentative | NA | NA |
| metab_10833 | Unannotated feature | neg | 185.0562 | 1.046 | 4.558 | 4.598 | 0.039 | 0.002 | 1.000 | 1.000 | Unannotated | NA | NA |
| metab_1711 | (-)-trans-3,4-Dihydro-4,8-dihydroxy-3-methyl-1H-2-benzopyran-1-one | pos | 406.1509 | 3.934 | 3.446 | 3.357 | -0.089 | -0.002 | 1.000 | 1.000 | Unannotated | NA | NA |
| metab_1651 | Unannotated feature | pos | 407.2263 | 4.579 | 3.712 | 3.591 | -0.120 | 0.002 | 1.000 | 1.000 | Unannotated | NA | NA |
| metab_14969 | Unannotated feature | neg | 227.0382 | 4.266 | 4.372 | 4.378 | 0.006 | 0.002 | 1.000 | 1.000 | Unannotated | NA | NA |
| metab_1119 | Unannotated feature | pos | 307.2251 | 6.630 | 4.764 | 4.765 | 0.001 | -0.002 | 1.000 | 1.000 | Unannotated | NA | NA |
| metab_11240 | Unannotated feature | neg | 214.0330 | 0.636 | 5.049 | 5.137 | 0.088 | -0.002 | 1.000 | 1.000 | Unannotated | NA | NA |
| metab_8124 | P-Hydroxyl-ethotoin | pos | 238.1190 | 2.732 | 3.662 | 3.580 | -0.082 | 0.002 | 1.000 | 1.000 | Unannotated | NA | NA |
| metab_18353 | Unannotated feature | neg | 307.1296 | 6.170 | 5.620 | 5.710 | 0.090 | 0.002 | 1.000 | 1.000 | Unannotated | NA | NA |
| metab_3382 | Unannotated feature | pos | 109.1017 | 4.344 | 3.970 | 3.992 | 0.023 | -0.002 | 1.000 | 1.000 | Unannotated | NA | NA |
| metab_16789 | Unannotated feature | neg | 311.9436 | 7.702 | 5.194 | 5.195 | 0.001 | 0.002 | 1.000 | 1.000 | Unannotated | NA | NA |
| metab_127 | Unannotated feature | pos | 254.0132 | 0.625 | 3.579 | 4.067 | 0.488 | 0.002 | 1.000 | 1.000 | Unannotated | NA | NA |
| metab_124 | L-Alanine | pos | 90.0555 | 0.633 | 6.249 | 6.250 | 0.001 | -0.002 | 1.000 | 1.000 | Moderate putative | NA | NA |
| metab_7142 | Paclobutrazol | pos | 335.1608 | 4.564 | 4.040 | 3.959 | -0.081 | 0.002 | 1.000 | 1.000 | Tentative | NA | NA |
| metab_5673 | Picrocrocin | pos | 295.1550 | 6.143 | 3.686 | 3.796 | 0.110 | 0.002 | 1.000 | 1.000 | Tentative | NA | NA |
| metab_1656 | Unannotated feature | pos | 265.1051 | 4.559 | 4.501 | 4.753 | 0.252 | -0.002 | 1.000 | 1.000 | Unannotated | NA | NA |
| metab_12740 | Unannotated feature | neg | 363.0994 | 3.658 | 4.461 | 4.536 | 0.075 | 0.002 | 1.000 | 1.000 | Unannotated | NA | NA |
| metab_16081 | Unannotated feature | neg | 128.0346 | 1.149 | 6.619 | 6.575 | -0.044 | 0.002 | 1.000 | 1.000 | Unannotated | NA | NA |
| metab_16061 | Citrusin C | neg | 347.1099 | 1.135 | 5.133 | 5.177 | 0.044 | -0.002 | 1.000 | 1.000 | Unannotated | NA | NA |
| metab_16163 | Unannotated feature | neg | 188.0559 | 1.258 | 7.281 | 7.238 | -0.043 | -0.002 | 1.000 | 1.000 | Unannotated | NA | NA |
| metab_16196 | Deoxycholylglycine | neg | 448.3072 | 6.404 | 6.322 | 6.346 | 0.024 | 0.002 | 1.000 | 1.000 | Tentative | NA | NA |
| metab_12624 | Unannotated feature | neg | 343.1766 | 6.007 | 5.547 | 5.439 | -0.108 | 0.002 | 1.000 | 1.000 | Unannotated | NA | NA |
| metab_5587 | Unannotated feature | pos | 265.1799 | 6.247 | 4.802 | 4.718 | -0.084 | -0.002 | 1.000 | 1.000 | Unannotated | NA | NA |
| metab_11120 | Arabinogalactose | neg | 357.1046 | 0.714 | 5.313 | 5.371 | 0.058 | -0.002 | 1.000 | 1.000 | Tentative | NA | NA |
| metab_18555 | Unannotated feature | neg | 229.0029 | 3.082 | 3.781 | 4.060 | 0.279 | -0.002 | 1.000 | 1.000 | Unannotated | NA | NA |
| metab_16184 | Unannotated feature | neg | 684.7401 | 0.533 | 5.078 | 5.079 | 0.001 | -0.002 | 1.000 | 1.000 | Unannotated | NA | NA |
| metab_19671 | Unannotated feature | neg | 333.1460 | 4.603 | 4.339 | 4.810 | 0.472 | 0.002 | 1.000 | 1.000 | Unannotated | NA | NA |
| metab_19667 | Unannotated feature | neg | 266.0180 | 4.613 | 3.201 | 3.295 | 0.095 | 0.002 | 1.000 | 1.000 | Unannotated | NA | NA |
| metab_15374 | Equol | neg | 287.0929 | 5.029 | 4.712 | 4.567 | -0.145 | 0.002 | 1.000 | 1.000 | Tentative | NA | NA |
| metab_12081 | Unannotated feature | neg | 567.3913 | 6.436 | 6.159 | 6.346 | 0.187 | -0.002 | 1.000 | 1.000 | Unannotated | NA | NA |
| metab_7110 | 5-Deoxymyricanone | pos | 363.1558 | 4.620 | 4.218 | 4.813 | 0.595 | -0.002 | 1.000 | 1.000 | Unannotated | NA | NA |
| metab_19842 | 4-Hydroxycinnamic acid | neg | 387.1092 | 5.087 | 4.086 | 4.469 | 0.382 | -0.002 | 1.000 | 1.000 | Tentative | NA | NA |
| metab_4704 | Unannotated feature | pos | 106.0462 | 7.602 | 5.067 | 5.085 | 0.018 | 0.002 | 1.000 | 1.000 | Unannotated | NA | NA |
| metab_3421 | N-(2,3,4-Trihydroxybutyl)-L-valine | pos | 186.1128 | 4.487 | 4.468 | 4.553 | 0.085 | 0.002 | 1.000 | 1.000 | Unannotated | NA | NA |
| metab_9509 | Unannotated feature | neg | 215.1286 | 4.118 | 4.486 | 4.478 | -0.008 | 0.002 | 1.000 | 1.000 | Unannotated | NA | NA |

The table contains all 11,837 fecal metabolomic features retained after quality-control filtering. Values are medians of log10-transformed, sum-normalized feature abundances. Median difference was calculated as CKM stages 3-4 minus stages 1-2; it is not a log2 fold change. P values were obtained using two-sided Mann-Whitney U tests. BH-FDR denotes the Benjamini-Hochberg false-discovery rate calculated across all retained features. Metabolite annotations are putative because authentic-standard retention-time matching was unavailable. CKM, cardiovascular-kidney-metabolic; RT, retention time.
