# Supplementary material for: Paired metabolomics identify associations between fecal and serum metabolites in cardiovascular-kidney-metabolic syndrome
Source: Front Endocrinol (Lausanne). 2026 Oct 27;17:1982623. doi: 10.3389/fendo.2026.1982623 (PMC13747780; doi:10.3389/fendo.2026.1982623)
Supplement: Supplementary file 4 [file Table5.docx]

**Supplementary Table S4. Clinical correlations of metabolite features identified by paired metabolomics**

| **Data type** | **Metabolite ID** | **Metabolite feature** | **Annotation confidence** | **Clinical indicator** | **Unadjusted rho** | **Unadjusted *P* value** | **Unadjusted BH-FDR** | **CKM-adjusted partial rho** | **Adjusted *P* value** | **Adjusted BH-FDR** |
| --- | --- | --- | --- | --- | --- | --- | --- | --- | --- | --- |
| Fecal | metab_5297 | 1-Palmitoyl Lysophosphatidic Acid | Moderate | eGFR | 0.3969 | 0.0076 | 0.0324 | 0.1891 | 0.2245 | 0.6188 |
| Fecal | metab_5297 | 1-Palmitoyl Lysophosphatidic Acid | Moderate | Serum creatinine | -0.3474 | 0.0209 | 0.0598 | -0.1180 | 0.4512 | 0.7521 |
| Fecal | metab_5297 | 1-Palmitoyl Lysophosphatidic Acid | Moderate | Blood urea nitrogen | -0.3237 | 0.0320 | 0.0783 | -0.0838 | 0.5933 | 0.8402 |
| Fecal | metab_5297 | 1-Palmitoyl Lysophosphatidic Acid | Moderate | Albumin | 0.3115 | 0.0396 | 0.0903 | 0.0542 | 0.7299 | 0.8921 |
| Fecal | metab_5297 | 1-Palmitoyl Lysophosphatidic Acid | Moderate | Hemoglobin | 0.3463 | 0.0213 | 0.0598 | 0.1824 | 0.2418 | 0.6188 |
| Fecal | metab_5297 | 1-Palmitoyl Lysophosphatidic Acid | Moderate | Systolic blood pressure | -0.3266 | 0.0305 | 0.0774 | -0.1396 | 0.3721 | 0.7016 |
| Fecal | metab_5297 | 1-Palmitoyl Lysophosphatidic Acid | Moderate | Diastolic blood pressure | -0.2373 | 0.1209 | 0.2020 | -0.0676 | 0.6665 | 0.8542 |
| Fecal | metab_5297 | 1-Palmitoyl Lysophosphatidic Acid | Moderate | Total cholesterol | 0.0867 | 0.5758 | 0.6787 | 0.1866 | 0.2309 | 0.6188 |
| Fecal | metab_5297 | 1-Palmitoyl Lysophosphatidic Acid | Moderate | Triglycerides | 0.2878 | 0.0581 | 0.1163 | 0.3081 | 0.0444 | 0.3092 |
| Fecal | metab_5297 | 1-Palmitoyl Lysophosphatidic Acid | Moderate | LDL cholesterol | 0.0878 | 0.5708 | 0.6787 | 0.1067 | 0.4957 | 0.7689 |
| Fecal | metab_5297 | 1-Palmitoyl Lysophosphatidic Acid | Moderate | BMI | 0.2993 | 0.0484 | 0.1047 | 0.2464 | 0.1112 | 0.5060 |
| Fecal | metab_5297 | 1-Palmitoyl Lysophosphatidic Acid | Moderate | Diabetes history | -0.1905 | 0.2155 | 0.3232 | -0.0419 | 0.7899 | 0.9093 |
| Fecal | metab_8430 | Ile Arg | Moderate | eGFR | 0.3639 | 0.0152 | 0.0513 | 0.1098 | 0.4833 | 0.7643 |
| Fecal | metab_8430 | Ile Arg | Moderate | Serum creatinine | -0.3641 | 0.0151 | 0.0513 | -0.1055 | 0.5009 | 0.7689 |
| Fecal | metab_8430 | Ile Arg | Moderate | Blood urea nitrogen | -0.3557 | 0.0178 | 0.0559 | -0.0913 | 0.5602 | 0.8126 |
| Fecal | metab_8430 | Ile Arg | Moderate | Albumin | 0.4779 | 0.0010 | 0.0097 | 0.2531 | 0.1015 | 0.5060 |
| Fecal | metab_8430 | Ile Arg | Moderate | Hemoglobin | 0.2907 | 0.0556 | 0.1163 | 0.0883 | 0.5734 | 0.8227 |
| Fecal | metab_8430 | Ile Arg | Moderate | Systolic blood pressure | -0.4048 | 0.0064 | 0.0314 | -0.2162 | 0.1639 | 0.5787 |
| Fecal | metab_8430 | Ile Arg | Moderate | Diastolic blood pressure | -0.3980 | 0.0075 | 0.0324 | -0.2485 | 0.1081 | 0.5060 |
| Fecal | metab_8430 | Ile Arg | Moderate | Total cholesterol | -0.0183 | 0.9060 | 0.9114 | 0.0784 | 0.6171 | 0.8463 |
| Fecal | metab_8430 | Ile Arg | Moderate | Triglycerides | -0.1434 | 0.3532 | 0.4571 | -0.1869 | 0.2302 | 0.6188 |
| Fecal | metab_8430 | Ile Arg | Moderate | LDL cholesterol | 0.1528 | 0.3220 | 0.4338 | 0.1870 | 0.2300 | 0.6188 |
| Fecal | metab_8430 | Ile Arg | Moderate | BMI | 0.1963 | 0.2015 | 0.3081 | 0.1215 | 0.4378 | 0.7521 |
| Fecal | metab_8430 | Ile Arg | Moderate | Diabetes history | -0.4170 | 0.0049 | 0.0291 | -0.3026 | 0.0486 | 0.3092 |
| Fecal | metab_17858 | PE(16:0/0:0) | High | eGFR | 0.4107 | 0.0056 | 0.0297 | 0.2259 | 0.1452 | 0.5547 |
| Fecal | metab_17858 | PE(16:0/0:0) | High | Serum creatinine | -0.3655 | 0.0147 | 0.0513 | -0.1622 | 0.2987 | 0.6359 |
| Fecal | metab_17858 | PE(16:0/0:0) | High | Blood urea nitrogen | -0.3727 | 0.0127 | 0.0480 | -0.1703 | 0.2749 | 0.6256 |
| Fecal | metab_17858 | PE(16:0/0:0) | High | Albumin | 0.3078 | 0.0421 | 0.0941 | 0.0716 | 0.6482 | 0.8542 |
| Fecal | metab_17858 | PE(16:0/0:0) | High | Hemoglobin | 0.3590 | 0.0167 | 0.0544 | 0.2105 | 0.1754 | 0.5787 |
| Fecal | metab_17858 | PE(16:0/0:0) | High | Systolic blood pressure | -0.3021 | 0.0463 | 0.1018 | -0.1235 | 0.4302 | 0.7521 |
| Fecal | metab_17858 | PE(16:0/0:0) | High | Diastolic blood pressure | -0.1848 | 0.2297 | 0.3369 | -0.0169 | 0.9146 | 0.9506 |
| Fecal | metab_17858 | PE(16:0/0:0) | High | Total cholesterol | 0.0974 | 0.5294 | 0.6470 | 0.1903 | 0.2216 | 0.6188 |
| Fecal | metab_17858 | PE(16:0/0:0) | High | Triglycerides | 0.3691 | 0.0137 | 0.0502 | 0.3945 | 0.0089 | 0.1698 |
| Fecal | metab_17858 | PE(16:0/0:0) | High | LDL cholesterol | 0.0383 | 0.8048 | 0.8590 | 0.0496 | 0.7522 | 0.9093 |
| Fecal | metab_17858 | PE(16:0/0:0) | High | BMI | 0.1875 | 0.2228 | 0.3305 | 0.1221 | 0.4353 | 0.7521 |
| Fecal | metab_17858 | PE(16:0/0:0) | High | Diabetes history | -0.1582 | 0.3052 | 0.4196 | -0.0145 | 0.9267 | 0.9557 |
| Fecal | metab_15647 | Gossypetin | High | eGFR | -0.3519 | 0.0191 | 0.0578 | -0.0826 | 0.5983 | 0.8402 |
| Fecal | metab_15647 | Gossypetin | High | Serum creatinine | 0.3451 | 0.0218 | 0.0599 | 0.0679 | 0.6655 | 0.8542 |
| Fecal | metab_15647 | Gossypetin | High | Blood urea nitrogen | 0.2699 | 0.0764 | 0.1401 | -0.0421 | 0.7889 | 0.9093 |
| Fecal | metab_15647 | Gossypetin | High | Albumin | -0.3956 | 0.0079 | 0.0324 | -0.1235 | 0.4301 | 0.7521 |
| Fecal | metab_15647 | Gossypetin | High | Hemoglobin | -0.2879 | 0.0581 | 0.1163 | -0.0774 | 0.6219 | 0.8463 |
| Fecal | metab_15647 | Gossypetin | High | Systolic blood pressure | 0.2715 | 0.0747 | 0.1401 | 0.0315 | 0.8409 | 0.9174 |
| Fecal | metab_15647 | Gossypetin | High | Diastolic blood pressure | 0.1145 | 0.4593 | 0.5719 | -0.1195 | 0.4453 | 0.7521 |
| Fecal | metab_15647 | Gossypetin | High | Total cholesterol | 0.0520 | 0.7374 | 0.8045 | -0.0418 | 0.7899 | 0.9093 |
| Fecal | metab_15647 | Gossypetin | High | Triglycerides | 0.0639 | 0.6802 | 0.7609 | 0.0957 | 0.5416 | 0.8025 |
| Fecal | metab_15647 | Gossypetin | High | LDL cholesterol | -0.1494 | 0.3330 | 0.4358 | -0.1852 | 0.2345 | 0.6188 |
| Fecal | metab_15647 | Gossypetin | High | BMI | -0.0644 | 0.6778 | 0.7609 | 0.0386 | 0.8060 | 0.9093 |
| Fecal | metab_15647 | Gossypetin | High | Diabetes history | 0.3163 | 0.0365 | 0.0875 | 0.1738 | 0.2650 | 0.6188 |
| Fecal | metab_3085 | Asp Ala Phe | Moderate | eGFR | 0.3813 | 0.0107 | 0.0414 | 0.1866 | 0.2309 | 0.6188 |
| Fecal | metab_3085 | Asp Ala Phe | Moderate | Serum creatinine | -0.4072 | 0.0061 | 0.0309 | -0.2183 | 0.1597 | 0.5787 |
| Fecal | metab_3085 | Asp Ala Phe | Moderate | Blood urea nitrogen | -0.4000 | 0.0071 | 0.0324 | -0.2071 | 0.1826 | 0.5880 |
| Fecal | metab_3085 | Asp Ala Phe | Moderate | Albumin | 0.4365 | 0.0031 | 0.0205 | 0.2482 | 0.1086 | 0.5060 |
| Fecal | metab_3085 | Asp Ala Phe | Moderate | Hemoglobin | 0.3113 | 0.0397 | 0.0903 | 0.1517 | 0.3314 | 0.6634 |
| Fecal | metab_3085 | Asp Ala Phe | Moderate | Systolic blood pressure | -0.3835 | 0.0102 | 0.0407 | -0.2259 | 0.1453 | 0.5547 |
| Fecal | metab_3085 | Asp Ala Phe | Moderate | Diastolic blood pressure | -0.3144 | 0.0377 | 0.0888 | -0.1734 | 0.2662 | 0.6188 |
| Fecal | metab_3085 | Asp Ala Phe | Moderate | Total cholesterol | -0.0330 | 0.8317 | 0.8783 | 0.0435 | 0.7819 | 0.9093 |
| Fecal | metab_3085 | Asp Ala Phe | Moderate | Triglycerides | -0.0708 | 0.6481 | 0.7439 | -0.0944 | 0.5472 | 0.8025 |
| Fecal | metab_3085 | Asp Ala Phe | Moderate | LDL cholesterol | -0.0249 | 0.8726 | 0.8963 | -0.0206 | 0.8954 | 0.9420 |
| Fecal | metab_3085 | Asp Ala Phe | Moderate | BMI | 0.1810 | 0.2397 | 0.3477 | 0.1147 | 0.4639 | 0.7521 |
| Fecal | metab_3085 | Asp Ala Phe | Moderate | Diabetes history | -0.4134 | 0.0053 | 0.0291 | -0.3153 | 0.0394 | 0.3079 |
| Fecal | metab_7538 | Sinapic Acid | High | eGFR | -0.3504 | 0.0197 | 0.0578 | -0.1406 | 0.3686 | 0.7016 |
| Fecal | metab_7538 | Sinapic Acid | High | Serum creatinine | 0.3333 | 0.0270 | 0.0702 | 0.1140 | 0.4665 | 0.7521 |
| Fecal | metab_7538 | Sinapic Acid | High | Blood urea nitrogen | 0.3511 | 0.0195 | 0.0578 | 0.1363 | 0.3835 | 0.7038 |
| Fecal | metab_7538 | Sinapic Acid | High | Albumin | -0.1510 | 0.3280 | 0.4358 | 0.1501 | 0.3367 | 0.6634 |
| Fecal | metab_7538 | Sinapic Acid | High | Hemoglobin | -0.3332 | 0.0271 | 0.0702 | -0.1755 | 0.2603 | 0.6188 |
| Fecal | metab_7538 | Sinapic Acid | High | Systolic blood pressure | 0.1587 | 0.3034 | 0.4196 | -0.0612 | 0.6965 | 0.8611 |
| Fecal | metab_7538 | Sinapic Acid | High | Diastolic blood pressure | -0.0234 | 0.8801 | 0.8963 | -0.2390 | 0.1227 | 0.5224 |
| Fecal | metab_7538 | Sinapic Acid | High | Total cholesterol | -0.0237 | 0.8787 | 0.8963 | -0.1090 | 0.4864 | 0.7643 |
| Fecal | metab_7538 | Sinapic Acid | High | Triglycerides | -0.2291 | 0.1346 | 0.2167 | -0.2396 | 0.1217 | 0.5224 |
| Fecal | metab_7538 | Sinapic Acid | High | LDL cholesterol | -0.0259 | 0.8673 | 0.8963 | -0.0361 | 0.8184 | 0.9125 |
| Fecal | metab_7538 | Sinapic Acid | High | BMI | -0.0229 | 0.8827 | 0.8963 | 0.0656 | 0.6758 | 0.8578 |
| Fecal | metab_7538 | Sinapic Acid | High | Diabetes history | 0.2085 | 0.1744 | 0.2741 | 0.0712 | 0.6500 | 0.8542 |
| Serum | metab_5108 | Uridine | High | eGFR | 0.4919 | 0.0007 | 0.0077 | 0.3323 | 0.0295 | 0.2781 |
| Serum | metab_5108 | Uridine | High | Serum creatinine | -0.5328 | 0.0002 | 0.0028 | -0.3849 | 0.0108 | 0.1698 |
| Serum | metab_5108 | Uridine | High | Blood urea nitrogen | -0.4479 | 0.0023 | 0.0168 | -0.2694 | 0.0806 | 0.4255 |
| Serum | metab_5108 | Uridine | High | Albumin | 0.4137 | 0.0053 | 0.0291 | 0.2144 | 0.1675 | 0.5787 |
| Serum | metab_5108 | Uridine | High | Hemoglobin | 0.4598 | 0.0017 | 0.0131 | 0.3334 | 0.0289 | 0.2781 |
| Serum | metab_5108 | Uridine | High | Systolic blood pressure | -0.2699 | 0.0764 | 0.1401 | -0.0811 | 0.6052 | 0.8409 |
| Serum | metab_5108 | Uridine | High | Diastolic blood pressure | -0.2890 | 0.0571 | 0.1163 | -0.1413 | 0.3663 | 0.7016 |
| Serum | metab_5108 | Uridine | High | Total cholesterol | -0.2352 | 0.1243 | 0.2050 | -0.1840 | 0.2376 | 0.6188 |
| Serum | metab_5108 | Uridine | High | Triglycerides | -0.0787 | 0.6115 | 0.7143 | -0.1036 | 0.5087 | 0.7718 |
| Serum | metab_5108 | Uridine | High | LDL cholesterol | -0.1957 | 0.2031 | 0.3081 | -0.2107 | 0.1750 | 0.5787 |
| Serum | metab_5108 | Uridine | High | BMI | 0.3584 | 0.0169 | 0.0544 | 0.3150 | 0.0396 | 0.3079 |
| Serum | metab_5108 | Uridine | High | Diabetes history | -0.2013 | 0.1901 | 0.2953 | -0.0640 | 0.6835 | 0.8592 |
| Serum | metab_4952 | L-Carnitine | High | eGFR | 0.7515 | 0.0000 | 0.0000 | 0.6155 | 0.0000 | 0.0013 |
| Serum | metab_4952 | L-Carnitine | High | Serum creatinine | -0.7461 | 0.0000 | 0.0000 | -0.6024 | 0.0000 | 0.0013 |
| Serum | metab_4952 | L-Carnitine | High | Blood urea nitrogen | -0.7239 | 0.0000 | 0.0000 | -0.5643 | 0.0001 | 0.0036 |
| Serum | metab_4952 | L-Carnitine | High | Albumin | 0.5621 | 0.0001 | 0.0017 | 0.2828 | 0.0661 | 0.3635 |
| Serum | metab_4952 | L-Carnitine | High | Hemoglobin | 0.5439 | 0.0001 | 0.0026 | 0.3807 | 0.0118 | 0.1698 |
| Serum | metab_4952 | L-Carnitine | High | Systolic blood pressure | -0.5600 | 0.0001 | 0.0017 | -0.3764 | 0.0129 | 0.1698 |
| Serum | metab_4952 | L-Carnitine | High | Diastolic blood pressure | -0.4015 | 0.0069 | 0.0324 | -0.2041 | 0.1893 | 0.5949 |
| Serum | metab_4952 | L-Carnitine | High | Total cholesterol | -0.0379 | 0.8070 | 0.8590 | 0.0975 | 0.5339 | 0.8009 |
| Serum | metab_4952 | L-Carnitine | High | Triglycerides | 0.0173 | 0.9114 | 0.9114 | -0.0059 | 0.9699 | 0.9773 |
| Serum | metab_4952 | L-Carnitine | High | LDL cholesterol | -0.0995 | 0.5204 | 0.6420 | -0.1206 | 0.4411 | 0.7521 |
| Serum | metab_4952 | L-Carnitine | High | BMI | 0.2319 | 0.1298 | 0.2115 | 0.1505 | 0.3354 | 0.6634 |
| Serum | metab_4952 | L-Carnitine | High | Diabetes history | -0.5356 | 0.0002 | 0.0028 | -0.4445 | 0.0028 | 0.0745 |
| Serum | metab_640 | Muramic acid | Moderate | eGFR | 0.4879 | 0.0008 | 0.0079 | 0.3044 | 0.0471 | 0.3092 |
| Serum | metab_640 | Muramic acid | Moderate | Serum creatinine | -0.4324 | 0.0034 | 0.0212 | -0.2249 | 0.1471 | 0.5547 |
| Serum | metab_640 | Muramic acid | Moderate | Blood urea nitrogen | -0.4992 | 0.0006 | 0.0067 | -0.3156 | 0.0393 | 0.3079 |
| Serum | metab_640 | Muramic acid | Moderate | Albumin | 0.2584 | 0.0904 | 0.1570 | -0.0320 | 0.8385 | 0.9174 |
| Serum | metab_640 | Muramic acid | Moderate | Hemoglobin | 0.4357 | 0.0031 | 0.0205 | 0.2891 | 0.0601 | 0.3447 |
| Serum | metab_640 | Muramic acid | Moderate | Systolic blood pressure | -0.2714 | 0.0747 | 0.1401 | -0.0609 | 0.6980 | 0.8611 |
| Serum | metab_640 | Muramic acid | Moderate | Diastolic blood pressure | -0.0959 | 0.5356 | 0.6486 | 0.1139 | 0.4672 | 0.7521 |
| Serum | metab_640 | Muramic acid | Moderate | Total cholesterol | -0.0896 | 0.5628 | 0.6754 | -0.0126 | 0.9360 | 0.9577 |
| Serum | metab_640 | Muramic acid | Moderate | Triglycerides | 0.1674 | 0.2775 | 0.3896 | 0.1730 | 0.2672 | 0.6188 |
| Serum | metab_640 | Muramic acid | Moderate | LDL cholesterol | -0.0727 | 0.6392 | 0.7402 | -0.0749 | 0.6331 | 0.8528 |
| Serum | metab_640 | Muramic acid | Moderate | BMI | 0.1178 | 0.4465 | 0.5613 | 0.0352 | 0.8226 | 0.9125 |
| Serum | metab_640 | Muramic acid | Moderate | Diabetes history | -0.1546 | 0.3165 | 0.4306 | 0.0071 | 0.9640 | 0.9773 |
| Serum | metab_3488 | L-2-Aminoadipate | Moderate | eGFR | -0.2223 | 0.1470 | 0.2338 | 0.0284 | 0.8566 | 0.9268 |
| Serum | metab_3488 | L-2-Aminoadipate | Moderate | Serum creatinine | 0.2760 | 0.0697 | 0.1374 | 0.0394 | 0.8018 | 0.9093 |
| Serum | metab_3488 | L-2-Aminoadipate | Moderate | Blood urea nitrogen | 0.2630 | 0.0846 | 0.1501 | 0.0199 | 0.8992 | 0.9420 |
| Serum | metab_3488 | L-2-Aminoadipate | Moderate | Albumin | -0.2551 | 0.0947 | 0.1623 | 0.0037 | 0.9814 | 0.9814 |
| Serum | metab_3488 | L-2-Aminoadipate | Moderate | Hemoglobin | -0.3245 | 0.0316 | 0.0783 | -0.1664 | 0.2863 | 0.6298 |
| Serum | metab_3488 | L-2-Aminoadipate | Moderate | Systolic blood pressure | 0.1725 | 0.2627 | 0.3729 | -0.0416 | 0.7913 | 0.9093 |
| Serum | metab_3488 | L-2-Aminoadipate | Moderate | Diastolic blood pressure | 0.0411 | 0.7913 | 0.8562 | -0.1588 | 0.3091 | 0.6476 |
| Serum | metab_3488 | L-2-Aminoadipate | Moderate | Total cholesterol | -0.2496 | 0.1022 | 0.1730 | -0.3631 | 0.0167 | 0.2005 |
| Serum | metab_3488 | L-2-Aminoadipate | Moderate | Triglycerides | -0.1493 | 0.3335 | 0.4358 | -0.1503 | 0.3359 | 0.6634 |
| Serum | metab_3488 | L-2-Aminoadipate | Moderate | LDL cholesterol | -0.2629 | 0.0847 | 0.1501 | -0.2997 | 0.0509 | 0.3092 |
| Serum | metab_3488 | L-2-Aminoadipate | Moderate | BMI | -0.0581 | 0.7081 | 0.7854 | 0.0249 | 0.8738 | 0.9378 |
| Serum | metab_3488 | L-2-Aminoadipate | Moderate | Diabetes history | 0.2624 | 0.0853 | 0.1501 | 0.1362 | 0.3839 | 0.7038 |
| Serum | metab_9222 | 4-Methylcatechol | Moderate | eGFR | -0.5301 | 0.0002 | 0.0028 | -0.3872 | 0.0103 | 0.1698 |
| Serum | metab_9222 | 4-Methylcatechol | Moderate | Serum creatinine | 0.4657 | 0.0014 | 0.0119 | 0.2989 | 0.0515 | 0.3092 |
| Serum | metab_9222 | 4-Methylcatechol | Moderate | Blood urea nitrogen | 0.6086 | 0.0000 | 0.0004 | 0.4899 | 0.0009 | 0.0282 |
| Serum | metab_9222 | 4-Methylcatechol | Moderate | Albumin | -0.2700 | 0.0763 | 0.1401 | -0.0226 | 0.8855 | 0.9420 |
| Serum | metab_9222 | 4-Methylcatechol | Moderate | Hemoglobin | -0.4702 | 0.0013 | 0.0112 | -0.3487 | 0.0219 | 0.2410 |
| Serum | metab_9222 | 4-Methylcatechol | Moderate | Systolic blood pressure | 0.3359 | 0.0258 | 0.0695 | 0.1678 | 0.2820 | 0.6298 |
| Serum | metab_9222 | 4-Methylcatechol | Moderate | Diastolic blood pressure | 0.1359 | 0.3789 | 0.4856 | -0.0404 | 0.7970 | 0.9093 |
| Serum | metab_9222 | 4-Methylcatechol | Moderate | Total cholesterol | -0.1302 | 0.3995 | 0.5070 | -0.2262 | 0.1447 | 0.5547 |
| Serum | metab_9222 | 4-Methylcatechol | Moderate | Triglycerides | -0.1733 | 0.2606 | 0.3729 | -0.1767 | 0.2571 | 0.6188 |
| Serum | metab_9222 | 4-Methylcatechol | Moderate | LDL cholesterol | -0.0560 | 0.7179 | 0.7897 | -0.0691 | 0.6599 | 0.8542 |
| Serum | metab_9222 | 4-Methylcatechol | Moderate | BMI | 0.0658 | 0.6715 | 0.7609 | 0.1630 | 0.2963 | 0.6359 |
| Serum | metab_9222 | 4-Methylcatechol | Moderate | Diabetes history | 0.2947 | 0.0521 | 0.1109 | 0.1766 | 0.2573 | 0.6188 |

# 1. Supplementary Table S5. Clinical correlations of metabolite features identified by paired metabolomics

Unadjusted values are Spearman rank correlations. Adjusted values are partial Spearman correlations after residualizing both ranked variables for CKM group. BH correction was applied separately to the 132 unadjusted tests and the 132 CKM-adjusted tests. Diabetes history was coded as a binary variable.
