# Supplementary material for: Paired metabolomics identify associations between fecal and serum metabolites in cardiovascular-kidney-metabolic syndrome
Source: Front Endocrinol (Lausanne). 2026 Oct 27;17:1982623. doi: 10.3389/fendo.2026.1982623 (PMC13747780; doi:10.3389/fendo.2026.1982623)
Supplement: Supplementary file 5 [file Table6.docx]

**Supplementary Table S5. Differential serum proteins**

| **Accession** | **Protein** | **Direction in stages 3-4** | **Mean difference** | **Rank-biserial effect** | **P value** | **BH-FDR** | **KEGG annotation** |
| --- | --- | --- | --- | --- | --- | --- | --- |
| A0A8Q3WKW0 | CFD | CKM stages 3-4 | 0.3831 | 0.8627 | 1.424e-05 | 0.0198 | hsa04080(Neuroactive ligand-receptor interaction); hsa04610(Complement and coagulation cascades); hsa05171(Coronavirus disease - COVID-19); hsa05150(Staphylococcus aureus infection) |
| P41222 | PTGDS | CKM stages 3-4 | 0.5451 | 0.8497 | 1.919e-05 | 0.0198 | hsa00590(Arachidonic acid metabolism); hsa01100(Metabolic pathways); hsa04657(IL-17 signaling pathway) |
| P18065 | IGFBP2 | CKM stages 3-4 | 0.6825 | 0.8431 | 2.224e-05 | 0.0198 | hsa04218(Cellular senescence); hsa04115(p53 signaling pathway); hsa05202(Transcriptional misregulation in cancer); hsa04935(Growth hormone synthesis, secretion and action) |
| A0A5C2G4U1 | A0A5C2G4U1_HUMAN | CKM stages 1-2 | -0.3768 | -0.8301 | 2.978e-05 | 0.0198 |  |
| P24592 | IGFBP6 | CKM stages 3-4 | 0.5498 | 0.8301 | 2.978e-05 | 0.0198 | hsa04218(Cellular senescence); hsa04115(p53 signaling pathway); hsa05202(Transcriptional misregulation in cancer); hsa04935(Growth hormone synthesis, secretion and action) |
| Q96S96 | PEBP4 | CKM stages 3-4 | 0.3796 | 0.8235 | 3.441e-05 | 0.0198 |  |
| P07998 | RNASE1 | CKM stages 3-4 | 0.4019 | 0.8235 | 3.441e-05 | 0.0198 | hsa05014(Amyotrophic lateral sclerosis) |
| A0A024R2W4 | DAG1 | CKM stages 3-4 | 0.3844 | 0.8170 | 3.972e-05 | 0.0198 | hsa05410(Hypertrophic cardiomyopathy); hsa04512(ECM-receptor interaction); hsa05412(Arrhythmogenic right ventricular cardiomyopathy); hsa05414(Dilated cardiomyopathy); hsa05416(Viral myocarditis) |
| A0A172WBW8 | CD55 | CKM stages 3-4 | 0.3306 | 0.8105 | 4.580e-05 | 0.0198 | hsa05133(Pertussis); hsa04610(Complement and coagulation cascades); hsa04613(Neutrophil extracellular trap formation); hsa05134(Legionellosis); hsa04662(B cell receptor signaling pathway); hsa05140(Leishmaniasis); hsa05169(Epstein-Barr virus infection); hsa04640(Hematopoietic cell lineage); hsa05152(Tuberculosis); hsa05416(Viral myocarditis); hsa05144(Malaria) |
| P06727 | APOA4 | CKM stages 3-4 | 0.3420 | 0.8039 | 5.275e-05 | 0.0198 | hsa04979(Cholesterol metabolism); hsa04977(Vitamin digestion and absorption); hsa04975(Fat digestion and absorption); hsa05417(Lipid and atherosclerosis) |
| A0A140VJJ6 | A0A140VJJ6_HUMAN | CKM stages 3-4 | 0.4828 | 0.7974 | 6.069e-05 | 0.0198 | hsa04613(Neutrophil extracellular trap formation); hsa04611(Platelet activation); hsa04610(Complement and coagulation cascades); hsa05171(Coronavirus disease - COVID-19); hsa05150(Staphylococcus aureus infection) |
| A0A5C2H1M0 | A0A5C2H1M0_HUMAN | CKM stages 1-2 | -0.4845 | -0.7974 | 6.069e-05 | 0.0198 |  |
| P02671 | FGA | CKM stages 3-4 | 0.2948 | 0.7974 | 6.069e-05 | 0.0198 | hsa04613(Neutrophil extracellular trap formation); hsa04611(Platelet activation); hsa04610(Complement and coagulation cascades); hsa05171(Coronavirus disease - COVID-19); hsa05150(Staphylococcus aureus infection) |
| A0A5C2G9T9 | A0A5C2G9T9_HUMAN | CKM stages 1-2 | -0.3018 | -0.7908 | 6.976e-05 | 0.0198 | hsa05130(Pathogenic Escherichia coli infection); hsa04666(Fc gamma R-mediated phagocytosis); hsa04613(Neutrophil extracellular trap formation); hsa04650(Natural killer cell mediated cytotoxicity); hsa04020(Calcium signaling pathway); hsa05330(Allograft rejection); hsa04672(Intestinal immune network for IgA production); hsa04145(Phagosome); hsa05310(Asthma); hsa04064(NF-kappa B signaling pathway); hsa05414(Dilated cardiomyopathy); hsa05416(Viral myocarditis); hsa05135(Yersinia infection); hsa05143(African trypanosomiasis); hsa05140(Leishmaniasis); hsa05146(Amoebiasis); hsa05169(Epstein-Barr virus infection); hsa05322(Systemic lupus erythematosus); hsa05323(Rheumatoid arthritis); hsa05320(Autoimmune thyroid disease); hsa05202(Transcriptional misregulation in cancer); hsa04662(B cell receptor signaling pathway); hsa04072(Phospholipase D signaling pathway); hsa04664(Fc epsilon RI signaling pathway); hsa04640(Hematopoietic cell lineage); hsa05340(Primary immunodeficiency); hsa04151(PI3K-Akt signaling pathway); hsa05150(Staphylococcus aureus infection); hsa05152(Tuberculosis); hsa05171(Coronavirus disease - COVID-19) |
| A0A7S5C0Q0 | A0A7S5C0Q0_HUMAN | CKM stages 1-2 | -0.7347 | -0.7908 | 6.976e-05 | 0.0198 | hsa05130(Pathogenic Escherichia coli infection); hsa04666(Fc gamma R-mediated phagocytosis); hsa04613(Neutrophil extracellular trap formation); hsa04650(Natural killer cell mediated cytotoxicity); hsa04020(Calcium signaling pathway); hsa05330(Allograft rejection); hsa04672(Intestinal immune network for IgA production); hsa04145(Phagosome); hsa05310(Asthma); hsa04064(NF-kappa B signaling pathway); hsa05414(Dilated cardiomyopathy); hsa05416(Viral myocarditis); hsa05135(Yersinia infection); hsa05143(African trypanosomiasis); hsa05140(Leishmaniasis); hsa05146(Amoebiasis); hsa05169(Epstein-Barr virus infection); hsa05322(Systemic lupus erythematosus); hsa05323(Rheumatoid arthritis); hsa05320(Autoimmune thyroid disease); hsa05202(Transcriptional misregulation in cancer); hsa04662(B cell receptor signaling pathway); hsa04072(Phospholipase D signaling pathway); hsa04664(Fc epsilon RI signaling pathway); hsa04640(Hematopoietic cell lineage); hsa05340(Primary immunodeficiency); hsa04151(PI3K-Akt signaling pathway); hsa05150(Staphylococcus aureus infection); hsa05152(Tuberculosis); hsa05171(Coronavirus disease - COVID-19) |
| A8KAJ3 | A8KAJ3_HUMAN | CKM stages 3-4 | 0.2420 | 0.7908 | 6.976e-05 | 0.0198 |  |
| O15204 | ADAMDEC1 | CKM stages 3-4 | 0.3274 | 0.7908 | 6.976e-05 | 0.0198 |  |
| P10645 | CHGA | CKM stages 3-4 | 0.4942 | 0.7908 | 6.976e-05 | 0.0198 |  |
| A0A5C2G6Y8 | A0A5C2G6Y8_HUMAN | CKM stages 1-2 | -0.4775 | -0.7843 | 8.010e-05 | 0.0198 | hsa05135(Yersinia infection); hsa04612(Antigen processing and presentation); hsa04660(T cell receptor signaling pathway); hsa04514(Cell adhesion molecules); hsa04640(Hematopoietic cell lineage); hsa05340(Primary immunodeficiency) |
| Q13508 | ART3 | CKM stages 3-4 | 0.4045 | 0.7843 | 8.010e-05 | 0.0198 |  |
| Q16627 | CCL14 | CKM stages 3-4 | 0.2708 | 0.7843 | 8.010e-05 | 0.0198 | hsa05163(Human cytomegalovirus infection); hsa05323(Rheumatoid arthritis); hsa04620(Toll-like receptor signaling pathway); hsa04623(Cytosolic DNA-sensing pathway); hsa05142(Chagas disease); hsa04064(NF-kappa B signaling pathway); hsa04061(Viral protein interaction with cytokine and cytokine receptor); hsa04062(Chemokine signaling pathway); hsa04060(Cytokine-cytokine receptor interaction); hsa05417(Lipid and atherosclerosis) |
| P01034 | CST3 | CKM stages 3-4 | 0.3443 | 0.7778 | 9.188e-05 | 0.0198 | hsa04970(Salivary secretion) |
| A0A5C2G2J4 | A0A5C2G2J4_HUMAN | CKM stages 1-2 | -0.4548 | -0.7712 | 1.053e-04 | 0.0198 |  |
| A0A5C2GMH8 | A0A5C2GMH8_HUMAN | CKM stages 1-2 | -0.4851 | -0.7712 | 1.053e-04 | 0.0198 |  |
| A0A5C2GPI8 | A0A5C2GPI8_HUMAN | CKM stages 1-2 | -0.5543 | -0.7712 | 1.053e-04 | 0.0198 | hsa05130(Pathogenic Escherichia coli infection); hsa04666(Fc gamma R-mediated phagocytosis); hsa04613(Neutrophil extracellular trap formation); hsa04650(Natural killer cell mediated cytotoxicity); hsa04020(Calcium signaling pathway); hsa05330(Allograft rejection); hsa04672(Intestinal immune network for IgA production); hsa04145(Phagosome); hsa05310(Asthma); hsa04064(NF-kappa B signaling pathway); hsa05414(Dilated cardiomyopathy); hsa05416(Viral myocarditis); hsa05135(Yersinia infection); hsa05143(African trypanosomiasis); hsa05140(Leishmaniasis); hsa05146(Amoebiasis); hsa05169(Epstein-Barr virus infection); hsa05322(Systemic lupus erythematosus); hsa05323(Rheumatoid arthritis); hsa05320(Autoimmune thyroid disease); hsa05202(Transcriptional misregulation in cancer); hsa04662(B cell receptor signaling pathway); hsa04072(Phospholipase D signaling pathway); hsa04664(Fc epsilon RI signaling pathway); hsa04640(Hematopoietic cell lineage); hsa05340(Primary immunodeficiency); hsa04151(PI3K-Akt signaling pathway); hsa05150(Staphylococcus aureus infection); hsa05152(Tuberculosis); hsa05171(Coronavirus disease - COVID-19) |
| A0A7S5EYV5 | A0A7S5EYV5_HUMAN | CKM stages 1-2 | -0.2197 | -0.7712 | 1.053e-04 | 0.0198 | hsa05130(Pathogenic Escherichia coli infection); hsa04666(Fc gamma R-mediated phagocytosis); hsa04613(Neutrophil extracellular trap formation); hsa04650(Natural killer cell mediated cytotoxicity); hsa04020(Calcium signaling pathway); hsa05330(Allograft rejection); hsa04672(Intestinal immune network for IgA production); hsa04145(Phagosome); hsa05310(Asthma); hsa04064(NF-kappa B signaling pathway); hsa05414(Dilated cardiomyopathy); hsa05416(Viral myocarditis); hsa05135(Yersinia infection); hsa05143(African trypanosomiasis); hsa05140(Leishmaniasis); hsa05146(Amoebiasis); hsa05169(Epstein-Barr virus infection); hsa05322(Systemic lupus erythematosus); hsa05323(Rheumatoid arthritis); hsa05320(Autoimmune thyroid disease); hsa05202(Transcriptional misregulation in cancer); hsa04662(B cell receptor signaling pathway); hsa04072(Phospholipase D signaling pathway); hsa04664(Fc epsilon RI signaling pathway); hsa04640(Hematopoietic cell lineage); hsa05340(Primary immunodeficiency); hsa04151(PI3K-Akt signaling pathway); hsa05150(Staphylococcus aureus infection); hsa05152(Tuberculosis); hsa05171(Coronavirus disease - COVID-19) |
| A0A384MDU2 | COL1A2 | CKM stages 3-4 | 0.2087 | 0.7712 | 1.053e-04 | 0.0198 | hsa04611(Platelet activation); hsa04974(Protein digestion and absorption); hsa05205(Proteoglycans in cancer); hsa04926(Relaxin signaling pathway); hsa04512(ECM-receptor interaction); hsa04510(Focal adhesion); hsa05165(Human papillomavirus infection); hsa05146(Amoebiasis); hsa04933(AGE-RAGE signaling pathway in diabetic complications); hsa05415(Diabetic cardiomyopathy); hsa04151(PI3K-Akt signaling pathway) |
| A0A5C2G7P9 | A0A5C2G7P9_HUMAN | CKM stages 1-2 | -0.4408 | -0.7647 | 1.205e-04 | 0.0198 |  |
| A0A5C2H0E8 | A0A5C2H0E8_HUMAN | CKM stages 1-2 | -0.3603 | -0.7647 | 1.205e-04 | 0.0198 |  |
| P25311 | AZGP1 | CKM stages 3-4 | 0.2234 | 0.7647 | 1.205e-04 | 0.0198 | hsa05163(Human cytomegalovirus infection); hsa04612(Antigen processing and presentation); hsa04650(Natural killer cell mediated cytotoxicity); hsa05320(Autoimmune thyroid disease); hsa05330(Allograft rejection); hsa05203(Viral carcinogenesis); hsa05332(Graft-versus-host disease); hsa04940(Type I diabetes mellitus); hsa04218(Cellular senescence); hsa04514(Cell adhesion molecules); hsa05168(Herpes simplex virus 1 infection); hsa04144(Endocytosis); hsa05165(Human papillomavirus infection); hsa04145(Phagosome); hsa05166(Human T-cell leukemia virus 1 infection); hsa05167(Kaposi sarcoma-associated herpesvirus infection); hsa05169(Epstein-Barr virus infection); hsa05416(Viral myocarditis); hsa05170(Human immunodeficiency virus 1 infection) |
| P22352 | GPX3 | CKM stages 1-2 | -0.2340 | -0.7647 | 1.205e-04 | 0.0198 | hsa00590(Arachidonic acid metabolism); hsa01100(Metabolic pathways); hsa00480(Glutathione metabolism); hsa05022(Pathways of neurodegeneration - multiple diseases); hsa05014(Amyotrophic lateral sclerosis); hsa05016(Huntington disease); hsa04918(Thyroid hormone synthesis) |
| Q3ZCW2 | LGALSL | CKM stages 1-2 | -0.2227 | -0.7647 | 1.205e-04 | 0.0198 |  |
| P60174 | TPI1 | CKM stages 1-2 | -0.3233 | -0.7647 | 1.205e-04 | 0.0198 | hsa00562(Inositol phosphate metabolism); hsa01100(Metabolic pathways); hsa01200(Carbon metabolism); hsa00051(Fructose and mannose metabolism); hsa01230(Biosynthesis of amino acids); hsa00010(Glycolysis / Gluconeogenesis) |
| A0A5C2G9J9 | A0A5C2G9J9_HUMAN | CKM stages 1-2 | -0.4623 | -0.7582 | 1.378e-04 | 0.0198 |  |
| A0A5C2GGJ7 | A0A5C2GGJ7_HUMAN | CKM stages 3-4 | 0.4667 | 0.7582 | 1.378e-04 | 0.0198 | hsa05130(Pathogenic Escherichia coli infection); hsa04666(Fc gamma R-mediated phagocytosis); hsa04613(Neutrophil extracellular trap formation); hsa04650(Natural killer cell mediated cytotoxicity); hsa04020(Calcium signaling pathway); hsa05330(Allograft rejection); hsa04672(Intestinal immune network for IgA production); hsa04145(Phagosome); hsa05310(Asthma); hsa04064(NF-kappa B signaling pathway); hsa05414(Dilated cardiomyopathy); hsa05416(Viral myocarditis); hsa05135(Yersinia infection); hsa05143(African trypanosomiasis); hsa05140(Leishmaniasis); hsa05146(Amoebiasis); hsa05169(Epstein-Barr virus infection); hsa05322(Systemic lupus erythematosus); hsa05323(Rheumatoid arthritis); hsa05320(Autoimmune thyroid disease); hsa05202(Transcriptional misregulation in cancer); hsa04662(B cell receptor signaling pathway); hsa04072(Phospholipase D signaling pathway); hsa04664(Fc epsilon RI signaling pathway); hsa04640(Hematopoietic cell lineage); hsa05340(Primary immunodeficiency); hsa04151(PI3K-Akt signaling pathway); hsa05150(Staphylococcus aureus infection); hsa05152(Tuberculosis); hsa05171(Coronavirus disease - COVID-19) |
| P0CG39 | POTEJ | CKM stages 3-4 | 0.4614 | 0.7582 | 1.378e-04 | 0.0198 | hsa05132(Salmonella infection); hsa05131(Shigellosis); hsa05130(Pathogenic Escherichia coli infection); hsa04611(Platelet activation); hsa05135(Yersinia infection); hsa05418(Fluid shear stress and atherosclerosis); hsa04670(Leukocyte transendothelial migration); hsa04145(Phagosome); hsa04510(Focal adhesion); hsa05410(Hypertrophic cardiomyopathy); hsa05412(Arrhythmogenic right ventricular cardiomyopathy); hsa05414(Dilated cardiomyopathy); hsa05416(Viral myocarditis); hsa04613(Neutrophil extracellular trap formation); hsa04921(Oxytocin signaling pathway); hsa05164(Influenza A); hsa04210(Apoptosis); hsa05100(Bacterial invasion of epithelial cells); hsa04971(Gastric acid secretion); hsa05205(Proteoglycans in cancer); hsa04015(Rap1 signaling pathway); hsa04520(Adherens junction); hsa04390(Hippo signaling pathway); hsa04919(Thyroid hormone signaling pathway); hsa04714(Thermogenesis); hsa04810(Regulation of actin cytoskeleton); hsa04530(Tight junction); hsa05225(Hepatocellular carcinoma); hsa05014(Amyotrophic lateral sclerosis); hsa05110(Vibrio cholerae infection) |
| A8KAG5 | A8KAG5_HUMAN | CKM stages 3-4 | 0.4128 | 0.9412 | 1.387e-04 | 0.0198 | hsa04514(Cell adhesion molecules); hsa04640(Hematopoietic cell lineage) |
| A0A5C2FW41 | A0A5C2FW41_HUMAN | CKM stages 1-2 | -0.4436 | -0.7516 | 1.574e-04 | 0.0198 |  |
| A0A5C2G429 | A0A5C2G429_HUMAN | CKM stages 3-4 | 0.5081 | 0.7516 | 1.574e-04 | 0.0198 | hsa05135(Yersinia infection); hsa04612(Antigen processing and presentation); hsa04660(T cell receptor signaling pathway); hsa04514(Cell adhesion molecules); hsa04640(Hematopoietic cell lineage); hsa05340(Primary immunodeficiency) |
| A0A5C2GCU8 | A0A5C2GCU8_HUMAN | CKM stages 1-2 | -0.5578 | -0.7516 | 1.574e-04 | 0.0198 | hsa05130(Pathogenic Escherichia coli infection); hsa04666(Fc gamma R-mediated phagocytosis); hsa04613(Neutrophil extracellular trap formation); hsa04650(Natural killer cell mediated cytotoxicity); hsa04020(Calcium signaling pathway); hsa05330(Allograft rejection); hsa04672(Intestinal immune network for IgA production); hsa04145(Phagosome); hsa05310(Asthma); hsa04064(NF-kappa B signaling pathway); hsa05414(Dilated cardiomyopathy); hsa05416(Viral myocarditis); hsa05135(Yersinia infection); hsa05143(African trypanosomiasis); hsa05140(Leishmaniasis); hsa05146(Amoebiasis); hsa05169(Epstein-Barr virus infection); hsa05322(Systemic lupus erythematosus); hsa05323(Rheumatoid arthritis); hsa05320(Autoimmune thyroid disease); hsa05202(Transcriptional misregulation in cancer); hsa04662(B cell receptor signaling pathway); hsa04072(Phospholipase D signaling pathway); hsa04664(Fc epsilon RI signaling pathway); hsa04640(Hematopoietic cell lineage); hsa05340(Primary immunodeficiency); hsa04151(PI3K-Akt signaling pathway); hsa05150(Staphylococcus aureus infection); hsa05152(Tuberculosis); hsa05171(Coronavirus disease - COVID-19) |
| A0A5C2GHT7 | A0A5C2GHT7_HUMAN | CKM stages 1-2 | -0.3700 | -0.7516 | 1.574e-04 | 0.0198 | hsa05130(Pathogenic Escherichia coli infection); hsa04666(Fc gamma R-mediated phagocytosis); hsa04613(Neutrophil extracellular trap formation); hsa04650(Natural killer cell mediated cytotoxicity); hsa04020(Calcium signaling pathway); hsa05330(Allograft rejection); hsa04672(Intestinal immune network for IgA production); hsa04145(Phagosome); hsa05310(Asthma); hsa04064(NF-kappa B signaling pathway); hsa05414(Dilated cardiomyopathy); hsa05416(Viral myocarditis); hsa05135(Yersinia infection); hsa05143(African trypanosomiasis); hsa05140(Leishmaniasis); hsa05146(Amoebiasis); hsa05169(Epstein-Barr virus infection); hsa05322(Systemic lupus erythematosus); hsa05323(Rheumatoid arthritis); hsa05320(Autoimmune thyroid disease); hsa05202(Transcriptional misregulation in cancer); hsa04662(B cell receptor signaling pathway); hsa04072(Phospholipase D signaling pathway); hsa04664(Fc epsilon RI signaling pathway); hsa04640(Hematopoietic cell lineage); hsa05340(Primary immunodeficiency); hsa04151(PI3K-Akt signaling pathway); hsa05150(Staphylococcus aureus infection); hsa05152(Tuberculosis); hsa05171(Coronavirus disease - COVID-19) |
| A0A5C2GPG7 | A0A5C2GPG7_HUMAN | CKM stages 1-2 | -0.3625 | -0.7516 | 1.574e-04 | 0.0198 | hsa05130(Pathogenic Escherichia coli infection); hsa04666(Fc gamma R-mediated phagocytosis); hsa04613(Neutrophil extracellular trap formation); hsa04650(Natural killer cell mediated cytotoxicity); hsa04020(Calcium signaling pathway); hsa05330(Allograft rejection); hsa04672(Intestinal immune network for IgA production); hsa04145(Phagosome); hsa05310(Asthma); hsa04064(NF-kappa B signaling pathway); hsa05414(Dilated cardiomyopathy); hsa05416(Viral myocarditis); hsa05135(Yersinia infection); hsa05143(African trypanosomiasis); hsa05140(Leishmaniasis); hsa05146(Amoebiasis); hsa05169(Epstein-Barr virus infection); hsa05322(Systemic lupus erythematosus); hsa05323(Rheumatoid arthritis); hsa05320(Autoimmune thyroid disease); hsa05202(Transcriptional misregulation in cancer); hsa04662(B cell receptor signaling pathway); hsa04072(Phospholipase D signaling pathway); hsa04664(Fc epsilon RI signaling pathway); hsa04640(Hematopoietic cell lineage); hsa05340(Primary immunodeficiency); hsa04151(PI3K-Akt signaling pathway); hsa05150(Staphylococcus aureus infection); hsa05152(Tuberculosis); hsa05171(Coronavirus disease - COVID-19) |
| A0A5C2GWT5 | A0A5C2GWT5_HUMAN | CKM stages 1-2 | -0.3256 | -0.7516 | 1.574e-04 | 0.0198 |  |
| A0A8Q3SI99 | CFH | CKM stages 1-2 | -0.6032 | -0.7516 | 1.574e-04 | 0.0198 | hsa04610(Complement and coagulation cascades); hsa05150(Staphylococcus aureus infection) |
| P17900 | GM2A | CKM stages 3-4 | 0.4287 | 0.7516 | 1.574e-04 | 0.0198 | hsa04142(Lysosome) |
| P22692 | IGFBP4 | CKM stages 3-4 | 0.2378 | 0.7516 | 1.574e-04 | 0.0198 | hsa04218(Cellular senescence); hsa04115(p53 signaling pathway); hsa05202(Transcriptional misregulation in cancer); hsa04935(Growth hormone synthesis, secretion and action) |
| O95998 | IL18BP | CKM stages 3-4 | 0.3790 | 0.7516 | 1.574e-04 | 0.0198 |  |
| C9JMK5 | PIK3IP1 | CKM stages 3-4 | 0.4542 | 0.7516 | 1.574e-04 | 0.0198 | hsa05218(Melanoma); hsa04610(Complement and coagulation cascades); hsa04020(Calcium signaling pathway); hsa05211(Renal cell carcinoma); hsa05215(Prostate cancer); hsa05418(Fluid shear stress and atherosclerosis); hsa04510(Focal adhesion); hsa01521(EGFR tyrosine kinase inhibitor resistance); hsa05144(Malaria); hsa05164(Influenza A); hsa05208(Chemical carcinogenesis - reactive oxygen species); hsa04371(Apelin signaling pathway); hsa05205(Proteoglycans in cancer); hsa05200(Pathways in cancer); hsa05202(Transcriptional misregulation in cancer); hsa05226(Gastric cancer); hsa05225(Hepatocellular carcinoma); hsa04014(Ras signaling pathway); hsa05223(Non-small cell lung cancer); hsa04010(MAPK signaling pathway); hsa04151(PI3K-Akt signaling pathway); hsa04015(Rap1 signaling pathway); hsa05150(Staphylococcus aureus infection); hsa04080(Neuroactive ligand-receptor interaction) |
| A0A5C2GHI1 | A0A5C2GHI1_HUMAN | CKM stages 1-2 | -0.8609 | -0.7451 | 1.796e-04 | 0.0204 |  |
| P07307 | ASGR2 | CKM stages 3-4 | 0.3159 | 0.7451 | 1.796e-04 | 0.0204 | hsa04918(Thyroid hormone synthesis); hsa04145(Phagosome) |
| Q9ULI3 | HEG1 | CKM stages 3-4 | 0.2706 | 0.7451 | 1.796e-04 | 0.0204 | hsa04512(ECM-receptor interaction); hsa04390(Hippo signaling pathway); hsa04610(Complement and coagulation cascades) |
| A0A087X054 | HYOU1 | CKM stages 3-4 | 0.2439 | 0.7451 | 1.796e-04 | 0.0204 | hsa04530(Tight junction); hsa03040(Spliceosome); hsa04612(Antigen processing and presentation); hsa05020(Prion disease); hsa04010(MAPK signaling pathway); hsa05134(Legionellosis); hsa04141(Protein processing in endoplasmic reticulum); hsa04144(Endocytosis); hsa04915(Estrogen signaling pathway); hsa04213(Longevity regulating pathway - multiple species); hsa05145(Toxoplasmosis); hsa05162(Measles); hsa05417(Lipid and atherosclerosis) |
| A0A024R1G3 | MB | CKM stages 3-4 | 0.3826 | 0.7451 | 1.796e-04 | 0.0204 |  |
| A0A5C2GII9 | A0A5C2GII9_HUMAN | CKM stages 3-4 | 0.4889 | 0.8833 | 1.836e-04 | 0.0205 |  |
| A0A5C2FUU0 | A0A5C2FUU0_HUMAN | CKM stages 1-2 | -0.6276 | -0.7386 | 2.048e-04 | 0.0209 |  |
| A0A7S5C0H2 | A0A7S5C0H2_HUMAN | CKM stages 1-2 | -0.2014 | -0.7386 | 2.048e-04 | 0.0209 | hsa05130(Pathogenic Escherichia coli infection); hsa04666(Fc gamma R-mediated phagocytosis); hsa04613(Neutrophil extracellular trap formation); hsa04650(Natural killer cell mediated cytotoxicity); hsa04020(Calcium signaling pathway); hsa05330(Allograft rejection); hsa04672(Intestinal immune network for IgA production); hsa04145(Phagosome); hsa05310(Asthma); hsa04064(NF-kappa B signaling pathway); hsa05414(Dilated cardiomyopathy); hsa05416(Viral myocarditis); hsa05135(Yersinia infection); hsa05143(African trypanosomiasis); hsa05140(Leishmaniasis); hsa05146(Amoebiasis); hsa05169(Epstein-Barr virus infection); hsa05322(Systemic lupus erythematosus); hsa05323(Rheumatoid arthritis); hsa05320(Autoimmune thyroid disease); hsa05202(Transcriptional misregulation in cancer); hsa04662(B cell receptor signaling pathway); hsa04072(Phospholipase D signaling pathway); hsa04664(Fc epsilon RI signaling pathway); hsa04640(Hematopoietic cell lineage); hsa05340(Primary immunodeficiency); hsa04151(PI3K-Akt signaling pathway); hsa05150(Staphylococcus aureus infection); hsa05152(Tuberculosis); hsa05171(Coronavirus disease - COVID-19) |
| A0A7T0PWU2 | A0A7T0PWU2_HUMAN | CKM stages 1-2 | -0.3033 | -0.7386 | 2.048e-04 | 0.0209 | hsa05130(Pathogenic Escherichia coli infection); hsa04666(Fc gamma R-mediated phagocytosis); hsa04613(Neutrophil extracellular trap formation); hsa04650(Natural killer cell mediated cytotoxicity); hsa04020(Calcium signaling pathway); hsa05330(Allograft rejection); hsa04672(Intestinal immune network for IgA production); hsa04145(Phagosome); hsa05310(Asthma); hsa04064(NF-kappa B signaling pathway); hsa05414(Dilated cardiomyopathy); hsa05416(Viral myocarditis); hsa05135(Yersinia infection); hsa05143(African trypanosomiasis); hsa05140(Leishmaniasis); hsa05146(Amoebiasis); hsa05169(Epstein-Barr virus infection); hsa05322(Systemic lupus erythematosus); hsa05323(Rheumatoid arthritis); hsa05320(Autoimmune thyroid disease); hsa05202(Transcriptional misregulation in cancer); hsa04662(B cell receptor signaling pathway); hsa04072(Phospholipase D signaling pathway); hsa04664(Fc epsilon RI signaling pathway); hsa04640(Hematopoietic cell lineage); hsa05340(Primary immunodeficiency); hsa04151(PI3K-Akt signaling pathway); hsa05150(Staphylococcus aureus infection); hsa05152(Tuberculosis); hsa05171(Coronavirus disease - COVID-19) |
| Q15828 | CST6 | CKM stages 3-4 | 0.4078 | 0.7386 | 2.048e-04 | 0.0209 | hsa04970(Salivary secretion) |
| A0A024QZW7 | NUP153 | CKM stages 3-4 | 0.8419 | 0.7386 | 2.048e-04 | 0.0209 | hsa05014(Amyotrophic lateral sclerosis); hsa03250(Viral life cycle - HIV-1); hsa03013(Nucleocytoplasmic transport) |
| A0A5C2FXX9 | A0A5C2FXX9_HUMAN | CKM stages 1-2 | -0.6024 | -0.7320 | 2.332e-04 | 0.0220 |  |
| A0A5C2FZA5 | A0A5C2FZA5_HUMAN | CKM stages 1-2 | -0.4836 | -0.7320 | 2.332e-04 | 0.0220 |  |
| A0A5C2GCN9 | A0A5C2GCN9_HUMAN | CKM stages 1-2 | -0.3274 | -0.7320 | 2.332e-04 | 0.0220 |  |
| A0A5C2GJH6 | A0A5C2GJH6_HUMAN | CKM stages 1-2 | -0.3884 | -0.7320 | 2.332e-04 | 0.0220 | hsa04380(Osteoclast differentiation) |
| Q6UWP8 | SBSN | CKM stages 3-4 | 0.3108 | 0.7320 | 2.332e-04 | 0.0220 |  |
| A0A5C2GKZ2 | A0A5C2GKZ2_HUMAN | CKM stages 1-2 | -0.4575 | -0.7255 | 2.653e-04 | 0.0235 | hsa05130(Pathogenic Escherichia coli infection); hsa04666(Fc gamma R-mediated phagocytosis); hsa04613(Neutrophil extracellular trap formation); hsa04650(Natural killer cell mediated cytotoxicity); hsa04020(Calcium signaling pathway); hsa05330(Allograft rejection); hsa04672(Intestinal immune network for IgA production); hsa04145(Phagosome); hsa05310(Asthma); hsa04064(NF-kappa B signaling pathway); hsa05414(Dilated cardiomyopathy); hsa05416(Viral myocarditis); hsa05135(Yersinia infection); hsa05143(African trypanosomiasis); hsa05140(Leishmaniasis); hsa05146(Amoebiasis); hsa05169(Epstein-Barr virus infection); hsa05322(Systemic lupus erythematosus); hsa05323(Rheumatoid arthritis); hsa05320(Autoimmune thyroid disease); hsa05202(Transcriptional misregulation in cancer); hsa04662(B cell receptor signaling pathway); hsa04072(Phospholipase D signaling pathway); hsa04664(Fc epsilon RI signaling pathway); hsa04640(Hematopoietic cell lineage); hsa05340(Primary immunodeficiency); hsa04151(PI3K-Akt signaling pathway); hsa05150(Staphylococcus aureus infection); hsa05152(Tuberculosis); hsa05171(Coronavirus disease - COVID-19) |
| A0A5C2GVZ1 | A0A5C2GVZ1_HUMAN | CKM stages 1-2 | -0.5777 | -0.7255 | 2.653e-04 | 0.0235 |  |
| Q15485 | FCN2 | CKM stages 3-4 | 0.2977 | 0.7255 | 2.653e-04 | 0.0235 |  |
| P19957 | PI3 | CKM stages 3-4 | 0.5154 | 0.7255 | 2.653e-04 | 0.0235 |  |
| A0A5C2GGJ4 | A0A5C2GGJ4_HUMAN | CKM stages 1-2 | -0.3704 | -0.7190 | 3.015e-04 | 0.0256 |  |
| A0A5C2GNF1 | A0A5C2GNF1_HUMAN | CKM stages 3-4 | 0.4627 | 0.7190 | 3.015e-04 | 0.0256 | hsa05130(Pathogenic Escherichia coli infection); hsa04666(Fc gamma R-mediated phagocytosis); hsa04613(Neutrophil extracellular trap formation); hsa04650(Natural killer cell mediated cytotoxicity); hsa04020(Calcium signaling pathway); hsa05330(Allograft rejection); hsa04672(Intestinal immune network for IgA production); hsa04145(Phagosome); hsa05310(Asthma); hsa04064(NF-kappa B signaling pathway); hsa05414(Dilated cardiomyopathy); hsa05416(Viral myocarditis); hsa05135(Yersinia infection); hsa05143(African trypanosomiasis); hsa05140(Leishmaniasis); hsa05146(Amoebiasis); hsa05169(Epstein-Barr virus infection); hsa05322(Systemic lupus erythematosus); hsa05323(Rheumatoid arthritis); hsa05320(Autoimmune thyroid disease); hsa05202(Transcriptional misregulation in cancer); hsa04662(B cell receptor signaling pathway); hsa04072(Phospholipase D signaling pathway); hsa04664(Fc epsilon RI signaling pathway); hsa04640(Hematopoietic cell lineage); hsa05340(Primary immunodeficiency); hsa04151(PI3K-Akt signaling pathway); hsa05150(Staphylococcus aureus infection); hsa05152(Tuberculosis); hsa05171(Coronavirus disease - COVID-19) |
| Q59EB6 | Q59EB6_HUMAN | CKM stages 3-4 | 0.3190 | 0.7190 | 3.015e-04 | 0.0256 | hsa04610(Complement and coagulation cascades); hsa04979(Cholesterol metabolism); hsa05418(Fluid shear stress and atherosclerosis); hsa05144(Malaria); hsa05010(Alzheimer disease); hsa04933(AGE-RAGE signaling pathway in diabetic complications) |
| A0A5C2FWX9 | A0A5C2FWX9_HUMAN | CKM stages 1-2 | -0.5902 | -0.7124 | 3.422e-04 | 0.0268 |  |
| A0A5C2GBU1 | A0A5C2GBU1_HUMAN | CKM stages 1-2 | -0.4883 | -0.7124 | 3.422e-04 | 0.0268 |  |
| A0A5C2GZS9 | A0A5C2GZS9_HUMAN | CKM stages 1-2 | -0.1756 | -0.7124 | 3.422e-04 | 0.0268 |  |
| A0A7S5C0T5 | A0A7S5C0T5_HUMAN | CKM stages 1-2 | -0.4391 | -0.7124 | 3.422e-04 | 0.0268 | hsa05130(Pathogenic Escherichia coli infection); hsa04666(Fc gamma R-mediated phagocytosis); hsa04613(Neutrophil extracellular trap formation); hsa04650(Natural killer cell mediated cytotoxicity); hsa04020(Calcium signaling pathway); hsa05330(Allograft rejection); hsa04672(Intestinal immune network for IgA production); hsa04145(Phagosome); hsa05310(Asthma); hsa04064(NF-kappa B signaling pathway); hsa05414(Dilated cardiomyopathy); hsa05416(Viral myocarditis); hsa05135(Yersinia infection); hsa05143(African trypanosomiasis); hsa05140(Leishmaniasis); hsa05146(Amoebiasis); hsa05169(Epstein-Barr virus infection); hsa05322(Systemic lupus erythematosus); hsa05323(Rheumatoid arthritis); hsa05320(Autoimmune thyroid disease); hsa05202(Transcriptional misregulation in cancer); hsa04662(B cell receptor signaling pathway); hsa04072(Phospholipase D signaling pathway); hsa04664(Fc epsilon RI signaling pathway); hsa04640(Hematopoietic cell lineage); hsa05340(Primary immunodeficiency); hsa04151(PI3K-Akt signaling pathway); hsa05150(Staphylococcus aureus infection); hsa05152(Tuberculosis); hsa05171(Coronavirus disease - COVID-19) |
| A0A7S5EYB3 | A0A7S5EYB3_HUMAN | CKM stages 1-2 | -0.4513 | -0.7124 | 3.422e-04 | 0.0268 | hsa05130(Pathogenic Escherichia coli infection); hsa04666(Fc gamma R-mediated phagocytosis); hsa04613(Neutrophil extracellular trap formation); hsa04650(Natural killer cell mediated cytotoxicity); hsa04020(Calcium signaling pathway); hsa05330(Allograft rejection); hsa04672(Intestinal immune network for IgA production); hsa04145(Phagosome); hsa05310(Asthma); hsa04064(NF-kappa B signaling pathway); hsa05414(Dilated cardiomyopathy); hsa05416(Viral myocarditis); hsa05135(Yersinia infection); hsa05143(African trypanosomiasis); hsa05140(Leishmaniasis); hsa05146(Amoebiasis); hsa05169(Epstein-Barr virus infection); hsa05322(Systemic lupus erythematosus); hsa05323(Rheumatoid arthritis); hsa05320(Autoimmune thyroid disease); hsa05202(Transcriptional misregulation in cancer); hsa04662(B cell receptor signaling pathway); hsa04072(Phospholipase D signaling pathway); hsa04664(Fc epsilon RI signaling pathway); hsa04640(Hematopoietic cell lineage); hsa05340(Primary immunodeficiency); hsa04151(PI3K-Akt signaling pathway); hsa05150(Staphylococcus aureus infection); hsa05152(Tuberculosis); hsa05171(Coronavirus disease - COVID-19) |
| B3GN61 | CDH1 | CKM stages 3-4 | 0.2337 | 0.7124 | 3.422e-04 | 0.0268 | hsa05218(Melanoma); hsa05219(Bladder cancer); hsa05100(Bacterial invasion of epithelial cells); hsa04371(Apelin signaling pathway); hsa05213(Endometrial cancer); hsa05216(Thyroid cancer); hsa05200(Pathways in cancer); hsa05226(Gastric cancer); hsa04514(Cell adhesion molecules); hsa04520(Adherens junction); hsa04015(Rap1 signaling pathway); hsa04390(Hippo signaling pathway); hsa05412(Arrhythmogenic right ventricular cardiomyopathy) |
| A0A5C2GBH9 | A0A5C2GBH9_HUMAN | CKM stages 3-4 | 0.6129 | 0.8642 | 3.506e-04 | 0.0271 | hsa05130(Pathogenic Escherichia coli infection); hsa04666(Fc gamma R-mediated phagocytosis); hsa04613(Neutrophil extracellular trap formation); hsa04650(Natural killer cell mediated cytotoxicity); hsa04020(Calcium signaling pathway); hsa05330(Allograft rejection); hsa04672(Intestinal immune network for IgA production); hsa04145(Phagosome); hsa05310(Asthma); hsa04064(NF-kappa B signaling pathway); hsa05414(Dilated cardiomyopathy); hsa05416(Viral myocarditis); hsa05135(Yersinia infection); hsa05143(African trypanosomiasis); hsa05140(Leishmaniasis); hsa05146(Amoebiasis); hsa05169(Epstein-Barr virus infection); hsa05322(Systemic lupus erythematosus); hsa05323(Rheumatoid arthritis); hsa05320(Autoimmune thyroid disease); hsa05202(Transcriptional misregulation in cancer); hsa04662(B cell receptor signaling pathway); hsa04072(Phospholipase D signaling pathway); hsa04664(Fc epsilon RI signaling pathway); hsa04640(Hematopoietic cell lineage); hsa05340(Primary immunodeficiency); hsa04151(PI3K-Akt signaling pathway); hsa05150(Staphylococcus aureus infection); hsa05152(Tuberculosis); hsa05171(Coronavirus disease - COVID-19) |
| A0A5C2G0Q6 | A0A5C2G0Q6_HUMAN | CKM stages 1-2 | -0.3684 | -0.7059 | 3.881e-04 | 0.0275 |  |
| A0A5C2G6X9 | A0A5C2G6X9_HUMAN | CKM stages 1-2 | -0.2874 | -0.7059 | 3.881e-04 | 0.0275 | hsa05130(Pathogenic Escherichia coli infection); hsa04666(Fc gamma R-mediated phagocytosis); hsa04613(Neutrophil extracellular trap formation); hsa04650(Natural killer cell mediated cytotoxicity); hsa04020(Calcium signaling pathway); hsa05330(Allograft rejection); hsa04672(Intestinal immune network for IgA production); hsa04145(Phagosome); hsa05310(Asthma); hsa04064(NF-kappa B signaling pathway); hsa05414(Dilated cardiomyopathy); hsa05416(Viral myocarditis); hsa05135(Yersinia infection); hsa05143(African trypanosomiasis); hsa05140(Leishmaniasis); hsa05146(Amoebiasis); hsa05169(Epstein-Barr virus infection); hsa05322(Systemic lupus erythematosus); hsa05323(Rheumatoid arthritis); hsa05320(Autoimmune thyroid disease); hsa05202(Transcriptional misregulation in cancer); hsa04662(B cell receptor signaling pathway); hsa04072(Phospholipase D signaling pathway); hsa04664(Fc epsilon RI signaling pathway); hsa04640(Hematopoietic cell lineage); hsa05340(Primary immunodeficiency); hsa04151(PI3K-Akt signaling pathway); hsa05150(Staphylococcus aureus infection); hsa05152(Tuberculosis); hsa05171(Coronavirus disease - COVID-19) |
| A0A5C2GBV5 | A0A5C2GBV5_HUMAN | CKM stages 1-2 | -0.3241 | -0.7059 | 3.881e-04 | 0.0275 |  |
| P02760 | AMBP | CKM stages 3-4 | 0.1430 | 0.7059 | 3.881e-04 | 0.0275 | hsa04610(Complement and coagulation cascades) |
| B4DV10 | B4DV10_HUMAN | CKM stages 3-4 | 0.2649 | 0.7059 | 3.881e-04 | 0.0275 | hsa04979(Cholesterol metabolism); hsa04142(Lysosome) |
| P80723 | BASP1 | CKM stages 3-4 | 0.1899 | 0.7059 | 3.881e-04 | 0.0275 |  |
| Q71U36 | TUBA1A | CKM stages 1-2 | -0.2604 | -0.7059 | 3.881e-04 | 0.0275 | hsa05132(Salmonella infection); hsa05130(Pathogenic Escherichia coli infection); hsa04540(Gap junction); hsa04530(Tight junction); hsa04145(Phagosome); hsa05020(Prion disease); hsa05022(Pathways of neurodegeneration - multiple diseases); hsa05014(Amyotrophic lateral sclerosis); hsa04210(Apoptosis); hsa05016(Huntington disease); hsa05010(Alzheimer disease); hsa05012(Parkinson disease) |
| A0A5C2G2U8 | A0A5C2G2U8_HUMAN | CKM stages 1-2 | -0.3348 | -0.6993 | 4.397e-04 | 0.0291 |  |
| A0A5C2GBN2 | A0A5C2GBN2_HUMAN | CKM stages 1-2 | -0.4049 | -0.6993 | 4.397e-04 | 0.0291 | hsa05130(Pathogenic Escherichia coli infection); hsa04666(Fc gamma R-mediated phagocytosis); hsa04613(Neutrophil extracellular trap formation); hsa04650(Natural killer cell mediated cytotoxicity); hsa04020(Calcium signaling pathway); hsa05330(Allograft rejection); hsa04672(Intestinal immune network for IgA production); hsa04145(Phagosome); hsa05310(Asthma); hsa04064(NF-kappa B signaling pathway); hsa05414(Dilated cardiomyopathy); hsa05416(Viral myocarditis); hsa05135(Yersinia infection); hsa05143(African trypanosomiasis); hsa05140(Leishmaniasis); hsa05146(Amoebiasis); hsa05169(Epstein-Barr virus infection); hsa05322(Systemic lupus erythematosus); hsa05323(Rheumatoid arthritis); hsa05320(Autoimmune thyroid disease); hsa05202(Transcriptional misregulation in cancer); hsa04662(B cell receptor signaling pathway); hsa04072(Phospholipase D signaling pathway); hsa04664(Fc epsilon RI signaling pathway); hsa04640(Hematopoietic cell lineage); hsa05340(Primary immunodeficiency); hsa04151(PI3K-Akt signaling pathway); hsa05150(Staphylococcus aureus infection); hsa05152(Tuberculosis); hsa05171(Coronavirus disease - COVID-19) |
| A0A5C2GGH2 | A0A5C2GGH2_HUMAN | CKM stages 1-2 | -0.2181 | -0.6993 | 4.397e-04 | 0.0291 |  |
| P02675 | FGB | CKM stages 3-4 | 0.3465 | 0.6993 | 4.397e-04 | 0.0291 | hsa04613(Neutrophil extracellular trap formation); hsa04611(Platelet activation); hsa04610(Complement and coagulation cascades); hsa05171(Coronavirus disease - COVID-19); hsa05150(Staphylococcus aureus infection) |
| Q99969 | RARRES2 | CKM stages 3-4 | 0.1818 | 0.6993 | 4.397e-04 | 0.0291 |  |
| A0A5C2G0H8 | A0A5C2G0H8_HUMAN | CKM stages 1-2 | -0.3208 | -0.6928 | 4.976e-04 | 0.0291 |  |
| A0A5C2G0T0 | A0A5C2G0T0_HUMAN | CKM stages 3-4 | 0.5617 | 0.6928 | 4.976e-04 | 0.0291 |  |
| A0A5C2G5D3 | A0A5C2G5D3_HUMAN | CKM stages 1-2 | -0.4892 | -0.6928 | 4.976e-04 | 0.0291 | hsa05130(Pathogenic Escherichia coli infection); hsa04666(Fc gamma R-mediated phagocytosis); hsa04613(Neutrophil extracellular trap formation); hsa04650(Natural killer cell mediated cytotoxicity); hsa04020(Calcium signaling pathway); hsa05330(Allograft rejection); hsa04672(Intestinal immune network for IgA production); hsa04145(Phagosome); hsa05310(Asthma); hsa04064(NF-kappa B signaling pathway); hsa05414(Dilated cardiomyopathy); hsa05416(Viral myocarditis); hsa05135(Yersinia infection); hsa05143(African trypanosomiasis); hsa05140(Leishmaniasis); hsa05146(Amoebiasis); hsa05169(Epstein-Barr virus infection); hsa05322(Systemic lupus erythematosus); hsa05323(Rheumatoid arthritis); hsa05320(Autoimmune thyroid disease); hsa05202(Transcriptional misregulation in cancer); hsa04662(B cell receptor signaling pathway); hsa04072(Phospholipase D signaling pathway); hsa04664(Fc epsilon RI signaling pathway); hsa04640(Hematopoietic cell lineage); hsa05340(Primary immunodeficiency); hsa04151(PI3K-Akt signaling pathway); hsa05150(Staphylococcus aureus infection); hsa05152(Tuberculosis); hsa05171(Coronavirus disease - COVID-19) |
| A0A5C2G7E1 | A0A5C2G7E1_HUMAN | CKM stages 1-2 | -0.3228 | -0.6928 | 4.976e-04 | 0.0291 |  |
| A0A5C2GF52 | A0A5C2GF52_HUMAN | CKM stages 1-2 | -0.3402 | -0.6928 | 4.976e-04 | 0.0291 | hsa05130(Pathogenic Escherichia coli infection); hsa04666(Fc gamma R-mediated phagocytosis); hsa04613(Neutrophil extracellular trap formation); hsa04650(Natural killer cell mediated cytotoxicity); hsa04020(Calcium signaling pathway); hsa05330(Allograft rejection); hsa04672(Intestinal immune network for IgA production); hsa04145(Phagosome); hsa05310(Asthma); hsa04064(NF-kappa B signaling pathway); hsa05414(Dilated cardiomyopathy); hsa05416(Viral myocarditis); hsa05135(Yersinia infection); hsa05143(African trypanosomiasis); hsa05140(Leishmaniasis); hsa05146(Amoebiasis); hsa05169(Epstein-Barr virus infection); hsa05322(Systemic lupus erythematosus); hsa05323(Rheumatoid arthritis); hsa05320(Autoimmune thyroid disease); hsa05202(Transcriptional misregulation in cancer); hsa04662(B cell receptor signaling pathway); hsa04072(Phospholipase D signaling pathway); hsa04664(Fc epsilon RI signaling pathway); hsa04640(Hematopoietic cell lineage); hsa05340(Primary immunodeficiency); hsa04151(PI3K-Akt signaling pathway); hsa05150(Staphylococcus aureus infection); hsa05152(Tuberculosis); hsa05171(Coronavirus disease - COVID-19) |
| A0A5C2GR82 | A0A5C2GR82_HUMAN | CKM stages 1-2 | -0.2477 | -0.6928 | 4.976e-04 | 0.0291 | hsa05130(Pathogenic Escherichia coli infection); hsa04666(Fc gamma R-mediated phagocytosis); hsa04613(Neutrophil extracellular trap formation); hsa04650(Natural killer cell mediated cytotoxicity); hsa04020(Calcium signaling pathway); hsa05330(Allograft rejection); hsa04672(Intestinal immune network for IgA production); hsa04145(Phagosome); hsa05310(Asthma); hsa04064(NF-kappa B signaling pathway); hsa05414(Dilated cardiomyopathy); hsa05416(Viral myocarditis); hsa05135(Yersinia infection); hsa05143(African trypanosomiasis); hsa05140(Leishmaniasis); hsa05146(Amoebiasis); hsa05169(Epstein-Barr virus infection); hsa05322(Systemic lupus erythematosus); hsa05323(Rheumatoid arthritis); hsa05320(Autoimmune thyroid disease); hsa05202(Transcriptional misregulation in cancer); hsa04662(B cell receptor signaling pathway); hsa04072(Phospholipase D signaling pathway); hsa04664(Fc epsilon RI signaling pathway); hsa04640(Hematopoietic cell lineage); hsa05340(Primary immunodeficiency); hsa04151(PI3K-Akt signaling pathway); hsa05150(Staphylococcus aureus infection); hsa05152(Tuberculosis); hsa05171(Coronavirus disease - COVID-19) |
| A0A5C2GST5 | A0A5C2GST5_HUMAN | CKM stages 1-2 | -0.5082 | -0.6928 | 4.976e-04 | 0.0291 |  |
| A0A5C2GVL1 | A0A5C2GVL1_HUMAN | CKM stages 1-2 | -0.7073 | -0.6928 | 4.976e-04 | 0.0291 |  |
| A8K7T4 | A8K7T4_HUMAN | CKM stages 3-4 | 0.2014 | 0.6928 | 4.976e-04 | 0.0291 | hsa04141(Protein processing in endoplasmic reticulum) |
| P02652 | APOA2 | CKM stages 1-2 | -0.2359 | -0.6928 | 4.976e-04 | 0.0291 | hsa04979(Cholesterol metabolism); hsa03320(PPAR signaling pathway) |
| A0A087WTY6 | NBL1 | CKM stages 3-4 | 0.3824 | 0.6928 | 4.976e-04 | 0.0291 | hsa04350(TGF-beta signaling pathway) |
| P19652 | ORM2 | CKM stages 3-4 | 0.1134 | 0.6928 | 4.976e-04 | 0.0291 |  |
| A0A7I2V3L2 | UVSSA | CKM stages 1-2 | -0.4194 | -0.6928 | 4.976e-04 | 0.0291 |  |
| A0A7S5C0R5 | A0A7S5C0R5_HUMAN | CKM stages 1-2 | -0.6545 | -0.7812 | 5.430e-04 | 0.0314 | hsa05130(Pathogenic Escherichia coli infection); hsa04666(Fc gamma R-mediated phagocytosis); hsa04613(Neutrophil extracellular trap formation); hsa04650(Natural killer cell mediated cytotoxicity); hsa04020(Calcium signaling pathway); hsa05330(Allograft rejection); hsa04672(Intestinal immune network for IgA production); hsa04145(Phagosome); hsa05310(Asthma); hsa04064(NF-kappa B signaling pathway); hsa05414(Dilated cardiomyopathy); hsa05416(Viral myocarditis); hsa05135(Yersinia infection); hsa05143(African trypanosomiasis); hsa05140(Leishmaniasis); hsa05146(Amoebiasis); hsa05169(Epstein-Barr virus infection); hsa05322(Systemic lupus erythematosus); hsa05323(Rheumatoid arthritis); hsa05320(Autoimmune thyroid disease); hsa05202(Transcriptional misregulation in cancer); hsa04662(B cell receptor signaling pathway); hsa04072(Phospholipase D signaling pathway); hsa04664(Fc epsilon RI signaling pathway); hsa04640(Hematopoietic cell lineage); hsa05340(Primary immunodeficiency); hsa04151(PI3K-Akt signaling pathway); hsa05150(Staphylococcus aureus infection); hsa05152(Tuberculosis); hsa05171(Coronavirus disease - COVID-19) |
| A0A5C2GG88 | A0A5C2GG88_HUMAN | CKM stages 1-2 | -0.4978 | -0.6863 | 5.626e-04 | 0.0314 | hsa05130(Pathogenic Escherichia coli infection); hsa04666(Fc gamma R-mediated phagocytosis); hsa04613(Neutrophil extracellular trap formation); hsa04650(Natural killer cell mediated cytotoxicity); hsa04020(Calcium signaling pathway); hsa05330(Allograft rejection); hsa04672(Intestinal immune network for IgA production); hsa04145(Phagosome); hsa05310(Asthma); hsa04064(NF-kappa B signaling pathway); hsa05414(Dilated cardiomyopathy); hsa05416(Viral myocarditis); hsa05135(Yersinia infection); hsa05143(African trypanosomiasis); hsa05140(Leishmaniasis); hsa05146(Amoebiasis); hsa05169(Epstein-Barr virus infection); hsa05322(Systemic lupus erythematosus); hsa05323(Rheumatoid arthritis); hsa05320(Autoimmune thyroid disease); hsa05202(Transcriptional misregulation in cancer); hsa04662(B cell receptor signaling pathway); hsa04072(Phospholipase D signaling pathway); hsa04664(Fc epsilon RI signaling pathway); hsa04640(Hematopoietic cell lineage); hsa05340(Primary immunodeficiency); hsa04151(PI3K-Akt signaling pathway); hsa05150(Staphylococcus aureus infection); hsa05152(Tuberculosis); hsa05171(Coronavirus disease - COVID-19) |
| P02747 | C1QC | CKM stages 1-2 | -0.1013 | -0.6863 | 5.626e-04 | 0.0314 | hsa05133(Pertussis); hsa04610(Complement and coagulation cascades); hsa05322(Systemic lupus erythematosus); hsa04152(AMPK signaling pathway); hsa03320(PPAR signaling pathway); hsa05142(Chagas disease); hsa04932(Non-alcoholic fatty liver disease); hsa05150(Staphylococcus aureus infection); hsa04211(Longevity regulating pathway); hsa04936(Alcoholic liver disease); hsa04920(Adipocytokine signaling pathway); hsa04930(Type II diabetes mellitus); hsa05171(Coronavirus disease - COVID-19); hsa05020(Prion disease) |
| A0A7P0T861 | CALR | CKM stages 3-4 | 0.2510 | 0.6863 | 5.626e-04 | 0.0314 | hsa04141(Protein processing in endoplasmic reticulum); hsa04612(Antigen processing and presentation); hsa05142(Chagas disease); hsa05168(Herpes simplex virus 1 infection); hsa05169(Epstein-Barr virus infection); hsa04145(Phagosome); hsa05166(Human T-cell leukemia virus 1 infection); hsa05163(Human cytomegalovirus infection); hsa04918(Thyroid hormone synthesis); hsa05170(Human immunodeficiency virus 1 infection) |
| P02763 | ORM1 | CKM stages 3-4 | 0.1440 | 0.6863 | 5.626e-04 | 0.0314 |  |
| A0A5C2FXP5 | A0A5C2FXP5_HUMAN | CKM stages 1-2 | -0.4966 | -0.6797 | 6.355e-04 | 0.0336 |  |
| A0A5C2GPH5 | A0A5C2GPH5_HUMAN | CKM stages 1-2 | -0.2848 | -0.6797 | 6.355e-04 | 0.0336 | hsa05130(Pathogenic Escherichia coli infection); hsa04666(Fc gamma R-mediated phagocytosis); hsa04613(Neutrophil extracellular trap formation); hsa04650(Natural killer cell mediated cytotoxicity); hsa04020(Calcium signaling pathway); hsa05330(Allograft rejection); hsa04672(Intestinal immune network for IgA production); hsa04145(Phagosome); hsa05310(Asthma); hsa04064(NF-kappa B signaling pathway); hsa05414(Dilated cardiomyopathy); hsa05416(Viral myocarditis); hsa05135(Yersinia infection); hsa05143(African trypanosomiasis); hsa05140(Leishmaniasis); hsa05146(Amoebiasis); hsa05169(Epstein-Barr virus infection); hsa05322(Systemic lupus erythematosus); hsa05323(Rheumatoid arthritis); hsa05320(Autoimmune thyroid disease); hsa05202(Transcriptional misregulation in cancer); hsa04662(B cell receptor signaling pathway); hsa04072(Phospholipase D signaling pathway); hsa04664(Fc epsilon RI signaling pathway); hsa04640(Hematopoietic cell lineage); hsa05340(Primary immunodeficiency); hsa04151(PI3K-Akt signaling pathway); hsa05150(Staphylococcus aureus infection); hsa05152(Tuberculosis); hsa05171(Coronavirus disease - COVID-19) |
| A0A7S5C006 | A0A7S5C006_HUMAN | CKM stages 1-2 | -0.4342 | -0.6797 | 6.355e-04 | 0.0336 | hsa05130(Pathogenic Escherichia coli infection); hsa04666(Fc gamma R-mediated phagocytosis); hsa04613(Neutrophil extracellular trap formation); hsa04650(Natural killer cell mediated cytotoxicity); hsa04020(Calcium signaling pathway); hsa05330(Allograft rejection); hsa04672(Intestinal immune network for IgA production); hsa04145(Phagosome); hsa05310(Asthma); hsa04064(NF-kappa B signaling pathway); hsa05414(Dilated cardiomyopathy); hsa05416(Viral myocarditis); hsa05135(Yersinia infection); hsa05143(African trypanosomiasis); hsa05140(Leishmaniasis); hsa05146(Amoebiasis); hsa05169(Epstein-Barr virus infection); hsa05322(Systemic lupus erythematosus); hsa05323(Rheumatoid arthritis); hsa05320(Autoimmune thyroid disease); hsa05202(Transcriptional misregulation in cancer); hsa04662(B cell receptor signaling pathway); hsa04072(Phospholipase D signaling pathway); hsa04664(Fc epsilon RI signaling pathway); hsa04640(Hematopoietic cell lineage); hsa05340(Primary immunodeficiency); hsa04151(PI3K-Akt signaling pathway); hsa05150(Staphylococcus aureus infection); hsa05152(Tuberculosis); hsa05171(Coronavirus disease - COVID-19) |
| P36222 | CHI3L1 | CKM stages 3-4 | 0.3200 | 0.6797 | 6.355e-04 | 0.0336 | hsa01100(Metabolic pathways); hsa00520(Amino sugar and nucleotide sugar metabolism) |
| A0A087WT78 | HSP90B1 | CKM stages 3-4 | 0.1838 | 0.6797 | 6.355e-04 | 0.0336 | hsa04659(Th17 cell differentiation); hsa05132(Salmonella infection); hsa04612(Antigen processing and presentation); hsa05207(Chemical carcinogenesis - receptor activation); hsa05022(Pathways of neurodegeneration - multiple diseases); hsa05200(Pathways in cancer); hsa04657(IL-17 signaling pathway); hsa05215(Prostate cancer); hsa04141(Protein processing in endoplasmic reticulum); hsa04621(NOD-like receptor signaling pathway); hsa05418(Fluid shear stress and atherosclerosis); hsa05012(Parkinson disease); hsa04914(Progesterone-mediated oocyte maturation); hsa04915(Estrogen signaling pathway); hsa04918(Thyroid hormone synthesis); hsa04151(PI3K-Akt signaling pathway); hsa04217(Necroptosis); hsa05417(Lipid and atherosclerosis) |
| B2ZDQ1 | NGAL | CKM stages 3-4 | 0.2035 | 0.6797 | 6.355e-04 | 0.0336 | hsa00590(Arachidonic acid metabolism); hsa01100(Metabolic pathways); hsa04657(IL-17 signaling pathway) |
| A0A5C2FX63 | A0A5C2FX63_HUMAN | CKM stages 1-2 | -0.4718 | -0.6732 | 7.170e-04 | 0.0349 |  |
| A0A5C2G7L7 | A0A5C2G7L7_HUMAN | CKM stages 1-2 | -0.4210 | -0.6732 | 7.170e-04 | 0.0349 |  |
| A0A5C2G945 | A0A5C2G945_HUMAN | CKM stages 1-2 | -0.5234 | -0.6732 | 7.170e-04 | 0.0349 |  |
| A0A7S5EWB1 | A0A7S5EWB1_HUMAN | CKM stages 1-2 | -0.3715 | -0.6732 | 7.170e-04 | 0.0349 | hsa05130(Pathogenic Escherichia coli infection); hsa04666(Fc gamma R-mediated phagocytosis); hsa04613(Neutrophil extracellular trap formation); hsa04650(Natural killer cell mediated cytotoxicity); hsa04020(Calcium signaling pathway); hsa05330(Allograft rejection); hsa04672(Intestinal immune network for IgA production); hsa04145(Phagosome); hsa05310(Asthma); hsa04064(NF-kappa B signaling pathway); hsa05414(Dilated cardiomyopathy); hsa05416(Viral myocarditis); hsa05135(Yersinia infection); hsa05143(African trypanosomiasis); hsa05140(Leishmaniasis); hsa05146(Amoebiasis); hsa05169(Epstein-Barr virus infection); hsa05322(Systemic lupus erythematosus); hsa05323(Rheumatoid arthritis); hsa05320(Autoimmune thyroid disease); hsa05202(Transcriptional misregulation in cancer); hsa04662(B cell receptor signaling pathway); hsa04072(Phospholipase D signaling pathway); hsa04664(Fc epsilon RI signaling pathway); hsa04640(Hematopoietic cell lineage); hsa05340(Primary immunodeficiency); hsa04151(PI3K-Akt signaling pathway); hsa05150(Staphylococcus aureus infection); hsa05152(Tuberculosis); hsa05171(Coronavirus disease - COVID-19) |
| A0A7S5EWH5 | A0A7S5EWH5_HUMAN | CKM stages 1-2 | -0.3733 | -0.6732 | 7.170e-04 | 0.0349 | hsa05130(Pathogenic Escherichia coli infection); hsa04666(Fc gamma R-mediated phagocytosis); hsa04613(Neutrophil extracellular trap formation); hsa04650(Natural killer cell mediated cytotoxicity); hsa04020(Calcium signaling pathway); hsa05330(Allograft rejection); hsa04672(Intestinal immune network for IgA production); hsa04145(Phagosome); hsa05310(Asthma); hsa04064(NF-kappa B signaling pathway); hsa05414(Dilated cardiomyopathy); hsa05416(Viral myocarditis); hsa05135(Yersinia infection); hsa05143(African trypanosomiasis); hsa05140(Leishmaniasis); hsa05146(Amoebiasis); hsa05169(Epstein-Barr virus infection); hsa05322(Systemic lupus erythematosus); hsa05323(Rheumatoid arthritis); hsa05320(Autoimmune thyroid disease); hsa05202(Transcriptional misregulation in cancer); hsa04662(B cell receptor signaling pathway); hsa04072(Phospholipase D signaling pathway); hsa04664(Fc epsilon RI signaling pathway); hsa04640(Hematopoietic cell lineage); hsa05340(Primary immunodeficiency); hsa04151(PI3K-Akt signaling pathway); hsa05150(Staphylococcus aureus infection); hsa05152(Tuberculosis); hsa05171(Coronavirus disease - COVID-19) |
| A0A7T0PWX7 | A0A7T0PWX7_HUMAN | CKM stages 1-2 | -0.4112 | -0.6732 | 7.170e-04 | 0.0349 | hsa05130(Pathogenic Escherichia coli infection); hsa04666(Fc gamma R-mediated phagocytosis); hsa04613(Neutrophil extracellular trap formation); hsa04650(Natural killer cell mediated cytotoxicity); hsa04020(Calcium signaling pathway); hsa05330(Allograft rejection); hsa04672(Intestinal immune network for IgA production); hsa04145(Phagosome); hsa05310(Asthma); hsa04064(NF-kappa B signaling pathway); hsa05414(Dilated cardiomyopathy); hsa05416(Viral myocarditis); hsa05135(Yersinia infection); hsa05143(African trypanosomiasis); hsa05140(Leishmaniasis); hsa05146(Amoebiasis); hsa05169(Epstein-Barr virus infection); hsa05322(Systemic lupus erythematosus); hsa05323(Rheumatoid arthritis); hsa05320(Autoimmune thyroid disease); hsa05202(Transcriptional misregulation in cancer); hsa04662(B cell receptor signaling pathway); hsa04072(Phospholipase D signaling pathway); hsa04664(Fc epsilon RI signaling pathway); hsa04640(Hematopoietic cell lineage); hsa05340(Primary immunodeficiency); hsa04151(PI3K-Akt signaling pathway); hsa05150(Staphylococcus aureus infection); hsa05152(Tuberculosis); hsa05171(Coronavirus disease - COVID-19) |
| A8K2P8 | A8K2P8_HUMAN | CKM stages 3-4 | 0.1949 | 0.6732 | 7.170e-04 | 0.0349 | hsa05412(Arrhythmogenic right ventricular cardiomyopathy); hsa04514(Cell adhesion molecules) |
| A0A0A0N0N2 | CNTFR | CKM stages 3-4 | 0.2601 | 0.6732 | 7.170e-04 | 0.0349 | hsa04659(Th17 cell differentiation); hsa05133(Pertussis); hsa04658(Th1 and Th2 cell differentiation); hsa05330(Allograft rejection); hsa04630(JAK-STAT signaling pathway); hsa04940(Type I diabetes mellitus); hsa04060(Cytokine-cytokine receptor interaction); hsa05417(Lipid and atherosclerosis); hsa05134(Legionellosis); hsa05142(Chagas disease); hsa05143(African trypanosomiasis); hsa05140(Leishmaniasis); hsa05168(Herpes simplex virus 1 infection); hsa05145(Toxoplasmosis); hsa05164(Influenza A); hsa05146(Amoebiasis); hsa05162(Measles); hsa05205(Proteoglycans in cancer); hsa04625(C-type lectin receptor signaling pathway); hsa05321(Inflammatory bowel disease); hsa04620(Toll-like receptor signaling pathway); hsa05200(Pathways in cancer); hsa04622(RIG-I-like receptor signaling pathway); hsa04640(Hematopoietic cell lineage); hsa04936(Alcoholic liver disease); hsa05152(Tuberculosis); hsa05171(Coronavirus disease - COVID-19) |
| E7EX60 | NRP1 | CKM stages 3-4 | 0.1615 | 0.6732 | 7.170e-04 | 0.0349 | hsa04360(Axon guidance); hsa05166(Human T-cell leukemia virus 1 infection); hsa04610(Complement and coagulation cascades); hsa05171(Coronavirus disease - COVID-19) |
| P01009 | SERPINA1 | CKM stages 3-4 | 0.1074 | 0.6732 | 7.170e-04 | 0.0349 | hsa04610(Complement and coagulation cascades) |
| A0A5C2FUV9 | A0A5C2FUV9_HUMAN | CKM stages 3-4 | 0.7012 | 0.9630 | 7.992e-04 | 0.0358 |  |
| A0A5C2G839 | A0A5C2G839_HUMAN | CKM stages 1-2 | -0.4918 | -0.6667 | 8.081e-04 | 0.0358 | hsa05130(Pathogenic Escherichia coli infection); hsa04666(Fc gamma R-mediated phagocytosis); hsa04613(Neutrophil extracellular trap formation); hsa04650(Natural killer cell mediated cytotoxicity); hsa04020(Calcium signaling pathway); hsa05330(Allograft rejection); hsa04672(Intestinal immune network for IgA production); hsa04145(Phagosome); hsa05310(Asthma); hsa04064(NF-kappa B signaling pathway); hsa05414(Dilated cardiomyopathy); hsa05416(Viral myocarditis); hsa05135(Yersinia infection); hsa05143(African trypanosomiasis); hsa05140(Leishmaniasis); hsa05146(Amoebiasis); hsa05169(Epstein-Barr virus infection); hsa05322(Systemic lupus erythematosus); hsa05323(Rheumatoid arthritis); hsa05320(Autoimmune thyroid disease); hsa05202(Transcriptional misregulation in cancer); hsa04662(B cell receptor signaling pathway); hsa04072(Phospholipase D signaling pathway); hsa04664(Fc epsilon RI signaling pathway); hsa04640(Hematopoietic cell lineage); hsa05340(Primary immunodeficiency); hsa04151(PI3K-Akt signaling pathway); hsa05150(Staphylococcus aureus infection); hsa05152(Tuberculosis); hsa05171(Coronavirus disease - COVID-19) |
| A0A5C2GEW0 | A0A5C2GEW0_HUMAN | CKM stages 1-2 | -0.3819 | -0.6667 | 8.081e-04 | 0.0358 | hsa05130(Pathogenic Escherichia coli infection); hsa04666(Fc gamma R-mediated phagocytosis); hsa04613(Neutrophil extracellular trap formation); hsa04650(Natural killer cell mediated cytotoxicity); hsa04020(Calcium signaling pathway); hsa05330(Allograft rejection); hsa04672(Intestinal immune network for IgA production); hsa04145(Phagosome); hsa05310(Asthma); hsa04064(NF-kappa B signaling pathway); hsa05414(Dilated cardiomyopathy); hsa05416(Viral myocarditis); hsa05135(Yersinia infection); hsa05143(African trypanosomiasis); hsa05140(Leishmaniasis); hsa05146(Amoebiasis); hsa05169(Epstein-Barr virus infection); hsa05322(Systemic lupus erythematosus); hsa05323(Rheumatoid arthritis); hsa05320(Autoimmune thyroid disease); hsa05202(Transcriptional misregulation in cancer); hsa04662(B cell receptor signaling pathway); hsa04072(Phospholipase D signaling pathway); hsa04664(Fc epsilon RI signaling pathway); hsa04640(Hematopoietic cell lineage); hsa05340(Primary immunodeficiency); hsa04151(PI3K-Akt signaling pathway); hsa05150(Staphylococcus aureus infection); hsa05152(Tuberculosis); hsa05171(Coronavirus disease - COVID-19) |
| A0A5C2GFX4 | A0A5C2GFX4_HUMAN | CKM stages 1-2 | -0.2632 | -0.6667 | 8.081e-04 | 0.0358 |  |
| A0A5C2GUV3 | A0A5C2GUV3_HUMAN | CKM stages 1-2 | -0.4236 | -0.6667 | 8.081e-04 | 0.0358 | hsa05130(Pathogenic Escherichia coli infection); hsa04666(Fc gamma R-mediated phagocytosis); hsa04613(Neutrophil extracellular trap formation); hsa04650(Natural killer cell mediated cytotoxicity); hsa04020(Calcium signaling pathway); hsa05330(Allograft rejection); hsa04672(Intestinal immune network for IgA production); hsa04145(Phagosome); hsa05310(Asthma); hsa04064(NF-kappa B signaling pathway); hsa05414(Dilated cardiomyopathy); hsa05416(Viral myocarditis); hsa05135(Yersinia infection); hsa05143(African trypanosomiasis); hsa05140(Leishmaniasis); hsa05146(Amoebiasis); hsa05169(Epstein-Barr virus infection); hsa05322(Systemic lupus erythematosus); hsa05323(Rheumatoid arthritis); hsa05320(Autoimmune thyroid disease); hsa05202(Transcriptional misregulation in cancer); hsa04662(B cell receptor signaling pathway); hsa04072(Phospholipase D signaling pathway); hsa04664(Fc epsilon RI signaling pathway); hsa04640(Hematopoietic cell lineage); hsa05340(Primary immunodeficiency); hsa04151(PI3K-Akt signaling pathway); hsa05150(Staphylococcus aureus infection); hsa05152(Tuberculosis); hsa05171(Coronavirus disease - COVID-19) |
| A0A5C2GXA1 | A0A5C2GXA1_HUMAN | CKM stages 1-2 | -0.4114 | -0.6667 | 8.081e-04 | 0.0358 |  |
| A0A7S5BXY2 | A0A7S5BXY2_HUMAN | CKM stages 1-2 | -0.5320 | -0.6667 | 8.081e-04 | 0.0358 | hsa05130(Pathogenic Escherichia coli infection); hsa04666(Fc gamma R-mediated phagocytosis); hsa04613(Neutrophil extracellular trap formation); hsa04650(Natural killer cell mediated cytotoxicity); hsa04020(Calcium signaling pathway); hsa05330(Allograft rejection); hsa04672(Intestinal immune network for IgA production); hsa04145(Phagosome); hsa05310(Asthma); hsa04064(NF-kappa B signaling pathway); hsa05414(Dilated cardiomyopathy); hsa05416(Viral myocarditis); hsa05135(Yersinia infection); hsa05143(African trypanosomiasis); hsa05140(Leishmaniasis); hsa05146(Amoebiasis); hsa05169(Epstein-Barr virus infection); hsa05322(Systemic lupus erythematosus); hsa05323(Rheumatoid arthritis); hsa05320(Autoimmune thyroid disease); hsa05202(Transcriptional misregulation in cancer); hsa04662(B cell receptor signaling pathway); hsa04072(Phospholipase D signaling pathway); hsa04664(Fc epsilon RI signaling pathway); hsa04640(Hematopoietic cell lineage); hsa05340(Primary immunodeficiency); hsa04151(PI3K-Akt signaling pathway); hsa05150(Staphylococcus aureus infection); hsa05152(Tuberculosis); hsa05171(Coronavirus disease - COVID-19) |
| A0A7S5C300 | A0A7S5C300_HUMAN | CKM stages 1-2 | -0.5069 | -0.6667 | 8.081e-04 | 0.0358 | hsa05130(Pathogenic Escherichia coli infection); hsa04666(Fc gamma R-mediated phagocytosis); hsa04613(Neutrophil extracellular trap formation); hsa04650(Natural killer cell mediated cytotoxicity); hsa04020(Calcium signaling pathway); hsa05330(Allograft rejection); hsa04672(Intestinal immune network for IgA production); hsa04145(Phagosome); hsa05310(Asthma); hsa04064(NF-kappa B signaling pathway); hsa05414(Dilated cardiomyopathy); hsa05416(Viral myocarditis); hsa05135(Yersinia infection); hsa05143(African trypanosomiasis); hsa05140(Leishmaniasis); hsa05146(Amoebiasis); hsa05169(Epstein-Barr virus infection); hsa05322(Systemic lupus erythematosus); hsa05323(Rheumatoid arthritis); hsa05320(Autoimmune thyroid disease); hsa05202(Transcriptional misregulation in cancer); hsa04662(B cell receptor signaling pathway); hsa04072(Phospholipase D signaling pathway); hsa04664(Fc epsilon RI signaling pathway); hsa04640(Hematopoietic cell lineage); hsa05340(Primary immunodeficiency); hsa04151(PI3K-Akt signaling pathway); hsa05150(Staphylococcus aureus infection); hsa05152(Tuberculosis); hsa05171(Coronavirus disease - COVID-19) |
| Q8N3K9 | CMYA5 | CKM stages 3-4 | 0.2699 | 0.6667 | 8.081e-04 | 0.0358 | hsa04120(Ubiquitin mediated proteolysis) |
| A0A024RC29 | DSC3 | CKM stages 3-4 | 0.2791 | 0.6667 | 8.081e-04 | 0.0358 | hsa05412(Arrhythmogenic right ventricular cardiomyopathy); hsa04514(Cell adhesion molecules) |
| P29966 | MARCKS | CKM stages 3-4 | 0.2310 | 0.6667 | 8.081e-04 | 0.0358 | hsa05140(Leishmaniasis); hsa05206(MicroRNAs in cancer); hsa04666(Fc gamma R-mediated phagocytosis) |
| P10720 | PF4V1 | CKM stages 1-2 | -0.3436 | -0.6667 | 8.081e-04 | 0.0358 | hsa05133(Pertussis); hsa05323(Rheumatoid arthritis); hsa04657(IL-17 signaling pathway); hsa04668(TNF signaling pathway); hsa04062(Chemokine signaling pathway); hsa04060(Cytokine-cytokine receptor interaction); hsa04061(Viral protein interaction with cytokine and cytokine receptor) |
| A0A5C2GF02 | A0A5C2GF02_HUMAN | CKM stages 1-2 | -0.2782 | -0.7500 | 9.024e-04 | 0.0381 | hsa05130(Pathogenic Escherichia coli infection); hsa04666(Fc gamma R-mediated phagocytosis); hsa04613(Neutrophil extracellular trap formation); hsa04650(Natural killer cell mediated cytotoxicity); hsa04020(Calcium signaling pathway); hsa05330(Allograft rejection); hsa04672(Intestinal immune network for IgA production); hsa04145(Phagosome); hsa05310(Asthma); hsa04064(NF-kappa B signaling pathway); hsa05414(Dilated cardiomyopathy); hsa05416(Viral myocarditis); hsa05135(Yersinia infection); hsa05143(African trypanosomiasis); hsa05140(Leishmaniasis); hsa05146(Amoebiasis); hsa05169(Epstein-Barr virus infection); hsa05322(Systemic lupus erythematosus); hsa05323(Rheumatoid arthritis); hsa05320(Autoimmune thyroid disease); hsa05202(Transcriptional misregulation in cancer); hsa04662(B cell receptor signaling pathway); hsa04072(Phospholipase D signaling pathway); hsa04664(Fc epsilon RI signaling pathway); hsa04640(Hematopoietic cell lineage); hsa05340(Primary immunodeficiency); hsa04151(PI3K-Akt signaling pathway); hsa05150(Staphylococcus aureus infection); hsa05152(Tuberculosis); hsa05171(Coronavirus disease - COVID-19) |
| A0A5C2FW89 | A0A5C2FW89_HUMAN | CKM stages 1-2 | -0.4468 | -0.6601 | 9.099e-04 | 0.0381 |  |
| A0A5C2G7K3 | A0A5C2G7K3_HUMAN | CKM stages 3-4 | 0.2554 | 0.6601 | 9.099e-04 | 0.0381 |  |
| A0A5C2GUW4 | A0A5C2GUW4_HUMAN | CKM stages 1-2 | -0.2042 | -0.6601 | 9.099e-04 | 0.0381 | hsa05130(Pathogenic Escherichia coli infection); hsa04666(Fc gamma R-mediated phagocytosis); hsa04613(Neutrophil extracellular trap formation); hsa04650(Natural killer cell mediated cytotoxicity); hsa04020(Calcium signaling pathway); hsa05330(Allograft rejection); hsa04672(Intestinal immune network for IgA production); hsa04145(Phagosome); hsa05310(Asthma); hsa04064(NF-kappa B signaling pathway); hsa05414(Dilated cardiomyopathy); hsa05416(Viral myocarditis); hsa05135(Yersinia infection); hsa05143(African trypanosomiasis); hsa05140(Leishmaniasis); hsa05146(Amoebiasis); hsa05169(Epstein-Barr virus infection); hsa05322(Systemic lupus erythematosus); hsa05323(Rheumatoid arthritis); hsa05320(Autoimmune thyroid disease); hsa05202(Transcriptional misregulation in cancer); hsa04662(B cell receptor signaling pathway); hsa04072(Phospholipase D signaling pathway); hsa04664(Fc epsilon RI signaling pathway); hsa04640(Hematopoietic cell lineage); hsa05340(Primary immunodeficiency); hsa04151(PI3K-Akt signaling pathway); hsa05150(Staphylococcus aureus infection); hsa05152(Tuberculosis); hsa05171(Coronavirus disease - COVID-19) |
| A0A7S5C3I4 | A0A7S5C3I4_HUMAN | CKM stages 1-2 | -0.2859 | -0.6601 | 9.099e-04 | 0.0381 | hsa05130(Pathogenic Escherichia coli infection); hsa04666(Fc gamma R-mediated phagocytosis); hsa04613(Neutrophil extracellular trap formation); hsa04650(Natural killer cell mediated cytotoxicity); hsa04020(Calcium signaling pathway); hsa05330(Allograft rejection); hsa04672(Intestinal immune network for IgA production); hsa04145(Phagosome); hsa05310(Asthma); hsa04064(NF-kappa B signaling pathway); hsa05414(Dilated cardiomyopathy); hsa05416(Viral myocarditis); hsa05135(Yersinia infection); hsa05143(African trypanosomiasis); hsa05140(Leishmaniasis); hsa05146(Amoebiasis); hsa05169(Epstein-Barr virus infection); hsa05322(Systemic lupus erythematosus); hsa05323(Rheumatoid arthritis); hsa05320(Autoimmune thyroid disease); hsa05202(Transcriptional misregulation in cancer); hsa04662(B cell receptor signaling pathway); hsa04072(Phospholipase D signaling pathway); hsa04664(Fc epsilon RI signaling pathway); hsa04640(Hematopoietic cell lineage); hsa05340(Primary immunodeficiency); hsa04151(PI3K-Akt signaling pathway); hsa05150(Staphylococcus aureus infection); hsa05152(Tuberculosis); hsa05171(Coronavirus disease - COVID-19) |
| A0A7S5C4G7 | A0A7S5C4G7_HUMAN | CKM stages 1-2 | -0.3449 | -0.6601 | 9.099e-04 | 0.0381 | hsa05130(Pathogenic Escherichia coli infection); hsa04666(Fc gamma R-mediated phagocytosis); hsa04613(Neutrophil extracellular trap formation); hsa04650(Natural killer cell mediated cytotoxicity); hsa04020(Calcium signaling pathway); hsa05330(Allograft rejection); hsa04672(Intestinal immune network for IgA production); hsa04145(Phagosome); hsa05310(Asthma); hsa04064(NF-kappa B signaling pathway); hsa05414(Dilated cardiomyopathy); hsa05416(Viral myocarditis); hsa05135(Yersinia infection); hsa05143(African trypanosomiasis); hsa05140(Leishmaniasis); hsa05146(Amoebiasis); hsa05169(Epstein-Barr virus infection); hsa05322(Systemic lupus erythematosus); hsa05323(Rheumatoid arthritis); hsa05320(Autoimmune thyroid disease); hsa05202(Transcriptional misregulation in cancer); hsa04662(B cell receptor signaling pathway); hsa04072(Phospholipase D signaling pathway); hsa04664(Fc epsilon RI signaling pathway); hsa04640(Hematopoietic cell lineage); hsa05340(Primary immunodeficiency); hsa04151(PI3K-Akt signaling pathway); hsa05150(Staphylococcus aureus infection); hsa05152(Tuberculosis); hsa05171(Coronavirus disease - COVID-19) |
| A0A7S5EWS4 | A0A7S5EWS4_HUMAN | CKM stages 1-2 | -0.4401 | -0.6601 | 9.099e-04 | 0.0381 | hsa05130(Pathogenic Escherichia coli infection); hsa04666(Fc gamma R-mediated phagocytosis); hsa04613(Neutrophil extracellular trap formation); hsa04650(Natural killer cell mediated cytotoxicity); hsa04020(Calcium signaling pathway); hsa05330(Allograft rejection); hsa04672(Intestinal immune network for IgA production); hsa04145(Phagosome); hsa05310(Asthma); hsa04064(NF-kappa B signaling pathway); hsa05414(Dilated cardiomyopathy); hsa05416(Viral myocarditis); hsa05135(Yersinia infection); hsa05143(African trypanosomiasis); hsa05140(Leishmaniasis); hsa05146(Amoebiasis); hsa05169(Epstein-Barr virus infection); hsa05322(Systemic lupus erythematosus); hsa05323(Rheumatoid arthritis); hsa05320(Autoimmune thyroid disease); hsa05202(Transcriptional misregulation in cancer); hsa04662(B cell receptor signaling pathway); hsa04072(Phospholipase D signaling pathway); hsa04664(Fc epsilon RI signaling pathway); hsa04640(Hematopoietic cell lineage); hsa05340(Primary immunodeficiency); hsa04151(PI3K-Akt signaling pathway); hsa05150(Staphylococcus aureus infection); hsa05152(Tuberculosis); hsa05171(Coronavirus disease - COVID-19) |
| P04004 | VTN | CKM stages 1-2 | -0.1256 | -0.6601 | 9.099e-04 | 0.0381 | hsa04610(Complement and coagulation cascades); hsa05205(Proteoglycans in cancer); hsa04926(Relaxin signaling pathway); hsa04657(IL-17 signaling pathway); hsa04912(GnRH signaling pathway); hsa04512(ECM-receptor interaction); hsa04510(Focal adhesion); hsa04928(Parathyroid hormone synthesis, secretion and action); hsa04668(TNF signaling pathway); hsa05165(Human papillomavirus infection); hsa04151(PI3K-Akt signaling pathway) |
| A0A5C2GMZ2 | A0A5C2GMZ2_HUMAN | CKM stages 1-2 | -0.1799 | -0.6536 | 0.0010 | 0.0398 | hsa05130(Pathogenic Escherichia coli infection); hsa04666(Fc gamma R-mediated phagocytosis); hsa04613(Neutrophil extracellular trap formation); hsa04650(Natural killer cell mediated cytotoxicity); hsa04020(Calcium signaling pathway); hsa05330(Allograft rejection); hsa04672(Intestinal immune network for IgA production); hsa04145(Phagosome); hsa05310(Asthma); hsa04064(NF-kappa B signaling pathway); hsa05414(Dilated cardiomyopathy); hsa05416(Viral myocarditis); hsa05135(Yersinia infection); hsa05143(African trypanosomiasis); hsa05140(Leishmaniasis); hsa05146(Amoebiasis); hsa05169(Epstein-Barr virus infection); hsa05322(Systemic lupus erythematosus); hsa05323(Rheumatoid arthritis); hsa05320(Autoimmune thyroid disease); hsa05202(Transcriptional misregulation in cancer); hsa04662(B cell receptor signaling pathway); hsa04072(Phospholipase D signaling pathway); hsa04664(Fc epsilon RI signaling pathway); hsa04640(Hematopoietic cell lineage); hsa05340(Primary immunodeficiency); hsa04151(PI3K-Akt signaling pathway); hsa05150(Staphylococcus aureus infection); hsa05152(Tuberculosis); hsa05171(Coronavirus disease - COVID-19) |
| A0A5C2GX76 | A0A5C2GX76_HUMAN | CKM stages 1-2 | -0.5548 | -0.6536 | 0.0010 | 0.0398 |  |
| A0A7S5C3N2 | A0A7S5C3N2_HUMAN | CKM stages 1-2 | -0.5774 | -0.6536 | 0.0010 | 0.0398 | hsa05130(Pathogenic Escherichia coli infection); hsa04666(Fc gamma R-mediated phagocytosis); hsa04613(Neutrophil extracellular trap formation); hsa04650(Natural killer cell mediated cytotoxicity); hsa04020(Calcium signaling pathway); hsa05330(Allograft rejection); hsa04672(Intestinal immune network for IgA production); hsa04145(Phagosome); hsa05310(Asthma); hsa04064(NF-kappa B signaling pathway); hsa05414(Dilated cardiomyopathy); hsa05416(Viral myocarditis); hsa05135(Yersinia infection); hsa05143(African trypanosomiasis); hsa05140(Leishmaniasis); hsa05146(Amoebiasis); hsa05169(Epstein-Barr virus infection); hsa05322(Systemic lupus erythematosus); hsa05323(Rheumatoid arthritis); hsa05320(Autoimmune thyroid disease); hsa05202(Transcriptional misregulation in cancer); hsa04662(B cell receptor signaling pathway); hsa04072(Phospholipase D signaling pathway); hsa04664(Fc epsilon RI signaling pathway); hsa04640(Hematopoietic cell lineage); hsa05340(Primary immunodeficiency); hsa04151(PI3K-Akt signaling pathway); hsa05150(Staphylococcus aureus infection); hsa05152(Tuberculosis); hsa05171(Coronavirus disease - COVID-19) |
| A0A7S5C4A7 | A0A7S5C4A7_HUMAN | CKM stages 1-2 | -0.3871 | -0.6536 | 0.0010 | 0.0398 | hsa05130(Pathogenic Escherichia coli infection); hsa04666(Fc gamma R-mediated phagocytosis); hsa04613(Neutrophil extracellular trap formation); hsa04650(Natural killer cell mediated cytotoxicity); hsa04020(Calcium signaling pathway); hsa05330(Allograft rejection); hsa04672(Intestinal immune network for IgA production); hsa04145(Phagosome); hsa05310(Asthma); hsa04064(NF-kappa B signaling pathway); hsa05414(Dilated cardiomyopathy); hsa05416(Viral myocarditis); hsa05135(Yersinia infection); hsa05143(African trypanosomiasis); hsa05140(Leishmaniasis); hsa05146(Amoebiasis); hsa05169(Epstein-Barr virus infection); hsa05322(Systemic lupus erythematosus); hsa05323(Rheumatoid arthritis); hsa05320(Autoimmune thyroid disease); hsa05202(Transcriptional misregulation in cancer); hsa04662(B cell receptor signaling pathway); hsa04072(Phospholipase D signaling pathway); hsa04664(Fc epsilon RI signaling pathway); hsa04640(Hematopoietic cell lineage); hsa05340(Primary immunodeficiency); hsa04151(PI3K-Akt signaling pathway); hsa05150(Staphylococcus aureus infection); hsa05152(Tuberculosis); hsa05171(Coronavirus disease - COVID-19) |
| A0A7S5ETQ8 | A0A7S5ETQ8_HUMAN | CKM stages 1-2 | -0.3435 | -0.6536 | 0.0010 | 0.0398 | hsa05130(Pathogenic Escherichia coli infection); hsa04666(Fc gamma R-mediated phagocytosis); hsa04613(Neutrophil extracellular trap formation); hsa04650(Natural killer cell mediated cytotoxicity); hsa04020(Calcium signaling pathway); hsa05330(Allograft rejection); hsa04672(Intestinal immune network for IgA production); hsa04145(Phagosome); hsa05310(Asthma); hsa04064(NF-kappa B signaling pathway); hsa05414(Dilated cardiomyopathy); hsa05416(Viral myocarditis); hsa05135(Yersinia infection); hsa05143(African trypanosomiasis); hsa05140(Leishmaniasis); hsa05146(Amoebiasis); hsa05169(Epstein-Barr virus infection); hsa05322(Systemic lupus erythematosus); hsa05323(Rheumatoid arthritis); hsa05320(Autoimmune thyroid disease); hsa05202(Transcriptional misregulation in cancer); hsa04662(B cell receptor signaling pathway); hsa04072(Phospholipase D signaling pathway); hsa04664(Fc epsilon RI signaling pathway); hsa04640(Hematopoietic cell lineage); hsa05340(Primary immunodeficiency); hsa04151(PI3K-Akt signaling pathway); hsa05150(Staphylococcus aureus infection); hsa05152(Tuberculosis); hsa05171(Coronavirus disease - COVID-19) |
| A0A7S5EW96 | A0A7S5EW96_HUMAN | CKM stages 1-2 | -0.2961 | -0.6536 | 0.0010 | 0.0398 | hsa05130(Pathogenic Escherichia coli infection); hsa04666(Fc gamma R-mediated phagocytosis); hsa04613(Neutrophil extracellular trap formation); hsa04650(Natural killer cell mediated cytotoxicity); hsa04020(Calcium signaling pathway); hsa05330(Allograft rejection); hsa04672(Intestinal immune network for IgA production); hsa04145(Phagosome); hsa05310(Asthma); hsa04064(NF-kappa B signaling pathway); hsa05414(Dilated cardiomyopathy); hsa05416(Viral myocarditis); hsa05135(Yersinia infection); hsa05143(African trypanosomiasis); hsa05140(Leishmaniasis); hsa05146(Amoebiasis); hsa05169(Epstein-Barr virus infection); hsa05322(Systemic lupus erythematosus); hsa05323(Rheumatoid arthritis); hsa05320(Autoimmune thyroid disease); hsa05202(Transcriptional misregulation in cancer); hsa04662(B cell receptor signaling pathway); hsa04072(Phospholipase D signaling pathway); hsa04664(Fc epsilon RI signaling pathway); hsa04640(Hematopoietic cell lineage); hsa05340(Primary immunodeficiency); hsa04151(PI3K-Akt signaling pathway); hsa05150(Staphylococcus aureus infection); hsa05152(Tuberculosis); hsa05171(Coronavirus disease - COVID-19) |
| A0A7T0LNG0 | A0A7T0LNG0_HUMAN | CKM stages 1-2 | -0.4161 | -0.6536 | 0.0010 | 0.0398 | hsa05130(Pathogenic Escherichia coli infection); hsa04666(Fc gamma R-mediated phagocytosis); hsa04613(Neutrophil extracellular trap formation); hsa04650(Natural killer cell mediated cytotoxicity); hsa04020(Calcium signaling pathway); hsa05330(Allograft rejection); hsa04672(Intestinal immune network for IgA production); hsa04145(Phagosome); hsa05310(Asthma); hsa04064(NF-kappa B signaling pathway); hsa05414(Dilated cardiomyopathy); hsa05416(Viral myocarditis); hsa05135(Yersinia infection); hsa05143(African trypanosomiasis); hsa05140(Leishmaniasis); hsa05146(Amoebiasis); hsa05169(Epstein-Barr virus infection); hsa05322(Systemic lupus erythematosus); hsa05323(Rheumatoid arthritis); hsa05320(Autoimmune thyroid disease); hsa05202(Transcriptional misregulation in cancer); hsa04662(B cell receptor signaling pathway); hsa04072(Phospholipase D signaling pathway); hsa04664(Fc epsilon RI signaling pathway); hsa04640(Hematopoietic cell lineage); hsa05340(Primary immunodeficiency); hsa04151(PI3K-Akt signaling pathway); hsa05150(Staphylococcus aureus infection); hsa05152(Tuberculosis); hsa05171(Coronavirus disease - COVID-19) |
| A0A024R035 | C9 | CKM stages 3-4 | 0.1929 | 0.6536 | 0.0010 | 0.0398 | hsa05322(Systemic lupus erythematosus); hsa05146(Amoebiasis); hsa04610(Complement and coagulation cascades); hsa05171(Coronavirus disease - COVID-19); hsa05020(Prion disease) |
| O14498 | ISLR | CKM stages 3-4 | 0.1667 | 0.6536 | 0.0010 | 0.0398 | hsa04613(Neutrophil extracellular trap formation); hsa05022(Pathways of neurodegeneration - multiple diseases); hsa04933(AGE-RAGE signaling pathway in diabetic complications); hsa05010(Alzheimer disease); hsa05415(Diabetic cardiomyopathy); hsa05417(Lipid and atherosclerosis) |
| P17931 | LGALS3 | CKM stages 3-4 | 0.1953 | 0.6536 | 0.0010 | 0.0398 |  |
| P23470 | PTPRG | CKM stages 3-4 | 0.1651 | 0.6536 | 0.0010 | 0.0398 | hsa05120(Epithelial cell signaling in Helicobacter pylori infection); hsa04514(Cell adhesion molecules) |
| A0A5C2FW65 | A0A5C2FW65_HUMAN | CKM stages 3-4 | 0.4400 | 0.6471 | 0.0012 | 0.0420 |  |
| A0A5C2G647 | A0A5C2G647_HUMAN | CKM stages 1-2 | -0.2028 | -0.6471 | 0.0012 | 0.0420 | hsa05130(Pathogenic Escherichia coli infection); hsa04666(Fc gamma R-mediated phagocytosis); hsa04613(Neutrophil extracellular trap formation); hsa04650(Natural killer cell mediated cytotoxicity); hsa04020(Calcium signaling pathway); hsa05330(Allograft rejection); hsa04672(Intestinal immune network for IgA production); hsa04145(Phagosome); hsa05310(Asthma); hsa04064(NF-kappa B signaling pathway); hsa05414(Dilated cardiomyopathy); hsa05416(Viral myocarditis); hsa05135(Yersinia infection); hsa05143(African trypanosomiasis); hsa05140(Leishmaniasis); hsa05146(Amoebiasis); hsa05169(Epstein-Barr virus infection); hsa05322(Systemic lupus erythematosus); hsa05323(Rheumatoid arthritis); hsa05320(Autoimmune thyroid disease); hsa05202(Transcriptional misregulation in cancer); hsa04662(B cell receptor signaling pathway); hsa04072(Phospholipase D signaling pathway); hsa04664(Fc epsilon RI signaling pathway); hsa04640(Hematopoietic cell lineage); hsa05340(Primary immunodeficiency); hsa04151(PI3K-Akt signaling pathway); hsa05150(Staphylococcus aureus infection); hsa05152(Tuberculosis); hsa05171(Coronavirus disease - COVID-19) |
| A0A5C2G7T7 | A0A5C2G7T7_HUMAN | CKM stages 1-2 | -0.4333 | -0.6471 | 0.0012 | 0.0420 |  |
| A0A5C2GET5 | A0A5C2GET5_HUMAN | CKM stages 1-2 | -0.4662 | -0.6471 | 0.0012 | 0.0420 | hsa05130(Pathogenic Escherichia coli infection); hsa04666(Fc gamma R-mediated phagocytosis); hsa04613(Neutrophil extracellular trap formation); hsa04650(Natural killer cell mediated cytotoxicity); hsa04020(Calcium signaling pathway); hsa05330(Allograft rejection); hsa04672(Intestinal immune network for IgA production); hsa04145(Phagosome); hsa05310(Asthma); hsa04064(NF-kappa B signaling pathway); hsa05414(Dilated cardiomyopathy); hsa05416(Viral myocarditis); hsa05135(Yersinia infection); hsa05143(African trypanosomiasis); hsa05140(Leishmaniasis); hsa05146(Amoebiasis); hsa05169(Epstein-Barr virus infection); hsa05322(Systemic lupus erythematosus); hsa05323(Rheumatoid arthritis); hsa05320(Autoimmune thyroid disease); hsa05202(Transcriptional misregulation in cancer); hsa04662(B cell receptor signaling pathway); hsa04072(Phospholipase D signaling pathway); hsa04664(Fc epsilon RI signaling pathway); hsa04640(Hematopoietic cell lineage); hsa05340(Primary immunodeficiency); hsa04151(PI3K-Akt signaling pathway); hsa05150(Staphylococcus aureus infection); hsa05152(Tuberculosis); hsa05171(Coronavirus disease - COVID-19) |
| A0A5C2GGY7 | A0A5C2GGY7_HUMAN | CKM stages 1-2 | -0.3307 | -0.6471 | 0.0012 | 0.0420 | hsa05130(Pathogenic Escherichia coli infection); hsa04666(Fc gamma R-mediated phagocytosis); hsa04613(Neutrophil extracellular trap formation); hsa04650(Natural killer cell mediated cytotoxicity); hsa04020(Calcium signaling pathway); hsa05330(Allograft rejection); hsa04672(Intestinal immune network for IgA production); hsa04145(Phagosome); hsa05310(Asthma); hsa04064(NF-kappa B signaling pathway); hsa05414(Dilated cardiomyopathy); hsa05416(Viral myocarditis); hsa05135(Yersinia infection); hsa05143(African trypanosomiasis); hsa05140(Leishmaniasis); hsa05146(Amoebiasis); hsa05169(Epstein-Barr virus infection); hsa05322(Systemic lupus erythematosus); hsa05323(Rheumatoid arthritis); hsa05320(Autoimmune thyroid disease); hsa05202(Transcriptional misregulation in cancer); hsa04662(B cell receptor signaling pathway); hsa04072(Phospholipase D signaling pathway); hsa04664(Fc epsilon RI signaling pathway); hsa04640(Hematopoietic cell lineage); hsa05340(Primary immunodeficiency); hsa04151(PI3K-Akt signaling pathway); hsa05150(Staphylococcus aureus infection); hsa05152(Tuberculosis); hsa05171(Coronavirus disease - COVID-19) |
| A0A5C2GJJ6 | A0A5C2GJJ6_HUMAN | CKM stages 1-2 | -0.3296 | -0.6471 | 0.0012 | 0.0420 | hsa04640(Hematopoietic cell lineage); hsa04380(Osteoclast differentiation) |
| A0A5C2GJT4 | A0A5C2GJT4_HUMAN | CKM stages 1-2 | -0.3660 | -0.6471 | 0.0012 | 0.0420 | hsa05130(Pathogenic Escherichia coli infection); hsa04666(Fc gamma R-mediated phagocytosis); hsa04613(Neutrophil extracellular trap formation); hsa04650(Natural killer cell mediated cytotoxicity); hsa04020(Calcium signaling pathway); hsa05330(Allograft rejection); hsa04672(Intestinal immune network for IgA production); hsa04145(Phagosome); hsa05310(Asthma); hsa04064(NF-kappa B signaling pathway); hsa05414(Dilated cardiomyopathy); hsa05416(Viral myocarditis); hsa05135(Yersinia infection); hsa05143(African trypanosomiasis); hsa05140(Leishmaniasis); hsa05146(Amoebiasis); hsa05169(Epstein-Barr virus infection); hsa05322(Systemic lupus erythematosus); hsa05323(Rheumatoid arthritis); hsa05320(Autoimmune thyroid disease); hsa05202(Transcriptional misregulation in cancer); hsa04662(B cell receptor signaling pathway); hsa04072(Phospholipase D signaling pathway); hsa04664(Fc epsilon RI signaling pathway); hsa04640(Hematopoietic cell lineage); hsa05340(Primary immunodeficiency); hsa04151(PI3K-Akt signaling pathway); hsa05150(Staphylococcus aureus infection); hsa05152(Tuberculosis); hsa05171(Coronavirus disease - COVID-19) |
| A0A7S5EW73 | A0A7S5EW73_HUMAN | CKM stages 1-2 | -0.4521 | -0.6471 | 0.0012 | 0.0420 | hsa05130(Pathogenic Escherichia coli infection); hsa04666(Fc gamma R-mediated phagocytosis); hsa04613(Neutrophil extracellular trap formation); hsa04650(Natural killer cell mediated cytotoxicity); hsa04020(Calcium signaling pathway); hsa05330(Allograft rejection); hsa04672(Intestinal immune network for IgA production); hsa04145(Phagosome); hsa05310(Asthma); hsa04064(NF-kappa B signaling pathway); hsa05414(Dilated cardiomyopathy); hsa05416(Viral myocarditis); hsa05135(Yersinia infection); hsa05143(African trypanosomiasis); hsa05140(Leishmaniasis); hsa05146(Amoebiasis); hsa05169(Epstein-Barr virus infection); hsa05322(Systemic lupus erythematosus); hsa05323(Rheumatoid arthritis); hsa05320(Autoimmune thyroid disease); hsa05202(Transcriptional misregulation in cancer); hsa04662(B cell receptor signaling pathway); hsa04072(Phospholipase D signaling pathway); hsa04664(Fc epsilon RI signaling pathway); hsa04640(Hematopoietic cell lineage); hsa05340(Primary immunodeficiency); hsa04151(PI3K-Akt signaling pathway); hsa05150(Staphylococcus aureus infection); hsa05152(Tuberculosis); hsa05171(Coronavirus disease - COVID-19) |
| A0A024RAB6 | HSPG2 | CKM stages 3-4 | 0.1434 | 0.6471 | 0.0012 | 0.0420 | hsa05205(Proteoglycans in cancer); hsa05145(Toxoplasmosis); hsa05200(Pathways in cancer); hsa04512(ECM-receptor interaction); hsa05222(Small cell lung cancer); hsa04510(Focal adhesion); hsa05165(Human papillomavirus infection); hsa05410(Hypertrophic cardiomyopathy); hsa05146(Amoebiasis); hsa05412(Arrhythmogenic right ventricular cardiomyopathy); hsa05414(Dilated cardiomyopathy); hsa05161(Hepatitis B); hsa05416(Viral myocarditis); hsa04151(PI3K-Akt signaling pathway) |
| Q15113 | PCOLCE | CKM stages 3-4 | 0.2354 | 0.6471 | 0.0012 | 0.0420 |  |
| A0A5C2FUI3 | A0A5C2FUI3_HUMAN | CKM stages 1-2 | -0.4541 | -0.6405 | 0.0013 | 0.0440 |  |
| A0A5C2FZ57 | A0A5C2FZ57_HUMAN | CKM stages 3-4 | 0.2331 | 0.6405 | 0.0013 | 0.0440 |  |
| A0A5C2G450 | A0A5C2G450_HUMAN | CKM stages 3-4 | 0.3022 | 0.6405 | 0.0013 | 0.0440 |  |
| A0A5C2G9G2 | A0A5C2G9G2_HUMAN | CKM stages 1-2 | -0.3139 | -0.6405 | 0.0013 | 0.0440 | hsa04640(Hematopoietic cell lineage) |
| A0A5C2GA26 | A0A5C2GA26_HUMAN | CKM stages 1-2 | -0.4374 | -0.6405 | 0.0013 | 0.0440 |  |
| A0A5C2GFE8 | A0A5C2GFE8_HUMAN | CKM stages 1-2 | -0.1602 | -0.6405 | 0.0013 | 0.0440 | hsa05130(Pathogenic Escherichia coli infection); hsa04666(Fc gamma R-mediated phagocytosis); hsa04613(Neutrophil extracellular trap formation); hsa04650(Natural killer cell mediated cytotoxicity); hsa04020(Calcium signaling pathway); hsa05330(Allograft rejection); hsa04672(Intestinal immune network for IgA production); hsa04145(Phagosome); hsa05310(Asthma); hsa04064(NF-kappa B signaling pathway); hsa05414(Dilated cardiomyopathy); hsa05416(Viral myocarditis); hsa05135(Yersinia infection); hsa05143(African trypanosomiasis); hsa05140(Leishmaniasis); hsa05146(Amoebiasis); hsa05169(Epstein-Barr virus infection); hsa05322(Systemic lupus erythematosus); hsa05323(Rheumatoid arthritis); hsa05320(Autoimmune thyroid disease); hsa05202(Transcriptional misregulation in cancer); hsa04662(B cell receptor signaling pathway); hsa04072(Phospholipase D signaling pathway); hsa04664(Fc epsilon RI signaling pathway); hsa04640(Hematopoietic cell lineage); hsa05340(Primary immunodeficiency); hsa04151(PI3K-Akt signaling pathway); hsa05150(Staphylococcus aureus infection); hsa05152(Tuberculosis); hsa05171(Coronavirus disease - COVID-19) |
| A0A5C2GRK2 | A0A5C2GRK2_HUMAN | CKM stages 1-2 | -0.4210 | -0.6405 | 0.0013 | 0.0440 | hsa04380(Osteoclast differentiation) |
| A0A5C2GU79 | A0A5C2GU79_HUMAN | CKM stages 1-2 | -0.1391 | -0.6405 | 0.0013 | 0.0440 |  |
| Q5SZC9 | ABRACL | CKM stages 1-2 | -0.3133 | -0.6405 | 0.0013 | 0.0440 |  |
| Q92520 | FAM3C | CKM stages 3-4 | 0.2615 | 0.6405 | 0.0013 | 0.0440 |  |
| Q02747 | GUCA2A | CKM stages 3-4 | 0.3311 | 0.6405 | 0.0013 | 0.0440 |  |
| E9PN95 | SCGB1A1 | CKM stages 3-4 | 0.5241 | 0.6405 | 0.0013 | 0.0440 |  |
| A0A7T0LNS4 | A0A7T0LNS4_HUMAN | CKM stages 1-2 | -0.5704 | -0.7157 | 0.0013 | 0.0449 |  |
| A0A5C2FU92 | A0A5C2FU92_HUMAN | CKM stages 1-2 | -0.3285 | -0.6340 | 0.0014 | 0.0462 |  |
| A0A5C2G4I0 | A0A5C2G4I0_HUMAN | CKM stages 1-2 | -0.3260 | -0.6340 | 0.0014 | 0.0462 |  |
| A0A5C2G8G6 | A0A5C2G8G6_HUMAN | CKM stages 3-4 | 0.3902 | 0.6340 | 0.0014 | 0.0462 |  |
| A0A5C2GE65 | A0A5C2GE65_HUMAN | CKM stages 1-2 | -0.2539 | -0.6340 | 0.0014 | 0.0462 | hsa05130(Pathogenic Escherichia coli infection); hsa04666(Fc gamma R-mediated phagocytosis); hsa04613(Neutrophil extracellular trap formation); hsa04650(Natural killer cell mediated cytotoxicity); hsa04020(Calcium signaling pathway); hsa05330(Allograft rejection); hsa04672(Intestinal immune network for IgA production); hsa04145(Phagosome); hsa05310(Asthma); hsa04064(NF-kappa B signaling pathway); hsa05414(Dilated cardiomyopathy); hsa05416(Viral myocarditis); hsa05135(Yersinia infection); hsa05143(African trypanosomiasis); hsa05140(Leishmaniasis); hsa05146(Amoebiasis); hsa05169(Epstein-Barr virus infection); hsa05322(Systemic lupus erythematosus); hsa05323(Rheumatoid arthritis); hsa05320(Autoimmune thyroid disease); hsa05202(Transcriptional misregulation in cancer); hsa04662(B cell receptor signaling pathway); hsa04072(Phospholipase D signaling pathway); hsa04664(Fc epsilon RI signaling pathway); hsa04640(Hematopoietic cell lineage); hsa05340(Primary immunodeficiency); hsa04151(PI3K-Akt signaling pathway); hsa05150(Staphylococcus aureus infection); hsa05152(Tuberculosis); hsa05171(Coronavirus disease - COVID-19) |
| A0A5C2GF05 | A0A5C2GF05_HUMAN | CKM stages 1-2 | -0.3303 | -0.6340 | 0.0014 | 0.0462 | hsa05130(Pathogenic Escherichia coli infection); hsa04666(Fc gamma R-mediated phagocytosis); hsa04613(Neutrophil extracellular trap formation); hsa04650(Natural killer cell mediated cytotoxicity); hsa04020(Calcium signaling pathway); hsa05330(Allograft rejection); hsa04672(Intestinal immune network for IgA production); hsa04145(Phagosome); hsa05310(Asthma); hsa04064(NF-kappa B signaling pathway); hsa05414(Dilated cardiomyopathy); hsa05416(Viral myocarditis); hsa05135(Yersinia infection); hsa05143(African trypanosomiasis); hsa05140(Leishmaniasis); hsa05146(Amoebiasis); hsa05169(Epstein-Barr virus infection); hsa05322(Systemic lupus erythematosus); hsa05323(Rheumatoid arthritis); hsa05320(Autoimmune thyroid disease); hsa05202(Transcriptional misregulation in cancer); hsa04662(B cell receptor signaling pathway); hsa04072(Phospholipase D signaling pathway); hsa04664(Fc epsilon RI signaling pathway); hsa04640(Hematopoietic cell lineage); hsa05340(Primary immunodeficiency); hsa04151(PI3K-Akt signaling pathway); hsa05150(Staphylococcus aureus infection); hsa05152(Tuberculosis); hsa05171(Coronavirus disease - COVID-19) |
| A0A5C2GMW9 | A0A5C2GMW9_HUMAN | CKM stages 1-2 | -0.3863 | -0.6340 | 0.0014 | 0.0462 |  |
| A0A5C2GP39 | A0A5C2GP39_HUMAN | CKM stages 1-2 | -0.5022 | -0.6340 | 0.0014 | 0.0462 | hsa05130(Pathogenic Escherichia coli infection); hsa04666(Fc gamma R-mediated phagocytosis); hsa04613(Neutrophil extracellular trap formation); hsa04650(Natural killer cell mediated cytotoxicity); hsa04020(Calcium signaling pathway); hsa05330(Allograft rejection); hsa04672(Intestinal immune network for IgA production); hsa04145(Phagosome); hsa05310(Asthma); hsa04064(NF-kappa B signaling pathway); hsa05414(Dilated cardiomyopathy); hsa05416(Viral myocarditis); hsa05135(Yersinia infection); hsa05143(African trypanosomiasis); hsa05140(Leishmaniasis); hsa05146(Amoebiasis); hsa05169(Epstein-Barr virus infection); hsa05322(Systemic lupus erythematosus); hsa05323(Rheumatoid arthritis); hsa05320(Autoimmune thyroid disease); hsa05202(Transcriptional misregulation in cancer); hsa04662(B cell receptor signaling pathway); hsa04072(Phospholipase D signaling pathway); hsa04664(Fc epsilon RI signaling pathway); hsa04640(Hematopoietic cell lineage); hsa05340(Primary immunodeficiency); hsa04151(PI3K-Akt signaling pathway); hsa05150(Staphylococcus aureus infection); hsa05152(Tuberculosis); hsa05171(Coronavirus disease - COVID-19) |
| A0A7S5BZB0 | A0A7S5BZB0_HUMAN | CKM stages 1-2 | -0.4332 | -0.6340 | 0.0014 | 0.0462 | hsa05130(Pathogenic Escherichia coli infection); hsa04666(Fc gamma R-mediated phagocytosis); hsa04613(Neutrophil extracellular trap formation); hsa04650(Natural killer cell mediated cytotoxicity); hsa04020(Calcium signaling pathway); hsa05330(Allograft rejection); hsa04672(Intestinal immune network for IgA production); hsa04145(Phagosome); hsa05310(Asthma); hsa04064(NF-kappa B signaling pathway); hsa05414(Dilated cardiomyopathy); hsa05416(Viral myocarditis); hsa05135(Yersinia infection); hsa05143(African trypanosomiasis); hsa05140(Leishmaniasis); hsa05146(Amoebiasis); hsa05169(Epstein-Barr virus infection); hsa05322(Systemic lupus erythematosus); hsa05323(Rheumatoid arthritis); hsa05320(Autoimmune thyroid disease); hsa05202(Transcriptional misregulation in cancer); hsa04662(B cell receptor signaling pathway); hsa04072(Phospholipase D signaling pathway); hsa04664(Fc epsilon RI signaling pathway); hsa04640(Hematopoietic cell lineage); hsa05340(Primary immunodeficiency); hsa04151(PI3K-Akt signaling pathway); hsa05150(Staphylococcus aureus infection); hsa05152(Tuberculosis); hsa05171(Coronavirus disease - COVID-19) |
| A0A7S5C3X1 | A0A7S5C3X1_HUMAN | CKM stages 1-2 | -0.3077 | -0.6340 | 0.0014 | 0.0462 | hsa05130(Pathogenic Escherichia coli infection); hsa04666(Fc gamma R-mediated phagocytosis); hsa04613(Neutrophil extracellular trap formation); hsa04650(Natural killer cell mediated cytotoxicity); hsa04020(Calcium signaling pathway); hsa05330(Allograft rejection); hsa04672(Intestinal immune network for IgA production); hsa04145(Phagosome); hsa05310(Asthma); hsa04064(NF-kappa B signaling pathway); hsa05414(Dilated cardiomyopathy); hsa05416(Viral myocarditis); hsa05135(Yersinia infection); hsa05143(African trypanosomiasis); hsa05140(Leishmaniasis); hsa05146(Amoebiasis); hsa05169(Epstein-Barr virus infection); hsa05322(Systemic lupus erythematosus); hsa05323(Rheumatoid arthritis); hsa05320(Autoimmune thyroid disease); hsa05202(Transcriptional misregulation in cancer); hsa04662(B cell receptor signaling pathway); hsa04072(Phospholipase D signaling pathway); hsa04664(Fc epsilon RI signaling pathway); hsa04640(Hematopoietic cell lineage); hsa05340(Primary immunodeficiency); hsa04151(PI3K-Akt signaling pathway); hsa05150(Staphylococcus aureus infection); hsa05152(Tuberculosis); hsa05171(Coronavirus disease - COVID-19) |
| P04080 | CSTB | CKM stages 3-4 | 0.3352 | 0.6340 | 0.0014 | 0.0462 |  |
| Q6N092 | DKFZp686K18196 | CKM stages 1-2 | -0.8030 | -0.6340 | 0.0014 | 0.0462 | hsa05130(Pathogenic Escherichia coli infection); hsa04666(Fc gamma R-mediated phagocytosis); hsa04613(Neutrophil extracellular trap formation); hsa04650(Natural killer cell mediated cytotoxicity); hsa04020(Calcium signaling pathway); hsa04380(Osteoclast differentiation); hsa05330(Allograft rejection); hsa04672(Intestinal immune network for IgA production); hsa04145(Phagosome); hsa05310(Asthma); hsa04064(NF-kappa B signaling pathway); hsa05414(Dilated cardiomyopathy); hsa05416(Viral myocarditis); hsa05135(Yersinia infection); hsa05143(African trypanosomiasis); hsa05140(Leishmaniasis); hsa05146(Amoebiasis); hsa05169(Epstein-Barr virus infection); hsa05322(Systemic lupus erythematosus); hsa05323(Rheumatoid arthritis); hsa05320(Autoimmune thyroid disease); hsa05202(Transcriptional misregulation in cancer); hsa04662(B cell receptor signaling pathway); hsa04072(Phospholipase D signaling pathway); hsa04664(Fc epsilon RI signaling pathway); hsa04640(Hematopoietic cell lineage); hsa05340(Primary immunodeficiency); hsa04151(PI3K-Akt signaling pathway); hsa05150(Staphylococcus aureus infection); hsa05152(Tuberculosis); hsa05171(Coronavirus disease - COVID-19) |
| A0A5C2FZQ2 | A0A5C2FZQ2_HUMAN | CKM stages 1-2 | -0.2667 | -0.8036 | 0.0015 | 0.0463 |  |
| A0A7S5BY02 | A0A7S5BY02_HUMAN | CKM stages 1-2 | -0.9037 | -0.8933 | 0.0015 | 0.0463 | hsa05130(Pathogenic Escherichia coli infection); hsa04666(Fc gamma R-mediated phagocytosis); hsa04613(Neutrophil extracellular trap formation); hsa04650(Natural killer cell mediated cytotoxicity); hsa04020(Calcium signaling pathway); hsa05330(Allograft rejection); hsa04672(Intestinal immune network for IgA production); hsa04145(Phagosome); hsa05310(Asthma); hsa04064(NF-kappa B signaling pathway); hsa05414(Dilated cardiomyopathy); hsa05416(Viral myocarditis); hsa05135(Yersinia infection); hsa05143(African trypanosomiasis); hsa05140(Leishmaniasis); hsa05146(Amoebiasis); hsa05169(Epstein-Barr virus infection); hsa05322(Systemic lupus erythematosus); hsa05323(Rheumatoid arthritis); hsa05320(Autoimmune thyroid disease); hsa05202(Transcriptional misregulation in cancer); hsa04662(B cell receptor signaling pathway); hsa04072(Phospholipase D signaling pathway); hsa04664(Fc epsilon RI signaling pathway); hsa04640(Hematopoietic cell lineage); hsa05340(Primary immunodeficiency); hsa04151(PI3K-Akt signaling pathway); hsa05150(Staphylococcus aureus infection); hsa05152(Tuberculosis); hsa05171(Coronavirus disease - COVID-19) |
| A0A5C2GM54 | A0A5C2GM54_HUMAN | CKM stages 1-2 | -0.2971 | -0.6723 | 0.0016 | 0.0463 | hsa05130(Pathogenic Escherichia coli infection); hsa04666(Fc gamma R-mediated phagocytosis); hsa04613(Neutrophil extracellular trap formation); hsa04650(Natural killer cell mediated cytotoxicity); hsa04020(Calcium signaling pathway); hsa05330(Allograft rejection); hsa04672(Intestinal immune network for IgA production); hsa04145(Phagosome); hsa05310(Asthma); hsa04064(NF-kappa B signaling pathway); hsa05414(Dilated cardiomyopathy); hsa05416(Viral myocarditis); hsa05135(Yersinia infection); hsa05143(African trypanosomiasis); hsa05140(Leishmaniasis); hsa05146(Amoebiasis); hsa05169(Epstein-Barr virus infection); hsa05322(Systemic lupus erythematosus); hsa05323(Rheumatoid arthritis); hsa05320(Autoimmune thyroid disease); hsa05202(Transcriptional misregulation in cancer); hsa04662(B cell receptor signaling pathway); hsa04072(Phospholipase D signaling pathway); hsa04664(Fc epsilon RI signaling pathway); hsa04640(Hematopoietic cell lineage); hsa05340(Primary immunodeficiency); hsa04151(PI3K-Akt signaling pathway); hsa05150(Staphylococcus aureus infection); hsa05152(Tuberculosis); hsa05171(Coronavirus disease - COVID-19) |
| A0A5C2FTH5 | A0A5C2FTH5_HUMAN | CKM stages 1-2 | -0.5525 | -0.6275 | 0.0016 | 0.0463 |  |
| A0A5C2G3L5 | A0A5C2G3L5_HUMAN | CKM stages 1-2 | -0.2242 | -0.6275 | 0.0016 | 0.0463 |  |
| A0A5C2G469 | A0A5C2G469_HUMAN | CKM stages 1-2 | -0.1751 | -0.6275 | 0.0016 | 0.0463 | hsa05135(Yersinia infection); hsa04612(Antigen processing and presentation); hsa04660(T cell receptor signaling pathway); hsa04514(Cell adhesion molecules); hsa04640(Hematopoietic cell lineage); hsa05340(Primary immunodeficiency) |
| A0A5C2G5S2 | A0A5C2G5S2_HUMAN | CKM stages 1-2 | -0.2648 | -0.6275 | 0.0016 | 0.0463 | hsa04660(T cell receptor signaling pathway); hsa05235(PD-L1 expression and PD-1 checkpoint pathway in cancer); hsa04514(Cell adhesion molecules) |
| A0A5C2GB19 | A0A5C2GB19_HUMAN | CKM stages 1-2 | -0.2558 | -0.6275 | 0.0016 | 0.0463 | hsa05130(Pathogenic Escherichia coli infection); hsa04666(Fc gamma R-mediated phagocytosis); hsa04613(Neutrophil extracellular trap formation); hsa04650(Natural killer cell mediated cytotoxicity); hsa04020(Calcium signaling pathway); hsa05330(Allograft rejection); hsa04672(Intestinal immune network for IgA production); hsa04145(Phagosome); hsa05310(Asthma); hsa04064(NF-kappa B signaling pathway); hsa05414(Dilated cardiomyopathy); hsa05416(Viral myocarditis); hsa05135(Yersinia infection); hsa05143(African trypanosomiasis); hsa05140(Leishmaniasis); hsa05146(Amoebiasis); hsa05169(Epstein-Barr virus infection); hsa05322(Systemic lupus erythematosus); hsa05323(Rheumatoid arthritis); hsa05320(Autoimmune thyroid disease); hsa05202(Transcriptional misregulation in cancer); hsa04662(B cell receptor signaling pathway); hsa04072(Phospholipase D signaling pathway); hsa04664(Fc epsilon RI signaling pathway); hsa04640(Hematopoietic cell lineage); hsa05340(Primary immunodeficiency); hsa04151(PI3K-Akt signaling pathway); hsa05150(Staphylococcus aureus infection); hsa05152(Tuberculosis); hsa05171(Coronavirus disease - COVID-19) |
| A0A5C2GLK9 | A0A5C2GLK9_HUMAN | CKM stages 1-2 | -0.2846 | -0.6275 | 0.0016 | 0.0463 |  |
| A0A5C2GRL8 | A0A5C2GRL8_HUMAN | CKM stages 1-2 | -0.4565 | -0.6275 | 0.0016 | 0.0463 |  |
| A0A7S5BYC4 | A0A7S5BYC4_HUMAN | CKM stages 1-2 | -0.4220 | -0.6275 | 0.0016 | 0.0463 | hsa05130(Pathogenic Escherichia coli infection); hsa04666(Fc gamma R-mediated phagocytosis); hsa04613(Neutrophil extracellular trap formation); hsa04650(Natural killer cell mediated cytotoxicity); hsa04020(Calcium signaling pathway); hsa05330(Allograft rejection); hsa04672(Intestinal immune network for IgA production); hsa04145(Phagosome); hsa05310(Asthma); hsa04064(NF-kappa B signaling pathway); hsa05414(Dilated cardiomyopathy); hsa05416(Viral myocarditis); hsa05135(Yersinia infection); hsa05143(African trypanosomiasis); hsa05140(Leishmaniasis); hsa05146(Amoebiasis); hsa05169(Epstein-Barr virus infection); hsa05322(Systemic lupus erythematosus); hsa05323(Rheumatoid arthritis); hsa05320(Autoimmune thyroid disease); hsa05202(Transcriptional misregulation in cancer); hsa04662(B cell receptor signaling pathway); hsa04072(Phospholipase D signaling pathway); hsa04664(Fc epsilon RI signaling pathway); hsa04640(Hematopoietic cell lineage); hsa05340(Primary immunodeficiency); hsa04151(PI3K-Akt signaling pathway); hsa05150(Staphylococcus aureus infection); hsa05152(Tuberculosis); hsa05171(Coronavirus disease - COVID-19) |
| A0A7S5BZA7 | A0A7S5BZA7_HUMAN | CKM stages 1-2 | -0.3157 | -0.6275 | 0.0016 | 0.0463 | hsa05130(Pathogenic Escherichia coli infection); hsa04666(Fc gamma R-mediated phagocytosis); hsa04613(Neutrophil extracellular trap formation); hsa04650(Natural killer cell mediated cytotoxicity); hsa04020(Calcium signaling pathway); hsa05330(Allograft rejection); hsa04672(Intestinal immune network for IgA production); hsa04145(Phagosome); hsa05310(Asthma); hsa04064(NF-kappa B signaling pathway); hsa05414(Dilated cardiomyopathy); hsa05416(Viral myocarditis); hsa05135(Yersinia infection); hsa05143(African trypanosomiasis); hsa05140(Leishmaniasis); hsa05146(Amoebiasis); hsa05169(Epstein-Barr virus infection); hsa05322(Systemic lupus erythematosus); hsa05323(Rheumatoid arthritis); hsa05320(Autoimmune thyroid disease); hsa05202(Transcriptional misregulation in cancer); hsa04662(B cell receptor signaling pathway); hsa04072(Phospholipase D signaling pathway); hsa04664(Fc epsilon RI signaling pathway); hsa04640(Hematopoietic cell lineage); hsa05340(Primary immunodeficiency); hsa04151(PI3K-Akt signaling pathway); hsa05150(Staphylococcus aureus infection); hsa05152(Tuberculosis); hsa05171(Coronavirus disease - COVID-19) |
| A0A7S5C3K5 | A0A7S5C3K5_HUMAN | CKM stages 1-2 | -0.2532 | -0.6275 | 0.0016 | 0.0463 | hsa05130(Pathogenic Escherichia coli infection); hsa04666(Fc gamma R-mediated phagocytosis); hsa04613(Neutrophil extracellular trap formation); hsa04650(Natural killer cell mediated cytotoxicity); hsa04020(Calcium signaling pathway); hsa05330(Allograft rejection); hsa04672(Intestinal immune network for IgA production); hsa04145(Phagosome); hsa05310(Asthma); hsa04064(NF-kappa B signaling pathway); hsa05414(Dilated cardiomyopathy); hsa05416(Viral myocarditis); hsa05135(Yersinia infection); hsa05143(African trypanosomiasis); hsa05140(Leishmaniasis); hsa05146(Amoebiasis); hsa05169(Epstein-Barr virus infection); hsa05322(Systemic lupus erythematosus); hsa05323(Rheumatoid arthritis); hsa05320(Autoimmune thyroid disease); hsa05202(Transcriptional misregulation in cancer); hsa04662(B cell receptor signaling pathway); hsa04072(Phospholipase D signaling pathway); hsa04664(Fc epsilon RI signaling pathway); hsa04640(Hematopoietic cell lineage); hsa05340(Primary immunodeficiency); hsa04151(PI3K-Akt signaling pathway); hsa05150(Staphylococcus aureus infection); hsa05152(Tuberculosis); hsa05171(Coronavirus disease - COVID-19) |
| A0A7S5C3N7 | A0A7S5C3N7_HUMAN | CKM stages 1-2 | -0.2465 | -0.6275 | 0.0016 | 0.0463 | hsa05130(Pathogenic Escherichia coli infection); hsa04666(Fc gamma R-mediated phagocytosis); hsa04613(Neutrophil extracellular trap formation); hsa04650(Natural killer cell mediated cytotoxicity); hsa04020(Calcium signaling pathway); hsa05330(Allograft rejection); hsa04672(Intestinal immune network for IgA production); hsa04145(Phagosome); hsa05310(Asthma); hsa04064(NF-kappa B signaling pathway); hsa05414(Dilated cardiomyopathy); hsa05416(Viral myocarditis); hsa05135(Yersinia infection); hsa05143(African trypanosomiasis); hsa05140(Leishmaniasis); hsa05146(Amoebiasis); hsa05169(Epstein-Barr virus infection); hsa05322(Systemic lupus erythematosus); hsa05323(Rheumatoid arthritis); hsa05320(Autoimmune thyroid disease); hsa05202(Transcriptional misregulation in cancer); hsa04662(B cell receptor signaling pathway); hsa04072(Phospholipase D signaling pathway); hsa04664(Fc epsilon RI signaling pathway); hsa04640(Hematopoietic cell lineage); hsa05340(Primary immunodeficiency); hsa04151(PI3K-Akt signaling pathway); hsa05150(Staphylococcus aureus infection); hsa05152(Tuberculosis); hsa05171(Coronavirus disease - COVID-19) |
| A0A7S5C3Y5 | A0A7S5C3Y5_HUMAN | CKM stages 1-2 | -0.2305 | -0.6275 | 0.0016 | 0.0463 | hsa05130(Pathogenic Escherichia coli infection); hsa04666(Fc gamma R-mediated phagocytosis); hsa04613(Neutrophil extracellular trap formation); hsa04650(Natural killer cell mediated cytotoxicity); hsa04020(Calcium signaling pathway); hsa05330(Allograft rejection); hsa04672(Intestinal immune network for IgA production); hsa04145(Phagosome); hsa05310(Asthma); hsa04064(NF-kappa B signaling pathway); hsa05414(Dilated cardiomyopathy); hsa05416(Viral myocarditis); hsa05135(Yersinia infection); hsa05143(African trypanosomiasis); hsa05140(Leishmaniasis); hsa05146(Amoebiasis); hsa05169(Epstein-Barr virus infection); hsa05322(Systemic lupus erythematosus); hsa05323(Rheumatoid arthritis); hsa05320(Autoimmune thyroid disease); hsa05202(Transcriptional misregulation in cancer); hsa04662(B cell receptor signaling pathway); hsa04072(Phospholipase D signaling pathway); hsa04664(Fc epsilon RI signaling pathway); hsa04640(Hematopoietic cell lineage); hsa05340(Primary immunodeficiency); hsa04151(PI3K-Akt signaling pathway); hsa05150(Staphylococcus aureus infection); hsa05152(Tuberculosis); hsa05171(Coronavirus disease - COVID-19) |
| A0A7S5EYA8 | A0A7S5EYA8_HUMAN | CKM stages 1-2 | -0.3565 | -0.6275 | 0.0016 | 0.0463 | hsa05130(Pathogenic Escherichia coli infection); hsa04666(Fc gamma R-mediated phagocytosis); hsa04613(Neutrophil extracellular trap formation); hsa04650(Natural killer cell mediated cytotoxicity); hsa04020(Calcium signaling pathway); hsa05330(Allograft rejection); hsa04672(Intestinal immune network for IgA production); hsa04145(Phagosome); hsa05310(Asthma); hsa04064(NF-kappa B signaling pathway); hsa05414(Dilated cardiomyopathy); hsa05416(Viral myocarditis); hsa05135(Yersinia infection); hsa05143(African trypanosomiasis); hsa05140(Leishmaniasis); hsa05146(Amoebiasis); hsa05169(Epstein-Barr virus infection); hsa05322(Systemic lupus erythematosus); hsa05323(Rheumatoid arthritis); hsa05320(Autoimmune thyroid disease); hsa05202(Transcriptional misregulation in cancer); hsa04662(B cell receptor signaling pathway); hsa04072(Phospholipase D signaling pathway); hsa04664(Fc epsilon RI signaling pathway); hsa04640(Hematopoietic cell lineage); hsa05340(Primary immunodeficiency); hsa04151(PI3K-Akt signaling pathway); hsa05150(Staphylococcus aureus infection); hsa05152(Tuberculosis); hsa05171(Coronavirus disease - COVID-19) |
| B2R892 | B2R892_HUMAN | CKM stages 1-2 | -0.2986 | -0.6275 | 0.0016 | 0.0463 | hsa01100(Metabolic pathways); hsa00330(Arginine and proline metabolism) |
| B2RA39 | B2RA39_HUMAN | CKM stages 3-4 | 0.1479 | 0.6275 | 0.0016 | 0.0463 | hsa04610(Complement and coagulation cascades); hsa05150(Staphylococcus aureus infection) |
| Q6MZL2 | DKFZp686M0562 | CKM stages 1-2 | -0.1434 | -0.6275 | 0.0016 | 0.0463 | hsa05133(Pertussis); hsa04610(Complement and coagulation cascades); hsa05322(Systemic lupus erythematosus); hsa04145(Phagosome); hsa05150(Staphylococcus aureus infection); hsa05171(Coronavirus disease - COVID-19) |
| A0A8Q3WL37 | F12 | CKM stages 1-2 | -0.2436 | -0.6275 | 0.0016 | 0.0463 | hsa05219(Bladder cancer); hsa04610(Complement and coagulation cascades); hsa05161(Hepatitis B); hsa05205(Proteoglycans in cancer); hsa01522(Endocrine resistance); hsa05206(MicroRNAs in cancer); hsa05200(Pathways in cancer); hsa04657(IL-17 signaling pathway); hsa05202(Transcriptional misregulation in cancer); hsa05418(Fluid shear stress and atherosclerosis); hsa04670(Leukocyte transendothelial migration); hsa04915(Estrogen signaling pathway); hsa04668(TNF signaling pathway); hsa04926(Relaxin signaling pathway); hsa05215(Prostate cancer); hsa05415(Diabetic cardiomyopathy); hsa05417(Lipid and atherosclerosis) |
| A0A5E4DMZ9 | ITGB3 | CKM stages 1-2 | -0.2470 | -0.6275 | 0.0016 | 0.0463 | hsa05133(Pertussis); hsa05131(Shigellosis); hsa05130(Pathogenic Escherichia coli infection); hsa04611(Platelet activation); hsa04610(Complement and coagulation cascades); hsa04613(Neutrophil extracellular trap formation); hsa05134(Legionellosis); hsa04650(Natural killer cell mediated cytotoxicity); hsa05418(Fluid shear stress and atherosclerosis); hsa04672(Intestinal immune network for IgA production); hsa04514(Cell adhesion molecules); hsa04670(Leukocyte transendothelial migration); hsa04145(Phagosome); hsa04510(Focal adhesion); hsa04380(Osteoclast differentiation); hsa05410(Hypertrophic cardiomyopathy); hsa05412(Arrhythmogenic right ventricular cardiomyopathy); hsa05414(Dilated cardiomyopathy); hsa05416(Viral myocarditis); hsa05135(Yersinia infection); hsa05140(Leishmaniasis); hsa05168(Herpes simplex virus 1 infection); hsa05144(Malaria); hsa05145(Toxoplasmosis); hsa05165(Human papillomavirus infection); hsa05166(Human T-cell leukemia virus 1 infection); hsa04151(PI3K-Akt signaling pathway); hsa05163(Human cytomegalovirus infection); hsa05100(Bacterial invasion of epithelial cells); hsa05205(Proteoglycans in cancer); hsa05323(Rheumatoid arthritis); hsa05206(MicroRNAs in cancer); hsa05200(Pathways in cancer); hsa05202(Transcriptional misregulation in cancer); hsa04015(Rap1 signaling pathway); hsa04390(Hippo signaling pathway); hsa05222(Small cell lung cancer); hsa04640(Hematopoietic cell lineage); hsa04360(Axon guidance); hsa04919(Thyroid hormone signaling pathway); hsa04810(Regulation of actin cytoskeleton); hsa04530(Tight junction); hsa05146(Amoebiasis); hsa05150(Staphylococcus aureus infection); hsa05152(Tuberculosis); hsa04512(ECM-receptor interaction) |
| P02750 | LRG1 | CKM stages 3-4 | 0.1838 | 0.6275 | 0.0016 | 0.0463 |  |
| A0A5C2FTC1 | A0A5C2FTC1_HUMAN | CKM stages 1-2 | -0.4621 | -0.6209 | 0.0018 | 0.0495 |  |
| A0A5C2FUU9 | A0A5C2FUU9_HUMAN | CKM stages 1-2 | -0.3101 | -0.6209 | 0.0018 | 0.0495 | hsa04380(Osteoclast differentiation) |
| A0A5C2FV93 | A0A5C2FV93_HUMAN | CKM stages 1-2 | -0.2567 | -0.6209 | 0.0018 | 0.0495 |  |
| A0A5C2GDK6 | A0A5C2GDK6_HUMAN | CKM stages 1-2 | -0.2715 | -0.6209 | 0.0018 | 0.0495 |  |
| A0A5C2GKT3 | A0A5C2GKT3_HUMAN | CKM stages 1-2 | -0.1759 | -0.6209 | 0.0018 | 0.0495 |  |
| A0A5C2GLF7 | A0A5C2GLF7_HUMAN | CKM stages 1-2 | -0.3654 | -0.6209 | 0.0018 | 0.0495 | hsa05130(Pathogenic Escherichia coli infection); hsa04666(Fc gamma R-mediated phagocytosis); hsa04613(Neutrophil extracellular trap formation); hsa04650(Natural killer cell mediated cytotoxicity); hsa04020(Calcium signaling pathway); hsa05330(Allograft rejection); hsa04672(Intestinal immune network for IgA production); hsa04145(Phagosome); hsa05310(Asthma); hsa04064(NF-kappa B signaling pathway); hsa05414(Dilated cardiomyopathy); hsa05416(Viral myocarditis); hsa05135(Yersinia infection); hsa05143(African trypanosomiasis); hsa05140(Leishmaniasis); hsa05146(Amoebiasis); hsa05169(Epstein-Barr virus infection); hsa05322(Systemic lupus erythematosus); hsa05323(Rheumatoid arthritis); hsa05320(Autoimmune thyroid disease); hsa05202(Transcriptional misregulation in cancer); hsa04662(B cell receptor signaling pathway); hsa04072(Phospholipase D signaling pathway); hsa04664(Fc epsilon RI signaling pathway); hsa04640(Hematopoietic cell lineage); hsa05340(Primary immunodeficiency); hsa04151(PI3K-Akt signaling pathway); hsa05150(Staphylococcus aureus infection); hsa05152(Tuberculosis); hsa05171(Coronavirus disease - COVID-19) |
| A0A5C2GM07 | A0A5C2GM07_HUMAN | CKM stages 1-2 | -0.3302 | -0.6209 | 0.0018 | 0.0495 |  |
| Q14126 | DSG2 | CKM stages 3-4 | 0.1842 | 0.6209 | 0.0018 | 0.0495 | hsa05412(Arrhythmogenic right ventricular cardiomyopathy); hsa05150(Staphylococcus aureus infection); hsa04514(Cell adhesion molecules) |
| A0A0K0K1H8 | HEL | CKM stages 1-2 | -0.1422 | -0.6209 | 0.0018 | 0.0495 | hsa04066(HIF-1 signaling pathway); hsa04978(Mineral absorption); hsa04216(Ferroptosis) |
| Q6ZW64 | Q6ZW64_HUMAN | CKM stages 1-2 | -0.4368 | -0.6209 | 0.0018 | 0.0495 | hsa05130(Pathogenic Escherichia coli infection); hsa04666(Fc gamma R-mediated phagocytosis); hsa04613(Neutrophil extracellular trap formation); hsa04650(Natural killer cell mediated cytotoxicity); hsa04020(Calcium signaling pathway); hsa04380(Osteoclast differentiation); hsa05330(Allograft rejection); hsa04672(Intestinal immune network for IgA production); hsa04145(Phagosome); hsa05310(Asthma); hsa04064(NF-kappa B signaling pathway); hsa05414(Dilated cardiomyopathy); hsa05416(Viral myocarditis); hsa05135(Yersinia infection); hsa05143(African trypanosomiasis); hsa05140(Leishmaniasis); hsa05146(Amoebiasis); hsa05169(Epstein-Barr virus infection); hsa05322(Systemic lupus erythematosus); hsa05323(Rheumatoid arthritis); hsa05320(Autoimmune thyroid disease); hsa05202(Transcriptional misregulation in cancer); hsa04662(B cell receptor signaling pathway); hsa04072(Phospholipase D signaling pathway); hsa04664(Fc epsilon RI signaling pathway); hsa04640(Hematopoietic cell lineage); hsa05340(Primary immunodeficiency); hsa04151(PI3K-Akt signaling pathway); hsa05150(Staphylococcus aureus infection); hsa05152(Tuberculosis); hsa05171(Coronavirus disease - COVID-19) |
